# Current dichotomous metrics obscure trends in severe and extreme child growth failure

**DOI:** 10.1126/sciadv.abm8954

**Published:** 2022-05-20

**Authors:** Ryan Fitzgerald, Helena Manguerra, Michael B. Arndt, William M. Gardner, Ya-Yin Chang, Bethany Zigler, Heather Jean Taylor, Kelly Bienhoff, David L. Smith, Christopher J. L. Murray, Simon I. Hay, Robert C. Reiner, Nicholas J. Kassebaum

**Affiliations:** 1 Institute for Health Metrics and Evaluation, University of Washington, Seattle, WA, USA.; 2 Department of Global Health, University of Washington, Seattle, WA, USA.; 3 Department of Health Metrics Sciences, School of Medicine, University of Washington, Seattle, WA, USA.; 4 Department of Anesthesiology and Pain Medicine, School of Medicine, University of Washington, Seattle, WA, USA.

## Abstract

Historically, the prevalence of child growth failure (CGF) has been tracked dichotomously as the proportion of children more than 2 SDs below the median of the World Health Organization growth standards. However, this conventional “thresholding” approach fails to recognize child growth as a spectrum and obscures trends in populations with the highest rates of CGF. Our analysis presents the first ever estimates of entire distributions of HAZ, WHZ, and WAZ for each of 204 countries and territories from 1990 to 2020 for children less than 5 years old by age group and sex. This approach reflects the continuous nature of CGF, allows us to more comprehensively assess shrinking or widening disparities over time, and reveals otherwise hidden trends that disproportionately affect the most vulnerable populations.

## Teaser

Comprehensive assessment of child growth distributions reveals trends hidden by conventional dichotomous monitoring metrics.

## INTRODUCTION

Child growth failure (CGF) is characterized by deficiencies in height-for-age (HAZ; stunting), weight-for-height (WHZ; wasting), and weight-for-age (WAZ; underweight) and measured as SDs (*z* scores) from the medians of age- and sex-specific World Health Organization (WHO) growth standards (*1*). Suboptimal development in the form of CGF is associated with an increased risk of acquiring communicable diseases such as diarrheal diseases, lower respiratory infections (LRIs), and measles, in addition to developing noncommunicable conditions including cognitive impairments, cardiovascular disease, and metabolic disorders (*2*, *3*). Chronic nutritional deficiencies early in life may also increase the risk of an individual developing adverse health conditions later in life (*3*). This makes CGF an important risk factor for addressing morbidity and mortality globally.

International CGF surveillance efforts have historically only monitored the proportion of children below 2 SDs from the median of age- and sex-specific WHO growth standards without consideration for trends in those with more severe growth failure (*4*). The Global Burden of Diseases, Injuries, and Risk Factors Study (GBD) 2020, in contrast, estimated the prevalence of multiple severities of CGF while also quantifying associated relative risks for LRIs, diarrhea, measles, and protein-energy malnutrition. Several large pooled meta-analyses have identified that these relative risks grow exponentially with worsening CGF (*5*, *6*). Consequently, CGF-attributable disability and deaths are disproportionately linked to severe CGF, meaning that they occur in children who are more than 3 SDs below the median of the WHO growth standard curves.

While tracking overall CGF can provide insight into a country’s general progress toward reducing child malnutrition, this singular metric often obscures more nuanced trends that affect the most vulnerable populations. To ensure that CGF progress is equitable and that the most vulnerable groups are benefitting from efforts to reduce CGF, it is necessary to actively monitor more severe forms of CGF in addition to overall CGF. To that end, this analysis assesses CGF at multiple cutoffs to more comprehensively capture how the distribution of CGF has changed over time.

## RESULTS

### The case for monitoring severe and extreme CGF

**Fig. 1.**
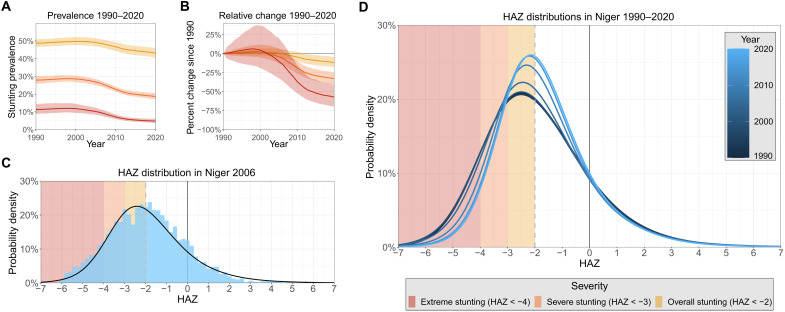
Stunting trends and distribution curves in Niger. Trends in each severity of stunting in Niger from 1990 to 2020 (**A**). The yellow line for overall stunting reflects the conventional stunting monitoring metric; relative changes in each severity since 1990 (**B**). A step in our methodology by which we incorporate surveys with individual measurements of HAZ to paramaterize characteristic curves of HAZ. Here, individual anthropometric measurements from the 2006 Demographic and Health Survey in Niger are overlaid with the curve of final HAZ estimates for Niger in 2006 (**C**). This process is completed for all years, yielding a sequence of changing HAZ curves over time (**D**).

When a continuous distribution of child growth is assessed, as compared to only point prevalence values of overall CGF, a more complete understanding of progress emerges. In this analysis, completed as part of the GBD 2020, characteristic shapes of HAZ, WHZ, and WAZ curves were parameterized by fitting an ensemble of distribution families to anthropometric measurements from studies that reported individual-level data (ensemble weight sets shown in table S5) (*7*). These curve shapes were further optimized to align with results from spatiotemporal regressions that incorporated evidence from surveys that only reported point prevalences of overall and severe CGF (data file S1 shows regression input data and results; fig. S1 shows regression covariates). This process allowed us to leverage the wide breadth of input data that only report point prevalences while still incorporating the nuances that can only be derived from surveys that report individual anthropometric measurements (data landscape maps are shown in fig. S2). The advantages of this approach are highlighted in Fig. 1 in the representative example of stunting in Niger. Corresponding curves for all 204 countries and territories can be found in data file S1.

The country of Niger saw only modest overall stunting progress in recent decades, as prevalence decreased from 48.8% (46.8 to 51.0%) in 1990 to 43.2% (40.6 to 45.9%) in 2020. This marks an 11.7% (4.51 to 17.7%) relative decrease in overall stunting (HAZ < −2SD), whereas severe (HAZ < −3SD) and extreme (HAZ < −4SD) stunting saw much larger 33.0% (25.3 to 40.8%) and 57.3% (41.6 to 70.2%) relative decreases, respectively, over the same 30-year span. These larger improvements in severe and extreme stunting are more easily observed when the time series of entire distributions are considered. The mean of the curves shifted to the right over recent decades from an HAZ value of −1.88 (−1.97 to −1.78) to −1.65 (−1.74 to −1.54), reflecting Niger’s slight overall stunting progress. The spread of the curves also decreased over this time, contributing to greater progress in severe and extreme stunting as compared to overall stunting in Niger. These metrics reveal a dynamic progression of stunting in Niger that is otherwise undetectable by tracking only the comparatively static prevalence of overall stunting through time.

This example also highlights that in high-burden countries, extreme CGF is more common than might be expected considering that we are unaware of any systematic reporting or tracking of it in any previous global nutrition assessment. In 1990, 11.3% (8.79 to 14.3%) of children less than 5 years old in Niger were extremely stunted, with an estimated 23.2% (17.7 to 29.7%) of all stunted children in the country considered to be experiencing extreme stunting. By 2020, 4.80% (3.77 to 6.11%) of all children less than 5 years old in Niger were extremely stunted, with 11.1% (8.62 to 14.5%) of all stunted children experiencing extreme stunting. While overall stunting rates in Niger have only experienced slight decreases since 1990, this analysis reveals that the risk profile of those cases has shifted markedly. Dichotomous estimation of CGF fails to detect this progress and functionally assumes that all stunted children are at the same risk for developing adverse health outcomes. This assumption is an oversimplification that obscures the true risk of the most vulnerable populations that experience severe and extreme CGF.

### Worldwide CGF exposure

**Fig. 2.**
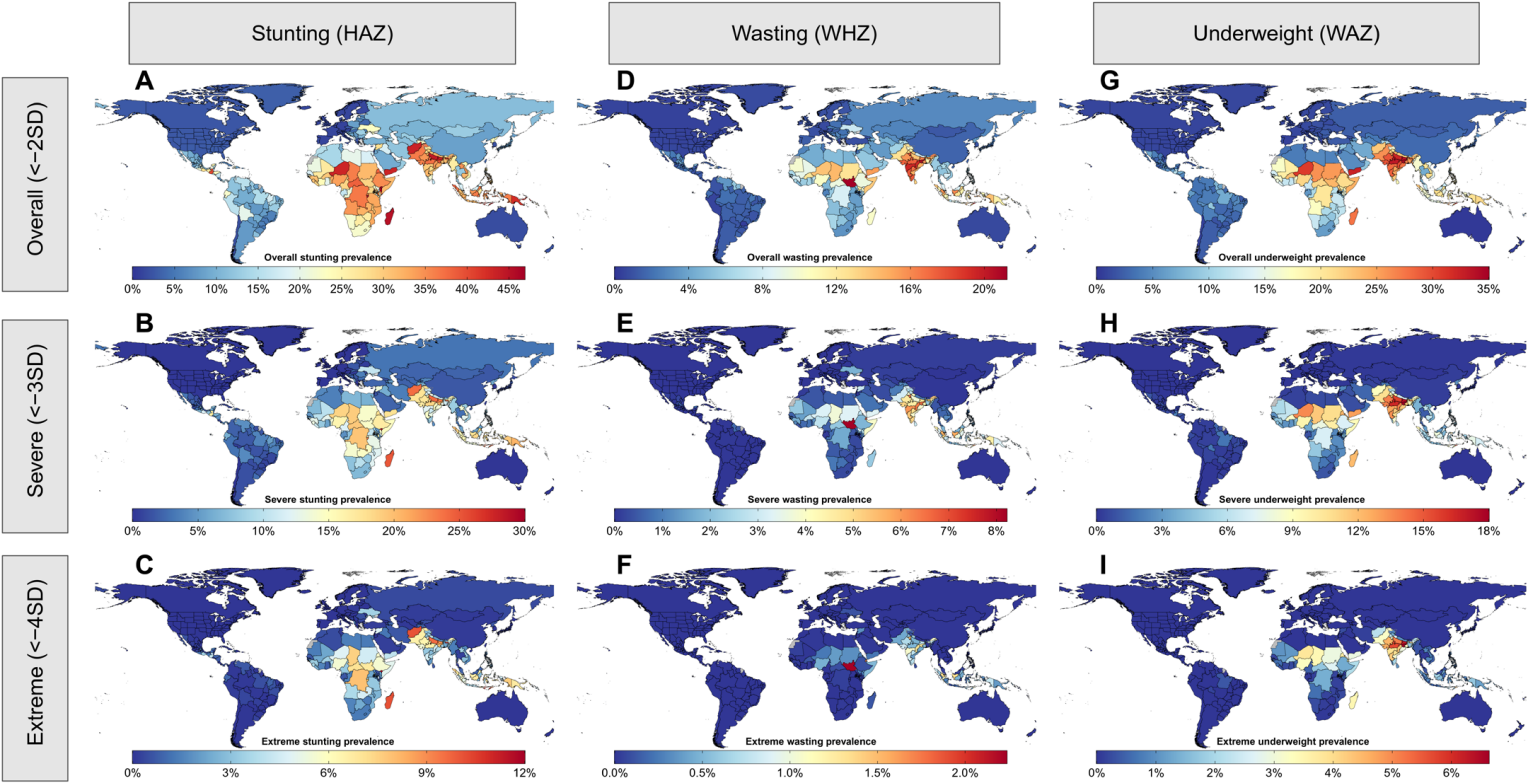
Maps of stunting, wasting, and underweight prevalences in 2020. Maps of overall (**A**), severe (**B**), and extreme (**C**) stunting prevalences; overall (**D**), severe (**E**), and extreme (**F**) wasting prevalences; and overall (**G**), severe (**H**), and extreme (**I**) underweight prevalences. All maps shown are for children less than 5 years old, both sexes, in 2020.

Of the three forms of CGF, stunting was most prevalent worldwide in 2020, with an estimated global prevalence of 24.1% (24.0 to 24.3%) in children less than 5 years old (Fig. 2). Stunting prevalence was highest in South Asia [35.6% (35.2 to 35.9%)], Sub-Saharan Africa [32.3% (32.0 to 32.6%)], and Southeast Asia [27.3% (27.0 to 27.7%)]. Of the estimated 158.3 (157.3 to 159.4) million children worldwide who were stunted in 2020, approximately 66.2 (65.1 to 67.3) million were severely stunted and 20.3 (19.2 to 21.4) million were extremely stunted. Global wasting prevalence was lower, at 7.41% (7.35 to 7.46%), and was also most common in South Asia [14.8% (14.6 to 15.0%)], Southeast Asia [8.02% (7.90 to 8.14%)], and countries in the Sahel region of Sub-Saharan Africa such as South Sudan [21.2% (20.1 to 22.4%)] and Chad [14.3% (13.6 to 15.0%)]. Worldwide, approximately 48.6 (48.2 to 49.0) million children were wasted in 2020, of which 18.1 (17.9 to 18.3) million were in India. Relatively fewer wasting cases were severe and extreme compared with stunting cases, as 10.9 (10.8 to 11.1) million children globally were severely wasted and 1.32 (1.26 to 1.38) million were extremely wasted. The worldwide prevalence of underweight among children was approximately 14.7% (14.6 to 14.8%) and was highest in South Asia [28.1% (27.9 to 28.3%)], Southeast Asia [15.8% (15.6 to 16.0%)], and Sub-Saharan Africa [18.0% (17.9 to 18.2%)]. Of the 96.6 (96.1 to 97.1) million underweight children around the world, approximately 35.7 million (35.2 to 36.2) were severely underweight and 9.51 (9.13 to 9.87) million were extremely underweight. Prevalence estimates for all forms and severities of CGF in 204 countries and territories in 1990, 2000, 2010, and 2020 are included in table S8, and additional maps for 1990, 2000, and 2010 are shown in fig. S3.

### Assessing progress across CGF severities

**Fig. 3.**
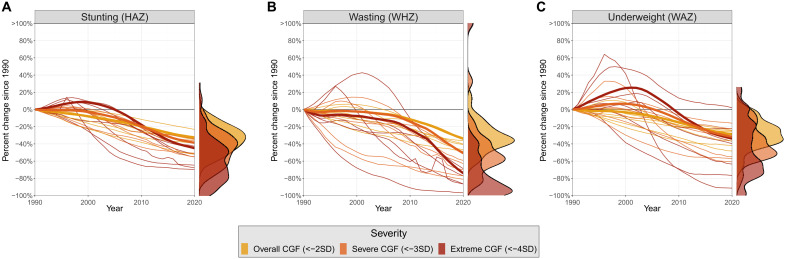
Relative changes in stunting, wasting, and underweight prevalences since 1990. Relative changes in stunting (**A**), wasting (**B**), and underweight (**C**) prevalences since 1990. Bold lines reflect global trends in CGF prevalence, while thin lines represent trends for seven super regions. Density plots on the right display the range of percent change values estimated in 2020 for countries with extreme CGF prevalences greater than one per million and populations larger than 300,000.

All severities of CGF saw notable progress from 1990 to 2020, but not all severities experienced the same magnitude of progress. Severe and extreme CGF have generally experienced greater relative progress than overall CGF in recent decades. Globally, relative improvements in severe stunting, wasting, and underweight have respectively been 1.19 (1.14 to 1.24), 1.49 (1.47 to 1.51), and 1.11 (1.06 to 1.15) times larger than those of overall CGF since 1990. Similarly, relative improvements in extreme stunting, wasting, and underweight have respectively been 1.38 (1.25 to 1.51), 2.17 (2.13 to 2.22), and 1.18 (1.03 to 1.34) times larger than relative improvements in overall CGF globally since 1990. While the strength of this trend may vary, it remains consistently observable across GBD super regions, locations, and forms of CGF, as seen in Fig. 3 (see fig. S4 for super-region disaggregation).

Locations with the highest rates of CGF often exhibited severe and extreme CGF improvements that exceeded overall CGF improvements by the largest margins. In Sub-Saharan Africa, for example, severe and extreme stunting have respectively improved 1.49 (1.39 to 1.59) and 1.98 (1.70 to 2.25) times more than overall stunting. This is the highest margin of any super region in the world and significantly greater than the global average. Similarly, in countries with some of the highest wasting rates in the world, such as Yemen and South Sudan, severe and extreme wasting have improved much more than overall wasting. Severe wasting prevalence in Yemen and South Sudan has respectively improved 3.57 (2.78 to 4.86) and 2.32 (1.71 to 3.44) times more than overall wasting since 1990, while extreme wasting has improved 7.03 (4.86 to 10.53) and 4.19 (2.71 to 7.39) times more than overall wasting. This pattern is similarly mirrored in underweight, as the high-burden countries of Niger and Burundi have seen severe and extreme underweight rates improve faster than the global average. Severe underweight has respectively improved 1.98 (1.48 to 2.53) and 2.00 (1.43 to 2.56) times more than overall underweight in Niger and Burundi, while extreme underweight has improved 3.16 (2.09 to 4.17) and 3.25 (1.91 to 4.44) times more than overall underweight since 1990. These representative examples indicate that in key high-burden regions where CGF progress has often been considered static, undetected progress is most notable. While this trend is particularly strong in the highlighted regions with the highest CGF rates, it is also widely observed across countries for all three dimensions of CGF.

### Health system strength and equitable progress

**Fig. 4.**
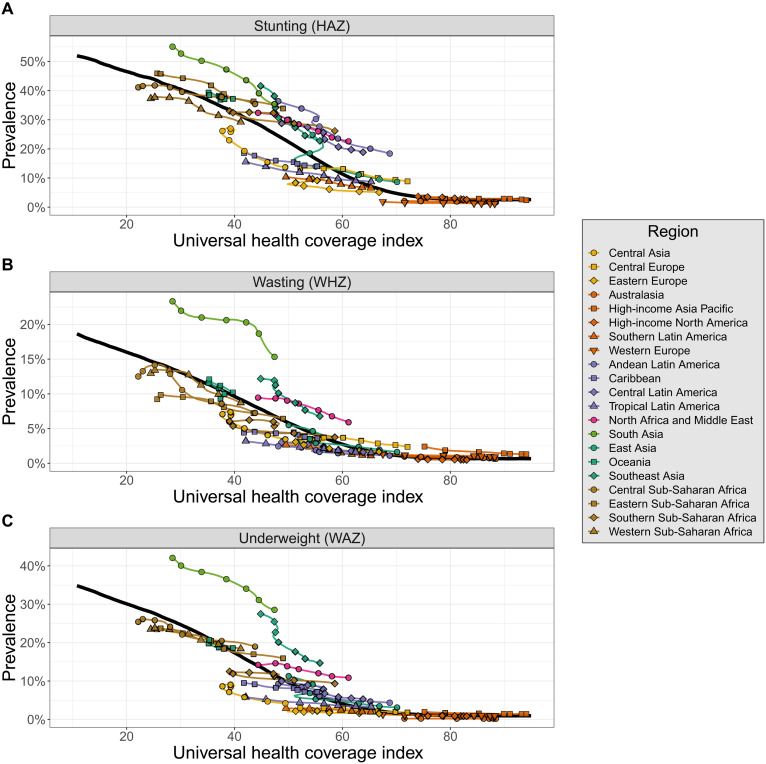
Estimated overall stunting, wasting, and underweight prevalences by region compared to what would be expected based on the UHC index. The expected values of overall stunting (**A**), wasting (**B**), and underweight (**C**) based on the UHC index are represented by the solid black lines. Estimated values of overall CGF are shown for each region and colored by super region. Points are shown every 5 years from 1990 to 2020.

To understand factors associated with differential progress across CGF severities, we compared overall, severe, and extreme CGF prevalences to expected prevalences for a location based on its universal health coverage (UHC) index (Fig. 4) (*7*). This index synthesizes estimates of intervention coverage and health outcomes to estimate access to quality health care, making it a useful indicator of health system strength. We ran meta-regression–Bayesian, regularized, trimmed (MR-BRT) models to capture relationships that we expected might be heterogeneous over the domain of the UHC index (*8*). (Age-sex specific model fits are shown in data file S2, with weighted ensemble knot placements shown in data file S3.)

Health system performance, as measured by the UHC index, was found to be strongly inversely associated with prevalence of all forms and severities of CGF. However, there remains notable regional variation in CGF prevalences compared with values that would be expected based on the UHC index. This can be seen in Fig. 4 (A to C), which compares estimated overall stunting, wasting, and underweight prevalences by region compared to what would be expected based on the UHC index (see fig. S5 for region designations). Regional variation in comparison to expected CGF prevalences is generally consistent across CGF severities (fig. S6). Some regions, such as Central Asia and Tropical Latin America, generally see lower CGF prevalences than what would be expected based on the UHC index. In contrast, other regions such as South Asia, Southeast Asia, and North Africa and the Middle East generally have CGF prevalences that exceed expected values based on the UHC index. South Asia, in particular, has wasting and underweight prevalences that far exceed what would be expected based on the UHC index. Performance of individual countries and territories compared with expected values can be seen for all severities and forms of CGF in fig. S7.

**Fig. 5.**
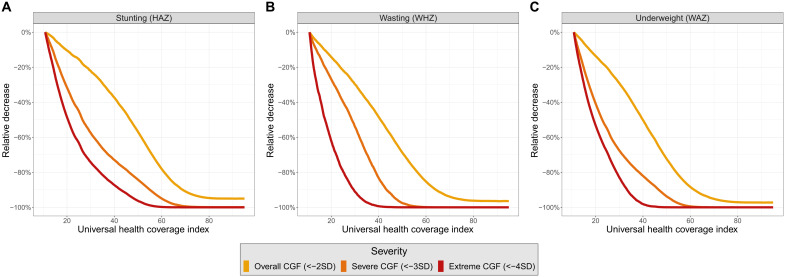
Expected values of overall, severe, and extreme CGF scaled to show how trajectories change as the UHC index improves. Expected values of overall, severe, and extreme CGF scaled to show how trajectories in stunting (**A**), wasting (**B**), and underweight (**C**) change as the UHC index improves. Prevalences have been scaled to demonstrate expected relative changes as the UHC index increases from the minimum observed UHC index value.

While UHC improvements are associated with decreases in all severities of CGF, expected decreases are sharper for severe and extreme CGF. To compare the expected relative progress across severities of CGF as the UHC index increases, the expected trajectories of overall, severe, and extreme CGF have been scaled in Fig. 5. Although overall CGF is inherently more common than severe and extreme CGF—meaning that absolute reductions in overall CGF over time may be larger—this scaled analysis reveals that expected relative decreases are larger for severe and extreme CGF. Notably, expected severe and extreme CGF prevalences approach zero substantially earlier than overall CGF prevalence. The sharper decline in expected severe and extreme CGF prevalences as the UHC index improves suggests that even slight UHC index changes are associated with marked consequences for severe and extreme CGF prevalences. The relationship between the UHC index and CGF prevalence is strong, but likely interconnected with other factors such as household income, food security, and immunization coverage. For this reason, we completed additional MR-BRT models that included sociodemographic index (SDI) as a covariate. While expected CGF prevalences varied considerably across a range of SDI values, the tendency for severe and extreme CGF to be more sensitive to UHC index change remained consistent across a wide range of SDI values (see data file S4 for age- and sex-specific MR-BRT model fits with SDI covariate; see fig. S8 for scaled relationships across SDI range). Considering the disproportionate burden of severe and extreme CGF, for locations with any given SDI, strengthening health systems with the goal of specifically targeting severe and extreme CGF may have the greatest effect on total CGF burden.

### Health system disruptions and uneven setbacks

**Fig. 6.**
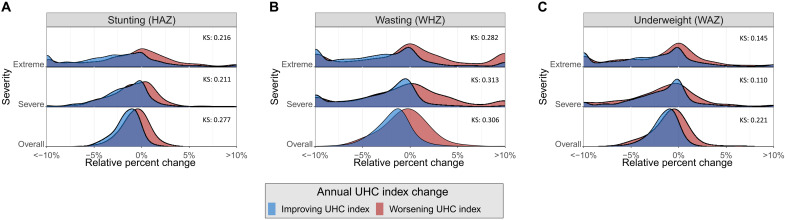
Comparison of annual relative changes across severities for each form of CGF for country-years that experienced UHC index improvements and setbacks. Annual relative changes for each severity of stunting (**A**), wasting (**B**), and underweight (**C**), categorized by whether the UHC index improved or worsened in that year.

While the UHC index has generally improved steadily over the past 30 years, there have been notable exceptions, in which wars, natural disasters, recessions, epidemics, and other factors have contributed toward worsening UHC index. Several of these setbacks are visible at the region level in Fig. 4 (see fig. S5 for region disaggregation). For example, worsening UHC indexes in Oceania from 1994 to 2007 and in Central Asia from 1993 to 2000 are likely due to political instability and violence in key populous countries such as Papua New Guinea, Uzbekistan, and Kazakhstan during those time periods (*9*, *10*). Similarly, the decreasing UHC index in Southern Sub-Saharan Africa from 1993 to 2006 likely reflects the strain placed on health care systems during the height of the HIV/AIDS epidemic (*11*). Considering the ongoing COVID-19 pandemic has likely disrupted health care systems around the world, understanding the effects of similar disruptions on child growth is an urgent issue (*12*). To assess the historical relationship between UHC index disruptions and CGF, we analyzed all country-years from 1991 to 2019 and categorized them according to whether the UHC index increased or decreased. We then compared annual changes across severities of each form of CGF for country-years that experienced UHC index improvements and country-years that experienced UHC index setbacks (Fig. 6).

We found that country-years that experienced UHC index improvements tended to have larger annual improvements in CGF as compared with country-years that experienced UHC index setbacks. We also found that annual changes are more variable in severe and extreme CGF as compared with overall CGF. We calculated Kolmogorov-Smirnov (KS) statistics for distributions of annual change by CGF type and severity and found all to be significant. Notably, KS statistics for wasting are higher than those for stunting and underweight. This trend is expected as acute malnutrition may be most sensitive to annual UHC index changes. Chronic malnutrition in the forms of stunting and underweight may better reflect longitudinal trends in the UHC index.

Annual changes that may seem small in magnitude have the potential to accumulate if UHC index disruptions are prolonged. For example, the UHC index worsened in Djibouti every year from 1993 to 2001, likely as a consequence of a civil war and other concurrent crises throughout the region (*13*, *14*). In Djibouti from 1993 to 2001, overall, severe, and extreme wasting respectively experienced relative increases of 10.7% (5.58 to 15.8%), 17.1% (7.73 to 27.3%), and 27.0% (11.4 to 46.2%). Considering that most CGF surveillance strategies only monitor overall CGF at present, this example highlights that the true magnitude of increases in CGF prevalence may not be easily detectable using conventional metrics. Interventions aimed at combatting this increase were therefore likely designed without granularity in wasting trends that could have been valuable in ensuring that the most vulnerable populations were prioritized.

## DISCUSSION

In the analyses that we conducted, a central theme is that trends are heightened in severe and extreme CGF as compared with overall CGF. Since 1990, severe and extreme CGF have almost universally experienced greater improvements than overall CGF. This trend has mainly been driven by improvements since 2000, as the 1990s saw relatively static trends in CGF prevalence. In some cases, the 1990s actually saw worsening CGF trends, and in these cases, severe and extreme CGF often experienced exaggerated setbacks as compared with overall CGF. Relationships identified with the UHC index revealed that as a location’s UHC index improves, severe and extreme CGF are expected to improve more markedly than overall CGF. In many cases, this implies that there is progress that has gone undetected as current metrics do not capture trends in severe and extreme CGF. However, our final analysis reveals that in times of UHC index disruption, setbacks are similarly amplified in severe and extreme CGF. Thus, the utilization of simplistic dichotomous metrics potentially conceals concerning trends in child nutrition in times of crisis.

These trends are identified in our analyses through the utilization of nationally representative data sources. Localized evidence is invaluable in highlighting how these trends manifest and is crucial for informing targeted interventions. Vulnerable subsets of a country’s population may experience marked swings in CGF prevalence, in part explaining widening or shrinking disparities on a national scale. For example, in the wake of the 2008 financial crisis, China’s nationwide stunting rate continued to decline, but prevalence in certain poorer rural areas sharply increased, doubling in children less than 12 months of age (*15*). Local trends in severe and extreme stunting therefore may be more relevant than national trends in severe and extreme stunting. As another example, findings in India from 2009 show that neighborhoods that experienced large floods in 2006 and 2008 had wasting rates about twice as high as nearby neighborhoods that experienced less severe flooding even after adjusting for several sociodemographic variables (*16*). CGF monitoring efforts should take these vulnerable communities into account, specifically tracking severe and extreme CGF prevalences among high-risk populations.

The need to monitor severe and extreme CGF is urgent, as COVID-19 disrupts health systems around the world. Preliminary projections and studies aimed at forecasting increases in CGF as a result of the pandemic paint a dire picture (*17*, *18*). Quickly adopting a comprehensive monitoring strategy that includes severe and extreme CGF has the potential to identify pandemic-related setbacks that historically would not have been detected by traditional metrics. COVID-19–associated disruptions are also connected to rising food insecurity rates throughout the United States—which, as evidence suggests, has uniquely affected racial/ethnic minorities—demonstrating that this type of monitoring is also important in high-income countries (*19*).

While we have highlighted the importance of severe and extreme CGF in this analysis, it is imperative to acknowledge the importance of monitoring overall CGF as well. Children experiencing all forms of CGF are at elevated risks for developing adverse health outcomes, even if their CGF is not severe or extreme. We have chosen to emphasize severe and extreme CGF as these forms of CGF are often overlooked in the field and have particularly high relative risks. However, because of its inherently higher prevalence, overall CGF remains a pressing global health issue. Overall CGF also lingers in high- and middle-income regions—albeit at substantially lower rates—demonstrating that vulnerable communities face a double burden of malnutrition via both under- and overnutrition (*20*). Thus, overall CGF should not be ignored or deprioritized as a global health metric but, instead, viewed as one useful way of considering the spectrum of child growth and development. The thresholds of −2SD, −3SD, and −4SD are all inherently arbitrary cut points for defining CGF that fail to individually capture information reflected in the entire distributions of HAZ, WHZ, and WAZ. In highlighting severe and extreme CGF, we hope to illustrate that the use of multiple cut points is preferable to one and that several cut points can be used in tandem to glean insights contained in continuous distributions.

Continuous estimation of HAZ, WHZ, and WAZ is a key step forward in enhancing our understanding of CGF progress, and future methodological advances have the potential to further improve this approach. Jointly estimating height and weight in a continuous fashion may provide a modeling strategy that better accounts for the relationships between anthropometric variables, allowing us to overcome limitations brought on by utilizing the distinct constructs of HAZ, WHZ, and WAZ. In addition, a continuous risk assessment paired with our estimates of complete CGF distributions could further highlight the importance of addressing extreme CGF, as currently in the GBD, our most severe risk category includes all children with *z* scores below −3SD. We anticipate that relative risks for children with more extreme forms of CGF will likely be higher, and a continuous risk assessment will elucidate what portion of CGF burden is attributable to extreme and severe CGF.

Particularly relevant for interventions aimed at addressing severe and extreme CGF, future work should aim to relate the health consequences of CGF to low birthweight and short gestational age. Children who are born preterm are more likely to experience CGF even years after birth (*21*, *22*). Work linking severe and extreme CGF to preterm birth may identify that the most effective interventions take place upstream, aimed at preventing CGF instead of treating it (*23*). These may manifest as interventions aimed at improving maternal nutrition, which has shown promise in contributing toward lower CGF rates, but studies linking this specifically to severe and extreme CGF are rare (*24*). Continuous estimation of CGF is a substantial step forward in reflecting the complex nature of child nutrition, but these advances could elucidate even more nuanced discoveries with direct policy implications.

In the field of health metrics, there are many conditions that are continuums that have historically been dichotomized to make the process of monitoring and evaluating them simpler. CGF represents one such condition, as for decades, international surveillance efforts have relied on one metric to reflect an entire spectrum of measurements. The use of this dichotomous metric often incorrectly implies that all children experiencing CGF are at the same risk for developing and dying from adverse health outcomes. We have presented a monitoring approach that overcomes that limitation, allowing for estimation of entire distributions of HAZ, WHZ, and WAZ. By estimating these entire distributions, we are able to highlight the most severe cases of CGF, capture progress that was previously undetectable, and more easily view trajectories of change—whether that be improvements or setbacks. An enhanced ability to recognize nuanced trends in more severe forms of CGF has direct implications for policies and interventions aimed at combatting CGF in the most vulnerable populations. Our analysis demonstrates that assessments of policy and intervention effectiveness may reach different conclusions if they utilize dichotomous definitions of CGF as compared to more holistic metrics. The measurement of entire spectrums of conditions as opposed to dichotomous constructs is a key step forward in achieving this goal. The development of more comprehensive metrics to assess other health conditions may reveal similarly nuanced findings that are now obscured by dichotomized constructs. While the simplicity of these categorical approaches may be appealing, the insights garnered from more comprehensive analyses are remarkable.

## MATERIALS AND METHODS

This analysis was completed as part of the GBD 2020 study. GBD is a comprehensive, systematic effort to estimate comparative health loss by age, sex, geography, and time for a comparative set of health states, disease, and injuries across all populations. What follows is a description of the methodological approach for the GBD 2020 analysis of CGF (stunting, wasting, and underweight). Further technical detail is available in the Supplementary Materials.

### Input data and data cleaning

We included data from population-representative surveys, administrative data sources, and published scientific literature. Data sources can be categorized into three main types: (i) age- and sex-specific microdata from population surveys, (ii) tabulated reports, and (iii) the WHO Global Database on Child Growth and Malnutrition. Here, microdata refer to a cross-sectional data source with individual-level measurements of height, weight, and age. Tabulated reports contain sample sizes and prevalences of categorical forms of CGF, which may be reported in an age- and sex-specific fashion or collectively for children less than 5 years of age. These are often Demographic and Health Surveys and Multiple Indicator Cluster Surveys. The WHO Global Database on Child Growth and Malnutrition contains a large collection of tabulation sources that also report sample sizes and prevalences of categorical forms of CGF. In total, we respectively incorporated 1762, 1782, and 1771 stunting, wasting, and underweight data sources into our models. Maps of input data source counts can be seen in fig. S2. A list of sources used for each location for stunting, wasting, and underweight models can be seen in data file S1, while a comprehensive list of input sources for all locations can be seen in table S4.

Studies were excluded if they were not representative of a geography’s entire population, included self-reported height or weight values, recruited specific subgroups, administered interventions that could alter measurements, or presented other undue biases. Microdata underwent additional cleaning steps to ensure that incorporated data were standardized according to the age- and sex-specific WHO 2006 child growth standards. This included dropping impossible values and adjusting older data based on previous 1978 National Center for Health Statistics growth standards. Last, age- and sex-specific data, including microdata, were used to split age- and sex-aggregated data into distinct age groups for children less than 5 years of age (0 to 6 days, 7 to 27 days, 1 to 5 months, 6 to 11 months, 12 to 23 months, and 2 to 4 years), which are the age groups utilized in subsequent modeling processes.

### Estimating *z* score distributions and prevalence of CGF

#### Ensemble weight fitting

All microdata sources were included in the process of fitting characteristic HAZ, WHZ, and WAZ curve shapes by creating an ensemble of several distribution families. Ten distributions were fit simultaneously to microdata sources: normal, log-normal, log-logistic, exponential, gamma, mirrored gamma, inverse gamma, Gumbel, mirrored Gumbel, and Weibull. All component distributions were parameterized using “methods of moments,” meaning that each could be described as a function of the mean and variance of the CGF *z* score distribution. A subplex optimization (a variant of the Nelder-Mead approach) was used to calculate the weight of each component distribution that minimized the error in predicting CGF prevalence <−1SD, <−2SD, and <−3SD, given the mean *z* score of a microdata source. The predictive error in these three portions of the curve was minimized across all microdata sources simultaneously, with each input source weighted evenly. One hundred sets of initial component distribution weights were entered into the optimization algorithm, and the final global stunting, wasting, and underweight ensemble weights sets are shown in table S5.

#### Spatiotemporal Gaussian process regression

Spatiotemporal Gaussian process regression (ST-GPR) is a common modeling framework used across the GBD that leverages strength of evidence across space and time to produce estimates for each age group, sex, year, and location. The first step is an ensemble mixed-effects linear regression. We identified potentially predictive covariates from the GBD database and regressed them against all of our input data, incorporating nested random effects at the super region, region, and location levels. Models with coefficients that were not statistically significant (*P* < 0.05) or not in the a priori expected direction were dropped. The remaining models were then ranked by their out-of-sample root mean square error, with a weighted ensemble of the top performing models ultimately used as the first stage prior in ST-GPR. Ultimately, the covariates that were selected included maternal care and immunization, health care access and quality index, age-standardized prevalence of severe anemia, SDI, age- and sex-specific unsafe sanitation summary exposure value, and all age energy unadjusted (kilocalories per person per day available from food supplies) (see fig S1). The second step of ST-GPR is a regression that incorporates evidence from neighboring locations, proximate years, and similar age groups to smooth residuals in the estimate from the first stage prior. The spatiotemporal smoothing is controlled by three hyperparameters (ζ = space, λ = time, and Ω = age), which were adjusted to maximize out-of-sample predictive validity throughout the entire time series. The third step of ST-GPR is a Gaussian process regression that further smoothens the residuals between input data and the stage 2 estimate, estimating uncertainty in the final modeled estimate by incorporating uncertainty in the data itself as well as the difference between the first two stages. We take 1000 samples from the final Gaussian process distribution, calculating the 2.5th and 97.5th percentiles to define the uncertainty interval of estimates.

#### Variance modeling and integration

We synthesized results from the previous two modeling steps to estimate continuous HAZ, WHZ, and WAZ curves. We ran an optim optimization (another variant of the Nelder-Mead approach) to calculate the SD of curves—in the functional form defined from our ensemble weight fitting—that best aligns with ST-GPR modeled CGF prevalence below −2SD and −3SD while anchoring the mean of the curve at the mean value estimated by ST-GPR. Using the methods of moments equation for each component of the ensemble distribution, a probability density function was calculated 1000 times for each age, sex, year, and location using the 1000 estimates from ST-GPR. Area under the curve between various thresholds was integrated to determine the final prevalences of CGF, with the mean, 2.5th, and 97.5th percentiles of these 1000 estimates representing the final mean and uncertainty intervals.

### Relative change over time

We calculated change in CGF prevalence since 1990 using 1000 draws of each age- and sex-specific CGF prevalence and dividing it by the prevalence in the same demographic in 1990. These age- and sex-specific, draw-level relative change values were then aggregated up to relative change values for children of both sexes, less than 5 years old, using age- and sex-specific GBD population estimates. While other estimates in this report reflect the mean of 1000 draw-level estimates with a 95% uncertainty interval, relative change calculations reflect the median with a 95% uncertainty interval. This decision was made because locations with low CGF prevalences had some draws of prevalence that approached or were equal to zero, leading to unrealistically high values for relative change over time when these draws were used in percent change calculations. These draws led to skewed distributions for estimates of relative change, in which the mean was often not representative of the overall distribution of draw values. The median was chosen to better represent the most commonly estimated values of relative change in CGF prevalence.

### Epidemiological transition analysis

Relationships between the UHC index and CGF prevalence were assessed using MR-BRT models. Twenty submodels—each composed of five component quadratic models across the domain of the UHC index—were weighted according to their predictive validity to generate the final model fit (see data file S3). Models were fit in logit space to standard GBD locations (all 204 countries and territories plus subnational locations for India, China, the United States, and Brazil), with all locations weighted evenly. In addition, splines were fit in an age- and sex-specific manner and later aggregated using GBD population estimates to generate a spline for both sexes and all children less than 5 year old (see data file S2). Models were provided with a decreasing monotonicity prior, with the lowest and highest 0.5% of input estimates relative to the spline trimmed from the model.

The final aggregated splines were scaled to aid in interpretation of improvement trajectories across severities. To accomplish this, a ratio of the splines for different severities was calculated at the spline value that corresponded to the lowest UHC index value. In this way, scaled splines were all anchored to the same starting point at the lowest UHC index value. The splines were then multiplied by this scalar for all values across the domain of UHC index values.

Additional MR-BRT models were run using identical specifications except for the addition of a linear covariate of SDI. This covariate was included to highlight the interconnectedness of factors such as the UHC index and SDI. Expected values of CGF prevalence for a given location with SDI values of 20, 40, 60, and 80 were generated (see fig. S8). These splines were scaled in the same manner as previously described to assess trajectories across severities of CGF (see data S4).

#### Annual changes and the UHC index

All location-years from 1991 to 2019 were included in a secondary analysis assessing the relationship between annual UHC index changes and relative annual CGF prevalence changes. In this analysis, 1990 was not included because there were no 1989 reference estimates that could be used to calculate annual percentage changes. In addition, 2020 was not included in this analysis because the effects of the COVID-19 pandemic on the UHC index remain less certain at this time than UHC index estimates for previous years. This analysis instead focuses on the historical relationship between the UHC index and annual changes in CGF prevalence.

Location-years were grouped by whether UHC increased or decreased in comparison to the previous year. Relative percentage changes in overall (*z* score < −2SD), severe (*z* score < −3SD), and extreme (*z* score < −4SD) CGF were calculated using the same methodology as previously described, except in this case, the reference year was the prior year instead of 1990. Only location-years with CGF prevalences greater than one per million and populations greater than 300,000 were included in this analysis to minimize the influence of locations with low CGF prevalence or volatile CGF trends. KS statistics were calculated to assess differences in the curves of relative CGF change for each CGF type and severity.

## Supplementary Materials

### This PDF file includes:

Methods - Additional Detail Tables S1 to S8 Figs. S1 to S8 References
