# Supplementary material for: Current dichotomous metrics obscure trends in severe and extreme child growth failure
Source: Sci Adv. 2022 May 20;8(20):eabm8954. doi: 10.1126/sciadv.abm8954 (PMC9122330; doi:10.1126/sciadv.abm8954)
Supplement: Supplementary file 1 — Methods - Additional Detail Tables S1 to S8 Figs. S1 to S8 References [file sciadv.abm8954_sm.pdf]

Science Advances
AAAS

Supplementary Materials for

Current dichotomous metrics obscure trends in severe and extreme child growth failure

Ryan Fitzgerald, Helena Manguerra, Michael B. Arndt, William M. Gardner, Ya-Yin Chang, Bethany Zigler, Heather Jean Taylor, Kelly Bienhoff, David L. Smith, Christopher J. L. Murray, Simon I. Hay, Robert C. Reiner, Jr. Nicholas J. Kassebaum*

*Corresponding author. Email: nickjk@uw.edu

Published 20 May 2022, *Sci. Adv.* **8**, eabm8954 (2022)
DOI: 10.1126/sciadv.abm8954

The PDF file includes:

Methods - Additional Detail
Tables S1 to S8
Figs. S1 to S8
Legends for data S1 to S4
References

Other Supplementary Material for this manuscript includes the following:

Data S1 to S4

Methods – Additional Detail
This study complies with the Guidelines for Accurate and Transparent Health Estimates Reporting (GATHER) (*26*) as detailed in Table S1, and International Committee of Medical Journal Editors (ICMJE) recommendations have been followed.

Overview
We incorporated representative stunting, wasting, and underweight data in ST-GPR (spatiotemporal Gaussian process regression) models to estimate the prevalence of CGF below -2 and -3 standard deviations from the WHO Growth Standards median (*1, 2*) (See fig. S3 for data landscape maps; See fig. S1 for ST-GPR results and location-specific data sources). We then leveraged surveys that included individual-level stunting, wasting, and underweight observations in order to parameterize characteristic HAZ, WHZ, and WAZ curve shapes using an ensemble distribution modeling strategy. By optimizing characteristically shaped curves to align with age-, sex-, location-, and year-specific regression output, we estimated age- and sex-specific continuous HAZ, WHZ, and WAZ distributions for 204 countries and territories from 1990 to 2020 (See fig. S1 for CGF distributions for all locations). Final estimates of overall (< -2SD), severe (< -3SD), and extreme (< -4SD) CGF reflect the prevalence of each severity as integrated from the estimated continuous distributions.

Definitions
Child growth failure (CGF) is estimated using three indicators – stunting (height-for-age; HAZ), wasting (weight-for-height; WHZ), and underweight (weight-for-age; WAZ) – which are defined using the age- and sex-specific WHO growth standards for children aged 0-59 months (*1*). These standards were developed in the 2006 WHO Multicenter Growth Reference Study, which followed an international cohort of ideally nourished children (*1*). Here, HAZ, WHZ, and WAZ are measured in terms of Z-scores from these growth standards curve medians. Categorical terms are used to describe the severity of CGF represented by these Z-scores, as shown in Table S2. Here, overall CGF is inclusive of both severe and extreme CGF and severe CGF is inclusive of extreme CGF. In this analysis, the term overall CGF is used to refer to CGF below -2SD, and is therefore distinct from the term moderate CGF, which is exclusively used to refer to CGF Z-scores between -3SD and -2SD.

Input data
We included data from population-representative surveys, administrative data sources, and published scientific literature. These sources can be categorized into three main data types: 1) age- and sex-specific microdata from population surveys, 2) tabulated reports, and 3) the WHO Global Database on Child Growth and Malnutrition (*27*). Here, microdata refers to a cross-sectional data source with individual-level observations of height, weight, and age. Tabulated reports contain sample sizes and prevalences of categorical forms of CGF, which may be reported in an age- and sex-specific fashion, or collectively for children under 5 years of age. These are often Demographic and Health Surveys and Multiple Indicator Cluster Surveys. The WHO Global Database on Child Growth and Malnutrition contains a large collection of tabulation sources which also contain sample sizes and prevalences of categorical forms of CGF. Studies that were not representative of a geography's population were excluded. Any study with self-reported height and weight values (as opposed to measurements from examinations) was also excluded. Final counts of sources included for stunting, wasting, and underweight

estimation are included in Table S3. Maps of input source counts can be seen in fig. S3. Sources used in each location for stunting, wasting, and underweight models can be seen in fig. S1, and a comprehensive list of input data sources used in this CGF analysis can be seen in Table S4.

Data processing
Only data sources that were representative of a location's entire population were included in the analysis to avoid sampling bias. Clinical trials that recruited specific populations, studies that implemented interventions that could have affected measurements, or analyses that were in specific non-representative locations were excluded. Four data cleaning steps were performed to maximize internal consistency of the modeling dataset. First, microdata that reported height, weight, and age were converted into stunting, wasting, and underweight Z-scores using the 2006 Child Growth Standards and the LMS method (*28*). Any data with impossible values (negative height or weight) were dropped, and any Z-scores above 6SD or below -6SD were also dropped. Second, any tabulation data that were reported using the National Center for Health Statistics (NCHS) 1978 growth standards were transformed to corresponding values on the WHO 2006 Growth Standards curves. This was done using an OLS linear regression model and informed by a study that evaluated concordance between the two growth standards (*29*). Only overall (Z-score < -2SD) CGF prevalences were transformed, as this is where concordance was highest. Third, for any study that lacked a measure of mean Z-score for stunting, wasting, or underweight, we predicted a mean value for that study. To accomplish this, an ordinary-least-squares regression of mean Z-score versus overall (Z-score < -2SD) CGF prevalence was conducted for all sources where both were reported. Lastly, any data that were reported as both sexes combined, or for age groups broader than the GBD 2020 age groups for children under 5 (0-6 days, 7-27 days, 1-5 months, 6-11 months, 12-23 months, 2-4 years), were split into these most detailed groups. This process was conducted by applying the age and sex pattern from an ST-GPR (spatiotemporal Gaussian process regression) model which only included sources with age- and sex-specific data. Any data with sample sizes less than 5 after age sex disaggregation were excluded.

Modeling
A four-step modeling strategy was employed and applied in parallel to stunting, wasting, and underweight. These four steps are described in detail below.

Ensemble weight fitting
All microdata sources were included in the process of fitting ensemble weights to parameterize characteristic HAZ, WHZ, and WAZ curve shapes. Ten distributions were fit simultaneously to microdata sources: normal, log-normal, log-logistic, exponential, gamma, mirrored gamma, inverse gamma, Gumbel, mirrored Gumbel, and Weibull. All component distributions were parameterized using "methods of moments," meaning that each could be described as a function of the mean and variance of the CGF Z-score distribution. Ensemble weights were assigned to each distribution, and the resulting ensemble distribution was the weighted sum of these individual distributions. Previous iterations of GBD have optimized ensemble weight sets to minimize predictive error across the entire distribution of CGF Z-scores. However, GBD 2020 methodology includes advancements that allow for ensemble weight sets to specifically minimize predictive error at the most relevant portions of the curves, the tails of the distribution associated with disease, namely Z-scores of -3SD, -2SD, and -1SD. The optimization process

weighted the fit evenly at these three portions of the curve. The predictive error in these three portions of the curve was minimized across all input microdata sources simultaneously, with each input source weighted evenly. One hundred sets of initial weights were entered into an optimization algorithm that aimed to reduce this targeted predictive error across all input microdata sources, with final stunting, wasting, and underweight ensemble weight sets shown in Table S5.

ST-GPR (spatiotemporal Gaussian process regression)

All microdata were collapsed to calculate mean Z-score, overall (CGF < -2SD) prevalence, and severe (CGF < -3SD) prevalence which was added to all tabulation data, including the WHO Global Database on Child Growth and Malnutrition, to be used in ST-GPR (spatiotemporal Gaussian process regression). ST-GPR is a common modeling framework used across GBD that leverages strength of evidence across space and time to produce estimates for each age group, sex, year, and location (*2*).

The first step of the ST-GPR process is an ensemble mixed-effects linear regression. For each indicator, we identified potentially predictive covariates from the GBD covariates database and tested every combination of those covariates in a mixed-effects linear regression. In this analysis, indicator data points were regressed on the predictive covariates with nested random effects at the super-region, region, and location levels (See fig. S8 for region and super-region designations). Models where estimated coefficients were not statistically significant ($p < 0.05$) or were not in the a priori expected direction were dropped. The remaining models were then ranked by their out-of-sample root-mean-square error (RMSE), with a weighted ensemble of the top-performing models ultimately used as the first-stage prior in ST-GPR. The covariates that were tested in CGF ensemble mixed-effects linear regression are shown in Table S6, with covariates that were ultimately selected shown with a star. Histograms in fig. S2 show the frequency with which selected covariates were included in ensemble priors, as well as the standardized betas of covariates in those models. Selected covariates were consistent across all ST-GPR models for stunting, wasting, and underweight.

The second step of ST-GPR is a regression that incorporates evidence from neighboring locations, proximate years, and similar age groups to smooth residuals in the estimate from the ensemble mixed-effects linear regression created in step 1. The spatiotemporal smoothing is controlled by three hyper-parameters (ζ = space, λ = time, Ω = age), which were adjusted to maximize out-of-sample predictive validity throughout the entire time series.

The third step of ST-GPR is a Gaussian process regression that further smooths the residuals between observed data and the stage two estimate and estimates uncertainty in the final modeled estimate. The Gaussian process regression incorporates uncertainty of input data points as well as difference between the stage two estimate and the ensemble mixed-effects linear regression created in step one to estimate model uncertainty. We obtained 1000 samples from the final Gaussian process distributions for every location, age, sex, and year, from which we calculated 95% uncertainty intervals as the 2.5th and 97.5th percentiles of these sample distributions. In countries that are modeled subnationally, national-level estimates may reflect either sums of estimates from subnational locations (a process called aggregation), or subnational estimates may reflect the proportionally split national-level estimate (a process called raking). This decision is

based on availability of subnational data, concordance of subnational and national-level data, and levels of uncertainty in subnational and national-level data. In cases with more high-quality subnational-level data, aggregation is preferred. Raking allows for national-level estimates to have a larger influence on subnational estimates. Table S7 shows whether a country was raked or aggregated if that country is modeled subnationally. Raking and aggregating decisions were consistent across stunting, wasting, and underweight.

Variance modeling
The third modeling step is an optimization process that grounds the distribution at the mean Z-score estimate modeled from ST-GPR and calculates the variance value that minimizes the predictive error on ST-GPR estimates of severe (Z-score < -3SD) and overall (Z-score < -2SD) CGF prevalence. The form of this distribution does not change in this optimization process, as the distribution maintains the characteristic shape determined by the weight optimization in modeling step 1. In this way, the spread of the characteristic curve shape for stunting, wasting, and underweight is optimized to best align with ST-GPR estimates of key portions of the distribution. Using the "method of moments" equation for each component of the ensemble distribution, a probability density function is calculated for each age, sex, year, and location.

Integration
The probability density functions produced from variance modeling are integrated to determine the prevalence of overall CGF (Z-score < -2SD), mild CGF (-2SD < Z-score < -1SD), moderate CGF (-3SD < Z-score < -2SD), severe CGF (Z-score < -3SD), and extreme CGF (Z-score < -4SD). The categorical exposures to mild, moderate, and severe CGF are used for subsequent risk analysis in GBD.

Relative change over time
The relative change in CGF prevalence from 1990 to 2020 was calculated for overall, severe, and extreme stunting, wasting, and underweight. To accomplish this, we started with 1,000 draws of each age-sex specific CGF prevalence from our models. We then calculated the relative change of each draw to the corresponding draw of the same demographic in 1990. These age-sex specific, draw-level relative change values were then aggregated up to relative change values for children of both sex, under age 5 using age-sex specific GBD population estimates.

While other estimates in this report reflect the mean of 1,000 draw-level estimates with a 95% uncertainty interval, relative change calculations reflect the median with a 95% uncertainty interval. This decision was made because locations with low CGF prevalences had some draws of prevalence that approached or were equal to zero, leading to unrealistically high values for relative change over time when these draws were used in percent change calculations. These draws led to skewed distributions for estimates of relative change, in which the mean was often not representative of the overall distribution of draw values. The median was chosen to better represent the most commonly estimated values of relative change in CGF prevalence. The results are shown in Fig. 3 at the global level, the super-region level, and the location level for locations CGF prevalences greater than 1 per million and populations greater than 300,000. Results are more clearly shown by super region in fig. S5 which shows separate plots for each super region. (Notably this does not include extreme wasting in the high-income super region because the prevalence of extreme wasting was estimated to be lower than 1 per million).

To make statements about how much larger severe and extreme CGF improvements have been in comparison to overall CGF improvements, we began with 1,000 draws of severe/extreme CGF relative change since 1990 in 2020. We then divided these values by the corresponding 1,000 draws of the relative change in overall CGF since 1990 in 2020. The mean, 2.5th percentile, and 97.5th percentile of these ratio draws were used to reflect the relative magnitude of severe and extreme CGF in comparison to overall CGF.

Epidemiological transition analysis

To investigate relationships between CGF prevalences and health systems, MR-BRT (meta-regression—Bayesian, regularized, trimmed) splines were fit on universal health coverage index (UHC index). The UHC index reflects a population's access to quality health care by incorporating intervention coverage and outcome-based measures, and is ultimately transformed to a 0-100 scale (*8*). Populations with higher UHC index have increased access to quality health care. Splines fit on UHC index for overall (Z-score < -2SD) CGF, severe (Z-score < -3SD), and extreme (Z-score < -4SD) CGF reflect the expected values of those severities of CGF based on UHC index.

Quadratic splines were fit in logit space to standard GBD locations, weighting each location evenly by setting the standard errors equal to 1 for all locations in logit space. Standard GBD locations refer to all 204 countries and territories plus subnational locations for India, China, the United States, and Brazil. Six knots were set in the model using an ensemble knot placement method, which places one knot at the lowest UHC index value observed from 1990 to 2020, one knot at the highest UHC index value observed from 1990 to 2020, and four knots spaced between these knots. Twenty combinations of knot placements were utilized for each model fit, with each weighted according to its predictive power after running for a maximum of 30 iterations. Figure S6 shows the knot placements and weight given to each sub-model. Models were fit with decreasing monotonicity prior, meaning that the models were forced to show decreasing CGF prevalence as UHC index improved. The lowest and highest 0.5% of input estimates relative to the spline were trimmed from the models. Splines were fit in an age- and sex-specific manner (males and females; 0-6 days, 7-27 days, 1-5 months, 6-11 months, 12-23 months, 2-4 years), and later a weighted aggregate for both sexes and all children under 5 was calculated using GBD population estimates. Age- and sex-specific splines for overall, severe, and extreme stunting, wasting, and underweight can be seen in both logit space and normal space in fig. S6.

Expected values of CGF prevalence for children under age 5, both sexes are shown at the region level in Fig. 4. Results for individual countries and territories can be seen in fig. S10.

Splines were scaled to aid in interpretation of improvement trajectories across severities. To accomplish this, a ratio of the splines for different severities was calculated at the spline value that corresponded to the lowest UHC index value. In this way, scaled splines were all anchored to the same starting point at the lowest UHC index value. The spline values for all other values of UHC index were then multiplied by this ratio to calculate the scaled splines. Severe (Z-score < -3SD) CGF and extreme (Z-score < -4SD) CGF splines were scaled to overall (Z-score < -

2SD) CGF splines. These scaled splines can be viewed in Fig. 5 showing the expected trajectory of CGF prevalence improvement by severity as UHC index improves.

Additional MR-BRT models were run using identical specifications except for the addition of a linear covariate of Socio-demographic index (SDI). This covariate was included to highlight the interconnectedness of factors such as UHC index and SDI. Expected values of CGF prevalence for a given location with SDI values of 20, 40, 60, and 80 are shown in fig. S11, analogous to fig. S6, which did not include the linear covariate of SDI. These splines were scaled in the same manner as previously described to assess trajectories across severities. Fig. S12 shows the relative trajectories of expected progress as UHC index improves for a given location with SDI values of 20, 40, 60, and 80.

Annual changes and UHC index

All location-years from 1991 to 2019 were included in a secondary analysis assessing the relationship between annual UHC index changes and relative annual CGF prevalence changes. In this analysis, 1990 was not included because there were no 1989 reference estimates that could be used to calculate annual percentage changes. Additionally, 2020 was not included in this analysis because the effects of the COVID-19 pandemic on UHC index remain less certain at this time than UHC index estimates for previous years. Limited data availability and ongoing volatility in the pandemic led 2020 to be excluded. This analysis instead focuses on the historical relationship between UHC index and annual changes in CGF prevalence.

Location-years were grouped by whether UHC increased or decreased in comparison to the previous year. Relative percentage changes in mild (Z-score < -1SD), overall (Z-score < -2SD), severe (Z-score < -3SD), and extreme (Z-score < -4SD) CGF were calculated using the same methodology as in Fig 3, except in this case the reference year was the prior year instead of 1990. Only location-years with CGF prevalences greater than 1 per million were included to minimize the influence of locations with the lowest absolute CGF prevalence, in which miniscule prevalence changes could be overwhelming in relative space. Similarly, location-years with populations lower than 300,000 were excluded from this analysis.

After creating density plots showing the spread of CGF annual relative change for location-years in which UHC increased and decreased, the Kolmogorov-Smirnov (KS) test statistic was calculated to assess differences in the curves using the ks.test function in the stats package of R.

Table S1. Guidelines for Accurate and Transparent Health Estimates Reporting (GATHER) checklist.

#	GATHER checklist item	Description of compliance	Reference
**Objectives and funding**			
**1**	Define the indicator(s), populations (including age, sex, and geographic entities), and time period(s) for which estimates were made.	Description of indicators, definitions, relevant time periods, and populations in paper and appendix.	Main manuscript, Abstract and Main text sections; and Supplementary Materials, Methods – Additional Detail, Overview and Definitions sections, pp. 2
**2**	List the funding sources for the work.	Funding sources listed in paper.	Main manuscript, Funding section
**Data inputs**			
For all data inputs from multiple sources that are synthesized as part of the study:			
**3**	Describe how the data were identified and how the data were accessed.	Narrative description of data seeking methodology provided.	Supplementary Materials, Methods – Additional Detail, Input data section, pp. 2
**4**	Specify the inclusion and exclusion criteria. Identify all ad-hoc exclusions.	Narrative about inclusion and exclusion criteria by data type provided in linked materials.	Supplementary Materials, Methods – Additional Detail, Data Processing section, pp. 3
**5**	Provide information on all included data sources and their main characteristics. For each data source used, report reference information or contact name/institution, population represented, data collection method, year(s) of data collection, sex and age range, diagnostic criteria or measurement method, and sample size, as relevant.	An interactive, online data source tool that provides metadata for data sources by component, geography, cause, risk, or impairment.	Main manuscript, and Supplementary Materials, Table S4
**6**	Identify and describe any categories of input data that have potentially important biases (e.g., based on characteristics listed in item 5).	Summary of known biases included in paper	Supplementary Materials, Methods – Additional Detail, Data Processing section, pp. 3
For all data inputs that contribute to the analysis but were not synthesized as part of the study:			
**7**	Describe and give sources for any other data inputs.	An interactive, online data source tool that provides metadata for data sources by component, geography, cause, risk, or impairment.	Supplementary Materials, Methods – Additional Detail, Data Processing section, pp. 3
For all data inputs:			
**8**	Provide all data inputs in a file format from which data can be efficiently extracted (e.g., a spreadsheet as opposed to a PDF), including all relevant meta-data listed in item 5. For any data inputs that cannot be shared due to ethical or legal reasons, such as third-party ownership, provide a contact name or the name of the institution that retains the right to the data.	Downloads of input data are available through online tools, including data visualization tools and data query tools. Input data is not available in tools but can be made available upon request.	Manuscript main text, and Supplementary Materials, Table S4
**Data analysis**			
**9**	Provide a conceptual overview of the data analysis method. A diagram may be helpful.	Flow diagrams of the overall methodological processes, as well as cause-specific modelling processes have been provided.	Supplementary Materials, Methods – Additional Detail, Data processing and Modeling sections, pp. 3-5
**10**	Provide a detailed description of all steps of the analysis, including mathematical formulae. This description should cover, as relevant, data cleaning, data pre-processing, data adjustments and weighting of data sources, and mathematical or statistical model(s).	Provided in the methodological write-ups.	Supplementary Materials, Methods – Additional Detail, Modeling section, pp. 3-5
**11**	Describe how candidate models were evaluated and how the final model(s) were selected.	Provided in the methodological write-ups.	Supplementary Materials, Methods – Additional Detail, pp. 3 and pp. 6

**12**	Provide the results of an evaluation of model performance, if done, as well as the results of any relevant sensitivity analysis.	Provided in the methodological write-ups.	Supplementary Materials, Data S1, Data S2 and Data S4
**13**	Describe methods for calculating uncertainty of the estimates. State which sources of uncertainty were, and were not, accounted for in the uncertainty analysis.	Provided in the methodological write-ups.	Supplementary Materials, Methods – Additional Detail pp. 4
**14**	State how analytic or statistical source code used to generate estimates can be accessed.	Access statement provided.	Main manuscript, Methods – Additional Detail, Data and materials availability section
**Results and Discussion**			
**15**	Provide published estimates in a file format from which data can be efficiently extracted.	GBD 2020 results are available through online data visualization tools, the Global Health Data Exchange, and the online data query tool.	Available pending publication at http://ghdx.healthdata.org/gbd-2020
**16**	Report a quantitative measure of the uncertainty of the estimates (e.g., uncertainty intervals).	Uncertainty intervals are provided with all results.	Main manuscript; and Supplementary Materials, Data S1
**17**	Interpret results in light of existing evidence. If updating a previous set of estimates, describe the reasons for changes in estimates.		Main manuscript
**18**	Discuss limitations of the estimates. Include a discussion of any modelling assumptions or data limitations that affect interpretation of the estimates.	Discussion of limitations provided in the main text.	Main manuscript, Caveats section

Table S2. Categorical terms and Z-score definitions for describing the severity of CGF.

Term	Definition
Overall CGF	Z-score < -2SD
Mild CGF	-2SD < Z-score < -1SD
Moderate CGF	-3SD < Z-score < -2SD
Severe CGF	Z-score < -3SD
Extreme CGF	Z-score < -4SD

Table S3. Final counts of input data sources included for stunting, wasting, and underweight estimation.

CGF dimension	Country-years of source data	Countries with sources (out of 204)
**Stunting**	1762	160
**Wasting**	1782	160
**Underweight**	1771	159

Table S4. Comprehensive list of input data sources used in this CGF analysis. All data underlying the study are available on the Global Health Data Exchange (GHDx), a catalog of demographic and global health data created and supported by IHME. It includes population census data, surveys, registries, indicators and estimates, administrative health data, and financial data related to health. Each dataset is assigned a record to catalogue general metadata, citation, and information about where to obtain the dataset or access the files. Each data source record is assigned an NID (a unique and persistent identifier) and the full list of NIDs included in this child growth failure analysis is included in this table. To access the GHDx record for each NID, use the following URL: “https://ghdx.healthdata.org/node/<insert NID here>”. There are no restrictions on data availability such as an MTA. While the GHDx does not host or distribute most data sources, each record includes a URL to the data provider wherever possible.

**Input data source NIDs (778 total sources; Indexed from https://ghdx.healthdata.org)**																
Row 1	595	7401	12320	19198	20103	20829	23289	45283	76707	129884	152720	200617	233917	267785	308316	409558
Row 2	608	7440	12399	19211	20120	20841	23565	45376	76708	129905	152783	200636	234733	270404	310085	411301
Row 3	627	7465	12584	19274	20132	20852	23786	45464	76709	129913	153062	200707	235215	270469	311265	413663
Row 4	687	7540	12608	19292	20145	20865	25914	45549	76850	130075	153643	200838	235348	270470	316243	413741
Row 5	881	7618	12732	19305	20154	20875	26433	45718	76878	130085	153674	203654	236156	270471	316438	416595
Row 6	1039	7629	12896	19315	20167	20887	26826	45852	77384	130486	154897	203663	236205	270627	316440	417560
Row 7	1089	7721	12940	19324	20191	20888	26842	45854	77385	131467	155335	203664	236266	274160	316442	422512
Row 8	1385	7843	13064	19333	20202	20896	26855	45856	77388	133731	156596	204667	244455	274707	316444	423654
Row 9	1404	7919	13226	19350	20212	20909	26866	45989	77390	133732	157021	206075	244458	274952	316736	424884
Row 10	1855	8115	13445	19359	20223	20917	26919	46317	77515	134371	157024	209930	244463	275090	322237	425072
Row 11	1912	8618	13465	19370	20235	20926	26930	46517	77516	135258	157025	210182	244464	275122	324470	425208
Row 12	1927	8777	13708	19381	20252	20936	26989	46563	77517	135416	157026	210231	244465	275123	326837	426238
Row 13	1970	8788	13719	19391	20263	20947	26998	46682	77518	135720	157027	210614	244467	281993	327852	427778
Row 14	1994	8819	13973	19400	20274	20954	27022	46881	77521	135721	157031	214640	244468	283269	327857	427952
Row 15	2039	8932	14015	19410	20283	20964	27031	46924	77819	135773	157050	218035	244469	283272	330624	427983
Row 16	2063	9212	14027	19421	20301	20976	27044	47478	79839	135825	157057	218555	244471	283406	331377	428683
Row 17	2196	9270	14063	19444	20315	20993	27055	47962	80626	135826	157058	218563	244472	283811	332558	428990
Row 18	2209	9310	14105	19456	20322	21014	27069	48332	80731	136038	157061	218565	244473	284177	334706	428993
Row 19	2223	9370	14122	19472	20339	21033	27234	48361	81004	136055	157063	218568	244480	284410	334707	428995
Row 20	2244	9422	14340	19482	20347	21068	27551	49205	81005	139614	157064	218581	246145	285874	334709	428998
Row 21	3114	9439	14341	19493	20361	21079	27563	49502	81203	140116	157066	218582	246209	285880	334710	429000
Row 22	3150	9506	18499	19511	20371	21090	27590	55956	81509	140118	159617	218592	246246	285887	334953	429463
Row 23	3161	9516	18519	19521	20382	21102	27599	55975	81748	140200	159868	218593	246248	286053	335994	429991
Row 24	3404	9989	18531	19529	20394	21117	27606	55992	82832	141521	159873	218611	246249	286131	336042	431951
Row 25	3455	9999	18533	19539	20404	21126	27630	56021	90696	141572	160053	218613	246250	286211	336401	436764
Row 26	3655	10001	18834	19546	20417	21139	27885	56040	91324	141910	160103	218619	246254	286232	340363	437724

Row 27	3922	10023	18843	19557	20428	21151	30037	56063	93806	141948	160576	219201	248224	286277	341838	437993
Row 28	3935	10224	18854	19571	20437	21163	30325	56151	93807	142934	160781	222494	249499	286657	355051	438014
Row 29	3970	10460	18865	19579	20450	21188	30368	56153	93848	142935	161590	222676	256541	286766	374851	
Row 30	4012	11214	18878	19587	20462	21198	30379	56241	95440	146860	161662	222752	257050	286772	374865	
Row 31	4043	11238	18902	19604	20478	21206	30431	56420	103973	148649	162031	223566	257052	286780	374889	
Row 32	4075	11239	18913	19614	20487	21240	30963	56883	104236	149821	162283	224096	257841	286782	374958	
Row 33	4694	11240	18938	19627	20499	21258	30991	57999	106158	149860	162317	224223	260403	286783	375026	
Row 34	4779	11271	18950	19637	20518	21274	31050	58185	110300	149906	163056	224233	261683	286785	375074	
Row 35	4808	11299	18959	19647	20537	21281	31142	58188	111432	150382	165390	224236	261873	286802	375078	
Row 36	4818	11300	18971	19656	20552	21301	31143	58419	111815	150526	165498	224239	261875	293979	375090	
Row 37	4916	11301	18979	19670	20567	21311	31750	58660	112222	150866	165645	224240	261879	293980	375093	
Row 38	4926	11302	18990	19683	20573	21331	31831	60942	112500	150870	165892	224248	261880	293981	375098	
Row 39	5009	11551	19001	19695	20584	21348	32189	63993	112656	151086	169975	224251	261883	293982	375129	
Row 40	5583	11639	19016	19708	20596	21365	32421	65118	115896	151436	174049	224848	261887	294482	375362	
Row 41	5827	11649	19027	19720	20608	21382	33019	69761	120208	151724	189045	224849	261889	296646	375813	
Row 42	6111	11774	19046	19728	20626	21393	33196	74393	124408	151725	189048	224853	262810	296715	384608	
Row 43	6464	11928	19064	19787	20638	21409	34072	74460	124467	151727	189092	224854	262834	296882	385708	
Row 44	6935	12013	19076	19950	20649	21412	34085	76699	125591	151729	189153	224855	264583	297069	387266	
Row 45	7028	12101	19088	19963	20711	21433	35493	76700	126331	151732	189463	224856	264590	299307	387395	
Row 46	7054	12201	19133	20051	20722	22125	40021	76701	126909	151797	191270	230882	264910	303663	387410	
Row 47	7222	12232	19142	20060	20740	22882	40028	76702	126911	152424	192017	231832	264956	303664	393876	
Row 48	7340	12243	19156	20073	20749	22950	44126	76703	126952	152561	194011	231835	264959	306329	396957	
Row 49	7375	12280	19167	20083	20767	23017	44586	76704	129770	152562	194016	232534	265153	307776	408484	
Row 50	7387	12289	19188	20092	20806	23219	44645	76706	129783	152563	200598	232763	267343	307779	409169	

Table S5. Final stunting, wasting, and underweight ensemble weight sets used in the ST-GPR models.

	Normal	Log-normal	Log-logistic	Exponential	Gamma	Mirrored gamma	Inverse gamma	Gumbel	Mirrored Gumbel	Weibull
**HAZ**	0	0.425449	0.371227	0	0	0	0	0	0.05795	0.145373
**WHZ**	0	0	0.542213	0	0.048224	0	0	0	0	0.409563
**WAZ**	0	0	0.195735	0	0	0.087365	0.138334	0.290187	0.288379	0

Table S6. List of covariates tested in CGF ensemble mixed-effects linear regression. Covariates ultimately selected are shown with a star (*).

Covariate	
Energy unadjusted (kcal)*	Short gestation for birthweight SEV
Healthcare Access and Quality index*	Low birthweight for gestation SEV
Maternal care and immunization*	Protein-energy malnutrition mortality rate
Prevalence of severe anemia*	Mortality rate due to war and terrorism
Socio-demographic index*	Toilet access
Unsafe sanitation SEV*	GDP per capita base 2010 international dollars

Table S7. List of countries modeled subnationally in the GBD, and raking and aggregating decisions for this CGF analysis. Raking and aggregating decisions were consistent across stunting, wasting, and underweight estimation.

Country	Raked or aggregated	Country	Raked or aggregated
Brazil	Aggregated	New Zealand	Raked
China	Aggregated	Nigeria	Raked
Ethiopia	Aggregated	Norway	Raked
India	Aggregated	Pakistan	Raked
Indonesia	Aggregated	Philippines	Raked
Iran	Raked	Poland	Raked
Italy	Raked	Russia	Raked
Japan	Aggregated	South Africa	Aggregated
Kenya	Aggregated	United Kingdom	Raked
Mexico	Aggregated	United States	Raked

Table S8a. Estimated overall, severe, and extreme **stunting** prevalence (%) in children under 5 years, both sexes, in 1990, 2000, 2010, and 2020.

Location	Total				Severe				Extreme			
	1990	2000	2010	2020	1990	2000	2010	2020	1990	2000	2010	2020
Global	35.68%<br>(35.34% - 36.05%)	32.88%<br>(32.65% - 33.11%)	28.70%<br>(28.53% - 28.86%)	24.12%<br>(23.96% - 24.29%)	16.43%<br>(16.08% - 16.77%)	15.95%<br>(15.73% - 16.18%)	13.21%<br>(13.06% - 13.36%)	10.08%<br>(9.92% - 10.25%)	5.59%<br>(5.32% - 5.89%)	6.06%<br>(5.78% - 6.40%)	4.60%<br>(4.44% - 4.78%)	3.09%<br>(2.93% - 3.25%)
Central Europe, Eastern Europe, and Central Asia	19.58%<br>(19.15% - 19.99%)	18.89%<br>(18.56% - 19.22%)	14.91%<br>(14.54% - 15.29%)	12.14%<br>(11.73% - 12.53%)	6.70%<br>(6.35% - 7.07%)	6.53%<br>(6.26% - 6.81%)	4.48%<br>(4.24% - 4.74%)	3.25%<br>(3.00% - 3.50%)	1.54%<br>(1.36% - 1.72%)	1.50%<br>(1.36% - 1.67%)	0.82%<br>(0.72% - 0.93%)	0.50%<br>(0.41% - 0.59%)
**Central Asia**	25.82%<br>(25.25% - 26.38%)	26.14%<br>(25.63% - 26.70%)	19.25%<br>(18.71% - 19.77%)	13.69%<br>(13.16% - 14.27%)	9.62%<br>(9.04% - 10.20%)	9.80%<br>(9.23% - 10.38%)	5.57%<br>(5.14% - 5.99%)	3.09%<br>(2.76% - 3.42%)	2.36%<br>(1.98% - 2.78%)	2.36%<br>(2.02% - 2.74%)	0.84%<br>(0.69% - 1.02%)	0.27%<br>(0.20% - 0.37%)
Armenia	17.15%<br>(15.94% - 18.31%)	18.03%<br>(16.95% - 19.02%)	15.10%<br>(14.15% - 16.01%)	11.59%<br>(10.65% - 12.55%)	4.66%<br>(3.86% - 5.47%)	5.13%<br>(4.37% - 5.94%)	4.15%<br>(3.60% - 4.76%)	2.66%<br>(2.23% - 3.18%)	0.60%<br>(0.35% - 0.91%)	0.74%<br>(0.47% - 1.06%)	0.55%<br>(0.36% - 0.77%)	0.21%<br>(0.11% - 0.34%)
Azerbaijan	25.45%<br>(24.27% - 26.63%)	25.52%<br>(24.70% - 26.36%)	19.38%<br>(18.66% - 20.12%)	16.54%<br>(15.44% - 17.57%)	9.54%<br>(8.30% - 10.83%)	9.88%<br>(8.99% - 10.77%)	6.63%<br>(5.98% - 7.25%)	5.21%<br>(4.42% - 5.95%)	2.25%<br>(1.67% - 2.93%)	2.43%<br>(1.96% - 2.92%)	1.35%<br>(1.04% - 1.68%)	0.92%<br>(0.63% - 1.22%)
Georgia	14.89%<br>(13.65% - 16.13%)	14.26%<br>(13.33% - 15.24%)	11.84%<br>(11.03% - 12.71%)	8.14%<br>(7.38% - 9.05%)	3.36%<br>(2.70% - 4.05%)	3.36%<br>(2.85% - 3.90%)	2.66%<br>(2.25% - 3.11%)	1.29%<br>(0.98% - 1.66%)	0.26%<br>(0.11% - 0.44%)	0.28%<br>(0.16% - 0.43%)	0.17%<br>(0.07% - 0.29%)	0.01%<br>(0.00% - 0.05%)
Kazakhstan	17.31%<br>(16.25% - 18.33%)	16.65%<br>(15.74% - 17.62%)	14.03%<br>(13.34% - 14.84%)	10.01%<br>(9.21% - 10.88%)	4.80%<br>(4.08% - 5.54%)	4.42%<br>(3.74% - 5.04%)	3.63%<br>(3.20% - 4.12%)	2.24%<br>(1.83% - 2.68%)	0.67%<br>(0.45% - 0.95%)	0.57%<br>(0.38% - 0.78%)	0.43%<br>(0.30% - 0.58%)	0.14%<br>(0.06% - 0.25%)
Kyrgyzstan	27.11%<br>(25.81% - 28.49%)	25.70%<br>(24.59% - 26.82%)	18.80%<br>(17.82% - 19.72%)	15.70%<br>(14.52% - 16.84%)	9.89%<br>(8.47% - 11.33%)	9.32%<br>(8.18% - 10.45%)	5.46%<br>(4.82% - 6.22%)	4.03%<br>(3.23% - 4.86%)	2.25%<br>(1.52% - 3.04%)	2.11%<br>(1.56% - 2.71%)	0.83%<br>(0.59% - 1.14%)	0.47%<br>(0.22% - 0.77%)
Mongolia	31.02%<br>(29.34% - 32.97%)	30.08%<br>(29.02% - 31.49%)	17.92%<br>(16.95% - 18.89%)	12.63%<br>(11.66% - 13.67%)	8.94%<br>(7.13% - 10.62%)	9.82%<br>(8.62% - 10.98%)	4.17%<br>(3.47% - 4.87%)	2.22%<br>(1.66% - 2.84%)	1.25%<br>(0.66% - 2.03%)	1.75%<br>(1.20% - 2.39%)	0.39%<br>(0.20% - 0.62%)	0.07%<br>(0.01% - 0.20%)
Tajikistan	34.17%<br>(32.81% - 35.57%)	35.85%<br>(34.82% - 36.87%)	30.41%<br>(29.23% - 31.68%)	22.96%<br>(21.53% - 24.39%)	12.78%<br>(11.12% - 14.43%)	14.14%<br>(12.88% - 15.41%)	10.22%<br>(8.96% - 11.49%)	6.39%<br>(5.17% - 7.70%)	2.95%<br>(2.15% - 3.91%)	3.53%<br>(2.84% - 4.24%)	1.96%<br>(1.39% - 2.61%)	0.89%<br>(0.48% - 1.41%)
Turkmenistan	24.67%<br>(23.28% - 26.03%)	24.13%<br>(23.36% - 25.00%)	16.14%<br>(15.37% - 16.86%)	11.25%<br>(10.41% - 12.08%)	7.09%<br>(5.83% - 8.28%)	7.51%<br>(6.75% - 8.34%)	3.90%<br>(3.40% - 4.41%)	2.12%<br>(1.72% - 2.55%)	1.17%<br>(0.79% - 1.59%)	1.39%<br>(1.12% - 1.72%)	0.39%<br>(0.25% - 0.55%)	0.07%<br>(0.02% - 0.16%)
Uzbekistan	30.26%<br>(28.89% - 31.60%)	28.32%<br>(27.24% - 29.59%)	19.67%<br>(18.57% - 20.78%)	12.75%<br>(11.60% - 13.97%)	13.31%<br>(11.87% - 14.78%)	11.61%<br>(10.47% - 12.85%)	5.52%<br>(4.54% - 6.46%)	2.36%<br>(1.77% - 3.05%)	3.99%<br>(3.06% - 5.08%)	3.12%<br>(2.41% - 3.94%)	0.77%<br>(0.44% - 1.14%)	0.08%<br>(0.00% - 0.24%)
**Central Europe**	13.07%<br>(12.64% - 13.52%)	12.59%<br>(12.21% - 12.98%)	9.79%<br>(9.45% - 10.13%)	8.65%<br>(8.32% - 9.01%)	3.49%<br>(3.25% - 3.78%)	3.62%<br>(3.40% - 3.83%)	2.45%<br>(2.27% - 2.63%)	1.97%<br>(1.81% - 2.15%)	0.59%<br>(0.49% - 0.69%)	0.68%<br>(0.60% - 0.77%)	0.31%<br>(0.25% - 0.37%)	0.19%<br>(0.14% - 0.25%)
Albania	37.89%<br>(36.48% - 39.38%)	35.27%<br>(34.14% - 36.48%)	23.87%<br>(22.81% - 24.91%)	18.26%<br>(17.11% - 19.47%)	19.47%<br>(17.71% - 21.46%)	18.71%<br>(17.36% - 20.21%)	10.32%<br>(9.39% - 11.26%)	6.34%<br>(5.52% - 7.23%)	7.26%<br>(5.83% - 8.99%)	7.42%<br>(6.27% - 8.72%)	3.17%<br>(2.66% - 3.73%)	1.37%<br>(1.05% - 1.76%)
Bosnia and Herzegovina	15.71%<br>(14.60% - 16.84%)	12.52%<br>(11.66% - 13.37%)	9.69%<br>(9.02% - 10.41%)	9.13%<br>(8.38% - 9.94%)	4.35%<br>(3.65% - 5.04%)	3.41%<br>(2.98% - 3.93%)	2.37%<br>(2.05% - 2.69%)	2.13%<br>(1.81% - 2.49%)	0.61%<br>(0.39% - 0.85%)	0.44%<br>(0.30% - 0.62%)	0.23%<br>(0.16% - 0.32%)	0.19%<br>(0.13% - 0.28%)
Bulgaria	11.82%<br>(10.84% - 12.92%)	13.51%<br>(12.54% - 14.64%)	11.50%<br>(10.60% - 12.46%)	10.31%<br>(9.40% - 11.26%)	3.12%<br>(2.56% - 3.76%)	4.04%<br>(3.43% - 4.76%)	3.20%<br>(2.72% - 3.74%)	2.73%<br>(2.26% - 3.24%)	0.35%<br>(0.19% - 0.57%)	0.64%<br>(0.43% - 0.92%)	0.42%<br>(0.27% - 0.61%)	0.30%<br>(0.17% - 0.47%)
Croatia	11.96%<br>(10.89% - 12.99%)	12.22%<br>(11.17% - 13.27%)	9.60%<br>(8.74% - 10.47%)	8.90%<br>(8.16% - 9.71%)	2.82%<br>(2.23% - 3.41%)	3.18%<br>(2.61% - 3.75%)	2.33%<br>(1.91% - 2.76%)	2.03%<br>(1.69% - 2.44%)	0.23%<br>(0.09% - 0.41%)	0.36%<br>(0.20% - 0.56%)	0.19%<br>(0.09% - 0.32%)	0.13%<br>(0.06% - 0.23%)
Czechia	3.16%<br>(2.87% - 3.48%)	2.68%<br>(2.41% - 2.98%)	2.52%<br>(2.26% - 2.78%)	2.46%<br>(2.21% - 2.71%)	0.13%<br>(0.09% - 0.19%)	0.13%<br>(0.09% - 0.18%)	0.13%<br>(0.09% - 0.19%)	0.13%<br>(0.09% - 0.18%)	0.00%<br>(0.00% - 0.00%)	0.00%<br>(0.00% - 0.00%)	0.00%<br>(0.00% - 0.00%)	0.00%<br>(0.00% - 0.00%)
Hungary	4.02%<br>(3.36% - 4.67%)	4.12%<br>(3.53% - 4.72%)	4.31%<br>(3.82% - 4.84%)	4.23%<br>(3.76% - 4.72%)	0.12%<br>(0.03% - 0.25%)	0.21%<br>(0.09% - 0.36%)	0.35%<br>(0.21% - 0.51%)	0.34%<br>(0.21% - 0.49%)	0.00%<br>(0.00% - 0.00%)	0.00%<br>(0.00% - 0.00%)	0.00%<br>(0.00% - 0.00%)	0.00%<br>(0.00% - 0.00%)
North Macedonia	11.43%<br>(10.45% - 12.46%)	10.46%<br>(9.60% - 11.37%)	8.26%<br>(7.60% - 8.93%)	7.46%<br>(6.82% - 8.15%)	2.20%<br>(1.71% - 2.67%)	2.16%<br>(1.75% - 2.61%)	1.58%<br>(1.31% - 1.87%)	1.33%<br>(1.08% - 1.61%)	0.11%<br>(0.03% - 0.24%)	0.13%<br>(0.05% - 0.25%)	0.05%<br>(0.01% - 0.11%)	0.02%<br>(0.00% - 0.06%)
Montenegro	6.56%<br>(5.96% - 7.21%)	7.91%<br>(7.22% - 8.66%)	9.07%<br>(8.37% - 9.80%)	9.74%<br>(8.93% - 10.64%)	0.76%<br>(0.57% - 0.98%)	1.15%<br>(0.89% - 1.43%)	1.68%<br>(1.36% - 2.00%)	1.87%<br>(1.51% - 2.29%)	0.00%<br>(0.00% - 0.00%)	0.01%<br>(0.00% - 0.03%)	0.04%<br>(0.01% - 0.10%)	0.09%<br>(0.03% - 0.17%)
Poland	12.54%<br>(11.49% - 13.64%)	11.48%<br>(10.52% - 12.52%)	9.26%<br>(8.43% - 10.12%)	8.26%<br>(7.50% - 9.06%)	2.63%<br>(2.04% - 3.28%)	2.70%<br>(2.16% - 3.26%)	2.07%<br>(1.71% - 2.46%)	1.70%<br>(1.40% - 2.07%)	0.17%<br>(0.06% - 0.34%)	0.24%<br>(0.12% - 0.40%)	0.14%<br>(0.07% - 0.24%)	0.08%<br>(0.04% - 0.15%)

Table S8a. Estimated overall, severe, and extreme **stunting** prevalence (%) in children under 5 years, both sexes, in 1990, 2000, 2010, and 2020.												
**Location**	**Total**				**Severe**				**Extreme**			
	**1990**	**2000**	**2010**	**2020**	**1990**	**2000**	**2010**	**2020**	**1990**	**2000**	**2010**	**2020**
Romania	16.63%	17.74%	14.69%	13.13%	4.69%	5.32%	4.13%	3.58%	0.67%	0.91%	0.62%	0.50%
	(15.64% - 17.62%)	(17.06% - 18.50%)	(13.65% - 15.77%)	(12.00% - 14.28%)	(4.04% - 5.37%)	(4.80% - 5.85%)	(3.48% - 4.80%)	(2.89% - 4.29%)	(0.46% - 0.93%)	(0.68% - 1.17%)	(0.38% - 0.90%)	(0.26% - 0.80%)
Serbia	11.02%	10.48%	9.19%	7.97%	2.20%	2.23%	2.05%	1.66%	0.09%	0.11%	0.12%	0.07%
	(10.13% - 11.95%)	(9.68% - 11.31%)	(8.59% - 9.89%)	(7.29% - 8.67%)	(1.80% - 2.64%)	(1.88% - 2.64%)	(1.76% - 2.38%)	(1.37% - 2.01%)	(0.03% - 0.19%)	(0.05% - 0.21%)	(0.06% - 0.20%)	(0.03% - 0.13%)
Slovakia	12.38%	12.29%	10.27%	9.35%	2.81%	3.12%	2.49%	2.09%	0.21%	0.33%	0.21%	0.13%
	(11.25% - 13.51%)	(11.22% - 13.41%)	(9.44% - 11.21%)	(8.48% - 10.22%)	(2.21% - 3.43%)	(2.55% - 3.77%)	(2.08% - 2.98%)	(1.71% - 2.50%)	(0.07% - 0.40%)	(0.17% - 0.54%)	(0.12% - 0.34%)	(0.06% - 0.24%)
Slovenia	10.85%	10.70%	8.54%	7.94%	2.49%	2.68%	2.06%	1.81%	0.18%	0.26%	0.16%	0.10%
	(9.79% - 11.87%)	(9.77% - 11.73%)	(7.80% - 9.33%)	(7.20% - 8.66%)	(1.99% - 3.04%)	(2.21% - 3.23%)	(1.72% - 2.44%)	(1.47% - 2.14%)	(0.06% - 0.33%)	(0.13% - 0.43%)	(0.08% - 0.26%)	(0.05% - 0.19%)
**Eastern Europe**	19.51%	16.94%	14.25%	12.50%	6.77%	5.70%	4.70%	3.99%	1.57%	1.32%	1.06%	0.83%
	(18.74% - 20.28%)	(16.32% - 17.56%)	(13.49% - 14.96%)	(11.71% - 13.30%)	(6.15% - 7.40%)	(5.25% - 6.15%)	(4.23% - 5.19%)	(3.53% - 4.45%)	(1.29% - 1.88%)	(1.11% - 1.54%)	(0.86% - 1.28%)	(0.65% - 1.03%)
Belarus	5.29%	5.02%	4.37%	3.74%	0.48%	0.50%	0.45%	0.38%	0.00%	0.01%	0.00%	0.00%
	(4.79% - 5.82%)	(4.53% - 5.51%)	(3.98% - 4.81%)	(3.37% - 4.17%)	(0.36% - 0.63%)	(0.37% - 0.64%)	(0.34% - 0.57%)	(0.28% - 0.48%)	(0.00% - 0.02%)	(0.00% - 0.02%)	(0.00% - 0.02%)	(0.00% - 0.01%)
Estonia	15.38%	11.53%	7.20%	6.37%	4.19%	2.84%	1.41%	1.18%	0.54%	0.26%	0.04%	0.02%
	(14.12% - 16.64%)	(10.46% - 12.59%)	(6.52% - 7.96%)	(5.72% - 7.09%)	(3.38% - 5.05%)	(2.29% - 3.49%)	(1.08% - 1.76%)	(0.91% - 1.50%)	(0.28% - 0.86%)	(0.11% - 0.47%)	(0.00% - 0.12%)	(0.00% - 0.08%)
Latvia	15.38%	12.70%	8.42%	7.06%	4.12%	3.19%	1.63%	1.27%	0.52%	0.32%	0.04%	0.02%
	(14.17% - 16.70%)	(11.64% - 13.79%)	(7.58% - 9.42%)	(6.33% - 7.80%)	(3.32% - 5.02%)	(2.56% - 3.84%)	(1.27% - 2.10%)	(0.96% - 1.65%)	(0.26% - 0.85%)	(0.14% - 0.54%)	(0.00% - 0.15%)	(0.00% - 0.09%)
Lithuania	14.32%	11.17%	7.48%	6.45%	3.75%	2.70%	1.37%	1.12%	0.44%	0.23%	0.02%	0.01%
	(13.21% - 15.54%)	(10.09% - 12.23%)	(6.80% - 8.22%)	(5.84% - 7.20%)	(3.02% - 4.52%)	(2.15% - 3.29%)	(1.07% - 1.73%)	(0.86% - 1.44%)	(0.21% - 0.70%)	(0.08% - 0.42%)	(0.00% - 0.09%)	(0.00% - 0.07%)
Republic of Moldova	10.59%	10.49%	8.23%	6.44%	1.85%	1.95%	1.25%	0.91%	0.03%	0.05%	0.00%	0.00%
	(9.59% - 11.63%)	(9.62% - 11.40%)	(7.58% - 8.91%)	(5.86% - 7.12%)	(1.43% - 2.31%)	(1.59% - 2.37%)	(1.01% - 1.54%)	(0.70% - 1.16%)	(0.00% - 0.10%)	(0.01% - 0.12%)	(0.00% - 0.02%)	(0.00% - 0.00%)
Russian Federation	19.16%	15.45%	12.36%	11.08%	5.93%	4.28%	3.30%	2.99%	1.03%	0.59%	0.41%	0.37%
	(18.07% - 20.23%)	(14.59% - 16.35%)	(11.39% - 13.35%)	(10.06% - 12.13%)	(5.06% - 6.84%)	(3.67% - 4.89%)	(2.73% - 3.87%)	(2.41% - 3.58%)	(0.70% - 1.40%)	(0.39% - 0.82%)	(0.24% - 0.61%)	(0.19% - 0.59%)
Ukraine	25.39%	25.61%	24.13%	22.97%	11.73%	12.02%	11.08%	10.38%	3.95%	4.14%	3.71%	3.41%
	(24.12% - 26.67%)	(24.52% - 26.64%)	(22.87% - 25.36%)	(21.53% - 24.30%)	(10.40% - 13.00%)	(10.97% - 13.06%)	(9.92% - 12.23%)	(9.17% - 11.62%)	(3.12% - 4.82%)	(3.41% - 4.87%)	(3.01% - 4.51%)	(2.67% - 4.22%)
High-income	4.12%	3.57%	3.03%	2.79%	0.29%	0.28%	0.22%	0.20%	0.01%	0.01%	0.00%	0.00%
	(3.99% - 4.25%)	(3.46% - 3.68%)	(2.94% - 3.13%)	(2.69% - 2.90%)	(0.26% - 0.33%)	(0.26% - 0.31%)	(0.19% - 0.24%)	(0.17% - 0.22%)	(0.01% - 0.01%)	(0.00% - 0.01%)	(0.00% - 0.00%)	(0.00% - 0.00%)
**Australasia**	2.35%	2.09%	1.91%	1.79%	0.04%	0.03%	0.01%	0.01%	0.00%	0.00%	0.00%	0.00%
	(2.15% - 2.58%)	(1.90% - 2.32%)	(1.74% - 2.10%)	(1.63% - 1.97%)	(0.03% - 0.06%)	(0.02% - 0.04%)	(0.01% - 0.02%)	(0.01% - 0.02%)	(0.00% - 0.00%)	(0.00% - 0.00%)	(0.00% - 0.00%)	(0.00% - 0.00%)
Australia	2.12%	1.95%	1.81%	1.70%	0.01%	0.00%	0.00%	0.00%	0.00%	0.00%	0.00%	0.00%
	(1.90% - 2.40%)	(1.72% - 2.20%)	(1.60% - 2.04%)	(1.52% - 1.91%)	(0.00% - 0.02%)	(0.00% - 0.01%)	(0.00% - 0.01%)	(0.00% - 0.01%)	(0.00% - 0.00%)	(0.00% - 0.00%)	(0.00% - 0.00%)	(0.00% - 0.00%)
New Zealand	3.34%	2.74%	2.42%	2.22%	0.21%	0.12%	0.07%	0.06%	0.00%	0.00%	0.00%	0.00%
	(3.01% - 3.67%)	(2.47% - 3.03%)	(2.20% - 2.70%)	(2.01% - 2.45%)	(0.16% - 0.26%)	(0.08% - 0.16%)	(0.04% - 0.09%)	(0.04% - 0.09%)	(0.00% - 0.00%)	(0.00% - 0.00%)	(0.00% - 0.00%)	(0.00% - 0.00%)
**High-income Asia Pacific**	7.30%	6.44%	6.41%	5.99%	0.54%	0.57%	0.64%	0.62%	0.01%	0.01%	0.01%	0.01%
	(7.13% - 7.48%)	(6.30% - 6.58%)	(6.33% - 6.49%)	(5.88% - 6.11%)	(0.50% - 0.58%)	(0.54% - 0.60%)	(0.61% - 0.67%)	(0.59% - 0.66%)	(0.01% - 0.01%)	(0.01% - 0.01%)	(0.01% - 0.01%)	(0.01% - 0.01%)
Brunei Darussalam	21.63%	19.56%	18.34%	17.51%	7.40%	6.66%	6.26%	6.01%	1.64%	1.48%	1.41%	1.37%
	(20.34% - 22.91%)	(18.44% - 20.77%)	(17.39% - 19.30%)	(16.34% - 18.66%)	(6.37% - 8.46%)	(5.86% - 7.61%)	(5.55% - 6.93%)	(5.24% - 6.85%)	(1.22% - 2.11%)	(1.14% - 1.91%)	(1.10% - 1.75%)	(1.05% - 1.75%)
Japan	9.43%	8.48%	8.04%	7.67%	0.78%	0.84%	0.88%	0.86%	0.00%	0.00%	0.00%	0.00%
	(9.24% - 9.62%)	(8.33% - 8.63%)	(7.95% - 8.13%)	(7.53% - 7.82%)	(0.73% - 0.84%)	(0.79% - 0.88%)	(0.84% - 0.91%)	(0.81% - 0.91%)	(0.00% - 0.00%)	(0.00% - 0.00%)	(0.00% - 0.00%)	(0.00% - 0.00%)
Hokkaidō	9.90%	8.81%	8.43%	8.10%	0.76%	0.84%	0.91%	0.91%	0.00%	0.00%	0.00%	0.00%
	(8.86% - 11.06%)	(8.05% - 9.64%)	(8.20% - 8.67%)	(7.37% - 8.87%)	(0.47% - 1.10%)	(0.60% - 1.13%)	(0.75% - 1.07%)	(0.68% - 1.18%)	(0.00% - 0.01%)	(0.00% - 0.01%)	(0.00% - 0.00%)	(0.00% - 0.01%)
Aomori	10.13%	9.11%	8.50%	8.04%	1.03%	1.09%	1.13%	1.10%	0.00%	0.00%	0.00%	0.00%
	(9.10% - 11.21%)	(8.17% - 10.06%)	(7.64% - 9.44%)	(7.23% - 8.87%)	(0.68% - 1.43%)	(0.76% - 1.47%)	(0.84% - 1.48%)	(0.83% - 1.42%)	(0.00% - 0.00%)	(0.00% - 0.01%)	(0.00% - 0.01%)	(0.00% - 0.01%)
Iwate	12.57%	11.30%	10.79%	10.17%	1.78%	1.84%	1.86%	1.77%	0.03%	0.04%	0.04%	0.04%
	(11.44% - 13.73%)	(10.26% - 12.40%)	(9.84% - 11.82%)	(9.27% - 11.21%)	(1.35% - 2.32%)	(1.42% - 2.30%)	(1.48% - 2.31%)	(1.39% - 2.20%)	(0.00% - 0.07%)	(0.01% - 0.09%)	(0.01% - 0.10%)	(0.01% - 0.10%)

Table S8a. Estimated overall, severe, and extreme **stunting** prevalence (%) in children under 5 years, both sexes, in 1990, 2000, 2010, and 2020.

Location	Total				Severe				Extreme			
	1990	2000	2010	2020	1990	2000	2010	2020	1990	2000	2010	2020
Miyagi	9.36% (8.40% - 10.35%)	8.44% (7.61% - 9.30%)	8.08% (7.26% - 8.82%)	7.70% (6.89% - 8.43%)	1.13% (0.82% - 1.49%)	1.14% (0.87% - 1.47%)	1.18% (0.91% - 1.45%)	1.12% (0.86% - 1.41%)	0.00% (0.00% - 0.01%)	0.00% (0.00% - 0.01%)	0.00% (0.00% - 0.01%)	0.00% (0.00% - 0.01%)
Akita	9.61% (8.71% - 10.60%)	8.67% (7.87% - 9.54%)	8.33% (7.50% - 9.18%)	7.97% (7.23% - 8.82%)	1.21% (0.87% - 1.59%)	1.23% (0.94% - 1.58%)	1.25% (0.97% - 1.58%)	1.21% (0.95% - 1.50%)	0.00% (0.00% - 0.02%)	0.00% (0.00% - 0.02%)	0.00% (0.00% - 0.02%)	0.00% (0.00% - 0.02%)
Yamagata	9.25% (8.35% - 10.30%)	8.57% (7.69% - 9.47%)	8.21% (7.37% - 9.08%)	7.88% (7.13% - 8.73%)	0.75% (0.49% - 1.10%)	0.84% (0.58% - 1.15%)	0.91% (0.64% - 1.20%)	0.89% (0.64% - 1.16%)	0.00% (0.00% - 0.00%)	0.00% (0.00% - 0.00%)	0.00% (0.00% - 0.00%)	0.00% (0.00% - 0.00%)
Fukushima	6.59% (5.90% - 7.36%)	5.90% (5.30% - 6.55%)	5.56% (4.97% - 6.14%)	5.30% (4.75% - 5.87%)	0.17% (0.07% - 0.32%)	0.22% (0.11% - 0.36%)	0.26% (0.15% - 0.40%)	0.27% (0.15% - 0.40%)	0.00% (0.00% - 0.00%)	0.00% (0.00% - 0.00%)	0.00% (0.00% - 0.00%)	0.00% (0.00% - 0.00%)
Ibaraki	9.63% (8.61% - 10.80%)	8.59% (7.73% - 9.50%)	8.13% (7.34% - 8.94%)	7.75% (6.96% - 8.58%)	0.65% (0.37% - 1.00%)	0.74% (0.49% - 1.07%)	0.80% (0.56% - 1.06%)	0.79% (0.54% - 1.10%)	0.00% (0.00% - 0.00%)	0.00% (0.00% - 0.00%)	0.00% (0.00% - 0.00%)	0.00% (0.00% - 0.00%)
Tochigi	10.54% (9.52% - 11.67%)	9.41% (8.55% - 10.38%)	8.90% (8.00% - 9.89%)	8.51% (7.64% - 9.48%)	0.68% (0.40% - 1.04%)	0.80% (0.54% - 1.12%)	0.87% (0.62% - 1.18%)	0.88% (0.61% - 1.20%)	0.00% (0.00% - 0.00%)	0.00% (0.00% - 0.00%)	0.00% (0.00% - 0.00%)	0.00% (0.00% - 0.00%)
Gunma	12.68% (11.59% - 13.87%)	11.51% (10.48% - 12.60%)	11.00% (9.98% - 11.99%)	10.49% (9.49% - 11.50%)	1.40% (0.96% - 1.92%)	1.48% (1.08% - 1.94%)	1.56% (1.19% - 1.97%)	1.53% (1.15% - 1.95%)	0.01% (0.00% - 0.03%)	0.01% (0.00% - 0.03%)	0.01% (0.00% - 0.03%)	0.01% (0.00% - 0.04%)
Saitama	10.01% (8.98% - 11.10%)	9.00% (8.27% - 9.84%)	8.58% (8.39% - 8.76%)	8.24% (7.56% - 8.98%)	0.64% (0.39% - 0.99%)	0.74% (0.51% - 1.01%)	0.81% (0.67% - 0.97%)	0.82% (0.60% - 1.06%)	0.00% (0.00% - 0.00%)	0.00% (0.00% - 0.00%)	0.00% (0.00% - 0.00%)	0.00% (0.00% - 0.00%)
Chiba	8.03% (7.23% - 8.89%)	7.29% (6.59% - 8.04%)	6.96% (6.35% - 7.66%)	6.73% (6.04% - 7.41%)	0.47% (0.27% - 0.68%)	0.52% (0.35% - 0.72%)	0.58% (0.42% - 0.76%)	0.57% (0.41% - 0.77%)	0.00% (0.00% - 0.00%)	0.00% (0.00% - 0.00%)	0.00% (0.00% - 0.00%)	0.00% (0.00% - 0.00%)
Tōkyō	7.39% (6.62% - 8.22%)	6.72% (6.11% - 7.32%)	6.38% (6.19% - 6.57%)	6.19% (5.64% - 6.84%)	0.32% (0.19% - 0.51%)	0.36% (0.24% - 0.52%)	0.41% (0.32% - 0.50%)	0.43% (0.29% - 0.60%)	0.00% (0.00% - 0.00%)	0.00% (0.00% - 0.00%)	0.00% (0.00% - 0.00%)	0.00% (0.00% - 0.00%)
Kanagawa	8.94% (8.04% - 9.93%)	8.12% (7.39% - 8.81%)	7.77% (7.58% - 7.96%)	7.54% (6.88% - 8.25%)	0.63% (0.42% - 0.91%)	0.70% (0.50% - 0.93%)	0.75% (0.62% - 0.88%)	0.76% (0.56% - 0.98%)	0.00% (0.00% - 0.00%)	0.00% (0.00% - 0.00%)	0.00% (0.00% - 0.00%)	0.00% (0.00% - 0.00%)
Niigata	11.82% (10.64% - 13.07%)	10.78% (9.79% - 11.94%)	10.40% (9.45% - 11.33%)	9.98% (8.99% - 11.02%)	1.27% (0.85% - 1.78%)	1.34% (0.95% - 1.80%)	1.41% (1.05% - 1.80%)	1.38% (1.01% - 1.81%)	0.00% (0.00% - 0.02%)	0.01% (0.00% - 0.02%)	0.01% (0.00% - 0.02%)	0.01% (0.00% - 0.02%)
Toyama	8.98% (8.04% - 10.00%)	8.20% (7.39% - 9.04%)	7.88% (7.14% - 8.67%)	7.55% (6.82% - 8.33%)	1.10% (0.79% - 1.46%)	1.14% (0.86% - 1.46%)	1.16% (0.90% - 1.45%)	1.13% (0.87% - 1.41%)	0.00% (0.00% - 0.01%)	0.00% (0.00% - 0.01%)	0.00% (0.00% - 0.01%)	0.00% (0.00% - 0.01%)
Ishikawa	9.94% (9.01% - 10.94%)	9.01% (8.18% - 9.91%)	8.68% (7.88% - 9.55%)	8.38% (7.62% - 9.28%)	1.24% (0.91% - 1.61%)	1.25% (0.96% - 1.60%)	1.30% (1.02% - 1.65%)	1.27% (0.99% - 1.60%)	0.00% (0.00% - 0.02%)	0.00% (0.00% - 0.02%)	0.01% (0.00% - 0.03%)	0.01% (0.00% - 0.02%)
Fukui	8.54% (7.75% - 9.51%)	7.70% (6.95% - 8.53%)	7.34% (6.58% - 8.14%)	7.08% (6.42% - 7.89%)	0.93% (0.67% - 1.26%)	0.94% (0.70% - 1.23%)	0.97% (0.72% - 1.26%)	0.95% (0.73% - 1.23%)	0.00% (0.00% - 0.00%)	0.00% (0.00% - 0.00%)	0.00% (0.00% - 0.00%)	0.00% (0.00% - 0.00%)
Yamanashi	9.20% (8.28% - 10.24%)	8.46% (7.71% - 9.41%)	8.06% (7.28% - 8.89%)	7.74% (6.99% - 8.58%)	0.69% (0.41% - 1.04%)	0.81% (0.55% - 1.11%)	0.86% (0.61% - 1.14%)	0.85% (0.63% - 1.15%)	0.00% (0.00% - 0.00%)	0.00% (0.00% - 0.00%)	0.00% (0.00% - 0.00%)	0.00% (0.00% - 0.00%)
Nagano	8.67% (7.78% - 9.57%)	7.91% (7.15% - 8.73%)	7.65% (6.84% - 8.48%)	7.39% (6.63% - 8.18%)	0.87% (0.59% - 1.17%)	0.90% (0.67% - 1.19%)	0.94% (0.68% - 1.21%)	0.91% (0.67% - 1.19%)	0.00% (0.00% - 0.00%)	0.00% (0.00% - 0.00%)	0.00% (0.00% - 0.00%)	0.00% (0.00% - 0.00%)
Gifu	8.69% (7.82% - 9.61%)	7.89% (7.13% - 8.65%)	7.53% (6.74% - 8.32%)	7.23% (6.53% - 8.02%)	1.00% (0.71% - 1.32%)	1.00% (0.75% - 1.27%)	1.03% (0.79% - 1.31%)	1.00% (0.78% - 1.28%)	0.00% (0.00% - 0.01%)	0.00% (0.00% - 0.01%)	0.00% (0.00% - 0.01%)	0.00% (0.00% - 0.01%)
Shizuoka	12.38% (11.20% - 13.61%)	11.16% (10.15% - 12.16%)	10.64% (9.76% - 11.51%)	10.22% (9.22% - 11.21%)	1.41% (0.93% - 1.95%)	1.50% (1.10% - 1.94%)	1.54% (1.20% - 1.93%)	1.50% (1.11% - 1.93%)	0.01% (0.00% - 0.03%)	0.01% (0.00% - 0.03%)	0.01% (0.00% - 0.03%)	0.01% (0.00% - 0.04%)
Aichi	8.21% (7.36% - 9.13%)	7.40% (6.76% - 8.09%)	6.92% (6.74% - 7.10%)	6.57% (5.99% - 7.18%)	0.45% (0.28% - 0.70%)	0.51% (0.34% - 0.70%)	0.54% (0.43% - 0.64%)	0.55% (0.40% - 0.71%)	0.00% (0.00% - 0.00%)	0.00% (0.00% - 0.00%)	0.00% (0.00% - 0.00%)	0.00% (0.00% - 0.00%)
Mie	8.73% (7.88% - 9.70%)	7.89% (7.07% - 8.77%)	7.47% (6.70% - 8.26%)	7.10% (6.42% - 7.85%)	0.98% (0.71% - 1.34%)	0.98% (0.74% - 1.31%)	1.01% (0.77% - 1.29%)	0.97% (0.74% - 1.24%)	0.00% (0.00% - 0.00%)	0.00% (0.00% - 0.00%)	0.00% (0.00% - 0.01%)	0.00% (0.00% - 0.00%)
Shiga	10.36% (9.31% - 11.53%)	9.25% (8.34% - 10.22%)	8.85% (8.04% - 9.78%)	8.52% (7.66% - 9.44%)	0.86% (0.54% - 1.23%)	0.95% (0.66% - 1.30%)	1.02% (0.75% - 1.34%)	1.01% (0.74% - 1.33%)	0.00% (0.00% - 0.00%)	0.00% (0.00% - 0.01%)	0.00% (0.00% - 0.01%)	0.00% (0.00% - 0.01%)

Table S8a. Estimated overall, severe, and extreme **stunting** prevalence (%) in children under 5 years, both sexes, in 1990, 2000, 2010, and 2020.

Location	Total				Severe				Extreme			
	1990	2000	2010	2020	1990	2000	2010	2020	1990	2000	2010	2020
Kyōto	10.58%	9.50%	9.06%	8.71%	0.87%	0.97%	1.05%	1.04%	0.00%	0.00%	0.00%	0.00%
	(9.53% - 11.76%)	(8.56% - 10.53%)	(8.17% - 10.02%)	(7.87% - 9.70%)	(0.56% - 1.29%)	(0.66% - 1.31%)	(0.76% - 1.39%)	(0.75% - 1.39%)	(0.00% - 0.01%)	(0.00% - 0.01%)	(0.00% - 0.01%)	(0.00% - 0.01%)
Ōsaka	9.60%	8.53%	8.06%	7.68%	0.67%	0.74%	0.80%	0.80%	0.00%	0.00%	0.00%	0.00%
	(8.68% - 10.62%)	(7.86% - 9.30%)	(7.89% - 8.22%)	(7.03% - 8.37%)	(0.42% - 0.97%)	(0.54% - 0.97%)	(0.66% - 0.93%)	(0.60% - 1.03%)	(0.00% - 0.00%)	(0.00% - 0.00%)	(0.00% - 0.00%)	(0.00% - 0.00%)
Hyōgo	8.49%	7.55%	7.21%	6.91%	0.54%	0.59%	0.66%	0.65%	0.00%	0.00%	0.00%	0.00%
	(7.61% - 9.46%)	(6.74% - 8.35%)	(6.54% - 7.90%)	(6.22% - 7.60%)	(0.31% - 0.81%)	(0.40% - 0.83%)	(0.47% - 0.87%)	(0.46% - 0.88%)	(0.00% - 0.00%)	(0.00% - 0.00%)	(0.00% - 0.00%)	(0.00% - 0.00%)
	9.25%	8.44%	8.16%	7.89%	0.76%	0.87%	0.94%	0.93%	0.00%	0.00%	0.00%	0.00%
Nara	(8.32% - 10.28%)	(7.59% - 9.42%)	(7.35% - 9.13%)	(7.06% - 8.77%)	(0.48% - 1.07%)	(0.60% - 1.20%)	(0.68% - 1.27%)	(0.68% - 1.22%)	(0.00% - 0.00%)	(0.00% - 0.00%)	(0.00% - 0.00%)	(0.00% - 0.00%)
	9.53%	8.67%	8.19%	7.84%	0.87%	0.96%	0.99%	0.97%	0.00%	0.00%	0.00%	0.00%
Wakayama	(8.57% - 10.63%)	(7.78% - 9.66%)	(7.40% - 9.08%)	(7.06% - 8.60%)	(0.56% - 1.27%)	(0.68% - 1.30%)	(0.73% - 1.30%)	(0.73% - 1.25%)	(0.00% - 0.00%)	(0.00% - 0.00%)	(0.00% - 0.00%)	(0.00% - 0.00%)
	9.34%	8.51%	8.22%	7.92%	1.09%	1.14%	1.17%	1.15%	0.00%	0.00%	0.00%	0.00%
Tottori	(8.42% - 10.30%)	(7.70% - 9.41%)	(7.47% - 9.11%)	(7.16% - 8.82%)	(0.76% - 1.44%)	(0.87% - 1.46%)	(0.91% - 1.51%)	(0.88% - 1.47%)	(0.00% - 0.01%)	(0.00% - 0.01%)	(0.00% - 0.01%)	(0.00% - 0.01%)
	9.45%	8.59%	8.19%	7.81%	1.00%	1.06%	1.10%	1.06%	0.00%	0.00%	0.00%	0.00%
Shimane	(8.47% - 10.49%)	(7.70% - 9.50%)	(7.36% - 9.08%)	(7.03% - 8.65%)	(0.68% - 1.37%)	(0.77% - 1.39%)	(0.81% - 1.40%)	(0.80% - 1.37%)	(0.00% - 0.00%)	(0.00% - 0.01%)	(0.00% - 0.01%)	(0.00% - 0.01%)
	6.22%	5.61%	5.35%	5.11%	0.13%	0.17%	0.20%	0.20%	0.00%	0.00%	0.00%	0.00%
Okayama	(5.53% - 6.99%)	(4.98% - 6.24%)	(4.80% - 5.94%)	(4.59% - 5.70%)	(0.07% - 0.23%)	(0.10% - 0.28%)	(0.12% - 0.30%)	(0.12% - 0.32%)	(0.00% - 0.00%)	(0.00% - 0.00%)	(0.00% - 0.00%)	(0.00% - 0.00%)
	6.46%	5.85%	5.63%	5.41%	0.15%	0.20%	0.25%	0.25%	0.00%	0.00%	0.00%	0.00%
Hiroshima	(5.76% - 7.25%)	(5.23% - 6.51%)	(5.09% - 6.25%)	(4.85% - 5.99%)	(0.06% - 0.30%)	(0.09% - 0.35%)	(0.14% - 0.39%)	(0.14% - 0.39%)	(0.00% - 0.00%)	(0.00% - 0.00%)	(0.00% - 0.00%)	(0.00% - 0.00%)
	11.75%	10.73%	10.20%	9.71%	1.79%	1.83%	1.83%	1.75%	0.03%	0.04%	0.05%	0.04%
Yamaguchi	(10.72% - 12.84%)	(9.81% - 11.74%)	(9.32% - 11.15%)	(8.85% - 10.64%)	(1.37% - 2.27%)	(1.45% - 2.28%)	(1.47% - 2.25%)	(1.41% - 2.12%)	(0.00% - 0.08%)	(0.01% - 0.10%)	(0.01% - 0.10%)	(0.01% - 0.10%)
	9.64%	8.76%	8.24%	7.81%	0.81%	0.91%	0.95%	0.92%	0.00%	0.00%	0.00%	0.00%
Tokushima	(8.68% - 10.71%)	(7.84% - 9.72%)	(7.38% - 9.08%)	(7.02% - 8.63%)	(0.50% - 1.18%)	(0.62% - 1.26%)	(0.69% - 1.27%)	(0.67% - 1.22%)	(0.00% - 0.00%)	(0.00% - 0.00%)	(0.00% - 0.00%)	(0.00% - 0.00%)
	9.11%	8.37%	8.02%	7.68%	1.04%	1.09%	1.12%	1.09%	0.00%	0.00%	0.00%	0.00%
Kagawa	(8.28% - 10.11%)	(7.57% - 9.23%)	(7.21% - 8.90%)	(6.95% - 8.48%)	(0.75% - 1.39%)	(0.82% - 1.41%)	(0.82% - 1.44%)	(0.83% - 1.39%)	(0.00% - 0.01%)	(0.00% - 0.01%)	(0.00% - 0.01%)	(0.00% - 0.01%)
	9.78%	8.81%	8.41%	8.03%	1.24%	1.24%	1.28%	1.23%	0.00%	0.00%	0.01%	0.00%
Ehime	(8.87% - 10.81%)	(7.96% - 9.72%)	(7.64% - 9.24%)	(7.25% - 8.86%)	(0.91% - 1.62%)	(0.94% - 1.59%)	(1.00% - 1.59%)	(0.95% - 1.52%)	(0.00% - 0.02%)	(0.00% - 0.02%)	(0.00% - 0.02%)	(0.00% - 0.02%)
Kōchi	9.77%	8.85%	8.41%	7.92%	1.27%	1.29%	1.31%	1.25%	0.00%	0.00%	0.01%	0.00%
	(8.77% - 10.88%)	(8.02% - 9.72%)	(7.61% - 9.32%)	(7.23% - 8.78%)	(0.91% - 1.68%)	(0.97% - 1.66%)	(1.03% - 1.67%)	(0.99% - 1.60%)	(0.00% - 0.02%)	(0.00% - 0.02%)	(0.00% - 0.03%)	(0.00% - 0.03%)
	13.85%	12.50%	11.92%	11.38%	1.25%	1.45%	1.57%	1.55%	0.00%	0.01%	0.00%	0.01%
Fukuoka	(12.51% - 15.21%)	(11.44% - 13.48%)	(11.71% - 12.15%)	(10.45% - 12.30%)	(0.79% - 1.82%)	(1.05% - 1.90%)	(1.32% - 1.81%)	(1.16% - 2.00%)	(0.00% - 0.02%)	(0.00% - 0.02%)	(0.00% - 0.01%)	(0.00% - 0.03%)
	10.79%	9.73%	9.18%	8.81%	1.57%	1.57%	1.57%	1.52%	0.02%	0.02%	0.03%	0.02%
Saga	(9.80% - 11.87%)	(8.88% - 10.64%)	(8.31% - 10.15%)	(8.01% - 9.70%)	(1.21% - 2.01%)	(1.22% - 1.95%)	(1.25% - 1.94%)	(1.22% - 1.85%)	(0.00% - 0.05%)	(0.00% - 0.06%)	(0.00% - 0.07%)	(0.00% - 0.06%)
	12.92%	11.42%	10.81%	10.30%	1.10%	1.25%	1.35%	1.34%	0.00%	0.00%	0.00%	0.00%
Nagasaki	(11.68% - 14.29%)	(10.37% - 12.58%)	(9.93% - 11.74%)	(9.25% - 11.36%)	(0.66% - 1.63%)	(0.86% - 1.73%)	(1.01% - 1.73%)	(0.99% - 1.74%)	(0.00% - 0.01%)	(0.00% - 0.02%)	(0.00% - 0.02%)	(0.00% - 0.02%)
	8.87%	7.99%	7.63%	7.33%	0.95%	0.99%	1.02%	0.99%	0.00%	0.00%	0.00%	0.00%
Kumamoto	(8.00% - 9.80%)	(7.17% - 8.85%)	(6.84% - 8.41%)	(6.60% - 8.13%)	(0.66% - 1.27%)	(0.73% - 1.30%)	(0.76% - 1.31%)	(0.74% - 1.27%)	(0.00% - 0.00%)	(0.00% - 0.00%)	(0.00% - 0.00%)	(0.00% - 0.01%)
Ōita	10.16%	9.04%	8.62%	8.29%	1.31%	1.31%	1.34%	1.30%	0.00%	0.01%	0.01%	0.01%
	(9.18% - 11.18%)	(8.17% - 9.95%)	(7.85% - 9.51%)	(7.50% - 9.08%)	(0.94% - 1.71%)	(1.01% - 1.66%)	(1.07% - 1.68%)	(1.02% - 1.61%)	(0.00% - 0.02%)	(0.00% - 0.02%)	(0.00% - 0.03%)	(0.00% - 0.03%)
	7.89%	6.98%	6.61%	6.31%	0.58%	0.63%	0.67%	0.65%	0.00%	0.00%	0.00%	0.00%
Miyazaki	(7.08% - 8.75%)	(6.26% - 7.78%)	(5.92% - 7.35%)	(5.68% - 7.01%)	(0.36% - 0.85%)	(0.42% - 0.87%)	(0.46% - 0.89%)	(0.45% - 0.87%)	(0.00% - 0.00%)	(0.00% - 0.00%)	(0.00% - 0.00%)	(0.00% - 0.00%)
	7.84%	6.90%	6.47%	6.13%	0.61%	0.63%	0.67%	0.65%	0.00%	0.00%	0.00%	0.00%
Kagoshima	(7.05% - 8.75%)	(6.24% - 7.65%)	(5.84% - 7.17%)	(5.51% - 6.80%)	(0.38% - 0.88%)	(0.44% - 0.88%)	(0.48% - 0.90%)	(0.47% - 0.88%)	(0.00% - 0.00%)	(0.00% - 0.00%)	(0.00% - 0.00%)	(0.00% - 0.00%)
	9.41%	8.58%	8.03%	7.57%	1.04%	1.07%	1.09%	1.03%	0.00%	0.00%	0.00%	0.00%
Okinawa	(8.47% - 10.43%)	(7.80% - 9.52%)	(7.22% - 8.88%)	(6.81% - 8.38%)	(0.73% - 1.39%)	(0.80% - 1.38%)	(0.82% - 1.39%)	(0.78% - 1.33%)	(0.00% - 0.01%)	(0.00% - 0.01%)	(0.00% - 0.01%)	(0.00% - 0.01%)

Table S8a. Estimated overall, severe, and extreme **stunting** prevalence (%) in children under 5 years, both sexes, in 1990, 2000, 2010, and 2020.

Location	Total				Severe				Extreme			
	1990	2000	2010	2020	1990	2000	2010	2020	1990	2000	2010	2020
Republic of Korea	2.98% (2.65% - 3.32%)	2.59% (2.33% - 2.88%)	2.59% (2.42% - 2.77%)	1.96% (1.78% - 2.19%)	0.00% (0.00% - 0.01%)	0.01% (0.00% - 0.03%)	0.03% (0.01% - 0.07%)	0.01% (0.00% - 0.03%)	0.00% (0.00% - 0.00%)	0.00% (0.00% - 0.00%)	0.00% (0.00% - 0.00%)	0.00% (0.00% - 0.00%)
Singapore	5.72% (5.21% - 6.24%)	4.44% (4.11% - 4.81%)	3.78% (3.44% - 4.17%)	3.50% (3.15% - 3.92%)	0.25% (0.14% - 0.37%)	0.23% (0.15% - 0.33%)	0.22% (0.14% - 0.32%)	0.21% (0.12% - 0.31%)	0.00% (0.00% - 0.00%)	0.00% (0.00% - 0.00%)	0.00% (0.00% - 0.00%)	0.00% (0.00% - 0.00%)
**High-income North America**	3.63% (3.33% - 3.96%)	3.36% (3.10% - 3.61%)	2.76% (2.55% - 2.99%)	2.58% (2.35% - 2.85%)	0.09% (0.04% - 0.16%)	0.13% (0.08% - 0.19%)	0.07% (0.04% - 0.12%)	0.06% (0.02% - 0.10%)	0.00% (0.00% - 0.00%)	0.00% (0.00% - 0.00%)	0.00% (0.00% - 0.00%)	0.00% (0.00% - 0.00%)
Canada	3.42% (3.07% - 3.78%)	3.04% (2.73% - 3.37%)	2.53% (2.28% - 2.81%)	2.46% (2.21% - 2.73%)	0.09% (0.03% - 0.17%)	0.10% (0.05% - 0.17%)	0.05% (0.01% - 0.10%)	0.05% (0.02% - 0.11%)	0.00% (0.00% - 0.00%)	0.00% (0.00% - 0.00%)	0.00% (0.00% - 0.00%)	0.00% (0.00% - 0.00%)
United States of America	3.64% (3.31% - 4.00%)	3.39% (3.10% - 3.67%)	2.78% (2.55% - 3.04%)	2.60% (2.34% - 2.89%)	0.09% (0.04% - 0.17%)	0.13% (0.08% - 0.20%)	0.07% (0.03% - 0.13%)	0.06% (0.02% - 0.11%)	0.00% (0.00% - 0.00%)	0.00% (0.00% - 0.00%)	0.00% (0.00% - 0.00%)	0.00% (0.00% - 0.00%)
Alabama	3.93% (3.37% - 4.53%)	3.69% (3.23% - 4.22%)	3.02% (2.62% - 3.48%)	2.87% (2.49% - 3.34%)	0.10% (0.02% - 0.23%)	0.13% (0.05% - 0.24%)	0.07% (0.02% - 0.16%)	0.05% (0.01% - 0.14%)	0.00% (0.00% - 0.00%)	0.00% (0.00% - 0.00%)	0.00% (0.00% - 0.00%)	0.00% (0.00% - 0.00%)
Alaska	3.70% (3.18% - 4.28%)	3.42% (2.98% - 3.92%)	2.84% (2.48% - 3.24%)	2.62% (2.24% - 3.05%)	0.09% (0.02% - 0.21%)	0.13% (0.05% - 0.24%)	0.07% (0.02% - 0.16%)	0.06% (0.01% - 0.14%)	0.00% (0.00% - 0.00%)	0.00% (0.00% - 0.00%)	0.00% (0.00% - 0.00%)	0.00% (0.00% - 0.00%)
Arizona	3.78% (3.20% - 4.37%)	3.56% (3.07% - 4.08%)	2.85% (2.45% - 3.29%)	2.67% (2.30% - 3.16%)	0.10% (0.02% - 0.22%)	0.14% (0.05% - 0.25%)	0.08% (0.02% - 0.17%)	0.06% (0.01% - 0.15%)	0.00% (0.00% - 0.00%)	0.00% (0.00% - 0.00%)	0.00% (0.00% - 0.00%)	0.00% (0.00% - 0.00%)
Arkansas	3.97% (3.42% - 4.57%)	3.75% (3.26% - 4.27%)	3.09% (2.71% - 3.53%)	2.89% (2.48% - 3.37%)	0.10% (0.02% - 0.22%)	0.13% (0.05% - 0.26%)	0.07% (0.02% - 0.17%)	0.06% (0.01% - 0.14%)	0.00% (0.00% - 0.00%)	0.00% (0.00% - 0.00%)	0.00% (0.00% - 0.00%)	0.00% (0.00% - 0.00%)
California	3.69% (3.22% - 4.25%)	3.32% (2.90% - 3.78%)	2.68% (2.35% - 3.04%)	2.47% (2.14% - 2.84%)	0.09% (0.02% - 0.20%)	0.13% (0.05% - 0.23%)	0.07% (0.02% - 0.15%)	0.05% (0.01% - 0.12%)	0.00% (0.00% - 0.00%)	0.00% (0.00% - 0.00%)	0.00% (0.00% - 0.00%)	0.00% (0.00% - 0.00%)
Colorado	3.45% (2.99% - 4.00%)	3.29% (2.88% - 3.75%)	2.71% (2.34% - 3.11%)	2.51% (2.15% - 2.94%)	0.10% (0.02% - 0.20%)	0.13% (0.05% - 0.24%)	0.07% (0.02% - 0.16%)	0.06% (0.01% - 0.13%)	0.00% (0.00% - 0.00%)	0.00% (0.00% - 0.00%)	0.00% (0.00% - 0.00%)	0.00% (0.00% - 0.00%)
Connecticut	3.21% (2.78% - 3.69%)	3.04% (2.66% - 3.47%)	2.48% (2.15% - 2.87%)	2.34% (2.02% - 2.75%)	0.09% (0.02% - 0.19%)	0.12% (0.05% - 0.23%)	0.07% (0.02% - 0.15%)	0.05% (0.01% - 0.13%)	0.00% (0.00% - 0.00%)	0.00% (0.00% - 0.00%)	0.00% (0.00% - 0.00%)	0.00% (0.00% - 0.00%)
Delaware	3.57% (3.04% - 4.13%)	3.32% (2.87% - 3.79%)	2.72% (2.36% - 3.10%)	2.56% (2.20% - 2.99%)	0.09% (0.01% - 0.20%)	0.12% (0.04% - 0.23%)	0.07% (0.02% - 0.15%)	0.06% (0.01% - 0.13%)	0.00% (0.00% - 0.00%)	0.00% (0.00% - 0.00%)	0.00% (0.00% - 0.00%)	0.00% (0.00% - 0.00%)
District of Columbia	4.37% (3.79% - 5.06%)	3.65% (3.18% - 4.15%)	2.88% (2.50% - 3.29%)	2.64% (2.27% - 3.07%)	0.07% (0.01% - 0.20%)	0.11% (0.04% - 0.22%)	0.06% (0.01% - 0.15%)	0.05% (0.01% - 0.12%)	0.00% (0.00% - 0.00%)	0.00% (0.00% - 0.00%)	0.00% (0.00% - 0.00%)	0.00% (0.00% - 0.00%)
Florida	3.62% (3.13% - 4.17%)	3.33% (2.89% - 3.76%)	2.74% (2.40% - 3.13%)	2.60% (2.21% - 3.01%)	0.10% (0.02% - 0.22%)	0.13% (0.05% - 0.24%)	0.07% (0.02% - 0.16%)	0.06% (0.01% - 0.14%)	0.00% (0.00% - 0.00%)	0.00% (0.00% - 0.00%)	0.00% (0.00% - 0.00%)	0.00% (0.00% - 0.00%)
Georgia	3.89% (3.35% - 4.50%)	3.61% (3.12% - 4.10%)	2.90% (2.51% - 3.32%)	2.72% (2.35% - 3.15%)	0.09% (0.02% - 0.20%)	0.13% (0.05% - 0.24%)	0.07% (0.02% - 0.16%)	0.06% (0.01% - 0.13%)	0.00% (0.00% - 0.00%)	0.00% (0.00% - 0.00%)	0.00% (0.00% - 0.00%)	0.00% (0.00% - 0.00%)
Hawaii	3.49% (3.00% - 4.02%)	3.27% (2.84% - 3.75%)	2.72% (2.36% - 3.14%)	2.56% (2.19% - 2.97%)	0.09% (0.02% - 0.21%)	0.13% (0.05% - 0.23%)	0.08% (0.02% - 0.17%)	0.06% (0.01% - 0.14%)	0.00% (0.00% - 0.00%)	0.00% (0.00% - 0.00%)	0.00% (0.00% - 0.00%)	0.00% (0.00% - 0.00%)
Idaho	3.79% (3.24% - 4.37%)	3.52% (3.05% - 4.07%)	2.87% (2.49% - 3.28%)	2.70% (2.33% - 3.13%)	0.10% (0.02% - 0.22%)	0.14% (0.05% - 0.26%)	0.08% (0.02% - 0.17%)	0.06% (0.01% - 0.15%)	0.00% (0.00% - 0.00%)	0.00% (0.00% - 0.00%)	0.00% (0.00% - 0.00%)	0.00% (0.00% - 0.00%)
Illinois	3.64% (3.11% - 4.18%)	3.34% (2.93% - 3.80%)	2.70% (2.34% - 3.10%)	2.51% (2.15% - 2.93%)	0.09% (0.02% - 0.21%)	0.13% (0.04% - 0.23%)	0.07% (0.02% - 0.15%)	0.05% (0.01% - 0.14%)	0.00% (0.00% - 0.00%)	0.00% (0.00% - 0.00%)	0.00% (0.00% - 0.00%)	0.00% (0.00% - 0.00%)
Indiana	3.67% (3.15% - 4.26%)	3.46% (3.05% - 3.96%)	2.89% (2.53% - 3.34%)	2.71% (2.34% - 3.18%)	0.10% (0.02% - 0.22%)	0.13% (0.05% - 0.24%)	0.07% (0.02% - 0.16%)	0.06% (0.01% - 0.14%)	0.00% (0.00% - 0.00%)	0.00% (0.00% - 0.00%)	0.00% (0.00% - 0.00%)	0.00% (0.00% - 0.00%)
Iowa	3.44% (2.96% - 3.95%)	3.31% (2.90% - 3.77%)	2.73% (2.39% - 3.16%)	2.54% (2.20% - 2.95%)	0.10% (0.02% - 0.22%)	0.13% (0.05% - 0.24%)	0.07% (0.02% - 0.16%)	0.06% (0.01% - 0.14%)	0.00% (0.00% - 0.00%)	0.00% (0.00% - 0.00%)	0.00% (0.00% - 0.00%)	0.00% (0.00% - 0.00%)
Kansas	3.53% (3.07% - 4.11%)	3.36% (2.95% - 3.85%)	2.83% (2.46% - 3.23%)	2.62% (2.26% - 3.06%)	0.10% (0.02% - 0.23%)	0.13% (0.05% - 0.23%)	0.07% (0.02% - 0.17%)	0.06% (0.01% - 0.14%)	0.00% (0.00% - 0.00%)	0.00% (0.00% - 0.00%)	0.00% (0.00% - 0.00%)	0.00% (0.00% - 0.00%)

Table S8a. Estimated overall, severe, and extreme **stunting** prevalence (%) in children under 5 years, both sexes, in 1990, 2000, 2010, and 2020.

Location	Total				Severe				Extreme			
	1990	2000	2010	2020	1990	2000	2010	2020	1990	2000	2010	2020
Kentucky	3.76% (3.26% - 4.33%)	3.54% (3.08% - 4.03%)	2.97% (2.58% - 3.41%)	2.75% (2.36% - 3.18%)	0.10% (0.02% - 0.23%)	0.14% (0.05% - 0.25%)	0.07% (0.02% - 0.17%)	0.06% (0.01% - 0.14%)	0.00% (0.00% - 0.00%)	0.00% (0.00% - 0.00%)	0.00% (0.00% - 0.00%)	0.00% (0.00% - 0.00%)
Louisiana	3.98% (3.38% - 4.58%)	3.71% (3.22% - 4.23%)	2.95% (2.58% - 3.36%)	2.78% (2.40% - 3.24%)	0.10% (0.02% - 0.22%)	0.13% (0.04% - 0.24%)	0.07% (0.01% - 0.17%)	0.05% (0.01% - 0.14%)	0.00% (0.00% - 0.00%)	0.00% (0.00% - 0.00%)	0.00% (0.00% - 0.00%)	0.00% (0.00% - 0.00%)
Maine	3.43% (2.93% - 3.95%)	3.26% (2.81% - 3.69%)	2.71% (2.37% - 3.09%)	2.57% (2.20% - 2.95%)	0.10% (0.02% - 0.20%)	0.13% (0.05% - 0.24%)	0.07% (0.02% - 0.16%)	0.06% (0.01% - 0.14%)	0.00% (0.00% - 0.00%)	0.00% (0.00% - 0.00%)	0.00% (0.00% - 0.00%)	0.00% (0.00% - 0.00%)
Maryland	3.49% (3.02% - 3.99%)	3.28% (2.85% - 3.75%)	2.65% (2.28% - 3.03%)	2.52% (2.16% - 2.95%)	0.09% (0.02% - 0.20%)	0.12% (0.05% - 0.23%)	0.07% (0.02% - 0.15%)	0.05% (0.01% - 0.14%)	0.00% (0.00% - 0.00%)	0.00% (0.00% - 0.00%)	0.00% (0.00% - 0.00%)	0.00% (0.00% - 0.00%)
Massachusetts	3.20% (2.77% - 3.71%)	3.00% (2.61% - 3.46%)	2.48% (2.13% - 2.84%)	2.31% (1.98% - 2.66%)	0.09% (0.02% - 0.20%)	0.13% (0.05% - 0.23%)	0.07% (0.02% - 0.15%)	0.05% (0.01% - 0.12%)	0.00% (0.00% - 0.00%)	0.00% (0.00% - 0.00%)	0.00% (0.00% - 0.00%)	0.00% (0.00% - 0.00%)
Michigan	3.66% (3.16% - 4.19%)	3.39% (2.95% - 3.87%)	2.85% (2.48% - 3.29%)	2.63% (2.27% - 3.06%)	0.09% (0.02% - 0.21%)	0.13% (0.05% - 0.23%)	0.07% (0.02% - 0.16%)	0.05% (0.01% - 0.13%)	0.00% (0.00% - 0.00%)	0.00% (0.00% - 0.00%)	0.00% (0.00% - 0.00%)	0.00% (0.00% - 0.00%)
Minnesota	3.26% (2.80% - 3.76%)	3.11% (2.72% - 3.57%)	2.57% (2.23% - 2.97%)	2.40% (2.06% - 2.82%)	0.10% (0.03% - 0.21%)	0.13% (0.05% - 0.24%)	0.08% (0.02% - 0.16%)	0.06% (0.01% - 0.13%)	0.00% (0.00% - 0.00%)	0.00% (0.00% - 0.00%)	0.00% (0.00% - 0.00%)	0.00% (0.00% - 0.00%)
Mississippi	4.11% (3.57% - 4.79%)	3.89% (3.36% - 4.47%)	3.17% (2.75% - 3.66%)	2.97% (2.55% - 3.45%)	0.10% (0.02% - 0.22%)	0.13% (0.04% - 0.24%)	0.07% (0.01% - 0.16%)	0.05% (0.01% - 0.14%)	0.00% (0.00% - 0.00%)	0.00% (0.00% - 0.00%)	0.00% (0.00% - 0.00%)	0.00% (0.00% - 0.00%)
Missouri	3.67% (3.18% - 4.22%)	3.43% (2.99% - 3.92%)	2.86% (2.49% - 3.27%)	2.72% (2.33% - 3.16%)	0.10% (0.02% - 0.22%)	0.13% (0.05% - 0.23%)	0.07% (0.02% - 0.16%)	0.06% (0.01% - 0.14%)	0.00% (0.00% - 0.00%)	0.00% (0.00% - 0.00%)	0.00% (0.00% - 0.00%)	0.00% (0.00% - 0.00%)
Montana	3.67% (3.16% - 4.25%)	3.44% (3.00% - 3.92%)	2.87% (2.51% - 3.28%)	2.64% (2.27% - 3.04%)	0.10% (0.02% - 0.22%)	0.13% (0.05% - 0.24%)	0.07% (0.02% - 0.16%)	0.06% (0.01% - 0.14%)	0.00% (0.00% - 0.00%)	0.00% (0.00% - 0.00%)	0.00% (0.00% - 0.00%)	0.00% (0.00% - 0.00%)
Nebraska	3.46% (2.98% - 3.97%)	3.30% (2.87% - 3.79%)	2.71% (2.35% - 3.10%)	2.50% (2.16% - 2.89%)	0.10% (0.02% - 0.21%)	0.14% (0.06% - 0.26%)	0.08% (0.02% - 0.16%)	0.06% (0.01% - 0.14%)	0.00% (0.00% - 0.00%)	0.00% (0.00% - 0.00%)	0.00% (0.00% - 0.00%)	0.00% (0.00% - 0.00%)
Nevada	3.85% (3.30% - 4.45%)	3.59% (3.14% - 4.08%)	2.90% (2.52% - 3.33%)	2.74% (2.35% - 3.19%)	0.09% (0.02% - 0.21%)	0.13% (0.05% - 0.23%)	0.07% (0.02% - 0.16%)	0.06% (0.01% - 0.14%)	0.00% (0.00% - 0.00%)	0.00% (0.00% - 0.00%)	0.00% (0.00% - 0.00%)	0.00% (0.00% - 0.00%)
New Hampshire	3.29% (2.83% - 3.82%)	3.08% (2.70% - 3.51%)	2.53% (2.23% - 2.88%)	2.39% (2.06% - 2.76%)	0.09% (0.02% - 0.21%)	0.12% (0.05% - 0.23%)	0.07% (0.02% - 0.14%)	0.06% (0.01% - 0.13%)	0.00% (0.00% - 0.00%)	0.00% (0.00% - 0.00%)	0.00% (0.00% - 0.00%)	0.00% (0.00% - 0.00%)
New Jersey	3.35% (2.91% - 3.88%)	3.13% (2.72% - 3.60%)	2.56% (2.23% - 2.93%)	2.43% (2.09% - 2.82%)	0.09% (0.02% - 0.20%)	0.13% (0.04% - 0.22%)	0.07% (0.02% - 0.15%)	0.06% (0.01% - 0.13%)	0.00% (0.00% - 0.00%)	0.00% (0.00% - 0.00%)	0.00% (0.00% - 0.00%)	0.00% (0.00% - 0.00%)
New Mexico	4.00% (3.45% - 4.61%)	3.69% (3.16% - 4.21%)	3.00% (2.62% - 3.44%)	2.83% (2.44% - 3.31%)	0.10% (0.02% - 0.23%)	0.13% (0.05% - 0.25%)	0.07% (0.02% - 0.16%)	0.06% (0.01% - 0.14%)	0.00% (0.00% - 0.00%)	0.00% (0.00% - 0.00%)	0.00% (0.00% - 0.00%)	0.00% (0.00% - 0.00%)
New York	3.55% (3.08% - 4.09%)	3.18% (2.80% - 3.64%)	2.62% (2.29% - 2.99%)	2.45% (2.11% - 2.83%)	0.09% (0.02% - 0.19%)	0.12% (0.05% - 0.23%)	0.07% (0.02% - 0.15%)	0.05% (0.01% - 0.13%)	0.00% (0.00% - 0.00%)	0.00% (0.00% - 0.00%)	0.00% (0.00% - 0.00%)	0.00% (0.00% - 0.00%)
North Carolina	3.78% (3.25% - 4.40%)	3.50% (3.06% - 3.98%)	2.86% (2.48% - 3.25%)	2.69% (2.30% - 3.10%)	0.09% (0.02% - 0.21%)	0.13% (0.05% - 0.24%)	0.07% (0.02% - 0.16%)	0.06% (0.01% - 0.13%)	0.00% (0.00% - 0.00%)	0.00% (0.00% - 0.00%)	0.00% (0.00% - 0.00%)	0.00% (0.00% - 0.00%)
North Dakota	3.51% (3.00% - 4.05%)	3.29% (2.86% - 3.76%)	2.78% (2.43% - 3.19%)	2.50% (2.17% - 2.89%)	0.10% (0.02% - 0.22%)	0.13% (0.05% - 0.25%)	0.08% (0.02% - 0.16%)	0.05% (0.01% - 0.13%)	0.00% (0.00% - 0.00%)	0.00% (0.00% - 0.00%)	0.00% (0.00% - 0.00%)	0.00% (0.00% - 0.00%)
Ohio	3.63% (3.11% - 4.18%)	3.42% (2.98% - 3.91%)	2.84% (2.46% - 3.26%)	2.74% (2.34% - 3.18%)	0.10% (0.02% - 0.21%)	0.13% (0.05% - 0.24%)	0.07% (0.02% - 0.16%)	0.06% (0.01% - 0.14%)	0.00% (0.00% - 0.00%)	0.00% (0.00% - 0.00%)	0.00% (0.00% - 0.00%)	0.00% (0.00% - 0.00%)
Oklahoma	3.72% (3.19% - 4.34%)	3.66% (3.18% - 4.17%)	3.02% (2.62% - 3.44%)	2.79% (2.40% - 3.26%)	0.10% (0.02% - 0.22%)	0.13% (0.05% - 0.24%)	0.07% (0.01% - 0.15%)	0.05% (0.01% - 0.14%)	0.00% (0.00% - 0.00%)	0.00% (0.00% - 0.00%)	0.00% (0.00% - 0.00%)	0.00% (0.00% - 0.00%)
Oregon	3.53% (3.03% - 4.04%)	3.28% (2.88% - 3.76%)	2.73% (2.38% - 3.11%)	2.54% (2.19% - 2.95%)	0.10% (0.03% - 0.22%)	0.13% (0.05% - 0.24%)	0.08% (0.02% - 0.17%)	0.06% (0.01% - 0.14%)	0.00% (0.00% - 0.00%)	0.00% (0.00% - 0.00%)	0.00% (0.00% - 0.00%)	0.00% (0.00% - 0.00%)
Pennsylvania	3.54% (3.06% - 4.09%)	3.27% (2.87% - 3.73%)	2.71% (2.38% - 3.10%)	2.54% (2.20% - 2.96%)	0.10% (0.02% - 0.21%)	0.13% (0.05% - 0.22%)	0.07% (0.02% - 0.15%)	0.05% (0.01% - 0.14%)	0.00% (0.00% - 0.00%)	0.00% (0.00% - 0.00%)	0.00% (0.00% - 0.00%)	0.00% (0.00% - 0.00%)

Table S8a. Estimated overall, severe, and extreme **stunting** prevalence (%) in children under 5 years, both sexes, in 1990, 2000, 2010, and 2020.

Location	Total				Severe				Extreme			
	1990	2000	2010	2020	1990	2000	2010	2020	1990	2000	2010	2020
Rhode Island	3.38% (2.93% - 3.95%)	3.17% (2.74% - 3.63%)	2.61% (2.27% - 3.02%)	2.44% (2.10% - 2.82%)	0.09% (0.02% - 0.20%)	0.13% (0.05% - 0.24%)	0.07% (0.02% - 0.15%)	0.06% (0.01% - 0.13%)	0.00% (0.00% - 0.00%)	0.00% (0.00% - 0.00%)	0.00% (0.00% - 0.00%)	0.00% (0.00% - 0.00%)
South Carolina	3.97% (3.41% - 4.60%)	3.64% (3.15% - 4.17%)	2.98% (2.58% - 3.44%)	2.80% (2.40% - 3.27%)	0.09% (0.02% - 0.22%)	0.13% (0.05% - 0.24%)	0.07% (0.02% - 0.17%)	0.06% (0.01% - 0.14%)	0.00% (0.00% - 0.00%)	0.00% (0.00% - 0.00%)	0.00% (0.00% - 0.00%)	0.00% (0.00% - 0.00%)
South Dakota	3.66% (3.17% - 4.26%)	3.40% (2.96% - 3.87%)	2.83% (2.47% - 3.28%)	2.66% (2.30% - 3.09%)	0.10% (0.02% - 0.23%)	0.13% (0.05% - 0.24%)	0.07% (0.02% - 0.16%)	0.06% (0.01% - 0.13%)	0.00% (0.00% - 0.00%)	0.00% (0.00% - 0.00%)	0.00% (0.00% - 0.00%)	0.00% (0.00% - 0.00%)
Tennessee	3.86% (3.33% - 4.51%)	3.58% (3.10% - 4.07%)	3.09% (2.70% - 3.53%)	2.78% (2.40% - 3.22%)	0.10% (0.02% - 0.23%)	0.13% (0.05% - 0.25%)	0.07% (0.01% - 0.16%)	0.06% (0.01% - 0.14%)	0.00% (0.00% - 0.00%)	0.00% (0.00% - 0.00%)	0.00% (0.00% - 0.00%)	0.00% (0.00% - 0.00%)
Texas	3.82% (3.33% - 4.40%)	3.58% (3.13% - 4.08%)	2.88% (2.52% - 3.29%)	2.67% (2.32% - 3.06%)	0.10% (0.02% - 0.20%)	0.13% (0.06% - 0.26%)	0.07% (0.02% - 0.15%)	0.05% (0.01% - 0.14%)	0.00% (0.00% - 0.00%)	0.00% (0.00% - 0.00%)	0.00% (0.00% - 0.00%)	0.00% (0.00% - 0.00%)
Utah	3.77% (3.22% - 4.38%)	3.50% (3.04% - 3.97%)	2.84% (2.47% - 3.25%)	2.67% (2.30% - 3.13%)	0.10% (0.02% - 0.23%)	0.14% (0.06% - 0.25%)	0.08% (0.02% - 0.17%)	0.06% (0.01% - 0.15%)	0.00% (0.00% - 0.00%)	0.00% (0.00% - 0.00%)	0.00% (0.00% - 0.00%)	0.00% (0.00% - 0.00%)
Vermont	3.47% (2.96% - 4.01%)	3.21% (2.77% - 3.70%)	2.64% (2.30% - 3.05%)	2.47% (2.11% - 2.86%)	0.09% (0.02% - 0.21%)	0.13% (0.05% - 0.24%)	0.07% (0.02% - 0.17%)	0.06% (0.01% - 0.13%)	0.00% (0.00% - 0.00%)	0.00% (0.00% - 0.00%)	0.00% (0.00% - 0.00%)	0.00% (0.00% - 0.00%)
Virginia	3.55% (3.07% - 4.11%)	3.28% (2.86% - 3.77%)	2.65% (2.32% - 3.05%)	2.50% (2.13% - 2.89%)	0.09% (0.02% - 0.20%)	0.13% (0.05% - 0.24%)	0.07% (0.02% - 0.15%)	0.06% (0.01% - 0.13%)	0.00% (0.00% - 0.00%)	0.00% (0.00% - 0.00%)	0.00% (0.00% - 0.00%)	0.00% (0.00% - 0.00%)
Washington	3.48% (3.00% - 4.05%)	3.22% (2.82% - 3.70%)	2.66% (2.29% - 3.04%)	2.46% (2.10% - 2.89%)	0.10% (0.02% - 0.21%)	0.13% (0.05% - 0.24%)	0.08% (0.02% - 0.17%)	0.06% (0.01% - 0.15%)	0.00% (0.00% - 0.00%)	0.00% (0.00% - 0.00%)	0.00% (0.00% - 0.00%)	0.00% (0.00% - 0.00%)
West Virginia	3.86% (3.33% - 4.44%)	3.61% (3.15% - 4.14%)	3.08% (2.68% - 3.50%)	2.86% (2.45% - 3.36%)	0.10% (0.02% - 0.24%)	0.13% (0.05% - 0.24%)	0.07% (0.02% - 0.16%)	0.06% (0.01% - 0.14%)	0.00% (0.00% - 0.00%)	0.00% (0.00% - 0.00%)	0.00% (0.00% - 0.00%)	0.00% (0.00% - 0.00%)
Wisconsin	3.41% (2.97% - 3.94%)	3.24% (2.83% - 3.73%)	2.70% (2.33% - 3.12%)	2.55% (2.18% - 2.99%)	0.10% (0.02% - 0.22%)	0.13% (0.05% - 0.25%)	0.08% (0.02% - 0.17%)	0.06% (0.01% - 0.14%)	0.00% (0.00% - 0.00%)	0.00% (0.00% - 0.00%)	0.00% (0.00% - 0.00%)	0.00% (0.00% - 0.00%)
Wyoming	3.63% (3.14% - 4.25%)	3.45% (2.98% - 3.95%)	2.81% (2.43% - 3.21%)	2.60% (2.24% - 3.03%)	0.10% (0.02% - 0.22%)	0.13% (0.04% - 0.24%)	0.07% (0.02% - 0.16%)	0.05% (0.01% - 0.13%)	0.00% (0.00% - 0.00%)	0.00% (0.00% - 0.00%)	0.00% (0.00% - 0.00%)	0.00% (0.00% - 0.00%)
Greenland	5.04% (4.55% - 5.61%)	4.49% (4.03% - 5.01%)	3.53% (3.18% - 3.90%)	3.12% (2.80% - 3.46%)	0.03% (0.00% - 0.10%)	0.06% (0.01% - 0.14%)	0.02% (0.00% - 0.07%)	0.03% (0.00% - 0.08%)	0.00% (0.00% - 0.00%)	0.00% (0.00% - 0.00%)	0.00% (0.00% - 0.00%)	0.00% (0.00% - 0.00%)
**Southern Latin America**	10.36% (9.73% - 11.04%)	8.89% (8.43% - 9.43%)	7.21% (6.73% - 7.74%)	6.61% (6.07% - 7.13%)	1.87% (1.59% - 2.19%)	1.61% (1.41% - 1.83%)	1.15% (0.97% - 1.35%)	1.05% (0.86% - 1.26%)	0.10% (0.06% - 0.15%)	0.07% (0.05% - 0.10%)	0.03% (0.01% - 0.05%)	0.02% (0.01% - 0.04%)
Argentina	12.76% (11.88% - 13.74%)	10.71% (10.03% - 11.47%)	8.51% (7.86% - 9.25%)	7.82% (7.08% - 8.54%)	2.44% (2.02% - 2.93%)	2.02% (1.74% - 2.35%)	1.41% (1.16% - 1.69%)	1.28% (1.02% - 1.57%)	0.10% (0.04% - 0.18%)	0.06% (0.03% - 0.11%)	0.02% (0.00% - 0.04%)	0.01% (0.00% - 0.03%)
Chile	3.50% (3.11% - 3.94%)	2.76% (2.59% - 2.95%)	2.37% (2.22% - 2.56%)	1.97% (1.78% - 2.19%)	0.02% (0.00% - 0.07%)	0.01% (0.00% - 0.04%)	0.02% (0.01% - 0.05%)	0.02% (0.00% - 0.05%)	0.00% (0.00% - 0.00%)	0.00% (0.00% - 0.00%)	0.00% (0.00% - 0.00%)	0.00% (0.00% - 0.00%)
Uruguay	16.20% (15.21% - 17.15%)	14.07% (13.08% - 15.05%)	12.58% (11.54% - 13.65%)	11.37% (10.33% - 12.40%)	4.44% (3.82% - 5.09%)	3.66% (3.10% - 4.21%)	3.02% (2.50% - 3.64%)	2.69% (2.20% - 3.23%)	0.60% (0.41% - 0.82%)	0.44% (0.28% - 0.62%)	0.29% (0.15% - 0.45%)	0.23% (0.12% - 0.39%)
**Western Europe**	1.88% (1.82% - 1.95%)	1.41% (1.36% - 1.46%)	1.30% (1.26% - 1.34%)	1.22% (1.18% - 1.26%)	0.04% (0.03% - 0.04%)	0.02% (0.02% - 0.03%)	0.02% (0.02% - 0.03%)	0.02% (0.02% - 0.03%)	0.00% (0.00% - 0.00%)	0.00% (0.00% - 0.00%)	0.00% (0.00% - 0.00%)	0.00% (0.00% - 0.00%)
Monaco	1.38% (1.25% - 1.53%)	1.11% (1.00% - 1.23%)	1.10% (1.00% - 1.23%)	1.05% (0.94% - 1.16%)	0.02% (0.01% - 0.04%)	0.01% (0.00% - 0.02%)	0.01% (0.01% - 0.02%)	0.01% (0.01% - 0.02%)	0.00% (0.00% - 0.00%)	0.00% (0.00% - 0.00%)	0.00% (0.00% - 0.00%)	0.00% (0.00% - 0.00%)
San Marino	1.50% (1.35% - 1.66%)	1.23% (1.11% - 1.36%)	1.20% (1.07% - 1.33%)	1.23% (1.10% - 1.37%)	0.03% (0.01% - 0.04%)	0.02% (0.01% - 0.03%)	0.02% (0.01% - 0.03%)	0.02% (0.01% - 0.03%)	0.00% (0.00% - 0.00%)	0.00% (0.00% - 0.00%)	0.00% (0.00% - 0.00%)	0.00% (0.00% - 0.00%)
Andorra	1.55% (1.40% - 1.72%)	1.20% (1.08% - 1.34%)	1.12% (1.00% - 1.24%)	1.13% (1.01% - 1.26%)	0.02% (0.01% - 0.04%)	0.01% (0.01% - 0.02%)	0.01% (0.01% - 0.02%)	0.02% (0.01% - 0.03%)	0.00% (0.00% - 0.00%)	0.00% (0.00% - 0.00%)	0.00% (0.00% - 0.00%)	0.00% (0.00% - 0.00%)
Austria	1.97% (1.78% - 2.20%)	1.47% (1.31% - 1.64%)	1.35% (1.21% - 1.51%)	1.25% (1.12% - 1.40%)	0.04% (0.02% - 0.06%)	0.02% (0.01% - 0.03%)	0.02% (0.01% - 0.03%)	0.02% (0.01% - 0.03%)	0.00% (0.00% - 0.00%)	0.00% (0.00% - 0.00%)	0.00% (0.00% - 0.00%)	0.00% (0.00% - 0.00%)

Table S8a. Estimated overall, severe, and extreme **stunting** prevalence (%) in children under 5 years, both sexes, in 1990, 2000, 2010, and 2020.

Location	Total				Severe				Extreme			
	1990	2000	2010	2020	1990	2000	2010	2020	1990	2000	2010	2020
Belgium	1.87% (1.68% - 2.08%)	1.45% (1.30% - 1.62%)	1.29% (1.15% - 1.44%)	1.21% (1.09% - 1.35%)	0.03% (0.02% - 0.05%)	0.02% (0.01% - 0.03%)	0.02% (0.01% - 0.03%)	0.02% (0.01% - 0.03%)	0.00% (0.00% - 0.00%)	0.00% (0.00% - 0.00%)	0.00% (0.00% - 0.00%)	0.00% (0.00% - 0.00%)
Cyprus	2.41% (2.17% - 2.67%)	1.78% (1.60% - 1.98%)	1.59% (1.44% - 1.78%)	1.50% (1.36% - 1.68%)	0.05% (0.02% - 0.07%)	0.03% (0.01% - 0.04%)	0.03% (0.02% - 0.05%)	0.03% (0.02% - 0.05%)	0.00% (0.00% - 0.00%)	0.00% (0.00% - 0.00%)	0.00% (0.00% - 0.00%)	0.00% (0.00% - 0.00%)
Denmark	1.96% (1.76% - 2.18%)	1.48% (1.32% - 1.66%)	1.41% (1.27% - 1.57%)	1.32% (1.19% - 1.46%)	0.04% (0.02% - 0.06%)	0.02% (0.01% - 0.03%)	0.02% (0.01% - 0.04%)	0.02% (0.01% - 0.04%)	0.00% (0.00% - 0.00%)	0.00% (0.00% - 0.00%)	0.00% (0.00% - 0.00%)	0.00% (0.00% - 0.00%)
Finland	1.96% (1.76% - 2.16%)	1.53% (1.38% - 1.71%)	1.41% (1.28% - 1.57%)	1.36% (1.23% - 1.52%)	0.04% (0.02% - 0.06%)	0.02% (0.01% - 0.04%)	0.03% (0.01% - 0.04%)	0.03% (0.01% - 0.04%)	0.00% (0.00% - 0.00%)	0.00% (0.00% - 0.00%)	0.00% (0.00% - 0.00%)	0.00% (0.00% - 0.00%)
France	1.91% (1.72% - 2.12%)	1.44% (1.30% - 1.61%)	1.34% (1.22% - 1.48%)	1.25% (1.12% - 1.39%)	0.03% (0.02% - 0.05%)	0.02% (0.01% - 0.03%)	0.02% (0.01% - 0.03%)	0.02% (0.01% - 0.03%)	0.00% (0.00% - 0.00%)	0.00% (0.00% - 0.00%)	0.00% (0.00% - 0.00%)	0.00% (0.00% - 0.00%)
Germany	1.44% (1.30% - 1.59%)	1.17% (1.06% - 1.29%)	1.09% (0.99% - 1.19%)	1.04% (0.95% - 1.14%)	0.03% (0.01% - 0.04%)	0.02% (0.01% - 0.04%)	0.03% (0.01% - 0.04%)	0.02% (0.01% - 0.04%)	0.00% (0.00% - 0.00%)	0.00% (0.00% - 0.00%)	0.00% (0.00% - 0.00%)	0.00% (0.00% - 0.00%)
Greece	2.68% (2.39% - 2.97%)	2.28% (2.05% - 2.50%)	2.13% (1.92% - 2.35%)	2.14% (1.91% - 2.39%)	0.18% (0.11% - 0.26%)	0.15% (0.09% - 0.21%)	0.13% (0.08% - 0.19%)	0.13% (0.08% - 0.20%)	0.00% (0.00% - 0.00%)	0.00% (0.00% - 0.00%)	0.00% (0.00% - 0.00%)	0.00% (0.00% - 0.00%)
Iceland	1.65% (1.49% - 1.82%)	1.37% (1.22% - 1.52%)	1.33% (1.21% - 1.46%)	1.27% (1.15% - 1.41%)	0.03% (0.02% - 0.05%)	0.02% (0.01% - 0.03%)	0.02% (0.01% - 0.04%)	0.02% (0.01% - 0.04%)	0.00% (0.00% - 0.00%)	0.00% (0.00% - 0.00%)	0.00% (0.00% - 0.00%)	0.00% (0.00% - 0.00%)
Ireland	1.93% (1.74% - 2.16%)	1.48% (1.34% - 1.65%)	1.29% (1.17% - 1.43%)	1.16% (1.04% - 1.29%)	0.03% (0.01% - 0.05%)	0.02% (0.01% - 0.03%)	0.02% (0.01% - 0.03%)	0.02% (0.01% - 0.03%)	0.00% (0.00% - 0.00%)	0.00% (0.00% - 0.00%)	0.00% (0.00% - 0.00%)	0.00% (0.00% - 0.00%)
Israel	1.79% (1.61% - 2.00%)	1.38% (1.24% - 1.54%)	1.29% (1.16% - 1.42%)	1.21% (1.08% - 1.35%)	0.03% (0.01% - 0.05%)	0.01% (0.00% - 0.03%)	0.02% (0.01% - 0.03%)	0.02% (0.01% - 0.03%)	0.00% (0.00% - 0.00%)	0.00% (0.00% - 0.00%)	0.00% (0.00% - 0.00%)	0.00% (0.00% - 0.00%)
Italy	1.96% (1.77% - 2.17%)	1.40% (1.25% - 1.55%)	1.26% (1.13% - 1.39%)	1.21% (1.09% - 1.36%)	0.04% (0.02% - 0.06%)	0.02% (0.01% - 0.03%)	0.02% (0.01% - 0.03%)	0.02% (0.01% - 0.03%)	0.00% (0.00% - 0.00%)	0.00% (0.00% - 0.00%)	0.00% (0.00% - 0.00%)	0.00% (0.00% - 0.00%)
Piemonte	1.85% (1.59% - 2.14%)	1.35% (1.18% - 1.58%)	1.23% (1.06% - 1.42%)	1.18% (1.02% - 1.36%)	0.04% (0.01% - 0.06%)	0.02% (0.00% - 0.04%)	0.02% (0.01% - 0.04%)	0.02% (0.01% - 0.04%)	0.00% (0.00% - 0.00%)	0.00% (0.00% - 0.00%)	0.00% (0.00% - 0.00%)	0.00% (0.00% - 0.00%)
Valle d'Aosta	1.87% (1.60% - 2.18%)	1.37% (1.18% - 1.58%)	1.23% (1.05% - 1.42%)	1.18% (1.02% - 1.39%)	0.04% (0.01% - 0.07%)	0.02% (0.00% - 0.04%)	0.02% (0.00% - 0.04%)	0.02% (0.00% - 0.04%)	0.00% (0.00% - 0.00%)	0.00% (0.00% - 0.00%)	0.00% (0.00% - 0.00%)	0.00% (0.00% - 0.00%)
Liguria	1.83% (1.59% - 2.11%)	1.34% (1.15% - 1.57%)	1.22% (1.04% - 1.42%)	1.18% (1.02% - 1.39%)	0.04% (0.01% - 0.07%)	0.02% (0.00% - 0.04%)	0.02% (0.01% - 0.04%)	0.02% (0.01% - 0.04%)	0.00% (0.00% - 0.00%)	0.00% (0.00% - 0.00%)	0.00% (0.00% - 0.00%)	0.00% (0.00% - 0.00%)
Lombardia	1.75% (1.52% - 2.02%)	1.29% (1.12% - 1.47%)	1.18% (1.02% - 1.35%)	1.14% (0.99% - 1.31%)	0.03% (0.01% - 0.06%)	0.02% (0.01% - 0.03%)	0.02% (0.01% - 0.03%)	0.02% (0.01% - 0.03%)	0.00% (0.00% - 0.00%)	0.00% (0.00% - 0.00%)	0.00% (0.00% - 0.00%)	0.00% (0.00% - 0.00%)
Provincia autonoma di Bolzano	1.78% (1.53% - 2.07%)	1.29% (1.10% - 1.50%)	1.18% (1.03% - 1.37%)	1.14% (0.97% - 1.35%)	0.03% (0.01% - 0.06%)	0.02% (0.00% - 0.04%)	0.02% (0.00% - 0.04%)	0.02% (0.01% - 0.04%)	0.00% (0.00% - 0.00%)	0.00% (0.00% - 0.00%)	0.00% (0.00% - 0.00%)	0.00% (0.00% - 0.00%)
Provincia autonoma di Trento	1.75% (1.51% - 2.03%)	1.30% (1.11% - 1.52%)	1.19% (1.03% - 1.39%)	1.15% (0.99% - 1.33%)	0.03% (0.01% - 0.06%)	0.02% (0.00% - 0.04%)	0.02% (0.01% - 0.04%)	0.02% (0.01% - 0.03%)	0.00% (0.00% - 0.00%)	0.00% (0.00% - 0.00%)	0.00% (0.00% - 0.00%)	0.00% (0.00% - 0.00%)
Veneto	1.82% (1.56% - 2.12%)	1.33% (1.15% - 1.51%)	1.21% (1.05% - 1.39%)	1.17% (1.01% - 1.36%)	0.04% (0.01% - 0.06%)	0.02% (0.00% - 0.04%)	0.02% (0.01% - 0.04%)	0.02% (0.01% - 0.04%)	0.00% (0.00% - 0.00%)	0.00% (0.00% - 0.00%)	0.00% (0.00% - 0.00%)	0.00% (0.00% - 0.00%)
Friuli-Venezia Giulia	1.84% (1.58% - 2.14%)	1.34% (1.15% - 1.55%)	1.22% (1.04% - 1.41%)	1.18% (1.01% - 1.39%)	0.04% (0.01% - 0.07%)	0.02% (0.01% - 0.04%)	0.02% (0.01% - 0.04%)	0.02% (0.00% - 0.04%)	0.00% (0.00% - 0.00%)	0.00% (0.00% - 0.00%)	0.00% (0.00% - 0.00%)	0.00% (0.00% - 0.00%)
Emilia-Romagna	1.86% (1.61% - 2.13%)	1.31% (1.13% - 1.51%)	1.20% (1.04% - 1.38%)	1.15% (1.00% - 1.33%)	0.04% (0.01% - 0.07%)	0.02% (0.01% - 0.04%)	0.02% (0.01% - 0.04%)	0.02% (0.01% - 0.03%)	0.00% (0.00% - 0.00%)	0.00% (0.00% - 0.00%)	0.00% (0.00% - 0.00%)	0.00% (0.00% - 0.00%)
Toscana	1.82% (1.56% - 2.10%)	1.34% (1.15% - 1.54%)	1.21% (1.05% - 1.40%)	1.17% (1.01% - 1.35%)	0.04% (0.01% - 0.06%)	0.02% (0.01% - 0.04%)	0.02% (0.01% - 0.04%)	0.02% (0.01% - 0.03%)	0.00% (0.00% - 0.00%)	0.00% (0.00% - 0.00%)	0.00% (0.00% - 0.00%)	0.00% (0.00% - 0.00%)
Umbria	1.85% (1.58% - 2.14%)	1.36% (1.18% - 1.59%)	1.24% (1.07% - 1.44%)	1.21% (1.03% - 1.42%)	0.04% (0.01% - 0.07%)	0.02% (0.01% - 0.04%)	0.02% (0.01% - 0.04%)	0.02% (0.01% - 0.04%)	0.00% (0.00% - 0.00%)	0.00% (0.00% - 0.00%)	0.00% (0.00% - 0.00%)	0.00% (0.00% - 0.00%)

Table S8a. Estimated overall, severe, and extreme **stunting** prevalence (%) in children under 5 years, both sexes, in 1990, 2000, 2010, and 2020.

Location	Total				Severe				Extreme			
	1990	2000	2010	2020	1990	2000	2010	2020	1990	2000	2010	2020
Marche	1.88% (1.62% - 2.16%)	1.36% (1.17% - 1.58%)	1.24% (1.07% - 1.44%)	1.20% (1.03% - 1.40%)	0.04% (0.02% - 0.07%)	0.02% (0.01% - 0.04%)	0.02% (0.01% - 0.04%)	0.02% (0.01% - 0.04%)	0.00% (0.00% - 0.00%)	0.00% (0.00% - 0.00%)	0.00% (0.00% - 0.00%)	0.00% (0.00% - 0.00%)
Lazio	1.80% (1.56% - 2.07%)	1.32% (1.13% - 1.53%)	1.20% (1.04% - 1.39%)	1.16% (1.01% - 1.35%)	0.03% (0.01% - 0.06%)	0.02% (0.00% - 0.04%)	0.02% (0.00% - 0.04%)	0.02% (0.01% - 0.03%)	0.00% (0.00% - 0.00%)	0.00% (0.00% - 0.00%)	0.00% (0.00% - 0.00%)	0.00% (0.00% - 0.00%)
Abruzzo	1.89% (1.61% - 2.21%)	1.37% (1.18% - 1.60%)	1.27% (1.08% - 1.47%)	1.22% (1.05% - 1.41%)	0.04% (0.01% - 0.07%)	0.02% (0.01% - 0.04%)	0.02% (0.01% - 0.04%)	0.02% (0.01% - 0.04%)	0.00% (0.00% - 0.00%)	0.00% (0.00% - 0.00%)	0.00% (0.00% - 0.00%)	0.00% (0.00% - 0.00%)
Molise	2.01% (1.72% - 2.33%)	1.44% (1.24% - 1.68%)	1.31% (1.12% - 1.51%)	1.27% (1.08% - 1.50%)	0.04% (0.01% - 0.07%)	0.02% (0.00% - 0.04%)	0.02% (0.01% - 0.04%)	0.02% (0.01% - 0.04%)	0.00% (0.00% - 0.00%)	0.00% (0.00% - 0.00%)	0.00% (0.00% - 0.00%)	0.00% (0.00% - 0.00%)
Campania	2.16% (1.87% - 2.48%)	1.53% (1.33% - 1.77%)	1.36% (1.17% - 1.57%)	1.32% (1.14% - 1.53%)	0.04% (0.02% - 0.07%)	0.02% (0.01% - 0.04%)	0.02% (0.01% - 0.04%)	0.02% (0.01% - 0.04%)	0.00% (0.00% - 0.00%)	0.00% (0.00% - 0.00%)	0.00% (0.00% - 0.00%)	0.00% (0.00% - 0.00%)
Puglia	2.13% (1.84% - 2.43%)	1.50% (1.30% - 1.75%)	1.35% (1.17% - 1.55%)	1.30% (1.13% - 1.54%)	0.04% (0.02% - 0.07%)	0.02% (0.01% - 0.04%)	0.02% (0.01% - 0.04%)	0.02% (0.01% - 0.04%)	0.00% (0.00% - 0.00%)	0.00% (0.00% - 0.00%)	0.00% (0.00% - 0.00%)	0.00% (0.00% - 0.00%)
Basilicata	2.07% (1.77% - 2.42%)	1.46% (1.25% - 1.70%)	1.33% (1.15% - 1.54%)	1.28% (1.11% - 1.51%)	0.04% (0.01% - 0.07%)	0.02% (0.01% - 0.04%)	0.02% (0.01% - 0.04%)	0.02% (0.01% - 0.04%)	0.00% (0.00% - 0.00%)	0.00% (0.00% - 0.00%)	0.00% (0.00% - 0.00%)	0.00% (0.00% - 0.00%)
Calabria	2.12% (1.83% - 2.49%)	1.51% (1.29% - 1.76%)	1.36% (1.17% - 1.58%)	1.32% (1.14% - 1.55%)	0.04% (0.01% - 0.07%)	0.02% (0.01% - 0.04%)	0.02% (0.01% - 0.04%)	0.02% (0.01% - 0.04%)	0.00% (0.00% - 0.00%)	0.00% (0.00% - 0.00%)	0.00% (0.00% - 0.00%)	0.00% (0.00% - 0.00%)
Sicilia	2.19% (1.90% - 2.51%)	1.53% (1.32% - 1.77%)	1.36% (1.17% - 1.58%)	1.33% (1.15% - 1.53%)	0.04% (0.01% - 0.07%)	0.02% (0.01% - 0.04%)	0.02% (0.01% - 0.04%)	0.02% (0.01% - 0.04%)	0.00% (0.00% - 0.00%)	0.00% (0.00% - 0.00%)	0.00% (0.00% - 0.00%)	0.00% (0.00% - 0.00%)
Sardegna	2.02% (1.73% - 2.34%)	1.46% (1.24% - 1.70%)	1.32% (1.14% - 1.52%)	1.27% (1.08% - 1.48%)	0.04% (0.01% - 0.07%)	0.02% (0.01% - 0.04%)	0.02% (0.01% - 0.04%)	0.02% (0.01% - 0.04%)	0.00% (0.00% - 0.00%)	0.00% (0.00% - 0.00%)	0.00% (0.00% - 0.00%)	0.00% (0.00% - 0.00%)
Luxembourg	2.03% (1.82% - 2.25%)	1.55% (1.40% - 1.74%)	1.44% (1.30% - 1.59%)	1.35% (1.22% - 1.50%)	0.04% (0.02% - 0.06%)	0.02% (0.01% - 0.04%)	0.03% (0.02% - 0.04%)	0.03% (0.02% - 0.04%)	0.00% (0.00% - 0.00%)	0.00% (0.00% - 0.00%)	0.00% (0.00% - 0.00%)	0.00% (0.00% - 0.00%)
Malta	2.21% (2.00% - 2.46%)	1.65% (1.49% - 1.84%)	1.55% (1.40% - 1.73%)	1.37% (1.23% - 1.52%)	0.04% (0.02% - 0.06%)	0.02% (0.01% - 0.04%)	0.03% (0.01% - 0.04%)	0.02% (0.01% - 0.04%)	0.00% (0.00% - 0.00%)	0.00% (0.00% - 0.00%)	0.00% (0.00% - 0.00%)	0.00% (0.00% - 0.00%)
Netherlands	1.50% (1.36% - 1.66%)	1.42% (1.28% - 1.56%)	1.31% (1.19% - 1.45%)	1.28% (1.16% - 1.42%)	0.05% (0.03% - 0.08%)	0.05% (0.03% - 0.08%)	0.05% (0.03% - 0.07%)	0.05% (0.03% - 0.07%)	0.00% (0.00% - 0.00%)	0.00% (0.00% - 0.00%)	0.00% (0.00% - 0.00%)	0.00% (0.00% - 0.00%)
Norway	1.80% (1.62% - 1.99%)	1.37% (1.24% - 1.51%)	1.30% (1.17% - 1.44%)	1.22% (1.09% - 1.36%)	0.04% (0.02% - 0.06%)	0.02% (0.01% - 0.03%)	0.02% (0.01% - 0.04%)	0.02% (0.01% - 0.03%)	0.00% (0.00% - 0.00%)	0.00% (0.00% - 0.00%)	0.00% (0.00% - 0.00%)	0.00% (0.00% - 0.00%)
Portugal	2.40% (2.16% - 2.66%)	1.75% (1.57% - 1.95%)	1.53% (1.38% - 1.70%)	1.44% (1.30% - 1.59%)	0.04% (0.02% - 0.06%)	0.02% (0.01% - 0.04%)	0.03% (0.01% - 0.04%)	0.03% (0.01% - 0.04%)	0.00% (0.00% - 0.00%)	0.00% (0.00% - 0.00%)	0.00% (0.00% - 0.00%)	0.00% (0.00% - 0.00%)
Spain	2.44% (2.16% - 2.76%)	1.31% (1.17% - 1.47%)	1.15% (1.02% - 1.29%)	1.12% (0.98% - 1.26%)	0.03% (0.00% - 0.07%)	0.00% (0.00% - 0.00%)	0.00% (0.00% - 0.00%)	0.00% (0.00% - 0.00%)	0.00% (0.00% - 0.00%)	0.00% (0.00% - 0.00%)	0.00% (0.00% - 0.00%)	0.00% (0.00% - 0.00%)
Sweden	1.76% (1.59% - 1.97%)	1.37% (1.24% - 1.53%)	1.28% (1.16% - 1.42%)	1.22% (1.11% - 1.36%)	0.04% (0.02% - 0.06%)	0.02% (0.01% - 0.03%)	0.02% (0.01% - 0.04%)	0.02% (0.01% - 0.03%)	0.00% (0.00% - 0.00%)	0.00% (0.00% - 0.00%)	0.00% (0.00% - 0.00%)	0.00% (0.00% - 0.00%)
Sweden except Stockholm	1.75% (1.58% - 1.98%)	1.36% (1.22% - 1.53%)	1.28% (1.14% - 1.43%)	1.22% (1.10% - 1.36%)	0.04% (0.02% - 0.06%)	0.02% (0.01% - 0.03%)	0.02% (0.01% - 0.04%)	0.02% (0.01% - 0.03%)	0.00% (0.00% - 0.00%)	0.00% (0.00% - 0.00%)	0.00% (0.00% - 0.00%)	0.00% (0.00% - 0.00%)
Stockholm	1.79% (1.54% - 2.08%)	1.40% (1.19% - 1.64%)	1.30% (1.11% - 1.51%)	1.24% (1.07% - 1.45%)	0.04% (0.02% - 0.08%)	0.02% (0.01% - 0.05%)	0.03% (0.01% - 0.05%)	0.02% (0.01% - 0.04%)	0.00% (0.00% - 0.00%)	0.00% (0.00% - 0.00%)	0.00% (0.00% - 0.00%)	0.00% (0.00% - 0.00%)
Switzerland	1.61% (1.46% - 1.79%)	1.31% (1.17% - 1.46%)	1.23% (1.11% - 1.37%)	1.18% (1.06% - 1.32%)	0.03% (0.02% - 0.05%)	0.02% (0.01% - 0.03%)	0.02% (0.01% - 0.03%)	0.02% (0.01% - 0.03%)	0.00% (0.00% - 0.00%)	0.00% (0.00% - 0.00%)	0.00% (0.00% - 0.00%)	0.00% (0.00% - 0.00%)
United Kingdom	1.96% (1.76% - 2.17%)	1.48% (1.33% - 1.65%)	1.39% (1.26% - 1.54%)	1.31% (1.19% - 1.46%)	0.04% (0.02% - 0.06%)	0.02% (0.01% - 0.04%)	0.03% (0.01% - 0.04%)	0.02% (0.01% - 0.04%)	0.00% (0.00% - 0.00%)	0.00% (0.00% - 0.00%)	0.00% (0.00% - 0.00%)	0.00% (0.00% - 0.00%)
Northern Ireland	2.05% (1.72% - 2.43%)	1.51% (1.27% - 1.79%)	1.45% (1.23% - 1.74%)	1.35% (1.14% - 1.60%)	0.04% (0.01% - 0.09%)	0.02% (0.01% - 0.05%)	0.03% (0.01% - 0.06%)	0.03% (0.01% - 0.05%)	0.00% (0.00% - 0.00%)	0.00% (0.00% - 0.00%)	0.00% (0.00% - 0.00%)	0.00% (0.00% - 0.00%)

Table S8a. Estimated overall, severe, and extreme **stunting** prevalence (%) in children under 5 years, both sexes, in 1990, 2000, 2010, and 2020.

Location	Total				Severe				Extreme			
	1990	2000	2010	2020	1990	2000	2010	2020	1990	2000	2010	2020
	2.03%	1.51%	1.43%	1.34%	0.04%	0.02%	0.03%	0.02%	0.00%	0.00%	0.00%	0.00%
Scotland	(1.73% - 2.39%)	(1.28% - 1.81%)	(1.20% - 1.69%)	(1.14% - 1.61%)	(0.02% - 0.09%)	(0.00% - 0.05%)	(0.01% - 0.05%)	(0.01% - 0.05%)	(0.00% - 0.00%)	(0.00% - 0.00%)	(0.00% - 0.00%)	(0.00% - 0.00%)
	2.08%	1.57%	1.48%	1.38%	0.05%	0.02%	0.03%	0.03%	0.00%	0.00%	0.00%	0.00%
Wales	(1.76% - 2.47%)	(1.32% - 1.88%)	(1.25% - 1.76%)	(1.15% - 1.65%)	(0.02% - 0.09%)	(0.01% - 0.06%)	(0.01% - 0.06%)	(0.01% - 0.05%)	(0.00% - 0.00%)	(0.00% - 0.00%)	(0.00% - 0.00%)	(0.00% - 0.00%)
	1.94%	1.48%	1.38%	1.30%	0.04%	0.02%	0.03%	0.02%	0.00%	0.00%	0.00%	0.00%
England	(1.75% - 2.15%)	(1.32% - 1.64%)	(1.24% - 1.53%)	(1.18% - 1.45%)	(0.02% - 0.06%)	(0.01% - 0.04%)	(0.01% - 0.04%)	(0.01% - 0.04%)	(0.00% - 0.00%)	(0.00% - 0.00%)	(0.00% - 0.00%)	(0.00% - 0.00%)
	2.08%	1.56%	1.43%	1.36%	0.04%	0.02%	0.03%	0.03%	0.00%	0.00%	0.00%	0.00%
North East England	(1.82% - 2.42%)	(1.34% - 1.81%)	(1.22% - 1.66%)	(1.17% - 1.58%)	(0.02% - 0.08%)	(0.01% - 0.05%)	(0.01% - 0.05%)	(0.01% - 0.05%)	(0.00% - 0.00%)	(0.00% - 0.00%)	(0.00% - 0.00%)	(0.00% - 0.00%)
	1.98%	1.51%	1.41%	1.34%	0.04%	0.02%	0.03%	0.02%	0.00%	0.00%	0.00%	0.00%
North West England	(1.73% - 2.27%)	(1.31% - 1.74%)	(1.21% - 1.61%)	(1.16% - 1.55%)	(0.02% - 0.07%)	(0.01% - 0.04%)	(0.01% - 0.04%)	(0.01% - 0.04%)	(0.00% - 0.00%)	(0.00% - 0.00%)	(0.00% - 0.00%)	(0.00% - 0.00%)
	1.99%	1.51%	1.42%	1.34%	0.04%	0.02%	0.03%	0.02%	0.00%	0.00%	0.00%	0.00%
Yorkshire and the Humber	(1.71% - 2.28%)	(1.30% - 1.77%)	(1.24% - 1.64%)	(1.16% - 1.57%)	(0.02% - 0.08%)	(0.01% - 0.04%)	(0.01% - 0.05%)	(0.01% - 0.04%)	(0.00% - 0.00%)	(0.00% - 0.00%)	(0.00% - 0.00%)	(0.00% - 0.00%)
	1.95%	1.48%	1.40%	1.33%	0.04%	0.02%	0.03%	0.02%	0.00%	0.00%	0.00%	0.00%
East Midlands	(1.70% - 2.25%)	(1.27% - 1.71%)	(1.22% - 1.61%)	(1.15% - 1.54%)	(0.02% - 0.07%)	(0.01% - 0.04%)	(0.01% - 0.05%)	(0.01% - 0.04%)	(0.00% - 0.00%)	(0.00% - 0.00%)	(0.00% - 0.00%)	(0.00% - 0.00%)
	2.06%	1.56%	1.43%	1.37%	0.04%	0.02%	0.03%	0.02%	0.00%	0.00%	0.00%	0.00%
West Midlands	(1.78% - 2.36%)	(1.33% - 1.83%)	(1.25% - 1.64%)	(1.19% - 1.57%)	(0.02% - 0.08%)	(0.01% - 0.04%)	(0.01% - 0.05%)	(0.01% - 0.05%)	(0.00% - 0.00%)	(0.00% - 0.00%)	(0.00% - 0.00%)	(0.00% - 0.00%)
	1.86%	1.42%	1.34%	1.28%	0.04%	0.02%	0.03%	0.02%	0.00%	0.00%	0.00%	0.00%
East of England	(1.61% - 2.14%)	(1.21% - 1.64%)	(1.17% - 1.54%)	(1.11% - 1.46%)	(0.02% - 0.07%)	(0.01% - 0.04%)	(0.01% - 0.05%)	(0.01% - 0.04%)	(0.00% - 0.00%)	(0.00% - 0.00%)	(0.00% - 0.00%)	(0.00% - 0.00%)
	1.91%	1.46%	1.33%	1.23%	0.04%	0.02%	0.02%	0.02%	0.00%	0.00%	0.00%	0.00%
Greater London	(1.67% - 2.18%)	(1.26% - 1.69%)	(1.16% - 1.52%)	(1.08% - 1.42%)	(0.02% - 0.07%)	(0.01% - 0.04%)	(0.01% - 0.04%)	(0.01% - 0.04%)	(0.00% - 0.00%)	(0.00% - 0.00%)	(0.00% - 0.00%)	(0.00% - 0.00%)
	1.82%	1.40%	1.32%	1.27%	0.04%	0.02%	0.02%	0.02%	0.00%	0.00%	0.00%	0.00%
South East England	(1.59% - 2.12%)	(1.20% - 1.61%)	(1.14% - 1.52%)	(1.10% - 1.47%)	(0.02% - 0.07%)	(0.01% - 0.04%)	(0.01% - 0.04%)	(0.01% - 0.04%)	(0.00% - 0.00%)	(0.00% - 0.00%)	(0.00% - 0.00%)	(0.00% - 0.00%)
	1.90%	1.46%	1.38%	1.32%	0.04%	0.02%	0.03%	0.03%	0.00%	0.00%	0.00%	0.00%
South West England	(1.64% - 2.19%)	(1.23% - 1.72%)	(1.20% - 1.60%)	(1.13% - 1.53%)	(0.02% - 0.07%)	(0.01% - 0.05%)	(0.01% - 0.05%)	(0.01% - 0.04%)	(0.00% - 0.00%)	(0.00% - 0.00%)	(0.00% - 0.00%)	(0.00% - 0.00%)
Latin America and Caribbean	22.82%	19.14%	15.51%	13.90%	7.17%	5.71%	4.04%	3.53%	1.40%	1.05%	0.59%	0.50%
	(22.61% - 23.03%)	(18.98% - 19.31%)	(15.36% - 15.68%)	(13.70% - 14.09%)	(7.00% - 7.34%)	(5.57% - 5.85%)	(3.93% - 4.15%)	(3.40% - 3.67%)	(1.30% - 1.52%)	(0.96% - 1.15%)	(0.54% - 0.66%)	(0.43% - 0.57%)
	36.37%	30.26%	23.50%	18.39%	12.75%	9.60%	5.83%	4.13%	2.50%	1.63%	0.66%	0.39%
**Andean Latin America**	(35.38% - 37.51%)	(29.60% - 30.99%)	(22.92% - 24.05%)	(17.67% - 19.14%)	(11.94% - 13.58%)	(8.92% - 10.25%)	(5.31% - 6.37%)	(3.61% - 4.65%)	(1.99% - 3.10%)	(1.31% - 1.96%)	(0.49% - 0.85%)	(0.25% - 0.56%)
	39.09%	33.97%	25.64%	19.79%	15.84%	11.28%	6.14%	4.13%	3.89%	2.08%	0.65%	0.31%
Bolivia (Plurinational State of)	(37.36% - 41.02%)	(32.71% - 35.55%)	(24.40% - 26.83%)	(18.44% - 21.15%)	(14.53% - 17.22%)	(10.15% - 12.42%)	(4.98% - 7.18%)	(3.00% - 5.18%)	(2.95% - 5.09%)	(1.54% - 2.68%)	(0.37% - 1.00%)	(0.11% - 0.58%)
	34.41%	28.05%	24.65%	22.26%	10.41%	8.67%	7.08%	6.21%	1.55%	1.39%	1.06%	0.90%
Ecuador	(33.07% - 36.06%)	(26.99% - 29.17%)	(23.48% - 25.83%)	(20.87% - 23.64%)	(9.04% - 11.83%)	(7.46% - 9.83%)	(5.88% - 8.25%)	(5.01% - 7.44%)	(1.07% - 2.19%)	(0.95% - 1.92%)	(0.64% - 1.57%)	(0.52% - 1.43%)
	36.29%	30.00%	21.90%	15.63%	12.70%	9.44%	4.95%	2.94%	2.43%	1.58%	0.42%	0.13%
Peru	(34.85% - 38.17%)	(28.97% - 31.09%)	(21.17% - 22.66%)	(14.60% - 16.72%)	(11.52% - 13.97%)	(8.40% - 10.42%)	(4.33% - 5.58%)	(2.38% - 3.58%)	(1.66% - 3.42%)	(1.08% - 2.13%)	(0.24% - 0.63%)	(0.03% - 0.28%)
	18.52%	15.97%	14.90%	14.07%	6.57%	4.89%	4.10%	3.75%	1.72%	0.97%	0.62%	0.53%
**Caribbean**	(18.17% - 18.91%)	(15.60% - 16.32%)	(14.47% - 15.32%)	(13.48% - 14.62%)	(6.18% - 6.99%)	(4.51% - 5.23%)	(3.72% - 4.47%)	(3.26% - 4.22%)	(1.43% - 2.08%)	(0.75% - 1.19%)	(0.46% - 0.82%)	(0.33% - 0.74%)
	9.89%	8.59%	7.88%	7.50%	1.95%	1.63%	1.50%	1.42%	0.12%	0.07%	0.05%	0.04%
Antigua and Barbuda	(8.98% - 10.89%)	(7.78% - 9.43%)	(7.12% - 8.67%)	(6.79% - 8.21%)	(1.56% - 2.40%)	(1.30% - 2.01%)	(1.19% - 1.82%)	(1.15% - 1.75%)	(0.06% - 0.22%)	(0.02% - 0.14%)	(0.01% - 0.10%)	(0.01% - 0.09%)
	10.90%	9.02%	8.65%	8.36%	2.41%	1.83%	1.75%	1.67%	0.17%	0.09%	0.07%	0.06%
Bahamas	(9.96% - 11.97%)	(8.12% - 9.92%)	(7.83% - 9.56%)	(7.56% - 9.16%)	(1.95% - 2.95%)	(1.45% - 2.26%)	(1.39% - 2.14%)	(1.33% - 2.04%)	(0.08% - 0.31%)	(0.03% - 0.19%)	(0.02% - 0.17%)	(0.02% - 0.14%)
	9.35%	9.62%	10.64%	10.18%	1.74%	1.98%	2.58%	2.50%	0.08%	0.11%	0.25%	0.25%
Barbados	(8.50% - 10.24%)	(8.81% - 10.53%)	(9.70% - 11.64%)	(9.31% - 11.09%)	(1.35% - 2.14%)	(1.62% - 2.37%)	(2.09% - 3.10%)	(2.04% - 3.00%)	(0.02% - 0.17%)	(0.04% - 0.20%)	(0.12% - 0.41%)	(0.12% - 0.42%)
	25.22%	22.67%	18.71%	15.99%	7.14%	6.62%	5.23%	4.23%	1.08%	1.06%	0.75%	0.51%
Belize	(23.69% - 26.70%)	(21.47% - 23.90%)	(18.01% - 19.43%)	(15.15% - 16.79%)	(5.61% - 8.51%)	(5.41% - 7.73%)	(4.64% - 5.80%)	(3.70% - 4.78%)	(0.61% - 1.63%)	(0.67% - 1.52%)	(0.55% - 0.97%)	(0.36% - 0.70%)

Table S8a. Estimated overall, severe, and extreme **stunting** prevalence (%) in children under 5 years, both sexes, in 1990, 2000, 2010, and 2020.

Location	Total				Severe				Extreme			
	1990	2000	2010	2020	1990	2000	2010	2020	1990	2000	2010	2020
	5.14%	6.03%	5.86%	5.89%	0.39%	0.61%	0.71%	0.83%	0.00%	0.00%	0.00%	0.01%
Cuba	(4.85% - 5.45%)	(5.70% - 6.36%)	(5.36% - 6.37%)	(5.39% - 6.46%)	(0.32% - 0.49%)	(0.52% - 0.71%)	(0.55% - 0.87%)	(0.65% - 1.04%)	(0.00% - 0.00%)	(0.00% - 0.00%)	(0.00% - 0.01%)	(0.00% - 0.02%)
	11.71%	9.04%	8.57%	8.41%	2.52%	1.75%	1.62%	1.58%	0.17%	0.08%	0.05%	0.05%
Dominica	(10.64% - 12.75%)	(8.21% - 9.91%)	(7.76% - 9.40%)	(7.67% - 9.28%)	(2.00% - 3.02%)	(1.40% - 2.15%)	(1.29% - 1.99%)	(1.25% - 1.94%)	(0.08% - 0.30%)	(0.02% - 0.16%)	(0.01% - 0.12%)	(0.01% - 0.11%)
	19.45%	12.75%	10.07%	8.49%	5.37%	2.67%	1.86%	1.41%	0.75%	0.16%	0.06%	0.03%
Dominican Republic	(18.61% - 20.34%)	(12.12% - 13.40%)	(9.44% - 10.70%)	(7.78% - 9.27%)	(4.70% - 6.09%)	(2.31% - 3.04%)	(1.59% - 2.16%)	(1.13% - 1.73%)	(0.51% - 1.05%)	(0.08% - 0.26%)	(0.02% - 0.12%)	(0.00% - 0.07%)
	14.44%	10.69%	10.08%	9.19%	3.23%	2.22%	2.05%	1.83%	0.27%	0.13%	0.10%	0.08%
Grenada	(13.24% - 15.69%)	(9.78% - 11.70%)	(9.17% - 10.98%)	(8.35% - 10.09%)	(2.55% - 3.94%)	(1.78% - 2.76%)	(1.63% - 2.47%)	(1.46% - 2.25%)	(0.14% - 0.46%)	(0.06% - 0.26%)	(0.04% - 0.19%)	(0.02% - 0.16%)
	19.72%	15.99%	16.05%	12.43%	5.19%	4.01%	4.16%	2.89%	0.65%	0.44%	0.50%	0.24%
Guyana	(18.56% - 20.91%)	(14.94% - 16.99%)	(15.17% - 16.95%)	(11.45% - 13.37%)	(4.32% - 6.03%)	(3.39% - 4.67%)	(3.62% - 4.77%)	(2.39% - 3.44%)	(0.40% - 0.93%)	(0.27% - 0.64%)	(0.33% - 0.69%)	(0.12% - 0.40%)
	35.55%	30.43%	25.70%	22.96%	16.81%	12.03%	8.46%	7.16%	5.55%	3.04%	1.59%	1.23%
Haiti	(34.56% - 36.63%)	(29.42% - 31.45%)	(24.69% - 26.73%)	(21.73% - 24.23%)	(15.48% - 18.30%)	(10.84% - 13.16%)	(7.46% - 9.45%)	(6.03% - 8.29%)	(4.44% - 6.88%)	(2.29% - 3.79%)	(1.14% - 2.11%)	(0.75% - 1.72%)
	12.99%	9.76%	8.32%	7.47%	2.98%	2.00%	1.55%	1.32%	0.26%	0.11%	0.05%	0.04%
Jamaica	(12.16% - 13.82%)	(9.11% - 10.44%)	(7.66% - 9.00%)	(6.78% - 8.16%)	(2.57% - 3.41%)	(1.72% - 2.31%)	(1.29% - 1.82%)	(1.07% - 1.59%)	(0.16% - 0.37%)	(0.07% - 0.18%)	(0.02% - 0.09%)	(0.01% - 0.07%)
	6.69%	4.90%	3.49%	3.22%	0.78%	0.43%	0.20%	0.16%	0.02%	0.00%	0.00%	0.00%
Saint Lucia	(6.04% - 7.41%)	(4.42% - 5.43%)	(3.13% - 3.93%)	(2.87% - 3.65%)	(0.60% - 1.00%)	(0.32% - 0.56%)	(0.14% - 0.27%)	(0.11% - 0.23%)	(0.00% - 0.06%)	(0.00% - 0.01%)	(0.00% - 0.00%)	(0.00% - 0.00%)
	13.46%	10.81%	9.53%	8.83%	2.98%	2.15%	1.83%	1.67%	0.24%	0.12%	0.07%	0.05%
Saint Vincent and the Grenadines	(12.35% - 14.73%)	(9.78% - 11.83%)	(8.63% - 10.52%)	(8.07% - 9.72%)	(2.39% - 3.64%)	(1.71% - 2.65%)	(1.46% - 2.28%)	(1.33% - 2.07%)	(0.11% - 0.41%)	(0.04% - 0.23%)	(0.02% - 0.15%)	(0.01% - 0.13%)
	13.66%	13.08%	9.72%	8.56%	2.27%	2.38%	1.52%	1.24%	0.10%	0.11%	0.03%	0.01%
Suriname	(12.57% - 14.80%)	(12.22% - 13.94%)	(9.00% - 10.45%)	(7.77% - 9.41%)	(1.77% - 2.89%)	(1.96% - 2.85%)	(1.21% - 1.84%)	(0.95% - 1.59%)	(0.03% - 0.20%)	(0.04% - 0.19%)	(0.00% - 0.07%)	(0.00% - 0.04%)
	6.86%	7.61%	9.31%	8.55%	0.91%	1.41%	2.18%	1.97%	0.00%	0.07%	0.20%	0.16%
Trinidad and Tobago	(6.23% - 7.51%)	(6.91% - 8.32%)	(8.61% - 10.03%)	(7.80% - 9.31%)	(0.69% - 1.19%)	(1.10% - 1.74%)	(1.85% - 2.59%)	(1.62% - 2.36%)	(0.00% - 0.02%)	(0.02% - 0.16%)	(0.10% - 0.32%)	(0.07% - 0.29%)
	8.41%	6.96%	6.50%	6.02%	1.84%	1.47%	1.38%	1.27%	0.11%	0.07%	0.06%	0.04%
Bermuda	(7.65% - 9.25%)	(6.33% - 7.65%)	(5.88% - 7.18%)	(5.46% - 6.57%)	(1.49% - 2.20%)	(1.22% - 1.79%)	(1.12% - 1.67%)	(1.03% - 1.50%)	(0.05% - 0.21%)	(0.02% - 0.15%)	(0.02% - 0.12%)	(0.01% - 0.10%)
	8.64%	6.94%	6.27%	5.83%	1.84%	1.36%	1.22%	1.13%	0.11%	0.05%	0.03%	0.02%
Puerto Rico	(7.82% - 9.47%)	(6.29% - 7.63%)	(5.66% - 6.96%)	(5.32% - 6.47%)	(1.47% - 2.26%)	(1.09% - 1.65%)	(0.99% - 1.50%)	(0.91% - 1.39%)	(0.04% - 0.21%)	(0.01% - 0.11%)	(0.01% - 0.08%)	(0.00% - 0.07%)
	12.26%	9.41%	8.67%	8.08%	2.73%	1.92%	1.75%	1.60%	0.21%	0.10%	0.07%	0.06%
Saint Kitts and Nevis	(11.19% - 13.40%)	(8.56% - 10.29%)	(7.84% - 9.55%)	(7.35% - 8.93%)	(2.19% - 3.35%)	(1.54% - 2.34%)	(1.40% - 2.15%)	(1.28% - 1.96%)	(0.10% - 0.37%)	(0.04% - 0.20%)	(0.02% - 0.15%)	(0.01% - 0.13%)
	10.46%	8.10%	7.31%	7.22%	2.25%	1.58%	1.38%	1.36%	0.15%	0.06%	0.04%	0.04%
United States Virgin Islands	(9.49% - 11.48%)	(7.32% - 8.93%)	(6.61% - 8.05%)	(6.56% - 7.97%)	(1.82% - 2.78%)	(1.26% - 1.94%)	(1.11% - 1.69%)	(1.10% - 1.68%)	(0.06% - 0.28%)	(0.02% - 0.14%)	(0.01% - 0.09%)	(0.01% - 0.09%)
	26.10%	22.42%	17.96%	16.41%	8.50%	7.09%	5.01%	4.54%	1.75%	1.46%	0.85%	0.76%
**Central Latin America**	(25.75% - 26.43%)	(22.16% - 22.69%)	(17.72% - 18.25%)	(16.10% - 16.73%)	(8.21% - 8.79%)	(6.87% - 7.32%)	(4.83% - 5.20%)	(4.30% - 4.77%)	(1.57% - 1.94%)	(1.30% - 1.65%)	(0.75% - 0.98%)	(0.64% - 0.90%)
	22.39%	18.27%	14.01%	11.77%	5.65%	3.78%	2.39%	2.05%	0.64%	0.25%	0.07%	0.06%
Colombia	(21.50% - 23.31%)	(17.51% - 18.97%)	(13.40% - 14.69%)	(10.82% - 12.82%)	(4.87% - 6.49%)	(3.17% - 4.34%)	(2.02% - 2.81%)	(1.59% - 2.53%)	(0.41% - 0.95%)	(0.11% - 0.42%)	(0.02% - 0.16%)	(0.01% - 0.17%)
	10.51%	9.16%	7.10%	6.30%	1.61%	1.30%	0.95%	0.84%	0.02%	0.01%	0.00%	0.00%
Costa Rica	(9.29% - 11.69%)	(8.24% - 10.25%)	(6.40% - 7.87%)	(5.68% - 6.99%)	(1.11% - 2.18%)	(0.94% - 1.75%)	(0.73% - 1.22%)	(0.62% - 1.08%)	(0.00% - 0.09%)	(0.00% - 0.04%)	(0.00% - 0.01%)	(0.00% - 0.01%)
	33.92%	26.66%	18.93%	15.44%	10.81%	7.78%	4.08%	3.01%	1.78%	1.15%	0.31%	0.17%
El Salvador	(32.48% - 35.70%)	(25.66% - 27.69%)	(17.96% - 19.93%)	(14.26% - 16.64%)	(9.49% - 12.06%)	(6.77% - 8.76%)	(3.30% - 4.88%)	(2.31% - 3.81%)	(1.18% - 2.45%)	(0.73% - 1.62%)	(0.12% - 0.53%)	(0.04% - 0.36%)
	58.56%	49.62%	43.11%	40.06%	29.27%	25.24%	18.81%	16.48%	8.39%	8.52%	5.12%	4.15%
Guatemala	(55.87% - 60.88%)	(47.74% - 51.95%)	(41.47% - 45.12%)	(38.44% - 42.11%)	(26.73% - 32.02%)	(23.44% - 27.10%)	(17.29% - 20.57%)	(14.80% - 18.34%)	(6.21% - 10.96%)	(6.67% - 10.74%)	(3.85% - 6.67%)	(2.94% - 5.68%)
	41.77%	36.30%	26.29%	23.36%	16.53%	13.03%	7.52%	6.37%	3.74%	2.51%	1.06%	0.84%
Honduras	(39.96% - 43.96%)	(35.12% - 37.79%)	(25.09% - 27.51%)	(21.92% - 24.83%)	(14.97% - 18.12%)	(11.80% - 14.19%)	(6.26% - 8.84%)	(4.93% - 7.67%)	(2.88% - 4.83%)	(1.89% - 3.19%)	(0.61% - 1.63%)	(0.40% - 1.40%)
	23.65%	19.35%	15.30%	13.85%	6.80%	5.29%	3.74%	3.36%	1.31%	0.86%	0.53%	0.49%
Mexico	(23.27% - 24.00%)	(19.07% - 19.63%)	(15.04% - 15.53%)	(13.61% - 14.10%)	(6.52% - 7.08%)	(5.08% - 5.49%)	(3.57% - 3.90%)	(3.19% - 3.54%)	(1.19% - 1.45%)	(0.78% - 0.95%)	(0.46% - 0.60%)	(0.42% - 0.56%)

Table S8a. Estimated overall, severe, and extreme **stunting** prevalence (%) in children under 5 years, both sexes, in 1990, 2000, 2010, and 2020.

Location	Total				Severe				Extreme			
	1990	2000	2010	2020	1990	2000	2010	2020	1990	2000	2010	2020
	14.93%	12.21%	11.20%	10.49%	2.94%	2.07%	1.99%	1.86%	0.27%	0.09%	0.05%	0.04%
Aguascalientes	(13.63% - 16.26%)	(11.13% - 13.37%)	(10.22% - 12.23%)	(9.51% - 11.49%)	(2.29% - 3.68%)	(1.59% - 2.60%)	(1.55% - 2.48%)	(1.44% - 2.32%)	(0.16% - 0.44%)	(0.04% - 0.17%)	(0.01% - 0.13%)	(0.01% - 0.11%)
	11.91%	9.00%	7.93%	7.34%	2.45%	1.52%	1.22%	1.10%	0.22%	0.07%	0.02%	0.02%
Baja California	(10.82% - 12.98%)	(8.15% - 9.88%)	(7.22% - 8.69%)	(6.65% - 8.06%)	(1.89% - 3.05%)	(1.14% - 1.97%)	(0.95% - 1.54%)	(0.83% - 1.43%)	(0.07% - 0.42%)	(0.00% - 0.21%)	(0.00% - 0.08%)	(0.00% - 0.09%)
	20.42%	17.95%	16.49%	15.35%	7.07%	5.67%	4.79%	4.41%	1.51%	1.03%	0.73%	0.66%
Baja California Sur	(19.20% - 21.73%)	(16.92% - 19.03%)	(15.45% - 17.49%)	(14.16% - 16.50%)	(6.12% - 8.18%)	(4.92% - 6.45%)	(4.14% - 5.49%)	(3.69% - 5.14%)	(1.08% - 2.05%)	(0.73% - 1.36%)	(0.50% - 1.00%)	(0.41% - 0.93%)
	38.60%	31.09%	22.93%	20.74%	16.70%	10.60%	6.04%	5.76%	4.55%	1.99%	0.90%	0.92%
Campeche	(36.63% - 40.83%)	(28.51% - 33.68%)	(20.88% - 24.71%)	(18.90% - 22.39%)	(14.34% - 18.97%)	(8.01% - 12.75%)	(4.41% - 7.54%)	(4.43% - 7.06%)	(2.97% - 6.36%)	(1.19% - 3.01%)	(0.53% - 1.36%)	(0.55% - 1.42%)
	13.81%	11.10%	9.46%	8.76%	2.61%	1.94%	1.50%	1.30%	0.27%	0.12%	0.08%	0.06%
Coahuila	(12.62% - 15.07%)	(10.13% - 12.15%)	(8.67% - 10.37%)	(7.94% - 9.63%)	(2.08% - 3.22%)	(1.53% - 2.41%)	(1.20% - 1.86%)	(1.03% - 1.63%)	(0.16% - 0.40%)	(0.06% - 0.20%)	(0.04% - 0.13%)	(0.02% - 0.11%)
	7.71%	5.40%	4.75%	4.63%	0.38%	0.28%	0.25%	0.25%	0.00%	0.00%	0.00%	0.00%
Colima	(7.00% - 8.59%)	(4.86% - 5.98%)	(4.25% - 5.28%)	(4.12% - 5.13%)	(0.22% - 0.59%)	(0.17% - 0.43%)	(0.14% - 0.37%)	(0.14% - 0.37%)	(0.00% - 0.00%)	(0.00% - 0.00%)	(0.00% - 0.00%)	(0.00% - 0.00%)
	40.80%	36.68%	29.95%	26.20%	18.76%	13.92%	9.10%	7.59%	5.61%	3.03%	1.51%	1.22%
Chiapas	(39.18% - 42.80%)	(34.82% - 38.87%)	(28.46% - 31.51%)	(24.62% - 27.81%)	(17.41% - 20.22%)	(12.49% - 15.39%)	(7.35% - 10.58%)	(6.12% - 9.03%)	(4.64% - 6.86%)	(2.25% - 3.98%)	(0.97% - 2.16%)	(0.74% - 1.82%)
	15.20%	13.86%	10.17%	9.41%	2.84%	2.70%	1.61%	1.57%	0.32%	0.41%	0.06%	0.06%
Chihuahua	(14.03% - 16.48%)	(12.99% - 14.84%)	(9.41% - 10.96%)	(8.58% - 10.28%)	(2.30% - 3.45%)	(2.31% - 3.16%)	(1.31% - 1.94%)	(1.24% - 1.95%)	(0.22% - 0.45%)	(0.32% - 0.51%)	(0.03% - 0.11%)	(0.02% - 0.14%)
	15.84%	13.33%	12.75%	11.50%	3.09%	2.62%	2.45%	2.26%	0.28%	0.22%	0.14%	0.13%
Mexico City	(14.60% - 17.16%)	(12.35% - 14.36%)	(11.73% - 13.83%)	(10.49% - 12.58%)	(2.42% - 3.86%)	(2.07% - 3.19%)	(1.97% - 3.04%)	(1.78% - 2.85%)	(0.12% - 0.48%)	(0.11% - 0.38%)	(0.07% - 0.27%)	(0.05% - 0.28%)
	17.63%	13.32%	11.15%	10.01%	4.86%	3.10%	2.47%	2.23%	0.73%	0.30%	0.20%	0.18%
Durango	(16.42% - 18.91%)	(12.33% - 14.41%)	(10.24% - 11.99%)	(9.11% - 10.91%)	(4.02% - 5.77%)	(2.53% - 3.72%)	(2.01% - 2.95%)	(1.78% - 2.72%)	(0.43% - 1.12%)	(0.13% - 0.50%)	(0.08% - 0.36%)	(0.06% - 0.34%)
	20.45%	15.20%	12.42%	10.58%	3.83%	3.01%	2.34%	1.91%	0.30%	0.18%	0.11%	0.09%
Guanajuato	(18.83% - 21.92%)	(14.20% - 16.23%)	(11.63% - 13.33%)	(9.59% - 11.52%)	(2.85% - 4.82%)	(2.45% - 3.61%)	(1.96% - 2.82%)	(1.49% - 2.36%)	(0.16% - 0.49%)	(0.09% - 0.32%)	(0.04% - 0.20%)	(0.02% - 0.20%)
	40.08%	33.56%	25.53%	21.36%	18.55%	12.93%	8.23%	6.57%	5.71%	3.04%	1.58%	1.21%
Guerrero	(38.80% - 41.67%)	(32.55% - 34.89%)	(24.57% - 26.58%)	(20.12% - 22.65%)	(17.42% - 19.68%)	(11.84% - 14.08%)	(7.25% - 9.19%)	(5.56% - 7.64%)	(4.68% - 6.84%)	(2.39% - 3.81%)	(1.18% - 2.05%)	(0.84% - 1.65%)
	35.86%	27.64%	22.48%	20.27%	11.58%	8.61%	7.20%	7.02%	2.11%	1.61%	1.36%	1.58%
Hidalgo	(32.92% - 38.68%)	(26.25% - 29.06%)	(21.23% - 23.81%)	(18.96% - 21.61%)	(9.45% - 13.79%)	(7.10% - 10.07%)	(6.16% - 8.28%)	(5.98% - 8.12%)	(1.20% - 3.22%)	(0.97% - 2.36%)	(0.92% - 1.90%)	(1.07% - 2.13%)
	13.99%	10.85%	8.98%	8.33%	2.40%	1.91%	1.39%	1.17%	0.11%	0.08%	0.02%	0.01%
Jalisco	(12.78% - 15.21%)	(9.95% - 11.82%)	(8.21% - 9.85%)	(7.53% - 9.16%)	(1.76% - 3.11%)	(1.46% - 2.44%)	(1.07% - 1.75%)	(0.87% - 1.54%)	(0.02% - 0.30%)	(0.01% - 0.20%)	(0.00% - 0.07%)	(0.00% - 0.04%)
	25.99%	20.73%	14.66%	12.87%	6.15%	4.93%	2.88%	2.39%	0.89%	0.59%	0.30%	0.24%
México	(24.37% - 27.60%)	(19.48% - 21.94%)	(13.58% - 15.71%)	(11.80% - 14.00%)	(4.77% - 7.41%)	(3.93% - 5.89%)	(2.24% - 3.53%)	(1.80% - 3.05%)	(0.60% - 1.22%)	(0.38% - 0.85%)	(0.16% - 0.51%)	(0.12% - 0.44%)
	23.19%	18.41%	15.00%	13.20%	5.63%	4.57%	3.55%	3.01%	0.64%	0.51%	0.34%	0.26%
Michoacán de Ocampo	(21.74% - 24.70%)	(17.15% - 19.58%)	(14.02% - 16.05%)	(12.18% - 14.26%)	(4.44% - 6.89%)	(3.76% - 5.43%)	(2.95% - 4.17%)	(2.43% - 3.63%)	(0.34% - 1.05%)	(0.29% - 0.77%)	(0.19% - 0.53%)	(0.14% - 0.44%)
	17.43%	13.69%	11.17%	10.19%	3.22%	2.63%	2.06%	1.86%	0.23%	0.15%	0.11%	0.08%
Morelos	(15.95% - 18.77%)	(12.53% - 14.80%)	(10.27% - 12.20%)	(9.28% - 11.15%)	(2.41% - 4.09%)	(2.03% - 3.26%)	(1.65% - 2.52%)	(1.47% - 2.28%)	(0.11% - 0.42%)	(0.07% - 0.27%)	(0.05% - 0.20%)	(0.04% - 0.16%)
	11.88%	8.80%	7.32%	6.84%	0.96%	0.81%	0.71%	0.68%	0.00%	0.00%	0.00%	0.00%
Nayarit	(10.75% - 13.16%)	(7.99% - 9.78%)	(6.63% - 8.08%)	(6.13% - 7.63%)	(0.60% - 1.40%)	(0.55% - 1.10%)	(0.51% - 0.96%)	(0.48% - 0.91%)	(0.00% - 0.01%)	(0.00% - 0.00%)	(0.00% - 0.00%)	(0.00% - 0.00%)
	13.10%	10.00%	8.60%	8.14%	2.11%	1.29%	1.20%	1.20%	0.11%	0.01%	0.01%	0.01%
Nuevo León	(11.91% - 14.33%)	(9.06% - 11.05%)	(7.81% - 9.47%)	(7.32% - 8.94%)	(1.62% - 2.64%)	(0.96% - 1.69%)	(0.88% - 1.57%)	(0.89% - 1.54%)	(0.06% - 0.17%)	(0.00% - 0.03%)	(0.00% - 0.06%)	(0.00% - 0.07%)
	41.78%	36.31%	29.82%	27.74%	18.54%	15.71%	12.57%	11.67%	5.34%	4.54%	3.67%	3.45%
Oaxaca	(40.16% - 43.87%)	(35.05% - 37.57%)	(28.56% - 31.08%)	(26.41% - 29.17%)	(16.34% - 20.73%)	(14.18% - 17.33%)	(11.19% - 14.15%)	(10.03% - 13.29%)	(3.49% - 7.54%)	(3.33% - 5.97%)	(2.66% - 4.90%)	(2.36% - 4.60%)
	31.97%	26.02%	21.16%	20.92%	9.00%	7.30%	5.86%	6.08%	1.23%	1.01%	0.80%	0.92%
Puebla	(30.20% - 34.17%)	(24.64% - 27.46%)	(19.81% - 22.43%)	(19.46% - 22.28%)	(7.07% - 10.75%)	(5.90% - 8.69%)	(4.76% - 6.95%)	(4.90% - 7.25%)	(0.59% - 2.05%)	(0.51% - 1.62%)	(0.45% - 1.23%)	(0.47% - 1.46%)
	18.96%	14.73%	13.09%	12.05%	4.48%	2.99%	2.63%	2.46%	0.55%	0.19%	0.13%	0.12%
Querétaro	(17.54% - 20.33%)	(13.55% - 16.01%)	(12.04% - 14.27%)	(10.95% - 13.10%)	(3.48% - 5.46%)	(2.34% - 3.73%)	(2.11% - 3.23%)	(1.94% - 3.03%)	(0.36% - 0.78%)	(0.10% - 0.35%)	(0.06% - 0.26%)	(0.04% - 0.25%)
	26.59%	20.59%	16.65%	15.51%	6.71%	5.17%	3.90%	3.78%	0.83%	0.60%	0.36%	0.39%

Table S8a. Estimated overall, severe, and extreme **stunting** prevalence (%) in children under 5 years, both sexes, in 1990, 2000, 2010, and 2020.

Location	Total				Severe				Extreme			
	1990	2000	2010	2020	1990	2000	2010	2020	1990	2000	2010	2020
Quintana Roo	(24.96% - 28.67%)	(19.27% - 21.87%)	(15.43% - 17.80%)	(14.35% - 16.77%)	(5.18% - 8.18%)	(4.02% - 6.26%)	(3.12% - 4.69%)	(3.04% - 4.63%)	(0.33% - 1.50%)	(0.24% - 1.04%)	(0.16% - 0.62%)	(0.17% - 0.67%)
	24.86%	18.85%	12.04%	9.97%	7.64%	4.66%	2.31%	2.09%	1.57%	0.64%	0.18%	0.22%
San Luis Potosí	(23.01% - 26.61%)	(17.41% - 20.29%)	(11.07% - 13.06%)	(9.12% - 10.90%)	(6.06% - 9.30%)	(3.72% - 5.73%)	(1.85% - 2.84%)	(1.67% - 2.60%)	(0.91% - 2.37%)	(0.30% - 1.08%)	(0.06% - 0.35%)	(0.08% - 0.41%)
	22.29%	18.33%	11.91%	11.15%	7.86%	5.24%	2.36%	2.21%	2.07%	0.94%	0.20%	0.17%
Sinaloa	(21.00% - 23.59%)	(17.21% - 19.52%)	(11.02% - 12.83%)	(10.17% - 12.20%)	(6.65% - 9.16%)	(4.33% - 6.13%)	(1.86% - 2.94%)	(1.69% - 2.83%)	(1.38% - 2.92%)	(0.56% - 1.40%)	(0.06% - 0.39%)	(0.03% - 0.38%)
	15.42%	13.07%	12.73%	11.48%	3.95%	3.06%	3.37%	3.06%	0.54%	0.35%	0.58%	0.54%
Sonora	(14.31% - 16.59%)	(12.05% - 14.10%)	(11.77% - 13.72%)	(10.44% - 12.53%)	(3.20% - 4.77%)	(2.47% - 3.68%)	(2.75% - 4.15%)	(2.42% - 3.81%)	(0.28% - 0.85%)	(0.16% - 0.57%)	(0.32% - 0.95%)	(0.27% - 0.92%)
	30.23%	23.69%	18.03%	16.36%	9.00%	6.84%	4.71%	4.40%	1.52%	1.08%	0.59%	0.60%
Tabasco	(28.49% - 32.12%)	(22.30% - 25.09%)	(16.81% - 19.32%)	(15.03% - 17.69%)	(7.13% - 10.72%)	(5.38% - 8.31%)	(3.83% - 5.64%)	(3.51% - 5.37%)	(0.79% - 2.39%)	(0.57% - 1.73%)	(0.34% - 0.92%)	(0.32% - 0.94%)
	11.54%	8.72%	7.00%	6.65%	1.45%	1.03%	0.65%	0.59%	0.01%	0.00%	0.00%	0.00%
Tamaulipas	(10.35% - 12.74%)	(7.88% - 9.64%)	(6.28% - 7.81%)	(5.96% - 7.47%)	(0.98% - 1.99%)	(0.73% - 1.40%)	(0.44% - 0.90%)	(0.39% - 0.86%)	(0.00% - 0.05%)	(0.00% - 0.01%)	(0.00% - 0.00%)	(0.00% - 0.00%)
	22.46%	17.55%	14.35%	13.29%	2.73%	2.56%	2.82%	2.99%	0.11%	0.08%	0.14%	0.24%
Tlaxcala	(20.37% - 24.57%)	(16.00% - 19.11%)	(13.13% - 15.53%)	(12.19% - 14.52%)	(1.56% - 4.07%)	(1.74% - 3.49%)	(2.17% - 3.53%)	(2.38% - 3.66%)	(0.02% - 0.28%)	(0.01% - 0.24%)	(0.03% - 0.31%)	(0.08% - 0.45%)
	22.36%	19.01%	12.60%	9.99%	5.19%	4.67%	2.37%	1.52%	0.60%	0.53%	0.17%	0.09%
Veracruz de Ignacio de la Llave	(20.70% - 24.08%)	(17.78% - 20.24%)	(11.79% - 13.52%)	(9.13% - 10.85%)	(3.98% - 6.44%)	(3.82% - 5.53%)	(1.98% - 2.82%)	(1.21% - 1.88%)	(0.35% - 0.95%)	(0.32% - 0.79%)	(0.11% - 0.26%)	(0.05% - 0.15%)
	38.03%	30.80%	26.63%	25.12%	11.17%	9.61%	8.37%	8.01%	1.59%	1.60%	1.49%	1.47%
Yucatán	(35.80% - 41.04%)	(29.33% - 32.41%)	(25.32% - 27.89%)	(23.65% - 26.54%)	(9.44% - 12.93%)	(8.03% - 11.09%)	(6.85% - 9.80%)	(6.46% - 9.47%)	(0.87% - 2.59%)	(0.95% - 2.41%)	(0.95% - 2.16%)	(0.87% - 2.14%)
	17.29%	14.61%	12.34%	11.39%	3.35%	2.57%	2.05%	1.82%	0.29%	0.13%	0.08%	0.05%
Zacatecas	(15.82% - 18.93%)	(13.40% - 15.86%)	(11.23% - 13.50%)	(10.27% - 12.57%)	(2.53% - 4.24%)	(1.90% - 3.28%)	(1.57% - 2.59%)	(1.35% - 2.30%)	(0.18% - 0.46%)	(0.06% - 0.23%)	(0.03% - 0.14%)	(0.01% - 0.12%)
	28.42%	25.47%	19.95%	16.58%	11.86%	8.94%	5.95%	4.68%	3.34%	1.89%	0.95%	0.68%
Nicaragua	(27.39% - 29.48%)	(24.70% - 26.26%)	(18.99% - 20.89%)	(15.44% - 17.73%)	(10.72% - 13.03%)	(8.04% - 9.86%)	(5.17% - 6.77%)	(3.86% - 5.54%)	(2.65% - 4.14%)	(1.43% - 2.41%)	(0.65% - 1.31%)	(0.39% - 1.03%)
	18.89%	20.10%	19.54%	15.42%	5.99%	6.71%	5.93%	4.20%	1.08%	1.32%	0.98%	0.55%
Panama	(17.84% - 20.05%)	(19.28% - 20.97%)	(18.63% - 20.45%)	(14.19% - 16.63%)	(5.12% - 6.96%)	(5.96% - 7.46%)	(5.17% - 6.64%)	(3.39% - 5.02%)	(0.74% - 1.49%)	(0.97% - 1.68%)	(0.69% - 1.29%)	(0.30% - 0.84%)
	19.98%	19.46%	15.92%	15.43%	6.24%	6.19%	4.56%	4.43%	1.06%	1.10%	0.67%	0.66%
Venezuela (Bolivarian Republic of)	(19.21% - 20.77%)	(18.78% - 20.18%)	(15.02% - 16.85%)	(14.29% - 16.58%)	(5.66% - 6.90%)	(5.69% - 6.73%)	(4.00% - 5.17%)	(3.70% - 5.16%)	(0.85% - 1.31%)	(0.92% - 1.30%)	(0.49% - 0.87%)	(0.42% - 0.94%)
	15.56%	11.92%	9.76%	8.73%	3.92%	2.81%	2.14%	1.88%	0.55%	0.33%	0.21%	0.17%
**Tropical Latin America**	(15.30% - 15.82%)	(11.71% - 12.14%)	(9.55% - 9.97%)	(8.55% - 8.93%)	(3.75% - 4.09%)	(2.69% - 2.93%)	(2.03% - 2.26%)	(1.77% - 1.99%)	(0.49% - 0.62%)	(0.29% - 0.37%)	(0.18% - 0.25%)	(0.14% - 0.21%)
	15.44%	11.78%	9.67%	8.79%	3.90%	2.77%	2.13%	1.91%	0.55%	0.33%	0.21%	0.18%
Brazil	(15.18% - 15.71%)	(11.56% - 11.99%)	(9.46% - 9.89%)	(8.59% - 8.99%)	(3.72% - 4.07%)	(2.65% - 2.90%)	(2.02% - 2.25%)	(1.80% - 2.02%)	(0.49% - 0.62%)	(0.29% - 0.37%)	(0.18% - 0.26%)	(0.14% - 0.22%)
	25.11%	14.31%	10.54%	9.53%	2.89%	0.54%	0.36%	0.46%	0.09%	0.00%	0.00%	0.00%
Acre	(20.85% - 30.42%)	(12.70% - 16.07%)	(9.33% - 11.87%)	(8.46% - 10.55%)	(0.99% - 5.11%)	(0.09% - 1.15%)	(0.08% - 0.75%)	(0.18% - 0.82%)	(0.00% - 0.33%)	(0.00% - 0.00%)	(0.00% - 0.00%)	(0.00% - 0.00%)
	20.86%	15.97%	13.02%	11.83%	5.83%	4.00%	3.05%	2.71%	0.97%	0.56%	0.34%	0.27%
Alagoas	(19.47% - 22.18%)	(14.80% - 17.11%)	(11.95% - 14.17%)	(10.80% - 12.96%)	(4.79% - 6.90%)	(3.27% - 4.75%)	(2.50% - 3.68%)	(2.20% - 3.31%)	(0.70% - 1.32%)	(0.39% - 0.76%)	(0.22% - 0.50%)	(0.16% - 0.42%)
	16.86%	12.33%	10.62%	9.58%	3.13%	2.27%	1.87%	1.67%	0.26%	0.15%	0.09%	0.06%
Amazonas	(15.52% - 18.28%)	(11.25% - 13.42%)	(9.59% - 11.68%)	(8.67% - 10.54%)	(2.16% - 4.14%)	(1.68% - 2.86%)	(1.39% - 2.44%)	(1.24% - 2.19%)	(0.03% - 0.57%)	(0.02% - 0.32%)	(0.00% - 0.23%)	(0.00% - 0.20%)
	15.82%	11.54%	9.10%	8.71%	3.99%	2.42%	1.64%	1.60%	0.44%	0.12%	0.03%	0.03%
Amapá	(14.50% - 17.16%)	(10.47% - 12.62%)	(8.23% - 10.05%)	(7.84% - 9.62%)	(3.21% - 4.84%)	(1.91% - 2.98%)	(1.25% - 2.08%)	(1.25% - 2.01%)	(0.23% - 0.72%)	(0.04% - 0.25%)	(0.00% - 0.12%)	(0.00% - 0.11%)
	20.11%	16.88%	14.05%	12.72%	6.24%	5.00%	3.91%	3.45%	1.10%	0.84%	0.56%	0.46%
Bahia	(18.92% - 21.24%)	(15.75% - 18.05%)	(13.04% - 15.13%)	(11.70% - 13.85%)	(5.37% - 7.17%)	(4.27% - 5.76%)	(3.27% - 4.60%)	(2.85% - 4.06%)	(0.78% - 1.50%)	(0.57% - 1.15%)	(0.36% - 0.81%)	(0.28% - 0.69%)
	24.10%	17.55%	9.57%	7.74%	6.88%	4.57%	1.82%	1.36%	1.00%	0.54%	0.10%	0.04%
Ceará	(22.81% - 25.29%)	(16.56% - 18.48%)	(8.71% - 10.48%)	(6.95% - 8.61%)	(5.71% - 8.01%)	(3.83% - 5.30%)	(1.39% - 2.33%)	(1.03% - 1.76%)	(0.62% - 1.45%)	(0.30% - 0.81%)	(0.00% - 0.24%)	(0.00% - 0.13%)
	10.45%	7.17%	5.48%	4.97%	1.77%	0.98%	0.65%	0.61%	0.06%	0.00%	0.00%	0.00%
Distrito Federal	(9.50% - 11.41%)	(6.53% - 7.85%)	(4.90% - 6.08%)	(4.44% - 5.50%)	(1.38% - 2.22%)	(0.74% - 1.24%)	(0.47% - 0.85%)	(0.45% - 0.79%)	(0.02% - 0.13%)	(0.00% - 0.01%)	(0.00% - 0.00%)	(0.00% - 0.00%)
	12.85%	8.85%	6.89%	6.28%	2.65%	1.55%	1.06%	0.99%	0.15%	0.02%	0.00%	0.00%
Espírito Santo	(11.72% - 14.05%)	(8.00% - 9.74%)	(6.20% - 7.65%)	(5.65% - 6.95%)	(2.07% - 3.34%)	(1.20% - 1.96%)	(0.82% - 1.34%)	(0.76% - 1.26%)	(0.06% - 0.31%)	(0.00% - 0.07%)	(0.00% - 0.02%)	(0.00% - 0.01%)

Table S8a. Estimated overall, severe, and extreme **stunting** prevalence (%) in children under 5 years, both sexes, in 1990, 2000, 2010, and 2020.

Location	Total				Severe				Extreme			
	1990	2000	2010	2020	1990	2000	2010	2020	1990	2000	2010	2020
	17.75%	11.69%	8.77%	7.99%	4.35%	2.46%	1.56%	1.45%	0.46%	0.14%	0.03%	0.02%
Goiás	(16.45% - 19.03%)	(10.64% - 12.87%)	(7.95% - 9.73%)	(7.19% - 8.81%)	(3.50% - 5.26%)	(1.99% - 3.07%)	(1.22% - 1.98%)	(1.16% - 1.83%)	(0.23% - 0.74%)	(0.04% - 0.29%)	(0.00% - 0.10%)	(0.00% - 0.09%)
	31.04%	23.77%	19.69%	18.03%	11.14%	7.66%	6.04%	5.37%	2.36%	1.40%	1.03%	0.86%
Maranhão	(29.82% - 32.36%)	(22.48% - 25.00%)	(18.46% - 21.00%)	(16.70% - 19.43%)	(9.76% - 12.53%)	(6.51% - 8.78%)	(5.05% - 7.15%)	(4.33% - 6.39%)	(1.72% - 3.17%)	(0.97% - 1.94%)	(0.64% - 1.51%)	(0.51% - 1.27%)
	10.56%	7.72%	6.40%	5.73%	1.59%	1.18%	0.94%	0.84%	0.08%	0.05%	0.03%	0.02%
Minas Gerais	(9.54% - 11.53%)	(7.04% - 8.45%)	(5.81% - 7.03%)	(5.19% - 6.30%)	(1.25% - 1.97%)	(0.95% - 1.45%)	(0.75% - 1.16%)	(0.66% - 1.04%)	(0.03% - 0.15%)	(0.02% - 0.10%)	(0.00% - 0.07%)	(0.00% - 0.05%)
	17.55%	13.31%	10.52%	9.40%	5.33%	3.64%	2.55%	2.25%	0.91%	0.49%	0.24%	0.18%
Mato Grosso do Sul	(16.34% - 18.79%)	(12.25% - 14.43%)	(9.57% - 11.57%)	(8.59% - 10.29%)	(4.45% - 6.25%)	(3.04% - 4.43%)	(2.06% - 3.14%)	(1.83% - 2.71%)	(0.59% - 1.28%)	(0.29% - 0.78%)	(0.10% - 0.42%)	(0.06% - 0.33%)
	14.82%	10.61%	8.46%	7.62%	3.25%	2.21%	1.66%	1.49%	0.26%	0.13%	0.07%	0.06%
Mato Grosso	(13.68% - 16.02%)	(9.69% - 11.58%)	(7.71% - 9.29%)	(6.90% - 8.43%)	(2.57% - 4.05%)	(1.78% - 2.71%)	(1.31% - 2.04%)	(1.17% - 1.85%)	(0.08% - 0.51%)	(0.03% - 0.28%)	(0.00% - 0.18%)	(0.00% - 0.16%)
	25.86%	20.08%	16.13%	14.75%	7.56%	5.66%	4.22%	3.84%	1.17%	0.83%	0.54%	0.48%
Pará	(24.39% - 27.53%)	(18.79% - 21.39%)	(14.91% - 17.32%)	(13.55% - 15.92%)	(5.94% - 8.97%)	(4.65% - 6.75%)	(3.36% - 5.11%)	(3.04% - 4.65%)	(0.62% - 1.81%)	(0.46% - 1.27%)	(0.26% - 0.89%)	(0.22% - 0.79%)
	21.41%	16.45%	13.37%	12.23%	6.59%	4.59%	3.49%	3.15%	1.12%	0.65%	0.42%	0.35%
Paraíba	(20.16% - 22.54%)	(15.37% - 17.52%)	(12.32% - 14.38%)	(11.24% - 13.26%)	(5.67% - 7.60%)	(3.88% - 5.37%)	(2.89% - 4.13%)	(2.61% - 3.76%)	(0.79% - 1.56%)	(0.42% - 0.95%)	(0.23% - 0.63%)	(0.19% - 0.55%)
	9.44%	7.58%	6.40%	5.69%	1.40%	1.12%	0.93%	0.83%	0.01%	0.00%	0.00%	0.00%
Paraná	(8.55% - 10.42%)	(6.84% - 8.36%)	(5.82% - 7.07%)	(5.17% - 6.31%)	(1.07% - 1.77%)	(0.86% - 1.42%)	(0.72% - 1.18%)	(0.64% - 1.05%)	(0.00% - 0.04%)	(0.00% - 0.01%)	(0.00% - 0.01%)	(0.00% - 0.00%)
	20.83%	15.34%	12.45%	11.10%	6.14%	4.15%	3.20%	2.79%	1.00%	0.56%	0.37%	0.29%
Pernambuco	(19.60% - 21.99%)	(14.31% - 16.36%)	(11.47% - 13.43%)	(10.20% - 12.01%)	(5.30% - 7.05%)	(3.52% - 4.79%)	(2.69% - 3.75%)	(2.33% - 3.28%)	(0.71% - 1.34%)	(0.38% - 0.78%)	(0.22% - 0.54%)	(0.16% - 0.44%)
	25.35%	19.54%	15.89%	14.22%	8.49%	5.87%	4.44%	3.88%	1.82%	1.08%	0.69%	0.56%
Piaui	(24.04% - 26.55%)	(18.16% - 20.77%)	(14.57% - 17.06%)	(13.03% - 15.36%)	(7.25% - 9.70%)	(4.89% - 6.86%)	(3.65% - 5.23%)	(3.18% - 4.64%)	(1.37% - 2.32%)	(0.77% - 1.46%)	(0.46% - 0.97%)	(0.36% - 0.81%)
	9.62%	6.96%	5.89%	5.40%	1.57%	1.09%	0.89%	0.81%	0.04%	0.01%	0.00%	0.00%
Rio de Janeiro	(8.70% - 10.61%)	(6.34% - 7.61%)	(5.36% - 6.48%)	(4.94% - 5.90%)	(1.21% - 1.95%)	(0.88% - 1.34%)	(0.71% - 1.12%)	(0.63% - 1.00%)	(0.01% - 0.08%)	(0.00% - 0.03%)	(0.00% - 0.02%)	(0.00% - 0.01%)
	16.98%	12.79%	10.19%	9.34%	4.21%	3.01%	2.23%	2.03%	0.47%	0.27%	0.16%	0.13%
Rio Grande do Norte	(15.69% - 18.25%)	(11.75% - 13.86%)	(9.36% - 11.16%)	(8.54% - 10.22%)	(3.39% - 5.07%)	(2.43% - 3.60%)	(1.81% - 2.71%)	(1.63% - 2.52%)	(0.24% - 0.76%)	(0.12% - 0.47%)	(0.05% - 0.30%)	(0.03% - 0.27%)
	19.11%	13.11%	9.89%	9.02%	3.62%	2.18%	1.41%	1.32%	0.47%	0.17%	0.05%	0.04%
Rondônia	(17.44% - 20.95%)	(11.84% - 14.39%)	(8.95% - 10.93%)	(8.11% - 10.04%)	(2.88% - 4.49%)	(1.73% - 2.76%)	(1.07% - 1.81%)	(0.99% - 1.68%)	(0.25% - 0.74%)	(0.06% - 0.32%)	(0.00% - 0.14%)	(0.00% - 0.12%)
	17.33%	12.00%	9.14%	8.61%	5.88%	3.25%	2.11%	1.99%	1.24%	0.42%	0.14%	0.12%
Roraima	(16.10% - 18.64%)	(11.02% - 13.01%)	(8.29% - 10.03%)	(7.88% - 9.45%)	(5.02% - 6.82%)	(2.72% - 3.83%)	(1.72% - 2.54%)	(1.63% - 2.39%)	(0.88% - 1.65%)	(0.25% - 0.63%)	(0.05% - 0.26%)	(0.04% - 0.24%)
	12.89%	9.57%	7.56%	7.01%	3.24%	2.14%	1.52%	1.42%	0.40%	0.17%	0.07%	0.06%
Rio Grande do Sul	(11.78% - 14.05%)	(8.75% - 10.49%)	(6.83% - 8.32%)	(6.34% - 7.77%)	(2.66% - 3.92%)	(1.75% - 2.60%)	(1.22% - 1.86%)	(1.13% - 1.76%)	(0.22% - 0.65%)	(0.07% - 0.31%)	(0.01% - 0.16%)	(0.00% - 0.15%)
	9.45%	6.90%	5.29%	4.82%	2.03%	1.20%	0.77%	0.71%	0.20%	0.05%	0.01%	0.00%
Santa Catarina	(8.51% - 10.39%)	(6.20% - 7.65%)	(4.76% - 5.89%)	(4.30% - 5.36%)	(1.57% - 2.55%)	(0.91% - 1.56%)	(0.57% - 1.00%)	(0.54% - 0.93%)	(0.06% - 0.37%)	(0.00% - 0.15%)	(0.00% - 0.04%)	(0.00% - 0.04%)
	19.12%	13.40%	9.67%	8.95%	4.53%	2.65%	1.64%	1.56%	0.46%	0.13%	0.03%	0.03%
Sergipe	(17.63% - 20.58%)	(12.24% - 14.59%)	(8.76% - 10.64%)	(8.12% - 9.84%)	(3.58% - 5.57%)	(2.09% - 3.29%)	(1.25% - 2.05%)	(1.22% - 1.95%)	(0.23% - 0.77%)	(0.03% - 0.28%)	(0.00% - 0.11%)	(0.00% - 0.09%)
	8.54%	6.52%	5.48%	5.06%	1.23%	0.94%	0.76%	0.70%	0.03%	0.01%	0.00%	0.00%
São Paulo	(7.79% - 9.27%)	(5.95% - 7.10%)	(5.00% - 6.01%)	(4.62% - 5.56%)	(0.97% - 1.50%)	(0.75% - 1.14%)	(0.60% - 0.93%)	(0.55% - 0.86%)	(0.01% - 0.07%)	(0.00% - 0.04%)	(0.00% - 0.02%)	(0.00% - 0.02%)
	19.42%	13.77%	9.92%	8.96%	6.99%	4.01%	2.41%	2.16%	1.64%	0.63%	0.21%	0.16%
Tocantins	(18.14% - 20.68%)	(12.67% - 14.96%)	(9.04% - 10.91%)	(8.11% - 9.94%)	(6.03% - 8.08%)	(3.36% - 4.76%)	(1.97% - 2.94%)	(1.77% - 2.60%)	(1.22% - 2.15%)	(0.41% - 0.91%)	(0.09% - 0.37%)	(0.07% - 0.30%)
	18.87%	15.86%	11.95%	7.60%	4.68%	3.84%	2.45%	1.13%	0.53%	0.39%	0.15%	0.01%
Paraguay	(17.98% - 19.74%)	(14.94% - 16.82%)	(11.26% - 12.66%)	(6.98% - 8.23%)	(4.07% - 5.36%)	(3.25% - 4.44%)	(2.14% - 2.78%)	(0.90% - 1.36%)	(0.34% - 0.77%)	(0.22% - 0.57%)	(0.08% - 0.23%)	(0.00% - 0.03%)

Table S8a. Estimated overall, severe, and extreme **stunting** prevalence (%) in children under 5 years, both sexes, in 1990, 2000, 2010, and 2020.

Location	Total				Severe				Extreme			
	1990	2000	2010	2020	1990	2000	2010	2020	1990	2000	2010	2020
North Africa and Middle East	31.27% (30.87% - 31.68%)	28.82% (28.51% - 29.14%)	24.72% (24.39% - 25.04%)	21.40% (21.02% - 21.80%)	13.38% (12.96% - 13.81%)	13.03% (12.69% - 13.37%)	10.50% (10.17% - 10.84%)	8.49% (8.10% - 8.87%)	4.23% (3.90% - 4.56%)	4.64% (4.30% - 4.99%)	3.34% (3.02% - 3.66%)	2.43% (2.11% - 2.76%)
**North Africa and Middle East**	31.27% (30.87% - 31.68%)	28.82% (28.51% - 29.14%)	24.72% (24.39% - 25.04%)	21.40% (21.02% - 21.80%)	13.38% (12.96% - 13.81%)	13.03% (12.69% - 13.37%)	10.50% (10.17% - 10.84%)	8.49% (8.10% - 8.87%)	4.23% (3.90% - 4.56%)	4.64% (4.30% - 4.99%)	3.34% (3.02% - 3.66%)	2.43% (2.11% - 2.76%)
Algeria	31.00% (29.59% - 32.41%)	26.81% (25.63% - 27.98%)	17.34% (16.22% - 18.41%)	13.96% (12.78% - 15.04%)	11.16% (9.60% - 12.64%)	9.56% (8.44% - 10.79%)	4.88% (4.12% - 5.65%)	3.63% (2.95% - 4.34%)	2.38% (1.64% - 3.24%)	2.06% (1.54% - 2.69%)	0.69% (0.43% - 0.97%)	0.42% (0.21% - 0.65%)
Bahrain	15.61% (14.41% - 16.73%)	12.98% (11.94% - 14.06%)	10.65% (9.73% - 11.61%)	9.65% (8.73% - 10.58%)	4.04% (3.30% - 4.80%)	3.26% (2.67% - 3.88%)	2.64% (2.14% - 3.15%)	2.36% (1.91% - 2.86%)	0.47% (0.28% - 0.69%)	0.34% (0.18% - 0.52%)	0.24% (0.12% - 0.40%)	0.20% (0.09% - 0.35%)
Egypt	33.92% (32.96% - 34.86%)	28.95% (28.02% - 29.85%)	23.65% (22.70% - 24.66%)	18.29% (17.20% - 19.47%)	17.00% (15.75% - 18.27%)	12.29% (11.29% - 13.30%)	9.14% (8.30% - 10.02%)	6.28% (5.49% - 7.17%)	6.24% (5.20% - 7.34%)	3.53% (2.94% - 4.18%)	2.34% (1.90% - 2.81%)	1.34% (0.99% - 1.74%)
Iran (Islamic Republic of)	26.05% (24.86% - 27.24%)	19.21% (18.40% - 20.02%)	13.97% (13.04% - 14.83%)	11.88% (10.87% - 12.91%)	9.33% (8.22% - 10.44%)	6.62% (6.00% - 7.27%)	4.37% (3.82% - 4.94%)	3.49% (2.89% - 4.10%)	2.50% (1.93% - 3.15%)	1.60% (1.31% - 1.91%)	0.90% (0.69% - 1.16%)	0.64% (0.44% - 0.87%)
Iraq	29.09% (27.99% - 30.10%)	28.41% (27.78% - 29.18%)	24.25% (23.28% - 25.23%)	18.04% (16.90% - 19.20%)	11.39% (10.21% - 12.64%)	11.70% (10.96% - 12.62%)	9.55% (8.68% - 10.50%)	6.10% (5.26% - 6.93%)	2.90% (2.31% - 3.58%)	3.20% (2.81% - 3.70%)	2.50% (2.06% - 2.99%)	1.27% (0.94% - 1.64%)
Jordan	18.71% (17.74% - 19.69%)	13.20% (12.38% - 14.12%)	9.85% (9.11% - 10.63%)	8.84% (8.00% - 9.77%)	4.12% (3.41% - 4.82%)	2.41% (1.95% - 2.89%)	1.65% (1.32% - 1.98%)	1.42% (1.11% - 1.81%)	0.32% (0.16% - 0.52%)	0.08% (0.02% - 0.17%)	0.02% (0.00% - 0.07%)	0.02% (0.00% - 0.07%)
Kuwait	8.66% (8.04% - 9.35%)	5.88% (5.52% - 6.25%)	4.53% (4.26% - 4.83%)	4.13% (3.78% - 4.51%)	1.28% (1.05% - 1.53%)	0.69% (0.58% - 0.81%)	0.44% (0.37% - 0.53%)	0.41% (0.31% - 0.50%)	0.01% (0.00% - 0.03%)	0.00% (0.00% - 0.00%)	0.00% (0.00% - 0.01%)	0.00% (0.00% - 0.01%)
Lebanon	20.92% (19.61% - 22.15%)	16.67% (15.71% - 17.65%)	13.84% (12.83% - 14.73%)	11.84% (10.78% - 12.85%)	6.52% (5.48% - 7.50%)	4.87% (4.17% - 5.55%)	3.49% (2.91% - 4.06%)	2.88% (2.35% - 3.41%)	1.15% (0.79% - 1.55%)	0.75% (0.52% - 1.01%)	0.38% (0.23% - 0.56%)	0.27% (0.14% - 0.43%)
Libya	27.73% (26.34% - 29.16%)	22.59% (21.42% - 23.74%)	20.13% (18.99% - 21.30%)	19.89% (18.61% - 21.30%)	8.98% (7.42% - 10.37%)	7.57% (6.60% - 8.67%)	6.96% (6.01% - 7.86%)	6.74% (5.66% - 7.86%)	1.86% (1.35% - 2.49%)	1.62% (1.23% - 2.06%)	1.54% (1.17% - 1.96%)	1.44% (1.05% - 1.93%)
Morocco	30.99% (29.91% - 32.28%)	26.01% (25.03% - 27.02%)	19.05% (18.09% - 20.04%)	15.00% (13.97% - 16.08%)	12.27% (11.04% - 13.45%)	9.49% (8.50% - 10.50%)	5.46% (4.68% - 6.19%)	4.02% (3.38% - 4.70%)	3.04% (2.25% - 3.90%)	2.14% (1.63% - 2.71%)	0.81% (0.56% - 1.11%)	0.51% (0.31% - 0.75%)
Palestine	15.94% (14.89% - 16.91%)	14.06% (13.32% - 14.80%)	12.52% (11.85% - 13.23%)	8.86% (8.13% - 9.62%)	3.20% (2.54% - 3.87%)	3.02% (2.58% - 3.47%)	2.61% (2.20% - 3.09%)	1.56% (1.24% - 1.88%)	0.20% (0.09% - 0.34%)	0.21% (0.12% - 0.32%)	0.15% (0.07% - 0.27%)	0.03% (0.00% - 0.09%)
Oman	23.01% (22.17% - 23.92%)	14.31% (13.70% - 14.90%)	12.74% (12.17% - 13.33%)	12.62% (11.79% - 13.52%)	6.34% (5.54% - 7.14%)	3.24% (2.85% - 3.65%)	2.82% (2.49% - 3.19%)	3.08% (2.63% - 3.56%)	0.89% (0.61% - 1.21%)	0.27% (0.16% - 0.39%)	0.19% (0.11% - 0.30%)	0.29% (0.16% - 0.45%)
Qatar	14.95% (13.75% - 16.21%)	12.45% (11.41% - 13.54%)	10.15% (9.24% - 11.10%)	8.88% (8.04% - 9.77%)	3.71% (2.96% - 4.49%)	3.04% (2.48% - 3.65%)	2.45% (2.00% - 2.94%)	2.15% (1.74% - 2.62%)	0.38% (0.19% - 0.61%)	0.28% (0.14% - 0.46%)	0.19% (0.08% - 0.34%)	0.16% (0.06% - 0.30%)
Saudi Arabia	24.60% (23.35% - 25.70%)	16.24% (15.28% - 17.27%)	11.32% (10.42% - 12.31%)	9.43% (8.54% - 10.40%)	8.05% (6.93% - 9.23%)	4.44% (3.74% - 5.12%)	2.70% (2.22% - 3.22%)	2.23% (1.79% - 2.70%)	1.55% (1.13% - 2.06%)	0.60% (0.39% - 0.83%)	0.24% (0.12% - 0.39%)	0.18% (0.07% - 0.31%)
Syrian Arab Republic	35.82% (34.57% - 37.04%)	32.23% (31.25% - 33.26%)	28.29% (27.21% - 29.36%)	28.12% (26.72% - 29.53%)	18.31% (16.75% - 20.00%)	16.63% (15.38% - 18.03%)	13.67% (12.49% - 14.73%)	13.57% (12.08% - 15.09%)	6.87% (5.68% - 8.27%)	6.41% (5.50% - 7.47%)	4.85% (4.06% - 5.62%)	4.81% (3.82% - 5.88%)
Tunisia	23.55% (22.68% - 24.46%)	16.20% (15.69% - 16.75%)	10.81% (10.25% - 11.40%)	9.14% (8.50% - 9.81%)	7.99% (7.11% - 8.83%)	4.51% (4.09% - 4.92%)	2.34% (2.04% - 2.67%)	1.85% (1.56% - 2.16%)	1.56% (1.14% - 1.98%)	0.61% (0.47% - 0.76%)	0.13% (0.07% - 0.21%)	0.07% (0.03% - 0.13%)
Turkey	23.88% (22.57% - 25.28%)	18.47% (17.55% - 19.44%)	12.27% (11.40% - 13.15%)	10.11% (9.19% - 11.06%)	6.37% (5.05% - 7.67%)	4.64% (3.84% - 5.46%)	2.86% (2.34% - 3.42%)	2.29% (1.81% - 2.81%)	0.78% (0.35% - 1.35%)	0.50% (0.24% - 0.81%)	0.23% (0.09% - 0.42%)	0.16% (0.03% - 0.32%)
United Arab Emirates	21.54% (20.14% - 22.90%)	18.10% (16.96% - 19.31%)	15.57% (14.44% - 16.76%)	13.91% (12.79% - 15.22%)	6.79% (5.65% - 7.95%)	5.53% (4.67% - 6.47%)	4.56% (3.85% - 5.36%)	3.98% (3.33% - 4.79%)	1.22% (0.81% - 1.71%)	0.94% (0.64% - 1.34%)	0.72% (0.47% - 1.03%)	0.59% (0.37% - 0.87%)
Yemen	52.36% (50.26% - 54.86%)	52.30% (50.61% - 54.17%)	48.14% (46.41% - 50.13%)	43.27% (41.30% - 45.68%)	28.51% (26.35% - 30.69%)	31.00% (29.21% - 32.73%)	23.70% (22.58% - 24.87%)	17.67% (16.23% - 19.26%)	10.49% (8.20% - 13.08%)	13.65% (11.32% - 16.42%)	7.42% (6.15% - 8.86%)	4.10% (3.09% - 5.41%)
Afghanistan	50.86% (49.54% - 52.47%)	52.11% (51.15% - 53.07%)	48.55% (47.37% - 49.88%)	43.45% (42.00% - 45.06%)	31.01% (28.62% - 33.64%)	34.12% (31.50% - 36.60%)	29.33% (27.01% - 31.54%)	23.97% (21.84% - 26.22%)	15.33% (12.18% - 18.81%)	18.87% (15.25% - 22.52%)	14.22% (11.36% - 17.15%)	10.07% (7.99% - 12.58%)

Table S8a. Estimated overall, severe, and extreme **stunting** prevalence (%) in children under 5 years, both sexes, in 1990, 2000, 2010, and 2020.

Location	Total				Severe				Extreme			
	1990	2000	2010	2020	1990	2000	2010	2020	1990	2000	2010	2020
Sudan	41.09% (39.51% - 42.91%)	40.39% (39.06% - 41.95%)	36.07% (34.93% - 37.37%)	31.70% (30.50% - 32.93%)	21.14% (19.39% - 23.03%)	21.57% (19.91% - 23.35%)	17.06% (15.52% - 18.52%)	14.39% (12.90% - 15.92%)	7.75% (6.00% - 9.74%)	8.40% (6.67% - 10.36%)	5.55% (4.18% - 7.01%)	4.50% (3.31% - 5.89%)
South Asia	54.77% (54.15% - 55.34%)	49.82% (49.29% - 50.35%)	43.42% (43.10% - 43.77%)	35.57% (35.20% - 35.94%)	30.49% (29.87% - 31.10%)	27.98% (27.41% - 28.57%)	22.94% (22.56% - 23.34%)	16.81% (16.39% - 17.26%)	12.01% (11.16% - 12.94%)	11.91% (11.00% - 13.08%)	9.00% (8.48% - 9.56%)	5.76% (5.30% - 6.24%)
**South Asia**	54.77% (54.15% - 55.34%)	49.82% (49.29% - 50.35%)	43.42% (43.10% - 43.77%)	35.57% (35.20% - 35.94%)	30.49% (29.87% - 31.10%)	27.98% (27.41% - 28.57%)	22.94% (22.56% - 23.34%)	16.81% (16.39% - 17.26%)	12.01% (11.16% - 12.94%)	11.91% (11.00% - 13.08%)	9.00% (8.48% - 9.56%)	5.76% (5.30% - 6.24%)
Bangladesh	57.07% (54.72% - 59.45%)	46.66% (45.78% - 47.66%)	38.13% (37.17% - 39.25%)	31.71% (30.40% - 32.99%)	27.25% (24.99% - 29.63%)	24.78% (23.07% - 26.65%)	16.01% (14.90% - 17.22%)	11.28% (9.81% - 12.70%)	7.01% (5.19% - 9.44%)	9.31% (7.57% - 11.40%)	4.21% (3.38% - 5.19%)	2.32% (1.55% - 3.17%)
Bhutan	55.68% (53.04% - 58.10%)	45.09% (42.28% - 48.02%)	31.59% (30.11% - 33.57%)	23.72% (22.22% - 25.23%)	22.98% (20.00% - 26.49%)	16.98% (15.12% - 19.19%)	10.52% (9.23% - 11.77%)	7.23% (5.82% - 8.67%)	4.35% (3.00% - 6.36%)	3.19% (2.36% - 4.21%)	1.88% (1.29% - 2.59%)	1.17% (0.63% - 1.83%)
India	54.70% (54.04% - 55.28%)	50.69% (50.09% - 51.25%)	44.33% (44.04% - 44.66%)	35.94% (35.63% - 36.26%)	31.50% (30.83% - 32.12%)	29.31% (28.72% - 30.05%)	24.09% (23.70% - 24.50%)	17.48% (17.09% - 17.93%)	13.20% (12.16% - 14.34%)	13.11% (11.99% - 14.60%)	9.73% (9.15% - 10.31%)	6.09% (5.65% - 6.59%)
Other Union Territories	42.51% (40.76% - 44.38%)	37.45% (36.09% - 38.92%)	29.51% (28.50% - 30.52%)	23.48% (22.51% - 24.45%)	17.28% (15.96% - 18.59%)	14.89% (13.63% - 16.16%)	11.35% (10.25% - 12.36%)	8.48% (7.61% - 9.32%)	4.74% (3.91% - 5.60%)	4.02% (3.26% - 4.85%)	2.93% (2.36% - 3.50%)	2.00% (1.60% - 2.44%)
Andhra Pradesh	50.74% (48.52% - 53.13%)	44.92% (43.48% - 46.58%)	38.70% (38.06% - 39.36%)	30.61% (29.84% - 31.42%)	22.97% (21.40% - 24.90%)	19.40% (18.20% - 20.64%)	17.87% (17.02% - 18.75%)	13.07% (12.11% - 14.08%)	5.92% (4.69% - 7.65%)	4.88% (4.03% - 5.94%)	5.52% (4.82% - 6.31%)	3.76% (3.11% - 4.48%)
Arunachal Pradesh	48.74% (47.53% - 50.17%)	42.86% (41.84% - 43.90%)	38.42% (37.63% - 39.20%)	31.96% (30.94% - 33.02%)	30.87% (28.33% - 33.37%)	25.02% (23.35% - 26.86%)	21.90% (20.62% - 23.25%)	16.21% (14.97% - 17.56%)	15.67% (12.04% - 19.36%)	11.27% (9.28% - 13.57%)	9.68% (8.37% - 11.06%)	6.10% (5.16% - 7.25%)
Assam	53.49% (51.90% - 55.63%)	50.31% (48.92% - 52.05%)	43.06% (42.09% - 44.24%)	33.78% (32.63% - 34.84%)	34.16% (31.73% - 36.75%)	30.55% (28.69% - 32.37%)	21.56% (20.44% - 22.66%)	14.87% (13.75% - 16.06%)	17.26% (13.29% - 21.84%)	14.51% (11.86% - 17.19%)	7.43% (6.38% - 8.60%)	4.41% (3.59% - 5.26%)
Bihar	59.23% (57.15% - 62.02%)	56.33% (54.81% - 58.38%)	51.99% (50.71% - 53.51%)	43.71% (42.65% - 45.01%)	42.40% (39.23% - 45.47%)	38.31% (35.80% - 40.52%)	30.64% (29.30% - 32.03%)	23.34% (21.86% - 24.93%)	25.58% (18.57% - 32.66%)	21.41% (16.67% - 25.68%)	13.13% (11.12% - 15.29%)	8.92% (7.31% - 10.77%)
Chhattisgarh	54.83% (52.38% - 57.33%)	52.57% (50.58% - 54.77%)	46.84% (45.61% - 48.20%)	34.21% (33.36% - 35.16%)	30.30% (27.98% - 32.69%)	29.35% (27.41% - 31.35%)	23.71% (22.63% - 24.83%)	15.06% (13.96% - 16.07%)	11.69% (9.07% - 14.81%)	11.86% (9.52% - 14.63%)	8.24% (7.10% - 9.51%)	4.44% (3.66% - 5.24%)
Delhi	53.55% (51.30% - 55.93%)	49.42% (47.37% - 51.76%)	39.51% (38.40% - 40.73%)	30.95% (29.85% - 32.15%)	29.28% (26.98% - 31.67%)	26.42% (24.53% - 28.54%)	20.22% (18.88% - 21.67%)	14.26% (12.82% - 15.82%)	10.53% (8.13% - 13.09%)	9.56% (7.74% - 11.73%)	7.39% (6.19% - 8.82%)	4.58% (3.55% - 5.77%)
Goa	40.79% (39.52% - 42.36%)	32.04% (31.00% - 33.06%)	24.66% (23.91% - 25.40%)	20.66% (19.90% - 21.48%)	18.45% (17.14% - 19.92%)	12.47% (11.40% - 13.61%)	9.05% (8.37% - 9.79%)	7.20% (6.55% - 7.88%)	5.39% (4.23% - 6.77%)	3.01% (2.41% - 3.74%)	2.05% (1.71% - 2.42%)	1.51% (1.22% - 1.84%)
Gujarat	56.54% (54.70% - 58.48%)	52.11% (50.71% - 53.70%)	45.44% (44.60% - 46.27%)	36.01% (35.25% - 36.79%)	33.44% (31.57% - 35.47%)	28.75% (27.45% - 30.26%)	23.41% (22.53% - 24.34%)	16.84% (15.80% - 17.95%)	13.82% (11.28% - 17.19%)	10.93% (9.52% - 12.55%)	8.46% (7.57% - 9.43%)	5.50% (4.68% - 6.49%)
Haryana	55.92% (53.21% - 58.63%)	51.39% (49.65% - 53.50%)	41.89% (41.24% - 42.64%)	33.04% (32.26% - 33.87%)	29.39% (27.20% - 31.71%)	28.33% (26.60% - 30.10%)	21.66% (20.79% - 22.55%)	15.29% (14.36% - 16.25%)	9.47% (7.19% - 13.13%)	10.98% (8.58% - 13.73%)	7.97% (7.14% - 8.81%)	4.88% (4.20% - 5.62%)
Himachal Pradesh	51.38% (49.36% - 53.90%)	46.31% (45.03% - 47.92%)	39.06% (38.19% - 40.05%)	29.33% (28.32% - 30.34%)	27.26% (24.99% - 29.81%)	24.48% (22.76% - 26.34%)	18.95% (17.87% - 20.04%)	11.92% (10.76% - 13.05%)	10.17% (7.69% - 13.33%)	9.52% (7.47% - 11.80%)	6.50% (5.54% - 7.50%)	3.19% (2.51% - 3.97%)
Jammu & Kashmir and Ladakh	47.60% (45.98% - 49.62%)	43.07% (41.96% - 44.33%)	34.75% (33.93% - 35.56%)	27.16% (26.22% - 28.16%)	25.03% (23.03% - 27.08%)	22.89% (21.33% - 24.50%)	15.86% (14.82% - 17.00%)	11.07% (10.09% - 12.08%)	9.21% (6.80% - 12.11%)	8.92% (7.18% - 10.92%)	4.93% (4.12% - 5.81%)	2.97% (2.41% - 3.60%)
Jharkhand	54.42% (52.32% - 56.85%)	52.40% (50.85% - 54.36%)	48.80% (47.83% - 49.92%)	39.51% (38.58% - 40.42%)	32.21% (29.71% - 34.97%)	31.48% (29.30% - 33.95%)	28.38% (27.04% - 29.88%)	20.50% (19.22% - 21.83%)	13.96% (10.35% - 18.83%)	14.30% (11.02% - 18.46%)	12.34% (10.53% - 14.44%)	7.75% (6.44% - 9.13%)
Karnataka	51.96% (50.42% - 53.95%)	46.50% (45.52% - 47.62%)	39.30% (38.72% - 39.90%)	31.96% (31.18% - 32.75%)	30.25% (28.25% - 32.46%)	25.55% (24.26% - 27.01%)	20.29% (19.46% - 21.17%)	14.51% (13.55% - 15.48%)	13.36% (9.92% - 17.45%)	10.32% (8.58% - 12.25%)	7.54% (6.75% - 8.46%)	4.54% (3.85% - 5.25%)
Kerala	41.65% (39.81% - 44.13%)	34.22% (33.30% - 35.27%)	24.48% (23.84% - 25.11%)	19.43% (18.66% - 20.20%)	16.04% (14.75% - 17.45%)	12.98% (12.02% - 13.93%)	8.53% (7.94% - 9.13%)	6.40% (5.81% - 6.98%)	3.43% (2.58% - 4.54%)	2.93% (2.32% - 3.61%)	1.77% (1.51% - 2.06%)	1.25% (1.02% - 1.51%)
Madhya Pradesh	58.00% (55.61% - 60.32%)	54.59% (53.30% - 56.42%)	47.47% (46.72% - 48.28%)	37.24% (36.46% - 38.05%)	35.93% (33.64% - 38.51%)	35.22% (33.50% - 37.22%)	27.14% (26.13% - 28.23%)	18.56% (17.46% - 19.76%)	16.27% (12.56% - 21.60%)	17.92% (14.65% - 21.73%)	11.47% (10.23% - 12.87%)	6.54% (5.56% - 7.63%)

Table S8a. Estimated overall, severe, and extreme **stunting** prevalence (%) in children under 5 years, both sexes, in 1990, 2000, 2010, and 2020.

Location	Total				Severe				Extreme			
	1990	2000	2010	2020	1990	2000	2010	2020	1990	2000	2010	2020
Maharashtra	53.66% (51.43% - 55.99%)	47.29% (45.74% - 49.01%)	38.36% (37.71% - 39.10%)	30.16% (29.41% - 30.91%)	27.99% (26.19% - 29.87%)	21.45% (20.36% - 22.62%)	16.50% (15.77% - 17.24%)	12.24% (11.37% - 13.11%)	9.23% (7.12% - 12.30%)	5.74% (4.82% - 6.76%)	4.41% (3.84% - 5.04%)	3.19% (2.67% - 3.76%)
Manipur	44.37% (42.60% - 46.58%)	39.85% (38.57% - 41.33%)	35.15% (34.36% - 35.91%)	29.37% (28.44% - 30.22%)	21.11% (19.38% - 22.96%)	18.45% (16.99% - 19.95%)	15.86% (14.94% - 16.87%)	12.04% (10.98% - 12.95%)	6.59% (4.72% - 8.85%)	5.85% (4.50% - 7.41%)	4.83% (4.12% - 5.65%)	3.22% (2.57% - 3.86%)
Meghalaya	56.66% (54.55% - 58.98%)	54.41% (52.24% - 56.78%)	49.86% (48.34% - 51.75%)	43.44% (42.27% - 44.74%)	38.82% (36.23% - 41.56%)	33.89% (31.68% - 36.23%)	30.66% (29.01% - 32.35%)	25.19% (23.49% - 26.97%)	21.45% (16.68% - 27.34%)	15.73% (12.87% - 19.34%)	14.41% (12.10% - 16.98%)	11.13% (9.08% - 13.41%)
Mizoram	47.83% (45.84% - 50.16%)	41.56% (40.48% - 42.89%)	34.89% (34.16% - 35.60%)	28.08% (27.25% - 28.98%)	23.38% (21.52% - 25.35%)	21.48% (19.95% - 23.05%)	17.16% (16.17% - 18.19%)	12.05% (11.12% - 13.08%)	7.57% (5.69% - 10.67%)	8.19% (6.61% - 9.98%)	6.11% (5.21% - 7.03%)	3.54% (2.90% - 4.28%)
Nagaland	41.14% (39.66% - 42.86%)	38.74% (37.55% - 40.03%)	35.26% (34.42% - 36.12%)	29.64% (28.84% - 30.47%)	20.47% (18.62% - 22.33%)	19.65% (18.13% - 21.17%)	17.75% (16.61% - 18.92%)	13.15% (12.18% - 14.17%)	7.19% (5.40% - 9.34%)	7.36% (5.86% - 9.15%)	6.54% (5.49% - 7.69%)	4.07% (3.29% - 4.88%)
Odisha	53.27% (51.32% - 55.76%)	50.80% (49.11% - 52.70%)	43.33% (42.38% - 44.45%)	32.77% (31.88% - 33.64%)	29.84% (27.82% - 32.09%)	26.90% (25.41% - 28.29%)	21.60% (20.58% - 22.64%)	13.87% (12.78% - 14.93%)	11.63% (8.89% - 15.05%)	9.75% (7.92% - 11.60%)	7.41% (6.33% - 8.59%)	3.85% (3.09% - 4.58%)
Punjab	48.01% (46.55% - 49.60%)	44.25% (43.27% - 45.40%)	36.41% (35.81% - 37.00%)	27.98% (27.21% - 28.75%)	24.20% (22.72% - 25.65%)	22.68% (21.40% - 23.98%)	17.31% (16.58% - 18.06%)	11.23% (10.37% - 12.04%)	8.02% (6.45% - 9.77%)	8.02% (6.70% - 9.36%)	5.69% (5.07% - 6.37%)	2.92% (2.43% - 3.44%)
Rajasthan	53.08% (51.96% - 54.59%)	51.10% (50.33% - 51.90%)	43.46% (42.88% - 44.07%)	34.46% (33.61% - 35.26%)	36.27% (34.26% - 38.15%)	35.48% (33.72% - 37.21%)	26.07% (24.89% - 27.27%)	17.35% (16.19% - 18.62%)	20.81% (17.11% - 24.24%)	21.24% (18.17% - 24.00%)	12.41% (11.00% - 13.90%)	6.39% (5.42% - 7.51%)
Sikkim	49.02% (46.87% - 51.59%)	44.46% (42.71% - 46.64%)	35.02% (34.23% - 35.81%)	27.16% (26.30% - 28.02%)	26.13% (23.86% - 28.49%)	22.64% (20.83% - 24.54%)	15.66% (14.75% - 16.57%)	11.20% (10.27% - 12.02%)	9.61% (7.15% - 12.52%)	7.93% (6.07% - 9.94%)	4.75% (4.06% - 5.46%)	3.12% (2.61% - 3.62%)
Tamil Nadu	42.52% (40.72% - 44.69%)	35.36% (34.55% - 36.31%)	28.84% (28.31% - 29.40%)	22.13% (21.44% - 22.87%)	17.11% (15.75% - 18.53%)	14.75% (13.78% - 15.66%)	11.96% (11.35% - 12.58%)	8.20% (7.56% - 8.85%)	3.99% (3.08% - 5.05%)	3.94% (3.26% - 4.65%)	3.29% (2.92% - 3.67%)	1.95% (1.63% - 2.29%)
Telangana	49.55% (47.67% - 51.83%)	44.63% (43.33% - 46.08%)	37.56% (36.79% - 38.37%)	28.12% (27.19% - 28.98%)	22.54% (20.85% - 24.54%)	19.49% (17.90% - 21.08%)	16.06% (14.94% - 17.10%)	11.12% (10.12% - 12.11%)	6.31% (4.80% - 8.39%)	5.37% (4.08% - 6.99%)	4.35% (3.59% - 5.18%)	2.81% (2.24% - 3.44%)
Tripura	49.76% (47.51% - 52.75%)	43.22% (41.92% - 44.85%)	34.99% (34.21% - 35.80%)	28.09% (27.13% - 29.10%)	25.55% (23.30% - 27.97%)	22.22% (20.53% - 23.95%)	15.83% (14.92% - 16.76%)	11.27% (10.32% - 12.31%)	9.07% (6.76% - 12.12%)	8.19% (6.46% - 10.21%)	4.88% (4.18% - 5.63%)	2.96% (2.40% - 3.61%)
Uttar Pradesh	64.63% (61.88% - 66.53%)	58.96% (56.32% - 61.54%)	52.79% (51.72% - 54.18%)	44.25% (43.23% - 45.42%)	41.23% (38.66% - 43.82%)	38.33% (36.05% - 41.42%)	32.64% (31.16% - 34.12%)	24.14% (22.67% - 25.65%)	17.49% (14.27% - 23.53%)	19.23% (14.80% - 26.85%)	15.48% (13.18% - 17.96%)	9.61% (7.95% - 11.39%)
Uttarakhand	50.44% (48.58% - 52.65%)	47.77% (46.44% - 49.40%)	39.92% (39.17% - 40.72%)	30.19% (29.35% - 31.07%)	27.44% (25.22% - 29.82%)	26.96% (25.17% - 28.88%)	19.83% (18.78% - 20.85%)	13.13% (12.23% - 14.10%)	10.51% (7.86% - 13.82%)	11.47% (9.09% - 14.28%)	6.89% (5.95% - 7.91%)	3.87% (3.27% - 4.55%)
West Bengal	50.97% (48.82% - 53.28%)	46.32% (45.00% - 47.95%)	39.10% (38.48% - 39.78%)	30.80% (29.98% - 31.59%)	23.69% (21.95% - 25.66%)	21.86% (20.68% - 23.25%)	18.44% (17.59% - 19.32%)	12.89% (11.93% - 13.87%)	6.54% (5.04% - 8.34%)	6.63% (5.37% - 8.11%)	5.94% (5.20% - 6.70%)	3.56% (2.92% - 4.24%)
Nepal	57.41% (54.77% - 59.80%)	52.35% (49.76% - 54.82%)	40.89% (38.72% - 43.68%)	32.20% (30.41% - 34.42%)	30.75% (27.85% - 34.16%)	25.76% (23.68% - 27.95%)	16.04% (14.49% - 17.81%)	10.53% (9.01% - 11.97%)	9.72% (6.93% - 13.78%)	7.25% (5.49% - 9.35%)	3.36% (2.42% - 4.63%)	1.76% (1.09% - 2.64%)
Pakistan	52.62% (50.93% - 54.54%)	47.38% (45.54% - 49.62%)	42.78% (41.53% - 44.17%)	36.35% (35.02% - 37.77%)	27.40% (25.42% - 29.58%)	23.80% (22.08% - 25.61%)	22.66% (21.29% - 24.07%)	17.56% (16.12% - 19.19%)	9.87% (7.54% - 12.45%)	8.36% (6.51% - 10.67%)	9.19% (7.44% - 11.22%)	6.53% (5.01% - 8.36%)
Southeast Asia, East Asia, and Oceania	34.30% (33.25% - 35.43%)	26.48% (25.72% - 27.23%)	21.21% (20.72% - 21.79%)	16.49% (16.03% - 17.00%)	12.28% (11.29% - 13.33%)	9.56% (9.05% - 10.08%)	7.88% (7.59% - 8.19%)	5.53% (5.28% - 5.81%)	2.89% (2.54% - 3.38%)	2.48% (2.31% - 2.67%)	2.17% (2.07% - 2.28%)	1.29% (1.19% - 1.39%)
**East Asia**	30.00% (28.44% - 31.71%)	18.45% (17.19% - 19.73%)	11.07% (10.17% - 12.08%)	8.63% (7.87% - 9.48%)	8.78% (7.34% - 10.32%)	4.30% (3.45% - 5.19%)	2.20% (1.75% - 2.70%)	1.69% (1.35% - 2.09%)	1.44% (0.94% - 2.16%)	0.49% (0.28% - 0.77%)	0.17% (0.09% - 0.28%)	0.10% (0.05% - 0.19%)
China	30.01% (28.40% - 31.77%)	17.92% (16.60% - 19.25%)	10.67% (9.75% - 11.72%)	8.43% (7.63% - 9.30%)	8.73% (7.25% - 10.32%)	3.96% (3.06% - 4.92%)	2.04% (1.57% - 2.56%)	1.63% (1.28% - 2.04%)	1.42% (0.90% - 2.18%)	0.39% (0.18% - 0.69%)	0.13% (0.05% - 0.26%)	0.09% (0.03% - 0.18%)
Democratic People's Republic of Korea	47.07% (44.50% - 50.11%)	45.43% (43.59% - 47.71%)	30.21% (29.21% - 31.29%)	23.57% (22.25% - 24.79%)	17.60% (15.97% - 19.58%)	17.83% (16.32% - 19.47%)	9.59% (8.41% - 10.81%)	5.79% (4.58% - 6.88%)	3.50% (2.57% - 4.82%)	3.91% (2.99% - 5.14%)	1.62% (1.07% - 2.30%)	0.60% (0.27% - 0.98%)
Taiwan (Province of China)	4.74% (4.27% - 5.21%)	3.87% (3.49% - 4.28%)	3.50% (3.11% - 3.86%)	3.39% (3.03% - 3.77%)	0.41% (0.28% - 0.56%)	0.36% (0.25% - 0.49%)	0.35% (0.24% - 0.46%)	0.35% (0.24% - 0.47%)	0.00% (0.00% - 0.00%)	0.00% (0.00% - 0.00%)	0.00% (0.00% - 0.00%)	0.00% (0.00% - 0.00%)

Table S8a. Estimated overall, severe, and extreme **stunting** prevalence (%) in children under 5 years, both sexes, in 1990, 2000, 2010, and 2020.

Location	Total				Severe				Extreme			
	1990	2000	2010	2020	1990	2000	2010	2020	1990	2000	2010	2020
**Oceania**	36.89% (35.32% - 38.90%)	38.18% (36.74% - 39.89%)	38.39% (37.55% - 39.43%)	37.20% (36.01% - 38.84%)	15.83% (14.50% - 17.32%)	16.87% (15.76% - 18.02%)	18.49% (17.82% - 19.25%)	17.54% (16.28% - 18.90%)	4.37% (3.19% - 5.98%)	4.82% (3.87% - 5.97%)	6.12% (5.38% - 6.95%)	5.62% (4.45% - 7.04%)
Fiji	6.74% (5.89% - 7.60%)	7.22% (6.37% - 8.11%)	7.46% (6.63% - 8.44%)	6.68% (5.87% - 7.63%)	0.83% (0.53% - 1.20%)	1.14% (0.77% - 1.61%)	1.31% (0.90% - 1.80%)	1.13% (0.78% - 1.59%)	0.01% (0.00% - 0.07%)	0.04% (0.00% - 0.13%)	0.06% (0.00% - 0.18%)	0.03% (0.00% - 0.14%)
Kiribati	28.44% (27.20% - 29.63%)	22.57% (21.00% - 23.96%)	17.14% (15.76% - 18.53%)	15.29% (14.15% - 16.48%)	8.50% (7.32% - 9.76%)	5.88% (4.72% - 7.16%)	3.57% (2.65% - 4.53%)	2.96% (2.19% - 3.76%)	1.43% (1.04% - 1.92%)	0.76% (0.43% - 1.21%)	0.24% (0.06% - 0.51%)	0.15% (0.03% - 0.36%)
Marshall Islands	18.30% (16.99% - 19.51%)	17.15% (15.78% - 18.57%)	15.21% (13.88% - 16.54%)	13.56% (12.39% - 14.81%)	2.68% (1.99% - 3.44%)	2.41% (1.75% - 3.20%)	2.51% (1.87% - 3.17%)	2.42% (1.85% - 3.05%)	0.10% (0.04% - 0.18%)	0.08% (0.03% - 0.16%)	0.09% (0.03% - 0.18%)	0.10% (0.04% - 0.20%)
Micronesia (Federated States of)	20.91% (19.35% - 22.43%)	22.01% (20.55% - 23.45%)	21.04% (19.69% - 22.44%)	18.82% (17.52% - 20.09%)	3.96% (2.91% - 5.09%)	5.69% (4.54% - 6.98%)	6.40% (5.22% - 7.65%)	5.54% (4.57% - 6.60%)	0.21% (0.05% - 0.49%)	0.67% (0.31% - 1.12%)	1.05% (0.63% - 1.61%)	0.86% (0.50% - 1.34%)
Papua New Guinea	46.19% (43.91% - 49.09%)	46.10% (44.12% - 48.48%)	45.08% (44.02% - 46.43%)	42.46% (41.00% - 44.53%)	20.91% (19.01% - 23.07%)	21.25% (19.74% - 22.86%)	22.59% (21.72% - 23.61%)	20.58% (19.00% - 22.30%)	5.98% (4.23% - 8.33%)	6.26% (4.96% - 7.85%)	7.70% (6.73% - 8.81%)	6.73% (5.25% - 8.52%)
Samoa	7.29% (6.51% - 8.12%)	6.53% (5.85% - 7.26%)	5.48% (4.89% - 6.09%)	4.84% (4.27% - 5.45%)	0.80% (0.56% - 1.10%)	0.74% (0.53% - 0.98%)	0.55% (0.37% - 0.76%)	0.46% (0.29% - 0.67%)	0.00% (0.00% - 0.00%)	0.00% (0.00% - 0.00%)	0.00% (0.00% - 0.00%)	0.00% (0.00% - 0.00%)
Solomon Islands	32.59% (31.36% - 34.02%)	30.78% (29.77% - 31.80%)	29.80% (28.97% - 30.71%)	26.82% (25.76% - 27.94%)	11.84% (10.42% - 13.16%)	11.54% (10.31% - 12.81%)	11.68% (10.82% - 12.60%)	10.89% (9.77% - 12.08%)	2.59% (1.70% - 3.54%)	2.63% (1.95% - 3.41%)	2.89% (2.35% - 3.52%)	2.96% (2.28% - 3.76%)
Tonga	2.50% (2.24% - 2.80%)	5.07% (4.39% - 5.73%)	8.51% (7.71% - 9.32%)	7.92% (7.17% - 8.75%)	0.00% (0.00% - 0.01%)	0.29% (0.14% - 0.47%)	1.24% (0.97% - 1.54%)	1.21% (0.94% - 1.53%)	0.00% (0.00% - 0.00%)	0.00% (0.00% - 0.00%)	0.00% (0.00% - 0.02%)	0.01% (0.00% - 0.04%)
American Samoa	12.99% (11.82% - 14.18%)	14.42% (13.22% - 15.67%)	14.80% (13.66% - 16.04%)	13.21% (12.08% - 14.33%)	2.63% (2.02% - 3.28%)	3.63% (2.92% - 4.44%)	4.19% (3.43% - 5.03%)	3.61% (2.88% - 4.33%)	0.13% (0.02% - 0.30%)	0.38% (0.17% - 0.65%)	0.60% (0.33% - 0.92%)	0.47% (0.24% - 0.75%)
Vanuatu	26.39% (25.00% - 27.72%)	25.66% (24.39% - 26.86%)	26.10% (24.98% - 27.20%)	25.01% (23.70% - 26.36%)	9.26% (7.80% - 10.63%)	9.16% (7.92% - 10.45%)	9.53% (8.39% - 10.70%)	9.10% (7.77% - 10.53%)	1.94% (1.32% - 2.64%)	1.98% (1.39% - 2.70%)	2.13% (1.53% - 2.77%)	2.04% (1.36% - 2.81%)
Cook Islands	11.16% (10.20% - 12.22%)	11.24% (10.27% - 12.31%)	11.06% (10.03% - 12.10%)	9.68% (8.64% - 10.69%)	2.32% (1.85% - 2.90%)	2.79% (2.26% - 3.40%)	3.04% (2.46% - 3.65%)	2.57% (2.07% - 3.11%)	0.12% (0.02% - 0.27%)	0.27% (0.10% - 0.47%)	0.39% (0.21% - 0.62%)	0.30% (0.14% - 0.50%)
Guam	9.31% (8.44% - 10.28%)	9.89% (8.94% - 10.85%)	10.41% (9.51% - 11.36%)	9.71% (8.87% - 10.65%)	1.88% (1.51% - 2.33%)	2.38% (1.90% - 2.90%)	2.76% (2.25% - 3.30%)	2.51% (2.03% - 3.07%)	0.07% (0.01% - 0.18%)	0.20% (0.06% - 0.36%)	0.32% (0.16% - 0.53%)	0.27% (0.12% - 0.46%)
Nauru	18.60% (17.29% - 19.99%)	22.73% (21.36% - 24.09%)	21.55% (20.29% - 22.76%)	16.34% (15.05% - 17.56%)	4.69% (3.74% - 5.67%)	6.51% (5.30% - 7.77%)	6.83% (5.82% - 7.97%)	4.90% (4.06% - 5.76%)	0.53% (0.26% - 0.88%)	0.96% (0.56% - 1.50%)	1.22% (0.82% - 1.75%)	0.79% (0.48% - 1.15%)
Niue	12.79% (11.56% - 14.04%)	13.70% (12.46% - 14.95%)	13.67% (12.52% - 14.89%)	12.27% (11.16% - 13.35%)	2.69% (2.07% - 3.42%)	3.49% (2.74% - 4.29%)	3.88% (3.15% - 4.69%)	3.36% (2.74% - 4.02%)	0.16% (0.03% - 0.35%)	0.38% (0.15% - 0.66%)	0.55% (0.31% - 0.88%)	0.44% (0.22% - 0.69%)
Northern Mariana Islands	9.23% (8.37% - 10.12%)	9.64% (8.84% - 10.51%)	11.04% (10.04% - 12.03%)	10.11% (9.13% - 11.00%)	1.86% (1.49% - 2.29%)	2.31% (1.89% - 2.77%)	2.99% (2.44% - 3.59%)	2.65% (2.14% - 3.19%)	0.07% (0.00% - 0.17%)	0.19% (0.08% - 0.33%)	0.37% (0.19% - 0.60%)	0.30% (0.13% - 0.49%)
Palau	11.83% (10.73% - 12.92%)	12.67% (11.60% - 13.71%)	14.08% (12.95% - 15.26%)	12.24% (11.15% - 13.37%)	2.47% (1.96% - 3.07%)	3.19% (2.57% - 3.86%)	4.00% (3.26% - 4.79%)	3.33% (2.69% - 4.03%)	0.13% (0.03% - 0.30%)	0.33% (0.15% - 0.57%)	0.58% (0.33% - 0.89%)	0.43% (0.21% - 0.69%)
Tokelau	17.12% (15.69% - 18.53%)	17.34% (15.95% - 18.62%)	16.10% (14.83% - 17.25%)	13.43% (12.16% - 14.63%)	3.48% (2.59% - 4.46%)	4.50% (3.62% - 5.42%)	4.69% (3.88% - 5.59%)	3.75% (3.04% - 4.53%)	0.21% (0.05% - 0.46%)	0.53% (0.25% - 0.88%)	0.72% (0.43% - 1.10%)	0.52% (0.28% - 0.83%)
Tuvalu	14.50% (13.19% - 15.92%)	12.56% (11.22% - 14.08%)	11.54% (10.32% - 12.80%)	10.23% (9.13% - 11.40%)	1.37% (0.93% - 1.99%)	1.39% (0.93% - 2.05%)	1.54% (1.05% - 2.10%)	1.47% (1.04% - 1.96%)	0.01% (0.00% - 0.04%)	0.01% (0.00% - 0.04%)	0.02% (0.00% - 0.05%)	0.02% (0.00% - 0.05%)
**Southeast Asia**	43.38% (42.77% - 44.03%)	37.24% (36.84% - 37.67%)	32.44% (32.17% - 32.75%)	27.31% (26.98% - 27.66%)	19.65% (19.16% - 20.19%)	16.61% (16.27% - 16.96%)	14.13% (13.83% - 14.42%)	10.77% (10.37% - 11.14%)	5.96% (5.58% - 6.38%)	5.16% (4.88% - 5.45%)	4.38% (4.18% - 4.58%)	2.89% (2.68% - 3.12%)
Cambodia	51.83% (49.33% - 54.47%)	48.55% (46.94% - 50.72%)	39.30% (37.83% - 41.02%)	31.83% (30.41% - 33.40%)	29.01% (26.31% - 31.65%)	27.45% (25.52% - 29.32%)	17.69% (16.35% - 19.13%)	12.39% (10.71% - 14.04%)	11.20% (8.26% - 14.61%)	11.11% (8.66% - 13.63%)	5.07% (3.92% - 6.38%)	2.96% (1.97% - 4.04%)
Indonesia	46.99% (46.23% - 47.83%)	41.49% (41.07% - 41.91%)	37.92% (37.71% - 38.13%)	32.76% (32.45% - 33.07%)	22.98% (22.40% - 23.58%)	20.96% (20.50% - 21.40%)	19.51% (19.26% - 19.76%)	14.88% (14.48% - 15.27%)	7.81% (7.27% - 8.39%)	7.69% (7.27% - 8.10%)	7.36% (7.13% - 7.61%)	4.77% (4.50% - 5.05%)

Table S8a. Estimated overall, severe, and extreme **stunting** prevalence (%) in children under 5 years, both sexes, in 1990, 2000, 2010, and 2020.												
**Location**	**Total**				**Severe**				**Extreme**			
	1990	2000	2010	2020	1990	2000	2010	2020	1990	2000	2010	2020
	50.02%	47.17%	43.33%	38.71%	30.57%	28.71%	24.72%	20.26%	14.22%	13.46%	10.52%	7.68%
Aceh	(48.43% - 52.16%)	(45.95% - 48.63%)	(42.41% - 44.28%)	(37.45% - 40.05%)	(28.02% - 33.27%)	(26.53% - 30.86%)	(23.39% - 26.15%)	(18.49% - 22.12%)	(10.92% - 18.07%)	(10.89% - 16.24%)	(9.18% - 12.09%)	(6.14% - 9.46%)
	52.04%	47.29%	42.84%	37.20%	28.39%	27.34%	25.03%	18.85%	10.77%	11.80%	11.23%	6.90%
North Sumatra	(49.38% - 55.14%)	(45.87% - 48.94%)	(42.26% - 43.46%)	(36.06% - 38.35%)	(26.05% - 30.84%)	(25.53% - 29.27%)	(24.07% - 25.98%)	(17.23% - 20.48%)	(8.33% - 13.67%)	(9.71% - 14.17%)	(10.20% - 12.33%)	(5.56% - 8.32%)
	45.47%	41.15%	37.81%	32.41%	21.32%	19.51%	18.00%	13.24%	6.46%	6.17%	5.83%	3.50%
West Sumatra	(43.46% - 47.99%)	(39.69% - 42.79%)	(36.95% - 38.69%)	(31.08% - 33.63%)	(19.42% - 23.49%)	(17.79% - 21.28%)	(16.89% - 19.08%)	(11.79% - 14.78%)	(4.85% - 8.45%)	(4.72% - 7.83%)	(4.84% - 6.79%)	(2.68% - 4.48%)
	40.38%	36.41%	34.15%	31.11%	22.44%	19.54%	18.06%	15.58%	9.44%	7.92%	7.23%	5.81%
Riau	(38.98% - 41.79%)	(35.20% - 37.68%)	(33.35% - 34.98%)	(29.92% - 32.37%)	(20.38% - 24.62%)	(17.97% - 21.13%)	(17.09% - 19.04%)	(14.19% - 17.09%)	(7.59% - 11.71%)	(6.60% - 9.38%)	(6.44% - 8.07%)	(4.74% - 6.97%)
	44.52%	40.45%	36.99%	31.93%	26.05%	23.09%	20.11%	15.84%	11.65%	10.11%	8.26%	5.75%
Jambi	(43.09% - 46.10%)	(39.28% - 41.70%)	(36.29% - 37.68%)	(30.73% - 33.12%)	(23.71% - 28.42%)	(21.40% - 24.85%)	(19.20% - 21.03%)	(14.37% - 17.28%)	(9.08% - 14.30%)	(8.41% - 11.98%)	(7.36% - 9.27%)	(4.65% - 6.88%)
	52.45%	45.49%	39.92%	32.63%	28.48%	26.29%	22.90%	15.44%	10.41%	11.37%	10.08%	5.15%
South Sumatra	(49.84% - 55.74%)	(44.28% - 46.97%)	(39.20% - 40.68%)	(31.29% - 33.84%)	(25.94% - 31.23%)	(24.35% - 28.44%)	(21.81% - 24.05%)	(13.88% - 16.98%)	(7.65% - 13.88%)	(9.26% - 13.95%)	(8.90% - 11.29%)	(4.04% - 6.28%)
	43.96%	40.14%	36.11%	31.47%	26.31%	23.18%	19.94%	16.34%	12.27%	10.35%	8.42%	6.39%
Bengkulu	(42.57% - 45.39%)	(38.91% - 41.37%)	(35.30% - 36.88%)	(30.20% - 32.70%)	(24.07% - 28.69%)	(21.42% - 25.13%)	(18.87% - 21.00%)	(14.81% - 17.81%)	(9.89% - 15.18%)	(8.57% - 12.35%)	(7.38% - 9.50%)	(5.20% - 7.60%)
	50.06%	43.04%	38.48%	32.74%	24.87%	23.15%	21.22%	15.65%	8.15%	9.13%	8.84%	5.36%
Lampung	(47.45% - 53.19%)	(41.64% - 44.66%)	(37.58% - 39.37%)	(31.49% - 34.01%)	(22.55% - 27.41%)	(21.34% - 25.07%)	(19.94% - 22.47%)	(14.15% - 17.13%)	(6.26% - 10.67%)	(7.21% - 11.15%)	(7.59% - 10.13%)	(4.32% - 6.48%)
	42.83%	38.77%	32.94%	27.27%	20.98%	18.89%	14.51%	11.31%	6.96%	6.37%	4.26%	3.17%
Bangka-Belitung Islands	(41.28% - 44.75%)	(37.50% - 40.16%)	(32.06% - 33.83%)	(26.02% - 28.56%)	(19.02% - 23.05%)	(17.32% - 20.54%)	(13.52% - 15.49%)	(9.92% - 12.55%)	(5.30% - 8.97%)	(5.04% - 7.90%)	(3.54% - 5.00%)	(2.40% - 3.96%)
	37.19%	30.76%	28.05%	24.36%	14.42%	11.64%	10.36%	8.55%	3.36%	2.73%	2.36%	1.82%
Riau Islands	(35.32% - 39.47%)	(29.33% - 32.23%)	(27.05% - 29.13%)	(23.07% - 25.72%)	(12.50% - 16.42%)	(10.04% - 13.24%)	(9.22% - 11.49%)	(7.29% - 9.76%)	(2.34% - 4.70%)	(1.93% - 3.68%)	(1.82% - 2.97%)	(1.28% - 2.42%)
	50.92%	45.18%	40.67%	36.65%	28.45%	24.96%	21.28%	18.17%	11.24%	10.05%	7.99%	6.41%
North Kalimantan	(48.82% - 53.57%)	(43.82% - 47.00%)	(39.75% - 41.74%)	(35.42% - 37.87%)	(26.02% - 31.21%)	(23.07% - 26.95%)	(20.06% - 22.37%)	(16.43% - 19.85%)	(8.55% - 14.57%)	(7.98% - 12.39%)	(6.80% - 9.15%)	(5.00% - 7.98%)
	37.16%	30.91%	28.25%	24.55%	15.61%	12.99%	12.45%	9.52%	4.18%	3.60%	3.73%	2.37%
Jakarta	(35.63% - 39.05%)	(29.89% - 32.00%)	(27.66% - 28.90%)	(23.40% - 25.73%)	(13.93% - 17.35%)	(11.74% - 14.26%)	(11.77% - 13.12%)	(8.38% - 10.66%)	(3.05% - 5.43%)	(2.81% - 4.43%)	(3.26% - 4.23%)	(1.79% - 2.99%)
	46.60%	39.52%	36.34%	31.88%	20.07%	17.41%	17.38%	13.35%	5.38%	4.99%	5.76%	3.72%
West Java	(44.07% - 49.62%)	(38.07% - 41.16%)	(35.71% - 36.98%)	(30.86% - 33.02%)	(18.03% - 22.13%)	(16.02% - 18.90%)	(16.61% - 18.19%)	(12.02% - 14.72%)	(3.85% - 7.51%)	(3.96% - 6.19%)	(5.15% - 6.40%)	(2.92% - 4.64%)
	48.36%	41.39%	37.14%	31.18%	20.68%	19.38%	18.59%	13.34%	5.44%	6.15%	6.59%	3.85%
Central Java	(45.44% - 51.63%)	(40.04% - 42.96%)	(36.59% - 37.73%)	(30.05% - 32.28%)	(18.78% - 22.72%)	(18.00% - 20.93%)	(17.99% - 19.25%)	(12.02% - 14.53%)	(4.15% - 7.19%)	(4.98% - 7.49%)	(6.01% - 7.24%)	(3.02% - 4.66%)
	40.13%	32.75%	29.23%	25.52%	16.50%	12.88%	11.29%	9.18%	4.49%	3.21%	2.71%	2.00%
Yogyakarta	(37.88% - 42.97%)	(31.52% - 34.16%)	(28.49% - 30.03%)	(24.38% - 26.69%)	(14.65% - 18.38%)	(11.52% - 14.39%)	(10.44% - 12.16%)	(8.06% - 10.31%)	(3.32% - 5.83%)	(2.40% - 4.20%)	(2.22% - 3.27%)	(1.45% - 2.60%)
	43.94%	39.67%	37.02%	31.63%	21.11%	19.26%	18.94%	13.78%	6.92%	6.55%	6.98%	4.03%
East Java	(42.04% - 46.53%)	(38.36% - 41.18%)	(36.22% - 37.76%)	(30.41% - 32.85%)	(19.12% - 23.10%)	(17.60% - 20.74%)	(17.93% - 19.89%)	(12.35% - 15.28%)	(5.39% - 8.59%)	(5.29% - 7.90%)	(6.13% - 7.89%)	(3.11% - 5.10%)
	45.91%	41.74%	37.52%	31.93%	24.46%	22.58%	19.27%	14.14%	9.26%	9.01%	7.21%	4.34%
Banten	(44.03% - 48.20%)	(40.36% - 43.33%)	(36.69% - 38.42%)	(30.65% - 33.21%)	(22.21% - 26.77%)	(20.64% - 24.52%)	(18.14% - 20.37%)	(12.62% - 15.65%)	(7.05% - 11.86%)	(7.14% - 10.96%)	(6.20% - 8.24%)	(3.33% - 5.43%)
	44.38%	36.51%	32.85%	27.65%	22.34%	16.95%	15.49%	11.14%	7.73%	5.32%	5.09%	2.93%
Bali	(42.68% - 46.29%)	(35.29% - 37.74%)	(32.17% - 33.61%)	(26.47% - 28.88%)	(20.33% - 24.46%)	(15.52% - 18.54%)	(14.72% - 16.35%)	(9.92% - 12.30%)	(5.88% - 10.00%)	(4.21% - 6.60%)	(4.46% - 5.80%)	(2.23% - 3.65%)
	57.01%	52.42%	46.21%	40.47%	35.88%	31.71%	26.45%	20.03%	16.80%	14.30%	11.25%	6.97%
West Nusa Tenggara	(54.90% - 59.25%)	(50.57% - 54.66%)	(45.10% - 47.56%)	(39.21% - 41.86%)	(32.71% - 39.18%)	(29.22% - 34.29%)	(24.90% - 27.97%)	(18.06% - 21.74%)	(12.18% - 21.68%)	(11.00% - 18.02%)	(9.37% - 13.25%)	(5.41% - 8.59%)
	52.40%	49.44%	46.63%	41.94%	32.78%	30.70%	28.30%	24.07%	15.91%	14.89%	13.34%	10.60%
East Nusa Tenggara	(50.52% - 54.90%)	(48.02% - 51.11%)	(45.52% - 47.94%)	(40.68% - 43.29%)	(29.71% - 36.07%)	(28.38% - 33.28%)	(26.62% - 30.14%)	(22.10% - 26.12%)	(11.76% - 20.78%)	(11.72% - 18.42%)	(11.29% - 15.84%)	(8.60% - 12.83%)
	48.54%	44.64%	41.17%	34.90%	28.19%	25.55%	23.07%	17.78%	12.13%	10.96%	9.70%	6.59%
West Kalimantan	(46.71% - 50.73%)	(43.32% - 46.10%)	(40.37% - 41.98%)	(33.68% - 36.16%)	(25.72% - 30.91%)	(23.62% - 27.51%)	(22.07% - 24.14%)	(16.15% - 19.42%)	(9.30% - 15.70%)	(8.87% - 13.28%)	(8.56% - 10.89%)	(5.27% - 8.11%)
	49.16%	45.29%	42.25%	36.70%	28.82%	26.66%	23.61%	18.55%	12.59%	11.98%	9.85%	6.76%
Central Kalimantan	(47.36% - 51.26%)	(44.11% - 46.67%)	(41.38% - 43.12%)	(35.43% - 37.95%)	(26.34% - 31.54%)	(24.79% - 28.56%)	(22.36% - 24.86%)	(16.83% - 20.25%)	(9.62% - 16.30%)	(9.78% - 14.32%)	(8.52% - 11.31%)	(5.38% - 8.35%)
	52.45%	47.75%	44.86%	36.94%	22.96%	20.84%	17.14%	12.49%	5.80%	5.60%	3.77%	2.53%

Table S8a. Estimated overall, severe, and extreme **stunting** prevalence (%) in children under 5 years, both sexes, in 1990, 2000, 2010, and 2020.												
**Location**	**Total**				**Severe**				**Extreme**			
	1990	2000	2010	2020	1990	2000	2010	2020	1990	2000	2010	2020
South Kalimantan	(49.41% - 56.05%)	(45.15% - 50.80%)	(42.45% - 47.86%)	(35.17% - 39.09%)	(20.73% - 25.54%)	(18.74% - 22.92%)	(15.39% - 19.11%)	(10.52% - 14.37%)	(4.32% - 8.08%)	(4.04% - 7.64%)	(2.61% - 5.38%)	(1.54% - 3.63%)
	41.69%	37.85%	32.31%	26.97%	20.55%	18.57%	14.40%	10.81%	6.93%	6.37%	4.32%	2.82%
East Kalimantan	(40.17% - 43.53%)	(36.62% - 39.11%)	(31.46% - 33.11%)	(25.74% - 28.26%)	(18.45% - 22.66%)	(16.95% - 20.24%)	(13.39% - 15.38%)	(9.49% - 12.10%)	(5.21% - 9.05%)	(5.05% - 7.83%)	(3.68% - 5.00%)	(2.12% - 3.59%)
	41.88%	35.90%	33.92%	29.89%	19.34%	15.79%	14.72%	12.46%	5.81%	4.58%	4.22%	3.43%
North Sulawesi	(40.15% - 44.12%)	(34.51% - 37.26%)	(33.00% - 35.02%)	(28.55% - 31.18%)	(17.30% - 21.40%)	(14.15% - 17.38%)	(13.66% - 15.78%)	(11.04% - 13.94%)	(4.33% - 7.57%)	(3.51% - 5.77%)	(3.46% - 4.99%)	(2.59% - 4.47%)
	48.56%	44.94%	41.94%	35.64%	28.81%	26.26%	24.00%	17.89%	12.89%	11.65%	10.40%	6.47%
Central Sulawesi	(46.89% - 50.65%)	(43.70% - 46.34%)	(41.04% - 42.82%)	(34.35% - 37.00%)	(26.29% - 31.14%)	(24.42% - 28.36%)	(22.59% - 25.40%)	(16.26% - 19.63%)	(9.84% - 15.94%)	(9.58% - 14.35%)	(8.98% - 11.96%)	(5.16% - 8.03%)
	50.30%	42.41%	38.55%	32.81%	19.46%	17.69%	17.57%	13.31%	4.04%	4.77%	5.38%	3.51%
South Sulawesi	(46.75% - 53.88%)	(40.39% - 45.22%)	(37.24% - 40.15%)	(31.36% - 34.35%)	(17.16% - 22.41%)	(16.07% - 19.46%)	(16.02% - 19.26%)	(11.60% - 14.94%)	(3.05% - 5.32%)	(3.66% - 6.19%)	(4.19% - 6.79%)	(2.52% - 4.58%)
	46.50%	43.73%	40.05%	35.29%	27.32%	25.48%	22.28%	18.26%	12.20%	11.38%	9.37%	6.98%
Southeast Sulawesi	(44.83% - 48.21%)	(42.48% - 45.10%)	(39.19% - 40.93%)	(34.05% - 36.46%)	(24.93% - 29.79%)	(23.51% - 27.43%)	(21.03% - 23.55%)	(16.70% - 19.94%)	(9.41% - 15.36%)	(9.31% - 13.77%)	(8.13% - 10.75%)	(5.68% - 8.44%)
	46.85%	44.28%	41.97%	34.75%	26.23%	24.97%	23.20%	16.81%	10.69%	10.46%	9.50%	5.76%
Gorontalo	(45.05% - 48.86%)	(42.87% - 45.71%)	(40.93% - 43.11%)	(33.34% - 36.02%)	(23.91% - 28.69%)	(23.00% - 27.13%)	(21.62% - 24.87%)	(15.07% - 18.46%)	(8.14% - 13.72%)	(8.34% - 12.90%)	(7.90% - 11.23%)	(4.47% - 7.11%)
	49.28%	46.80%	44.22%	37.92%	30.70%	29.38%	26.64%	20.60%	14.94%	14.65%	12.52%	8.44%
West Sulawesi	(47.78% - 51.16%)	(45.57% - 48.15%)	(43.27% - 45.27%)	(36.71% - 39.13%)	(28.17% - 33.44%)	(27.43% - 31.68%)	(25.35% - 28.01%)	(18.82% - 22.46%)	(11.68% - 18.81%)	(12.07% - 17.67%)	(10.89% - 14.27%)	(6.73% - 10.31%)
	45.20%	43.25%	42.53%	36.93%	24.86%	24.38%	23.33%	18.66%	9.95%	10.31%	9.42%	6.79%
Maluku	(43.52% - 47.36%)	(41.97% - 44.73%)	(41.59% - 43.60%)	(35.62% - 38.21%)	(22.63% - 27.18%)	(22.54% - 26.32%)	(22.02% - 24.63%)	(16.99% - 20.41%)	(7.62% - 12.71%)	(8.29% - 12.61%)	(8.03% - 10.78%)	(5.43% - 8.37%)
	44.67%	41.35%	39.15%	34.75%	23.92%	22.05%	19.73%	16.22%	9.11%	8.50%	7.00%	5.23%
North Maluku	(42.90% - 46.82%)	(39.99% - 42.87%)	(38.10% - 40.25%)	(33.45% - 36.11%)	(21.63% - 26.10%)	(20.43% - 23.81%)	(18.67% - 20.83%)	(14.73% - 17.74%)	(7.00% - 11.52%)	(6.95% - 10.22%)	(5.95% - 8.03%)	(4.09% - 6.48%)
	45.90%	42.00%	39.13%	33.39%	22.55%	20.60%	19.15%	14.91%	7.36%	6.90%	6.51%	4.52%
West Papua	(43.87% - 48.58%)	(40.39% - 43.92%)	(37.53% - 40.80%)	(31.99% - 34.78%)	(20.35% - 24.82%)	(18.64% - 22.76%)	(17.28% - 21.15%)	(13.16% - 16.65%)	(5.56% - 9.84%)	(5.16% - 9.00%)	(5.03% - 8.29%)	(3.39% - 5.78%)
	43.44%	40.71%	40.45%	36.11%	24.26%	22.57%	22.94%	19.16%	10.08%	9.40%	9.91%	7.58%
Papua	(42.09% - 44.86%)	(39.51% - 41.98%)	(39.47% - 41.37%)	(34.83% - 37.36%)	(22.17% - 26.57%)	(20.71% - 24.63%)	(21.41% - 24.46%)	(17.41% - 21.05%)	(7.85% - 12.72%)	(7.55% - 11.52%)	(8.35% - 11.52%)	(6.05% - 9.32%)
	53.22%	50.37%	43.28%	34.69%	28.14%	25.40%	19.34%	13.92%	9.52%	8.04%	5.25%	3.41%
Lao People's Democratic Republic	(51.00% - 55.58%)	(48.21% - 52.58%)	(41.38% - 45.74%)	(33.16% - 36.46%)	(25.71% - 30.74%)	(23.60% - 27.50%)	(17.88% - 20.96%)	(12.26% - 15.60%)	(7.18% - 12.51%)	(6.41% - 10.04%)	(3.99% - 6.84%)	(2.31% - 4.70%)
	34.11%	23.77%	19.84%	18.28%	12.84%	7.16%	5.76%	5.41%	2.88%	1.16%	0.87%	0.85%
Malaysia	(32.99% - 35.32%)	(22.72% - 24.91%)	(18.85% - 20.85%)	(17.31% - 19.29%)	(11.53% - 14.16%)	(6.06% - 8.24%)	(4.91% - 6.58%)	(4.70% - 6.18%)	(2.14% - 3.69%)	(0.79% - 1.60%)	(0.59% - 1.17%)	(0.62% - 1.15%)
	42.49%	33.83%	21.19%	17.73%	21.64%	15.59%	6.87%	4.81%	7.82%	5.01%	1.33%	0.67%
Maldives	(40.94% - 44.19%)	(32.60% - 35.13%)	(20.08% - 22.33%)	(16.54% - 18.89%)	(19.85% - 23.66%)	(14.13% - 17.14%)	(5.93% - 7.80%)	(4.03% - 5.68%)	(6.28% - 9.86%)	(4.01% - 6.16%)	(0.96% - 1.74%)	(0.42% - 0.96%)
	45.25%	41.14%	33.51%	26.07%	18.32%	16.35%	11.81%	8.26%	3.93%	3.58%	2.25%	1.41%
Myanmar	(42.56% - 48.18%)	(39.19% - 43.40%)	(32.29% - 34.99%)	(24.70% - 27.43%)	(16.18% - 20.49%)	(14.98% - 17.82%)	(10.52% - 13.15%)	(6.85% - 9.59%)	(2.81% - 5.52%)	(2.75% - 4.67%)	(1.58% - 3.09%)	(0.80% - 2.11%)
	39.76%	35.43%	31.50%	26.51%	16.13%	13.88%	12.11%	9.79%	4.40%	3.47%	2.97%	2.35%
Philippines	(38.69% - 40.86%)	(34.45% - 36.55%)	(30.65% - 32.59%)	(25.45% - 27.66%)	(14.97% - 17.44%)	(12.92% - 14.99%)	(11.16% - 13.12%)	(8.51% - 11.03%)	(3.71% - 5.22%)	(2.86% - 4.23%)	(2.29% - 3.71%)	(1.61% - 3.09%)
	45.27%	39.08%	32.74%	27.07%	18.20%	14.83%	12.06%	9.41%	4.61%	3.36%	2.67%	2.00%
Mountain Province	(42.17% - 48.72%)	(36.79% - 42.19%)	(31.16% - 34.82%)	(25.29% - 28.87%)	(15.85% - 20.91%)	(12.73% - 17.01%)	(10.52% - 13.60%)	(7.57% - 11.36%)	(3.40% - 6.15%)	(2.27% - 4.81%)	(1.69% - 3.86%)	(1.09% - 3.13%)
	45.51%	37.68%	32.55%	26.68%	18.21%	14.25%	11.97%	9.34%	4.58%	3.24%	2.65%	2.02%
Ifugao	(42.48% - 49.10%)	(35.68% - 40.33%)	(31.04% - 34.67%)	(24.92% - 28.38%)	(15.98% - 20.91%)	(12.23% - 16.30%)	(10.26% - 13.54%)	(7.47% - 11.13%)	(3.35% - 6.20%)	(2.14% - 4.57%)	(1.63% - 3.76%)	(1.13% - 3.10%)
	42.50%	36.66%	32.48%	27.68%	16.94%	14.01%	12.22%	9.87%	4.37%	3.27%	2.81%	2.18%
Benguet	(39.88% - 45.62%)	(34.61% - 39.12%)	(31.05% - 34.41%)	(25.82% - 29.46%)	(14.64% - 19.28%)	(12.03% - 16.10%)	(10.65% - 13.78%)	(7.94% - 11.70%)	(3.13% - 5.96%)	(2.19% - 4.63%)	(1.74% - 3.95%)	(1.22% - 3.27%)
	41.72%	37.53%	32.74%	27.60%	16.73%	14.47%	12.20%	9.71%	4.37%	3.40%	2.76%	2.10%
Abra	(39.18% - 44.95%)	(35.44% - 40.15%)	(31.14% - 34.75%)	(25.78% - 29.42%)	(14.58% - 18.97%)	(12.49% - 16.56%)	(10.48% - 13.71%)	(7.62% - 11.64%)	(3.21% - 5.80%)	(2.33% - 4.80%)	(1.68% - 3.93%)	(1.10% - 3.19%)
	46.80%	40.69%	33.32%	27.11%	19.10%	15.39%	12.18%	9.46%	4.97%	3.43%	2.65%	2.02%
Apayao	(43.76% - 50.47%)	(38.32% - 43.77%)	(31.71% - 35.43%)	(25.36% - 29.12%)	(16.79% - 21.77%)	(13.40% - 17.56%)	(10.52% - 13.88%)	(7.45% - 11.38%)	(3.76% - 6.59%)	(2.42% - 4.90%)	(1.61% - 3.83%)	(1.07% - 3.13%)
	45.95%	38.39%	32.74%	27.64%	18.23%	14.50%	12.03%	9.67%	4.50%	3.28%	2.65%	2.07%
Kalinga	(42.78% - 49.74%)	(36.20% - 41.09%)	(31.19% - 34.79%)	(25.75% - 29.47%)	(15.88% - 20.86%)	(12.41% - 16.72%)	(10.45% - 13.61%)	(7.56% - 11.59%)	(3.31% - 5.99%)	(2.15% - 4.80%)	(1.68% - 3.78%)	(1.11% - 3.16%)

Table S8a. Estimated overall, severe, and extreme **stunting** prevalence (%) in children under 5 years, both sexes, in 1990, 2000, 2010, and 2020.

Location	Total				Severe				Extreme			
	1990	2000	2010	2020	1990	2000	2010	2020	1990	2000	2010	2020
La Union	35.63% (33.54% - 38.26%)	31.25% (29.53% - 33.11%)	27.79% (26.50% - 29.27%)	23.24% (21.53% - 24.95%)	12.55% (10.44% - 14.68%)	10.59% (8.67% - 12.57%)	9.05% (7.42% - 10.49%)	7.32% (5.54% - 8.97%)	2.85% (1.96% - 3.97%)	2.10% (1.26% - 3.16%)	1.67% (0.90% - 2.53%)	1.34% (0.63% - 2.14%)
Ilocos Norte	33.20% (31.36% - 35.23%)	30.04% (28.34% - 31.89%)	27.27% (25.86% - 28.80%)	24.62% (22.83% - 26.47%)	11.35% (9.31% - 13.34%)	9.87% (7.76% - 11.69%)	8.80% (7.19% - 10.34%)	7.59% (5.65% - 9.40%)	2.52% (1.77% - 3.48%)	1.90% (1.13% - 2.79%)	1.61% (0.89% - 2.45%)	1.33% (0.58% - 2.20%)
Ilocos Sur	36.02% (33.91% - 38.68%)	30.68% (29.02% - 32.45%)	27.82% (26.52% - 29.41%)	22.90% (21.14% - 24.66%)	12.54% (10.22% - 14.72%)	10.32% (8.36% - 12.22%)	9.03% (7.53% - 10.50%)	7.17% (5.56% - 8.88%)	2.80% (1.96% - 3.99%)	2.03% (1.21% - 3.09%)	1.65% (0.94% - 2.52%)	1.30% (0.62% - 2.20%)
Pangasinan	36.49% (34.47% - 39.22%)	32.41% (30.55% - 34.53%)	27.91% (26.57% - 29.45%)	22.64% (20.81% - 24.48%)	12.81% (10.58% - 14.86%)	10.91% (8.98% - 12.79%)	9.11% (7.50% - 10.62%)	7.07% (5.41% - 8.73%)	2.89% (1.97% - 3.96%)	2.13% (1.32% - 3.14%)	1.69% (0.91% - 2.56%)	1.27% (0.62% - 2.09%)
Nueva Vizcaya	36.12% (34.23% - 38.39%)	32.24% (30.52% - 34.08%)	28.71% (27.45% - 30.14%)	24.97% (23.27% - 26.61%)	13.71% (11.48% - 15.88%)	11.86% (9.88% - 13.76%)	10.43% (8.93% - 11.97%)	8.78% (7.00% - 10.43%)	3.43% (2.39% - 4.65%)	2.69% (1.69% - 3.83%)	2.33% (1.46% - 3.40%)	1.92% (1.06% - 2.90%)
Cagayan	38.34% (36.24% - 41.17%)	33.56% (31.91% - 35.43%)	29.19% (27.96% - 30.71%)	24.27% (22.50% - 26.01%)	14.49% (12.39% - 16.76%)	12.51% (10.55% - 14.70%)	10.69% (9.07% - 12.18%)	8.52% (6.63% - 10.23%)	3.58% (2.46% - 5.03%)	2.87% (1.88% - 4.25%)	2.41% (1.46% - 3.42%)	1.87% (1.01% - 2.83%)
Isabela	38.25% (36.18% - 40.97%)	33.73% (32.00% - 35.67%)	29.04% (27.77% - 30.57%)	24.48% (22.83% - 26.16%)	14.46% (12.39% - 16.58%)	12.58% (10.58% - 14.43%)	10.59% (9.03% - 12.06%)	8.57% (6.94% - 10.23%)	3.59% (2.55% - 4.95%)	2.90% (1.89% - 4.17%)	2.37% (1.45% - 3.35%)	1.86% (1.04% - 2.79%)
Quirino	41.29% (38.92% - 44.44%)	34.82% (33.11% - 36.85%)	29.59% (28.20% - 31.12%)	24.27% (22.53% - 25.95%)	16.08% (14.06% - 18.39%)	13.25% (11.27% - 15.40%)	10.89% (9.26% - 12.47%)	8.63% (6.95% - 10.44%)	4.07% (2.94% - 5.45%)	3.10% (2.04% - 4.48%)	2.47% (1.54% - 3.53%)	1.93% (1.10% - 2.97%)
Batanes	39.43% (37.31% - 42.44%)	34.54% (32.64% - 36.70%)	29.02% (27.71% - 30.43%)	26.10% (24.30% - 27.91%)	15.29% (13.19% - 17.49%)	12.88% (10.98% - 14.85%)	10.65% (9.10% - 12.06%)	9.17% (7.21% - 11.12%)	3.96% (2.86% - 5.45%)	2.94% (1.89% - 4.26%)	2.41% (1.53% - 3.36%)	2.00% (1.08% - 3.15%)
Bataan	29.85% (28.02% - 31.62%)	25.25% (23.59% - 26.94%)	22.22% (20.98% - 23.52%)	18.68% (17.09% - 20.24%)	10.29% (8.22% - 12.26%)	8.24% (6.68% - 9.83%)	6.94% (5.70% - 8.20%)	5.56% (4.28% - 6.93%)	2.26% (1.53% - 3.19%)	1.56% (0.94% - 2.39%)	1.22% (0.68% - 1.87%)	0.92% (0.44% - 1.56%)
Zambales	27.97% (26.35% - 29.60%)	24.70% (23.17% - 26.27%)	22.24% (21.04% - 23.52%)	18.03% (16.44% - 19.88%)	9.53% (7.76% - 11.35%)	7.99% (6.38% - 9.56%)	6.94% (5.70% - 8.14%)	5.39% (4.13% - 6.76%)	2.06% (1.39% - 2.93%)	1.50% (0.86% - 2.28%)	1.22% (0.67% - 1.81%)	0.90% (0.42% - 1.50%)
Tarlac	29.67% (27.94% - 31.57%)	25.14% (23.62% - 26.81%)	21.88% (20.63% - 23.09%)	18.60% (16.93% - 20.30%)	9.95% (8.06% - 11.95%)	8.06% (6.53% - 9.64%)	6.70% (5.53% - 7.85%)	5.50% (4.16% - 6.96%)	2.12% (1.41% - 3.03%)	1.49% (0.89% - 2.25%)	1.14% (0.63% - 1.72%)	0.90% (0.41% - 1.53%)
Pampanga	29.27% (27.53% - 31.11%)	24.97% (23.35% - 26.68%)	21.58% (20.39% - 22.87%)	19.23% (17.63% - 20.87%)	9.77% (7.96% - 11.53%)	7.92% (6.38% - 9.52%)	6.59% (5.40% - 7.82%)	5.64% (4.24% - 7.06%)	2.07% (1.42% - 2.90%)	1.44% (0.83% - 2.20%)	1.12% (0.61% - 1.73%)	0.91% (0.40% - 1.55%)
Bulacan	29.04% (27.28% - 30.89%)	24.43% (22.90% - 25.98%)	22.05% (20.84% - 23.31%)	17.80% (16.18% - 19.34%)	9.89% (8.08% - 11.75%)	7.78% (6.36% - 9.35%)	6.84% (5.66% - 8.03%)	5.23% (3.96% - 6.48%)	2.14% (1.48% - 3.02%)	1.42% (0.87% - 2.15%)	1.19% (0.66% - 1.81%)	0.85% (0.37% - 1.41%)
Nueva Ecija	28.94% (27.13% - 30.70%)	25.37% (23.80% - 26.99%)	22.11% (20.90% - 23.31%)	17.40% (15.88% - 18.95%)	9.81% (8.04% - 11.60%)	8.18% (6.67% - 9.80%)	6.85% (5.62% - 8.04%)	5.10% (3.92% - 6.41%)	2.11% (1.44% - 2.95%)	1.52% (0.91% - 2.28%)	1.19% (0.65% - 1.82%)	0.82% (0.38% - 1.41%)
Aurora	31.16% (29.35% - 33.04%)	27.20% (25.61% - 29.00%)	23.03% (21.72% - 24.33%)	18.45% (16.79% - 19.99%)	11.22% (9.06% - 13.29%)	9.23% (7.42% - 10.95%)	7.47% (6.09% - 8.88%)	5.62% (4.18% - 6.97%)	2.60% (1.73% - 3.70%)	1.85% (1.10% - 2.78%)	1.40% (0.76% - 2.18%)	0.97% (0.42% - 1.61%)
Rizal	31.76% (30.12% - 33.53%)	28.49% (26.94% - 30.07%)	26.02% (24.71% - 27.48%)	21.43% (19.78% - 23.08%)	11.84% (10.03% - 13.65%)	10.06% (8.32% - 11.67%)	8.91% (7.46% - 10.27%)	6.98% (5.38% - 8.50%)	2.94% (2.10% - 3.95%)	2.16% (1.37% - 3.09%)	1.81% (1.07% - 2.60%)	1.36% (0.67% - 2.21%)
Cavite	30.78% (28.94% - 32.55%)	28.52% (26.99% - 30.10%)	25.83% (24.61% - 27.23%)	20.98% (19.37% - 22.59%)	11.29% (9.32% - 13.12%)	10.05% (8.37% - 11.77%)	8.81% (7.49% - 10.09%)	6.82% (5.41% - 8.29%)	2.73% (1.90% - 3.74%)	2.15% (1.39% - 3.10%)	1.78% (1.09% - 2.56%)	1.32% (0.72% - 2.08%)
Laguna	31.66% (29.96% - 33.33%)	29.04% (27.46% - 30.53%)	25.66% (24.51% - 27.05%)	20.84% (19.15% - 22.60%)	11.54% (9.73% - 13.46%)	10.20% (8.56% - 11.81%)	8.73% (7.38% - 10.12%)	6.76% (5.27% - 8.20%)	2.78% (1.97% - 3.76%)	2.18% (1.41% - 3.05%)	1.76% (1.07% - 2.59%)	1.31% (0.68% - 2.03%)
Batangas	33.79% (32.00% - 35.86%)	30.59% (28.91% - 32.24%)	25.56% (24.38% - 26.83%)	21.15% (19.60% - 22.79%)	12.29% (10.25% - 14.27%)	10.64% (8.78% - 12.58%)	8.66% (7.27% - 10.06%)	6.86% (5.42% - 8.33%)	2.93% (2.07% - 3.99%)	2.21% (1.38% - 3.27%)	1.74% (1.03% - 2.52%)	1.33% (0.71% - 2.08%)
Quezon	33.57% (31.87% - 35.38%)	29.67% (28.05% - 31.40%)	26.30% (25.00% - 27.61%)	21.49% (19.83% - 23.28%)	12.61% (10.59% - 14.47%)	10.61% (8.81% - 12.33%)	9.17% (7.77% - 10.59%)	7.08% (5.46% - 8.74%)	3.10% (2.14% - 4.21%)	2.30% (1.43% - 3.24%)	1.93% (1.15% - 2.80%)	1.40% (0.72% - 2.22%)
Occidental Mindoro	40.50% (38.55% - 43.13%)	36.43% (34.73% - 38.35%)	33.52% (32.13% - 35.22%)	29.16% (27.38% - 30.98%)	17.55% (15.41% - 19.94%)	15.47% (13.51% - 17.49%)	13.96% (12.47% - 15.58%)	11.60% (9.60% - 13.68%)	5.24% (3.92% - 7.10%)	4.31% (2.95% - 5.85%)	3.79% (2.61% - 5.11%)	3.04% (1.85% - 4.50%)

Table S8a. Estimated overall, severe, and extreme **stunting** prevalence (%) in children under 5 years, both sexes, in 1990, 2000, 2010, and 2020.

Location	Total				Severe				Extreme			
	1990	2000	2010	2020	1990	2000	2010	2020	1990	2000	2010	2020
Oriental Mindoro	42.53%<br>(40.22% - 45.59%)	38.20%<br>(36.51% - 40.48%)	33.79%<br>(32.38% - 35.49%)	28.00%<br>(26.40% - 29.69%)	18.57%<br>(16.31% - 20.93%)	16.31%<br>(14.18% - 18.43%)	14.23%<br>(12.60% - 15.92%)	11.16%<br>(9.35% - 13.11%)	5.56%<br>(4.07% - 7.43%)	4.54%<br>(3.15% - 6.13%)	3.93%<br>(2.73% - 5.33%)	2.96%<br>(1.82% - 4.31%)
Romblon	43.53%<br>(41.27% - 46.56%)	39.01%<br>(37.09% - 41.39%)	33.87%<br>(32.47% - 35.64%)	28.07%<br>(26.41% - 29.84%)	19.13%<br>(16.81% - 21.56%)	16.64%<br>(14.49% - 18.87%)	14.09%<br>(12.45% - 15.72%)	11.14%<br>(9.24% - 13.08%)	5.74%<br>(4.24% - 7.58%)	4.59%<br>(3.16% - 6.23%)	3.82%<br>(2.59% - 5.24%)	2.93%<br>(1.80% - 4.22%)
Palawan	41.62%<br>(39.47% - 44.50%)	37.33%<br>(35.59% - 39.44%)	33.77%<br>(32.41% - 35.45%)	26.90%<br>(25.35% - 28.69%)	18.12%<br>(15.98% - 20.45%)	15.93%<br>(13.87% - 18.19%)	14.03%<br>(12.35% - 15.57%)	10.56%<br>(8.64% - 12.43%)	5.45%<br>(4.06% - 7.16%)	4.45%<br>(3.12% - 6.10%)	3.78%<br>(2.62% - 5.07%)	2.74%<br>(1.66% - 3.96%)
Marinduque	42.93%<br>(40.67% - 45.75%)	37.92%<br>(36.17% - 40.05%)	33.90%<br>(32.47% - 35.74%)	28.70%<br>(26.88% - 30.45%)	18.64%<br>(16.36% - 21.05%)	16.15%<br>(14.04% - 18.35%)	14.09%<br>(12.48% - 15.66%)	11.30%<br>(9.31% - 13.24%)	5.50%<br>(4.02% - 7.40%)	4.47%<br>(3.00% - 6.14%)	3.81%<br>(2.59% - 5.07%)	2.93%<br>(1.70% - 4.25%)
Catanduanes	43.76%<br>(41.05% - 47.10%)	38.30%<br>(36.31% - 41.11%)	34.07%<br>(32.49% - 36.05%)	29.72%<br>(27.96% - 31.63%)	17.69%<br>(15.67% - 20.11%)	15.00%<br>(13.06% - 17.16%)	13.20%<br>(11.44% - 14.93%)	11.04%<br>(9.02% - 13.09%)	4.63%<br>(3.41% - 6.24%)	3.61%<br>(2.50% - 5.01%)	3.16%<br>(1.95% - 4.51%)	2.58%<br>(1.48% - 3.88%)
Camarines Norte	41.68%<br>(39.42% - 44.92%)	36.97%<br>(35.14% - 39.31%)	34.46%<br>(32.83% - 36.57%)	28.05%<br>(26.32% - 29.83%)	16.97%<br>(14.93% - 19.15%)	14.50%<br>(12.54% - 16.60%)	13.22%<br>(11.44% - 14.87%)	10.27%<br>(8.20% - 12.31%)	4.57%<br>(3.41% - 6.09%)	3.53%<br>(2.36% - 4.98%)	3.10%<br>(1.89% - 4.36%)	2.37%<br>(1.32% - 3.60%)
Sorsogon	44.74%<br>(41.93% - 48.43%)	39.22%<br>(37.15% - 41.98%)	34.49%<br>(32.92% - 36.49%)	28.81%<br>(26.99% - 30.63%)	18.33%<br>(16.06% - 20.93%)	15.40%<br>(13.51% - 17.49%)	13.38%<br>(11.64% - 15.10%)	10.68%<br>(8.50% - 12.81%)	4.86%<br>(3.60% - 6.61%)	3.69%<br>(2.52% - 5.21%)	3.19%<br>(2.00% - 4.46%)	2.51%<br>(1.34% - 3.83%)
Albay	43.59%<br>(40.87% - 47.16%)	39.31%<br>(37.13% - 42.38%)	34.38%<br>(32.78% - 36.42%)	27.87%<br>(26.15% - 29.61%)	17.86%<br>(15.59% - 20.32%)	15.70%<br>(13.79% - 17.74%)	13.48%<br>(11.83% - 15.14%)	10.34%<br>(8.32% - 12.32%)	4.79%<br>(3.53% - 6.55%)	3.88%<br>(2.69% - 5.42%)	3.28%<br>(2.14% - 4.56%)	2.44%<br>(1.40% - 3.73%)
Masbate	42.32%<br>(39.99% - 45.48%)	39.19%<br>(37.13% - 42.17%)	34.41%<br>(32.75% - 36.76%)	28.19%<br>(26.44% - 29.92%)	17.36%<br>(15.13% - 19.62%)	15.51%<br>(13.46% - 17.89%)	13.21%<br>(11.64% - 14.87%)	10.33%<br>(8.32% - 12.26%)	4.73%<br>(3.39% - 6.34%)	3.79%<br>(2.56% - 5.49%)	3.10%<br>(2.08% - 4.33%)	2.39%<br>(1.34% - 3.63%)
Camarines Sur	42.60%<br>(40.20% - 45.88%)	38.23%<br>(36.18% - 40.96%)	34.31%<br>(32.74% - 36.53%)	28.22%<br>(26.50% - 29.90%)	17.49%<br>(15.13% - 19.77%)	15.27%<br>(13.25% - 17.39%)	13.34%<br>(11.56% - 15.07%)	10.48%<br>(8.59% - 12.33%)	4.76%<br>(3.40% - 6.37%)	3.80%<br>(2.57% - 5.32%)	3.21%<br>(2.02% - 4.52%)	2.47%<br>(1.41% - 3.74%)
Capiz	46.23%<br>(43.24% - 50.33%)	41.73%<br>(39.33% - 45.06%)	36.41%<br>(34.85% - 38.82%)	30.16%<br>(28.42% - 32.03%)	19.16%<br>(16.64% - 21.75%)	16.80%<br>(14.63% - 19.13%)	14.61%<br>(12.93% - 16.41%)	11.76%<br>(9.75% - 13.90%)	5.21%<br>(3.87% - 6.90%)	4.19%<br>(2.97% - 6.03%)	3.68%<br>(2.34% - 5.15%)	2.99%<br>(1.79% - 4.50%)
Aklan	46.11%<br>(43.01% - 50.08%)	41.84%<br>(39.45% - 45.25%)	36.38%<br>(34.71% - 38.73%)	31.99%<br>(30.07% - 34.01%)	19.19%<br>(16.82% - 21.94%)	16.86%<br>(14.75% - 19.34%)	14.70%<br>(12.96% - 16.51%)	12.46%<br>(10.20% - 14.65%)	5.22%<br>(3.75% - 7.28%)	4.19%<br>(2.91% - 6.08%)	3.74%<br>(2.45% - 5.30%)	3.12%<br>(1.84% - 4.71%)
Antique	45.87%<br>(43.16% - 49.51%)	40.91%<br>(38.82% - 43.76%)	36.63%<br>(34.90% - 39.03%)	30.40%<br>(28.48% - 32.25%)	19.01%<br>(16.71% - 21.56%)	16.57%<br>(14.45% - 18.87%)	14.57%<br>(12.90% - 16.44%)	11.76%<br>(9.78% - 13.77%)	5.16%<br>(3.81% - 6.92%)	4.16%<br>(2.78% - 5.85%)	3.60%<br>(2.36% - 5.21%)	2.94%<br>(1.78% - 4.27%)
Negros Occidental	44.34%<br>(41.81% - 47.79%)	40.18%<br>(38.10% - 42.98%)	36.17%<br>(34.50% - 38.51%)	32.24%<br>(30.50% - 34.31%)	18.16%<br>(15.88% - 20.79%)	16.14%<br>(14.10% - 18.36%)	14.50%<br>(12.82% - 16.30%)	12.54%<br>(10.33% - 14.70%)	4.90%<br>(3.53% - 6.71%)	4.05%<br>(2.74% - 5.67%)	3.65%<br>(2.37% - 5.09%)	3.12%<br>(1.82% - 4.61%)
Iloilo	43.32%<br>(40.70% - 46.78%)	40.31%<br>(38.17% - 43.16%)	36.38%<br>(34.80% - 38.59%)	31.26%<br>(29.45% - 33.26%)	18.24%<br>(16.24% - 20.62%)	16.64%<br>(14.56% - 19.15%)	14.76%<br>(12.87% - 16.69%)	12.24%<br>(10.26% - 14.20%)	5.14%<br>(3.84% - 6.92%)	4.34%<br>(3.00% - 6.33%)	3.78%<br>(2.41% - 5.39%)	3.11%<br>(1.83% - 4.51%)
Guimaras	51.34%<br>(47.50% - 55.38%)	45.02%<br>(42.22% - 48.90%)	36.66%<br>(35.00% - 39.12%)	31.53%<br>(29.69% - 33.48%)	21.57%<br>(19.05% - 24.71%)	17.97%<br>(15.88% - 20.52%)	14.67%<br>(12.84% - 16.51%)	12.24%<br>(10.19% - 14.38%)	5.69%<br>(4.24% - 7.70%)	4.32%<br>(3.07% - 6.27%)	3.67%<br>(2.28% - 5.19%)	3.05%<br>(1.80% - 4.56%)
Negros Oriental	44.63%<br>(41.94% - 48.19%)	39.96%<br>(37.72% - 43.23%)	36.23%<br>(34.37% - 38.62%)	30.21%<br>(28.33% - 32.21%)	18.57%<br>(16.27% - 21.09%)	16.12%<br>(14.04% - 18.31%)	14.34%<br>(12.62% - 16.01%)	11.34%<br>(9.25% - 13.36%)	5.04%<br>(3.66% - 6.78%)	4.03%<br>(2.79% - 5.74%)	3.50%<br>(2.23% - 4.88%)	2.69%<br>(1.55% - 3.98%)
Cebu	45.87%<br>(43.07% - 49.33%)	41.84%<br>(39.41% - 45.33%)	35.98%<br>(34.29% - 38.21%)	30.50%<br>(28.70% - 32.54%)	19.38%<br>(16.96% - 21.94%)	17.00%<br>(14.92% - 19.38%)	14.30%<br>(12.63% - 15.98%)	11.47%<br>(9.28% - 13.58%)	5.29%<br>(3.83% - 7.09%)	4.22%<br>(2.92% - 5.96%)	3.51%<br>(2.30% - 4.87%)	2.72%<br>(1.57% - 4.09%)
Bohol	47.41%<br>(44.32% - 50.86%)	41.78%<br>(39.47% - 45.21%)	36.19%<br>(34.45% - 38.57%)	30.89%<br>(29.11% - 32.97%)	20.42%<br>(17.87% - 23.04%)	17.40%<br>(15.26% - 19.68%)	14.42%<br>(12.72% - 16.15%)	11.77%<br>(9.55% - 13.88%)	5.69%<br>(4.25% - 7.67%)	4.52%<br>(3.15% - 6.24%)	3.57%<br>(2.33% - 5.01%)	2.85%<br>(1.62% - 4.26%)
Siquijor	49.77%<br>(46.42% - 53.55%)	42.46%<br>(40.00% - 45.75%)	36.25%<br>(34.49% - 38.61%)	31.53%<br>(29.66% - 33.40%)	21.13%<br>(18.48% - 24.19%)	16.97%<br>(14.81% - 19.18%)	14.23%<br>(12.39% - 15.97%)	11.84%<br>(9.49% - 13.88%)	5.66%<br>(4.18% - 7.50%)	4.10%<br>(2.82% - 5.71%)	3.42%<br>(2.17% - 4.77%)	2.80%<br>(1.57% - 4.15%)
Southern Leyte	47.66%<br>(44.65% - 51.55%)	42.12%<br>(39.76% - 45.01%)	37.57%<br>(35.76% - 39.88%)	32.86%<br>(30.99% - 34.90%)	20.12%<br>(17.68% - 22.87%)	17.31%<br>(15.07% - 19.70%)	15.42%<br>(13.58% - 17.29%)	13.02%<br>(10.65% - 15.19%)	5.46%<br>(4.00% - 7.39%)	4.41%<br>(2.97% - 6.21%)	3.98%<br>(2.56% - 5.60%)	3.32%<br>(1.95% - 4.91%)
Eastern Samar	49.48%<br>(46.07% - 53.21%)	43.98%<br>(41.44% - 47.46%)	37.74%<br>(35.99% - 40.18%)	31.24%<br>(29.53% - 33.15%)	20.98%<br>(18.30% - 23.83%)	18.09%<br>(15.94% - 20.29%)	15.29%<br>(13.52% - 17.12%)	12.23%<br>(9.86% - 14.36%)	5.64%<br>(4.11% - 7.74%)	4.55%<br>(3.20% - 6.28%)	3.86%<br>(2.50% - 5.41%)	3.10%<br>(1.78% - 4.47%)

Table S8a. Estimated overall, severe, and extreme **stunting** prevalence (%) in children under 5 years, both sexes, in 1990, 2000, 2010, and 2020.

Location	Total				Severe				Extreme			
	1990	2000	2010	2020	1990	2000	2010	2020	1990	2000	2010	2020
Northern Samar	46.73% (43.63% - 50.42%)	42.63% (40.28% - 45.90%)	38.06% (36.17% - 40.75%)	30.61% (28.84% - 32.46%)	19.52% (17.06% - 21.97%)	17.52% (15.40% - 19.72%)	15.46% (13.59% - 17.32%)	11.96% (9.85% - 13.96%)	5.32% (3.88% - 7.09%)	4.46% (3.15% - 6.18%)	3.91% (2.52% - 5.55%)	3.03% (1.82% - 4.41%)
Samar (Western Samar)	47.39% (44.20% - 51.23%)	42.68% (40.30% - 46.10%)	37.59% (35.83% - 40.04%)	31.10% (29.34% - 32.94%)	19.73% (17.30% - 22.45%)	17.52% (15.45% - 19.73%)	15.33% (13.51% - 17.15%)	12.20% (10.01% - 14.27%)	5.31% (3.85% - 7.29%)	4.46% (3.15% - 6.24%)	3.93% (2.57% - 5.54%)	3.11% (1.85% - 4.57%)
Leyte	49.91% (46.34% - 53.61%)	44.70% (41.93% - 48.41%)	38.31% (36.45% - 40.87%)	30.74% (29.05% - 32.48%)	21.34% (18.66% - 24.25%)	18.67% (16.57% - 21.17%)	15.74% (13.90% - 17.58%)	12.27% (10.22% - 14.28%)	5.78% (4.23% - 7.75%)	4.80% (3.38% - 6.84%)	4.06% (2.67% - 5.74%)	3.21% (1.95% - 4.62%)
Biliran	50.15% (46.55% - 53.94%)	43.63% (41.10% - 47.23%)	37.66% (35.93% - 40.26%)	33.75% (32.01% - 35.92%)	21.42% (18.80% - 24.42%)	17.86% (15.57% - 20.28%)	15.38% (13.58% - 17.16%)	13.30% (11.05% - 15.64%)	5.82% (4.25% - 7.89%)	4.47% (3.04% - 6.26%)	3.94% (2.51% - 5.51%)	3.36% (2.01% - 5.08%)
Zamboanga Sibugay	46.96% (44.25% - 50.24%)	44.22% (41.77% - 47.21%)	39.11% (37.29% - 41.68%)	31.73% (30.05% - 33.55%)	21.34% (18.94% - 24.32%)	19.60% (17.44% - 22.29%)	16.99% (15.23% - 18.82%)	13.04% (10.85% - 15.21%)	6.52% (4.82% - 8.98%)	5.54% (3.98% - 7.79%)	4.77% (3.29% - 6.65%)	3.56% (2.18% - 5.33%)
Zamboanga Del Norte	47.16% (44.43% - 50.80%)	43.06% (40.77% - 45.95%)	38.67% (36.90% - 41.14%)	33.17% (31.37% - 35.21%)	21.43% (18.77% - 24.33%)	19.21% (16.97% - 21.53%)	16.96% (15.07% - 18.73%)	13.73% (11.39% - 15.95%)	6.54% (4.78% - 8.95%)	5.54% (3.93% - 7.59%)	4.86% (3.25% - 6.59%)	3.75% (2.21% - 5.43%)
Zamboanga Del Sur	47.28% (44.50% - 50.70%)	42.87% (40.50% - 45.87%)	39.01% (37.26% - 41.45%)	32.67% (30.96% - 34.58%)	21.42% (18.95% - 24.19%)	19.14% (17.03% - 21.51%)	17.17% (15.37% - 19.15%)	13.60% (11.34% - 15.71%)	6.49% (4.81% - 8.82%)	5.54% (3.92% - 7.69%)	4.92% (3.38% - 6.77%)	3.76% (2.26% - 5.34%)
Misamis Occidental	42.06% (39.79% - 45.33%)	37.59% (35.63% - 40.02%)	34.49% (33.00% - 36.37%)	28.78% (27.04% - 30.66%)	17.77% (15.45% - 20.07%)	15.37% (13.28% - 17.69%)	13.78% (12.13% - 15.47%)	10.93% (8.91% - 12.87%)	5.03% (3.66% - 6.69%)	3.98% (2.78% - 5.70%)	3.46% (2.31% - 4.79%)	2.67% (1.54% - 3.91%)
Bukidnon	44.01% (41.57% - 47.39%)	39.36% (37.29% - 42.25%)	34.80% (33.31% - 36.79%)	27.40% (25.66% - 29.17%)	18.47% (16.25% - 20.92%)	16.06% (13.97% - 18.22%)	13.92% (12.18% - 15.56%)	10.34% (8.38% - 12.12%)	5.15% (3.83% - 6.88%)	4.11% (2.80% - 5.68%)	3.49% (2.26% - 4.83%)	2.51% (1.46% - 3.65%)
Lanao Del Norte	41.29% (39.02% - 44.15%)	37.54% (35.67% - 39.98%)	34.25% (32.67% - 36.15%)	29.15% (27.57% - 31.13%)	17.39% (15.23% - 19.66%)	15.46% (13.36% - 17.58%)	13.69% (12.02% - 15.26%)	11.12% (8.97% - 13.20%)	4.95% (3.64% - 6.54%)	4.07% (2.79% - 5.57%)	3.44% (2.26% - 4.71%)	2.73% (1.56% - 4.03%)
Misamis Oriental	43.44% (40.87% - 46.73%)	39.42% (37.37% - 41.99%)	34.15% (32.64% - 35.92%)	29.75% (27.97% - 31.59%)	18.60% (16.34% - 21.22%)	16.34% (14.25% - 18.33%)	13.81% (12.21% - 15.46%)	11.50% (9.38% - 13.65%)	5.32% (3.99% - 7.17%)	4.28% (2.90% - 5.83%)	3.54% (2.38% - 4.83%)	2.87% (1.72% - 4.23%)
Camiguin	46.60% (43.53% - 50.15%)	40.73% (38.56% - 43.58%)	34.76% (33.22% - 36.62%)	29.38% (27.70% - 31.22%)	20.10% (17.56% - 22.75%)	16.66% (14.51% - 19.05%)	13.89% (12.32% - 15.59%)	11.34% (9.31% - 13.25%)	5.75% (4.29% - 7.71%)	4.26% (2.93% - 6.10%)	3.49% (2.30% - 4.80%)	2.83% (1.70% - 4.13%)
Davao Oriental	42.77% (40.12% - 46.22%)	37.57% (35.62% - 40.38%)	33.35% (31.74% - 35.27%)	27.35% (25.61% - 29.12%)	17.20% (14.90% - 19.62%)	14.54% (12.40% - 16.65%)	12.52% (10.86% - 14.17%)	9.86% (8.00% - 11.66%)	4.53% (3.29% - 6.19%)	3.45% (2.30% - 4.93%)	2.86% (1.74% - 4.05%)	2.23% (1.25% - 3.32%)
Davao de Oro	43.48% (40.77% - 46.80%)	38.48% (36.46% - 41.23%)	33.02% (31.54% - 34.73%)	27.03% (25.35% - 28.82%)	17.37% (15.12% - 19.66%)	14.86% (12.98% - 16.87%)	12.53% (10.89% - 14.19%)	9.81% (7.79% - 11.69%)	4.51% (3.29% - 6.05%)	3.50% (2.39% - 4.84%)	2.92% (1.89% - 4.07%)	2.23% (1.22% - 3.42%)
Davao Del Sur	43.29% (40.68% - 46.60%)	38.08% (36.17% - 40.55%)	32.94% (31.40% - 34.65%)	27.16% (25.38% - 28.91%)	17.41% (15.31% - 19.72%)	14.85% (12.83% - 16.93%)	12.54% (10.91% - 14.20%)	9.94% (7.94% - 11.83%)	4.58% (3.37% - 6.10%)	3.56% (2.38% - 4.97%)	2.93% (1.90% - 4.20%)	2.30% (1.28% - 3.43%)
Davao Occidental	40.81% (38.47% - 43.85%)	37.28% (35.25% - 39.90%)	32.88% (31.32% - 34.94%)	27.06% (25.27% - 28.83%)	16.22% (14.05% - 18.35%)	14.25% (12.35% - 16.32%)	12.29% (10.57% - 13.87%)	9.64% (7.82% - 11.50%)	4.26% (3.13% - 5.67%)	3.33% (2.26% - 4.80%)	2.79% (1.69% - 3.96%)	2.14% (1.20% - 3.23%)
Davao Del Norte	44.70% (41.86% - 48.34%)	39.08% (36.94% - 41.86%)	33.25% (31.70% - 35.09%)	28.38% (26.46% - 30.25%)	18.12% (15.73% - 20.86%)	15.33% (13.17% - 17.64%)	12.66% (10.94% - 14.34%)	10.49% (8.48% - 12.61%)	4.72% (3.47% - 6.38%)	3.68% (2.50% - 5.29%)	2.96% (1.86% - 4.13%)	2.44% (1.37% - 3.74%)
South Cotabato	46.72% (43.84% - 50.19%)	42.06% (39.89% - 44.99%)	38.72% (36.93% - 41.31%)	32.37% (30.62% - 34.48%)	20.73% (18.31% - 23.40%)	18.19% (15.89% - 20.48%)	16.50% (14.65% - 18.42%)	13.22% (11.06% - 15.34%)	6.13% (4.57% - 8.22%)	5.04% (3.55% - 6.98%)	4.50% (3.02% - 6.27%)	3.56% (2.17% - 5.18%)
Sultan Kudarat	46.89% (43.96% - 50.38%)	42.77% (40.51% - 46.00%)	38.31% (36.52% - 40.82%)	33.16% (31.38% - 35.35%)	20.46% (18.05% - 23.08%)	18.31% (16.16% - 20.68%)	16.18% (14.40% - 18.05%)	13.37% (11.17% - 15.61%)	5.92% (4.42% - 8.02%)	4.98% (3.56% - 6.91%)	4.37% (2.96% - 6.05%)	3.51% (2.08% - 5.19%)
Cotabato (North Cotabato)	44.66% (41.96% - 48.16%)	41.24% (39.10% - 44.27%)	38.47% (36.63% - 41.32%)	32.81% (30.88% - 34.96%)	19.29% (17.09% - 21.90%)	17.61% (15.47% - 19.76%)	16.28% (14.38% - 18.14%)	13.27% (11.08% - 15.42%)	5.58% (4.16% - 7.61%)	4.80% (3.39% - 6.64%)	4.40% (2.85% - 6.15%)	3.51% (2.11% - 5.11%)
Sarangani	46.28% (43.46% - 49.64%)	42.35% (40.00% - 45.41%)	39.31% (37.38% - 42.25%)	33.53% (31.59% - 35.93%)	20.15% (17.62% - 23.00%)	18.09% (15.81% - 20.54%)	16.68% (14.68% - 18.74%)	13.54% (11.28% - 15.88%)	5.78% (4.07% - 7.93%)	4.89% (3.32% - 6.99%)	4.50% (2.86% - 6.43%)	3.56% (2.05% - 5.36%)
Agusan Del Norte	42.02% (39.52% - 45.38%)	37.82% (35.82% - 40.40%)	34.53% (32.90% - 36.72%)	27.71% (25.96% - 29.47%)	16.53% (14.45% - 18.80%)	14.49% (12.51% - 16.81%)	12.85% (11.21% - 14.65%)	10.16% (8.20% - 12.06%)	4.27% (3.10% - 5.73%)	3.40% (2.28% - 5.02%)	2.87% (1.78% - 4.19%)	2.35% (1.32% - 3.60%)

Table S8a. Estimated overall, severe, and extreme **stunting** prevalence (%) in children under 5 years, both sexes, in 1990, 2000, 2010, and 2020.

Location	Total				Severe				Extreme			
	1990	2000	2010	2020	1990	2000	2010	2020	1990	2000	2010	2020
Agusan Del Sur	41.91% (39.40% - 45.26%)	37.63% (35.61% - 40.33%)	34.06% (32.46% - 36.33%)	29.11% (27.47% - 31.01%)	16.39% (14.20% - 18.60%)	14.34% (12.28% - 16.64%)	12.64% (10.97% - 14.42%)	10.51% (8.26% - 12.49%)	4.22% (3.08% - 5.68%)	3.35% (2.23% - 4.81%)	2.82% (1.73% - 4.13%)	2.36% (1.29% - 3.54%)
Surigao Del Sur	45.32% (42.37% - 49.26%)	40.31% (37.95% - 43.58%)	34.47% (32.76% - 36.58%)	27.83% (26.08% - 29.74%)	17.80% (15.46% - 20.25%)	15.39% (13.28% - 17.59%)	12.90% (11.18% - 14.71%)	10.12% (8.13% - 12.05%)	4.45% (3.24% - 6.18%)	3.53% (2.37% - 4.97%)	2.92% (1.82% - 4.24%)	2.32% (1.31% - 3.51%)
Surigao Del Norte	45.05% (42.00% - 48.80%)	39.92% (37.78% - 43.26%)	34.28% (32.63% - 36.62%)	28.51% (26.76% - 30.35%)	17.56% (15.45% - 20.06%)	15.09% (13.15% - 17.19%)	12.76% (11.02% - 14.38%)	10.35% (8.27% - 12.46%)	4.36% (3.21% - 5.94%)	3.40% (2.30% - 4.84%)	2.85% (1.75% - 4.06%)	2.35% (1.28% - 3.60%)
Dinagat Islands	47.36% (44.13% - 51.40%)	41.32% (38.92% - 44.57%)	34.85% (33.14% - 37.12%)	28.21% (26.44% - 29.99%)	18.69% (16.25% - 21.45%)	15.66% (13.50% - 17.90%)	12.94% (11.24% - 14.76%)	10.19% (8.39% - 12.12%)	4.66% (3.47% - 6.36%)	3.53% (2.42% - 5.08%)	2.88% (1.82% - 4.24%)	2.29% (1.33% - 3.47%)
Tawi-Tawi	44.43% (42.44% - 46.62%)	42.54% (40.88% - 44.45%)	40.34% (38.79% - 42.11%)	34.94% (33.16% - 36.84%)	23.48% (20.66% - 26.66%)	22.09% (19.58% - 24.56%)	20.80% (18.90% - 22.74%)	16.83% (14.48% - 19.20%)	9.18% (6.65% - 12.39%)	8.22% (5.97% - 10.88%)	7.76% (5.79% - 9.91%)	5.83% (3.92% - 7.96%)
Basilan	48.70% (46.24% - 51.57%)	44.57% (42.61% - 47.19%)	40.17% (38.71% - 41.99%)	35.25% (33.69% - 36.95%)	25.65% (22.71% - 28.79%)	23.16% (20.65% - 25.93%)	20.87% (18.87% - 22.84%)	17.07% (14.75% - 19.50%)	9.78% (7.13% - 13.09%)	8.57% (6.21% - 11.54%)	7.90% (5.73% - 10.14%)	5.96% (4.04% - 8.19%)
Sulu	44.49% (42.71% - 46.78%)	42.02% (40.28% - 44.17%)	40.17% (38.66% - 41.88%)	35.33% (33.47% - 37.24%)	23.74% (21.12% - 26.52%)	21.88% (19.44% - 24.52%)	20.78% (19.00% - 22.73%)	17.06% (14.49% - 19.56%)	9.43% (7.11% - 12.20%)	8.21% (5.99% - 10.81%)	7.79% (5.88% - 9.96%)	5.91% (3.90% - 8.22%)
Maguindanao	46.56% (44.50% - 48.87%)	43.26% (41.46% - 45.51%)	40.15% (38.68% - 41.98%)	35.01% (33.25% - 36.87%)	24.81% (22.00% - 27.69%)	22.64% (20.28% - 25.22%)	20.76% (18.93% - 22.80%)	16.83% (14.52% - 19.34%)	9.77% (7.14% - 12.85%)	8.53% (6.17% - 11.30%)	7.79% (5.90% - 10.07%)	5.80% (3.96% - 8.00%)
Lanao Del Sur	46.50% (44.40% - 49.07%)	43.22% (41.45% - 45.41%)	39.85% (38.32% - 41.63%)	36.49% (34.65% - 38.50%)	24.62% (21.73% - 27.48%)	22.48% (20.15% - 25.13%)	20.55% (18.75% - 22.38%)	17.87% (15.58% - 20.35%)	9.60% (7.11% - 12.55%)	8.38% (6.19% - 11.05%)	7.68% (5.76% - 9.75%)	6.30% (4.29% - 8.68%)
National Capital Region	30.27% (28.79% - 31.76%)	27.68% (26.24% - 29.12%)	25.43% (24.25% - 26.70%)	21.29% (19.67% - 22.83%)	10.55% (8.87% - 12.13%)	9.04% (7.57% - 10.54%)	7.99% (6.83% - 9.08%)	6.51% (5.04% - 8.02%)	2.38% (1.71% - 3.12%)	1.70% (1.10% - 2.44%)	1.38% (0.85% - 1.99%)	1.12% (0.55% - 1.84%)
Sri Lanka	30.89% (30.00% - 31.77%)	25.05% (24.28% - 25.78%)	18.50% (17.62% - 19.34%)	15.83% (14.90% - 16.80%)	11.04% (10.05% - 12.00%)	8.47% (7.75% - 9.18%)	5.23% (4.57% - 5.90%)	4.33% (3.70% - 4.98%)	2.37% (1.87% - 2.88%)	1.71% (1.41% - 2.02%)	0.79% (0.57% - 1.04%)	0.61% (0.42% - 0.83%)
Thailand	24.77% (23.81% - 25.75%)	17.91% (16.86% - 18.98%)	14.38% (13.43% - 15.31%)	12.40% (11.43% - 13.29%)	7.65% (6.75% - 8.65%)	4.86% (4.16% - 5.61%)	3.55% (3.04% - 4.08%)	2.79% (2.33% - 3.29%)	1.28% (0.94% - 1.70%)	0.66% (0.45% - 0.91%)	0.37% (0.23% - 0.52%)	0.20% (0.10% - 0.33%)
Mauritius	20.98% (20.12% - 21.84%)	16.07% (15.07% - 17.14%)	16.82% (15.67% - 17.97%)	15.85% (14.61% - 17.07%)	6.84% (6.07% - 7.60%)	4.71% (3.96% - 5.40%)	5.72% (4.95% - 6.53%)	5.30% (4.51% - 6.09%)	1.27% (0.97% - 1.58%)	0.73% (0.50% - 0.97%)	1.28% (0.98% - 1.61%)	1.13% (0.85% - 1.46%)
Seychelles	12.22% (10.86% - 13.65%)	11.61% (10.35% - 12.85%)	10.69% (9.65% - 11.85%)	9.57% (8.60% - 10.58%)	2.91% (2.17% - 3.76%)	2.76% (2.11% - 3.49%)	2.27% (1.72% - 2.90%)	1.88% (1.43% - 2.35%)	0.25% (0.08% - 0.51%)	0.23% (0.07% - 0.44%)	0.12% (0.02% - 0.27%)	0.06% (0.01% - 0.16%)
Timor-Leste	56.13% (53.49% - 58.83%)	54.32% (52.16% - 56.78%)	48.54% (46.91% - 50.36%)	44.69% (43.19% - 46.58%)	33.94% (31.11% - 36.94%)	33.44% (31.11% - 35.76%)	27.07% (25.31% - 28.97%)	23.93% (21.89% - 26.19%)	14.59% (10.79% - 19.22%)	15.15% (12.17% - 19.03%)	10.78% (8.76% - 13.19%)	9.12% (7.17% - 11.78%)
Viet Nam	50.35% (47.73% - 53.31%)	39.88% (38.35% - 41.78%)	29.81% (28.89% - 30.81%)	24.29% (22.90% - 25.54%)	23.54% (21.61% - 25.50%)	16.54% (15.33% - 17.87%)	10.63% (9.59% - 11.72%)	8.27% (7.00% - 9.45%)	6.93% (5.39% - 8.86%)	4.22% (3.37% - 5.31%)	2.26% (1.72% - 2.91%)	1.68% (1.11% - 2.24%)
Sub-Saharan Africa	42.06% (41.67% - 42.49%)	41.56% (41.22% - 41.91%)	37.23% (36.92% - 37.55%)	32.32% (32.00% - 32.63%)	21.64% (21.21% - 22.09%)	21.85% (21.45% - 22.27%)	17.67% (17.36% - 17.98%)	14.18% (13.83% - 14.57%)	8.18% (7.66% - 8.77%)	8.67% (8.17% - 9.22%)	6.05% (5.71% - 6.38%)	4.42% (4.08% - 4.81%)
**Central Sub-Saharan Africa**	41.09% (40.15% - 42.05%)	41.75% (40.98% - 42.63%)	39.56% (38.70% - 40.57%)	35.40% (34.46% - 36.43%)	24.04% (22.78% - 25.27%)	24.72% (23.62% - 25.86%)	20.97% (19.82% - 22.06%)	17.62% (16.30% - 19.02%)	11.11% (9.56% - 12.56%)	11.73% (10.27% - 13.20%)	8.42% (7.08% - 9.83%)	6.50% (5.15% - 7.98%)
Angola	50.73% (48.79% - 53.05%)	47.62% (45.86% - 49.73%)	40.59% (38.76% - 42.75%)	34.57% (33.13% - 36.15%)	28.22% (25.93% - 30.48%)	25.66% (23.89% - 27.59%)	17.59% (15.96% - 19.29%)	14.17% (12.56% - 15.85%)	10.99% (8.24% - 14.12%)	9.61% (7.43% - 11.88%)	4.74% (3.43% - 6.25%)	3.70% (2.58% - 4.99%)
Central African Republic	41.18% (39.98% - 42.40%)	42.13% (41.05% - 43.29%)	39.43% (38.29% - 40.77%)	38.29% (36.83% - 39.91%)	23.48% (21.49% - 25.52%)	24.60% (22.93% - 26.25%)	20.66% (18.99% - 22.26%)	19.33% (17.30% - 21.43%)	10.42% (7.88% - 13.07%)	11.19% (8.91% - 13.43%)	7.90% (5.97% - 9.97%)	7.03% (4.87% - 9.40%)
Congo	25.03% (23.90% - 26.23%)	28.51% (27.46% - 29.57%)	25.42% (24.62% - 26.25%)	21.34% (20.14% - 22.51%)	10.24% (9.08% - 11.44%)	12.43% (11.28% - 13.60%)	9.62% (8.78% - 10.43%)	7.23% (6.15% - 8.13%)	2.79% (2.17% - 3.49%)	3.75% (2.96% - 4.59%)	2.32% (1.82% - 2.82%)	1.44% (0.99% - 1.90%)
Democratic Republic of the Congo	39.53% (38.31% - 40.62%)	40.94% (39.95% - 41.96%)	40.50% (39.45% - 41.80%)	36.66% (35.33% - 38.07%)	24.01% (22.25% - 25.73%)	25.47% (23.86% - 27.06%)	23.23% (21.72% - 24.81%)	19.64% (17.66% - 21.57%)	11.89% (9.91% - 13.75%)	13.12% (11.03% - 15.18%)	10.32% (8.37% - 12.39%)	7.93% (5.97% - 10.12%)

Table S8a. Estimated overall, severe, and extreme **stunting** prevalence (%) in children under 5 years, both sexes, in 1990, 2000, 2010, and 2020.

Location	Total				Severe				Extreme			
	1990	2000	2010	2020	1990	2000	2010	2020	1990	2000	2010	2020
Equatorial Guinea	49.89%<br>(47.14% - 52.69%)	42.45%<br>(40.81% - 44.42%)	27.80%<br>(26.96% - 28.64%)	23.15%<br>(21.86% - 24.42%)	25.08%<br>(22.71% - 27.84%)	21.26%<br>(20.01% - 22.63%)	10.79%<br>(9.83% - 11.79%)	8.43%<br>(7.27% - 9.66%)	7.85%<br>(5.90% - 10.29%)	7.19%<br>(5.94% - 8.59%)	2.73%<br>(2.22% - 3.29%)	1.95%<br>(1.43% - 2.57%)
Gabon	27.20%<br>(25.85% - 28.60%)	25.53%<br>(24.37% - 26.73%)	20.05%<br>(18.91% - 21.19%)	16.26%<br>(15.04% - 17.39%)	8.77%<br>(7.34% - 10.25%)	8.33%<br>(7.19% - 9.45%)	5.79%<br>(4.96% - 6.66%)	4.51%<br>(3.73% - 5.34%)	1.59%<br>(1.00% - 2.31%)	1.56%<br>(1.05% - 2.15%)	0.87%<br>(0.56% - 1.22%)	0.62%<br>(0.36% - 0.95%)
**Eastern Sub-Saharan Africa**	46.08%<br>(45.57% - 46.59%)	44.55%<br>(44.09% - 45.04%)	38.94%<br>(38.55% - 39.36%)	33.70%<br>(33.31% - 34.08%)	23.93%<br>(23.40% - 24.49%)	23.13%<br>(22.68% - 23.61%)	18.33%<br>(17.97% - 18.71%)	14.63%<br>(14.21% - 15.07%)	8.77%<br>(8.15% - 9.43%)	8.67%<br>(8.14% - 9.27%)	6.12%<br>(5.76% - 6.50%)	4.41%<br>(4.06% - 4.77%)
Burundi	47.75%<br>(46.49% - 49.13%)	51.01%<br>(49.56% - 52.69%)	48.78%<br>(46.86% - 51.27%)	46.90%<br>(45.02% - 49.48%)	29.06%<br>(26.96% - 31.22%)	31.78%<br>(29.66% - 34.08%)	25.70%<br>(23.80% - 27.68%)	23.34%<br>(21.13% - 25.59%)	13.76%<br>(11.06% - 16.68%)	15.40%<br>(12.27% - 18.95%)	9.31%<br>(7.24% - 11.82%)	7.72%<br>(5.36% - 10.47%)
Comoros	44.39%<br>(42.83% - 46.21%)	43.38%<br>(41.95% - 45.13%)	35.55%<br>(34.20% - 36.98%)	31.65%<br>(30.22% - 32.99%)	23.18%<br>(21.10% - 25.26%)	23.85%<br>(22.08% - 25.76%)	16.94%<br>(15.23% - 18.54%)	14.18%<br>(12.48% - 15.84%)	8.48%<br>(6.52% - 10.74%)	9.71%<br>(7.85% - 11.77%)	5.68%<br>(4.48% - 6.99%)	4.38%<br>(3.31% - 5.59%)
Djibouti	33.17%<br>(31.93% - 34.45%)	33.60%<br>(32.58% - 34.71%)	32.88%<br>(31.99% - 33.83%)	29.64%<br>(28.44% - 30.87%)	15.35%<br>(13.97% - 16.78%)	16.62%<br>(15.34% - 17.93%)	17.25%<br>(16.08% - 18.44%)	14.63%<br>(13.23% - 16.11%)	4.95%<br>(3.99% - 5.95%)	6.00%<br>(5.02% - 7.08%)	6.93%<br>(5.95% - 7.98%)	5.37%<br>(4.33% - 6.57%)
Eritrea	50.14%<br>(47.96% - 52.37%)	46.25%<br>(44.05% - 48.72%)	42.74%<br>(40.52% - 45.41%)	38.33%<br>(36.70% - 40.39%)	28.63%<br>(26.21% - 31.01%)	23.59%<br>(21.70% - 25.59%)	19.98%<br>(17.79% - 22.36%)	17.95%<br>(15.79% - 20.28%)	11.94%<br>(8.73% - 15.56%)	7.96%<br>(5.90% - 10.43%)	6.01%<br>(3.80% - 8.83%)	5.70%<br>(3.62% - 8.26%)
Ethiopia	50.50%<br>(49.35% - 51.77%)	48.66%<br>(47.65% - 49.83%)	41.71%<br>(40.93% - 42.57%)	35.28%<br>(34.59% - 35.98%)	27.93%<br>(26.65% - 29.24%)	27.50%<br>(26.34% - 28.64%)	21.62%<br>(20.79% - 22.53%)	16.77%<br>(15.94% - 17.61%)	10.82%<br>(9.25% - 12.66%)	11.30%<br>(9.73% - 12.91%)	8.04%<br>(7.09% - 9.12%)	5.62%<br>(4.88% - 6.41%)
Kenya	36.73%<br>(36.08% - 37.43%)	36.41%<br>(35.71% - 37.18%)	33.43%<br>(32.71% - 34.17%)	28.67%<br>(28.02% - 29.36%)	15.80%<br>(15.14% - 16.46%)	15.00%<br>(14.34% - 15.66%)	12.44%<br>(11.82% - 13.07%)	10.09%<br>(9.46% - 10.79%)	4.87%<br>(4.35% - 5.44%)	4.30%<br>(3.82% - 4.82%)	3.05%<br>(2.64% - 3.48%)	2.42%<br>(2.08% - 2.79%)
Baringo	38.98%<br>(36.60% - 42.29%)	37.42%<br>(34.68% - 40.78%)	34.54%<br>(31.66% - 38.38%)	28.46%<br>(25.95% - 31.49%)	15.80%<br>(13.42% - 18.52%)	14.52%<br>(12.09% - 17.13%)	11.16%<br>(8.62% - 13.92%)	8.41%<br>(5.90% - 10.78%)	4.13%<br>(2.48% - 6.38%)	3.45%<br>(2.02% - 5.30%)	2.01%<br>(1.06% - 3.34%)	1.39%<br>(0.63% - 2.52%)
Bomet	44.76%<br>(42.13% - 48.13%)	41.35%<br>(38.81% - 44.73%)	34.89%<br>(32.57% - 37.49%)	30.17%<br>(28.10% - 32.51%)	21.35%<br>(17.62% - 25.25%)	17.70%<br>(14.46% - 21.27%)	13.88%<br>(11.20% - 16.71%)	11.32%<br>(8.85% - 13.73%)	7.95%<br>(4.05% - 12.75%)	5.51%<br>(2.69% - 9.17%)	4.12%<br>(2.00% - 6.72%)	3.03%<br>(1.43% - 4.87%)
Bungoma	42.18%<br>(40.15% - 44.52%)	39.63%<br>(37.78% - 41.75%)	33.97%<br>(32.00% - 36.26%)	28.08%<br>(26.33% - 29.97%)	21.16%<br>(18.43% - 24.23%)	18.58%<br>(16.52% - 20.78%)	13.59%<br>(11.54% - 15.76%)	9.72%<br>(7.50% - 11.77%)	7.54%<br>(5.11% - 10.81%)	5.93%<br>(4.06% - 8.14%)	3.58%<br>(2.01% - 5.40%)	2.14%<br>(1.01% - 3.48%)
Busia	40.73%<br>(38.41% - 44.04%)	38.28%<br>(35.74% - 41.77%)	34.66%<br>(31.59% - 38.44%)	27.16%<br>(24.56% - 29.88%)	17.57%<br>(14.64% - 20.56%)	14.64%<br>(11.86% - 17.52%)	11.52%<br>(8.75% - 14.16%)	7.54%<br>(5.18% - 9.89%)	5.72%<br>(2.87% - 9.33%)	3.80%<br>(1.78% - 6.65%)	2.18%<br>(1.00% - 3.94%)	1.14%<br>(0.37% - 2.31%)
Elgeyo-Marakwet	41.74%<br>(38.72% - 45.46%)	40.95%<br>(37.51% - 45.21%)	38.28%<br>(33.19% - 44.10%)	29.29%<br>(26.51% - 32.49%)	15.09%<br>(12.58% - 17.86%)	12.62%<br>(10.09% - 15.10%)	8.84%<br>(6.14% - 11.66%)	6.59%<br>(3.86% - 9.21%)	3.18%<br>(1.73% - 5.05%)	2.12%<br>(1.06% - 3.80%)	0.93%<br>(0.38% - 1.81%)	0.76%<br>(0.22% - 1.62%)
Embu	22.48%<br>(20.20% - 24.65%)	21.21%<br>(19.12% - 23.33%)	22.90%<br>(20.63% - 25.07%)	20.30%<br>(18.19% - 22.26%)	5.64%<br>(3.77% - 7.43%)	5.42%<br>(3.83% - 7.13%)	5.80%<br>(3.80% - 7.68%)	5.03%<br>(3.29% - 6.62%)	0.88%<br>(0.37% - 1.58%)	0.84%<br>(0.33% - 1.51%)	0.80%<br>(0.27% - 1.56%)	0.66%<br>(0.16% - 1.28%)
Garissa	44.03%<br>(40.89% - 47.56%)	43.97%<br>(40.39% - 47.74%)	39.71%<br>(35.44% - 44.07%)	36.67%<br>(33.70% - 40.13%)	17.31%<br>(14.56% - 20.20%)	16.49%<br>(13.81% - 19.55%)	12.45%<br>(9.44% - 15.73%)	12.41%<br>(9.88% - 14.78%)	3.93%<br>(2.23% - 6.34%)	3.38%<br>(2.01% - 5.50%)	2.03%<br>(1.06% - 3.58%)	2.43%<br>(1.25% - 4.05%)
HomaBay	40.50%<br>(38.40% - 43.02%)	37.11%<br>(34.95% - 39.59%)	30.38%<br>(28.45% - 32.36%)	24.36%<br>(22.42% - 26.33%)	19.04%<br>(16.16% - 21.99%)	16.74%<br>(14.17% - 19.31%)	11.71%<br>(9.36% - 14.09%)	8.06%<br>(6.01% - 10.08%)	6.63%<br>(4.00% - 9.70%)	5.68%<br>(3.34% - 8.43%)	3.40%<br>(1.71% - 5.39%)	1.73%<br>(0.72% - 2.98%)
Isiolo	18.20%<br>(15.92% - 20.40%)	17.39%<br>(15.25% - 19.54%)	18.62%<br>(16.49% - 20.82%)	16.33%<br>(14.46% - 18.17%)	4.51%<br>(2.85% - 6.09%)	3.91%<br>(2.50% - 5.32%)	4.42%<br>(2.74% - 6.22%)	4.19%<br>(2.89% - 5.63%)	0.69%<br>(0.20% - 1.35%)	0.50%<br>(0.13% - 1.06%)	0.55%<br>(0.12% - 1.19%)	0.57%<br>(0.18% - 1.15%)
Kajiado	29.96%<br>(28.12% - 31.63%)	28.93%<br>(26.96% - 30.60%)	24.49%<br>(22.54% - 26.37%)	20.10%<br>(18.19% - 22.03%)	14.27%<br>(11.86% - 16.67%)	13.26%<br>(10.97% - 15.51%)	9.24%<br>(6.97% - 11.33%)	6.87%<br>(5.09% - 8.70%)	5.42%<br>(3.37% - 7.78%)	4.70%<br>(2.87% - 6.76%)	2.33%<br>(1.13% - 3.68%)	1.48%<br>(0.70% - 2.48%)
Kakamega	36.44%<br>(34.57% - 38.82%)	39.06%<br>(36.64% - 42.08%)	38.22%<br>(36.00% - 40.94%)	33.70%<br>(31.76% - 35.70%)	15.12%<br>(12.92% - 17.27%)	15.50%<br>(13.22% - 17.97%)	15.52%<br>(13.53% - 17.72%)	13.50%<br>(11.15% - 15.68%)	4.21%<br>(2.86% - 5.85%)	4.06%<br>(2.76% - 5.78%)	4.07%<br>(2.84% - 5.78%)	3.58%<br>(2.25% - 5.15%)
Kericho	43.22%<br>(40.70% - 46.39%)	42.41%<br>(39.63% - 45.73%)	39.58%<br>(36.71% - 43.35%)	31.88%<br>(29.43% - 34.48%)	21.00%<br>(18.56% - 23.52%)	18.97%<br>(16.57% - 21.44%)	15.15%<br>(12.74% - 17.79%)	10.83%<br>(8.29% - 13.32%)	7.33%<br>(5.44% - 9.62%)	5.62%<br>(3.97% - 7.73%)	3.49%<br>(2.09% - 5.37%)	2.18%<br>(1.06% - 3.58%)
Kiambu	37.00%<br>(35.37% - 38.78%)	35.07%<br>(33.45% - 36.69%)	29.92%<br>(28.29% - 31.52%)	24.70%<br>(23.13% - 26.26%)	17.47%<br>(15.13% - 19.96%)	15.78%<br>(13.62% - 17.86%)	12.29%<br>(10.29% - 14.39%)	9.31%<br>(7.69% - 10.92%)	5.82%<br>(3.89% - 8.19%)	4.96%<br>(3.34% - 6.84%)	3.48%<br>(2.25% - 5.12%)	2.36%<br>(1.44% - 3.41%)

Table S8a. Estimated overall, severe, and extreme **stunting** prevalence (%) in children under 5 years, both sexes, in 1990, 2000, 2010, and 2020.

Location	Total				Severe				Extreme			
	1990	2000	2010	2020	1990	2000	2010	2020	1990	2000	2010	2020
Kilifi	44.69%<br>(42.61% - 47.96%)	43.21%<br>(41.02% - 46.40%)	36.80%<br>(34.58% - 39.81%)	32.07%<br>(30.27% - 34.10%)	22.04%<br>(19.57% - 24.92%)	20.14%<br>(17.95% - 22.81%)	14.39%<br>(12.23% - 16.71%)	12.59%<br>(10.45% - 14.79%)	8.10%<br>(5.65% - 11.01%)	6.81%<br>(4.69% - 9.53%)	3.82%<br>(2.47% - 5.53%)	3.31%<br>(2.03% - 4.82%)
Kirinyaga	21.78%<br>(19.02% - 24.31%)	19.82%<br>(17.56% - 22.02%)	17.82%<br>(15.63% - 20.19%)	16.16%<br>(14.09% - 18.44%)	4.45%<br>(2.47% - 6.41%)	3.71%<br>(2.00% - 5.38%)	3.32%<br>(1.88% - 4.95%)	3.11%<br>(1.70% - 4.60%)	0.40%<br>(0.10% - 0.92%)	0.27%<br>(0.03% - 0.67%)	0.25%<br>(0.03% - 0.67%)	0.24%<br>(0.02% - 0.67%)
Kisii	36.54%<br>(34.38% - 39.51%)	37.48%<br>(35.00% - 40.46%)	34.09%<br>(32.14% - 36.28%)	29.45%<br>(27.43% - 31.44%)	14.13%<br>(11.86% - 16.44%)	13.75%<br>(11.54% - 16.05%)	11.64%<br>(9.43% - 13.96%)	9.61%<br>(7.39% - 11.66%)	3.47%<br>(2.10% - 5.15%)	3.02%<br>(1.79% - 4.53%)	2.45%<br>(1.26% - 3.89%)	1.97%<br>(0.93% - 3.24%)
Kisumu	36.09%<br>(34.37% - 37.94%)	37.32%<br>(35.30% - 39.27%)	31.92%<br>(29.61% - 34.49%)	24.97%<br>(22.84% - 27.18%)	17.97%<br>(15.54% - 20.25%)	18.83%<br>(16.36% - 21.20%)	12.62%<br>(10.46% - 14.72%)	8.53%<br>(6.44% - 10.53%)	6.65%<br>(4.65% - 8.77%)	7.07%<br>(4.89% - 9.39%)	3.32%<br>(2.15% - 4.68%)	1.86%<br>(1.03% - 2.82%)
Kitui	44.54%<br>(42.32% - 47.60%)	42.82%<br>(40.59% - 45.92%)	39.17%<br>(37.02% - 41.81%)	35.69%<br>(33.79% - 37.73%)	22.24%<br>(19.01% - 25.47%)	20.14%<br>(17.45% - 23.15%)	16.71%<br>(14.17% - 19.28%)	15.05%<br>(12.45% - 17.44%)	8.36%<br>(4.94% - 12.38%)	6.85%<br>(4.29% - 10.28%)	4.99%<br>(2.78% - 7.76%)	4.44%<br>(2.53% - 6.73%)
Kwale	38.64%<br>(36.38% - 41.47%)	38.22%<br>(35.85% - 41.04%)	34.04%<br>(31.91% - 36.42%)	28.39%<br>(26.49% - 30.47%)	15.55%<br>(12.78% - 18.34%)	15.82%<br>(13.20% - 18.65%)	13.16%<br>(10.71% - 15.63%)	10.04%<br>(7.84% - 12.28%)	4.15%<br>(2.07% - 6.65%)	4.38%<br>(2.35% - 7.01%)	3.44%<br>(1.86% - 5.45%)	2.33%<br>(1.18% - 3.79%)
Laikipia	28.65%<br>(26.15% - 31.20%)	27.59%<br>(25.06% - 30.26%)	27.77%<br>(25.25% - 30.74%)	23.65%<br>(21.44% - 25.93%)	9.06%<br>(6.63% - 11.40%)	8.13%<br>(6.15% - 10.33%)	7.95%<br>(5.75% - 10.12%)	6.85%<br>(4.85% - 9.00%)	2.14%<br>(1.09% - 3.58%)	1.74%<br>(0.85% - 2.97%)	1.38%<br>(0.57% - 2.53%)	1.21%<br>(0.41% - 2.25%)
Lamu	19.57%<br>(17.18% - 21.95%)	19.91%<br>(17.47% - 22.60%)	23.33%<br>(20.87% - 25.82%)	21.61%<br>(19.40% - 23.84%)	3.55%<br>(2.04% - 5.19%)	3.35%<br>(1.79% - 5.08%)	5.52%<br>(3.57% - 7.51%)	5.83%<br>(4.01% - 7.78%)	0.37%<br>(0.14% - 0.79%)	0.33%<br>(0.11% - 0.71%)	0.83%<br>(0.22% - 1.68%)	1.02%<br>(0.30% - 1.95%)
Machakos	40.88%<br>(38.95% - 43.62%)	40.36%<br>(38.24% - 43.35%)	34.65%<br>(32.25% - 37.87%)	29.64%<br>(27.64% - 31.82%)	19.90%<br>(17.43% - 22.79%)	18.76%<br>(16.30% - 21.51%)	13.05%<br>(10.71% - 15.36%)	10.79%<br>(8.41% - 12.99%)	6.92%<br>(4.67% - 9.90%)	5.97%<br>(3.83% - 8.84%)	3.03%<br>(1.70% - 4.74%)	2.43%<br>(1.30% - 3.77%)
Makueni	37.49%<br>(35.17% - 40.28%)	38.58%<br>(35.98% - 41.25%)	35.46%<br>(33.02% - 38.28%)	28.72%<br>(26.34% - 31.01%)	13.88%<br>(11.34% - 16.64%)	14.28%<br>(11.47% - 17.00%)	12.69%<br>(10.18% - 15.20%)	9.20%<br>(6.96% - 11.43%)	3.64%<br>(1.91% - 5.73%)	3.43%<br>(1.74% - 5.52%)	2.84%<br>(1.43% - 4.79%)	1.81%<br>(0.78% - 3.05%)
Mandera	45.96%<br>(44.37% - 47.45%)	46.02%<br>(44.39% - 47.83%)	45.34%<br>(43.41% - 47.98%)	47.04%<br>(45.22% - 49.26%)	29.53%<br>(26.32% - 32.57%)	28.77%<br>(25.43% - 32.32%)	24.25%<br>(21.58% - 27.11%)	29.21%<br>(26.09% - 32.53%)	15.83%<br>(11.98% - 19.48%)	14.74%<br>(10.96% - 19.11%)	9.65%<br>(6.78% - 12.89%)	14.71%<br>(10.76% - 19.03%)
Marsabit	21.39%<br>(19.17% - 23.53%)	20.57%<br>(18.24% - 22.74%)	23.24%<br>(21.05% - 25.54%)	20.75%<br>(18.62% - 22.69%)	5.50%<br>(3.58% - 7.34%)	4.88%<br>(3.17% - 6.69%)	6.05%<br>(3.89% - 8.13%)	4.75%<br>(2.92% - 6.51%)	0.78%<br>(0.23% - 1.54%)	0.61%<br>(0.16% - 1.32%)	0.85%<br>(0.20% - 1.78%)	0.50%<br>(0.10% - 1.14%)
Meru	34.37%<br>(32.13% - 36.78%)	35.32%<br>(33.07% - 37.98%)	33.24%<br>(30.90% - 36.06%)	28.81%<br>(26.57% - 31.04%)	13.77%<br>(11.17% - 15.94%)	13.77%<br>(11.56% - 16.06%)	11.65%<br>(9.15% - 14.06%)	9.71%<br>(7.41% - 11.91%)	3.65%<br>(2.42% - 5.16%)	3.43%<br>(2.27% - 4.88%)	2.45%<br>(1.38% - 3.91%)	1.99%<br>(1.03% - 3.22%)
Migori	43.37%<br>(40.16% - 46.98%)	38.27%<br>(35.52% - 41.89%)	36.59%<br>(33.83% - 39.72%)	29.79%<br>(27.09% - 32.14%)	18.61%<br>(15.70% - 21.66%)	15.79%<br>(13.28% - 18.55%)	14.11%<br>(11.77% - 16.79%)	10.53%<br>(7.70% - 12.83%)	5.54%<br>(3.34% - 8.42%)	4.64%<br>(2.68% - 6.97%)	3.35%<br>(1.92% - 5.31%)	2.49%<br>(1.24% - 3.99%)
Mombasa	26.78%<br>(24.80% - 28.70%)	25.76%<br>(23.87% - 27.76%)	25.33%<br>(23.39% - 27.29%)	21.22%<br>(19.36% - 23.08%)	10.11%<br>(7.83% - 12.08%)	9.11%<br>(6.93% - 11.15%)	8.55%<br>(6.52% - 10.60%)	6.61%<br>(4.89% - 8.32%)	2.50%<br>(1.34% - 3.80%)	2.02%<br>(1.04% - 3.26%)	1.75%<br>(0.83% - 2.93%)	1.23%<br>(0.51% - 2.10%)
Murang'a	33.75%<br>(31.95% - 35.62%)	33.94%<br>(32.11% - 35.93%)	28.15%<br>(26.06% - 30.09%)	24.17%<br>(22.00% - 26.24%)	15.23%<br>(12.84% - 17.37%)	15.18%<br>(12.75% - 17.50%)	10.27%<br>(8.24% - 12.48%)	7.87%<br>(5.93% - 9.95%)	4.87%<br>(3.17% - 6.70%)	4.83%<br>(2.96% - 6.94%)	2.46%<br>(1.32% - 3.91%)	1.61%<br>(0.74% - 2.81%)
Nairobi	24.42%<br>(23.07% - 25.77%)	24.32%<br>(22.89% - 25.65%)	21.88%<br>(20.50% - 23.24%)	19.78%<br>(18.29% - 21.16%)	9.79%<br>(8.48% - 11.20%)	9.33%<br>(8.01% - 10.82%)	7.63%<br>(6.38% - 8.95%)	6.65%<br>(5.39% - 7.86%)	2.66%<br>(1.90% - 3.49%)	2.35%<br>(1.63% - 3.22%)	1.65%<br>(1.07% - 2.34%)	1.36%<br>(0.83% - 2.02%)
Nakuru	38.56%<br>(36.55% - 41.00%)	40.84%<br>(38.49% - 44.12%)	37.21%<br>(34.70% - 40.23%)	30.12%<br>(28.10% - 32.36%)	17.66%<br>(15.42% - 20.32%)	18.06%<br>(15.62% - 20.42%)	14.25%<br>(11.95% - 16.67%)	10.65%<br>(8.24% - 12.94%)	5.97%<br>(4.27% - 8.22%)	5.52%<br>(3.78% - 7.50%)	3.34%<br>(1.93% - 5.06%)	2.32%<br>(1.17% - 3.75%)
Nandi	36.27%<br>(33.92% - 39.04%)	36.48%<br>(33.87% - 39.39%)	33.39%<br>(31.21% - 35.87%)	26.87%<br>(24.70% - 28.94%)	15.40%<br>(12.78% - 17.85%)	14.99%<br>(12.30% - 17.58%)	12.68%<br>(9.95% - 15.11%)	9.39%<br>(7.16% - 11.49%)	5.03%<br>(3.46% - 6.89%)	4.87%<br>(3.22% - 6.75%)	3.24%<br>(1.85% - 4.95%)	2.13%<br>(1.02% - 3.44%)
Narok	36.69%<br>(34.16% - 39.69%)	36.69%<br>(34.00% - 39.73%)	34.19%<br>(31.44% - 37.51%)	29.42%<br>(27.04% - 31.99%)	13.59%<br>(11.10% - 16.07%)	13.79%<br>(11.38% - 16.28%)	10.86%<br>(8.53% - 13.24%)	8.76%<br>(6.24% - 11.12%)	3.26%<br>(1.69% - 5.38%)	3.35%<br>(1.72% - 5.38%)	2.01%<br>(0.98% - 3.39%)	1.57%<br>(0.56% - 2.79%)
Nyamira	31.55%<br>(29.58% - 33.64%)	32.15%<br>(30.00% - 34.44%)	34.04%<br>(31.70% - 37.19%)	29.82%<br>(27.89% - 32.04%)	12.27%<br>(10.01% - 14.61%)	11.94%<br>(9.46% - 14.34%)	13.12%<br>(10.66% - 15.65%)	11.81%<br>(9.42% - 14.18%)	3.19%<br>(2.02% - 4.73%)	2.93%<br>(1.79% - 4.32%)	3.34%<br>(2.03% - 5.08%)	3.13%<br>(1.82% - 4.73%)
Nyandarua	27.72%<br>(25.73% - 29.83%)	29.41%<br>(27.13% - 32.31%)	30.28%<br>(27.79% - 33.73%)	28.26%<br>(26.07% - 31.16%)	10.13%<br>(7.92% - 12.30%)	10.35%<br>(8.07% - 12.45%)	10.29%<br>(8.03% - 12.46%)	9.55%<br>(6.85% - 11.84%)	2.60%<br>(1.60% - 3.81%)	2.49%<br>(1.48% - 3.71%)	2.19%<br>(1.21% - 3.46%)	2.01%<br>(0.97% - 3.30%)

Table S8a. Estimated overall, severe, and extreme **stunting** prevalence (%) in children under 5 years, both sexes, in 1990, 2000, 2010, and 2020.

Location	Total				Severe				Extreme			
	1990	2000	2010	2020	1990	2000	2010	2020	1990	2000	2010	2020
Nyeri	29.27%<br>(27.37% - 31.18%)	28.27%<br>(26.33% - 30.37%)	24.35%<br>(22.05% - 26.64%)	21.12%<br>(18.74% - 23.36%)	12.22%<br>(10.08% - 14.50%)	10.51%<br>(8.38% - 12.76%)	7.48%<br>(5.28% - 9.52%)	5.94%<br>(4.07% - 7.81%)	3.70%<br>(2.33% - 5.36%)	2.59%<br>(1.51% - 3.97%)	1.41%<br>(0.60% - 2.40%)	0.95%<br>(0.34% - 1.74%)
Samburu	37.91%<br>(34.39% - 42.02%)	38.15%<br>(34.09% - 42.37%)	37.27%<br>(32.31% - 42.28%)	30.99%<br>(27.29% - 35.76%)	12.43%<br>(10.01% - 15.30%)	11.67%<br>(8.94% - 14.85%)	10.45%<br>(7.86% - 13.76%)	7.57%<br>(4.78% - 10.09%)	2.42%<br>(1.21% - 4.02%)	1.93%<br>(0.92% - 3.42%)	1.39%<br>(0.65% - 2.64%)	0.89%<br>(0.32% - 1.86%)
Siaya	44.18%<br>(40.87% - 48.17%)	44.04%<br>(40.30% - 48.01%)	40.64%<br>(37.19% - 44.79%)	30.88%<br>(28.26% - 33.82%)	18.00%<br>(15.73% - 20.65%)	16.48%<br>(14.05% - 19.56%)	13.72%<br>(11.09% - 16.57%)	9.01%<br>(6.61% - 11.35%)	4.40%<br>(3.05% - 6.20%)	3.31%<br>(2.18% - 4.79%)	2.42%<br>(1.37% - 4.00%)	1.41%<br>(0.51% - 2.67%)
TaitaTaveta	34.65%<br>(31.73% - 38.48%)	33.47%<br>(29.76% - 38.32%)	28.68%<br>(24.62% - 34.39%)	21.65%<br>(18.98% - 23.99%)	11.74%<br>(9.51% - 14.26%)	9.33%<br>(6.65% - 12.37%)	6.49%<br>(3.74% - 9.67%)	4.85%<br>(2.72% - 6.97%)	2.42%<br>(1.35% - 3.83%)	1.50%<br>(0.72% - 2.56%)	0.83%<br>(0.35% - 1.59%)	0.58%<br>(0.22% - 1.23%)
TanaRiver	29.57%<br>(26.86% - 32.93%)	31.66%<br>(28.82% - 35.08%)	30.58%<br>(27.68% - 34.34%)	22.81%<br>(19.83% - 26.30%)	9.73%<br>(6.80% - 12.29%)	10.26%<br>(7.33% - 12.75%)	9.71%<br>(6.83% - 12.34%)	4.75%<br>(2.29% - 7.07%)	1.86%<br>(0.75% - 3.31%)	1.85%<br>(0.80% - 3.31%)	1.71%<br>(0.70% - 3.09%)	0.46%<br>(0.07% - 1.13%)
TharakaNithi	29.40%<br>(26.79% - 32.71%)	30.57%<br>(27.71% - 35.17%)	29.46%<br>(26.50% - 33.56%)	23.75%<br>(21.25% - 26.03%)	8.07%<br>(5.52% - 10.64%)	7.40%<br>(4.79% - 9.85%)	7.03%<br>(4.19% - 9.49%)	5.54%<br>(3.42% - 7.47%)	1.31%<br>(0.56% - 2.37%)	1.02%<br>(0.45% - 1.85%)	0.86%<br>(0.28% - 1.79%)	0.65%<br>(0.14% - 1.47%)
TransNzoia	38.47%<br>(35.92% - 41.83%)	37.43%<br>(34.66% - 40.73%)	36.02%<br>(32.96% - 39.71%)	29.87%<br>(27.01% - 33.40%)	14.45%<br>(12.17% - 17.03%)	13.06%<br>(10.87% - 15.76%)	11.54%<br>(9.03% - 14.29%)	9.02%<br>(6.85% - 11.55%)	3.24%<br>(1.88% - 5.20%)	2.55%<br>(1.43% - 4.24%)	1.92%<br>(1.05% - 3.36%)	1.50%<br>(0.66% - 2.92%)
Turkana	27.70%<br>(25.78% - 29.63%)	26.18%<br>(24.40% - 28.04%)	26.83%<br>(25.06% - 28.65%)	23.40%<br>(21.51% - 25.37%)	12.92%<br>(10.56% - 15.43%)	12.05%<br>(9.90% - 14.31%)	11.04%<br>(8.91% - 13.06%)	7.57%<br>(5.47% - 9.60%)	4.56%<br>(2.82% - 6.78%)	4.14%<br>(2.61% - 5.88%)	3.14%<br>(1.78% - 4.66%)	1.46%<br>(0.64% - 2.53%)
UasinGishu	33.96%<br>(32.06% - 36.06%)	33.16%<br>(31.21% - 35.30%)	32.01%<br>(30.05% - 34.16%)	28.04%<br>(26.03% - 30.01%)	15.39%<br>(13.06% - 17.75%)	14.60%<br>(12.41% - 16.86%)	13.11%<br>(10.95% - 15.35%)	11.40%<br>(9.42% - 13.58%)	5.04%<br>(3.42% - 6.93%)	4.61%<br>(3.09% - 6.50%)	3.95%<br>(2.54% - 5.66%)	3.47%<br>(2.24% - 5.03%)
Vihiga	34.81%<br>(31.55% - 38.76%)	33.72%<br>(29.95% - 38.39%)	32.27%<br>(28.14% - 37.88%)	26.72%<br>(23.87% - 29.50%)	9.78%<br>(7.07% - 12.33%)	8.20%<br>(5.07% - 10.99%)	6.61%<br>(3.19% - 9.47%)	5.76%<br>(2.90% - 8.23%)	1.44%<br>(0.60% - 2.68%)	1.01%<br>(0.34% - 1.98%)	0.62%<br>(0.16% - 1.42%)	0.61%<br>(0.15% - 1.38%)
Wajir	55.06%<br>(51.03% - 58.44%)	55.52%<br>(51.89% - 58.48%)	50.43%<br>(46.60% - 53.85%)	46.57%<br>(41.95% - 50.30%)	22.71%<br>(17.80% - 28.26%)	24.31%<br>(19.60% - 29.61%)	20.79%<br>(16.12% - 25.84%)	14.47%<br>(9.26% - 20.43%)	4.81%<br>(2.28% - 9.12%)	5.85%<br>(2.77% - 10.18%)	4.63%<br>(2.10% - 8.93%)	2.00%<br>(0.50% - 4.98%)
WestPokot	51.90%<br>(48.33% - 57.60%)	51.43%<br>(47.10% - 56.69%)	47.82%<br>(44.36% - 52.14%)	42.99%<br>(40.31% - 46.46%)	23.19%<br>(19.26% - 27.67%)	21.08%<br>(17.71% - 25.02%)	21.91%<br>(18.65% - 25.25%)	20.42%<br>(17.64% - 23.32%)	7.11%<br>(4.00% - 12.05%)	5.46%<br>(3.11% - 9.40%)	6.57%<br>(4.05% - 9.94%)	6.72%<br>(4.34% - 9.77%)
Madagascar	51.36%<br>(49.90% - 52.99%)	50.96%<br>(49.29% - 53.08%)	47.89%<br>(46.25% - 50.07%)	45.19%<br>(43.58% - 47.06%)	31.65%<br>(29.36% - 33.90%)	30.93%<br>(28.92% - 33.15%)	27.53%<br>(25.65% - 29.50%)	25.08%<br>(22.88% - 27.37%)	15.12%<br>(12.20% - 18.72%)	14.33%<br>(11.68% - 17.68%)	11.68%<br>(9.44% - 14.22%)	10.15%<br>(7.78% - 13.01%)
Malawi	49.01%<br>(46.81% - 51.64%)	47.09%<br>(45.16% - 49.64%)	38.21%<br>(36.95% - 39.78%)	30.14%<br>(28.91% - 31.43%)	23.87%<br>(21.71% - 26.19%)	23.07%<br>(21.37% - 24.90%)	16.30%<br>(14.82% - 17.86%)	10.58%<br>(9.10% - 11.98%)	7.34%<br>(5.44% - 10.00%)	7.38%<br>(5.62% - 9.53%)	4.42%<br>(3.34% - 5.74%)	2.18%<br>(1.44% - 2.94%)
Mozambique	50.64%<br>(48.31% - 53.03%)	45.34%<br>(43.47% - 47.60%)	38.59%<br>(36.94% - 40.71%)	33.13%<br>(31.54% - 35.02%)	24.96%<br>(22.67% - 27.42%)	21.51%<br>(19.75% - 23.45%)	15.52%<br>(14.01% - 17.15%)	12.27%<br>(10.70% - 13.91%)	7.73%<br>(5.55% - 10.51%)	6.60%<br>(4.93% - 8.65%)	3.74%<br>(2.69% - 5.05%)	2.69%<br>(1.82% - 3.80%)
Rwanda	46.58%<br>(44.93% - 48.61%)	46.21%<br>(44.62% - 48.17%)	39.98%<br>(38.56% - 41.93%)	35.14%<br>(33.64% - 36.74%)	25.00%<br>(22.96% - 27.16%)	24.87%<br>(23.17% - 26.67%)	18.09%<br>(16.59% - 19.71%)	14.27%<br>(12.47% - 15.97%)	9.57%<br>(7.18% - 12.56%)	9.55%<br>(7.43% - 12.00%)	5.34%<br>(4.02% - 6.86%)	3.64%<br>(2.48% - 4.85%)
Somalia	33.00%<br>(31.62% - 34.43%)	32.46%<br>(31.23% - 33.57%)	32.59%<br>(31.51% - 33.76%)	31.55%<br>(30.21% - 32.95%)	16.12%<br>(14.44% - 17.85%)	16.67%<br>(15.06% - 18.33%)	16.20%<br>(14.60% - 17.75%)	14.84%<br>(13.15% - 16.55%)	5.71%<br>(4.42% - 7.23%)	6.43%<br>(5.14% - 7.95%)	5.91%<br>(4.61% - 7.42%)	4.97%<br>(3.73% - 6.49%)
United Republic of Tanzania	45.78%<br>(44.17% - 47.70%)	43.91%<br>(42.45% - 45.71%)	38.45%<br>(37.20% - 39.95%)	33.24%<br>(32.02% - 34.61%)	21.67%<br>(20.14% - 23.32%)	20.29%<br>(18.91% - 21.71%)	16.13%<br>(14.92% - 17.40%)	12.91%<br>(11.44% - 14.44%)	6.55%<br>(5.12% - 8.38%)	5.99%<br>(4.71% - 7.50%)	4.41%<br>(3.49% - 5.52%)	3.15%<br>(2.25% - 4.21%)
Uganda	43.91%<br>(42.32% - 45.85%)	41.63%<br>(40.19% - 43.56%)	34.42%<br>(33.33% - 35.64%)	28.82%<br>(27.69% - 30.07%)	21.18%<br>(19.55% - 22.87%)	19.09%<br>(17.54% - 20.53%)	13.98%<br>(12.89% - 15.10%)	10.46%<br>(9.10% - 11.85%)	6.82%<br>(5.15% - 8.83%)	5.72%<br>(4.23% - 7.23%)	3.59%<br>(2.80% - 4.48%)	2.30%<br>(1.59% - 3.10%)
Zambia	46.84%<br>(45.00% - 49.07%)	48.66%<br>(47.06% - 50.46%)	43.84%<br>(42.65% - 45.22%)	32.39%<br>(31.05% - 33.75%)	25.10%<br>(23.04% - 27.30%)	28.77%<br>(27.02% - 30.61%)	24.75%<br>(23.16% - 26.30%)	14.67%<br>(13.13% - 16.27%)	9.47%<br>(7.15% - 12.43%)	12.90%<br>(10.52% - 15.68%)	10.55%<br>(8.78% - 12.39%)	4.58%<br>(3.53% - 5.78%)
South Sudan	37.39%<br>(36.09% - 38.65%)	37.09%<br>(36.17% - 38.01%)	30.74%<br>(30.03% - 31.45%)	28.99%<br>(27.83% - 30.17%)	21.23%<br>(19.32% - 23.10%)	22.13%<br>(20.83% - 23.49%)	16.40%<br>(15.57% - 17.26%)	14.38%<br>(12.96% - 15.81%)	9.42%<br>(7.47% - 11.39%)	10.73%<br>(9.39% - 12.12%)	6.82%<br>(6.09% - 7.57%)	5.32%<br>(4.18% - 6.45%)

Table S8a. Estimated overall, severe, and extreme **stunting** prevalence (%) in children under 5 years, both sexes, in 1990, 2000, 2010, and 2020.

Location	Total				Severe				Extreme			
	1990	2000	2010	2020	1990	2000	2010	2020	1990	2000	2010	2020
**Southern Sub-Saharan Africa**	31.03% (30.55% - 31.52%)	30.87% (30.41% - 31.32%)	28.45% (28.00% - 28.88%)	23.73% (23.28% - 24.23%)	11.41% (10.91% - 11.93%)	11.79% (11.33% - 12.24%)	10.03% (9.63% - 10.45%)	7.40% (6.97% - 7.85%)	2.67% (2.41% - 2.98%)	2.89% (2.63% - 3.17%)	2.18% (1.95% - 2.42%)	1.38% (1.20% - 1.58%)
Botswana	33.50% (31.98% - 35.33%)	31.53% (30.30% - 32.88%)	30.03% (28.81% - 31.34%)	26.19% (24.78% - 27.58%)	13.09% (11.46% - 14.74%)	12.30% (10.82% - 13.66%)	11.95% (10.61% - 13.28%)	9.86% (8.53% - 11.29%)	3.26% (2.39% - 4.37%)	3.08% (2.29% - 3.98%)	3.15% (2.41% - 4.02%)	2.40% (1.73% - 3.17%)
Lesotho	40.77% (39.56% - 42.06%)	43.09% (41.67% - 44.75%)	39.11% (37.32% - 41.35%)	33.35% (31.91% - 35.02%)	21.14% (19.35% - 22.97%)	21.61% (19.82% - 23.54%)	15.63% (13.98% - 17.33%)	11.89% (10.24% - 13.48%)	8.01% (6.54% - 9.68%)	7.70% (6.07% - 9.65%)	3.78% (2.64% - 5.18%)	2.47% (1.61% - 3.41%)
Namibia	32.22% (31.20% - 33.37%)	30.03% (28.92% - 31.20%)	27.01% (25.98% - 28.00%)	22.17% (20.83% - 23.44%)	13.13% (11.89% - 14.47%)	11.26% (10.02% - 12.45%)	9.93% (8.78% - 11.03%)	7.54% (6.40% - 8.68%)	3.49% (2.71% - 4.38%)	2.64% (1.92% - 3.39%)	2.29% (1.69% - 2.97%)	1.53% (1.01% - 2.11%)
South Africa	30.17% (29.61% - 30.74%)	29.92% (29.46% - 30.43%)	26.18% (25.69% - 26.66%)	22.23% (21.68% - 22.78%)	10.79% (10.16% - 11.42%)	11.06% (10.53% - 11.58%)	8.97% (8.55% - 9.43%)	6.83% (6.43% - 7.27%)	2.44% (2.14% - 2.79%)	2.59% (2.33% - 2.87%)	1.93% (1.73% - 2.15%)	1.32% (1.16% - 1.49%)
Eswatini	36.14% (34.49% - 38.61%)	35.28% (33.66% - 37.17%)	31.89% (30.45% - 33.36%)	26.67% (25.26% - 28.10%)	12.88% (11.18% - 14.74%)	12.87% (11.43% - 14.35%)	10.69% (9.30% - 12.08%)	8.54% (7.03% - 10.05%)	2.62% (1.75% - 3.76%)	2.73% (1.98% - 3.68%)	1.99% (1.33% - 2.78%)	1.55% (0.91% - 2.34%)
Zimbabwe	31.08% (29.78% - 32.41%)	31.45% (30.25% - 32.68%)	32.77% (31.67% - 34.04%)	26.02% (24.78% - 27.33%)	11.16% (9.80% - 12.66%)	12.38% (11.06% - 13.69%)	11.85% (10.69% - 13.13%)	7.93% (6.62% - 9.16%)	2.38% (1.60% - 3.27%)	3.10% (2.25% - 4.02%)	2.54% (1.82% - 3.38%)	1.30% (0.78% - 1.88%)
**Western Sub-Saharan Africa**	40.56% (39.84% - 41.36%)	40.31% (39.66% - 41.06%)	36.18% (35.66% - 36.75%)	31.25% (30.67% - 31.84%)	20.73% (19.91% - 21.62%)	21.40% (20.60% - 22.27%)	17.13% (16.56% - 17.67%)	13.61% (12.97% - 14.30%)	7.86% (6.80% - 9.19%)	8.73% (7.71% - 9.92%)	5.81% (5.22% - 6.46%)	4.19% (3.58% - 4.89%)
Benin	38.41% (37.32% - 39.60%)	38.75% (37.47% - 40.20%)	38.44% (37.17% - 39.86%)	33.15% (31.79% - 34.57%)	19.84% (18.15% - 21.51%)	19.19% (17.69% - 20.89%)	19.06% (17.69% - 20.43%)	13.95% (12.45% - 15.47%)	7.41% (5.88% - 9.18%)	6.62% (5.06% - 8.35%)	6.55% (5.27% - 7.94%)	3.72% (2.68% - 4.89%)
Burkina Faso	40.88% (39.29% - 42.77%)	42.57% (41.42% - 43.97%)	35.35% (34.38% - 36.28%)	30.35% (29.14% - 31.54%)	19.88% (17.99% - 21.72%)	24.14% (22.73% - 25.65%)	16.95% (15.81% - 18.22%)	12.51% (11.01% - 13.91%)	6.51% (4.75% - 8.57%)	10.36% (8.62% - 12.33%)	5.70% (4.73% - 6.83%)	3.34% (2.40% - 4.33%)
Cameroon	37.85% (36.03% - 39.73%)	39.62% (38.13% - 41.37%)	34.25% (33.08% - 35.67%)	29.09% (27.78% - 30.50%)	17.25% (15.65% - 18.79%)	19.87% (18.35% - 21.39%)	15.23% (13.88% - 16.62%)	11.91% (10.45% - 13.39%)	5.08% (3.74% - 6.56%)	6.95% (5.46% - 8.65%)	4.47% (3.35% - 5.70%)	3.16% (2.29% - 4.19%)
Cabo Verde	24.64% (23.39% - 25.86%)	20.86% (19.61% - 22.31%)	15.50% (14.40% - 16.71%)	12.98% (11.88% - 14.16%)	9.89% (8.63% - 11.18%)	7.69% (6.62% - 9.01%)	5.02% (4.20% - 5.86%)	3.99% (3.30% - 4.81%)	2.60% (1.92% - 3.40%)	1.78% (1.28% - 2.48%)	0.93% (0.62% - 1.30%)	0.66% (0.42% - 0.99%)
Chad	39.70% (38.41% - 41.21%)	40.46% (39.34% - 41.82%)	39.06% (37.93% - 40.37%)	36.35% (35.17% - 37.76%)	21.75% (19.75% - 23.91%)	23.16% (21.51% - 24.85%)	22.04% (20.49% - 23.72%)	19.21% (17.52% - 21.00%)	9.01% (6.63% - 11.58%)	10.27% (8.22% - 12.67%)	9.58% (7.74% - 11.66%)	7.57% (5.71% - 9.66%)
Côte d'Ivoire	28.63% (27.47% - 29.86%)	32.60% (31.50% - 33.81%)	30.85% (29.73% - 32.04%)	23.80% (22.50% - 25.05%)	11.30% (10.02% - 12.48%)	14.26% (12.97% - 15.62%)	12.68% (11.41% - 13.97%)	7.93% (6.67% - 9.24%)	2.85% (2.15% - 3.60%)	4.14% (3.14% - 5.27%)	3.36% (2.54% - 4.25%)	1.53% (1.00% - 2.17%)
Gambia	30.30% (28.89% - 31.93%)	28.10% (27.07% - 29.29%)	24.63% (23.62% - 25.67%)	20.77% (19.55% - 21.97%)	9.78% (8.29% - 11.27%)	9.33% (8.13% - 10.60%)	8.34% (7.35% - 9.32%)	6.12% (5.10% - 7.13%)	1.73% (1.10% - 2.46%)	1.75% (1.16% - 2.40%)	1.67% (1.19% - 2.16%)	0.97% (0.58% - 1.44%)
Ghana	35.32% (34.17% - 36.60%)	32.37% (31.22% - 33.58%)	26.22% (25.18% - 27.29%)	19.36% (18.15% - 20.59%)	15.61% (14.34% - 16.90%)	12.89% (11.67% - 14.18%)	8.72% (7.63% - 9.72%)	5.50% (4.56% - 6.51%)	4.51% (3.50% - 5.67%)	3.17% (2.36% - 4.17%)	1.65% (1.16% - 2.16%)	0.78% (0.47% - 1.19%)
Guinea	36.81% (35.54% - 38.29%)	37.60% (36.42% - 38.85%)	35.40% (34.10% - 36.89%)	31.18% (29.82% - 32.61%)	16.95% (15.29% - 18.82%)	17.41% (15.94% - 18.92%)	15.42% (14.14% - 16.68%)	12.70% (11.23% - 14.13%)	5.22% (3.94% - 6.84%)	5.38% (4.22% - 6.66%)	4.32% (3.38% - 5.39%)	3.29% (2.37% - 4.27%)
Guinea-Bissau	37.59% (36.06% - 39.31%)	36.86% (35.62% - 38.25%)	33.27% (32.30% - 34.27%)	28.59% (27.28% - 29.92%)	16.32% (14.60% - 18.16%)	16.74% (15.20% - 18.38%)	13.84% (12.56% - 15.04%)	10.37% (8.97% - 11.76%)	4.56% (3.36% - 5.99%)	5.12% (4.00% - 6.45%)	3.71% (2.93% - 4.53%)	2.26% (1.52% - 3.06%)
Liberia	45.93% (43.83% - 48.34%)	45.62% (43.66% - 47.83%)	36.07% (34.25% - 38.17%)	29.65% (28.10% - 31.42%)	23.46% (21.09% - 26.12%)	24.19% (22.07% - 26.45%)	15.00% (13.61% - 16.46%)	11.00% (9.30% - 12.71%)	7.93% (5.95% - 10.36%)	8.82% (6.89% - 10.94%)	3.79% (2.87% - 4.86%)	2.43% (1.58% - 3.50%)
Mali	36.27% (35.21% - 37.28%)	35.78% (34.85% - 36.79%)	29.96% (29.02% - 30.95%)	26.64% (25.46% - 27.87%)	19.87% (18.35% - 21.36%)	19.24% (18.02% - 20.63%)	12.58% (11.52% - 13.62%)	9.94% (8.71% - 11.23%)	8.42% (6.89% - 9.97%)	7.93% (6.63% - 9.36%)	3.47% (2.71% - 4.24%)	2.28% (1.61% - 3.04%)
Mauritania	41.52% (40.32% - 42.80%)	34.43% (33.42% - 35.46%)	25.56% (24.70% - 26.44%)	20.58% (19.40% - 21.79%)	23.39% (21.75% - 25.11%)	17.31% (15.96% - 18.77%)	9.86% (9.01% - 10.80%)	6.85% (5.93% - 7.88%)	10.12% (8.22% - 12.21%)	6.43% (5.24% - 7.80%)	2.48% (2.03% - 3.00%)	1.37% (0.97% - 1.86%)
Niger	48.83% (46.80% - 51.03%)	49.77% (47.63% - 52.26%)	46.76% (44.45% - 49.32%)	43.17% (40.64% - 45.93%)	27.93% (25.83% - 30.05%)	28.63% (26.77% - 30.47%)	22.77% (21.38% - 24.24%)	18.67% (17.07% - 20.56%)	11.31% (8.79% - 14.27%)	11.84% (9.47% - 14.55%)	7.14% (5.92% - 8.54%)	4.80% (3.77% - 6.11%)

Table S8a. Estimated overall, severe, and extreme **stunting** prevalence (%) in children under 5 years, both sexes, in 1990, 2000, 2010, and 2020.

Location	Total				Severe				Extreme			
	1990	2000	2010	2020	1990	2000	2010	2020	1990	2000	2010	2020
Nigeria	44.63% (43.02% - 46.42%)	43.43% (42.09% - 44.98%)	38.97% (37.96% - 40.07%)	33.73% (32.60% - 34.87%)	24.15% (22.48% - 26.09%)	24.51% (22.85% - 26.24%)	19.55% (18.43% - 20.62%)	15.97% (14.67% - 17.33%)	10.05% (7.76% - 12.87%)	11.03% (8.94% - 13.55%)	7.23% (6.02% - 8.55%)	5.59% (4.33% - 7.08%)
Sao Tome and Principe	33.89% (32.66% - 35.15%)	33.20% (32.13% - 34.32%)	26.94% (26.02% - 27.94%)	20.07% (18.91% - 21.23%)	13.77% (12.32% - 15.33%)	13.37% (12.10% - 14.53%)	9.56% (8.71% - 10.48%)	6.17% (5.28% - 7.09%)	3.59% (2.77% - 4.52%)	3.45% (2.72% - 4.21%)	2.09% (1.69% - 2.55%)	1.06% (0.72% - 1.47%)
Senegal	32.54% (31.21% - 34.00%)	28.44% (27.34% - 29.66%)	23.07% (22.33% - 23.89%)	18.20% (17.23% - 19.19%)	13.04% (11.77% - 14.33%)	10.39% (9.29% - 11.51%)	7.20% (6.40% - 8.01%)	4.83% (4.01% - 5.70%)	3.25% (2.31% - 4.32%)	2.28% (1.63% - 2.99%)	1.24% (0.86% - 1.65%)	0.62% (0.33% - 0.97%)
Sierra Leone	40.76% (39.74% - 41.80%)	39.98% (38.75% - 41.32%)	38.48% (37.35% - 39.78%)	31.14% (29.92% - 32.42%)	20.49% (18.53% - 22.55%)	20.91% (19.32% - 22.67%)	18.98% (17.62% - 20.31%)	13.10% (11.68% - 14.64%)	7.22% (5.65% - 9.06%)	7.87% (6.29% - 9.82%)	6.50% (5.25% - 7.88%)	3.63% (2.70% - 4.70%)

Table S8b. Estimated overall, severe, and extreme **wasting** prevalence (%) in children under 5 years, both sexes, in 1990, 2000, 2010, and 2020.

Location	Total				Severe				Extreme			
	1990	2000	2010	2020	1990	2000	2010	2020	1990	2000	2010	2020
Global	11.23% (11.14% - 11.33%)	10.97% (10.90% - 11.03%)	9.96% (9.91% - 10.01%)	7.41% (7.35% - 7.46%)	3.38% (3.34% - 3.42%)	3.30% (3.26% - 3.34%)	2.90% (2.87% - 2.93%)	1.66% (1.64% - 1.69%)	0.78% (0.76% - 0.80%)	0.72% (0.70% - 0.74%)	0.60% (0.58% - 0.61%)	0.20% (0.19% - 0.21%)
Central Europe, Eastern Europe, and Central Asia	5.51% (5.36% - 5.68%)	5.62% (5.49% - 5.76%)	4.30% (4.17% - 4.43%)	3.26% (3.15% - 3.39%)	0.90% (0.85% - 0.97%)	0.94% (0.89% - 1.00%)	0.53% (0.49% - 0.57%)	0.27% (0.24% - 0.30%)	0.06% (0.05% - 0.08%)	0.06% (0.05% - 0.07%)	0.02% (0.02% - 0.03%)	0.01% (0.00% - 0.01%)
**Central Asia**	6.86% (6.63% - 7.12%)	7.07% (6.84% - 7.32%)	5.03% (4.86% - 5.22%)	3.46% (3.32% - 3.61%)	1.41% (1.30% - 1.52%)	1.42% (1.33% - 1.53%)	0.68% (0.63% - 0.75%)	0.27% (0.24% - 0.30%)	0.11% (0.08% - 0.14%)	0.10% (0.07% - 0.12%)	0.02% (0.02% - 0.03%)	0.00% (0.00% - 0.01%)
Armenia	2.81% (2.60% - 3.00%)	3.44% (3.21% - 3.69%)	3.48% (3.17% - 3.81%)	3.30% (3.00% - 3.65%)	0.16% (0.12% - 0.20%)	0.22% (0.18% - 0.27%)	0.14% (0.10% - 0.20%)	0.14% (0.07% - 0.22%)	0.00% (0.00% - 0.00%)	0.00% (0.00% - 0.00%)	0.00% (0.00% - 0.00%)	0.00% (0.00% - 0.00%)
Azerbaijan	7.84% (7.36% - 8.33%)	8.53% (8.09% - 9.02%)	6.17% (5.83% - 6.54%)	4.89% (4.53% - 5.27%)	1.56% (1.36% - 1.78%)	1.85% (1.65% - 2.05%)	1.00% (0.87% - 1.15%)	0.58% (0.46% - 0.72%)	0.07% (0.04% - 0.11%)	0.08% (0.05% - 0.11%)	0.00% (0.00% - 0.00%)	0.00% (0.00% - 0.00%)
Georgia	2.31% (2.09% - 2.53%)	2.20% (2.01% - 2.40%)	1.64% (1.51% - 1.79%)	0.89% (0.75% - 0.98%)	0.01% (0.00% - 0.03%)	0.01% (0.00% - 0.02%)	0.01% (0.00% - 0.02%)	0.00% (0.00% - 0.01%)	0.00% (0.00% - 0.00%)	0.00% (0.00% - 0.00%)	0.00% (0.00% - 0.00%)	0.00% (0.00% - 0.00%)
Kazakhstan	3.68% (3.42% - 3.96%)	4.09% (3.76% - 4.39%)	4.04% (3.74% - 4.35%)	2.70% (2.51% - 2.90%)	0.39% (0.32% - 0.45%)	0.43% (0.36% - 0.52%)	0.37% (0.30% - 0.44%)	0.13% (0.10% - 0.16%)	0.02% (0.01% - 0.04%)	0.03% (0.01% - 0.05%)	0.01% (0.00% - 0.03%)	0.00% (0.00% - 0.00%)
Kyrgyzstan	4.69% (4.31% - 5.09%)	4.71% (4.34% - 5.08%)	3.14% (2.89% - 3.41%)	2.39% (2.20% - 2.61%)	0.43% (0.32% - 0.56%)	0.44% (0.33% - 0.56%)	0.11% (0.08% - 0.16%)	0.03% (0.02% - 0.05%)	0.00% (0.00% - 0.00%)	0.00% (0.00% - 0.00%)	0.00% (0.00% - 0.00%)	0.00% (0.00% - 0.00%)
Mongolia	3.92% (3.69% - 4.18%)	3.98% (3.76% - 4.21%)	1.94% (1.82% - 2.08%)	1.19% (1.10% - 1.30%)	0.28% (0.24% - 0.34%)	0.33% (0.28% - 0.38%)	0.04% (0.02% - 0.05%)	0.00% (0.00% - 0.01%)	0.00% (0.00% - 0.01%)	0.01% (0.00% - 0.02%)	0.00% (0.00% - 0.00%)	0.00% (0.00% - 0.00%)
Tajikistan	9.78% (9.24% - 10.31%)	11.09% (10.59% - 11.62%)	9.11% (8.68% - 9.57%)	6.76% (6.39% - 7.15%)	2.24% (1.98% - 2.49%)	2.86% (2.61% - 3.14%)	2.06% (1.87% - 2.27%)	1.13% (0.98% - 1.29%)	0.04% (0.01% - 0.09%)	0.17% (0.10% - 0.26%)	0.12% (0.09% - 0.16%)	0.02% (0.01% - 0.05%)
Turkmenistan	8.71% (8.13% - 9.32%)	7.82% (7.36% - 8.26%)	5.99% (5.65% - 6.35%)	4.37% (4.01% - 4.73%)	1.71% (1.47% - 1.98%)	1.38% (1.20% - 1.57%)	0.82% (0.70% - 0.95%)	0.35% (0.28% - 0.44%)	0.03% (0.01% - 0.06%)	0.01% (0.00% - 0.01%)	0.01% (0.00% - 0.01%)	0.00% (0.00% - 0.00%)
Uzbekistan	9.03% (8.45% - 9.69%)	7.76% (7.29% - 8.28%)	4.87% (4.49% - 5.27%)	3.10% (2.82% - 3.41%)	2.29% (2.02% - 2.61%)	1.70% (1.50% - 1.94%)	0.59% (0.49% - 0.72%)	0.12% (0.08% - 0.17%)	0.26% (0.18% - 0.34%)	0.15% (0.10% - 0.20%)	0.02% (0.00% - 0.04%)	0.00% (0.00% - 0.00%)
**Central Europe**	3.50% (3.36% - 3.65%)	3.44% (3.30% - 3.57%)	2.77% (2.66% - 2.88%)	2.25% (2.16% - 2.35%)	0.30% (0.27% - 0.33%)	0.29% (0.27% - 0.32%)	0.13% (0.12% - 0.14%)	0.05% (0.04% - 0.06%)	0.03% (0.02% - 0.04%)	0.02% (0.01% - 0.03%)	0.00% (0.00% - 0.00%)	0.00% (0.00% - 0.00%)
Albania	12.70% (11.91% - 13.48%)	11.23% (10.62% - 11.84%)	7.16% (6.71% - 7.65%)	3.85% (3.49% - 4.19%)	4.05% (3.57% - 4.54%)	3.34% (3.00% - 3.71%)	1.45% (1.24% - 1.67%)	0.28% (0.17% - 0.40%)	0.66% (0.46% - 0.88%)	0.42% (0.29% - 0.58%)	0.02% (0.00% - 0.03%)	0.00% (0.00% - 0.00%)
Bosnia and Herzegovina	3.19% (2.96% - 3.44%)	3.93% (3.55% - 4.33%)	2.71% (2.50% - 2.94%)	2.39% (2.18% - 2.61%)	0.33% (0.27% - 0.40%)	0.41% (0.33% - 0.49%)	0.14% (0.11% - 0.17%)	0.09% (0.06% - 0.12%)	0.01% (0.00% - 0.03%)	0.03% (0.01% - 0.05%)	0.00% (0.00% - 0.00%)	0.00% (0.00% - 0.00%)
Bulgaria	2.93% (2.69% - 3.19%)	3.31% (3.06% - 3.58%)	2.92% (2.68% - 3.17%)	2.48% (2.27% - 2.72%)	0.27% (0.20% - 0.34%)	0.36% (0.28% - 0.44%)	0.25% (0.19% - 0.33%)	0.15% (0.10% - 0.21%)	0.00% (0.00% - 0.00%)	0.00% (0.00% - 0.00%)	0.00% (0.00% - 0.00%)	0.00% (0.00% - 0.00%)
Croatia	2.72% (2.46% - 3.02%)	3.21% (2.85% - 3.63%)	2.74% (2.46% - 3.05%)	2.28% (2.05% - 2.53%)	0.07% (0.04% - 0.10%)	0.12% (0.08% - 0.19%)	0.07% (0.04% - 0.10%)	0.03% (0.01% - 0.05%)	0.00% (0.00% - 0.00%)	0.00% (0.00% - 0.00%)	0.00% (0.00% - 0.00%)	0.00% (0.00% - 0.00%)
Czechia	3.23% (2.92% - 3.56%)	3.11% (2.84% - 3.40%)	2.81% (2.57% - 3.12%)	2.59% (2.34% - 2.88%)	0.08% (0.04% - 0.15%)	0.19% (0.15% - 0.26%)	0.14% (0.11% - 0.20%)	0.09% (0.06% - 0.13%)	0.00% (0.00% - 0.00%)	0.00% (0.00% - 0.00%)	0.00% (0.00% - 0.00%)	0.00% (0.00% - 0.00%)
Hungary	2.10% (1.92% - 2.29%)	1.79% (1.63% - 1.97%)	1.69% (1.53% - 1.88%)	1.54% (1.39% - 1.71%)	0.11% (0.08% - 0.14%)	0.06% (0.03% - 0.08%)	0.03% (0.01% - 0.05%)	0.02% (0.01% - 0.04%)	0.00% (0.00% - 0.00%)	0.00% (0.00% - 0.00%)	0.00% (0.00% - 0.00%)	0.00% (0.00% - 0.00%)
North Macedonia	3.97% (3.67% - 4.30%)	3.66% (3.39% - 3.95%)	3.55% (3.29% - 3.85%)	2.93% (2.70% - 3.18%)	0.35% (0.28% - 0.42%)	0.30% (0.24% - 0.36%)	0.24% (0.18% - 0.30%)	0.14% (0.10% - 0.19%)	0.00% (0.00% - 0.02%)	0.00% (0.00% - 0.01%)	0.00% (0.00% - 0.00%)	0.00% (0.00% - 0.00%)
Montenegro	2.29% (2.06% - 2.56%)	3.21% (2.89% - 3.55%)	3.12% (2.85% - 3.41%)	2.42% (2.21% - 2.64%)	0.01% (0.00% - 0.02%)	0.09% (0.05% - 0.16%)	0.12% (0.08% - 0.19%)	0.07% (0.05% - 0.10%)	0.00% (0.00% - 0.00%)	0.00% (0.00% - 0.00%)	0.00% (0.00% - 0.00%)	0.00% (0.00% - 0.00%)
Poland	2.94% (2.61% - 3.30%)	2.90% (2.58% - 3.24%)	2.58% (2.31% - 2.86%)	2.02% (1.80% - 2.25%)	0.10% (0.07% - 0.14%)	0.10% (0.06% - 0.14%)	0.06% (0.04% - 0.09%)	0.02% (0.01% - 0.04%)	0.00% (0.00% - 0.00%)	0.00% (0.00% - 0.00%)	0.00% (0.00% - 0.00%)	0.00% (0.00% - 0.00%)
Romania	3.57% (3.22% - 3.95%)	3.16% (2.91% - 3.47%)	2.39% (2.18% - 2.60%)	2.07% (1.88% - 2.29%)	0.12% (0.07% - 0.19%)	0.11% (0.09% - 0.15%)	0.04% (0.03% - 0.06%)	0.02% (0.01% - 0.03%)	0.00% (0.00% - 0.00%)	0.00% (0.00% - 0.00%)	0.00% (0.00% - 0.00%)	0.00% (0.00% - 0.00%)

Table S8b. Estimated overall, severe, and extreme **wasting** prevalence (%) in children under 5 years, both sexes, in 1990, 2000, 2010, and 2020.

Location	Total				Severe				Extreme			
	1990	2000	2010	2020	1990	2000	2010	2020	1990	2000	2010	2020
Serbia	3.37% (3.06% - 3.73%)	3.73% (3.37% - 4.10%)	3.44% (3.14% - 3.79%)	2.94% (2.69% - 3.25%)	0.08% (0.05% - 0.14%)	0.17% (0.12% - 0.25%)	0.16% (0.13% - 0.22%)	0.10% (0.07% - 0.13%)	0.00% (0.00% - 0.00%)	0.00% (0.00% - 0.00%)	0.00% (0.00% - 0.00%)	0.00% (0.00% - 0.00%)
Slovakia	2.88% (2.60% - 3.18%)	3.24% (2.87% - 3.66%)	3.06% (2.73% - 3.46%)	2.44% (2.20% - 2.72%)	0.09% (0.05% - 0.12%)	0.13% (0.08% - 0.19%)	0.10% (0.06% - 0.15%)	0.04% (0.02% - 0.07%)	0.00% (0.00% - 0.00%)	0.00% (0.00% - 0.00%)	0.00% (0.00% - 0.00%)	0.00% (0.00% - 0.00%)
Slovenia	2.35% (2.12% - 2.64%)	2.64% (2.37% - 2.96%)	2.33% (2.10% - 2.59%)	1.94% (1.73% - 2.18%)	0.04% (0.02% - 0.07%)	0.06% (0.04% - 0.10%)	0.04% (0.02% - 0.06%)	0.01% (0.00% - 0.03%)	0.00% (0.00% - 0.00%)	0.00% (0.00% - 0.00%)	0.00% (0.00% - 0.00%)	0.00% (0.00% - 0.00%)
**Eastern Europe**	5.81% (5.52% - 6.11%)	5.82% (5.56% - 6.09%)	4.52% (4.28% - 4.79%)	3.59% (3.35% - 3.84%)	0.94% (0.84% - 1.06%)	0.97% (0.88% - 1.07%)	0.62% (0.54% - 0.70%)	0.38% (0.32% - 0.44%)	0.05% (0.03% - 0.07%)	0.06% (0.04% - 0.07%)	0.04% (0.02% - 0.05%)	0.02% (0.01% - 0.03%)
Belarus	2.18% (1.93% - 2.45%)	2.09% (1.87% - 2.31%)	1.72% (1.54% - 1.91%)	1.38% (1.23% - 1.57%)	0.05% (0.03% - 0.08%)	0.06% (0.04% - 0.08%)	0.04% (0.02% - 0.06%)	0.01% (0.00% - 0.03%)	0.00% (0.00% - 0.00%)	0.00% (0.00% - 0.00%)	0.00% (0.00% - 0.00%)	0.00% (0.00% - 0.00%)
Estonia	4.41% (3.93% - 4.91%)	3.53% (3.07% - 3.97%)	1.92% (1.71% - 2.16%)	1.64% (1.46% - 1.86%)	0.34% (0.22% - 0.50%)	0.16% (0.09% - 0.25%)	0.01% (0.00% - 0.02%)	0.00% (0.00% - 0.01%)	0.00% (0.00% - 0.00%)	0.00% (0.00% - 0.00%)	0.00% (0.00% - 0.00%)	0.00% (0.00% - 0.00%)
Latvia	4.36% (3.87% - 4.85%)	4.01% (3.54% - 4.45%)	2.36% (2.08% - 2.69%)	1.88% (1.67% - 2.12%)	0.33% (0.21% - 0.49%)	0.25% (0.15% - 0.37%)	0.02% (0.01% - 0.04%)	0.01% (0.00% - 0.02%)	0.00% (0.00% - 0.00%)	0.00% (0.00% - 0.00%)	0.00% (0.00% - 0.00%)	0.00% (0.00% - 0.00%)
Lithuania	3.98% (3.51% - 4.44%)	3.29% (2.88% - 3.73%)	2.06% (1.83% - 2.31%)	1.66% (1.47% - 1.88%)	0.25% (0.16% - 0.38%)	0.12% (0.06% - 0.20%)	0.01% (0.00% - 0.03%)	0.00% (0.00% - 0.01%)	0.00% (0.00% - 0.00%)	0.00% (0.00% - 0.00%)	0.00% (0.00% - 0.00%)	0.00% (0.00% - 0.00%)
Republic of Moldova	4.56% (4.15% - 5.02%)	4.53% (4.12% - 4.96%)	3.08% (2.79% - 3.40%)	2.13% (1.92% - 2.38%)	0.38% (0.26% - 0.53%)	0.38% (0.25% - 0.52%)	0.07% (0.04% - 0.12%)	0.01% (0.00% - 0.03%)	0.00% (0.00% - 0.00%)	0.00% (0.00% - 0.00%)	0.00% (0.00% - 0.00%)	0.00% (0.00% - 0.00%)
Russian Federation	5.20% (4.81% - 5.59%)	5.18% (4.85% - 5.53%)	3.68% (3.39% - 3.98%)	2.92% (2.67% - 3.23%)	0.61% (0.50% - 0.73%)	0.62% (0.52% - 0.74%)	0.25% (0.19% - 0.31%)	0.11% (0.07% - 0.16%)	0.00% (0.00% - 0.00%)	0.00% (0.00% - 0.00%)	0.00% (0.00% - 0.00%)	0.00% (0.00% - 0.00%)
Ukraine	8.89% (8.24% - 9.62%)	9.10% (8.56% - 9.65%)	8.43% (7.83% - 9.12%)	7.71% (7.06% - 8.43%)	2.33% (2.02% - 2.68%)	2.41% (2.14% - 2.69%)	2.12% (1.85% - 2.45%)	1.80% (1.51% - 2.14%)	0.23% (0.15% - 0.33%)	0.25% (0.18% - 0.33%)	0.18% (0.11% - 0.26%)	0.11% (0.06% - 0.17%)
High-income	1.41% (1.37% - 1.44%)	1.15% (1.11% - 1.18%)	0.98% (0.96% - 1.01%)	0.90% (0.87% - 0.93%)	0.03% (0.02% - 0.03%)	0.01% (0.01% - 0.01%)	0.01% (0.01% - 0.01%)	0.01% (0.01% - 0.01%)	0.00% (0.00% - 0.00%)	0.00% (0.00% - 0.00%)	0.00% (0.00% - 0.00%)	0.00% (0.00% - 0.00%)
**Australasia**	0.98% (0.90% - 1.08%)	0.86% (0.79% - 0.95%)	0.74% (0.67% - 0.82%)	0.66% (0.59% - 0.74%)	0.00% (0.00% - 0.00%)	0.00% (0.00% - 0.00%)	0.00% (0.00% - 0.00%)	0.00% (0.00% - 0.00%)	0.00% (0.00% - 0.00%)	0.00% (0.00% - 0.00%)	0.00% (0.00% - 0.00%)	0.00% (0.00% - 0.00%)
Australia	0.97% (0.87% - 1.08%)	0.86% (0.77% - 0.95%)	0.73% (0.65% - 0.82%)	0.66% (0.57% - 0.74%)	0.00% (0.00% - 0.00%)	0.00% (0.00% - 0.00%)	0.00% (0.00% - 0.00%)	0.00% (0.00% - 0.00%)	0.00% (0.00% - 0.00%)	0.00% (0.00% - 0.00%)	0.00% (0.00% - 0.00%)	0.00% (0.00% - 0.00%)
New Zealand	1.06% (0.95% - 1.17%)	0.90% (0.80% - 1.00%)	0.78% (0.68% - 0.88%)	0.67% (0.54% - 0.76%)	0.00% (0.00% - 0.00%)	0.00% (0.00% - 0.00%)	0.00% (0.00% - 0.00%)	0.00% (0.00% - 0.00%)	0.00% (0.00% - 0.00%)	0.00% (0.00% - 0.00%)	0.00% (0.00% - 0.00%)	0.00% (0.00% - 0.00%)
**High-income Asia Pacific**	2.65% (2.58% - 2.73%)	2.23% (2.17% - 2.28%)	2.13% (2.08% - 2.17%)	1.99% (1.95% - 2.04%)	0.07% (0.07% - 0.08%)	0.05% (0.05% - 0.06%)	0.05% (0.05% - 0.06%)	0.04% (0.04% - 0.04%)	0.00% (0.00% - 0.00%)	0.00% (0.00% - 0.00%)	0.00% (0.00% - 0.00%)	0.00% (0.00% - 0.00%)
Brunei Darussalam	3.72% (3.35% - 4.11%)	3.05% (2.75% - 3.38%)	2.66% (2.41% - 2.94%)	2.45% (2.20% - 2.71%)	0.24% (0.16% - 0.34%)	0.14% (0.08% - 0.20%)	0.09% (0.04% - 0.14%)	0.07% (0.02% - 0.12%)	0.00% (0.00% - 0.00%)	0.00% (0.00% - 0.00%)	0.00% (0.00% - 0.00%)	0.00% (0.00% - 0.00%)
Japan	2.78% (2.73% - 2.84%)	2.57% (2.53% - 2.62%)	2.48% (2.44% - 2.52%)	2.31% (2.26% - 2.35%)	0.09% (0.08% - 0.10%)	0.08% (0.07% - 0.09%)	0.08% (0.07% - 0.08%)	0.06% (0.05% - 0.06%)	0.00% (0.00% - 0.00%)	0.00% (0.00% - 0.00%)	0.00% (0.00% - 0.00%)	0.00% (0.00% - 0.00%)
Hokkaidō	2.69% (2.46% - 2.96%)	2.38% (2.16% - 2.60%)	2.30% (2.11% - 2.51%)	2.15% (1.96% - 2.35%)	0.08% (0.05% - 0.12%)	0.06% (0.03% - 0.09%)	0.05% (0.03% - 0.08%)	0.04% (0.02% - 0.06%)	0.00% (0.00% - 0.00%)	0.00% (0.00% - 0.00%)	0.00% (0.00% - 0.00%)	0.00% (0.00% - 0.00%)
Aomori	2.66% (2.41% - 2.91%)	2.58% (2.35% - 2.82%)	2.43% (2.20% - 2.66%)	2.24% (2.04% - 2.46%)	0.03% (0.01% - 0.06%)	0.05% (0.02% - 0.08%)	0.04% (0.02% - 0.07%)	0.03% (0.01% - 0.06%)	0.00% (0.00% - 0.00%)	0.00% (0.00% - 0.00%)	0.00% (0.00% - 0.00%)	0.00% (0.00% - 0.00%)
Iwate	2.52% (2.28% - 2.74%)	2.45% (2.23% - 2.66%)	2.38% (2.18% - 2.61%)	2.19% (1.98% - 2.40%)	0.02% (0.01% - 0.05%)	0.04% (0.02% - 0.07%)	0.04% (0.02% - 0.07%)	0.03% (0.01% - 0.05%)	0.00% (0.00% - 0.00%)	0.00% (0.00% - 0.00%)	0.00% (0.00% - 0.00%)	0.00% (0.00% - 0.00%)
Miyagi	2.30% (2.10% - 2.54%)	2.32% (2.10% - 2.55%)	2.26% (2.06% - 2.48%)	2.10% (1.91% - 2.31%)	0.01% (0.00% - 0.04%)	0.03% (0.01% - 0.06%)	0.03% (0.01% - 0.06%)	0.02% (0.01% - 0.04%)	0.00% (0.00% - 0.00%)	0.00% (0.00% - 0.00%)	0.00% (0.00% - 0.00%)	0.00% (0.00% - 0.00%)
Akita	2.47% (2.23% - 2.71%)	2.40% (2.19% - 2.61%)	2.36% (2.15% - 2.57%)	2.23% (2.04% - 2.44%)	0.03% (0.01% - 0.06%)	0.05% (0.03% - 0.08%)	0.05% (0.03% - 0.08%)	0.04% (0.02% - 0.07%)	0.00% (0.00% - 0.00%)	0.00% (0.00% - 0.00%)	0.00% (0.00% - 0.00%)	0.00% (0.00% - 0.00%)

Table S8b. Estimated overall, severe, and extreme **wasting** prevalence (%) in children under 5 years, both sexes, in 1990, 2000, 2010, and 2020.												
**Location**	**Total**				**Severe**				**Extreme**			
	1990	2000	2010	2020	1990	2000	2010	2020	1990	2000	2010	2020
Yamagata	2.32% (2.10% - 2.56%)	2.36% (2.15% - 2.59%)	2.29% (2.10% - 2.52%)	2.17% (1.97% - 2.37%)	0.01% (0.00% - 0.03%)	0.03% (0.01% - 0.05%)	0.03% (0.01% - 0.05%)	0.02% (0.00% - 0.04%)	0.00% (0.00% - 0.00%)	0.00% (0.00% - 0.00%)	0.00% (0.00% - 0.00%)	0.00% (0.00% - 0.00%)
Fukushima	2.76% (2.50% - 3.01%)	2.45% (2.22% - 2.67%)	2.30% (2.09% - 2.53%)	2.16% (1.97% - 2.35%)	0.08% (0.05% - 0.12%)	0.06% (0.03% - 0.09%)	0.05% (0.02% - 0.07%)	0.03% (0.01% - 0.06%)	0.00% (0.00% - 0.00%)	0.00% (0.00% - 0.00%)	0.00% (0.00% - 0.00%)	0.00% (0.00% - 0.00%)
Ibaraki	1.98% (1.80% - 2.16%)	1.76% (1.61% - 1.92%)	1.65% (1.51% - 1.81%)	1.53% (1.40% - 1.69%)	0.03% (0.01% - 0.06%)	0.02% (0.00% - 0.04%)	0.01% (0.00% - 0.03%)	0.01% (0.00% - 0.02%)	0.00% (0.00% - 0.00%)	0.00% (0.00% - 0.00%)	0.00% (0.00% - 0.00%)	0.00% (0.00% - 0.00%)
Tochigi	3.04% (2.81% - 3.32%)	2.77% (2.56% - 3.00%)	2.61% (2.40% - 2.82%)	2.42% (2.23% - 2.63%)	0.21% (0.15% - 0.28%)	0.16% (0.10% - 0.22%)	0.13% (0.09% - 0.19%)	0.11% (0.06% - 0.15%)	0.00% (0.00% - 0.00%)	0.00% (0.00% - 0.00%)	0.00% (0.00% - 0.00%)	0.00% (0.00% - 0.00%)
Gunma	1.92% (1.76% - 2.09%)	1.73% (1.59% - 1.88%)	1.65% (1.51% - 1.80%)	1.53% (1.40% - 1.66%)	0.04% (0.02% - 0.07%)	0.03% (0.01% - 0.05%)	0.02% (0.01% - 0.04%)	0.01% (0.00% - 0.03%)	0.00% (0.00% - 0.00%)	0.00% (0.00% - 0.00%)	0.00% (0.00% - 0.00%)	0.00% (0.00% - 0.00%)
Saitama	2.68% (2.45% - 2.95%)	2.39% (2.17% - 2.62%)	2.29% (2.08% - 2.49%)	2.15% (1.96% - 2.36%)	0.08% (0.05% - 0.12%)	0.06% (0.03% - 0.09%)	0.06% (0.03% - 0.08%)	0.04% (0.02% - 0.07%)	0.00% (0.00% - 0.00%)	0.00% (0.00% - 0.00%)	0.00% (0.00% - 0.00%)	0.00% (0.00% - 0.00%)
Chiba	1.23% (1.13% - 1.35%)	1.15% (1.05% - 1.25%)	1.10% (1.01% - 1.21%)	1.03% (0.95% - 1.12%)	0.01% (0.00% - 0.03%)	0.01% (0.00% - 0.02%)	0.00% (0.00% - 0.02%)	0.00% (0.00% - 0.01%)	0.00% (0.00% - 0.00%)	0.00% (0.00% - 0.00%)	0.00% (0.00% - 0.00%)	0.00% (0.00% - 0.00%)
Tōkyō	2.36% (2.18% - 2.59%)	2.18% (2.00% - 2.37%)	2.14% (1.99% - 2.30%)	1.95% (1.80% - 2.11%)	0.06% (0.03% - 0.09%)	0.05% (0.03% - 0.08%)	0.06% (0.05% - 0.08%)	0.03% (0.01% - 0.06%)	0.00% (0.00% - 0.00%)	0.00% (0.00% - 0.00%)	0.00% (0.00% - 0.00%)	0.00% (0.00% - 0.00%)
Kanagawa	3.12% (2.85% - 3.42%)	2.86% (2.63% - 3.11%)	2.79% (2.59% - 3.01%)	2.61% (2.39% - 2.83%)	0.13% (0.09% - 0.17%)	0.11% (0.08% - 0.15%)	0.12% (0.11% - 0.14%)	0.09% (0.06% - 0.12%)	0.00% (0.00% - 0.00%)	0.00% (0.00% - 0.00%)	0.00% (0.00% - 0.00%)	0.00% (0.00% - 0.00%)
Niigata	4.26% (3.82% - 4.72%)	3.84% (3.47% - 4.26%)	3.74% (3.38% - 4.12%)	3.53% (3.20% - 3.89%)	0.21% (0.15% - 0.29%)	0.16% (0.11% - 0.22%)	0.16% (0.11% - 0.21%)	0.13% (0.09% - 0.18%)	0.00% (0.00% - 0.00%)	0.00% (0.00% - 0.00%)	0.00% (0.00% - 0.00%)	0.00% (0.00% - 0.00%)
Toyama	2.25% (2.04% - 2.45%)	2.26% (2.06% - 2.47%)	2.19% (2.00% - 2.40%)	2.07% (1.90% - 2.27%)	0.02% (0.01% - 0.05%)	0.05% (0.02% - 0.07%)	0.05% (0.02% - 0.07%)	0.04% (0.02% - 0.06%)	0.00% (0.00% - 0.00%)	0.00% (0.00% - 0.00%)	0.00% (0.00% - 0.00%)	0.00% (0.00% - 0.00%)
Ishikawa	2.27% (2.06% - 2.49%)	2.27% (2.07% - 2.48%)	2.23% (2.05% - 2.45%)	2.13% (1.94% - 2.33%)	0.03% (0.01% - 0.05%)	0.05% (0.02% - 0.08%)	0.05% (0.02% - 0.08%)	0.04% (0.02% - 0.07%)	0.00% (0.00% - 0.00%)	0.00% (0.00% - 0.00%)	0.00% (0.00% - 0.00%)	0.00% (0.00% - 0.00%)
Fukui	2.27% (2.06% - 2.51%)	2.27% (2.07% - 2.48%)	2.21% (2.02% - 2.42%)	2.11% (1.92% - 2.31%)	0.02% (0.00% - 0.04%)	0.04% (0.02% - 0.07%)	0.04% (0.02% - 0.06%)	0.03% (0.01% - 0.05%)	0.00% (0.00% - 0.00%)	0.00% (0.00% - 0.00%)	0.00% (0.00% - 0.00%)	0.00% (0.00% - 0.00%)
Yamanashi	2.32% (2.09% - 2.56%)	2.31% (2.10% - 2.54%)	2.24% (2.04% - 2.46%)	2.12% (1.94% - 2.34%)	0.02% (0.00% - 0.04%)	0.04% (0.02% - 0.06%)	0.04% (0.01% - 0.06%)	0.03% (0.01% - 0.05%)	0.00% (0.00% - 0.00%)	0.00% (0.00% - 0.00%)	0.00% (0.00% - 0.00%)	0.00% (0.00% - 0.00%)
Nagano	2.20% (2.00% - 2.41%)	2.23% (2.03% - 2.44%)	2.18% (1.99% - 2.40%)	2.08% (1.90% - 2.28%)	0.01% (0.00% - 0.02%)	0.02% (0.00% - 0.04%)	0.02% (0.00% - 0.04%)	0.02% (0.00% - 0.04%)	0.00% (0.00% - 0.00%)	0.00% (0.00% - 0.00%)	0.00% (0.00% - 0.00%)	0.00% (0.00% - 0.00%)
Gifu	2.30% (2.10% - 2.53%)	2.32% (2.12% - 2.54%)	2.25% (2.05% - 2.48%)	2.12% (1.92% - 2.35%)	0.02% (0.00% - 0.04%)	0.04% (0.02% - 0.07%)	0.04% (0.01% - 0.07%)	0.03% (0.01% - 0.05%)	0.00% (0.00% - 0.00%)	0.00% (0.00% - 0.00%)	0.00% (0.00% - 0.00%)	0.00% (0.00% - 0.00%)
Shizuoka	3.59% (3.25% - 3.95%)	3.20% (2.93% - 3.49%)	3.06% (2.78% - 3.34%)	2.88% (2.62% - 3.17%)	0.15% (0.11% - 0.20%)	0.11% (0.08% - 0.15%)	0.10% (0.07% - 0.14%)	0.08% (0.06% - 0.12%)	0.00% (0.00% - 0.00%)	0.00% (0.00% - 0.00%)	0.00% (0.00% - 0.00%)	0.00% (0.00% - 0.00%)
Aichi	3.21% (2.93% - 3.51%)	2.91% (2.68% - 3.18%)	2.78% (2.57% - 3.02%)	2.53% (2.31% - 2.76%)	0.11% (0.08% - 0.15%)	0.10% (0.07% - 0.13%)	0.10% (0.09% - 0.12%)	0.07% (0.04% - 0.10%)	0.00% (0.00% - 0.00%)	0.00% (0.00% - 0.00%)	0.00% (0.00% - 0.00%)	0.00% (0.00% - 0.00%)
Mie	2.33% (2.12% - 2.56%)	2.34% (2.15% - 2.55%)	2.24% (2.04% - 2.44%)	2.08% (1.91% - 2.26%)	0.03% (0.01% - 0.06%)	0.06% (0.03% - 0.09%)	0.05% (0.03% - 0.08%)	0.04% (0.02% - 0.06%)	0.00% (0.00% - 0.00%)	0.00% (0.00% - 0.00%)	0.00% (0.00% - 0.00%)	0.00% (0.00% - 0.00%)
Shiga	3.36% (3.10% - 3.65%)	3.05% (2.82% - 3.30%)	2.93% (2.69% - 3.17%)	2.75% (2.53% - 2.97%)	0.26% (0.20% - 0.33%)	0.21% (0.14% - 0.27%)	0.19% (0.13% - 0.25%)	0.16% (0.10% - 0.21%)	0.00% (0.00% - 0.00%)	0.00% (0.00% - 0.00%)	0.00% (0.00% - 0.00%)	0.00% (0.00% - 0.00%)
Kyōto	4.73% (4.31% - 5.18%)	4.27% (3.90% - 4.69%)	4.07% (3.73% - 4.42%)	3.86% (3.53% - 4.21%)	0.41% (0.33% - 0.50%)	0.34% (0.27% - 0.43%)	0.31% (0.24% - 0.39%)	0.28% (0.21% - 0.35%)	0.00% (0.00% - 0.00%)	0.00% (0.00% - 0.00%)	0.00% (0.00% - 0.00%)	0.00% (0.00% - 0.00%)
Ōsaka	3.26% (2.97% - 3.60%)	2.84% (2.60% - 3.13%)	2.70% (2.47% - 2.94%)	2.53% (2.30% - 2.75%)	0.13% (0.09% - 0.17%)	0.10% (0.06% - 0.13%)	0.09% (0.06% - 0.12%)	0.07% (0.04% - 0.10%)	0.00% (0.00% - 0.00%)	0.00% (0.00% - 0.00%)	0.00% (0.00% - 0.00%)	0.00% (0.00% - 0.00%)
Hyōgo	3.94% (3.59% - 4.33%)	3.48% (3.16% - 3.79%)	3.35% (3.07% - 3.66%)	3.14% (2.87% - 3.44%)	0.20% (0.15% - 0.26%)	0.15% (0.11% - 0.20%)	0.15% (0.10% - 0.19%)	0.12% (0.08% - 0.16%)	0.00% (0.00% - 0.00%)	0.00% (0.00% - 0.00%)	0.00% (0.00% - 0.00%)	0.00% (0.00% - 0.00%)

Table S8b. Estimated overall, severe, and extreme **wasting** prevalence (%) in children under 5 years, both sexes, in 1990, 2000, 2010, and 2020.												
**Location**	**Total**				**Severe**				**Extreme**			
	**1990**	**2000**	**2010**	**2020**	**1990**	**2000**	**2010**	**2020**	**1990**	**2000**	**2010**	**2020**
	2.35%	2.35%	2.32%	2.19%	0.01%	0.02%	0.02%	0.02%	0.00%	0.00%	0.00%	0.00%
Nara	(2.12% - 2.58%)	(2.14% - 2.57%)	(2.11% - 2.55%)	(1.99% - 2.41%)	(0.00% - 0.02%)	(0.00% - 0.05%)	(0.01% - 0.05%)	(0.00% - 0.04%)	(0.00% - 0.00%)	(0.00% - 0.00%)	(0.00% - 0.00%)	(0.00% - 0.00%)
	2.44%	2.43%	2.33%	2.16%	0.02%	0.05%	0.04%	0.03%	0.00%	0.00%	0.00%	0.00%
Wakayama	(2.22% - 2.69%)	(2.21% - 2.66%)	(2.13% - 2.56%)	(1.97% - 2.38%)	(0.01% - 0.05%)	(0.02% - 0.07%)	(0.02% - 0.07%)	(0.01% - 0.05%)	(0.00% - 0.00%)	(0.00% - 0.00%)	(0.00% - 0.00%)	(0.00% - 0.00%)
	2.37%	2.37%	2.35%	2.22%	0.02%	0.05%	0.05%	0.04%	0.00%	0.00%	0.00%	0.00%
Tottori	(2.16% - 2.62%)	(2.16% - 2.58%)	(2.15% - 2.56%)	(2.03% - 2.44%)	(0.01% - 0.05%)	(0.02% - 0.07%)	(0.02% - 0.08%)	(0.02% - 0.07%)	(0.00% - 0.00%)	(0.00% - 0.00%)	(0.00% - 0.00%)	(0.00% - 0.00%)
	2.39%	2.37%	2.31%	2.15%	0.02%	0.04%	0.04%	0.03%	0.00%	0.00%	0.00%	0.00%
Shimane	(2.17% - 2.64%)	(2.17% - 2.58%)	(2.10% - 2.53%)	(1.96% - 2.35%)	(0.00% - 0.04%)	(0.02% - 0.06%)	(0.02% - 0.06%)	(0.01% - 0.05%)	(0.00% - 0.00%)	(0.00% - 0.00%)	(0.00% - 0.00%)	(0.00% - 0.00%)
	0.90%	0.82%	0.80%	0.75%	0.00%	0.00%	0.00%	0.00%	0.00%	0.00%	0.00%	0.00%
Okayama	(0.73% - 1.02%)	(0.68% - 0.93%)	(0.67% - 0.90%)	(0.65% - 0.84%)	(0.00% - 0.01%)	(0.00% - 0.01%)	(0.00% - 0.00%)	(0.00% - 0.00%)	(0.00% - 0.00%)	(0.00% - 0.00%)	(0.00% - 0.00%)	(0.00% - 0.00%)
	2.86%	2.58%	2.50%	2.33%	0.09%	0.07%	0.06%	0.05%	0.00%	0.00%	0.00%	0.00%
Hiroshima	(2.61% - 3.13%)	(2.35% - 2.83%)	(2.29% - 2.75%)	(2.13% - 2.56%)	(0.06% - 0.13%)	(0.04% - 0.10%)	(0.04% - 0.09%)	(0.02% - 0.07%)	(0.00% - 0.00%)	(0.00% - 0.00%)	(0.00% - 0.00%)	(0.00% - 0.00%)
	2.34%	2.34%	2.26%	2.10%	0.02%	0.05%	0.04%	0.03%	0.00%	0.00%	0.00%	0.00%
Yamaguchi	(2.11% - 2.58%)	(2.13% - 2.56%)	(2.06% - 2.47%)	(1.91% - 2.30%)	(0.01% - 0.05%)	(0.02% - 0.08%)	(0.02% - 0.07%)	(0.01% - 0.06%)	(0.00% - 0.00%)	(0.00% - 0.00%)	(0.00% - 0.00%)	(0.00% - 0.00%)
	2.48%	2.44%	2.33%	2.16%	0.04%	0.07%	0.06%	0.05%	0.00%	0.00%	0.00%	0.00%
Tokushima	(2.24% - 2.74%)	(2.23% - 2.66%)	(2.12% - 2.55%)	(1.96% - 2.36%)	(0.02% - 0.07%)	(0.04% - 0.10%)	(0.03% - 0.09%)	(0.02% - 0.07%)	(0.00% - 0.00%)	(0.00% - 0.00%)	(0.00% - 0.00%)	(0.00% - 0.00%)
	2.29%	2.33%	2.27%	2.14%	0.03%	0.05%	0.05%	0.04%	0.00%	0.00%	0.00%	0.00%
Kagawa	(2.08% - 2.52%)	(2.12% - 2.55%)	(2.06% - 2.49%)	(1.95% - 2.33%)	(0.01% - 0.05%)	(0.02% - 0.08%)	(0.02% - 0.08%)	(0.02% - 0.07%)	(0.00% - 0.00%)	(0.00% - 0.00%)	(0.00% - 0.00%)	(0.00% - 0.00%)
	2.42%	2.40%	2.34%	2.18%	0.02%	0.04%	0.04%	0.03%	0.00%	0.00%	0.00%	0.00%
Ehime	(2.19% - 2.66%)	(2.20% - 2.62%)	(2.14% - 2.57%)	(1.98% - 2.39%)	(0.00% - 0.04%)	(0.02% - 0.07%)	(0.02% - 0.07%)	(0.01% - 0.05%)	(0.00% - 0.00%)	(0.00% - 0.00%)	(0.00% - 0.00%)	(0.00% - 0.00%)
Kōchi	2.52%	2.49%	2.41%	2.23%	0.04%	0.06%	0.06%	0.04%	0.00%	0.00%	0.00%	0.00%
	(2.27% - 2.79%)	(2.27% - 2.72%)	(2.19% - 2.64%)	(2.03% - 2.44%)	(0.02% - 0.06%)	(0.03% - 0.09%)	(0.03% - 0.09%)	(0.02% - 0.07%)	(0.00% - 0.00%)	(0.00% - 0.00%)	(0.00% - 0.00%)	(0.00% - 0.00%)
	3.97%	3.56%	3.46%	3.20%	0.18%	0.15%	0.15%	0.11%	0.00%	0.00%	0.00%	0.00%
Fukuoka	(3.62% - 4.38%)	(3.25% - 3.91%)	(3.18% - 3.74%)	(2.90% - 3.50%)	(0.13% - 0.24%)	(0.11% - 0.19%)	(0.13% - 0.17%)	(0.08% - 0.15%)	(0.00% - 0.00%)	(0.00% - 0.00%)	(0.00% - 0.00%)	(0.00% - 0.00%)
	2.44%	2.43%	2.35%	2.20%	0.02%	0.03%	0.03%	0.02%	0.00%	0.00%	0.00%	0.00%
Saga	(2.22% - 2.70%)	(2.23% - 2.66%)	(2.15% - 2.57%)	(2.01% - 2.42%)	(0.00% - 0.04%)	(0.01% - 0.06%)	(0.01% - 0.06%)	(0.01% - 0.05%)	(0.00% - 0.00%)	(0.00% - 0.00%)	(0.00% - 0.00%)	(0.00% - 0.00%)
	3.24%	2.87%	2.76%	2.55%	0.25%	0.18%	0.16%	0.13%	0.00%	0.00%	0.00%	0.00%
Nagasaki	(2.99% - 3.52%)	(2.63% - 3.11%)	(2.55% - 3.00%)	(2.34% - 2.78%)	(0.19% - 0.32%)	(0.12% - 0.25%)	(0.11% - 0.22%)	(0.08% - 0.19%)	(0.00% - 0.00%)	(0.00% - 0.00%)	(0.00% - 0.00%)	(0.00% - 0.00%)
	2.38%	2.39%	2.37%	2.20%	0.01%	0.03%	0.03%	0.02%	0.00%	0.00%	0.00%	0.00%
Kumamoto	(2.15% - 2.65%)	(2.16% - 2.63%)	(2.16% - 2.60%)	(1.98% - 2.41%)	(0.00% - 0.03%)	(0.01% - 0.06%)	(0.01% - 0.06%)	(0.01% - 0.05%)	(0.00% - 0.00%)	(0.00% - 0.00%)	(0.00% - 0.00%)	(0.00% - 0.00%)
Ōita	2.38%	2.34%	2.29%	2.14%	0.04%	0.06%	0.06%	0.05%	0.00%	0.00%	0.00%	0.00%
	(2.15% - 2.64%)	(2.13% - 2.56%)	(2.10% - 2.51%)	(1.96% - 2.35%)	(0.01% - 0.06%)	(0.03% - 0.09%)	(0.03% - 0.09%)	(0.02% - 0.07%)	(0.00% - 0.00%)	(0.00% - 0.00%)	(0.00% - 0.00%)	(0.00% - 0.00%)
	2.52%	2.46%	2.41%	2.23%	0.03%	0.05%	0.05%	0.04%	0.00%	0.00%	0.00%	0.00%
Miyazaki	(2.28% - 2.79%)	(2.24% - 2.70%)	(2.19% - 2.64%)	(2.01% - 2.44%)	(0.01% - 0.06%)	(0.02% - 0.08%)	(0.02% - 0.08%)	(0.02% - 0.06%)	(0.00% - 0.00%)	(0.00% - 0.00%)	(0.00% - 0.00%)	(0.00% - 0.00%)
	2.59%	2.53%	2.43%	2.23%	0.03%	0.05%	0.04%	0.03%	0.00%	0.00%	0.00%	0.00%
Kagoshima	(2.35% - 2.83%)	(2.31% - 2.76%)	(2.21% - 2.66%)	(2.03% - 2.46%)	(0.01% - 0.05%)	(0.02% - 0.08%)	(0.02% - 0.07%)	(0.01% - 0.05%)	(0.00% - 0.00%)	(0.00% - 0.00%)	(0.00% - 0.00%)	(0.00% - 0.00%)
	2.61%	2.66%	2.55%	2.33%	0.02%	0.05%	0.05%	0.03%	0.00%	0.00%	0.00%	0.00%
Okinawa	(2.36% - 2.87%)	(2.42% - 2.90%)	(2.33% - 2.80%)	(2.12% - 2.56%)	(0.01% - 0.04%)	(0.02% - 0.08%)	(0.02% - 0.08%)	(0.01% - 0.06%)	(0.00% - 0.00%)	(0.00% - 0.00%)	(0.00% - 0.00%)	(0.00% - 0.00%)
	2.23%	1.46%	1.19%	1.07%	0.02%	0.00%	0.00%	0.00%	0.00%	0.00%	0.00%	0.00%
Republic of Korea	(2.04% - 2.44%)	(1.33% - 1.60%)	(1.08% - 1.32%)	(0.96% - 1.20%)	(0.01% - 0.04%)	(0.00% - 0.01%)	(0.00% - 0.00%)	(0.00% - 0.00%)	(0.00% - 0.00%)	(0.00% - 0.00%)	(0.00% - 0.00%)	(0.00% - 0.00%)
	5.05%	3.67%	2.96%	2.69%	0.37%	0.07%	0.01%	0.01%	0.00%	0.00%	0.00%	0.00%
Singapore	(4.56% - 5.56%)	(3.37% - 3.98%)	(2.66% - 3.29%)	(2.40% - 3.05%)	(0.24% - 0.53%)	(0.03% - 0.13%)	(0.00% - 0.04%)	(0.00% - 0.03%)	(0.00% - 0.00%)	(0.00% - 0.00%)	(0.00% - 0.00%)	(0.00% - 0.00%)
	0.59%	0.53%	0.50%	0.48%	0.00%	0.00%	0.00%	0.00%	0.00%	0.00%	0.00%	0.00%
**High-income North America**	(0.53% - 0.65%)	(0.45% - 0.60%)	(0.44% - 0.55%)	(0.42% - 0.54%)	(0.00% - 0.00%)	(0.00% - 0.00%)	(0.00% - 0.00%)	(0.00% - 0.00%)	(0.00% - 0.00%)	(0.00% - 0.00%)	(0.00% - 0.00%)	(0.00% - 0.00%)
	0.62%	0.50%	0.46%	0.44%	0.00%	0.00%	0.00%	0.00%	0.00%	0.00%	0.00%	0.00%
Canada	(0.55% - 0.71%)	(0.31% - 0.63%)	(0.32% - 0.57%)	(0.31% - 0.56%)	(0.00% - 0.00%)	(0.00% - 0.00%)	(0.00% - 0.00%)	(0.00% - 0.00%)	(0.00% - 0.00%)	(0.00% - 0.00%)	(0.00% - 0.00%)	(0.00% - 0.00%)

Table S8b. Estimated overall, severe, and extreme **wasting** prevalence (%) in children under 5 years, both sexes, in 1990, 2000, 2010, and 2020.

Location	Total				Severe				Extreme			
	1990	2000	2010	2020	1990	2000	2010	2020	1990	2000	2010	2020
United States of America	0.59% (0.52% - 0.65%)	0.53% (0.45% - 0.61%)	0.50% (0.44% - 0.56%)	0.49% (0.42% - 0.55%)	0.00% (0.00% - 0.00%)	0.00% (0.00% - 0.00%)	0.00% (0.00% - 0.00%)	0.00% (0.00% - 0.00%)	0.00% (0.00% - 0.00%)	0.00% (0.00% - 0.00%)	0.00% (0.00% - 0.00%)	0.00% (0.00% - 0.00%)
Alabama	0.67% (0.59% - 0.75%)	0.64% (0.50% - 0.73%)	0.59% (0.49% - 0.72%)	0.60% (0.50% - 0.72%)	0.00% (0.00% - 0.00%)	0.00% (0.00% - 0.00%)	0.00% (0.00% - 0.00%)	0.00% (0.00% - 0.00%)	0.00% (0.00% - 0.00%)	0.00% (0.00% - 0.00%)	0.00% (0.00% - 0.00%)	0.00% (0.00% - 0.00%)
Alaska	0.61% (0.47% - 0.71%)	0.55% (0.41% - 0.67%)	0.53% (0.38% - 0.66%)	0.49% (0.34% - 0.61%)	0.00% (0.00% - 0.00%)	0.00% (0.00% - 0.00%)	0.00% (0.00% - 0.00%)	0.00% (0.00% - 0.00%)	0.00% (0.00% - 0.00%)	0.00% (0.00% - 0.00%)	0.00% (0.00% - 0.00%)	0.00% (0.00% - 0.00%)
Arizona	0.63% (0.51% - 0.73%)	0.60% (0.47% - 0.70%)	0.53% (0.40% - 0.65%)	0.51% (0.37% - 0.64%)	0.00% (0.00% - 0.00%)	0.00% (0.00% - 0.00%)	0.00% (0.00% - 0.00%)	0.00% (0.00% - 0.00%)	0.00% (0.00% - 0.00%)	0.00% (0.00% - 0.00%)	0.00% (0.00% - 0.00%)	0.00% (0.00% - 0.00%)
Arkansas	0.67% (0.59% - 0.75%)	0.65% (0.52% - 0.75%)	0.62% (0.51% - 0.75%)	0.60% (0.50% - 0.72%)	0.00% (0.00% - 0.00%)	0.00% (0.00% - 0.00%)	0.00% (0.00% - 0.00%)	0.00% (0.00% - 0.00%)	0.00% (0.00% - 0.00%)	0.00% (0.00% - 0.00%)	0.00% (0.00% - 0.00%)	0.00% (0.00% - 0.00%)
California	0.61% (0.48% - 0.70%)	0.50% (0.32% - 0.63%)	0.47% (0.32% - 0.58%)	0.44% (0.31% - 0.56%)	0.00% (0.00% - 0.00%)	0.00% (0.00% - 0.00%)	0.00% (0.00% - 0.00%)	0.00% (0.00% - 0.00%)	0.00% (0.00% - 0.00%)	0.00% (0.00% - 0.00%)	0.00% (0.00% - 0.00%)	0.00% (0.00% - 0.00%)
Colorado	0.52% (0.35% - 0.63%)	0.49% (0.32% - 0.62%)	0.48% (0.33% - 0.60%)	0.46% (0.32% - 0.58%)	0.00% (0.00% - 0.00%)	0.00% (0.00% - 0.00%)	0.00% (0.00% - 0.00%)	0.00% (0.00% - 0.00%)	0.00% (0.00% - 0.00%)	0.00% (0.00% - 0.00%)	0.00% (0.00% - 0.00%)	0.00% (0.00% - 0.00%)
Connecticut	0.44% (0.29% - 0.59%)	0.39% (0.24% - 0.56%)	0.39% (0.26% - 0.52%)	0.40% (0.28% - 0.53%)	0.00% (0.00% - 0.00%)	0.00% (0.00% - 0.00%)	0.00% (0.00% - 0.00%)	0.00% (0.00% - 0.00%)	0.00% (0.00% - 0.00%)	0.00% (0.00% - 0.00%)	0.00% (0.00% - 0.00%)	0.00% (0.00% - 0.00%)
Delaware	0.58% (0.44% - 0.68%)	0.51% (0.33% - 0.63%)	0.49% (0.34% - 0.60%)	0.48% (0.34% - 0.59%)	0.00% (0.00% - 0.00%)	0.00% (0.00% - 0.00%)	0.00% (0.00% - 0.00%)	0.00% (0.00% - 0.00%)	0.00% (0.00% - 0.00%)	0.00% (0.00% - 0.00%)	0.00% (0.00% - 0.00%)	0.00% (0.00% - 0.00%)
District of Columbia	0.80% (0.72% - 0.90%)	0.63% (0.49% - 0.73%)	0.56% (0.42% - 0.68%)	0.52% (0.37% - 0.64%)	0.00% (0.00% - 0.00%)	0.00% (0.00% - 0.00%)	0.00% (0.00% - 0.00%)	0.00% (0.00% - 0.00%)	0.00% (0.00% - 0.00%)	0.00% (0.00% - 0.00%)	0.00% (0.00% - 0.00%)	0.00% (0.00% - 0.00%)
Florida	0.58% (0.45% - 0.68%)	0.51% (0.33% - 0.63%)	0.49% (0.34% - 0.61%)	0.48% (0.34% - 0.59%)	0.00% (0.00% - 0.00%)	0.00% (0.00% - 0.00%)	0.00% (0.00% - 0.00%)	0.00% (0.00% - 0.00%)	0.00% (0.00% - 0.00%)	0.00% (0.00% - 0.00%)	0.00% (0.00% - 0.00%)	0.00% (0.00% - 0.00%)
Georgia	0.66% (0.55% - 0.75%)	0.61% (0.47% - 0.71%)	0.54% (0.45% - 0.68%)	0.53% (0.38% - 0.66%)	0.00% (0.00% - 0.00%)	0.00% (0.00% - 0.00%)	0.00% (0.00% - 0.00%)	0.00% (0.00% - 0.00%)	0.00% (0.00% - 0.00%)	0.00% (0.00% - 0.00%)	0.00% (0.00% - 0.00%)	0.00% (0.00% - 0.00%)
Hawaii	0.55% (0.41% - 0.66%)	0.48% (0.31% - 0.62%)	0.48% (0.34% - 0.61%)	0.47% (0.33% - 0.60%)	0.00% (0.00% - 0.00%)	0.00% (0.00% - 0.00%)	0.00% (0.00% - 0.00%)	0.00% (0.00% - 0.00%)	0.00% (0.00% - 0.00%)	0.00% (0.00% - 0.00%)	0.00% (0.00% - 0.00%)	0.00% (0.00% - 0.00%)
Idaho	0.63% (0.51% - 0.71%)	0.59% (0.45% - 0.69%)	0.54% (0.45% - 0.66%)	0.52% (0.37% - 0.66%)	0.00% (0.00% - 0.00%)	0.00% (0.00% - 0.00%)	0.00% (0.00% - 0.00%)	0.00% (0.00% - 0.00%)	0.00% (0.00% - 0.00%)	0.00% (0.00% - 0.00%)	0.00% (0.00% - 0.00%)	0.00% (0.00% - 0.00%)
Illinois	0.60% (0.47% - 0.69%)	0.51% (0.33% - 0.64%)	0.47% (0.32% - 0.59%)	0.46% (0.32% - 0.57%)	0.00% (0.00% - 0.00%)	0.00% (0.00% - 0.00%)	0.00% (0.00% - 0.00%)	0.00% (0.00% - 0.00%)	0.00% (0.00% - 0.00%)	0.00% (0.00% - 0.00%)	0.00% (0.00% - 0.00%)	0.00% (0.00% - 0.00%)
Indiana	0.60% (0.47% - 0.69%)	0.56% (0.43% - 0.68%)	0.54% (0.45% - 0.65%)	0.53% (0.37% - 0.65%)	0.00% (0.00% - 0.00%)	0.00% (0.00% - 0.00%)	0.00% (0.00% - 0.00%)	0.00% (0.00% - 0.00%)	0.00% (0.00% - 0.00%)	0.00% (0.00% - 0.00%)	0.00% (0.00% - 0.00%)	0.00% (0.00% - 0.00%)
Iowa	0.53% (0.36% - 0.64%)	0.50% (0.32% - 0.63%)	0.48% (0.33% - 0.61%)	0.46% (0.32% - 0.59%)	0.00% (0.00% - 0.00%)	0.00% (0.00% - 0.00%)	0.00% (0.00% - 0.00%)	0.00% (0.00% - 0.00%)	0.00% (0.00% - 0.00%)	0.00% (0.00% - 0.00%)	0.00% (0.00% - 0.00%)	0.00% (0.00% - 0.00%)
Kansas	0.56% (0.43% - 0.66%)	0.53% (0.35% - 0.65%)	0.52% (0.38% - 0.64%)	0.50% (0.35% - 0.62%)	0.00% (0.00% - 0.00%)	0.00% (0.00% - 0.00%)	0.00% (0.00% - 0.00%)	0.00% (0.00% - 0.00%)	0.00% (0.00% - 0.00%)	0.00% (0.00% - 0.00%)	0.00% (0.00% - 0.00%)	0.00% (0.00% - 0.00%)
Kentucky	0.62% (0.50% - 0.71%)	0.59% (0.46% - 0.70%)	0.58% (0.47% - 0.70%)	0.55% (0.40% - 0.67%)	0.00% (0.00% - 0.00%)	0.00% (0.00% - 0.00%)	0.00% (0.00% - 0.00%)	0.00% (0.00% - 0.00%)	0.00% (0.00% - 0.00%)	0.00% (0.00% - 0.00%)	0.00% (0.00% - 0.00%)	0.00% (0.00% - 0.00%)
Louisiana	0.67% (0.59% - 0.76%)	0.65% (0.51% - 0.75%)	0.57% (0.48% - 0.69%)	0.56% (0.47% - 0.69%)	0.00% (0.00% - 0.00%)	0.00% (0.00% - 0.00%)	0.00% (0.00% - 0.00%)	0.00% (0.00% - 0.00%)	0.00% (0.00% - 0.00%)	0.00% (0.00% - 0.00%)	0.00% (0.00% - 0.00%)	0.00% (0.00% - 0.00%)
Maine	0.52% (0.38% - 0.64%)	0.48% (0.31% - 0.61%)	0.47% (0.32% - 0.59%)	0.47% (0.33% - 0.60%)	0.00% (0.00% - 0.00%)	0.00% (0.00% - 0.00%)	0.00% (0.00% - 0.00%)	0.00% (0.00% - 0.00%)	0.00% (0.00% - 0.00%)	0.00% (0.00% - 0.00%)	0.00% (0.00% - 0.00%)	0.00% (0.00% - 0.00%)
Maryland	0.55% (0.43% - 0.65%)	0.49% (0.31% - 0.63%)	0.46% (0.32% - 0.58%)	0.46% (0.33% - 0.58%)	0.00% (0.00% - 0.00%)	0.00% (0.00% - 0.00%)	0.00% (0.00% - 0.00%)	0.00% (0.00% - 0.00%)	0.00% (0.00% - 0.00%)	0.00% (0.00% - 0.00%)	0.00% (0.00% - 0.00%)	0.00% (0.00% - 0.00%)
Massachusetts	0.44% (0.29% - 0.58%)	0.38% (0.25% - 0.54%)	0.40% (0.27% - 0.54%)	0.38% (0.27% - 0.51%)	0.00% (0.00% - 0.00%)	0.00% (0.00% - 0.00%)	0.00% (0.00% - 0.00%)	0.00% (0.00% - 0.00%)	0.00% (0.00% - 0.00%)	0.00% (0.00% - 0.00%)	0.00% (0.00% - 0.00%)	0.00% (0.00% - 0.00%)

Table S8b. Estimated overall, severe, and extreme **wasting** prevalence (%) in children under 5 years, both sexes, in 1990, 2000, 2010, and 2020.												
**Location**	**Total**				**Severe**				**Extreme**			
	**1990**	**2000**	**2010**	**2020**	**1990**	**2000**	**2010**	**2020**	**1990**	**2000**	**2010**	**2020**
Michigan	0.60%	0.53%	0.52%	0.50%	0.00%	0.00%	0.00%	0.00%	0.00%	0.00%	0.00%	0.00%
	(0.47% - 0.70%)	(0.36% - 0.66%)	(0.39% - 0.65%)	(0.35% - 0.63%)	(0.00% - 0.00%)	(0.00% - 0.00%)	(0.00% - 0.00%)	(0.00% - 0.00%)	(0.00% - 0.00%)	(0.00% - 0.00%)	(0.00% - 0.00%)	(0.00% - 0.00%)
Minnesota	0.46%	0.42%	0.43%	0.41%	0.00%	0.00%	0.00%	0.00%	0.00%	0.00%	0.00%	0.00%
	(0.29% - 0.59%)	(0.27% - 0.58%)	(0.29% - 0.56%)	(0.29% - 0.54%)	(0.00% - 0.00%)	(0.00% - 0.00%)	(0.00% - 0.00%)	(0.00% - 0.00%)	(0.00% - 0.00%)	(0.00% - 0.00%)	(0.00% - 0.00%)	(0.00% - 0.00%)
Mississippi	0.70%	0.69%	0.65%	0.64%	0.00%	0.00%	0.00%	0.00%	0.00%	0.00%	0.00%	0.00%
	(0.63% - 0.79%)	(0.57% - 0.78%)	(0.53% - 0.76%)	(0.52% - 0.76%)	(0.00% - 0.00%)	(0.00% - 0.00%)	(0.00% - 0.00%)	(0.00% - 0.00%)	(0.00% - 0.00%)	(0.00% - 0.00%)	(0.00% - 0.00%)	(0.00% - 0.00%)
Missouri	0.60%	0.55%	0.53%	0.54%	0.00%	0.00%	0.00%	0.00%	0.00%	0.00%	0.00%	0.00%
	(0.47% - 0.69%)	(0.41% - 0.66%)	(0.39% - 0.66%)	(0.39% - 0.66%)	(0.00% - 0.00%)	(0.00% - 0.00%)	(0.00% - 0.00%)	(0.00% - 0.00%)	(0.00% - 0.00%)	(0.00% - 0.00%)	(0.00% - 0.00%)	(0.00% - 0.00%)
Montana	0.59%	0.56%	0.53%	0.50%	0.00%	0.00%	0.00%	0.00%	0.00%	0.00%	0.00%	0.00%
	(0.46% - 0.69%)	(0.42% - 0.67%)	(0.43% - 0.66%)	(0.34% - 0.63%)	(0.00% - 0.00%)	(0.00% - 0.00%)	(0.00% - 0.00%)	(0.00% - 0.00%)	(0.00% - 0.00%)	(0.00% - 0.00%)	(0.00% - 0.00%)	(0.00% - 0.00%)
Nebraska	0.53%	0.50%	0.48%	0.45%	0.00%	0.00%	0.00%	0.00%	0.00%	0.00%	0.00%	0.00%
	(0.39% - 0.65%)	(0.33% - 0.63%)	(0.33% - 0.60%)	(0.32% - 0.57%)	(0.00% - 0.00%)	(0.00% - 0.00%)	(0.00% - 0.00%)	(0.00% - 0.00%)	(0.00% - 0.00%)	(0.00% - 0.00%)	(0.00% - 0.00%)	(0.00% - 0.00%)
Nevada	0.65%	0.61%	0.55%	0.55%	0.00%	0.00%	0.00%	0.00%	0.00%	0.00%	0.00%	0.00%
	(0.54% - 0.74%)	(0.47% - 0.71%)	(0.45% - 0.68%)	(0.42% - 0.68%)	(0.00% - 0.00%)	(0.00% - 0.00%)	(0.00% - 0.00%)	(0.00% - 0.00%)	(0.00% - 0.00%)	(0.00% - 0.00%)	(0.00% - 0.00%)	(0.00% - 0.00%)
New Hampshire	0.47%	0.41%	0.41%	0.41%	0.00%	0.00%	0.00%	0.00%	0.00%	0.00%	0.00%	0.00%
	(0.29% - 0.60%)	(0.26% - 0.55%)	(0.28% - 0.53%)	(0.29% - 0.55%)	(0.00% - 0.00%)	(0.00% - 0.00%)	(0.00% - 0.00%)	(0.00% - 0.00%)	(0.00% - 0.00%)	(0.00% - 0.00%)	(0.00% - 0.00%)	(0.00% - 0.00%)
New Jersey	0.50%	0.43%	0.43%	0.43%	0.00%	0.00%	0.00%	0.00%	0.00%	0.00%	0.00%	0.00%
	(0.32% - 0.63%)	(0.27% - 0.58%)	(0.29% - 0.56%)	(0.30% - 0.55%)	(0.00% - 0.00%)	(0.00% - 0.00%)	(0.00% - 0.00%)	(0.00% - 0.00%)	(0.00% - 0.00%)	(0.00% - 0.00%)	(0.00% - 0.00%)	(0.00% - 0.00%)
New Mexico	0.67%	0.64%	0.58%	0.58%	0.00%	0.00%	0.00%	0.00%	0.00%	0.00%	0.00%	0.00%
	(0.60% - 0.75%)	(0.49% - 0.75%)	(0.49% - 0.70%)	(0.49% - 0.71%)	(0.00% - 0.00%)	(0.00% - 0.00%)	(0.00% - 0.00%)	(0.00% - 0.00%)	(0.00% - 0.00%)	(0.00% - 0.00%)	(0.00% - 0.00%)	(0.00% - 0.00%)
New York	0.57%	0.45%	0.45%	0.44%	0.00%	0.00%	0.00%	0.00%	0.00%	0.00%	0.00%	0.00%
	(0.44% - 0.67%)	(0.29% - 0.59%)	(0.32% - 0.57%)	(0.31% - 0.57%)	(0.00% - 0.00%)	(0.00% - 0.00%)	(0.00% - 0.00%)	(0.00% - 0.00%)	(0.00% - 0.00%)	(0.00% - 0.00%)	(0.00% - 0.00%)	(0.00% - 0.00%)
North Carolina	0.64%	0.58%	0.53%	0.52%	0.00%	0.00%	0.00%	0.00%	0.00%	0.00%	0.00%	0.00%
	(0.51% - 0.73%)	(0.44% - 0.69%)	(0.39% - 0.65%)	(0.37% - 0.66%)	(0.00% - 0.00%)	(0.00% - 0.00%)	(0.00% - 0.00%)	(0.00% - 0.00%)	(0.00% - 0.00%)	(0.00% - 0.00%)	(0.00% - 0.00%)	(0.00% - 0.00%)
North Dakota	0.55%	0.50%	0.51%	0.45%	0.00%	0.00%	0.00%	0.00%	0.00%	0.00%	0.00%	0.00%
	(0.41% - 0.65%)	(0.32% - 0.64%)	(0.36% - 0.63%)	(0.31% - 0.58%)	(0.00% - 0.00%)	(0.00% - 0.00%)	(0.00% - 0.00%)	(0.00% - 0.00%)	(0.00% - 0.00%)	(0.00% - 0.00%)	(0.00% - 0.00%)	(0.00% - 0.00%)
Ohio	0.59%	0.55%	0.53%	0.55%	0.00%	0.00%	0.00%	0.00%	0.00%	0.00%	0.00%	0.00%
	(0.45% - 0.68%)	(0.42% - 0.66%)	(0.38% - 0.65%)	(0.45% - 0.67%)	(0.00% - 0.00%)	(0.00% - 0.00%)	(0.00% - 0.00%)	(0.00% - 0.00%)	(0.00% - 0.00%)	(0.00% - 0.00%)	(0.00% - 0.00%)	(0.00% - 0.00%)
Oklahoma	0.62%	0.64%	0.60%	0.56%	0.00%	0.00%	0.00%	0.00%	0.00%	0.00%	0.00%	0.00%
	(0.50% - 0.71%)	(0.50% - 0.74%)	(0.50% - 0.71%)	(0.46% - 0.69%)	(0.00% - 0.00%)	(0.00% - 0.00%)	(0.00% - 0.00%)	(0.00% - 0.00%)	(0.00% - 0.00%)	(0.00% - 0.00%)	(0.00% - 0.00%)	(0.00% - 0.00%)
Oregon	0.56%	0.50%	0.49%	0.46%	0.00%	0.00%	0.00%	0.00%	0.00%	0.00%	0.00%	0.00%
	(0.42% - 0.66%)	(0.32% - 0.63%)	(0.33% - 0.61%)	(0.32% - 0.58%)	(0.00% - 0.00%)	(0.00% - 0.00%)	(0.00% - 0.00%)	(0.00% - 0.00%)	(0.00% - 0.00%)	(0.00% - 0.00%)	(0.00% - 0.00%)	(0.00% - 0.00%)
Pennsylvania	0.56%	0.49%	0.48%	0.47%	0.00%	0.00%	0.00%	0.00%	0.00%	0.00%	0.00%	0.00%
	(0.43% - 0.67%)	(0.31% - 0.62%)	(0.33% - 0.59%)	(0.33% - 0.59%)	(0.00% - 0.00%)	(0.00% - 0.00%)	(0.00% - 0.00%)	(0.00% - 0.00%)	(0.00% - 0.00%)	(0.00% - 0.00%)	(0.00% - 0.00%)	(0.00% - 0.00%)
Rhode Island	0.51%	0.45%	0.44%	0.43%	0.00%	0.00%	0.00%	0.00%	0.00%	0.00%	0.00%	0.00%
	(0.34% - 0.62%)	(0.28% - 0.59%)	(0.31% - 0.57%)	(0.31% - 0.57%)	(0.00% - 0.00%)	(0.00% - 0.00%)	(0.00% - 0.00%)	(0.00% - 0.00%)	(0.00% - 0.00%)	(0.00% - 0.00%)	(0.00% - 0.00%)	(0.00% - 0.00%)
South Carolina	0.68%	0.63%	0.58%	0.57%	0.00%	0.00%	0.00%	0.00%	0.00%	0.00%	0.00%	0.00%
	(0.60% - 0.76%)	(0.48% - 0.73%)	(0.48% - 0.70%)	(0.48% - 0.69%)	(0.00% - 0.00%)	(0.00% - 0.00%)	(0.00% - 0.00%)	(0.00% - 0.00%)	(0.00% - 0.00%)	(0.00% - 0.00%)	(0.00% - 0.00%)	(0.00% - 0.00%)
South Dakota	0.59%	0.54%	0.52%	0.51%	0.00%	0.00%	0.00%	0.00%	0.00%	0.00%	0.00%	0.00%
	(0.46% - 0.69%)	(0.36% - 0.66%)	(0.38% - 0.65%)	(0.36% - 0.63%)	(0.00% - 0.00%)	(0.00% - 0.00%)	(0.00% - 0.00%)	(0.00% - 0.00%)	(0.00% - 0.00%)	(0.00% - 0.00%)	(0.00% - 0.00%)	(0.00% - 0.00%)
Tennessee	0.65%	0.61%	0.63%	0.56%	0.00%	0.00%	0.00%	0.00%	0.00%	0.00%	0.00%	0.00%
	(0.55% - 0.74%)	(0.47% - 0.71%)	(0.52% - 0.75%)	(0.43% - 0.68%)	(0.00% - 0.00%)	(0.00% - 0.00%)	(0.00% - 0.00%)	(0.00% - 0.00%)	(0.00% - 0.00%)	(0.00% - 0.00%)	(0.00% - 0.00%)	(0.00% - 0.00%)
Texas	0.64%	0.61%	0.54%	0.51%	0.00%	0.00%	0.00%	0.00%	0.00%	0.00%	0.00%	0.00%
	(0.52% - 0.73%)	(0.48% - 0.72%)	(0.45% - 0.66%)	(0.36% - 0.63%)	(0.00% - 0.00%)	(0.00% - 0.00%)	(0.00% - 0.00%)	(0.00% - 0.00%)	(0.00% - 0.00%)	(0.00% - 0.00%)	(0.00% - 0.00%)	(0.00% - 0.00%)
Utah	0.62%	0.58%	0.52%	0.52%	0.00%	0.00%	0.00%	0.00%	0.00%	0.00%	0.00%	0.00%
	(0.49% - 0.72%)	(0.43% - 0.69%)	(0.39% - 0.64%)	(0.37% - 0.65%)	(0.00% - 0.00%)	(0.00% - 0.00%)	(0.00% - 0.00%)	(0.00% - 0.00%)	(0.00% - 0.00%)	(0.00% - 0.00%)	(0.00% - 0.00%)	(0.00% - 0.00%)
	0.54%	0.46%	0.45%	0.44%	0.00%	0.00%	0.00%	0.00%	0.00%	0.00%	0.00%	0.00%

Table S8b. Estimated overall, severe, and extreme **wasting** prevalence (%) in children under 5 years, both sexes, in 1990, 2000, 2010, and 2020.

Location	Total				Severe				Extreme			
	1990	2000	2010	2020	1990	2000	2010	2020	1990	2000	2010	2020
Vermont	(0.41% - 0.65%)	(0.29% - 0.60%)	(0.30% - 0.57%)	(0.31% - 0.57%)	(0.00% - 0.00%)	(0.00% - 0.00%)	(0.00% - 0.00%)	(0.00% - 0.00%)	(0.00% - 0.00%)	(0.00% - 0.00%)	(0.00% - 0.00%)	(0.00% - 0.00%)
	0.57%	0.49%	0.47%	0.46%	0.00%	0.00%	0.00%	0.00%	0.00%	0.00%	0.00%	0.00%
Virginia	(0.43% - 0.68%)	(0.32% - 0.63%)	(0.32% - 0.59%)	(0.32% - 0.59%)	(0.00% - 0.00%)	(0.00% - 0.00%)	(0.00% - 0.00%)	(0.00% - 0.00%)	(0.00% - 0.00%)	(0.00% - 0.00%)	(0.00% - 0.00%)	(0.00% - 0.00%)
	0.54%	0.47%	0.46%	0.43%	0.00%	0.00%	0.00%	0.00%	0.00%	0.00%	0.00%	0.00%
Washington	(0.40% - 0.66%)	(0.30% - 0.60%)	(0.32% - 0.58%)	(0.31% - 0.56%)	(0.00% - 0.00%)	(0.00% - 0.00%)	(0.00% - 0.00%)	(0.00% - 0.00%)	(0.00% - 0.00%)	(0.00% - 0.00%)	(0.00% - 0.00%)	(0.00% - 0.00%)
	0.64%	0.61%	0.62%	0.59%	0.00%	0.00%	0.00%	0.00%	0.00%	0.00%	0.00%	0.00%
West Virginia	(0.55% - 0.73%)	(0.48% - 0.71%)	(0.51% - 0.74%)	(0.49% - 0.71%)	(0.00% - 0.00%)	(0.00% - 0.00%)	(0.00% - 0.00%)	(0.00% - 0.00%)	(0.00% - 0.00%)	(0.00% - 0.00%)	(0.00% - 0.00%)	(0.00% - 0.00%)
	0.51%	0.47%	0.47%	0.47%	0.00%	0.00%	0.00%	0.00%	0.00%	0.00%	0.00%	0.00%
Wisconsin	(0.35% - 0.63%)	(0.30% - 0.61%)	(0.33% - 0.59%)	(0.33% - 0.59%)	(0.00% - 0.00%)	(0.00% - 0.00%)	(0.00% - 0.00%)	(0.00% - 0.00%)	(0.00% - 0.00%)	(0.00% - 0.00%)	(0.00% - 0.00%)	(0.00% - 0.00%)
	0.58%	0.55%	0.51%	0.48%	0.00%	0.00%	0.00%	0.00%	0.00%	0.00%	0.00%	0.00%
Wyoming	(0.44% - 0.67%)	(0.41% - 0.66%)	(0.36% - 0.63%)	(0.33% - 0.61%)	(0.00% - 0.00%)	(0.00% - 0.00%)	(0.00% - 0.00%)	(0.00% - 0.00%)	(0.00% - 0.00%)	(0.00% - 0.00%)	(0.00% - 0.00%)	(0.00% - 0.00%)
	1.09%	0.94%	0.86%	0.72%	0.00%	0.00%	0.00%	0.00%	0.00%	0.00%	0.00%	0.00%
Greenland	(0.99% - 1.20%)	(0.85% - 1.05%)	(0.77% - 0.95%)	(0.60% - 0.83%)	(0.00% - 0.00%)	(0.00% - 0.00%)	(0.00% - 0.00%)	(0.00% - 0.00%)	(0.00% - 0.00%)	(0.00% - 0.00%)	(0.00% - 0.00%)	(0.00% - 0.00%)
	2.64%	1.91%	1.30%	1.09%	0.01%	0.00%	0.00%	0.00%	0.00%	0.00%	0.00%	0.00%
**Southern Latin America**	(2.46% - 2.86%)	(1.79% - 2.03%)	(1.20% - 1.41%)	(0.99% - 1.20%)	(0.00% - 0.04%)	(0.00% - 0.00%)	(0.00% - 0.00%)	(0.00% - 0.00%)	(0.00% - 0.00%)	(0.00% - 0.00%)	(0.00% - 0.00%)	(0.00% - 0.00%)
	3.18%	2.26%	1.50%	1.31%	0.02%	0.00%	0.00%	0.00%	0.00%	0.00%	0.00%	0.00%
Argentina	(2.91% - 3.51%)	(2.09% - 2.44%)	(1.36% - 1.66%)	(1.18% - 1.45%)	(0.00% - 0.05%)	(0.00% - 0.00%)	(0.00% - 0.00%)	(0.00% - 0.00%)	(0.00% - 0.00%)	(0.00% - 0.00%)	(0.00% - 0.00%)	(0.00% - 0.00%)
	1.25%	0.84%	0.57%	0.32%	0.00%	0.00%	0.00%	0.00%	0.00%	0.00%	0.00%	0.00%
Chile	(1.13% - 1.38%)	(0.76% - 0.93%)	(0.43% - 0.66%)	(0.14% - 0.45%)	(0.00% - 0.00%)	(0.00% - 0.00%)	(0.00% - 0.00%)	(0.00% - 0.00%)	(0.00% - 0.00%)	(0.00% - 0.00%)	(0.00% - 0.00%)	(0.00% - 0.00%)
	3.14%	2.40%	1.95%	1.62%	0.04%	0.00%	0.00%	0.00%	0.00%	0.00%	0.00%	0.00%
Uruguay	(2.88% - 3.43%)	(2.18% - 2.62%)	(1.77% - 2.14%)	(1.46% - 1.78%)	(0.03% - 0.07%)	(0.00% - 0.01%)	(0.00% - 0.00%)	(0.00% - 0.00%)	(0.00% - 0.00%)	(0.00% - 0.00%)	(0.00% - 0.00%)	(0.00% - 0.00%)
	1.36%	1.13%	1.01%	0.92%	0.03%	0.01%	0.00%	0.00%	0.00%	0.00%	0.00%	0.00%
**Western Europe**	(1.32% - 1.41%)	(1.09% - 1.18%)	(0.97% - 1.05%)	(0.88% - 0.97%)	(0.03% - 0.04%)	(0.01% - 0.01%)	(0.00% - 0.01%)	(0.00% - 0.00%)	(0.00% - 0.00%)	(0.00% - 0.00%)	(0.00% - 0.00%)	(0.00% - 0.00%)
	0.95%	0.81%	0.75%	0.70%	0.01%	0.00%	0.00%	0.00%	0.00%	0.00%	0.00%	0.00%
Monaco	(0.80% - 1.08%)	(0.67% - 0.97%)	(0.61% - 0.89%)	(0.57% - 0.83%)	(0.00% - 0.02%)	(0.00% - 0.00%)	(0.00% - 0.00%)	(0.00% - 0.00%)	(0.00% - 0.00%)	(0.00% - 0.00%)	(0.00% - 0.00%)	(0.00% - 0.00%)
	1.04%	0.93%	0.87%	0.90%	0.01%	0.00%	0.00%	0.00%	0.00%	0.00%	0.00%	0.00%
San Marino	(0.92% - 1.14%)	(0.76% - 1.09%)	(0.71% - 1.00%)	(0.74% - 1.03%)	(0.00% - 0.03%)	(0.00% - 0.01%)	(0.00% - 0.00%)	(0.00% - 0.00%)	(0.00% - 0.00%)	(0.00% - 0.00%)	(0.00% - 0.00%)	(0.00% - 0.00%)
	1.08%	0.91%	0.77%	0.78%	0.02%	0.00%	0.00%	0.00%	0.00%	0.00%	0.00%	0.00%
Andorra	(0.97% - 1.19%)	(0.74% - 1.04%)	(0.62% - 0.92%)	(0.63% - 0.92%)	(0.01% - 0.03%)	(0.00% - 0.01%)	(0.00% - 0.00%)	(0.00% - 0.00%)	(0.00% - 0.00%)	(0.00% - 0.00%)	(0.00% - 0.00%)	(0.00% - 0.00%)
	1.47%	1.18%	1.02%	0.92%	0.04%	0.01%	0.00%	0.00%	0.00%	0.00%	0.00%	0.00%
Austria	(1.33% - 1.60%)	(1.07% - 1.31%)	(0.89% - 1.15%)	(0.74% - 1.06%)	(0.02% - 0.06%)	(0.00% - 0.02%)	(0.00% - 0.01%)	(0.00% - 0.01%)	(0.00% - 0.00%)	(0.00% - 0.00%)	(0.00% - 0.00%)	(0.00% - 0.00%)
	1.37%	1.15%	0.95%	0.86%	0.03%	0.01%	0.00%	0.00%	0.00%	0.00%	0.00%	0.00%
Belgium	(1.25% - 1.50%)	(1.04% - 1.28%)	(0.79% - 1.07%)	(0.70% - 1.00%)	(0.02% - 0.05%)	(0.00% - 0.02%)	(0.00% - 0.01%)	(0.00% - 0.00%)	(0.00% - 0.00%)	(0.00% - 0.00%)	(0.00% - 0.00%)	(0.00% - 0.00%)
	1.93%	1.51%	1.32%	1.24%	0.09%	0.02%	0.01%	0.01%	0.00%	0.00%	0.00%	0.00%
Cyprus	(1.75% - 2.10%)	(1.37% - 1.66%)	(1.20% - 1.45%)	(1.12% - 1.37%)	(0.06% - 0.12%)	(0.01% - 0.04%)	(0.00% - 0.02%)	(0.00% - 0.02%)	(0.00% - 0.00%)	(0.00% - 0.00%)	(0.00% - 0.00%)	(0.00% - 0.00%)
	1.49%	1.21%	1.11%	1.01%	0.04%	0.01%	0.00%	0.00%	0.00%	0.00%	0.00%	0.00%
Denmark	(1.36% - 1.64%)	(1.10% - 1.35%)	(1.00% - 1.22%)	(0.88% - 1.14%)	(0.03% - 0.07%)	(0.00% - 0.02%)	(0.00% - 0.01%)	(0.00% - 0.01%)	(0.00% - 0.00%)	(0.00% - 0.00%)	(0.00% - 0.00%)	(0.00% - 0.00%)
	1.47%	1.26%	1.11%	1.05%	0.04%	0.01%	0.00%	0.00%	0.00%	0.00%	0.00%	0.00%
Finland	(1.35% - 1.60%)	(1.13% - 1.39%)	(1.00% - 1.23%)	(0.94% - 1.18%)	(0.02% - 0.07%)	(0.00% - 0.02%)	(0.00% - 0.01%)	(0.00% - 0.01%)	(0.00% - 0.00%)	(0.00% - 0.00%)	(0.00% - 0.00%)	(0.00% - 0.00%)
	1.40%	1.15%	1.01%	0.91%	0.04%	0.01%	0.00%	0.00%	0.00%	0.00%	0.00%	0.00%
France	(1.27% - 1.54%)	(1.04% - 1.28%)	(0.90% - 1.12%)	(0.75% - 1.05%)	(0.02% - 0.06%)	(0.00% - 0.02%)	(0.00% - 0.01%)	(0.00% - 0.01%)	(0.00% - 0.00%)	(0.00% - 0.00%)	(0.00% - 0.00%)	(0.00% - 0.00%)
	1.22%	0.91%	0.79%	0.74%	0.02%	0.01%	0.00%	0.00%	0.00%	0.00%	0.00%	0.00%
Germany	(1.11% - 1.33%)	(0.77% - 1.03%)	(0.69% - 0.93%)	(0.63% - 0.88%)	(0.01% - 0.03%)	(0.00% - 0.01%)	(0.00% - 0.01%)	(0.00% - 0.01%)	(0.00% - 0.00%)	(0.00% - 0.00%)	(0.00% - 0.00%)	(0.00% - 0.00%)
	0.99%	0.77%	0.70%	0.70%	0.00%	0.00%	0.00%	0.00%	0.00%	0.00%	0.00%	0.00%
Greece	(0.89% - 1.11%)	(0.68% - 0.87%)	(0.56% - 0.80%)	(0.56% - 0.81%)	(0.00% - 0.00%)	(0.00% - 0.00%)	(0.00% - 0.00%)	(0.00% - 0.00%)	(0.00% - 0.00%)	(0.00% - 0.00%)	(0.00% - 0.00%)	(0.00% - 0.00%)
	1.18%	1.10%	1.02%	0.95%	0.02%	0.00%	0.00%	0.00%	0.00%	0.00%	0.00%	0.00%
Iceland	(1.07% - 1.31%)	(0.99% - 1.22%)	(0.90% - 1.14%)	(0.79% - 1.07%)	(0.01% - 0.04%)	(0.00% - 0.01%)	(0.00% - 0.01%)	(0.00% - 0.01%)	(0.00% - 0.00%)	(0.00% - 0.00%)	(0.00% - 0.00%)	(0.00% - 0.00%)

Table S8b. Estimated overall, severe, and extreme **wasting** prevalence (%) in children under 5 years, both sexes, in 1990, 2000, 2010, and 2020.

Location	Total				Severe				Extreme			
	1990	2000	2010	2020	1990	2000	2010	2020	1990	2000	2010	2020
	1.41%	1.20%	0.96%	0.81%	0.04%	0.01%	0.00%	0.00%	0.00%	0.00%	0.00%	0.00%
Ireland	(1.28% - 1.55%)	(1.08% - 1.33%)	(0.80% - 1.10%)	(0.67% - 0.95%)	(0.02% - 0.06%)	(0.00% - 0.02%)	(0.00% - 0.01%)	(0.00% - 0.00%)	(0.00% - 0.00%)	(0.00% - 0.00%)	(0.00% - 0.00%)	(0.00% - 0.00%)
	1.32%	1.10%	0.95%	0.86%	0.03%	0.00%	0.00%	0.00%	0.00%	0.00%	0.00%	0.00%
Israel	(1.20% - 1.45%)	(0.98% - 1.24%)	(0.78% - 1.08%)	(0.71% - 1.00%)	(0.02% - 0.05%)	(0.00% - 0.02%)	(0.00% - 0.01%)	(0.00% - 0.00%)	(0.00% - 0.00%)	(0.00% - 0.00%)	(0.00% - 0.00%)	(0.00% - 0.00%)
	1.45%	1.11%	0.91%	0.86%	0.04%	0.01%	0.00%	0.00%	0.00%	0.00%	0.00%	0.00%
Italy	(1.33% - 1.59%)	(0.99% - 1.23%)	(0.80% - 1.03%)	(0.75% - 0.98%)	(0.03% - 0.06%)	(0.00% - 0.01%)	(0.00% - 0.01%)	(0.00% - 0.00%)	(0.00% - 0.00%)	(0.00% - 0.00%)	(0.00% - 0.00%)	(0.00% - 0.00%)
	1.35%	1.07%	0.88%	0.83%	0.04%	0.00%	0.00%	0.00%	0.00%	0.00%	0.00%	0.00%
Piemonte	(1.18% - 1.53%)	(0.90% - 1.24%)	(0.70% - 1.05%)	(0.66% - 1.00%)	(0.01% - 0.06%)	(0.00% - 0.02%)	(0.00% - 0.01%)	(0.00% - 0.01%)	(0.00% - 0.00%)	(0.00% - 0.00%)	(0.00% - 0.00%)	(0.00% - 0.00%)
	1.38%	1.08%	0.89%	0.83%	0.04%	0.01%	0.00%	0.00%	0.00%	0.00%	0.00%	0.00%
Valle d'Aosta	(1.21% - 1.57%)	(0.90% - 1.26%)	(0.70% - 1.08%)	(0.65% - 1.00%)	(0.02% - 0.07%)	(0.00% - 0.02%)	(0.00% - 0.01%)	(0.00% - 0.01%)	(0.00% - 0.00%)	(0.00% - 0.00%)	(0.00% - 0.00%)	(0.00% - 0.00%)
	1.34%	1.05%	0.87%	0.83%	0.03%	0.00%	0.00%	0.00%	0.00%	0.00%	0.00%	0.00%
Liguria	(1.17% - 1.53%)	(0.87% - 1.23%)	(0.69% - 1.07%)	(0.66% - 1.02%)	(0.01% - 0.06%)	(0.00% - 0.02%)	(0.00% - 0.01%)	(0.00% - 0.01%)	(0.00% - 0.00%)	(0.00% - 0.00%)	(0.00% - 0.00%)	(0.00% - 0.00%)
	1.27%	1.00%	0.83%	0.79%	0.03%	0.00%	0.00%	0.00%	0.00%	0.00%	0.00%	0.00%
Lombardia	(1.11% - 1.44%)	(0.82% - 1.18%)	(0.66% - 1.00%)	(0.61% - 0.96%)	(0.01% - 0.05%)	(0.00% - 0.01%)	(0.00% - 0.01%)	(0.00% - 0.01%)	(0.00% - 0.00%)	(0.00% - 0.00%)	(0.00% - 0.00%)	(0.00% - 0.00%)
	1.29%	0.99%	0.83%	0.78%	0.03%	0.00%	0.00%	0.00%	0.00%	0.00%	0.00%	0.00%
Provincia autonoma di Bolzano	(1.13% - 1.49%)	(0.80% - 1.19%)	(0.65% - 1.02%)	(0.62% - 0.96%)	(0.01% - 0.06%)	(0.00% - 0.02%)	(0.00% - 0.01%)	(0.00% - 0.01%)	(0.00% - 0.00%)	(0.00% - 0.00%)	(0.00% - 0.00%)	(0.00% - 0.00%)
	1.26%	1.00%	0.85%	0.80%	0.03%	0.00%	0.00%	0.00%	0.00%	0.00%	0.00%	0.00%
Provincia autonoma di Trento	(1.10% - 1.45%)	(0.79% - 1.18%)	(0.66% - 1.02%)	(0.62% - 0.97%)	(0.01% - 0.05%)	(0.00% - 0.02%)	(0.00% - 0.01%)	(0.00% - 0.01%)	(0.00% - 0.00%)	(0.00% - 0.00%)	(0.00% - 0.00%)	(0.00% - 0.00%)
	1.33%	1.04%	0.87%	0.83%	0.03%	0.00%	0.00%	0.00%	0.00%	0.00%	0.00%	0.00%
Veneto	(1.17% - 1.53%)	(0.86% - 1.22%)	(0.69% - 1.04%)	(0.65% - 1.00%)	(0.01% - 0.06%)	(0.00% - 0.02%)	(0.00% - 0.01%)	(0.00% - 0.01%)	(0.00% - 0.00%)	(0.00% - 0.00%)	(0.00% - 0.00%)	(0.00% - 0.00%)
	1.35%	1.05%	0.88%	0.82%	0.03%	0.00%	0.00%	0.00%	0.00%	0.00%	0.00%	0.00%
Friuli-Venezia Giulia	(1.17% - 1.53%)	(0.88% - 1.23%)	(0.69% - 1.06%)	(0.64% - 1.00%)	(0.01% - 0.06%)	(0.00% - 0.02%)	(0.00% - 0.01%)	(0.00% - 0.01%)	(0.00% - 0.00%)	(0.00% - 0.00%)	(0.00% - 0.00%)	(0.00% - 0.00%)
	1.39%	1.02%	0.85%	0.80%	0.04%	0.00%	0.00%	0.00%	0.00%	0.00%	0.00%	0.00%
Emilia-Romagna	(1.22% - 1.57%)	(0.84% - 1.22%)	(0.67% - 1.02%)	(0.62% - 0.98%)	(0.01% - 0.07%)	(0.00% - 0.02%)	(0.00% - 0.01%)	(0.00% - 0.01%)	(0.00% - 0.00%)	(0.00% - 0.00%)	(0.00% - 0.00%)	(0.00% - 0.00%)
	1.33%	1.05%	0.87%	0.82%	0.03%	0.00%	0.00%	0.00%	0.00%	0.00%	0.00%	0.00%
Toscana	(1.17% - 1.51%)	(0.87% - 1.22%)	(0.69% - 1.05%)	(0.64% - 0.99%)	(0.01% - 0.06%)	(0.00% - 0.02%)	(0.00% - 0.01%)	(0.00% - 0.01%)	(0.00% - 0.00%)	(0.00% - 0.00%)	(0.00% - 0.00%)	(0.00% - 0.00%)
	1.36%	1.07%	0.90%	0.86%	0.04%	0.01%	0.00%	0.00%	0.00%	0.00%	0.00%	0.00%
Umbria	(1.19% - 1.54%)	(0.91% - 1.25%)	(0.71% - 1.10%)	(0.68% - 1.06%)	(0.01% - 0.06%)	(0.00% - 0.02%)	(0.00% - 0.01%)	(0.00% - 0.01%)	(0.00% - 0.00%)	(0.00% - 0.00%)	(0.00% - 0.00%)	(0.00% - 0.00%)
	1.39%	1.07%	0.90%	0.85%	0.04%	0.01%	0.00%	0.00%	0.00%	0.00%	0.00%	0.00%
Marche	(1.22% - 1.58%)	(0.88% - 1.24%)	(0.72% - 1.09%)	(0.66% - 1.02%)	(0.01% - 0.07%)	(0.00% - 0.02%)	(0.00% - 0.01%)	(0.00% - 0.01%)	(0.00% - 0.00%)	(0.00% - 0.00%)	(0.00% - 0.00%)	(0.00% - 0.00%)
	1.30%	1.02%	0.85%	0.80%	0.03%	0.00%	0.00%	0.00%	0.00%	0.00%	0.00%	0.00%
Lazio	(1.14% - 1.49%)	(0.83% - 1.19%)	(0.69% - 1.02%)	(0.64% - 0.97%)	(0.01% - 0.06%)	(0.00% - 0.02%)	(0.00% - 0.01%)	(0.00% - 0.01%)	(0.00% - 0.00%)	(0.00% - 0.00%)	(0.00% - 0.00%)	(0.00% - 0.00%)
	1.38%	1.08%	0.92%	0.87%	0.04%	0.01%	0.00%	0.00%	0.00%	0.00%	0.00%	0.00%
Abruzzo	(1.20% - 1.57%)	(0.92% - 1.26%)	(0.73% - 1.09%)	(0.68% - 1.07%)	(0.02% - 0.06%)	(0.00% - 0.02%)	(0.00% - 0.01%)	(0.00% - 0.01%)	(0.00% - 0.00%)	(0.00% - 0.00%)	(0.00% - 0.00%)	(0.00% - 0.00%)
	1.49%	1.15%	0.98%	0.94%	0.05%	0.01%	0.00%	0.00%	0.00%	0.00%	0.00%	0.00%
Molise	(1.30% - 1.71%)	(0.99% - 1.32%)	(0.79% - 1.16%)	(0.75% - 1.13%)	(0.02% - 0.08%)	(0.00% - 0.02%)	(0.00% - 0.01%)	(0.00% - 0.01%)	(0.00% - 0.00%)	(0.00% - 0.00%)	(0.00% - 0.00%)	(0.00% - 0.00%)
	1.64%	1.24%	1.03%	0.98%	0.06%	0.01%	0.00%	0.00%	0.00%	0.00%	0.00%	0.00%
Campania	(1.46% - 1.85%)	(1.08% - 1.41%)	(0.85% - 1.20%)	(0.79% - 1.18%)	(0.03% - 0.10%)	(0.00% - 0.03%)	(0.00% - 0.01%)	(0.00% - 0.01%)	(0.00% - 0.00%)	(0.00% - 0.00%)	(0.00% - 0.00%)	(0.00% - 0.00%)
	1.61%	1.22%	1.02%	0.97%	0.06%	0.01%	0.00%	0.00%	0.00%	0.00%	0.00%	0.00%
Puglia	(1.41% - 1.81%)	(1.06% - 1.39%)	(0.84% - 1.18%)	(0.79% - 1.15%)	(0.03% - 0.09%)	(0.00% - 0.03%)	(0.00% - 0.01%)	(0.00% - 0.01%)	(0.00% - 0.00%)	(0.00% - 0.00%)	(0.00% - 0.00%)	(0.00% - 0.00%)
	1.53%	1.18%	1.00%	0.94%	0.05%	0.01%	0.00%	0.00%	0.00%	0.00%	0.00%	0.00%
Basilicata	(1.34% - 1.75%)	(1.03% - 1.37%)	(0.82% - 1.15%)	(0.75% - 1.12%)	(0.02% - 0.08%)	(0.00% - 0.03%)	(0.00% - 0.01%)	(0.00% - 0.01%)	(0.00% - 0.00%)	(0.00% - 0.00%)	(0.00% - 0.00%)	(0.00% - 0.00%)
	1.58%	1.21%	1.02%	0.97%	0.05%	0.01%	0.00%	0.00%	0.00%	0.00%	0.00%	0.00%
Calabria	(1.39% - 1.79%)	(1.05% - 1.39%)	(0.84% - 1.20%)	(0.80% - 1.17%)	(0.03% - 0.09%)	(0.00% - 0.02%)	(0.00% - 0.01%)	(0.00% - 0.01%)	(0.00% - 0.00%)	(0.00% - 0.00%)	(0.00% - 0.00%)	(0.00% - 0.00%)
	1.66%	1.24%	1.03%	0.99%	0.06%	0.01%	0.00%	0.00%	0.00%	0.00%	0.00%	0.00%
Sicilia	(1.47% - 1.88%)	(1.08% - 1.41%)	(0.86% - 1.22%)	(0.81% - 1.16%)	(0.03% - 0.10%)	(0.00% - 0.03%)	(0.00% - 0.01%)	(0.00% - 0.01%)	(0.00% - 0.00%)	(0.00% - 0.00%)	(0.00% - 0.00%)	(0.00% - 0.00%)

Table S8b. Estimated overall, severe, and extreme **wasting** prevalence (%) in children under 5 years, both sexes, in 1990, 2000, 2010, and 2020.												
**Location**	**Total**				**Severe**				**Extreme**			
	**1990**	**2000**	**2010**	**2020**	**1990**	**2000**	**2010**	**2020**	**1990**	**2000**	**2010**	**2020**
	1.49%	1.18%	0.98%	0.93%	0.05%	0.01%	0.00%	0.00%	0.00%	0.00%	0.00%	0.00%
Sardegna	(1.30% - 1.71%)	(1.02% - 1.37%)	(0.80% - 1.15%)	(0.73% - 1.09%)	(0.02% - 0.08%)	(0.00% - 0.02%)	(0.00% - 0.01%)	(0.00% - 0.01%)	(0.00% - 0.00%)	(0.00% - 0.00%)	(0.00% - 0.00%)	(0.00% - 0.00%)
	1.55%	1.31%	1.16%	1.06%	0.05%	0.01%	0.01%	0.00%	0.00%	0.00%	0.00%	0.00%
Luxembourg	(1.41% - 1.69%)	(1.18% - 1.44%)	(1.05% - 1.28%)	(0.94% - 1.18%)	(0.03% - 0.07%)	(0.00% - 0.03%)	(0.00% - 0.02%)	(0.00% - 0.01%)	(0.00% - 0.00%)	(0.00% - 0.00%)	(0.00% - 0.00%)	(0.00% - 0.00%)
	1.68%	1.37%	1.24%	1.05%	0.06%	0.01%	0.01%	0.00%	0.00%	0.00%	0.00%	0.00%
Malta	(1.54% - 1.85%)	(1.23% - 1.50%)	(1.12% - 1.36%)	(0.95% - 1.17%)	(0.04% - 0.09%)	(0.00% - 0.03%)	(0.00% - 0.02%)	(0.00% - 0.01%)	(0.00% - 0.00%)	(0.00% - 0.00%)	(0.00% - 0.00%)	(0.00% - 0.00%)
	1.21%	1.08%	0.93%	0.88%	0.08%	0.06%	0.04%	0.04%	0.00%	0.00%	0.00%	0.00%
Netherlands	(1.06% - 1.33%)	(0.93% - 1.21%)	(0.80% - 1.07%)	(0.70% - 1.03%)	(0.06% - 0.11%)	(0.04% - 0.08%)	(0.02% - 0.06%)	(0.02% - 0.05%)	(0.00% - 0.00%)	(0.00% - 0.00%)	(0.00% - 0.00%)	(0.00% - 0.00%)
	1.32%	1.09%	0.98%	0.90%	0.03%	0.01%	0.00%	0.00%	0.00%	0.00%	0.00%	0.00%
Norway	(1.21% - 1.45%)	(0.98% - 1.21%)	(0.87% - 1.09%)	(0.78% - 1.01%)	(0.02% - 0.05%)	(0.00% - 0.01%)	(0.00% - 0.01%)	(0.00% - 0.01%)	(0.00% - 0.00%)	(0.00% - 0.00%)	(0.00% - 0.00%)	(0.00% - 0.00%)
	1.86%	1.45%	1.21%	1.10%	0.08%	0.02%	0.01%	0.00%	0.00%	0.00%	0.00%	0.00%
Portugal	(1.70% - 2.04%)	(1.32% - 1.61%)	(1.09% - 1.32%)	(0.99% - 1.23%)	(0.05% - 0.11%)	(0.01% - 0.03%)	(0.00% - 0.02%)	(0.00% - 0.01%)	(0.00% - 0.00%)	(0.00% - 0.00%)	(0.00% - 0.00%)	(0.00% - 0.00%)
	1.34%	1.48%	1.43%	1.37%	0.00%	0.00%	0.00%	0.00%	0.00%	0.00%	0.00%	0.00%
Spain	(1.19% - 1.51%)	(1.32% - 1.66%)	(1.27% - 1.62%)	(1.21% - 1.54%)	(0.00% - 0.00%)	(0.00% - 0.00%)	(0.00% - 0.00%)	(0.00% - 0.00%)	(0.00% - 0.00%)	(0.00% - 0.00%)	(0.00% - 0.00%)	(0.00% - 0.00%)
	1.30%	1.10%	0.97%	0.90%	0.03%	0.01%	0.00%	0.00%	0.00%	0.00%	0.00%	0.00%
Sweden	(1.19% - 1.43%)	(0.99% - 1.22%)	(0.83% - 1.10%)	(0.75% - 1.02%)	(0.02% - 0.05%)	(0.00% - 0.02%)	(0.00% - 0.01%)	(0.00% - 0.01%)	(0.00% - 0.00%)	(0.00% - 0.00%)	(0.00% - 0.00%)	(0.00% - 0.00%)
	1.29%	1.09%	0.96%	0.89%	0.03%	0.00%	0.00%	0.00%	0.00%	0.00%	0.00%	0.00%
Sweden except Stockholm	(1.17% - 1.42%)	(0.98% - 1.23%)	(0.81% - 1.11%)	(0.72% - 1.01%)	(0.01% - 0.05%)	(0.00% - 0.02%)	(0.00% - 0.01%)	(0.00% - 0.00%)	(0.00% - 0.00%)	(0.00% - 0.00%)	(0.00% - 0.00%)	(0.00% - 0.00%)
	1.35%	1.15%	0.99%	0.93%	0.04%	0.01%	0.00%	0.00%	0.00%	0.00%	0.00%	0.00%
Stockholm	(1.17% - 1.55%)	(0.97% - 1.33%)	(0.81% - 1.16%)	(0.73% - 1.10%)	(0.01% - 0.07%)	(0.00% - 0.02%)	(0.00% - 0.01%)	(0.00% - 0.01%)	(0.00% - 0.00%)	(0.00% - 0.00%)	(0.00% - 0.00%)	(0.00% - 0.00%)
	1.16%	1.04%	0.92%	0.86%	0.02%	0.00%	0.00%	0.00%	0.00%	0.00%	0.00%	0.00%
Switzerland	(1.05% - 1.28%)	(0.93% - 1.16%)	(0.76% - 1.04%)	(0.70% - 0.99%)	(0.01% - 0.04%)	(0.00% - 0.01%)	(0.00% - 0.01%)	(0.00% - 0.00%)	(0.00% - 0.00%)	(0.00% - 0.00%)	(0.00% - 0.00%)	(0.00% - 0.00%)
	1.46%	1.19%	1.06%	0.98%	0.04%	0.01%	0.00%	0.00%	0.00%	0.00%	0.00%	0.00%
United Kingdom	(1.33% - 1.60%)	(1.08% - 1.32%)	(0.95% - 1.17%)	(0.87% - 1.08%)	(0.03% - 0.07%)	(0.00% - 0.02%)	(0.00% - 0.01%)	(0.00% - 0.01%)	(0.00% - 0.00%)	(0.00% - 0.00%)	(0.00% - 0.00%)	(0.00% - 0.00%)
	1.54%	1.22%	1.12%	1.02%	0.05%	0.01%	0.01%	0.00%	0.00%	0.00%	0.00%	0.00%
Northern Ireland	(1.31% - 1.79%)	(1.03% - 1.44%)	(0.94% - 1.32%)	(0.81% - 1.23%)	(0.02% - 0.09%)	(0.00% - 0.03%)	(0.00% - 0.02%)	(0.00% - 0.02%)	(0.00% - 0.00%)	(0.00% - 0.00%)	(0.00% - 0.00%)	(0.00% - 0.00%)
	1.53%	1.23%	1.10%	1.02%	0.05%	0.01%	0.01%	0.00%	0.00%	0.00%	0.00%	0.00%
Scotland	(1.31% - 1.76%)	(1.04% - 1.43%)	(0.91% - 1.30%)	(0.83% - 1.20%)	(0.02% - 0.09%)	(0.00% - 0.03%)	(0.00% - 0.02%)	(0.00% - 0.02%)	(0.00% - 0.00%)	(0.00% - 0.00%)	(0.00% - 0.00%)	(0.00% - 0.00%)
	1.58%	1.27%	1.16%	1.05%	0.05%	0.01%	0.01%	0.00%	0.00%	0.00%	0.00%	0.00%
Wales	(1.35% - 1.84%)	(1.07% - 1.52%)	(0.96% - 1.35%)	(0.86% - 1.25%)	(0.02% - 0.10%)	(0.00% - 0.03%)	(0.00% - 0.03%)	(0.00% - 0.02%)	(0.00% - 0.00%)	(0.00% - 0.00%)	(0.00% - 0.00%)	(0.00% - 0.00%)
	1.44%	1.18%	1.05%	0.97%	0.04%	0.01%	0.00%	0.00%	0.00%	0.00%	0.00%	0.00%
England	(1.31% - 1.58%)	(1.07% - 1.31%)	(0.94% - 1.16%)	(0.86% - 1.08%)	(0.03% - 0.06%)	(0.00% - 0.02%)	(0.00% - 0.01%)	(0.00% - 0.01%)	(0.00% - 0.00%)	(0.00% - 0.00%)	(0.00% - 0.00%)	(0.00% - 0.00%)
	1.57%	1.26%	1.10%	1.02%	0.05%	0.01%	0.01%	0.00%	0.00%	0.00%	0.00%	0.00%
North East England	(1.36% - 1.79%)	(1.10% - 1.46%)	(0.94% - 1.27%)	(0.87% - 1.17%)	(0.03% - 0.09%)	(0.00% - 0.03%)	(0.00% - 0.02%)	(0.00% - 0.01%)	(0.00% - 0.00%)	(0.00% - 0.00%)	(0.00% - 0.00%)	(0.00% - 0.00%)
	1.47%	1.22%	1.09%	1.01%	0.05%	0.01%	0.01%	0.00%	0.00%	0.00%	0.00%	0.00%
North West England	(1.29% - 1.66%)	(1.06% - 1.38%)	(0.95% - 1.24%)	(0.86% - 1.15%)	(0.02% - 0.08%)	(0.00% - 0.03%)	(0.00% - 0.02%)	(0.00% - 0.01%)	(0.00% - 0.00%)	(0.00% - 0.00%)	(0.00% - 0.00%)	(0.00% - 0.00%)
	1.48%	1.22%	1.09%	1.01%	0.05%	0.01%	0.01%	0.00%	0.00%	0.00%	0.00%	0.00%
Yorkshire and the Humber	(1.30% - 1.68%)	(1.06% - 1.39%)	(0.94% - 1.25%)	(0.85% - 1.17%)	(0.02% - 0.08%)	(0.00% - 0.03%)	(0.00% - 0.02%)	(0.00% - 0.01%)	(0.00% - 0.00%)	(0.00% - 0.00%)	(0.00% - 0.00%)	(0.00% - 0.00%)
	1.45%	1.19%	1.07%	0.99%	0.04%	0.01%	0.01%	0.00%	0.00%	0.00%	0.00%	0.00%
East Midlands	(1.26% - 1.65%)	(1.03% - 1.36%)	(0.91% - 1.23%)	(0.83% - 1.15%)	(0.02% - 0.08%)	(0.00% - 0.02%)	(0.00% - 0.02%)	(0.00% - 0.01%)	(0.00% - 0.00%)	(0.00% - 0.00%)	(0.00% - 0.00%)	(0.00% - 0.00%)
	1.55%	1.27%	1.11%	1.03%	0.05%	0.01%	0.01%	0.00%	0.00%	0.00%	0.00%	0.00%
West Midlands	(1.37% - 1.75%)	(1.11% - 1.45%)	(0.95% - 1.28%)	(0.87% - 1.19%)	(0.03% - 0.08%)	(0.00% - 0.03%)	(0.00% - 0.02%)	(0.00% - 0.02%)	(0.00% - 0.00%)	(0.00% - 0.00%)	(0.00% - 0.00%)	(0.00% - 0.00%)
	1.36%	1.12%	1.01%	0.93%	0.04%	0.01%	0.00%	0.00%	0.00%	0.00%	0.00%	0.00%
East of England	(1.19% - 1.55%)	(0.97% - 1.29%)	(0.85% - 1.16%)	(0.78% - 1.09%)	(0.02% - 0.06%)	(0.00% - 0.02%)	(0.00% - 0.02%)	(0.00% - 0.01%)	(0.00% - 0.00%)	(0.00% - 0.00%)	(0.00% - 0.00%)	(0.00% - 0.00%)
	1.41%	1.17%	0.99%	0.89%	0.04%	0.01%	0.00%	0.00%	0.00%	0.00%	0.00%	0.00%
Greater London	(1.26% - 1.58%)	(1.02% - 1.33%)	(0.85% - 1.15%)	(0.75% - 1.03%)	(0.02% - 0.07%)	(0.00% - 0.02%)	(0.00% - 0.02%)	(0.00% - 0.01%)	(0.00% - 0.00%)	(0.00% - 0.00%)	(0.00% - 0.00%)	(0.00% - 0.00%)

Table S8b. Estimated overall, severe, and extreme **wasting** prevalence (%) in children under 5 years, both sexes, in 1990, 2000, 2010, and 2020.

Location	Total				Severe				Extreme			
	1990	2000	2010	2020	1990	2000	2010	2020	1990	2000	2010	2020
South East England	1.33% (1.17% - 1.50%)	1.11% (0.96% - 1.29%)	1.00% (0.85% - 1.15%)	0.94% (0.80% - 1.07%)	0.03% (0.01% - 0.06%)	0.01% (0.00% - 0.02%)	0.00% (0.00% - 0.01%)	0.00% (0.00% - 0.01%)	0.00% (0.00% - 0.00%)	0.00% (0.00% - 0.00%)	0.00% (0.00% - 0.00%)	0.00% (0.00% - 0.00%)
South West England	1.44% (1.26% - 1.65%)	1.19% (1.03% - 1.38%)	1.08% (0.94% - 1.24%)	1.01% (0.87% - 1.16%)	0.04% (0.02% - 0.07%)	0.01% (0.00% - 0.02%)	0.00% (0.00% - 0.02%)	0.00% (0.00% - 0.01%)	0.00% (0.00% - 0.00%)	0.00% (0.00% - 0.00%)	0.00% (0.00% - 0.00%)	0.00% (0.00% - 0.00%)
Latin America and Caribbean	3.49% (3.45% - 3.53%)	2.62% (2.59% - 2.65%)	2.16% (2.13% - 2.19%)	1.80% (1.77% - 1.83%)	0.31% (0.29% - 0.32%)	0.15% (0.15% - 0.16%)	0.10% (0.09% - 0.10%)	0.06% (0.05% - 0.06%)	0.02% (0.02% - 0.02%)	0.01% (0.00% - 0.01%)	0.00% (0.00% - 0.00%)	0.00% (0.00% - 0.00%)
**Andean Latin America**	2.45% (2.36% - 2.55%)	1.86% (1.80% - 1.93%)	1.45% (1.37% - 1.52%)	1.10% (1.04% - 1.18%)	0.08% (0.06% - 0.09%)	0.07% (0.06% - 0.08%)	0.04% (0.03% - 0.05%)	0.02% (0.01% - 0.02%)	0.00% (0.00% - 0.00%)	0.00% (0.00% - 0.00%)	0.00% (0.00% - 0.00%)	0.00% (0.00% - 0.00%)
Bolivia (Plurinational State of)	2.95% (2.78% - 3.15%)	2.36% (2.20% - 2.52%)	1.89% (1.76% - 2.05%)	1.50% (1.38% - 1.63%)	0.16% (0.12% - 0.19%)	0.10% (0.08% - 0.13%)	0.06% (0.05% - 0.08%)	0.03% (0.02% - 0.04%)	0.00% (0.00% - 0.00%)	0.00% (0.00% - 0.00%)	0.00% (0.00% - 0.00%)	0.00% (0.00% - 0.00%)
Ecuador	3.20% (2.98% - 3.41%)	2.62% (2.46% - 2.79%)	2.32% (2.18% - 2.48%)	1.91% (1.78% - 2.07%)	0.18% (0.14% - 0.23%)	0.16% (0.13% - 0.20%)	0.10% (0.07% - 0.12%)	0.04% (0.03% - 0.06%)	0.00% (0.00% - 0.00%)	0.00% (0.00% - 0.00%)	0.00% (0.00% - 0.00%)	0.00% (0.00% - 0.00%)
Peru	1.94% (1.82% - 2.07%)	1.26% (1.18% - 1.34%)	0.73% (0.61% - 0.80%)	0.49% (0.39% - 0.59%)	0.00% (0.00% - 0.01%)	0.00% (0.00% - 0.00%)	0.00% (0.00% - 0.00%)	0.00% (0.00% - 0.00%)	0.00% (0.00% - 0.00%)	0.00% (0.00% - 0.00%)	0.00% (0.00% - 0.00%)	0.00% (0.00% - 0.00%)
**Caribbean**	4.41% (4.28% - 4.55%)	4.33% (4.19% - 4.46%)	4.15% (3.99% - 4.30%)	3.62% (3.45% - 3.79%)	0.62% (0.57% - 0.68%)	0.62% (0.57% - 0.68%)	0.55% (0.49% - 0.61%)	0.37% (0.32% - 0.43%)	0.03% (0.02% - 0.04%)	0.02% (0.02% - 0.03%)	0.01% (0.00% - 0.01%)	0.00% (0.00% - 0.00%)
Antigua and Barbuda	7.28% (6.61% - 7.95%)	5.42% (4.91% - 5.97%)	3.90% (3.48% - 4.31%)	3.48% (3.10% - 3.90%)	1.42% (1.15% - 1.70%)	0.67% (0.49% - 0.86%)	0.23% (0.13% - 0.34%)	0.14% (0.07% - 0.23%)	0.03% (0.00% - 0.07%)	0.00% (0.00% - 0.00%)	0.00% (0.00% - 0.00%)	0.00% (0.00% - 0.00%)
Bahamas	2.70% (2.42% - 3.02%)	2.64% (2.36% - 2.96%)	2.60% (2.34% - 2.93%)	2.54% (2.27% - 2.87%)	0.05% (0.02% - 0.08%)	0.03% (0.01% - 0.06%)	0.03% (0.01% - 0.06%)	0.02% (0.01% - 0.05%)	0.00% (0.00% - 0.00%)	0.00% (0.00% - 0.00%)	0.00% (0.00% - 0.00%)	0.00% (0.00% - 0.00%)
Barbados	4.88% (4.46% - 5.32%)	4.18% (3.78% - 4.61%)	3.95% (3.56% - 4.36%)	3.66% (3.29% - 4.05%)	0.45% (0.32% - 0.59%)	0.23% (0.13% - 0.35%)	0.17% (0.09% - 0.29%)	0.12% (0.05% - 0.21%)	0.00% (0.00% - 0.00%)	0.00% (0.00% - 0.00%)	0.00% (0.00% - 0.00%)	0.00% (0.00% - 0.00%)
Belize	5.01% (4.53% - 5.47%)	4.24% (3.83% - 4.62%)	3.43% (3.12% - 3.77%)	3.15% (2.86% - 3.48%)	0.52% (0.37% - 0.67%)	0.32% (0.22% - 0.44%)	0.15% (0.09% - 0.22%)	0.11% (0.08% - 0.17%)	0.00% (0.00% - 0.00%)	0.00% (0.00% - 0.00%)	0.00% (0.00% - 0.00%)	0.00% (0.00% - 0.00%)
Cuba	1.80% (1.69% - 1.91%)	1.92% (1.79% - 2.08%)	1.53% (1.41% - 1.68%)	1.39% (1.25% - 1.55%)	0.02% (0.01% - 0.04%)	0.00% (0.00% - 0.00%)	0.00% (0.00% - 0.01%)	0.00% (0.00% - 0.01%)	0.00% (0.00% - 0.00%)	0.00% (0.00% - 0.00%)	0.00% (0.00% - 0.00%)	0.00% (0.00% - 0.00%)
Dominica	2.99% (2.68% - 3.33%)	2.65% (2.38% - 2.96%)	2.54% (2.26% - 2.86%)	2.55% (2.29% - 2.86%)	0.07% (0.04% - 0.12%)	0.04% (0.01% - 0.07%)	0.03% (0.01% - 0.05%)	0.02% (0.01% - 0.05%)	0.00% (0.00% - 0.00%)	0.00% (0.00% - 0.00%)	0.00% (0.00% - 0.00%)	0.00% (0.00% - 0.00%)
Dominican Republic	2.38% (2.23% - 2.56%)	2.14% (2.00% - 2.29%)	1.97% (1.83% - 2.10%)	1.63% (1.50% - 1.76%)	0.09% (0.07% - 0.12%)	0.06% (0.05% - 0.09%)	0.03% (0.02% - 0.04%)	0.01% (0.00% - 0.02%)	0.00% (0.00% - 0.00%)	0.00% (0.00% - 0.00%)	0.00% (0.00% - 0.00%)	0.00% (0.00% - 0.00%)
Grenada	4.00% (3.56% - 4.45%)	3.39% (3.04% - 3.81%)	3.23% (2.87% - 3.61%)	2.88% (2.57% - 3.25%)	0.20% (0.13% - 0.31%)	0.11% (0.06% - 0.18%)	0.09% (0.04% - 0.15%)	0.05% (0.02% - 0.09%)	0.00% (0.00% - 0.00%)	0.00% (0.00% - 0.00%)	0.00% (0.00% - 0.00%)	0.00% (0.00% - 0.00%)
Guyana	12.82% (12.07% - 13.62%)	10.32% (9.71% - 10.94%)	7.36% (6.90% - 7.85%)	5.64% (5.25% - 6.05%)	3.54% (3.13% - 3.98%)	2.47% (2.16% - 2.78%)	1.28% (1.11% - 1.49%)	0.69% (0.55% - 0.83%)	0.38% (0.24% - 0.55%)	0.13% (0.05% - 0.22%)	0.01% (0.00% - 0.03%)	0.00% (0.00% - 0.00%)
Haiti	8.35% (7.89% - 8.81%)	8.27% (7.84% - 8.70%)	7.29% (6.89% - 7.69%)	5.90% (5.52% - 6.29%)	1.69% (1.51% - 1.88%)	1.73% (1.56% - 1.92%)	1.33% (1.17% - 1.49%)	0.82% (0.69% - 0.96%)	0.06% (0.03% - 0.09%)	0.07% (0.04% - 0.10%)	0.02% (0.00% - 0.04%)	0.00% (0.00% - 0.00%)
Jamaica	4.26% (3.97% - 4.54%)	3.31% (3.09% - 3.53%)	2.91% (2.69% - 3.14%)	2.59% (2.39% - 2.80%)	0.22% (0.16% - 0.31%)	0.08% (0.06% - 0.11%)	0.03% (0.02% - 0.06%)	0.02% (0.00% - 0.03%)	0.00% (0.00% - 0.00%)	0.00% (0.00% - 0.00%)	0.00% (0.00% - 0.00%)	0.00% (0.00% - 0.00%)
Saint Lucia	4.30% (3.84% - 4.79%)	3.85% (3.44% - 4.31%)	3.40% (3.01% - 3.82%)	3.25% (2.86% - 3.65%)	0.30% (0.17% - 0.45%)	0.19% (0.08% - 0.32%)	0.11% (0.04% - 0.22%)	0.08% (0.02% - 0.18%)	0.00% (0.00% - 0.00%)	0.00% (0.00% - 0.00%)	0.00% (0.00% - 0.00%)	0.00% (0.00% - 0.00%)
Saint Vincent and the Grenadines	3.63% (3.24% - 4.03%)	3.40% (3.04% - 3.79%)	2.94% (2.63% - 3.30%)	2.71% (2.44% - 3.05%)	0.15% (0.09% - 0.23%)	0.11% (0.06% - 0.18%)	0.06% (0.02% - 0.10%)	0.03% (0.01% - 0.07%)	0.00% (0.00% - 0.00%)	0.00% (0.00% - 0.00%)	0.00% (0.00% - 0.00%)	0.00% (0.00% - 0.00%)
Suriname	7.59% (7.03% - 8.22%)	6.86% (6.42% - 7.34%)	5.50% (5.13% - 5.90%)	5.07% (4.65% - 5.48%)	1.15% (0.93% - 1.38%)	0.92% (0.76% - 1.09%)	0.58% (0.46% - 0.71%)	0.47% (0.35% - 0.60%)	0.00% (0.00% - 0.02%)	0.00% (0.00% - 0.01%)	0.00% (0.00% - 0.00%)	0.00% (0.00% - 0.00%)
Trinidad and Tobago	5.10% (4.61% - 5.64%)	5.42% (4.96% - 5.94%)	5.83% (5.37% - 6.33%)	5.26% (4.80% - 5.77%)	0.41% (0.26% - 0.58%)	0.54% (0.39% - 0.72%)	0.78% (0.61% - 0.96%)	0.61% (0.45% - 0.79%)	0.00% (0.00% - 0.00%)	0.00% (0.00% - 0.00%)	0.00% (0.00% - 0.00%)	0.00% (0.00% - 0.00%)

Table S8b. Estimated overall, severe, and extreme **wasting** prevalence (%) in children under 5 years, both sexes, in 1990, 2000, 2010, and 2020.

Location	Total				Severe				Extreme			
	1990	2000	2010	2020	1990	2000	2010	2020	1990	2000	2010	2020
Bermuda	1.83%	1.83%	1.74%	1.62%	0.01%	0.00%	0.00%	0.00%	0.00%	0.00%	0.00%	0.00%
	(1.65% - 2.05%)	(1.64% - 2.05%)	(1.55% - 1.96%)	(1.43% - 1.82%)	(0.00% - 0.02%)	(0.00% - 0.01%)	(0.00% - 0.01%)	(0.00% - 0.00%)	(0.00% - 0.00%)	(0.00% - 0.00%)	(0.00% - 0.00%)	(0.00% - 0.00%)
Puerto Rico	1.89%	1.76%	1.57%	1.46%	0.01%	0.00%	0.00%	0.00%	0.00%	0.00%	0.00%	0.00%
	(1.68% - 2.11%)	(1.56% - 1.98%)	(1.39% - 1.77%)	(1.30% - 1.65%)	(0.00% - 0.02%)	(0.00% - 0.01%)	(0.00% - 0.00%)	(0.00% - 0.00%)	(0.00% - 0.00%)	(0.00% - 0.00%)	(0.00% - 0.00%)	(0.00% - 0.00%)
Saint Kitts and Nevis	3.31%	2.86%	2.61%	2.40%	0.11%	0.05%	0.03%	0.02%	0.00%	0.00%	0.00%	0.00%
	(2.96% - 3.67%)	(2.56% - 3.17%)	(2.35% - 2.90%)	(2.14% - 2.69%)	(0.07% - 0.17%)	(0.03% - 0.09%)	(0.01% - 0.06%)	(0.00% - 0.04%)	(0.00% - 0.00%)	(0.00% - 0.00%)	(0.00% - 0.00%)	(0.00% - 0.00%)
United States Virgin Islands	2.46%	2.20%	1.97%	2.00%	0.03%	0.01%	0.00%	0.00%	0.00%	0.00%	0.00%	0.00%
	(2.20% - 2.77%)	(1.97% - 2.46%)	(1.77% - 2.21%)	(1.77% - 2.24%)	(0.01% - 0.05%)	(0.00% - 0.03%)	(0.00% - 0.02%)	(0.00% - 0.01%)	(0.00% - 0.00%)	(0.00% - 0.00%)	(0.00% - 0.00%)	(0.00% - 0.00%)
**Central Latin America**	3.77%	2.72%	2.24%	1.86%	0.36%	0.15%	0.08%	0.04%	0.04%	0.01%	0.00%	0.00%
	(3.72% - 3.82%)	(2.67% - 2.76%)	(2.19% - 2.28%)	(1.82% - 1.91%)	(0.34% - 0.38%)	(0.14% - 0.16%)	(0.07% - 0.09%)	(0.04% - 0.05%)	(0.03% - 0.04%)	(0.01% - 0.01%)	(0.00% - 0.00%)	(0.00% - 0.00%)
Colombia	2.27%	1.58%	1.26%	0.99%	0.02%	0.01%	0.00%	0.00%	0.00%	0.00%	0.00%	0.00%
	(2.11% - 2.42%)	(1.48% - 1.69%)	(1.17% - 1.36%)	(0.90% - 1.20%)	(0.01% - 0.03%)	(0.00% - 0.01%)	(0.00% - 0.01%)	(0.00% - 0.00%)	(0.00% - 0.00%)	(0.00% - 0.00%)	(0.00% - 0.00%)	(0.00% - 0.00%)
Costa Rica	1.53%	1.37%	1.10%	0.88%	0.01%	0.01%	0.00%	0.00%	0.00%	0.00%	0.00%	0.00%
	(1.36% - 1.69%)	(1.19% - 1.55%)	(0.97% - 1.26%)	(0.77% - 1.01%)	(0.00% - 0.03%)	(0.00% - 0.03%)	(0.00% - 0.01%)	(0.00% - 0.00%)	(0.00% - 0.00%)	(0.00% - 0.00%)	(0.00% - 0.00%)	(0.00% - 0.00%)
El Salvador	2.78%	1.74%	1.52%	1.41%	0.08%	0.01%	0.01%	0.02%	0.00%	0.00%	0.00%	0.00%
	(2.59% - 2.99%)	(1.62% - 1.88%)	(1.41% - 1.66%)	(1.31% - 1.55%)	(0.05% - 0.10%)	(0.00% - 0.02%)	(0.01% - 0.03%)	(0.01% - 0.03%)	(0.00% - 0.00%)	(0.00% - 0.00%)	(0.00% - 0.00%)	(0.00% - 0.00%)
Guatemala	3.64%	3.00%	1.45%	1.02%	0.22%	0.17%	0.00%	0.00%	0.00%	0.00%	0.00%	0.00%
	(3.40% - 3.89%)	(2.83% - 3.16%)	(1.34% - 1.58%)	(0.91% - 1.23%)	(0.18% - 0.27%)	(0.14% - 0.20%)	(0.00% - 0.00%)	(0.00% - 0.00%)	(0.00% - 0.00%)	(0.00% - 0.00%)	(0.00% - 0.00%)	(0.00% - 0.00%)
Honduras	2.34%	1.83%	1.42%	1.24%	0.00%	0.00%	0.00%	0.00%	0.00%	0.00%	0.00%	0.00%
	(2.19% - 2.50%)	(1.71% - 1.95%)	(1.32% - 1.52%)	(1.14% - 1.42%)	(0.00% - 0.00%)	(0.00% - 0.01%)	(0.00% - 0.01%)	(0.00% - 0.01%)	(0.00% - 0.00%)	(0.00% - 0.00%)	(0.00% - 0.00%)	(0.00% - 0.00%)
Mexico	4.46%	3.03%	2.54%	2.15%	0.59%	0.23%	0.15%	0.08%	0.07%	0.02%	0.01%	0.00%
	(4.38% - 4.54%)	(2.98% - 3.09%)	(2.48% - 2.60%)	(2.10% - 2.22%)	(0.56% - 0.62%)	(0.21% - 0.25%)	(0.13% - 0.16%)	(0.07% - 0.09%)	(0.06% - 0.09%)	(0.01% - 0.03%)	(0.00% - 0.01%)	(0.00% - 0.01%)
Aguascalientes	5.40%	3.86%	3.29%	2.88%	0.60%	0.26%	0.18%	0.12%	0.00%	0.00%	0.00%	0.00%
	(5.00% - 5.78%)	(3.56% - 4.20%)	(3.06% - 3.58%)	(2.66% - 3.10%)	(0.49% - 0.73%)	(0.21% - 0.32%)	(0.14% - 0.22%)	(0.09% - 0.15%)	(0.00% - 0.01%)	(0.00% - 0.00%)	(0.00% - 0.00%)	(0.00% - 0.00%)
Baja California	4.76%	3.32%	2.88%	2.47%	0.41%	0.09%	0.02%	0.00%	0.00%	0.00%	0.00%	0.00%
	(4.40% - 5.13%)	(3.03% - 3.60%)	(2.63% - 3.17%)	(2.25% - 2.72%)	(0.30% - 0.53%)	(0.04% - 0.14%)	(0.00% - 0.05%)	(0.00% - 0.01%)	(0.00% - 0.00%)	(0.00% - 0.00%)	(0.00% - 0.00%)	(0.00% - 0.00%)
Baja California Sur	1.52%	1.08%	0.92%	0.82%	0.07%	0.02%	0.01%	0.00%	0.00%	0.00%	0.00%	0.00%
	(1.39% - 1.63%)	(1.00% - 1.18%)	(0.85% - 0.99%)	(0.75% - 0.95%)	(0.05% - 0.09%)	(0.01% - 0.04%)	(0.00% - 0.02%)	(0.00% - 0.01%)	(0.00% - 0.00%)	(0.00% - 0.00%)	(0.00% - 0.00%)	(0.00% - 0.00%)
Campeche	3.96%	2.62%	2.13%	1.74%	0.42%	0.15%	0.07%	0.03%	0.00%	0.00%	0.00%	0.00%
	(3.68% - 4.24%)	(2.45% - 2.83%)	(1.99% - 2.30%)	(1.60% - 1.89%)	(0.34% - 0.52%)	(0.11% - 0.19%)	(0.05% - 0.10%)	(0.01% - 0.05%)	(0.00% - 0.00%)	(0.00% - 0.00%)	(0.00% - 0.00%)	(0.00% - 0.00%)
Coahuila	6.21%	4.23%	3.54%	3.22%	0.94%	0.35%	0.26%	0.20%	0.00%	0.00%	0.00%	0.00%
	(5.79% - 6.65%)	(3.88% - 4.62%)	(3.25% - 3.86%)	(2.94% - 3.54%)	(0.78% - 1.12%)	(0.25% - 0.46%)	(0.18% - 0.36%)	(0.12% - 0.30%)	(0.00% - 0.01%)	(0.00% - 0.00%)	(0.00% - 0.00%)	(0.00% - 0.00%)
Colima	3.27%	1.99%	1.72%	1.53%	0.29%	0.07%	0.04%	0.02%	0.00%	0.00%	0.00%	0.00%
	(3.02% - 3.54%)	(1.81% - 2.18%)	(1.57% - 1.90%)	(1.39% - 1.70%)	(0.22% - 0.36%)	(0.05% - 0.10%)	(0.02% - 0.06%)	(0.01% - 0.04%)	(0.00% - 0.00%)	(0.00% - 0.00%)	(0.00% - 0.00%)	(0.00% - 0.00%)
Chiapas	6.25%	3.20%	2.03%	1.55%	1.01%	0.25%	0.06%	0.02%	0.02%	0.00%	0.00%	0.00%
	(5.90% - 6.58%)	(3.02% - 3.40%)	(1.90% - 2.16%)	(1.39% - 1.68%)	(0.88% - 1.13%)	(0.21% - 0.29%)	(0.04% - 0.09%)	(0.01% - 0.03%)	(0.00% - 0.04%)	(0.00% - 0.00%)	(0.00% - 0.00%)	(0.00% - 0.00%)
Chihuahua	4.12%	3.47%	2.55%	2.27%	0.34%	0.22%	0.09%	0.05%	0.00%	0.00%	0.00%	0.00%
	(3.85% - 4.39%)	(3.25% - 3.72%)	(2.39% - 2.74%)	(2.12% - 2.45%)	(0.27% - 0.41%)	(0.19% - 0.26%)	(0.07% - 0.11%)	(0.04% - 0.08%)	(0.00% - 0.00%)	(0.00% - 0.00%)	(0.00% - 0.00%)	(0.00% - 0.00%)
Mexico City	3.64%	2.54%	1.62%	1.38%	0.30%	0.11%	0.01%	0.00%	0.00%	0.00%	0.00%	0.00%
	(3.40% - 3.91%)	(2.38% - 2.73%)	(1.50% - 1.76%)	(1.28% - 1.50%)	(0.24% - 0.36%)	(0.08% - 0.15%)	(0.00% - 0.03%)	(0.00% - 0.01%)	(0.00% - 0.00%)	(0.00% - 0.00%)	(0.00% - 0.00%)	(0.00% - 0.00%)
Durango	3.38%	1.88%	1.26%	1.11%	0.17%	0.01%	0.00%	0.00%	0.00%	0.00%	0.00%	0.00%
	(2.95% - 3.70%)	(1.69% - 2.08%)	(1.14% - 1.40%)	(0.99% - 1.25%)	(0.08% - 0.27%)	(0.00% - 0.02%)	(0.00% - 0.01%)	(0.00% - 0.00%)	(0.00% - 0.00%)	(0.00% - 0.00%)	(0.00% - 0.00%)	(0.00% - 0.00%)
Guanajuato	4.72%	3.00%	2.89%	2.52%	0.33%	0.04%	0.04%	0.01%	0.00%	0.00%	0.00%	0.00%
	(4.35% - 5.12%)	(2.72% - 3.27%)	(2.65% - 3.14%)	(2.30% - 2.75%)	(0.23% - 0.45%)	(0.00% - 0.10%)	(0.00% - 0.10%)	(0.00% - 0.06%)	(0.00% - 0.00%)	(0.00% - 0.00%)	(0.00% - 0.00%)	(0.00% - 0.00%)
Guerrero	4.06%	2.35%	1.68%	1.56%	0.38%	0.07%	0.01%	0.00%	0.00%	0.00%	0.00%	0.00%
	(3.91% - 4.24%)	(2.22% - 2.50%)	(1.55% - 1.81%)	(1.43% - 1.70%)	(0.33% - 0.43%)	(0.05% - 0.09%)	(0.00% - 0.03%)	(0.00% - 0.00%)	(0.00% - 0.00%)	(0.00% - 0.00%)	(0.00% - 0.00%)	(0.00% - 0.00%)

Table S8b. Estimated overall, severe, and extreme **wasting** prevalence (%) in children under 5 years, both sexes, in 1990, 2000, 2010, and 2020.												
**Location**	**Total**				**Severe**				**Extreme**			
	**1990**	**2000**	**2010**	**2020**	**1990**	**2000**	**2010**	**2020**	**1990**	**2000**	**2010**	**2020**
Hidalgo	3.99% (3.71% - 4.29%)	2.47% (2.29% - 2.69%)	2.02% (1.86% - 2.18%)	1.64% (1.51% - 1.78%)	0.24% (0.18% - 0.31%)	0.05% (0.03% - 0.07%)	0.03% (0.01% - 0.04%)	0.01% (0.00% - 0.02%)	0.00% (0.00% - 0.00%)	0.00% (0.00% - 0.00%)	0.00% (0.00% - 0.00%)	0.00% (0.00% - 0.00%)
Jalisco	2.35% (2.08% - 2.60%)	1.49% (1.33% - 1.67%)	1.21% (1.08% - 1.36%)	1.12% (1.00% - 1.25%)	0.04% (0.00% - 0.11%)	0.00% (0.00% - 0.00%)	0.00% (0.00% - 0.00%)	0.00% (0.00% - 0.00%)	0.00% (0.00% - 0.00%)	0.00% (0.00% - 0.00%)	0.00% (0.00% - 0.00%)	0.00% (0.00% - 0.00%)
México	3.58% (3.25% - 3.91%)	2.74% (2.47% - 3.00%)	2.99% (2.69% - 3.33%)	2.26% (1.99% - 2.66%)	0.22% (0.12% - 0.32%)	0.10% (0.03% - 0.17%)	0.14% (0.07% - 0.24%)	0.01% (0.00% - 0.04%)	0.00% (0.00% - 0.00%)	0.00% (0.00% - 0.00%)	0.00% (0.00% - 0.00%)	0.00% (0.00% - 0.00%)
Michoacán de Ocampo	3.19% (2.92% - 3.47%)	2.23% (1.90% - 2.47%)	2.54% (2.31% - 2.80%)	2.10% (1.88% - 2.34%)	0.27% (0.18% - 0.36%)	0.08% (0.03% - 0.14%)	0.18% (0.11% - 0.26%)	0.08% (0.03% - 0.15%)	0.00% (0.00% - 0.00%)	0.00% (0.00% - 0.00%)	0.00% (0.00% - 0.00%)	0.00% (0.00% - 0.00%)
Morelos	9.73% (9.07% - 10.46%)	7.34% (6.74% - 7.90%)	6.61% (6.11% - 7.18%)	5.92% (5.37% - 6.49%)	2.72% (2.27% - 3.24%)	1.74% (1.41% - 2.09%)	1.45% (1.17% - 1.79%)	1.21% (0.91% - 1.51%)	0.57% (0.35% - 0.85%)	0.29% (0.15% - 0.45%)	0.21% (0.09% - 0.36%)	0.13% (0.02% - 0.25%)
Nayarit	3.74% (3.47% - 4.08%)	2.38% (2.18% - 2.60%)	1.92% (1.77% - 2.08%)	1.68% (1.56% - 1.83%)	0.25% (0.20% - 0.32%)	0.09% (0.06% - 0.12%)	0.05% (0.04% - 0.08%)	0.04% (0.02% - 0.05%)	0.00% (0.00% - 0.00%)	0.00% (0.00% - 0.00%)	0.00% (0.00% - 0.00%)	0.00% (0.00% - 0.00%)
Nuevo León	3.50% (3.18% - 3.77%)	2.86% (2.50% - 3.13%)	3.02% (2.75% - 3.28%)	2.70% (2.48% - 2.94%)	0.24% (0.17% - 0.32%)	0.12% (0.06% - 0.18%)	0.18% (0.10% - 0.26%)	0.11% (0.05% - 0.19%)	0.00% (0.00% - 0.00%)	0.00% (0.00% - 0.00%)	0.00% (0.00% - 0.00%)	0.00% (0.00% - 0.00%)
Oaxaca	3.20% (2.97% - 3.43%)	2.65% (2.46% - 2.84%)	2.57% (2.38% - 2.83%)	2.11% (1.95% - 2.29%)	0.15% (0.12% - 0.18%)	0.09% (0.07% - 0.12%)	0.07% (0.05% - 0.10%)	0.03% (0.01% - 0.05%)	0.00% (0.00% - 0.00%)	0.00% (0.00% - 0.00%)	0.00% (0.00% - 0.00%)	0.00% (0.00% - 0.00%)
Puebla	2.80% (2.60% - 3.03%)	1.87% (1.73% - 2.02%)	1.29% (1.20% - 1.42%)	1.37% (1.24% - 1.50%)	0.08% (0.05% - 0.10%)	0.01% (0.00% - 0.02%)	0.00% (0.00% - 0.01%)	0.00% (0.00% - 0.00%)	0.00% (0.00% - 0.00%)	0.00% (0.00% - 0.00%)	0.00% (0.00% - 0.00%)	0.00% (0.00% - 0.00%)
Querétaro	4.60% (4.27% - 4.93%)	1.60% (1.47% - 1.73%)	0.88% (0.80% - 0.96%)	0.78% (0.70% - 0.86%)	0.85% (0.73% - 0.98%)	0.04% (0.02% - 0.06%)	0.00% (0.00% - 0.00%)	0.00% (0.00% - 0.00%)	0.16% (0.11% - 0.21%)	0.00% (0.00% - 0.00%)	0.00% (0.00% - 0.00%)	0.00% (0.00% - 0.00%)
Quintana Roo	1.43% (1.33% - 1.53%)	0.88% (0.76% - 0.97%)	0.66% (0.50% - 0.78%)	0.50% (0.33% - 0.65%)	0.05% (0.03% - 0.07%)	0.01% (0.00% - 0.02%)	0.00% (0.00% - 0.01%)	0.00% (0.00% - 0.00%)	0.00% (0.00% - 0.00%)	0.00% (0.00% - 0.00%)	0.00% (0.00% - 0.00%)	0.00% (0.00% - 0.00%)
San Luis Potosí	14.33% (13.36% - 15.31%)	8.45% (7.69% - 9.25%)	5.22% (4.73% - 5.76%)	4.31% (3.86% - 4.81%)	5.86% (5.05% - 6.72%)	2.44% (1.95% - 2.98%)	0.91% (0.69% - 1.20%)	0.59% (0.41% - 0.82%)	1.91% (1.42% - 2.46%)	0.46% (0.25% - 0.73%)	0.02% (0.00% - 0.11%)	0.00% (0.00% - 0.00%)
Sinaloa	3.67% (3.39% - 3.96%)	2.51% (2.20% - 2.78%)	1.93% (1.76% - 2.22%)	1.73% (1.58% - 1.90%)	0.26% (0.17% - 0.36%)	0.07% (0.03% - 0.13%)	0.03% (0.01% - 0.04%)	0.01% (0.00% - 0.02%)	0.00% (0.00% - 0.00%)	0.00% (0.00% - 0.00%)	0.00% (0.00% - 0.00%)	0.00% (0.00% - 0.00%)
Sonora	4.84% (4.48% - 5.25%)	3.58% (3.31% - 3.86%)	3.62% (3.35% - 3.91%)	3.08% (2.78% - 3.38%)	0.52% (0.41% - 0.63%)	0.22% (0.15% - 0.29%)	0.22% (0.16% - 0.30%)	0.12% (0.07% - 0.18%)	0.00% (0.00% - 0.00%)	0.00% (0.00% - 0.00%)	0.00% (0.00% - 0.00%)	0.00% (0.00% - 0.00%)
Tabasco	1.16% (1.03% - 1.30%)	0.58% (0.47% - 0.72%)	0.23% (0.17% - 0.34%)	0.19% (0.02% - 0.47%)	0.00% (0.00% - 0.00%)	0.00% (0.00% - 0.00%)	0.00% (0.00% - 0.00%)	0.00% (0.00% - 0.00%)	0.00% (0.00% - 0.00%)	0.00% (0.00% - 0.00%)	0.00% (0.00% - 0.00%)	0.00% (0.00% - 0.00%)
Tamaulipas	4.89% (4.57% - 5.23%)	3.62% (3.37% - 3.87%)	3.18% (2.96% - 3.42%)	2.77% (2.57% - 2.99%)	0.69% (0.58% - 0.82%)	0.33% (0.25% - 0.41%)	0.21% (0.17% - 0.26%)	0.13% (0.10% - 0.17%)	0.00% (0.00% - 0.01%)	0.00% (0.00% - 0.00%)	0.00% (0.00% - 0.00%)	0.00% (0.00% - 0.00%)
Tlaxcala	5.42% (5.04% - 5.80%)	3.75% (3.43% - 4.05%)	2.90% (2.69% - 3.15%)	2.56% (2.36% - 2.78%)	0.62% (0.49% - 0.75%)	0.18% (0.13% - 0.24%)	0.08% (0.05% - 0.10%)	0.05% (0.03% - 0.07%)	0.00% (0.00% - 0.01%)	0.00% (0.00% - 0.00%)	0.00% (0.00% - 0.00%)	0.00% (0.00% - 0.00%)
Veracruz de Ignacio de la Llave	6.03% (5.65% - 6.45%)	4.48% (4.17% - 4.80%)	3.40% (3.16% - 3.66%)	2.96% (2.73% - 3.20%)	0.87% (0.73% - 1.02%)	0.40% (0.31% - 0.50%)	0.19% (0.13% - 0.27%)	0.11% (0.07% - 0.16%)	0.01% (0.00% - 0.03%)	0.00% (0.00% - 0.00%)	0.00% (0.00% - 0.00%)	0.00% (0.00% - 0.00%)
Yucatán	8.50% (7.85% - 9.17%)	6.34% (5.81% - 6.94%)	5.59% (5.11% - 6.09%)	4.95% (4.52% - 5.43%)	2.21% (1.82% - 2.64%)	1.38% (1.10% - 1.71%)	1.11% (0.88% - 1.39%)	0.89% (0.69% - 1.13%)	0.43% (0.24% - 0.64%)	0.19% (0.07% - 0.34%)	0.11% (0.02% - 0.23%)	0.05% (0.00% - 0.14%)
Zacatecas	5.79% (5.44% - 6.16%)	4.61% (4.30% - 4.92%)	4.04% (3.78% - 4.31%)	3.33% (3.13% - 3.55%)	0.76% (0.65% - 0.89%)	0.48% (0.40% - 0.56%)	0.36% (0.30% - 0.43%)	0.24% (0.20% - 0.29%)	0.00% (0.00% - 0.01%)	0.00% (0.00% - 0.00%)	0.00% (0.00% - 0.00%)	0.00% (0.00% - 0.00%)
Nicaragua	2.98% (2.81% - 3.16%)	2.34% (2.20% - 2.49%)	1.57% (1.47% - 1.71%)	1.23% (1.14% - 1.32%)	0.19% (0.16% - 0.22%)	0.09% (0.07% - 0.11%)	0.01% (0.00% - 0.02%)	0.00% (0.00% - 0.00%)	0.00% (0.00% - 0.00%)	0.00% (0.00% - 0.00%)	0.00% (0.00% - 0.00%)	0.00% (0.00% - 0.00%)
Panama	2.48% (2.28% - 2.68%)	1.49% (1.36% - 1.63%)	1.18% (1.07% - 1.31%)	0.86% (0.72% - 0.94%)	0.04% (0.02% - 0.06%)	0.00% (0.00% - 0.00%)	0.00% (0.00% - 0.00%)	0.00% (0.00% - 0.00%)	0.00% (0.00% - 0.00%)	0.00% (0.00% - 0.00%)	0.00% (0.00% - 0.00%)	0.00% (0.00% - 0.00%)
Venezuela (Bolivarian Republic of)	4.57% (4.40% - 4.73%)	4.08% (3.92% - 4.25%)	3.76% (3.53% - 3.99%)	3.39% (3.13% - 3.67%)	0.24% (0.18% - 0.29%)	0.13% (0.08% - 0.18%)	0.07% (0.02% - 0.13%)	0.02% (0.00% - 0.07%)	0.00% (0.00% - 0.00%)	0.00% (0.00% - 0.00%)	0.00% (0.00% - 0.00%)	0.00% (0.00% - 0.00%)

Table S8b. Estimated overall, severe, and extreme **wasting** prevalence (%) in children under 5 years, both sexes, in 1990, 2000, 2010, and 2020.

Location	Total				Severe				Extreme			
	1990	2000	2010	2020	1990	2000	2010	2020	1990	2000	2010	2020
**Tropical Latin America**	3.21%	2.33%	1.83%	1.52%	0.24%	0.08%	0.03%	0.01%	0.00%	0.00%	0.00%	0.00%
	(3.14% - 3.29%)	(2.27% - 2.39%)	(1.79% - 1.87%)	(1.48% - 1.56%)	(0.22% - 0.25%)	(0.07% - 0.10%)	(0.03% - 0.04%)	(0.01% - 0.02%)	(0.00% - 0.01%)	(0.00% - 0.00%)	(0.00% - 0.00%)	(0.00% - 0.00%)
Brazil	3.31%	2.38%	1.83%	1.54%	0.24%	0.09%	0.03%	0.01%	0.00%	0.00%	0.00%	0.00%
	(3.24% - 3.38%)	(2.32% - 2.44%)	(1.79% - 1.88%)	(1.50% - 1.58%)	(0.22% - 0.26%)	(0.08% - 0.10%)	(0.02% - 0.04%)	(0.01% - 0.02%)	(0.00% - 0.01%)	(0.00% - 0.00%)	(0.00% - 0.00%)	(0.00% - 0.00%)
Acre	3.47%	2.81%	2.11%	1.68%	0.19%	0.10%	0.02%	0.00%	0.00%	0.00%	0.00%	0.00%
	(3.19% - 3.75%)	(2.60% - 3.06%)	(1.94% - 2.30%)	(1.55% - 1.84%)	(0.14% - 0.23%)	(0.07% - 0.13%)	(0.01% - 0.04%)	(0.00% - 0.01%)	(0.00% - 0.00%)	(0.00% - 0.00%)	(0.00% - 0.00%)	(0.00% - 0.00%)
Alagoas	7.51%	5.56%	4.06%	3.42%	1.65%	0.90%	0.47%	0.31%	0.17%	0.01%	0.00%	0.00%
	(6.91% - 8.14%)	(5.10% - 6.02%)	(3.72% - 4.45%)	(3.09% - 3.78%)	(1.35% - 2.00%)	(0.71% - 1.11%)	(0.34% - 0.62%)	(0.20% - 0.44%)	(0.05% - 0.33%)	(0.00% - 0.05%)	(0.00% - 0.00%)	(0.00% - 0.00%)
Amazonas	4.17%	2.92%	2.33%	1.95%	0.34%	0.15%	0.08%	0.04%	0.00%	0.00%	0.00%	0.00%
	(3.79% - 4.56%)	(2.71% - 3.19%)	(2.15% - 2.52%)	(1.80% - 2.12%)	(0.27% - 0.42%)	(0.12% - 0.18%)	(0.05% - 0.10%)	(0.02% - 0.06%)	(0.00% - 0.00%)	(0.00% - 0.00%)	(0.00% - 0.00%)	(0.00% - 0.00%)
Amapá	2.24%	2.08%	1.71%	1.53%	0.05%	0.03%	0.00%	0.00%	0.00%	0.00%	0.00%	0.00%
	(2.07% - 2.44%)	(1.91% - 2.27%)	(1.57% - 1.89%)	(1.40% - 1.68%)	(0.03% - 0.07%)	(0.02% - 0.05%)	(0.00% - 0.01%)	(0.00% - 0.00%)	(0.00% - 0.00%)	(0.00% - 0.00%)	(0.00% - 0.00%)	(0.00% - 0.00%)
Bahia	4.47%	2.95%	2.26%	1.86%	0.58%	0.18%	0.05%	0.01%	0.00%	0.00%	0.00%	0.00%
	(4.13% - 4.81%)	(2.70% - 3.21%)	(2.06% - 2.48%)	(1.66% - 2.05%)	(0.46% - 0.72%)	(0.11% - 0.26%)	(0.01% - 0.12%)	(0.00% - 0.04%)	(0.00% - 0.00%)	(0.00% - 0.00%)	(0.00% - 0.00%)	(0.00% - 0.00%)
Ceará	4.72%	3.08%	1.92%	1.56%	0.39%	0.07%	0.00%	0.00%	0.00%	0.00%	0.00%	0.00%
	(4.34% - 5.11%)	(2.78% - 3.38%)	(1.73% - 2.17%)	(1.40% - 1.75%)	(0.28% - 0.52%)	(0.02% - 0.14%)	(0.00% - 0.02%)	(0.00% - 0.00%)	(0.00% - 0.00%)	(0.00% - 0.00%)	(0.00% - 0.00%)	(0.00% - 0.00%)
Distrito Federal	3.78%	2.86%	2.18%	1.82%	0.42%	0.19%	0.08%	0.04%	0.00%	0.00%	0.00%	0.00%
	(3.43% - 4.16%)	(2.57% - 3.14%)	(1.97% - 2.41%)	(1.61% - 2.04%)	(0.30% - 0.56%)	(0.11% - 0.30%)	(0.02% - 0.16%)	(0.00% - 0.10%)	(0.00% - 0.00%)	(0.00% - 0.00%)	(0.00% - 0.00%)	(0.00% - 0.00%)
Espírito Santo	2.42%	2.01%	1.57%	1.28%	0.07%	0.03%	0.00%	0.00%	0.00%	0.00%	0.00%	0.00%
	(2.23% - 2.61%)	(1.84% - 2.19%)	(1.45% - 1.71%)	(1.17% - 1.38%)	(0.04% - 0.09%)	(0.01% - 0.04%)	(0.00% - 0.01%)	(0.00% - 0.00%)	(0.00% - 0.00%)	(0.00% - 0.00%)	(0.00% - 0.00%)	(0.00% - 0.00%)
Goiás	2.78%	2.12%	1.65%	1.36%	0.09%	0.03%	0.00%	0.00%	0.00%	0.00%	0.00%	0.00%
	(2.57% - 2.99%)	(1.96% - 2.30%)	(1.52% - 1.80%)	(1.26% - 1.49%)	(0.06% - 0.12%)	(0.01% - 0.05%)	(0.00% - 0.01%)	(0.00% - 0.00%)	(0.00% - 0.00%)	(0.00% - 0.00%)	(0.00% - 0.00%)	(0.00% - 0.00%)
Maranhão	5.45%	3.71%	2.73%	2.25%	0.62%	0.20%	0.07%	0.02%	0.00%	0.00%	0.00%	0.00%
	(5.02% - 5.87%)	(3.39% - 4.08%)	(2.50% - 2.96%)	(2.07% - 2.46%)	(0.49% - 0.76%)	(0.15% - 0.27%)	(0.04% - 0.10%)	(0.01% - 0.04%)	(0.00% - 0.00%)	(0.00% - 0.00%)	(0.00% - 0.00%)	(0.00% - 0.00%)
Minas Gerais	2.34%	1.58%	1.28%	1.04%	0.02%	0.00%	0.00%	0.00%	0.00%	0.00%	0.00%	0.00%
	(2.13% - 2.56%)	(1.44% - 1.73%)	(1.17% - 1.40%)	(0.95% - 1.14%)	(0.01% - 0.03%)	(0.00% - 0.01%)	(0.00% - 0.00%)	(0.00% - 0.00%)	(0.00% - 0.00%)	(0.00% - 0.00%)	(0.00% - 0.00%)	(0.00% - 0.00%)
Mato Grosso do Sul	2.97%	2.37%	1.86%	1.48%	0.07%	0.03%	0.00%	0.00%	0.00%	0.00%	0.00%	0.00%
	(2.73% - 3.24%)	(2.17% - 2.59%)	(1.70% - 2.03%)	(1.36% - 1.62%)	(0.04% - 0.10%)	(0.01% - 0.05%)	(0.00% - 0.01%)	(0.00% - 0.00%)	(0.00% - 0.00%)	(0.00% - 0.00%)	(0.00% - 0.00%)	(0.00% - 0.00%)
Mato Grosso	2.96%	2.33%	1.75%	1.44%	0.08%	0.03%	0.00%	0.00%	0.00%	0.00%	0.00%	0.00%
	(2.75% - 3.20%)	(2.15% - 2.51%)	(1.61% - 1.89%)	(1.32% - 1.56%)	(0.05% - 0.11%)	(0.02% - 0.05%)	(0.00% - 0.01%)	(0.00% - 0.00%)	(0.00% - 0.00%)	(0.00% - 0.00%)	(0.00% - 0.00%)	(0.00% - 0.00%)
Pará	2.98%	2.06%	1.66%	1.37%	0.10%	0.03%	0.01%	0.00%	0.00%	0.00%	0.00%	0.00%
	(2.67% - 3.38%)	(1.89% - 2.25%)	(1.54% - 1.80%)	(1.26% - 1.50%)	(0.06% - 0.15%)	(0.01% - 0.05%)	(0.00% - 0.02%)	(0.00% - 0.00%)	(0.00% - 0.00%)	(0.00% - 0.00%)	(0.00% - 0.00%)	(0.00% - 0.00%)
Paraíba	3.25%	2.74%	2.07%	1.71%	0.17%	0.10%	0.02%	0.00%	0.00%	0.00%	0.00%	0.00%
	(3.00% - 3.51%)	(2.53% - 2.97%)	(1.91% - 2.25%)	(1.57% - 1.87%)	(0.13% - 0.21%)	(0.07% - 0.13%)	(0.01% - 0.04%)	(0.00% - 0.01%)	(0.00% - 0.00%)	(0.00% - 0.00%)	(0.00% - 0.00%)	(0.00% - 0.00%)
Paraná	0.74%	0.47%	0.36%	0.28%	0.01%	0.00%	0.00%	0.00%	0.00%	0.00%	0.00%	0.00%
	(0.65% - 0.85%)	(0.40% - 0.55%)	(0.31% - 0.42%)	(0.25% - 0.31%)	(0.00% - 0.02%)	(0.00% - 0.00%)	(0.00% - 0.00%)	(0.00% - 0.00%)	(0.00% - 0.00%)	(0.00% - 0.00%)	(0.00% - 0.00%)	(0.00% - 0.00%)
Pernambuco	2.63%	1.90%	1.36%	1.12%	0.12%	0.02%	0.00%	0.00%	0.00%	0.00%	0.00%	0.00%
	(2.45% - 2.82%)	(1.75% - 2.06%)	(1.26% - 1.47%)	(1.03% - 1.21%)	(0.09% - 0.14%)	(0.01% - 0.04%)	(0.00% - 0.00%)	(0.00% - 0.00%)	(0.00% - 0.00%)	(0.00% - 0.00%)	(0.00% - 0.00%)	(0.00% - 0.00%)
Piaui	2.40%	1.90%	1.41%	1.15%	0.08%	0.01%	0.00%	0.00%	0.00%	0.00%	0.00%	0.00%
	(2.23% - 2.60%)	(1.71% - 2.10%)	(1.28% - 1.56%)	(1.04% - 1.26%)	(0.06% - 0.10%)	(0.00% - 0.02%)	(0.00% - 0.00%)	(0.00% - 0.00%)	(0.00% - 0.00%)	(0.00% - 0.00%)	(0.00% - 0.00%)	(0.00% - 0.00%)
Rio de Janeiro	4.94%	3.41%	2.72%	2.34%	0.45%	0.15%	0.05%	0.02%	0.00%	0.00%	0.00%	0.00%
	(4.58% - 5.30%)	(3.16% - 3.70%)	(2.51% - 2.95%)	(2.16% - 2.56%)	(0.35% - 0.56%)	(0.10% - 0.19%)	(0.02% - 0.09%)	(0.00% - 0.04%)	(0.00% - 0.00%)	(0.00% - 0.00%)	(0.00% - 0.00%)	(0.00% - 0.00%)
Rio Grande do Norte	2.07%	1.68%	1.25%	1.07%	0.06%	0.01%	0.00%	0.00%	0.00%	0.00%	0.00%	0.00%
	(1.92% - 2.23%)	(1.55% - 1.84%)	(1.14% - 1.37%)	(0.97% - 1.18%)	(0.04% - 0.08%)	(0.00% - 0.02%)	(0.00% - 0.00%)	(0.00% - 0.00%)	(0.00% - 0.00%)	(0.00% - 0.00%)	(0.00% - 0.00%)	(0.00% - 0.00%)
Rondônia	3.08%	2.50%	1.95%	1.60%	0.12%	0.06%	0.01%	0.00%	0.00%	0.00%	0.00%	0.00%
	(2.86% - 3.34%)	(2.31% - 2.70%)	(1.79% - 2.12%)	(1.47% - 1.74%)	(0.09% - 0.15%)	(0.04% - 0.08%)	(0.00% - 0.03%)	(0.00% - 0.01%)	(0.00% - 0.00%)	(0.00% - 0.00%)	(0.00% - 0.00%)	(0.00% - 0.00%)

Table S8b. Estimated overall, severe, and extreme **wasting** prevalence (%) in children under 5 years, both sexes, in 1990, 2000, 2010, and 2020.

Location	Total				Severe				Extreme			
	1990	2000	2010	2020	1990	2000	2010	2020	1990	2000	2010	2020
Roraima	2.89%<br>(2.67% - 3.11%)	2.31%<br>(2.14% - 2.50%)	1.79%<br>(1.65% - 1.94%)	1.54%<br>(1.41% - 1.68%)	0.14%<br>(0.10% - 0.17%)	0.07%<br>(0.04% - 0.09%)	0.01%<br>(0.00% - 0.03%)	0.00%<br>(0.00% - 0.01%)	0.00%<br>(0.00% - 0.00%)	0.00%<br>(0.00% - 0.00%)	0.00%<br>(0.00% - 0.00%)	0.00%<br>(0.00% - 0.00%)
Rio Grande do Sul	1.87%<br>(1.73% - 2.03%)	1.63%<br>(1.50% - 1.78%)	1.30%<br>(1.20% - 1.41%)	1.11%<br>(1.02% - 1.20%)	0.02%<br>(0.01% - 0.03%)	0.01%<br>(0.00% - 0.02%)	0.00%<br>(0.00% - 0.00%)	0.00%<br>(0.00% - 0.00%)	0.00%<br>(0.00% - 0.00%)	0.00%<br>(0.00% - 0.00%)	0.00%<br>(0.00% - 0.00%)	0.00%<br>(0.00% - 0.00%)
Santa Catarina	1.89%<br>(1.74% - 2.04%)	1.70%<br>(1.55% - 1.86%)	1.31%<br>(1.20% - 1.41%)	1.07%<br>(0.98% - 1.17%)	0.02%<br>(0.01% - 0.04%)	0.01%<br>(0.00% - 0.02%)	0.00%<br>(0.00% - 0.00%)	0.00%<br>(0.00% - 0.00%)	0.00%<br>(0.00% - 0.00%)	0.00%<br>(0.00% - 0.00%)	0.00%<br>(0.00% - 0.00%)	0.00%<br>(0.00% - 0.00%)
Sergipe	5.25%<br>(4.84% - 5.71%)	3.49%<br>(3.22% - 3.79%)	2.50%<br>(2.30% - 2.72%)	2.11%<br>(1.95% - 2.29%)	0.42%<br>(0.32% - 0.53%)	0.12%<br>(0.08% - 0.17%)	0.02%<br>(0.00% - 0.04%)	0.00%<br>(0.00% - 0.01%)	0.00%<br>(0.00% - 0.00%)	0.00%<br>(0.00% - 0.00%)	0.00%<br>(0.00% - 0.00%)	0.00%<br>(0.00% - 0.00%)
São Paulo	3.18%<br>(2.95% - 3.46%)	2.26%<br>(2.09% - 2.47%)	1.77%<br>(1.61% - 1.94%)	1.53%<br>(1.40% - 1.67%)	0.17%<br>(0.12% - 0.23%)	0.06%<br>(0.03% - 0.09%)	0.01%<br>(0.00% - 0.03%)	0.00%<br>(0.00% - 0.02%)	0.00%<br>(0.00% - 0.00%)	0.00%<br>(0.00% - 0.00%)	0.00%<br>(0.00% - 0.00%)	0.00%<br>(0.00% - 0.00%)
Tocantins	3.41%<br>(3.15% - 3.69%)	2.86%<br>(2.65% - 3.08%)	2.05%<br>(1.90% - 2.23%)	1.64%<br>(1.51% - 1.78%)	0.17%<br>(0.13% - 0.22%)	0.11%<br>(0.08% - 0.14%)	0.02%<br>(0.01% - 0.03%)	0.00%<br>(0.00% - 0.01%)	0.00%<br>(0.00% - 0.00%)	0.00%<br>(0.00% - 0.00%)	0.00%<br>(0.00% - 0.00%)	0.00%<br>(0.00% - 0.00%)
Paraguay	0.54%<br>(0.36% - 0.65%)	0.99%<br>(0.90% - 1.08%)	1.70%<br>(1.58% - 1.82%)	1.12%<br>(1.01% - 1.29%)	0.00%<br>(0.00% - 0.00%)	0.00%<br>(0.00% - 0.00%)	0.07%<br>(0.06% - 0.09%)	0.04%<br>(0.03% - 0.05%)	0.00%<br>(0.00% - 0.00%)	0.00%<br>(0.00% - 0.00%)	0.00%<br>(0.00% - 0.00%)	0.00%<br>(0.00% - 0.00%)
North Africa and Middle East	9.42%<br>(9.26% - 9.59%)	8.69%<br>(8.56% - 8.82%)	7.31%<br>(7.19% - 7.44%)	5.64%<br>(5.52% - 5.77%)	2.51%<br>(2.41% - 2.59%)	2.23%<br>(2.15% - 2.29%)	1.60%<br>(1.54% - 1.66%)	0.93%<br>(0.88% - 0.98%)	0.40%<br>(0.37% - 0.43%)	0.32%<br>(0.29% - 0.35%)	0.17%<br>(0.15% - 0.19%)	0.05%<br>(0.04% - 0.07%)
**North Africa and Middle East**	9.42%<br>(9.26% - 9.59%)	8.69%<br>(8.56% - 8.82%)	7.31%<br>(7.19% - 7.44%)	5.64%<br>(5.52% - 5.77%)	2.51%<br>(2.41% - 2.59%)	2.23%<br>(2.15% - 2.29%)	1.60%<br>(1.54% - 1.66%)	0.93%<br>(0.88% - 0.98%)	0.40%<br>(0.37% - 0.43%)	0.32%<br>(0.29% - 0.35%)	0.17%<br>(0.15% - 0.19%)	0.05%<br>(0.04% - 0.07%)
Algeria	10.82%<br>(10.22% - 11.44%)	8.85%<br>(8.40% - 9.27%)	5.91%<br>(5.58% - 6.26%)	4.29%<br>(3.93% - 4.62%)	2.94%<br>(2.65% - 3.25%)	2.06%<br>(1.86% - 2.26%)	0.94%<br>(0.82% - 1.08%)	0.42%<br>(0.34% - 0.51%)	0.32%<br>(0.25% - 0.40%)	0.11%<br>(0.09% - 0.14%)	0.04%<br>(0.03% - 0.06%)	0.01%<br>(0.00% - 0.03%)
Bahrain	6.69%<br>(6.12% - 7.31%)	4.97%<br>(4.49% - 5.49%)	3.58%<br>(3.21% - 3.94%)	2.90%<br>(2.60% - 3.29%)	1.18%<br>(0.96% - 1.44%)	0.58%<br>(0.41% - 0.76%)	0.14%<br>(0.07% - 0.24%)	0.03%<br>(0.01% - 0.08%)	0.00%<br>(0.00% - 0.00%)	0.00%<br>(0.00% - 0.00%)	0.00%<br>(0.00% - 0.00%)	0.00%<br>(0.00% - 0.00%)
Egypt	7.35%<br>(6.94% - 7.80%)	6.47%<br>(6.14% - 6.80%)	6.25%<br>(5.95% - 6.59%)	4.55%<br>(4.18% - 4.88%)	1.36%<br>(1.19% - 1.54%)	1.12%<br>(0.99% - 1.25%)	1.14%<br>(1.02% - 1.27%)	0.54%<br>(0.45% - 0.64%)	0.05%<br>(0.03% - 0.07%)	0.05%<br>(0.03% - 0.07%)	0.08%<br>(0.06% - 0.10%)	0.01%<br>(0.00% - 0.03%)
Iran (Islamic Republic of)	9.29%<br>(8.74% - 9.91%)	5.95%<br>(5.64% - 6.26%)	4.48%<br>(4.14% - 4.83%)	3.52%<br>(3.21% - 3.87%)	2.31%<br>(2.02% - 2.62%)	0.99%<br>(0.87% - 1.10%)	0.54%<br>(0.44% - 0.64%)	0.29%<br>(0.23% - 0.38%)	0.27%<br>(0.20% - 0.36%)	0.04%<br>(0.02% - 0.05%)	0.01%<br>(0.01% - 0.02%)	0.00%<br>(0.00% - 0.00%)
Iraq	9.63%<br>(9.12% - 10.15%)	9.52%<br>(9.08% - 9.97%)	6.92%<br>(6.51% - 7.37%)	4.58%<br>(4.26% - 4.89%)	2.37%<br>(2.13% - 2.62%)	2.39%<br>(2.18% - 2.61%)	1.30%<br>(1.13% - 1.48%)	0.47%<br>(0.36% - 0.57%)	0.11%<br>(0.07% - 0.15%)	0.12%<br>(0.09% - 0.16%)	0.02%<br>(0.01% - 0.04%)	0.00%<br>(0.00% - 0.00%)
Jordan	4.14%<br>(3.82% - 4.42%)	3.13%<br>(2.91% - 3.39%)	2.44%<br>(2.26% - 2.64%)	2.07%<br>(1.89% - 2.26%)	0.35%<br>(0.28% - 0.43%)	0.13%<br>(0.10% - 0.16%)	0.03%<br>(0.02% - 0.05%)	0.01%<br>(0.00% - 0.02%)	0.00%<br>(0.00% - 0.00%)	0.00%<br>(0.00% - 0.00%)	0.00%<br>(0.00% - 0.00%)	0.00%<br>(0.00% - 0.00%)
Kuwait	4.34%<br>(4.03% - 4.65%)	2.98%<br>(2.78% - 3.20%)	2.12%<br>(1.97% - 2.26%)	1.81%<br>(1.66% - 1.95%)	0.30%<br>(0.21% - 0.40%)	0.05%<br>(0.03% - 0.07%)	0.02%<br>(0.01% - 0.03%)	0.01%<br>(0.00% - 0.01%)	0.00%<br>(0.00% - 0.00%)	0.00%<br>(0.00% - 0.00%)	0.00%<br>(0.00% - 0.00%)	0.00%<br>(0.00% - 0.00%)
Lebanon	7.01%<br>(6.43% - 7.58%)	5.41%<br>(5.00% - 5.83%)	4.39%<br>(4.00% - 4.79%)	3.49%<br>(3.12% - 3.86%)	1.20%<br>(0.97% - 1.45%)	0.68%<br>(0.53% - 0.84%)	0.38%<br>(0.25% - 0.51%)	0.16%<br>(0.07% - 0.27%)	0.01%<br>(0.00% - 0.04%)	0.00%<br>(0.00% - 0.00%)	0.00%<br>(0.00% - 0.00%)	0.00%<br>(0.00% - 0.00%)
Libya	6.05%<br>(5.55% - 6.60%)	4.99%<br>(4.57% - 5.40%)	5.13%<br>(4.70% - 5.55%)	4.98%<br>(4.45% - 5.45%)	0.87%<br>(0.69% - 1.06%)	0.55%<br>(0.43% - 0.69%)	0.61%<br>(0.48% - 0.76%)	0.50%<br>(0.37% - 0.65%)	0.01%<br>(0.00% - 0.03%)	0.00%<br>(0.00% - 0.01%)	0.01%<br>(0.00% - 0.02%)	0.00%<br>(0.00% - 0.01%)
Morocco	5.22%<br>(4.88% - 5.53%)	6.89%<br>(6.51% - 7.27%)	4.63%<br>(4.36% - 4.94%)	2.67%<br>(2.47% - 2.93%)	0.62%<br>(0.51% - 0.73%)	1.30%<br>(1.14% - 1.46%)	0.47%<br>(0.38% - 0.58%)	0.09%<br>(0.07% - 0.11%)	0.01%<br>(0.00% - 0.02%)	0.04%<br>(0.02% - 0.06%)	0.00%<br>(0.00% - 0.01%)	0.00%<br>(0.00% - 0.00%)
Palestine	5.67%<br>(5.36% - 6.00%)	5.30%<br>(5.08% - 5.55%)	3.77%<br>(3.52% - 4.01%)	2.03%<br>(1.88% - 2.19%)	0.74%<br>(0.63% - 0.86%)	0.65%<br>(0.57% - 0.74%)	0.19%<br>(0.15% - 0.24%)	0.01%<br>(0.00% - 0.02%)	0.00%<br>(0.00% - 0.00%)	0.00%<br>(0.00% - 0.00%)	0.00%<br>(0.00% - 0.00%)	0.00%<br>(0.00% - 0.00%)
Oman	10.85%<br>(10.22% - 11.52%)	7.89%<br>(7.45% - 8.31%)	6.71%<br>(6.28% - 7.18%)	5.66%<br>(5.20% - 6.13%)	2.70%<br>(2.39% - 3.05%)	1.43%<br>(1.26% - 1.61%)	1.09%<br>(0.93% - 1.28%)	0.75%<br>(0.59% - 0.92%)	0.15%<br>(0.05% - 0.27%)	0.00%<br>(0.00% - 0.01%)	0.01%<br>(0.00% - 0.02%)	0.00%<br>(0.00% - 0.01%)
Qatar	2.90%<br>(2.65% - 3.17%)	2.24%<br>(2.05% - 2.44%)	1.79%<br>(1.62% - 1.96%)	1.40%<br>(1.26% - 1.56%)	0.16%<br>(0.12% - 0.21%)	0.07%<br>(0.05% - 0.10%)	0.02%<br>(0.00% - 0.04%)	0.00%<br>(0.00% - 0.01%)	0.00%<br>(0.00% - 0.00%)	0.00%<br>(0.00% - 0.00%)	0.00%<br>(0.00% - 0.00%)	0.00%<br>(0.00% - 0.00%)
Saudi Arabia	13.39%<br>(12.36% - 14.42%)	9.94%<br>(9.15% - 10.74%)	7.39%<br>(6.76% - 8.05%)	5.52%<br>(4.98% - 6.04%)	4.53%<br>(3.95% - 5.12%)	2.76%<br>(2.37% - 3.19%)	1.55%<br>(1.29% - 1.84%)	0.79%<br>(0.61% - 1.00%)	0.95%<br>(0.70% - 1.23%)	0.32%<br>(0.21% - 0.47%)	0.07%<br>(0.04% - 0.12%)	0.01%<br>(0.00% - 0.03%)

Table S8b. Estimated overall, severe, and extreme **wasting** prevalence (%) in children under 5 years, both sexes, in 1990, 2000, 2010, and 2020.

Location	Total				Severe				Extreme			
	1990	2000	2010	2020	1990	2000	2010	2020	1990	2000	2010	2020
Syrian Arab Republic	13.45% (12.62% - 14.31%)	11.00% (10.30% - 11.67%)	10.36% (9.72% - 11.01%)	10.42% (9.71% - 11.20%)	4.44% (3.93% - 4.96%)	3.18% (2.82% - 3.55%)	2.99% (2.64% - 3.35%)	2.98% (2.60% - 3.41%)	0.83% (0.62% - 1.06%)	0.36% (0.24% - 0.49%)	0.34% (0.23% - 0.47%)	0.34% (0.21% - 0.49%)
Tunisia	5.96% (5.55% - 6.38%)	4.40% (4.07% - 4.71%)	2.93% (2.73% - 3.15%)	2.25% (2.07% - 2.43%)	0.84% (0.70% - 0.99%)	0.33% (0.25% - 0.43%)	0.15% (0.11% - 0.18%)	0.07% (0.05% - 0.09%)	0.00% (0.00% - 0.00%)	0.00% (0.00% - 0.00%)	0.00% (0.00% - 0.00%)	0.00% (0.00% - 0.00%)
Turkey	3.71% (3.46% - 4.00%)	2.31% (2.15% - 2.47%)	1.13% (0.97% - 1.25%)	0.88% (0.73% - 0.96%)	0.31% (0.25% - 0.37%)	0.10% (0.08% - 0.13%)	0.01% (0.00% - 0.02%)	0.00% (0.00% - 0.01%)	0.00% (0.00% - 0.01%)	0.00% (0.00% - 0.00%)	0.00% (0.00% - 0.00%)	0.00% (0.00% - 0.00%)
United Arab Emirates	13.34% (12.35% - 14.38%)	10.04% (9.26% - 10.87%)	7.27% (6.63% - 7.91%)	5.58% (5.07% - 6.16%)	3.92% (3.37% - 4.51%)	2.32% (1.97% - 2.73%)	1.19% (0.96% - 1.45%)	0.62% (0.46% - 0.82%)	0.55% (0.35% - 0.78%)	0.09% (0.04% - 0.17%)	0.00% (0.00% - 0.02%)	0.00% (0.00% - 0.00%)
Yemen	17.49% (16.85% - 18.18%)	17.43% (16.92% - 17.98%)	16.04% (15.60% - 16.49%)	15.41% (14.75% - 16.06%)	5.64% (5.25% - 6.05%)	5.25% (4.93% - 5.58%)	3.87% (3.64% - 4.11%)	3.32% (3.00% - 3.67%)	1.15% (0.99% - 1.33%)	0.94% (0.82% - 1.07%)	0.41% (0.35% - 0.48%)	0.23% (0.17% - 0.31%)
Afghanistan	10.15% (9.61% - 10.76%)	10.68% (10.27% - 11.14%)	8.27% (7.87% - 8.69%)	6.08% (5.66% - 6.48%)	2.67% (2.38% - 2.99%)	2.94% (2.72% - 3.19%)	1.80% (1.63% - 2.00%)	0.95% (0.79% - 1.11%)	0.14% (0.07% - 0.25%)	0.23% (0.15% - 0.32%)	0.01% (0.00% - 0.03%)	0.00% (0.00% - 0.00%)
Sudan	19.77% (19.16% - 20.40%)	19.36% (18.61% - 20.09%)	16.43% (15.66% - 17.12%)	12.41% (11.69% - 13.08%)	8.01% (7.55% - 8.51%)	7.42% (6.90% - 7.96%)	5.47% (5.04% - 5.91%)	3.29% (2.92% - 3.65%)	2.39% (2.15% - 2.65%)	2.03% (1.78% - 2.30%)	1.13% (0.95% - 1.33%)	0.33% (0.24% - 0.45%)
South Asia	22.56% (22.38% - 22.75%)	20.48% (20.31% - 20.65%)	19.70% (19.55% - 19.85%)	14.81% (14.64% - 14.97%)	8.38% (8.25% - 8.53%)	7.15% (7.03% - 7.27%)	6.85% (6.75% - 6.95%)	4.06% (3.97% - 4.16%)	2.29% (2.22% - 2.37%)	1.75% (1.70% - 1.81%)	1.62% (1.58% - 1.67%)	0.57% (0.54% - 0.60%)
**South Asia**	22.56% (22.38% - 22.75%)	20.48% (20.31% - 20.65%)	19.70% (19.55% - 19.85%)	14.81% (14.64% - 14.97%)	8.38% (8.25% - 8.53%)	7.15% (7.03% - 7.27%)	6.85% (6.75% - 6.95%)	4.06% (3.97% - 4.16%)	2.29% (2.22% - 2.37%)	1.75% (1.70% - 1.81%)	1.62% (1.58% - 1.67%)	0.57% (0.54% - 0.60%)
Bangladesh	18.44% (17.84% - 19.00%)	15.10% (14.71% - 15.48%)	14.83% (14.45% - 15.19%)	11.14% (10.59% - 11.71%)	6.30% (5.91% - 6.68%)	4.92% (4.70% - 5.16%)	4.32% (4.10% - 4.54%)	2.12% (1.90% - 2.37%)	1.40% (1.22% - 1.57%)	0.96% (0.87% - 1.07%)	0.61% (0.52% - 0.70%)	0.02% (0.00% - 0.04%)
Bhutan	8.24% (7.65% - 8.88%)	6.68% (6.29% - 7.13%)	5.80% (5.49% - 6.12%)	3.80% (3.54% - 4.11%)	1.33% (1.11% - 1.58%)	0.94% (0.80% - 1.12%)	0.85% (0.74% - 0.97%)	0.30% (0.24% - 0.36%)	0.00% (0.00% - 0.01%)	0.01% (0.00% - 0.03%)	0.04% (0.02% - 0.06%)	0.00% (0.00% - 0.00%)
India	24.31% (24.12% - 24.51%)	22.11% (21.94% - 22.28%)	21.26% (21.10% - 21.42%)	16.06% (15.90% - 16.22%)	9.31% (9.14% - 9.48%)	7.95% (7.82% - 8.08%)	7.72% (7.60% - 7.84%)	4.64% (4.54% - 4.74%)	2.68% (2.59% - 2.78%)	2.05% (1.98% - 2.12%)	1.93% (1.87% - 1.99%)	0.69% (0.66% - 0.73%)
Other Union Territories	35.95% (35.09% - 36.88%)	31.93% (31.14% - 32.76%)	24.59% (23.77% - 25.45%)	18.18% (17.41% - 18.95%)	19.06% (17.93% - 20.33%)	14.88% (13.93% - 15.93%)	8.80% (8.12% - 9.53%)	4.75% (4.28% - 5.21%)	8.45% (7.45% - 9.63%)	5.54% (4.80% - 6.37%)	2.19% (1.81% - 2.61%)	0.55% (0.39% - 0.73%)
Andhra Pradesh	25.22% (24.40% - 26.04%)	22.56% (21.97% - 23.16%)	20.23% (19.80% - 20.70%)	14.83% (14.33% - 15.34%)	9.74% (9.03% - 10.46%)	7.93% (7.47% - 8.47%)	6.96% (6.64% - 7.31%)	3.89% (3.63% - 4.17%)	2.75% (2.38% - 3.15%)	1.88% (1.66% - 2.15%)	1.54% (1.40% - 1.69%)	0.38% (0.30% - 0.47%)
Arunachal Pradesh	19.84% (18.93% - 20.79%)	19.81% (18.99% - 20.68%)	20.49% (19.83% - 21.18%)	16.42% (15.77% - 17.08%)	7.81% (7.19% - 8.48%)	7.48% (6.92% - 8.10%)	7.94% (7.47% - 8.47%)	5.43% (5.00% - 5.87%)	2.32% (2.02% - 2.67%)	2.03% (1.77% - 2.33%)	2.19% (1.95% - 2.46%)	1.08% (0.90% - 1.28%)
Assam	16.72% (15.90% - 17.61%)	20.08% (19.23% - 20.93%)	20.08% (19.45% - 20.78%)	14.98% (14.38% - 15.61%)	5.00% (4.51% - 5.51%)	7.34% (6.74% - 8.00%)	7.86% (7.33% - 8.45%)	4.72% (4.32% - 5.14%)	0.90% (0.72% - 1.08%)	1.83% (1.54% - 2.16%)	2.19% (1.90% - 2.54%)	0.81% (0.64% - 0.99%)
Bihar	25.81% (24.95% - 26.71%)	24.47% (23.65% - 25.24%)	22.04% (21.45% - 22.67%)	16.21% (15.61% - 16.81%)	10.88% (10.09% - 11.64%)	9.97% (9.32% - 10.59%)	8.19% (7.74% - 8.65%)	4.77% (4.42% - 5.12%)	3.51% (3.08% - 3.95%)	3.05% (2.71% - 3.40%)	2.16% (1.94% - 2.39%)	0.75% (0.62% - 0.88%)
Chhattisgarh	27.19% (26.32% - 28.14%)	24.87% (24.01% - 25.75%)	25.24% (24.59% - 25.92%)	19.44% (18.77% - 20.09%)	11.21% (10.47% - 12.06%)	9.48% (8.81% - 10.17%)	10.15% (9.59% - 10.73%)	6.40% (5.96% - 6.86%)	3.74% (3.35% - 4.21%)	2.82% (2.50% - 3.16%)	3.08% (2.78% - 3.40%)	1.32% (1.13% - 1.52%)
Delhi	20.78% (19.97% - 21.61%)	19.41% (18.68% - 20.22%)	17.59% (16.90% - 18.29%)	13.02% (12.35% - 13.67%)	7.39% (6.82% - 8.02%)	6.97% (6.47% - 7.55%)	6.08% (5.62% - 6.56%)	3.54% (3.18% - 3.91%)	1.76% (1.49% - 2.08%)	1.67% (1.42% - 1.95%)	1.34% (1.13% - 1.56%)	0.37% (0.25% - 0.50%)
Goa	20.32% (19.58% - 21.04%)	18.33% (17.66% - 19.01%)	18.12% (17.45% - 18.81%)	16.43% (15.64% - 17.28%)	6.15% (5.63% - 6.68%)	5.47% (5.00% - 5.98%)	6.10% (5.59% - 6.66%)	5.34% (4.76% - 5.95%)	1.06% (0.83% - 1.29%)	0.91% (0.71% - 1.14%)	1.30% (1.05% - 1.57%)	1.03% (0.76% - 1.34%)
Gujarat	27.19% (26.41% - 27.94%)	24.97% (24.36% - 25.61%)	22.28% (21.79% - 22.75%)	17.27% (16.72% - 17.78%)	10.65% (9.95% - 11.41%)	9.13% (8.61% - 9.68%)	7.84% (7.47% - 8.23%)	5.09% (4.78% - 5.42%)	3.05% (2.67% - 3.45%)	2.30% (2.05% - 2.58%)	1.87% (1.70% - 2.06%)	0.83% (0.71% - 0.95%)
Haryana	13.69% (13.07% - 14.31%)	15.80% (15.18% - 16.48%)	22.60% (22.00% - 23.18%)	18.19% (17.60% - 18.79%)	3.69% (3.37% - 4.01%)	4.90% (4.54% - 5.28%)	9.44% (8.97% - 9.95%)	6.47% (6.05% - 6.90%)	0.57% (0.48% - 0.67%)	0.90% (0.77% - 1.05%)	2.91% (2.64% - 3.21%)	1.49% (1.30% - 1.71%)
Himachal Pradesh	22.81% (21.84% - 23.78%)	19.16% (18.35% - 20.01%)	16.38% (15.62% - 17.19%)	11.57% (10.95% - 12.21%)	9.09% (8.32% - 9.95%)	6.48% (5.90% - 7.11%)	5.01% (4.54% - 5.50%)	2.76% (2.45% - 3.10%)	2.63% (2.21% - 3.14%)	1.38% (1.13% - 1.69%)	0.81% (0.62% - 1.02%)	0.12% (0.05% - 0.22%)

Table S8b. Estimated overall, severe, and extreme **wasting** prevalence (%) in children under 5 years, both sexes, in 1990, 2000, 2010, and 2020.												
**Location**	**Total**				**Severe**				**Extreme**			
	**1990**	**2000**	**2010**	**2020**	**1990**	**2000**	**2010**	**2020**	**1990**	**2000**	**2010**	**2020**
Jammu & Kashmir and Ladakh	18.14% (17.41% - 18.91%)	14.85% (14.25% - 15.45%)	12.34% (11.82% - 12.88%)	9.99% (9.56% - 10.46%)	5.74% (5.28% - 6.25%)	4.28% (3.94% - 4.59%)	3.36% (3.09% - 3.66%)	2.46% (2.24% - 2.69%)	1.09% (0.91% - 1.31%)	0.60% (0.49% - 0.71%)	0.32% (0.24% - 0.42%)	0.14% (0.09% - 0.19%)
Jharkhand	36.95% (35.96% - 38.05%)	34.90% (33.99% - 35.82%)	28.90% (28.07% - 29.73%)	20.59% (19.85% - 21.40%)	19.64% (18.22% - 21.14%)	17.49% (16.33% - 18.73%)	12.38% (11.52% - 13.22%)	6.53% (6.00% - 7.07%)	8.61% (7.53% - 9.82%)	7.11% (6.26% - 8.03%)	4.04% (3.54% - 4.54%)	1.26% (1.04% - 1.48%)
Karnataka	21.98% (21.31% - 22.65%)	21.15% (20.55% - 21.73%)	22.42% (21.93% - 22.87%)	17.69% (17.12% - 18.27%)	6.96% (6.52% - 7.41%)	6.82% (6.43% - 7.23%)	8.18% (7.82% - 8.55%)	5.32% (4.94% - 5.70%)	1.47% (1.29% - 1.67%)	1.46% (1.29% - 1.63%)	2.03% (1.86% - 2.21%)	0.86% (0.72% - 1.02%)
Kerala	17.27% (16.61% - 17.95%)	16.33% (15.79% - 16.89%)	17.95% (17.49% - 18.44%)	15.20% (14.65% - 15.79%)	5.13% (4.75% - 5.53%)	4.79% (4.49% - 5.15%)	5.88% (5.57% - 6.22%)	4.40% (4.06% - 4.77%)	0.90% (0.76% - 1.06%)	0.75% (0.64% - 0.89%)	1.17% (1.03% - 1.32%)	0.60% (0.47% - 0.75%)
Madhya Pradesh	27.99% (27.14% - 28.79%)	26.32% (25.73% - 26.95%)	26.02% (25.54% - 26.51%)	17.32% (16.78% - 17.90%)	10.61% (9.84% - 11.39%)	9.32% (8.82% - 9.86%)	9.63% (9.17% - 10.08%)	4.55% (4.22% - 4.91%)	2.86% (2.50% - 3.27%)	2.24% (2.00% - 2.51%)	2.44% (2.21% - 2.68%)	0.51% (0.40% - 0.63%)
Maharashtra	27.31% (26.53% - 28.02%)	26.46% (25.88% - 27.04%)	25.06% (24.56% - 25.58%)	19.47% (18.89% - 20.07%)	9.57% (8.91% - 10.21%)	9.63% (9.12% - 10.23%)	9.86% (9.39% - 10.37%)	6.04% (5.64% - 6.46%)	2.29% (1.97% - 2.61%)	2.39% (2.13% - 2.71%)	2.72% (2.46% - 3.00%)	1.06% (0.89% - 1.24%)
Manipur	11.08% (10.61% - 11.57%)	10.25% (9.79% - 10.69%)	10.93% (10.49% - 11.36%)	8.42% (8.08% - 8.84%)	2.61% (2.38% - 2.86%)	2.28% (2.07% - 2.49%)	2.77% (2.54% - 2.99%)	1.73% (1.57% - 1.92%)	0.26% (0.20% - 0.33%)	0.13% (0.08% - 0.17%)	0.16% (0.09% - 0.23%)	0.02% (0.00% - 0.06%)
Meghalaya	28.07% (27.10% - 29.13%)	25.77% (24.82% - 26.82%)	22.76% (21.92% - 23.62%)	16.01% (15.35% - 16.75%)	13.69% (12.71% - 14.76%)	11.18% (10.29% - 12.19%)	9.11% (8.40% - 9.84%)	4.93% (4.51% - 5.41%)	5.39% (4.68% - 6.19%)	3.74% (3.18% - 4.40%)	2.67% (2.29% - 3.08%)	0.84% (0.67% - 1.05%)
Mizoram	14.42% (13.81% - 15.06%)	14.09% (13.58% - 14.67%)	14.08% (13.71% - 14.48%)	10.03% (9.66% - 10.41%)	5.19% (4.73% - 5.67%)	4.57% (4.23% - 4.94%)	4.49% (4.28% - 4.75%)	2.43% (2.25% - 2.61%)	1.49% (1.25% - 1.76%)	0.96% (0.82% - 1.13%)	0.82% (0.73% - 0.92%)	0.14% (0.11% - 0.19%)
Nagaland	17.84% (17.02% - 18.72%)	15.24% (14.51% - 16.01%)	13.89% (13.37% - 14.42%)	11.20% (10.72% - 11.69%)	6.29% (5.71% - 6.94%)	4.76% (4.35% - 5.25%)	4.16% (3.83% - 4.48%)	2.87% (2.62% - 3.13%)	1.43% (1.16% - 1.76%)	0.80% (0.63% - 1.01%)	0.60% (0.47% - 0.73%)	0.21% (0.14% - 0.30%)
Odisha	23.49% (22.63% - 24.33%)	21.53% (20.84% - 22.21%)	19.94% (19.32% - 20.51%)	14.72% (14.15% - 15.30%)	8.46% (7.82% - 9.08%)	7.25% (6.74% - 7.73%)	6.59% (6.20% - 6.99%)	3.86% (3.56% - 4.19%)	2.19% (1.89% - 2.48%)	1.66% (1.43% - 1.87%)	1.42% (1.25% - 1.61%)	0.44% (0.36% - 0.54%)
Punjab	17.82% (17.14% - 18.47%)	14.89% (14.33% - 15.47%)	16.38% (15.91% - 16.86%)	13.18% (12.69% - 13.70%)	6.19% (5.76% - 6.63%)	4.86% (4.53% - 5.21%)	5.82% (5.51% - 6.14%)	3.89% (3.61% - 4.20%)	1.48% (1.28% - 1.70%)	1.04% (0.92% - 1.18%)	1.37% (1.23% - 1.53%)	0.56% (0.46% - 0.68%)
Rajasthan	23.61% (22.83% - 24.41%)	20.01% (19.36% - 20.70%)	19.73% (19.18% - 20.26%)	15.64% (15.06% - 16.21%)	9.08% (8.47% - 9.76%)	6.44% (6.00% - 6.92%)	6.82% (6.43% - 7.20%)	4.66% (4.32% - 5.00%)	2.46% (2.15% - 2.81%)	1.26% (1.07% - 1.47%)	1.52% (1.35% - 1.70%)	0.70% (0.57% - 0.84%)
Sikkim	14.40% (13.71% - 15.11%)	12.26% (11.66% - 12.89%)	11.97% (11.44% - 12.50%)	9.11% (8.68% - 9.57%)	4.91% (4.49% - 5.33%)	3.65% (3.34% - 3.99%)	3.23% (2.95% - 3.52%)	1.96% (1.76% - 2.18%)	1.14% (0.98% - 1.33%)	0.63% (0.52% - 0.74%)	0.33% (0.24% - 0.43%)	0.04% (0.01% - 0.10%)
Tamil Nadu	29.68% (28.96% - 30.38%)	24.35% (23.78% - 24.94%)	23.25% (22.79% - 23.69%)	17.69% (17.16% - 18.25%)	12.42% (11.72% - 13.16%)	8.49% (8.03% - 8.96%)	8.41% (8.02% - 8.76%)	5.10% (4.76% - 5.46%)	3.90% (3.52% - 4.33%)	1.99% (1.77% - 2.22%)	2.04% (1.84% - 2.22%)	0.73% (0.60% - 0.87%)
Telangana	33.27% (32.33% - 34.17%)	28.90% (28.01% - 29.84%)	25.78% (25.08% - 26.58%)	16.83% (16.27% - 17.44%)	17.67% (16.57% - 18.83%)	13.59% (12.63% - 14.66%)	11.26% (10.55% - 12.06%)	4.95% (4.59% - 5.33%)	7.67% (6.83% - 8.60%)	5.02% (4.35% - 5.78%)	3.69% (3.27% - 4.22%)	0.73% (0.60% - 0.88%)
Tripura	26.39% (25.41% - 27.41%)	24.46% (23.61% - 25.34%)	20.92% (20.36% - 21.53%)	16.26% (15.70% - 16.81%)	10.25% (9.45% - 11.10%)	9.03% (8.35% - 9.76%)	7.26% (6.85% - 7.73%)	4.78% (4.43% - 5.13%)	2.98% (2.62% - 3.38%)	2.35% (2.02% - 2.71%)	1.63% (1.44% - 1.86%)	0.70% (0.57% - 0.84%)
Uttar Pradesh	19.79% (19.17% - 20.46%)	16.89% (16.35% - 17.43%)	17.37% (16.93% - 17.83%)	13.88% (13.41% - 14.36%)	6.57% (6.15% - 6.98%)	5.10% (4.79% - 5.41%)	5.58% (5.30% - 5.86%)	3.72% (3.48% - 3.96%)	1.52% (1.34% - 1.72%)	1.02% (0.90% - 1.15%)	1.10% (1.00% - 1.22%)	0.47% (0.41% - 0.54%)
Uttarakhand	25.31% (24.34% - 26.38%)	22.97% (22.09% - 23.88%)	18.73% (18.16% - 19.33%)	13.55% (13.04% - 14.13%)	10.68% (9.84% - 11.65%)	9.08% (8.34% - 9.87%)	6.95% (6.54% - 7.38%)	4.08% (3.77% - 4.41%)	3.42% (2.91% - 4.02%)	2.60% (2.21% - 3.04%)	1.77% (1.56% - 2.00%)	0.60% (0.47% - 0.74%)
West Bengal	24.03% (23.16% - 24.89%)	22.32% (21.66% - 23.01%)	21.29% (20.79% - 21.82%)	15.53% (14.91% - 16.10%)	8.59% (7.89% - 9.28%)	7.59% (7.08% - 8.12%)	7.62% (7.23% - 8.05%)	4.33% (3.99% - 4.69%)	2.13% (1.82% - 2.46%)	1.70% (1.47% - 1.94%)	1.82% (1.62% - 2.03%)	0.53% (0.41% - 0.66%)
Nepal	15.81% (14.98% - 16.62%)	12.68% (12.01% - 13.34%)	10.54% (9.99% - 11.13%)	7.87% (7.41% - 8.34%)	4.00% (3.58% - 4.45%)	2.69% (2.40% - 2.98%)	1.86% (1.64% - 2.10%)	1.00% (0.84% - 1.17%)	0.43% (0.31% - 0.54%)	0.18% (0.13% - 0.25%)	0.04% (0.02% - 0.08%)	0.00% (0.00% - 0.01%)
Pakistan	16.95% (16.26% - 17.63%)	17.30% (16.59% - 18.02%)	16.61% (16.07% - 17.16%)	12.61% (12.05% - 13.19%)	5.48% (5.05% - 5.92%)	5.36% (4.93% - 5.81%)	5.04% (4.73% - 5.39%)	3.14% (2.84% - 3.46%)	1.08% (0.90% - 1.28%)	1.04% (0.87% - 1.21%)	1.03% (0.90% - 1.18%)	0.44% (0.34% - 0.55%)

Table S8b. Estimated overall, severe, and extreme **wasting** prevalence (%) in children under 5 years, both sexes, in 1990, 2000, 2010, and 2020.												
**Location**	**Total**				**Severe**				**Extreme**			
	**1990**	**2000**	**2010**	**2020**	**1990**	**2000**	**2010**	**2020**	**1990**	**2000**	**2010**	**2020**
Southeast Asia, East Asia, and Oceania	8.08%	7.12%	6.09%	4.30%	1.73%	1.58%	1.29%	0.70%	0.27%	0.24%	0.18%	0.06%
	(7.83% - 8.34%)	(6.98% - 7.27%)	(5.99% - 6.20%)	(4.22% - 4.39%)	(1.66% - 1.80%)	(1.54% - 1.61%)	(1.26% - 1.31%)	(0.68% - 0.72%)	(0.26% - 0.29%)	(0.23% - 0.25%)	(0.17% - 0.19%)	(0.06% - 0.07%)
**East Asia**	5.50%	3.51%	2.36%	1.63%	0.60%	0.24%	0.07%	0.02%	0.02%	0.01%	0.00%	0.00%
	(5.14% - 5.89%)	(3.28% - 3.74%)	(2.19% - 2.53%)	(1.51% - 1.75%)	(0.51% - 0.70%)	(0.21% - 0.28%)	(0.06% - 0.09%)	(0.01% - 0.02%)	(0.01% - 0.03%)	(0.01% - 0.02%)	(0.00% - 0.00%)	(0.00% - 0.00%)
China	5.36%	3.24%	2.27%	1.59%	0.54%	0.15%	0.06%	0.02%	0.01%	0.00%	0.00%	0.00%
	(4.99% - 5.76%)	(3.00% - 3.49%)	(2.10% - 2.45%)	(1.47% - 1.72%)	(0.45% - 0.65%)	(0.12% - 0.19%)	(0.04% - 0.08%)	(0.01% - 0.02%)	(0.00% - 0.02%)	(0.00% - 0.00%)	(0.00% - 0.00%)	(0.00% - 0.00%)
Democratic People's Republic of Korea	13.97%	12.91%	5.87%	3.69%	3.92%	3.55%	0.58%	0.03%	0.48%	0.38%	0.00%	0.00%
	(13.16% - 14.82%)	(12.31% - 13.50%)	(5.45% - 6.31%)	(3.36% - 4.08%)	(3.46% - 4.42%)	(3.20% - 3.88%)	(0.45% - 0.73%)	(0.00% - 0.09%)	(0.31% - 0.68%)	(0.24% - 0.51%)	(0.00% - 0.00%)	(0.00% - 0.00%)
Taiwan (Province of China)	3.77%	2.48%	1.88%	1.58%	0.11%	0.01%	0.00%	0.00%	0.00%	0.00%	0.00%	0.00%
	(3.38% - 4.19%)	(2.24% - 2.76%)	(1.71% - 2.08%)	(1.43% - 1.76%)	(0.05% - 0.19%)	(0.00% - 0.03%)	(0.00% - 0.01%)	(0.00% - 0.00%)	(0.00% - 0.00%)	(0.00% - 0.00%)	(0.00% - 0.00%)	(0.00% - 0.00%)
**Oceania**	9.27%	10.77%	12.09%	10.20%	2.08%	2.84%	3.68%	2.73%	0.12%	0.32%	0.66%	0.30%
	(8.78% - 9.76%)	(10.26% - 11.33%)	(11.53% - 12.68%)	(9.61% - 10.78%)	(1.86% - 2.32%)	(2.58% - 3.13%)	(3.37% - 4.02%)	(2.44% - 3.03%)	(0.08% - 0.18%)	(0.24% - 0.42%)	(0.53% - 0.80%)	(0.21% - 0.39%)
Fiji	8.65%	6.41%	5.52%	4.62%	1.85%	1.00%	0.71%	0.46%	0.03%	0.01%	0.00%	0.00%
	(7.97% - 9.40%)	(5.92% - 6.93%)	(5.10% - 5.98%)	(4.22% - 5.02%)	(1.57% - 2.18%)	(0.82% - 1.20%)	(0.57% - 0.86%)	(0.36% - 0.58%)	(0.00% - 0.08%)	(0.00% - 0.03%)	(0.00% - 0.01%)	(0.00% - 0.00%)
Kiribati	10.56%	6.84%	4.27%	3.51%	2.59%	1.09%	0.42%	0.30%	0.11%	0.00%	0.00%	0.00%
	(9.76% - 11.42%)	(6.26% - 7.40%)	(3.92% - 4.67%)	(3.24% - 3.81%)	(2.20% - 3.03%)	(0.87% - 1.30%)	(0.33% - 0.51%)	(0.23% - 0.38%)	(0.03% - 0.24%)	(0.00% - 0.01%)	(0.00% - 0.00%)	(0.00% - 0.00%)
Marshall Islands	0.31%	0.29%	0.25%	0.12%	0.00%	0.00%	0.00%	0.00%	0.00%	0.00%	0.00%	0.00%
	(0.25% - 0.36%)	(0.21% - 0.34%)	(0.11% - 0.35%)	(0.06% - 0.23%)	(0.00% - 0.00%)	(0.00% - 0.00%)	(0.00% - 0.00%)	(0.00% - 0.00%)	(0.00% - 0.00%)	(0.00% - 0.00%)	(0.00% - 0.00%)	(0.00% - 0.00%)
Micronesia (Federated States of)	4.77%	5.43%	5.05%	4.44%	0.31%	0.54%	0.49%	0.37%	0.00%	0.00%	0.00%	0.00%
	(4.24% - 5.30%)	(4.89% - 5.98%)	(4.52% - 5.58%)	(3.97% - 4.88%)	(0.18% - 0.47%)	(0.38% - 0.73%)	(0.34% - 0.65%)	(0.26% - 0.49%)	(0.00% - 0.00%)	(0.00% - 0.00%)	(0.00% - 0.00%)	(0.00% - 0.00%)
Papua New Guinea	10.58%	12.83%	14.27%	11.49%	2.51%	3.64%	4.64%	3.24%	0.17%	0.44%	0.87%	0.37%
	(9.90% - 11.26%)	(12.12% - 13.58%)	(13.54% - 15.03%)	(10.76% - 12.19%)	(2.19% - 2.82%)	(3.28% - 4.04%)	(4.22% - 5.09%)	(2.87% - 3.62%)	(0.10% - 0.25%)	(0.33% - 0.58%)	(0.70% - 1.06%)	(0.27% - 0.49%)
Samoa	5.70%	4.97%	4.60%	4.14%	0.93%	0.70%	0.61%	0.51%	0.04%	0.02%	0.01%	0.01%
	(5.20% - 6.26%)	(4.54% - 5.43%)	(4.23% - 5.04%)	(3.78% - 4.52%)	(0.76% - 1.12%)	(0.55% - 0.86%)	(0.49% - 0.76%)	(0.40% - 0.63%)	(0.01% - 0.07%)	(0.00% - 0.04%)	(0.00% - 0.03%)	(0.00% - 0.02%)
Solomon Islands	7.17%	6.29%	7.00%	7.61%	1.19%	0.88%	1.17%	1.52%	0.00%	0.00%	0.00%	0.00%
	(6.71% - 7.65%)	(5.85% - 6.74%)	(6.61% - 7.39%)	(7.11% - 8.10%)	(1.01% - 1.38%)	(0.72% - 1.04%)	(1.02% - 1.34%)	(1.31% - 1.75%)	(0.00% - 0.01%)	(0.00% - 0.00%)	(0.00% - 0.00%)	(0.00% - 0.02%)
Tonga	1.42%	2.56%	4.99%	4.57%	0.00%	0.01%	0.53%	0.45%	0.00%	0.00%	0.00%	0.00%
	(1.27% - 1.59%)	(2.21% - 3.05%)	(4.53% - 5.50%)	(4.20% - 4.96%)	(0.00% - 0.00%)	(0.00% - 0.06%)	(0.37% - 0.71%)	(0.32% - 0.60%)	(0.00% - 0.00%)	(0.00% - 0.00%)	(0.00% - 0.00%)	(0.00% - 0.00%)
American Samoa	2.23%	2.71%	2.80%	2.55%	0.00%	0.03%	0.06%	0.05%	0.00%	0.00%	0.00%	0.00%
	(2.00% - 2.48%)	(2.42% - 3.04%)	(2.50% - 3.12%)	(2.29% - 2.85%)	(0.00% - 0.01%)	(0.00% - 0.06%)	(0.03% - 0.10%)	(0.02% - 0.09%)	(0.00% - 0.00%)	(0.00% - 0.00%)	(0.00% - 0.00%)	(0.00% - 0.00%)
Vanuatu	7.02%	6.11%	5.26%	4.50%	1.05%	0.77%	0.50%	0.29%	0.00%	0.00%	0.00%	0.00%
	(6.52% - 7.57%)	(5.69% - 6.54%)	(4.89% - 5.69%)	(4.08% - 4.93%)	(0.86% - 1.27%)	(0.62% - 0.92%)	(0.38% - 0.65%)	(0.18% - 0.42%)	(0.00% - 0.00%)	(0.00% - 0.00%)	(0.00% - 0.00%)	(0.00% - 0.00%)
Cook Islands	1.72%	1.81%	1.71%	1.49%	0.00%	0.00%	0.00%	0.00%	0.00%	0.00%	0.00%	0.00%
	(1.53% - 1.93%)	(1.62% - 2.05%)	(1.52% - 1.94%)	(1.34% - 1.67%)	(0.00% - 0.00%)	(0.00% - 0.00%)	(0.00% - 0.01%)	(0.00% - 0.00%)	(0.00% - 0.00%)	(0.00% - 0.00%)	(0.00% - 0.00%)	(0.00% - 0.00%)
Guam	1.36%	1.53%	1.66%	1.58%	0.00%	0.00%	0.00%	0.00%	0.00%	0.00%	0.00%	0.00%
	(1.22% - 1.52%)	(1.37% - 1.73%)	(1.50% - 1.89%)	(1.42% - 1.74%)	(0.00% - 0.00%)	(0.00% - 0.00%)	(0.00% - 0.00%)	(0.00% - 0.01%)	(0.00% - 0.00%)	(0.00% - 0.00%)	(0.00% - 0.00%)	(0.00% - 0.00%)
Nauru	1.56%	1.83%	1.60%	1.21%	0.00%	0.01%	0.01%	0.01%	0.00%	0.00%	0.00%	0.00%
	(1.41% - 1.71%)	(1.66% - 2.00%)	(1.47% - 1.76%)	(1.09% - 1.34%)	(0.00% - 0.01%)	(0.00% - 0.03%)	(0.00% - 0.03%)	(0.00% - 0.02%)	(0.00% - 0.00%)	(0.00% - 0.00%)	(0.00% - 0.00%)	(0.00% - 0.00%)
Niue	2.16%	2.56%	2.48%	2.24%	0.00%	0.02%	0.03%	0.02%	0.00%	0.00%	0.00%	0.00%
	(1.92% - 2.43%)	(2.30% - 2.88%)	(2.22% - 2.79%)	(2.00% - 2.48%)	(0.00% - 0.01%)	(0.00% - 0.05%)	(0.01% - 0.06%)	(0.00% - 0.05%)	(0.00% - 0.00%)	(0.00% - 0.00%)	(0.00% - 0.00%)	(0.00% - 0.00%)
Northern Mariana Islands	1.38%	1.64%	1.78%	1.67%	0.00%	0.00%	0.00%	0.00%	0.00%	0.00%	0.00%	0.00%
	(1.22% - 1.56%)	(1.48% - 1.80%)	(1.59% - 1.98%)	(1.51% - 1.87%)	(0.00% - 0.00%)	(0.00% - 0.00%)	(0.00% - 0.01%)	(0.00% - 0.01%)	(0.00% - 0.00%)	(0.00% - 0.00%)	(0.00% - 0.00%)	(0.00% - 0.00%)
Palau	1.95%	2.25%	2.62%	2.19%	0.00%	0.01%	0.04%	0.02%	0.00%	0.00%	0.00%	0.00%
	(1.74% - 2.17%)	(2.00% - 2.52%)	(2.35% - 2.92%)	(1.95% - 2.46%)	(0.00% - 0.00%)	(0.00% - 0.02%)	(0.01% - 0.08%)	(0.00% - 0.05%)	(0.00% - 0.00%)	(0.00% - 0.00%)	(0.00% - 0.00%)	(0.00% - 0.00%)
Tokelau	3.48%	3.84%	3.35%	2.57%	0.07%	0.17%	0.13%	0.05%	0.00%	0.00%	0.00%	0.00%
	(3.13% - 3.87%)	(3.46% - 4.25%)	(3.01% - 3.79%)	(2.28% - 2.87%)	(0.03% - 0.14%)	(0.10% - 0.26%)	(0.07% - 0.19%)	(0.02% - 0.09%)	(0.00% - 0.00%)	(0.00% - 0.00%)	(0.00% - 0.00%)	(0.00% - 0.00%)

Table S8b. Estimated overall, severe, and extreme **wasting** prevalence (%) in children under 5 years, both sexes, in 1990, 2000, 2010, and 2020.

Location	Total				Severe				Extreme			
	1990	2000	2010	2020	1990	2000	2010	2020	1990	2000	2010	2020
	5.41%	3.99%	3.28%	2.76%	0.50%	0.13%	0.04%	0.01%	0.00%	0.00%	0.00%	0.00%
Tuvalu	(4.93% - 5.97%)	(3.51% - 4.43%)	(2.90% - 3.73%)	(2.45% - 3.11%)	(0.35% - 0.69%)	(0.04% - 0.25%)	(0.00% - 0.12%)	(0.00% - 0.05%)	(0.00% - 0.00%)	(0.00% - 0.00%)	(0.00% - 0.00%)	(0.00% - 0.00%)
	13.53%	12.00%	10.24%	8.02%	4.12%	3.38%	2.62%	1.65%	0.82%	0.56%	0.37%	0.15%
**Southeast Asia**	(13.36% - 13.69%)	(11.86% - 12.13%)	(10.12% - 10.36%)	(7.90% - 8.14%)	(4.03% - 4.21%)	(3.31% - 3.44%)	(2.56% - 2.67%)	(1.60% - 1.70%)	(0.79% - 0.86%)	(0.54% - 0.58%)	(0.36% - 0.39%)	(0.14% - 0.16%)
	18.71%	16.99%	11.21%	8.08%	5.93%	5.00%	2.28%	1.14%	1.13%	0.76%	0.06%	0.00%
Cambodia	(17.85% - 19.59%)	(16.32% - 17.69%)	(10.71% - 11.72%)	(7.59% - 8.62%)	(5.37% - 6.52%)	(4.57% - 5.42%)	(2.05% - 2.51%)	(0.97% - 1.34%)	(0.90% - 1.38%)	(0.60% - 0.92%)	(0.04% - 0.09%)	(0.00% - 0.01%)
	17.19%	14.64%	12.69%	10.07%	6.34%	4.97%	4.03%	2.66%	1.80%	1.20%	0.83%	0.36%
Indonesia	(16.97% - 17.42%)	(14.45% - 14.82%)	(12.52% - 12.85%)	(9.92% - 10.22%)	(6.20% - 6.49%)	(4.86% - 5.08%)	(3.94% - 4.12%)	(2.60% - 2.74%)	(1.73% - 1.87%)	(1.16% - 1.25%)	(0.80% - 0.86%)	(0.34% - 0.38%)
	20.63%	18.28%	16.14%	13.86%	8.95%	7.31%	5.89%	4.45%	3.00%	2.14%	1.44%	0.79%
Aceh	(19.63% - 21.71%)	(17.43% - 19.11%)	(15.48% - 16.79%)	(13.16% - 14.62%)	(8.15% - 9.81%)	(6.69% - 7.93%)	(5.45% - 6.35%)	(4.04% - 4.92%)	(2.56% - 3.50%)	(1.83% - 2.48%)	(1.23% - 1.67%)	(0.62% - 1.00%)
	15.50%	14.78%	13.82%	10.85%	5.55%	5.23%	4.65%	3.04%	1.67%	1.45%	1.05%	0.53%
North Sumatra	(14.73% - 16.26%)	(14.05% - 15.48%)	(13.16% - 14.52%)	(10.30% - 11.44%)	(5.06% - 6.03%)	(4.82% - 5.65%)	(4.28% - 5.06%)	(2.74% - 3.35%)	(1.43% - 1.93%)	(1.24% - 1.66%)	(0.90% - 1.21%)	(0.42% - 0.65%)
	14.08%	13.72%	11.41%	8.64%	5.07%	4.60%	3.22%	1.90%	1.50%	1.16%	0.58%	0.23%
West Sumatra	(13.35% - 14.90%)	(13.01% - 14.48%)	(10.83% - 12.04%)	(8.13% - 9.17%)	(4.64% - 5.59%)	(4.16% - 5.08%)	(2.90% - 3.54%)	(1.69% - 2.15%)	(1.28% - 1.77%)	(0.97% - 1.38%)	(0.47% - 0.70%)	(0.16% - 0.32%)
	24.79%	21.85%	18.99%	15.58%	12.53%	10.18%	8.05%	5.59%	5.19%	3.75%	2.55%	1.31%
Riau	(23.58% - 26.01%)	(20.84% - 22.88%)	(18.23% - 19.84%)	(14.76% - 16.48%)	(11.46% - 13.67%)	(9.32% - 11.07%)	(7.51% - 8.68%)	(5.07% - 6.17%)	(4.51% - 5.94%)	(3.24% - 4.30%)	(2.26% - 2.90%)	(1.07% - 1.58%)
	24.88%	21.40%	18.26%	14.51%	12.43%	9.66%	7.36%	5.02%	5.12%	3.42%	2.15%	1.06%
Jambi	(23.74% - 26.07%)	(20.54% - 22.31%)	(17.62% - 18.92%)	(13.80% - 15.34%)	(11.42% - 13.51%)	(8.96% - 10.41%)	(6.86% - 7.86%)	(4.58% - 5.55%)	(4.48% - 5.82%)	(3.02% - 3.88%)	(1.89% - 2.41%)	(0.87% - 1.30%)
	20.36%	17.73%	14.61%	10.75%	7.92%	6.54%	5.03%	2.88%	2.34%	1.70%	1.07%	0.26%
South Sumatra	(19.39% - 21.40%)	(16.94% - 18.52%)	(14.11% - 15.18%)	(10.21% - 11.35%)	(7.18% - 8.71%)	(5.99% - 7.10%)	(4.71% - 5.39%)	(2.61% - 3.18%)	(1.96% - 2.76%)	(1.44% - 1.98%)	(0.93% - 1.23%)	(0.19% - 0.35%)
	23.24%	19.84%	16.81%	13.51%	11.53%	8.93%	6.82%	4.78%	4.66%	3.11%	2.00%	1.04%
Bengkulu	(22.23% - 24.31%)	(18.90% - 20.83%)	(16.09% - 17.57%)	(12.77% - 14.25%)	(10.64% - 12.47%)	(8.18% - 9.71%)	(6.32% - 7.38%)	(4.33% - 5.24%)	(4.09% - 5.28%)	(2.68% - 3.57%)	(1.74% - 2.28%)	(0.84% - 1.25%)
	19.70%	16.36%	14.24%	11.20%	7.16%	5.63%	4.87%	3.07%	1.88%	1.27%	1.01%	0.35%
Lampung	(18.80% - 20.61%)	(15.63% - 17.20%)	(13.55% - 14.89%)	(10.58% - 11.92%)	(6.52% - 7.84%)	(5.15% - 6.17%)	(4.47% - 5.28%)	(2.76% - 3.43%)	(1.57% - 2.22%)	(1.06% - 1.51%)	(0.84% - 1.20%)	(0.27% - 0.45%)
	12.85%	10.87%	8.32%	6.77%	3.93%	2.98%	1.86%	1.28%	0.69%	0.43%	0.20%	0.10%
Bangka-Belitung Islands	(12.16% - 13.59%)	(10.24% - 11.47%)	(7.88% - 8.82%)	(6.36% - 7.21%)	(3.56% - 4.33%)	(2.69% - 3.26%)	(1.67% - 2.06%)	(1.12% - 1.45%)	(0.56% - 0.82%)	(0.34% - 0.51%)	(0.16% - 0.24%)	(0.06% - 0.13%)
	11.02%	8.57%	7.39%	6.19%	2.88%	1.84%	1.42%	0.99%	0.40%	0.19%	0.13%	0.07%
Riau Islands	(10.40% - 11.66%)	(8.06% - 9.08%)	(6.99% - 7.83%)	(5.81% - 6.58%)	(2.59% - 3.18%)	(1.63% - 2.06%)	(1.24% - 1.59%)	(0.85% - 1.13%)	(0.32% - 0.49%)	(0.14% - 0.25%)	(0.10% - 0.18%)	(0.04% - 0.11%)
	23.32%	19.21%	15.95%	12.94%	10.62%	7.56%	5.53%	3.87%	3.84%	2.15%	1.20%	0.53%
North Kalimantan	(22.27% - 24.41%)	(18.28% - 20.12%)	(15.26% - 16.67%)	(12.25% - 13.63%)	(9.73% - 11.56%)	(6.91% - 8.22%)	(5.08% - 5.98%)	(3.48% - 4.26%)	(3.31% - 4.40%)	(1.81% - 2.50%)	(1.00% - 1.41%)	(0.39% - 0.69%)
	19.20%	14.82%	11.96%	9.22%	6.95%	4.90%	3.65%	2.32%	1.79%	1.10%	0.70%	0.30%
Jakarta	(18.29% - 20.18%)	(14.12% - 15.60%)	(11.35% - 12.56%)	(8.68% - 9.76%)	(6.31% - 7.62%)	(4.48% - 5.39%)	(3.34% - 3.98%)	(2.07% - 2.59%)	(1.48% - 2.13%)	(0.93% - 1.30%)	(0.60% - 0.82%)	(0.23% - 0.39%)
	13.54%	11.15%	8.80%	6.67%	3.73%	2.88%	2.09%	1.21%	0.58%	0.41%	0.27%	0.07%
West Java	(12.79% - 14.32%)	(10.51% - 11.79%)	(8.33% - 9.28%)	(6.28% - 7.07%)	(3.32% - 4.16%)	(2.56% - 3.19%)	(1.88% - 2.30%)	(1.06% - 1.36%)	(0.47% - 0.72%)	(0.31% - 0.50%)	(0.21% - 0.34%)	(0.04% - 0.11%)
	12.52%	11.27%	11.15%	9.34%	3.64%	3.09%	3.24%	2.30%	0.75%	0.51%	0.50%	0.18%
Central Java	(11.86% - 13.25%)	(10.63% - 11.90%)	(10.59% - 11.76%)	(8.75% - 9.92%)	(3.28% - 4.06%)	(2.77% - 3.40%)	(2.95% - 3.55%)	(2.04% - 2.56%)	(0.61% - 0.91%)	(0.41% - 0.61%)	(0.41% - 0.61%)	(0.13% - 0.23%)
	15.09%	11.42%	8.24%	7.12%	5.13%	3.18%	1.78%	1.34%	1.11%	0.38%	0.10%	0.06%
Yogyakarta	(14.24% - 15.94%)	(10.74% - 12.12%)	(7.79% - 8.72%)	(6.67% - 7.56%)	(4.59% - 5.68%)	(2.84% - 3.56%)	(1.59% - 1.99%)	(1.17% - 1.51%)	(0.87% - 1.39%)	(0.26% - 0.52%)	(0.07% - 0.13%)	(0.03% - 0.09%)
	17.61%	13.47%	10.99%	8.57%	6.18%	4.02%	3.19%	1.99%	1.47%	0.64%	0.51%	0.20%
East Java	(16.62% - 18.59%)	(12.74% - 14.21%)	(10.38% - 11.60%)	(8.07% - 9.12%)	(5.53% - 6.85%)	(3.62% - 4.42%)	(2.90% - 3.50%)	(1.78% - 2.23%)	(1.20% - 1.78%)	(0.50% - 0.78%)	(0.43% - 0.59%)	(0.15% - 0.26%)
	22.49%	18.74%	16.34%	13.27%	10.67%	7.86%	6.25%	4.44%	4.03%	2.45%	1.64%	0.84%
Banten	(21.45% - 23.65%)	(17.81% - 19.68%)	(15.71% - 17.08%)	(12.53% - 14.06%)	(9.76% - 11.66%)	(7.16% - 8.64%)	(5.81% - 6.80%)	(3.97% - 4.92%)	(3.47% - 4.65%)	(2.06% - 2.89%)	(1.42% - 1.93%)	(0.64% - 1.06%)
	14.10%	10.06%	8.40%	5.78%	5.00%	2.97%	2.05%	1.11%	1.46%	0.70%	0.38%	0.08%
Bali	(13.18% - 15.01%)	(9.48% - 10.68%)	(7.61% - 9.05%)	(5.43% - 6.17%)	(4.52% - 5.56%)	(2.68% - 3.29%)	(1.84% - 2.28%)	(0.98% - 1.24%)	(1.24% - 1.72%)	(0.59% - 0.82%)	(0.31% - 0.47%)	(0.04% - 0.12%)
	23.05%	19.74%	14.69%	11.05%	9.85%	7.66%	4.69%	2.74%	3.33%	2.21%	0.86%	0.25%
West Nusa Tenggara	(22.02% - 24.11%)	(18.81% - 20.67%)	(13.95% - 15.49%)	(10.39% - 11.75%)	(9.03% - 10.74%)	(7.05% - 8.36%)	(4.26% - 5.16%)	(2.43% - 3.07%)	(2.90% - 3.84%)	(1.89% - 2.56%)	(0.70% - 1.05%)	(0.19% - 0.31%)

Table S8b. Estimated overall, severe, and extreme **wasting** prevalence (%) in children under 5 years, both sexes, in 1990, 2000, 2010, and 2020.

Location	Total				Severe				Extreme			
	1990	2000	2010	2020	1990	2000	2010	2020	1990	2000	2010	2020
	23.86%	21.27%	18.29%	14.10%	10.10%	8.15%	6.16%	3.84%	3.25%	2.22%	1.28%	0.42%
East Nusa Tenggara	(22.73% - 25.01%)	(20.25% - 22.29%)	(17.42% - 19.23%)	(13.23% - 15.05%)	(9.16% - 11.09%)	(7.39% - 8.94%)	(5.58% - 6.79%)	(3.37% - 4.39%)	(2.72% - 3.78%)	(1.85% - 2.62%)	(1.05% - 1.56%)	(0.27% - 0.62%)
	26.11%	22.65%	20.10%	15.97%	13.26%	10.40%	8.46%	5.69%	5.49%	3.73%	2.64%	1.30%
West Kalimantan	(24.91% - 27.30%)	(21.62% - 23.75%)	(19.26% - 20.94%)	(15.14% - 16.84%)	(12.13% - 14.48%)	(9.53% - 11.37%)	(7.78% - 9.16%)	(5.15% - 6.27%)	(4.72% - 6.34%)	(3.23% - 4.33%)	(2.27% - 3.05%)	(1.05% - 1.58%)
	21.05%	18.01%	15.47%	12.22%	9.09%	7.00%	5.38%	3.62%	3.01%	1.93%	1.17%	0.46%
Central Kalimantan	(20.01% - 22.08%)	(17.19% - 18.79%)	(14.97% - 15.98%)	(11.52% - 12.89%)	(8.30% - 9.93%)	(6.44% - 7.54%)	(5.07% - 5.73%)	(3.24% - 4.00%)	(2.57% - 3.50%)	(1.65% - 2.21%)	(1.03% - 1.33%)	(0.32% - 0.61%)
	16.93%	14.55%	12.43%	8.36%	4.77%	3.48%	2.70%	1.18%	0.68%	0.34%	0.26%	0.03%
South Kalimantan	(15.93% - 17.85%)	(13.76% - 15.44%)	(11.71% - 13.14%)	(7.78% - 8.88%)	(4.21% - 5.33%)	(3.08% - 3.95%)	(2.39% - 3.04%)	(0.98% - 1.37%)	(0.51% - 0.88%)	(0.25% - 0.45%)	(0.19% - 0.35%)	(0.01% - 0.06%)
	19.75%	17.35%	14.14%	11.33%	8.48%	6.87%	4.89%	3.37%	2.78%	1.95%	1.03%	0.42%
East Kalimantan	(18.77% - 20.75%)	(16.51% - 18.18%)	(13.48% - 14.86%)	(10.63% - 12.00%)	(7.75% - 9.26%)	(6.30% - 7.46%)	(4.48% - 5.35%)	(2.99% - 3.75%)	(2.38% - 3.21%)	(1.66% - 2.26%)	(0.85% - 1.23%)	(0.30% - 0.56%)
	14.80%	11.75%	9.84%	7.75%	4.76%	3.23%	2.38%	1.57%	0.90%	0.34%	0.14%	0.03%
North Sulawesi	(13.97% - 15.55%)	(11.12% - 12.44%)	(9.26% - 10.38%)	(7.27% - 8.23%)	(4.30% - 5.22%)	(2.91% - 3.58%)	(2.13% - 2.63%)	(1.37% - 1.77%)	(0.71% - 1.09%)	(0.25% - 0.44%)	(0.10% - 0.18%)	(0.01% - 0.06%)
	22.02%	19.06%	15.67%	11.55%	9.18%	7.12%	5.06%	2.98%	2.84%	1.82%	0.93%	0.20%
Central Sulawesi	(20.96% - 23.12%)	(18.07% - 20.07%)	(14.89% - 16.42%)	(10.86% - 12.30%)	(8.35% - 10.07%)	(6.46% - 7.82%)	(4.59% - 5.56%)	(2.62% - 3.37%)	(2.40% - 3.33%)	(1.51% - 2.17%)	(0.74% - 1.14%)	(0.11% - 0.31%)
	15.85%	11.99%	11.96%	9.85%	5.28%	3.46%	3.19%	2.17%	1.32%	0.79%	0.48%	0.15%
South Sulawesi	(15.06% - 16.71%)	(11.36% - 12.65%)	(11.29% - 12.64%)	(9.25% - 10.51%)	(4.81% - 5.83%)	(3.15% - 3.81%)	(2.86% - 3.54%)	(1.91% - 2.46%)	(1.11% - 1.56%)	(0.67% - 0.95%)	(0.38% - 0.58%)	(0.10% - 0.22%)
	21.82%	19.47%	16.43%	12.67%	9.61%	7.92%	5.96%	3.82%	3.26%	2.36%	1.42%	0.53%
Southeast Sulawesi	(20.72% - 23.01%)	(18.58% - 20.37%)	(15.72% - 17.18%)	(11.94% - 13.39%)	(8.74% - 10.55%)	(7.26% - 8.59%)	(5.50% - 6.44%)	(3.43% - 4.25%)	(2.80% - 3.80%)	(2.02% - 2.71%)	(1.21% - 1.64%)	(0.39% - 0.70%)
	20.57%	18.68%	16.17%	12.04%	8.08%	6.80%	5.33%	3.14%	2.30%	1.68%	1.05%	0.27%
Gorontalo	(19.57% - 21.58%)	(17.84% - 19.56%)	(15.32% - 17.05%)	(11.34% - 12.83%)	(7.36% - 8.85%)	(6.23% - 7.41%)	(4.80% - 5.89%)	(2.78% - 3.56%)	(1.94% - 2.69%)	(1.40% - 1.97%)	(0.82% - 1.29%)	(0.18% - 0.39%)
	20.61%	18.40%	15.43%	11.24%	8.76%	7.24%	5.32%	3.04%	2.85%	2.07%	1.15%	0.26%
West Sulawesi	(19.62% - 21.55%)	(17.60% - 19.19%)	(14.99% - 15.88%)	(10.62% - 11.88%)	(8.03% - 9.50%)	(6.71% - 7.80%)	(5.03% - 5.61%)	(2.72% - 3.37%)	(2.45% - 3.28%)	(1.81% - 2.35%)	(1.02% - 1.28%)	(0.18% - 0.36%)
	19.71%	17.94%	18.12%	14.83%	7.59%	6.41%	6.41%	4.46%	2.06%	1.50%	1.46%	0.67%
Maluku	(18.66% - 20.66%)	(17.20% - 18.70%)	(17.60% - 18.72%)	(14.12% - 15.57%)	(6.86% - 8.32%)	(5.90% - 6.97%)	(6.02% - 6.86%)	(4.04% - 4.91%)	(1.71% - 2.45%)	(1.27% - 1.77%)	(1.28% - 1.67%)	(0.50% - 0.84%)
	20.42%	17.83%	16.36%	13.05%	8.20%	6.48%	5.49%	3.65%	2.40%	1.57%	1.11%	0.40%
North Maluku	(19.48% - 21.41%)	(17.05% - 18.59%)	(15.87% - 16.91%)	(12.35% - 13.75%)	(7.49% - 8.95%)	(5.95% - 7.02%)	(5.16% - 5.85%)	(3.29% - 4.05%)	(2.05% - 2.80%)	(1.33% - 1.83%)	(0.97% - 1.27%)	(0.27% - 0.55%)
	16.38%	14.89%	14.36%	10.73%	5.47%	4.73%	4.59%	2.76%	1.16%	0.87%	0.83%	0.26%
West Papua	(15.51% - 17.30%)	(14.07% - 15.73%)	(13.57% - 15.17%)	(10.09% - 11.44%)	(4.95% - 6.03%)	(4.25% - 5.24%)	(4.13% - 5.06%)	(2.46% - 3.11%)	(0.93% - 1.41%)	(0.68% - 1.09%)	(0.64% - 1.03%)	(0.19% - 0.35%)
	19.27%	16.82%	16.57%	14.07%	7.62%	6.03%	5.95%	4.56%	2.16%	1.40%	1.38%	0.82%
Papua	(18.28% - 20.20%)	(16.02% - 17.65%)	(16.03% - 17.15%)	(13.37% - 14.76%)	(6.91% - 8.32%)	(5.51% - 6.60%)	(5.58% - 6.33%)	(4.15% - 4.97%)	(1.81% - 2.52%)	(1.16% - 1.67%)	(1.22% - 1.56%)	(0.65% - 0.99%)
	13.20%	12.77%	8.37%	6.60%	3.68%	3.20%	1.31%	0.76%	0.40%	0.22%	0.00%	0.00%
Lao People's Democratic Republic	(12.55% - 13.87%)	(12.30% - 13.31%)	(7.99% - 8.82%)	(6.21% - 7.02%)	(3.32% - 4.07%)	(2.94% - 3.47%)	(1.16% - 1.48%)	(0.62% - 0.90%)	(0.27% - 0.54%)	(0.15% - 0.31%)	(0.00% - 0.01%)	(0.00% - 0.00%)
	12.14%	11.97%	9.75%	8.36%	3.63%	3.61%	2.41%	1.79%	0.50%	0.51%	0.10%	0.03%
Malaysia	(11.45% - 12.76%)	(11.34% - 12.66%)	(9.16% - 10.38%)	(7.83% - 8.94%)	(3.26% - 3.99%)	(3.27% - 4.03%)	(2.14% - 2.73%)	(1.57% - 2.05%)	(0.36% - 0.65%)	(0.37% - 0.68%)	(0.05% - 0.18%)	(0.01% - 0.06%)
	18.02%	13.87%	9.08%	7.72%	5.76%	3.72%	1.59%	1.08%	1.08%	0.38%	0.00%	0.00%
Maldives	(17.02% - 19.03%)	(13.00% - 14.68%)	(8.42% - 9.72%)	(7.09% - 8.41%)	(5.06% - 6.45%)	(3.24% - 4.21%)	(1.32% - 1.88%)	(0.83% - 1.35%)	(0.78% - 1.39%)	(0.22% - 0.56%)	(0.00% - 0.01%)	(0.00% - 0.00%)
	14.06%	11.71%	8.47%	5.73%	3.25%	2.32%	1.21%	0.43%	0.18%	0.03%	0.00%	0.00%
Myanmar	(13.17% - 15.01%)	(11.21% - 12.25%)	(8.07% - 8.90%)	(5.26% - 6.18%)	(2.84% - 3.74%)	(2.08% - 2.57%)	(1.06% - 1.38%)	(0.31% - 0.56%)	(0.09% - 0.30%)	(0.01% - 0.06%)	(0.00% - 0.00%)	(0.00% - 0.00%)
	8.55%	8.23%	8.35%	6.81%	1.84%	1.69%	1.76%	1.18%	0.05%	0.05%	0.10%	0.03%
Philippines	(8.20% - 8.93%)	(7.85% - 8.60%)	(8.00% - 8.72%)	(6.44% - 7.18%)	(1.68% - 2.01%)	(1.53% - 1.85%)	(1.62% - 1.91%)	(1.04% - 1.33%)	(0.03% - 0.08%)	(0.03% - 0.07%)	(0.08% - 0.13%)	(0.02% - 0.05%)
	9.03%	8.30%	7.93%	6.24%	2.35%	2.02%	1.89%	1.20%	0.11%	0.08%	0.13%	0.03%
Mountain Province	(8.36% - 9.76%)	(7.68% - 8.95%)	(7.36% - 8.51%)	(5.72% - 6.78%)	(2.02% - 2.73%)	(1.74% - 2.32%)	(1.65% - 2.16%)	(0.99% - 1.42%)	(0.04% - 0.22%)	(0.03% - 0.14%)	(0.07% - 0.21%)	(0.00% - 0.07%)
	8.80%	7.63%	7.56%	6.04%	2.23%	1.72%	1.72%	1.12%	0.08%	0.04%	0.10%	0.02%
Ifugao	(8.15% - 9.47%)	(7.03% - 8.24%)	(6.98% - 8.10%)	(5.59% - 6.58%)	(1.92% - 2.58%)	(1.47% - 2.00%)	(1.45% - 1.97%)	(0.93% - 1.34%)	(0.02% - 0.18%)	(0.01% - 0.09%)	(0.05% - 0.16%)	(0.00% - 0.05%)
	7.00%	6.46%	6.60%	5.53%	1.43%	1.22%	1.29%	0.90%	0.00%	0.00%	0.04%	0.01%
Benguet	(6.42% - 7.58%)	(5.92% - 6.99%)	(6.08% - 7.15%)	(5.08% - 6.04%)	(1.19% - 1.70%)	(1.00% - 1.44%)	(1.09% - 1.53%)	(0.72% - 1.11%)	(0.00% - 0.02%)	(0.00% - 0.02%)	(0.01% - 0.08%)	(0.00% - 0.03%)

Table S8b. Estimated overall, severe, and extreme **wasting** prevalence (%) in children under 5 years, both sexes, in 1990, 2000, 2010, and 2020.

Location	Total				Severe				Extreme			
	1990	2000	2010	2020	1990	2000	2010	2020	1990	2000	2010	2020
Abra	7.07%	6.92%	6.85%	5.64%	1.47%	1.41%	1.40%	0.94%	0.00%	0.01%	0.05%	0.01%
	(6.50% - 7.66%)	(6.39% - 7.51%)	(6.31% - 7.42%)	(5.19% - 6.16%)	(1.24% - 1.73%)	(1.18% - 1.66%)	(1.18% - 1.64%)	(0.77% - 1.15%)	(0.00% - 0.03%)	(0.00% - 0.04%)	(0.02% - 0.09%)	(0.00% - 0.03%)
Apayao	10.07%	9.34%	8.44%	6.56%	2.93%	2.56%	2.18%	1.38%	0.30%	0.22%	0.21%	0.06%
	(9.32% - 10.88%)	(8.63% - 10.11%)	(7.82% - 9.07%)	(5.97% - 7.13%)	(2.53% - 3.38%)	(2.21% - 2.95%)	(1.89% - 2.49%)	(1.14% - 1.62%)	(0.18% - 0.48%)	(0.13% - 0.33%)	(0.14% - 0.30%)	(0.02% - 0.11%)
Kalinga	10.24%	8.92%	8.54%	6.97%	2.98%	2.34%	2.20%	1.52%	0.31%	0.15%	0.21%	0.07%
	(9.49% - 11.02%)	(8.27% - 9.58%)	(7.93% - 9.18%)	(6.38% - 7.57%)	(2.59% - 3.40%)	(2.03% - 2.67%)	(1.90% - 2.52%)	(1.26% - 1.79%)	(0.17% - 0.48%)	(0.08% - 0.24%)	(0.13% - 0.30%)	(0.03% - 0.13%)
La Union	7.60%	7.21%	7.52%	6.21%	1.43%	1.27%	1.39%	0.94%	0.00%	0.00%	0.04%	0.01%
	(6.99% - 8.27%)	(6.61% - 7.82%)	(6.99% - 8.11%)	(5.66% - 6.77%)	(1.17% - 1.71%)	(1.04% - 1.52%)	(1.19% - 1.66%)	(0.74% - 1.14%)	(0.00% - 0.02%)	(0.00% - 0.02%)	(0.01% - 0.08%)	(0.00% - 0.03%)
Ilocos Norte	5.84%	5.91%	6.25%	5.66%	0.77%	0.78%	0.89%	0.69%	0.00%	0.00%	0.00%	0.00%
	(5.32% - 6.40%)	(5.41% - 6.44%)	(5.75% - 6.76%)	(5.10% - 6.22%)	(0.58% - 0.98%)	(0.61% - 0.97%)	(0.72% - 1.08%)	(0.53% - 0.89%)	(0.00% - 0.00%)	(0.00% - 0.00%)	(0.00% - 0.01%)	(0.00% - 0.01%)
Ilocos Sur	7.50%	6.85%	7.33%	5.86%	1.38%	1.13%	1.31%	0.81%	0.00%	0.00%	0.03%	0.00%
	(6.92% - 8.15%)	(6.29% - 7.45%)	(6.78% - 7.91%)	(5.33% - 6.43%)	(1.14% - 1.66%)	(0.92% - 1.37%)	(1.10% - 1.54%)	(0.63% - 1.02%)	(0.00% - 0.02%)	(0.00% - 0.02%)	(0.00% - 0.06%)	(0.00% - 0.02%)
Pangasinan	8.47%	8.22%	8.16%	6.45%	1.79%	1.67%	1.67%	1.04%	0.01%	0.02%	0.07%	0.01%
	(7.82% - 9.21%)	(7.53% - 8.88%)	(7.59% - 8.82%)	(5.90% - 7.06%)	(1.50% - 2.12%)	(1.38% - 1.96%)	(1.42% - 1.94%)	(0.84% - 1.27%)	(0.00% - 0.05%)	(0.00% - 0.06%)	(0.03% - 0.12%)	(0.00% - 0.04%)
Nueva Vizcaya	7.80%	7.47%	7.67%	6.57%	1.31%	1.18%	1.25%	0.90%	0.00%	0.00%	0.02%	0.01%
	(7.15% - 8.51%)	(6.84% - 8.11%)	(7.06% - 8.29%)	(5.90% - 7.23%)	(1.06% - 1.62%)	(0.95% - 1.43%)	(1.02% - 1.49%)	(0.69% - 1.13%)	(0.00% - 0.02%)	(0.00% - 0.02%)	(0.00% - 0.06%)	(0.00% - 0.02%)
Cagayan	9.74%	8.92%	8.65%	6.95%	2.10%	1.74%	1.65%	1.06%	0.03%	0.02%	0.06%	0.01%
	(8.95% - 10.60%)	(8.22% - 9.68%)	(8.00% - 9.33%)	(6.34% - 7.59%)	(1.75% - 2.52%)	(1.44% - 2.06%)	(1.38% - 1.94%)	(0.84% - 1.30%)	(0.00% - 0.10%)	(0.00% - 0.07%)	(0.02% - 0.11%)	(0.00% - 0.04%)
Isabela	9.23%	8.53%	8.28%	6.74%	1.87%	1.56%	1.49%	0.97%	0.02%	0.02%	0.05%	0.01%
	(8.50% - 10.02%)	(7.85% - 9.32%)	(7.69% - 8.91%)	(6.13% - 7.36%)	(1.54% - 2.23%)	(1.29% - 1.89%)	(1.26% - 1.74%)	(0.76% - 1.20%)	(0.00% - 0.06%)	(0.00% - 0.05%)	(0.01% - 0.09%)	(0.00% - 0.03%)
Quirino	9.99%	8.79%	8.33%	6.51%	2.16%	1.68%	1.53%	0.92%	0.03%	0.02%	0.05%	0.00%
	(9.27% - 10.82%)	(8.11% - 9.55%)	(7.71% - 9.04%)	(5.89% - 7.09%)	(1.84% - 2.54%)	(1.40% - 1.99%)	(1.28% - 1.81%)	(0.72% - 1.13%)	(0.00% - 0.08%)	(0.00% - 0.05%)	(0.01% - 0.10%)	(0.00% - 0.02%)
Batanes	8.11%	7.47%	6.99%	6.13%	1.44%	1.20%	1.06%	0.79%	0.01%	0.01%	0.03%	0.01%
	(7.43% - 8.81%)	(6.83% - 8.16%)	(6.42% - 7.58%)	(5.49% - 6.75%)	(1.16% - 1.74%)	(0.95% - 1.48%)	(0.86% - 1.28%)	(0.60% - 0.99%)	(0.00% - 0.05%)	(0.00% - 0.04%)	(0.01% - 0.07%)	(0.00% - 0.04%)
Bataan	7.26%	6.81%	7.09%	5.97%	1.32%	1.15%	1.26%	0.88%	0.00%	0.00%	0.03%	0.00%
	(6.72% - 7.88%)	(6.29% - 7.37%)	(6.57% - 7.63%)	(5.44% - 6.51%)	(1.10% - 1.57%)	(0.94% - 1.37%)	(1.06% - 1.48%)	(0.69% - 1.08%)	(0.00% - 0.01%)	(0.00% - 0.01%)	(0.00% - 0.06%)	(0.00% - 0.02%)
Zambales	7.27%	7.31%	7.92%	6.35%	1.36%	1.36%	1.61%	1.04%	0.00%	0.01%	0.07%	0.01%
	(6.67% - 7.88%)	(6.73% - 7.88%)	(7.31% - 8.55%)	(5.82% - 6.92%)	(1.11% - 1.63%)	(1.14% - 1.60%)	(1.37% - 1.88%)	(0.84% - 1.26%)	(0.00% - 0.01%)	(0.00% - 0.03%)	(0.03% - 0.12%)	(0.00% - 0.04%)
Tarlac	6.80%	6.35%	6.53%	5.57%	1.12%	0.96%	1.04%	0.72%	0.00%	0.00%	0.01%	0.00%
	(6.25% - 7.35%)	(5.80% - 6.91%)	(6.02% - 7.09%)	(5.08% - 6.08%)	(0.91% - 1.34%)	(0.77% - 1.18%)	(0.85% - 1.24%)	(0.56% - 0.91%)	(0.00% - 0.00%)	(0.00% - 0.00%)	(0.00% - 0.03%)	(0.00% - 0.01%)
Pampanga	6.21%	5.87%	5.95%	5.45%	0.90%	0.78%	0.82%	0.66%	0.00%	0.00%	0.00%	0.00%
	(5.70% - 6.78%)	(5.40% - 6.33%)	(5.48% - 6.43%)	(4.95% - 5.99%)	(0.72% - 1.12%)	(0.62% - 0.95%)	(0.66% - 1.00%)	(0.50% - 0.85%)	(0.00% - 0.00%)	(0.00% - 0.00%)	(0.00% - 0.02%)	(0.00% - 0.01%)
Bulacan	6.81%	6.29%	6.76%	5.45%	1.14%	0.96%	1.13%	0.70%	0.00%	0.00%	0.02%	0.00%
	(6.28% - 7.39%)	(5.80% - 6.84%)	(6.23% - 7.33%)	(4.95% - 5.91%)	(0.93% - 1.38%)	(0.78% - 1.16%)	(0.93% - 1.35%)	(0.54% - 0.86%)	(0.00% - 0.00%)	(0.00% - 0.00%)	(0.00% - 0.04%)	(0.00% - 0.01%)
Nueva Ecija	7.03%	6.94%	7.17%	5.53%	1.23%	1.19%	1.29%	0.74%	0.00%	0.00%	0.03%	0.00%
	(6.49% - 7.65%)	(6.40% - 7.51%)	(6.62% - 7.73%)	(5.06% - 6.01%)	(1.02% - 1.50%)	(0.98% - 1.42%)	(1.07% - 1.50%)	(0.58% - 0.91%)	(0.00% - 0.00%)	(0.00% - 0.02%)	(0.00% - 0.06%)	(0.00% - 0.01%)
Aurora	9.67%	9.43%	9.37%	7.33%	2.44%	2.32%	2.32%	1.47%	0.11%	0.12%	0.22%	0.06%
	(8.92% - 10.44%)	(8.75% - 10.13%)	(8.70% - 10.07%)	(6.68% - 7.96%)	(2.08% - 2.84%)	(2.00% - 2.67%)	(1.99% - 2.65%)	(1.21% - 1.74%)	(0.04% - 0.22%)	(0.06% - 0.21%)	(0.14% - 0.31%)	(0.02% - 0.11%)
Rizal	6.50%	6.36%	6.80%	5.60%	1.13%	1.07%	1.23%	0.82%	0.00%	0.00%	0.02%	0.00%
	(5.99% - 7.10%)	(5.89% - 6.93%)	(6.28% - 7.36%)	(5.12% - 6.12%)	(0.93% - 1.39%)	(0.89% - 1.30%)	(1.02% - 1.45%)	(0.65% - 1.01%)	(0.00% - 0.00%)	(0.00% - 0.01%)	(0.00% - 0.05%)	(0.00% - 0.02%)
Cavite	6.04%	6.26%	6.64%	5.30%	0.96%	1.02%	1.17%	0.71%	0.00%	0.00%	0.02%	0.00%
	(5.52% - 6.59%)	(5.73% - 6.83%)	(6.14% - 7.16%)	(4.80% - 5.77%)	(0.77% - 1.19%)	(0.82% - 1.26%)	(0.97% - 1.37%)	(0.55% - 0.89%)	(0.00% - 0.00%)	(0.00% - 0.01%)	(0.00% - 0.05%)	(0.00% - 0.01%)
Laguna	6.41%	6.58%	6.81%	5.41%	1.10%	1.14%	1.23%	0.75%	0.00%	0.00%	0.02%	0.00%
	(5.94% - 6.94%)	(6.09% - 7.09%)	(6.31% - 7.35%)	(4.98% - 5.90%)	(0.90% - 1.32%)	(0.94% - 1.35%)	(1.03% - 1.45%)	(0.59% - 0.94%)	(0.00% - 0.00%)	(0.00% - 0.01%)	(0.00% - 0.06%)	(0.00% - 0.01%)
Batangas	6.79%	6.78%	6.40%	5.24%	1.21%	1.19%	1.07%	0.68%	0.00%	0.00%	0.01%	0.00%
	(6.28% - 7.39%)	(6.24% - 7.34%)	(5.92% - 6.91%)	(4.78% - 5.67%)	(1.01% - 1.46%)	(0.98% - 1.43%)	(0.89% - 1.28%)	(0.53% - 0.84%)	(0.00% - 0.00%)	(0.00% - 0.02%)	(0.00% - 0.04%)	(0.00% - 0.01%)

Table S8b. Estimated overall, severe, and extreme **wasting** prevalence (%) in children under 5 years, both sexes, in 1990, 2000, 2010, and 2020.												
**Location**	**Total**				**Severe**				**Extreme**			
	**1990**	**2000**	**2010**	**2020**	**1990**	**2000**	**2010**	**2020**	**1990**	**2000**	**2010**	**2020**
Quezon	8.81% (8.08% - 9.54%)	8.54% (7.86% - 9.31%)	8.80% (8.15% - 9.51%)	6.99% (6.40% - 7.63%)	2.14% (1.78% - 2.51%)	2.01% (1.70% - 2.36%)	2.12% (1.82% - 2.46%)	1.38% (1.13% - 1.65%)	0.05% (0.00% - 0.15%)	0.06% (0.02% - 0.11%)	0.15% (0.09% - 0.22%)	0.04% (0.01% - 0.08%)
Occidental Mindoro	10.42% (9.66% - 11.22%)	10.06% (9.28% - 10.88%)	10.66% (9.92% - 11.43%)	8.90% (8.16% - 9.66%)	2.37% (2.02% - 2.76%)	2.19% (1.84% - 2.57%)	2.45% (2.11% - 2.80%)	1.73% (1.42% - 2.05%)	0.05% (0.00% - 0.15%)	0.06% (0.01% - 0.12%)	0.18% (0.11% - 0.27%)	0.06% (0.02% - 0.12%)
Oriental Mindoro	10.17% (9.41% - 11.01%)	9.55% (8.79% - 10.31%)	9.44% (8.71% - 10.13%)	7.33% (6.71% - 8.00%)	2.19% (1.83% - 2.57%)	1.91% (1.58% - 2.26%)	1.88% (1.60% - 2.18%)	1.13% (0.91% - 1.38%)	0.03% (0.00% - 0.10%)	0.03% (0.00% - 0.07%)	0.08% (0.03% - 0.14%)	0.01% (0.00% - 0.04%)
Romblon	10.94% (10.13% - 11.83%)	10.33% (9.60% - 11.16%)	9.94% (9.24% - 10.76%)	7.70% (7.08% - 8.42%)	2.52% (2.12% - 2.97%)	2.24% (1.90% - 2.62%)	2.10% (1.81% - 2.44%)	1.27% (1.03% - 1.55%)	0.07% (0.01% - 0.18%)	0.06% (0.02% - 0.13%)	0.12% (0.06% - 0.19%)	0.02% (0.00% - 0.05%)
Palawan	11.50% (10.64% - 12.39%)	10.86% (10.04% - 11.69%)	11.23% (10.44% - 12.08%)	8.22% (7.50% - 8.92%)	2.87% (2.45% - 3.34%)	2.54% (2.16% - 2.94%)	2.71% (2.36% - 3.11%)	1.51% (1.23% - 1.81%)	0.15% (0.05% - 0.30%)	0.11% (0.04% - 0.19%)	0.24% (0.15% - 0.34%)	0.04% (0.01% - 0.09%)
Marinduque	10.73% (9.92% - 11.60%)	9.88% (9.15% - 10.69%)	9.92% (9.21% - 10.69%)	8.03% (7.37% - 8.76%)	2.45% (2.08% - 2.91%)	2.07% (1.75% - 2.44%)	2.09% (1.79% - 2.41%)	1.39% (1.14% - 1.69%)	0.06% (0.01% - 0.17%)	0.04% (0.01% - 0.10%)	0.11% (0.06% - 0.18%)	0.03% (0.00% - 0.07%)
Catanduanes	8.49% (7.80% - 9.24%)	8.01% (7.35% - 8.67%)	8.18% (7.59% - 8.80%)	6.85% (6.28% - 7.47%)	1.57% (1.29% - 1.89%)	1.39% (1.15% - 1.67%)	1.47% (1.24% - 1.73%)	1.02% (0.81% - 1.25%)	0.00% (0.00% - 0.02%)	0.00% (0.00% - 0.02%)	0.04% (0.01% - 0.08%)	0.01% (0.00% - 0.03%)
Camarines Norte	9.04% (8.37% - 9.75%)	8.59% (7.93% - 9.34%)	9.39% (8.68% - 10.12%)	7.26% (6.67% - 7.94%)	1.85% (1.57% - 2.17%)	1.66% (1.39% - 1.98%)	2.00% (1.72% - 2.32%)	1.22% (0.99% - 1.48%)	0.01% (0.00% - 0.05%)	0.02% (0.00% - 0.05%)	0.12% (0.06% - 0.19%)	0.02% (0.00% - 0.05%)
Sorsogon	8.95% (8.21% - 9.69%)	8.29% (7.66% - 9.03%)	8.17% (7.58% - 8.80%)	6.60% (6.02% - 7.24%)	1.75% (1.43% - 2.08%)	1.49% (1.24% - 1.80%)	1.47% (1.23% - 1.72%)	0.94% (0.73% - 1.17%)	0.01% (0.00% - 0.04%)	0.01% (0.00% - 0.03%)	0.04% (0.01% - 0.08%)	0.01% (0.00% - 0.02%)
Albay	8.61% (8.02% - 9.31%)	8.35% (7.67% - 9.08%)	8.18% (7.55% - 8.80%)	6.32% (5.77% - 6.90%)	1.62% (1.37% - 1.92%)	1.50% (1.24% - 1.79%)	1.47% (1.22% - 1.73%)	0.86% (0.67% - 1.07%)	0.00% (0.00% - 0.02%)	0.01% (0.00% - 0.03%)	0.04% (0.01% - 0.09%)	0.00% (0.00% - 0.02%)
Masbate	10.95% (10.17% - 11.83%)	11.18% (10.35% - 12.06%)	11.29% (10.49% - 12.07%)	8.89% (8.18% - 9.64%)	2.74% (2.36% - 3.19%)	2.83% (2.43% - 3.27%)	2.92% (2.56% - 3.32%)	1.92% (1.61% - 2.25%)	0.15% (0.05% - 0.28%)	0.20% (0.11% - 0.32%)	0.33% (0.23% - 0.44%)	0.11% (0.05% - 0.18%)
Camarines Sur	8.80% (8.07% - 9.52%)	8.49% (7.79% - 9.19%)	8.71% (8.09% - 9.39%)	6.89% (6.32% - 7.56%)	1.72% (1.42% - 2.03%)	1.59% (1.31% - 1.87%)	1.70% (1.45% - 1.99%)	1.07% (0.86% - 1.31%)	0.01% (0.00% - 0.03%)	0.01% (0.00% - 0.04%)	0.07% (0.03% - 0.12%)	0.01% (0.00% - 0.04%)
Capiz	9.14% (8.40% - 9.84%)	8.80% (8.11% - 9.54%)	8.75% (8.06% - 9.42%)	6.69% (6.14% - 7.30%)	2.08% (1.74% - 2.40%)	1.93% (1.63% - 2.27%)	1.94% (1.66% - 2.22%)	1.16% (0.95% - 1.40%)	0.04% (0.00% - 0.11%)	0.05% (0.01% - 0.10%)	0.12% (0.06% - 0.19%)	0.02% (0.00% - 0.05%)
Aklan	7.57% (7.00% - 8.21%)	7.37% (6.79% - 8.02%)	7.16% (6.61% - 7.74%)	6.08% (5.57% - 6.63%)	1.39% (1.16% - 1.66%)	1.31% (1.08% - 1.56%)	1.25% (1.04% - 1.48%)	0.89% (0.70% - 1.10%)	0.00% (0.00% - 0.01%)	0.00% (0.00% - 0.02%)	0.02% (0.00% - 0.06%)	0.00% (0.00% - 0.02%)
Antique	8.88% (8.21% - 9.60%)	8.41% (7.77% - 9.11%)	8.68% (8.07% - 9.35%)	6.63% (6.05% - 7.22%)	1.97% (1.67% - 2.31%)	1.77% (1.50% - 2.07%)	1.91% (1.64% - 2.21%)	1.13% (0.92% - 1.37%)	0.03% (0.00% - 0.08%)	0.03% (0.00% - 0.08%)	0.11% (0.06% - 0.18%)	0.02% (0.00% - 0.05%)
Negros Occidental	8.74% (8.07% - 9.44%)	8.56% (7.89% - 9.21%)	8.88% (8.23% - 9.51%)	7.74% (7.14% - 8.37%)	1.94% (1.65% - 2.26%)	1.85% (1.57% - 2.14%)	2.02% (1.73% - 2.31%)	1.56% (1.31% - 1.84%)	0.02% (0.00% - 0.07%)	0.04% (0.01% - 0.09%)	0.14% (0.07% - 0.21%)	0.06% (0.02% - 0.11%)
Iloilo	7.05% (6.49% - 7.63%)	7.18% (6.61% - 7.78%)	7.45% (6.87% - 8.03%)	6.15% (5.63% - 6.69%)	1.21% (0.99% - 1.45%)	1.25% (1.02% - 1.49%)	1.37% (1.16% - 1.61%)	0.92% (0.74% - 1.13%)	0.00% (0.00% - 0.00%)	0.00% (0.00% - 0.02%)	0.04% (0.01% - 0.07%)	0.01% (0.00% - 0.03%)
Guimaras	8.57% (7.89% - 9.27%)	7.96% (7.32% - 8.65%)	6.99% (6.44% - 7.55%)	5.67% (5.17% - 6.23%)	1.81% (1.52% - 2.13%)	1.56% (1.30% - 1.85%)	1.23% (1.02% - 1.46%)	0.78% (0.62% - 0.98%)	0.02% (0.00% - 0.05%)	0.02% (0.00% - 0.05%)	0.03% (0.00% - 0.07%)	0.00% (0.00% - 0.02%)
Negros Oriental	9.50% (8.74% - 10.29%)	9.11% (8.39% - 9.84%)	9.44% (8.79% - 10.13%)	7.57% (6.94% - 8.21%)	2.22% (1.87% - 2.60%)	2.04% (1.73% - 2.39%)	2.21% (1.93% - 2.53%)	1.47% (1.22% - 1.74%)	0.05% (0.01% - 0.13%)	0.06% (0.02% - 0.12%)	0.17% (0.10% - 0.25%)	0.05% (0.01% - 0.10%)
Cebu	8.57% (7.94% - 9.28%)	8.52% (7.84% - 9.19%)	8.17% (7.59% - 8.78%)	6.69% (6.14% - 7.27%)	1.76% (1.49% - 2.09%)	1.73% (1.45% - 2.02%)	1.63% (1.39% - 1.88%)	1.10% (0.89% - 1.32%)	0.01% (0.00% - 0.03%)	0.02% (0.00% - 0.06%)	0.06% (0.03% - 0.11%)	0.01% (0.00% - 0.04%)
Bohol	9.20% (8.51% - 9.96%)	8.54% (7.87% - 9.22%)	8.17% (7.57% - 8.86%)	6.75% (6.18% - 7.37%)	2.01% (1.70% - 2.36%)	1.73% (1.46% - 2.03%)	1.62% (1.38% - 1.89%)	1.11% (0.91% - 1.35%)	0.02% (0.00% - 0.07%)	0.02% (0.00% - 0.07%)	0.06% (0.02% - 0.11%)	0.01% (0.00% - 0.04%)
Siquijor	9.47% (8.75% - 10.25%)	8.41% (7.73% - 9.14%)	7.99% (7.40% - 8.60%)	6.65% (6.10% - 7.23%)	2.19% (1.87% - 2.57%)	1.75% (1.47% - 2.05%)	1.61% (1.37% - 1.86%)	1.11% (0.89% - 1.33%)	0.06% (0.01% - 0.12%)	0.04% (0.01% - 0.09%)	0.08% (0.03% - 0.13%)	0.02% (0.00% - 0.06%)
Southern Leyte	8.33% (7.65% - 9.03%)	7.74% (7.11% - 8.38%)	7.73% (7.15% - 8.36%)	6.49% (5.88% - 7.13%)	1.50% (1.21% - 1.81%)	1.27% (1.03% - 1.53%)	1.28% (1.07% - 1.53%)	0.87% (0.67% - 1.09%)	0.00% (0.00% - 0.01%)	0.00% (0.00% - 0.01%)	0.02% (0.00% - 0.05%)	0.00% (0.00% - 0.02%)

Table S8b. Estimated overall, severe, and extreme **wasting** prevalence (%) in children under 5 years, both sexes, in 1990, 2000, 2010, and 2020.

Location	Total				Severe				Extreme			
	1990	2000	2010	2020	1990	2000	2010	2020	1990	2000	2010	2020
Eastern Samar	11.07%	10.40%	9.85%	7.72%	2.72%	2.40%	2.18%	1.39%	0.12%	0.08%	0.13%	0.02%
	(10.22% - 11.96%)	(9.61% - 11.33%)	(9.13% - 10.61%)	(7.07% - 8.36%)	(2.29% - 3.18%)	(2.02% - 2.82%)	(1.88% - 2.52%)	(1.14% - 1.66%)	(0.03% - 0.26%)	(0.02% - 0.17%)	(0.07% - 0.20%)	(0.00% - 0.06%)
Northern Samar	11.49%	11.33%	11.34%	8.49%	3.00%	2.89%	2.90%	1.72%	0.21%	0.19%	0.29%	0.06%
	(10.62% - 12.36%)	(10.45% - 12.21%)	(10.61% - 12.18%)	(7.82% - 9.25%)	(2.57% - 3.47%)	(2.47% - 3.33%)	(2.55% - 3.30%)	(1.45% - 2.04%)	(0.08% - 0.38%)	(0.10% - 0.33%)	(0.20% - 0.39%)	(0.02% - 0.12%)
Samar (Western Samar)	11.22%	10.83%	10.69%	8.37%	2.85%	2.63%	2.59%	1.66%	0.16%	0.13%	0.21%	0.05%
	(10.39% - 12.10%)	(10.03% - 11.75%)	(9.98% - 11.43%)	(7.68% - 9.13%)	(2.45% - 3.31%)	(2.25% - 3.09%)	(2.25% - 2.94%)	(1.36% - 1.99%)	(0.05% - 0.32%)	(0.06% - 0.24%)	(0.14% - 0.31%)	(0.01% - 0.11%)
Leyte	10.69%	10.04%	9.41%	7.18%	2.52%	2.20%	1.98%	1.19%	0.07%	0.05%	0.09%	0.01%
	(9.88% - 11.51%)	(9.22% - 10.89%)	(8.70% - 10.15%)	(6.55% - 7.82%)	(2.12% - 2.91%)	(1.84% - 2.62%)	(1.68% - 2.30%)	(0.95% - 1.44%)	(0.01% - 0.18%)	(0.01% - 0.11%)	(0.04% - 0.16%)	(0.00% - 0.04%)
Biliran	8.50%	7.69%	7.30%	6.34%	1.61%	1.30%	1.18%	0.86%	0.00%	0.00%	0.02%	0.01%
	(7.84% - 9.20%)	(7.03% - 8.37%)	(6.77% - 7.89%)	(5.75% - 6.94%)	(1.32% - 1.93%)	(1.04% - 1.57%)	(0.98% - 1.42%)	(0.66% - 1.08%)	(0.00% - 0.02%)	(0.00% - 0.02%)	(0.00% - 0.05%)	(0.00% - 0.02%)
Zamboanga Sibugay	10.01%	10.29%	9.93%	7.50%	2.55%	2.64%	2.49%	1.51%	0.12%	0.16%	0.21%	0.04%
	(9.27% - 10.85%)	(9.47% - 11.15%)	(9.17% - 10.72%)	(6.88% - 8.17%)	(2.19% - 2.98%)	(2.26% - 3.06%)	(2.14% - 2.86%)	(1.24% - 1.80%)	(0.03% - 0.26%)	(0.08% - 0.29%)	(0.14% - 0.30%)	(0.01% - 0.10%)
Zamboanga Del Norte	10.17%	9.90%	9.84%	8.00%	2.63%	2.47%	2.45%	1.69%	0.14%	0.12%	0.20%	0.06%
	(9.39% - 10.97%)	(9.18% - 10.73%)	(9.15% - 10.62%)	(7.31% - 8.70%)	(2.24% - 3.05%)	(2.15% - 2.88%)	(2.14% - 2.80%)	(1.40% - 2.00%)	(0.04% - 0.28%)	(0.05% - 0.22%)	(0.12% - 0.29%)	(0.02% - 0.12%)
Zamboanga Del Sur	10.10%	9.67%	9.75%	7.81%	2.59%	2.36%	2.41%	1.62%	0.13%	0.10%	0.19%	0.05%
	(9.35% - 10.88%)	(8.96% - 10.39%)	(9.00% - 10.48%)	(7.17% - 8.48%)	(2.21% - 2.99%)	(2.04% - 2.72%)	(2.06% - 2.76%)	(1.35% - 1.92%)	(0.03% - 0.26%)	(0.04% - 0.19%)	(0.12% - 0.28%)	(0.02% - 0.11%)
Misamis Occidental	8.67%	8.37%	8.93%	7.18%	1.78%	1.65%	1.86%	1.23%	0.01%	0.01%	0.08%	0.01%
	(7.98% - 9.37%)	(7.72% - 9.08%)	(8.29% - 9.65%)	(6.60% - 7.79%)	(1.50% - 2.12%)	(1.37% - 1.96%)	(1.61% - 2.16%)	(1.01% - 1.48%)	(0.00% - 0.04%)	(0.00% - 0.04%)	(0.03% - 0.14%)	(0.00% - 0.04%)
Bukidnon	10.78%	10.30%	10.17%	7.46%	2.73%	2.48%	2.43%	1.39%	0.14%	0.11%	0.18%	0.03%
	(9.96% - 11.63%)	(9.59% - 11.10%)	(9.41% - 10.92%)	(6.81% - 8.11%)	(2.32% - 3.18%)	(2.15% - 2.88%)	(2.09% - 2.76%)	(1.12% - 1.66%)	(0.04% - 0.29%)	(0.04% - 0.20%)	(0.11% - 0.27%)	(0.00% - 0.06%)
Lanao Del Norte	9.24%	9.17%	9.56%	7.77%	2.05%	1.99%	2.16%	1.45%	0.02%	0.03%	0.13%	0.03%
	(8.51% - 9.97%)	(8.47% - 9.92%)	(8.85% - 10.31%)	(7.10% - 8.45%)	(1.71% - 2.40%)	(1.70% - 2.33%)	(1.85% - 2.48%)	(1.18% - 1.74%)	(0.00% - 0.09%)	(0.00% - 0.08%)	(0.07% - 0.20%)	(0.00% - 0.07%)
Misamis Oriental	8.57%	8.38%	8.11%	7.02%	1.70%	1.61%	1.52%	1.15%	0.00%	0.01%	0.04%	0.01%
	(7.85% - 9.31%)	(7.69% - 9.13%)	(7.49% - 8.72%)	(6.41% - 7.66%)	(1.40% - 2.04%)	(1.31% - 1.91%)	(1.28% - 1.77%)	(0.92% - 1.40%)	(0.00% - 0.03%)	(0.00% - 0.04%)	(0.01% - 0.07%)	(0.00% - 0.04%)
Camiguin	8.95%	8.25%	7.76%	6.26%	1.89%	1.60%	1.43%	0.90%	0.01%	0.01%	0.04%	0.01%
	(8.28% - 9.69%)	(7.64% - 8.96%)	(7.13% - 8.41%)	(5.67% - 6.82%)	(1.58% - 2.24%)	(1.36% - 1.89%)	(1.19% - 1.69%)	(0.71% - 1.11%)	(0.00% - 0.05%)	(0.00% - 0.04%)	(0.01% - 0.07%)	(0.00% - 0.02%)
Davao Oriental	10.75%	10.04%	10.11%	8.11%	2.77%	2.43%	2.50%	1.69%	0.17%	0.13%	0.23%	0.08%
	(9.98% - 11.69%)	(9.31% - 10.84%)	(9.36% - 10.89%)	(7.43% - 8.86%)	(2.39% - 3.26%)	(2.09% - 2.80%)	(2.17% - 2.87%)	(1.41% - 2.01%)	(0.07% - 0.32%)	(0.05% - 0.21%)	(0.15% - 0.33%)	(0.03% - 0.14%)
Davao de Oro	9.88%	9.45%	9.04%	7.10%	2.32%	2.12%	1.99%	1.25%	0.06%	0.07%	0.12%	0.03%
	(9.14% - 10.77%)	(8.66% - 10.26%)	(8.37% - 9.73%)	(6.52% - 7.76%)	(1.98% - 2.74%)	(1.79% - 2.49%)	(1.70% - 2.28%)	(1.03% - 1.50%)	(0.01% - 0.15%)	(0.02% - 0.14%)	(0.07% - 0.19%)	(0.00% - 0.06%)
Davao Del Sur	10.08%	9.45%	9.08%	7.25%	2.42%	2.13%	2.01%	1.32%	0.08%	0.07%	0.13%	0.03%
	(9.33% - 10.82%)	(8.69% - 10.24%)	(8.41% - 9.80%)	(6.64% - 7.89%)	(2.06% - 2.79%)	(1.80% - 2.51%)	(1.73% - 2.32%)	(1.08% - 1.58%)	(0.02% - 0.18%)	(0.02% - 0.14%)	(0.07% - 0.20%)	(0.00% - 0.07%)
Davao Occidental	9.79%	9.86%	9.94%	7.75%	2.33%	2.34%	2.41%	1.52%	0.07%	0.11%	0.21%	0.05%
	(9.03% - 10.59%)	(9.13% - 10.67%)	(9.25% - 10.73%)	(7.14% - 8.47%)	(1.98% - 2.73%)	(2.02% - 2.74%)	(2.09% - 2.76%)	(1.28% - 1.81%)	(0.01% - 0.16%)	(0.05% - 0.19%)	(0.13% - 0.31%)	(0.01% - 0.11%)
Davao Del Norte	9.64%	8.93%	8.48%	7.17%	2.16%	1.86%	1.73%	1.26%	0.04%	0.04%	0.08%	0.03%
	(8.92% - 10.37%)	(8.20% - 9.65%)	(7.83% - 9.14%)	(6.56% - 7.87%)	(1.84% - 2.51%)	(1.55% - 2.16%)	(1.45% - 2.01%)	(1.03% - 1.54%)	(0.00% - 0.10%)	(0.01% - 0.08%)	(0.04% - 0.14%)	(0.00% - 0.06%)
South Cotabato	9.45%	9.03%	9.45%	7.55%	2.09%	1.90%	2.08%	1.36%	0.02%	0.03%	0.12%	0.03%
	(8.74% - 10.20%)	(8.29% - 9.76%)	(8.80% - 10.16%)	(6.90% - 8.26%)	(1.75% - 2.47%)	(1.58% - 2.24%)	(1.79% - 2.39%)	(1.11% - 1.66%)	(0.00% - 0.09%)	(0.00% - 0.07%)	(0.06% - 0.18%)	(0.00% - 0.06%)
Sultan Kudarat	10.02%	9.82%	9.88%	8.01%	2.36%	2.24%	2.28%	1.53%	0.06%	0.06%	0.15%	0.04%
	(9.26% - 10.84%)	(9.11% - 10.60%)	(9.15% - 10.62%)	(7.35% - 8.73%)	(2.01% - 2.76%)	(1.91% - 2.63%)	(1.96% - 2.61%)	(1.26% - 1.84%)	(0.00% - 0.16%)	(0.02% - 0.13%)	(0.08% - 0.23%)	(0.01% - 0.09%)
Cotabato (North Cotabato)	9.40%	9.44%	10.06%	8.12%	2.12%	2.11%	2.36%	1.58%	0.03%	0.05%	0.17%	0.04%
	(8.75% - 10.18%)	(8.70% - 10.20%)	(9.33% - 10.84%)	(7.46% - 8.80%)	(1.82% - 2.50%)	(1.78% - 2.45%)	(2.05% - 2.72%)	(1.32% - 1.87%)	(0.00% - 0.10%)	(0.01% - 0.11%)	(0.10% - 0.25%)	(0.01% - 0.09%)
Sarangani	9.61%	9.28%	9.43%	7.81%	2.19%	2.02%	2.07%	1.45%	0.03%	0.04%	0.12%	0.03%
	(8.82% - 10.40%)	(8.53% - 10.08%)	(8.66% - 10.28%)	(7.09% - 8.57%)	(1.84% - 2.58%)	(1.69% - 2.40%)	(1.74% - 2.44%)	(1.17% - 1.76%)	(0.00% - 0.12%)	(0.01% - 0.09%)	(0.06% - 0.18%)	(0.01% - 0.08%)
Agusan Del Norte	9.92%	9.65%	10.23%	7.96%	2.23%	2.09%	2.34%	1.49%	0.03%	0.04%	0.16%	0.03%
	(9.18% - 10.69%)	(8.88% - 10.46%)	(9.49% - 11.01%)	(7.35% - 8.68%)	(1.89% - 2.60%)	(1.75% - 2.45%)	(2.02% - 2.69%)	(1.25% - 1.79%)	(0.00% - 0.11%)	(0.01% - 0.10%)	(0.10% - 0.24%)	(0.00% - 0.08%)

Table S8b. Estimated overall, severe, and extreme **wasting** prevalence (%) in children under 5 years, both sexes, in 1990, 2000, 2010, and 2020.												
**Location**	**Total**				**Severe**				**Extreme**			
	**1990**	**2000**	**2010**	**2020**	**1990**	**2000**	**2010**	**2020**	**1990**	**2000**	**2010**	**2020**
Agusan Del Sur	10.45% (9.70% - 11.31%)	10.21% (9.42% - 10.99%)	10.58% (9.81% - 11.31%)	8.69% (7.97% - 9.44%)	2.49% (2.12% - 2.94%)	2.35% (2.00% - 2.73%)	2.53% (2.18% - 2.88%)	1.75% (1.46% - 2.07%)	0.08% (0.01% - 0.20%)	0.08% (0.02% - 0.15%)	0.20% (0.12% - 0.29%)	0.06% (0.02% - 0.12%)
Surigao Del Sur	10.22% (9.45% - 11.09%)	9.67% (8.89% - 10.47%)	9.21% (8.49% - 9.98%)	7.10% (6.49% - 7.76%)	2.30% (1.92% - 2.74%)	2.05% (1.70% - 2.41%)	1.88% (1.59% - 2.22%)	1.14% (0.91% - 1.40%)	0.04% (0.00% - 0.13%)	0.04% (0.00% - 0.09%)	0.08% (0.04% - 0.14%)	0.01% (0.00% - 0.04%)
Surigao Del Norte	10.89% (10.08% - 11.76%)	10.27% (9.46% - 11.11%)	9.79% (9.09% - 10.56%)	7.86% (7.23% - 8.62%)	2.65% (2.25% - 3.07%)	2.34% (1.96% - 2.73%)	2.15% (1.85% - 2.51%)	1.43% (1.18% - 1.72%)	0.10% (0.02% - 0.22%)	0.08% (0.02% - 0.15%)	0.12% (0.06% - 0.20%)	0.03% (0.00% - 0.07%)
Dinagat Islands	9.34% (8.59% - 10.16%)	8.57% (7.90% - 9.29%)	7.83% (7.25% - 8.48%)	6.00% (5.42% - 6.53%)	1.94% (1.62% - 2.32%)	1.62% (1.35% - 1.93%)	1.36% (1.14% - 1.62%)	0.78% (0.60% - 0.97%)	0.02% (0.00% - 0.06%)	0.02% (0.00% - 0.05%)	0.03% (0.01% - 0.07%)	0.00% (0.00% - 0.02%)
Tawi-Tawi	10.65% (9.85% - 11.55%)	11.21% (10.35% - 12.13%)	11.76% (10.95% - 12.65%)	9.80% (8.99% - 10.62%)	2.72% (2.31% - 3.19%)	2.94% (2.54% - 3.41%)	3.21% (2.83% - 3.65%)	2.32% (1.95% - 2.70%)	0.14% (0.04% - 0.30%)	0.22% (0.12% - 0.36%)	0.38% (0.28% - 0.50%)	0.17% (0.09% - 0.26%)
Basilan	11.13% (10.32% - 12.02%)	10.41% (9.60% - 11.24%)	9.87% (9.17% - 10.62%)	8.39% (7.72% - 9.07%)	2.86% (2.44% - 3.36%)	2.50% (2.10% - 2.89%)	2.26% (1.95% - 2.62%)	1.67% (1.39% - 1.96%)	0.17% (0.06% - 0.34%)	0.12% (0.05% - 0.21%)	0.16% (0.10% - 0.24%)	0.06% (0.02% - 0.12%)
Sulu	12.33% (11.45% - 13.21%)	12.50% (11.57% - 13.45%)	13.26% (12.40% - 14.21%)	11.29% (10.42% - 12.21%)	3.59% (3.12% - 4.10%)	3.63% (3.13% - 4.18%)	4.04% (3.55% - 4.55%)	3.06% (2.63% - 3.53%)	0.44% (0.25% - 0.65%)	0.46% (0.29% - 0.67%)	0.65% (0.48% - 0.85%)	0.34% (0.23% - 0.47%)
Maguindanao	12.63% (11.71% - 13.58%)	12.35% (11.45% - 13.30%)	12.41% (11.56% - 13.37%)	10.50% (9.69% - 11.38%)	3.71% (3.20% - 4.25%)	3.52% (3.04% - 4.05%)	3.56% (3.14% - 4.04%)	2.66% (2.28% - 3.07%)	0.47% (0.27% - 0.69%)	0.41% (0.25% - 0.61%)	0.48% (0.35% - 0.63%)	0.24% (0.15% - 0.34%)
Lanao Del Sur	11.95% (11.12% - 12.82%)	11.70% (10.90% - 12.59%)	11.82% (11.02% - 12.66%)	10.69% (9.83% - 11.54%)	3.35% (2.93% - 3.84%)	3.19% (2.79% - 3.67%)	3.25% (2.86% - 3.68%)	2.72% (2.32% - 3.13%)	0.34% (0.19% - 0.53%)	0.30% (0.17% - 0.47%)	0.39% (0.29% - 0.52%)	0.25% (0.16% - 0.36%)
National Capital Region	5.46% (5.04% - 5.92%)	5.65% (5.20% - 6.09%)	6.24% (5.78% - 6.73%)	5.23% (4.79% - 5.65%)	0.81% (0.65% - 0.99%)	0.87% (0.70% - 1.04%)	1.10% (0.92% - 1.29%)	0.75% (0.59% - 0.91%)	0.00% (0.00% - 0.00%)	0.00% (0.00% - 0.00%)	0.02% (0.00% - 0.04%)	0.00% (0.00% - 0.01%)
Sri Lanka	17.31% (16.73% - 17.94%)	18.12% (17.43% - 18.80%)	15.18% (14.69% - 15.66%)	13.35% (12.72% - 13.98%)	4.75% (4.37% - 5.16%)	5.10% (4.64% - 5.58%)	3.71% (3.44% - 4.00%)	2.96% (2.65% - 3.30%)	0.58% (0.44% - 0.74%)	0.68% (0.50% - 0.88%)	0.26% (0.16% - 0.37%)	0.07% (0.01% - 0.17%)
Thailand	7.26% (6.80% - 7.74%)	6.24% (5.84% - 6.63%)	5.94% (5.57% - 6.32%)	5.34% (4.98% - 5.73%)	1.09% (0.92% - 1.27%)	0.88% (0.73% - 1.03%)	0.85% (0.72% - 0.99%)	0.64% (0.51% - 0.77%)	0.00% (0.00% - 0.00%)	0.00% (0.00% - 0.00%)	0.00% (0.00% - 0.01%)	0.00% (0.00% - 0.00%)
Mauritius	14.01% (13.53% - 14.53%)	12.01% (11.34% - 12.76%)	9.31% (8.48% - 10.14%)	8.07% (7.39% - 8.80%)	4.18% (3.91% - 4.48%)	3.23% (2.87% - 3.64%)	2.02% (1.67% - 2.42%)	1.53% (1.25% - 1.85%)	0.62% (0.52% - 0.75%)	0.29% (0.16% - 0.44%)	0.02% (0.00% - 0.08%)	0.00% (0.00% - 0.01%)
Seychelles	3.09% (2.79% - 3.46%)	3.56% (3.14% - 4.01%)	4.21% (3.72% - 4.76%)	3.77% (3.37% - 4.30%)	0.00% (0.00% - 0.01%)	0.01% (0.00% - 0.05%)	0.09% (0.03% - 0.20%)	0.04% (0.01% - 0.13%)	0.00% (0.00% - 0.00%)	0.00% (0.00% - 0.00%)	0.00% (0.00% - 0.00%)	0.00% (0.00% - 0.00%)
Timor-Leste	24.15% (23.04% - 25.27%)	21.72% (20.80% - 22.68%)	20.50% (19.67% - 21.34%)	19.69% (18.68% - 20.72%)	8.91% (8.02% - 9.85%)	7.25% (6.54% - 8.00%)	6.44% (5.83% - 7.03%)	6.09% (5.38% - 6.81%)	2.30% (1.87% - 2.75%)	1.55% (1.25% - 1.89%)	1.19% (0.95% - 1.46%)	1.08% (0.81% - 1.38%)
Viet Nam	11.37% (10.99% - 11.76%)	10.59% (10.31% - 10.86%)	7.58% (7.31% - 7.88%)	5.49% (5.14% - 5.84%)	2.82% (2.63% - 3.02%)	2.43% (2.30% - 2.58%)	1.27% (1.16% - 1.40%)	0.56% (0.45% - 0.68%)	0.13% (0.08% - 0.19%)	0.03% (0.01% - 0.06%)	0.00% (0.00% - 0.00%)	0.00% (0.00% - 0.00%)
Sub-Saharan Africa	11.78% (11.65% - 11.92%)	12.46% (12.31% - 12.60%)	10.40% (10.28% - 10.51%)	7.93% (7.80% - 8.05%)	3.30% (3.22% - 3.38%)	3.77% (3.68% - 3.87%)	2.81% (2.74% - 2.88%)	1.65% (1.60% - 1.71%)	0.54% (0.51% - 0.58%)	0.77% (0.73% - 0.81%)	0.49% (0.46% - 0.52%)	0.17% (0.15% - 0.19%)
**Central Sub-Saharan Africa**	12.50% (12.03% - 13.02%)	14.16% (13.71% - 14.64%)	10.55% (10.15% - 10.96%)	7.25% (6.91% - 7.61%)	3.28% (3.03% - 3.55%)	4.43% (4.16% - 4.74%)	2.68% (2.48% - 2.89%)	1.29% (1.15% - 1.45%)	0.29% (0.21% - 0.39%)	0.81% (0.70% - 0.94%)	0.27% (0.21% - 0.35%)	0.03% (0.01% - 0.05%)
Angola	14.20% (13.37% - 15.04%)	11.15% (10.57% - 11.75%)	7.41% (6.97% - 7.89%)	5.07% (4.67% - 5.45%)	3.33% (2.92% - 3.77%)	2.39% (2.13% - 2.67%)	1.16% (1.00% - 1.35%)	0.47% (0.36% - 0.59%)	0.19% (0.10% - 0.31%)	0.10% (0.06% - 0.16%)	0.00% (0.00% - 0.01%)	0.00% (0.00% - 0.00%)
Central African Republic	9.80% (9.24% - 10.43%)	11.20% (10.59% - 11.80%)	10.42% (9.87% - 10.98%)	9.36% (8.79% - 9.99%)	2.46% (2.19% - 2.77%)	3.21% (2.89% - 3.53%)	2.67% (2.40% - 2.96%)	2.15% (1.89% - 2.45%)	0.15% (0.08% - 0.24%)	0.36% (0.26% - 0.47%)	0.20% (0.14% - 0.28%)	0.09% (0.04% - 0.15%)
Congo	6.88% (6.49% - 7.30%)	8.45% (8.01% - 8.94%)	7.02% (6.68% - 7.40%)	5.58% (5.21% - 5.99%)	1.02% (0.87% - 1.17%)	1.74% (1.55% - 1.96%)	1.24% (1.11% - 1.39%)	0.73% (0.62% - 0.87%)	0.00% (0.00% - 0.00%)	0.06% (0.03% - 0.09%)	0.02% (0.00% - 0.03%)	0.00% (0.00% - 0.00%)
Democratic Republic of the Congo	12.70% (12.10% - 13.39%)	15.88% (15.27% - 16.53%)	12.10% (11.52% - 12.68%)	8.16% (7.69% - 8.71%)	3.50% (3.17% - 3.88%)	5.41% (5.02% - 5.82%)	3.39% (3.08% - 3.70%)	1.62% (1.42% - 1.86%)	0.34% (0.23% - 0.49%)	1.13% (0.96% - 1.31%)	0.40% (0.30% - 0.51%)	0.04% (0.01% - 0.06%)
Equatorial Guinea	11.82% (11.03% - 12.65%)	8.29% (7.78% - 8.84%)	3.74% (3.32% - 4.13%)	2.68% (2.42% - 2.99%)	3.49% (3.06% - 3.95%)	1.94% (1.71% - 2.18%)	0.28% (0.18% - 0.40%)	0.06% (0.03% - 0.13%)	0.47% (0.32% - 0.65%)	0.13% (0.09% - 0.18%)	0.00% (0.00% - 0.00%)	0.00% (0.00% - 0.00%)

Location	Total				Severe				Extreme			
	1990	2000	2010	2020	1990	2000	2010	2020	1990	2000	2010	2020
Gabon	5.15%	4.39%	3.61%	2.65%	0.65%	0.47%	0.21%	0.07%	0.01%	0.00%	0.00%	0.00%
	(4.71% - 5.59%)	(4.07% - 4.73%)	(3.30% - 3.96%)	(2.41% - 2.91%)	(0.54% - 0.79%)	(0.39% - 0.55%)	(0.16% - 0.28%)	(0.05% - 0.10%)	(0.00% - 0.04%)	(0.00% - 0.02%)	(0.00% - 0.00%)	(0.00% - 0.00%)
**Eastern Sub-Saharan Africa**	10.90%	10.66%	8.78%	6.92%	2.90%	2.78%	2.04%	1.33%	0.45%	0.42%	0.28%	0.13%
	(10.76% - 11.05%)	(10.52% - 10.79%)	(8.66% - 8.89%)	(6.81% - 7.04%)	(2.82% - 2.98%)	(2.71% - 2.85%)	(1.99% - 2.10%)	(1.28% - 1.39%)	(0.42% - 0.48%)	(0.39% - 0.44%)	(0.26% - 0.30%)	(0.12% - 0.15%)
Burundi	10.42%	9.44%	6.90%	5.45%	2.58%	2.16%	1.10%	0.54%	0.11%	0.05%	0.00%	0.00%
	(9.91% - 10.94%)	(8.97% - 9.93%)	(6.53% - 7.30%)	(5.09% - 5.80%)	(2.34% - 2.84%)	(1.95% - 2.39%)	(0.95% - 1.24%)	(0.43% - 0.66%)	(0.06% - 0.18%)	(0.02% - 0.09%)	(0.00% - 0.01%)	(0.00% - 0.00%)
Comoros	13.25%	13.33%	10.71%	9.01%	4.39%	4.55%	3.07%	2.26%	0.82%	0.91%	0.36%	0.18%
	(12.34% - 14.22%)	(12.55% - 14.07%)	(10.08% - 11.42%)	(8.42% - 9.66%)	(3.88% - 4.98%)	(4.09% - 5.02%)	(2.73% - 3.45%)	(1.97% - 2.58%)	(0.60% - 1.07%)	(0.72% - 1.12%)	(0.27% - 0.47%)	(0.12% - 0.25%)
Djibouti	20.90%	24.09%	22.94%	18.45%	8.96%	11.41%	10.09%	6.75%	2.93%	4.33%	3.43%	1.68%
	(19.76% - 22.08%)	(23.04% - 25.18%)	(22.04% - 23.92%)	(17.39% - 19.54%)	(8.10% - 9.91%)	(10.47% - 12.37%)	(9.33% - 10.91%)	(6.03% - 7.52%)	(2.46% - 3.46%)	(3.77% - 4.94%)	(3.01% - 3.90%)	(1.36% - 2.03%)
Eritrea	18.94%	16.23%	15.06%	11.75%	6.53%	4.82%	4.33%	2.72%	1.46%	0.80%	0.69%	0.24%
	(17.97% - 19.87%)	(15.44% - 16.99%)	(14.25% - 15.86%)	(10.97% - 12.52%)	(5.91% - 7.15%)	(4.38% - 5.28%)	(3.90% - 4.78%)	(2.37% - 3.10%)	(1.19% - 1.74%)	(0.65% - 0.99%)	(0.56% - 0.85%)	(0.16% - 0.35%)
Ethiopia	16.20%	14.83%	12.13%	9.40%	4.80%	4.11%	2.91%	1.83%	0.84%	0.63%	0.29%	0.09%
	(15.80% - 16.59%)	(14.47% - 15.22%)	(11.80% - 12.45%)	(9.13% - 9.68%)	(4.58% - 5.02%)	(3.93% - 4.31%)	(2.75% - 3.06%)	(1.72% - 1.95%)	(0.77% - 0.93%)	(0.57% - 0.69%)	(0.25% - 0.33%)	(0.07% - 0.12%)
Kenya	7.22%	6.69%	5.25%	3.89%	1.45%	1.25%	0.82%	0.46%	0.14%	0.11%	0.06%	0.02%
	(7.03% - 7.41%)	(6.52% - 6.87%)	(5.11% - 5.40%)	(3.78% - 4.00%)	(1.37% - 1.53%)	(1.18% - 1.32%)	(0.77% - 0.87%)	(0.43% - 0.49%)	(0.12% - 0.15%)	(0.10% - 0.13%)	(0.06% - 0.07%)	(0.02% - 0.03%)
Baringo	11.30%	10.84%	7.36%	4.83%	2.82%	2.54%	1.27%	0.50%	0.33%	0.28%	0.05%	0.00%
	(10.36% - 12.26%)	(9.92% - 11.78%)	(6.68% - 8.10%)	(4.32% - 5.40%)	(2.34% - 3.30%)	(2.09% - 3.06%)	(1.00% - 1.60%)	(0.34% - 0.70%)	(0.20% - 0.51%)	(0.14% - 0.46%)	(0.01% - 0.11%)	(0.00% - 0.01%)
Bomet	5.16%	4.31%	2.56%	1.59%	0.69%	0.43%	0.13%	0.03%	0.00%	0.00%	0.00%	0.00%
	(4.65% - 5.69%)	(3.88% - 4.77%)	(2.32% - 2.85%)	(1.42% - 1.78%)	(0.52% - 0.90%)	(0.30% - 0.57%)	(0.09% - 0.19%)	(0.01% - 0.06%)	(0.00% - 0.03%)	(0.00% - 0.00%)	(0.00% - 0.00%)	(0.00% - 0.00%)
Bungoma	6.67%	6.49%	5.99%	4.33%	1.01%	0.96%	0.80%	0.31%	0.00%	0.00%	0.00%	0.00%
	(6.08% - 7.29%)	(5.96% - 7.01%)	(5.48% - 6.53%)	(3.91% - 4.71%)	(0.79% - 1.24%)	(0.77% - 1.16%)	(0.62% - 1.00%)	(0.20% - 0.43%)	(0.00% - 0.02%)	(0.00% - 0.00%)	(0.00% - 0.00%)	(0.00% - 0.00%)
Busia	8.59%	7.96%	5.71%	3.65%	1.79%	1.53%	0.78%	0.23%	0.06%	0.02%	0.00%	0.00%
	(7.76% - 9.47%)	(7.25% - 8.73%)	(5.16% - 6.32%)	(3.29% - 4.06%)	(1.40% - 2.22%)	(1.21% - 1.91%)	(0.57% - 1.04%)	(0.13% - 0.36%)	(0.00% - 0.18%)	(0.00% - 0.11%)	(0.00% - 0.00%)	(0.00% - 0.00%)
Elgeyo-Marakwet	8.73%	7.11%	5.26%	3.56%	1.79%	1.26%	0.82%	0.37%	0.09%	0.03%	0.03%	0.00%
	(7.97% - 9.59%)	(6.46% - 7.79%)	(4.72% - 5.85%)	(3.17% - 3.97%)	(1.46% - 2.21%)	(1.00% - 1.54%)	(0.63% - 1.08%)	(0.26% - 0.52%)	(0.02% - 0.21%)	(0.00% - 0.07%)	(0.01% - 0.07%)	(0.00% - 0.01%)
Embu	3.85%	3.46%	2.62%	1.66%	0.21%	0.14%	0.08%	0.01%	0.00%	0.00%	0.00%	0.00%
	(3.44% - 4.27%)	(3.09% - 3.85%)	(2.35% - 2.90%)	(1.49% - 1.87%)	(0.12% - 0.33%)	(0.08% - 0.22%)	(0.05% - 0.11%)	(0.00% - 0.02%)	(0.00% - 0.00%)	(0.00% - 0.00%)	(0.00% - 0.00%)	(0.00% - 0.00%)
Garissa	11.82%	11.14%	7.77%	5.84%	3.27%	2.97%	1.76%	1.01%	0.46%	0.38%	0.12%	0.00%
	(10.88% - 12.76%)	(10.20% - 12.04%)	(6.97% - 8.59%)	(5.28% - 6.47%)	(2.74% - 3.85%)	(2.47% - 3.53%)	(1.40% - 2.15%)	(0.79% - 1.27%)	(0.24% - 0.72%)	(0.19% - 0.64%)	(0.01% - 0.26%)	(0.00% - 0.02%)
HomaBay	6.86%	6.37%	4.72%	3.23%	1.06%	1.05%	0.66%	0.24%	0.04%	0.04%	0.01%	0.00%
	(6.24% - 7.44%)	(5.86% - 6.92%)	(4.35% - 5.15%)	(2.94% - 3.55%)	(0.85% - 1.29%)	(0.85% - 1.26%)	(0.53% - 0.80%)	(0.17% - 0.32%)	(0.00% - 0.09%)	(0.00% - 0.10%)	(0.00% - 0.03%)	(0.00% - 0.00%)
Isiolo	15.51%	14.11%	12.14%	7.45%	4.14%	3.44%	2.62%	0.96%	0.55%	0.34%	0.13%	0.00%
	(14.22% - 16.89%)	(12.95% - 15.28%)	(11.11% - 13.30%)	(6.71% - 8.19%)	(3.48% - 4.92%)	(2.89% - 4.07%)	(2.15% - 3.16%)	(0.73% - 1.22%)	(0.34% - 0.81%)	(0.21% - 0.51%)	(0.06% - 0.22%)	(0.00% - 0.02%)
Kajiado	8.48%	7.28%	3.83%	2.12%	1.49%	1.07%	0.19%	0.01%	0.03%	0.00%	0.00%	0.00%
	(7.63% - 9.29%)	(6.55% - 8.12%)	(3.34% - 4.26%)	(1.89% - 2.36%)	(1.15% - 1.87%)	(0.78% - 1.41%)	(0.09% - 0.30%)	(0.00% - 0.03%)	(0.00% - 0.14%)	(0.00% - 0.03%)	(0.00% - 0.00%)	(0.00% - 0.00%)
Kakamega	10.52%	10.21%	6.63%	4.70%	2.85%	2.70%	1.20%	0.60%	0.35%	0.34%	0.05%	0.00%
	(9.86% - 11.25%)	(9.47% - 10.89%)	(6.11% - 7.19%)	(4.33% - 5.10%)	(2.51% - 3.22%)	(2.36% - 3.04%)	(1.02% - 1.42%)	(0.49% - 0.73%)	(0.25% - 0.46%)	(0.24% - 0.46%)	(0.02% - 0.10%)	(0.00% - 0.02%)
Kericho	9.38%	7.40%	6.15%	4.44%	2.33%	1.57%	1.23%	0.68%	0.18%	0.09%	0.11%	0.02%
	(8.64% - 10.14%)	(6.81% - 8.04%)	(5.59% - 6.83%)	(4.03% - 4.86%)	(1.98% - 2.73%)	(1.34% - 1.86%)	(1.00% - 1.47%)	(0.53% - 0.85%)	(0.09% - 0.32%)	(0.03% - 0.16%)	(0.05% - 0.18%)	(0.00% - 0.06%)
Kiambu	5.91%	5.84%	4.29%	3.14%	0.87%	0.86%	0.43%	0.15%	0.00%	0.00%	0.00%	0.00%
	(5.43% - 6.39%)	(5.38% - 6.30%)	(3.93% - 4.65%)	(2.87% - 3.41%)	(0.69% - 1.06%)	(0.70% - 1.04%)	(0.32% - 0.55%)	(0.10% - 0.21%)	(0.00% - 0.00%)	(0.00% - 0.01%)	(0.00% - 0.00%)	(0.00% - 0.00%)
Kilifi	7.57%	6.66%	5.31%	3.69%	1.38%	1.08%	0.71%	0.28%	0.01%	0.00%	0.00%	0.00%
	(7.02% - 8.14%)	(6.11% - 7.16%)	(4.88% - 5.77%)	(3.36% - 4.01%)	(1.16% - 1.64%)	(0.88% - 1.27%)	(0.57% - 0.86%)	(0.21% - 0.37%)	(0.00% - 0.03%)	(0.00% - 0.01%)	(0.00% - 0.00%)	(0.00% - 0.00%)
Kirinyaga	1.70%	1.40%	1.09%	0.83%	0.00%	0.00%	0.00%	0.00%	0.00%	0.00%	0.00%	0.00%
	(1.50% - 1.91%)	(1.26% - 1.57%)	(0.98% - 1.23%)	(0.66% - 0.95%)	(0.00% - 0.02%)	(0.00% - 0.00%)	(0.00% - 0.00%)	(0.00% - 0.00%)	(0.00% - 0.00%)	(0.00% - 0.00%)	(0.00% - 0.00%)	(0.00% - 0.00%)

Table S8b. Estimated overall, severe, and extreme **wasting** prevalence (%) in children under 5 years, both sexes, in 1990, 2000, 2010, and 2020.

Table S8b. Estimated overall, severe, and extreme **wasting** prevalence (%) in children under 5 years, both sexes, in 1990, 2000, 2010, and 2020.

Location	Total				Severe				Extreme			
	1990	2000	2010	2020	1990	2000	2010	2020	1990	2000	2010	2020
Kisii	6.84%	5.38%	3.73%	2.56%	1.13%	0.60%	0.32%	0.13%	0.01%	0.00%	0.00%	0.00%
	(6.21% - 7.51%)	(4.83% - 5.96%)	(3.33% - 4.19%)	(2.23% - 2.92%)	(0.87% - 1.44%)	(0.42% - 0.82%)	(0.19% - 0.47%)	(0.04% - 0.23%)	(0.00% - 0.10%)	(0.00% - 0.00%)	(0.00% - 0.00%)	(0.00% - 0.00%)
Kisumu	5.21%	5.36%	2.79%	1.57%	0.77%	0.79%	0.09%	0.00%	0.01%	0.01%	0.00%	0.00%
	(4.67% - 5.75%)	(4.82% - 5.93%)	(2.49% - 3.14%)	(1.38% - 1.79%)	(0.57% - 0.98%)	(0.58% - 1.02%)	(0.04% - 0.15%)	(0.00% - 0.02%)	(0.00% - 0.05%)	(0.00% - 0.06%)	(0.00% - 0.00%)	(0.00% - 0.00%)
Kitui	7.40%	7.32%	5.09%	3.00%	1.16%	1.11%	0.56%	0.13%	0.00%	0.00%	0.00%	0.00%
	(6.74% - 8.12%)	(6.74% - 7.92%)	(4.65% - 5.58%)	(2.71% - 3.31%)	(0.92% - 1.44%)	(0.90% - 1.35%)	(0.43% - 0.71%)	(0.08% - 0.19%)	(0.00% - 0.01%)	(0.00% - 0.01%)	(0.00% - 0.00%)	(0.00% - 0.00%)
Kwale	4.81%	4.55%	3.22%	1.94%	0.43%	0.38%	0.15%	0.01%	0.00%	0.00%	0.00%	0.00%
	(4.36% - 5.29%)	(4.10% - 5.02%)	(2.87% - 3.57%)	(1.70% - 2.19%)	(0.31% - 0.59%)	(0.26% - 0.54%)	(0.08% - 0.25%)	(0.00% - 0.04%)	(0.00% - 0.00%)	(0.00% - 0.00%)	(0.00% - 0.00%)	(0.00% - 0.00%)
Laikipia	6.14%	5.65%	3.82%	2.47%	0.92%	0.75%	0.20%	0.02%	0.00%	0.00%	0.00%	0.00%
	(5.59% - 6.75%)	(5.09% - 6.23%)	(3.43% - 4.24%)	(2.20% - 2.80%)	(0.72% - 1.16%)	(0.56% - 0.96%)	(0.11% - 0.33%)	(0.00% - 0.05%)	(0.00% - 0.01%)	(0.00% - 0.00%)	(0.00% - 0.00%)	(0.00% - 0.00%)
Lamu	10.26%	9.33%	5.05%	3.19%	3.41%	2.91%	0.69%	0.17%	0.97%	0.73%	0.00%	0.00%
	(9.31% - 11.29%)	(8.44% - 10.27%)	(4.45% - 5.69%)	(2.81% - 3.61%)	(2.82% - 4.04%)	(2.38% - 3.52%)	(0.48% - 0.94%)	(0.08% - 0.29%)	(0.69% - 1.28%)	(0.50% - 1.02%)	(0.00% - 0.02%)	(0.00% - 0.00%)
Machakos	5.54%	5.56%	4.71%	3.23%	0.86%	0.87%	0.67%	0.27%	0.02%	0.02%	0.00%	0.00%
	(5.07% - 6.06%)	(5.09% - 6.07%)	(4.29% - 5.15%)	(2.91% - 3.56%)	(0.68% - 1.06%)	(0.70% - 1.07%)	(0.53% - 0.83%)	(0.19% - 0.36%)	(0.00% - 0.06%)	(0.00% - 0.06%)	(0.00% - 0.01%)	(0.00% - 0.00%)
Makueni	5.26%	4.81%	3.09%	1.67%	0.62%	0.49%	0.15%	0.01%	0.00%	0.00%	0.00%	0.00%
	(4.72% - 5.82%)	(4.37% - 5.30%)	(2.75% - 3.44%)	(1.49% - 1.88%)	(0.44% - 0.82%)	(0.34% - 0.64%)	(0.09% - 0.23%)	(0.00% - 0.02%)	(0.00% - 0.00%)	(0.00% - 0.00%)	(0.00% - 0.00%)	(0.00% - 0.00%)
Mandera	10.58%	10.09%	9.07%	7.96%	2.50%	2.32%	1.93%	1.52%	0.58%	0.54%	0.39%	0.28%
	(9.81% - 11.45%)	(9.32% - 10.94%)	(8.37% - 9.83%)	(7.27% - 8.71%)	(2.16% - 2.85%)	(2.00% - 2.67%)	(1.68% - 2.22%)	(1.26% - 1.79%)	(0.43% - 0.75%)	(0.41% - 0.70%)	(0.29% - 0.51%)	(0.19% - 0.39%)
Marsabit	17.52%	16.28%	16.03%	12.18%	6.11%	5.43%	5.23%	2.99%	1.40%	1.12%	1.04%	0.22%
	(16.26% - 18.80%)	(15.13% - 17.52%)	(15.00% - 17.15%)	(11.42% - 12.99%)	(5.26% - 7.01%)	(4.72% - 6.22%)	(4.57% - 5.92%)	(2.59% - 3.44%)	(1.01% - 1.84%)	(0.81% - 1.49%)	(0.74% - 1.33%)	(0.12% - 0.35%)
Meru	8.25%	6.10%	3.62%	1.74%	1.82%	1.05%	0.18%	0.00%	0.16%	0.03%	0.00%	0.00%
	(7.58% - 8.96%)	(5.56% - 6.69%)	(3.17% - 4.01%)	(1.55% - 1.99%)	(1.50% - 2.18%)	(0.84% - 1.28%)	(0.10% - 0.27%)	(0.00% - 0.00%)	(0.07% - 0.28%)	(0.00% - 0.08%)	(0.00% - 0.00%)	(0.00% - 0.00%)
Migori	15.51%	13.23%	10.77%	7.07%	5.74%	4.40%	3.00%	1.38%	1.53%	0.98%	0.47%	0.11%
	(14.58% - 16.52%)	(12.39% - 14.12%)	(10.05% - 11.52%)	(6.61% - 7.60%)	(5.12% - 6.42%)	(3.88% - 4.94%)	(2.64% - 3.39%)	(1.18% - 1.60%)	(1.24% - 1.88%)	(0.75% - 1.24%)	(0.35% - 0.59%)	(0.06% - 0.17%)
Mombasa	6.78%	6.32%	3.99%	2.66%	1.34%	1.15%	0.36%	0.13%	0.13%	0.09%	0.00%	0.00%
	(6.21% - 7.36%)	(5.76% - 6.85%)	(3.58% - 4.41%)	(2.38% - 2.96%)	(1.11% - 1.59%)	(0.93% - 1.38%)	(0.27% - 0.47%)	(0.08% - 0.18%)	(0.07% - 0.20%)	(0.04% - 0.15%)	(0.00% - 0.01%)	(0.00% - 0.00%)
Murang'a	6.32%	5.27%	3.77%	2.58%	1.03%	0.65%	0.24%	0.05%	0.00%	0.00%	0.00%	0.00%
	(5.83% - 6.86%)	(4.79% - 5.77%)	(3.39% - 4.20%)	(2.30% - 2.90%)	(0.84% - 1.24%)	(0.48% - 0.83%)	(0.16% - 0.36%)	(0.01% - 0.08%)	(0.00% - 0.01%)	(0.00% - 0.00%)	(0.00% - 0.00%)	(0.00% - 0.00%)
Nairobi	3.12%	3.11%	2.40%	1.86%	0.11%	0.12%	0.05%	0.01%	0.00%	0.00%	0.00%	0.00%
	(2.87% - 3.39%)	(2.86% - 3.37%)	(2.21% - 2.59%)	(1.72% - 2.02%)	(0.07% - 0.17%)	(0.07% - 0.18%)	(0.02% - 0.07%)	(0.00% - 0.03%)	(0.00% - 0.00%)	(0.00% - 0.00%)	(0.00% - 0.00%)	(0.00% - 0.00%)
Nakuru	3.36%	3.27%	4.12%	3.30%	0.21%	0.24%	0.56%	0.36%	0.00%	0.00%	0.00%	0.00%
	(3.05% - 3.69%)	(2.99% - 3.59%)	(3.77% - 4.51%)	(3.03% - 3.63%)	(0.14% - 0.28%)	(0.18% - 0.32%)	(0.45% - 0.69%)	(0.28% - 0.45%)	(0.00% - 0.00%)	(0.00% - 0.00%)	(0.00% - 0.01%)	(0.00% - 0.00%)
Nandi	8.08%	7.15%	5.85%	3.48%	1.65%	1.33%	0.88%	0.26%	0.04%	0.02%	0.02%	0.00%
	(7.43% - 8.81%)	(6.54% - 7.79%)	(5.29% - 6.44%)	(3.12% - 3.87%)	(1.38% - 1.97%)	(1.10% - 1.59%)	(0.70% - 1.10%)	(0.19% - 0.35%)	(0.00% - 0.09%)	(0.00% - 0.07%)	(0.00% - 0.06%)	(0.00% - 0.00%)
Narok	9.23%	8.43%	5.06%	3.01%	1.92%	1.63%	0.47%	0.10%	0.07%	0.03%	0.00%	0.00%
	(8.48% - 10.08%)	(7.76% - 9.22%)	(4.55% - 5.58%)	(2.70% - 3.34%)	(1.59% - 2.31%)	(1.34% - 1.99%)	(0.33% - 0.63%)	(0.06% - 0.15%)	(0.01% - 0.16%)	(0.00% - 0.09%)	(0.00% - 0.00%)	(0.00% - 0.00%)
Nyamira	10.19%	9.76%	9.21%	6.67%	3.68%	3.31%	2.72%	1.43%	1.13%	0.94%	0.57%	0.08%
	(9.25% - 11.16%)	(8.87% - 10.75%)	(8.30% - 10.33%)	(5.86% - 7.54%)	(3.03% - 4.43%)	(2.71% - 3.99%)	(2.18% - 3.42%)	(1.04% - 1.89%)	(0.80% - 1.55%)	(0.64% - 1.29%)	(0.32% - 0.91%)	(0.00% - 0.25%)
Nyandarua	5.07%	5.31%	3.26%	2.13%	0.53%	0.62%	0.24%	0.11%	0.00%	0.00%	0.00%	0.00%
	(4.59% - 5.59%)	(4.79% - 5.91%)	(2.94% - 3.57%)	(1.90% - 2.36%)	(0.39% - 0.69%)	(0.46% - 0.82%)	(0.17% - 0.32%)	(0.06% - 0.16%)	(0.00% - 0.00%)	(0.00% - 0.00%)	(0.00% - 0.00%)	(0.00% - 0.00%)
Nyeri	5.59%	4.65%	4.28%	3.55%	0.79%	0.54%	0.43%	0.25%	0.00%	0.00%	0.00%	0.00%
	(5.08% - 6.17%)	(4.17% - 5.15%)	(3.85% - 4.76%)	(3.17% - 3.94%)	(0.61% - 0.99%)	(0.40% - 0.71%)	(0.31% - 0.58%)	(0.16% - 0.37%)	(0.00% - 0.01%)	(0.00% - 0.00%)	(0.00% - 0.00%)	(0.00% - 0.00%)
Samburu	14.40%	12.95%	12.53%	10.67%	3.47%	2.82%	2.53%	1.77%	0.37%	0.25%	0.13%	0.02%
	(13.22% - 15.54%)	(11.87% - 14.08%)	(11.45% - 13.60%)	(9.79% - 11.65%)	(2.89% - 4.07%)	(2.31% - 3.35%)	(2.04% - 3.08%)	(1.41% - 2.17%)	(0.22% - 0.57%)	(0.13% - 0.41%)	(0.03% - 0.29%)	(0.00% - 0.06%)
Siaya	5.76%	4.64%	2.15%	1.10%	0.74%	0.40%	0.01%	0.00%	0.00%	0.00%	0.00%	0.00%
	(5.22% - 6.34%)	(4.17% - 5.16%)	(1.89% - 2.45%)	(0.91% - 1.32%)	(0.56% - 0.94%)	(0.26% - 0.56%)	(0.00% - 0.04%)	(0.00% - 0.00%)	(0.00% - 0.02%)	(0.00% - 0.00%)	(0.00% - 0.00%)	(0.00% - 0.00%)

Table S8b. Estimated overall, severe, and extreme **wasting** prevalence (%) in children under 5 years, both sexes, in 1990, 2000, 2010, and 2020.												
**Location**	**Total**				**Severe**				**Extreme**			
	**1990**	**2000**	**2010**	**2020**	**1990**	**2000**	**2010**	**2020**	**1990**	**2000**	**2010**	**2020**
TaitaTaveta	9.30%	8.03%	7.25%	5.46%	2.18%	1.70%	1.46%	0.78%	0.13%	0.07%	0.02%	0.00%
	(8.53% - 10.14%)	(7.31% - 8.81%)	(6.58% - 7.97%)	(4.90% - 6.10%)	(1.82% - 2.58%)	(1.40% - 2.05%)	(1.16% - 1.78%)	(0.58% - 1.03%)	(0.05% - 0.23%)	(0.02% - 0.14%)	(0.00% - 0.06%)	(0.00% - 0.01%)
TanaRiver	7.20%	7.97%	7.17%	7.22%	1.26%	1.48%	1.29%	1.26%	0.15%	0.20%	0.13%	0.06%
	(6.55% - 7.86%)	(7.31% - 8.65%)	(6.57% - 7.84%)	(6.59% - 7.86%)	(1.03% - 1.52%)	(1.23% - 1.76%)	(1.07% - 1.55%)	(1.04% - 1.50%)	(0.08% - 0.23%)	(0.12% - 0.29%)	(0.07% - 0.21%)	(0.02% - 0.11%)
TharakaNithi	9.41%	9.48%	6.87%	3.88%	2.70%	2.74%	1.52%	0.52%	0.64%	0.68%	0.23%	0.01%
	(8.68% - 10.20%)	(8.76% - 10.19%)	(6.27% - 7.45%)	(3.52% - 4.25%)	(2.29% - 3.14%)	(2.37% - 3.13%)	(1.29% - 1.78%)	(0.41% - 0.64%)	(0.46% - 0.85%)	(0.51% - 0.88%)	(0.14% - 0.34%)	(0.00% - 0.03%)
TransNzoia	5.41%	4.62%	3.33%	2.15%	0.79%	0.61%	0.36%	0.15%	0.02%	0.01%	0.00%	0.00%
	(4.94% - 5.94%)	(4.21% - 5.03%)	(3.02% - 3.68%)	(1.94% - 2.38%)	(0.61% - 0.99%)	(0.47% - 0.77%)	(0.27% - 0.48%)	(0.09% - 0.22%)	(0.00% - 0.06%)	(0.00% - 0.04%)	(0.00% - 0.02%)	(0.00% - 0.00%)
Turkana	9.75%	8.40%	6.72%	5.12%	2.23%	1.69%	1.05%	0.55%	0.11%	0.04%	0.00%	0.00%
	(9.01% - 10.54%)	(7.70% - 9.15%)	(6.11% - 7.33%)	(4.65% - 5.62%)	(1.90% - 2.61%)	(1.39% - 2.01%)	(0.84% - 1.29%)	(0.42% - 0.71%)	(0.04% - 0.20%)	(0.00% - 0.09%)	(0.00% - 0.02%)	(0.00% - 0.00%)
UasinGishu	7.92%	7.86%	4.95%	2.93%	1.59%	1.62%	0.57%	0.13%	0.02%	0.02%	0.00%	0.00%
	(7.27% - 8.57%)	(7.18% - 8.61%)	(4.46% - 5.46%)	(2.62% - 3.34%)	(1.30% - 1.89%)	(1.34% - 1.96%)	(0.42% - 0.74%)	(0.08% - 0.20%)	(0.00% - 0.07%)	(0.00% - 0.06%)	(0.00% - 0.00%)	(0.00% - 0.00%)
Vihiga	4.19%	3.89%	2.84%	2.12%	0.35%	0.35%	0.19%	0.10%	0.00%	0.00%	0.00%	0.00%
	(3.69% - 4.64%)	(3.45% - 4.37%)	(2.55% - 3.16%)	(1.88% - 2.39%)	(0.24% - 0.48%)	(0.24% - 0.46%)	(0.12% - 0.27%)	(0.05% - 0.15%)	(0.00% - 0.00%)	(0.00% - 0.01%)	(0.00% - 0.00%)	(0.00% - 0.00%)
Wajir	13.46%	13.38%	10.92%	10.01%	4.46%	4.43%	3.07%	2.51%	0.88%	0.87%	0.37%	0.25%
	(12.57% - 14.39%)	(12.54% - 14.29%)	(10.16% - 11.63%)	(9.38% - 10.70%)	(3.96% - 5.03%)	(3.93% - 4.96%)	(2.68% - 3.46%)	(2.22% - 2.85%)	(0.66% - 1.14%)	(0.65% - 1.10%)	(0.26% - 0.51%)	(0.17% - 0.36%)
WestPokot	11.39%	10.70%	10.91%	8.60%	2.60%	2.31%	2.51%	1.51%	0.21%	0.20%	0.17%	0.02%
	(10.56% - 12.31%)	(9.85% - 11.57%)	(10.05% - 11.78%)	(7.85% - 9.42%)	(2.21% - 3.05%)	(1.91% - 2.73%)	(2.08% - 2.97%)	(1.21% - 1.87%)	(0.12% - 0.33%)	(0.11% - 0.32%)	(0.08% - 0.30%)	(0.00% - 0.06%)
Madagascar	8.74%	12.66%	12.68%	10.21%	1.61%	3.38%	3.35%	2.18%	0.02%	0.37%	0.38%	0.12%
	(8.28% - 9.25%)	(12.08% - 13.25%)	(12.01% - 13.36%)	(9.56% - 10.86%)	(1.43% - 1.83%)	(3.07% - 3.70%)	(3.00% - 3.71%)	(1.89% - 2.49%)	(0.00% - 0.04%)	(0.27% - 0.48%)	(0.27% - 0.51%)	(0.06% - 0.18%)
Malawi	6.68%	7.48%	5.20%	3.96%	1.13%	1.51%	0.65%	0.29%	0.01%	0.05%	0.00%	0.00%
	(6.34% - 7.05%)	(7.15% - 7.82%)	(4.92% - 5.47%)	(3.72% - 4.27%)	(0.99% - 1.27%)	(1.37% - 1.64%)	(0.56% - 0.74%)	(0.23% - 0.36%)	(0.00% - 0.03%)	(0.03% - 0.08%)	(0.00% - 0.00%)	(0.00% - 0.00%)
Mozambique	9.88%	7.85%	5.50%	4.01%	2.38%	1.57%	0.76%	0.34%	0.13%	0.03%	0.00%	0.00%
	(9.32% - 10.46%)	(7.43% - 8.27%)	(5.17% - 5.85%)	(3.69% - 4.33%)	(2.11% - 2.66%)	(1.39% - 1.74%)	(0.65% - 0.88%)	(0.28% - 0.43%)	(0.08% - 0.19%)	(0.01% - 0.06%)	(0.00% - 0.01%)	(0.00% - 0.00%)
Rwanda	6.91%	7.82%	3.77%	2.47%	1.29%	1.67%	0.34%	0.09%	0.08%	0.14%	0.00%	0.00%
	(6.54% - 7.29%)	(7.43% - 8.19%)	(3.56% - 4.00%)	(2.31% - 2.64%)	(1.15% - 1.45%)	(1.51% - 1.84%)	(0.30% - 0.39%)	(0.06% - 0.12%)	(0.05% - 0.12%)	(0.10% - 0.18%)	(0.00% - 0.00%)	(0.00% - 0.00%)
Somalia	18.07%	17.29%	14.60%	13.61%	6.45%	6.08%	4.49%	3.94%	1.54%	1.39%	0.72%	0.51%
	(17.25% - 18.89%)	(16.73% - 17.87%)	(13.90% - 15.30%)	(12.82% - 14.42%)	(5.88% - 7.04%)	(5.69% - 6.49%)	(4.09% - 4.91%)	(3.51% - 4.41%)	(1.27% - 1.82%)	(1.22% - 1.59%)	(0.56% - 0.90%)	(0.35% - 0.69%)
United Republic of Tanzania	7.97%	6.76%	4.95%	3.69%	1.64%	1.15%	0.55%	0.26%	0.04%	0.01%	0.00%	0.00%
	(7.63% - 8.32%)	(6.43% - 7.08%)	(4.68% - 5.22%)	(3.46% - 3.96%)	(1.50% - 1.79%)	(1.03% - 1.28%)	(0.48% - 0.63%)	(0.21% - 0.30%)	(0.02% - 0.06%)	(0.00% - 0.02%)	(0.00% - 0.00%)	(0.00% - 0.00%)
Uganda	4.73%	6.26%	5.34%	3.89%	0.55%	1.07%	0.81%	0.42%	0.00%	0.06%	0.03%	0.00%
	(4.48% - 4.98%)	(5.96% - 6.53%)	(5.11% - 5.59%)	(3.67% - 4.11%)	(0.48% - 0.62%)	(0.97% - 1.18%)	(0.74% - 0.89%)	(0.36% - 0.49%)	(0.00% - 0.00%)	(0.03% - 0.08%)	(0.01% - 0.04%)	(0.00% - 0.00%)
Zambia	6.38%	7.21%	6.55%	4.29%	1.08%	1.42%	1.16%	0.41%	0.00%	0.04%	0.01%	0.00%
	(6.06% - 6.71%)	(6.92% - 7.53%)	(6.22% - 6.85%)	(3.98% - 4.59%)	(0.95% - 1.21%)	(1.31% - 1.56%)	(1.03% - 1.29%)	(0.32% - 0.50%)	(0.00% - 0.02%)	(0.02% - 0.07%)	(0.00% - 0.02%)	(0.00% - 0.00%)
South Sudan	22.90%	23.91%	24.24%	21.19%	9.80%	10.65%	10.60%	8.21%	3.15%	3.62%	3.49%	2.23%
	(21.76% - 24.14%)	(23.06% - 24.82%)	(23.64% - 24.83%)	(20.13% - 22.38%)	(8.87% - 10.82%)	(9.89% - 11.45%)	(10.06% - 11.16%)	(7.37% - 9.21%)	(2.62% - 3.73%)	(3.18% - 4.11%)	(3.15% - 3.82%)	(1.82% - 2.77%)
**Southern Sub-Saharan Africa**	5.91%	5.91%	4.73%	3.46%	0.99%	0.94%	0.56%	0.28%	0.07%	0.04%	0.01%	0.00%
	(5.77% - 6.04%)	(5.79% - 6.04%)	(4.61% - 4.85%)	(3.36% - 3.55%)	(0.94% - 1.04%)	(0.89% - 0.99%)	(0.53% - 0.59%)	(0.26% - 0.30%)	(0.06% - 0.08%)	(0.04% - 0.05%)	(0.01% - 0.02%)	(0.00% - 0.00%)
Botswana	8.50%	7.50%	6.71%	5.32%	1.93%	1.57%	1.29%	0.76%	0.14%	0.11%	0.07%	0.02%
	(7.91% - 9.13%)	(7.04% - 7.95%)	(6.25% - 7.14%)	(4.92% - 5.74%)	(1.67% - 2.21%)	(1.38% - 1.77%)	(1.11% - 1.47%)	(0.63% - 0.91%)	(0.09% - 0.19%)	(0.08% - 0.14%)	(0.05% - 0.11%)	(0.00% - 0.04%)
Lesotho	8.91%	7.36%	4.69%	3.07%	1.85%	1.43%	0.49%	0.13%	0.21%	0.11%	0.00%	0.00%
	(8.48% - 9.35%)	(7.03% - 7.68%)	(4.43% - 4.96%)	(2.87% - 3.29%)	(1.68% - 2.04%)	(1.30% - 1.55%)	(0.41% - 0.57%)	(0.10% - 0.17%)	(0.17% - 0.24%)	(0.08% - 0.13%)	(0.00% - 0.00%)	(0.00% - 0.00%)
Namibia	10.41%	9.75%	8.37%	6.21%	2.56%	2.16%	1.64%	0.87%	0.15%	0.11%	0.05%	0.00%
	(9.82% - 11.01%)	(9.23% - 10.30%)	(7.94% - 8.84%)	(5.77% - 6.67%)	(2.29% - 2.86%)	(1.93% - 2.39%)	(1.45% - 1.83%)	(0.73% - 1.04%)	(0.08% - 0.22%)	(0.07% - 0.16%)	(0.02% - 0.08%)	(0.00% - 0.02%)
South Africa	6.22%	5.73%	4.36%	3.27%	1.09%	0.87%	0.46%	0.25%	0.08%	0.05%	0.01%	0.00%
	(6.05% - 6.39%)	(5.58% - 5.89%)	(4.23% - 4.50%)	(3.16% - 3.37%)	(1.03% - 1.17%)	(0.82% - 0.93%)	(0.42% - 0.49%)	(0.22% - 0.28%)	(0.06% - 0.10%)	(0.04% - 0.07%)	(0.01% - 0.02%)	(0.00% - 0.01%)

Table S8b. Estimated overall, severe, and extreme **wasting** prevalence (%) in children under 5 years, both sexes, in 1990, 2000, 2010, and 2020.												
**Location**	**Total**				**Severe**				**Extreme**			
	**1990**	**2000**	**2010**	**2020**	**1990**	**2000**	**2010**	**2020**	**1990**	**2000**	**2010**	**2020**
Eswatini	2.31%	2.22%	1.90%	1.53%	0.02%	0.02%	0.01%	0.00%	0.00%	0.00%	0.00%	0.00%
	(2.13% - 2.49%)	(2.06% - 2.38%)	(1.75% - 2.04%)	(1.40% - 1.73%)	(0.01% - 0.05%)	(0.00% - 0.04%)	(0.00% - 0.03%)	(0.00% - 0.01%)	(0.00% - 0.00%)	(0.00% - 0.00%)	(0.00% - 0.00%)	(0.00% - 0.00%)
Zimbabwe	4.07%	5.77%	5.13%	3.49%	0.34%	0.89%	0.65%	0.25%	0.00%	0.00%	0.00%	0.00%
	(3.77% - 4.39%)	(5.48% - 6.07%)	(4.85% - 5.43%)	(3.27% - 3.73%)	(0.28% - 0.41%)	(0.79% - 1.00%)	(0.57% - 0.74%)	(0.20% - 0.30%)	(0.00% - 0.00%)	(0.00% - 0.02%)	(0.00% - 0.00%)	(0.00% - 0.00%)
**Western Sub-Saharan Africa**	13.66%	14.79%	12.52%	9.41%	4.19%	5.02%	3.82%	2.16%	0.81%	1.22%	0.80%	0.25%
	(13.40% - 13.92%)	(14.52% - 15.06%)	(12.34% - 12.72%)	(9.19% - 9.62%)	(4.03% - 4.37%)	(4.82% - 5.22%)	(3.69% - 3.95%)	(2.06% - 2.28%)	(0.75% - 0.89%)	(1.13% - 1.32%)	(0.74% - 0.86%)	(0.22% - 0.29%)
Benin	10.92%	11.23%	9.73%	7.07%	2.85%	3.04%	2.41%	1.22%	0.27%	0.38%	0.16%	0.01%
	(10.39% - 11.45%)	(10.74% - 11.73%)	(9.27% - 10.22%)	(6.67% - 7.50%)	(2.59% - 3.13%)	(2.80% - 3.29%)	(2.19% - 2.65%)	(1.06% - 1.39%)	(0.20% - 0.34%)	(0.31% - 0.45%)	(0.11% - 0.22%)	(0.00% - 0.03%)
Burkina Faso	18.95%	21.44%	17.00%	12.24%	6.46%	8.46%	5.44%	2.87%	1.39%	2.41%	0.98%	0.11%
	(18.18% - 19.70%)	(20.78% - 22.12%)	(16.39% - 17.65%)	(11.61% - 12.90%)	(5.94% - 6.96%)	(7.98% - 8.94%)	(5.05% - 5.86%)	(2.54% - 3.18%)	(1.16% - 1.63%)	(2.16% - 2.65%)	(0.82% - 1.15%)	(0.04% - 0.21%)
Cameroon	6.44%	7.60%	6.27%	4.64%	1.09%	1.64%	1.10%	0.52%	0.02%	0.12%	0.02%	0.00%
	(6.07% - 6.84%)	(7.20% - 8.01%)	(5.95% - 6.60%)	(4.29% - 4.95%)	(0.96% - 1.25%)	(1.47% - 1.81%)	(0.98% - 1.23%)	(0.44% - 0.62%)	(0.00% - 0.04%)	(0.08% - 0.17%)	(0.01% - 0.05%)	(0.00% - 0.00%)
Cabo Verde	5.73%	4.37%	2.59%	1.91%	0.51%	0.18%	0.02%	0.00%	0.00%	0.00%	0.00%	0.00%
	(5.43% - 6.05%)	(3.91% - 4.87%)	(2.35% - 2.87%)	(1.72% - 2.10%)	(0.41% - 0.61%)	(0.11% - 0.28%)	(0.00% - 0.04%)	(0.00% - 0.01%)	(0.00% - 0.00%)	(0.00% - 0.00%)	(0.00% - 0.00%)	(0.00% - 0.00%)
Chad	17.81%	17.93%	17.12%	14.32%	5.93%	5.95%	5.34%	3.75%	1.20%	1.20%	0.93%	0.38%
	(17.04% - 18.58%)	(17.25% - 18.56%)	(16.46% - 17.82%)	(13.63% - 15.04%)	(5.46% - 6.46%)	(5.51% - 6.37%)	(4.95% - 5.78%)	(3.39% - 4.16%)	(0.98% - 1.44%)	(1.01% - 1.39%)	(0.77% - 1.10%)	(0.26% - 0.51%)
Côte d'Ivoire	9.64%	9.23%	8.32%	5.77%	2.10%	1.99%	1.67%	0.69%	0.10%	0.11%	0.12%	0.00%
	(9.20% - 10.10%)	(8.81% - 9.68%)	(7.91% - 8.73%)	(5.43% - 6.18%)	(1.90% - 2.32%)	(1.80% - 2.19%)	(1.51% - 1.84%)	(0.58% - 0.82%)	(0.06% - 0.14%)	(0.08% - 0.16%)	(0.08% - 0.16%)	(0.00% - 0.00%)
Gambia	11.64%	10.74%	9.89%	7.76%	2.65%	2.28%	1.80%	1.07%	0.23%	0.15%	0.07%	0.00%
	(10.95% - 12.36%)	(10.15% - 11.37%)	(9.43% - 10.39%)	(7.28% - 8.28%)	(2.35% - 2.98%)	(2.02% - 2.55%)	(1.61% - 2.00%)	(0.92% - 1.24%)	(0.15% - 0.31%)	(0.10% - 0.22%)	(0.03% - 0.10%)	(0.00% - 0.02%)
Ghana	9.98%	10.60%	9.26%	6.19%	2.23%	2.52%	1.93%	0.79%	0.13%	0.21%	0.07%	0.00%
	(9.54% - 10.49%)	(10.19% - 11.11%)	(8.85% - 9.77%)	(5.82% - 6.57%)	(2.03% - 2.47%)	(2.32% - 2.75%)	(1.75% - 2.13%)	(0.67% - 0.92%)	(0.08% - 0.18%)	(0.16% - 0.26%)	(0.04% - 0.11%)	(0.00% - 0.01%)
Guinea	12.35%	11.68%	10.11%	8.42%	3.56%	3.25%	2.44%	1.68%	0.50%	0.41%	0.19%	0.02%
	(11.66% - 13.12%)	(11.16% - 12.23%)	(9.67% - 10.60%)	(7.93% - 8.91%)	(3.20% - 3.98%)	(2.98% - 3.53%)	(2.23% - 2.67%)	(1.47% - 1.89%)	(0.39% - 0.63%)	(0.34% - 0.50%)	(0.14% - 0.25%)	(0.00% - 0.06%)
Guinea-Bissau	12.23%	11.76%	7.86%	6.11%	3.45%	3.30%	1.52%	0.82%	0.45%	0.42%	0.02%	0.00%
	(11.47% - 13.03%)	(11.22% - 12.38%)	(7.43% - 8.30%)	(5.67% - 6.59%)	(3.07% - 3.86%)	(3.02% - 3.62%)	(1.35% - 1.71%)	(0.67% - 0.99%)	(0.35% - 0.57%)	(0.35% - 0.50%)	(0.00% - 0.04%)	(0.00% - 0.00%)
Liberia	8.55%	8.41%	6.11%	4.64%	1.66%	1.60%	0.83%	0.40%	0.02%	0.01%	0.00%	0.00%
	(7.99% - 9.16%)	(7.93% - 8.97%)	(5.73% - 6.50%)	(4.28% - 5.01%)	(1.42% - 1.93%)	(1.40% - 1.84%)	(0.70% - 0.97%)	(0.31% - 0.49%)	(0.00% - 0.05%)	(0.00% - 0.04%)	(0.00% - 0.01%)	(0.00% - 0.00%)
Mali	17.86%	17.01%	12.16%	9.07%	6.17%	5.72%	2.87%	1.47%	1.37%	1.20%	0.25%	0.00%
	(17.17% - 18.55%)	(16.41% - 17.64%)	(11.63% - 12.64%)	(8.61% - 9.63%)	(5.75% - 6.63%)	(5.34% - 6.11%)	(2.61% - 3.10%)	(1.30% - 1.69%)	(1.17% - 1.58%)	(1.05% - 1.37%)	(0.19% - 0.31%)	(0.00% - 0.02%)
Mauritania	18.06%	15.93%	13.69%	9.93%	6.14%	4.79%	3.87%	2.16%	1.28%	0.73%	0.46%	0.04%
	(17.40% - 18.71%)	(15.33% - 16.57%)	(13.27% - 14.12%)	(9.40% - 10.44%)	(5.68% - 6.59%)	(4.43% - 5.20%)	(3.64% - 4.12%)	(1.93% - 2.39%)	(1.07% - 1.50%)	(0.59% - 0.90%)	(0.38% - 0.55%)	(0.01% - 0.07%)
Niger	21.01%	20.08%	17.07%	13.95%	7.04%	6.71%	4.99%	3.24%	1.52%	1.45%	0.75%	0.24%
	(20.30% - 21.71%)	(19.41% - 20.70%)	(16.54% - 17.61%)	(13.26% - 14.62%)	(6.57% - 7.53%)	(6.28% - 7.12%)	(4.69% - 5.32%)	(2.91% - 3.58%)	(1.32% - 1.76%)	(1.26% - 1.65%)	(0.63% - 0.89%)	(0.17% - 0.32%)
Nigeria	14.52%	16.41%	13.93%	10.26%	4.80%	6.22%	4.92%	2.78%	1.09%	1.80%	1.32%	0.47%
	(13.98% - 15.14%)	(15.85% - 16.99%)	(13.55% - 14.33%)	(9.80% - 10.70%)	(4.47% - 5.21%)	(5.82% - 6.65%)	(4.66% - 5.19%)	(2.56% - 3.02%)	(0.95% - 1.26%)	(1.60% - 2.02%)	(1.20% - 1.45%)	(0.40% - 0.55%)
Sao Tome and Principe	7.06%	6.89%	7.21%	4.34%	1.18%	1.19%	1.34%	0.40%	0.00%	0.00%	0.00%	0.00%
	(6.56% - 7.59%)	(6.43% - 7.36%)	(6.76% - 7.66%)	(4.00% - 4.69%)	(0.98% - 1.38%)	(1.02% - 1.38%)	(1.17% - 1.53%)	(0.31% - 0.49%)	(0.00% - 0.01%)	(0.00% - 0.01%)	(0.00% - 0.02%)	(0.00% - 0.00%)
Senegal	10.38%	10.54%	8.69%	6.38%	2.21%	2.13%	1.27%	0.57%	0.14%	0.13%	0.01%	0.00%
	(9.89% - 10.89%)	(10.06% - 11.03%)	(8.32% - 9.06%)	(6.00% - 6.78%)	(2.00% - 2.43%)	(1.93% - 2.34%)	(1.13% - 1.41%)	(0.46% - 0.70%)	(0.09% - 0.18%)	(0.09% - 0.16%)	(0.00% - 0.02%)	(0.00% - 0.00%)
Sierra Leone	11.29%	13.03%	10.48%	6.58%	3.04%	3.95%	2.78%	1.13%	0.23%	0.58%	0.27%	0.02%
	(10.72% - 11.83%)	(12.49% - 13.57%)	(10.06% - 10.93%)	(6.23% - 6.94%)	(2.75% - 3.34%)	(3.64% - 4.26%)	(2.57% - 3.01%)	(0.99% - 1.28%)	(0.13% - 0.33%)	(0.47% - 0.70%)	(0.22% - 0.32%)	(0.00% - 0.04%)

Table S8c. Estimated overall, severe, and extreme **underweight** prevalence (%) in children under 5 years, both sexes, in 1990, 2000, 2010, and 2020.

Location	Total				Severe				Extreme			
	1990	2000	2010	2020	1990	2000	2010	2020	1990	2000	2010	2020
Global	20.63%	19.96%	17.51%	14.71%	7.98%	8.51%	7.06%	5.45%	2.19%	2.73%	2.08%	1.45%
	(20.46% - 20.80%)	(19.87% - 20.05%)	(17.44% - 17.57%)	(14.64% - 14.79%)	(7.84% - 8.10%)	(8.42% - 8.60%)	(6.98% - 7.13%)	(5.37% - 5.52%)	(2.09% - 2.31%)	(2.63% - 2.84%)	(2.01% - 2.16%)	(1.39% - 1.50%)
Central Europe, Eastern Europe, and Central Asia	4.41%	4.78%	3.44%	2.80%	0.53%	0.67%	0.33%	0.21%	0.03%	0.05%	0.01%	0.00%
	(4.30% - 4.52%)	(4.67% - 4.89%)	(3.35% - 3.52%)	(2.72% - 2.89%)	(0.50% - 0.57%)	(0.63% - 0.71%)	(0.31% - 0.35%)	(0.19% - 0.23%)	(0.03% - 0.04%)	(0.04% - 0.05%)	(0.01% - 0.01%)	(0.00% - 0.01%)
**Central Asia**	8.46%	8.62%	5.82%	4.21%	1.63%	1.70%	0.82%	0.46%	0.12%	0.13%	0.03%	0.01%
	(8.22% - 8.72%)	(8.39% - 8.86%)	(5.66% - 6.00%)	(4.07% - 4.35%)	(1.50% - 1.75%)	(1.59% - 1.81%)	(0.76% - 0.89%)	(0.41% - 0.51%)	(0.09% - 0.14%)	(0.11% - 0.16%)	(0.02% - 0.04%)	(0.00% - 0.01%)
Armenia	3.27%	3.40%	3.07%	2.42%	0.13%	0.17%	0.19%	0.11%	0.00%	0.00%	0.00%	0.00%
	(2.99% - 3.54%)	(3.14% - 3.66%)	(2.87% - 3.31%)	(2.23% - 2.63%)	(0.09% - 0.17%)	(0.13% - 0.22%)	(0.15% - 0.24%)	(0.08% - 0.14%)	(0.00% - 0.00%)	(0.00% - 0.00%)	(0.00% - 0.00%)	(0.00% - 0.00%)
Azerbaijan	11.78%	12.61%	7.32%	6.11%	2.75%	3.20%	1.18%	0.90%	0.24%	0.34%	0.01%	0.00%
	(11.09% - 12.41%)	(11.99% - 13.21%)	(6.88% - 7.74%)	(5.67% - 6.57%)	(2.40% - 3.10%)	(2.87% - 3.54%)	(1.02% - 1.35%)	(0.75% - 1.07%)	(0.16% - 0.32%)	(0.26% - 0.44%)	(0.00% - 0.02%)	(0.00% - 0.01%)
Georgia	2.22%	2.15%	1.47%	1.39%	0.03%	0.03%	0.01%	0.01%	0.00%	0.00%	0.00%	0.00%
	(2.05% - 2.40%)	(1.99% - 2.32%)	(1.37% - 1.58%)	(1.29% - 1.51%)	(0.02% - 0.04%)	(0.02% - 0.04%)	(0.00% - 0.01%)	(0.00% - 0.01%)	(0.00% - 0.00%)	(0.00% - 0.00%)	(0.00% - 0.00%)	(0.00% - 0.00%)
Kazakhstan	4.38%	4.20%	3.58%	2.40%	0.31%	0.30%	0.27%	0.10%	0.00%	0.00%	0.00%	0.00%
	(4.07% - 4.72%)	(3.90% - 4.53%)	(3.34% - 3.83%)	(2.21% - 2.60%)	(0.25% - 0.39%)	(0.24% - 0.37%)	(0.22% - 0.33%)	(0.07% - 0.13%)	(0.00% - 0.00%)	(0.00% - 0.00%)	(0.00% - 0.01%)	(0.00% - 0.00%)
Kyrgyzstan	5.77%	5.35%	3.61%	3.00%	0.62%	0.52%	0.19%	0.12%	0.01%	0.00%	0.00%	0.00%
	(5.38% - 6.21%)	(4.98% - 5.73%)	(3.36% - 3.87%)	(2.75% - 3.26%)	(0.50% - 0.74%)	(0.42% - 0.62%)	(0.14% - 0.24%)	(0.08% - 0.18%)	(0.00% - 0.02%)	(0.00% - 0.01%)	(0.00% - 0.00%)	(0.00% - 0.00%)
Mongolia	8.21%	8.62%	3.84%	2.22%	1.00%	1.29%	0.25%	0.03%	0.00%	0.01%	0.00%	0.00%
	(7.67% - 8.81%)	(8.19% - 9.07%)	(3.59% - 4.09%)	(2.01% - 2.43%)	(0.81% - 1.23%)	(1.11% - 1.48%)	(0.19% - 0.31%)	(0.01% - 0.07%)	(0.00% - 0.01%)	(0.00% - 0.02%)	(0.00% - 0.00%)	(0.00% - 0.00%)
Tajikistan	16.21%	16.90%	13.46%	10.29%	4.37%	4.70%	3.03%	1.94%	0.54%	0.63%	0.24%	0.07%
	(15.41% - 17.00%)	(16.21% - 17.61%)	(12.81% - 14.09%)	(9.63% - 10.94%)	(3.87% - 4.94%)	(4.20% - 5.26%)	(2.68% - 3.40%)	(1.64% - 2.26%)	(0.39% - 0.73%)	(0.48% - 0.81%)	(0.16% - 0.34%)	(0.03% - 0.13%)
Turkmenistan	10.58%	10.29%	6.78%	4.36%	1.99%	2.01%	0.96%	0.41%	0.09%	0.09%	0.01%	0.00%
	(9.93% - 11.25%)	(9.87% - 10.67%)	(6.45% - 7.11%)	(4.06% - 4.65%)	(1.71% - 2.30%)	(1.82% - 2.20%)	(0.85% - 1.08%)	(0.34% - 0.49%)	(0.05% - 0.13%)	(0.06% - 0.12%)	(0.00% - 0.02%)	(0.00% - 0.00%)
Uzbekistan	9.34%	8.16%	5.24%	3.64%	1.89%	1.45%	0.61%	0.28%	0.10%	0.05%	0.00%	0.00%
	(8.76% - 9.92%)	(7.68% - 8.69%)	(4.90% - 5.60%)	(3.38% - 3.93%)	(1.63% - 2.16%)	(1.26% - 1.66%)	(0.51% - 0.72%)	(0.22% - 0.35%)	(0.06% - 0.16%)	(0.03% - 0.08%)	(0.00% - 0.01%)	(0.00% - 0.00%)
**Central Europe**	2.70%	2.59%	1.96%	1.71%	0.16%	0.18%	0.06%	0.04%	0.01%	0.01%	0.00%	0.00%
	(2.61% - 2.78%)	(2.51% - 2.67%)	(1.90% - 2.03%)	(1.65% - 1.77%)	(0.14% - 0.18%)	(0.17% - 0.20%)	(0.06% - 0.07%)	(0.03% - 0.04%)	(0.00% - 0.01%)	(0.01% - 0.02%)	(0.00% - 0.00%)	(0.00% - 0.00%)
Albania	11.76%	11.25%	5.99%	3.67%	2.44%	2.66%	0.90%	0.31%	0.14%	0.24%	0.02%	0.00%
	(11.08% - 12.47%)	(10.65% - 11.80%)	(5.60% - 6.37%)	(3.41% - 3.94%)	(2.07% - 2.81%)	(2.34% - 2.97%)	(0.77% - 1.04%)	(0.25% - 0.38%)	(0.07% - 0.23%)	(0.15% - 0.33%)	(0.00% - 0.03%)	(0.00% - 0.00%)
Bosnia and Herzegovina	3.64%	2.83%	1.88%	1.76%	0.21%	0.16%	0.05%	0.04%	0.00%	0.00%	0.00%	0.00%
	(3.37% - 3.93%)	(2.62% - 3.04%)	(1.75% - 2.04%)	(1.63% - 1.92%)	(0.16% - 0.27%)	(0.12% - 0.20%)	(0.04% - 0.07%)	(0.03% - 0.05%)	(0.00% - 0.01%)	(0.00% - 0.01%)	(0.00% - 0.00%)	(0.00% - 0.00%)
Bulgaria	1.98%	2.82%	2.40%	2.10%	0.01%	0.10%	0.07%	0.05%	0.00%	0.00%	0.00%	0.00%
	(1.80% - 2.23%)	(2.49% - 3.11%)	(2.13% - 2.64%)	(1.85% - 2.38%)	(0.01% - 0.03%)	(0.06% - 0.14%)	(0.04% - 0.11%)	(0.03% - 0.08%)	(0.00% - 0.00%)	(0.00% - 0.00%)	(0.00% - 0.00%)	(0.00% - 0.00%)
Croatia	2.44%	2.67%	2.05%	1.90%	0.05%	0.11%	0.05%	0.04%	0.00%	0.00%	0.00%	0.00%
	(2.23% - 2.65%)	(2.45% - 2.90%)	(1.87% - 2.23%)	(1.74% - 2.08%)	(0.02% - 0.07%)	(0.07% - 0.15%)	(0.03% - 0.08%)	(0.02% - 0.06%)	(0.00% - 0.00%)	(0.00% - 0.00%)	(0.00% - 0.00%)	(0.00% - 0.00%)
Czechia	1.05%	1.25%	1.21%	1.13%	0.00%	0.02%	0.02%	0.01%	0.00%	0.00%	0.00%	0.00%
	(0.97% - 1.14%)	(1.16% - 1.34%)	(1.13% - 1.30%)	(1.05% - 1.23%)	(0.00% - 0.00%)	(0.01% - 0.02%)	(0.01% - 0.03%)	(0.01% - 0.02%)	(0.00% - 0.00%)	(0.00% - 0.00%)	(0.00% - 0.00%)	(0.00% - 0.00%)
Hungary	1.40%	1.37%	1.30%	1.23%	0.02%	0.02%	0.02%	0.01%	0.00%	0.00%	0.00%	0.00%
	(1.30% - 1.50%)	(1.27% - 1.48%)	(1.20% - 1.41%)	(1.14% - 1.34%)	(0.01% - 0.02%)	(0.01% - 0.03%)	(0.01% - 0.02%)	(0.01% - 0.02%)	(0.00% - 0.00%)	(0.00% - 0.00%)	(0.00% - 0.00%)	(0.00% - 0.00%)
North Macedonia	1.91%	1.78%	1.68%	1.59%	0.01%	0.01%	0.02%	0.02%	0.00%	0.00%	0.00%	0.00%
	(1.75% - 2.08%)	(1.63% - 1.96%)	(1.53% - 1.83%)	(1.42% - 1.75%)	(0.00% - 0.01%)	(0.00% - 0.02%)	(0.00% - 0.05%)	(0.00% - 0.04%)	(0.00% - 0.00%)	(0.00% - 0.00%)	(0.00% - 0.00%)	(0.00% - 0.00%)
Montenegro	1.13%	1.62%	1.91%	2.44%	0.00%	0.00%	0.01%	0.06%	0.00%	0.00%	0.00%	0.00%
	(1.03% - 1.23%)	(1.49% - 1.76%)	(1.75% - 2.07%)	(2.24% - 2.65%)	(0.00% - 0.00%)	(0.00% - 0.00%)	(0.00% - 0.03%)	(0.03% - 0.09%)	(0.00% - 0.00%)	(0.00% - 0.00%)	(0.00% - 0.00%)	(0.00% - 0.00%)
Poland	2.06%	2.10%	1.73%	1.53%	0.01%	0.04%	0.02%	0.01%	0.00%	0.00%	0.00%	0.00%
	(1.90% - 2.25%)	(1.92% - 2.28%)	(1.60% - 1.89%)	(1.41% - 1.65%)	(0.00% - 0.02%)	(0.02% - 0.06%)	(0.01% - 0.03%)	(0.00% - 0.02%)	(0.00% - 0.00%)	(0.00% - 0.00%)	(0.00% - 0.00%)	(0.00% - 0.00%)
Romania	3.78%	2.99%	2.42%	2.19%	0.17%	0.12%	0.09%	0.08%	0.00%	0.00%	0.00%	0.00%
	(3.50% - 4.04%)	(2.81% - 3.18%)	(2.26% - 2.60%)	(2.03% - 2.34%)	(0.13% - 0.21%)	(0.10% - 0.15%)	(0.07% - 0.12%)	(0.06% - 0.10%)	(0.00% - 0.00%)	(0.00% - 0.00%)	(0.00% - 0.00%)	(0.00% - 0.00%)

Table S8c. Estimated overall, severe, and extreme **underweight** prevalence (%) in children under 5 years, both sexes, in 1990, 2000, 2010, and 2020.												
**Location**	**Total**				**Severe**				**Extreme**			
	**1990**	**2000**	**2010**	**2020**	**1990**	**2000**	**2010**	**2020**	**1990**	**2000**	**2010**	**2020**
	0.77%	1.12%	1.61%	1.39%	0.00%	0.00%	0.01%	0.00%	0.00%	0.00%	0.00%	0.00%
Serbia	(0.71% - 0.84%)	(1.03% - 1.22%)	(1.49% - 1.73%)	(1.28% - 1.51%)	(0.00% - 0.00%)	(0.00% - 0.00%)	(0.00% - 0.01%)	(0.00% - 0.01%)	(0.00% - 0.00%)	(0.00% - 0.00%)	(0.00% - 0.00%)	(0.00% - 0.00%)
	2.37%	2.61%	2.26%	2.01%	0.03%	0.09%	0.07%	0.04%	0.00%	0.00%	0.00%	0.00%
Slovakia	(2.18% - 2.56%)	(2.40% - 2.83%)	(2.09% - 2.45%)	(1.86% - 2.19%)	(0.01% - 0.05%)	(0.06% - 0.13%)	(0.04% - 0.10%)	(0.02% - 0.06%)	(0.00% - 0.00%)	(0.00% - 0.00%)	(0.00% - 0.00%)	(0.00% - 0.00%)
	2.12%	2.23%	1.78%	1.64%	0.03%	0.07%	0.04%	0.03%	0.00%	0.00%	0.00%	0.00%
Slovenia	(1.94% - 2.32%)	(2.05% - 2.42%)	(1.62% - 1.94%)	(1.51% - 1.79%)	(0.01% - 0.05%)	(0.04% - 0.10%)	(0.02% - 0.06%)	(0.01% - 0.04%)	(0.00% - 0.00%)	(0.00% - 0.00%)	(0.00% - 0.00%)	(0.00% - 0.00%)
	3.06%	3.00%	2.38%	2.13%	0.12%	0.13%	0.09%	0.08%	0.00%	0.00%	0.00%	0.00%
**Eastern Europe**	(2.88% - 3.26%)	(2.83% - 3.18%)	(2.23% - 2.53%)	(1.98% - 2.29%)	(0.10% - 0.16%)	(0.11% - 0.17%)	(0.07% - 0.12%)	(0.06% - 0.11%)	(0.00% - 0.00%)	(0.00% - 0.00%)	(0.00% - 0.00%)	(0.00% - 0.00%)
	1.64%	1.69%	1.55%	1.27%	0.07%	0.09%	0.09%	0.07%	0.00%	0.00%	0.00%	0.00%
Belarus	(1.53% - 1.75%)	(1.59% - 1.81%)	(1.44% - 1.65%)	(1.18% - 1.36%)	(0.05% - 0.10%)	(0.07% - 0.12%)	(0.07% - 0.11%)	(0.05% - 0.09%)	(0.00% - 0.00%)	(0.00% - 0.00%)	(0.00% - 0.00%)	(0.00% - 0.00%)
	2.91%	2.35%	1.59%	1.47%	0.09%	0.06%	0.02%	0.01%	0.00%	0.00%	0.00%	0.00%
Estonia	(2.66% - 3.18%)	(2.15% - 2.57%)	(1.47% - 1.74%)	(1.35% - 1.61%)	(0.06% - 0.14%)	(0.04% - 0.09%)	(0.01% - 0.03%)	(0.01% - 0.02%)	(0.00% - 0.00%)	(0.00% - 0.00%)	(0.00% - 0.00%)	(0.00% - 0.00%)
	2.80%	2.62%	1.87%	1.63%	0.07%	0.08%	0.02%	0.01%	0.00%	0.00%	0.00%	0.00%
Latvia	(2.56% - 3.04%)	(2.39% - 2.88%)	(1.71% - 2.07%)	(1.49% - 1.79%)	(0.04% - 0.11%)	(0.05% - 0.12%)	(0.01% - 0.04%)	(0.01% - 0.03%)	(0.00% - 0.00%)	(0.00% - 0.00%)	(0.00% - 0.00%)	(0.00% - 0.00%)
	2.54%	2.14%	1.59%	1.40%	0.06%	0.04%	0.01%	0.01%	0.00%	0.00%	0.00%	0.00%
Lithuania	(2.31% - 2.78%)	(1.94% - 2.36%)	(1.45% - 1.74%)	(1.28% - 1.54%)	(0.03% - 0.09%)	(0.02% - 0.06%)	(0.01% - 0.02%)	(0.00% - 0.01%)	(0.00% - 0.00%)	(0.00% - 0.00%)	(0.00% - 0.00%)	(0.00% - 0.00%)
	2.82%	2.88%	2.45%	2.05%	0.06%	0.08%	0.05%	0.03%	0.00%	0.00%	0.00%	0.00%
Republic of Moldova	(2.55% - 3.10%)	(2.62% - 3.15%)	(2.26% - 2.68%)	(1.87% - 2.25%)	(0.02% - 0.12%)	(0.04% - 0.13%)	(0.02% - 0.09%)	(0.01% - 0.06%)	(0.00% - 0.00%)	(0.00% - 0.00%)	(0.00% - 0.00%)	(0.00% - 0.00%)
	3.49%	3.34%	2.61%	2.30%	0.15%	0.16%	0.12%	0.10%	0.00%	0.00%	0.00%	0.00%
Russian Federation	(3.23% - 3.78%)	(3.10% - 3.60%)	(2.41% - 2.82%)	(2.11% - 2.51%)	(0.11% - 0.21%)	(0.12% - 0.21%)	(0.08% - 0.15%)	(0.07% - 0.13%)	(0.00% - 0.00%)	(0.00% - 0.00%)	(0.00% - 0.00%)	(0.00% - 0.00%)
	2.14%	2.38%	1.84%	1.65%	0.05%	0.08%	0.03%	0.02%	0.00%	0.00%	0.00%	0.00%
Ukraine	(1.99% - 2.32%)	(2.20% - 2.58%)	(1.69% - 2.02%)	(1.53% - 1.80%)	(0.04% - 0.07%)	(0.06% - 0.11%)	(0.02% - 0.05%)	(0.01% - 0.04%)	(0.00% - 0.00%)	(0.00% - 0.00%)	(0.00% - 0.00%)	(0.00% - 0.00%)
High-income	1.60%	1.43%	1.28%	1.21%	0.03%	0.03%	0.03%	0.02%	0.00%	0.00%	0.00%	0.00%
	(1.57% - 1.63%)	(1.40% - 1.46%)	(1.26% - 1.31%)	(1.18% - 1.23%)	(0.03% - 0.03%)	(0.03% - 0.03%)	(0.02% - 0.03%)	(0.02% - 0.03%)	(0.00% - 0.00%)	(0.00% - 0.00%)	(0.00% - 0.00%)	(0.00% - 0.00%)
	0.31%	0.27%	0.24%	0.23%	0.00%	0.00%	0.00%	0.00%	0.00%	0.00%	0.00%	0.00%
**Australasia**	(0.26% - 0.34%)	(0.21% - 0.31%)	(0.18% - 0.27%)	(0.18% - 0.26%)	(0.00% - 0.00%)	(0.00% - 0.00%)	(0.00% - 0.00%)	(0.00% - 0.00%)	(0.00% - 0.00%)	(0.00% - 0.00%)	(0.00% - 0.00%)	(0.00% - 0.00%)
	0.23%	0.21%	0.19%	0.19%	0.00%	0.00%	0.00%	0.00%	0.00%	0.00%	0.00%	0.00%
Australia	(0.16% - 0.26%)	(0.13% - 0.25%)	(0.12% - 0.23%)	(0.13% - 0.22%)	(0.00% - 0.00%)	(0.00% - 0.00%)	(0.00% - 0.00%)	(0.00% - 0.00%)	(0.00% - 0.00%)	(0.00% - 0.00%)	(0.00% - 0.00%)	(0.00% - 0.00%)
	0.70%	0.58%	0.46%	0.43%	0.01%	0.00%	0.00%	0.00%	0.00%	0.00%	0.00%	0.00%
New Zealand	(0.65% - 0.75%)	(0.52% - 0.63%)	(0.41% - 0.50%)	(0.38% - 0.46%)	(0.00% - 0.01%)	(0.00% - 0.01%)	(0.00% - 0.00%)	(0.00% - 0.00%)	(0.00% - 0.00%)	(0.00% - 0.00%)	(0.00% - 0.00%)	(0.00% - 0.00%)
**High-income Asia Pacific**	3.27%	2.90%	2.93%	2.81%	0.08%	0.09%	0.11%	0.11%	0.00%	0.00%	0.00%	0.00%
	(3.22% - 3.33%)	(2.85% - 2.95%)	(2.88% - 2.98%)	(2.76% - 2.86%)	(0.08% - 0.09%)	(0.08% - 0.09%)	(0.10% - 0.11%)	(0.10% - 0.11%)	(0.00% - 0.00%)	(0.00% - 0.00%)	(0.00% - 0.00%)	(0.00% - 0.00%)
	10.54%	9.47%	8.83%	8.47%	2.00%	1.82%	1.71%	1.66%	0.20%	0.18%	0.17%	0.16%
Brunei Darussalam	(9.86% - 11.24%)	(8.89% - 10.10%)	(8.31% - 9.39%)	(7.94% - 9.05%)	(1.73% - 2.28%)	(1.59% - 2.06%)	(1.52% - 1.94%)	(1.47% - 1.89%)	(0.15% - 0.26%)	(0.13% - 0.23%)	(0.12% - 0.22%)	(0.12% - 0.21%)
	3.98%	3.67%	3.62%	3.47%	0.11%	0.12%	0.14%	0.14%	0.00%	0.00%	0.00%	0.00%
Japan	(3.91% - 4.04%)	(3.61% - 3.73%)	(3.57% - 3.68%)	(3.40% - 3.53%)	(0.10% - 0.12%)	(0.11% - 0.13%)	(0.13% - 0.15%)	(0.13% - 0.15%)	(0.00% - 0.00%)	(0.00% - 0.00%)	(0.00% - 0.00%)	(0.00% - 0.00%)
Hokkaidō	5.84%	5.31%	5.27%	5.05%	0.39%	0.40%	0.45%	0.43%	0.00%	0.00%	0.00%	0.00%
	(5.42% - 6.35%)	(4.88% - 5.73%)	(4.87% - 5.65%)	(4.66% - 5.44%)	(0.30% - 0.51%)	(0.31% - 0.51%)	(0.36% - 0.55%)	(0.34% - 0.54%)	(0.00% - 0.00%)	(0.00% - 0.00%)	(0.00% - 0.00%)	(0.00% - 0.00%)
	3.97%	3.69%	3.57%	3.36%	0.10%	0.13%	0.14%	0.14%	0.00%	0.00%	0.00%	0.00%
Aomori	(3.62% - 4.33%)	(3.38% - 4.02%)	(3.28% - 3.90%)	(3.08% - 3.64%)	(0.07% - 0.15%)	(0.09% - 0.17%)	(0.10% - 0.20%)	(0.10% - 0.19%)	(0.00% - 0.00%)	(0.00% - 0.00%)	(0.00% - 0.00%)	(0.00% - 0.00%)
	3.84%	3.55%	3.52%	3.31%	0.09%	0.11%	0.13%	0.12%	0.00%	0.00%	0.00%	0.00%
Iwate	(3.54% - 4.18%)	(3.26% - 3.87%)	(3.26% - 3.82%)	(3.03% - 3.59%)	(0.05% - 0.13%)	(0.07% - 0.16%)	(0.09% - 0.18%)	(0.08% - 0.17%)	(0.00% - 0.00%)	(0.00% - 0.00%)	(0.00% - 0.00%)	(0.00% - 0.00%)
	3.56%	3.37%	3.37%	3.19%	0.06%	0.08%	0.10%	0.10%	0.00%	0.00%	0.00%	0.00%
Miyagi	(3.24% - 3.87%)	(3.07% - 3.67%)	(3.09% - 3.67%)	(2.91% - 3.46%)	(0.04% - 0.10%)	(0.05% - 0.12%)	(0.07% - 0.15%)	(0.06% - 0.14%)	(0.00% - 0.00%)	(0.00% - 0.00%)	(0.00% - 0.00%)	(0.00% - 0.00%)
	3.75%	3.49%	3.47%	3.32%	0.05%	0.06%	0.08%	0.08%	0.00%	0.00%	0.00%	0.00%
Akita	(3.44% - 4.12%)	(3.19% - 3.83%)	(3.16% - 3.78%)	(3.02% - 3.62%)	(0.03% - 0.07%)	(0.04% - 0.10%)	(0.05% - 0.12%)	(0.05% - 0.11%)	(0.00% - 0.00%)	(0.00% - 0.00%)	(0.00% - 0.00%)	(0.00% - 0.00%)

Table S8c. Estimated overall, severe, and extreme **underweight** prevalence (%) in children under 5 years, both sexes, in 1990, 2000, 2010, and 2020.

Location	Total				Severe				Extreme			
	1990	2000	2010	2020	1990	2000	2010	2020	1990	2000	2010	2020
Yamagata	3.61%	3.45%	3.43%	3.28%	0.07%	0.09%	0.11%	0.11%	0.00%	0.00%	0.00%	0.00%
	(3.28% - 3.93%)	(3.16% - 3.77%)	(3.14% - 3.73%)	(3.01% - 3.57%)	(0.04% - 0.11%)	(0.06% - 0.13%)	(0.08% - 0.16%)	(0.07% - 0.16%)	(0.00% - 0.00%)	(0.00% - 0.00%)	(0.00% - 0.00%)	(0.00% - 0.00%)
Fukushima	1.89%	1.73%	1.68%	1.60%	0.00%	0.00%	0.01%	0.00%	0.00%	0.00%	0.00%	0.00%
	(1.75% - 2.06%)	(1.59% - 1.87%)	(1.56% - 1.82%)	(1.47% - 1.72%)	(0.00% - 0.01%)	(0.00% - 0.01%)	(0.00% - 0.01%)	(0.00% - 0.01%)	(0.00% - 0.00%)	(0.00% - 0.00%)	(0.00% - 0.00%)	(0.00% - 0.00%)
Ibaraki	3.43%	3.16%	3.08%	2.92%	0.04%	0.04%	0.06%	0.05%	0.00%	0.00%	0.00%	0.00%
	(3.11% - 3.81%)	(2.87% - 3.47%)	(2.78% - 3.37%)	(2.62% - 3.22%)	(0.02% - 0.07%)	(0.02% - 0.08%)	(0.03% - 0.09%)	(0.03% - 0.09%)	(0.00% - 0.00%)	(0.00% - 0.00%)	(0.00% - 0.00%)	(0.00% - 0.00%)
Tochigi	4.22%	3.89%	3.80%	3.60%	0.09%	0.11%	0.13%	0.12%	0.00%	0.00%	0.00%	0.00%
	(3.85% - 4.58%)	(3.55% - 4.22%)	(3.48% - 4.13%)	(3.32% - 3.92%)	(0.06% - 0.14%)	(0.07% - 0.16%)	(0.09% - 0.18%)	(0.08% - 0.18%)	(0.00% - 0.00%)	(0.00% - 0.00%)	(0.00% - 0.00%)	(0.00% - 0.00%)
Gunma	3.51%	3.25%	3.20%	3.02%	0.04%	0.04%	0.05%	0.05%	0.00%	0.00%	0.00%	0.00%
	(3.24% - 3.80%)	(2.97% - 3.56%)	(2.91% - 3.47%)	(2.77% - 3.30%)	(0.02% - 0.05%)	(0.02% - 0.06%)	(0.03% - 0.07%)	(0.03% - 0.07%)	(0.00% - 0.00%)	(0.00% - 0.00%)	(0.00% - 0.00%)	(0.00% - 0.00%)
Saitama	3.65%	3.35%	3.31%	3.16%	0.04%	0.04%	0.05%	0.05%	0.00%	0.00%	0.00%	0.00%
	(3.34% - 3.99%)	(3.09% - 3.66%)	(3.03% - 3.60%)	(2.91% - 3.45%)	(0.02% - 0.06%)	(0.03% - 0.06%)	(0.04% - 0.08%)	(0.03% - 0.08%)	(0.00% - 0.00%)	(0.00% - 0.00%)	(0.00% - 0.00%)	(0.00% - 0.00%)
Chiba	2.93%	2.72%	2.69%	2.58%	0.02%	0.02%	0.03%	0.03%	0.00%	0.00%	0.00%	0.00%
	(2.69% - 3.19%)	(2.51% - 2.97%)	(2.48% - 2.92%)	(2.36% - 2.80%)	(0.01% - 0.03%)	(0.01% - 0.04%)	(0.02% - 0.04%)	(0.02% - 0.04%)	(0.00% - 0.00%)	(0.00% - 0.00%)	(0.00% - 0.00%)	(0.00% - 0.00%)
Tōkyō	4.20%	3.95%	3.87%	3.72%	0.14%	0.16%	0.18%	0.18%	0.00%	0.00%	0.00%	0.00%
	(3.87% - 4.58%)	(3.64% - 4.25%)	(3.60% - 4.16%)	(3.43% - 4.00%)	(0.10% - 0.20%)	(0.11% - 0.21%)	(0.14% - 0.24%)	(0.13% - 0.23%)	(0.00% - 0.00%)	(0.00% - 0.00%)	(0.00% - 0.00%)	(0.00% - 0.00%)
Kanagawa	4.65%	4.33%	4.29%	4.14%	0.16%	0.18%	0.21%	0.21%	0.00%	0.00%	0.00%	0.00%
	(4.27% - 5.03%)	(3.98% - 4.67%)	(4.00% - 4.63%)	(3.82% - 4.50%)	(0.11% - 0.23%)	(0.13% - 0.24%)	(0.16% - 0.28%)	(0.15% - 0.28%)	(0.00% - 0.00%)	(0.00% - 0.00%)	(0.00% - 0.00%)	(0.00% - 0.00%)
Niigata	6.50%	6.02%	6.00%	5.75%	0.45%	0.47%	0.52%	0.50%	0.00%	0.00%	0.00%	0.00%
	(6.00% - 7.05%)	(5.55% - 6.51%)	(5.52% - 6.48%)	(5.27% - 6.24%)	(0.34% - 0.59%)	(0.35% - 0.60%)	(0.40% - 0.64%)	(0.38% - 0.64%)	(0.00% - 0.00%)	(0.00% - 0.00%)	(0.00% - 0.00%)	(0.00% - 0.00%)
Toyama	3.51%	3.31%	3.30%	3.15%	0.08%	0.11%	0.13%	0.12%	0.00%	0.00%	0.00%	0.00%
	(3.20% - 3.81%)	(3.03% - 3.61%)	(3.02% - 3.59%)	(2.89% - 3.43%)	(0.05% - 0.13%)	(0.07% - 0.16%)	(0.08% - 0.18%)	(0.07% - 0.17%)	(0.00% - 0.00%)	(0.00% - 0.00%)	(0.00% - 0.00%)	(0.00% - 0.00%)
Ishikawa	3.52%	3.33%	3.34%	3.22%	0.06%	0.08%	0.11%	0.10%	0.00%	0.00%	0.00%	0.00%
	(3.20% - 3.85%)	(3.03% - 3.62%)	(3.07% - 3.64%)	(2.93% - 3.51%)	(0.03% - 0.10%)	(0.05% - 0.13%)	(0.07% - 0.16%)	(0.06% - 0.16%)	(0.00% - 0.00%)	(0.00% - 0.00%)	(0.00% - 0.00%)	(0.00% - 0.00%)
Fukui	3.52%	3.33%	3.30%	3.19%	0.07%	0.09%	0.11%	0.11%	0.00%	0.00%	0.00%	0.00%
	(3.20% - 3.84%)	(3.05% - 3.61%)	(3.03% - 3.58%)	(2.92% - 3.48%)	(0.03% - 0.11%)	(0.05% - 0.13%)	(0.07% - 0.16%)	(0.07% - 0.16%)	(0.00% - 0.00%)	(0.00% - 0.00%)	(0.00% - 0.00%)	(0.00% - 0.00%)
Yamanashi	3.59%	3.41%	3.35%	3.22%	0.09%	0.11%	0.13%	0.13%	0.00%	0.00%	0.00%	0.00%
	(3.26% - 3.92%)	(3.13% - 3.70%)	(3.09% - 3.65%)	(2.96% - 3.49%)	(0.05% - 0.13%)	(0.07% - 0.16%)	(0.09% - 0.18%)	(0.08% - 0.18%)	(0.00% - 0.00%)	(0.00% - 0.00%)	(0.00% - 0.00%)	(0.00% - 0.00%)
Nagano	3.42%	3.27%	3.27%	3.14%	0.02%	0.03%	0.05%	0.04%	0.00%	0.00%	0.00%	0.00%
	(3.11% - 3.74%)	(2.96% - 3.57%)	(2.97% - 3.55%)	(2.85% - 3.45%)	(0.00% - 0.04%)	(0.01% - 0.06%)	(0.02% - 0.08%)	(0.02% - 0.08%)	(0.00% - 0.00%)	(0.00% - 0.00%)	(0.00% - 0.00%)	(0.00% - 0.00%)
Gifu	3.59%	3.42%	3.38%	3.23%	0.09%	0.12%	0.14%	0.13%	0.00%	0.00%	0.00%	0.00%
	(3.27% - 3.91%)	(3.14% - 3.72%)	(3.11% - 3.64%)	(2.95% - 3.52%)	(0.05% - 0.15%)	(0.08% - 0.17%)	(0.09% - 0.19%)	(0.09% - 0.19%)	(0.00% - 0.00%)	(0.00% - 0.00%)	(0.00% - 0.00%)	(0.00% - 0.00%)
Shizuoka	6.02%	5.53%	5.42%	5.20%	0.36%	0.38%	0.41%	0.40%	0.00%	0.00%	0.00%	0.00%
	(5.54% - 6.55%)	(5.11% - 6.00%)	(5.00% - 5.88%)	(4.78% - 5.62%)	(0.26% - 0.47%)	(0.29% - 0.49%)	(0.32% - 0.53%)	(0.31% - 0.50%)	(0.00% - 0.00%)	(0.00% - 0.00%)	(0.00% - 0.00%)	(0.00% - 0.00%)
Aichi	3.56%	3.33%	3.24%	3.05%	0.03%	0.04%	0.05%	0.04%	0.00%	0.00%	0.00%	0.00%
	(3.26% - 3.87%)	(3.07% - 3.63%)	(2.96% - 3.51%)	(2.79% - 3.31%)	(0.01% - 0.05%)	(0.02% - 0.06%)	(0.03% - 0.07%)	(0.03% - 0.07%)	(0.00% - 0.00%)	(0.00% - 0.00%)	(0.00% - 0.00%)	(0.00% - 0.00%)
Mie	3.62%	3.41%	3.33%	3.15%	0.09%	0.11%	0.13%	0.12%	0.00%	0.00%	0.00%	0.00%
	(3.30% - 3.93%)	(3.12% - 3.70%)	(3.05% - 3.61%)	(2.89% - 3.43%)	(0.05% - 0.14%)	(0.07% - 0.16%)	(0.08% - 0.19%)	(0.08% - 0.18%)	(0.00% - 0.00%)	(0.00% - 0.00%)	(0.00% - 0.00%)	(0.00% - 0.00%)
Shiga	2.70%	2.49%	2.46%	2.35%	0.02%	0.03%	0.03%	0.03%	0.00%	0.00%	0.00%	0.00%
	(2.49% - 2.94%)	(2.30% - 2.70%)	(2.28% - 2.67%)	(2.16% - 2.54%)	(0.01% - 0.04%)	(0.01% - 0.04%)	(0.02% - 0.05%)	(0.02% - 0.05%)	(0.00% - 0.00%)	(0.00% - 0.00%)	(0.00% - 0.00%)	(0.00% - 0.00%)
Kyōto	4.19%	3.88%	3.83%	3.66%	0.09%	0.10%	0.13%	0.12%	0.00%	0.00%	0.00%	0.00%
	(3.83% - 4.53%)	(3.56% - 4.22%)	(3.48% - 4.18%)	(3.35% - 3.99%)	(0.06% - 0.13%)	(0.07% - 0.15%)	(0.09% - 0.18%)	(0.08% - 0.17%)	(0.00% - 0.00%)	(0.00% - 0.00%)	(0.00% - 0.00%)	(0.00% - 0.00%)
Ōsaka	4.49%	4.08%	3.99%	3.82%	0.12%	0.14%	0.17%	0.16%	0.00%	0.00%	0.00%	0.00%
	(4.11% - 4.87%)	(3.75% - 4.41%)	(3.67% - 4.31%)	(3.50% - 4.12%)	(0.08% - 0.18%)	(0.09% - 0.20%)	(0.12% - 0.22%)	(0.11% - 0.22%)	(0.00% - 0.00%)	(0.00% - 0.00%)	(0.00% - 0.00%)	(0.00% - 0.00%)
Hyōgo	3.32%	3.02%	2.99%	2.86%	0.04%	0.05%	0.06%	0.05%	0.00%	0.00%	0.00%	0.00%
	(3.02% - 3.60%)	(2.77% - 3.26%)	(2.74% - 3.25%)	(2.62% - 3.12%)	(0.02% - 0.06%)	(0.03% - 0.07%)	(0.04% - 0.08%)	(0.04% - 0.08%)	(0.00% - 0.00%)	(0.00% - 0.00%)	(0.00% - 0.00%)	(0.00% - 0.00%)

Table S8c. Estimated overall, severe, and extreme **underweight** prevalence (%) in children under 5 years, both sexes, in 1990, 2000, 2010, and 2020.

Location	Total				Severe				Extreme			
	1990	2000	2010	2020	1990	2000	2010	2020	1990	2000	2010	2020
Nara	3.70% (3.39% - 4.03%)	3.49% (3.19% - 3.81%)	3.48% (3.17% - 3.77%)	3.34% (3.05% - 3.64%)	0.08% (0.05% - 0.12%)	0.11% (0.07% - 0.15%)	0.13% (0.08% - 0.17%)	0.12% (0.08% - 0.17%)	0.00% (0.00% - 0.00%)	0.00% (0.00% - 0.00%)	0.00% (0.00% - 0.00%)	0.00% (0.00% - 0.00%)
Wakayama	3.71% (3.37% - 4.07%)	3.50% (3.19% - 3.82%)	3.41% (3.12% - 3.71%)	3.24% (2.95% - 3.53%)	0.03% (0.01% - 0.07%)	0.05% (0.02% - 0.09%)	0.07% (0.03% - 0.11%)	0.06% (0.03% - 0.11%)	0.00% (0.00% - 0.00%)	0.00% (0.00% - 0.00%)	0.00% (0.00% - 0.00%)	0.00% (0.00% - 0.00%)
Tottori	3.68% (3.38% - 4.00%)	3.47% (3.19% - 3.77%)	3.48% (3.20% - 3.76%)	3.35% (3.08% - 3.65%)	0.10% (0.06% - 0.15%)	0.13% (0.08% - 0.18%)	0.15% (0.10% - 0.21%)	0.15% (0.10% - 0.21%)	0.00% (0.00% - 0.00%)	0.00% (0.00% - 0.00%)	0.00% (0.00% - 0.00%)	0.00% (0.00% - 0.00%)
Shimane	3.72% (3.41% - 4.06%)	3.49% (3.19% - 3.79%)	3.44% (3.18% - 3.74%)	3.27% (3.00% - 3.54%)	0.12% (0.08% - 0.17%)	0.14% (0.09% - 0.20%)	0.16% (0.12% - 0.22%)	0.15% (0.11% - 0.21%)	0.00% (0.00% - 0.00%)	0.00% (0.00% - 0.00%)	0.00% (0.00% - 0.00%)	0.00% (0.00% - 0.00%)
Okayama	1.15% (1.06% - 1.24%)	1.07% (0.99% - 1.18%)	1.06% (0.98% - 1.15%)	1.00% (0.93% - 1.08%)	0.00% (0.00% - 0.00%)	0.00% (0.00% - 0.00%)	0.00% (0.00% - 0.00%)	0.00% (0.00% - 0.00%)	0.00% (0.00% - 0.00%)	0.00% (0.00% - 0.00%)	0.00% (0.00% - 0.00%)	0.00% (0.00% - 0.00%)
Hiroshima	2.83% (2.58% - 3.08%)	2.62% (2.39% - 2.84%)	2.59% (2.39% - 2.81%)	2.47% (2.28% - 2.69%)	0.03% (0.01% - 0.04%)	0.03% (0.02% - 0.04%)	0.04% (0.03% - 0.05%)	0.03% (0.02% - 0.05%)	0.00% (0.00% - 0.00%)	0.00% (0.00% - 0.00%)	0.00% (0.00% - 0.00%)	0.00% (0.00% - 0.00%)
Yamaguchi	3.58% (3.26% - 3.92%)	3.38% (3.08% - 3.69%)	3.33% (3.05% - 3.61%)	3.16% (2.89% - 3.44%)	0.04% (0.02% - 0.07%)	0.06% (0.03% - 0.10%)	0.08% (0.04% - 0.12%)	0.07% (0.04% - 0.11%)	0.00% (0.00% - 0.00%)	0.00% (0.00% - 0.00%)	0.00% (0.00% - 0.00%)	0.00% (0.00% - 0.00%)
Tokushima	3.76% (3.43% - 4.10%)	3.52% (3.22% - 3.82%)	3.42% (3.14% - 3.73%)	3.22% (2.95% - 3.49%)	0.04% (0.02% - 0.07%)	0.06% (0.03% - 0.10%)	0.08% (0.04% - 0.12%)	0.07% (0.04% - 0.11%)	0.00% (0.00% - 0.00%)	0.00% (0.00% - 0.00%)	0.00% (0.00% - 0.00%)	0.00% (0.00% - 0.00%)
Kagawa	3.56% (3.26% - 3.89%)	3.39% (3.10% - 3.70%)	3.37% (3.10% - 3.64%)	3.21% (2.94% - 3.47%)	0.06% (0.03% - 0.11%)	0.09% (0.05% - 0.14%)	0.11% (0.07% - 0.15%)	0.10% (0.06% - 0.14%)	0.00% (0.00% - 0.00%)	0.00% (0.00% - 0.00%)	0.00% (0.00% - 0.00%)	0.00% (0.00% - 0.00%)
Ehime	3.71% (3.39% - 4.03%)	3.47% (3.17% - 3.76%)	3.45% (3.17% - 3.75%)	3.28% (3.01% - 3.55%)	0.07% (0.05% - 0.11%)	0.09% (0.06% - 0.14%)	0.11% (0.08% - 0.16%)	0.11% (0.07% - 0.15%)	0.00% (0.00% - 0.00%)	0.00% (0.00% - 0.00%)	0.00% (0.00% - 0.00%)	0.00% (0.00% - 0.00%)
Kōchi	3.86% (3.53% - 4.21%)	3.62% (3.32% - 3.97%)	3.57% (3.27% - 3.86%)	3.36% (3.06% - 3.65%)	0.11% (0.06% - 0.16%)	0.13% (0.09% - 0.19%)	0.16% (0.11% - 0.22%)	0.15% (0.10% - 0.21%)	0.00% (0.00% - 0.00%)	0.00% (0.00% - 0.00%)	0.00% (0.00% - 0.00%)	0.00% (0.00% - 0.00%)
Fukuoka	4.55% (4.16% - 4.94%)	4.16% (3.83% - 4.50%)	4.10% (3.79% - 4.43%)	3.92% (3.60% - 4.25%)	0.09% (0.06% - 0.14%)	0.11% (0.07% - 0.16%)	0.14% (0.10% - 0.20%)	0.14% (0.09% - 0.19%)	0.00% (0.00% - 0.00%)	0.00% (0.00% - 0.00%)	0.00% (0.00% - 0.00%)	0.00% (0.00% - 0.00%)
Saga	3.77% (3.46% - 4.11%)	3.53% (3.24% - 3.85%)	3.48% (3.22% - 3.77%)	3.32% (3.06% - 3.61%)	0.12% (0.08% - 0.18%)	0.15% (0.10% - 0.21%)	0.17% (0.13% - 0.24%)	0.16% (0.11% - 0.22%)	0.00% (0.00% - 0.00%)	0.00% (0.00% - 0.00%)	0.00% (0.00% - 0.00%)	0.00% (0.00% - 0.00%)
Nagasaki	5.40% (4.97% - 5.87%)	4.88% (4.50% - 5.28%)	4.78% (4.43% - 5.17%)	4.54% (4.19% - 4.91%)	0.23% (0.15% - 0.31%)	0.25% (0.18% - 0.33%)	0.29% (0.21% - 0.37%)	0.27% (0.21% - 0.36%)	0.00% (0.00% - 0.00%)	0.00% (0.00% - 0.00%)	0.00% (0.00% - 0.00%)	0.00% (0.00% - 0.00%)
Kumamoto	3.71% (3.39% - 4.02%)	3.51% (3.24% - 3.80%)	3.50% (3.22% - 3.81%)	3.33% (3.08% - 3.62%)	0.12% (0.07% - 0.17%)	0.15% (0.10% - 0.20%)	0.18% (0.12% - 0.24%)	0.16% (0.11% - 0.22%)	0.00% (0.00% - 0.00%)	0.00% (0.00% - 0.00%)	0.00% (0.00% - 0.00%)	0.00% (0.00% - 0.00%)
Ōita	3.67% (3.36% - 4.01%)	3.42% (3.13% - 3.72%)	3.39% (3.11% - 3.66%)	3.23% (2.94% - 3.52%)	0.06% (0.03% - 0.10%)	0.08% (0.05% - 0.13%)	0.10% (0.06% - 0.15%)	0.10% (0.06% - 0.14%)	0.00% (0.00% - 0.00%)	0.00% (0.00% - 0.00%)	0.00% (0.00% - 0.00%)	0.00% (0.00% - 0.00%)
Miyazaki	3.86% (3.54% - 4.21%)	3.58% (3.28% - 3.89%)	3.52% (3.24% - 3.82%)	3.35% (3.07% - 3.66%)	0.10% (0.05% - 0.15%)	0.12% (0.07% - 0.17%)	0.14% (0.10% - 0.20%)	0.14% (0.09% - 0.19%)	0.00% (0.00% - 0.00%)	0.00% (0.00% - 0.00%)	0.00% (0.00% - 0.00%)	0.00% (0.00% - 0.00%)
Kagoshima	3.92% (3.58% - 4.30%)	3.64% (3.32% - 4.00%)	3.54% (3.24% - 3.85%)	3.34% (3.03% - 3.63%)	0.08% (0.05% - 0.12%)	0.10% (0.06% - 0.15%)	0.12% (0.08% - 0.17%)	0.11% (0.07% - 0.16%)	0.00% (0.00% - 0.00%)	0.00% (0.00% - 0.00%)	0.00% (0.00% - 0.00%)	0.00% (0.00% - 0.00%)
Okinawa	3.98% (3.63% - 4.32%)	3.79% (3.49% - 4.14%)	3.69% (3.42% - 4.00%)	3.48% (3.21% - 3.77%)	0.14% (0.09% - 0.21%)	0.17% (0.12% - 0.23%)	0.20% (0.14% - 0.26%)	0.18% (0.13% - 0.24%)	0.00% (0.00% - 0.00%)	0.00% (0.00% - 0.00%)	0.00% (0.00% - 0.00%)	0.00% (0.00% - 0.00%)
Republic of Korea	1.73% (1.61% - 1.87%)	1.32% (1.23% - 1.43%)	1.18% (1.09% - 1.28%)	1.09% (1.00% - 1.18%)	0.00% (0.00% - 0.00%)	0.00% (0.00% - 0.01%)	0.00% (0.00% - 0.00%)	0.00% (0.00% - 0.00%)	0.00% (0.00% - 0.00%)	0.00% (0.00% - 0.00%)	0.00% (0.00% - 0.00%)	0.00% (0.00% - 0.00%)
Singapore	4.24% (3.96% - 4.56%)	3.35% (3.18% - 3.51%)	2.88% (2.67% - 3.09%)	2.68% (2.46% - 2.91%)	0.15% (0.10% - 0.21%)	0.12% (0.09% - 0.16%)	0.11% (0.08% - 0.15%)	0.10% (0.06% - 0.14%)	0.00% (0.00% - 0.00%)	0.00% (0.00% - 0.00%)	0.00% (0.00% - 0.00%)	0.00% (0.00% - 0.00%)
**High-income North America**	0.91% (0.84% - 0.97%)	0.96% (0.89% - 1.02%)	0.86% (0.80% - 0.93%)	0.84% (0.78% - 0.90%)	0.00% (0.00% - 0.00%)	0.00% (0.00% - 0.00%)	0.00% (0.00% - 0.00%)	0.00% (0.00% - 0.00%)	0.00% (0.00% - 0.00%)	0.00% (0.00% - 0.00%)	0.00% (0.00% - 0.00%)	0.00% (0.00% - 0.00%)
Canada	0.98% (0.91% - 1.07%)	0.98% (0.90% - 1.08%)	0.88% (0.80% - 0.96%)	0.85% (0.79% - 0.94%)	0.00% (0.00% - 0.00%)	0.00% (0.00% - 0.00%)	0.00% (0.00% - 0.00%)	0.00% (0.00% - 0.00%)	0.00% (0.00% - 0.00%)	0.00% (0.00% - 0.00%)	0.00% (0.00% - 0.00%)	0.00% (0.00% - 0.00%)

Table S8c. Estimated overall, severe, and extreme **underweight** prevalence (%) in children under 5 years, both sexes, in 1990, 2000, 2010, and 2020.

Location	Total				Severe				Extreme			
	1990	2000	2010	2020	1990	2000	2010	2020	1990	2000	2010	2020
United States of America	0.90% (0.83% - 0.97%)	0.96% (0.88% - 1.03%)	0.86% (0.80% - 0.93%)	0.84% (0.78% - 0.91%)	0.00% (0.00% - 0.00%)	0.00% (0.00% - 0.00%)	0.00% (0.00% - 0.00%)	0.00% (0.00% - 0.00%)	0.00% (0.00% - 0.00%)	0.00% (0.00% - 0.00%)	0.00% (0.00% - 0.00%)	0.00% (0.00% - 0.00%)
Alabama	0.97% (0.87% - 1.08%)	1.03% (0.92% - 1.15%)	0.93% (0.83% - 1.04%)	0.92% (0.82% - 1.02%)	0.00% (0.00% - 0.00%)	0.00% (0.00% - 0.00%)	0.00% (0.00% - 0.00%)	0.00% (0.00% - 0.00%)	0.00% (0.00% - 0.00%)	0.00% (0.00% - 0.00%)	0.00% (0.00% - 0.00%)	0.00% (0.00% - 0.00%)
Alaska	0.89% (0.80% - 1.00%)	0.96% (0.86% - 1.07%)	0.88% (0.79% - 0.99%)	0.84% (0.75% - 0.94%)	0.00% (0.00% - 0.00%)	0.00% (0.00% - 0.00%)	0.00% (0.00% - 0.01%)	0.00% (0.00% - 0.00%)	0.00% (0.00% - 0.00%)	0.00% (0.00% - 0.00%)	0.00% (0.00% - 0.00%)	0.00% (0.00% - 0.00%)
Arizona	0.94% (0.84% - 1.05%)	1.01% (0.90% - 1.12%)	0.88% (0.79% - 0.99%)	0.87% (0.77% - 0.96%)	0.00% (0.00% - 0.00%)	0.00% (0.00% - 0.00%)	0.00% (0.00% - 0.01%)	0.00% (0.00% - 0.01%)	0.00% (0.00% - 0.00%)	0.00% (0.00% - 0.00%)	0.00% (0.00% - 0.00%)	0.00% (0.00% - 0.00%)
Arkansas	0.98% (0.87% - 1.10%)	1.04% (0.94% - 1.16%)	0.95% (0.85% - 1.06%)	0.92% (0.82% - 1.03%)	0.00% (0.00% - 0.00%)	0.00% (0.00% - 0.00%)	0.00% (0.00% - 0.00%)	0.00% (0.00% - 0.00%)	0.00% (0.00% - 0.00%)	0.00% (0.00% - 0.00%)	0.00% (0.00% - 0.00%)	0.00% (0.00% - 0.00%)
California	0.90% (0.81% - 1.01%)	0.94% (0.84% - 1.05%)	0.83% (0.74% - 0.93%)	0.80% (0.72% - 0.89%)	0.00% (0.00% - 0.00%)	0.00% (0.00% - 0.00%)	0.00% (0.00% - 0.00%)	0.00% (0.00% - 0.00%)	0.00% (0.00% - 0.00%)	0.00% (0.00% - 0.00%)	0.00% (0.00% - 0.00%)	0.00% (0.00% - 0.00%)
Colorado	0.86% (0.76% - 0.96%)	0.93% (0.84% - 1.04%)	0.85% (0.75% - 0.95%)	0.81% (0.72% - 0.91%)	0.00% (0.00% - 0.00%)	0.00% (0.00% - 0.00%)	0.00% (0.00% - 0.01%)	0.00% (0.00% - 0.00%)	0.00% (0.00% - 0.00%)	0.00% (0.00% - 0.00%)	0.00% (0.00% - 0.00%)	0.00% (0.00% - 0.00%)
Connecticut	0.80% (0.71% - 0.90%)	0.86% (0.76% - 0.96%)	0.77% (0.68% - 0.86%)	0.75% (0.67% - 0.85%)	0.00% (0.00% - 0.00%)	0.00% (0.00% - 0.00%)	0.00% (0.00% - 0.00%)	0.00% (0.00% - 0.00%)	0.00% (0.00% - 0.00%)	0.00% (0.00% - 0.00%)	0.00% (0.00% - 0.00%)	0.00% (0.00% - 0.00%)
Delaware	0.88% (0.78% - 0.98%)	0.93% (0.83% - 1.05%)	0.84% (0.75% - 0.95%)	0.82% (0.73% - 0.92%)	0.00% (0.00% - 0.00%)	0.00% (0.00% - 0.00%)	0.00% (0.00% - 0.00%)	0.00% (0.00% - 0.00%)	0.00% (0.00% - 0.00%)	0.00% (0.00% - 0.00%)	0.00% (0.00% - 0.00%)	0.00% (0.00% - 0.00%)
District of Columbia	1.03% (0.92% - 1.16%)	0.99% (0.89% - 1.10%)	0.88% (0.79% - 0.97%)	0.83% (0.74% - 0.93%)	0.00% (0.00% - 0.00%)	0.00% (0.00% - 0.00%)	0.00% (0.00% - 0.00%)	0.00% (0.00% - 0.00%)	0.00% (0.00% - 0.00%)	0.00% (0.00% - 0.00%)	0.00% (0.00% - 0.00%)	0.00% (0.00% - 0.00%)
Florida	0.90% (0.80% - 1.01%)	0.94% (0.84% - 1.05%)	0.85% (0.76% - 0.96%)	0.84% (0.74% - 0.93%)	0.00% (0.00% - 0.00%)	0.00% (0.00% - 0.00%)	0.00% (0.00% - 0.00%)	0.00% (0.00% - 0.00%)	0.00% (0.00% - 0.00%)	0.00% (0.00% - 0.00%)	0.00% (0.00% - 0.00%)	0.00% (0.00% - 0.00%)
Georgia	0.95% (0.85% - 1.06%)	1.00% (0.90% - 1.12%)	0.89% (0.80% - 1.00%)	0.87% (0.78% - 0.98%)	0.00% (0.00% - 0.00%)	0.00% (0.00% - 0.00%)	0.00% (0.00% - 0.00%)	0.00% (0.00% - 0.00%)	0.00% (0.00% - 0.00%)	0.00% (0.00% - 0.00%)	0.00% (0.00% - 0.00%)	0.00% (0.00% - 0.00%)
Hawaii	0.86% (0.77% - 0.96%)	0.93% (0.83% - 1.04%)	0.85% (0.76% - 0.95%)	0.83% (0.74% - 0.94%)	0.00% (0.00% - 0.00%)	0.00% (0.00% - 0.00%)	0.00% (0.00% - 0.01%)	0.00% (0.00% - 0.00%)	0.00% (0.00% - 0.00%)	0.00% (0.00% - 0.00%)	0.00% (0.00% - 0.00%)	0.00% (0.00% - 0.00%)
Idaho	0.95% (0.84% - 1.06%)	1.00% (0.90% - 1.13%)	0.90% (0.80% - 1.00%)	0.87% (0.78% - 0.97%)	0.00% (0.00% - 0.00%)	0.00% (0.00% - 0.00%)	0.00% (0.00% - 0.01%)	0.00% (0.00% - 0.01%)	0.00% (0.00% - 0.00%)	0.00% (0.00% - 0.00%)	0.00% (0.00% - 0.00%)	0.00% (0.00% - 0.00%)
Illinois	0.90% (0.80% - 1.00%)	0.94% (0.84% - 1.04%)	0.84% (0.75% - 0.94%)	0.81% (0.72% - 0.91%)	0.00% (0.00% - 0.00%)	0.00% (0.00% - 0.00%)	0.00% (0.00% - 0.00%)	0.00% (0.00% - 0.00%)	0.00% (0.00% - 0.00%)	0.00% (0.00% - 0.00%)	0.00% (0.00% - 0.00%)	0.00% (0.00% - 0.00%)
Indiana	0.92% (0.82% - 1.02%)	0.98% (0.88% - 1.09%)	0.89% (0.79% - 1.01%)	0.87% (0.78% - 0.98%)	0.00% (0.00% - 0.00%)	0.00% (0.00% - 0.00%)	0.00% (0.00% - 0.01%)	0.00% (0.00% - 0.01%)	0.00% (0.00% - 0.00%)	0.00% (0.00% - 0.00%)	0.00% (0.00% - 0.00%)	0.00% (0.00% - 0.00%)
Iowa	0.86% (0.77% - 0.97%)	0.94% (0.84% - 1.06%)	0.85% (0.76% - 0.96%)	0.82% (0.73% - 0.93%)	0.00% (0.00% - 0.00%)	0.00% (0.00% - 0.00%)	0.00% (0.00% - 0.01%)	0.00% (0.00% - 0.00%)	0.00% (0.00% - 0.00%)	0.00% (0.00% - 0.00%)	0.00% (0.00% - 0.00%)	0.00% (0.00% - 0.00%)
Kansas	0.88% (0.78% - 0.99%)	0.96% (0.85% - 1.08%)	0.88% (0.78% - 0.99%)	0.85% (0.76% - 0.96%)	0.00% (0.00% - 0.00%)	0.00% (0.00% - 0.00%)	0.00% (0.00% - 0.01%)	0.00% (0.00% - 0.00%)	0.00% (0.00% - 0.00%)	0.00% (0.00% - 0.00%)	0.00% (0.00% - 0.00%)	0.00% (0.00% - 0.00%)
Kentucky	0.93% (0.84% - 1.05%)	1.00% (0.90% - 1.11%)	0.92% (0.82% - 1.02%)	0.88% (0.78% - 0.97%)	0.00% (0.00% - 0.00%)	0.00% (0.00% - 0.00%)	0.00% (0.00% - 0.01%)	0.00% (0.00% - 0.00%)	0.00% (0.00% - 0.00%)	0.00% (0.00% - 0.00%)	0.00% (0.00% - 0.00%)	0.00% (0.00% - 0.00%)
Louisiana	0.98% (0.88% - 1.10%)	1.04% (0.93% - 1.16%)	0.91% (0.81% - 1.02%)	0.89% (0.79% - 0.99%)	0.00% (0.00% - 0.00%)	0.00% (0.00% - 0.00%)	0.00% (0.00% - 0.01%)	0.00% (0.00% - 0.00%)	0.00% (0.00% - 0.00%)	0.00% (0.00% - 0.00%)	0.00% (0.00% - 0.00%)	0.00% (0.00% - 0.00%)
Maine	0.86% (0.77% - 0.97%)	0.93% (0.83% - 1.05%)	0.85% (0.75% - 0.96%)	0.83% (0.74% - 0.94%)	0.00% (0.00% - 0.00%)	0.00% (0.00% - 0.00%)	0.00% (0.00% - 0.01%)	0.00% (0.00% - 0.00%)	0.00% (0.00% - 0.00%)	0.00% (0.00% - 0.00%)	0.00% (0.00% - 0.00%)	0.00% (0.00% - 0.00%)
Maryland	0.86% (0.77% - 0.97%)	0.92% (0.82% - 1.03%)	0.82% (0.73% - 0.92%)	0.81% (0.72% - 0.91%)	0.00% (0.00% - 0.00%)	0.00% (0.00% - 0.00%)	0.00% (0.00% - 0.00%)	0.00% (0.00% - 0.00%)	0.00% (0.00% - 0.00%)	0.00% (0.00% - 0.00%)	0.00% (0.00% - 0.00%)	0.00% (0.00% - 0.00%)
Massachusetts	0.80% (0.72% - 0.90%)	0.85% (0.76% - 0.96%)	0.77% (0.69% - 0.87%)	0.75% (0.66% - 0.84%)	0.00% (0.00% - 0.00%)	0.00% (0.00% - 0.00%)	0.00% (0.00% - 0.01%)	0.00% (0.00% - 0.00%)	0.00% (0.00% - 0.00%)	0.00% (0.00% - 0.00%)	0.00% (0.00% - 0.00%)	0.00% (0.00% - 0.00%)

Table S8c. Estimated overall, severe, and extreme **underweight** prevalence (%) in children under 5 years, both sexes, in 1990, 2000, 2010, and 2020.												
**Location**	**Total**				**Severe**				**Extreme**			
	**1990**	**2000**	**2010**	**2020**	**1990**	**2000**	**2010**	**2020**	**1990**	**2000**	**2010**	**2020**
Michigan	0.91% (0.81% - 1.01%)	0.96% (0.85% - 1.07%)	0.88% (0.79% - 0.99%)	0.85% (0.76% - 0.95%)	0.00% (0.00% - 0.00%)	0.00% (0.00% - 0.00%)	0.00% (0.00% - 0.01%)	0.00% (0.00% - 0.00%)	0.00% (0.00% - 0.00%)	0.00% (0.00% - 0.00%)	0.00% (0.00% - 0.00%)	0.00% (0.00% - 0.00%)
Minnesota	0.82% (0.74% - 0.93%)	0.90% (0.79% - 1.01%)	0.81% (0.72% - 0.91%)	0.78% (0.69% - 0.87%)	0.00% (0.00% - 0.00%)	0.00% (0.00% - 0.00%)	0.00% (0.00% - 0.01%)	0.00% (0.00% - 0.00%)	0.00% (0.00% - 0.00%)	0.00% (0.00% - 0.00%)	0.00% (0.00% - 0.00%)	0.00% (0.00% - 0.00%)
Mississippi	1.00% (0.90% - 1.11%)	1.07% (0.96% - 1.20%)	0.96% (0.86% - 1.06%)	0.93% (0.83% - 1.05%)	0.00% (0.00% - 0.00%)	0.00% (0.00% - 0.00%)	0.00% (0.00% - 0.00%)	0.00% (0.00% - 0.00%)	0.00% (0.00% - 0.00%)	0.00% (0.00% - 0.00%)	0.00% (0.00% - 0.00%)	0.00% (0.00% - 0.00%)
Missouri	0.91% (0.81% - 1.01%)	0.97% (0.87% - 1.08%)	0.89% (0.79% - 1.00%)	0.88% (0.78% - 0.99%)	0.00% (0.00% - 0.00%)	0.00% (0.00% - 0.00%)	0.00% (0.00% - 0.00%)	0.00% (0.00% - 0.01%)	0.00% (0.00% - 0.00%)	0.00% (0.00% - 0.00%)	0.00% (0.00% - 0.00%)	0.00% (0.00% - 0.00%)
Montana	0.91% (0.81% - 1.03%)	0.98% (0.87% - 1.09%)	0.90% (0.81% - 1.00%)	0.85% (0.76% - 0.96%)	0.00% (0.00% - 0.00%)	0.00% (0.00% - 0.00%)	0.00% (0.00% - 0.01%)	0.00% (0.00% - 0.00%)	0.00% (0.00% - 0.00%)	0.00% (0.00% - 0.00%)	0.00% (0.00% - 0.00%)	0.00% (0.00% - 0.00%)
Nebraska	0.86% (0.77% - 0.97%)	0.94% (0.84% - 1.06%)	0.85% (0.76% - 0.96%)	0.81% (0.72% - 0.91%)	0.00% (0.00% - 0.00%)	0.00% (0.00% - 0.00%)	0.00% (0.00% - 0.01%)	0.00% (0.00% - 0.00%)	0.00% (0.00% - 0.00%)	0.00% (0.00% - 0.00%)	0.00% (0.00% - 0.00%)	0.00% (0.00% - 0.00%)
Nevada	0.95% (0.85% - 1.06%)	1.01% (0.90% - 1.12%)	0.90% (0.81% - 1.00%)	0.89% (0.80% - 1.00%)	0.00% (0.00% - 0.00%)	0.00% (0.00% - 0.00%)	0.00% (0.00% - 0.01%)	0.00% (0.00% - 0.00%)	0.00% (0.00% - 0.00%)	0.00% (0.00% - 0.00%)	0.00% (0.00% - 0.00%)	0.00% (0.00% - 0.00%)
New Hampshire	0.82% (0.73% - 0.93%)	0.88% (0.79% - 0.99%)	0.79% (0.70% - 0.89%)	0.77% (0.69% - 0.86%)	0.00% (0.00% - 0.00%)	0.00% (0.00% - 0.00%)	0.00% (0.00% - 0.00%)	0.00% (0.00% - 0.00%)	0.00% (0.00% - 0.00%)	0.00% (0.00% - 0.00%)	0.00% (0.00% - 0.00%)	0.00% (0.00% - 0.00%)
New Jersey	0.83% (0.75% - 0.94%)	0.89% (0.78% - 0.99%)	0.80% (0.71% - 0.90%)	0.79% (0.70% - 0.89%)	0.00% (0.00% - 0.00%)	0.00% (0.00% - 0.00%)	0.00% (0.00% - 0.00%)	0.00% (0.00% - 0.00%)	0.00% (0.00% - 0.00%)	0.00% (0.00% - 0.00%)	0.00% (0.00% - 0.00%)	0.00% (0.00% - 0.00%)
New Mexico	0.98% (0.88% - 1.11%)	1.03% (0.93% - 1.16%)	0.93% (0.83% - 1.04%)	0.91% (0.82% - 1.02%)	0.00% (0.00% - 0.00%)	0.00% (0.00% - 0.00%)	0.00% (0.00% - 0.01%)	0.00% (0.00% - 0.01%)	0.00% (0.00% - 0.00%)	0.00% (0.00% - 0.00%)	0.00% (0.00% - 0.00%)	0.00% (0.00% - 0.00%)
New York	0.88% (0.79% - 0.98%)	0.90% (0.80% - 1.00%)	0.82% (0.73% - 0.92%)	0.79% (0.71% - 0.89%)	0.00% (0.00% - 0.00%)	0.00% (0.00% - 0.00%)	0.00% (0.00% - 0.00%)	0.00% (0.00% - 0.00%)	0.00% (0.00% - 0.00%)	0.00% (0.00% - 0.00%)	0.00% (0.00% - 0.00%)	0.00% (0.00% - 0.00%)
North Carolina	0.93% (0.84% - 1.04%)	0.98% (0.88% - 1.09%)	0.88% (0.79% - 0.99%)	0.87% (0.77% - 0.98%)	0.00% (0.00% - 0.00%)	0.00% (0.00% - 0.00%)	0.00% (0.00% - 0.00%)	0.00% (0.00% - 0.00%)	0.00% (0.00% - 0.00%)	0.00% (0.00% - 0.00%)	0.00% (0.00% - 0.00%)	0.00% (0.00% - 0.00%)
North Dakota	0.88% (0.78% - 0.99%)	0.94% (0.83% - 1.06%)	0.87% (0.78% - 0.98%)	0.80% (0.70% - 0.89%)	0.00% (0.00% - 0.00%)	0.00% (0.00% - 0.00%)	0.00% (0.00% - 0.01%)	0.00% (0.00% - 0.00%)	0.00% (0.00% - 0.00%)	0.00% (0.00% - 0.00%)	0.00% (0.00% - 0.00%)	0.00% (0.00% - 0.00%)
Ohio	0.90% (0.81% - 1.02%)	0.97% (0.86% - 1.08%)	0.88% (0.79% - 0.98%)	0.88% (0.78% - 0.99%)	0.00% (0.00% - 0.00%)	0.00% (0.00% - 0.00%)	0.00% (0.00% - 0.00%)	0.00% (0.00% - 0.00%)	0.00% (0.00% - 0.00%)	0.00% (0.00% - 0.00%)	0.00% (0.00% - 0.00%)	0.00% (0.00% - 0.00%)
Oklahoma	0.93% (0.83% - 1.04%)	1.04% (0.93% - 1.15%)	0.93% (0.83% - 1.03%)	0.88% (0.79% - 0.98%)	0.00% (0.00% - 0.00%)	0.00% (0.00% - 0.00%)	0.00% (0.00% - 0.00%)	0.00% (0.00% - 0.00%)	0.00% (0.00% - 0.00%)	0.00% (0.00% - 0.00%)	0.00% (0.00% - 0.00%)	0.00% (0.00% - 0.00%)
Oregon	0.88% (0.79% - 0.99%)	0.94% (0.84% - 1.06%)	0.86% (0.77% - 0.96%)	0.83% (0.74% - 0.93%)	0.00% (0.00% - 0.00%)	0.00% (0.00% - 0.00%)	0.00% (0.00% - 0.01%)	0.00% (0.00% - 0.01%)	0.00% (0.00% - 0.00%)	0.00% (0.00% - 0.00%)	0.00% (0.00% - 0.00%)	0.00% (0.00% - 0.00%)
Pennsylvania	0.88% (0.79% - 0.99%)	0.93% (0.83% - 1.06%)	0.84% (0.75% - 0.95%)	0.82% (0.73% - 0.93%)	0.00% (0.00% - 0.00%)	0.00% (0.00% - 0.00%)	0.00% (0.00% - 0.00%)	0.00% (0.00% - 0.00%)	0.00% (0.00% - 0.00%)	0.00% (0.00% - 0.00%)	0.00% (0.00% - 0.00%)	0.00% (0.00% - 0.00%)
Rhode Island	0.84% (0.75% - 0.95%)	0.90% (0.80% - 1.02%)	0.82% (0.73% - 0.93%)	0.79% (0.70% - 0.89%)	0.00% (0.00% - 0.00%)	0.00% (0.00% - 0.00%)	0.00% (0.00% - 0.01%)	0.00% (0.00% - 0.00%)	0.00% (0.00% - 0.00%)	0.00% (0.00% - 0.00%)	0.00% (0.00% - 0.00%)	0.00% (0.00% - 0.00%)
South Carolina	0.97% (0.87% - 1.09%)	1.02% (0.90% - 1.14%)	0.92% (0.83% - 1.04%)	0.90% (0.80% - 1.00%)	0.00% (0.00% - 0.00%)	0.00% (0.00% - 0.00%)	0.00% (0.00% - 0.01%)	0.00% (0.00% - 0.00%)	0.00% (0.00% - 0.00%)	0.00% (0.00% - 0.00%)	0.00% (0.00% - 0.00%)	0.00% (0.00% - 0.00%)
South Dakota	0.91% (0.81% - 1.03%)	0.97% (0.86% - 1.08%)	0.88% (0.78% - 0.99%)	0.85% (0.77% - 0.96%)	0.00% (0.00% - 0.00%)	0.00% (0.00% - 0.00%)	0.00% (0.00% - 0.01%)	0.00% (0.00% - 0.00%)	0.00% (0.00% - 0.00%)	0.00% (0.00% - 0.00%)	0.00% (0.00% - 0.00%)	0.00% (0.00% - 0.00%)
Tennessee	0.95% (0.85% - 1.06%)	1.00% (0.90% - 1.12%)	0.96% (0.86% - 1.08%)	0.89% (0.79% - 0.99%)	0.00% (0.00% - 0.00%)	0.00% (0.00% - 0.00%)	0.00% (0.00% - 0.01%)	0.00% (0.00% - 0.00%)	0.00% (0.00% - 0.00%)	0.00% (0.00% - 0.00%)	0.00% (0.00% - 0.00%)	0.00% (0.00% - 0.00%)
Texas	0.94% (0.84% - 1.05%)	1.01% (0.90% - 1.12%)	0.89% (0.79% - 1.00%)	0.86% (0.77% - 0.96%)	0.00% (0.00% - 0.00%)	0.00% (0.00% - 0.00%)	0.00% (0.00% - 0.00%)	0.00% (0.00% - 0.00%)	0.00% (0.00% - 0.00%)	0.00% (0.00% - 0.00%)	0.00% (0.00% - 0.00%)	0.00% (0.00% - 0.00%)
Utah	0.94% (0.84% - 1.06%)	1.00% (0.90% - 1.12%)	0.89% (0.79% - 1.01%)	0.87% (0.78% - 0.98%)	0.00% (0.00% - 0.00%)	0.00% (0.00% - 0.00%)	0.00% (0.00% - 0.01%)	0.00% (0.00% - 0.01%)	0.00% (0.00% - 0.00%)	0.00% (0.00% - 0.00%)	0.00% (0.00% - 0.00%)	0.00% (0.00% - 0.00%)

Table S8c. Estimated overall, severe, and extreme **underweight** prevalence (%) in children under 5 years, both sexes, in 1990, 2000, 2010, and 2020.

Location	Total				Severe				Extreme			
	1990	2000	2010	2020	1990	2000	2010	2020	1990	2000	2010	2020
	0.87%	0.92%	0.82%	0.80%	0.00%	0.00%	0.00%	0.00%	0.00%	0.00%	0.00%	0.00%
Vermont	(0.78% - 0.99%)	(0.81% - 1.03%)	(0.74% - 0.93%)	(0.71% - 0.91%)	(0.00% - 0.00%)	(0.00% - 0.00%)	(0.00% - 0.00%)	(0.00% - 0.00%)	(0.00% - 0.00%)	(0.00% - 0.00%)	(0.00% - 0.00%)	(0.00% - 0.00%)
	0.88%	0.93%	0.83%	0.81%	0.00%	0.00%	0.00%	0.00%	0.00%	0.00%	0.00%	0.00%
Virginia	(0.79% - 0.99%)	(0.83% - 1.03%)	(0.74% - 0.93%)	(0.72% - 0.90%)	(0.00% - 0.00%)	(0.00% - 0.00%)	(0.00% - 0.00%)	(0.00% - 0.00%)	(0.00% - 0.00%)	(0.00% - 0.00%)	(0.00% - 0.00%)	(0.00% - 0.00%)
	0.87%	0.92%	0.83%	0.80%	0.00%	0.00%	0.00%	0.00%	0.00%	0.00%	0.00%	0.00%
Washington	(0.77% - 0.98%)	(0.82% - 1.05%)	(0.74% - 0.94%)	(0.71% - 0.89%)	(0.00% - 0.00%)	(0.00% - 0.00%)	(0.00% - 0.01%)	(0.00% - 0.00%)	(0.00% - 0.00%)	(0.00% - 0.00%)	(0.00% - 0.00%)	(0.00% - 0.00%)
	0.95%	1.02%	0.95%	0.91%	0.00%	0.00%	0.00%	0.00%	0.00%	0.00%	0.00%	0.00%
West Virginia	(0.85% - 1.07%)	(0.91% - 1.14%)	(0.85% - 1.06%)	(0.82% - 1.02%)	(0.00% - 0.00%)	(0.00% - 0.00%)	(0.00% - 0.01%)	(0.00% - 0.00%)	(0.00% - 0.00%)	(0.00% - 0.00%)	(0.00% - 0.00%)	(0.00% - 0.00%)
	0.85%	0.92%	0.85%	0.83%	0.00%	0.00%	0.00%	0.00%	0.00%	0.00%	0.00%	0.00%
Wisconsin	(0.77% - 0.96%)	(0.83% - 1.03%)	(0.75% - 0.96%)	(0.74% - 0.92%)	(0.00% - 0.00%)	(0.00% - 0.00%)	(0.00% - 0.01%)	(0.00% - 0.00%)	(0.00% - 0.00%)	(0.00% - 0.00%)	(0.00% - 0.00%)	(0.00% - 0.00%)
	0.89%	0.97%	0.86%	0.83%	0.00%	0.00%	0.00%	0.00%	0.00%	0.00%	0.00%	0.00%
Wyoming	(0.80% - 1.01%)	(0.87% - 1.08%)	(0.77% - 0.97%)	(0.74% - 0.93%)	(0.00% - 0.00%)	(0.00% - 0.00%)	(0.00% - 0.00%)	(0.00% - 0.00%)	(0.00% - 0.00%)	(0.00% - 0.00%)	(0.00% - 0.00%)	(0.00% - 0.00%)
	1.28%	1.29%	1.12%	0.98%	0.00%	0.00%	0.00%	0.00%	0.00%	0.00%	0.00%	0.00%
Greenland	(1.18% - 1.38%)	(1.18% - 1.40%)	(1.04% - 1.21%)	(0.91% - 1.06%)	(0.00% - 0.00%)	(0.00% - 0.00%)	(0.00% - 0.00%)	(0.00% - 0.00%)	(0.00% - 0.00%)	(0.00% - 0.00%)	(0.00% - 0.00%)	(0.00% - 0.00%)
	2.91%	2.63%	2.15%	1.92%	0.09%	0.09%	0.06%	0.04%	0.00%	0.00%	0.00%	0.00%
**Southern Latin America**	(2.74% - 3.08%)	(2.48% - 2.78%)	(2.03% - 2.30%)	(1.79% - 2.08%)	(0.07% - 0.12%)	(0.07% - 0.12%)	(0.04% - 0.08%)	(0.03% - 0.07%)	(0.00% - 0.00%)	(0.00% - 0.00%)	(0.00% - 0.00%)	(0.00% - 0.00%)
	3.43%	2.96%	2.39%	2.21%	0.11%	0.08%	0.04%	0.04%	0.00%	0.00%	0.00%	0.00%
Argentina	(3.18% - 3.68%)	(2.75% - 3.17%)	(2.22% - 2.58%)	(2.03% - 2.43%)	(0.07% - 0.15%)	(0.05% - 0.12%)	(0.02% - 0.08%)	(0.01% - 0.07%)	(0.00% - 0.00%)	(0.00% - 0.00%)	(0.00% - 0.00%)	(0.00% - 0.00%)
	1.31%	1.30%	1.05%	0.68%	0.00%	0.04%	0.02%	0.00%	0.00%	0.00%	0.00%	0.00%
Chile	(1.22% - 1.41%)	(1.22% - 1.40%)	(0.99% - 1.12%)	(0.63% - 0.74%)	(0.00% - 0.00%)	(0.03% - 0.05%)	(0.02% - 0.03%)	(0.00% - 0.00%)	(0.00% - 0.00%)	(0.00% - 0.00%)	(0.00% - 0.00%)	(0.00% - 0.00%)
	4.82%	4.63%	4.21%	3.72%	0.42%	0.48%	0.42%	0.35%	0.00%	0.00%	0.00%	0.00%
Uruguay	(4.50% - 5.15%)	(4.32% - 4.96%)	(3.91% - 4.55%)	(3.42% - 4.02%)	(0.35% - 0.50%)	(0.40% - 0.57%)	(0.35% - 0.52%)	(0.28% - 0.43%)	(0.00% - 0.00%)	(0.00% - 0.01%)	(0.00% - 0.00%)	(0.00% - 0.00%)
	1.30%	1.06%	1.01%	0.98%	0.02%	0.02%	0.02%	0.02%	0.00%	0.00%	0.00%	0.00%
**Western Europe**	(1.26% - 1.33%)	(1.04% - 1.09%)	(0.98% - 1.04%)	(0.95% - 1.01%)	(0.02% - 0.03%)	(0.01% - 0.02%)	(0.01% - 0.02%)	(0.01% - 0.02%)	(0.00% - 0.00%)	(0.00% - 0.00%)	(0.00% - 0.00%)	(0.00% - 0.00%)
	0.86%	0.78%	0.81%	0.77%	0.01%	0.00%	0.01%	0.01%	0.00%	0.00%	0.00%	0.00%
Monaco	(0.80% - 0.92%)	(0.73% - 0.84%)	(0.75% - 0.88%)	(0.72% - 0.83%)	(0.00% - 0.01%)	(0.00% - 0.01%)	(0.00% - 0.01%)	(0.00% - 0.01%)	(0.00% - 0.00%)	(0.00% - 0.00%)	(0.00% - 0.00%)	(0.00% - 0.00%)
	0.98%	0.90%	0.93%	0.97%	0.01%	0.01%	0.01%	0.01%	0.00%	0.00%	0.00%	0.00%
San Marino	(0.91% - 1.06%)	(0.84% - 0.98%)	(0.86% - 1.00%)	(0.90% - 1.05%)	(0.01% - 0.02%)	(0.00% - 0.01%)	(0.01% - 0.02%)	(0.01% - 0.02%)	(0.00% - 0.00%)	(0.00% - 0.00%)	(0.00% - 0.00%)	(0.00% - 0.00%)
	1.00%	0.88%	0.87%	0.88%	0.01%	0.01%	0.01%	0.01%	0.00%	0.00%	0.00%	0.00%
Andorra	(0.93% - 1.08%)	(0.81% - 0.96%)	(0.80% - 0.94%)	(0.81% - 0.96%)	(0.01% - 0.02%)	(0.00% - 0.01%)	(0.00% - 0.01%)	(0.01% - 0.02%)	(0.00% - 0.00%)	(0.00% - 0.00%)	(0.00% - 0.00%)	(0.00% - 0.00%)
	1.31%	1.09%	1.04%	0.97%	0.02%	0.01%	0.02%	0.01%	0.00%	0.00%	0.00%	0.00%
Austria	(1.22% - 1.42%)	(1.01% - 1.17%)	(0.97% - 1.13%)	(0.90% - 1.04%)	(0.02% - 0.04%)	(0.01% - 0.02%)	(0.01% - 0.02%)	(0.01% - 0.02%)	(0.00% - 0.00%)	(0.00% - 0.00%)	(0.00% - 0.00%)	(0.00% - 0.00%)
	1.22%	1.05%	0.99%	0.93%	0.02%	0.01%	0.01%	0.01%	0.00%	0.00%	0.00%	0.00%
Belgium	(1.13% - 1.31%)	(0.97% - 1.13%)	(0.91% - 1.06%)	(0.86% - 1.01%)	(0.01% - 0.03%)	(0.01% - 0.02%)	(0.01% - 0.02%)	(0.01% - 0.02%)	(0.00% - 0.00%)	(0.00% - 0.00%)	(0.00% - 0.00%)	(0.00% - 0.00%)
	1.67%	1.43%	1.42%	1.35%	0.04%	0.03%	0.04%	0.04%	0.00%	0.00%	0.00%	0.00%
Cyprus	(1.55% - 1.78%)	(1.32% - 1.54%)	(1.32% - 1.53%)	(1.25% - 1.45%)	(0.03% - 0.05%)	(0.02% - 0.04%)	(0.03% - 0.05%)	(0.03% - 0.05%)	(0.00% - 0.00%)	(0.00% - 0.00%)	(0.00% - 0.00%)	(0.00% - 0.00%)
	1.39%	1.16%	1.17%	1.10%	0.03%	0.02%	0.02%	0.02%	0.00%	0.00%	0.00%	0.00%
Denmark	(1.29% - 1.49%)	(1.08% - 1.25%)	(1.08% - 1.25%)	(1.02% - 1.19%)	(0.02% - 0.04%)	(0.01% - 0.02%)	(0.02% - 0.03%)	(0.01% - 0.03%)	(0.00% - 0.00%)	(0.00% - 0.00%)	(0.00% - 0.00%)	(0.00% - 0.00%)
	1.36%	1.21%	1.19%	1.15%	0.03%	0.02%	0.03%	0.02%	0.00%	0.00%	0.00%	0.00%
Finland	(1.26% - 1.47%)	(1.12% - 1.30%)	(1.11% - 1.28%)	(1.06% - 1.24%)	(0.02% - 0.04%)	(0.01% - 0.03%)	(0.02% - 0.03%)	(0.02% - 0.03%)	(0.00% - 0.00%)	(0.00% - 0.00%)	(0.00% - 0.00%)	(0.00% - 0.00%)
	1.21%	1.04%	1.03%	0.97%	0.02%	0.01%	0.01%	0.01%	0.00%	0.00%	0.00%	0.00%
France	(1.12% - 1.32%)	(0.96% - 1.12%)	(0.96% - 1.11%)	(0.90% - 1.06%)	(0.01% - 0.03%)	(0.00% - 0.02%)	(0.01% - 0.02%)	(0.01% - 0.02%)	(0.00% - 0.00%)	(0.00% - 0.00%)	(0.00% - 0.00%)	(0.00% - 0.00%)
	1.57%	1.30%	1.18%	1.13%	0.04%	0.03%	0.03%	0.03%	0.00%	0.00%	0.00%	0.00%
Germany	(1.45% - 1.68%)	(1.21% - 1.40%)	(1.10% - 1.27%)	(1.05% - 1.22%)	(0.03% - 0.05%)	(0.02% - 0.04%)	(0.02% - 0.04%)	(0.02% - 0.03%)	(0.00% - 0.00%)	(0.00% - 0.00%)	(0.00% - 0.00%)	(0.00% - 0.00%)
	0.79%	0.73%	0.71%	0.73%	0.00%	0.00%	0.01%	0.01%	0.00%	0.00%	0.00%	0.00%
Greece	(0.72% - 0.87%)	(0.67% - 0.80%)	(0.65% - 0.78%)	(0.66% - 0.80%)	(0.00% - 0.00%)	(0.00% - 0.01%)	(0.00% - 0.01%)	(0.00% - 0.01%)	(0.00% - 0.00%)	(0.00% - 0.00%)	(0.00% - 0.00%)	(0.00% - 0.00%)
	1.09%	1.06%	1.09%	1.04%	0.02%	0.01%	0.02%	0.02%	0.00%	0.00%	0.00%	0.00%

Table S8c. Estimated overall, severe, and extreme **underweight** prevalence (%) in children under 5 years, both sexes, in 1990, 2000, 2010, and 2020.

Location	Total				Severe				Extreme			
	1990	2000	2010	2020	1990	2000	2010	2020	1990	2000	2010	2020
Iceland	(1.01% - 1.17%)	(0.99% - 1.14%)	(1.00% - 1.18%)	(0.97% - 1.13%)	(0.01% - 0.03%)	(0.01% - 0.02%)	(0.01% - 0.03%)	(0.01% - 0.03%)	(0.00% - 0.00%)	(0.00% - 0.00%)	(0.00% - 0.00%)	(0.00% - 0.00%)
	1.20%	1.06%	0.99%	0.89%	0.02%	0.01%	0.01%	0.01%	0.00%	0.00%	0.00%	0.00%
Ireland	(1.11% - 1.30%)	(0.98% - 1.15%)	(0.93% - 1.08%)	(0.83% - 0.97%)	(0.01% - 0.03%)	(0.00% - 0.02%)	(0.01% - 0.02%)	(0.01% - 0.02%)	(0.00% - 0.00%)	(0.00% - 0.00%)	(0.00% - 0.00%)	(0.00% - 0.00%)
	1.08%	0.93%	0.93%	0.88%	0.01%	0.01%	0.01%	0.01%	0.00%	0.00%	0.00%	0.00%
Israel	(1.00% - 1.16%)	(0.86% - 1.01%)	(0.86% - 1.00%)	(0.82% - 0.96%)	(0.01% - 0.02%)	(0.00% - 0.01%)	(0.00% - 0.01%)	(0.00% - 0.01%)	(0.00% - 0.00%)	(0.00% - 0.00%)	(0.00% - 0.00%)	(0.00% - 0.00%)
	1.23%	0.98%	0.94%	0.91%	0.02%	0.01%	0.01%	0.01%	0.00%	0.00%	0.00%	0.00%
Italy	(1.15% - 1.33%)	(0.91% - 1.06%)	(0.87% - 1.00%)	(0.84% - 0.98%)	(0.01% - 0.03%)	(0.00% - 0.02%)	(0.01% - 0.02%)	(0.01% - 0.02%)	(0.00% - 0.00%)	(0.00% - 0.00%)	(0.00% - 0.00%)	(0.00% - 0.00%)
	1.16%	0.95%	0.91%	0.89%	0.02%	0.01%	0.01%	0.01%	0.00%	0.00%	0.00%	0.00%
Piemonte	(1.04% - 1.29%)	(0.85% - 1.06%)	(0.82% - 1.01%)	(0.80% - 0.99%)	(0.01% - 0.03%)	(0.00% - 0.02%)	(0.00% - 0.02%)	(0.00% - 0.02%)	(0.00% - 0.00%)	(0.00% - 0.00%)	(0.00% - 0.00%)	(0.00% - 0.00%)
	1.16%	0.95%	0.91%	0.88%	0.02%	0.01%	0.01%	0.01%	0.00%	0.00%	0.00%	0.00%
Valle d'Aosta	(1.04% - 1.29%)	(0.85% - 1.05%)	(0.82% - 1.01%)	(0.79% - 0.97%)	(0.01% - 0.03%)	(0.00% - 0.02%)	(0.00% - 0.02%)	(0.00% - 0.02%)	(0.00% - 0.00%)	(0.00% - 0.00%)	(0.00% - 0.00%)	(0.00% - 0.00%)
	1.16%	0.95%	0.91%	0.89%	0.02%	0.01%	0.01%	0.01%	0.00%	0.00%	0.00%	0.00%
Liguria	(1.05% - 1.30%)	(0.85% - 1.05%)	(0.82% - 1.01%)	(0.79% - 0.98%)	(0.01% - 0.03%)	(0.00% - 0.02%)	(0.00% - 0.02%)	(0.00% - 0.02%)	(0.00% - 0.00%)	(0.00% - 0.00%)	(0.00% - 0.00%)	(0.00% - 0.00%)
	1.10%	0.90%	0.87%	0.85%	0.02%	0.01%	0.01%	0.01%	0.00%	0.00%	0.00%	0.00%
Lombardia	(1.00% - 1.22%)	(0.82% - 1.00%)	(0.79% - 0.96%)	(0.77% - 0.93%)	(0.01% - 0.03%)	(0.00% - 0.01%)	(0.00% - 0.02%)	(0.00% - 0.02%)	(0.00% - 0.00%)	(0.00% - 0.00%)	(0.00% - 0.00%)	(0.00% - 0.00%)
	1.11%	0.90%	0.88%	0.85%	0.02%	0.01%	0.01%	0.01%	0.00%	0.00%	0.00%	0.00%
Provincia autonoma di Bolzano	(1.01% - 1.23%)	(0.81% - 1.00%)	(0.78% - 0.97%)	(0.77% - 0.95%)	(0.01% - 0.03%)	(0.00% - 0.02%)	(0.00% - 0.02%)	(0.00% - 0.02%)	(0.00% - 0.00%)	(0.00% - 0.00%)	(0.00% - 0.00%)	(0.00% - 0.00%)
	1.09%	0.91%	0.88%	0.86%	0.02%	0.01%	0.01%	0.01%	0.00%	0.00%	0.00%	0.00%
Provincia autonoma di Trento	(0.98% - 1.21%)	(0.81% - 1.01%)	(0.80% - 0.99%)	(0.77% - 0.96%)	(0.01% - 0.03%)	(0.00% - 0.02%)	(0.00% - 0.02%)	(0.00% - 0.02%)	(0.00% - 0.00%)	(0.00% - 0.00%)	(0.00% - 0.00%)	(0.00% - 0.00%)
	1.14%	0.93%	0.90%	0.87%	0.02%	0.01%	0.01%	0.01%	0.00%	0.00%	0.00%	0.00%
Veneto	(1.03% - 1.26%)	(0.83% - 1.03%)	(0.80% - 0.99%)	(0.79% - 0.96%)	(0.01% - 0.03%)	(0.00% - 0.02%)	(0.00% - 0.02%)	(0.00% - 0.02%)	(0.00% - 0.00%)	(0.00% - 0.00%)	(0.00% - 0.00%)	(0.00% - 0.00%)
	1.17%	0.95%	0.91%	0.88%	0.02%	0.01%	0.01%	0.01%	0.00%	0.00%	0.00%	0.00%
Friuli-Venezia Giulia	(1.05% - 1.31%)	(0.85% - 1.06%)	(0.80% - 1.01%)	(0.79% - 0.98%)	(0.01% - 0.03%)	(0.00% - 0.02%)	(0.00% - 0.02%)	(0.00% - 0.02%)	(0.00% - 0.00%)	(0.00% - 0.00%)	(0.00% - 0.00%)	(0.00% - 0.00%)
	1.18%	0.93%	0.89%	0.86%	0.02%	0.01%	0.01%	0.01%	0.00%	0.00%	0.00%	0.00%
Emilia-Romagna	(1.06% - 1.31%)	(0.84% - 1.02%)	(0.80% - 0.99%)	(0.77% - 0.96%)	(0.01% - 0.03%)	(0.00% - 0.02%)	(0.00% - 0.02%)	(0.00% - 0.02%)	(0.00% - 0.00%)	(0.00% - 0.00%)	(0.00% - 0.00%)	(0.00% - 0.00%)
	1.15%	0.94%	0.91%	0.88%	0.02%	0.01%	0.01%	0.01%	0.00%	0.00%	0.00%	0.00%
Toscana	(1.03% - 1.27%)	(0.86% - 1.05%)	(0.82% - 1.01%)	(0.79% - 0.99%)	(0.01% - 0.03%)	(0.00% - 0.02%)	(0.00% - 0.02%)	(0.00% - 0.02%)	(0.00% - 0.00%)	(0.00% - 0.00%)	(0.00% - 0.00%)	(0.00% - 0.00%)
	1.17%	0.96%	0.93%	0.92%	0.02%	0.01%	0.01%	0.01%	0.00%	0.00%	0.00%	0.00%
Umbria	(1.04% - 1.31%)	(0.86% - 1.07%)	(0.83% - 1.04%)	(0.82% - 1.02%)	(0.01% - 0.03%)	(0.00% - 0.02%)	(0.00% - 0.02%)	(0.00% - 0.02%)	(0.00% - 0.00%)	(0.00% - 0.00%)	(0.00% - 0.00%)	(0.00% - 0.00%)
	1.19%	0.96%	0.92%	0.90%	0.02%	0.01%	0.01%	0.01%	0.00%	0.00%	0.00%	0.00%
Marche	(1.07% - 1.32%)	(0.86% - 1.06%)	(0.83% - 1.03%)	(0.81% - 1.00%)	(0.01% - 0.03%)	(0.00% - 0.02%)	(0.00% - 0.02%)	(0.00% - 0.02%)	(0.00% - 0.00%)	(0.00% - 0.00%)	(0.00% - 0.00%)	(0.00% - 0.00%)
	1.13%	0.93%	0.89%	0.87%	0.02%	0.01%	0.01%	0.01%	0.00%	0.00%	0.00%	0.00%
Lazio	(1.02% - 1.26%)	(0.84% - 1.02%)	(0.81% - 0.99%)	(0.79% - 0.97%)	(0.01% - 0.03%)	(0.00% - 0.02%)	(0.00% - 0.02%)	(0.00% - 0.02%)	(0.00% - 0.00%)	(0.00% - 0.00%)	(0.00% - 0.00%)	(0.00% - 0.00%)
	1.19%	0.97%	0.95%	0.92%	0.02%	0.01%	0.01%	0.01%	0.00%	0.00%	0.00%	0.00%
Abruzzo	(1.08% - 1.33%)	(0.86% - 1.09%)	(0.85% - 1.06%)	(0.82% - 1.03%)	(0.01% - 0.03%)	(0.00% - 0.02%)	(0.00% - 0.02%)	(0.00% - 0.02%)	(0.00% - 0.00%)	(0.00% - 0.00%)	(0.00% - 0.00%)	(0.00% - 0.00%)
	1.27%	1.01%	0.98%	0.96%	0.02%	0.01%	0.01%	0.01%	0.00%	0.00%	0.00%	0.00%
Molise	(1.13% - 1.41%)	(0.91% - 1.13%)	(0.87% - 1.10%)	(0.86% - 1.08%)	(0.01% - 0.03%)	(0.00% - 0.02%)	(0.00% - 0.02%)	(0.01% - 0.02%)	(0.00% - 0.00%)	(0.00% - 0.00%)	(0.00% - 0.00%)	(0.00% - 0.00%)
	1.37%	1.08%	1.02%	1.00%	0.02%	0.01%	0.01%	0.01%	0.00%	0.00%	0.00%	0.00%
Campania	(1.24% - 1.51%)	(0.98% - 1.19%)	(0.92% - 1.13%)	(0.90% - 1.11%)	(0.01% - 0.04%)	(0.00% - 0.02%)	(0.01% - 0.02%)	(0.01% - 0.02%)	(0.00% - 0.00%)	(0.00% - 0.00%)	(0.00% - 0.00%)	(0.00% - 0.00%)
	1.34%	1.06%	1.01%	0.99%	0.02%	0.01%	0.01%	0.01%	0.00%	0.00%	0.00%	0.00%
Puglia	(1.21% - 1.48%)	(0.96% - 1.17%)	(0.91% - 1.12%)	(0.89% - 1.10%)	(0.01% - 0.04%)	(0.00% - 0.02%)	(0.01% - 0.02%)	(0.01% - 0.02%)	(0.00% - 0.00%)	(0.00% - 0.00%)	(0.00% - 0.00%)	(0.00% - 0.00%)
	1.30%	1.03%	0.99%	0.97%	0.02%	0.01%	0.01%	0.01%	0.00%	0.00%	0.00%	0.00%
Basilicata	(1.16% - 1.44%)	(0.93% - 1.15%)	(0.89% - 1.11%)	(0.86% - 1.08%)	(0.01% - 0.03%)	(0.00% - 0.02%)	(0.01% - 0.02%)	(0.01% - 0.02%)	(0.00% - 0.00%)	(0.00% - 0.00%)	(0.00% - 0.00%)	(0.00% - 0.00%)
	1.34%	1.07%	1.02%	1.00%	0.02%	0.01%	0.01%	0.01%	0.00%	0.00%	0.00%	0.00%
Calabria	(1.21% - 1.49%)	(0.95% - 1.19%)	(0.91% - 1.15%)	(0.90% - 1.12%)	(0.01% - 0.04%)	(0.00% - 0.02%)	(0.01% - 0.03%)	(0.01% - 0.02%)	(0.00% - 0.00%)	(0.00% - 0.00%)	(0.00% - 0.00%)	(0.00% - 0.00%)
	1.38%	1.08%	1.02%	1.01%	0.02%	0.01%	0.01%	0.01%	0.00%	0.00%	0.00%	0.00%
Sicilia	(1.25% - 1.51%)	(0.98% - 1.19%)	(0.92% - 1.13%)	(0.90% - 1.12%)	(0.01% - 0.04%)	(0.00% - 0.02%)	(0.01% - 0.02%)	(0.01% - 0.02%)	(0.00% - 0.00%)	(0.00% - 0.00%)	(0.00% - 0.00%)	(0.00% - 0.00%)

Table S8c. Estimated overall, severe, and extreme **underweight** prevalence (%) in children under 5 years, both sexes, in 1990, 2000, 2010, and 2020.

Location	Total				Severe				Extreme			
	1990	2000	2010	2020	1990	2000	2010	2020	1990	2000	2010	2020
Sardegna	1.27% (1.14% - 1.42%)	1.03% (0.93% - 1.14%)	0.99% (0.89% - 1.10%)	0.96% (0.86% - 1.07%)	0.02% (0.01% - 0.03%)	0.01% (0.00% - 0.02%)	0.01% (0.01% - 0.02%)	0.01% (0.01% - 0.02%)	0.00% (0.00% - 0.00%)	0.00% (0.00% - 0.00%)	0.00% (0.00% - 0.00%)	0.00% (0.00% - 0.00%)
Luxembourg	1.45% (1.35% - 1.57%)	1.28% (1.19% - 1.38%)	1.27% (1.18% - 1.37%)	1.17% (1.09% - 1.26%)	0.03% (0.02% - 0.05%)	0.02% (0.02% - 0.03%)	0.03% (0.02% - 0.04%)	0.03% (0.02% - 0.04%)	0.00% (0.00% - 0.00%)	0.00% (0.00% - 0.00%)	0.00% (0.00% - 0.00%)	0.00% (0.00% - 0.00%)
Malta	1.45% (1.35% - 1.57%)	1.23% (1.14% - 1.32%)	1.25% (1.16% - 1.34%)	1.09% (1.02% - 1.18%)	0.03% (0.02% - 0.04%)	0.02% (0.01% - 0.02%)	0.03% (0.02% - 0.04%)	0.02% (0.01% - 0.03%)	0.00% (0.00% - 0.00%)	0.00% (0.00% - 0.00%)	0.00% (0.00% - 0.00%)	0.00% (0.00% - 0.00%)
Netherlands	0.90% (0.84% - 0.97%)	0.86% (0.80% - 0.92%)	0.81% (0.75% - 0.86%)	0.78% (0.72% - 0.84%)	0.04% (0.03% - 0.06%)	0.04% (0.03% - 0.05%)	0.04% (0.03% - 0.05%)	0.04% (0.03% - 0.05%)	0.00% (0.00% - 0.00%)	0.00% (0.00% - 0.00%)	0.00% (0.00% - 0.00%)	0.00% (0.00% - 0.00%)
Norway	1.22% (1.14% - 1.30%)	1.05% (0.97% - 1.12%)	1.07% (0.99% - 1.15%)	1.01% (0.94% - 1.08%)	0.02% (0.02% - 0.03%)	0.01% (0.01% - 0.02%)	0.02% (0.01% - 0.03%)	0.02% (0.01% - 0.03%)	0.00% (0.00% - 0.00%)	0.00% (0.00% - 0.00%)	0.00% (0.00% - 0.00%)	0.00% (0.00% - 0.00%)
Portugal	1.50% (1.40% - 1.61%)	1.25% (1.16% - 1.34%)	1.18% (1.10% - 1.27%)	1.12% (1.03% - 1.21%)	0.02% (0.02% - 0.03%)	0.01% (0.01% - 0.02%)	0.02% (0.01% - 0.03%)	0.02% (0.01% - 0.03%)	0.00% (0.00% - 0.00%)	0.00% (0.00% - 0.00%)	0.00% (0.00% - 0.00%)	0.00% (0.00% - 0.00%)
Spain	1.32% (1.20% - 1.45%)	0.81% (0.74% - 0.90%)	0.75% (0.68% - 0.83%)	0.73% (0.66% - 0.81%)	0.00% (0.00% - 0.00%)	0.00% (0.00% - 0.00%)	0.00% (0.00% - 0.00%)	0.00% (0.00% - 0.00%)	0.00% (0.00% - 0.00%)	0.00% (0.00% - 0.00%)	0.00% (0.00% - 0.00%)	0.00% (0.00% - 0.00%)
Sweden	1.23% (1.15% - 1.33%)	1.08% (0.99% - 1.16%)	1.05% (0.97% - 1.13%)	1.00% (0.92% - 1.07%)	0.03% (0.02% - 0.04%)	0.02% (0.01% - 0.02%)	0.02% (0.01% - 0.03%)	0.02% (0.01% - 0.02%)	0.00% (0.00% - 0.00%)	0.00% (0.00% - 0.00%)	0.00% (0.00% - 0.00%)	0.00% (0.00% - 0.00%)
Sweden except Stockholm	1.22% (1.13% - 1.32%)	1.06% (0.97% - 1.15%)	1.04% (0.95% - 1.12%)	0.98% (0.91% - 1.06%)	0.02% (0.02% - 0.04%)	0.01% (0.01% - 0.02%)	0.02% (0.01% - 0.03%)	0.02% (0.01% - 0.02%)	0.00% (0.00% - 0.00%)	0.00% (0.00% - 0.00%)	0.00% (0.00% - 0.00%)	0.00% (0.00% - 0.00%)
Stockholm	1.29% (1.17% - 1.44%)	1.13% (1.02% - 1.27%)	1.09% (0.98% - 1.22%)	1.04% (0.93% - 1.15%)	0.03% (0.02% - 0.05%)	0.02% (0.01% - 0.03%)	0.02% (0.01% - 0.04%)	0.02% (0.01% - 0.03%)	0.00% (0.00% - 0.00%)	0.00% (0.00% - 0.00%)	0.00% (0.00% - 0.00%)	0.00% (0.00% - 0.00%)
Switzerland	1.12% (1.04% - 1.21%)	1.02% (0.95% - 1.10%)	1.02% (0.94% - 1.10%)	0.98% (0.91% - 1.06%)	0.02% (0.01% - 0.03%)	0.01% (0.01% - 0.02%)	0.02% (0.01% - 0.03%)	0.02% (0.01% - 0.03%)	0.00% (0.00% - 0.00%)	0.00% (0.00% - 0.00%)	0.00% (0.00% - 0.00%)	0.00% (0.00% - 0.00%)
United Kingdom	1.31% (1.23% - 1.40%)	1.10% (1.03% - 1.18%)	1.09% (1.02% - 1.17%)	1.04% (0.97% - 1.12%)	0.03% (0.02% - 0.04%)	0.01% (0.01% - 0.02%)	0.02% (0.01% - 0.03%)	0.02% (0.01% - 0.02%)	0.00% (0.00% - 0.00%)	0.00% (0.00% - 0.00%)	0.00% (0.00% - 0.00%)	0.00% (0.00% - 0.00%)
Northern Ireland	1.37% (1.22% - 1.54%)	1.12% (0.99% - 1.26%)	1.15% (1.01% - 1.29%)	1.08% (0.95% - 1.21%)	0.03% (0.01% - 0.04%)	0.01% (0.00% - 0.02%)	0.02% (0.01% - 0.04%)	0.02% (0.01% - 0.03%)	0.00% (0.00% - 0.00%)	0.00% (0.00% - 0.00%)	0.00% (0.00% - 0.00%)	0.00% (0.00% - 0.00%)
Scotland	1.34% (1.19% - 1.51%)	1.10% (0.97% - 1.24%)	1.12% (0.99% - 1.26%)	1.07% (0.95% - 1.20%)	0.02% (0.01% - 0.04%)	0.01% (0.00% - 0.02%)	0.02% (0.01% - 0.03%)	0.02% (0.01% - 0.03%)	0.00% (0.00% - 0.00%)	0.00% (0.00% - 0.00%)	0.00% (0.00% - 0.00%)	0.00% (0.00% - 0.00%)
Wales	1.42% (1.26% - 1.59%)	1.16% (1.03% - 1.31%)	1.17% (1.04% - 1.32%)	1.10% (0.97% - 1.22%)	0.03% (0.02% - 0.05%)	0.01% (0.01% - 0.03%)	0.02% (0.01% - 0.04%)	0.02% (0.01% - 0.03%)	0.00% (0.00% - 0.00%)	0.00% (0.00% - 0.00%)	0.00% (0.00% - 0.00%)	0.00% (0.00% - 0.00%)
England	1.30% (1.22% - 1.39%)	1.10% (1.02% - 1.18%)	1.09% (1.01% - 1.17%)	1.03% (0.96% - 1.11%)	0.03% (0.02% - 0.03%)	0.01% (0.01% - 0.02%)	0.02% (0.01% - 0.03%)	0.02% (0.01% - 0.02%)	0.00% (0.00% - 0.00%)	0.00% (0.00% - 0.00%)	0.00% (0.00% - 0.00%)	0.00% (0.00% - 0.00%)
North East England	1.41% (1.26% - 1.56%)	1.16% (1.03% - 1.29%)	1.14% (1.02% - 1.26%)	1.08% (0.97% - 1.21%)	0.03% (0.02% - 0.04%)	0.01% (0.01% - 0.03%)	0.02% (0.01% - 0.03%)	0.02% (0.01% - 0.03%)	0.00% (0.00% - 0.00%)	0.00% (0.00% - 0.00%)	0.00% (0.00% - 0.00%)	0.00% (0.00% - 0.00%)
North West England	1.34% (1.21% - 1.48%)	1.12% (1.01% - 1.25%)	1.12% (1.02% - 1.23%)	1.06% (0.96% - 1.17%)	0.03% (0.01% - 0.04%)	0.01% (0.01% - 0.02%)	0.02% (0.01% - 0.03%)	0.02% (0.01% - 0.03%)	0.00% (0.00% - 0.00%)	0.00% (0.00% - 0.00%)	0.00% (0.00% - 0.00%)	0.00% (0.00% - 0.00%)
Yorkshire and the Humber	1.34% (1.22% - 1.47%)	1.13% (1.02% - 1.25%)	1.12% (1.02% - 1.24%)	1.07% (0.97% - 1.18%)	0.03% (0.01% - 0.04%)	0.01% (0.01% - 0.02%)	0.02% (0.01% - 0.03%)	0.02% (0.01% - 0.03%)	0.00% (0.00% - 0.00%)	0.00% (0.00% - 0.00%)	0.00% (0.00% - 0.00%)	0.00% (0.00% - 0.00%)
East Midlands	1.32% (1.19% - 1.45%)	1.10% (1.00% - 1.22%)	1.11% (1.00% - 1.22%)	1.05% (0.95% - 1.17%)	0.03% (0.01% - 0.04%)	0.01% (0.01% - 0.02%)	0.02% (0.01% - 0.03%)	0.02% (0.01% - 0.03%)	0.00% (0.00% - 0.00%)	0.00% (0.00% - 0.00%)	0.00% (0.00% - 0.00%)	0.00% (0.00% - 0.00%)
West Midlands	1.37% (1.24% - 1.51%)	1.15% (1.05% - 1.27%)	1.13% (1.03% - 1.24%)	1.08% (0.97% - 1.19%)	0.03% (0.01% - 0.04%)	0.01% (0.01% - 0.02%)	0.02% (0.01% - 0.03%)	0.02% (0.01% - 0.03%)	0.00% (0.00% - 0.00%)	0.00% (0.00% - 0.00%)	0.00% (0.00% - 0.00%)	0.00% (0.00% - 0.00%)
East of England	1.26% (1.15% - 1.39%)	1.06% (0.96% - 1.18%)	1.07% (0.97% - 1.18%)	1.02% (0.91% - 1.12%)	0.02% (0.01% - 0.04%)	0.01% (0.01% - 0.02%)	0.02% (0.01% - 0.03%)	0.02% (0.01% - 0.03%)	0.00% (0.00% - 0.00%)	0.00% (0.00% - 0.00%)	0.00% (0.00% - 0.00%)	0.00% (0.00% - 0.00%)
Greater London	1.25% (1.15% - 1.38%)	1.07% (0.96% - 1.18%)	1.03% (0.94% - 1.13%)	0.97% (0.88% - 1.06%)	0.02% (0.01% - 0.04%)	0.01% (0.01% - 0.02%)	0.02% (0.01% - 0.03%)	0.01% (0.01% - 0.02%)	0.00% (0.00% - 0.00%)	0.00% (0.00% - 0.00%)	0.00% (0.00% - 0.00%)	0.00% (0.00% - 0.00%)

Table S8c. Estimated overall, severe, and extreme **underweight** prevalence (%) in children under 5 years, both sexes, in 1990, 2000, 2010, and 2020.												
**Location**	**Total**				**Severe**				**Extreme**			
	**1990**	**2000**	**2010**	**2020**	**1990**	**2000**	**2010**	**2020**	**1990**	**2000**	**2010**	**2020**
South East England	1.23%	1.05%	1.05%	1.01%	0.02%	0.01%	0.02%	0.02%	0.00%	0.00%	0.00%	0.00%
	(1.11% - 1.35%)	(0.95% - 1.16%)	(0.95% - 1.16%)	(0.92% - 1.12%)	(0.01% - 0.04%)	(0.01% - 0.02%)	(0.01% - 0.03%)	(0.01% - 0.03%)	(0.00% - 0.00%)	(0.00% - 0.00%)	(0.00% - 0.00%)	(0.00% - 0.00%)
South West England	1.29%	1.09%	1.10%	1.05%	0.03%	0.01%	0.02%	0.02%	0.00%	0.00%	0.00%	0.00%
	(1.17% - 1.42%)	(0.97% - 1.21%)	(1.00% - 1.21%)	(0.95% - 1.15%)	(0.01% - 0.04%)	(0.01% - 0.02%)	(0.01% - 0.03%)	(0.01% - 0.03%)	(0.00% - 0.00%)	(0.00% - 0.00%)	(0.00% - 0.00%)	(0.00% - 0.00%)
Latin America and Caribbean	7.59%	6.00%	4.83%	4.41%	1.22%	0.86%	0.59%	0.53%	0.09%	0.06%	0.03%	0.02%
	(7.52% - 7.67%)	(5.94% - 6.06%)	(4.77% - 4.88%)	(4.35% - 4.47%)	(1.19% - 1.26%)	(0.83% - 0.89%)	(0.57% - 0.61%)	(0.50% - 0.55%)	(0.08% - 0.10%)	(0.05% - 0.07%)	(0.02% - 0.03%)	(0.02% - 0.03%)
**Andean Latin America**	9.24%	7.04%	5.26%	4.35%	1.47%	0.91%	0.52%	0.40%	0.05%	0.02%	0.00%	0.00%
	(8.96% - 9.51%)	(6.81% - 7.26%)	(5.08% - 5.43%)	(4.17% - 4.54%)	(1.36% - 1.58%)	(0.84% - 0.98%)	(0.47% - 0.57%)	(0.35% - 0.45%)	(0.04% - 0.06%)	(0.01% - 0.03%)	(0.00% - 0.01%)	(0.00% - 0.00%)
Bolivia (Plurinational State of)	10.13%	7.37%	5.23%	4.27%	1.95%	1.02%	0.55%	0.41%	0.07%	0.01%	0.00%	0.00%
	(9.73% - 10.55%)	(6.98% - 7.80%)	(4.90% - 5.60%)	(3.97% - 4.59%)	(1.78% - 2.14%)	(0.89% - 1.17%)	(0.46% - 0.64%)	(0.33% - 0.50%)	(0.05% - 0.10%)	(0.00% - 0.03%)	(0.00% - 0.01%)	(0.00% - 0.00%)
Ecuador	11.74%	8.92%	6.71%	5.92%	2.17%	1.39%	0.80%	0.68%	0.13%	0.06%	0.01%	0.00%
	(11.12% - 12.40%)	(8.41% - 9.44%)	(6.30% - 7.16%)	(5.50% - 6.37%)	(1.89% - 2.47%)	(1.21% - 1.59%)	(0.66% - 0.95%)	(0.55% - 0.81%)	(0.09% - 0.17%)	(0.04% - 0.09%)	(0.00% - 0.02%)	(0.00% - 0.01%)
Peru	7.80%	5.89%	4.39%	3.48%	0.98%	0.61%	0.34%	0.23%	0.00%	0.00%	0.00%	0.00%
	(7.41% - 8.20%)	(5.58% - 6.18%)	(4.20% - 4.60%)	(3.26% - 3.73%)	(0.85% - 1.12%)	(0.52% - 0.71%)	(0.30% - 0.39%)	(0.18% - 0.29%)	(0.00% - 0.01%)	(0.00% - 0.00%)	(0.00% - 0.00%)	(0.00% - 0.00%)
**Caribbean**	9.50%	8.25%	7.68%	7.24%	2.65%	1.97%	1.64%	1.46%	0.53%	0.30%	0.18%	0.13%
	(9.28% - 9.70%)	(8.05% - 8.46%)	(7.43% - 7.91%)	(6.94% - 7.53%)	(2.48% - 2.84%)	(1.82% - 2.11%)	(1.49% - 1.79%)	(1.30% - 1.65%)	(0.45% - 0.62%)	(0.25% - 0.36%)	(0.14% - 0.23%)	(0.09% - 0.19%)
Antigua and Barbuda	7.32%	6.35%	5.32%	4.99%	1.46%	1.12%	0.82%	0.74%	0.13%	0.06%	0.02%	0.01%
	(6.84% - 7.79%)	(5.92% - 6.81%)	(4.92% - 5.72%)	(4.64% - 5.41%)	(1.27% - 1.66%)	(0.97% - 1.29%)	(0.69% - 0.95%)	(0.64% - 0.87%)	(0.09% - 0.17%)	(0.03% - 0.09%)	(0.00% - 0.03%)	(0.00% - 0.02%)
Bahamas	5.73%	4.97%	4.74%	4.62%	0.83%	0.65%	0.61%	0.59%	0.01%	0.00%	0.00%	0.00%
	(5.33% - 6.17%)	(4.59% - 5.38%)	(4.39% - 5.11%)	(4.27% - 4.98%)	(0.70% - 0.98%)	(0.54% - 0.78%)	(0.50% - 0.71%)	(0.49% - 0.71%)	(0.00% - 0.02%)	(0.00% - 0.01%)	(0.00% - 0.01%)	(0.00% - 0.01%)
Barbados	4.10%	3.89%	3.71%	3.52%	0.29%	0.27%	0.25%	0.23%	0.00%	0.00%	0.00%	0.00%
	(3.78% - 4.43%)	(3.61% - 4.19%)	(3.43% - 3.98%)	(3.26% - 3.80%)	(0.23% - 0.37%)	(0.21% - 0.34%)	(0.19% - 0.31%)	(0.17% - 0.30%)	(0.00% - 0.00%)	(0.00% - 0.00%)	(0.00% - 0.00%)	(0.00% - 0.00%)
Belize	6.25%	6.02%	5.68%	5.31%	0.66%	0.66%	0.63%	0.57%	0.00%	0.00%	0.00%	0.00%
	(5.91% - 6.62%)	(5.60% - 6.42%)	(5.30% - 6.07%)	(4.88% - 5.75%)	(0.56% - 0.78%)	(0.53% - 0.80%)	(0.51% - 0.76%)	(0.45% - 0.70%)	(0.00% - 0.00%)	(0.00% - 0.00%)	(0.00% - 0.00%)	(0.00% - 0.00%)
Cuba	1.86%	2.49%	2.16%	1.98%	0.01%	0.04%	0.03%	0.03%	0.00%	0.00%	0.00%	0.00%
	(1.73% - 2.00%)	(2.31% - 2.67%)	(1.99% - 2.36%)	(1.80% - 2.17%)	(0.01% - 0.02%)	(0.02% - 0.05%)	(0.02% - 0.05%)	(0.02% - 0.05%)	(0.00% - 0.00%)	(0.00% - 0.00%)	(0.00% - 0.00%)	(0.00% - 0.00%)
Dominica	5.51%	4.36%	4.09%	4.08%	0.68%	0.45%	0.41%	0.41%	0.00%	0.00%	0.00%	0.00%
	(5.11% - 5.95%)	(4.03% - 4.70%)	(3.77% - 4.42%)	(3.75% - 4.42%)	(0.57% - 0.81%)	(0.37% - 0.55%)	(0.32% - 0.50%)	(0.33% - 0.50%)	(0.00% - 0.01%)	(0.00% - 0.00%)	(0.00% - 0.00%)	(0.00% - 0.00%)
Dominican Republic	7.28%	4.93%	4.07%	3.57%	0.98%	0.46%	0.31%	0.26%	0.01%	0.00%	0.00%	0.00%
	(6.91% - 7.69%)	(4.65% - 5.22%)	(3.82% - 4.36%)	(3.29% - 3.85%)	(0.84% - 1.13%)	(0.39% - 0.54%)	(0.25% - 0.38%)	(0.20% - 0.33%)	(0.00% - 0.02%)	(0.00% - 0.00%)	(0.00% - 0.00%)	(0.00% - 0.00%)
Grenada	7.24%	5.76%	5.36%	4.91%	1.02%	0.75%	0.67%	0.60%	0.01%	0.01%	0.00%	0.00%
	(6.70% - 7.79%)	(5.35% - 6.21%)	(4.97% - 5.76%)	(4.55% - 5.32%)	(0.84% - 1.23%)	(0.62% - 0.90%)	(0.56% - 0.80%)	(0.49% - 0.72%)	(0.00% - 0.03%)	(0.00% - 0.02%)	(0.00% - 0.01%)	(0.00% - 0.01%)
Guyana	14.33%	11.15%	9.58%	7.86%	3.53%	2.41%	1.83%	1.40%	0.38%	0.17%	0.09%	0.05%
	(13.61% - 15.07%)	(10.52% - 11.74%)	(9.05% - 10.13%)	(7.34% - 8.41%)	(3.07% - 4.00%)	(2.11% - 2.73%)	(1.60% - 2.07%)	(1.18% - 1.62%)	(0.26% - 0.51%)	(0.11% - 0.25%)	(0.06% - 0.13%)	(0.02% - 0.07%)
Haiti	21.27%	17.68%	14.59%	12.76%	8.17%	5.70%	3.95%	3.18%	1.96%	1.02%	0.50%	0.32%
	(20.62% - 21.92%)	(17.08% - 18.34%)	(13.97% - 15.18%)	(12.09% - 13.49%)	(7.54% - 8.83%)	(5.22% - 6.21%)	(3.55% - 4.36%)	(2.79% - 3.64%)	(1.66% - 2.30%)	(0.83% - 1.23%)	(0.38% - 0.63%)	(0.22% - 0.45%)
Jamaica	5.43%	3.93%	3.36%	2.99%	0.56%	0.31%	0.20%	0.16%	0.00%	0.00%	0.00%	0.00%
	(5.08% - 5.78%)	(3.68% - 4.19%)	(3.12% - 3.60%)	(2.75% - 3.27%)	(0.47% - 0.67%)	(0.25% - 0.37%)	(0.16% - 0.26%)	(0.12% - 0.21%)	(0.00% - 0.00%)	(0.00% - 0.00%)	(0.00% - 0.00%)	(0.00% - 0.00%)
Saint Lucia	5.64%	4.68%	4.01%	3.83%	0.59%	0.46%	0.39%	0.36%	0.00%	0.00%	0.00%	0.00%
	(5.16% - 6.08%)	(4.33% - 5.08%)	(3.71% - 4.33%)	(3.55% - 4.16%)	(0.46% - 0.72%)	(0.37% - 0.57%)	(0.30% - 0.49%)	(0.29% - 0.45%)	(0.00% - 0.00%)	(0.00% - 0.00%)	(0.00% - 0.00%)	(0.00% - 0.00%)
Saint Vincent and the Grenadines	6.63%	5.42%	4.58%	4.30%	0.89%	0.62%	0.47%	0.43%	0.01%	0.00%	0.00%	0.00%
	(6.12% - 7.16%)	(5.01% - 5.88%)	(4.21% - 4.95%)	(3.96% - 4.64%)	(0.73% - 1.08%)	(0.50% - 0.76%)	(0.37% - 0.58%)	(0.34% - 0.53%)	(0.00% - 0.02%)	(0.00% - 0.01%)	(0.00% - 0.00%)	(0.00% - 0.00%)
Suriname	9.85%	9.43%	6.86%	6.41%	1.37%	1.39%	0.85%	0.79%	0.01%	0.01%	0.00%	0.00%
	(9.18% - 10.52%)	(8.89% - 9.94%)	(6.42% - 7.29%)	(5.92% - 6.92%)	(1.12% - 1.65%)	(1.19% - 1.62%)	(0.71% - 0.99%)	(0.64% - 0.95%)	(0.00% - 0.03%)	(0.00% - 0.03%)	(0.00% - 0.00%)	(0.00% - 0.00%)
Trinidad and Tobago	4.81%	4.72%	4.82%	4.45%	0.43%	0.46%	0.58%	0.52%	0.00%	0.00%	0.00%	0.00%
	(4.46% - 5.19%)	(4.39% - 5.05%)	(4.50% - 5.16%)	(4.14% - 4.80%)	(0.35% - 0.53%)	(0.38% - 0.56%)	(0.48% - 0.67%)	(0.44% - 0.62%)	(0.00% - 0.00%)	(0.00% - 0.00%)	(0.00% - 0.00%)	(0.00% - 0.00%)

Table S8c. Estimated overall, severe, and extreme **underweight** prevalence (%) in children under 5 years, both sexes, in 1990, 2000, 2010, and 2020.

Location	Total				Severe				Extreme			
	1990	2000	2010	2020	1990	2000	2010	2020	1990	2000	2010	2020
	4.43%	4.03%	3.76%	3.50%	0.60%	0.55%	0.51%	0.46%	0.00%	0.00%	0.00%	0.00%
Bermuda	(4.10% - 4.76%)	(3.74% - 4.32%)	(3.49% - 4.04%)	(3.24% - 3.77%)	(0.50% - 0.71%)	(0.46% - 0.64%)	(0.43% - 0.60%)	(0.38% - 0.55%)	(0.00% - 0.01%)	(0.00% - 0.01%)	(0.00% - 0.00%)	(0.00% - 0.00%)
	4.40%	3.60%	3.20%	3.02%	0.57%	0.40%	0.33%	0.31%	0.00%	0.00%	0.00%	0.00%
Puerto Rico	(4.06% - 4.75%)	(3.33% - 3.89%)	(2.93% - 3.46%)	(2.77% - 3.29%)	(0.47% - 0.68%)	(0.32% - 0.48%)	(0.27% - 0.40%)	(0.25% - 0.38%)	(0.00% - 0.00%)	(0.00% - 0.00%)	(0.00% - 0.00%)	(0.00% - 0.00%)
	5.96%	4.99%	4.68%	4.32%	0.79%	0.62%	0.58%	0.51%	0.00%	0.00%	0.00%	0.00%
Saint Kitts and Nevis	(5.52% - 6.45%)	(4.63% - 5.35%)	(4.32% - 5.04%)	(4.00% - 4.64%)	(0.65% - 0.94%)	(0.52% - 0.74%)	(0.48% - 0.69%)	(0.43% - 0.61%)	(0.00% - 0.01%)	(0.00% - 0.01%)	(0.00% - 0.00%)	(0.00% - 0.00%)
	5.20%	4.08%	3.54%	3.60%	0.70%	0.46%	0.36%	0.38%	0.00%	0.00%	0.00%	0.00%
United States Virgin Islands	(4.84% - 5.63%)	(3.78% - 4.42%)	(3.29% - 3.84%)	(3.32% - 3.89%)	(0.59% - 0.84%)	(0.38% - 0.56%)	(0.29% - 0.43%)	(0.31% - 0.46%)	(0.00% - 0.01%)	(0.00% - 0.00%)	(0.00% - 0.00%)	(0.00% - 0.00%)
	8.24%	6.55%	5.15%	4.75%	1.32%	0.95%	0.59%	0.53%	0.08%	0.05%	0.01%	0.01%
**Central Latin America**	(8.13% - 8.36%)	(6.46% - 6.65%)	(5.06% - 5.24%)	(4.65% - 4.85%)	(1.26% - 1.38%)	(0.90% - 1.00%)	(0.56% - 0.63%)	(0.50% - 0.58%)	(0.06% - 0.09%)	(0.04% - 0.07%)	(0.01% - 0.02%)	(0.00% - 0.02%)
	7.73%	5.47%	4.05%	3.44%	1.02%	0.50%	0.25%	0.20%	0.00%	0.00%	0.00%	0.00%
Colombia	(7.28% - 8.20%)	(5.16% - 5.79%)	(3.80% - 4.30%)	(3.18% - 3.70%)	(0.86% - 1.18%)	(0.41% - 0.59%)	(0.20% - 0.31%)	(0.15% - 0.26%)	(0.00% - 0.01%)	(0.00% - 0.00%)	(0.00% - 0.00%)	(0.00% - 0.00%)
	2.24%	1.92%	1.53%	1.37%	0.04%	0.03%	0.01%	0.01%	0.00%	0.00%	0.00%	0.00%
Costa Rica	(2.05% - 2.45%)	(1.75% - 2.12%)	(1.40% - 1.66%)	(1.24% - 1.50%)	(0.03% - 0.06%)	(0.02% - 0.04%)	(0.01% - 0.02%)	(0.00% - 0.02%)	(0.00% - 0.00%)	(0.00% - 0.00%)	(0.00% - 0.00%)	(0.00% - 0.00%)
	10.33%	7.47%	5.73%	4.89%	1.49%	0.91%	0.59%	0.47%	0.01%	0.00%	0.00%	0.00%
El Salvador	(9.82% - 10.83%)	(7.12% - 7.83%)	(5.39% - 6.09%)	(4.54% - 5.28%)	(1.28% - 1.73%)	(0.77% - 1.04%)	(0.50% - 0.70%)	(0.38% - 0.58%)	(0.00% - 0.03%)	(0.00% - 0.00%)	(0.00% - 0.00%)	(0.00% - 0.00%)
	23.24%	18.19%	13.09%	11.48%	6.27%	4.87%	2.62%	2.10%	0.74%	0.60%	0.14%	0.07%
Guatemala	(22.54% - 23.99%)	(17.55% - 18.79%)	(12.45% - 13.72%)	(10.81% - 12.17%)	(5.57% - 6.98%)	(4.37% - 5.40%)	(2.26% - 2.99%)	(1.75% - 2.45%)	(0.53% - 0.99%)	(0.44% - 0.78%)	(0.07% - 0.23%)	(0.02% - 0.14%)
	15.43%	12.12%	7.98%	7.17%	3.55%	2.17%	1.04%	0.91%	0.30%	0.06%	0.00%	0.00%
Honduras	(14.82% - 16.03%)	(11.57% - 12.70%)	(7.46% - 8.49%)	(6.64% - 7.73%)	(3.13% - 3.98%)	(1.89% - 2.47%)	(0.86% - 1.23%)	(0.73% - 1.11%)	(0.21% - 0.41%)	(0.02% - 0.12%)	(0.00% - 0.01%)	(0.00% - 0.01%)
	6.62%	5.26%	4.48%	4.17%	0.83%	0.58%	0.47%	0.43%	0.03%	0.01%	0.01%	0.01%
Mexico	(6.50% - 6.72%)	(5.15% - 5.36%)	(4.38% - 4.58%)	(4.07% - 4.26%)	(0.79% - 0.87%)	(0.54% - 0.61%)	(0.43% - 0.50%)	(0.40% - 0.47%)	(0.02% - 0.04%)	(0.01% - 0.01%)	(0.00% - 0.01%)	(0.00% - 0.01%)
	4.09%	3.31%	3.25%	3.09%	0.25%	0.17%	0.20%	0.20%	0.00%	0.00%	0.00%	0.00%
Aguascalientes	(3.81% - 4.41%)	(3.08% - 3.57%)	(3.01% - 3.52%)	(2.85% - 3.32%)	(0.19% - 0.31%)	(0.13% - 0.22%)	(0.15% - 0.26%)	(0.15% - 0.26%)	(0.00% - 0.00%)	(0.00% - 0.00%)	(0.00% - 0.00%)	(0.00% - 0.00%)
	4.32%	3.18%	2.75%	2.58%	0.30%	0.17%	0.11%	0.09%	0.00%	0.00%	0.00%	0.00%
Baja California	(3.95% - 4.70%)	(2.90% - 3.48%)	(2.47% - 3.03%)	(2.34% - 2.85%)	(0.21% - 0.41%)	(0.11% - 0.25%)	(0.05% - 0.16%)	(0.05% - 0.14%)	(0.00% - 0.00%)	(0.00% - 0.00%)	(0.00% - 0.00%)	(0.00% - 0.00%)
	4.80%	3.94%	3.57%	3.45%	0.23%	0.20%	0.20%	0.20%	0.00%	0.00%	0.00%	0.00%
Baja California Sur	(4.44% - 5.21%)	(3.65% - 4.26%)	(3.31% - 3.86%)	(3.21% - 3.72%)	(0.15% - 0.33%)	(0.14% - 0.27%)	(0.14% - 0.26%)	(0.15% - 0.27%)	(0.00% - 0.00%)	(0.00% - 0.00%)	(0.00% - 0.00%)	(0.00% - 0.00%)
	5.91%	4.62%	4.09%	3.65%	0.70%	0.51%	0.43%	0.35%	0.01%	0.00%	0.00%	0.00%
Campeche	(5.43% - 6.39%)	(4.28% - 4.99%)	(3.77% - 4.40%)	(3.34% - 3.96%)	(0.54% - 0.88%)	(0.41% - 0.63%)	(0.34% - 0.53%)	(0.27% - 0.45%)	(0.00% - 0.02%)	(0.00% - 0.00%)	(0.00% - 0.00%)	(0.00% - 0.00%)
	5.10%	3.96%	3.81%	3.68%	0.44%	0.29%	0.25%	0.24%	0.00%	0.00%	0.00%	0.00%
Coahuila	(4.71% - 5.52%)	(3.63% - 4.30%)	(3.50% - 4.14%)	(3.36% - 4.03%)	(0.33% - 0.58%)	(0.22% - 0.38%)	(0.19% - 0.34%)	(0.17% - 0.32%)	(0.00% - 0.00%)	(0.00% - 0.00%)	(0.00% - 0.00%)	(0.00% - 0.00%)
	1.21%	0.86%	0.79%	0.75%	0.01%	0.00%	0.00%	0.00%	0.00%	0.00%	0.00%	0.00%
Colima	(1.13% - 1.30%)	(0.80% - 0.92%)	(0.73% - 0.86%)	(0.69% - 0.81%)	(0.00% - 0.02%)	(0.00% - 0.01%)	(0.00% - 0.01%)	(0.00% - 0.01%)	(0.00% - 0.00%)	(0.00% - 0.00%)	(0.00% - 0.00%)	(0.00% - 0.00%)
	13.16%	10.04%	7.14%	6.19%	2.88%	1.55%	0.77%	0.55%	0.21%	0.02%	0.00%	0.00%
Chiapas	(12.72% - 13.59%)	(9.45% - 10.60%)	(6.66% - 7.68%)	(5.73% - 6.67%)	(2.61% - 3.13%)	(1.32% - 1.80%)	(0.63% - 0.94%)	(0.43% - 0.68%)	(0.16% - 0.27%)	(0.01% - 0.04%)	(0.00% - 0.01%)	(0.00% - 0.00%)
	5.73%	5.07%	4.49%	4.26%	0.71%	0.61%	0.48%	0.41%	0.01%	0.00%	0.00%	0.00%
Chihuahua	(5.32% - 6.14%)	(4.72% - 5.42%)	(4.18% - 4.81%)	(3.95% - 4.61%)	(0.59% - 0.84%)	(0.51% - 0.71%)	(0.40% - 0.57%)	(0.34% - 0.50%)	(0.00% - 0.02%)	(0.00% - 0.01%)	(0.00% - 0.00%)	(0.00% - 0.00%)
	2.95%	2.37%	2.14%	1.93%	0.16%	0.12%	0.10%	0.08%	0.00%	0.00%	0.00%	0.00%
Mexico City	(2.76% - 3.16%)	(2.21% - 2.53%)	(1.99% - 2.29%)	(1.80% - 2.07%)	(0.12% - 0.20%)	(0.09% - 0.15%)	(0.07% - 0.12%)	(0.06% - 0.11%)	(0.00% - 0.00%)	(0.00% - 0.00%)	(0.00% - 0.00%)	(0.00% - 0.00%)
	4.50%	3.30%	2.86%	2.60%	0.16%	0.11%	0.10%	0.09%	0.00%	0.00%	0.00%	0.00%
Durango	(4.16% - 4.87%)	(3.03% - 3.55%)	(2.64% - 3.11%)	(2.39% - 2.83%)	(0.10% - 0.24%)	(0.07% - 0.16%)	(0.07% - 0.15%)	(0.05% - 0.13%)	(0.00% - 0.00%)	(0.00% - 0.00%)	(0.00% - 0.00%)	(0.00% - 0.00%)
	6.68%	4.79%	3.97%	3.60%	0.70%	0.43%	0.26%	0.22%	0.00%	0.00%	0.00%	0.00%
Guanajuato	(6.19% - 7.20%)	(4.40% - 5.17%)	(3.65% - 4.27%)	(3.32% - 3.88%)	(0.54% - 0.89%)	(0.33% - 0.54%)	(0.20% - 0.32%)	(0.16% - 0.29%)	(0.00% - 0.02%)	(0.00% - 0.00%)	(0.00% - 0.00%)	(0.00% - 0.00%)
	12.13%	8.48%	5.40%	4.41%	2.57%	1.22%	0.53%	0.45%	0.16%	0.00%	0.00%	0.00%
Guerrero	(11.81% - 12.46%)	(8.03% - 8.94%)	(4.99% - 5.79%)	(4.08% - 4.77%)	(2.38% - 2.76%)	(1.04% - 1.42%)	(0.42% - 0.65%)	(0.36% - 0.56%)	(0.11% - 0.21%)	(0.00% - 0.02%)	(0.00% - 0.00%)	(0.00% - 0.00%)

Table S8c. Estimated overall, severe, and extreme **underweight** prevalence (%) in children under 5 years, both sexes, in 1990, 2000, 2010, and 2020.												
**Location**	**Total**				**Severe**				**Extreme**			
	**1990**	**2000**	**2010**	**2020**	**1990**	**2000**	**2010**	**2020**	**1990**	**2000**	**2010**	**2020**
	8.21%	6.36%	5.46%	4.93%	0.87%	0.69%	0.62%	0.58%	0.00%	0.00%	0.00%	0.00%
Hidalgo	(7.60% - 8.87%)	(5.92% - 6.85%)	(5.07% - 5.86%)	(4.56% - 5.32%)	(0.68% - 1.10%)	(0.55% - 0.83%)	(0.50% - 0.75%)	(0.47% - 0.71%)	(0.00% - 0.01%)	(0.00% - 0.01%)	(0.00% - 0.01%)	(0.00% - 0.01%)
	3.85%	3.13%	2.59%	2.44%	0.15%	0.12%	0.10%	0.09%	0.00%	0.00%	0.00%	0.00%
Jalisco	(3.41% - 4.28%)	(2.76% - 3.45%)	(2.38% - 2.80%)	(2.21% - 2.65%)	(0.10% - 0.22%)	(0.07% - 0.17%)	(0.07% - 0.14%)	(0.06% - 0.13%)	(0.00% - 0.00%)	(0.00% - 0.00%)	(0.00% - 0.00%)	(0.00% - 0.00%)
	9.49%	7.68%	7.80%	7.40%	1.40%	1.09%	1.29%	1.23%	0.01%	0.00%	0.03%	0.03%
México	(8.87% - 10.14%)	(7.12% - 8.28%)	(7.20% - 8.40%)	(6.81% - 7.98%)	(1.15% - 1.69%)	(0.89% - 1.31%)	(1.05% - 1.57%)	(1.00% - 1.50%)	(0.00% - 0.03%)	(0.00% - 0.01%)	(0.00% - 0.08%)	(0.00% - 0.08%)
	5.43%	3.96%	2.73%	2.43%	0.44%	0.25%	0.09%	0.07%	0.00%	0.00%	0.00%	0.00%
Michoacán de Ocampo	(4.97% - 5.91%)	(3.60% - 4.34%)	(2.47% - 2.98%)	(2.18% - 2.65%)	(0.31% - 0.61%)	(0.16% - 0.34%)	(0.05% - 0.13%)	(0.04% - 0.11%)	(0.00% - 0.00%)	(0.00% - 0.00%)	(0.00% - 0.00%)	(0.00% - 0.00%)
	5.73%	4.46%	3.86%	3.62%	0.55%	0.42%	0.36%	0.34%	0.00%	0.00%	0.00%	0.00%
Morelos	(5.25% - 6.18%)	(4.11% - 4.86%)	(3.55% - 4.18%)	(3.31% - 3.95%)	(0.39% - 0.74%)	(0.31% - 0.55%)	(0.27% - 0.48%)	(0.25% - 0.45%)	(0.00% - 0.00%)	(0.00% - 0.00%)	(0.00% - 0.00%)	(0.00% - 0.00%)
	5.65%	4.42%	3.82%	3.55%	0.48%	0.36%	0.30%	0.27%	0.00%	0.00%	0.00%	0.00%
Nayarit	(5.19% - 6.09%)	(4.06% - 4.81%)	(3.53% - 4.13%)	(3.26% - 3.87%)	(0.36% - 0.61%)	(0.28% - 0.46%)	(0.24% - 0.39%)	(0.21% - 0.35%)	(0.00% - 0.00%)	(0.00% - 0.00%)	(0.00% - 0.00%)	(0.00% - 0.00%)
	2.08%	1.75%	1.52%	1.43%	0.01%	0.01%	0.00%	0.00%	0.00%	0.00%	0.00%	0.00%
Nuevo León	(1.91% - 2.28%)	(1.60% - 1.92%)	(1.39% - 1.65%)	(1.31% - 1.57%)	(0.01% - 0.02%)	(0.00% - 0.01%)	(0.00% - 0.01%)	(0.00% - 0.01%)	(0.00% - 0.00%)	(0.00% - 0.00%)	(0.00% - 0.00%)	(0.00% - 0.00%)
	9.98%	8.11%	6.07%	5.41%	1.29%	1.00%	0.64%	0.55%	0.01%	0.00%	0.00%	0.00%
Oaxaca	(9.26% - 10.74%)	(7.57% - 8.72%)	(5.59% - 6.57%)	(4.99% - 5.92%)	(1.00% - 1.65%)	(0.80% - 1.23%)	(0.49% - 0.81%)	(0.42% - 0.71%)	(0.00% - 0.04%)	(0.00% - 0.01%)	(0.00% - 0.00%)	(0.00% - 0.00%)
	6.26%	5.31%	5.06%	5.87%	0.39%	0.39%	0.50%	0.82%	0.00%	0.00%	0.00%	0.03%
Puebla	(5.76% - 6.82%)	(4.89% - 5.79%)	(4.65% - 5.49%)	(5.37% - 6.38%)	(0.26% - 0.56%)	(0.28% - 0.54%)	(0.37% - 0.65%)	(0.59% - 1.09%)	(0.00% - 0.00%)	(0.00% - 0.00%)	(0.00% - 0.01%)	(0.00% - 0.10%)
	5.13%	3.59%	3.05%	2.87%	0.45%	0.23%	0.16%	0.15%	0.00%	0.00%	0.00%	0.00%
Querétaro	(4.71% - 5.54%)	(3.31% - 3.91%)	(2.82% - 3.32%)	(2.63% - 3.12%)	(0.35% - 0.56%)	(0.18% - 0.30%)	(0.12% - 0.21%)	(0.11% - 0.21%)	(0.00% - 0.00%)	(0.00% - 0.00%)	(0.00% - 0.00%)	(0.00% - 0.00%)
	3.10%	2.31%	2.04%	1.89%	0.08%	0.05%	0.03%	0.03%	0.00%	0.00%	0.00%	0.00%
Quintana Roo	(2.86% - 3.37%)	(2.12% - 2.50%)	(1.90% - 2.20%)	(1.75% - 2.06%)	(0.06% - 0.11%)	(0.03% - 0.06%)	(0.02% - 0.05%)	(0.02% - 0.04%)	(0.00% - 0.00%)	(0.00% - 0.00%)	(0.00% - 0.00%)	(0.00% - 0.00%)
	11.46%	8.79%	5.55%	4.85%	2.79%	2.02%	1.02%	0.87%	0.41%	0.30%	0.12%	0.08%
San Luis Potosí	(10.72% - 12.22%)	(8.20% - 9.48%)	(5.07% - 6.01%)	(4.38% - 5.31%)	(2.23% - 3.35%)	(1.65% - 2.46%)	(0.78% - 1.30%)	(0.64% - 1.12%)	(0.22% - 0.64%)	(0.16% - 0.46%)	(0.05% - 0.21%)	(0.02% - 0.15%)
	4.02%	3.20%	2.91%	2.73%	0.18%	0.11%	0.09%	0.07%	0.00%	0.00%	0.00%	0.00%
Sinaloa	(3.70% - 4.39%)	(2.93% - 3.49%)	(2.65% - 3.17%)	(2.48% - 2.99%)	(0.12% - 0.25%)	(0.07% - 0.16%)	(0.06% - 0.14%)	(0.04% - 0.11%)	(0.00% - 0.00%)	(0.00% - 0.00%)	(0.00% - 0.00%)	(0.00% - 0.00%)
	8.52%	7.50%	7.08%	6.58%	1.39%	1.02%	0.99%	0.94%	0.07%	0.01%	0.00%	0.00%
Sonora	(7.91% - 9.13%)	(6.97% - 8.06%)	(6.55% - 7.60%)	(6.10% - 7.13%)	(1.15% - 1.64%)	(0.84% - 1.24%)	(0.79% - 1.20%)	(0.77% - 1.14%)	(0.05% - 0.11%)	(0.00% - 0.02%)	(0.00% - 0.01%)	(0.00% - 0.01%)
	8.24%	6.03%	4.68%	4.20%	1.21%	0.74%	0.47%	0.41%	0.02%	0.00%	0.00%	0.00%
Tabasco	(7.67% - 8.83%)	(5.59% - 6.49%)	(4.33% - 5.03%)	(3.87% - 4.56%)	(0.99% - 1.44%)	(0.59% - 0.90%)	(0.38% - 0.57%)	(0.32% - 0.51%)	(0.01% - 0.06%)	(0.00% - 0.00%)	(0.00% - 0.00%)	(0.00% - 0.00%)
	3.48%	2.71%	2.41%	2.24%	0.16%	0.10%	0.07%	0.06%	0.00%	0.00%	0.00%	0.00%
Tamaulipas	(3.21% - 3.77%)	(2.50% - 2.93%)	(2.23% - 2.61%)	(2.07% - 2.43%)	(0.11% - 0.21%)	(0.07% - 0.14%)	(0.05% - 0.09%)	(0.05% - 0.08%)	(0.00% - 0.00%)	(0.00% - 0.00%)	(0.00% - 0.00%)	(0.00% - 0.00%)
	6.78%	5.29%	4.40%	4.17%	0.59%	0.45%	0.36%	0.37%	0.00%	0.00%	0.00%	0.00%
Tlaxcala	(6.28% - 7.29%)	(4.93% - 5.67%)	(4.08% - 4.74%)	(3.86% - 4.53%)	(0.47% - 0.73%)	(0.36% - 0.55%)	(0.29% - 0.44%)	(0.29% - 0.46%)	(0.00% - 0.00%)	(0.00% - 0.00%)	(0.00% - 0.00%)	(0.00% - 0.00%)
	5.20%	4.30%	3.26%	2.76%	0.35%	0.29%	0.19%	0.14%	0.00%	0.00%	0.00%	0.00%
Veracruz de Ignacio de la Llave	(4.83% - 5.60%)	(3.98% - 4.65%)	(3.02% - 3.51%)	(2.54% - 2.99%)	(0.27% - 0.44%)	(0.22% - 0.37%)	(0.15% - 0.24%)	(0.10% - 0.18%)	(0.00% - 0.00%)	(0.00% - 0.00%)	(0.00% - 0.00%)	(0.00% - 0.00%)
	5.96%	4.83%	5.17%	4.82%	0.40%	0.34%	0.52%	0.47%	0.00%	0.00%	0.00%	0.00%
Yucatán	(5.50% - 6.44%)	(4.44% - 5.27%)	(4.74% - 5.66%)	(4.41% - 5.25%)	(0.30% - 0.52%)	(0.25% - 0.44%)	(0.39% - 0.66%)	(0.35% - 0.61%)	(0.00% - 0.00%)	(0.00% - 0.00%)	(0.00% - 0.00%)	(0.00% - 0.00%)
	6.41%	5.49%	4.93%	4.53%	0.79%	0.65%	0.51%	0.41%	0.01%	0.00%	0.00%	0.00%
Zacatecas	(5.96% - 6.91%)	(5.09% - 5.90%)	(4.56% - 5.32%)	(4.17% - 4.90%)	(0.64% - 0.97%)	(0.54% - 0.79%)	(0.41% - 0.63%)	(0.32% - 0.52%)	(0.00% - 0.02%)	(0.00% - 0.01%)	(0.00% - 0.01%)	(0.00% - 0.01%)
	9.35%	7.92%	5.57%	4.67%	1.70%	1.23%	0.63%	0.50%	0.04%	0.01%	0.00%	0.00%
Nicaragua	(8.88% - 9.80%)	(7.54% - 8.33%)	(5.18% - 5.98%)	(4.29% - 5.04%)	(1.50% - 1.89%)	(1.07% - 1.40%)	(0.53% - 0.75%)	(0.40% - 0.61%)	(0.02% - 0.07%)	(0.00% - 0.02%)	(0.00% - 0.00%)	(0.00% - 0.00%)
	6.00%	4.72%	3.98%	3.17%	0.68%	0.44%	0.30%	0.21%	0.00%	0.00%	0.00%	0.00%
Panama	(5.65% - 6.40%)	(4.46% - 5.01%)	(3.73% - 4.24%)	(2.92% - 3.45%)	(0.58% - 0.79%)	(0.37% - 0.51%)	(0.25% - 0.36%)	(0.16% - 0.27%)	(0.00% - 0.00%)	(0.00% - 0.00%)	(0.00% - 0.00%)	(0.00% - 0.00%)
	5.94%	4.37%	3.73%	3.81%	0.65%	0.33%	0.25%	0.28%	0.00%	0.00%	0.00%	0.00%
Venezuela (Bolivarian Republic of)	(5.76% - 6.16%)	(4.18% - 4.57%)	(3.49% - 3.99%)	(3.53% - 4.11%)	(0.59% - 0.71%)	(0.28% - 0.37%)	(0.19% - 0.30%)	(0.22% - 0.36%)	(0.00% - 0.00%)	(0.00% - 0.00%)	(0.00% - 0.00%)	(0.00% - 0.00%)

Table S8c. Estimated overall, severe, and extreme **underweight** prevalence (%) in children under 5 years, both sexes, in 1990, 2000, 2010, and 2020.												
**Location**	**Total**				**Severe**				**Extreme**			
	**1990**	**2000**	**2010**	**2020**	**1990**	**2000**	**2010**	**2020**	**1990**	**2000**	**2010**	**2020**
**Tropical Latin America**	5.81%	4.41%	3.61%	3.27%	0.69%	0.47%	0.37%	0.33%	0.03%	0.02%	0.02%	0.02%
	(5.69% - 5.93%)	(4.33% - 4.49%)	(3.54% - 3.68%)	(3.20% - 3.34%)	(0.65% - 0.73%)	(0.45% - 0.49%)	(0.35% - 0.39%)	(0.31% - 0.35%)	(0.02% - 0.03%)	(0.02% - 0.02%)	(0.02% - 0.03%)	(0.02% - 0.02%)
Brazil	5.92%	4.47%	3.66%	3.35%	0.71%	0.48%	0.38%	0.34%	0.03%	0.02%	0.02%	0.02%
	(5.80% - 6.05%)	(4.38% - 4.56%)	(3.58% - 3.73%)	(3.28% - 3.42%)	(0.67% - 0.75%)	(0.46% - 0.51%)	(0.36% - 0.40%)	(0.32% - 0.36%)	(0.02% - 0.03%)	(0.02% - 0.02%)	(0.02% - 0.03%)	(0.02% - 0.02%)
Acre	10.03%	7.42%	5.96%	5.37%	1.44%	0.98%	0.74%	0.68%	0.01%	0.00%	0.00%	0.00%
	(9.28% - 10.80%)	(6.85% - 7.96%)	(5.51% - 6.43%)	(4.94% - 5.80%)	(1.15% - 1.77%)	(0.78% - 1.18%)	(0.59% - 0.90%)	(0.54% - 0.82%)	(0.00% - 0.06%)	(0.00% - 0.01%)	(0.00% - 0.00%)	(0.00% - 0.00%)
Alagoas	8.95%	6.62%	5.02%	4.60%	1.16%	0.75%	0.49%	0.45%	0.05%	0.02%	0.00%	0.00%
	(8.32% - 9.68%)	(6.14% - 7.13%)	(4.64% - 5.42%)	(4.23% - 4.99%)	(0.91% - 1.43%)	(0.59% - 0.93%)	(0.38% - 0.63%)	(0.34% - 0.58%)	(0.00% - 0.12%)	(0.00% - 0.05%)	(0.00% - 0.02%)	(0.00% - 0.02%)
Amazonas	6.20%	5.04%	4.48%	4.03%	0.46%	0.42%	0.40%	0.35%	0.00%	0.00%	0.00%	0.00%
	(5.68% - 6.76%)	(4.67% - 5.43%)	(4.13% - 4.85%)	(3.70% - 4.35%)	(0.33% - 0.62%)	(0.33% - 0.53%)	(0.31% - 0.50%)	(0.27% - 0.44%)	(0.00% - 0.00%)	(0.00% - 0.00%)	(0.00% - 0.00%)	(0.00% - 0.00%)
Amapá	5.24%	3.97%	3.21%	2.99%	0.54%	0.32%	0.20%	0.17%	0.00%	0.00%	0.00%	0.00%
	(4.84% - 5.67%)	(3.64% - 4.30%)	(2.95% - 3.49%)	(2.76% - 3.25%)	(0.43% - 0.66%)	(0.25% - 0.40%)	(0.14% - 0.26%)	(0.13% - 0.23%)	(0.00% - 0.00%)	(0.00% - 0.00%)	(0.00% - 0.00%)	(0.00% - 0.00%)
Bahia	7.57%	5.69%	4.48%	4.11%	0.94%	0.65%	0.47%	0.43%	0.01%	0.00%	0.00%	0.00%
	(7.06% - 8.15%)	(5.31% - 6.11%)	(4.15% - 4.83%)	(3.79% - 4.43%)	(0.79% - 1.11%)	(0.54% - 0.78%)	(0.38% - 0.56%)	(0.35% - 0.52%)	(0.00% - 0.03%)	(0.00% - 0.00%)	(0.00% - 0.00%)	(0.00% - 0.00%)
Ceará	7.78%	5.08%	2.38%	1.97%	0.97%	0.46%	0.05%	0.03%	0.00%	0.00%	0.00%	0.00%
	(7.18% - 8.41%)	(4.68% - 5.51%)	(2.14% - 2.63%)	(1.78% - 2.20%)	(0.77% - 1.21%)	(0.35% - 0.60%)	(0.02% - 0.10%)	(0.01% - 0.07%)	(0.00% - 0.00%)	(0.00% - 0.00%)	(0.00% - 0.00%)	(0.00% - 0.00%)
Distrito Federal	5.14%	3.67%	3.00%	2.72%	0.57%	0.33%	0.24%	0.21%	0.00%	0.00%	0.00%	0.00%
	(4.74% - 5.57%)	(3.40% - 3.96%)	(2.77% - 3.25%)	(2.51% - 2.96%)	(0.46% - 0.70%)	(0.26% - 0.41%)	(0.18% - 0.30%)	(0.16% - 0.27%)	(0.00% - 0.00%)	(0.00% - 0.00%)	(0.00% - 0.00%)	(0.00% - 0.00%)
Espírito Santo	4.94%	3.73%	3.10%	2.83%	0.45%	0.29%	0.21%	0.19%	0.00%	0.00%	0.00%	0.00%
	(4.57% - 5.32%)	(3.45% - 4.04%)	(2.87% - 3.34%)	(2.61% - 3.05%)	(0.35% - 0.55%)	(0.23% - 0.37%)	(0.16% - 0.27%)	(0.15% - 0.25%)	(0.00% - 0.00%)	(0.00% - 0.00%)	(0.00% - 0.00%)	(0.00% - 0.00%)
Goiás	5.87%	4.05%	3.08%	2.74%	0.56%	0.32%	0.18%	0.14%	0.00%	0.00%	0.00%	0.00%
	(5.45% - 6.32%)	(3.77% - 4.36%)	(2.83% - 3.34%)	(2.51% - 2.98%)	(0.45% - 0.68%)	(0.25% - 0.40%)	(0.12% - 0.24%)	(0.10% - 0.19%)	(0.00% - 0.00%)	(0.00% - 0.00%)	(0.00% - 0.00%)	(0.00% - 0.00%)
Maranhão	10.45%	6.45%	4.74%	4.34%	1.85%	0.84%	0.52%	0.46%	0.08%	0.00%	0.00%	0.00%
	(9.80% - 11.13%)	(6.00% - 6.93%)	(4.39% - 5.09%)	(4.00% - 4.68%)	(1.59% - 2.15%)	(0.71% - 1.01%)	(0.43% - 0.62%)	(0.38% - 0.55%)	(0.05% - 0.12%)	(0.00% - 0.01%)	(0.00% - 0.00%)	(0.00% - 0.00%)
Minas Gerais	5.01%	3.73%	3.09%	2.81%	0.44%	0.30%	0.22%	0.20%	0.00%	0.00%	0.00%	0.00%
	(4.63% - 5.39%)	(3.45% - 4.03%)	(2.85% - 3.35%)	(2.60% - 3.03%)	(0.35% - 0.55%)	(0.23% - 0.37%)	(0.17% - 0.28%)	(0.15% - 0.25%)	(0.00% - 0.00%)	(0.00% - 0.00%)	(0.00% - 0.00%)	(0.00% - 0.00%)
Mato Grosso do Sul	7.49%	5.79%	4.58%	4.10%	1.33%	0.92%	0.64%	0.56%	0.06%	0.02%	0.01%	0.00%
	(6.95% - 8.09%)	(5.32% - 6.30%)	(4.21% - 4.98%)	(3.76% - 4.48%)	(1.08% - 1.63%)	(0.73% - 1.15%)	(0.50% - 0.79%)	(0.44% - 0.71%)	(0.01% - 0.13%)	(0.00% - 0.07%)	(0.00% - 0.04%)	(0.00% - 0.03%)
Mato Grosso	6.29%	4.30%	3.16%	2.84%	0.75%	0.42%	0.23%	0.20%	0.00%	0.00%	0.00%	0.00%
	(5.84% - 6.81%)	(3.95% - 4.63%)	(2.92% - 3.42%)	(2.62% - 3.06%)	(0.61% - 0.92%)	(0.32% - 0.51%)	(0.18% - 0.30%)	(0.15% - 0.26%)	(0.00% - 0.00%)	(0.00% - 0.00%)	(0.00% - 0.00%)	(0.00% - 0.00%)
Pará	9.38%	8.10%	7.63%	6.97%	2.23%	2.03%	2.01%	1.79%	0.42%	0.39%	0.40%	0.35%
	(8.87% - 9.94%)	(7.68% - 8.54%)	(7.23% - 8.03%)	(6.57% - 7.36%)	(2.01% - 2.46%)	(1.84% - 2.25%)	(1.83% - 2.22%)	(1.61% - 1.97%)	(0.35% - 0.51%)	(0.32% - 0.47%)	(0.33% - 0.48%)	(0.28% - 0.41%)
Paraíba	5.51%	4.45%	3.61%	3.37%	0.49%	0.36%	0.26%	0.26%	0.00%	0.00%	0.00%	0.00%
	(5.09% - 5.97%)	(4.12% - 4.79%)	(3.34% - 3.90%)	(3.11% - 3.67%)	(0.39% - 0.63%)	(0.28% - 0.45%)	(0.20% - 0.34%)	(0.19% - 0.33%)	(0.00% - 0.00%)	(0.00% - 0.00%)	(0.00% - 0.00%)	(0.00% - 0.00%)
Paraná	2.96%	2.59%	2.24%	2.02%	0.09%	0.09%	0.07%	0.06%	0.00%	0.00%	0.00%	0.00%
	(2.73% - 3.22%)	(2.38% - 2.80%)	(2.06% - 2.44%)	(1.85% - 2.19%)	(0.05% - 0.13%)	(0.05% - 0.13%)	(0.04% - 0.11%)	(0.03% - 0.09%)	(0.00% - 0.00%)	(0.00% - 0.00%)	(0.00% - 0.00%)	(0.00% - 0.00%)
Pernambuco	5.83%	4.54%	3.64%	3.30%	0.65%	0.45%	0.32%	0.29%	0.00%	0.00%	0.00%	0.00%
	(5.43% - 6.26%)	(4.25% - 4.86%)	(3.39% - 3.92%)	(3.05% - 3.57%)	(0.53% - 0.77%)	(0.38% - 0.54%)	(0.26% - 0.39%)	(0.23% - 0.36%)	(0.00% - 0.00%)	(0.00% - 0.00%)	(0.00% - 0.00%)	(0.00% - 0.00%)
Piaui	9.19%	6.84%	5.40%	4.86%	1.63%	1.04%	0.73%	0.65%	0.05%	0.01%	0.00%	0.00%
	(8.60% - 9.76%)	(6.36% - 7.31%)	(5.02% - 5.79%)	(4.53% - 5.23%)	(1.40% - 1.87%)	(0.88% - 1.22%)	(0.62% - 0.86%)	(0.55% - 0.78%)	(0.03% - 0.09%)	(0.00% - 0.02%)	(0.00% - 0.00%)	(0.00% - 0.00%)
Rio de Janeiro	5.36%	3.98%	3.30%	3.08%	0.54%	0.37%	0.28%	0.26%	0.00%	0.00%	0.00%	0.00%
	(4.91% - 5.83%)	(3.64% - 4.33%)	(3.04% - 3.59%)	(2.81% - 3.35%)	(0.42% - 0.70%)	(0.28% - 0.47%)	(0.21% - 0.36%)	(0.19% - 0.34%)	(0.00% - 0.00%)	(0.00% - 0.00%)	(0.00% - 0.00%)	(0.00% - 0.00%)
Rio Grande do Norte	3.89%	2.97%	2.45%	2.21%	0.21%	0.14%	0.11%	0.09%	0.00%	0.00%	0.00%	0.00%
	(3.61% - 4.16%)	(2.75% - 3.19%)	(2.28% - 2.64%)	(2.04% - 2.39%)	(0.16% - 0.27%)	(0.11% - 0.19%)	(0.08% - 0.14%)	(0.06% - 0.12%)	(0.00% - 0.00%)	(0.00% - 0.00%)	(0.00% - 0.00%)	(0.00% - 0.00%)
Rondônia	10.87%	9.45%	10.01%	9.09%	2.50%	1.98%	2.08%	1.88%	0.43%	0.23%	0.17%	0.15%
	(10.22% - 11.56%)	(8.90% - 9.98%)	(9.44% - 10.63%)	(8.60% - 9.65%)	(2.23% - 2.81%)	(1.73% - 2.21%)	(1.82% - 2.36%)	(1.67% - 2.10%)	(0.34% - 0.53%)	(0.17% - 0.30%)	(0.11% - 0.23%)	(0.10% - 0.20%)

Table S8c. Estimated overall, severe, and extreme **underweight** prevalence (%) in children under 5 years, both sexes, in 1990, 2000, 2010, and 2020.												
**Location**	**Total**				**Severe**				**Extreme**			
	**1990**	**2000**	**2010**	**2020**	**1990**	**2000**	**2010**	**2020**	**1990**	**2000**	**2010**	**2020**
	6.01%	4.22%	3.25%	2.98%	0.59%	0.35%	0.21%	0.18%	0.00%	0.00%	0.00%	0.00%
Roraima	(5.60% - 6.48%)	(3.89% - 4.58%)	(2.99% - 3.53%)	(2.76% - 3.23%)	(0.47% - 0.73%)	(0.27% - 0.43%)	(0.16% - 0.27%)	(0.13% - 0.24%)	(0.00% - 0.00%)	(0.00% - 0.00%)	(0.00% - 0.00%)	(0.00% - 0.00%)
	4.05%	3.20%	2.68%	2.49%	0.30%	0.21%	0.15%	0.14%	0.00%	0.00%	0.00%	0.00%
Rio Grande do Sul	(3.76% - 4.37%)	(2.95% - 3.45%)	(2.46% - 2.89%)	(2.30% - 2.68%)	(0.24% - 0.38%)	(0.15% - 0.27%)	(0.11% - 0.20%)	(0.10% - 0.18%)	(0.00% - 0.00%)	(0.00% - 0.00%)	(0.00% - 0.00%)	(0.00% - 0.00%)
	4.44%	3.45%	2.64%	2.33%	0.41%	0.26%	0.15%	0.12%	0.00%	0.00%	0.00%	0.00%
Santa Catarina	(4.09% - 4.84%)	(3.17% - 3.74%)	(2.43% - 2.85%)	(2.14% - 2.53%)	(0.33% - 0.51%)	(0.20% - 0.34%)	(0.10% - 0.19%)	(0.08% - 0.16%)	(0.00% - 0.00%)	(0.00% - 0.00%)	(0.00% - 0.00%)	(0.00% - 0.00%)
	6.40%	4.67%	3.42%	3.07%	0.47%	0.28%	0.15%	0.13%	0.00%	0.00%	0.00%	0.00%
Sergipe	(5.93% - 6.93%)	(4.31% - 5.07%)	(3.15% - 3.75%)	(2.83% - 3.34%)	(0.37% - 0.60%)	(0.21% - 0.37%)	(0.11% - 0.21%)	(0.09% - 0.18%)	(0.00% - 0.00%)	(0.00% - 0.00%)	(0.00% - 0.00%)	(0.00% - 0.00%)
	4.43%	3.37%	2.81%	2.67%	0.39%	0.27%	0.20%	0.19%	0.00%	0.00%	0.00%	0.00%
São Paulo	(4.07% - 4.85%)	(3.10% - 3.66%)	(2.58% - 3.09%)	(2.41% - 2.94%)	(0.29% - 0.53%)	(0.19% - 0.36%)	(0.14% - 0.27%)	(0.13% - 0.27%)	(0.00% - 0.00%)	(0.00% - 0.00%)	(0.00% - 0.00%)	(0.00% - 0.00%)
	7.01%	4.97%	3.56%	3.13%	0.86%	0.49%	0.26%	0.21%	0.00%	0.00%	0.00%	0.00%
Tocantins	(6.46% - 7.57%)	(4.61% - 5.36%)	(3.30% - 3.85%)	(2.88% - 3.38%)	(0.69% - 1.04%)	(0.39% - 0.60%)	(0.20% - 0.33%)	(0.16% - 0.27%)	(0.00% - 0.00%)	(0.00% - 0.00%)	(0.00% - 0.00%)	(0.00% - 0.00%)
	2.81%	2.75%	2.52%	1.62%	0.12%	0.09%	0.06%	0.02%	0.00%	0.00%	0.00%	0.00%
Paraguay	(2.62% - 3.03%)	(2.55% - 2.97%)	(2.35% - 2.70%)	(1.52% - 1.74%)	(0.10% - 0.16%)	(0.07% - 0.12%)	(0.04% - 0.08%)	(0.01% - 0.03%)	(0.00% - 0.00%)	(0.00% - 0.00%)	(0.00% - 0.00%)	(0.00% - 0.00%)
North Africa and Middle East	14.09%	13.40%	11.57%	10.42%	3.96%	4.40%	3.70%	3.34%	0.71%	1.05%	0.81%	0.73%
	(13.89% - 14.29%)	(13.25% - 13.56%)	(11.43% - 11.71%)	(10.27% - 10.58%)	(3.83% - 4.09%)	(4.27% - 4.53%)	(3.57% - 3.83%)	(3.19% - 3.49%)	(0.64% - 0.78%)	(0.95% - 1.15%)	(0.72% - 0.89%)	(0.64% - 0.82%)
	14.09%	13.40%	11.57%	10.42%	3.96%	4.40%	3.70%	3.34%	0.71%	1.05%	0.81%	0.73%
**North Africa and Middle East**	(13.89% - 14.29%)	(13.25% - 13.56%)	(11.43% - 11.71%)	(10.27% - 10.58%)	(3.83% - 4.09%)	(4.27% - 4.53%)	(3.57% - 3.83%)	(3.19% - 3.49%)	(0.64% - 0.78%)	(0.95% - 1.15%)	(0.72% - 0.89%)	(0.64% - 0.82%)
	12.86%	10.63%	5.38%	3.98%	2.72%	2.04%	0.60%	0.36%	0.18%	0.08%	0.00%	0.00%
Algeria	(12.09% - 13.68%)	(9.97% - 11.34%)	(4.99% - 5.79%)	(3.64% - 4.32%)	(2.31% - 3.16%)	(1.74% - 2.41%)	(0.49% - 0.72%)	(0.28% - 0.44%)	(0.10% - 0.27%)	(0.04% - 0.15%)	(0.00% - 0.01%)	(0.00% - 0.00%)
	7.12%	6.15%	5.04%	4.61%	1.00%	0.87%	0.69%	0.63%	0.00%	0.00%	0.00%	0.00%
Bahrain	(6.61% - 7.64%)	(5.69% - 6.65%)	(4.69% - 5.43%)	(4.26% - 4.99%)	(0.83% - 1.19%)	(0.73% - 1.03%)	(0.58% - 0.82%)	(0.52% - 0.75%)	(0.00% - 0.01%)	(0.00% - 0.00%)	(0.00% - 0.00%)	(0.00% - 0.00%)
	10.59%	8.02%	5.92%	4.41%	2.23%	1.44%	0.89%	0.56%	0.17%	0.09%	0.03%	0.01%
Egypt	(10.06% - 11.18%)	(7.61% - 8.47%)	(5.57% - 6.30%)	(4.12% - 4.76%)	(1.98% - 2.48%)	(1.29% - 1.61%)	(0.78% - 1.02%)	(0.48% - 0.67%)	(0.13% - 0.22%)	(0.07% - 0.11%)	(0.02% - 0.04%)	(0.00% - 0.01%)
	13.54%	9.72%	7.71%	6.82%	3.37%	2.17%	1.61%	1.39%	0.45%	0.22%	0.14%	0.11%
Iran (Islamic Republic of)	(12.86% - 14.23%)	(9.22% - 10.19%)	(7.21% - 8.22%)	(6.33% - 7.35%)	(2.95% - 3.82%)	(1.93% - 2.42%)	(1.39% - 1.88%)	(1.19% - 1.64%)	(0.34% - 0.57%)	(0.17% - 0.28%)	(0.09% - 0.20%)	(0.07% - 0.17%)
	12.44%	12.01%	8.22%	5.75%	2.60%	2.60%	1.46%	0.83%	0.19%	0.21%	0.07%	0.02%
Iraq	(11.75% - 13.10%)	(11.42% - 12.62%)	(7.73% - 8.71%)	(5.35% - 6.21%)	(2.26% - 2.94%)	(2.34% - 2.89%)	(1.28% - 1.66%)	(0.71% - 0.97%)	(0.13% - 0.25%)	(0.17% - 0.27%)	(0.05% - 0.09%)	(0.01% - 0.03%)
	4.83%	3.73%	2.76%	2.61%	0.31%	0.17%	0.07%	0.07%	0.00%	0.00%	0.00%	0.00%
Jordan	(4.53% - 5.17%)	(3.48% - 3.99%)	(2.55% - 2.97%)	(2.37% - 2.84%)	(0.24% - 0.39%)	(0.12% - 0.22%)	(0.04% - 0.12%)	(0.03% - 0.12%)	(0.00% - 0.00%)	(0.00% - 0.00%)	(0.00% - 0.00%)	(0.00% - 0.00%)
	3.68%	2.36%	1.96%	1.86%	0.18%	0.07%	0.07%	0.07%	0.00%	0.00%	0.00%	0.00%
Kuwait	(3.41% - 3.97%)	(2.19% - 2.55%)	(1.82% - 2.11%)	(1.72% - 2.02%)	(0.13% - 0.24%)	(0.05% - 0.09%)	(0.05% - 0.08%)	(0.05% - 0.08%)	(0.00% - 0.00%)	(0.00% - 0.00%)	(0.00% - 0.00%)	(0.00% - 0.00%)
	5.74%	4.36%	3.75%	3.29%	0.43%	0.32%	0.27%	0.23%	0.00%	0.00%	0.00%	0.00%
Lebanon	(5.30% - 6.24%)	(4.03% - 4.72%)	(3.46% - 4.05%)	(3.00% - 3.57%)	(0.32% - 0.56%)	(0.24% - 0.41%)	(0.20% - 0.35%)	(0.16% - 0.30%)	(0.00% - 0.00%)	(0.00% - 0.00%)	(0.00% - 0.00%)	(0.00% - 0.00%)
	5.48%	4.70%	4.57%	4.78%	0.48%	0.49%	0.58%	0.59%	0.00%	0.00%	0.01%	0.01%
Libya	(5.10% - 5.87%)	(4.38% - 5.04%)	(4.26% - 4.90%)	(4.43% - 5.14%)	(0.39% - 0.58%)	(0.41% - 0.58%)	(0.49% - 0.68%)	(0.49% - 0.70%)	(0.00% - 0.00%)	(0.00% - 0.01%)	(0.00% - 0.02%)	(0.00% - 0.01%)
	9.47%	7.73%	4.57%	3.28%	1.65%	1.21%	0.43%	0.24%	0.04%	0.02%	0.00%	0.00%
Morocco	(8.97% - 9.94%)	(7.31% - 8.16%)	(4.26% - 4.90%)	(3.04% - 3.56%)	(1.44% - 1.87%)	(1.05% - 1.40%)	(0.35% - 0.52%)	(0.19% - 0.30%)	(0.02% - 0.07%)	(0.01% - 0.03%)	(0.00% - 0.00%)	(0.00% - 0.00%)
	4.99%	3.83%	3.23%	2.14%	0.32%	0.21%	0.15%	0.04%	0.00%	0.00%	0.00%	0.00%
Palestine	(4.64% - 5.34%)	(3.54% - 4.11%)	(3.00% - 3.48%)	(1.96% - 2.33%)	(0.25% - 0.41%)	(0.15% - 0.27%)	(0.10% - 0.20%)	(0.02% - 0.07%)	(0.00% - 0.00%)	(0.00% - 0.00%)	(0.00% - 0.00%)	(0.00% - 0.00%)
	18.03%	12.64%	9.42%	8.82%	4.79%	2.70%	1.63%	1.64%	0.58%	0.19%	0.02%	0.03%
Oman	(17.44% - 18.68%)	(12.14% - 13.15%)	(9.00% - 9.84%)	(8.26% - 9.40%)	(4.29% - 5.35%)	(2.40% - 3.03%)	(1.43% - 1.83%)	(1.41% - 1.89%)	(0.42% - 0.78%)	(0.11% - 0.27%)	(0.00% - 0.05%)	(0.01% - 0.08%)
	5.99%	5.01%	4.04%	3.68%	0.68%	0.57%	0.44%	0.42%	0.00%	0.00%	0.00%	0.00%
Qatar	(5.55% - 6.47%)	(4.64% - 5.41%)	(3.75% - 4.37%)	(3.40% - 4.00%)	(0.55% - 0.82%)	(0.46% - 0.69%)	(0.36% - 0.54%)	(0.34% - 0.50%)	(0.00% - 0.00%)	(0.00% - 0.00%)	(0.00% - 0.00%)	(0.00% - 0.00%)
	13.55%	8.58%	5.88%	4.97%	3.00%	1.51%	0.86%	0.71%	0.23%	0.03%	0.00%	0.00%
Saudi Arabia	(12.82% - 14.27%)	(8.08% - 9.17%)	(5.41% - 6.34%)	(4.55% - 5.41%)	(2.62% - 3.44%)	(1.29% - 1.77%)	(0.69% - 1.04%)	(0.58% - 0.87%)	(0.14% - 0.33%)	(0.00% - 0.07%)	(0.00% - 0.01%)	(0.00% - 0.00%)

Table S8c. Estimated overall, severe, and extreme **underweight** prevalence (%) in children under 5 years, both sexes, in 1990, 2000, 2010, and 2020.

Location	Total				Severe				Extreme			
	1990	2000	2010	2020	1990	2000	2010	2020	1990	2000	2010	2020
Syrian Arab Republic	12.62% (11.87% - 13.36%)	10.42% (9.88% - 10.99%)	9.25% (8.71% - 9.84%)	9.65% (9.06% - 10.28%)	2.89% (2.51% - 3.30%)	2.45% (2.18% - 2.76%)	2.18% (1.93% - 2.46%)	2.38% (2.08% - 2.70%)	0.29% (0.21% - 0.38%)	0.23% (0.17% - 0.30%)	0.20% (0.14% - 0.27%)	0.25% (0.17% - 0.33%)
Tunisia	7.01% (6.64% - 7.44%)	4.04% (3.81% - 4.27%)	2.47% (2.29% - 2.65%)	1.93% (1.80% - 2.08%)	0.90% (0.78% - 1.05%)	0.28% (0.23% - 0.33%)	0.09% (0.06% - 0.11%)	0.06% (0.05% - 0.08%)	0.00% (0.00% - 0.01%)	0.00% (0.00% - 0.00%)	0.00% (0.00% - 0.00%)	0.00% (0.00% - 0.00%)
Turkey	7.37% (6.86% - 7.89%)	5.04% (4.68% - 5.41%)	2.51% (2.30% - 2.74%)	2.12% (1.91% - 2.32%)	0.85% (0.69% - 1.02%)	0.45% (0.35% - 0.55%)	0.09% (0.04% - 0.13%)	0.06% (0.03% - 0.10%)	0.00% (0.00% - 0.00%)	0.00% (0.00% - 0.00%)	0.00% (0.00% - 0.00%)	0.00% (0.00% - 0.00%)
United Arab Emirates	6.70% (6.19% - 7.22%)	5.59% (5.15% - 6.02%)	5.05% (4.68% - 5.45%)	4.79% (4.42% - 5.20%)	0.97% (0.80% - 1.16%)	0.75% (0.62% - 0.90%)	0.62% (0.51% - 0.75%)	0.58% (0.47% - 0.71%)	0.00% (0.00% - 0.01%)	0.00% (0.00% - 0.00%)	0.00% (0.00% - 0.00%)	0.00% (0.00% - 0.00%)
Yemen	32.73% (31.83% - 33.71%)	35.20% (34.44% - 36.08%)	33.85% (33.09% - 34.67%)	32.51% (31.77% - 33.37%)	11.99% (10.97% - 13.03%)	15.66% (14.62% - 16.72%)	13.67% (12.79% - 14.56%)	12.96% (11.91% - 14.09%)	2.45% (1.96% - 3.06%)	4.29% (3.47% - 5.18%)	3.09% (2.50% - 3.77%)	2.92% (2.30% - 3.70%)
Afghanistan	27.61% (26.87% - 28.33%)	29.31% (28.77% - 29.92%)	25.91% (25.21% - 26.55%)	22.64% (21.85% - 23.41%)	12.18% (11.23% - 13.21%)	13.97% (13.21% - 14.87%)	10.98% (10.20% - 11.78%)	8.95% (8.14% - 9.83%)	3.52% (2.89% - 4.27%)	4.54% (3.94% - 5.28%)	2.96% (2.47% - 3.48%)	2.19% (1.75% - 2.68%)
Sudan	31.98% (31.18% - 32.76%)	30.70% (30.06% - 31.42%)	28.28% (27.69% - 28.91%)	25.60% (24.87% - 26.31%)	14.54% (13.59% - 15.55%)	14.24% (13.28% - 15.25%)	12.07% (11.20% - 12.97%)	10.85% (10.00% - 11.76%)	4.19% (3.49% - 4.99%)	4.32% (3.59% - 5.19%)	3.22% (2.61% - 3.87%)	2.93% (2.35% - 3.57%)
South Asia	40.98% (40.62% - 41.34%)	37.59% (37.40% - 37.80%)	33.54% (33.38% - 33.72%)	28.08% (27.89% - 28.28%)	19.27% (18.91% - 19.64%)	19.34% (19.05% - 19.65%)	16.38% (16.14% - 16.61%)	12.54% (12.28% - 12.80%)	6.12% (5.72% - 6.55%)	7.16% (6.80% - 7.52%)	5.57% (5.34% - 5.82%)	3.85% (3.65% - 4.06%)
**South Asia**	40.98% (40.62% - 41.34%)	37.59% (37.40% - 37.80%)	33.54% (33.38% - 33.72%)	28.08% (27.89% - 28.28%)	19.27% (18.91% - 19.64%)	19.34% (19.05% - 19.65%)	16.38% (16.14% - 16.61%)	12.54% (12.28% - 12.80%)	6.12% (5.72% - 6.55%)	7.16% (6.80% - 7.52%)	5.57% (5.34% - 5.82%)	3.85% (3.65% - 4.06%)
Bangladesh	45.87% (43.72% - 47.83%)	35.80% (35.29% - 36.29%)	29.77% (29.20% - 30.33%)	25.18% (24.52% - 25.90%)	15.38% (13.91% - 16.89%)	16.98% (15.83% - 18.18%)	12.27% (11.41% - 13.25%)	8.97% (8.14% - 9.87%)	2.47% (1.88% - 3.22%)	5.14% (4.26% - 6.18%)	3.04% (2.50% - 3.74%)	1.76% (1.35% - 2.26%)
Bhutan	23.22% (22.49% - 23.95%)	15.52% (14.74% - 16.30%)	11.21% (10.59% - 11.84%)	8.67% (8.03% - 9.30%)	5.95% (5.27% - 6.60%)	2.56% (2.06% - 3.06%)	1.77% (1.47% - 2.07%)	1.36% (1.11% - 1.62%)	0.64% (0.46% - 0.84%)	0.09% (0.03% - 0.17%)	0.03% (0.01% - 0.06%)	0.01% (0.00% - 0.04%)
India	41.61% (41.26% - 41.96%)	38.94% (38.74% - 39.17%)	34.79% (34.62% - 34.97%)	29.15% (28.97% - 29.33%)	20.62% (20.20% - 21.06%)	20.77% (20.46% - 21.10%)	17.68% (17.41% - 17.96%)	13.64% (13.37% - 13.90%)	6.96% (6.45% - 7.52%)	8.08% (7.64% - 8.55%)	6.28% (6.00% - 6.60%)	4.37% (4.15% - 4.59%)
Other Union Territories	36.54% (35.48% - 37.73%)	32.23% (31.41% - 33.13%)	25.77% (25.12% - 26.42%)	21.06% (20.38% - 21.71%)	11.19% (10.26% - 12.14%)	10.32% (9.52% - 11.11%)	8.52% (7.83% - 9.20%)	6.60% (5.97% - 7.24%)	1.33% (0.99% - 1.74%)	1.52% (1.15% - 1.94%)	1.51% (1.18% - 1.89%)	1.12% (0.87% - 1.42%)
Andhra Pradesh	38.85% (37.75% - 40.15%)	35.23% (34.58% - 36.00%)	30.13% (29.59% - 30.63%)	25.09% (24.53% - 25.68%)	16.29% (15.09% - 17.40%)	16.02% (15.08% - 17.00%)	13.84% (13.09% - 14.63%)	10.86% (10.13% - 11.65%)	3.77% (2.96% - 4.71%)	4.51% (3.69% - 5.40%)	4.24% (3.65% - 4.96%)	3.17% (2.65% - 3.79%)
Arunachal Pradesh	28.04% (27.18% - 28.89%)	24.85% (24.08% - 25.64%)	26.20% (25.52% - 26.83%)	22.59% (21.88% - 23.30%)	9.24% (8.25% - 10.17%)	7.81% (6.99% - 8.68%)	10.27% (9.45% - 11.11%)	8.57% (7.84% - 9.34%)	1.72% (1.31% - 2.18%)	1.35% (1.00% - 1.72%)	2.51% (2.07% - 3.01%)	2.04% (1.65% - 2.46%)
Assam	35.88% (35.20% - 36.57%)	33.23% (32.64% - 33.86%)	29.56% (28.94% - 30.14%)	24.09% (23.39% - 24.77%)	19.39% (18.20% - 20.81%)	16.84% (15.91% - 17.90%)	13.55% (12.70% - 14.45%)	9.93% (9.18% - 10.72%)	7.53% (6.31% - 9.16%)	5.94% (5.08% - 6.99%)	4.13% (3.49% - 4.86%)	2.63% (2.17% - 3.16%)
Bihar	43.85% (43.06% - 44.77%)	42.47% (41.83% - 43.35%)	39.57% (38.97% - 40.26%)	33.67% (32.99% - 34.33%)	28.43% (26.57% - 30.30%)	26.43% (25.22% - 27.70%)	22.28% (21.14% - 23.38%)	17.33% (16.28% - 18.42%)	14.64% (11.96% - 17.56%)	12.60% (10.73% - 14.59%)	9.04% (7.64% - 10.45%)	6.28% (5.30% - 7.33%)
Chhattisgarh	42.68% (41.37% - 44.29%)	40.48% (39.49% - 41.76%)	36.93% (36.27% - 37.66%)	29.92% (29.37% - 30.48%)	18.66% (17.24% - 20.25%)	18.77% (17.43% - 20.25%)	17.81% (16.74% - 18.95%)	13.91% (13.05% - 14.77%)	4.53% (3.43% - 6.14%)	5.27% (4.12% - 6.77%)	5.54% (4.53% - 6.63%)	4.33% (3.65% - 5.01%)
Delhi	35.47% (34.67% - 36.32%)	31.86% (31.24% - 32.53%)	27.71% (27.05% - 28.35%)	23.09% (22.37% - 23.85%)	15.40% (14.20% - 16.59%)	14.15% (13.26% - 15.11%)	12.17% (11.40% - 12.94%)	9.38% (8.58% - 10.18%)	3.99% (3.15% - 4.95%)	3.92% (3.27% - 4.66%)	3.44% (2.90% - 3.99%)	2.42% (1.96% - 2.91%)
Goa	29.84% (29.11% - 30.62%)	23.89% (23.32% - 24.54%)	20.15% (19.56% - 20.77%)	17.84% (17.20% - 18.49%)	10.22% (9.39% - 11.08%)	7.45% (6.75% - 8.16%)	7.12% (6.53% - 7.76%)	6.28% (5.64% - 6.93%)	1.82% (1.37% - 2.36%)	1.21% (0.90% - 1.55%)	1.55% (1.25% - 1.87%)	1.39% (1.06% - 1.74%)
Gujarat	44.40% (42.78% - 46.05%)	39.80% (38.88% - 40.90%)	34.73% (34.21% - 35.25%)	29.33% (28.76% - 29.88%)	18.56% (17.30% - 19.76%)	17.97% (17.11% - 18.92%)	16.91% (16.11% - 17.71%)	13.73% (12.86% - 14.68%)	3.92% (3.05% - 4.95%)	4.81% (3.99% - 5.89%)	5.79% (4.95% - 6.68%)	4.63% (3.88% - 5.50%)
Haryana	33.34% (32.65% - 34.07%)	31.95% (31.38% - 32.52%)	30.10% (29.55% - 30.62%)	25.44% (24.81% - 26.06%)	13.63% (12.63% - 14.67%)	14.06% (13.33% - 14.87%)	13.97% (13.23% - 14.68%)	11.03% (10.29% - 11.71%)	3.19% (2.53% - 4.02%)	3.85% (3.29% - 4.47%)	4.29% (3.73% - 4.83%)	3.12% (2.67% - 3.58%)
Himachal Pradesh	34.65% (33.82% - 35.56%)	31.34% (30.75% - 31.95%)	26.83% (26.23% - 27.42%)	20.54% (19.79% - 21.24%)	14.79% (13.63% - 16.08%)	13.67% (12.81% - 14.51%)	10.77% (10.04% - 11.54%)	7.04% (6.31% - 7.72%)	3.71% (2.88% - 4.77%)	3.73% (3.07% - 4.37%)	2.62% (2.16% - 3.12%)	1.36% (1.05% - 1.69%)

Table S8c. Estimated overall, severe, and extreme **underweight** prevalence (%) in children under 5 years, both sexes, in 1990, 2000, 2010, and 2020.												
**Location**	**Total**				**Severe**				**Extreme**			
	**1990**	**2000**	**2010**	**2020**	**1990**	**2000**	**2010**	**2020**	**1990**	**2000**	**2010**	**2020**
Jammu & Kashmir and Ladakh	31.89% (31.23% - 32.57%)	27.06% (26.43% - 27.73%)	21.15% (20.45% - 21.77%)	16.19% (15.49% - 16.88%)	13.91% (12.93% - 14.99%)	10.76% (9.91% - 11.54%)	7.44% (6.76% - 8.07%)	5.06% (4.52% - 5.62%)	3.79% (3.05% - 4.62%)	2.58% (2.08% - 3.07%)	1.52% (1.20% - 1.83%)	0.85% (0.66% - 1.09%)
Jharkhand	47.07% (45.49% - 49.00%)	45.12% (43.74% - 46.69%)	40.98% (40.14% - 42.00%)	34.26% (33.71% - 34.83%)	23.40% (21.76% - 25.16%)	23.77% (22.41% - 25.24%)	21.34% (19.95% - 22.73%)	17.91% (16.98% - 18.94%)	7.13% (5.44% - 9.38%)	8.31% (6.58% - 10.37%)	7.43% (5.95% - 9.10%)	6.64% (5.67% - 7.74%)
Karnataka	40.11% (39.43% - 40.86%)	37.70% (37.06% - 38.48%)	33.49% (33.02% - 33.97%)	28.76% (28.22% - 29.30%)	21.03% (19.87% - 22.22%)	18.44% (17.49% - 19.44%)	16.67% (15.99% - 17.36%)	13.41% (12.63% - 14.18%)	7.60% (6.25% - 9.12%)	5.81% (4.87% - 6.88%)	5.67% (5.04% - 6.34%)	4.28% (3.68% - 4.92%)
Kerala	30.44% (29.51% - 31.55%)	27.18% (26.51% - 27.87%)	22.13% (21.54% - 22.69%)	17.62% (16.94% - 18.25%)	8.95% (8.03% - 9.83%)	8.78% (8.02% - 9.54%)	7.15% (6.56% - 7.71%)	5.08% (4.53% - 5.63%)	1.17% (0.85% - 1.55%)	1.42% (1.10% - 1.79%)	1.23% (1.00% - 1.49%)	0.73% (0.56% - 0.94%)
Madhya Pradesh	47.71% (45.98% - 49.56%)	45.04% (43.90% - 46.45%)	40.87% (40.03% - 41.88%)	33.15% (32.52% - 33.75%)	23.87% (22.19% - 25.60%)	25.23% (23.88% - 26.61%)	21.23% (20.04% - 22.47%)	16.53% (15.64% - 17.43%)	7.05% (5.41% - 9.46%)	9.68% (7.80% - 11.86%)	7.36% (5.99% - 8.98%)	5.69% (4.90% - 6.58%)
Maharashtra	44.41% (42.93% - 46.07%)	39.06% (38.26% - 40.02%)	32.90% (32.41% - 33.37%)	28.15% (27.61% - 28.68%)	19.14% (17.89% - 20.39%)	18.11% (17.23% - 19.13%)	15.76% (15.03% - 16.50%)	12.83% (12.12% - 13.56%)	4.28% (3.28% - 5.57%)	5.10% (4.22% - 6.18%)	5.01% (4.34% - 5.70%)	3.91% (3.38% - 4.51%)
Manipur	21.49% (20.73% - 22.20%)	18.91% (18.22% - 19.61%)	18.78% (18.12% - 19.41%)	15.53% (14.89% - 16.17%)	5.83% (5.12% - 6.57%)	5.13% (4.55% - 5.75%)	5.88% (5.24% - 6.46%)	4.49% (3.99% - 5.02%)	0.82% (0.54% - 1.16%)	0.74% (0.51% - 1.00%)	1.04% (0.75% - 1.34%)	0.68% (0.48% - 0.89%)
Meghalaya	38.57% (37.61% - 39.72%)	37.05% (36.09% - 38.09%)	35.55% (34.84% - 36.23%)	29.89% (29.26% - 30.55%)	17.40% (16.18% - 18.59%)	16.28% (15.32% - 17.34%)	17.42% (16.46% - 18.45%)	13.59% (12.66% - 14.55%)	4.63% (3.62% - 5.73%)	4.39% (3.52% - 5.45%)	5.72% (4.86% - 6.79%)	4.05% (3.36% - 4.87%)
Mizoram	21.09% (20.32% - 21.83%)	18.29% (17.58% - 18.96%)	17.38% (16.80% - 17.98%)	13.60% (12.98% - 14.19%)	4.65% (3.92% - 5.38%)	3.86% (3.33% - 4.42%)	4.66% (4.15% - 5.20%)	3.33% (2.91% - 3.77%)	0.42% (0.28% - 0.63%)	0.31% (0.21% - 0.45%)	0.60% (0.42% - 0.80%)	0.36% (0.24% - 0.50%)
Nagaland	22.97% (22.19% - 23.81%)	20.56% (19.73% - 21.28%)	21.16% (20.49% - 21.84%)	18.07% (17.47% - 18.78%)	7.02% (6.11% - 7.91%)	5.85% (5.15% - 6.56%)	7.06% (6.41% - 7.74%)	5.62% (5.06% - 6.25%)	1.08% (0.71% - 1.49%)	0.77% (0.54% - 1.05%)	1.30% (0.99% - 1.65%)	0.94% (0.69% - 1.23%)
Odisha	41.19% (40.22% - 42.36%)	39.29% (38.56% - 40.09%)	34.06% (33.43% - 34.73%)	27.66% (26.98% - 28.36%)	21.26% (19.71% - 22.76%)	20.49% (19.55% - 21.51%)	15.22% (14.32% - 16.19%)	11.52% (10.62% - 12.47%)	7.21% (5.61% - 8.95%)	7.16% (6.02% - 8.31%)	4.19% (3.46% - 4.99%)	2.98% (2.42% - 3.60%)
Punjab	33.43% (32.86% - 34.08%)	31.68% (31.22% - 32.16%)	28.19% (27.75% - 28.66%)	22.26% (21.70% - 22.83%)	14.96% (14.17% - 15.79%)	15.70% (15.09% - 16.37%)	13.88% (13.30% - 14.47%)	9.04% (8.48% - 9.60%)	4.21% (3.65% - 4.81%)	5.41% (4.88% - 6.00%)	4.90% (4.44% - 5.39%)	2.37% (2.07% - 2.68%)
Rajasthan	42.14% (41.59% - 42.77%)	42.31% (41.89% - 42.73%)	36.36% (35.88% - 36.86%)	29.30% (28.73% - 29.87%)	26.84% (25.27% - 28.45%)	29.17% (28.11% - 30.16%)	20.28% (19.31% - 21.27%)	13.94% (13.12% - 14.86%)	13.49% (11.30% - 15.87%)	16.94% (15.22% - 18.48%)	8.29% (7.25% - 9.46%)	4.53% (3.87% - 5.32%)
Sikkim	21.52% (20.55% - 22.45%)	19.25% (18.33% - 20.09%)	17.87% (17.15% - 18.56%)	13.96% (13.34% - 14.56%)	5.04% (4.19% - 5.88%)	4.75% (4.02% - 5.49%)	5.19% (4.61% - 5.78%)	3.82% (3.41% - 4.27%)	0.55% (0.36% - 0.81%)	0.57% (0.39% - 0.81%)	0.80% (0.60% - 1.01%)	0.52% (0.39% - 0.68%)
Tamil Nadu	38.00% (36.98% - 39.32%)	33.05% (32.42% - 33.69%)	27.81% (27.29% - 28.31%)	22.52% (21.93% - 23.10%)	14.79% (13.84% - 15.81%)	14.26% (13.57% - 15.06%)	11.47% (10.89% - 12.16%)	8.50% (7.89% - 9.11%)	3.06% (2.47% - 3.73%)	3.72% (3.20% - 4.32%)	2.91% (2.54% - 3.35%)	1.96% (1.64% - 2.30%)
Telangana	39.05% (38.14% - 40.18%)	35.48% (34.93% - 36.07%)	30.92% (30.39% - 31.43%)	25.00% (24.43% - 25.60%)	17.07% (15.33% - 18.90%)	17.35% (16.12% - 18.72%)	15.93% (14.90% - 16.92%)	11.61% (10.84% - 12.44%)	4.67% (2.96% - 6.88%)	6.15% (4.71% - 7.86%)	6.37% (5.34% - 7.44%)	3.96% (3.38% - 4.64%)
Tripura	38.34% (37.36% - 39.44%)	34.17% (33.45% - 34.87%)	30.94% (30.35% - 31.55%)	26.04% (25.38% - 26.77%)	17.33% (15.94% - 18.93%)	15.19% (14.16% - 16.28%)	14.46% (13.64% - 15.33%)	11.01% (10.20% - 11.82%)	4.81% (3.72% - 6.24%)	4.22% (3.49% - 5.09%)	4.48% (3.85% - 5.18%)	2.98% (2.46% - 3.51%)
Uttar Pradesh	44.95% (43.75% - 46.40%)	41.80% (41.24% - 42.50%)	38.04% (37.52% - 38.63%)	32.23% (31.70% - 32.83%)	25.33% (23.76% - 27.16%)	25.47% (24.44% - 26.57%)	20.96% (20.15% - 21.87%)	15.69% (14.87% - 16.60%)	9.88% (7.82% - 12.49%)	11.69% (10.19% - 13.29%)	8.20% (7.23% - 9.26%)	5.14% (4.46% - 5.87%)
Uttarakhand	40.42% (39.07% - 42.42%)	37.89% (37.04% - 38.95%)	31.51% (30.95% - 32.14%)	24.24% (23.61% - 24.86%)	14.08% (12.69% - 15.48%)	15.41% (14.13% - 16.65%)	13.87% (12.93% - 14.85%)	9.86% (9.17% - 10.63%)	2.49% (1.77% - 3.44%)	3.50% (2.62% - 4.47%)	3.86% (3.19% - 4.57%)	2.56% (2.16% - 3.03%)
West Bengal	40.27% (38.91% - 41.94%)	37.01% (36.21% - 37.94%)	31.84% (31.29% - 32.45%)	26.63% (26.03% - 27.23%)	16.34% (15.14% - 17.61%)	16.46% (15.53% - 17.38%)	14.57% (13.78% - 15.44%)	11.49% (10.70% - 12.21%)	3.49% (2.69% - 4.51%)	4.38% (3.57% - 5.24%)	4.36% (3.69% - 5.08%)	3.26% (2.72% - 3.81%)
Nepal	38.02% (36.59% - 39.62%)	33.82% (32.85% - 34.87%)	27.28% (26.52% - 28.11%)	22.30% (21.51% - 23.13%)	14.18% (13.03% - 15.39%)	13.03% (12.06% - 14.13%)	9.08% (8.20% - 10.01%)	6.77% (5.93% - 7.63%)	2.75% (2.15% - 3.53%)	2.75% (2.20% - 3.46%)	1.51% (1.12% - 2.00%)	1.01% (0.71% - 1.36%)
Pakistan	33.04% (32.35% - 33.77%)	32.53% (31.80% - 33.32%)	30.95% (30.42% - 31.57%)	26.12% (25.38% - 26.85%)	15.58% (14.62% - 16.65%)	14.71% (13.84% - 15.65%)	13.84% (13.13% - 14.54%)	10.76% (9.95% - 11.61%)	5.03% (4.25% - 5.92%)	4.55% (3.87% - 5.37%)	4.36% (3.72% - 4.99%)	3.22% (2.69% - 3.84%)

Table S8c. Estimated overall, severe, and extreme **underweight** prevalence (%) in children under 5 years, both sexes, in 1990, 2000, 2010, and 2020.

Location	Total				Severe				Extreme			
	1990	2000	2010	2020	1990	2000	2010	2020	1990	2000	2010	2020
Southeast Asia, East Asia,	16.22%	13.54%	10.92%	8.39%	4.52%	3.87%	2.89%	2.03%	0.75%	0.68%	0.44%	0.27%
and Oceania	(15.72% - 16.73%)	(13.26% - 13.83%)	(10.75% - 11.11%)	(8.21% - 8.56%)	(4.28% - 4.80%)	(3.76% - 4.00%)	(2.81% - 2.97%)	(1.96% - 2.11%)	(0.69% - 0.83%)	(0.65% - 0.71%)	(0.41% - 0.46%)	(0.25% - 0.30%)
	11.27%	6.86%	4.12%	3.09%	2.22%	1.05%	0.44%	0.28%	0.23%	0.09%	0.02%	0.01%
**East Asia**	(10.54% - 12.04%)	(6.40% - 7.40%)	(3.81% - 4.46%)	(2.84% - 3.36%)	(1.88% - 2.61%)	(0.89% - 1.25%)	(0.36% - 0.54%)	(0.22% - 0.35%)	(0.16% - 0.32%)	(0.07% - 0.11%)	(0.01% - 0.02%)	(0.00% - 0.01%)
	11.03%	6.32%	3.79%	2.90%	2.09%	0.80%	0.34%	0.23%	0.20%	0.03%	0.01%	0.00%
China	(10.29% - 11.83%)	(5.84% - 6.89%)	(3.47% - 4.14%)	(2.65% - 3.18%)	(1.73% - 2.48%)	(0.64% - 1.01%)	(0.26% - 0.44%)	(0.17% - 0.30%)	(0.13% - 0.30%)	(0.02% - 0.05%)	(0.00% - 0.01%)	(0.00% - 0.01%)
	29.50%	26.99%	17.11%	13.57%	10.48%	9.83%	4.36%	2.74%	2.00%	2.00%	0.46%	0.14%
Democratic People's Republic of Korea	(28.73% - 30.29%)	(26.41% - 27.57%)	(16.52% - 17.70%)	(12.79% - 14.29%)	(9.41% - 11.57%)	(9.05% - 10.58%)	(3.90% - 4.84%)	(2.27% - 3.23%)	(1.49% - 2.63%)	(1.62% - 2.42%)	(0.32% - 0.62%)	(0.05% - 0.26%)
	2.76%	3.05%	2.95%	2.79%	0.10%	0.25%	0.28%	0.27%	0.00%	0.00%	0.00%	0.00%
Taiwan (Province of China)	(2.50% - 3.02%)	(2.75% - 3.34%)	(2.68% - 3.28%)	(2.54% - 3.07%)	(0.05% - 0.15%)	(0.18% - 0.34%)	(0.21% - 0.37%)	(0.19% - 0.35%)	(0.00% - 0.00%)	(0.00% - 0.00%)	(0.00% - 0.00%)	(0.00% - 0.00%)
	18.83%	18.69%	20.68%	18.59%	5.55%	5.68%	7.64%	5.89%	0.86%	0.95%	1.76%	1.00%
**Oceania**	(18.24% - 19.42%)	(18.08% - 19.29%)	(20.19% - 21.14%)	(17.89% - 19.22%)	(4.99% - 6.11%)	(5.07% - 6.19%)	(7.14% - 8.18%)	(5.28% - 6.50%)	(0.65% - 1.08%)	(0.73% - 1.18%)	(1.50% - 2.07%)	(0.79% - 1.26%)
	6.83%	5.19%	4.61%	4.16%	0.81%	0.48%	0.38%	0.35%	0.00%	0.00%	0.00%	0.00%
Fiji	(6.33% - 7.38%)	(4.79% - 5.62%)	(4.24% - 5.00%)	(3.82% - 4.52%)	(0.65% - 0.99%)	(0.37% - 0.60%)	(0.29% - 0.49%)	(0.26% - 0.45%)	(0.00% - 0.00%)	(0.00% - 0.00%)	(0.00% - 0.00%)	(0.00% - 0.00%)
	11.55%	11.83%	11.67%	8.40%	2.60%	2.83%	2.74%	1.36%	0.21%	0.28%	0.28%	0.04%
Kiribati	(10.94% - 12.18%)	(11.15% - 12.58%)	(11.08% - 12.32%)	(7.88% - 8.95%)	(2.30% - 2.95%)	(2.47% - 3.23%)	(2.42% - 3.08%)	(1.16% - 1.59%)	(0.14% - 0.29%)	(0.19% - 0.39%)	(0.20% - 0.38%)	(0.02% - 0.08%)
	16.77%	15.74%	13.81%	12.34%	5.18%	4.57%	3.83%	3.34%	0.86%	0.68%	0.52%	0.43%
Marshall Islands	(15.99% - 17.47%)	(14.95% - 16.47%)	(13.05% - 14.59%)	(11.56% - 13.13%)	(4.61% - 5.76%)	(4.00% - 5.10%)	(3.34% - 4.34%)	(2.89% - 3.82%)	(0.67% - 1.08%)	(0.50% - 0.86%)	(0.38% - 0.68%)	(0.31% - 0.58%)
	11.47%	8.59%	7.79%	7.37%	2.00%	1.10%	1.07%	1.07%	0.05%	0.00%	0.00%	0.00%
Micronesia (Federated States of)	(10.75% - 12.24%)	(7.94% - 9.24%)	(7.18% - 8.38%)	(6.80% - 7.93%)	(1.66% - 2.37%)	(0.89% - 1.34%)	(0.87% - 1.29%)	(0.87% - 1.27%)	(0.01% - 0.11%)	(0.00% - 0.01%)	(0.00% - 0.02%)	(0.00% - 0.02%)
	23.20%	22.69%	24.73%	21.35%	7.24%	7.22%	9.55%	6.95%	1.18%	1.25%	2.26%	1.21%
Papua New Guinea	(22.35% - 24.07%)	(21.88% - 23.50%)	(24.10% - 25.33%)	(20.48% - 22.14%)	(6.42% - 8.07%)	(6.39% - 7.93%)	(8.88% - 10.26%)	(6.20% - 7.72%)	(0.89% - 1.50%)	(0.95% - 1.57%)	(1.93% - 2.68%)	(0.94% - 1.53%)
	2.29%	2.05%	2.74%	2.83%	0.01%	0.00%	0.09%	0.13%	0.00%	0.00%	0.00%	0.00%
Samoa	(2.09% - 2.53%)	(1.87% - 2.27%)	(2.48% - 3.00%)	(2.57% - 3.09%)	(0.00% - 0.02%)	(0.00% - 0.01%)	(0.05% - 0.14%)	(0.08% - 0.19%)	(0.00% - 0.00%)	(0.00% - 0.00%)	(0.00% - 0.00%)	(0.00% - 0.00%)
	15.59%	13.59%	13.31%	12.68%	3.88%	3.58%	3.77%	3.79%	0.40%	0.42%	0.53%	0.61%
Solomon Islands	(14.80% - 16.40%)	(12.87% - 14.37%)	(12.68% - 13.97%)	(11.98% - 13.40%)	(3.32% - 4.45%)	(3.14% - 4.11%)	(3.36% - 4.21%)	(3.34% - 4.30%)	(0.23% - 0.58%)	(0.29% - 0.59%)	(0.40% - 0.68%)	(0.45% - 0.80%)
	2.33%	1.88%	1.85%	1.69%	0.01%	0.00%	0.00%	0.00%	0.00%	0.00%	0.00%	0.00%
Tonga	(2.13% - 2.55%)	(1.73% - 2.07%)	(1.69% - 2.04%)	(1.54% - 1.85%)	(0.00% - 0.03%)	(0.00% - 0.01%)	(0.00% - 0.01%)	(0.00% - 0.01%)	(0.00% - 0.00%)	(0.00% - 0.00%)	(0.00% - 0.00%)	(0.00% - 0.00%)
	6.88%	5.36%	5.15%	4.92%	1.08%	0.65%	0.64%	0.63%	0.00%	0.00%	0.00%	0.00%
American Samoa	(6.42% - 7.36%)	(4.94% - 5.80%)	(4.77% - 5.57%)	(4.56% - 5.32%)	(0.91% - 1.26%)	(0.52% - 0.78%)	(0.53% - 0.77%)	(0.52% - 0.76%)	(0.00% - 0.01%)	(0.00% - 0.00%)	(0.00% - 0.00%)	(0.00% - 0.00%)
	12.67%	10.70%	10.12%	9.53%	2.96%	2.07%	1.82%	1.74%	0.26%	0.09%	0.06%	0.05%
Vanuatu	(12.03% - 13.35%)	(10.07% - 11.28%)	(9.55% - 10.70%)	(8.93% - 10.15%)	(2.61% - 3.33%)	(1.79% - 2.36%)	(1.57% - 2.08%)	(1.47% - 2.01%)	(0.18% - 0.36%)	(0.05% - 0.14%)	(0.03% - 0.09%)	(0.02% - 0.09%)
	5.96%	4.14%	3.83%	3.54%	0.91%	0.46%	0.44%	0.42%	0.00%	0.00%	0.00%	0.00%
Cook Islands	(5.52% - 6.43%)	(3.82% - 4.47%)	(3.54% - 4.15%)	(3.27% - 3.85%)	(0.76% - 1.08%)	(0.37% - 0.56%)	(0.36% - 0.53%)	(0.34% - 0.51%)	(0.00% - 0.00%)	(0.00% - 0.00%)	(0.00% - 0.00%)	(0.00% - 0.00%)
	4.88%	3.59%	3.55%	3.47%	0.70%	0.38%	0.39%	0.39%	0.00%	0.00%	0.00%	0.00%
Guam	(4.52% - 5.26%)	(3.31% - 3.88%)	(3.28% - 3.84%)	(3.19% - 3.75%)	(0.58% - 0.82%)	(0.31% - 0.47%)	(0.32% - 0.47%)	(0.31% - 0.47%)	(0.00% - 0.00%)	(0.00% - 0.00%)	(0.00% - 0.00%)	(0.00% - 0.00%)
	5.41%	5.78%	5.28%	4.01%	0.48%	0.43%	0.41%	0.31%	0.00%	0.00%	0.00%	0.00%
Nauru	(5.04% - 5.80%)	(5.32% - 6.21%)	(4.90% - 5.65%)	(3.70% - 4.33%)	(0.39% - 0.59%)	(0.33% - 0.54%)	(0.32% - 0.50%)	(0.25% - 0.38%)	(0.00% - 0.00%)	(0.00% - 0.00%)	(0.00% - 0.00%)	(0.00% - 0.00%)
	6.78%	5.12%	4.82%	4.58%	1.03%	0.60%	0.59%	0.58%	0.00%	0.00%	0.00%	0.00%
Niue	(6.28% - 7.28%)	(4.75% - 5.55%)	(4.44% - 5.22%)	(4.23% - 4.94%)	(0.86% - 1.23%)	(0.48% - 0.73%)	(0.48% - 0.71%)	(0.46% - 0.69%)	(0.00% - 0.01%)	(0.00% - 0.00%)	(0.00% - 0.00%)	(0.00% - 0.00%)
	4.91%	3.69%	3.82%	3.72%	0.71%	0.41%	0.44%	0.44%	0.00%	0.00%	0.00%	0.00%
Northern Mariana Islands	(4.54% - 5.28%)	(3.44% - 3.97%)	(3.54% - 4.13%)	(3.44% - 4.01%)	(0.60% - 0.83%)	(0.34% - 0.49%)	(0.36% - 0.53%)	(0.36% - 0.52%)	(0.00% - 0.00%)	(0.00% - 0.00%)	(0.00% - 0.00%)	(0.00% - 0.00%)
	6.30%	4.70%	5.03%	4.55%	0.96%	0.54%	0.64%	0.58%	0.00%	0.00%	0.00%	0.00%
Palau	(5.83% - 6.74%)	(4.34% - 5.08%)	(4.68% - 5.40%)	(4.22% - 4.89%)	(0.81% - 1.12%)	(0.44% - 0.66%)	(0.54% - 0.77%)	(0.47% - 0.69%)	(0.00% - 0.01%)	(0.00% - 0.00%)	(0.00% - 0.00%)	(0.00% - 0.00%)
	9.35%	6.73%	5.83%	5.06%	1.53%	0.83%	0.74%	0.65%	0.02%	0.00%	0.00%	0.00%
Tokelau	(8.68% - 10.04%)	(6.26% - 7.25%)	(5.38% - 6.31%)	(4.64% - 5.49%)	(1.25% - 1.83%)	(0.68% - 1.00%)	(0.61% - 0.90%)	(0.53% - 0.79%)	(0.00% - 0.05%)	(0.00% - 0.00%)	(0.00% - 0.00%)	(0.00% - 0.00%)

Table S8c. Estimated overall, severe, and extreme **underweight** prevalence (%) in children under 5 years, both sexes, in 1990, 2000, 2010, and 2020.

Location	Total				Severe				Extreme			
	1990	2000	2010	2020	1990	2000	2010	2020	1990	2000	2010	2020
	2.60%	1.87%	1.65%	1.53%	0.00%	0.00%	0.00%	0.00%	0.00%	0.00%	0.00%	0.00%
Tuvalu	(2.41% - 2.80%)	(1.73% - 2.02%)	(1.51% - 1.80%)	(1.41% - 1.66%)	(0.00% - 0.01%)	(0.00% - 0.00%)	(0.00% - 0.00%)	(0.00% - 0.00%)	(0.00% - 0.00%)	(0.00% - 0.00%)	(0.00% - 0.00%)	(0.00% - 0.00%)
	26.69%	22.57%	18.49%	15.80%	9.40%	7.69%	5.58%	4.48%	1.86%	1.48%	0.88%	0.64%
**Southeast Asia**	(26.49% - 26.89%)	(22.42% - 22.73%)	(18.35% - 18.64%)	(15.58% - 16.02%)	(9.19% - 9.62%)	(7.54% - 7.85%)	(5.45% - 5.71%)	(4.32% - 4.65%)	(1.76% - 1.96%)	(1.41% - 1.56%)	(0.83% - 0.93%)	(0.59% - 0.70%)
	35.95%	33.47%	25.60%	21.07%	13.90%	13.37%	8.25%	6.20%	2.74%	2.92%	1.29%	0.86%
Cambodia	(34.74% - 37.30%)	(32.57% - 34.53%)	(24.94% - 26.36%)	(20.25% - 21.87%)	(12.75% - 15.14%)	(12.28% - 14.47%)	(7.57% - 9.02%)	(5.37% - 7.06%)	(2.01% - 3.67%)	(2.15% - 3.75%)	(0.97% - 1.68%)	(0.57% - 1.21%)
	26.48%	22.36%	19.15%	16.75%	9.60%	7.93%	6.11%	5.05%	2.07%	1.69%	1.09%	0.81%
Indonesia	(26.27% - 26.67%)	(22.20% - 22.50%)	(18.98% - 19.32%)	(16.52% - 16.96%)	(9.36% - 9.83%)	(7.78% - 8.08%)	(5.97% - 6.26%)	(4.89% - 5.21%)	(1.95% - 2.18%)	(1.63% - 1.76%)	(1.04% - 1.14%)	(0.75% - 0.86%)
	33.29%	30.22%	25.76%	23.19%	17.37%	15.75%	11.38%	9.57%	6.44%	5.91%	3.29%	2.50%
Aceh	(32.55% - 34.05%)	(29.50% - 30.92%)	(24.88% - 26.59%)	(22.32% - 24.05%)	(16.15% - 18.66%)	(14.69% - 16.85%)	(10.40% - 12.44%)	(8.60% - 10.58%)	(5.33% - 7.70%)	(5.03% - 6.91%)	(2.66% - 4.03%)	(1.96% - 3.12%)
	29.68%	26.27%	21.96%	18.57%	12.18%	10.93%	7.70%	5.76%	2.97%	2.83%	1.55%	0.95%
North Sumatra	(29.06% - 30.34%)	(25.75% - 26.79%)	(21.34% - 22.55%)	(17.74% - 19.33%)	(11.33% - 13.06%)	(10.25% - 11.60%)	(7.11% - 8.34%)	(5.14% - 6.43%)	(2.47% - 3.52%)	(2.46% - 3.24%)	(1.30% - 1.84%)	(0.74% - 1.21%)
	24.96%	21.60%	18.73%	16.06%	7.95%	6.71%	4.91%	3.94%	1.33%	1.08%	0.57%	0.39%
West Sumatra	(24.34% - 25.58%)	(21.09% - 22.14%)	(18.06% - 19.37%)	(15.18% - 16.91%)	(7.23% - 8.63%)	(6.20% - 7.22%)	(4.41% - 5.45%)	(3.33% - 4.55%)	(1.06% - 1.64%)	(0.91% - 1.27%)	(0.43% - 0.73%)	(0.26% - 0.57%)
	28.17%	22.56%	18.77%	16.38%	12.24%	8.82%	6.33%	5.13%	3.38%	2.15%	1.23%	0.88%
Riau	(27.43% - 28.87%)	(21.91% - 23.19%)	(18.08% - 19.49%)	(15.60% - 17.21%)	(11.28% - 13.20%)	(8.18% - 9.46%)	(5.76% - 6.92%)	(4.56% - 5.73%)	(2.75% - 4.05%)	(1.82% - 2.49%)	(1.01% - 1.47%)	(0.68% - 1.11%)
	24.25%	22.24%	19.31%	16.33%	7.98%	7.98%	6.34%	4.98%	1.39%	1.65%	1.14%	0.79%
Jambi	(23.51% - 24.96%)	(21.73% - 22.79%)	(18.66% - 19.94%)	(15.50% - 17.13%)	(7.22% - 8.75%)	(7.47% - 8.56%)	(5.81% - 6.89%)	(4.35% - 5.58%)	(1.09% - 1.73%)	(1.43% - 1.92%)	(0.93% - 1.36%)	(0.58% - 1.02%)
	27.84%	23.41%	19.81%	16.57%	11.18%	9.02%	6.81%	5.11%	2.87%	2.12%	1.33%	0.82%
South Sumatra	(27.06% - 28.58%)	(22.85% - 24.04%)	(19.17% - 20.47%)	(15.73% - 17.43%)	(10.17% - 12.23%)	(8.37% - 9.74%)	(6.26% - 7.43%)	(4.48% - 5.76%)	(2.25% - 3.56%)	(1.80% - 2.52%)	(1.10% - 1.61%)	(0.61% - 1.07%)
	21.67%	17.42%	15.09%	12.69%	7.06%	5.36%	4.11%	3.26%	1.24%	0.88%	0.54%	0.37%
Bengkulu	(20.94% - 22.38%)	(16.81% - 18.10%)	(14.42% - 15.81%)	(12.01% - 13.39%)	(6.38% - 7.78%)	(4.85% - 5.88%)	(3.63% - 4.63%)	(2.83% - 3.70%)	(0.98% - 1.54%)	(0.71% - 1.07%)	(0.40% - 0.70%)	(0.26% - 0.49%)
	24.30%	20.76%	16.59%	13.98%	7.98%	6.96%	4.56%	3.51%	1.43%	1.35%	0.61%	0.38%
Lampung	(23.65% - 24.96%)	(20.23% - 21.27%)	(15.98% - 17.22%)	(13.20% - 14.82%)	(7.32% - 8.67%)	(6.46% - 7.46%)	(4.11% - 5.02%)	(3.06% - 4.04%)	(1.18% - 1.73%)	(1.16% - 1.56%)	(0.49% - 0.76%)	(0.26% - 0.52%)
	22.52%	20.36%	16.39%	13.59%	7.02%	6.45%	4.46%	3.54%	1.14%	1.09%	0.58%	0.42%
Bangka-Belitung Islands	(21.62% - 23.43%)	(19.67% - 21.04%)	(15.76% - 17.01%)	(12.81% - 14.37%)	(6.14% - 7.94%)	(5.81% - 7.10%)	(4.01% - 4.91%)	(3.09% - 4.03%)	(0.83% - 1.52%)	(0.86% - 1.34%)	(0.44% - 0.73%)	(0.29% - 0.58%)
	20.64%	17.16%	15.65%	13.76%	6.13%	4.91%	4.29%	3.58%	0.90%	0.69%	0.55%	0.40%
Riau Islands	(19.64% - 21.60%)	(16.26% - 18.02%)	(14.92% - 16.39%)	(12.97% - 14.51%)	(5.25% - 6.99%)	(4.27% - 5.61%)	(3.77% - 4.84%)	(3.09% - 4.07%)	(0.62% - 1.22%)	(0.48% - 0.93%)	(0.39% - 0.73%)	(0.28% - 0.55%)
	27.38%	24.77%	23.15%	20.67%	9.03%	8.40%	7.96%	6.95%	1.55%	1.54%	1.51%	1.30%
North Kalimantan	(26.50% - 28.30%)	(24.02% - 25.51%)	(22.58% - 23.70%)	(19.88% - 21.48%)	(7.99% - 10.08%)	(7.59% - 9.28%)	(7.41% - 8.53%)	(6.20% - 7.69%)	(1.14% - 2.03%)	(1.20% - 1.93%)	(1.27% - 1.76%)	(1.01% - 1.64%)
	23.09%	17.84%	14.66%	13.17%	8.41%	5.91%	4.18%	3.62%	1.81%	1.12%	0.59%	0.48%
Jakarta	(22.52% - 23.66%)	(17.43% - 18.27%)	(14.08% - 15.27%)	(12.43% - 13.86%)	(7.83% - 9.03%)	(5.57% - 6.29%)	(3.79% - 4.62%)	(3.16% - 4.08%)	(1.54% - 2.11%)	(0.99% - 1.28%)	(0.47% - 0.74%)	(0.33% - 0.63%)
	26.47%	20.79%	16.80%	15.23%	9.78%	7.11%	4.77%	4.19%	2.05%	1.35%	0.64%	0.53%
West Java	(25.86% - 27.12%)	(20.25% - 21.33%)	(16.15% - 17.43%)	(14.44% - 16.02%)	(9.01% - 10.55%)	(6.61% - 7.63%)	(4.31% - 5.25%)	(3.65% - 4.78%)	(1.68% - 2.44%)	(1.16% - 1.58%)	(0.50% - 0.80%)	(0.37% - 0.73%)
	24.93%	20.35%	17.03%	14.56%	7.76%	6.04%	4.59%	3.72%	1.25%	0.92%	0.58%	0.41%
Central Java	(24.26% - 25.56%)	(19.93% - 20.79%)	(16.48% - 17.57%)	(13.81% - 15.32%)	(7.04% - 8.44%)	(5.64% - 6.44%)	(4.19% - 5.01%)	(3.24% - 4.20%)	(1.00% - 1.52%)	(0.78% - 1.06%)	(0.46% - 0.71%)	(0.28% - 0.55%)
	18.24%	15.15%	12.32%	11.04%	4.45%	3.66%	2.56%	2.29%	0.45%	0.35%	0.15%	0.13%
Yogyakarta	(17.56% - 18.92%)	(14.65% - 15.64%)	(11.73% - 12.95%)	(10.36% - 11.74%)	(3.95% - 5.02%)	(3.31% - 4.02%)	(2.24% - 2.91%)	(1.95% - 2.64%)	(0.32% - 0.60%)	(0.27% - 0.45%)	(0.09% - 0.23%)	(0.06% - 0.21%)
	26.51%	21.66%	17.86%	15.87%	9.45%	7.19%	5.29%	4.54%	1.90%	1.32%	0.79%	0.63%
East Java	(25.87% - 27.05%)	(21.16% - 22.15%)	(17.23% - 18.45%)	(15.08% - 16.58%)	(8.69% - 10.24%)	(6.71% - 7.69%)	(4.80% - 5.79%)	(3.98% - 5.11%)	(1.57% - 2.28%)	(1.13% - 1.52%)	(0.62% - 0.96%)	(0.45% - 0.83%)
	23.40%	20.67%	18.85%	16.12%	7.39%	6.62%	5.82%	4.67%	1.20%	1.13%	0.93%	0.67%
Banten	(22.55% - 24.35%)	(20.00% - 21.33%)	(18.19% - 19.44%)	(15.29% - 16.89%)	(6.46% - 8.33%)	(6.01% - 7.30%)	(5.27% - 6.36%)	(4.03% - 5.23%)	(0.88% - 1.59%)	(0.91% - 1.39%)	(0.73% - 1.15%)	(0.46% - 0.87%)
	19.03%	14.74%	12.74%	10.73%	5.20%	3.79%	2.96%	2.31%	0.69%	0.44%	0.26%	0.14%
Bali	(18.38% - 19.67%)	(14.23% - 15.25%)	(12.13% - 13.33%)	(10.02% - 11.41%)	(4.64% - 5.76%)	(3.47% - 4.10%)	(2.64% - 3.30%)	(1.97% - 2.64%)	(0.53% - 0.86%)	(0.35% - 0.52%)	(0.18% - 0.34%)	(0.08% - 0.22%)
	35.32%	30.24%	25.46%	22.70%	11.23%	9.57%	8.50%	7.48%	1.72%	1.64%	1.57%	1.36%
West Nusa Tenggara	(33.77% - 37.03%)	(29.27% - 31.41%)	(24.80% - 26.15%)	(21.91% - 23.52%)	(10.06% - 12.50%)	(8.57% - 10.60%)	(7.66% - 9.35%)	(6.68% - 8.33%)	(1.33% - 2.19%)	(1.28% - 2.04%)	(1.25% - 1.94%)	(1.05% - 1.75%)

Table S8c. Estimated overall, severe, and extreme **underweight** prevalence (%) in children under 5 years, both sexes, in 1990, 2000, 2010, and 2020.												
**Location**	**Total**				**Severe**				**Extreme**			
	**1990**	**2000**	**2010**	**2020**	**1990**	**2000**	**2010**	**2020**	**1990**	**2000**	**2010**	**2020**
East Nusa Tenggara	31.73% (30.79% - 32.77%)	29.99% (29.14% - 30.91%)	28.50% (27.80% - 29.25%)	25.36% (24.57% - 26.15%)	11.11% (9.97% - 12.25%)	10.99% (9.99% - 12.04%)	10.39% (9.47% - 11.29%)	9.16% (8.27% - 10.15%)	2.05% (1.53% - 2.66%)	2.22% (1.70% - 2.78%)	2.09% (1.66% - 2.58%)	1.86% (1.44% - 2.40%)
West Kalimantan	33.69% (33.06% - 34.33%)	28.66% (28.11% - 29.27%)	25.11% (24.39% - 25.79%)	21.22% (20.37% - 22.02%)	17.72% (16.59% - 19.07%)	13.68% (12.86% - 14.61%)	10.64% (9.82% - 11.49%)	8.19% (7.39% - 9.03%)	6.60% (5.55% - 7.98%)	4.42% (3.79% - 5.13%)	2.86% (2.35% - 3.39%)	1.92% (1.52% - 2.38%)
Central Kalimantan	28.33% (27.67% - 29.03%)	26.92% (26.37% - 27.45%)	23.95% (23.35% - 24.56%)	20.48% (19.63% - 21.33%)	11.23% (10.34% - 12.18%)	12.28% (11.59% - 13.02%)	9.42% (8.74% - 10.12%)	7.42% (6.68% - 8.24%)	2.61% (2.12% - 3.21%)	3.72% (3.25% - 4.25%)	2.26% (1.90% - 2.66%)	1.58% (1.24% - 1.99%)
South Kalimantan	29.94% (29.32% - 30.63%)	25.72% (25.18% - 26.23%)	24.31% (23.54% - 25.09%)	20.43% (19.49% - 21.29%)	11.94% (11.03% - 12.91%)	9.68% (9.03% - 10.30%)	8.86% (8.02% - 9.71%)	6.59% (5.78% - 7.47%)	2.85% (2.33% - 3.43%)	2.13% (1.81% - 2.47%)	1.85% (1.45% - 2.29%)	1.14% (0.82% - 1.52%)
East Kalimantan	22.93% (22.47% - 23.39%)	20.39% (20.02% - 20.77%)	16.98% (16.63% - 17.37%)	14.65% (13.94% - 15.33%)	6.74% (6.21% - 7.23%)	6.53% (6.16% - 6.88%)	4.92% (4.62% - 5.22%)	4.08% (3.60% - 4.58%)	0.95% (0.77% - 1.14%)	1.11% (0.96% - 1.26%)	0.70% (0.60% - 0.81%)	0.54% (0.40% - 0.71%)
North Sulawesi	22.54% (21.84% - 23.27%)	20.71% (20.13% - 21.33%)	16.48% (15.85% - 17.13%)	13.74% (13.00% - 14.52%)	7.77% (7.11% - 8.48%)	7.83% (7.21% - 8.43%)	4.77% (4.30% - 5.25%)	3.62% (3.13% - 4.13%)	1.47% (1.18% - 1.81%)	1.75% (1.46% - 2.06%)	0.66% (0.52% - 0.83%)	0.41% (0.28% - 0.57%)
Central Sulawesi	25.98% (25.27% - 26.66%)	23.99% (23.44% - 24.53%)	22.99% (22.38% - 23.63%)	18.95% (18.14% - 19.79%)	8.73% (7.89% - 9.60%)	8.71% (8.13% - 9.26%)	8.16% (7.50% - 8.83%)	6.13% (5.43% - 6.87%)	1.57% (1.22% - 1.96%)	1.82% (1.54% - 2.09%)	1.65% (1.37% - 1.95%)	1.08% (0.84% - 1.39%)
South Sulawesi	27.89% (27.10% - 28.73%)	24.26% (23.66% - 24.87%)	22.22% (21.50% - 22.98%)	19.17% (18.28% - 20.02%)	9.27% (8.41% - 10.11%)	8.42% (7.80% - 9.07%)	7.40% (6.71% - 8.14%)	5.92% (5.17% - 6.64%)	1.70% (1.37% - 2.05%)	1.72% (1.47% - 2.01%)	1.37% (1.10% - 1.67%)	0.95% (0.69% - 1.22%)
Southeast Sulawesi	24.28% (23.57% - 24.93%)	22.33% (21.73% - 22.88%)	21.33% (20.70% - 21.98%)	17.90% (17.08% - 18.71%)	7.97% (7.27% - 8.71%)	7.72% (7.14% - 8.32%)	7.43% (6.85% - 8.07%)	5.76% (5.13% - 6.44%)	1.38% (1.10% - 1.71%)	1.49% (1.26% - 1.77%)	1.47% (1.21% - 1.75%)	1.01% (0.79% - 1.28%)
Gorontalo	34.43% (33.65% - 35.19%)	33.63% (32.98% - 34.28%)	30.04% (29.28% - 30.78%)	24.78% (23.89% - 25.70%)	18.63% (17.35% - 20.08%)	18.74% (17.56% - 19.96%)	15.17% (14.14% - 16.30%)	10.88% (9.85% - 11.97%)	7.24% (6.06% - 8.67%)	7.71% (6.59% - 8.96%)	5.36% (4.50% - 6.37%)	3.10% (2.47% - 3.85%)
West Sulawesi	26.90% (25.96% - 27.83%)	24.81% (23.99% - 25.69%)	22.86% (22.11% - 23.62%)	19.39% (18.55% - 20.24%)	8.62% (7.54% - 9.64%)	8.17% (7.30% - 9.03%)	7.45% (6.69% - 8.24%)	6.06% (5.33% - 6.83%)	1.41% (1.04% - 1.86%)	1.43% (1.10% - 1.81%)	1.30% (1.01% - 1.62%)	1.00% (0.75% - 1.30%)
Maluku	28.84% (28.10% - 29.58%)	25.59% (24.92% - 26.25%)	26.34% (25.63% - 27.01%)	22.95% (22.06% - 23.77%)	13.42% (12.39% - 14.46%)	11.35% (10.57% - 12.16%)	11.30% (10.45% - 12.17%)	9.12% (8.16% - 10.00%)	4.12% (3.38% - 4.95%)	3.26% (2.78% - 3.81%)	3.08% (2.53% - 3.66%)	2.23% (1.76% - 2.73%)
North Maluku	23.16% (22.33% - 24.03%)	21.08% (20.41% - 21.73%)	21.97% (21.42% - 22.56%)	19.46% (18.64% - 20.26%)	7.96% (7.11% - 8.93%)	7.30% (6.68% - 7.98%)	7.77% (7.21% - 8.37%)	6.52% (5.82% - 7.24%)	1.54% (1.19% - 1.96%)	1.45% (1.19% - 1.74%)	1.58% (1.32% - 1.87%)	1.23% (0.96% - 1.55%)
West Papua	25.17% (24.24% - 26.06%)	22.76% (21.87% - 23.63%)	20.48% (19.57% - 21.40%)	17.12% (16.26% - 17.97%)	8.40% (7.43% - 9.41%)	7.79% (6.93% - 8.75%)	6.50% (5.68% - 7.38%)	5.06% (4.44% - 5.74%)	1.49% (1.13% - 1.94%)	1.48% (1.12% - 1.91%)	1.10% (0.79% - 1.43%)	0.76% (0.54% - 1.00%)
Papua	27.98% (27.26% - 28.67%)	25.72% (25.10% - 26.37%)	23.86% (23.20% - 24.54%)	20.66% (19.84% - 21.47%)	12.54% (11.55% - 13.44%)	11.76% (11.04% - 12.57%)	9.67% (8.96% - 10.37%)	7.70% (6.94% - 8.47%)	3.65% (2.99% - 4.31%)	3.59% (3.11% - 4.15%)	2.44% (2.03% - 2.84%)	1.72% (1.35% - 2.11%)
Lao People's Democratic Republic	32.95% (32.19% - 33.77%)	31.34% (30.60% - 32.09%)	25.76% (24.99% - 26.53%)	20.56% (19.65% - 21.39%)	14.32% (13.23% - 15.54%)	13.20% (12.16% - 14.24%)	8.60% (7.68% - 9.51%)	6.08% (5.21% - 6.95%)	3.80% (3.05% - 4.72%)	3.34% (2.65% - 4.12%)	1.47% (1.05% - 1.95%)	0.86% (0.56% - 1.21%)
Malaysia	21.27% (20.88% - 21.67%)	16.18% (15.74% - 16.63%)	13.62% (13.14% - 14.11%)	12.43% (11.89% - 12.97%)	7.16% (6.79% - 7.54%)	4.66% (4.33% - 4.99%)	3.46% (3.17% - 3.76%)	3.07% (2.77% - 3.39%)	1.31% (1.16% - 1.48%)	0.65% (0.54% - 0.77%)	0.37% (0.28% - 0.46%)	0.30% (0.22% - 0.39%)
Maldives	35.06% (34.17% - 36.08%)	27.46% (26.70% - 28.19%)	17.73% (16.95% - 18.57%)	15.45% (14.67% - 16.28%)	14.32% (13.11% - 15.67%)	10.62% (9.61% - 11.61%)	5.10% (4.45% - 5.82%)	3.99% (3.45% - 4.63%)	3.29% (2.41% - 4.44%)	2.41% (1.81% - 3.07%)	0.72% (0.50% - 0.99%)	0.45% (0.29% - 0.66%)
Myanmar	31.12% (30.20% - 32.12%)	27.55% (26.82% - 28.29%)	21.28% (20.54% - 22.10%)	16.58% (15.70% - 17.46%)	10.07% (9.05% - 11.09%)	8.76% (7.90% - 9.75%)	5.73% (4.88% - 6.56%)	3.89% (3.21% - 4.62%)	1.53% (1.13% - 2.01%)	1.32% (0.98% - 1.79%)	0.65% (0.40% - 0.94%)	0.33% (0.16% - 0.53%)
Philippines	25.01% (24.66% - 25.35%)	22.74% (22.36% - 23.13%)	18.89% (18.52% - 19.25%)	16.27% (15.61% - 16.93%)	9.02% (8.58% - 9.41%)	7.84% (7.45% - 8.26%)	5.68% (5.34% - 6.03%)	4.76% (4.26% - 5.27%)	1.90% (1.69% - 2.11%)	1.53% (1.34% - 1.75%)	0.88% (0.72% - 1.03%)	0.72% (0.54% - 0.92%)
Mountain Province	20.20% (19.15% - 21.18%)	17.12% (16.21% - 18.06%)	12.51% (11.94% - 13.10%)	10.40% (9.53% - 11.26%)	4.65% (3.87% - 5.45%)	3.61% (2.96% - 4.29%)	2.16% (1.82% - 2.51%)	1.80% (1.39% - 2.25%)	0.44% (0.28% - 0.65%)	0.25% (0.13% - 0.40%)	0.05% (0.01% - 0.12%)	0.04% (0.00% - 0.13%)
Ifugao	20.20% (19.13% - 21.18%)	16.47% (15.56% - 17.47%)	12.38% (11.81% - 12.98%)	10.29% (9.43% - 11.13%)	4.62% (3.82% - 5.46%)	3.50% (2.87% - 4.17%)	2.12% (1.77% - 2.51%)	1.78% (1.37% - 2.22%)	0.43% (0.28% - 0.64%)	0.25% (0.14% - 0.40%)	0.05% (0.01% - 0.12%)	0.04% (0.00% - 0.13%)
Benguet	19.12% (18.08% - 20.13%)	16.13% (15.15% - 17.08%)	12.42% (11.82% - 13.06%)	10.69% (9.84% - 11.59%)	4.56% (3.77% - 5.30%)	3.46% (2.85% - 4.12%)	2.18% (1.82% - 2.56%)	1.89% (1.46% - 2.32%)	0.46% (0.29% - 0.66%)	0.25% (0.14% - 0.40%)	0.06% (0.01% - 0.13%)	0.05% (0.00% - 0.13%)

Table S8c. Estimated overall, severe, and extreme **underweight** prevalence (%) in children under 5 years, both sexes, in 1990, 2000, 2010, and 2020.

Location	Total				Severe				Extreme			
	1990	2000	2010	2020	1990	2000	2010	2020	1990	2000	2010	2020
	18.68%	16.44%	12.44%	10.60%	4.43%	3.57%	2.15%	1.85%	0.44%	0.27%	0.05%	0.05%
Abra	(17.65% - 19.63%)	(15.48% - 17.40%)	(11.84% - 13.05%)	(9.78% - 11.47%)	(3.71% - 5.18%)	(2.95% - 4.25%)	(1.77% - 2.53%)	(1.47% - 2.30%)	(0.29% - 0.63%)	(0.14% - 0.43%)	(0.01% - 0.12%)	(0.00% - 0.13%)
	20.64%	17.54%	12.51%	10.28%	4.68%	3.59%	2.12%	1.78%	0.43%	0.23%	0.05%	0.04%
Apayao	(19.61% - 21.64%)	(16.51% - 18.51%)	(11.91% - 13.15%)	(9.44% - 11.19%)	(3.83% - 5.56%)	(2.92% - 4.27%)	(1.75% - 2.46%)	(1.39% - 2.27%)	(0.27% - 0.64%)	(0.13% - 0.38%)	(0.00% - 0.11%)	(0.00% - 0.13%)
	20.45%	16.79%	12.43%	10.64%	4.49%	3.46%	2.11%	1.87%	0.39%	0.23%	0.05%	0.05%
Kalinga	(19.41% - 21.46%)	(15.82% - 17.83%)	(11.82% - 13.04%)	(9.74% - 11.54%)	(3.68% - 5.32%)	(2.81% - 4.13%)	(1.72% - 2.50%)	(1.44% - 2.36%)	(0.25% - 0.58%)	(0.12% - 0.37%)	(0.01% - 0.12%)	(0.00% - 0.14%)
	24.33%	22.09%	18.48%	16.06%	9.21%	7.87%	5.67%	4.78%	2.12%	1.64%	0.91%	0.75%
La Union	(23.33% - 25.25%)	(21.18% - 22.92%)	(17.91% - 19.10%)	(15.06% - 16.99%)	(8.24% - 10.31%)	(6.99% - 8.80%)	(5.12% - 6.23%)	(4.00% - 5.58%)	(1.65% - 2.71%)	(1.23% - 2.12%)	(0.67% - 1.17%)	(0.47% - 1.07%)
	22.99%	21.32%	18.14%	16.89%	8.34%	7.33%	5.50%	5.16%	1.79%	1.43%	0.86%	0.84%
Ilocos Norte	(22.02% - 23.86%)	(20.40% - 22.17%)	(17.54% - 18.75%)	(15.84% - 17.89%)	(7.37% - 9.38%)	(6.44% - 8.23%)	(4.96% - 6.05%)	(4.33% - 5.99%)	(1.36% - 2.31%)	(1.07% - 1.85%)	(0.64% - 1.10%)	(0.54% - 1.19%)
	24.30%	21.67%	18.39%	15.76%	9.00%	7.59%	5.59%	4.63%	1.98%	1.53%	0.88%	0.70%
Ilocos Sur	(23.33% - 25.23%)	(20.71% - 22.55%)	(17.72% - 19.00%)	(14.68% - 16.69%)	(8.02% - 10.02%)	(6.71% - 8.46%)	(5.04% - 6.15%)	(3.90% - 5.41%)	(1.55% - 2.51%)	(1.16% - 1.94%)	(0.66% - 1.12%)	(0.45% - 1.01%)
	24.66%	22.67%	18.50%	15.56%	9.29%	8.11%	5.66%	4.50%	2.11%	1.68%	0.90%	0.66%
Pangasinan	(23.76% - 25.59%)	(21.78% - 23.61%)	(17.90% - 19.14%)	(14.55% - 16.52%)	(8.23% - 10.37%)	(7.21% - 9.07%)	(5.12% - 6.24%)	(3.76% - 5.23%)	(1.60% - 2.69%)	(1.27% - 2.13%)	(0.67% - 1.16%)	(0.42% - 0.94%)
	26.67%	24.63%	21.03%	18.79%	10.67%	9.35%	7.13%	6.23%	2.64%	2.12%	1.35%	1.17%
Nueva Vizcaya	(25.69% - 27.65%)	(23.77% - 25.56%)	(20.43% - 21.67%)	(17.69% - 19.76%)	(9.47% - 11.83%)	(8.41% - 10.37%)	(6.51% - 7.74%)	(5.31% - 7.22%)	(2.04% - 3.29%)	(1.66% - 2.68%)	(1.04% - 1.67%)	(0.79% - 1.63%)
	28.02%	25.59%	21.37%	18.19%	11.28%	9.98%	7.33%	5.92%	2.80%	2.35%	1.41%	1.08%
Cagayan	(27.10% - 29.01%)	(24.74% - 26.50%)	(20.74% - 22.00%)	(17.14% - 19.14%)	(10.08% - 12.59%)	(8.98% - 11.07%)	(6.67% - 7.94%)	(5.01% - 6.77%)	(2.16% - 3.54%)	(1.83% - 2.97%)	(1.10% - 1.75%)	(0.72% - 1.48%)
	27.98%	25.73%	21.38%	18.47%	11.40%	10.15%	7.35%	6.04%	2.89%	2.44%	1.42%	1.11%
Isabela	(27.07% - 28.89%)	(24.85% - 26.62%)	(20.74% - 22.02%)	(17.41% - 19.48%)	(10.21% - 12.62%)	(9.09% - 11.23%)	(6.74% - 7.96%)	(5.13% - 6.99%)	(2.22% - 3.66%)	(1.87% - 3.05%)	(1.11% - 1.74%)	(0.73% - 1.52%)
	29.30%	26.10%	21.48%	18.18%	11.87%	10.21%	7.33%	5.95%	2.96%	2.41%	1.40%	1.09%
Quirino	(28.39% - 30.20%)	(25.16% - 26.96%)	(20.89% - 22.11%)	(17.20% - 19.26%)	(10.66% - 13.20%)	(9.16% - 11.30%)	(6.71% - 7.98%)	(5.08% - 6.86%)	(2.28% - 3.80%)	(1.87% - 3.03%)	(1.08% - 1.76%)	(0.73% - 1.49%)
	28.57%	26.15%	21.36%	19.58%	12.03%	10.41%	7.43%	6.66%	3.22%	2.54%	1.47%	1.30%
Batanes	(27.65% - 29.47%)	(25.26% - 27.00%)	(20.75% - 22.00%)	(18.54% - 20.65%)	(10.80% - 13.25%)	(9.41% - 11.48%)	(6.78% - 8.09%)	(5.72% - 7.62%)	(2.55% - 4.00%)	(2.03% - 3.18%)	(1.16% - 1.83%)	(0.90% - 1.75%)
	20.79%	18.24%	14.93%	13.10%	7.04%	5.73%	4.00%	3.48%	1.37%	0.98%	0.50%	0.43%
Bataan	(19.86% - 21.73%)	(17.43% - 19.08%)	(14.38% - 15.53%)	(12.19% - 14.03%)	(6.21% - 7.92%)	(5.06% - 6.42%)	(3.55% - 4.42%)	(2.91% - 4.17%)	(1.04% - 1.76%)	(0.73% - 1.27%)	(0.34% - 0.65%)	(0.26% - 0.66%)
	19.86%	18.00%	15.01%	12.75%	6.67%	5.63%	4.04%	3.35%	1.29%	0.95%	0.51%	0.40%
Zambales	(18.90% - 20.72%)	(17.07% - 18.87%)	(14.44% - 15.63%)	(11.83% - 13.67%)	(5.85% - 7.46%)	(4.93% - 6.38%)	(3.62% - 4.49%)	(2.78% - 3.97%)	(0.99% - 1.63%)	(0.70% - 1.26%)	(0.36% - 0.67%)	(0.23% - 0.61%)
	21.06%	18.47%	14.95%	13.24%	7.24%	5.90%	4.05%	3.55%	1.44%	1.04%	0.51%	0.45%
Tarlac	(20.09% - 21.95%)	(17.64% - 19.36%)	(14.38% - 15.50%)	(12.34% - 14.14%)	(6.41% - 8.12%)	(5.21% - 6.69%)	(3.62% - 4.51%)	(2.96% - 4.14%)	(1.13% - 1.85%)	(0.80% - 1.37%)	(0.37% - 0.68%)	(0.27% - 0.65%)
	20.80%	18.35%	14.79%	13.70%	7.09%	5.81%	4.00%	3.72%	1.40%	1.00%	0.50%	0.49%
Pampanga	(19.91% - 21.80%)	(17.48% - 19.20%)	(14.25% - 15.37%)	(12.79% - 14.60%)	(6.22% - 8.01%)	(5.10% - 6.54%)	(3.57% - 4.40%)	(3.10% - 4.36%)	(1.05% - 1.80%)	(0.74% - 1.30%)	(0.36% - 0.66%)	(0.30% - 0.70%)
	20.60%	17.89%	14.97%	12.66%	7.01%	5.58%	4.06%	3.30%	1.38%	0.94%	0.52%	0.39%
Bulacan	(19.69% - 21.52%)	(17.04% - 18.74%)	(14.41% - 15.54%)	(11.79% - 13.57%)	(6.16% - 7.88%)	(4.93% - 6.26%)	(3.62% - 4.48%)	(2.76% - 3.92%)	(1.04% - 1.73%)	(0.71% - 1.21%)	(0.37% - 0.68%)	(0.23% - 0.60%)
	20.53%	18.54%	15.07%	12.42%	6.99%	5.93%	4.09%	3.20%	1.37%	1.04%	0.52%	0.37%
Nueva Ecija	(19.64% - 21.43%)	(17.71% - 19.39%)	(14.53% - 15.63%)	(11.59% - 13.24%)	(6.14% - 7.86%)	(5.21% - 6.71%)	(3.65% - 4.53%)	(2.67% - 3.77%)	(1.04% - 1.76%)	(0.77% - 1.36%)	(0.37% - 0.68%)	(0.21% - 0.56%)
	20.99%	18.84%	15.02%	12.61%	6.92%	5.81%	3.99%	3.26%	1.28%	0.95%	0.48%	0.38%
Aurora	(20.04% - 21.88%)	(17.92% - 19.68%)	(14.45% - 15.58%)	(11.70% - 13.50%)	(6.08% - 7.76%)	(5.08% - 6.60%)	(3.54% - 4.41%)	(2.69% - 3.81%)	(0.96% - 1.64%)	(0.70% - 1.26%)	(0.33% - 0.64%)	(0.21% - 0.56%)
	19.80%	17.77%	14.76%	12.45%	6.44%	5.30%	3.78%	3.05%	1.18%	0.82%	0.42%	0.30%
Rizal	(18.88% - 20.71%)	(16.88% - 18.65%)	(14.18% - 15.35%)	(11.54% - 13.42%)	(5.65% - 7.25%)	(4.65% - 6.05%)	(3.35% - 4.23%)	(2.49% - 3.62%)	(0.88% - 1.52%)	(0.60% - 1.09%)	(0.28% - 0.57%)	(0.15% - 0.48%)
	19.18%	17.84%	14.70%	12.21%	6.09%	5.33%	3.76%	2.98%	1.07%	0.82%	0.41%	0.29%
Cavite	(18.26% - 20.11%)	(16.94% - 18.73%)	(14.15% - 15.23%)	(11.27% - 13.07%)	(5.31% - 6.93%)	(4.62% - 6.10%)	(3.33% - 4.18%)	(2.45% - 3.50%)	(0.80% - 1.39%)	(0.59% - 1.12%)	(0.27% - 0.57%)	(0.15% - 0.45%)
	19.69%	18.21%	14.63%	12.15%	6.29%	5.52%	3.72%	2.95%	1.11%	0.88%	0.40%	0.29%
Laguna	(18.77% - 20.56%)	(17.30% - 19.12%)	(14.08% - 15.21%)	(11.28% - 13.10%)	(5.50% - 7.04%)	(4.82% - 6.27%)	(3.28% - 4.16%)	(2.43% - 3.55%)	(0.82% - 1.42%)	(0.64% - 1.16%)	(0.27% - 0.55%)	(0.15% - 0.47%)
	20.94%	19.10%	14.58%	12.36%	6.92%	5.89%	3.70%	3.04%	1.29%	0.96%	0.40%	0.30%
Batangas	(19.97% - 21.85%)	(18.23% - 20.02%)	(13.98% - 15.14%)	(11.48% - 13.23%)	(6.05% - 7.81%)	(5.20% - 6.71%)	(3.31% - 4.13%)	(2.52% - 3.62%)	(0.96% - 1.66%)	(0.71% - 1.27%)	(0.28% - 0.55%)	(0.16% - 0.49%)

Location	Total				Severe				Extreme			
	1990	2000	2010	2020	1990	2000	2010	2020	1990	2000	2010	2020
Quezon	20.42% (19.45% - 21.30%)	18.19% (17.31% - 19.07%)	14.72% (14.13% - 15.33%)	12.38% (11.49% - 13.28%)	6.50% (5.66% - 7.36%)	5.36% (4.65% - 6.11%)	3.72% (3.28% - 4.13%)	3.04% (2.50% - 3.60%)	1.14% (0.84% - 1.48%)	0.81% (0.57% - 1.08%)	0.39% (0.27% - 0.54%)	0.30% (0.16% - 0.48%)
Occidental Mindoro	27.65% (26.74% - 28.54%)	25.53% (24.71% - 26.35%)	22.24% (21.63% - 22.91%)	19.79% (18.65% - 20.78%)	10.57% (9.36% - 11.75%)	9.45% (8.47% - 10.61%)	7.37% (6.70% - 8.06%)	6.51% (5.50% - 7.47%)	2.40% (1.82% - 3.07%)	2.03% (1.54% - 2.65%)	1.32% (1.00% - 1.69%)	1.19% (0.79% - 1.63%)
Oriental Mindoro	28.74% (27.79% - 29.67%)	26.52% (25.65% - 27.35%)	22.39% (21.78% - 23.06%)	18.96% (17.93% - 19.99%)	11.07% (9.80% - 12.39%)	9.96% (8.85% - 11.19%)	7.51% (6.82% - 8.20%)	6.11% (5.18% - 6.98%)	2.53% (1.89% - 3.30%)	2.18% (1.62% - 2.89%)	1.37% (1.03% - 1.74%)	1.08% (0.71% - 1.49%)
Romblon	29.24% (28.34% - 30.10%)	26.98% (26.10% - 27.86%)	22.45% (21.81% - 23.15%)	19.05% (17.99% - 20.07%)	11.37% (10.17% - 12.63%)	10.15% (8.99% - 11.26%)	7.46% (6.77% - 8.20%)	6.15% (5.22% - 7.11%)	2.63% (2.00% - 3.40%)	2.21% (1.66% - 2.83%)	1.34% (1.00% - 1.71%)	1.10% (0.73% - 1.52%)
Palawan	28.37% (27.55% - 29.30%)	26.15% (25.27% - 27.04%)	22.42% (21.83% - 23.02%)	18.38% (17.40% - 19.34%)	10.95% (9.82% - 12.21%)	9.83% (8.83% - 10.90%)	7.45% (6.80% - 8.06%)	5.84% (4.96% - 6.76%)	2.52% (1.91% - 3.27%)	2.16% (1.64% - 2.77%)	1.33% (1.02% - 1.68%)	1.01% (0.67% - 1.40%)
Marinduque	28.86% (27.96% - 29.78%)	26.33% (25.48% - 27.27%)	22.40% (21.76% - 23.07%)	19.41% (18.45% - 20.40%)	10.96% (9.72% - 12.29%)	9.82% (8.72% - 10.96%)	7.44% (6.76% - 8.08%)	6.24% (5.38% - 7.19%)	2.44% (1.83% - 3.20%)	2.12% (1.58% - 2.74%)	1.33% (1.00% - 1.68%)	1.10% (0.72% - 1.52%)
Catanduanes	28.42% (27.53% - 29.33%)	25.97% (25.13% - 26.83%)	22.21% (21.59% - 22.81%)	19.87% (18.83% - 20.81%)	10.87% (9.71% - 12.08%)	9.67% (8.70% - 10.71%)	7.45% (6.82% - 8.09%)	6.57% (5.61% - 7.52%)	2.46% (1.86% - 3.18%)	2.09% (1.58% - 2.68%)	1.36% (1.05% - 1.72%)	1.21% (0.81% - 1.69%)
Camarines Norte	27.65% (26.75% - 28.51%)	25.43% (24.60% - 26.33%)	22.40% (21.76% - 23.05%)	18.83% (17.82% - 19.83%)	10.81% (9.66% - 12.10%)	9.59% (8.46% - 10.79%)	7.53% (6.85% - 8.26%)	6.05% (5.15% - 6.96%)	2.55% (1.98% - 3.27%)	2.13% (1.58% - 2.77%)	1.39% (1.05% - 1.77%)	1.07% (0.71% - 1.47%)
Sorsogon	29.00% (28.10% - 29.91%)	26.47% (25.61% - 27.33%)	22.41% (21.80% - 23.06%)	19.37% (18.33% - 20.41%)	11.33% (10.12% - 12.59%)	9.98% (8.89% - 11.09%)	7.57% (6.90% - 8.23%)	6.37% (5.37% - 7.29%)	2.64% (1.99% - 3.42%)	2.19% (1.66% - 2.83%)	1.40% (1.06% - 1.76%)	1.17% (0.77% - 1.59%)
Albay	28.60% (27.68% - 29.50%)	26.61% (25.75% - 27.51%)	22.42% (21.81% - 23.03%)	18.79% (17.84% - 19.83%)	11.27% (9.97% - 12.61%)	10.22% (9.09% - 11.41%)	7.66% (6.99% - 8.30%)	6.05% (5.18% - 7.00%)	2.68% (2.02% - 3.48%)	2.32% (1.76% - 2.99%)	1.45% (1.12% - 1.81%)	1.08% (0.74% - 1.49%)
Masbate	28.03% (27.16% - 28.87%)	26.57% (25.70% - 27.44%)	22.44% (21.83% - 23.05%)	19.04% (18.05% - 20.04%)	10.99% (9.79% - 12.16%)	10.06% (9.02% - 11.23%)	7.53% (6.85% - 8.21%)	6.14% (5.25% - 7.10%)	2.60% (2.01% - 3.28%)	2.23% (1.71% - 2.92%)	1.38% (1.05% - 1.74%)	1.09% (0.74% - 1.51%)
Camarines Sur	28.16% (27.32% - 29.09%)	26.11% (25.27% - 26.95%)	22.37% (21.75% - 23.02%)	19.01% (17.99% - 19.98%)	11.13% (10.09% - 12.22%)	10.05% (9.02% - 11.17%)	7.59% (6.93% - 8.25%)	6.17% (5.28% - 7.07%)	2.66% (2.08% - 3.35%)	2.30% (1.75% - 2.96%)	1.42% (1.09% - 1.78%)	1.11% (0.73% - 1.54%)
Capiz	28.13% (27.21% - 29.02%)	25.99% (25.14% - 26.87%)	21.56% (20.97% - 22.17%)	18.13% (17.11% - 19.11%)	10.75% (9.55% - 12.01%)	9.55% (8.53% - 10.67%)	6.99% (6.35% - 7.62%)	5.63% (4.81% - 6.57%)	2.43% (1.85% - 3.10%)	2.01% (1.52% - 2.66%)	1.21% (0.92% - 1.53%)	0.94% (0.63% - 1.35%)
Aklan	27.95% (27.08% - 28.87%)	25.95% (25.08% - 26.83%)	21.50% (20.88% - 22.08%)	19.06% (18.02% - 20.15%)	10.67% (9.54% - 11.90%)	9.52% (8.48% - 10.59%)	6.99% (6.38% - 7.64%)	5.99% (5.06% - 6.95%)	2.41% (1.85% - 3.12%)	2.00% (1.50% - 2.59%)	1.22% (0.93% - 1.54%)	1.01% (0.65% - 1.45%)
Antique	27.88% (26.94% - 28.77%)	25.57% (24.70% - 26.50%)	21.61% (21.00% - 22.21%)	18.22% (17.28% - 19.24%)	10.64% (9.47% - 11.86%)	9.37% (8.33% - 10.51%)	6.95% (6.32% - 7.63%)	5.67% (4.91% - 6.57%)	2.40% (1.81% - 3.13%)	1.97% (1.47% - 2.60%)	1.18% (0.89% - 1.54%)	0.95% (0.65% - 1.34%)
Negros Occidental	27.15% (26.26% - 28.05%)	25.16% (24.30% - 26.04%)	21.37% (20.78% - 22.00%)	19.32% (18.29% - 20.30%)	10.22% (9.06% - 11.32%)	9.10% (8.12% - 10.22%)	6.88% (6.26% - 7.51%)	6.14% (5.24% - 7.10%)	2.26% (1.72% - 2.89%)	1.88% (1.42% - 2.48%)	1.18% (0.89% - 1.49%)	1.06% (0.69% - 1.50%)
Iloilo	26.77% (25.89% - 27.64%)	25.31% (24.40% - 26.22%)	21.52% (20.93% - 22.18%)	18.75% (17.72% - 19.72%)	10.39% (9.23% - 11.56%)	9.50% (8.49% - 10.62%)	7.02% (6.35% - 7.67%)	5.90% (5.03% - 6.77%)	2.43% (1.86% - 3.11%)	2.09% (1.61% - 2.72%)	1.23% (0.91% - 1.55%)	1.00% (0.67% - 1.38%)
Guimaras	29.91% (28.97% - 30.88%)	27.30% (26.42% - 28.13%)	21.61% (20.99% - 22.22%)	18.83% (17.83% - 19.87%)	11.28% (10.11% - 12.54%)	10.03% (8.96% - 11.21%)	7.04% (6.43% - 7.75%)	5.97% (5.09% - 6.89%)	2.47% (1.85% - 3.18%)	2.09% (1.56% - 2.75%)	1.23% (0.92% - 1.58%)	1.03% (0.69% - 1.44%)
Negros Oriental	26.25% (25.32% - 27.20%)	23.92% (22.99% - 24.79%)	20.09% (19.46% - 20.78%)	17.07% (16.10% - 18.09%)	8.91% (7.85% - 10.05%)	7.83% (6.87% - 8.78%)	5.69% (5.06% - 6.31%)	4.71% (3.90% - 5.49%)	1.66% (1.22% - 2.18%)	1.36% (0.98% - 1.78%)	0.76% (0.53% - 1.03%)	0.62% (0.36% - 0.91%)
Cebu	26.80% (25.84% - 27.70%)	24.78% (23.85% - 25.68%)	20.00% (19.45% - 20.60%)	17.29% (16.26% - 18.28%)	9.34% (8.17% - 10.47%)	8.24% (7.26% - 9.15%)	5.71% (5.11% - 6.32%)	4.80% (4.00% - 5.58%)	1.81% (1.32% - 2.37%)	1.46% (1.06% - 1.89%)	0.78% (0.54% - 1.03%)	0.64% (0.40% - 0.93%)
Bohol	27.66% (26.74% - 28.54%)	24.97% (24.04% - 25.86%)	20.12% (19.47% - 20.76%)	17.49% (16.56% - 18.52%)	9.61% (8.57% - 10.67%)	8.49% (7.48% - 9.48%)	5.76% (5.14% - 6.37%)	4.94% (4.15% - 5.75%)	1.85% (1.41% - 2.40%)	1.57% (1.15% - 2.06%)	0.79% (0.55% - 1.04%)	0.68% (0.41% - 1.01%)
Siquijor	28.18% (27.23% - 29.27%)	24.75% (23.87% - 25.62%)	19.97% (19.33% - 20.60%)	17.72% (16.71% - 18.76%)	9.14% (8.01% - 10.36%)	7.88% (6.91% - 8.92%)	5.64% (5.01% - 6.21%)	5.01% (4.19% - 5.92%)	1.56% (1.13% - 2.11%)	1.30% (0.93% - 1.74%)	0.75% (0.51% - 0.99%)	0.69% (0.42% - 1.04%)
Southern Leyte	29.44% (28.59% - 30.41%)	26.96% (26.09% - 27.82%)	22.96% (22.31% - 23.63%)	20.41% (19.38% - 21.42%)	10.77% (9.57% - 12.05%)	9.66% (8.59% - 10.88%)	7.38% (6.67% - 8.10%)	6.51% (5.54% - 7.49%)	2.24% (1.66% - 2.94%)	1.93% (1.43% - 2.59%)	1.23% (0.90% - 1.61%)	1.12% (0.75% - 1.57%)

Table S8c. Estimated overall, severe, and extreme **underweight** prevalence (%) in children under 5 years, both sexes, in 1990, 2000, 2010, and 2020.

Table S8c. Estimated overall, severe, and extreme **underweight** prevalence (%) in children under 5 years, both sexes, in 1990, 2000, 2010, and 2020.

Location	Total				Severe				Extreme			
	1990	2000	2010	2020	1990	2000	2010	2020	1990	2000	2010	2020
	30.48%	28.01%	23.08%	19.43%	10.95%	10.02%	7.34%	5.98%	2.19%	1.98%	1.20%	0.97%
Eastern Samar	(29.51% - 31.48%)	(27.07% - 28.87%)	(22.46% - 23.71%)	(18.39% - 20.46%)	(9.85% - 12.27%)	(8.84% - 11.33%)	(6.61% - 8.02%)	(5.10% - 6.93%)	(1.66% - 2.90%)	(1.43% - 2.70%)	(0.87% - 1.55%)	(0.63% - 1.40%)
	29.23%	27.32%	23.22%	19.14%	10.62%	9.80%	7.41%	5.91%	2.18%	1.96%	1.22%	0.97%
Northern Samar	(28.30% - 30.23%)	(26.43% - 28.28%)	(22.57% - 23.87%)	(18.09% - 20.24%)	(9.39% - 11.81%)	(8.70% - 10.94%)	(6.68% - 8.13%)	(5.01% - 6.84%)	(1.63% - 2.81%)	(1.46% - 2.58%)	(0.89% - 1.59%)	(0.62% - 1.36%)
	29.50%	27.36%	23.03%	19.43%	10.62%	9.78%	7.34%	6.02%	2.15%	1.94%	1.20%	0.99%
Samar (Western Samar)	(28.59% - 30.49%)	(26.50% - 28.27%)	(22.37% - 23.64%)	(18.42% - 20.43%)	(9.46% - 11.89%)	(8.65% - 10.93%)	(6.64% - 8.04%)	(5.11% - 6.96%)	(1.60% - 2.84%)	(1.43% - 2.56%)	(0.89% - 1.57%)	(0.62% - 1.40%)
	30.55%	28.28%	23.37%	19.19%	11.19%	10.35%	7.57%	5.96%	2.31%	2.12%	1.28%	0.98%
Leyte	(29.63% - 31.51%)	(27.42% - 29.16%)	(22.67% - 24.08%)	(18.18% - 20.21%)	(10.07% - 12.38%)	(9.23% - 11.51%)	(6.84% - 8.30%)	(5.06% - 6.84%)	(1.76% - 2.96%)	(1.59% - 2.81%)	(0.94% - 1.65%)	(0.65% - 1.39%)
	30.38%	27.51%	22.91%	20.83%	11.00%	9.81%	7.43%	6.70%	2.23%	1.93%	1.27%	1.17%
Biliran	(29.44% - 31.43%)	(26.66% - 28.40%)	(22.28% - 23.53%)	(19.72% - 21.85%)	(9.72% - 12.22%)	(8.60% - 10.92%)	(6.69% - 8.18%)	(5.63% - 7.75%)	(1.66% - 2.90%)	(1.39% - 2.53%)	(0.91% - 1.67%)	(0.75% - 1.66%)
	27.85%	26.53%	22.21%	18.36%	10.76%	9.97%	7.41%	5.85%	2.48%	2.18%	1.35%	1.03%
Zamboanga Sibugay	(26.97% - 28.76%)	(25.67% - 27.37%)	(21.63% - 22.82%)	(17.32% - 19.39%)	(9.58% - 12.03%)	(8.87% - 11.20%)	(6.76% - 8.07%)	(4.98% - 6.77%)	(1.89% - 3.18%)	(1.64% - 2.91%)	(1.03% - 1.71%)	(0.69% - 1.43%)
	27.98%	26.06%	22.05%	19.22%	10.77%	9.87%	7.40%	6.28%	2.46%	2.20%	1.36%	1.15%
Zamboanga Del Norte	(27.15% - 28.86%)	(25.19% - 26.91%)	(21.43% - 22.65%)	(18.24% - 20.25%)	(9.63% - 11.88%)	(8.82% - 10.97%)	(6.77% - 8.10%)	(5.41% - 7.31%)	(1.89% - 3.13%)	(1.65% - 2.82%)	(1.04% - 1.74%)	(0.79% - 1.60%)
	28.04%	25.95%	22.17%	18.96%	10.80%	9.83%	7.46%	6.18%	2.47%	2.19%	1.37%	1.12%
Zamboanga Del Sur	(27.19% - 28.89%)	(25.04% - 26.90%)	(21.60% - 22.83%)	(17.93% - 19.97%)	(9.69% - 12.03%)	(8.75% - 11.00%)	(6.81% - 8.18%)	(5.32% - 7.10%)	(1.90% - 3.21%)	(1.66% - 2.88%)	(1.05% - 1.74%)	(0.77% - 1.53%)
	25.99%	23.72%	20.26%	17.19%	9.19%	8.00%	5.95%	4.86%	1.84%	1.47%	0.85%	0.67%
Misamis Occidental	(25.05% - 26.87%)	(22.85% - 24.66%)	(19.63% - 20.90%)	(16.19% - 18.25%)	(8.14% - 10.27%)	(7.06% - 8.93%)	(5.32% - 6.54%)	(4.06% - 5.71%)	(1.38% - 2.35%)	(1.08% - 1.90%)	(0.61% - 1.11%)	(0.40% - 1.00%)
	27.10%	24.76%	20.45%	16.39%	9.43%	8.33%	6.02%	4.55%	1.83%	1.52%	0.87%	0.61%
Bukidnon	(26.19% - 28.02%)	(23.85% - 25.69%)	(19.80% - 21.08%)	(15.37% - 17.39%)	(8.32% - 10.56%)	(7.28% - 9.37%)	(5.40% - 6.67%)	(3.78% - 5.40%)	(1.36% - 2.38%)	(1.10% - 2.02%)	(0.62% - 1.15%)	(0.37% - 0.91%)
	25.81%	23.86%	20.16%	17.55%	9.09%	8.08%	5.90%	5.11%	1.82%	1.50%	0.84%	0.75%
Lanao Del Norte	(24.83% - 26.69%)	(22.97% - 24.80%)	(19.56% - 20.80%)	(16.55% - 18.56%)	(7.93% - 10.30%)	(7.09% - 9.04%)	(5.25% - 6.53%)	(4.31% - 5.99%)	(1.35% - 2.42%)	(1.09% - 1.95%)	(0.59% - 1.12%)	(0.46% - 1.12%)
	26.80%	24.78%	20.11%	17.85%	9.76%	8.63%	5.93%	5.19%	2.05%	1.67%	0.86%	0.76%
Misamis Oriental	(25.84% - 27.69%)	(23.87% - 25.69%)	(19.51% - 20.72%)	(16.80% - 18.92%)	(8.58% - 10.94%)	(7.61% - 9.73%)	(5.35% - 6.55%)	(4.34% - 6.03%)	(1.52% - 2.68%)	(1.26% - 2.24%)	(0.63% - 1.14%)	(0.47% - 1.09%)
	27.95%	25.19%	20.34%	17.63%	9.78%	8.50%	6.01%	5.22%	1.91%	1.55%	0.87%	0.80%
Camiguin	(26.97% - 28.89%)	(24.28% - 26.06%)	(19.75% - 20.96%)	(16.60% - 18.71%)	(8.68% - 10.94%)	(7.48% - 9.50%)	(5.41% - 6.63%)	(4.35% - 6.15%)	(1.44% - 2.46%)	(1.12% - 2.02%)	(0.62% - 1.15%)	(0.49% - 1.15%)
	26.04%	23.32%	19.14%	16.10%	8.88%	7.54%	5.29%	4.36%	1.67%	1.29%	0.68%	0.56%
Davao Oriental	(25.09% - 26.93%)	(22.41% - 24.17%)	(18.52% - 19.74%)	(15.09% - 17.10%)	(7.85% - 10.02%)	(6.55% - 8.46%)	(4.67% - 5.87%)	(3.60% - 5.15%)	(1.24% - 2.18%)	(0.90% - 1.68%)	(0.45% - 0.92%)	(0.32% - 0.86%)
	26.25%	23.78%	19.06%	15.98%	8.88%	7.67%	5.31%	4.33%	1.65%	1.30%	0.69%	0.55%
Davao de Oro	(25.32% - 27.14%)	(22.89% - 24.72%)	(18.49% - 19.66%)	(14.93% - 16.98%)	(7.76% - 9.98%)	(6.73% - 8.63%)	(4.74% - 5.89%)	(3.64% - 5.08%)	(1.22% - 2.15%)	(0.94% - 1.72%)	(0.48% - 0.92%)	(0.33% - 0.82%)
	26.17%	23.59%	19.00%	16.11%	8.90%	7.69%	5.29%	4.42%	1.67%	1.33%	0.69%	0.58%
Davao Del Sur	(25.24% - 27.12%)	(22.69% - 24.49%)	(18.39% - 19.58%)	(15.17% - 17.14%)	(7.78% - 9.99%)	(6.80% - 8.64%)	(4.74% - 5.90%)	(3.74% - 5.20%)	(1.22% - 2.20%)	(0.98% - 1.74%)	(0.48% - 0.92%)	(0.36% - 0.87%)
	24.95%	23.07%	18.91%	15.91%	8.20%	7.24%	5.17%	4.28%	1.46%	1.17%	0.65%	0.54%
Davao Occidental	(24.00% - 25.84%)	(22.13% - 24.03%)	(18.28% - 19.52%)	(14.97% - 16.81%)	(7.16% - 9.27%)	(6.31% - 8.20%)	(4.63% - 5.72%)	(3.60% - 5.02%)	(1.07% - 1.93%)	(0.83% - 1.57%)	(0.45% - 0.86%)	(0.32% - 0.81%)
	26.85%	24.16%	19.13%	16.71%	9.25%	8.07%	5.36%	4.65%	1.77%	1.46%	0.70%	0.63%
Davao Del Norte	(25.89% - 27.79%)	(23.25% - 25.13%)	(18.51% - 19.75%)	(15.71% - 17.65%)	(8.08% - 10.36%)	(7.02% - 9.08%)	(4.72% - 5.95%)	(3.86% - 5.43%)	(1.29% - 2.32%)	(1.03% - 1.94%)	(0.48% - 0.94%)	(0.38% - 0.92%)
	28.25%	26.01%	22.45%	19.11%	10.42%	9.28%	7.07%	5.86%	2.21%	1.86%	1.14%	0.94%
South Cotabato	(27.33% - 29.19%)	(25.12% - 26.86%)	(21.83% - 23.13%)	(18.09% - 20.09%)	(9.29% - 11.62%)	(8.16% - 10.44%)	(6.35% - 7.74%)	(4.91% - 6.75%)	(1.68% - 2.89%)	(1.35% - 2.51%)	(0.83% - 1.47%)	(0.59% - 1.32%)
	28.25%	26.30%	22.25%	19.53%	10.12%	9.28%	6.96%	6.04%	2.05%	1.82%	1.11%	0.99%
Sultan Kudarat	(27.29% - 29.18%)	(25.49% - 27.16%)	(21.61% - 22.89%)	(18.43% - 20.60%)	(8.97% - 11.34%)	(8.19% - 10.36%)	(6.30% - 7.65%)	(5.05% - 7.04%)	(1.50% - 2.68%)	(1.30% - 2.38%)	(0.83% - 1.43%)	(0.61% - 1.43%)
	27.22%	25.58%	22.29%	19.32%	9.85%	8.99%	6.97%	5.95%	2.04%	1.76%	1.11%	0.97%
Cotabato (North Cotabato)	(26.34% - 28.09%)	(24.67% - 26.47%)	(21.69% - 22.93%)	(18.30% - 20.30%)	(8.74% - 11.04%)	(7.88% - 10.03%)	(6.30% - 7.60%)	(5.03% - 6.91%)	(1.52% - 2.65%)	(1.27% - 2.28%)	(0.81% - 1.43%)	(0.62% - 1.37%)
	27.97%	26.05%	22.61%	19.59%	10.11%	9.15%	7.11%	6.04%	2.06%	1.77%	1.14%	0.98%
Sarangani	(27.02% - 28.97%)	(25.13% - 26.97%)	(21.94% - 23.29%)	(18.51% - 20.71%)	(8.97% - 11.26%)	(8.14% - 10.25%)	(6.37% - 7.80%)	(5.08% - 7.06%)	(1.53% - 2.68%)	(1.30% - 2.38%)	(0.81% - 1.48%)	(0.61% - 1.42%)
	26.82%	24.77%	21.23%	17.50%	9.61%	8.57%	6.36%	5.04%	1.96%	1.64%	0.94%	0.73%
Agusan Del Norte	(25.95% - 27.79%)	(23.88% - 25.75%)	(20.62% - 21.88%)	(16.50% - 18.56%)	(8.53% - 10.89%)	(7.52% - 9.68%)	(5.73% - 7.00%)	(4.19% - 5.91%)	(1.47% - 2.63%)	(1.20% - 2.16%)	(0.69% - 1.23%)	(0.44% - 1.08%)

Table S8c. Estimated overall, severe, and extreme **underweight** prevalence (%) in children under 5 years, both sexes, in 1990, 2000, 2010, and 2020.

Location	Total				Severe				Extreme			
	1990	2000	2010	2020	1990	2000	2010	2020	1990	2000	2010	2020
	26.71%	24.65%	20.95%	18.35%	9.42%	8.40%	6.20%	5.43%	1.87%	1.56%	0.90%	0.82%
Agusan Del Sur	(25.78% - 27.57%)	(23.75% - 25.51%)	(20.33% - 21.55%)	(17.34% - 19.38%)	(8.30% - 10.57%)	(7.41% - 9.41%)	(5.57% - 6.84%)	(4.56% - 6.31%)	(1.41% - 2.44%)	(1.16% - 2.06%)	(0.65% - 1.19%)	(0.52% - 1.19%)
	28.21%	25.92%	21.16%	17.59%	9.96%	8.95%	6.35%	5.10%	1.96%	1.69%	0.95%	0.75%
Surigao Del Sur	(27.29% - 29.13%)	(25.00% - 26.86%)	(20.52% - 21.81%)	(16.50% - 18.59%)	(8.84% - 11.07%)	(7.90% - 10.01%)	(5.71% - 6.96%)	(4.28% - 5.96%)	(1.45% - 2.54%)	(1.21% - 2.22%)	(0.67% - 1.23%)	(0.46% - 1.09%)
	28.16%	25.76%	21.06%	17.98%	9.94%	8.77%	6.26%	5.26%	1.96%	1.62%	0.92%	0.78%
Surigao Del Norte	(27.28% - 29.08%)	(24.81% - 26.64%)	(20.43% - 21.66%)	(16.94% - 19.00%)	(8.76% - 11.11%)	(7.71% - 9.88%)	(5.61% - 6.91%)	(4.39% - 6.08%)	(1.45% - 2.56%)	(1.16% - 2.20%)	(0.66% - 1.21%)	(0.48% - 1.13%)
	28.91%	26.24%	21.22%	17.60%	10.06%	8.94%	6.42%	5.09%	1.92%	1.64%	0.97%	0.74%
Dinagat Islands	(27.97% - 29.81%)	(25.32% - 27.10%)	(20.56% - 21.87%)	(16.48% - 18.67%)	(8.88% - 11.19%)	(7.91% - 10.08%)	(5.76% - 7.11%)	(4.19% - 5.94%)	(1.43% - 2.51%)	(1.20% - 2.22%)	(0.69% - 1.27%)	(0.45% - 1.07%)
	28.27%	27.23%	24.05%	21.07%	11.39%	10.55%	8.34%	7.28%	2.82%	2.42%	1.59%	1.44%
Tawi-Tawi	(27.43% - 29.11%)	(26.34% - 28.07%)	(23.36% - 24.74%)	(20.04% - 22.10%)	(10.17% - 12.58%)	(9.43% - 11.65%)	(7.56% - 9.06%)	(6.27% - 8.31%)	(2.17% - 3.60%)	(1.83% - 3.06%)	(1.19% - 2.00%)	(0.98% - 1.96%)
	30.58%	28.31%	23.92%	21.24%	11.92%	10.84%	8.38%	7.33%	2.74%	2.41%	1.63%	1.44%
Basilan	(29.68% - 31.48%)	(27.46% - 29.22%)	(23.23% - 24.64%)	(20.28% - 22.21%)	(10.66% - 13.22%)	(9.67% - 12.00%)	(7.65% - 9.13%)	(6.36% - 8.37%)	(2.07% - 3.55%)	(1.83% - 3.11%)	(1.23% - 2.07%)	(1.02% - 1.97%)
	28.50%	27.04%	24.03%	21.37%	11.46%	10.52%	8.38%	7.38%	2.82%	2.44%	1.62%	1.45%
Sulu	(27.63% - 29.45%)	(26.18% - 27.90%)	(23.37% - 24.70%)	(20.34% - 22.34%)	(10.28% - 12.66%)	(9.47% - 11.57%)	(7.59% - 9.15%)	(6.32% - 8.43%)	(2.19% - 3.57%)	(1.88% - 3.05%)	(1.22% - 2.04%)	(0.96% - 1.99%)
	29.65%	27.76%	23.98%	21.17%	11.81%	10.80%	8.38%	7.30%	2.85%	2.49%	1.62%	1.44%
Maguindanao	(28.77% - 30.58%)	(26.83% - 28.65%)	(23.34% - 24.66%)	(20.08% - 22.18%)	(10.61% - 13.07%)	(9.69% - 11.93%)	(7.61% - 9.15%)	(6.25% - 8.31%)	(2.18% - 3.61%)	(1.89% - 3.13%)	(1.23% - 2.06%)	(0.98% - 1.94%)
	29.42%	27.59%	23.83%	22.01%	11.73%	10.71%	8.31%	7.73%	2.83%	2.46%	1.60%	1.56%
Lanao Del Sur	(28.54% - 30.36%)	(26.69% - 28.42%)	(23.15% - 24.46%)	(20.95% - 23.06%)	(10.56% - 13.07%)	(9.60% - 11.82%)	(7.56% - 9.05%)	(6.70% - 8.78%)	(2.21% - 3.63%)	(1.89% - 3.12%)	(1.21% - 2.01%)	(1.09% - 2.09%)
	18.90%	17.32%	14.42%	12.49%	6.30%	5.32%	3.77%	3.19%	1.21%	0.87%	0.43%	0.35%
National Capital Region	(18.07% - 19.85%)	(16.50% - 18.18%)	(13.88% - 14.95%)	(11.62% - 13.31%)	(5.61% - 7.11%)	(4.68% - 5.99%)	(3.38% - 4.15%)	(2.64% - 3.74%)	(0.95% - 1.54%)	(0.66% - 1.13%)	(0.31% - 0.57%)	(0.20% - 0.52%)
	27.63%	23.79%	20.41%	18.20%	9.15%	7.88%	6.68%	5.84%	1.53%	1.38%	1.19%	1.03%
Sri Lanka	(26.97% - 28.34%)	(23.19% - 24.40%)	(19.74% - 21.09%)	(17.41% - 19.00%)	(8.34% - 9.98%)	(7.23% - 8.56%)	(6.07% - 7.26%)	(5.21% - 6.53%)	(1.20% - 1.93%)	(1.11% - 1.68%)	(0.95% - 1.43%)	(0.80% - 1.31%)
	16.53%	10.89%	7.99%	7.08%	4.20%	2.13%	1.31%	1.13%	0.45%	0.09%	0.02%	0.01%
Thailand	(15.84% - 17.23%)	(10.26% - 11.61%)	(7.47% - 8.51%)	(6.63% - 7.57%)	(3.70% - 4.75%)	(1.81% - 2.50%)	(1.13% - 1.53%)	(0.96% - 1.30%)	(0.32% - 0.60%)	(0.04% - 0.16%)	(0.01% - 0.04%)	(0.00% - 0.02%)
	17.65%	13.27%	11.76%	11.15%	5.54%	3.56%	2.96%	2.80%	0.93%	0.43%	0.31%	0.29%
Mauritius	(17.19% - 18.15%)	(12.64% - 13.91%)	(11.00% - 12.48%)	(10.42% - 11.93%)	(5.15% - 5.97%)	(3.14% - 3.97%)	(2.54% - 3.38%)	(2.41% - 3.22%)	(0.78% - 1.10%)	(0.32% - 0.56%)	(0.19% - 0.43%)	(0.18% - 0.41%)
	6.77%	6.13%	4.91%	4.36%	0.92%	0.74%	0.41%	0.32%	0.00%	0.00%	0.00%	0.00%
Seychelles	(6.21% - 7.42%)	(5.64% - 6.68%)	(4.49% - 5.37%)	(3.98% - 4.74%)	(0.73% - 1.14%)	(0.58% - 0.93%)	(0.31% - 0.53%)	(0.23% - 0.41%)	(0.00% - 0.01%)	(0.00% - 0.00%)	(0.00% - 0.00%)	(0.00% - 0.00%)
	40.29%	38.17%	34.40%	32.45%	16.39%	16.73%	15.83%	14.81%	3.42%	4.20%	4.56%	4.27%
Timor-Leste	(38.63% - 42.11%)	(36.93% - 39.66%)	(33.66% - 35.28%)	(31.74% - 33.31%)	(15.06% - 17.88%)	(15.59% - 18.05%)	(14.52% - 17.14%)	(13.63% - 16.13%)	(2.60% - 4.56%)	(3.31% - 5.46%)	(3.50% - 5.73%)	(3.30% - 5.46%)
	31.10%	25.23%	17.88%	14.31%	11.19%	8.50%	5.14%	3.68%	2.15%	1.50%	0.72%	0.41%
Viet Nam	(30.34% - 31.93%)	(24.66% - 25.76%)	(17.30% - 18.41%)	(13.55% - 15.09%)	(10.20% - 12.24%)	(7.83% - 9.19%)	(4.69% - 5.59%)	(3.19% - 4.17%)	(1.67% - 2.74%)	(1.20% - 1.85%)	(0.57% - 0.88%)	(0.28% - 0.56%)
Sub-Saharan Africa	23.77%	23.55%	20.51%	18.00%	9.32%	9.34%	7.52%	6.26%	2.48%	2.57%	1.91%	1.49%
	(23.64% - 23.90%)	(23.40% - 23.68%)	(20.39% - 20.63%)	(17.86% - 18.16%)	(9.14% - 9.51%)	(9.16% - 9.51%)	(7.38% - 7.65%)	(6.11% - 6.42%)	(2.33% - 2.63%)	(2.43% - 2.71%)	(1.80% - 2.02%)	(1.39% - 1.60%)
	25.41%	25.81%	22.11%	18.95%	9.99%	10.36%	7.67%	6.19%	2.37%	2.57%	1.53%	1.13%
**Central Sub-Saharan Africa**	(24.96% - 25.86%)	(25.37% - 26.22%)	(21.67% - 22.51%)	(18.40% - 19.45%)	(9.47% - 10.57%)	(9.87% - 10.87%)	(7.25% - 8.13%)	(5.70% - 6.74%)	(2.07% - 2.73%)	(2.25% - 2.93%)	(1.30% - 1.79%)	(0.90% - 1.41%)
	29.94%	26.66%	20.28%	17.22%	12.31%	9.98%	5.71%	4.74%	3.02%	2.14%	0.76%	0.61%
Angola	(29.06% - 30.77%)	(25.87% - 27.41%)	(19.42% - 21.11%)	(16.38% - 17.97%)	(11.30% - 13.40%)	(9.11% - 10.86%)	(4.97% - 6.38%)	(4.10% - 5.35%)	(2.41% - 3.74%)	(1.72% - 2.62%)	(0.55% - 0.98%)	(0.43% - 0.82%)
	21.82%	22.00%	22.28%	21.59%	7.96%	8.13%	8.30%	7.88%	1.72%	1.79%	1.85%	1.71%
Central African Republic	(21.10% - 22.57%)	(21.38% - 22.65%)	(21.67% - 22.94%)	(20.81% - 22.37%)	(7.19% - 8.75%)	(7.46% - 8.77%)	(7.61% - 8.99%)	(7.06% - 8.70%)	(1.35% - 2.13%)	(1.46% - 2.13%)	(1.49% - 2.24%)	(1.29% - 2.15%)
	13.87%	13.54%	11.91%	10.71%	3.55%	2.94%	2.36%	2.17%	0.40%	0.21%	0.11%	0.11%
Congo	(13.18% - 14.54%)	(12.83% - 14.26%)	(11.28% - 12.53%)	(10.06% - 11.40%)	(3.16% - 4.00%)	(2.55% - 3.36%)	(2.06% - 2.67%)	(1.85% - 2.50%)	(0.30% - 0.53%)	(0.13% - 0.31%)	(0.06% - 0.18%)	(0.05% - 0.18%)
	25.47%	26.99%	23.81%	20.22%	10.11%	11.32%	8.84%	6.98%	2.41%	2.97%	1.91%	1.37%
Democratic Republic of the Congo	(24.87% - 26.08%)	(26.39% - 27.57%)	(23.21% - 24.35%)	(19.47% - 20.96%)	(9.42% - 10.85%)	(10.63% - 12.01%)	(8.24% - 9.44%)	(6.24% - 7.78%)	(2.02% - 2.89%)	(2.52% - 3.46%)	(1.57% - 2.29%)	(1.03% - 1.79%)
	18.21%	13.88%	6.30%	5.47%	3.95%	2.94%	0.80%	0.72%	0.35%	0.23%	0.00%	0.00%
Equatorial Guinea	(17.31% - 19.06%)	(13.38% - 14.38%)	(6.00% - 6.64%)	(5.06% - 5.86%)	(3.33% - 4.63%)	(2.64% - 3.26%)	(0.71% - 0.91%)	(0.60% - 0.85%)	(0.20% - 0.57%)	(0.17% - 0.31%)	(0.00% - 0.00%)	(0.00% - 0.00%)

Table S8c. Estimated overall, severe, and extreme **underweight** prevalence (%) in children under 5 years, both sexes, in 1990, 2000, 2010, and 2020.												
**Location**	**Total**				**Severe**				**Extreme**			
	**1990**	**2000**	**2010**	**2020**	**1990**	**2000**	**2010**	**2020**	**1990**	**2000**	**2010**	**2020**
Gabon	9.67% (9.09% - 10.29%)	8.72% (8.23% - 9.25%)	6.83% (6.39% - 7.27%)	5.66% (5.27% - 6.09%)	1.39% (1.16% - 1.66%)	1.25% (1.05% - 1.45%)	0.92% (0.76% - 1.10%)	0.77% (0.64% - 0.92%)	0.03% (0.01% - 0.06%)	0.02% (0.01% - 0.05%)	0.01% (0.00% - 0.02%)	0.00% (0.00% - 0.01%)
**Eastern Sub-Saharan Africa**	25.37% (25.17% - 25.56%)	24.36% (24.18% - 24.53%)	19.84% (19.68% - 20.01%)	16.83% (16.63% - 17.03%)	9.58% (9.36% - 9.80%)	9.22% (9.01% - 9.44%)	6.56% (6.40% - 6.74%)	5.18% (5.01% - 5.34%)	2.23% (2.09% - 2.37%)	2.23% (2.10% - 2.37%)	1.35% (1.26% - 1.44%)	0.96% (0.89% - 1.04%)
Burundi	29.21% (28.50% - 29.94%)	30.43% (29.75% - 31.12%)	26.42% (25.78% - 27.03%)	25.04% (24.27% - 25.80%)	12.04% (11.02% - 13.10%)	13.37% (12.39% - 14.48%)	9.80% (8.99% - 10.57%)	8.60% (7.73% - 9.51%)	3.00% (2.37% - 3.71%)	3.68% (3.02% - 4.43%)	2.08% (1.68% - 2.52%)	1.60% (1.22% - 2.05%)
Comoros	22.73% (21.91% - 23.59%)	22.00% (21.30% - 22.69%)	17.71% (16.96% - 18.40%)	15.69% (14.91% - 16.48%)	8.55% (7.73% - 9.44%)	8.66% (7.98% - 9.38%)	5.77% (5.20% - 6.35%)	4.78% (4.22% - 5.35%)	1.96% (1.58% - 2.43%)	2.16% (1.81% - 2.56%)	1.06% (0.85% - 1.29%)	0.78% (0.58% - 0.99%)
Djibouti	23.96% (23.18% - 24.70%)	25.61% (24.86% - 26.31%)	25.50% (24.81% - 26.12%)	23.38% (22.56% - 24.18%)	10.69% (9.77% - 11.53%)	12.13% (11.24% - 12.93%)	12.14% (11.42% - 12.91%)	10.65% (9.77% - 11.60%)	3.25% (2.72% - 3.77%)	4.09% (3.50% - 4.66%)	4.11% (3.60% - 4.66%)	3.38% (2.84% - 4.00%)
Eritrea	35.48% (34.76% - 36.23%)	32.53% (31.85% - 33.24%)	30.37% (29.66% - 31.09%)	27.34% (26.56% - 28.14%)	18.40% (16.97% - 19.89%)	15.76% (14.53% - 16.90%)	14.34% (13.23% - 15.50%)	12.31% (11.21% - 13.41%)	6.68% (5.19% - 8.42%)	5.15% (4.10% - 6.30%)	4.55% (3.63% - 5.63%)	3.69% (2.91% - 4.57%)
Ethiopia	33.16% (32.73% - 33.66%)	32.10% (31.70% - 32.54%)	26.08% (25.68% - 26.50%)	21.13% (20.67% - 21.56%)	13.85% (13.28% - 14.46%)	13.93% (13.37% - 14.53%)	9.78% (9.31% - 10.28%)	7.05% (6.63% - 7.49%)	3.51% (3.11% - 3.98%)	3.83% (3.43% - 4.28%)	2.16% (1.91% - 2.43%)	1.32% (1.14% - 1.51%)
Kenya	17.05% (16.73% - 17.40%)	16.27% (15.93% - 16.60%)	14.14% (13.81% - 14.48%)	11.87% (11.56% - 12.18%)	4.54% (4.29% - 4.81%)	4.15% (3.90% - 4.40%)	3.26% (3.05% - 3.48%)	2.57% (2.39% - 2.75%)	0.66% (0.58% - 0.75%)	0.56% (0.48% - 0.65%)	0.37% (0.31% - 0.44%)	0.26% (0.22% - 0.31%)
Baringo	20.88% (19.53% - 22.15%)	19.81% (18.34% - 21.18%)	16.91% (15.50% - 18.17%)	13.61% (12.56% - 14.74%)	5.36% (4.34% - 6.48%)	4.82% (3.73% - 5.87%)	3.79% (2.90% - 4.69%)	2.73% (2.13% - 3.37%)	0.75% (0.45% - 1.14%)	0.58% (0.33% - 0.86%)	0.37% (0.20% - 0.61%)	0.19% (0.09% - 0.34%)
Bomet	17.81% (16.58% - 19.01%)	16.00% (14.69% - 17.25%)	11.69% (10.60% - 12.74%)	8.94% (7.93% - 10.01%)	5.04% (3.92% - 6.20%)	4.24% (3.23% - 5.28%)	2.30% (1.68% - 3.07%)	1.45% (1.00% - 2.00%)	0.80% (0.41% - 1.31%)	0.58% (0.28% - 0.98%)	0.15% (0.02% - 0.37%)	0.04% (0.00% - 0.14%)
Bungoma	21.24% (19.98% - 22.36%)	19.82% (18.72% - 20.87%)	17.75% (16.71% - 18.92%)	14.39% (13.41% - 15.48%)	6.49% (5.34% - 7.63%)	5.85% (4.83% - 6.81%)	4.88% (4.05% - 5.84%)	3.49% (2.84% - 4.20%)	1.01% (0.61% - 1.51%)	0.86% (0.53% - 1.24%)	0.64% (0.38% - 1.01%)	0.34% (0.17% - 0.54%)
Busia	19.03% (17.84% - 20.17%)	17.34% (16.11% - 18.52%)	14.93% (13.78% - 16.10%)	11.77% (10.71% - 12.89%)	5.80% (4.73% - 6.94%)	4.77% (3.75% - 5.82%)	3.76% (3.00% - 4.67%)	2.63% (2.01% - 3.36%)	1.09% (0.63% - 1.66%)	0.70% (0.34% - 1.14%)	0.44% (0.22% - 0.78%)	0.25% (0.09% - 0.47%)
Elgeyo-Marakwet	19.67% (18.23% - 21.04%)	17.78% (16.42% - 19.10%)	13.34% (12.11% - 14.63%)	9.94% (8.89% - 11.02%)	4.11% (3.03% - 5.22%)	3.60% (2.63% - 4.64%)	2.19% (1.56% - 2.92%)	1.47% (1.03% - 1.97%)	0.32% (0.10% - 0.61%)	0.28% (0.08% - 0.59%)	0.12% (0.02% - 0.32%)	0.05% (0.00% - 0.16%)
Embu	7.64% (6.66% - 8.64%)	6.95% (6.05% - 7.88%)	7.51% (6.59% - 8.53%)	6.53% (5.66% - 7.40%)	0.81% (0.52% - 1.16%)	0.74% (0.48% - 1.05%)	0.86% (0.54% - 1.24%)	0.78% (0.49% - 1.12%)	0.00% (0.00% - 0.02%)	0.00% (0.00% - 0.01%)	0.00% (0.00% - 0.03%)	0.00% (0.00% - 0.03%)
Garissa	21.29% (20.05% - 22.55%)	20.16% (18.90% - 21.37%)	16.97% (15.82% - 18.17%)	15.55% (14.42% - 16.63%)	4.97% (3.90% - 6.04%)	4.65% (3.65% - 5.66%)	4.10% (3.23% - 5.03%)	3.60% (2.80% - 4.40%)	0.60% (0.27% - 1.06%)	0.55% (0.23% - 0.95%)	0.57% (0.26% - 1.00%)	0.44% (0.18% - 0.76%)
HomaBay	18.07% (16.89% - 19.22%)	16.90% (15.74% - 17.96%)	13.41% (12.36% - 14.56%)	10.43% (9.45% - 11.40%)	4.47% (3.59% - 5.40%)	4.54% (3.70% - 5.43%)	3.32% (2.65% - 4.12%)	2.25% (1.76% - 2.86%)	0.66% (0.35% - 1.02%)	0.70% (0.38% - 1.09%)	0.42% (0.21% - 0.72%)	0.21% (0.08% - 0.39%)
Isiolo	14.72% (13.25% - 16.14%)	13.75% (12.44% - 15.09%)	13.63% (12.36% - 15.01%)	10.66% (9.66% - 11.77%)	2.16% (1.47% - 2.92%)	1.97% (1.38% - 2.73%)	2.25% (1.62% - 3.02%)	1.70% (1.22% - 2.25%)	0.07% (0.01% - 0.18%)	0.08% (0.02% - 0.17%)	0.09% (0.03% - 0.21%)	0.04% (0.00% - 0.11%)
Kajiado	17.72% (16.49% - 18.88%)	15.79% (14.55% - 17.01%)	10.04% (9.01% - 11.17%)	7.06% (6.24% - 7.90%)	5.55% (4.38% - 6.74%)	4.50% (3.51% - 5.55%)	1.74% (1.24% - 2.36%)	1.00% (0.69% - 1.37%)	1.05% (0.55% - 1.63%)	0.71% (0.35% - 1.13%)	0.06% (0.00% - 0.18%)	0.01% (0.00% - 0.05%)
Kakamega	18.26% (17.14% - 19.39%)	17.59% (16.44% - 18.84%)	15.45% (14.37% - 16.52%)	12.50% (11.51% - 13.59%)	4.78% (4.00% - 5.63%)	4.14% (3.31% - 4.99%)	3.31% (2.64% - 4.04%)	2.44% (1.89% - 3.04%)	0.60% (0.40% - 0.85%)	0.40% (0.20% - 0.64%)	0.23% (0.10% - 0.41%)	0.13% (0.03% - 0.27%)
Kericho	18.49% (17.14% - 19.70%)	14.69% (13.41% - 15.90%)	12.84% (11.67% - 14.00%)	9.73% (8.78% - 10.68%)	4.80% (3.86% - 5.75%)	3.10% (2.41% - 3.85%)	2.59% (1.94% - 3.29%)	1.70% (1.29% - 2.18%)	0.59% (0.35% - 0.90%)	0.29% (0.15% - 0.49%)	0.21% (0.07% - 0.41%)	0.08% (0.02% - 0.19%)
Kiambu	17.75% (16.71% - 18.77%)	16.31% (15.30% - 17.31%)	12.56% (11.65% - 13.48%)	9.68% (8.90% - 10.49%)	5.69% (4.85% - 6.58%)	4.85% (4.09% - 5.66%)	3.06% (2.47% - 3.72%)	2.04% (1.64% - 2.50%)	1.03% (0.69% - 1.44%)	0.75% (0.48% - 1.07%)	0.31% (0.14% - 0.55%)	0.14% (0.04% - 0.27%)
Kilifi	21.57% (20.56% - 22.63%)	20.14% (19.06% - 21.19%)	15.53% (14.47% - 16.62%)	13.01% (12.03% - 13.99%)	6.39% (5.35% - 7.52%)	5.83% (4.83% - 6.88%)	3.58% (2.89% - 4.34%)	2.85% (2.27% - 3.45%)	0.93% (0.56% - 1.38%)	0.82% (0.50% - 1.23%)	0.32% (0.16% - 0.51%)	0.21% (0.10% - 0.37%)
Kirinyaga	9.78% (8.67% - 10.86%)	9.24% (8.27% - 10.26%)	9.83% (8.86% - 10.84%)	9.82% (8.87% - 10.83%)	1.22% (0.84% - 1.68%)	1.17% (0.82% - 1.56%)	1.57% (1.18% - 2.02%)	1.78% (1.37% - 2.23%)	0.01% (0.00% - 0.07%)	0.01% (0.00% - 0.05%)	0.06% (0.01% - 0.14%)	0.08% (0.02% - 0.16%)

Table S8c. Estimated overall, severe, and extreme **underweight** prevalence (%) in children under 5 years, both sexes, in 1990, 2000, 2010, and 2020.

Location	Total				Severe				Extreme			
	1990	2000	2010	2020	1990	2000	2010	2020	1990	2000	2010	2020
	16.62%	15.40%	12.86%	10.48%	3.59%	3.05%	2.49%	1.82%	0.30%	0.21%	0.15%	0.08%
Kisii	(15.57% - 17.82%)	(14.29% - 16.61%)	(11.75% - 14.01%)	(9.45% - 11.56%)	(2.72% - 4.53%)	(2.27% - 3.88%)	(1.86% - 3.23%)	(1.30% - 2.45%)	(0.09% - 0.58%)	(0.04% - 0.46%)	(0.03% - 0.34%)	(0.00% - 0.22%)
	16.27%	16.84%	13.05%	10.24%	4.66%	4.68%	2.63%	1.91%	0.75%	0.70%	0.22%	0.10%
Kisumu	(15.09% - 17.43%)	(15.63% - 17.99%)	(11.74% - 14.27%)	(9.18% - 11.29%)	(3.84% - 5.58%)	(3.78% - 5.59%)	(1.98% - 3.32%)	(1.42% - 2.44%)	(0.48% - 1.08%)	(0.44% - 1.02%)	(0.11% - 0.37%)	(0.03% - 0.20%)
	22.51%	21.73%	18.42%	15.43%	6.20%	5.79%	4.35%	3.52%	0.83%	0.71%	0.41%	0.30%
Kitui	(21.26% - 23.68%)	(20.56% - 22.84%)	(17.28% - 19.54%)	(14.34% - 16.52%)	(5.10% - 7.41%)	(4.67% - 7.02%)	(3.47% - 5.37%)	(2.79% - 4.29%)	(0.48% - 1.34%)	(0.37% - 1.17%)	(0.20% - 0.73%)	(0.13% - 0.53%)
	12.64%	12.83%	11.34%	8.92%	1.84%	2.10%	2.08%	1.50%	0.10%	0.14%	0.18%	0.11%
Kwale	(11.50% - 13.77%)	(11.75% - 13.98%)	(10.29% - 12.42%)	(8.06% - 9.85%)	(1.28% - 2.53%)	(1.47% - 2.81%)	(1.46% - 2.74%)	(1.03% - 2.01%)	(0.00% - 0.27%)	(0.01% - 0.34%)	(0.04% - 0.39%)	(0.01% - 0.26%)
	14.47%	14.11%	11.64%	9.13%	3.56%	3.67%	2.77%	1.96%	0.46%	0.52%	0.39%	0.23%
Laikipia	(13.27% - 15.72%)	(12.96% - 15.29%)	(10.62% - 12.66%)	(8.25% - 10.08%)	(2.89% - 4.35%)	(2.96% - 4.46%)	(2.16% - 3.44%)	(1.50% - 2.49%)	(0.28% - 0.69%)	(0.32% - 0.82%)	(0.21% - 0.63%)	(0.11% - 0.38%)
	14.23%	13.66%	10.92%	8.98%	4.10%	3.74%	2.00%	1.42%	0.73%	0.59%	0.10%	0.03%
Lamu	(13.17% - 15.31%)	(12.61% - 14.79%)	(9.81% - 12.06%)	(8.00% - 9.99%)	(3.35% - 4.90%)	(3.01% - 4.63%)	(1.45% - 2.68%)	(0.99% - 1.91%)	(0.45% - 1.11%)	(0.34% - 0.94%)	(0.02% - 0.25%)	(0.00% - 0.12%)
	16.02%	14.96%	12.26%	9.94%	3.71%	3.21%	2.45%	1.86%	0.39%	0.27%	0.16%	0.10%
Machakos	(14.82% - 17.26%)	(13.70% - 16.15%)	(11.23% - 13.36%)	(8.96% - 10.92%)	(2.92% - 4.63%)	(2.44% - 4.00%)	(1.87% - 3.12%)	(1.41% - 2.35%)	(0.21% - 0.64%)	(0.12% - 0.46%)	(0.06% - 0.32%)	(0.04% - 0.20%)
	13.98%	13.26%	11.69%	8.79%	2.60%	2.24%	2.05%	1.41%	0.18%	0.11%	0.11%	0.06%
Makueni	(12.83% - 15.26%)	(12.03% - 14.56%)	(10.58% - 12.86%)	(7.86% - 9.73%)	(1.95% - 3.45%)	(1.60% - 2.97%)	(1.46% - 2.71%)	(0.98% - 1.91%)	(0.03% - 0.41%)	(0.00% - 0.27%)	(0.00% - 0.29%)	(0.00% - 0.18%)
	22.26%	21.63%	20.71%	21.26%	5.24%	4.76%	4.24%	5.34%	0.68%	0.58%	0.46%	0.69%
Mandera	(20.79% - 23.70%)	(20.17% - 23.05%)	(19.25% - 22.06%)	(19.89% - 22.49%)	(4.02% - 6.42%)	(3.66% - 5.87%)	(3.24% - 5.25%)	(4.22% - 6.50%)	(0.44% - 0.97%)	(0.38% - 0.82%)	(0.30% - 0.65%)	(0.48% - 1.01%)
	20.08%	19.06%	20.93%	19.30%	6.33%	5.80%	7.22%	6.18%	1.09%	0.94%	1.45%	1.09%
Marsabit	(18.75% - 21.30%)	(17.75% - 20.32%)	(19.76% - 22.13%)	(18.20% - 20.41%)	(5.17% - 7.52%)	(4.69% - 6.94%)	(6.07% - 8.42%)	(5.12% - 7.14%)	(0.66% - 1.61%)	(0.55% - 1.40%)	(0.95% - 2.06%)	(0.69% - 1.51%)
	19.17%	17.58%	14.02%	10.50%	6.42%	5.76%	3.58%	2.21%	1.28%	1.19%	0.46%	0.19%
Meru	(18.08% - 20.27%)	(16.54% - 18.72%)	(12.93% - 15.14%)	(9.51% - 11.46%)	(5.50% - 7.34%)	(4.90% - 6.73%)	(2.88% - 4.30%)	(1.70% - 2.75%)	(0.91% - 1.72%)	(0.83% - 1.61%)	(0.26% - 0.70%)	(0.09% - 0.34%)
	22.50%	19.34%	16.65%	12.99%	7.05%	5.48%	4.00%	2.85%	1.16%	0.75%	0.49%	0.28%
Migori	(21.41% - 23.61%)	(18.21% - 20.47%)	(15.52% - 17.83%)	(12.09% - 13.99%)	(5.98% - 8.20%)	(4.53% - 6.45%)	(3.19% - 4.79%)	(2.32% - 3.43%)	(0.75% - 1.66%)	(0.45% - 1.10%)	(0.26% - 0.78%)	(0.15% - 0.45%)
	12.50%	11.61%	11.54%	9.42%	3.26%	3.01%	2.69%	2.02%	0.56%	0.52%	0.32%	0.19%
Mombasa	(11.42% - 13.49%)	(10.69% - 12.54%)	(10.59% - 12.51%)	(8.52% - 10.32%)	(2.71% - 3.90%)	(2.52% - 3.61%)	(2.18% - 3.29%)	(1.61% - 2.47%)	(0.38% - 0.79%)	(0.35% - 0.74%)	(0.20% - 0.49%)	(0.11% - 0.32%)
	16.11%	14.67%	11.09%	9.45%	4.41%	3.66%	2.28%	1.77%	0.61%	0.45%	0.17%	0.10%
Murang'a	(15.05% - 17.16%)	(13.51% - 15.82%)	(9.94% - 12.24%)	(8.42% - 10.42%)	(3.59% - 5.32%)	(2.84% - 4.50%)	(1.69% - 2.93%)	(1.31% - 2.28%)	(0.34% - 0.95%)	(0.21% - 0.74%)	(0.06% - 0.34%)	(0.03% - 0.20%)
	11.11%	10.68%	9.10%	8.11%	2.71%	2.47%	1.88%	1.62%	0.26%	0.21%	0.10%	0.07%
Nairobi	(10.40% - 11.84%)	(9.98% - 11.39%)	(8.49% - 9.73%)	(7.45% - 8.74%)	(2.31% - 3.13%)	(2.09% - 2.86%)	(1.59% - 2.21%)	(1.33% - 1.92%)	(0.16% - 0.39%)	(0.11% - 0.31%)	(0.04% - 0.18%)	(0.01% - 0.14%)
	10.94%	10.56%	9.10%	7.36%	2.31%	1.99%	1.47%	1.12%	0.25%	0.15%	0.06%	0.02%
Nakuru	(9.98% - 11.94%)	(9.56% - 11.62%)	(8.21% - 10.01%)	(6.60% - 8.17%)	(1.84% - 2.87%)	(1.56% - 2.48%)	(1.10% - 1.87%)	(0.82% - 1.44%)	(0.13% - 0.42%)	(0.06% - 0.27%)	(0.01% - 0.12%)	(0.00% - 0.07%)
	17.99%	16.34%	13.05%	9.18%	5.11%	4.11%	2.58%	1.51%	0.84%	0.54%	0.21%	0.06%
Nandi	(16.76% - 19.22%)	(15.21% - 17.53%)	(11.91% - 14.32%)	(8.22% - 10.24%)	(4.15% - 6.16%)	(3.28% - 4.97%)	(1.95% - 3.32%)	(1.12% - 1.97%)	(0.55% - 1.20%)	(0.33% - 0.82%)	(0.11% - 0.36%)	(0.02% - 0.14%)
	16.20%	15.95%	12.03%	9.08%	3.10%	3.49%	1.87%	1.15%	0.22%	0.33%	0.06%	0.01%
Narok	(14.88% - 17.44%)	(14.70% - 17.30%)	(10.90% - 13.12%)	(8.08% - 10.20%)	(2.20% - 4.06%)	(2.53% - 4.49%)	(1.26% - 2.57%)	(0.75% - 1.63%)	(0.04% - 0.47%)	(0.10% - 0.65%)	(0.00% - 0.20%)	(0.00% - 0.07%)
	19.20%	18.46%	16.30%	13.77%	5.71%	5.23%	4.09%	3.28%	0.97%	0.84%	0.61%	0.42%
Nyamira	(18.00% - 20.47%)	(17.32% - 19.64%)	(15.08% - 17.49%)	(12.62% - 14.92%)	(4.71% - 6.73%)	(4.32% - 6.13%)	(3.32% - 4.91%)	(2.62% - 4.04%)	(0.66% - 1.33%)	(0.56% - 1.15%)	(0.40% - 0.86%)	(0.25% - 0.64%)
	12.95%	13.52%	12.56%	11.64%	3.76%	3.80%	3.33%	3.01%	0.81%	0.78%	0.64%	0.56%
Nyandarua	(12.02% - 13.94%)	(12.54% - 14.54%)	(11.50% - 13.63%)	(10.65% - 12.74%)	(3.10% - 4.51%)	(3.14% - 4.60%)	(2.75% - 4.02%)	(2.48% - 3.62%)	(0.54% - 1.17%)	(0.52% - 1.11%)	(0.44% - 0.89%)	(0.38% - 0.78%)
	10.99%	10.47%	9.73%	9.31%	2.30%	2.07%	1.86%	1.84%	0.24%	0.16%	0.11%	0.10%
Nyeri	(9.96% - 12.09%)	(9.46% - 11.54%)	(8.70% - 10.67%)	(8.32% - 10.27%)	(1.79% - 2.85%)	(1.61% - 2.59%)	(1.43% - 2.36%)	(1.42% - 2.33%)	(0.13% - 0.38%)	(0.08% - 0.26%)	(0.05% - 0.21%)	(0.04% - 0.20%)
	28.05%	26.64%	24.71%	21.20%	8.73%	7.91%	6.74%	5.26%	1.73%	1.47%	1.07%	0.66%
Samburu	(26.62% - 29.63%)	(25.09% - 28.33%)	(23.21% - 26.23%)	(19.81% - 22.57%)	(7.12% - 10.18%)	(6.22% - 9.51%)	(5.20% - 8.31%)	(4.12% - 6.49%)	(1.13% - 2.47%)	(0.91% - 2.18%)	(0.65% - 1.67%)	(0.36% - 1.05%)
	18.76%	17.62%	16.14%	12.61%	4.35%	3.94%	3.73%	2.80%	0.40%	0.33%	0.32%	0.21%
Siaya	(17.58% - 19.99%)	(16.45% - 18.86%)	(14.88% - 17.44%)	(11.47% - 13.71%)	(3.49% - 5.34%)	(3.11% - 4.88%)	(2.84% - 4.67%)	(2.17% - 3.49%)	(0.20% - 0.68%)	(0.15% - 0.56%)	(0.12% - 0.59%)	(0.07% - 0.40%)

Location	Total				Severe				Extreme			
	1990	2000	2010	2020	1990	2000	2010	2020	1990	2000	2010	2020
TaitaTaveta	20.55% (19.33% - 21.87%)	16.93% (15.55% - 18.29%)	12.50% (11.21% - 13.82%)	9.34% (8.33% - 10.55%)	7.09% (5.93% - 8.28%)	4.54% (3.56% - 5.65%)	2.58% (1.84% - 3.41%)	1.67% (1.19% - 2.27%)	1.42% (0.96% - 1.96%)	0.58% (0.31% - 0.94%)	0.18% (0.06% - 0.37%)	0.08% (0.02% - 0.21%)
TanaRiver	17.60% (16.32% - 18.93%)	18.65% (17.30% - 19.96%)	18.13% (16.73% - 19.45%)	16.25% (15.05% - 17.37%)	4.21% (3.24% - 5.28%)	4.39% (3.27% - 5.59%)	4.39% (3.21% - 5.57%)	4.26% (3.40% - 5.14%)	0.48% (0.23% - 0.83%)	0.51% (0.25% - 0.87%)	0.56% (0.33% - 0.87%)	0.55% (0.33% - 0.83%)
TharakaNithi	14.18% (12.94% - 15.39%)	13.88% (12.71% - 15.06%)	12.69% (11.61% - 13.90%)	9.91% (9.05% - 10.91%)	2.96% (2.36% - 3.60%)	2.75% (2.22% - 3.40%)	2.60% (2.06% - 3.20%)	1.92% (1.52% - 2.38%)	0.35% (0.21% - 0.53%)	0.35% (0.21% - 0.52%)	0.30% (0.19% - 0.45%)	0.15% (0.07% - 0.27%)
TransNzoia	12.95% (11.82% - 14.13%)	12.22% (11.13% - 13.35%)	12.46% (11.40% - 13.55%)	10.55% (9.56% - 11.55%)	2.19% (1.66% - 2.79%)	2.16% (1.69% - 2.72%)	2.49% (2.00% - 3.03%)	2.02% (1.61% - 2.49%)	0.12% (0.03% - 0.25%)	0.12% (0.05% - 0.24%)	0.23% (0.13% - 0.35%)	0.14% (0.06% - 0.24%)
Turkana	27.98% (27.01% - 29.08%)	26.22% (25.10% - 27.31%)	24.41% (23.27% - 25.56%)	22.39% (21.29% - 23.52%)	10.31% (8.86% - 11.83%)	9.39% (8.03% - 10.82%)	8.06% (6.83% - 9.36%)	7.18% (6.09% - 8.44%)	2.22% (1.43% - 3.21%)	1.94% (1.24% - 2.75%)	1.48% (0.90% - 2.22%)	1.24% (0.77% - 1.86%)
UasinGishu	14.81% (13.69% - 15.99%)	13.13% (12.03% - 14.19%)	11.28% (10.35% - 12.27%)	8.98% (8.21% - 9.80%)	3.69% (2.93% - 4.54%)	3.16% (2.53% - 3.84%)	2.54% (1.96% - 3.16%)	1.86% (1.44% - 2.36%)	0.45% (0.24% - 0.78%)	0.38% (0.19% - 0.61%)	0.28% (0.13% - 0.48%)	0.16% (0.06% - 0.31%)
Vihiga	9.26% (8.20% - 10.36%)	8.71% (7.67% - 9.82%)	7.85% (6.93% - 8.85%)	6.66% (5.84% - 7.54%)	0.87% (0.47% - 1.33%)	0.82% (0.49% - 1.26%)	0.66% (0.44% - 0.96%)	0.58% (0.38% - 0.86%)	0.01% (0.00% - 0.06%)	0.01% (0.00% - 0.05%)	0.02% (0.00% - 0.05%)	0.01% (0.00% - 0.03%)
Wajir	24.55% (23.37% - 25.73%)	24.17% (23.00% - 25.35%)	21.14% (19.80% - 22.42%)	17.25% (15.83% - 18.78%)	6.02% (4.80% - 7.27%)	5.63% (4.37% - 6.78%)	3.98% (2.82% - 5.12%)	1.98% (1.24% - 2.85%)	0.62% (0.27% - 1.10%)	0.50% (0.19% - 0.88%)	0.24% (0.05% - 0.53%)	0.03% (0.00% - 0.13%)
WestPokot	30.38% (29.38% - 31.52%)	29.19% (28.03% - 30.32%)	27.80% (26.74% - 28.82%)	25.25% (24.14% - 26.32%)	11.90% (10.40% - 13.51%)	10.99% (9.48% - 12.50%)	11.86% (10.46% - 13.32%)	10.43% (9.14% - 11.77%)	2.90% (2.03% - 4.00%)	2.56% (1.68% - 3.58%)	3.29% (2.42% - 4.36%)	2.79% (2.03% - 3.73%)
Madagascar	28.78% (28.14% - 29.46%)	30.63% (29.98% - 31.29%)	29.60% (28.84% - 30.36%)	27.58% (26.76% - 28.40%)	12.90% (12.00% - 13.93%)	14.45% (13.47% - 15.46%)	13.56% (12.43% - 14.77%)	12.10% (11.07% - 13.23%)	3.83% (3.24% - 4.55%)	4.65% (3.92% - 5.45%)	4.18% (3.37% - 5.08%)	3.49% (2.84% - 4.28%)
Malawi	20.26% (19.54% - 21.01%)	17.92% (17.23% - 18.54%)	12.66% (12.06% - 13.24%)	10.52% (9.88% - 11.16%)	6.15% (5.48% - 6.81%)	4.98% (4.47% - 5.47%)	2.67% (2.36% - 2.98%)	1.95% (1.65% - 2.24%)	0.97% (0.74% - 1.20%)	0.67% (0.52% - 0.84%)	0.17% (0.11% - 0.24%)	0.07% (0.04% - 0.13%)
Mozambique	23.08% (22.28% - 23.89%)	20.54% (19.85% - 21.23%)	14.97% (14.32% - 15.62%)	11.86% (11.17% - 12.59%)	8.05% (7.30% - 8.92%)	6.60% (6.00% - 7.23%)	3.67% (3.26% - 4.10%)	2.57% (2.22% - 2.95%)	1.61% (1.27% - 1.99%)	1.18% (0.95% - 1.42%)	0.41% (0.30% - 0.54%)	0.20% (0.13% - 0.30%)
Rwanda	20.95% (20.40% - 21.57%)	20.61% (20.15% - 21.08%)	12.88% (12.44% - 13.31%)	9.70% (9.18% - 10.19%)	7.23% (6.65% - 7.85%)	6.74% (6.31% - 7.18%)	2.82% (2.54% - 3.07%)	1.69% (1.45% - 1.95%)	1.46% (1.21% - 1.73%)	1.24% (1.06% - 1.42%)	0.20% (0.14% - 0.27%)	0.04% (0.02% - 0.08%)
Somalia	25.18% (24.32% - 25.99%)	25.23% (24.57% - 25.94%)	25.03% (24.29% - 25.76%)	24.22% (23.33% - 25.03%)	10.13% (9.15% - 11.14%)	10.78% (9.91% - 11.66%)	9.86% (8.96% - 10.77%)	9.18% (8.18% - 10.19%)	2.52% (1.97% - 3.14%)	2.98% (2.46% - 3.56%)	2.38% (1.89% - 2.92%)	2.09% (1.59% - 2.63%)
United Republic of Tanzania	24.18% (23.63% - 24.74%)	21.31% (20.76% - 21.89%)	15.10% (14.54% - 15.65%)	12.49% (11.76% - 13.17%)	8.70% (8.08% - 9.34%)	6.60% (6.05% - 7.20%)	3.33% (2.96% - 3.69%)	2.54% (2.19% - 2.93%)	1.80% (1.50% - 2.11%)	1.06% (0.85% - 1.29%)	0.25% (0.16% - 0.34%)	0.14% (0.07% - 0.22%)
Uganda	18.83% (18.21% - 19.42%)	18.08% (17.51% - 18.64%)	14.47% (13.98% - 14.96%)	11.44% (10.85% - 12.07%)	5.36% (4.88% - 5.85%)	5.05% (4.63% - 5.51%)	3.44% (3.11% - 3.77%)	2.36% (2.07% - 2.68%)	0.74% (0.57% - 0.93%)	0.72% (0.57% - 0.87%)	0.33% (0.24% - 0.42%)	0.15% (0.09% - 0.22%)
Zambia	19.06% (18.40% - 19.68%)	19.30% (18.84% - 19.79%)	15.20% (14.65% - 15.75%)	10.76% (10.13% - 11.45%)	5.75% (5.20% - 6.31%)	5.82% (5.38% - 6.28%)	3.82% (3.45% - 4.25%)	2.34% (2.01% - 2.71%)	0.91% (0.70% - 1.13%)	0.94% (0.77% - 1.12%)	0.41% (0.30% - 0.53%)	0.15% (0.08% - 0.25%)
South Sudan	27.06% (26.22% - 27.89%)	27.90% (27.24% - 28.54%)	25.92% (25.33% - 26.52%)	23.75% (22.93% - 24.55%)	11.71% (10.69% - 12.77%)	12.65% (11.74% - 13.54%)	11.40% (10.72% - 12.13%)	9.87% (8.98% - 10.79%)	3.23% (2.57% - 3.98%)	3.74% (3.11% - 4.41%)	3.30% (2.85% - 3.83%)	2.65% (2.10% - 3.27%)
**Southern Sub-Saharan Africa**	10.35% (10.17% - 10.55%)	10.18% (10.00% - 10.37%)	8.98% (8.84% - 9.13%)	7.26% (7.11% - 7.44%)	2.18% (2.09% - 2.28%)	2.12% (2.03% - 2.21%)	1.64% (1.56% - 1.71%)	1.14% (1.07% - 1.21%)	0.17% (0.15% - 0.19%)	0.17% (0.14% - 0.19%)	0.09% (0.08% - 0.10%)	0.04% (0.04% - 0.05%)
Botswana	12.46% (11.72% - 13.17%)	11.50% (10.99% - 11.99%)	10.84% (10.33% - 11.35%)	9.37% (8.77% - 10.01%)	2.37% (2.02% - 2.76%)	2.19% (1.94% - 2.47%)	2.21% (1.95% - 2.48%)	1.86% (1.59% - 2.18%)	0.11% (0.07% - 0.18%)	0.09% (0.06% - 0.14%)	0.13% (0.08% - 0.18%)	0.09% (0.05% - 0.15%)
Lesotho	15.75% (15.07% - 16.44%)	14.79% (14.19% - 15.40%)	13.39% (12.76% - 14.04%)	10.82% (10.20% - 11.44%)	4.50% (4.02% - 5.01%)	3.93% (3.50% - 4.36%)	2.93% (2.58% - 3.34%)	2.06% (1.75% - 2.40%)	0.65% (0.50% - 0.82%)	0.49% (0.37% - 0.63%)	0.23% (0.14% - 0.33%)	0.10% (0.04% - 0.18%)
Namibia	19.96% (19.25% - 20.67%)	18.27% (17.61% - 18.94%)	15.21% (14.65% - 15.84%)	12.53% (11.84% - 13.23%)	6.27% (5.59% - 6.95%)	5.12% (4.55% - 5.69%)	3.77% (3.34% - 4.23%)	2.90% (2.45% - 3.37%)	1.04% (0.77% - 1.32%)	0.68% (0.49% - 0.89%)	0.38% (0.25% - 0.53%)	0.24% (0.13% - 0.38%)
South Africa	9.81% (9.57% - 10.06%)	9.06% (8.85% - 9.27%)	7.31% (7.14% - 7.49%)	5.98% (5.83% - 6.14%)	2.02% (1.91% - 2.14%)	1.79% (1.69% - 1.88%)	1.25% (1.19% - 1.32%)	0.90% (0.85% - 0.96%)	0.16% (0.13% - 0.18%)	0.14% (0.12% - 0.15%)	0.07% (0.06% - 0.08%)	0.04% (0.03% - 0.05%)

Table S8c. Estimated overall, severe, and extreme **underweight** prevalence (%) in children under 5 years, both sexes, in 1990, 2000, 2010, and 2020.

Table S8c. Estimated overall, severe, and extreme **underweight** prevalence (%) in children under 5 years, both sexes, in 1990, 2000, 2010, and 2020.												
**Location**	**Total**				**Severe**				**Extreme**			
	**1990**	**2000**	**2010**	**2020**	**1990**	**2000**	**2010**	**2020**	**1990**	**2000**	**2010**	**2020**
Eswatini	7.97% (7.46% - 8.56%)	7.93% (7.44% - 8.46%)	6.60% (6.19% - 7.02%)	5.54% (5.15% - 5.94%)	1.17% (0.99% - 1.36%)	1.21% (1.05% - 1.41%)	0.87% (0.76% - 1.00%)	0.71% (0.60% - 0.83%)	0.02% (0.00% - 0.04%)	0.02% (0.01% - 0.04%)	0.01% (0.00% - 0.02%)	0.00% (0.00% - 0.01%)
Zimbabwe	9.76% (9.32% - 10.21%)	11.47% (11.03% - 11.95%)	11.81% (11.46% - 12.17%)	9.02% (8.55% - 9.53%)	1.81% (1.61% - 2.00%)	2.43% (2.17% - 2.71%)	2.15% (1.95% - 2.36%)	1.31% (1.12% - 1.53%)	0.05% (0.03% - 0.08%)	0.15% (0.09% - 0.22%)	0.09% (0.05% - 0.13%)	0.02% (0.01% - 0.05%)
**Western Sub-Saharan Africa**	24.43% (24.19% - 24.70%)	24.27% (24.02% - 24.53%)	22.13% (21.95% - 22.33%)	19.85% (19.62% - 20.10%)	10.33% (9.96% - 10.70%)	10.34% (9.97% - 10.68%)	9.08% (8.83% - 9.33%)	7.72% (7.44% - 8.00%)	3.24% (2.94% - 3.56%)	3.30% (3.00% - 3.62%)	2.75% (2.54% - 2.98%)	2.19% (1.97% - 2.41%)
Benin	22.55% (21.97% - 23.11%)	21.27% (20.64% - 21.92%)	18.64% (18.08% - 19.23%)	16.47% (15.79% - 17.15%)	8.41% (7.78% - 9.00%)	7.06% (6.49% - 7.63%)	5.62% (5.17% - 6.09%)	4.46% (3.96% - 4.99%)	1.85% (1.56% - 2.18%)	1.29% (1.05% - 1.55%)	0.85% (0.69% - 1.04%)	0.55% (0.40% - 0.73%)
Burkina Faso	30.08% (29.39% - 30.78%)	31.82% (31.21% - 32.42%)	26.89% (26.33% - 27.45%)	23.01% (22.28% - 23.77%)	13.80% (12.87% - 14.74%)	16.60% (15.71% - 17.47%)	11.90% (11.26% - 12.55%)	8.70% (7.98% - 9.44%)	4.19% (3.51% - 4.94%)	6.29% (5.53% - 7.13%)	3.47% (3.05% - 3.94%)	1.97% (1.60% - 2.39%)
Cameroon	15.88% (15.17% - 16.58%)	16.50% (15.85% - 17.14%)	14.77% (14.21% - 15.36%)	12.65% (11.96% - 13.33%)	4.39% (3.89% - 4.91%)	5.02% (4.57% - 5.51%)	4.23% (3.84% - 4.66%)	3.43% (3.02% - 3.89%)	0.57% (0.42% - 0.74%)	0.81% (0.64% - 1.00%)	0.60% (0.46% - 0.75%)	0.42% (0.30% - 0.57%)
Cabo Verde	13.34% (12.67% - 14.01%)	11.23% (10.53% - 11.91%)	8.41% (7.84% - 9.04%)	7.09% (6.54% - 7.68%)	3.65% (3.25% - 4.10%)	2.81% (2.40% - 3.22%)	1.85% (1.57% - 2.16%)	1.47% (1.23% - 1.74%)	0.45% (0.34% - 0.59%)	0.28% (0.18% - 0.39%)	0.10% (0.04% - 0.17%)	0.05% (0.02% - 0.10%)
Chad	28.89% (28.20% - 29.59%)	29.24% (28.62% - 29.88%)	28.16% (27.48% - 28.79%)	26.09% (25.34% - 26.83%)	13.61% (12.62% - 14.74%)	14.06% (13.23% - 14.95%)	13.34% (12.47% - 14.26%)	11.65% (10.68% - 12.66%)	4.40% (3.65% - 5.37%)	4.70% (4.02% - 5.47%)	4.35% (3.70% - 5.16%)	3.47% (2.76% - 4.23%)
Côte d'Ivoire	14.98% (14.47% - 15.52%)	16.25% (15.81% - 16.71%)	15.39% (14.98% - 15.80%)	12.28% (11.75% - 12.78%)	3.71% (3.35% - 4.08%)	4.49% (4.13% - 4.86%)	4.12% (3.81% - 4.44%)	2.74% (2.45% - 3.05%)	0.38% (0.28% - 0.48%)	0.58% (0.46% - 0.70%)	0.49% (0.39% - 0.61%)	0.20% (0.13% - 0.27%)
Gambia	19.38% (18.53% - 20.27%)	18.02% (17.33% - 18.74%)	16.97% (16.35% - 17.61%)	14.74% (14.05% - 15.49%)	5.35% (4.68% - 6.07%)	4.79% (4.23% - 5.34%)	4.61% (4.12% - 5.10%)	3.54% (3.08% - 4.07%)	0.72% (0.51% - 0.95%)	0.59% (0.43% - 0.76%)	0.60% (0.44% - 0.76%)	0.34% (0.22% - 0.50%)
Ghana	22.41% (21.74% - 23.13%)	19.86% (19.21% - 20.48%)	15.84% (15.22% - 16.50%)	12.26% (11.57% - 12.96%)	7.58% (6.93% - 8.30%)	5.92% (5.33% - 6.54%)	4.07% (3.63% - 4.52%)	2.76% (2.38% - 3.17%)	1.40% (1.12% - 1.72%)	0.89% (0.69% - 1.11%)	0.45% (0.33% - 0.59%)	0.21% (0.13% - 0.31%)
Guinea	21.12% (20.46% - 21.77%)	22.29% (21.85% - 22.76%)	19.58% (19.16% - 20.00%)	16.58% (15.97% - 17.20%)	7.02% (6.42% - 7.65%)	8.06% (7.56% - 8.52%)	6.11% (5.69% - 6.55%)	4.58% (4.12% - 5.07%)	1.28% (1.05% - 1.54%)	1.73% (1.49% - 1.97%)	1.01% (0.84% - 1.19%)	0.61% (0.47% - 0.78%)
Guinea-Bissau	20.22% (19.34% - 21.08%)	19.46% (18.81% - 20.10%)	17.45% (16.91% - 17.98%)	15.75% (14.98% - 16.48%)	5.81% (5.04% - 6.55%)	5.63% (5.06% - 6.17%)	4.83% (4.39% - 5.28%)	4.11% (3.57% - 4.68%)	0.81% (0.59% - 1.06%)	0.81% (0.62% - 1.00%)	0.62% (0.48% - 0.77%)	0.47% (0.32% - 0.65%)
Liberia	20.79% (19.92% - 21.67%)	21.41% (20.75% - 22.14%)	16.74% (16.17% - 17.35%)	13.92% (13.21% - 14.71%)	5.76% (5.01% - 6.63%)	6.48% (5.83% - 7.16%)	4.42% (3.98% - 4.86%)	3.39% (2.94% - 3.87%)	0.73% (0.51% - 1.02%)	0.98% (0.75% - 1.25%)	0.53% (0.39% - 0.67%)	0.33% (0.22% - 0.47%)
Mali	28.52% (27.80% - 29.22%)	26.76% (26.09% - 27.35%)	21.41% (20.77% - 22.04%)	19.33% (18.59% - 20.07%)	14.38% (13.49% - 15.35%)	12.47% (11.68% - 13.30%)	7.53% (6.93% - 8.16%)	6.21% (5.59% - 6.86%)	5.23% (4.52% - 6.07%)	4.03% (3.47% - 4.64%)	1.53% (1.25% - 1.84%)	1.08% (0.84% - 1.35%)
Mauritania	28.69% (28.02% - 29.35%)	24.25% (23.61% - 24.87%)	20.00% (19.43% - 20.57%)	16.91% (16.19% - 17.64%)	13.58% (12.66% - 14.51%)	10.13% (9.40% - 10.89%)	7.36% (6.79% - 7.92%)	5.54% (4.96% - 6.14%)	4.39% (3.70% - 5.20%)	2.73% (2.28% - 3.22%)	1.64% (1.38% - 1.92%)	1.03% (0.81% - 1.28%)
Niger	36.48% (35.72% - 37.38%)	36.51% (35.81% - 37.27%)	33.12% (32.39% - 33.88%)	30.85% (30.06% - 31.65%)	19.52% (18.27% - 20.76%)	19.66% (18.38% - 20.90%)	15.92% (14.86% - 17.01%)	13.58% (12.50% - 14.70%)	7.44% (6.17% - 8.75%)	7.65% (6.35% - 8.96%)	5.13% (4.28% - 6.06%)	3.82% (3.10% - 4.66%)
Nigeria	25.61% (25.08% - 26.14%)	25.23% (24.76% - 25.77%)	23.76% (23.41% - 24.10%)	21.65% (21.18% - 22.13%)	11.59% (10.87% - 12.38%)	11.37% (10.65% - 12.09%)	10.86% (10.37% - 11.33%)	9.59% (9.02% - 10.16%)	4.13% (3.49% - 4.81%)	4.00% (3.41% - 4.63%)	3.87% (3.45% - 4.30%)	3.28% (2.84% - 3.74%)
Sao Tome and Principe	12.63% (11.94% - 13.36%)	10.87% (10.27% - 11.48%)	10.50% (9.93% - 11.06%)	8.46% (7.89% - 9.04%)	2.63% (2.28% - 3.02%)	2.02% (1.76% - 2.31%)	2.09% (1.83% - 2.37%)	1.56% (1.34% - 1.80%)	0.18% (0.12% - 0.26%)	0.12% (0.08% - 0.16%)	0.13% (0.08% - 0.18%)	0.08% (0.04% - 0.11%)
Senegal	20.29% (19.65% - 20.92%)	18.95% (18.41% - 19.51%)	16.10% (15.66% - 16.57%)	13.25% (12.67% - 13.88%)	5.82% (5.24% - 6.45%)	4.91% (4.41% - 5.41%)	3.56% (3.20% - 3.94%)	2.69% (2.32% - 3.05%)	0.78% (0.57% - 1.01%)	0.53% (0.39% - 0.69%)	0.26% (0.18% - 0.37%)	0.15% (0.08% - 0.24%)
Sierra Leone	23.14% (22.48% - 23.81%)	23.32% (22.65% - 23.98%)	20.25% (19.71% - 20.85%)	15.39% (14.70% - 16.11%)	8.87% (8.15% - 9.58%)	9.03% (8.34% - 9.74%)	6.80% (6.31% - 7.32%)	4.31% (3.85% - 4.79%)	2.11% (1.76% - 2.47%)	2.22% (1.87% - 2.60%)	1.32% (1.10% - 1.56%)	0.62% (0.47% - 0.78%)

Fig. S1. Standardized betas for ST−GPR stage 1 priors by sex for all covariates included in ensemble priors by ST-GPR model for stunting, wasting, and underweight. Histograms are shown for overall (A), severe (B), and mean (C) stunting; for overall (D), severe (E), and mean (F) wasting; and for overall (G), severe (H), and mean (I) underweight models. Note that because of a transformation to mean Z−score models, those relationships are in the opposite direction.

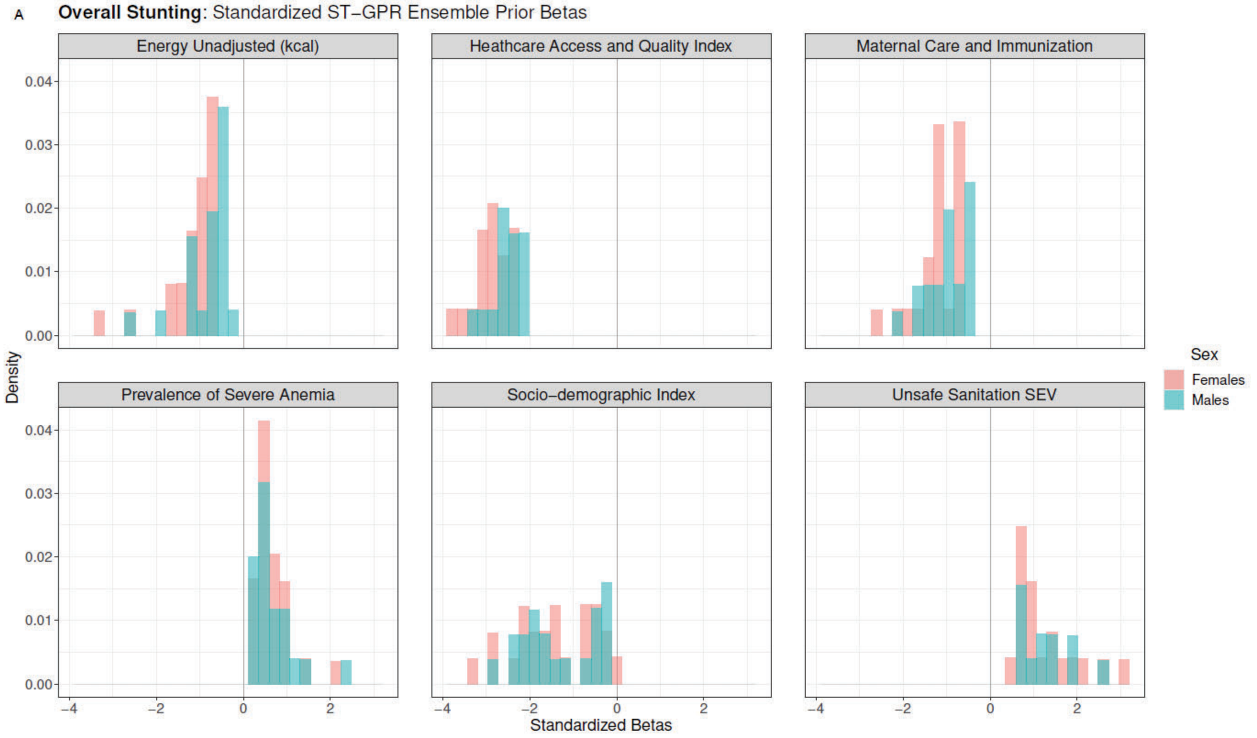

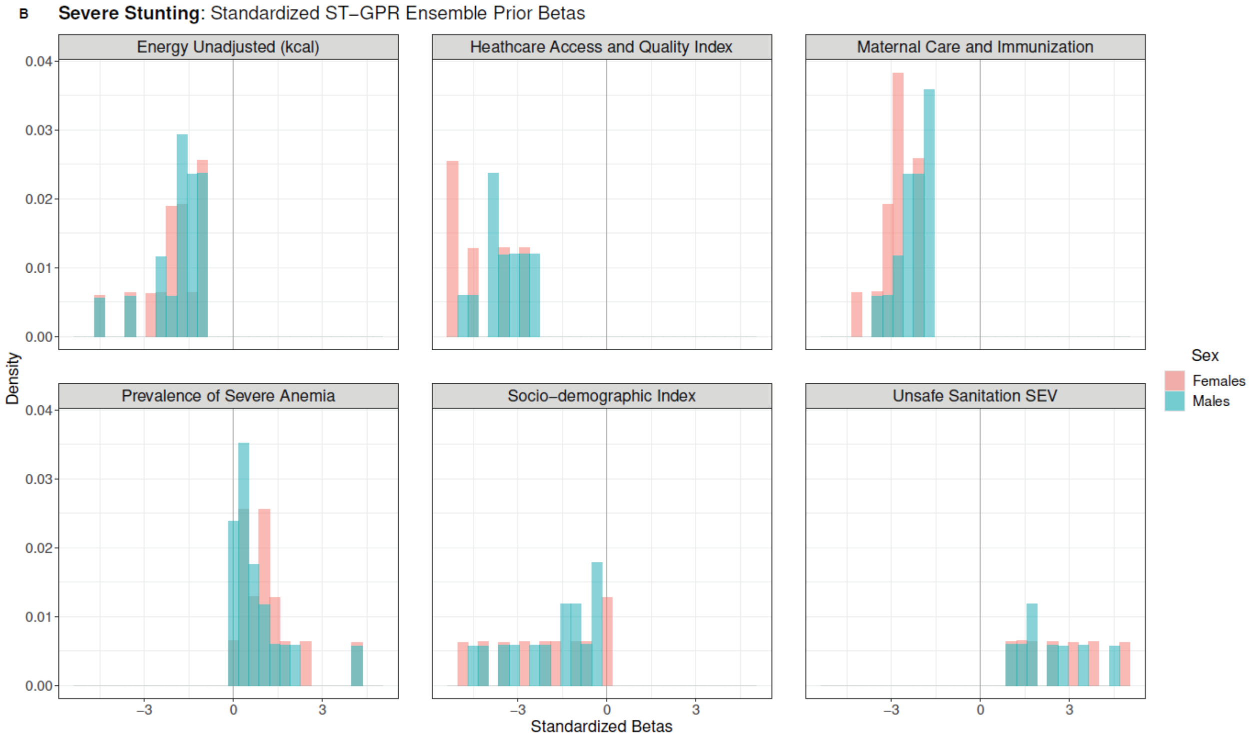

C **Mean Stunting**: Standardized ST–GPR Ensemble Prior Betas

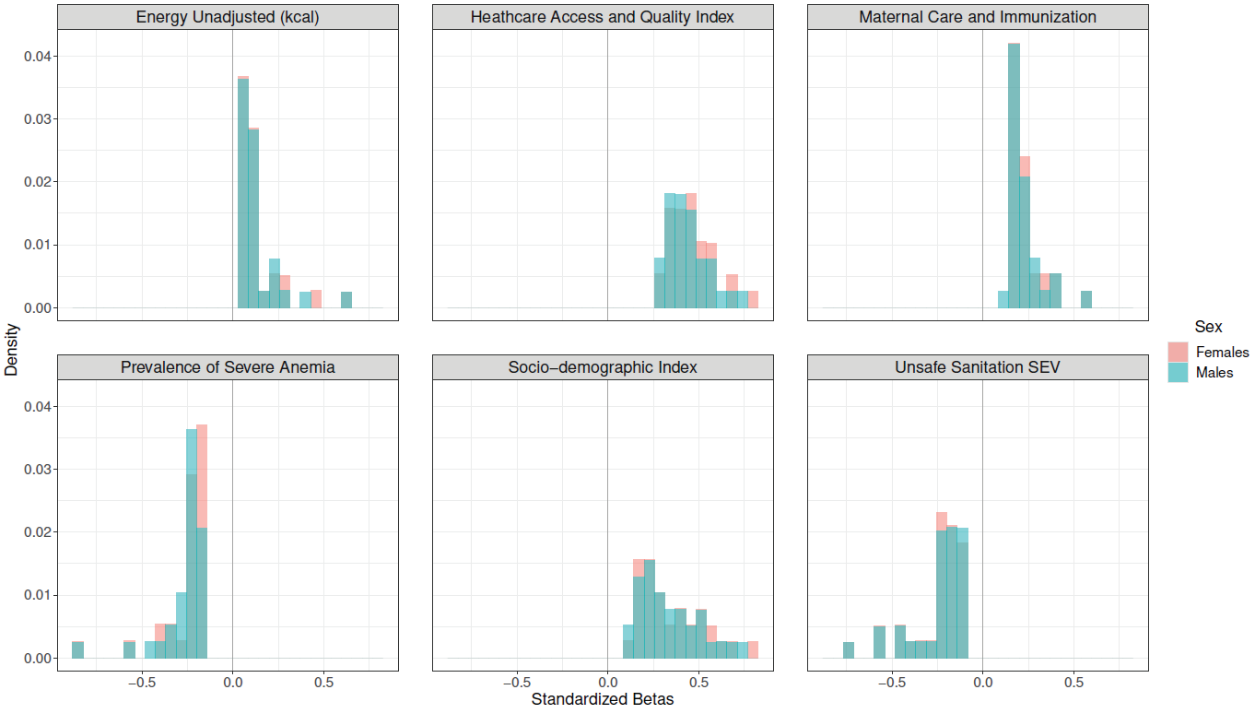

D **Overall Wasting**: Standardized ST–GPR Ensemble Prior Betas

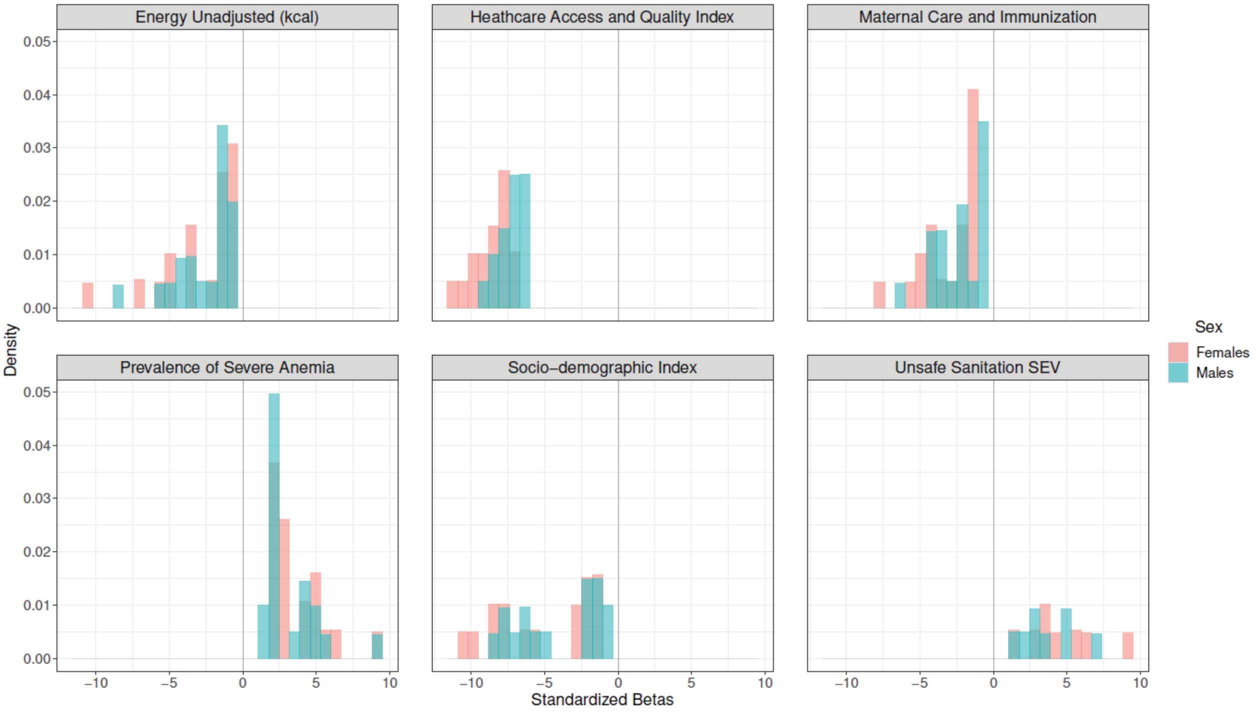

E **Severe Wasting**: Standardized ST–GPR Ensemble Prior Betas

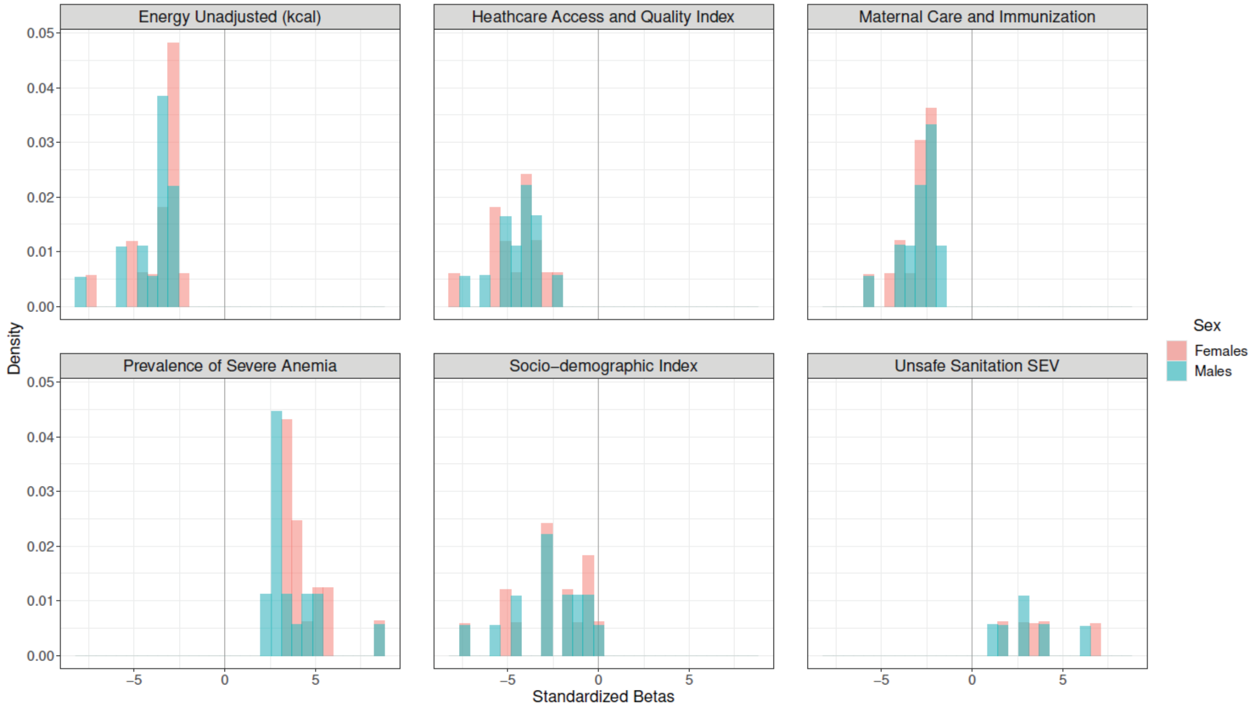

F **Mean Wasting**: Standardized ST–GPR Ensemble Prior Betas

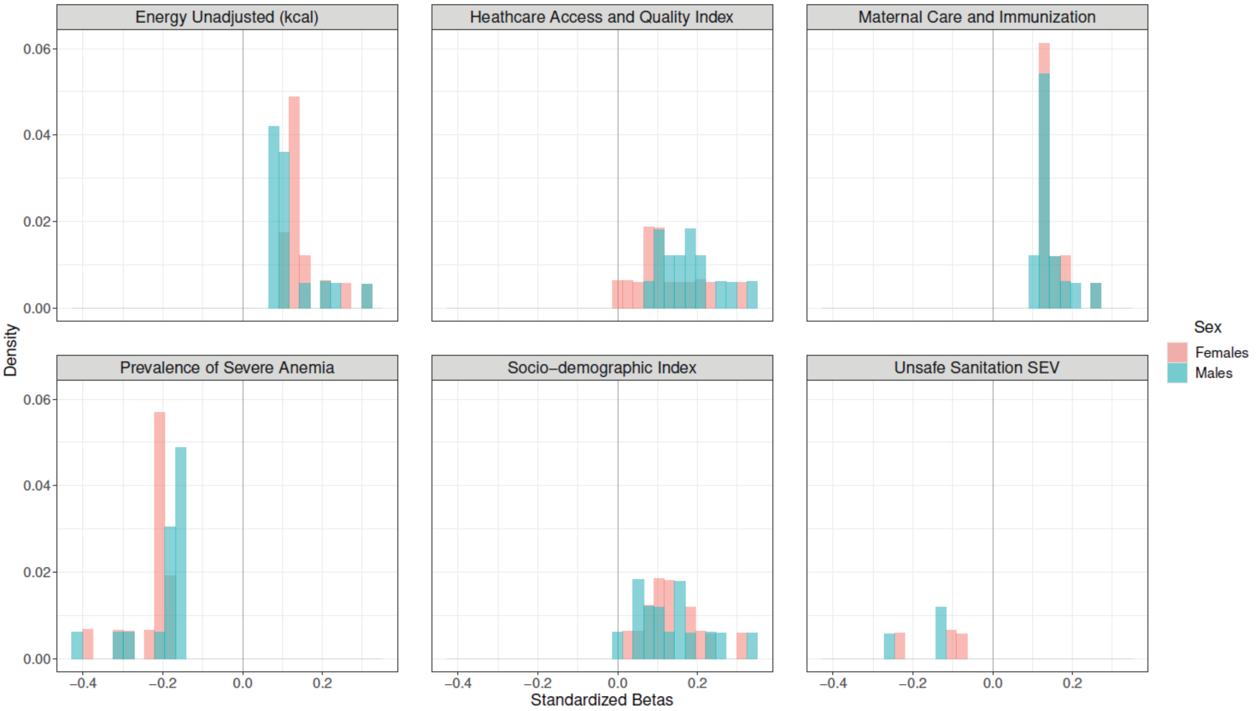

G **Overall Underweight**: Standardized ST–GPR Ensemble Prior Betas

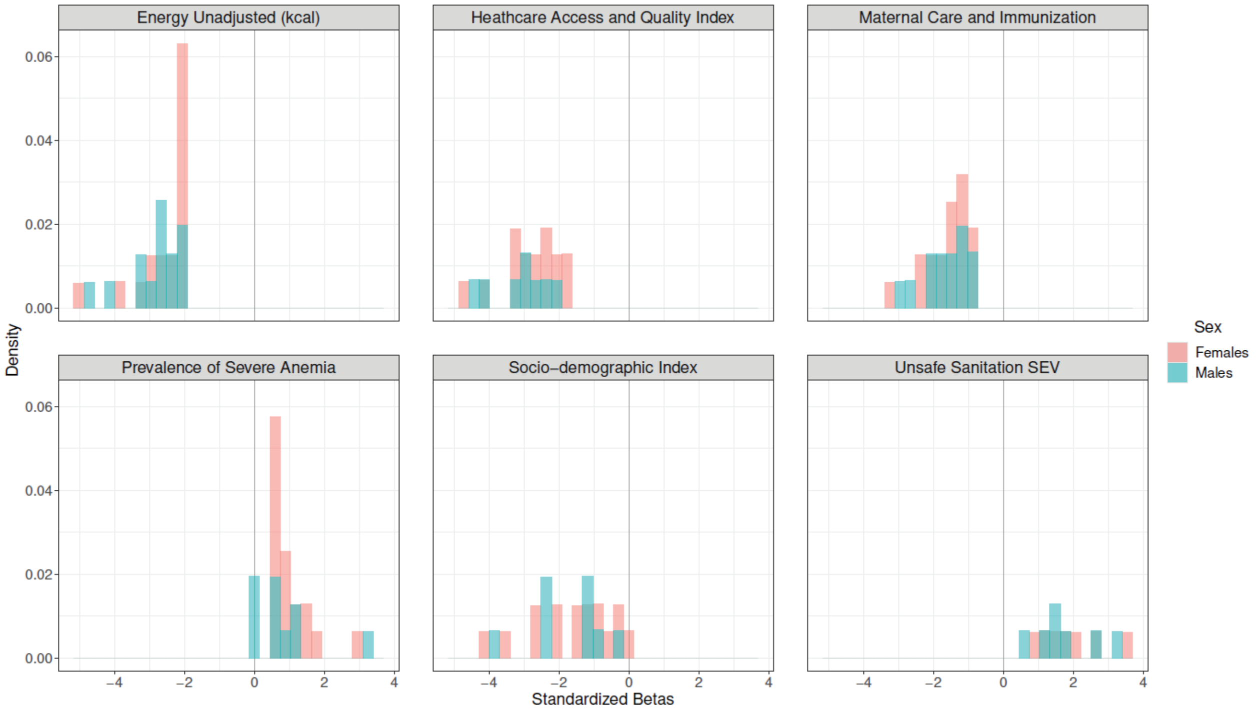

H **Severe Underweight**: Standardized ST–GPR Ensemble Prior Betas

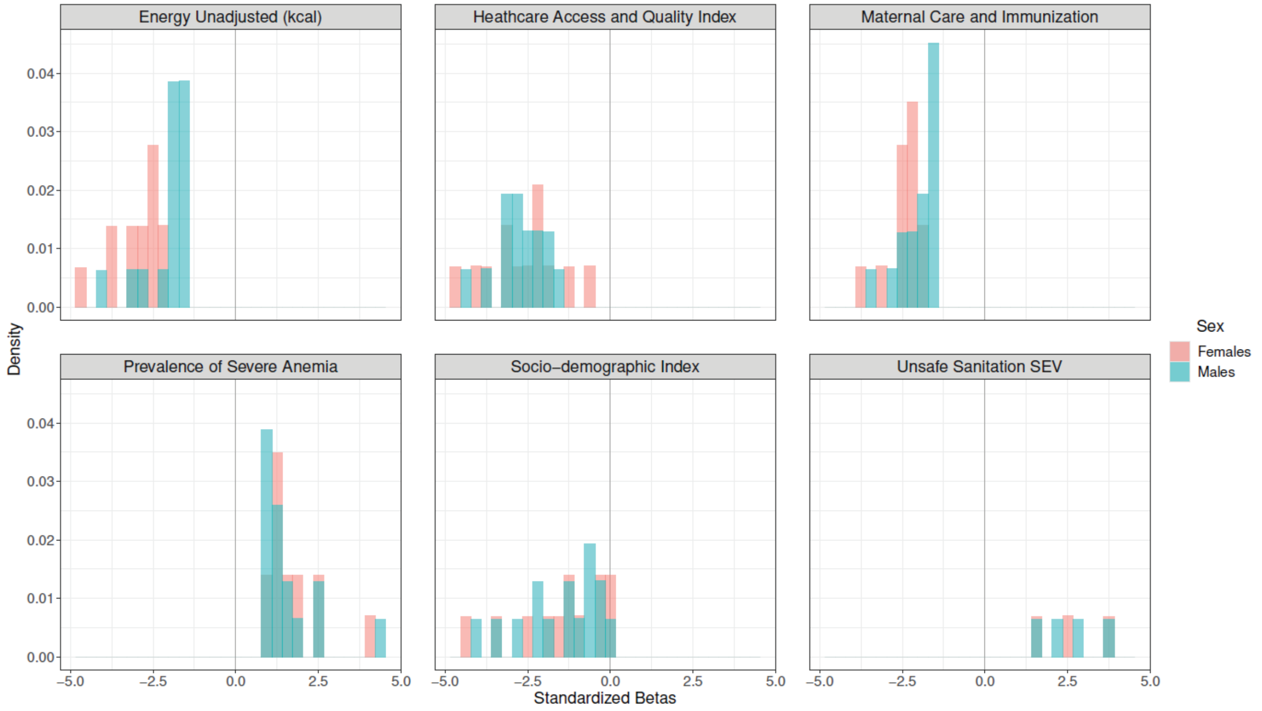

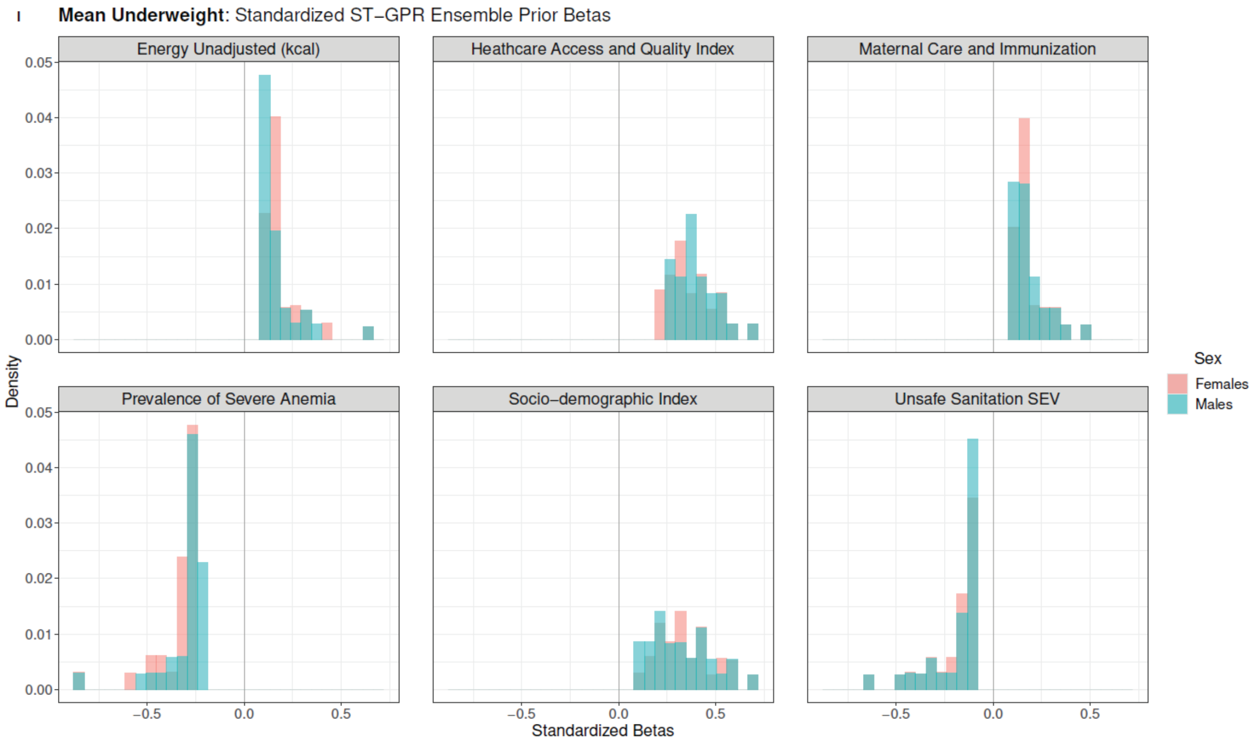
Mean Underweight: Standardized ST–GPR Ensemble Prior Betas
Energy Unadjusted (kcal)
Heathcare Access and Quality Index
Maternal Care and Immunization
Prevalence of Severe Anemia
Socio–demographic Index
Unsafe Sanitation SEV
Density
0.05
0.04
0.03
0.02
0.01
0.00
−0.5
0.0
0.5
Standardized Betas
Sex
Females
Males

Fig. S2. Map of source counts for GBD 2020 for stunting, wasting, and underweight. Map of source counts for GBD 2020 for stunting [HAZ] (A), wasting [WHZ] (B), and underweight [WAZ] (C).

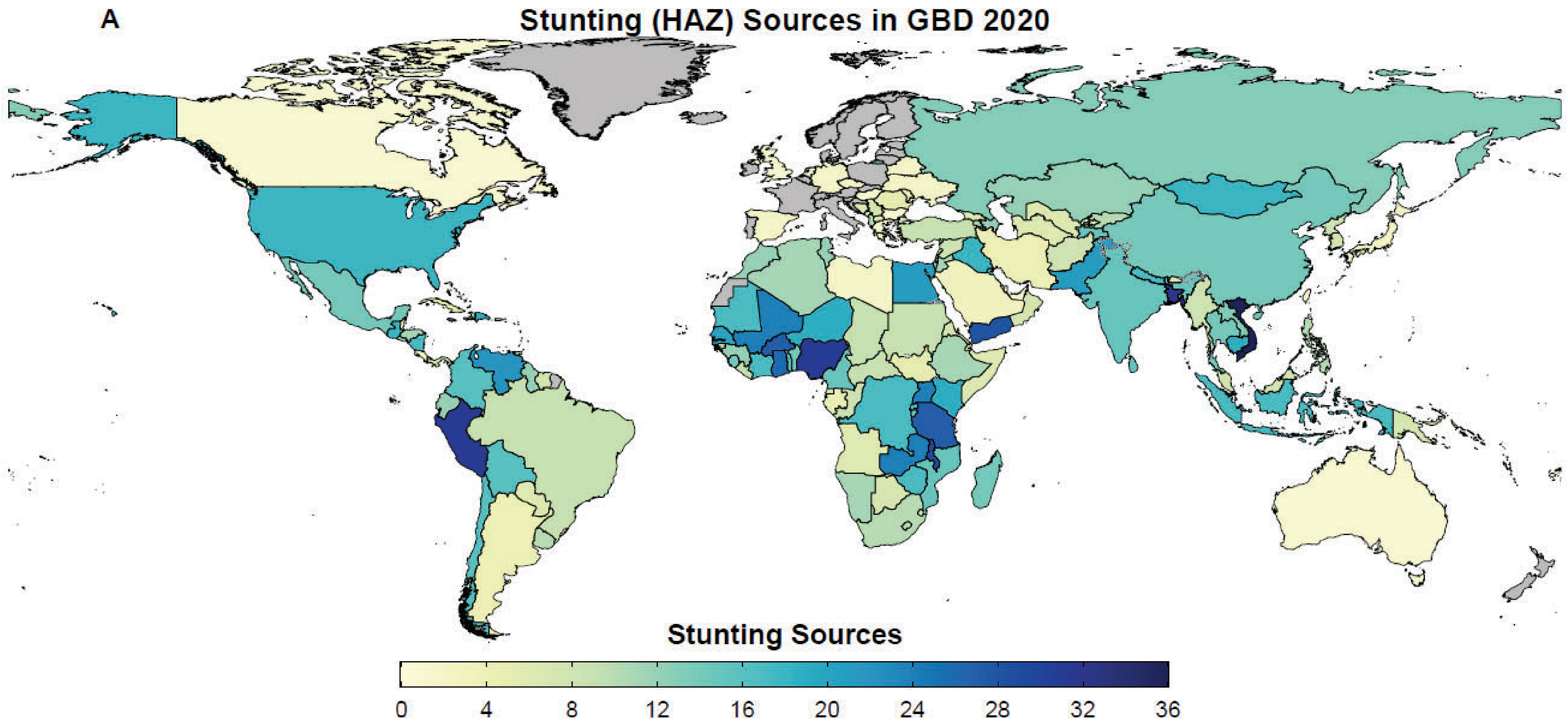

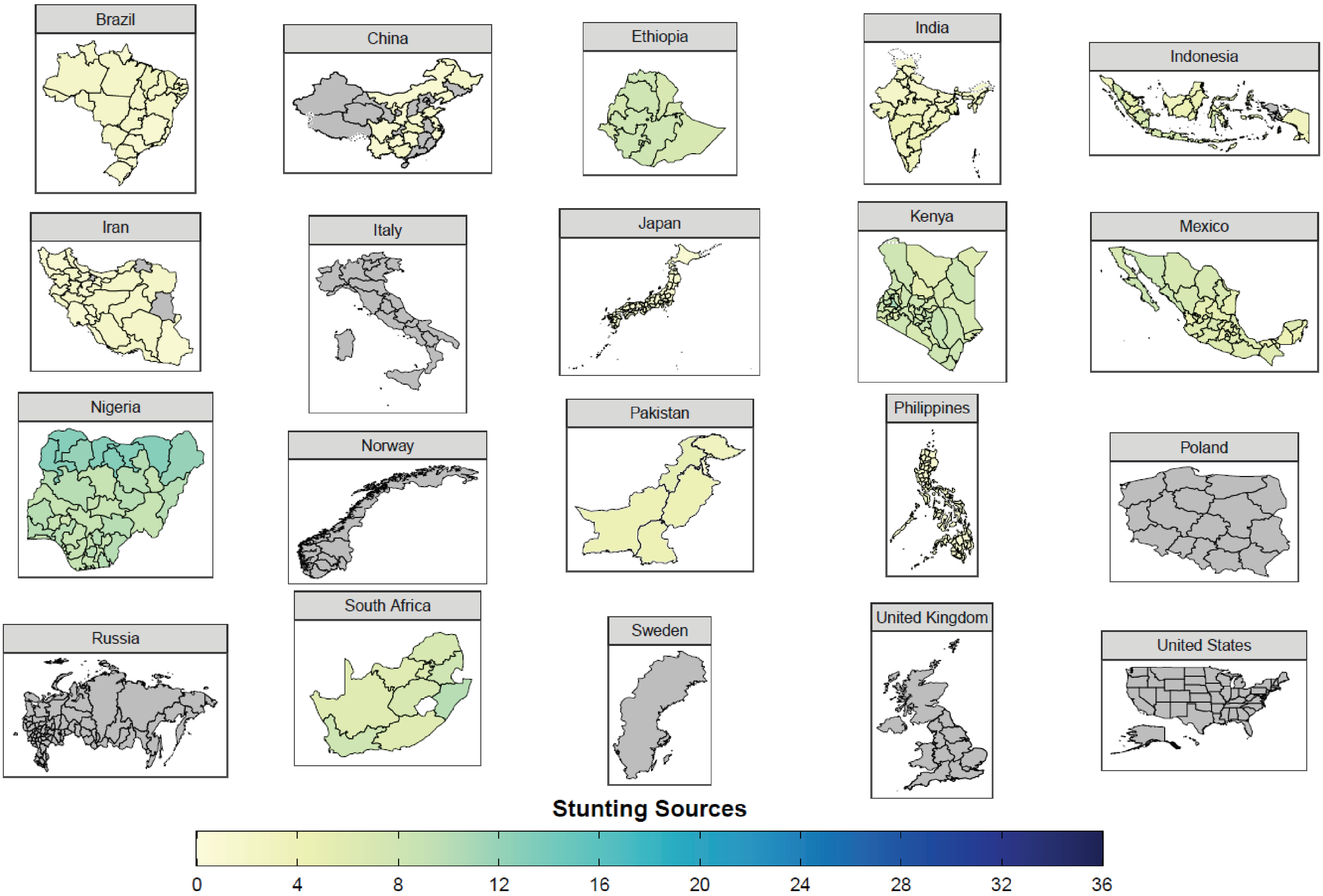
Brazil
China
Ethiopia
India
Indonesia
Iran
Italy
Japan
Kenya
Mexico
Nigeria
Norway
Pakistan
Philippines
Poland
Russia
South Africa
Sweden
United Kingdom
United States
Stunting Sources
0
4
8
12
16
20
24
28
32
36

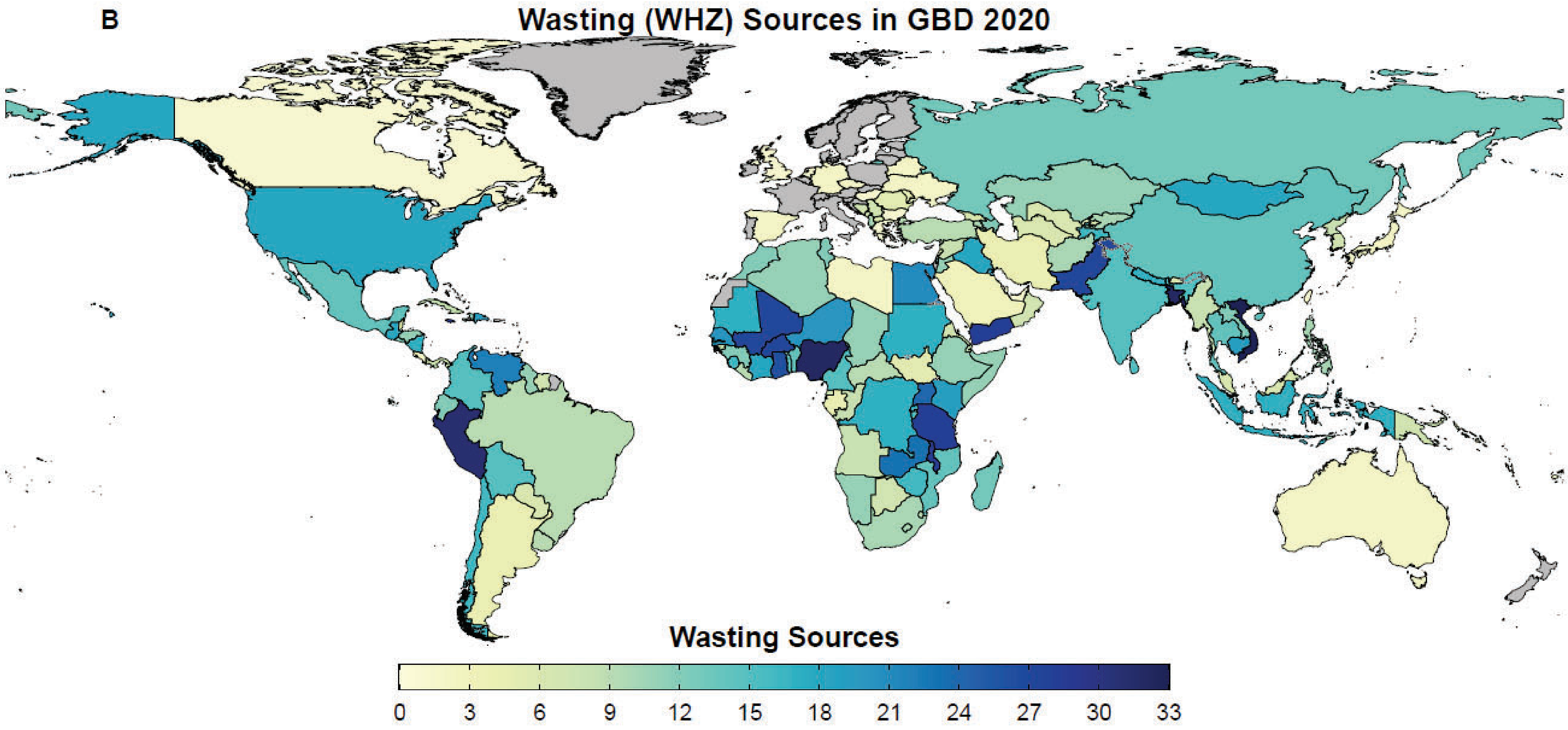
B
Wasting (WHZ) Sources in GBD 2020
Wasting Sources
0
3
6
9
12
15
18
21
24
27
30
33

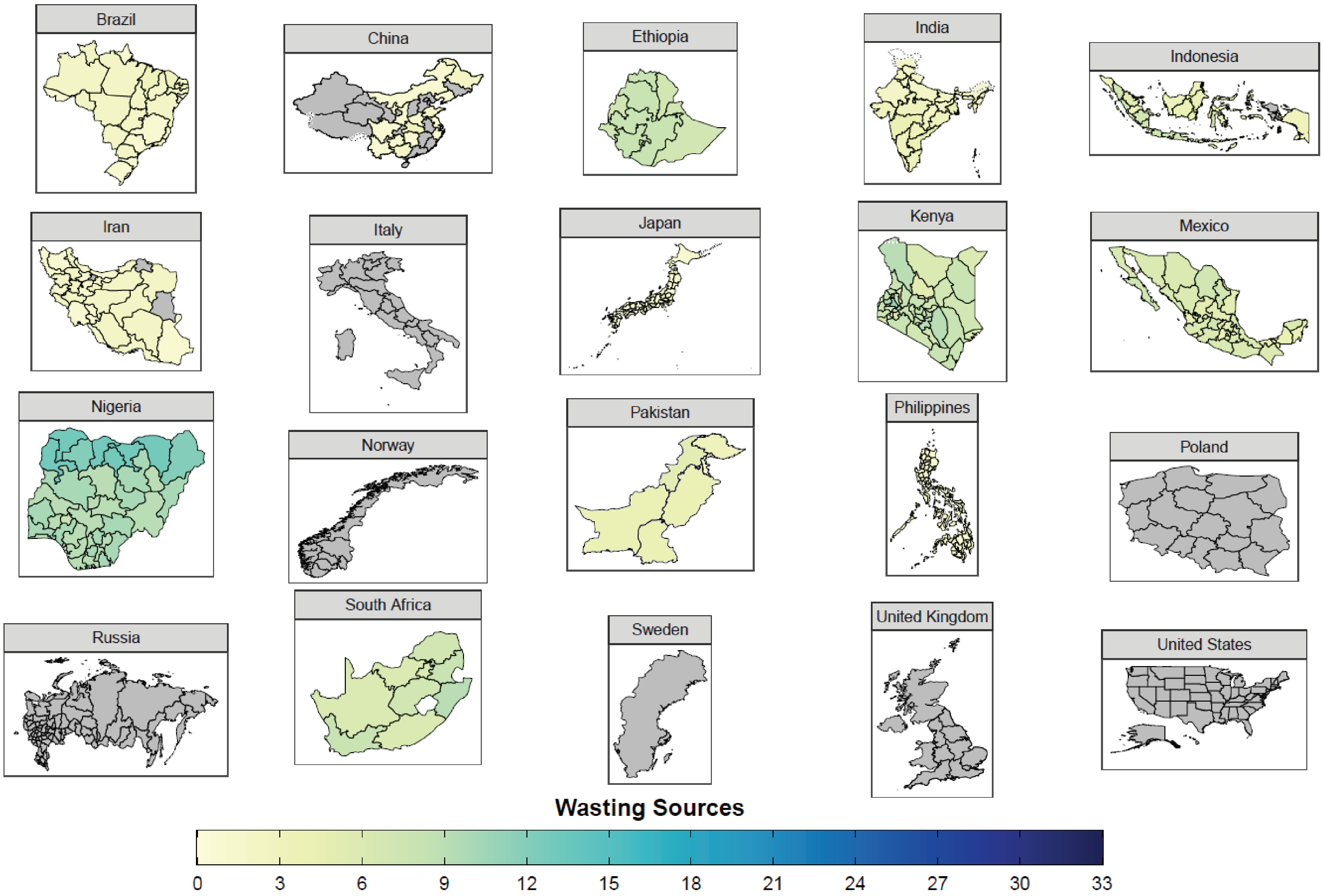
Brazil
China
Ethiopia
India
Indonesia
Iran
Italy
Japan
Kenya
Mexico
Nigeria
Norway
Pakistan
Philippines
Poland
Russia
South Africa
Sweden
United Kingdom
United States
Wasting Sources
0
3
6
9
12
15
18
21
24
27
30
33

C

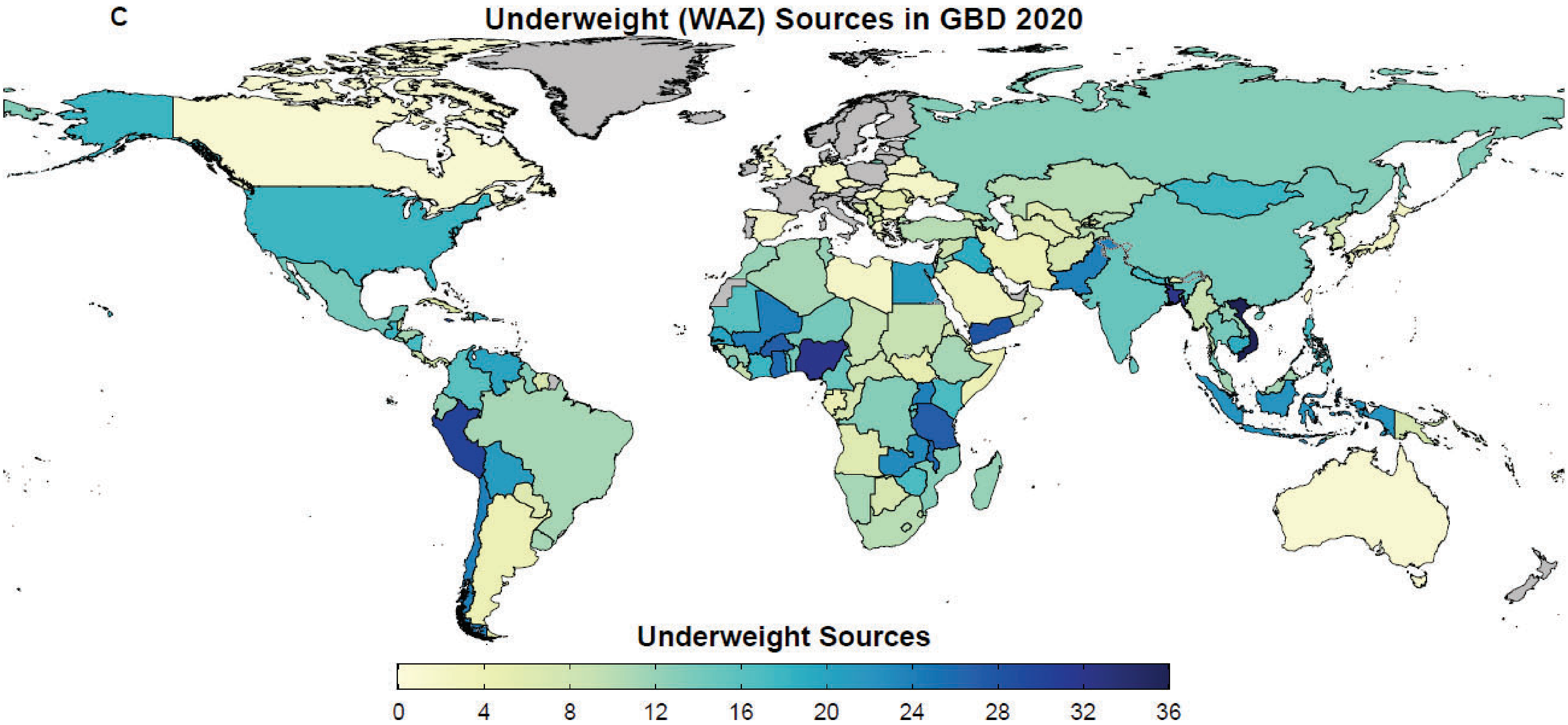
Underweight (WAZ) Sources in GBD 2020
Underweight Sources
0
4
8
12
16
20
24
28
32
36

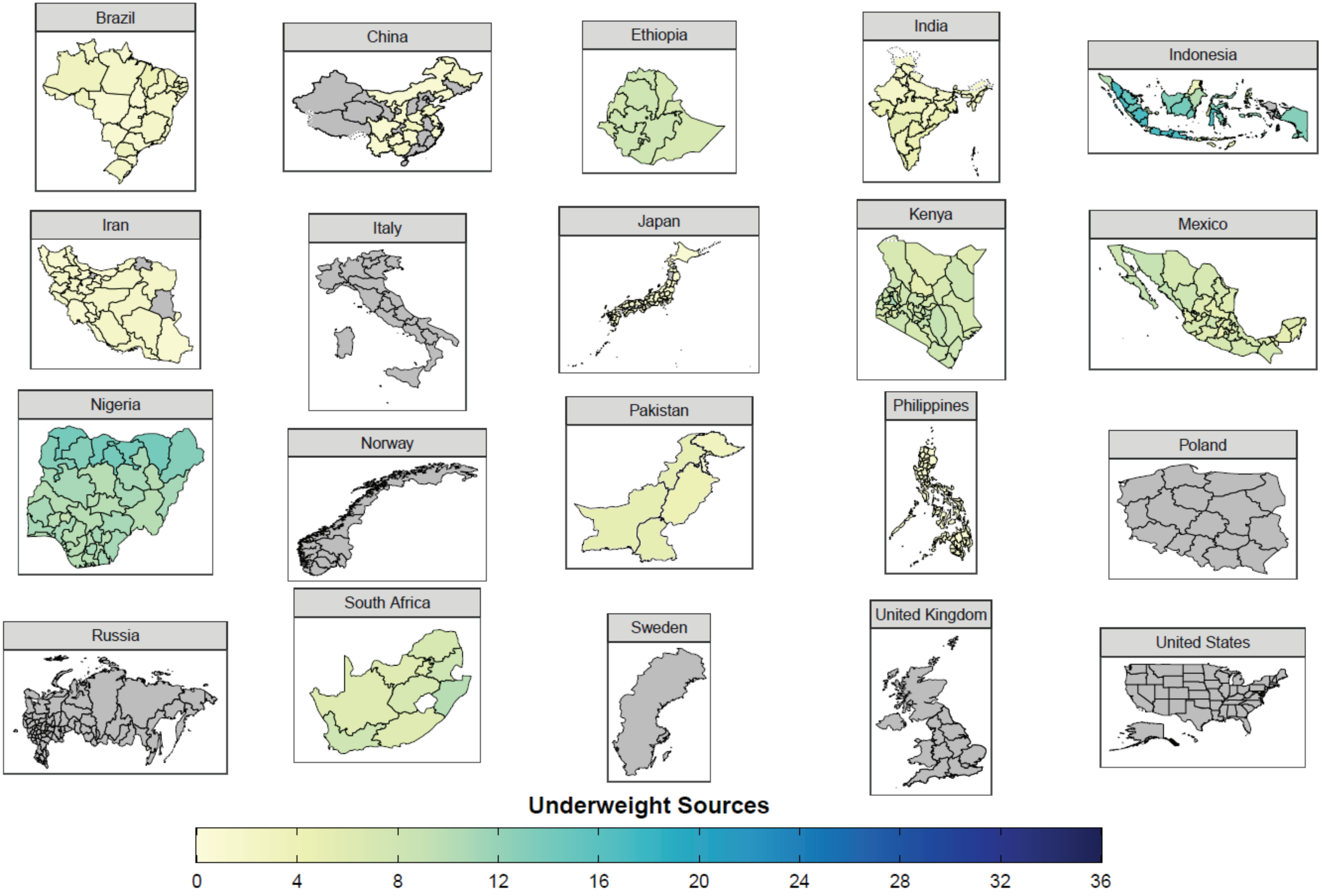
Brazil
China
Ethiopia
India
Indonesia
Iran
Italy
Japan
Kenya
Mexico
Nigeria
Norway
Pakistan
Philippines
Poland
Russia
South Africa
Sweden
United Kingdom
United States
Underweight Sources
0
4
8
12
16
20
24
28
32
36

Fig. S3. Estimated prevalence of overall, severe, and extreme CGF for all locations. Estimated prevalences of overall, severe, and extreme stunting (A–C), wasting (D–F), and underweight (G–I) among children under 5, both sexes, are shown in 1990. Additional maps are shown for 2000 (J–R), and 2010 (S-AA).

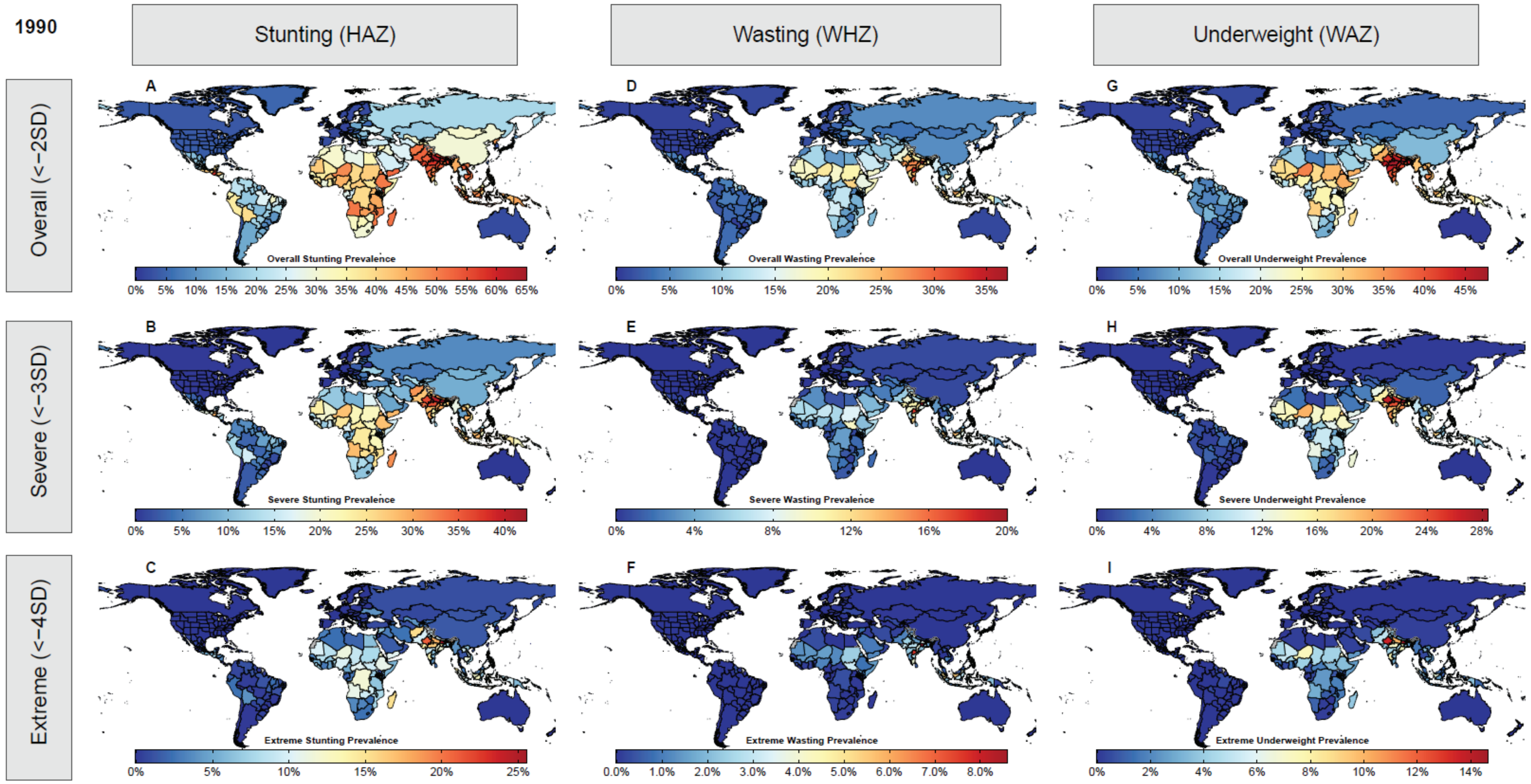

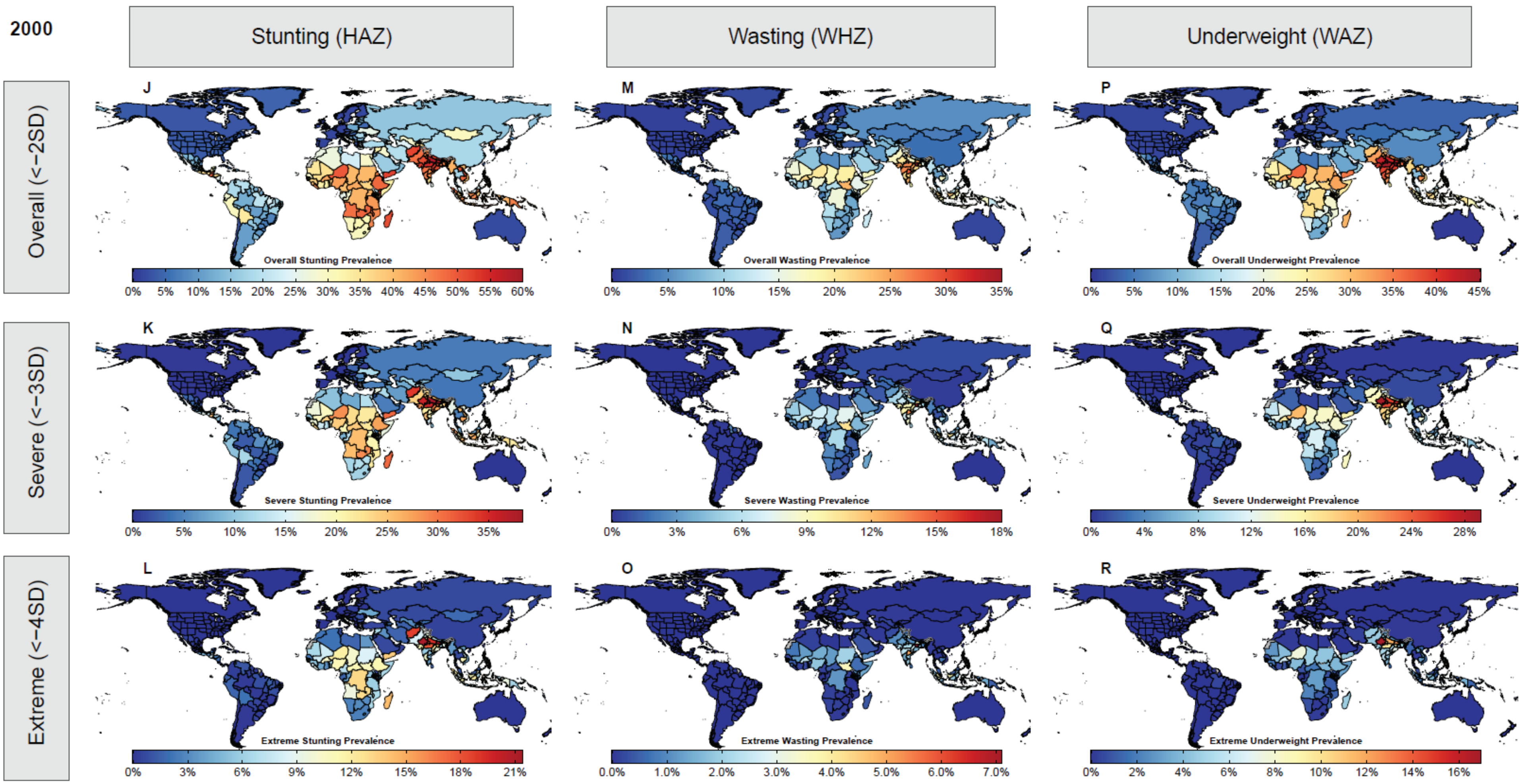
2000
Stunting (HAZ)
Wasting (WHZ)
Underweight (WAZ)
Overall (<−2SD)
Severe (<−3SD)
Extreme (<−4SD)
J
Overall Stunting Prevalence
0% 5% 10% 15% 20% 25% 30% 35% 40% 45% 50% 55% 60%
M
Overall Wasting Prevalence
0% 5% 10% 15% 20% 25% 30% 35%
P
Overall Underweight Prevalence
0% 5% 10% 15% 20% 25% 30% 35% 40% 45%
K
Severe Stunting Prevalence
0% 5% 10% 15% 20% 25% 30% 35%
N
Severe Wasting Prevalence
0% 3% 6% 9% 12% 15% 18%
Q
Severe Underweight Prevalence
0% 4% 8% 12% 16% 20% 24% 28%
L
Extreme Stunting Prevalence
0% 3% 6% 9% 12% 15% 18% 21%
O
Extreme Wasting Prevalence
0.0% 1.0% 2.0% 3.0% 4.0% 5.0% 6.0% 7.0%
R
Extreme Underweight Prevalence
0% 2% 4% 6% 8% 10% 12% 14% 16%

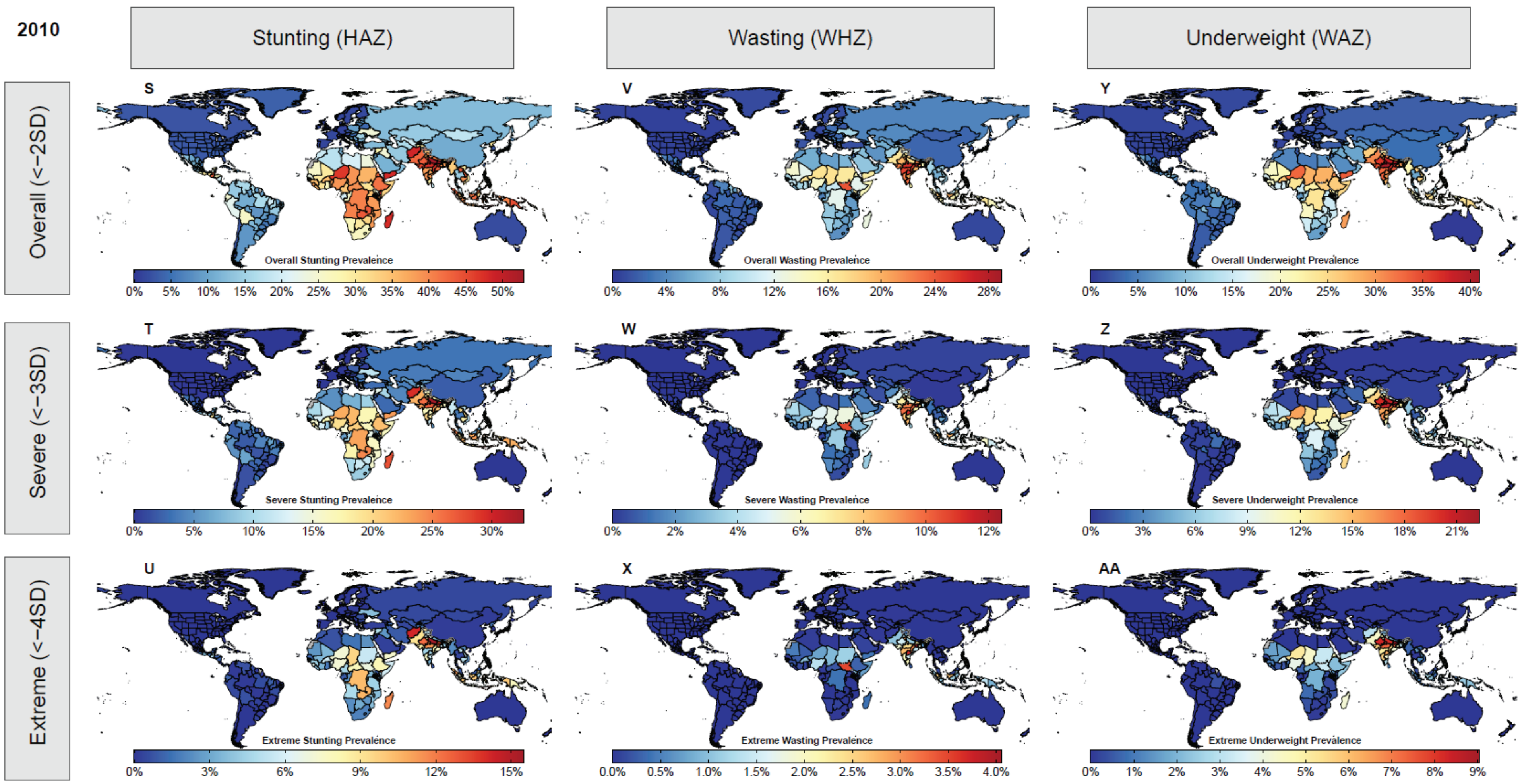
2010
Stunting (HAZ)
Wasting (WHZ)
Underweight (WAZ)
Overall (<−2SD)
Severe (<−3SD)
Extreme (<−4SD)
S
Overall Stunting Prevalence
0% 5% 10% 15% 20% 25% 30% 35% 40% 45% 50%
V
Overall Wasting Prevalence
0% 4% 8% 12% 16% 20% 24% 28%
Y
Overall Underweight Prevalence
0% 5% 10% 15% 20% 25% 30% 35% 40%
T
Severe Stunting Prevalence
0% 5% 10% 15% 20% 25% 30%
W
Severe Wasting Prevalence
0% 2% 4% 6% 8% 10% 12%
Z
Severe Underweight Prevalence
0% 3% 6% 9% 12% 15% 18% 21%
U
Extreme Stunting Prevalence
0% 3% 6% 9% 12% 15%
X
Extreme Wasting Prevalence
0.0% 0.5% 1.0% 1.5% 2.0% 2.5% 3.0% 3.5% 4.0%
AA
Extreme Underweight Prevalence
0% 1% 2% 3% 4% 5% 6% 7% 8% 9%

Fig. S4. Relative changes in stunting, wasting, and underweight prevalences since 1990, by GBD super-region. Relative changes in stunting, wasting, and underweight prevalences since 1990.

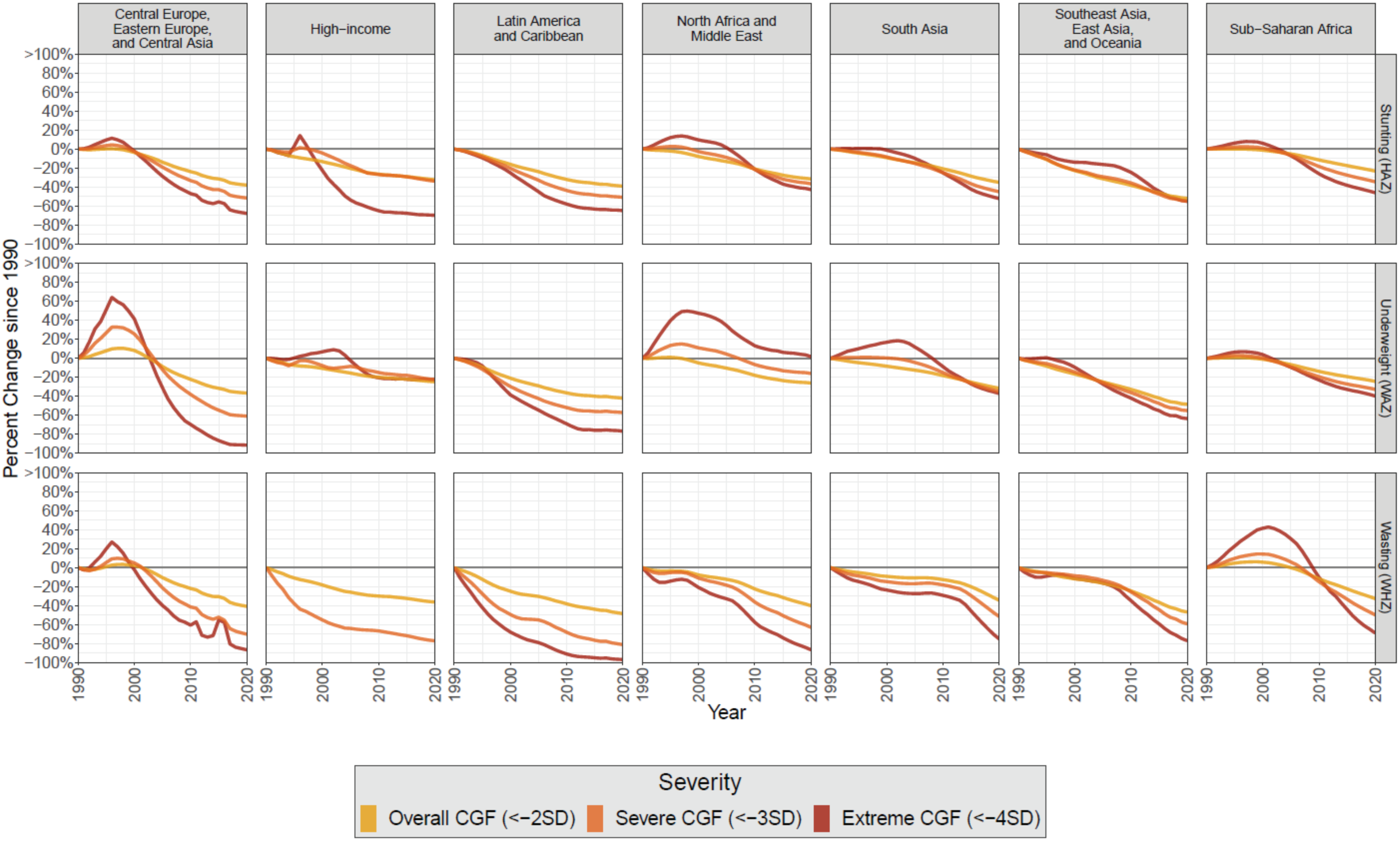

Fig. S5. Locations assigned to each region and super-region in GBD.

Locations assigned to each region (A) and super-region (B) in the GBD location hierarchy.

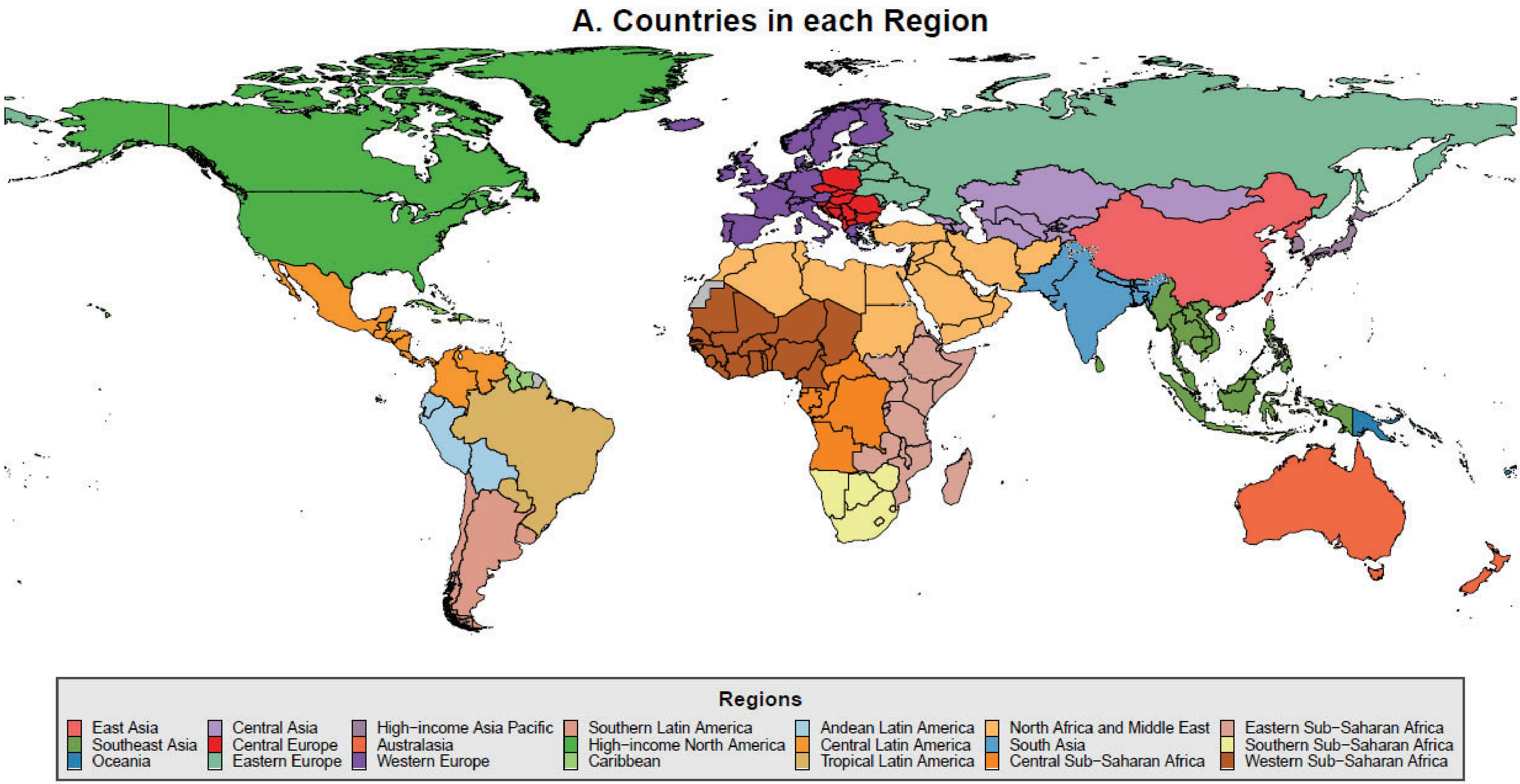

B. Countries in each Super Region

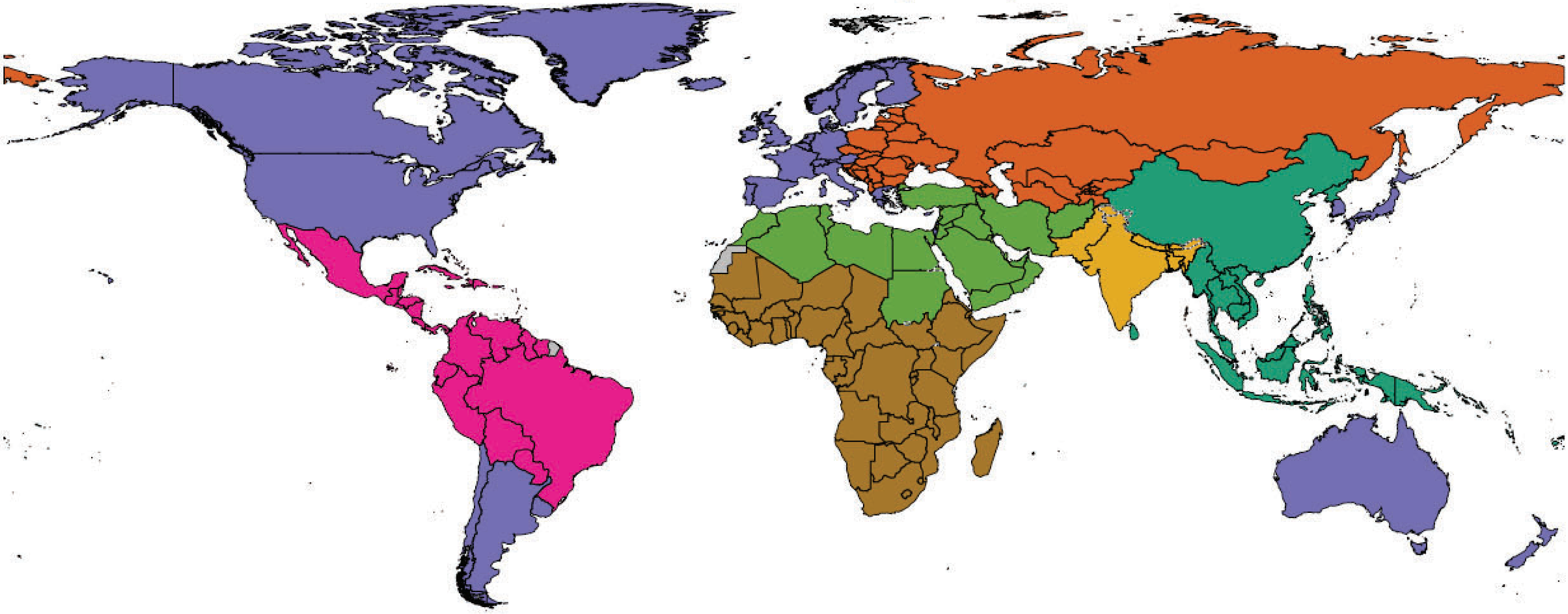

Super Regions

- Southeast Asia, East Asia, and Oceania
- Central Europe, Eastern Europe, and Central Asia
- High−income
- Latin America and Caribbean
- North Africa and Middle East
- South Asia
- Sub−Saharan Africa

Fig. S6. Epidemiological transition analysis of severe and extreme CGF by region. The expected values of severe stunting, wasting, and underweight based on UHC index are represented by the black lines in (A), (B), and (C). The expected values of extreme stunting, wasting, and underweight are shown in (D), (E), and (F). Estimated values are shown for each region, and colored by super region. Points are shown every five years from 1990−2020.

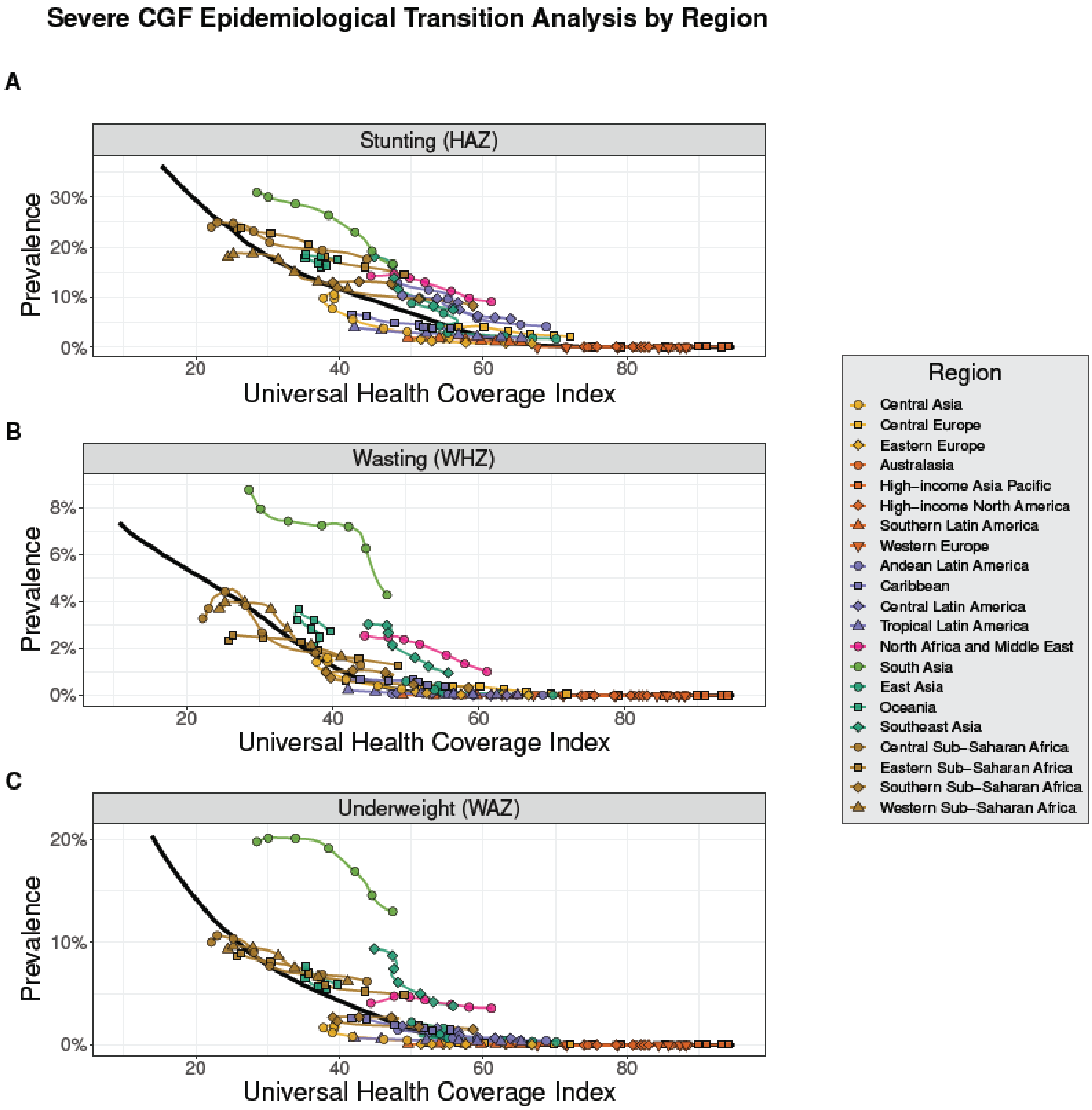

Extreme CGF Epidemiological Transition Analysis by Region

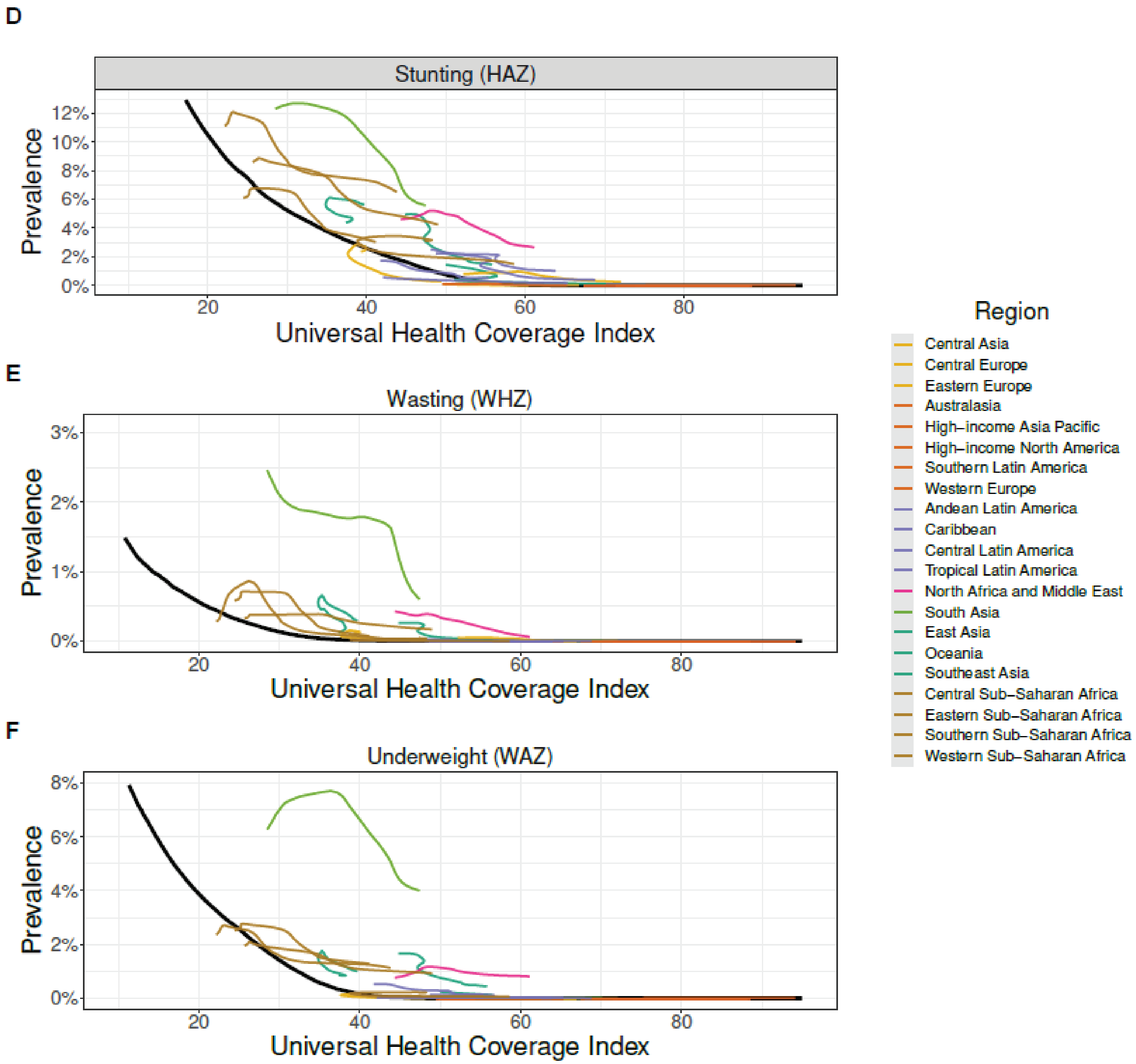

Fig. S7. Estimated values of all forms and severities of CGF compared to expected values based on UHC index for all locations within each region. Performance of individual countries and territories are shown for the following GBD regions: Central Asia (A), Central Europe (B), Eastern Europe (C), Australasia (D), High-income Asia Pacific (E), High-income North America (F), Southern Latin America (G), Western Europe (H), Andean Latin America (I), Caribbean (J), Central Latin America (K), Tropical Latin America (L), North Africa and Middle East (M), South Asia (N), East Asia (O), Oceania (P), Southeast Asia (Q), Central Sub-Saharan Africa (R), Eastern Sub-Saharan Africa (S), Southern Sub-Saharan Africa (T), and Western Sub-Saharan Africa (U). Points are shown for every five years from 1990–2020 with the grey line and grey squares representing the estimate for the aggregated region.

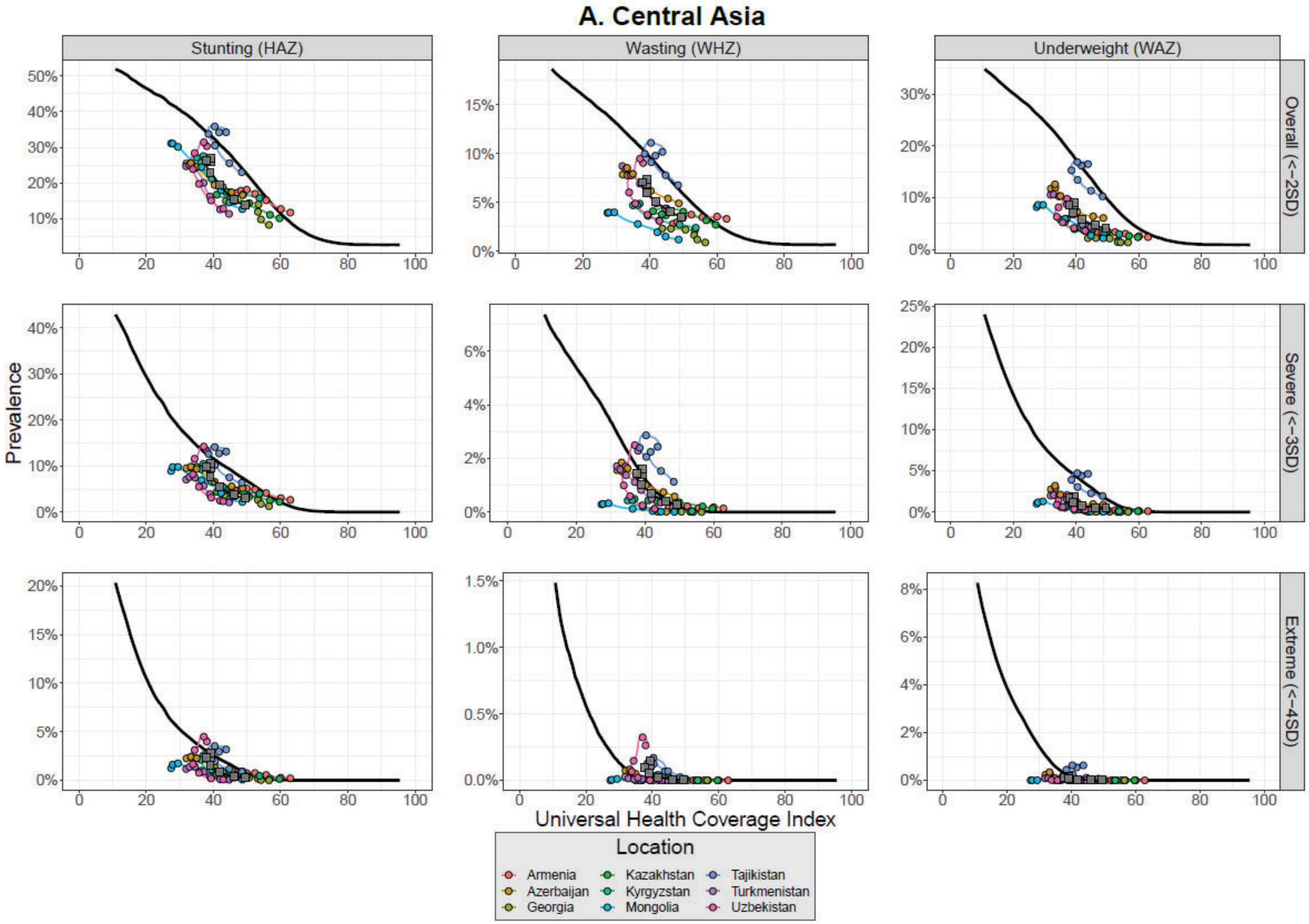

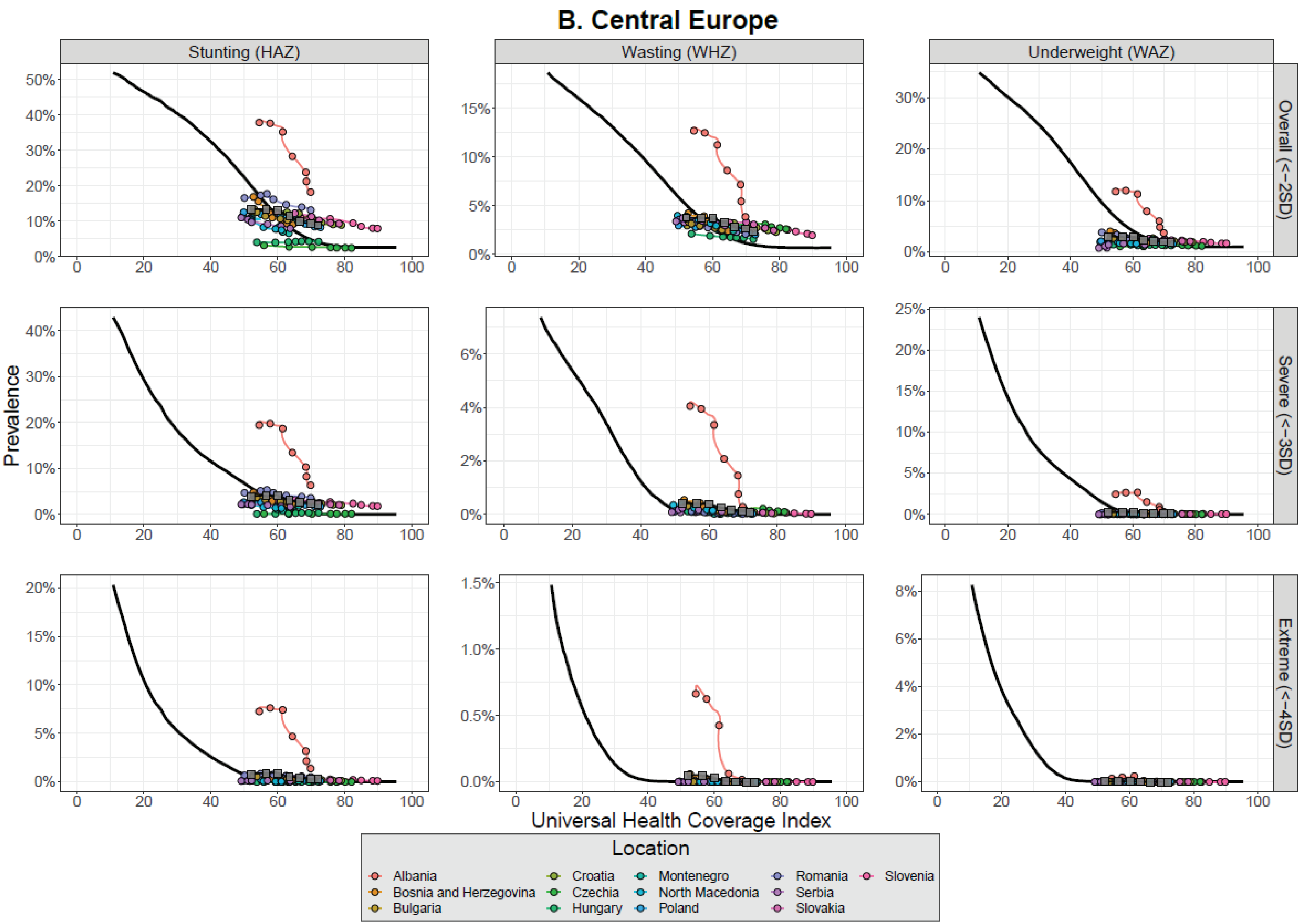

B. Central Europe
Stunting (HAZ)
Wasting (WHZ)
Underweight (WAZ)
Overall (<−2SD)
Severe (<−3SD)
Extreme (<−4SD)
Prevalence
Universal Health Coverage Index
Location
Albania
Bosnia and Herzegovina
Bulgaria
Croatia
Czechia
Hungary
Montenegro
North Macedonia
Poland
Romania
Serbia
Slovakia
Slovenia

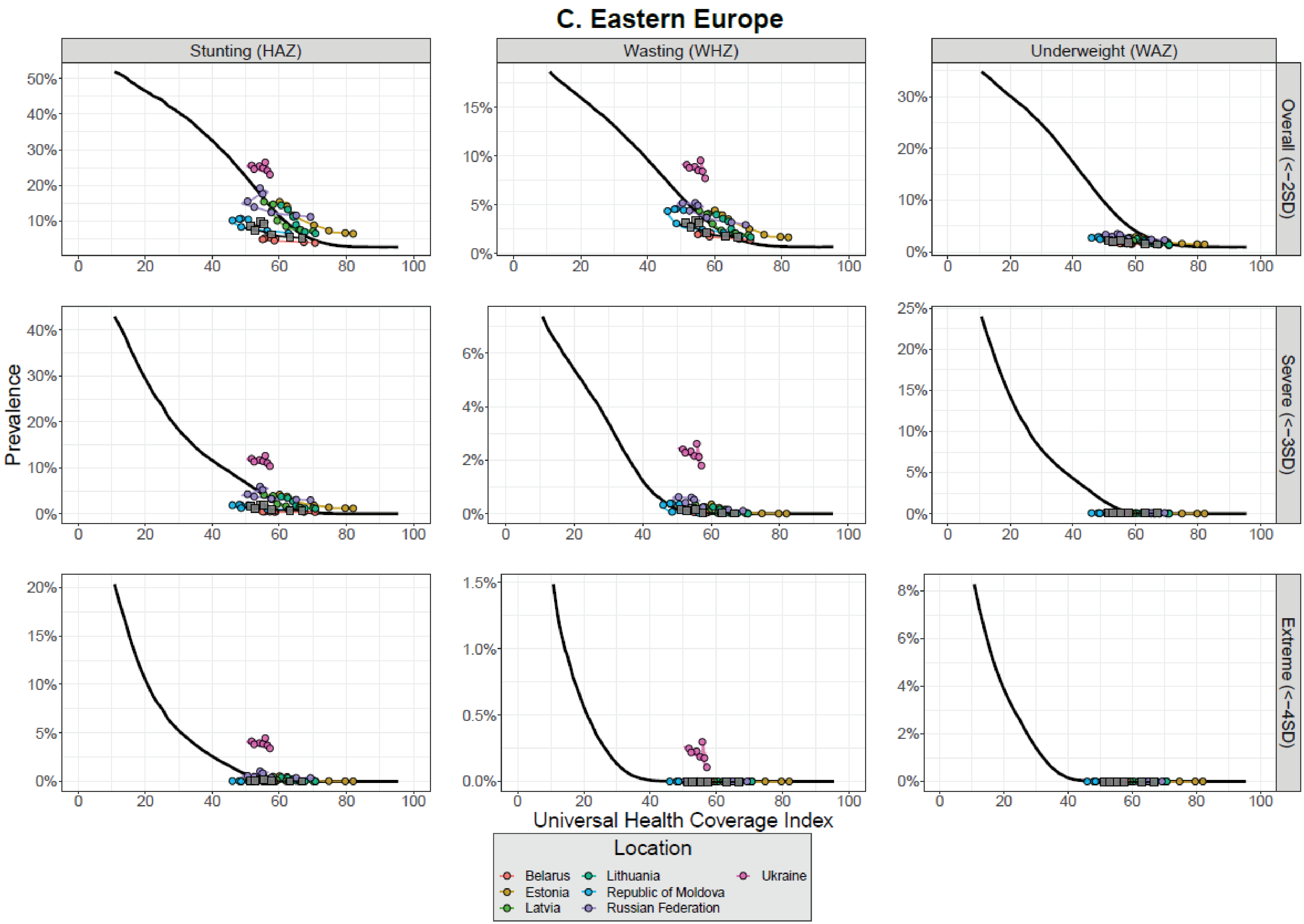
C. Eastern Europe
Stunting (HAZ)
Wasting (WHZ)
Underweight (WAZ)
Overall (<−2SD)
Severe (<−3SD)
Extreme (<−4SD)
Prevalence
Universal Health Coverage Index
Location
Belarus
Estonia
Latvia
Lithuania
Republic of Moldova
Russian Federation
Ukraine

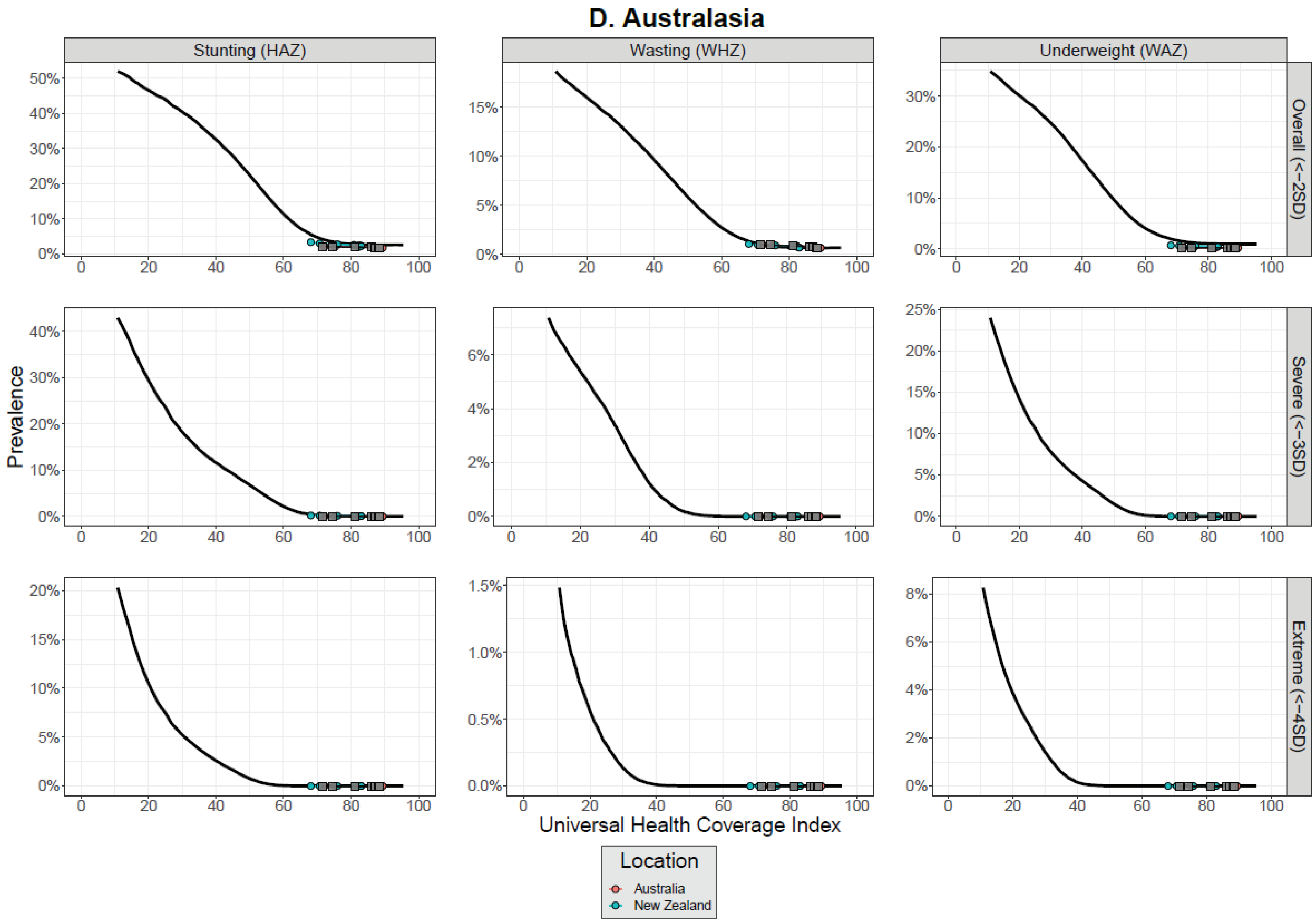

D. Australasia
Stunting (HAZ)
Wasting (WHZ)
Underweight (WAZ)
Overall (<−2SD)
Severe (<−3SD)
Extreme (<−4SD)
Prevalence
Universal Health Coverage Index
Location
Australia
New Zealand

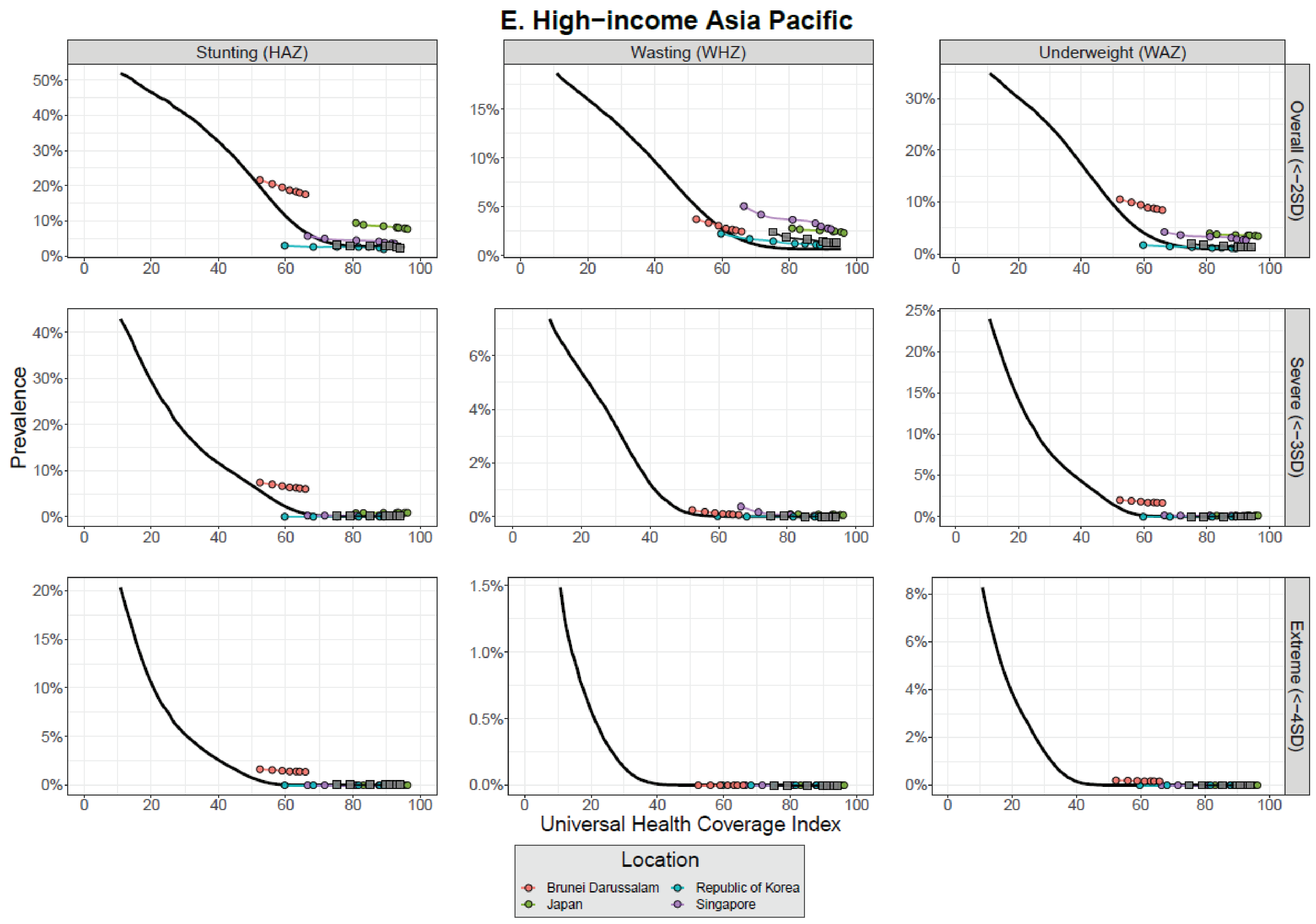
E. High−income Asia Pacific
Stunting (HAZ)
Wasting (WHZ)
Underweight (WAZ)
Overall (<−2SD)
Severe (<−3SD)
Extreme (<−4SD)
Prevalence
Universal Health Coverage Index
Location
Brunei Darussalam
Japan
Republic of Korea
Singapore

F. High−income North America

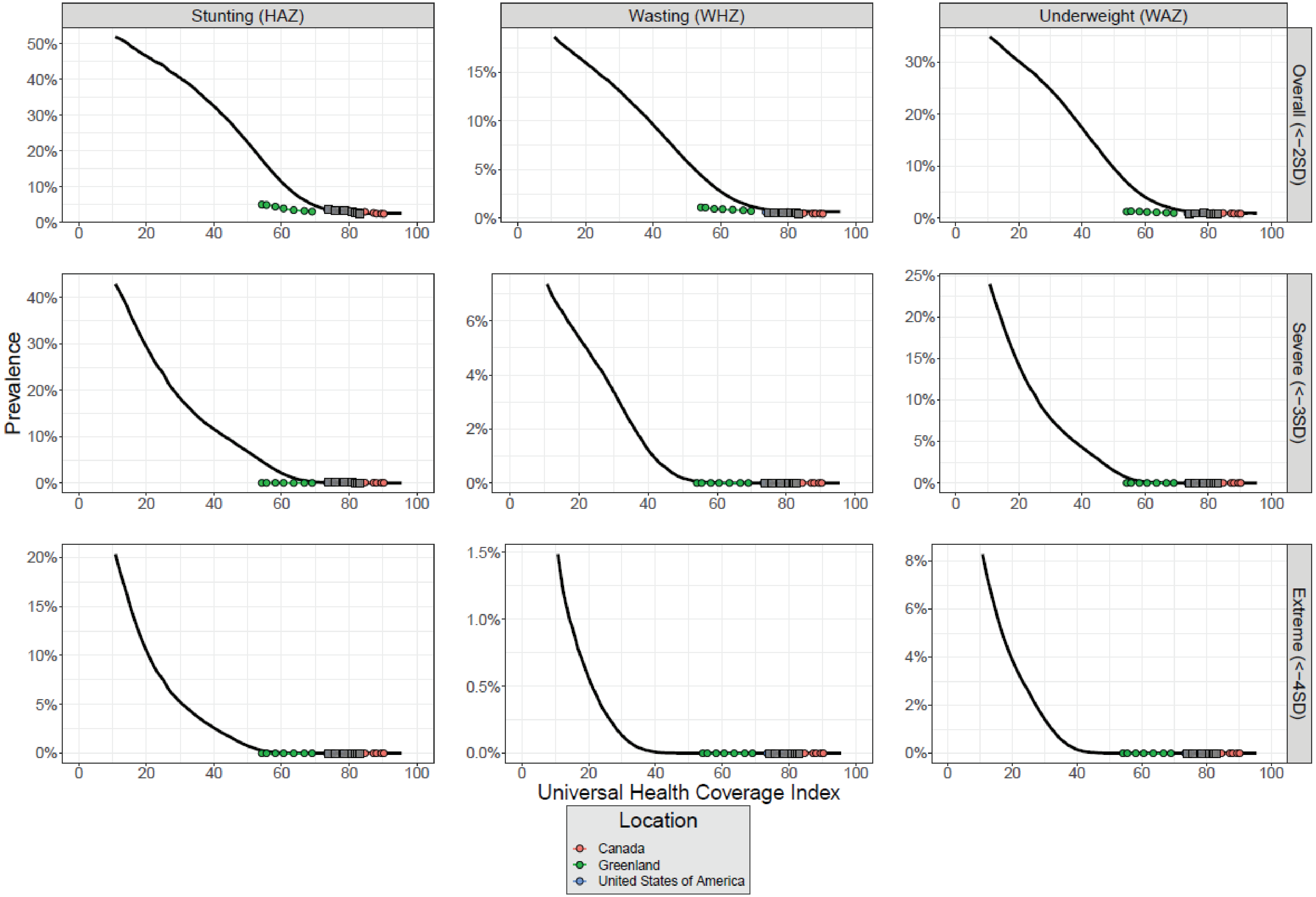

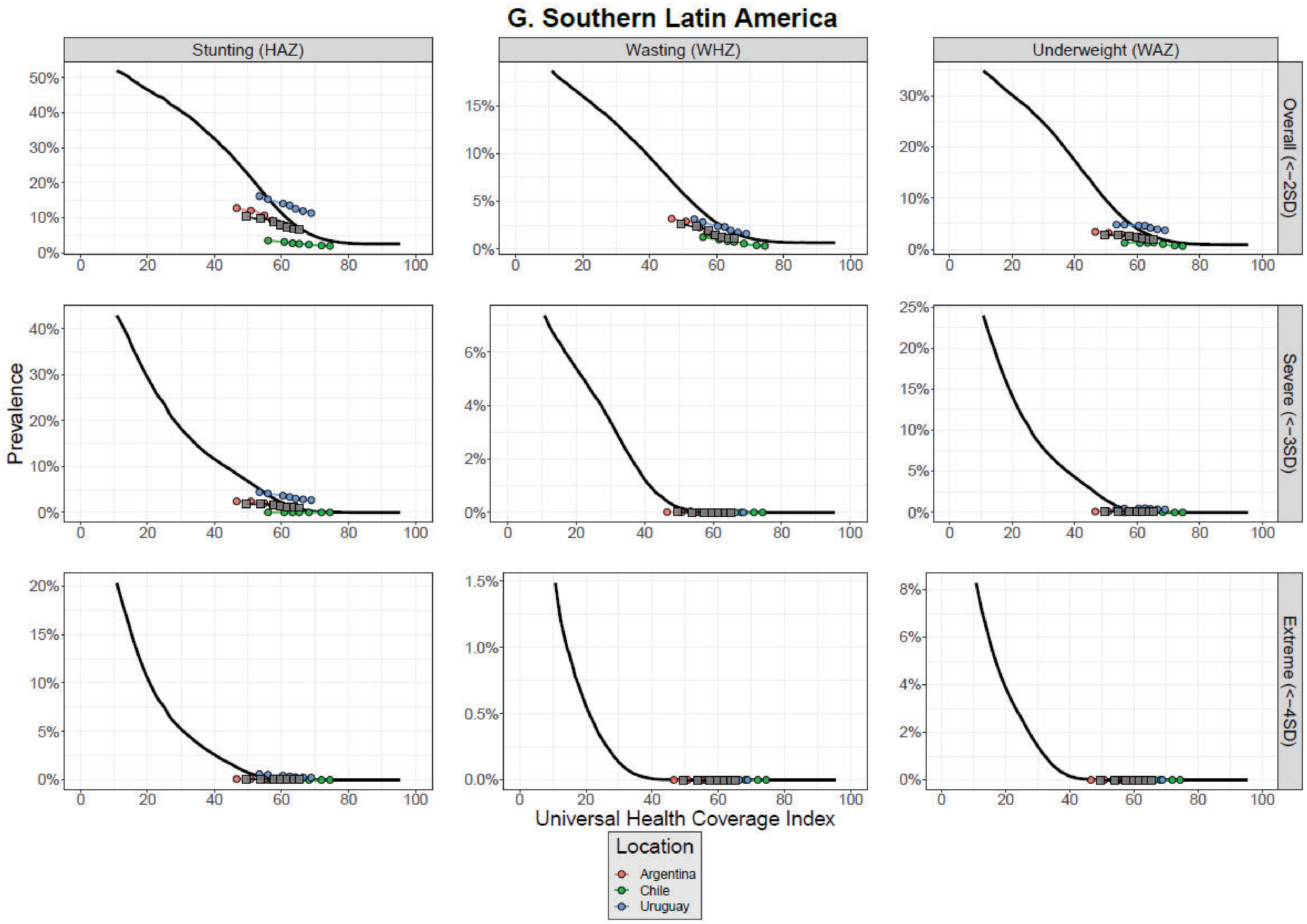
G. Southern Latin America
Stunting (HAZ)
Wasting (WHZ)
Underweight (WAZ)
Overall (<−2SD)
Severe (<−3SD)
Extreme (<−4SD)
Prevalence
Universal Health Coverage Index
Location
Argentina
Chile
Uruguay

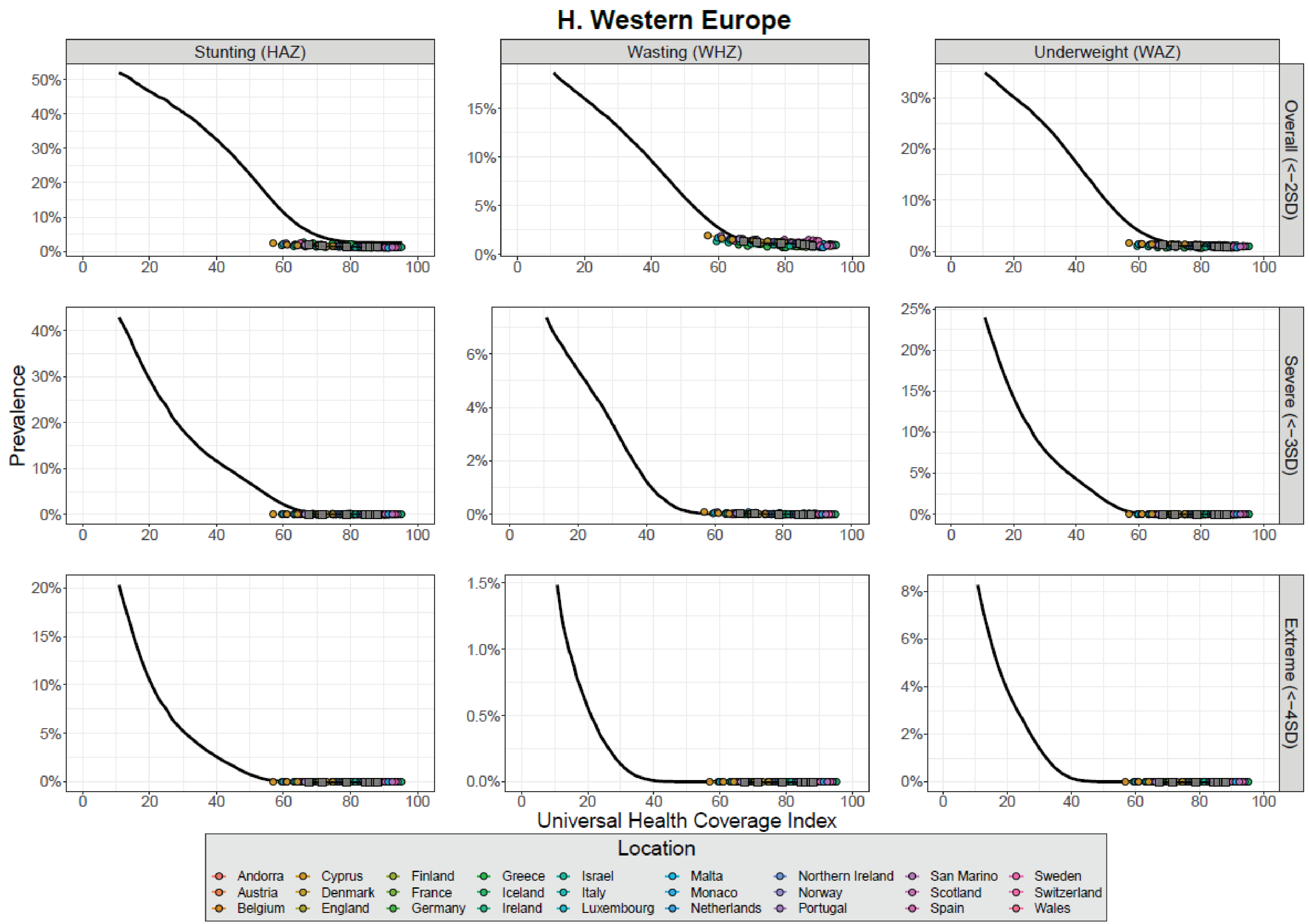
H. Western Europe
Stunting (HAZ)
Wasting (WHZ)
Underweight (WAZ)
Overall (<−2SD)
Severe (<−3SD)
Extreme (<−4SD)
Prevalence
Universal Health Coverage Index
Location
Andorra
Austria
Belgium
Cyprus
Denmark
England
Finland
France
Germany
Greece
Iceland
Ireland
Israel
Italy
Luxembourg
Malta
Monaco
Netherlands
Northern Ireland
Norway
Portugal
San Marino
Scotland
Spain
Sweden
Switzerland
Wales

I. Andean Latin America

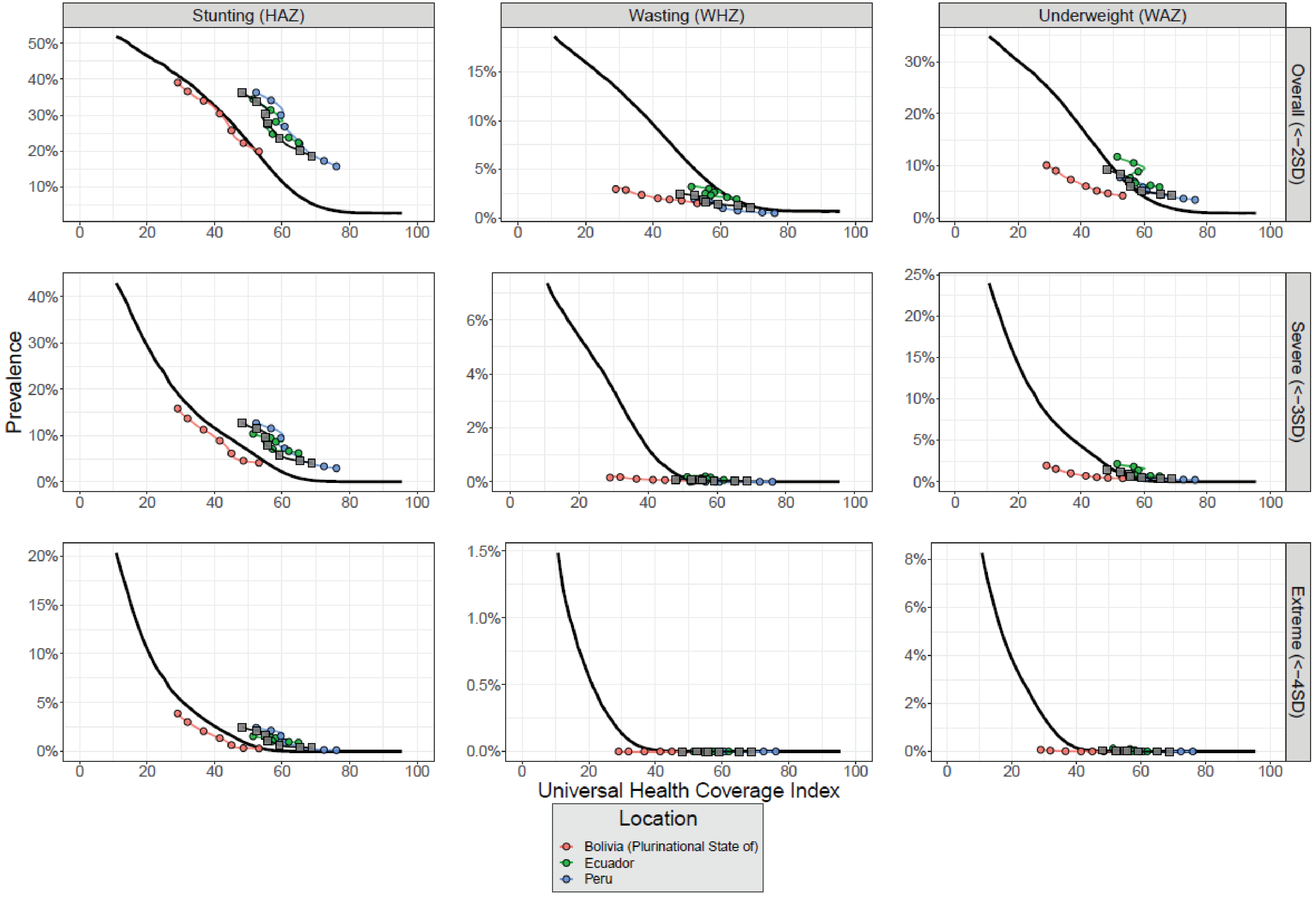

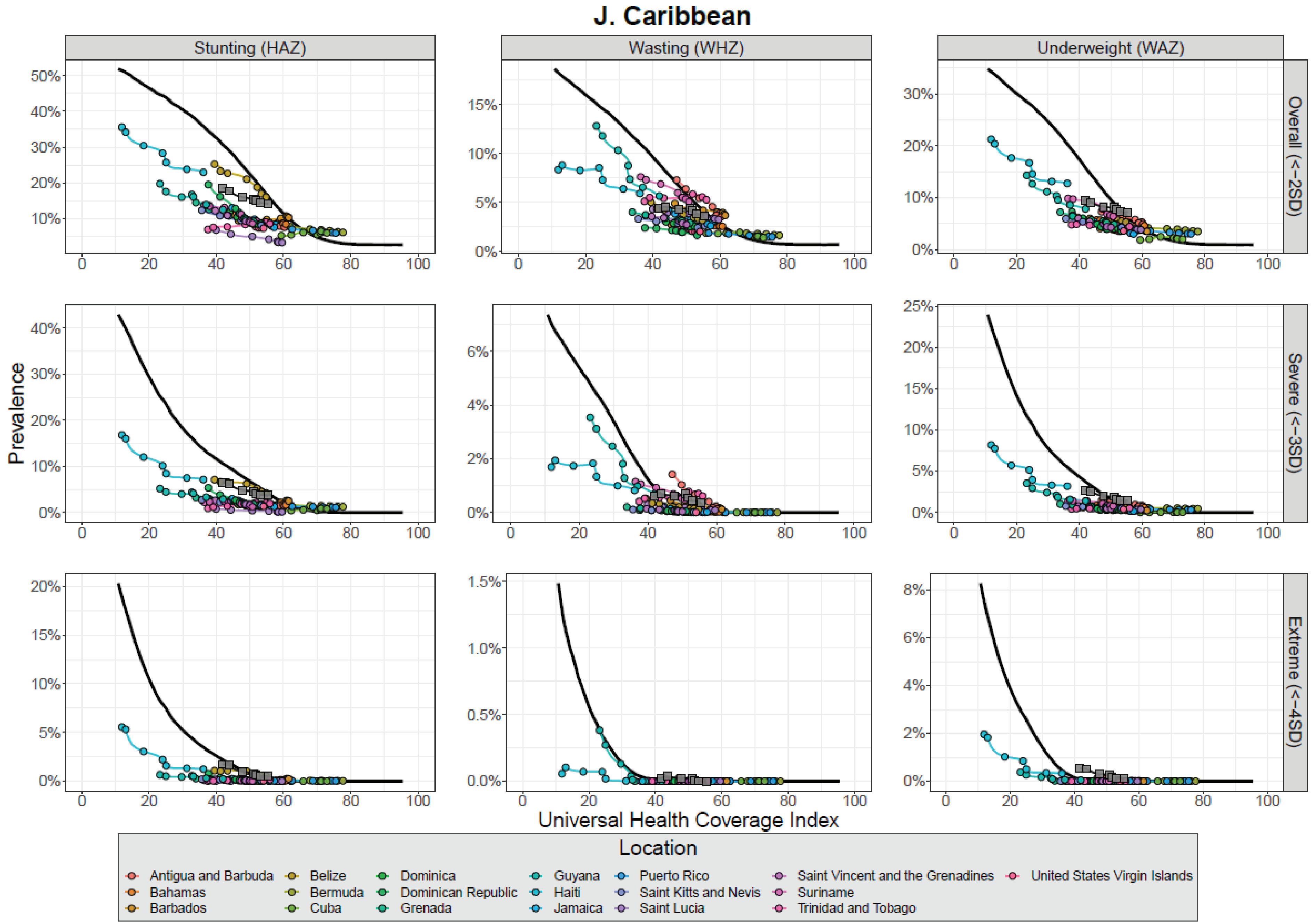
J. Caribbean
Stunting (HAZ)
Wasting (WHZ)
Underweight (WAZ)
Overall (<-2SD)
Severe (<-3SD)
Extreme (<-4SD)
Prevalence
Universal Health Coverage Index
Location
Antigua and Barbuda
Bahamas
Barbados
Belize
Bermuda
Cuba
Dominica
Dominican Republic
Grenada
Guyana
Haiti
Jamaica
Puerto Rico
Saint Kitts and Nevis
Saint Lucia
Saint Vincent and the Grenadines
Suriname
Trinidad and Tobago
United States Virgin Islands

K. Central Latin America

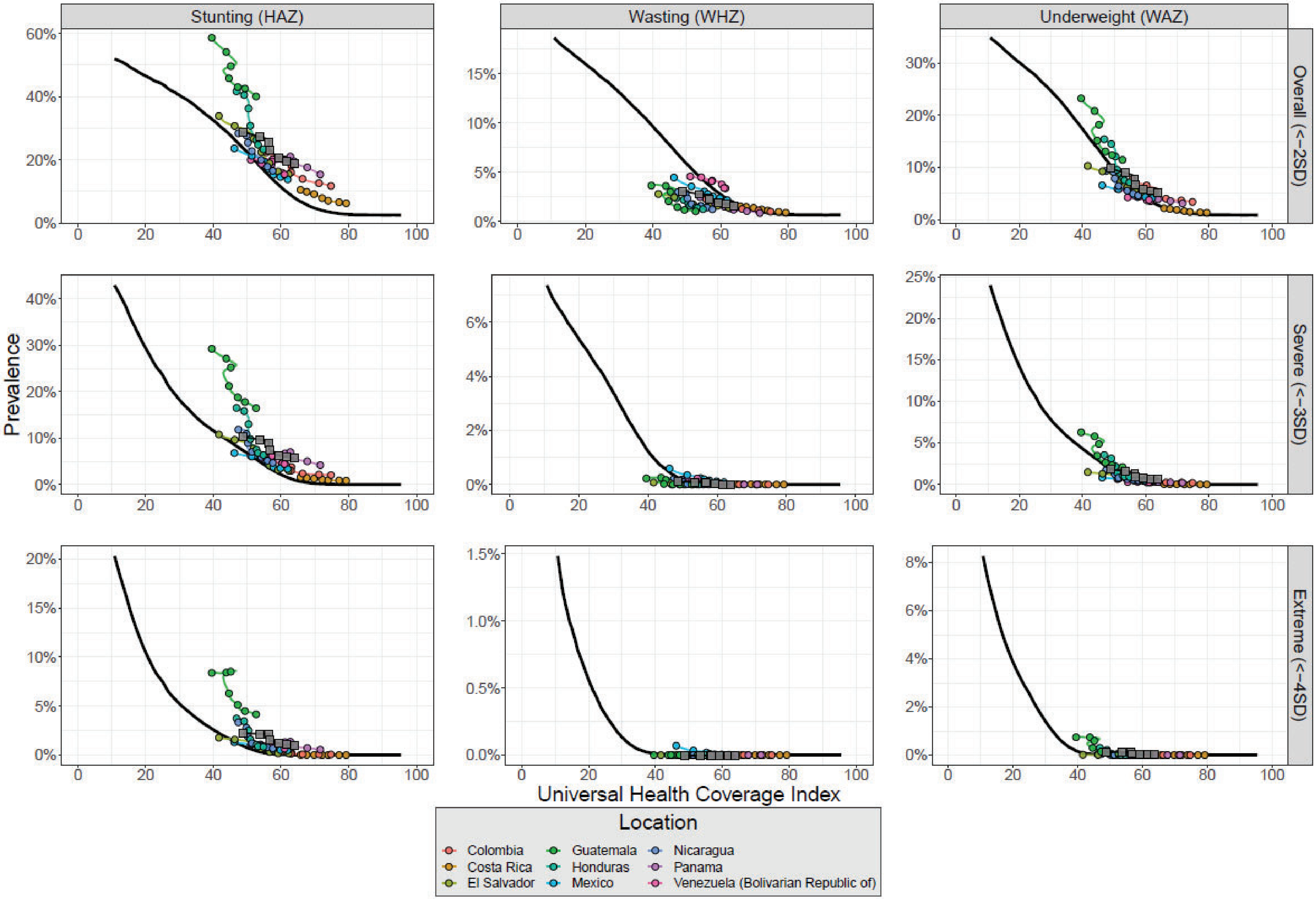

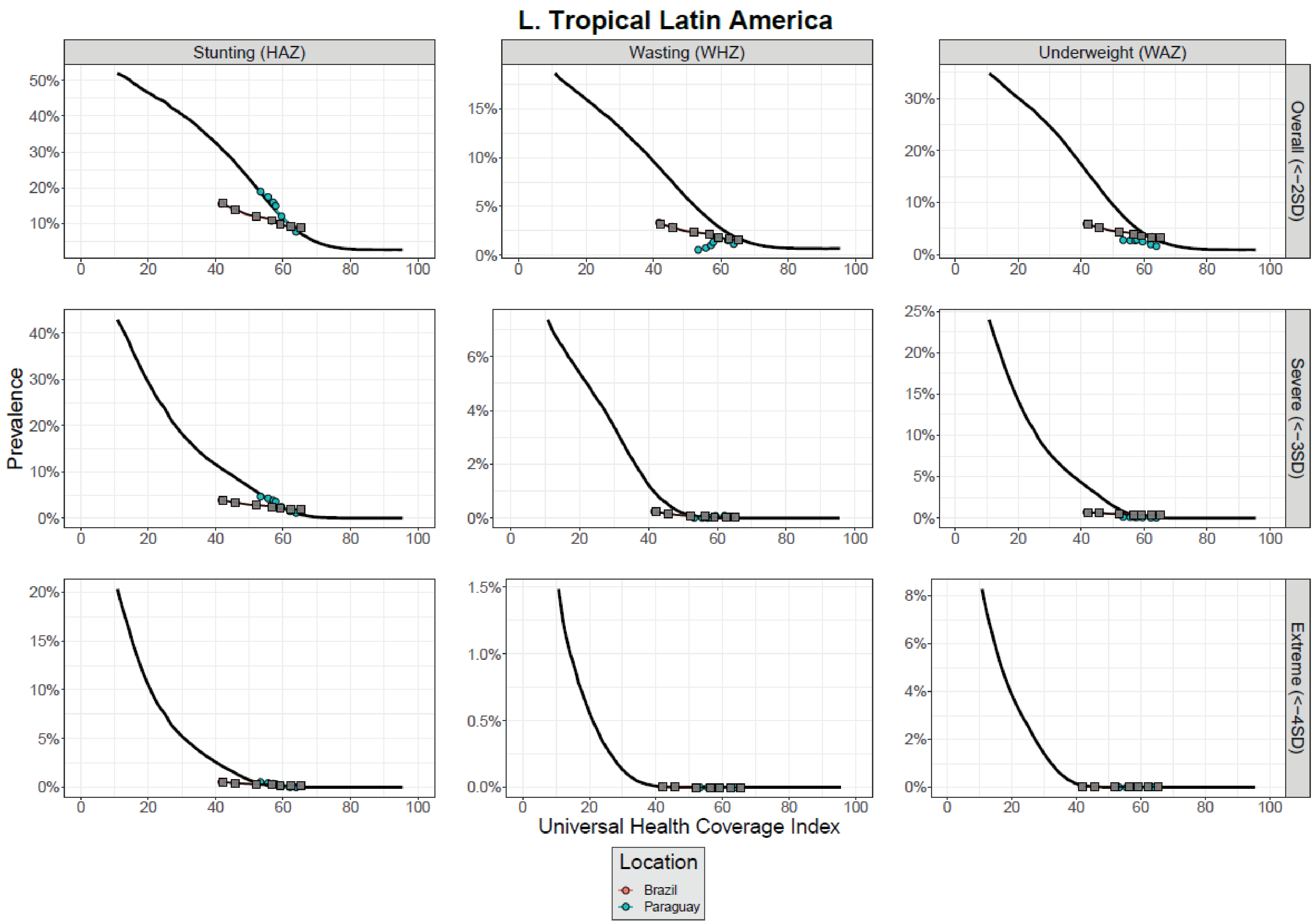

L. Tropical Latin America
Stunting (HAZ)
Wasting (WHZ)
Underweight (WAZ)
Overall (<−2SD)
Severe (<−3SD)
Extreme (<−4SD)
Prevalence
Universal Health Coverage Index
Location
Brazil
Paraguay

M. North Africa and Middle East

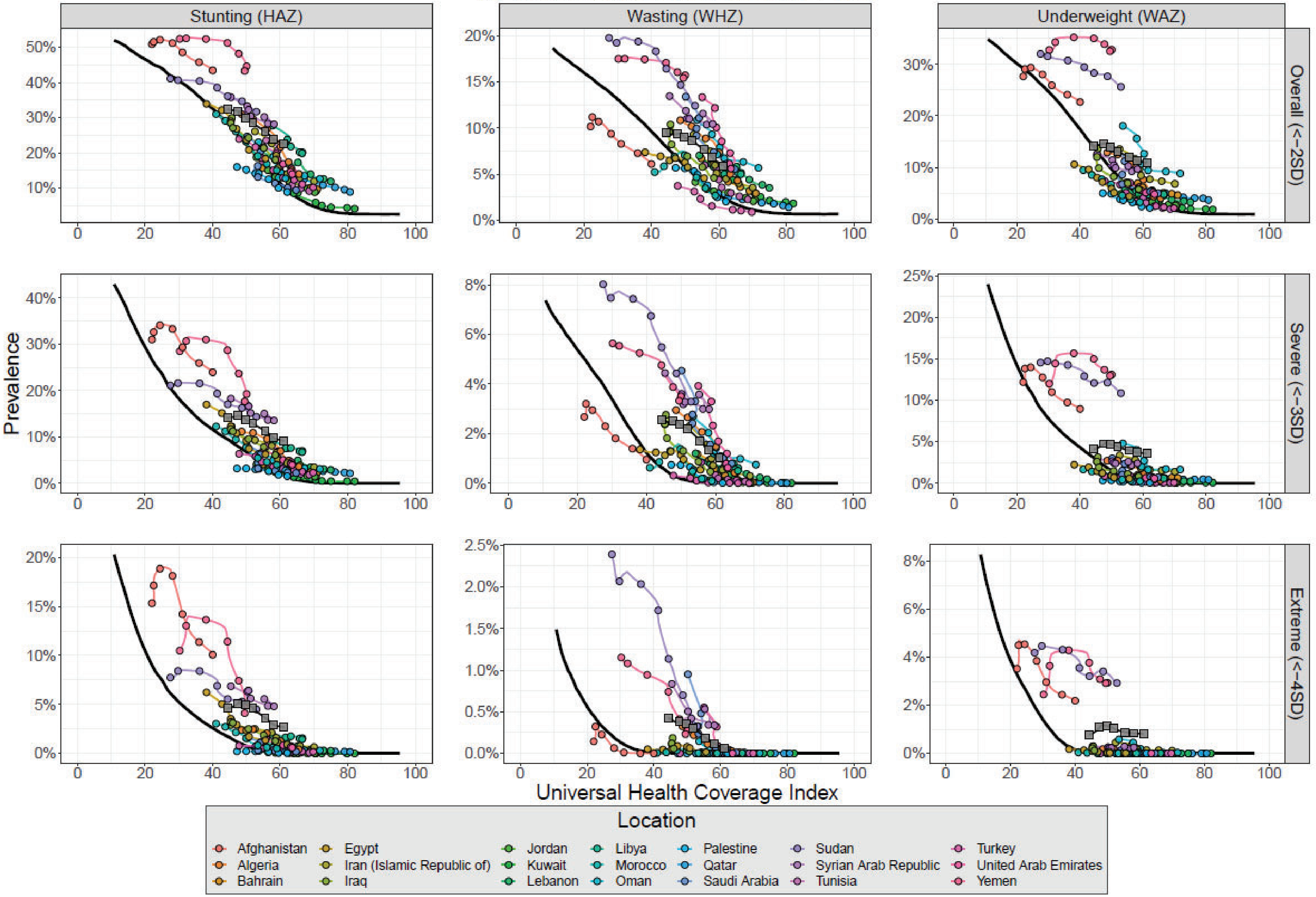

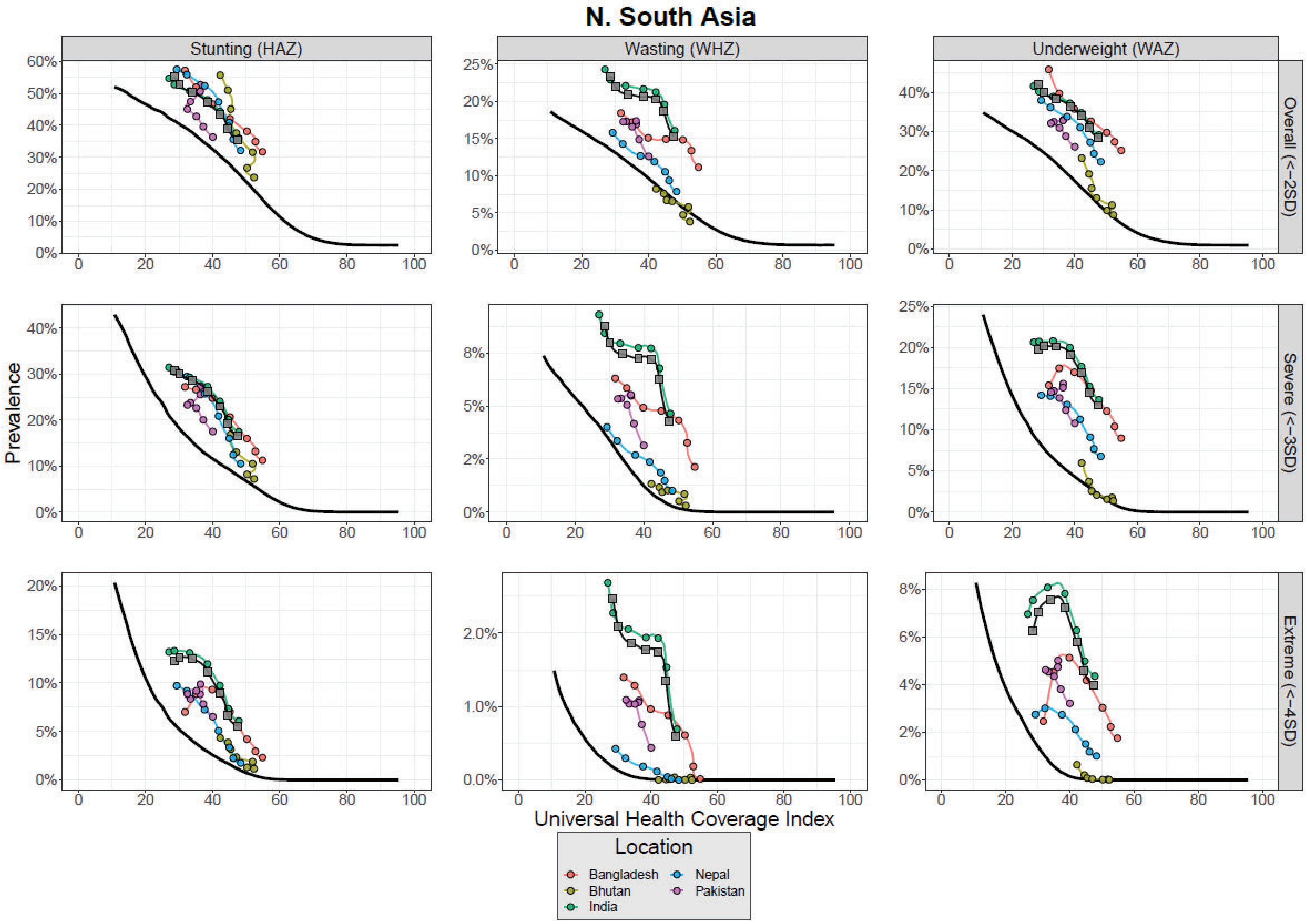

N. South Asia
Stunting (HAZ)
Wasting (WHZ)
Underweight (WAZ)
Overall (<−2SD)
Severe (<−3SD)
Extreme (<−4SD)
Prevalence
Universal Health Coverage Index
Location
Bangladesh
Bhutan
India
Nepal
Pakistan

O. East Asia

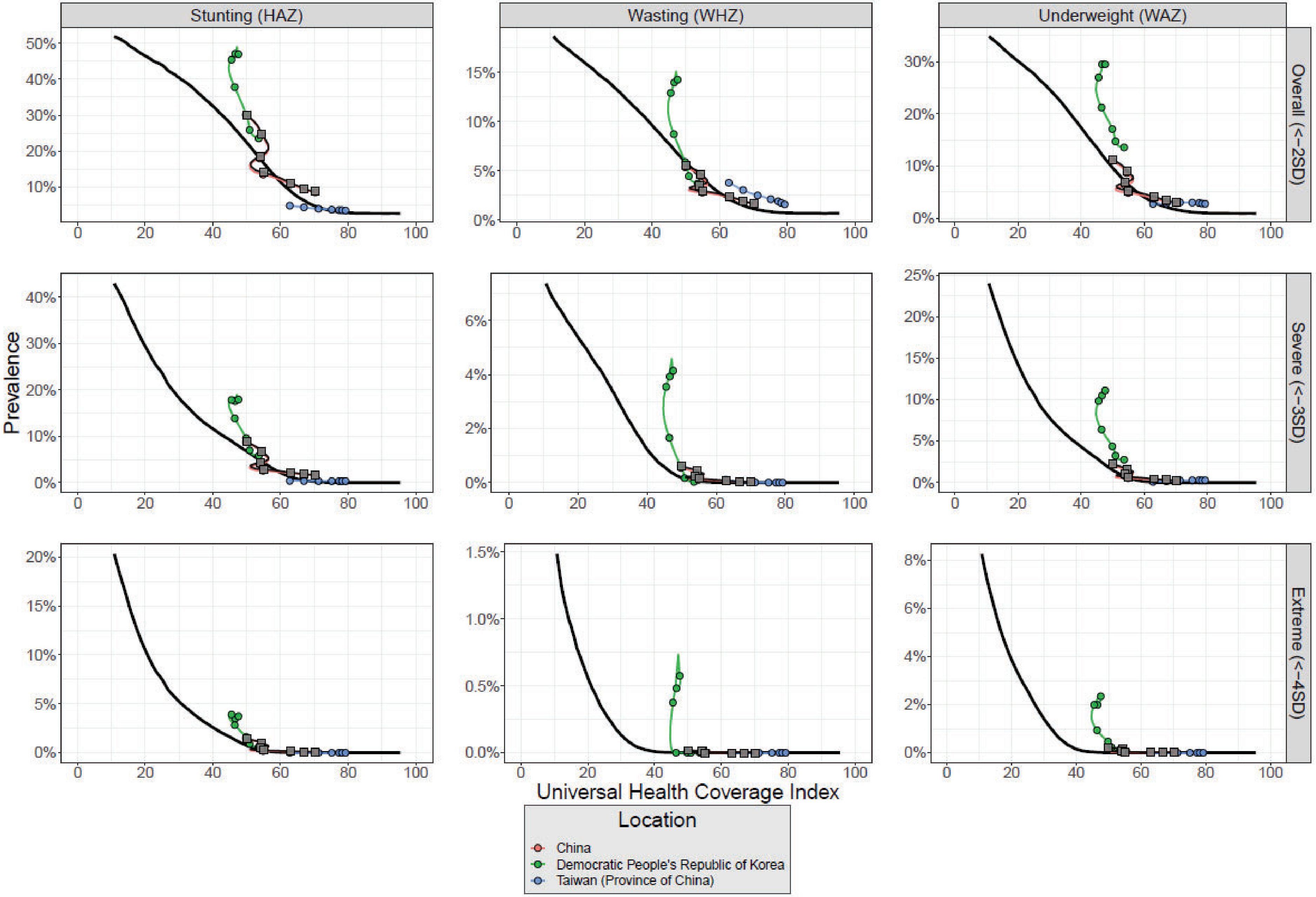

Stunting (HAZ)
Wasting (WHZ)
Underweight (WAZ)
Overall (<−2SD)
Severe (<−3SD)
Extreme (<−4SD)
Prevalence
Universal Health Coverage Index
Location
China
Democratic People's Republic of Korea
Taiwan (Province of China)

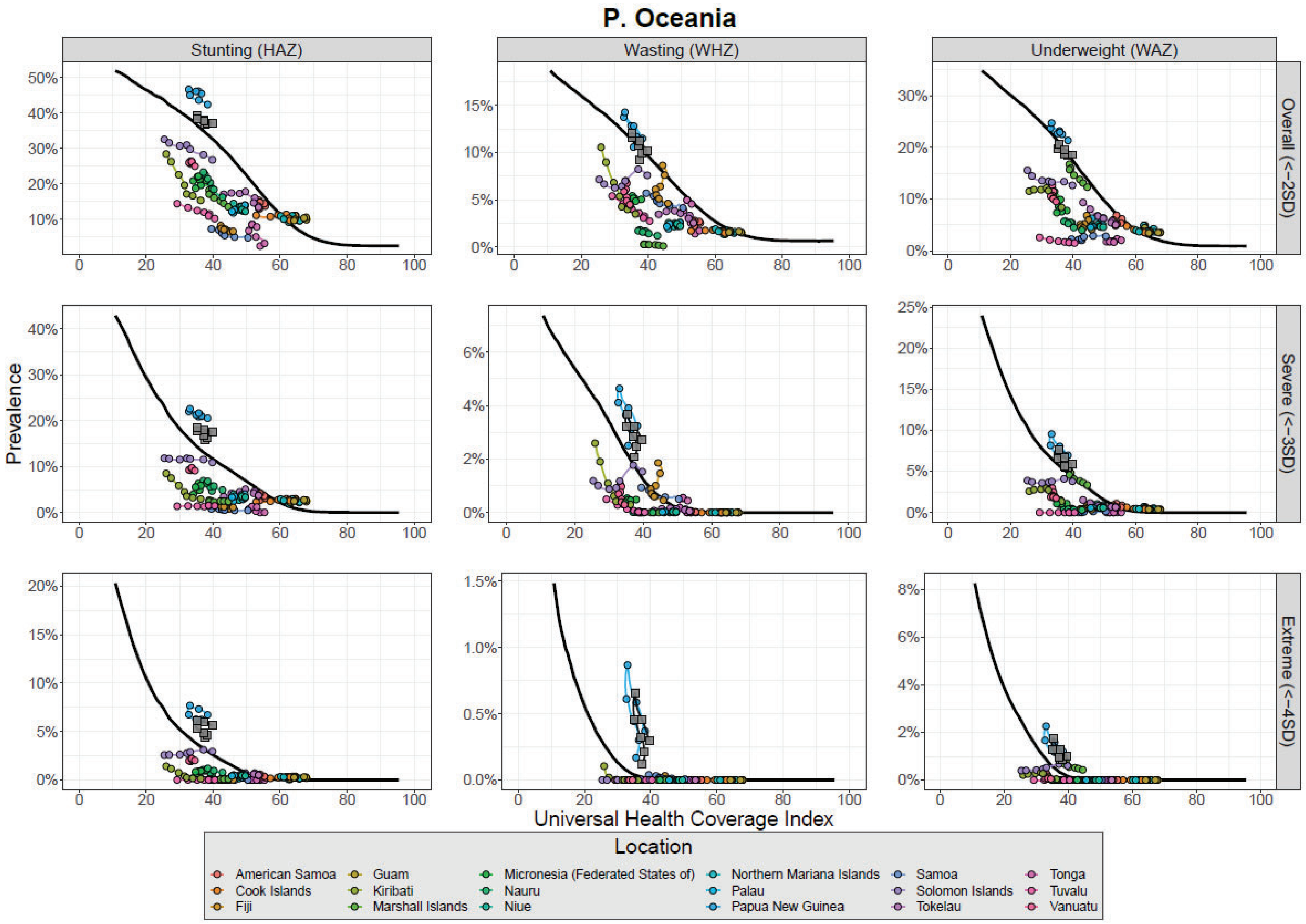
P. Oceania
Stunting (HAZ)
Wasting (WHZ)
Underweight (WAZ)
Overall (<−2SD)
Severe (<−3SD)
Extreme (<−4SD)
Prevalence
Universal Health Coverage Index
Location
American Samoa
Cook Islands
Fiji
Guam
Kiribati
Marshall Islands
Micronesia (Federated States of)
Nauru
Niue
Northern Mariana Islands
Palau
Papua New Guinea
Samoa
Solomon Islands
Tokelau
Tonga
Tuvalu
Vanuatu

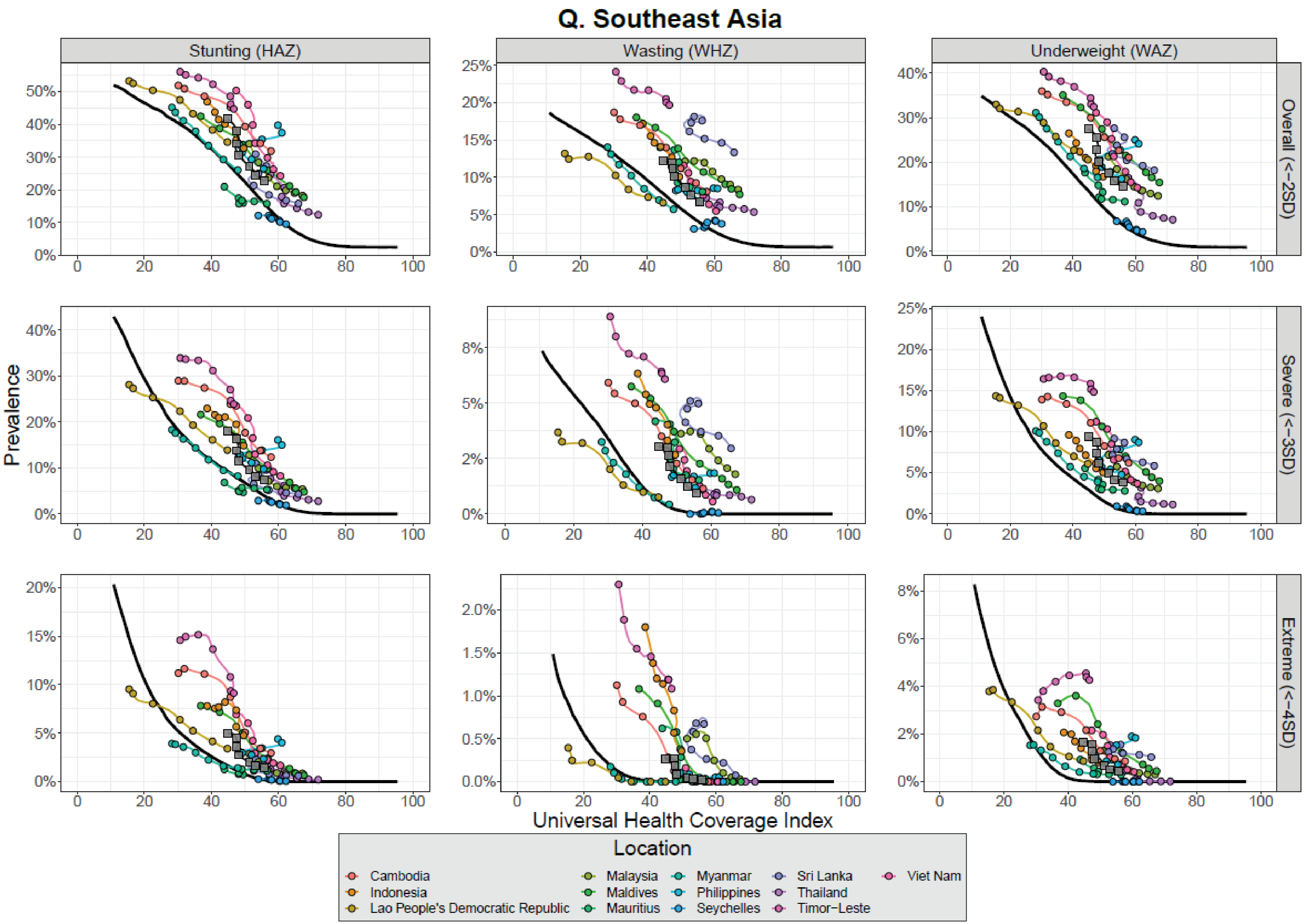

Q. Southeast Asia
Stunting (HAZ)
Wasting (WHZ)
Underweight (WAZ)
Overall (<−2SD)
Severe (<−3SD)
Extreme (<−4SD)
Prevalence
Universal Health Coverage Index
Location
Cambodia
Indonesia
Lao People's Democratic Republic
Malaysia
Maldives
Mauritius
Myanmar
Philippines
Seychelles
Sri Lanka
Thailand
Timor−Leste
Viet Nam

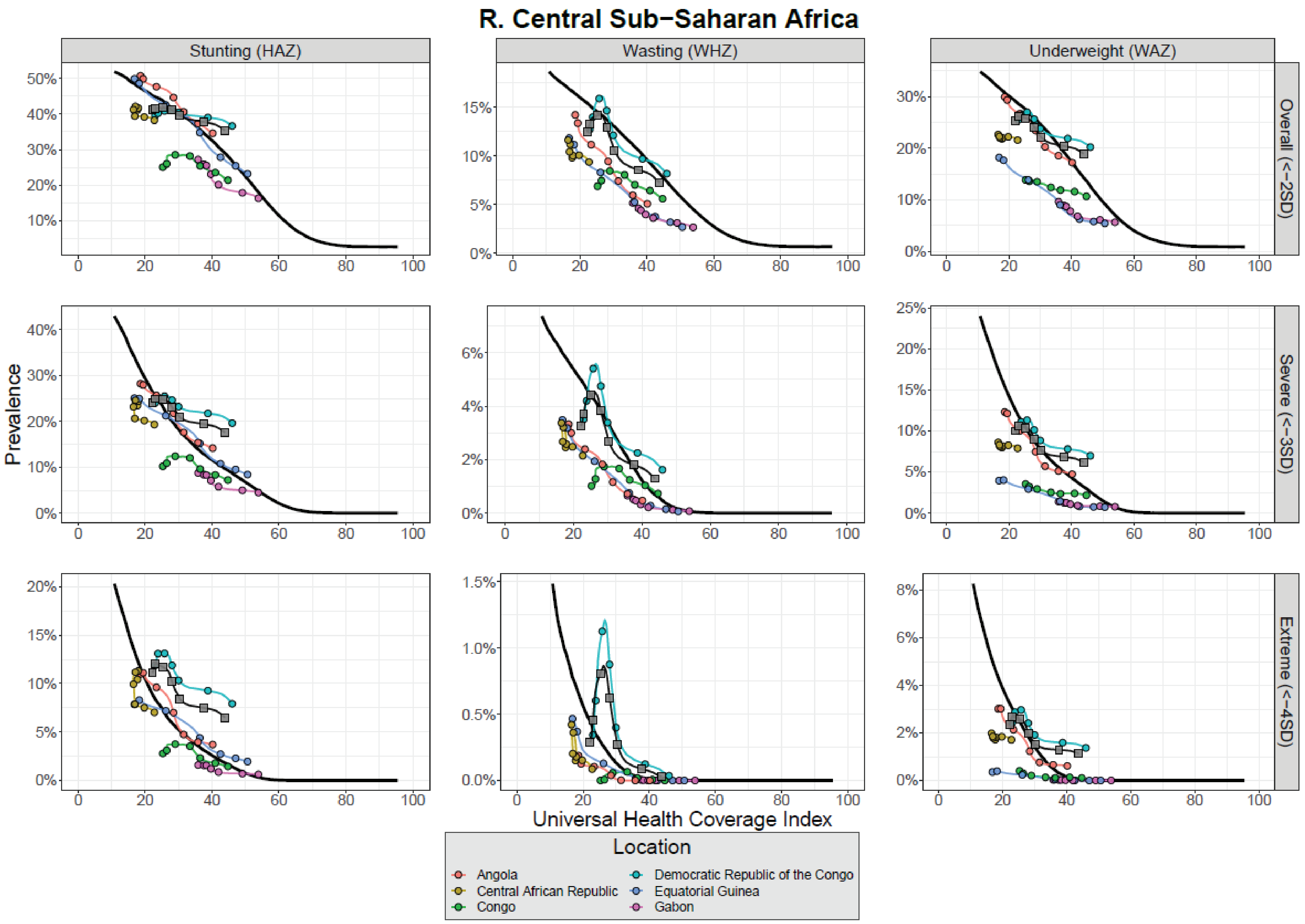
R. Central Sub-Saharan Africa
Stunting (HAZ)
Wasting (WHZ)
Underweight (WAZ)
Overall (<-2SD)
Severe (<-3SD)
Extreme (<-4SD)
Prevalence
Universal Health Coverage Index
Location
Angola
Central African Republic
Congo
Democratic Republic of the Congo
Equatorial Guinea
Gabon

S. Eastern Sub−Saharan Africa

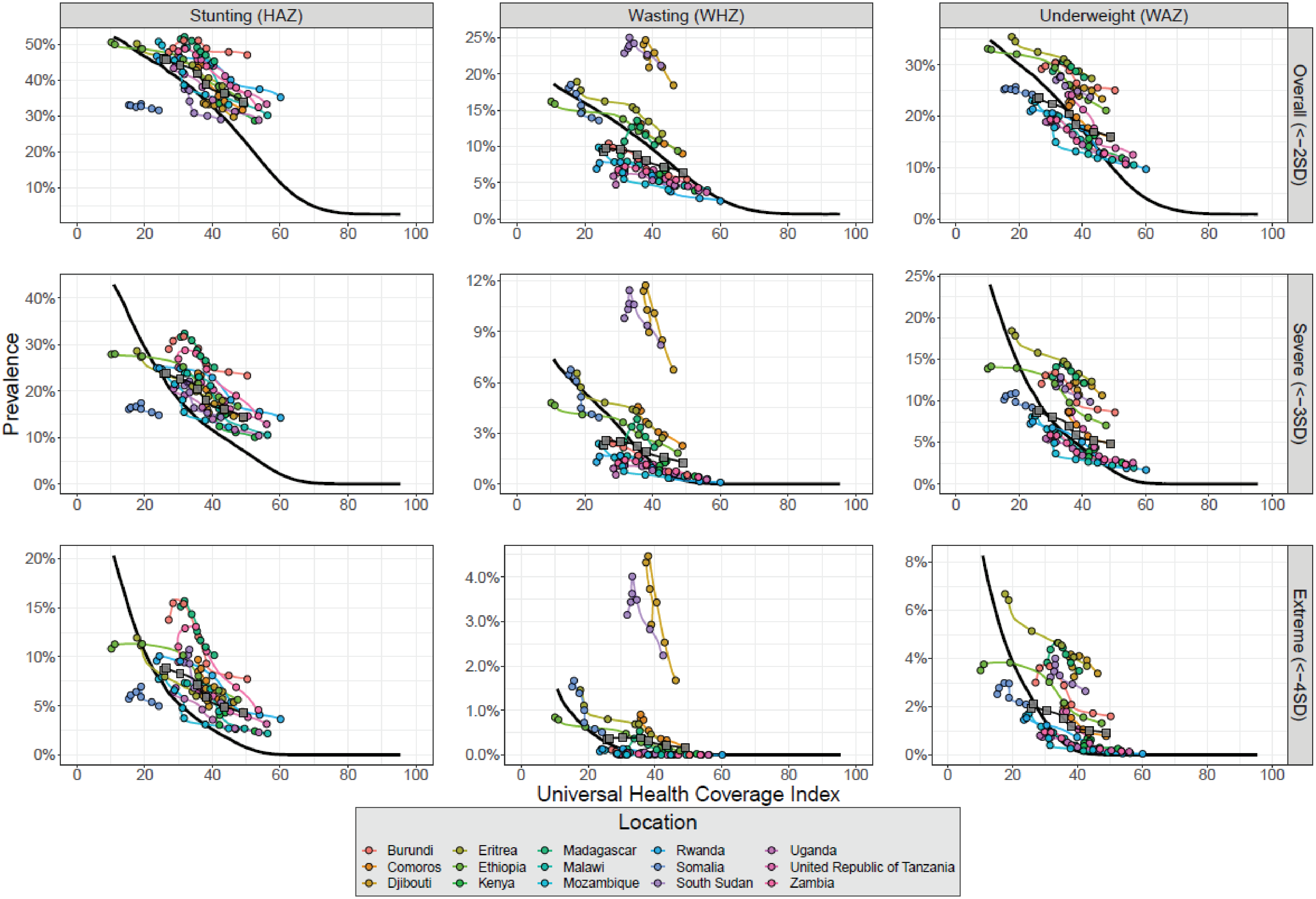

T. Southern Sub−Saharan Africa

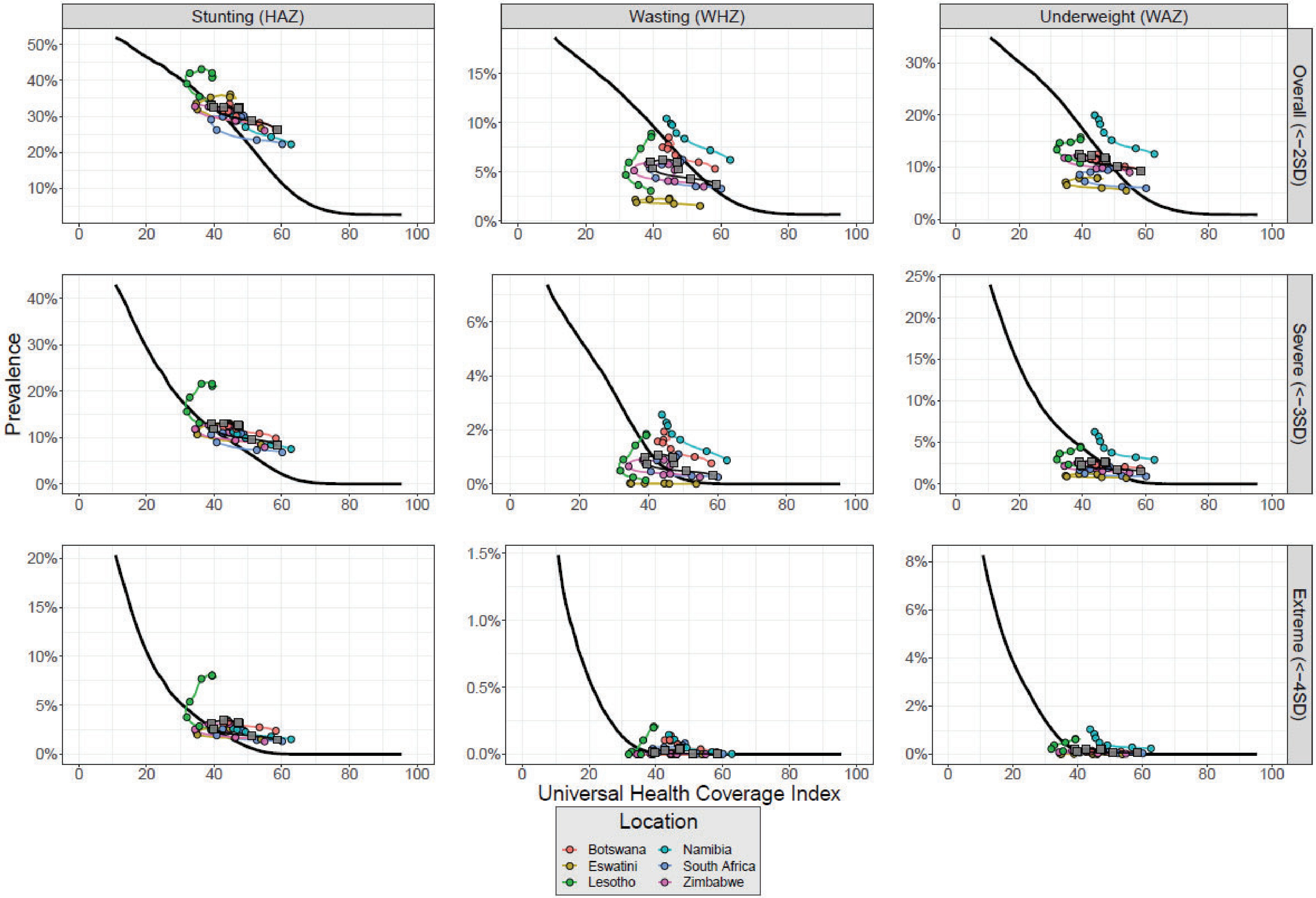

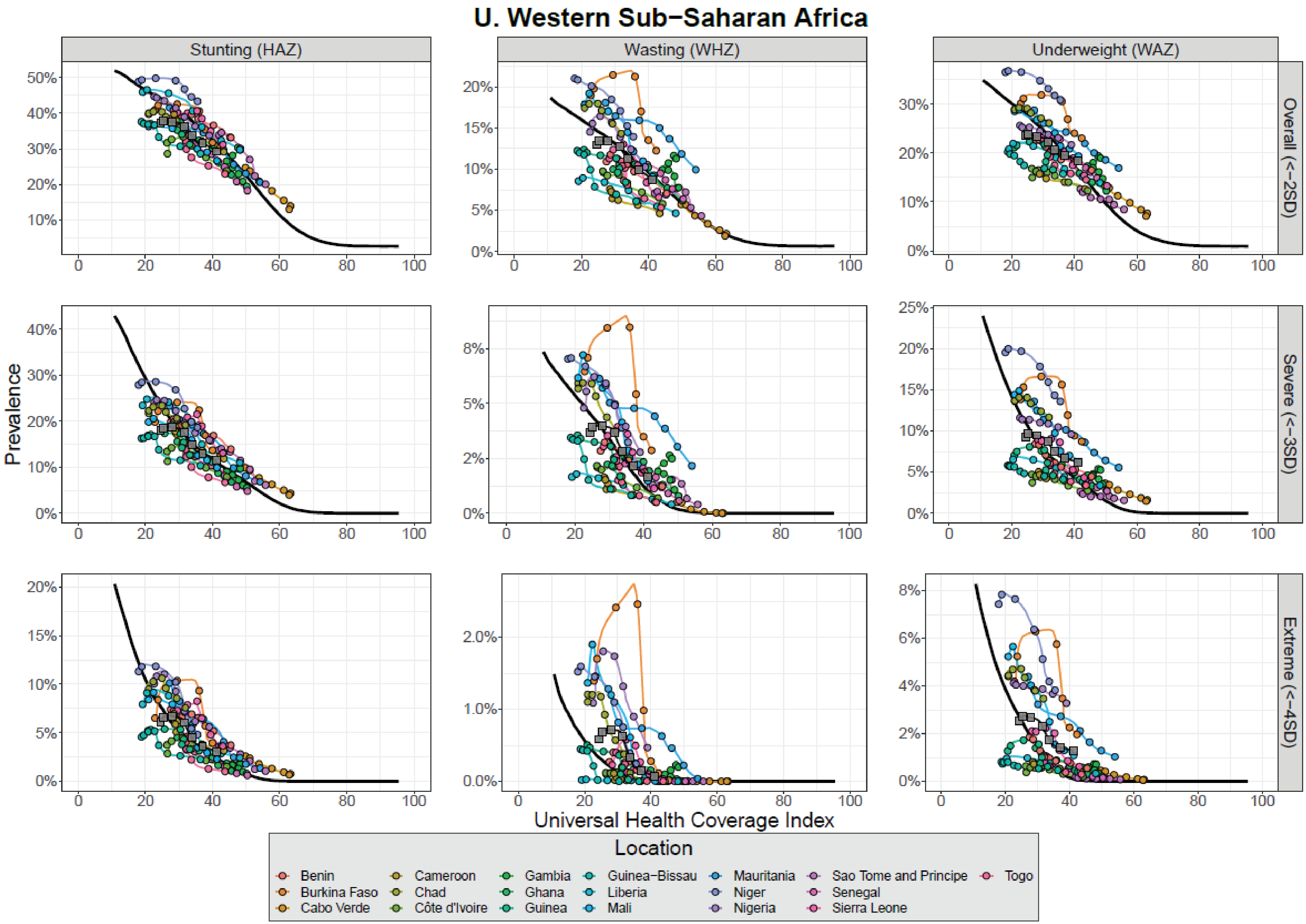
U. Western Sub−Saharan Africa
Stunting (HAZ)
Wasting (WHZ)
Underweight (WAZ)
Overall (<−2SD)
Severe (<−3SD)
Extreme (<−4SD)
Prevalence
Universal Health Coverage Index
Location
Benin
Burkina Faso
Cabo Verde
Cameroon
Chad
Côte d'Ivoire
Gambia
Ghana
Guinea
Guinea−Bissau
Liberia
Mali
Mauritania
Niger
Nigeria
Sao Tome and Principe
Senegal
Sierra Leone
Togo

Fig. S8. Expected relative CGF progress across a wide range of Socio-demographic Index. Expected values of overall, severe, and extreme CGF are scaled to show how trajectories of stunting, wasting, and underweight change as UHC index improves. Prevalences have been scaled to demonstrate expected relative changes as UHC index improves from the minimum observed UHC Index value. Expected values of CGF prevalence based on UHC Index are shown for SDI values of 20 (A, E, I), 40 (B,F, J), 60 (C, G, K) and 80 (D, H, L) for stunting, wasting, and underweight, respectively, to highlight that more extreme forms of CGF are sensitive to UHC Index changes across a wide range of SDI values.

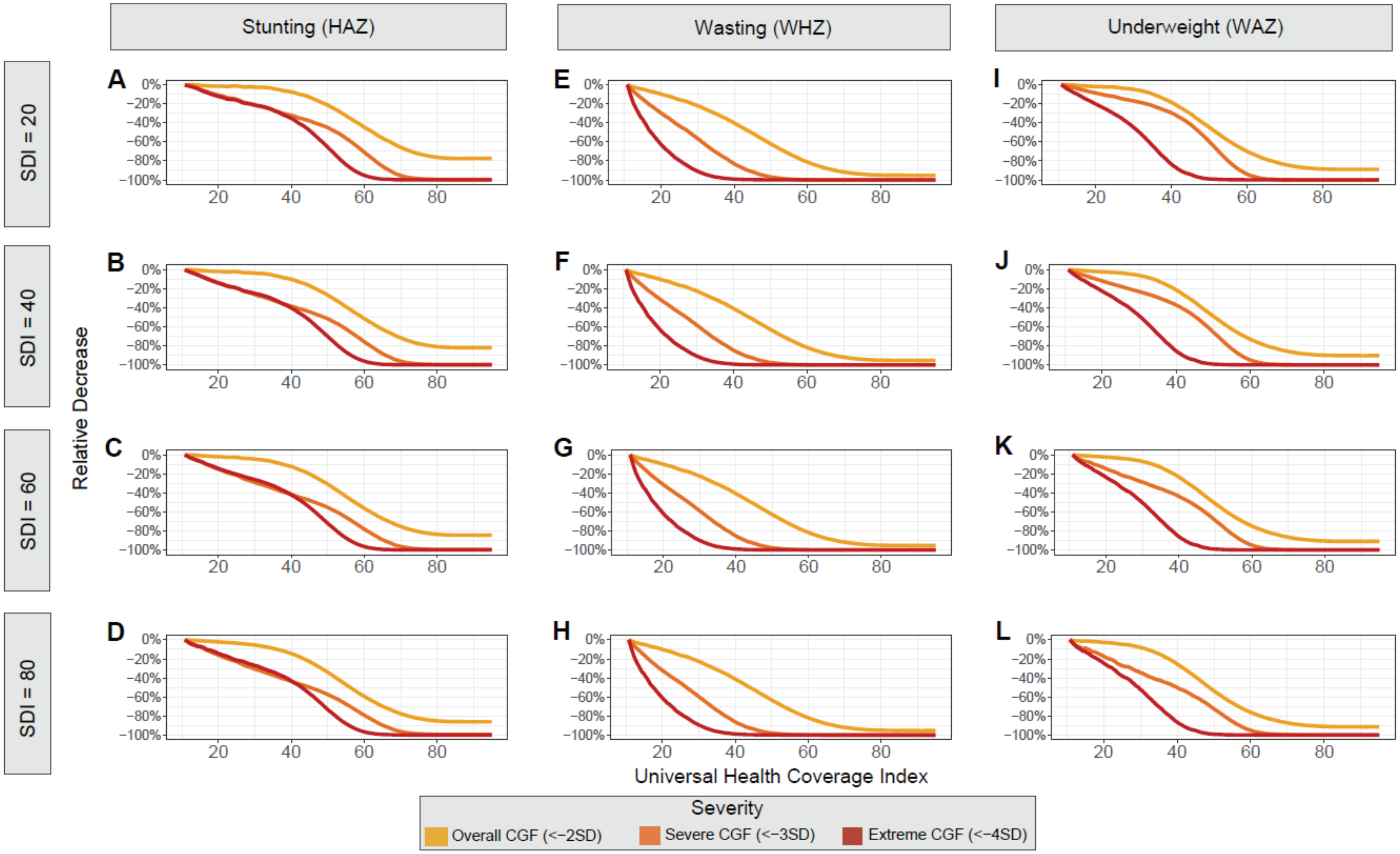

Data S1a-g. Spatio-temporal Gaussian Process Regression (ST-GPR) results for overall, severe, and mean CGF by location, including location-specific data sources; and distributions of stunting [HAZ], wasting [WHZ], and underweight [WAZ] for children under age five, both sexes, for every five years from 1990−2020. Country results are grouped by GBD super-region, including Central Europe, Eastern Europe, and Central Asia (S1a), High-income (S1b), Latin America and Caribbean (S1c), North Africa and Middle East (S1d), South Asia (S1e), Southeast Asia, East Asia, and Oceania (S1f), and Sub-Saharan Africa (S1g). Plots for each country include overall and severe stunting prevalence (A) and transformed mean stunting Z scores (B). A source list is shown which includes surveys included in the stunting models (C). Additional plots are shown for overall and severe wasting prevalence (D) and transformed mean wasting Z scores (E), followed by a source list with surveys included in the wasting models (F). Plots are then shown for overall and severe underweight prevalence (G), and transformed mean underweight Z scores (H), with a source list listing surveys included in the underweight models (I). Finally, distributions of stunting (J), wasting (K), and underweight (L) are shown for children under age five, both sexes, for every five years from 1990-2020. Surveys that were outliered are shown with X's on all plots. Surveys prior to 1990 may have been inputs to the models to inform trends, but estimates are only produced and shown for 1990−2020. For locations that are modeled nationally and subnationally, sources that are only included subnationally are not included in the plots of national level estimates. These sources were included in subnational models that influence national level models. Note that due to the transformation on mean Z scores, increasing values reflect improvements in mean Z score. Surveys conducted over a range of years were assigned to the midpoint year from that interval, which is the year reflected in the table and the plots. For the distributions of stunting, wasting, and underweight, the area under the curve reflects the estimated proportion of children experiencing that severity of CGF or worse. DHS is Demographic and Health Surveys. MICS is Multiple Indicator Cluster Survey. WHO CGM is the WHO Global Database on Child Growth and Malnutrition. SDNS is Survey of Diet and Nutritional Status. **The files are organized by GBD Super Region with ordering as follows:**

Data S1a: <u>Central Europe, Eastern Europe, and Central Asia</u>:

Central Asia: Armenia, Azerbaijan, Georgia, Kazakhstan, Kyrgyzstan, Mongolia, Tajikistan, Turkmenistan, Uzbekistan

Central Europe: Albania, Bosnia and Herzegovina, Bulgaria, Croatia, Czechia, Hungary, Montenegro, North Macedonia, Poland, Romania, Serbia, Slovakia, Slovenia,

Eastern Europe: Belarus, Estonia, Latvia, Lithuania, Republic of Moldova, Russian Federation, Ukraine

Data S1b: <u>High Income</u>:

Australasia: Australia, New Zealand

High-income Asia Pacific: Brunei Darussalam, Japan, Republic of Korea, Singapore

High-income North America: Canada, Georgia, United States of America

Southern Latin America: Argentina, Chile, Uruguay

Western Europe: Andorra, Austria, Belgium, Cyprus, Denmark, Finland, France, Germany, Greece, Iceland, Ireland, Israel, Italy, Luxembourg, Malta, Monaco, Netherlands, Norway, Portugal, San Marino, Spain, Sweden, Switzerland, United Kingdom

Data S1c: Latin America and the Caribbean:

Andean Latin America: Bolivia (Plurinational State of), Ecuador, Peru
Caribbean: Antigua and Barbuda, Bahamas, Barbados, Belize, Cuba, Dominica, Dominican Republic, Grenada, Guyana, Haiti, Jamaica, Saint Kitts and Nevis, Saint Lucia, Saint Vincent and the Grenadines, Suriname, Trinidad and Tobago
Central Latin America: Colombia, Costa Rica, El Salvador, Guatemala, Honduras, Mexico, Nicaragua, Panama, Venezuela (Bolivarian Republic of)
Tropical Latin America: Brazil, Paraguay

Data S1d: North Africa and the Middle East:

Algeria, Bahrain, Egypt, Iran (Islamic Republic of), Iraq, Jordan, Kuwait, Lebanon, Libya, Morocco, Oman, Palestine, Qatar, Saudi Arabia, Syrian Arab Republic, Tunisia, Turkey, United Arab Emirates, Yemen, Afghanistan, Sudan

Data S1e: South Asia:

Bangladesh, Bhutan, India, Nepal, Pakistan

Data S1f: Southeast Asia, East Asia, and Oceania:

East Asia: China, Democratic People's Republic of Korea, Taiwan (Province of China)
Oceania: Fiji, Kiribati, Marshall Islands, Micronesia (Federated States of), Nauru, Palau, Papua New Guinea, Samoa, Solomon Islands, Tonga, Tuvalu, Vanuatu
Southeast Asia: Cambodia, Indonesia, Lao People's Democratic Republic, Malaysia, Maldives, Mauritius, Myanmar, Philippines, Seychelles, Sri Lanka, Thailand, Timor-Leste, Viet Nam

Data S1g: sub-Saharan Africa:

Central sub-Saharan Africa: Angola, Central African Republic, Congo, Democratic Republic of the Congo, Equatorial Guinea, Gabon
Eastern Sub-Saharan Africa: Burundi, Comoros, Djibouti, Eritrea, Ethiopia, Kenya, Madagascar, Malawi, Mozambique, Rwanda, Somalia, South Sudan, Uganda, United Republic of Tanzania, Zambia
Southern Sub-Saharan Africa: Botswana, Eswatini, Lesotho, Namibia, South Africa, Zimbabwe
Western Sub-Saharan Africa: Benin, Burkina Faso, Cabo Verde, Cameroon, Chad, Côte d'Ivoire, Gambia, Ghana, Guinea, Guinea-Bissau, Liberia, Mali, Mauritania, Niger, Nigeria, Sao Tome and Principe, Senegal, Sierra Leone, Togo

Data S2. Age-sex specific fits of MR-BRT models for all forms and severities of CGF, 1990-2020. Prevalence models were fit in logit space, but are also shown in linear space for comparison. Figures shown include: overall stunting [HAZ < -2 standard deviations (SD)] prevalence shown in logit and linear space for males (A, B) and females (C, D); severe stunting [HAZ < -3 SD] for males (E, F) and females (G, H); extreme stunting [HAZ < -4 SD] for males (I, J) and females (K, L); overall wasting [WHZ < -2 SD] for males (M, N) and females (O, P); severe wasting [WHZ < -3 SD] for males (Q, R) and females (S, T); extreme wasting [WHZ < -4 SD] for males (U, V) and females (W, X); overall underweight [WAZ < -2 SD] for males (Y, Z) and females (AA, BB); severe underweight [WAZ < -3 SD] for males (CC, DD) and females (EE, FF); and extreme underweight [WAZ < -4 SD] for males (GG, HH) and females (II, JJ). Models were fit in logit space, but are also shown in linear space for comparison. All years were used as inputs to the models, but only 1990, 2000, 2010, and 2020 are shown here for simplicity.

Data S3. Ensemble MR-BRT knot placements and weight given to each sub-model, for all forms and severities of CGF, by sex. Twenty MR−BRT models with different knot placements were fit to each age−sex, weighted based by their predictive performance. Knots 1 and 6 were always respectively placed at the lowest and highest estimated UHC index values input to the model. Knot placements for overall stunting in males and females are shown in (A, B), followed by severe stunting (C, D), extreme stunting (E, F), overall wasting (G, H), severe wasting (I, J), extreme wasting (K, L), overall underweight (M, N), severe underweight (O, P), and extreme underweight (Q, R)

Data S4. Age-sex specific fits of MR-BRT models for all forms and severities of CGF, 1990-2020, with incorporation of SDI as a linear covariate. Prevalence models were fit in logit space, but are also shown in linear space for comparison. Figures shown include: overall stunting [HAZ < -2 standard deviations (SD)] prevalence shown in logit and linear space for males (A, B) and females (C, D); severe stunting [HAZ < -3 SD] for males (E, F) and females (G, H); extreme stunting [HAZ < -4 SD] for males (I, J) and females (K, L); overall wasting [WHZ < -2 SD] for males (M, N) and females (O, P); severe wasting [WHZ < -3 SD] for males (Q, R) and females (S, T); extreme wasting [WHZ < -4SD] for males (U, V) and females (W, X); overall underweight [WAZ < -2 SD] for males (Y, Z) and females (AA, BB); severe underweight [WAZ < -3 SD] for males (CC, DD) and females (EE, FF); and extreme underweight [WAZ < -4 SD] for males (GG, HH) and females (II, JJ). Models were fit in logit space, but are also shown in linear space for comparison. All years were used as inputs to the models, but only 1990, 2000, 2010, and 2020 are shown here for simplicity. In these MR-BRT models, a linear covariate of Socio-demographic index (SDI) wasincorporated, unlike the MR-BRT models shown in Fig. S6. The four black lines represent the expected values of CGF prevalence for a given location with SDI values of 20, 40, 60, and 80, with the highest SDI value having the lowest predicted CGF prevalence.
