# Supplementary material for: Current dichotomous metrics obscure trends in severe and extreme child growth failure
Source: Sci Adv. 2022 May 20;8(20):eabm8954. doi: 10.1126/sciadv.abm8954 (PMC9122330; doi:10.1126/sciadv.abm8954)

**Data S1a. Spatio-temporal Gaussian Process Regression (ST-GPR) results for overall, severe, and mean CGF by location, including location-specific data sources; and distributions of stunting [HAZ], wasting [WHZ], and underweight [WAZ] for children under age five, both sexes, for every five years from 1990−2020.** Country results are grouped by GBD super-region, including Central Europe, Eastern Europe, and Central Asia (S1a), High-income (S1b), Latin America and Caribbean (S1c), North Africa and Middle East (S1d), South Asia (S1e), Southeast Asia, East Asia, and Oceania (S1f), and Sub-Saharan Africa (S1g). Plots for each country include overall and severe stunting prevalence (A) and transformed mean stunting Z scores (B). A source list is shown which includes surveys included in the stunting models (C). Additional plots are shown for overall and severe wasting prevalence (D) and transformed mean wasting Z scores (E), followed by a source list with surveys included in the wasting models (F). Plots are then shown for overall and severe underweight prevalence (G), and transformed mean underweight Z scores (H), with a source list listing surveys included in the underweight models (I). Finally, distributions of stunting (J), wasting (K), and underweight (L) are shown for children under age five, both sexes, for every five years from 1990-2020. Surveys that were outliered are shown with X's on all plots. Surveys prior to 1990 may have been inputs to the models to inform trends, but estimates are only produced and shown for 1990−2020. For locations that are modeled nationally and subnationally, sources that are only included subnationally are not included in the plots of national level estimates. These sources were included in subnational models that influence national level models. Note that due to the transformation on mean Z scores, increasing values reflect improvements in mean Z score. Surveys conducted over a range of years were assigned to the midpoint year from that interval, which is the year reflected in the table and the plots. For the distributions of stunting, wasting, and underweight, the area under the curve reflects the estimated proportion of children experiencing that severity of CGF or worse. DHS is Demographic and Health Surveys. MICS is Multiple Indicator Cluster Survey. WHO CGM is the WHO Global Database on Child Growth and Malnutrition. SDNS is Survey of Diet and Nutritional Status.

**This file contains the above for the following locations in the GBD super region of Central Europe, Eastern Europe, and Central Asia, in the following order:**

**Central Asia:** Armenia, Azerbaijan, Georgia, Kazakhstan, Kyrgyzstan, Mongolia, Tajikistan, Turkmenistan, Uzbekistan

**Central Europe:** Albania, Bosnia and Herzegovina, Bulgaria, Croatia, Czechia, Hungary, Montenegro, North Macedonia, Poland, Romania, Serbia, Slovakia, Slovenia,

**Eastern Europe:** Belarus, Estonia, Latvia, Lithuania, Republic of Moldova, Russian Federation, Ukraine

**Armenia** – Stunting (HAZ)

**A**: Overall and Severe Stunting Prevalence

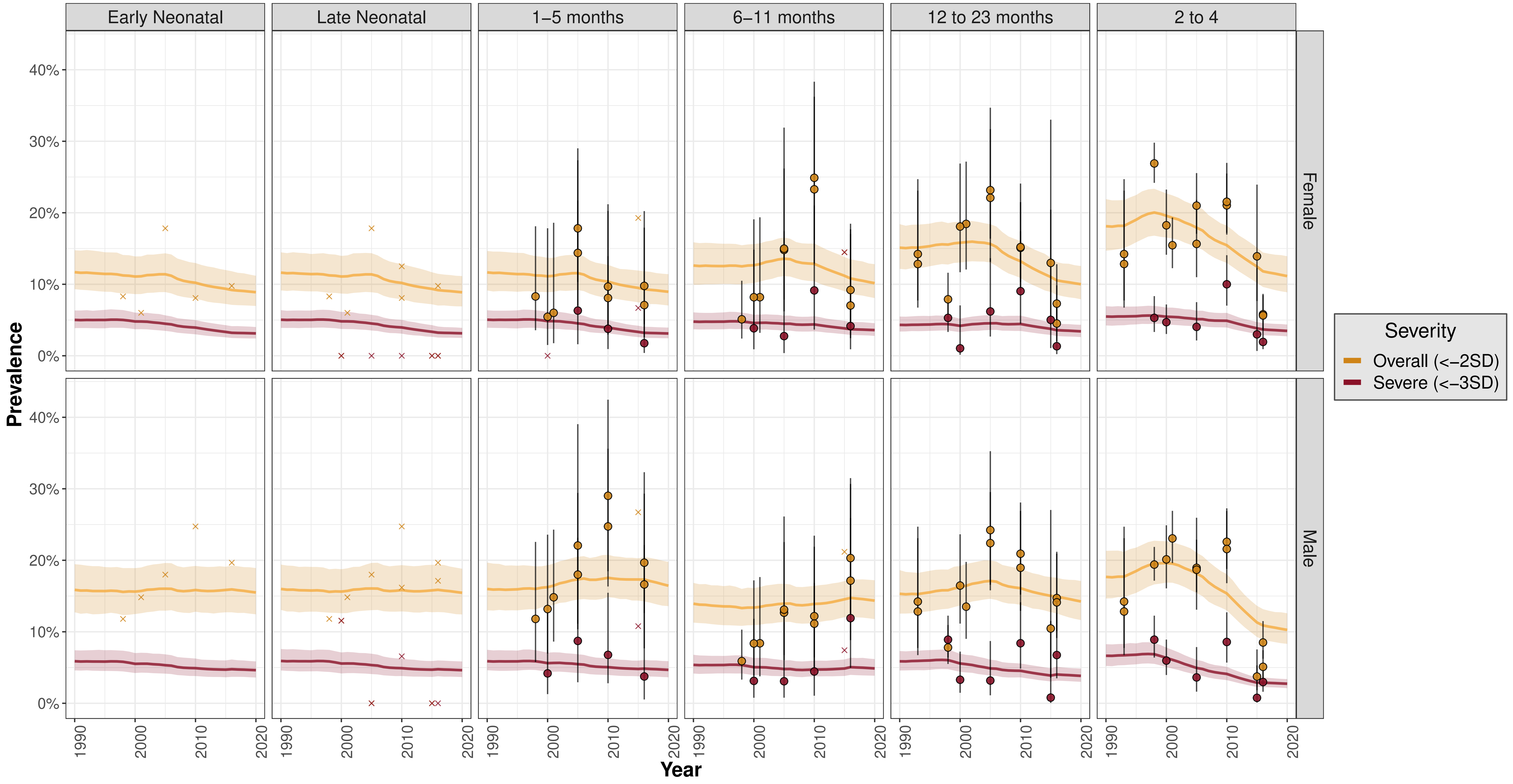


**B**: Transformed Mean Stunting Z Scores

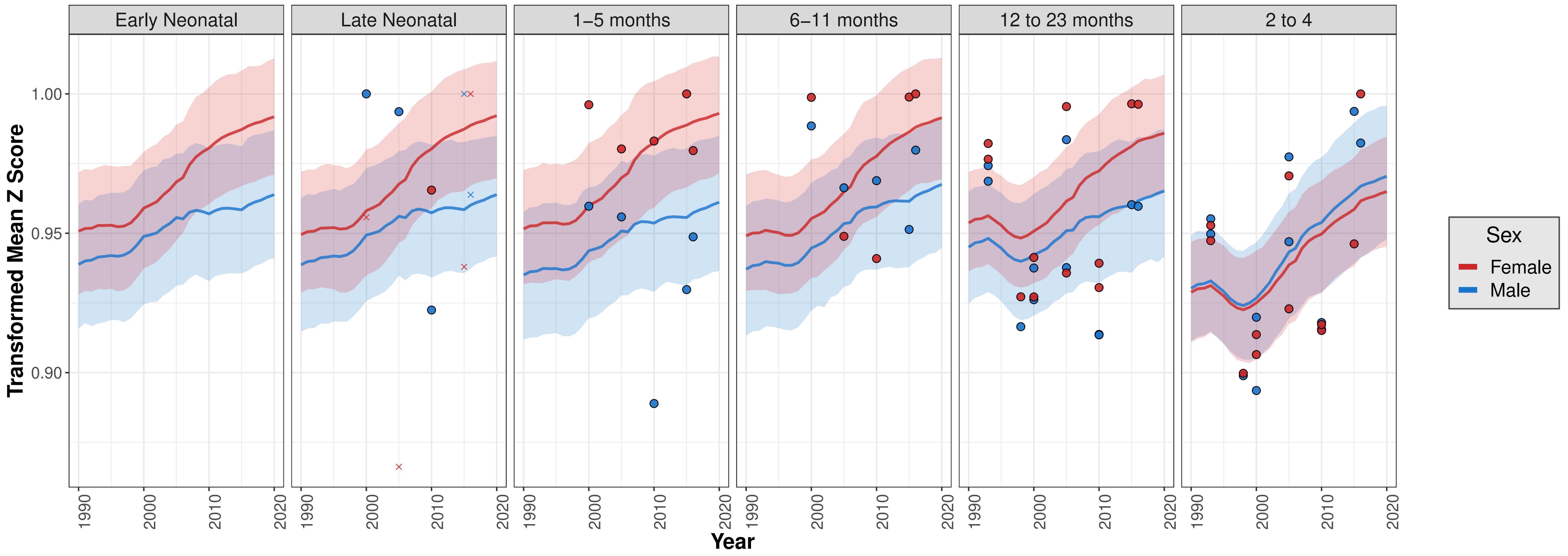


**C**

| Year | Source |
|---|---|
| 1993 | WHO CGM Database |
| 1998 | WHO CGM Database |
| 2000 | DHS |
| 2000 | WHO CGM Database |
| 2001 | WHO CGM Database |
| 2005 | DHS |
| 2005 | WHO CGM Database |
| 2010 | DHS |
| 2010 | WHO CGM Database |
| 2015 | DHS |
| 2016 | WHO CGM Database |
| 2016 | DHS |

# Armenia – Wasting (WHZ)

**D**: Overall and Severe Wasting Prevalence

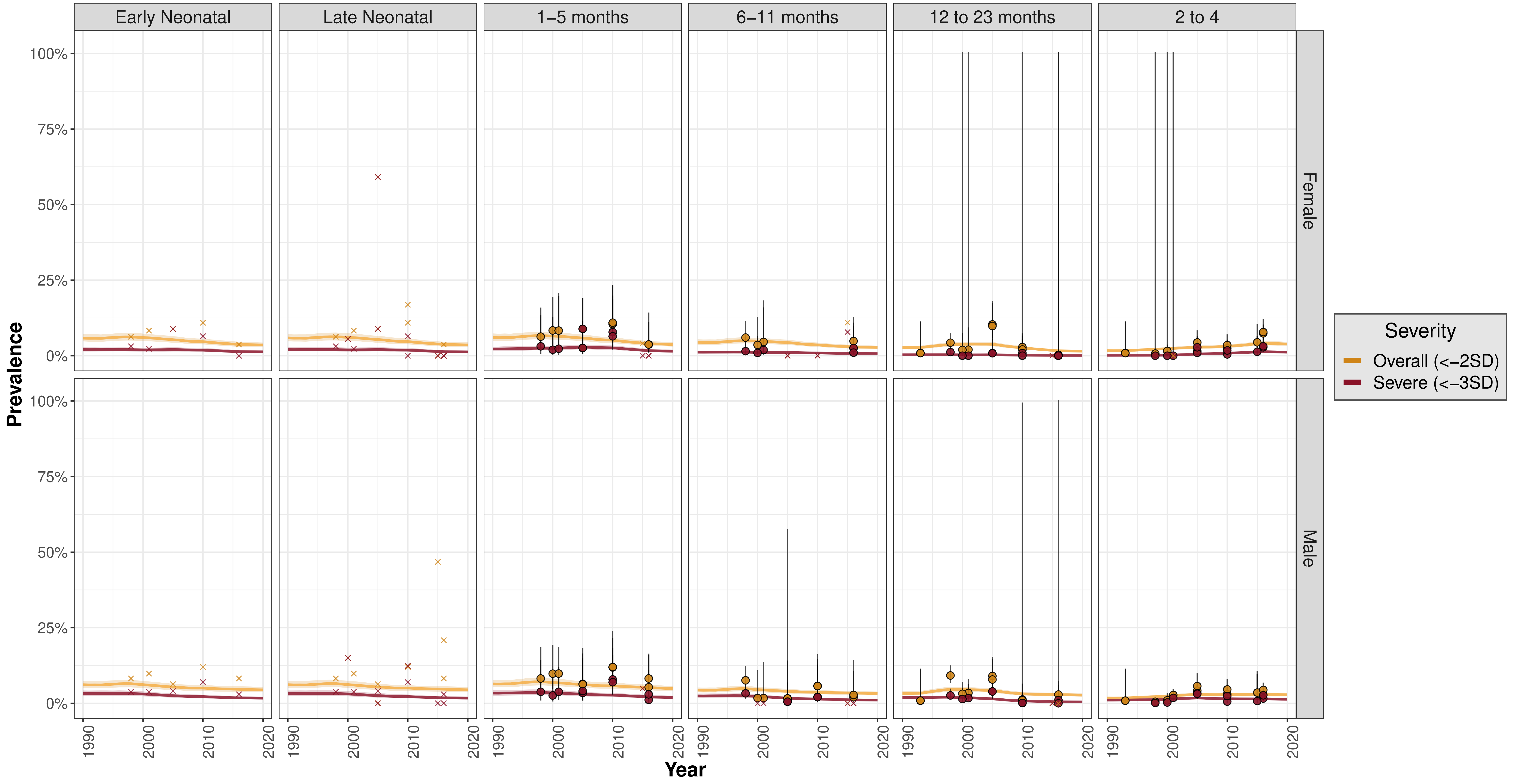


**E**: Transformed Mean Wasting Z Scores

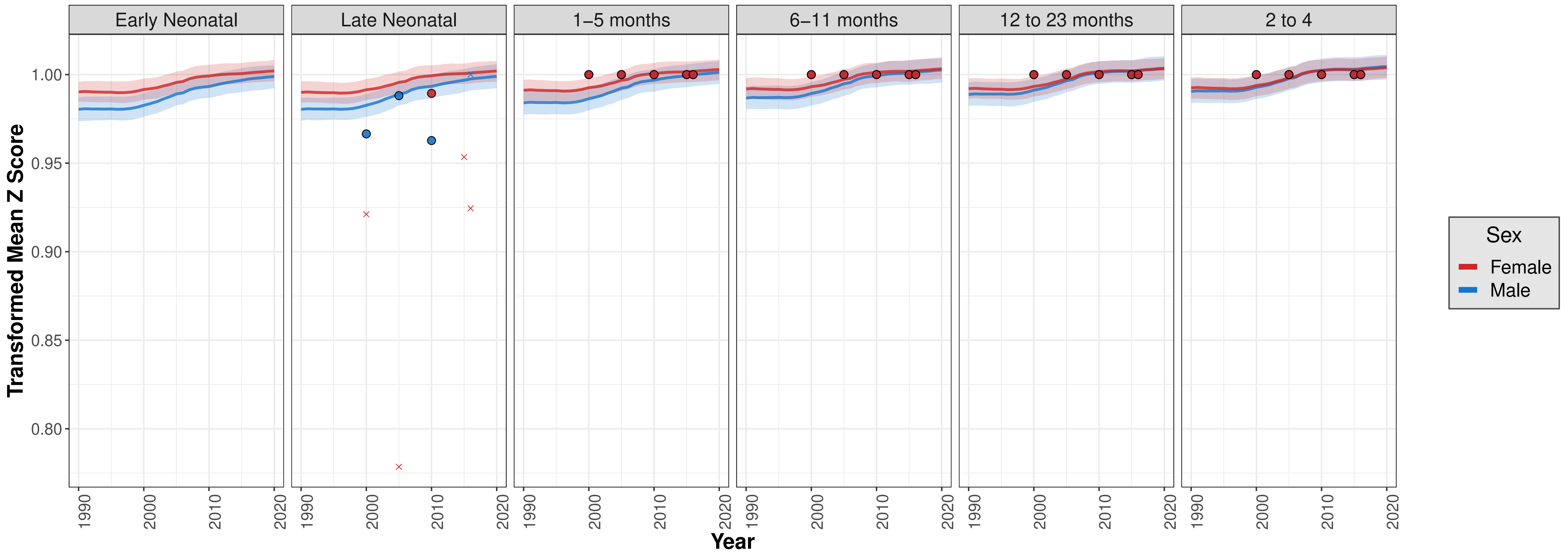


**F**

| Year | Source |
|---|---|
| 1993 | WHO CGM Database |
| 1998 | WHO CGM Database |
| 2000 | DHS |
| 2001 | WHO CGM Database |
| 2005 | DHS |
| 2005 | WHO CGM Database |
| 2010 | DHS |
| 2010 | WHO CGM Database |
| 2015 | DHS |
| 2016 | WHO CGM Database |
| 2016 | DHS |

# **Armenia** – Underweight (WAZ)

**G**: Overall and Severe Underweight Prevalence

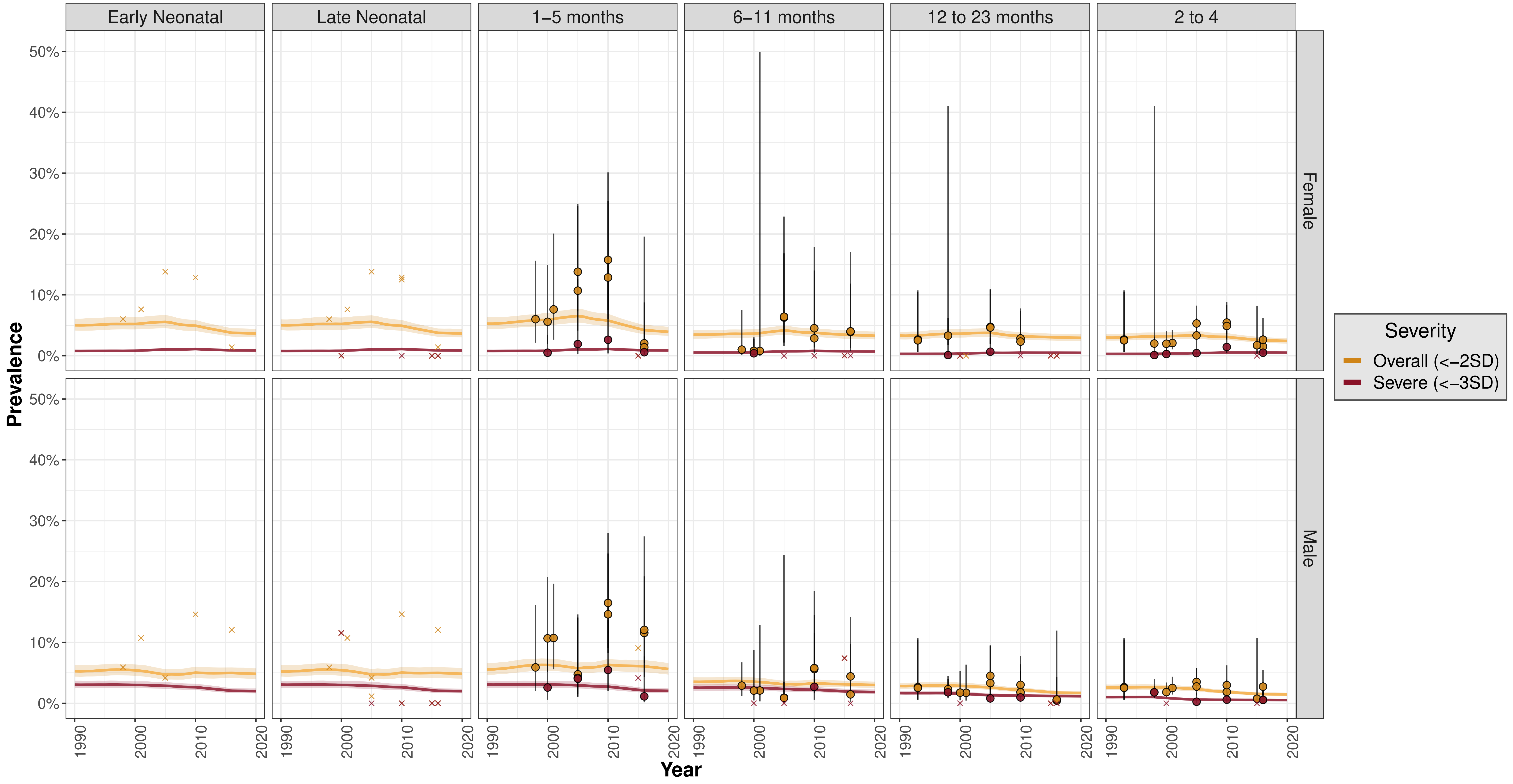


**H**: Transformed Mean Underweight Z Scores

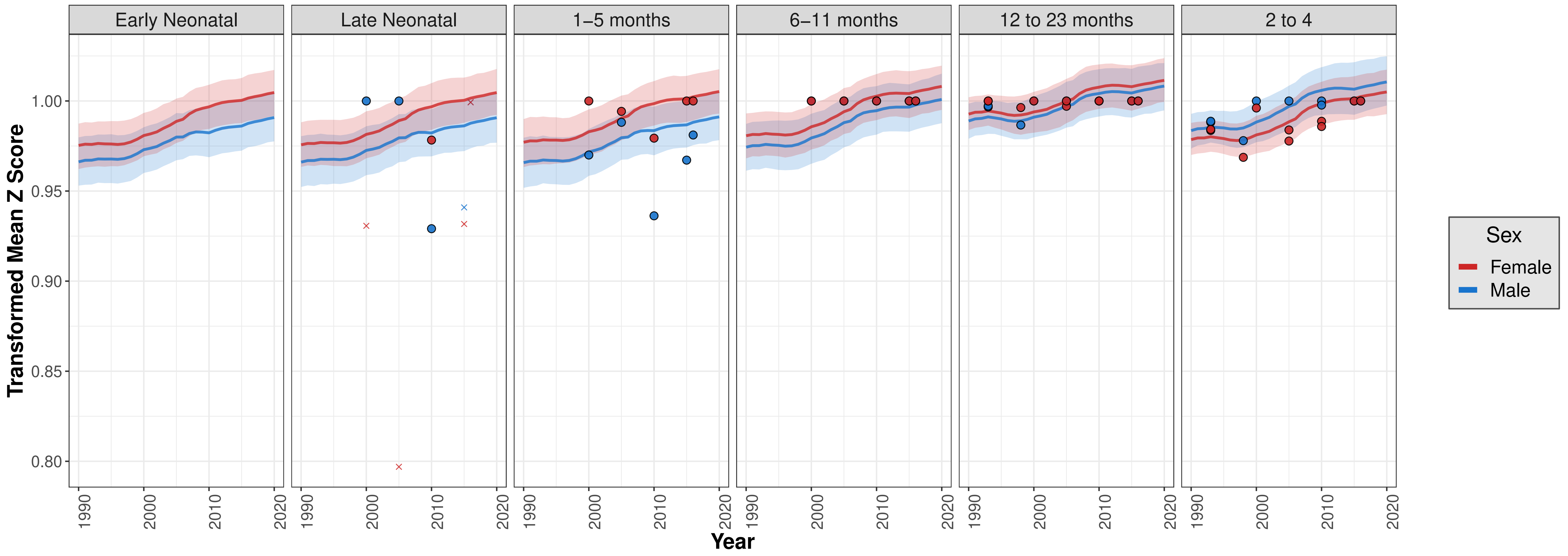


**I**

| Year | Source |
|---|---|
| 1993 | WHO CGM Database |
| 1998 | WHO CGM Database |
| 2000 | DHS |
| 2001 | WHO CGM Database |
| 2005 | DHS |
| 2005 | WHO CGM Database |
| 2010 | DHS |
| 2010 | WHO CGM Database |
| 2015 | DHS |
| 2016 | WHO CGM Database |
| 2016 | DHS |

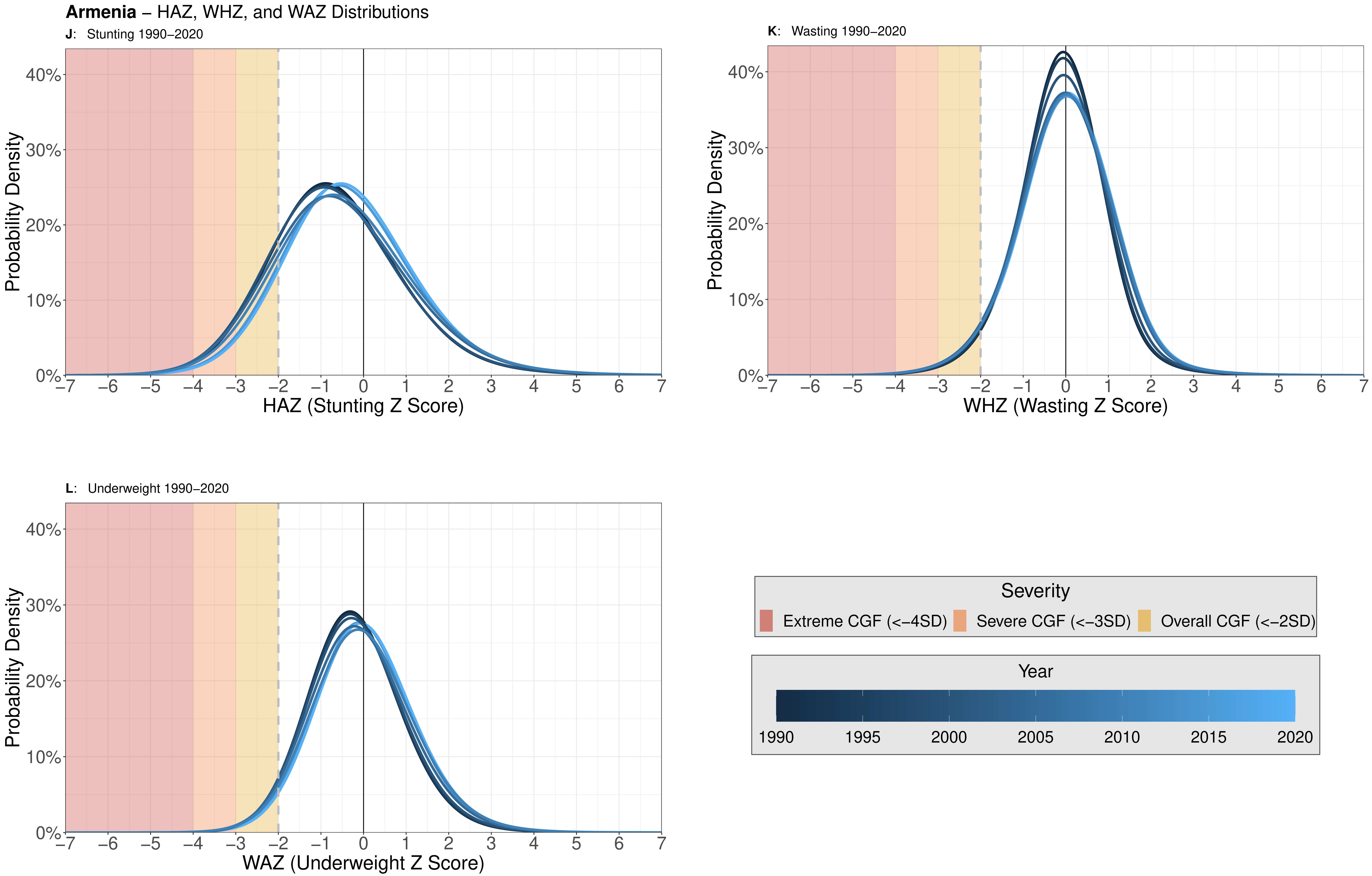
Armenia – HAZ, WHZ, and WAZ Distributions
J: Stunting 1990–2020
Probability Density
40%
30%
20%
10%
0%
−7 −6 −5 −4 −3 −2 −1 0 1 2 3 4 5 6 7
HAZ (Stunting Z Score)
K: Wasting 1990–2020
Probability Density
40%
30%
20%
10%
0%
−7 −6 −5 −4 −3 −2 −1 0 1 2 3 4 5 6 7
WHZ (Wasting Z Score)
L: Underweight 1990–2020
Probability Density
40%
30%
20%
10%
0%
−7 −6 −5 −4 −3 −2 −1 0 1 2 3 4 5 6 7
WAZ (Underweight Z Score)
Severity
Extreme CGF (<−4SD)
Severe CGF (<−3SD)
Overall CGF (<−2SD)
Year
1990
1995
2000
2005
2010
2015
2020

## **Azerbaijan** – Stunting (HAZ)

**A**: Overall and Severe Stunting Prevalence

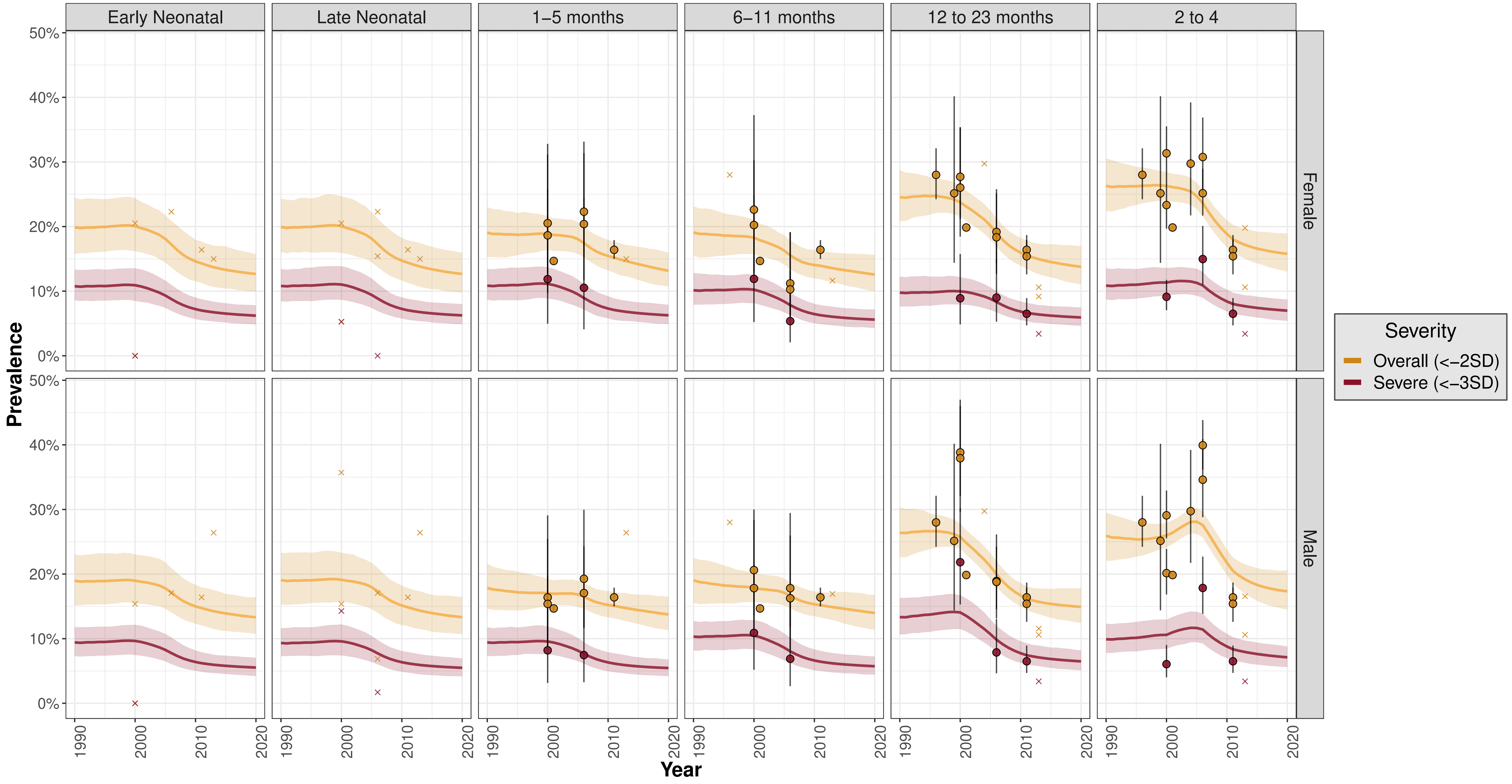


**B**: Transformed Mean Stunting Z Scores

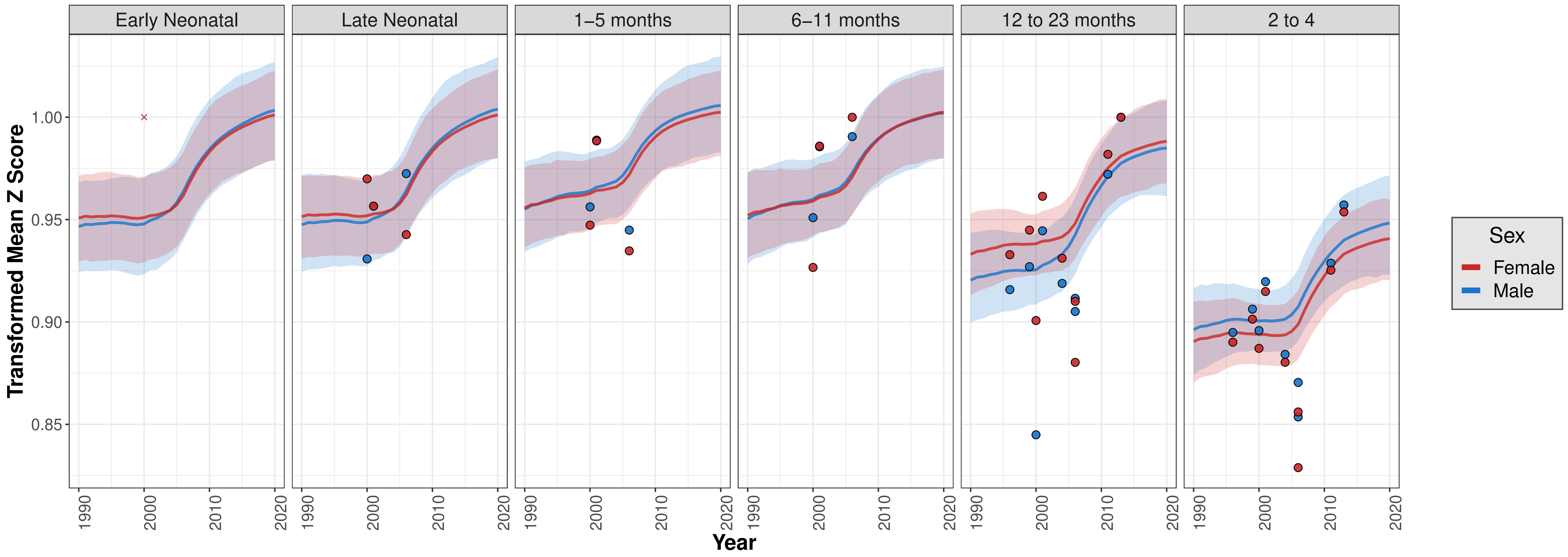


**C**

| Year | Source |
|---|---|
| 1996 | WHO CGM Database |
| 1999 | WHO CGM Database |
| 2000 | MICS |
| 2000 | WHO CGM Database |
| 2001 | WHO CGM Database |
| 2004 | Food Security and Nutrition Survey |
| 2006 | DHS |
| 2006 | WHO CGM Database |
| 2011 | WHO CGM Database |
| 2011 | DHS |
| 2013 | WHO CGM Database |
| 2013 | Nutrition Survey |

# **Azerbaijan** – Wasting (WHZ)

**D**: Overall and Severe Wasting Prevalence

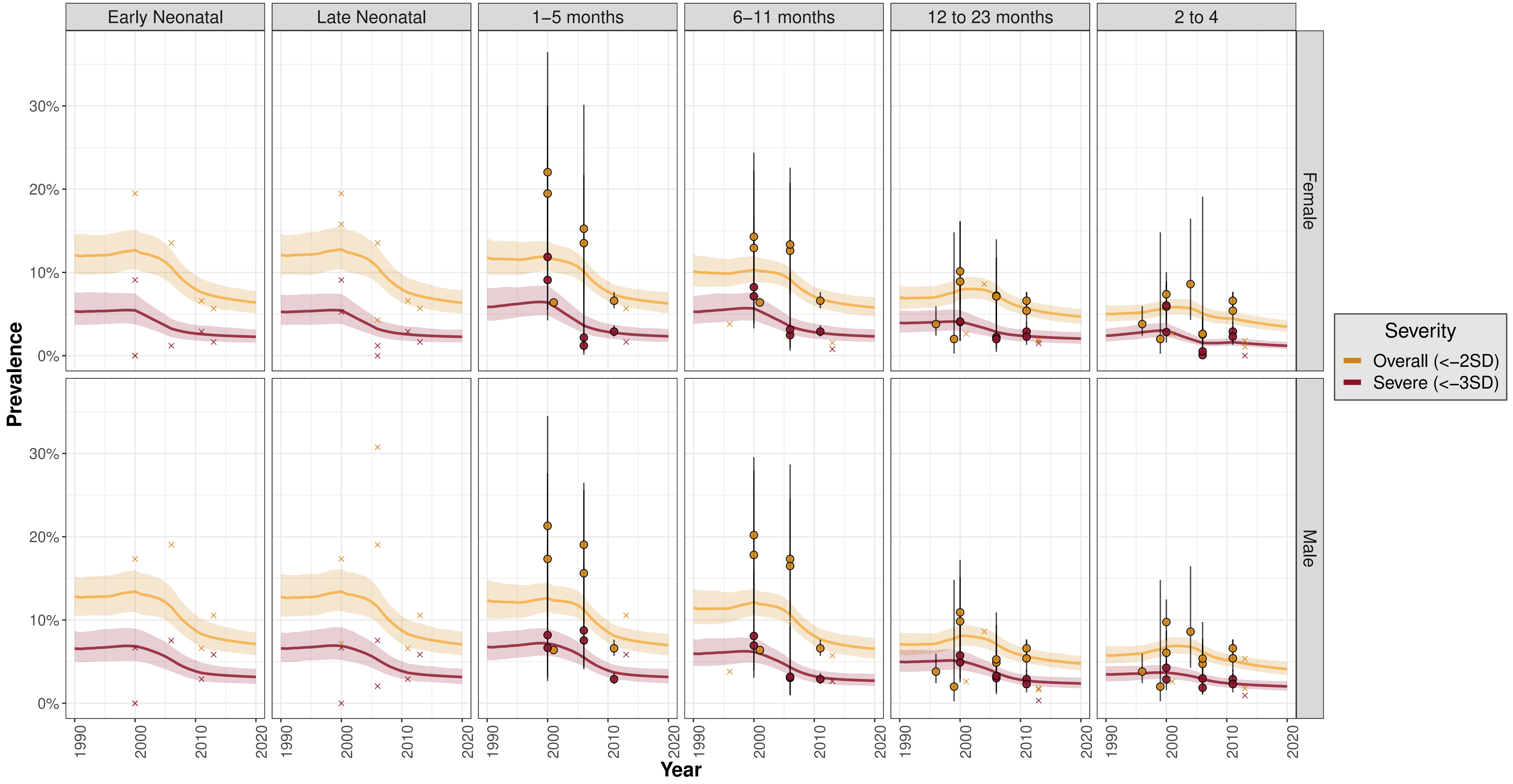


**E**: Transformed Mean Wasting Z Scores

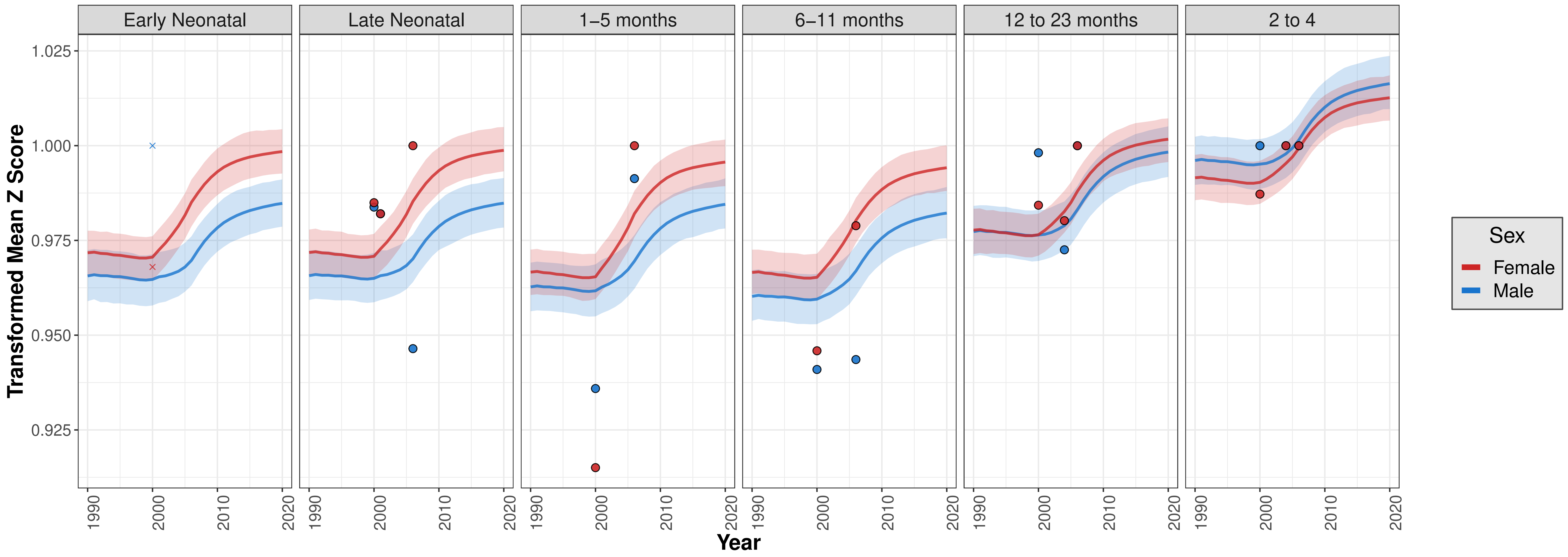


**F**

| Year | Source |
|---|---|
| 1996 | WHO CGM Database |
| 1999 | WHO CGM Database |
| 2000 | MICS |
| 2000 | WHO CGM Database |
| 2001 | WHO CGM Database |
| 2004 | Food Security and Nutrition Survey |
| 2006 | DHS |
| 2006 | WHO CGM Database |
| 2011 | WHO CGM Database |
| 2011 | DHS |
| 2013 | WHO CGM Database |
| 2013 | Nutrition Survey |

# **Azerbaijan** – Underweight (WAZ)

**G**: Overall and Severe Underweight Prevalence

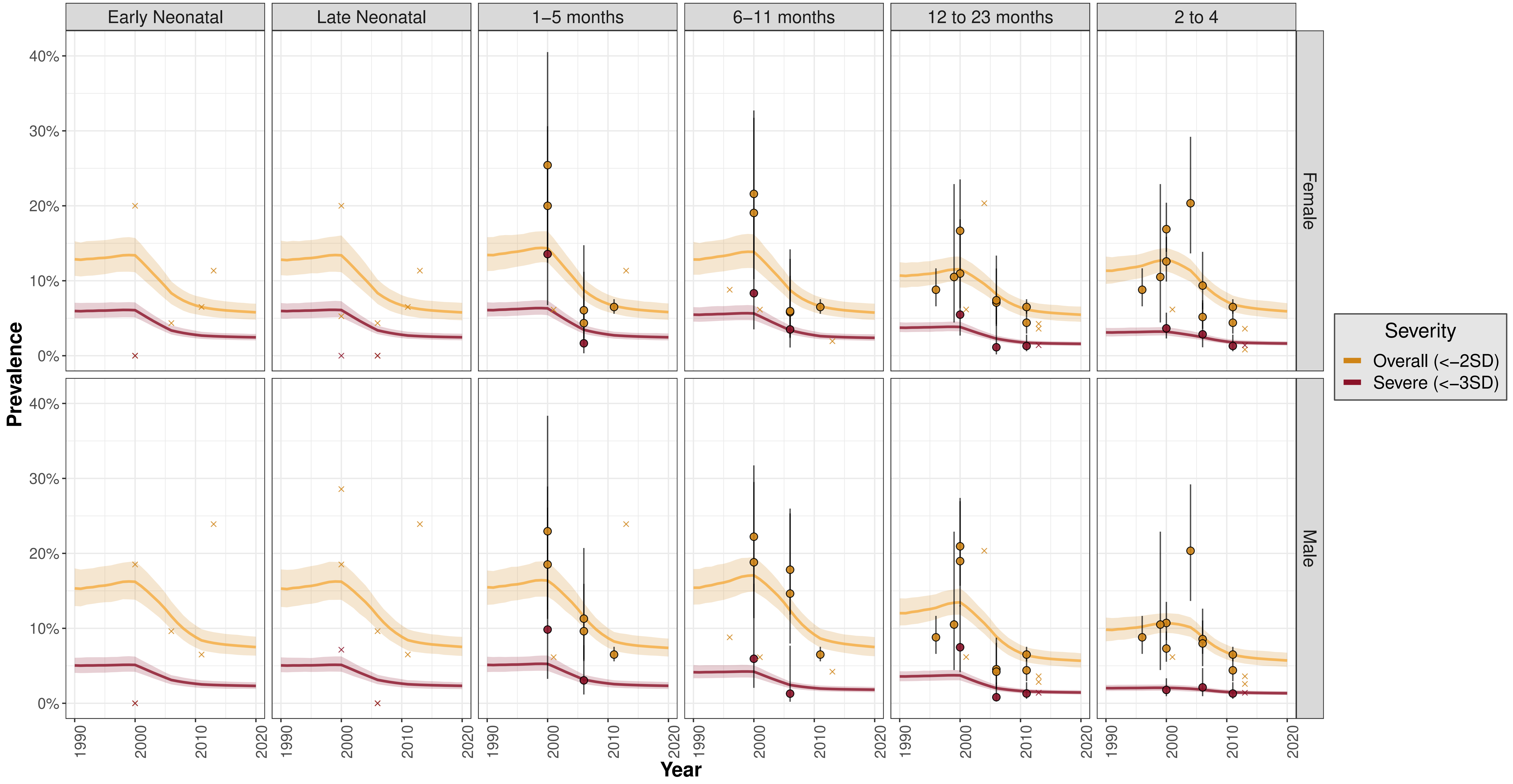


**H**: Transformed Mean Underweight Z Scores

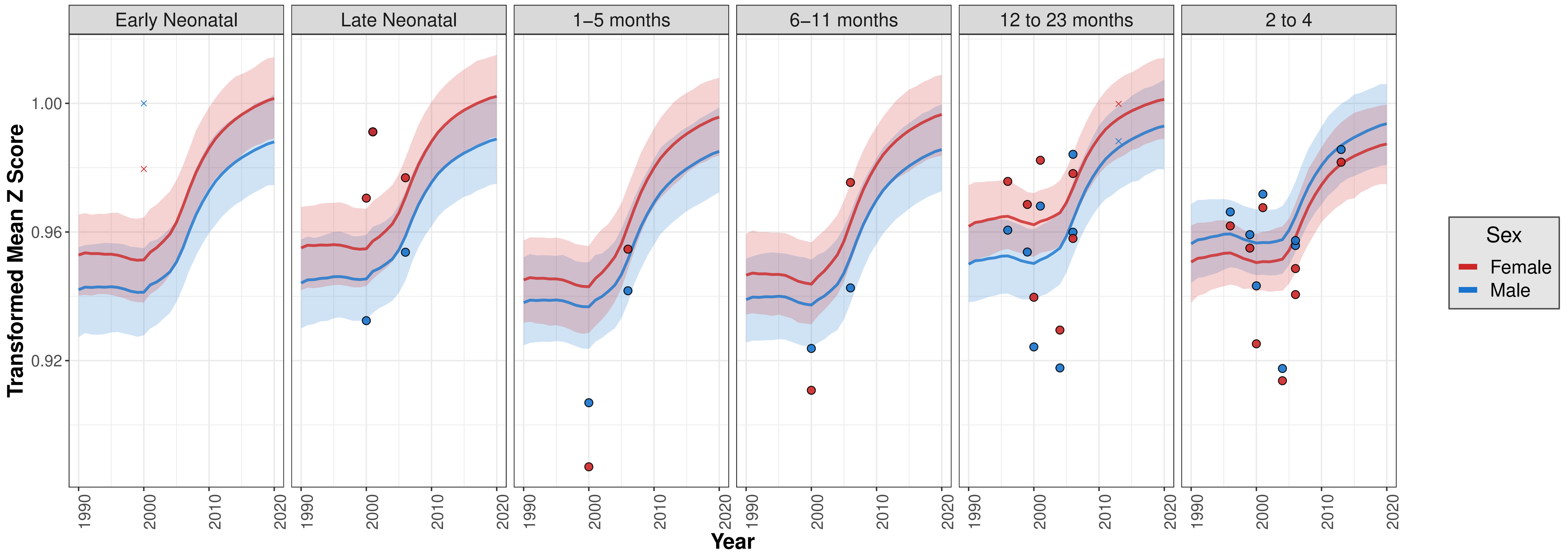


**I**

| Year | Source |
|---|---|
| 1996 | WHO CGM Database |
| 1999 | WHO CGM Database |
| 2000 | MICS |
| 2000 | WHO CGM Database |
| 2001 | WHO CGM Database |
| 2004 | Food Security and Nutrition Survey |
| 2006 | DHS |
| 2006 | WHO CGM Database |
| 2011 | WHO CGM Database |
| 2011 | DHS |
| 2013 | WHO CGM Database |
| 2013 | Nutrition Survey |

**Azerbaijan** – HAZ, WHZ, and WAZ Distributions

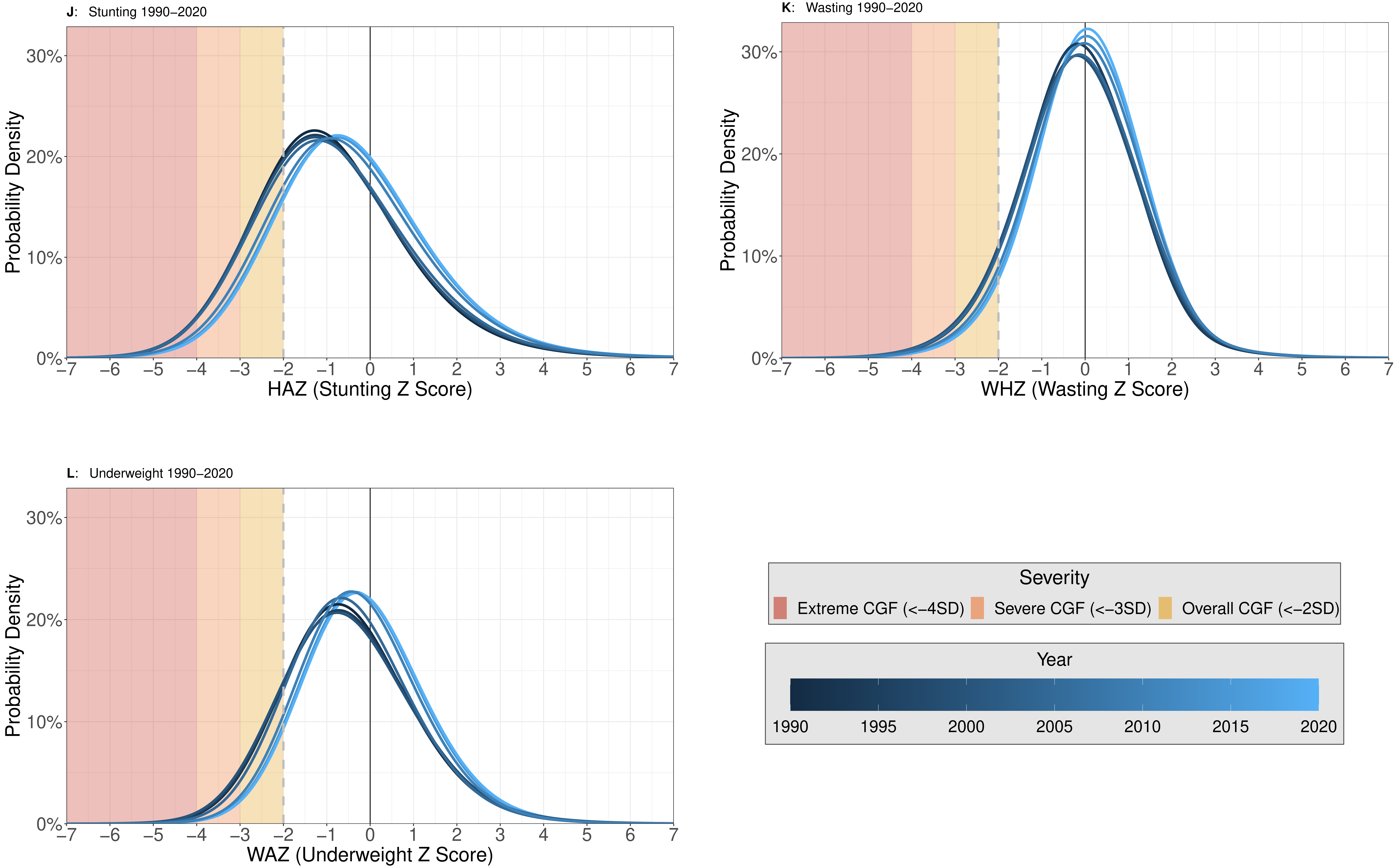

# **Georgia** – Stunting (HAZ)

**A**: Overall and Severe Stunting Prevalence

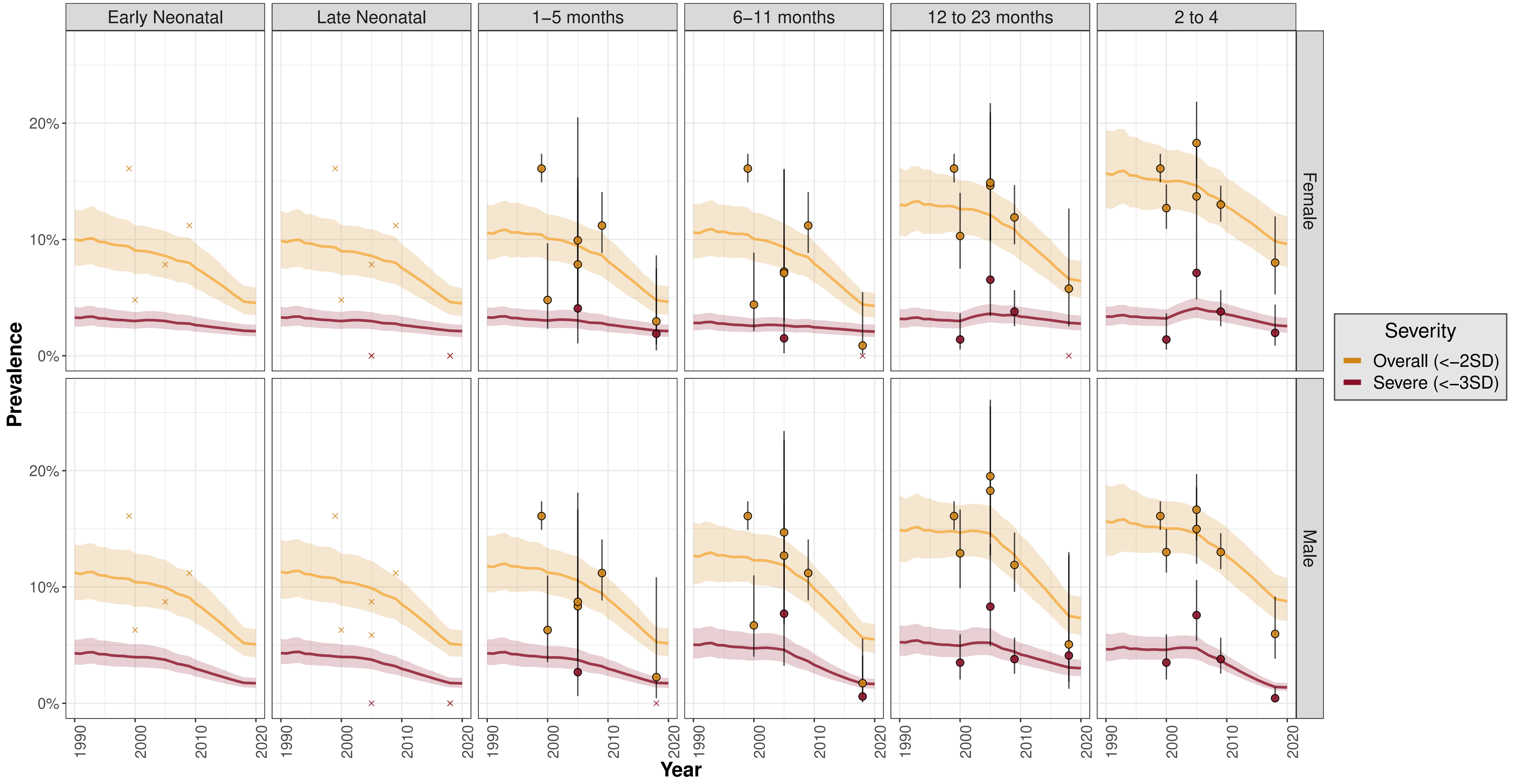


**B**: Transformed Mean Stunting Z Scores

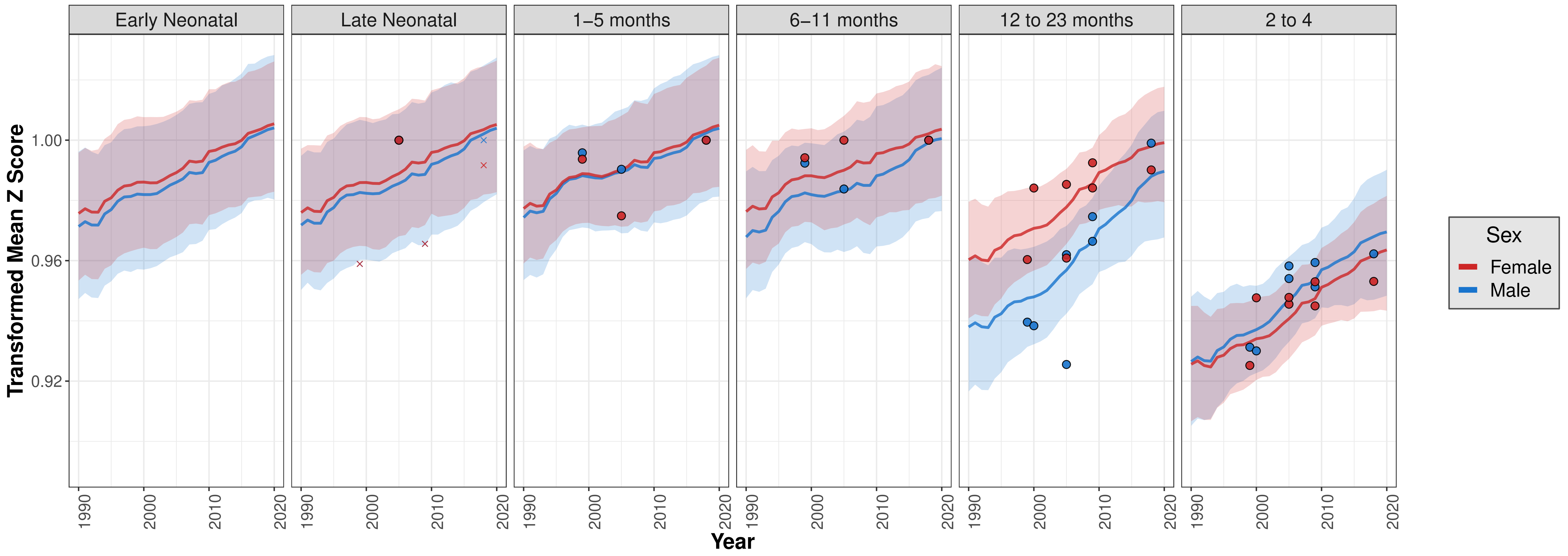


**C**

| Year | Source |
|---|---|
| 1999 | WHO CGM Database |
| 2000 | WHO CGM Database |
| 2005 | MICS |
| 2005 | WHO CGM Database |
| 2009 | WHO CGM Database |
| 2018 | MICS |

# **Georgia** – Wasting (WHZ)

**D**: Overall and Severe Wasting Prevalence

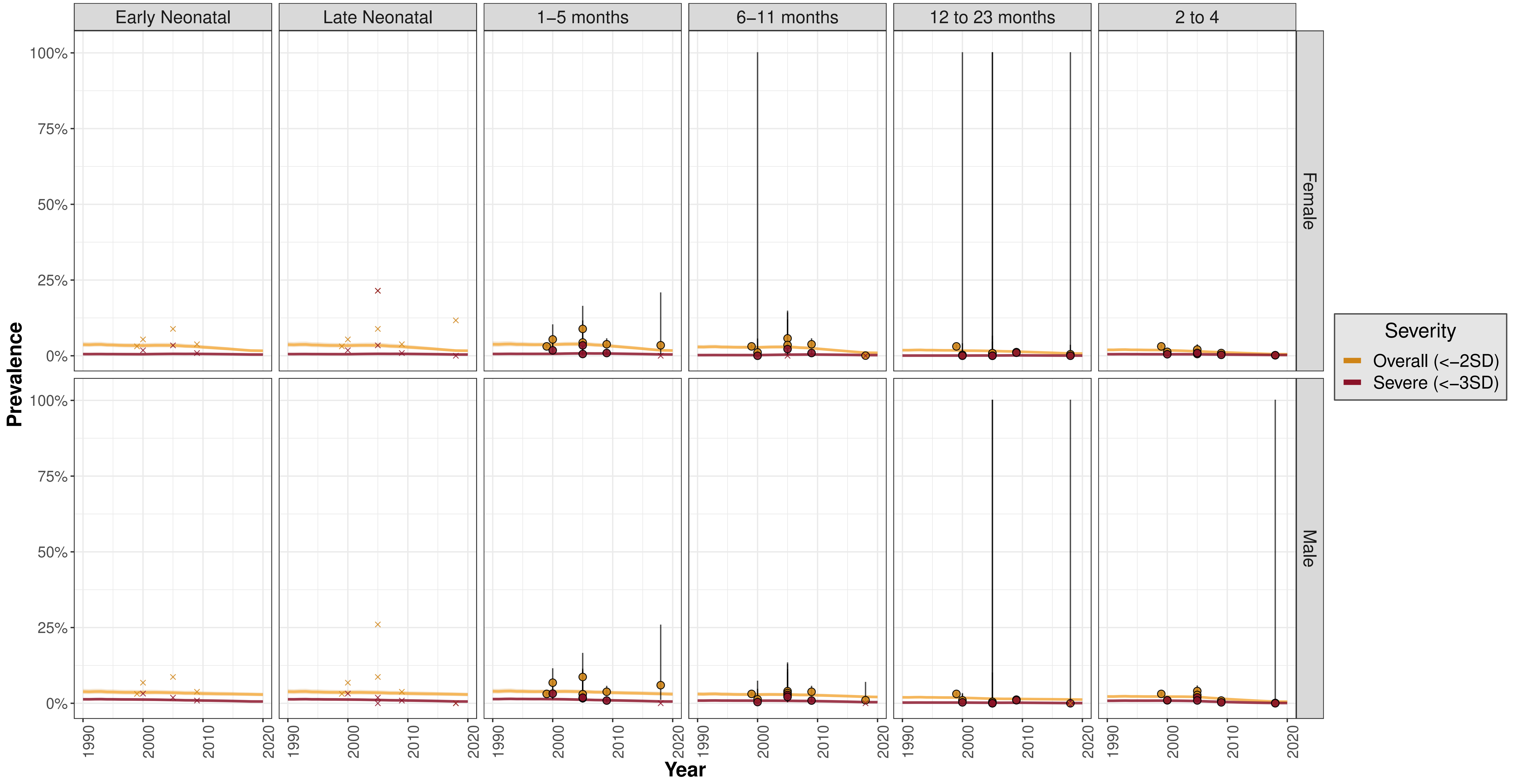


**E**: Transformed Mean Wasting Z Scores

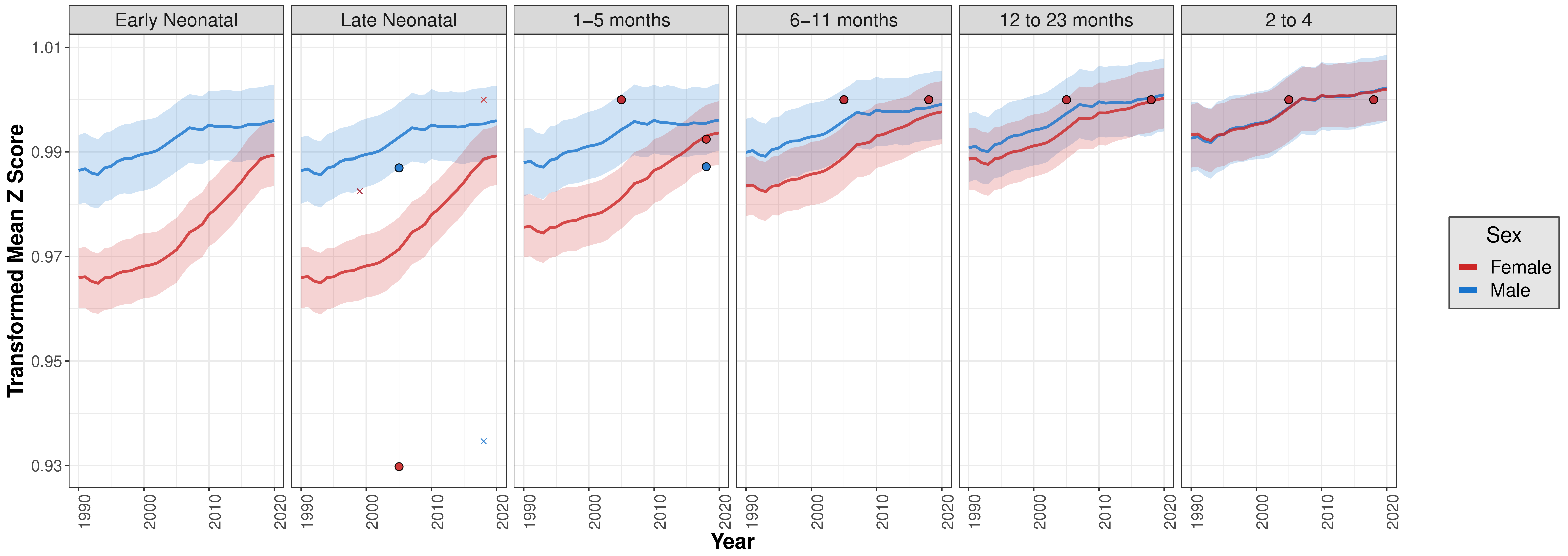


**F**

| Year | Source |
|---|---|
| 1999 | WHO CGM Database |
| 2000 | WHO CGM Database |
| 2005 | MICS |
| 2005 | WHO CGM Database |
| 2009 | WHO CGM Database |
| 2018 | MICS |

## **Georgia** – Underweight (WAZ)

**G**: Overall and Severe Underweight Prevalence

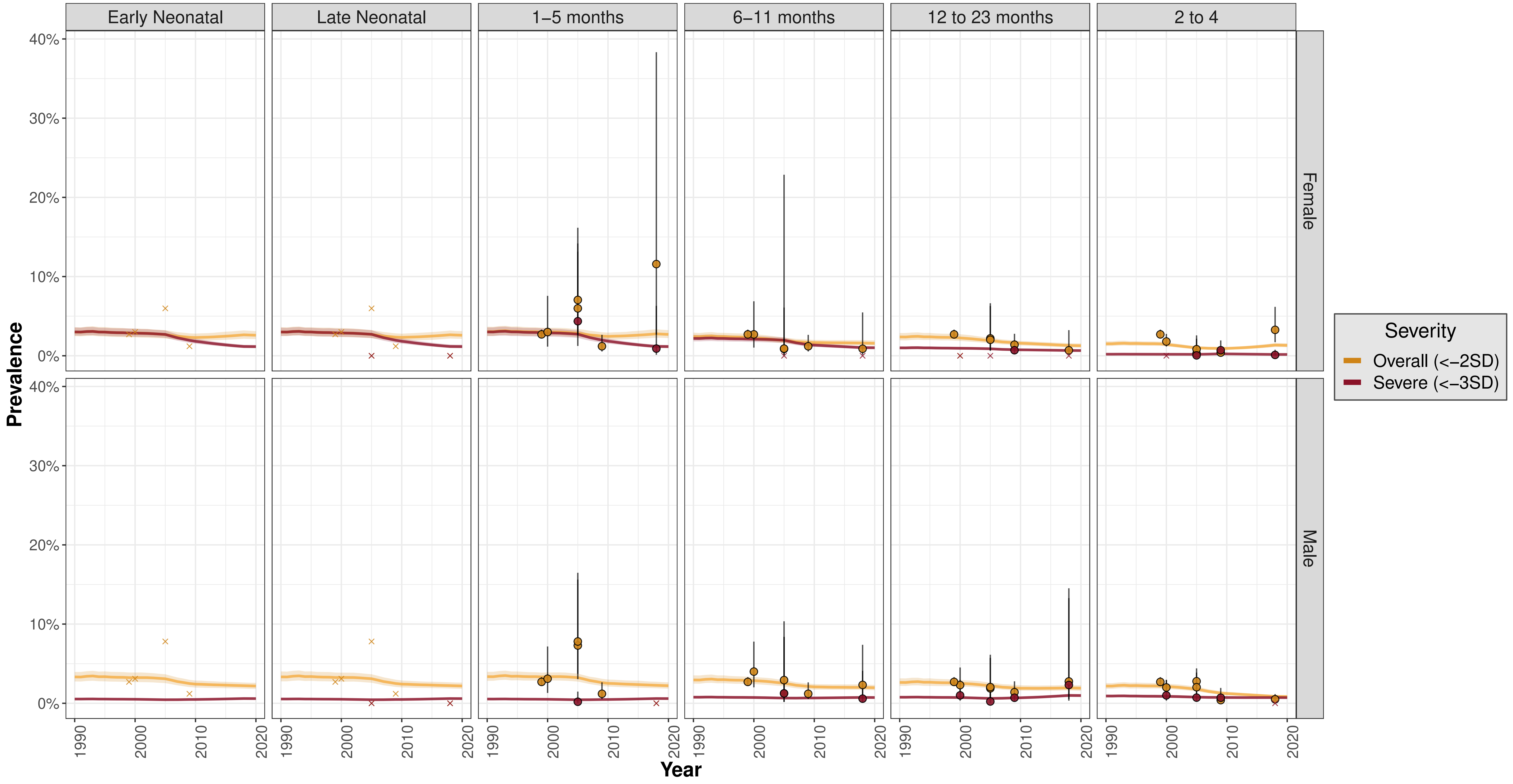


**H**: Transformed Mean Underweight Z Scores

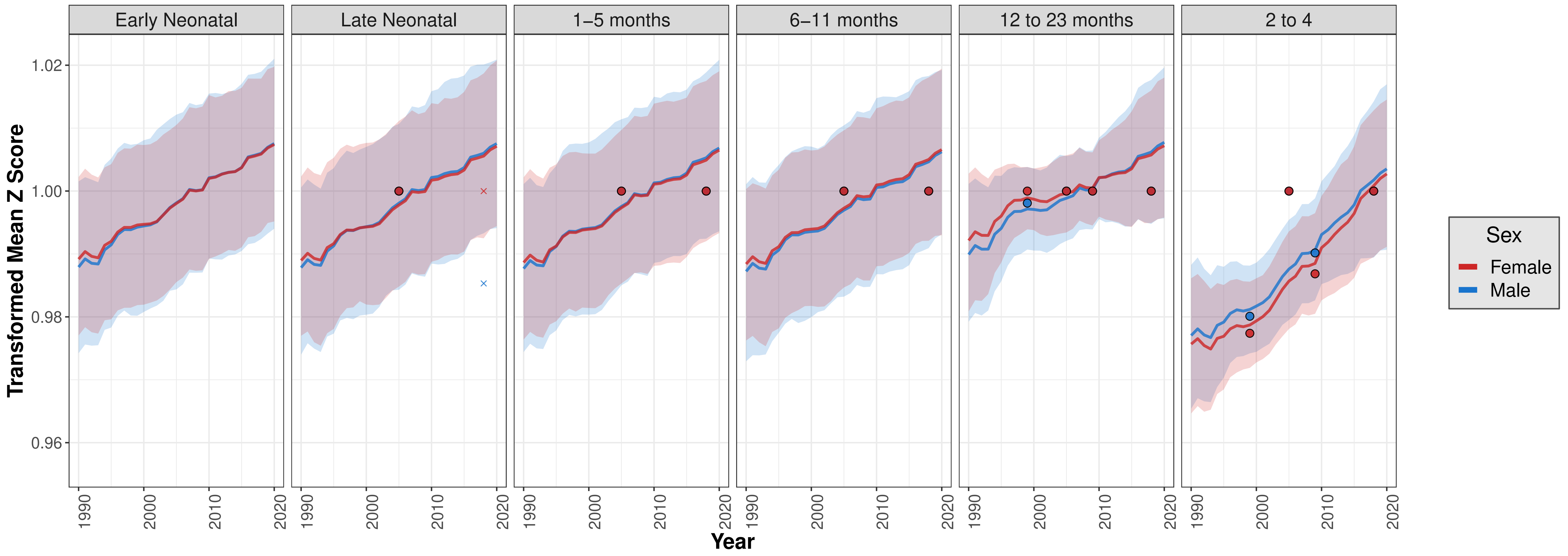


**I**

| Year | Source |
|---|---|
| 1999 | WHO CGM Database |
| 2000 | WHO CGM Database |
| 2005 | MICS |
| 2005 | WHO CGM Database |
| 2009 | WHO CGM Database |
| 2018 | MICS |

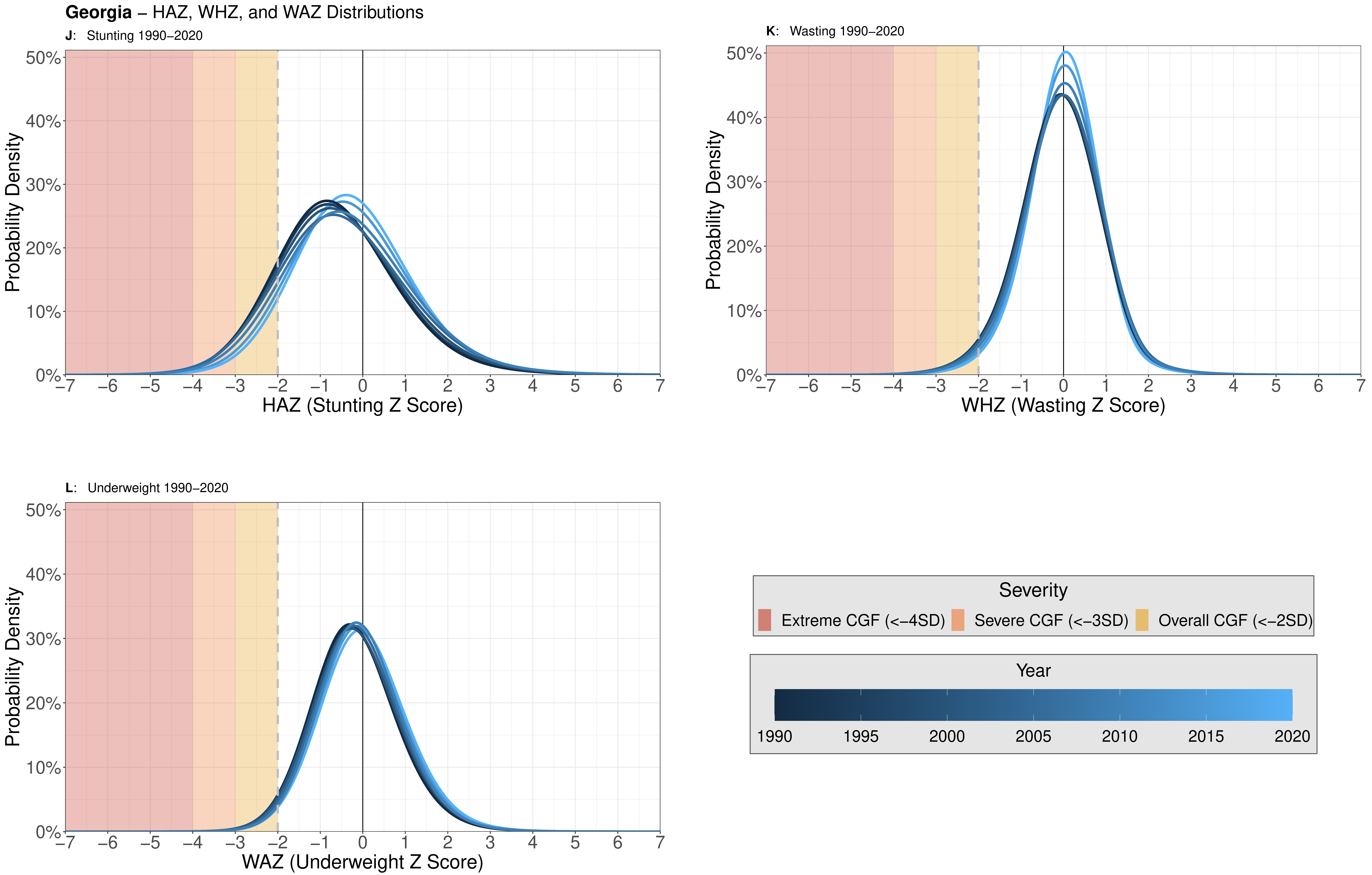

Georgia – HAZ, WHZ, and WAZ Distributions
J: Stunting 1990–2020
Probability Density
HAZ (Stunting Z Score)
K: Wasting 1990–2020
Probability Density
WHZ (Wasting Z Score)
L: Underweight 1990–2020
Probability Density
WAZ (Underweight Z Score)
0%
10%
20%
30%
40%
50%
−7
−6
−5
−4
−3
−2
−1
0
1
2
3
4
5
6
7
Severity
Extreme CGF (<−4SD)
Severe CGF (<−3SD)
Overall CGF (<−2SD)
Year
1990
1995
2000
2005
2010
2015
2020

# **Kazakhstan** – Stunting (HAZ)

**A**: Overall and Severe Stunting Prevalence

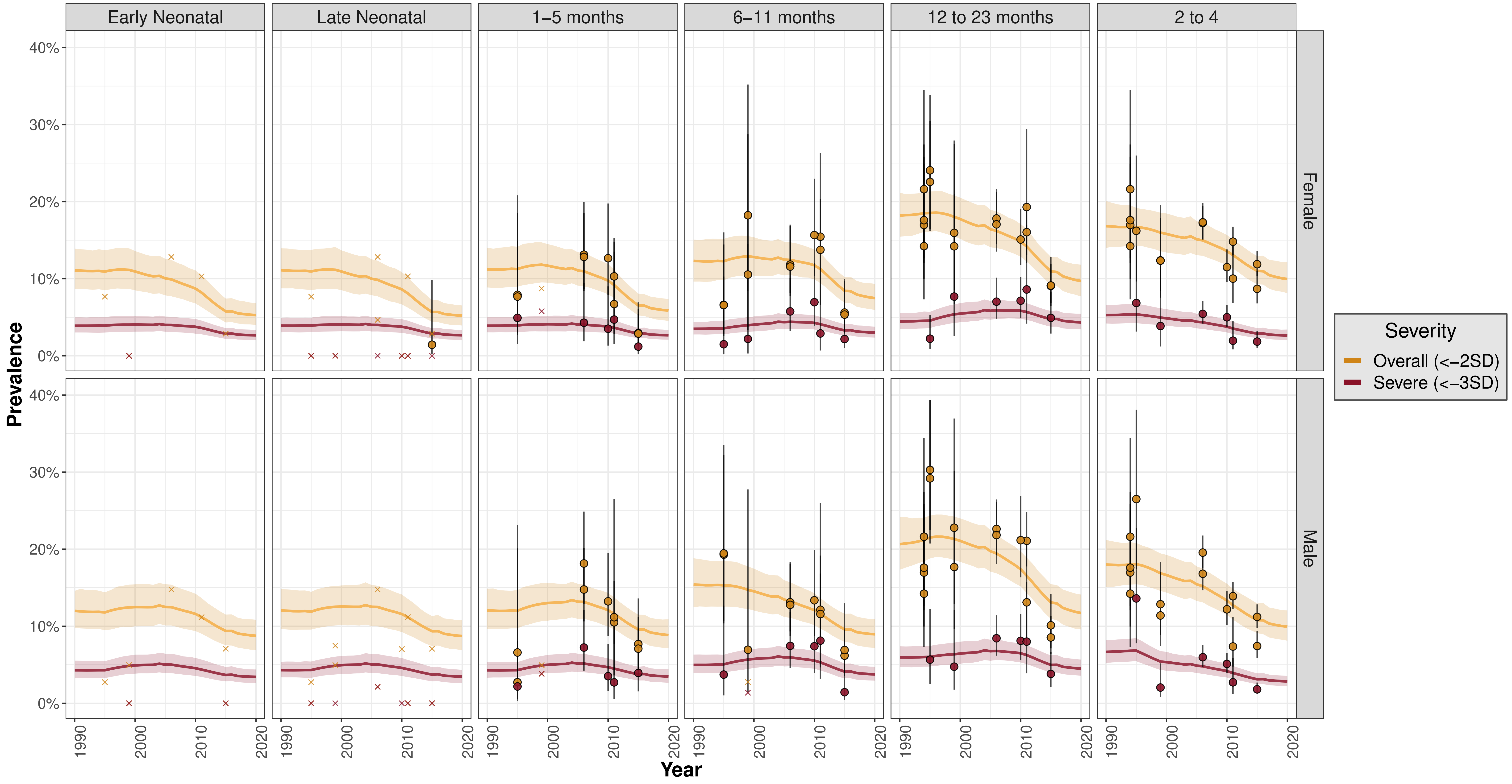


**B**: Transformed Mean Stunting Z Scores

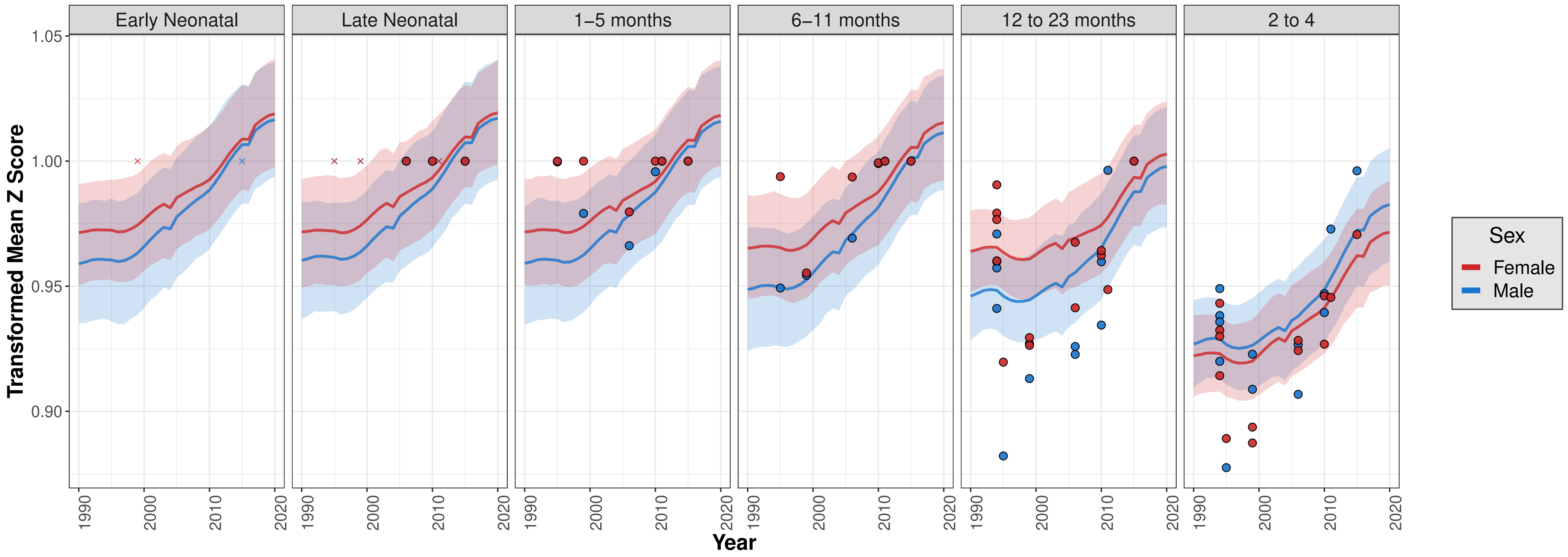


**C**

| Year | Source |
|---|---|
| 1994 | WHO CGM Database |
| 1995 | DHS |
| 1995 | WHO CGM Database |
| 1999 | DHS |
| 1999 | WHO CGM Database |
| 2006 | MICS |
| 2006 | WHO CGM Database |
| 2010 | MICS |
| 2010 | WHO CGM Database |
| 2011 | MICS |
| 2011 | WHO CGM Database |
| 2015 | WHO CGM Database |
| 2015 | MICS |

## **Kazakhstan** – Wasting (WHZ)

**D**: Overall and Severe Wasting Prevalence

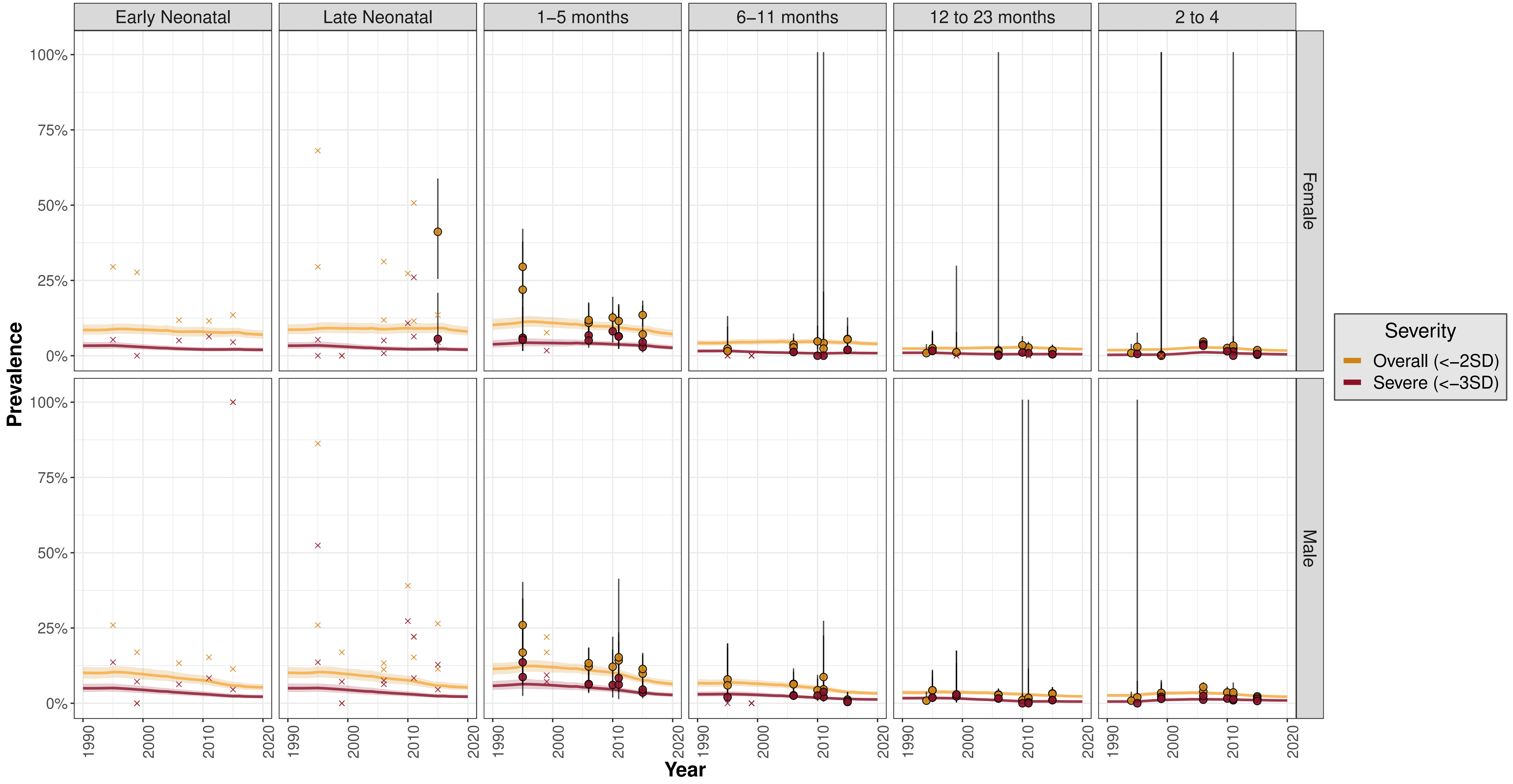


**E**: Transformed Mean Wasting Z Scores

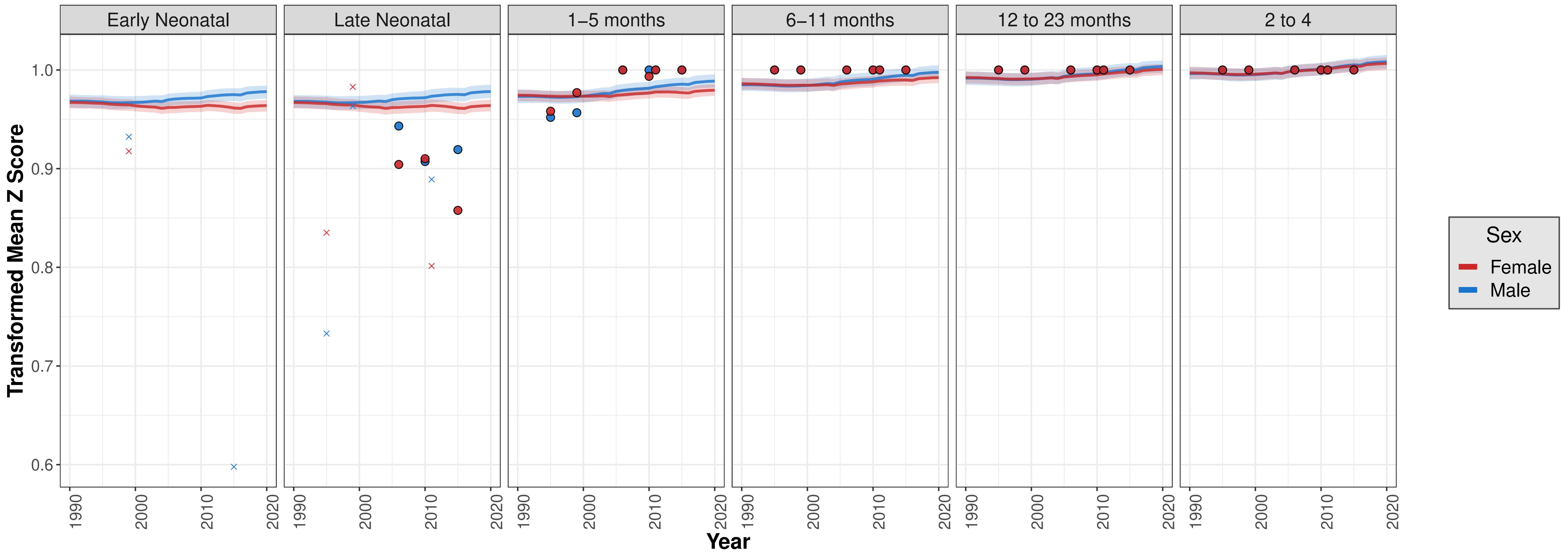


**F**

| Year | Source |
|---|---|
| 1994 | WHO CGM Database |
| 1995 | DHS |
| 1995 | WHO CGM Database |
| 1999 | DHS |
| 1999 | WHO CGM Database |
| 2006 | MICS |
| 2006 | WHO CGM Database |
| 2010 | MICS |
| 2011 | MICS |
| 2011 | WHO CGM Database |
| 2015 | WHO CGM Database |
| 2015 | MICS |

**Kazakhstan** – Underweight (WAZ)

**G**: Overall and Severe Underweight Prevalence

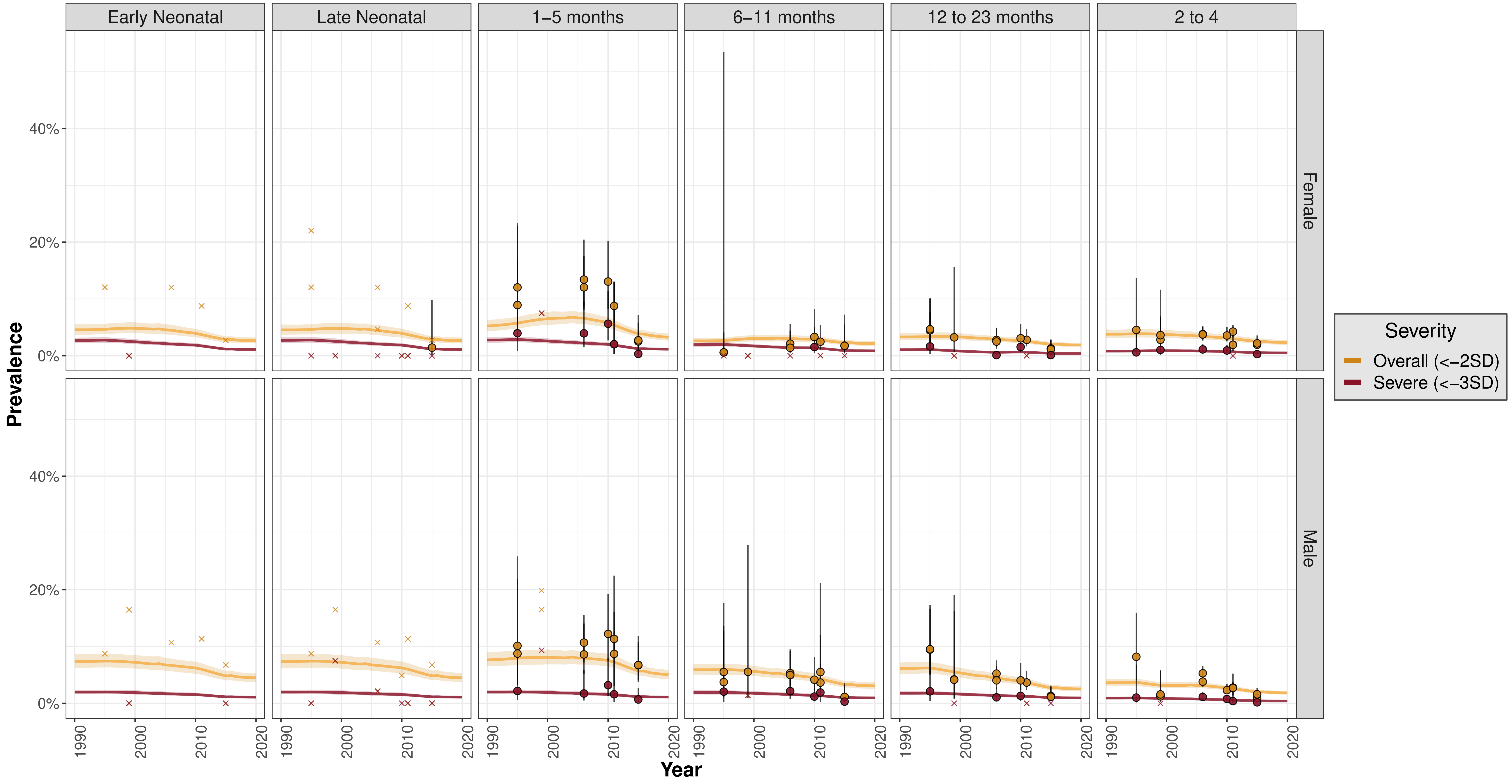


**H**: Transformed Mean Underweight Z Scores

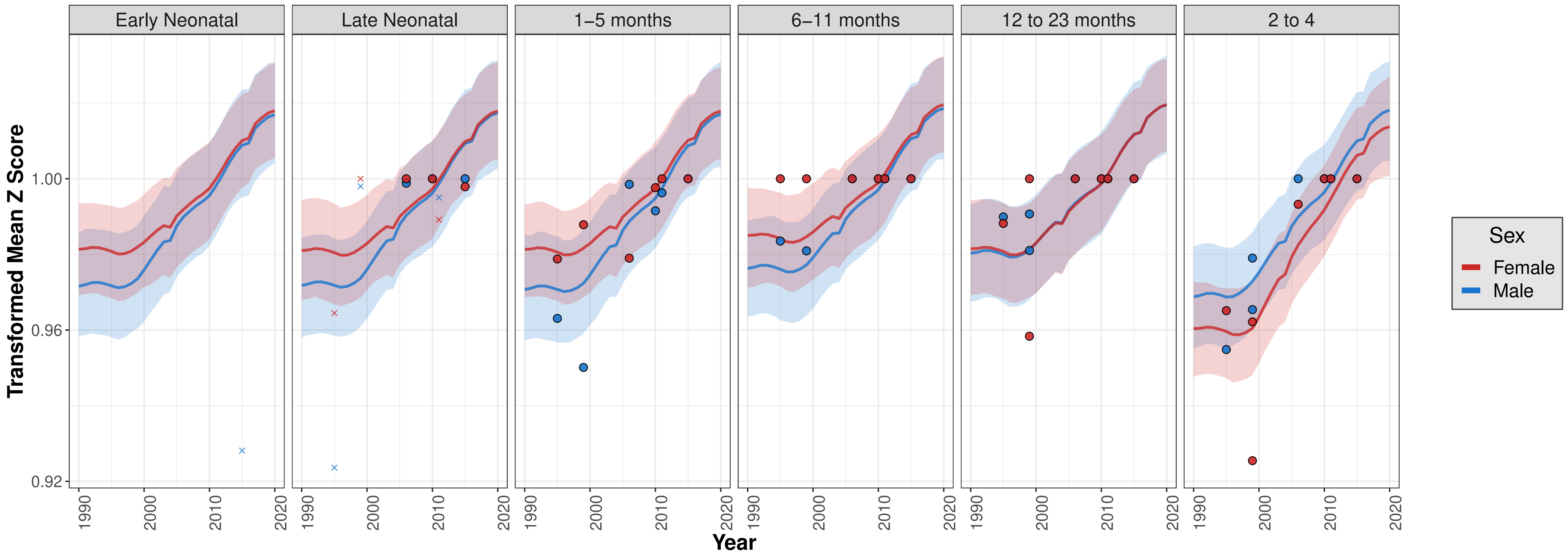


**I**

| Year | Source |
|---|---|
| 1995 | DHS |
| 1995 | WHO CGM Database |
| 1999 | DHS |
| 1999 | WHO CGM Database |
| 2006 | MICS |
| 2006 | WHO CGM Database |
| 2010 | MICS |
| 2011 | MICS |
| 2011 | WHO CGM Database |
| 2015 | WHO CGM Database |
| 2015 | MICS |

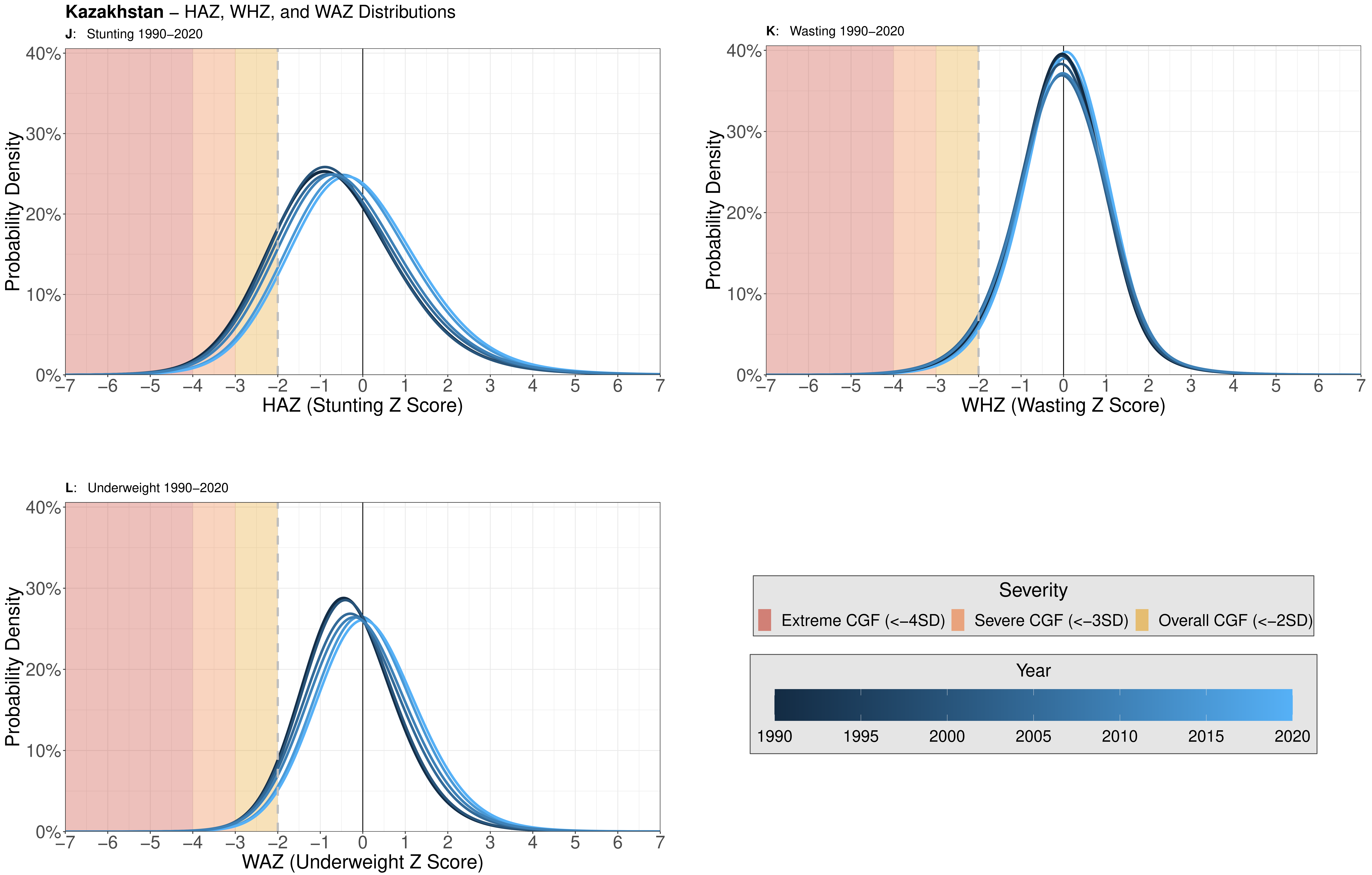
Kazakhstan – HAZ, WHZ, and WAZ Distributions
J: Stunting 1990–2020
Probability Density
40%
30%
20%
10%
0%
−7 −6 −5 −4 −3 −2 −1 0 1 2 3 4 5 6 7
HAZ (Stunting Z Score)
K: Wasting 1990–2020
Probability Density
WHZ (Wasting Z Score)
L: Underweight 1990–2020
Probability Density
WAZ (Underweight Z Score)
Severity
Extreme CGF (<−4SD)
Severe CGF (<−3SD)
Overall CGF (<−2SD)
Year
1990
1995
2000
2005
2010
2015
2020

## **Kyrgyzstan** – Stunting (HAZ)

**A**: Overall and Severe Stunting Prevalence

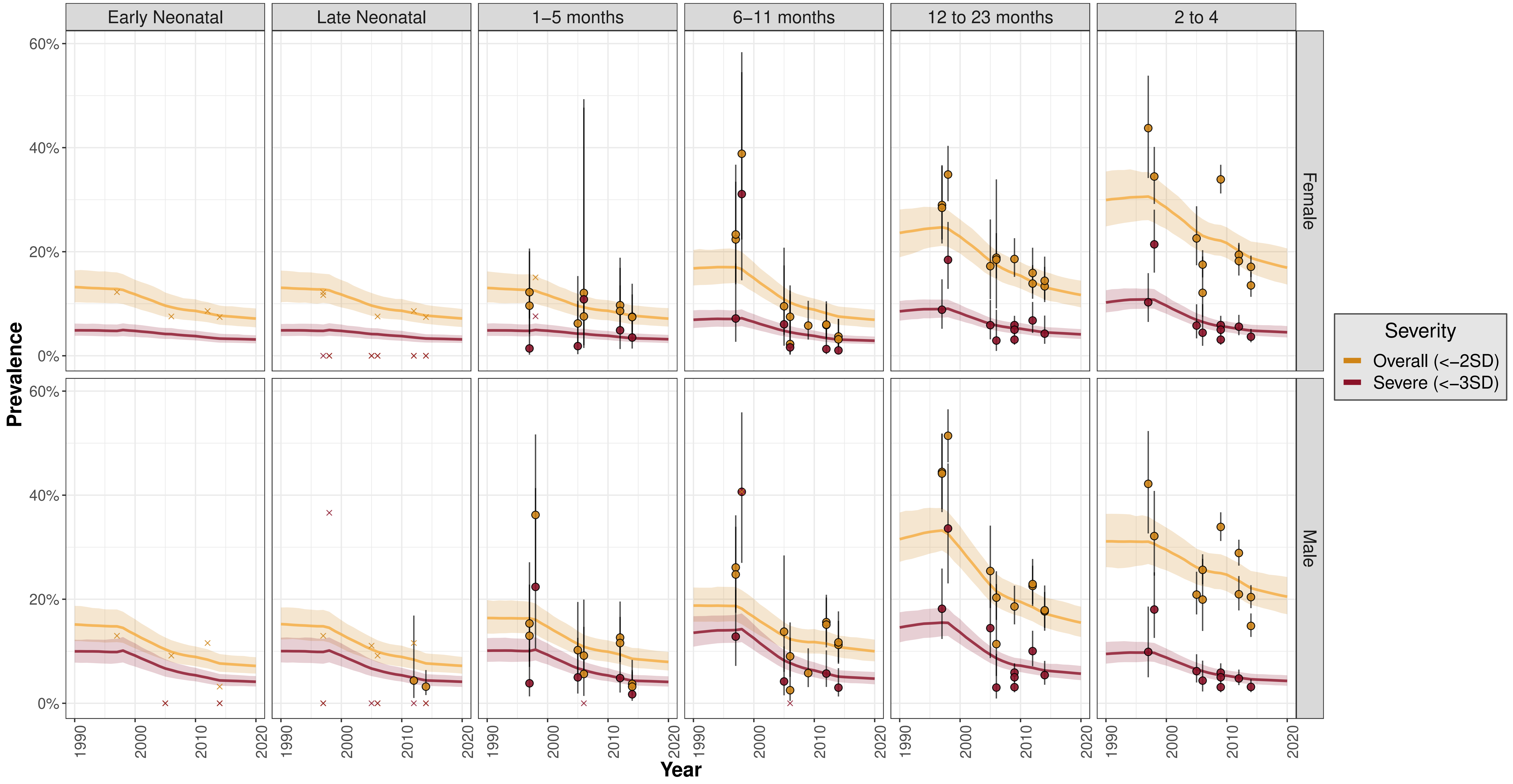


**B**: Transformed Mean Stunting Z Scores

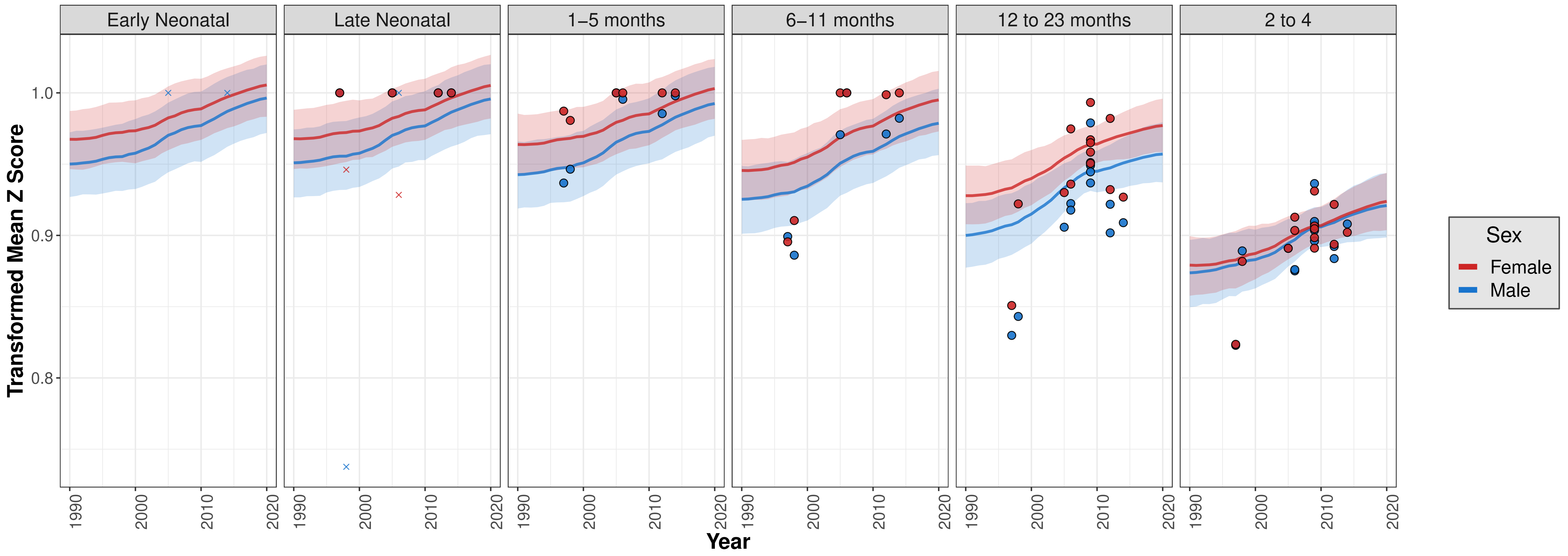


**C**

| Year | Source |
|---|---|
| 1997 | DHS |
| 1997 | WHO CGM Database |
| 1998 | Living Standards Measurement Survey |
| 2005 | MICS |
| 2006 | MICS |
| 2006 | WHO CGM Database |
| 2009 | WHO CGM Database |
| 2012 | DHS |
| 2012 | WHO CGM Database |
| 2014 | WHO CGM Database |
| 2014 | MICS |

# **Kyrgyzstan** – Wasting (WHZ)

**D**: Overall and Severe Wasting Prevalence

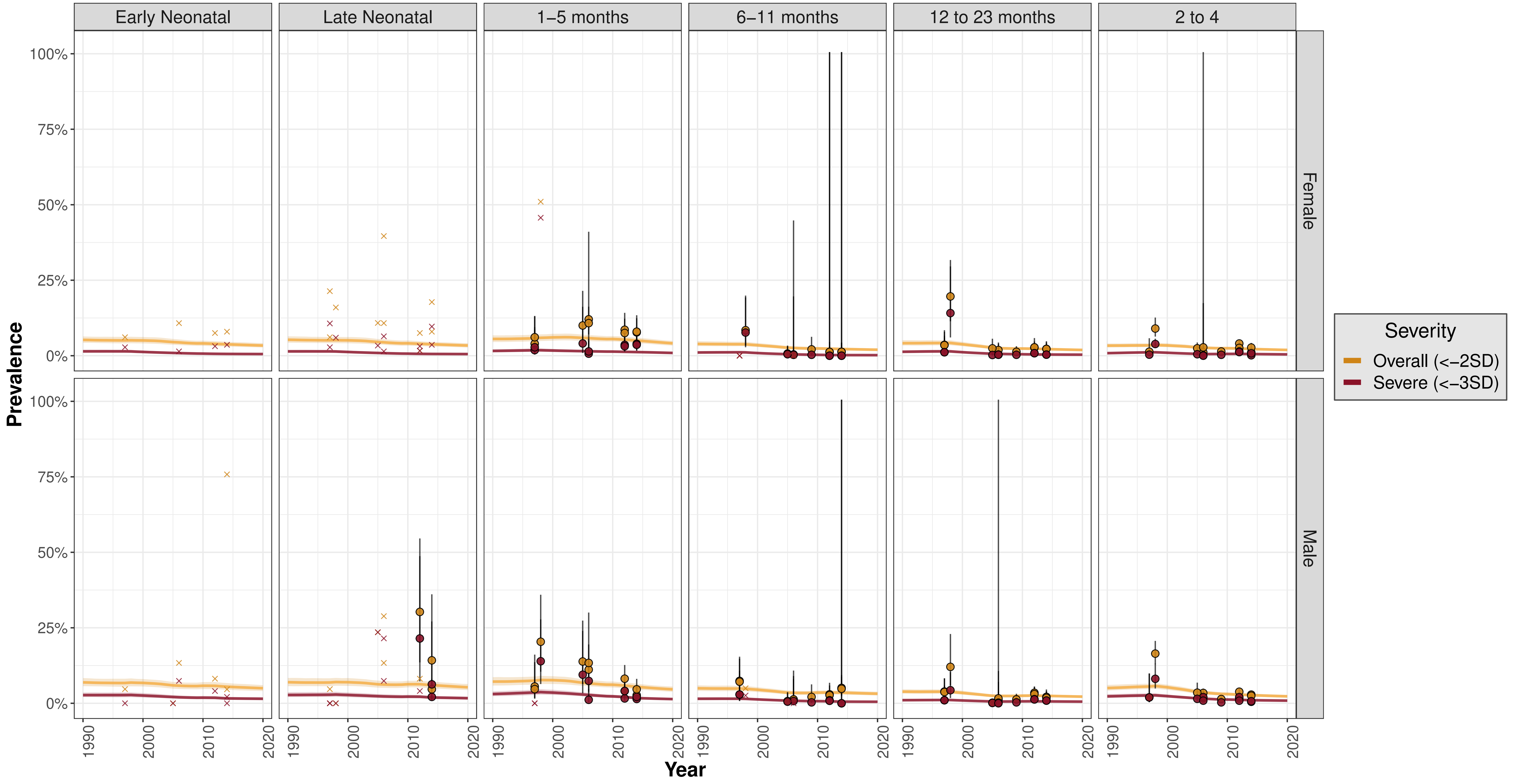


**E**: Transformed Mean Wasting Z Scores

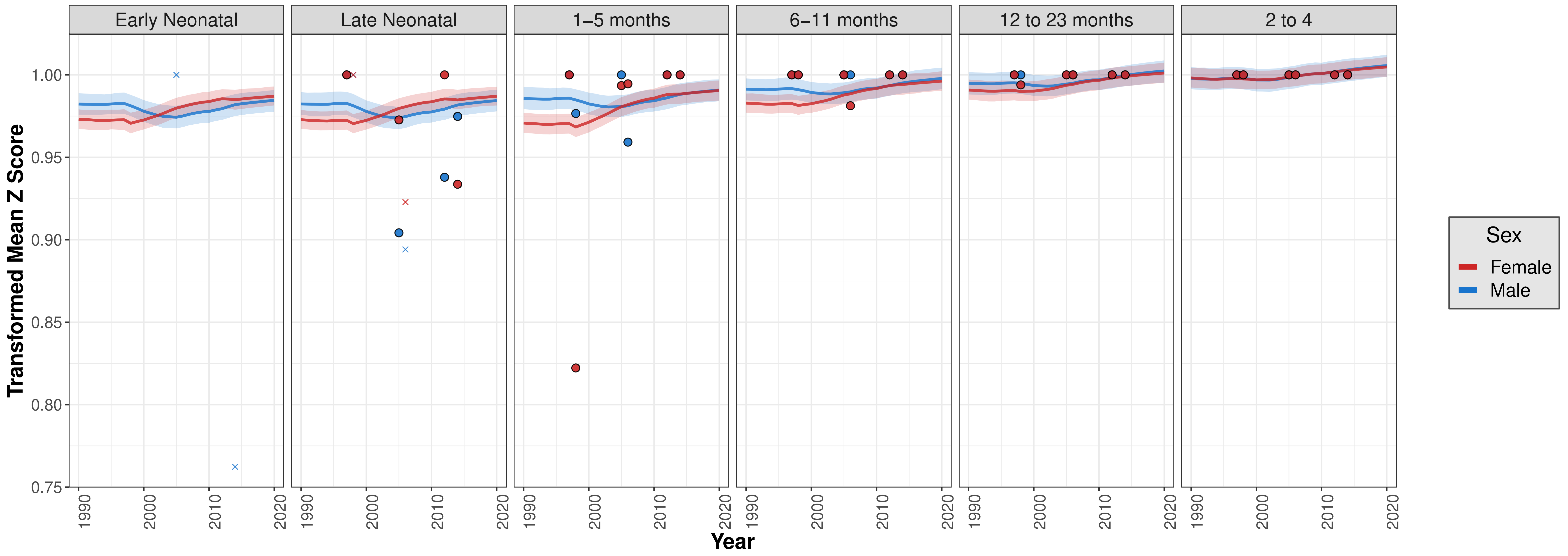


**F**

| Year | Source |
|---|---|
| 1997 | DHS |
| 1997 | WHO CGM Database |
| 1998 | Living Standards Measurement Survey |
| 2005 | MICS |
| 2006 | MICS |
| 2006 | WHO CGM Database |
| 2009 | WHO CGM Database |
| 2012 | DHS |
| 2012 | WHO CGM Database |
| 2014 | WHO CGM Database |
| 2014 | MICS |

**Kyrgyzstan** – Underweight (WAZ)

**G**: Overall and Severe Underweight Prevalence

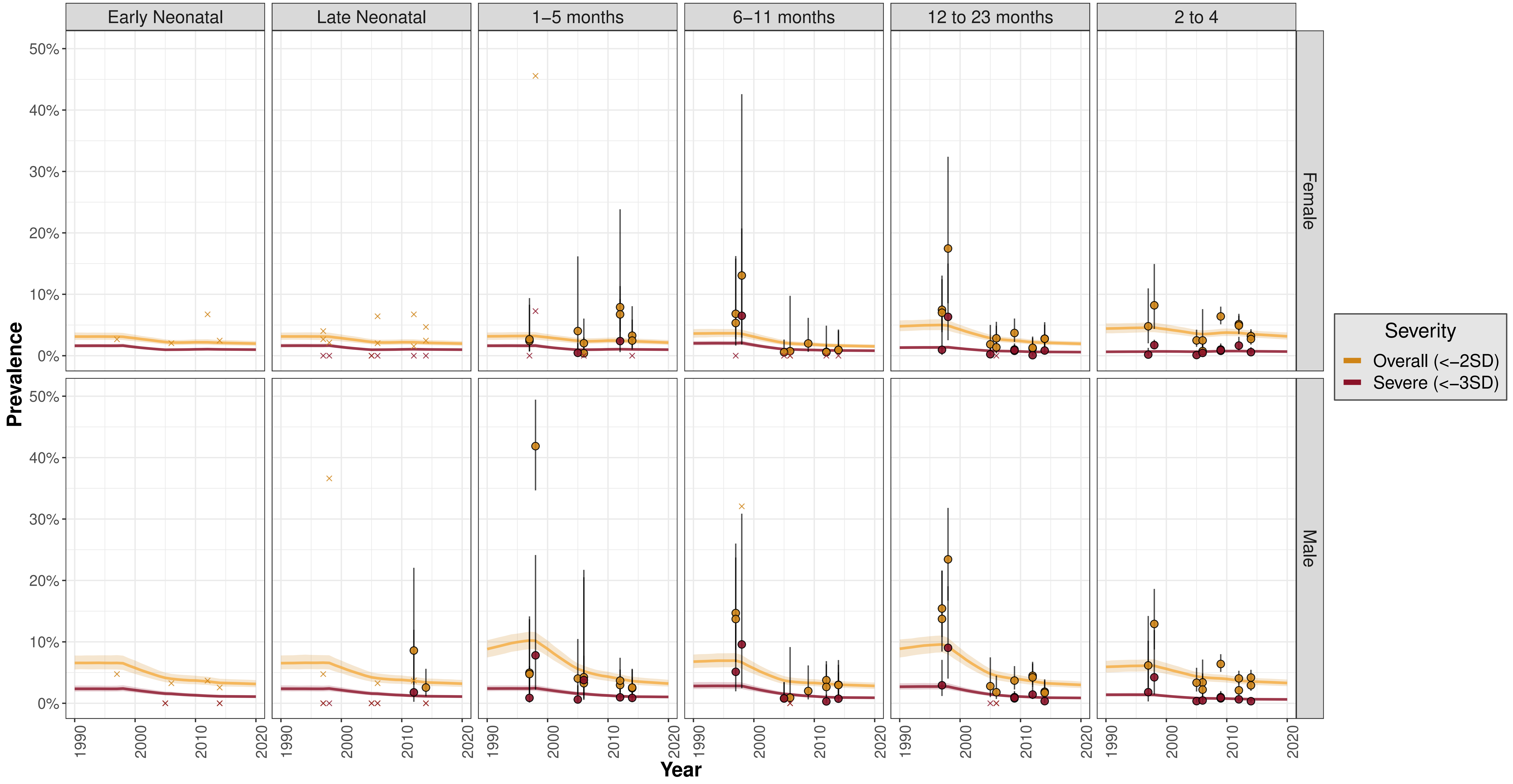


**H**: Transformed Mean Underweight Z Scores

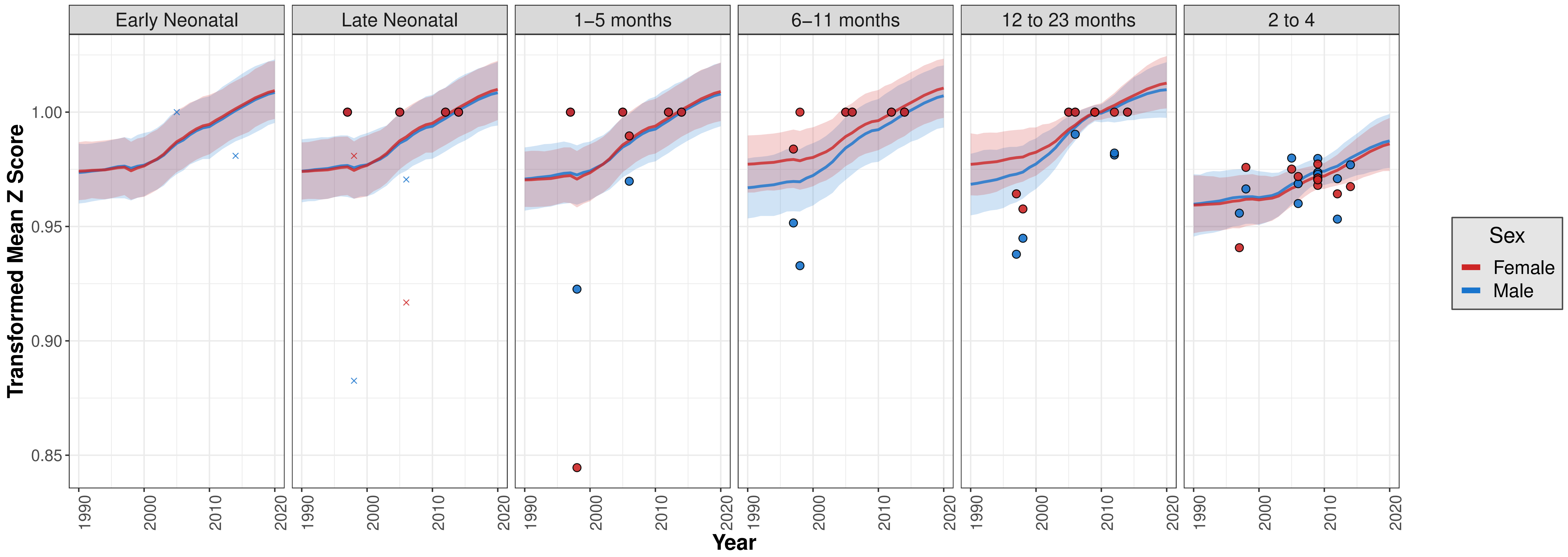


**I**

| Year | Source |
|---|---|
| 1997 | DHS |
| 1997 | WHO CGM Database |
| 1998 | Living Standards Measurement Survey |
| 2005 | MICS |
| 2006 | MICS |
| 2006 | WHO CGM Database |
| 2009 | WHO CGM Database |
| 2012 | DHS |
| 2012 | WHO CGM Database |
| 2014 | WHO CGM Database |
| 2014 | MICS |

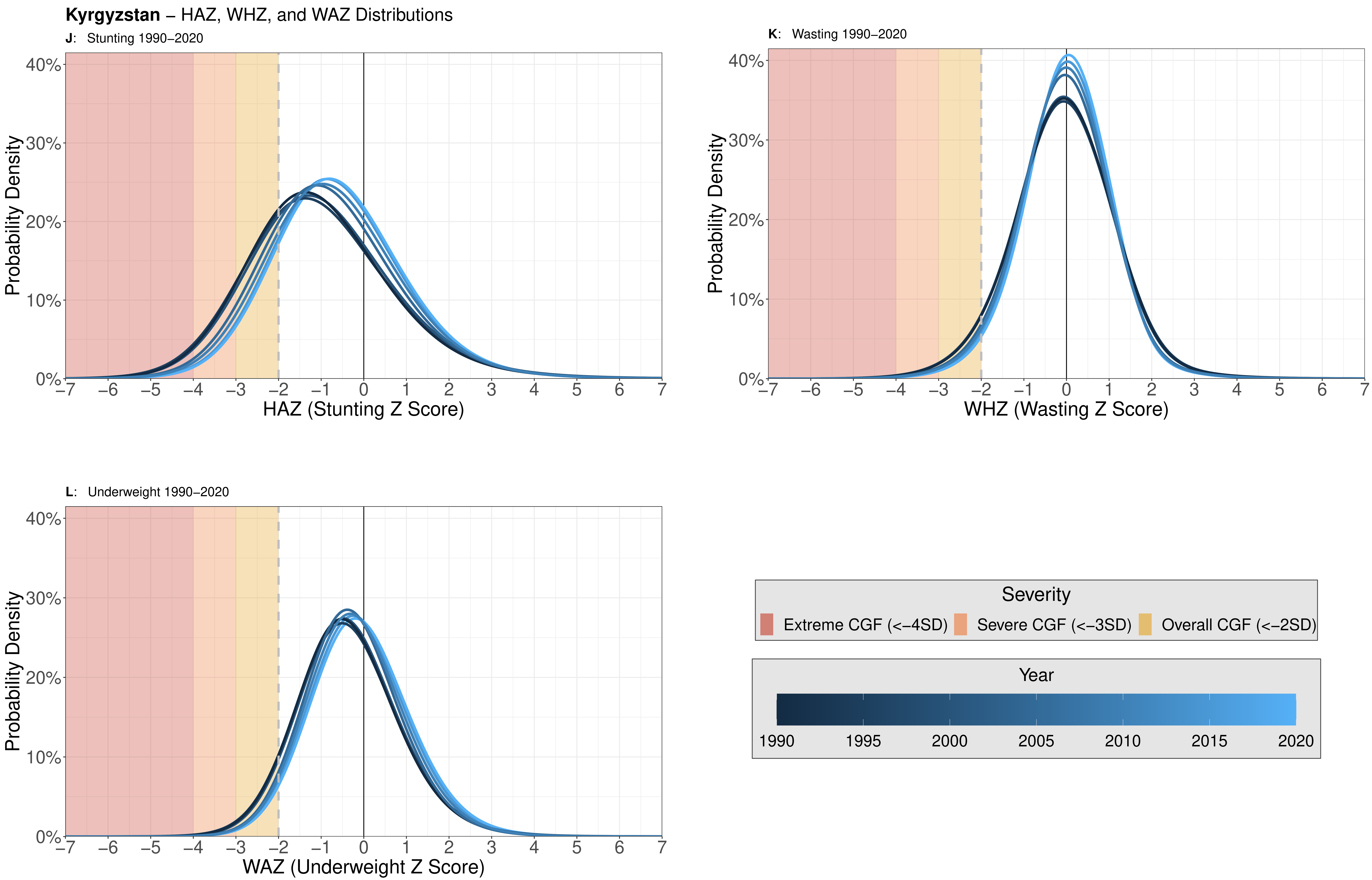

Kyrgyzstan – HAZ, WHZ, and WAZ Distributions
J: Stunting 1990–2020
K: Wasting 1990–2020
L: Underweight 1990–2020
Probability Density
0%
10%
20%
30%
40%
−7 −6 −5 −4 −3 −2 −1 0 1 2 3 4 5 6 7
HAZ (Stunting Z Score)
WHZ (Wasting Z Score)
WAZ (Underweight Z Score)
Severity
Extreme CGF (<−4SD)
Severe CGF (<−3SD)
Overall CGF (<−2SD)
Year
1990 1995 2000 2005 2010 2015 2020

**Mongolia** – Stunting (HAZ)

**A**: Overall and Severe Stunting Prevalence

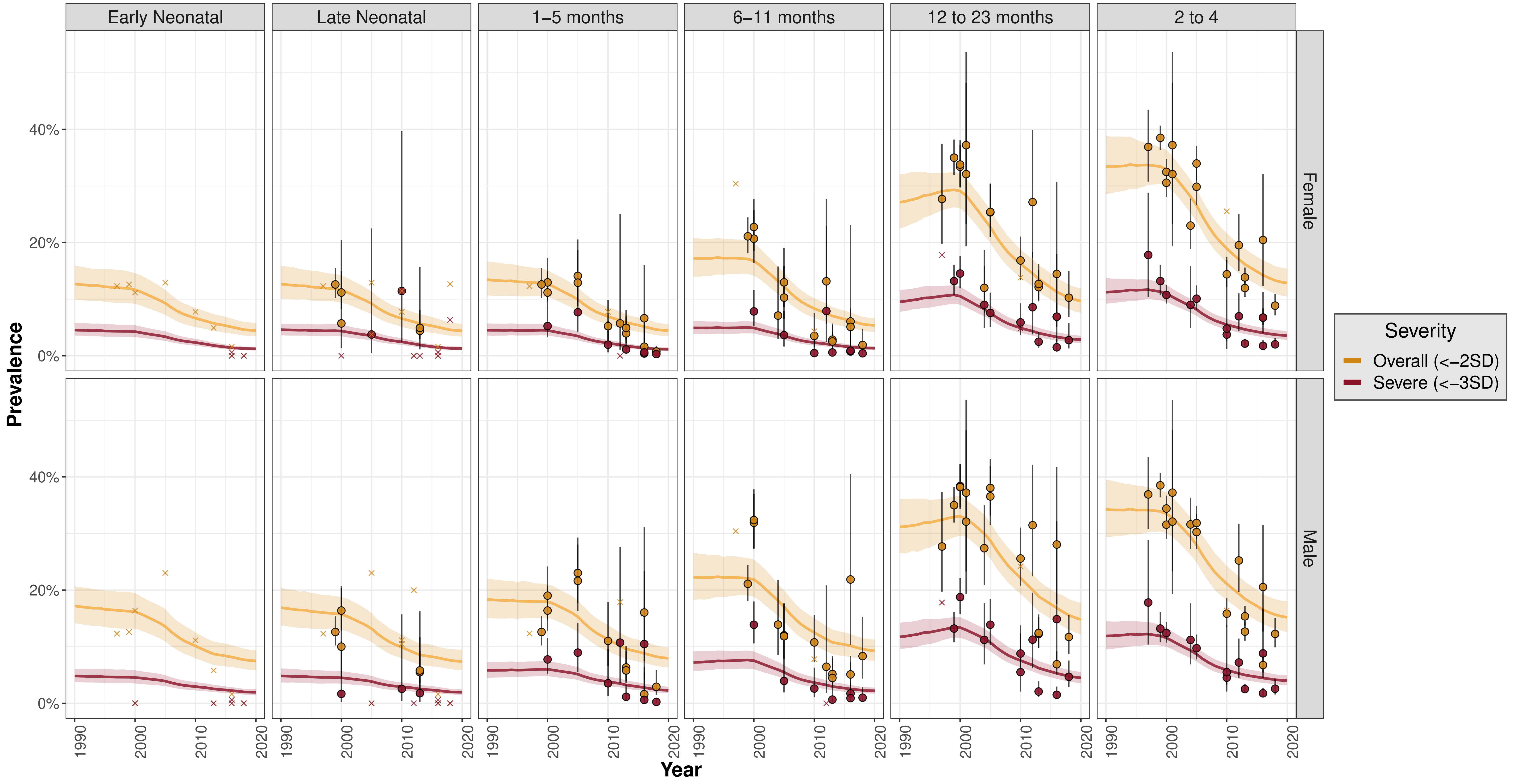


**B**: Transformed Mean Stunting Z Scores

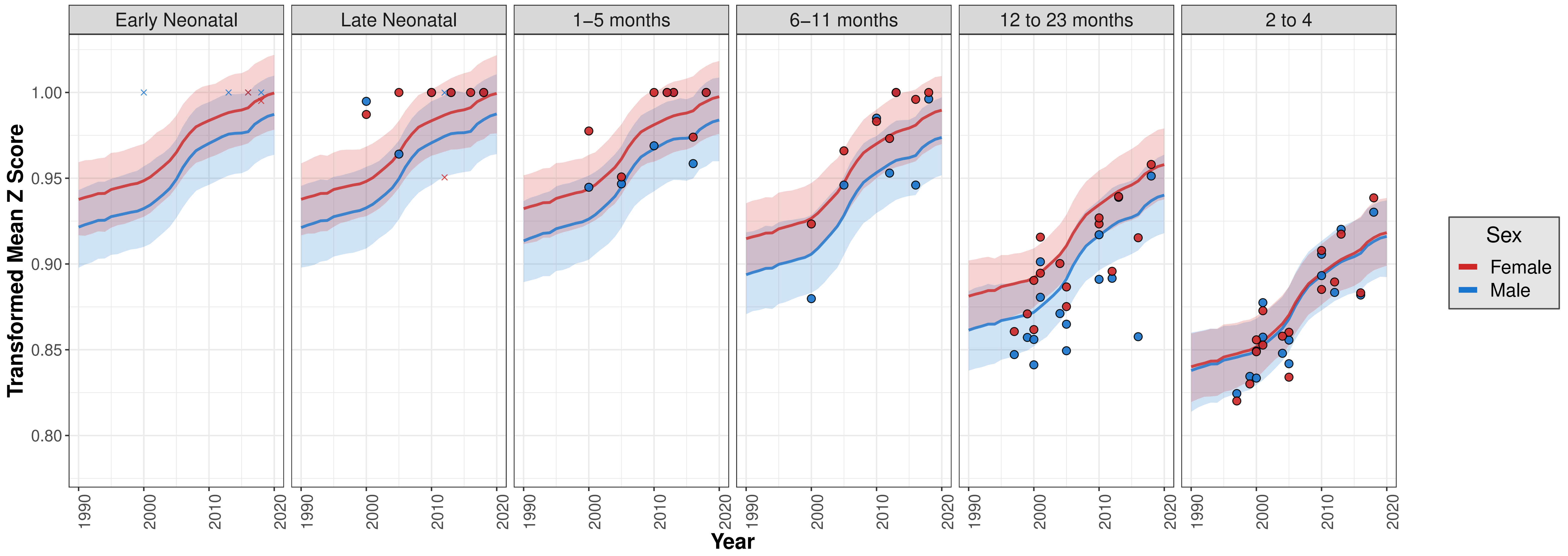


**C**

| Year | Source |
|---|---|
| 1997 | WHO CGM Database |
| 1999 | WHO CGM Database |
| 2000 | MICS |
| 2000 | WHO CGM Database |
| 2001 | WHO CGM Database |
| 2004 | WHO CGM Database |
| 2005 | MICS |
| 2005 | WHO CGM Database |
| 2010 | MICS |
| 2010 | WHO CGM Database |
| 2012 | Khuvsgul MICS |
| 2012 | Nalaikh District MICS |
| 2013 | WHO CGM Database |
| 2013 | MICS |
| 2016 | Khuvsgul MICS |
| 2016 | Nalaikh District MICS |
| 2016 | National Nutrition Survey |
| 2018 | MICS |

**Mongolia** – Wasting (WHZ)

**D**: Overall and Severe Wasting Prevalence

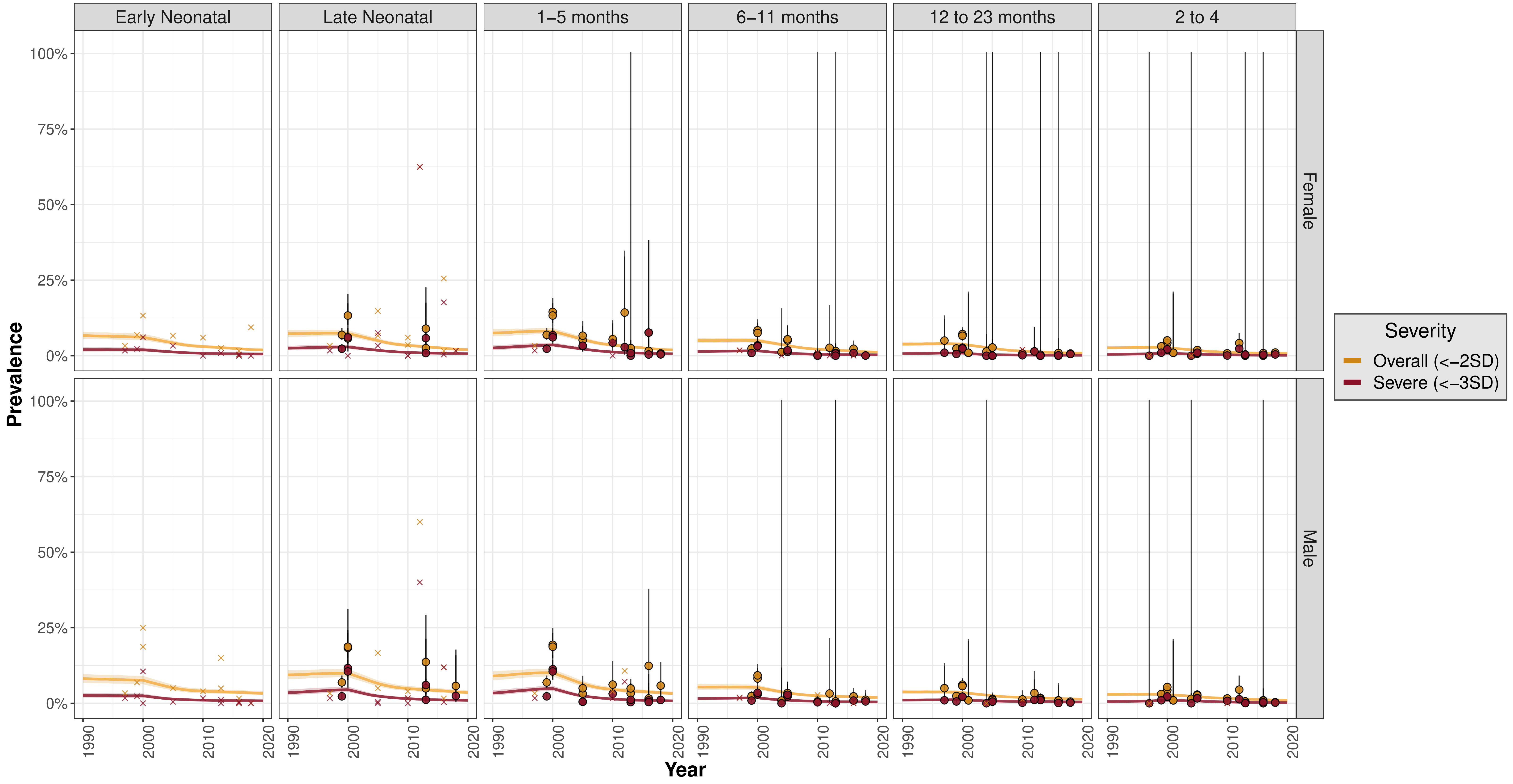


**E**: Transformed Mean Wasting Z Scores

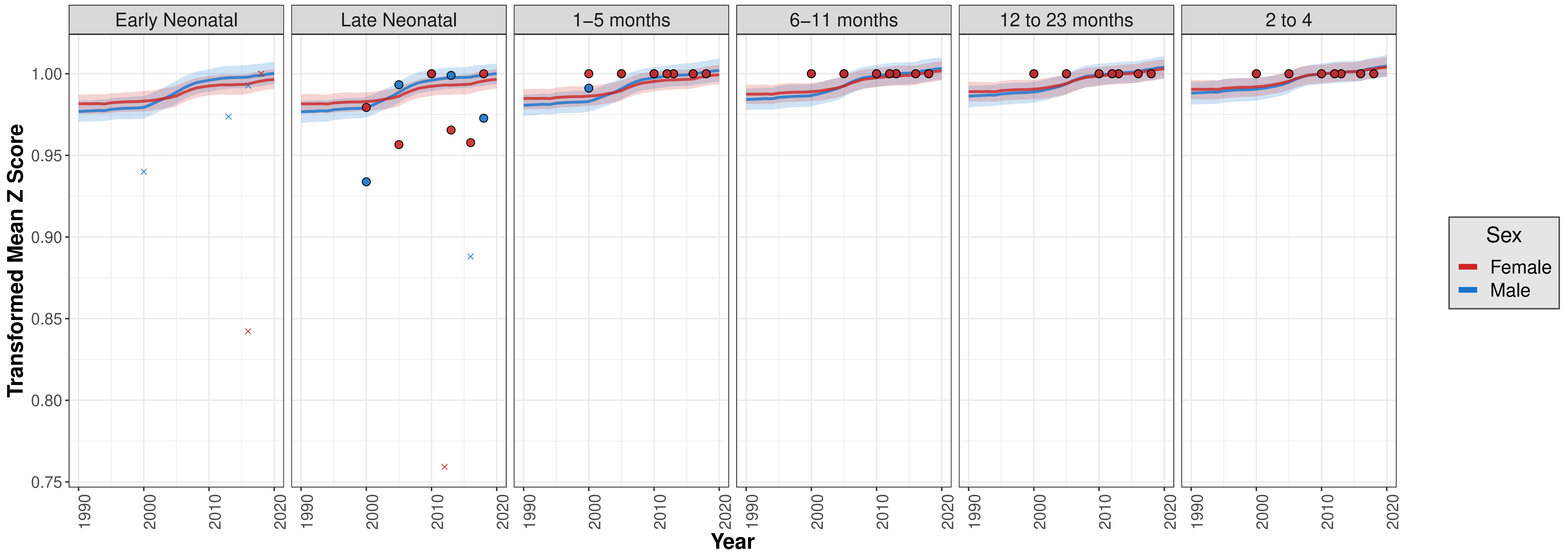


**F**

| Year | Source |
|---|---|
| 1997 | WHO CGM Database |
| 1999 | WHO CGM Database |
| 2000 | MICS |
| 2000 | WHO CGM Database |
| 2001 | WHO CGM Database |
| 2004 | WHO CGM Database |
| 2005 | MICS |
| 2005 | WHO CGM Database |
| 2010 | MICS |
| 2010 | WHO CGM Database |
| 2012 | Khuvsgul MICS |
| 2012 | Nalaikh District MICS |
| 2013 | WHO CGM Database |
| 2013 | MICS |
| 2016 | Khuvsgul MICS |
| 2016 | Nalaikh District MICS |
| 2016 | National Nutrition Survey |
| 2018 | MICS |

# **Mongolia** – Underweight (WAZ)

**G**: Overall and Severe Underweight Prevalence

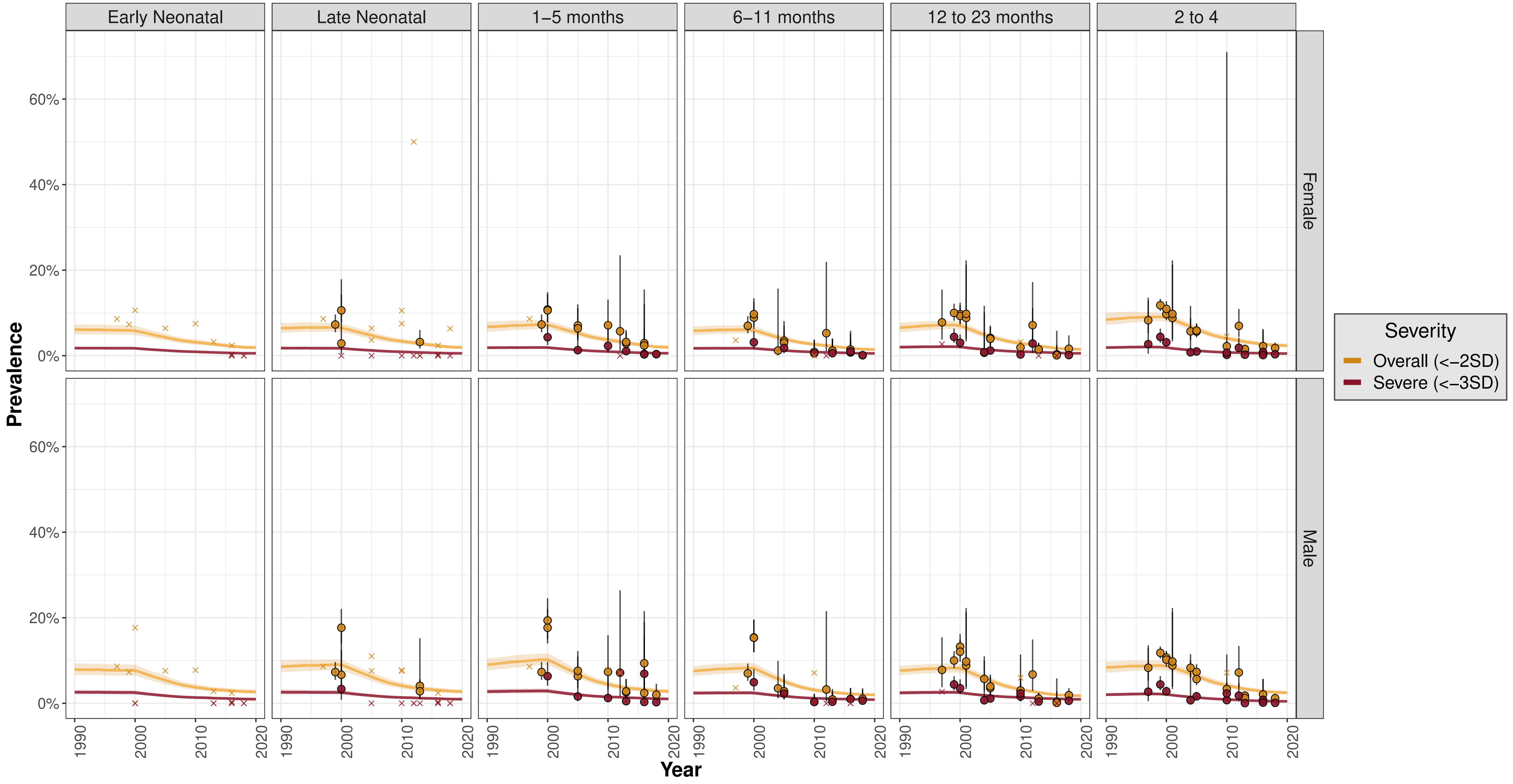


**H**: Transformed Mean Underweight Z Scores

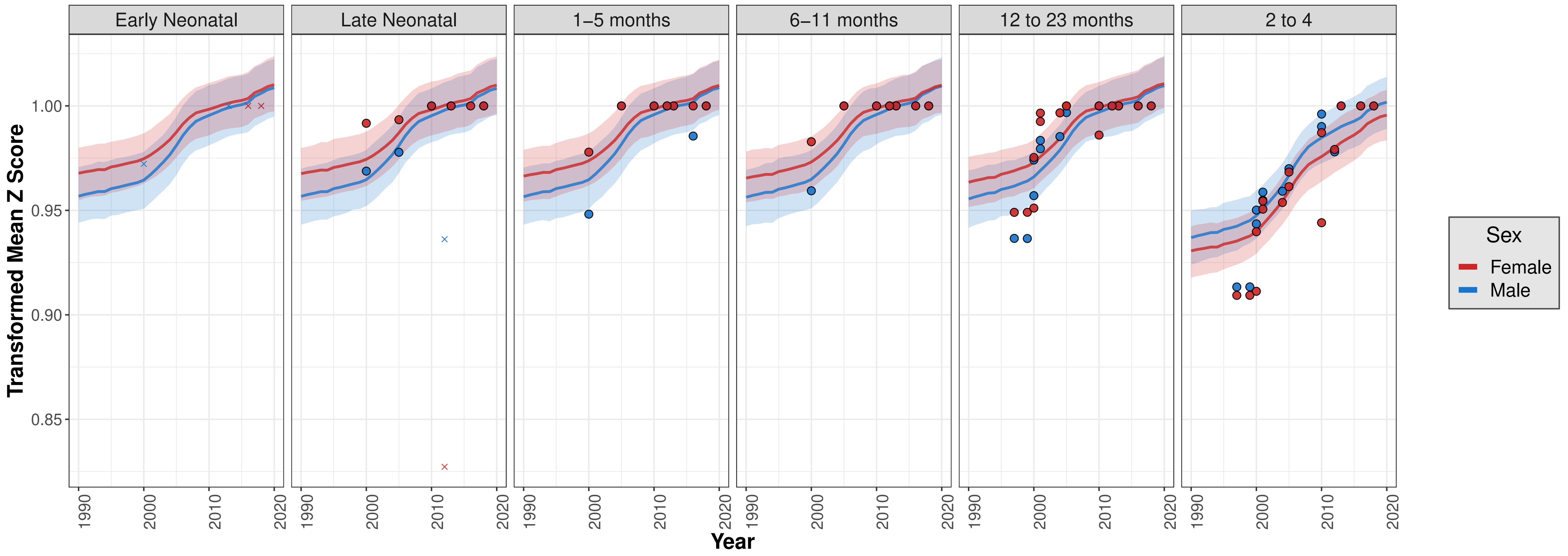


**I**

| Year | Source |
|---|---|
| 1997 | WHO CGM Database |
| 1999 | WHO CGM Database |
| 2000 | MICS |
| 2000 | WHO CGM Database |
| 2001 | WHO CGM Database |
| 2004 | WHO CGM Database |
| 2005 | MICS |
| 2005 | WHO CGM Database |
| 2010 | MICS |
| 2010 | WHO CGM Database |
| 2012 | Khuvsgul MICS |
| 2012 | Nalaikh District MICS |
| 2013 | WHO CGM Database |
| 2013 | MICS |
| 2016 | Khuvsgul MICS |
| 2016 | Nalaikh District MICS |
| 2016 | National Nutrition Survey |
| 2018 | MICS |

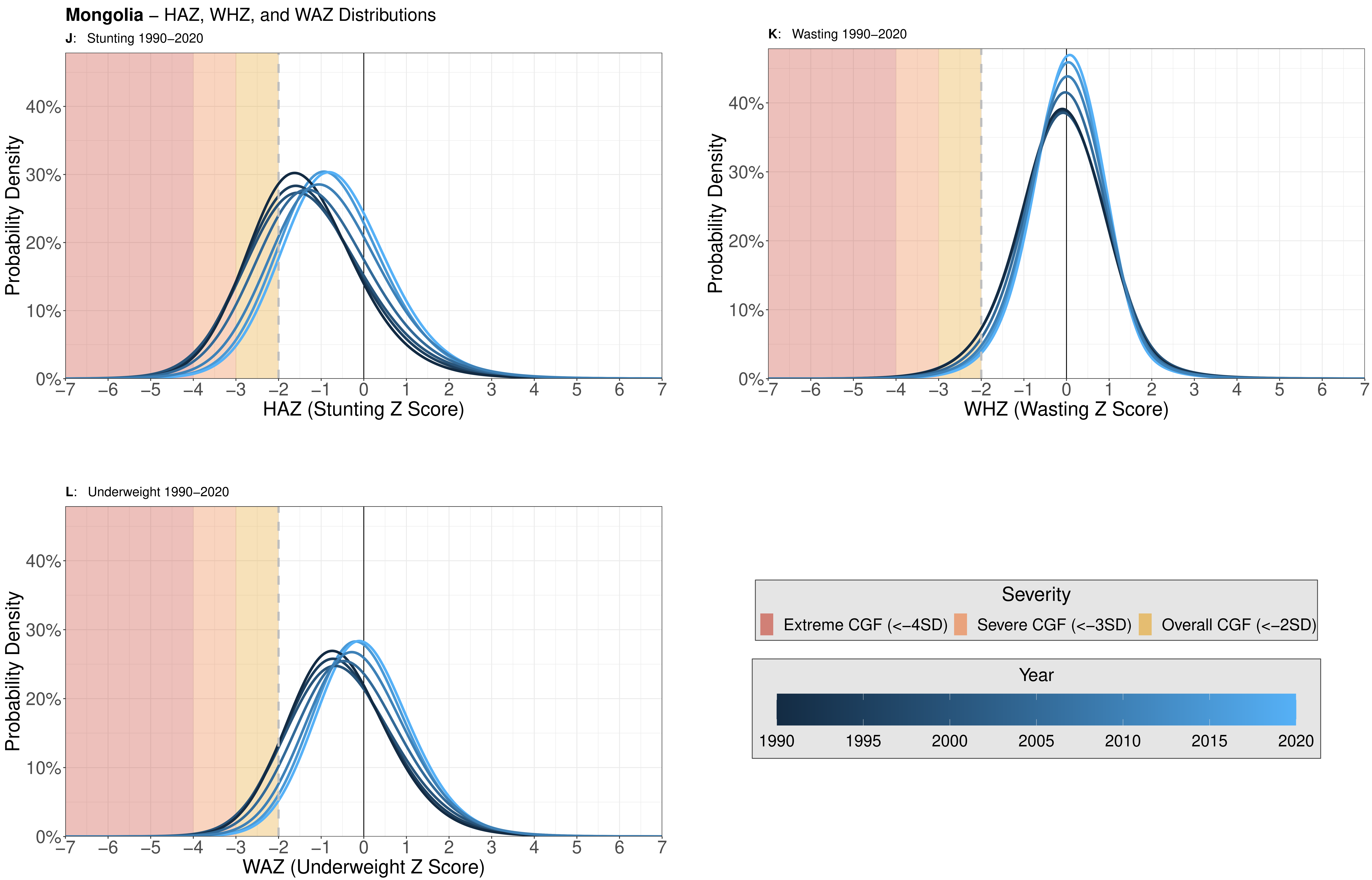
Mongolia – HAZ, WHZ, and WAZ Distributions
J: Stunting 1990–2020
Probability Density
0%
10%
20%
30%
40%
−7 −6 −5 −4 −3 −2 −1 0 1 2 3 4 5 6 7
HAZ (Stunting Z Score)
K: Wasting 1990–2020
Probability Density
WHZ (Wasting Z Score)
L: Underweight 1990–2020
Probability Density
WAZ (Underweight Z Score)
Severity
Extreme CGF (<−4SD)
Severe CGF (<−3SD)
Overall CGF (<−2SD)
Year
1990
1995
2000
2005
2010
2015
2020

**Tajikistan** – Stunting (HAZ)

**A**: Overall and Severe Stunting Prevalence

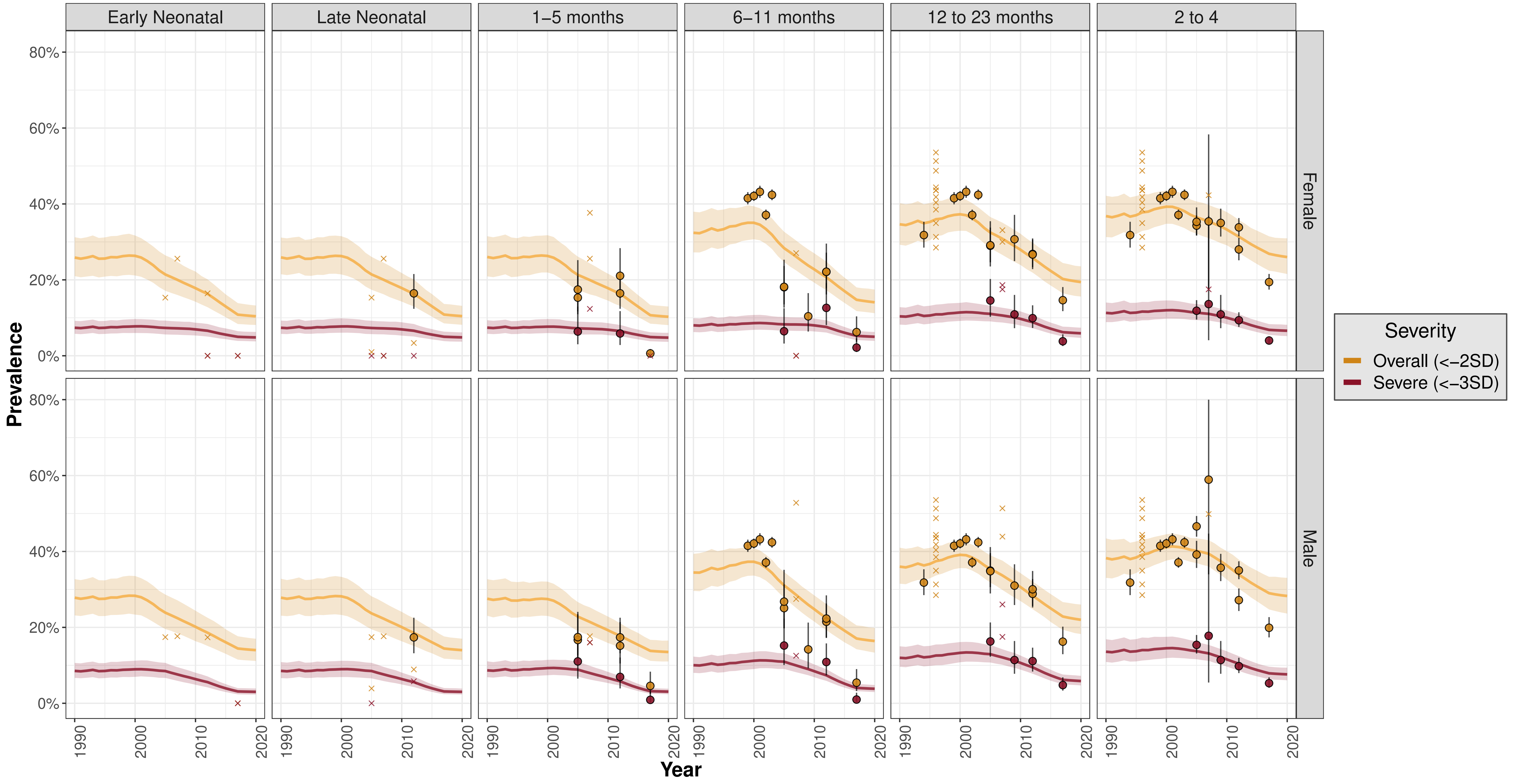


**B**: Transformed Mean Stunting Z Scores

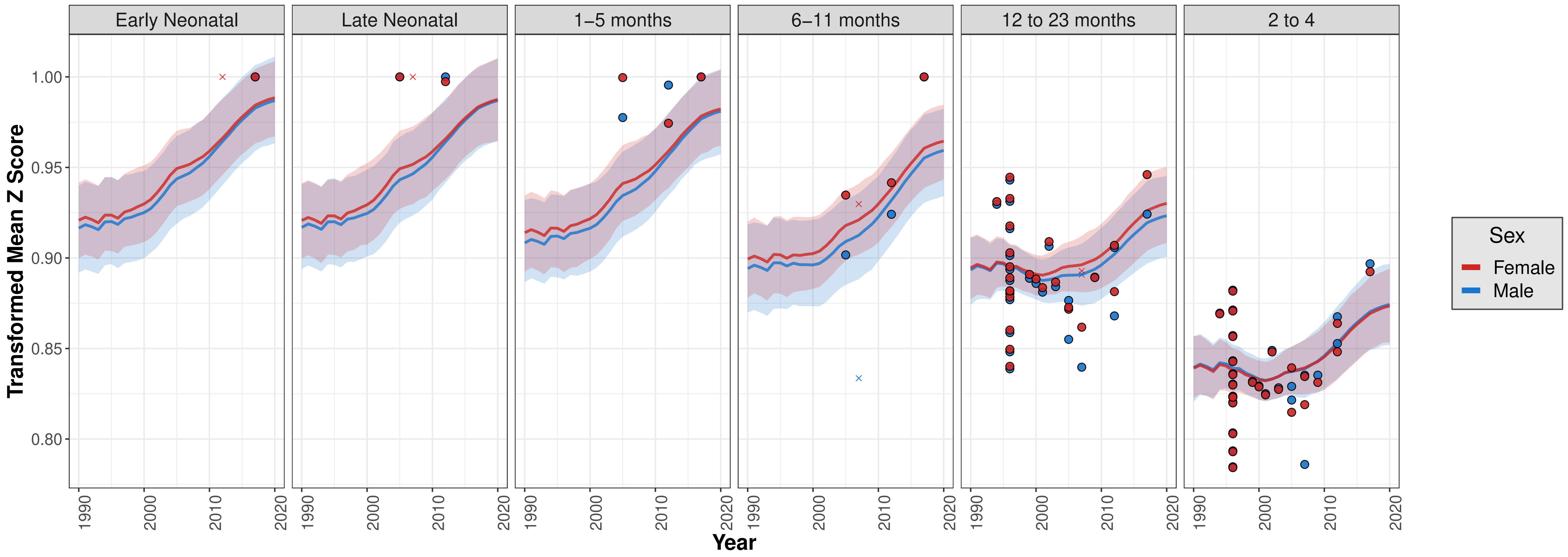


**C**

| Year | Source |
|---|---|
| 1994 | WHO CGM Database |
| 1996 | WHO CGM Database |
| 1999 | WHO CGM Database |
| 2000 | WHO CGM Database |
| 2001 | WHO CGM Database |
| 2002 | WHO CGM Database |
| 2003 | WHO CGM Database |
| 2005 | MICS |
| 2005 | WHO CGM Database |
| 2007 | Living Standards Measurement Survey |
| 2007 | WHO CGM Database |
| 2009 | WHO CGM Database |
| 2012 | DHS |
| 2012 | WHO CGM Database |
| 2017 | DHS |

# **Tajikistan** – Wasting (WHZ)

**D**: Overall and Severe Wasting Prevalence

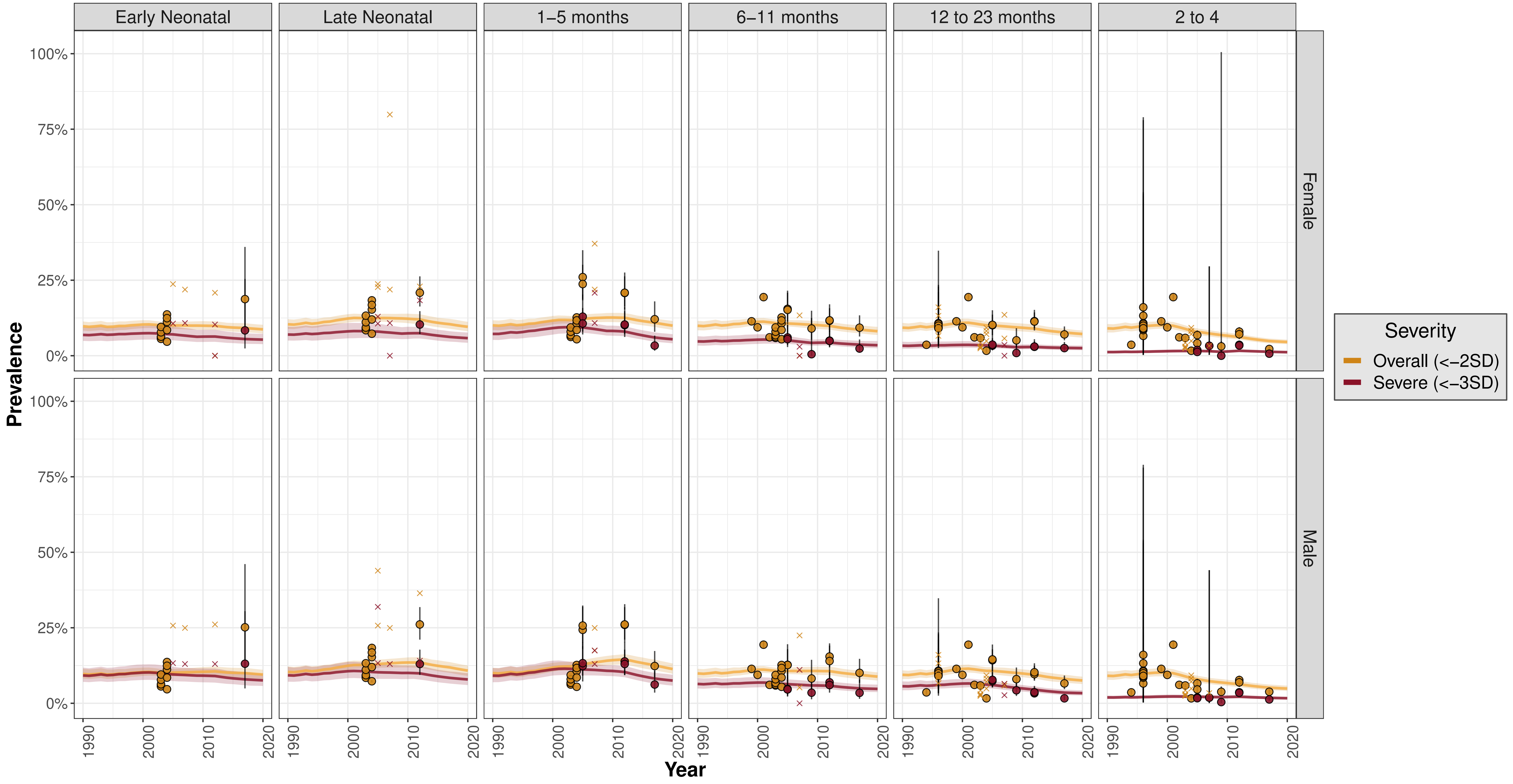


**E**: Transformed Mean Wasting Z Scores

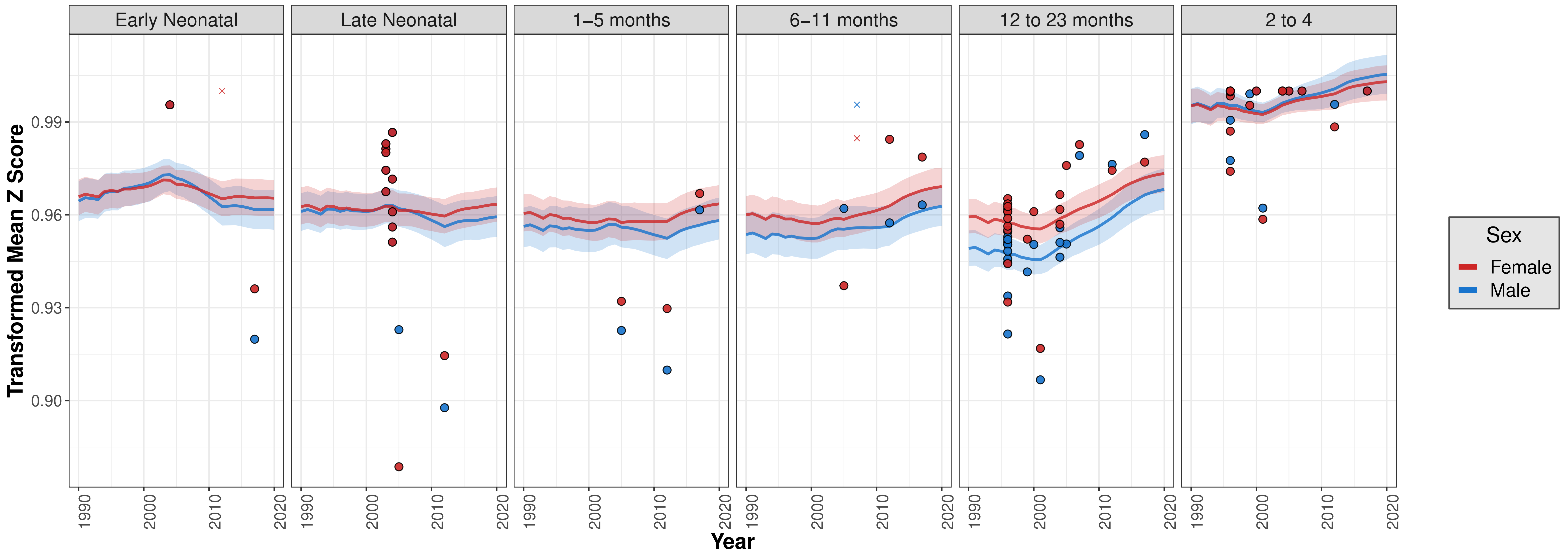


**F**

| Year | Source |
|---|---|
| 1994 | WHO CGM Database |
| 1996 | WHO CGM Database |
| 1999 | WHO CGM Database |
| 2000 | WHO CGM Database |
| 2001 | WHO CGM Database |
| 2002 | WHO CGM Database |
| 2003 | WHO CGM Database |
| 2003 | National Nutrition Survey |
| 2004 | National Nutrition Survey |
| 2005 | MICS |
| 2005 | WHO CGM Database |
| 2007 | Living Standards Measurement Survey |
| 2007 | WHO CGM Database |
| 2009 | WHO CGM Database |
| 2012 | DHS |
| 2012 | WHO CGM Database |
| 2017 | DHS |

**Tajikistan** – Underweight (WAZ)

**G**: Overall and Severe Underweight Prevalence

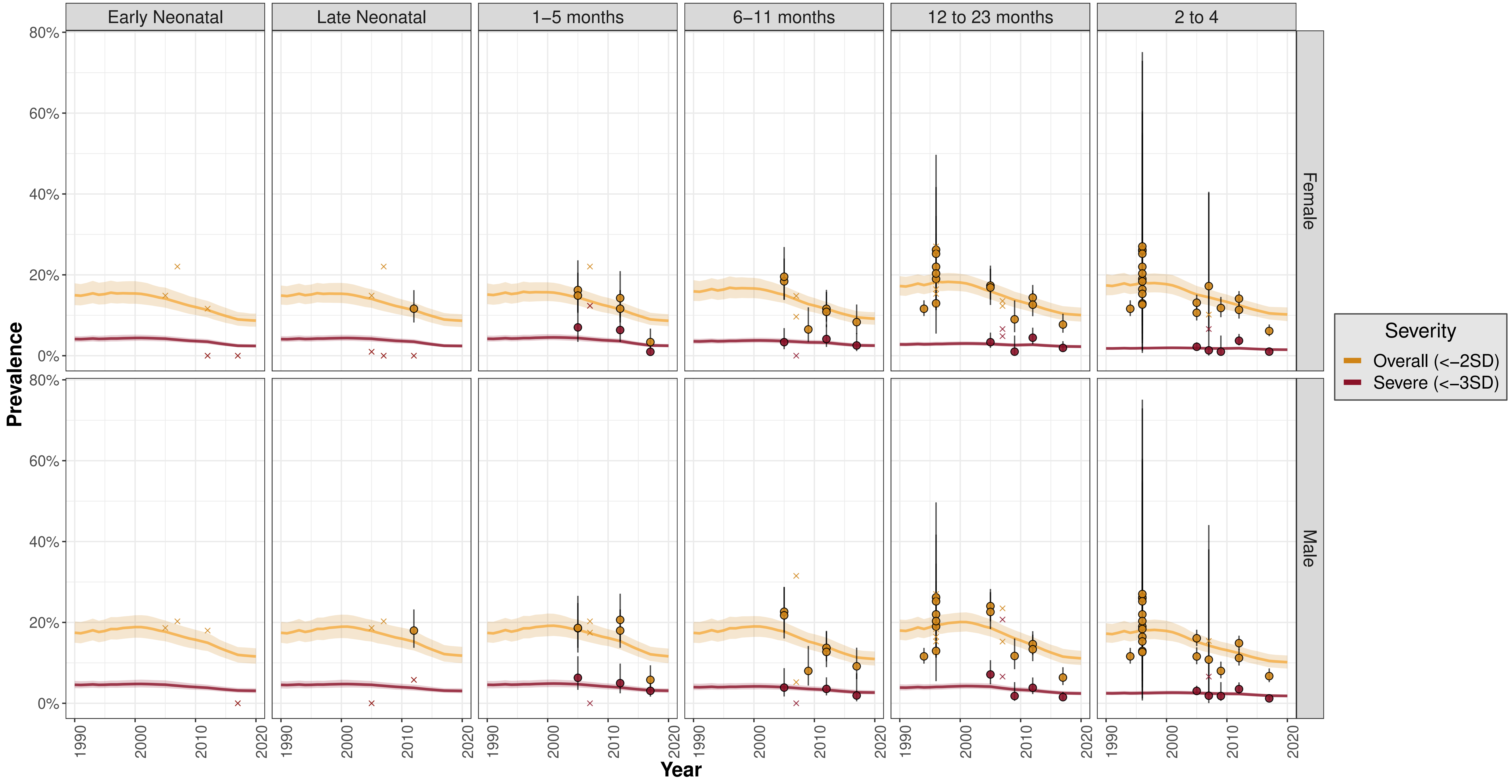


**H**: Transformed Mean Underweight Z Scores

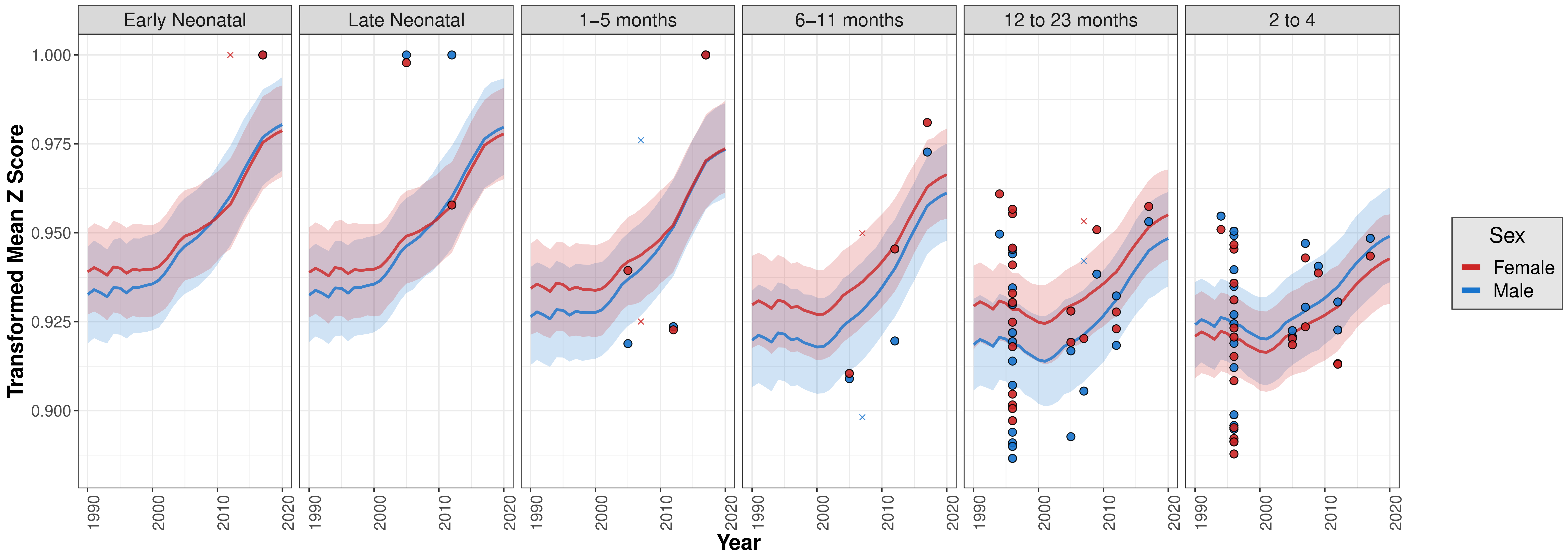


**I**

| Year | Source |
|---|---|
| 1994 | WHO CGM Database |
| 1996 | WHO CGM Database |
| 2005 | MICS |
| 2005 | WHO CGM Database |
| 2007 | Living Standards Measurement Survey |
| 2007 | WHO CGM Database |
| 2009 | WHO CGM Database |
| 2012 | DHS |
| 2012 | WHO CGM Database |
| 2017 | DHS |

**Tajikistan** – HAZ, WHZ, and WAZ Distributions

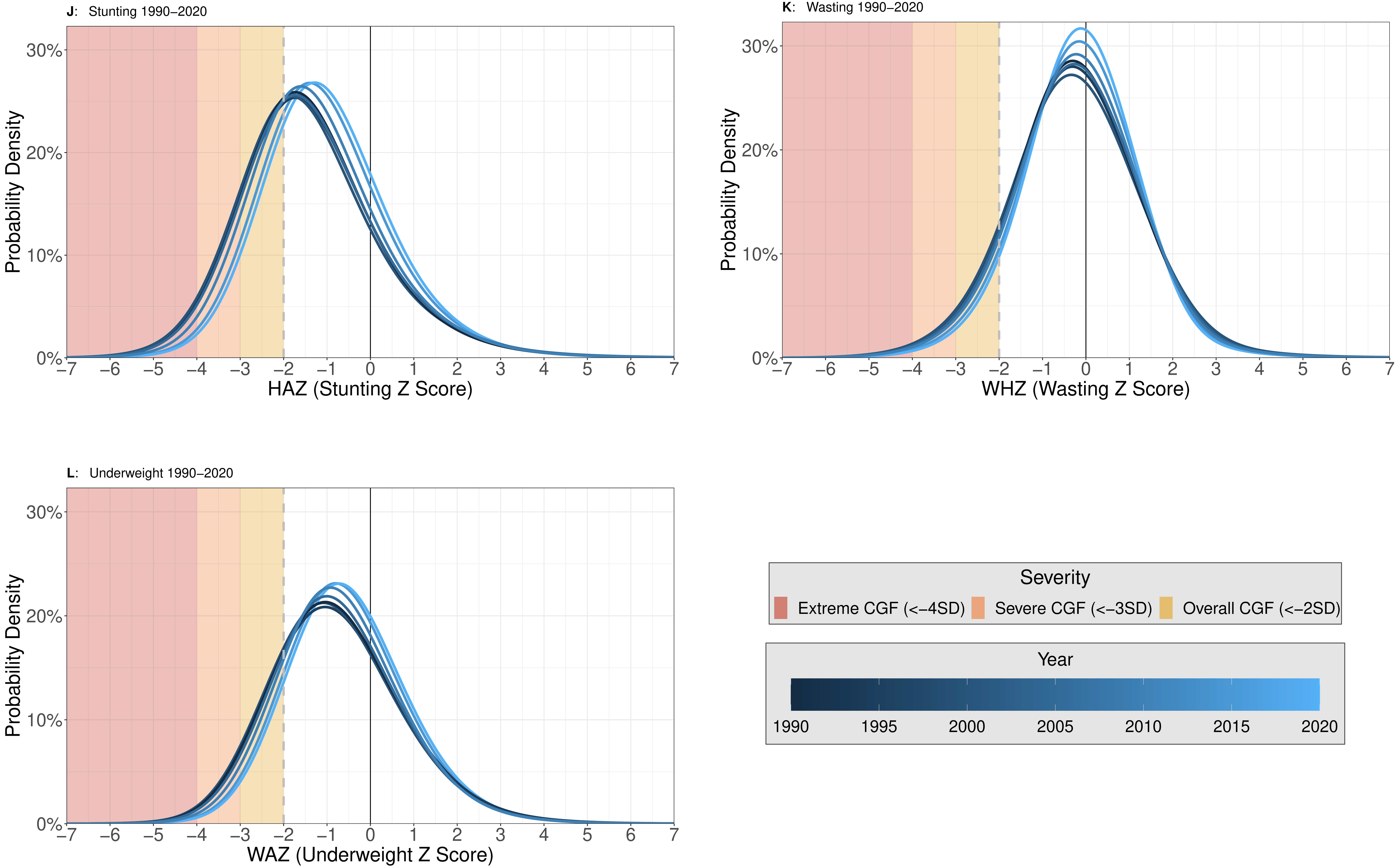

# **Turkmenistan** – Stunting (HAZ)

**A**: Overall and Severe Stunting Prevalence

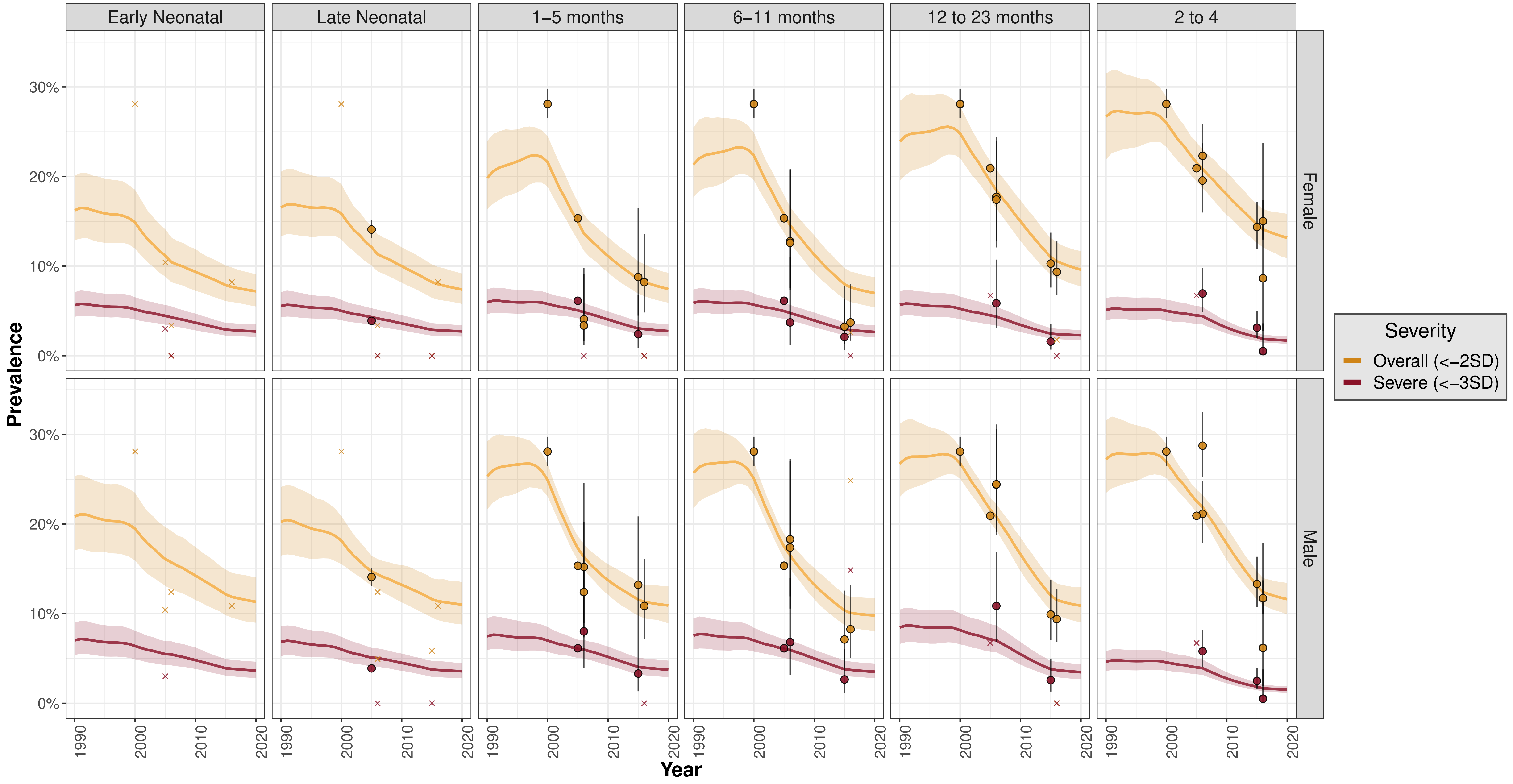


**B**: Transformed Mean Stunting Z Scores

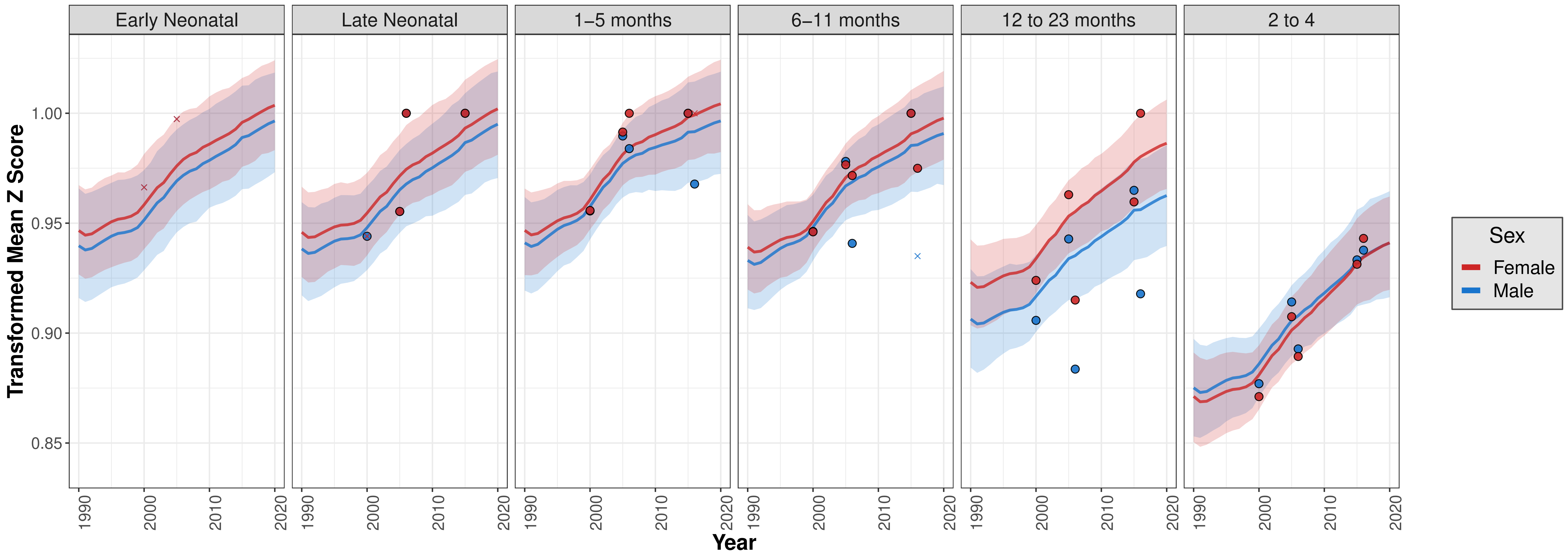


**C**

| Year | Source |
|---|---|
| 2000 | WHO CGM Database |
| 2005 | UNICEF Childinfo – Nutritional Status |
| 2006 | MICS |
| 2006 | WHO CGM Database |
| 2015 | MICS |
| 2016 | WHO CGM Database |
| 2016 | MICS |

# **Turkmenistan** – Wasting (WHZ)

**D**: Overall and Severe Wasting Prevalence

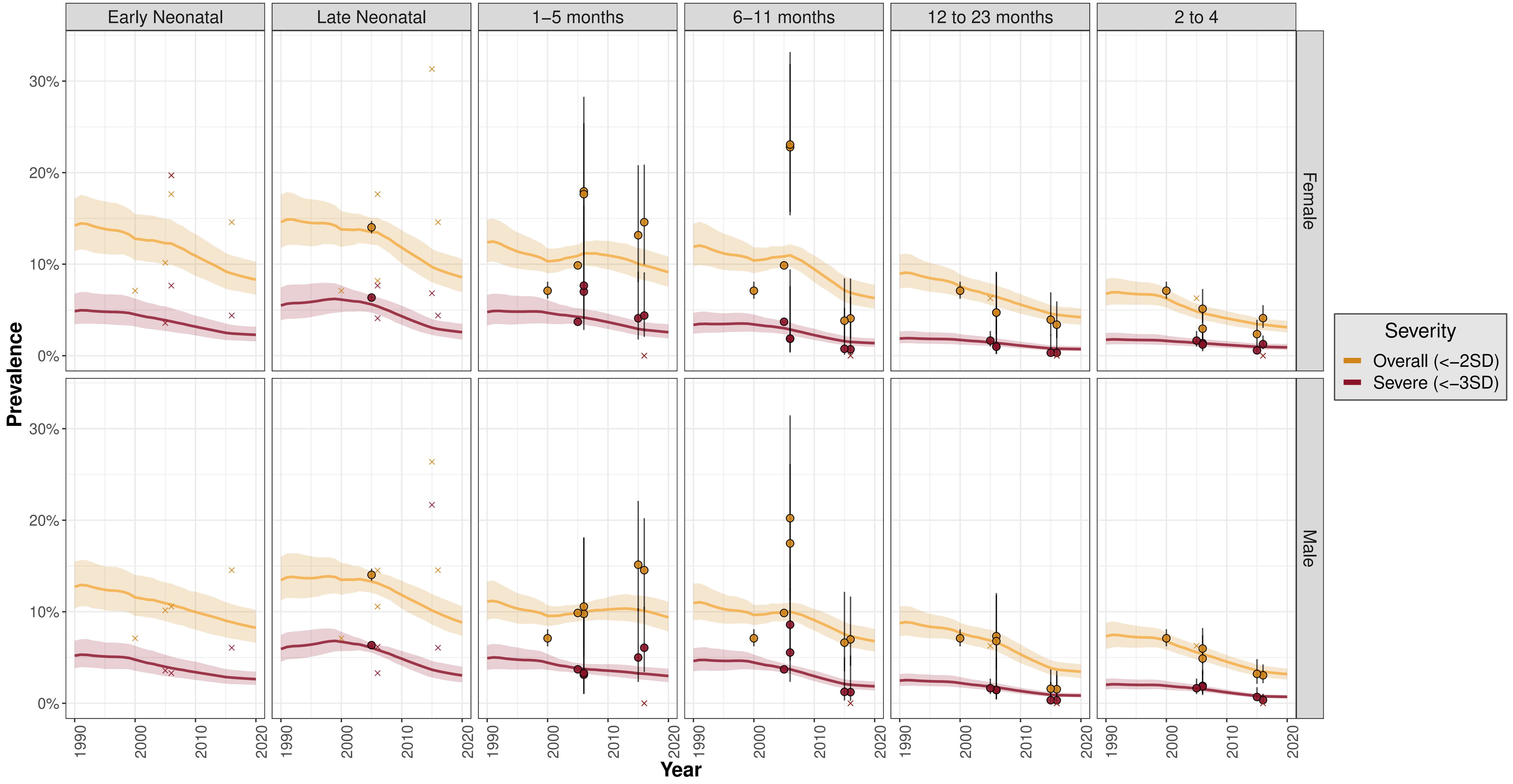


**E**: Transformed Mean Wasting Z Scores

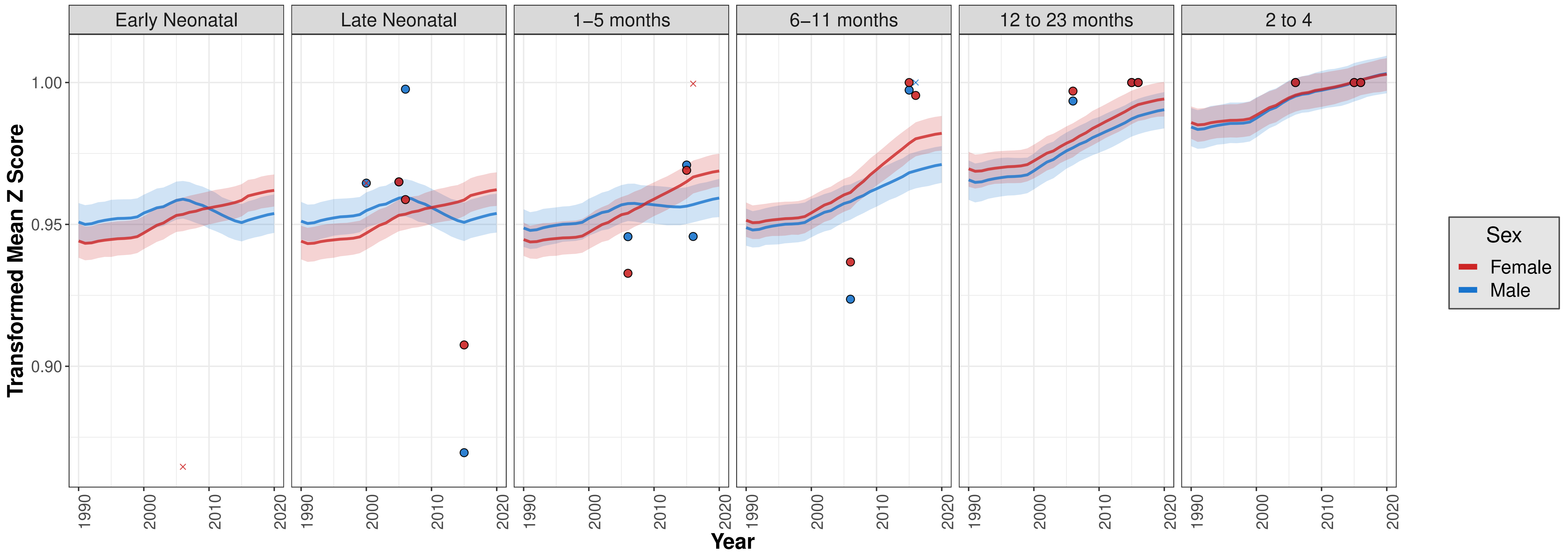


**F**

| Year | Source |
|---|---|
| 2000 | WHO CGM Database |
| 2005 | UNICEF Childinfo – Nutritional Status |
| 2006 | MICS |
| 2006 | WHO CGM Database |
| 2015 | MICS |
| 2016 | WHO CGM Database |
| 2016 | MICS |

# **Turkmenistan** – Underweight (WAZ)

**G**: Overall and Severe Underweight Prevalence

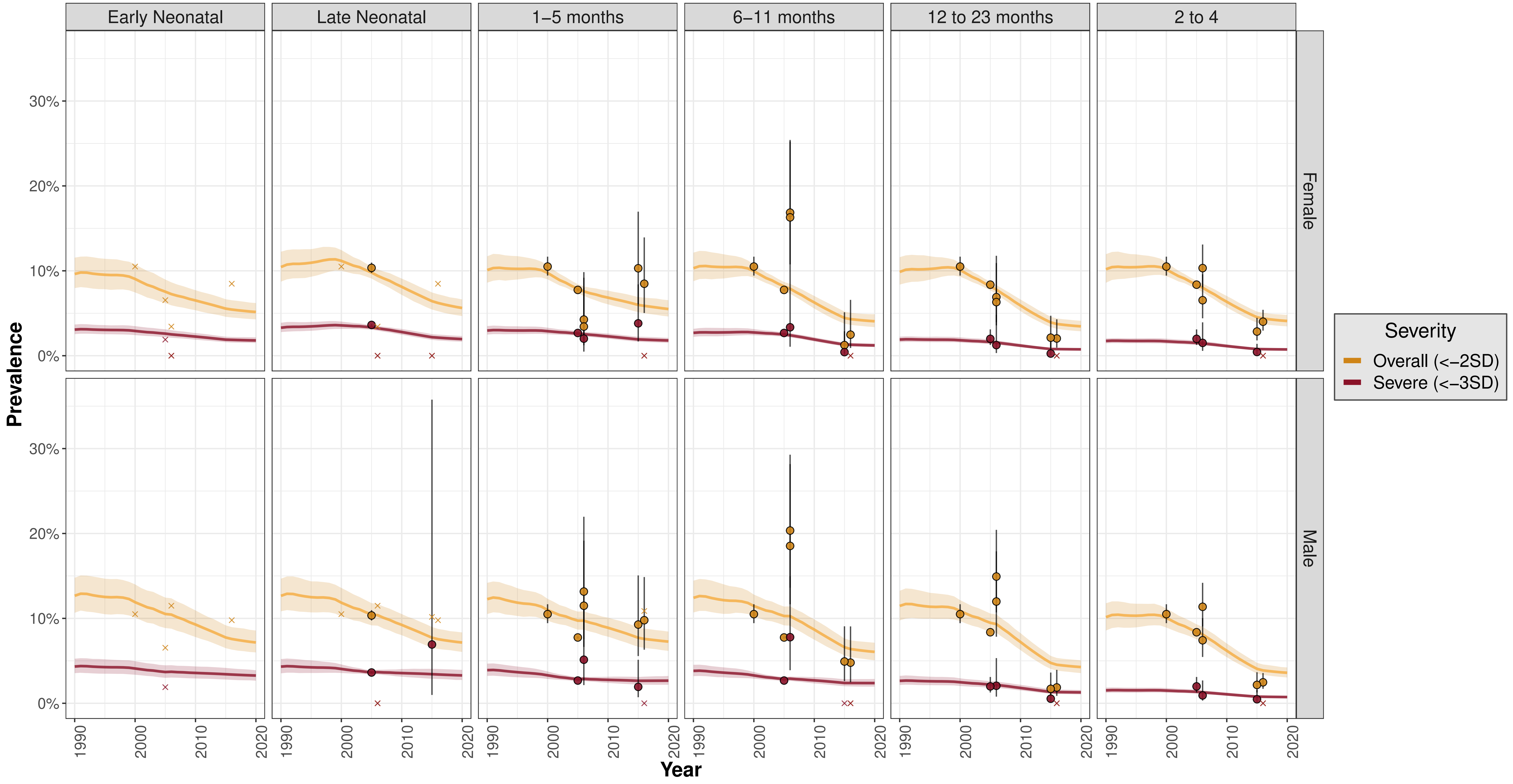


**H**: Transformed Mean Underweight Z Scores

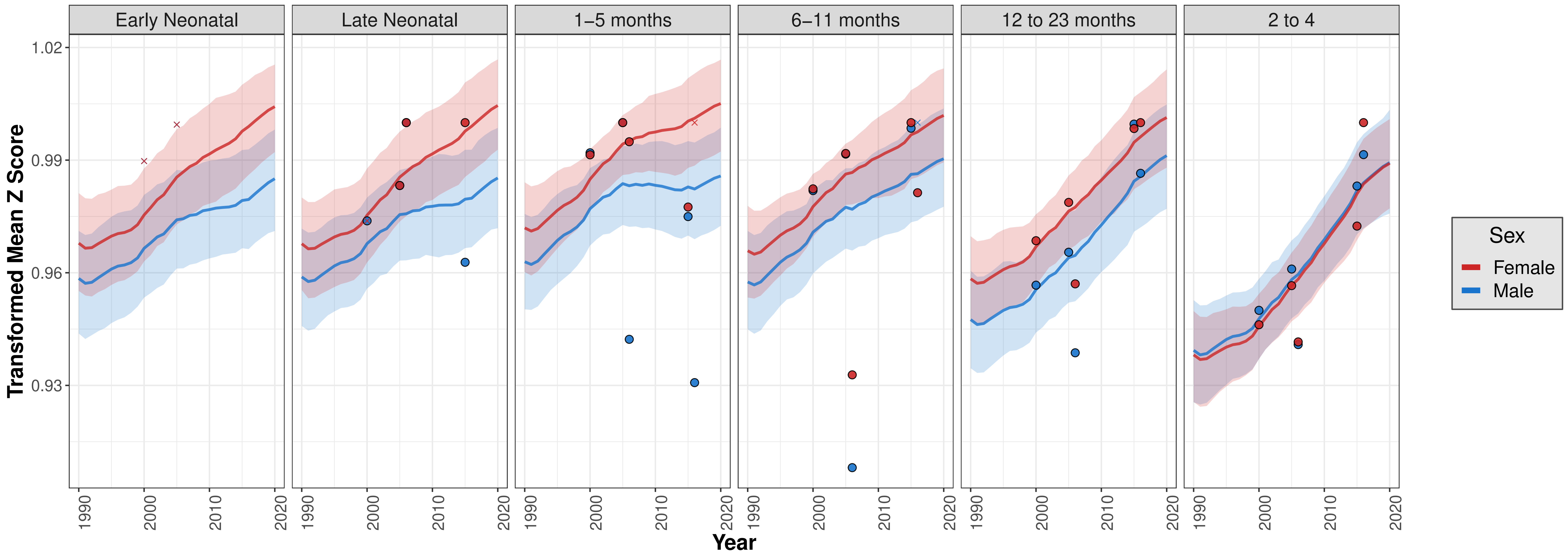


**I**

| Year | Source |
|---|---|
| 2000 | WHO CGM Database |
| 2005 | UNICEF Childinfo – Nutritional Status |
| 2006 | MICS |
| 2006 | WHO CGM Database |
| 2015 | MICS |
| 2016 | WHO CGM Database |
| 2016 | MICS |

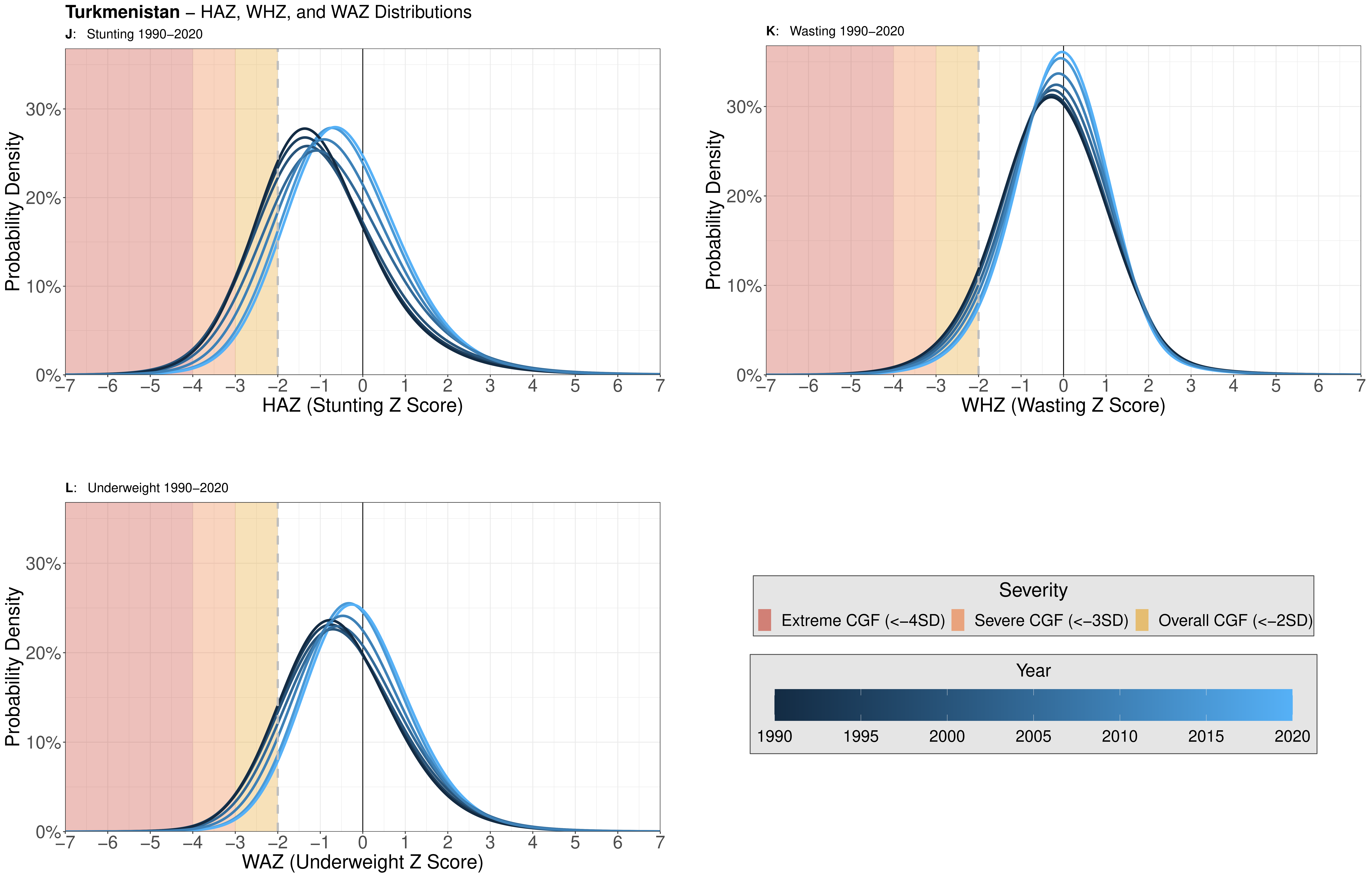
Turkmenistan – HAZ, WHZ, and WAZ Distributions
J: Stunting 1990–2020
Probability Density
30%
20%
10%
0%
−7 −6 −5 −4 −3 −2 −1 0 1 2 3 4 5 6 7
HAZ (Stunting Z Score)
K: Wasting 1990–2020
Probability Density
30%
20%
10%
0%
−7 −6 −5 −4 −3 −2 −1 0 1 2 3 4 5 6 7
WHZ (Wasting Z Score)
L: Underweight 1990–2020
Probability Density
30%
20%
10%
0%
−7 −6 −5 −4 −3 −2 −1 0 1 2 3 4 5 6 7
WAZ (Underweight Z Score)
Severity
Extreme CGF (<−4SD)
Severe CGF (<−3SD)
Overall CGF (<−2SD)
Year
1990
1995
2000
2005
2010
2015
2020

## **Uzbekistan** – Stunting (HAZ)

**A**: Overall and Severe Stunting Prevalence

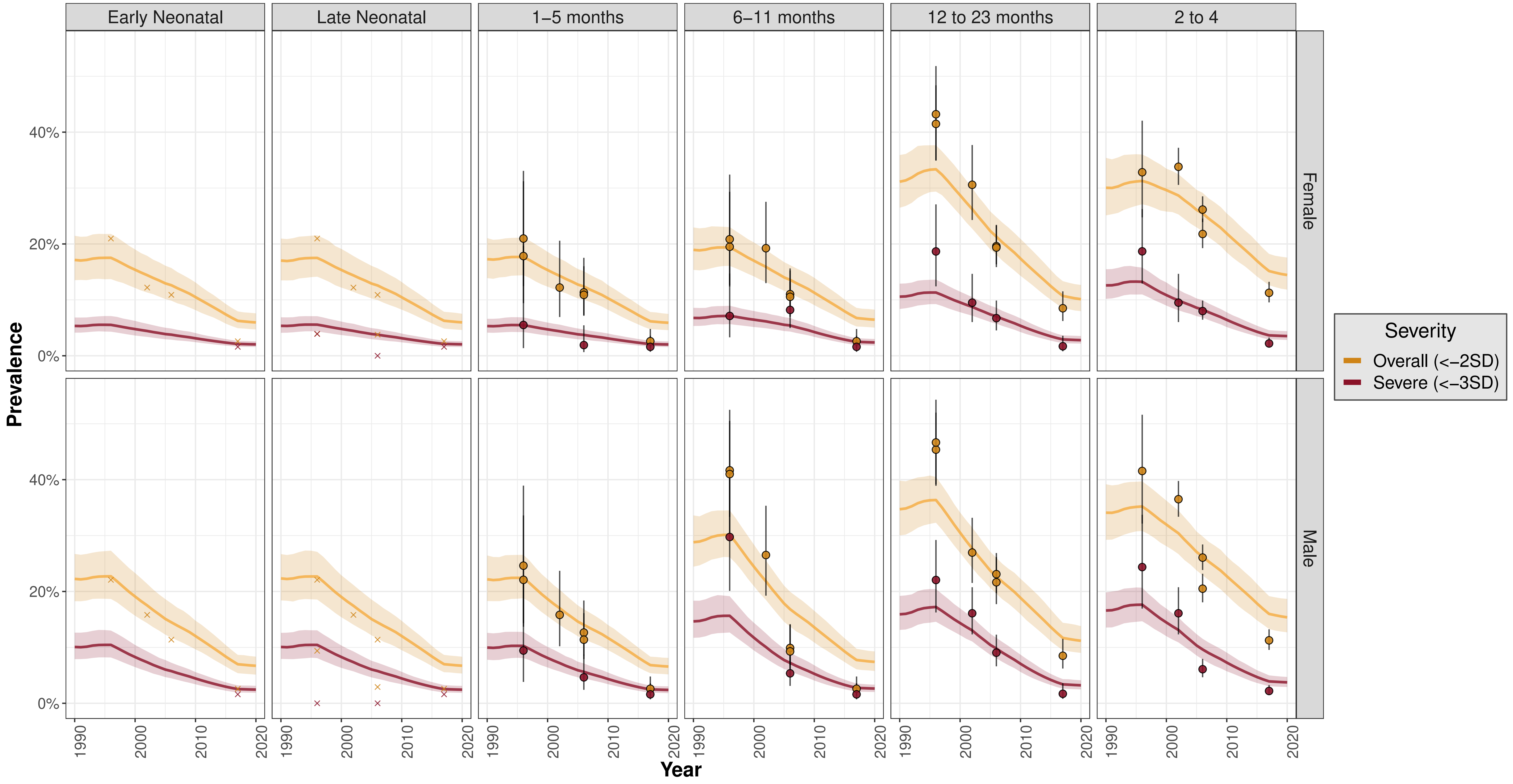


**B**: Transformed Mean Stunting Z Scores

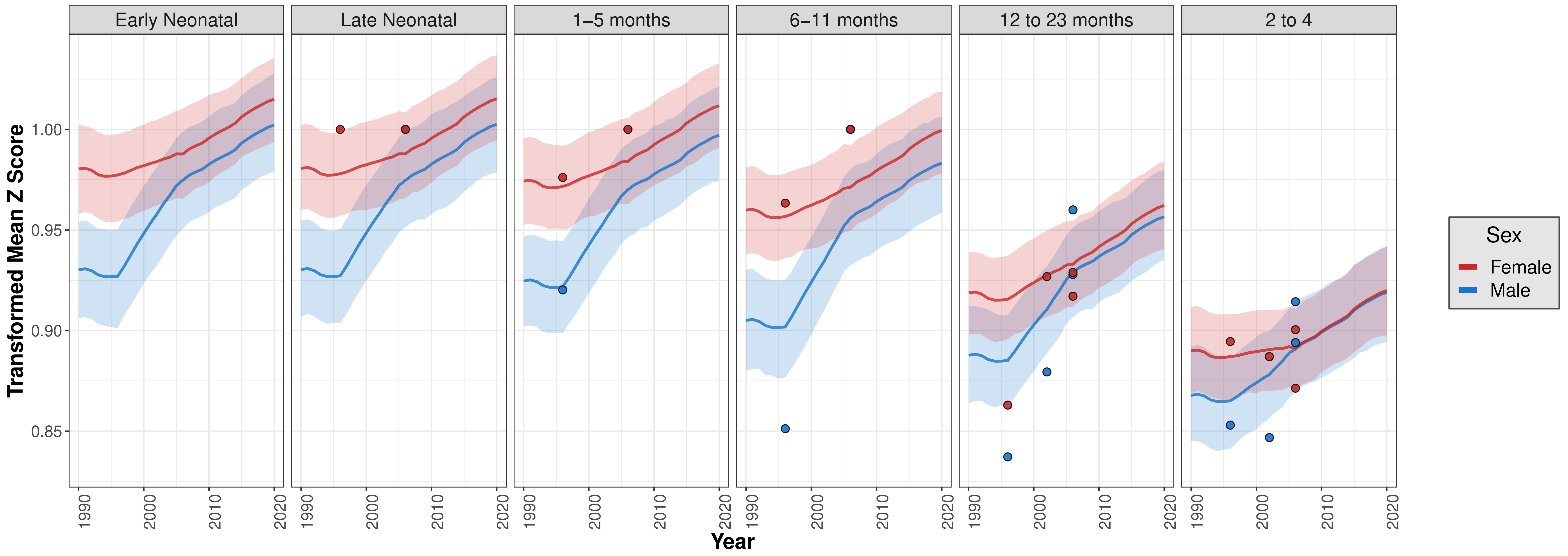


**C**

| Year | Source |
|---|---|
| 1996 | DHS |
| 1996 | WHO CGM Database |
| 2002 | WHO CGM Database |
| 2006 | MICS |
| 2006 | WHO CGM Database |
| 2017 | Nutrition Survey |

**Uzbekistan** – Wasting (WHZ)

**D**: Overall and Severe Wasting Prevalence

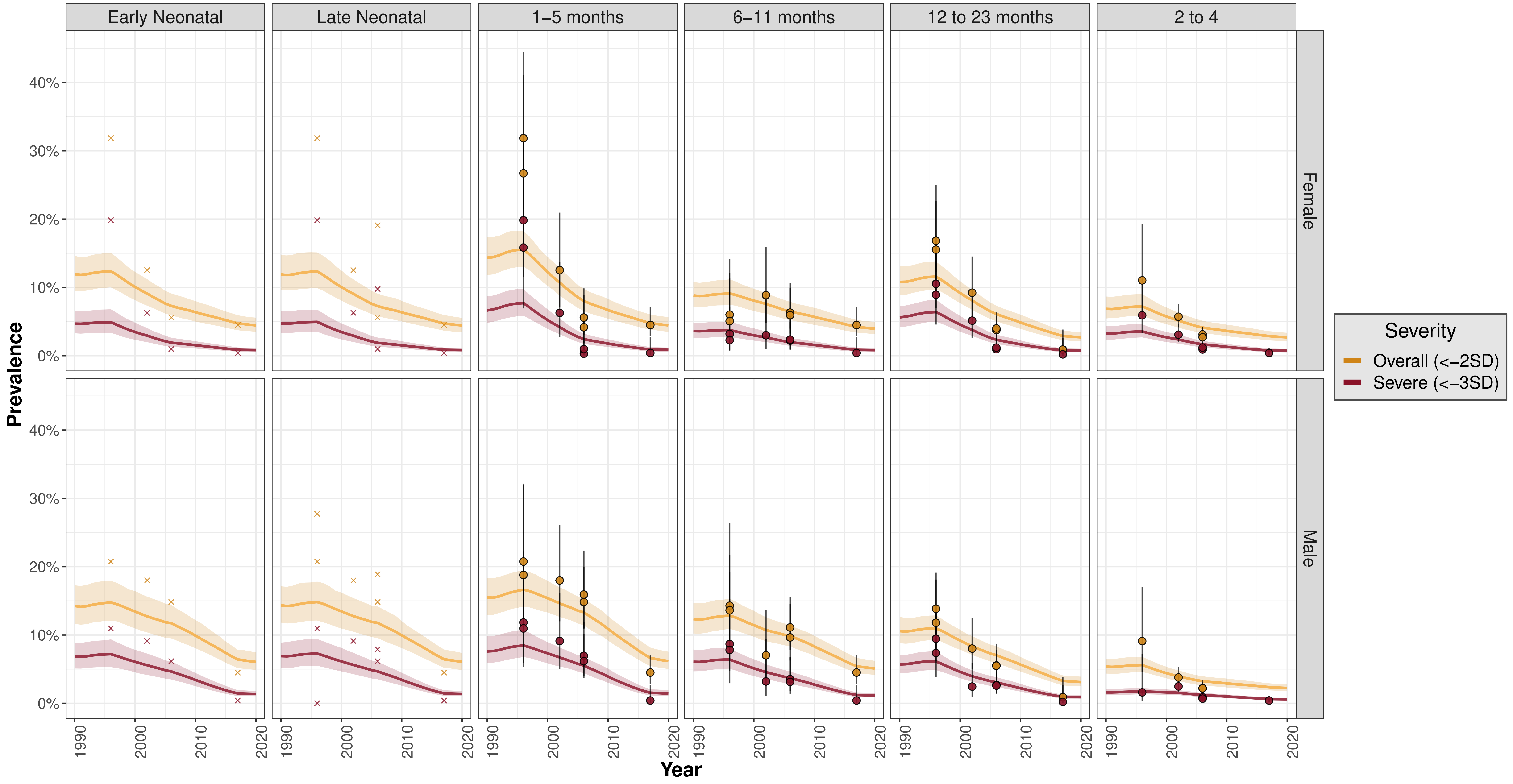


**F**

| Year | Source |
|---|---|
| 1996 | DHS |
| 1996 | WHO CGM Database |
| 2002 | WHO CGM Database |
| 2006 | MICS |
| 2006 | WHO CGM Database |
| 2017 | Nutrition Survey |

**E**: Transformed Mean Wasting Z Scores

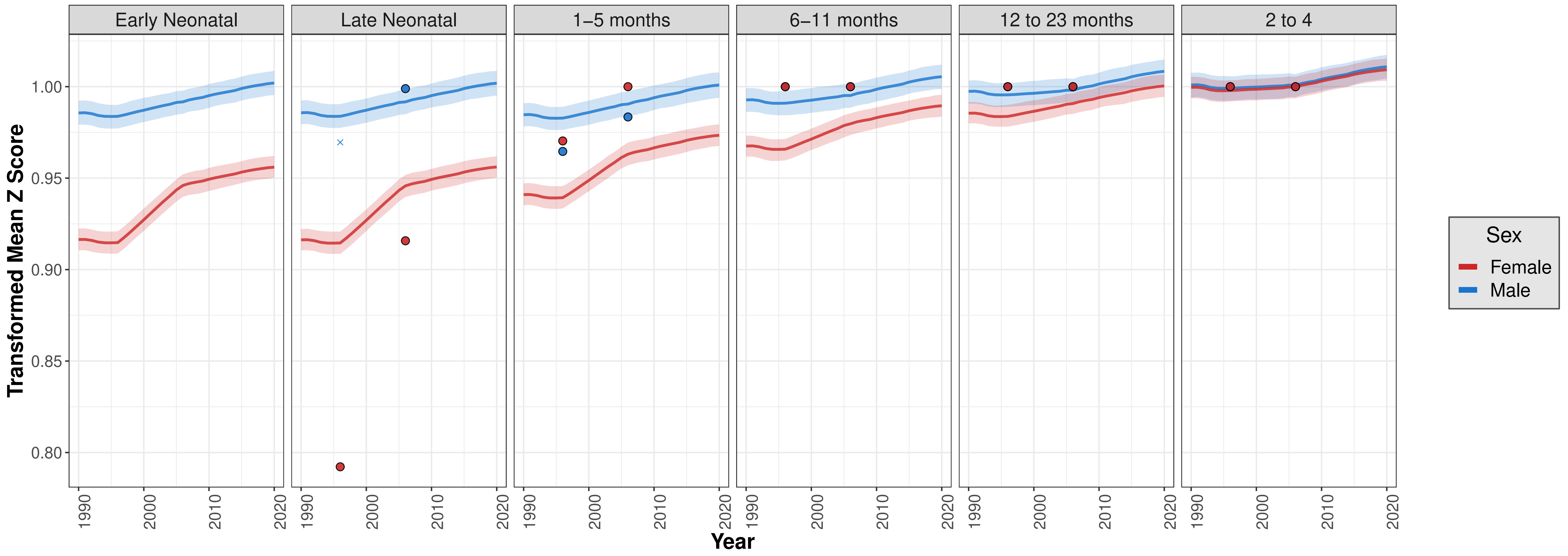

# **Uzbekistan** – Underweight (WAZ)

**G**: Overall and Severe Underweight Prevalence

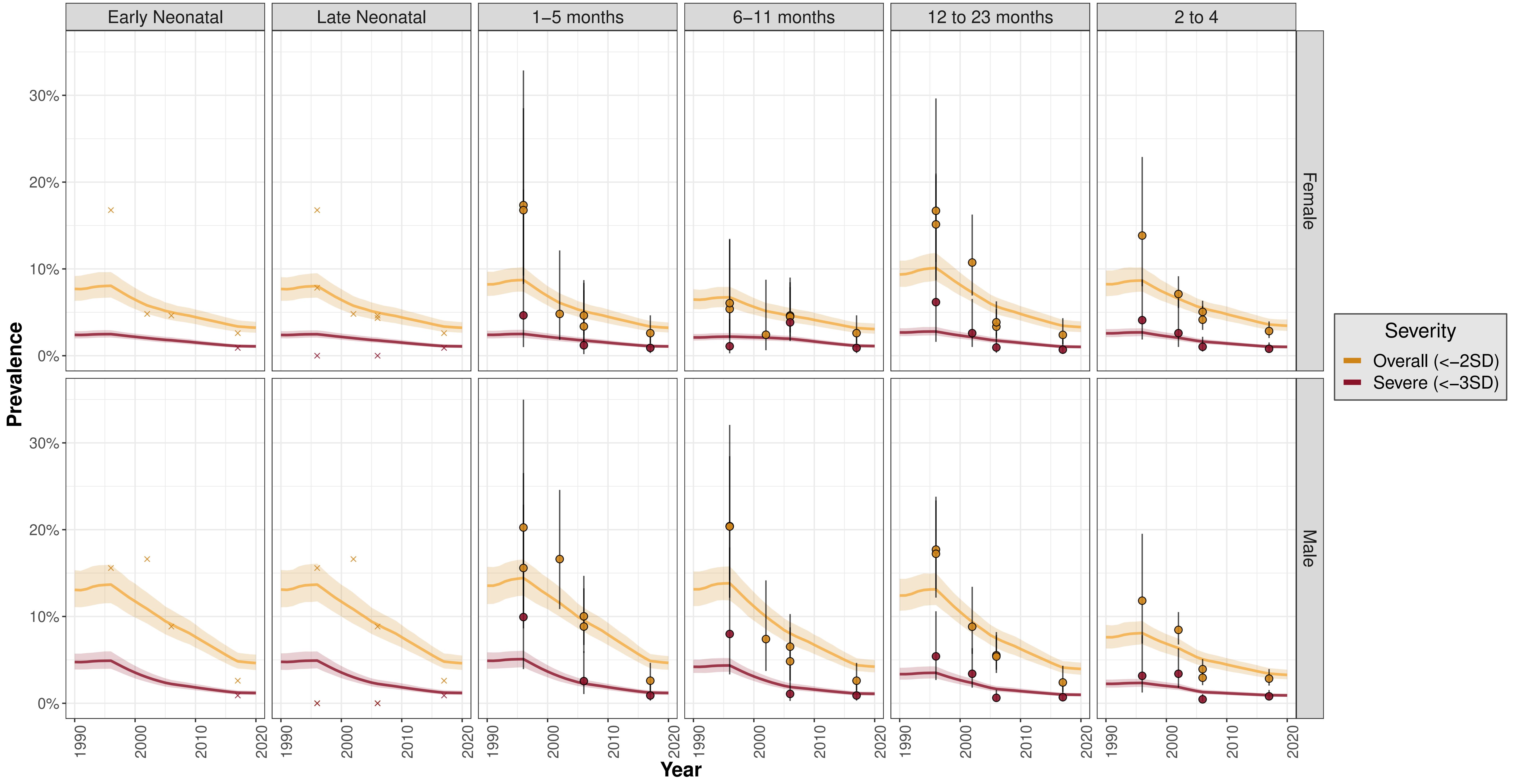


**H**: Transformed Mean Underweight Z Scores

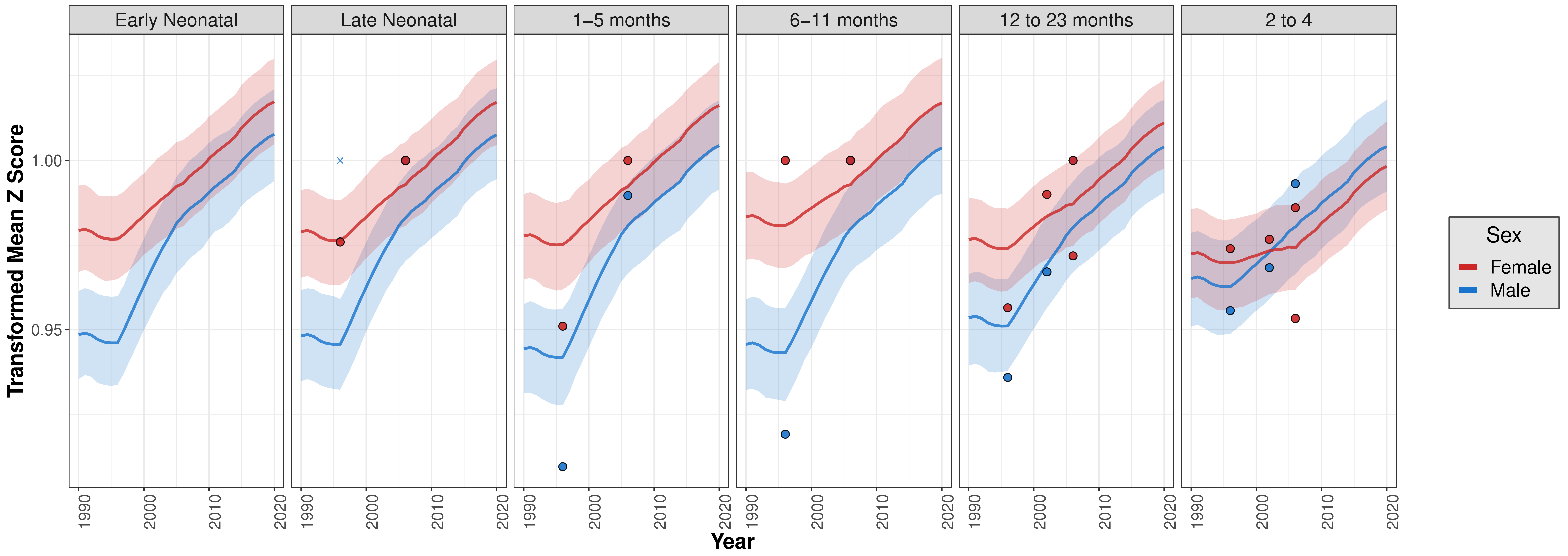


**I**

| Year | Source |
|---|---|
| 1996 | DHS |
| 1996 | WHO CGM Database |
| 2002 | WHO CGM Database |
| 2006 | MICS |
| 2006 | WHO CGM Database |
| 2017 | Nutrition Survey |

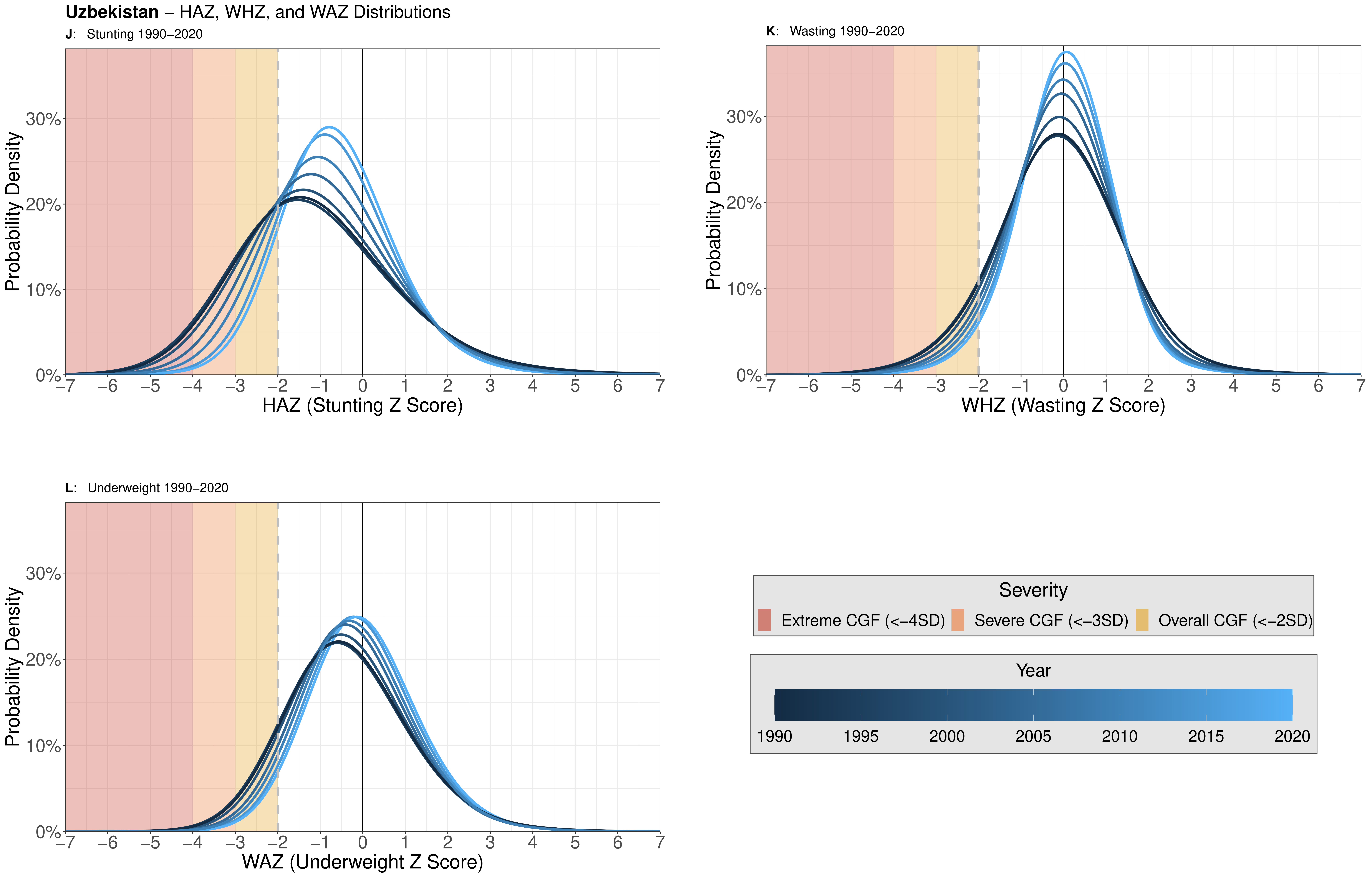

Uzbekistan – HAZ, WHZ, and WAZ Distributions
J: Stunting 1990–2020
Probability Density
0%
10%
20%
30%
−7 −6 −5 −4 −3 −2 −1 0 1 2 3 4 5 6 7
HAZ (Stunting Z Score)
K: Wasting 1990–2020
Probability Density
WHZ (Wasting Z Score)
L: Underweight 1990–2020
Probability Density
WAZ (Underweight Z Score)
Severity
Extreme CGF (<−4SD)
Severe CGF (<−3SD)
Overall CGF (<−2SD)
Year
1990
1995
2000
2005
2010
2015
2020

## **Albania** – Stunting (HAZ)

**A**: Overall and Severe Stunting Prevalence

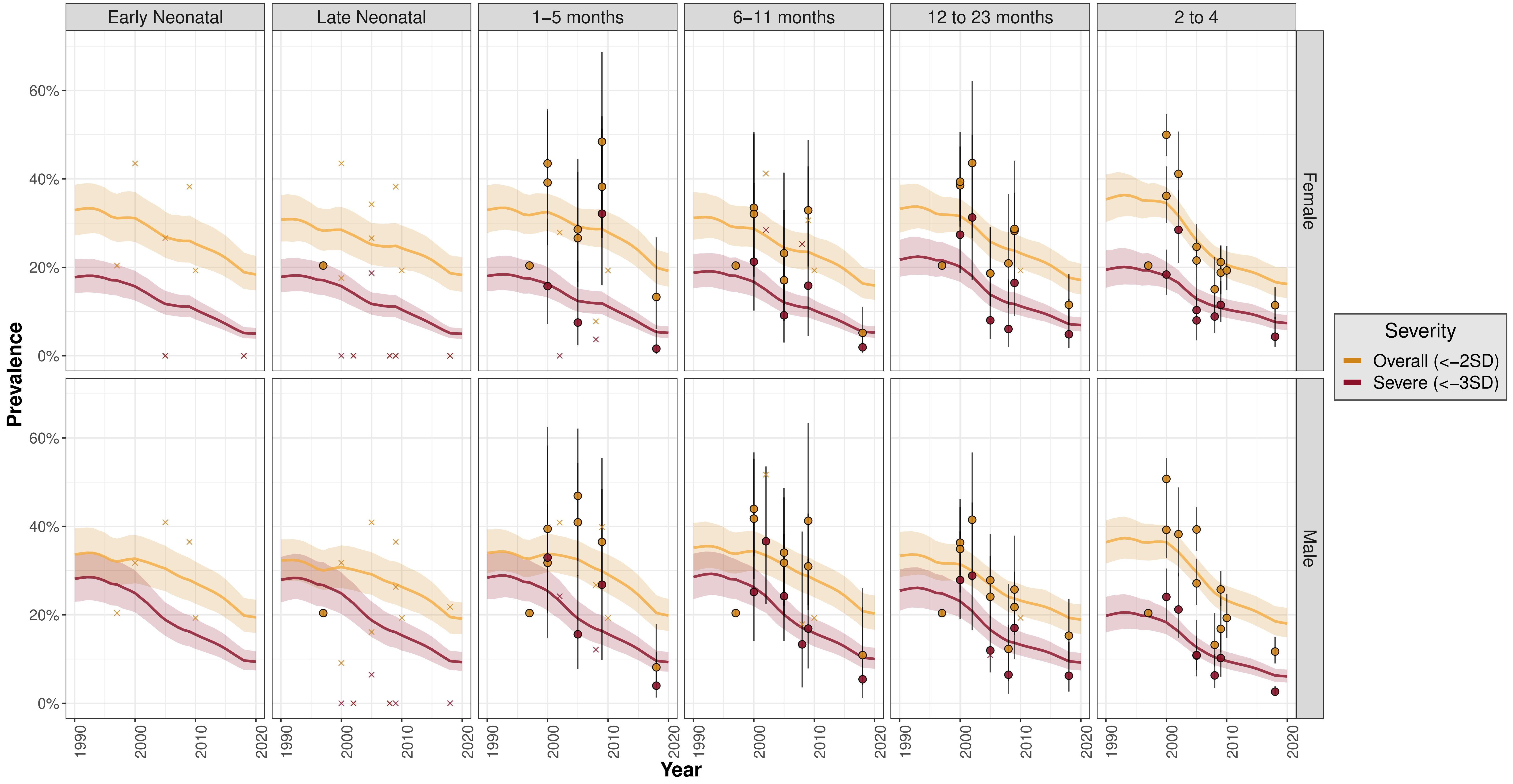


**B**: Transformed Mean Stunting Z Scores

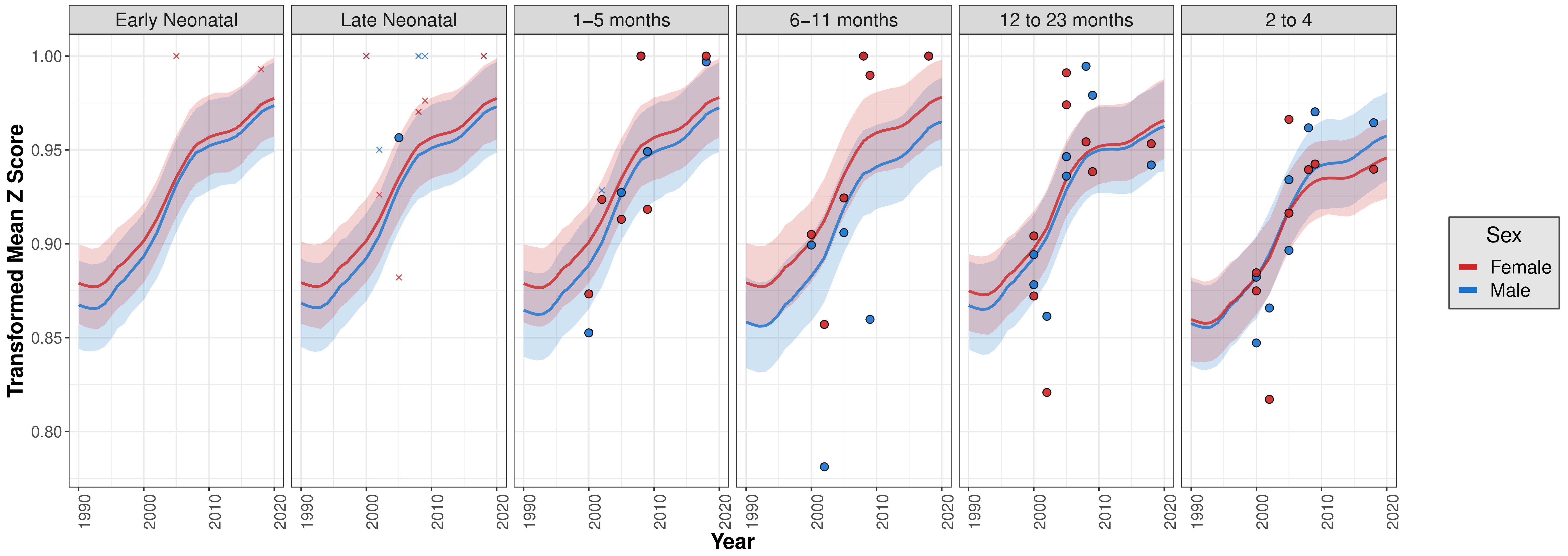


**C**

| Year | Source |
|---|---|
| 1997 | WHO CGM Database |
| 2000 | MICS |
| 2000 | WHO CGM Database |
| 2002 | Living Standards Measurement Survey |
| 2005 | MICS |
| 2005 | WHO CGM Database |
| 2008 | DHS |
| 2009 | DHS |
| 2009 | WHO CGM Database |
| 2010 | Baseline Nutrition and Food Security Survey |
| 2018 | DHS |

## **Albania** – Wasting (WHZ)

**D**: Overall and Severe Wasting Prevalence

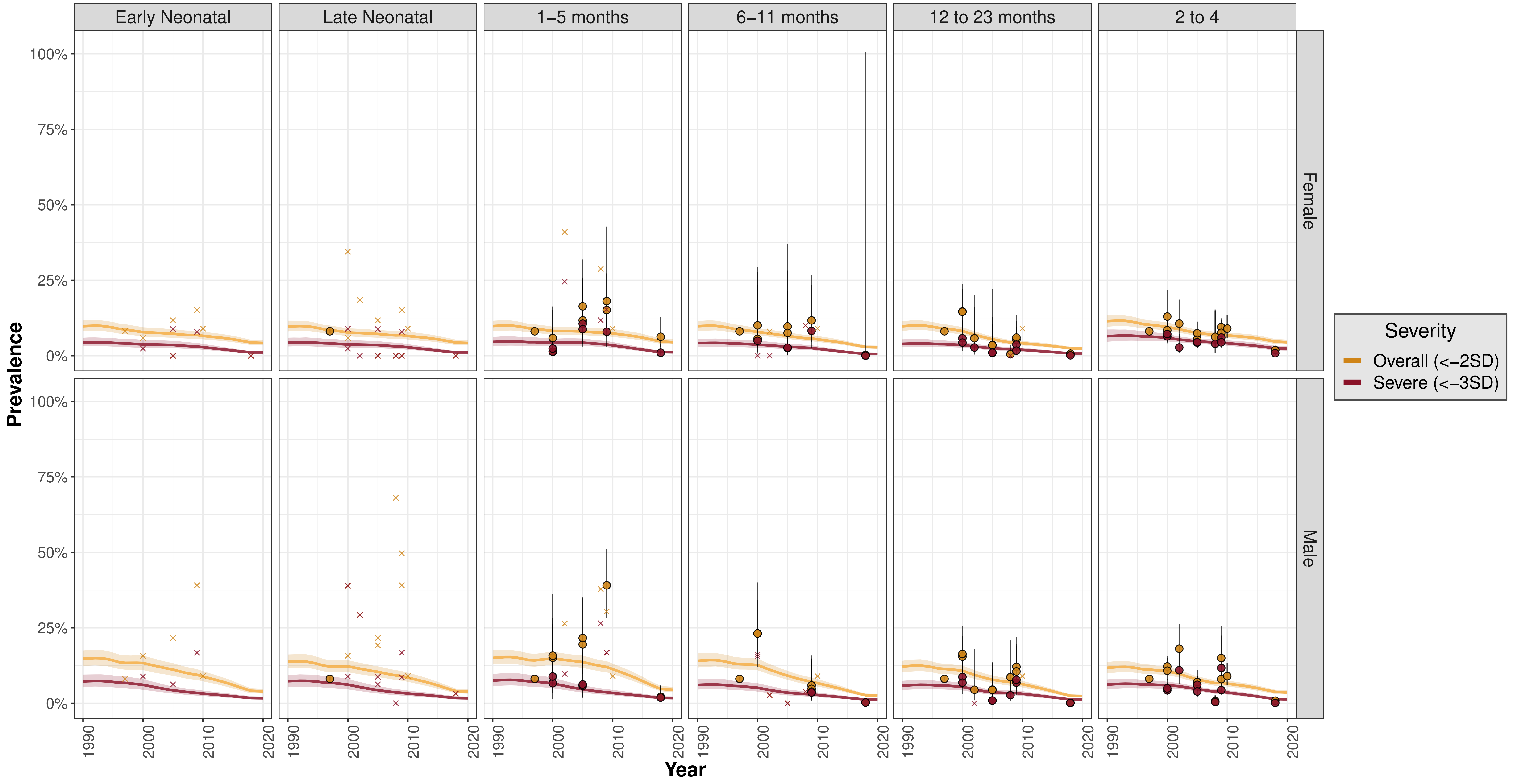


**E**: Transformed Mean Wasting Z Scores

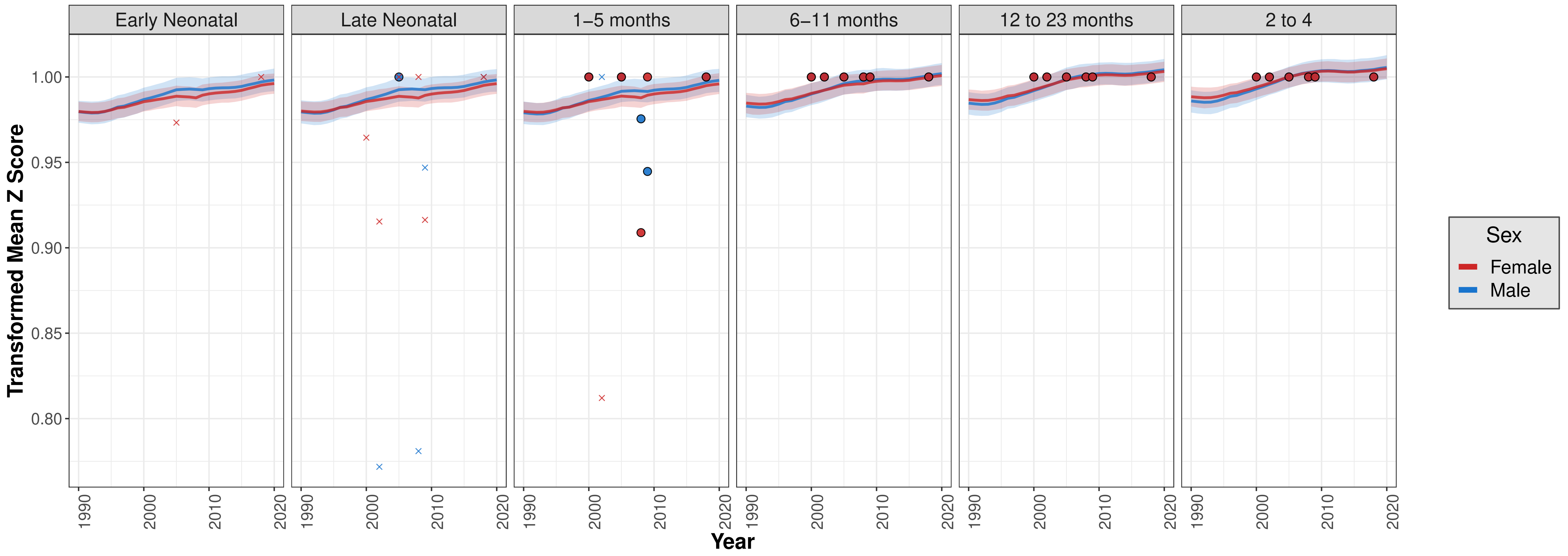


**F**

| Year | Source |
|---|---|
| 1997 | WHO CGM Database |
| 2000 | MICS |
| 2000 | WHO CGM Database |
| 2002 | Living Standards Measurement Survey |
| 2005 | MICS |
| 2005 | WHO CGM Database |
| 2008 | DHS |
| 2009 | DHS |
| 2009 | WHO CGM Database |
| 2010 | Baseline Nutrition and Food Security Survey |
| 2018 | DHS |

**Albania** – Underweight (WAZ)

**G**: Overall and Severe Underweight Prevalence

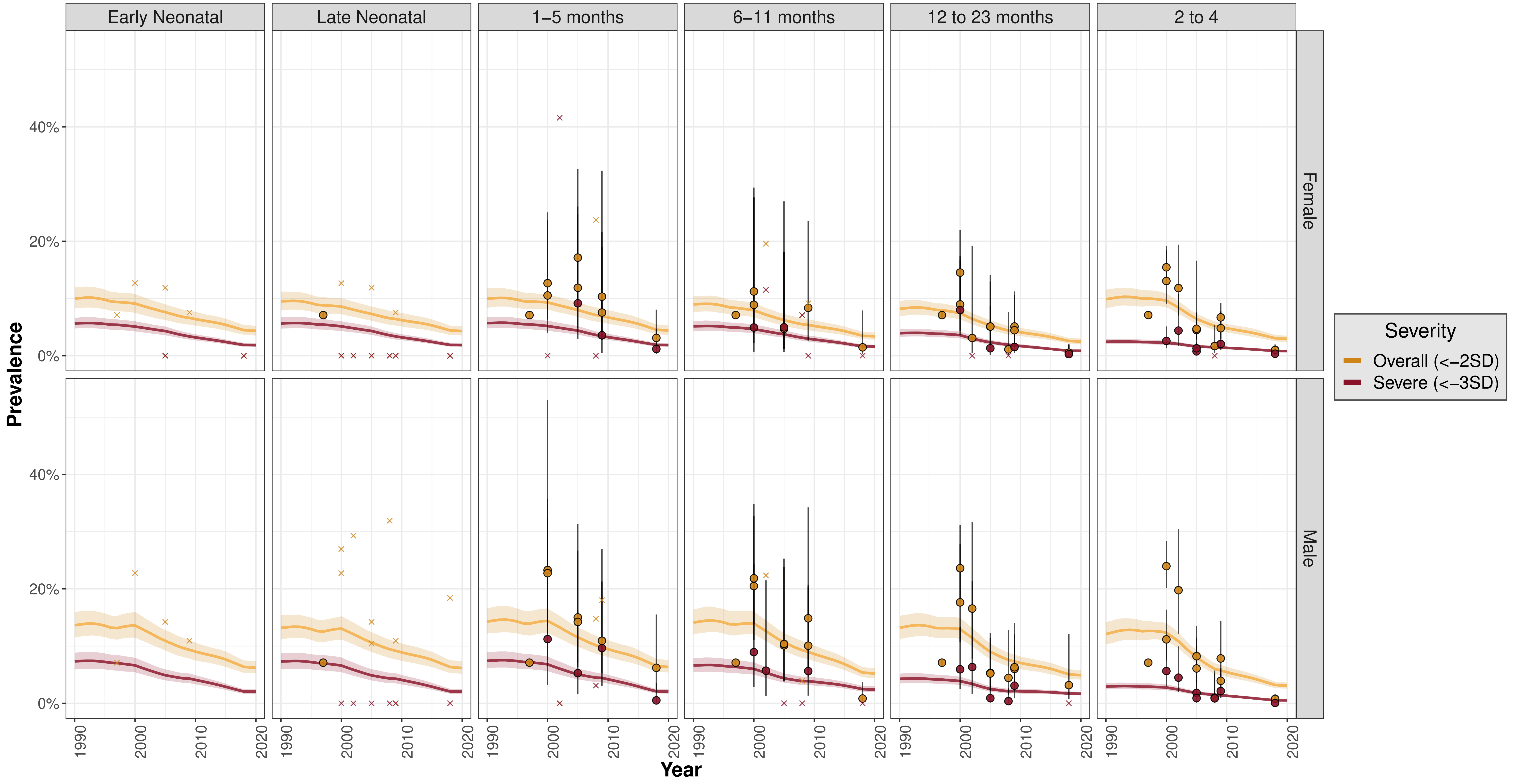


**H**: Transformed Mean Underweight Z Scores

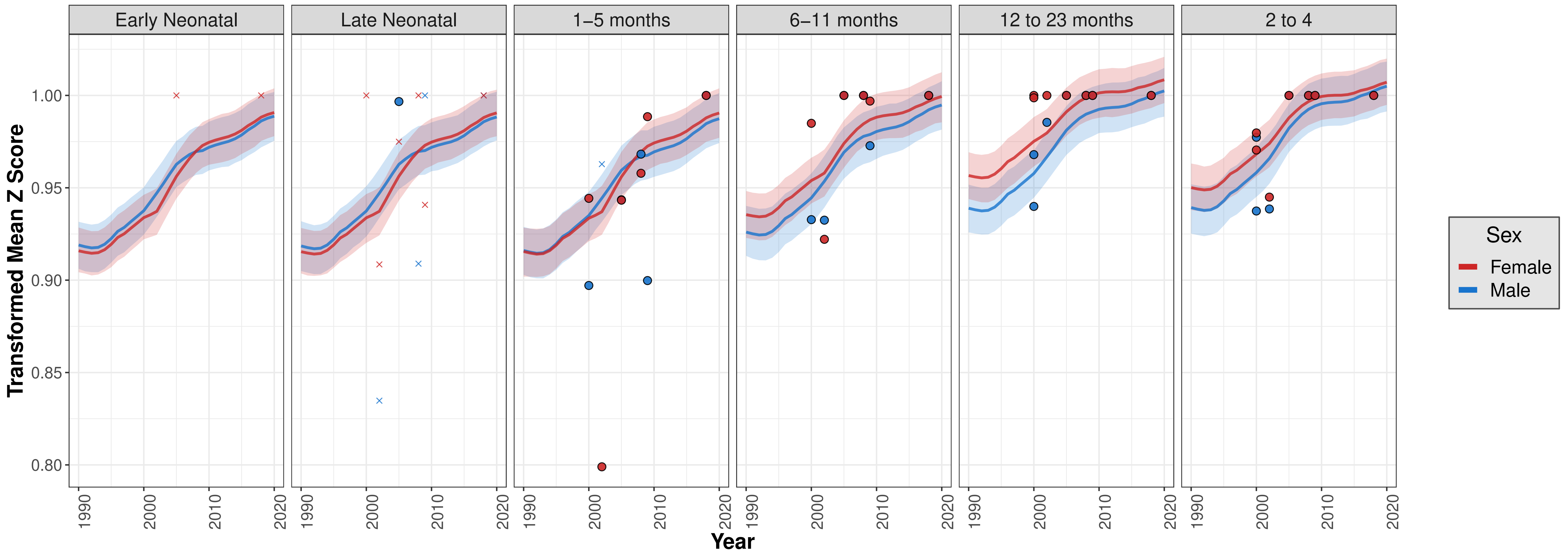


**I**

| Year | Source |
|---|---|
| 1997 | WHO CGM Database |
| 2000 | MICS |
| 2000 | WHO CGM Database |
| 2002 | Living Standards Measurement Survey |
| 2005 | MICS |
| 2005 | WHO CGM Database |
| 2008 | DHS |
| 2009 | DHS |
| 2009 | WHO CGM Database |
| 2018 | DHS |

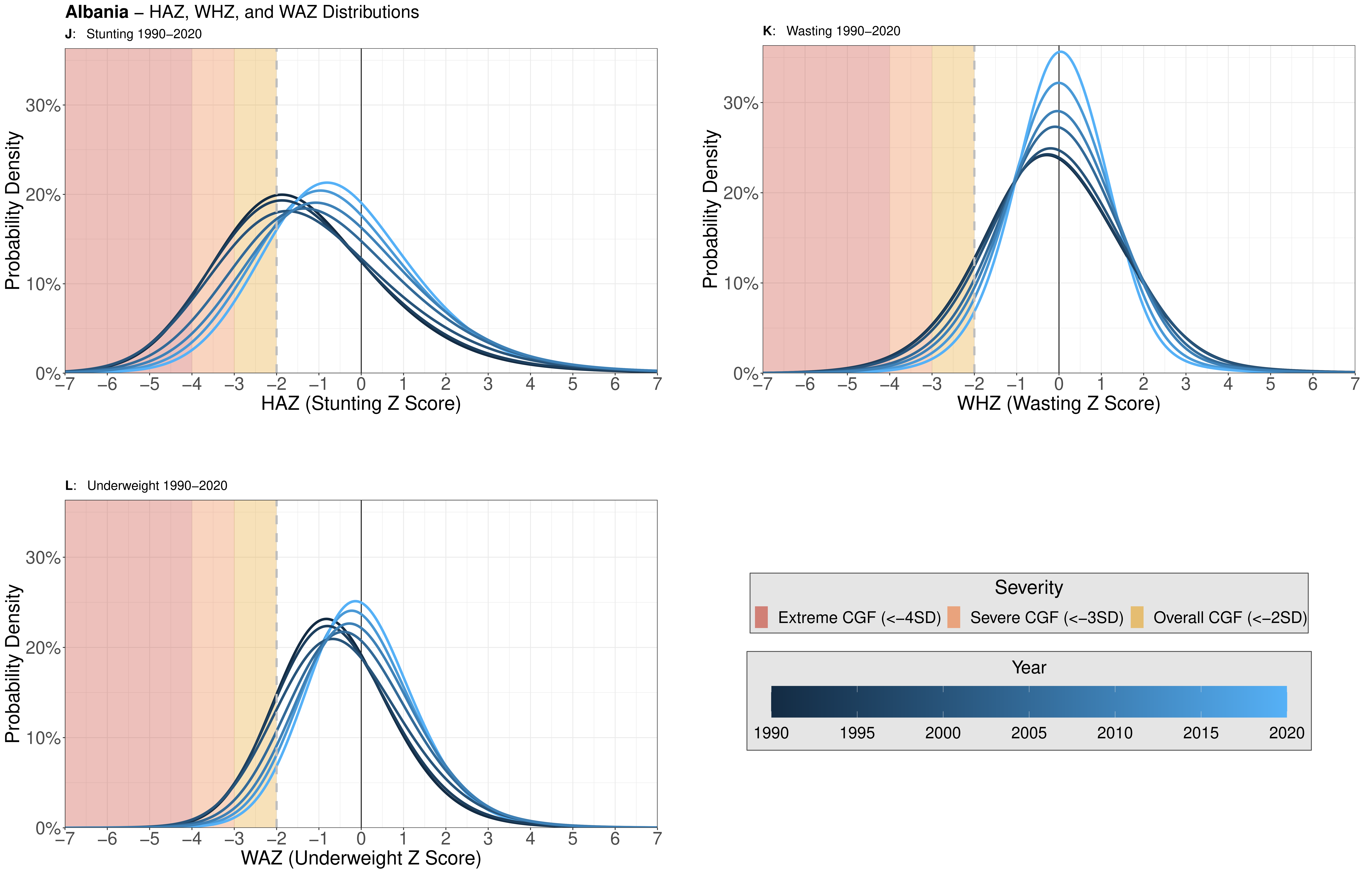

**Albania** – HAZ, WHZ, and WAZ Distributions
J: Stunting 1990–2020
K: Wasting 1990–2020
L: Underweight 1990–2020
Probability Density
0%
10%
20%
30%
−7 −6 −5 −4 −3 −2 −1 0 1 2 3 4 5 6 7
HAZ (Stunting Z Score)
WHZ (Wasting Z Score)
WAZ (Underweight Z Score)
Severity
Extreme CGF (<−4SD)
Severe CGF (<−3SD)
Overall CGF (<−2SD)
Year
1990
1995
2000
2005
2010
2015
2020

# **Bosnia and Herzegovina** – Stunting (HAZ)

**A**: Overall and Severe Stunting Prevalence

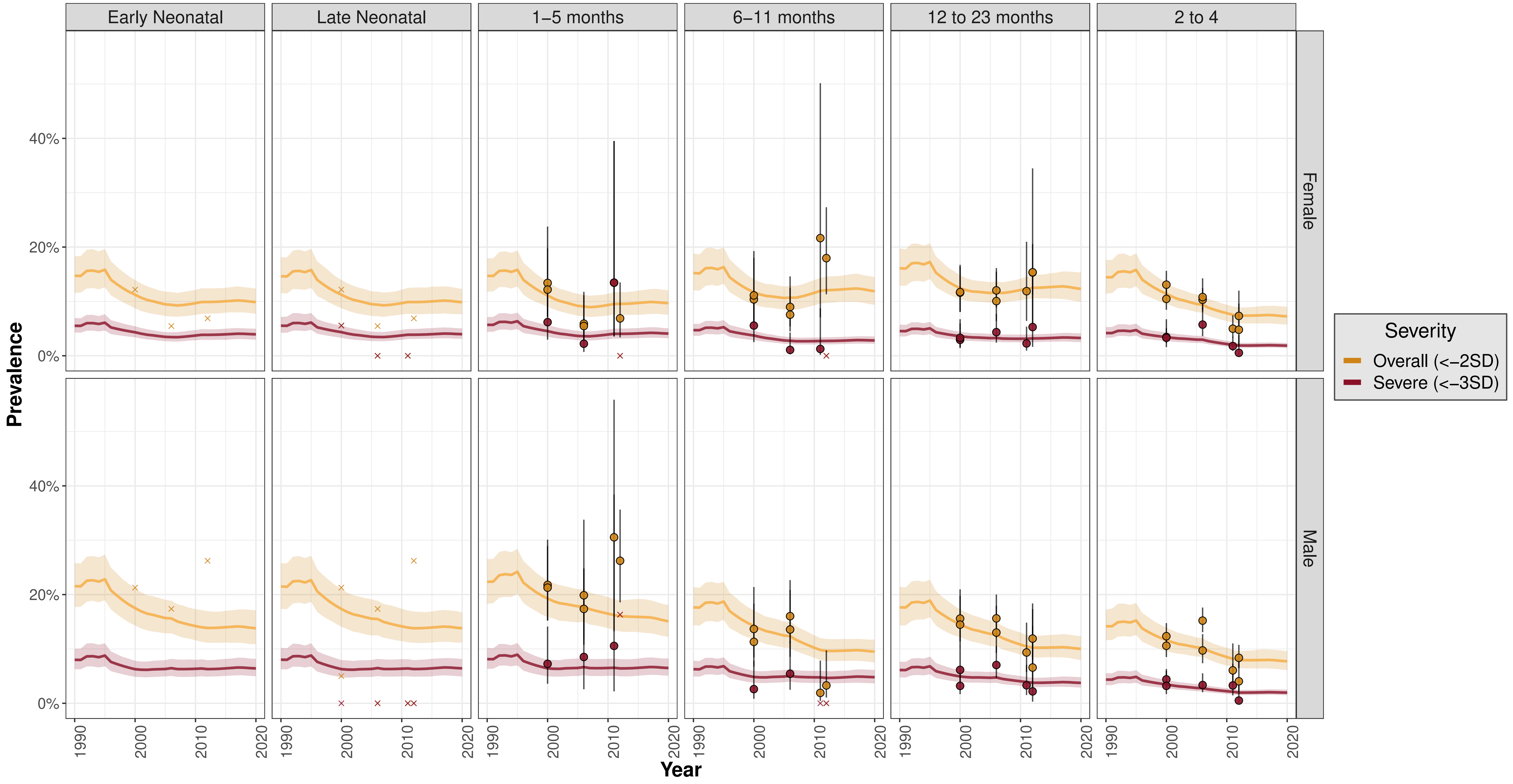


**B**: Transformed Mean Stunting Z Scores

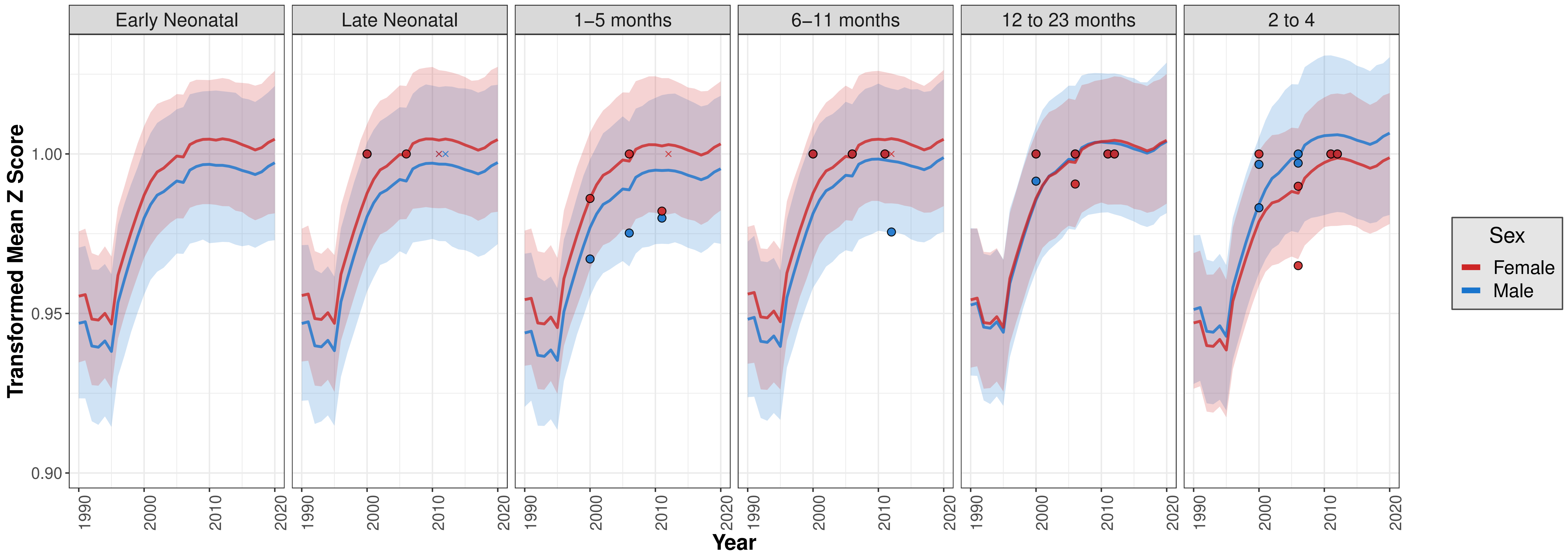


**C**

| Year | Source |
|---|---|
| 2000 | MICS |
| 2000 | WHO CGM Database |
| 2006 | MICS |
| 2006 | WHO CGM Database |
| 2011 | MICS |
| 2012 | MICS |
| 2012 | WHO CGM Database |

# **Bosnia and Herzegovina** – Wasting (WHZ)

**D**: Overall and Severe Wasting Prevalence

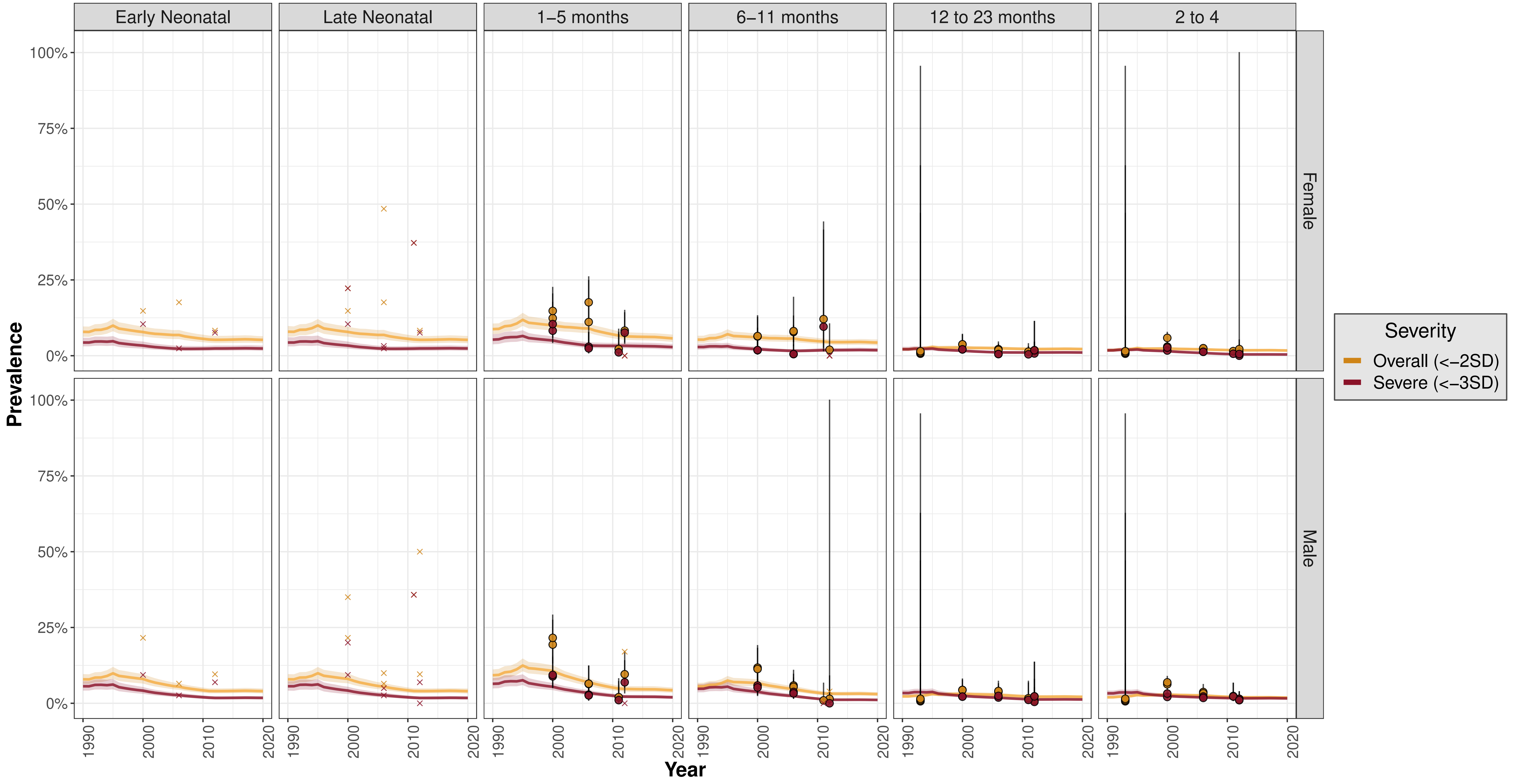


**E**: Transformed Mean Wasting Z Scores

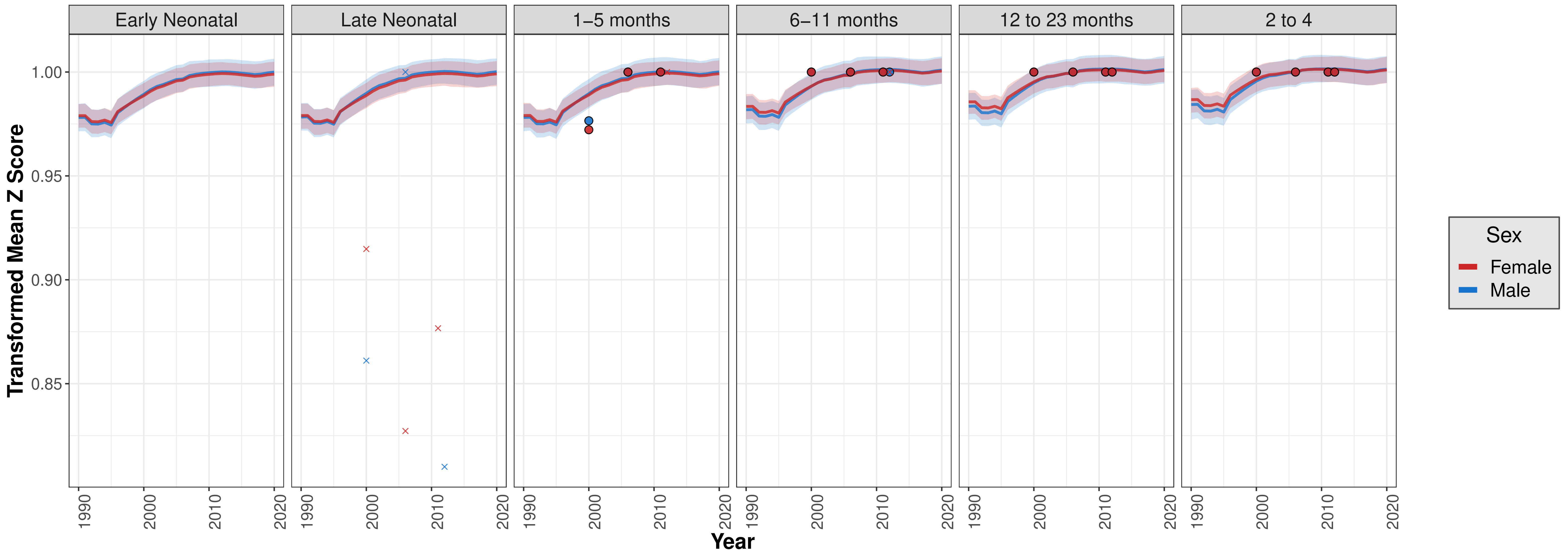


**F**

| Year | Source |
|---|---|
| 1993 | WHO CGM Database |
| 2000 | MICS |
| 2000 | WHO CGM Database |
| 2006 | MICS |
| 2006 | WHO CGM Database |
| 2011 | MICS |
| 2012 | MICS |
| 2012 | WHO CGM Database |

# **Bosnia and Herzegovina** – Underweight (WAZ)

**G**: Overall and Severe Underweight Prevalence

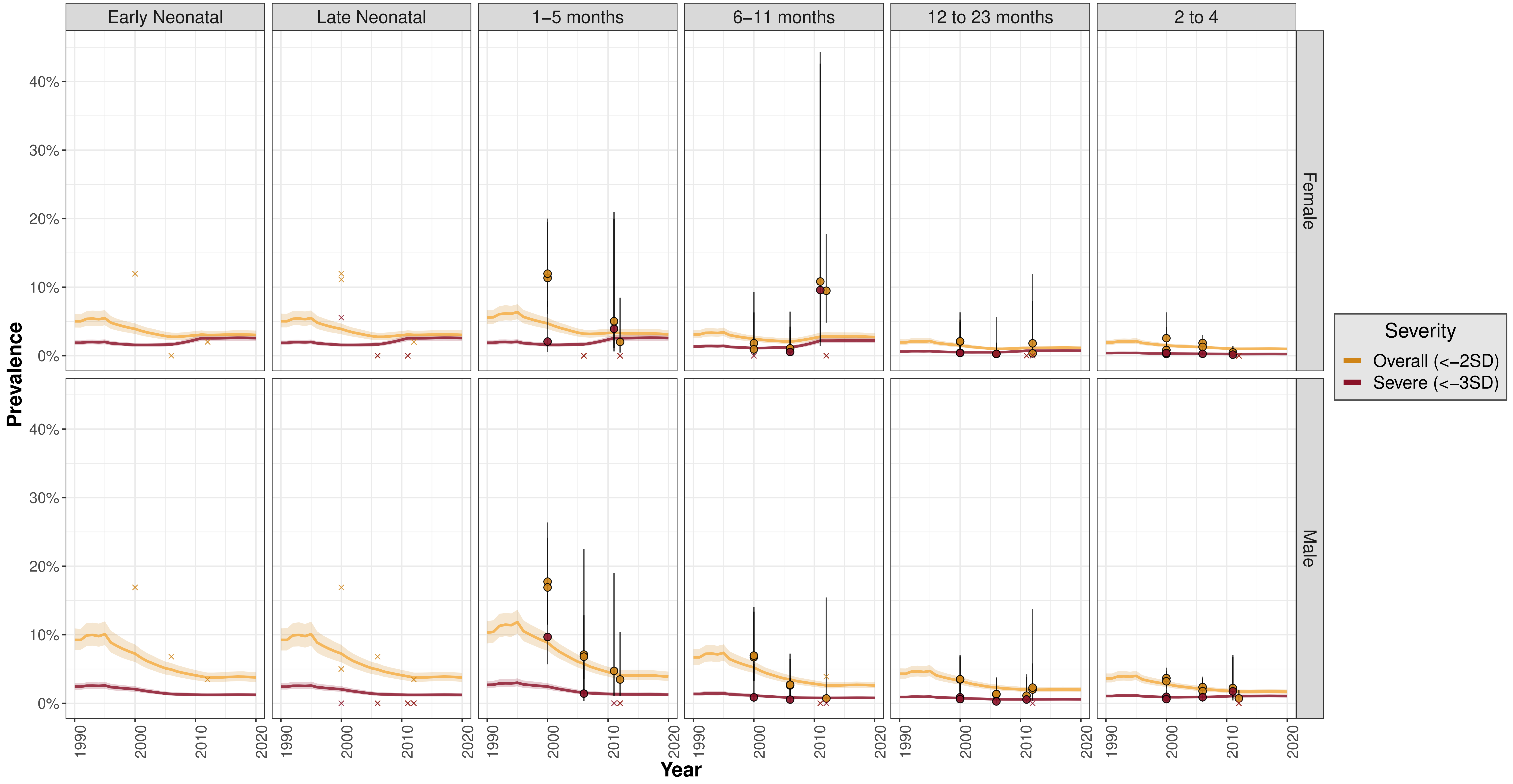


**H**: Transformed Mean Underweight Z Scores

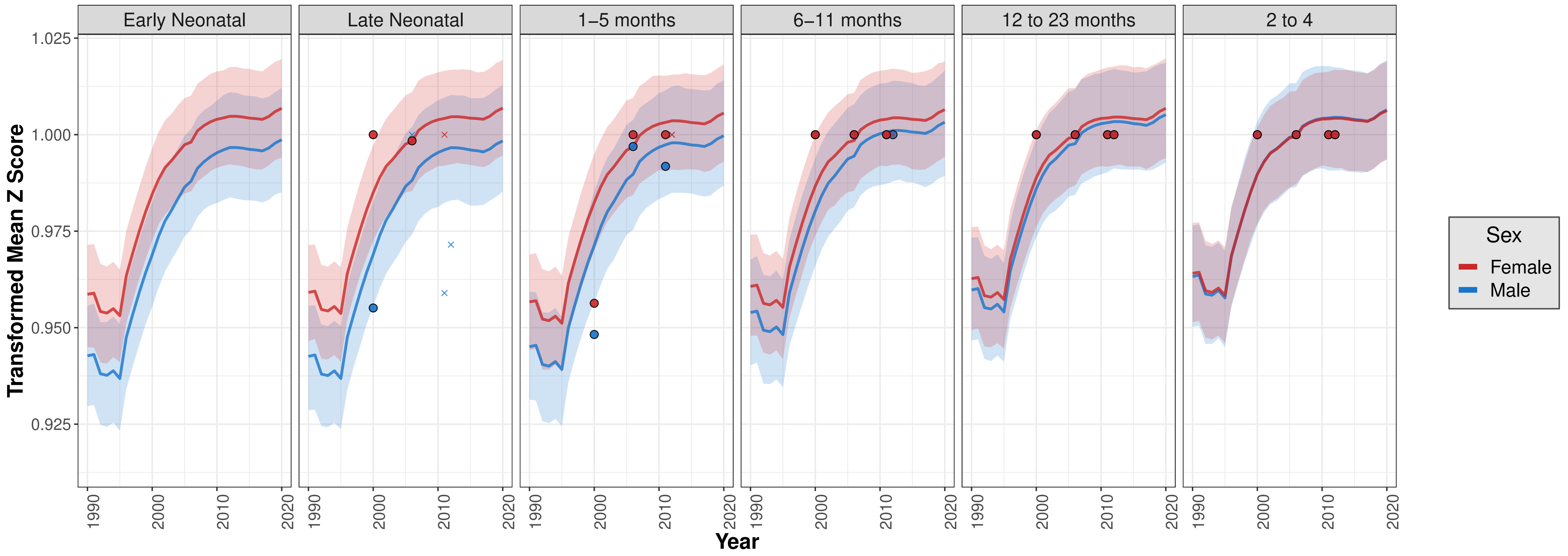


**I**

| Year | Source |
|---|---|
| 2000 | MICS |
| 2000 | WHO CGM Database |
| 2006 | MICS |
| 2006 | WHO CGM Database |
| 2011 | MICS |
| 2012 | MICS |
| 2012 | WHO CGM Database |

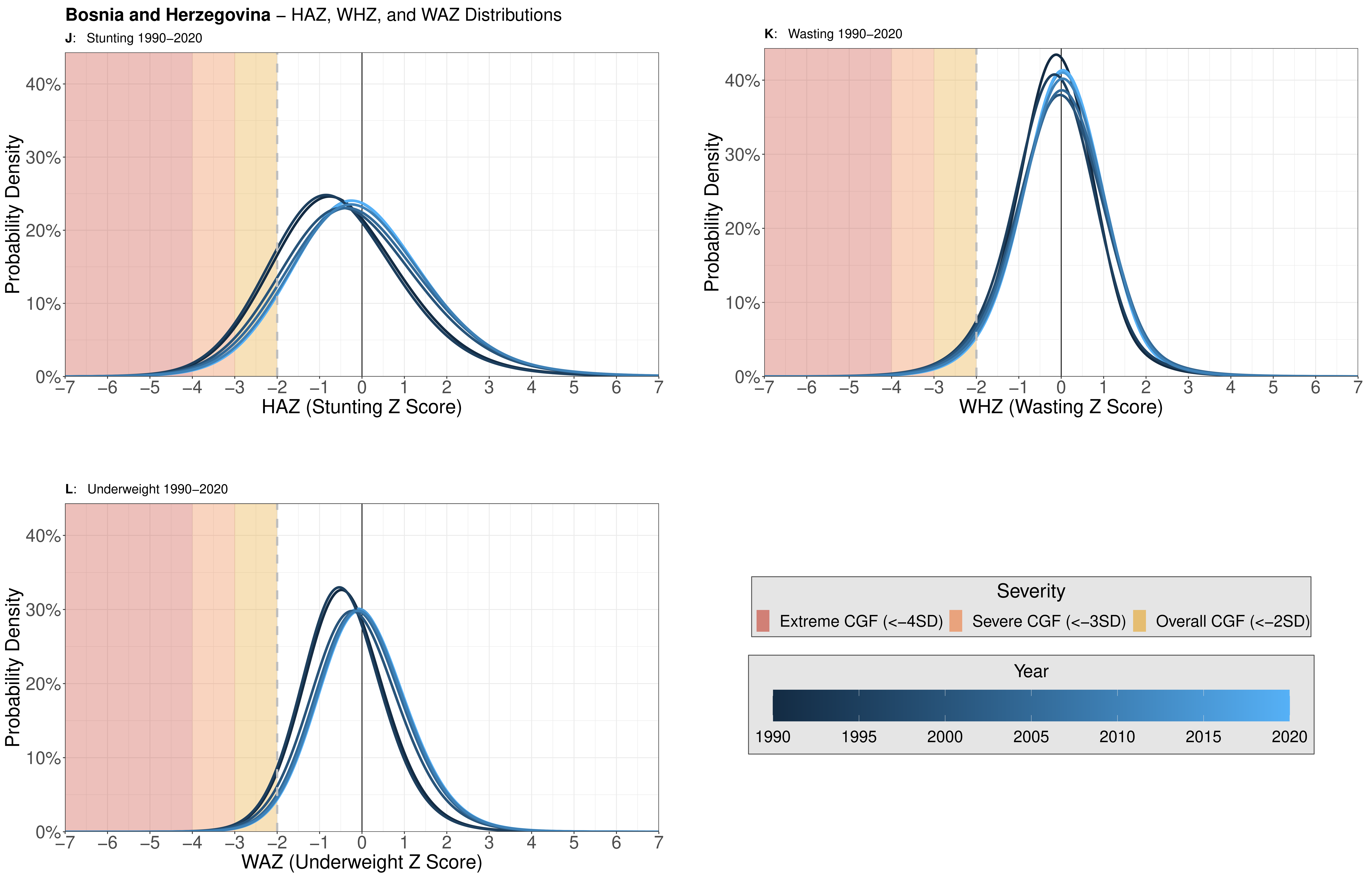
Bosnia and Herzegovina – HAZ, WHZ, and WAZ Distributions
J: Stunting 1990–2020
K: Wasting 1990–2020
L: Underweight 1990–2020
Probability Density
0%
10%
20%
30%
40%
−7 −6 −5 −4 −3 −2 −1 0 1 2 3 4 5 6 7
HAZ (Stunting Z Score)
WHZ (Wasting Z Score)
WAZ (Underweight Z Score)
Severity
Extreme CGF (<−4SD)
Severe CGF (<−3SD)
Overall CGF (<−2SD)
Year
1990
1995
2000
2005
2010
2015
2020

## **Bulgaria** – Stunting (HAZ)

**A**: Overall and Severe Stunting Prevalence

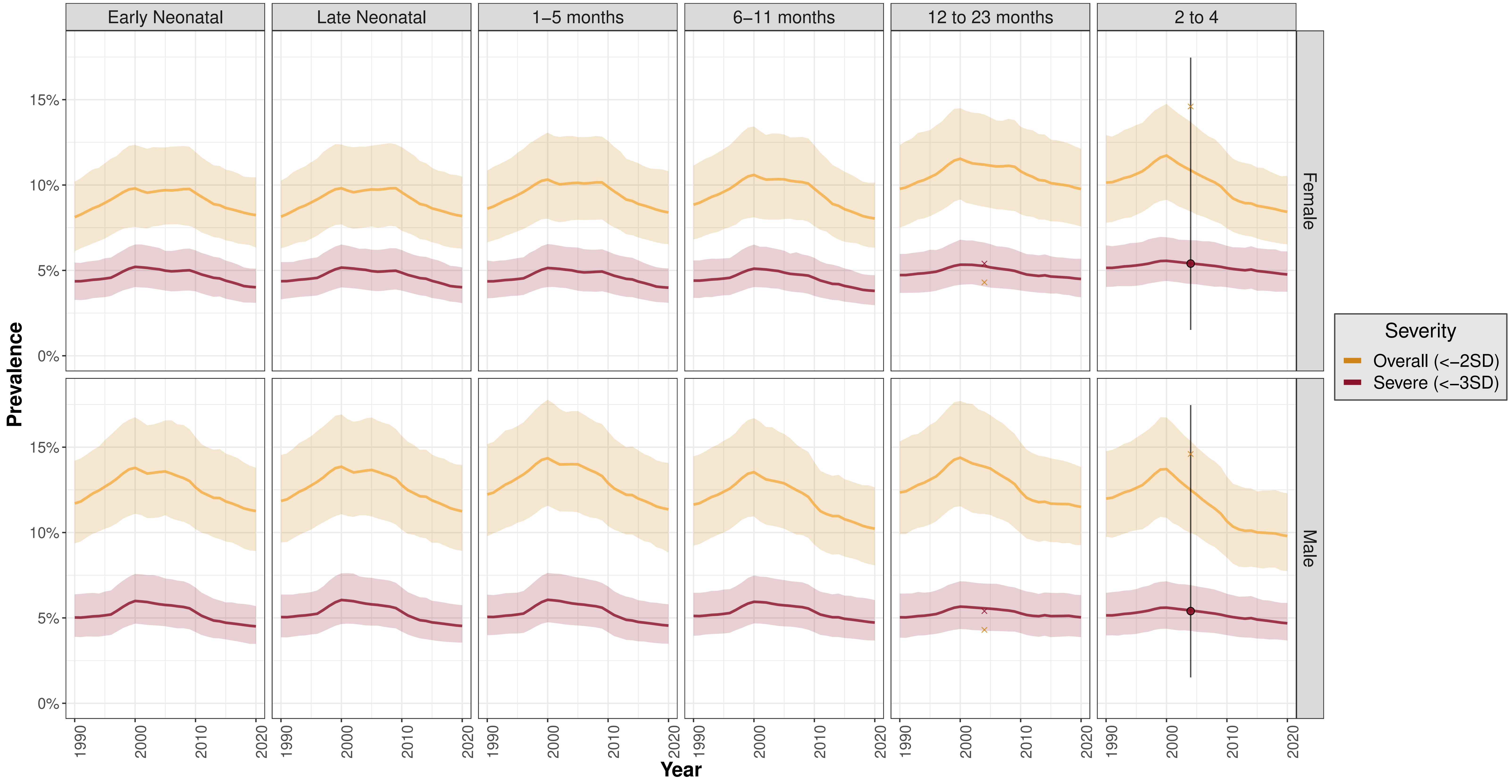


**B**: Transformed Mean Stunting Z Scores

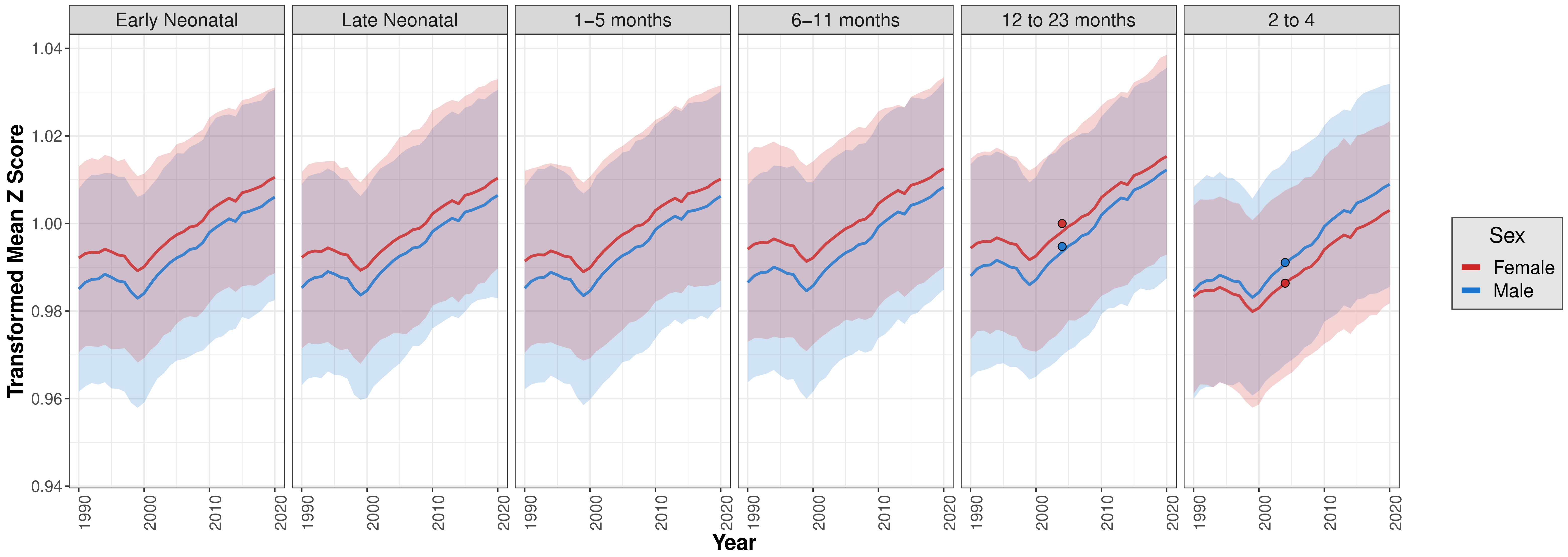


**C**

| Year | Source |
|---|---|
| 2004 | WHO CGM Database |

**Bulgaria** – Wasting (WHZ)

**D**: Overall and Severe Wasting Prevalence

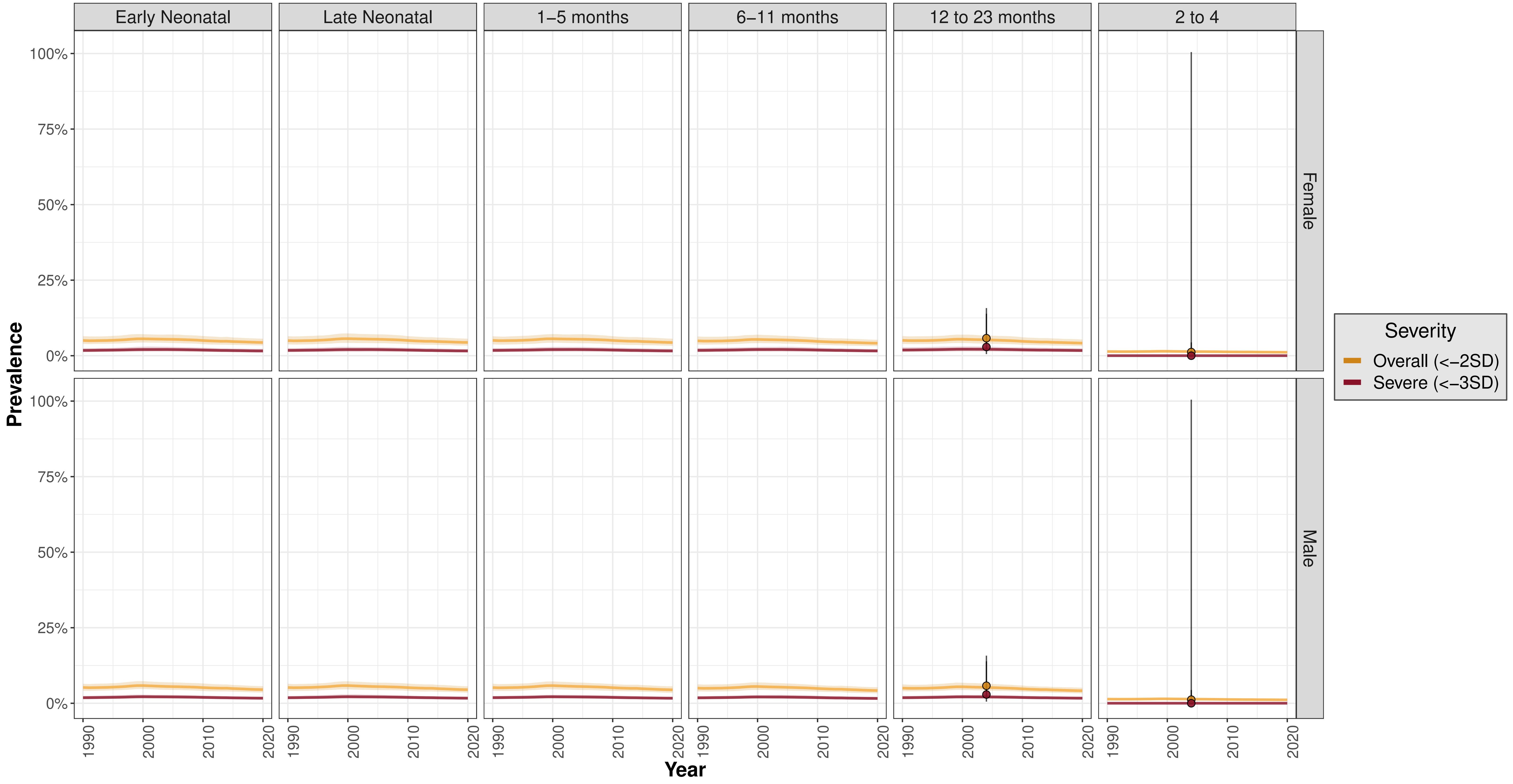


**E**: Transformed Mean Wasting Z Scores

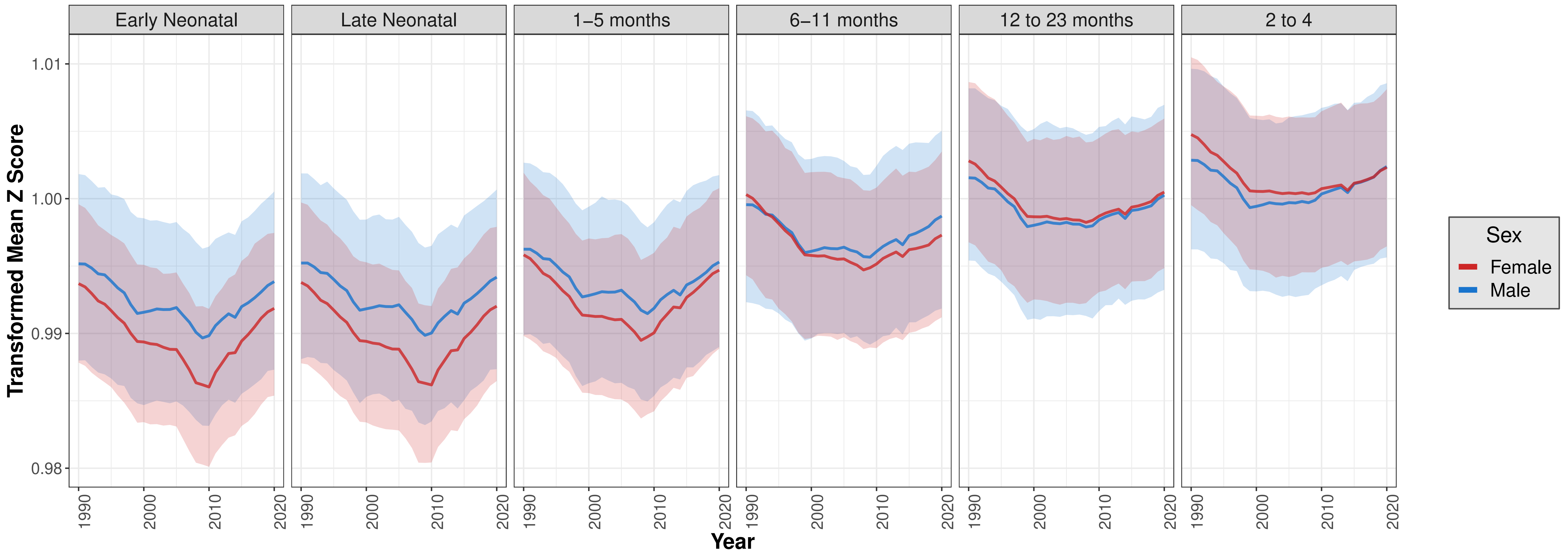


**F**

| Year | Source |
|---|---|
| 2004 | WHO CGM Database |

# **Bulgaria** – Underweight (WAZ)

**G**: Overall and Severe Underweight Prevalence

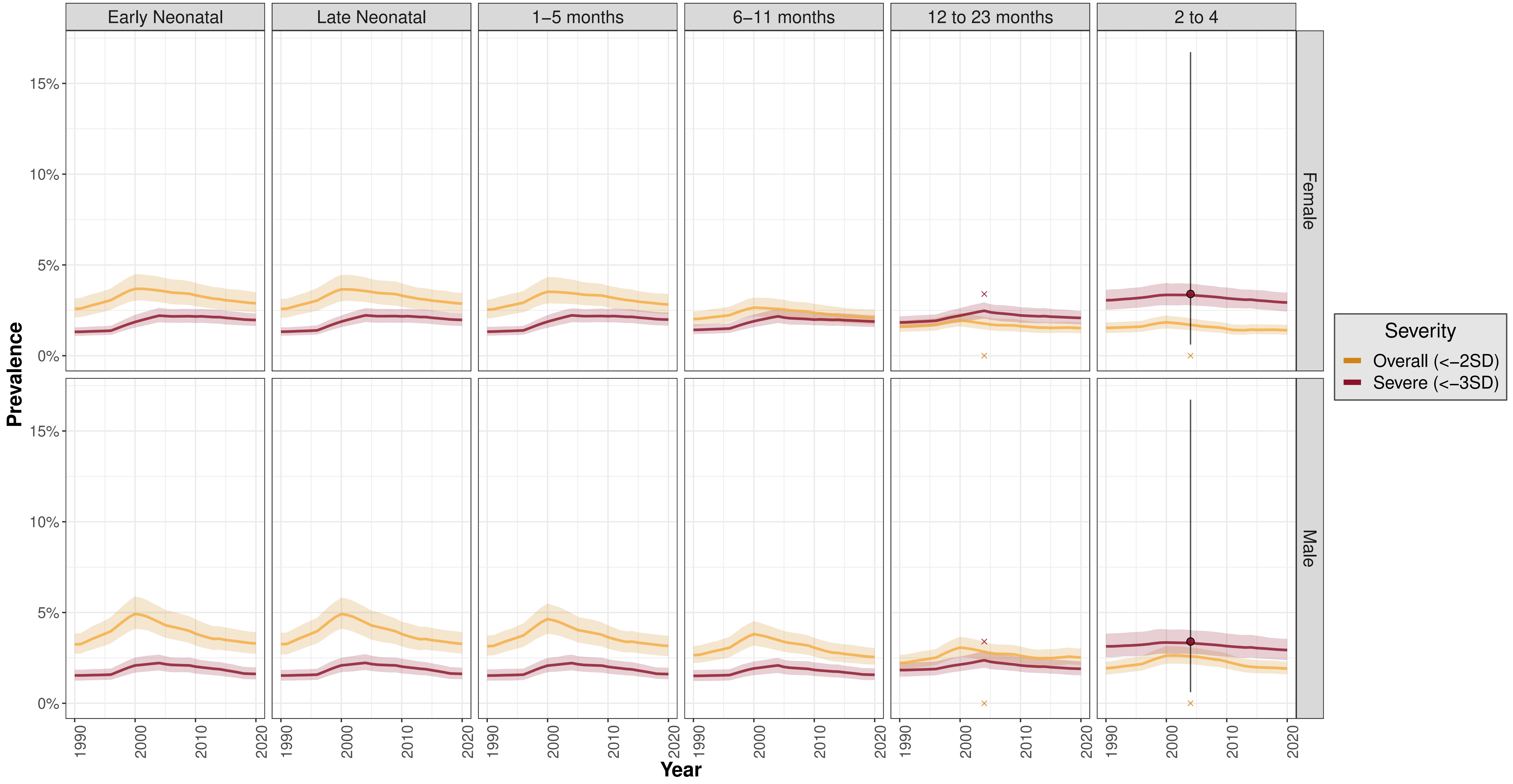


**H**: Transformed Mean Underweight Z Scores

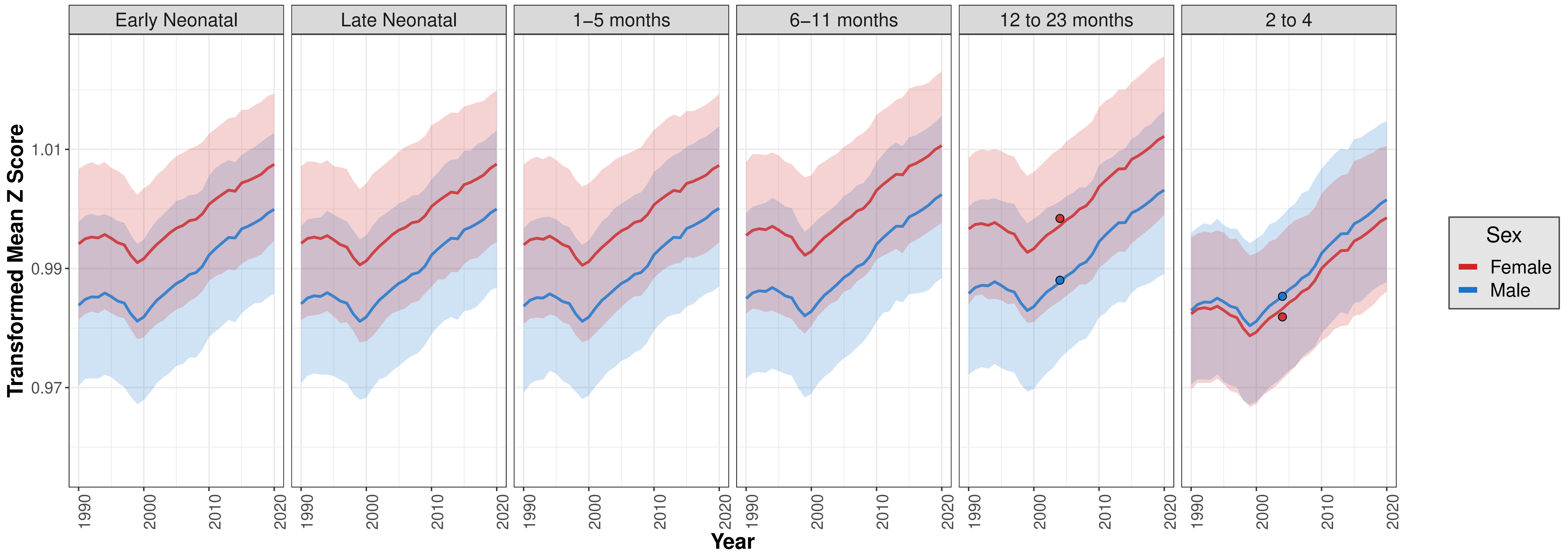


**I**

| Year | Source |
|---|---|
| 2004 | WHO CGM Database |

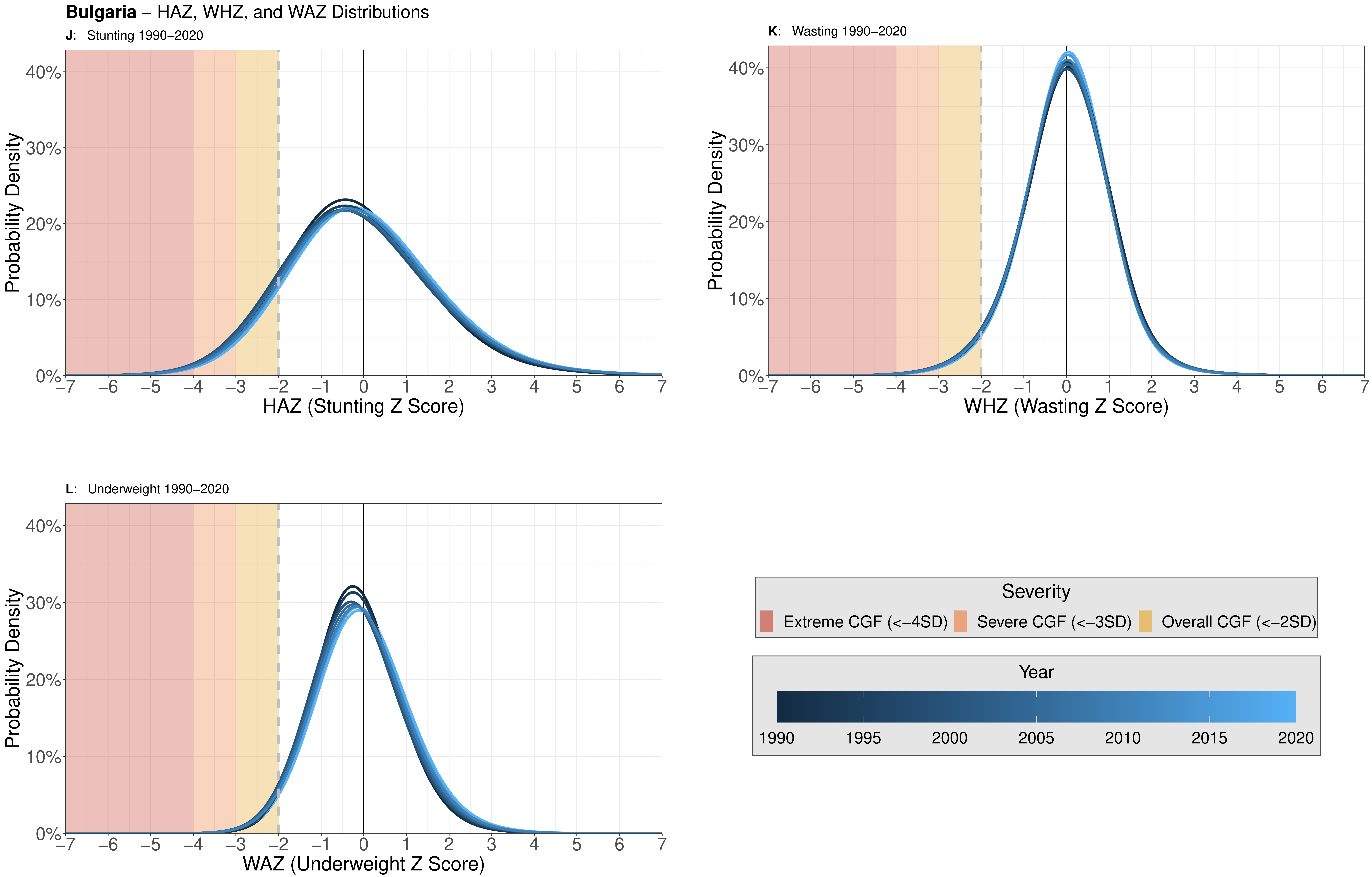
Bulgaria – HAZ, WHZ, and WAZ Distributions
J: Stunting 1990–2020
Probability Density
HAZ (Stunting Z Score)
K: Wasting 1990–2020
Probability Density
WHZ (Wasting Z Score)
L: Underweight 1990–2020
Probability Density
WAZ (Underweight Z Score)
0%
10%
20%
30%
40%
−7
−6
−5
−4
−3
−2
−1
0
1
2
3
4
5
6
7
Severity
Extreme CGF (<−4SD)
Severe CGF (<−3SD)
Overall CGF (<−2SD)
Year
1990
1995
2000
2005
2010
2015
2020

# **Croatia** – Stunting (HAZ)

**A**: Overall and Severe Stunting Prevalence

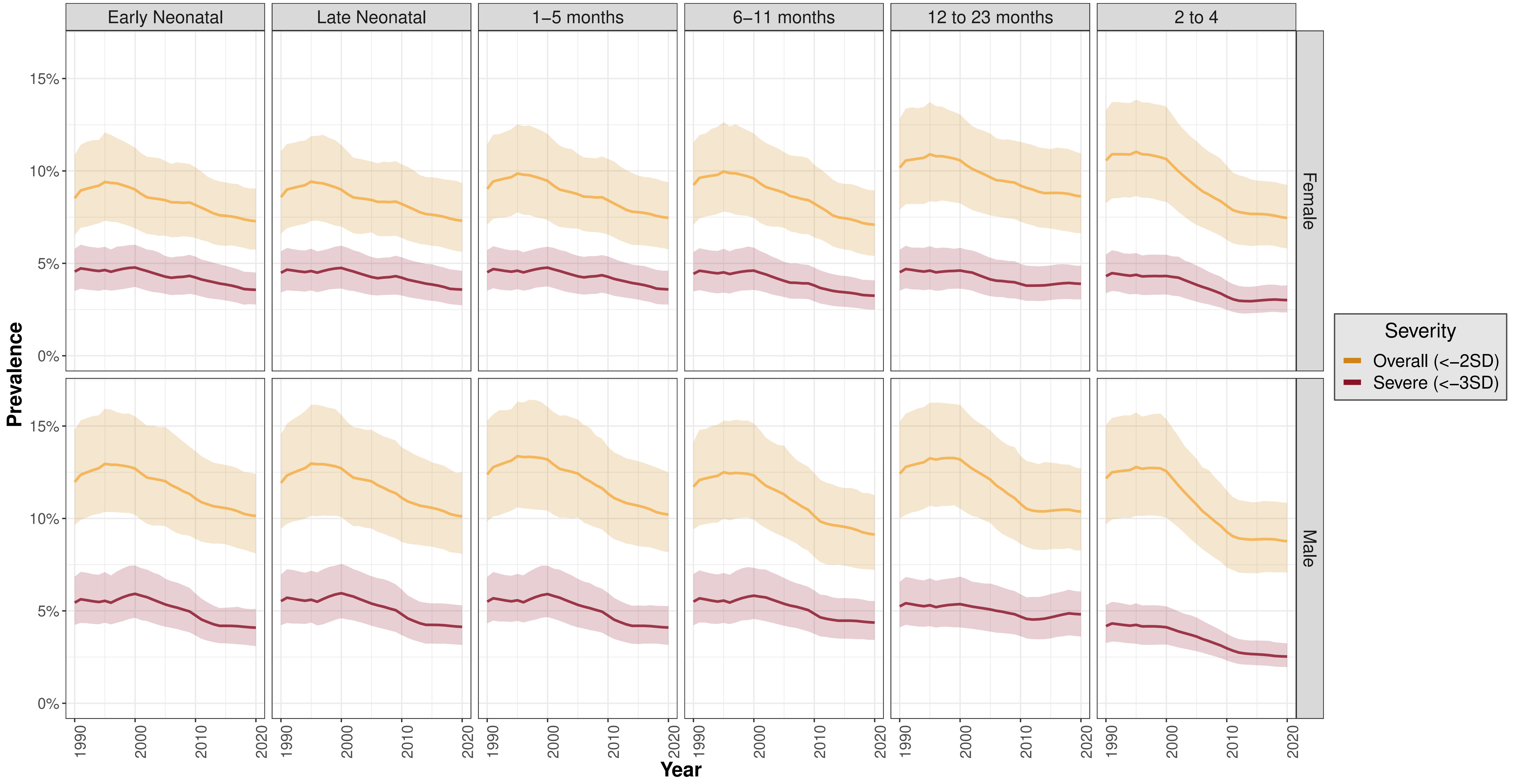


**B**: Transformed Mean Stunting Z Scores

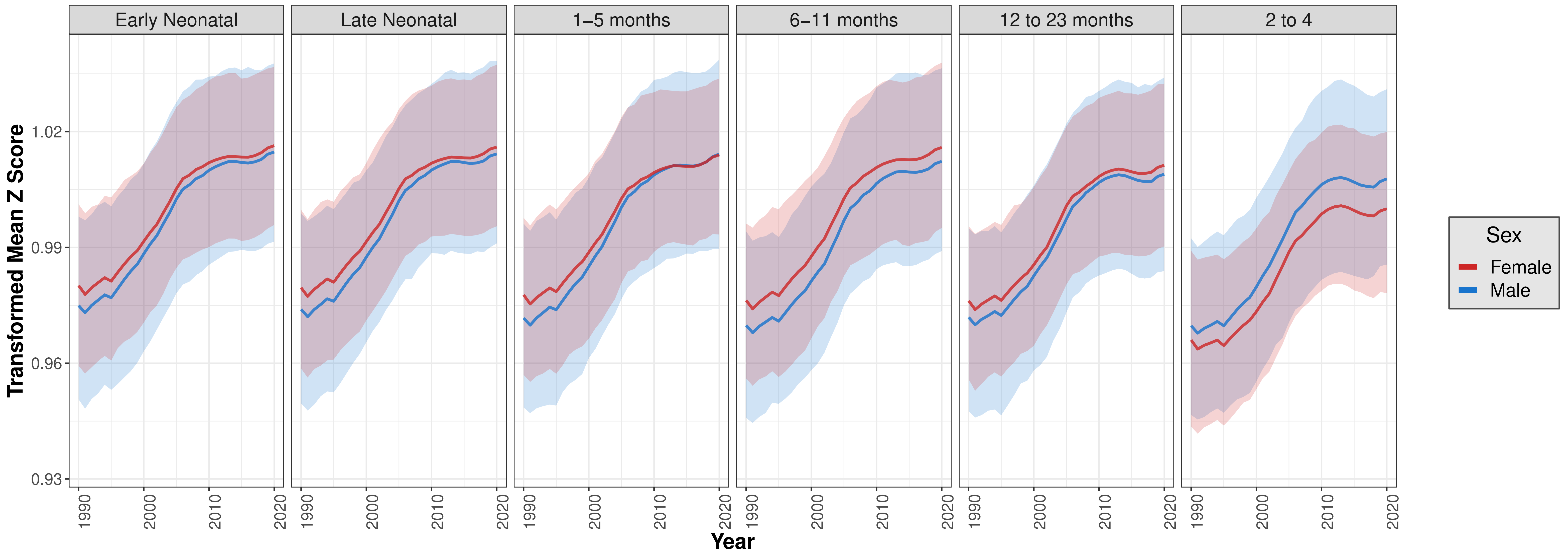


**C**

| Source |
| --- |
| No sources for this location |

**Croatia** – Wasting (WHZ)

**D:** Overall and Severe Wasting Prevalence

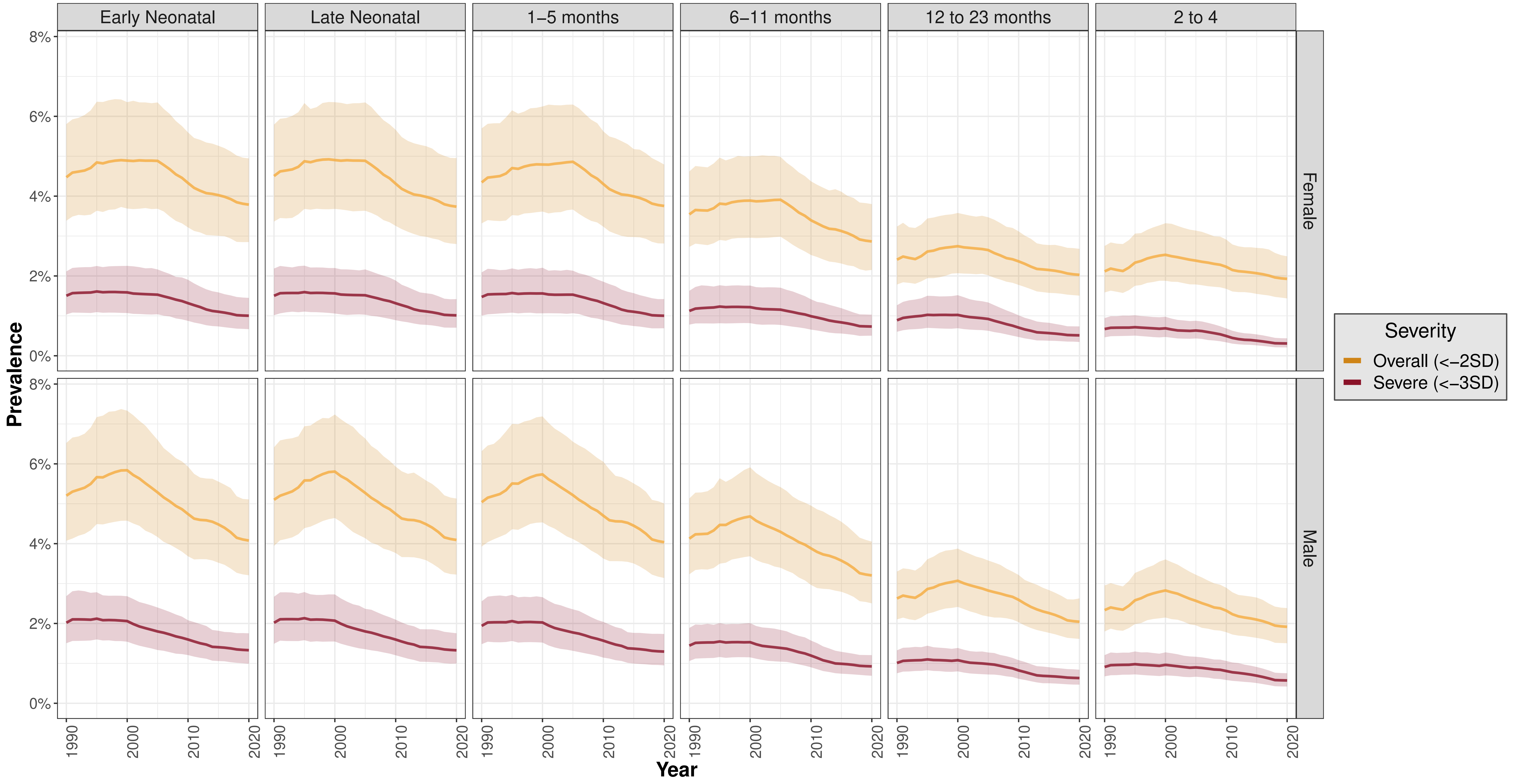


**E:** Transformed Mean Wasting Z Scores

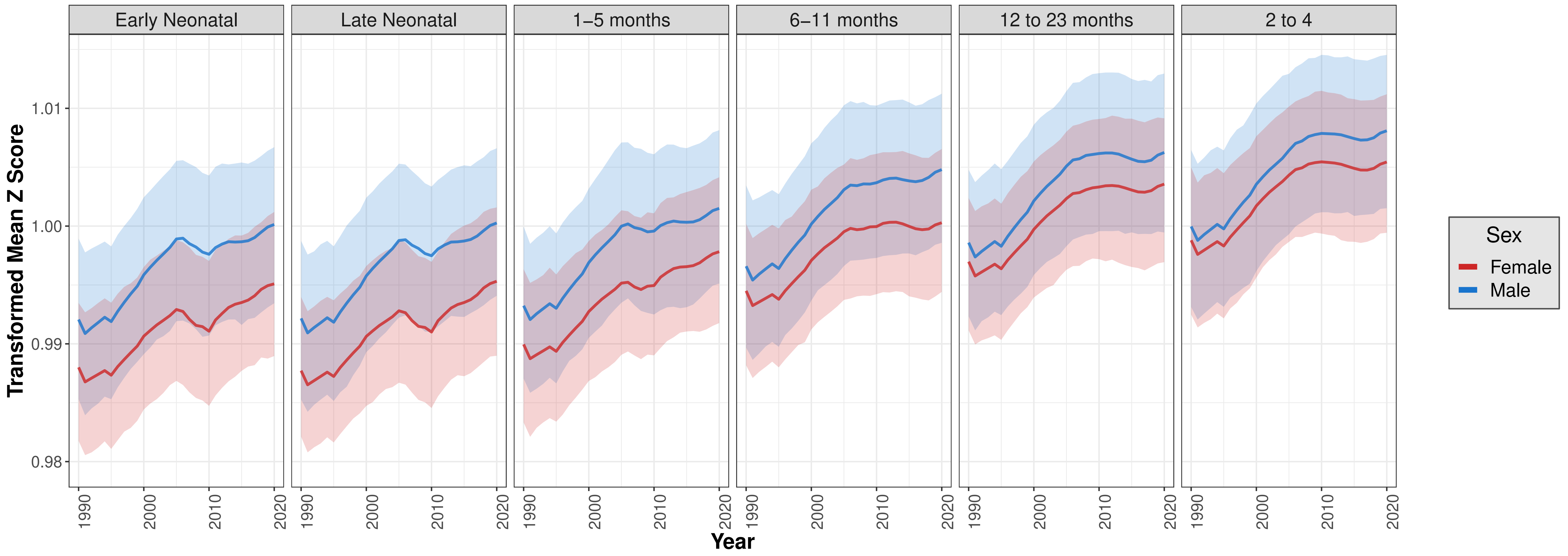


**F**

| Source |
|---|
| No sources for this location |

# **Croatia** – Underweight (WAZ)

**G:** Overall and Severe Underweight Prevalence

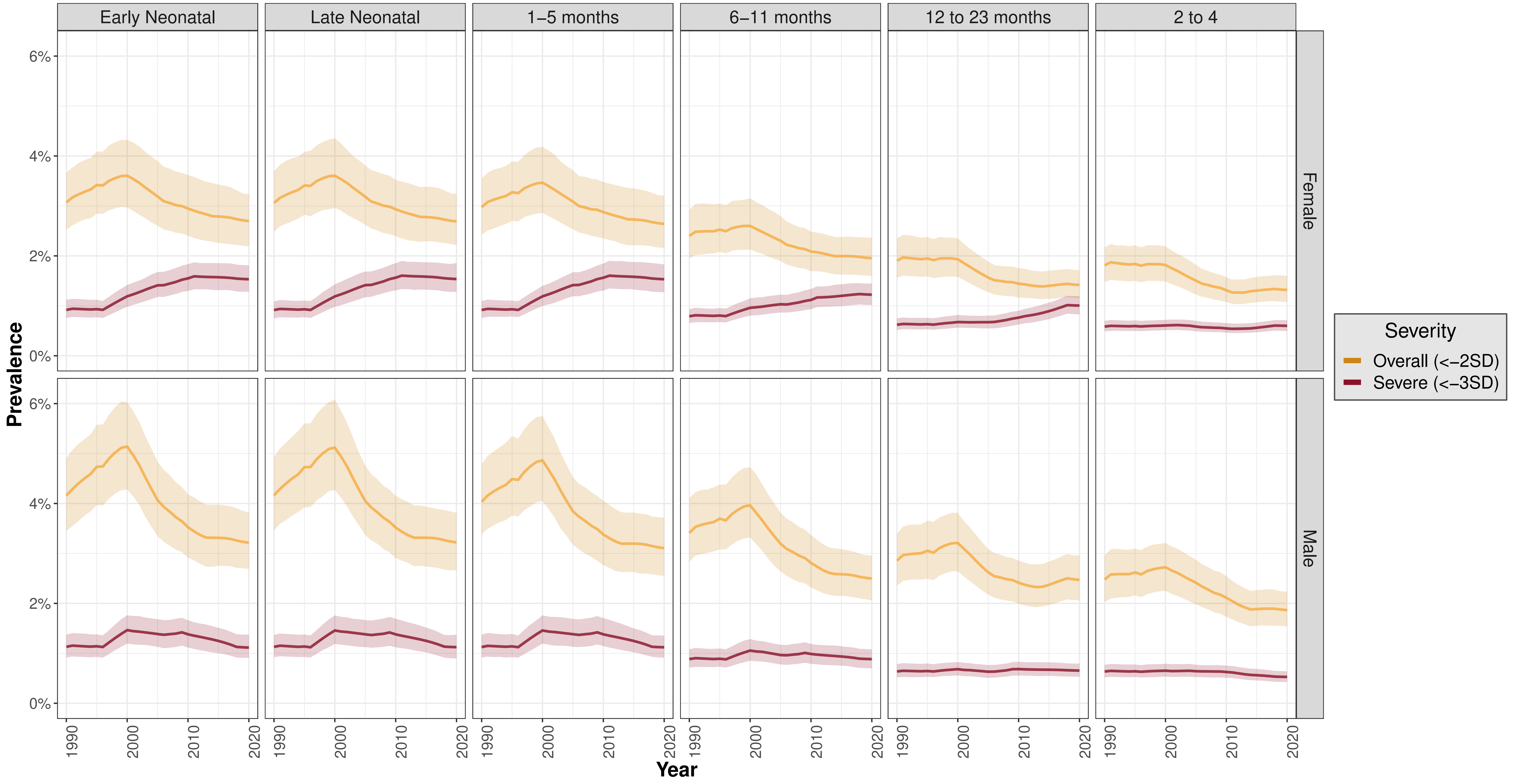


**H:** Transformed Mean Underweight Z Scores

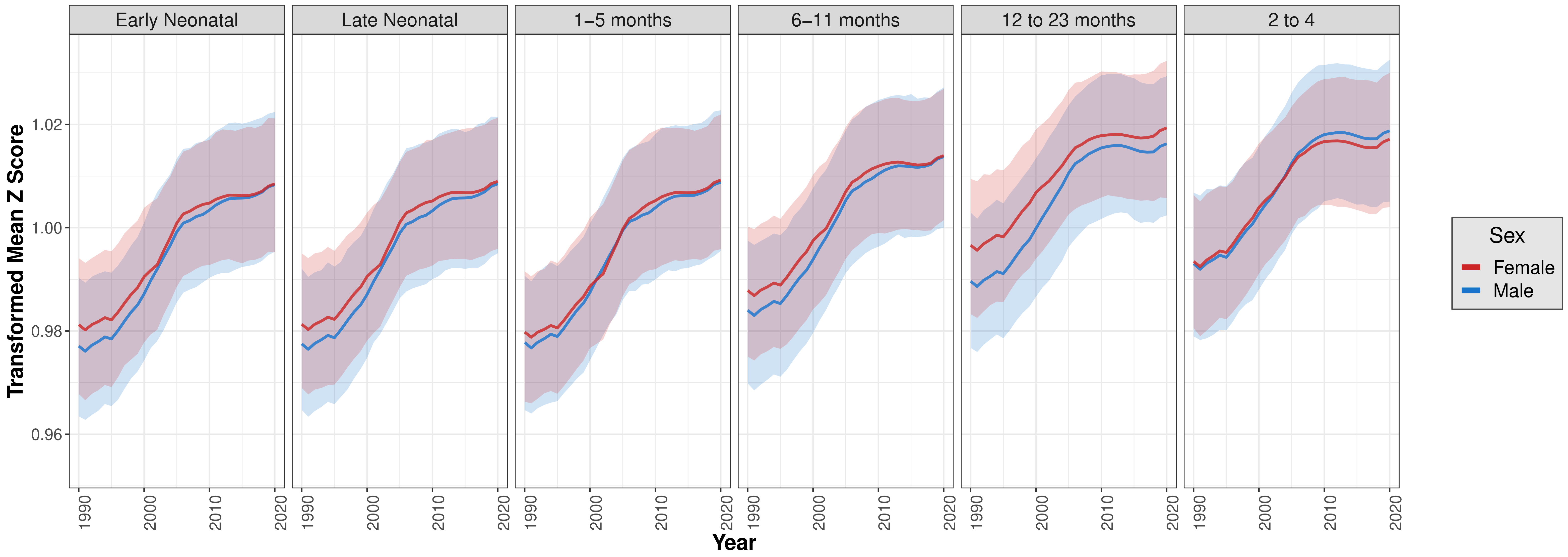


**I**

| Source |
| --- |
| No sources for this location |

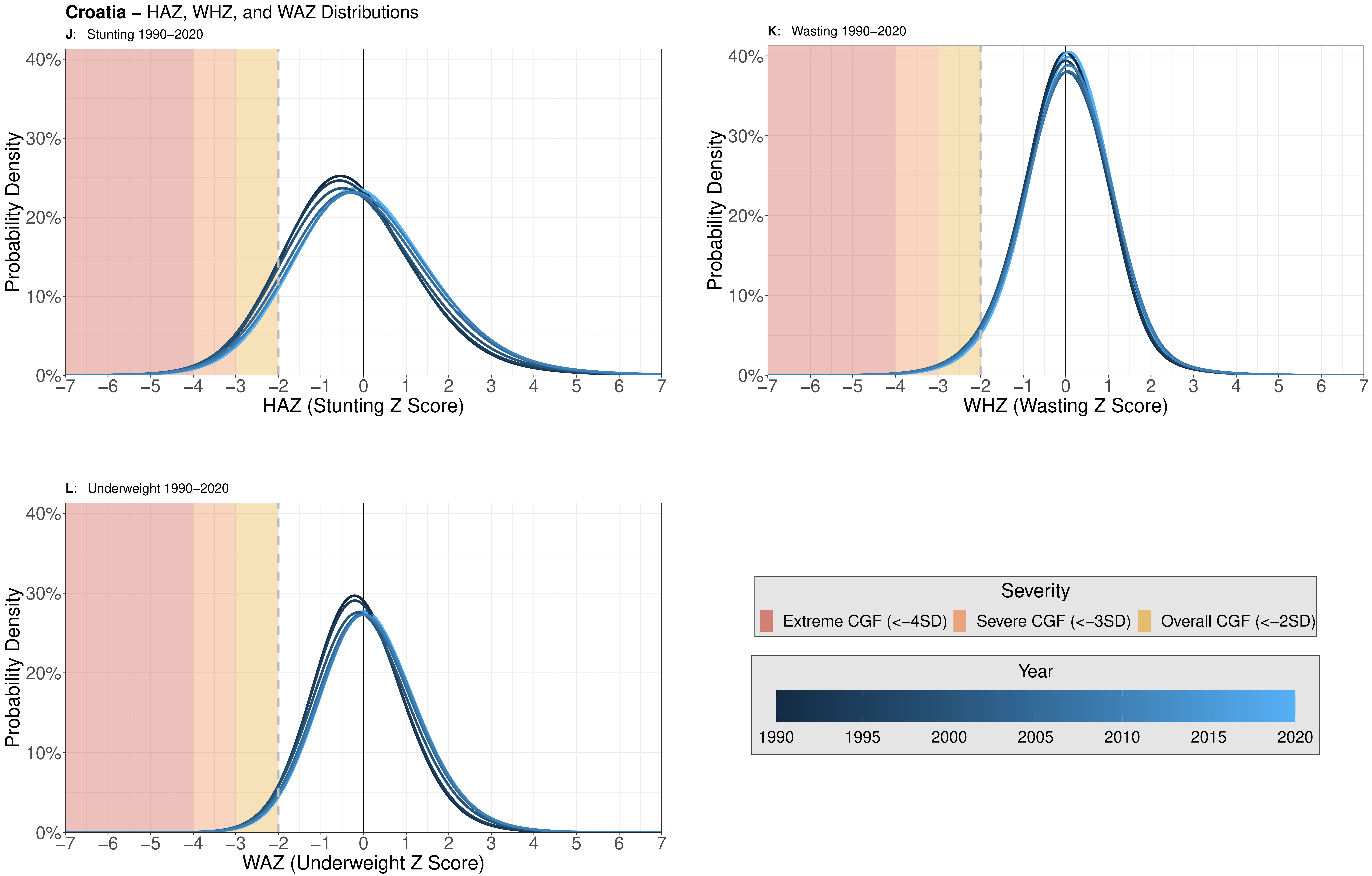

Croatia – HAZ, WHZ, and WAZ Distributions
J: Stunting 1990–2020
K: Wasting 1990–2020
L: Underweight 1990–2020
Probability Density
0%
10%
20%
30%
40%
−7
−6
−5
−4
−3
−2
−1
0
1
2
3
4
5
6
7
HAZ (Stunting Z Score)
WHZ (Wasting Z Score)
WAZ (Underweight Z Score)
Severity
Extreme CGF (<−4SD)
Severe CGF (<−3SD)
Overall CGF (<−2SD)
Year
1990
1995
2000
2005
2010
2015
2020

**Czechia** – Stunting (HAZ)

**A**: Overall and Severe Stunting Prevalence

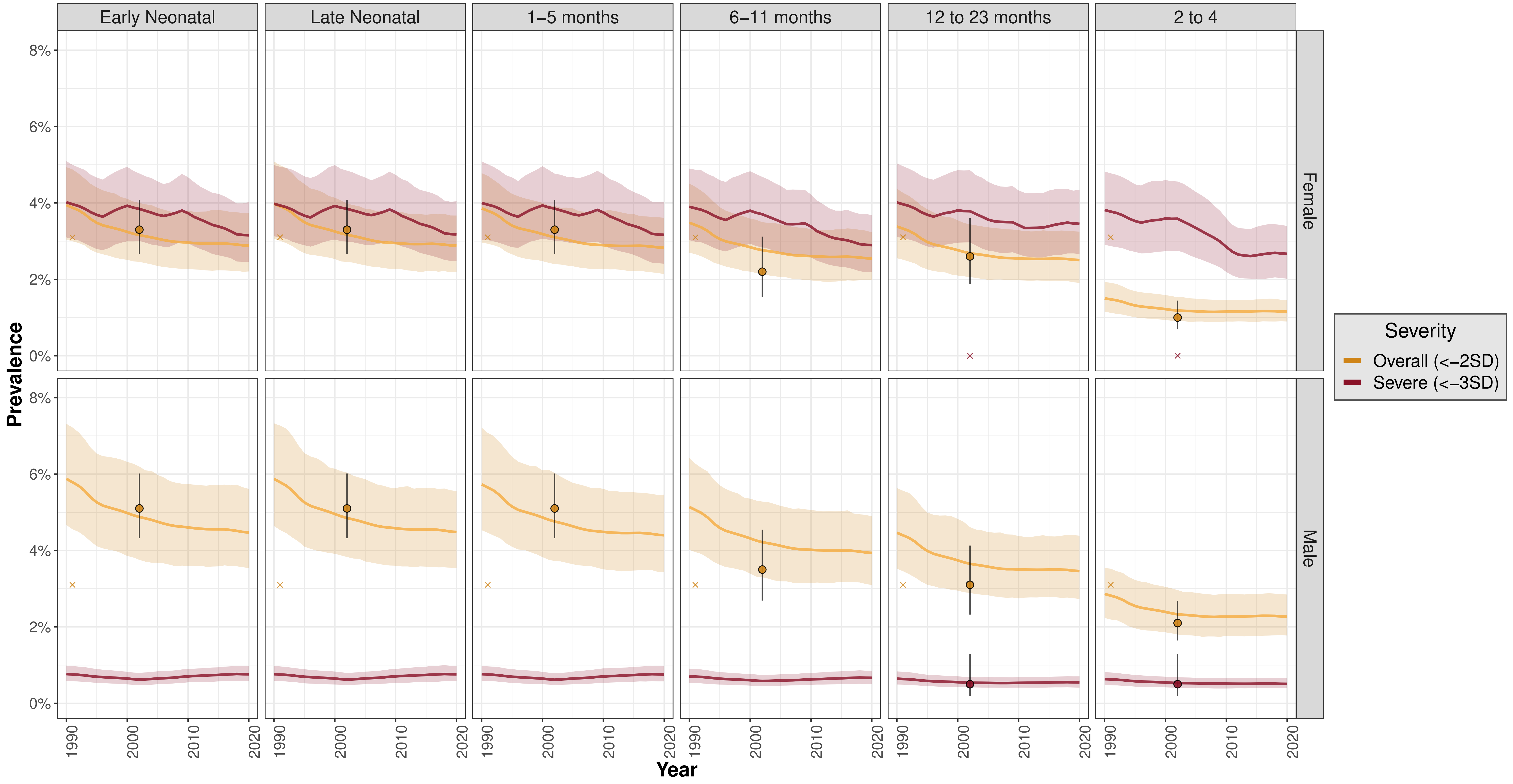


**B**: Transformed Mean Stunting Z Scores

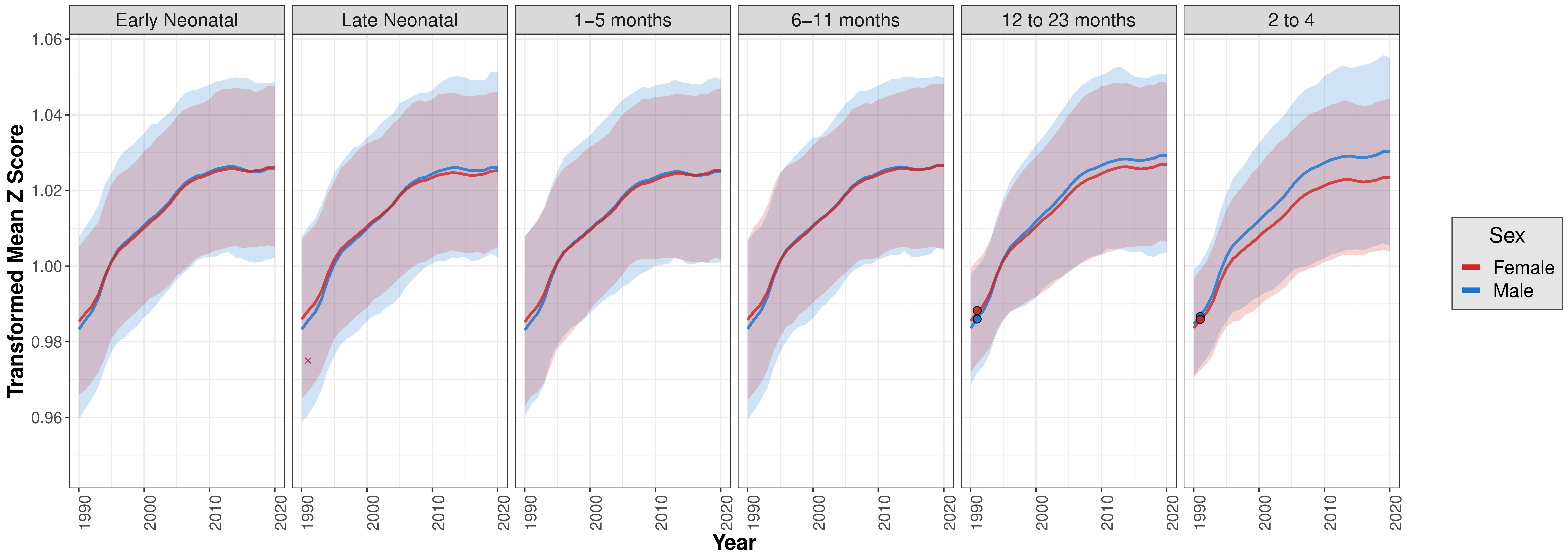


**C**

| Year | Source |
|---|---|
| 1991 | WHO CGM Database |
| 2002 | WHO CGM Database |

## **Czechia** – Wasting (WHZ)

**D**: Overall and Severe Wasting Prevalence

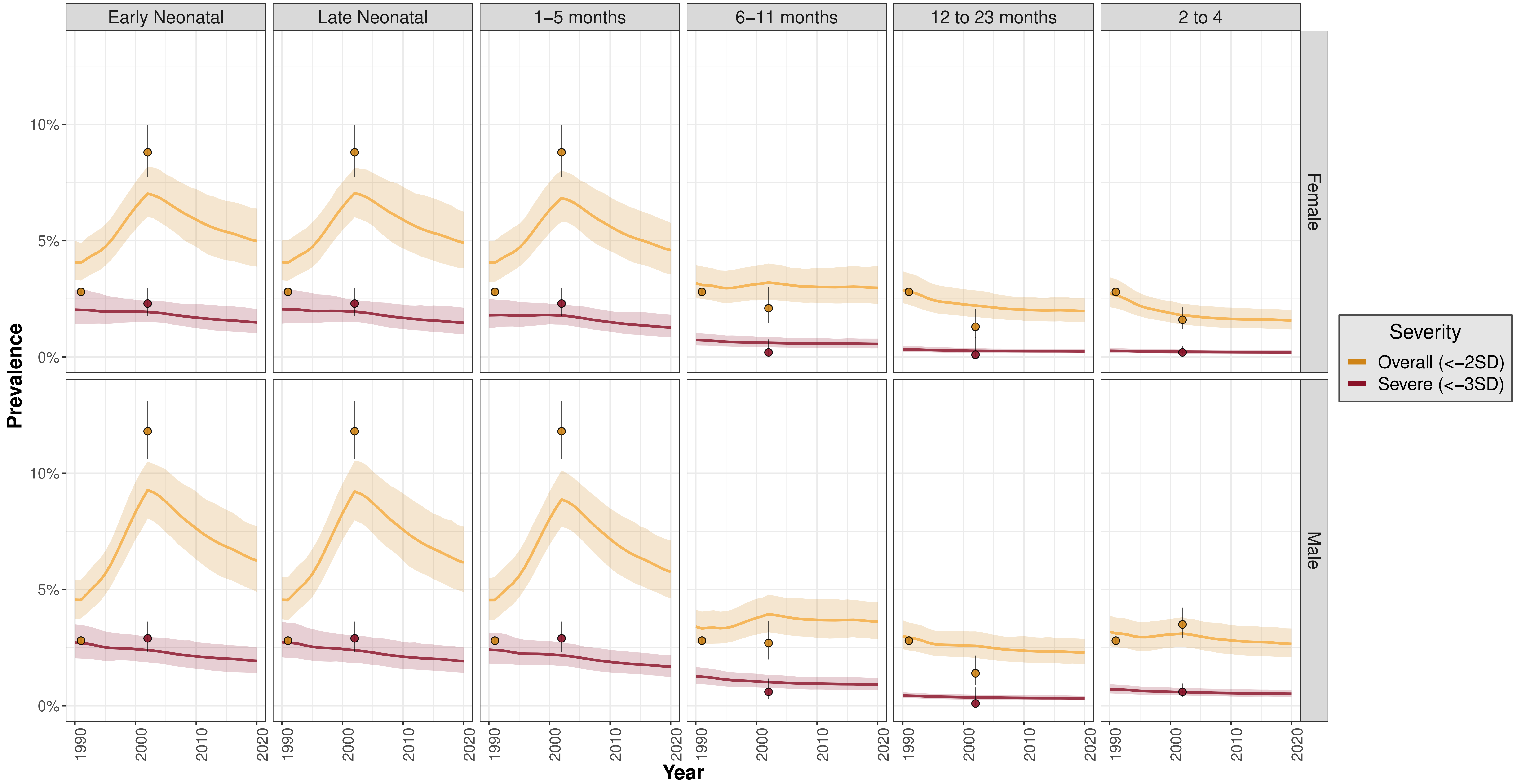


**E**: Transformed Mean Wasting Z Scores

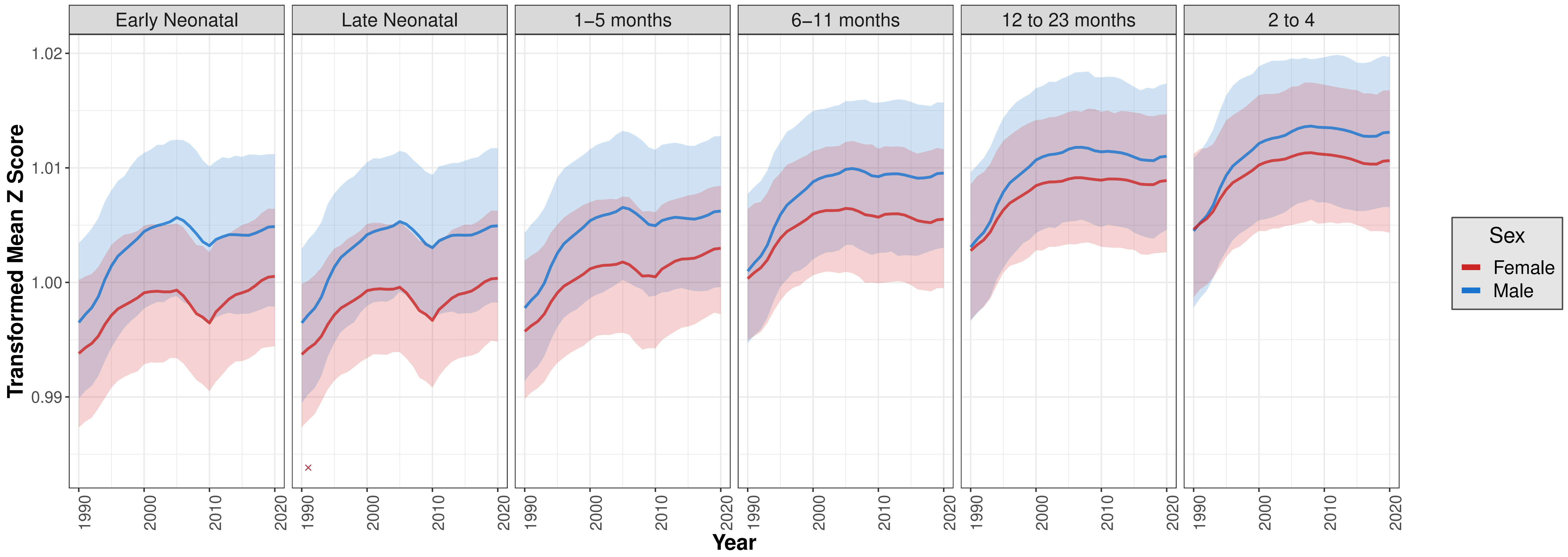


**F**

| Year | Source |
|---|---|
| 1991 | WHO CGM Database |
| 2002 | WHO CGM Database |

## **Czechia** – Underweight (WAZ)

**G:** Overall and Severe Underweight Prevalence

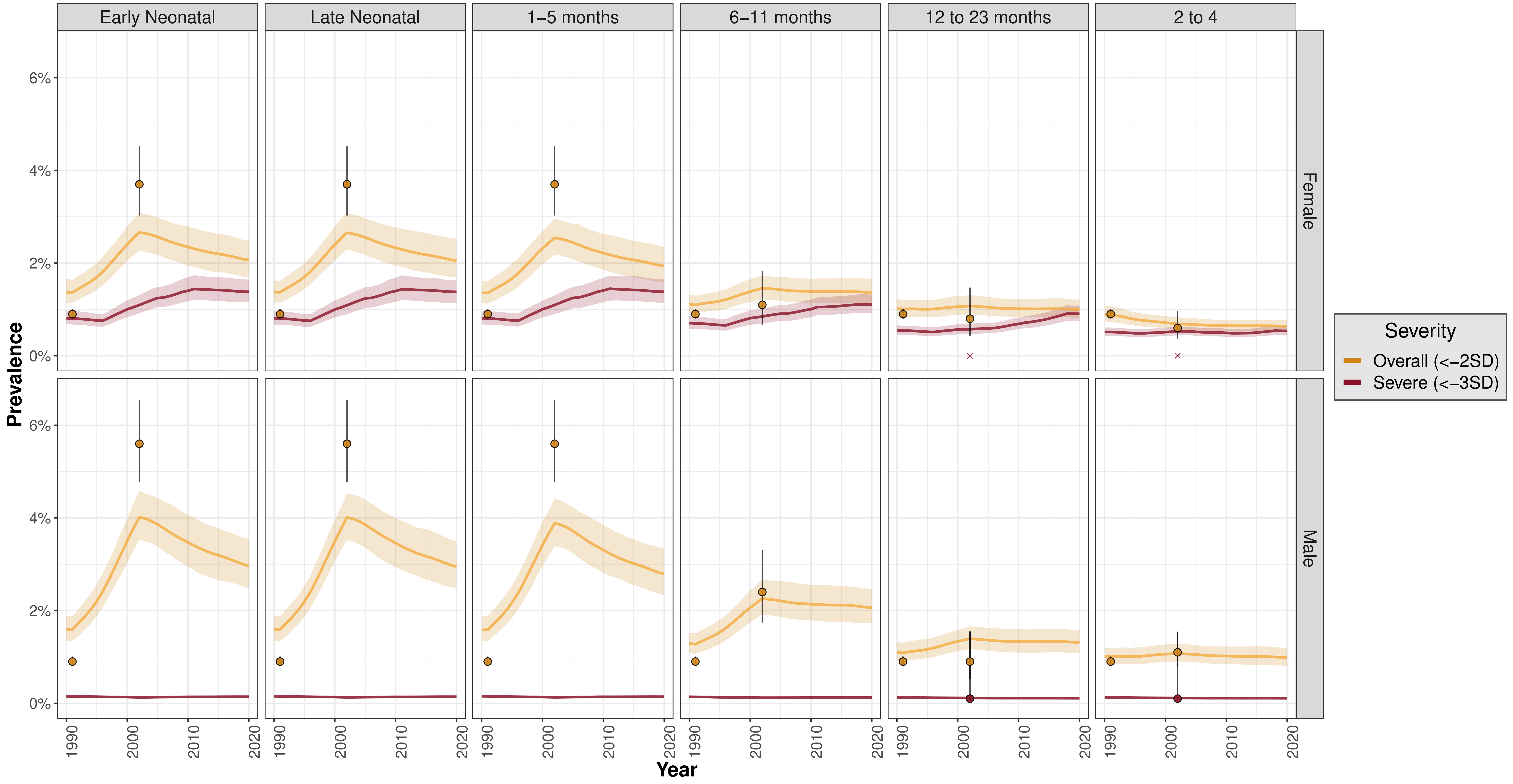


**H:** Transformed Mean Underweight Z Scores

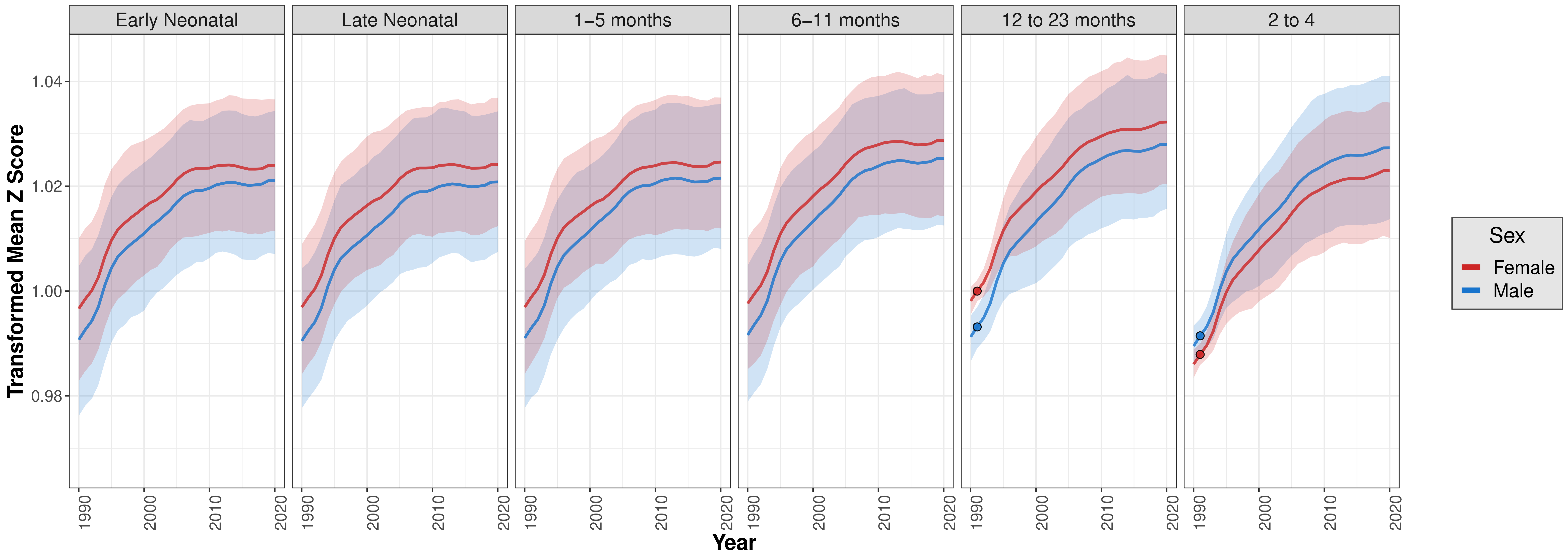


**I**

| Year | Source |
|---|---|
| 1991 | WHO CGM Database |
| 2002 | WHO CGM Database |

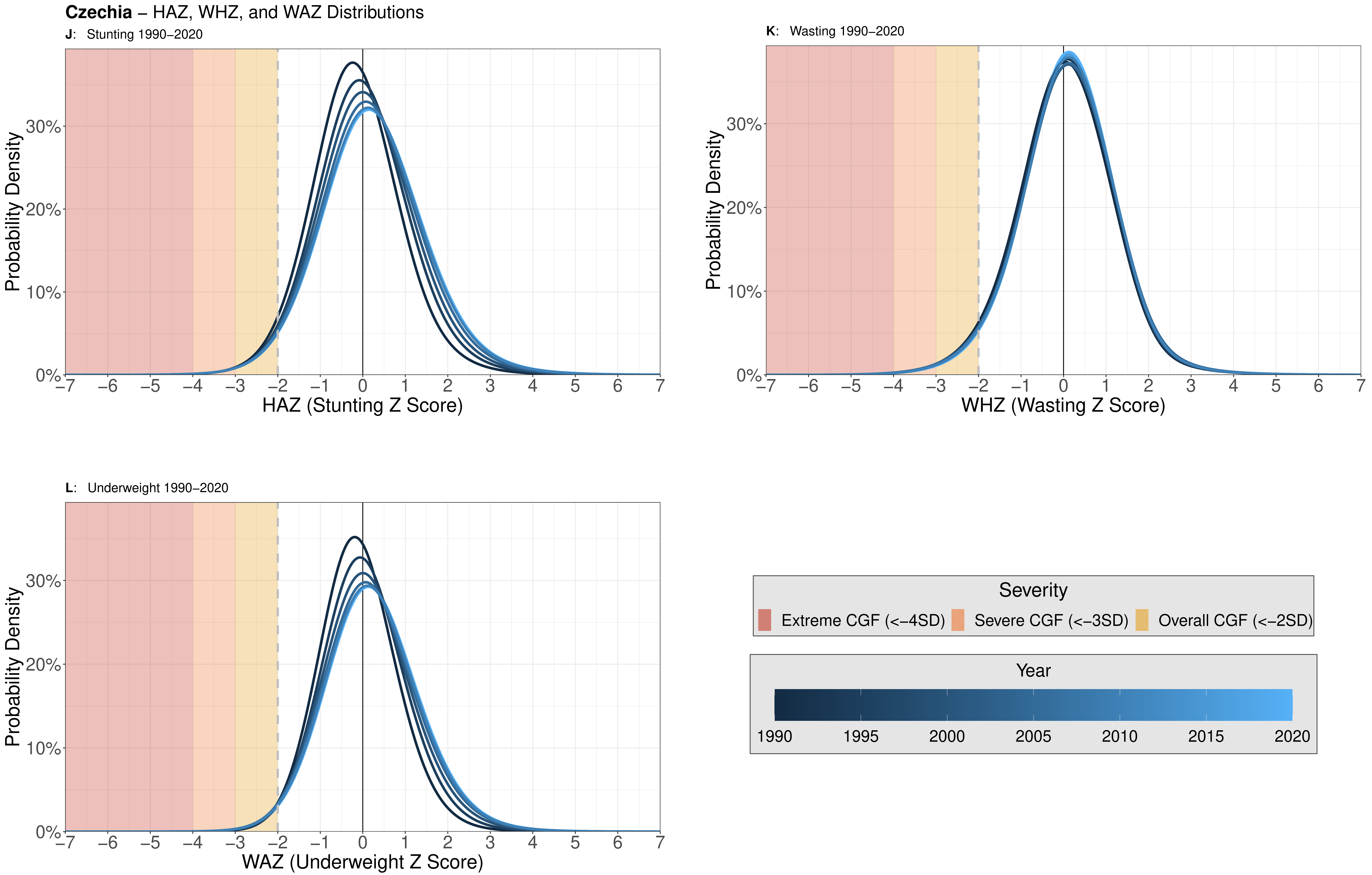

Czechia – HAZ, WHZ, and WAZ Distributions
J: Stunting 1990–2020
Probability Density
0%
10%
20%
30%
−7 −6 −5 −4 −3 −2 −1 0 1 2 3 4 5 6 7
HAZ (Stunting Z Score)
K: Wasting 1990–2020
Probability Density
0%
10%
20%
30%
−7 −6 −5 −4 −3 −2 −1 0 1 2 3 4 5 6 7
WHZ (Wasting Z Score)
L: Underweight 1990–2020
Probability Density
0%
10%
20%
30%
−7 −6 −5 −4 −3 −2 −1 0 1 2 3 4 5 6 7
WAZ (Underweight Z Score)
Severity
Extreme CGF (<−4SD)
Severe CGF (<−3SD)
Overall CGF (<−2SD)
Year
1990
1995
2000
2005
2010
2015
2020

# **Hungary** – Stunting (HAZ)

**A**: Overall and Severe Stunting Prevalence

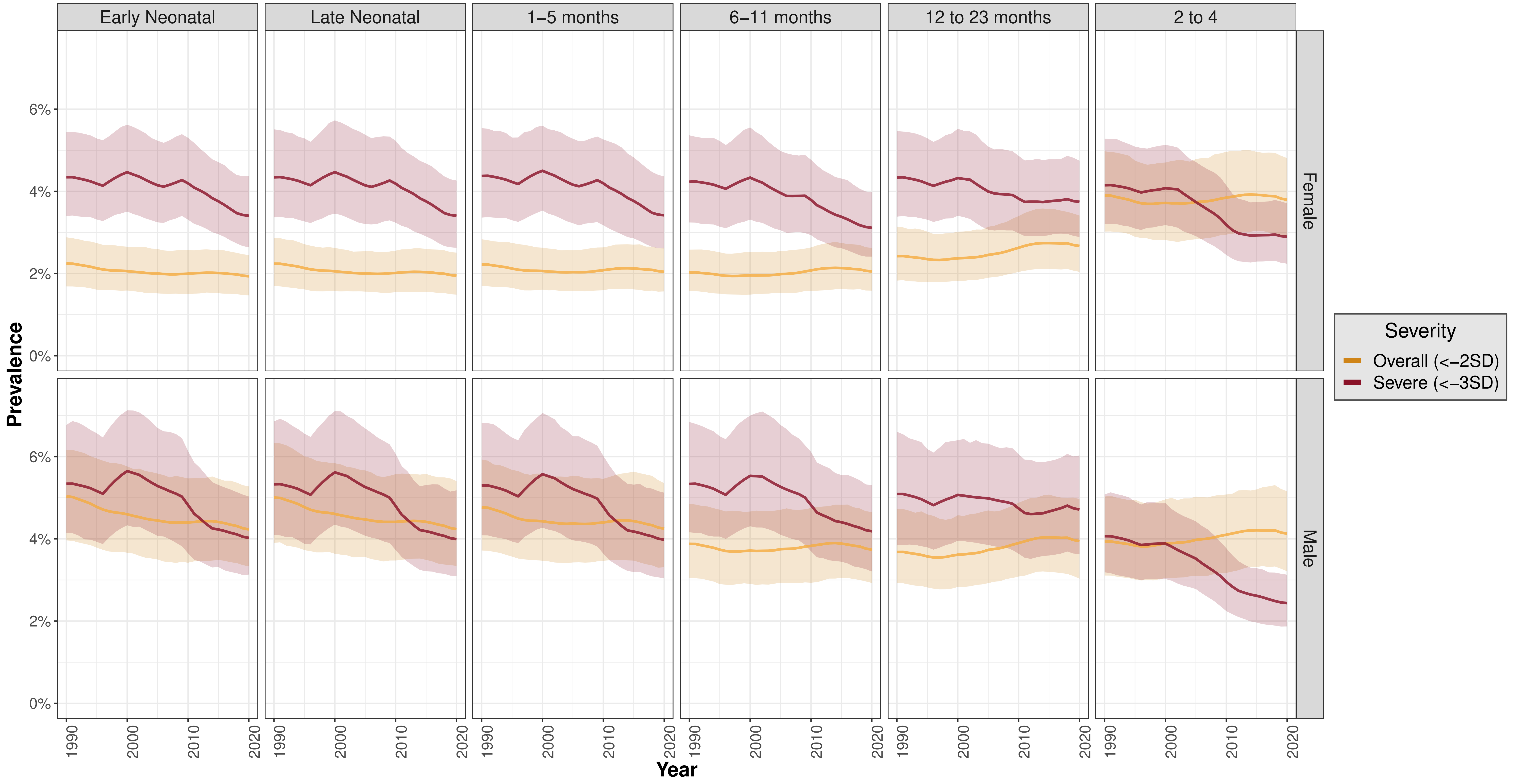


**B**: Transformed Mean Stunting Z Scores

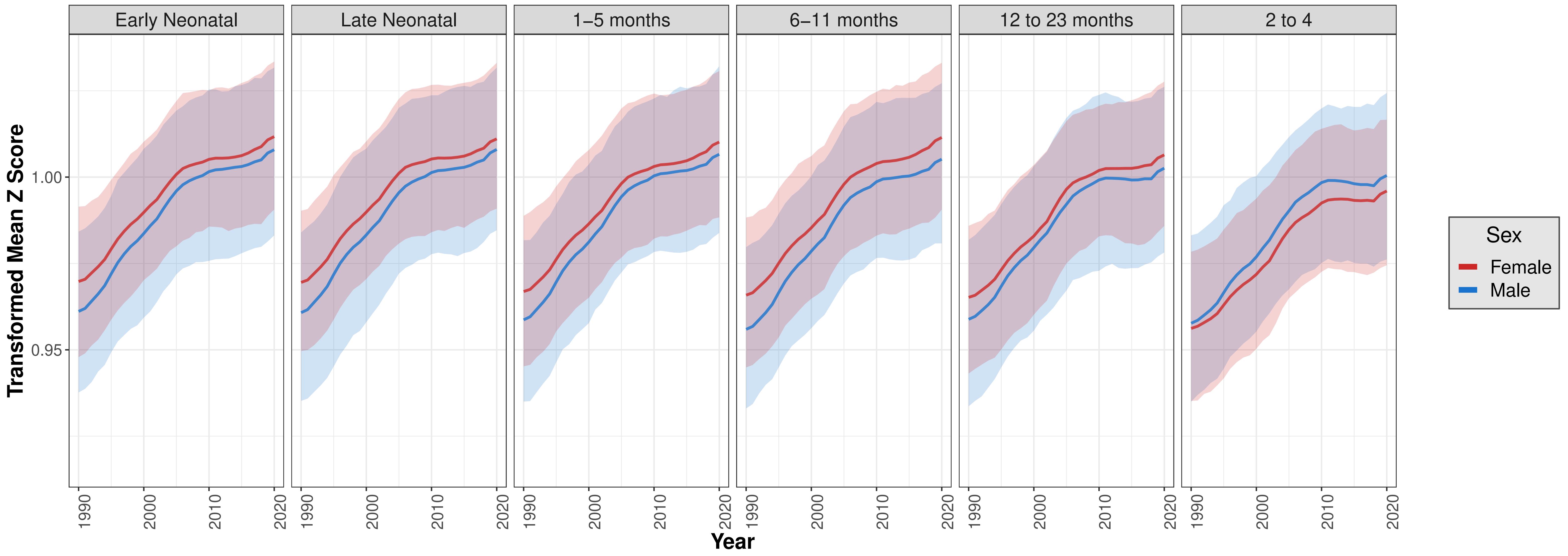


**C**

| Year | Source |
|---|---|
| 1984 | WHO CGM Database |

# **Hungary** – Wasting (WHZ)

**D**: Overall and Severe Wasting Prevalence

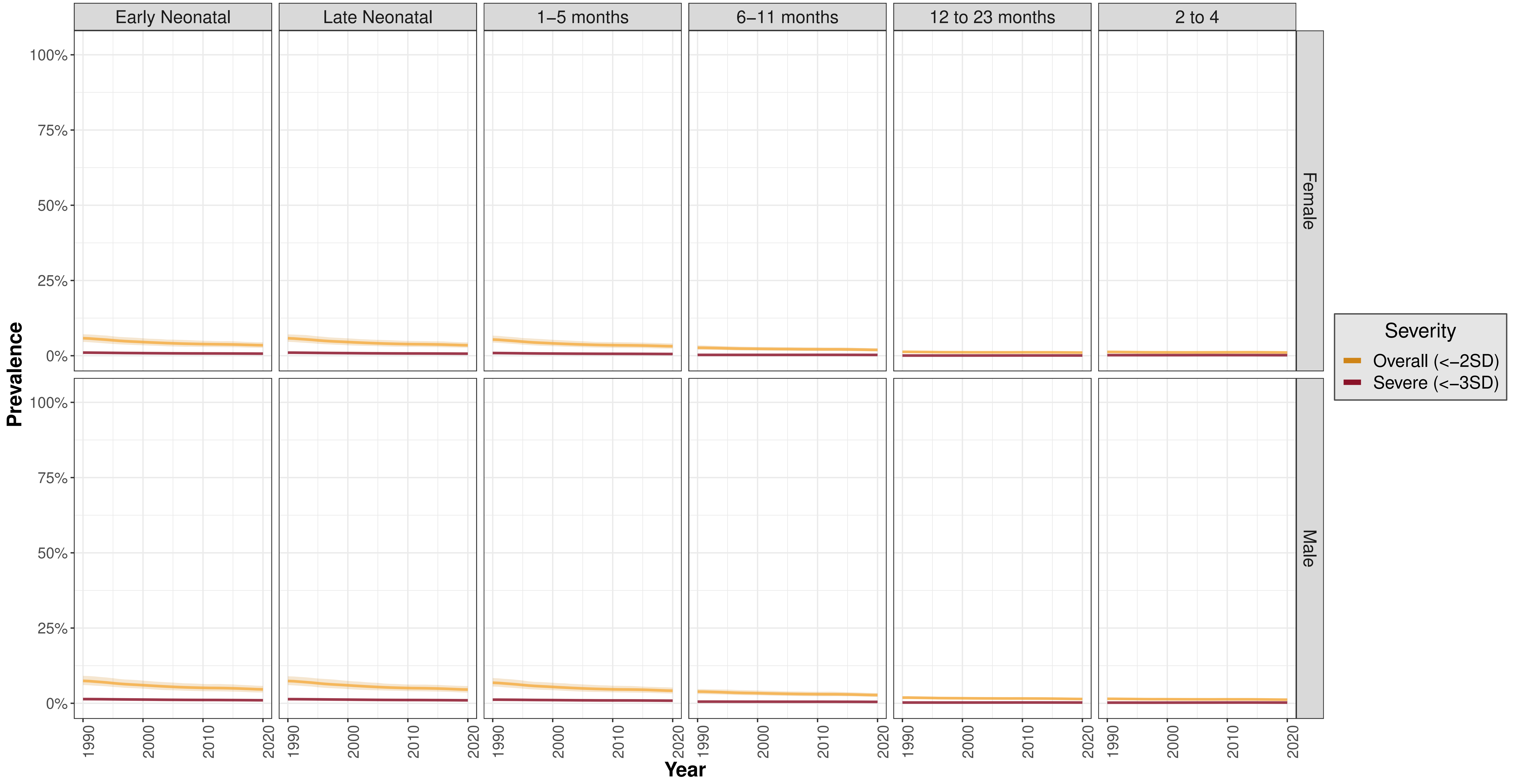


**E**: Transformed Mean Wasting Z Scores

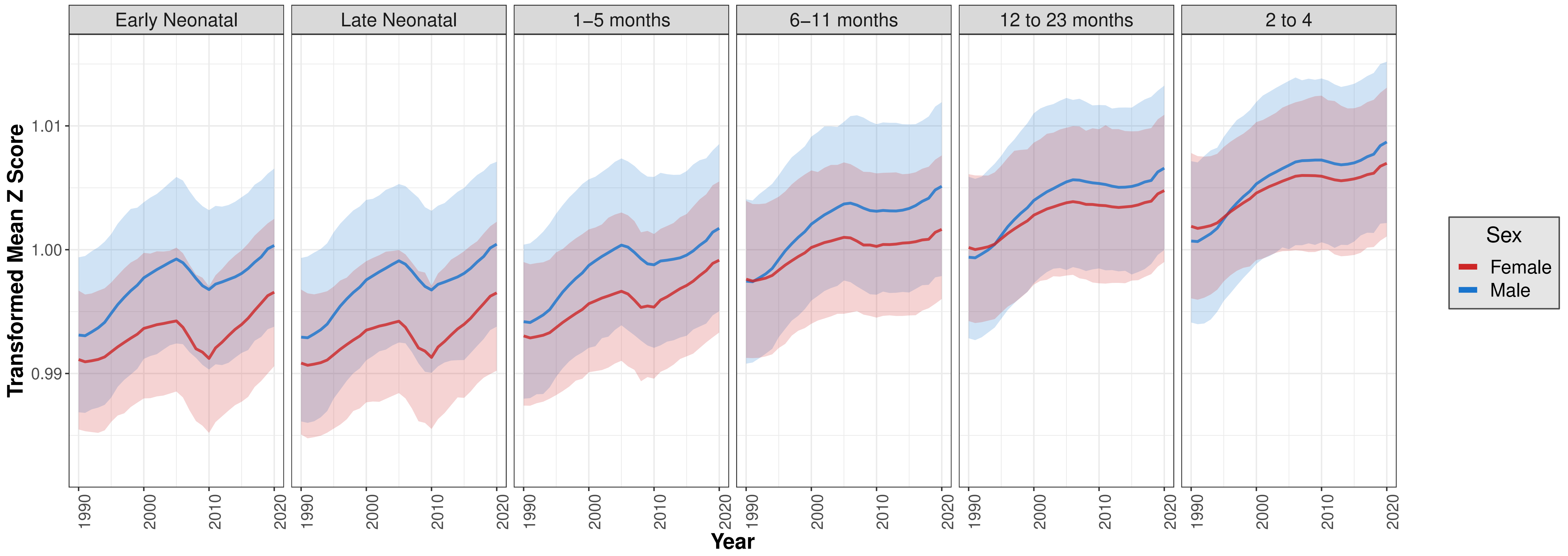


**F**

| Year | Source |
|---|---|
| 1984 | WHO CGM Database |

## **Hungary** – Underweight (WAZ)

**G:** Overall and Severe Underweight Prevalence

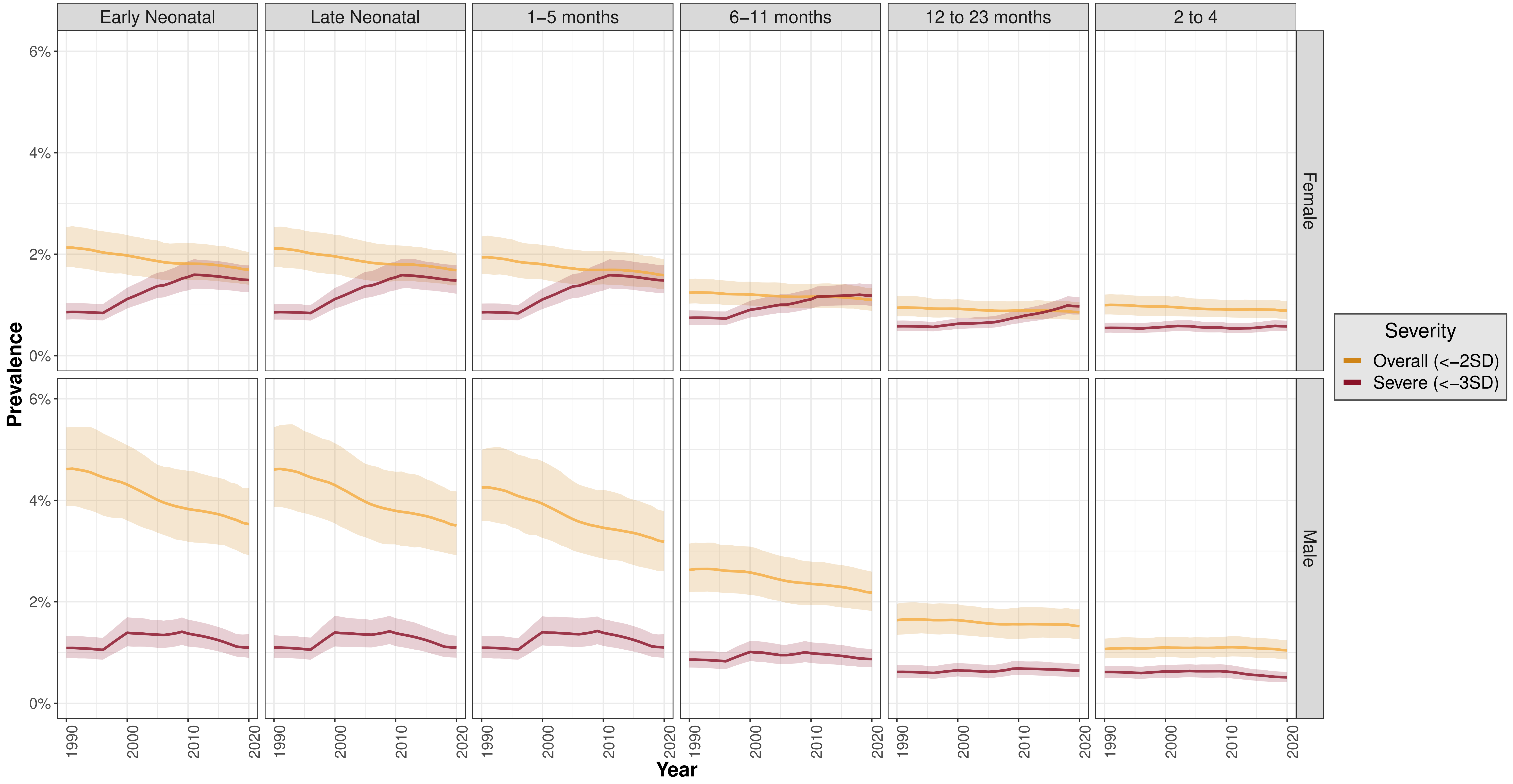


**H:** Transformed Mean Underweight Z Scores

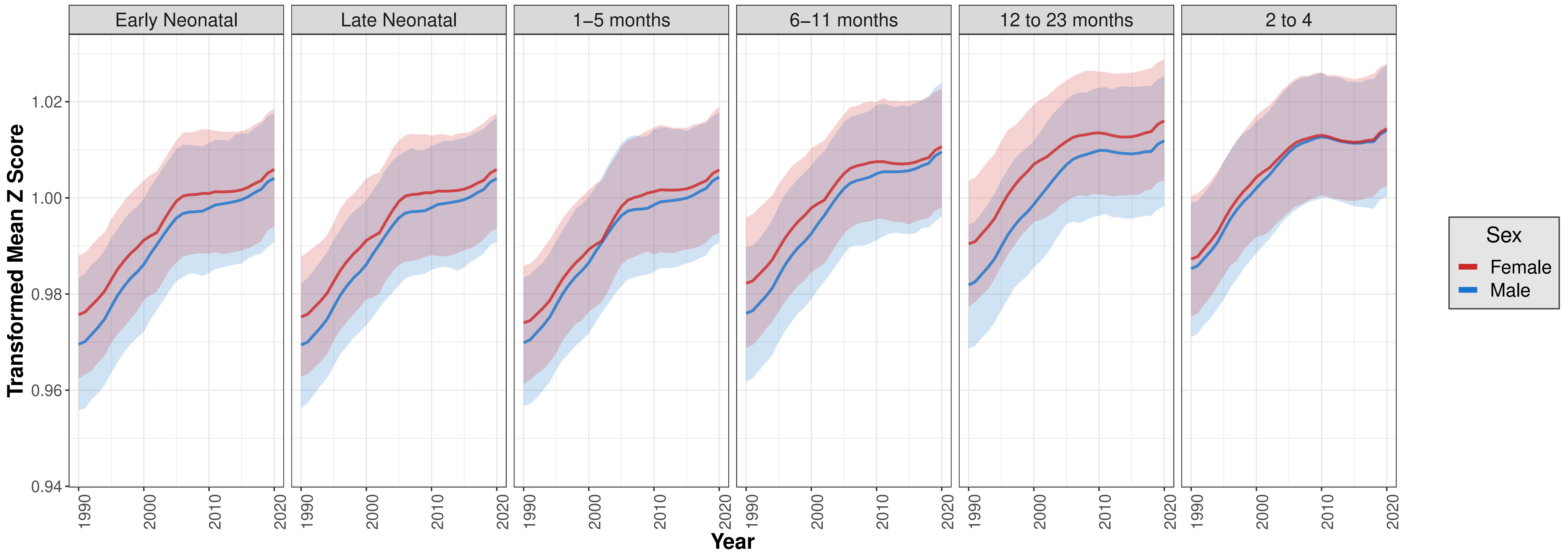


**I**

| Year | Source |
|---|---|
| 1984 | WHO CGM Database |

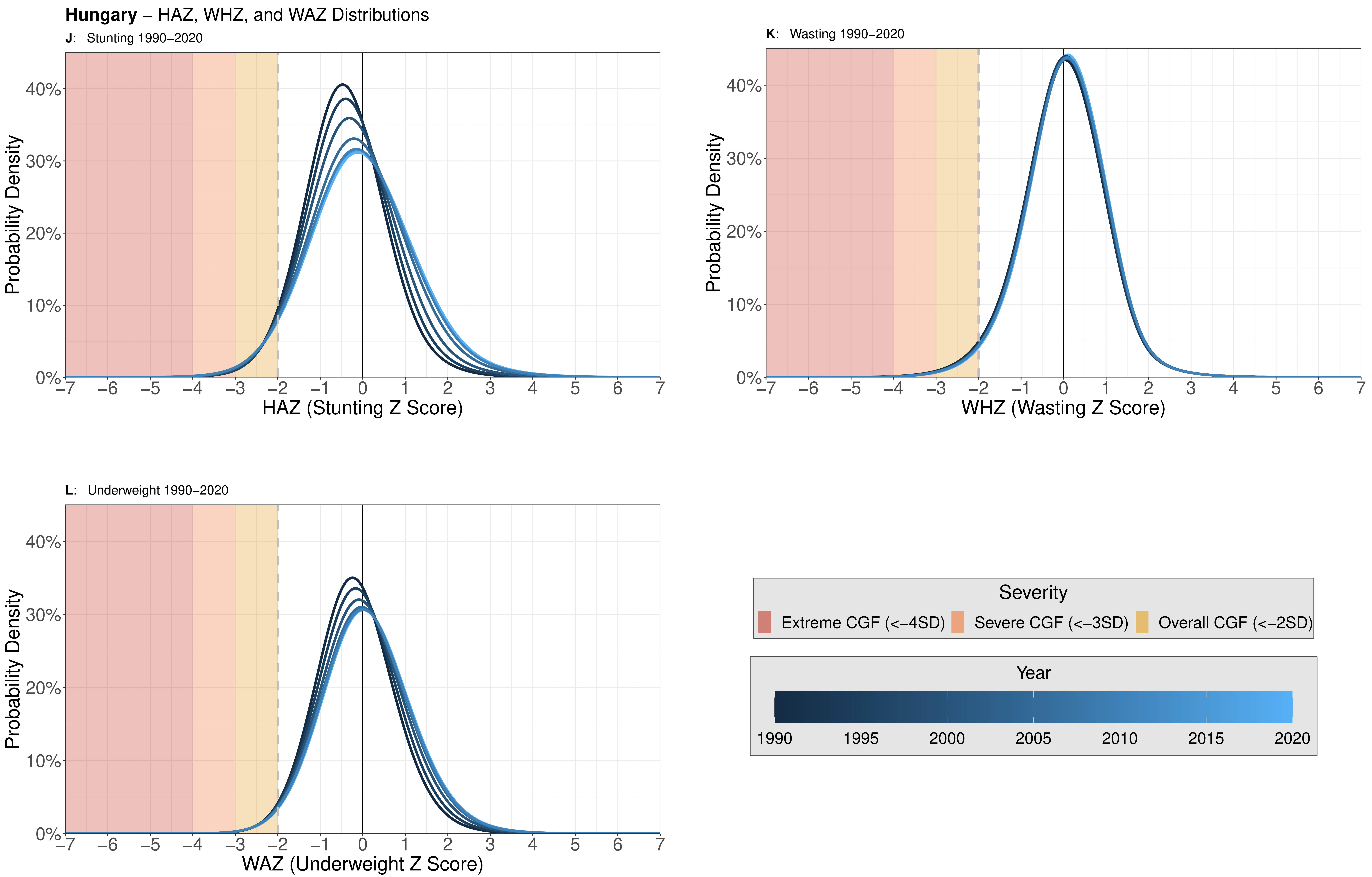
Hungary – HAZ, WHZ, and WAZ Distributions
J: Stunting 1990–2020
K: Wasting 1990–2020
L: Underweight 1990–2020
Probability Density
0%
10%
20%
30%
40%
−7 −6 −5 −4 −3 −2 −1 0 1 2 3 4 5 6 7
HAZ (Stunting Z Score)
WHZ (Wasting Z Score)
WAZ (Underweight Z Score)
Severity
Extreme CGF (<−4SD)
Severe CGF (<−3SD)
Overall CGF (<−2SD)
Year
1990
1995
2000
2005
2010
2015
2020

**North Macedonia** – Stunting (HAZ)

**A**: Overall and Severe Stunting Prevalence

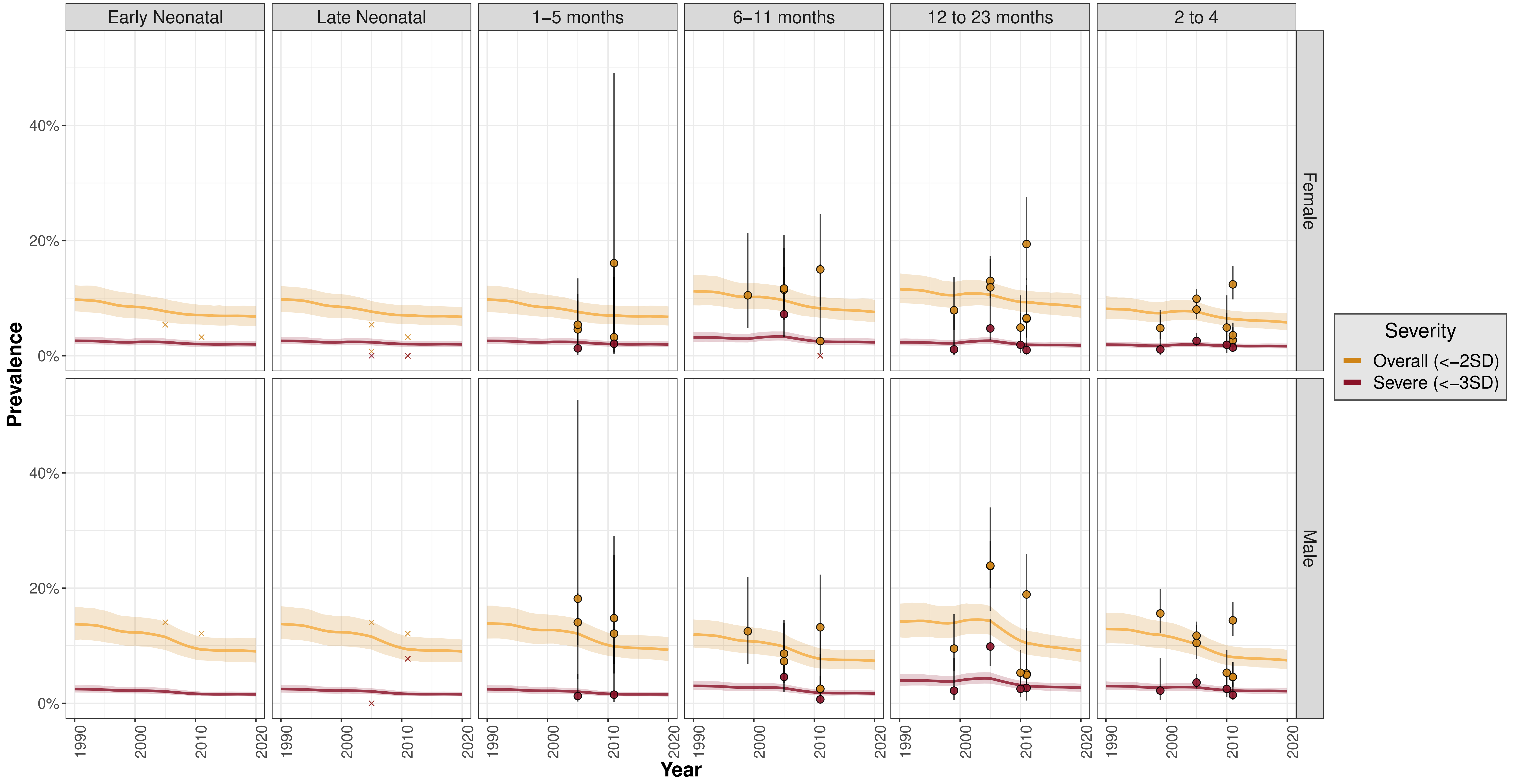


**B**: Transformed Mean Stunting Z Scores

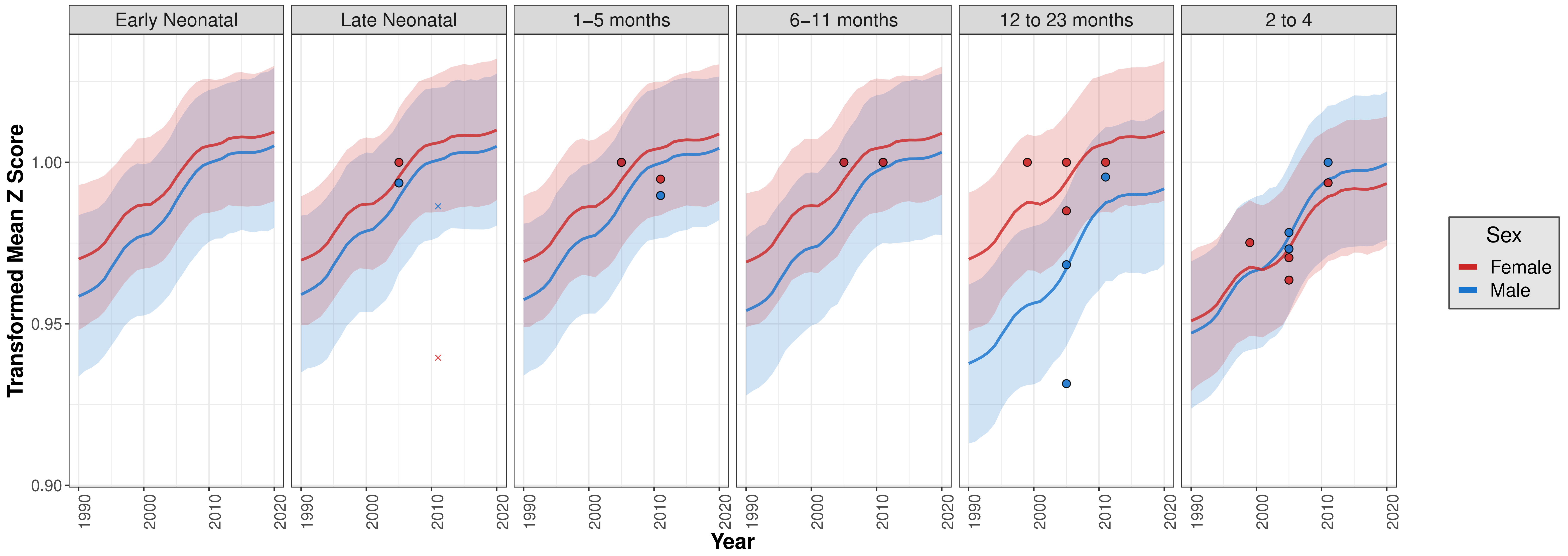


**C**

| Year | Source |
|---|---|
| 1999 | WHO CGM Database |
| 2005 | Macedonia MICS |
| 2005 | WHO CGM Database |
| 2010 | WHO CGM Database |
| 2011 | Macedonia MICS |
| 2011 | WHO CGM Database |

# North Macedonia – Wasting (WHZ)

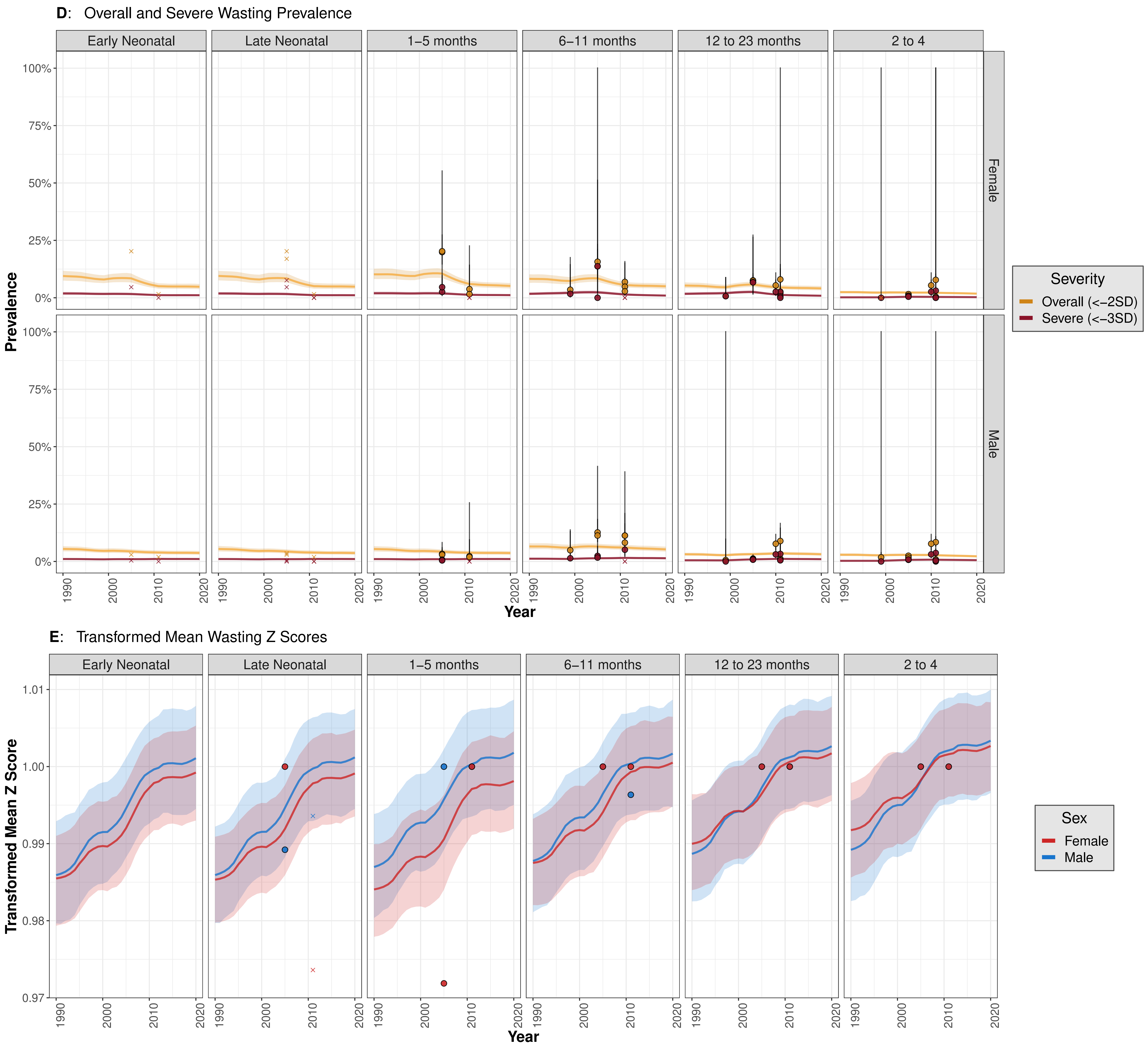


**F**

| Year | Source |
|---|---|
| 1999 | WHO CGM Database |
| 2005 | Macedonia MICS |
| 2005 | WHO CGM Database |
| 2010 | WHO CGM Database |
| 2011 | Macedonia MICS |
| 2011 | WHO CGM Database |

# **North Macedonia** – Underweight (WAZ)

**G**: Overall and Severe Underweight Prevalence

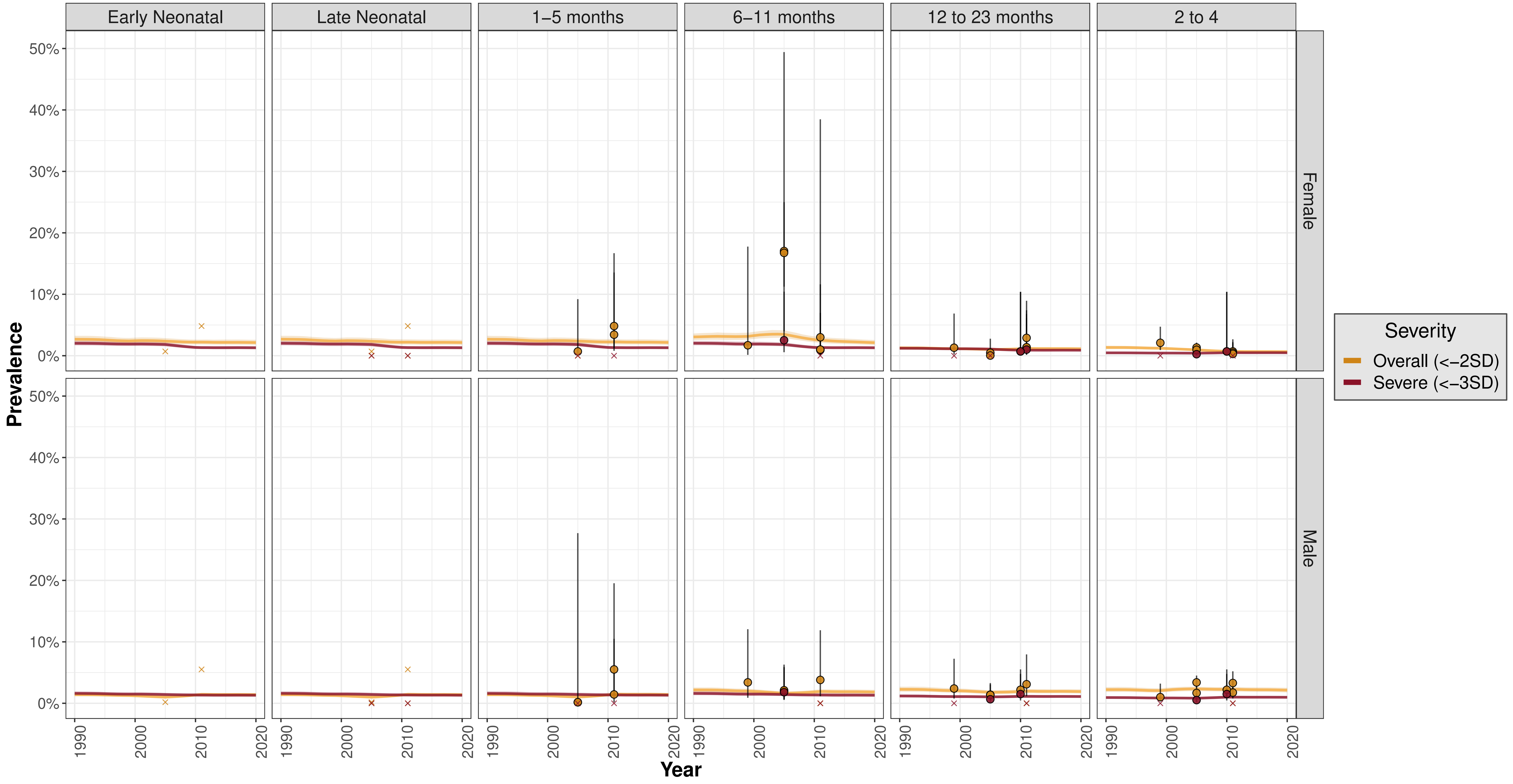


**H**: Transformed Mean Underweight Z Scores

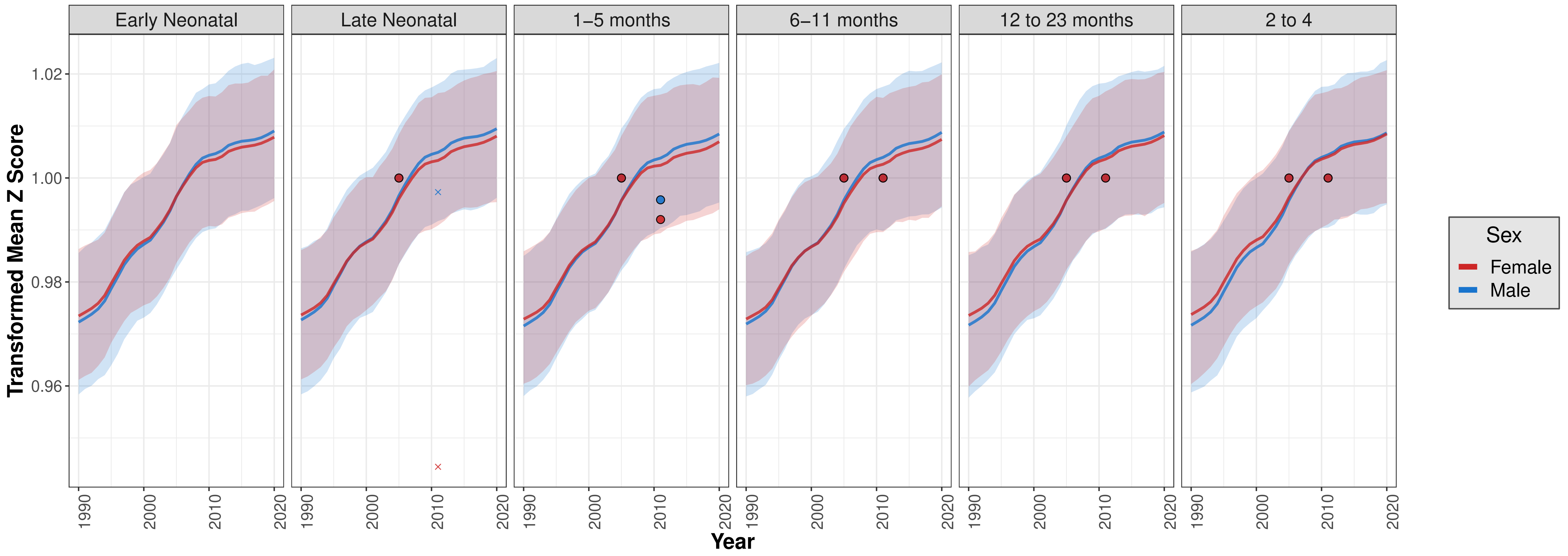


**I**

| Year | Source |
|---|---|
| 1999 | WHO CGM Database |
| 2005 | Macedonia MICS |
| 2005 | WHO CGM Database |
| 2010 | WHO CGM Database |
| 2011 | Macedonia MICS |
| 2011 | WHO CGM Database |

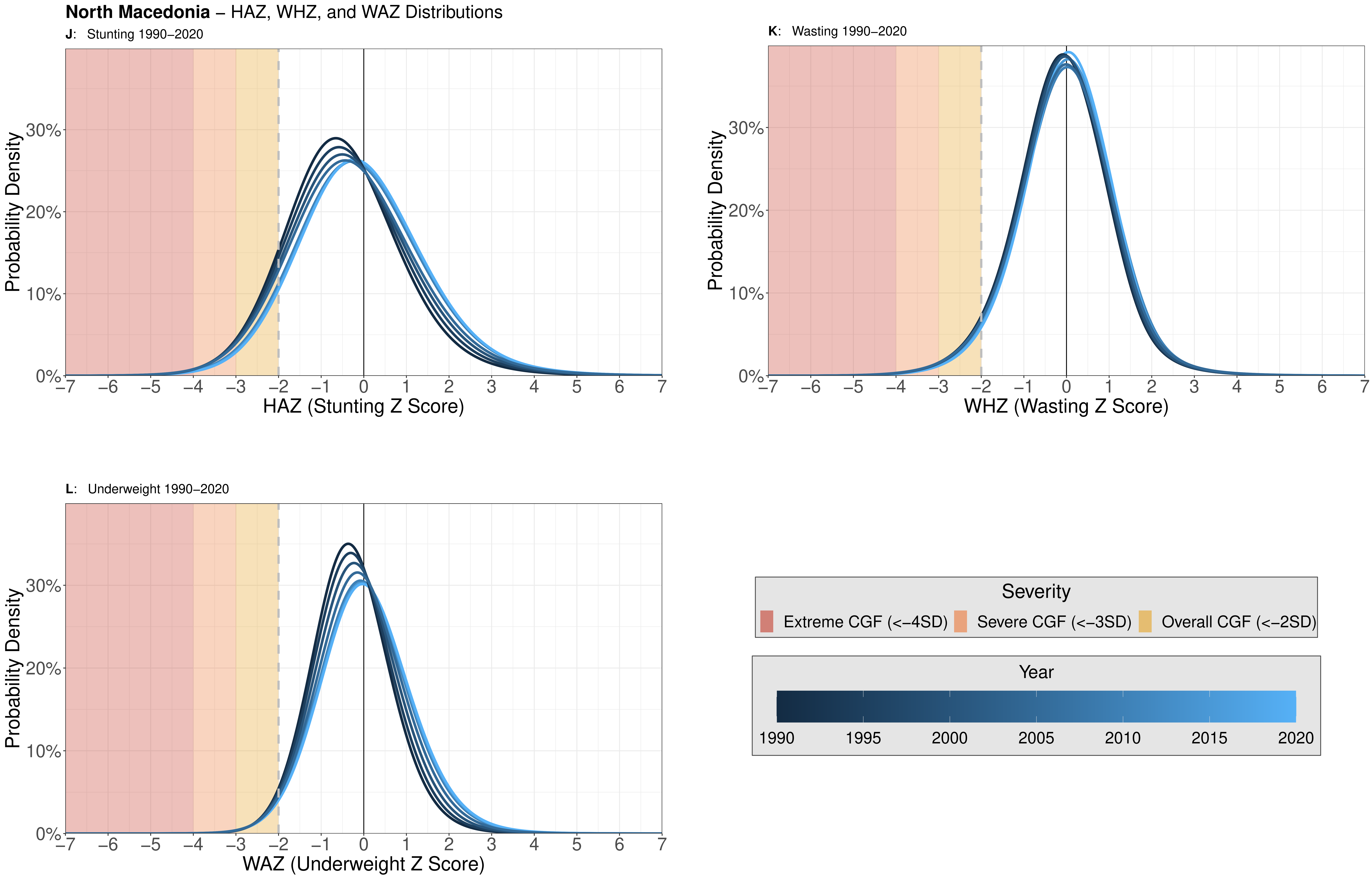
North Macedonia – HAZ, WHZ, and WAZ Distributions
J: Stunting 1990–2020
K: Wasting 1990–2020
L: Underweight 1990–2020
Probability Density
0%
10%
20%
30%
−7 −6 −5 −4 −3 −2 −1 0 1 2 3 4 5 6 7
HAZ (Stunting Z Score)
WHZ (Wasting Z Score)
WAZ (Underweight Z Score)
Severity
Extreme CGF (<−4SD)
Severe CGF (<−3SD)
Overall CGF (<−2SD)
Year
1990
1995
2000
2005
2010
2015
2020

# **Montenegro** – Stunting (HAZ)

**A**: Overall and Severe Stunting Prevalence

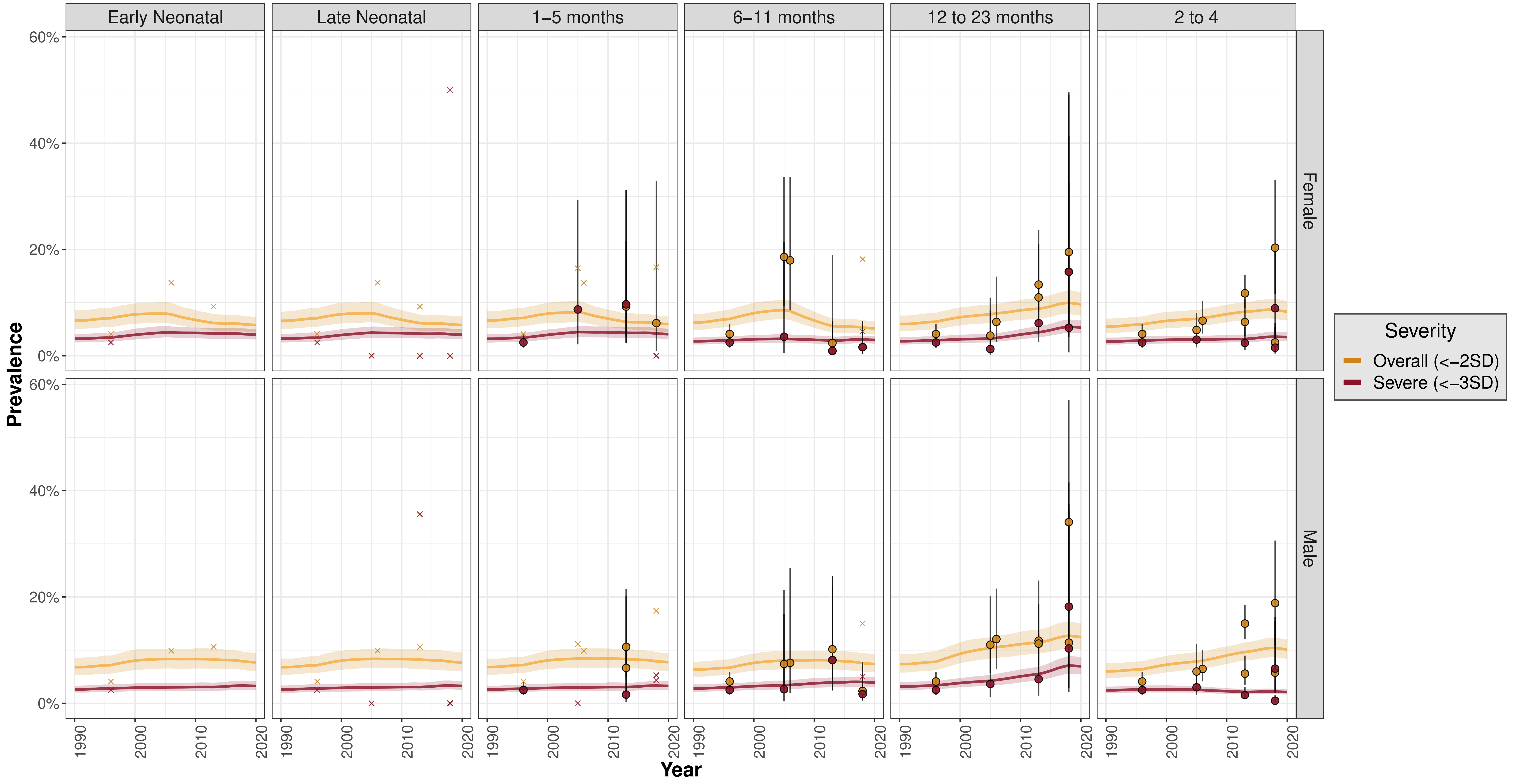


**B**: Transformed Mean Stunting Z Scores

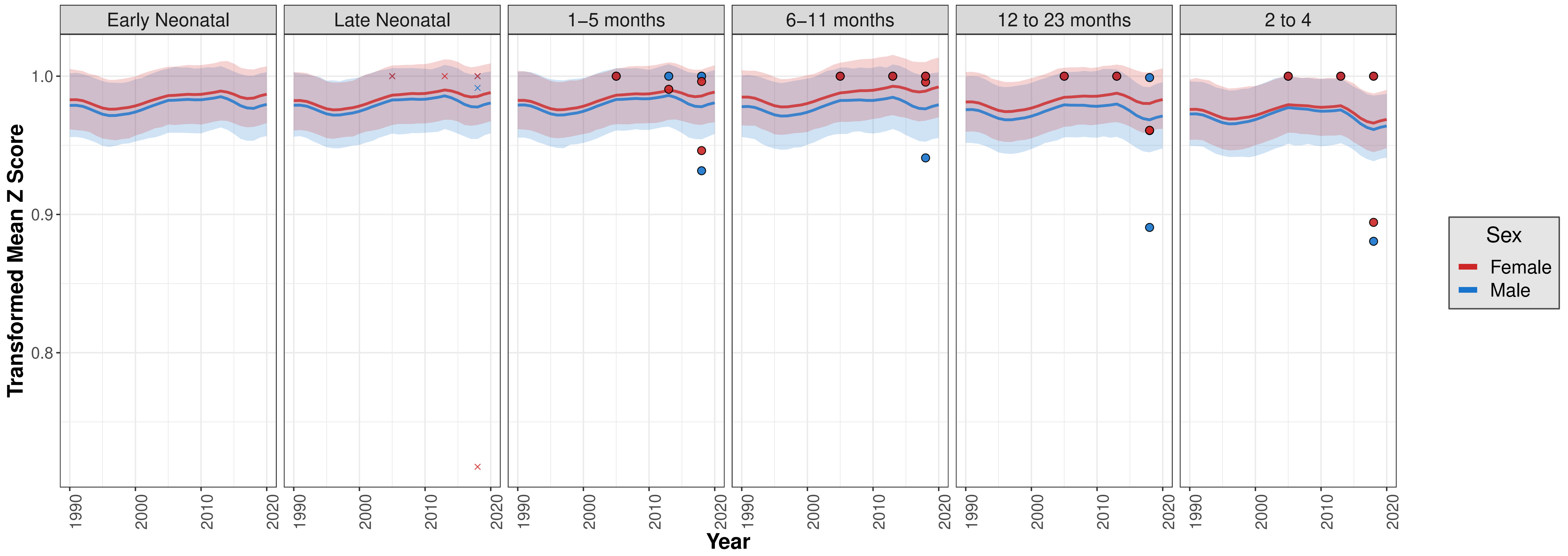


**C**

| Year | Source |
|---|---|
| 1996 | Yugoslavia, Federal Republic MICS |
| 2005 | MICS |
| 2006 | WHO CGM Database |
| 2013 | WHO CGM Database |
| 2013 | MICS |
| 2018 | Roma Settlements MICS |
| 2018 | MICS |

# **Montenegro** – Wasting (WHZ)

**D**: Overall and Severe Wasting Prevalence

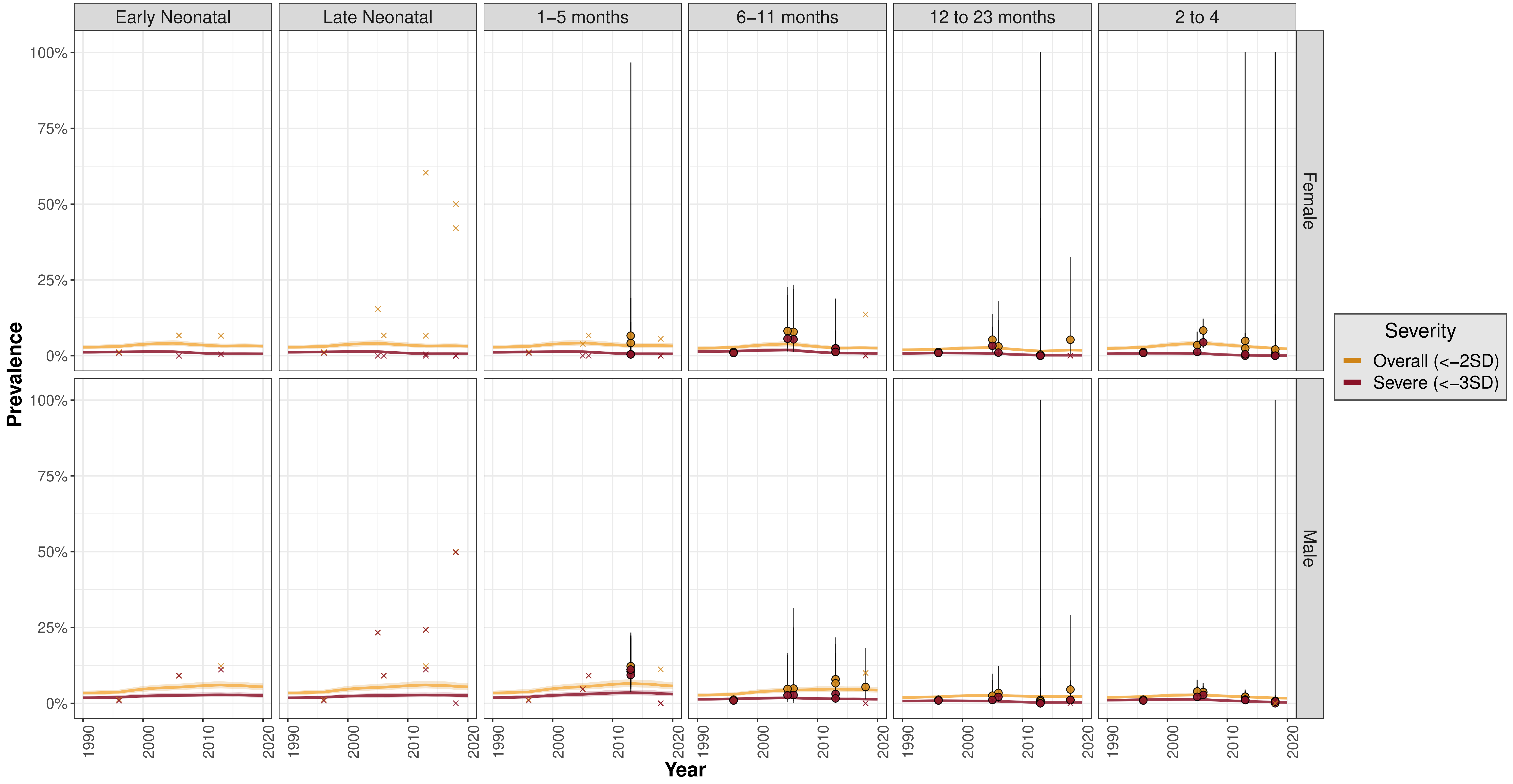


**E**: Transformed Mean Wasting Z Scores

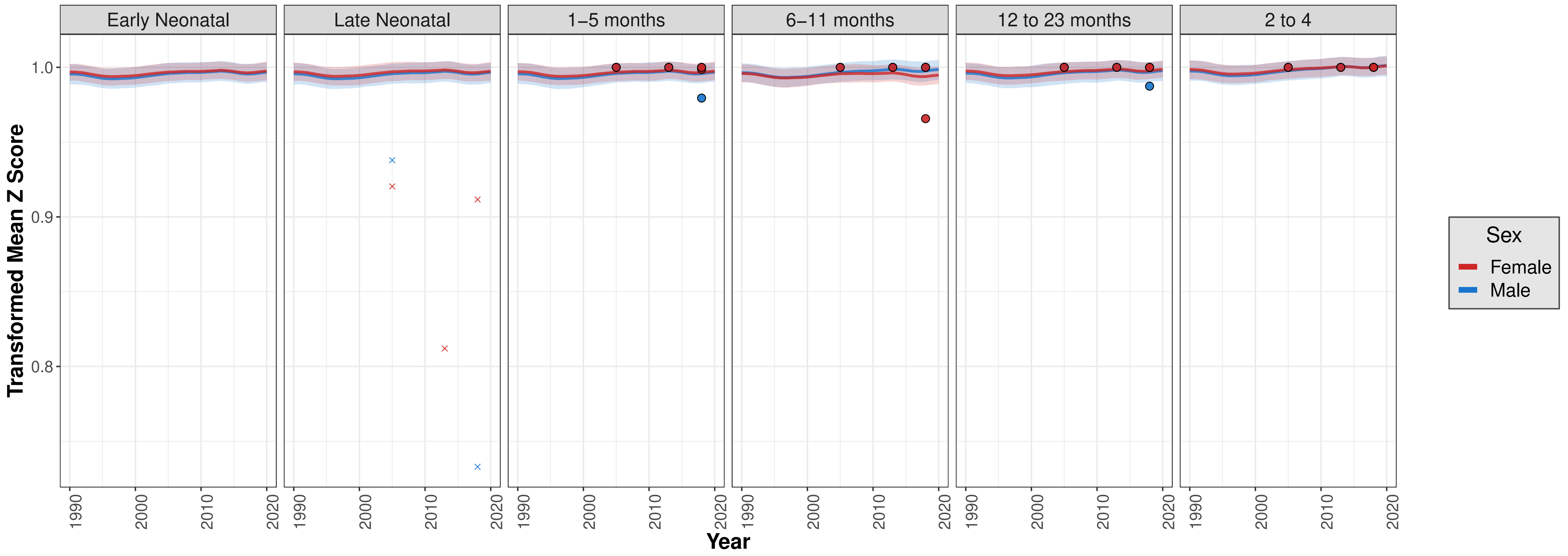


**F**

| Year | Source |
|---|---|
| 1996 | Yugoslavia, Federal Republic MICS |
| 2005 | MICS |
| 2006 | WHO CGM Database |
| 2013 | WHO CGM Database |
| 2013 | MICS |
| 2018 | Roma Settlements MICS |
| 2018 | MICS |

# **Montenegro** – Underweight (WAZ)

**G**: Overall and Severe Underweight Prevalence

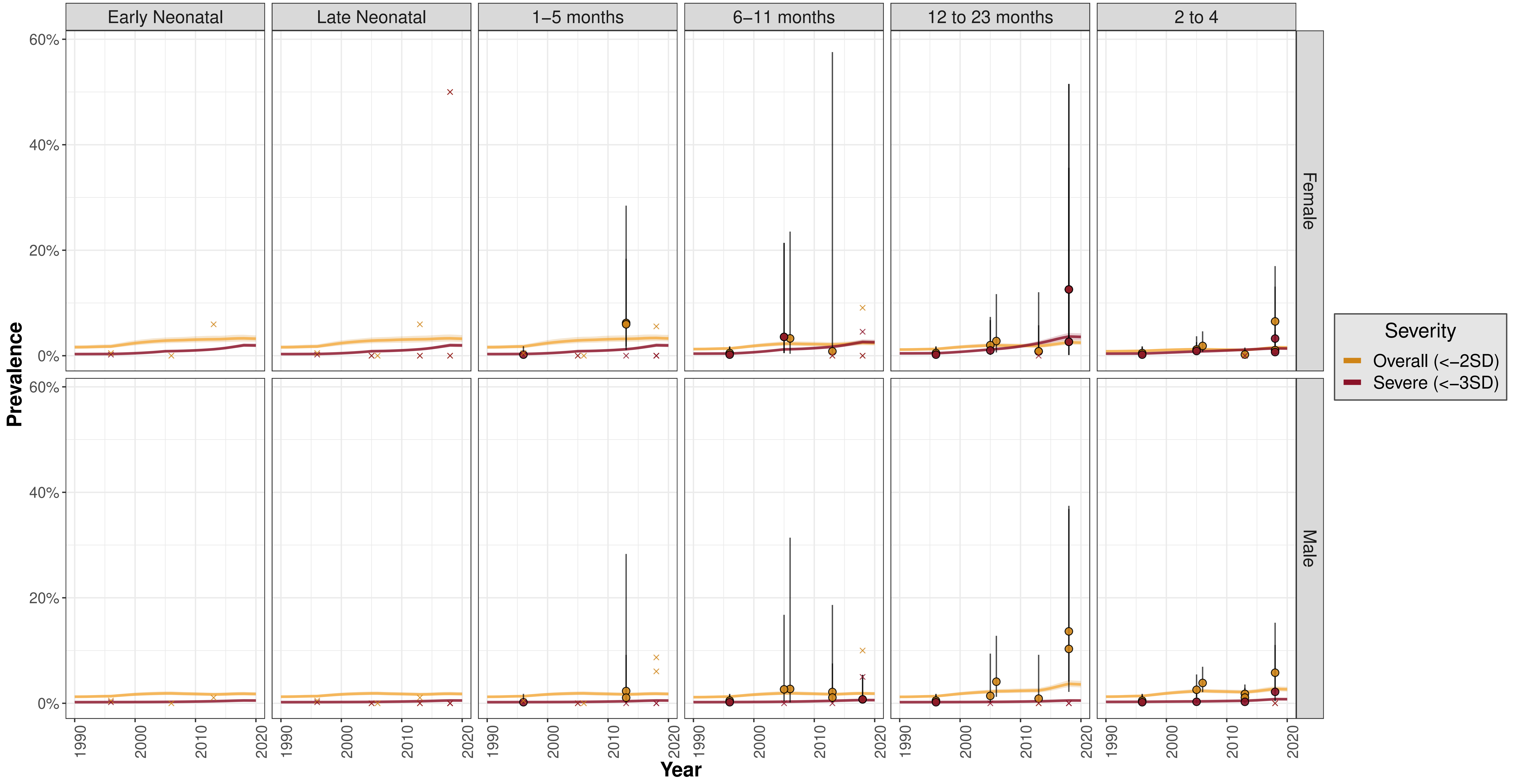


**H**: Transformed Mean Underweight Z Scores

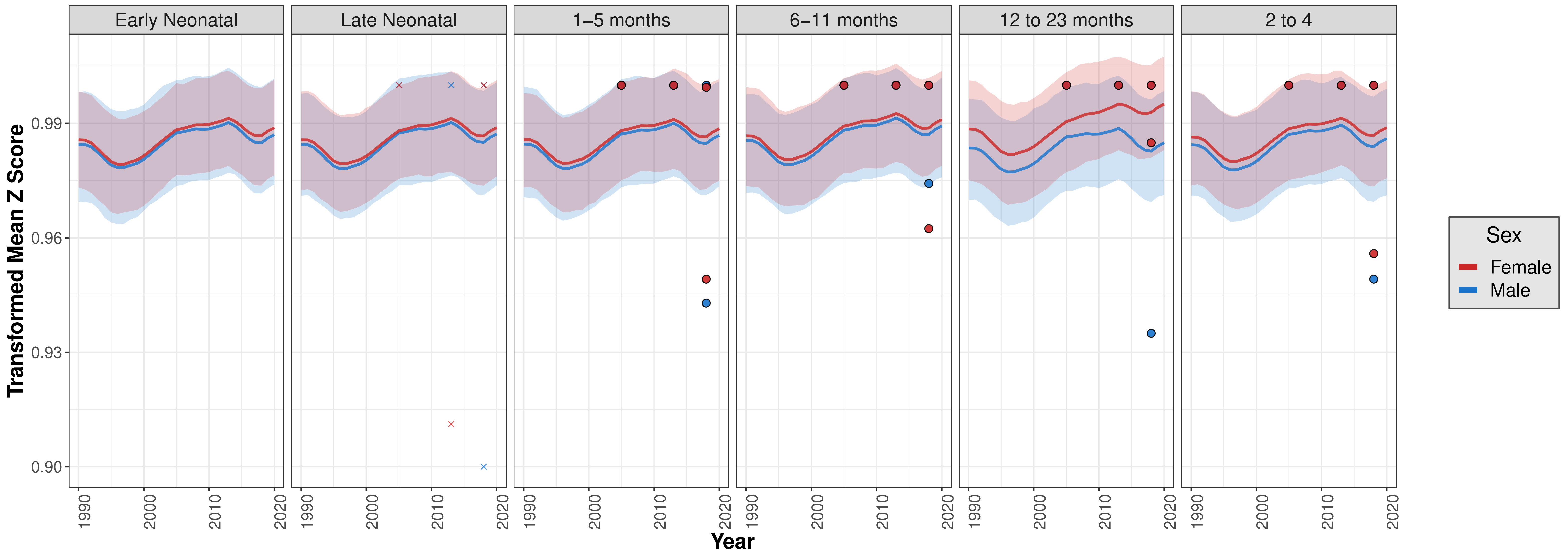


**I**

| Year | Source |
|---|---|
| 1996 | Yugoslavia, Federal Republic MICS |
| 2005 | MICS |
| 2006 | WHO CGM Database |
| 2013 | WHO CGM Database |
| 2013 | MICS |
| 2018 | Roma Settlements MICS |
| 2018 | MICS |

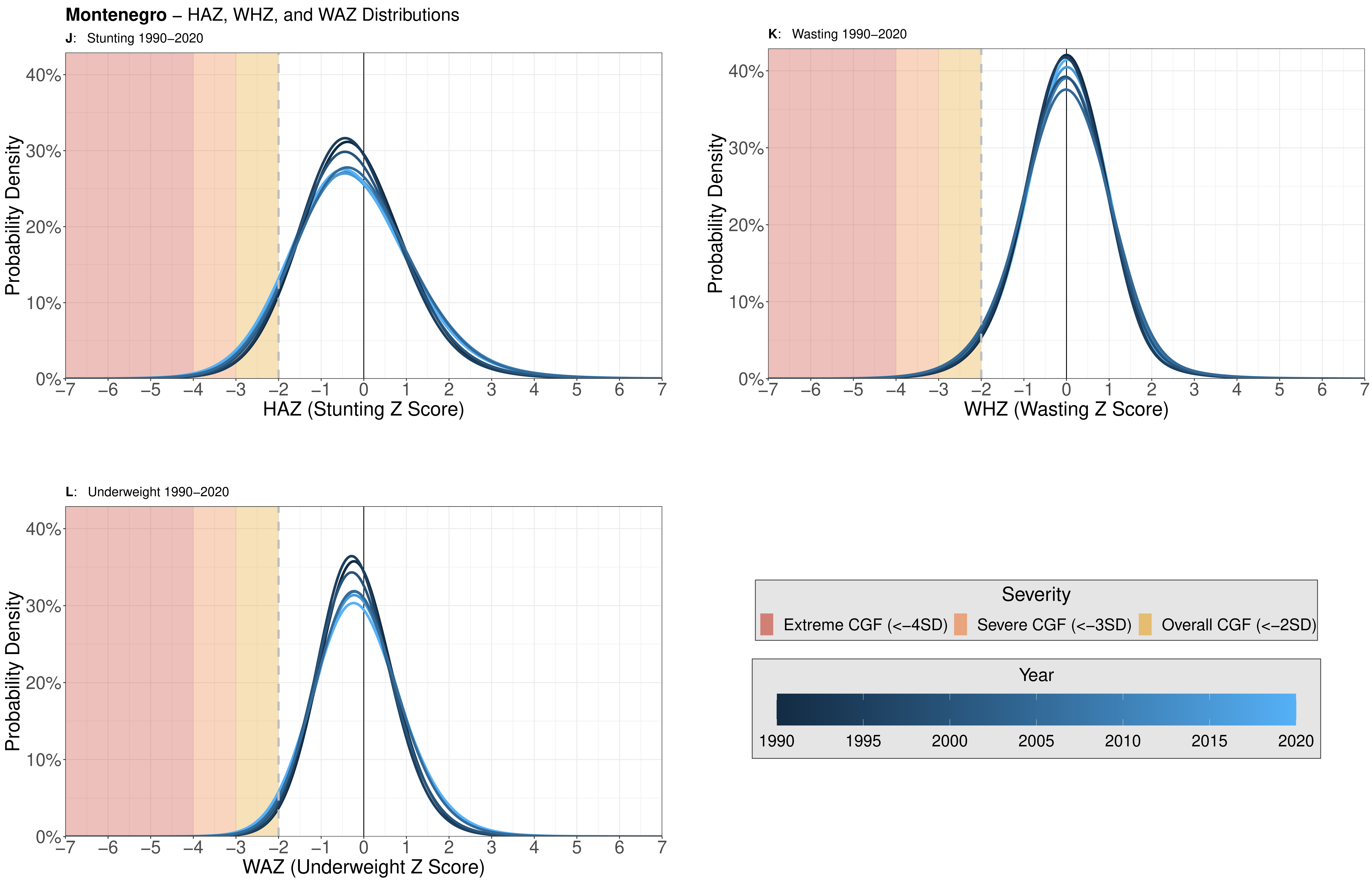

Montenegro – HAZ, WHZ, and WAZ Distributions
J: Stunting 1990–2020
Probability Density
0%
10%
20%
30%
40%
−7 −6 −5 −4 −3 −2 −1 0 1 2 3 4 5 6 7
HAZ (Stunting Z Score)
K: Wasting 1990–2020
Probability Density
0%
10%
20%
30%
40%
−7 −6 −5 −4 −3 −2 −1 0 1 2 3 4 5 6 7
WHZ (Wasting Z Score)
L: Underweight 1990–2020
Probability Density
0%
10%
20%
30%
40%
−7 −6 −5 −4 −3 −2 −1 0 1 2 3 4 5 6 7
WAZ (Underweight Z Score)
Severity
Extreme CGF (<−4SD)
Severe CGF (<−3SD)
Overall CGF (<−2SD)
Year
1990
1995
2000
2005
2010
2015
2020

# **Poland** – Stunting (HAZ)

**A**: Overall and Severe Stunting Prevalence

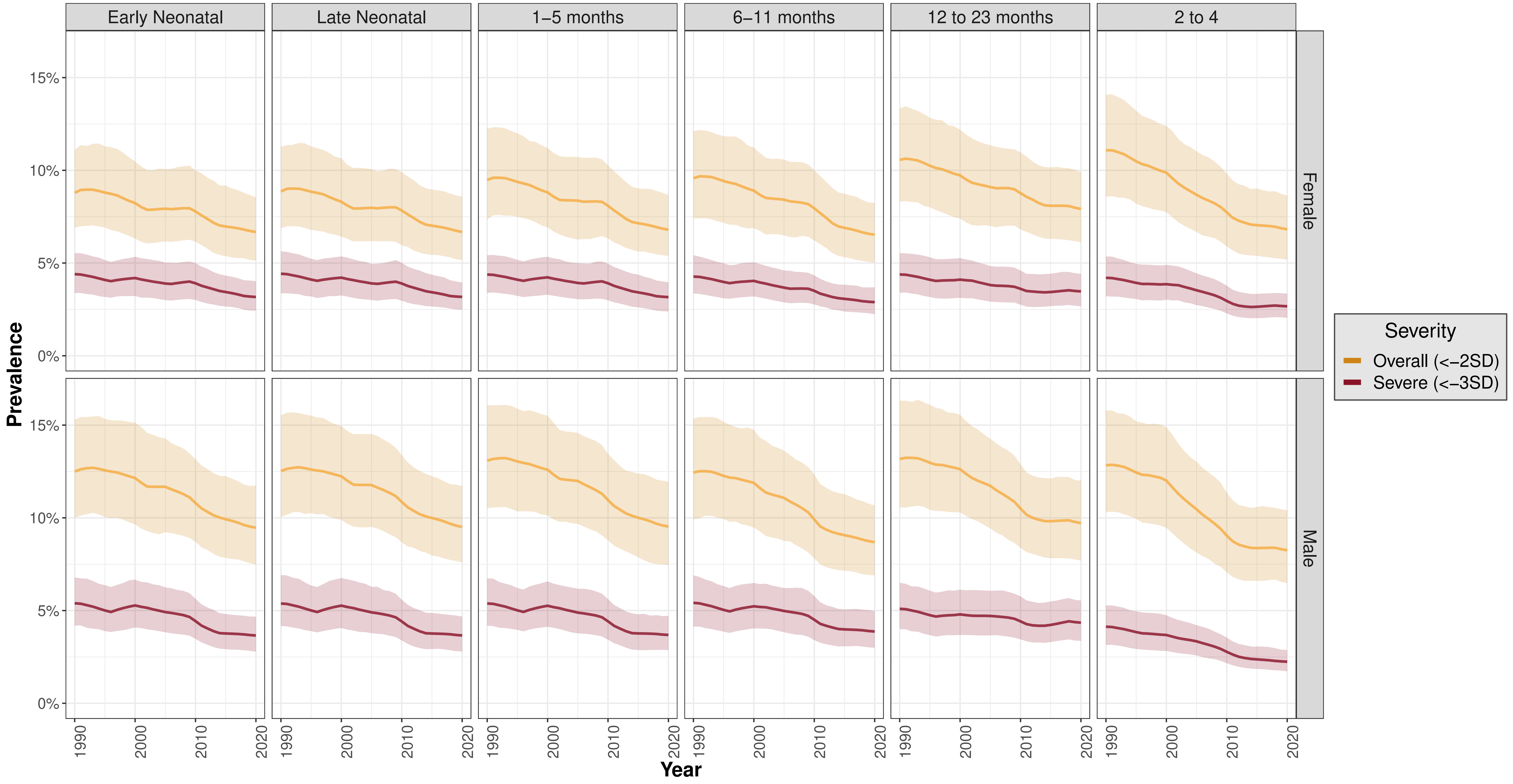


**B**: Transformed Mean Stunting Z Scores

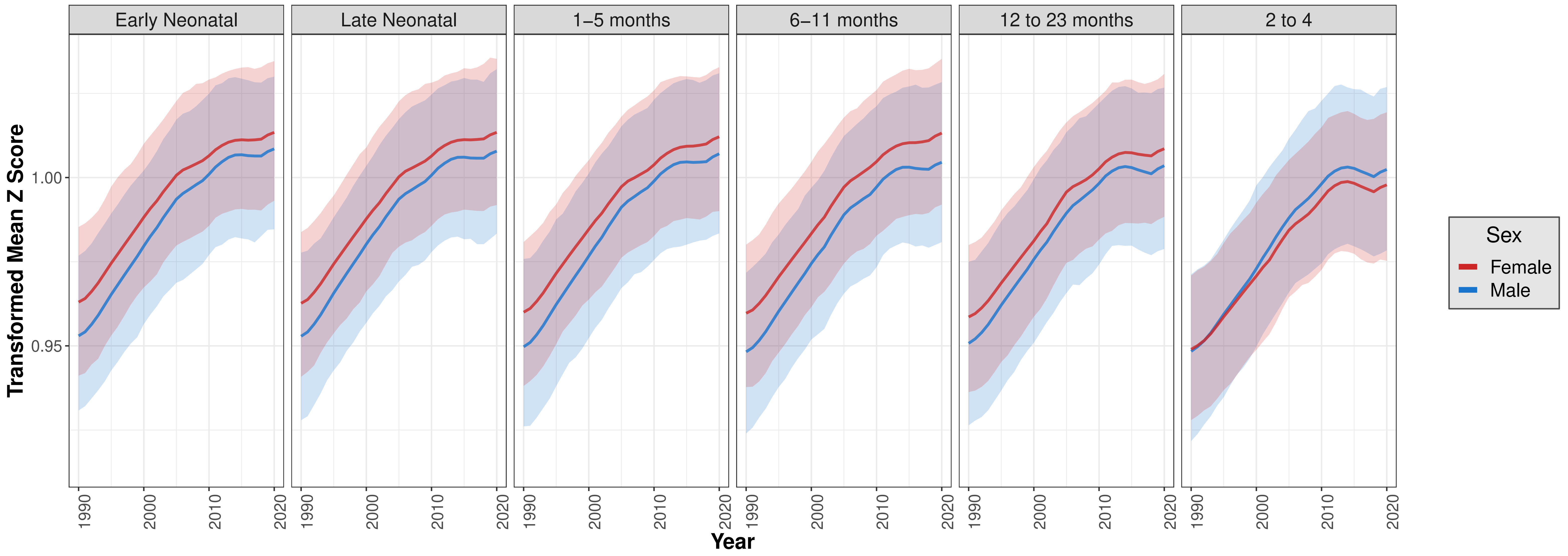


**C**

| Source |
|---|
| No national or subnational sources for this location |

## **Poland** – Wasting (WHZ)

**D**: Overall and Severe Wasting Prevalence

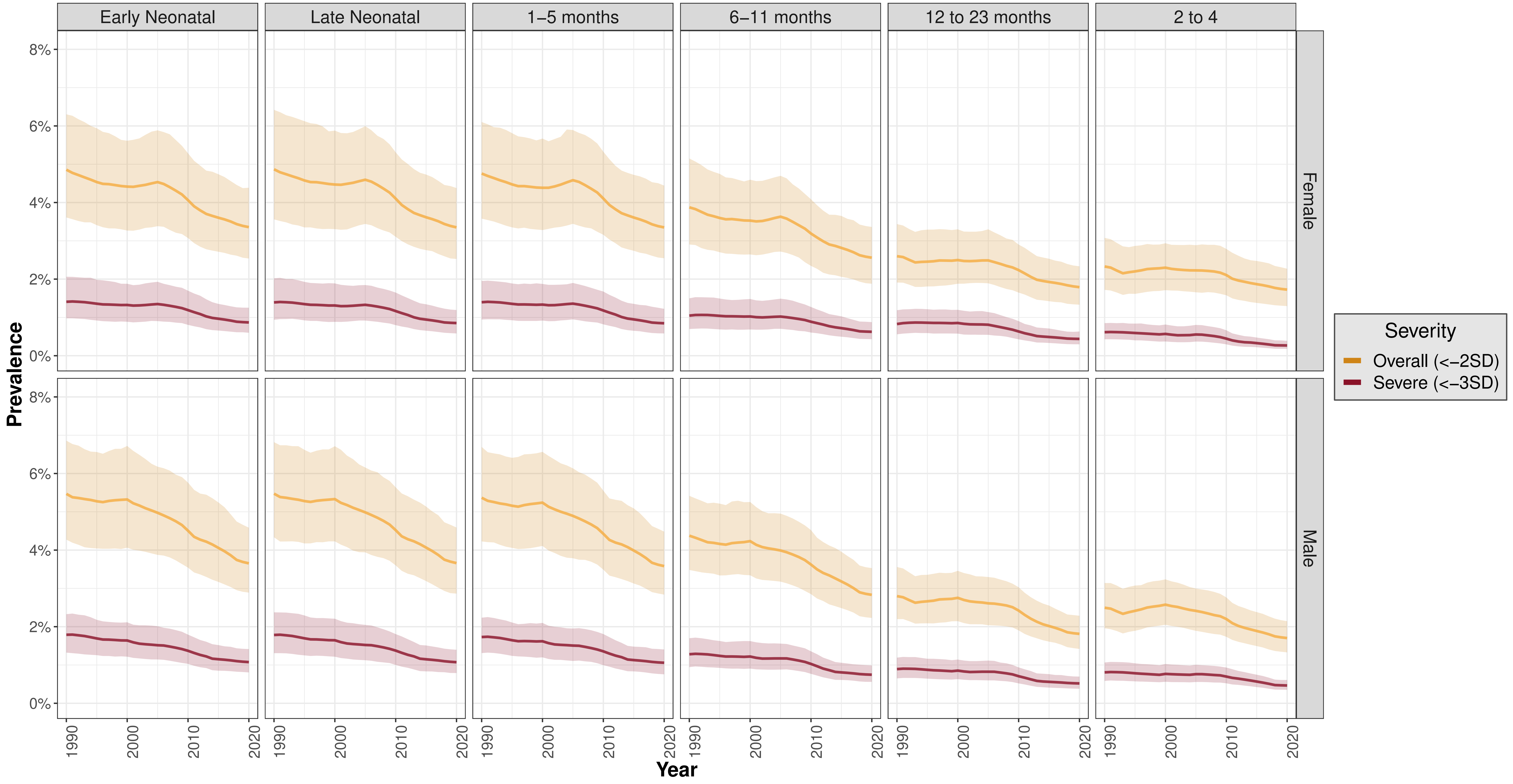


**E**: Transformed Mean Wasting Z Scores

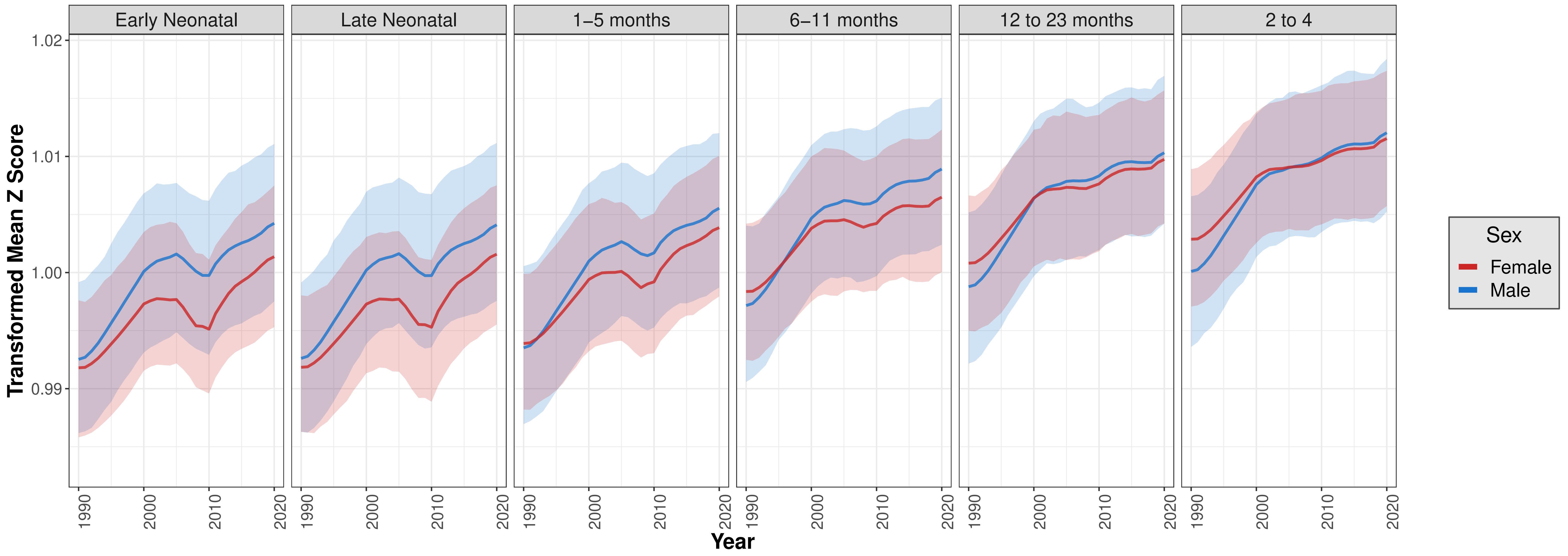


**F**

| Source |
| --- |
| No national or subnational sources for this location |

**Poland** – Underweight (WAZ)

**G:** Overall and Severe Underweight Prevalence

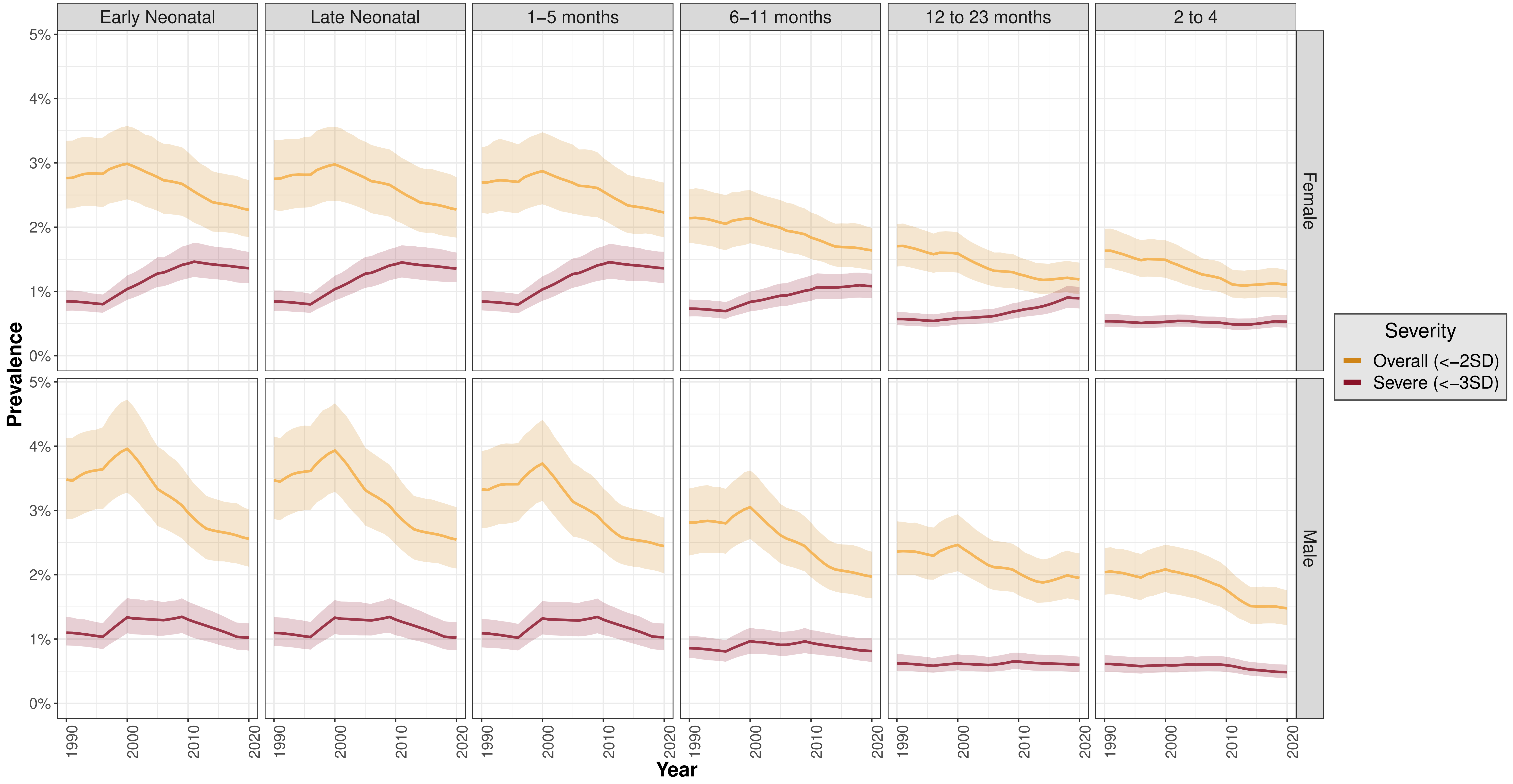


**H:** Transformed Mean Underweight Z Scores

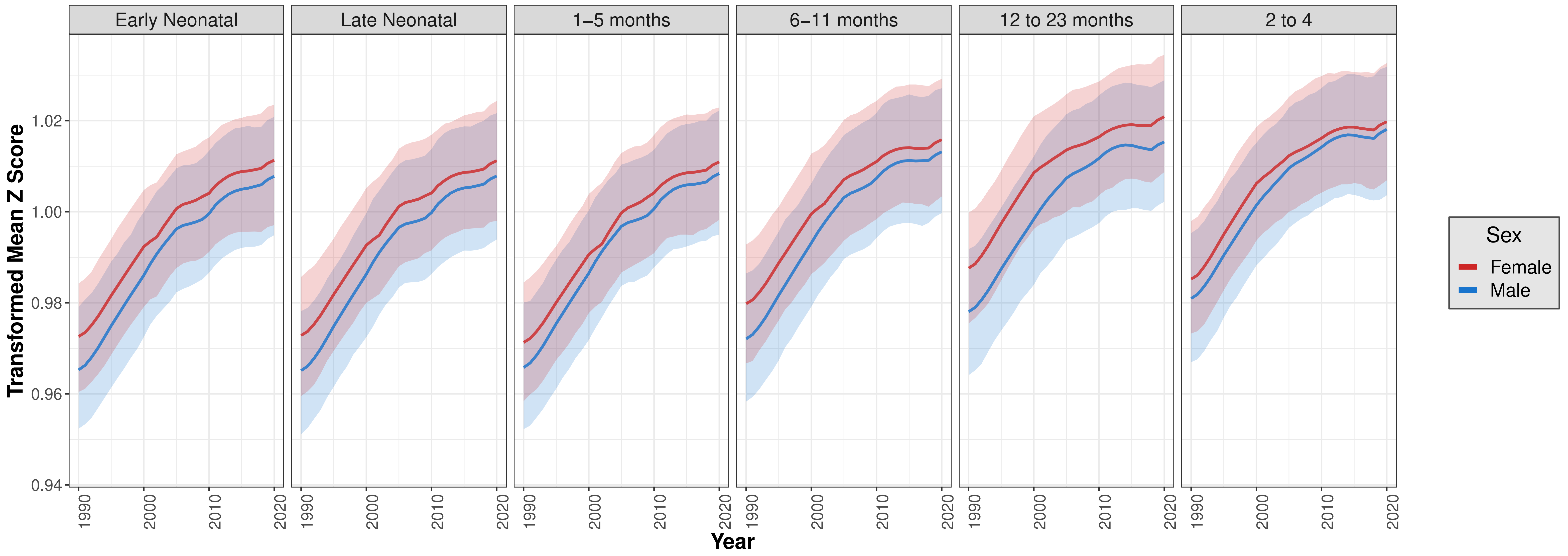


**I**

| Source |
| --- |
| No national or subnational sources for this location |

**Poland** – HAZ, WHZ, and WAZ Distributions

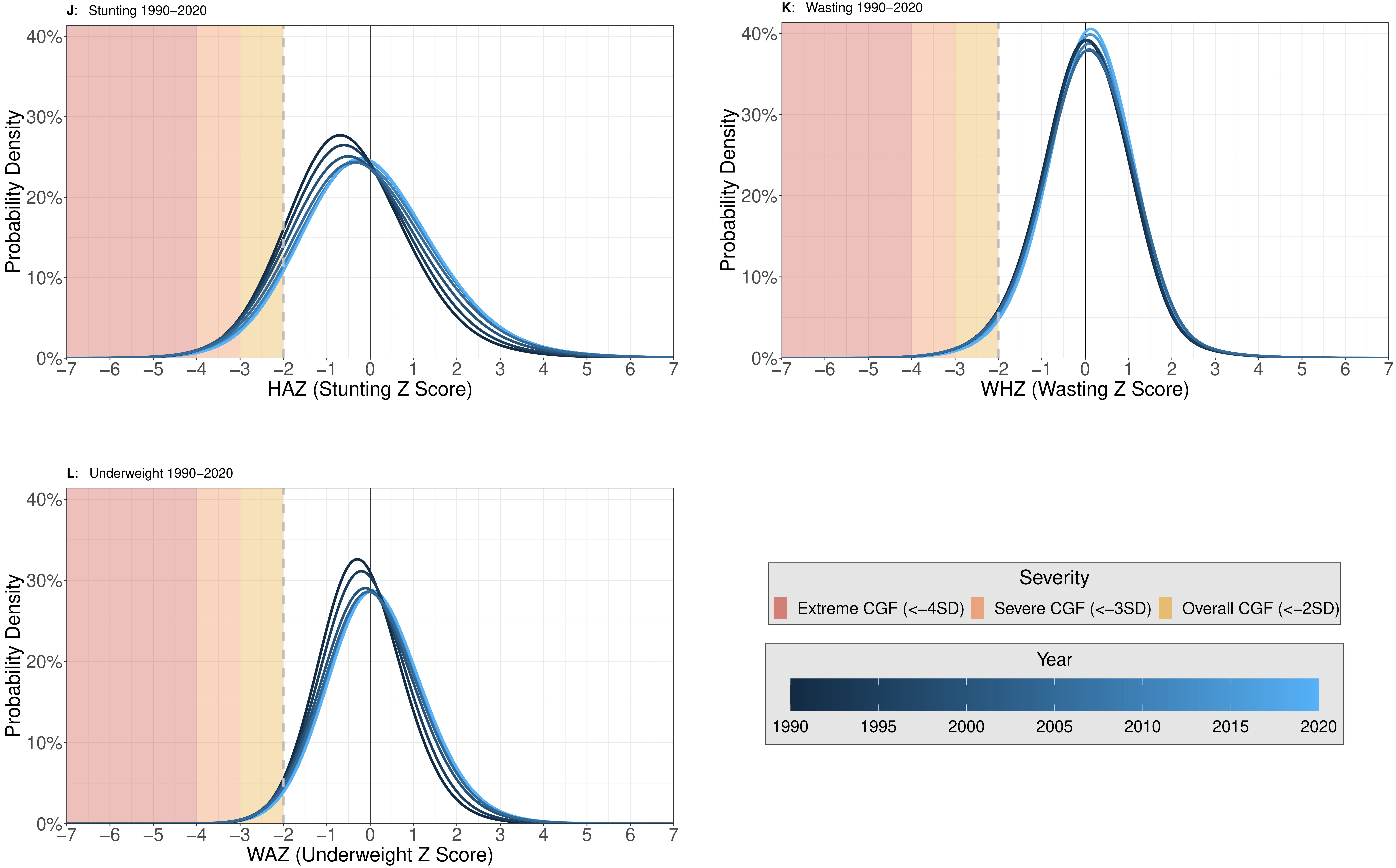

## **Romania** – Stunting (HAZ)

**A**: Overall and Severe Stunting Prevalence

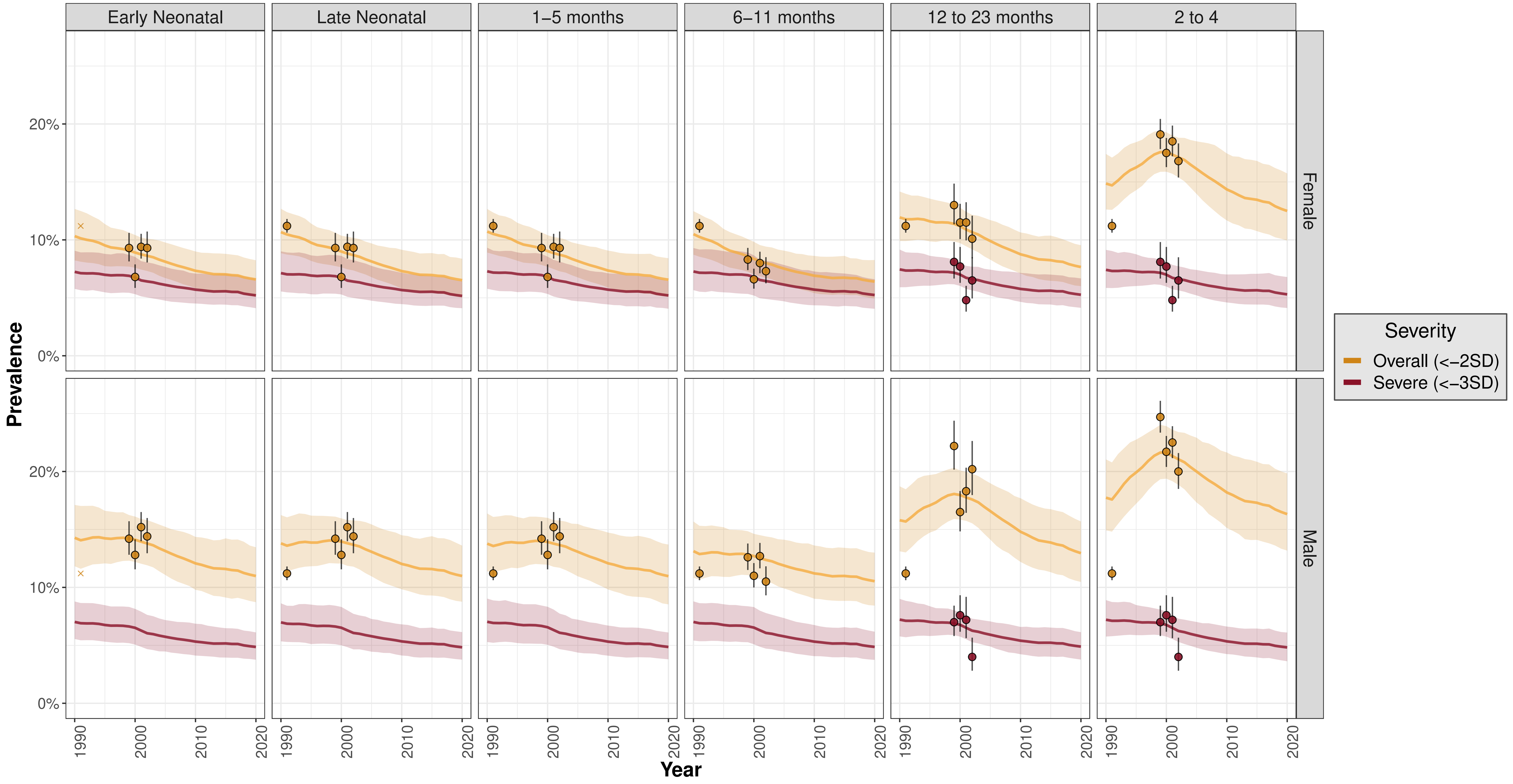


**B**: Transformed Mean Stunting Z Scores

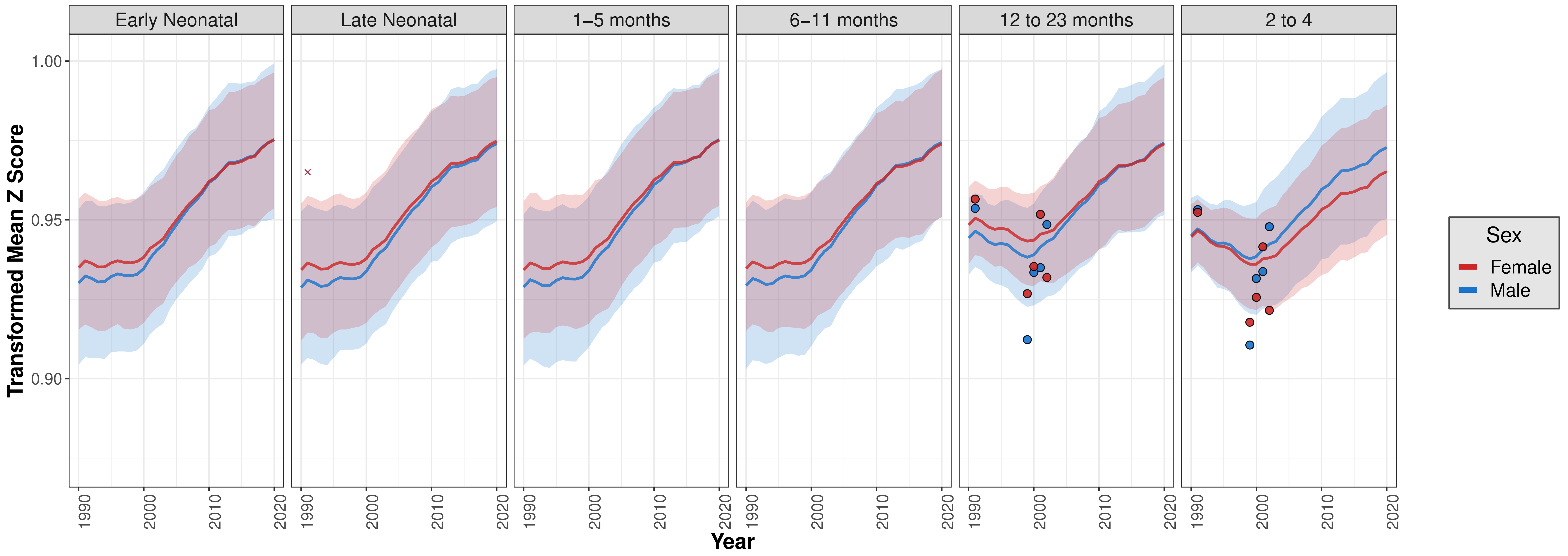


**C**

| Year | Source |
|---|---|
| 1991 | WHO CGM Database |
| 1999 | WHO CGM Database |
| 2000 | WHO CGM Database |
| 2001 | WHO CGM Database |
| 2002 | WHO CGM Database |

# **Romania** – Wasting (WHZ)

**D**: Overall and Severe Wasting Prevalence

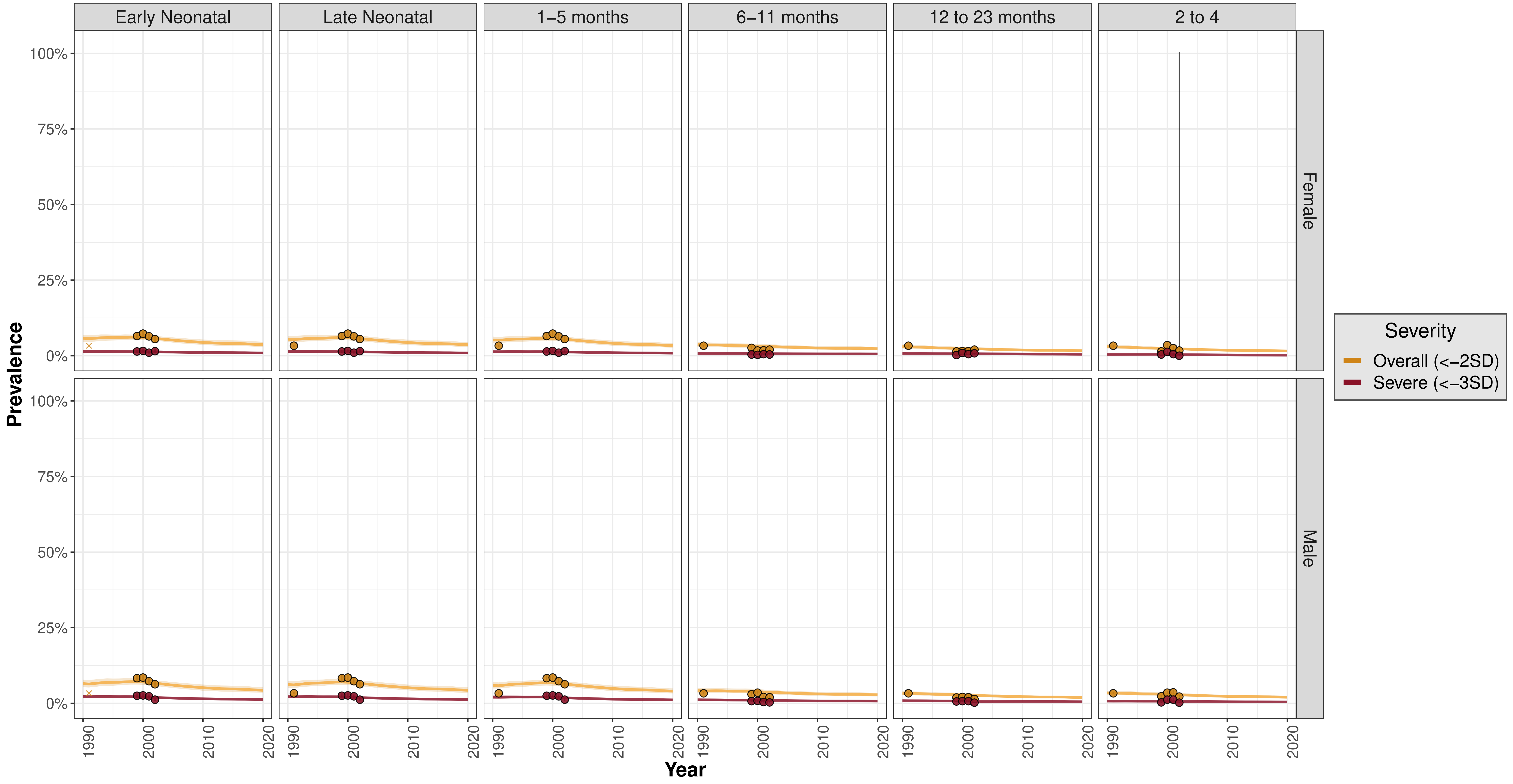


**E**: Transformed Mean Wasting Z Scores

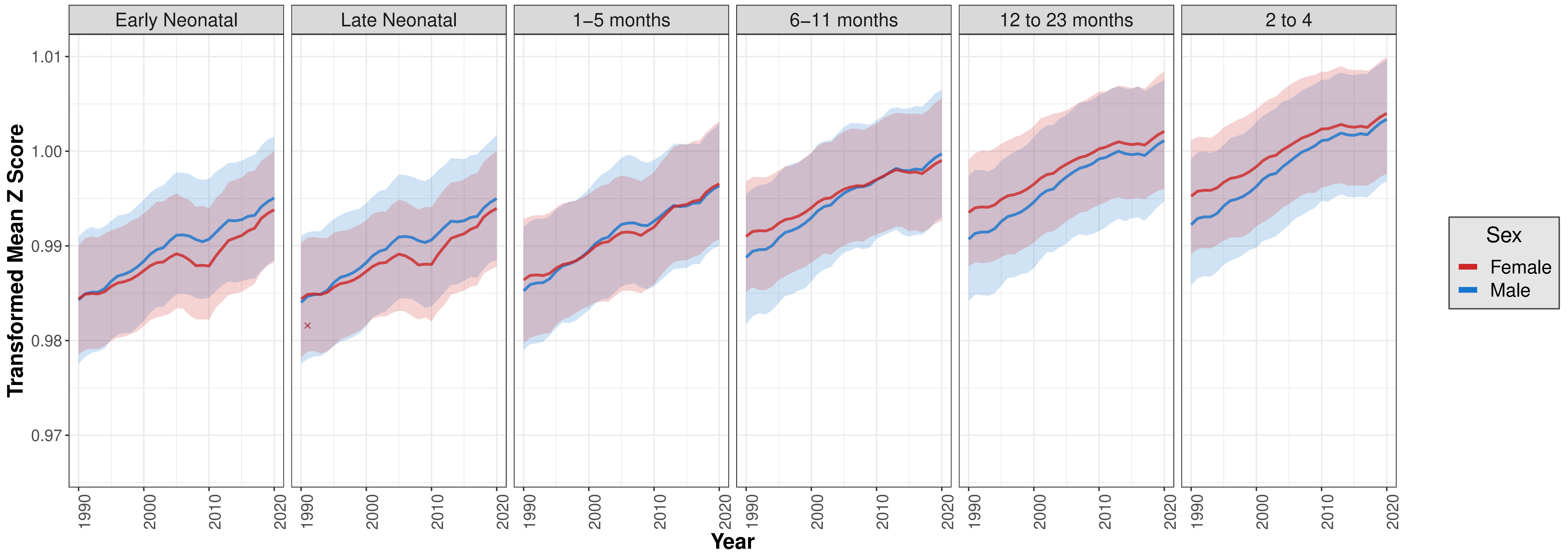


**F**

| Year | Source |
|---|---|
| 1991 | WHO CGM Database |
| 1999 | WHO CGM Database |
| 2000 | WHO CGM Database |
| 2001 | WHO CGM Database |
| 2002 | WHO CGM Database |

## Romania – Underweight (WAZ)

**G**: Overall and Severe Underweight Prevalence

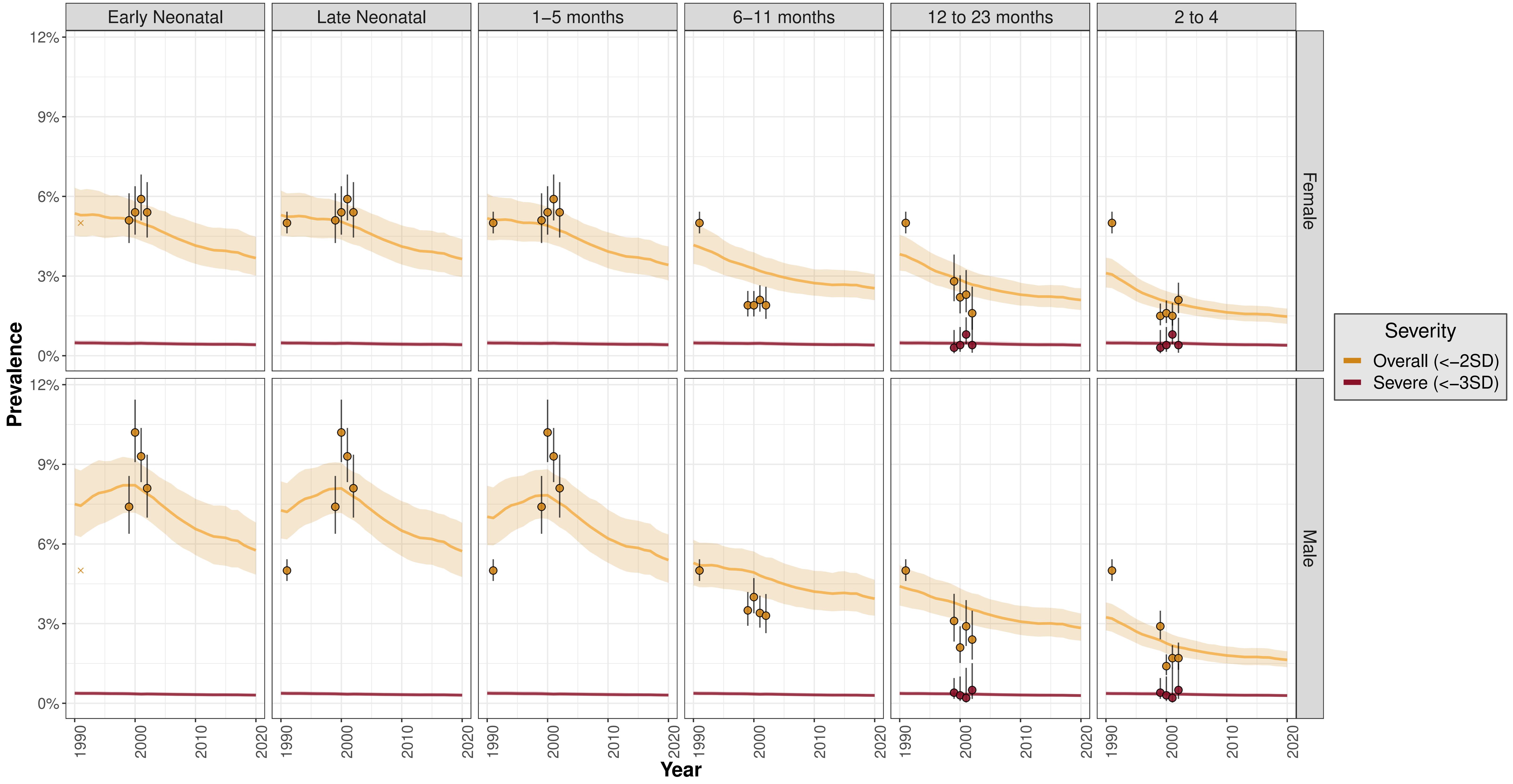


**H**: Transformed Mean Underweight Z Scores

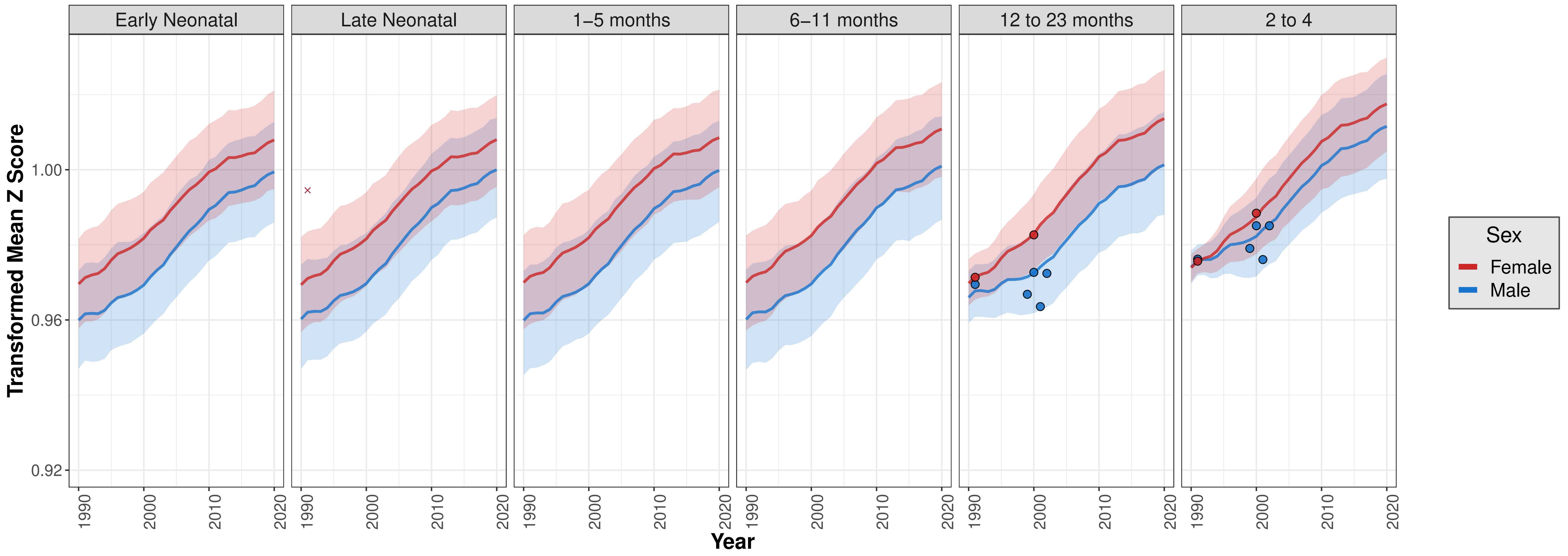


**I**

| Year | Source |
|---|---|
| 1991 | WHO CGM Database |
| 1999 | WHO CGM Database |
| 2000 | WHO CGM Database |
| 2001 | WHO CGM Database |
| 2002 | WHO CGM Database |

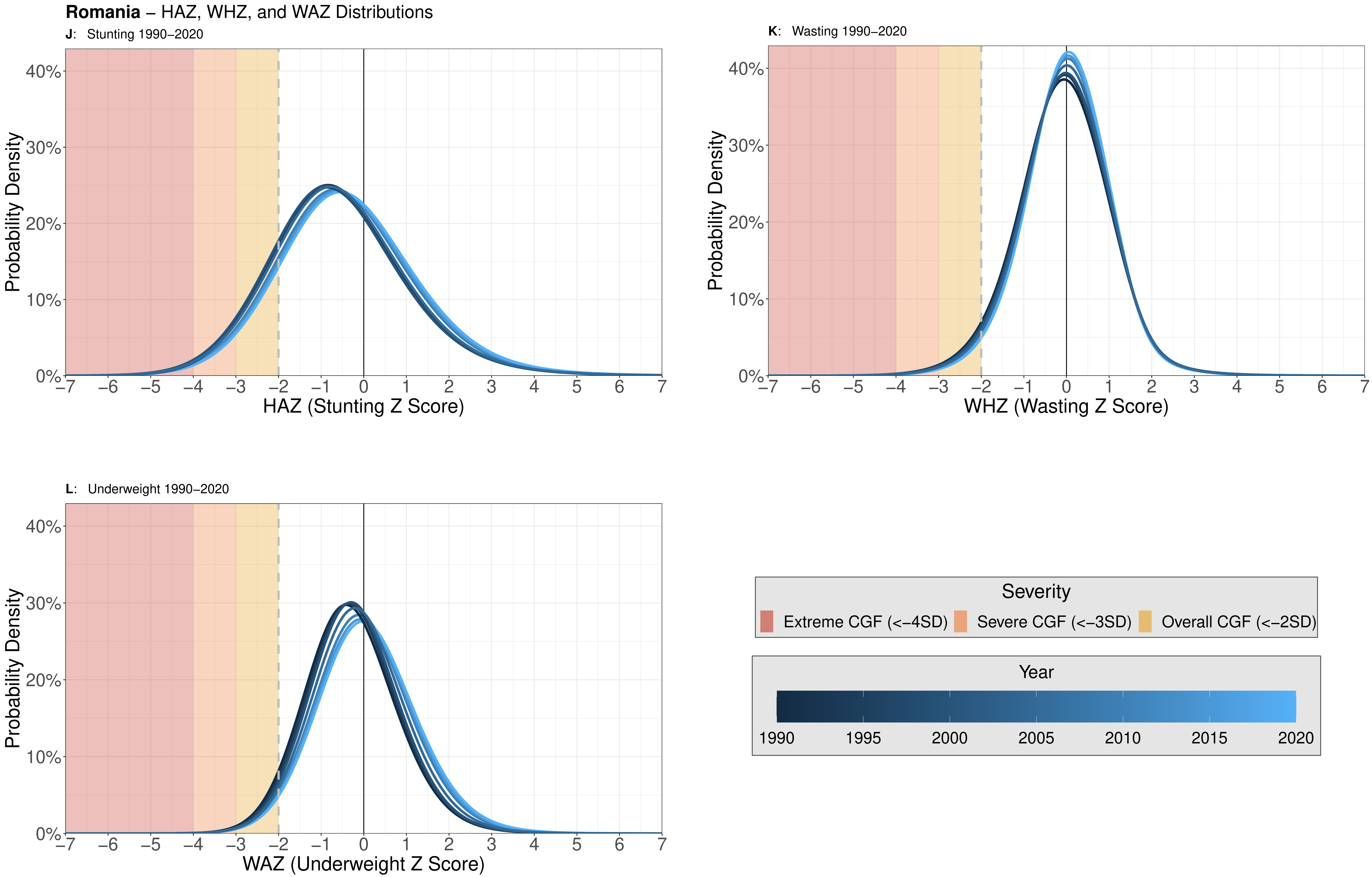
Romania – HAZ, WHZ, and WAZ Distributions
J: Stunting 1990–2020
K: Wasting 1990–2020
L: Underweight 1990–2020
Probability Density
0%
10%
20%
30%
40%
−7 −6 −5 −4 −3 −2 −1 0 1 2 3 4 5 6 7
HAZ (Stunting Z Score)
WHZ (Wasting Z Score)
WAZ (Underweight Z Score)
Severity
Extreme CGF (<−4SD)
Severe CGF (<−3SD)
Overall CGF (<−2SD)
Year
1990
1995
2000
2005
2010
2015
2020

# **Serbia** – Stunting (HAZ)

**A**: Overall and Severe Stunting Prevalence

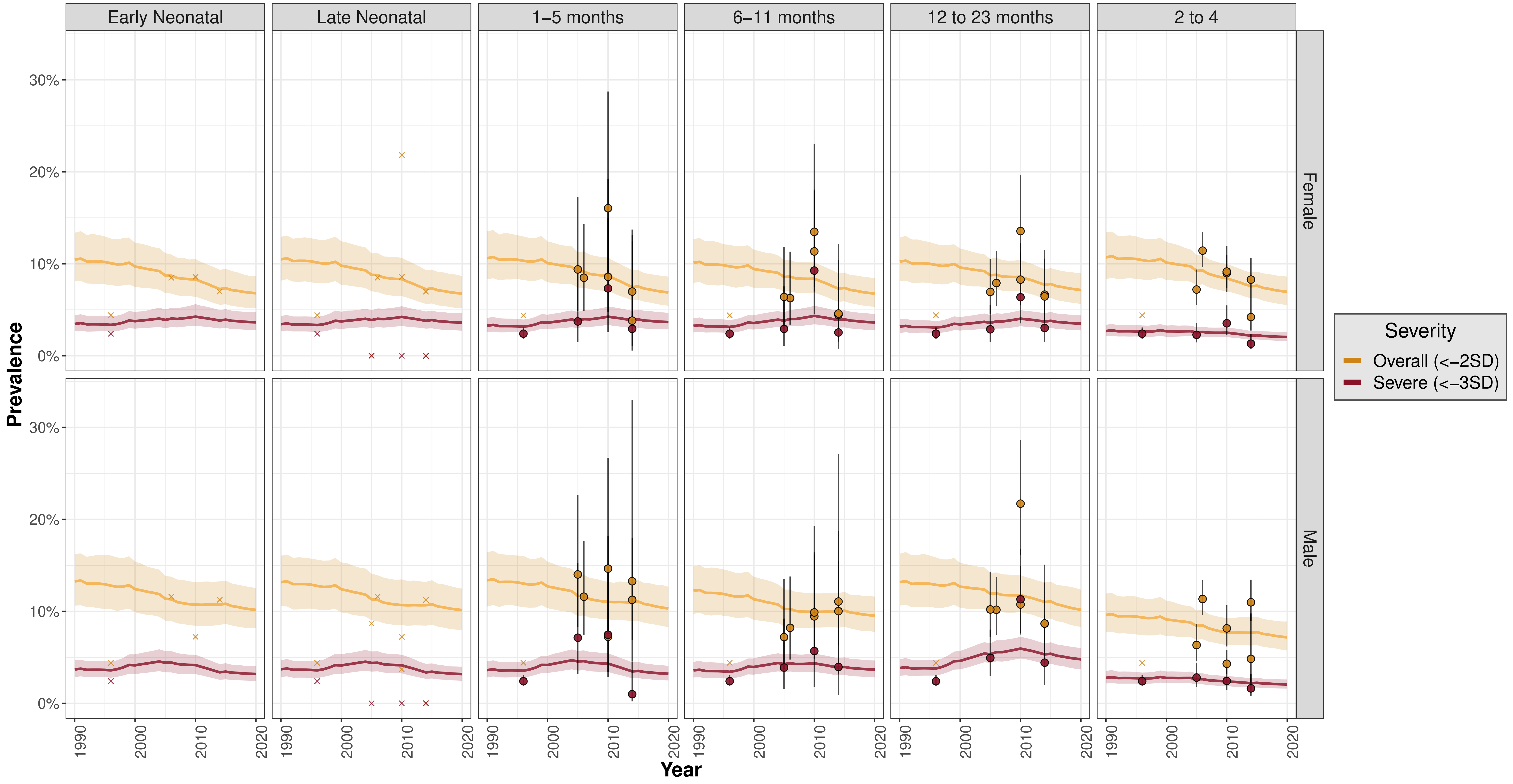


**B**: Transformed Mean Stunting Z Scores

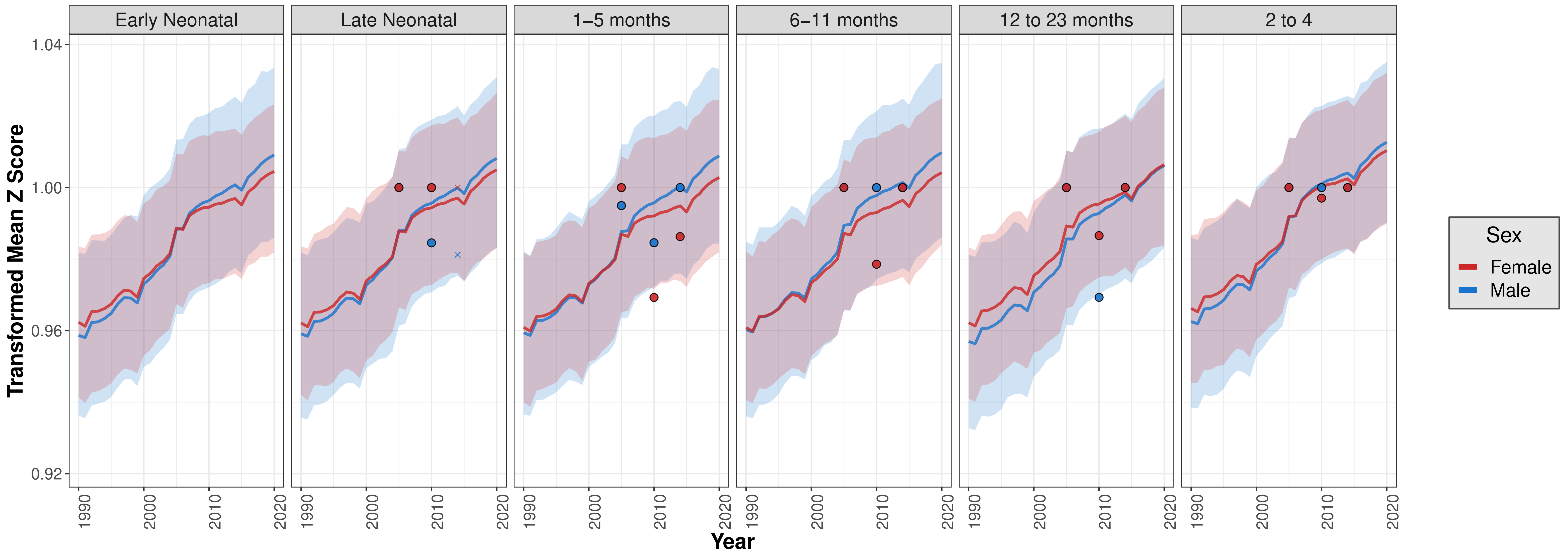


**C**

| Year | Source |
|---|---|
| 1996 | Yugoslavia, Federal Republic MICS |
| 2005 | MICS |
| 2006 | WHO CGM Database |
| 2010 | MICS |
| 2010 | WHO CGM Database |
| 2014 | WHO CGM Database |
| 2014 | MICS |

**Serbia** – Wasting (WHZ)

**D**: Overall and Severe Wasting Prevalence

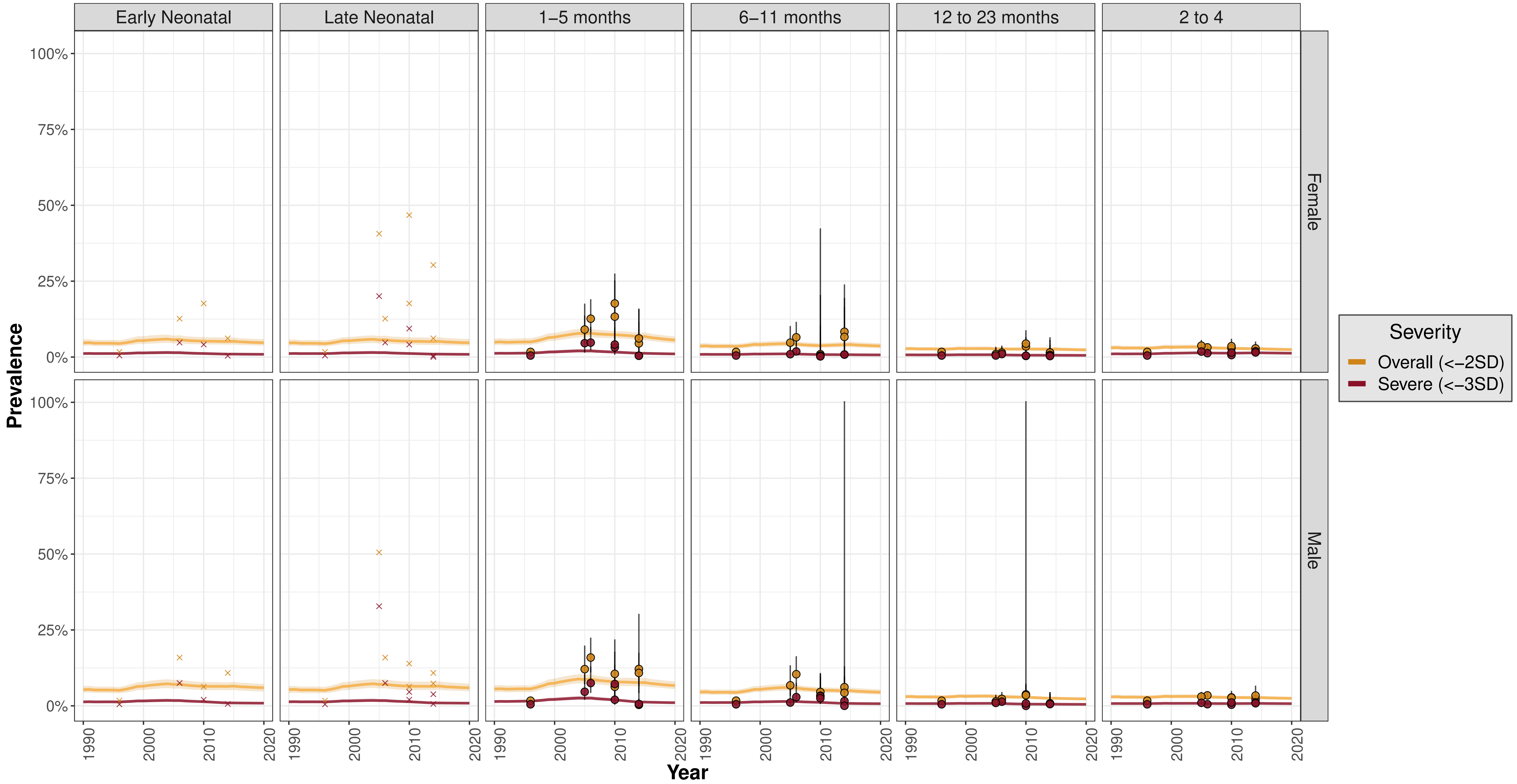


**E**: Transformed Mean Wasting Z Scores

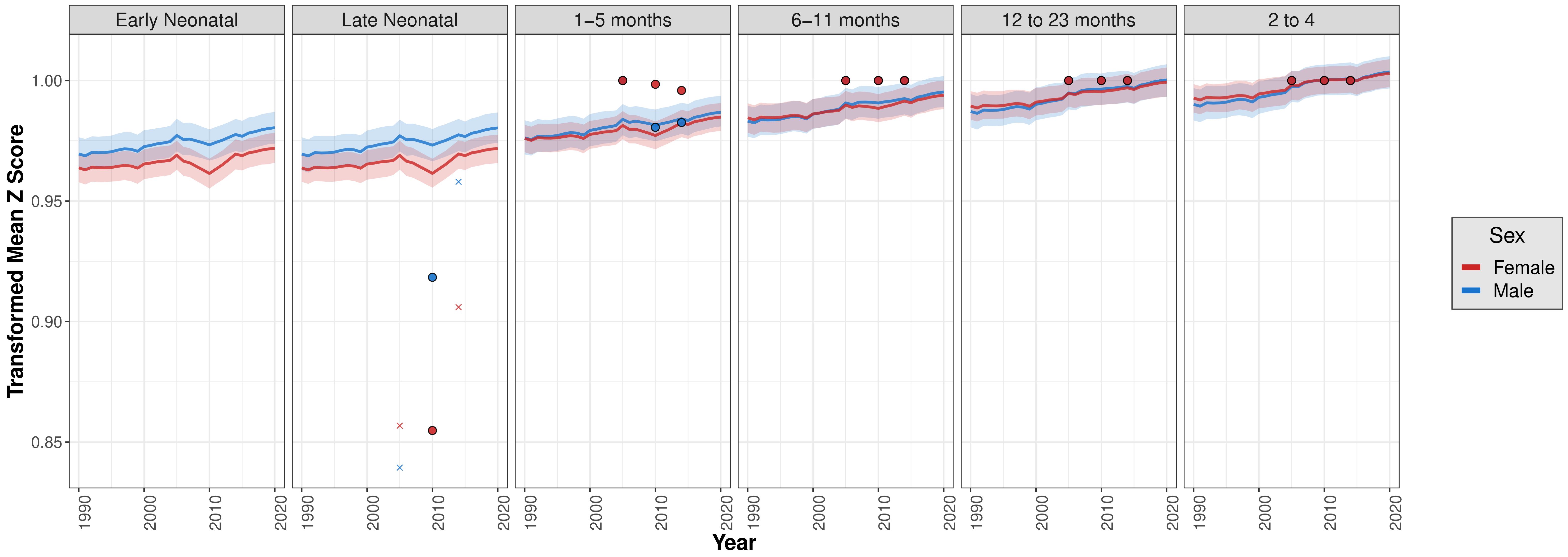


**F**

| Year | Source |
|---|---|
| 1996 | Yugoslavia, Federal Republic MICS |
| 2005 | MICS |
| 2006 | WHO CGM Database |
| 2010 | MICS |
| 2010 | WHO CGM Database |
| 2014 | WHO CGM Database |
| 2014 | MICS |

# **Serbia** – Underweight (WAZ)

**G**: Overall and Severe Underweight Prevalence

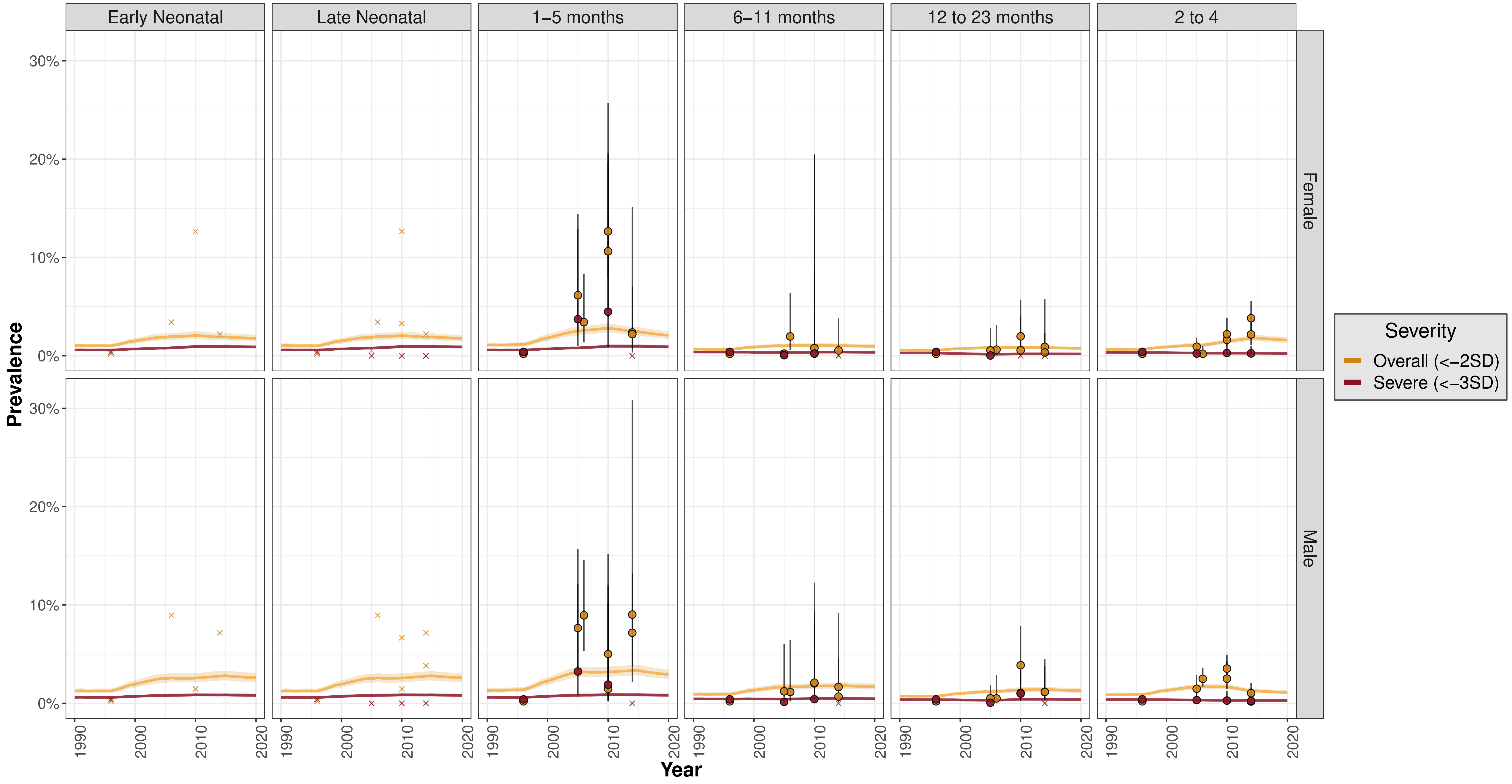


**H**: Transformed Mean Underweight Z Scores

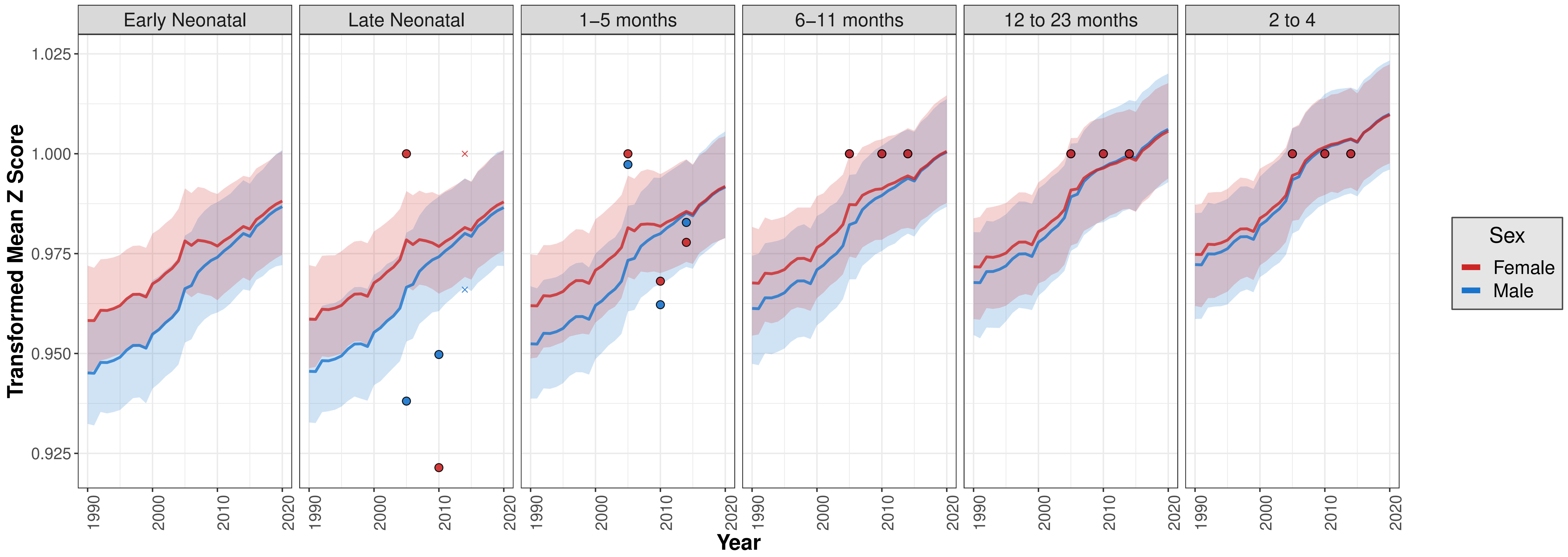


**I**

| Year | Source |
|---|---|
| 1996 | Yugoslavia, Federal Republic MICS |
| 2005 | MICS |
| 2006 | WHO CGM Database |
| 2010 | MICS |
| 2010 | WHO CGM Database |
| 2014 | WHO CGM Database |
| 2014 | MICS |

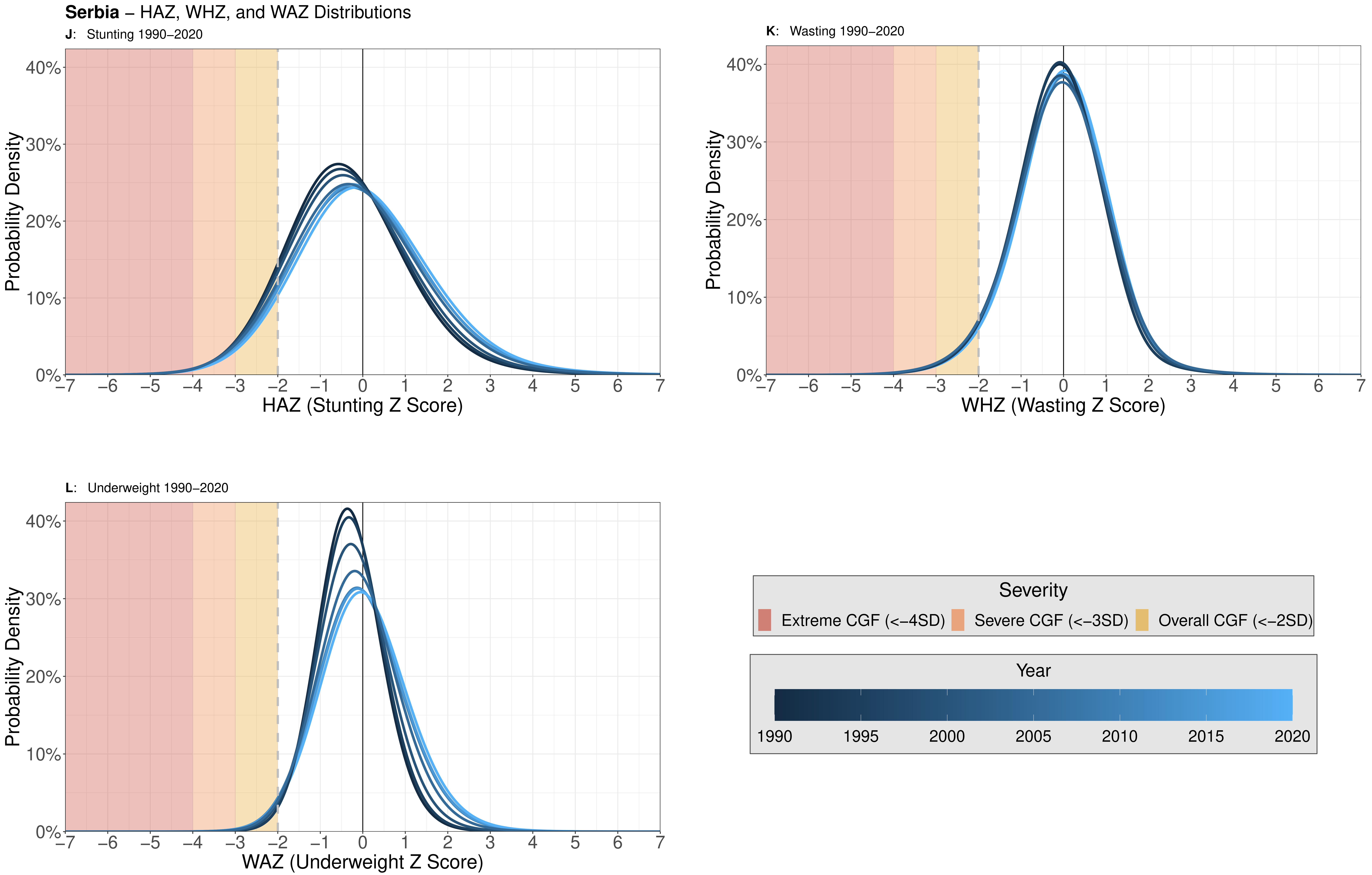

Serbia – HAZ, WHZ, and WAZ Distributions
J: Stunting 1990–2020
K: Wasting 1990–2020
L: Underweight 1990–2020
Probability Density
0%
10%
20%
30%
40%
−7
−6
−5
−4
−3
−2
−1
0
1
2
3
4
5
6
7
HAZ (Stunting Z Score)
WHZ (Wasting Z Score)
WAZ (Underweight Z Score)
Severity
Extreme CGF (<−4SD)
Severe CGF (<−3SD)
Overall CGF (<−2SD)
Year
1990
1995
2000
2005
2010
2015
2020

# **Slovakia** – Stunting (HAZ)

**A**: Overall and Severe Stunting Prevalence

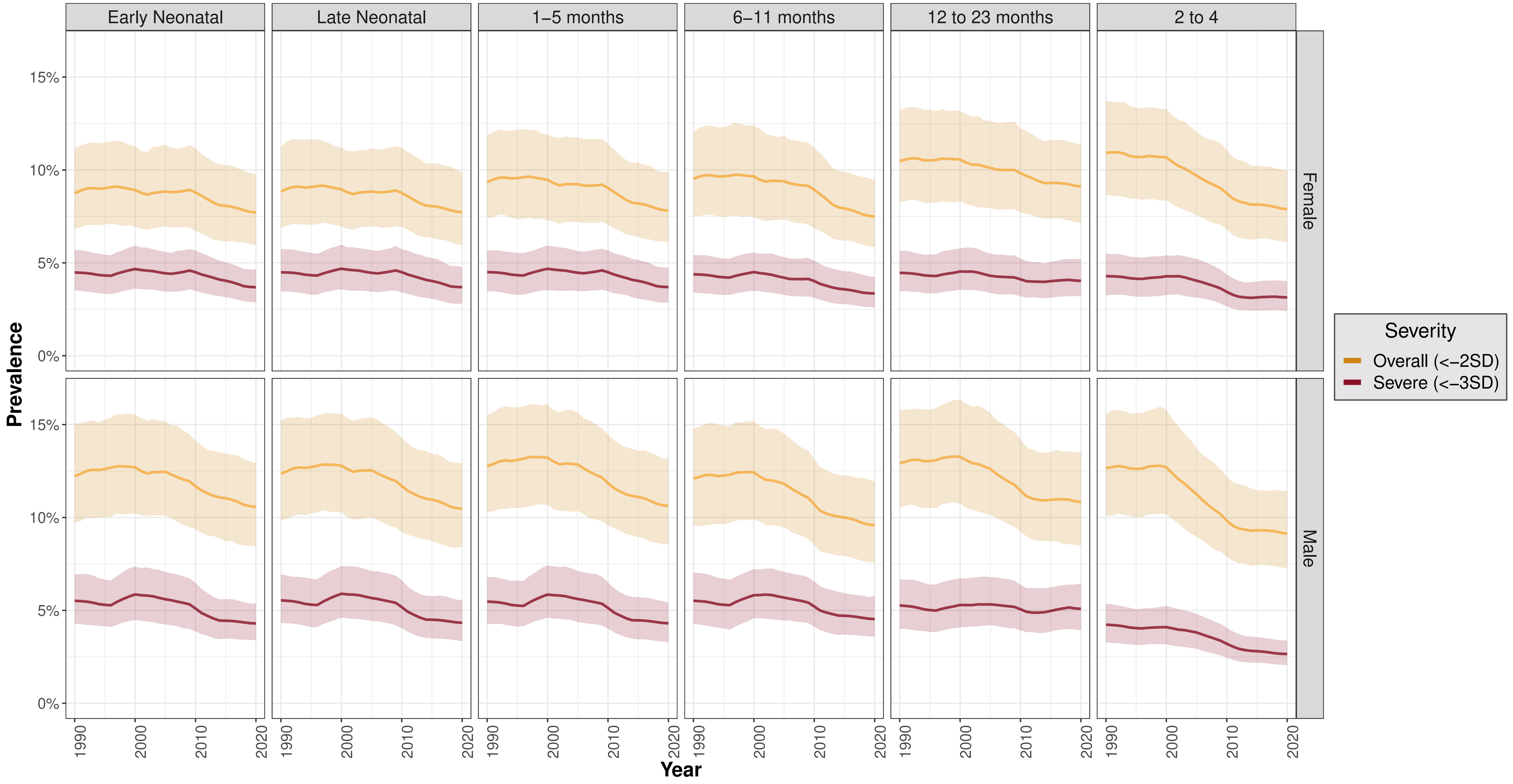


**B**: Transformed Mean Stunting Z Scores

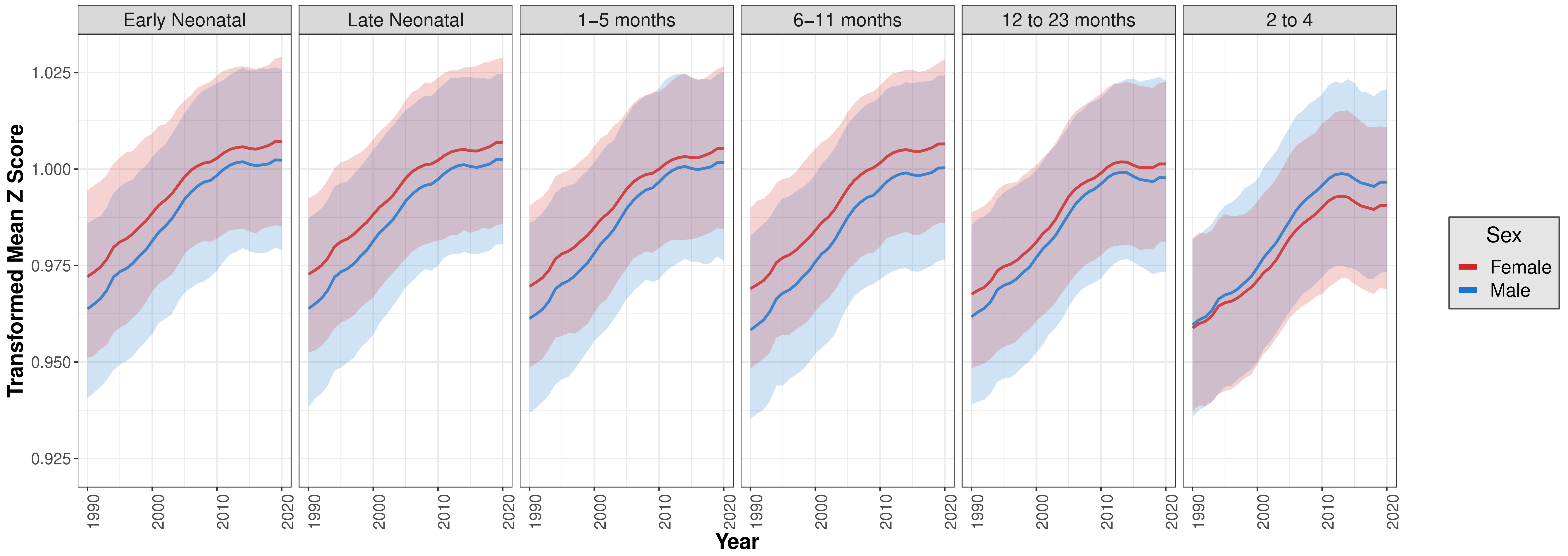


**C**

| Source |
| --- |
| No sources for this location |

# **Slovakia** – Wasting (WHZ)

**D**: Overall and Severe Wasting Prevalence

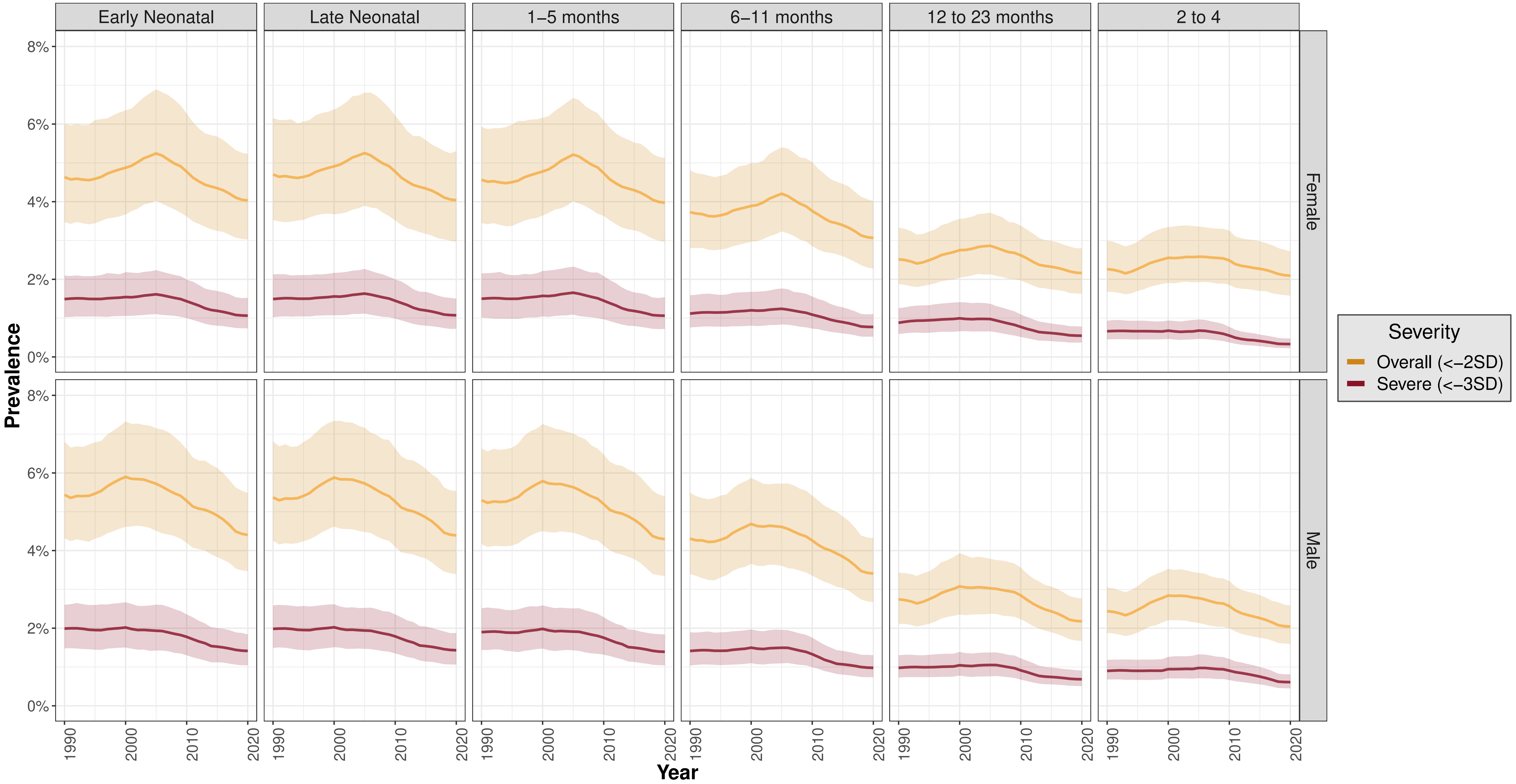


**E**: Transformed Mean Wasting Z Scores

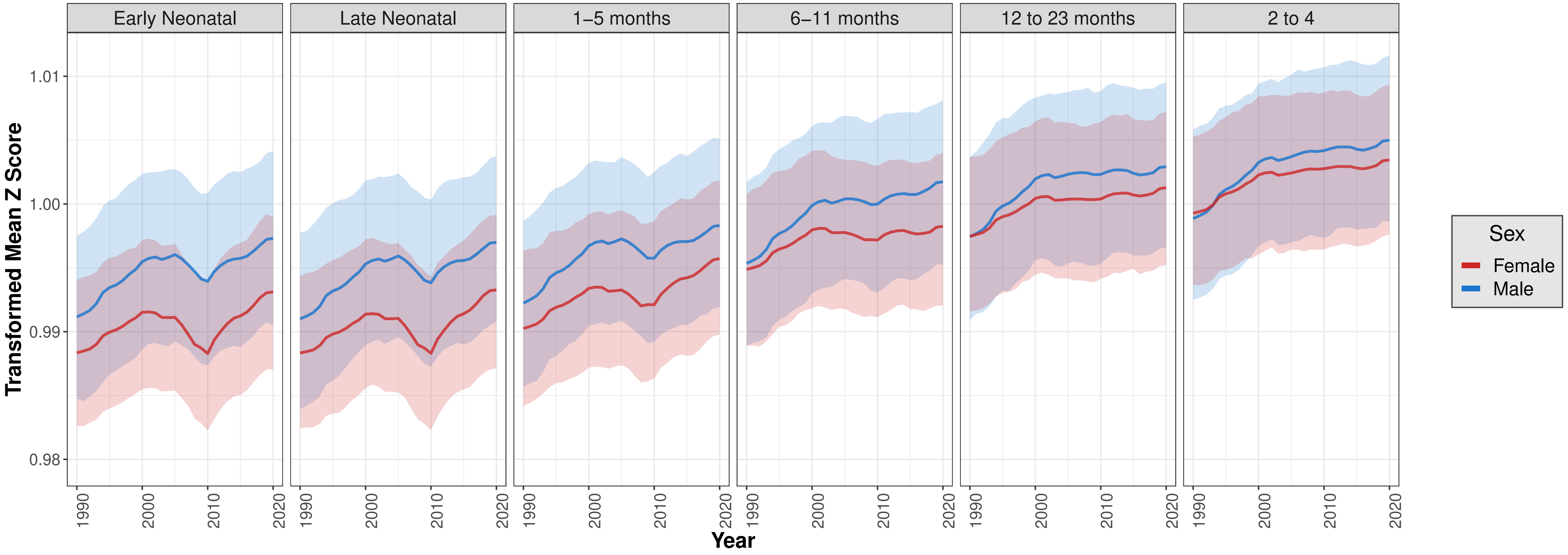


**F**

| Source |
|---|
| No sources for this location |

# **Slovakia** – Underweight (WAZ)

**G**: Overall and Severe Underweight Prevalence

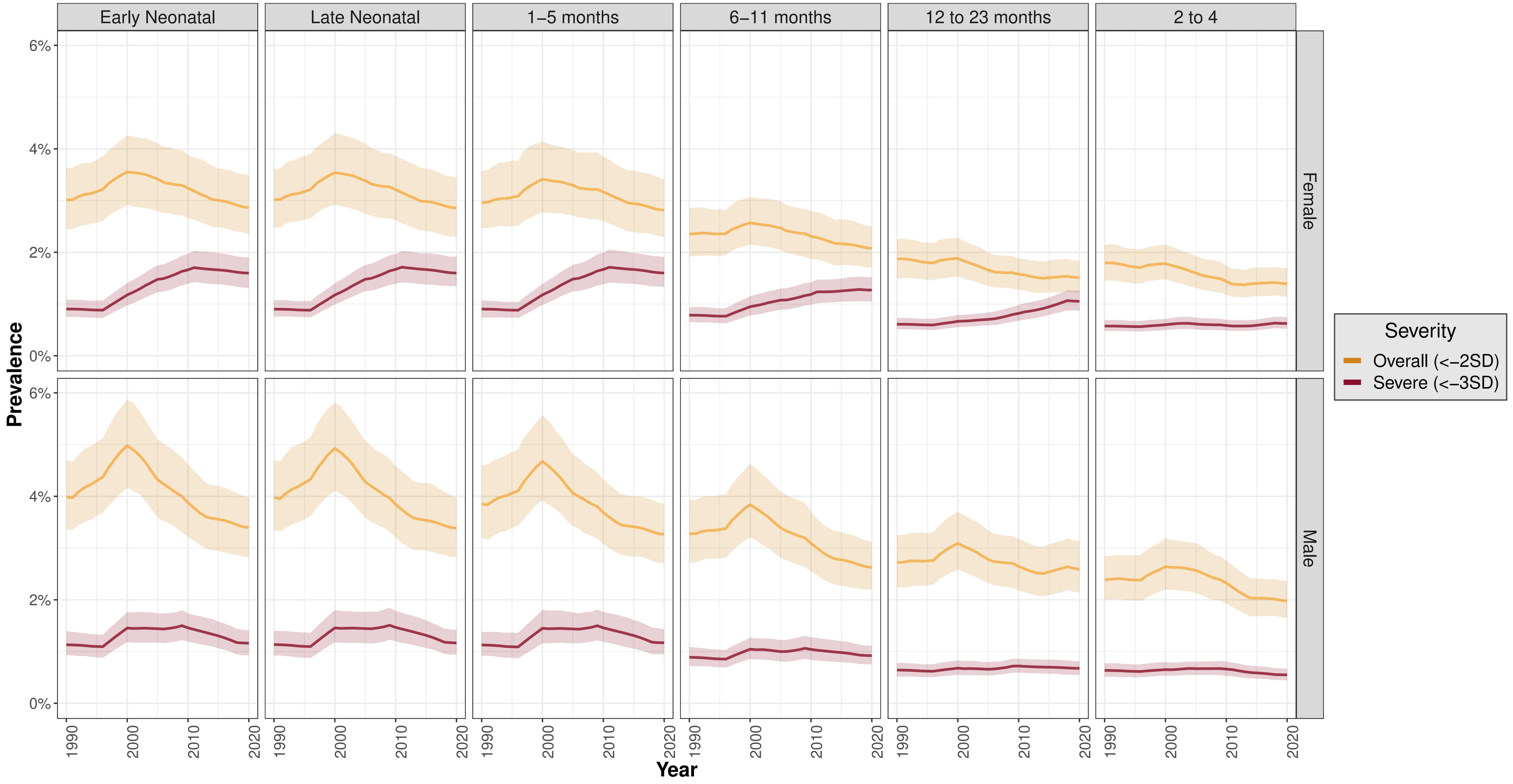


**I**

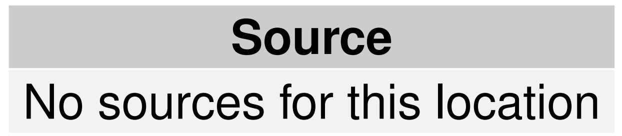


**H**: Transformed Mean Underweight Z Scores

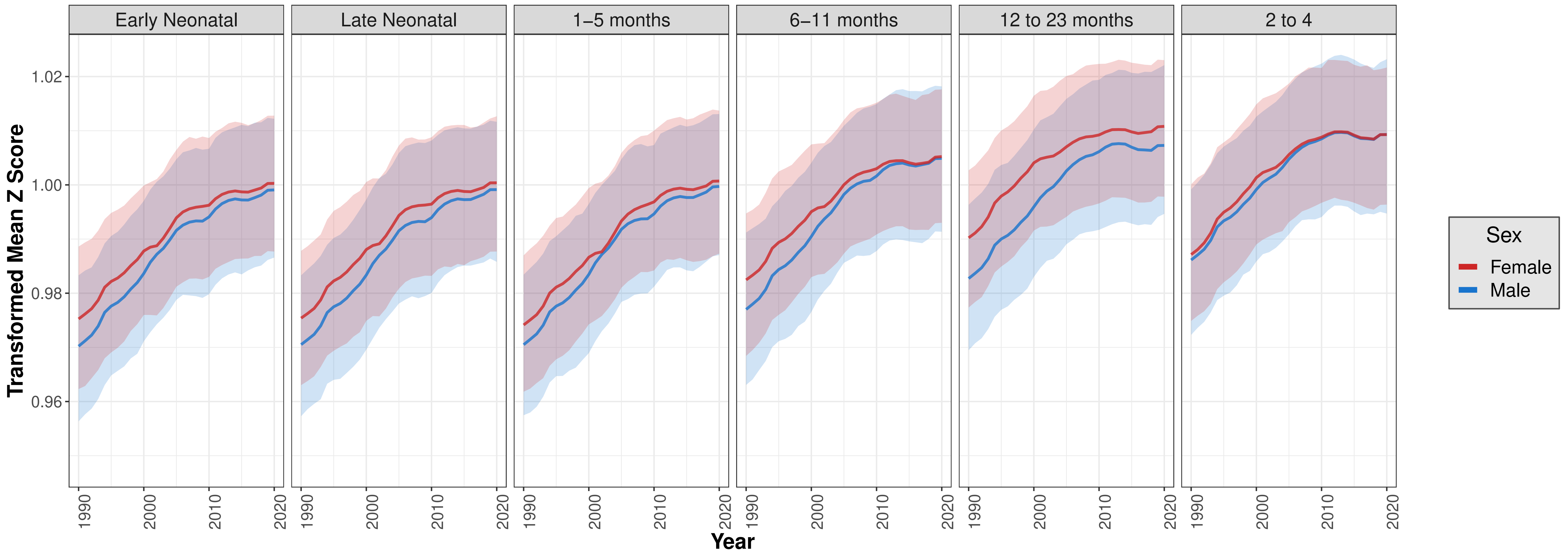

**Slovakia** – HAZ, WHZ, and WAZ Distributions

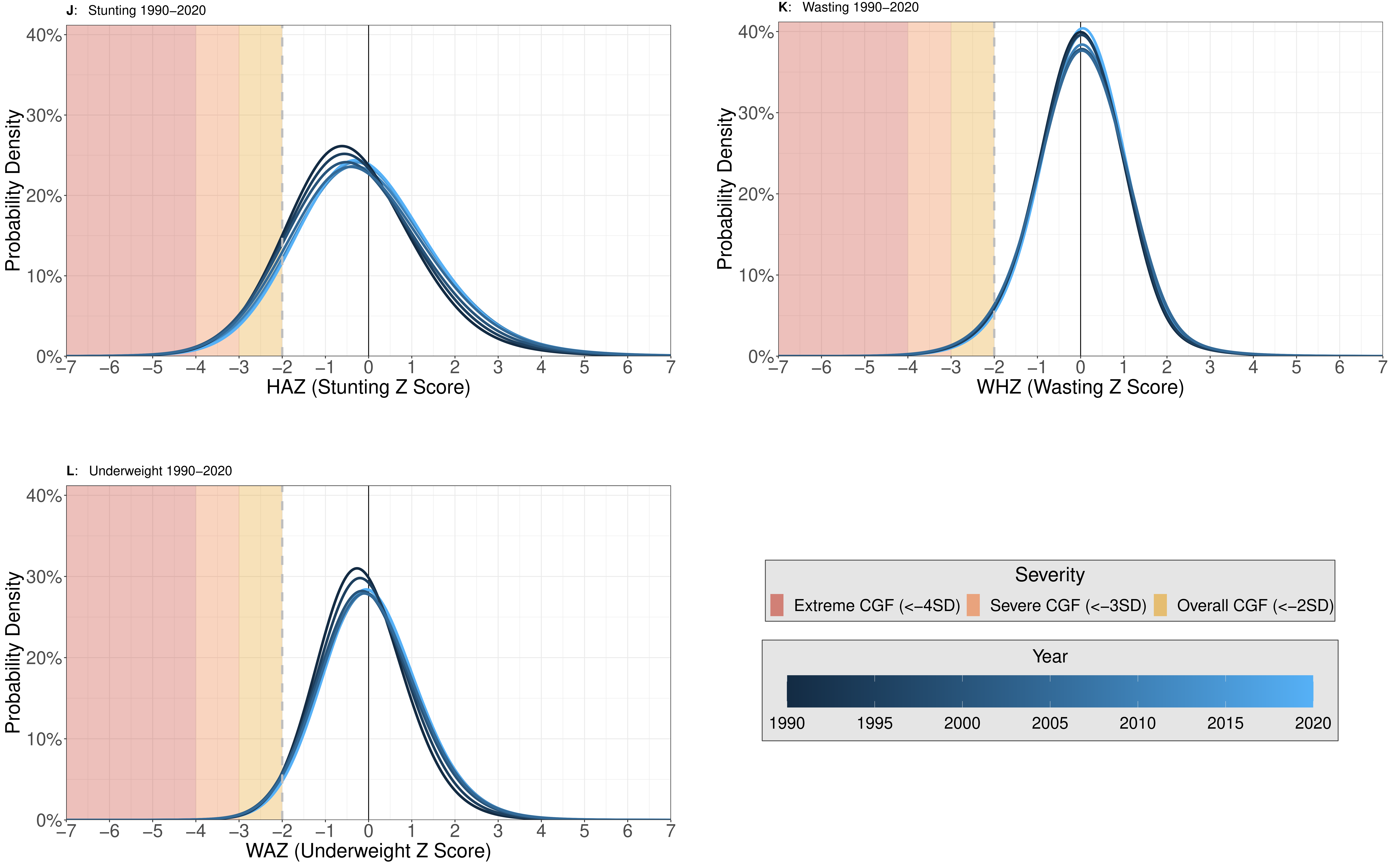

# **Slovenia** – Stunting (HAZ)

**A**: Overall and Severe Stunting Prevalence

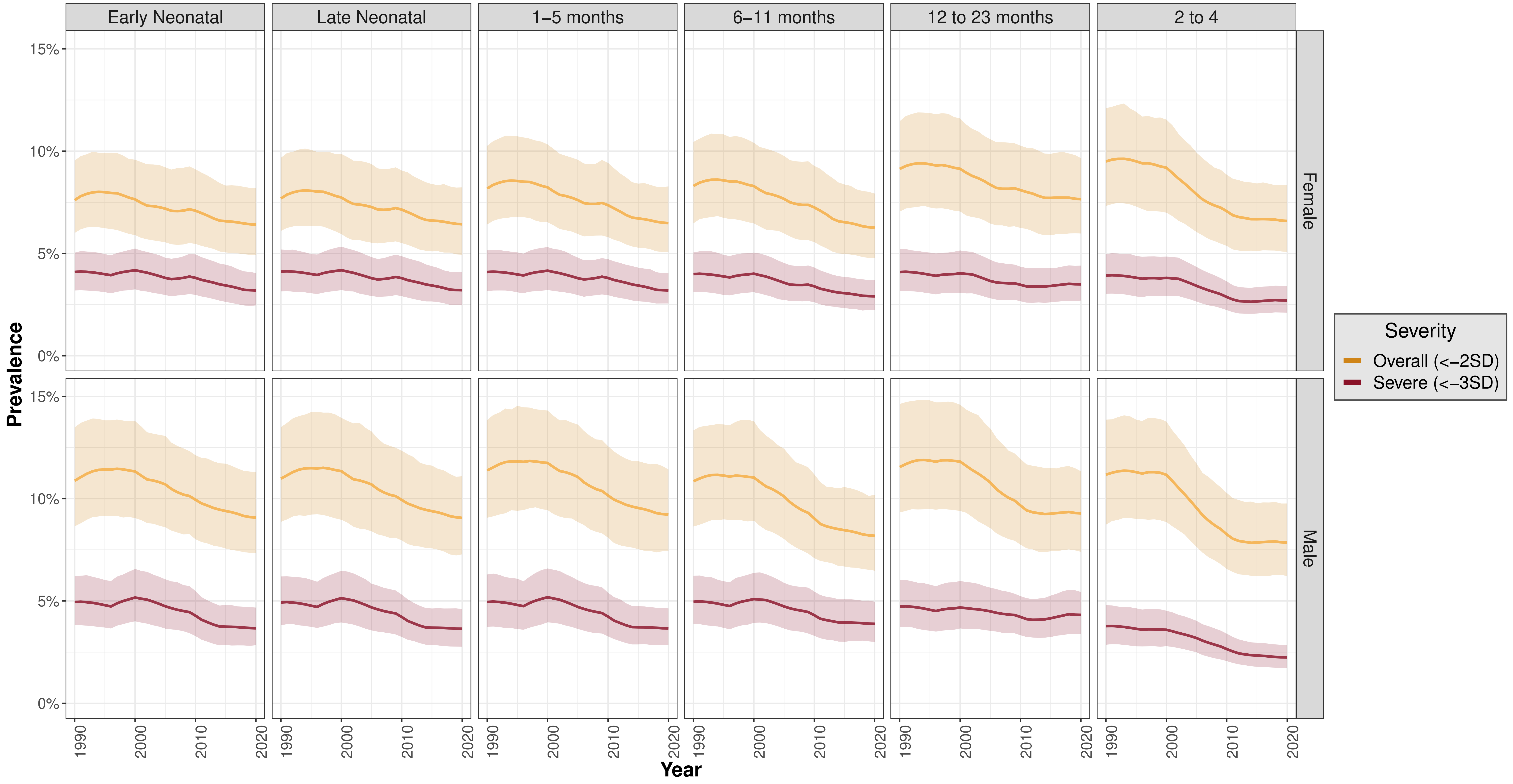


**B**: Transformed Mean Stunting Z Scores

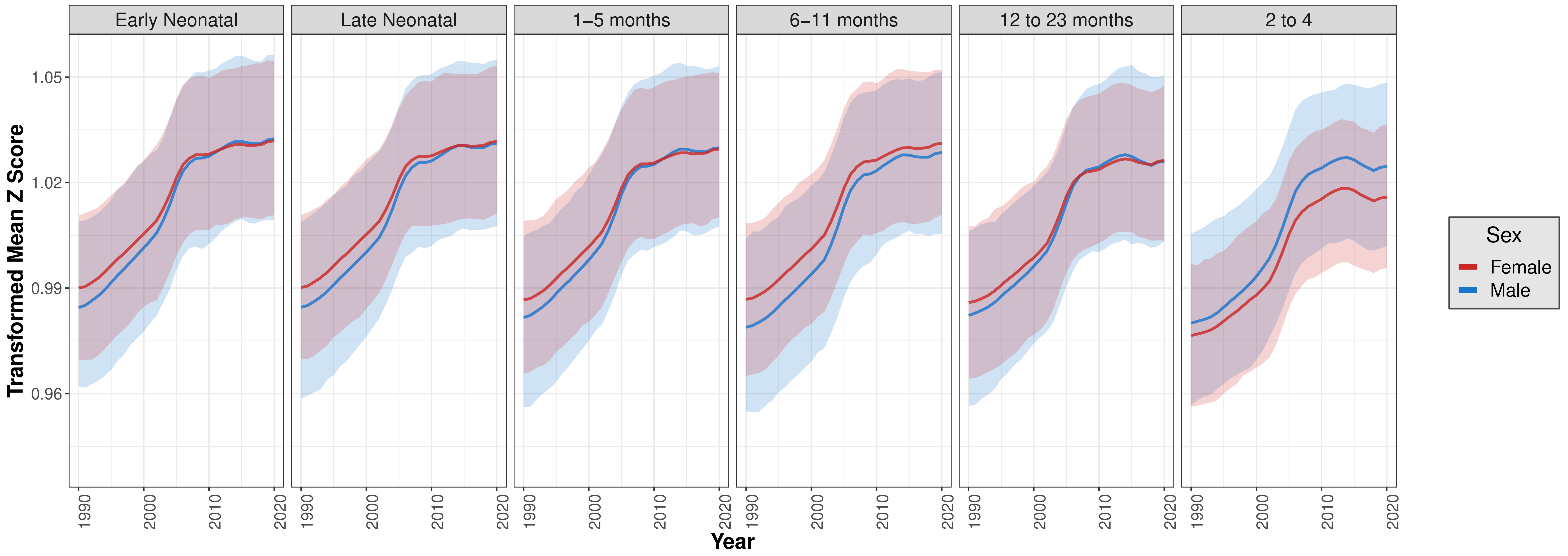


**C**

| Source |
|---|
| No sources for this location |

## **Slovenia** – Wasting (WHZ)

**D**: Overall and Severe Wasting Prevalence

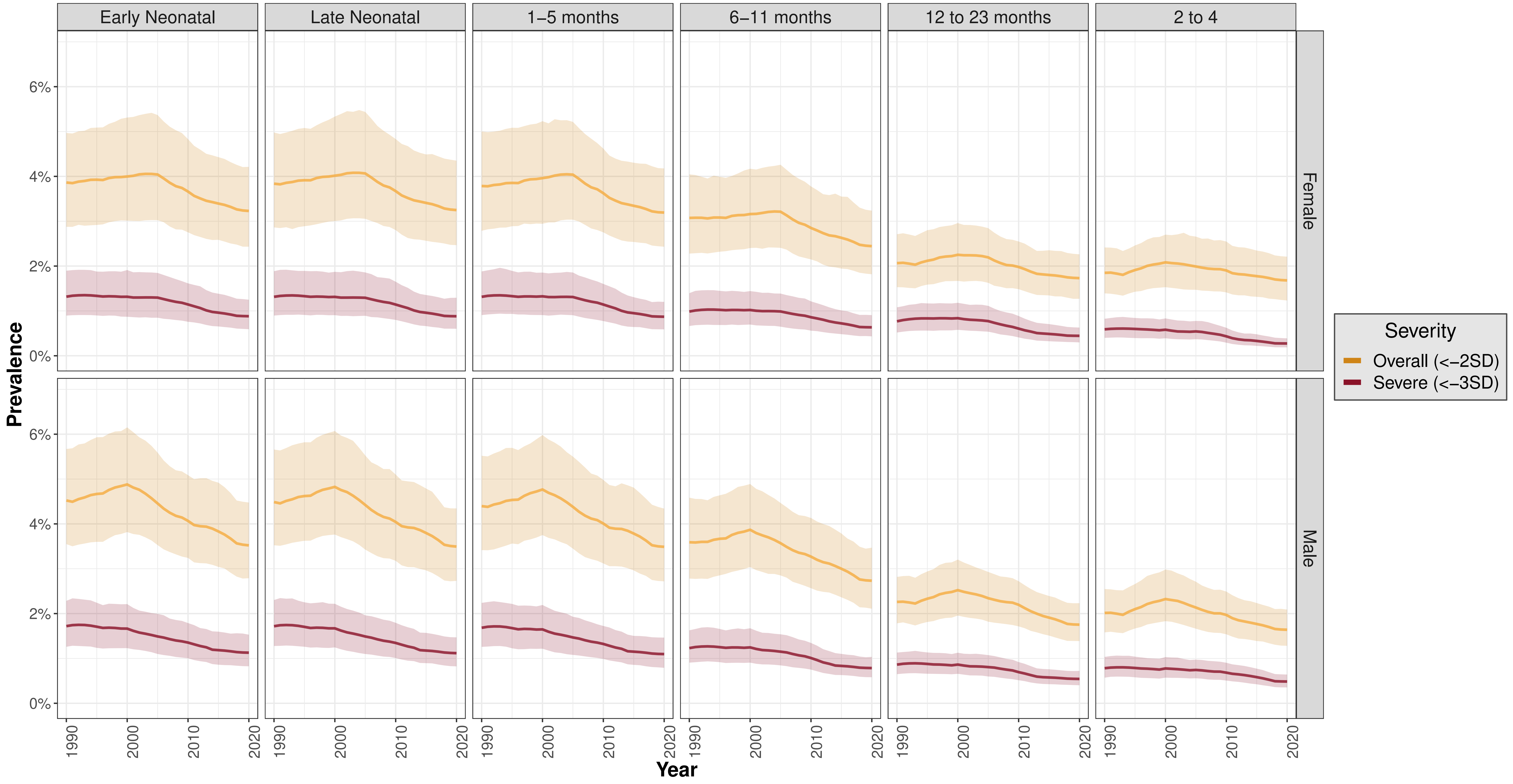


**E**: Transformed Mean Wasting Z Scores

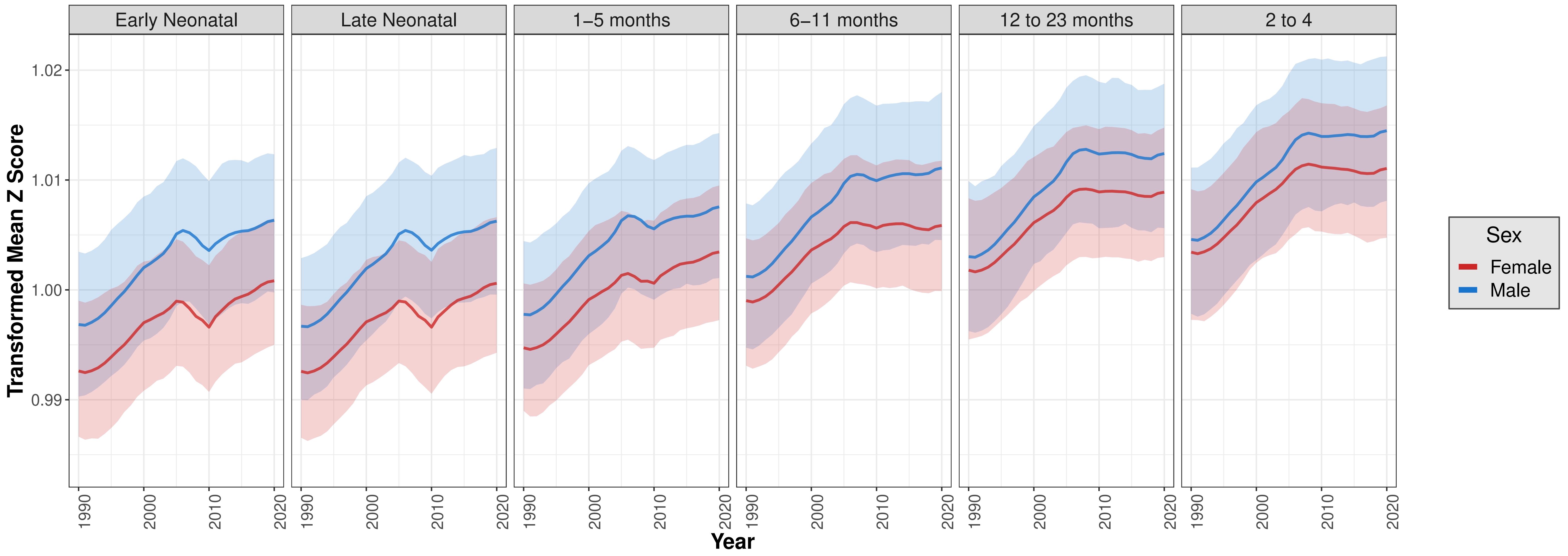


**F**

| Source |
|---|
| No sources for this location |

# **Slovenia** – Underweight (WAZ)

**G:** Overall and Severe Underweight Prevalence

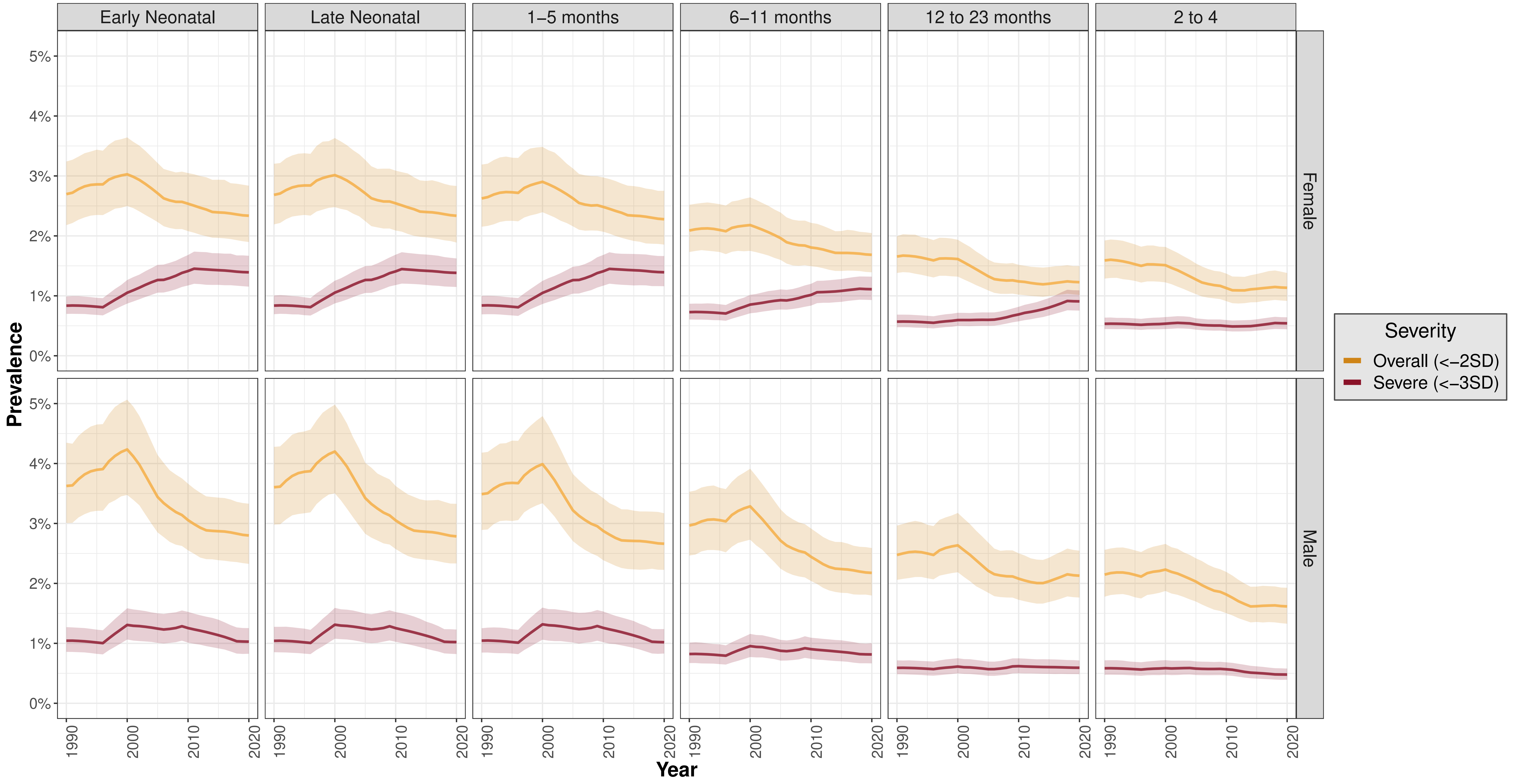


**H:** Transformed Mean Underweight Z Scores

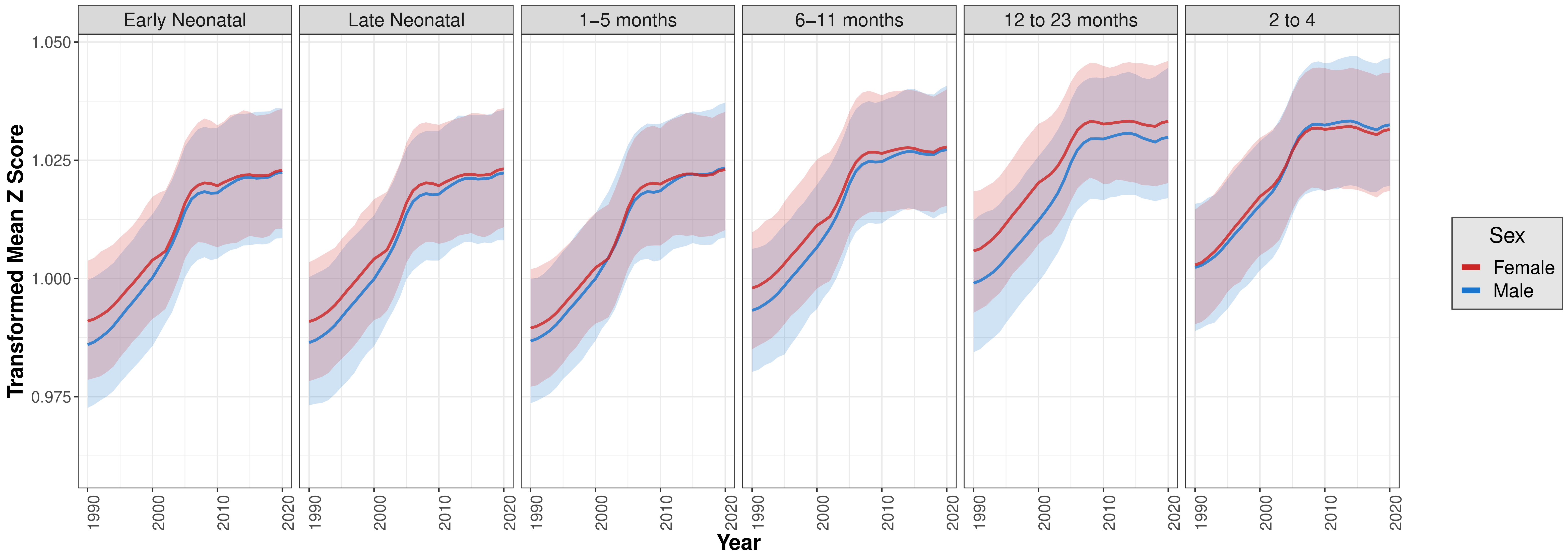


**I**

| **Source** |
| --- |
| No sources for this location |

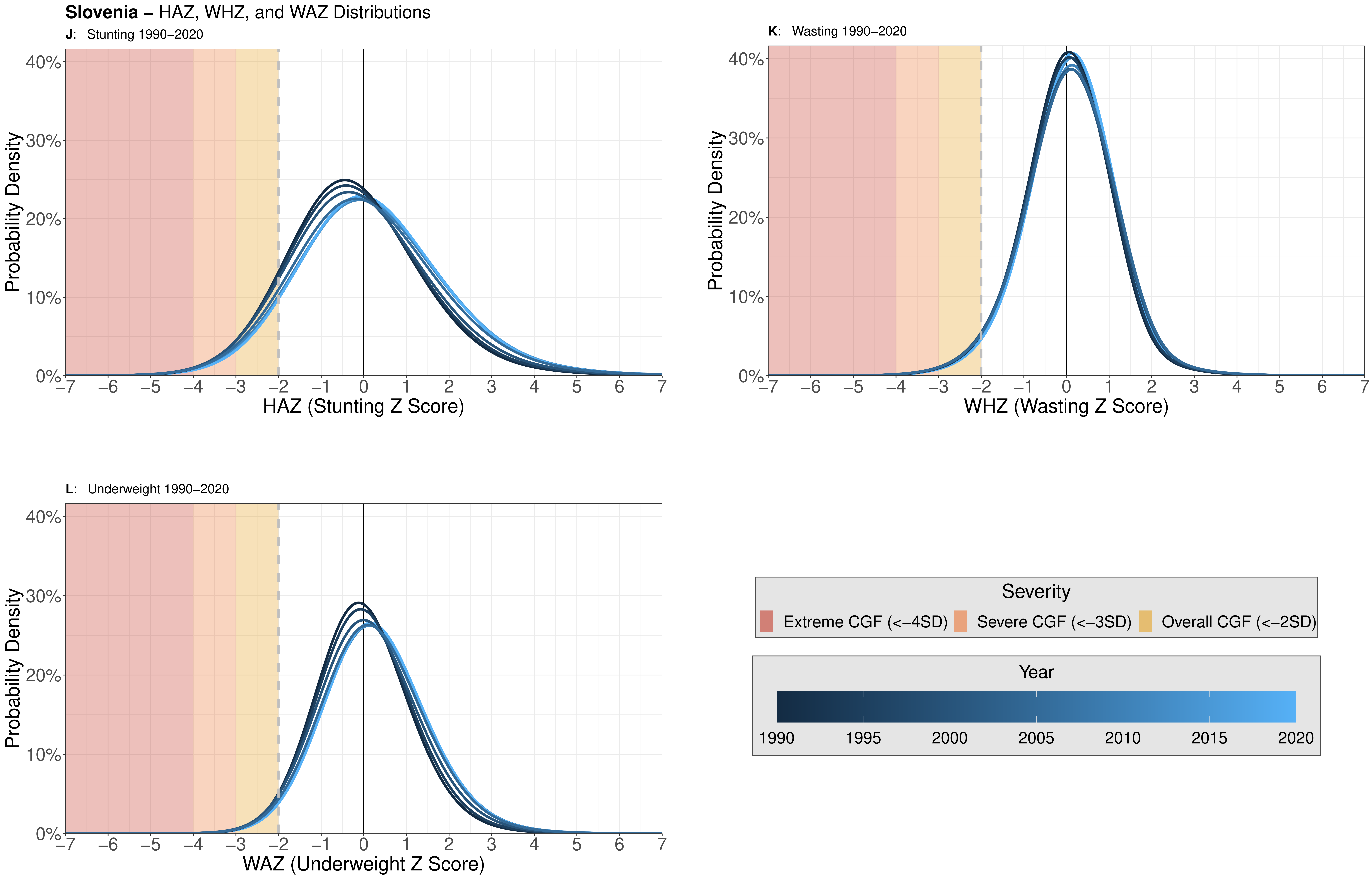
Slovenia – HAZ, WHZ, and WAZ Distributions
J: Stunting 1990–2020
K: Wasting 1990–2020
L: Underweight 1990–2020
Probability Density
40%
30%
20%
10%
0%
−7 −6 −5 −4 −3 −2 −1 0 1 2 3 4 5 6 7
HAZ (Stunting Z Score)
WHZ (Wasting Z Score)
WAZ (Underweight Z Score)
Severity
Extreme CGF (<−4SD)
Severe CGF (<−3SD)
Overall CGF (<−2SD)
Year
1990
1995
2000
2005
2010
2015
2020

# **Belarus** – Stunting (HAZ)

**A**: Overall and Severe Stunting Prevalence

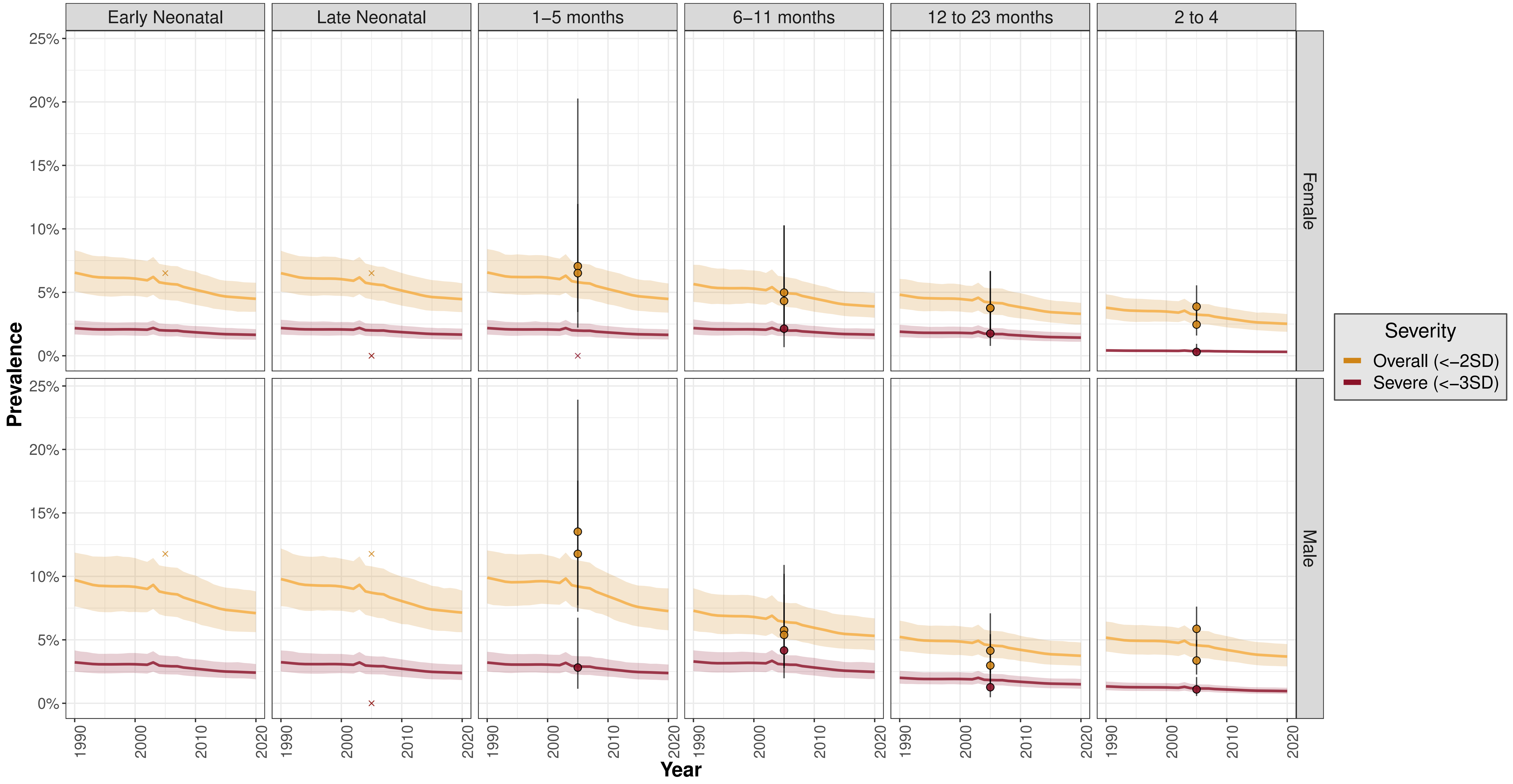


**B**: Transformed Mean Stunting Z Scores

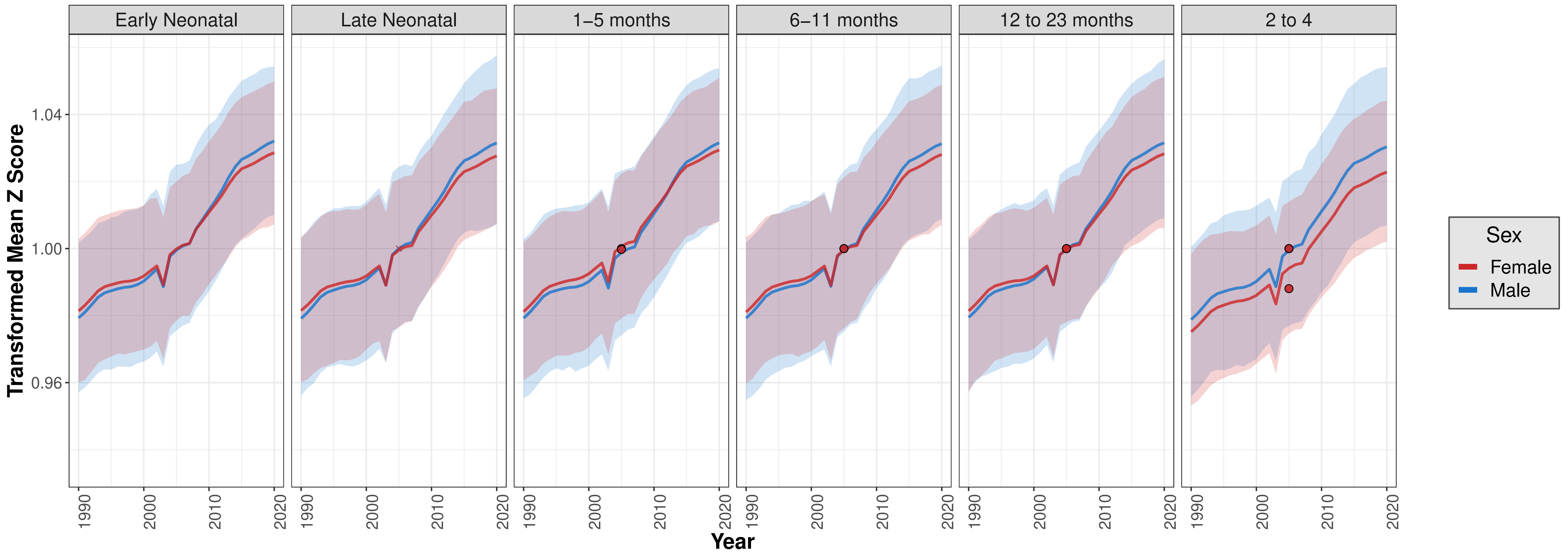


**C**

| Year | Source |
|---|---|
| 2005 | MICS |
| 2005 | WHO CGM Database |

# **Belarus** – Wasting (WHZ)

**D**: Overall and Severe Wasting Prevalence

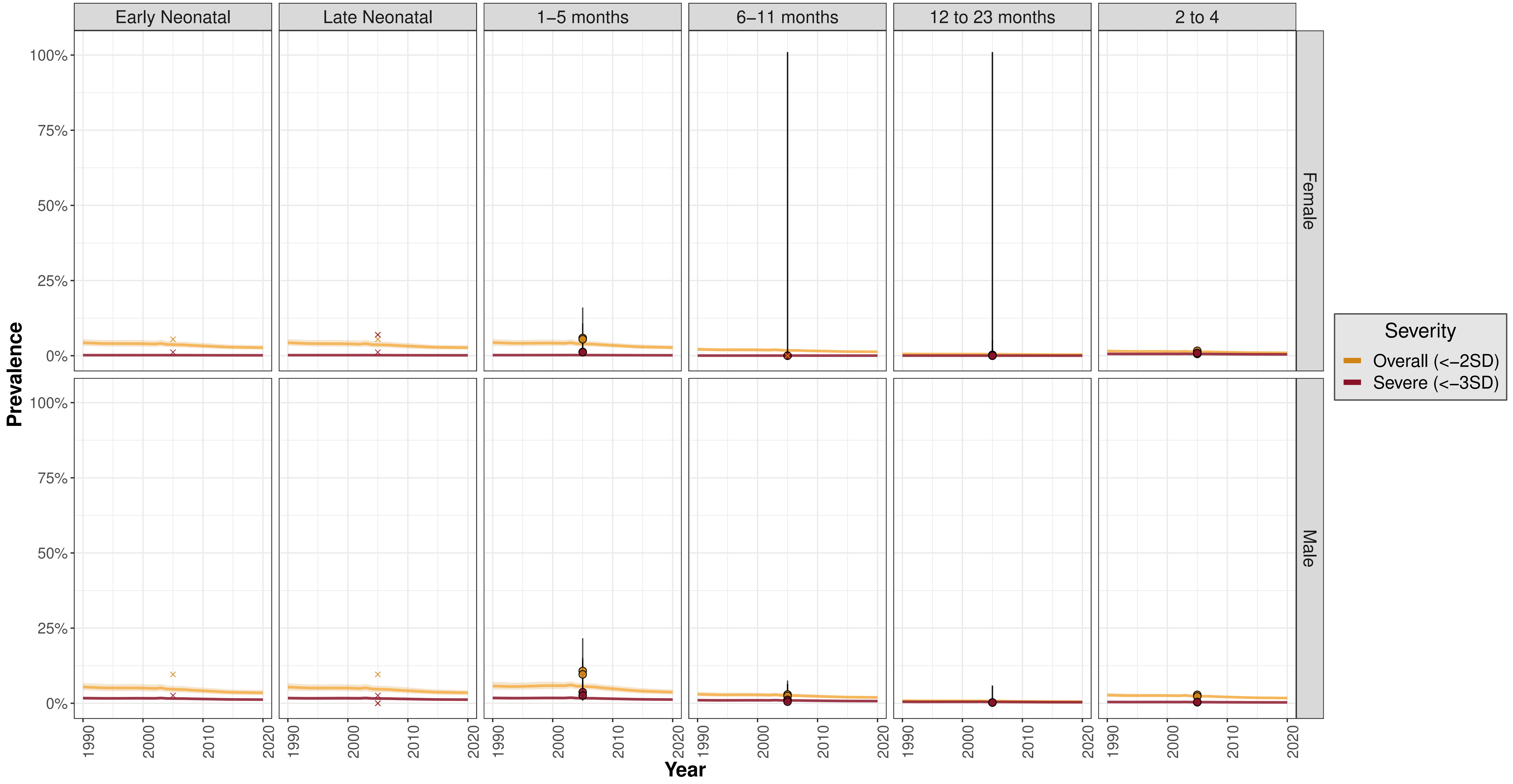


**E**: Transformed Mean Wasting Z Scores

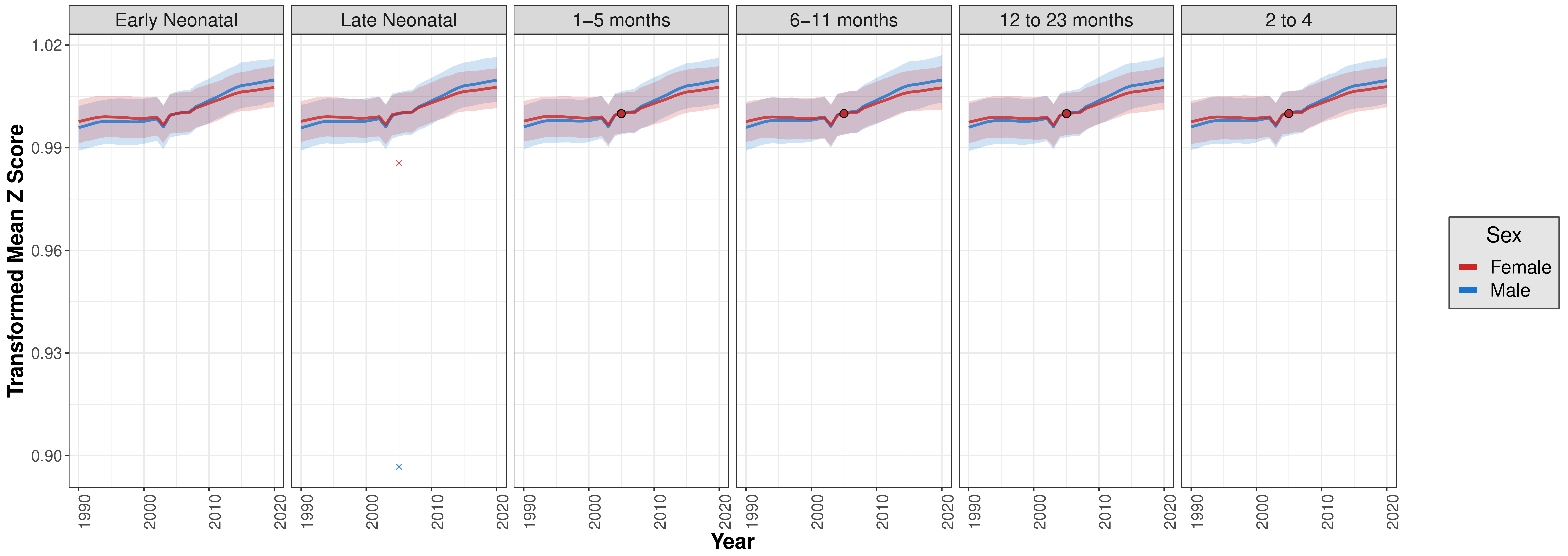


**F**

| Year | Source |
|---|---|
| 2005 | MICS |
| 2005 | WHO CGM Database |

# **Belarus** – Underweight (WAZ)

**G**: Overall and Severe Underweight Prevalence

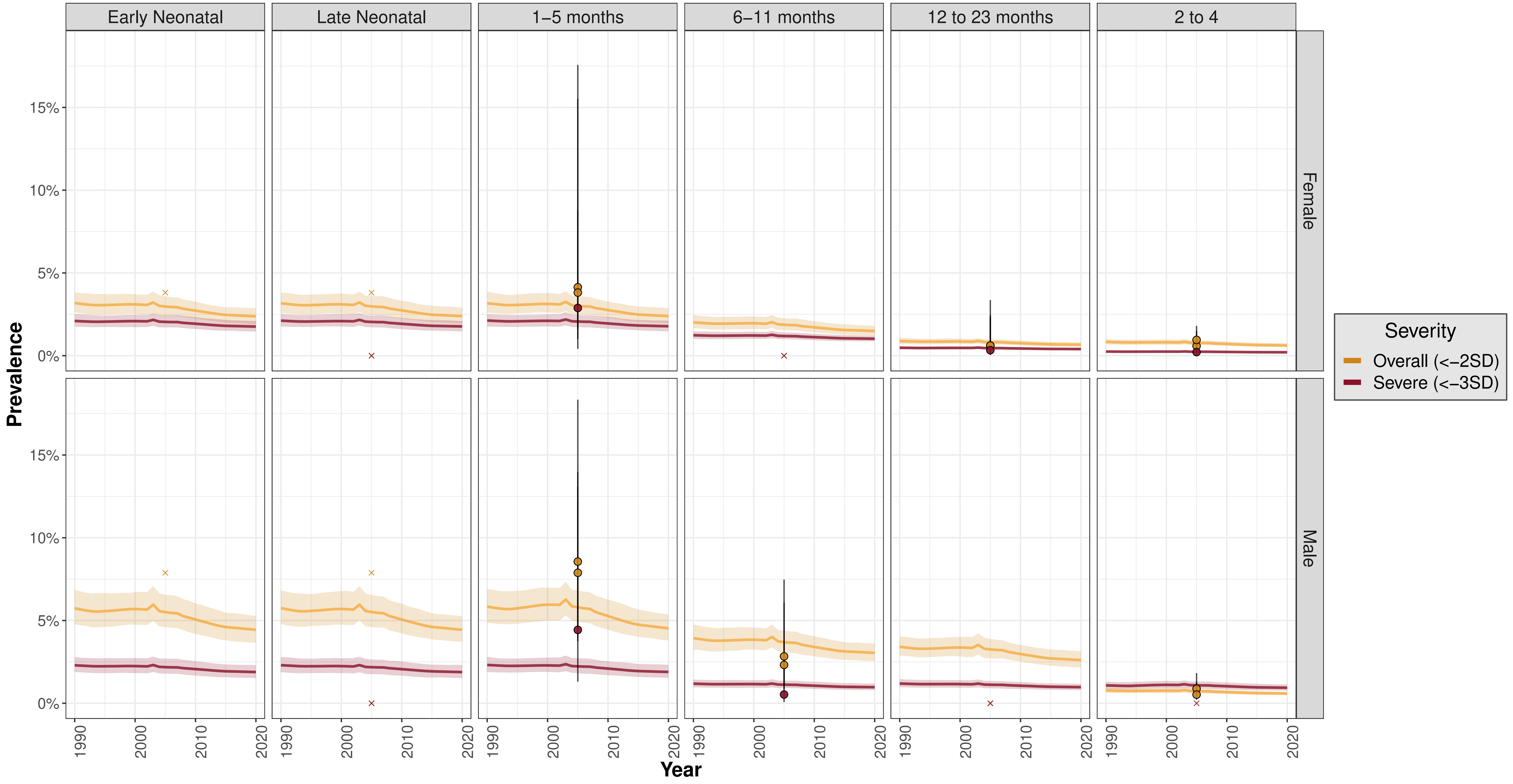


**H**: Transformed Mean Underweight Z Scores

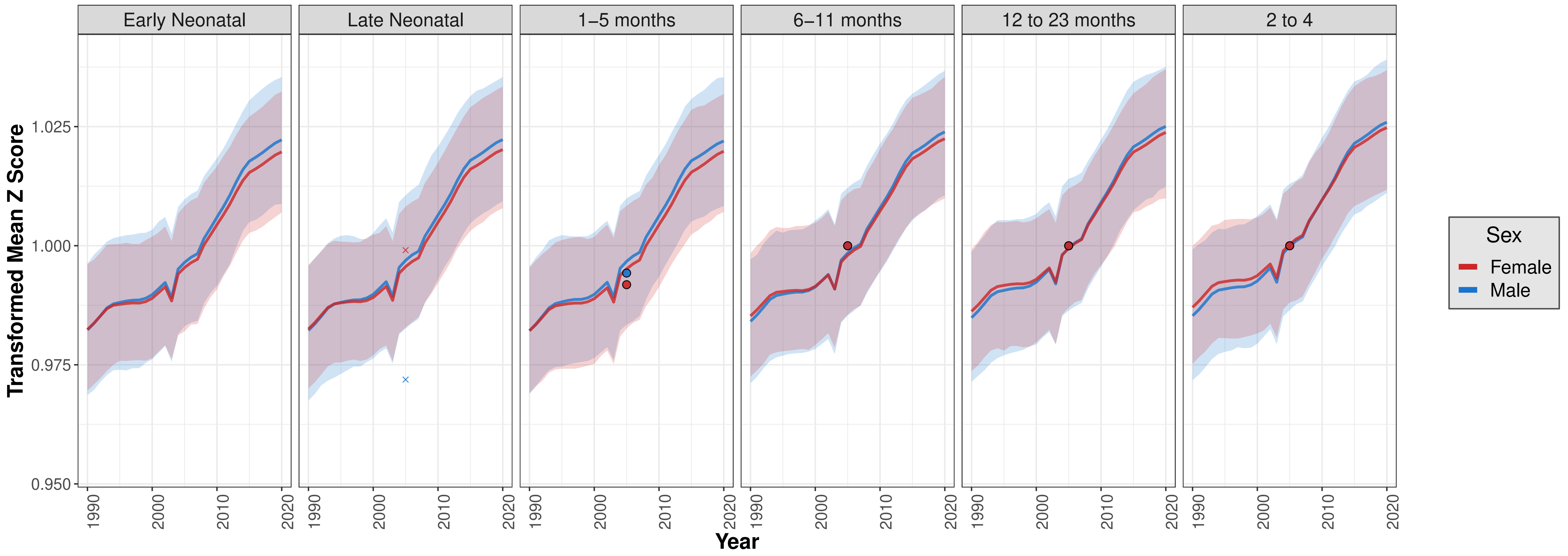


**I**

| Year | Source |
|---|---|
| 2005 | MICS |
| 2005 | WHO CGM Database |

**Belarus** – HAZ, WHZ, and WAZ Distributions

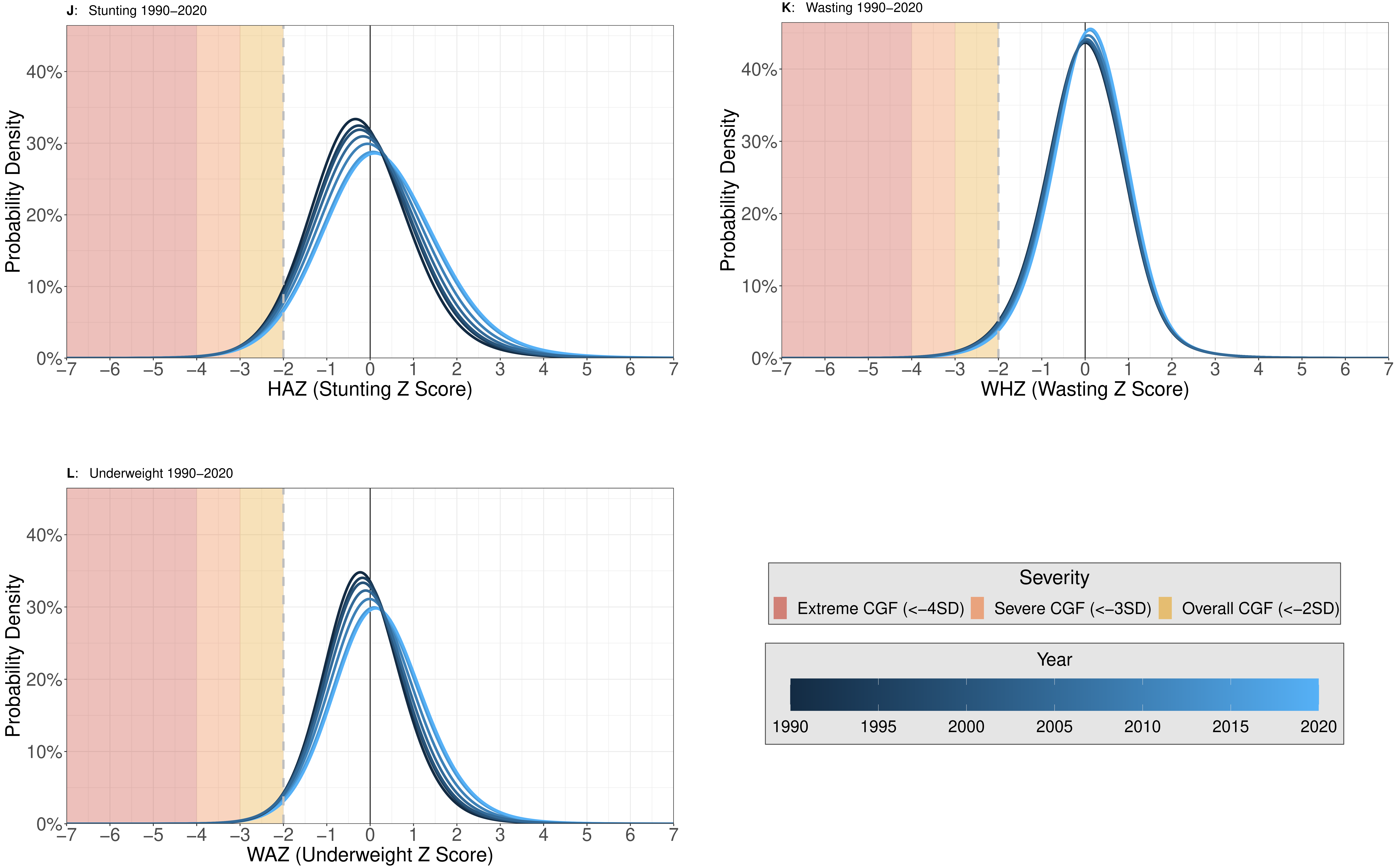

**Estonia** – Stunting (HAZ)

**A**: Overall and Severe Stunting Prevalence

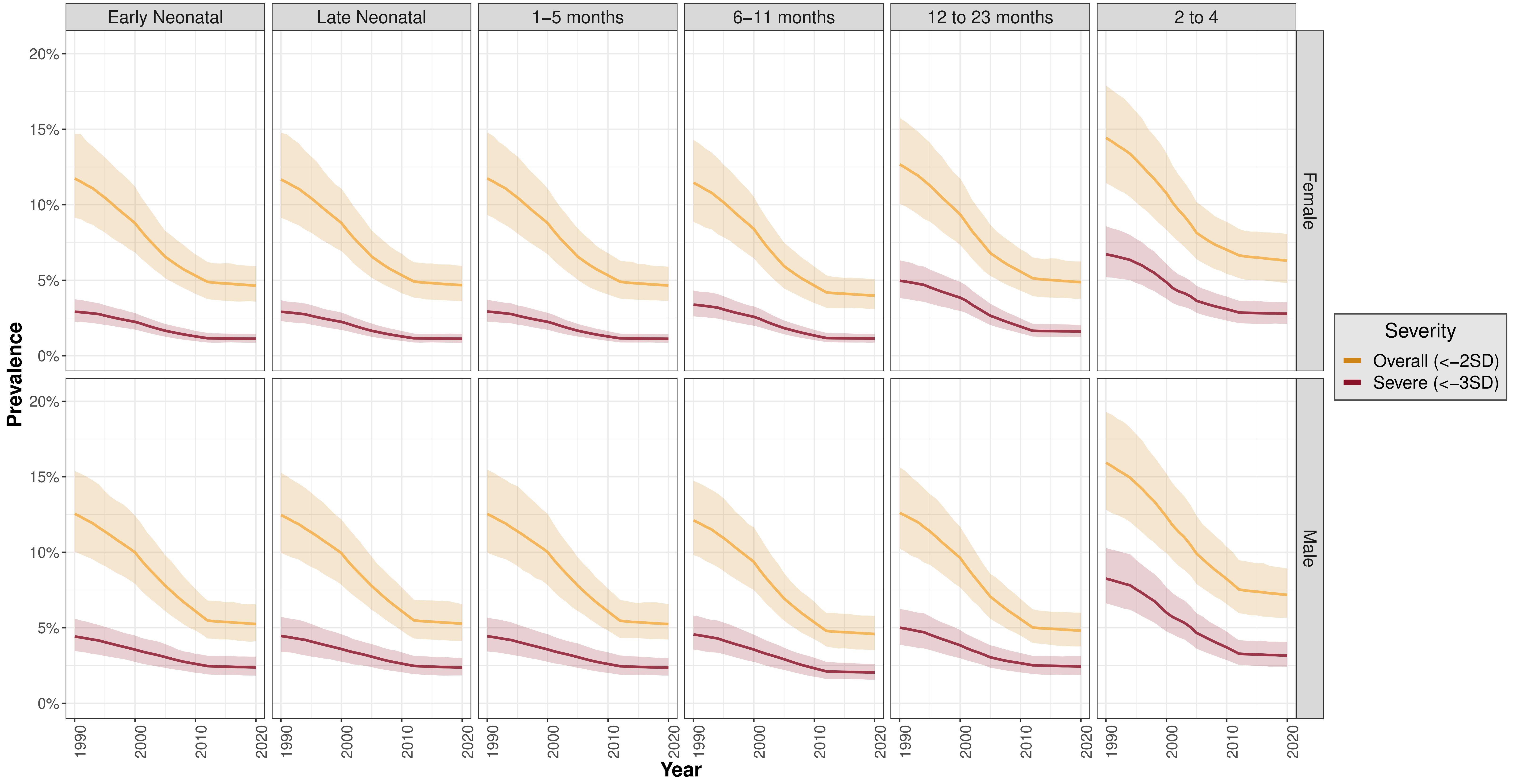


**B**: Transformed Mean Stunting Z Scores

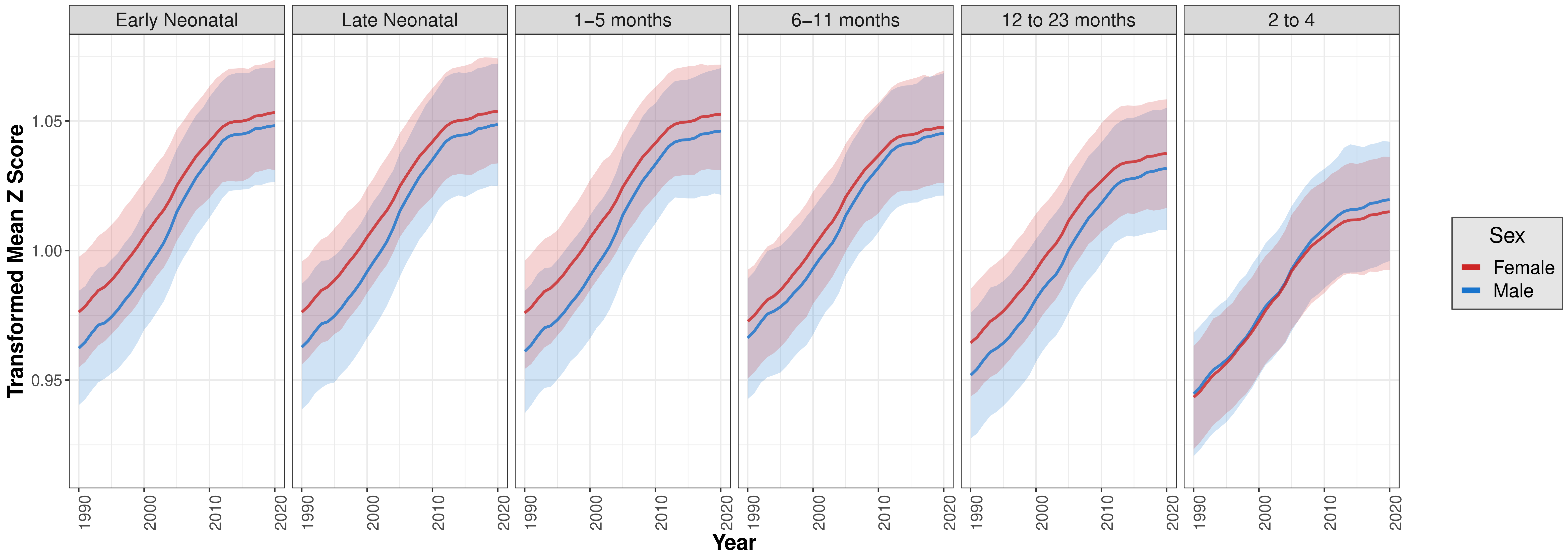


**C**

| Source |
| --- |
| No sources for this location |

# **Estonia** – Wasting (WHZ)

**D:** Overall and Severe Wasting Prevalence

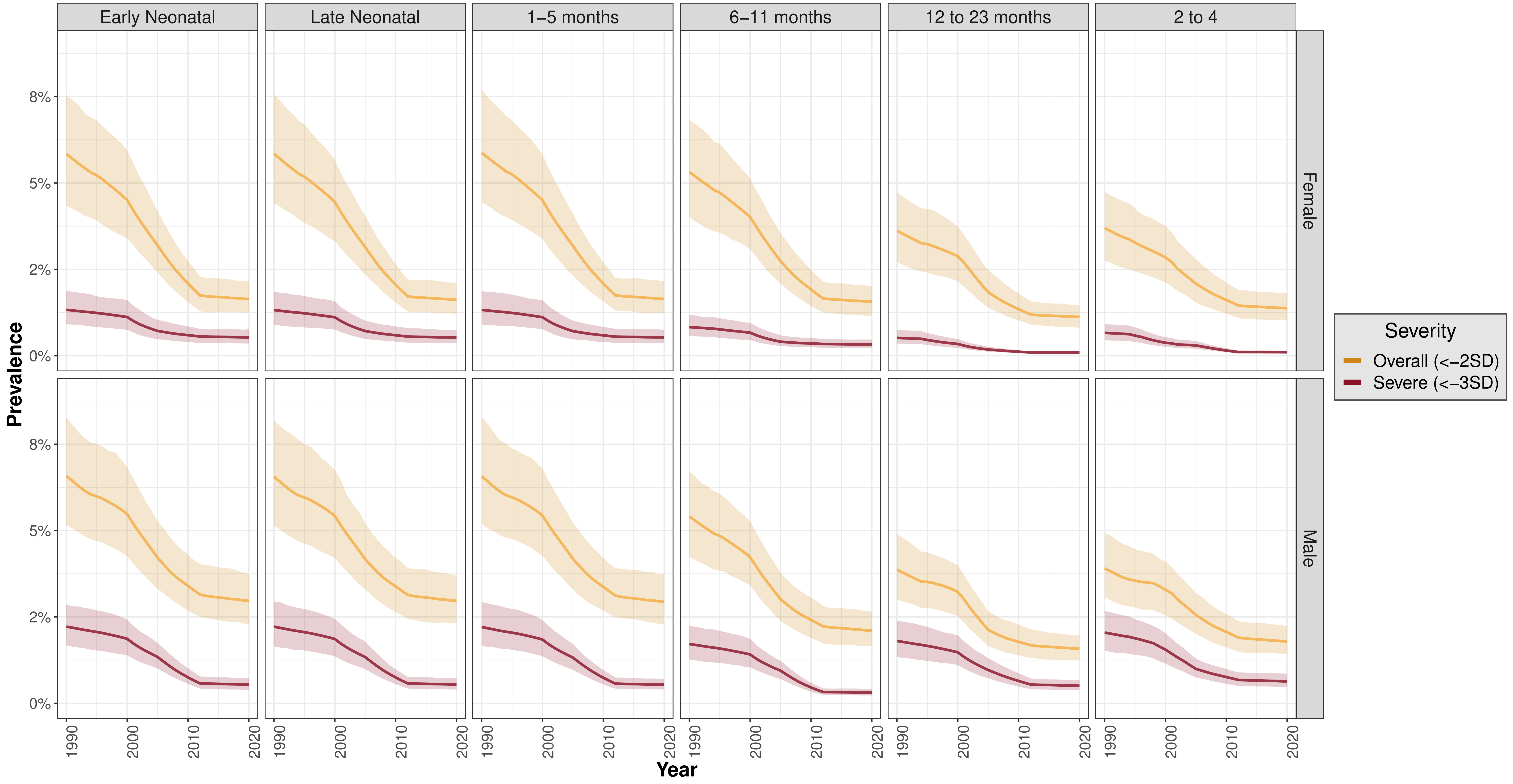


**E:** Transformed Mean Wasting Z Scores

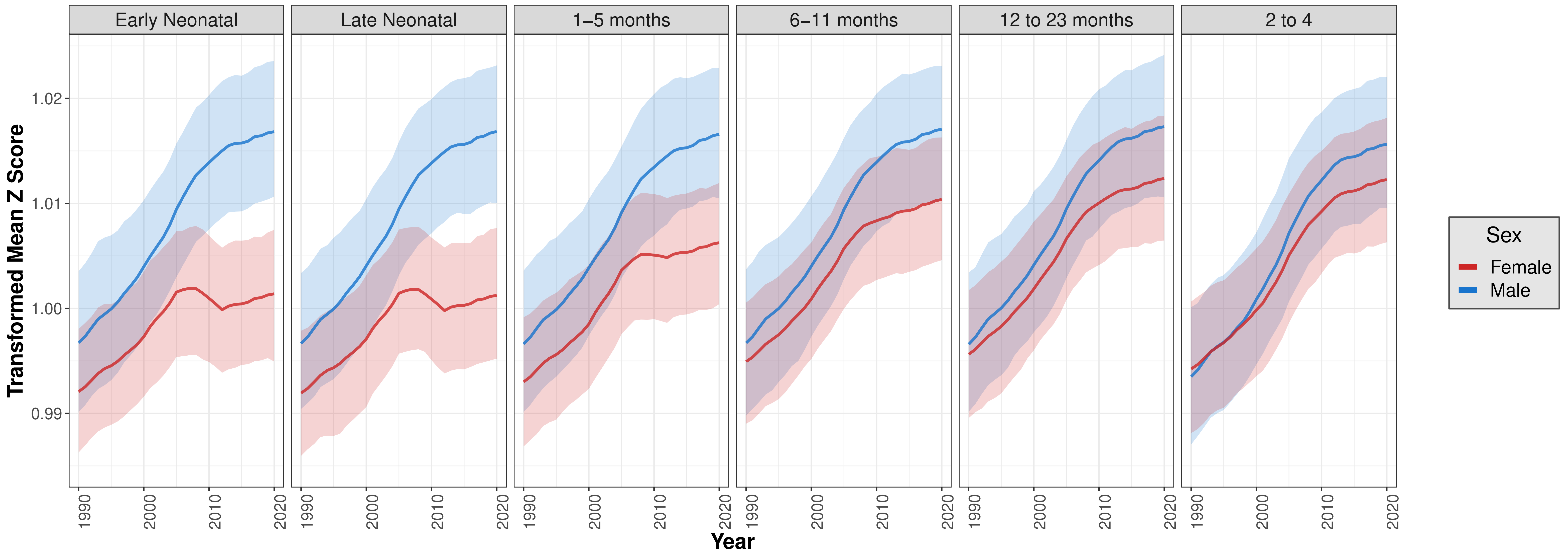


**F**

| Source |
| --- |
| No sources for this location |

## **Estonia** – Underweight (WAZ)

**G**: Overall and Severe Underweight Prevalence

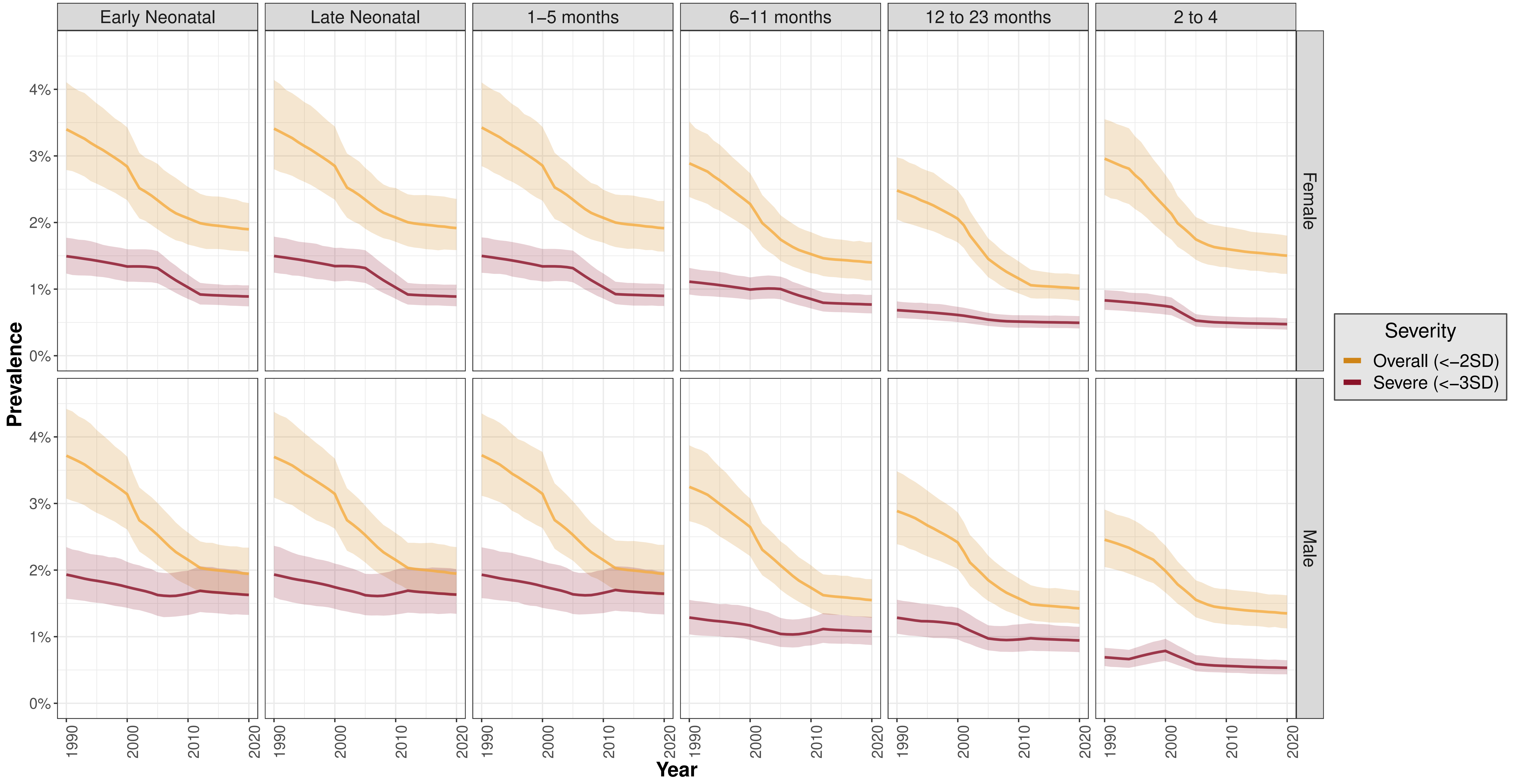


**H**: Transformed Mean Underweight Z Scores

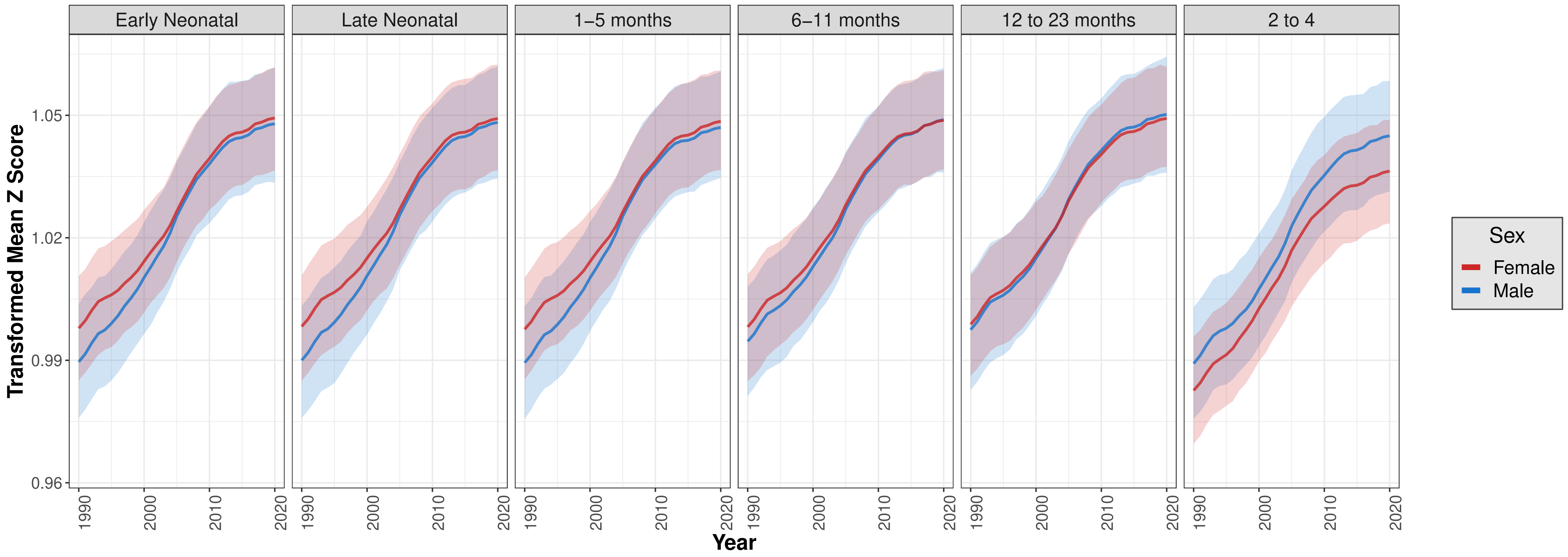


**I**

| Source |
| --- |
| No sources for this location |

**Estonia** – HAZ, WHZ, and WAZ Distributions

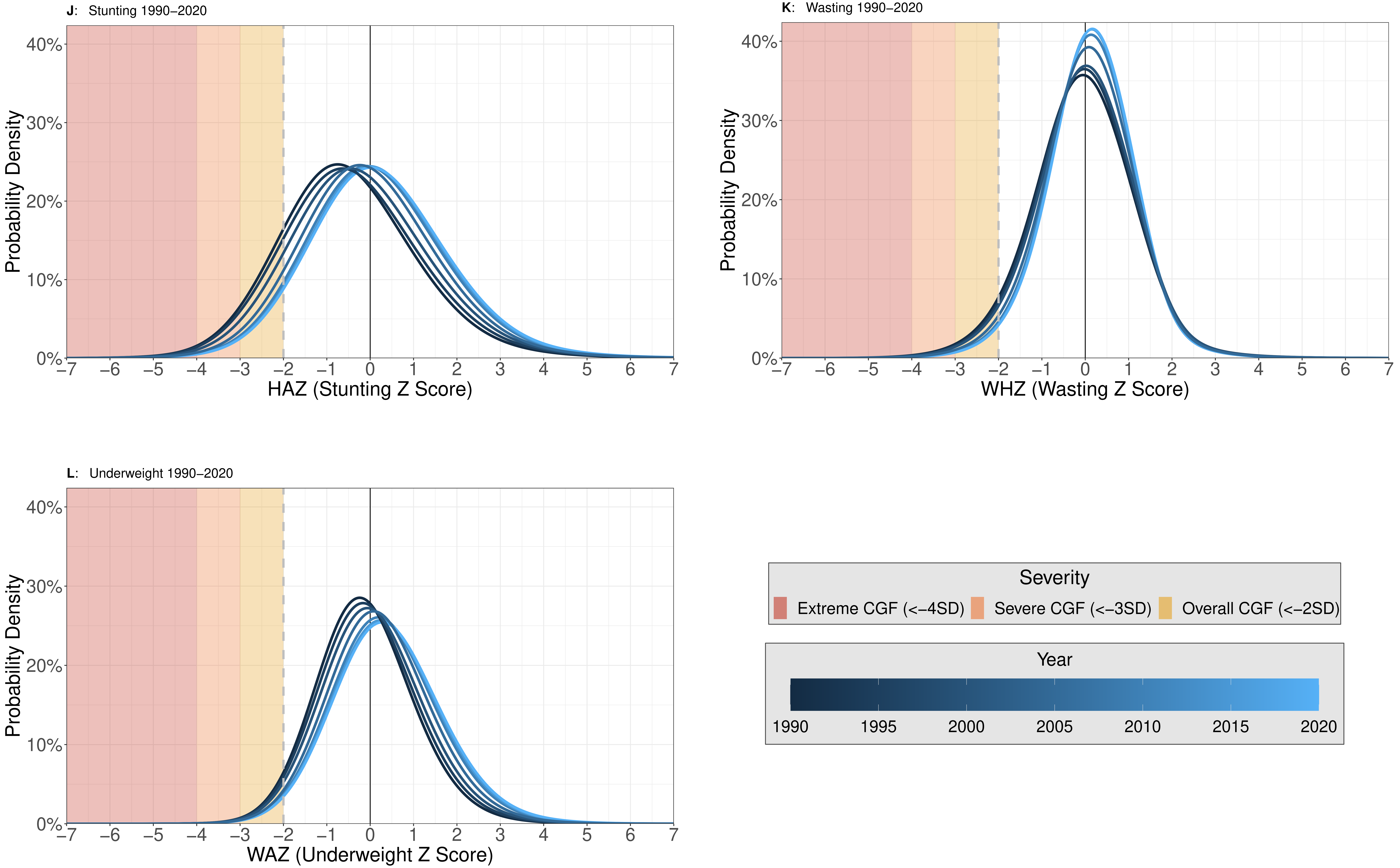

**Latvia** – Stunting (HAZ)

**A**: Overall and Severe Stunting Prevalence

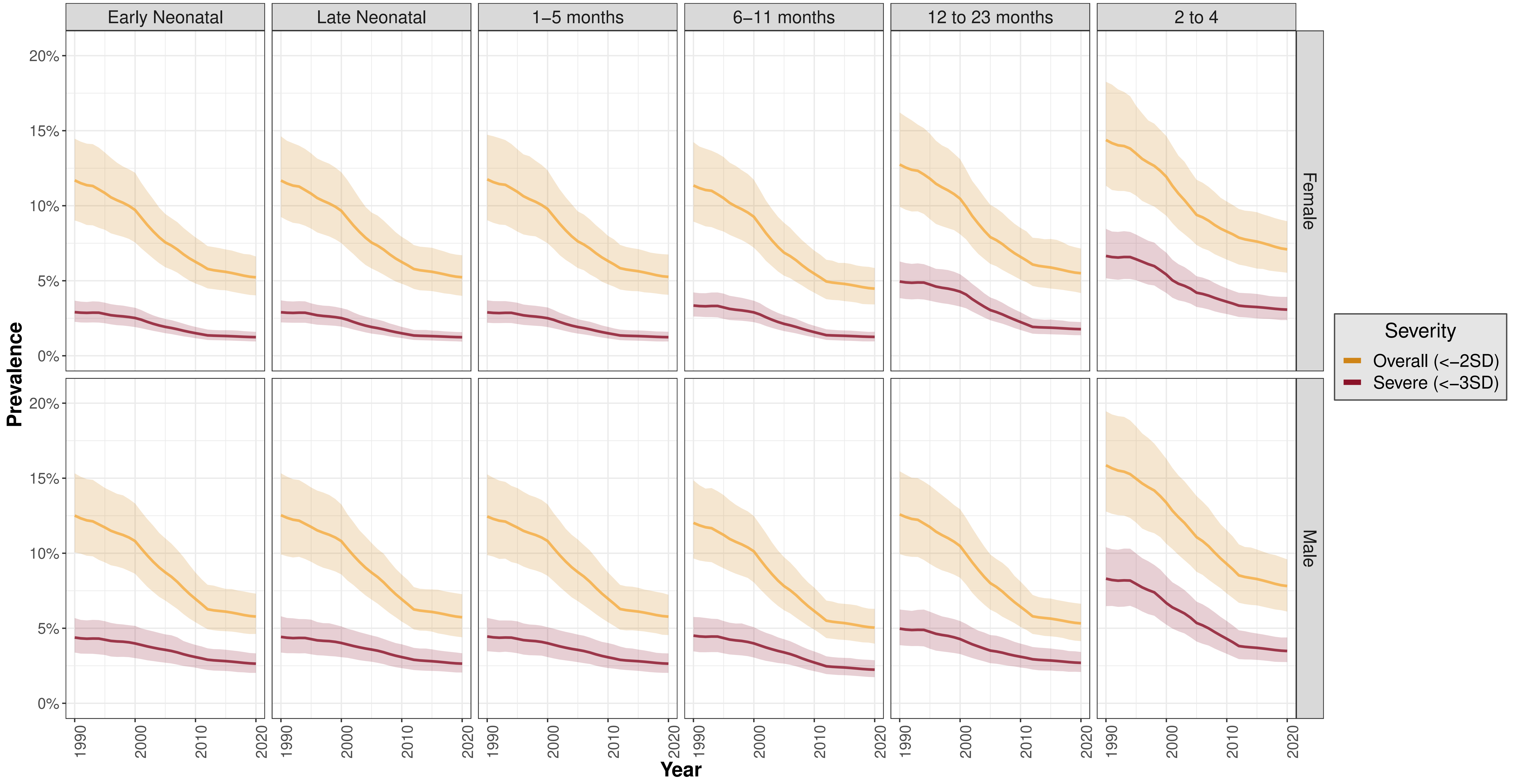


**B**: Transformed Mean Stunting Z Scores

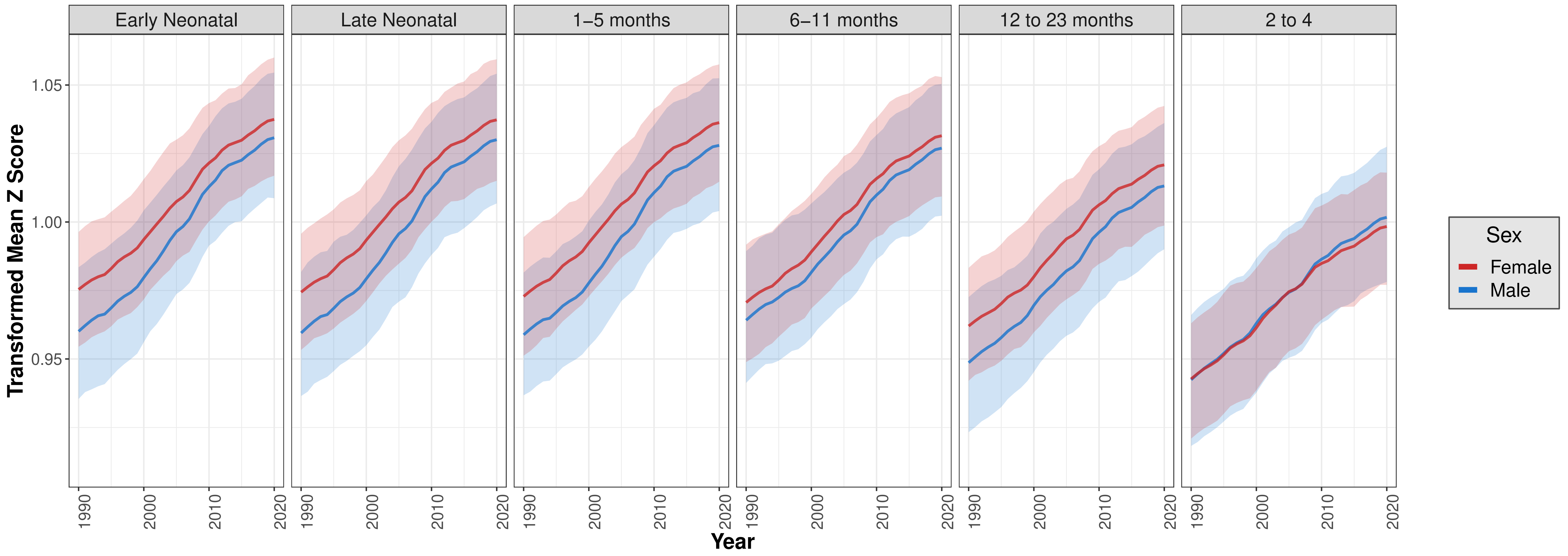


**C**

| Source |
| --- |
| No sources for this location |

# **Latvia** – Wasting (WHZ)

**D**: Overall and Severe Wasting Prevalence

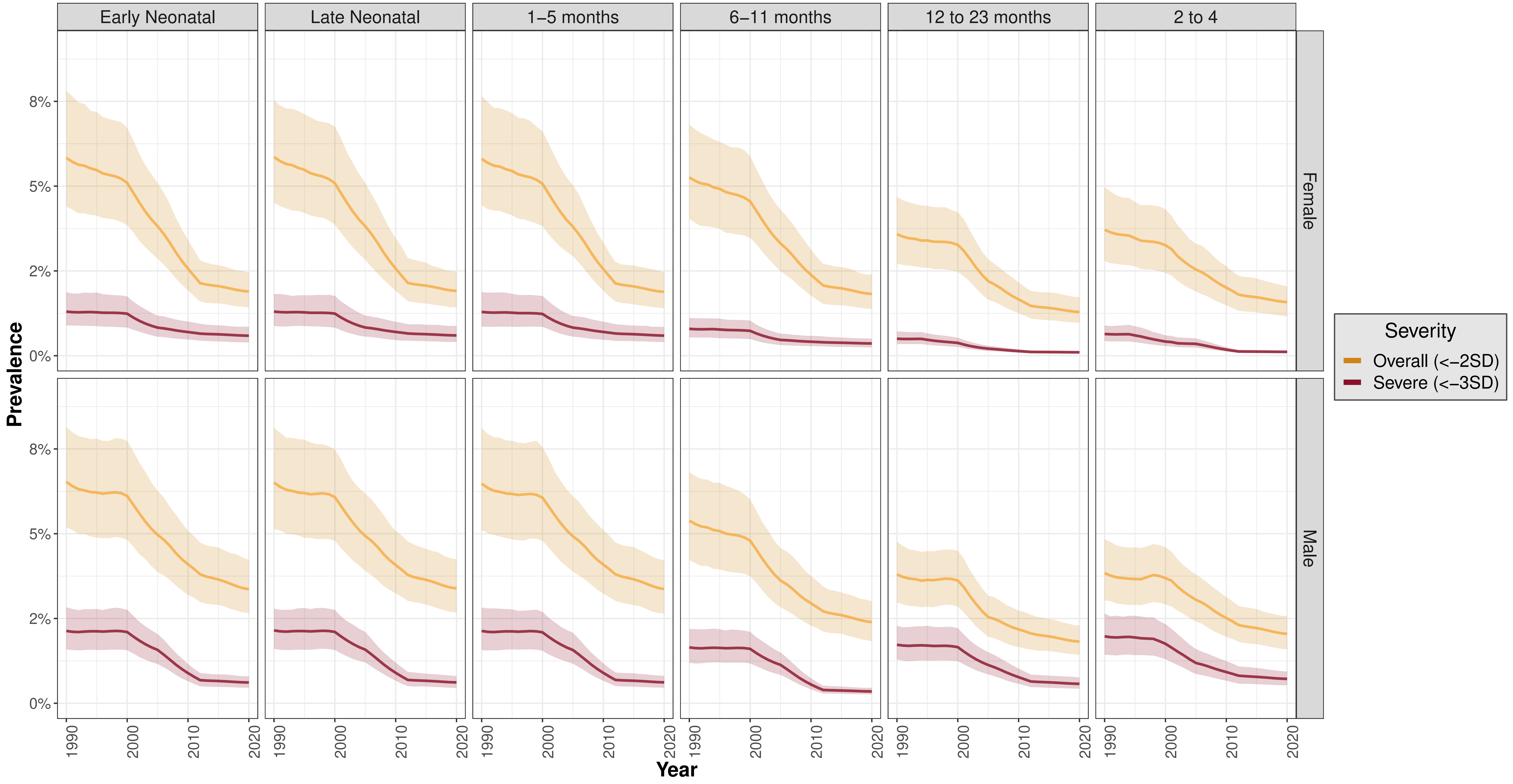


**E**: Transformed Mean Wasting Z Scores

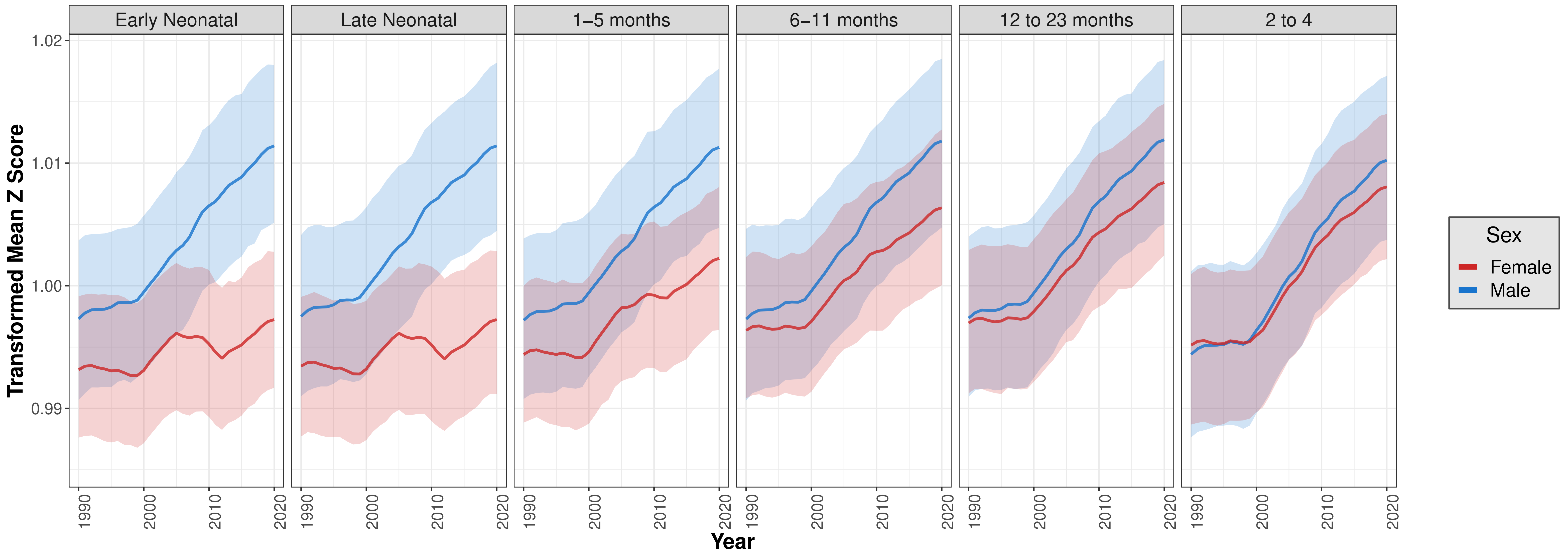


**F**

| Source |
| --- |
| No sources for this location |

**Latvia** – Underweight (WAZ)

**G**: Overall and Severe Underweight Prevalence

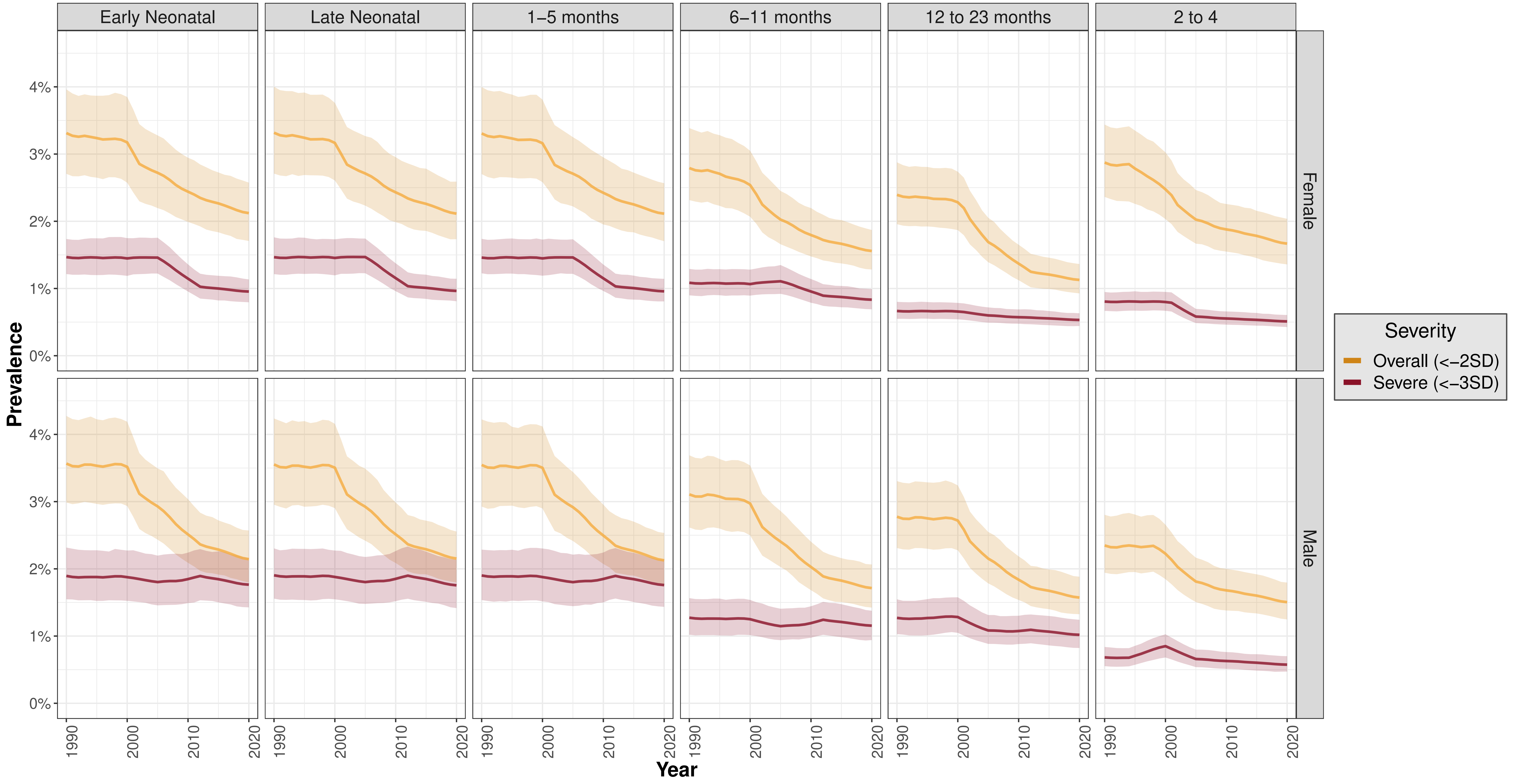


**H**: Transformed Mean Underweight Z Scores

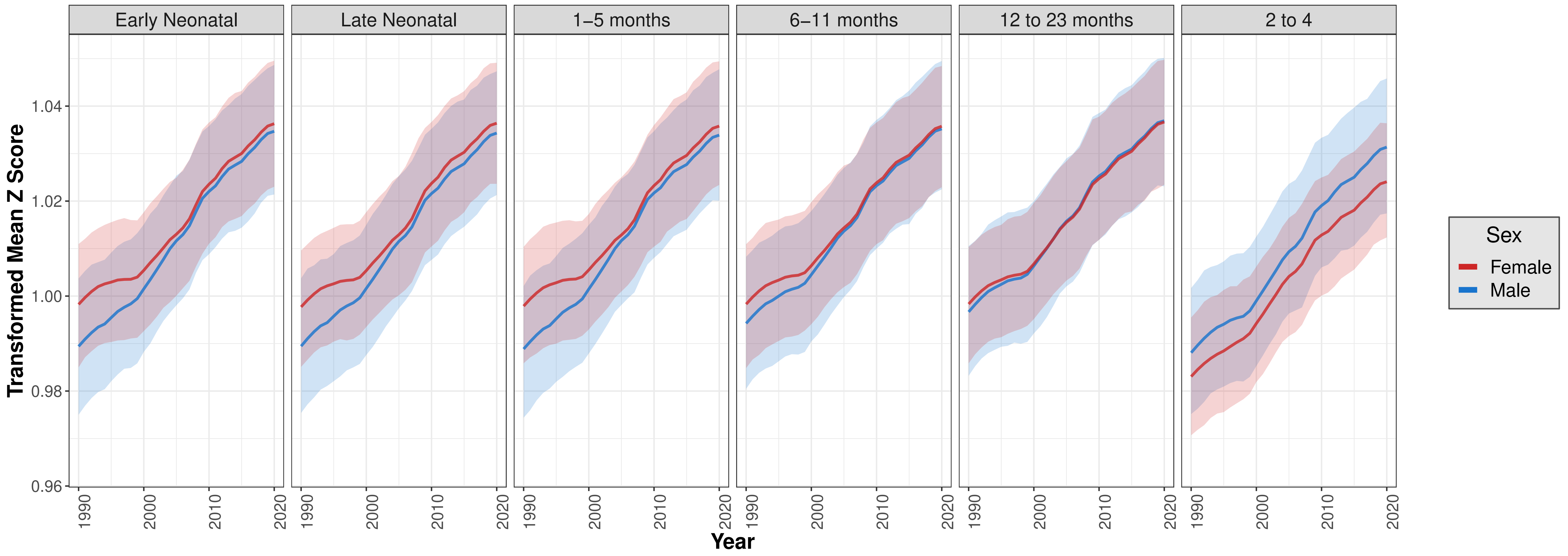


**I**

| Source |
|---|
| No sources for this location |

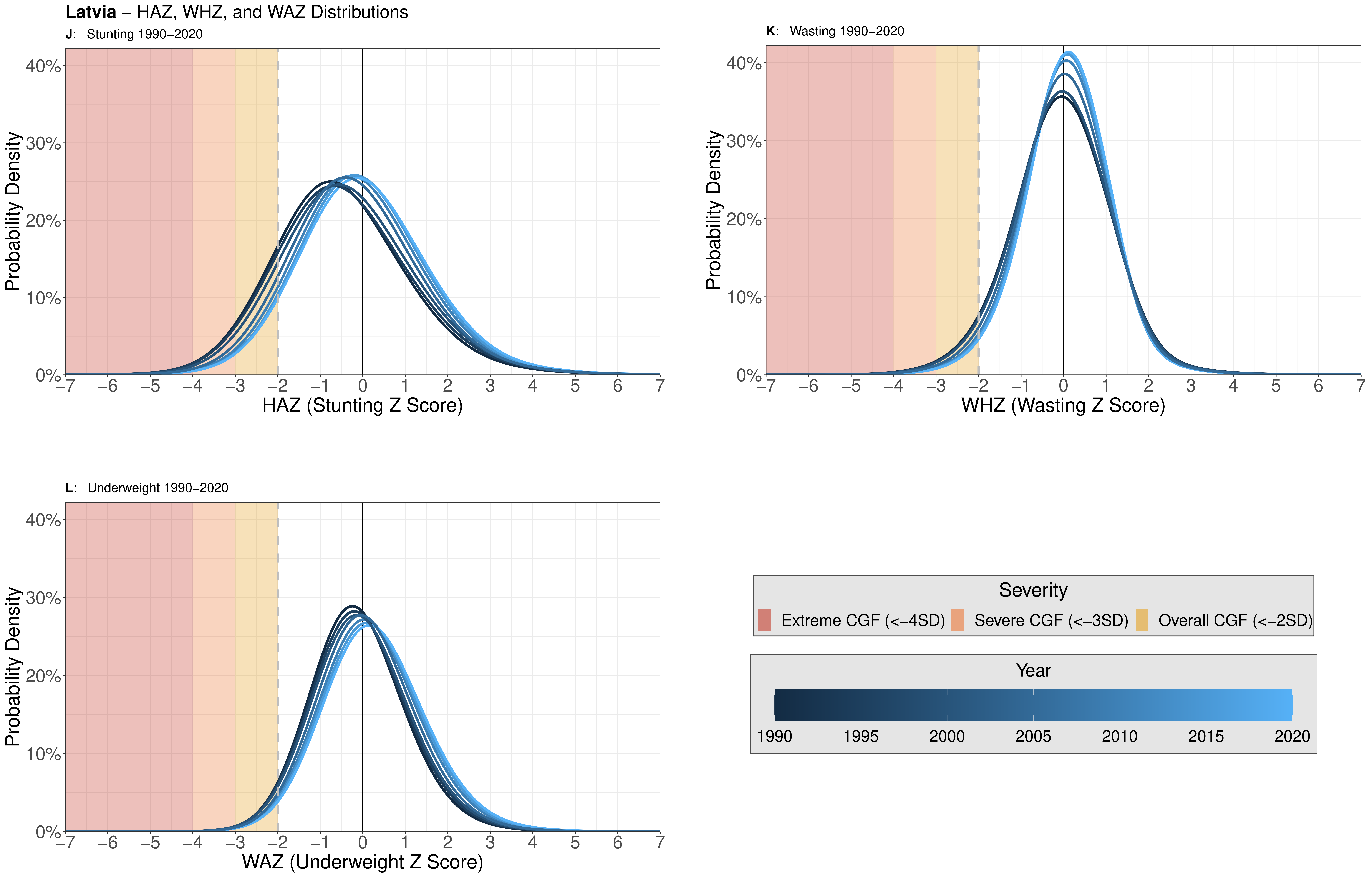

Latvia – HAZ, WHZ, and WAZ Distributions
J: Stunting 1990–2020
K: Wasting 1990–2020
L: Underweight 1990–2020
Probability Density
0%
10%
20%
30%
40%
−7
−6
−5
−4
−3
−2
−1
0
1
2
3
4
5
6
7
HAZ (Stunting Z Score)
WHZ (Wasting Z Score)
WAZ (Underweight Z Score)
Severity
Extreme CGF (<−4SD)
Severe CGF (<−3SD)
Overall CGF (<−2SD)
Year
1990
1995
2000
2005
2010
2015
2020

# **Lithuania** – Stunting (HAZ)

**A**: Overall and Severe Stunting Prevalence

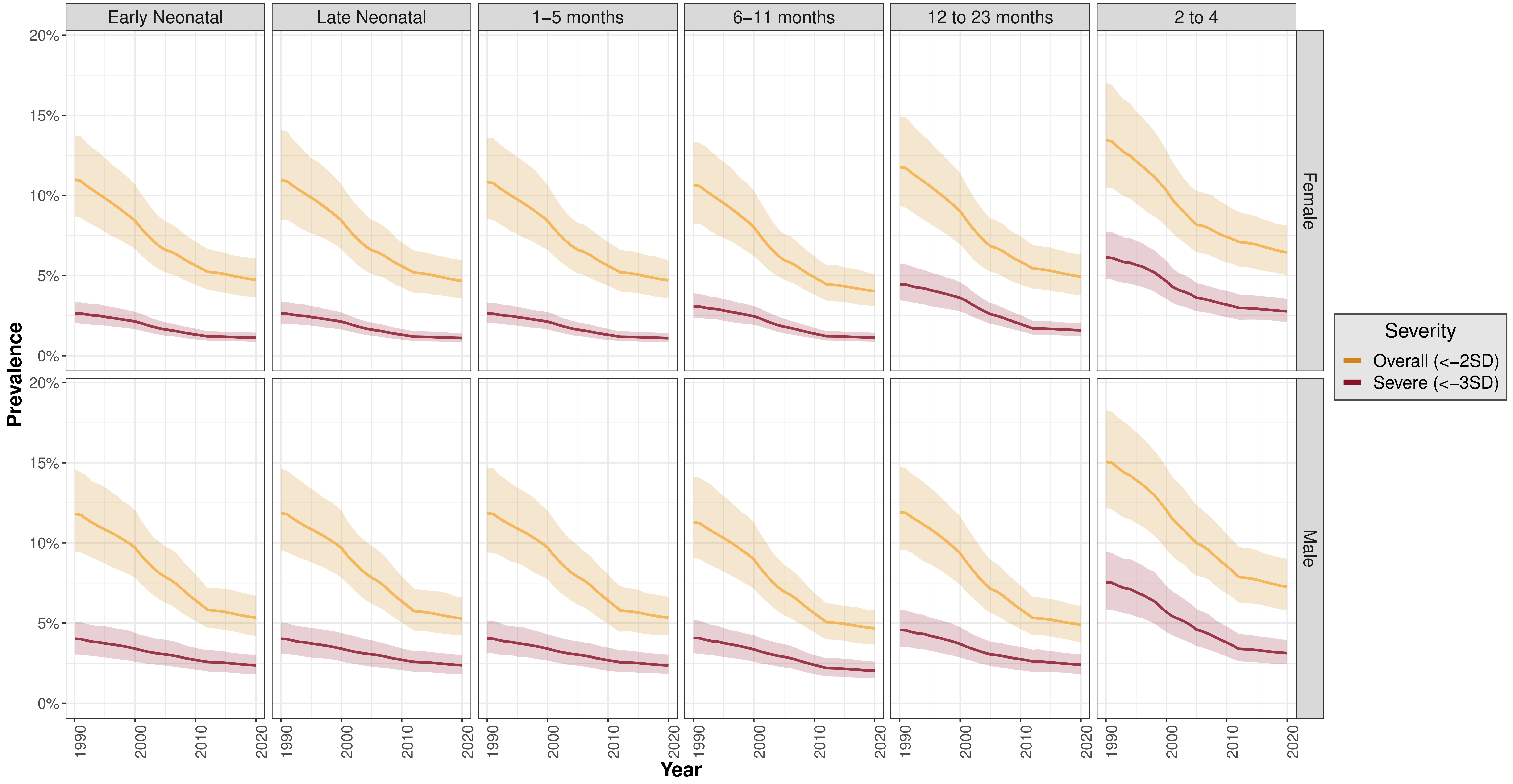


**B**: Transformed Mean Stunting Z Scores

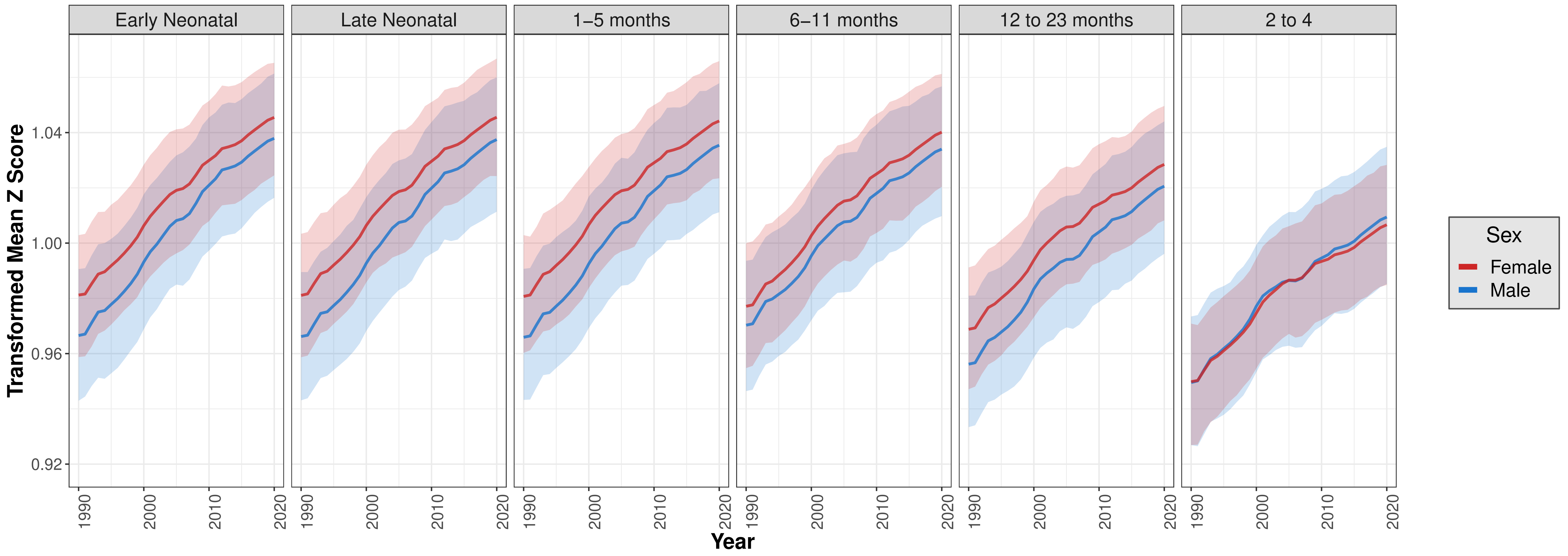


**C**

| Source |
| --- |
| No sources for this location |

# **Lithuania** – Wasting (WHZ)

**D**: Overall and Severe Wasting Prevalence

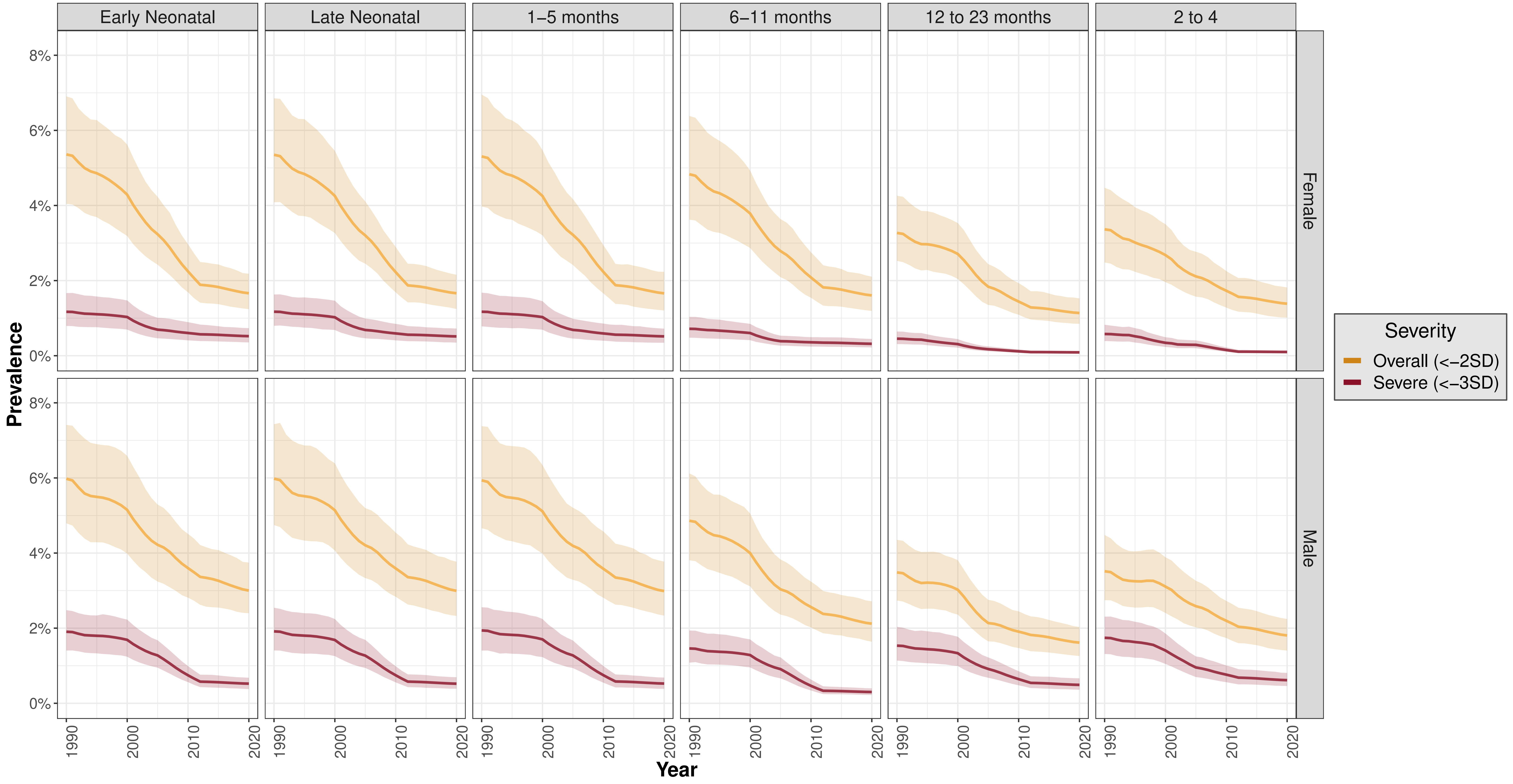


**E**: Transformed Mean Wasting Z Scores

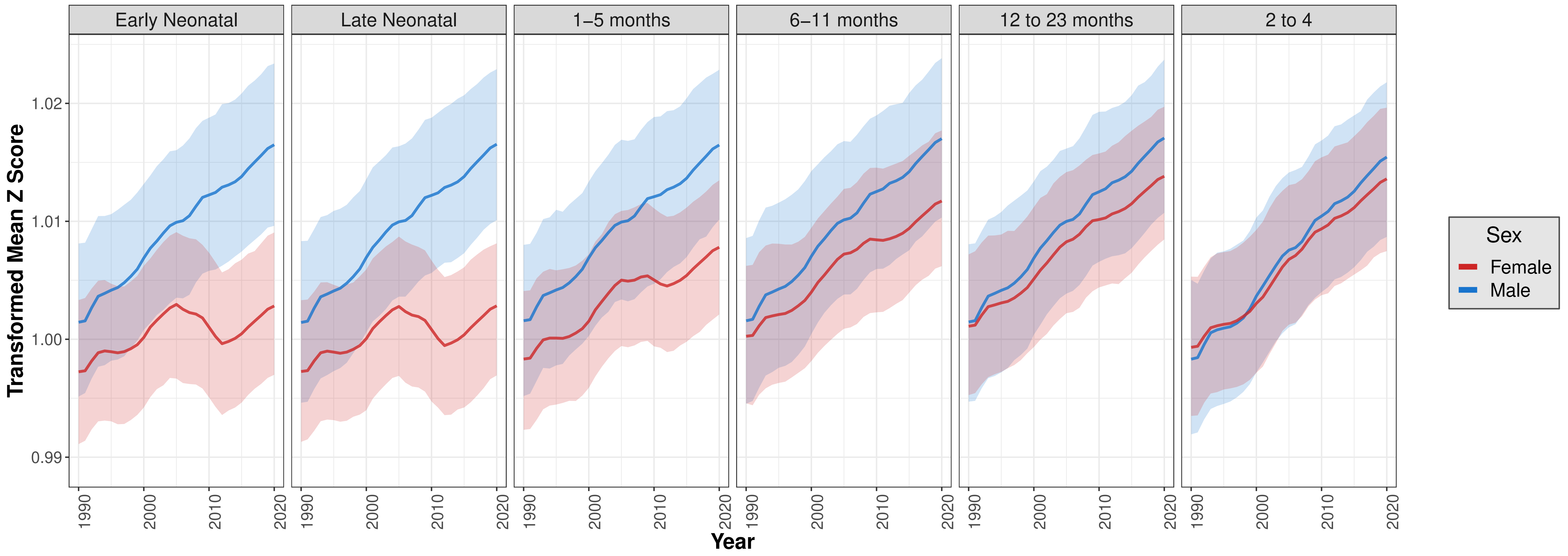


**F**

| Source |
|---|
| No sources for this location |

## **Lithuania** – Underweight (WAZ)

**G**: Overall and Severe Underweight Prevalence

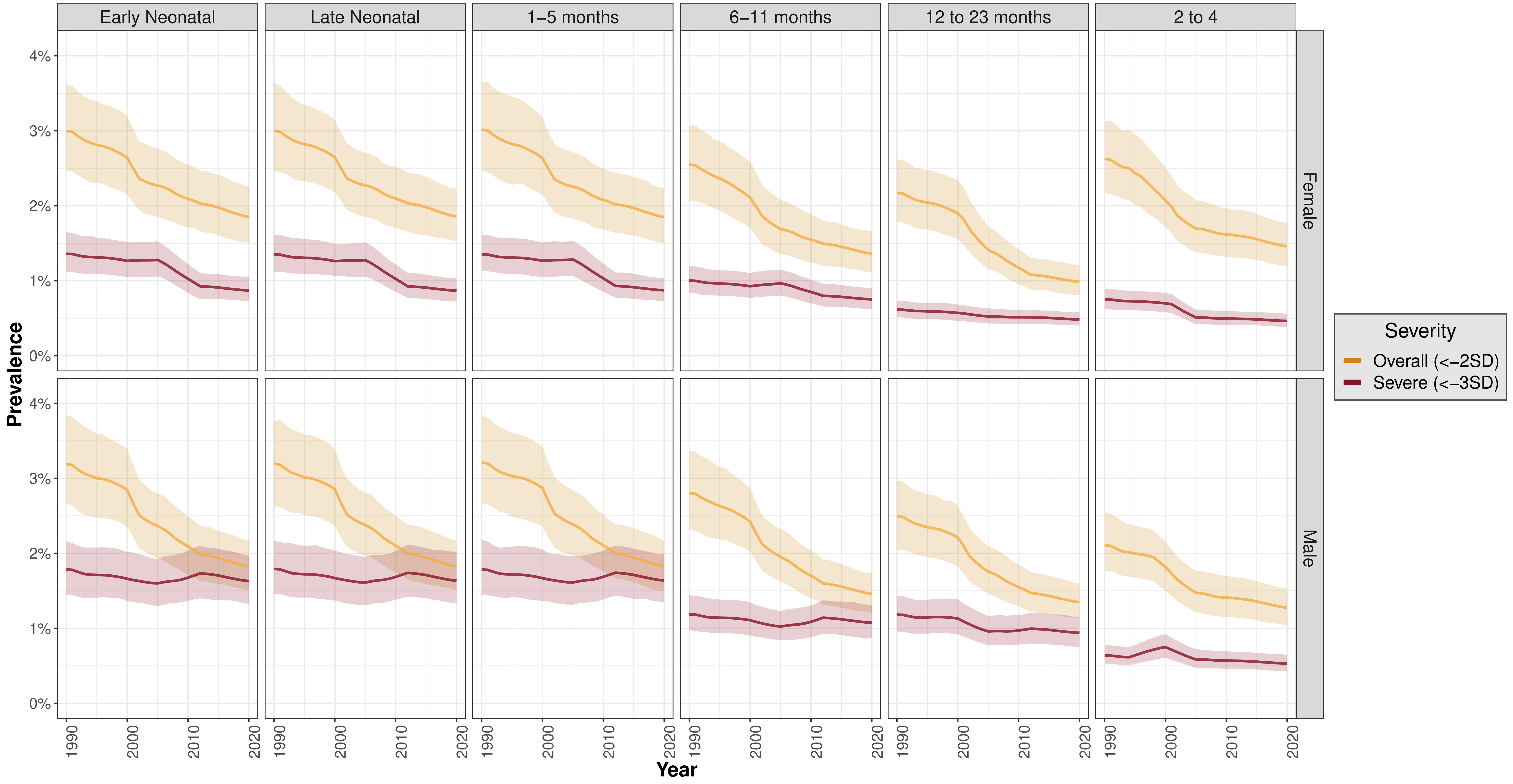


**H**: Transformed Mean Underweight Z Scores

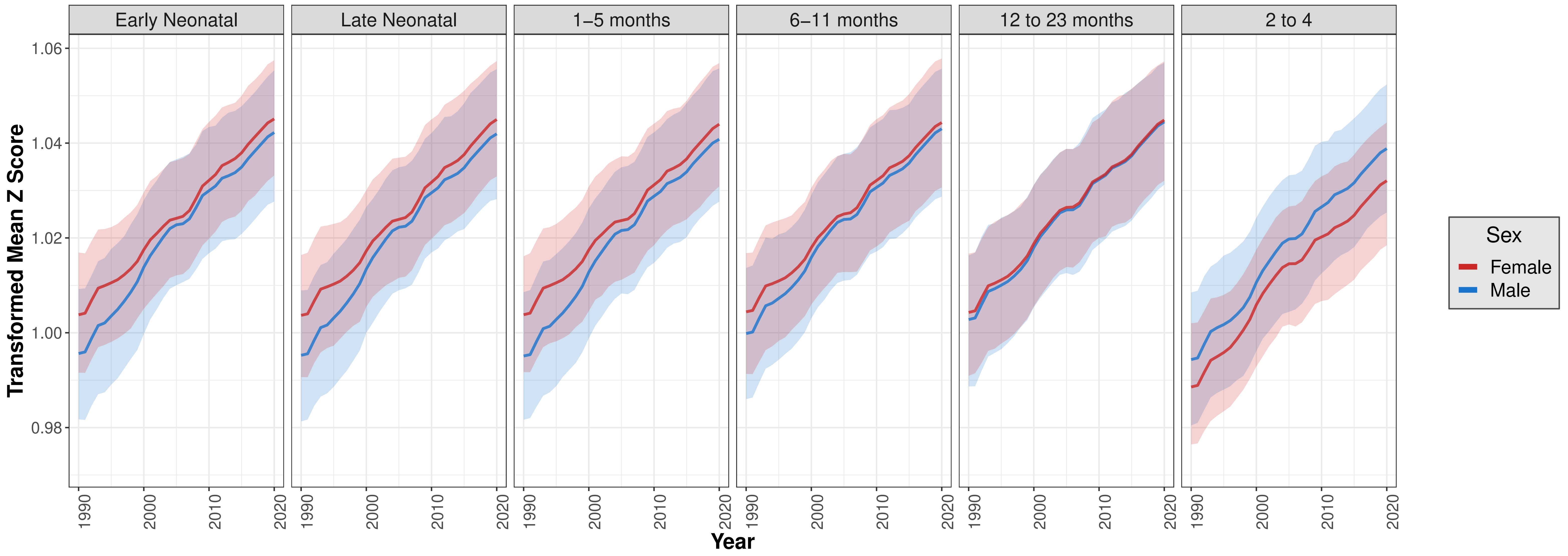


**I**

| Source |
| --- |
| No sources for this location |

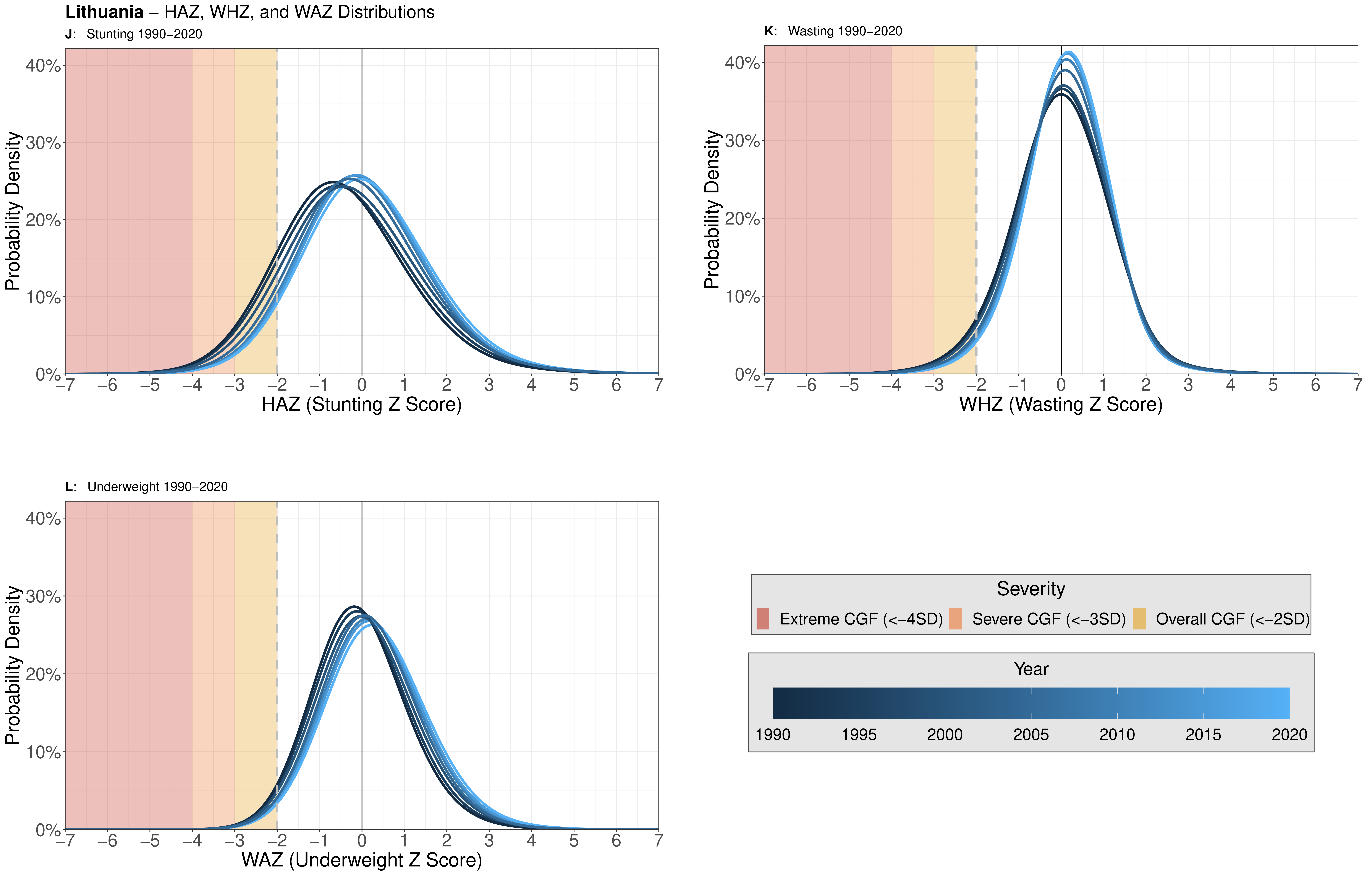

Lithuania – HAZ, WHZ, and WAZ Distributions
J: Stunting 1990–2020
K: Wasting 1990–2020
L: Underweight 1990–2020
Probability Density
0%
10%
20%
30%
40%
−7 −6 −5 −4 −3 −2 −1 0 1 2 3 4 5 6 7
HAZ (Stunting Z Score)
WHZ (Wasting Z Score)
WAZ (Underweight Z Score)
Severity
Extreme CGF (<−4SD)
Severe CGF (<−3SD)
Overall CGF (<−2SD)
Year
1990
1995
2000
2005
2010
2015
2020

# **Republic of Moldova** – Stunting (HAZ)

**A**: Overall and Severe Stunting Prevalence

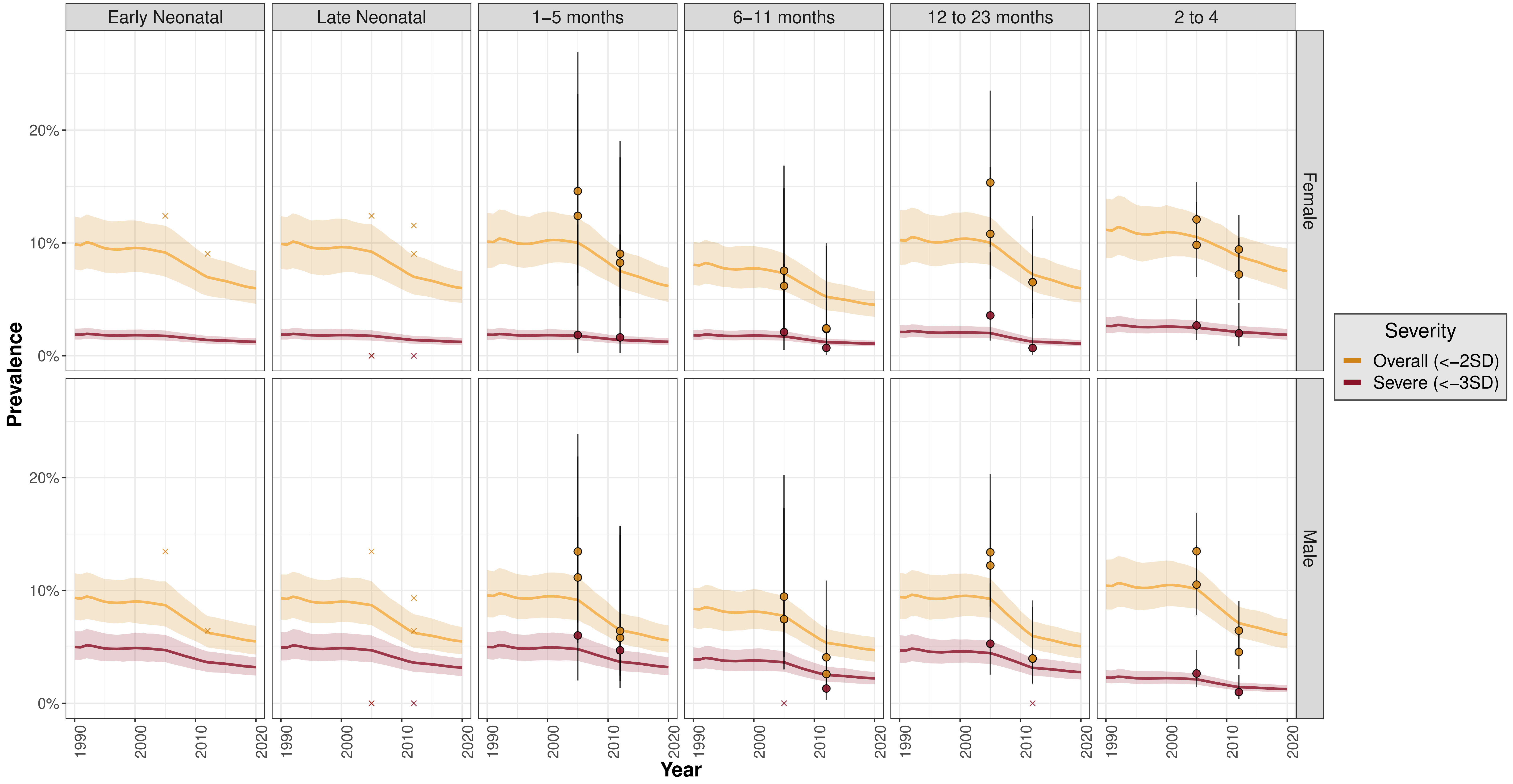


**B**: Transformed Mean Stunting Z Scores

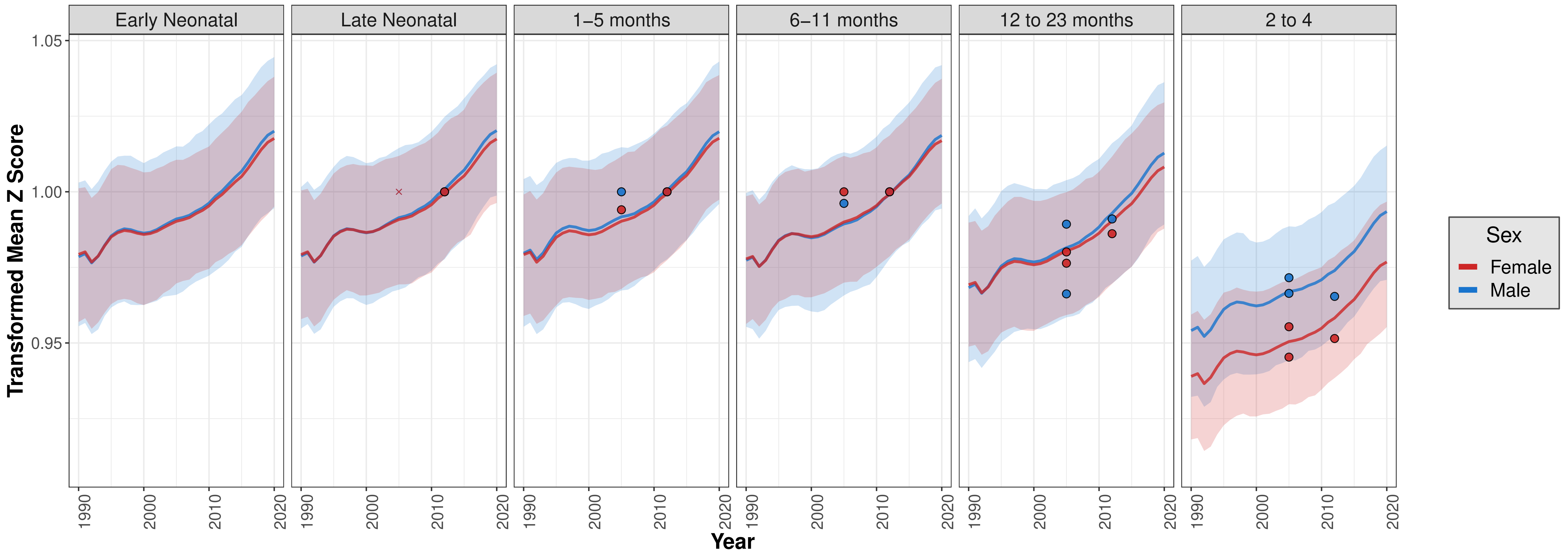


**C**

| Year | Source |
|---|---|
| 2005 | Moldova DHS |
| 2005 | WHO CGM Database |
| 2012 | WHO CGM Database |
| 2012 | Moldova MICS |

## **Republic of Moldova** – Wasting (WHZ)

**D**: Overall and Severe Wasting Prevalence

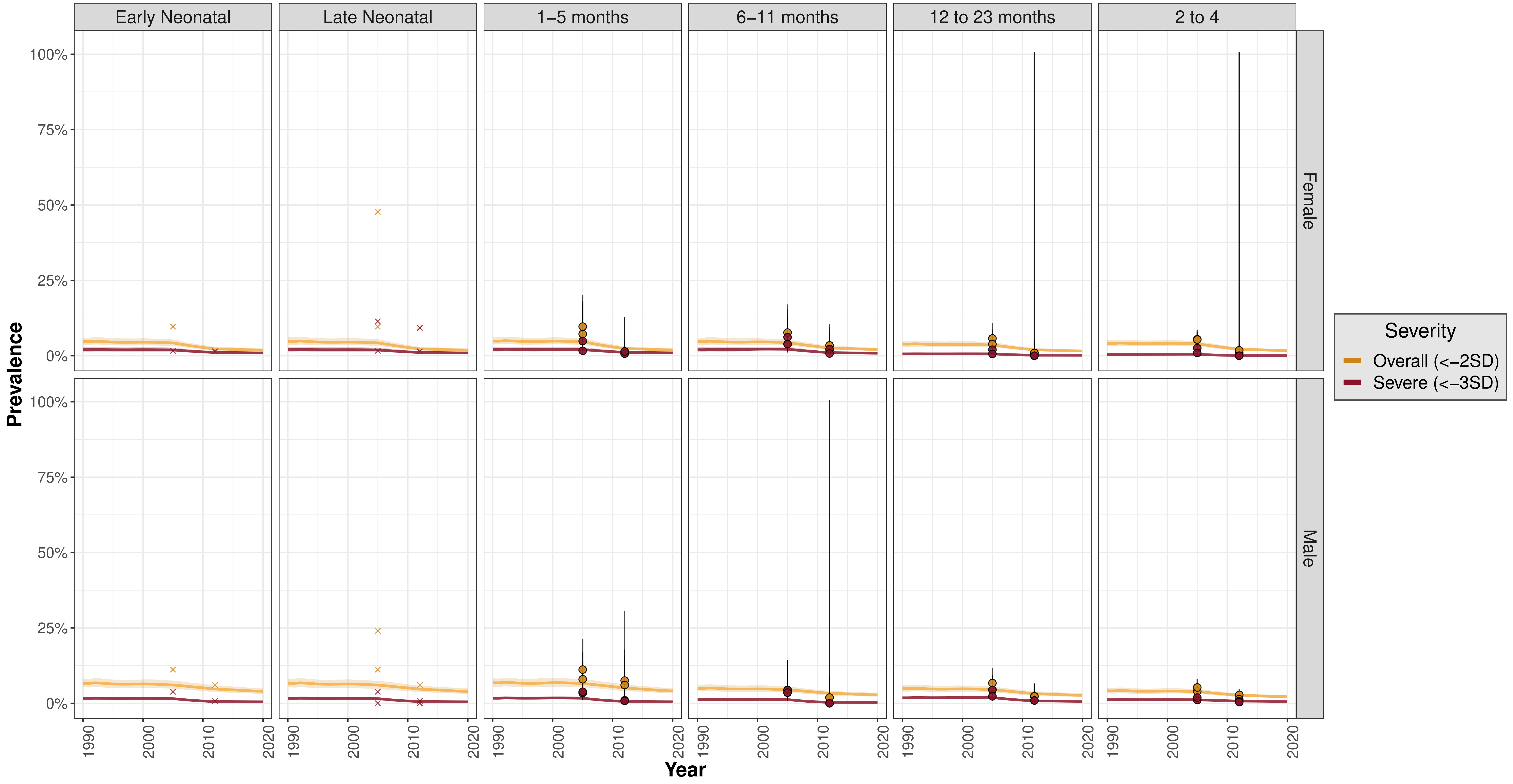


**E**: Transformed Mean Wasting Z Scores

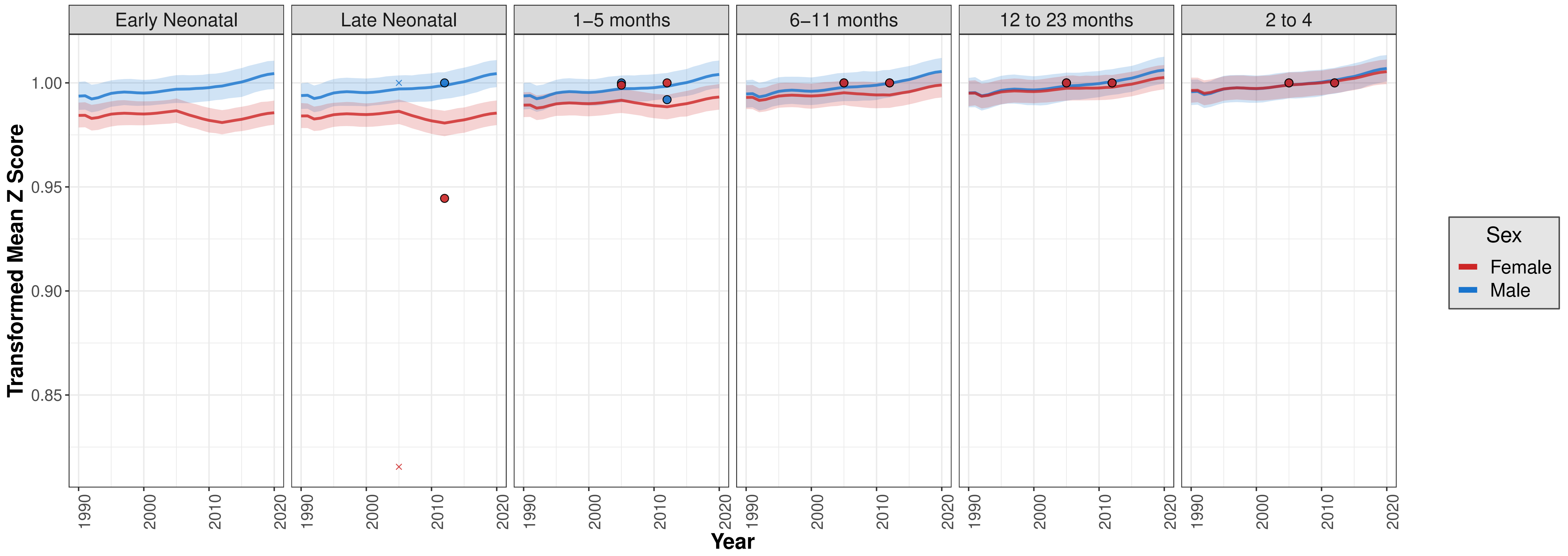


**F**

| Year | Source |
|---|---|
| 2005 | Moldova DHS |
| 2005 | WHO CGM Database |
| 2012 | WHO CGM Database |
| 2012 | Moldova MICS |

# **Republic of Moldova** – Underweight (WAZ)

**G**: Overall and Severe Underweight Prevalence

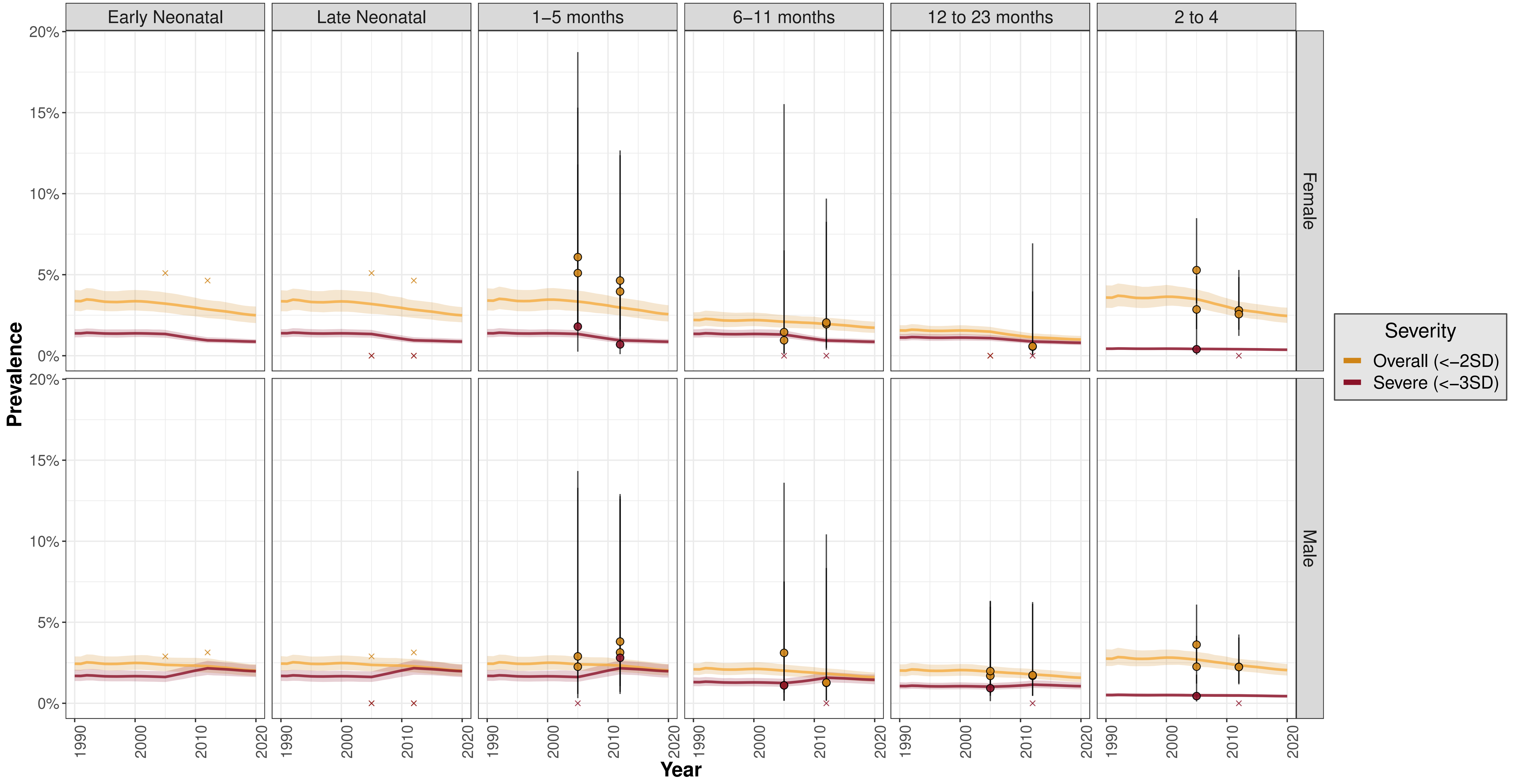


**H**: Transformed Mean Underweight Z Scores

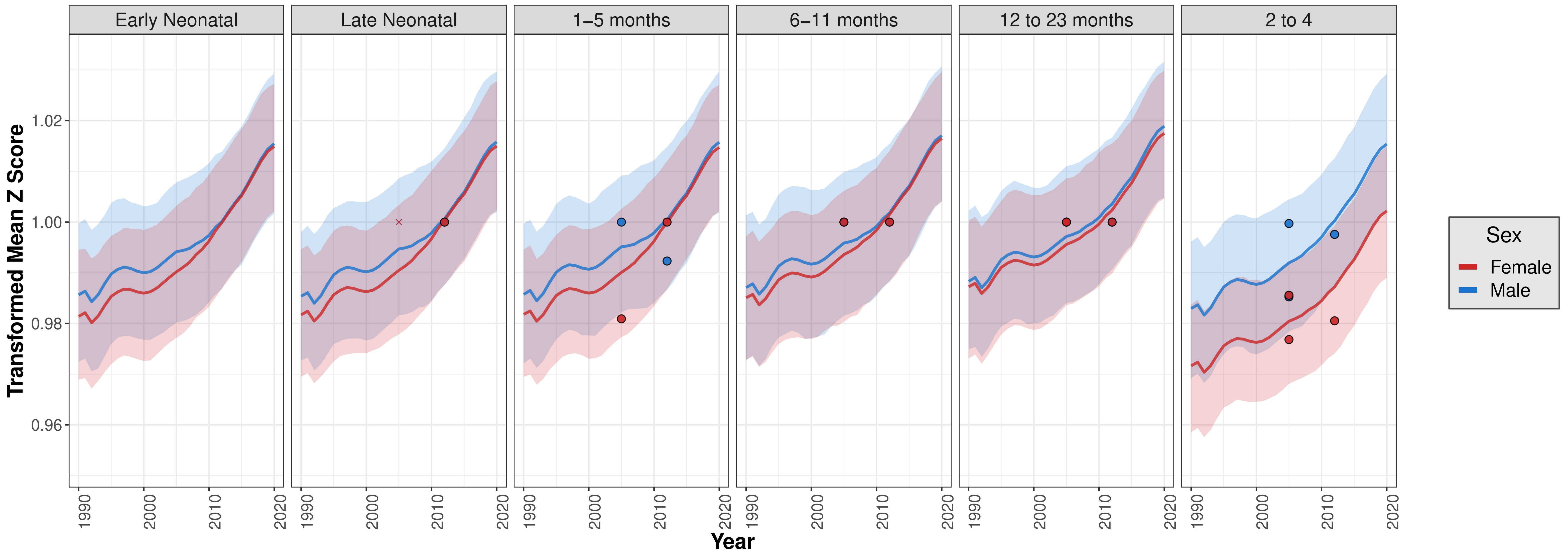


**I**

| Year | Source |
|---|---|
| 2005 | Moldova DHS |
| 2005 | WHO CGM Database |
| 2012 | WHO CGM Database |
| 2012 | Moldova MICS |

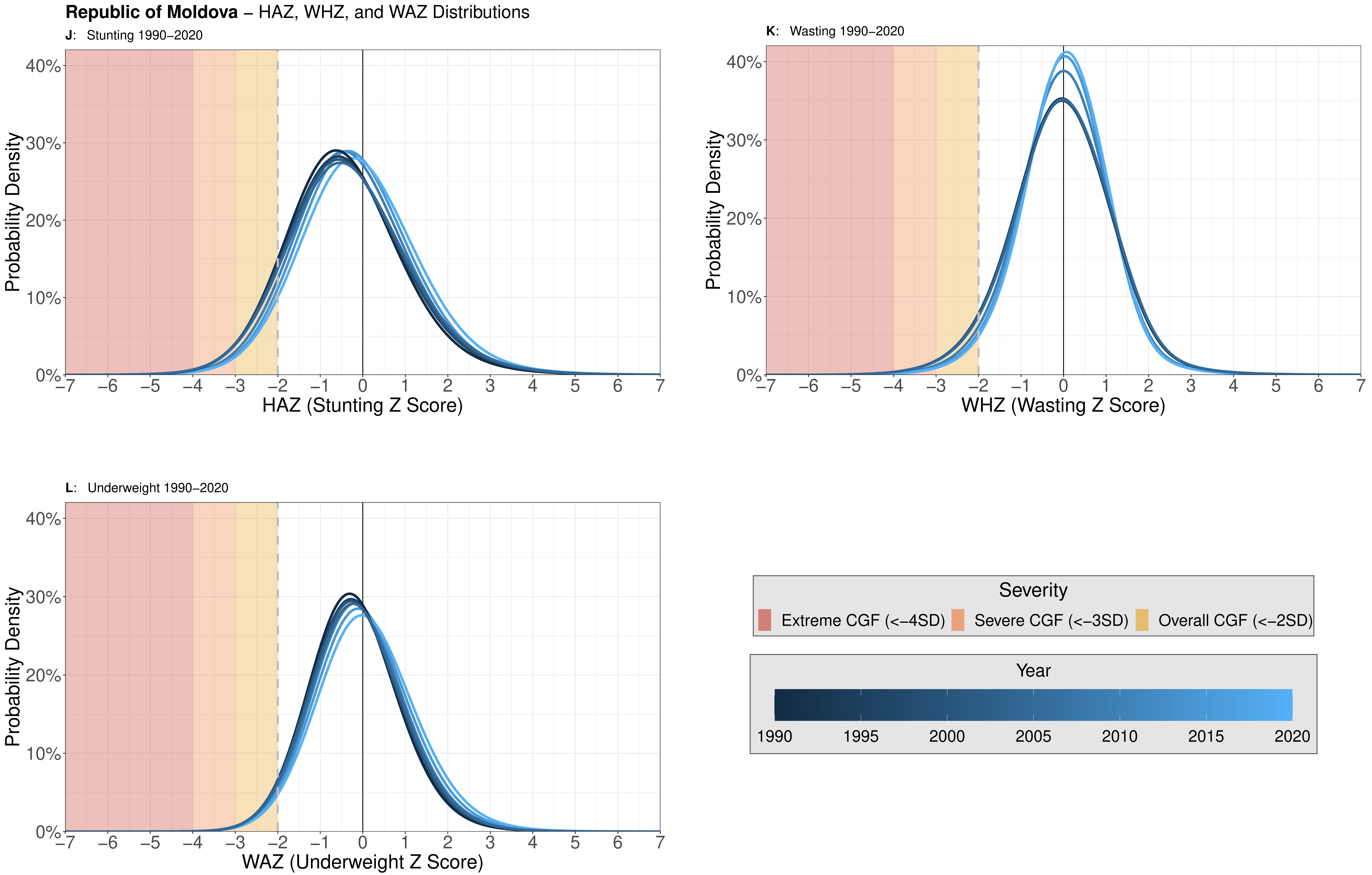

Republic of Moldova – HAZ, WHZ, and WAZ Distributions
J: Stunting 1990–2020
K: Wasting 1990–2020
L: Underweight 1990–2020
Probability Density
0%
10%
20%
30%
40%
−7 −6 −5 −4 −3 −2 −1 0 1 2 3 4 5 6 7
HAZ (Stunting Z Score)
WHZ (Wasting Z Score)
WAZ (Underweight Z Score)
Severity
Extreme CGF (<−4SD)
Severe CGF (<−3SD)
Overall CGF (<−2SD)
Year
1990
1995
2000
2005
2010
2015
2020

## **Russian Federation** – Stunting (HAZ)

**A**: Overall and Severe Stunting Prevalence

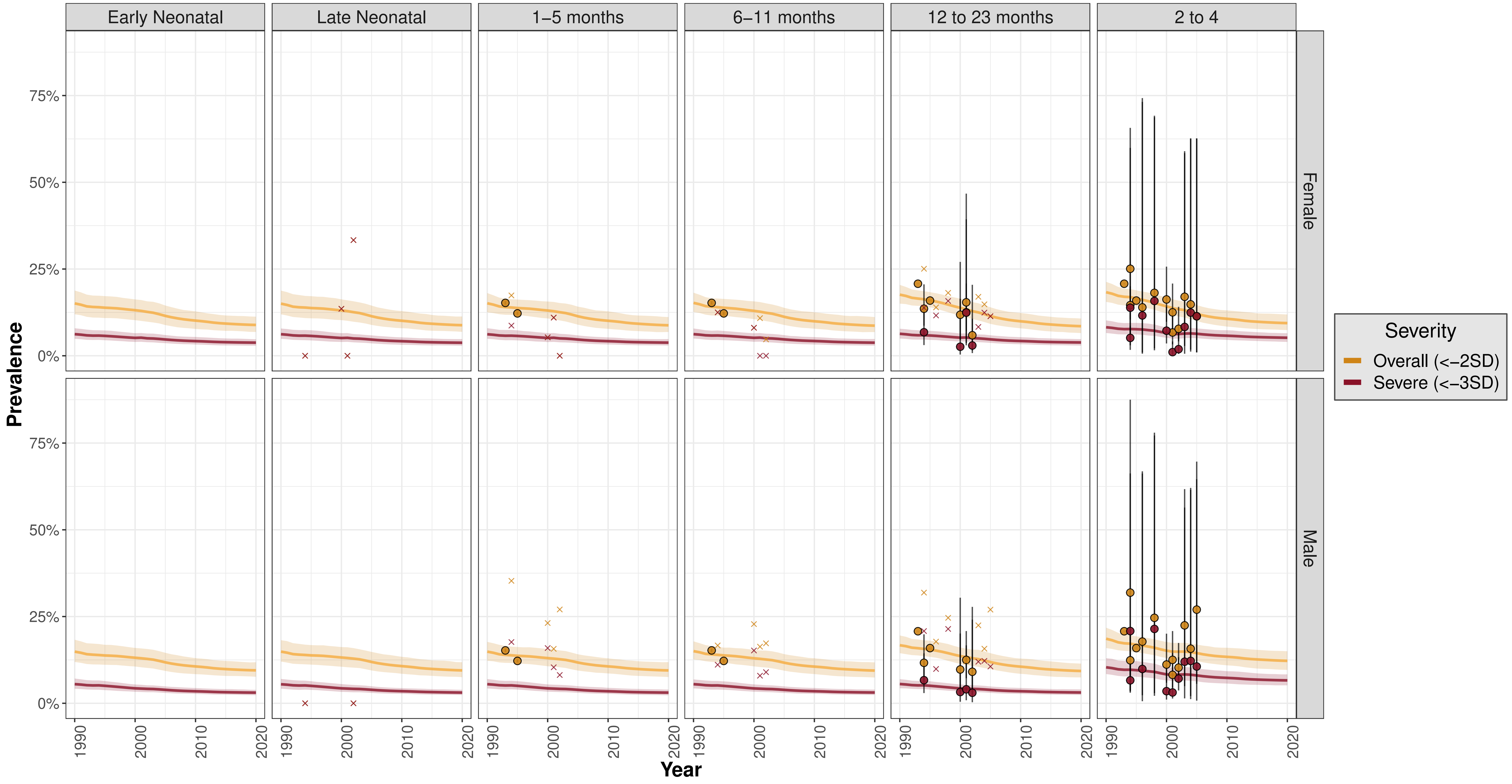


**B**: Transformed Mean Stunting Z Scores

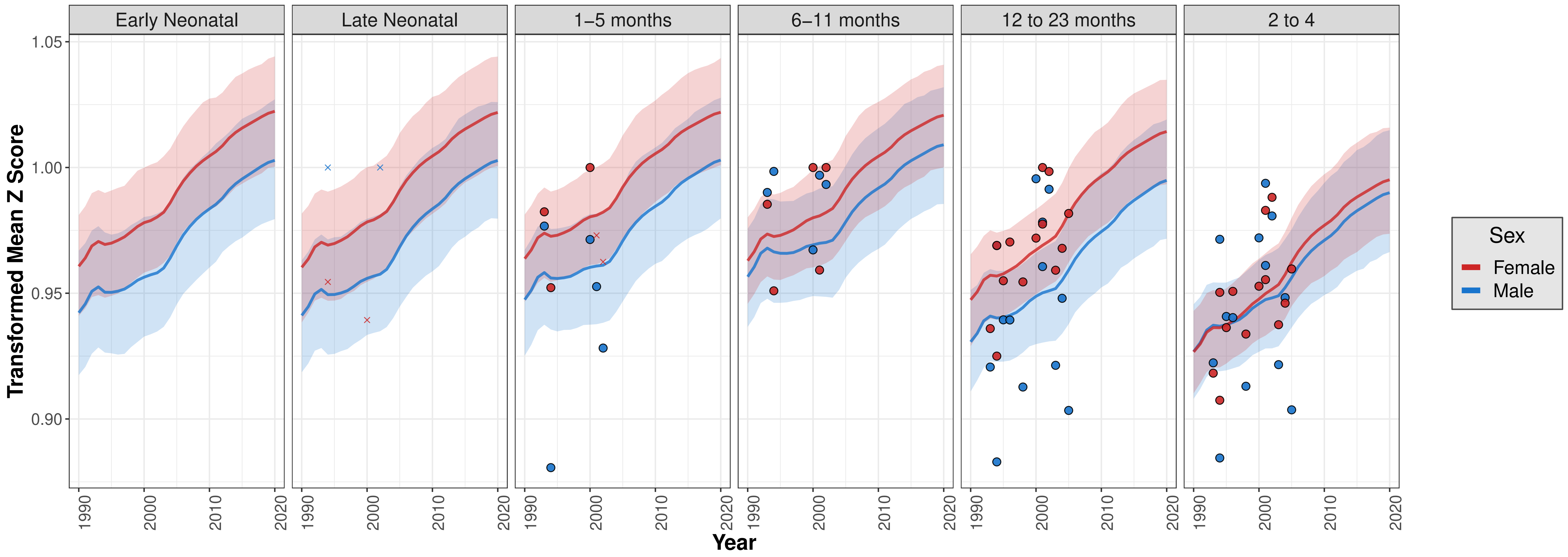


**C**

| Year | Source | National | Subnational |
|---|---|---|---|
| 1993 | WHO CGM Database | X | |
| 1994 | Russia Longitudinal Monitoring Survey of HSE, Round IV | X | |
| 1994 | Russia Longitudinal Monitoring Survey of HSE, Round V | X | |
| 1995 | WHO CGM Database | X | |
| 1996 | Russia Longitudinal Monitoring Survey of HSE, Round VII | X | |
| 1998 | Russia Longitudinal Monitoring Survey of HSE, Round VIII | X | |
| 2000 | Russia Longitudinal Monitoring Survey of HSE, Round IX | X | |
| 2001 | Russia Longitudinal Monitoring Survey of HSE, Round X | X | |
| 2001 | WHO CGM Database | X | |
| 2002 | Russia Longitudinal Monitoring Survey of HSE, Round XI | X | |
| 2003 | Russia Longitudinal Monitoring Survey of HSE, Round XII | X | |
| 2004 | Russia Longitudinal Monitoring Survey of HSE, Round XIII | X | |
| 2005 | Russia Longitudinal Monitoring Survey of HSE, Round XIV | X | |

# **Russian Federation** – Wasting (WHZ)

**D**: Overall and Severe Wasting Prevalence

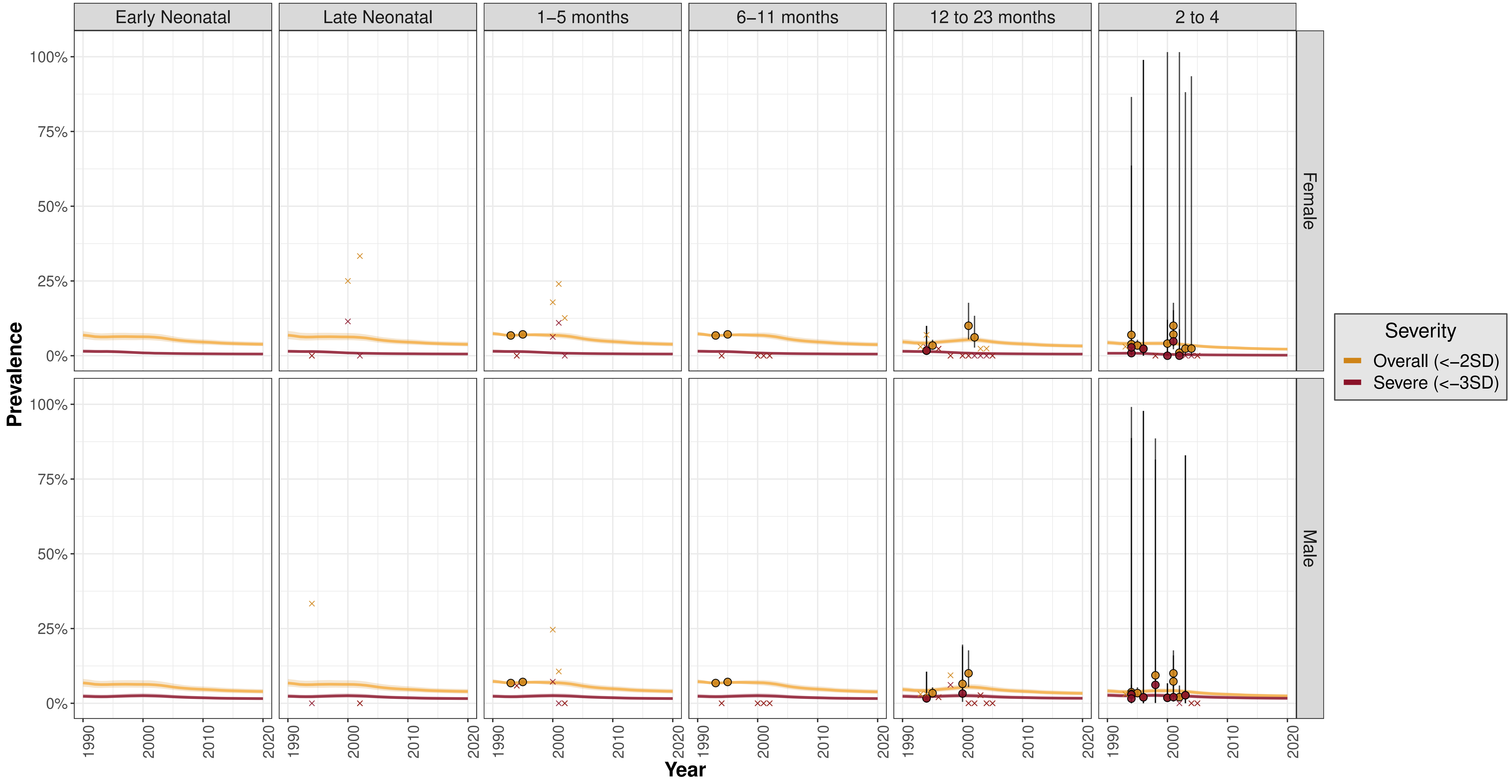


**E**: Transformed Mean Wasting Z Scores

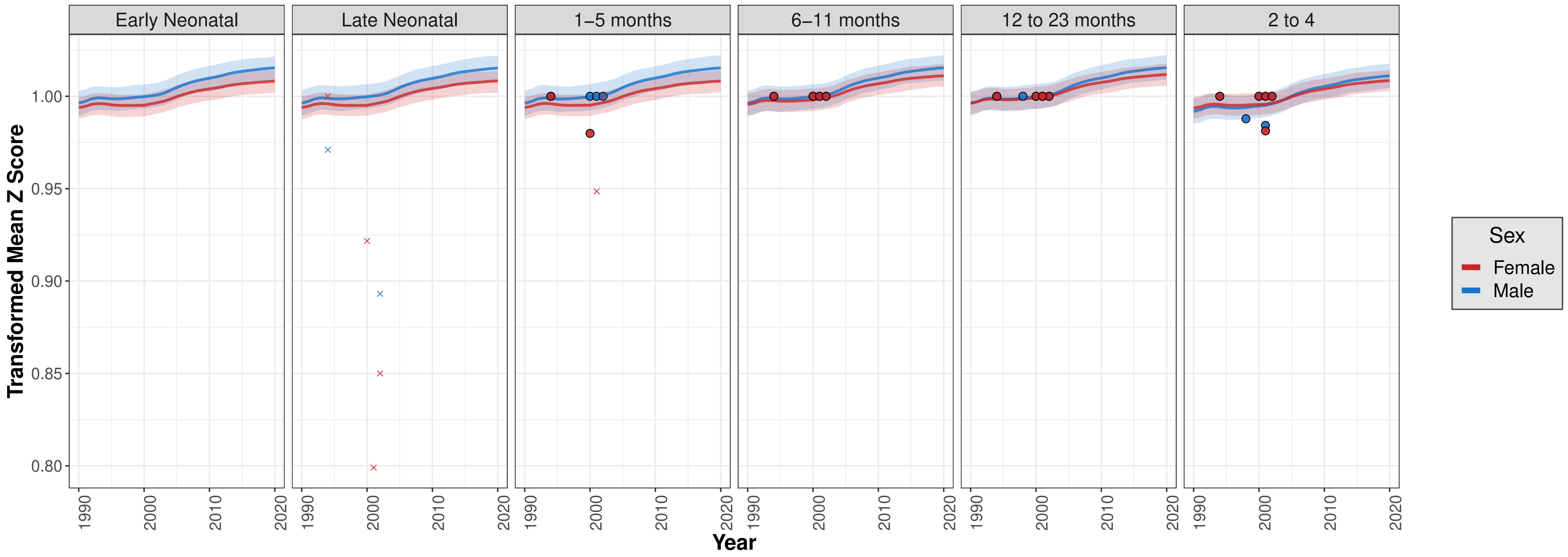


**F**

| Year | Source | National | Subnational |
|---|---|---|---|
| 1993 | WHO CGM Database | X | |
| 1994 | Russia Longitudinal Monitoring Survey of HSE, Round IV | X | |
| 1994 | Russia Longitudinal Monitoring Survey of HSE, Round V | X | |
| 1995 | WHO CGM Database | X | |
| 1996 | Russia Longitudinal Monitoring Survey of HSE, Round VII | X | |
| 1998 | Russia Longitudinal Monitoring Survey of HSE, Round VIII | X | |
| 2000 | Russia Longitudinal Monitoring Survey of HSE, Round IX | X | |
| 2001 | Russia Longitudinal Monitoring Survey of HSE, Round X | X | |
| 2001 | WHO CGM Database | X | |
| 2002 | Russia Longitudinal Monitoring Survey of HSE, Round XI | X | |
| 2003 | Russia Longitudinal Monitoring Survey of HSE, Round XII | X | |
| 2004 | Russia Longitudinal Monitoring Survey of HSE, Round XIII | X | |
| 2005 | Russia Longitudinal Monitoring Survey of HSE, Round XIV | X | |

# **Russian Federation** – Underweight (WAZ)

**G**: Overall and Severe Underweight Prevalence

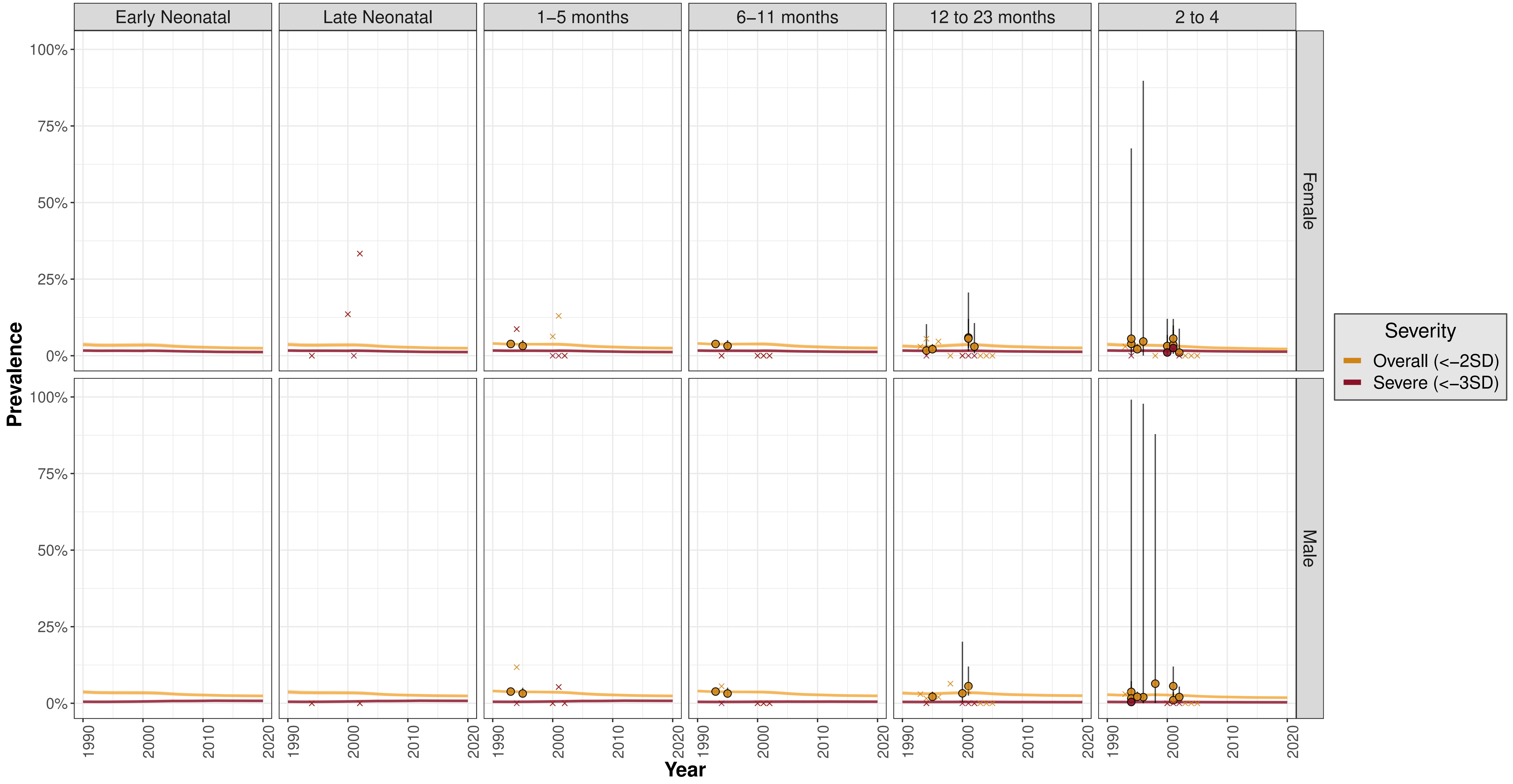


**H**: Transformed Mean Underweight Z Scores

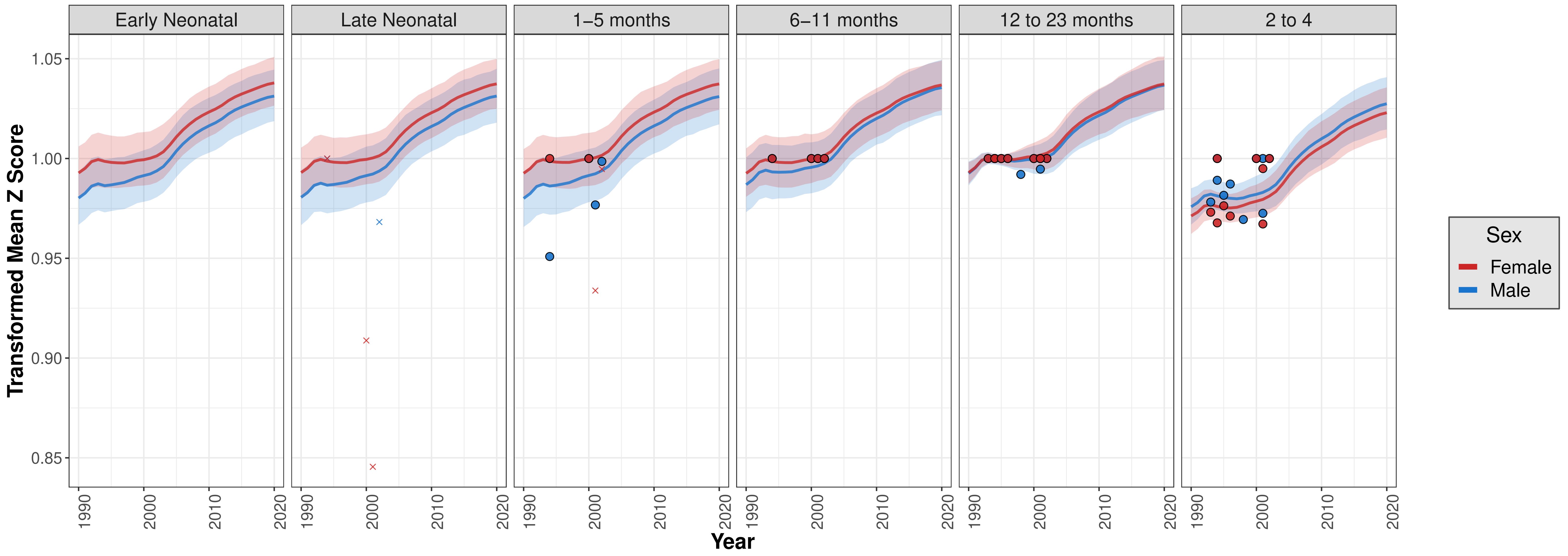


**I**

| Year | Source | National | Subnational |
|---|---|---|---|
| 1993 | WHO CGM Database | X | |
| 1994 | Russia Longitudinal Monitoring Survey of HSE, Round IV | X | |
| 1994 | Russia Longitudinal Monitoring Survey of HSE, Round V | X | |
| 1995 | WHO CGM Database | X | |
| 1996 | Russia Longitudinal Monitoring Survey of HSE, Round VII | X | |
| 1998 | Russia Longitudinal Monitoring Survey of HSE, Round VIII | X | |
| 2000 | Russia Longitudinal Monitoring Survey of HSE, Round IX | X | |
| 2001 | Russia Longitudinal Monitoring Survey of HSE, Round X | X | |
| 2001 | WHO CGM Database | X | |
| 2002 | Russia Longitudinal Monitoring Survey of HSE, Round XI | X | |
| 2003 | Russia Longitudinal Monitoring Survey of HSE, Round XII | X | |
| 2004 | Russia Longitudinal Monitoring Survey of HSE, Round XIII | X | |
| 2005 | Russia Longitudinal Monitoring Survey of HSE, Round XIV | X | |

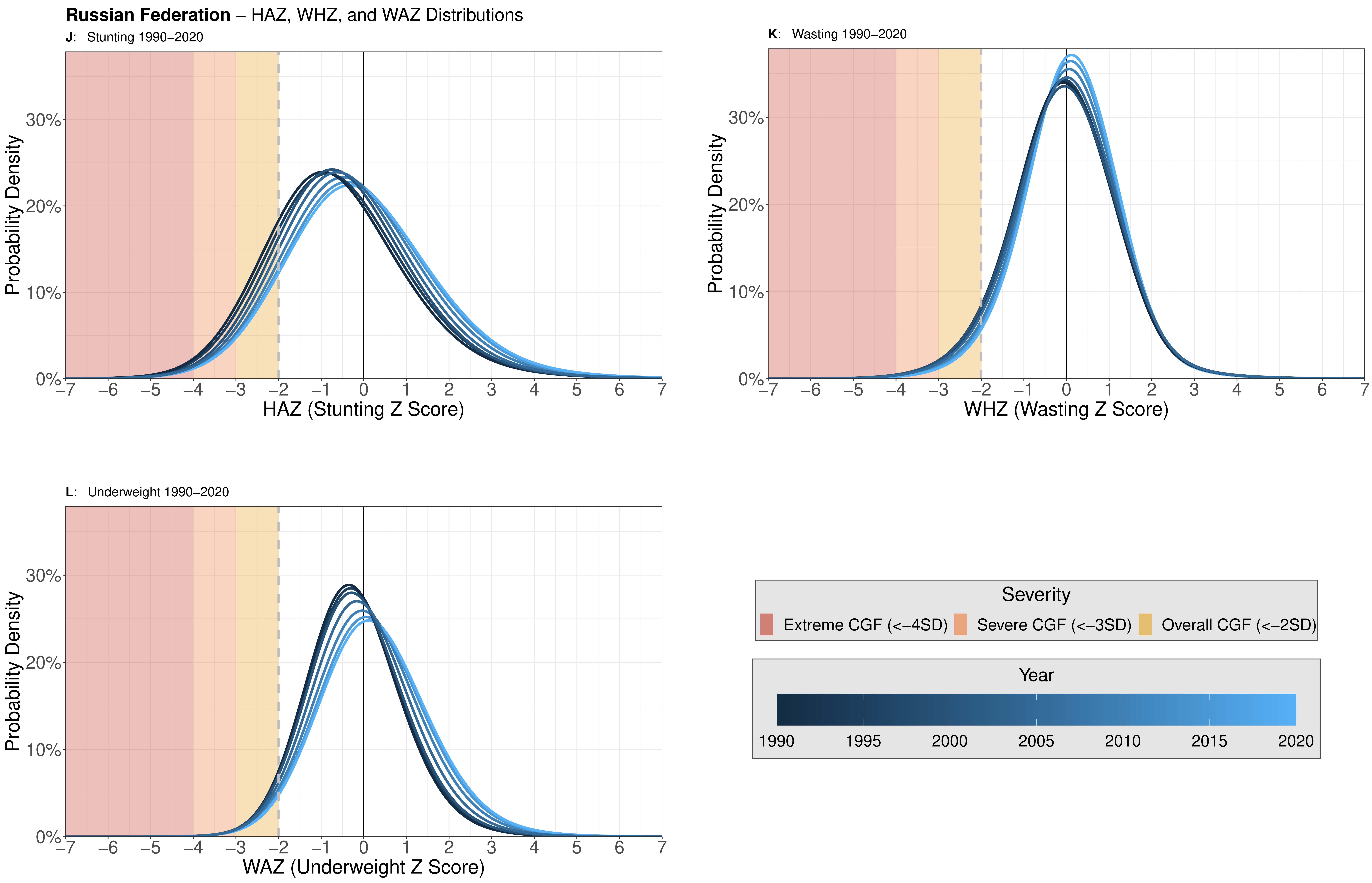
Russian Federation – HAZ, WHZ, and WAZ Distributions
J: Stunting 1990–2020
K: Wasting 1990–2020
L: Underweight 1990–2020
Probability Density
0%
10%
20%
30%
−7 −6 −5 −4 −3 −2 −1 0 1 2 3 4 5 6 7
HAZ (Stunting Z Score)
WHZ (Wasting Z Score)
WAZ (Underweight Z Score)
Severity
Extreme CGF (<−4SD)
Severe CGF (<−3SD)
Overall CGF (<−2SD)
Year
1990
1995
2000
2005
2010
2015
2020

**Ukraine** – Stunting (HAZ)

**A**: Overall and Severe Stunting Prevalence

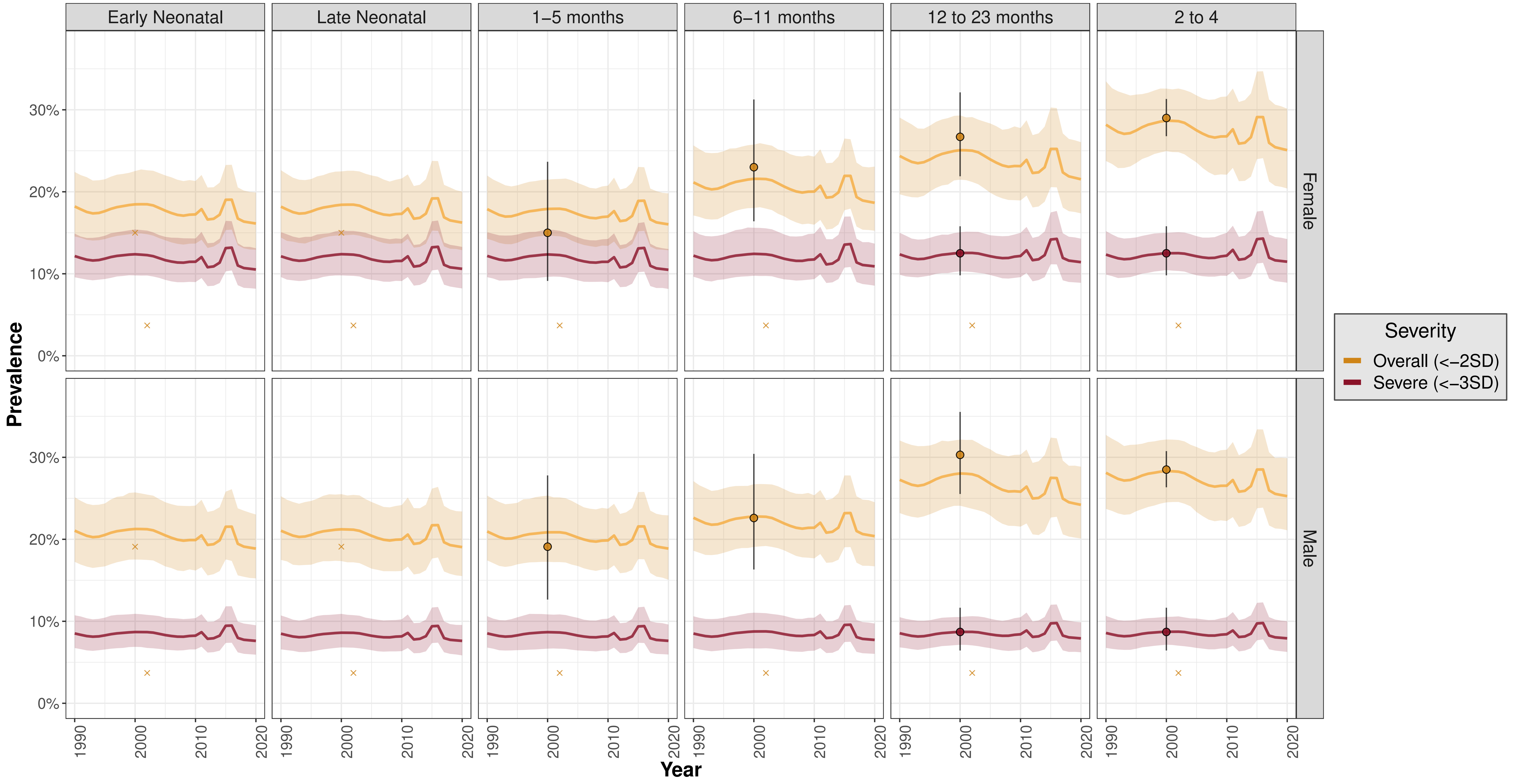


**B**: Transformed Mean Stunting Z Scores

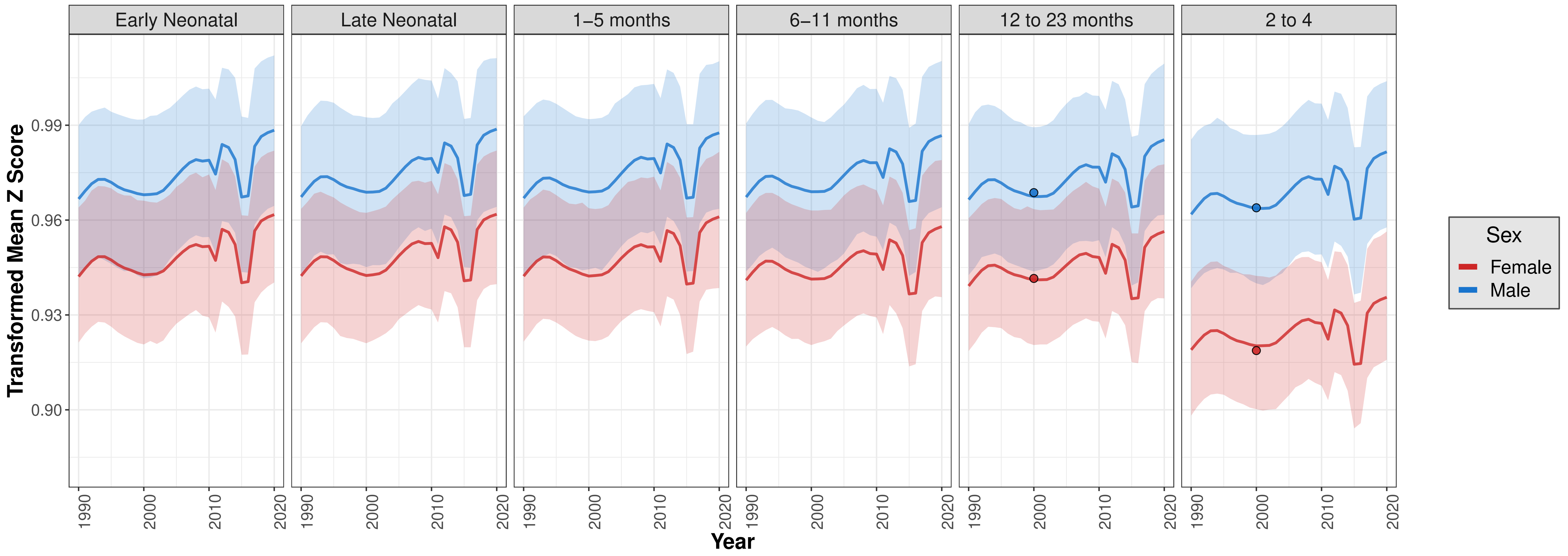


**C**

| Year | Source |
|---|---|
| 2000 | WHO CGM Database |
| 2002 | WHO CGM Database |

# **Ukraine** – Wasting (WHZ)

**D**:   Overall and Severe Wasting Prevalence

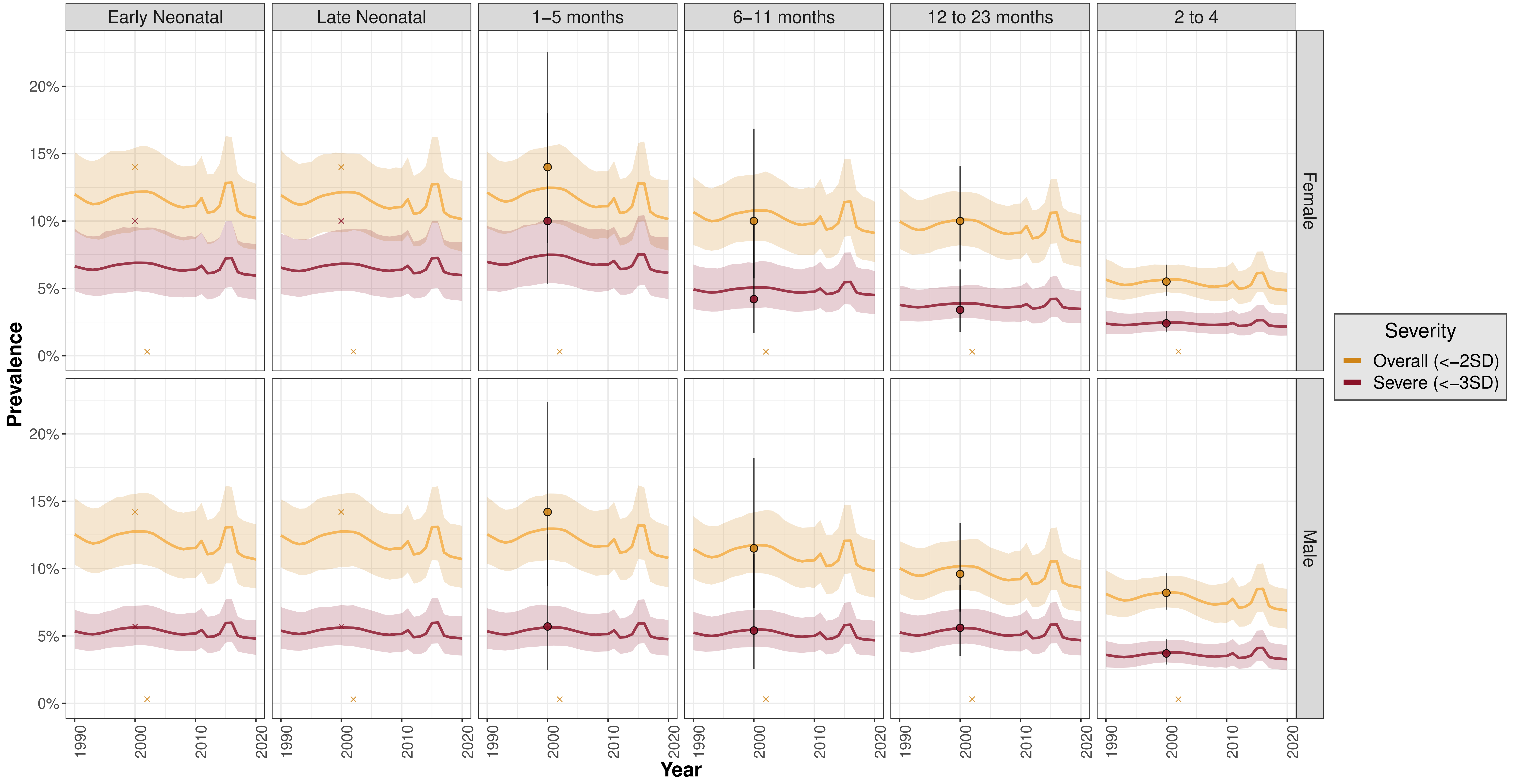


**E**:   Transformed Mean Wasting Z Scores

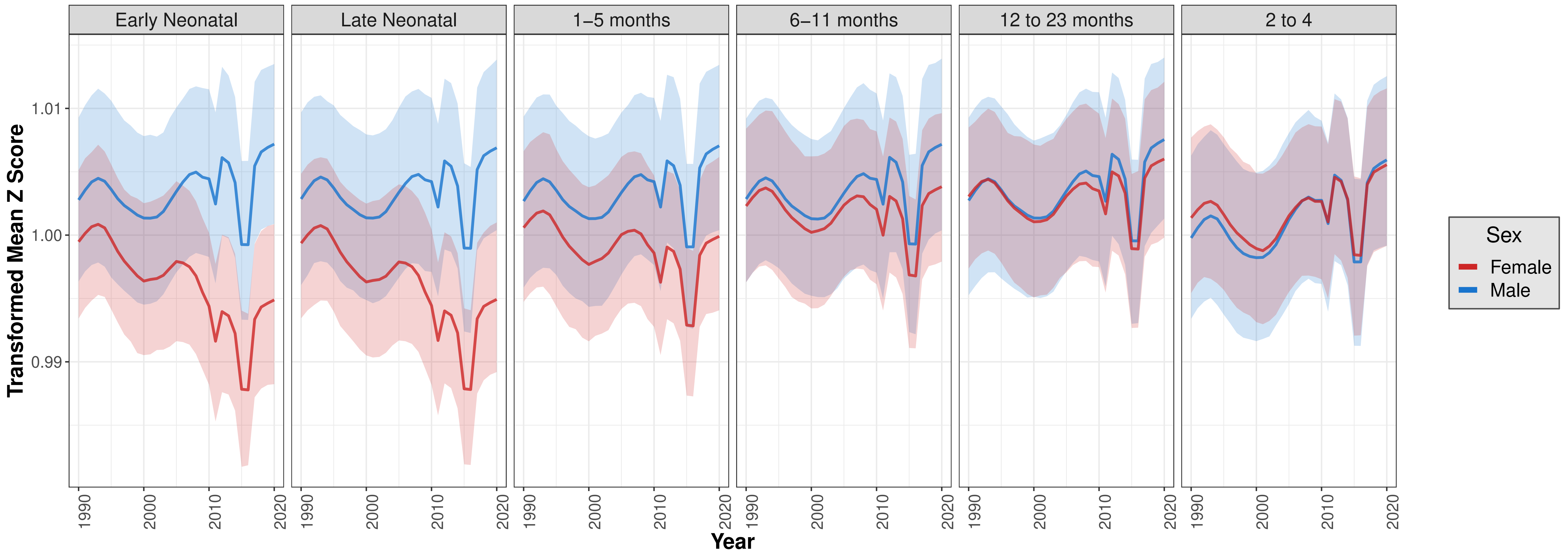


**F**

| Year | Source |
|---|---|
| 2000 | WHO CGM Database |
| 2002 | WHO CGM Database |

## **Ukraine** – Underweight (WAZ)

**G**: Overall and Severe Underweight Prevalence

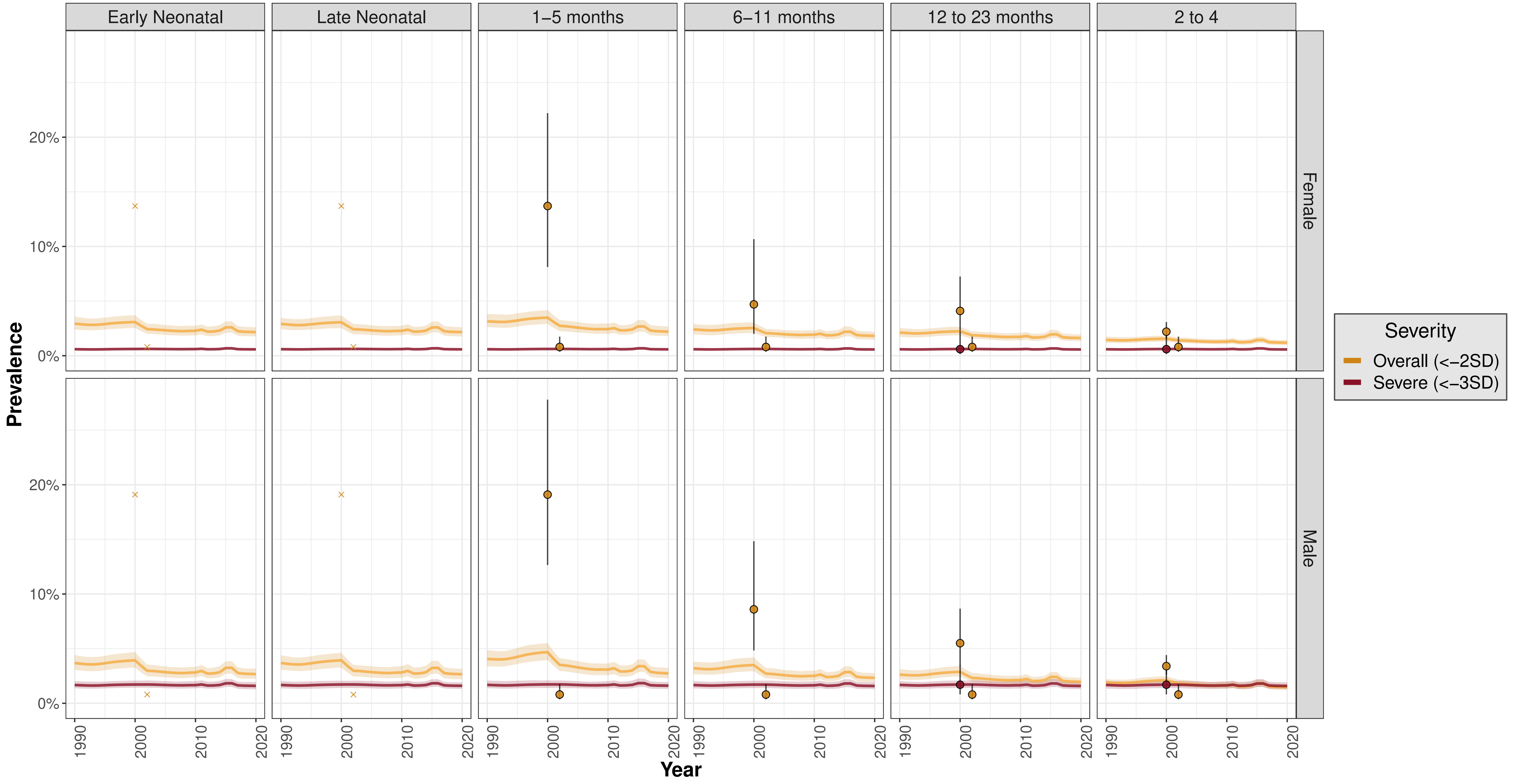


**H**: Transformed Mean Underweight Z Scores

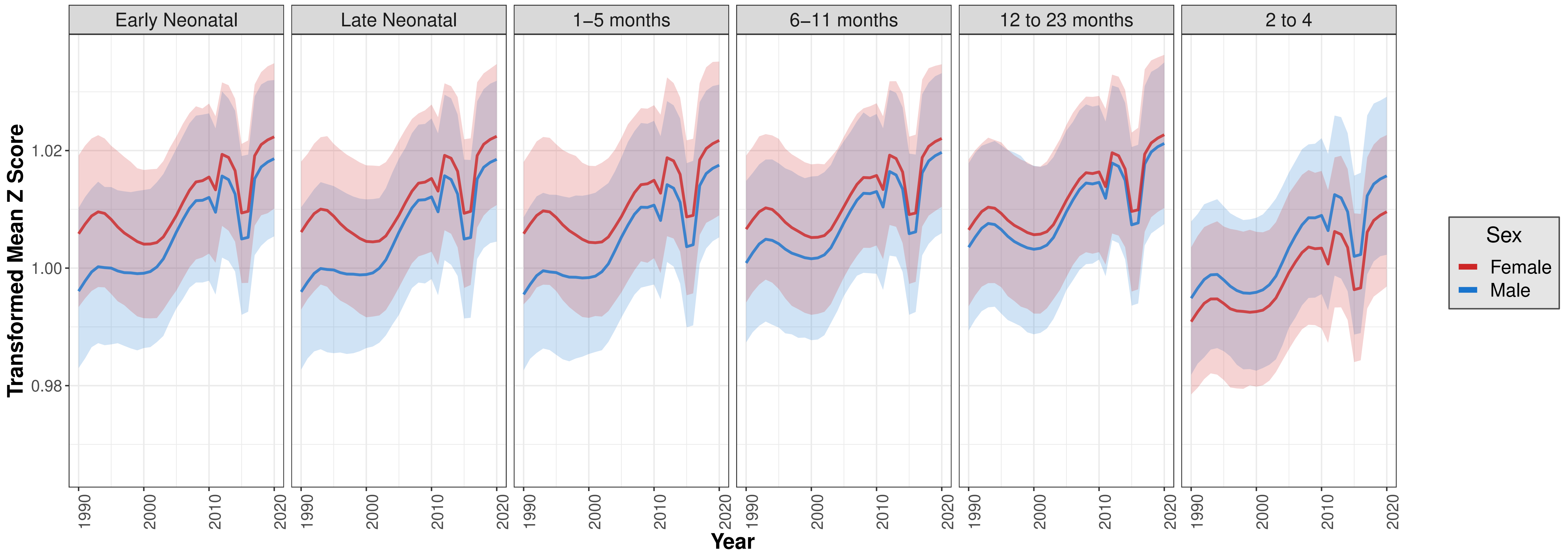


**I**

| Year | Source |
|---|---|
| 2000 | WHO CGM Database |
| 2002 | WHO CGM Database |

**Ukraine** – HAZ, WHZ, and WAZ Distributions

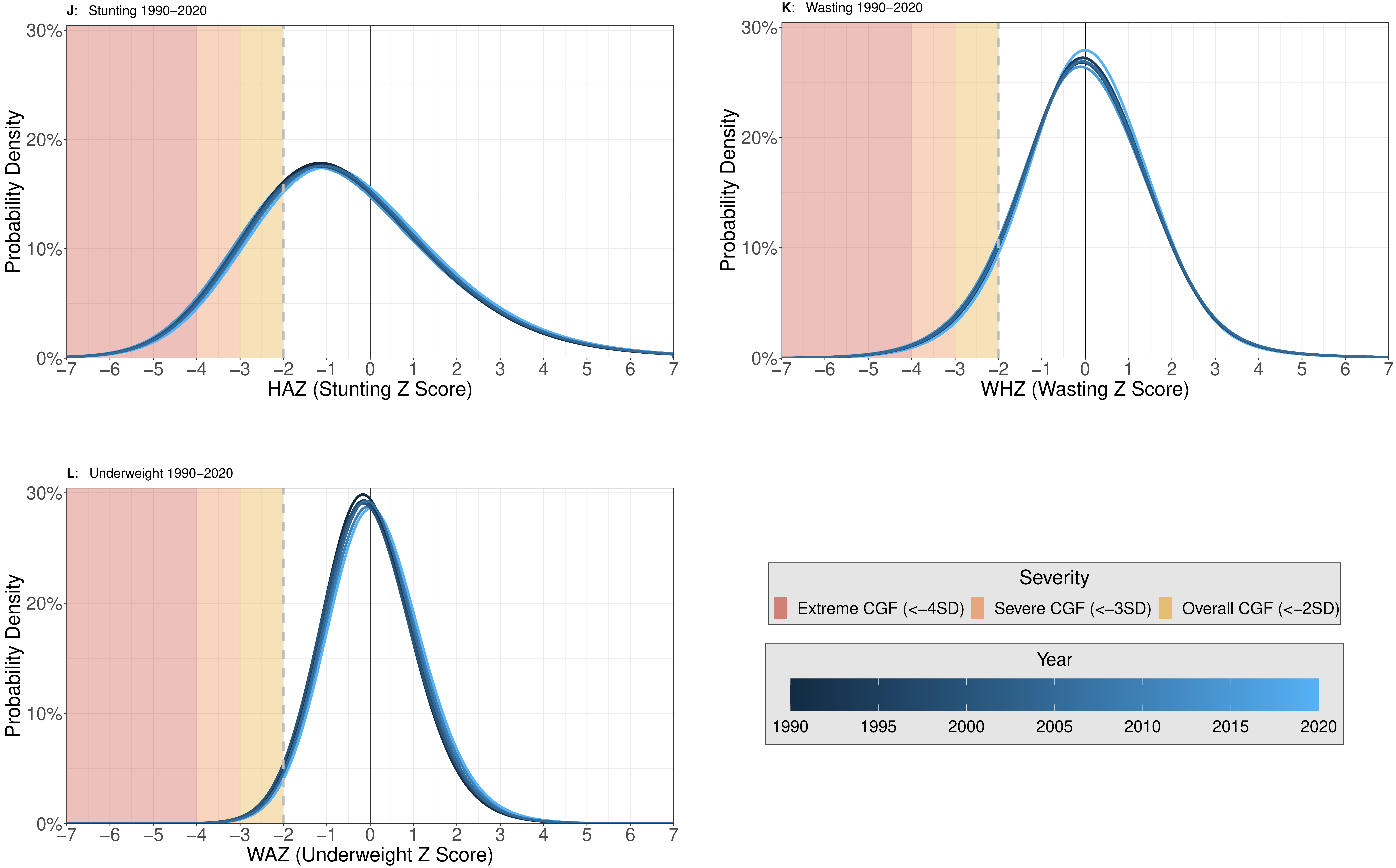

Supplement: Supplementary file 2 — Data S1 to S4 [file sciadv.abm8954_data_files_s1_to_s4.zip › sciadv.abm8954_data_file_s1a.pdf]
