# Supplementary material for: Current dichotomous metrics obscure trends in severe and extreme child growth failure
Source: Sci Adv. 2022 May 20;8(20):eabm8954. doi: 10.1126/sciadv.abm8954 (PMC9122330; doi:10.1126/sciadv.abm8954)
Supplement: Supplementary file 2 — Data S1 to S4 [file sciadv.abm8954_data_files_s1_to_s4.zip › sciadv.abm8954_data_file_s1b.pdf]

Data S1b. Spatio-temporal Gaussian Process Regression (ST-GPR) results for overall, severe, and mean CGF by location, including location-specific data sources; and distributions of stunting [HAZ], wasting [WHZ], and underweight [WAZ] for children under age five, both sexes, for every five years from 1990−2020. Country results are grouped by GBD super-region, including Central Europe, Eastern Europe, and Central Asia (S1a), High-income (S1b), Latin America and Caribbean (S1c), North Africa and Middle East (S1d), South Asia (S1e), Southeast Asia, East Asia, and Oceania (S1f), and Sub-Saharan Africa (S1g). Plots for each country include overall and severe stunting prevalence (A) and transformed mean stunting Z scores (B). A source list is shown which includes surveys included in the stunting models (C). Additional plots are shown for overall and severe wasting prevalence (D) and transformed mean wasting Z scores (E), followed by a source list with surveys included in the wasting models (F). Plots are then shown for overall and severe underweight prevalence (G), and transformed mean underweight Z scores (H), with a source list listing surveys included in the underweight models (I). Finally, distributions of stunting (J), wasting (K), and underweight (L) are shown for children under age five, both sexes, for every five years from 1990-2020. Surveys that were outliered are shown with X's on all plots. Surveys prior to 1990 may have been inputs to the models to inform trends, but estimates are only produced and shown for 1990−2020. For locations that are modeled nationally and subnationally, sources that are only included subnationally are not included in the plots of national level estimates. These sources were included in subnational models that influence national level models. Note that due to the transformation on mean Z scores, increasing values reflect improvements in mean Z score. Surveys conducted over a range of years were assigned to the midpoint year from that interval, which is the year reflected in the table and the plots. For the distributions of stunting, wasting, and underweight, the area under the curve reflects the estimated proportion of children experiencing that severity of CGF or worse. DHS is Demographic and Health Surveys. MICS is Multiple Indicator Cluster Survey. WHO CGM is the WHO Global Database on Child Growth and Malnutrition. SDNS is Survey of Diet and Nutritional Status.

This file contains the above for the following locations in the GBD super region of High Income, in the following order:

Australasia: Australia, New Zealand

High-income Asia Pacific: Brunei Darussalam, Japan, Republic of Korea, Singapore

High-income North America: Canada, Georgia, United States of America

Southern Latin America: Argentina, Chile, Uruguay

Western Europe: Andorra, Austria, Belgium, Cyprus, Denmark, Finland, France, Germany, Greece, Iceland, Ireland, Israel, Italy, Luxembourg, Malta, Monaco, Netherlands, Norway, Portugal, San Marino, Spain, Sweden, Switzerland, United Kingdom

Australia – Stunting (HAZ)

A: Overall and Severe Stunting Prevalence

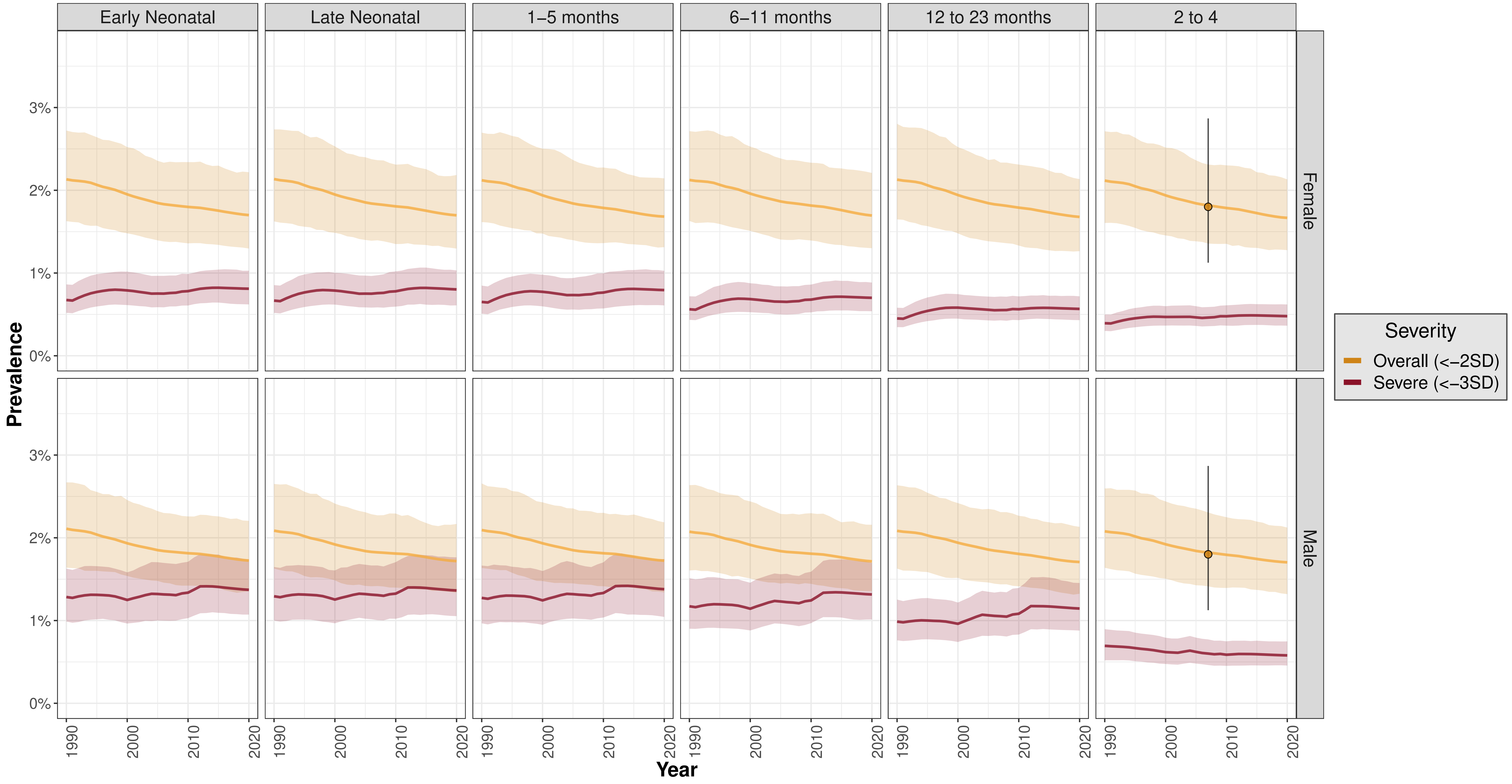

B: Transformed Mean Stunting Z Scores

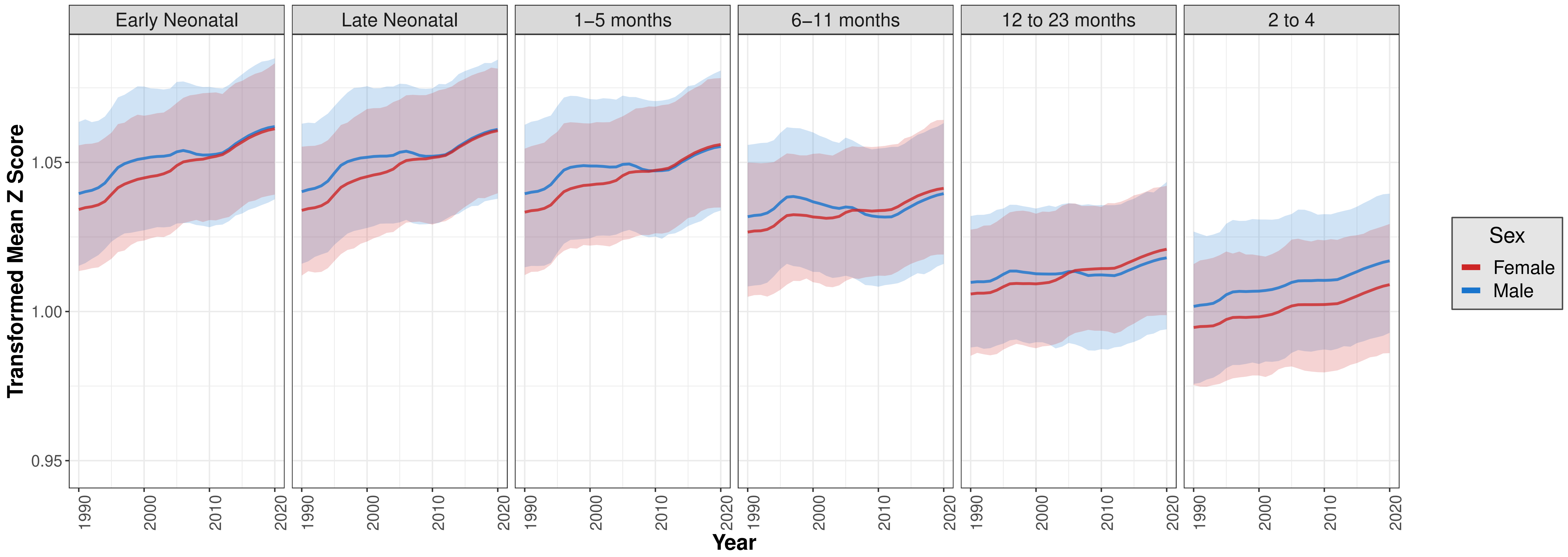

C

Year	Source
2007	WHO CGM Database

Australia – Wasting (WHZ)

D: Overall and Severe Wasting Prevalence

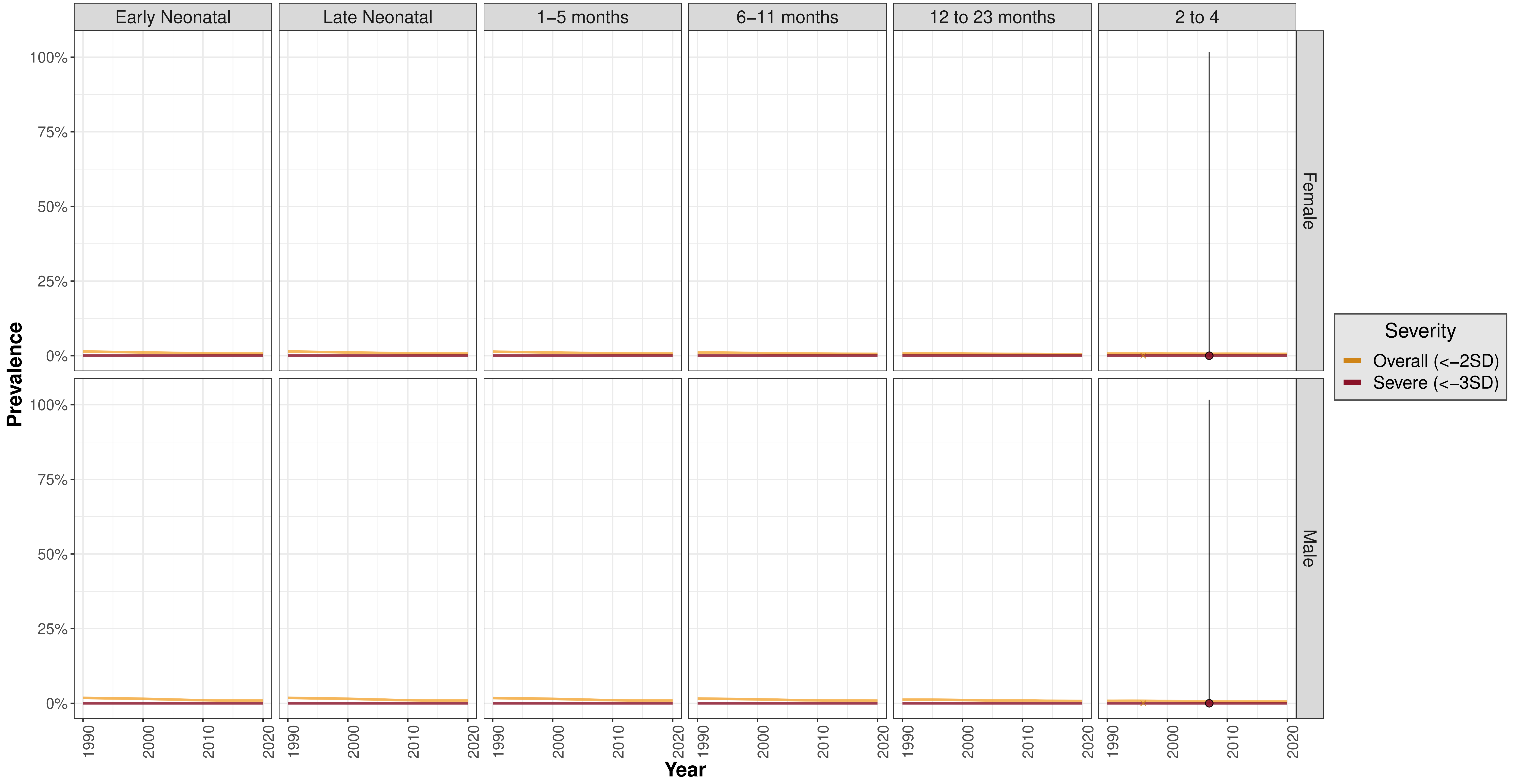

E: Transformed Mean Wasting Z Scores

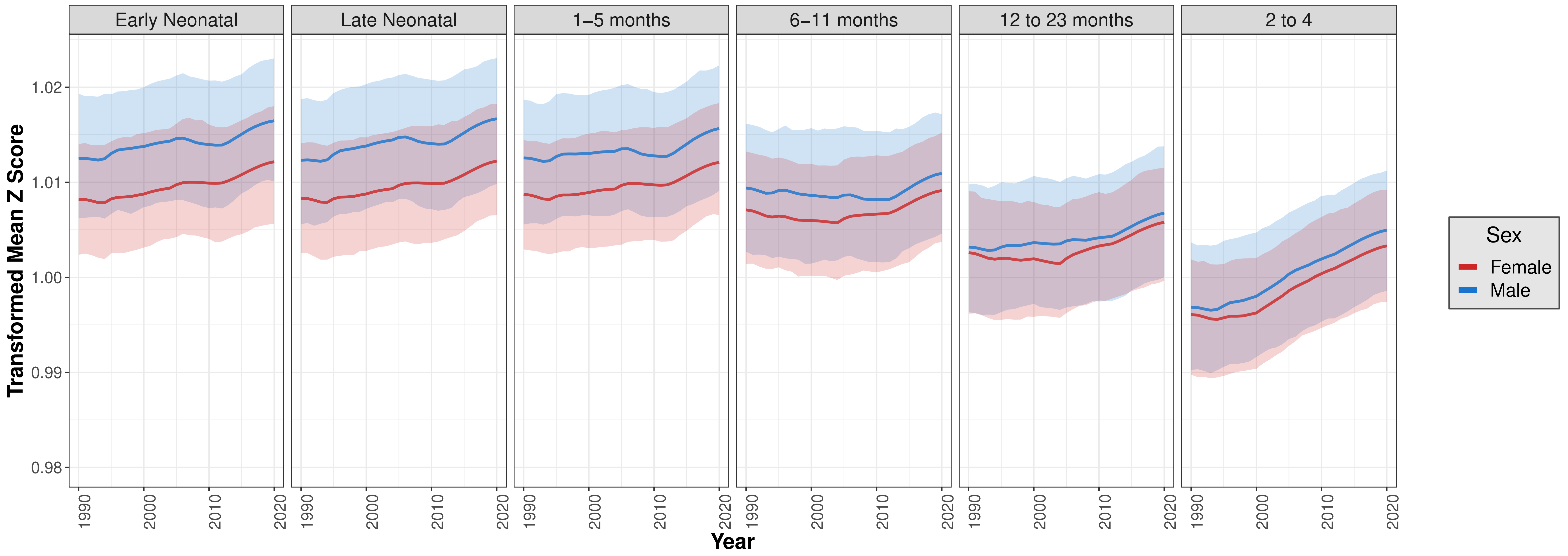

F

Year	Source
1996	WHO CGM Database
2007	WHO CGM Database

Australia – Underweight (WAZ)

G: Overall and Severe Underweight Prevalence

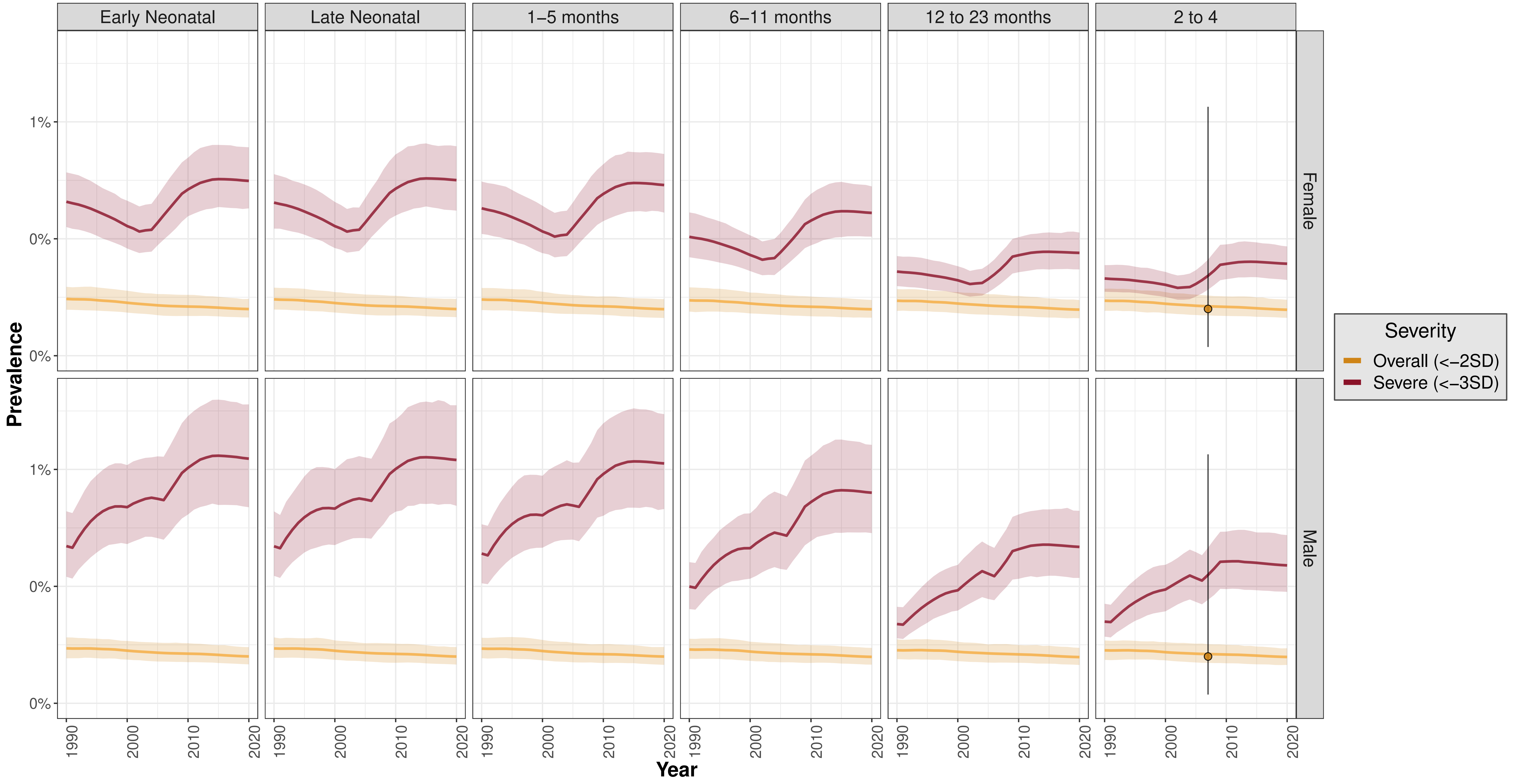

I

Year	Source
2007	WHO CGM Database

H: Transformed Mean Underweight Z Scores

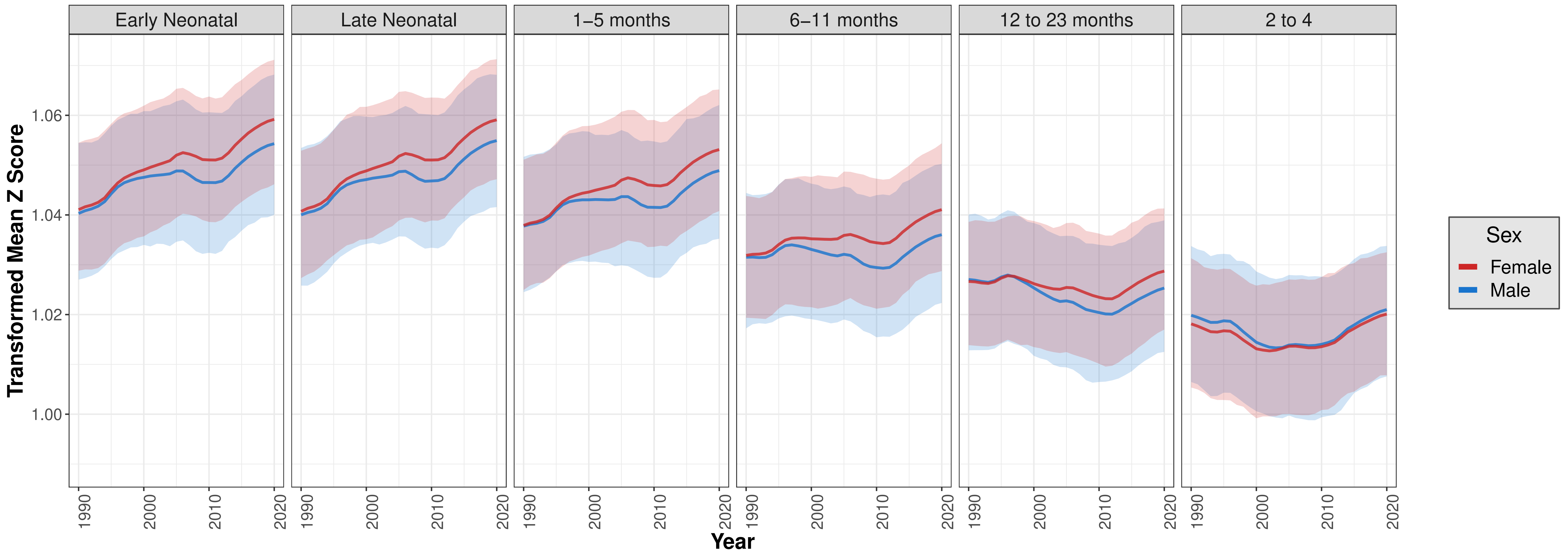

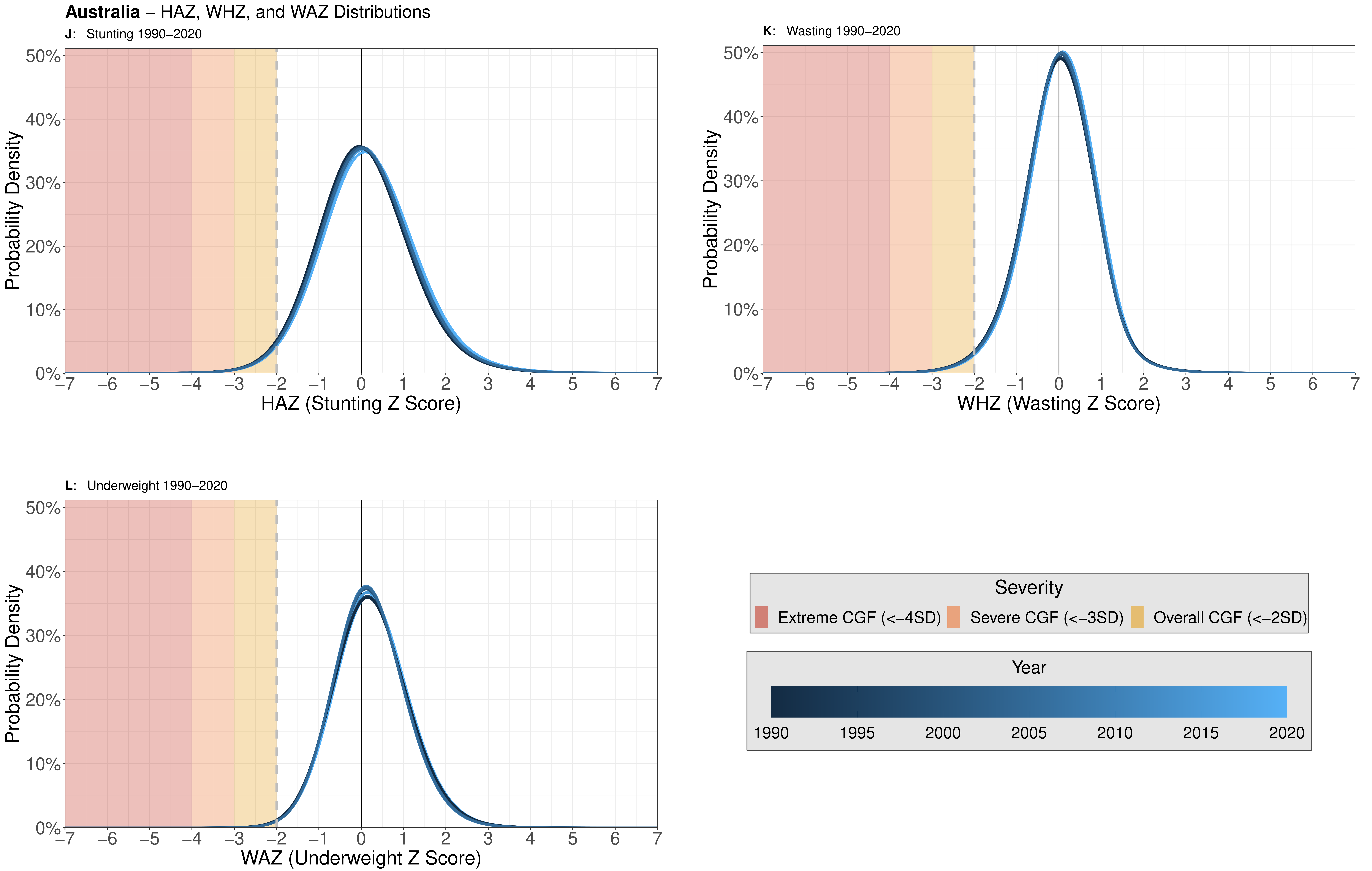
Australia – HAZ, WHZ, and WAZ Distributions
J: Stunting 1990–2020
K: Wasting 1990–2020
L: Underweight 1990–2020
Probability Density
0%
10%
20%
30%
40%
50%
−7 −6 −5 −4 −3 −2 −1 0 1 2 3 4 5 6 7
HAZ (Stunting Z Score)
WHZ (Wasting Z Score)
WAZ (Underweight Z Score)
Severity
Extreme CGF (<−4SD)
Severe CGF (<−3SD)
Overall CGF (<−2SD)
Year
1990
1995
2000
2005
2010
2015
2020

New Zealand – Stunting (HAZ)

A: Overall and Severe Stunting Prevalence

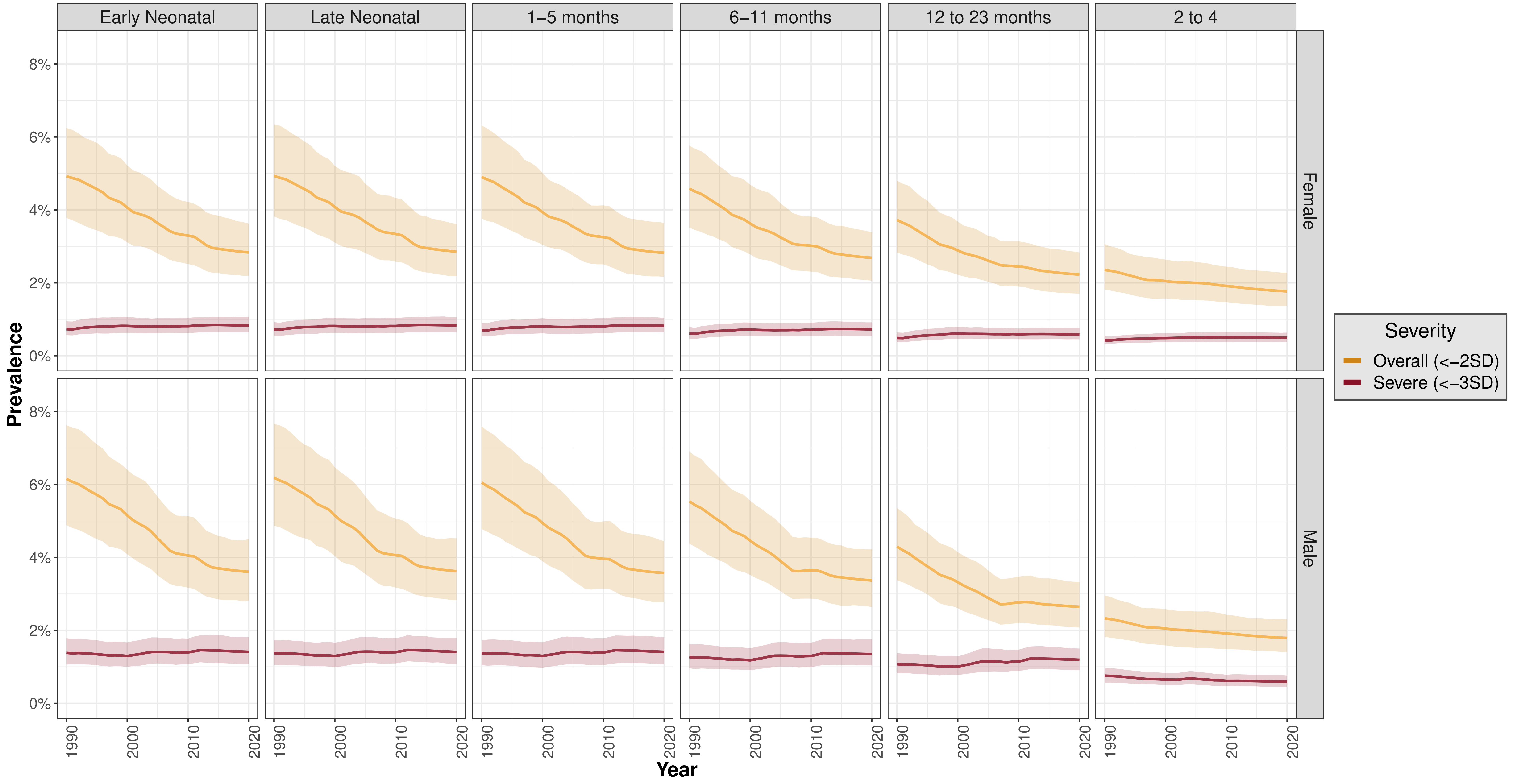

B: Transformed Mean Stunting Z Scores

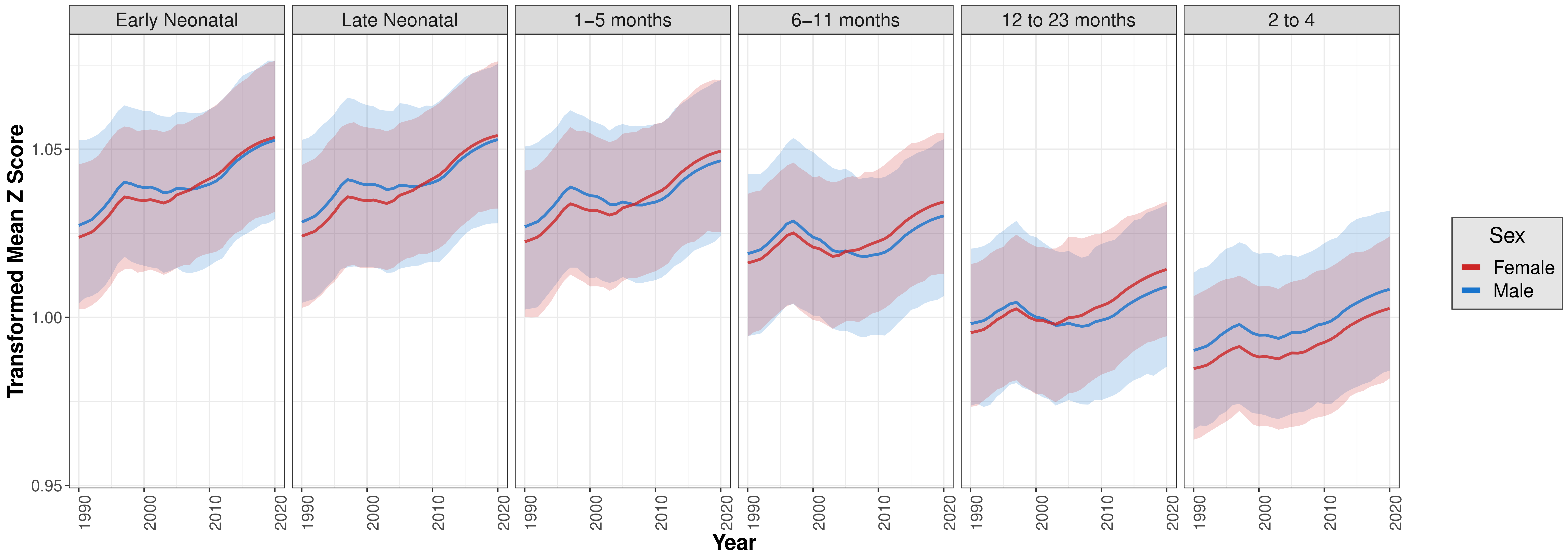

C

Source
No national or subnational sources for this location

New Zealand – Wasting (WHZ)

D: Overall and Severe Wasting Prevalence

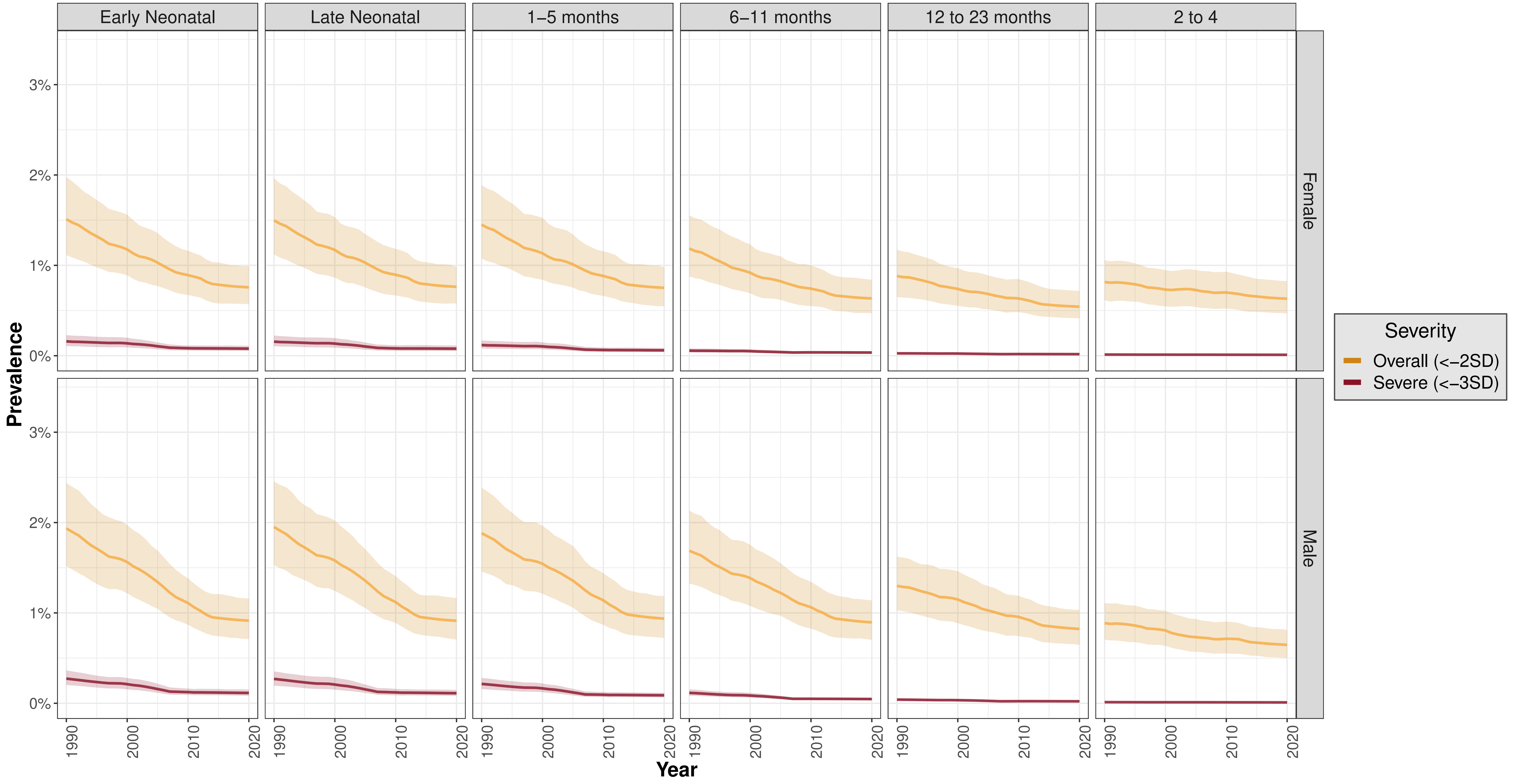

E: Transformed Mean Wasting Z Scores

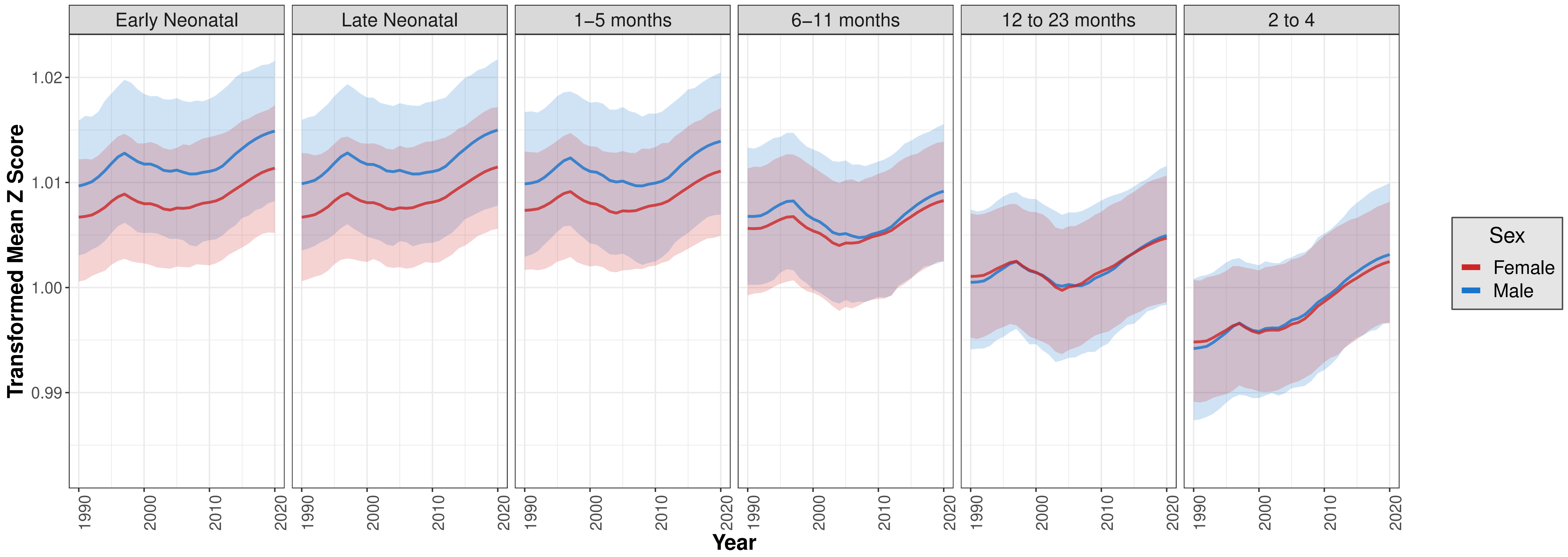

F

Source
No national or subnational sources for this location

New Zealand – Underweight (WAZ)

G: Overall and Severe Underweight Prevalence

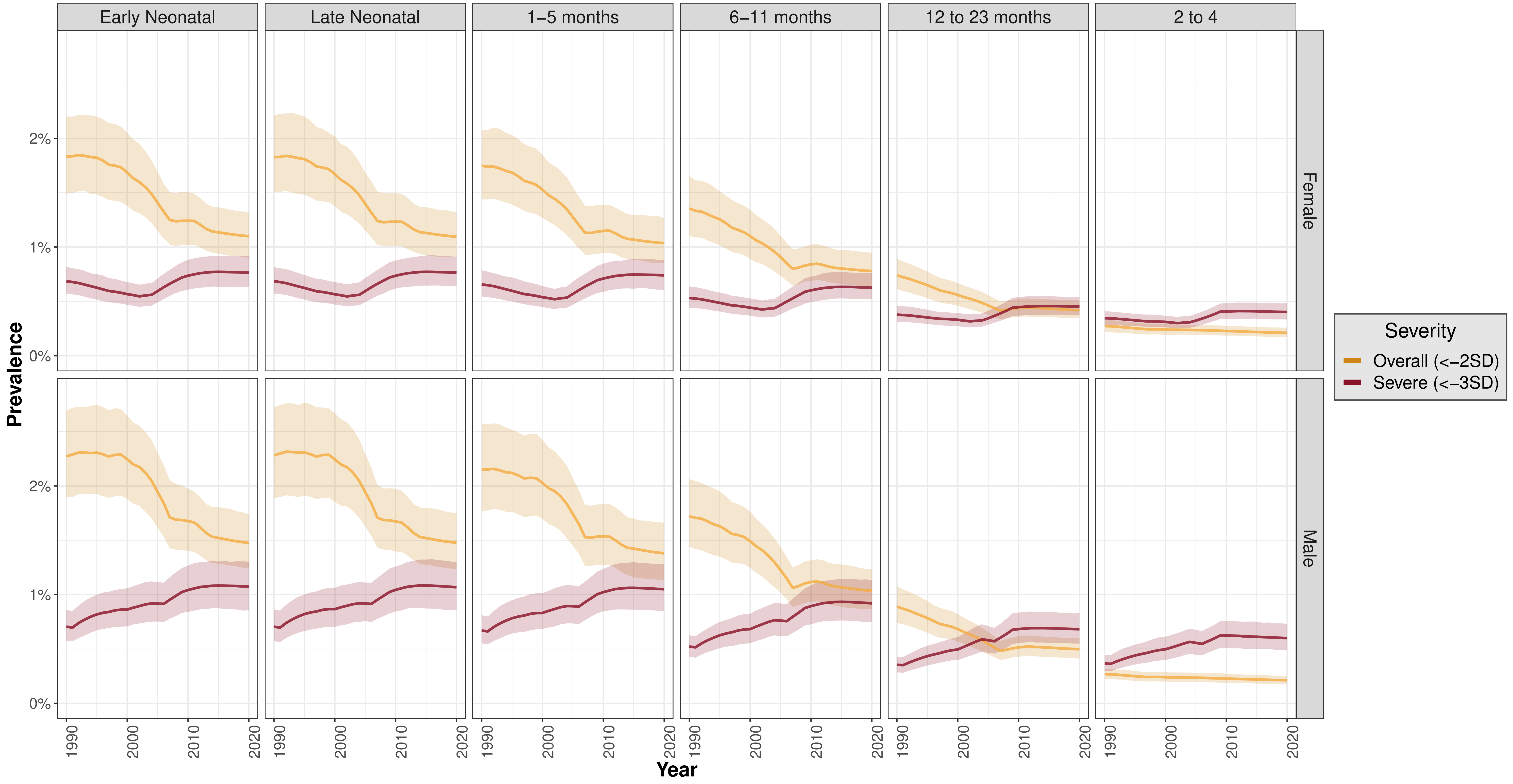

H: Transformed Mean Underweight Z Scores

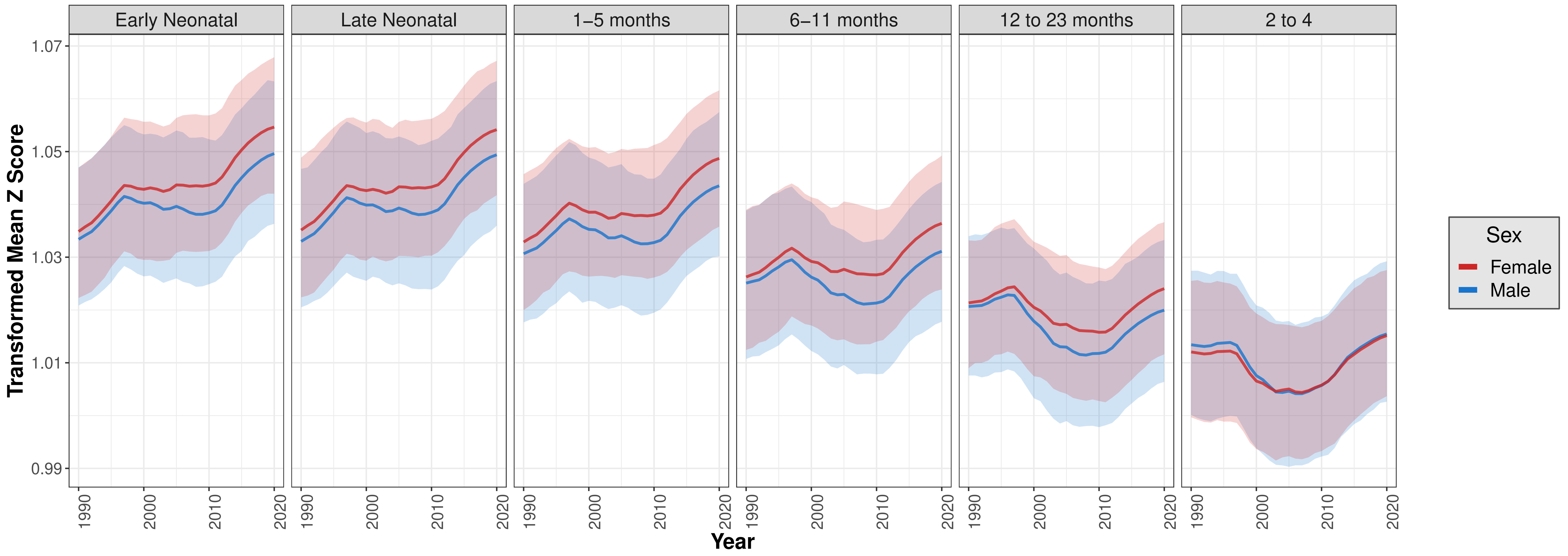

I

Source
No national or subnational sources for this location

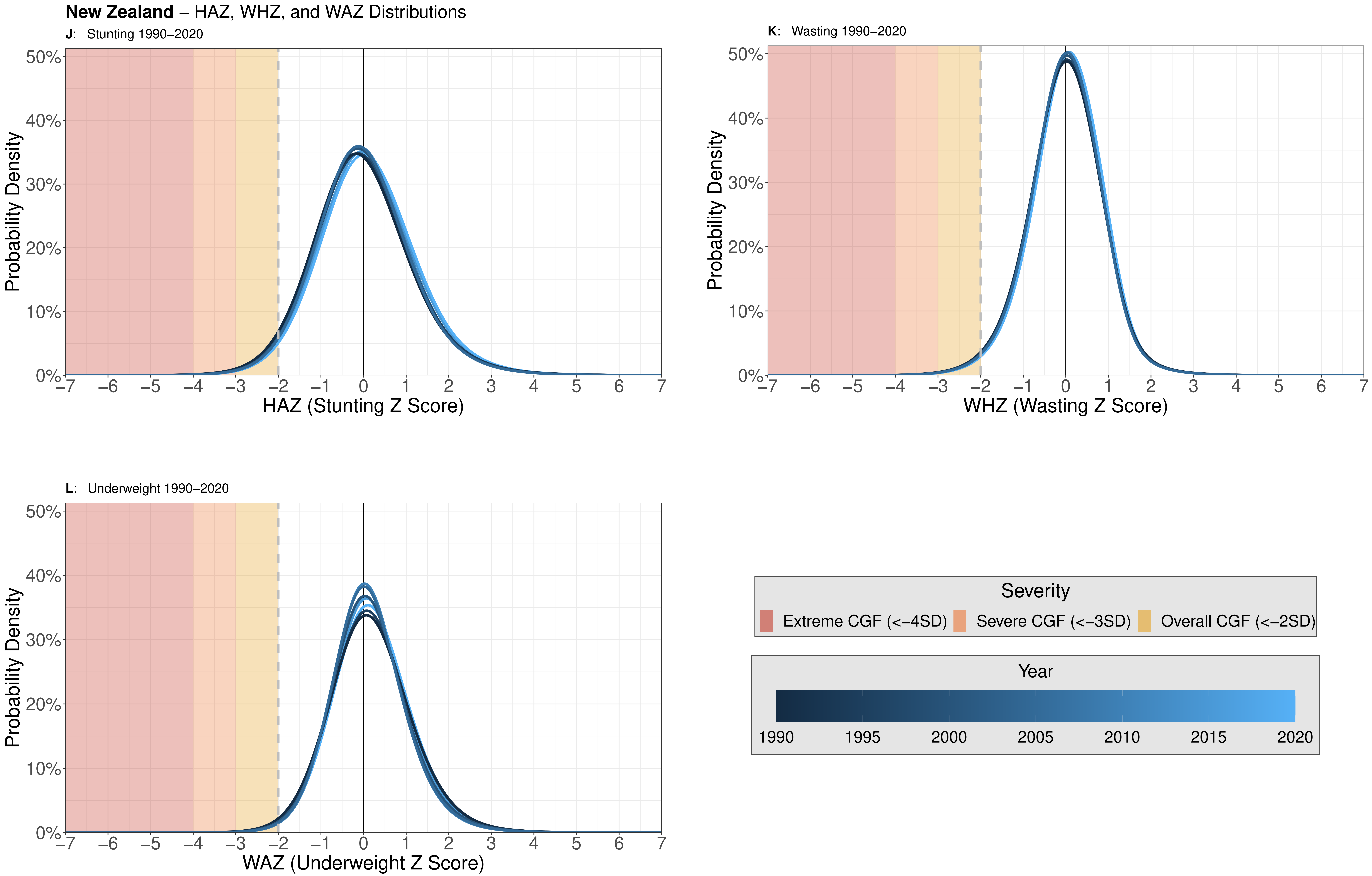

New Zealand – HAZ, WHZ, and WAZ Distributions
J: Stunting 1990–2020
Probability Density
50%
40%
30%
20%
10%
0%
−7 −6 −5 −4 −3 −2 −1 0 1 2 3 4 5 6 7
HAZ (Stunting Z Score)
K: Wasting 1990–2020
WHZ (Wasting Z Score)
L: Underweight 1990–2020
WAZ (Underweight Z Score)
Severity
Extreme CGF (<−4SD)
Severe CGF (<−3SD)
Overall CGF (<−2SD)
Year
1990
1995
2000
2005
2010
2015
2020

Brunei Darussalam – Stunting (HAZ)

A: Overall and Severe Stunting Prevalence

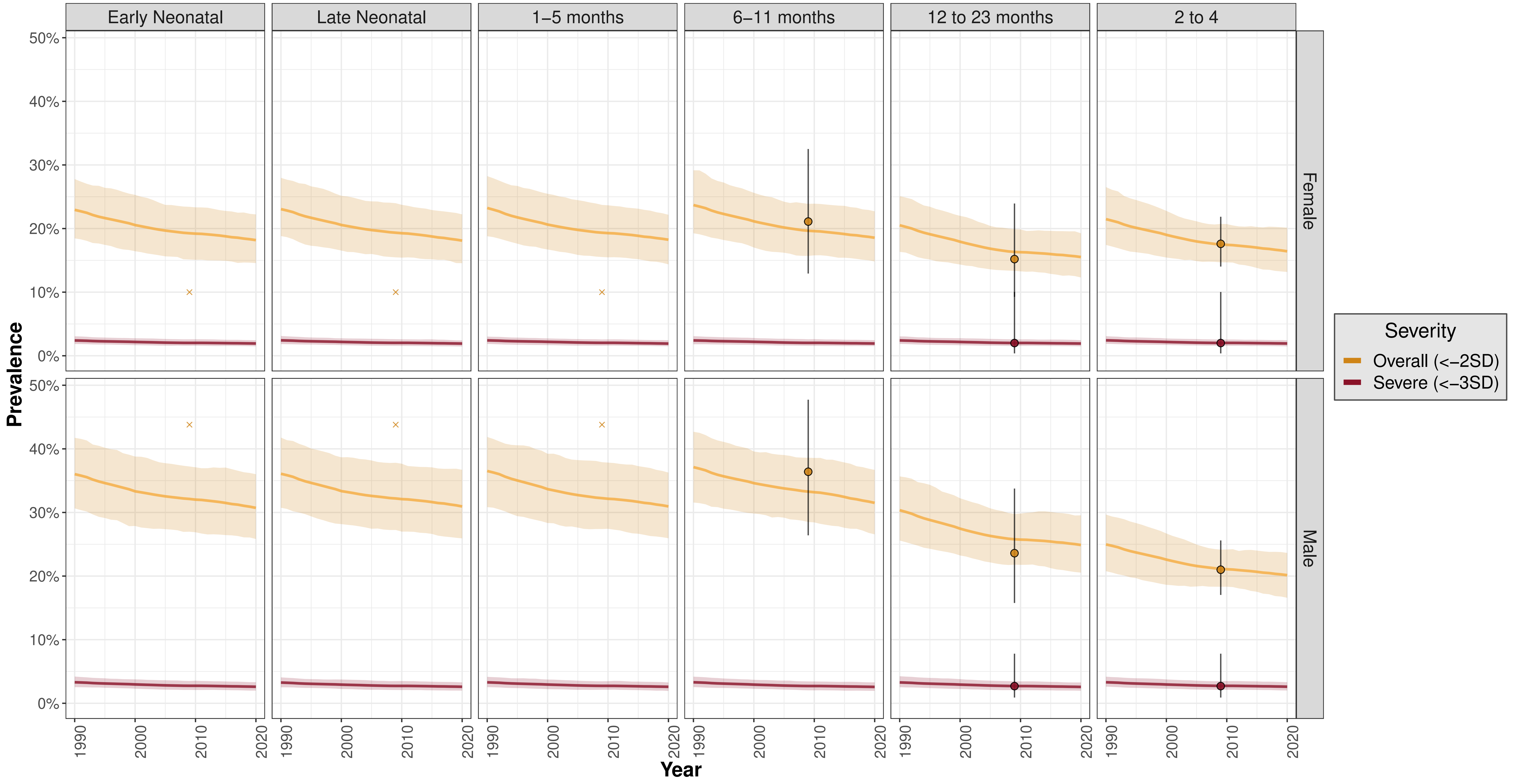

B: Transformed Mean Stunting Z Scores

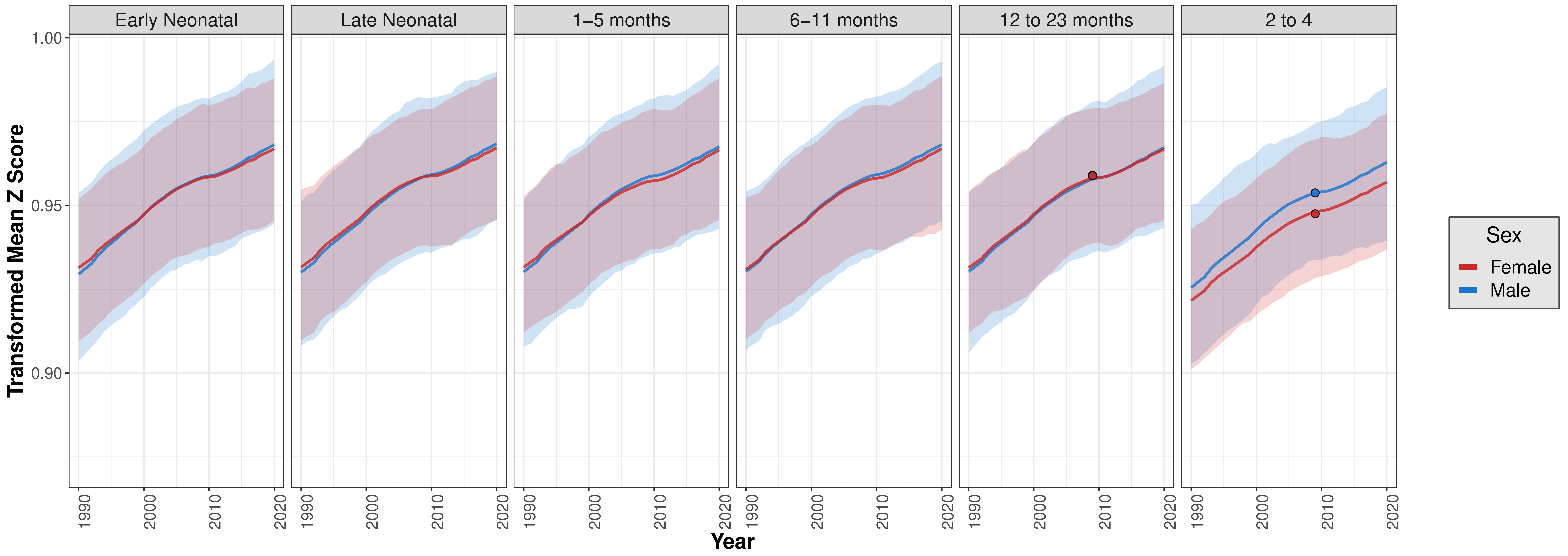

C

Year	Source
2009	WHO CGM Database

Brunei Darussalam – Wasting (WHZ)

D: Overall and Severe Wasting Prevalence

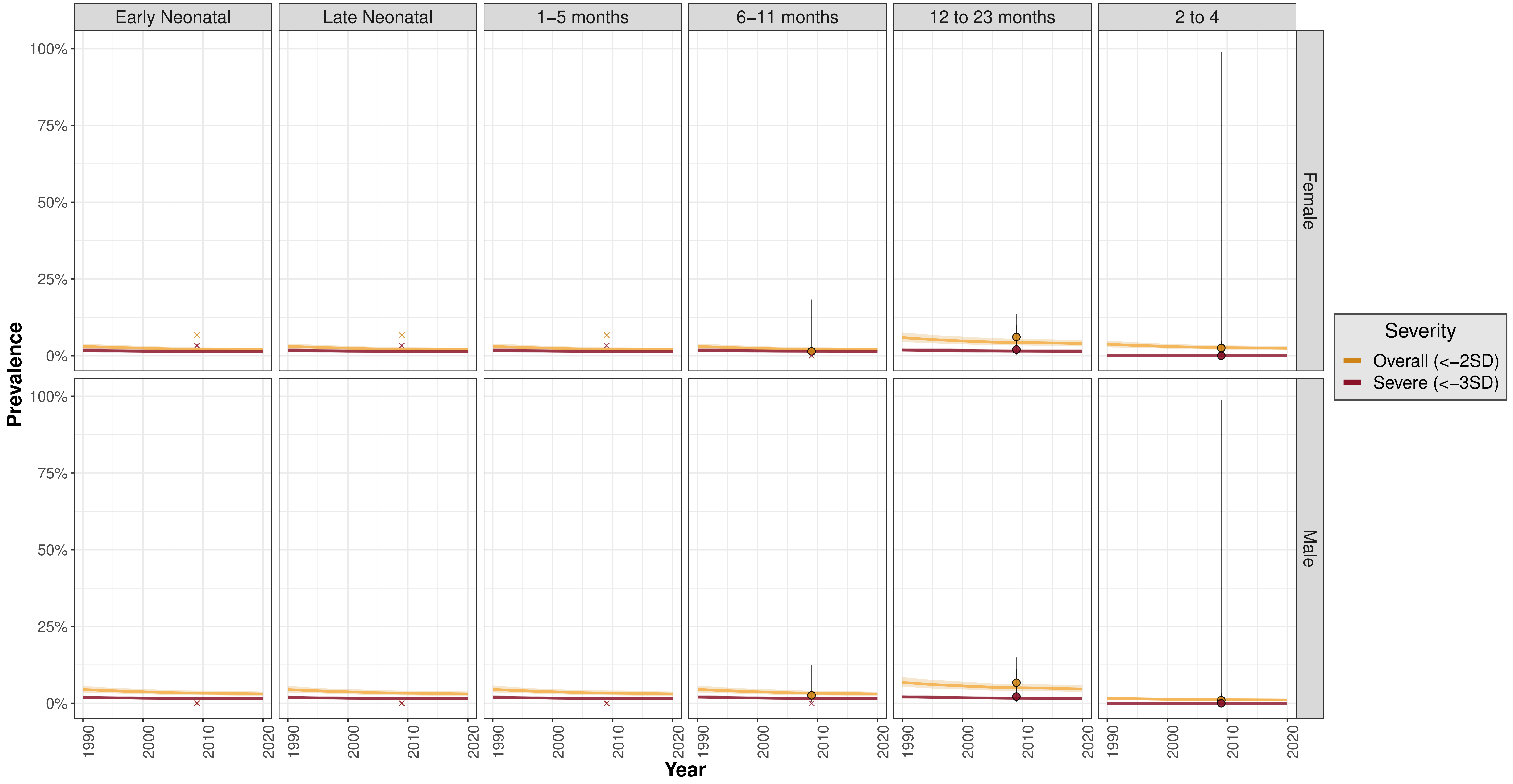

E: Transformed Mean Wasting Z Scores

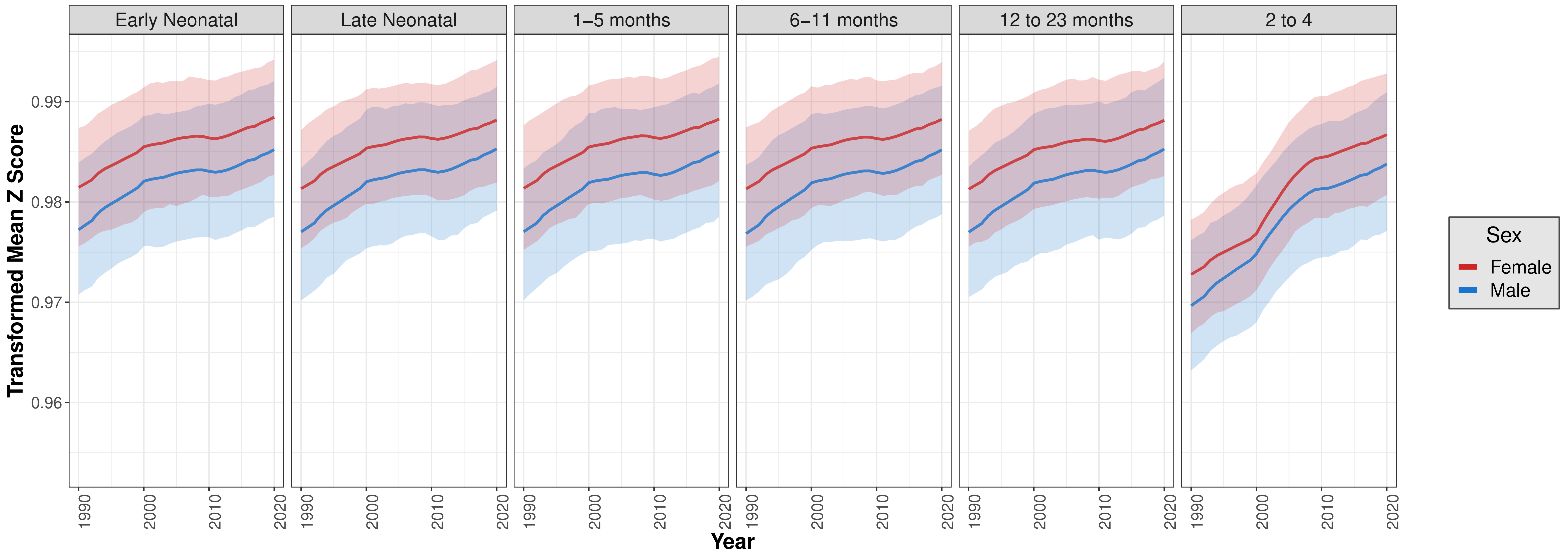

F

Year	Source
2009	WHO CGM Database

Brunei Darussalam – Underweight (WAZ)

G: Overall and Severe Underweight Prevalence

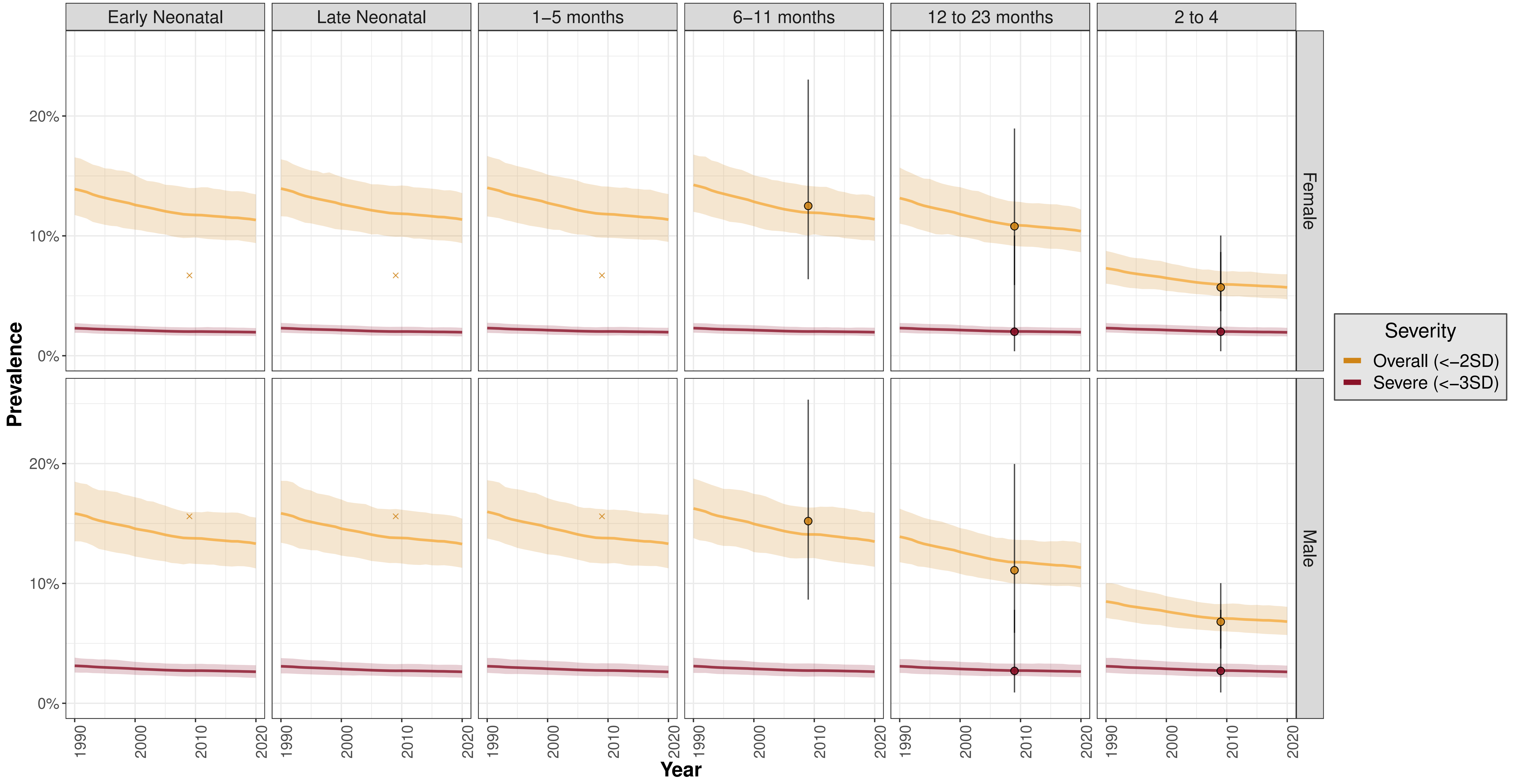

H: Transformed Mean Underweight Z Scores

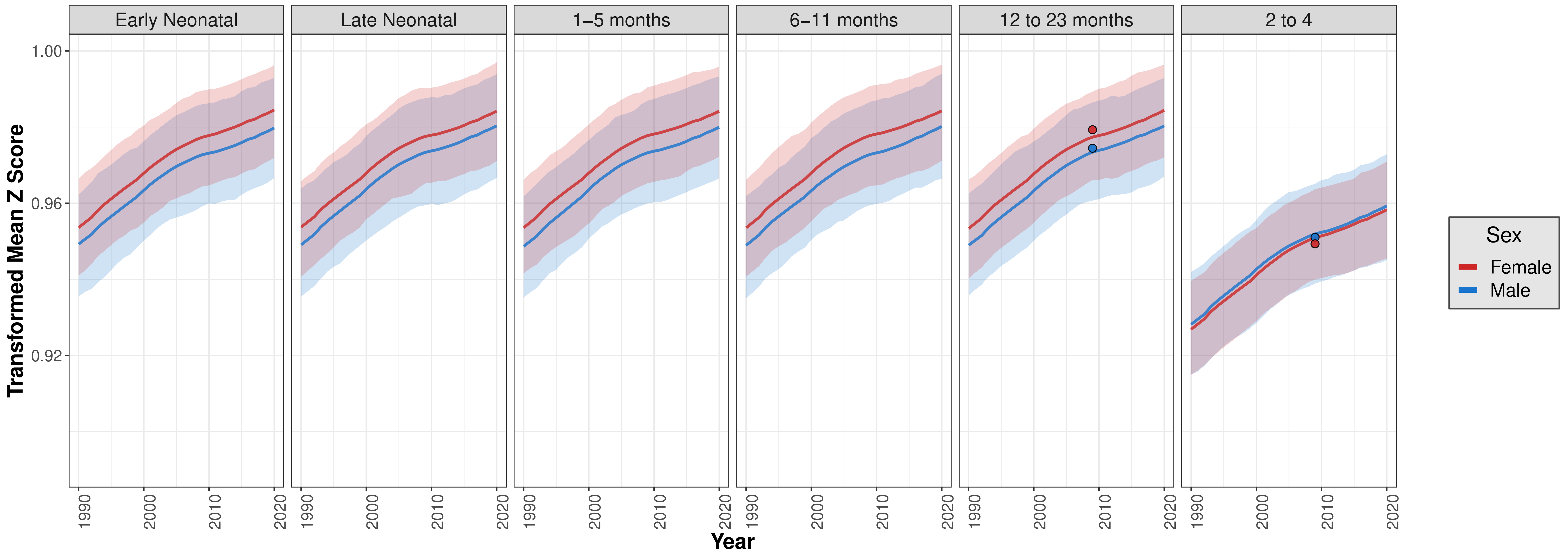

I

Year	Source
2009	WHO CGM Database

Brunei Darussalam – HAZ, WHZ, and WAZ Distributions

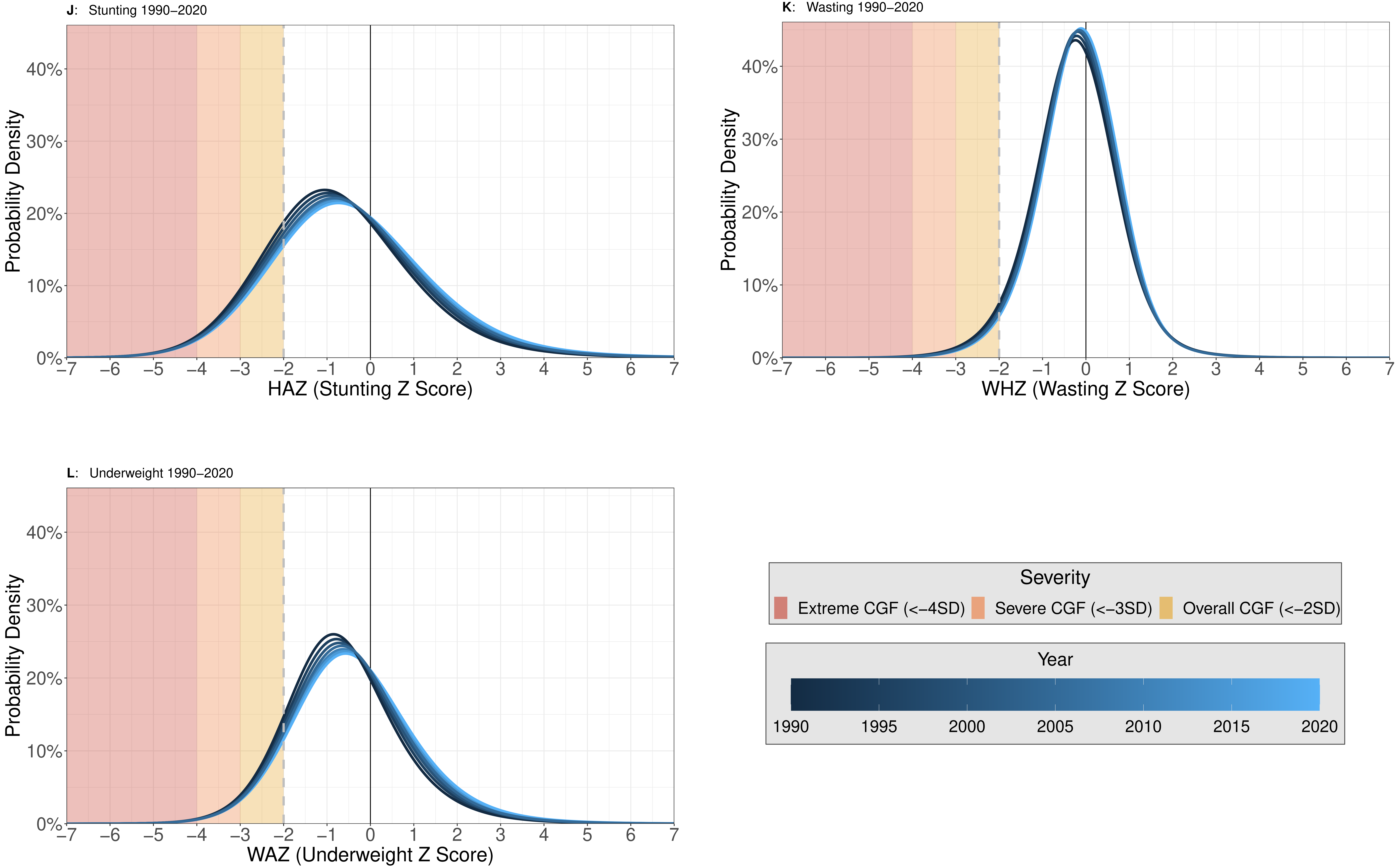

Japan – Stunting (HAZ)

A: Overall and Severe Stunting Prevalence

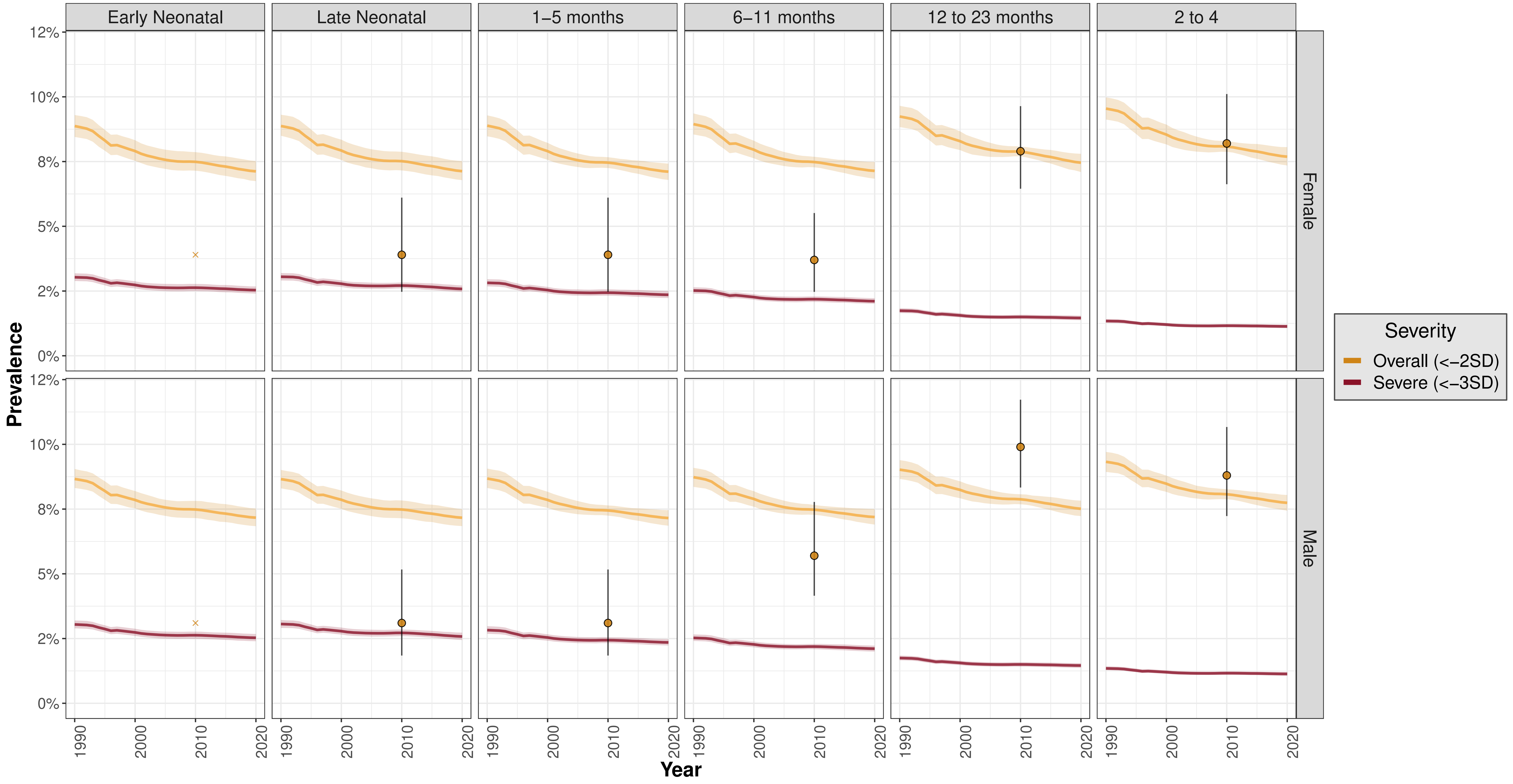

B: Transformed Mean Stunting Z Scores

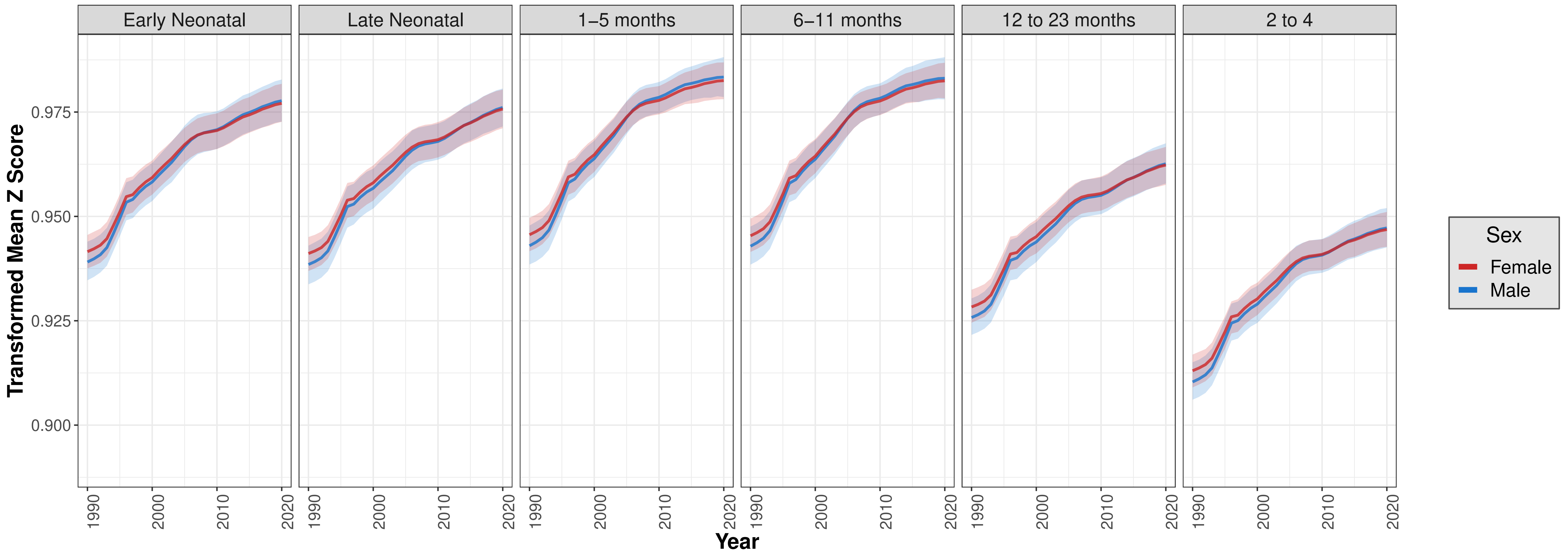

C

Year	Source	National	Subnational
1980	WHO CGM Database	X	
2010	WHO CGM Database	X	X

Japan – Wasting (WHZ)

D: Overall and Severe Wasting Prevalence

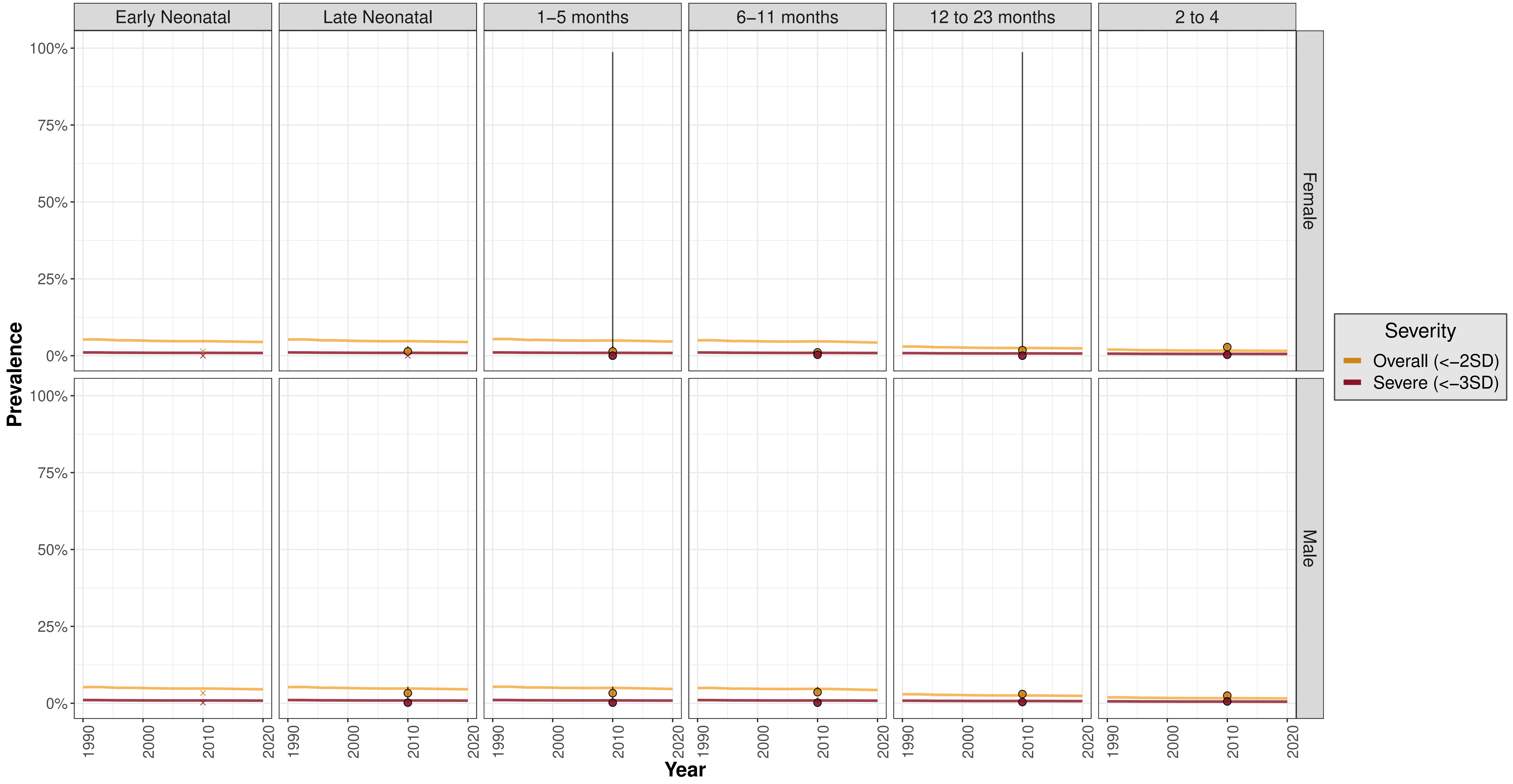

E: Transformed Mean Wasting Z Scores

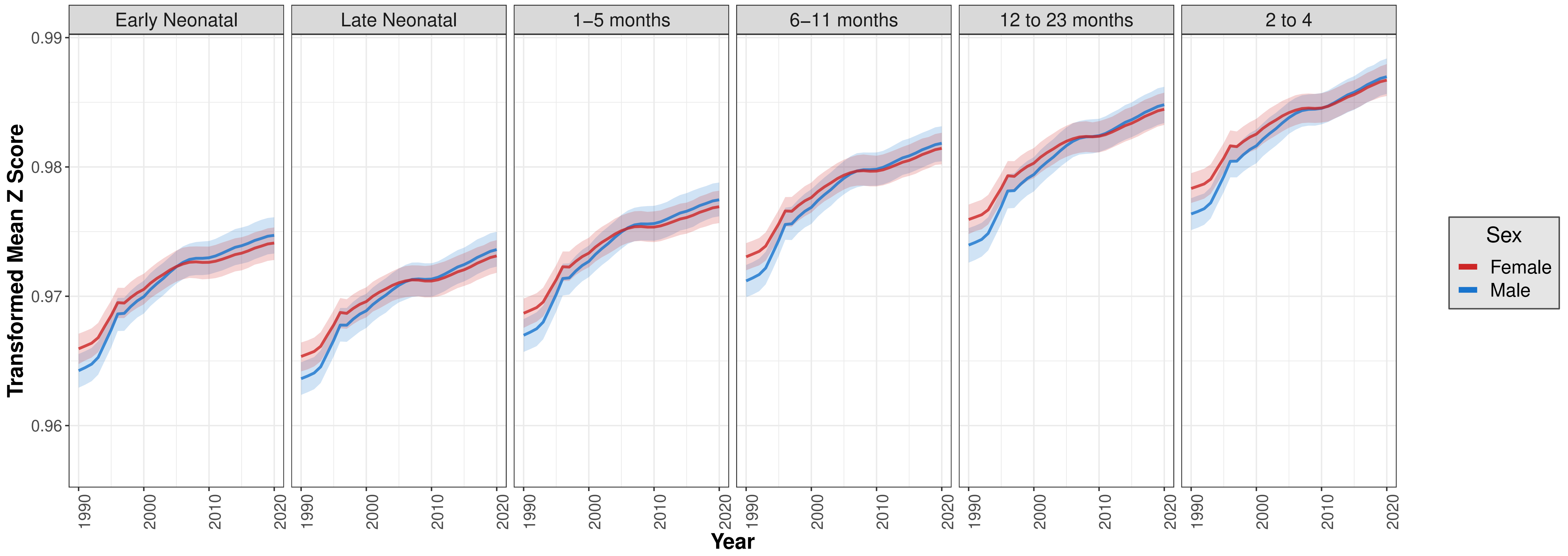

F

Year	Source	National	Subnational
1980	WHO CGM Database	X	
2010	WHO CGM Database	X	X

Japan – Underweight (WAZ)

G: Overall and Severe Underweight Prevalence

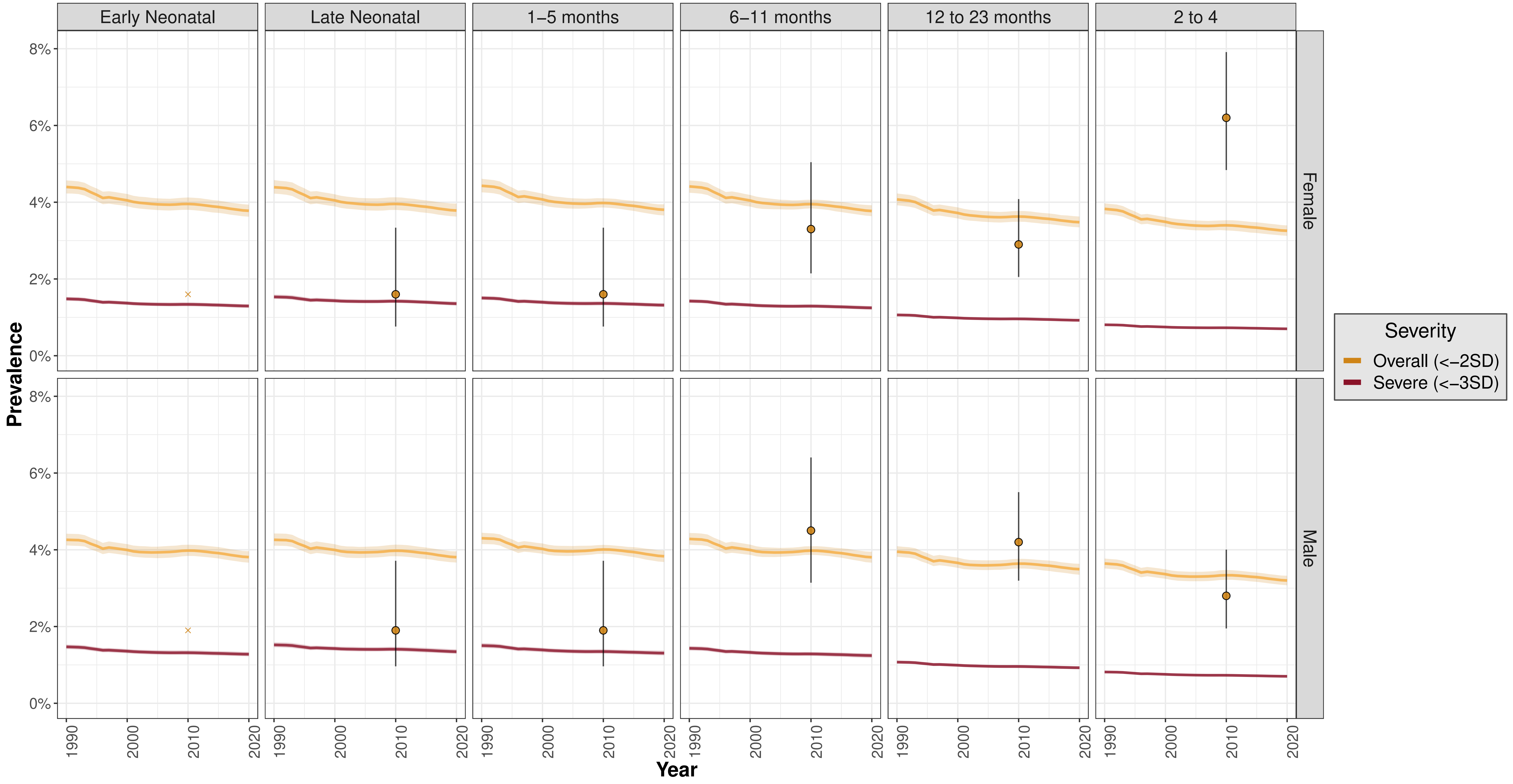

H: Transformed Mean Underweight Z Scores

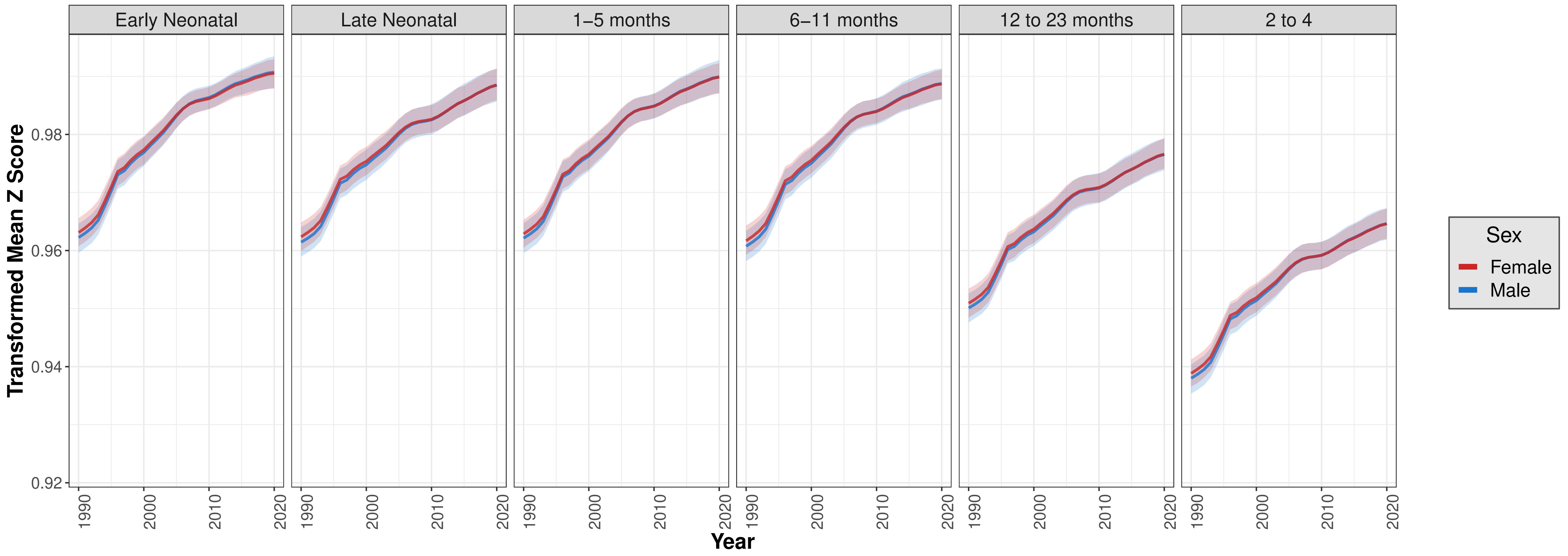

I

Year	Source	National	Subnational
1980	WHO CGM Database	X	
2010	WHO CGM Database	X	X

Japan – HAZ, WHZ, and WAZ Distributions

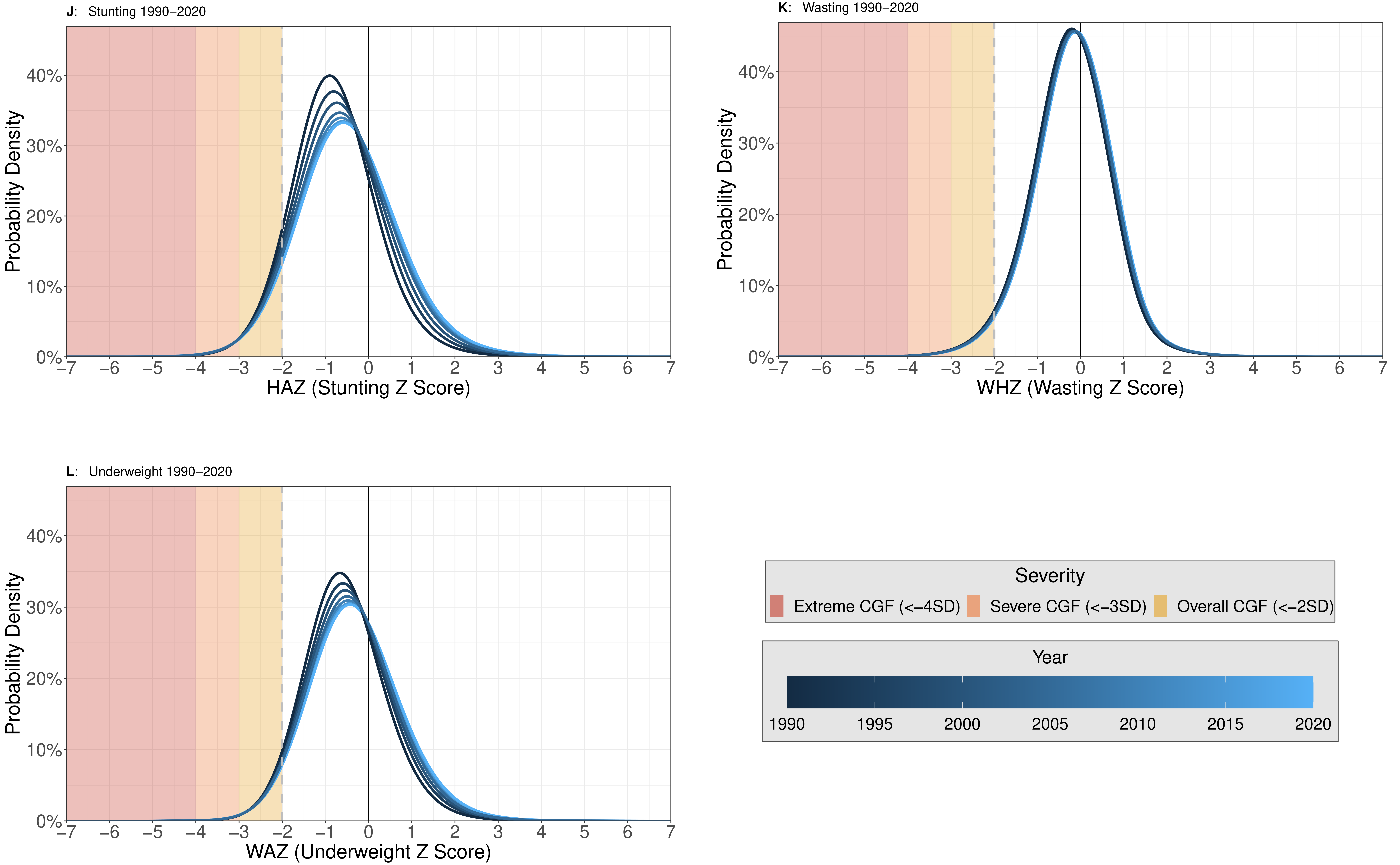

Republic of Korea – Stunting (HAZ)

A: Overall and Severe Stunting Prevalence

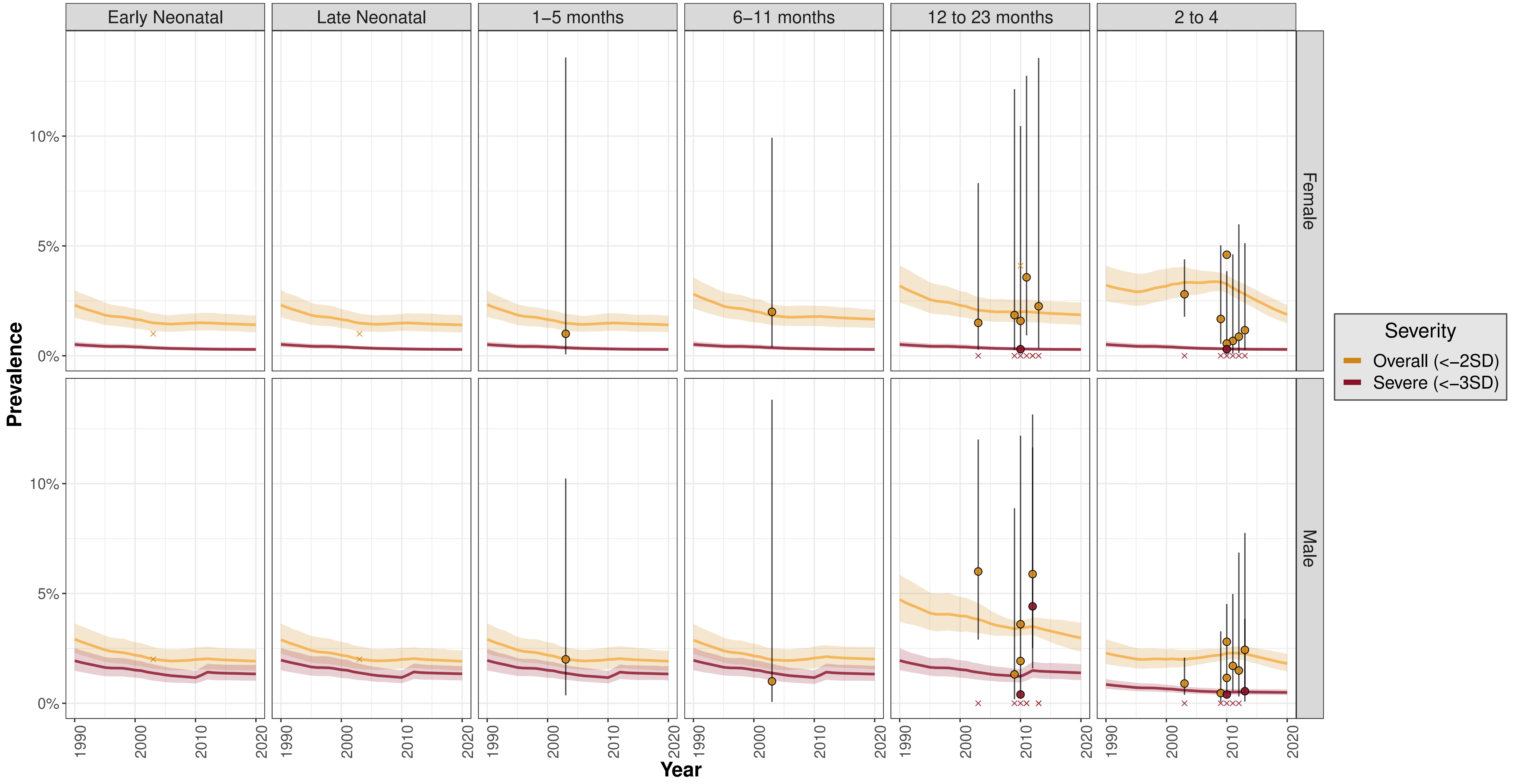

C

Year	Source
2003	WHO CGM Database
2009	National Health and Nutrition Examination Survey
2010	National Health and Nutrition Examination Survey
2010	WHO CGM Database
2011	National Health and Nutrition Examination Survey
2012	National Health and Nutrition Examination Survey
2013	National Health and Nutrition Examination Survey

B: Transformed Mean Stunting Z Scores

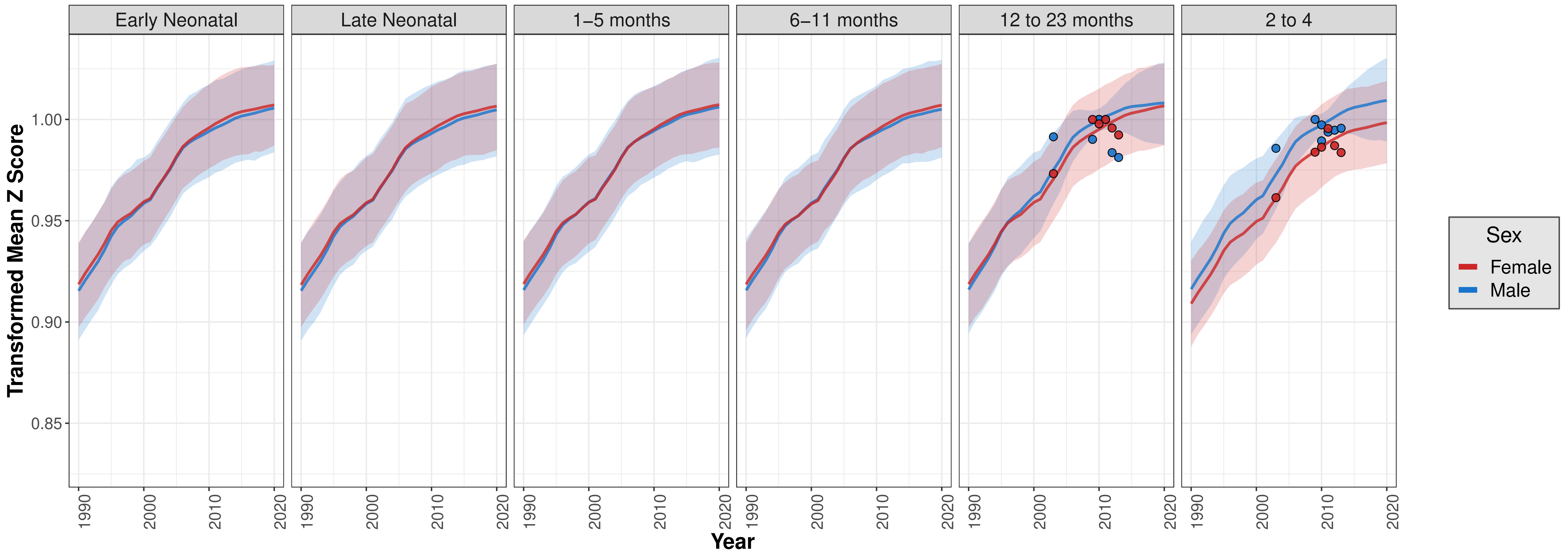

Republic of Korea – Wasting (WHZ)

D: Overall and Severe Wasting Prevalence

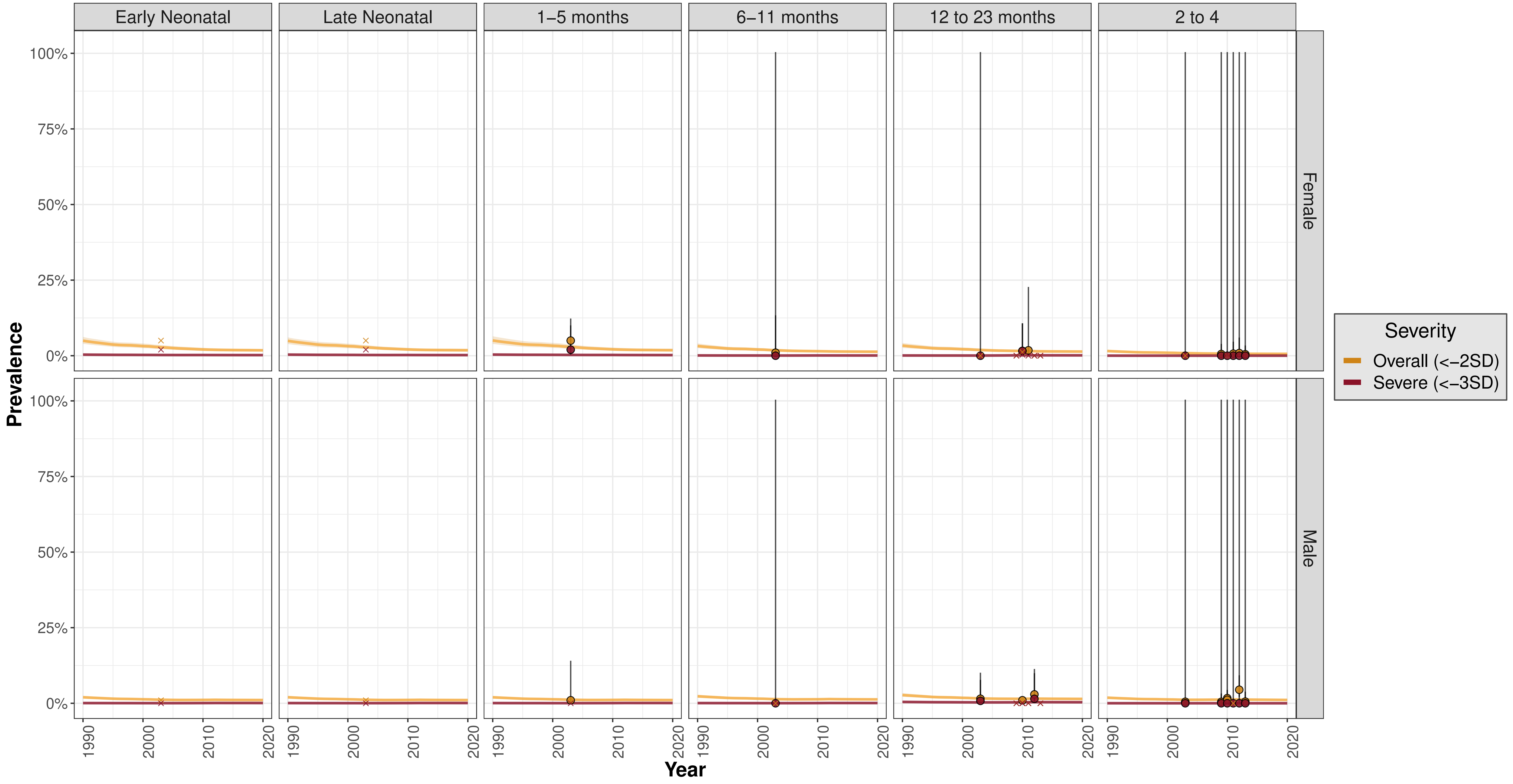

E: Transformed Mean Wasting Z Scores

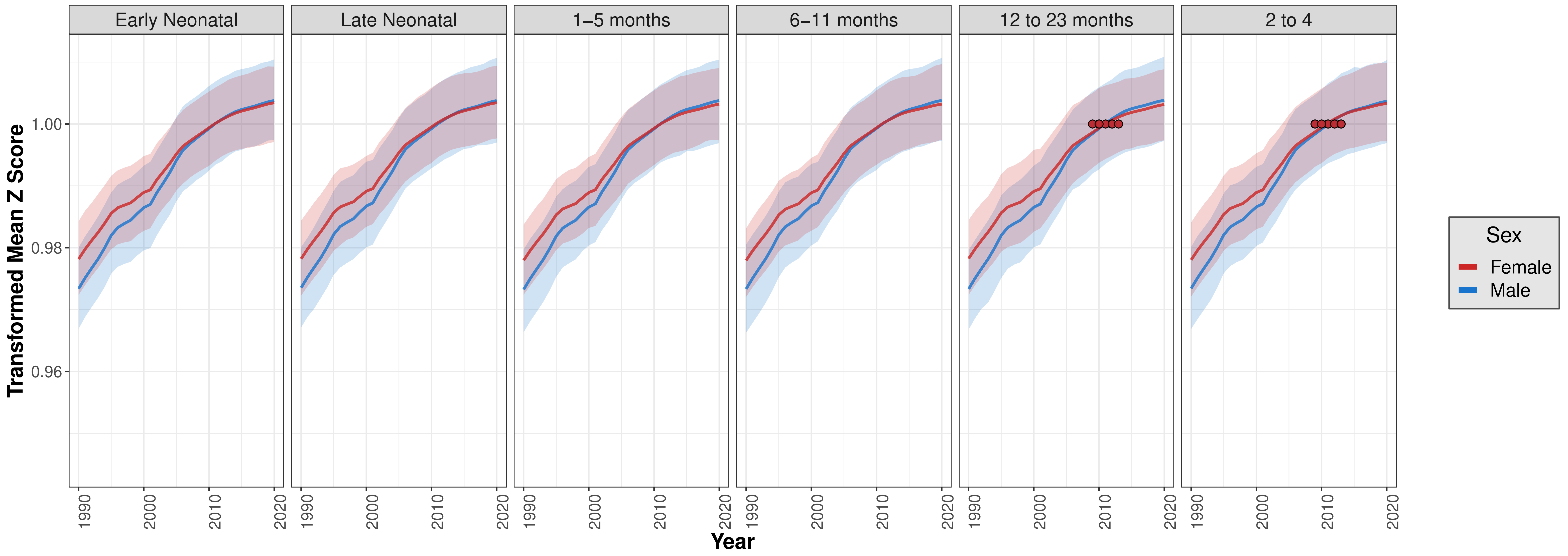

F

Year	Source
2003	WHO CGM Database
2009	National Health and Nutrition Examination Survey
2010	National Health and Nutrition Examination Survey
2010	WHO CGM Database
2011	National Health and Nutrition Examination Survey
2012	National Health and Nutrition Examination Survey
2013	National Health and Nutrition Examination Survey

Republic of Korea – Underweight (WAZ)

G: Overall and Severe Underweight Prevalence

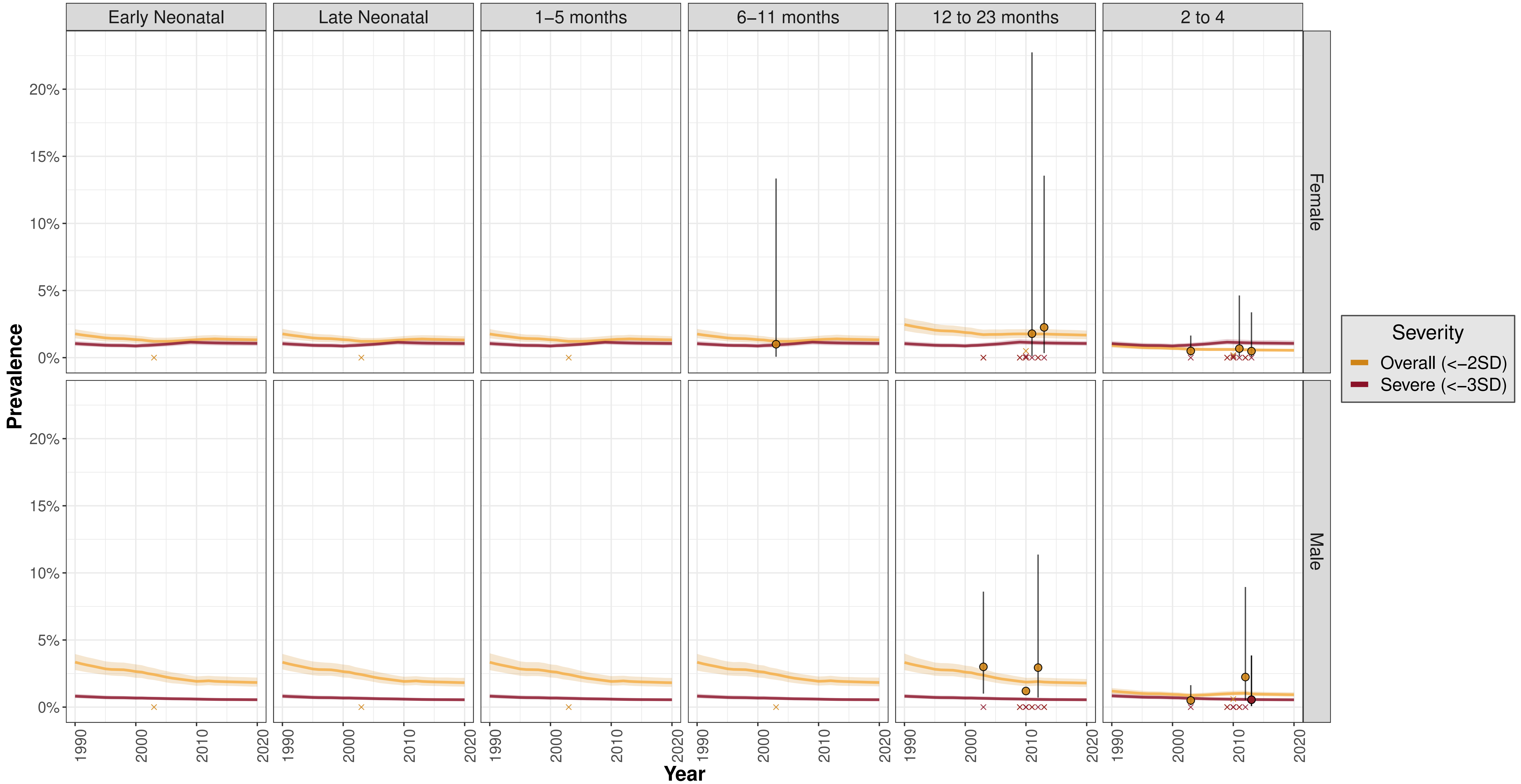

H: Transformed Mean Underweight Z Scores

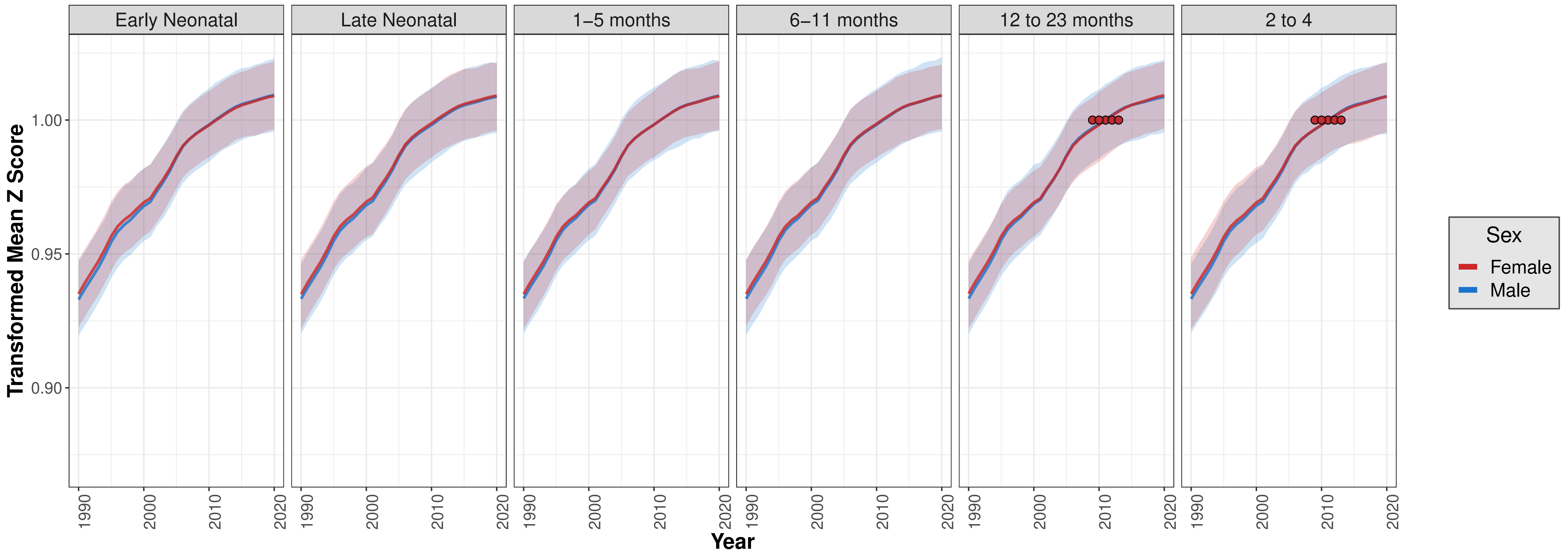

I

Year	Source
2003	WHO CGM Database
2009	National Health and Nutrition Examination Survey
2010	National Health and Nutrition Examination Survey
2010	WHO CGM Database
2011	National Health and Nutrition Examination Survey
2012	National Health and Nutrition Examination Survey
2013	National Health and Nutrition Examination Survey

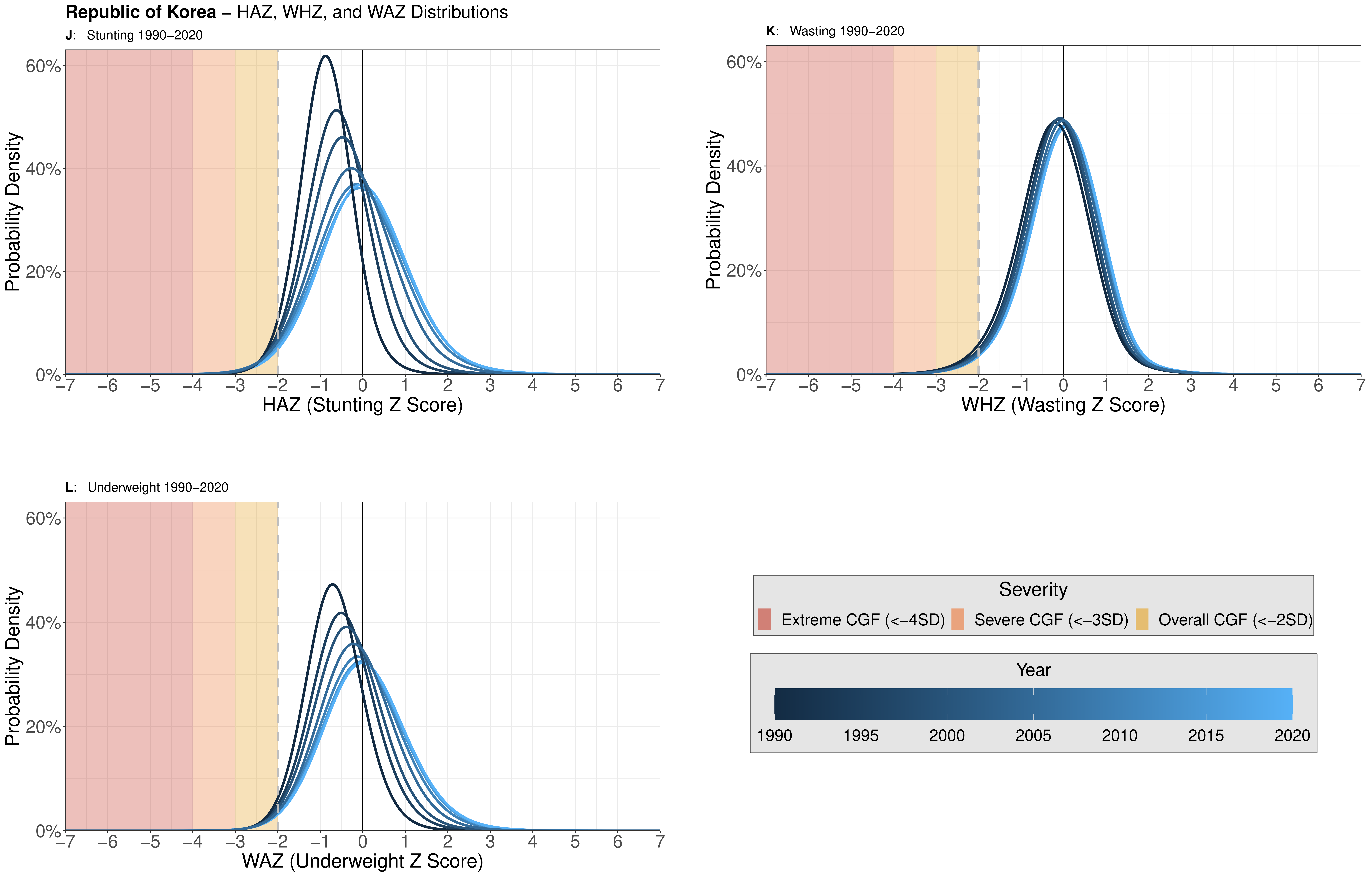

Republic of Korea – HAZ, WHZ, and WAZ Distributions
J: Stunting 1990–2020
Probability Density
HAZ (Stunting Z Score)
K: Wasting 1990–2020
WHZ (Wasting Z Score)
L: Underweight 1990–2020
WAZ (Underweight Z Score)
0%
20%
40%
60%
−7 −6 −5 −4 −3 −2 −1 0 1 2 3 4 5 6 7
Severity
Extreme CGF (<−4SD)
Severe CGF (<−3SD)
Overall CGF (<−2SD)
Year
1990 1995 2000 2005 2010 2015 2020

Singapore – Stunting (HAZ)

A: Overall and Severe Stunting Prevalence

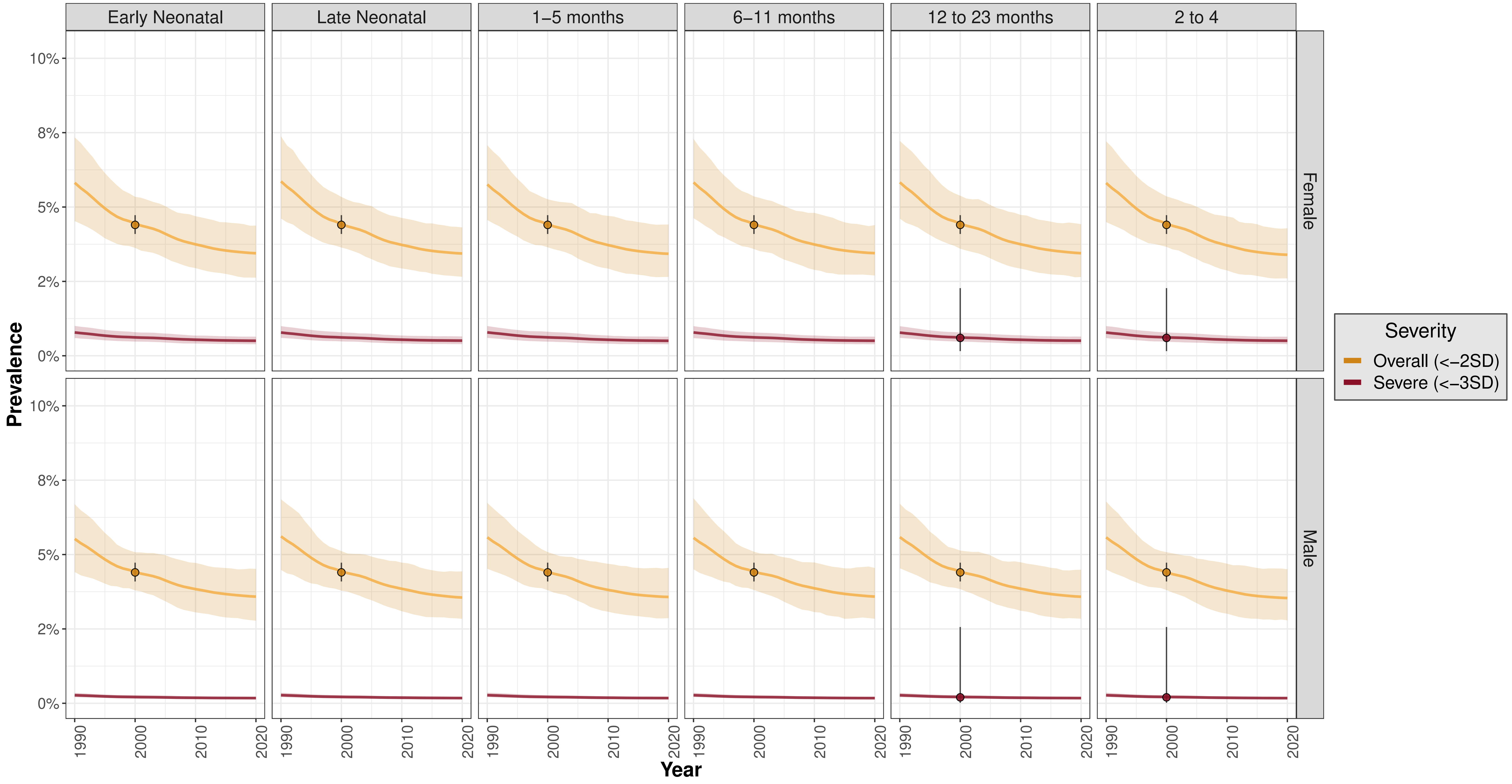

B: Transformed Mean Stunting Z Scores

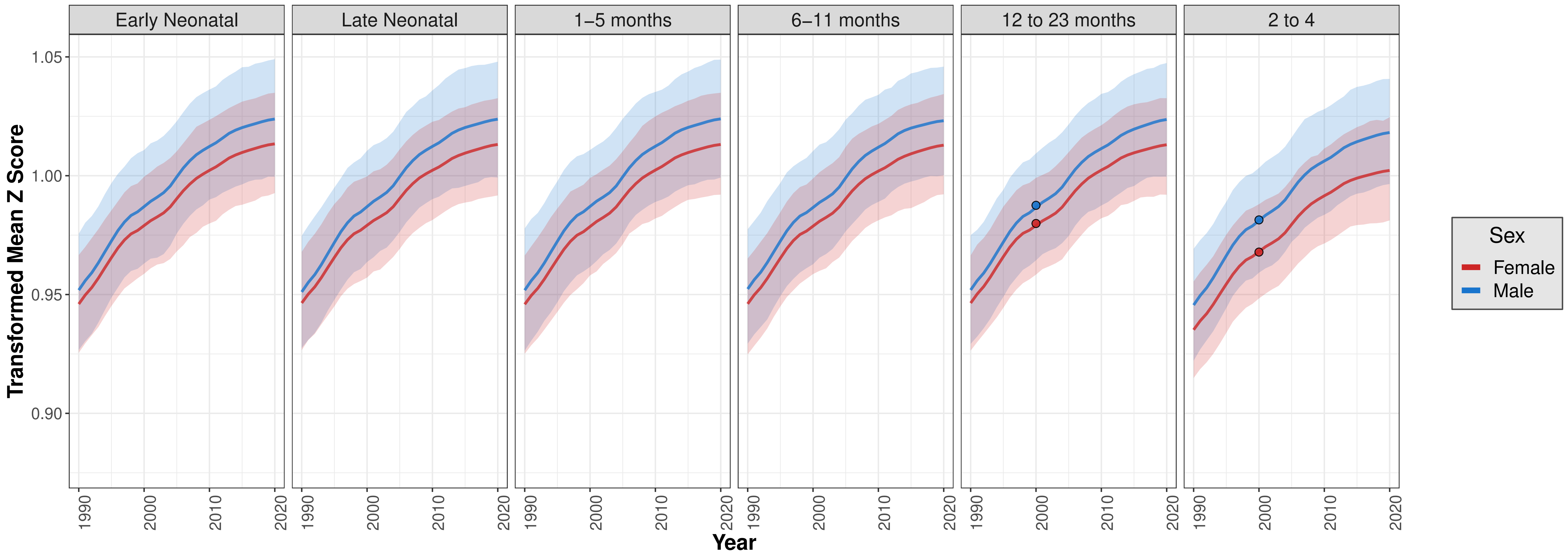

C

Year	Source
1974	WHO CGM Database
2000	WHO CGM Database

Singapore – Wasting (WHZ)

D: Overall and Severe Wasting Prevalence

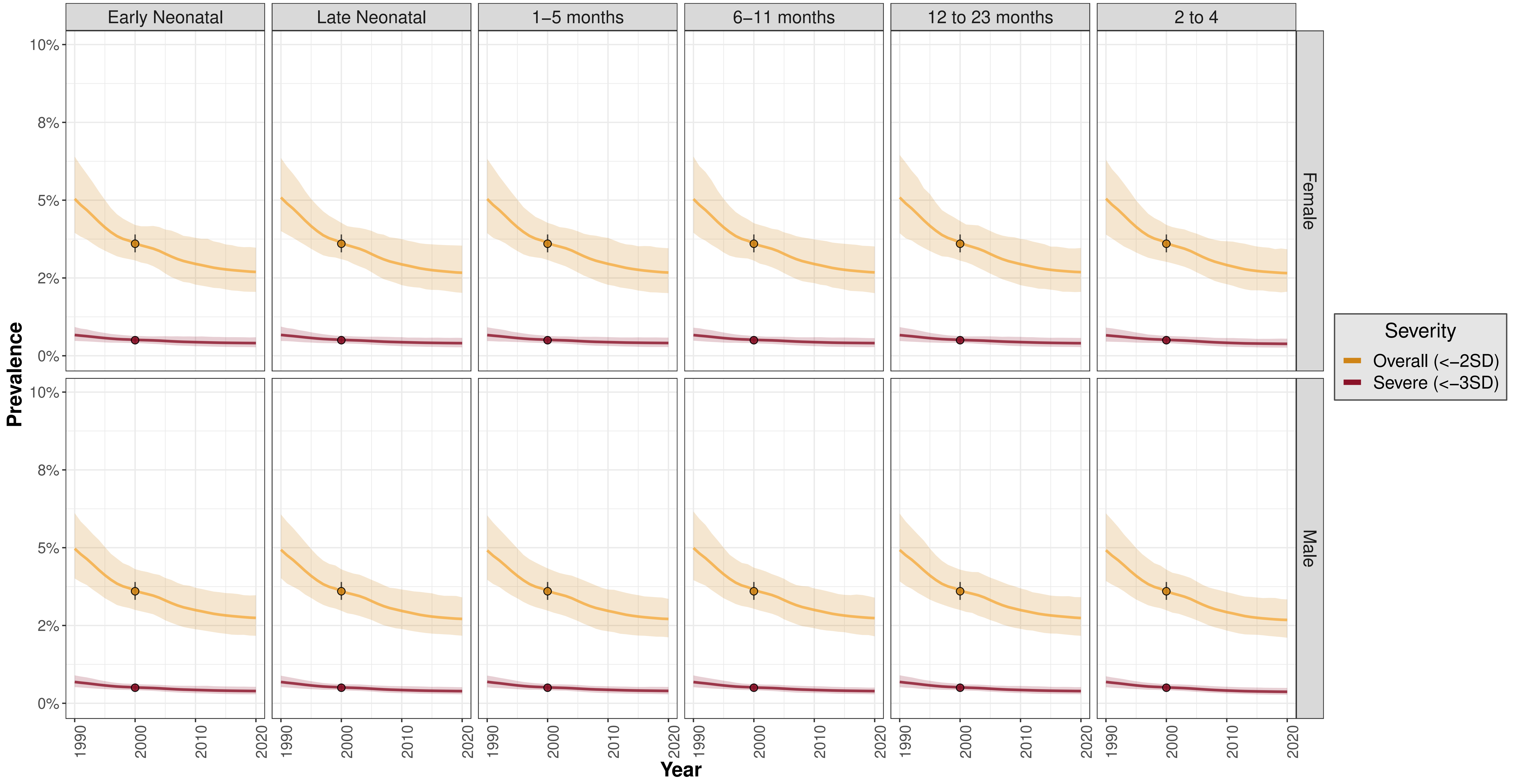

E: Transformed Mean Wasting Z Scores

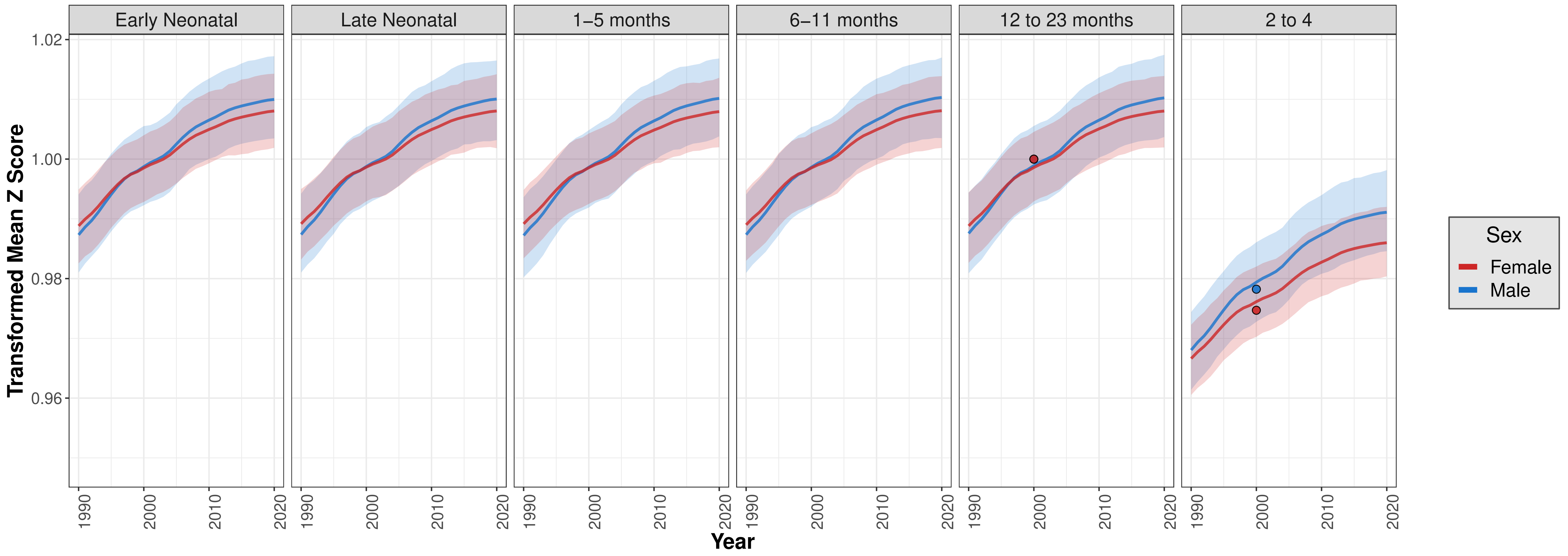

F

Year	Source
1974	WHO CGM Database
2000	WHO CGM Database

Singapore – Underweight (WAZ)

G: Overall and Severe Underweight Prevalence

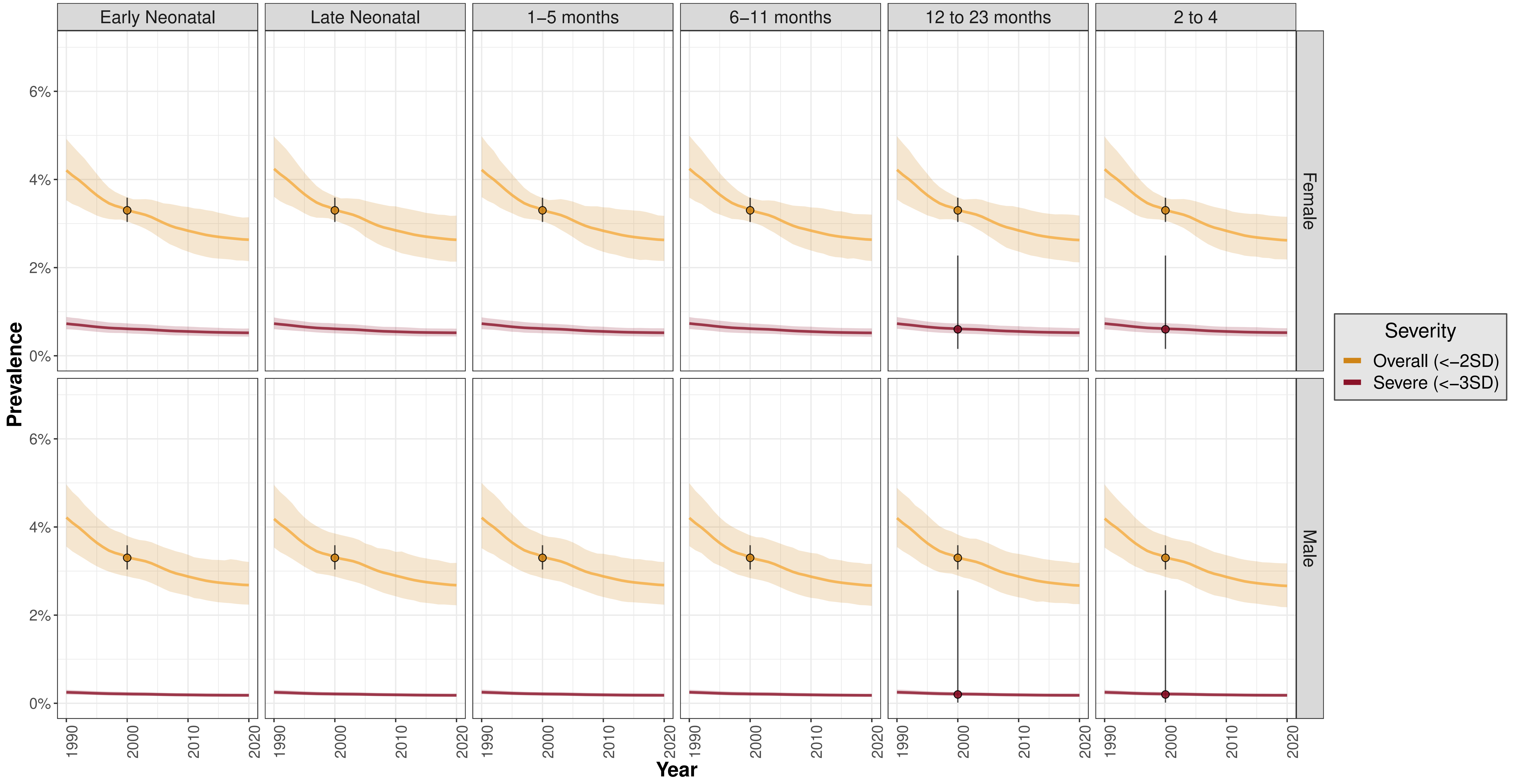

H: Transformed Mean Underweight Z Scores

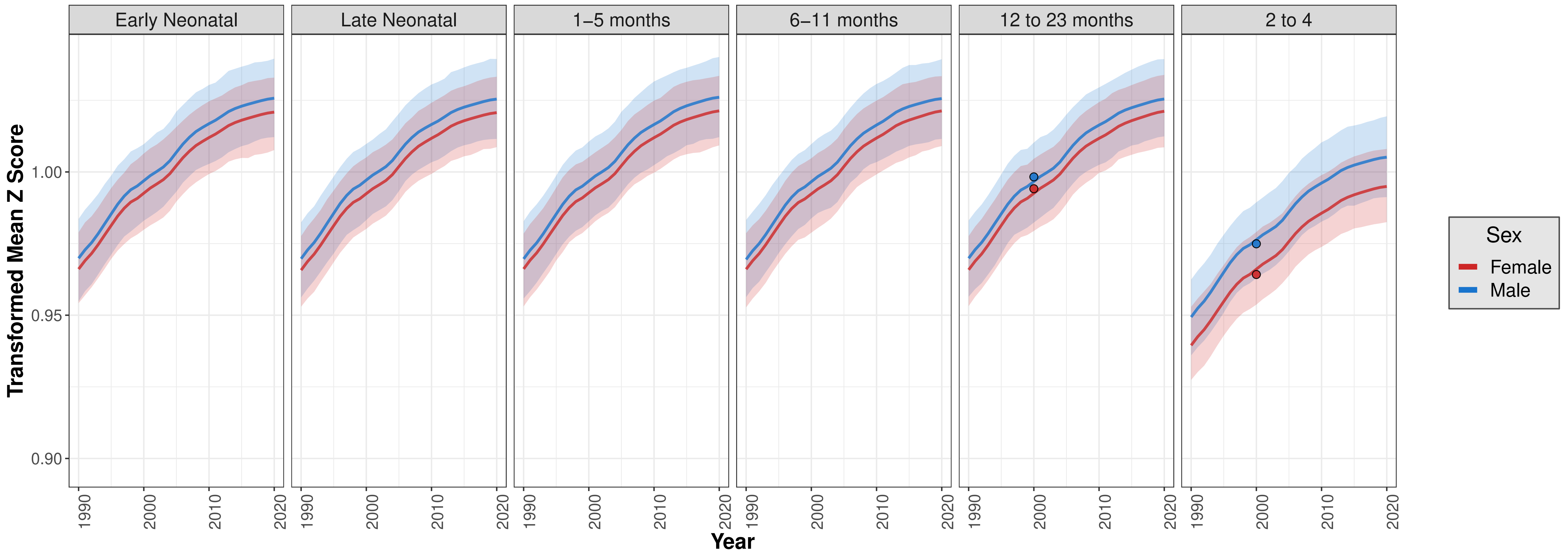

I

Year	Source
1974	WHO CGM Database
2000	WHO CGM Database

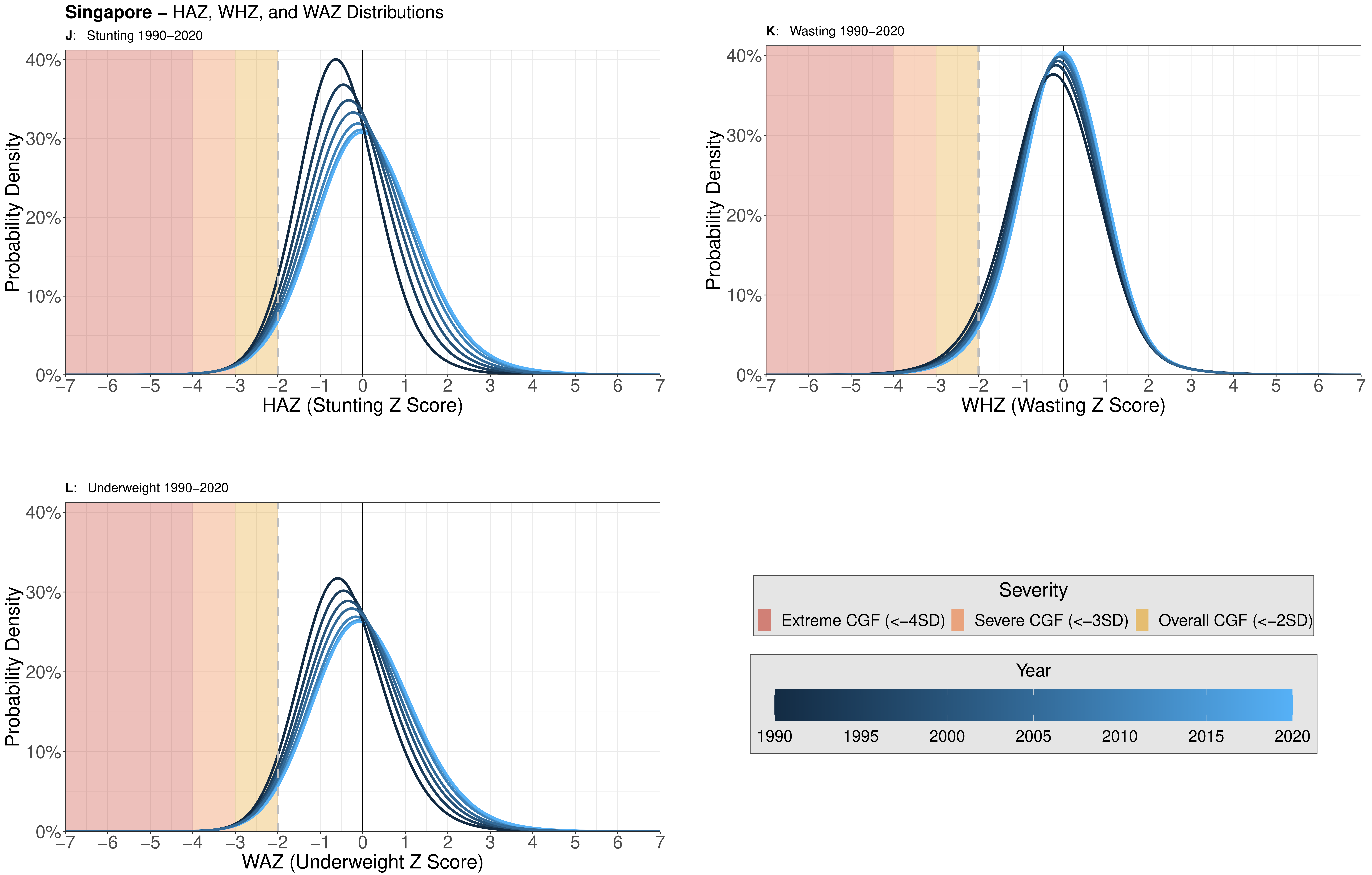
Singapore – HAZ, WHZ, and WAZ Distributions
J: Stunting 1990–2020
Probability Density
40%
30%
20%
10%
0%
−7 −6 −5 −4 −3 −2 −1 0 1 2 3 4 5 6 7
HAZ (Stunting Z Score)
K: Wasting 1990–2020
Probability Density
40%
30%
20%
10%
0%
−7 −6 −5 −4 −3 −2 −1 0 1 2 3 4 5 6 7
WHZ (Wasting Z Score)
L: Underweight 1990–2020
Probability Density
40%
30%
20%
10%
0%
−7 −6 −5 −4 −3 −2 −1 0 1 2 3 4 5 6 7
WAZ (Underweight Z Score)
Severity
Extreme CGF (<−4SD)
Severe CGF (<−3SD)
Overall CGF (<−2SD)
Year
1990
1995
2000
2005
2010
2015
2020

Canada – Stunting (HAZ)

A: Overall and Severe Stunting Prevalence

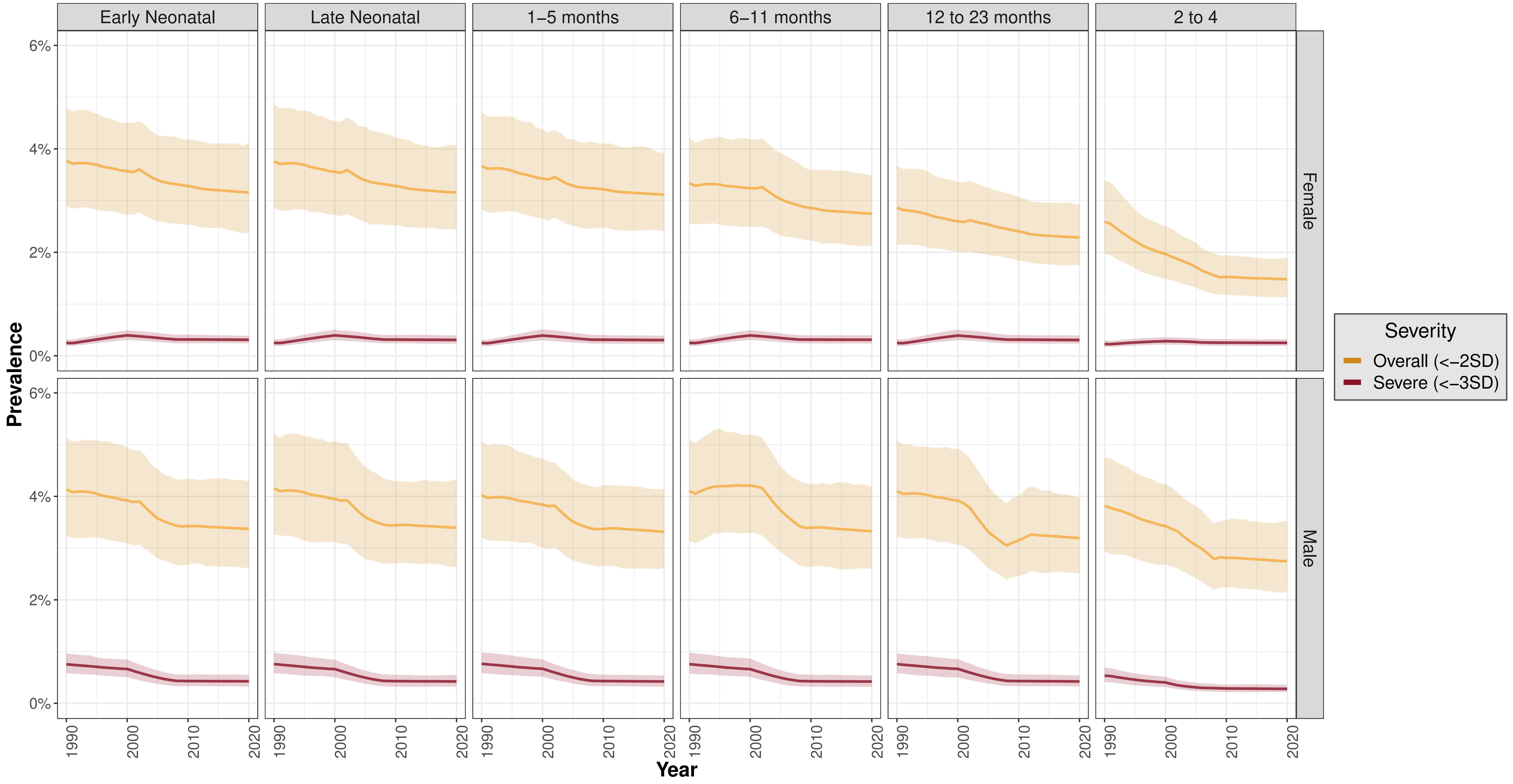

B: Transformed Mean Stunting Z Scores

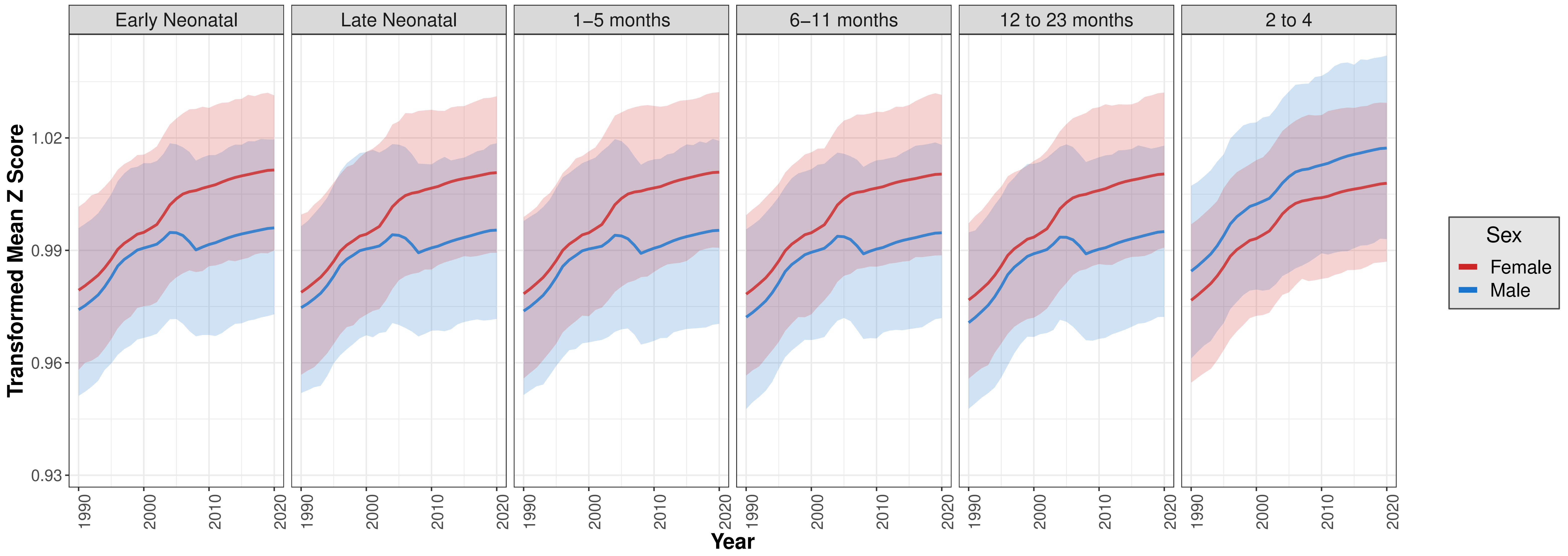

C

Year	Source
1971	WHO CGM Database

Canada – Wasting (WHZ)

D: Overall and Severe Wasting Prevalence

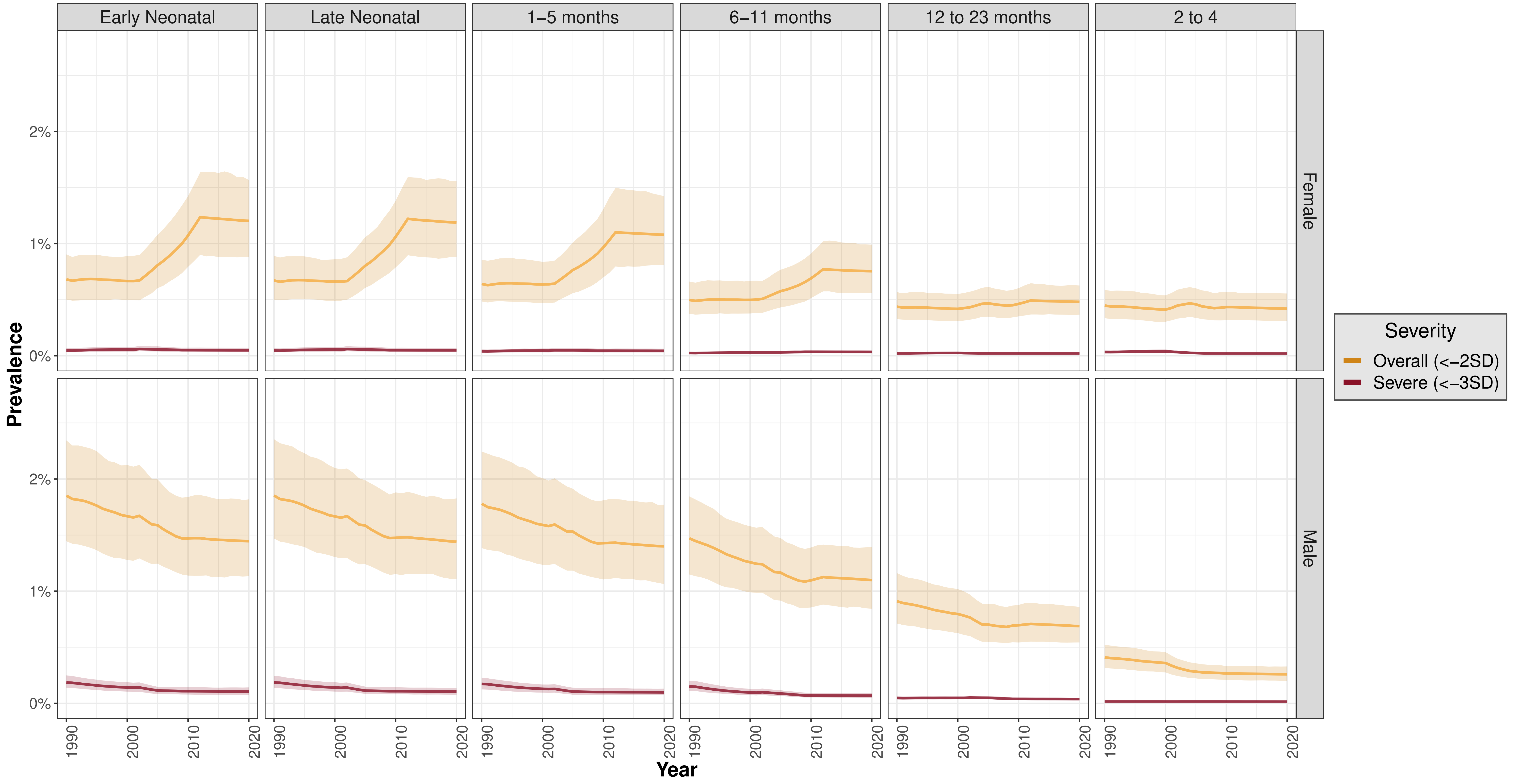

E: Transformed Mean Wasting Z Scores

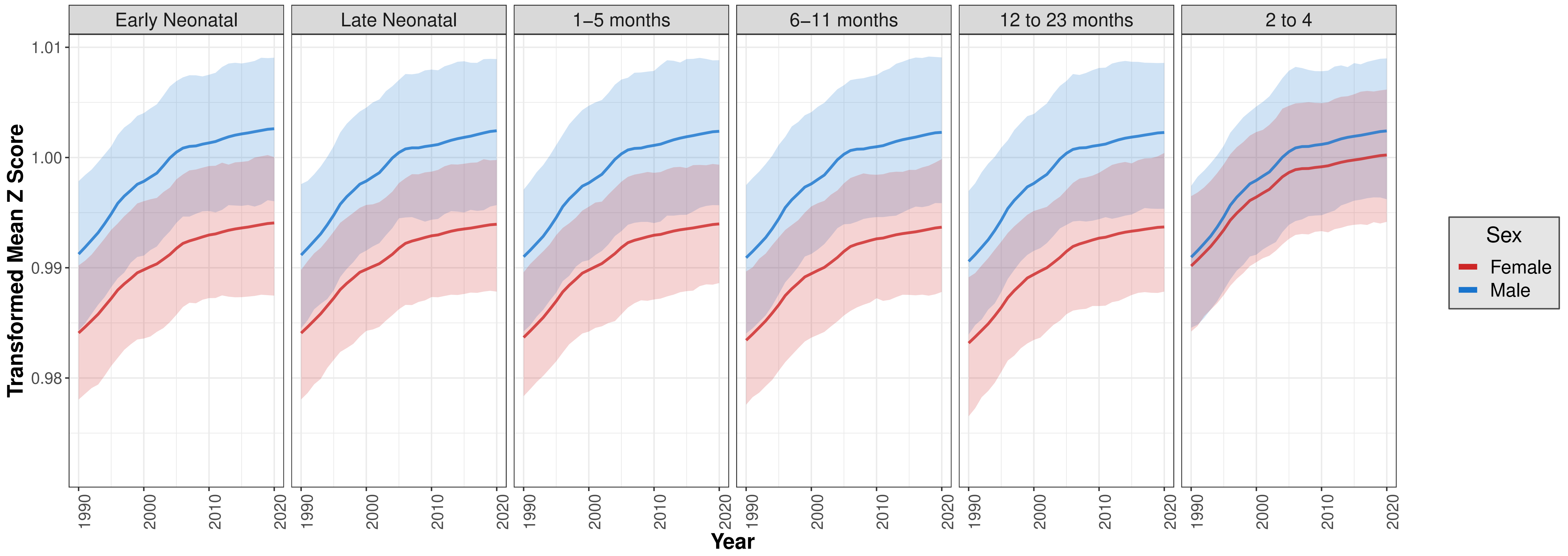

F

Year	Source
1971	WHO CGM Database

Canada – Underweight (WAZ)

G: Overall and Severe Underweight Prevalence

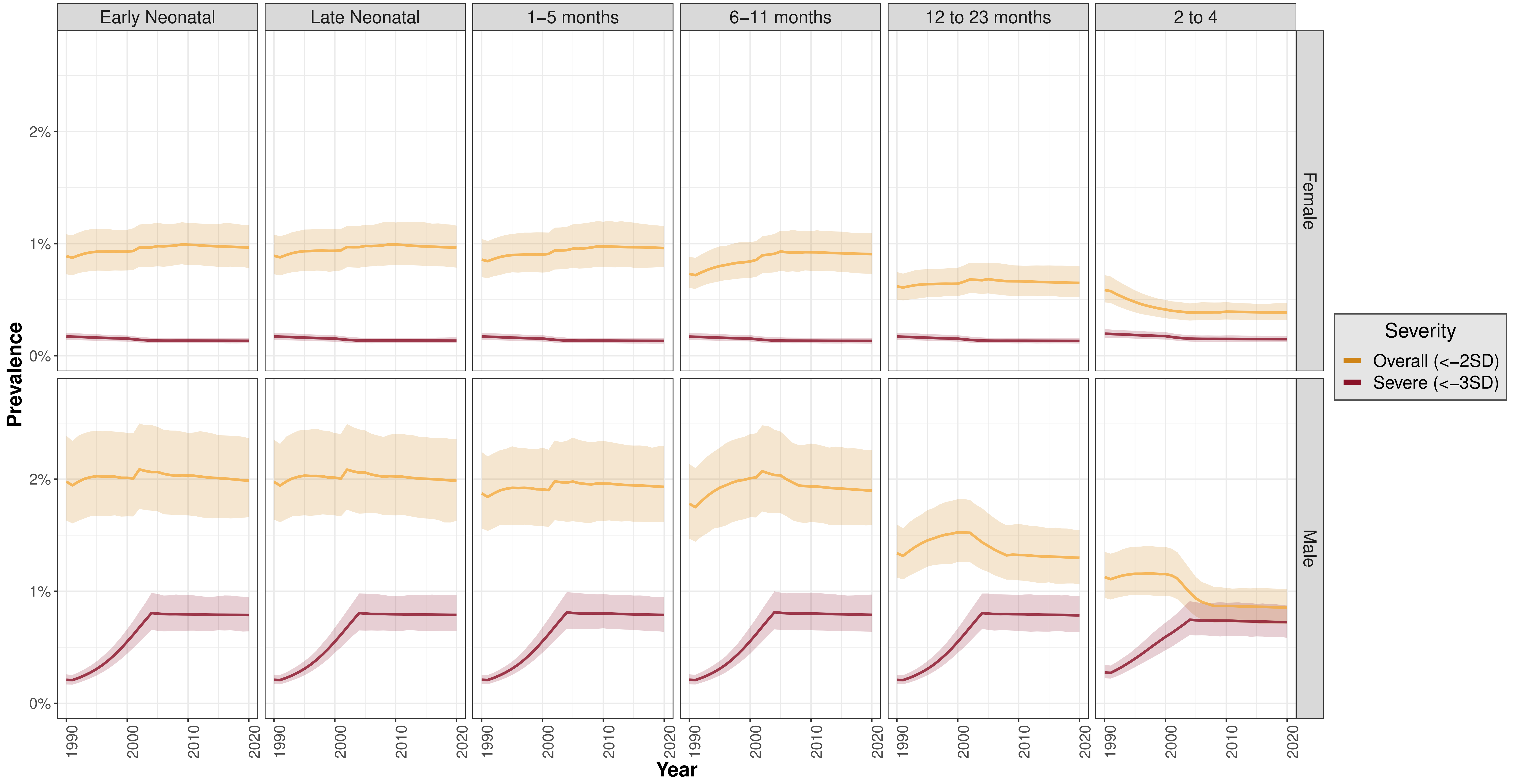

H: Transformed Mean Underweight Z Scores

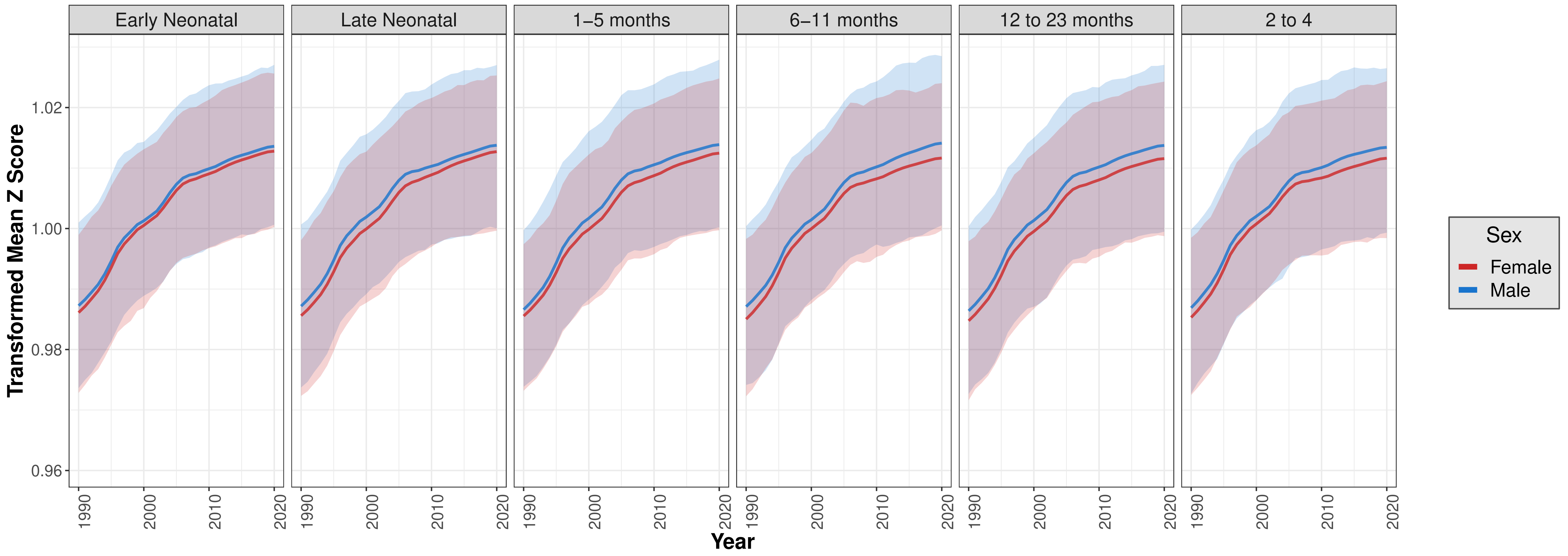

I

Year	Source
1971	WHO CGM Database

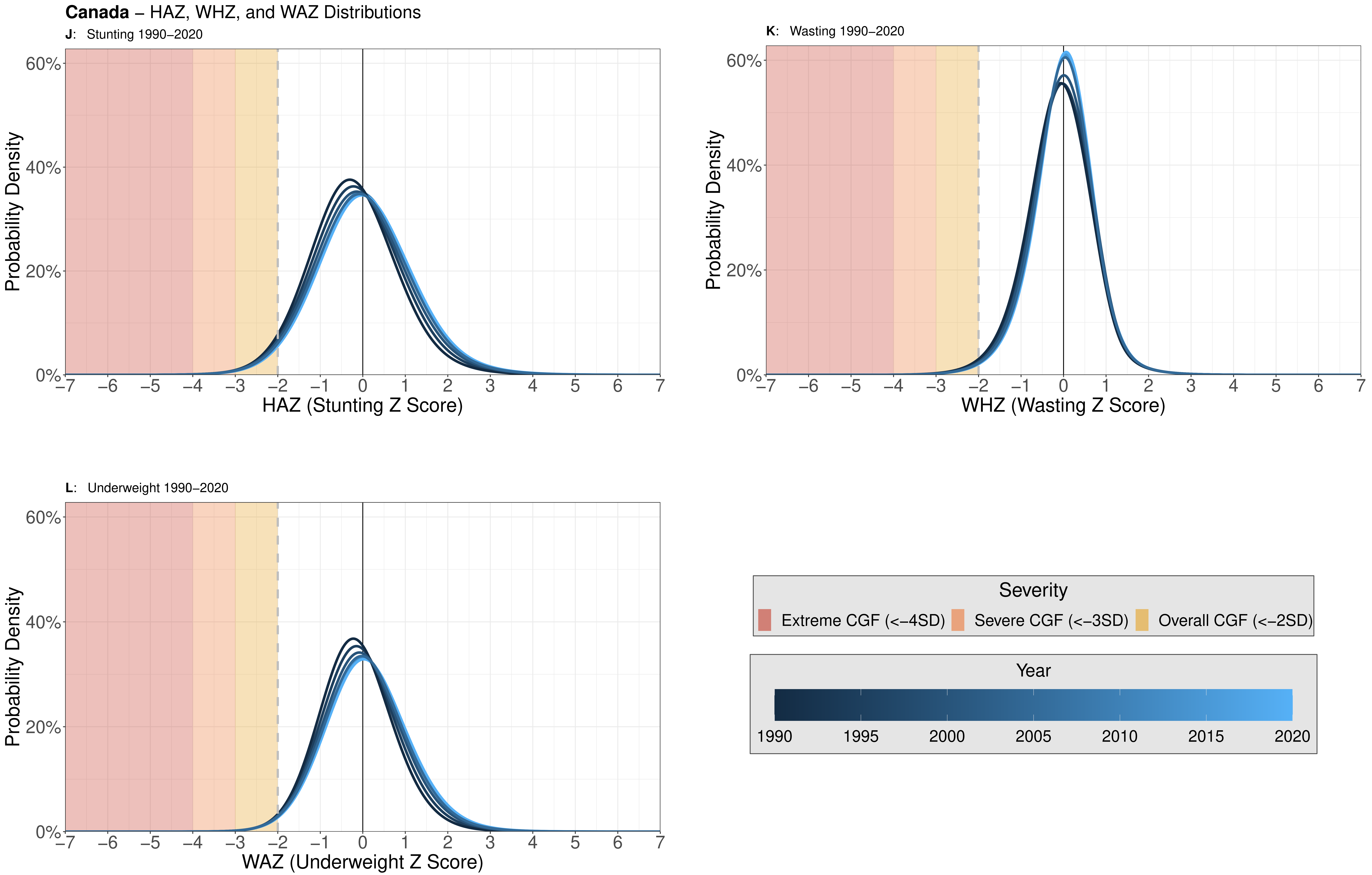
Canada – HAZ, WHZ, and WAZ Distributions
J: Stunting 1990–2020
K: Wasting 1990–2020
L: Underweight 1990–2020
Probability Density
0%
20%
40%
60%
−7 −6 −5 −4 −3 −2 −1 0 1 2 3 4 5 6 7
HAZ (Stunting Z Score)
WHZ (Wasting Z Score)
WAZ (Underweight Z Score)
Severity
Extreme CGF (<−4SD)
Severe CGF (<−3SD)
Overall CGF (<−2SD)
Year
1990
1995
2000
2005
2010
2015
2020

United States of America – Stunting (HAZ)

A: Overall and Severe Stunting Prevalence

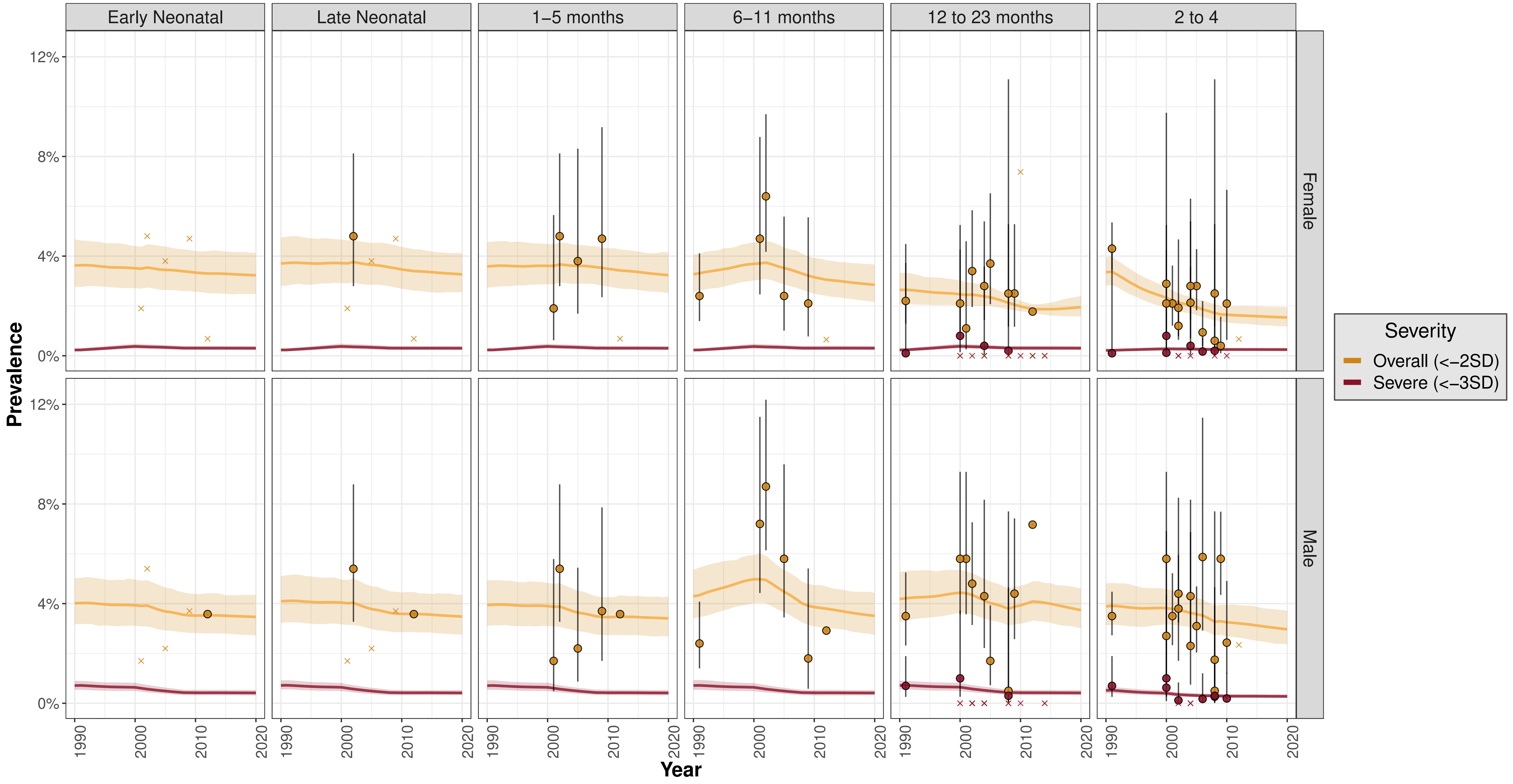

B: Transformed Mean Stunting Z Scores

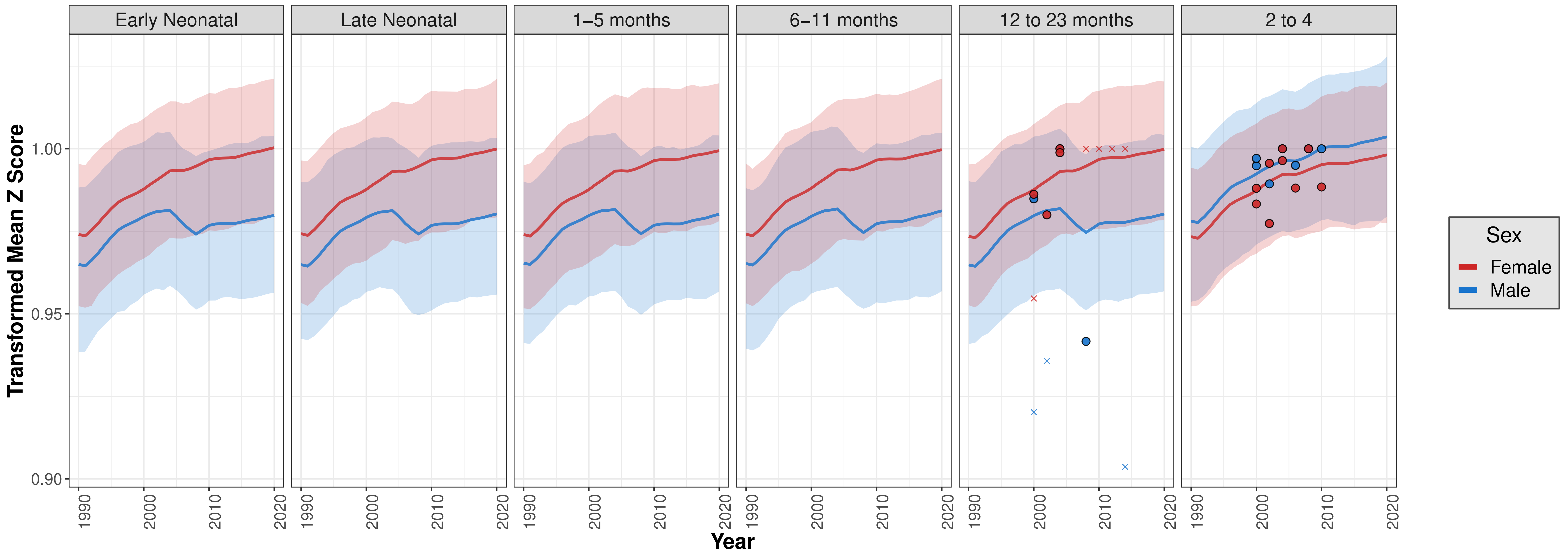

C

Year	Source	National	Subnational
1969	WHO CGM Database	X	
1991	WHO CGM Database	X	
2000	United States National Health and Nutrition Examination Survey	X	
2000	WHO CGM Database	X	
2001	WHO CGM Database	X	
2002	United States National Health and Nutrition Examination Survey	X	
2002	WHO CGM Database	X	
2004	United States National Health and Nutrition Examination Survey	X	
2004	WHO CGM Database	X	
2005	WHO CGM Database	X	
2006	United States National Health and Nutrition Examination Survey	X	
2008	United States National Health and Nutrition Examination Survey	X	
2008	WHO CGM Database	X	
2009	WHO CGM Database	X	
2010	United States National Health and Nutrition Examination Survey	X	
2012	United States National Health and Nutrition Examination Survey	X	
2012	WHO CGM Database	X	
2014	United States National Health and Nutrition Examination Survey	X	

United States of America – Wasting (WHZ)

D: Overall and Severe Wasting Prevalence

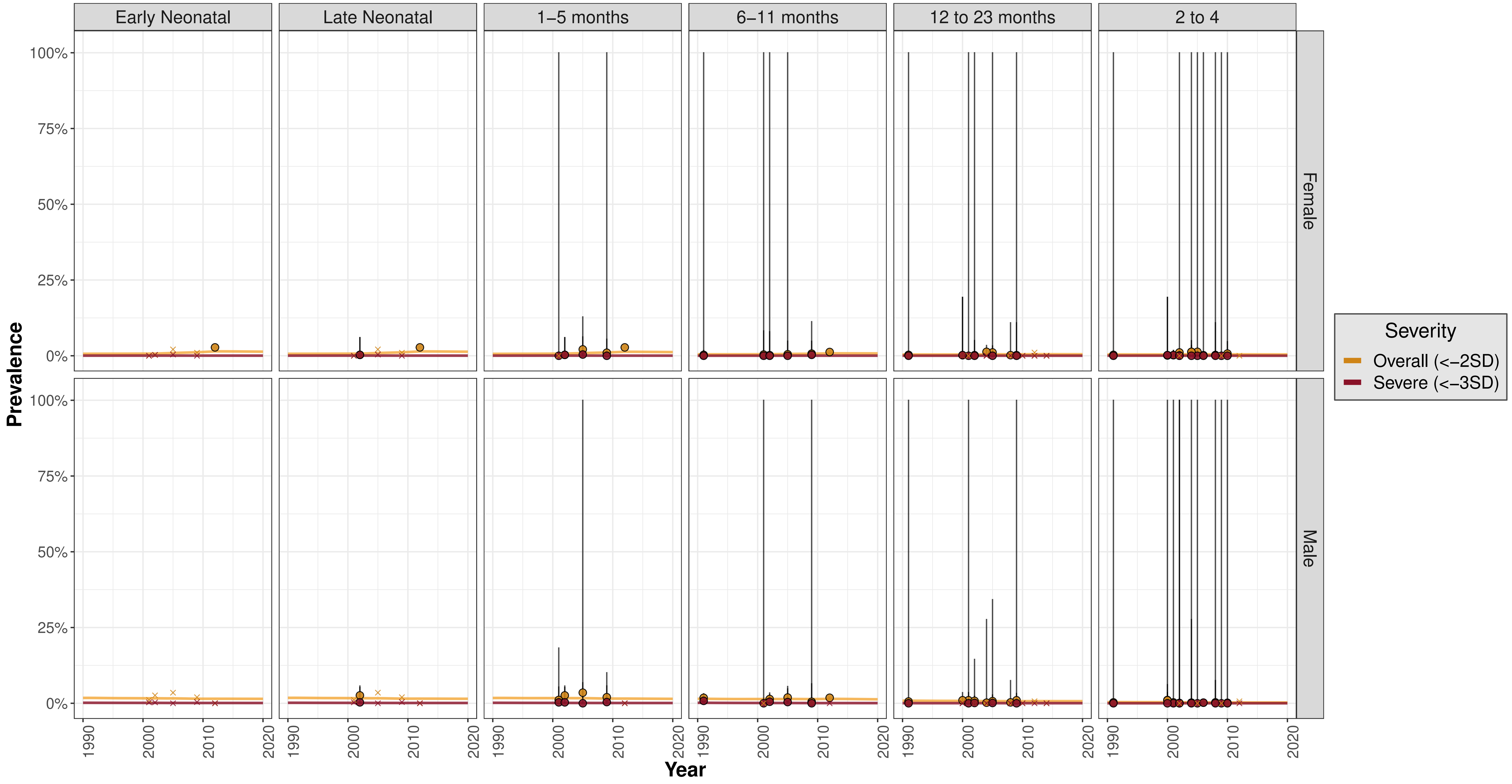

E: Transformed Mean Wasting Z Scores

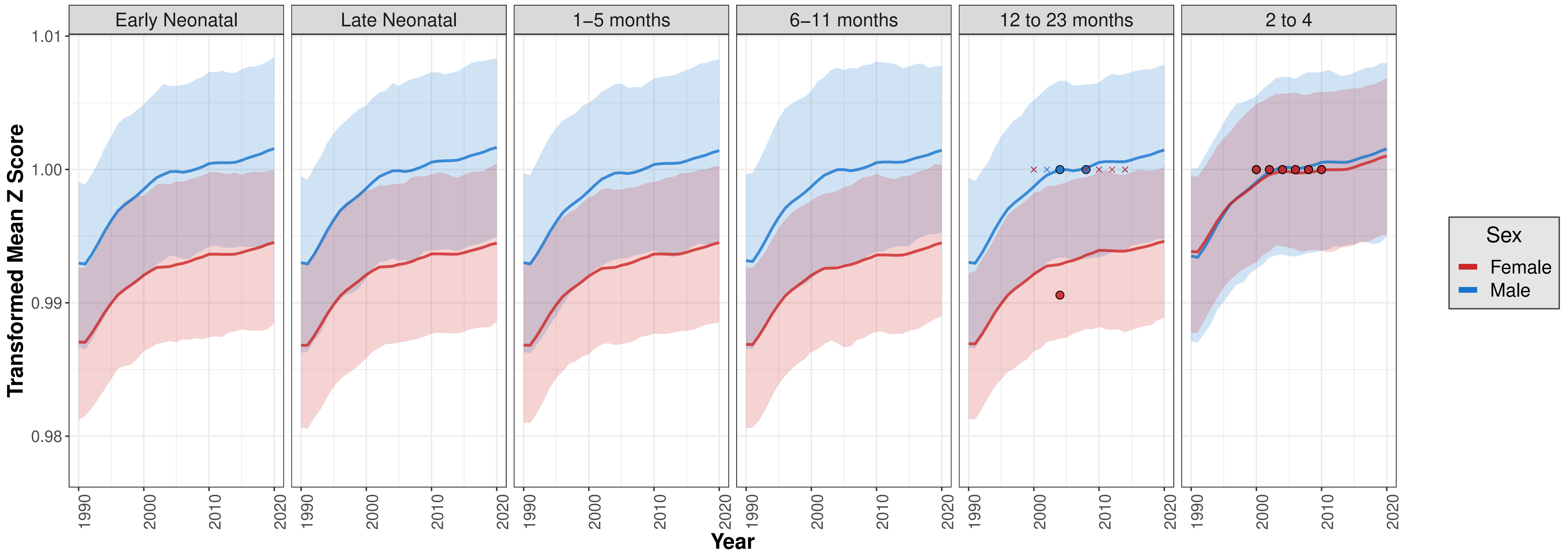

F

Year	Source	National	Subnational
1969	WHO CGM Database	X	
1991	WHO CGM Database	X	
2000	United States National Health and Nutrition Examination Survey	X	
2000	WHO CGM Database	X	
2001	WHO CGM Database	X	
2002	United States National Health and Nutrition Examination Survey	X	
2002	WHO CGM Database	X	
2004	United States National Health and Nutrition Examination Survey	X	
2004	WHO CGM Database	X	
2005	WHO CGM Database	X	
2006	United States National Health and Nutrition Examination Survey	X	
2008	United States National Health and Nutrition Examination Survey	X	
2008	WHO CGM Database	X	
2009	WHO CGM Database	X	
2010	United States National Health and Nutrition Examination Survey	X	
2012	United States National Health and Nutrition Examination Survey	X	
2012	WHO CGM Database	X	
2014	United States National Health and Nutrition Examination Survey	X	

United States of America – Underweight (WAZ)

G: Overall and Severe Underweight Prevalence

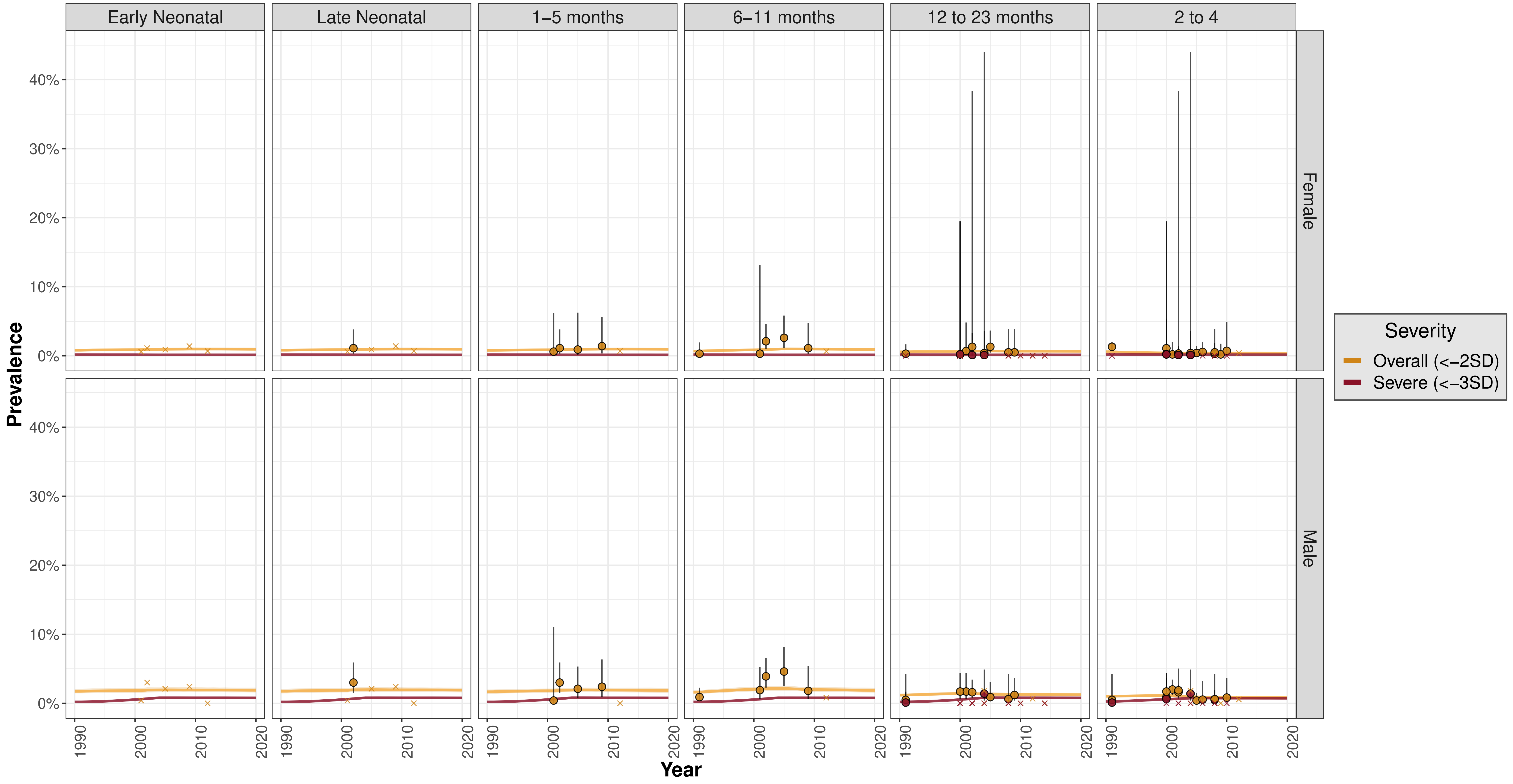

H: Transformed Mean Underweight Z Scores

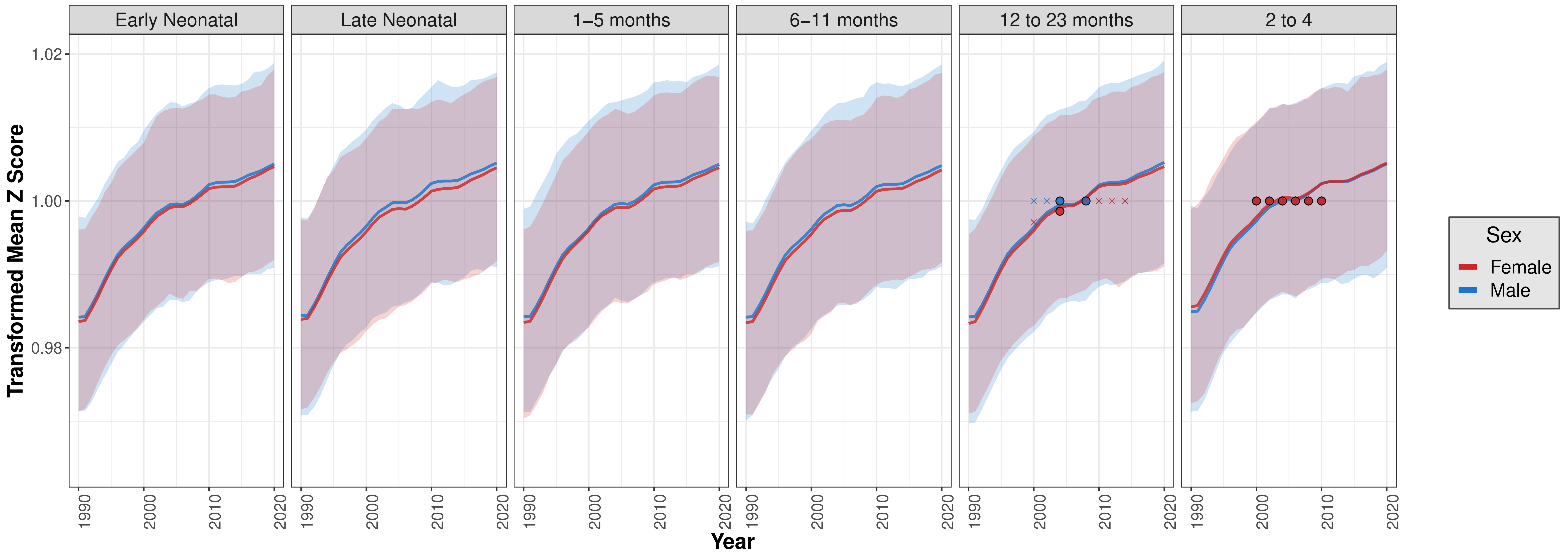

I

Year	Source	National	Subnational
1969	WHO CGM Database	X	
1991	WHO CGM Database	X	
2000	United States National Health and Nutrition Examination Survey	X	
2000	WHO CGM Database	X	
2001	WHO CGM Database	X	
2002	United States National Health and Nutrition Examination Survey	X	
2002	WHO CGM Database	X	
2004	United States National Health and Nutrition Examination Survey	X	
2004	WHO CGM Database	X	
2005	WHO CGM Database	X	
2006	United States National Health and Nutrition Examination Survey	X	
2008	United States National Health and Nutrition Examination Survey	X	
2008	WHO CGM Database	X	
2009	WHO CGM Database	X	
2010	United States National Health and Nutrition Examination Survey	X	
2012	United States National Health and Nutrition Examination Survey	X	
2012	WHO CGM Database	X	
2014	United States National Health and Nutrition Examination Survey	X	

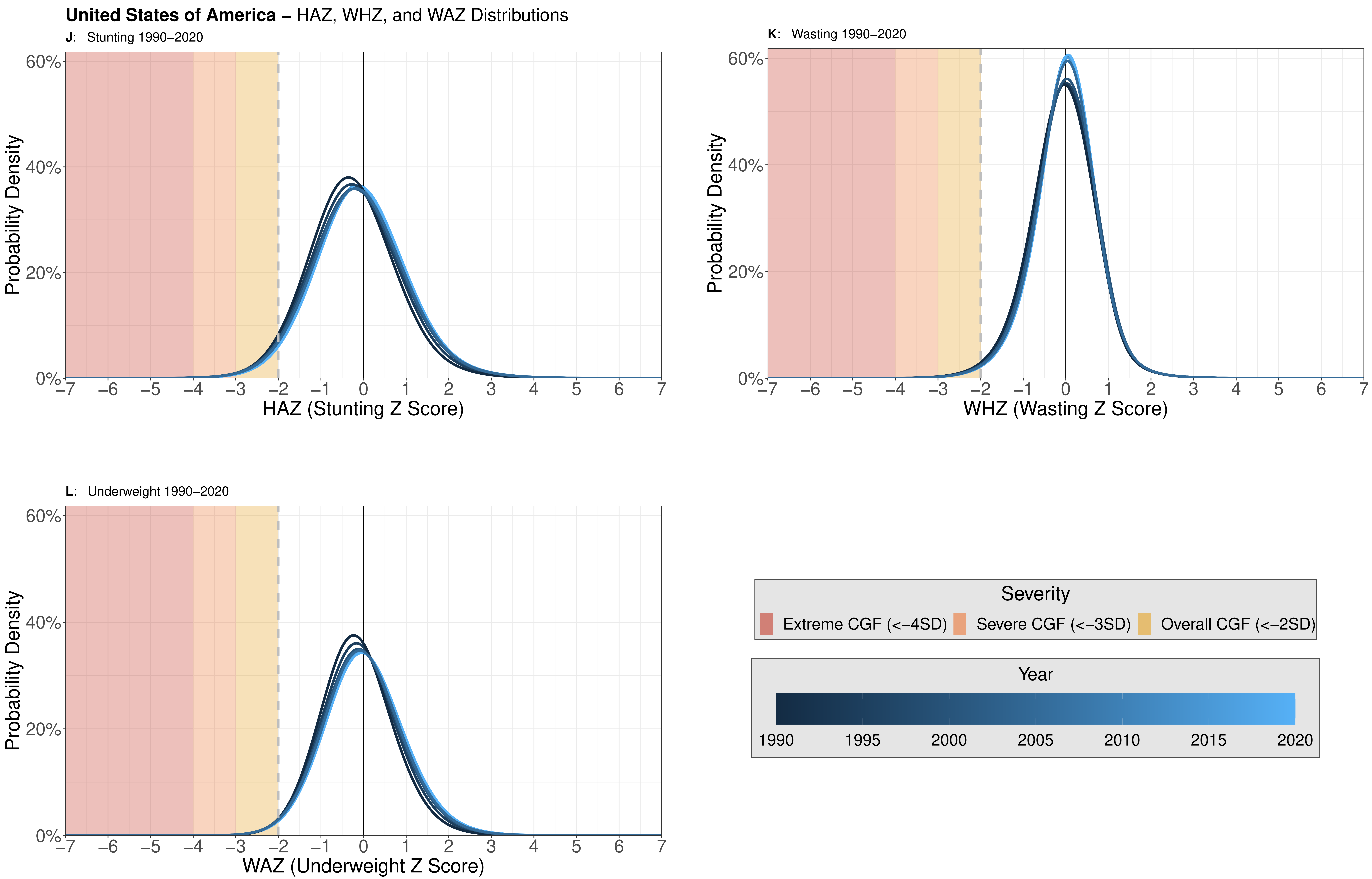
United States of America – HAZ, WHZ, and WAZ Distributions
J: Stunting 1990–2020
K: Wasting 1990–2020
L: Underweight 1990–2020
Probability Density
60%
40%
20%
0%
−7 −6 −5 −4 −3 −2 −1 0 1 2 3 4 5 6 7
HAZ (Stunting Z Score)
WHZ (Wasting Z Score)
WAZ (Underweight Z Score)
Severity
Extreme CGF (<−4SD)
Severe CGF (<−3SD)
Overall CGF (<−2SD)
Year
1990 1995 2000 2005 2010 2015 2020

Greenland – Stunting (HAZ)

A: Overall and Severe Stunting Prevalence

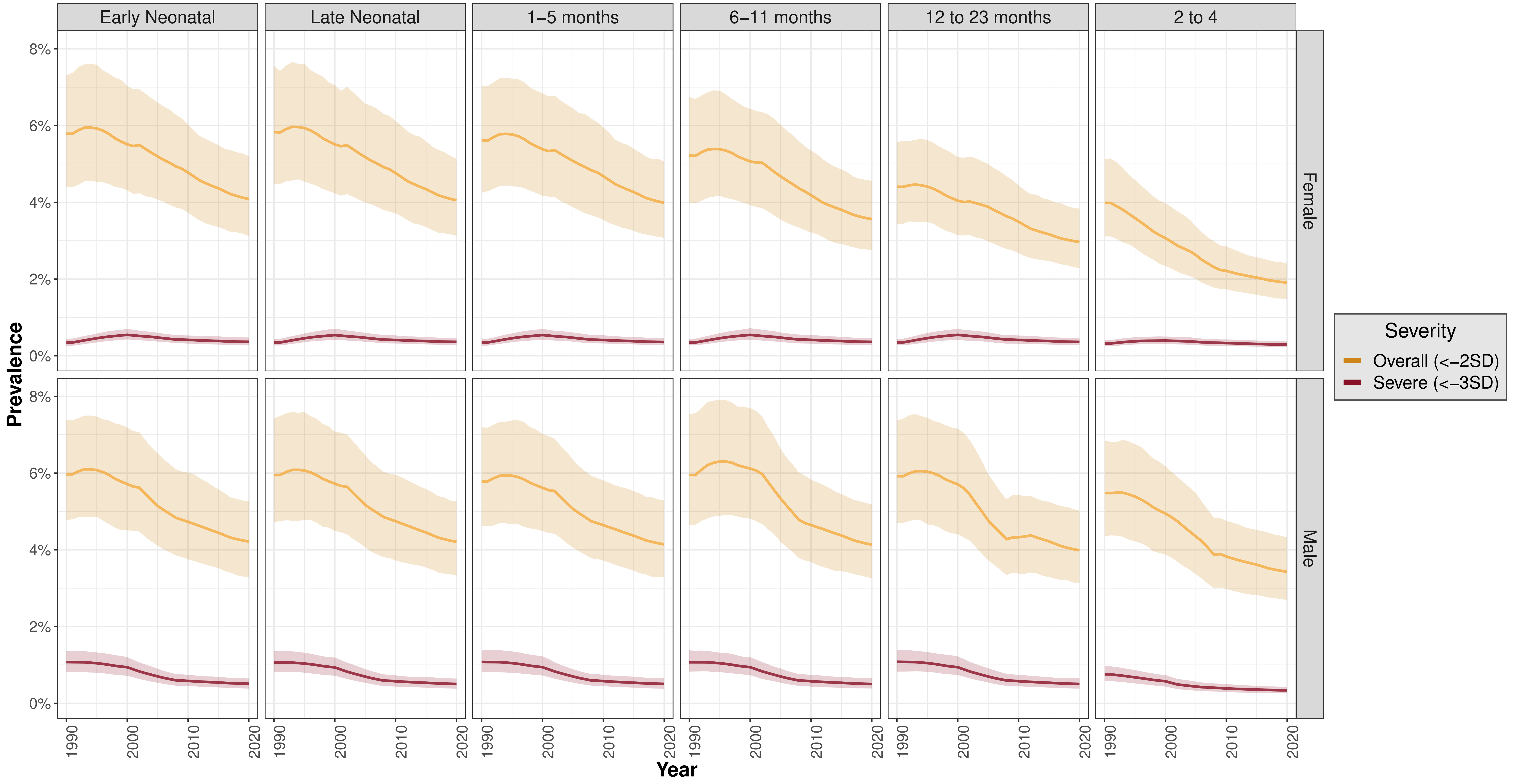

B: Transformed Mean Stunting Z Scores

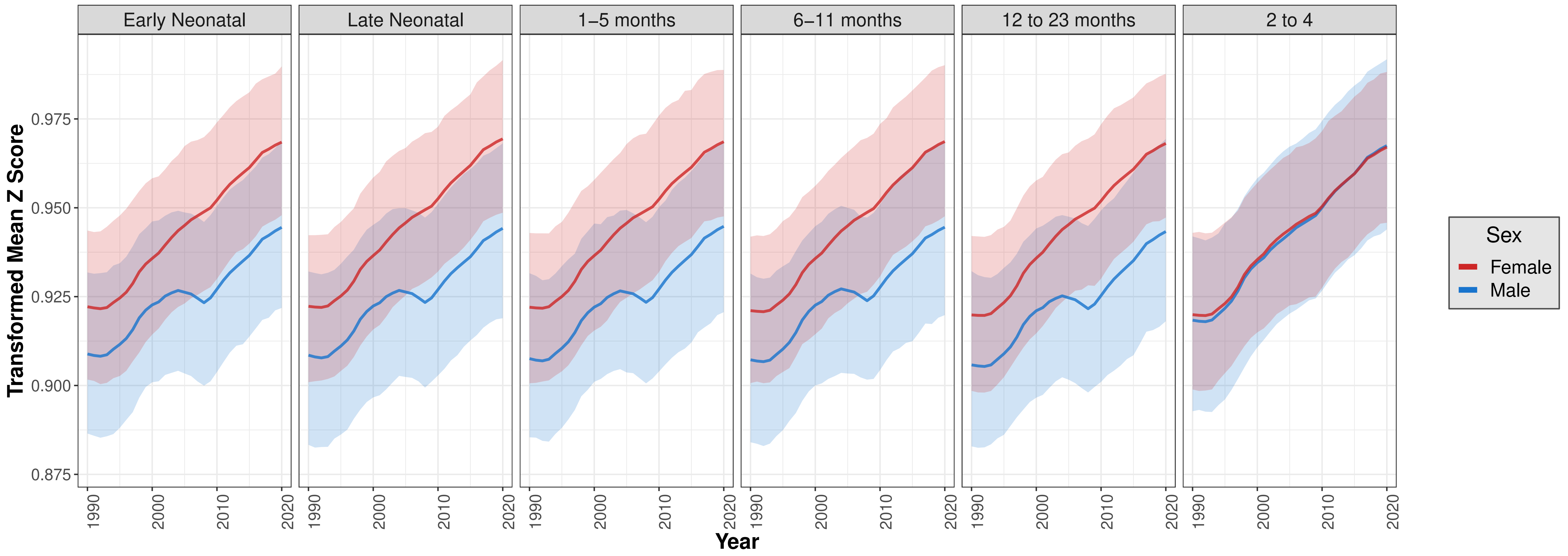

C

Source
No sources for this location

Greenland – Wasting (WHZ)

D: Overall and Severe Wasting Prevalence

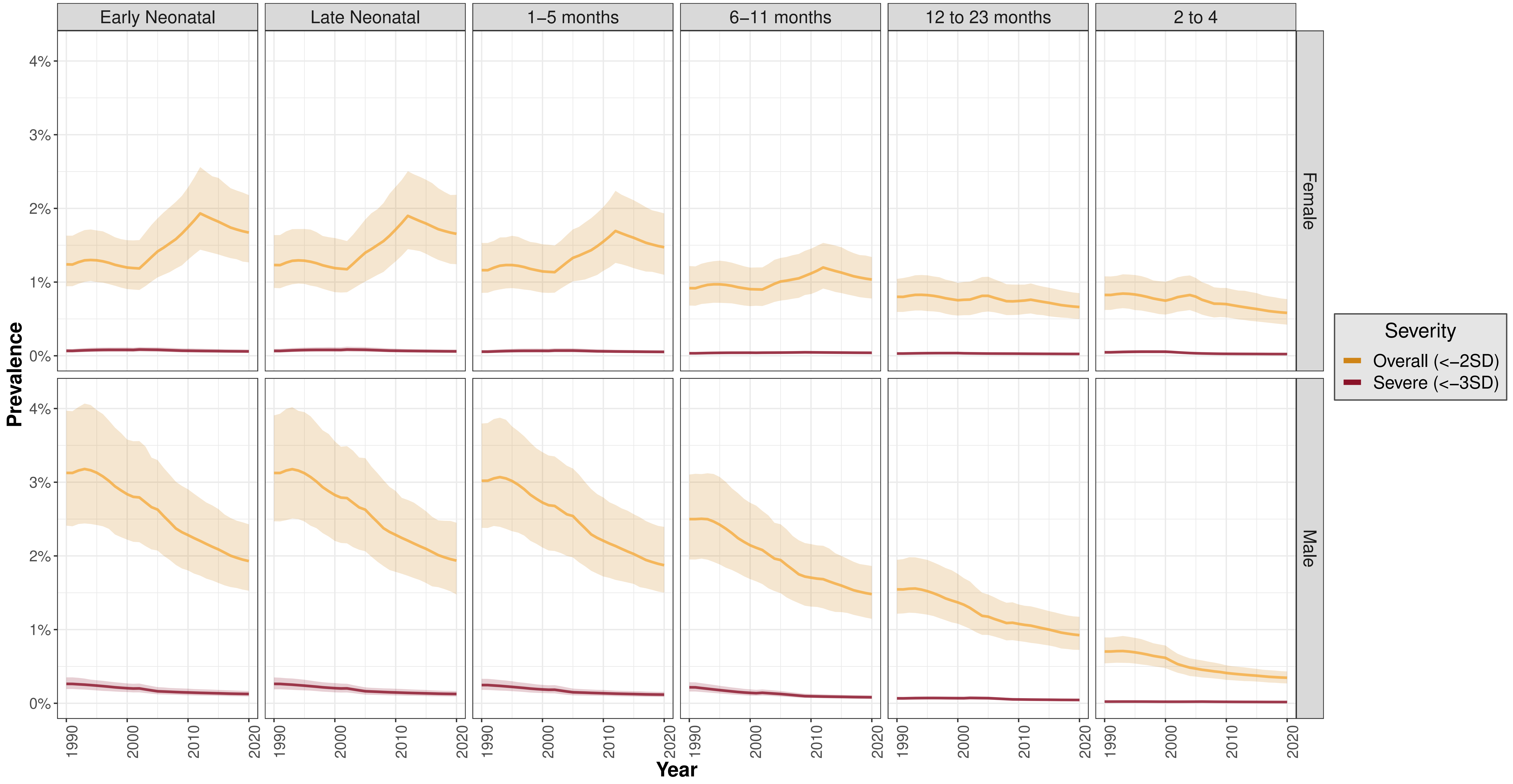

E: Transformed Mean Wasting Z Scores

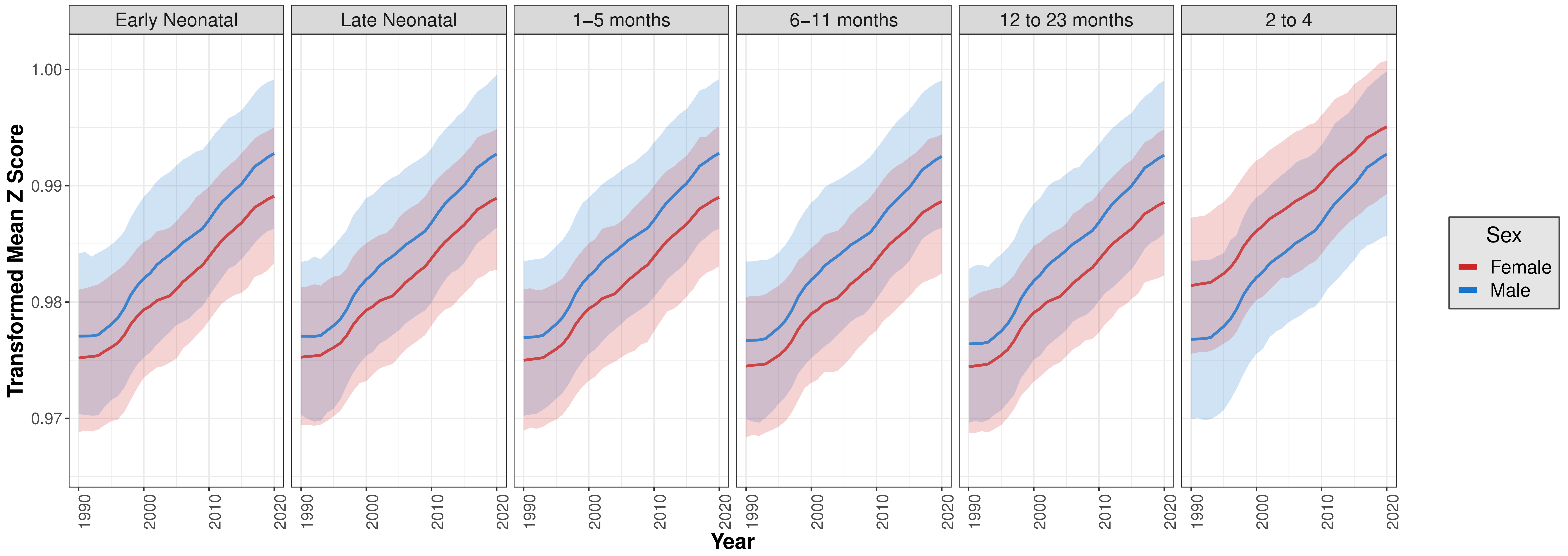

F

Source
No sources for this location

Greenland – Underweight (WAZ)

G: Overall and Severe Underweight Prevalence

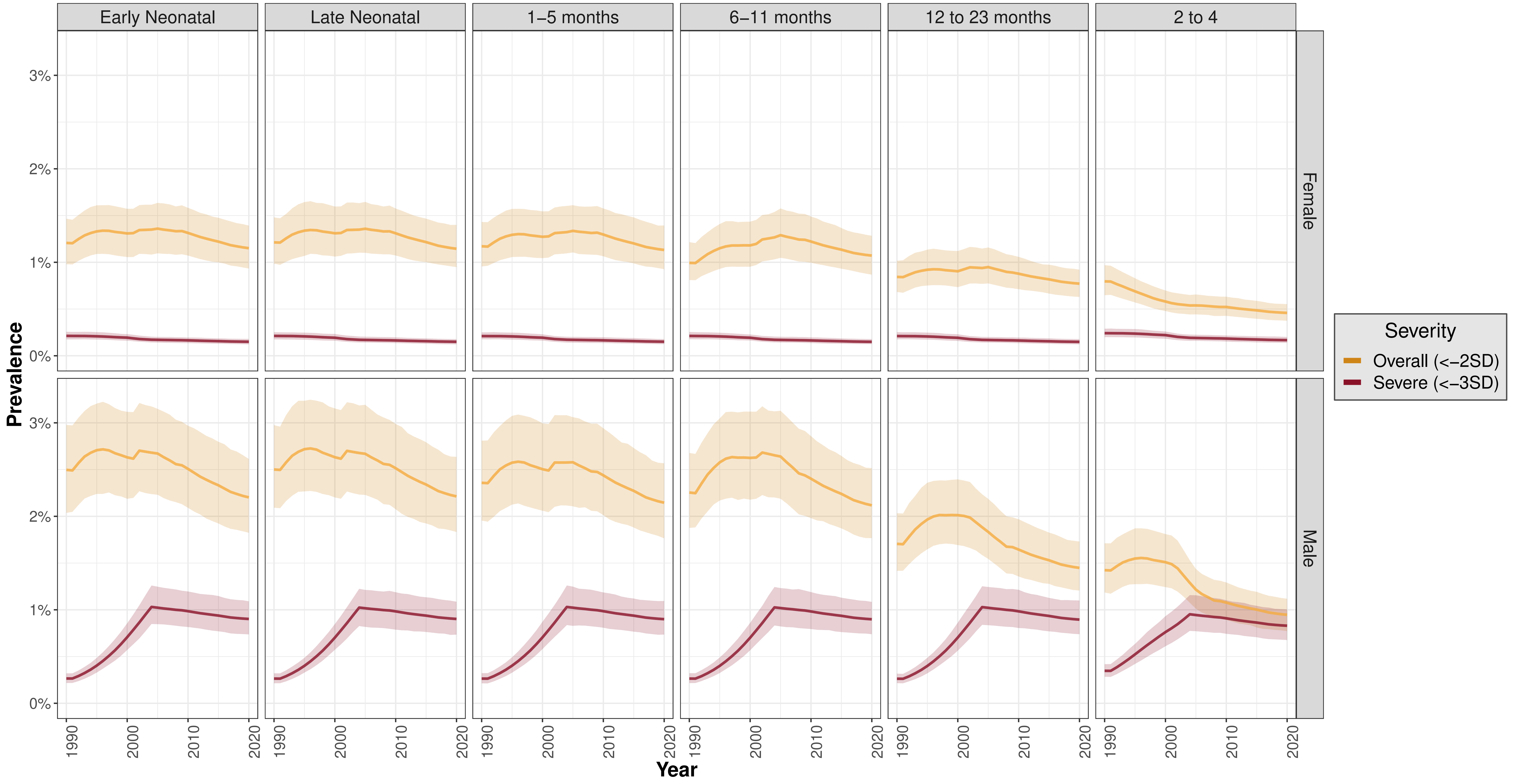

H: Transformed Mean Underweight Z Scores

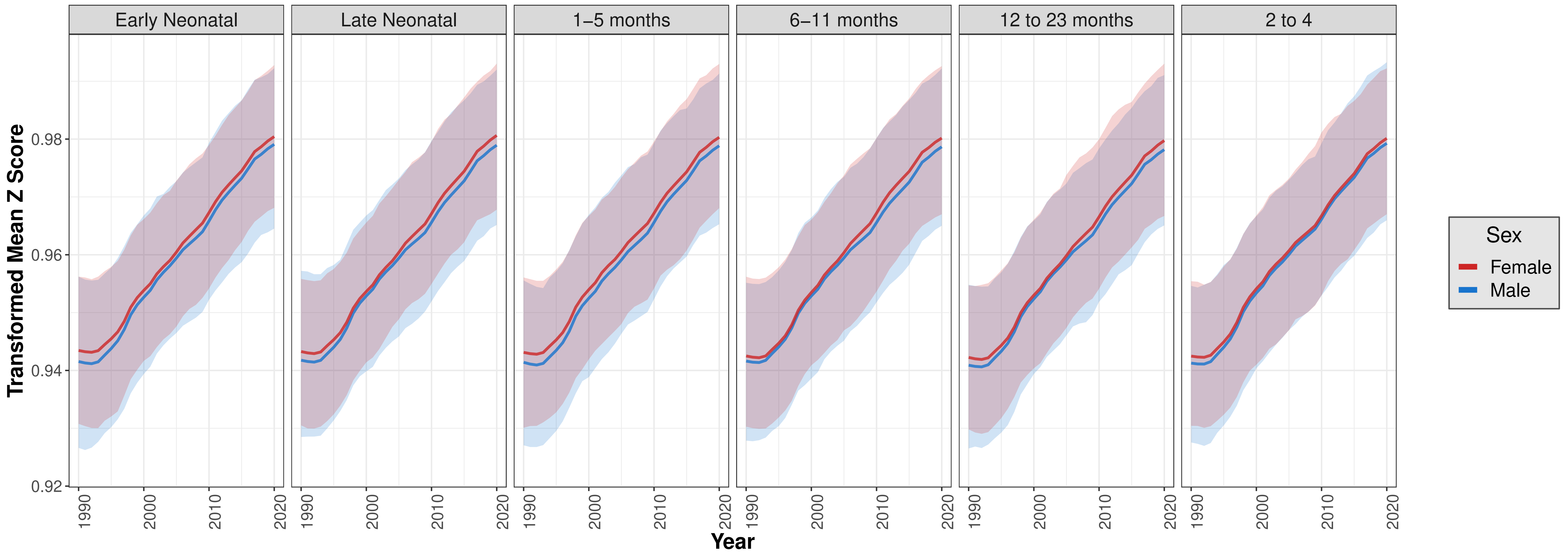

I

Source
No sources for this location

Greenland – HAZ, WHZ, and WAZ Distributions

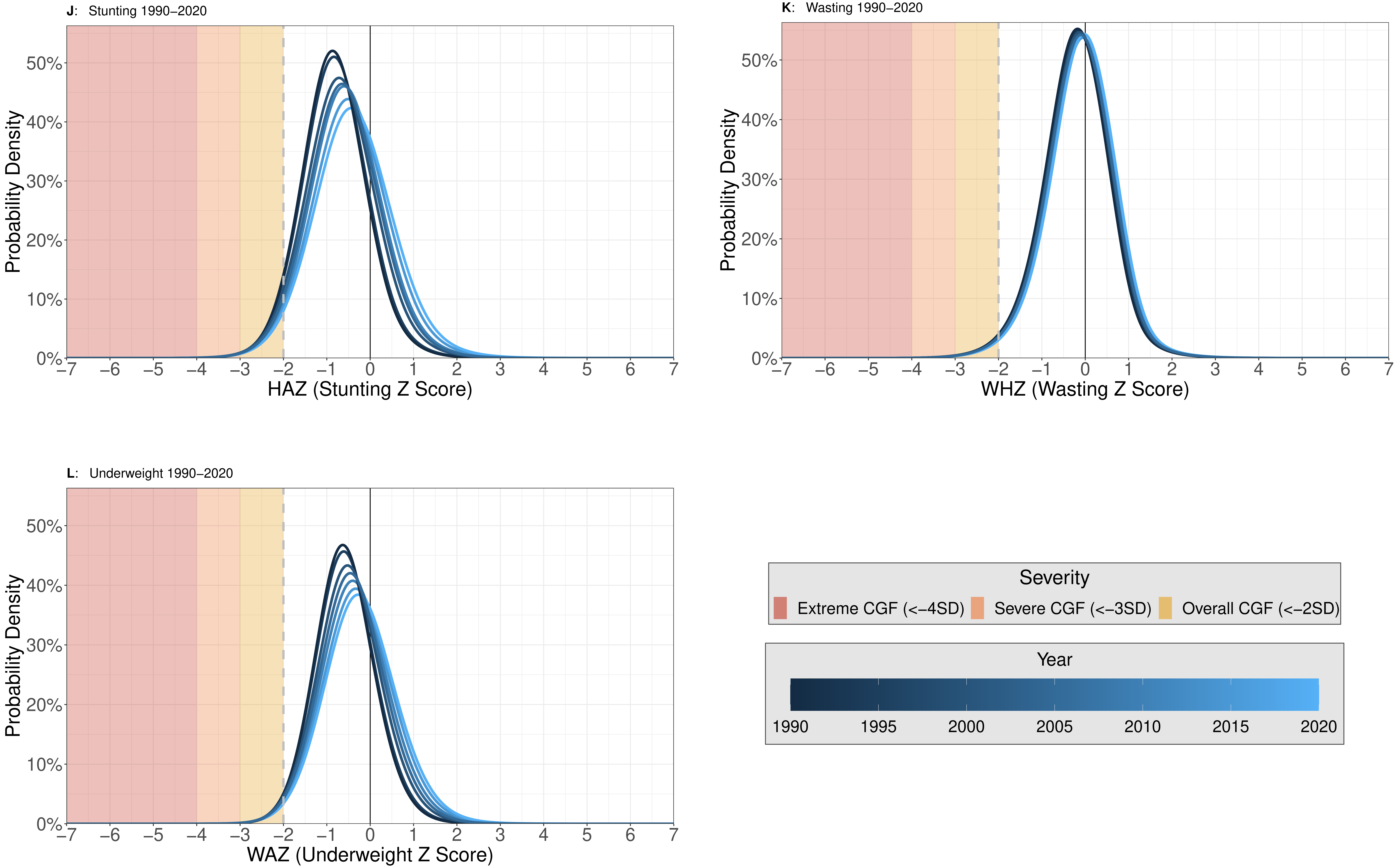

Argentina – Stunting (HAZ)

A: Overall and Severe Stunting Prevalence

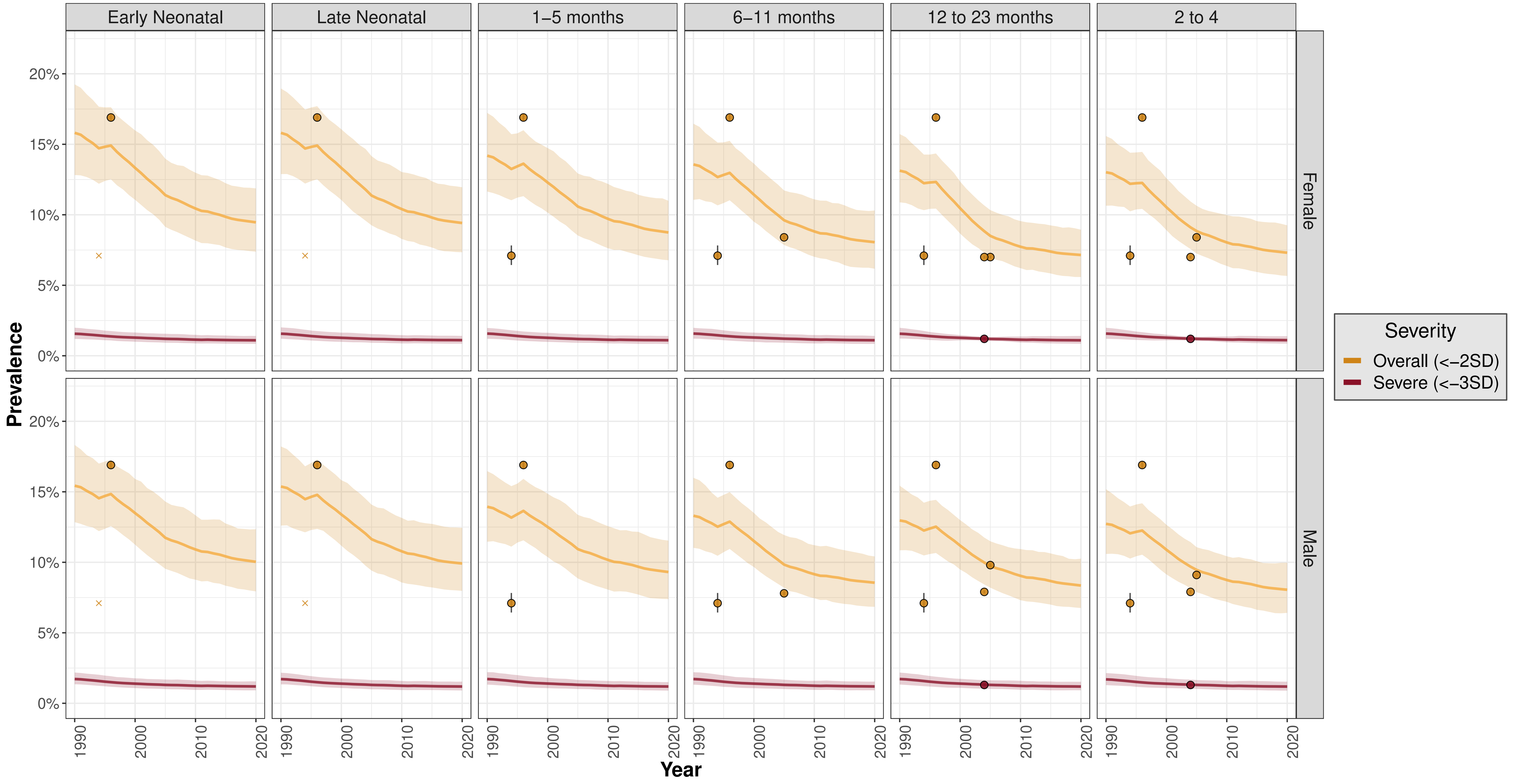

B: Transformed Mean Stunting Z Scores

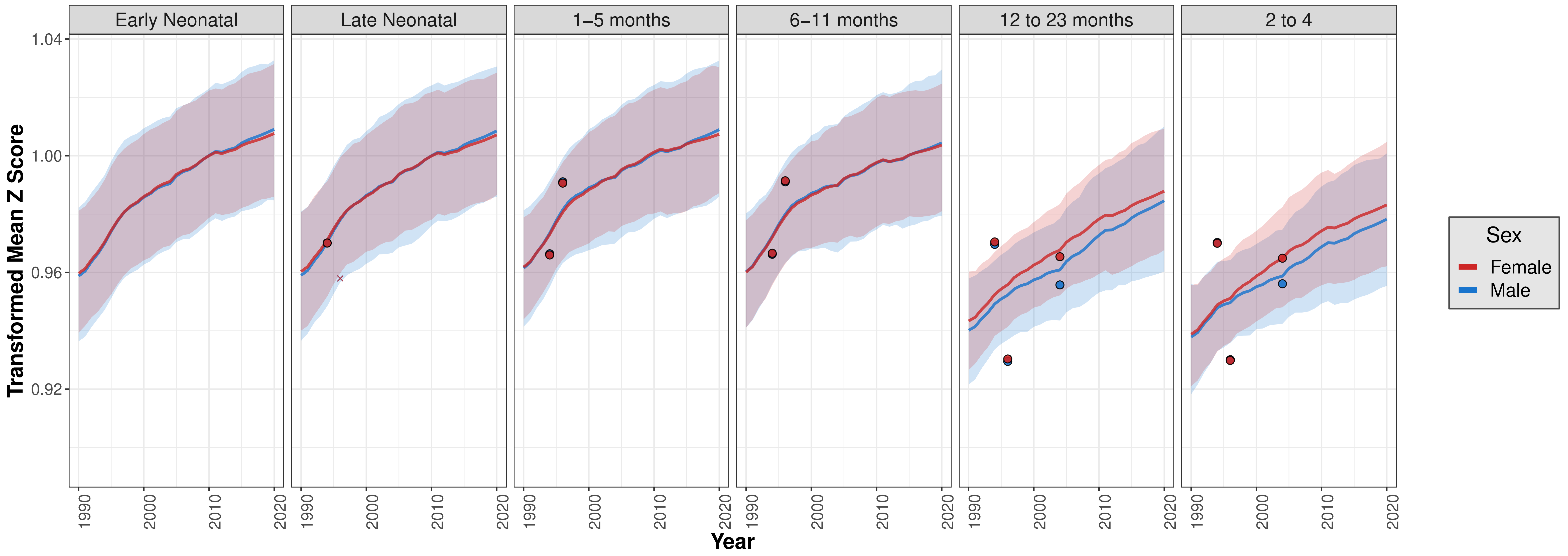

C

Year	Source
1994	WHO CGM Database
1996	WHO CGM Database
2004	WHO CGM Database
2005	WHO CGM Database

Argentina – Wasting (WHZ)

D: Overall and Severe Wasting Prevalence

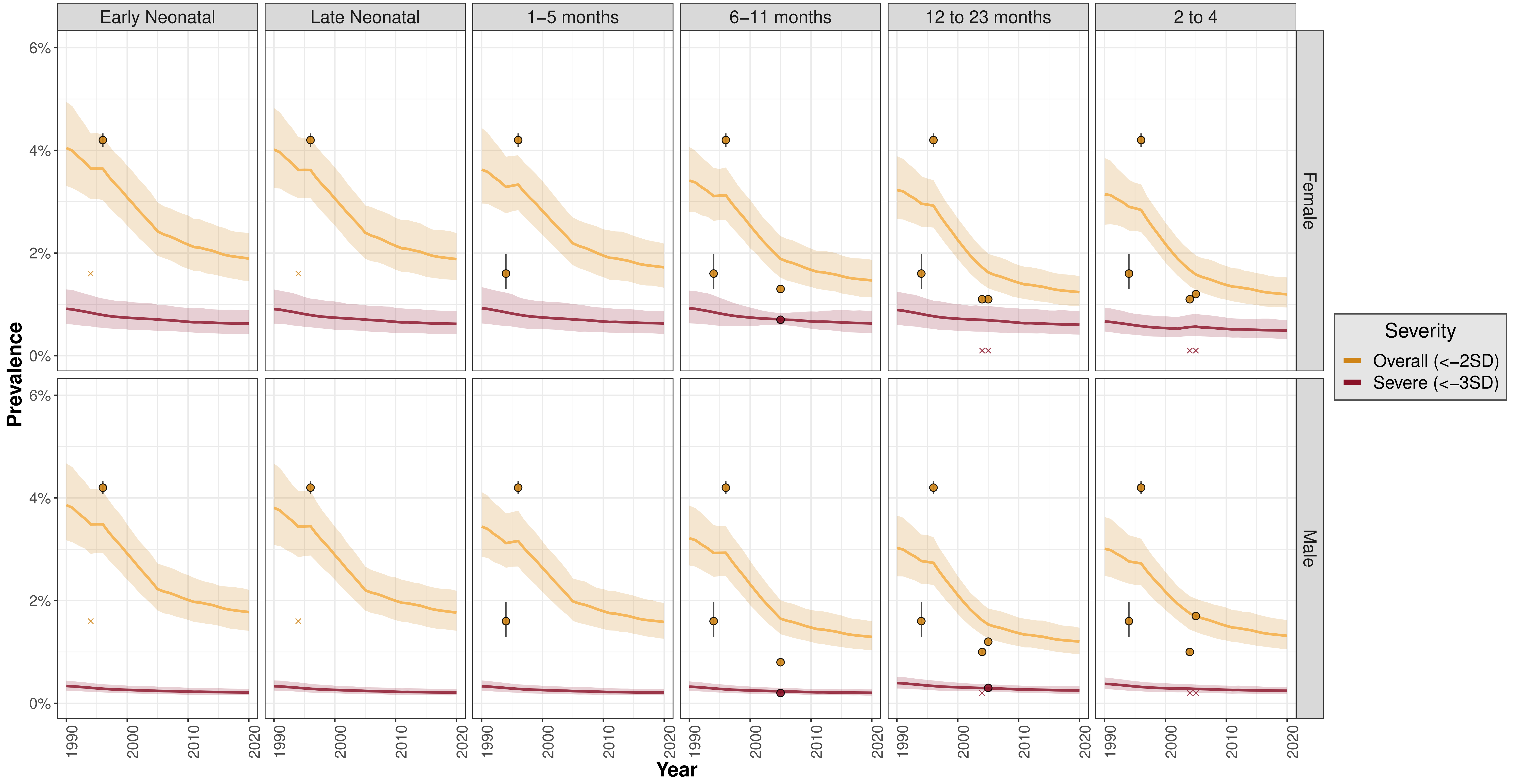

E: Transformed Mean Wasting Z Scores

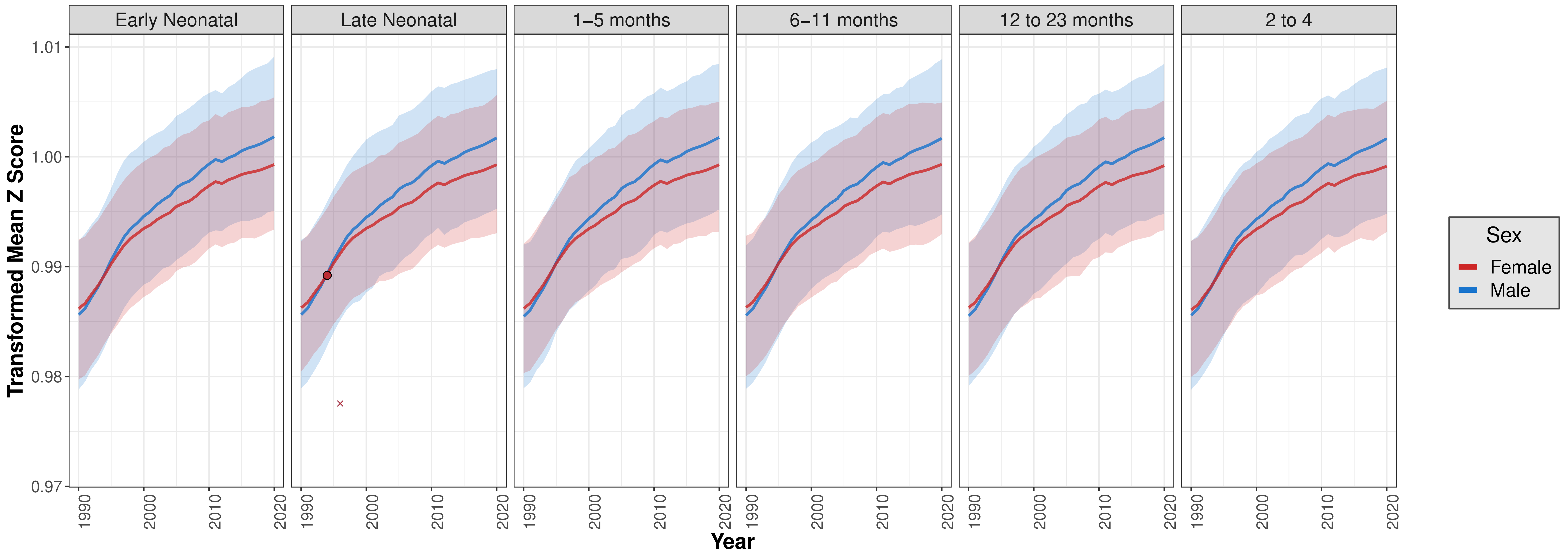

F

Year	Source
1994	WHO CGM Database
1996	WHO CGM Database
2004	WHO CGM Database
2005	WHO CGM Database

Argentina – Underweight (WAZ)

G: Overall and Severe Underweight Prevalence

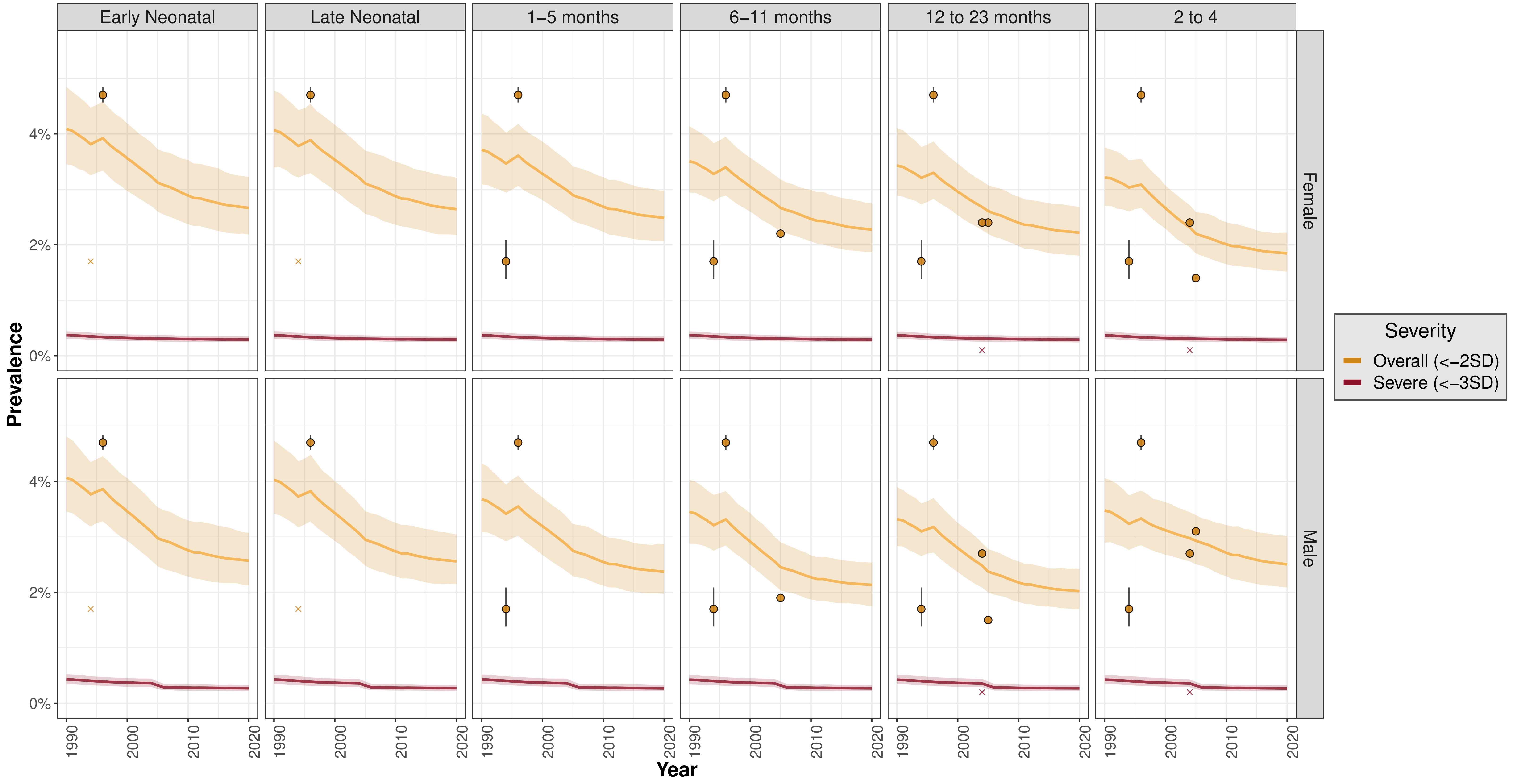

H: Transformed Mean Underweight Z Scores

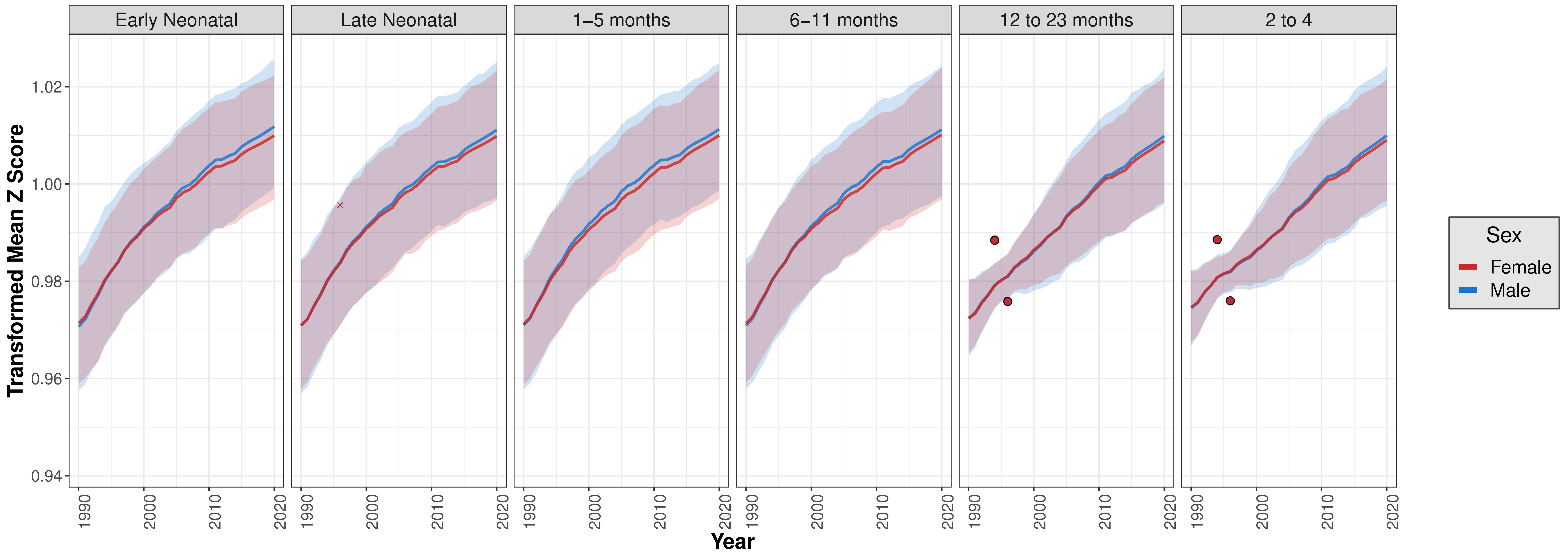

I

Year	Source
1994	WHO CGM Database
1996	WHO CGM Database
2004	WHO CGM Database
2005	WHO CGM Database

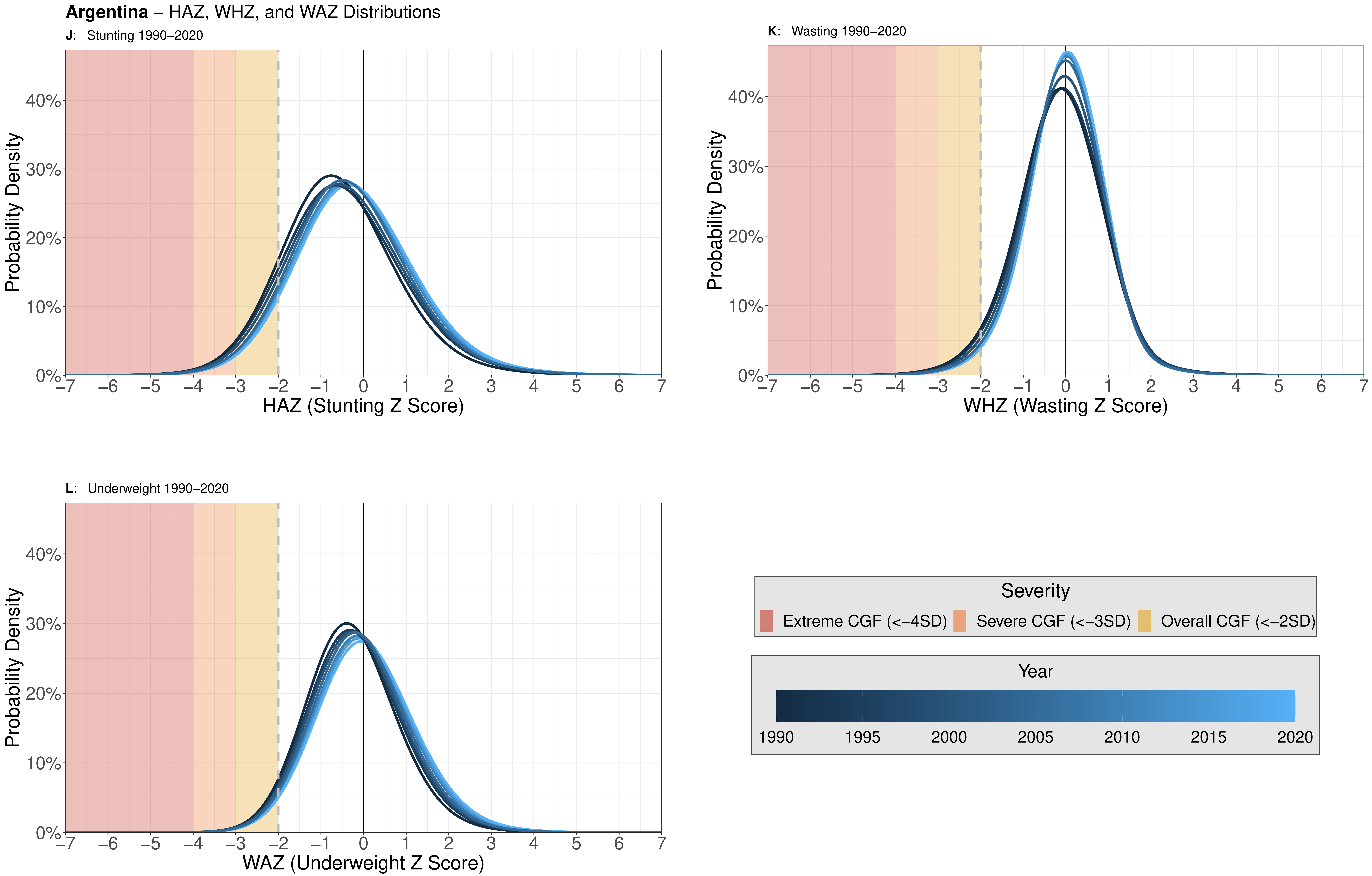
Argentina – HAZ, WHZ, and WAZ Distributions
J: Stunting 1990–2020
Probability Density
HAZ (Stunting Z Score)
K: Wasting 1990–2020
Probability Density
WHZ (Wasting Z Score)
L: Underweight 1990–2020
Probability Density
WAZ (Underweight Z Score)
0%
10%
20%
30%
40%
−7
−6
−5
−4
−3
−2
−1
0
1
2
3
4
5
6
7
Severity
Extreme CGF (<−4SD)
Severe CGF (<−3SD)
Overall CGF (<−2SD)
Year
1990
1995
2000
2005
2010
2015
2020

Chile – Stunting (HAZ)

A: Overall and Severe Stunting Prevalence

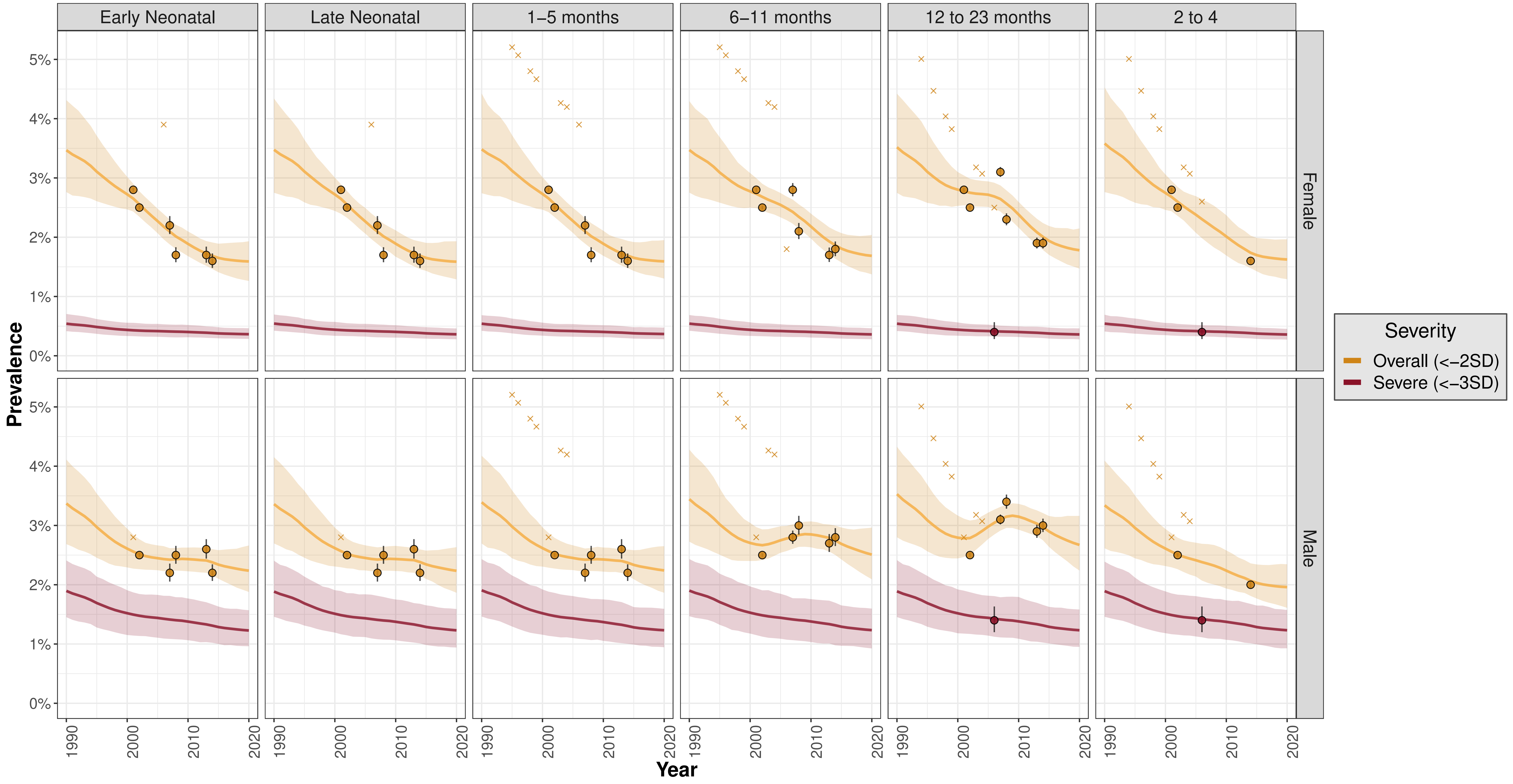

B: Transformed Mean Stunting Z Scores

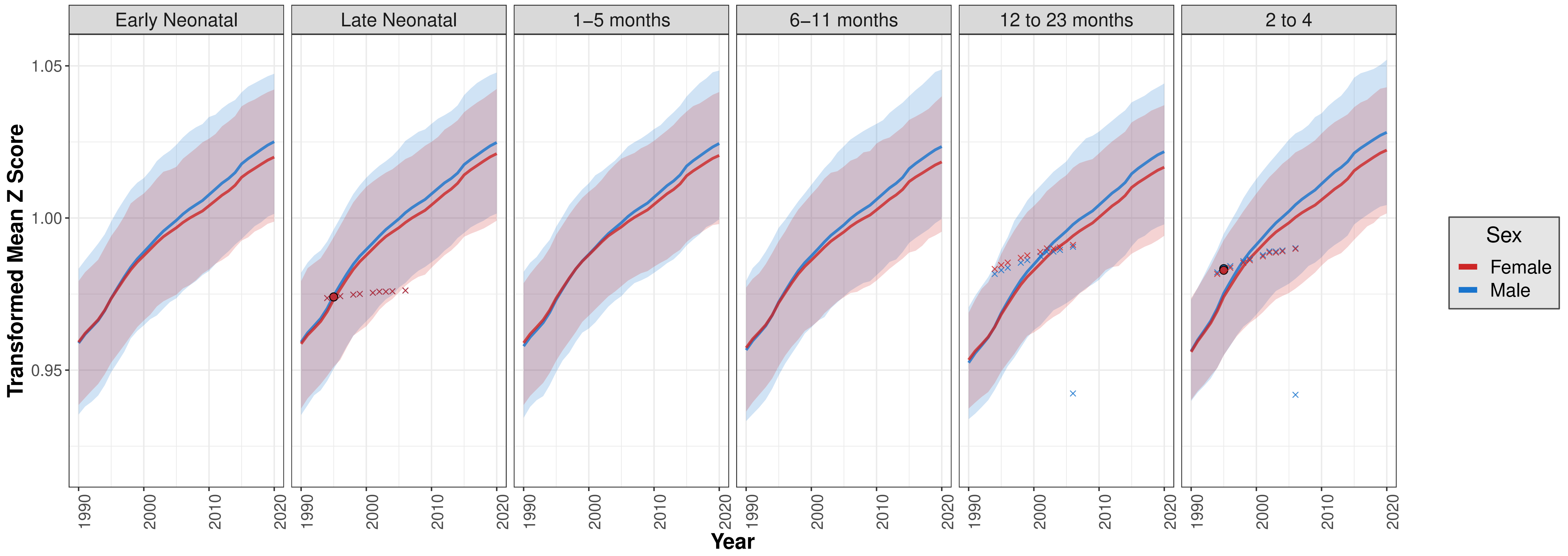

C

Year	Source
1985	WHO CGM Database
1986	WHO CGM Database
1994	WHO CGM Database
1995	WHO CGM Database
1996	WHO CGM Database
1998	WHO CGM Database
1999	WHO CGM Database
2001	WHO CGM Database
2002	WHO CGM Database
2003	WHO CGM Database
2004	WHO CGM Database
2006	WHO CGM Database
2007	WHO CGM Database
2008	WHO CGM Database
2013	WHO CGM Database
2014	WHO CGM Database

Chile – Wasting (WHZ)

D: Overall and Severe Wasting Prevalence

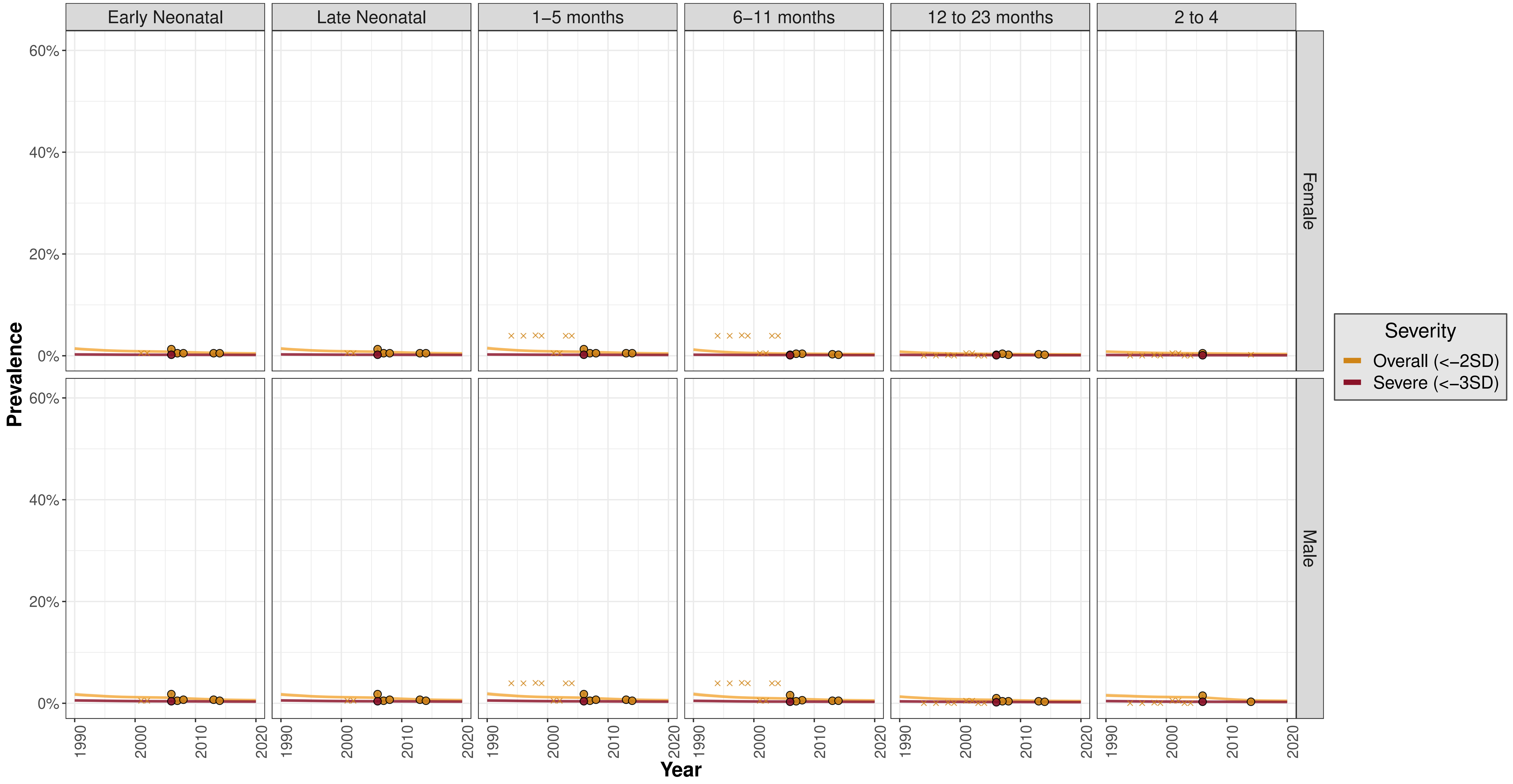

E: Transformed Mean Wasting Z Scores

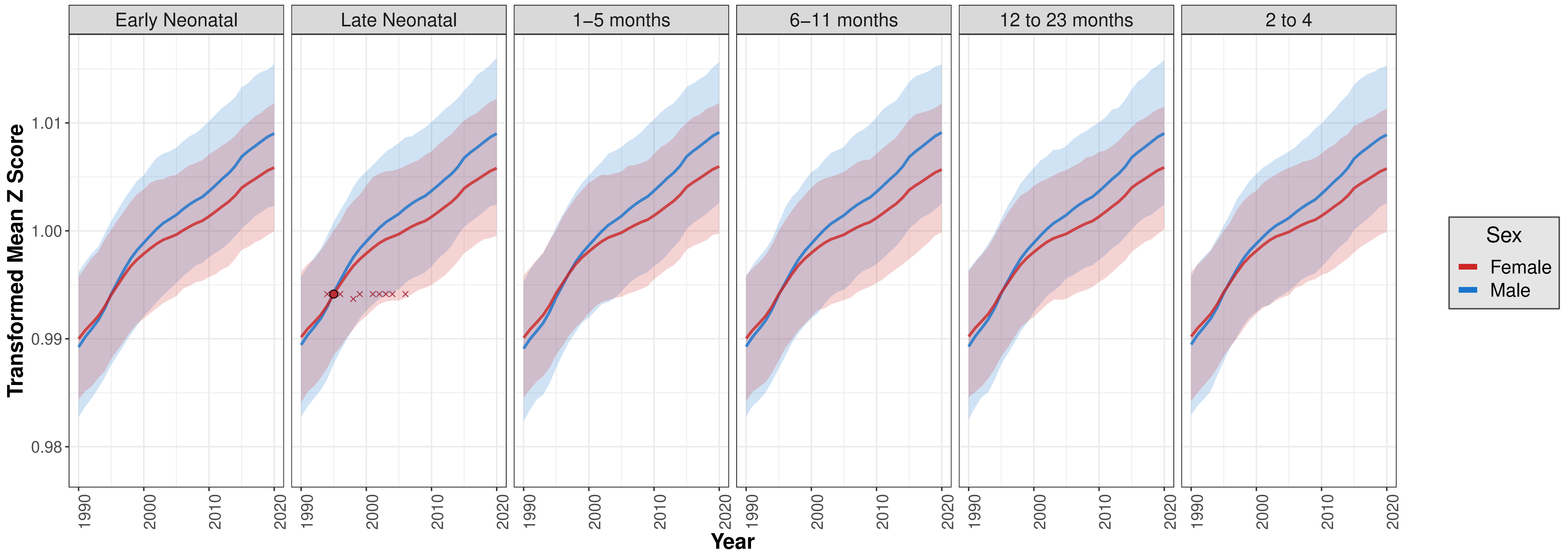

F

Year	Source
1985	WHO CGM Database
1986	WHO CGM Database
1994	WHO CGM Database
1995	WHO CGM Database
1996	WHO CGM Database
1998	WHO CGM Database
1999	WHO CGM Database
2001	WHO CGM Database
2002	WHO CGM Database
2003	WHO CGM Database
2004	WHO CGM Database
2006	WHO CGM Database
2007	WHO CGM Database
2008	WHO CGM Database
2013	WHO CGM Database
2014	WHO CGM Database

Chile – Underweight (WAZ)

G: Overall and Severe Underweight Prevalence

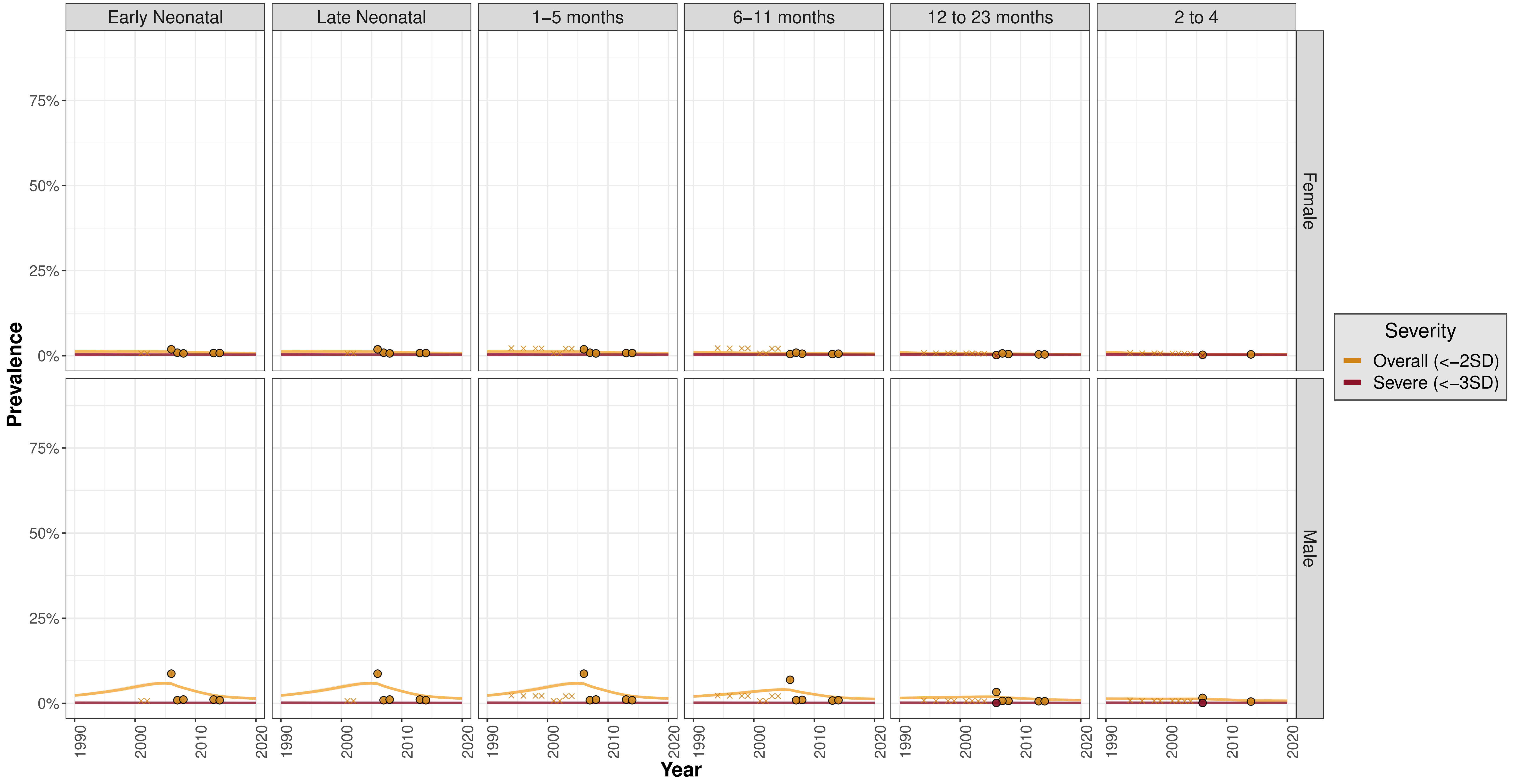

H: Transformed Mean Underweight Z Scores

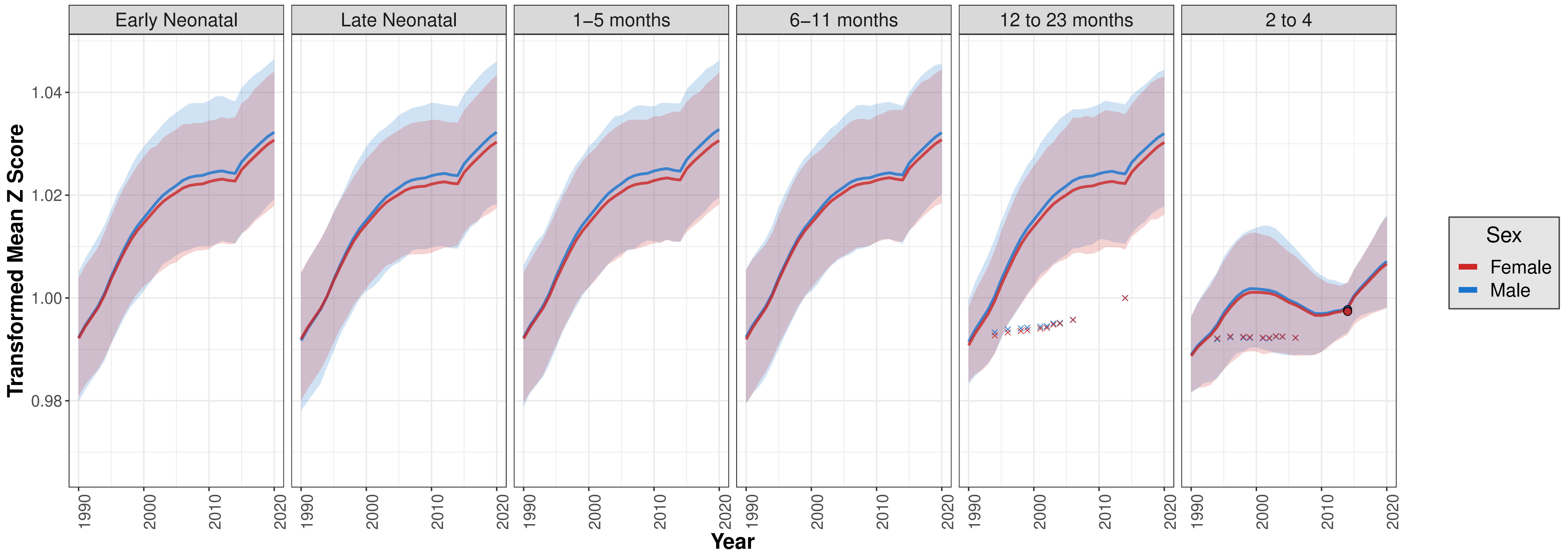

I

Year	Source
1976	WHO CGM Database
1977	WHO CGM Database
1978	WHO CGM Database
1979	WHO CGM Database
1980	WHO CGM Database
1981	WHO CGM Database
1982	WHO CGM Database
1983	WHO CGM Database
1984	WHO CGM Database
1985	WHO CGM Database
1986	WHO CGM Database
1994	WHO CGM Database
1996	WHO CGM Database
1998	WHO CGM Database
1999	WHO CGM Database
2001	WHO CGM Database
2002	WHO CGM Database
2003	WHO CGM Database
2004	WHO CGM Database
2006	WHO CGM Database
2007	WHO CGM Database
2008	WHO CGM Database
2013	WHO CGM Database
2014	WHO CGM Database

Chile – HAZ, WHZ, and WAZ Distributions

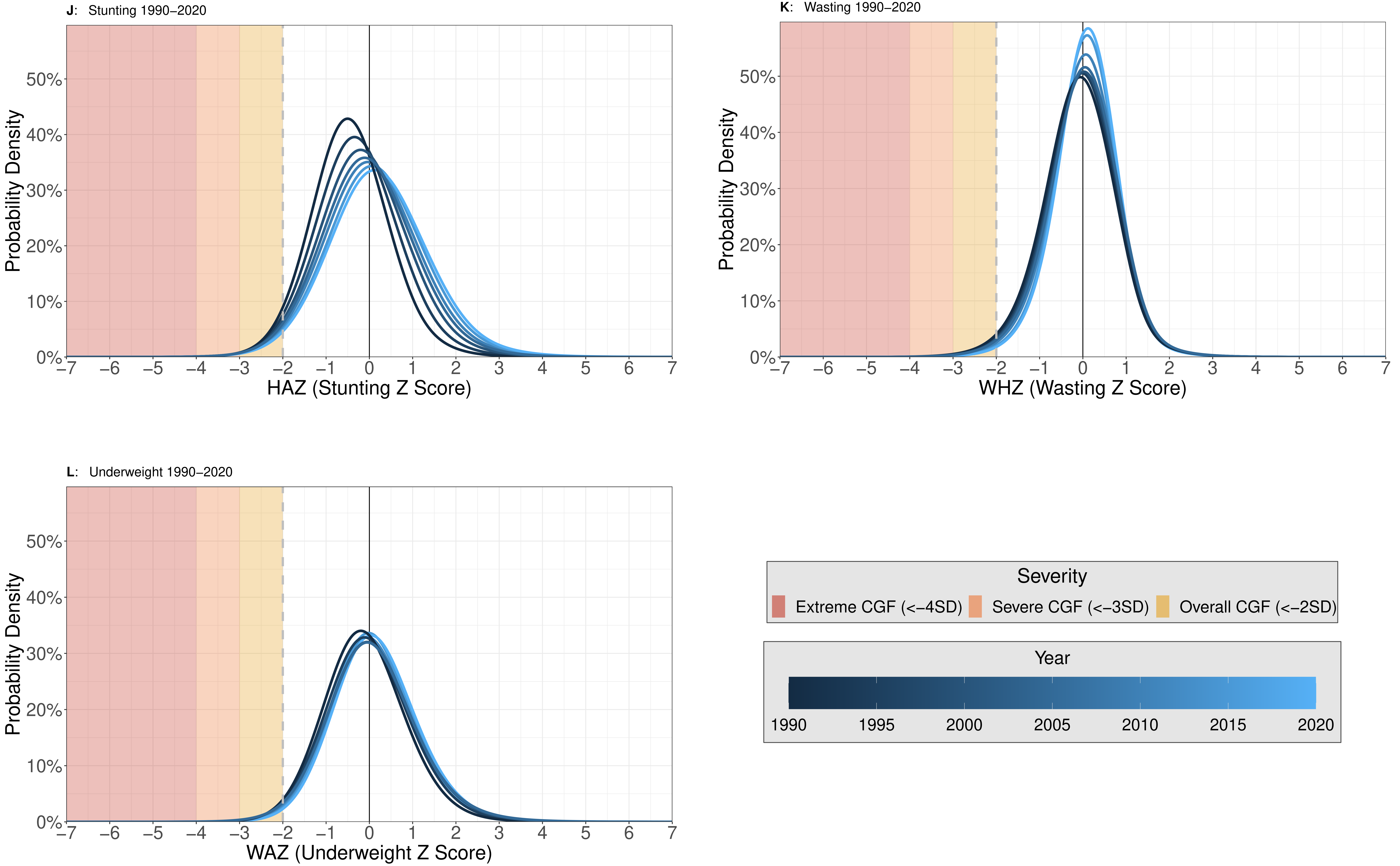

Uruguay – Stunting (HAZ)

A: Overall and Severe Stunting Prevalence

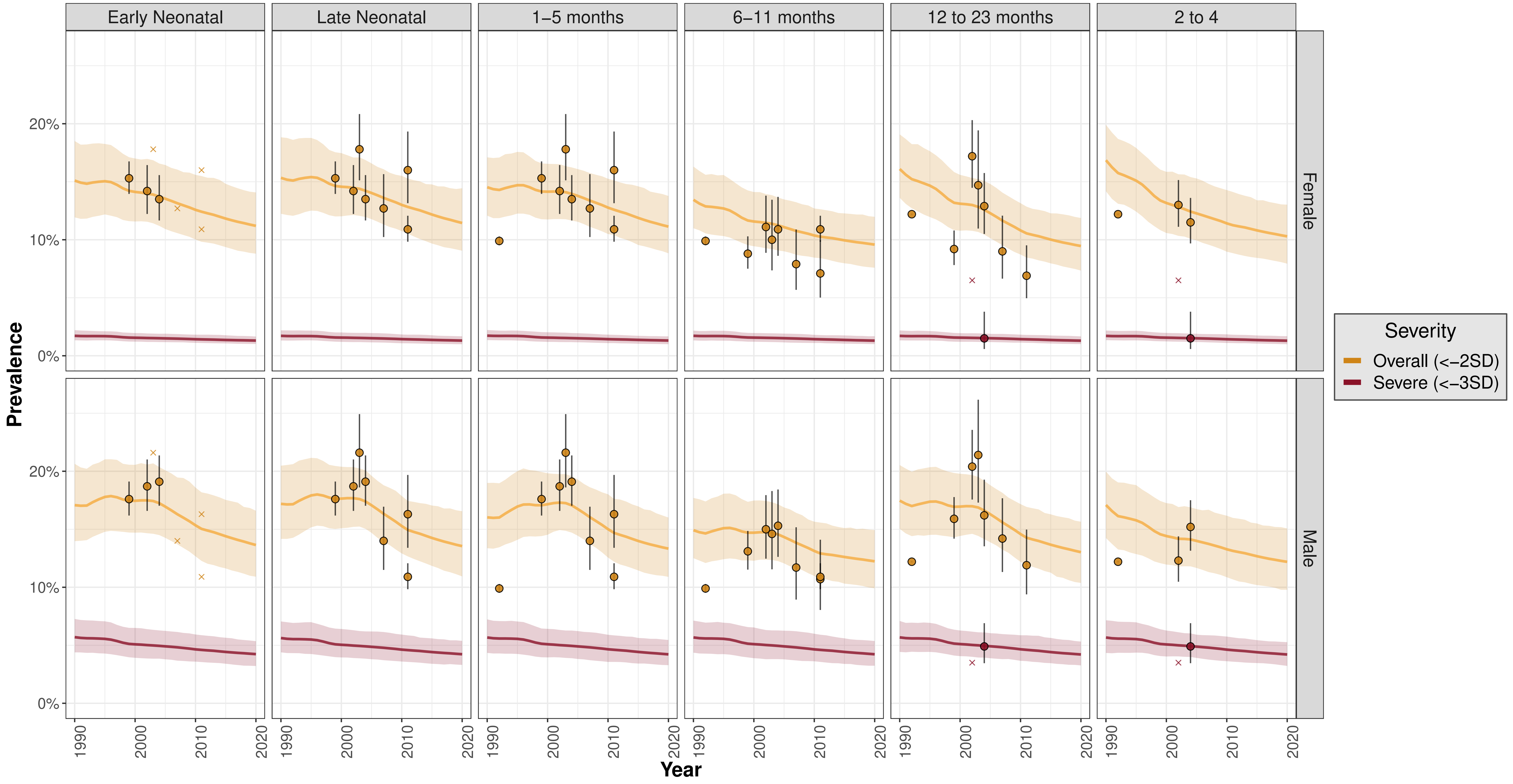

B: Transformed Mean Stunting Z Scores

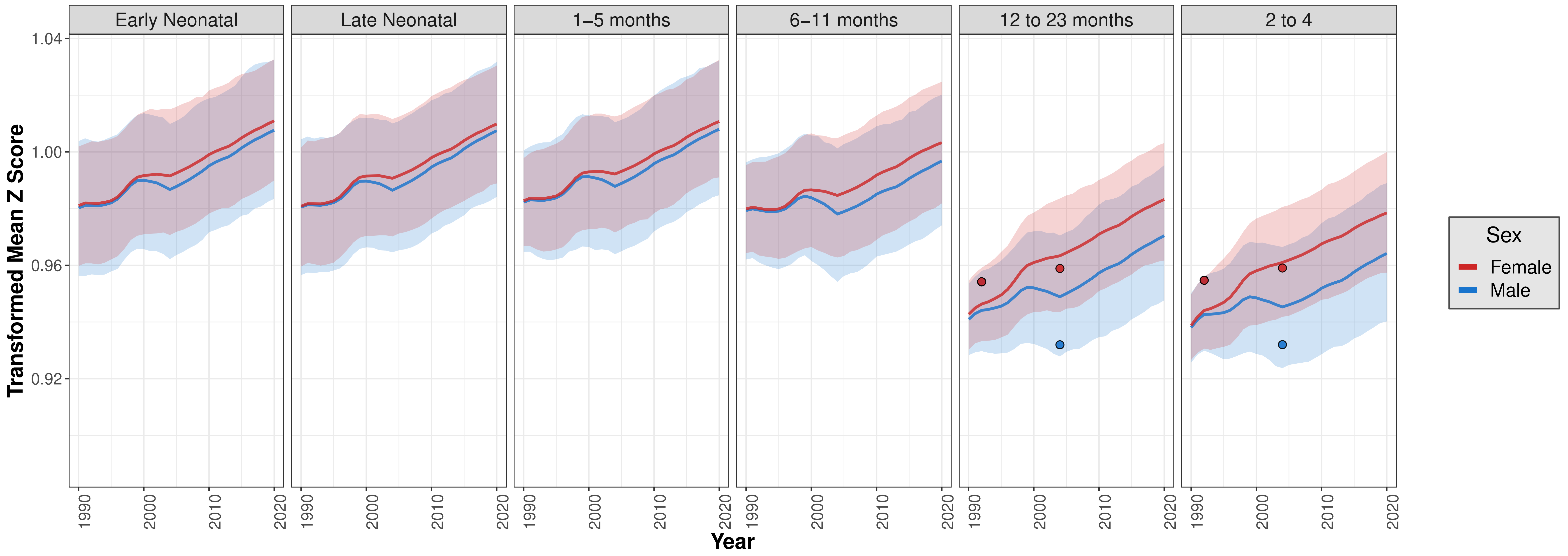

C

Year	Source
1987	WHO CGM Database
1988	WHO CGM Database
1992	WHO CGM Database
1999	WHO CGM Database
2002	WHO CGM Database
2003	WHO CGM Database
2004	WHO CGM Database
2007	WHO CGM Database
2011	WHO CGM Database
2011	National Survey on Nutritional Status, Feeding Practices and Anemia

Uruguay – Wasting (WHZ)

D: Overall and Severe Wasting Prevalence

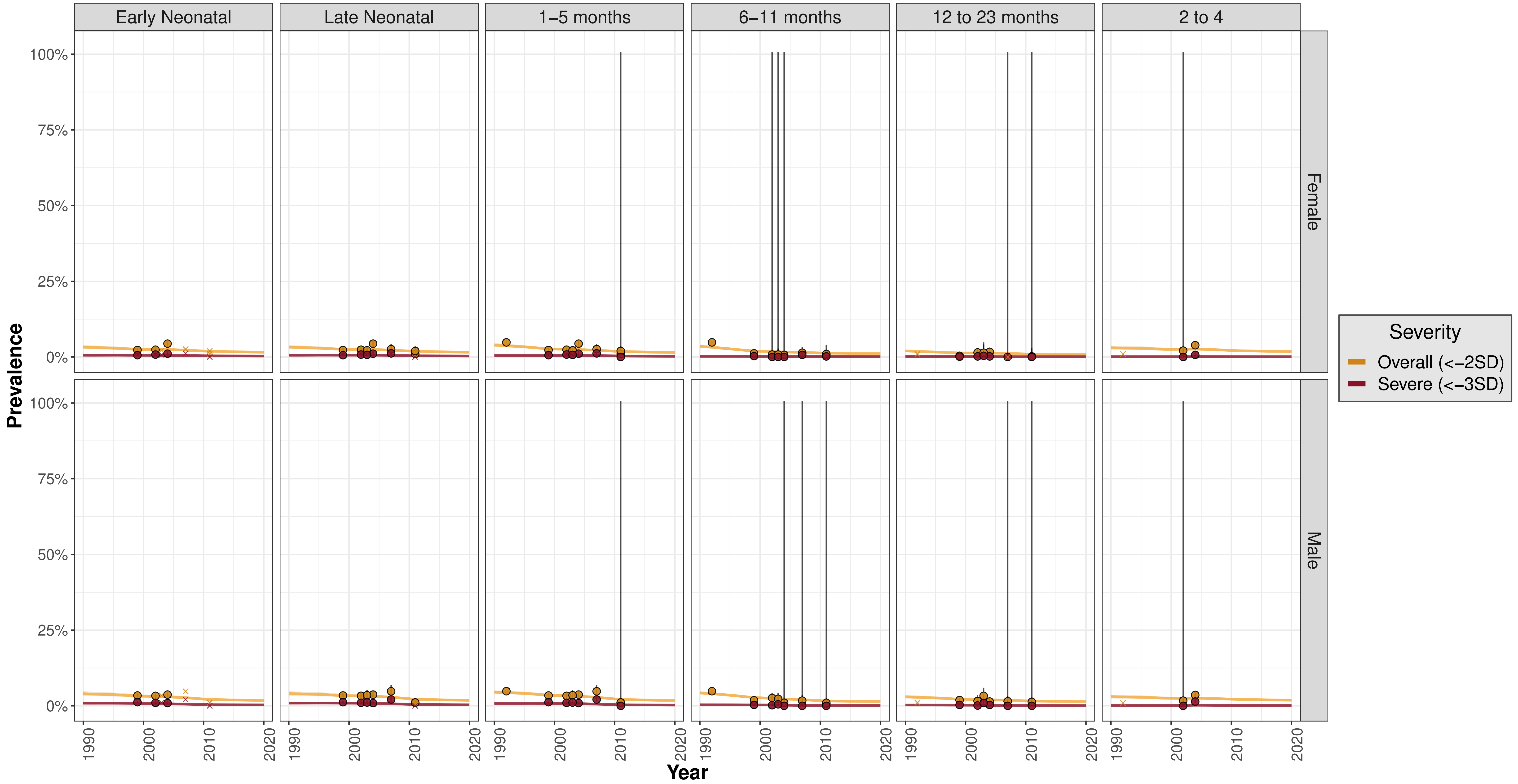

E: Transformed Mean Wasting Z Scores

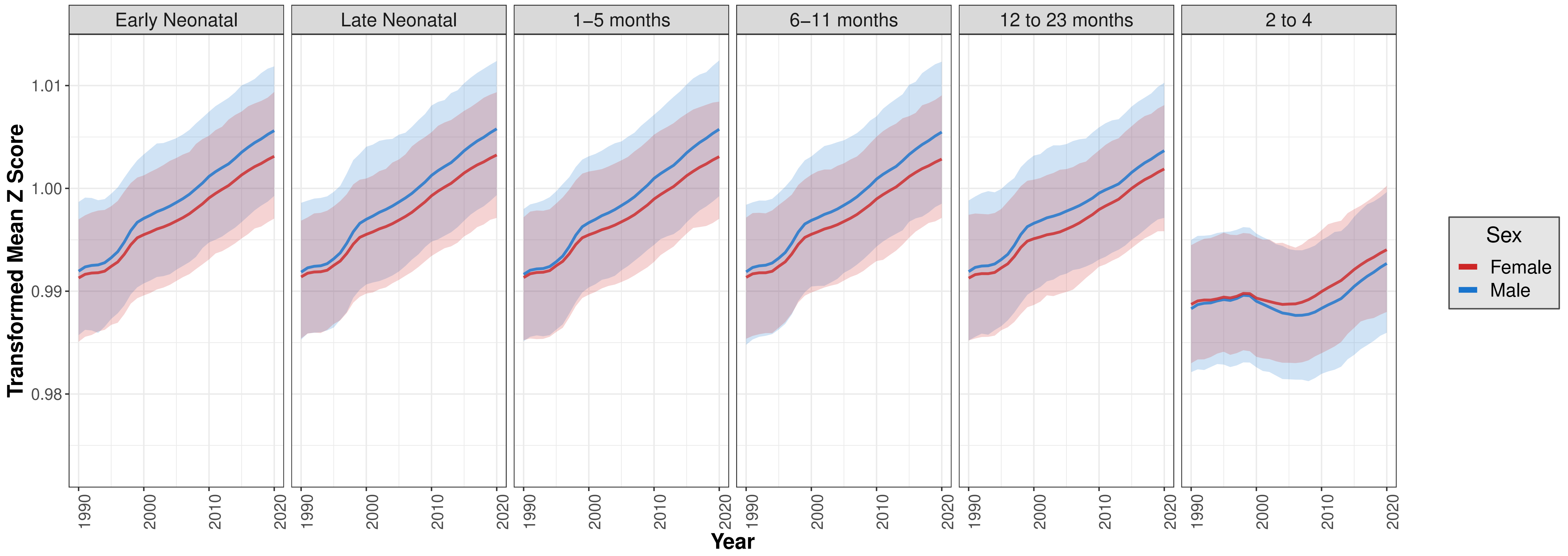

F

Year	Source
1988	WHO CGM Database
1992	WHO CGM Database
1999	WHO CGM Database
2002	WHO CGM Database
2003	WHO CGM Database
2004	WHO CGM Database
2007	WHO CGM Database
2011	WHO CGM Database
2011	National Survey on Nutritional Status, Feeding Practices and Anemia

Uruguay – Underweight (WAZ)

G: Overall and Severe Underweight Prevalence

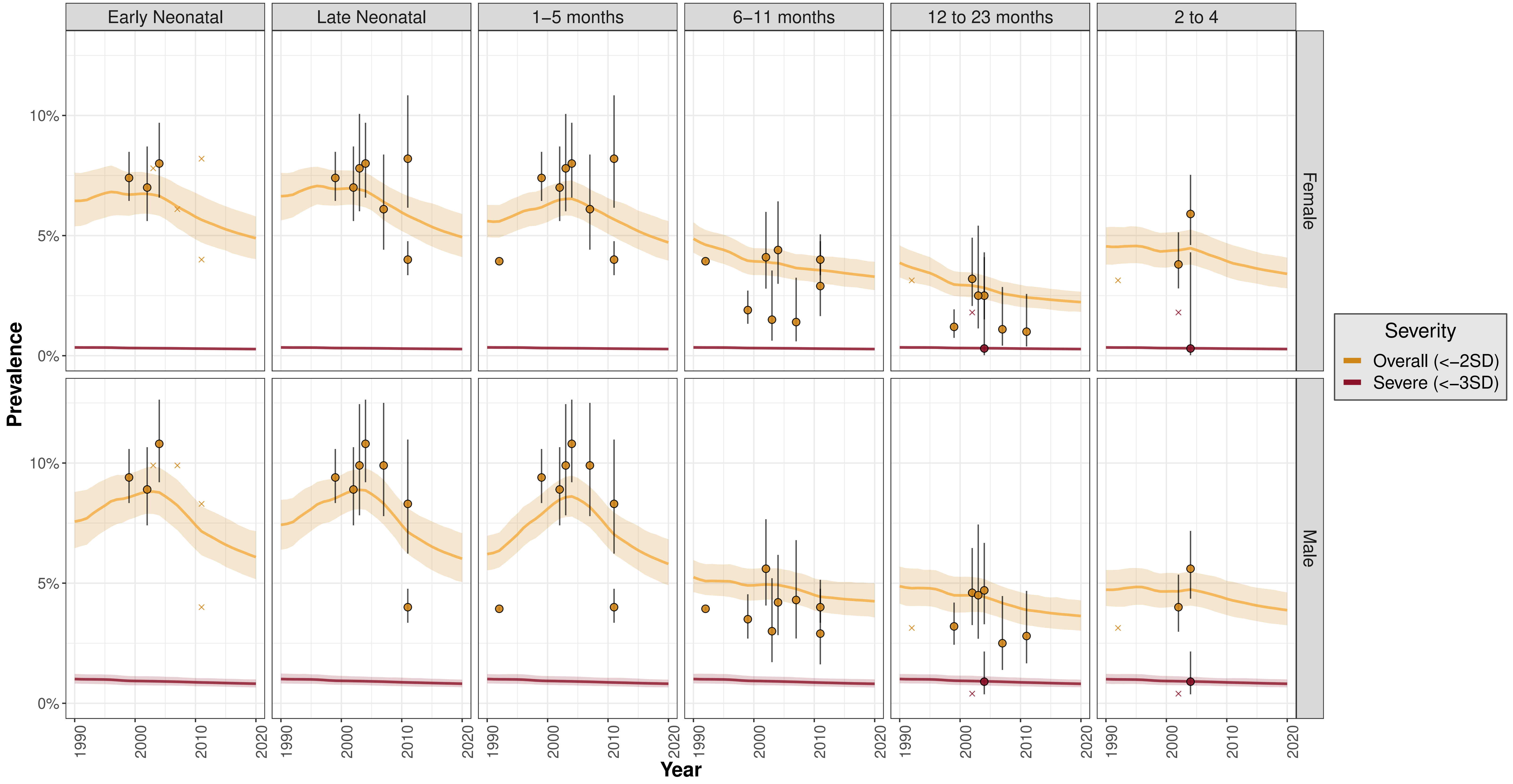

H: Transformed Mean Underweight Z Scores

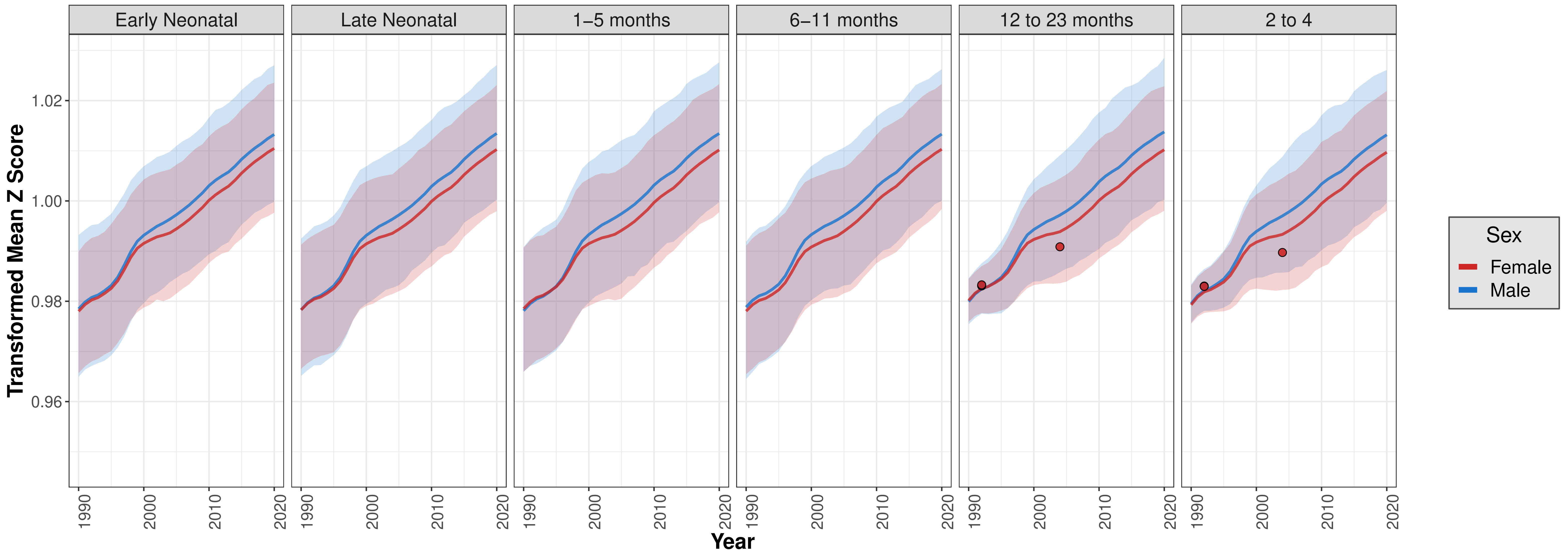

I

Year	Source
1987	WHO CGM Database
1988	WHO CGM Database
1992	WHO CGM Database
1999	WHO CGM Database
2002	WHO CGM Database
2003	WHO CGM Database
2004	WHO CGM Database
2007	WHO CGM Database
2011	WHO CGM Database
2011	National Survey on Nutritional Status, Feeding Practices and Anemia

Uruguay – HAZ, WHZ, and WAZ Distributions

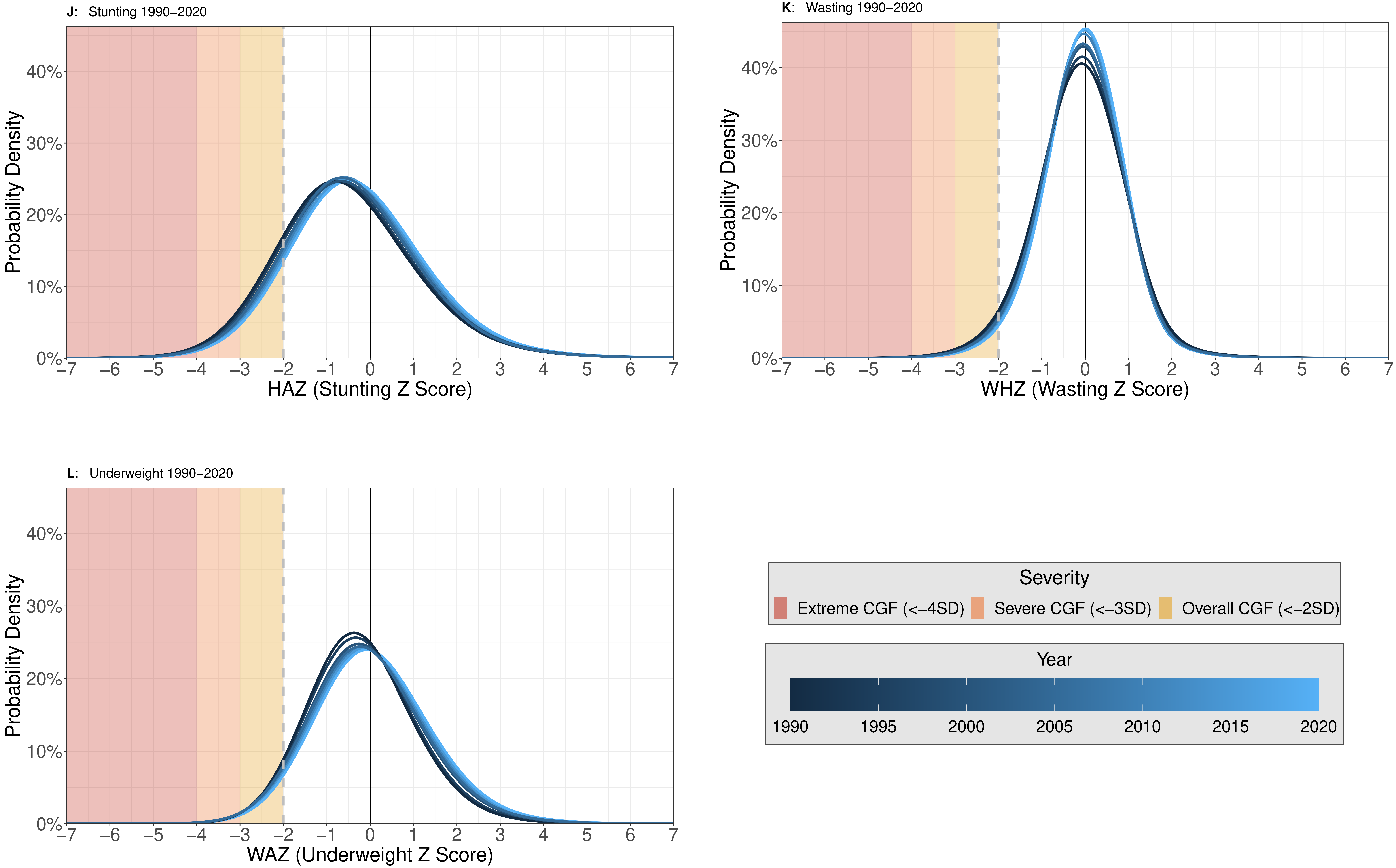

Monaco – Stunting (HAZ)

A: Overall and Severe Stunting Prevalence

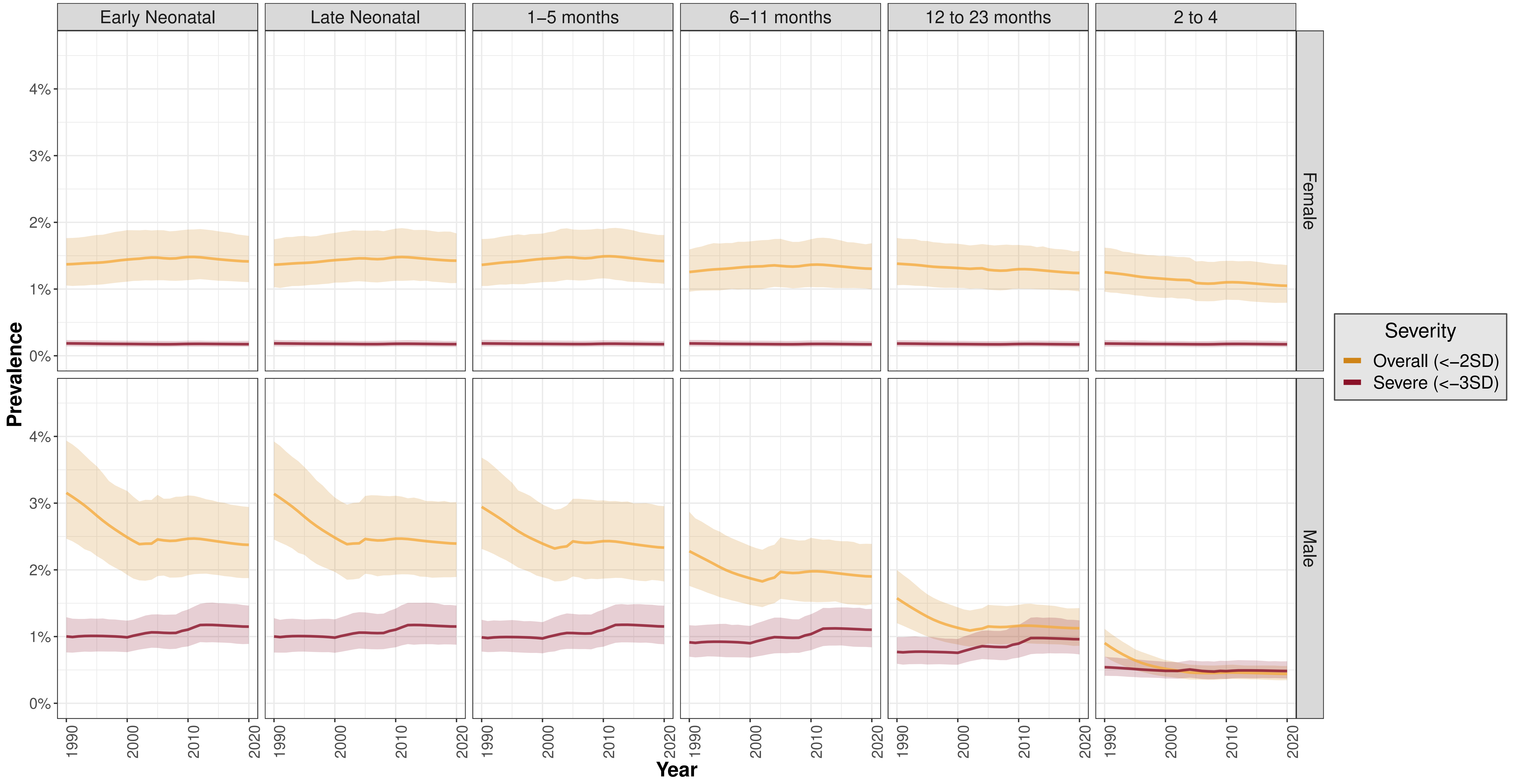

B: Transformed Mean Stunting Z Scores

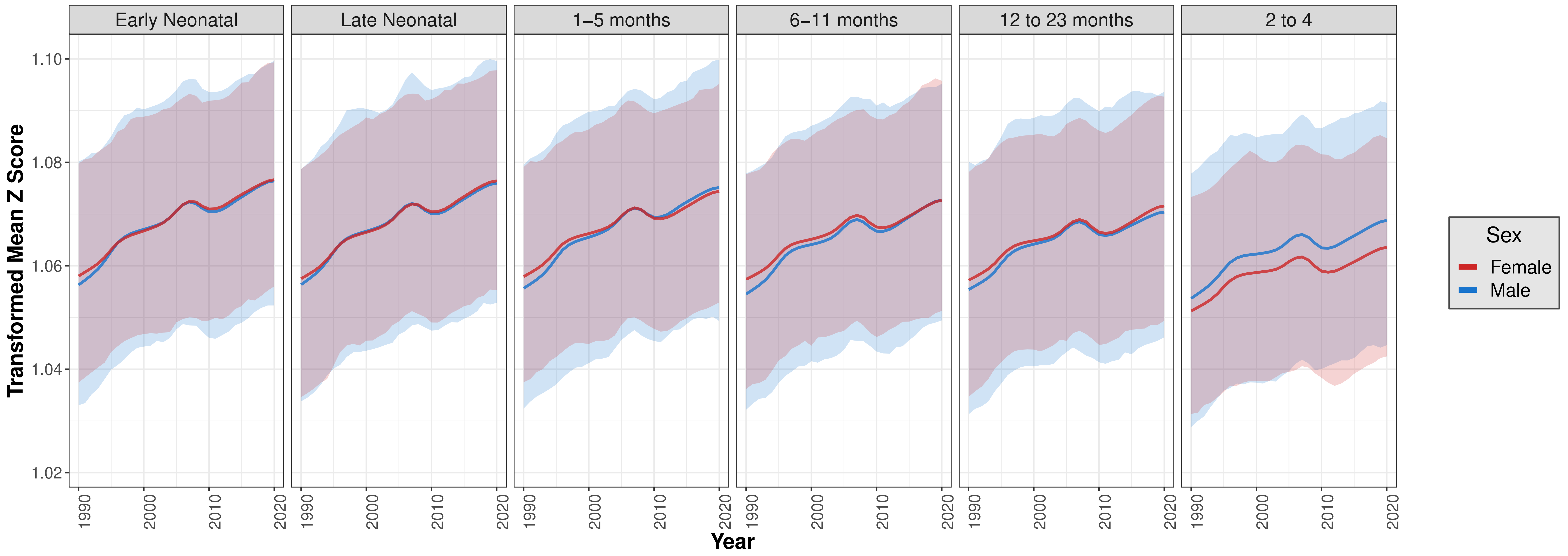

C

Source
No sources for this location

Monaco – Wasting (WHZ)

D: Overall and Severe Wasting Prevalence

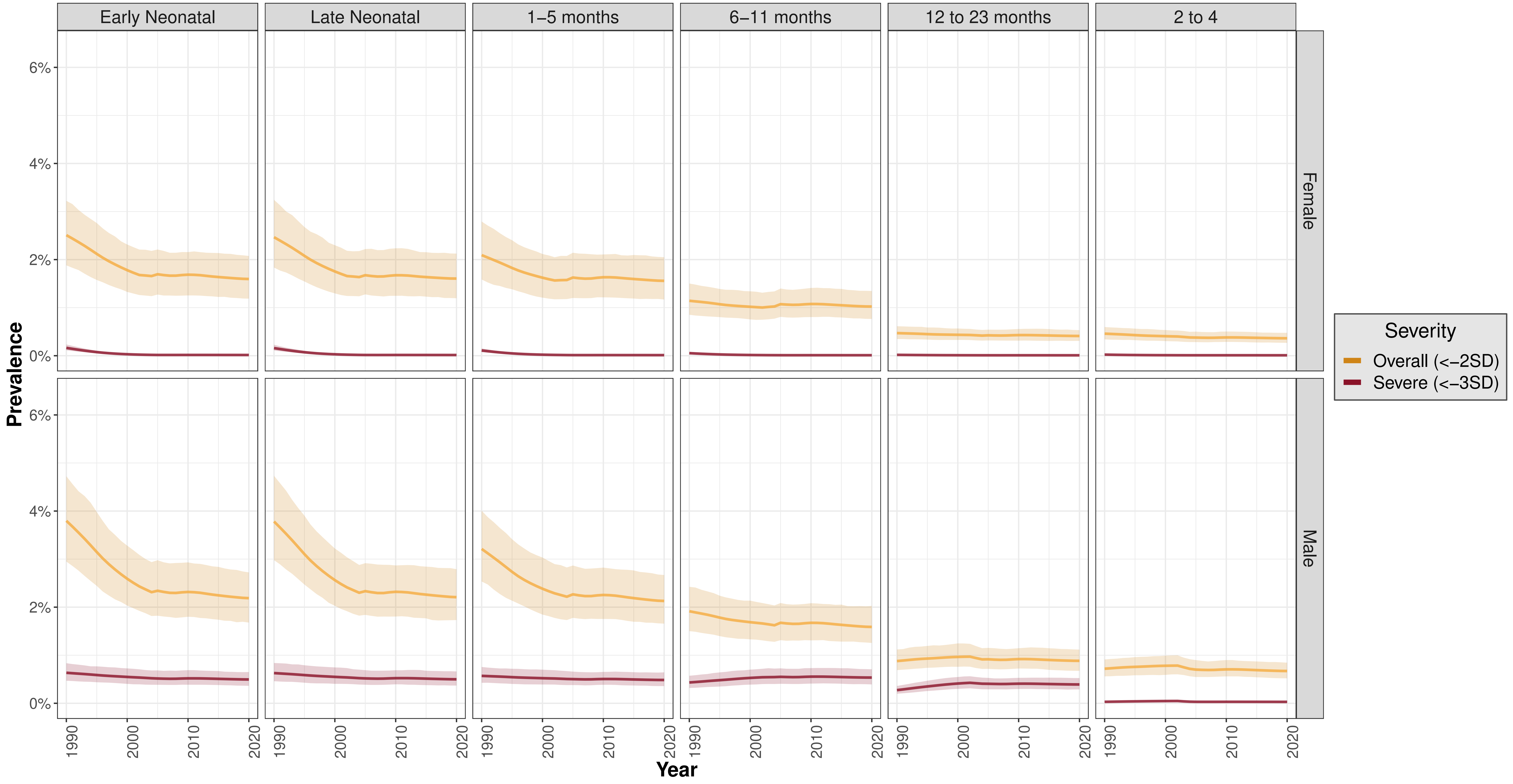

E: Transformed Mean Wasting Z Scores

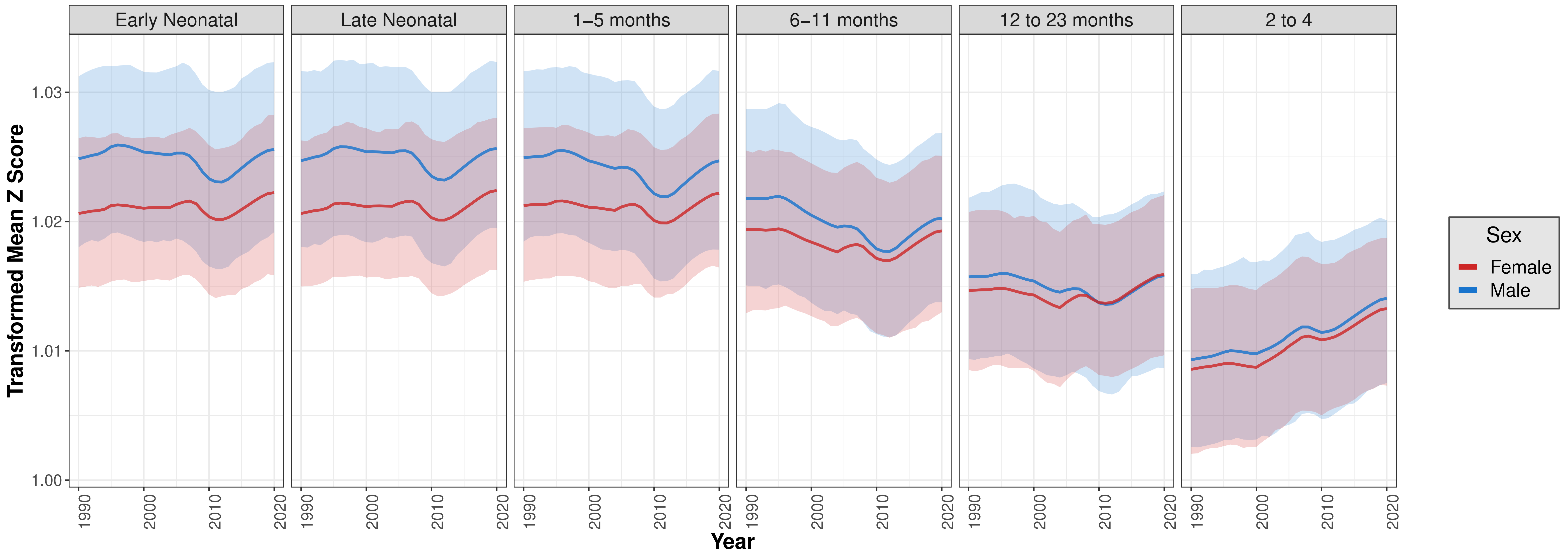

F

Source
No sources for this location

Monaco – Underweight (WAZ)

G: Overall and Severe Underweight Prevalence

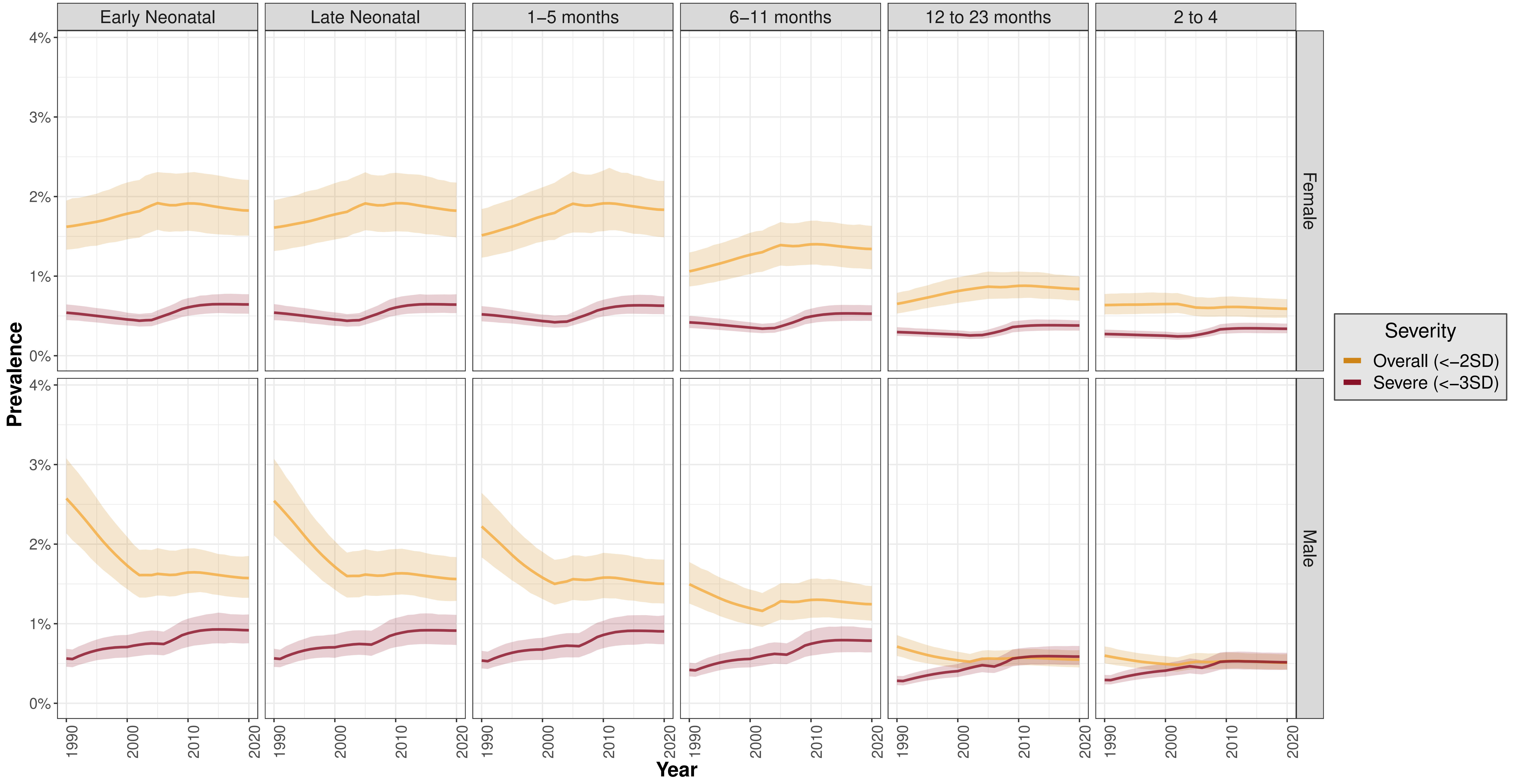

H: Transformed Mean Underweight Z Scores

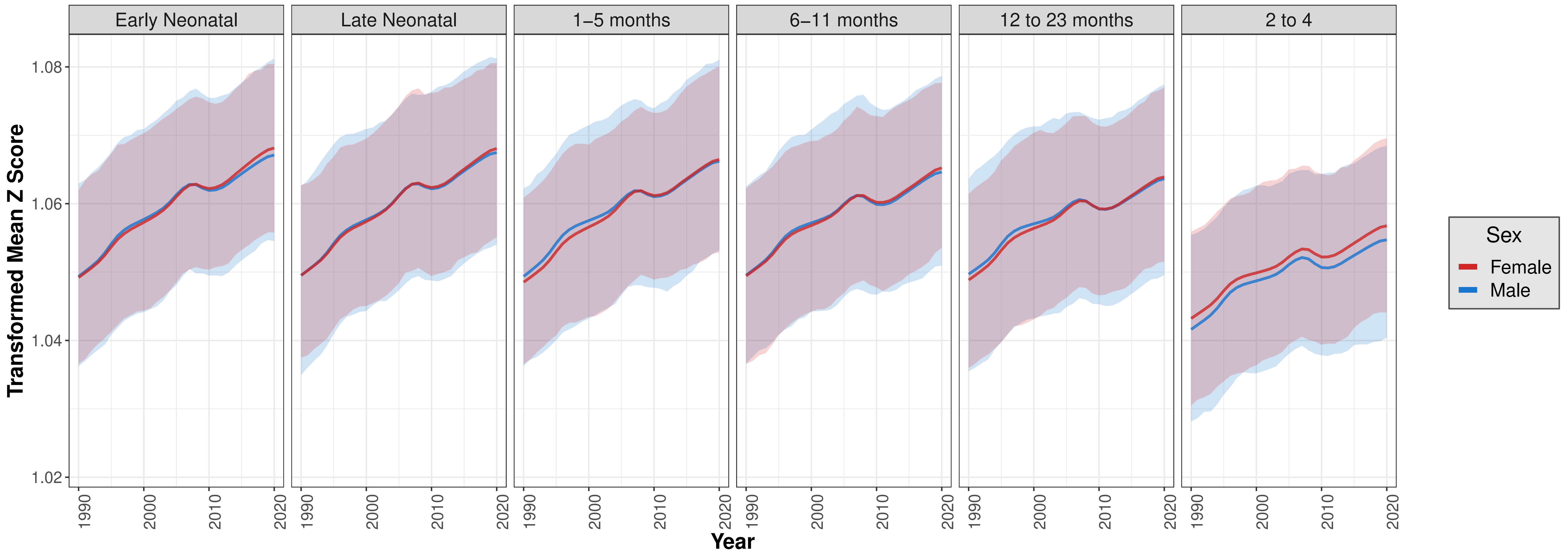

I

Source
No sources for this location

Monaco – HAZ, WHZ, and WAZ Distributions

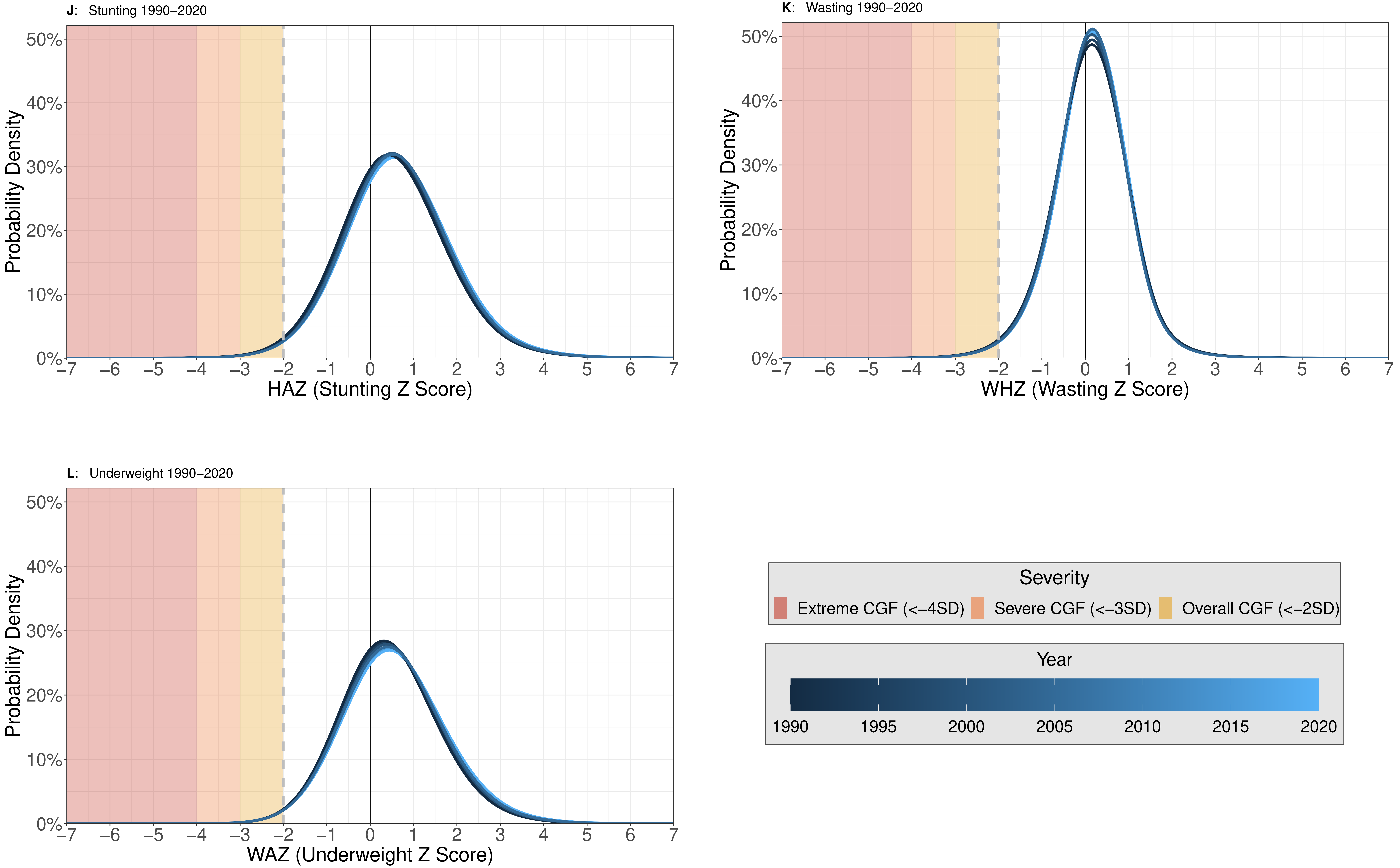

San Marino – Stunting (HAZ)

A: Overall and Severe Stunting Prevalence

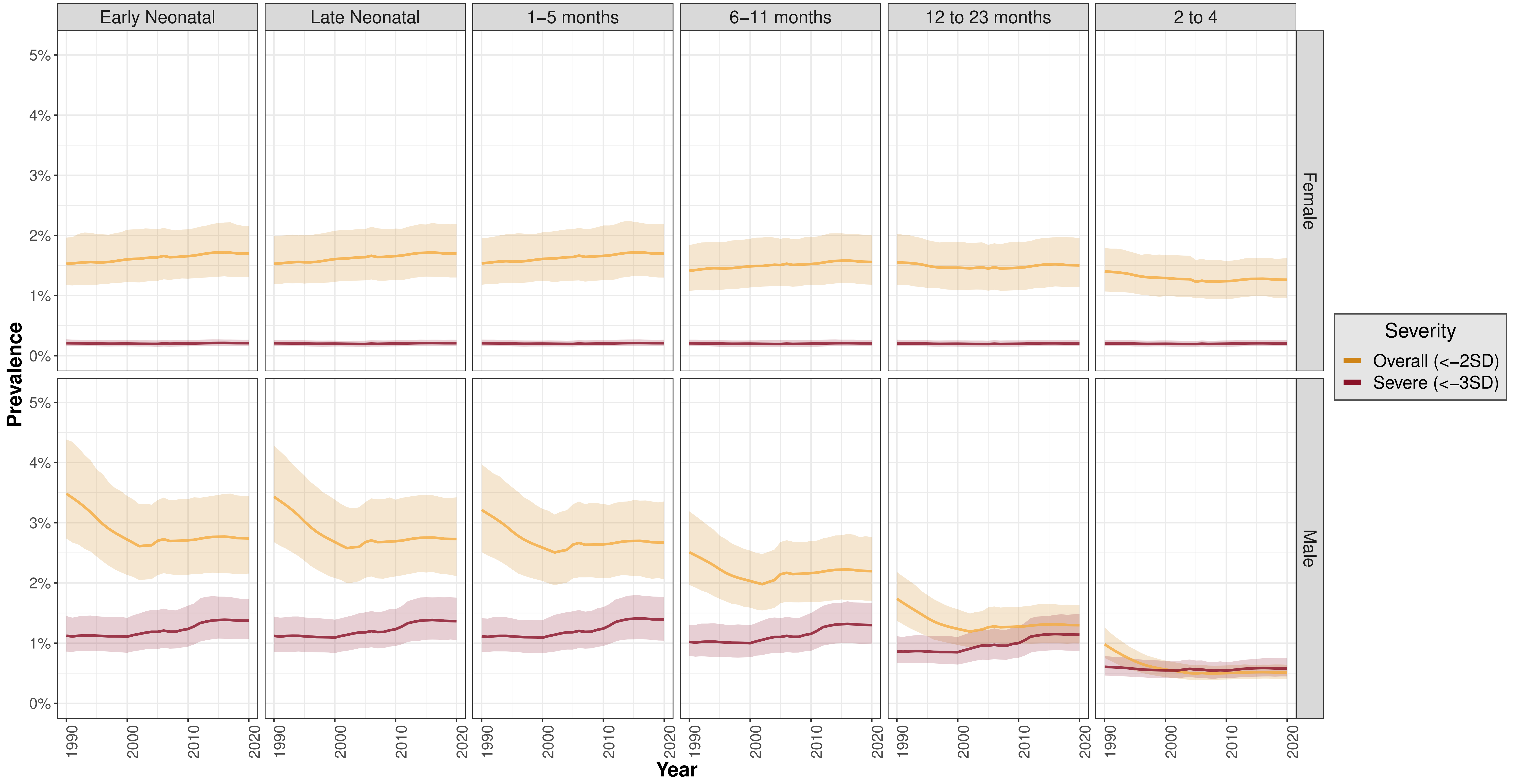

B: Transformed Mean Stunting Z Scores

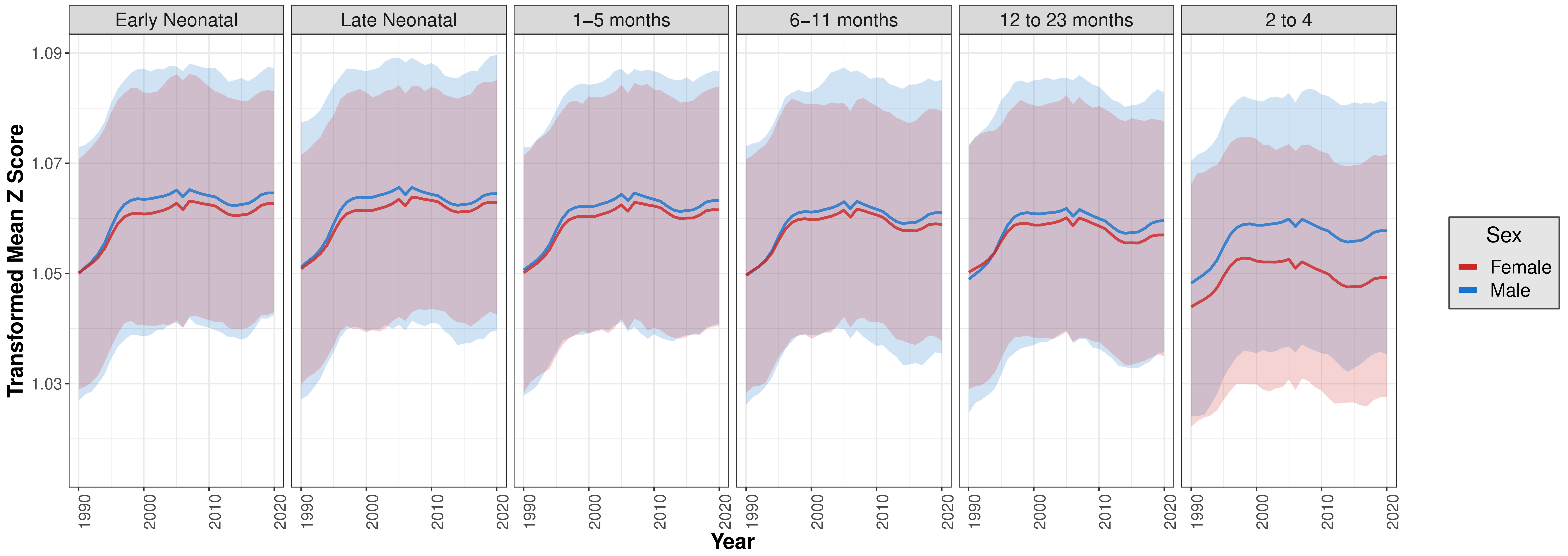

C

Source
No sources for this location

San Marino – Wasting (WHZ)

D: Overall and Severe Wasting Prevalence

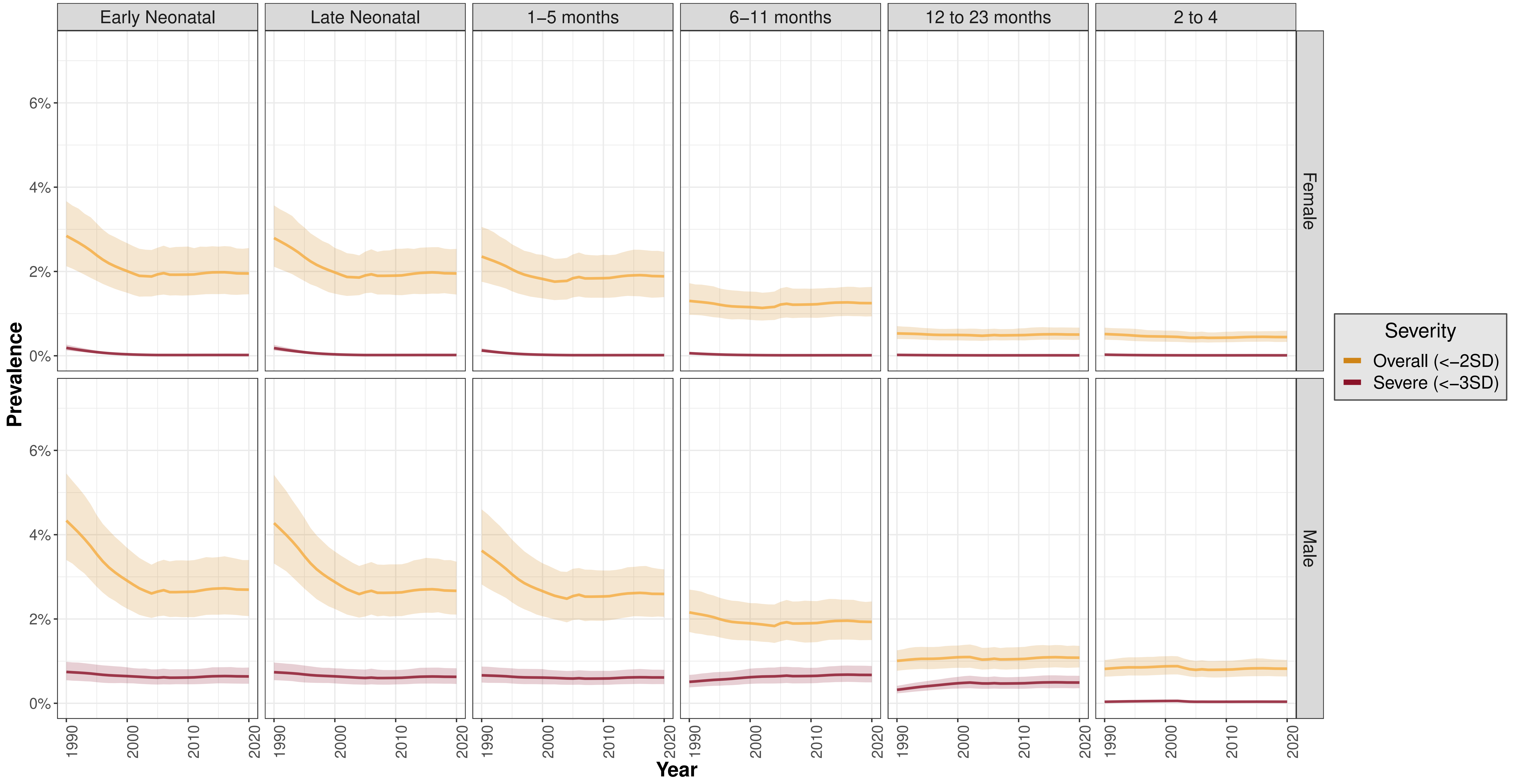

E: Transformed Mean Wasting Z Scores

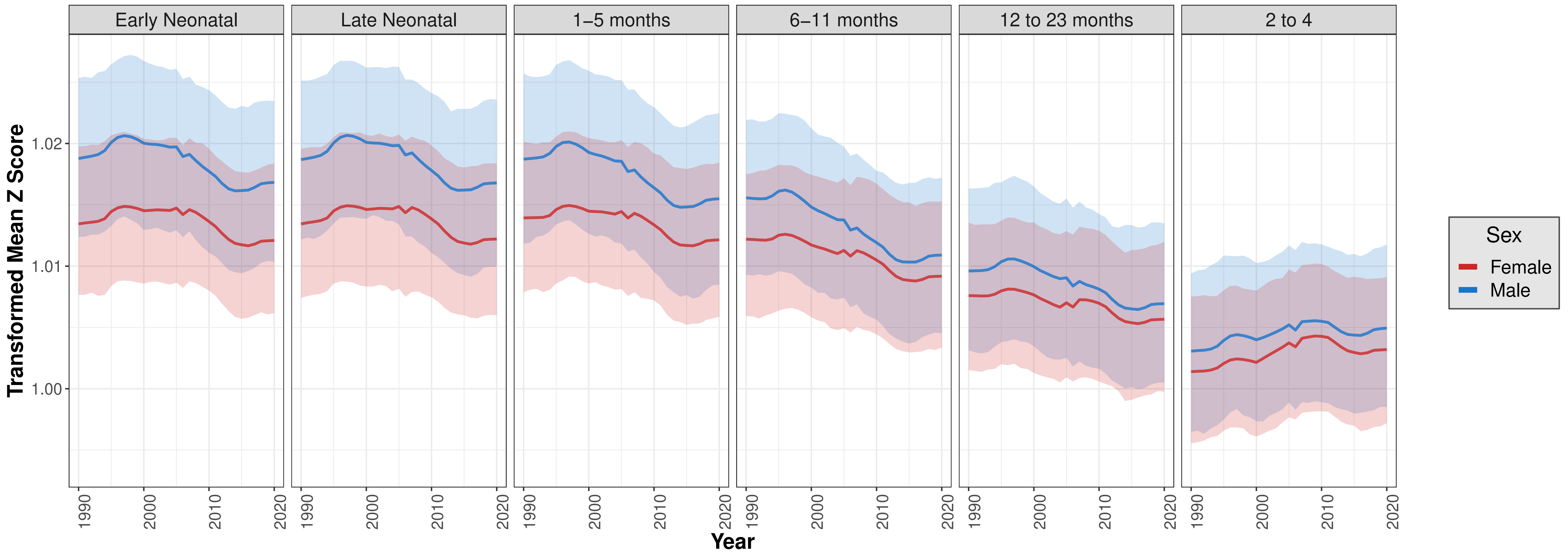

F

Source
No sources for this location

San Marino – Underweight (WAZ)

G: Overall and Severe Underweight Prevalence

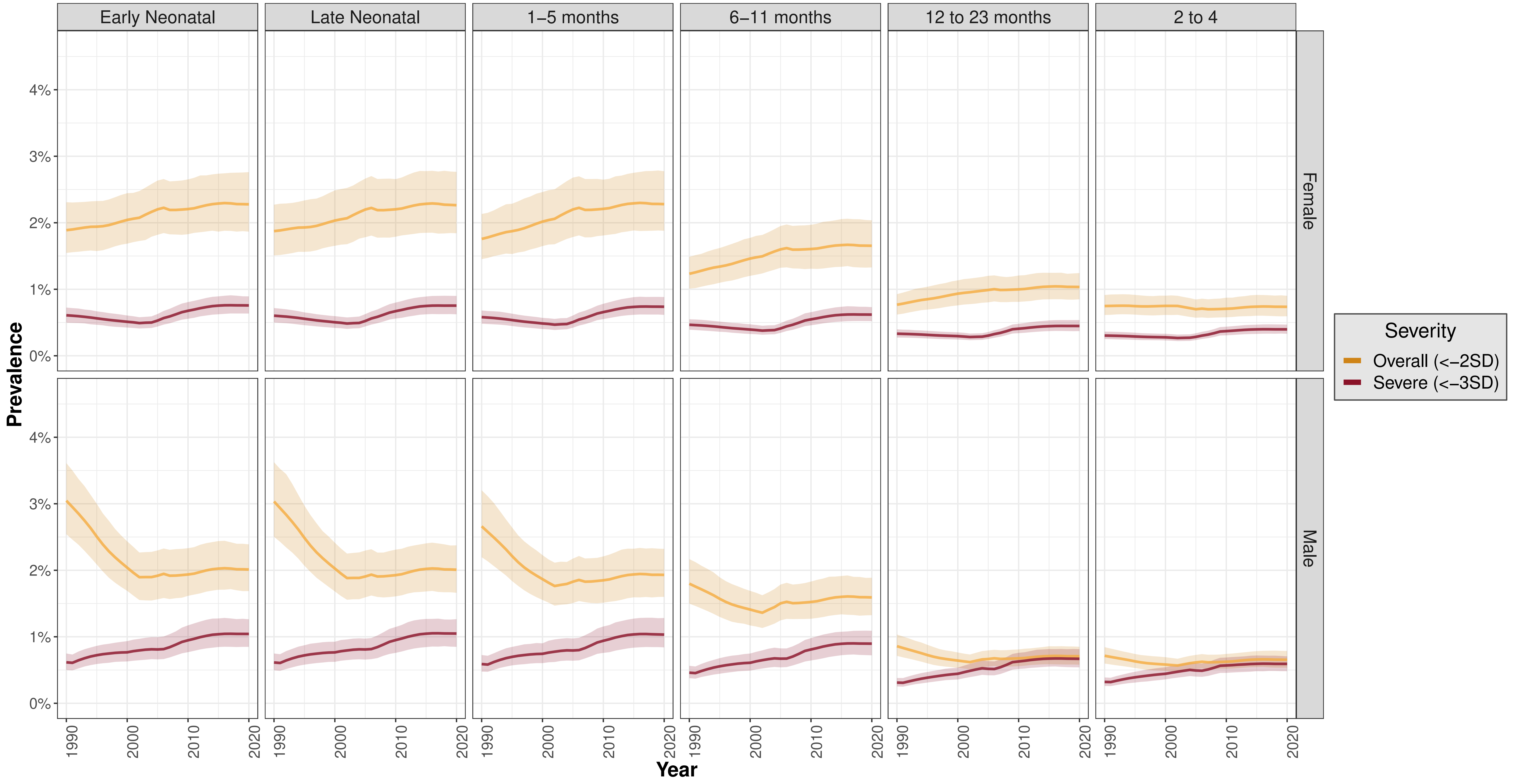

H: Transformed Mean Underweight Z Scores

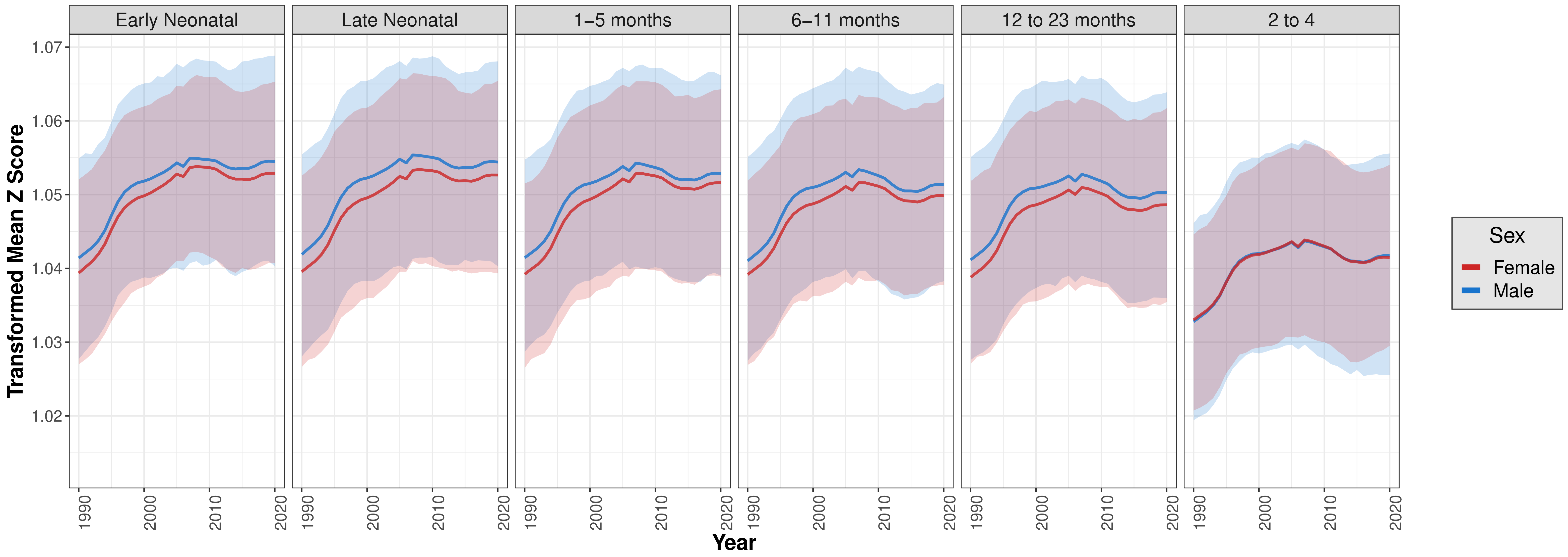

I

Source
No sources for this location

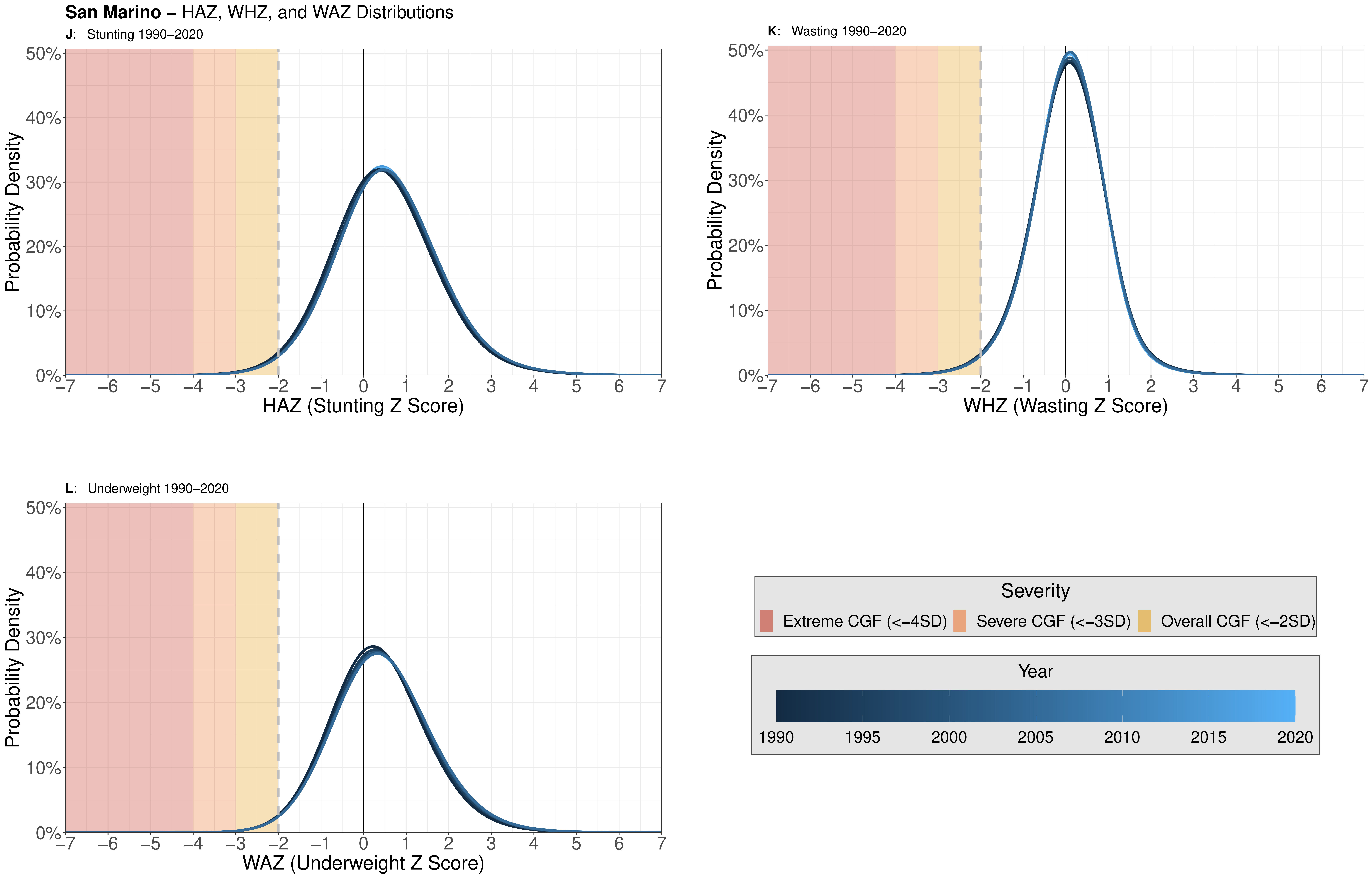
San Marino – HAZ, WHZ, and WAZ Distributions
J: Stunting 1990–2020
Probability Density
50%
40%
30%
20%
10%
0%
−7 −6 −5 −4 −3 −2 −1 0 1 2 3 4 5 6 7
HAZ (Stunting Z Score)
K: Wasting 1990–2020
Probability Density
50%
40%
30%
20%
10%
0%
−7 −6 −5 −4 −3 −2 −1 0 1 2 3 4 5 6 7
WHZ (Wasting Z Score)
L: Underweight 1990–2020
Probability Density
50%
40%
30%
20%
10%
0%
−7 −6 −5 −4 −3 −2 −1 0 1 2 3 4 5 6 7
WAZ (Underweight Z Score)
Severity
Extreme CGF (<−4SD)
Severe CGF (<−3SD)
Overall CGF (<−2SD)
Year
1990
1995
2000
2005
2010
2015
2020

Andorra – Stunting (HAZ)

A: Overall and Severe Stunting Prevalence

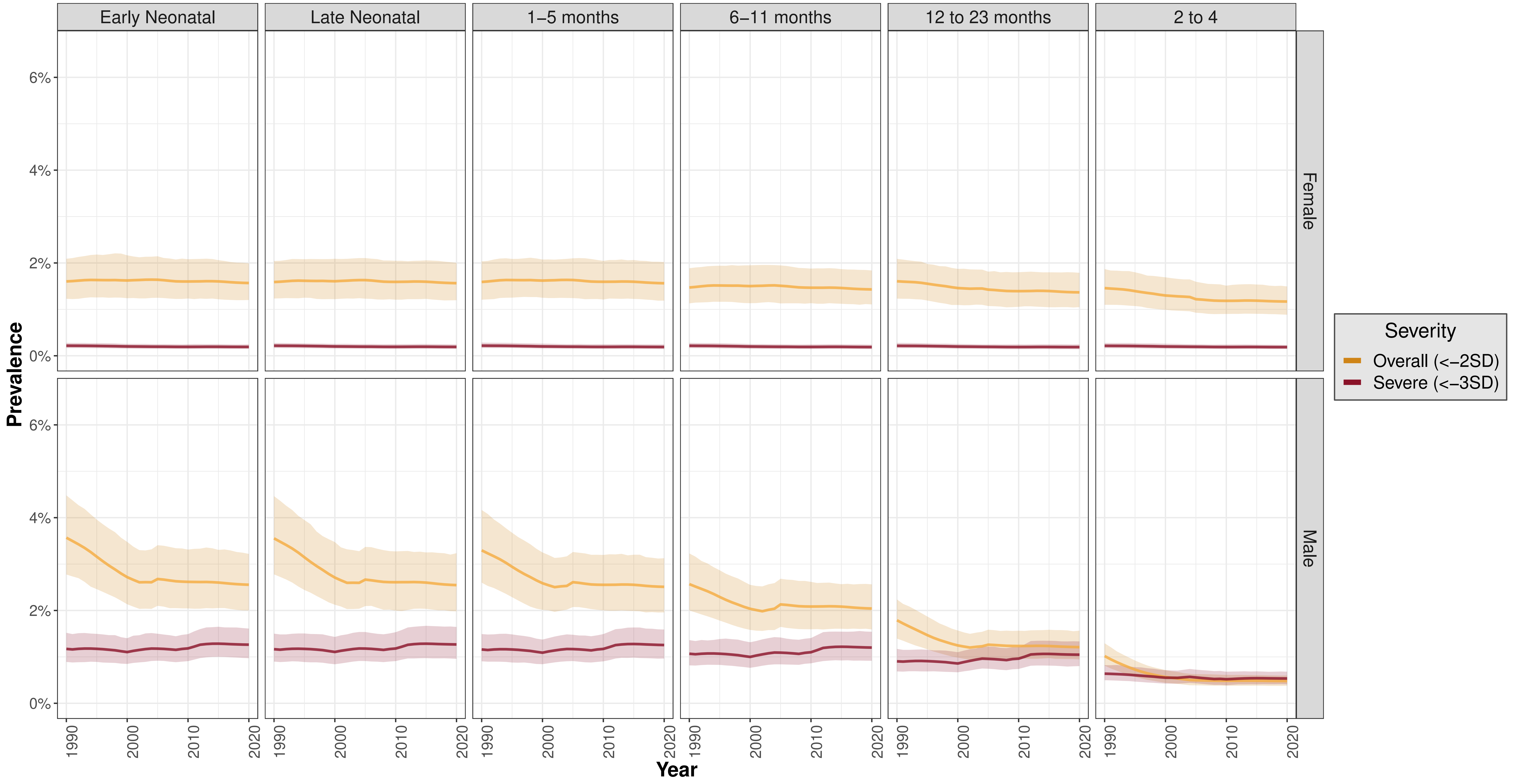

B: Transformed Mean Stunting Z Scores

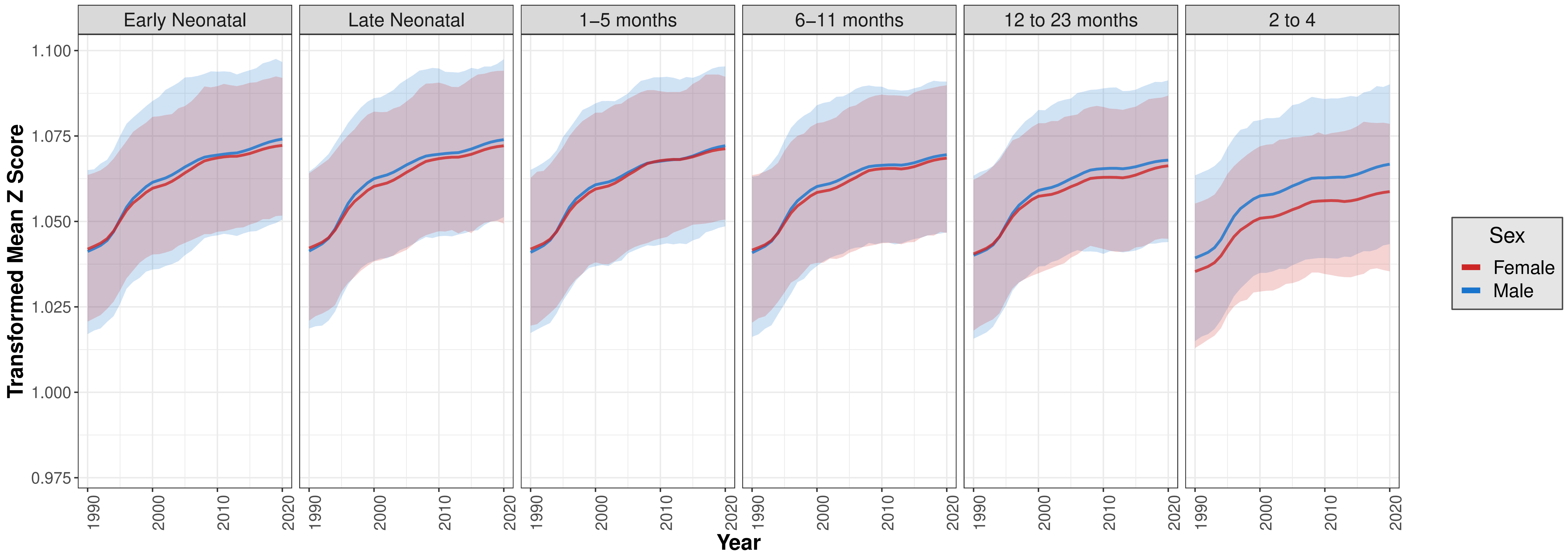

C

Source
No sources for this location

Andorra – Wasting (WHZ)

D: Overall and Severe Wasting Prevalence

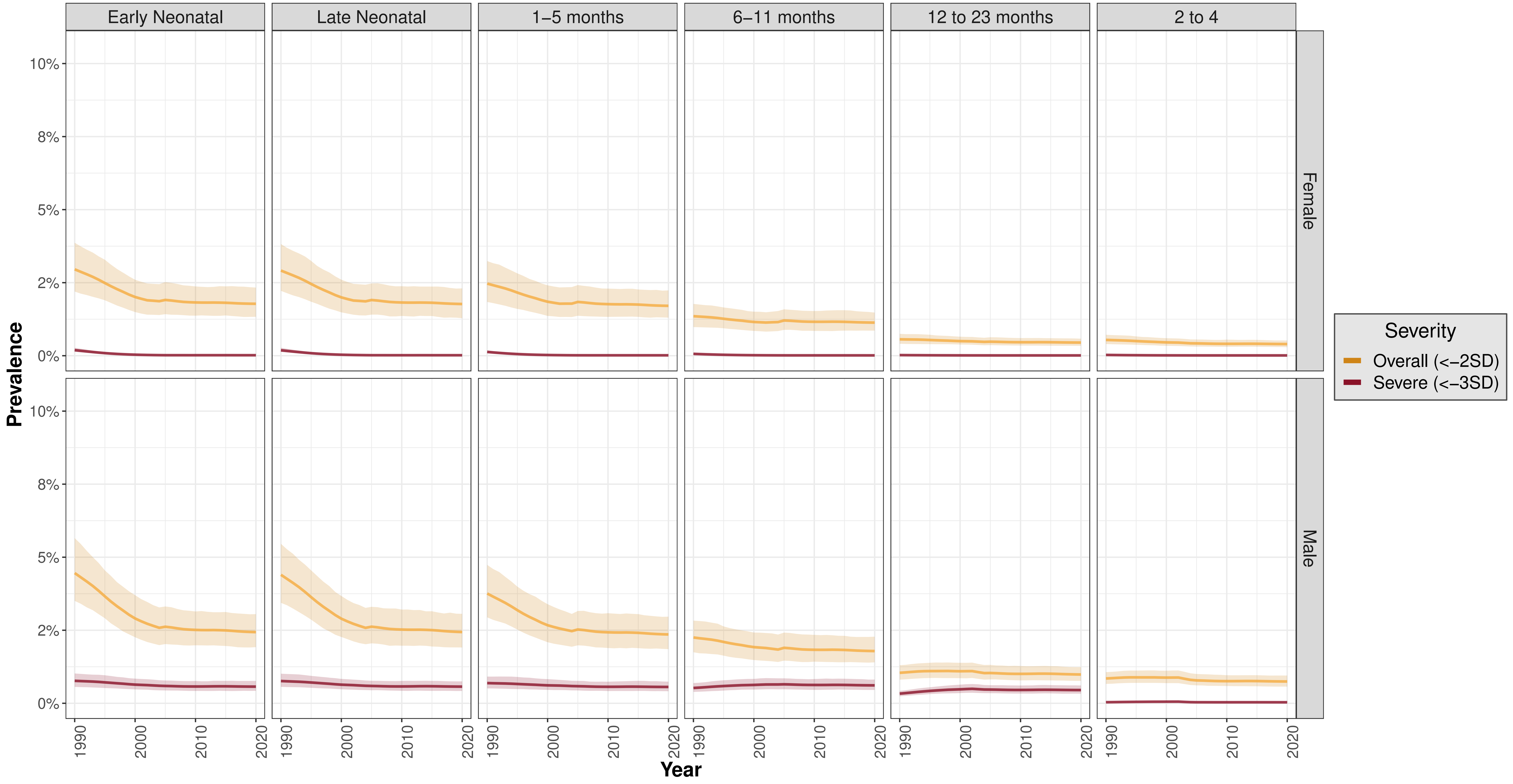

E: Transformed Mean Wasting Z Scores

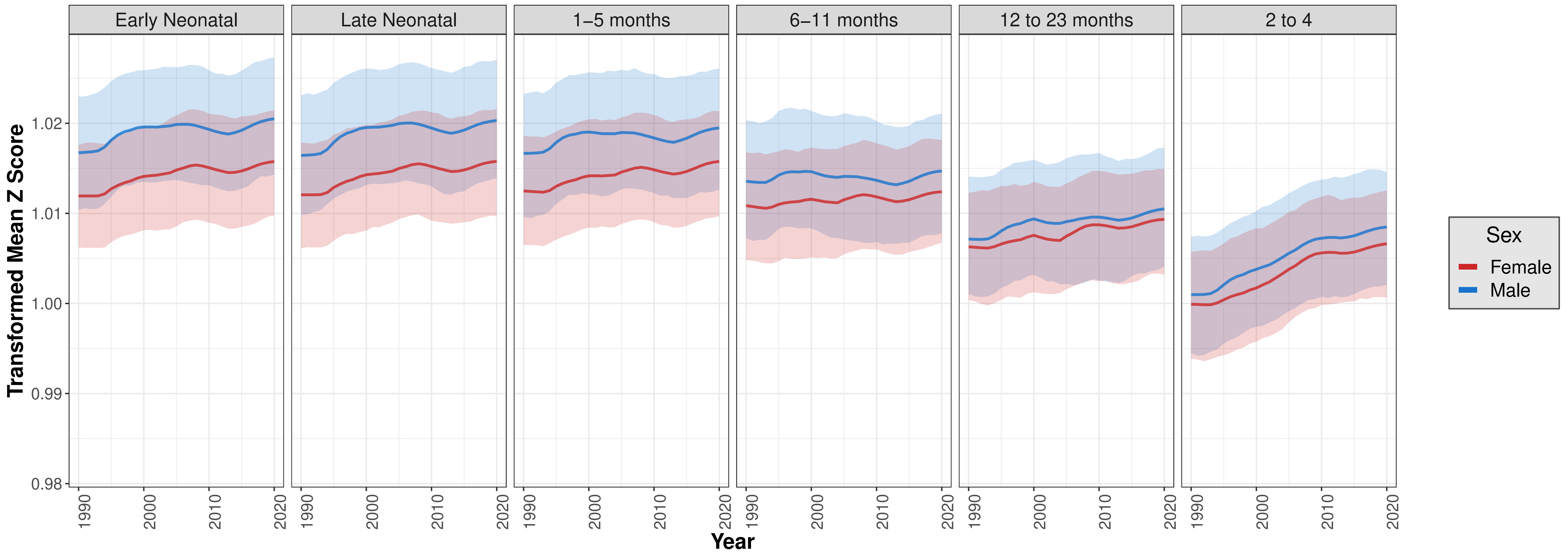

F

Source
No sources for this location

Andorra – Underweight (WAZ)

G: Overall and Severe Underweight Prevalence

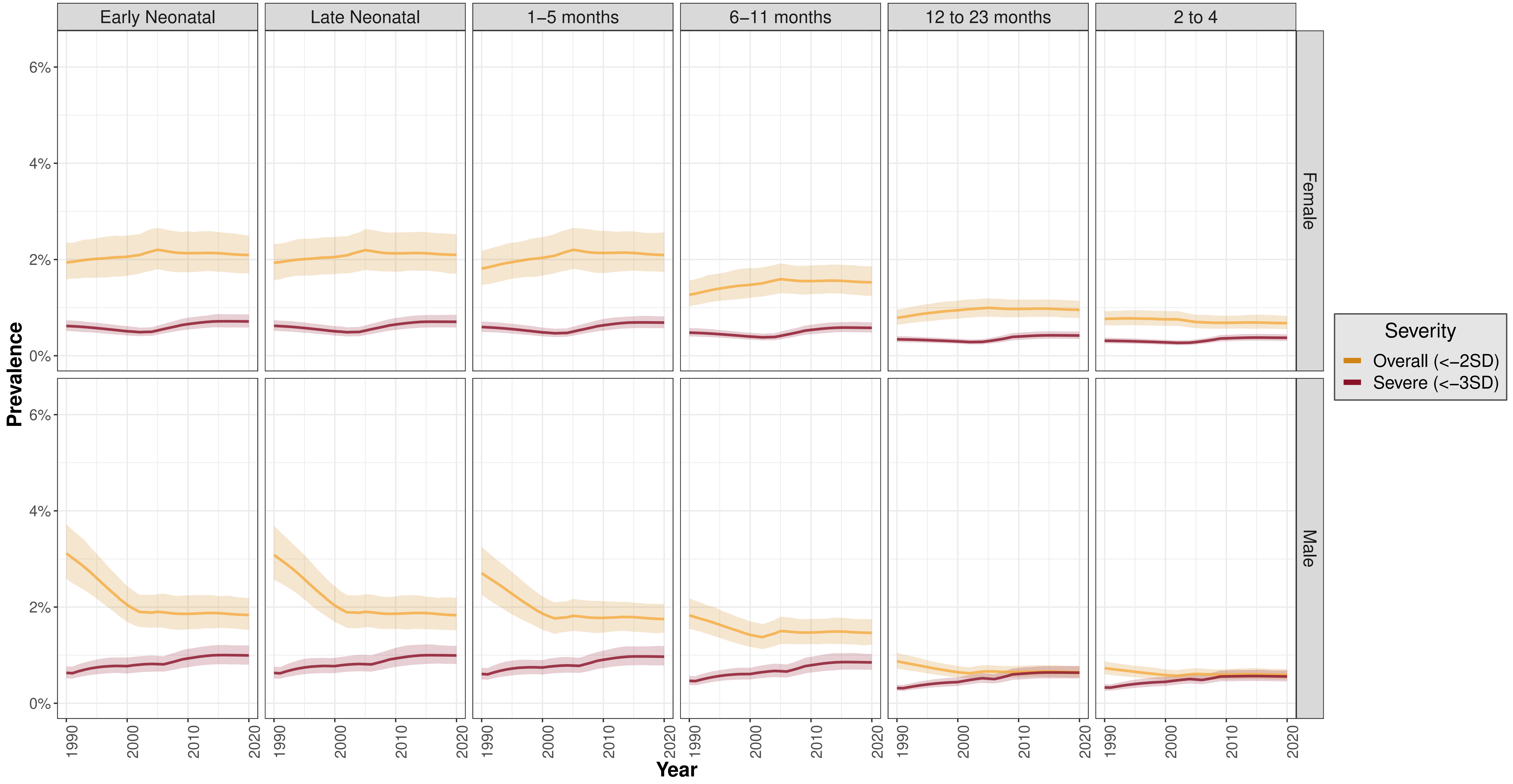

H: Transformed Mean Underweight Z Scores

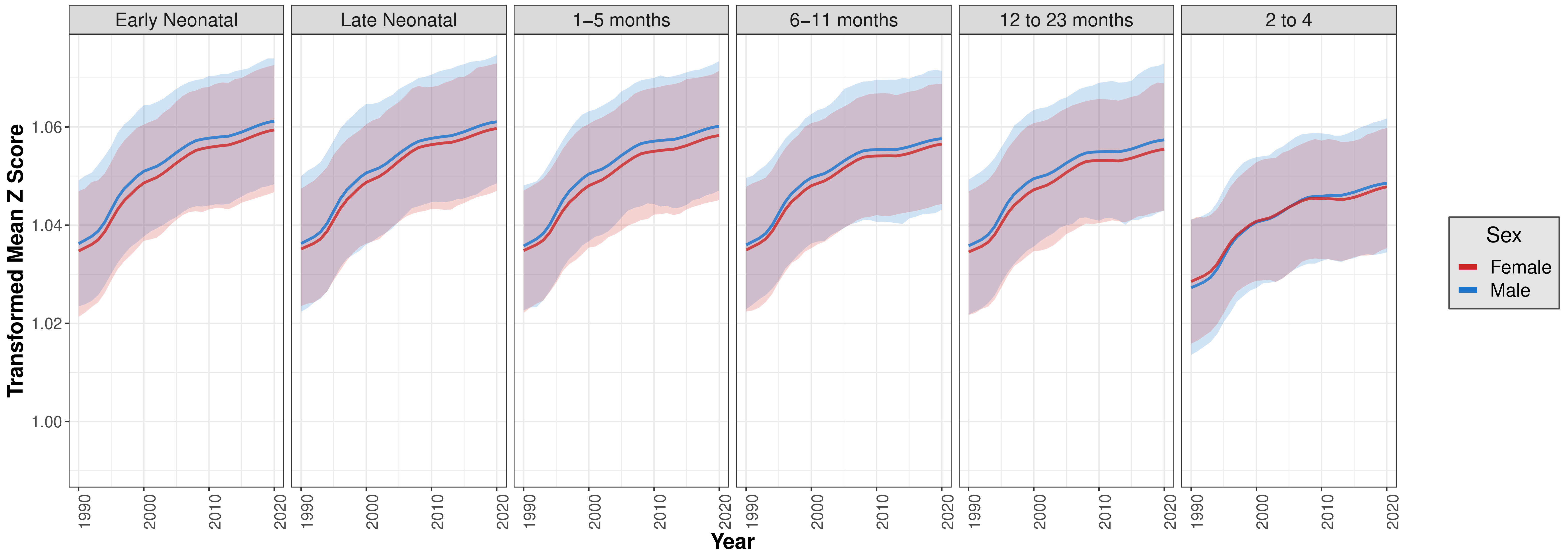

I

Source
No sources for this location

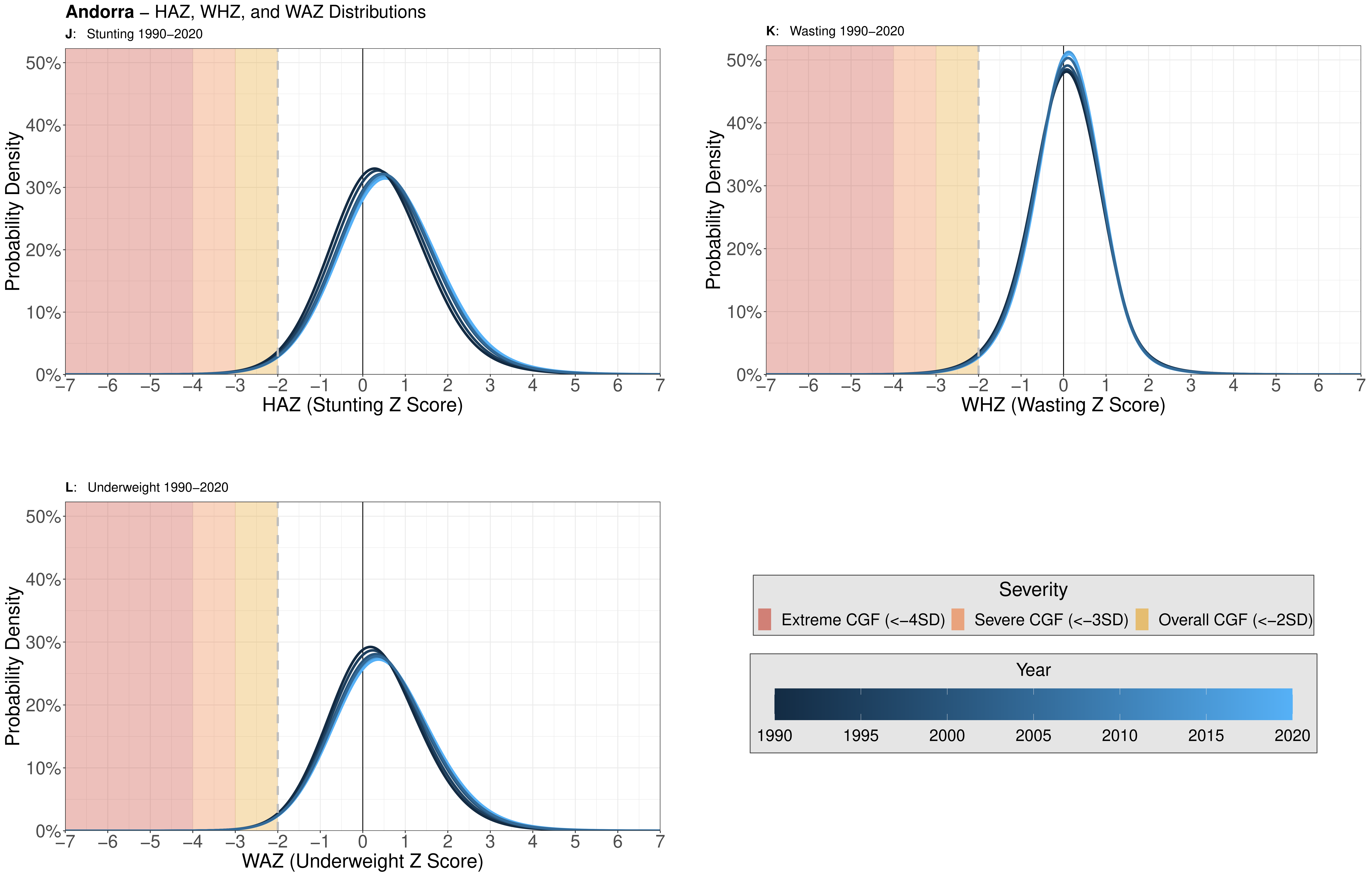

Andorra – HAZ, WHZ, and WAZ Distributions
J: Stunting 1990–2020
Probability Density
50%
40%
30%
20%
10%
0%
−7 −6 −5 −4 −3 −2 −1 0 1 2 3 4 5 6 7
HAZ (Stunting Z Score)
K: Wasting 1990–2020
Probability Density
WHZ (Wasting Z Score)
L: Underweight 1990–2020
Probability Density
WAZ (Underweight Z Score)
Severity
Extreme CGF (<−4SD)
Severe CGF (<−3SD)
Overall CGF (<−2SD)
Year
1990
1995
2000
2005
2010
2015
2020

Austria – Stunting (HAZ)

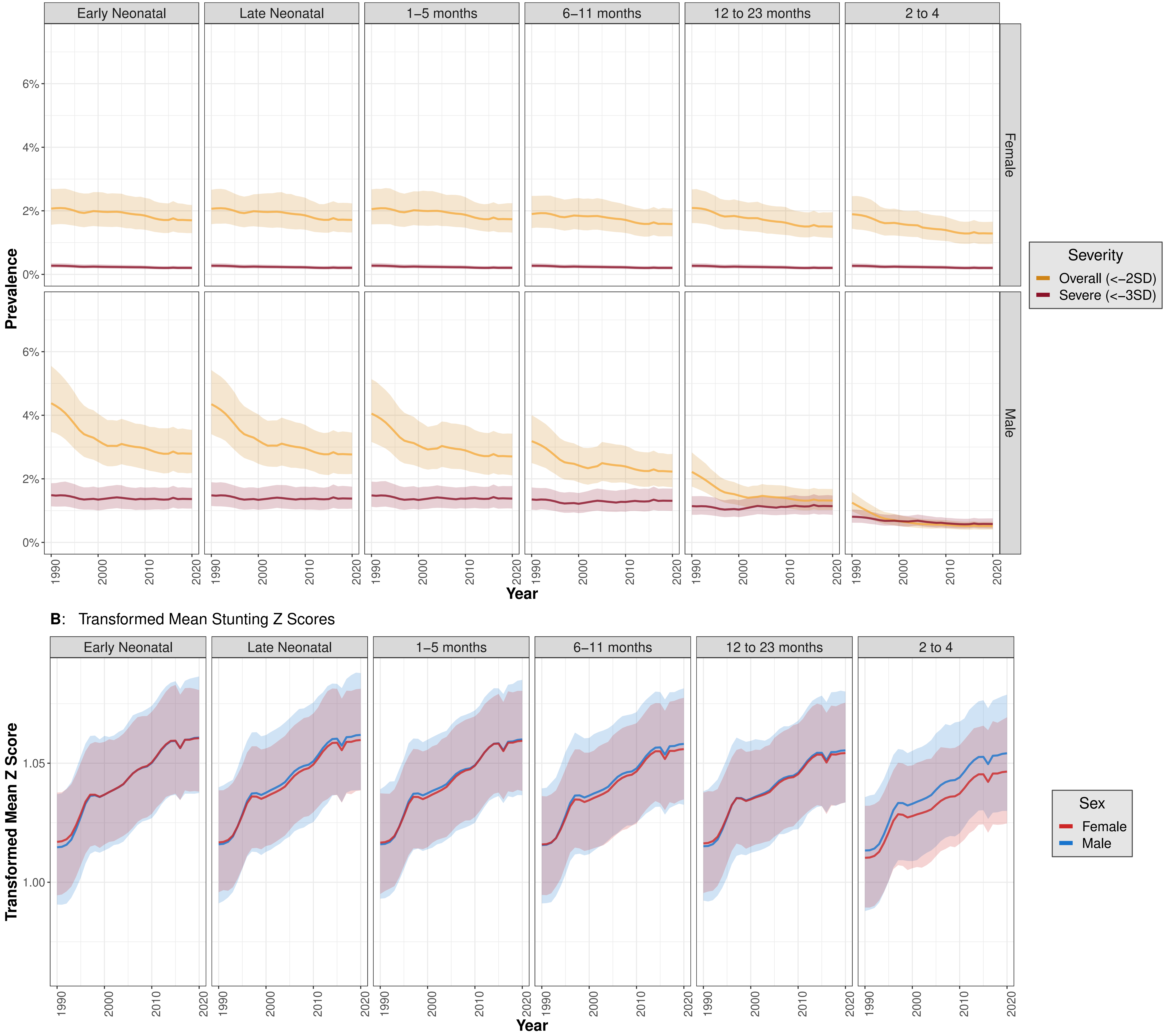

C

Source
No sources for this location

Austria – Wasting (WHZ)

D: Overall and Severe Wasting Prevalence

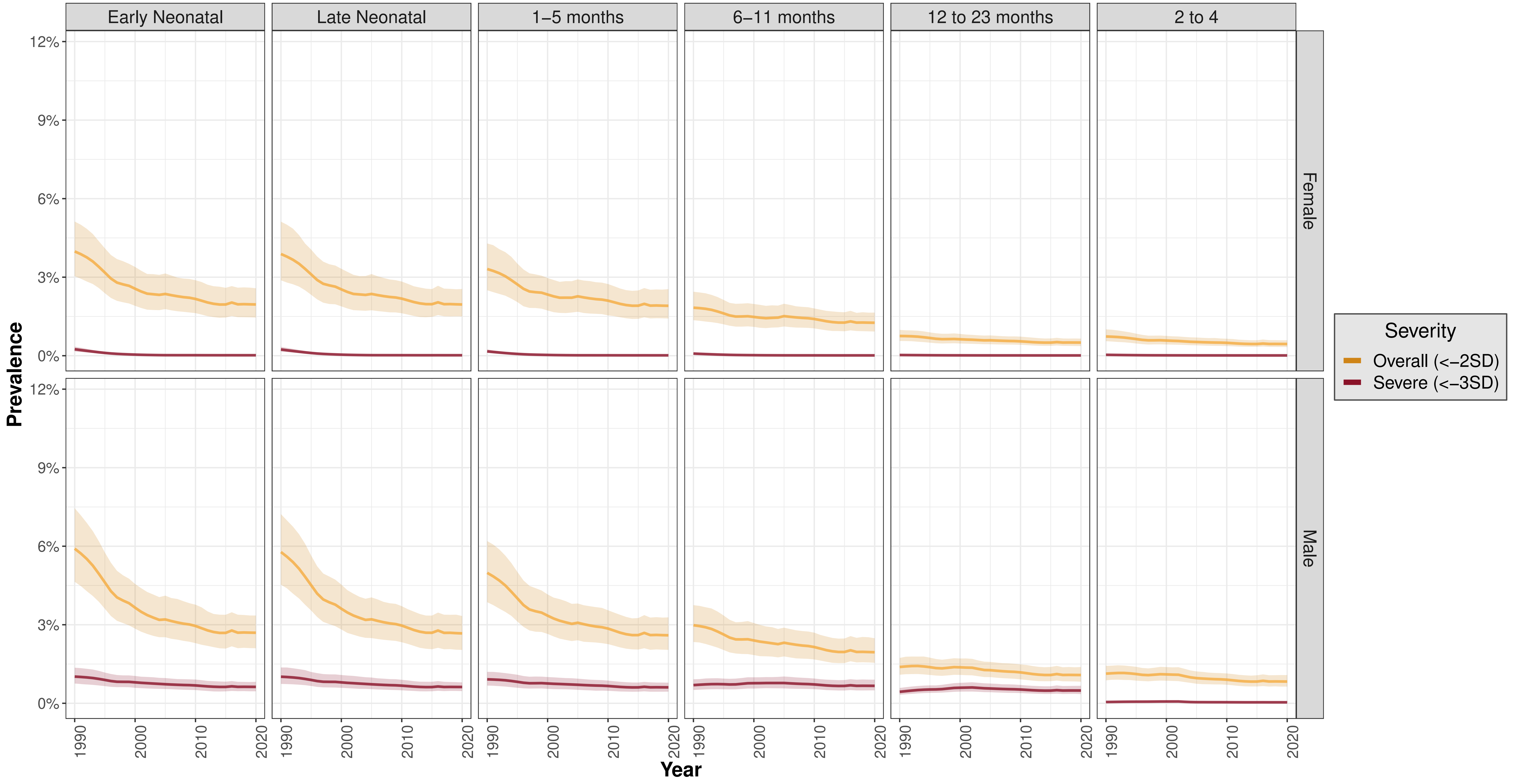

E: Transformed Mean Wasting Z Scores

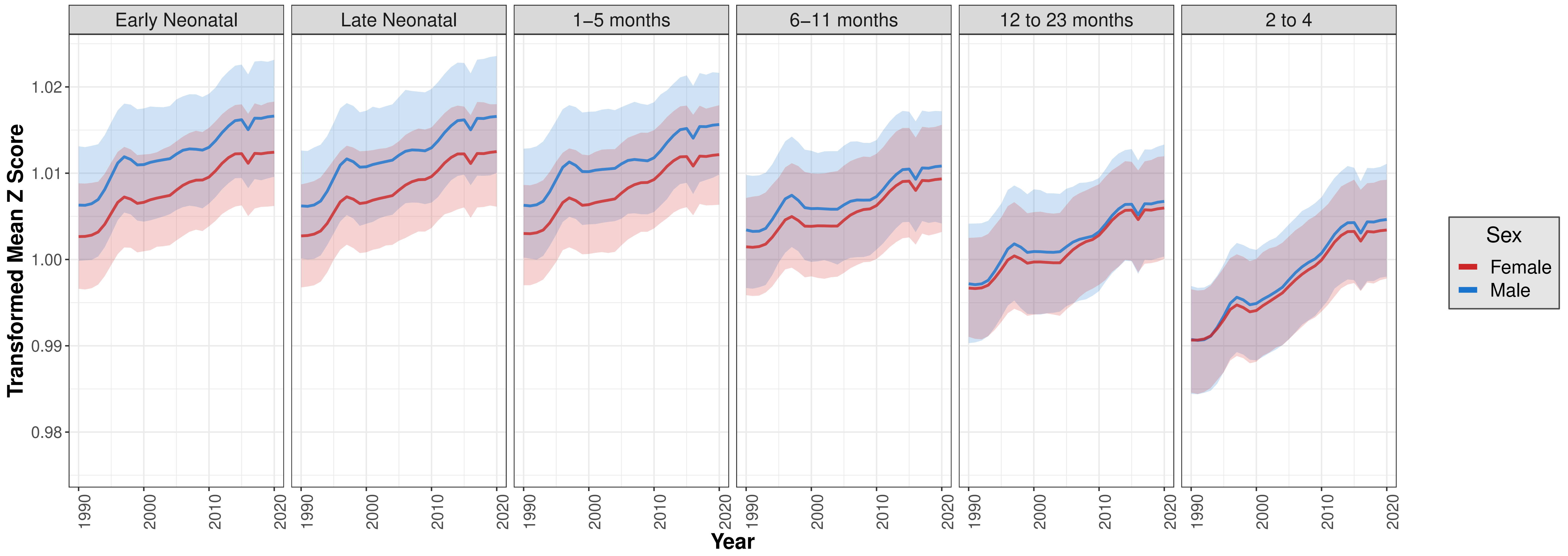

F

Source
No sources for this location

Austria – Underweight (WAZ)

G: Overall and Severe Underweight Prevalence

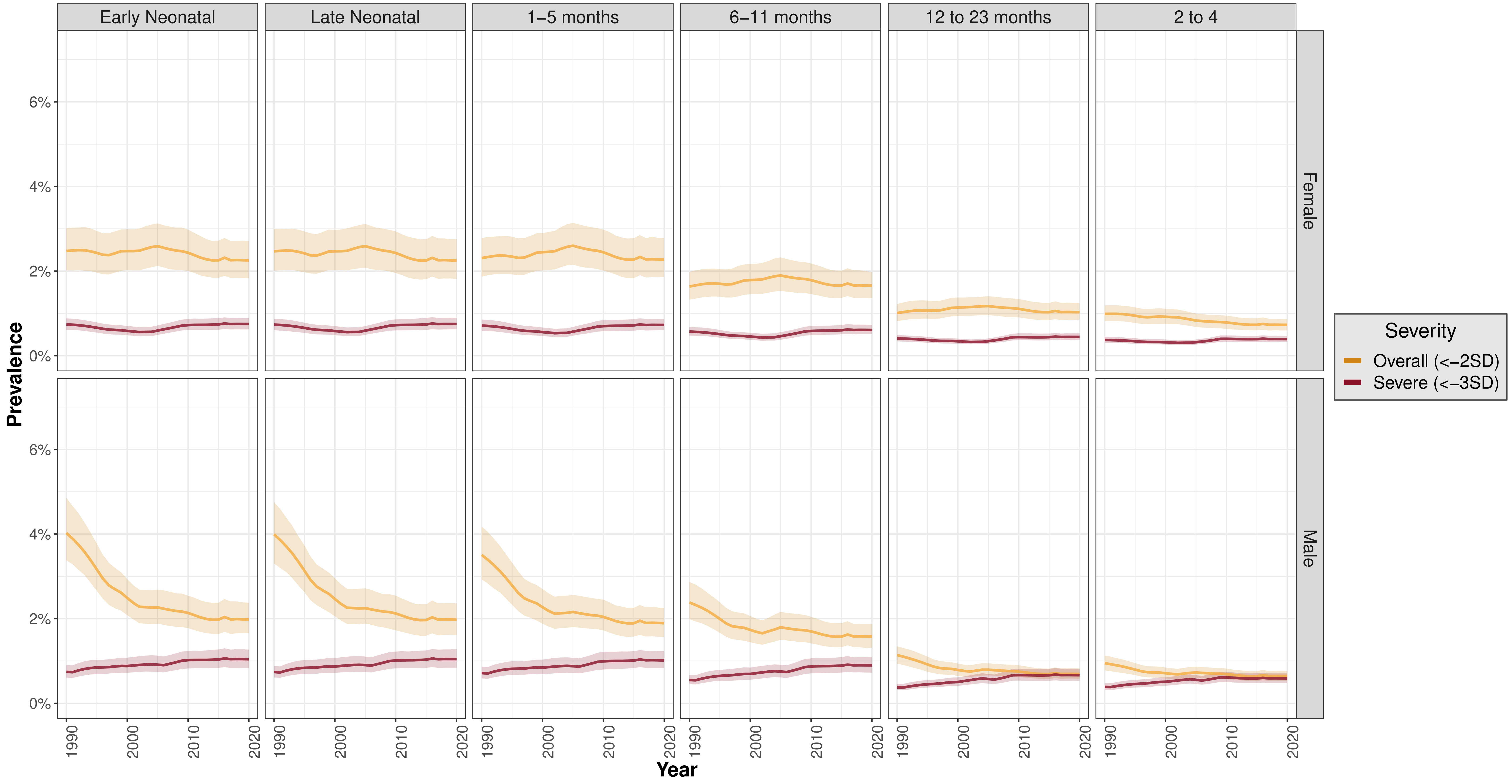

H: Transformed Mean Underweight Z Scores

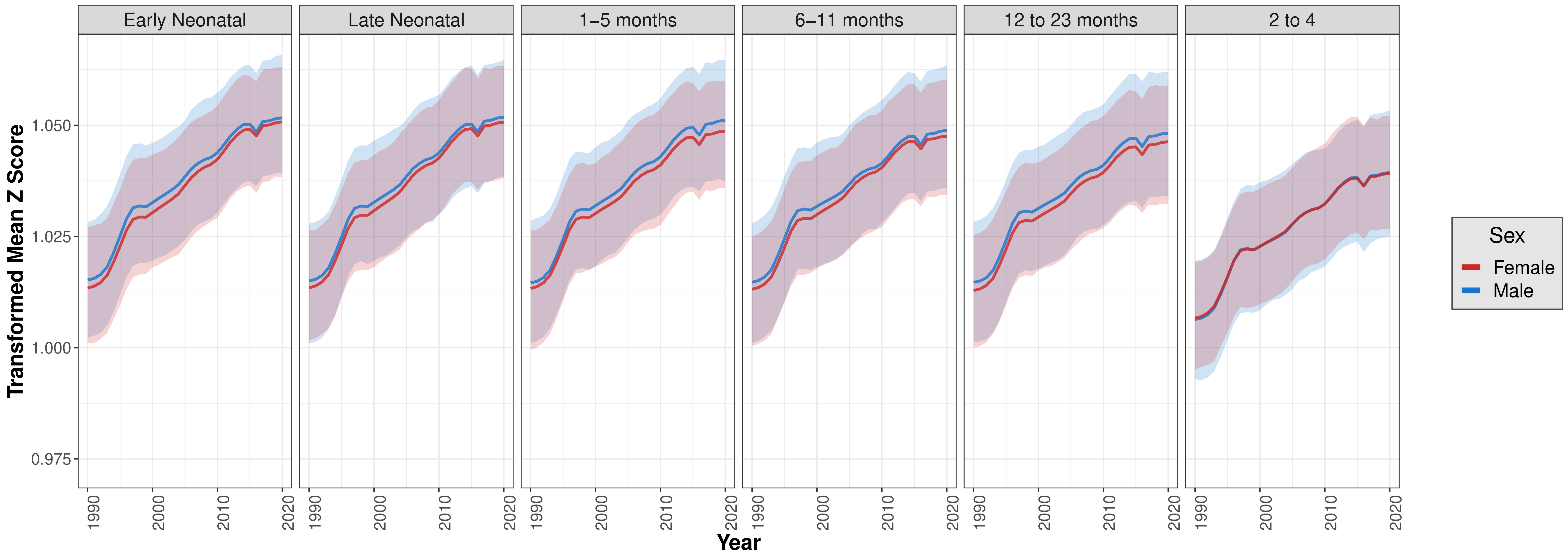

I

Source
No sources for this location

Austria – HAZ, WHZ, and WAZ Distributions

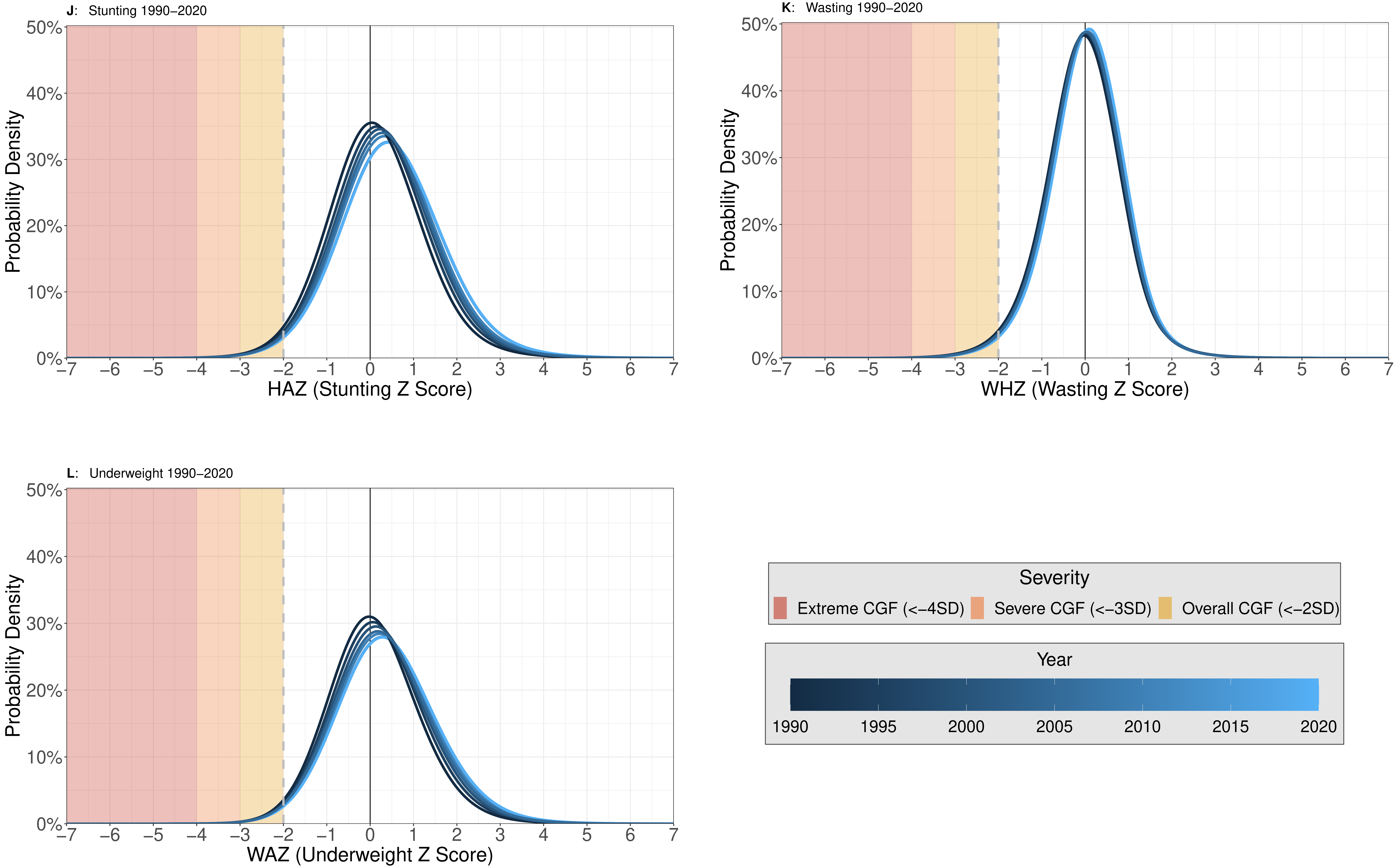

Belgium – Stunting (HAZ)

A: Overall and Severe Stunting Prevalence

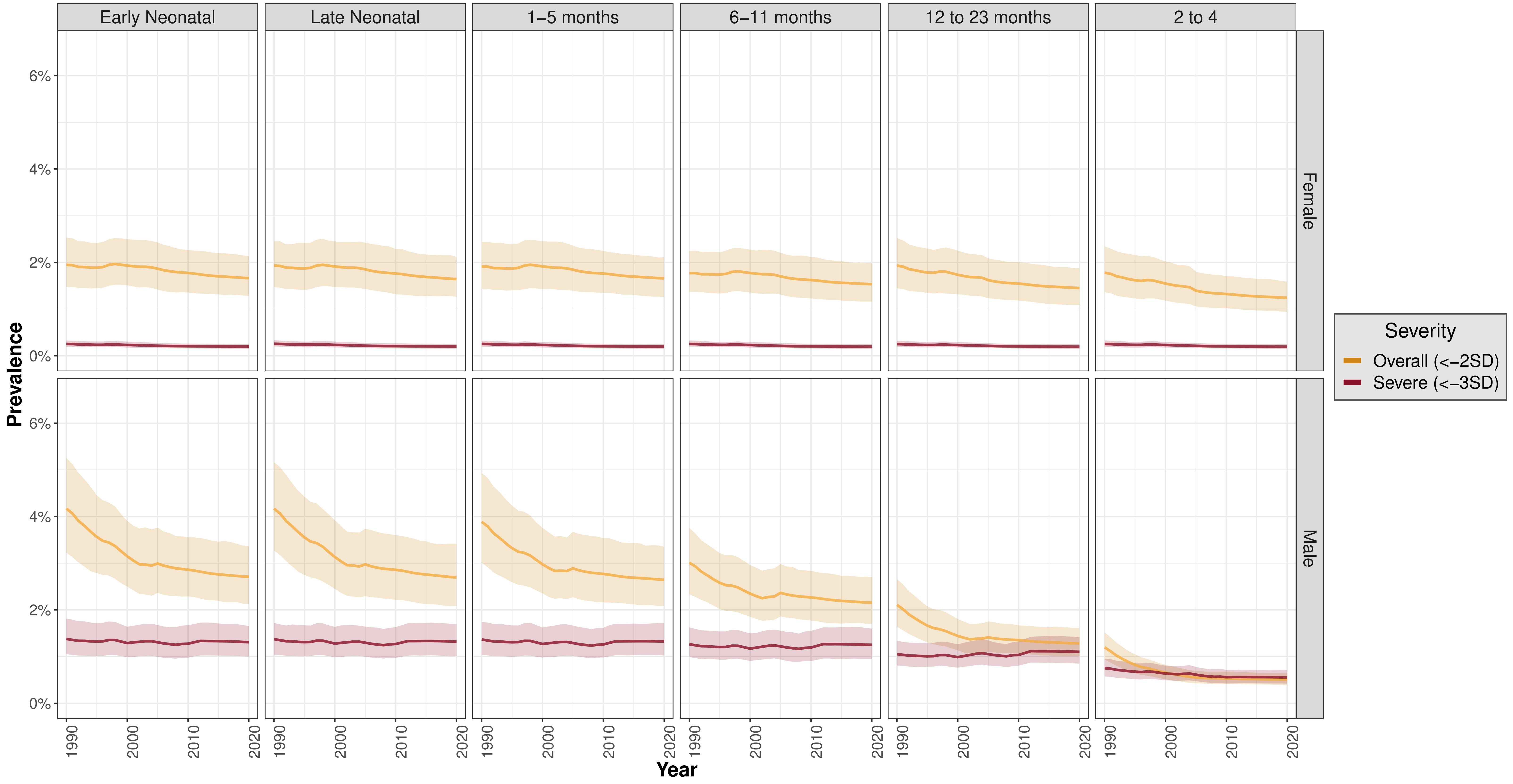

B: Transformed Mean Stunting Z Scores

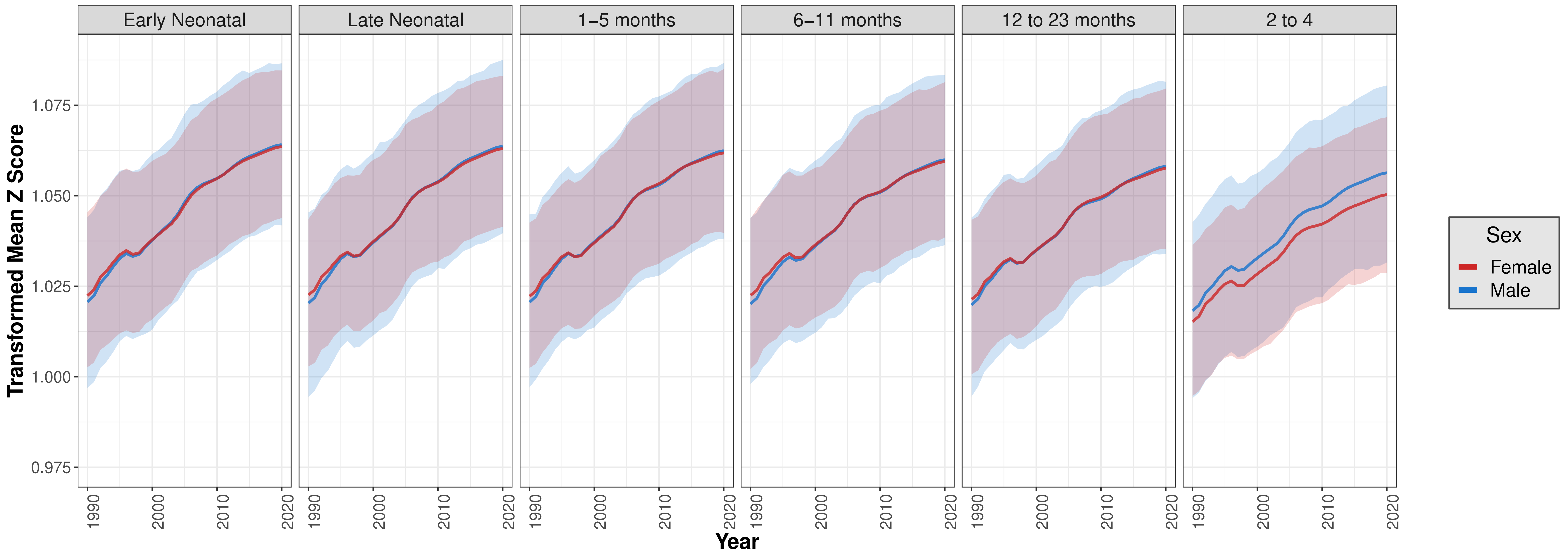

C

Source
No sources for this location

Belgium – Wasting (WHZ)

D: Overall and Severe Wasting Prevalence

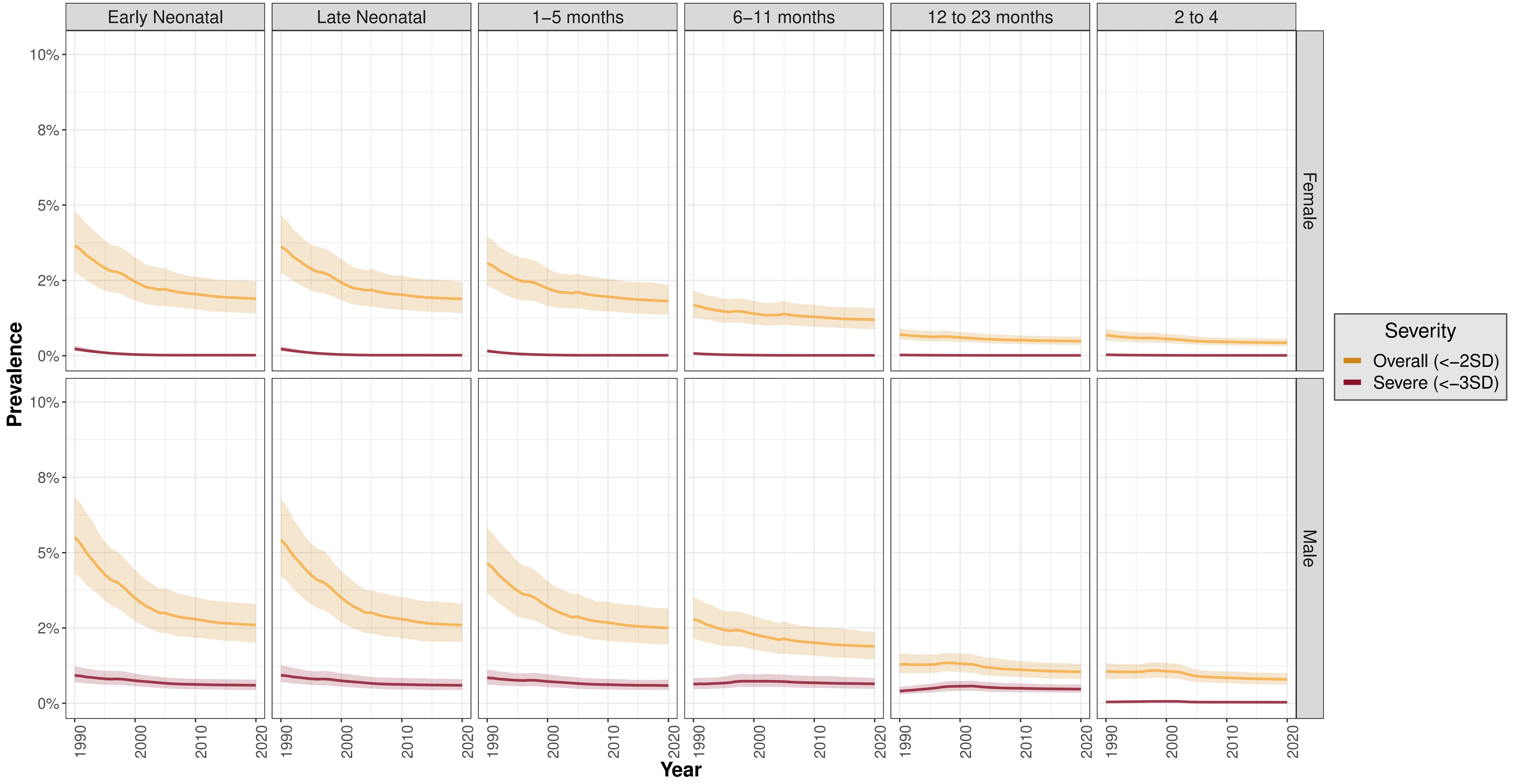

E: Transformed Mean Wasting Z Scores

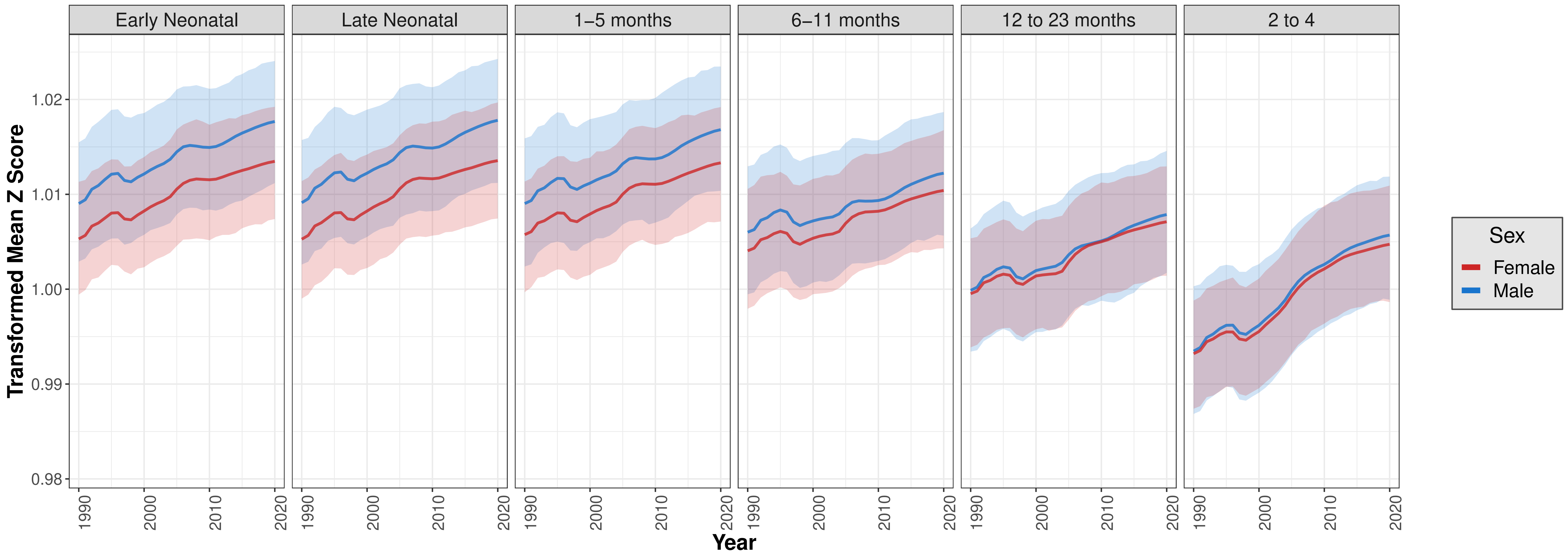

F

Source
No sources for this location

Belgium – Underweight (WAZ)

G: Overall and Severe Underweight Prevalence

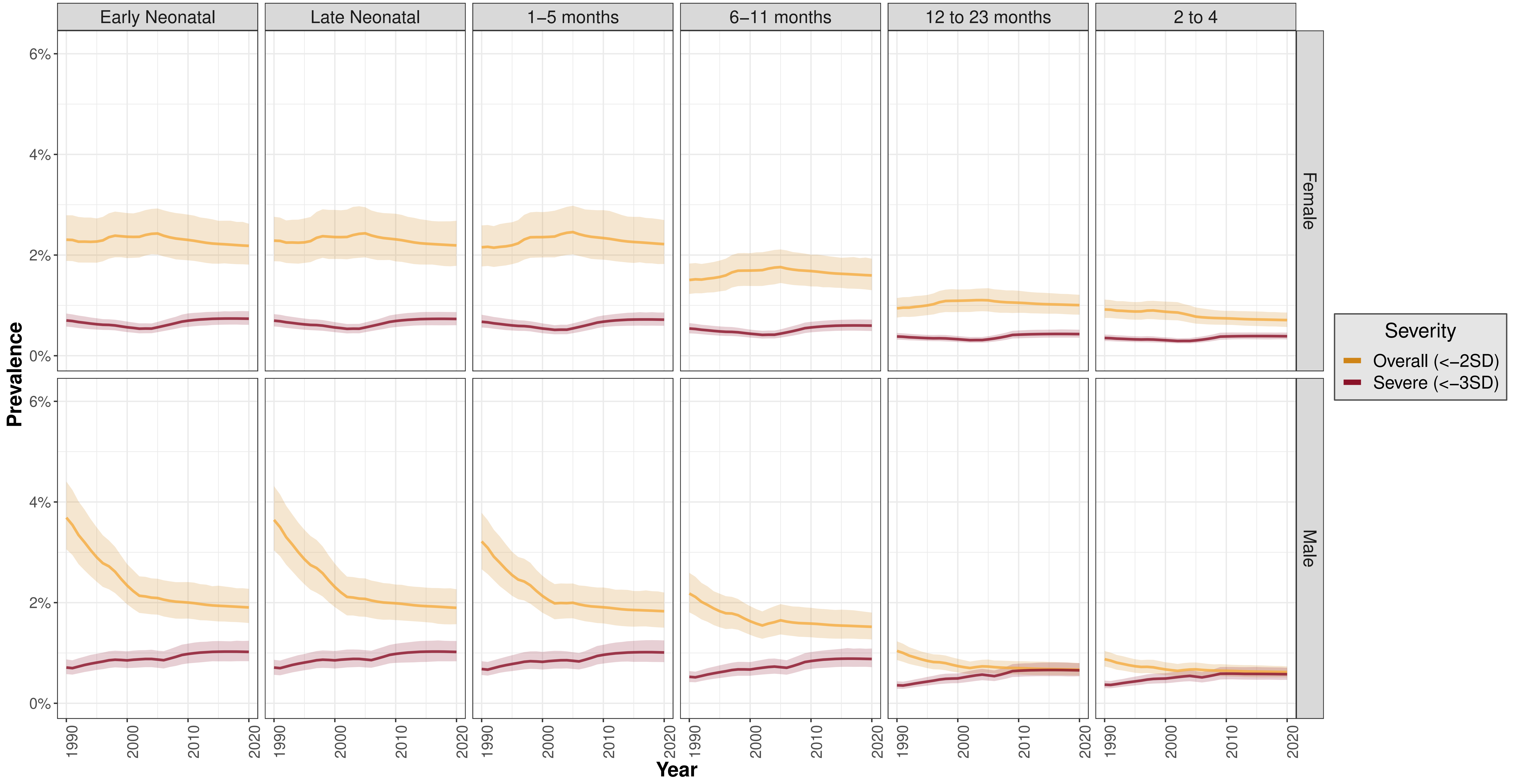

H: Transformed Mean Underweight Z Scores

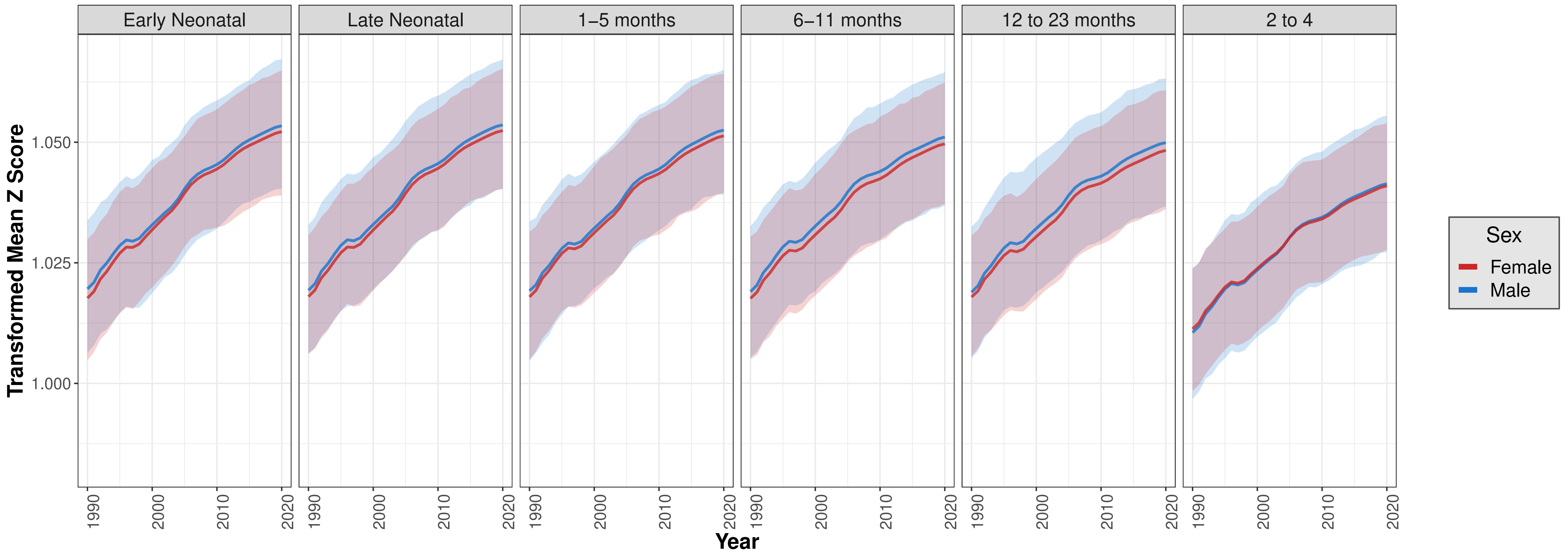

I

Source
No sources for this location

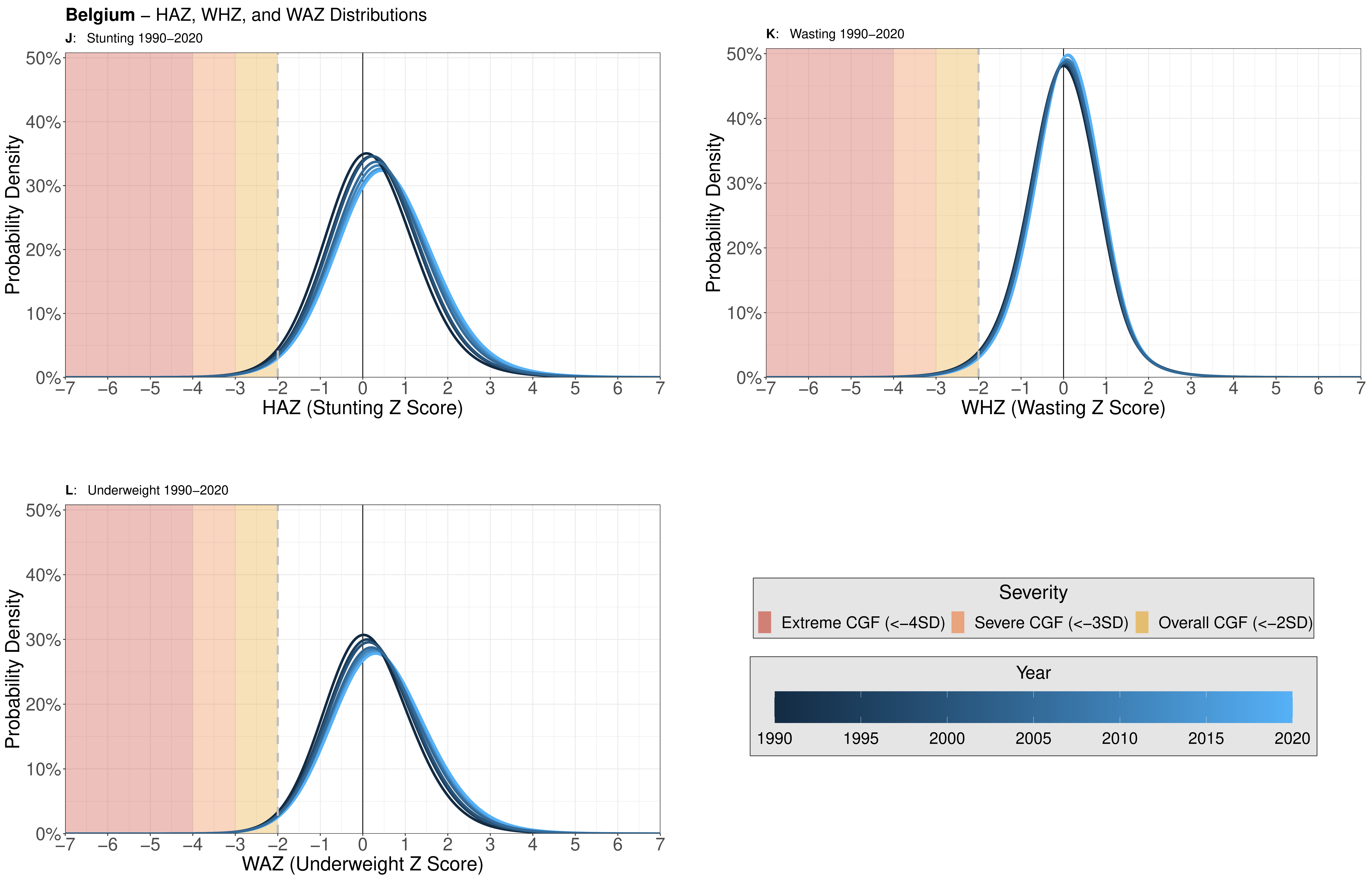

Belgium – HAZ, WHZ, and WAZ Distributions
J: Stunting 1990–2020
Probability Density
HAZ (Stunting Z Score)
K: Wasting 1990–2020
WHZ (Wasting Z Score)
L: Underweight 1990–2020
WAZ (Underweight Z Score)
Severity
Extreme CGF (<–4SD)
Severe CGF (<–3SD)
Overall CGF (<–2SD)
Year
1990
1995
2000
2005
2010
2015
2020

Cyprus – Stunting (HAZ)

A: Overall and Severe Stunting Prevalence

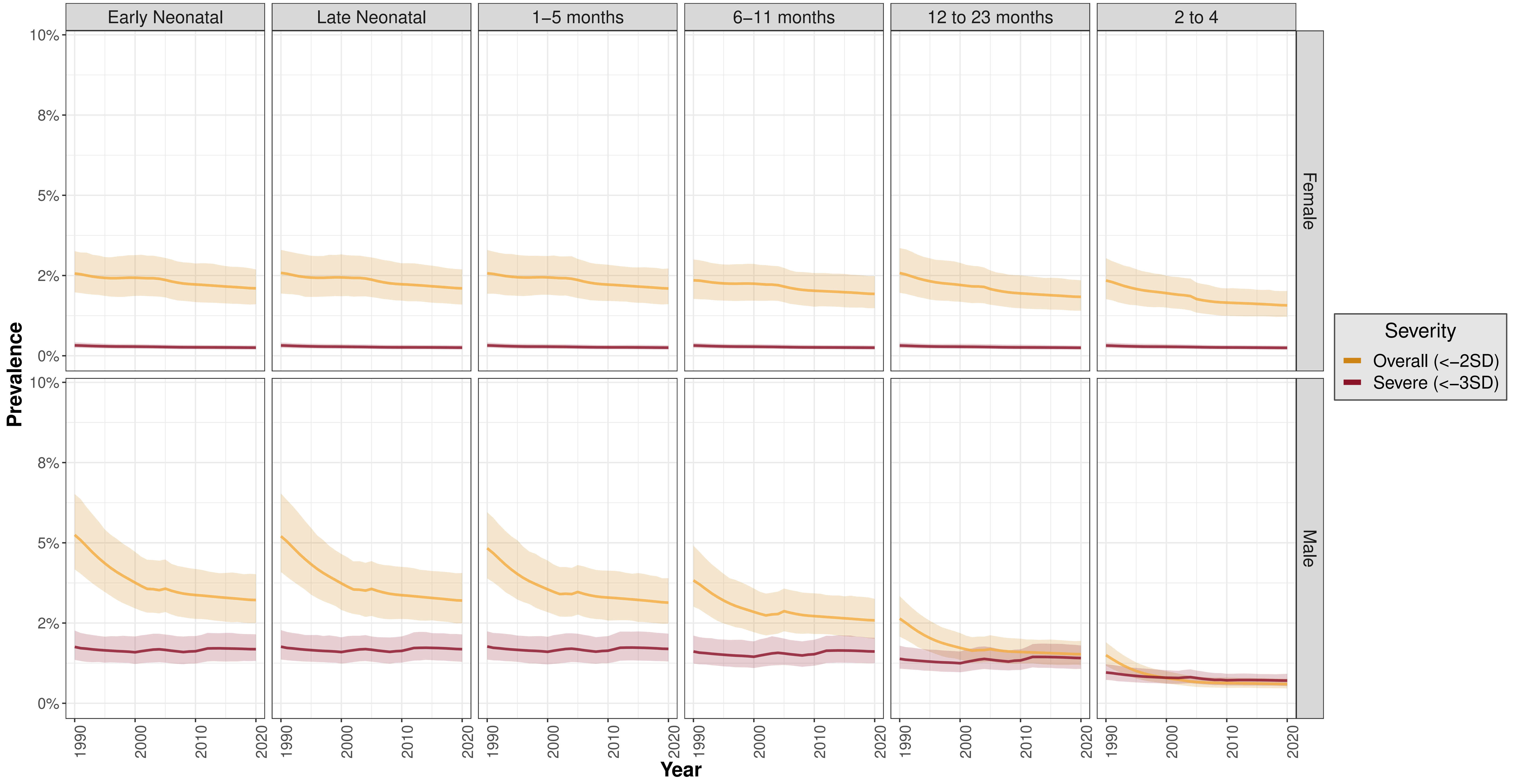

B: Transformed Mean Stunting Z Scores

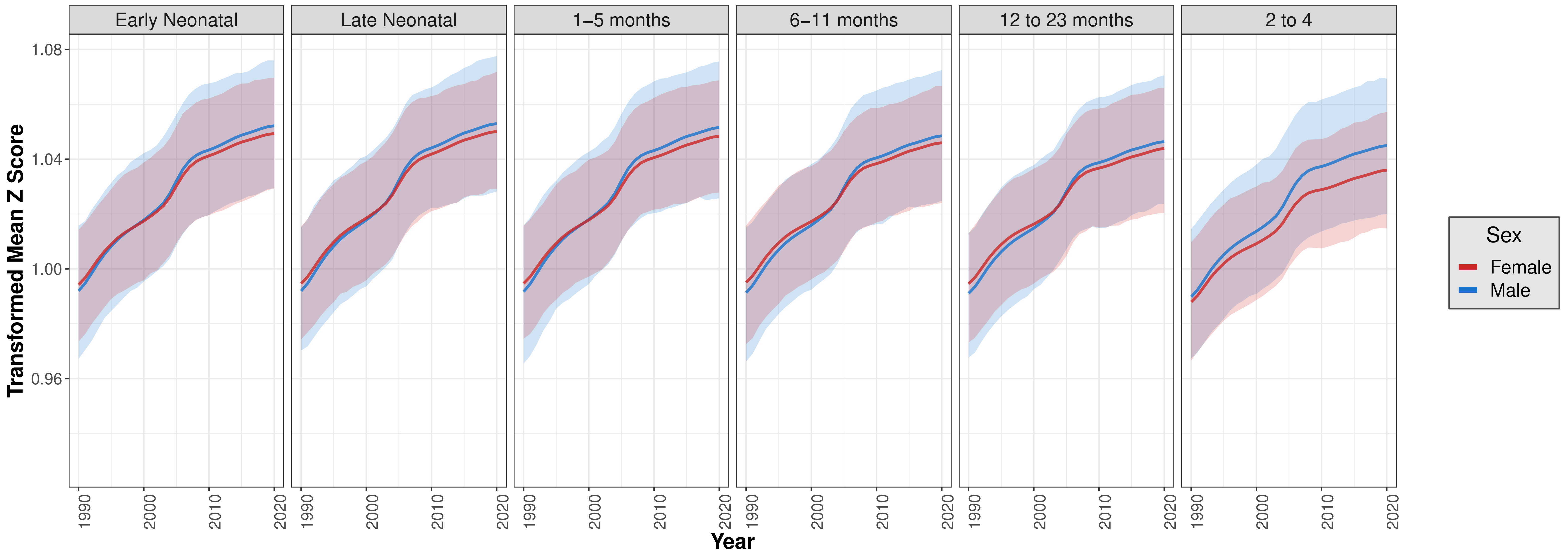

C

Source
No sources for this location

Cyprus – Wasting (WHZ)

D: Overall and Severe Wasting Prevalence

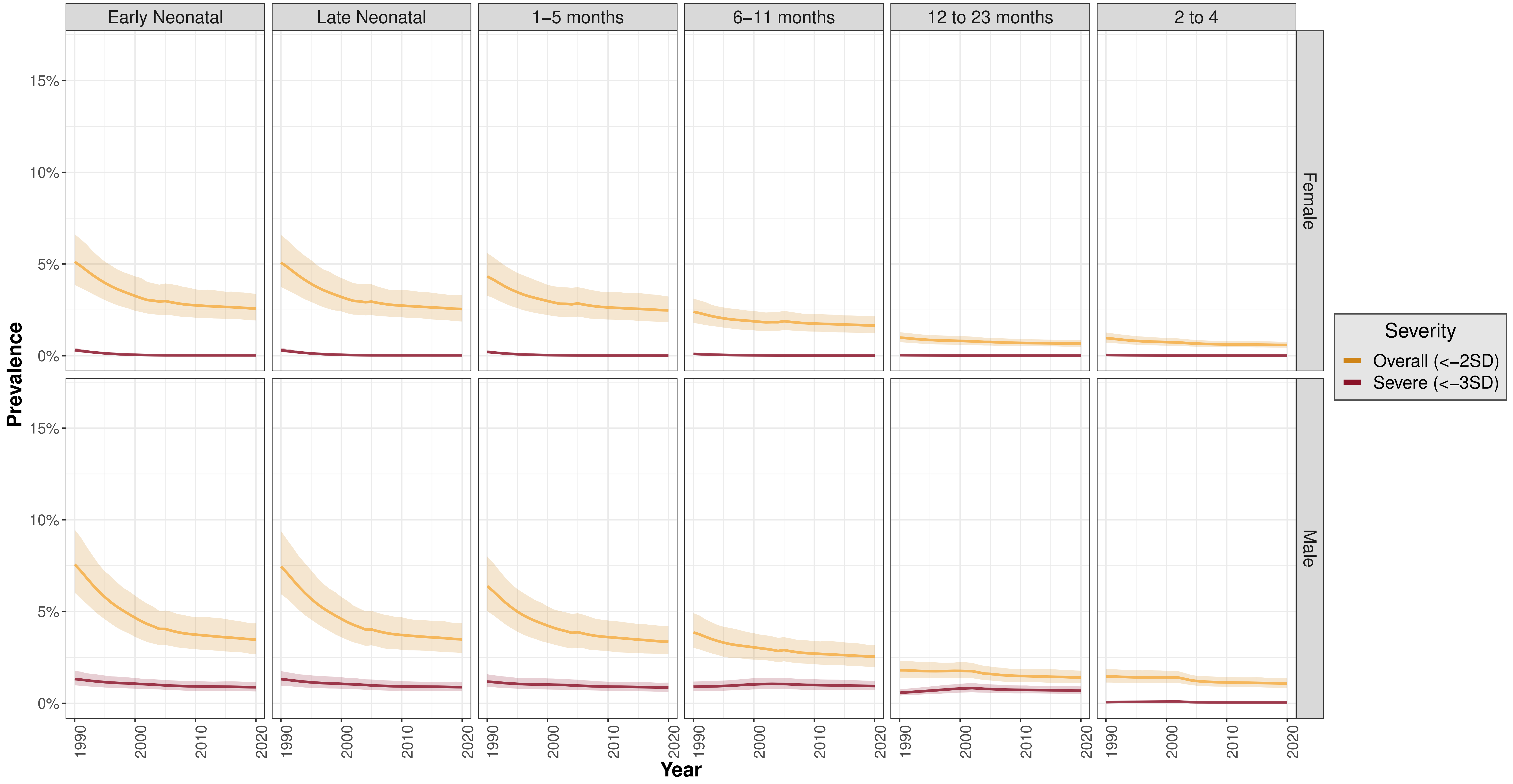

E: Transformed Mean Wasting Z Scores

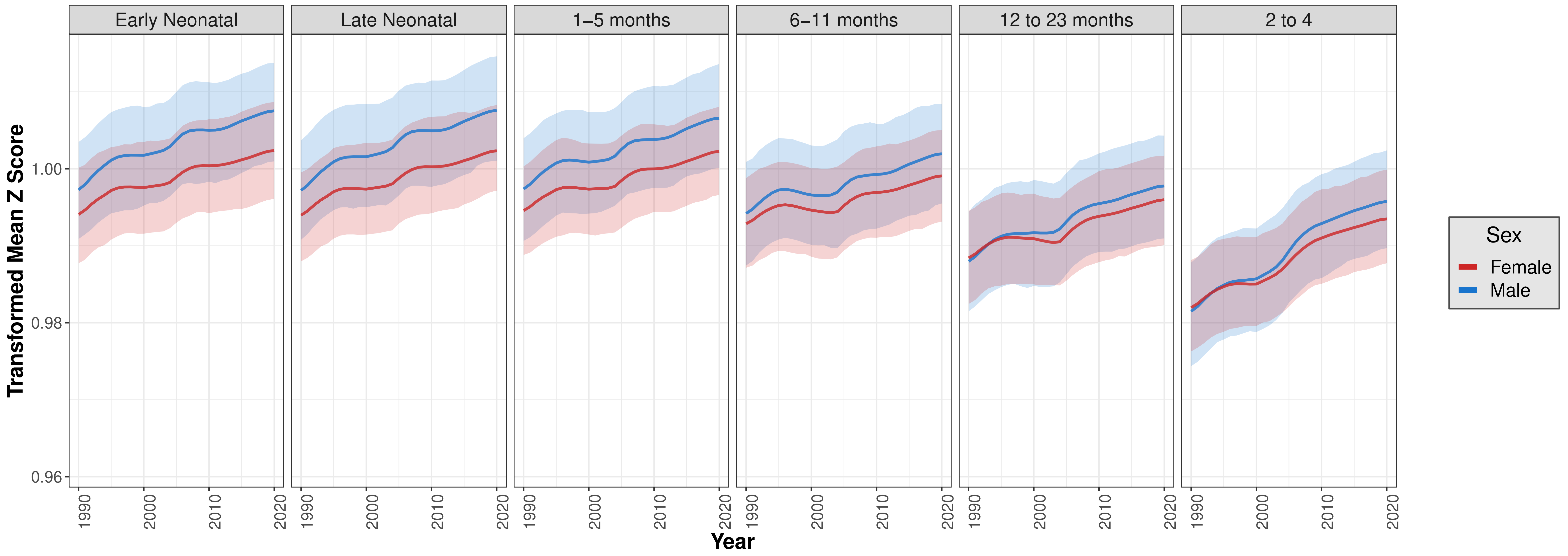

F

Source
No sources for this location

Cyprus – Underweight (WAZ)

G: Overall and Severe Underweight Prevalence

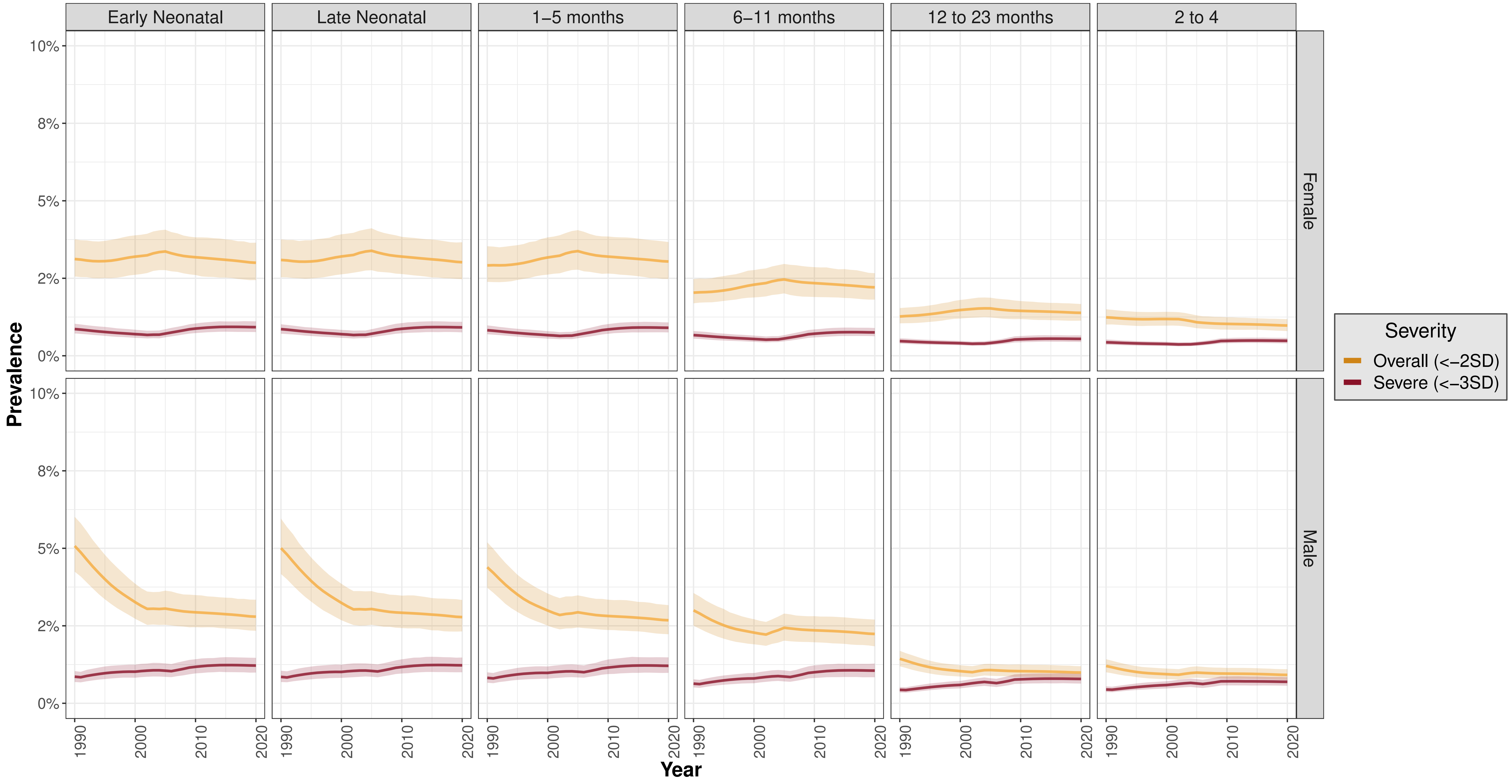

H: Transformed Mean Underweight Z Scores

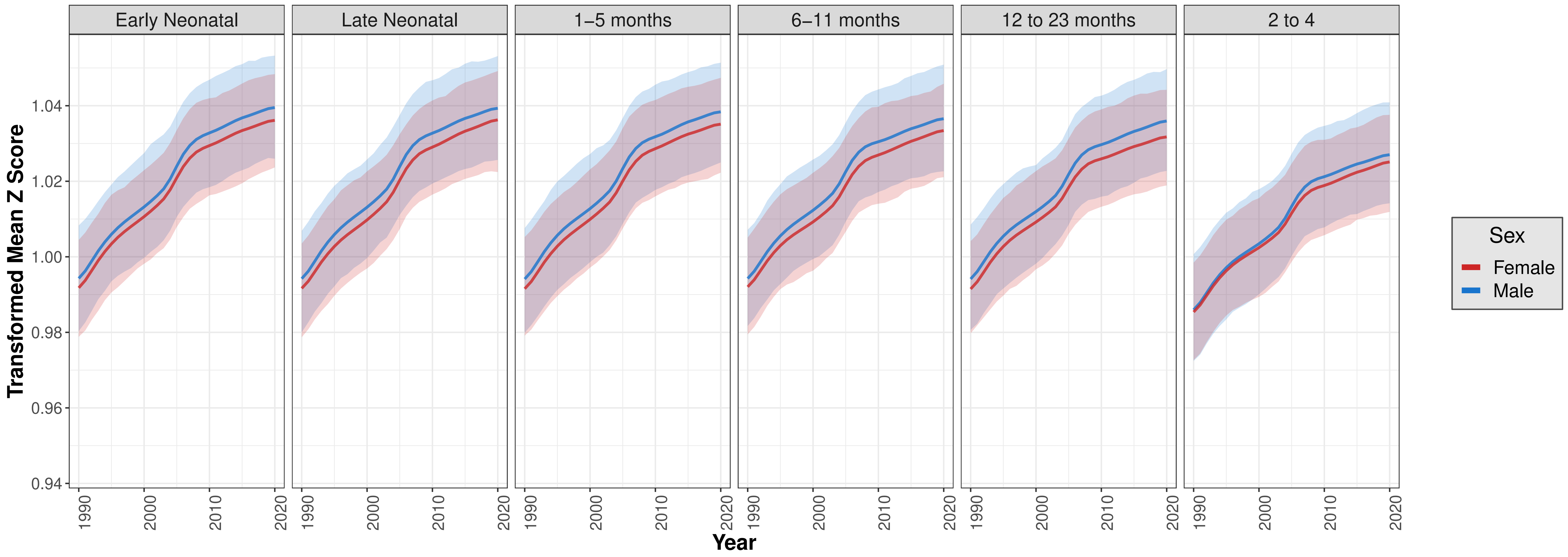

I

Source
No sources for this location

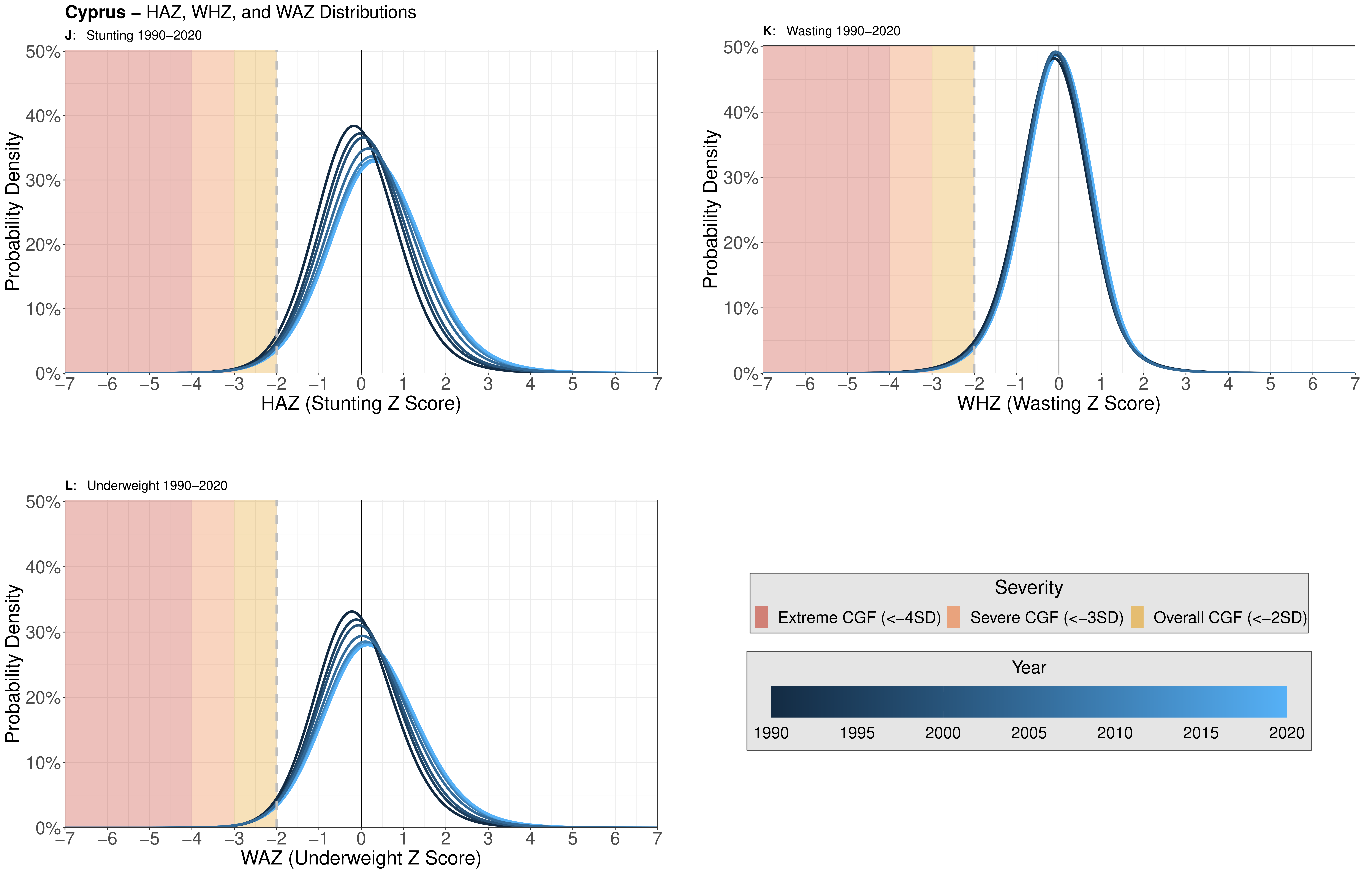

Cyprus – HAZ, WHZ, and WAZ Distributions
J: Stunting 1990–2020
K: Wasting 1990–2020
L: Underweight 1990–2020
Probability Density
0%
10%
20%
30%
40%
50%
−7 −6 −5 −4 −3 −2 −1 0 1 2 3 4 5 6 7
HAZ (Stunting Z Score)
WHZ (Wasting Z Score)
WAZ (Underweight Z Score)
Severity
Extreme CGF (<−4SD)
Severe CGF (<−3SD)
Overall CGF (<−2SD)
Year
1990
1995
2000
2005
2010
2015
2020

Denmark – Stunting (HAZ)

A: Overall and Severe Stunting Prevalence

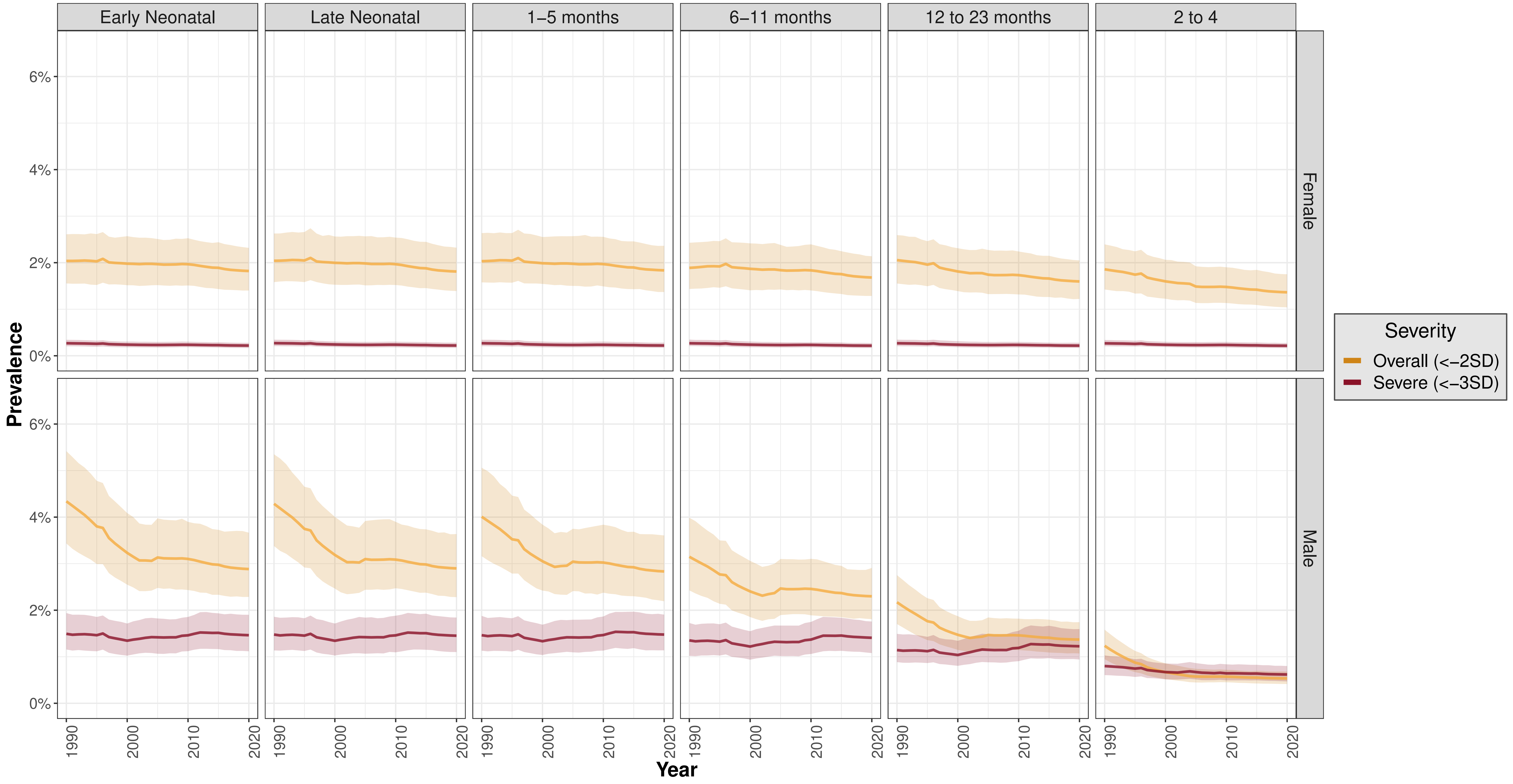

B: Transformed Mean Stunting Z Scores

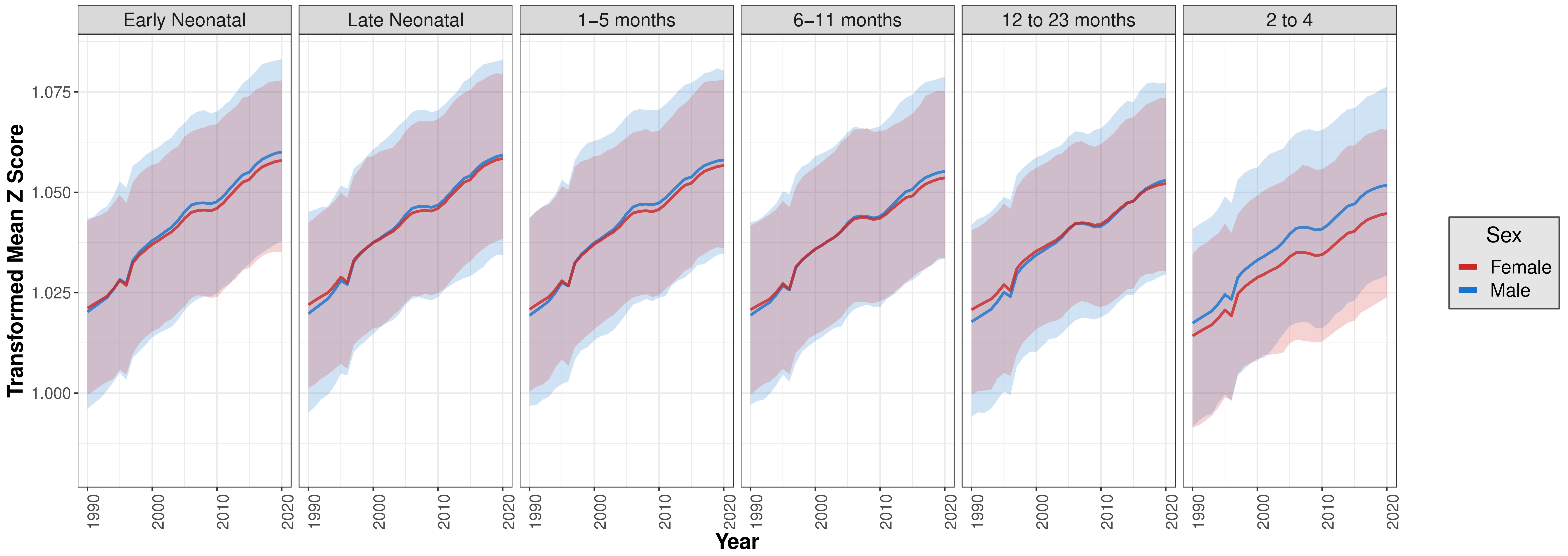

C

Source
No sources for this location

Denmark – Wasting (WHZ)

D: Overall and Severe Wasting Prevalence

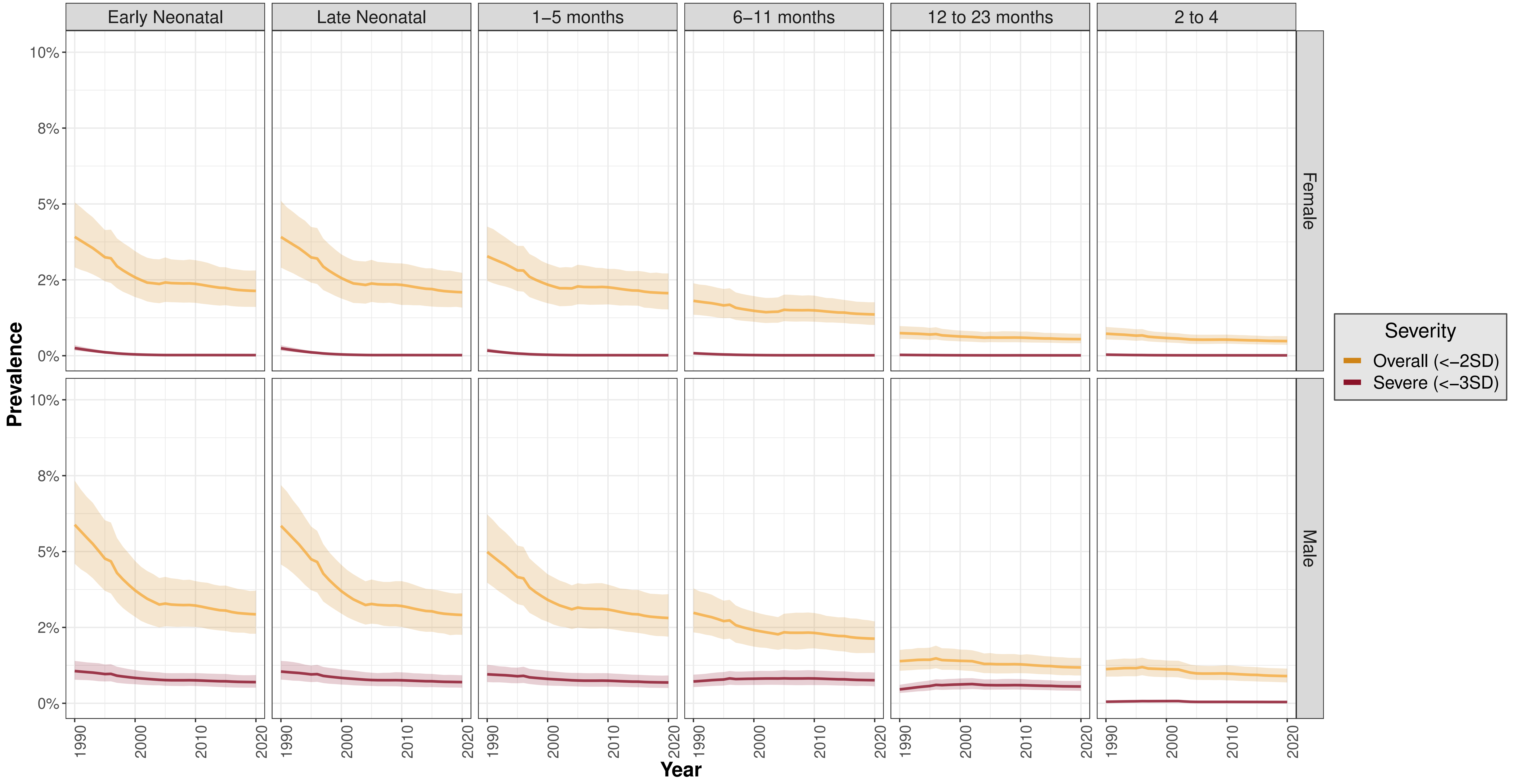

E: Transformed Mean Wasting Z Scores

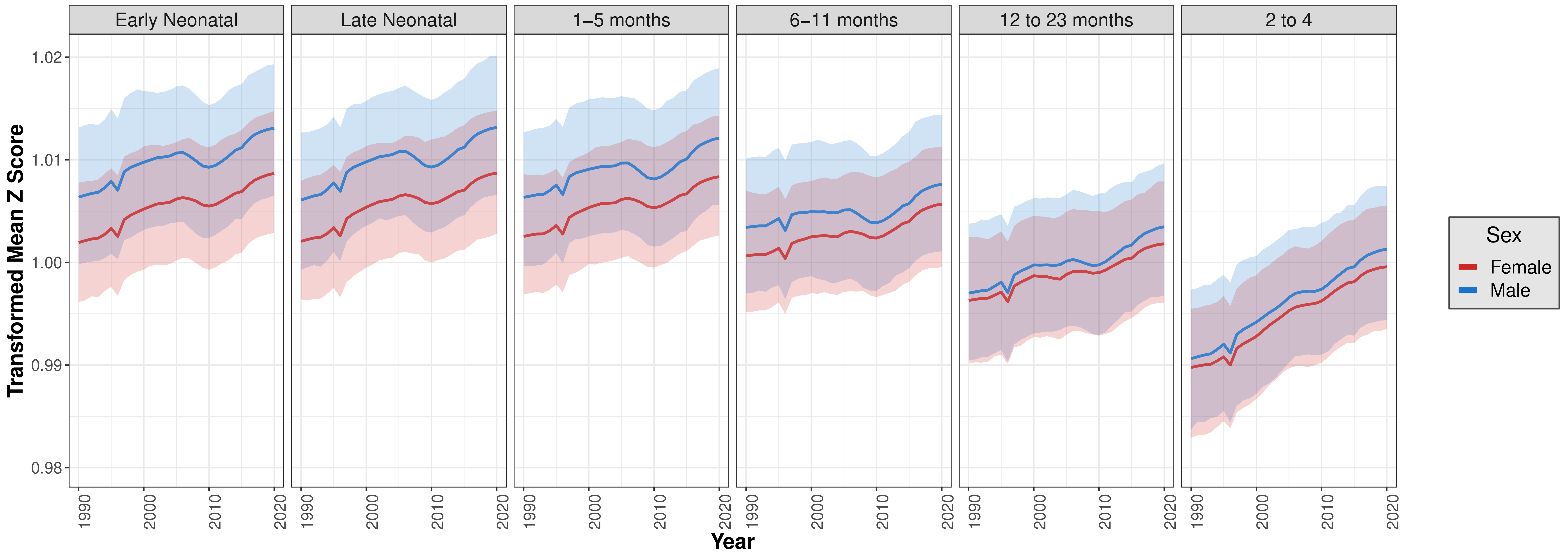

F

Source
No sources for this location

Denmark – Underweight (WAZ)

G: Overall and Severe Underweight Prevalence

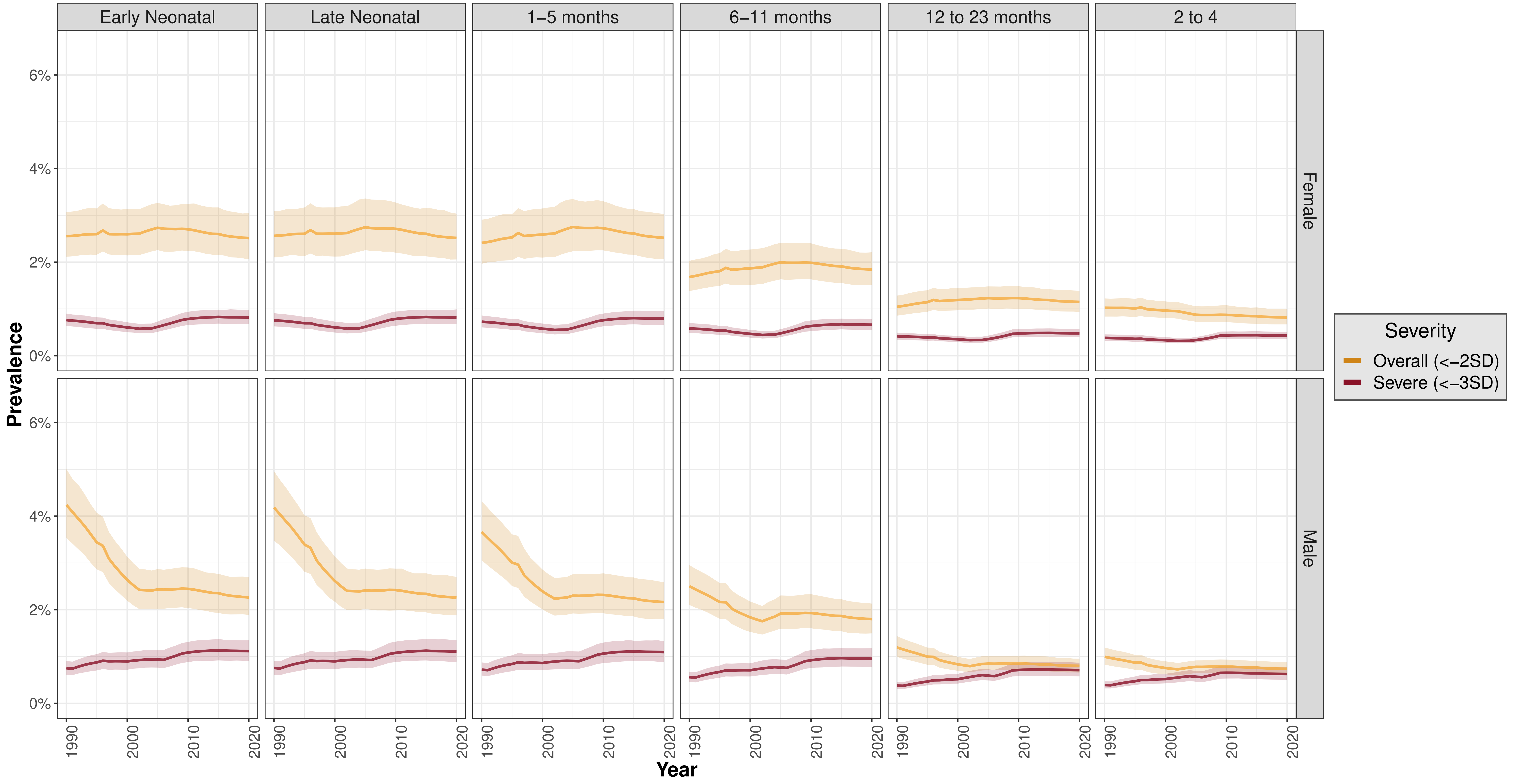

H: Transformed Mean Underweight Z Scores

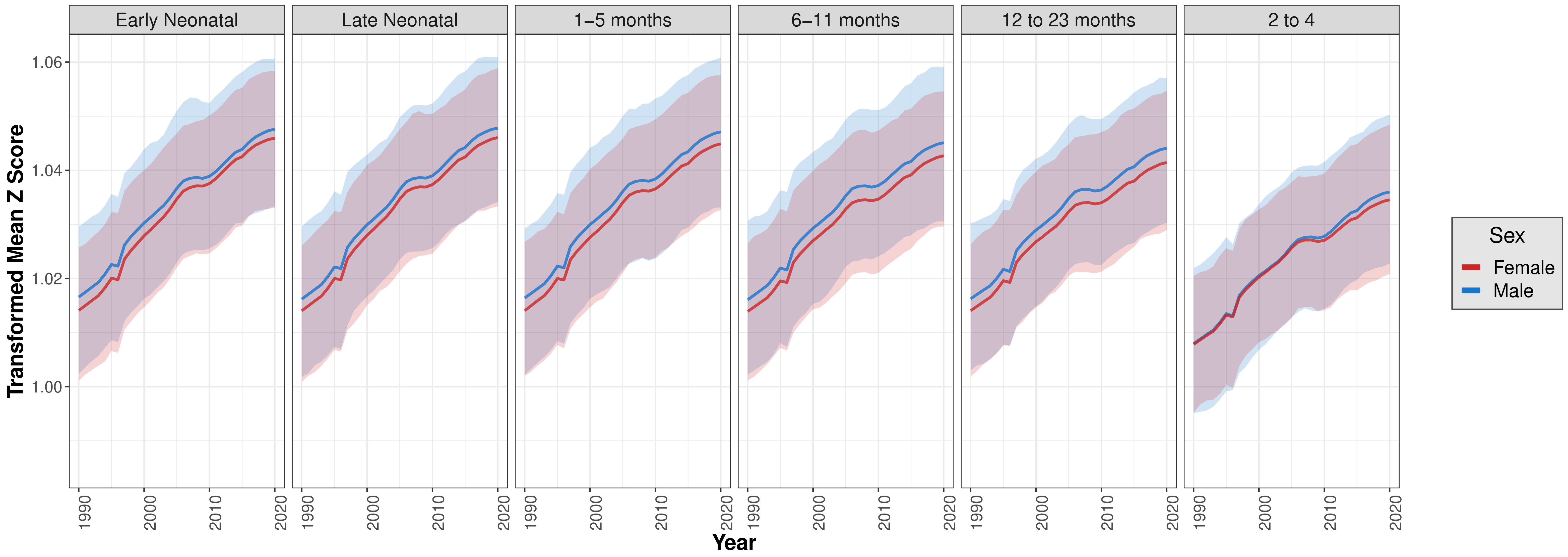

I

Source
No sources for this location

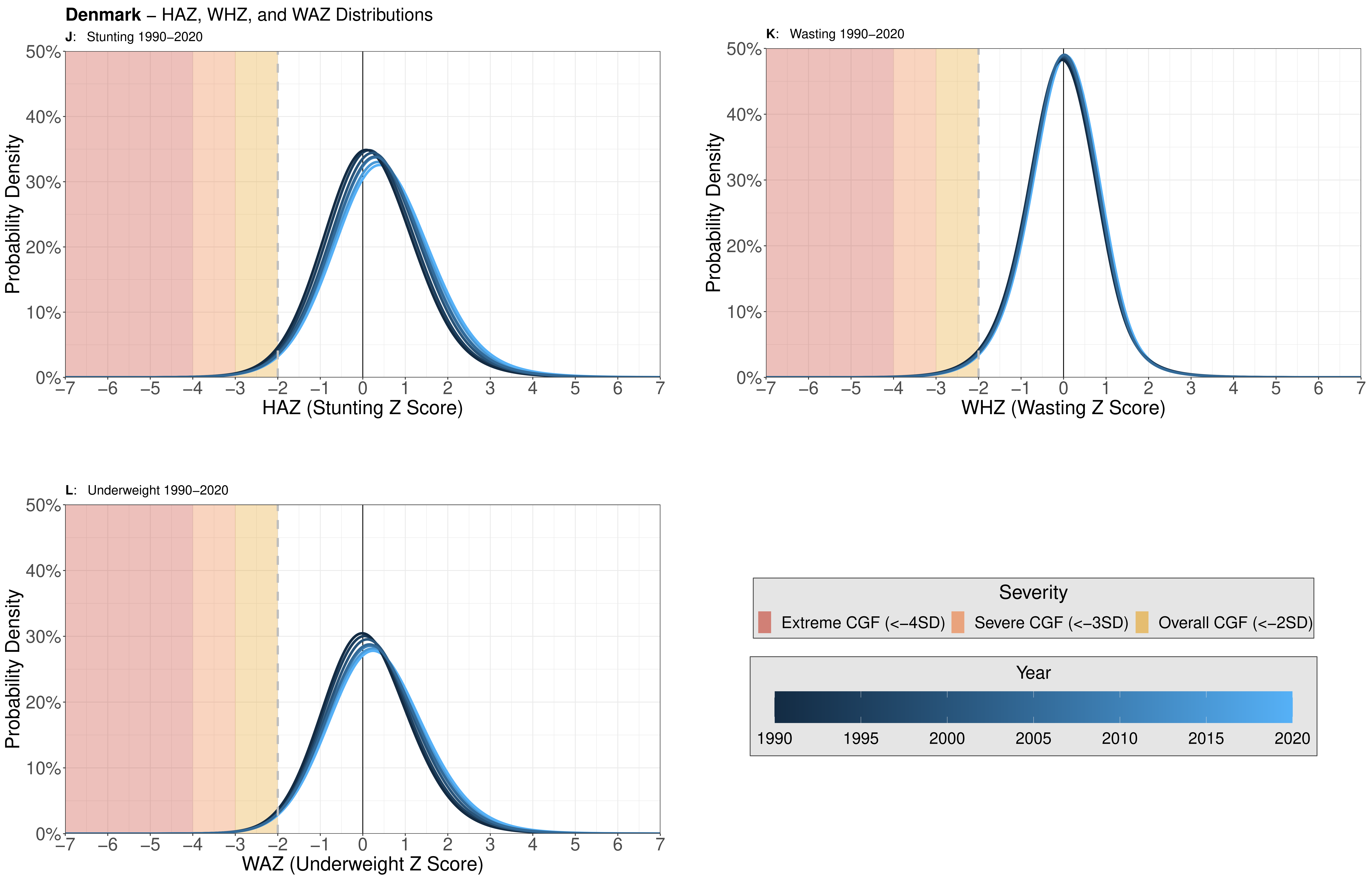
Denmark – HAZ, WHZ, and WAZ Distributions
J: Stunting 1990–2020
Probability Density
50%
40%
30%
20%
10%
0%
−7 −6 −5 −4 −3 −2 −1 0 1 2 3 4 5 6 7
HAZ (Stunting Z Score)
K: Wasting 1990–2020
Probability Density
50%
40%
30%
20%
10%
0%
−7 −6 −5 −4 −3 −2 −1 0 1 2 3 4 5 6 7
WHZ (Wasting Z Score)
L: Underweight 1990–2020
Probability Density
50%
40%
30%
20%
10%
0%
−7 −6 −5 −4 −3 −2 −1 0 1 2 3 4 5 6 7
WAZ (Underweight Z Score)
Severity
Extreme CGF (<−4SD)
Severe CGF (<−3SD)
Overall CGF (<−2SD)
Year
1990
1995
2000
2005
2010
2015
2020

Finland – Stunting (HAZ)

A: Overall and Severe Stunting Prevalence

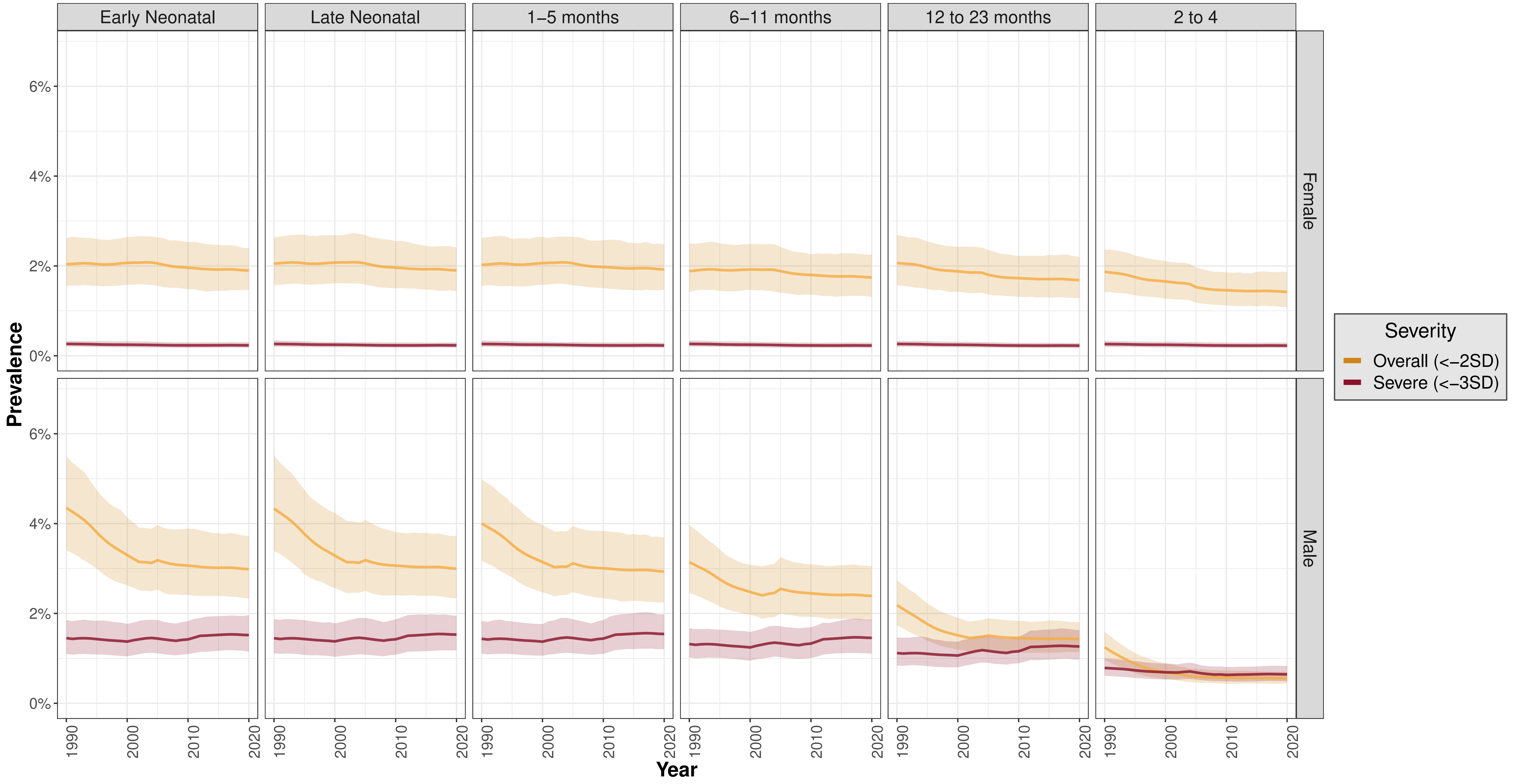

B: Transformed Mean Stunting Z Scores

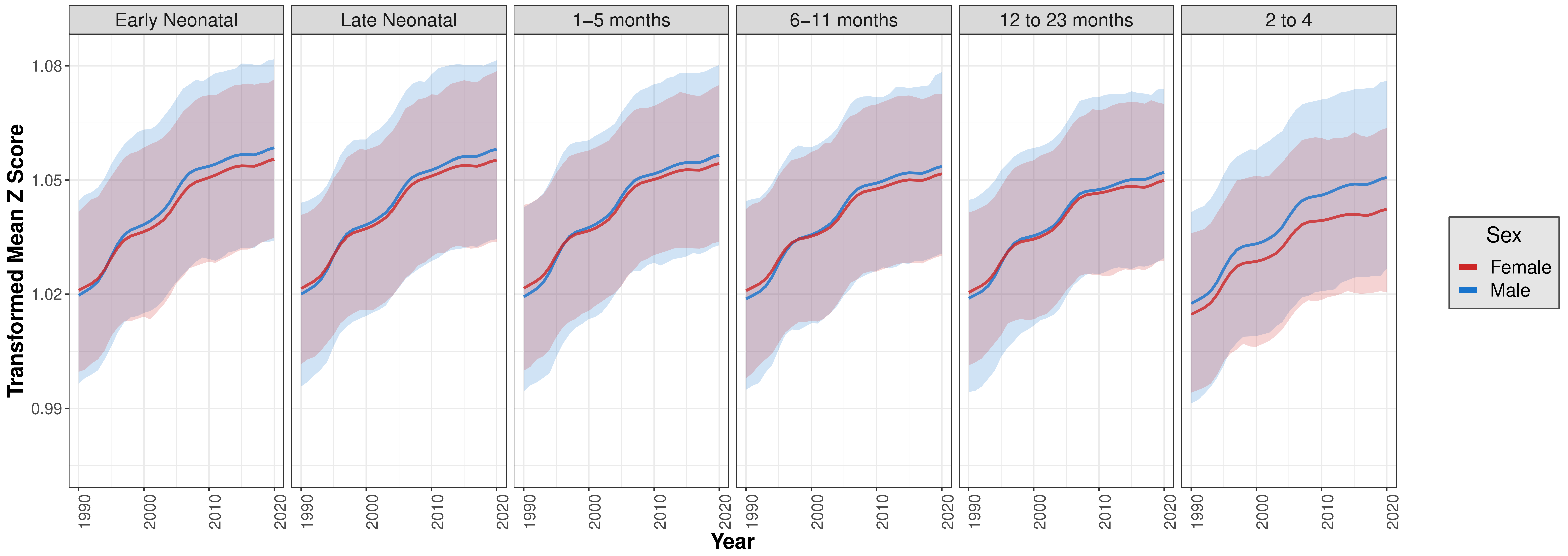

C

Source
No sources for this location

Finland – Wasting (WHZ)

D: Overall and Severe Wasting Prevalence

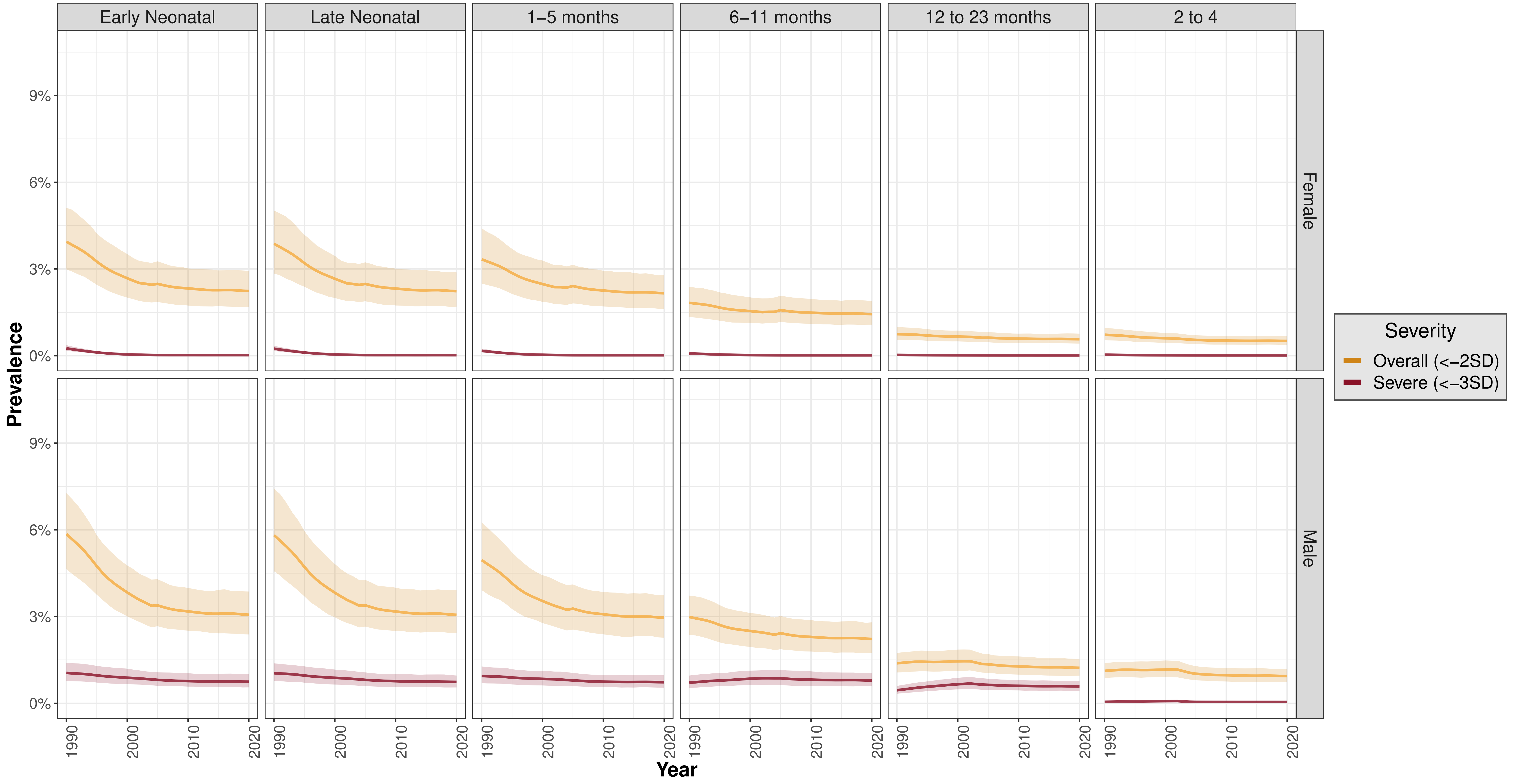

E: Transformed Mean Wasting Z Scores

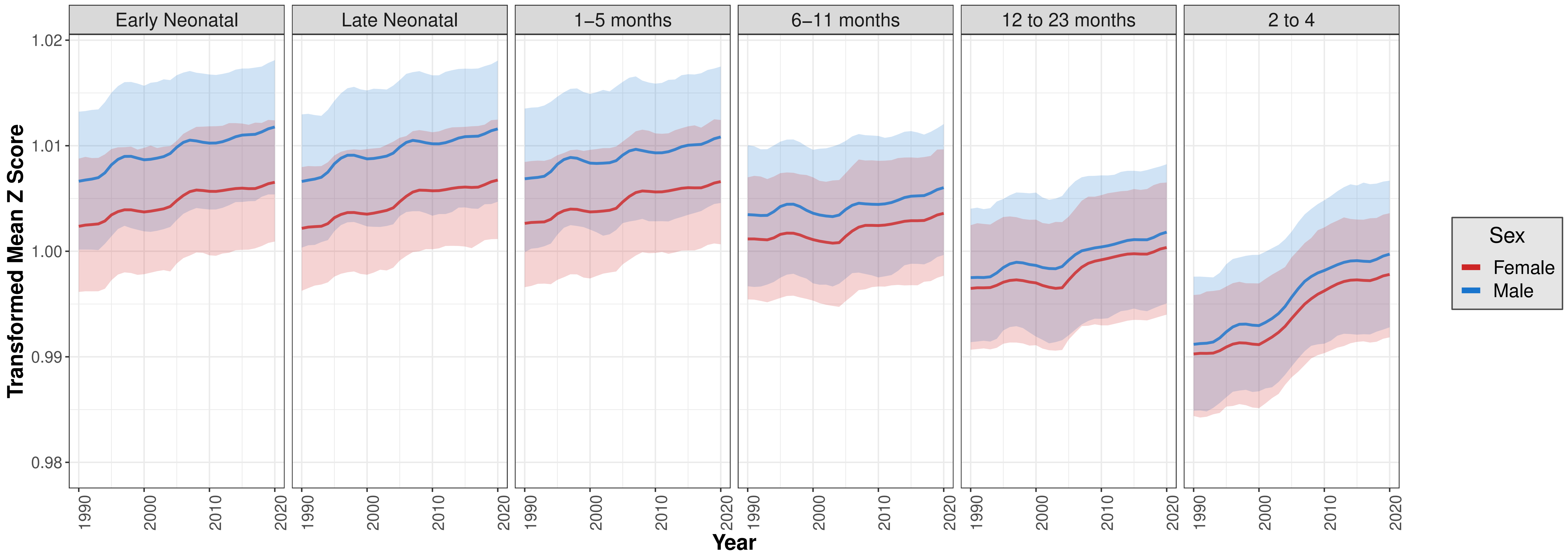

F

Source
No sources for this location

Finland – Underweight (WAZ)

G: Overall and Severe Underweight Prevalence

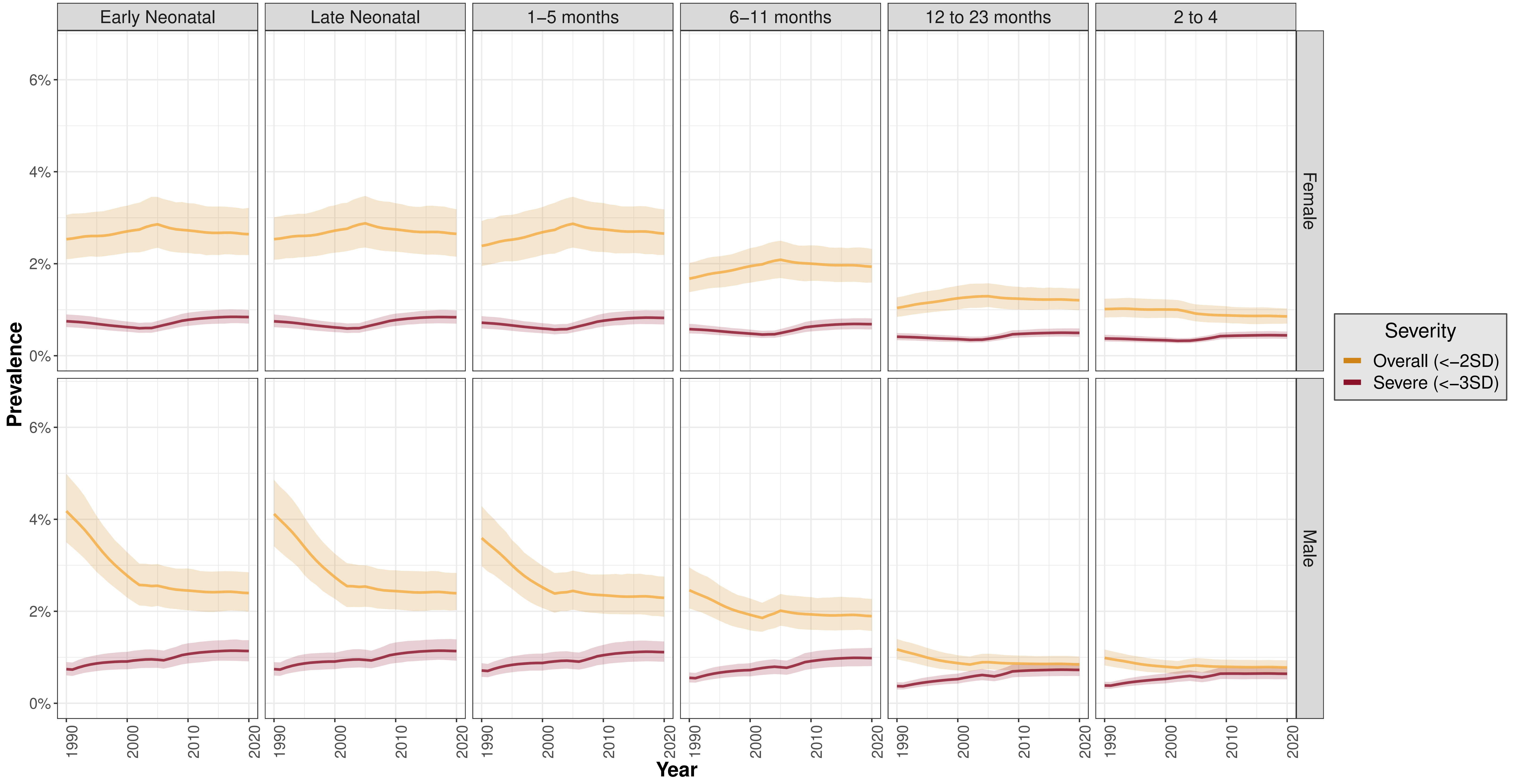

H: Transformed Mean Underweight Z Scores

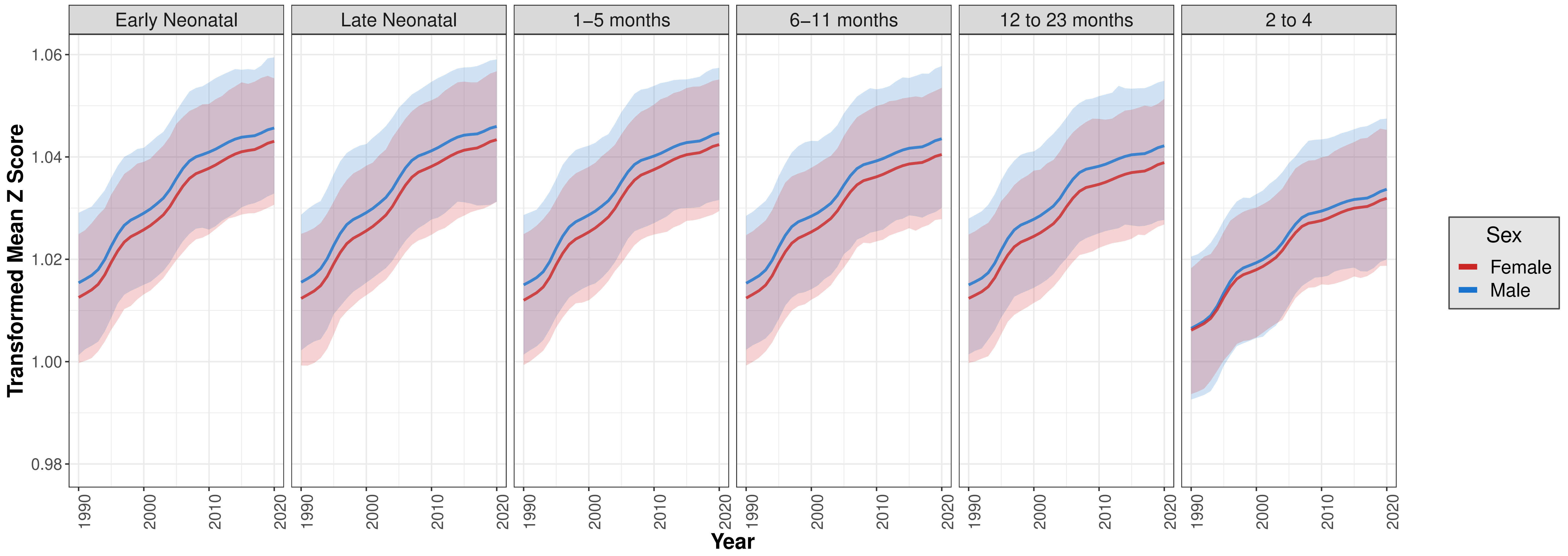

I

Source
No sources for this location

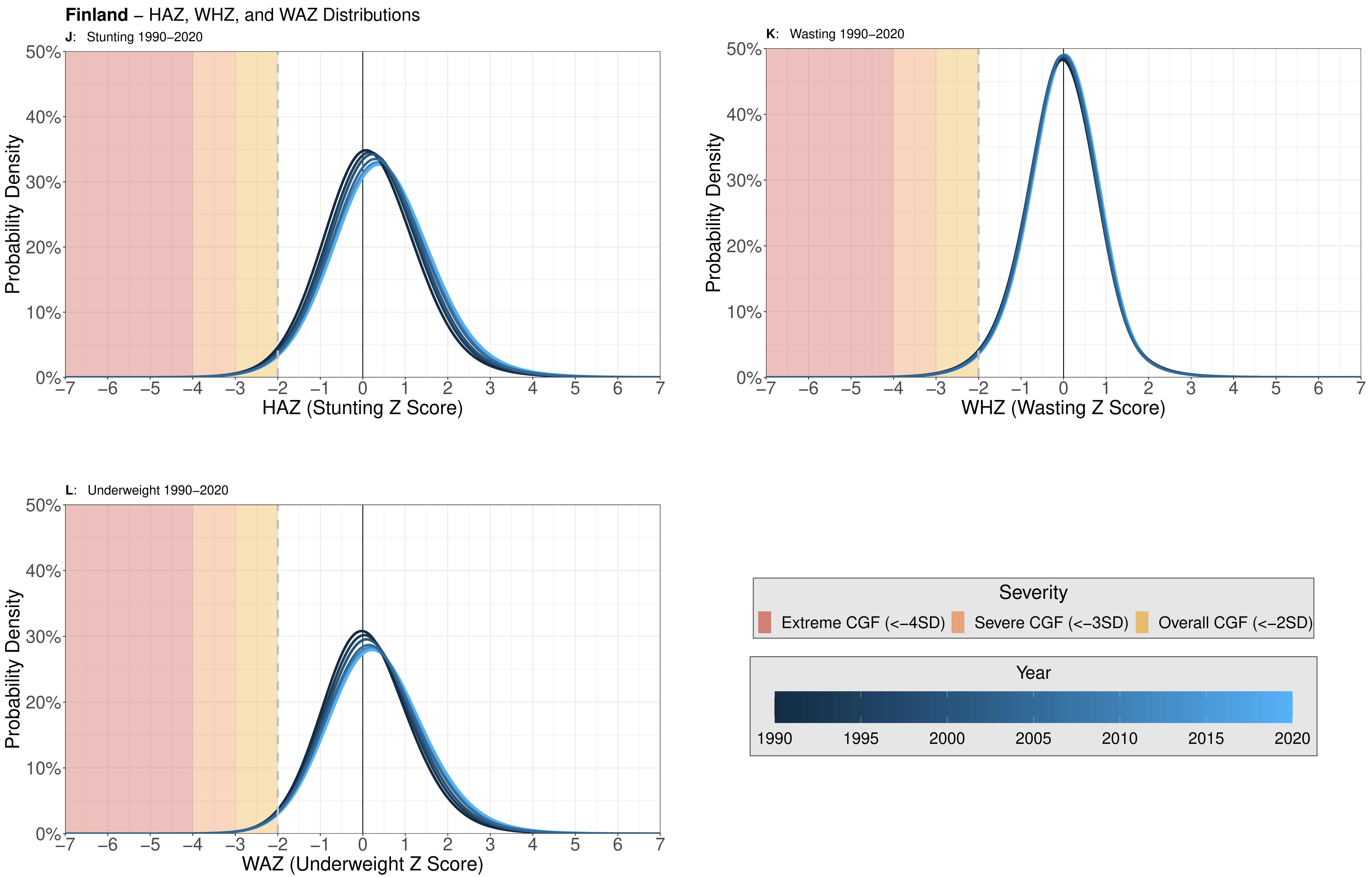
Finland – HAZ, WHZ, and WAZ Distributions
J: Stunting 1990–2020
K: Wasting 1990–2020
L: Underweight 1990–2020
Probability Density
0%
10%
20%
30%
40%
50%
−7 −6 −5 −4 −3 −2 −1 0 1 2 3 4 5 6 7
HAZ (Stunting Z Score)
WHZ (Wasting Z Score)
WAZ (Underweight Z Score)
Severity
Extreme CGF (<−4SD)
Severe CGF (<−3SD)
Overall CGF (<−2SD)
Year
1990
1995
2000
2005
2010
2015
2020

France – Stunting (HAZ)

A: Overall and Severe Stunting Prevalence

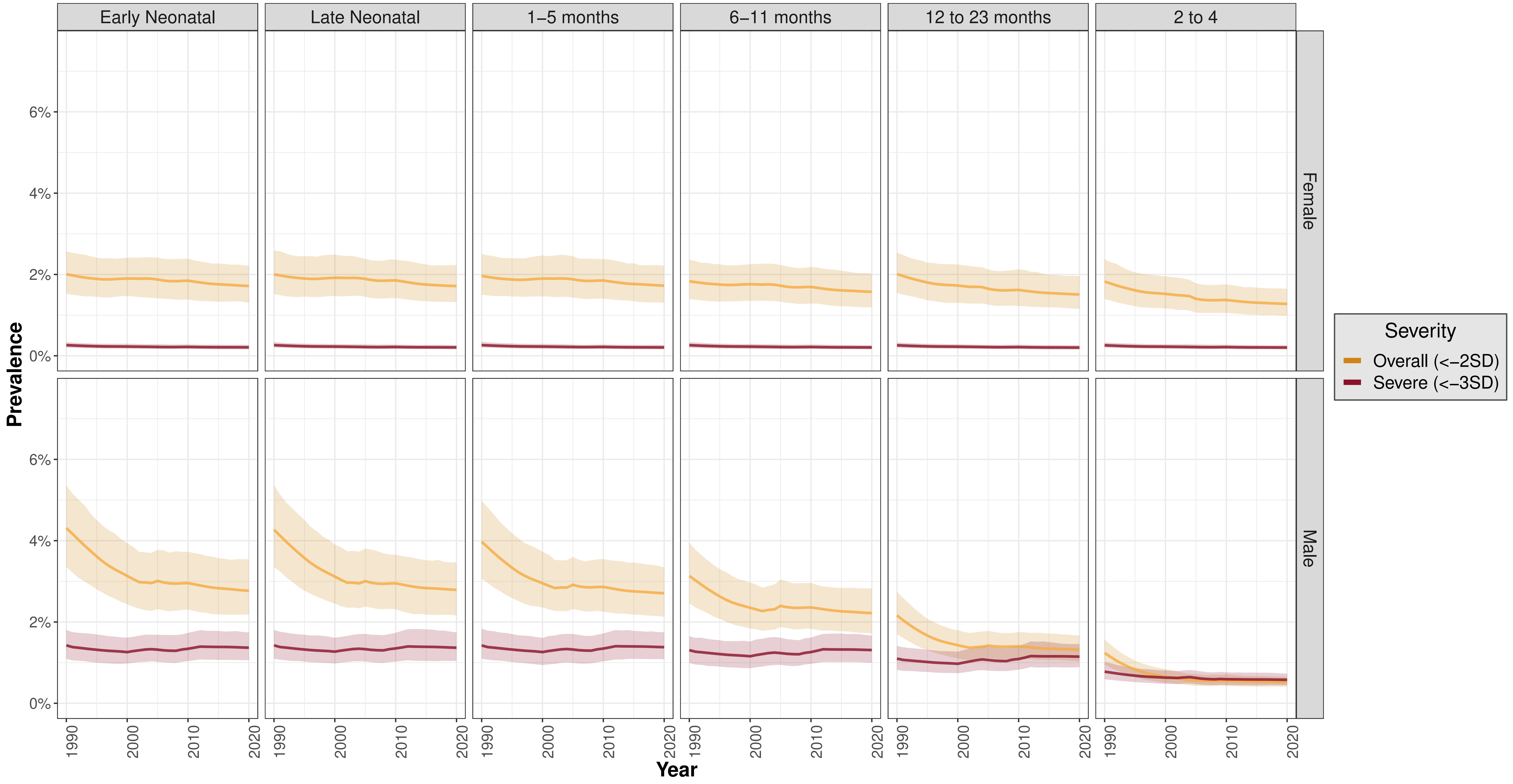

B: Transformed Mean Stunting Z Scores

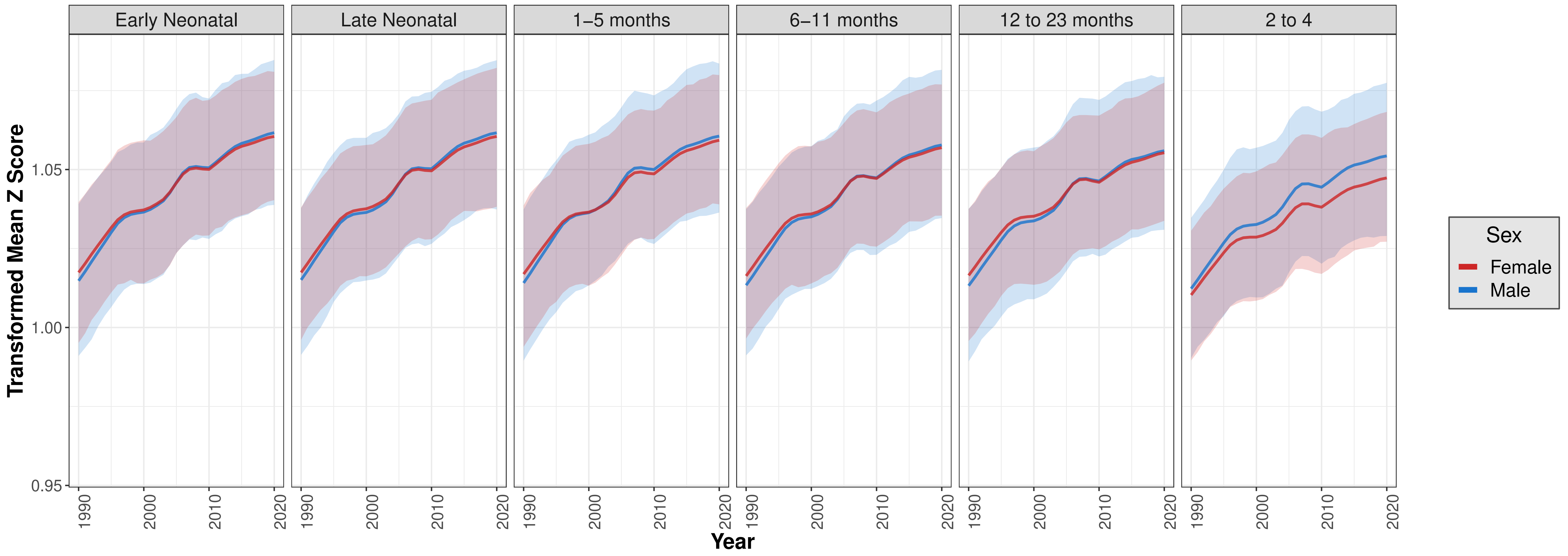

C

Source
No sources for this location

France – Wasting (WHZ)

D: Overall and Severe Wasting Prevalence

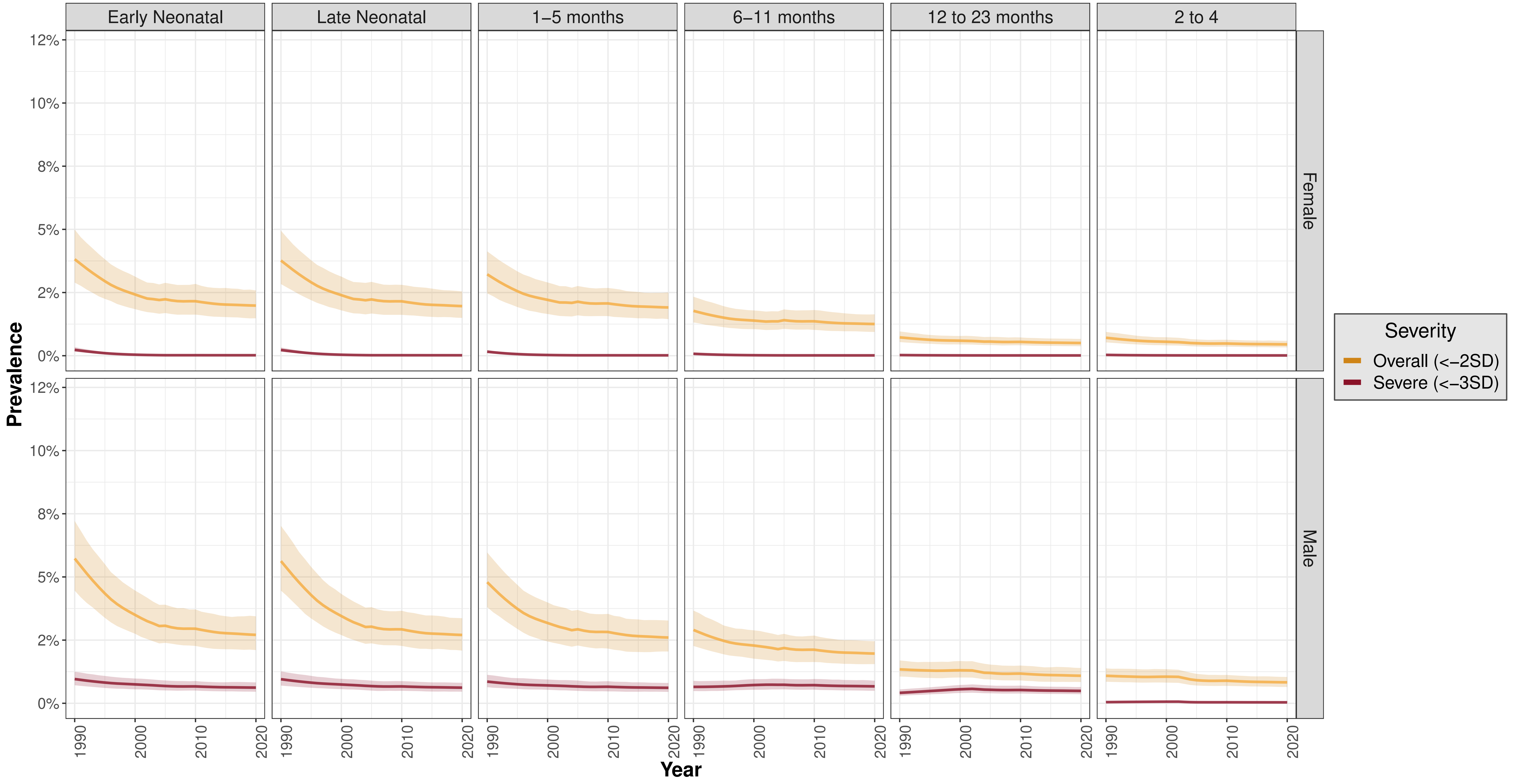

E: Transformed Mean Wasting Z Scores

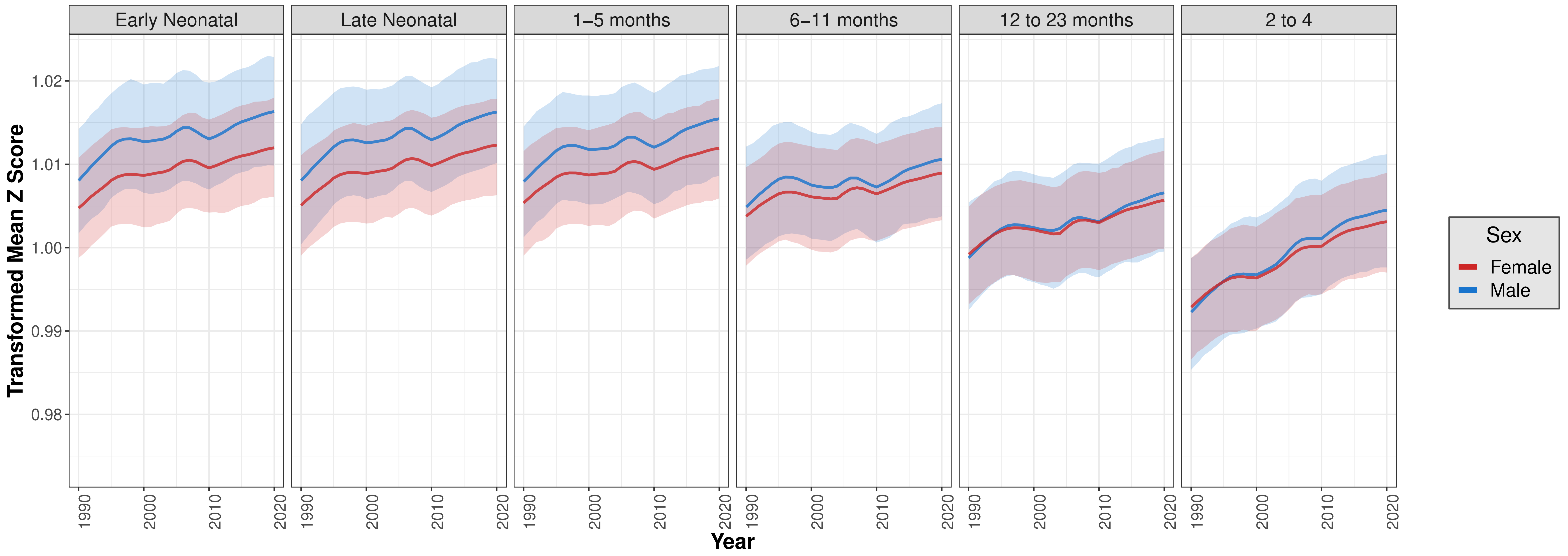

F

Source
No sources for this location

France – Underweight (WAZ)

G: Overall and Severe Underweight Prevalence

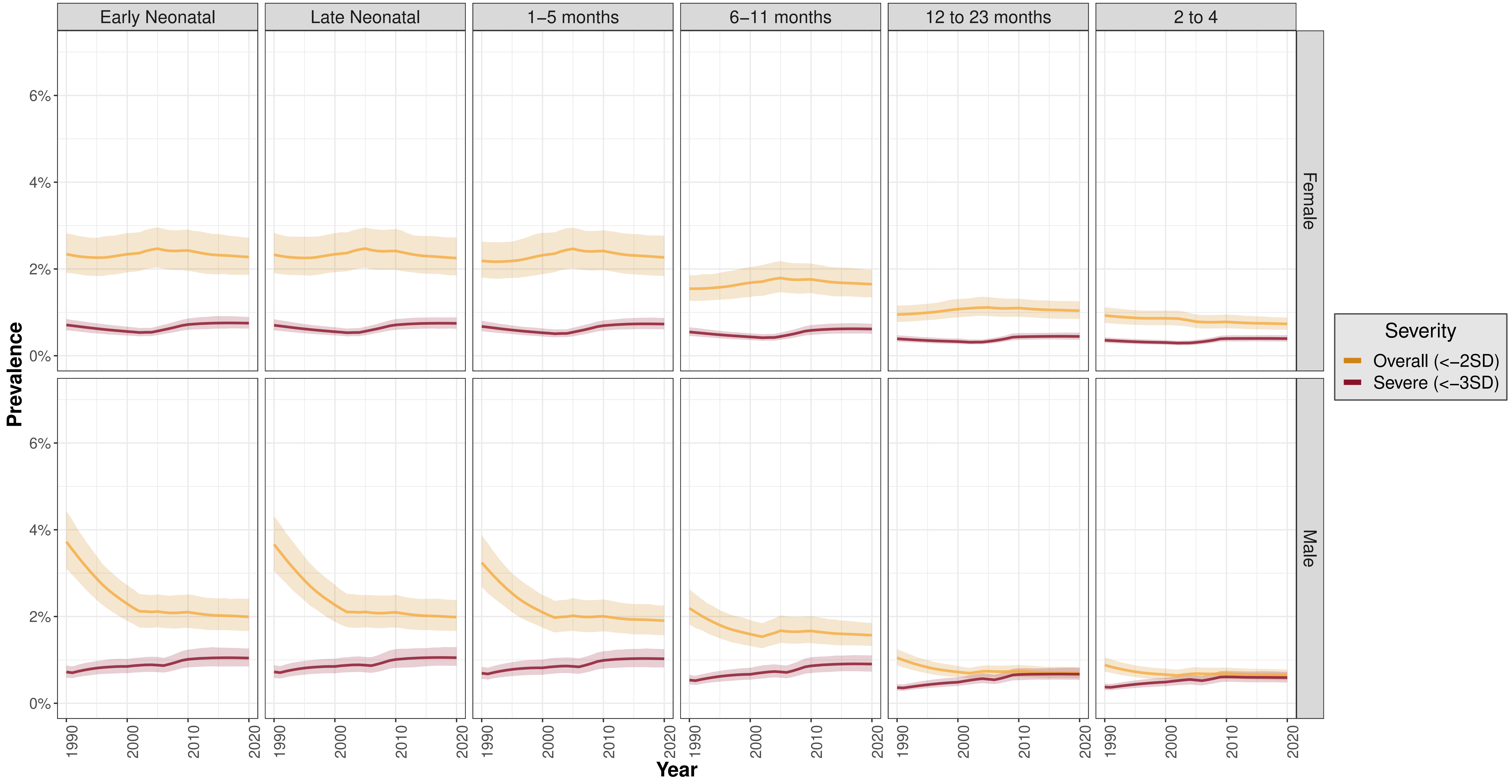

H: Transformed Mean Underweight Z Scores

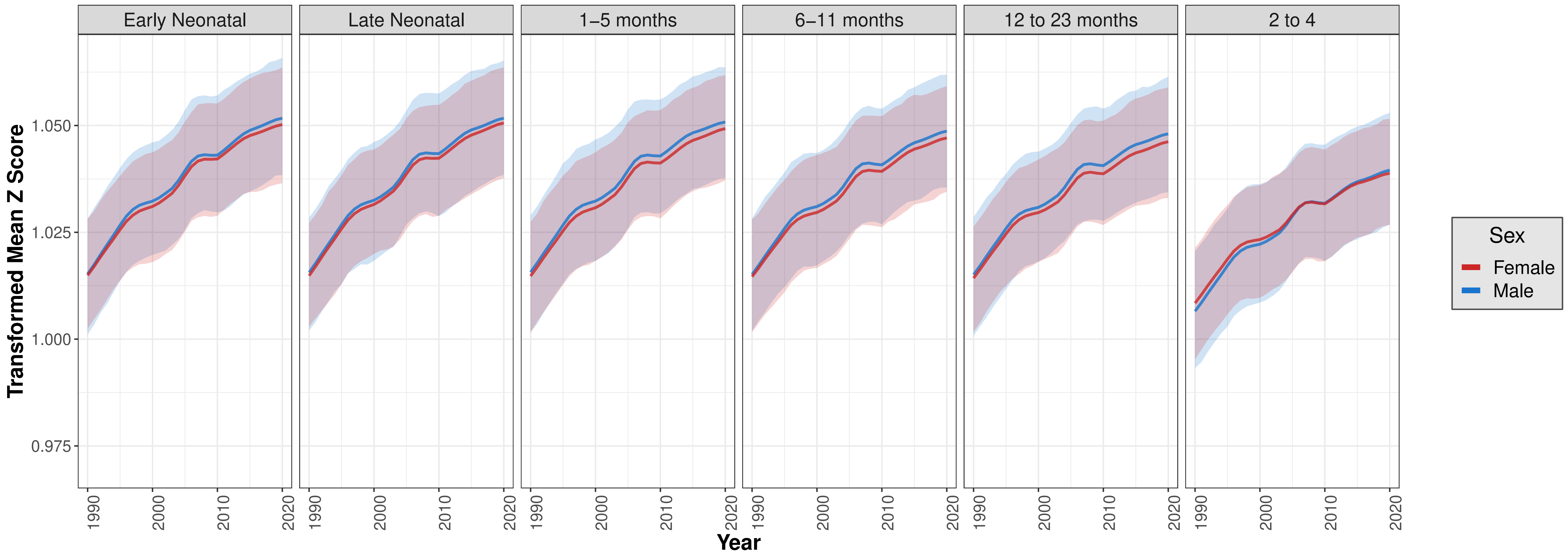

I

Source
No sources for this location

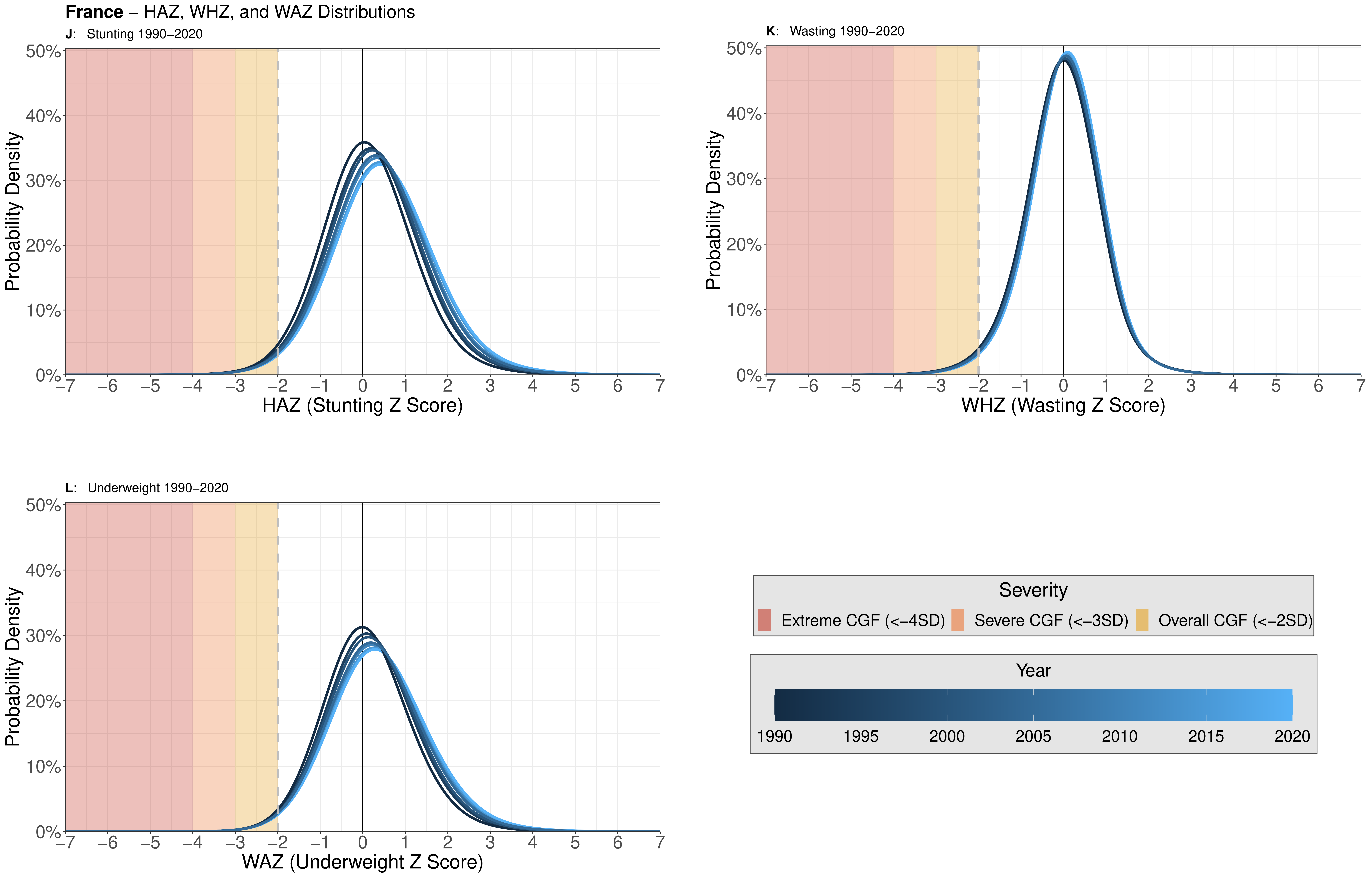
France – HAZ, WHZ, and WAZ Distributions
J: Stunting 1990–2020
K: Wasting 1990–2020
L: Underweight 1990–2020
Probability Density
0%
10%
20%
30%
40%
50%
−7
−6
−5
−4
−3
−2
−1
0
1
2
3
4
5
6
7
HAZ (Stunting Z Score)
WHZ (Wasting Z Score)
WAZ (Underweight Z Score)
Severity
Extreme CGF (<−4SD)
Severe CGF (<−3SD)
Overall CGF (<−2SD)
Year
1990
1995
2000
2005
2010
2015
2020

Germany – Stunting (HAZ)

A: Overall and Severe Stunting Prevalence

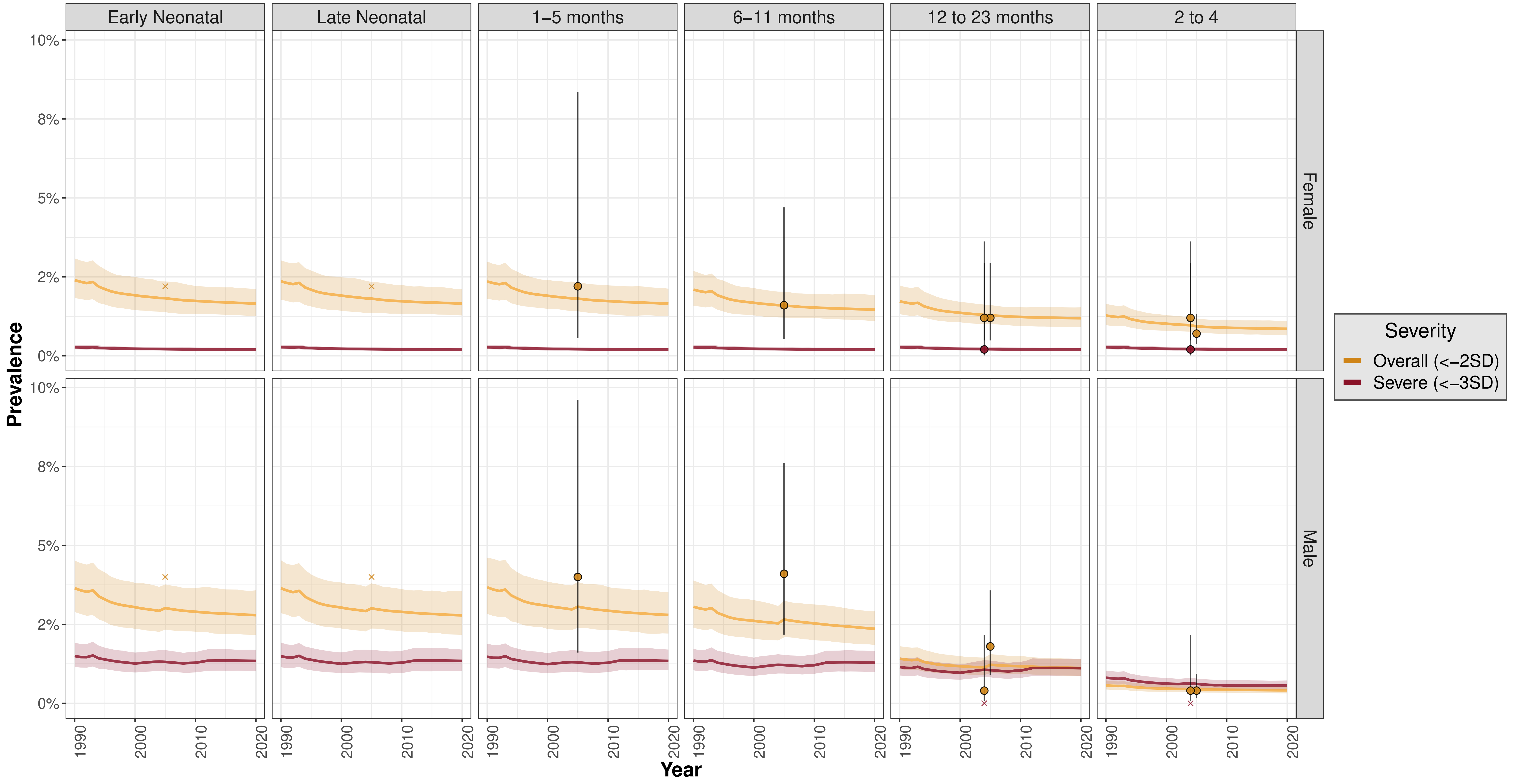

B: Transformed Mean Stunting Z Scores

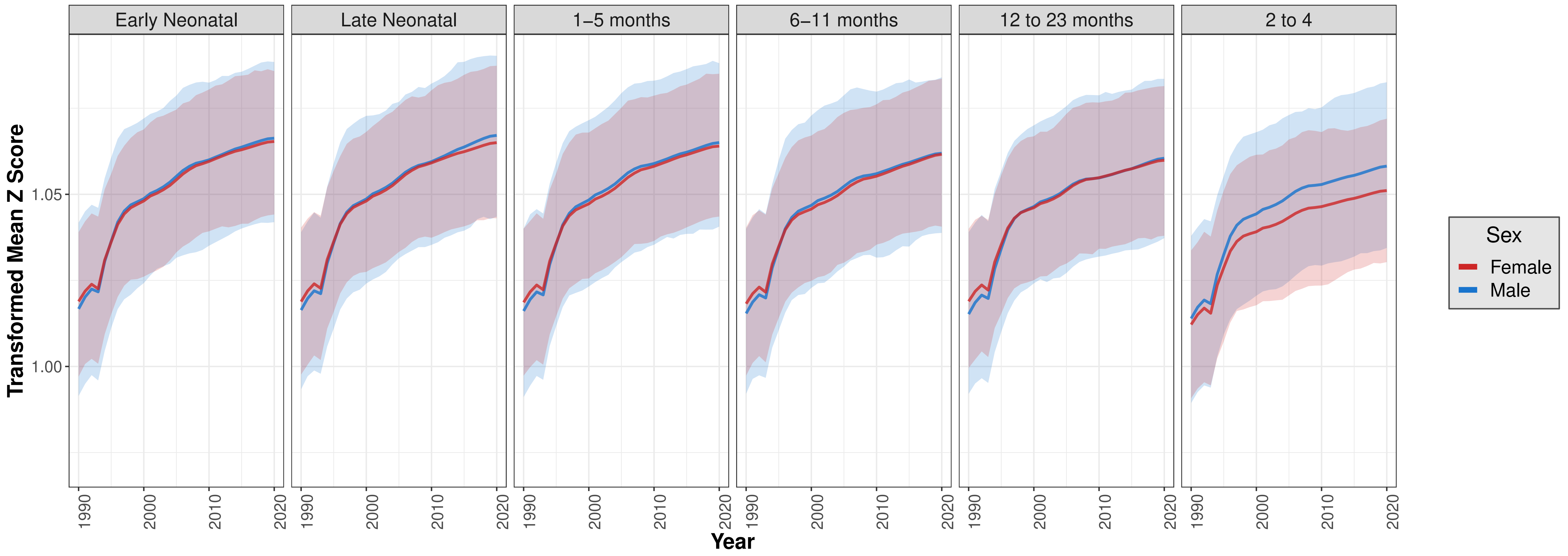

C

Year	Source
2004	WHO CGM Database
2005	WHO CGM Database

Germany – Wasting (WHZ)

D: Overall and Severe Wasting Prevalence

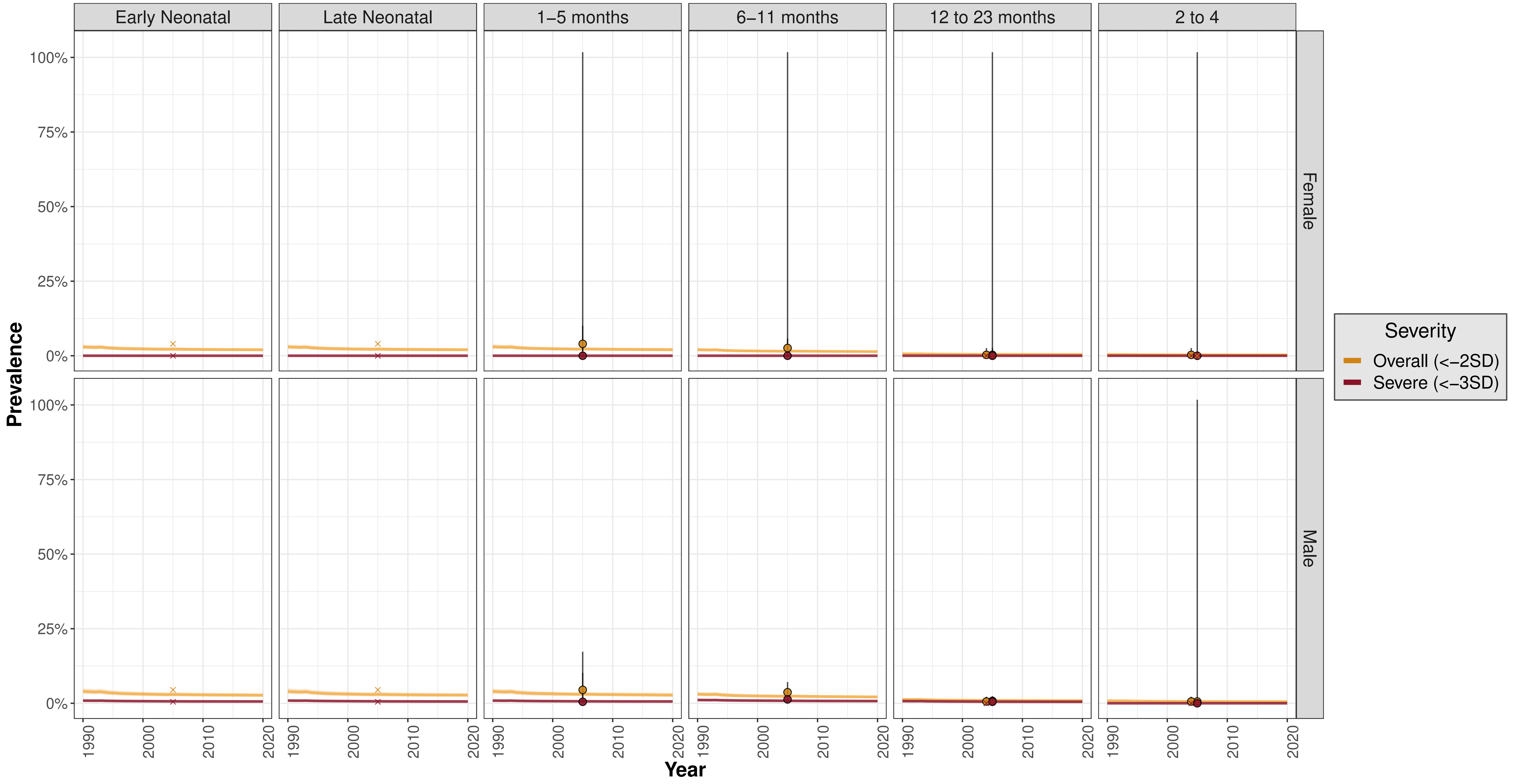

E: Transformed Mean Wasting Z Scores

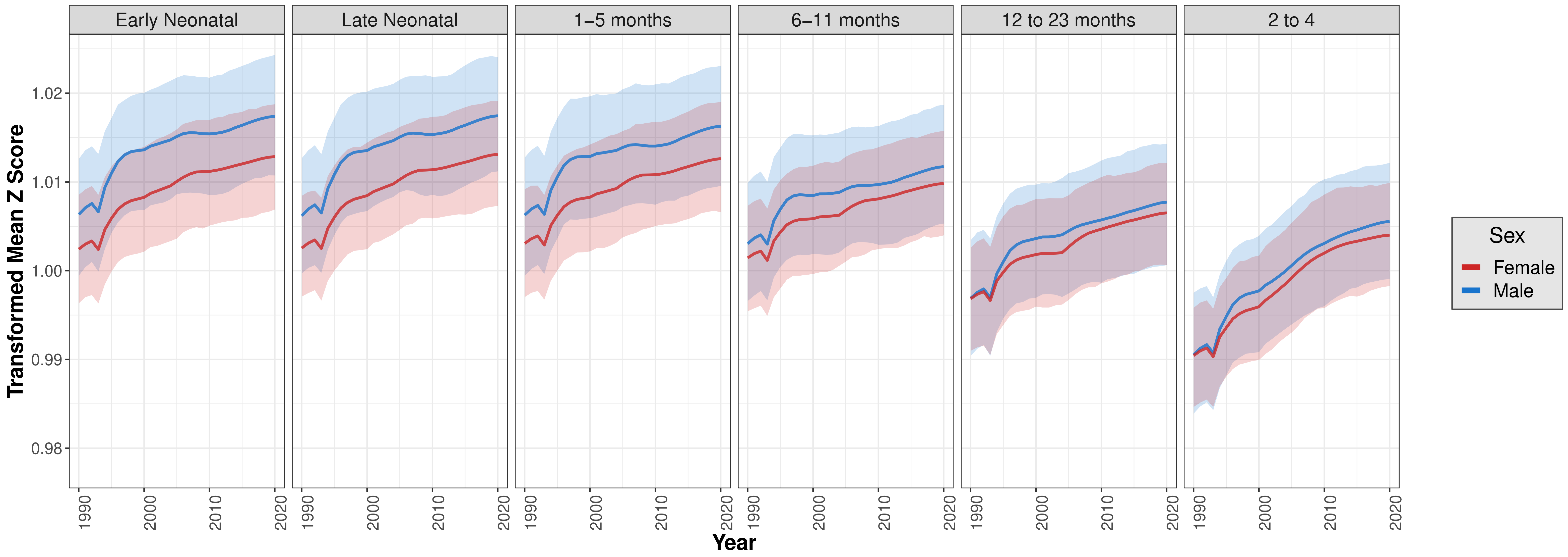

F

Year	Source
2004	WHO CGM Database
2005	WHO CGM Database

Germany – Underweight (WAZ)

G: Overall and Severe Underweight Prevalence

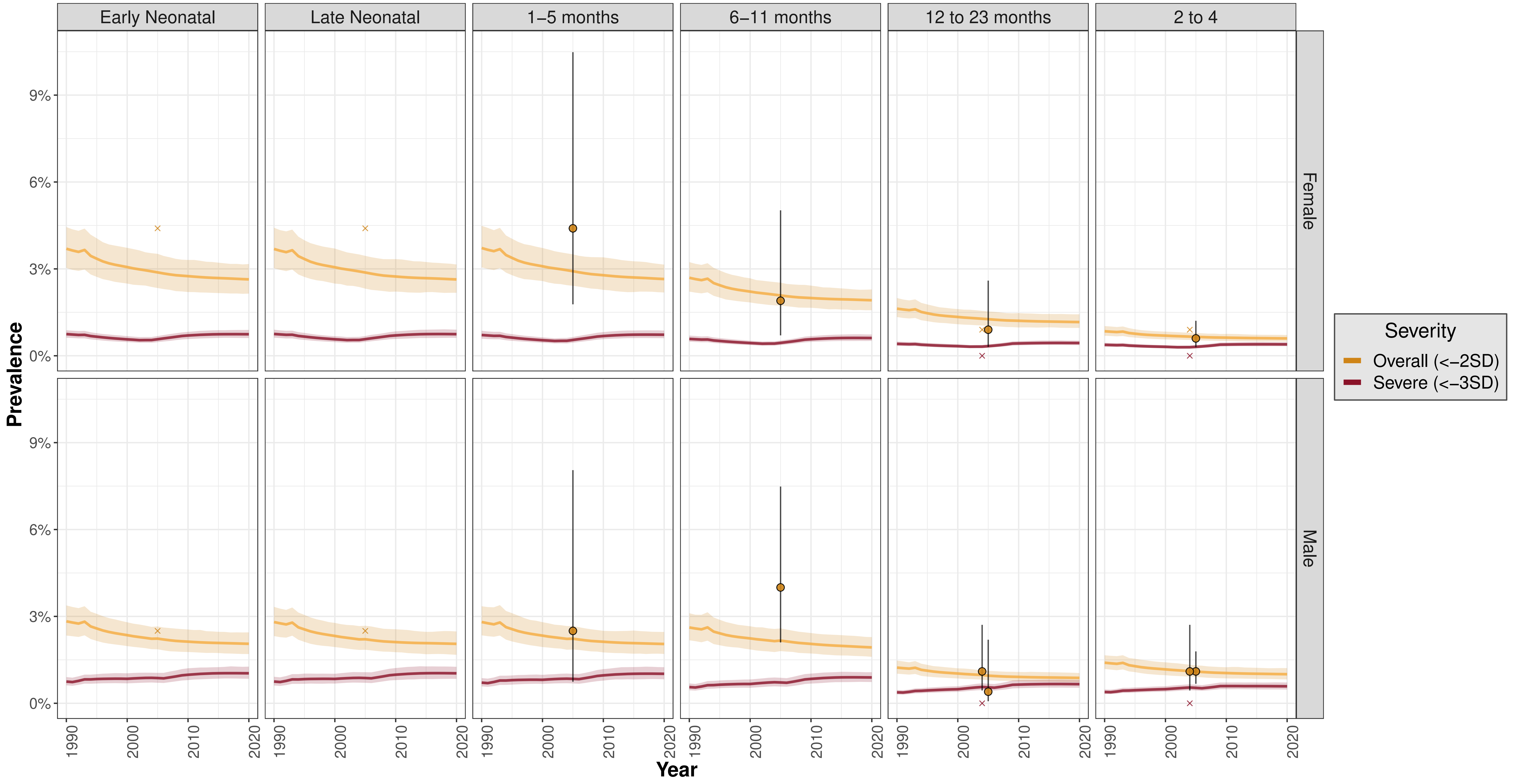

H: Transformed Mean Underweight Z Scores

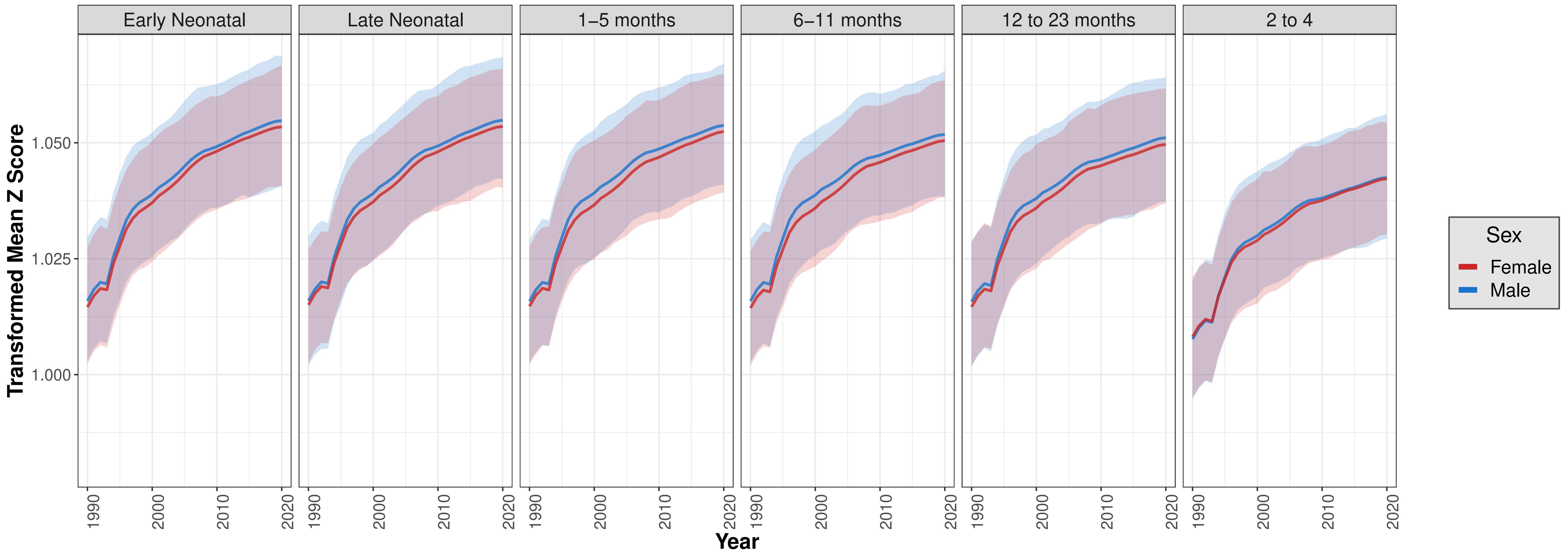

I

Year	Source
2004	WHO CGM Database
2005	WHO CGM Database

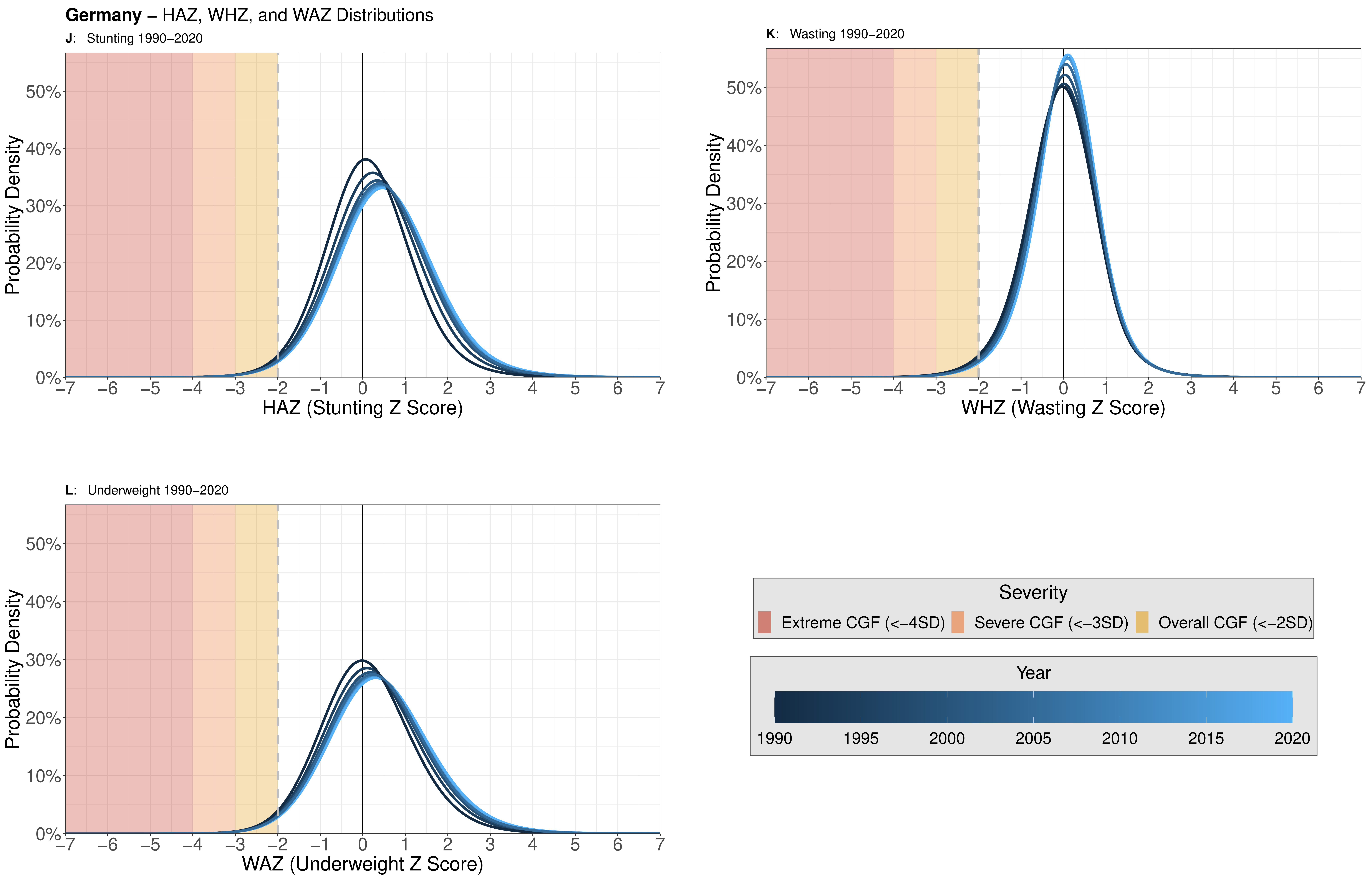
Germany – HAZ, WHZ, and WAZ Distributions
J: Stunting 1990–2020
Probability Density
0%
10%
20%
30%
40%
50%
−7 −6 −5 −4 −3 −2 −1 0 1 2 3 4 5 6 7
HAZ (Stunting Z Score)
K: Wasting 1990–2020
Probability Density
0%
10%
20%
30%
40%
50%
−7 −6 −5 −4 −3 −2 −1 0 1 2 3 4 5 6 7
WHZ (Wasting Z Score)
L: Underweight 1990–2020
Probability Density
0%
10%
20%
30%
40%
50%
−7 −6 −5 −4 −3 −2 −1 0 1 2 3 4 5 6 7
WAZ (Underweight Z Score)
Severity
Extreme CGF (<−4SD)
Severe CGF (<−3SD)
Overall CGF (<−2SD)
Year
1990
1995
2000
2005
2010
2015
2020

Greece – Stunting (HAZ)

A: Overall and Severe Stunting Prevalence

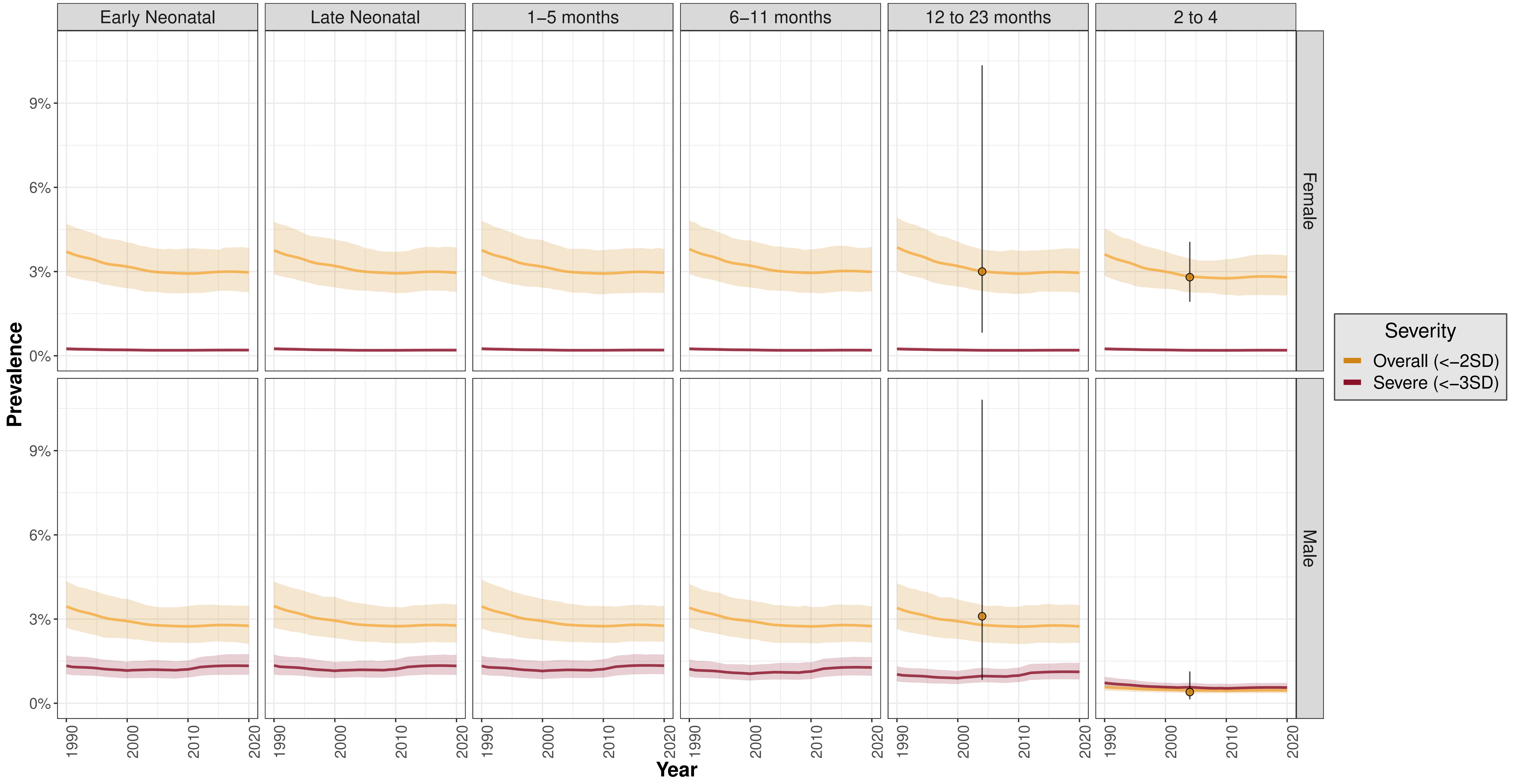

B: Transformed Mean Stunting Z Scores

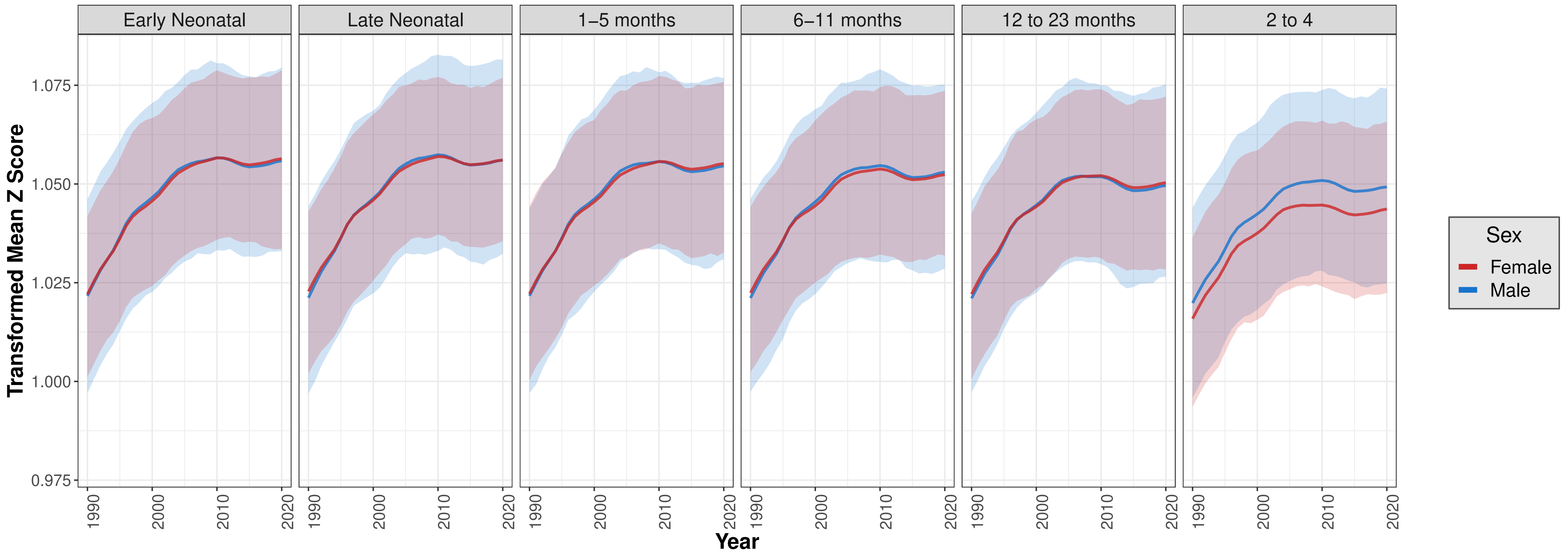

C

Year	Source
2004	WHO CGM Database

Greece – Wasting (WHZ)

D: Overall and Severe Wasting Prevalence

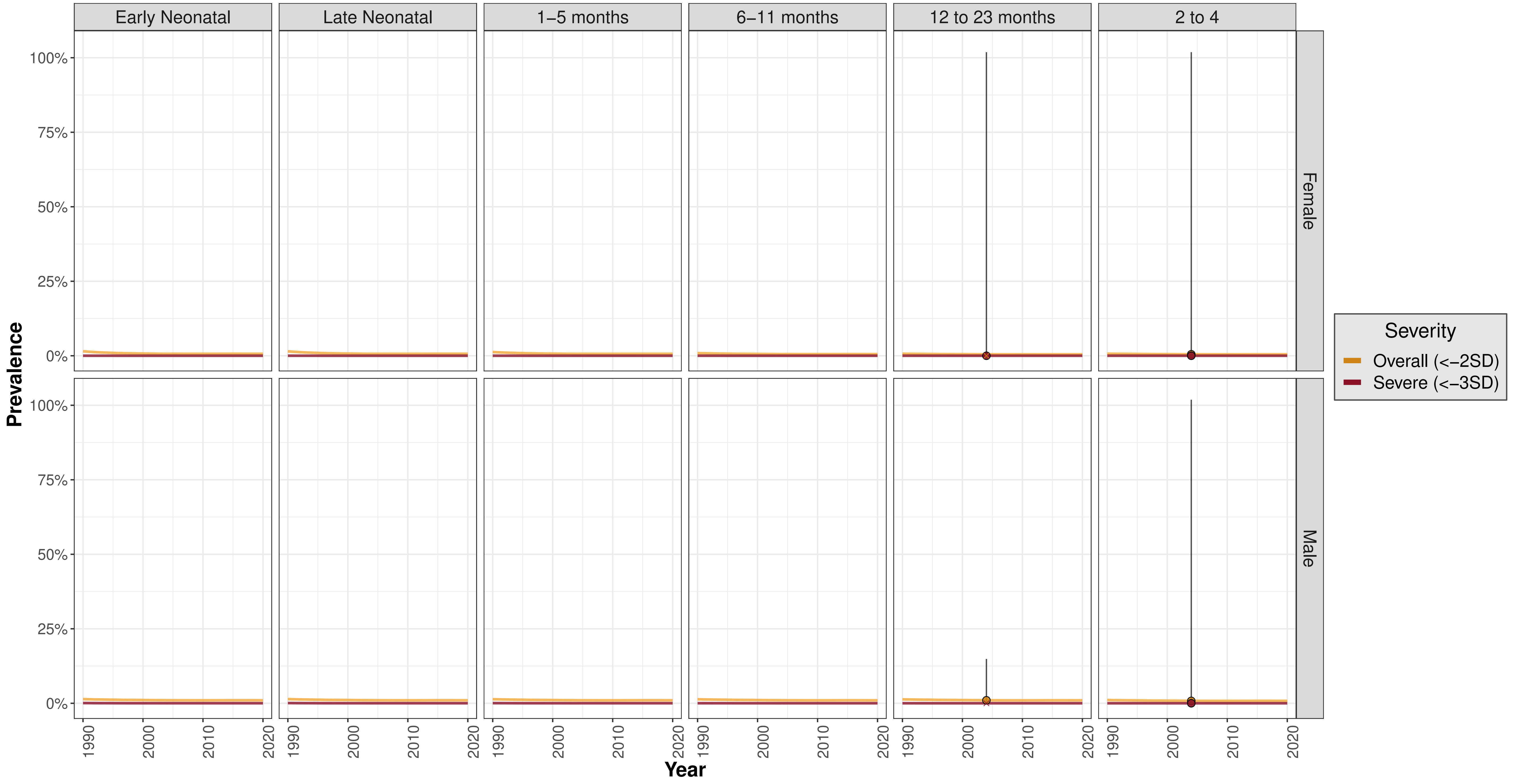

E: Transformed Mean Wasting Z Scores

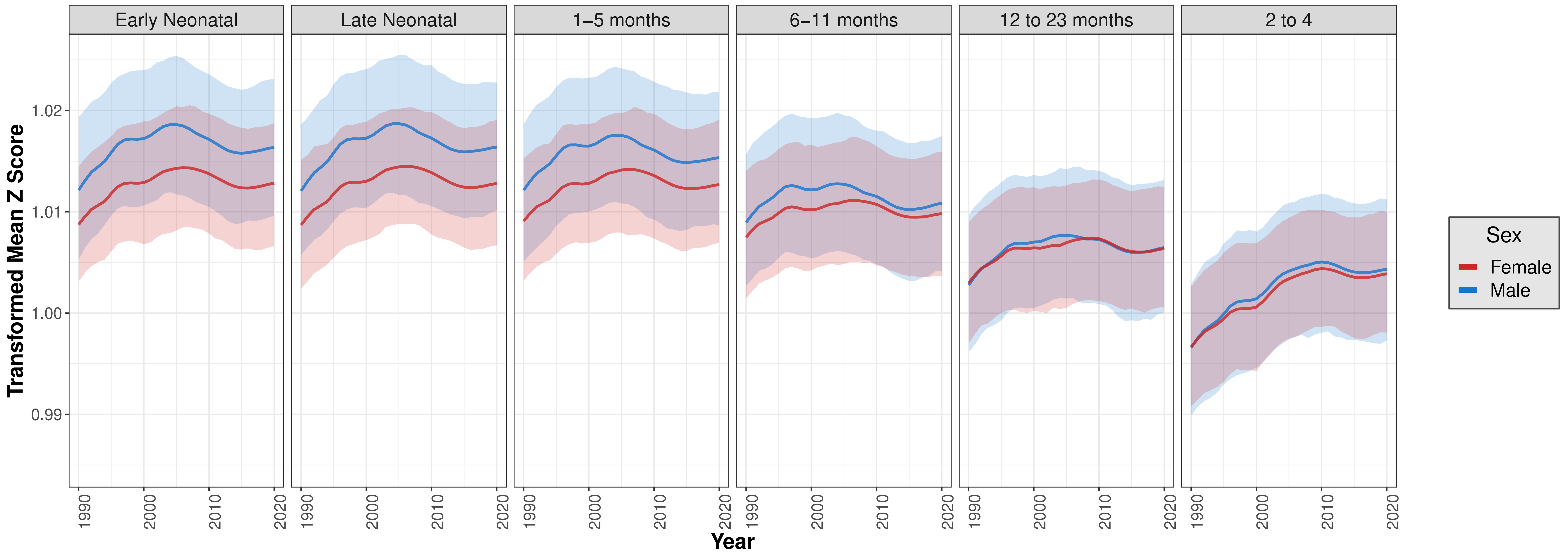

F

Year	Source
2004	WHO CGM Database

Greece – Underweight (WAZ)

G: Overall and Severe Underweight Prevalence

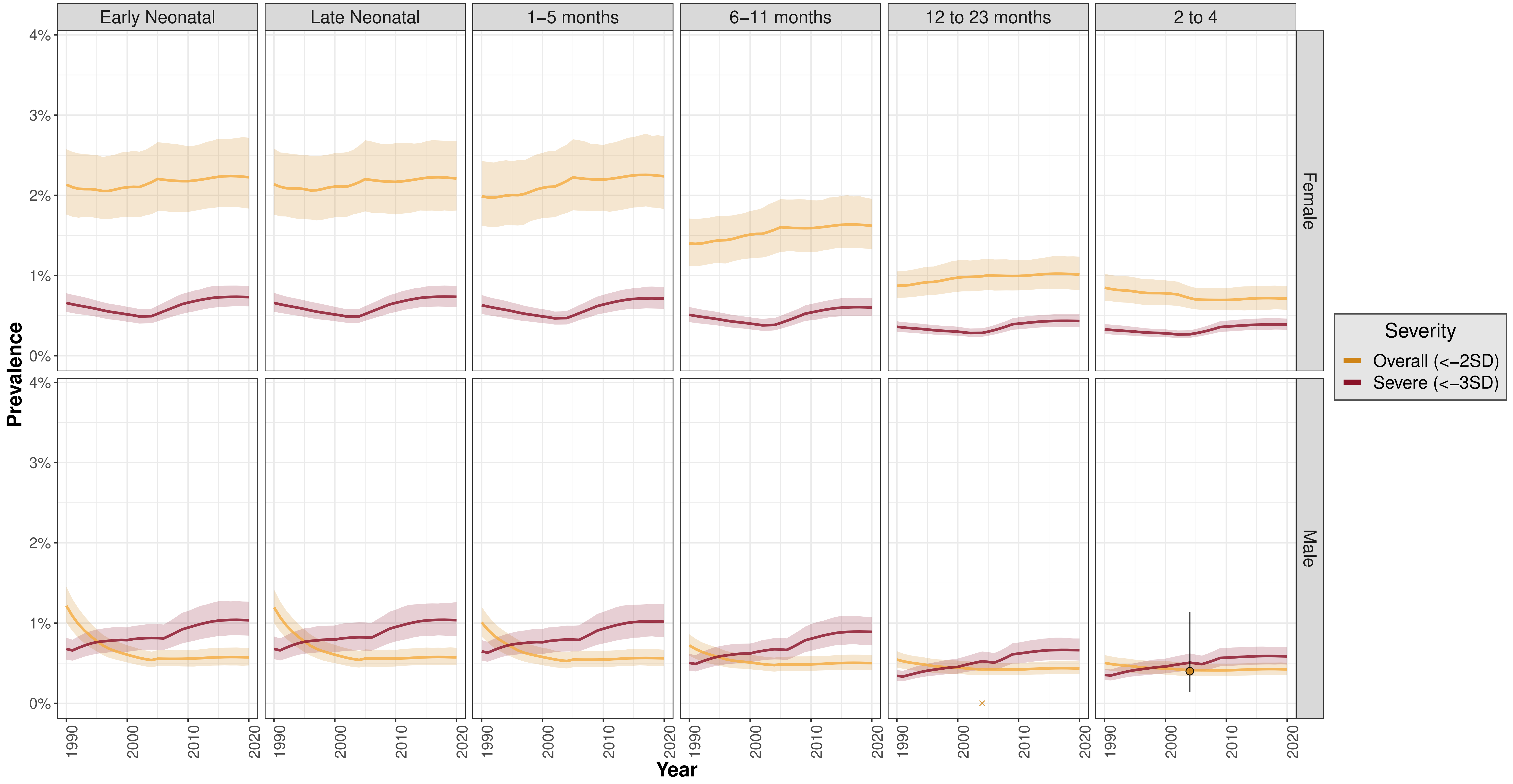

H: Transformed Mean Underweight Z Scores

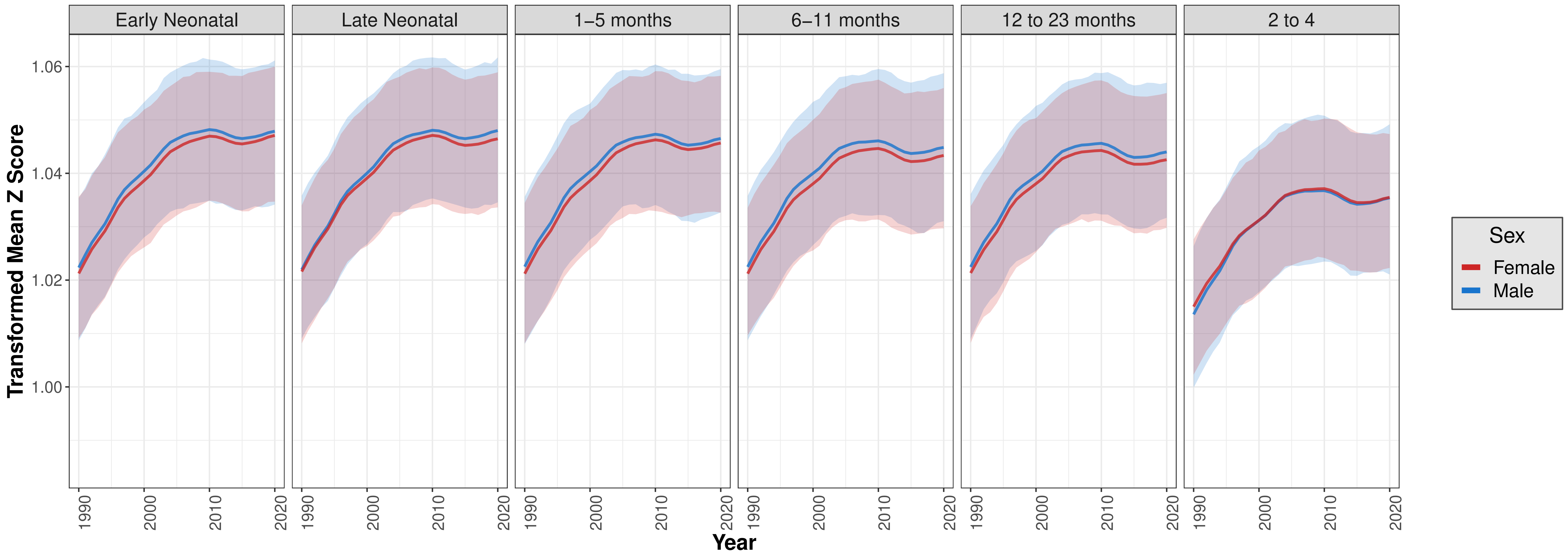

I

Year	Source
2004	WHO CGM Database

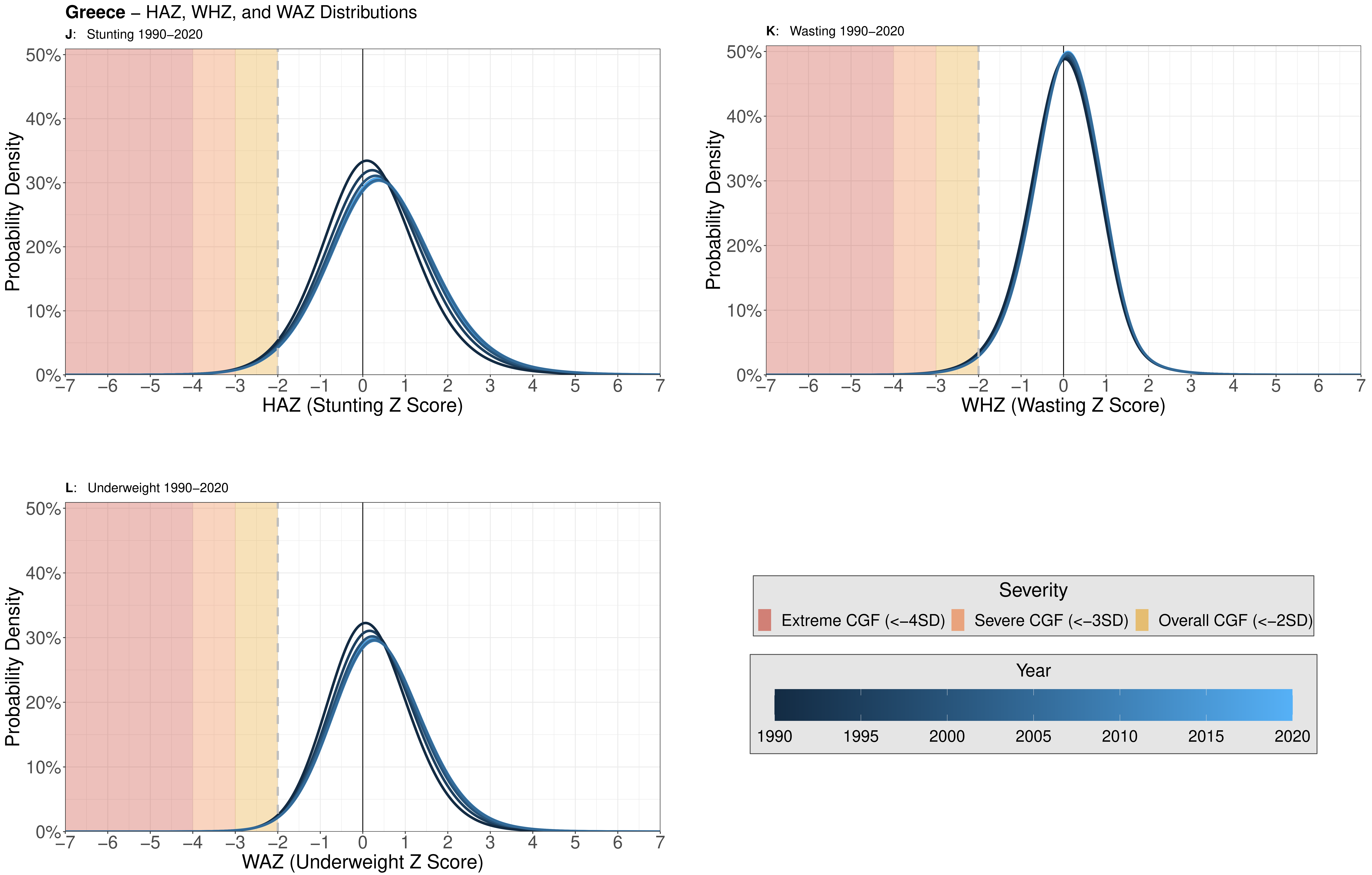
Greece – HAZ, WHZ, and WAZ Distributions
J: Stunting 1990–2020
K: Wasting 1990–2020
L: Underweight 1990–2020
Probability Density
0%
10%
20%
30%
40%
50%
−7
−6
−5
−4
−3
−2
−1
0
1
2
3
4
5
6
7
HAZ (Stunting Z Score)
WHZ (Wasting Z Score)
WAZ (Underweight Z Score)
Severity
Extreme CGF (<−4SD)
Severe CGF (<−3SD)
Overall CGF (<−2SD)
Year
1990
1995
2000
2005
2010
2015
2020

Iceland – Stunting (HAZ)

A: Overall and Severe Stunting Prevalence

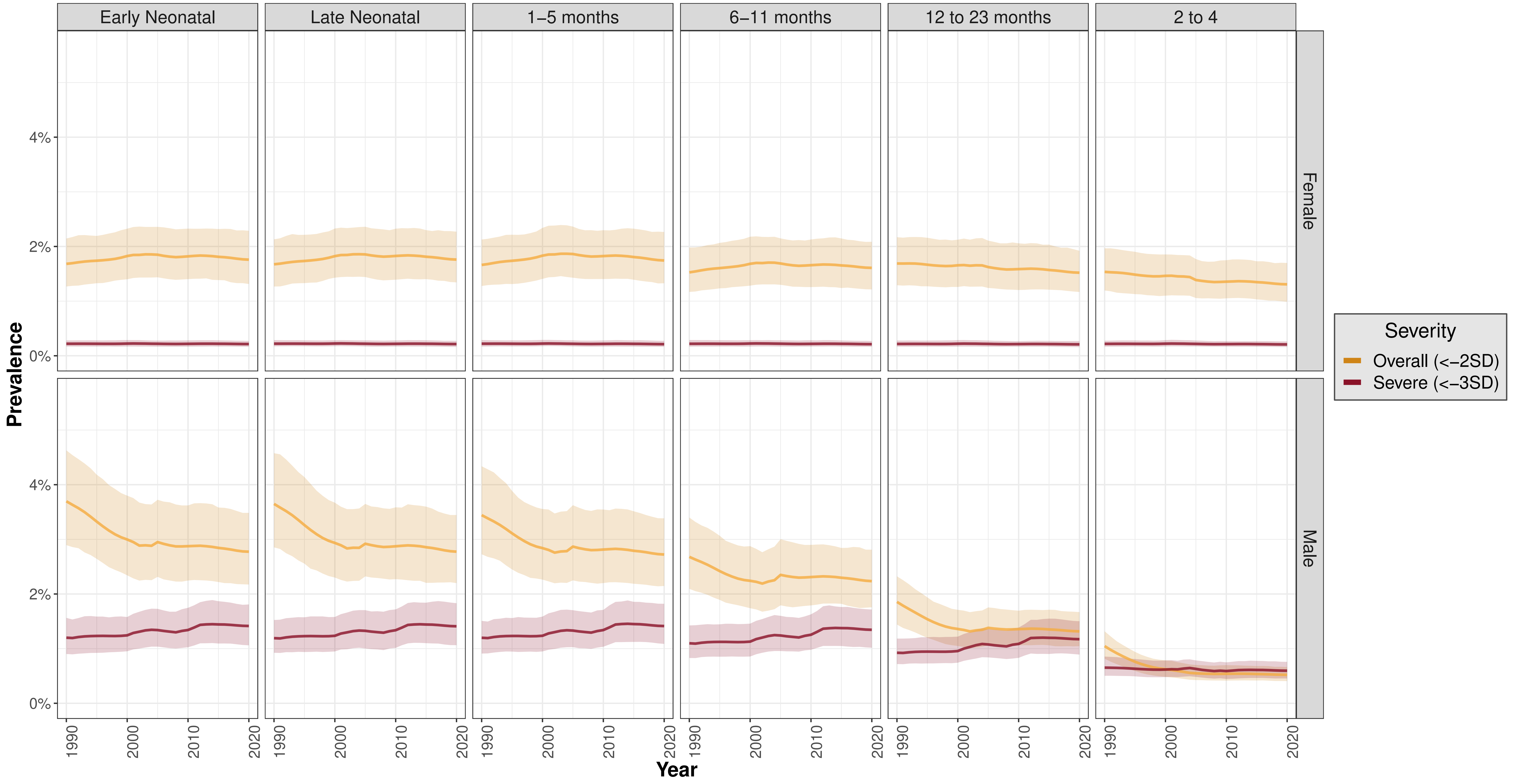

B: Transformed Mean Stunting Z Scores

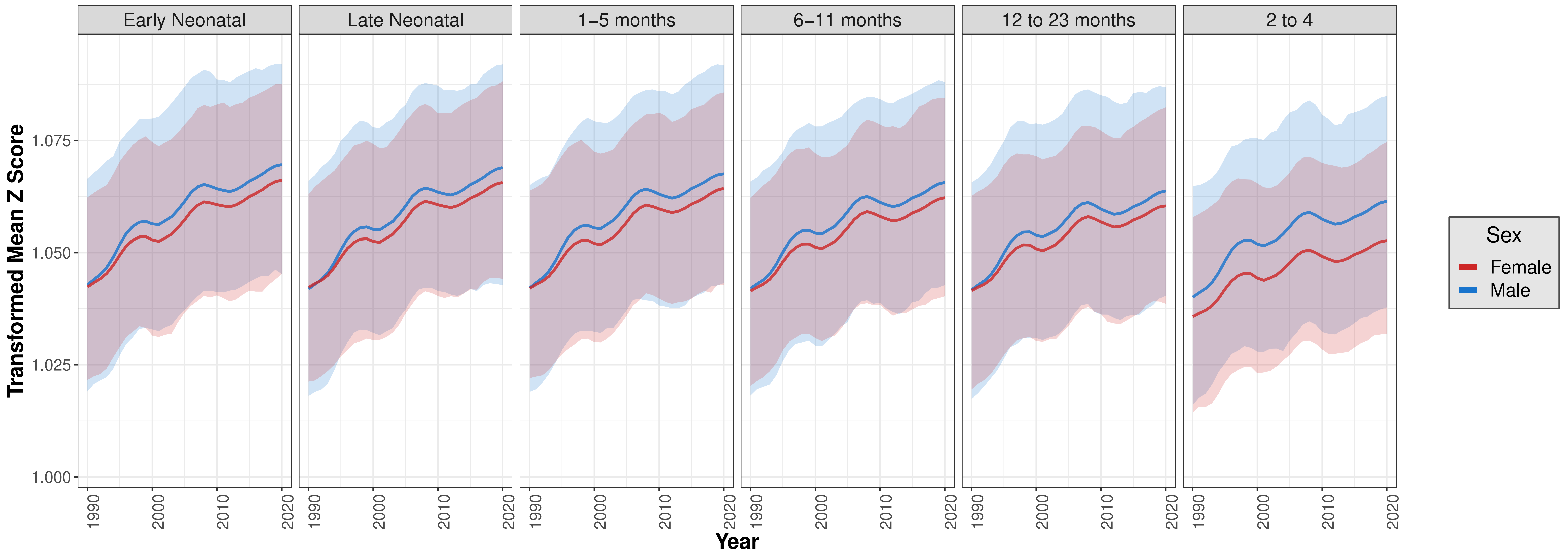

C

Source
No sources for this location

Iceland – Wasting (WHZ)

D: Overall and Severe Wasting Prevalence

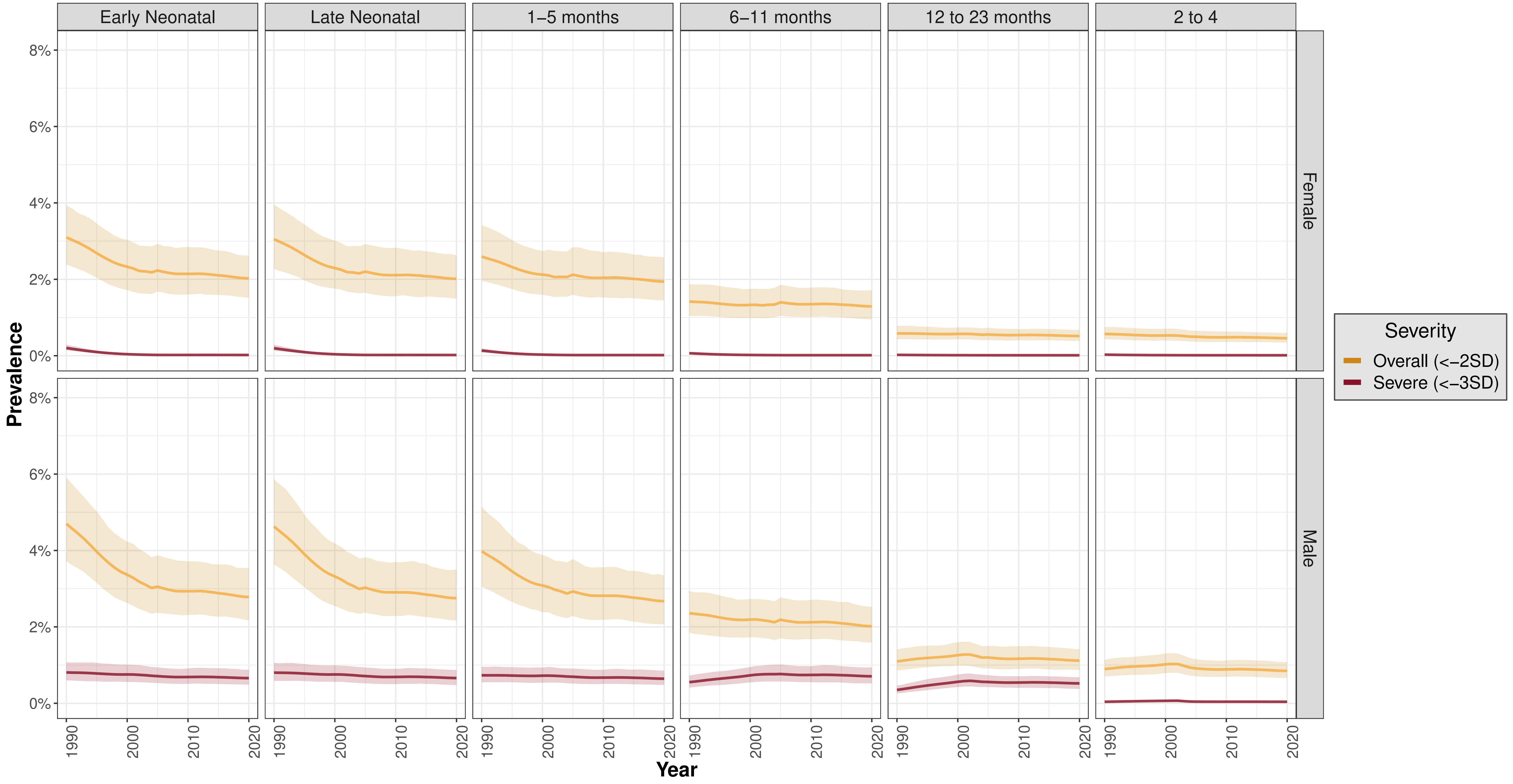

E: Transformed Mean Wasting Z Scores

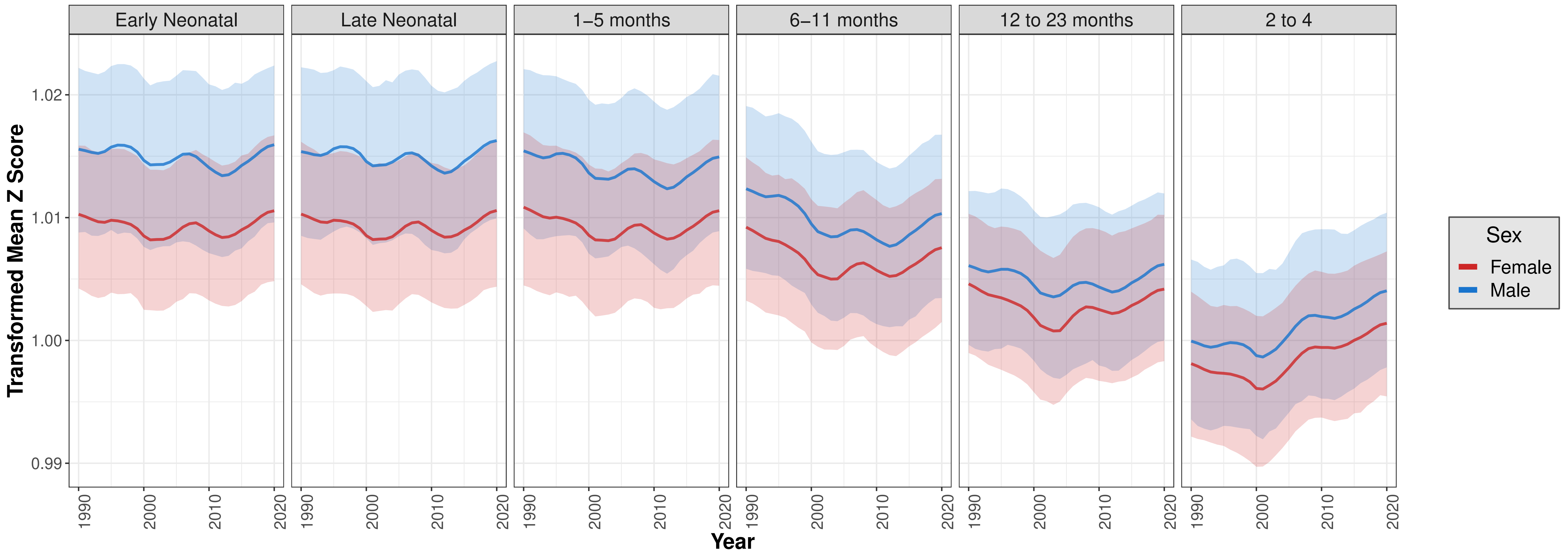

F

**Source**
No sources for this location

Iceland – Underweight (WAZ)

G: Overall and Severe Underweight Prevalence

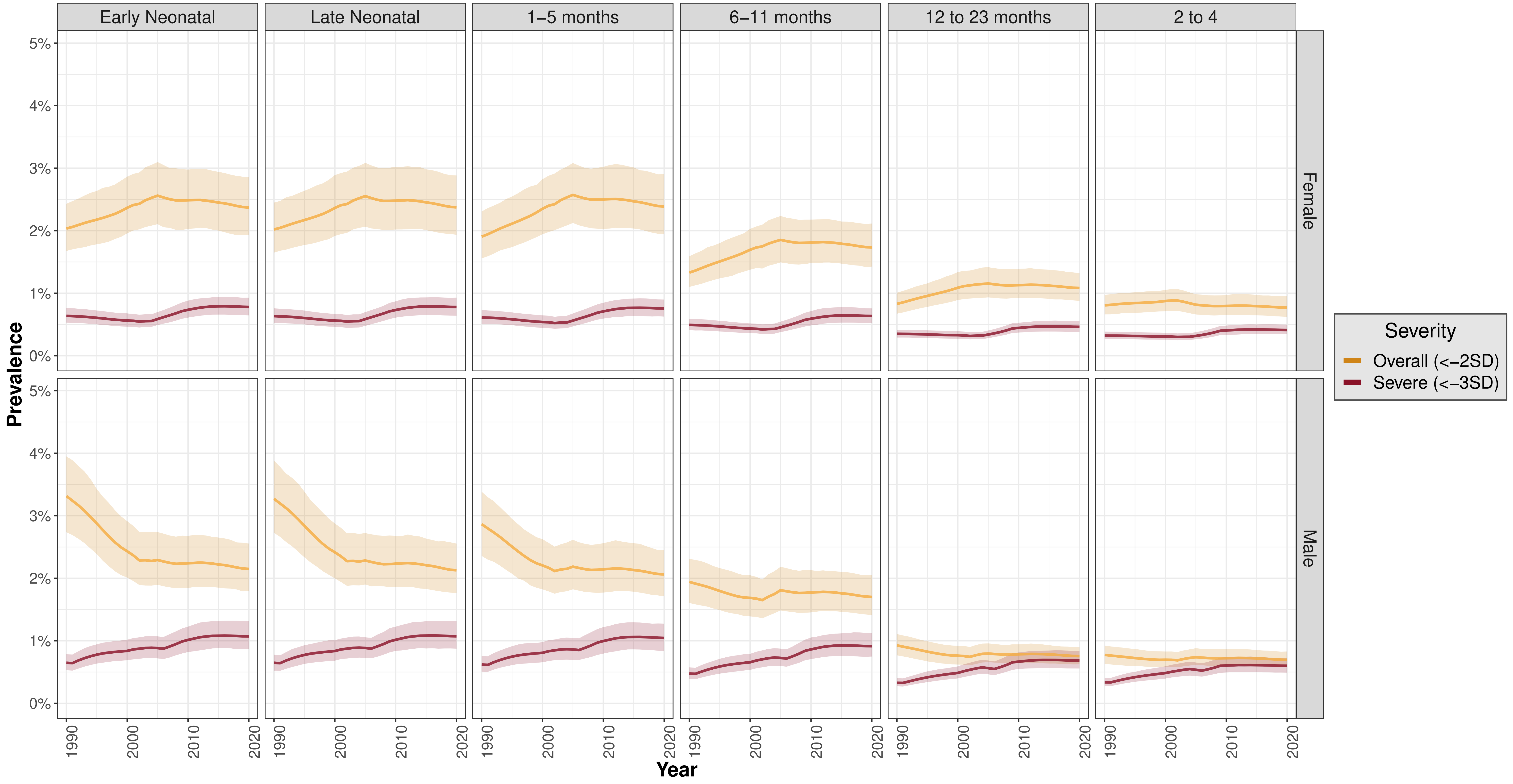

H: Transformed Mean Underweight Z Scores

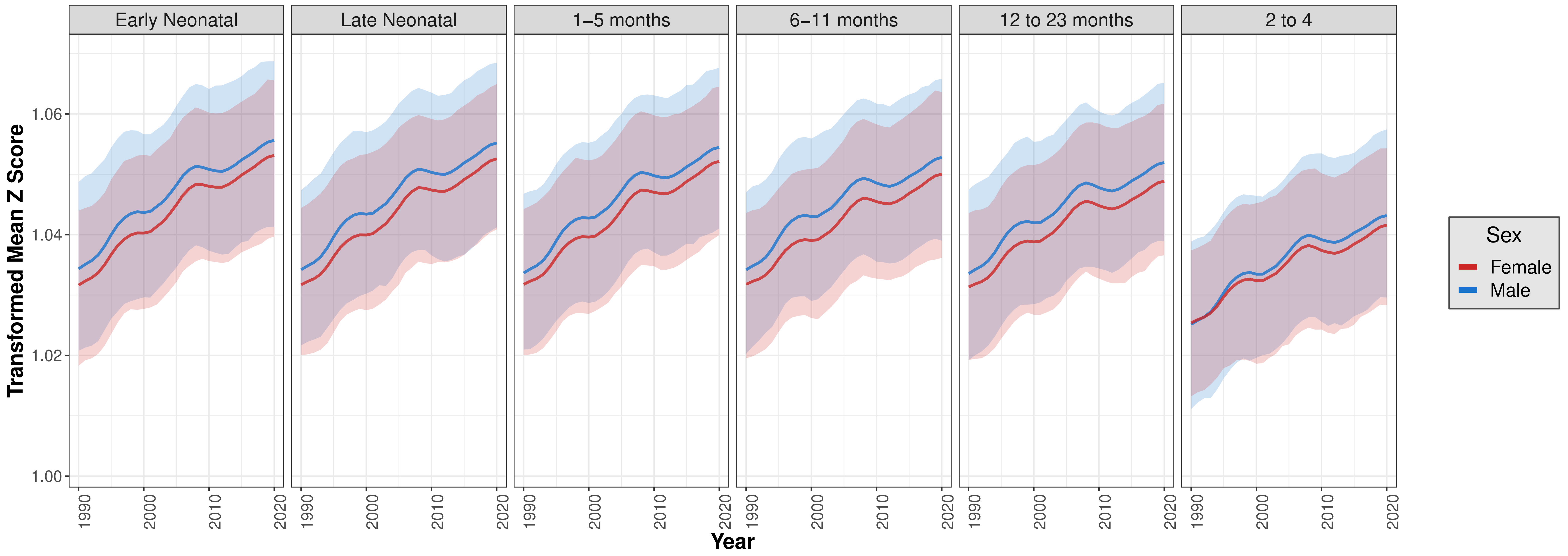

I

Source
No sources for this location

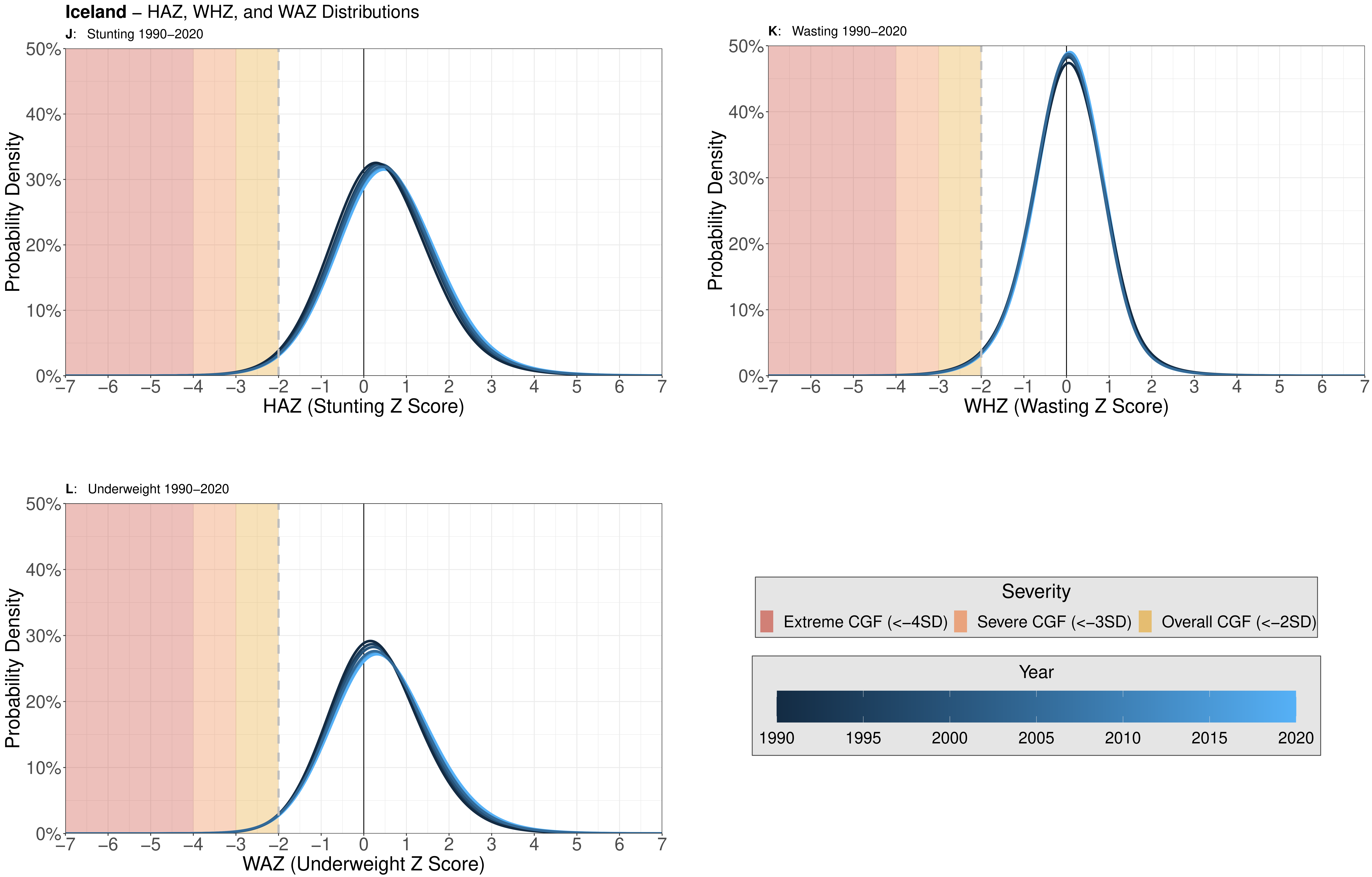
Iceland – HAZ, WHZ, and WAZ Distributions
J: Stunting 1990–2020
Probability Density
HAZ (Stunting Z Score)
K: Wasting 1990–2020
Probability Density
WHZ (Wasting Z Score)
L: Underweight 1990–2020
Probability Density
WAZ (Underweight Z Score)
0%
10%
20%
30%
40%
50%
−7
−6
−5
−4
−3
−2
−1
0
1
2
3
4
5
6
7
Severity
Extreme CGF (<−4SD)
Severe CGF (<−3SD)
Overall CGF (<−2SD)
Year
1990
1995
2000
2005
2010
2015
2020

Ireland – Stunting (HAZ)

A: Overall and Severe Stunting Prevalence

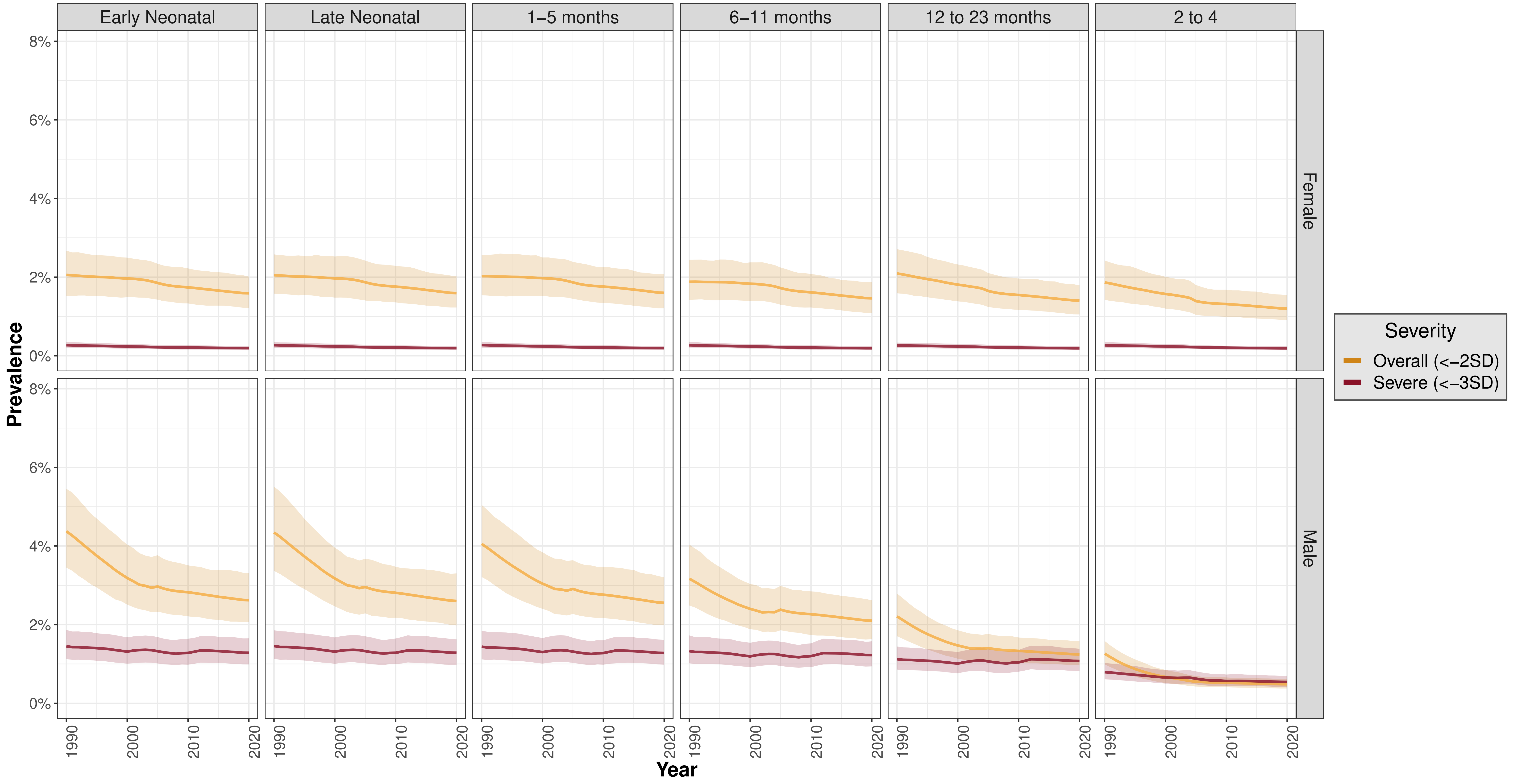

B: Transformed Mean Stunting Z Scores

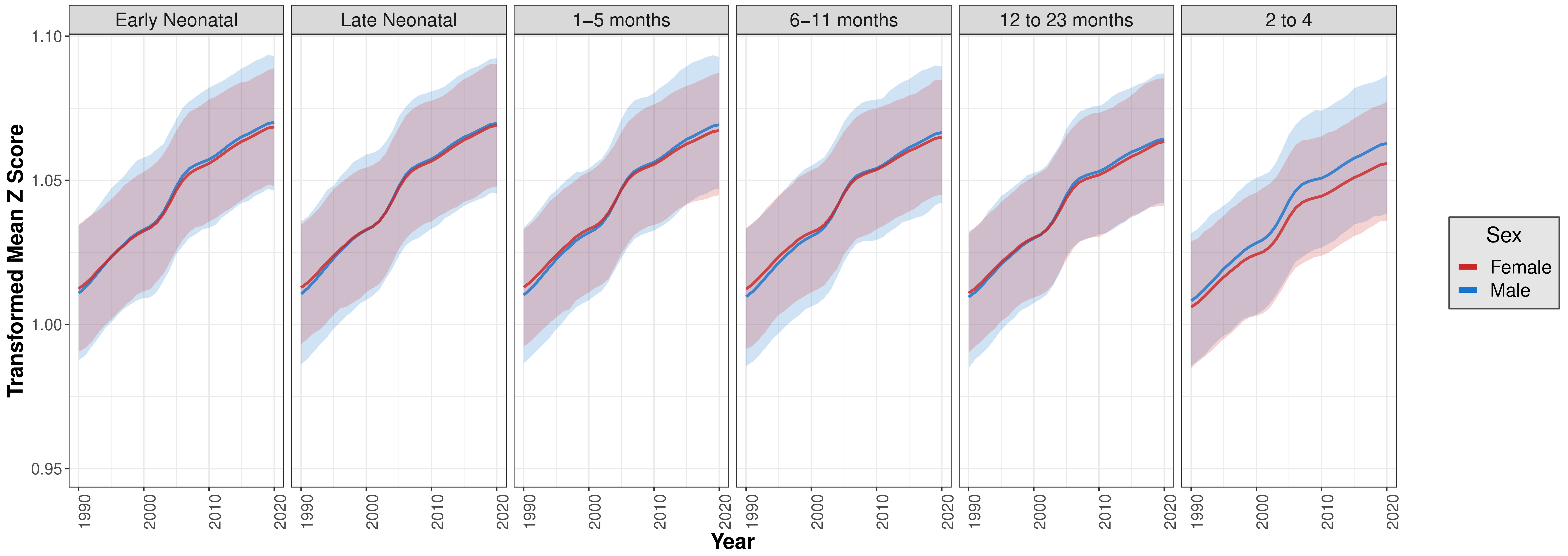

C

Source
No sources for this location

Ireland – Wasting (WHZ)

D: Overall and Severe Wasting Prevalence

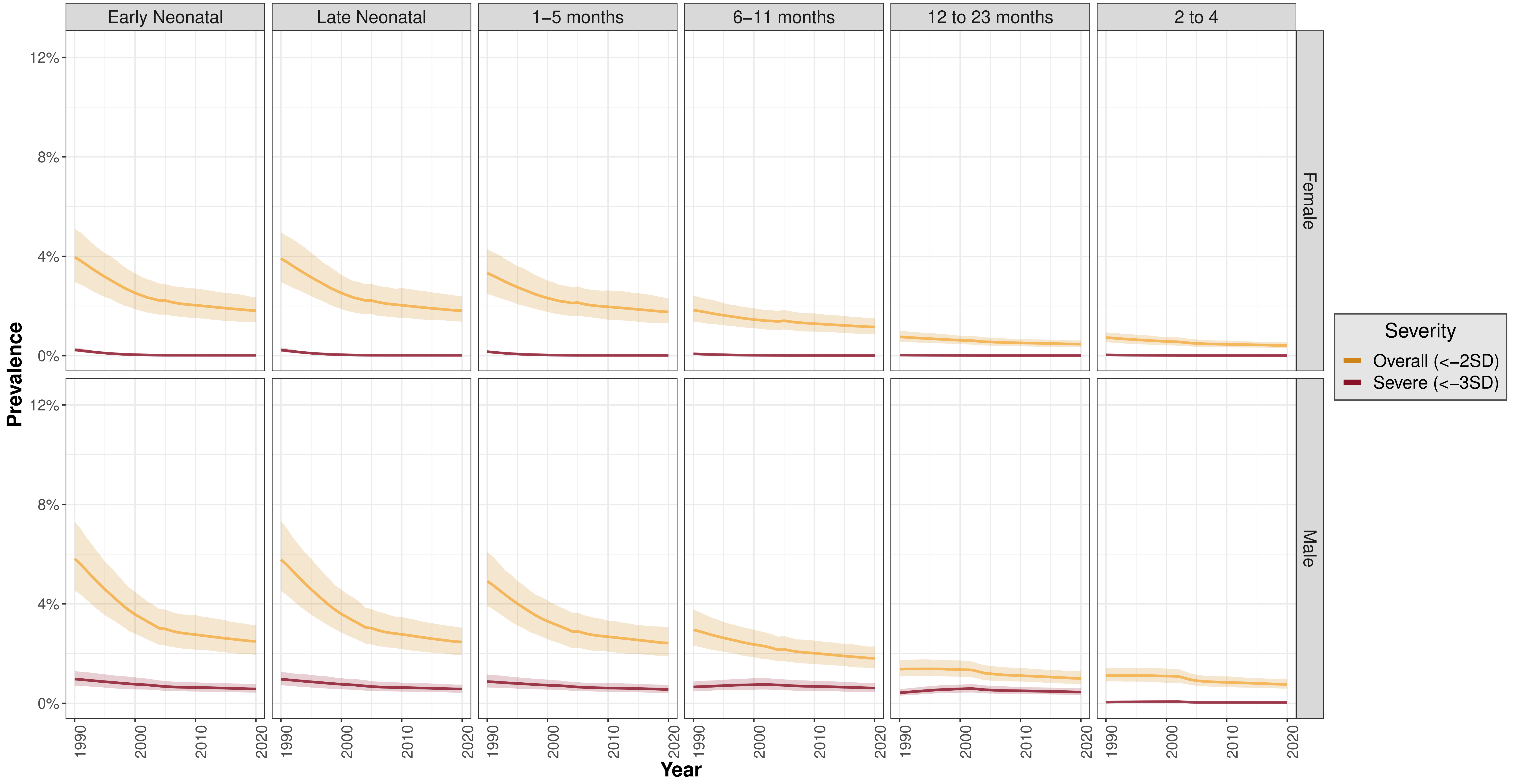

E: Transformed Mean Wasting Z Scores

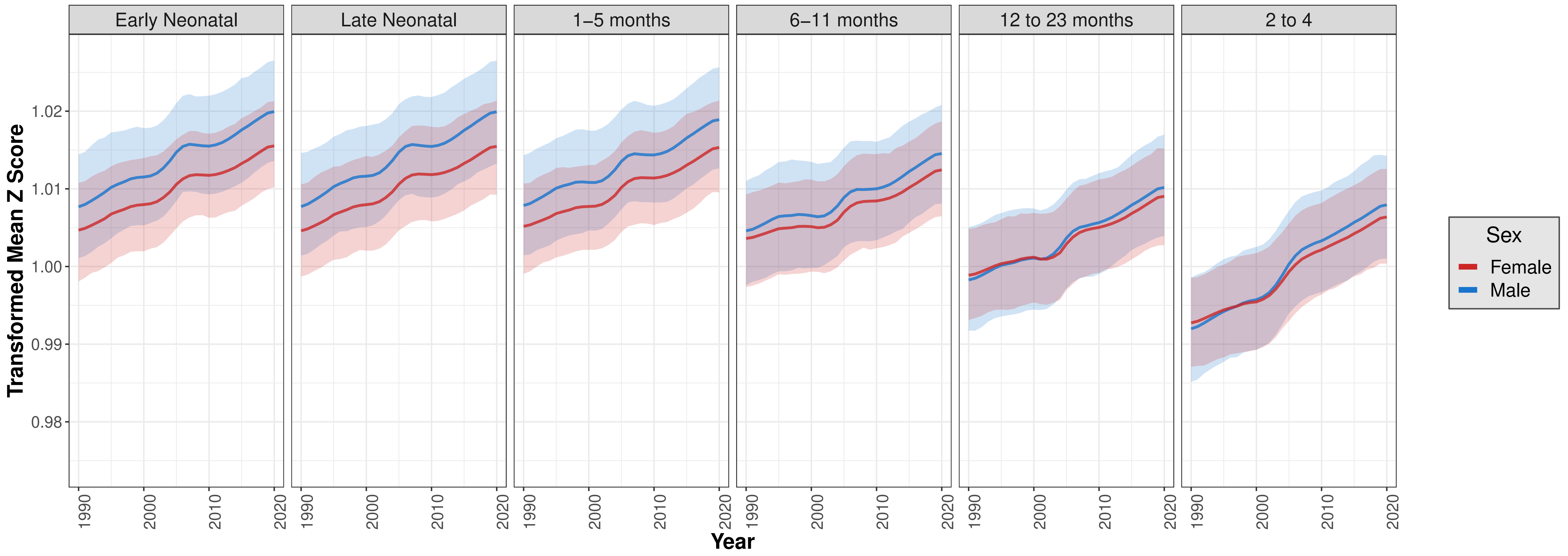

F

Source
No sources for this location

Ireland – Underweight (WAZ)

G: Overall and Severe Underweight Prevalence

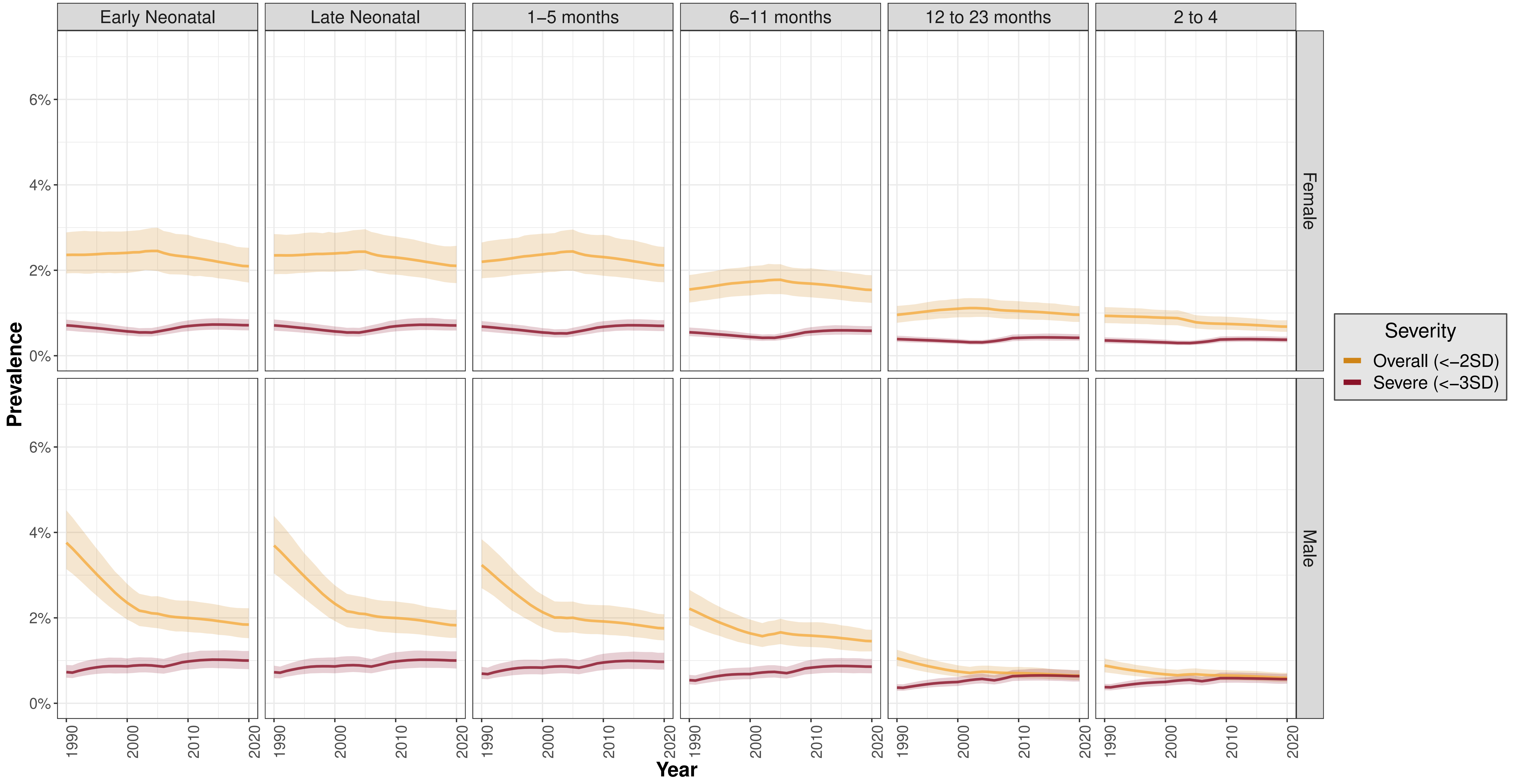

H: Transformed Mean Underweight Z Scores

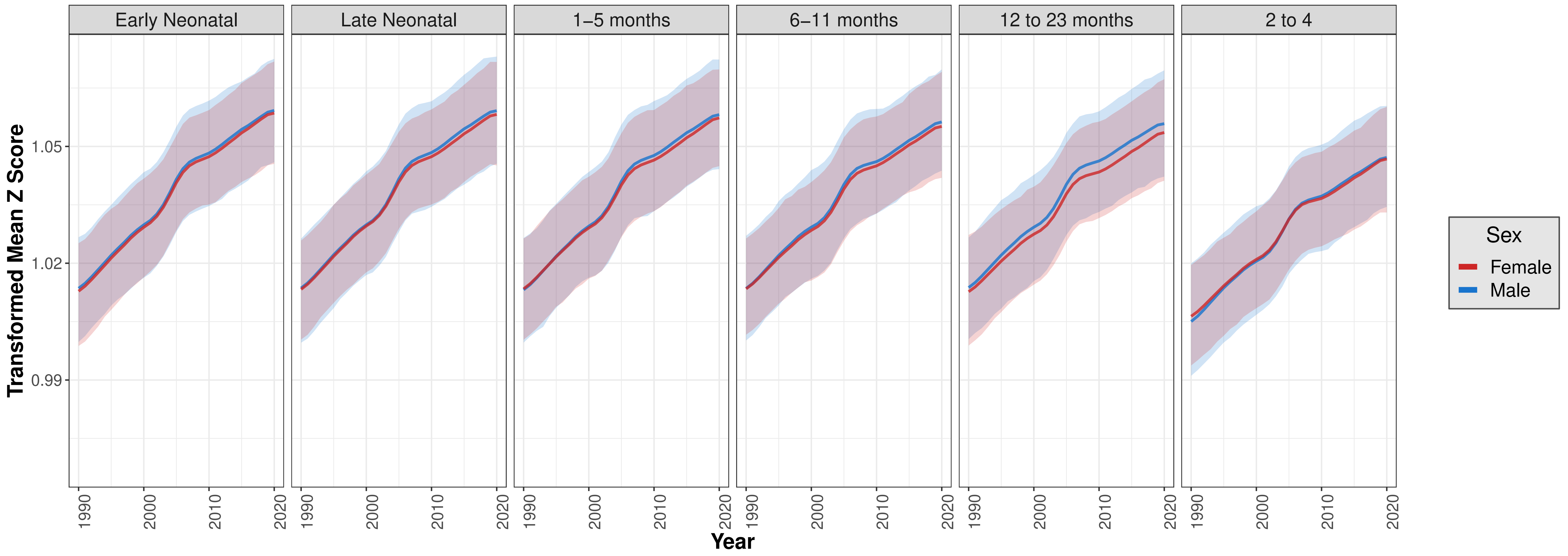

I

Source
No sources for this location

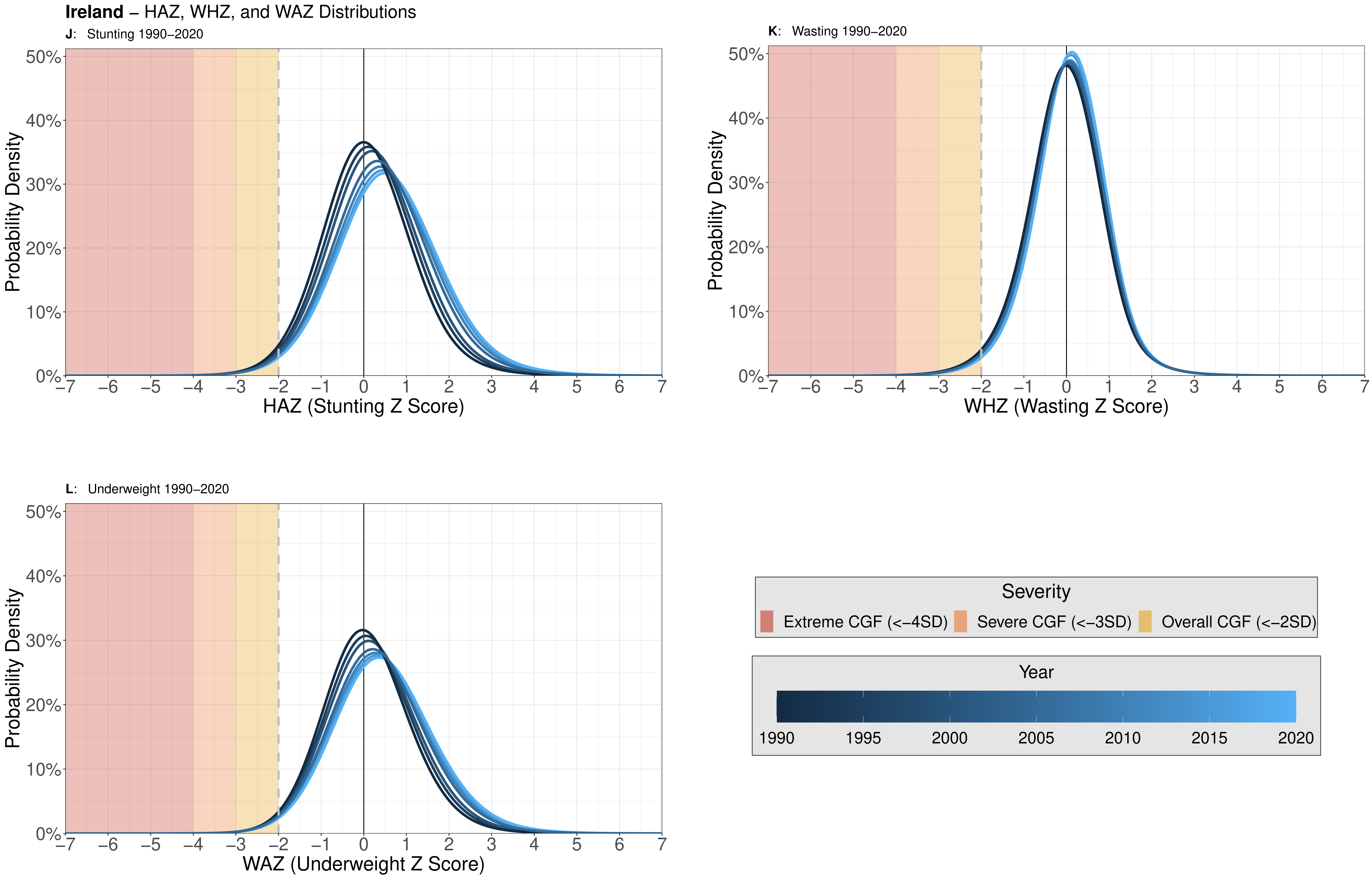
Ireland – HAZ, WHZ, and WAZ Distributions
J: Stunting 1990–2020
K: Wasting 1990–2020
L: Underweight 1990–2020
Probability Density
0%
10%
20%
30%
40%
50%
−7
−6
−5
−4
−3
−2
−1
0
1
2
3
4
5
6
7
HAZ (Stunting Z Score)
WHZ (Wasting Z Score)
WAZ (Underweight Z Score)
Severity
Extreme CGF (<−4SD)
Severe CGF (<−3SD)
Overall CGF (<−2SD)
Year
1990
1995
2000
2005
2010
2015
2020

Israel – Stunting (HAZ)

A: Overall and Severe Stunting Prevalence

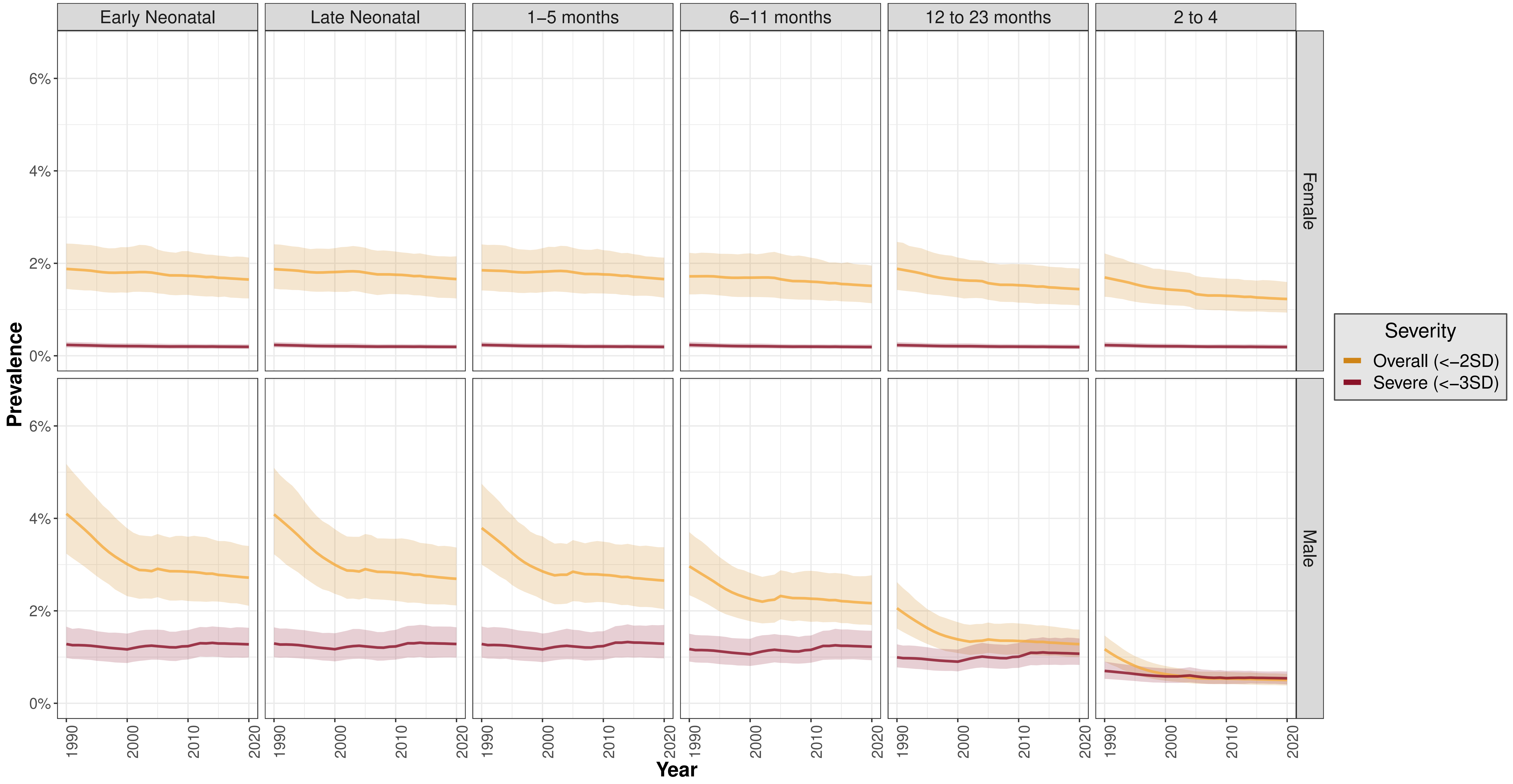

B: Transformed Mean Stunting Z Scores

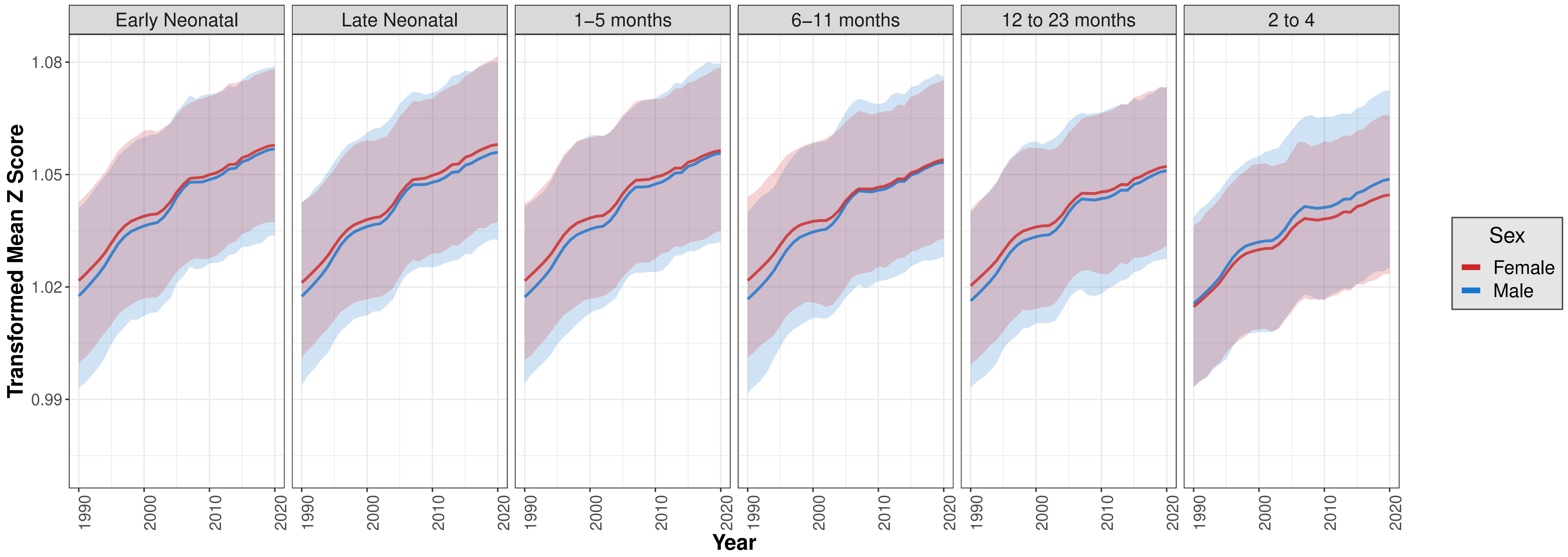

C

Source
No sources for this location

Israel – Wasting (WHZ)

D: Overall and Severe Wasting Prevalence

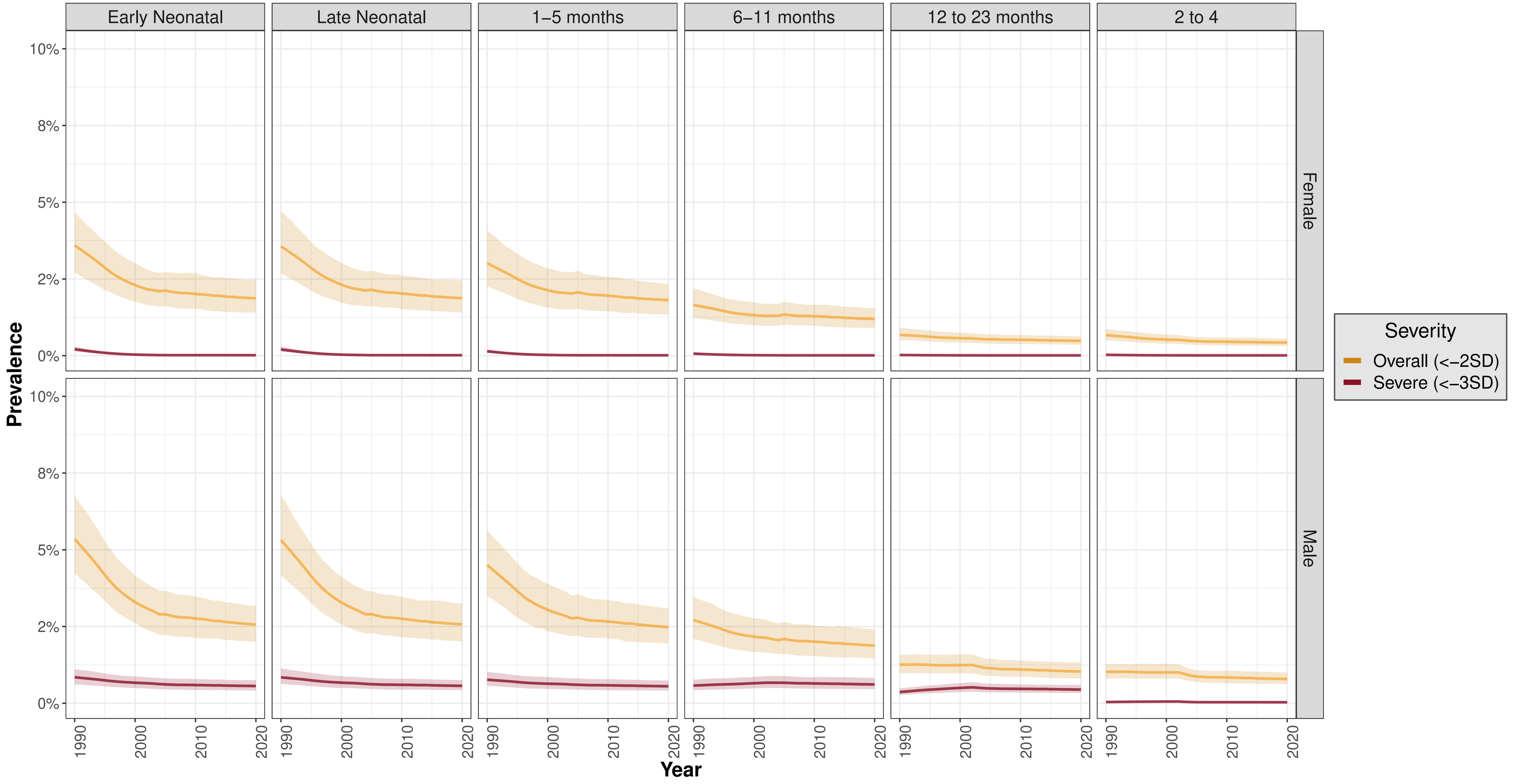

E: Transformed Mean Wasting Z Scores

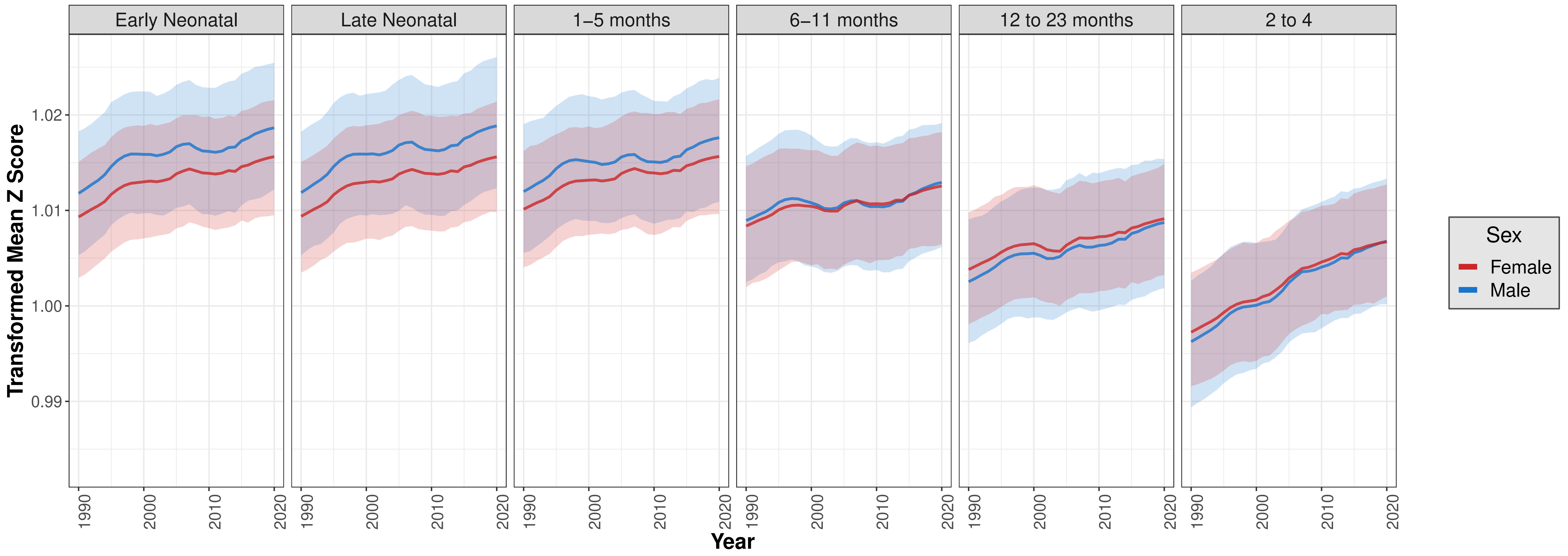

F

Source
No sources for this location

Israel – Underweight (WAZ)

G: Overall and Severe Underweight Prevalence

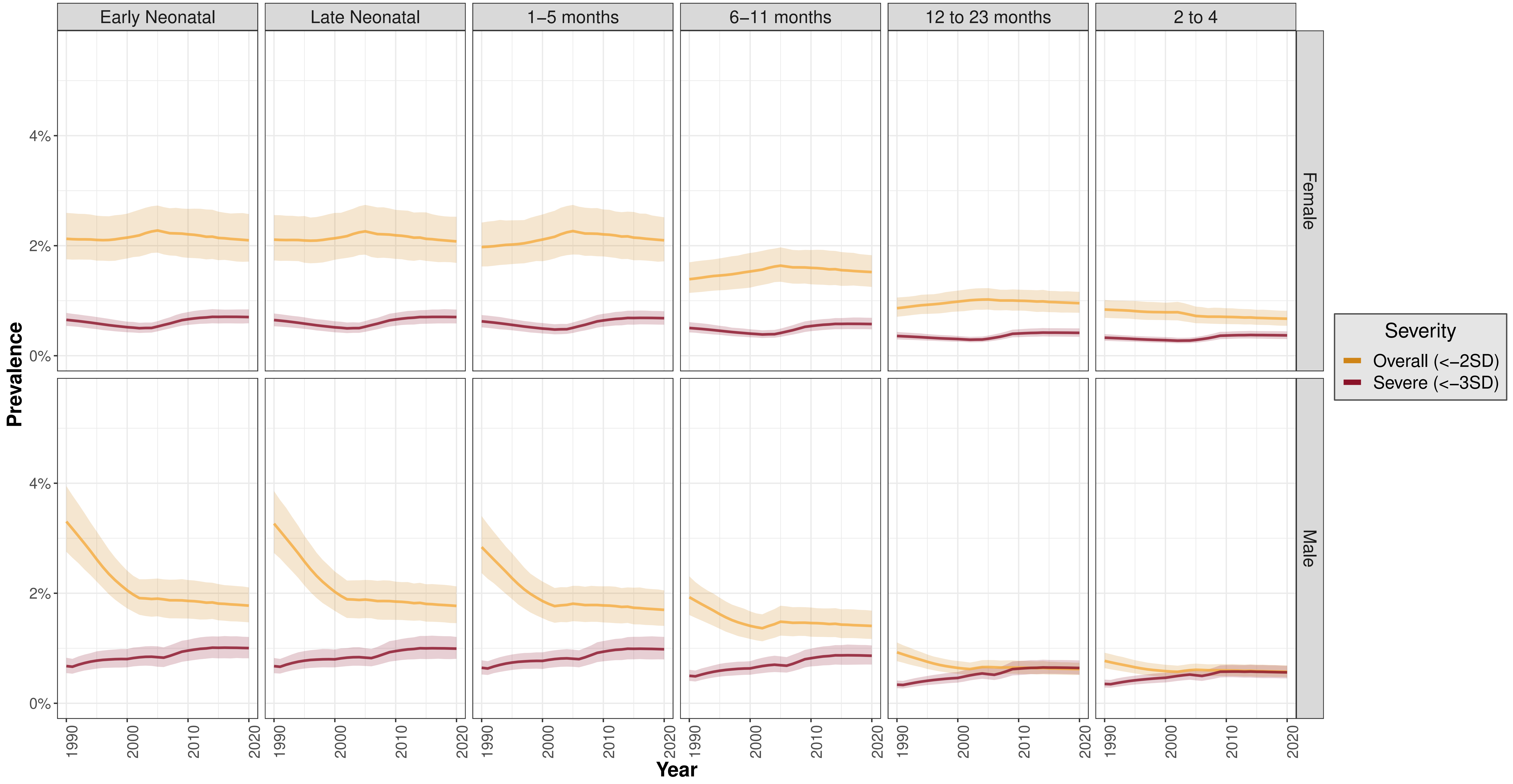

H: Transformed Mean Underweight Z Scores

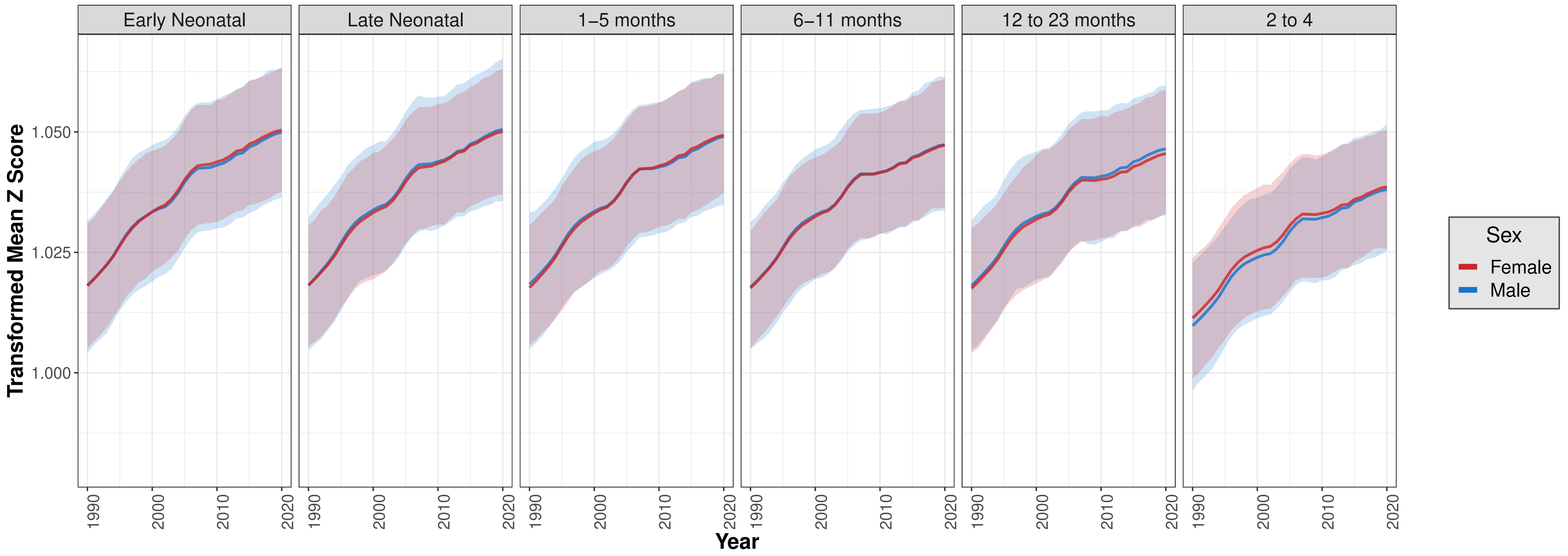

I

Source
No sources for this location

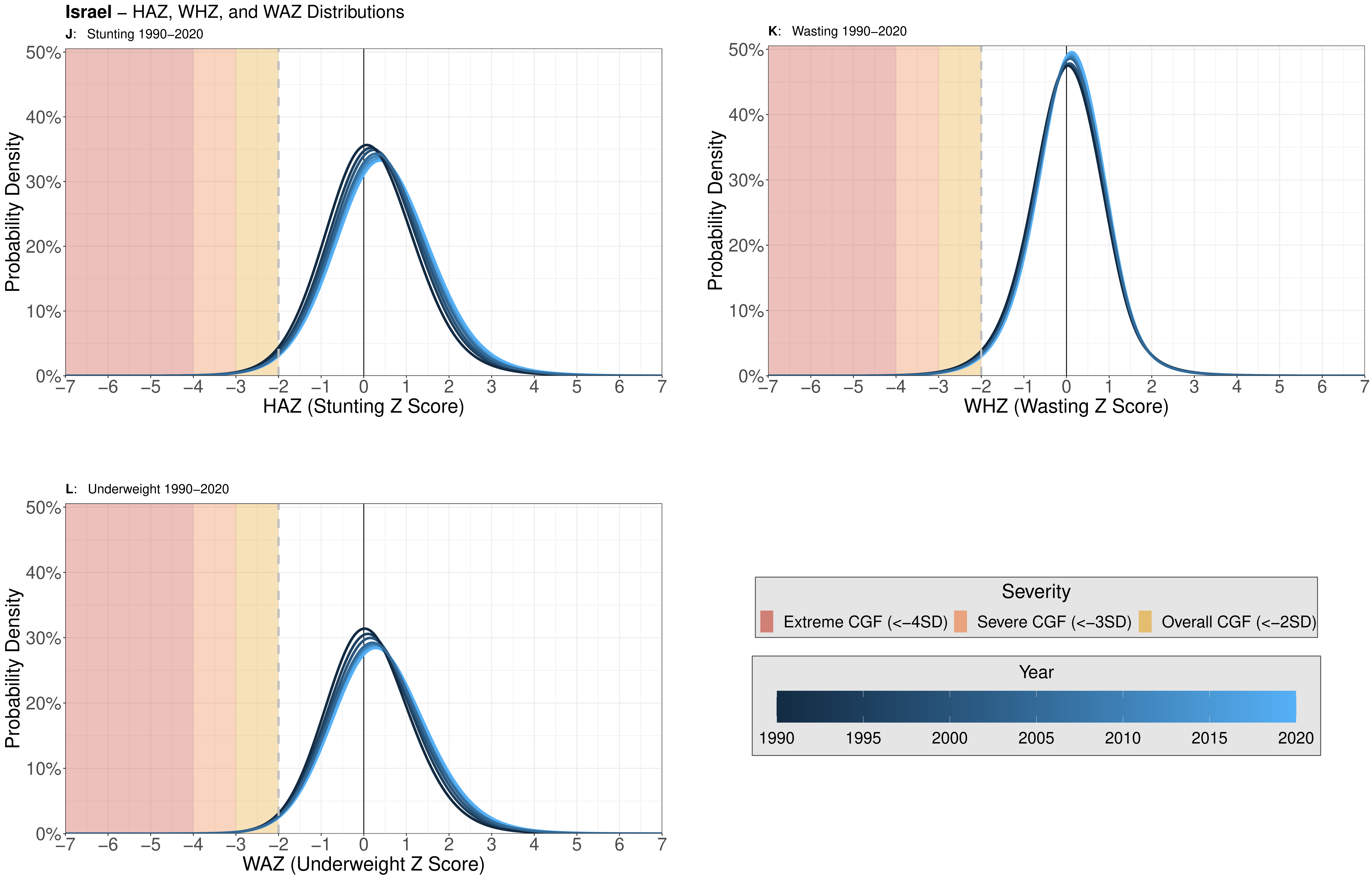
Israel – HAZ, WHZ, and WAZ Distributions
J: Stunting 1990–2020
Probability Density
50%
40%
30%
20%
10%
0%
−7 −6 −5 −4 −3 −2 −1 0 1 2 3 4 5 6 7
HAZ (Stunting Z Score)
K: Wasting 1990–2020
Probability Density
WHZ (Wasting Z Score)
L: Underweight 1990–2020
Probability Density
WAZ (Underweight Z Score)
Severity
Extreme CGF (<−4SD)
Severe CGF (<−3SD)
Overall CGF (<−2SD)
Year
1990
1995
2000
2005
2010
2015
2020

Italy – Stunting (HAZ)

A: Overall and Severe Stunting Prevalence

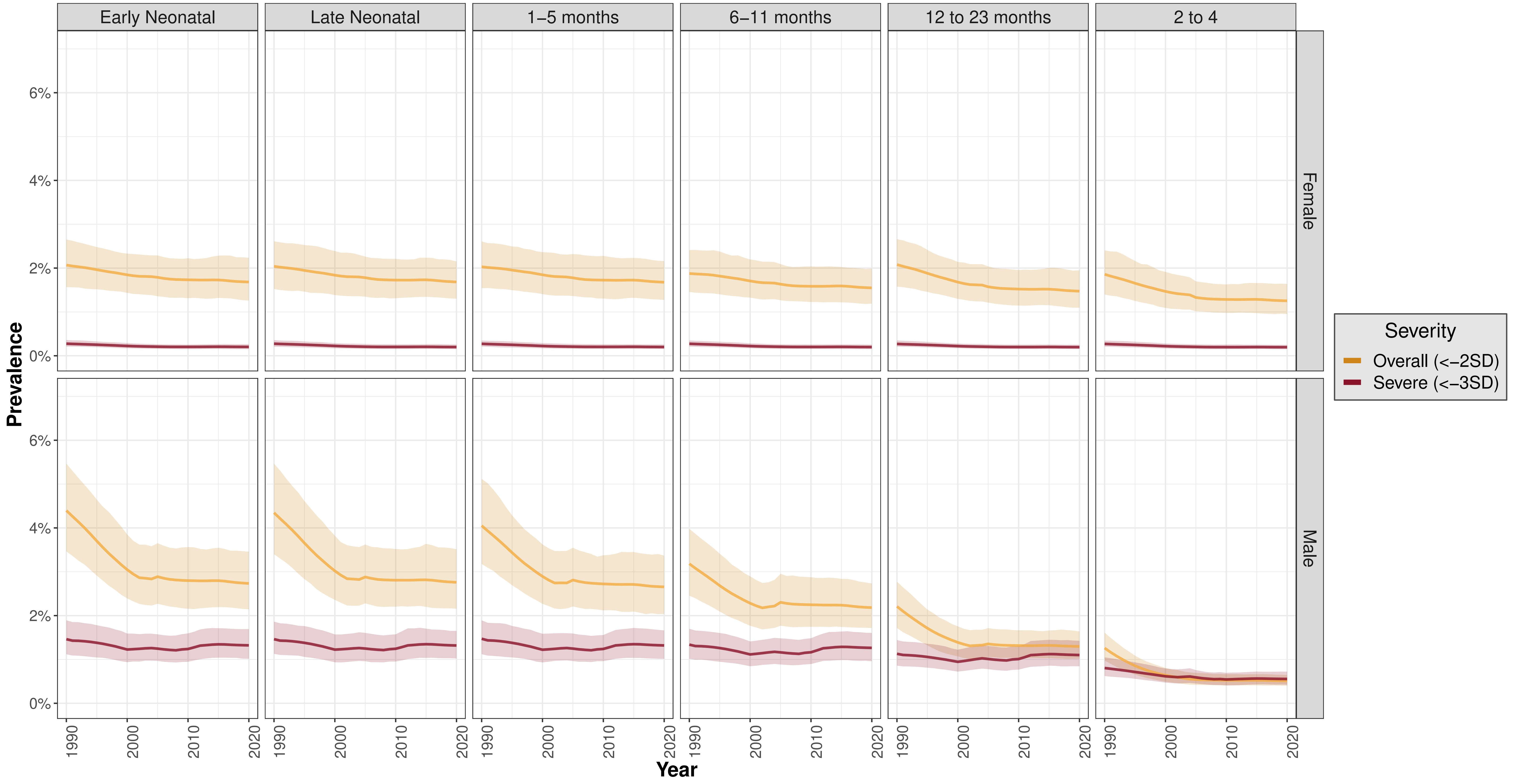

B: Transformed Mean Stunting Z Scores

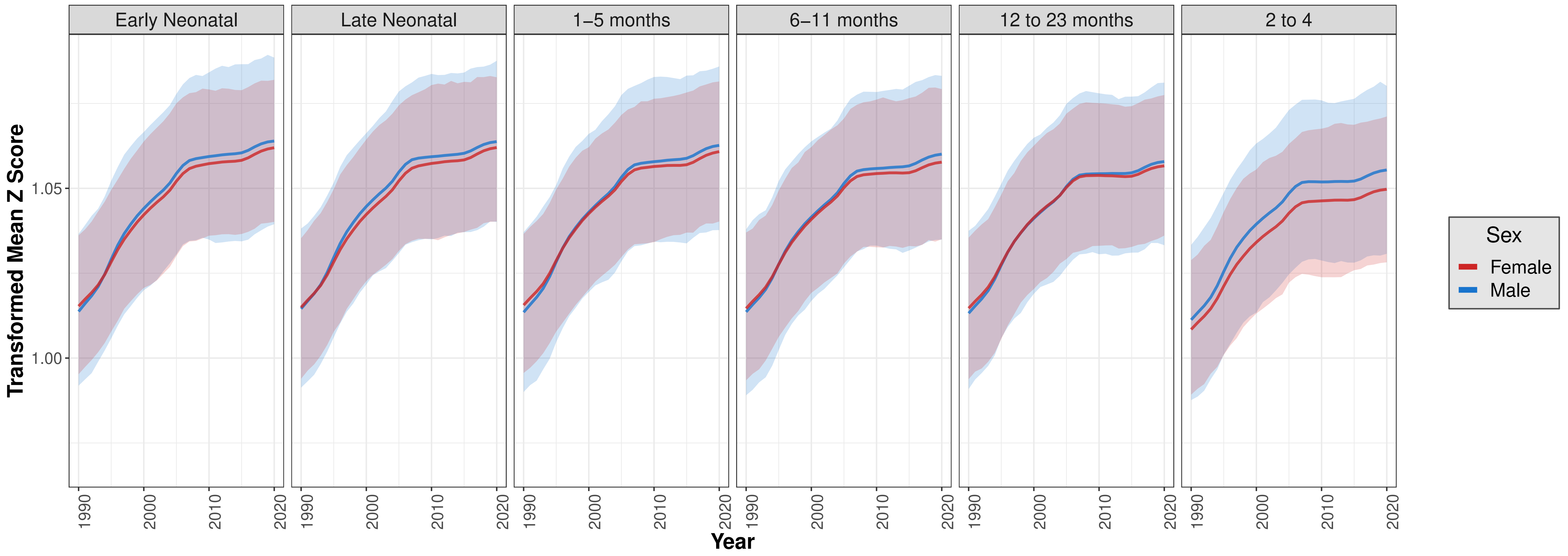

C

Source
No national or subnational sources for this location

Italy – Wasting (WHZ)

D: Overall and Severe Wasting Prevalence

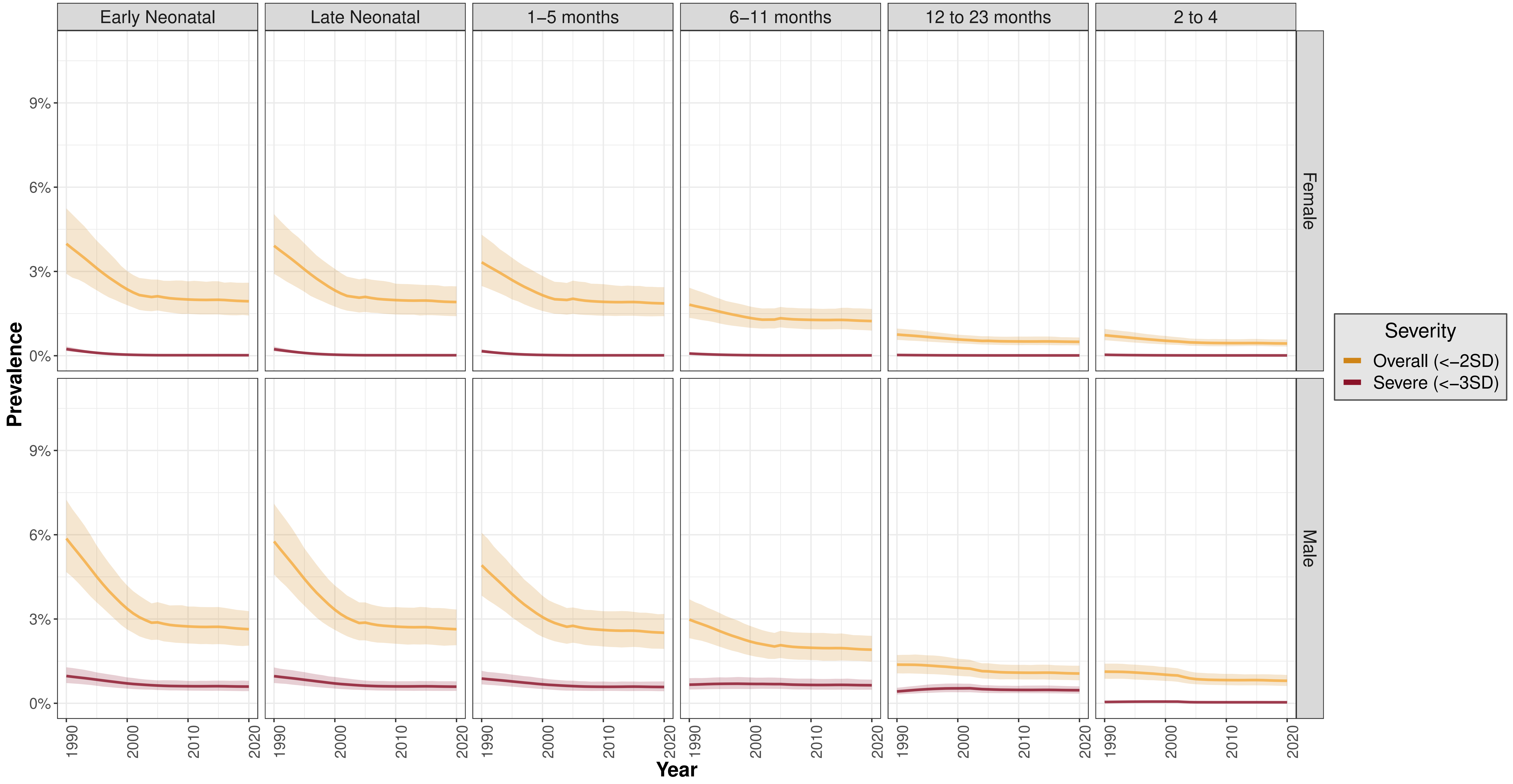

E: Transformed Mean Wasting Z Scores

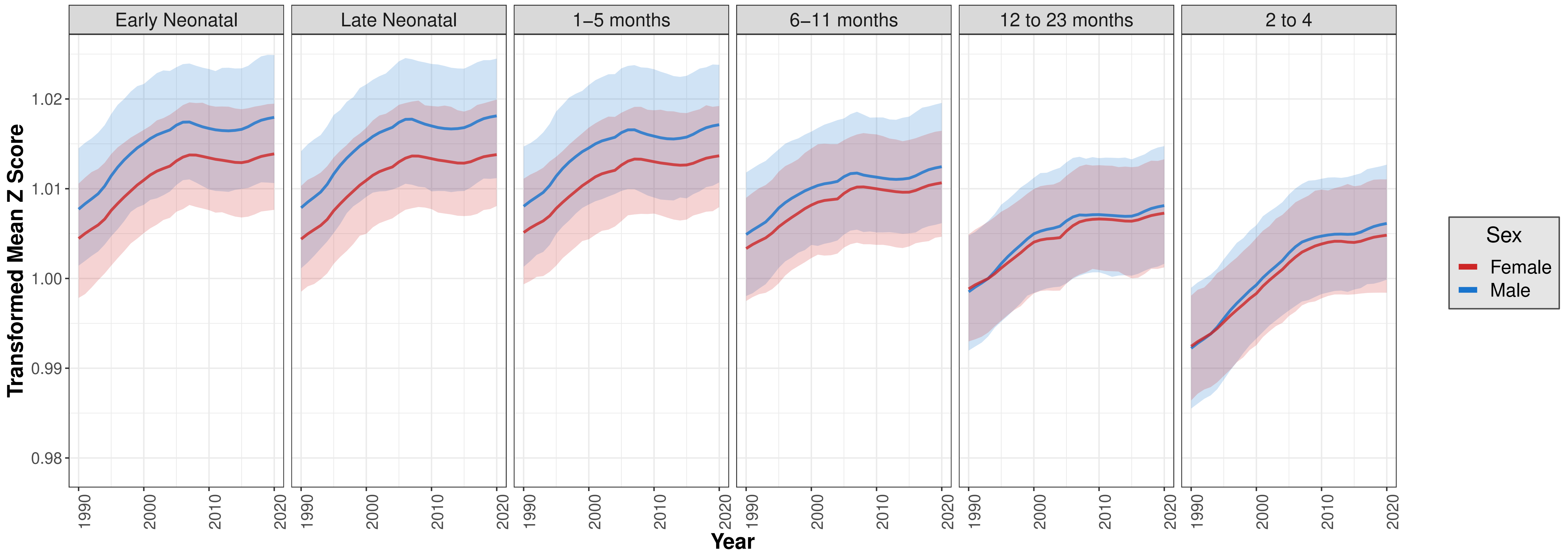

F

Source
No national or subnational sources for this location

Italy – Underweight (WAZ)

G: Overall and Severe Underweight Prevalence

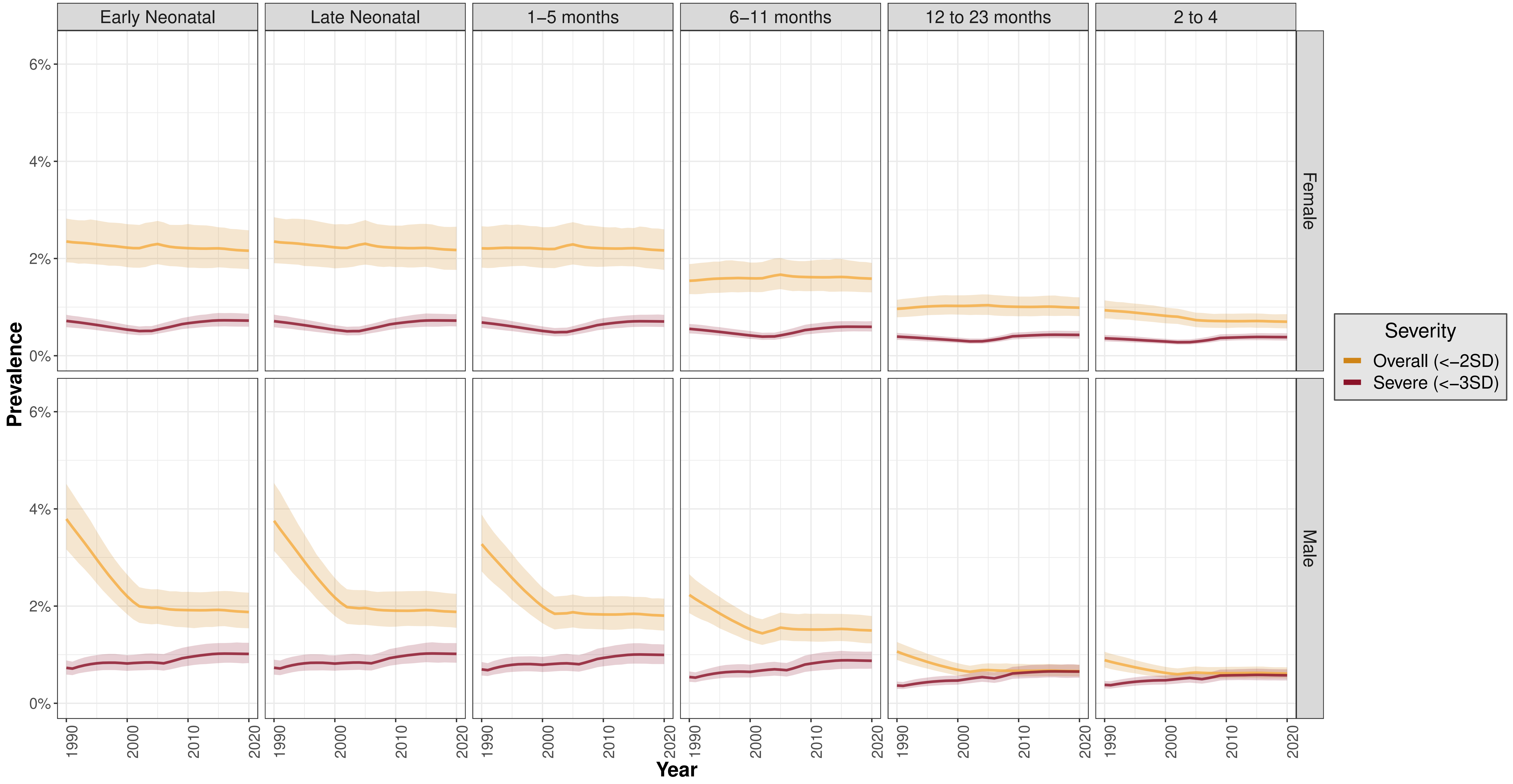

H: Transformed Mean Underweight Z Scores

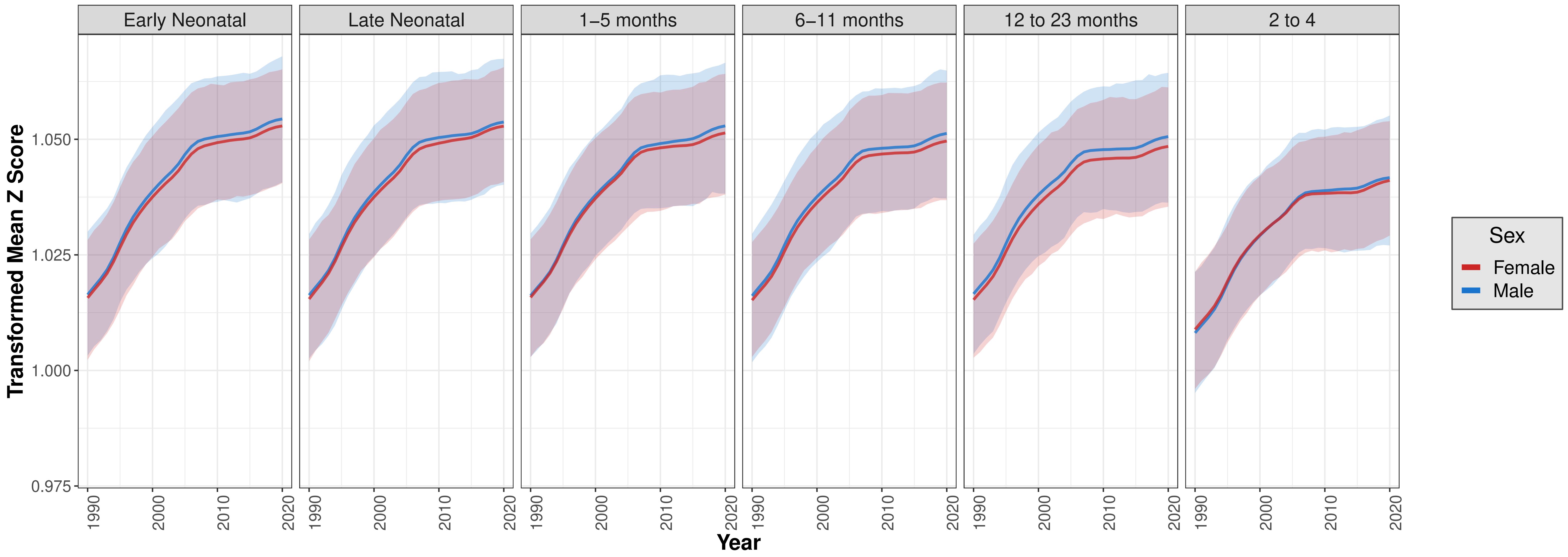

I

Source
No national or subnational sources for this location

Italy – HAZ, WHZ, and WAZ Distributions

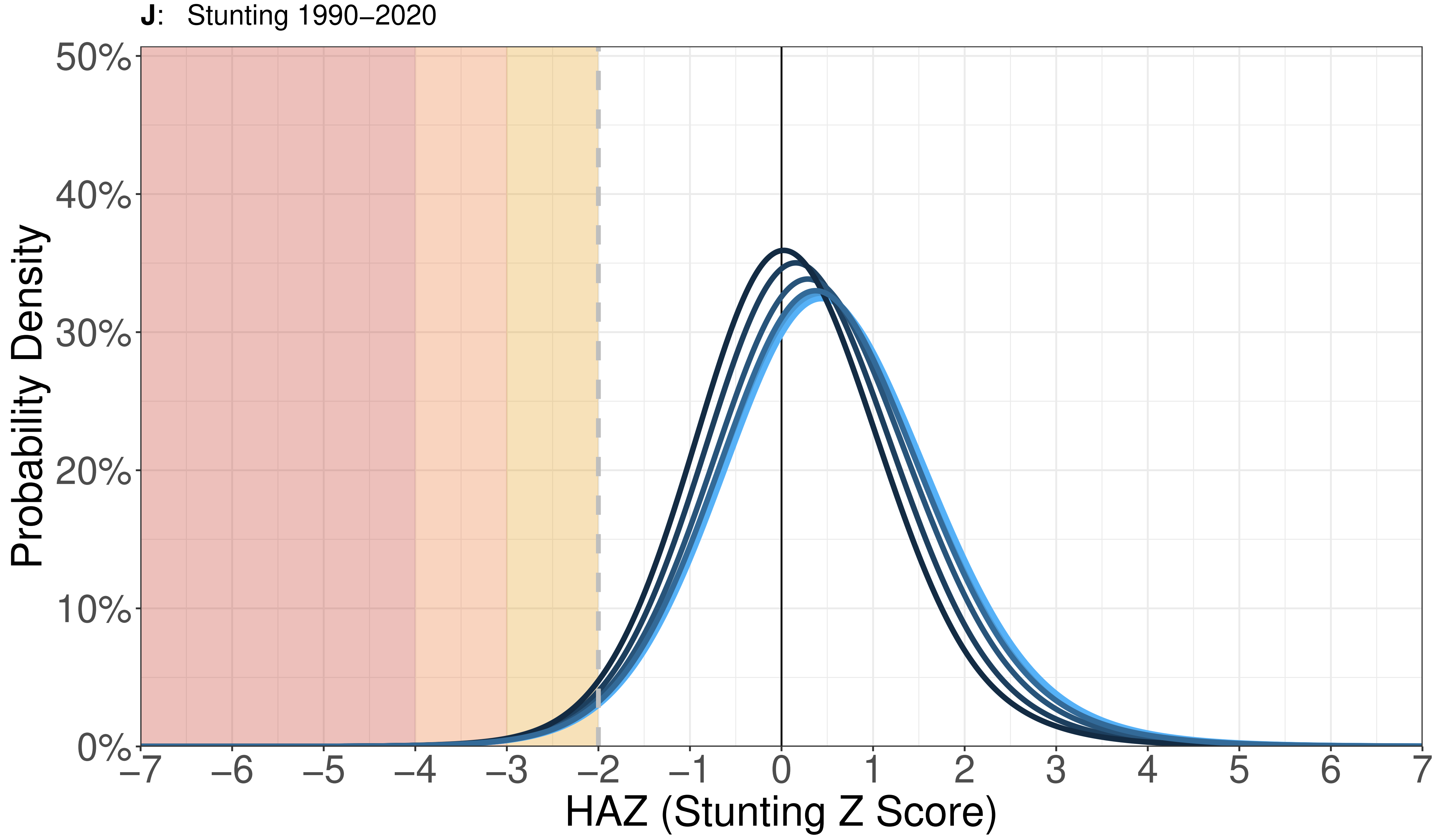

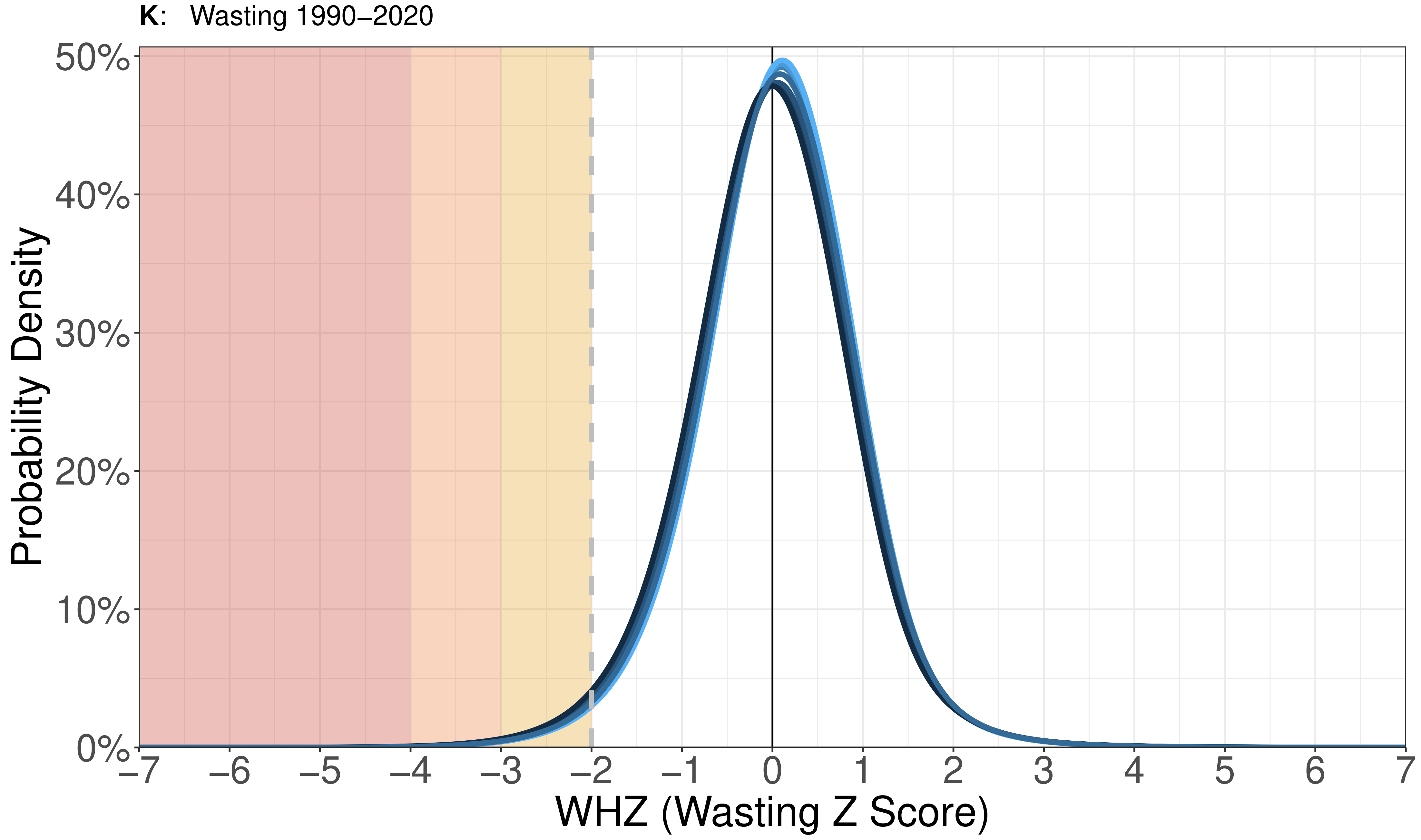

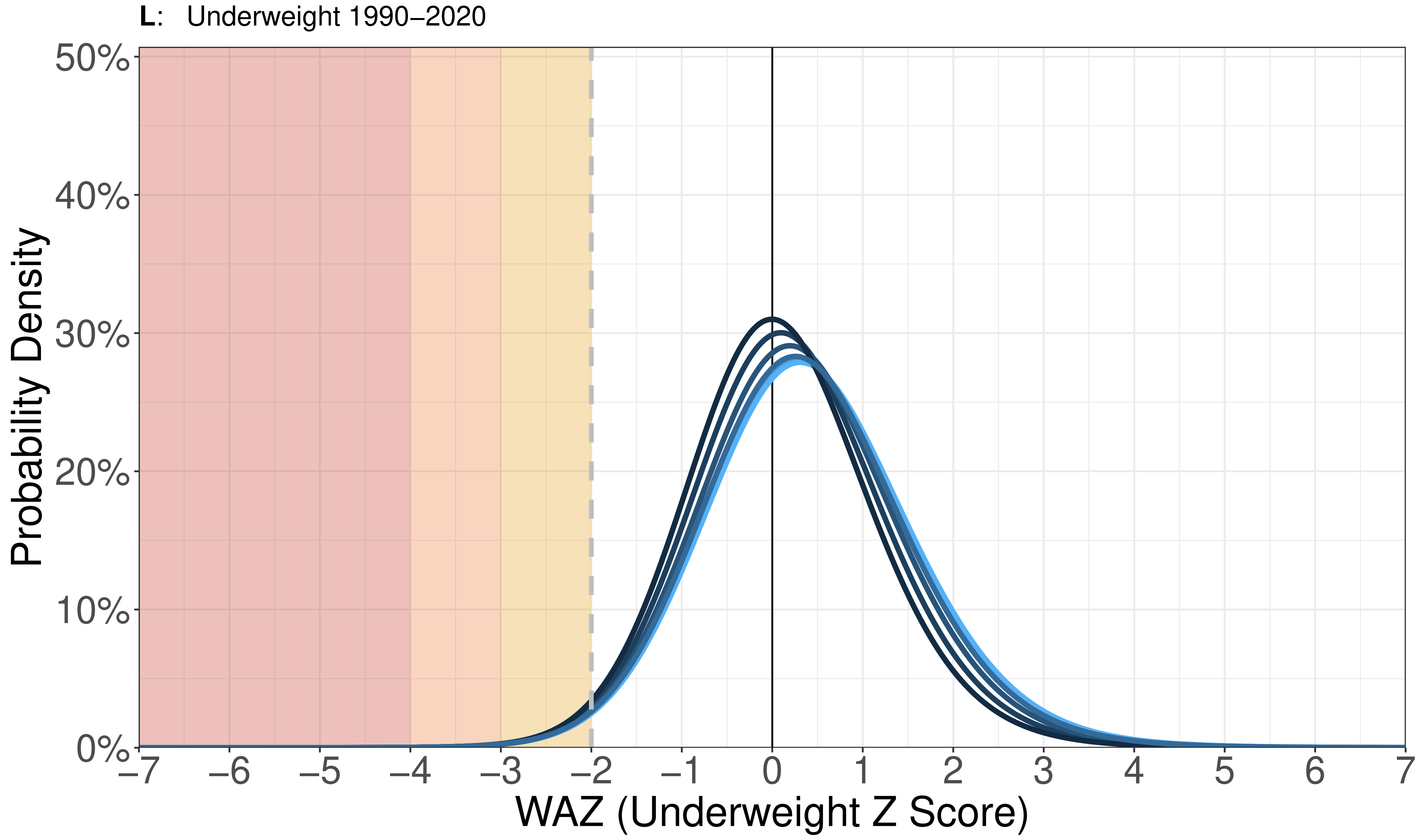

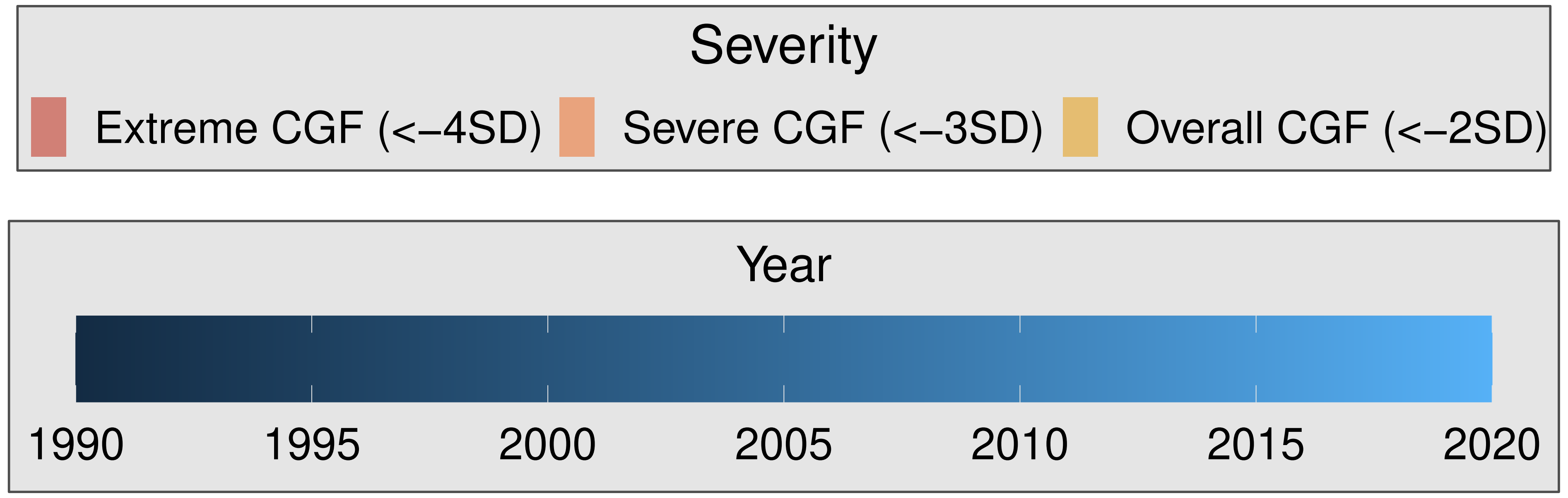

Luxembourg – Stunting (HAZ)

A: Overall and Severe Stunting Prevalence

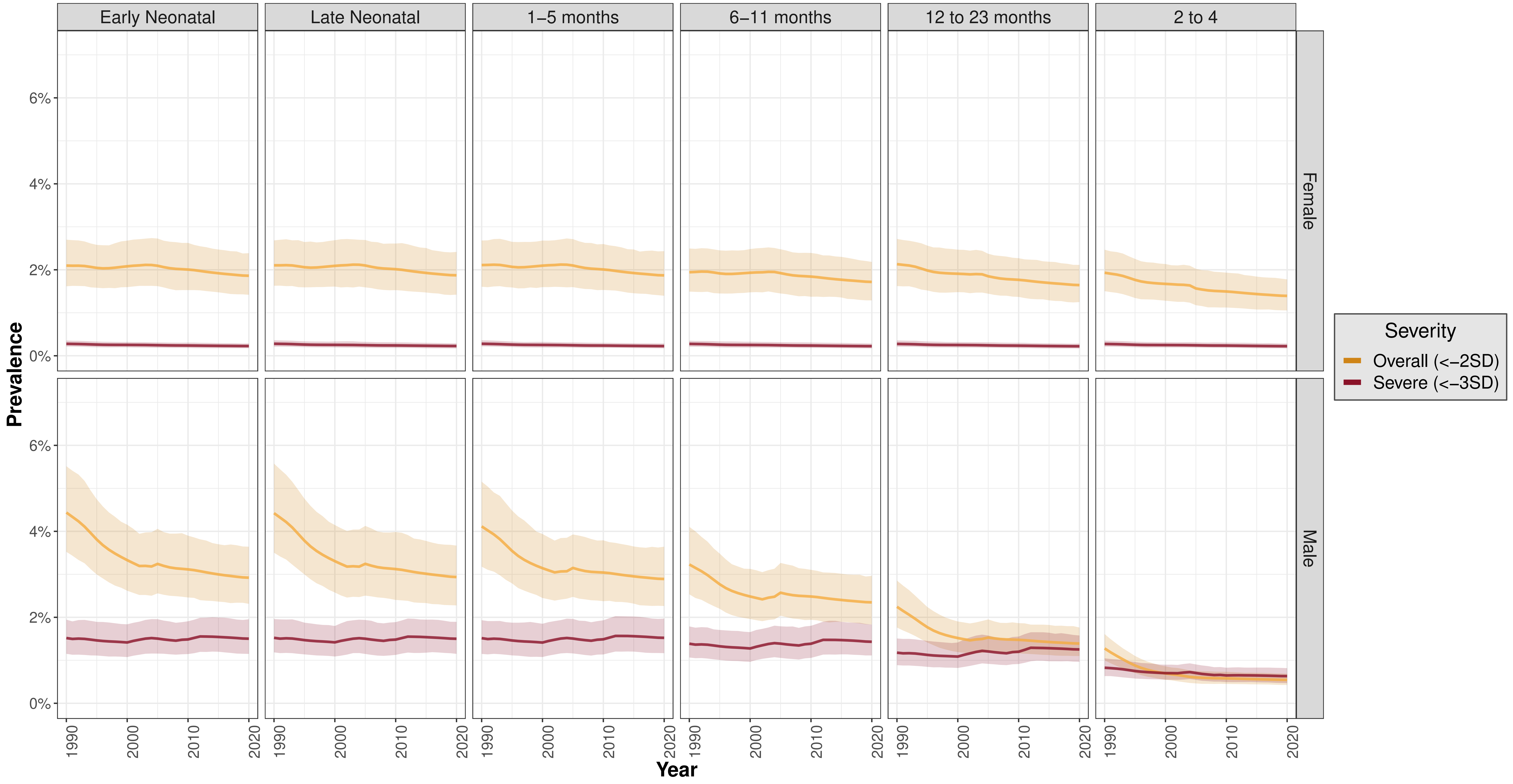

B: Transformed Mean Stunting Z Scores

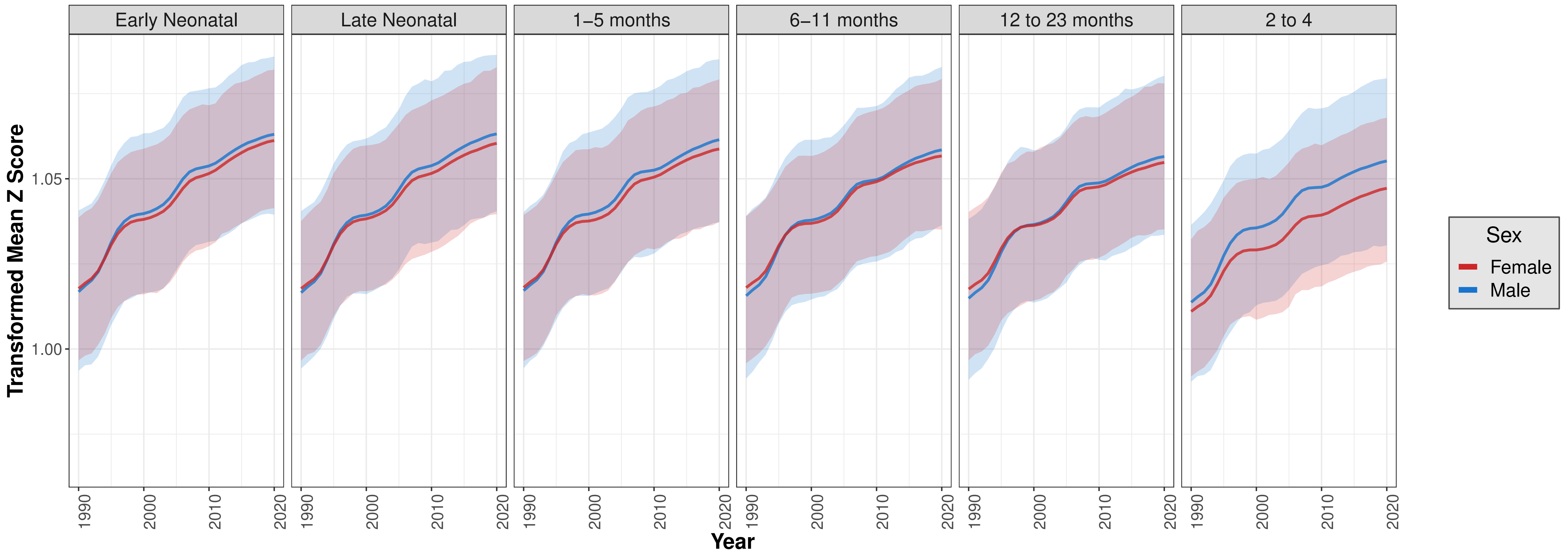

C

Source
No sources for this location

Luxembourg – Wasting (WHZ)

D: Overall and Severe Wasting Prevalence

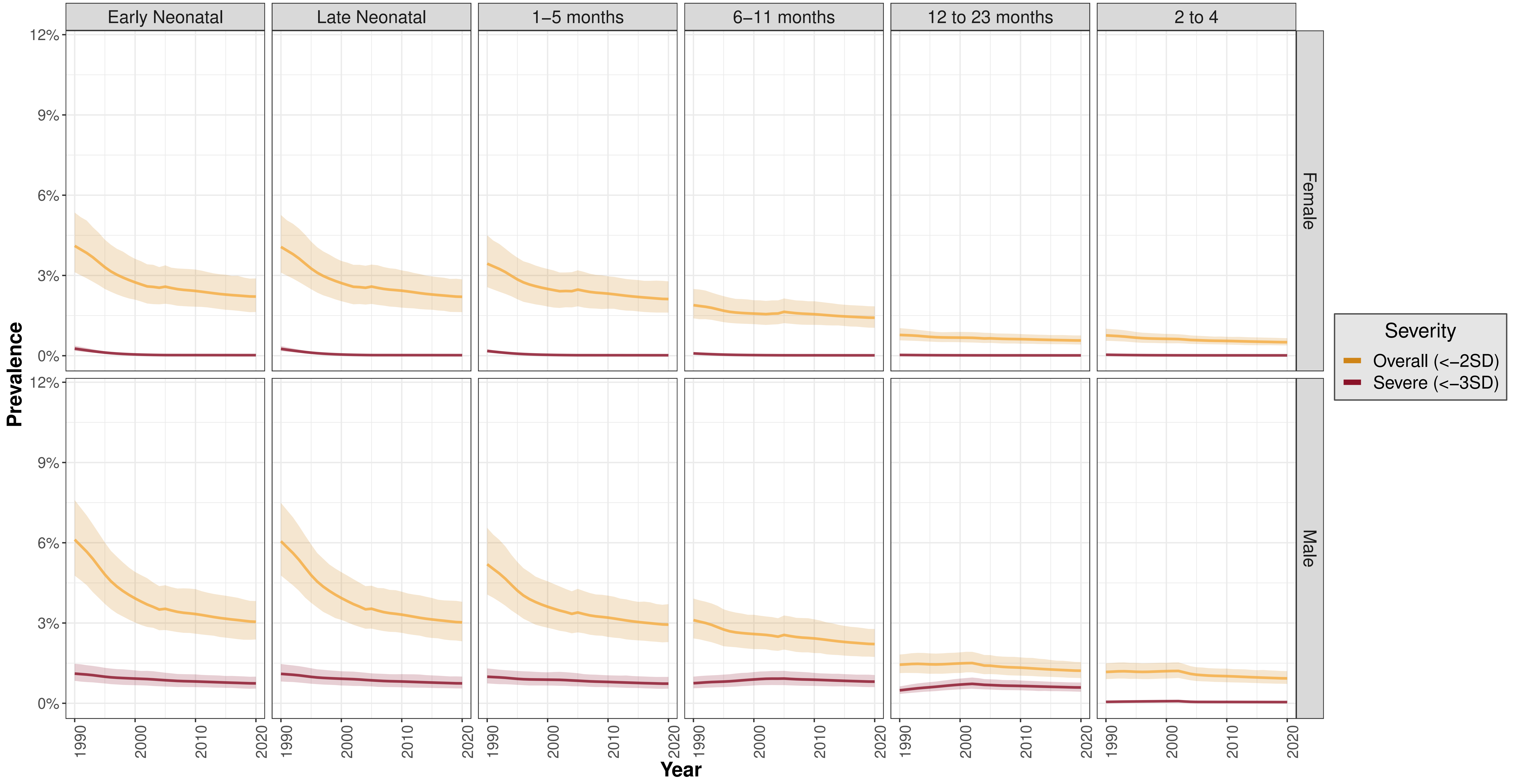

E: Transformed Mean Wasting Z Scores

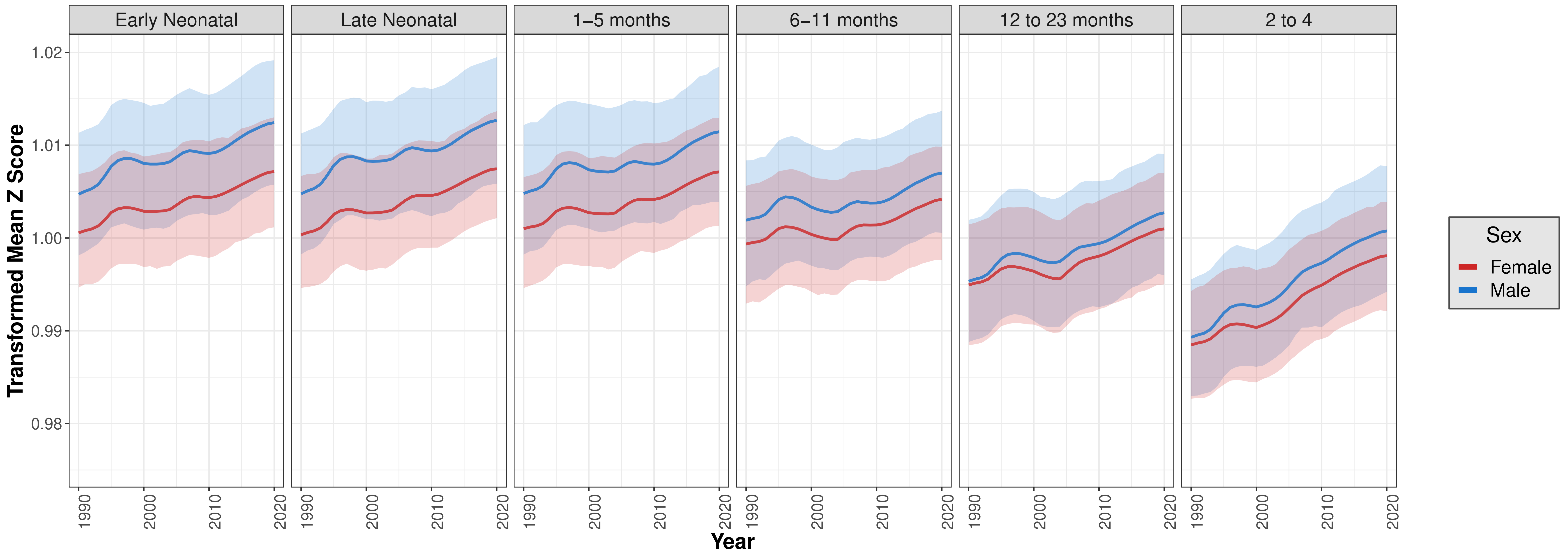

F

Source
No sources for this location

Luxembourg – Underweight (WAZ)

G: Overall and Severe Underweight Prevalence

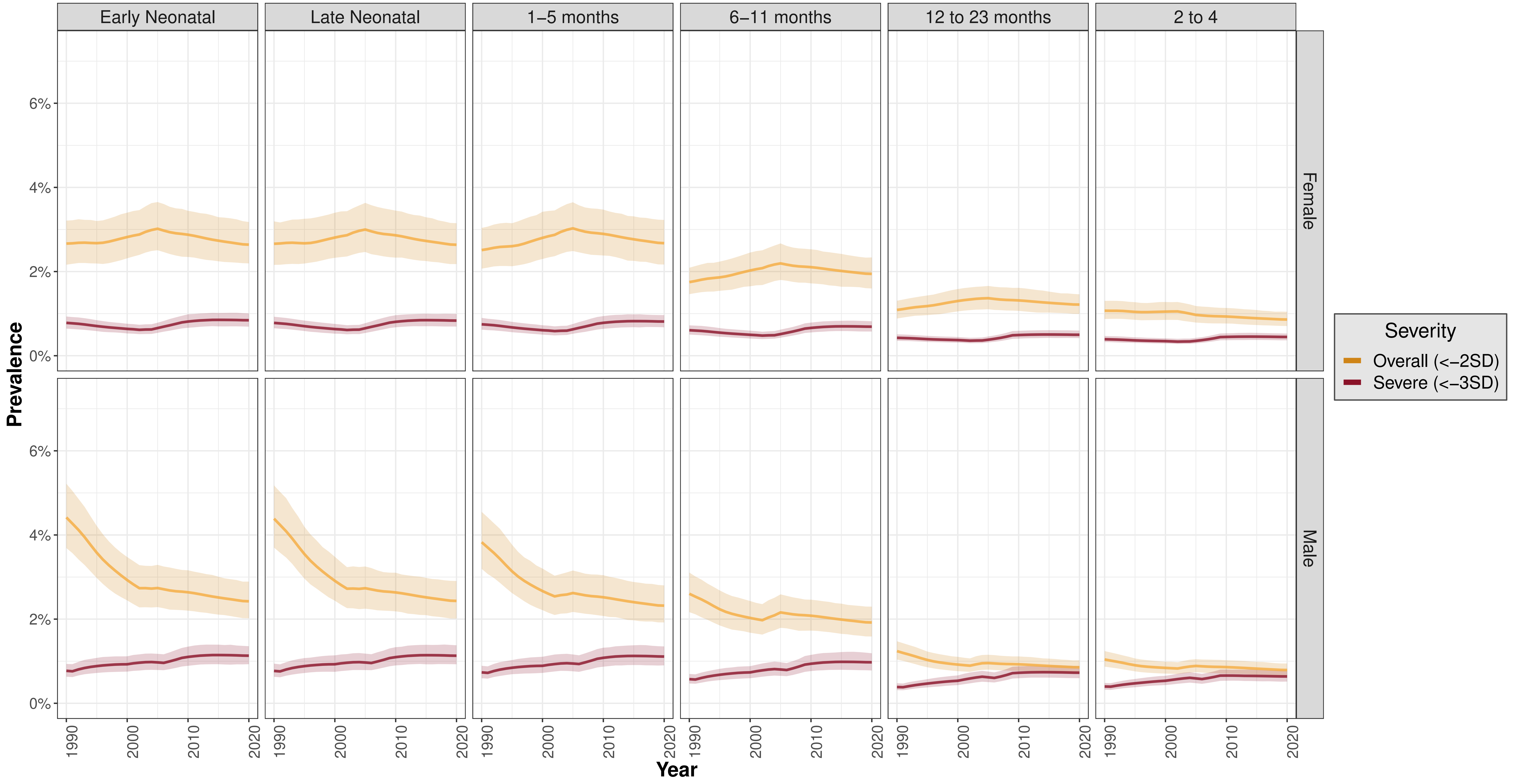

H: Transformed Mean Underweight Z Scores

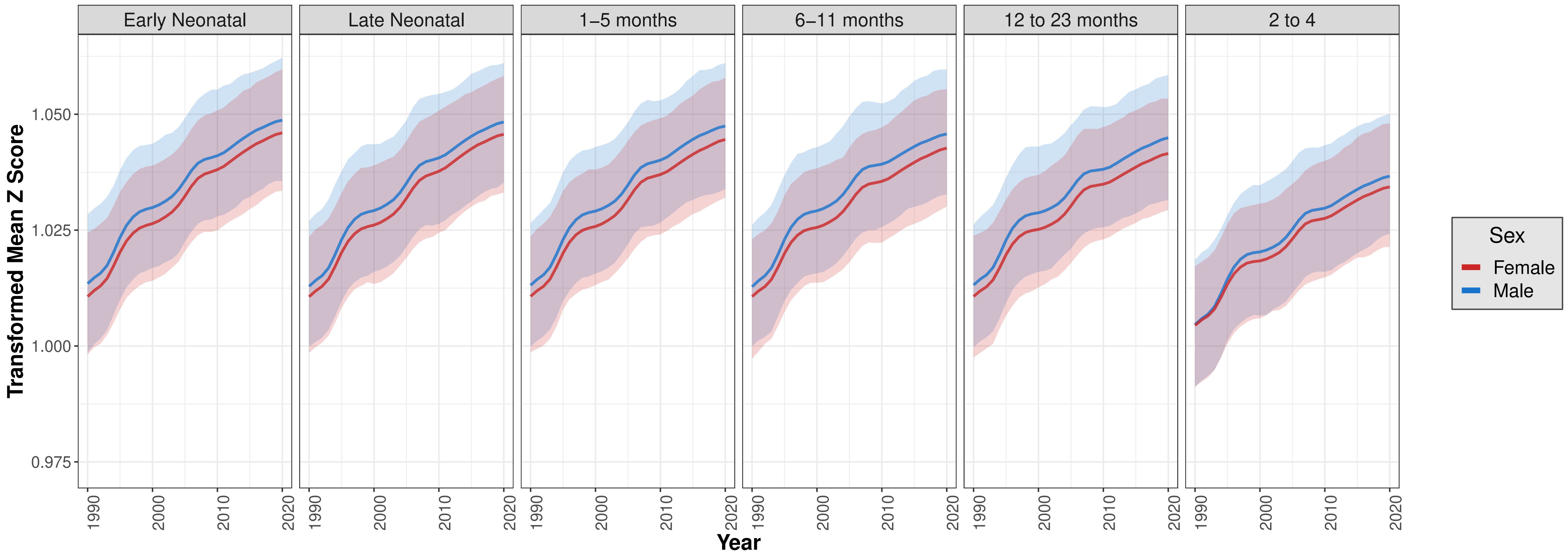

I

Source
No sources for this location

Luxembourg – HAZ, WHZ, and WAZ Distributions

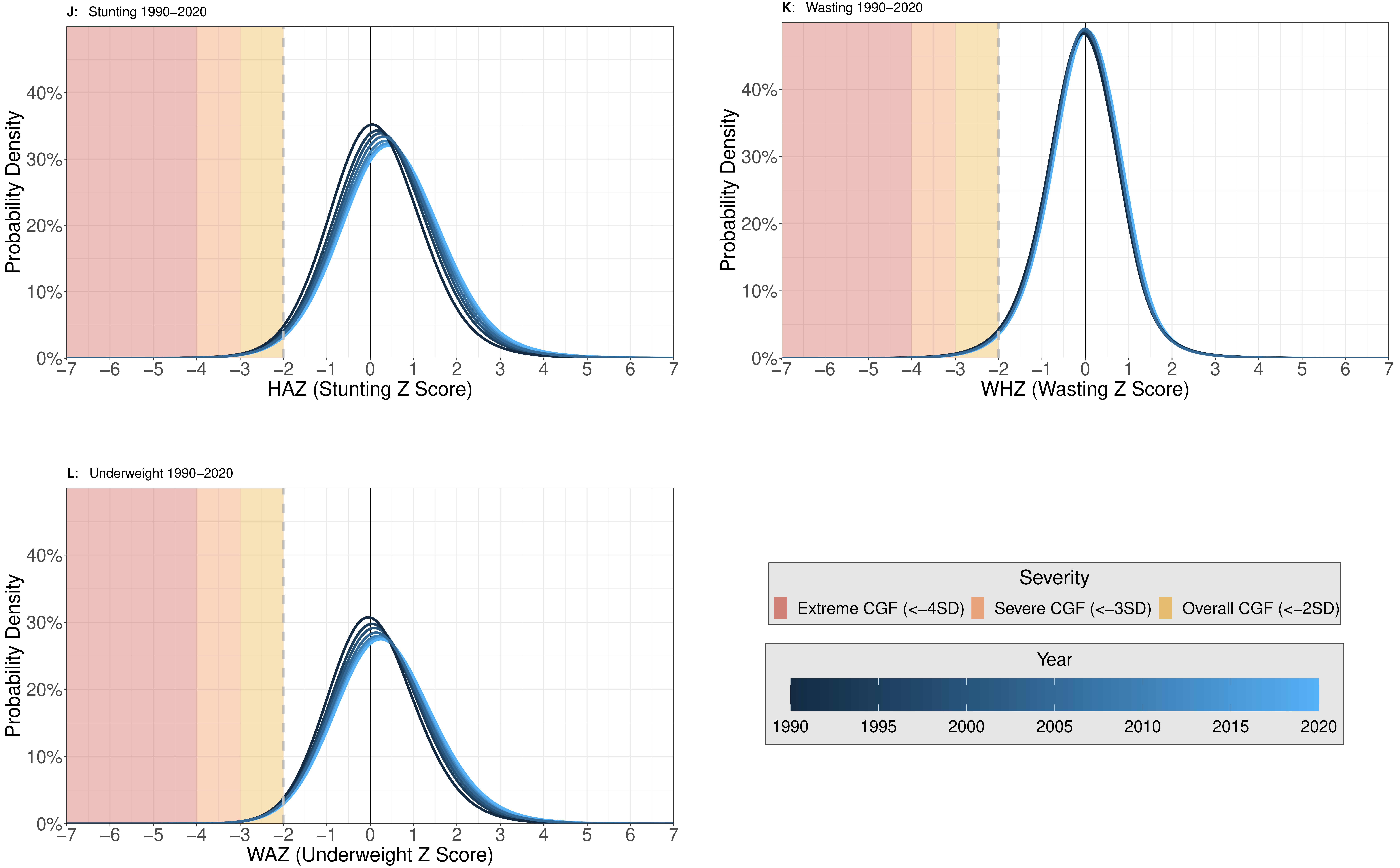

Malta – Stunting (HAZ)

A: Overall and Severe Stunting Prevalence

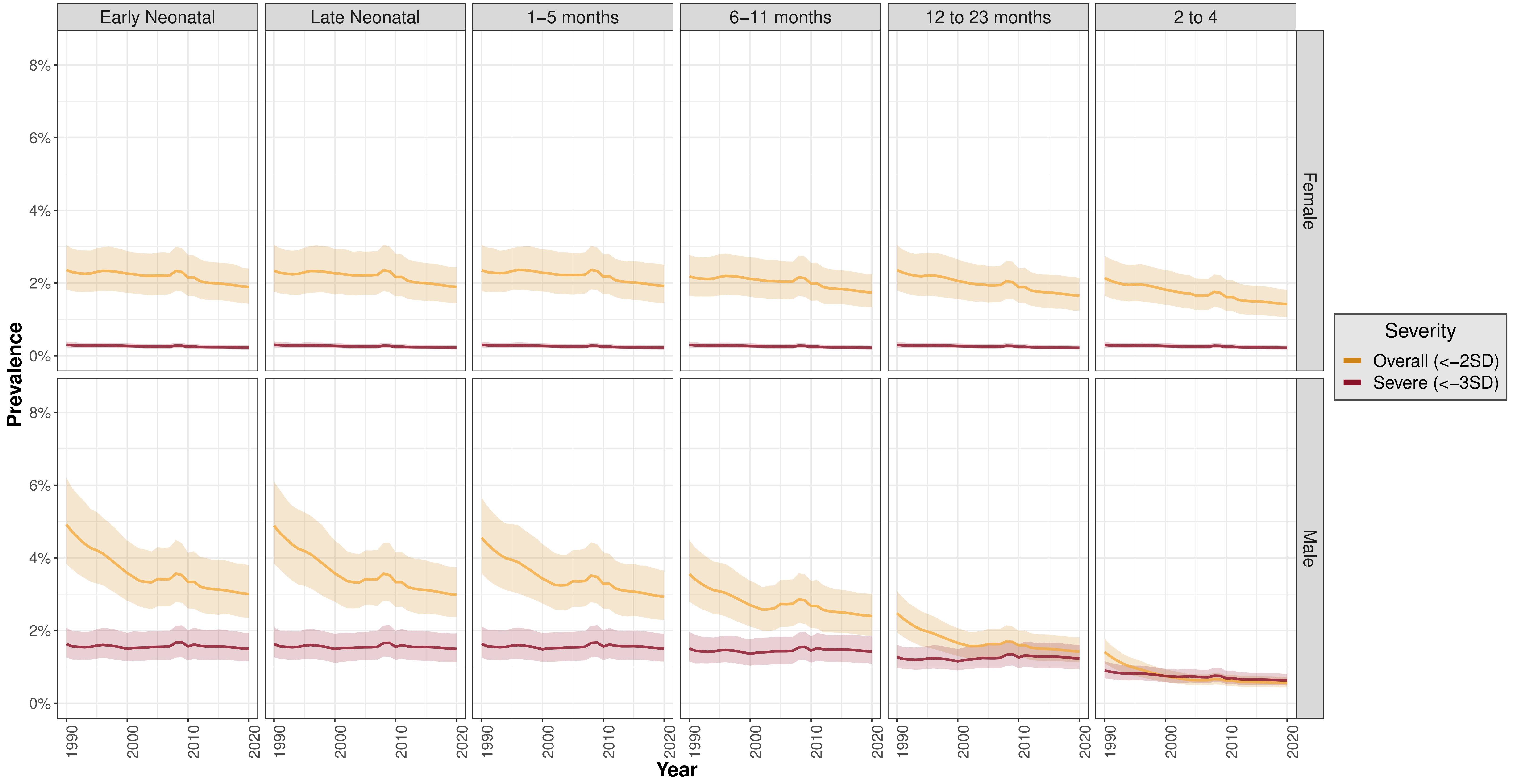

B: Transformed Mean Stunting Z Scores

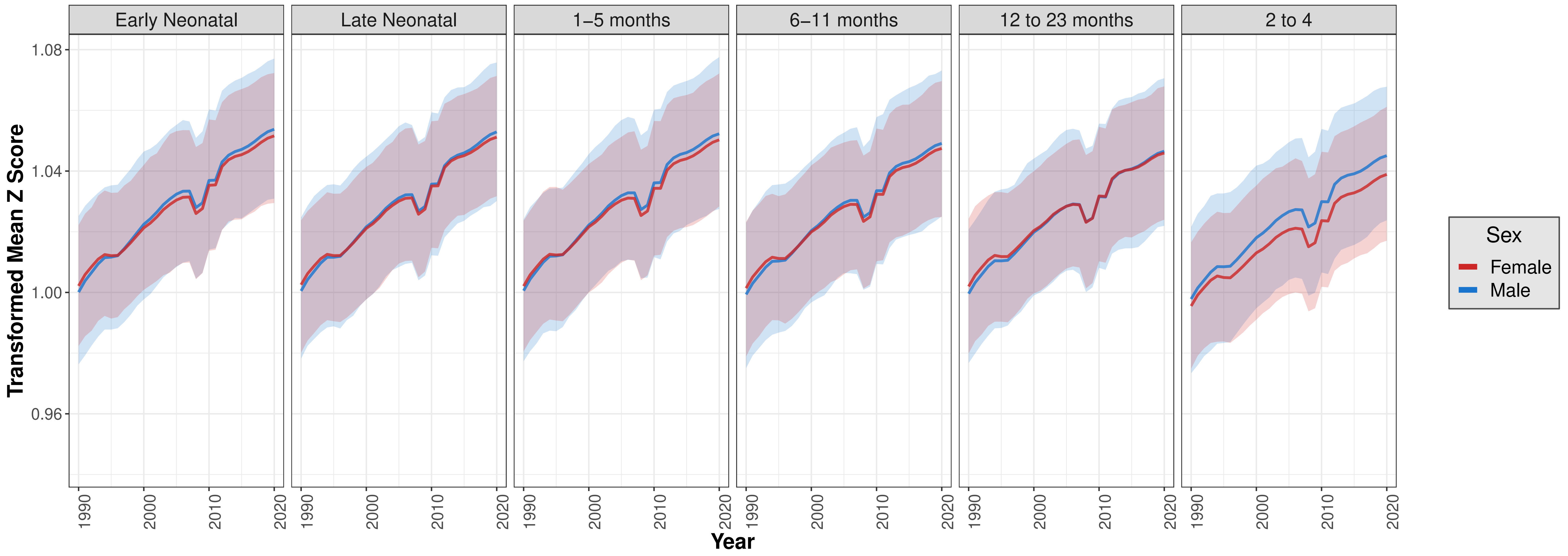

C

Source
No sources for this location

Malta – Wasting (WHZ)

D: Overall and Severe Wasting Prevalence

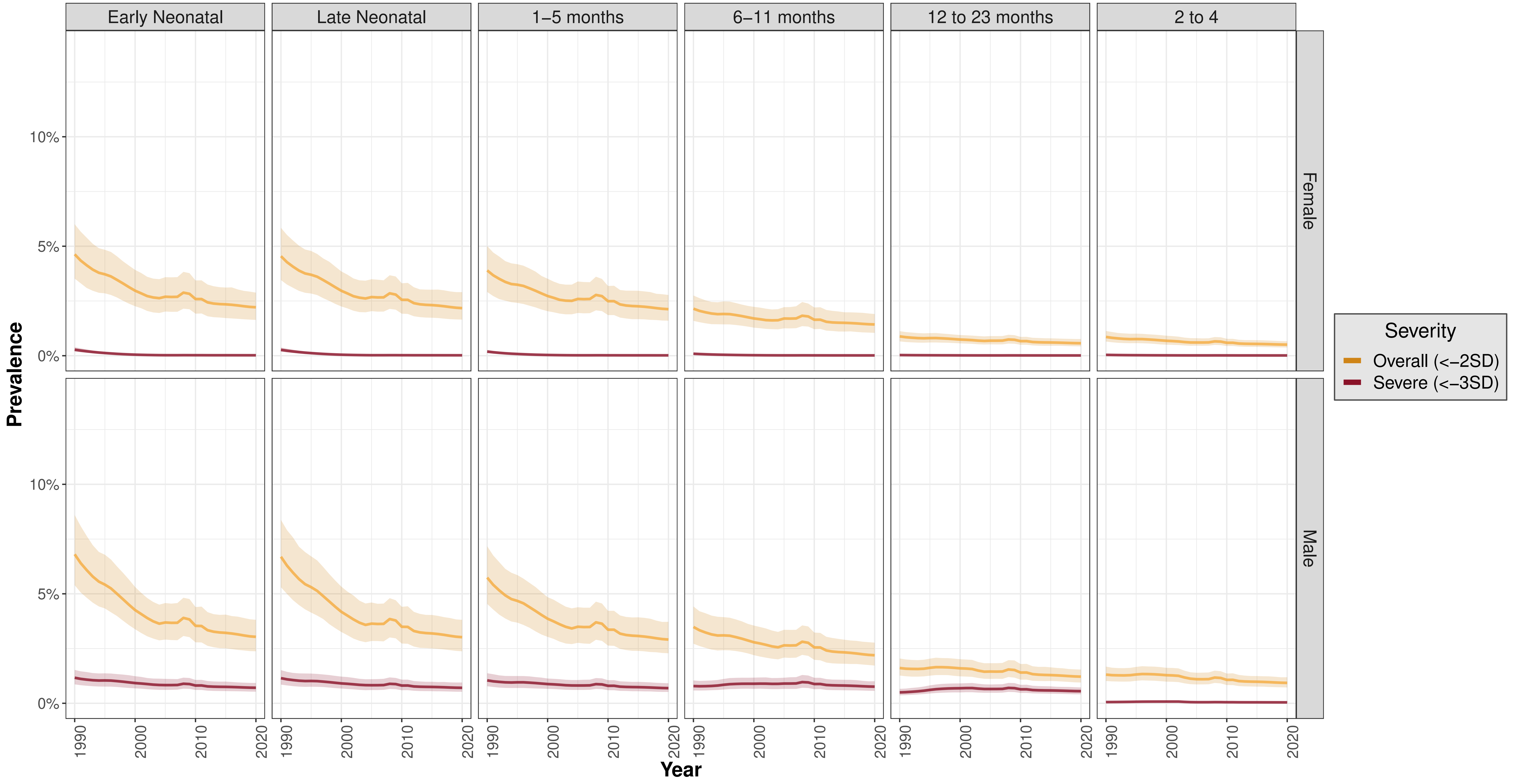

E: Transformed Mean Wasting Z Scores

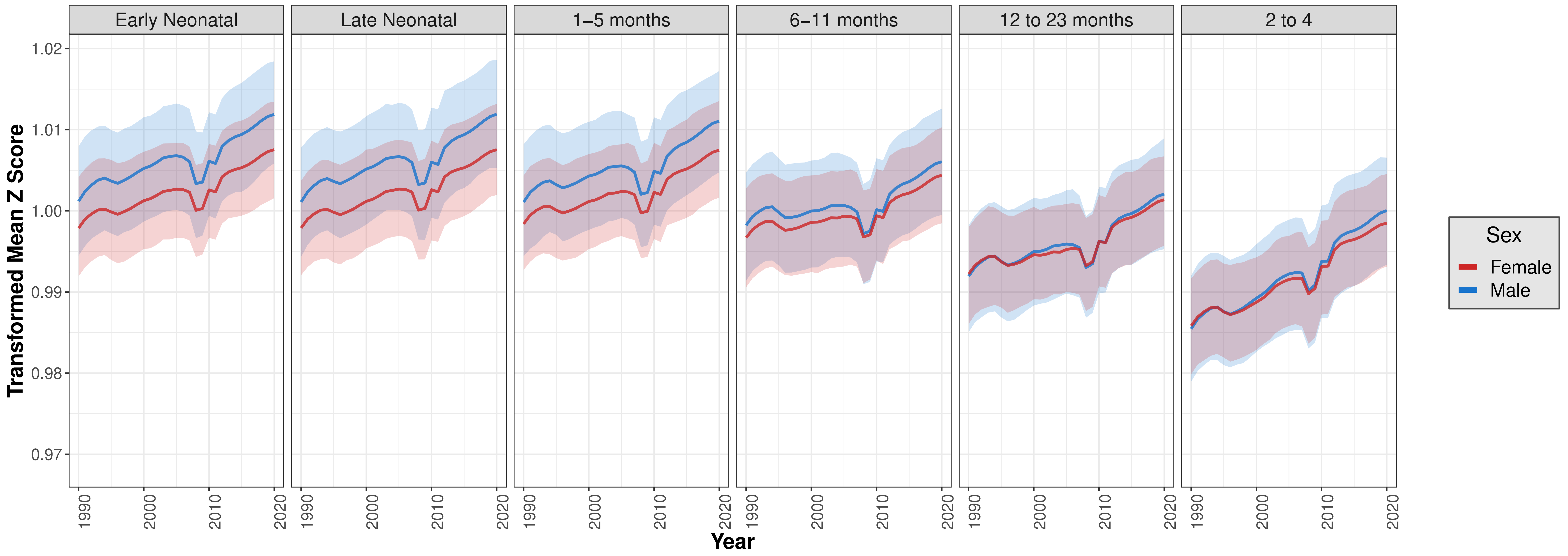

F

Source
No sources for this location

Malta – Underweight (WAZ)

G: Overall and Severe Underweight Prevalence

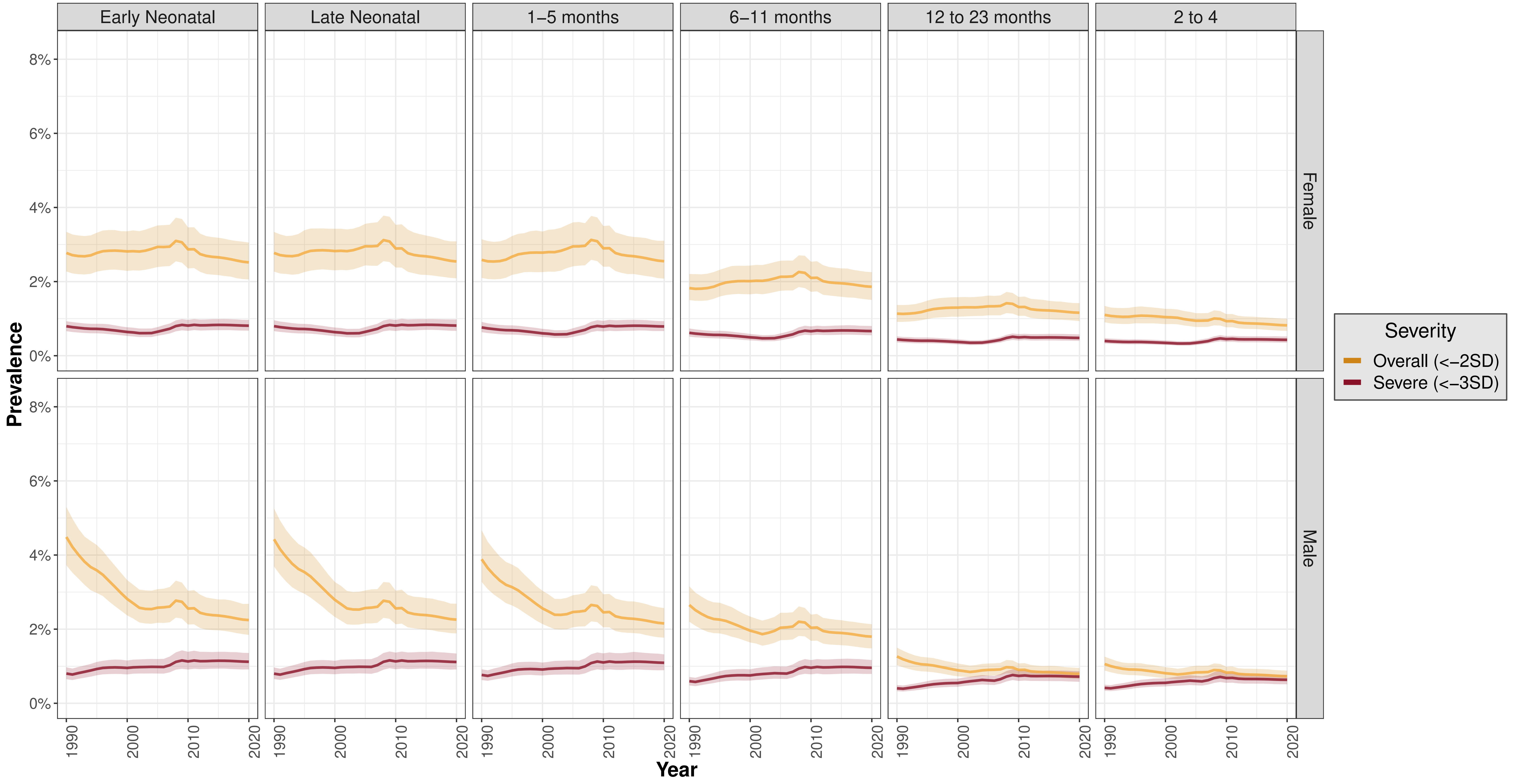

H: Transformed Mean Underweight Z Scores

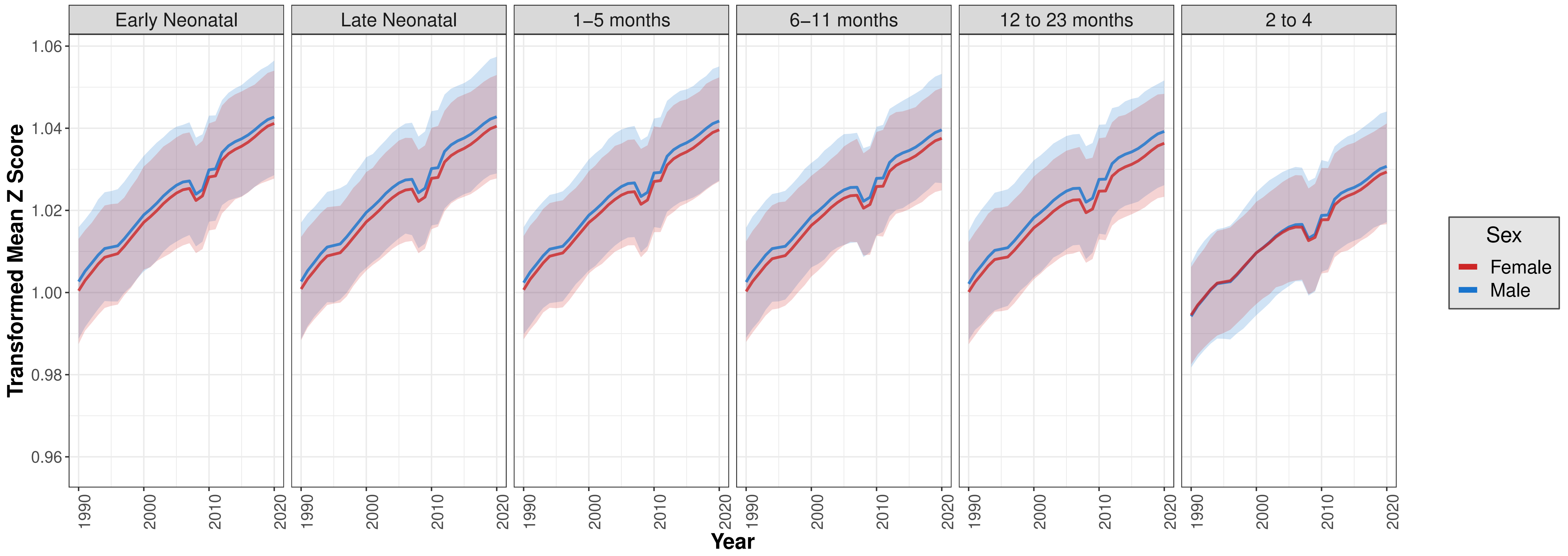

I

Source
No sources for this location

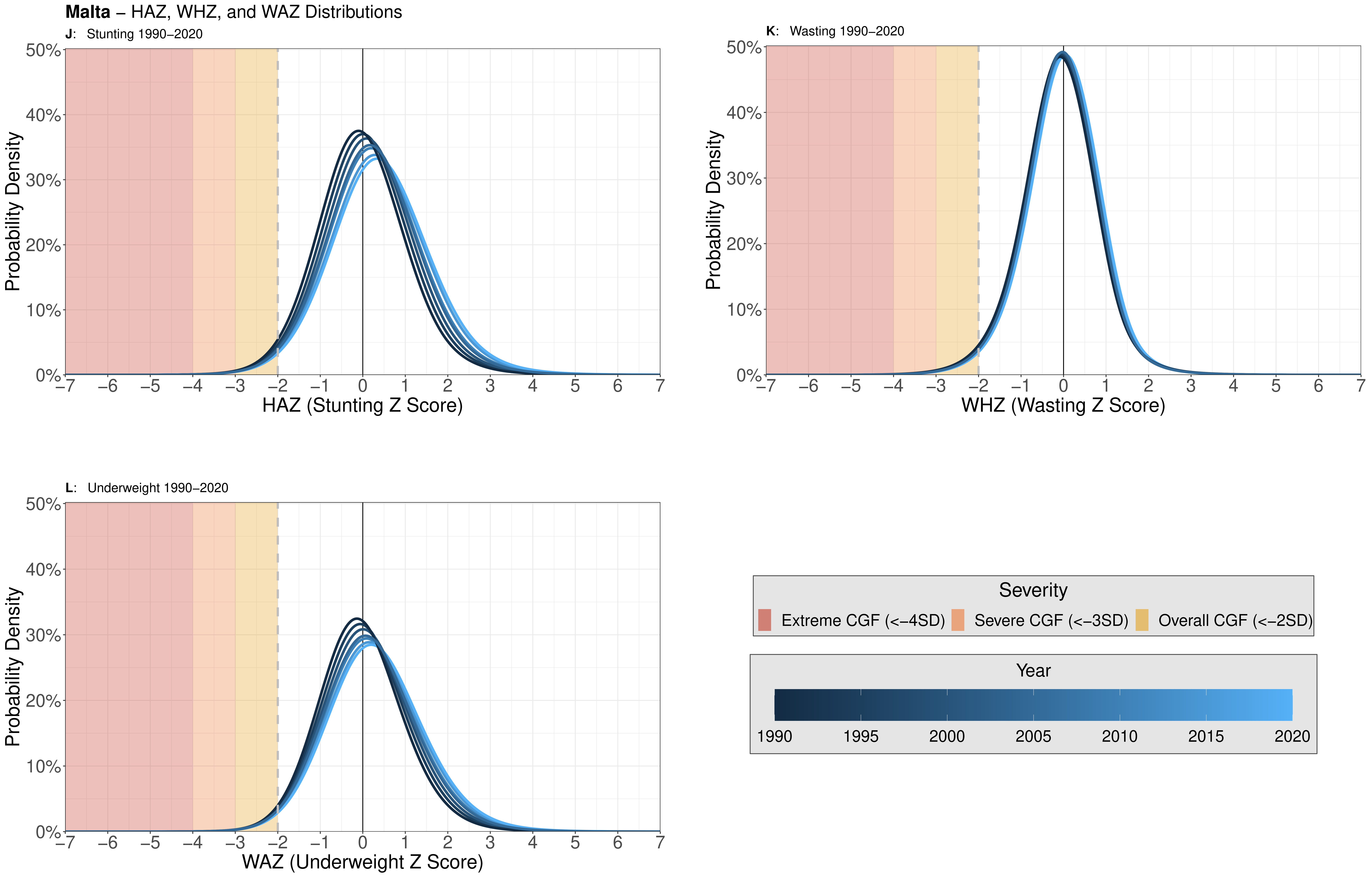
Malta – HAZ, WHZ, and WAZ Distributions
J: Stunting 1990–2020
K: Wasting 1990–2020
L: Underweight 1990–2020
Probability Density
0%
10%
20%
30%
40%
50%
−7
−6
−5
−4
−3
−2
−1
0
1
2
3
4
5
6
7
HAZ (Stunting Z Score)
WHZ (Wasting Z Score)
WAZ (Underweight Z Score)
Severity
Extreme CGF (<−4SD)
Severe CGF (<−3SD)
Overall CGF (<−2SD)
Year
1990
1995
2000
2005
2010
2015
2020

Netherlands – Stunting (HAZ)

A: Overall and Severe Stunting Prevalence

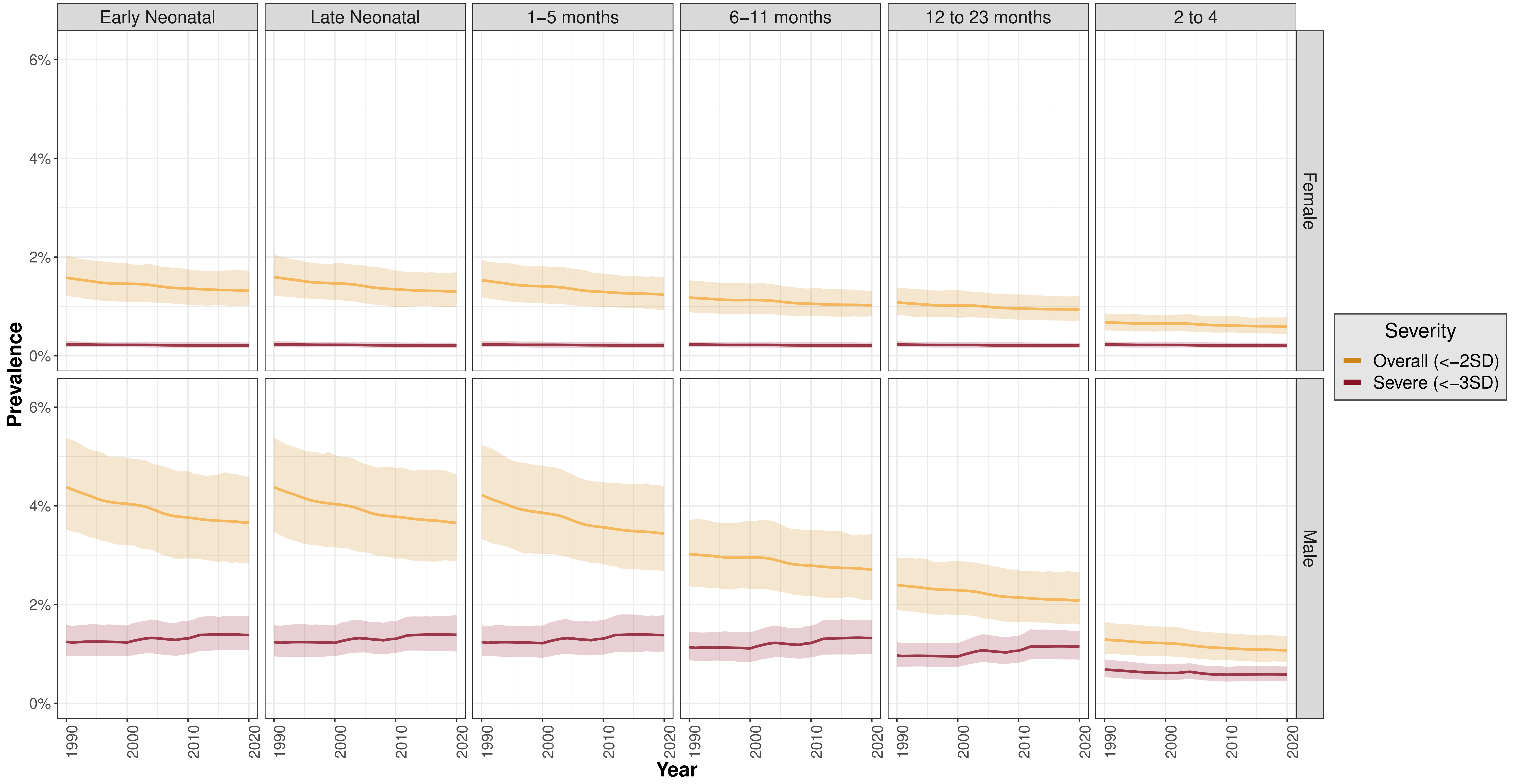

B: Transformed Mean Stunting Z Scores

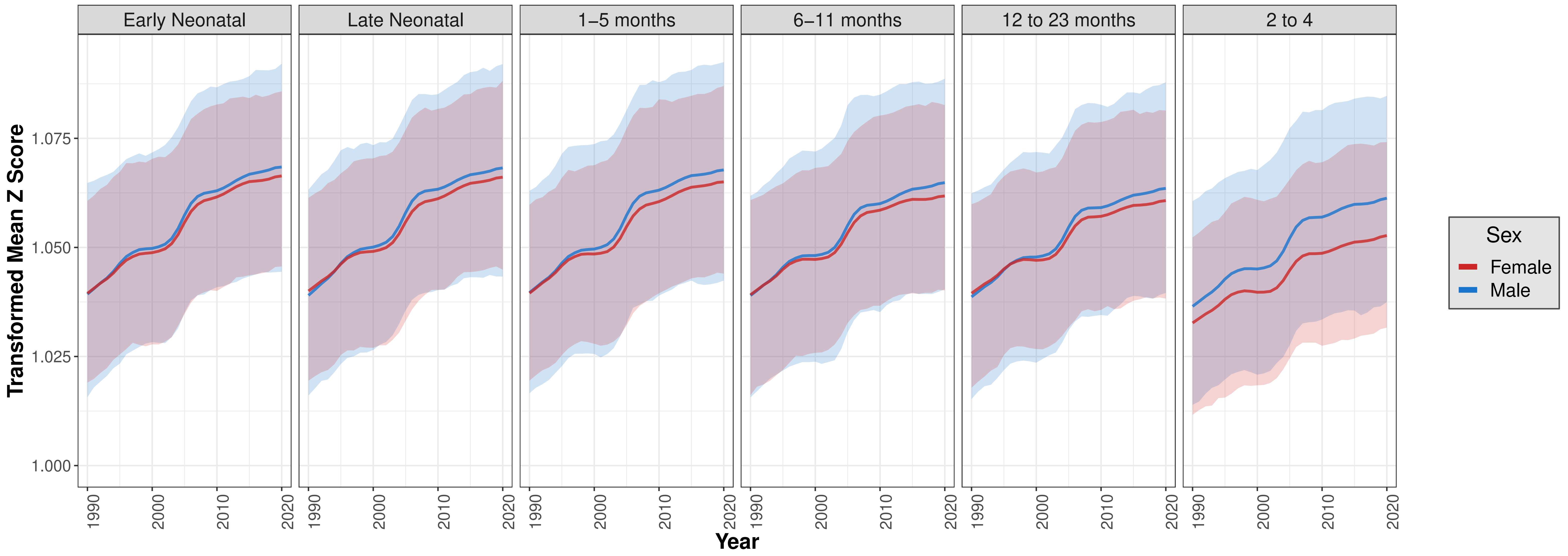

C

Year	Source
1980	WHO CGM Database

Netherlands – Wasting (WHZ)

D: Overall and Severe Wasting Prevalence

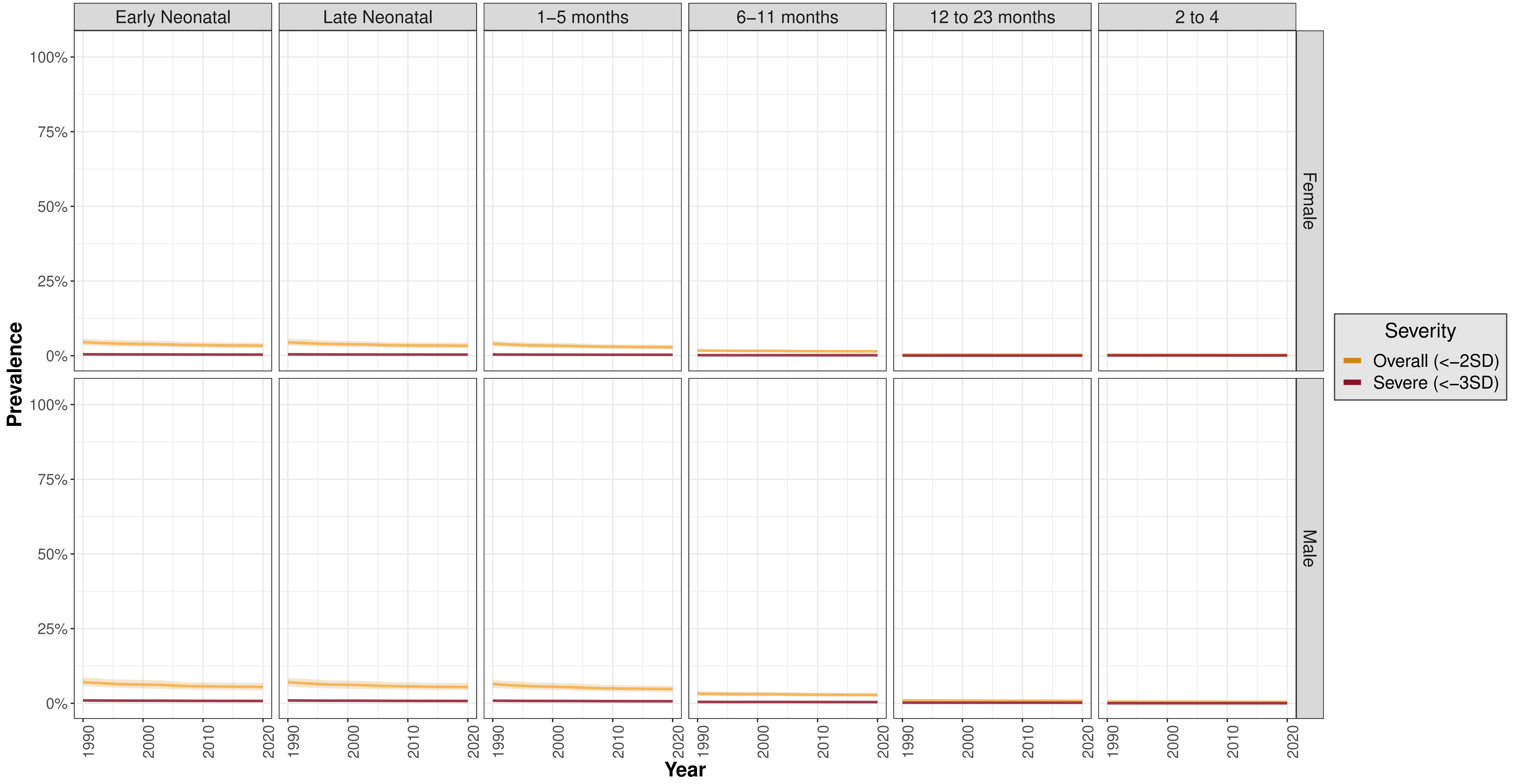

E: Transformed Mean Wasting Z Scores

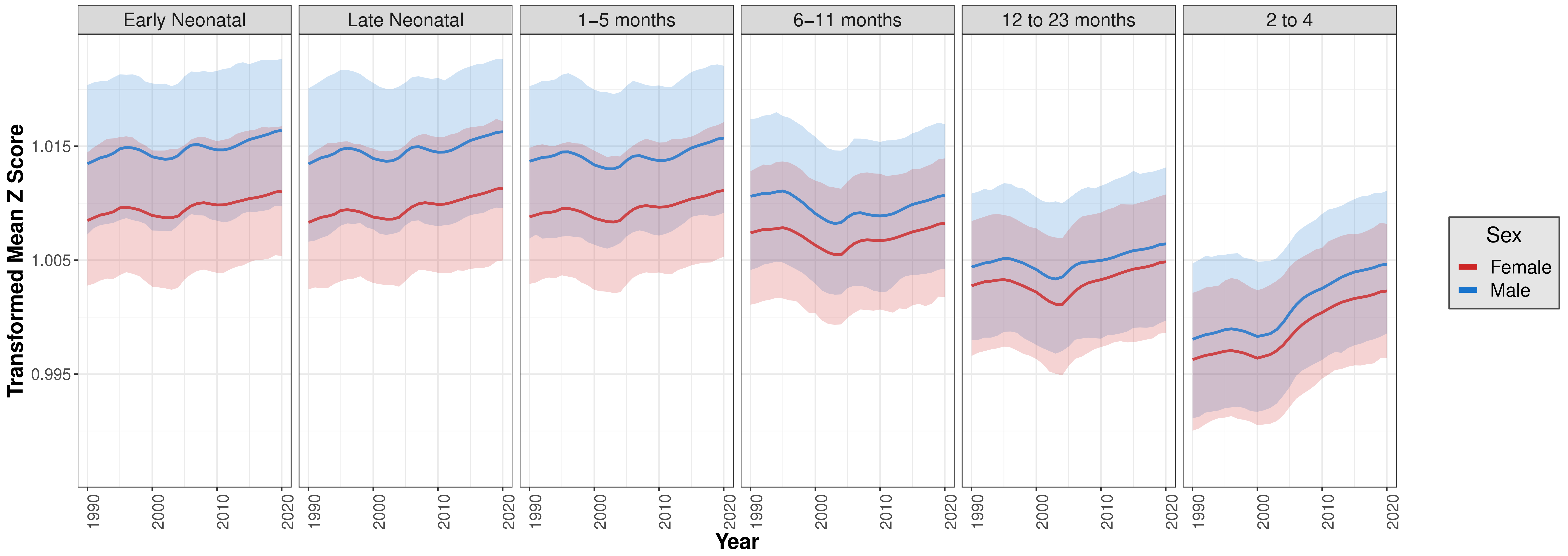

F

Year	Source
1980	WHO CGM Database

Netherlands – Underweight (WAZ)

G: Overall and Severe Underweight Prevalence

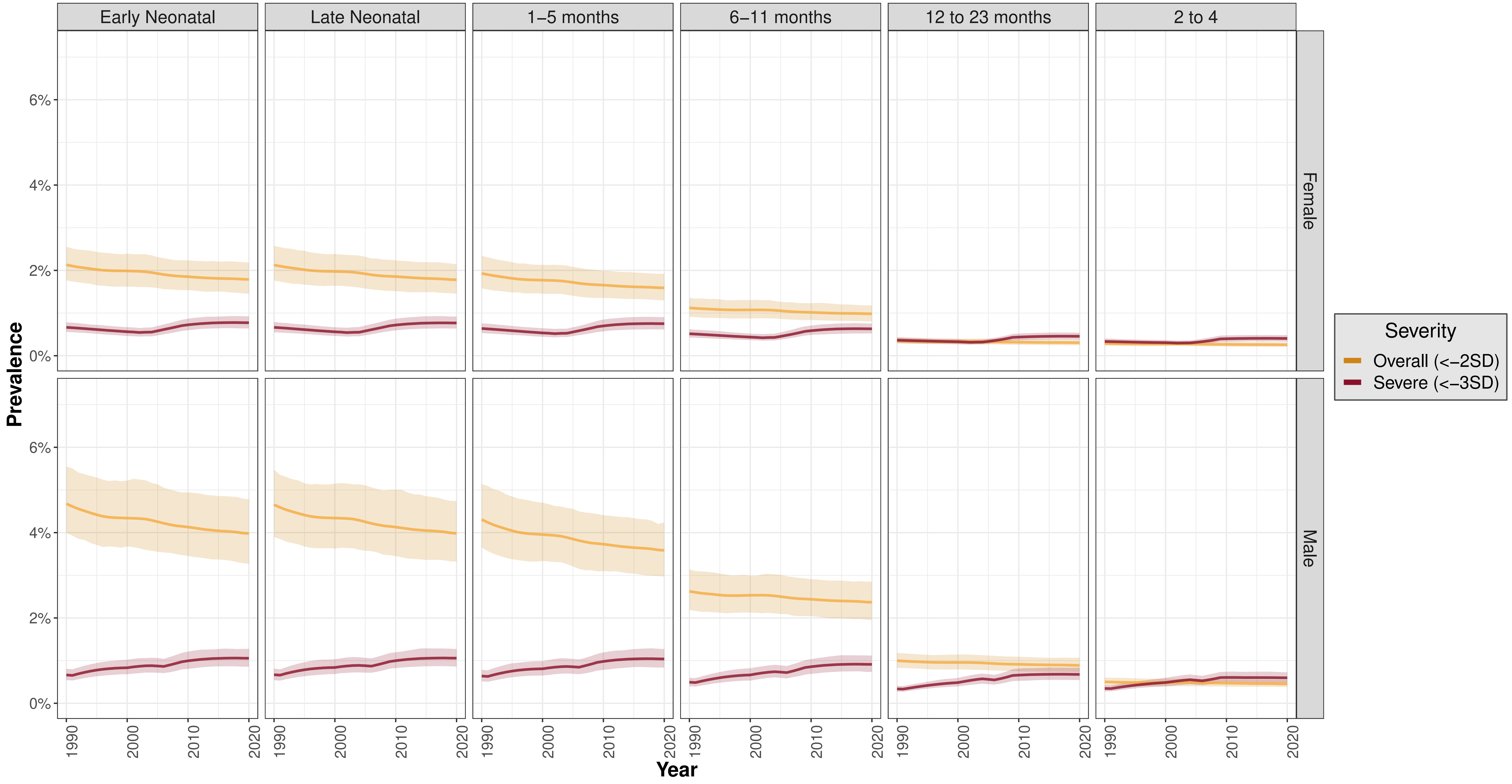

H: Transformed Mean Underweight Z Scores

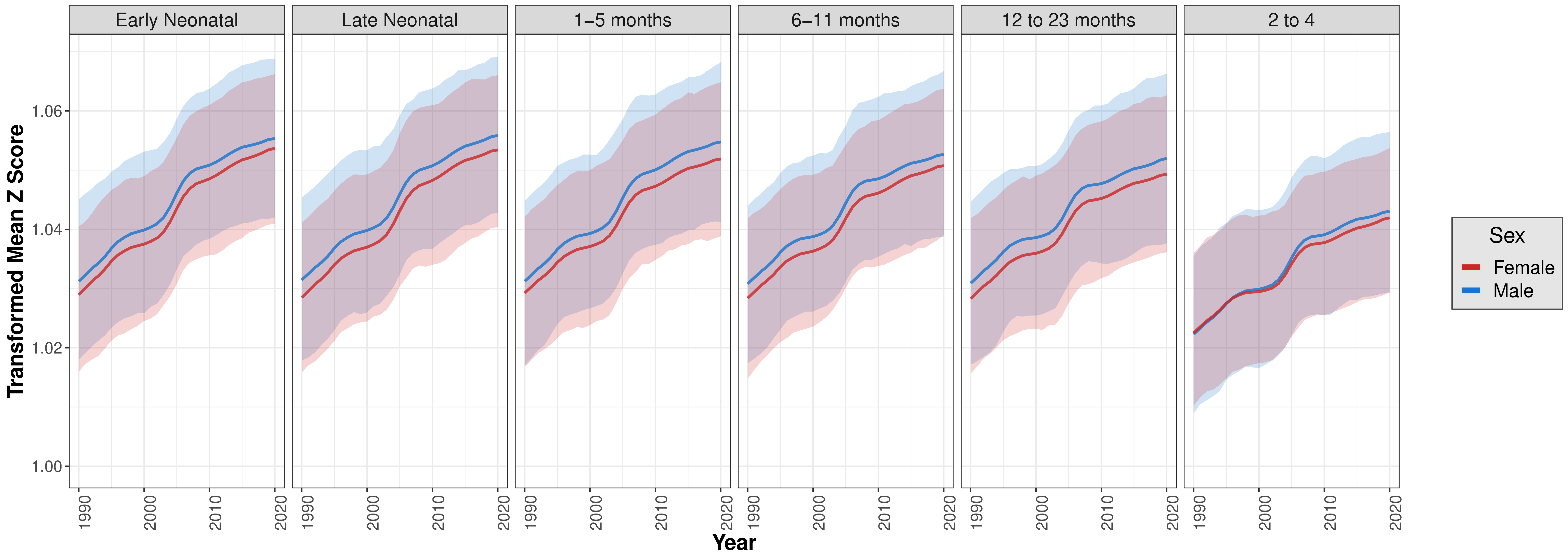

I

Year	Source
1980	WHO CGM Database

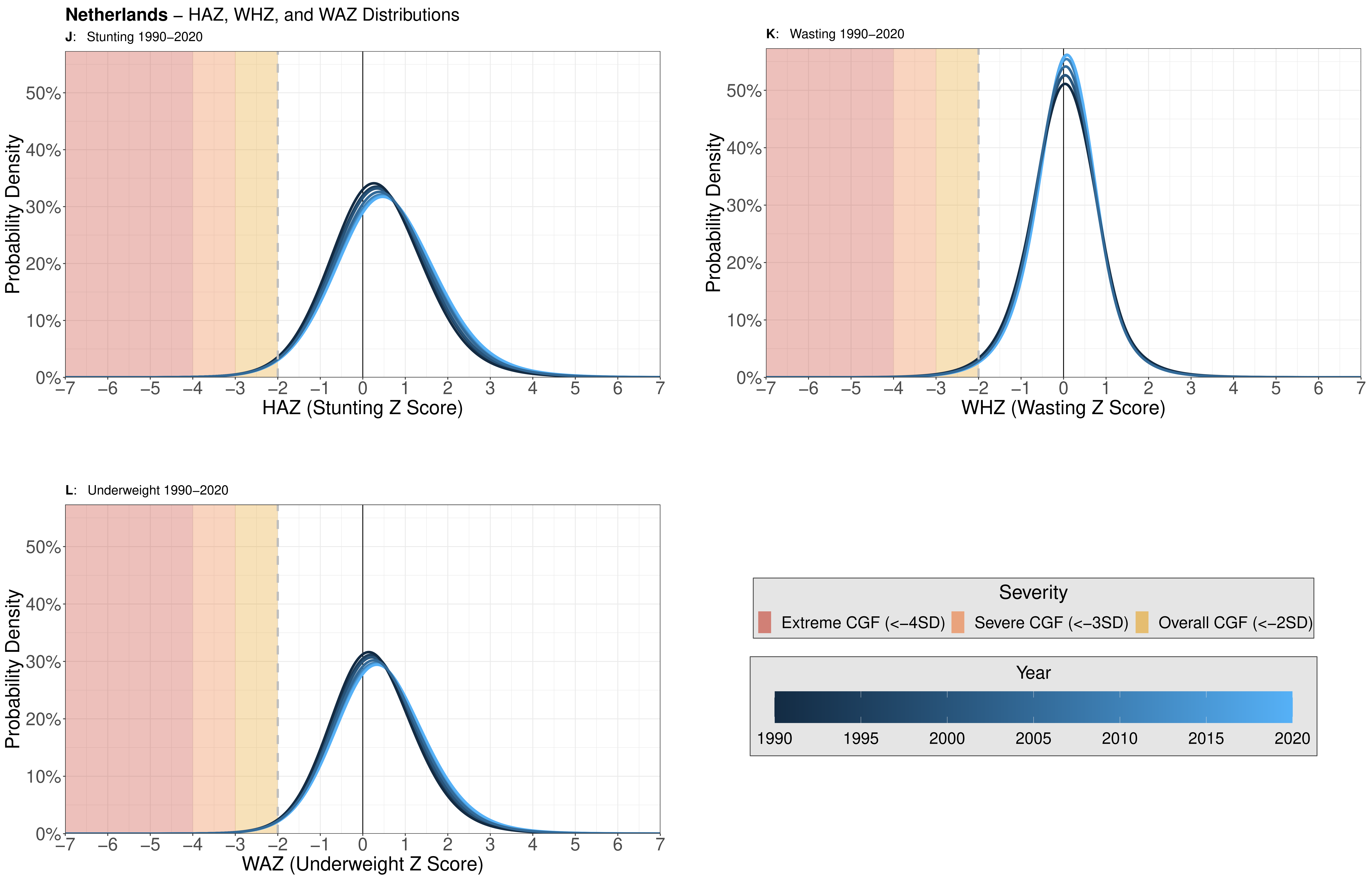

Netherlands – HAZ, WHZ, and WAZ Distributions
J: Stunting 1990–2020
K: Wasting 1990–2020
L: Underweight 1990–2020
Probability Density
0%
10%
20%
30%
40%
50%
−7
−6
−5
−4
−3
−2
−1
0
1
2
3
4
5
6
7
HAZ (Stunting Z Score)
WHZ (Wasting Z Score)
WAZ (Underweight Z Score)
Severity
Extreme CGF (<−4SD)
Severe CGF (<−3SD)
Overall CGF (<−2SD)
Year
1990
1995
2000
2005
2010
2015
2020

Norway – Stunting (HAZ)

A: Overall and Severe Stunting Prevalence

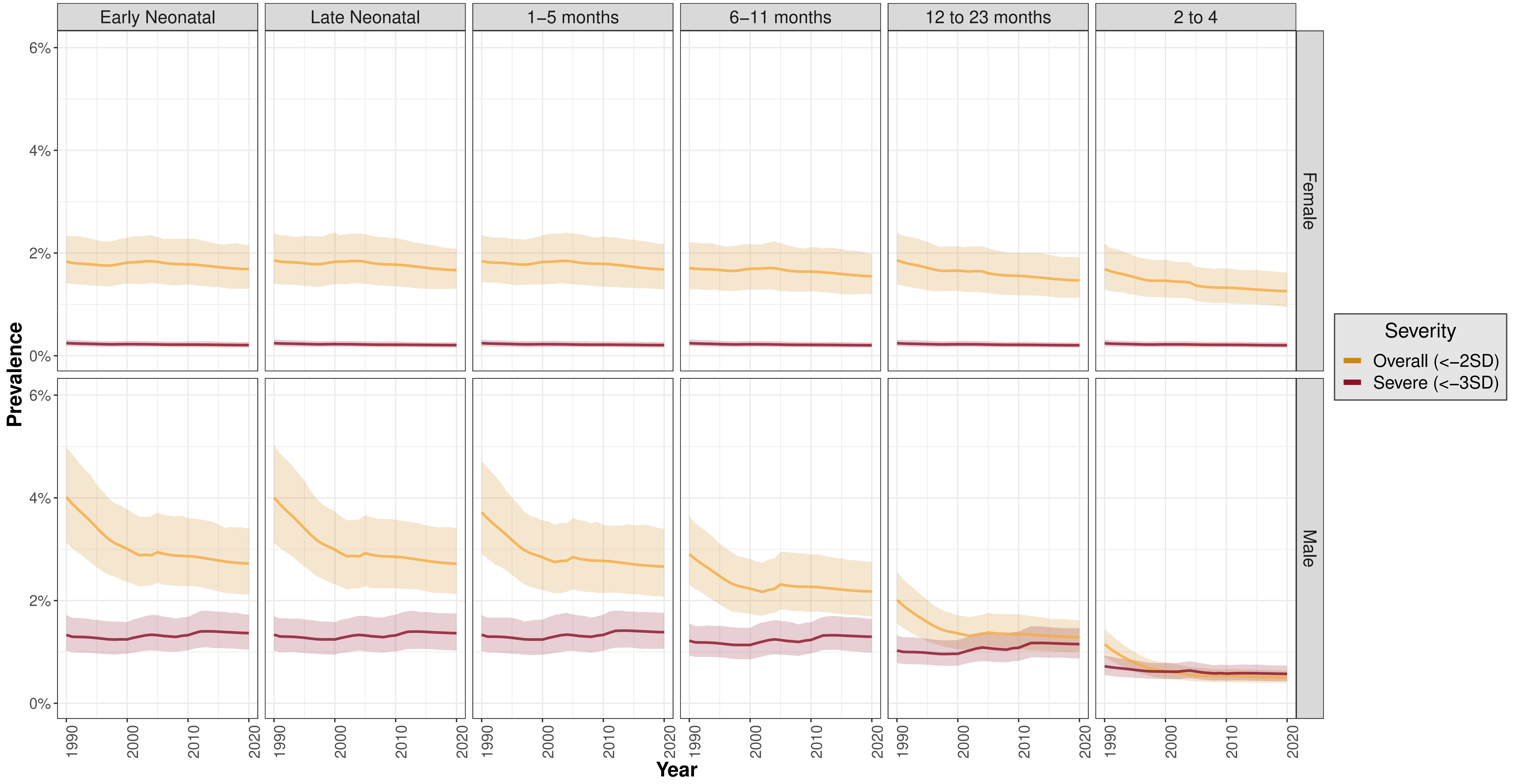

B: Transformed Mean Stunting Z Scores

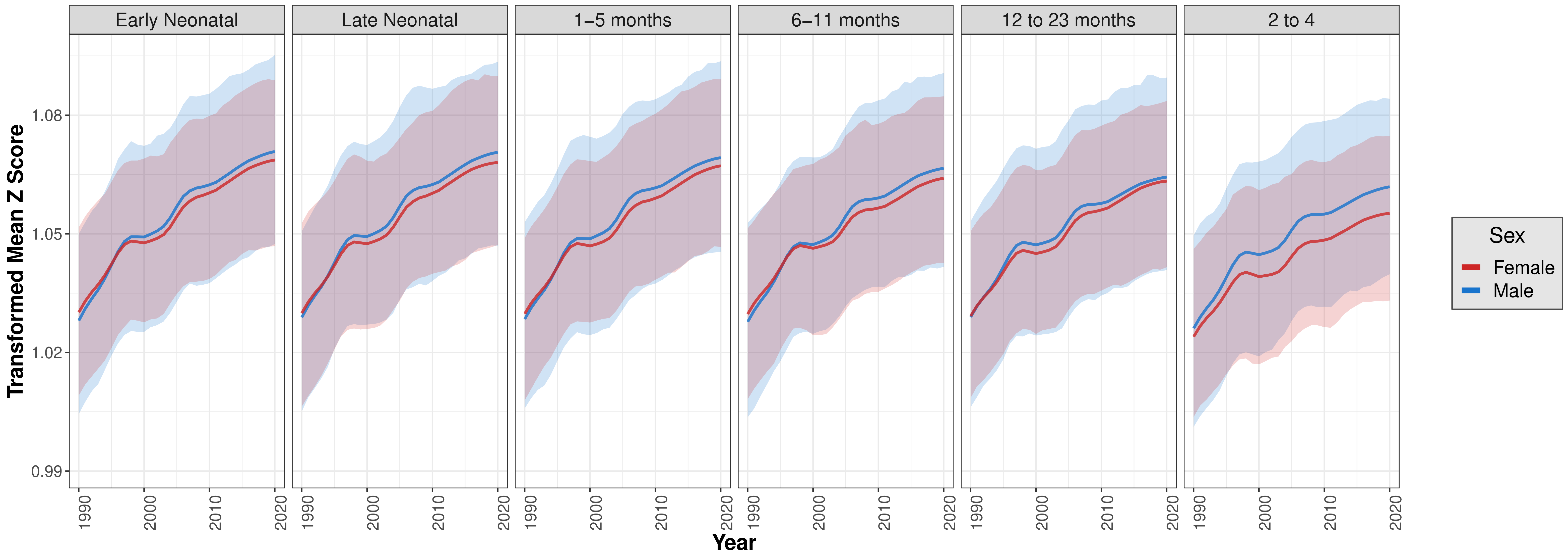

C

Source
No national or subnational sources for this location

Norway – Wasting (WHZ)

D: Overall and Severe Wasting Prevalence

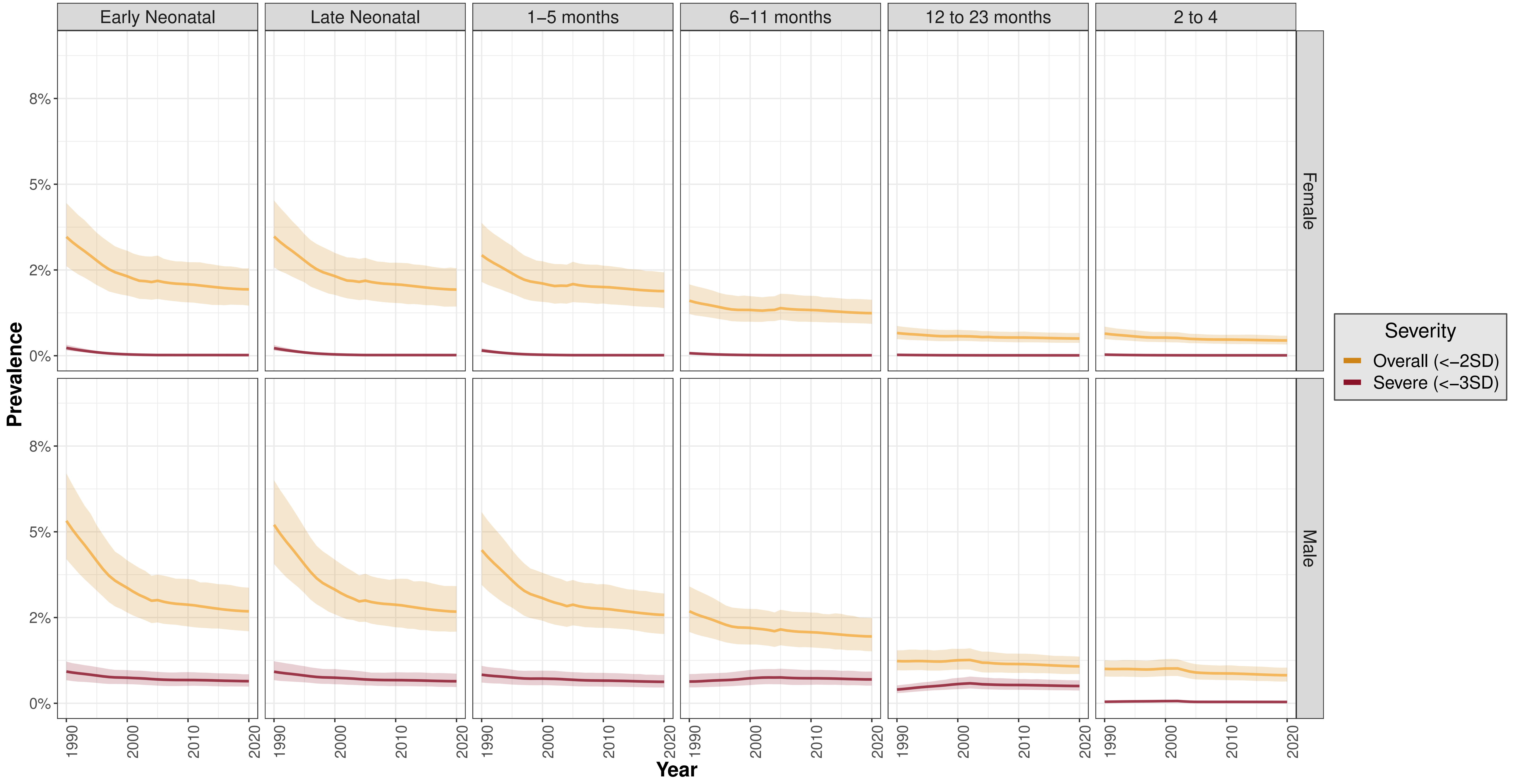

E: Transformed Mean Wasting Z Scores

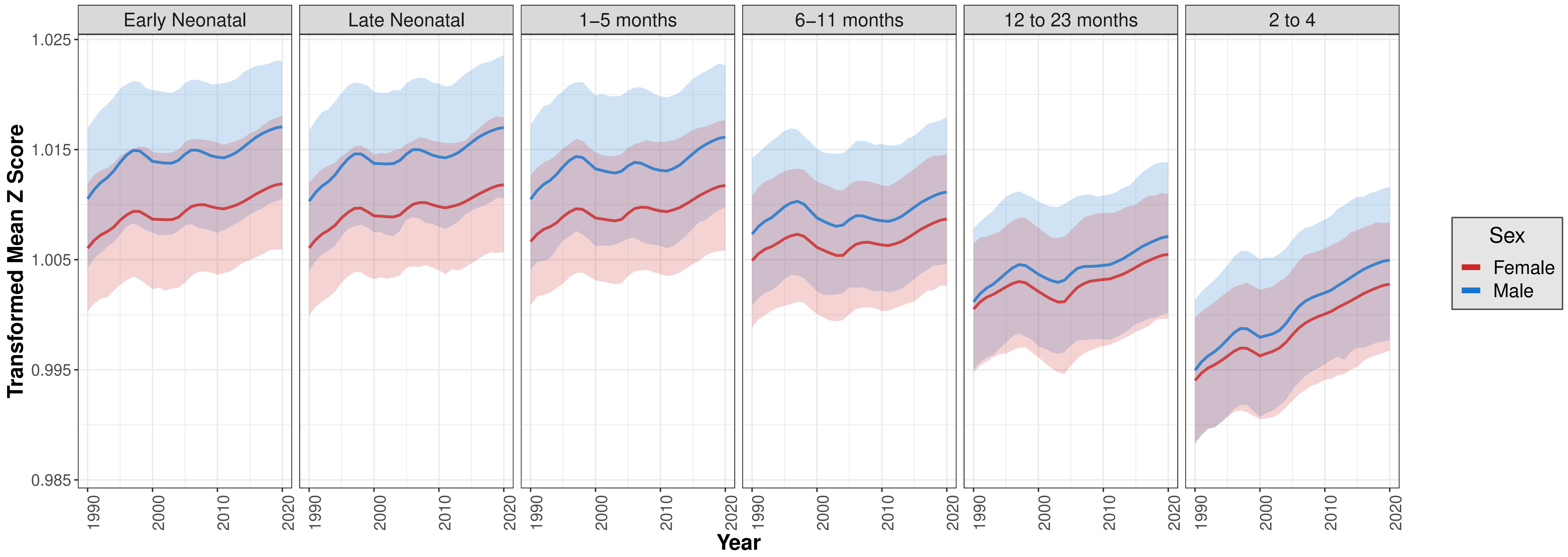

F

Source
No national or subnational sources for this location

Norway – Underweight (WAZ)

G: Overall and Severe Underweight Prevalence

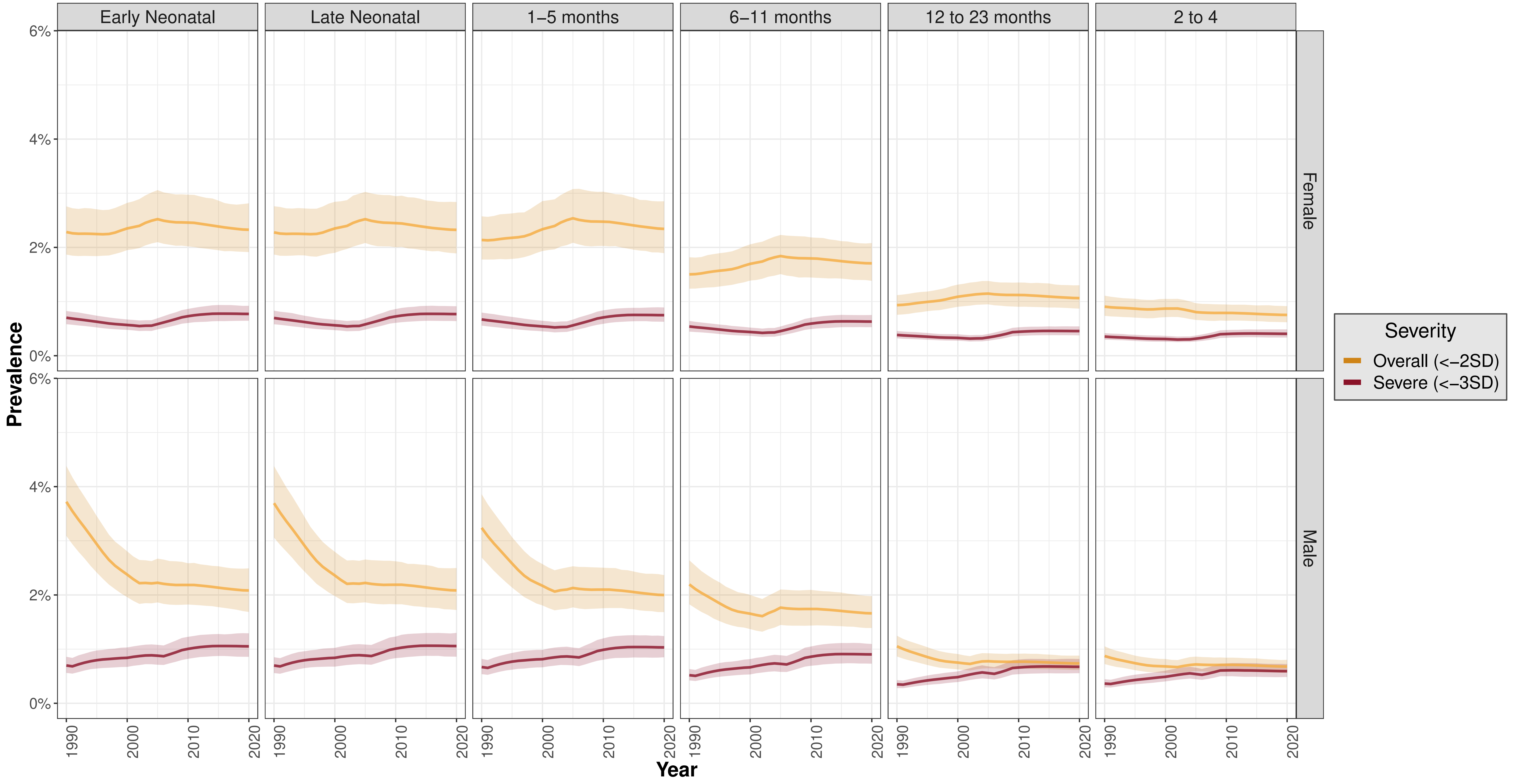

H: Transformed Mean Underweight Z Scores

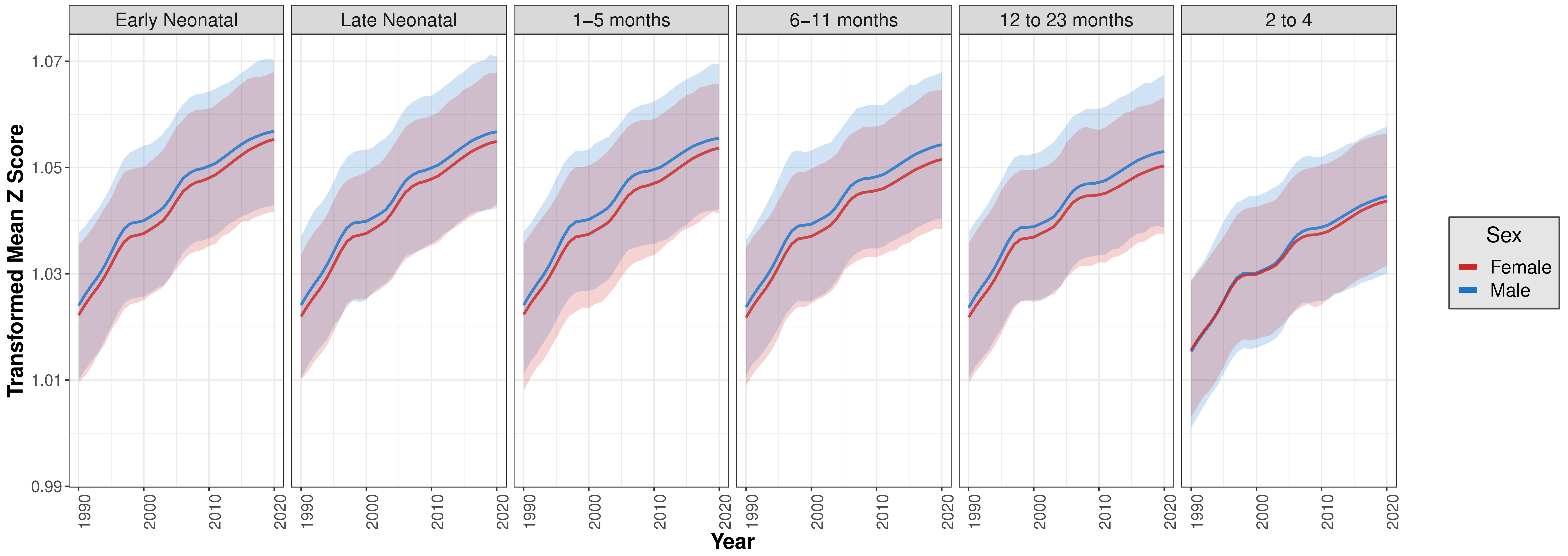

I

Source
No national or subnational sources for this location

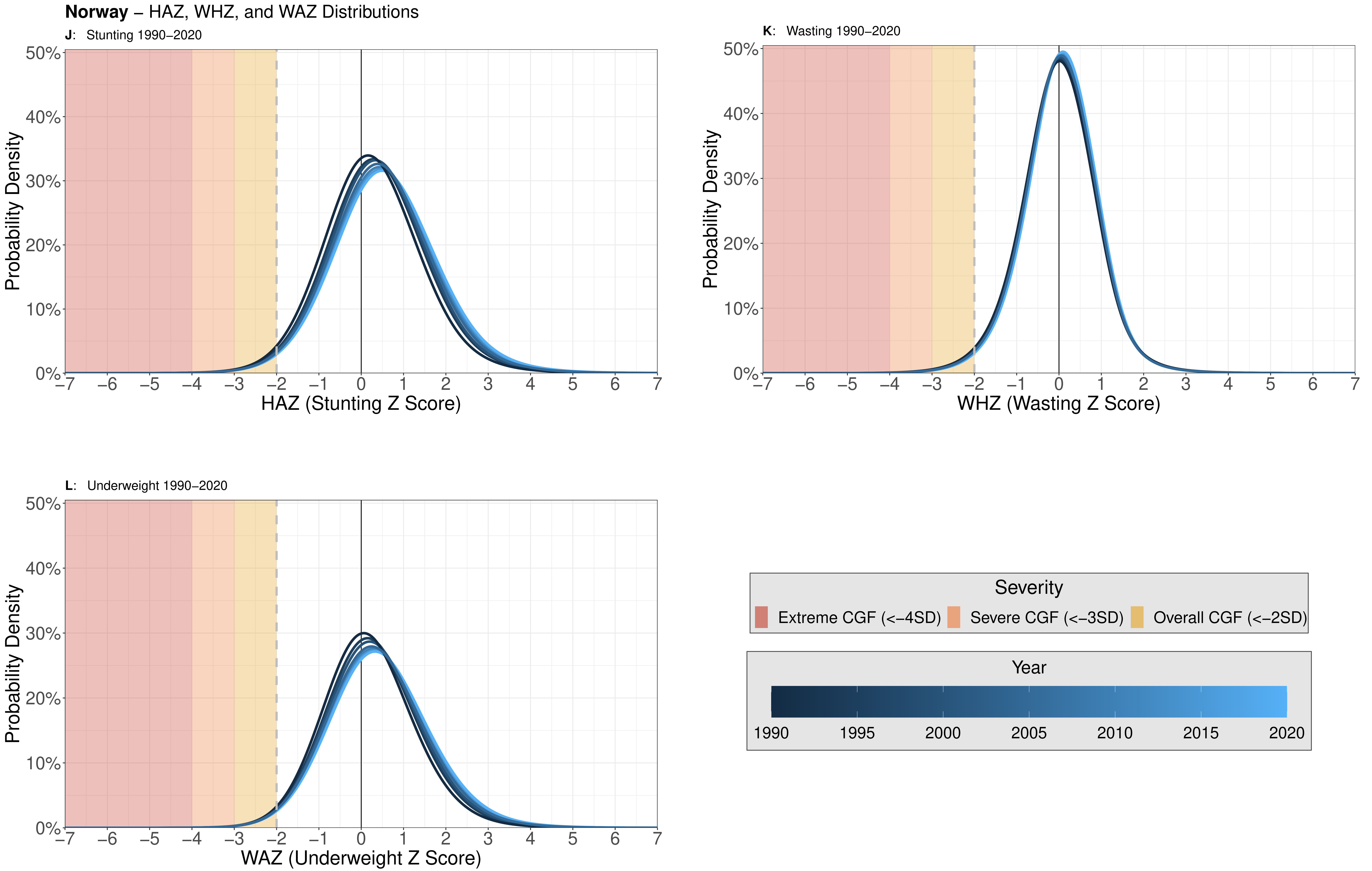

Norway – HAZ, WHZ, and WAZ Distributions
J: Stunting 1990–2020
Probability Density
50%
40%
30%
20%
10%
0%
−7 −6 −5 −4 −3 −2 −1 0 1 2 3 4 5 6 7
HAZ (Stunting Z Score)
K: Wasting 1990–2020
Probability Density
WHZ (Wasting Z Score)
L: Underweight 1990–2020
Probability Density
WAZ (Underweight Z Score)
Severity
Extreme CGF (<−4SD)
Severe CGF (<−3SD)
Overall CGF (<−2SD)
Year
1990
1995
2000
2005
2010
2015
2020

Portugal – Stunting (HAZ)

A: Overall and Severe Stunting Prevalence

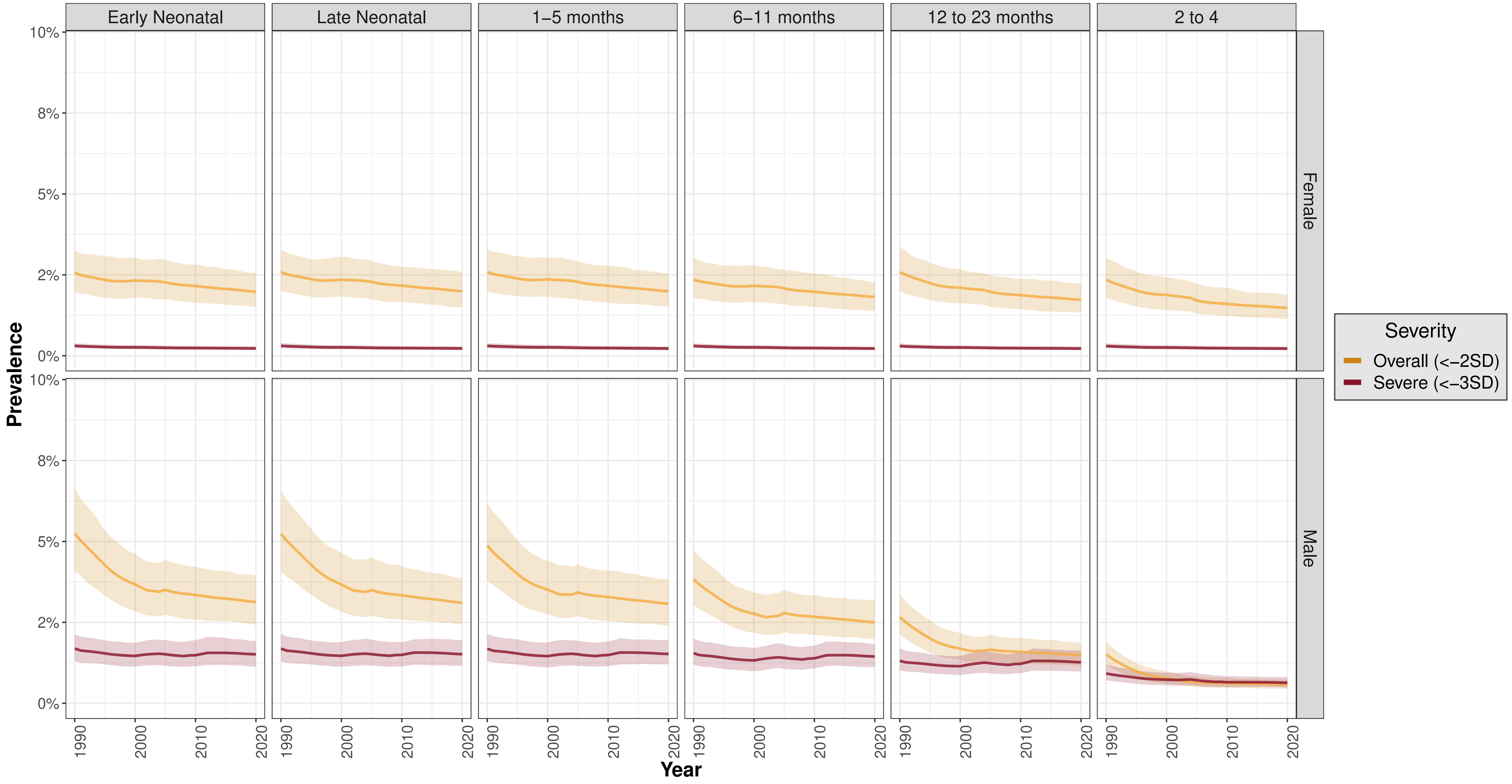

B: Transformed Mean Stunting Z Scores

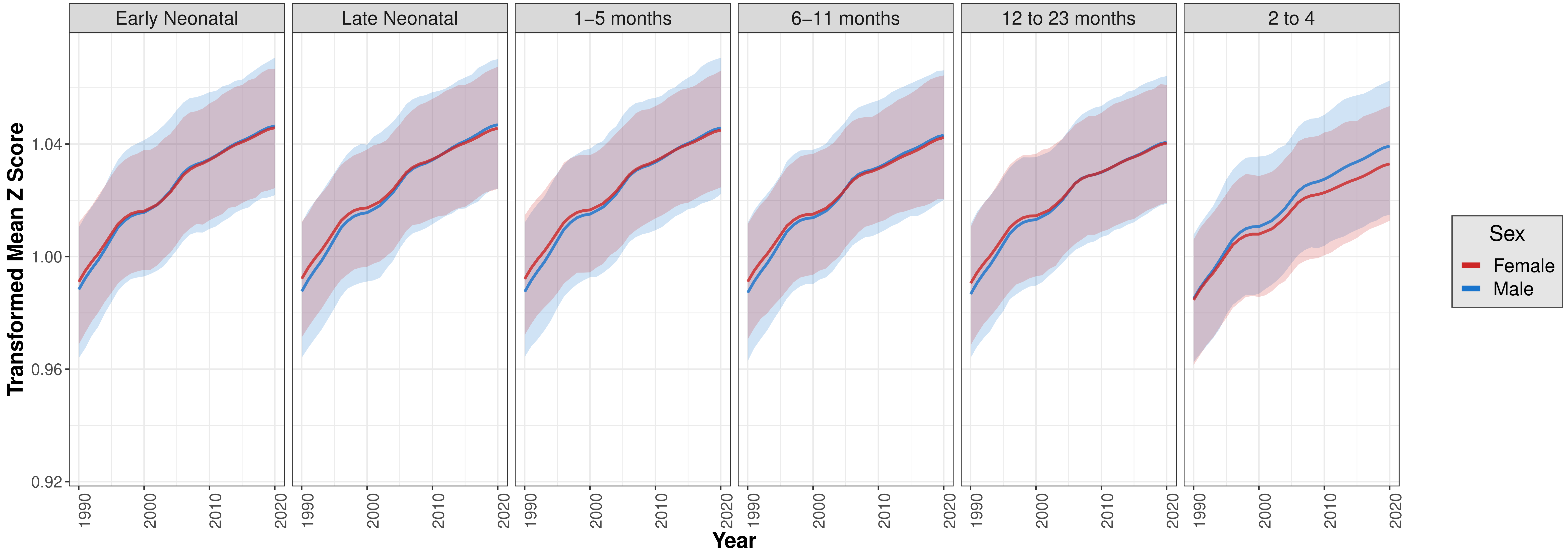

C

Source
No sources for this location

Portugal – Wasting (WHZ)

D: Overall and Severe Wasting Prevalence

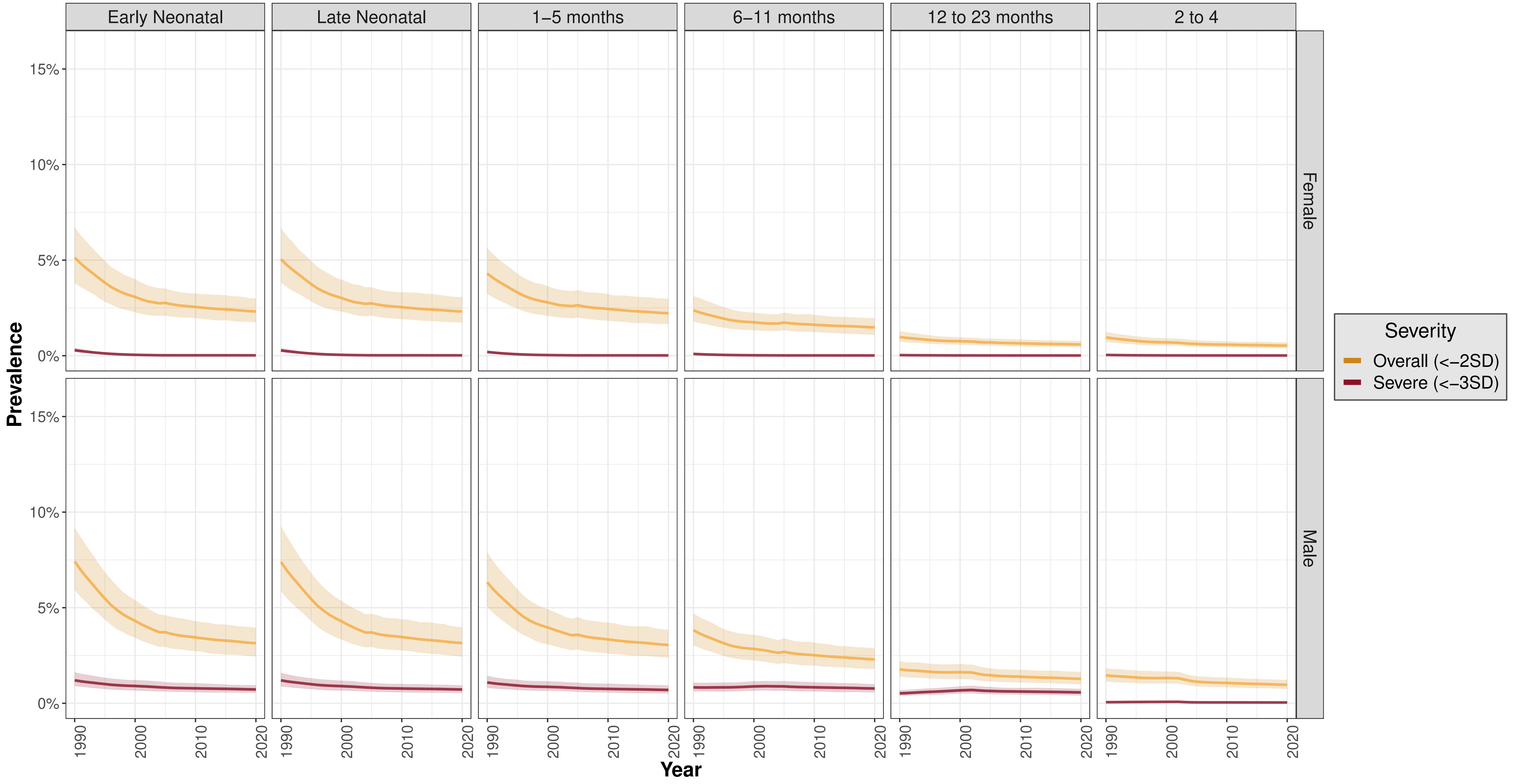

E: Transformed Mean Wasting Z Scores

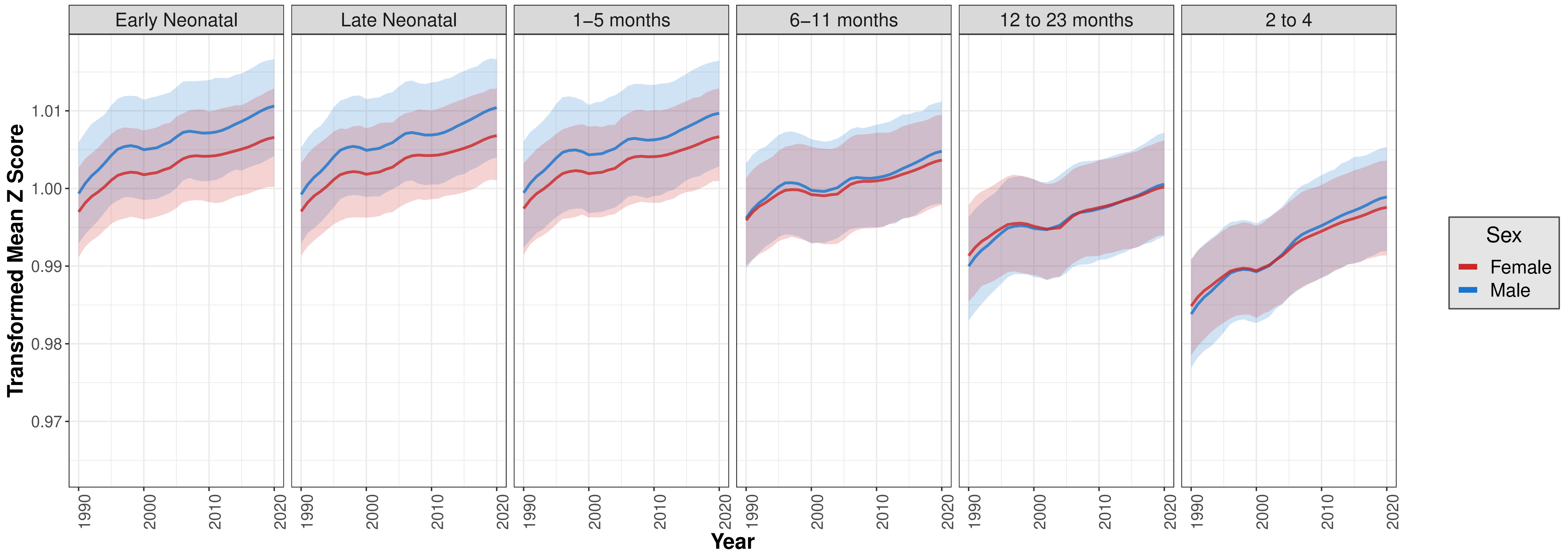

F

Source
No sources for this location

Portugal – Underweight (WAZ)

G: Overall and Severe Underweight Prevalence

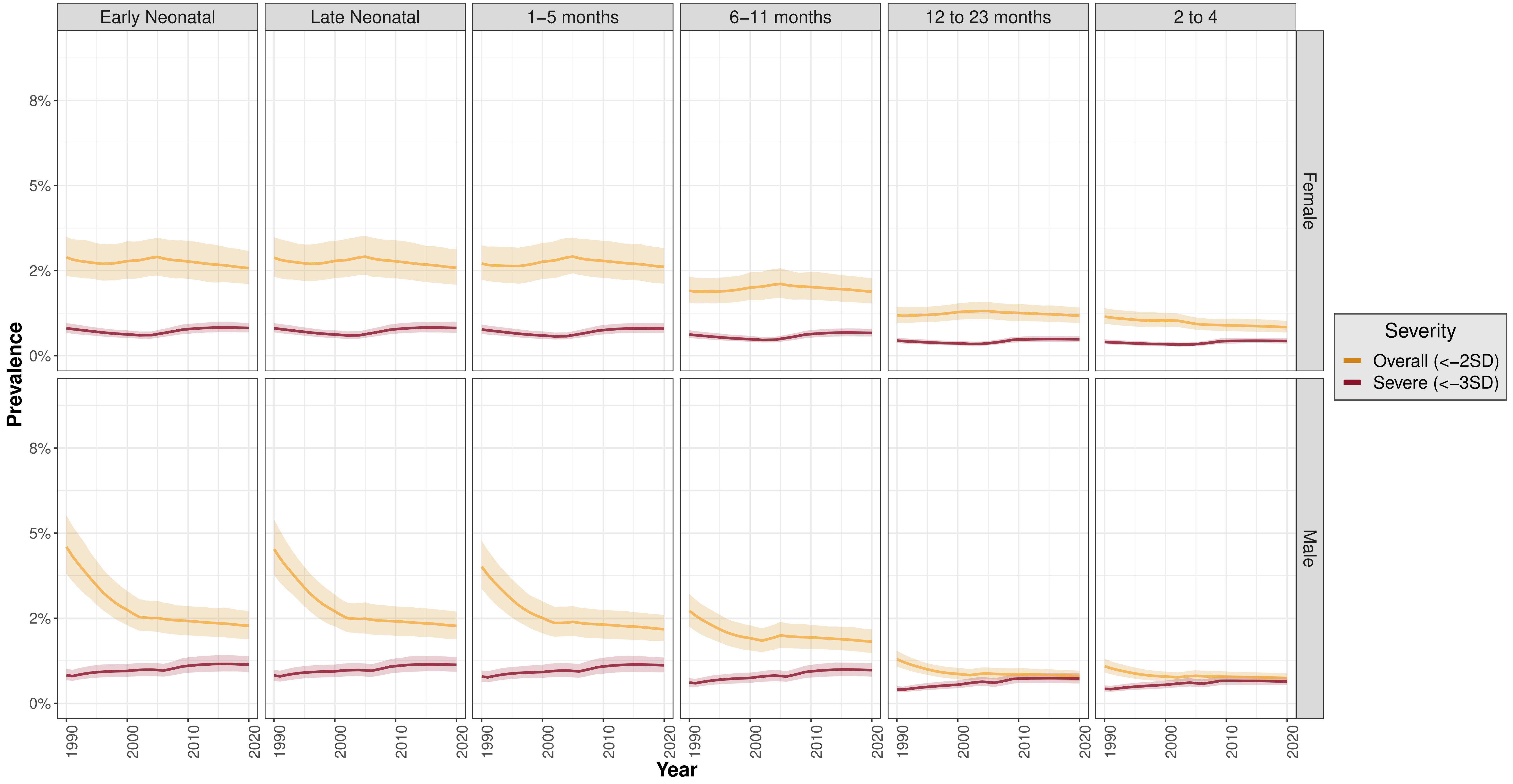

H: Transformed Mean Underweight Z Scores

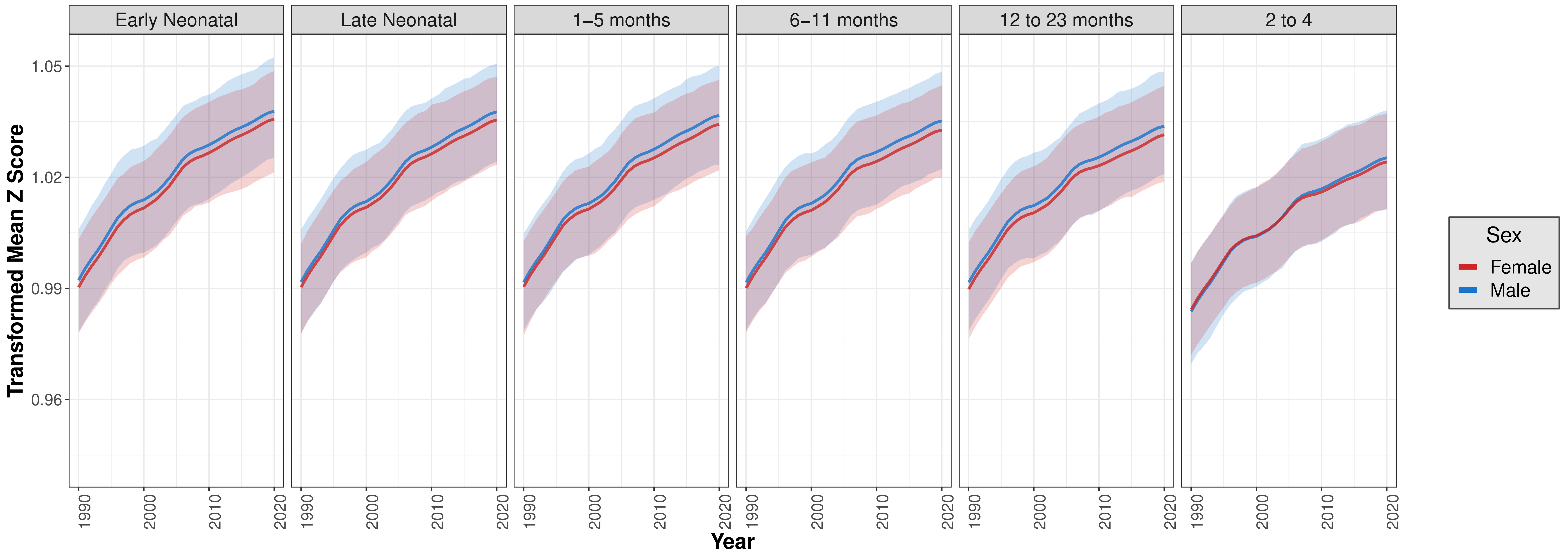

I

Source
No sources for this location

Portugal – HAZ, WHZ, and WAZ Distributions

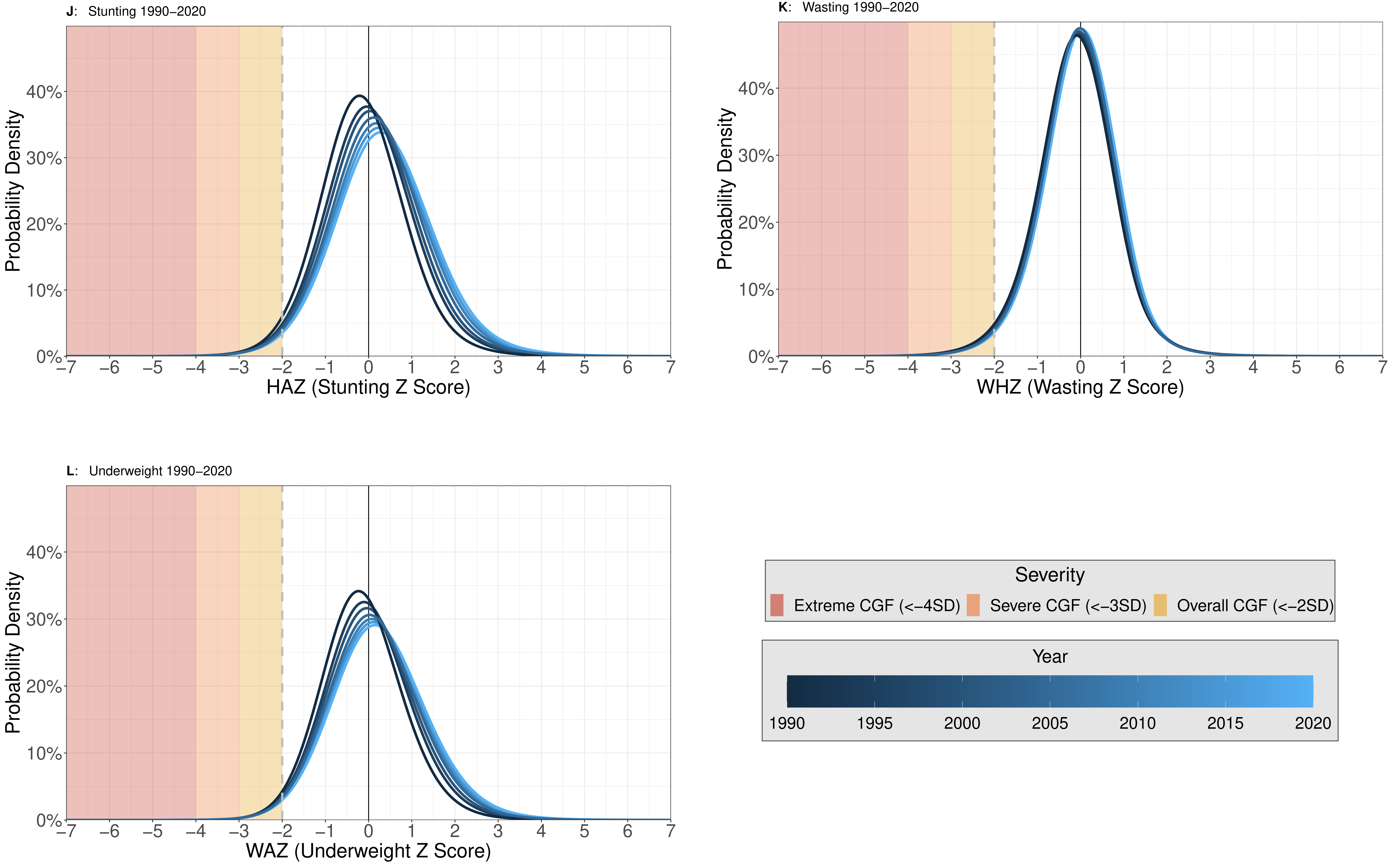

Spain – Stunting (HAZ)

A: Overall and Severe Stunting Prevalence

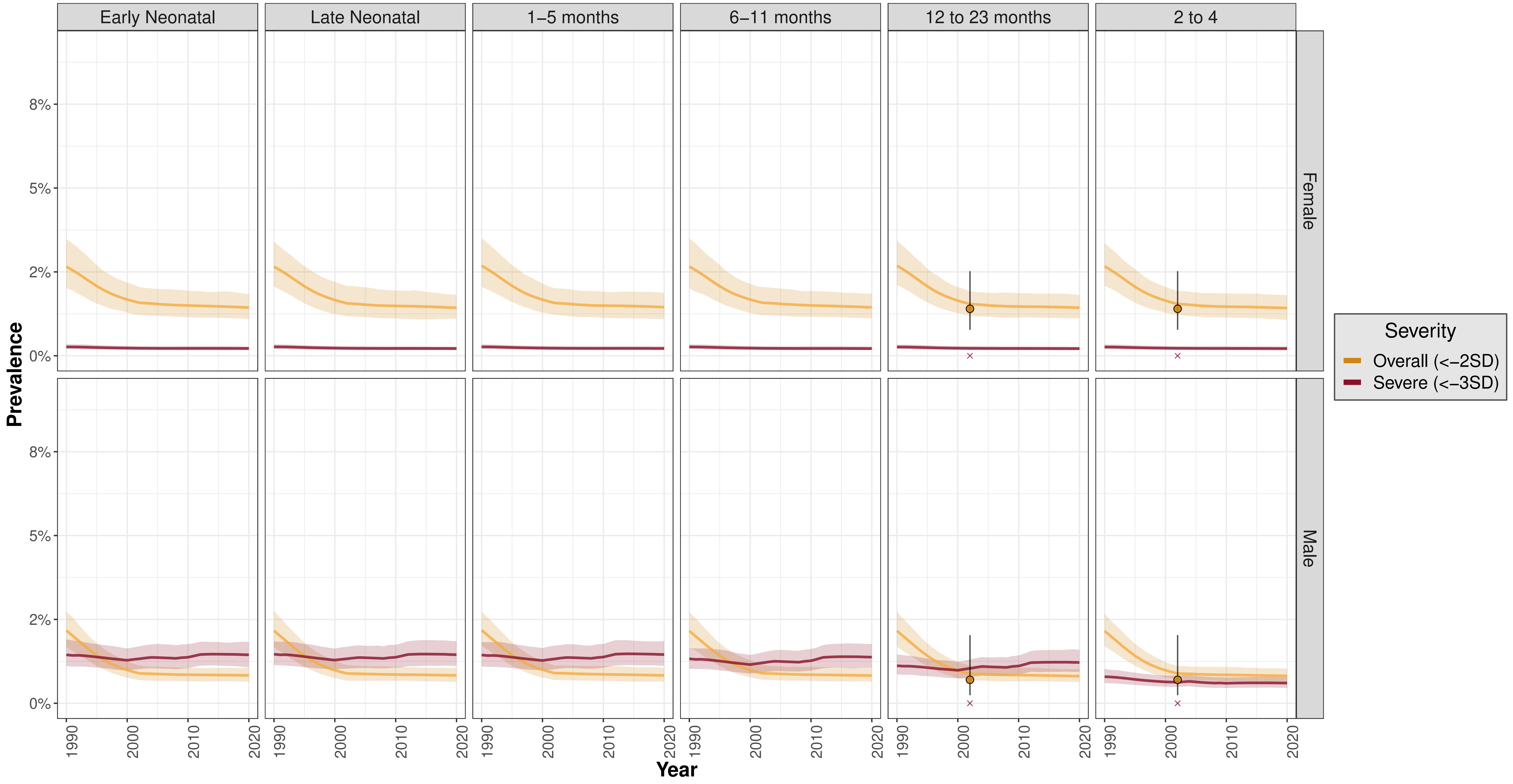

B: Transformed Mean Stunting Z Scores

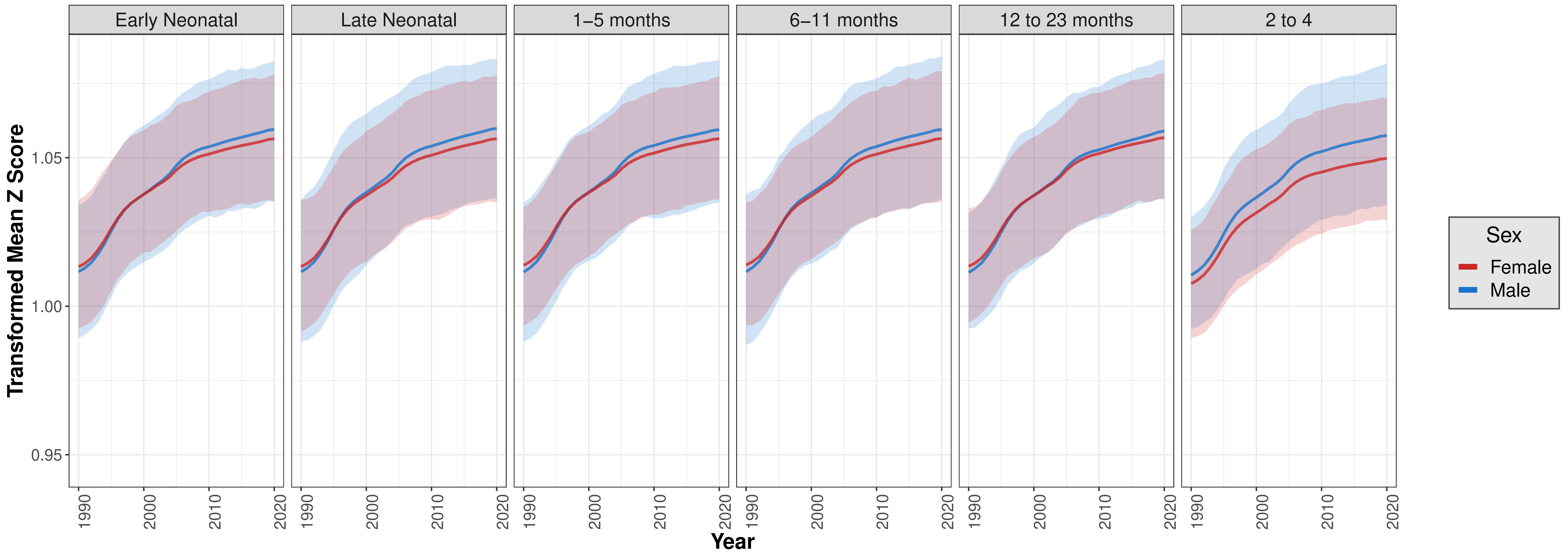

C

Year	Source
1985	WHO CGM Database
2002	WHO CGM Database

Spain – Wasting (WHZ)

D: Overall and Severe Wasting Prevalence

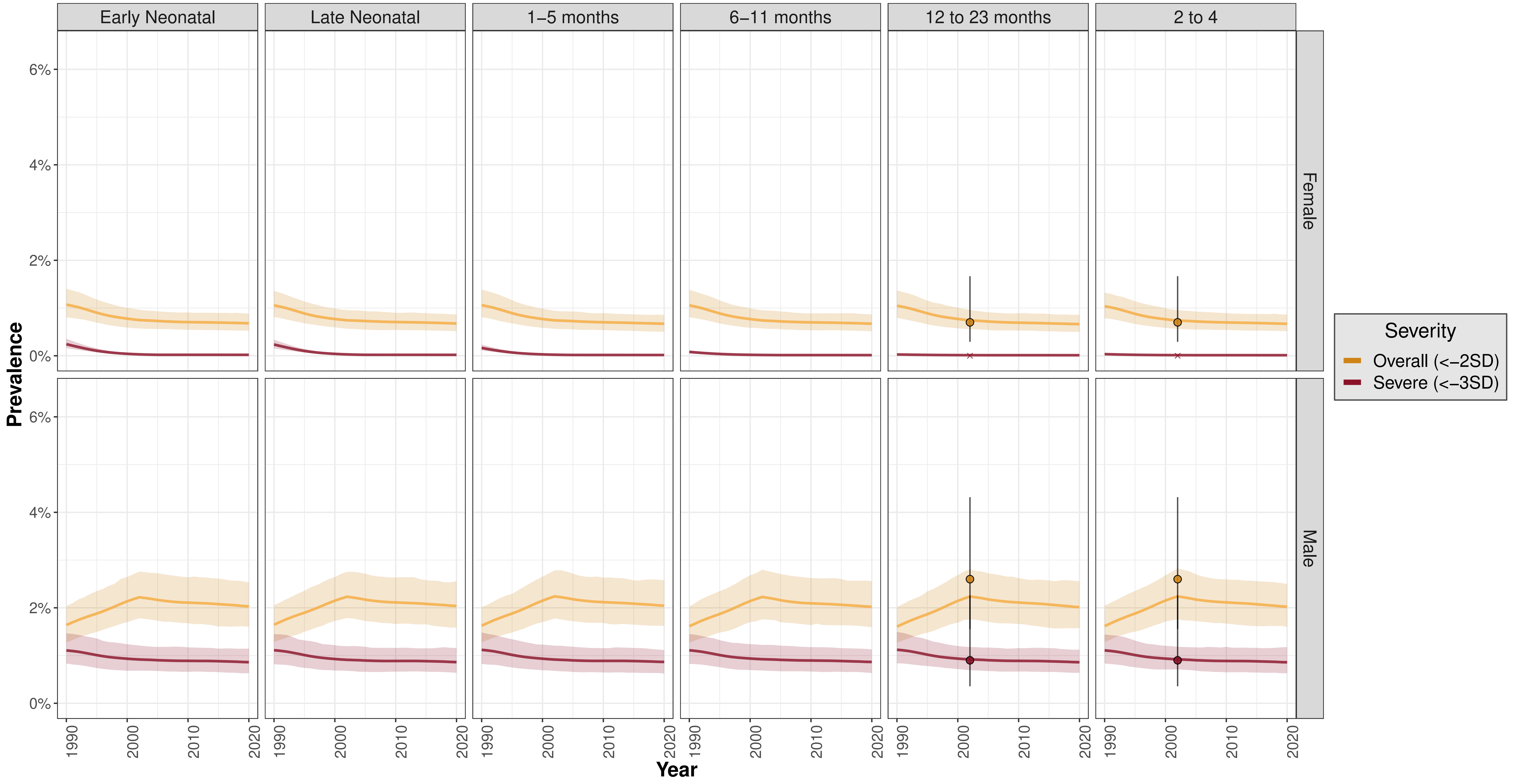

E: Transformed Mean Wasting Z Scores

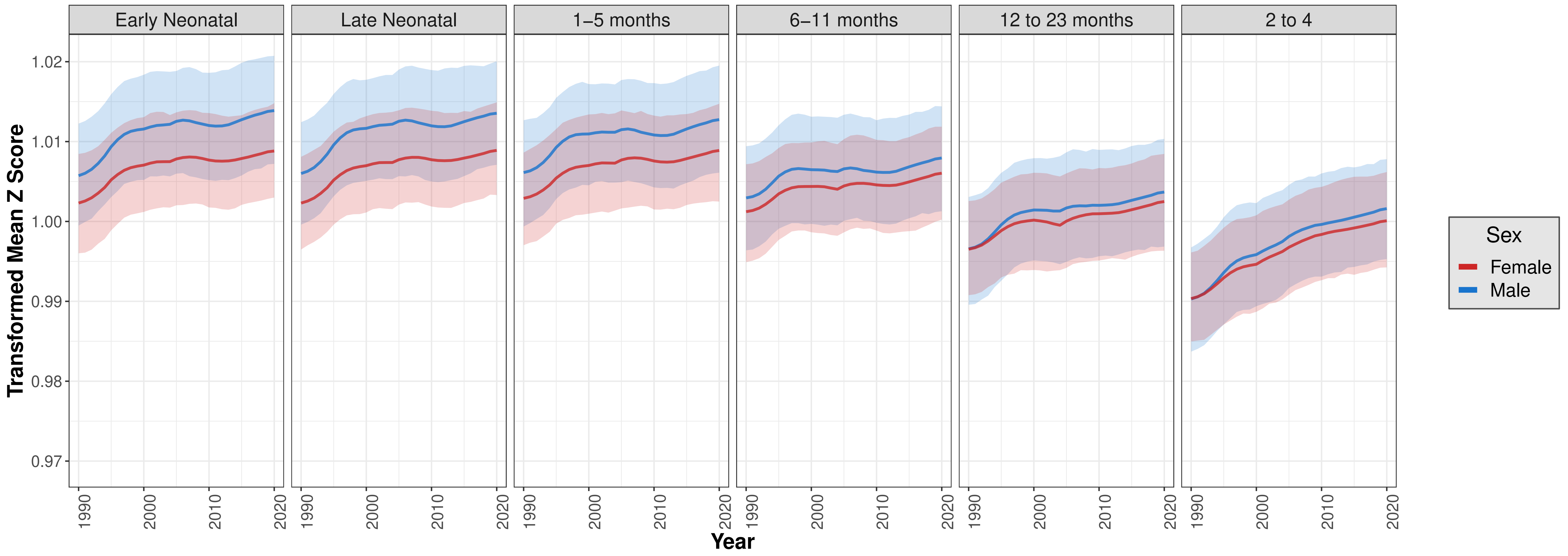

F

Year	Source
1985	WHO CGM Database
2002	WHO CGM Database

Spain – Underweight (WAZ)

G: Overall and Severe Underweight Prevalence

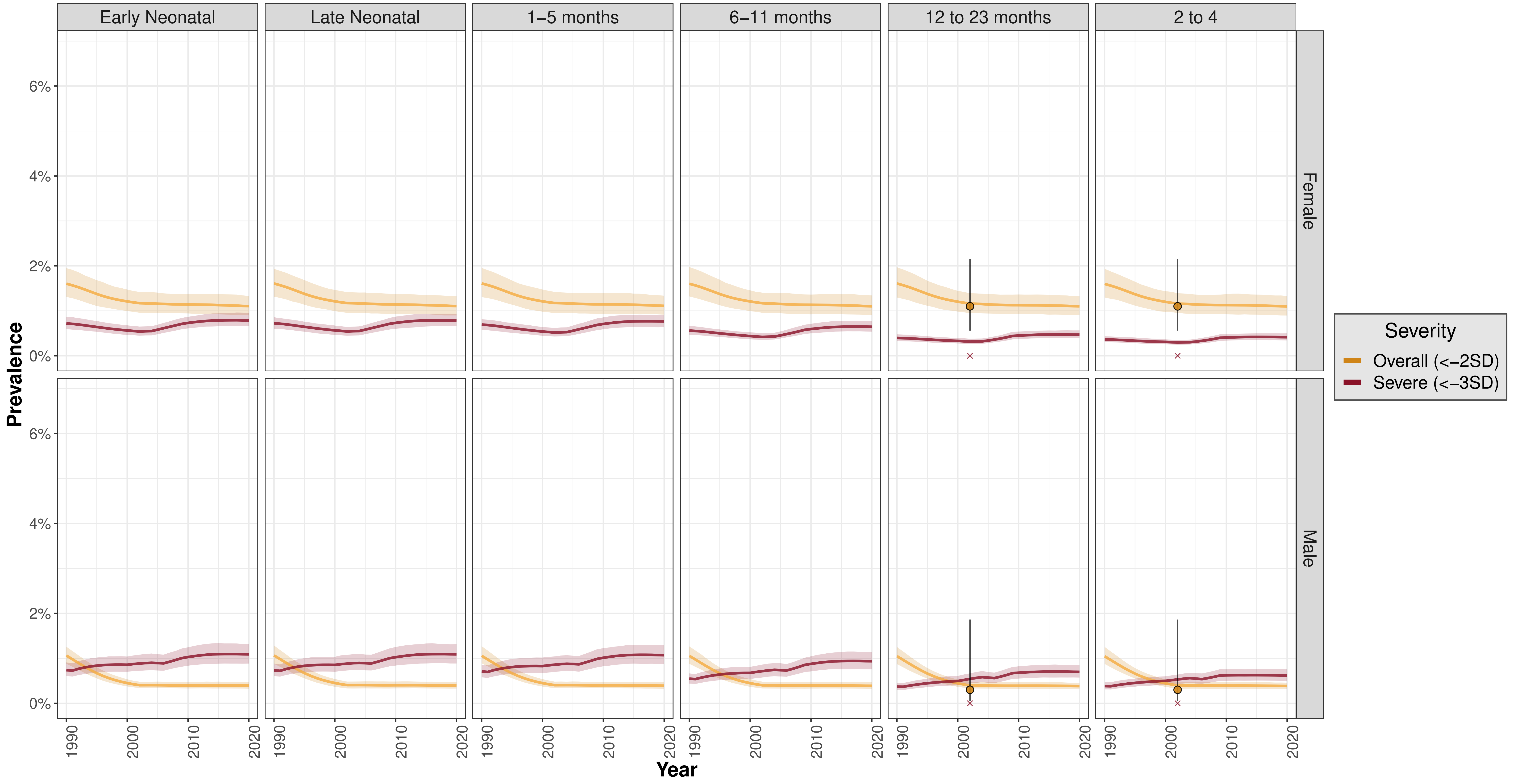

H: Transformed Mean Underweight Z Scores

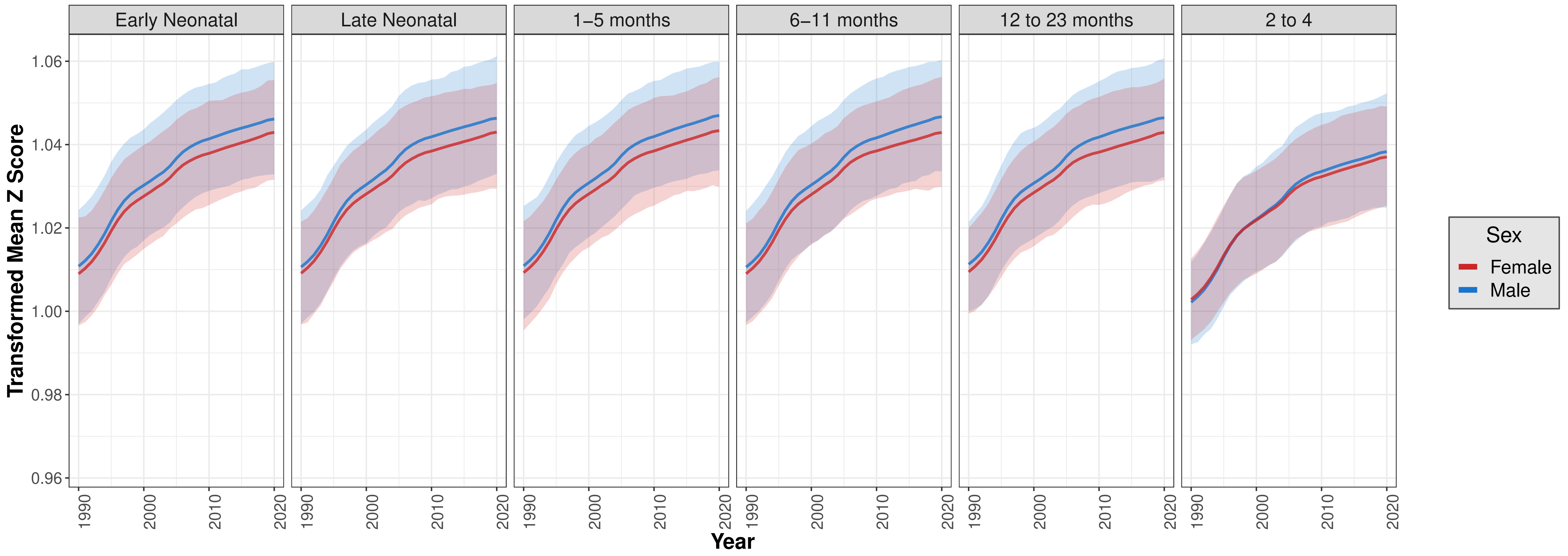

I

Year	Source
1985	WHO CGM Database
2002	WHO CGM Database

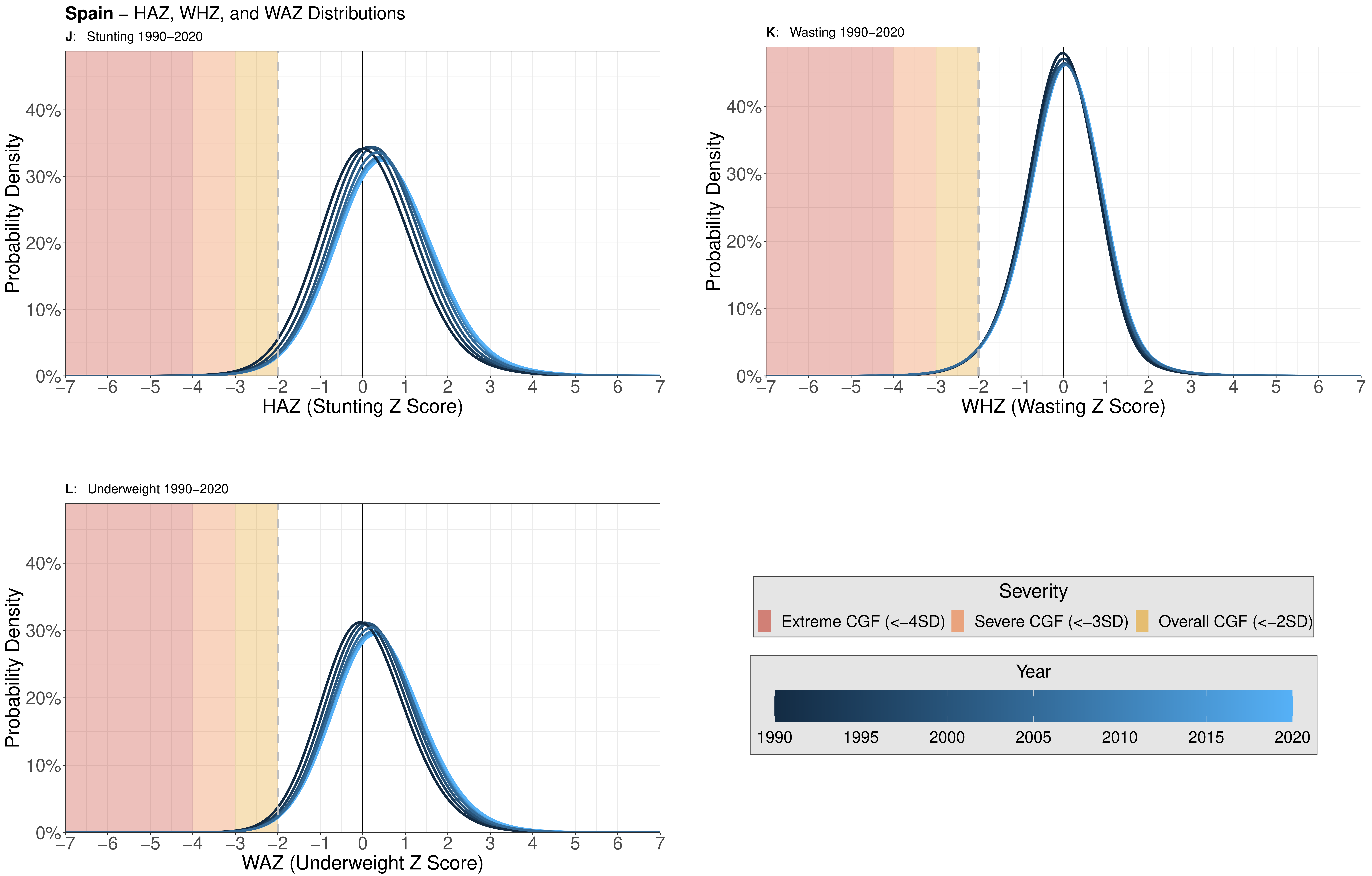

Spain – HAZ, WHZ, and WAZ Distributions
J: Stunting 1990–2020
Probability Density
0%
10%
20%
30%
40%
−7 −6 −5 −4 −3 −2 −1 0 1 2 3 4 5 6 7
HAZ (Stunting Z Score)
K: Wasting 1990–2020
Probability Density
0%
10%
20%
30%
40%
−7 −6 −5 −4 −3 −2 −1 0 1 2 3 4 5 6 7
WHZ (Wasting Z Score)
L: Underweight 1990–2020
Probability Density
0%
10%
20%
30%
40%
−7 −6 −5 −4 −3 −2 −1 0 1 2 3 4 5 6 7
WAZ (Underweight Z Score)
Severity
Extreme CGF (<−4SD)
Severe CGF (<−3SD)
Overall CGF (<−2SD)
Year
1990
1995
2000
2005
2010
2015
2020

Sweden – Stunting (HAZ)

A: Overall and Severe Stunting Prevalence

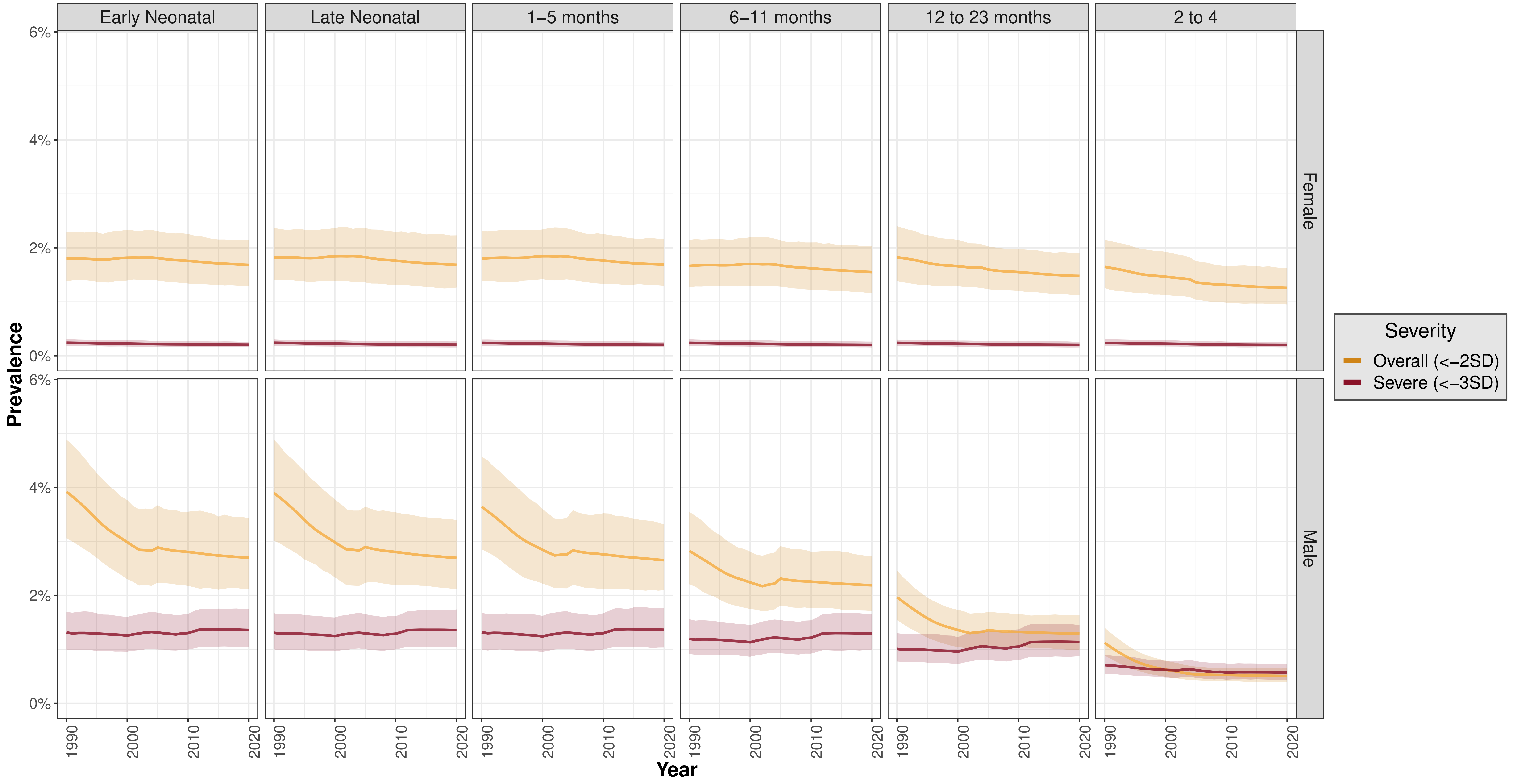

B: Transformed Mean Stunting Z Scores

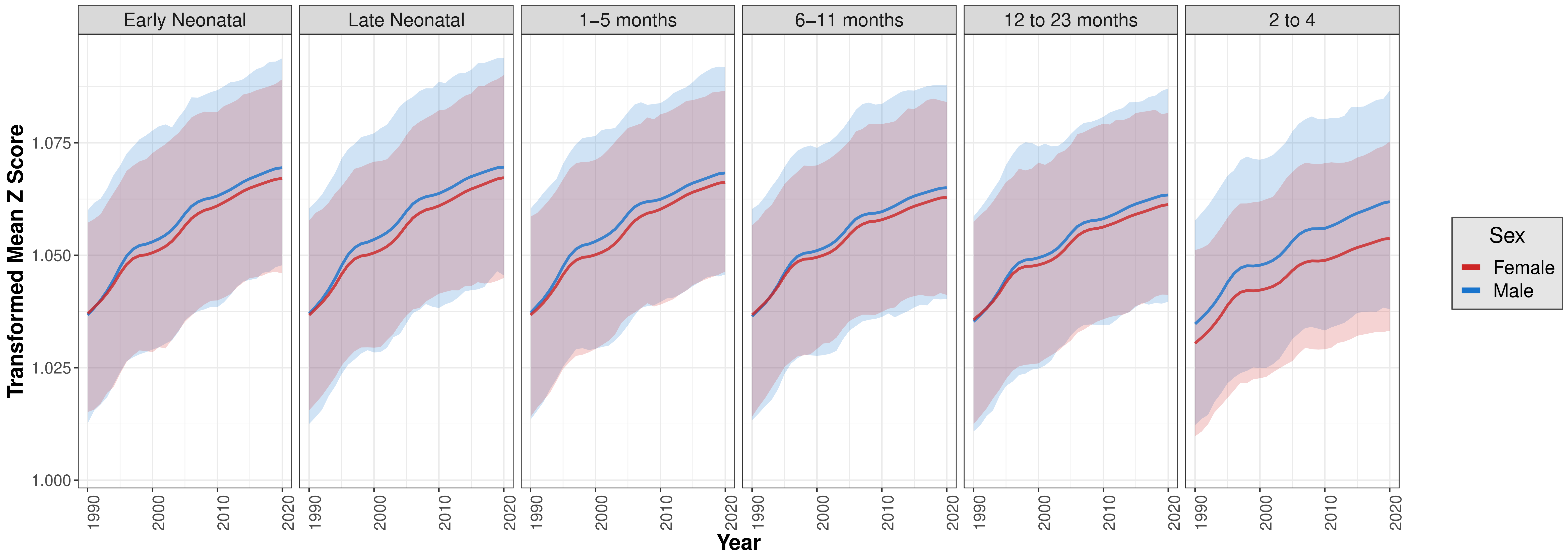

C

Source
No sources for this location

Sweden – Wasting (WHZ)

D: Overall and Severe Wasting Prevalence

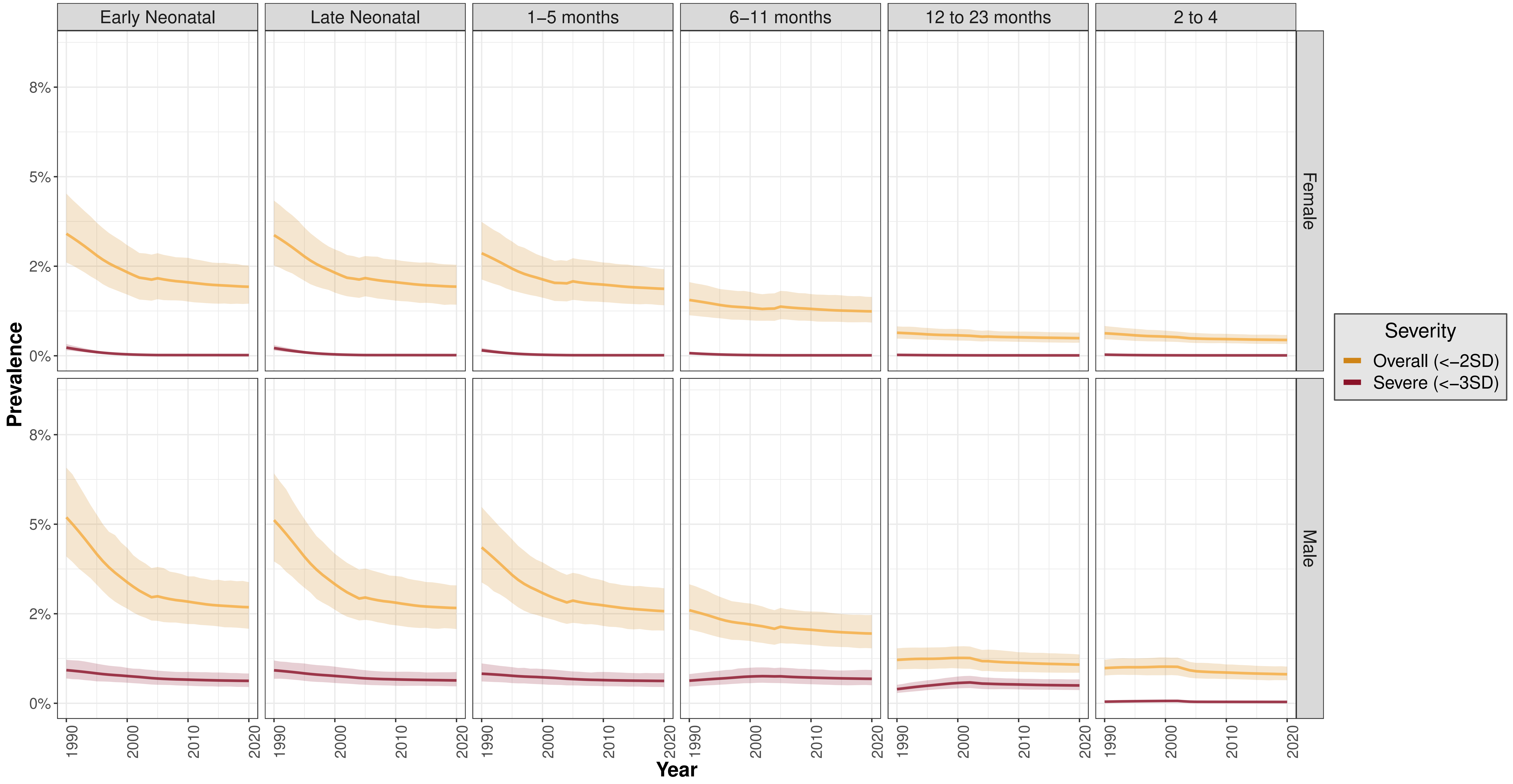

E: Transformed Mean Wasting Z Scores

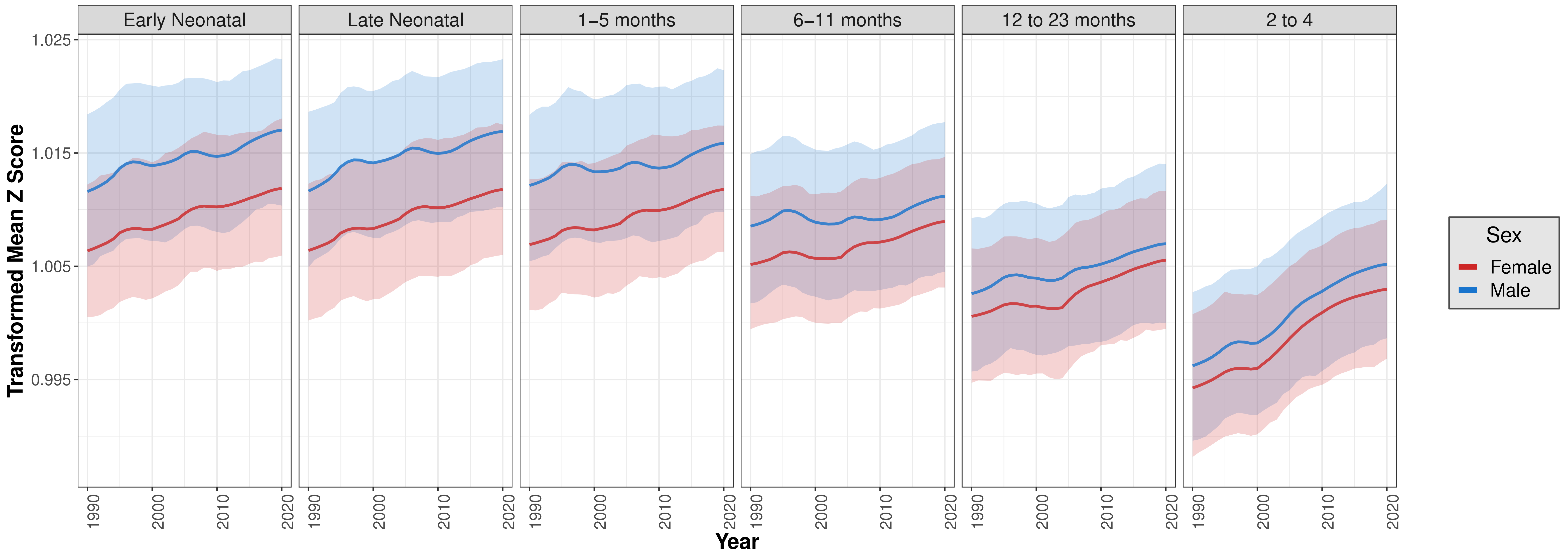

F

Source
No sources for this location

Sweden – Underweight (WAZ)

G: Overall and Severe Underweight Prevalence

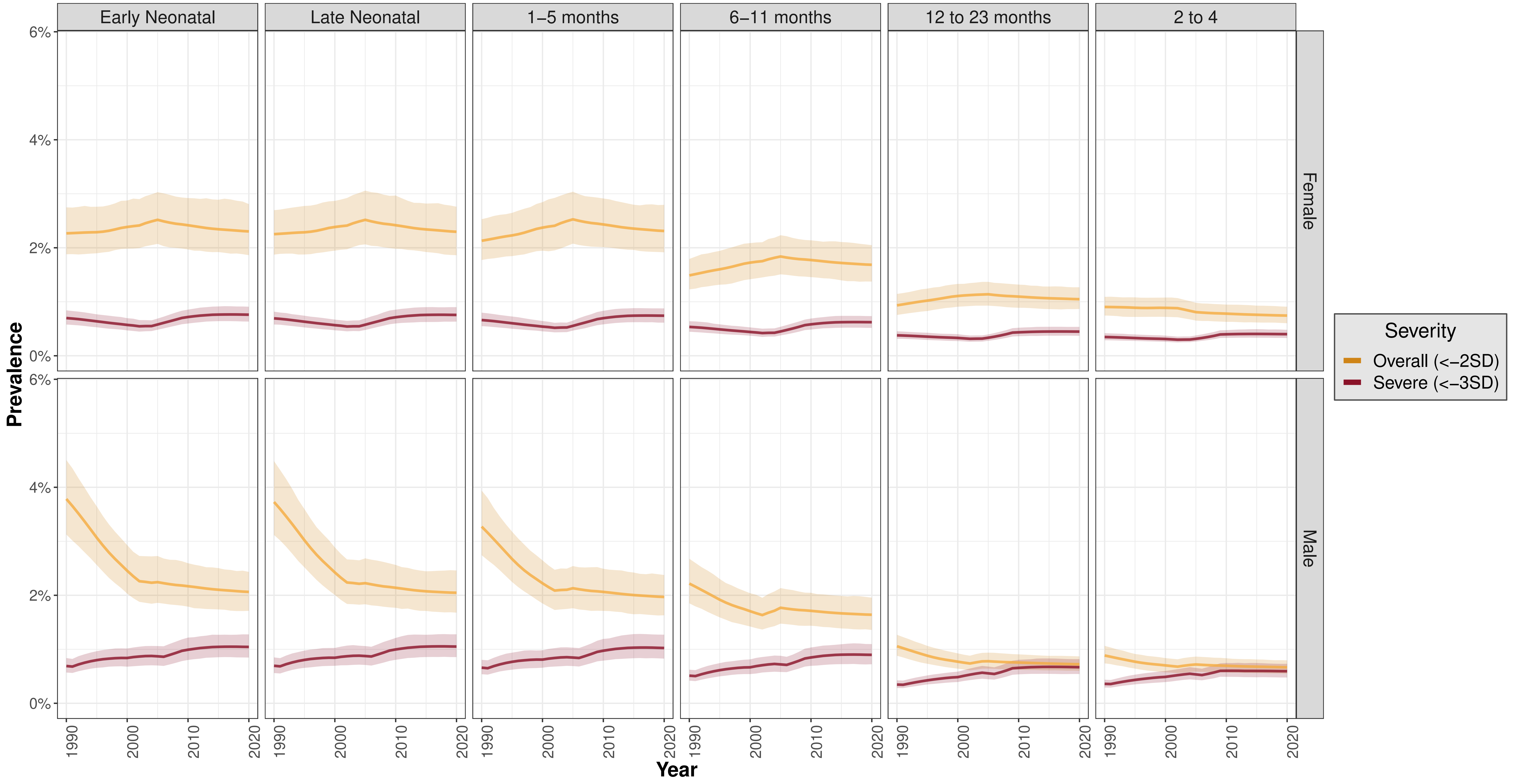

H: Transformed Mean Underweight Z Scores

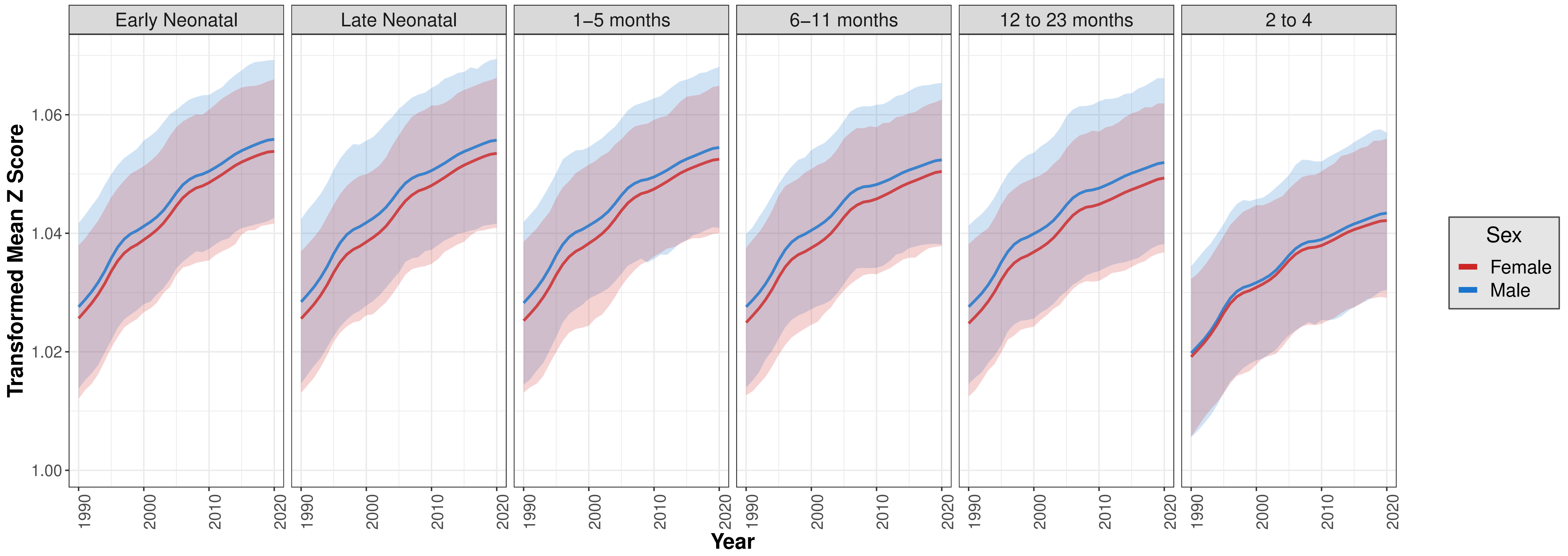

I

Source
No sources for this location

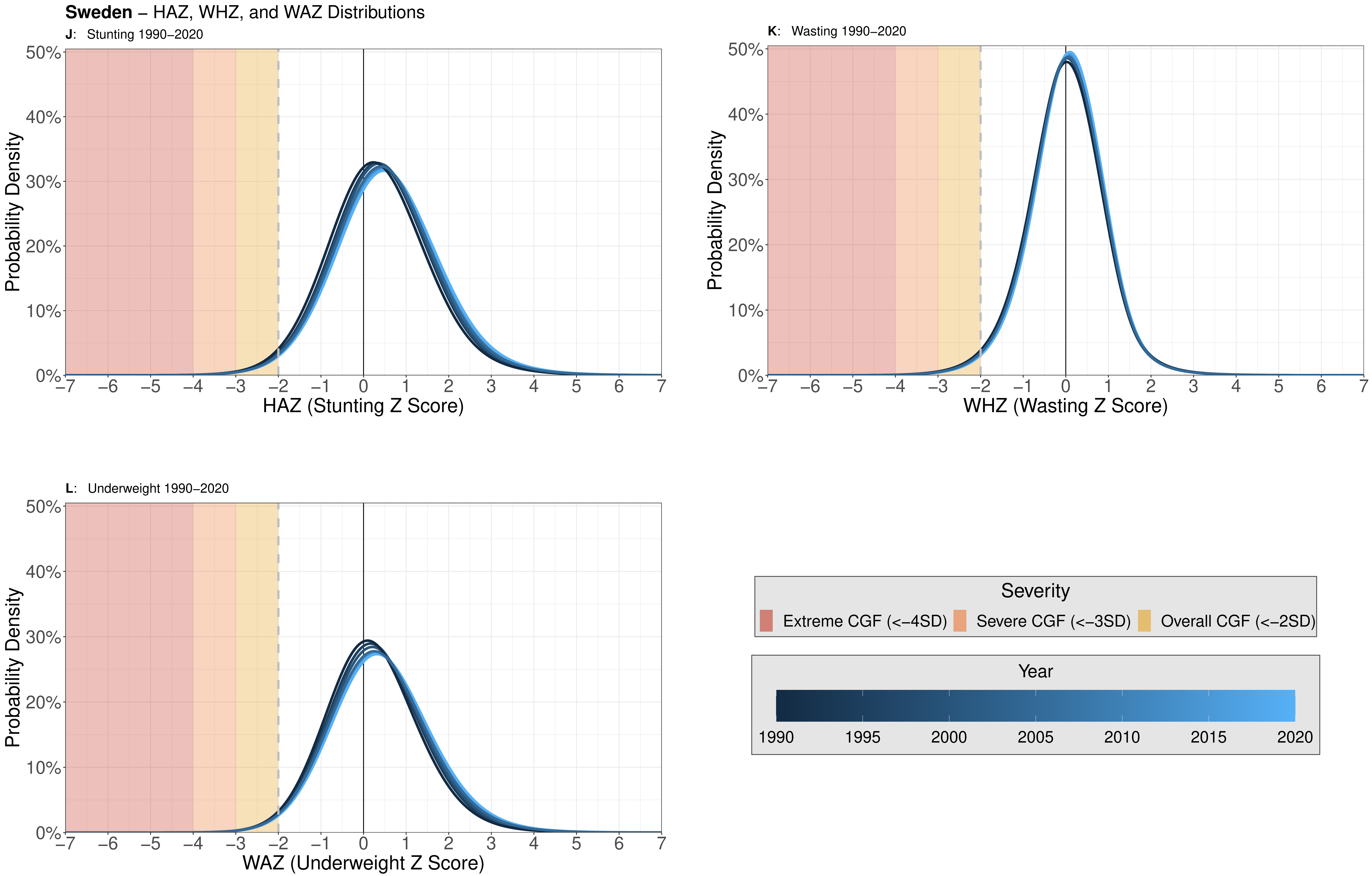

Sweden – HAZ, WHZ, and WAZ Distributions
J: Stunting 1990–2020
K: Wasting 1990–2020
L: Underweight 1990–2020
Probability Density
0%
10%
20%
30%
40%
50%
−7
−6
−5
−4
−3
−2
−1
0
1
2
3
4
5
6
7
HAZ (Stunting Z Score)
WHZ (Wasting Z Score)
WAZ (Underweight Z Score)
Severity
Extreme CGF (<−4SD)
Severe CGF (<−3SD)
Overall CGF (<−2SD)
Year
1990
1995
2000
2005
2010
2015
2020

Switzerland – Stunting (HAZ)

A: Overall and Severe Stunting Prevalence

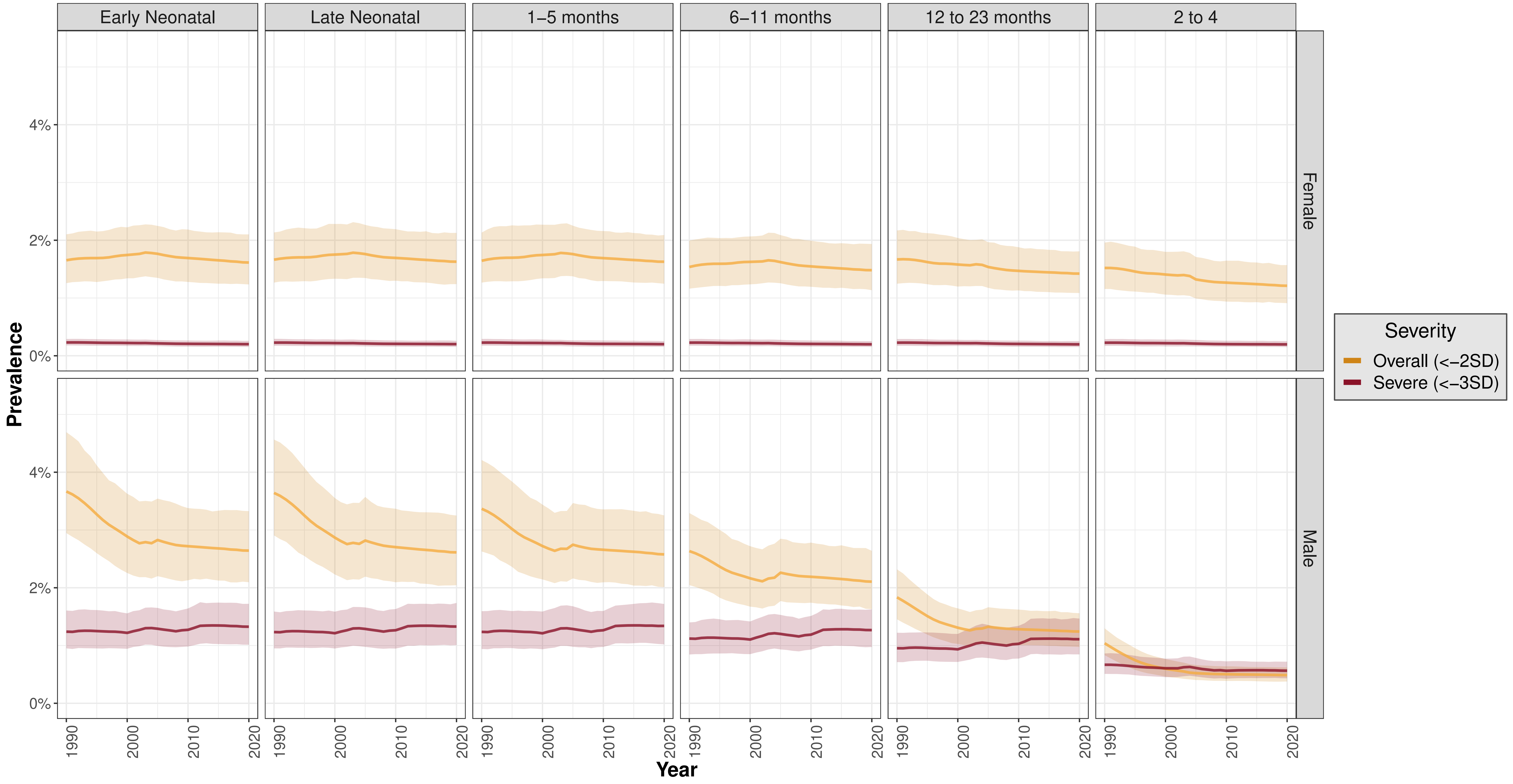

B: Transformed Mean Stunting Z Scores

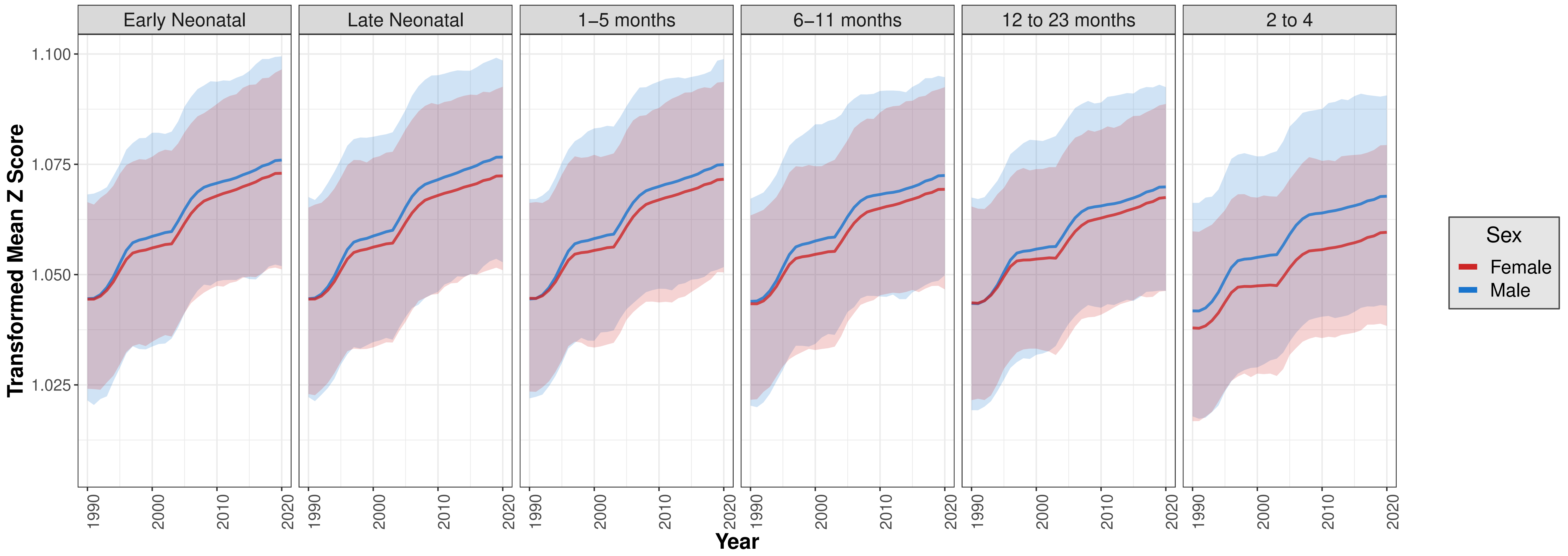

C

Source
No sources for this location

Switzerland – Wasting (WHZ)

D: Overall and Severe Wasting Prevalence

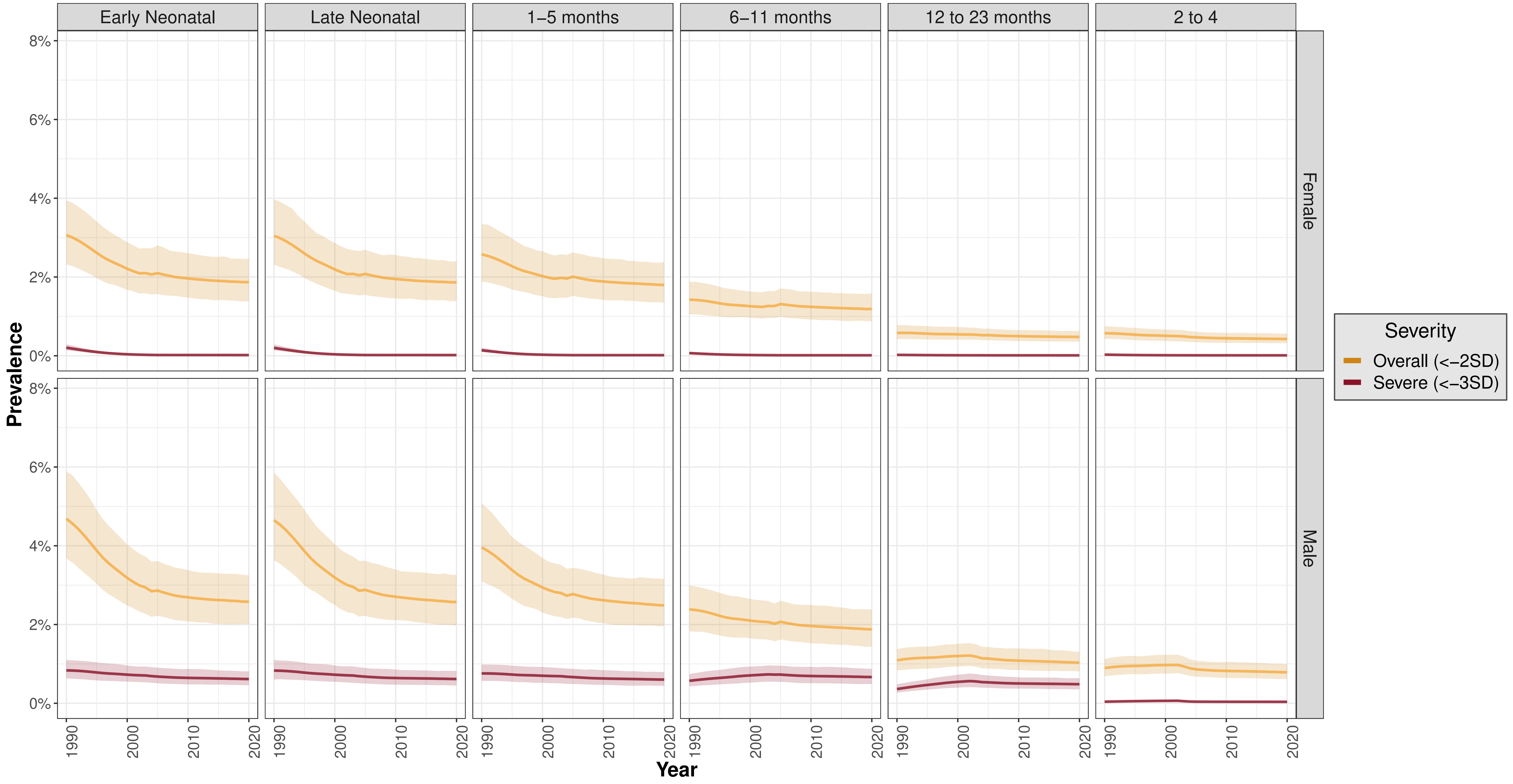

E: Transformed Mean Wasting Z Scores

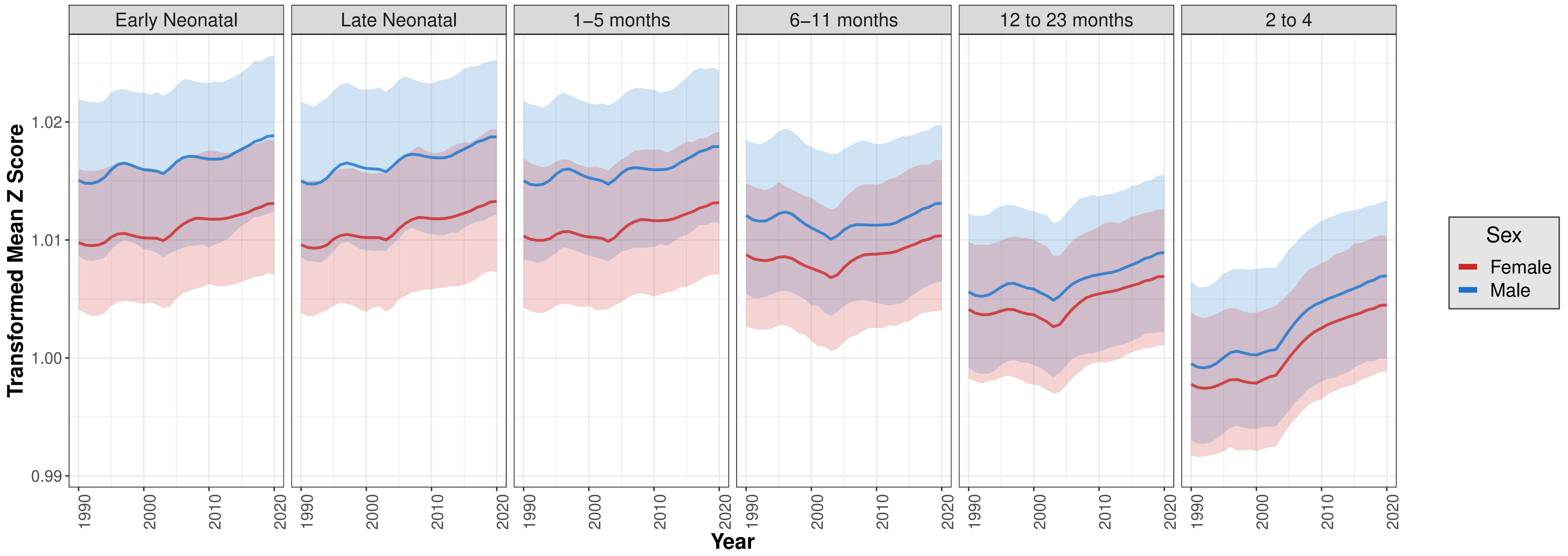

F

Source
No sources for this location

Switzerland – Underweight (WAZ)

G: Overall and Severe Underweight Prevalence

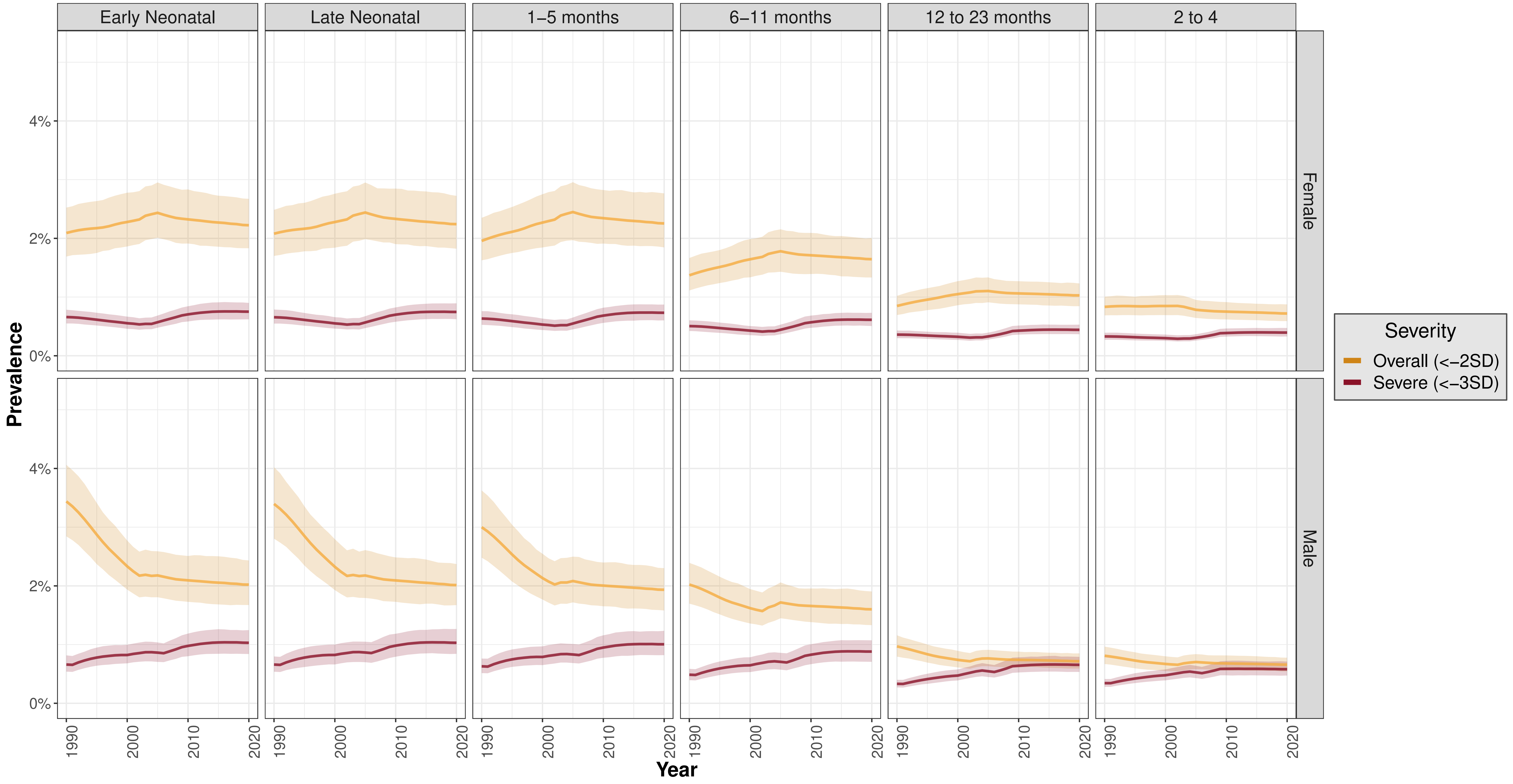

H: Transformed Mean Underweight Z Scores

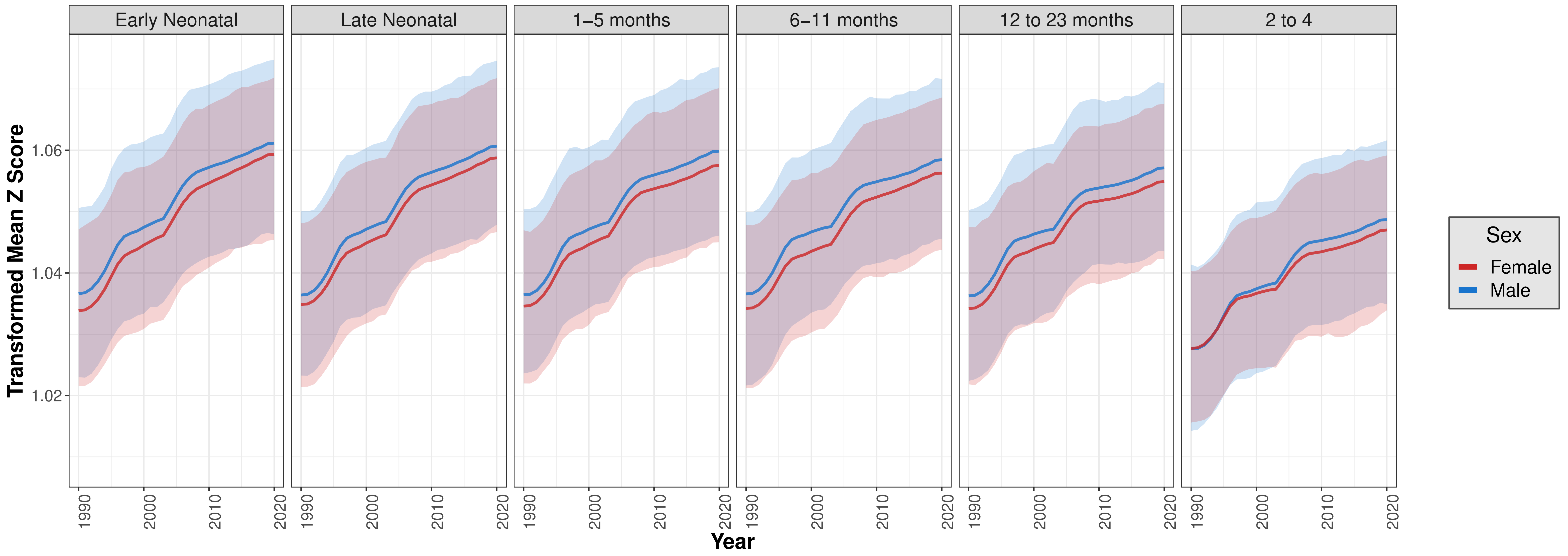

I

**Source**
No sources for this location

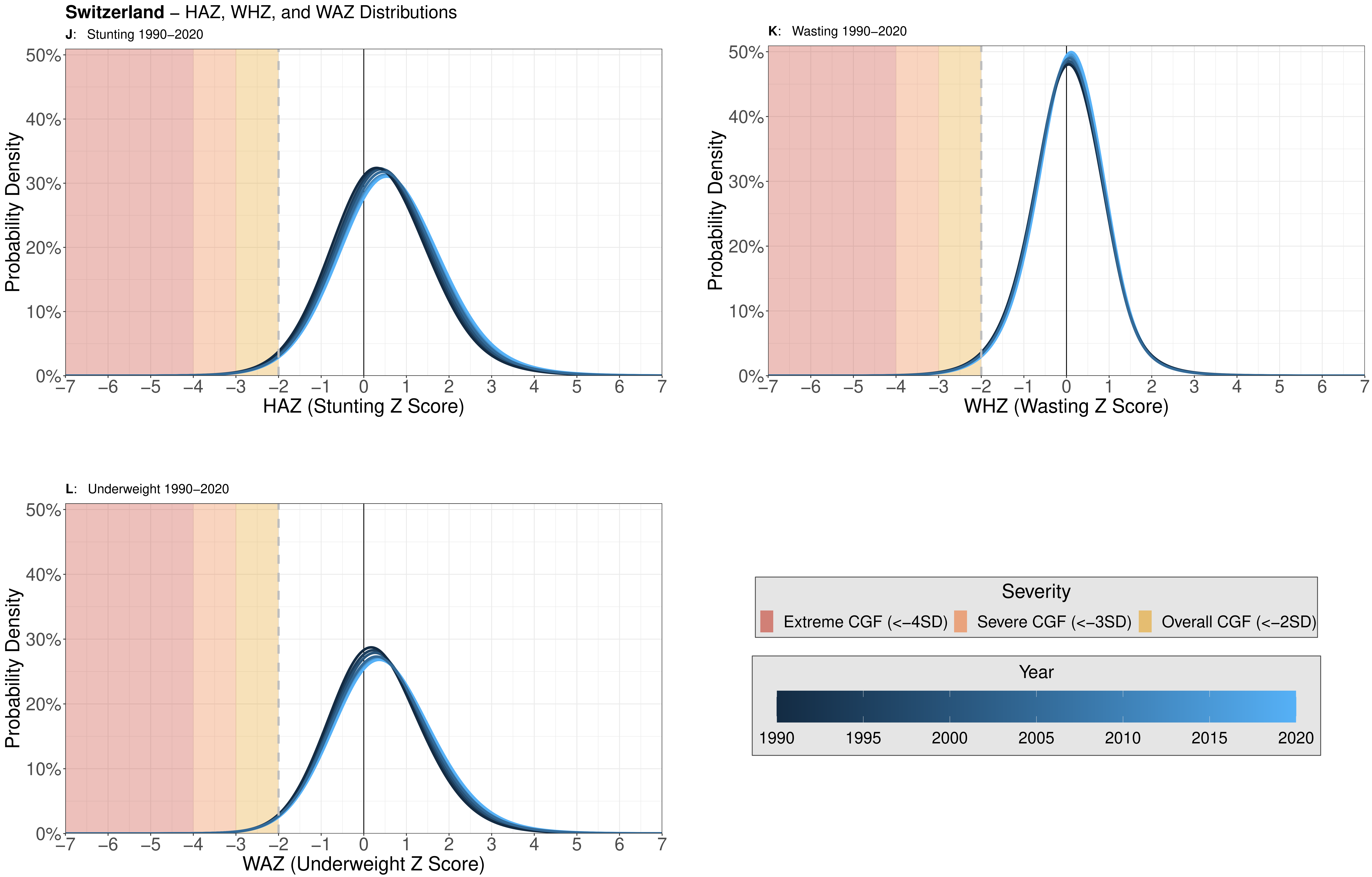

Switzerland – HAZ, WHZ, and WAZ Distributions
J: Stunting 1990–2020
Probability Density
50%
40%
30%
20%
10%
0%
−7 −6 −5 −4 −3 −2 −1 0 1 2 3 4 5 6 7
HAZ (Stunting Z Score)
K: Wasting 1990–2020
Probability Density
WHZ (Wasting Z Score)
L: Underweight 1990–2020
Probability Density
WAZ (Underweight Z Score)
Severity
Extreme CGF (<−4SD)
Severe CGF (<−3SD)
Overall CGF (<−2SD)
Year
1990
1995
2000
2005
2010
2015
2020

Northern Ireland – Stunting (HAZ)

A: Overall and Severe Stunting Prevalence

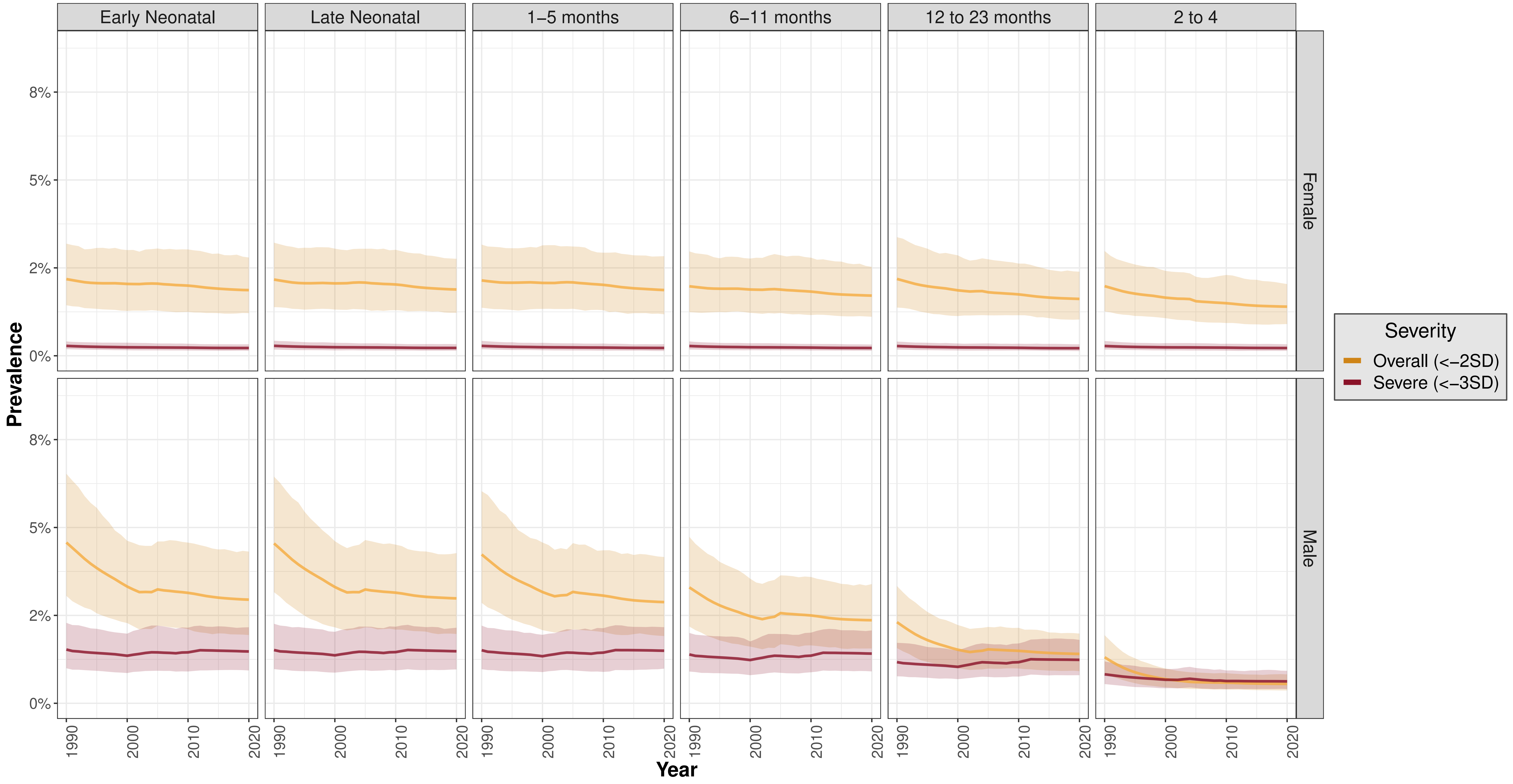

B: Transformed Mean Stunting Z Scores

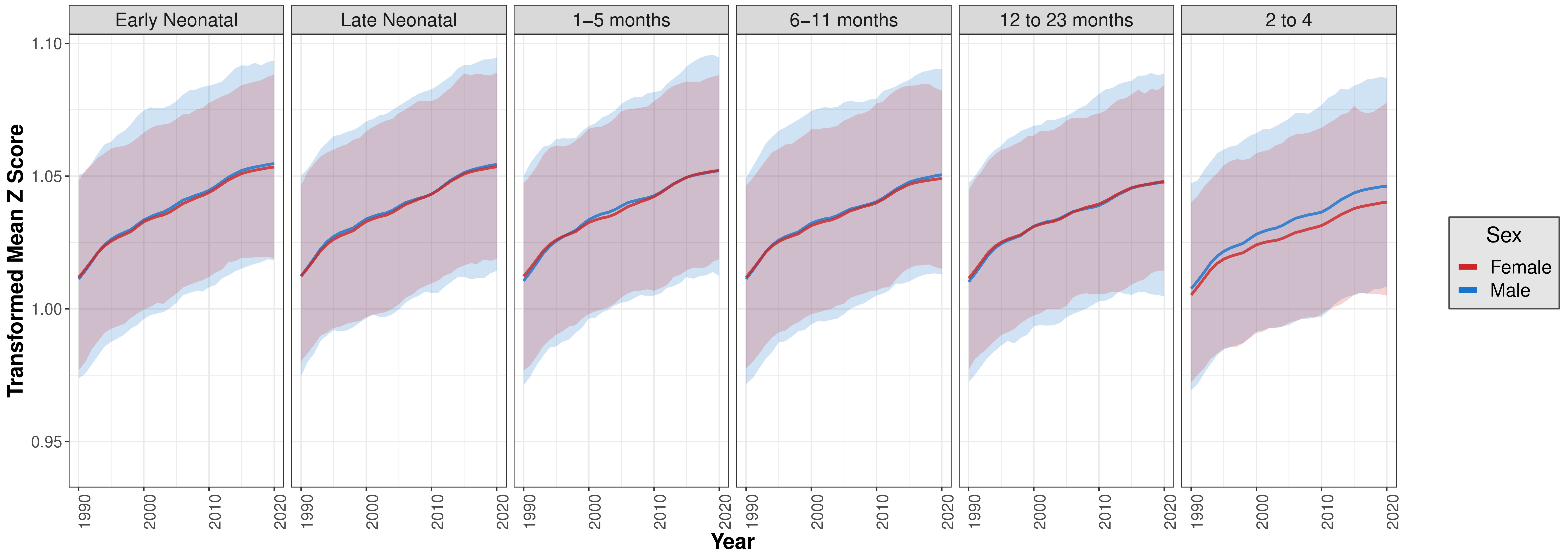

C

**Source**
No sources for this location

Northern Ireland – Wasting (WHZ)

D: Overall and Severe Wasting Prevalence

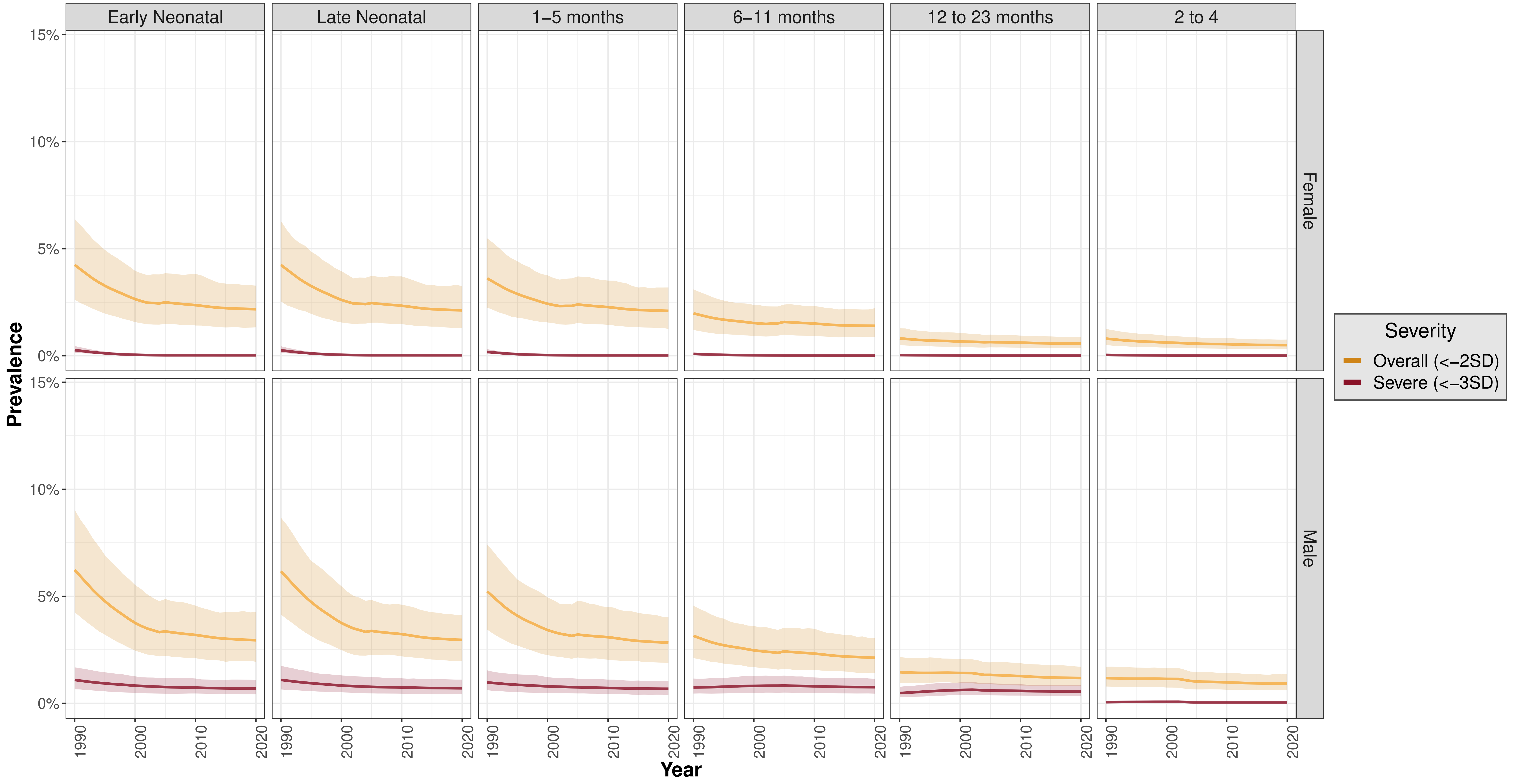

E: Transformed Mean Wasting Z Scores

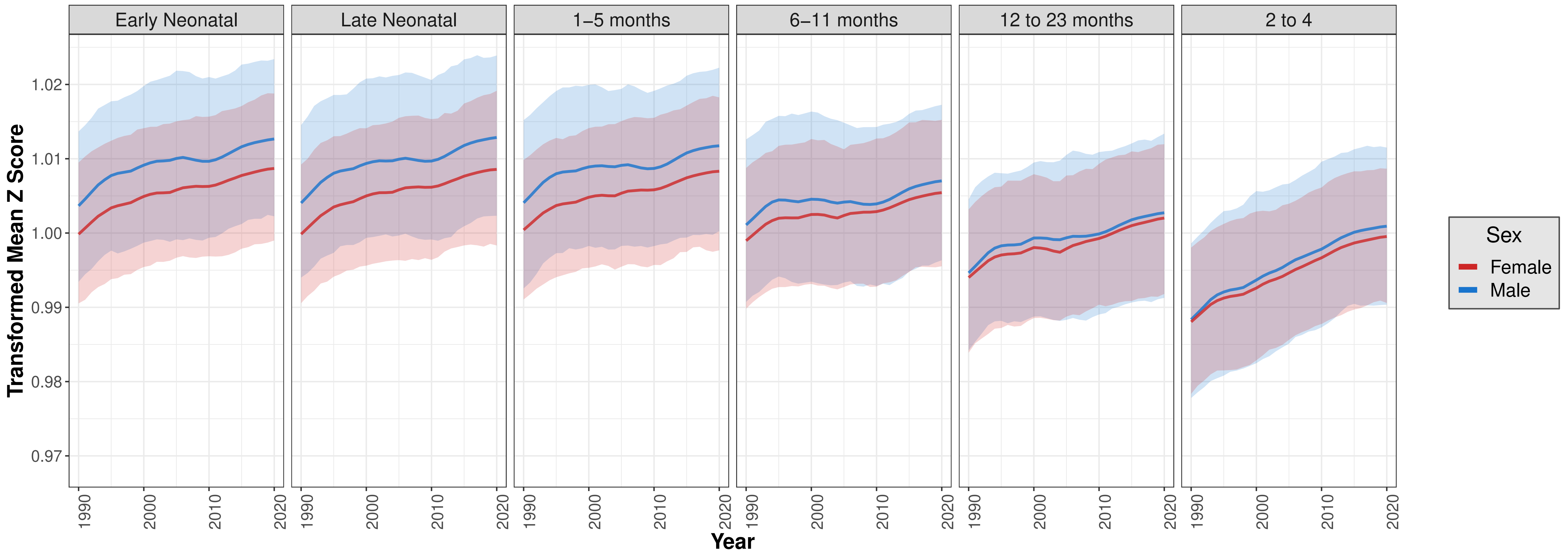

F

**Source**
No sources for this location

Northern Ireland – Underweight (WAZ)

G: Overall and Severe Underweight Prevalence

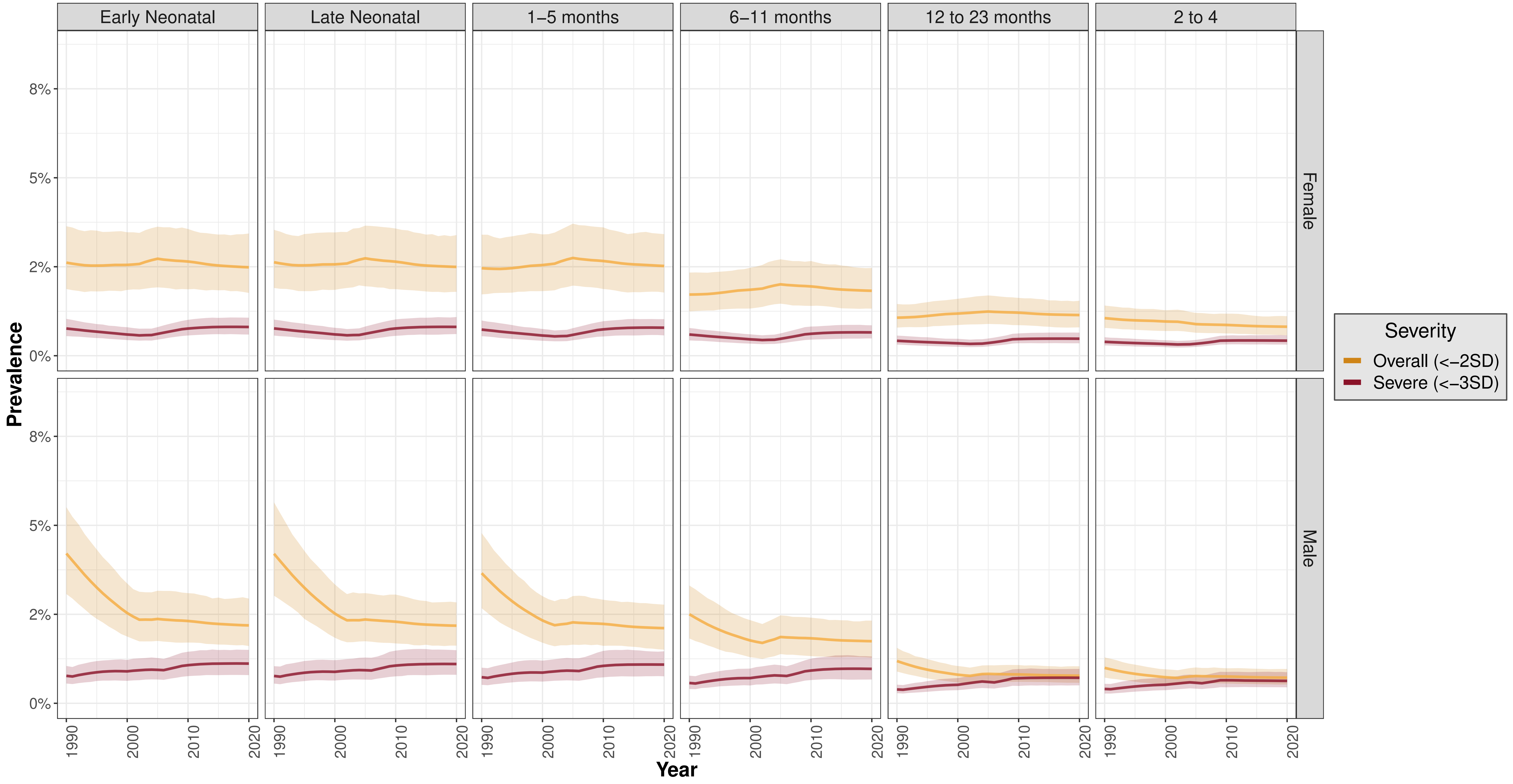

H: Transformed Mean Underweight Z Scores

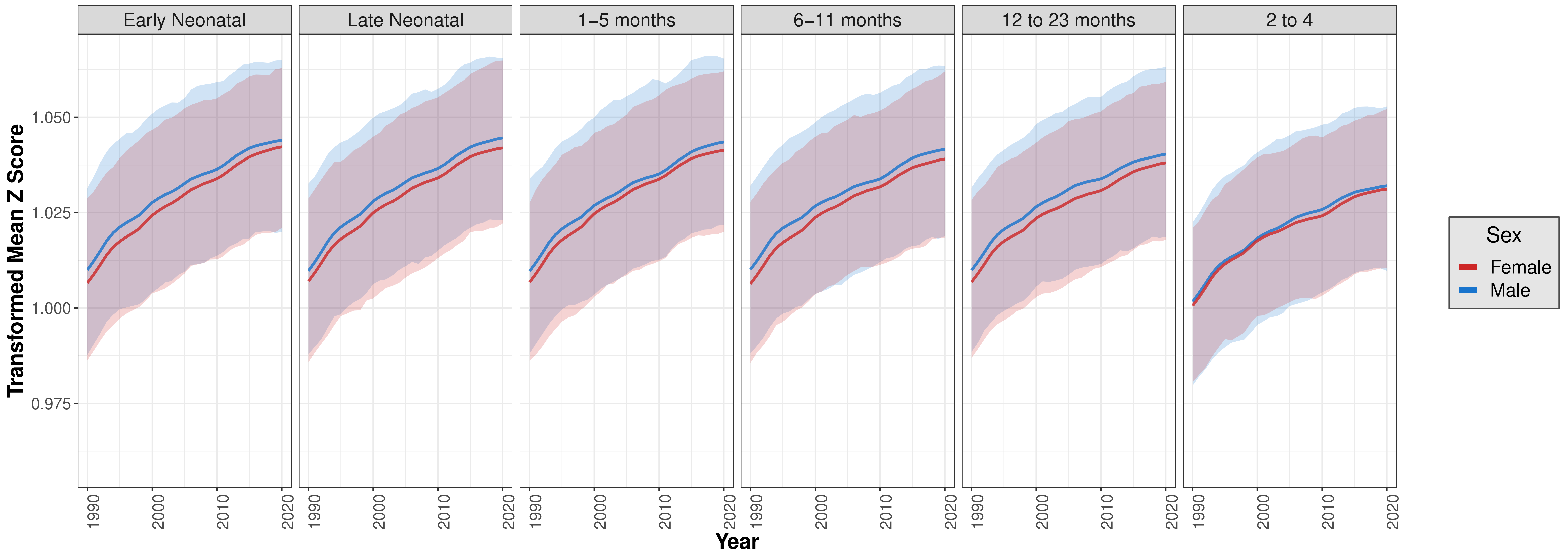

I

Source
No sources for this location

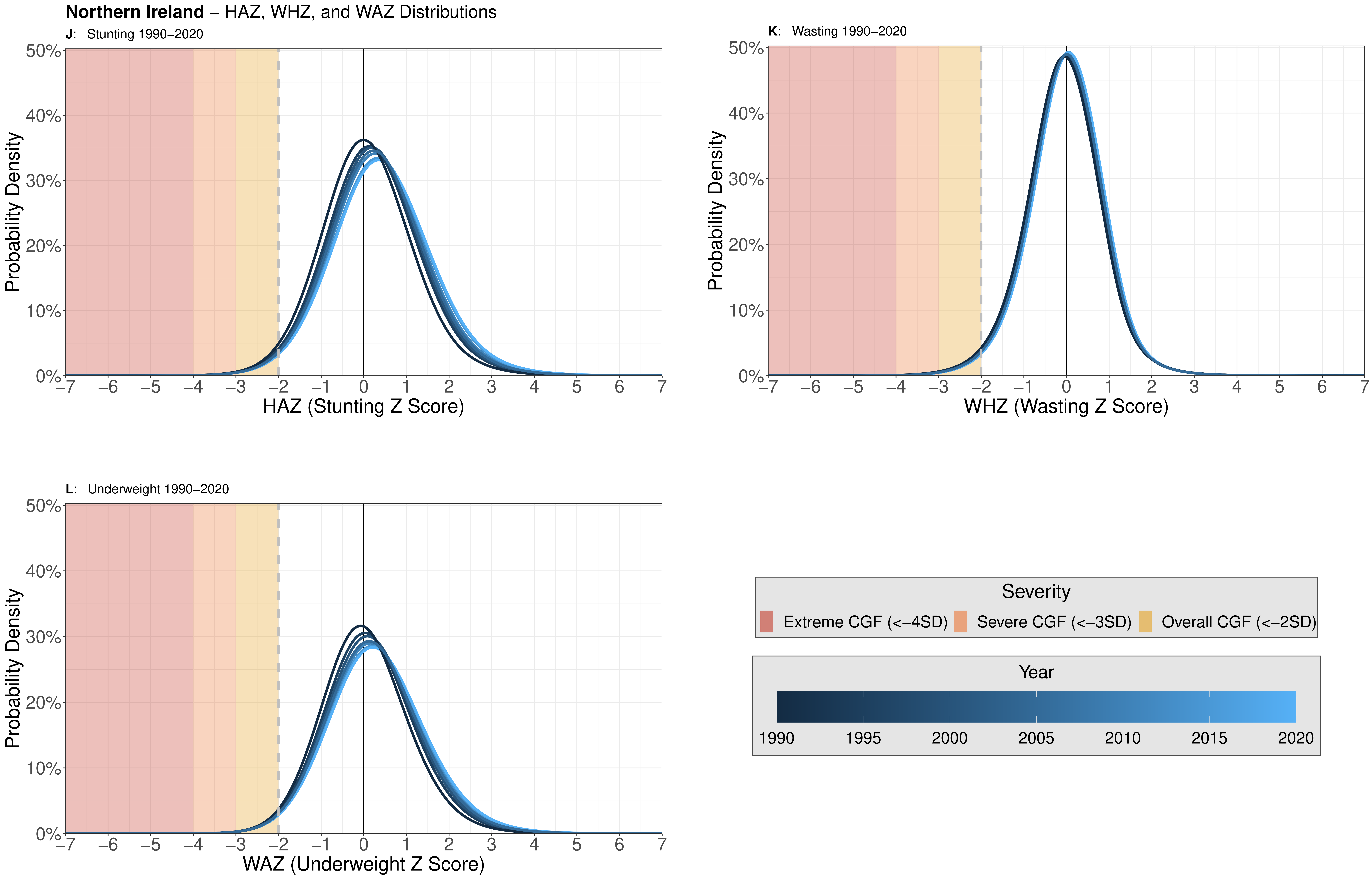

Northern Ireland – HAZ, WHZ, and WAZ Distributions
J: Stunting 1990–2020
K: Wasting 1990–2020
L: Underweight 1990–2020
Probability Density
0%
10%
20%
30%
40%
50%
−7 −6 −5 −4 −3 −2 −1 0 1 2 3 4 5 6 7
HAZ (Stunting Z Score)
WHZ (Wasting Z Score)
WAZ (Underweight Z Score)
Severity
Extreme CGF (<−4SD)
Severe CGF (<−3SD)
Overall CGF (<−2SD)
Year
1990 1995 2000 2005 2010 2015 2020

Scotland – Stunting (HAZ)

A: Overall and Severe Stunting Prevalence

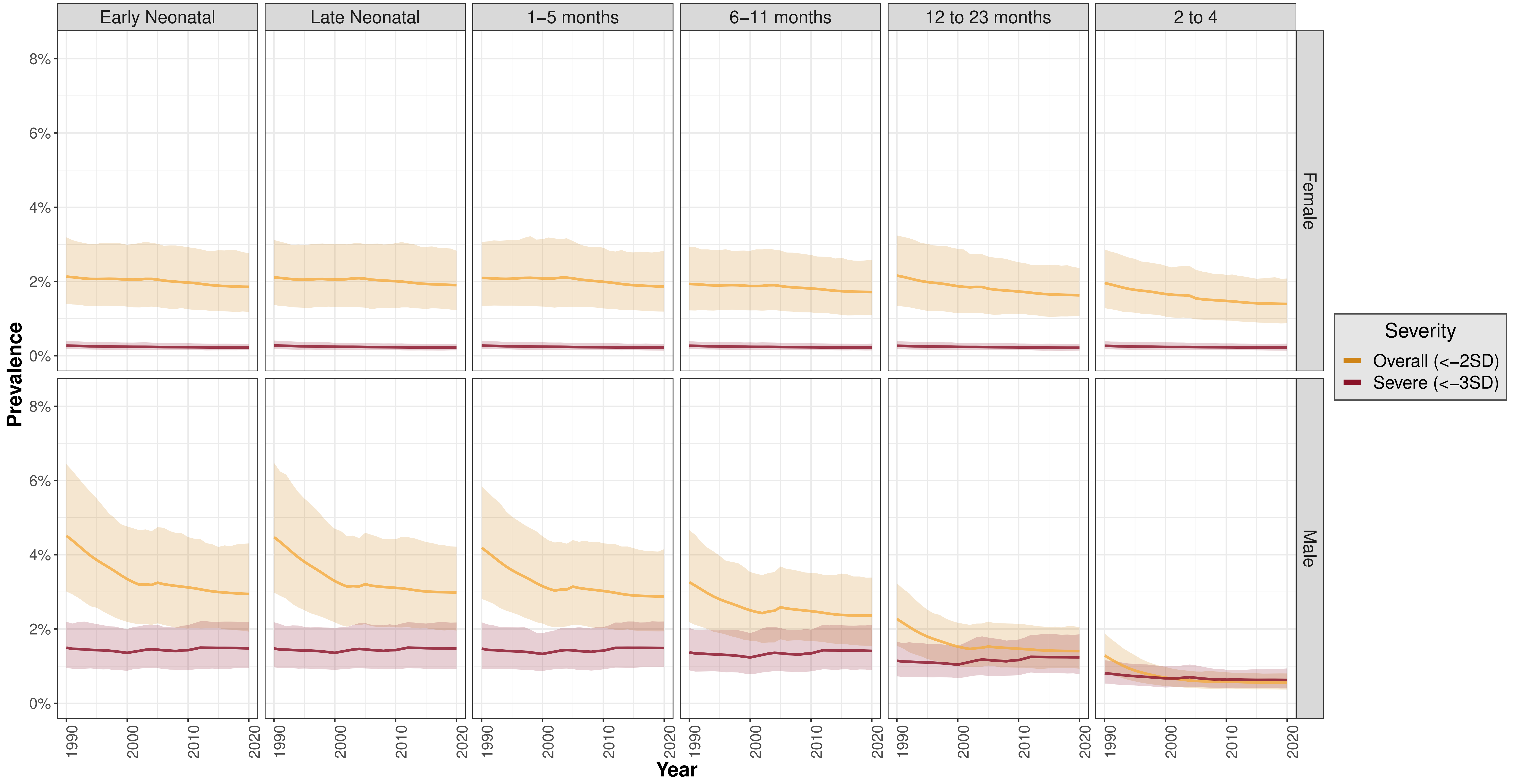

B: Transformed Mean Stunting Z Scores

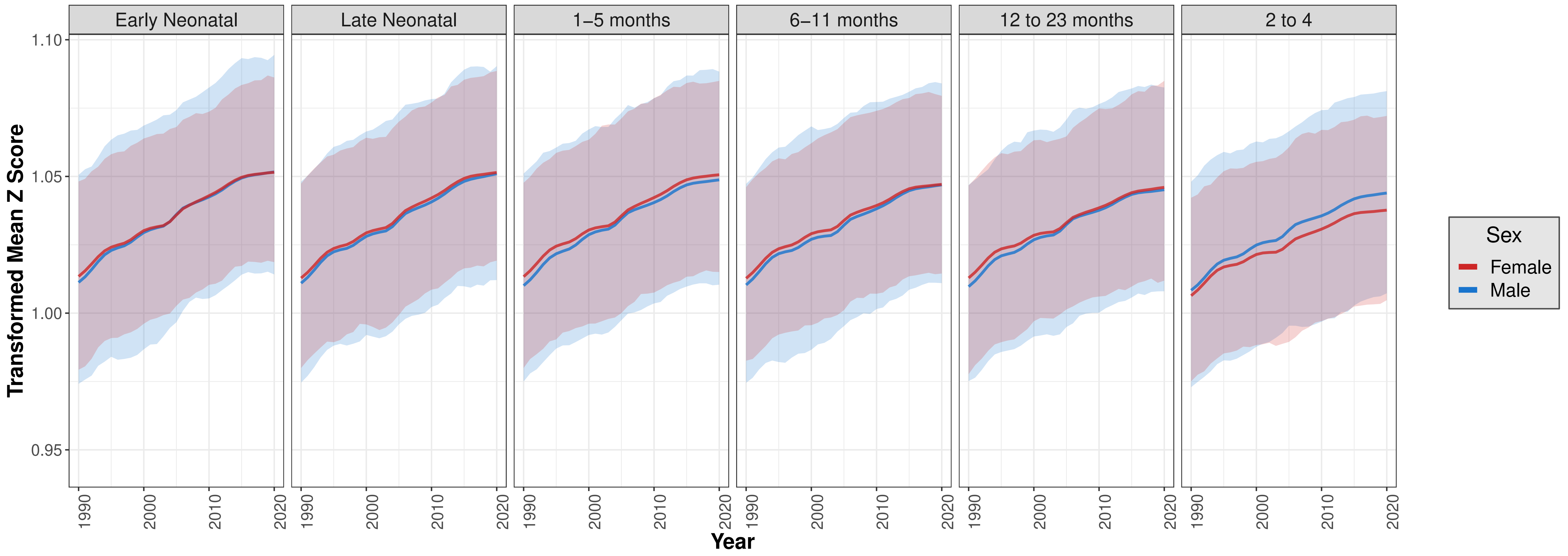

C

**Source**
No sources for this location

Scotland – Wasting (WHZ)

D: Overall and Severe Wasting Prevalence

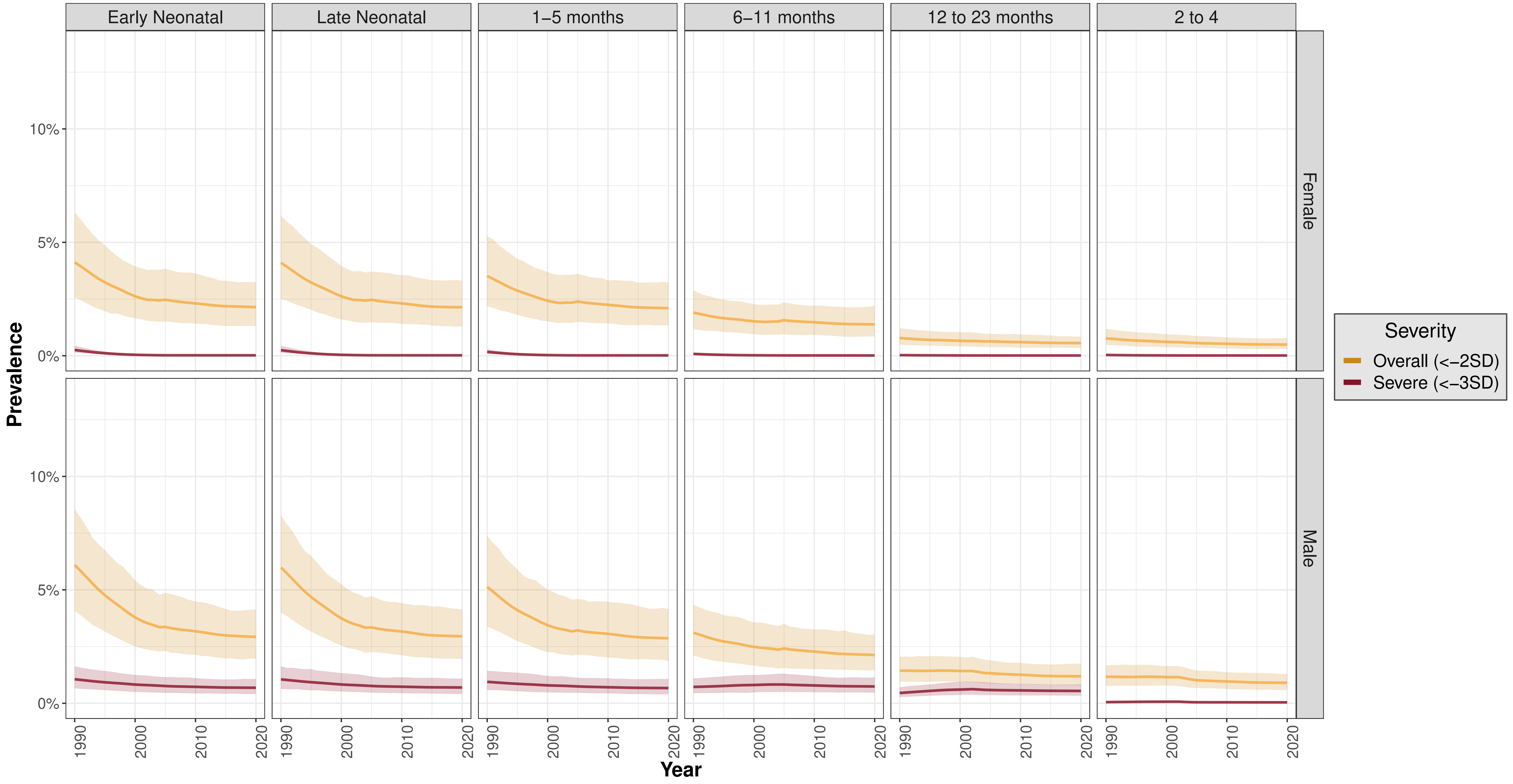

E: Transformed Mean Wasting Z Scores

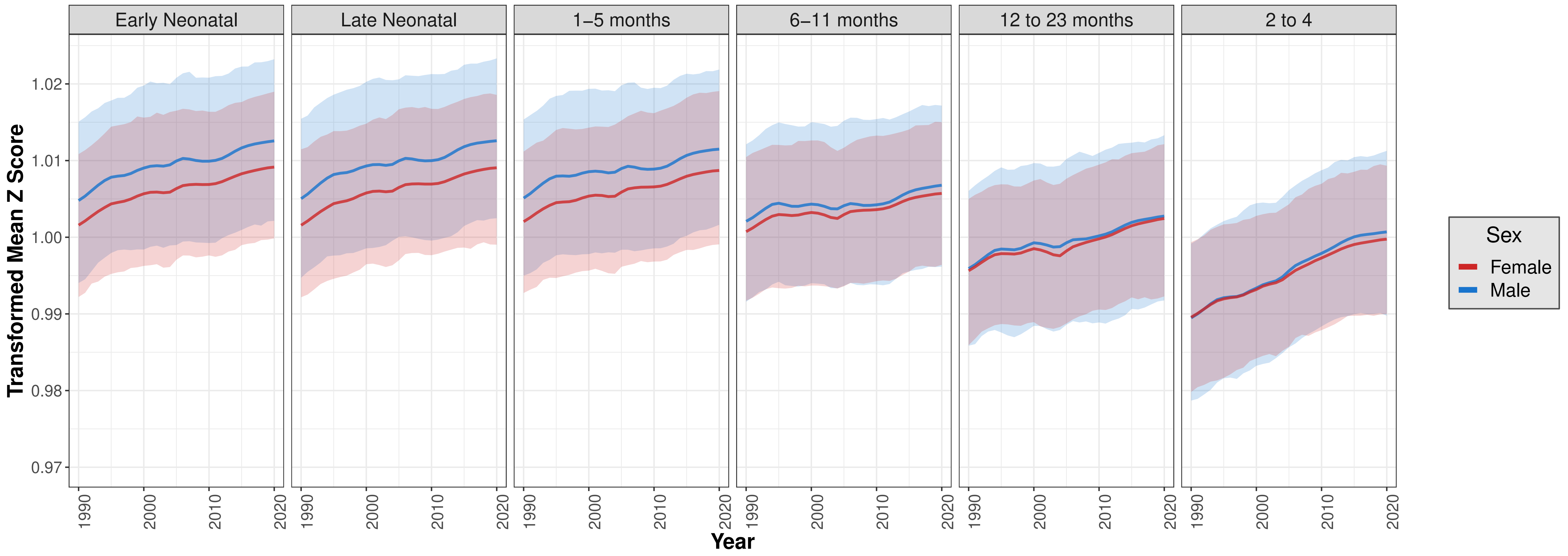

F

Source
No sources for this location

Scotland – Underweight (WAZ)

G: Overall and Severe Underweight Prevalence

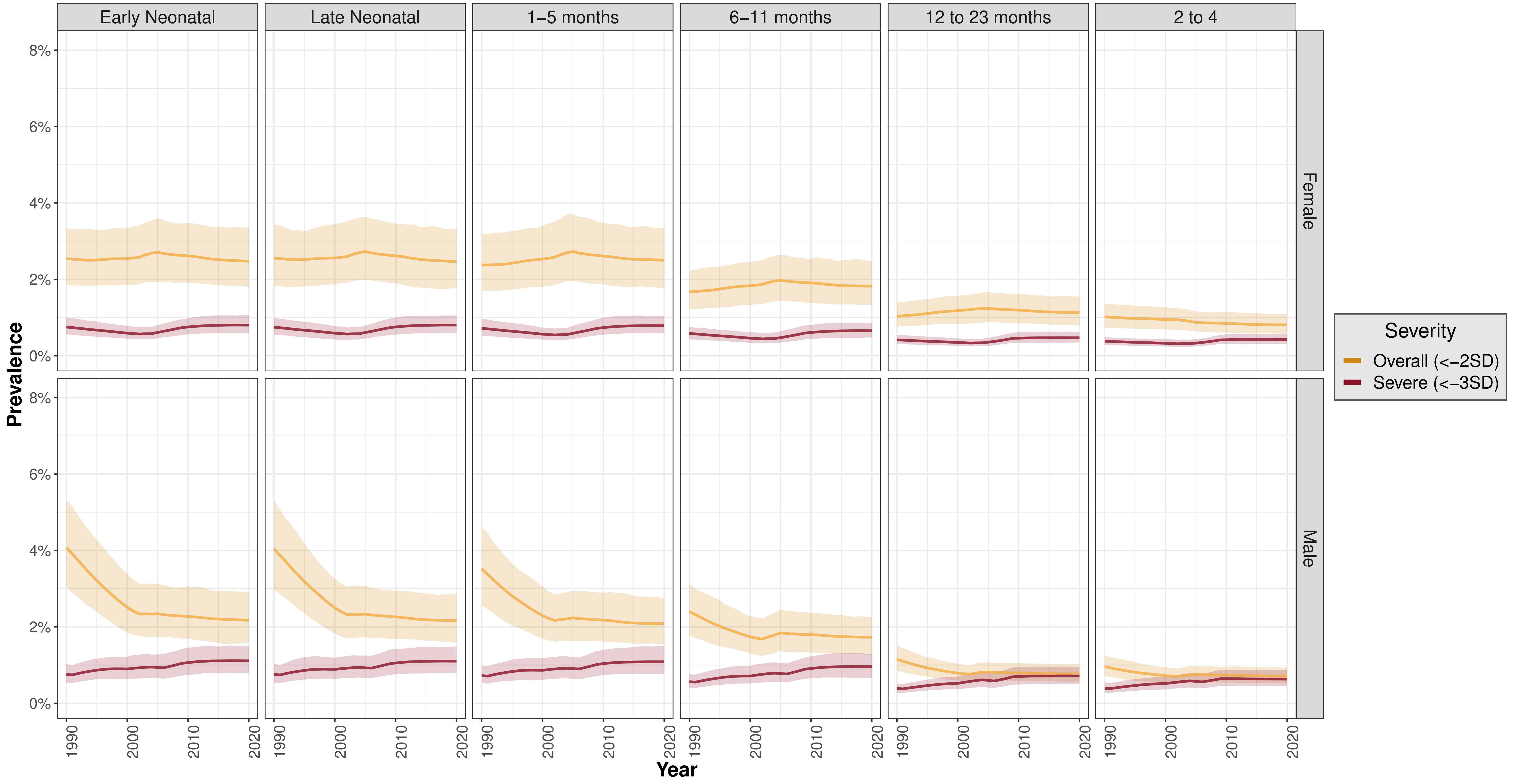

H: Transformed Mean Underweight Z Scores

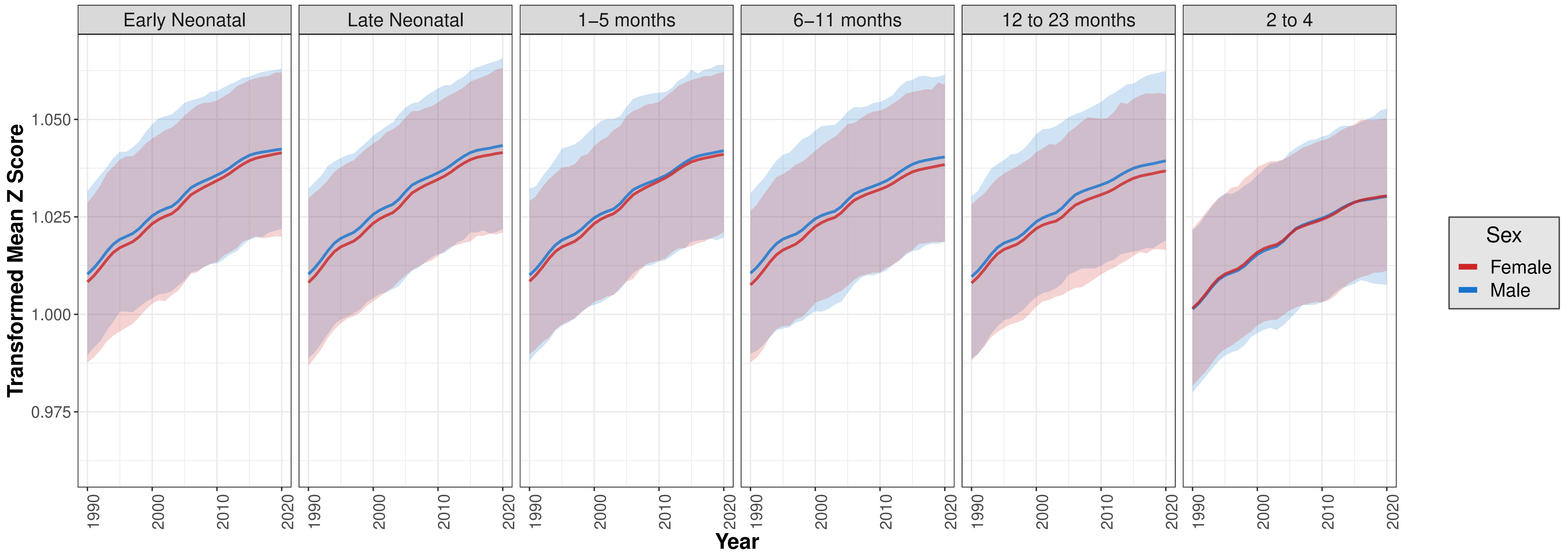

I

Source
No sources for this location

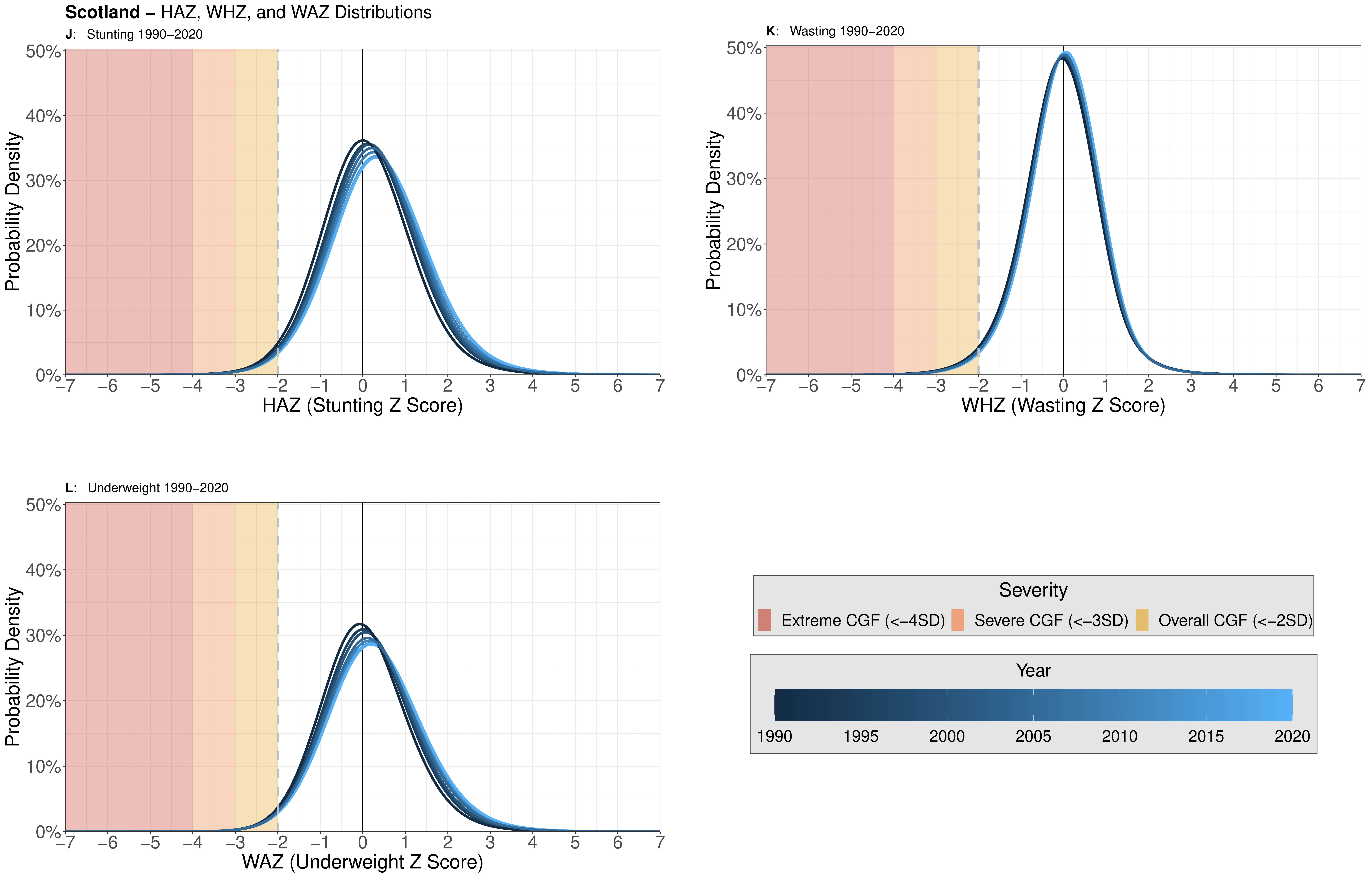
Scotland – HAZ, WHZ, and WAZ Distributions
J: Stunting 1990–2020
Probability Density
50%
40%
30%
20%
10%
0%
−7 −6 −5 −4 −3 −2 −1 0 1 2 3 4 5 6 7
HAZ (Stunting Z Score)
K: Wasting 1990–2020
Probability Density
WHZ (Wasting Z Score)
L: Underweight 1990–2020
Probability Density
WAZ (Underweight Z Score)
Severity
Extreme CGF (<−4SD)
Severe CGF (<−3SD)
Overall CGF (<−2SD)
Year
1990
1995
2000
2005
2010
2015
2020

Wales – Stunting (HAZ)

A: Overall and Severe Stunting Prevalence

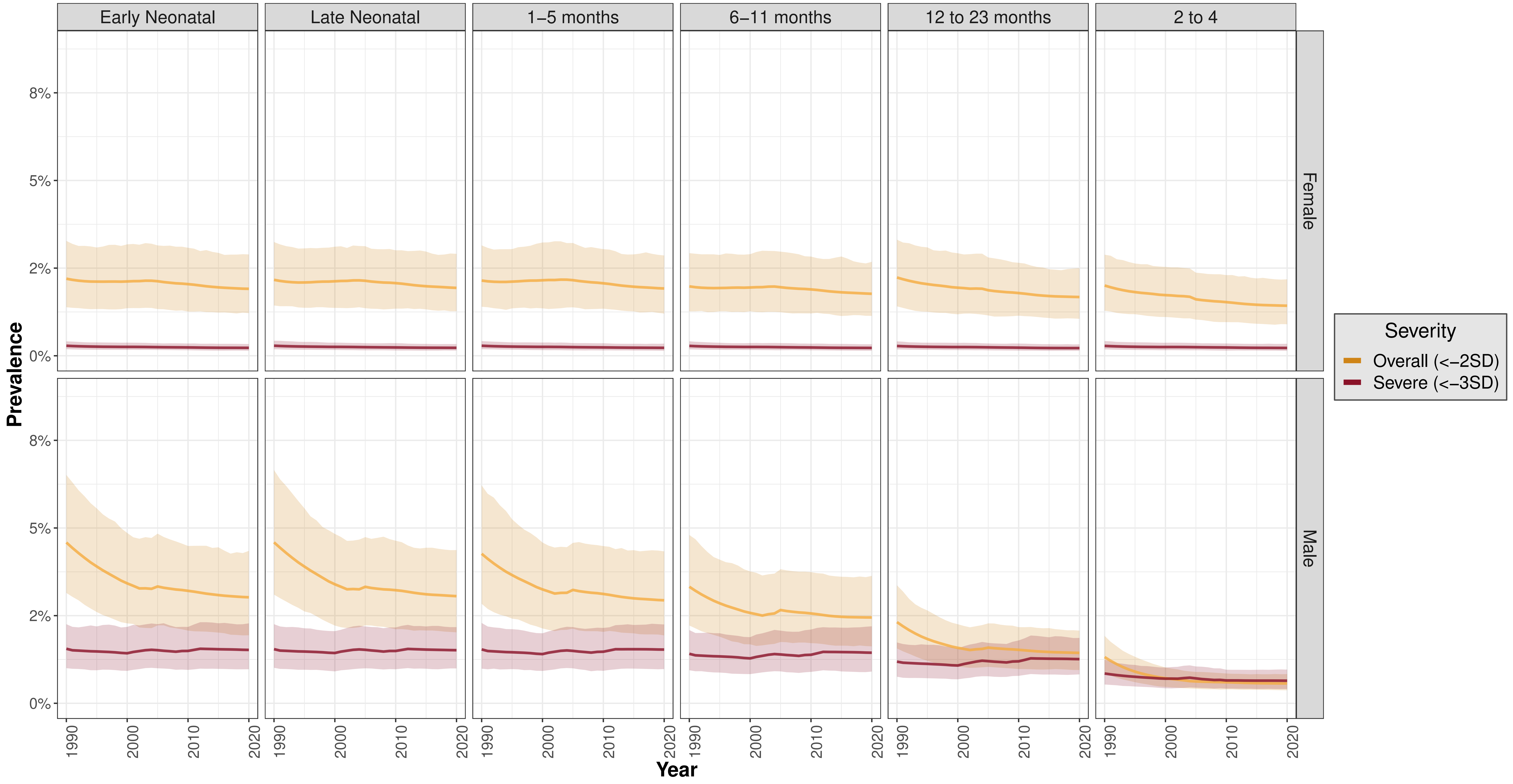

B: Transformed Mean Stunting Z Scores

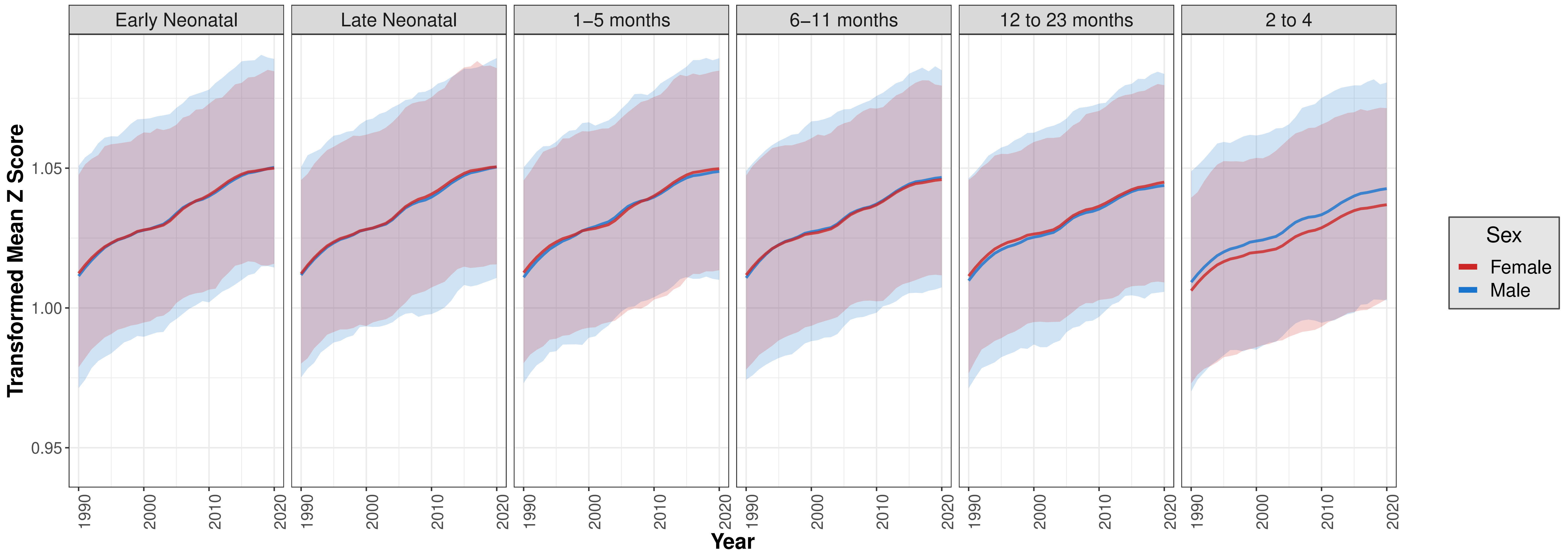

C

**Source**
No sources for this location

Wales – Wasting (WHZ)

D: Overall and Severe Wasting Prevalence

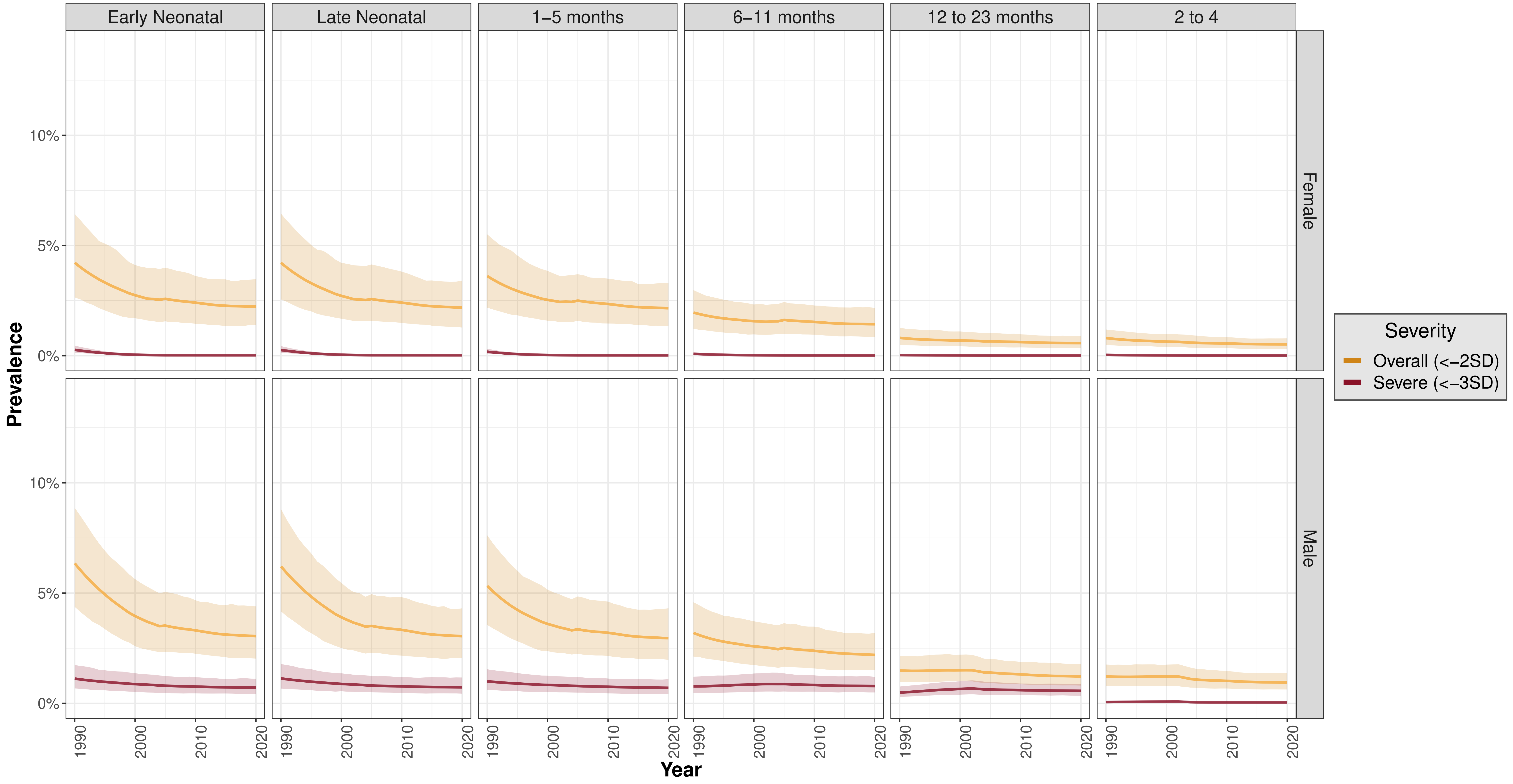

F

Source
No sources for this location

E: Transformed Mean Wasting Z Scores

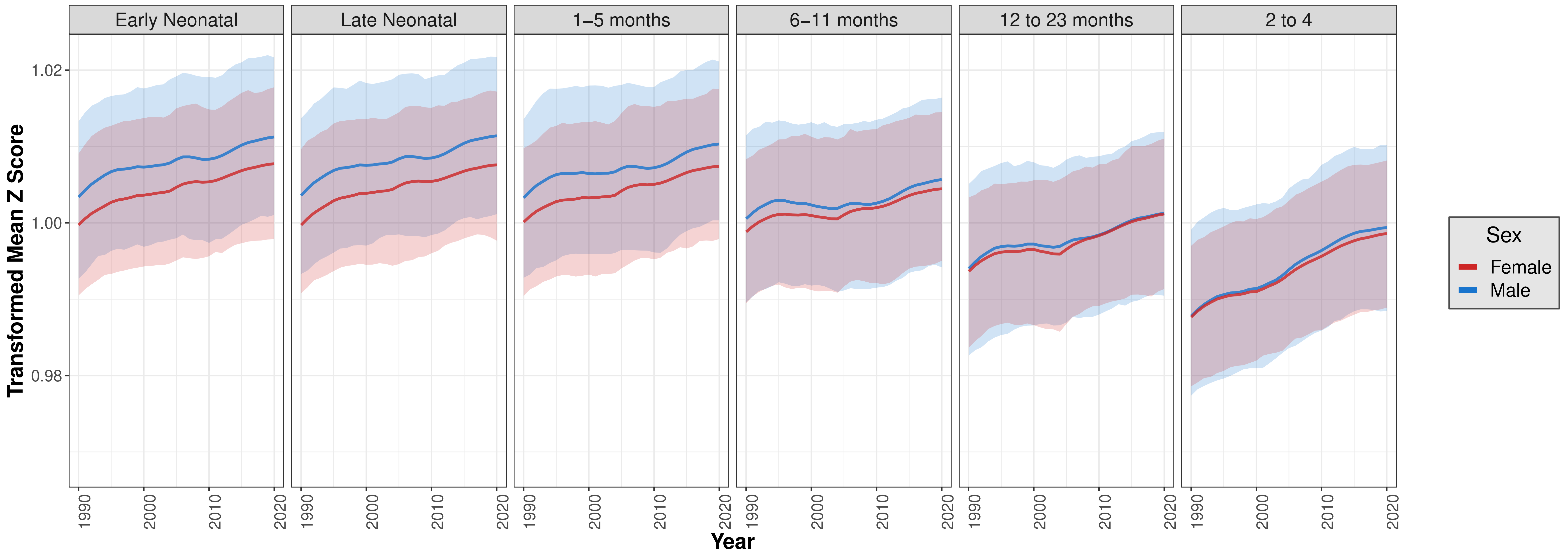

Wales – Underweight (WAZ)

G: Overall and Severe Underweight Prevalence

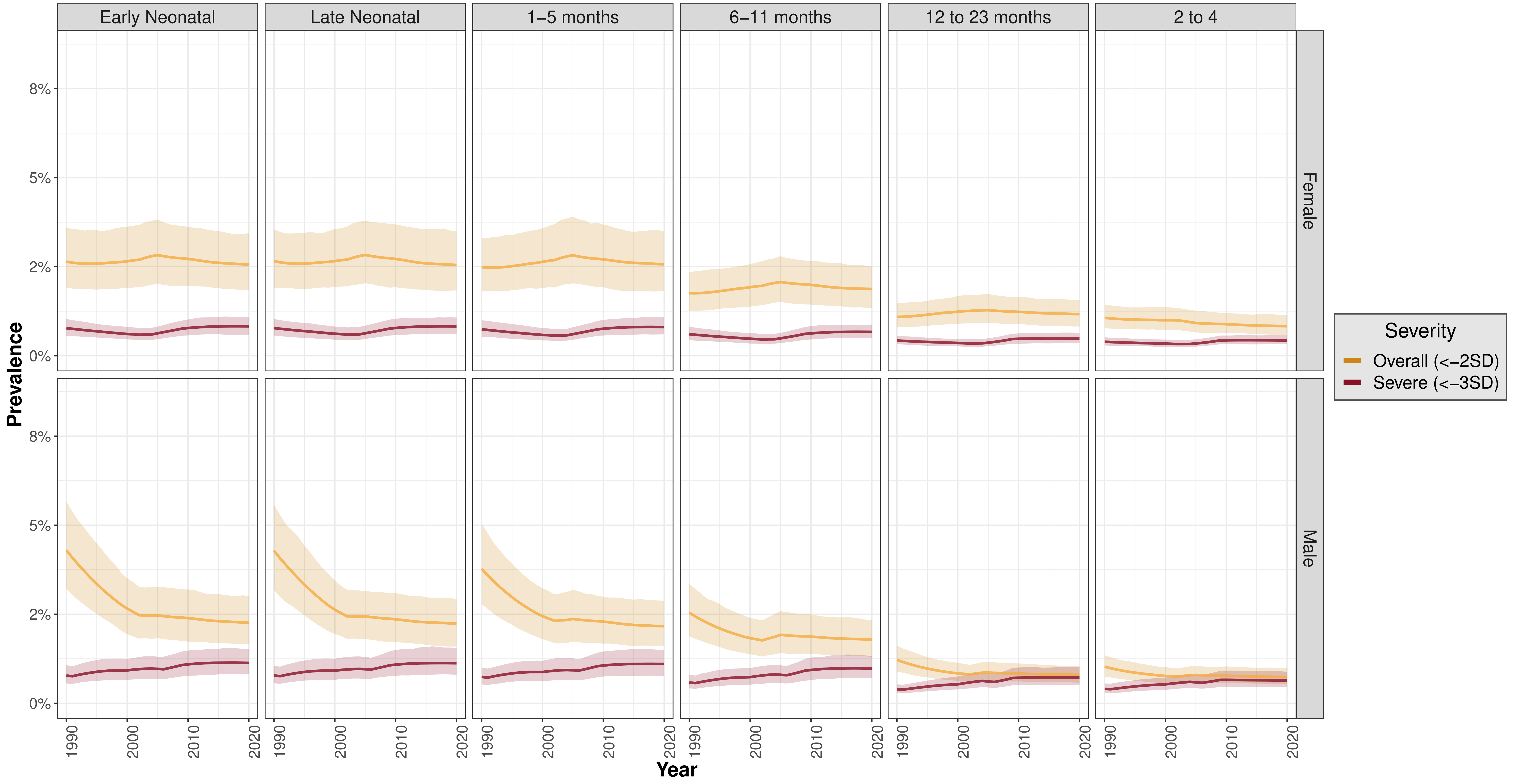

H: Transformed Mean Underweight Z Scores

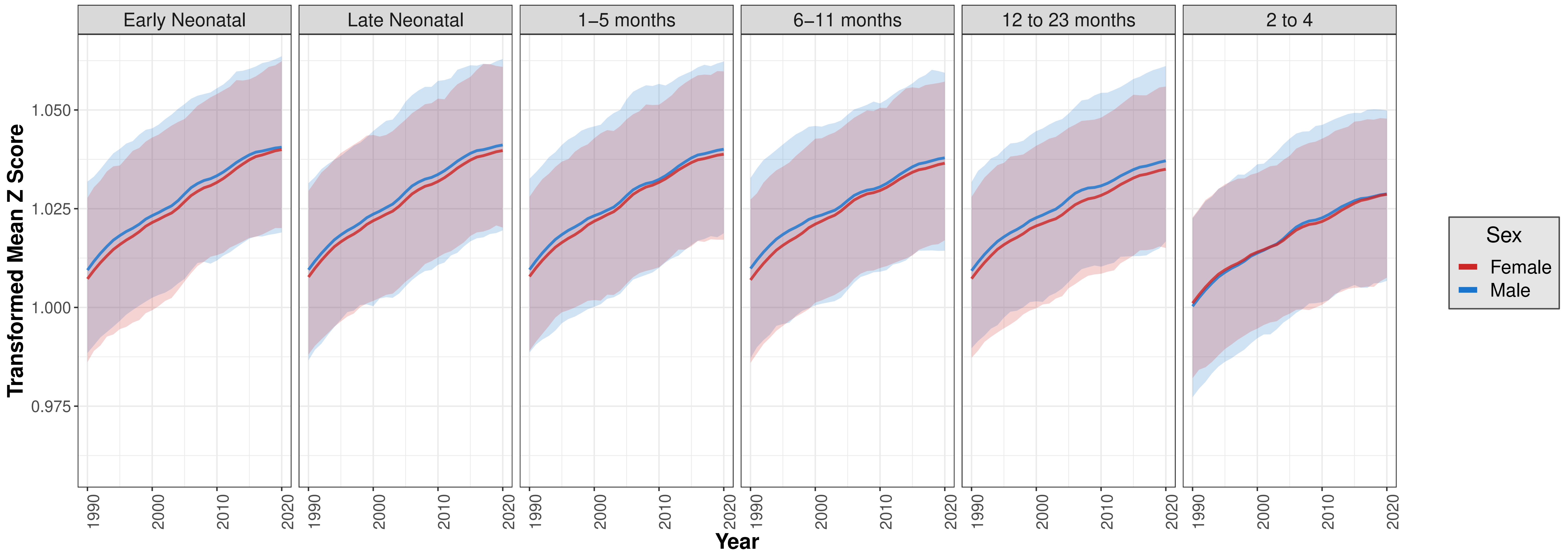

I

**Source**
No sources for this location

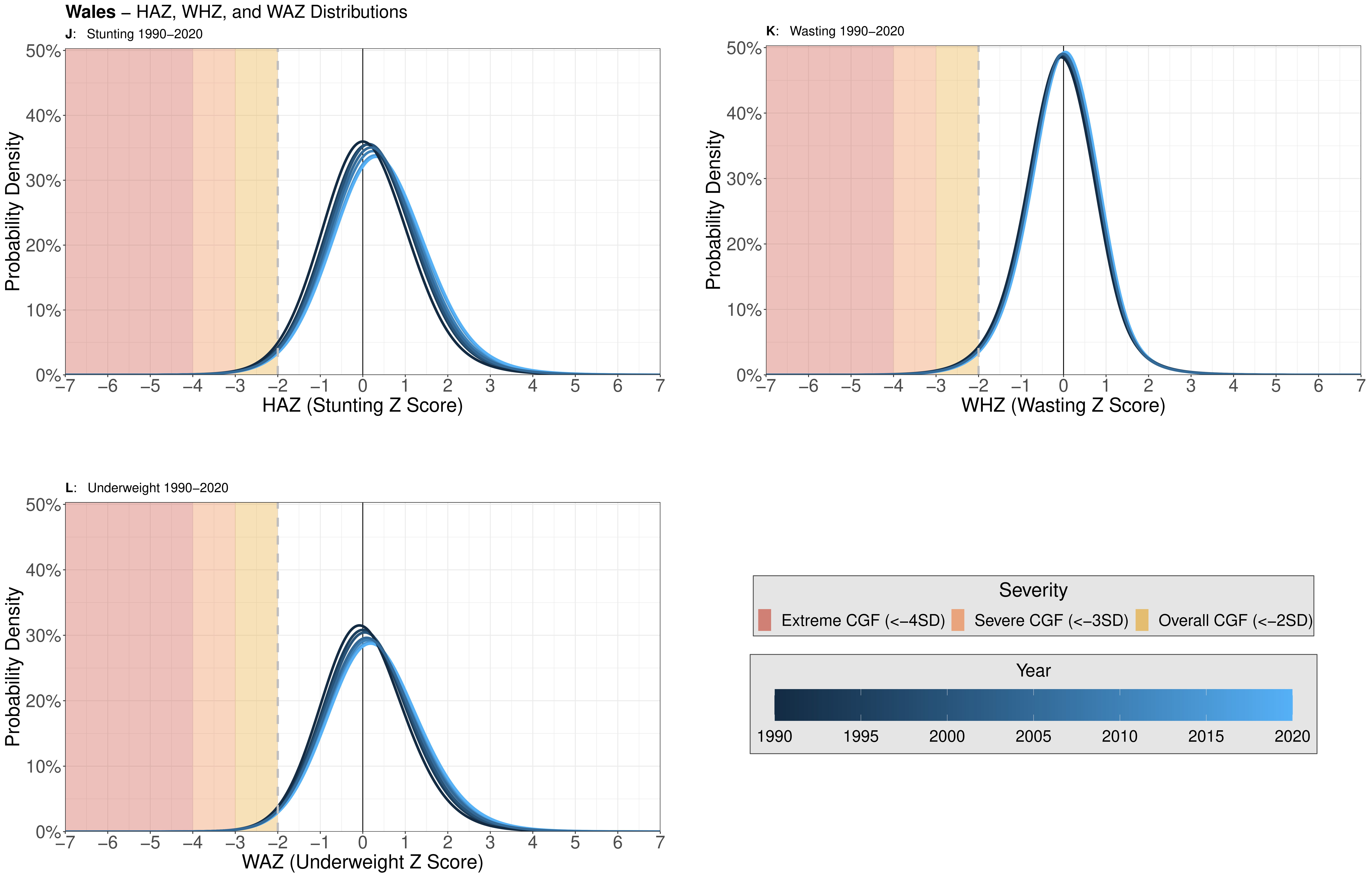
Wales – HAZ, WHZ, and WAZ Distributions
J: Stunting 1990–2020
K: Wasting 1990–2020
L: Underweight 1990–2020
Probability Density
50%
40%
30%
20%
10%
0%
−7 −6 −5 −4 −3 −2 −1 0 1 2 3 4 5 6 7
HAZ (Stunting Z Score)
WHZ (Wasting Z Score)
WAZ (Underweight Z Score)
Severity
Extreme CGF (<−4SD)
Severe CGF (<−3SD)
Overall CGF (<−2SD)
Year
1990
1995
2000
2005
2010
2015
2020

England – Stunting (HAZ)

A: Overall and Severe Stunting Prevalence

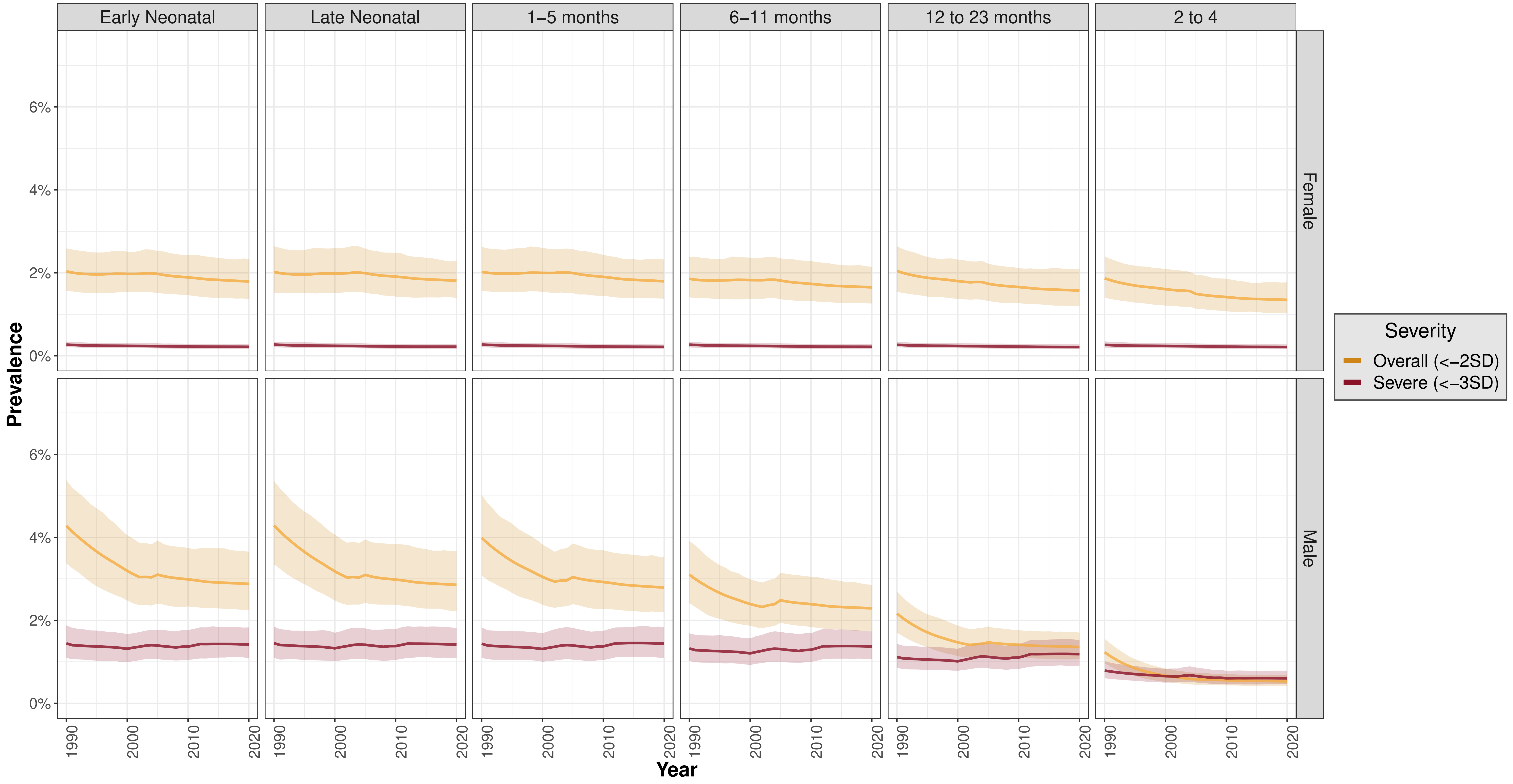

B: Transformed Mean Stunting Z Scores

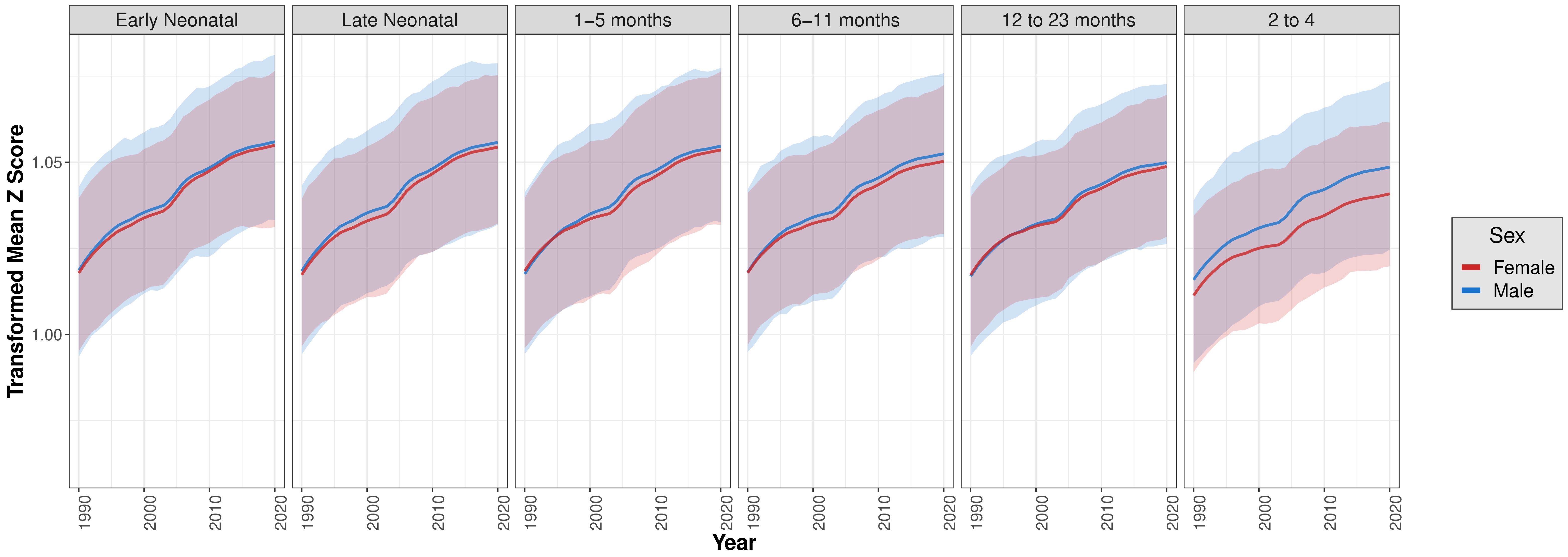

C

Source
No sources for this location

England – Wasting (WHZ)

D: Overall and Severe Wasting Prevalence

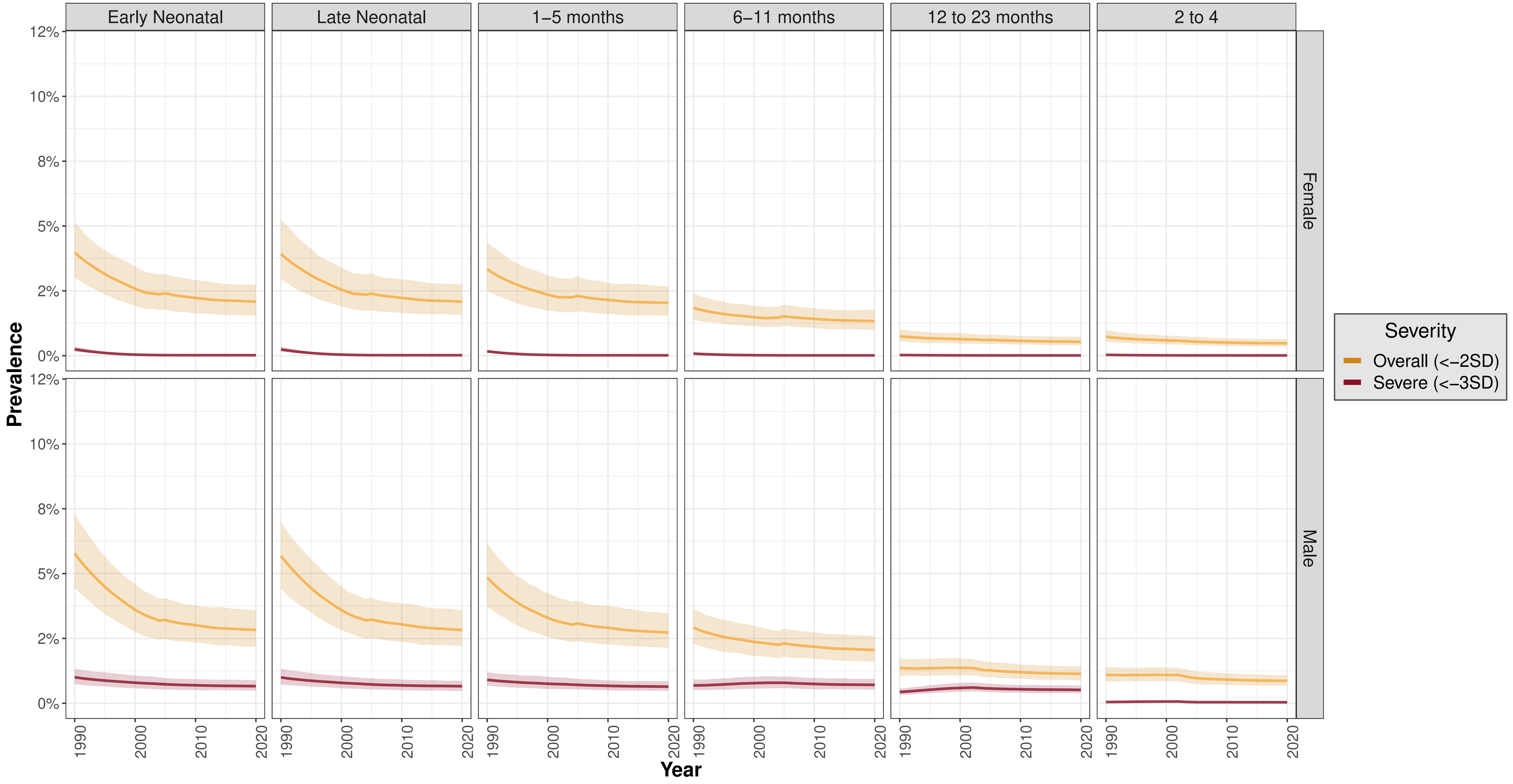

E: Transformed Mean Wasting Z Scores

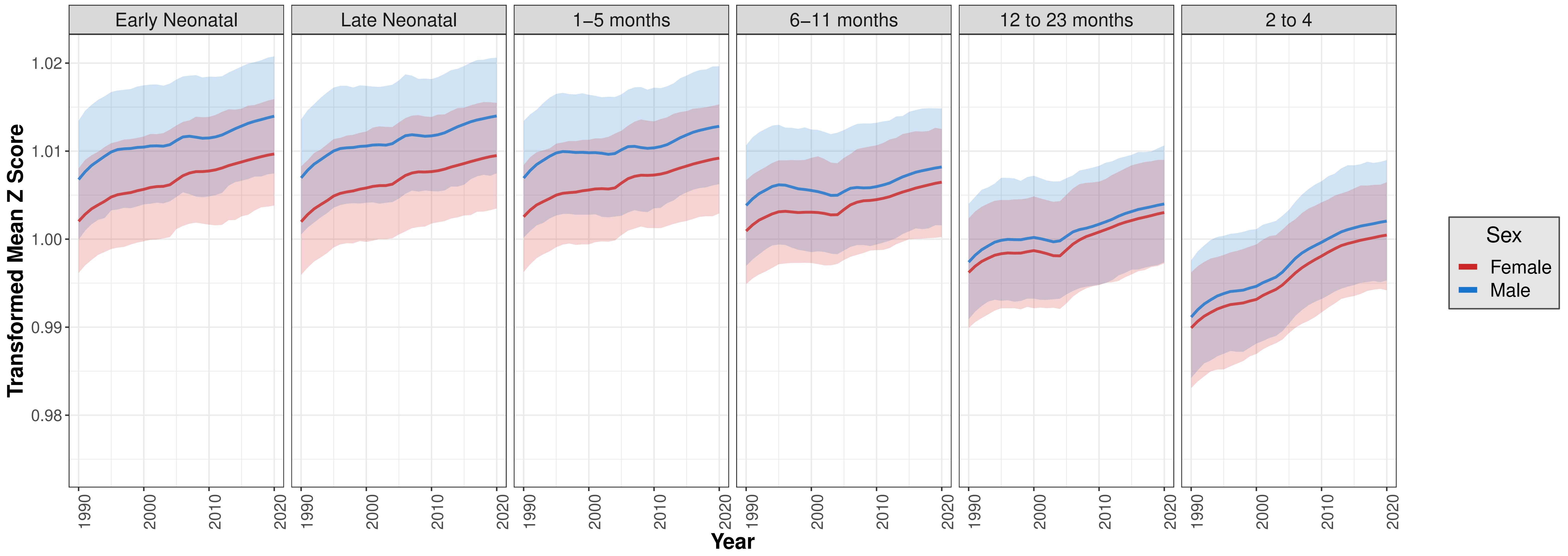

F

Source
No sources for this location

England – Underweight (WAZ)

G: Overall and Severe Underweight Prevalence

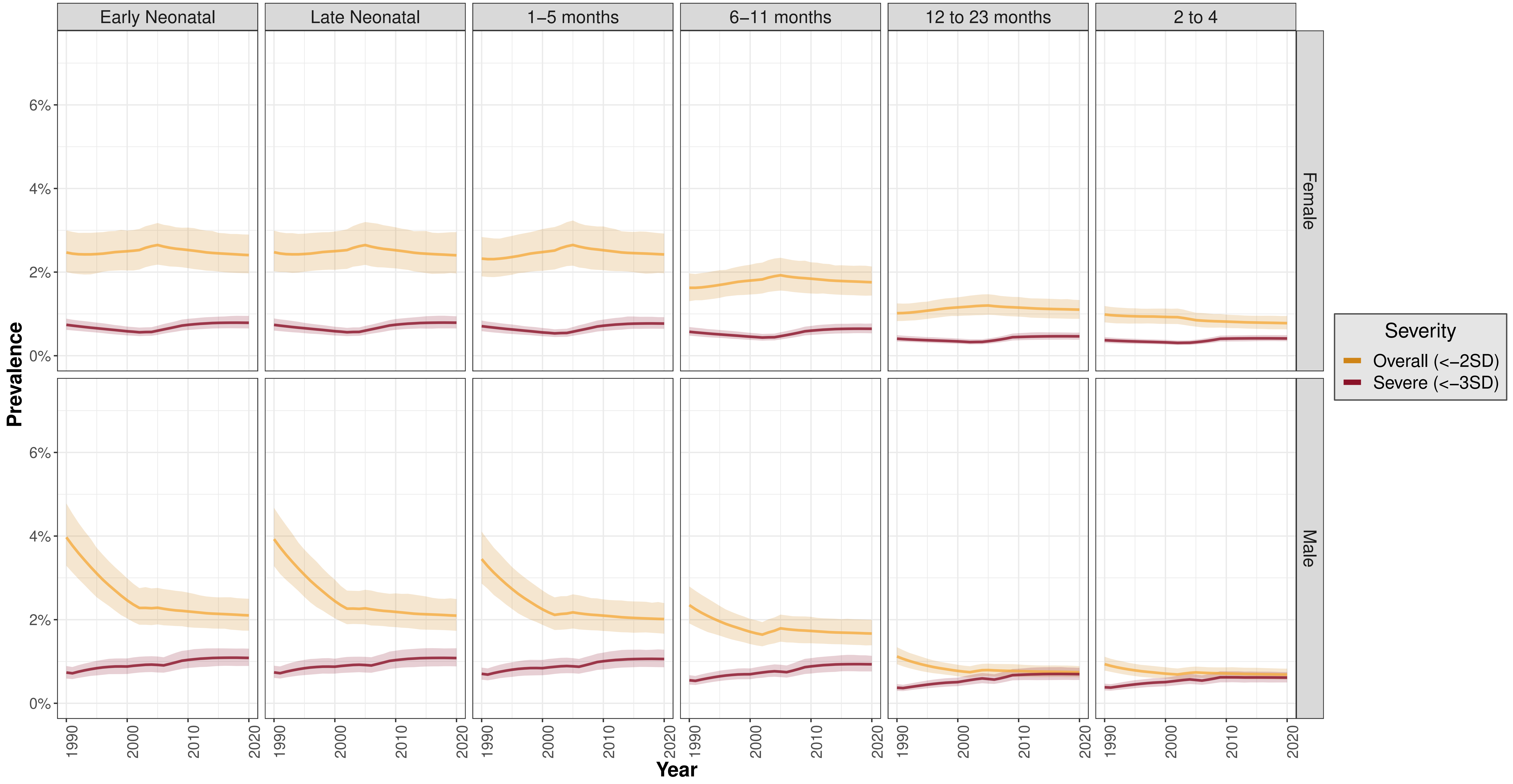

H: Transformed Mean Underweight Z Scores

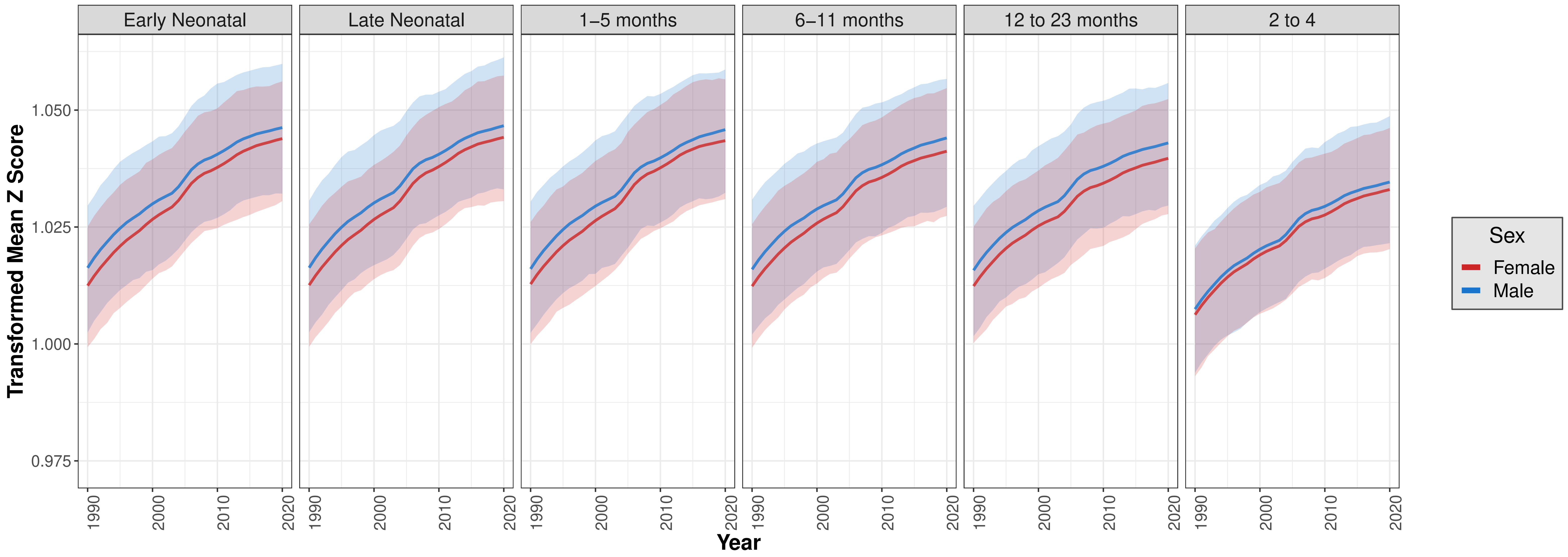

I

Source
No sources for this location

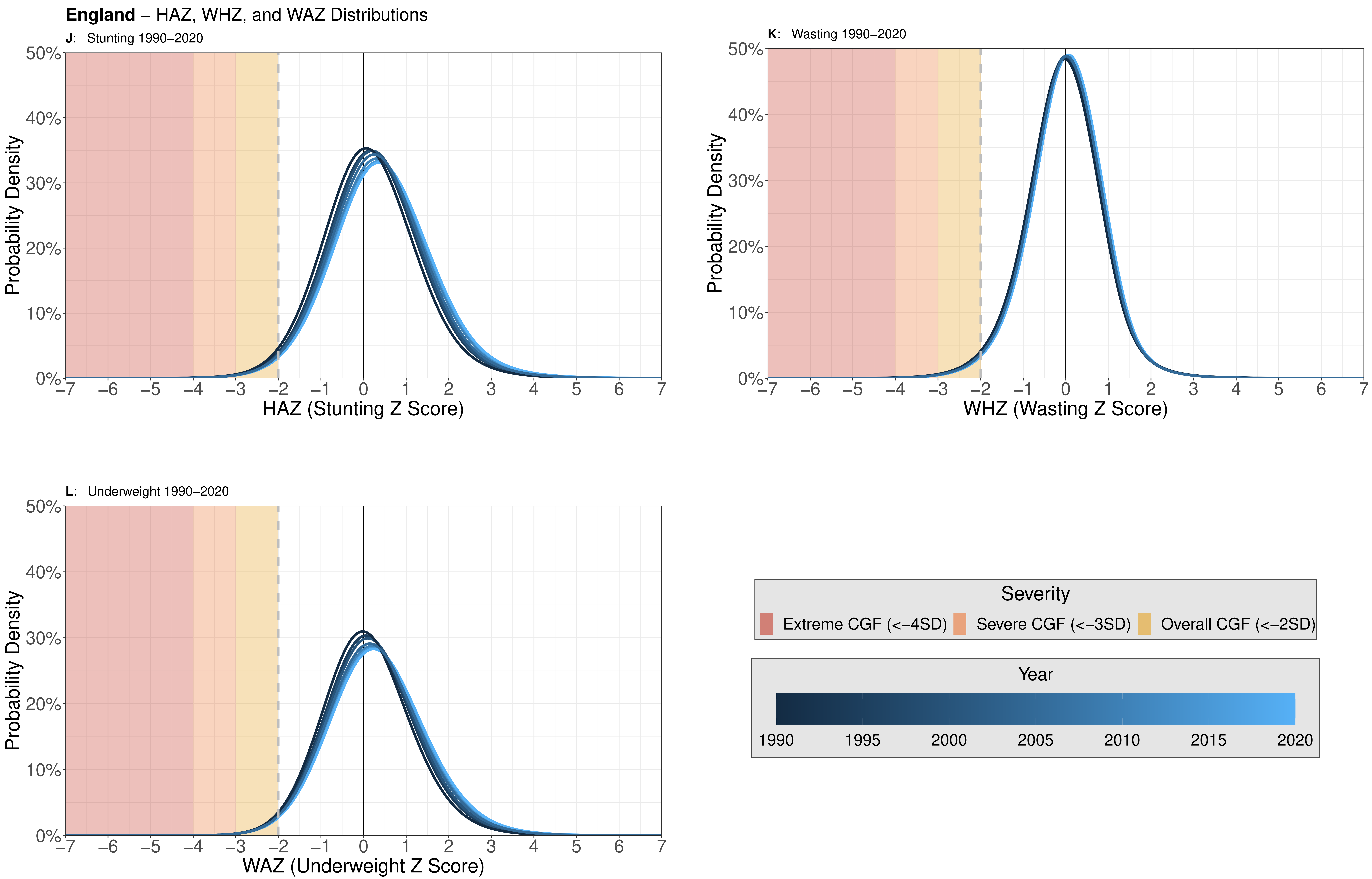
England – HAZ, WHZ, and WAZ Distributions
J: Stunting 1990–2020
K: Wasting 1990–2020
L: Underweight 1990–2020
Probability Density
50%
40%
30%
20%
10%
0%
−7 −6 −5 −4 −3 −2 −1 0 1 2 3 4 5 6 7
HAZ (Stunting Z Score)
WHZ (Wasting Z Score)
WAZ (Underweight Z Score)
Severity
Extreme CGF (<−4SD)
Severe CGF (<−3SD)
Overall CGF (<−2SD)
Year
1990
1995
2000
2005
2010
2015
2020
