# Supplementary material for: Current dichotomous metrics obscure trends in severe and extreme child growth failure
Source: Sci Adv. 2022 May 20;8(20):eabm8954. doi: 10.1126/sciadv.abm8954 (PMC9122330; doi:10.1126/sciadv.abm8954)

Data S1c. Spatio-temporal Gaussian Process Regression (ST-GPR) results for overall, severe, and mean CGF by location, including location-specific data sources; and distributions of stunting [HAZ], wasting [WHZ], and underweight [WAZ] for children under age five, both sexes, for every five years from 1990−2020. Country results are grouped by GBD super-region, including Central Europe, Eastern Europe, and Central Asia (S1a), High-income (S1b), Latin America and Caribbean (S1c), North Africa and Middle East (S1d), South Asia (S1e), Southeast Asia, East Asia, and Oceania (S1f), and Sub-Saharan Africa (S1g). Plots for each country include overall and severe stunting prevalence (A) and transformed mean stunting Z scores (B). A source list is shown which includes surveys included in the stunting models (C). Additional plots are shown for overall and severe wasting prevalence (D) and transformed mean wasting Z scores (E), followed by a source list with surveys included in the wasting models (F). Plots are then shown for overall and severe underweight prevalence (G), and transformed mean underweight Z scores (H), with a source list listing surveys included in the underweight models (I). Finally, distributions of stunting (J), wasting (K), and underweight (L) are shown for children under age five, both sexes, for every five years from 1990-2020. Surveys that were outliered are shown with X's on all plots. Surveys prior to 1990 may have been inputs to the models to inform trends, but estimates are only produced and shown for 1990−2020. For locations that are modeled nationally and subnationally, sources that are only included subnationally are not included in the plots of national level estimates. These sources were included in subnational models that influence national level models. Note that due to the transformation on mean Z scores, increasing values reflect improvements in mean Z score. Surveys conducted over a range of years were assigned to the midpoint year from that interval, which is the year reflected in the table and the plots. For the distributions of stunting, wasting, and underweight, the area under the curve reflects the estimated proportion of children experiencing that severity of CGF or worse. DHS is Demographic and Health Surveys. MICS is Multiple Indicator Cluster Survey. WHO CGM is the WHO Global Database on Child Growth and Malnutrition. SDNS is Survey of Diet and Nutritional Status.

This file contains the above for the following locations in the GBD super region of Latin America and the Caribbean, in the following order:

Andean Latin America: Bolivia (Plurinational State of), Ecuador, Peru

Caribbean: Antigua and Barbuda, Bahamas, Barbados, Belize, Cuba, Dominica, Dominican Republic, Grenada, Guyana, Haiti, Jamaica, Saint Kitts and Nevis, Saint Lucia, Saint Vincent and the Grenadines, Suriname, Trinidad and Tobago

Central Latin America: Colombia, Costa Rica, El Salvador, Guatemala, Honduras, Mexico, Nicaragua, Panama, Venezuela (Bolivarian Republic of)

Tropical Latin America: Brazil, Paraguay

Bolivia (Plurinational State of) – Stunting (HAZ)

A: Overall and Severe Stunting Prevalence

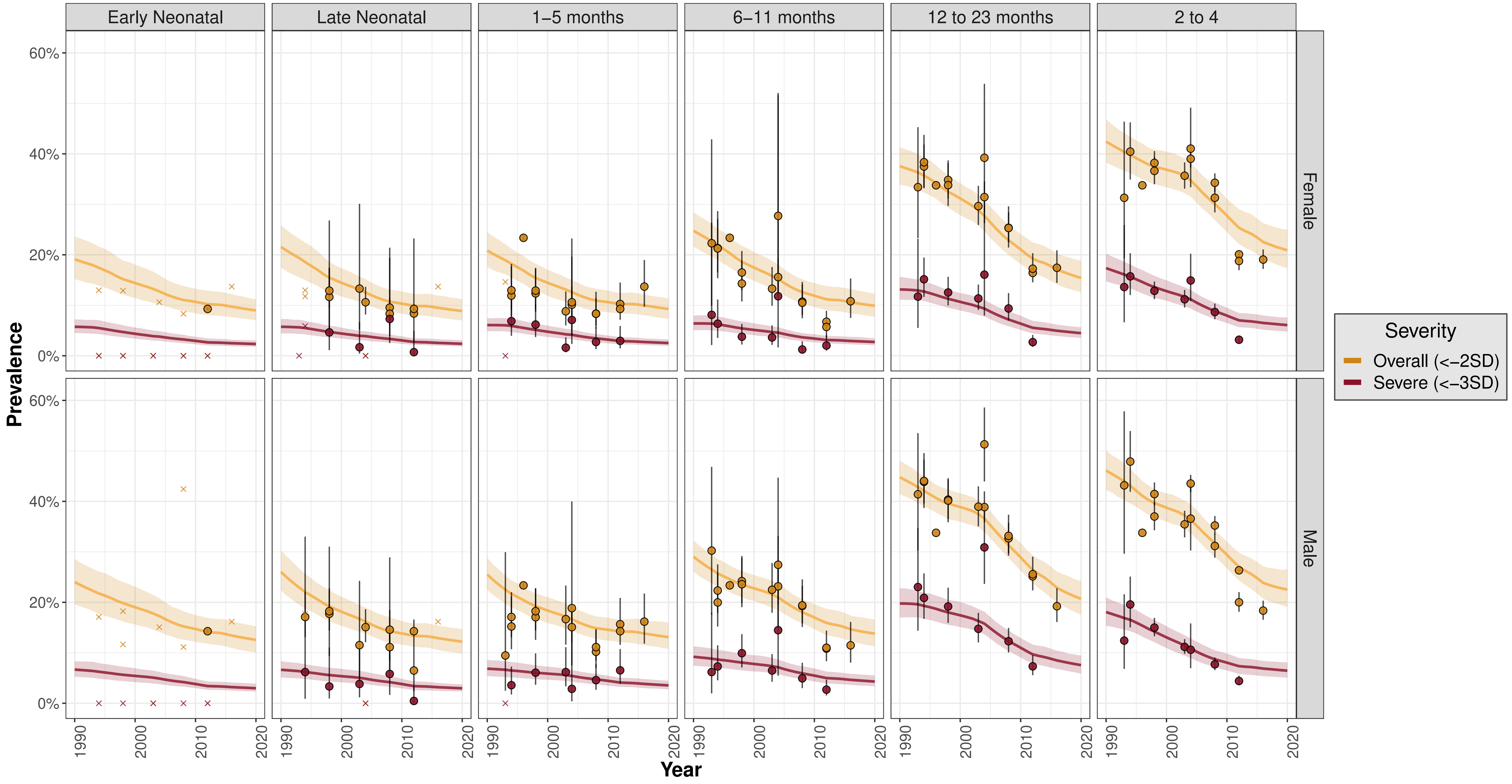

B: Transformed Mean Stunting Z Scores

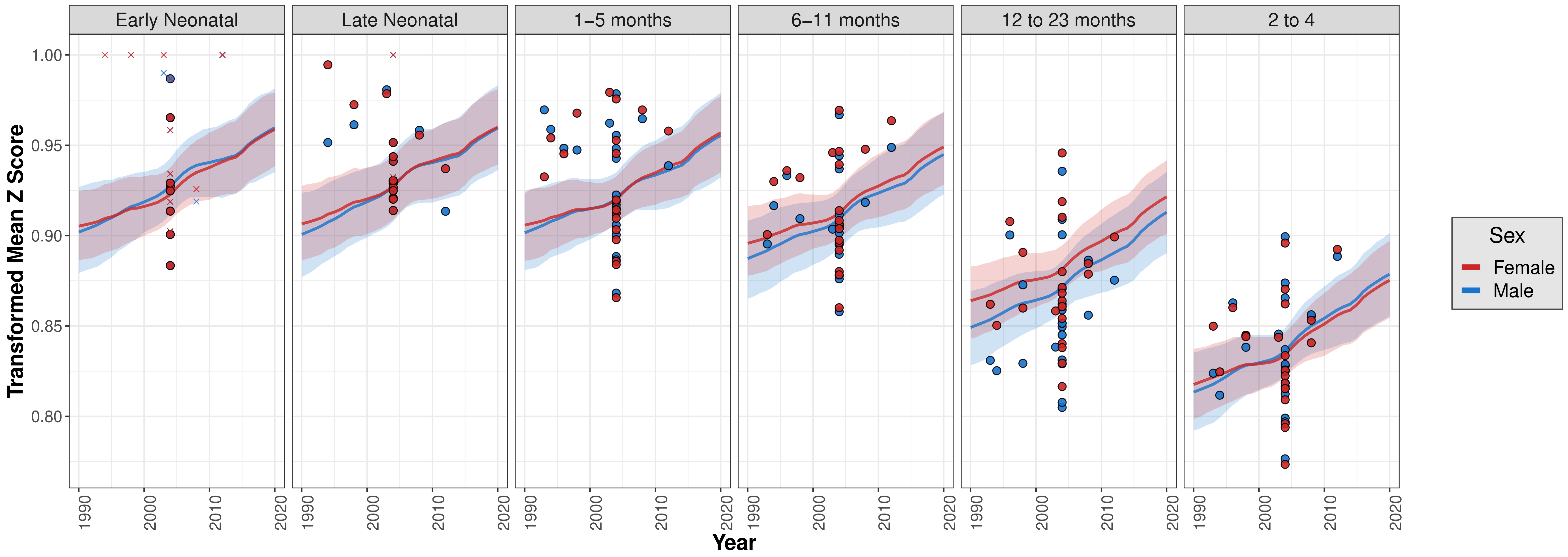

C

Year	Source
1981	WHO CGM Database
1986	WHO CGM Database
1989	DHS
1989	WHO CGM Database
1993	DHS
1994	DHS
1994	WHO CGM Database
1996	WHO CGM Database
1998	DHS
1998	WHO CGM Database
2003	DHS
2004	DHS
2004	WHO CGM Database
2008	DHS
2008	WHO CGM Database
2012	WHO CGM Database
2012	Health and Nutrition Assessment Survey
2016	WHO CGM Database

Bolivia (Plurinational State of) – Wasting (WHZ)

D: Overall and Severe Wasting Prevalence

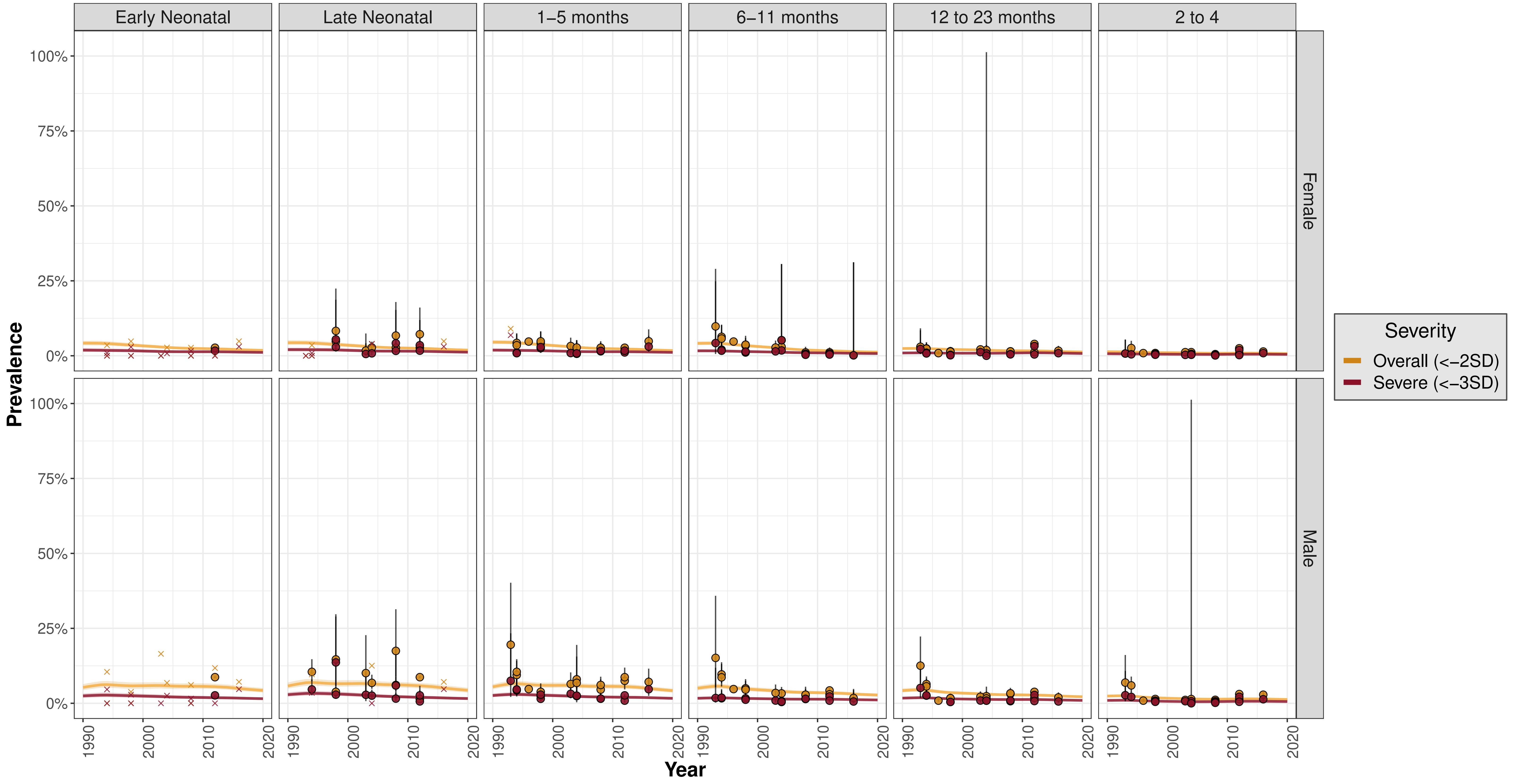

E: Transformed Mean Wasting Z Scores

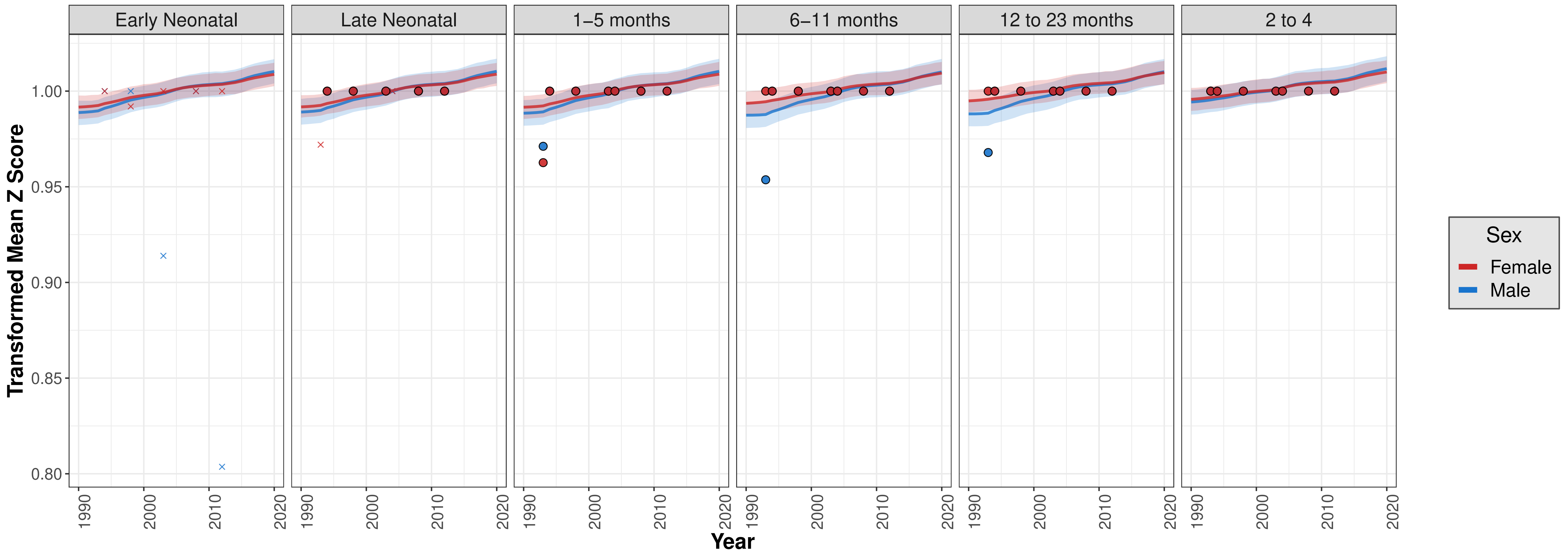

F

Year	Source
1981	WHO CGM Database
1989	DHS
1989	WHO CGM Database
1993	DHS
1994	DHS
1994	WHO CGM Database
1996	WHO CGM Database
1998	DHS
1998	WHO CGM Database
2003	DHS
2004	DHS
2004	WHO CGM Database
2008	DHS
2008	WHO CGM Database
2012	WHO CGM Database
2012	Health and Nutrition Assessment Survey
2016	WHO CGM Database

Bolivia (Plurinational State of) – Underweight (WAZ)

G: Overall and Severe Underweight Prevalence

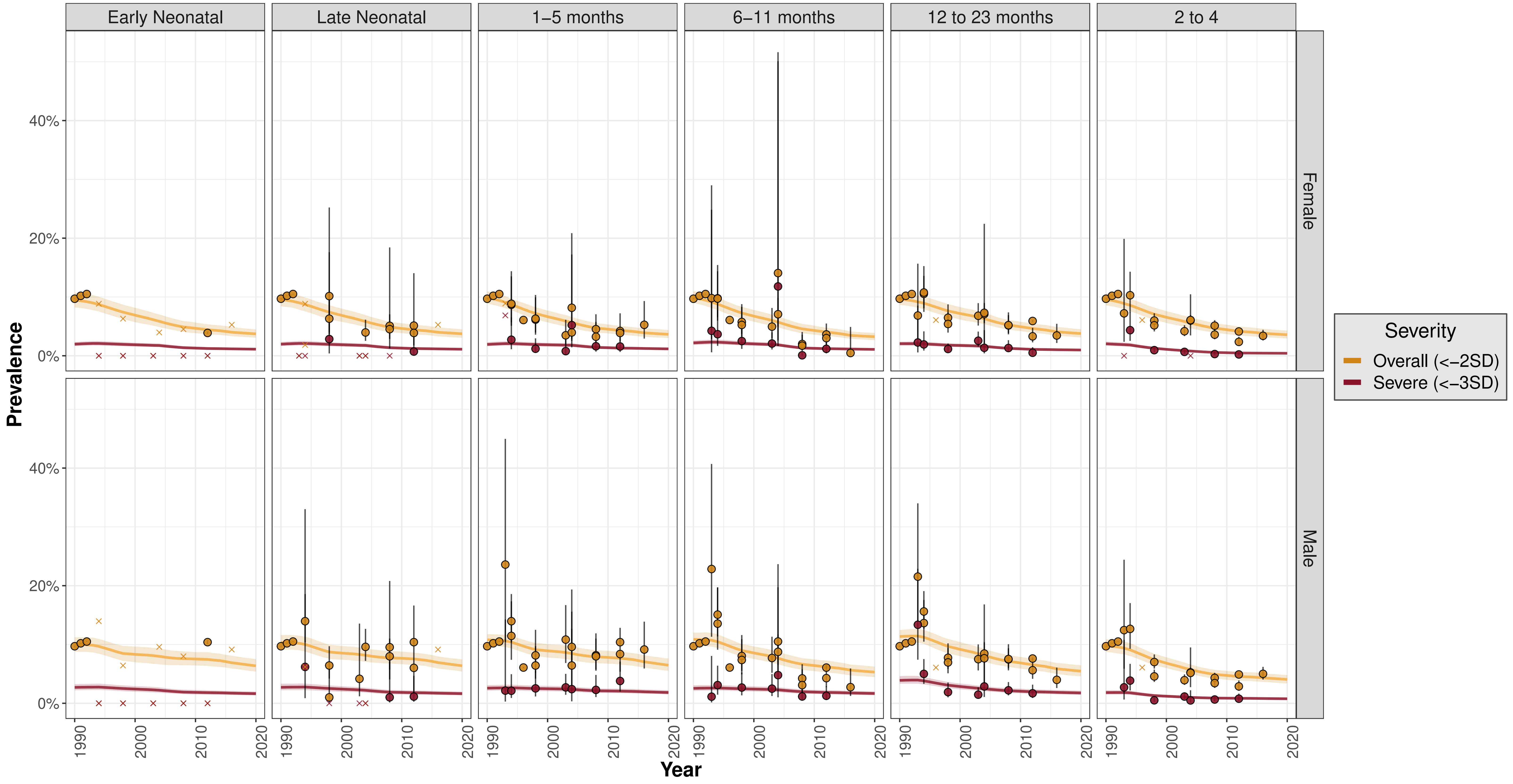

H: Transformed Mean Underweight Z Scores

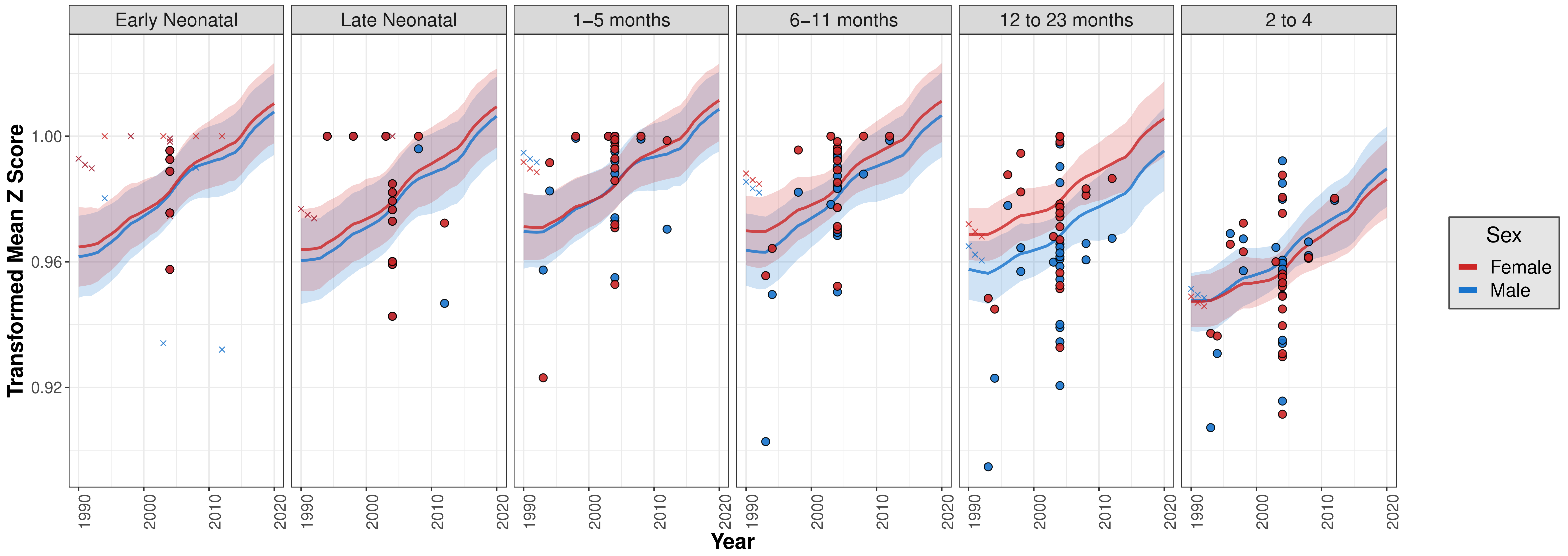

I

Year	Source
1981	WHO CGM Database
1986	WHO CGM Database
1987	WHO CGM Database
1988	WHO CGM Database
1989	DHS
1989	WHO CGM Database
1990	WHO CGM Database
1991	WHO CGM Database
1992	WHO CGM Database
1993	DHS
1994	DHS
1994	WHO CGM Database
1996	WHO CGM Database
1998	DHS
1998	WHO CGM Database
2003	DHS
2004	DHS
2004	WHO CGM Database
2008	DHS
2008	WHO CGM Database
2012	WHO CGM Database
2012	Health and Nutrition Assessment Survey
2016	WHO CGM Database

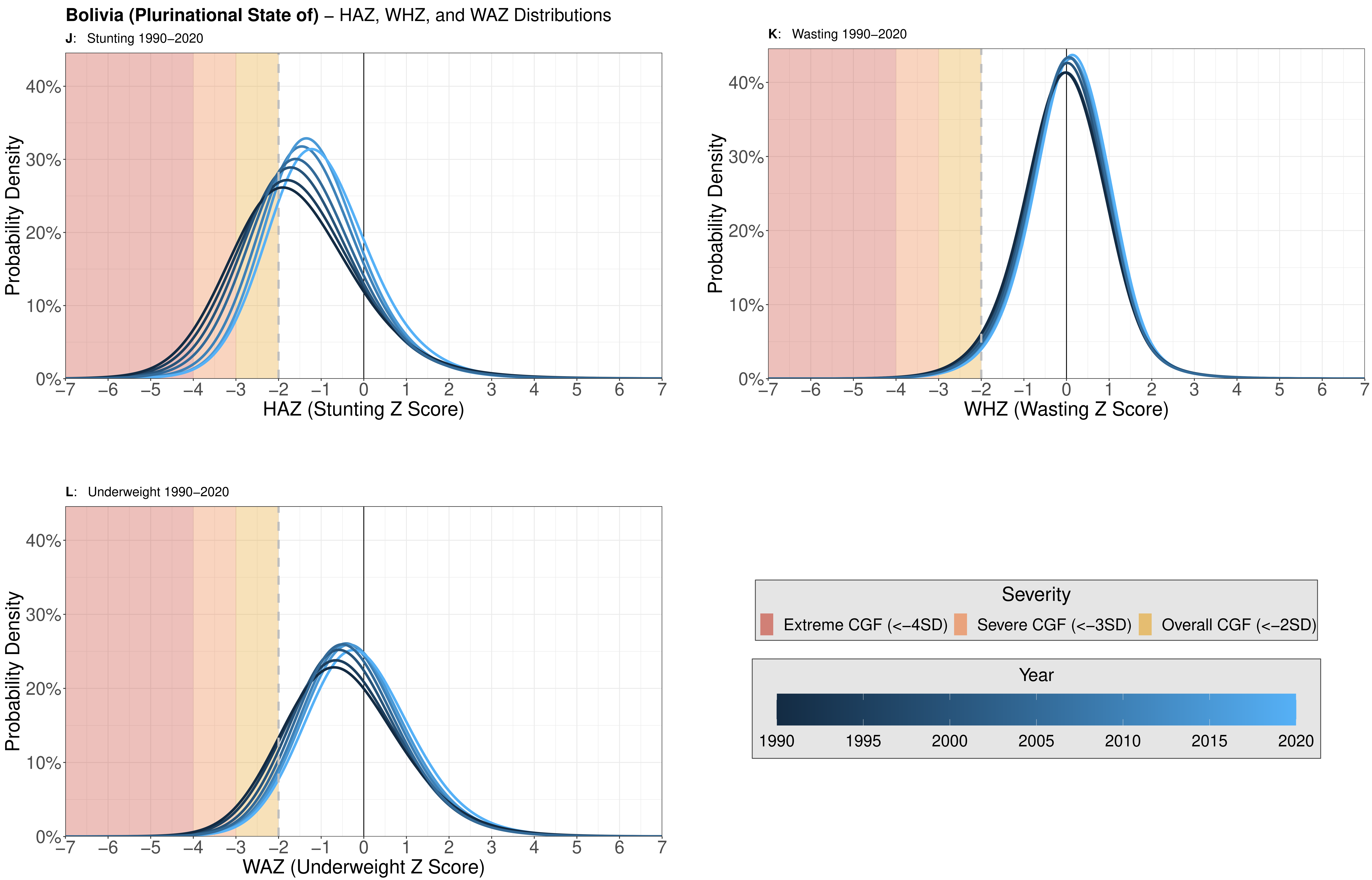
Bolivia (Plurinational State of) – HAZ, WHZ, and WAZ Distributions
J: Stunting 1990–2020
Probability Density
0%
10%
20%
30%
40%
−7 −6 −5 −4 −3 −2 −1 0 1 2 3 4 5 6 7
HAZ (Stunting Z Score)
K: Wasting 1990–2020
Probability Density
WHZ (Wasting Z Score)
L: Underweight 1990–2020
Probability Density
WAZ (Underweight Z Score)
Severity
Extreme CGF (<−4SD)
Severe CGF (<−3SD)
Overall CGF (<−2SD)
Year
1990
1995
2000
2005
2010
2015
2020

Ecuador – Stunting (HAZ)

A: Overall and Severe Stunting Prevalence

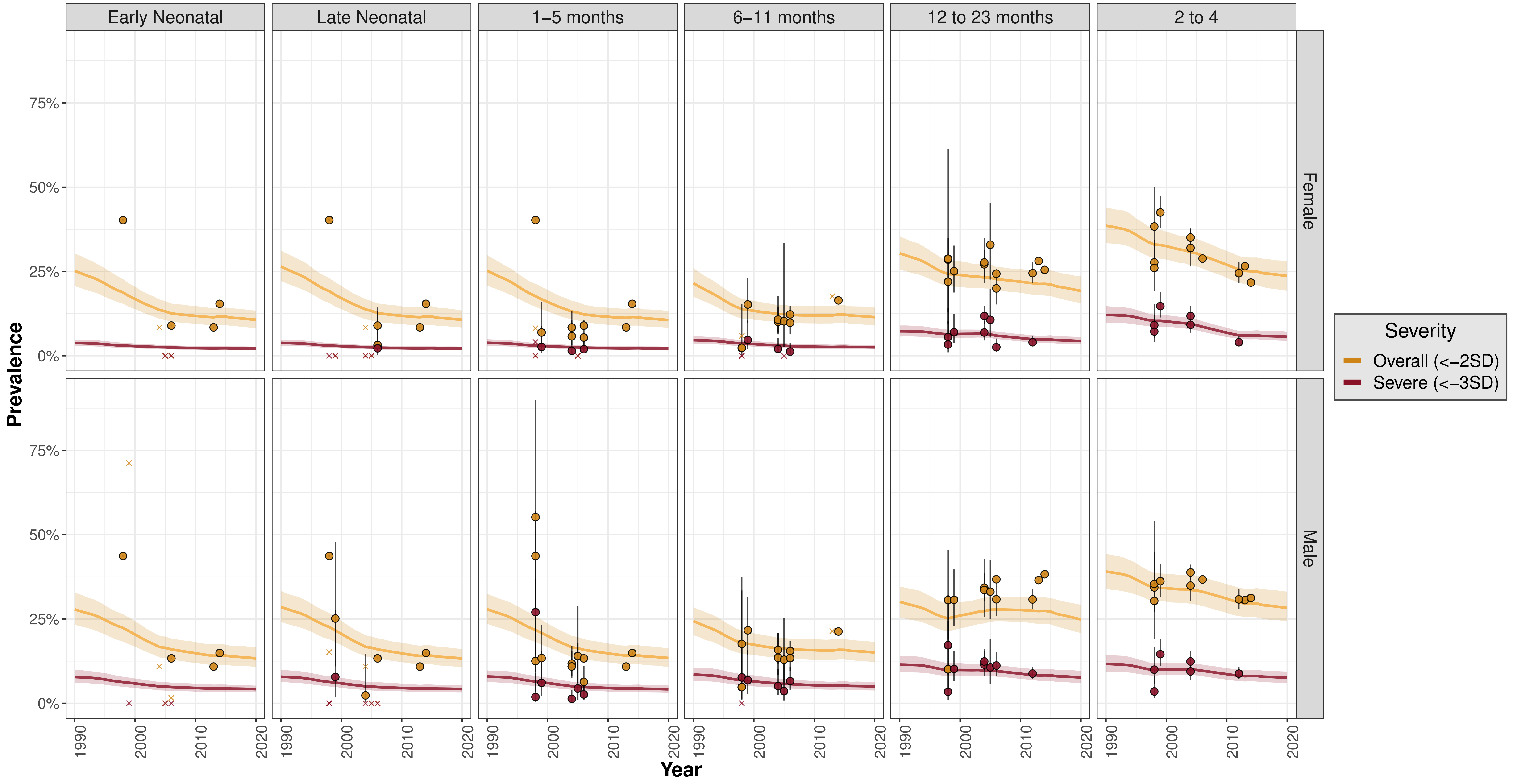

B: Transformed Mean Stunting Z Scores

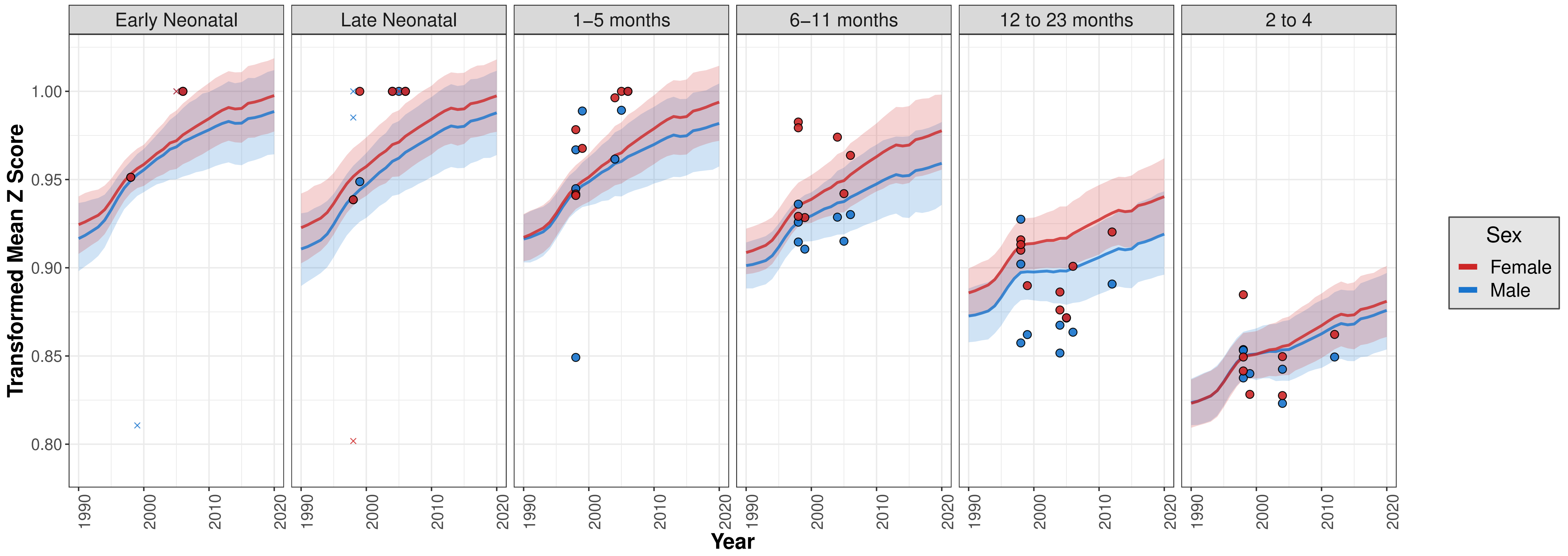

C

Year	Source
1986	WHO CGM Database
1998	Living Standards Measurement Survey
1998	Living Conditions Survey
1998	WHO CGM Database
1999	Living Conditions Survey
2004	Reproductive Health Survey
2004	WHO CGM Database
2005	Living Conditions Survey
2006	Living Conditions Survey
2006	WHO CGM Database
2012	WHO CGM Database
2012	National Health and Nutrition Survey
2013	WHO CGM Database
2014	WHO CGM Database

Ecuador – Wasting (WHZ)

D: Overall and Severe Wasting Prevalence

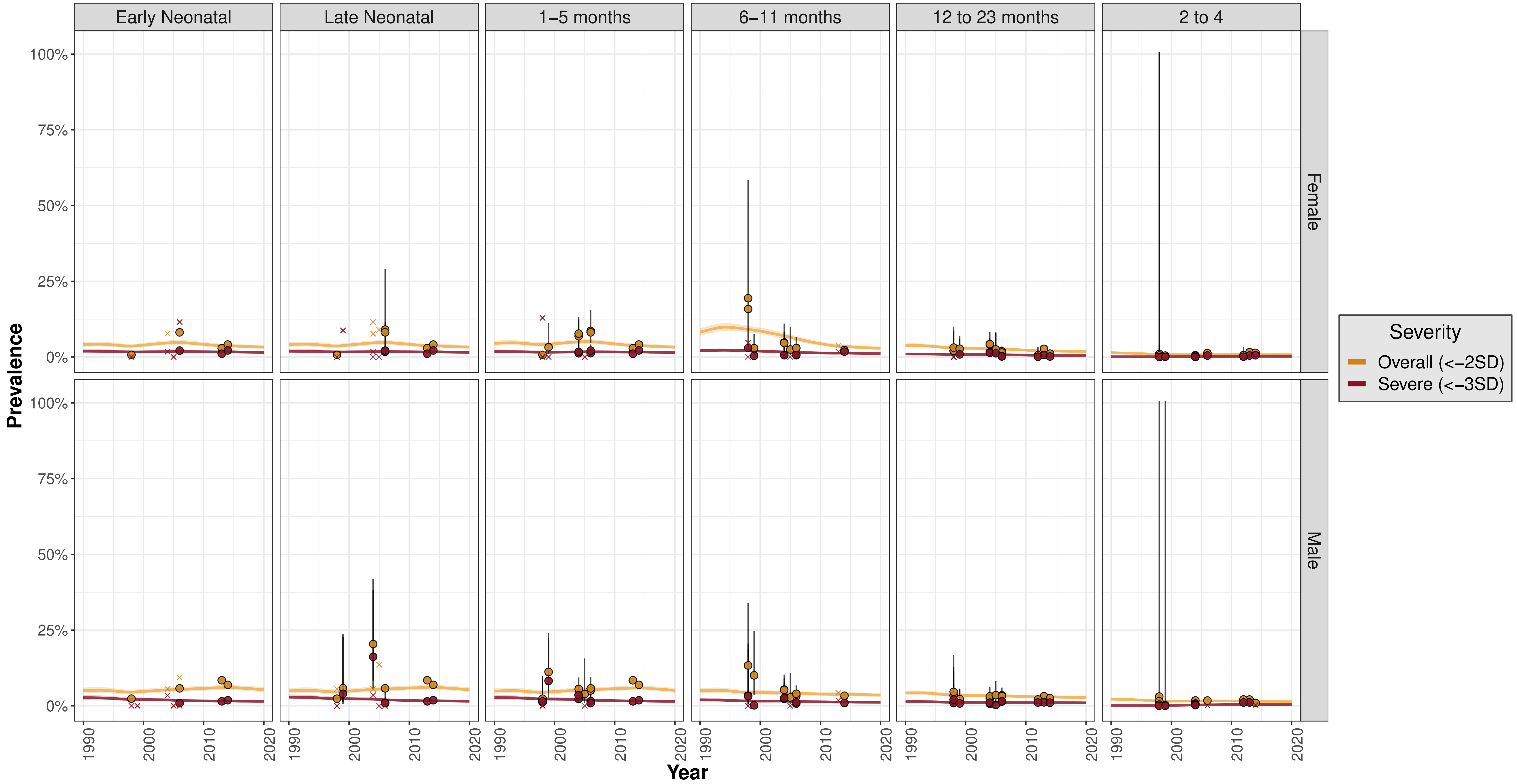

E: Transformed Mean Wasting Z Scores

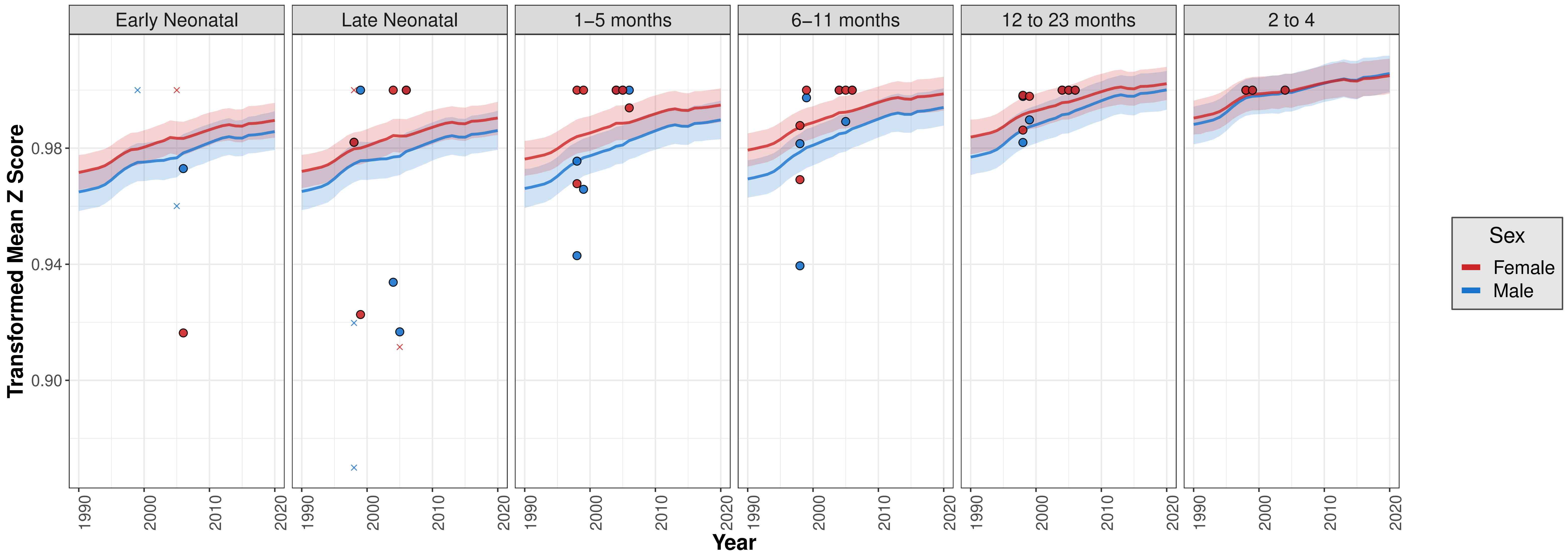

F

Year	Source
1986	WHO CGM Database
1998	Living Standards Measurement Survey
1998	Living Conditions Survey
1998	WHO CGM Database
1999	Living Conditions Survey
2004	Reproductive Health Survey
2004	WHO CGM Database
2005	Living Conditions Survey
2006	Living Conditions Survey
2006	WHO CGM Database
2012	WHO CGM Database
2012	National Health and Nutrition Survey
2013	WHO CGM Database
2014	WHO CGM Database

Ecuador – Underweight (WAZ)

G: Overall and Severe Underweight Prevalence

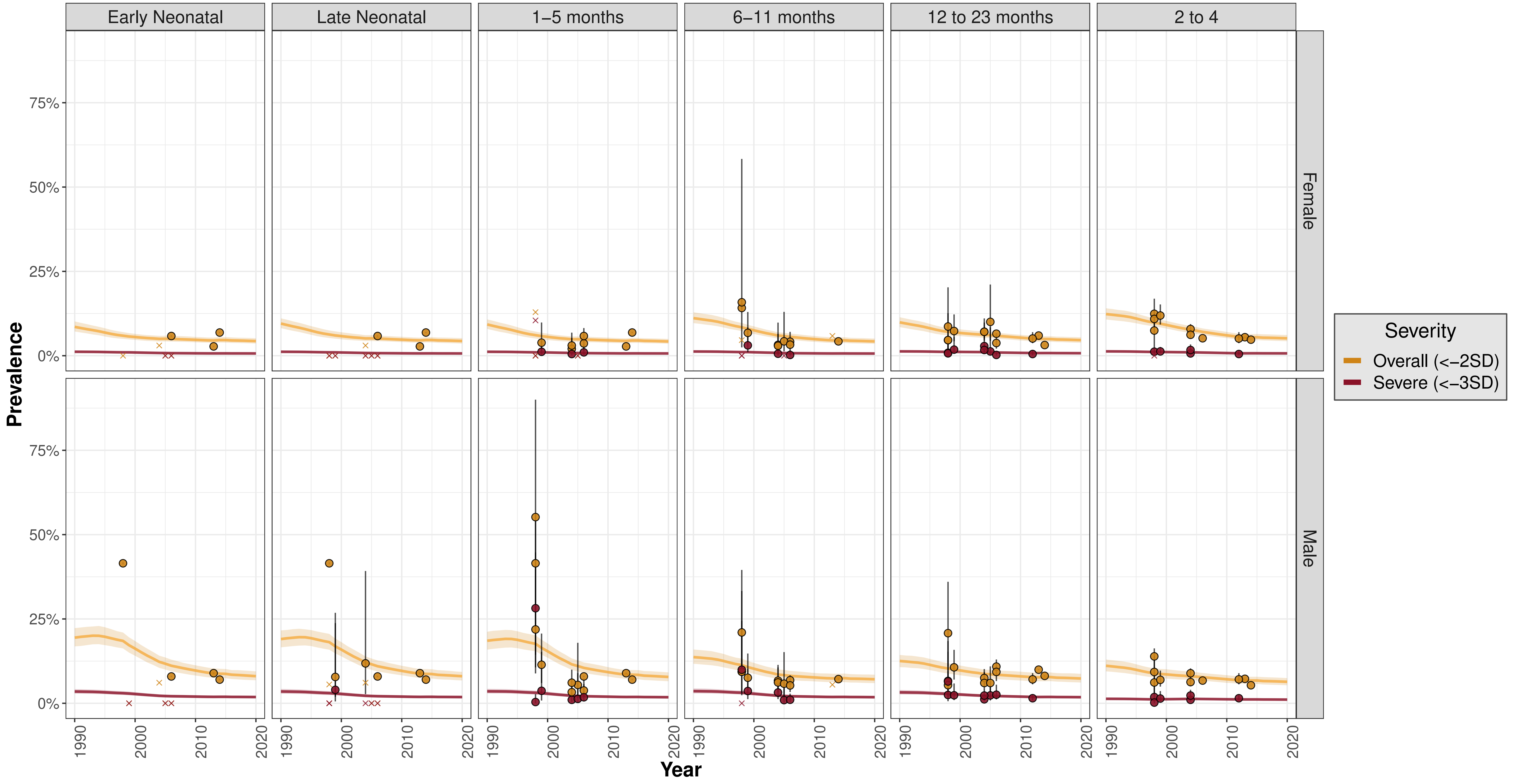

H: Transformed Mean Underweight Z Scores

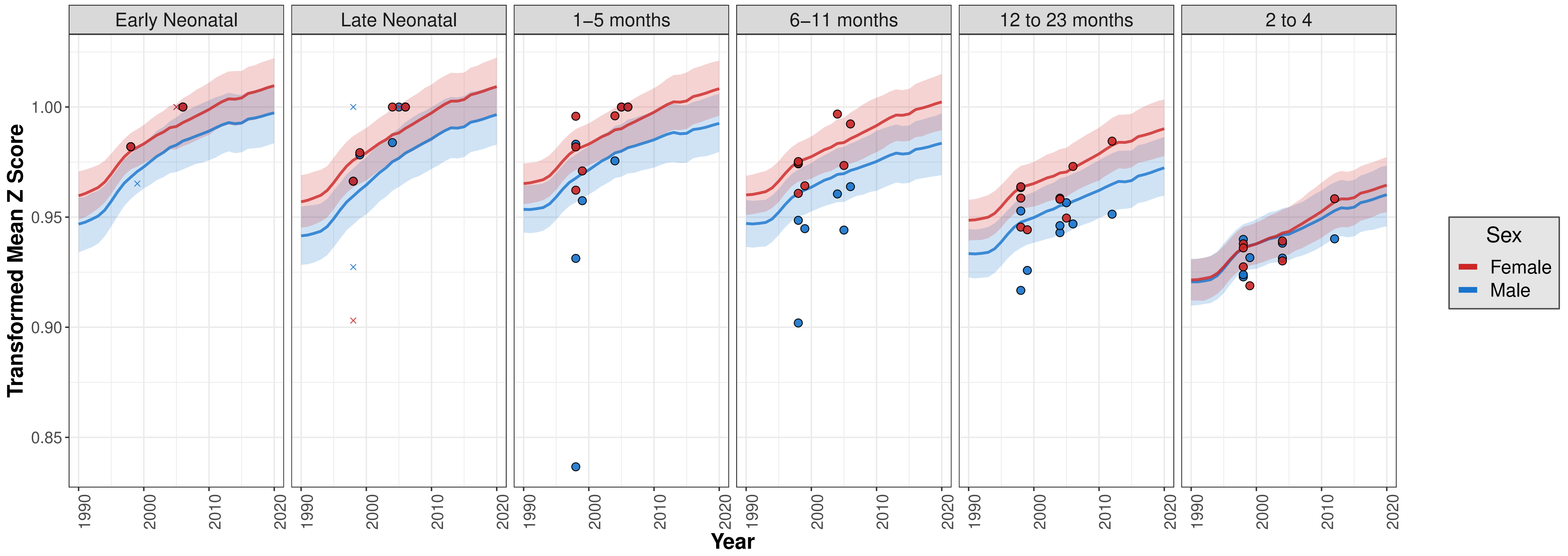

I

Year	Source
1986	WHO CGM Database
1998	Living Standards Measurement Survey
1998	Living Conditions Survey
1998	WHO CGM Database
1999	Living Conditions Survey
2004	Reproductive Health Survey
2004	WHO CGM Database
2005	Living Conditions Survey
2006	Living Conditions Survey
2006	WHO CGM Database
2012	WHO CGM Database
2012	National Health and Nutrition Survey
2013	WHO CGM Database
2014	WHO CGM Database

Ecuador – HAZ, WHZ, and WAZ Distributions

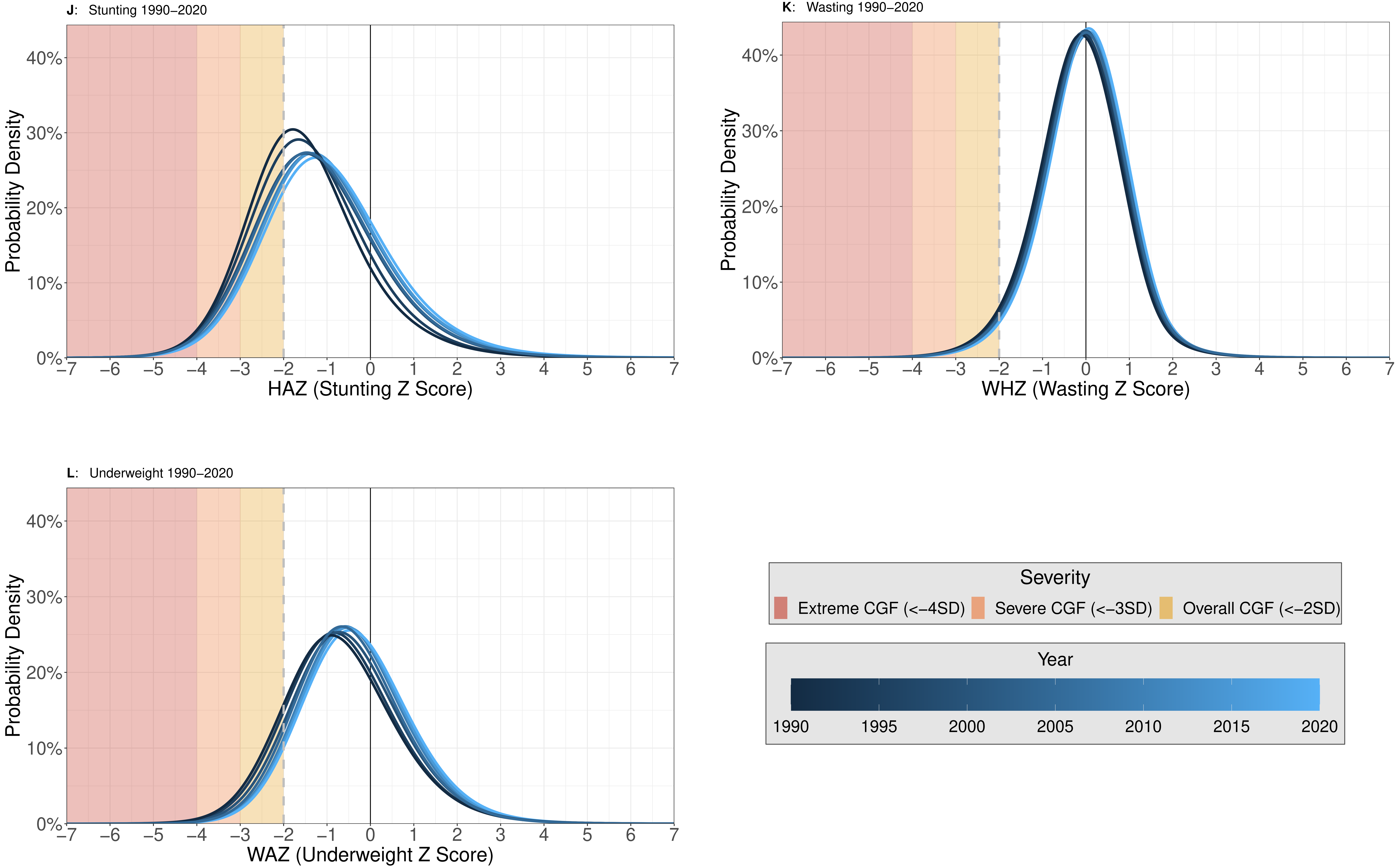

Peru – Stunting (HAZ)

A: Overall and Severe Stunting Prevalence

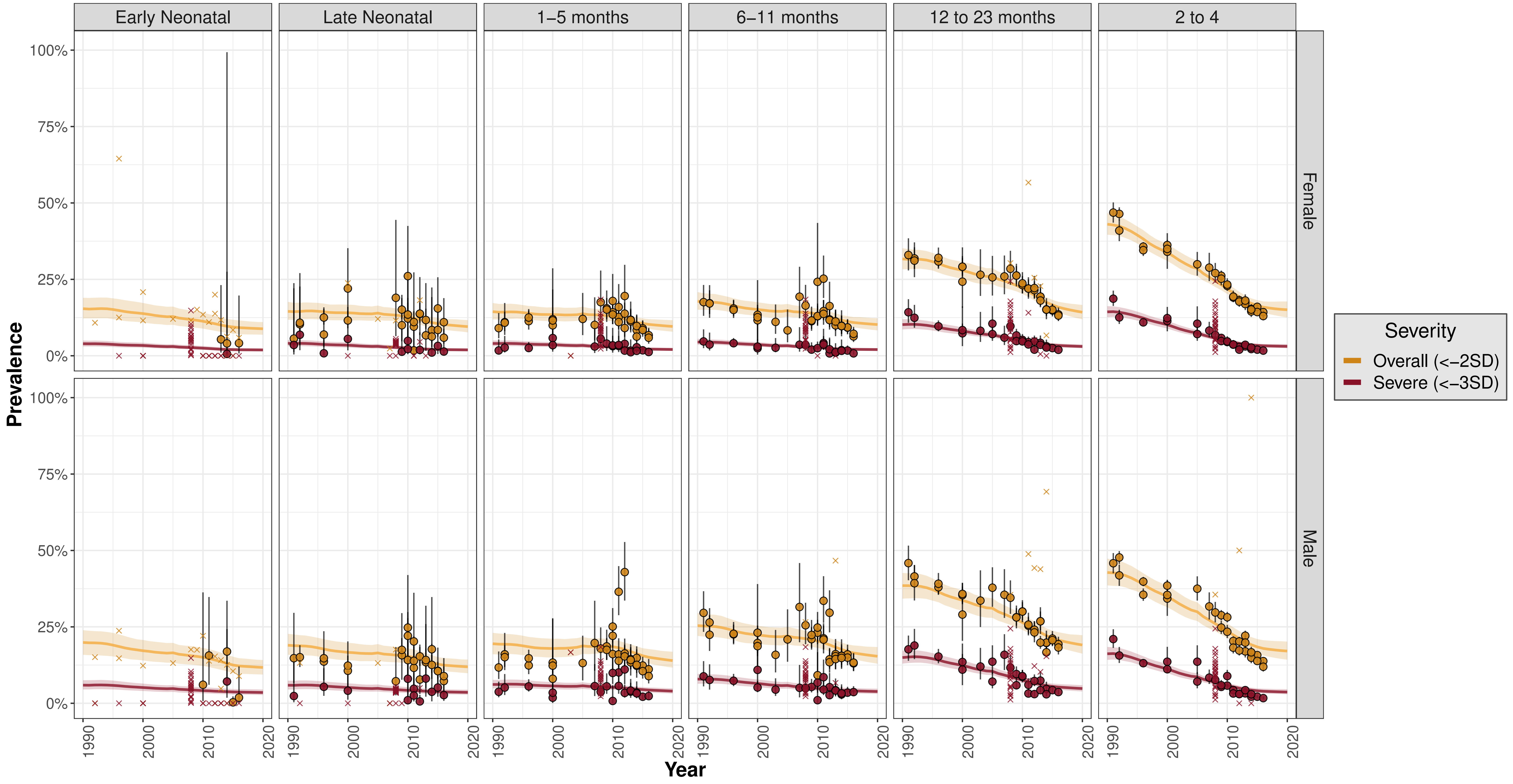

B: Transformed Mean Stunting Z Scores

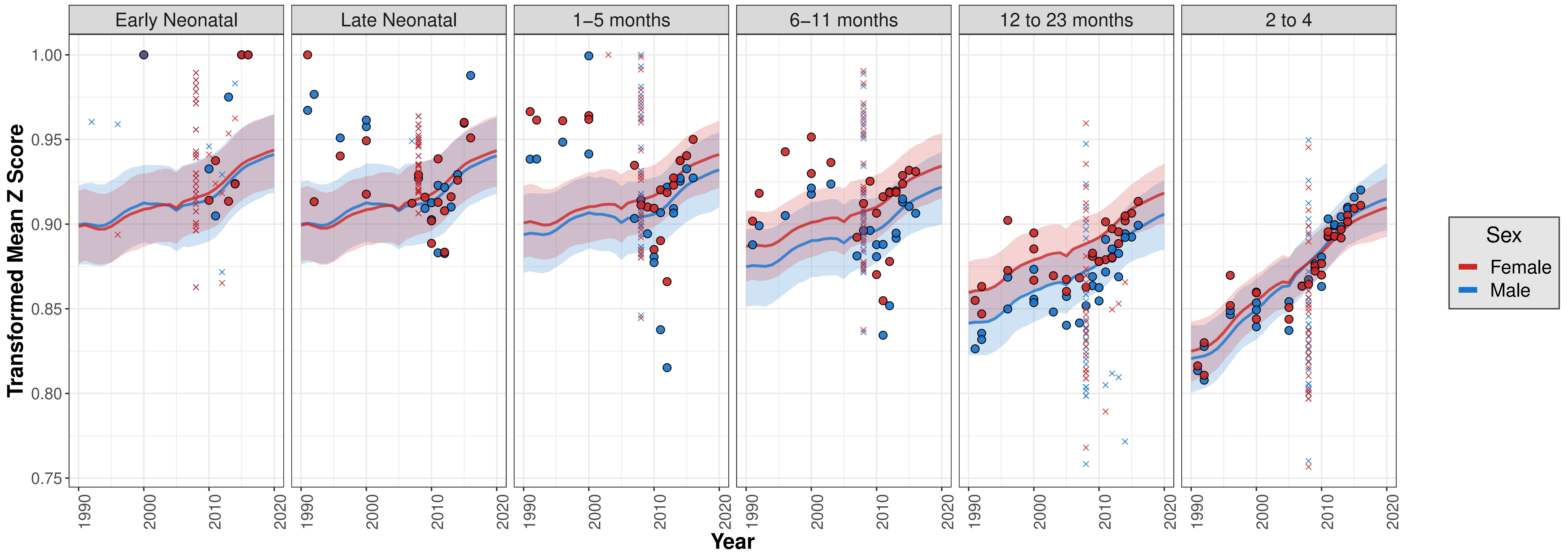

C

Year	Source
1985	WHO CGM Database
1991	DHS
1992	DHS
1992	WHO CGM Database
1996	DHS
1996	WHO CGM Database
2000	National Living Standards Measurement Survey
2000	DHS
2000	WHO CGM Database
2003	Young Lives: Household and Child Survey Round 1 – UK Data Service
2005	WHO CGM Database
2007	Continuous DHS
2008	WHO CGM Database
2008	Continuous DHS
2009	WHO CGM Database
2009	Continuous DHS
2010	WHO CGM Database
2010	Loreto Malnutrition and Enteric Disease Study
2010	Continuous DHS
2011	WHO CGM Database
2011	Loreto Malnutrition and Enteric Disease Study
2011	Continuous DHS
2012	WHO CGM Database
2012	Loreto Malnutrition and Enteric Disease Study
2012	Continuous DHS
2013	WHO CGM Database
2013	Continuous DHS
2013	Continuous DHS – INEI
2013	Loreto Malnutrition and Enteric Disease Study
2014	WHO CGM Database
2014	Continuous DHS
2014	Continuous DHS – INEI
2014	Loreto Malnutrition and Enteric Disease Study
2015	WHO CGM Database
2015	Demographic and Family Health Survey
2016	WHO CGM Database
2016	Demographic and Family Health Survey

Peru – Wasting (WHZ)

D: Overall and Severe Wasting Prevalence

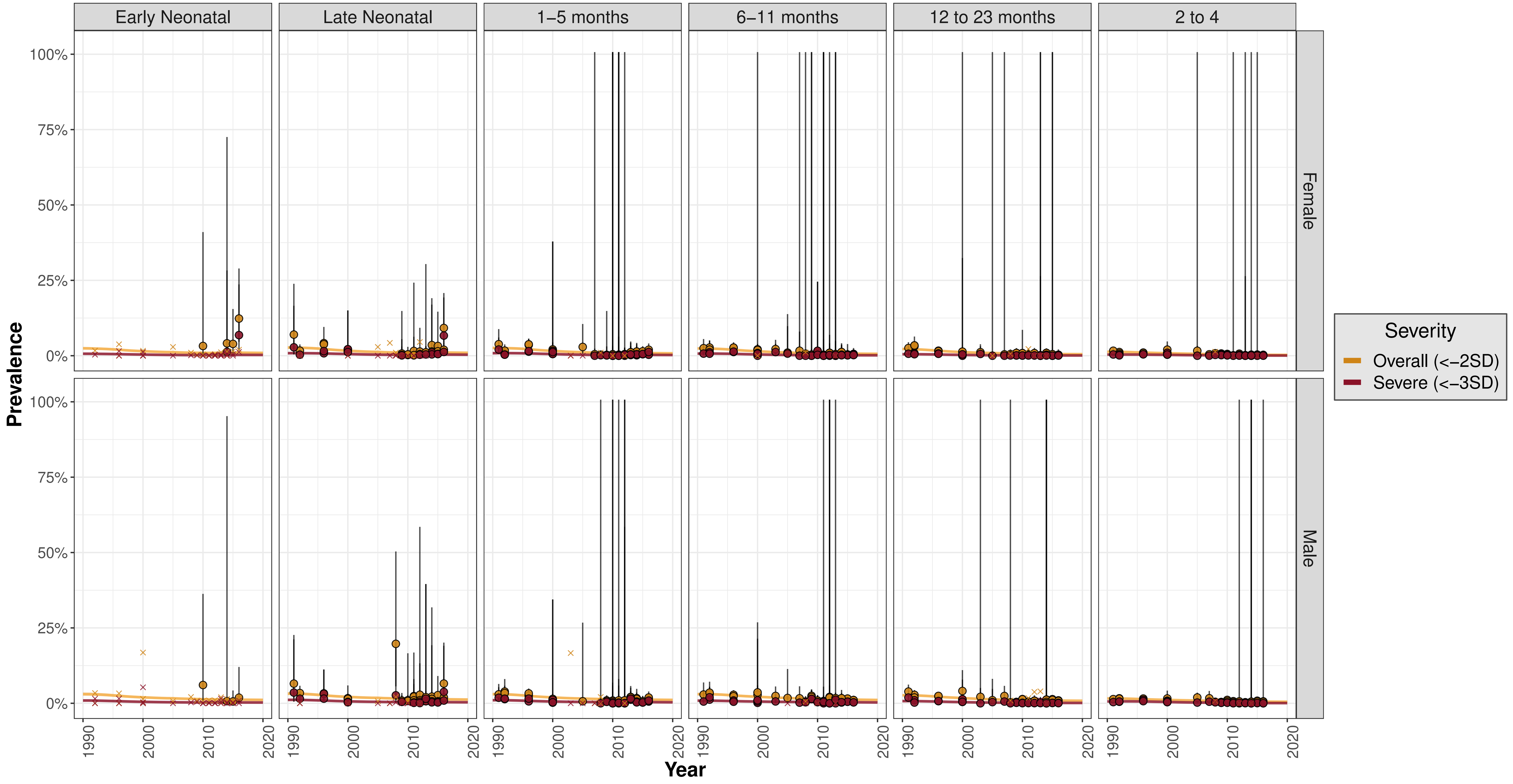

E: Transformed Mean Wasting Z Scores

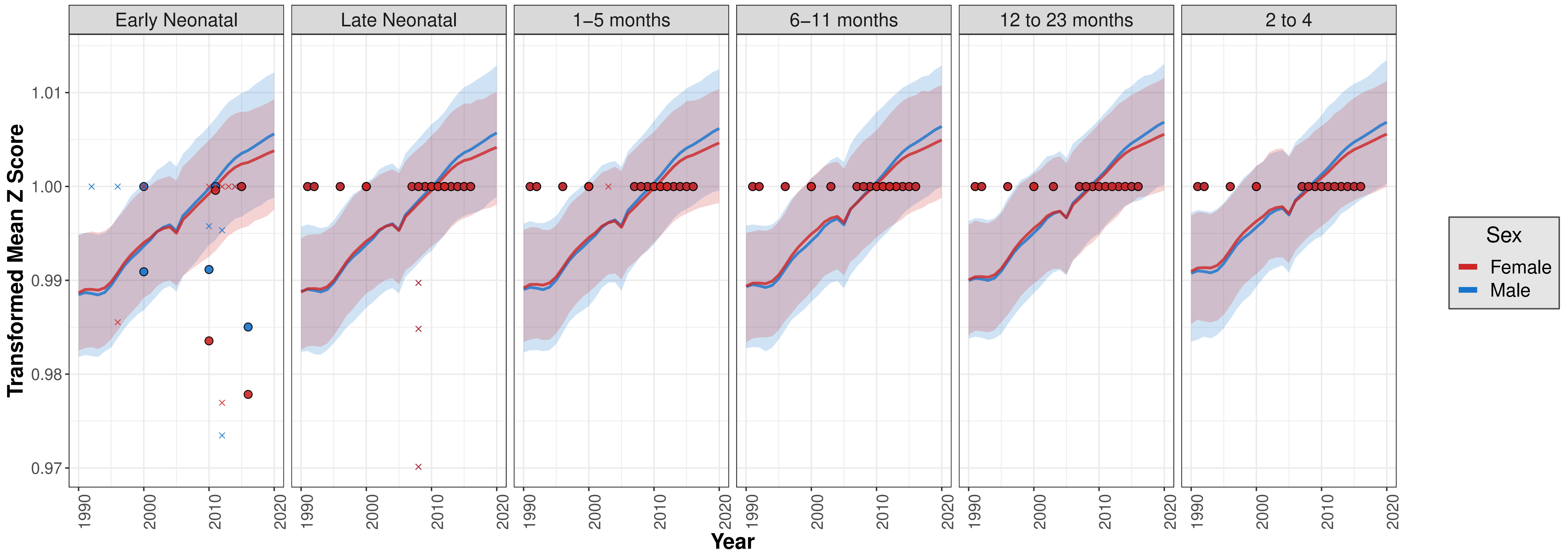

F

Year	Source
1985	WHO CGM Database
1991	DHS
1992	DHS
1992	WHO CGM Database
1996	DHS
1996	WHO CGM Database
2000	National Living Standards Measurement Survey
2000	DHS
2000	WHO CGM Database
2003	Young Lives: Household and Child Survey Round 1 – UK Data Service
2005	WHO CGM Database
2007	Continuous DHS
2008	WHO CGM Database
2008	Continuous DHS
2009	WHO CGM Database
2009	Continuous DHS
2010	WHO CGM Database
2010	Loreto Malnutrition and Enteric Disease Study
2010	Continuous DHS
2011	WHO CGM Database
2011	Loreto Malnutrition and Enteric Disease Study
2011	Continuous DHS
2012	WHO CGM Database
2012	Loreto Malnutrition and Enteric Disease Study
2012	Continuous DHS
2013	WHO CGM Database
2013	Continuous DHS
2013	Continuous DHS – INEI
2013	Loreto Malnutrition and Enteric Disease Study
2014	WHO CGM Database
2014	Continuous DHS
2014	Continuous DHS – INEI
2014	Loreto Malnutrition and Enteric Disease Study
2015	WHO CGM Database
2015	Demographic and Family Health Survey
2016	WHO CGM Database
2016	Demographic and Family Health Survey

Peru – Underweight (WAZ)

G: Overall and Severe Underweight Prevalence

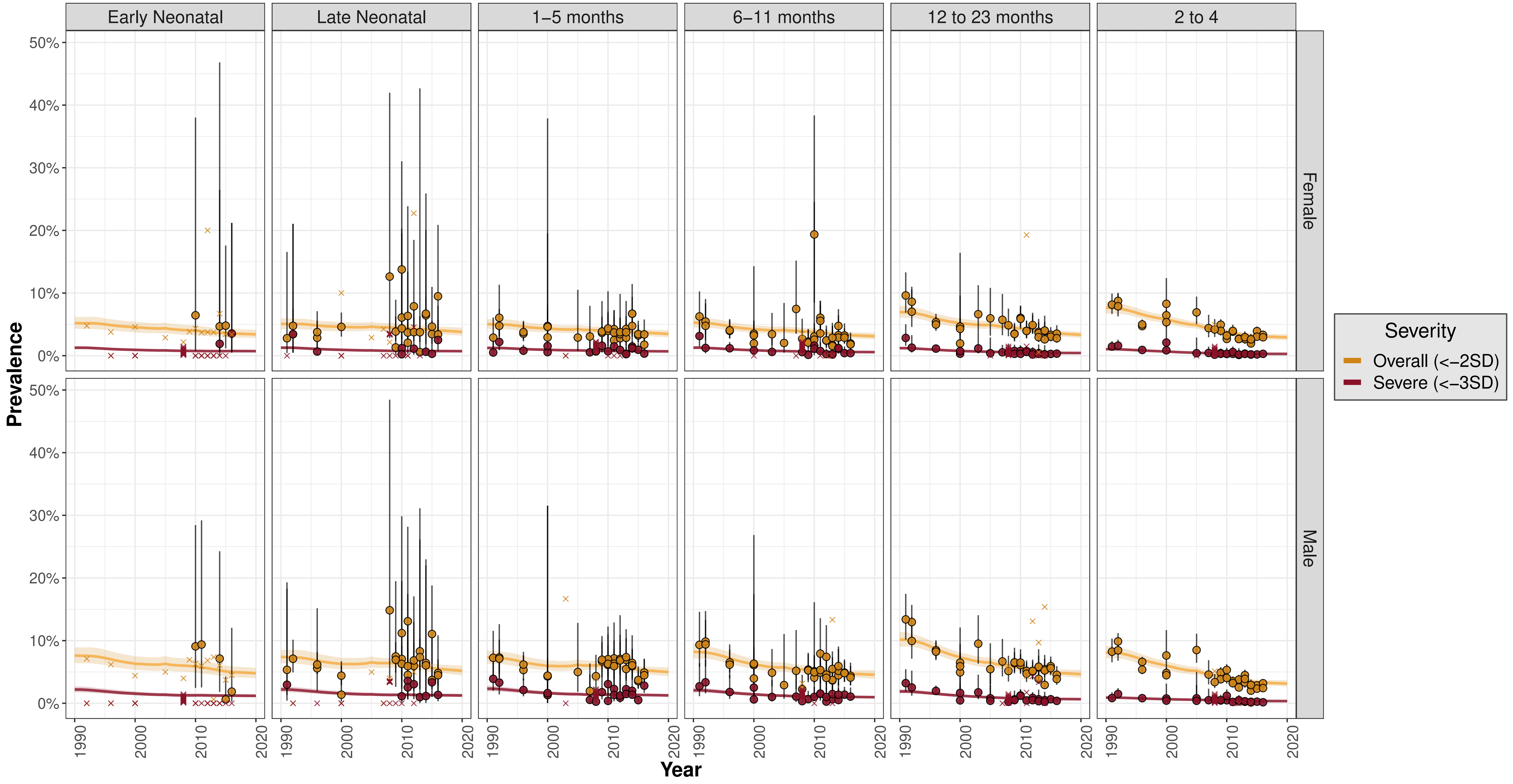

H: Transformed Mean Underweight Z Scores

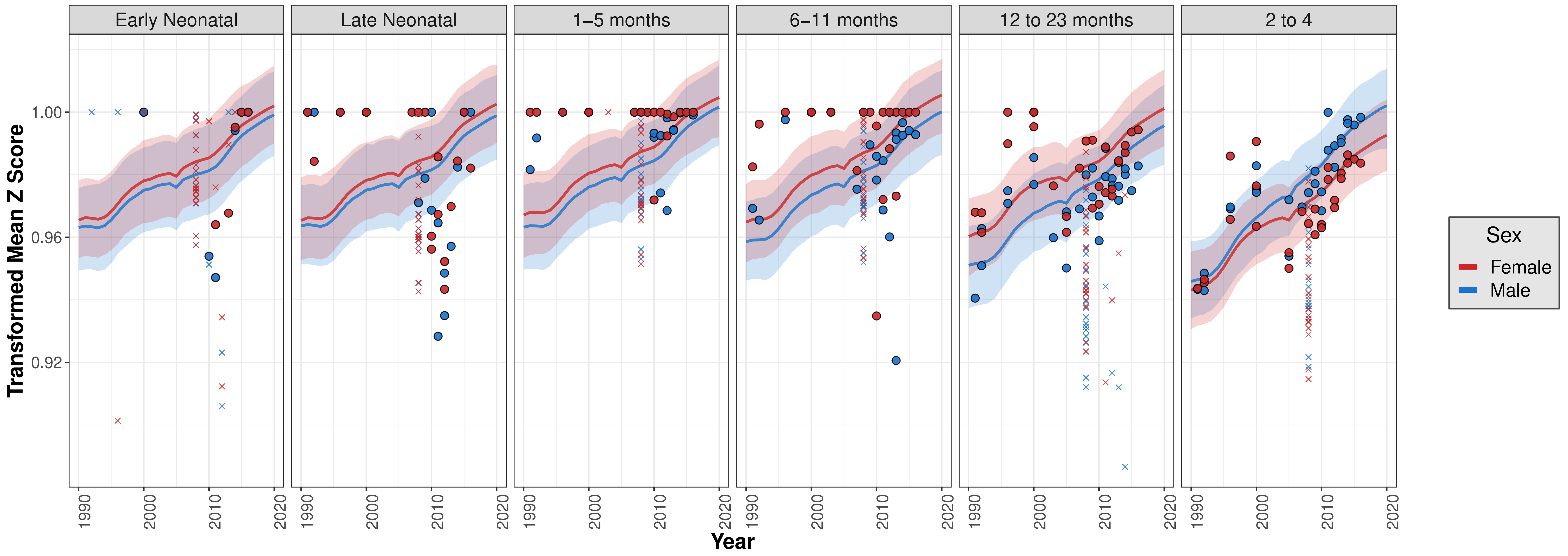

I

Year	Source
1991	DHS
1992	DHS
1992	WHO CGM Database
1996	DHS
1996	WHO CGM Database
2000	National Living Standards Measurement Survey
2000	DHS
2000	WHO CGM Database
2003	Young Lives: Household and Child Survey Round 1 – UK Data Service
2005	WHO CGM Database
2007	Continuous DHS
2008	WHO CGM Database
2008	Continuous DHS
2009	WHO CGM Database
2009	Continuous DHS
2010	WHO CGM Database
2010	Loreto Malnutrition and Enteric Disease Study
2010	Continuous DHS
2011	WHO CGM Database
2011	Loreto Malnutrition and Enteric Disease Study
2011	Continuous DHS
2012	WHO CGM Database
2012	Loreto Malnutrition and Enteric Disease Study
2012	Continuous DHS
2013	WHO CGM Database
2013	Continuous DHS
2013	Continuous DHS – INEI
2013	Loreto Malnutrition and Enteric Disease Study
2014	WHO CGM Database
2014	Continuous DHS
2014	Continuous DHS – INEI
2014	Loreto Malnutrition and Enteric Disease Study
2015	WHO CGM Database
2015	Demographic and Family Health Survey
2016	WHO CGM Database
2016	Demographic and Family Health Survey

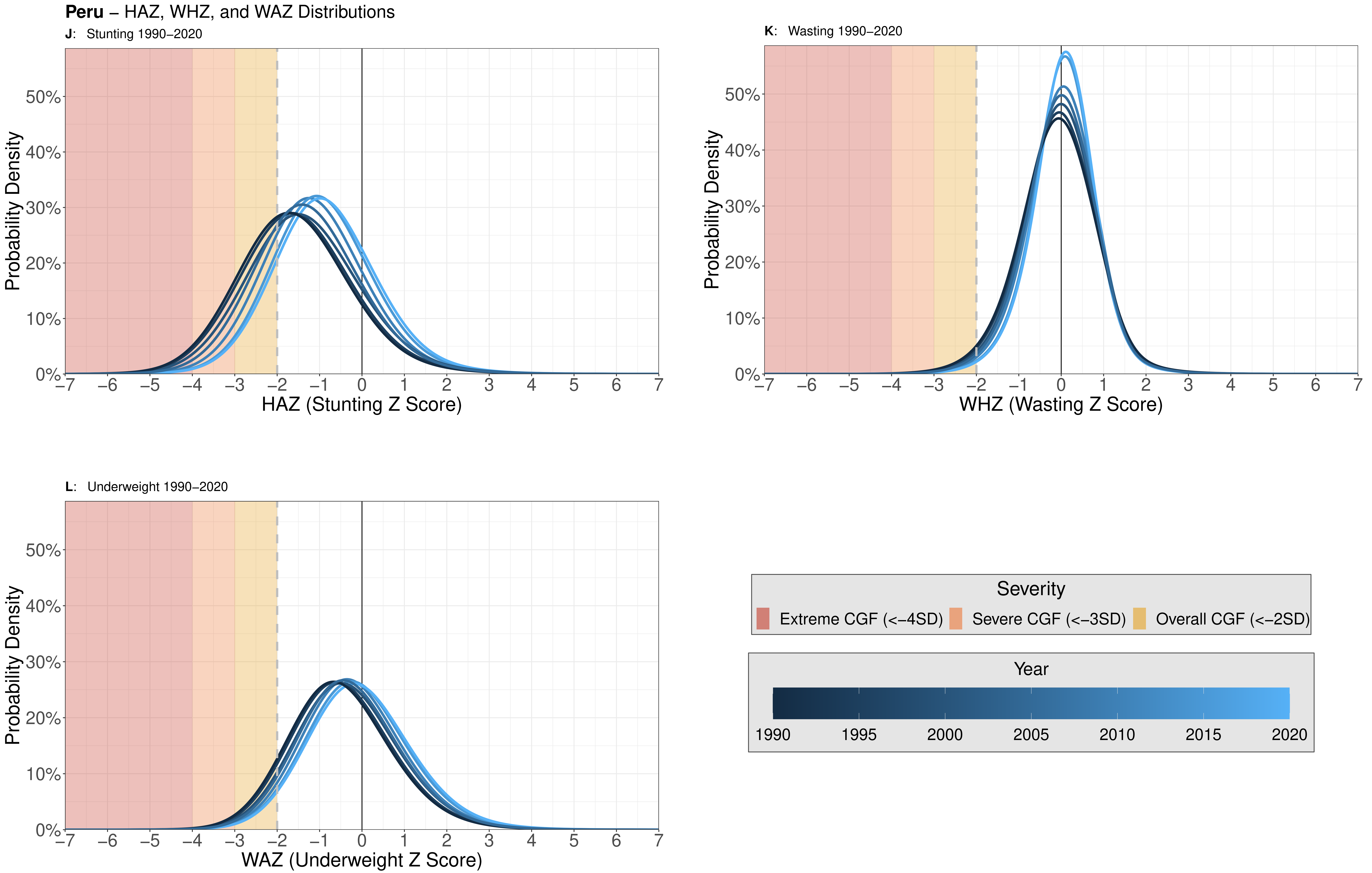

Peru – HAZ, WHZ, and WAZ Distributions
J: Stunting 1990–2020
Probability Density
50%
40%
30%
20%
10%
0%
−7 −6 −5 −4 −3 −2 −1 0 1 2 3 4 5 6 7
HAZ (Stunting Z Score)
K: Wasting 1990–2020
Probability Density
50%
40%
30%
20%
10%
0%
−7 −6 −5 −4 −3 −2 −1 0 1 2 3 4 5 6 7
WHZ (Wasting Z Score)
L: Underweight 1990–2020
Probability Density
50%
40%
30%
20%
10%
0%
−7 −6 −5 −4 −3 −2 −1 0 1 2 3 4 5 6 7
WAZ (Underweight Z Score)
Severity
Extreme CGF (<−4SD)
Severe CGF (<−3SD)
Overall CGF (<−2SD)
Year
1990
1995
2000
2005
2010
2015
2020

Antigua and Barbuda – Stunting (HAZ)

A: Overall and Severe Stunting Prevalence

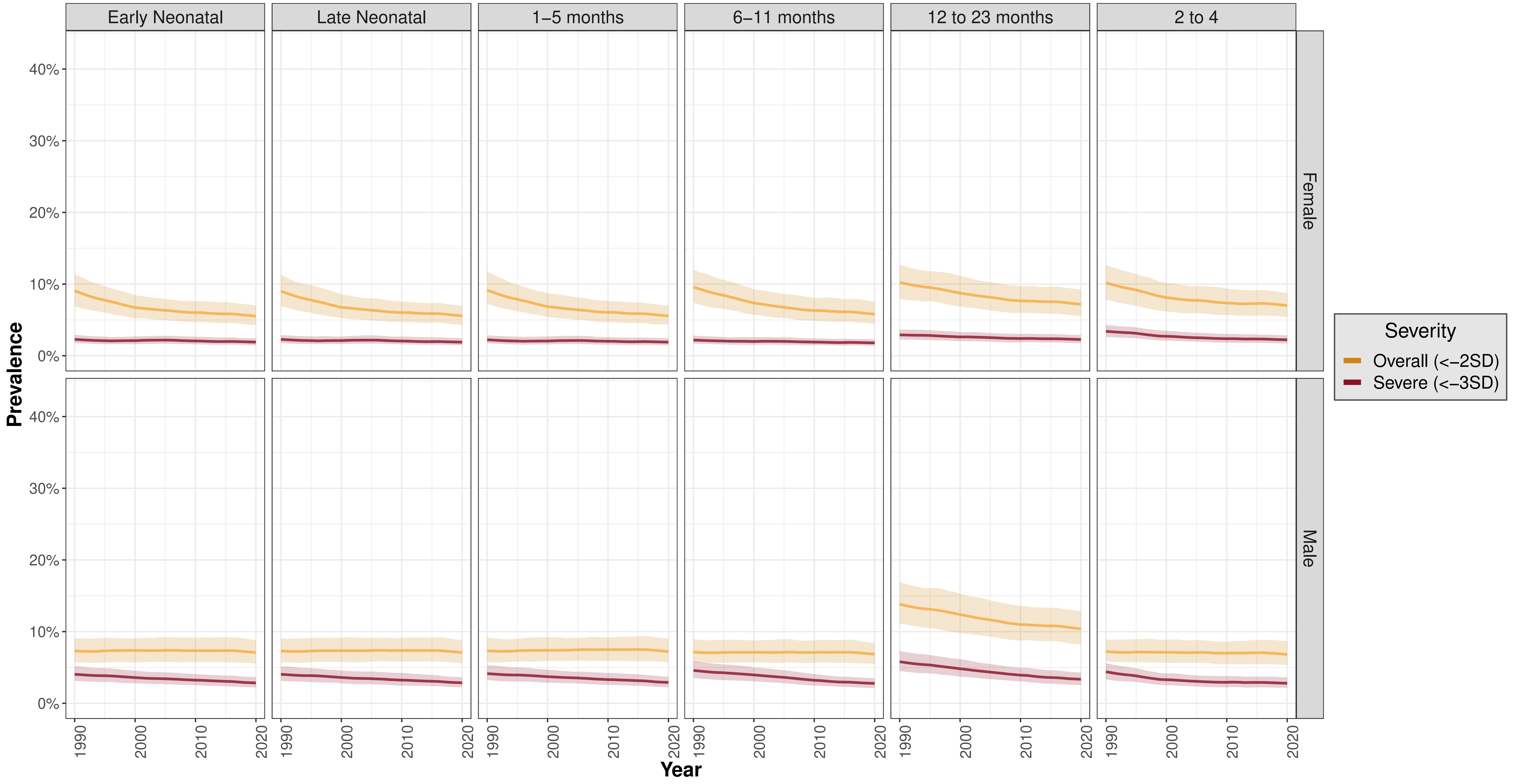

B: Transformed Mean Stunting Z Scores

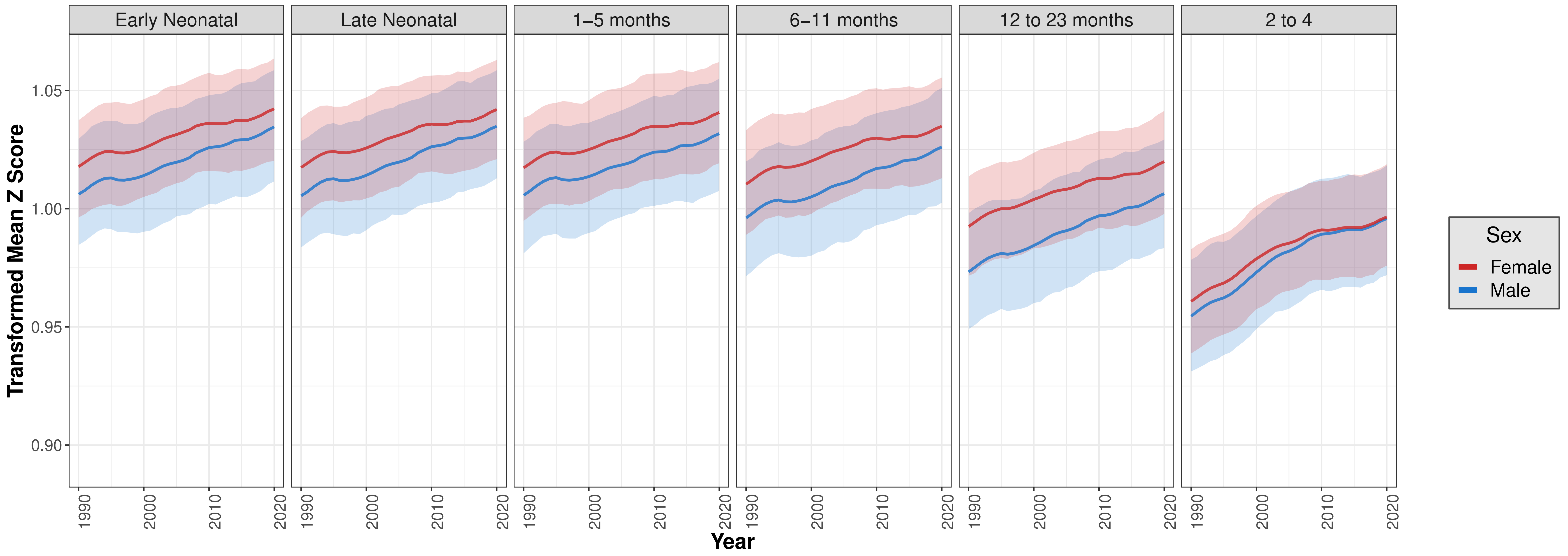

C

Year	Source
1981	WHO CGM Database

Antigua and Barbuda – Wasting (WHZ)

D: Overall and Severe Wasting Prevalence

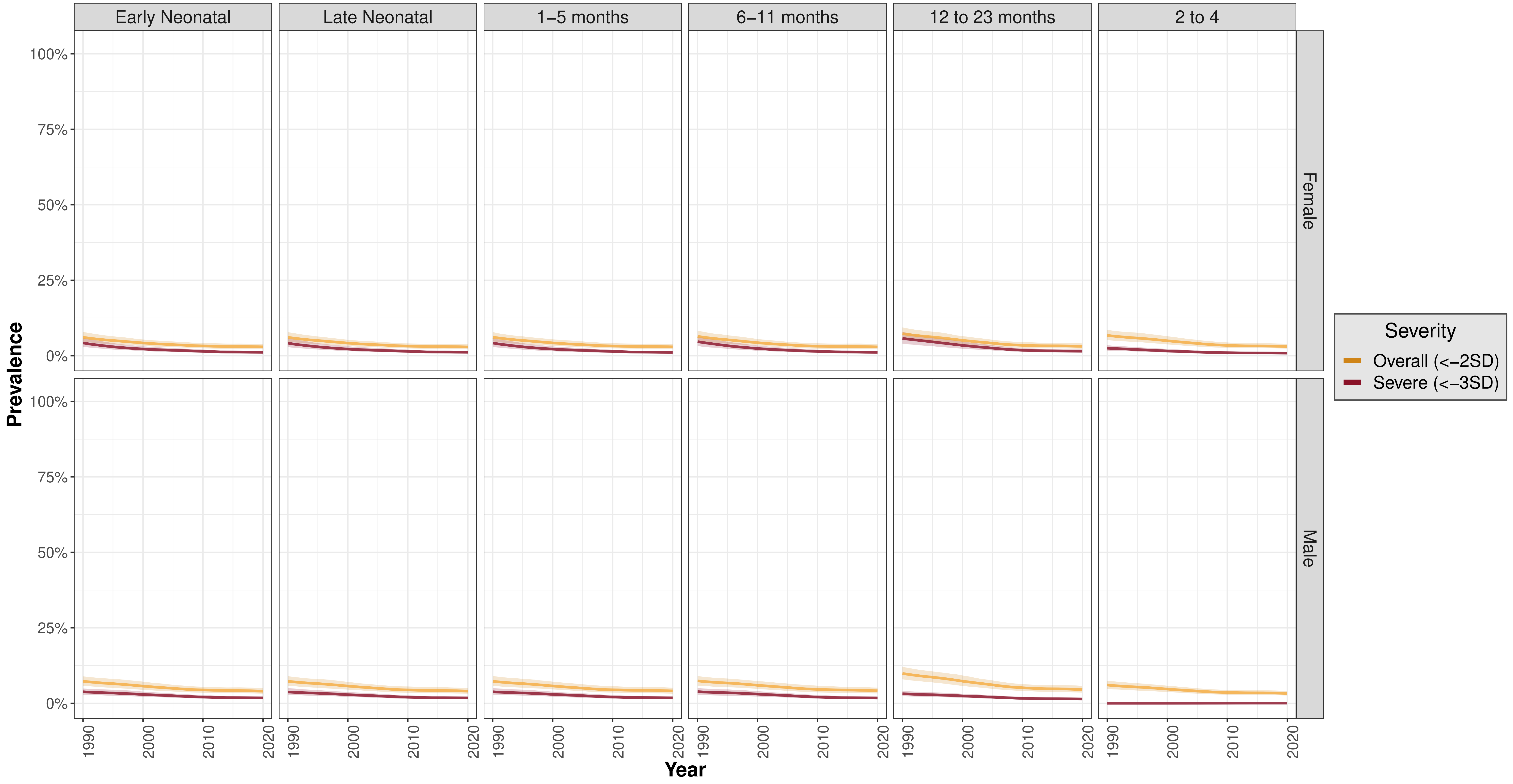

E: Transformed Mean Wasting Z Scores

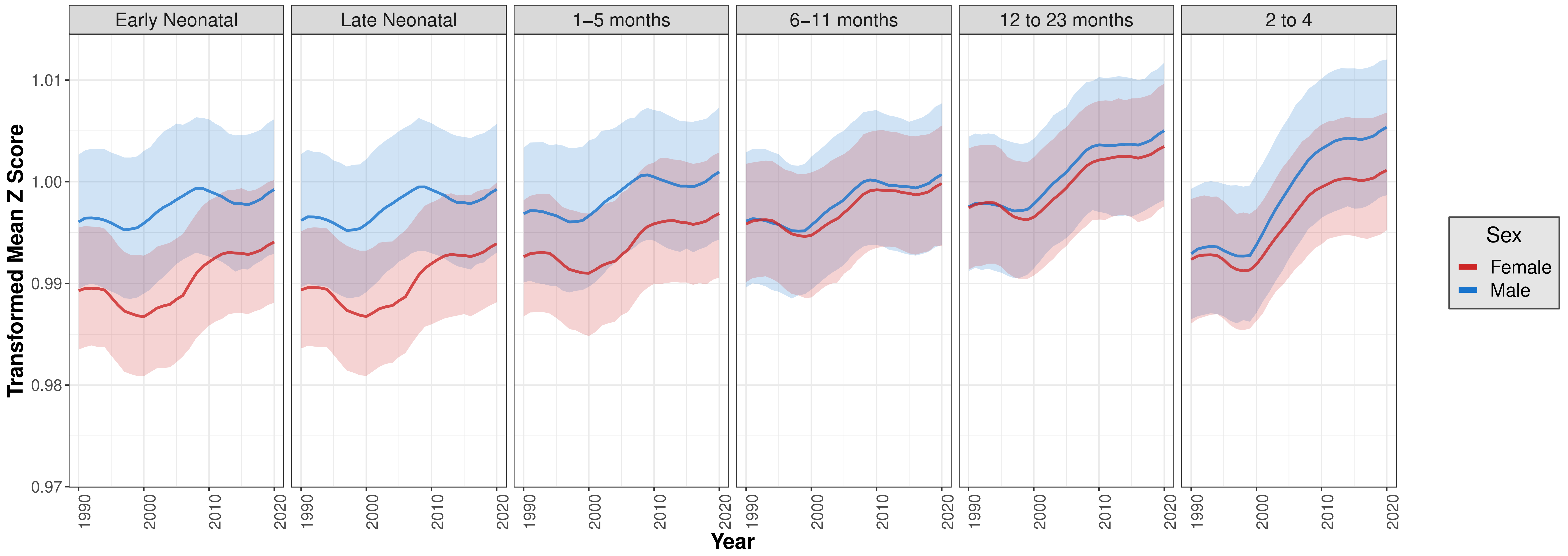

F

Year	Source
1981	WHO CGM Database

Antigua and Barbuda – Underweight (WAZ)

G: Overall and Severe Underweight Prevalence

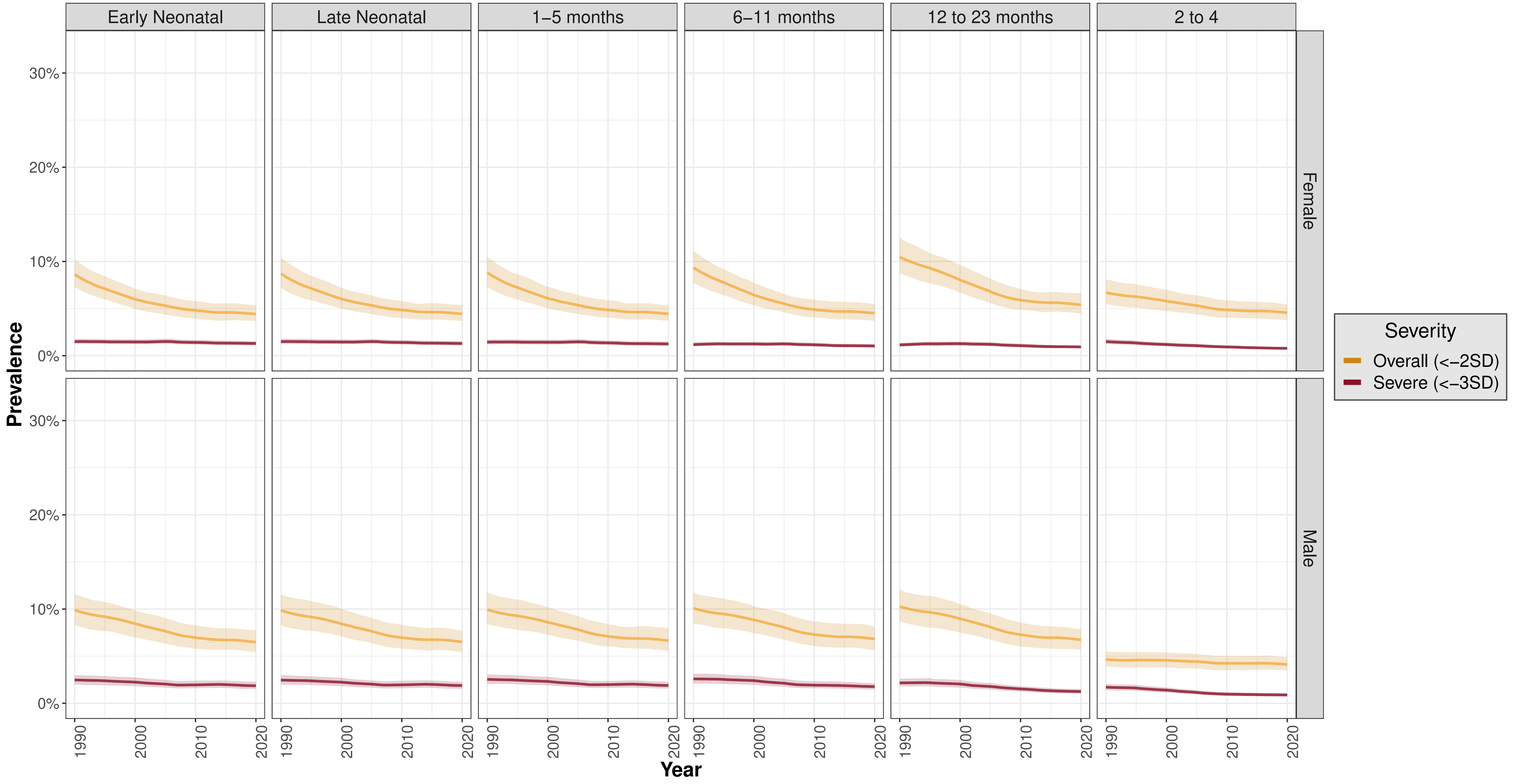

H: Transformed Mean Underweight Z Scores

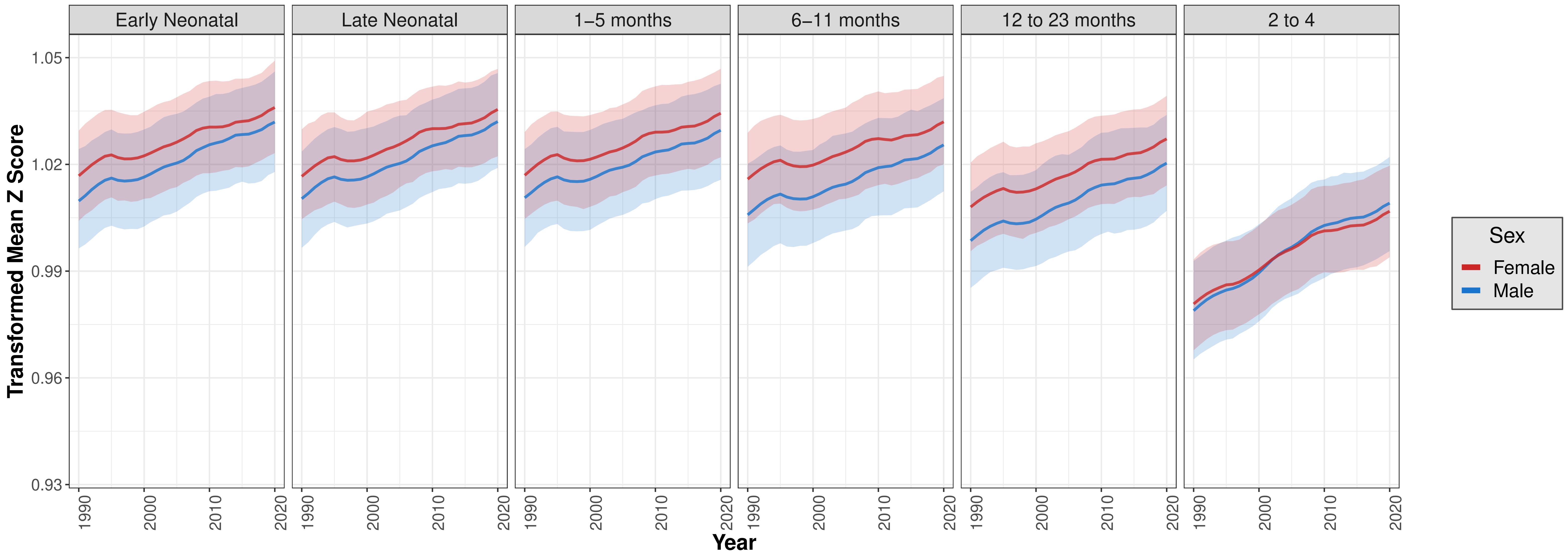

I

Year	Source
1981	WHO CGM Database

Antigua and Barbuda – HAZ, WHZ, and WAZ Distributions

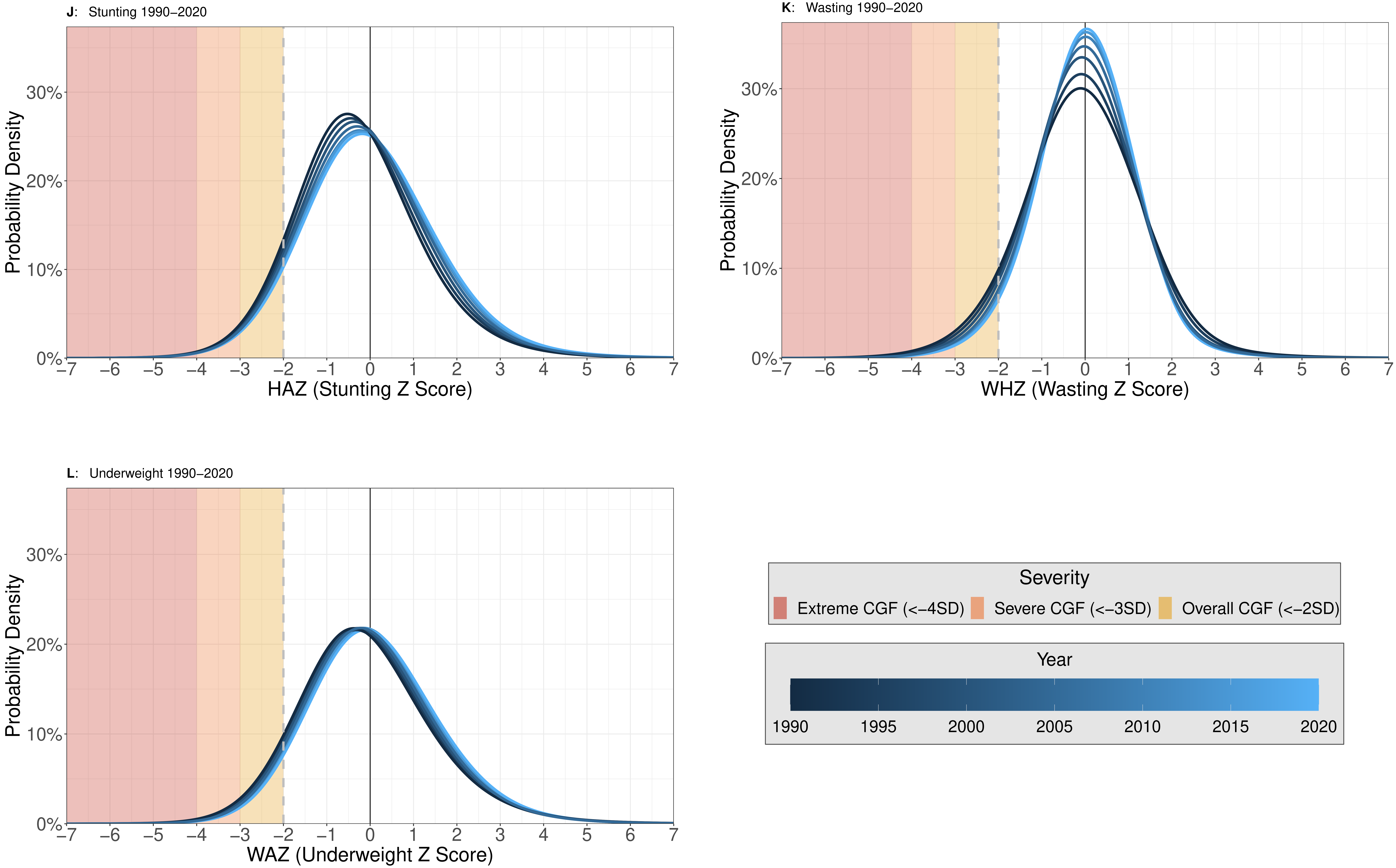

Bahamas – Stunting (HAZ)

A: Overall and Severe Stunting Prevalence

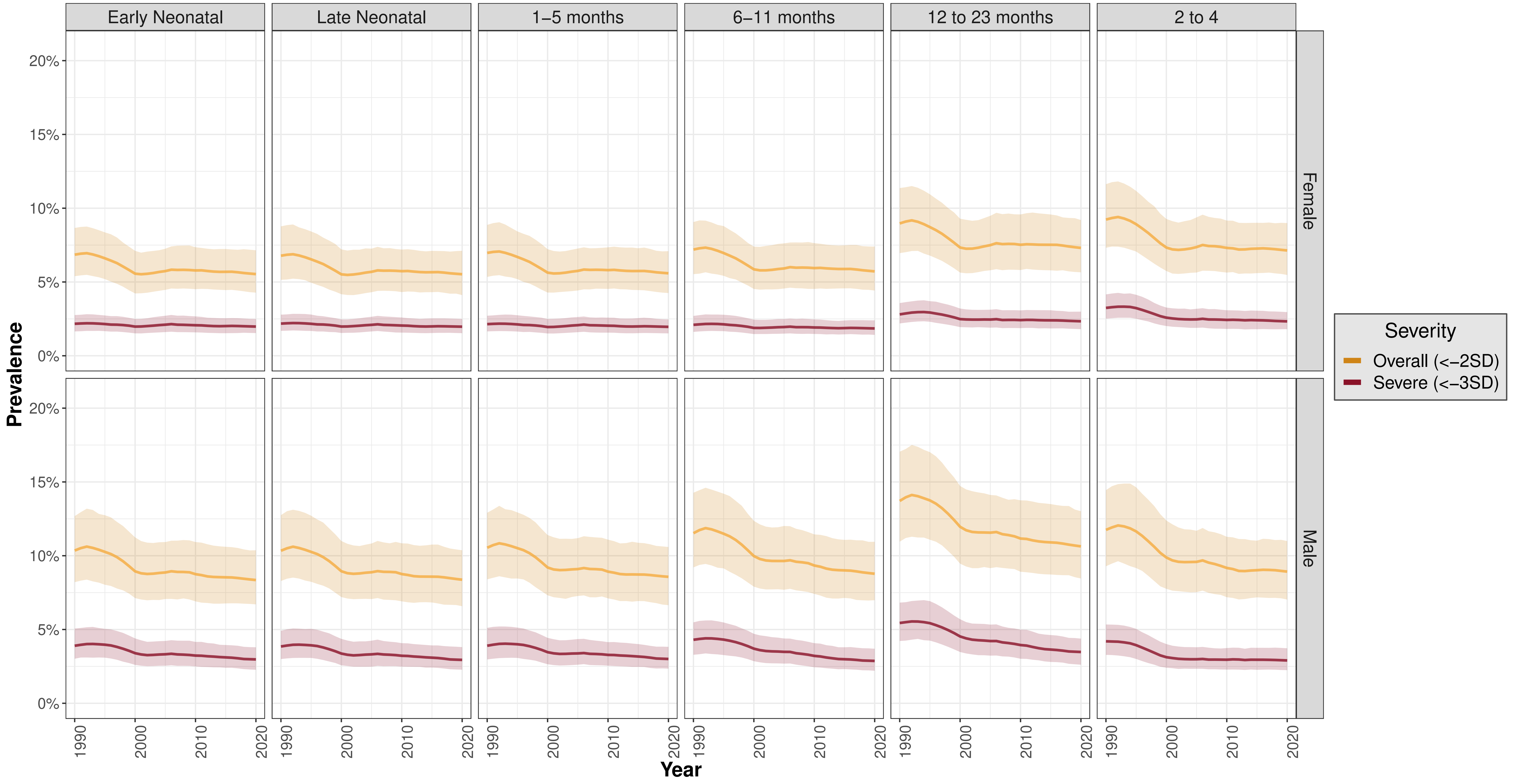

B: Transformed Mean Stunting Z Scores

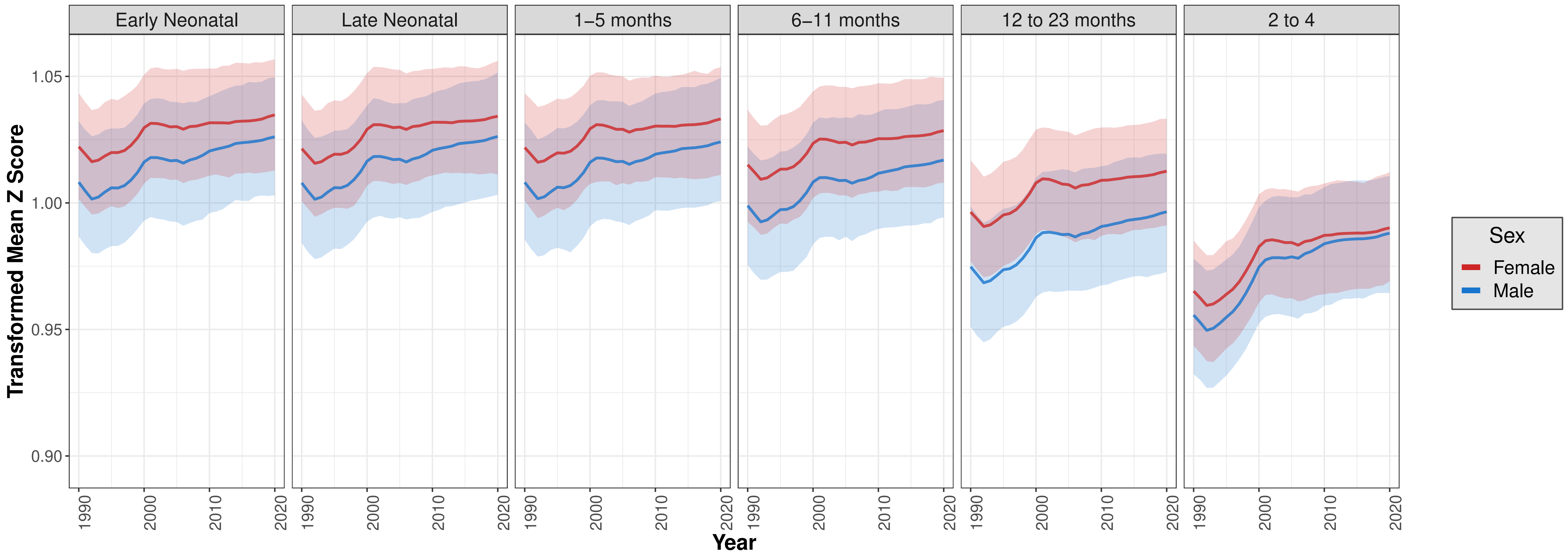

C

Source
No sources for this location

Bahamas – Wasting (WHZ)

D: Overall and Severe Wasting Prevalence

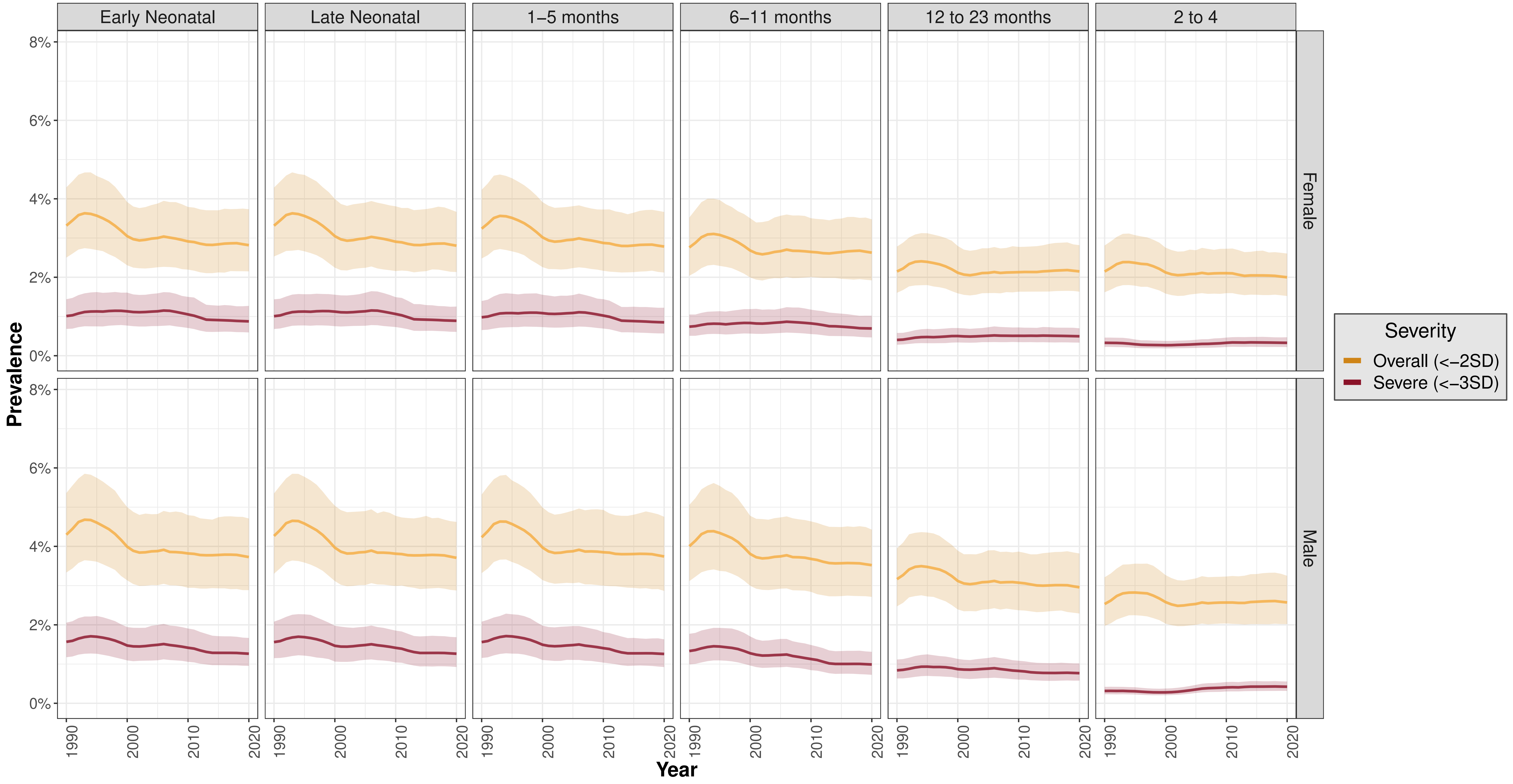

E: Transformed Mean Wasting Z Scores

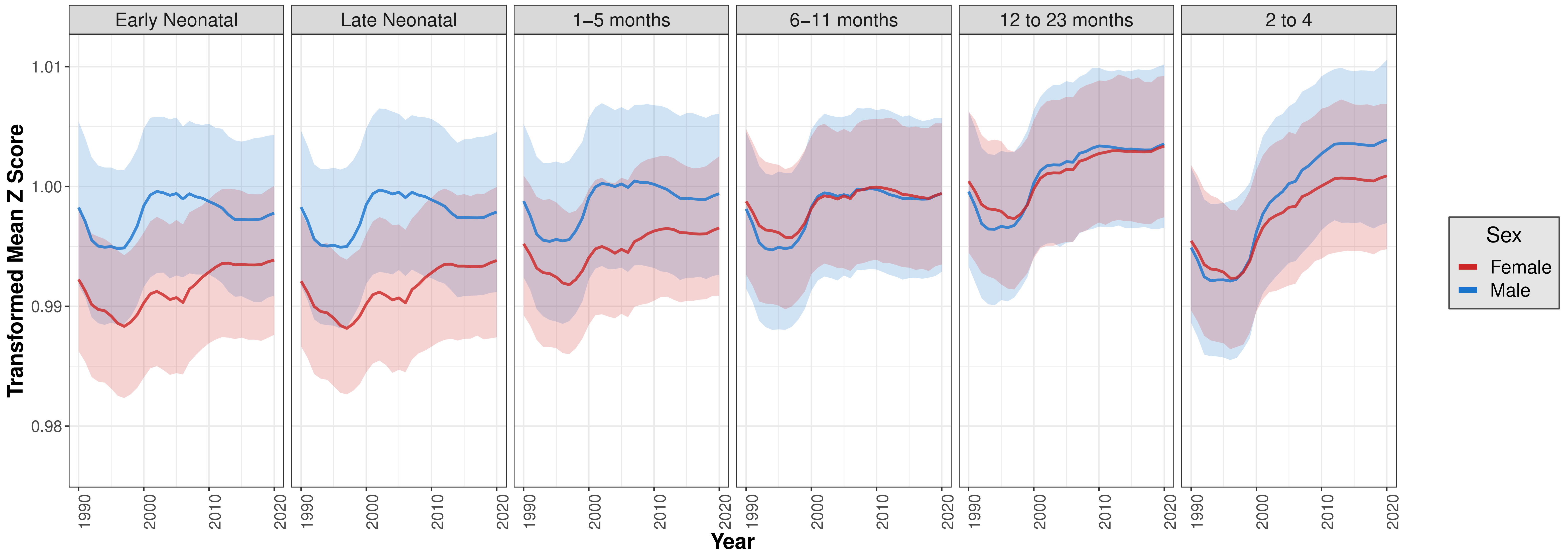

F

Source
No sources for this location

Bahamas – Underweight (WAZ)

G: Overall and Severe Underweight Prevalence

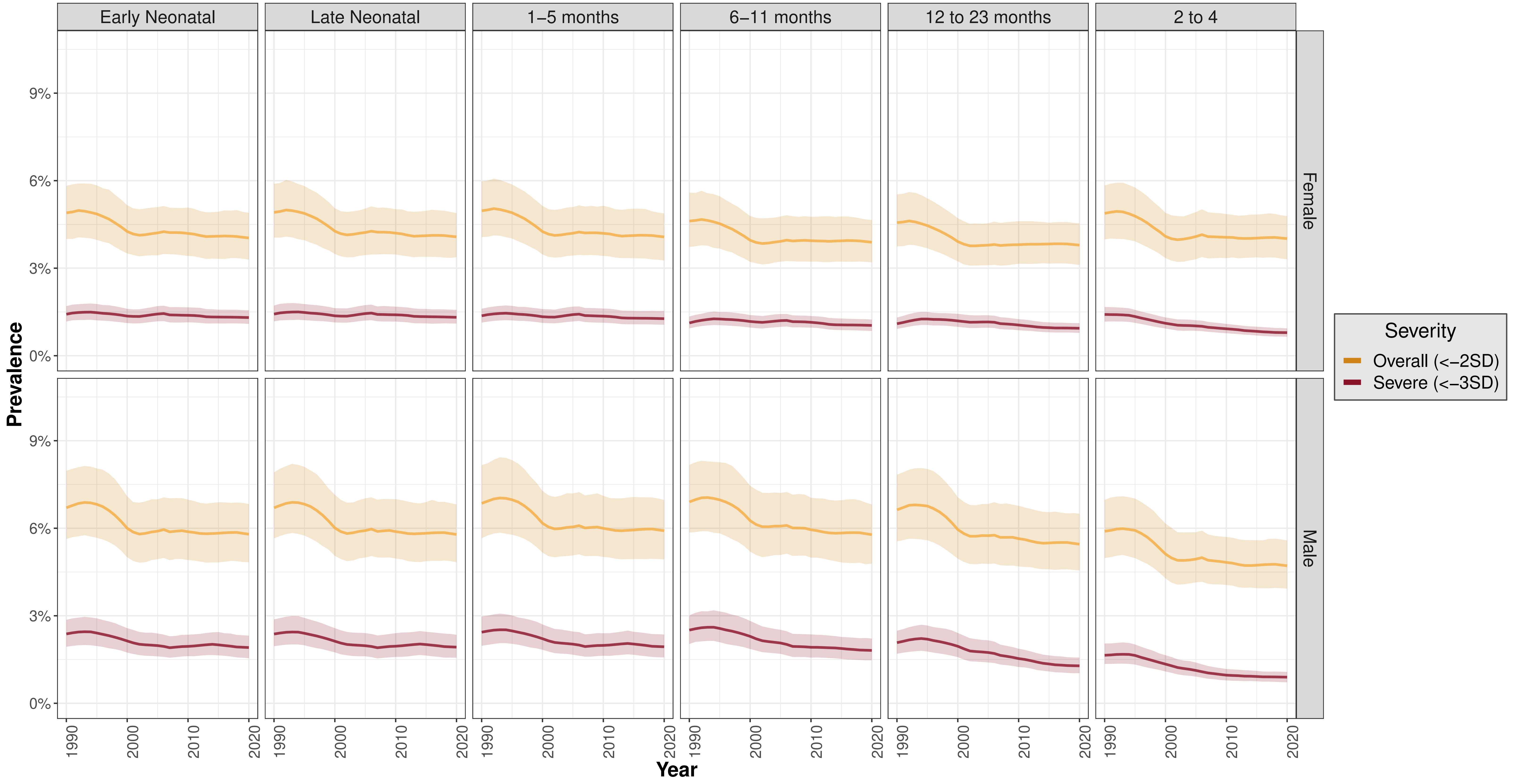

H: Transformed Mean Underweight Z Scores

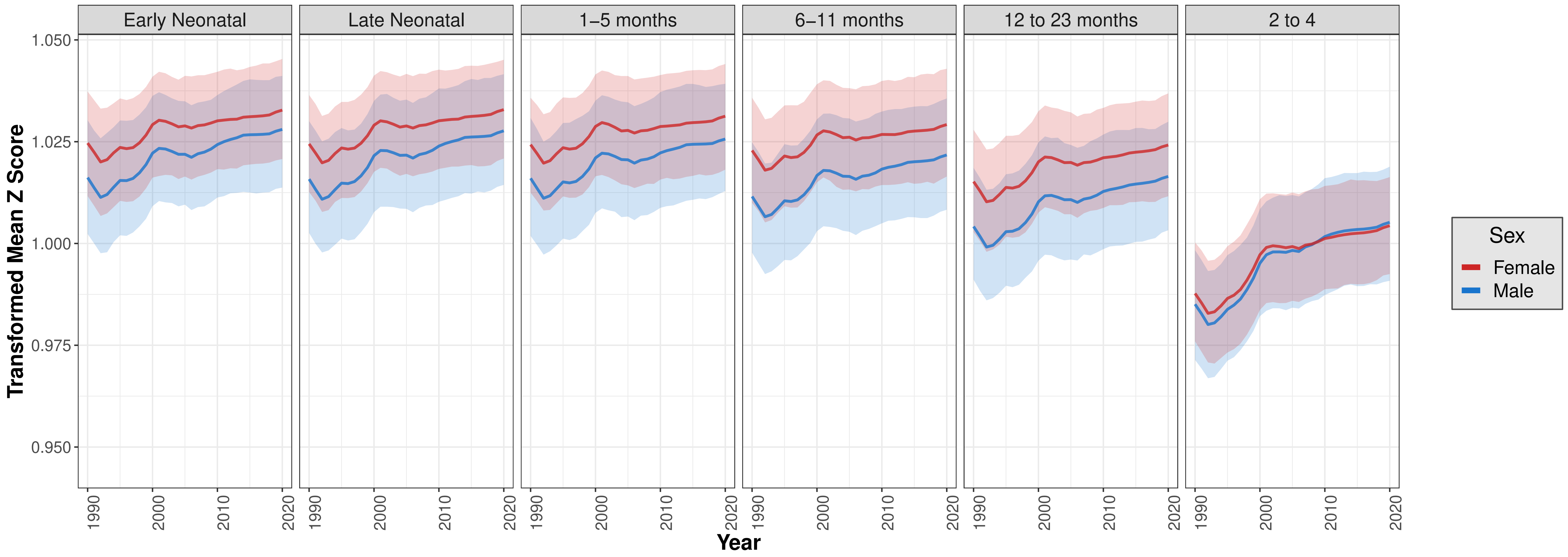

I

Source
No sources for this location

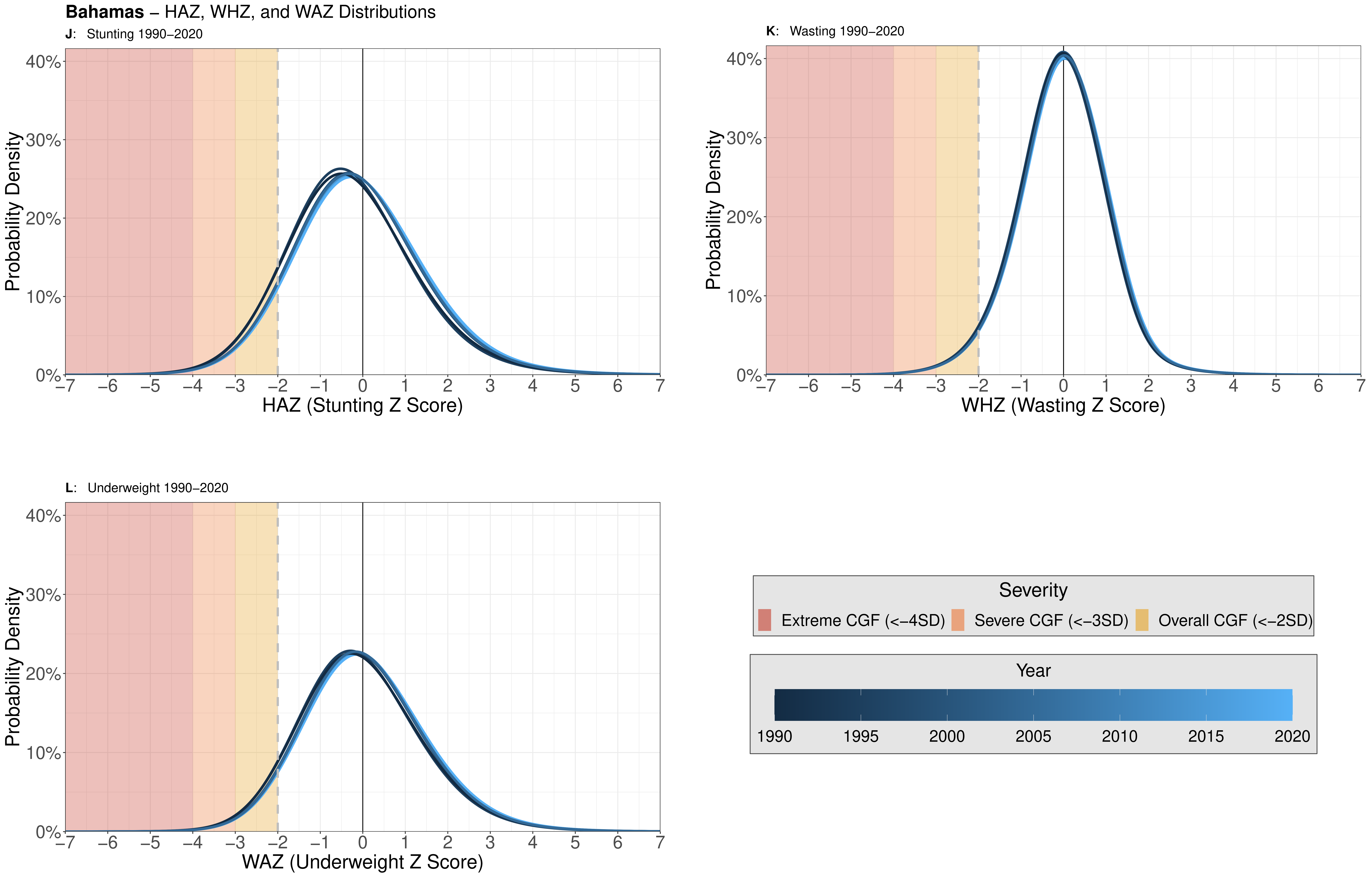

Bahamas – HAZ, WHZ, and WAZ Distributions
J: Stunting 1990–2020
Probability Density
HAZ (Stunting Z Score)
K: Wasting 1990–2020
WHZ (Wasting Z Score)
L: Underweight 1990–2020
WAZ (Underweight Z Score)
0%
10%
20%
30%
40%
−7
−6
−5
−4
−3
−2
−1
0
1
2
3
4
5
6
7
Severity
Extreme CGF (<−4SD)
Severe CGF (<−3SD)
Overall CGF (<−2SD)
Year
1990
1995
2000
2005
2010
2015
2020

Barbados – Stunting (HAZ)

A: Overall and Severe Stunting Prevalence

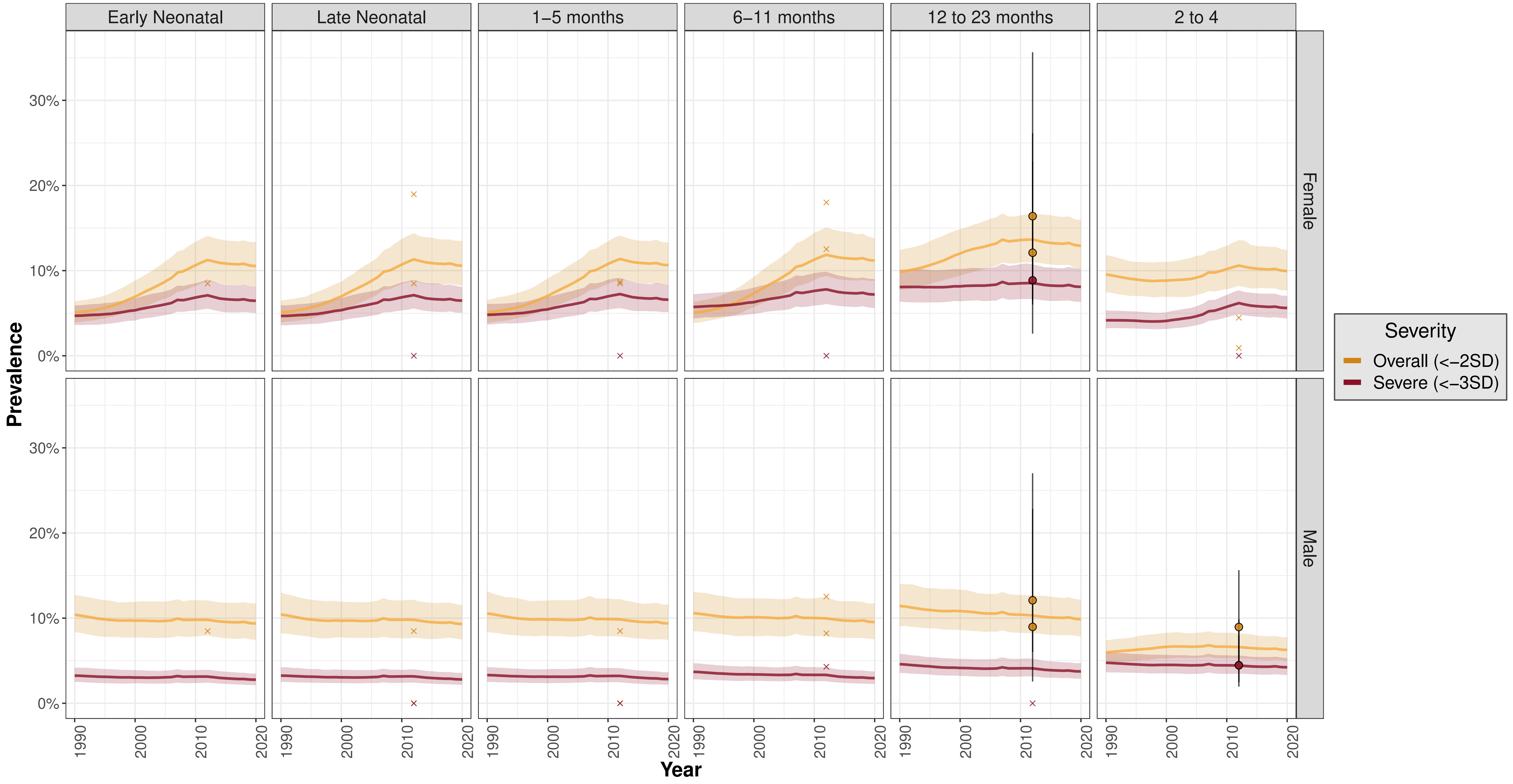

B: Transformed Mean Stunting Z Scores

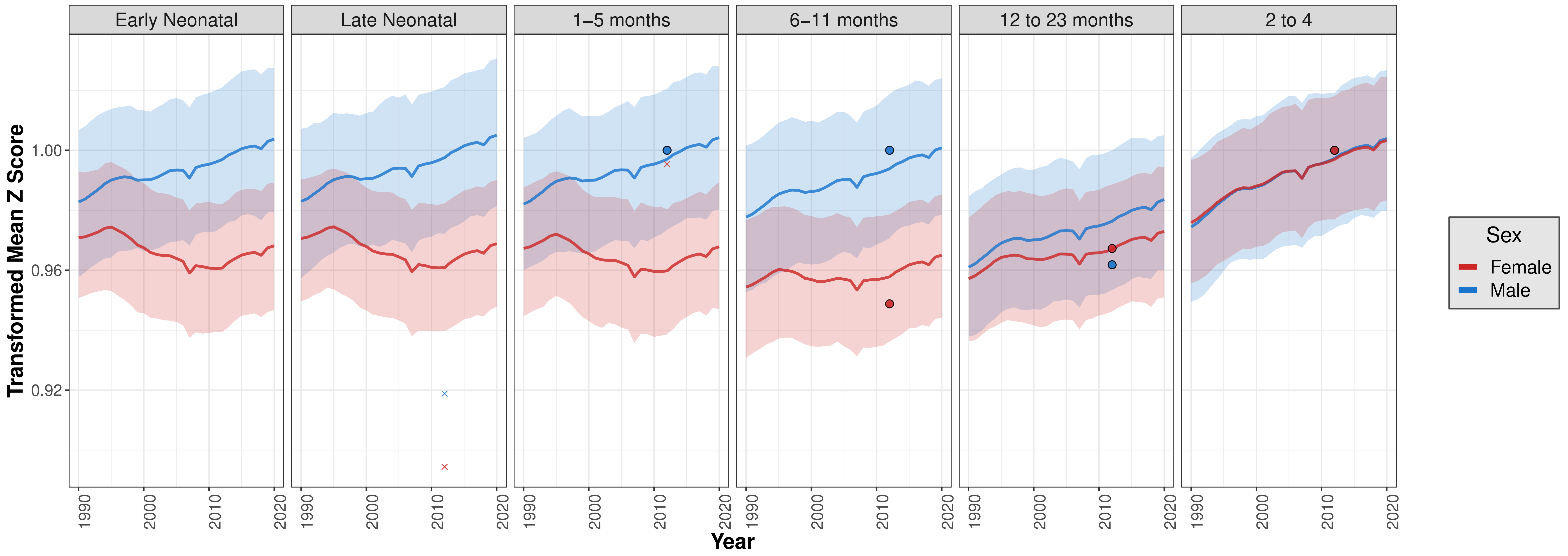

C

Year	Source
1981	WHO CGM Database
2012	WHO CGM Database
2012	MICS

Barbados – Wasting (WHZ)

D: Overall and Severe Wasting Prevalence

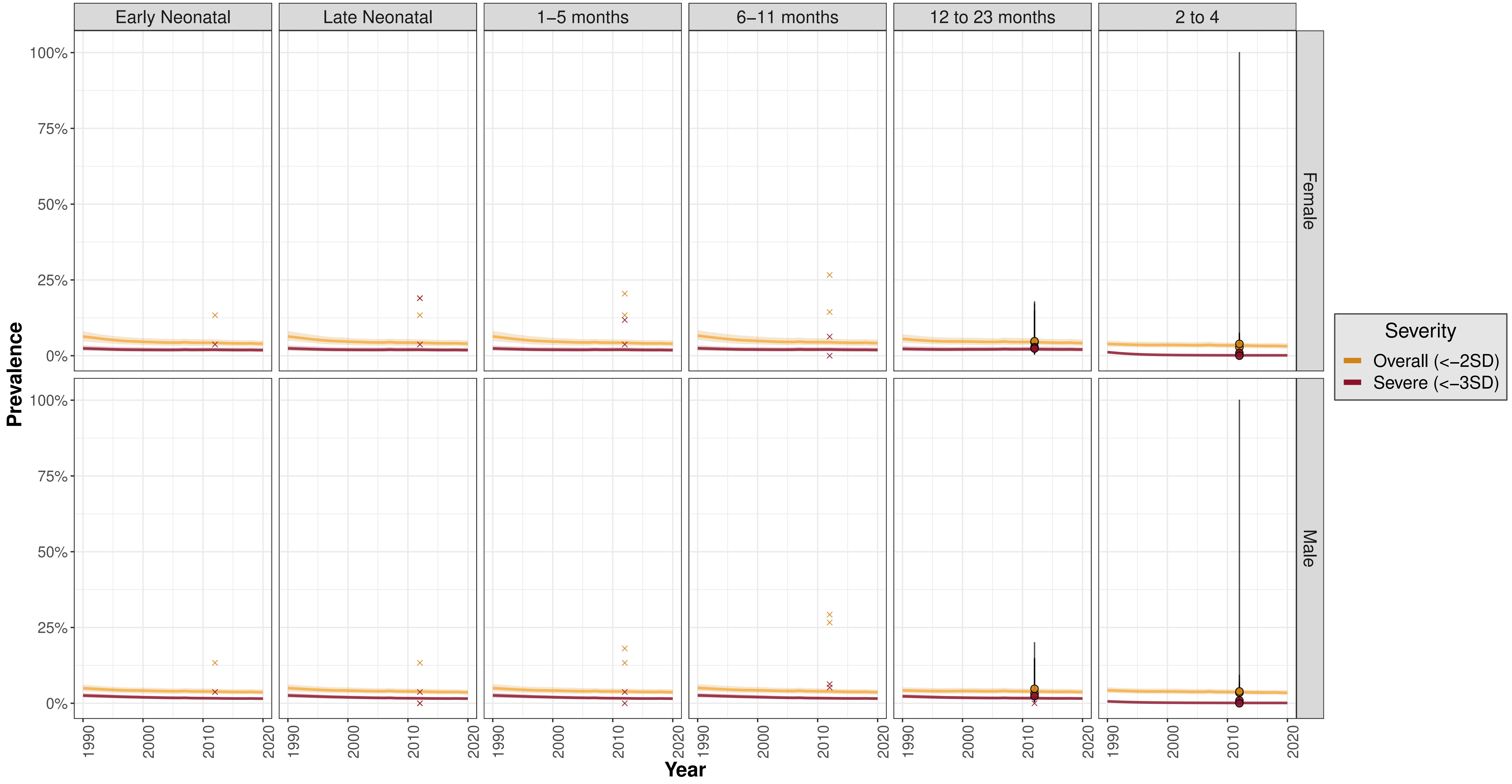

E: Transformed Mean Wasting Z Scores

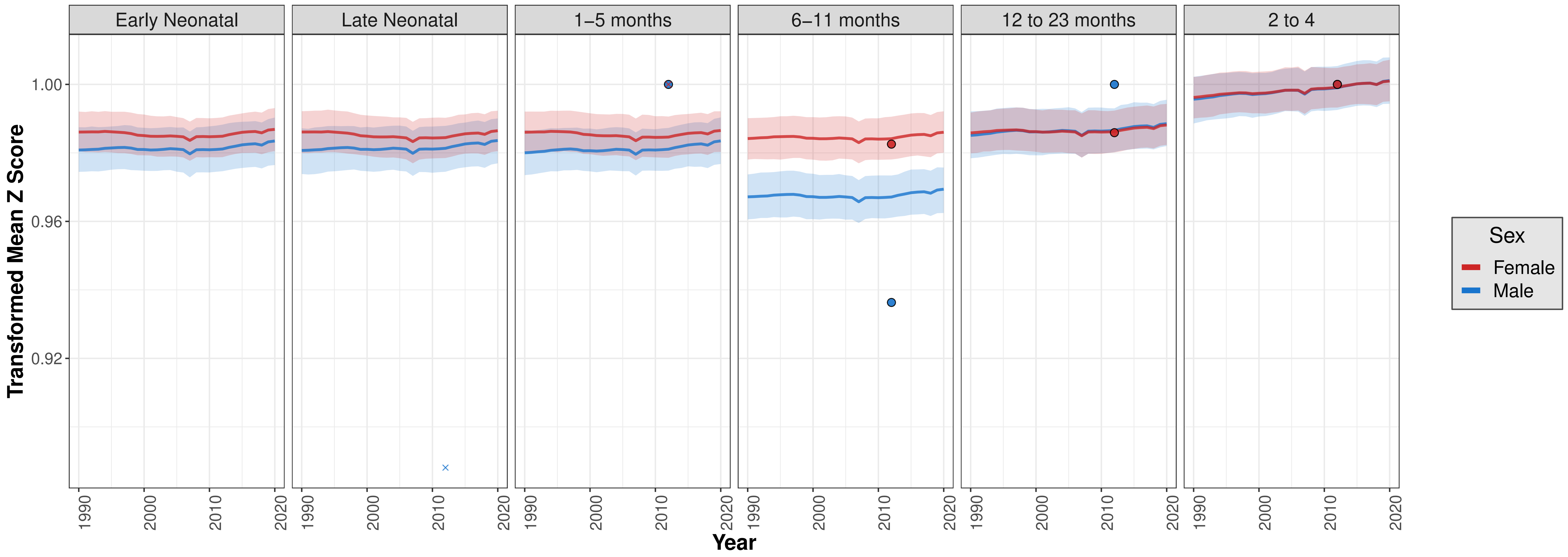

F

Year	Source
1981	WHO CGM Database
2012	WHO CGM Database
2012	MICS

Barbados – Underweight (WAZ)

G: Overall and Severe Underweight Prevalence

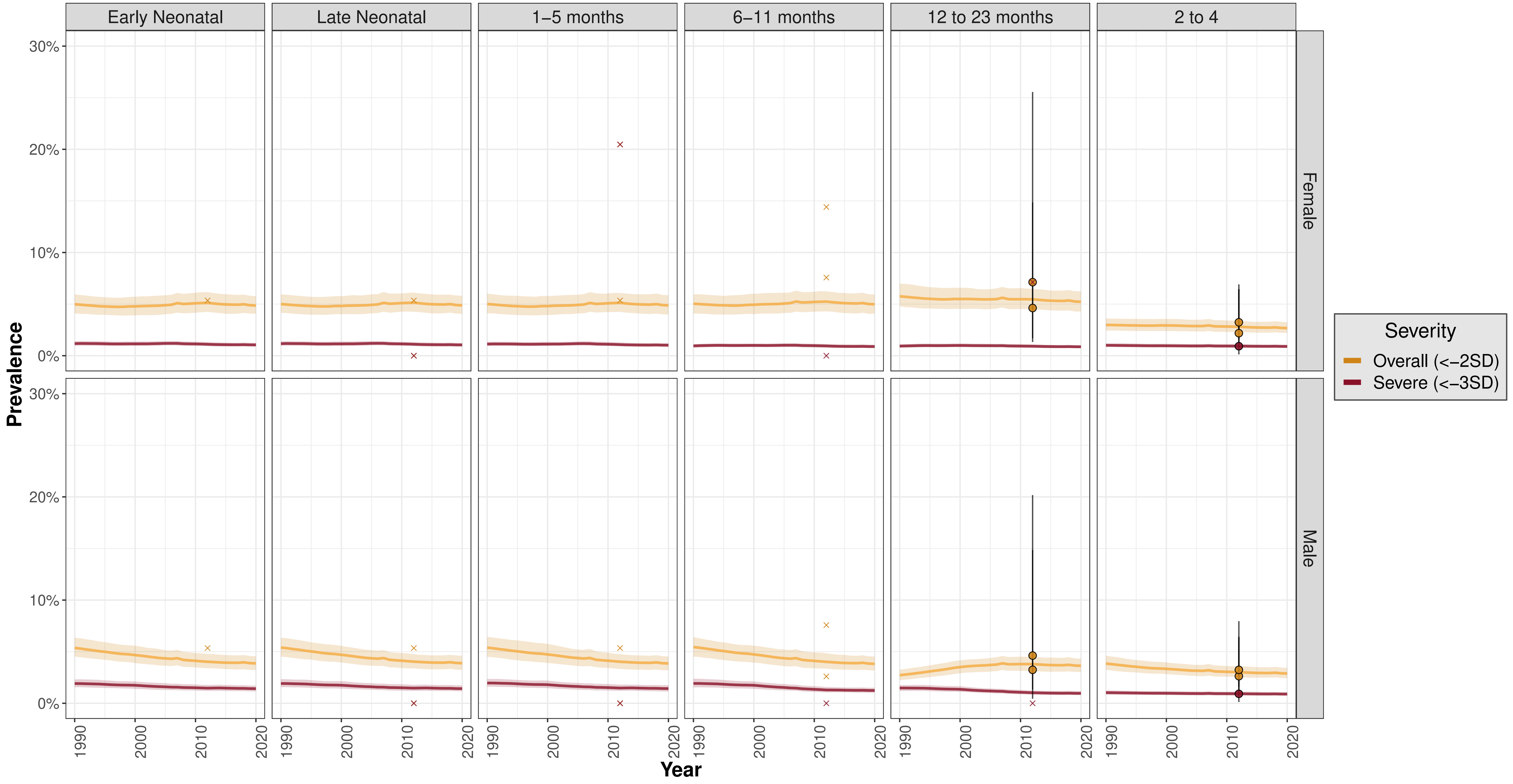

H: Transformed Mean Underweight Z Scores

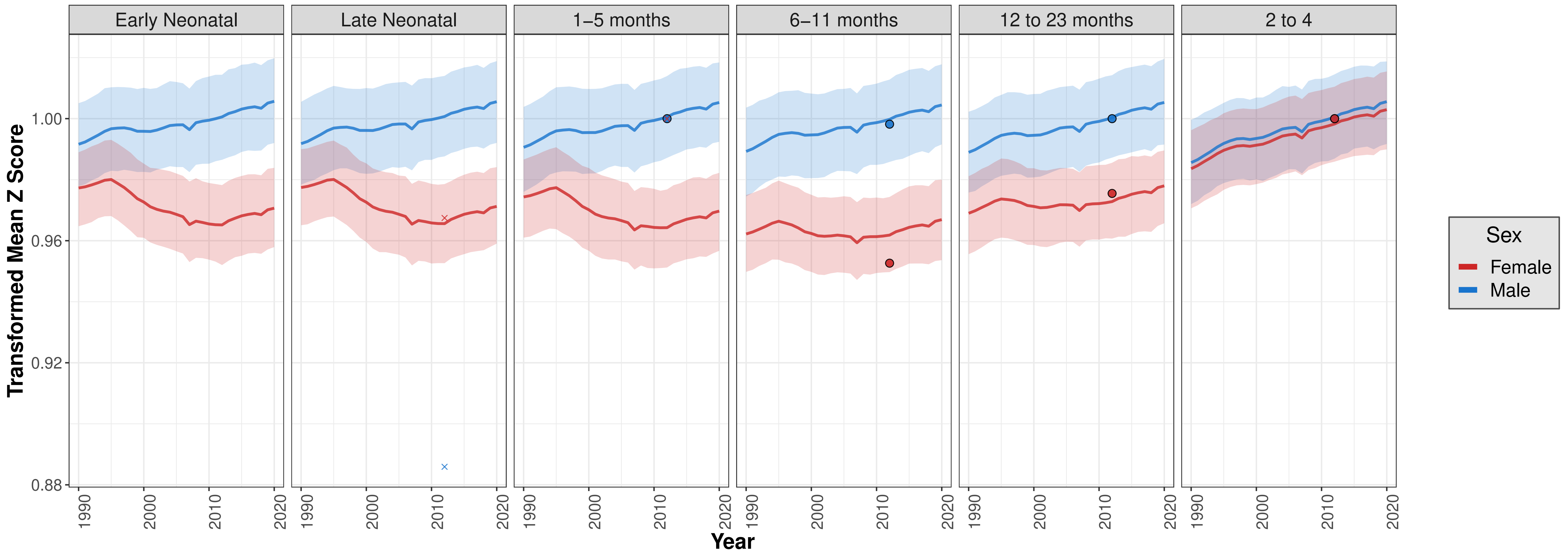

I

Year	Source
1981	WHO CGM Database
2012	WHO CGM Database
2012	MICS

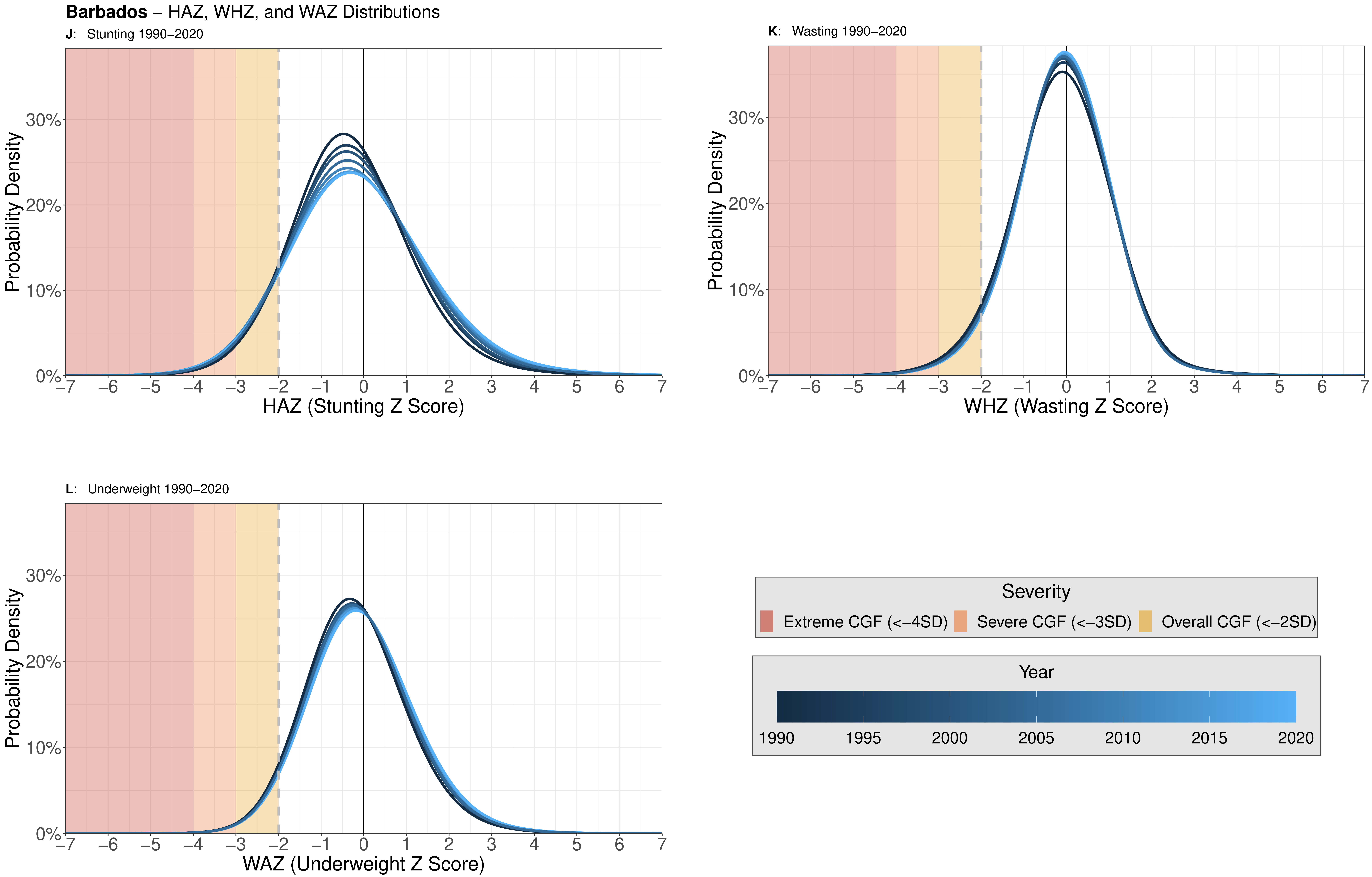

Barbados – HAZ, WHZ, and WAZ Distributions
J: Stunting 1990–2020
Probability Density
30%
20%
10%
0%
−7 −6 −5 −4 −3 −2 −1 0 1 2 3 4 5 6 7
HAZ (Stunting Z Score)
K: Wasting 1990–2020
Probability Density
30%
20%
10%
0%
−7 −6 −5 −4 −3 −2 −1 0 1 2 3 4 5 6 7
WHZ (Wasting Z Score)
L: Underweight 1990–2020
Probability Density
30%
20%
10%
0%
−7 −6 −5 −4 −3 −2 −1 0 1 2 3 4 5 6 7
WAZ (Underweight Z Score)
Severity
Extreme CGF (<−4SD)
Severe CGF (<−3SD)
Overall CGF (<−2SD)
Year
1990
1995
2000
2005
2010
2015
2020

Belize – Stunting (HAZ)

A: Overall and Severe Stunting Prevalence

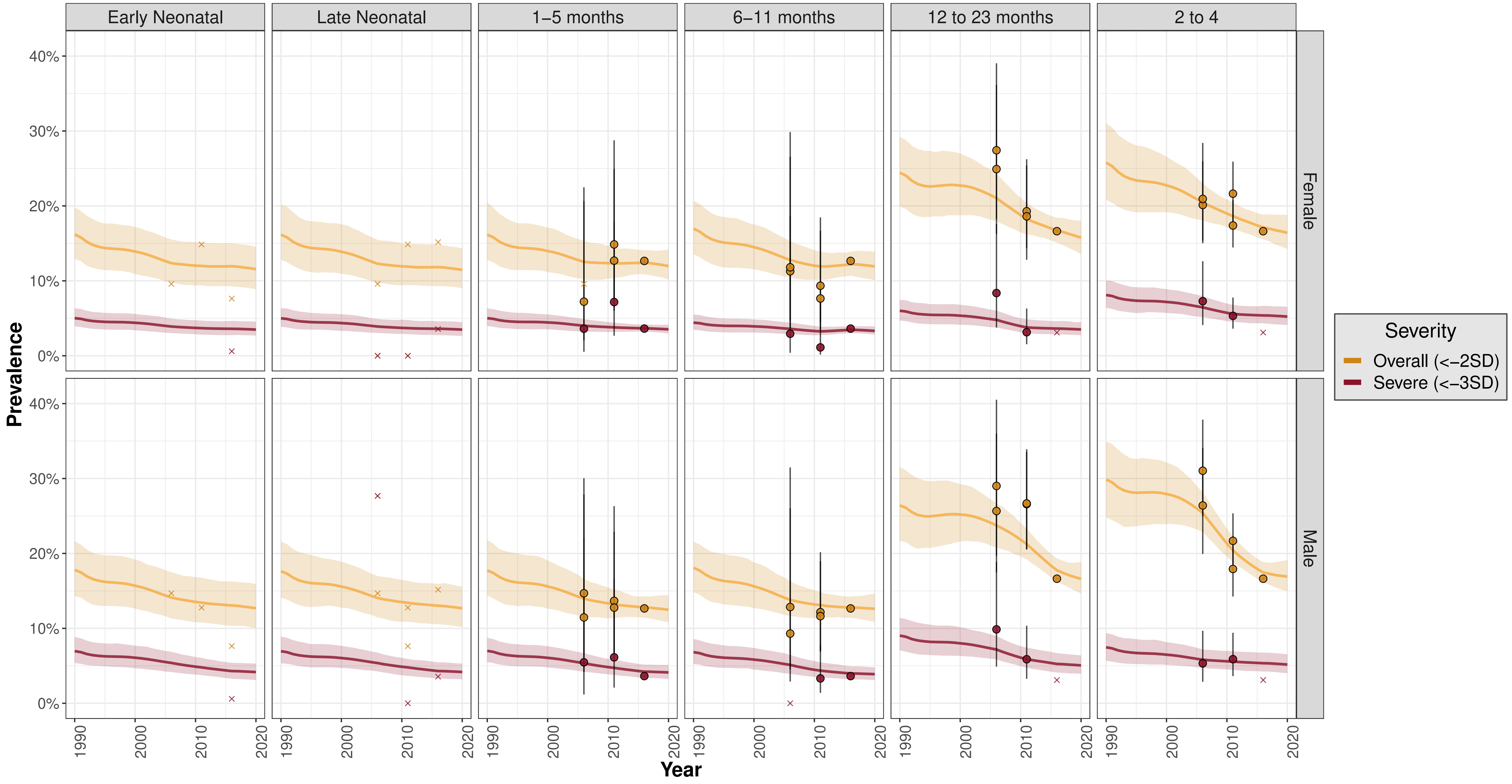

B: Transformed Mean Stunting Z Scores

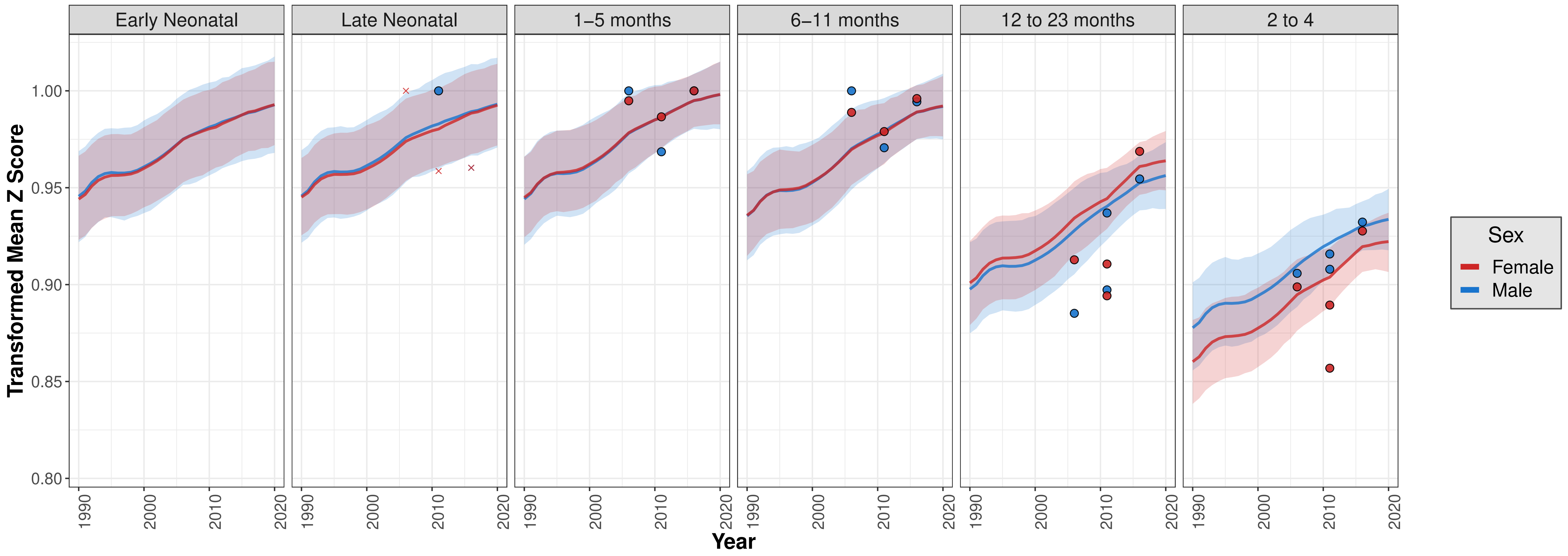

C

Year	Source
2006	MICS
2006	WHO CGM Database
2011	MICS
2011	WHO CGM Database
2016	MICS

Belize – Wasting (WHZ)

D: Overall and Severe Wasting Prevalence

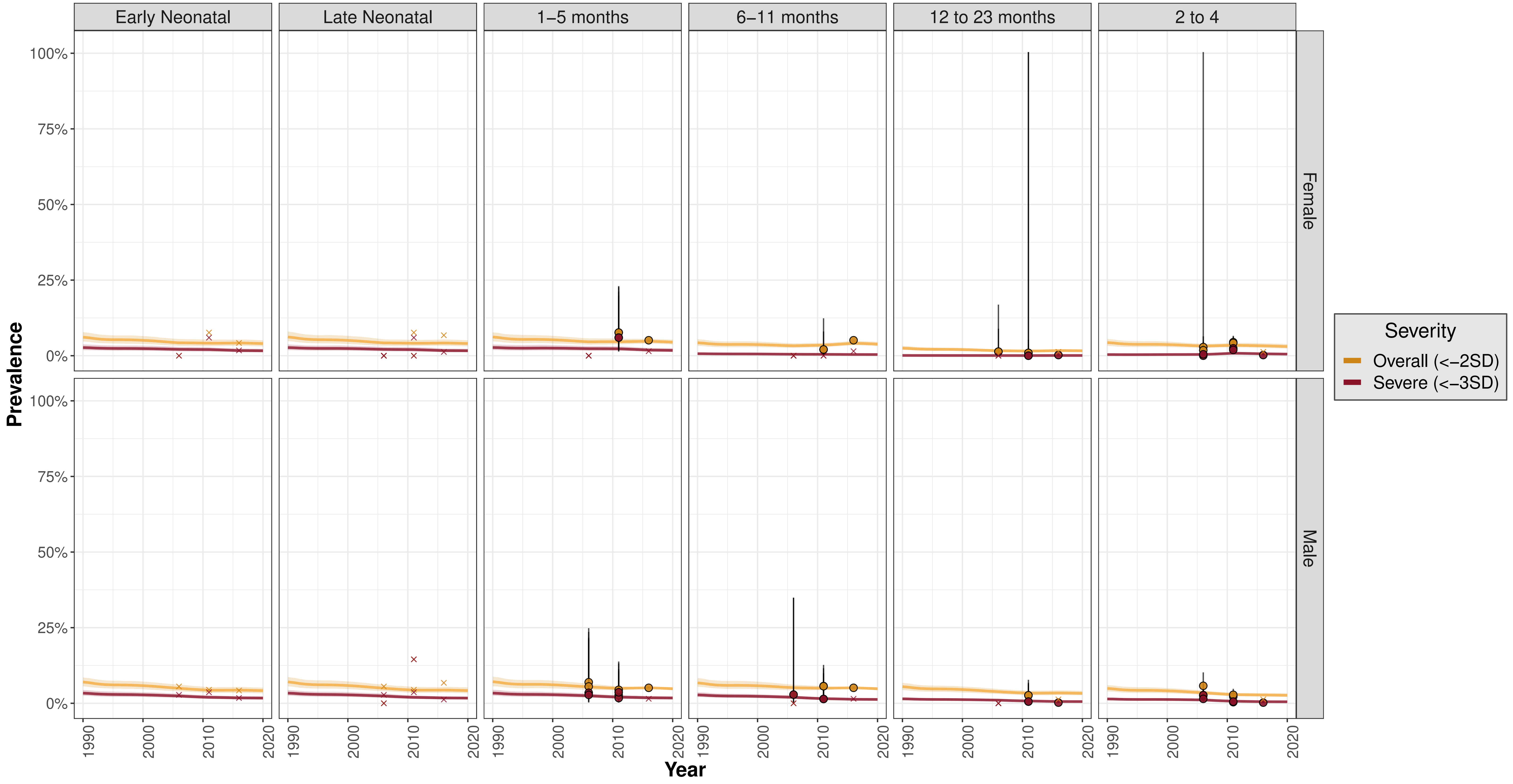

E: Transformed Mean Wasting Z Scores

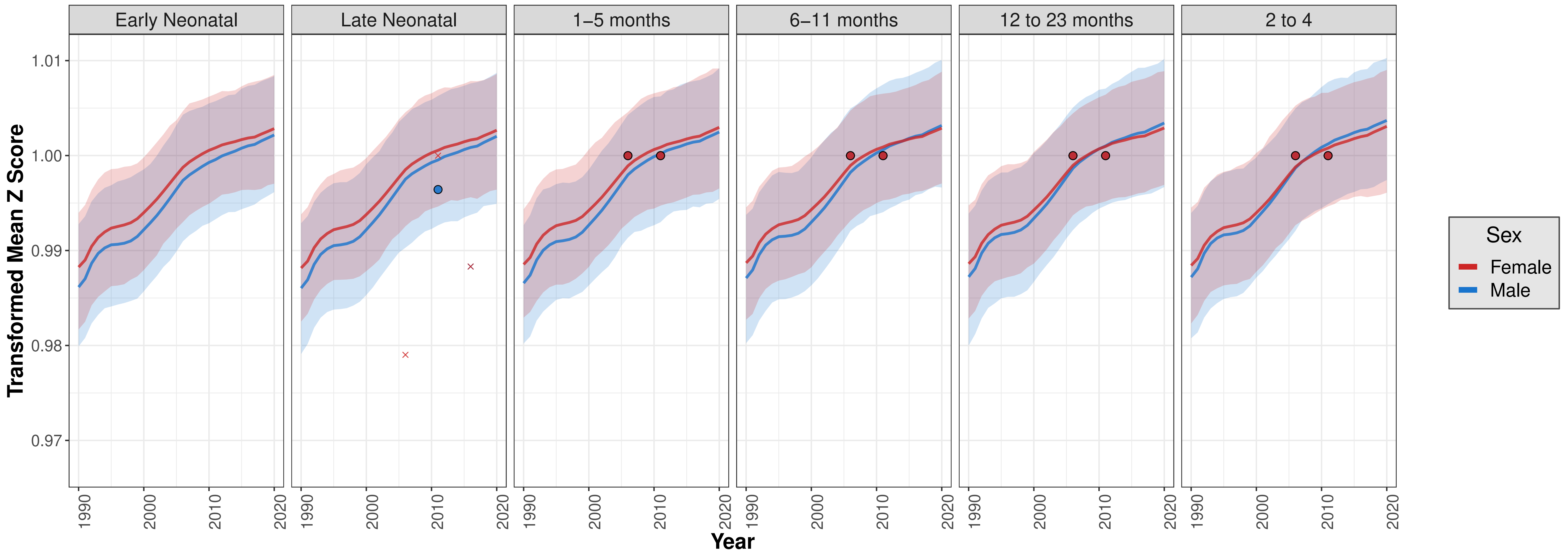

F

Year	Source
2006	MICS
2006	WHO CGM Database
2011	MICS
2011	WHO CGM Database
2016	MICS

Belize – Underweight (WAZ)

G: Overall and Severe Underweight Prevalence

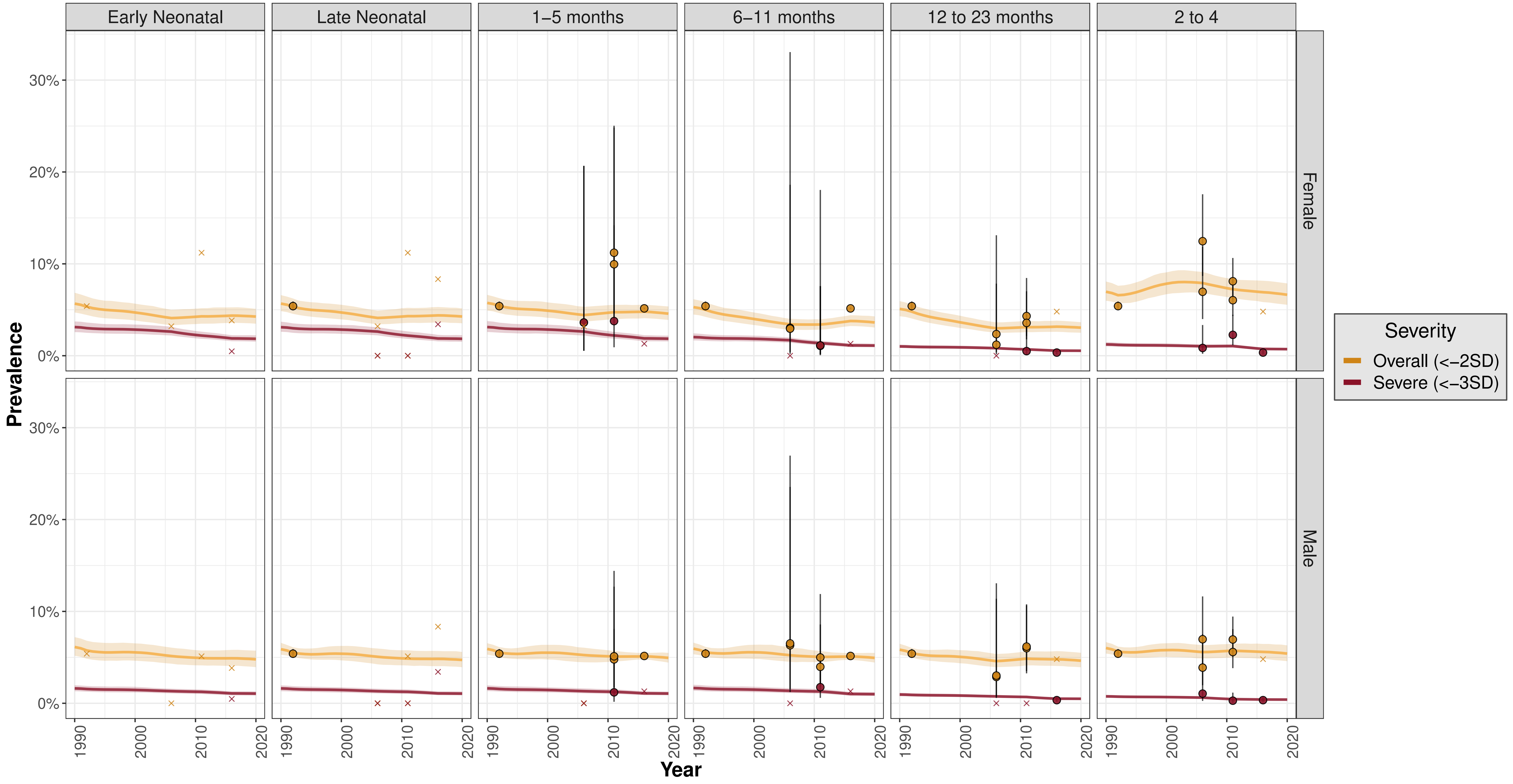

H: Transformed Mean Underweight Z Scores

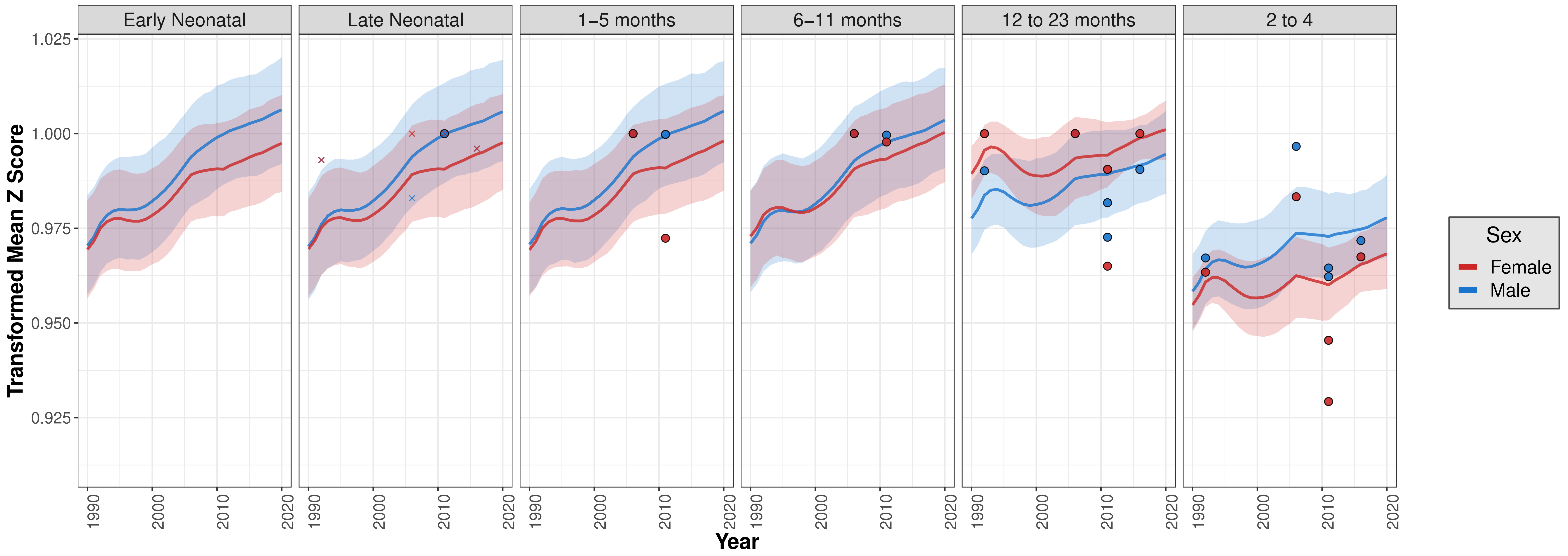

I

Year	Source
1992	WHO CGM Database
2006	MICS
2006	WHO CGM Database
2011	MICS
2011	WHO CGM Database
2016	MICS

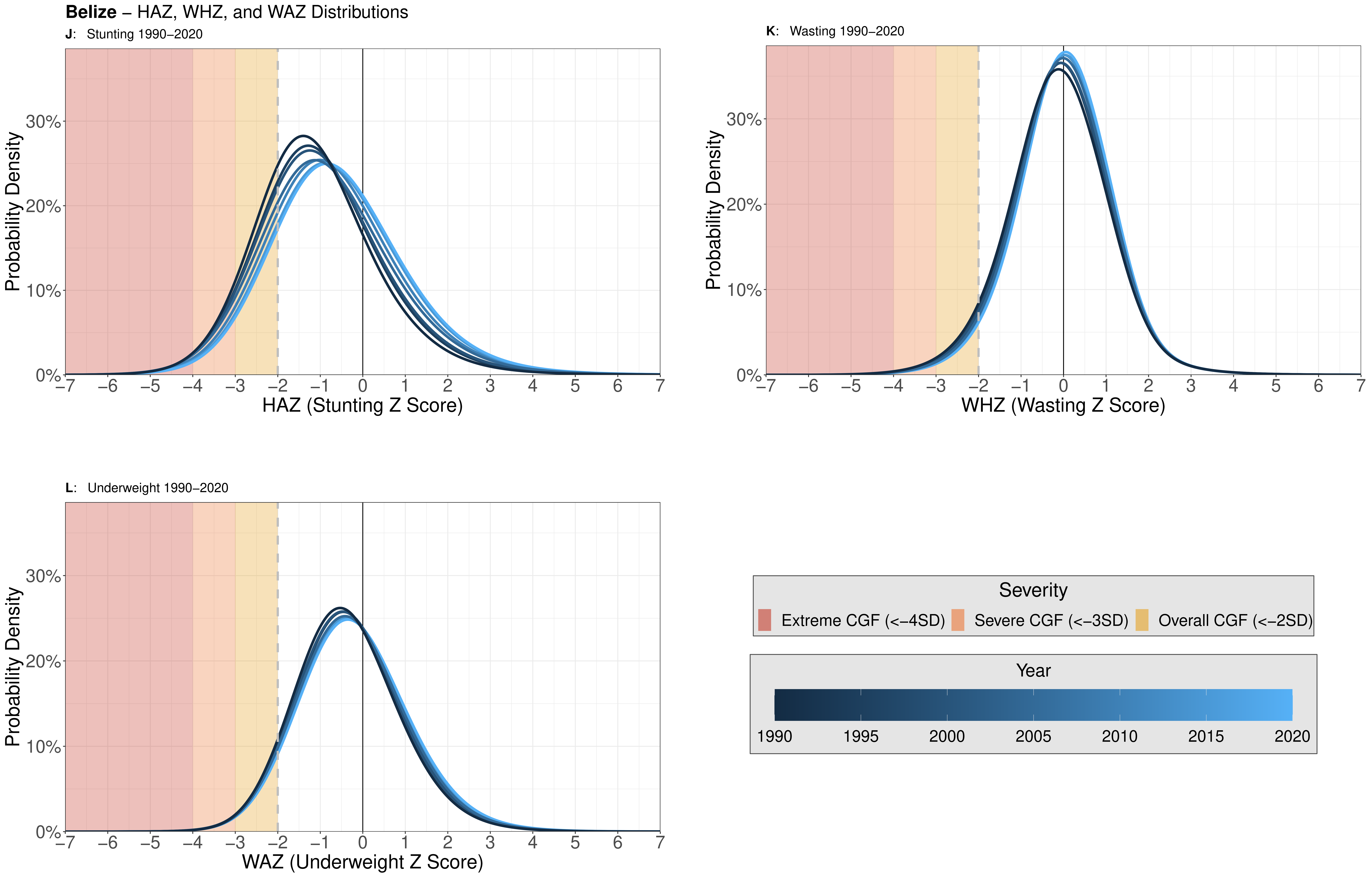
Belize – HAZ, WHZ, and WAZ Distributions
J: Stunting 1990–2020
Probability Density
HAZ (Stunting Z Score)
K: Wasting 1990–2020
WHZ (Wasting Z Score)
L: Underweight 1990–2020
WAZ (Underweight Z Score)
0%
10%
20%
30%
−7
−6
−5
−4
−3
−2
−1
0
1
2
3
4
5
6
7
Severity
Extreme CGF (<−4SD)
Severe CGF (<−3SD)
Overall CGF (<−2SD)
Year
1990
1995
2000
2005
2010
2015
2020

Cuba – Stunting (HAZ)

A: Overall and Severe Stunting Prevalence

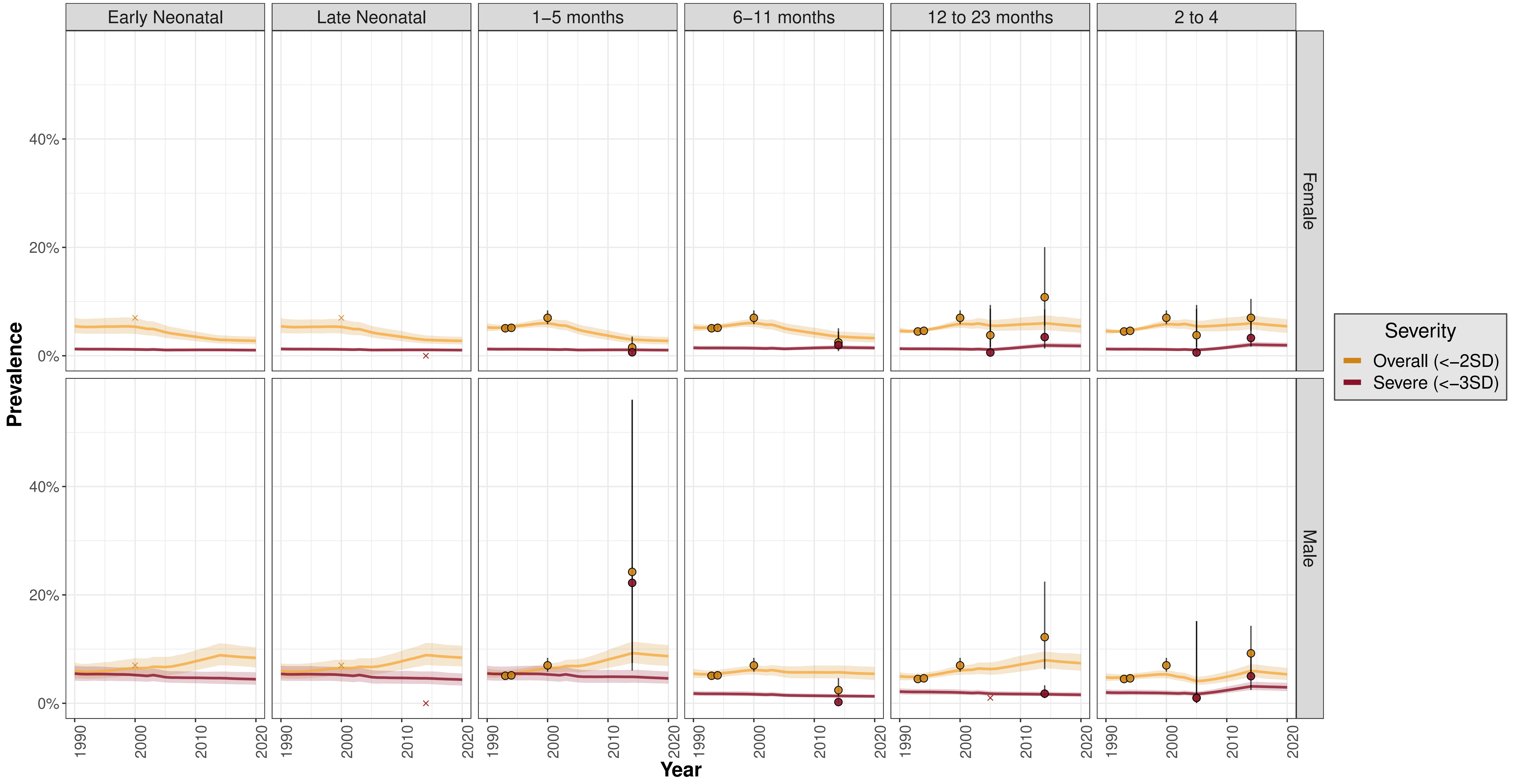

B: Transformed Mean Stunting Z Scores

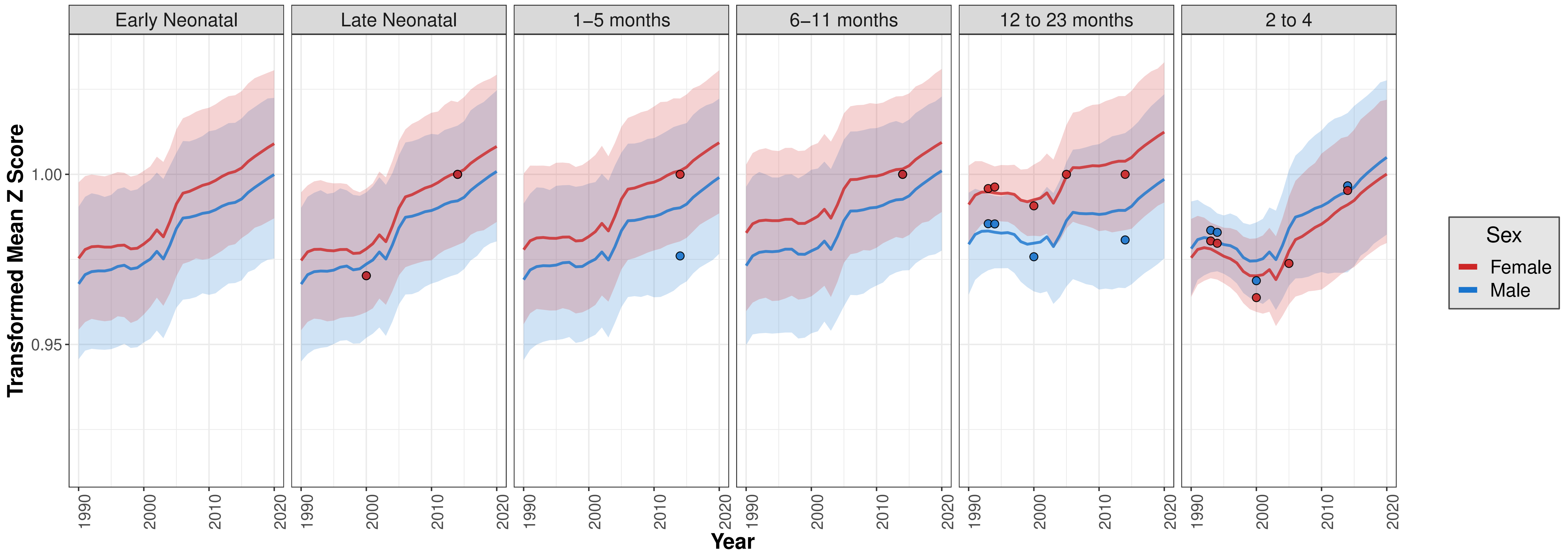

C

Year	Source
1993	WHO CGM Database
1994	WHO CGM Database
2000	WHO CGM Database
2005	WHO CGM Database
2014	MICS

Cuba – Wasting (WHZ)

D: Overall and Severe Wasting Prevalence

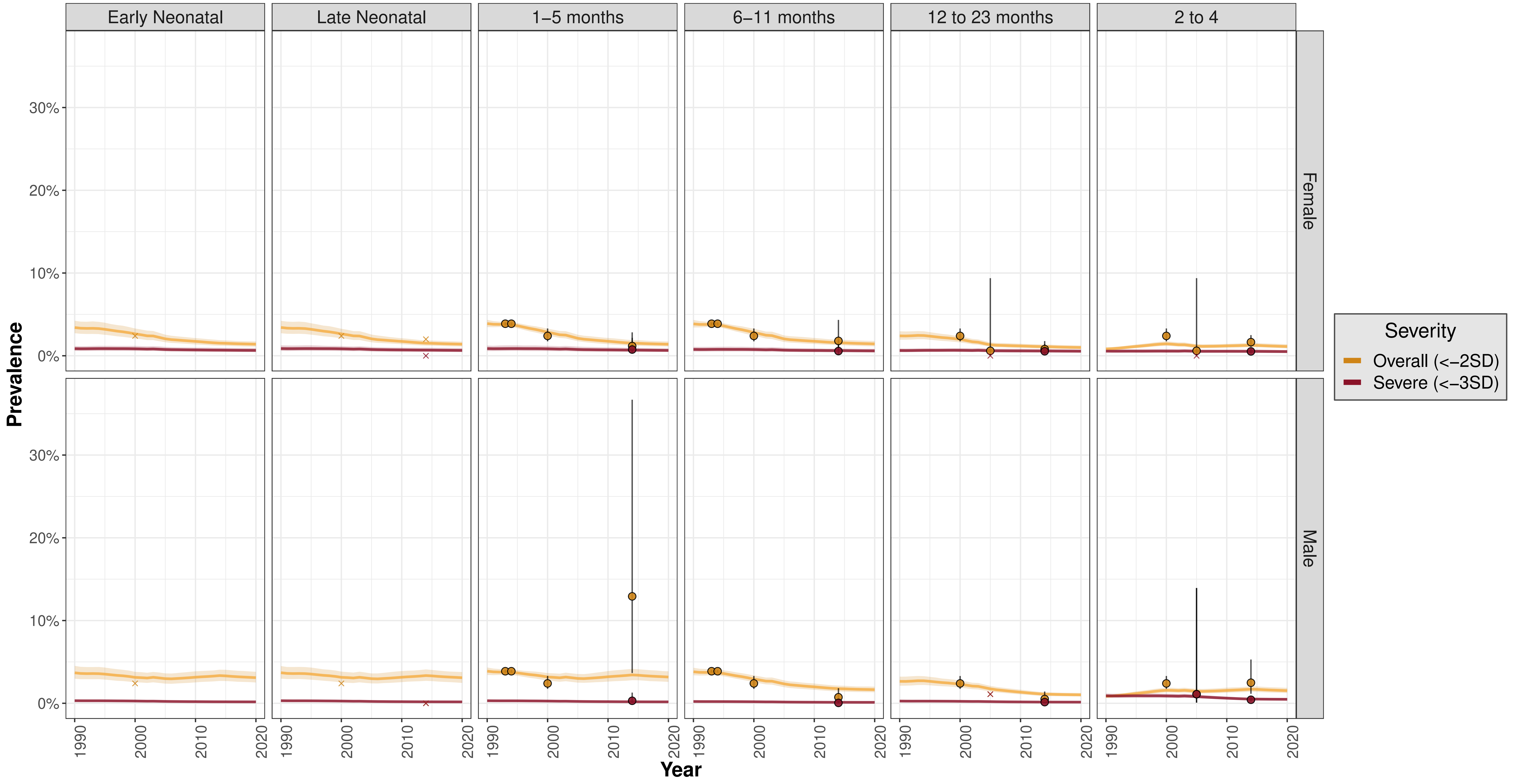

E: Transformed Mean Wasting Z Scores

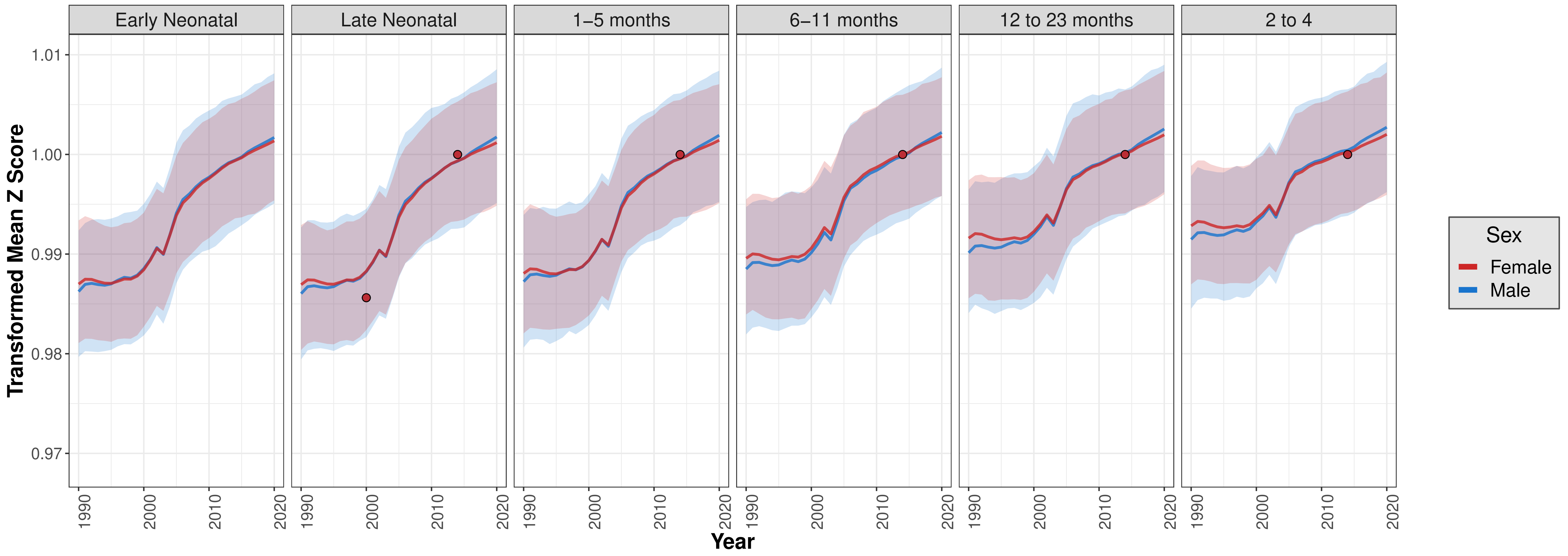

F

Year	Source
1985	WHO CGM Database
1986	WHO CGM Database
1987	WHO CGM Database
1993	WHO CGM Database
1994	WHO CGM Database
2000	WHO CGM Database
2005	WHO CGM Database
2014	MICS

Cuba – Underweight (WAZ)

G: Overall and Severe Underweight Prevalence

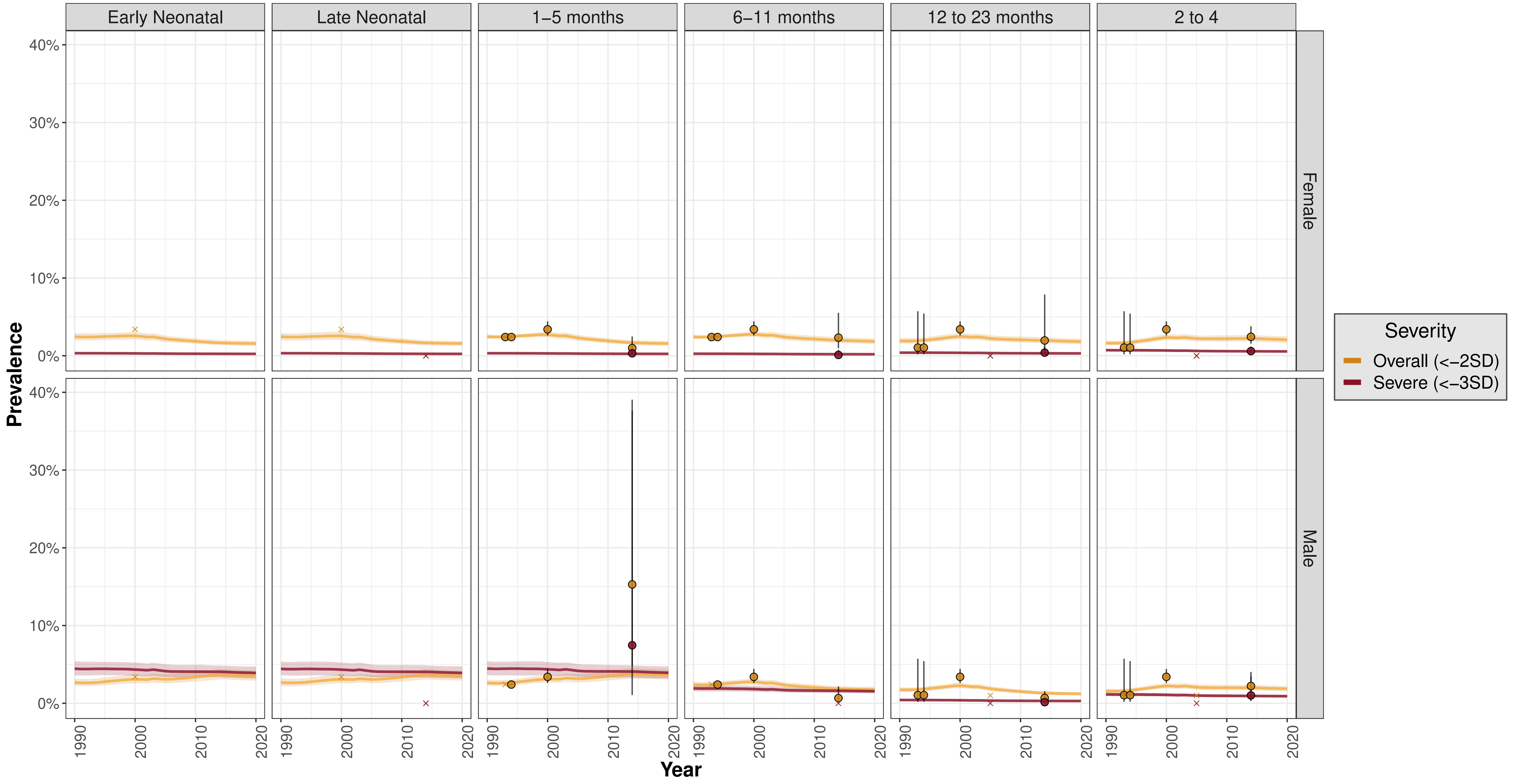

H: Transformed Mean Underweight Z Scores

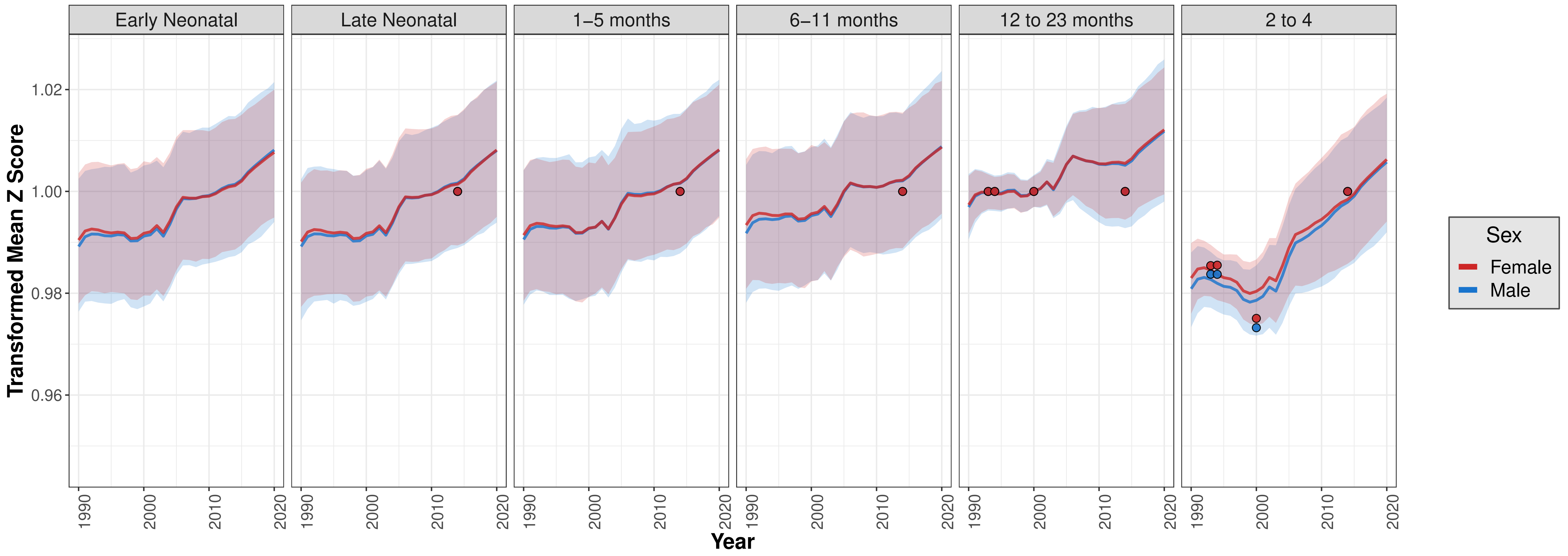

I

Year	Source
1993	WHO CGM Database
1994	WHO CGM Database
2000	WHO CGM Database
2005	WHO CGM Database
2014	MICS

Cuba – HAZ, WHZ, and WAZ Distributions

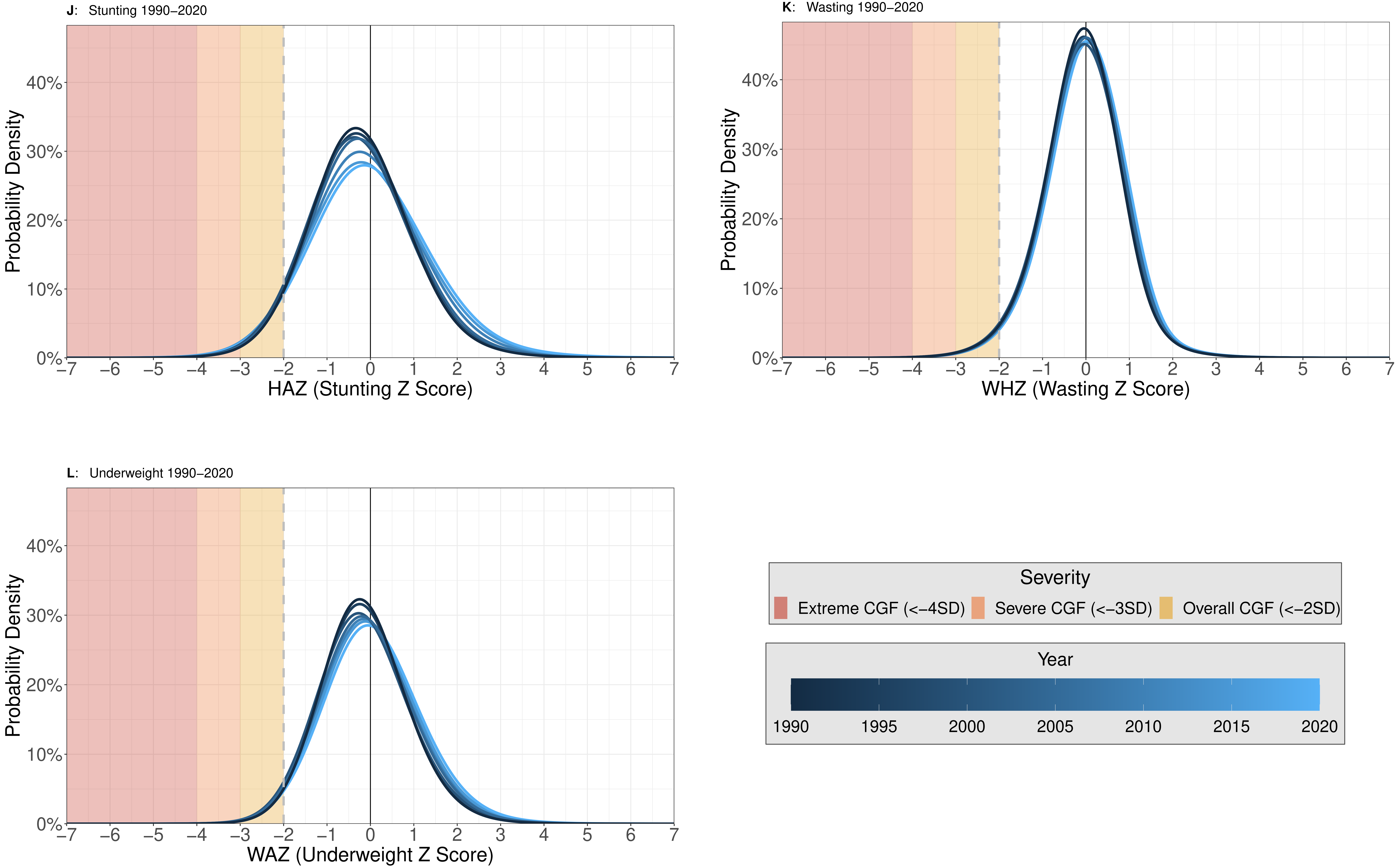

Dominica – Stunting (HAZ)

A: Overall and Severe Stunting Prevalence

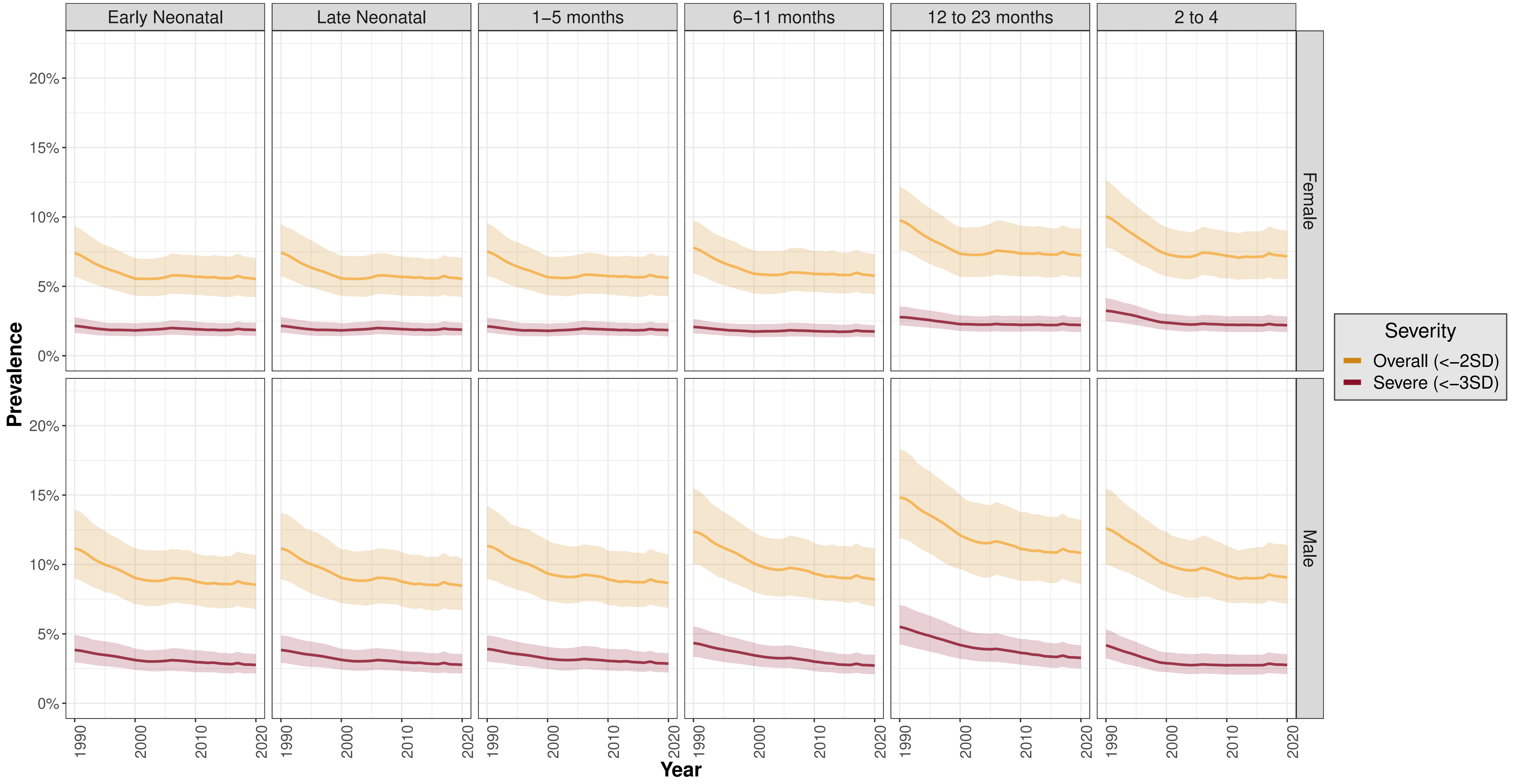

B: Transformed Mean Stunting Z Scores

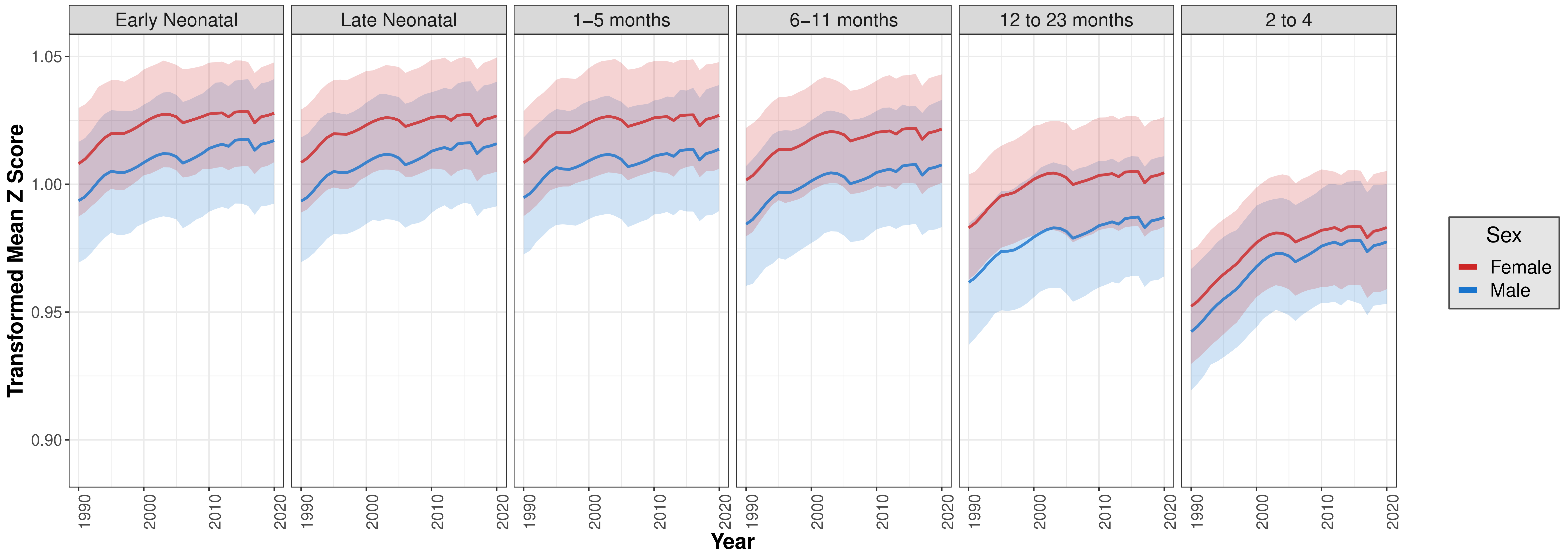

C

Source
No sources for this location

Dominica – Wasting (WHZ)

D: Overall and Severe Wasting Prevalence

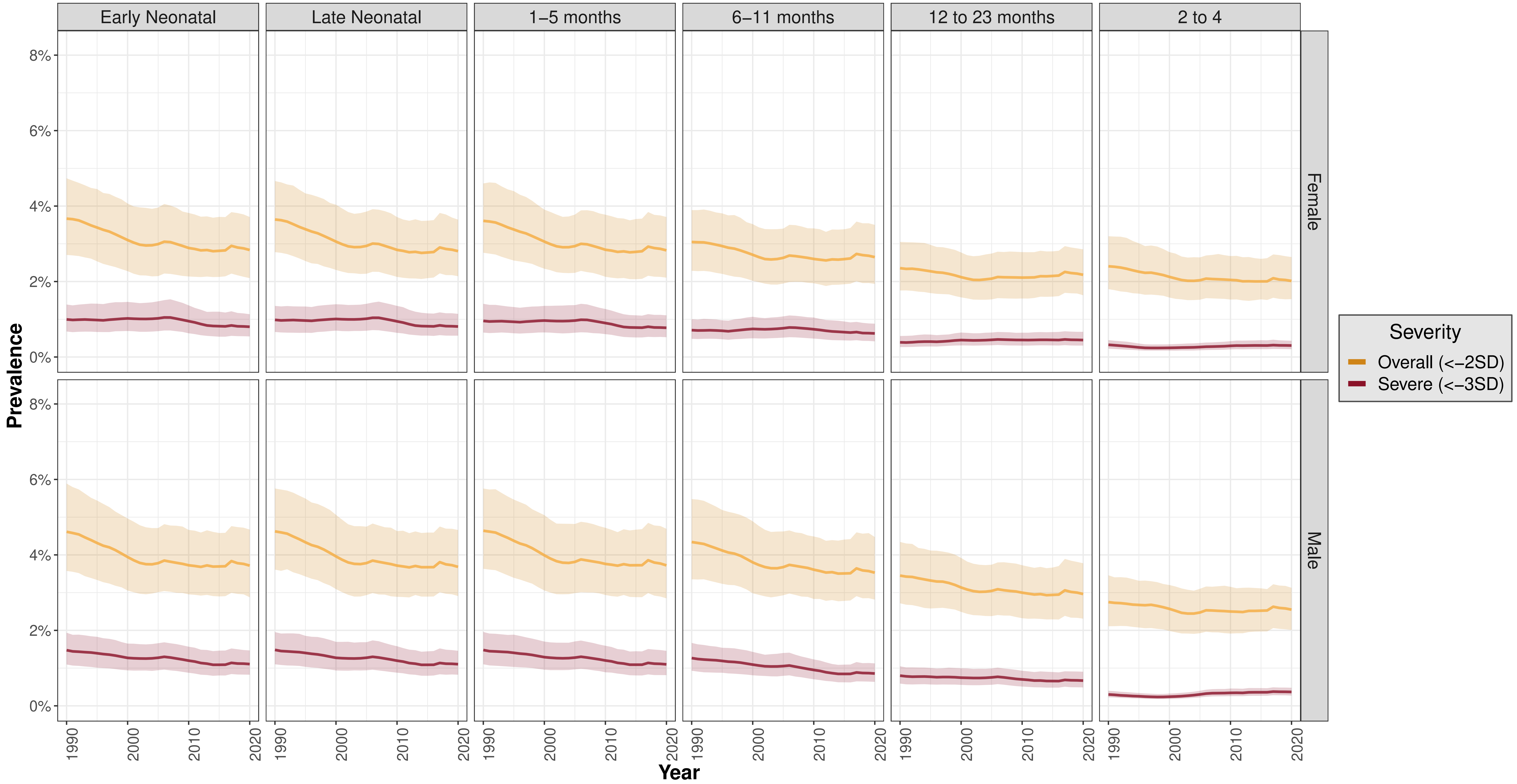

E: Transformed Mean Wasting Z Scores

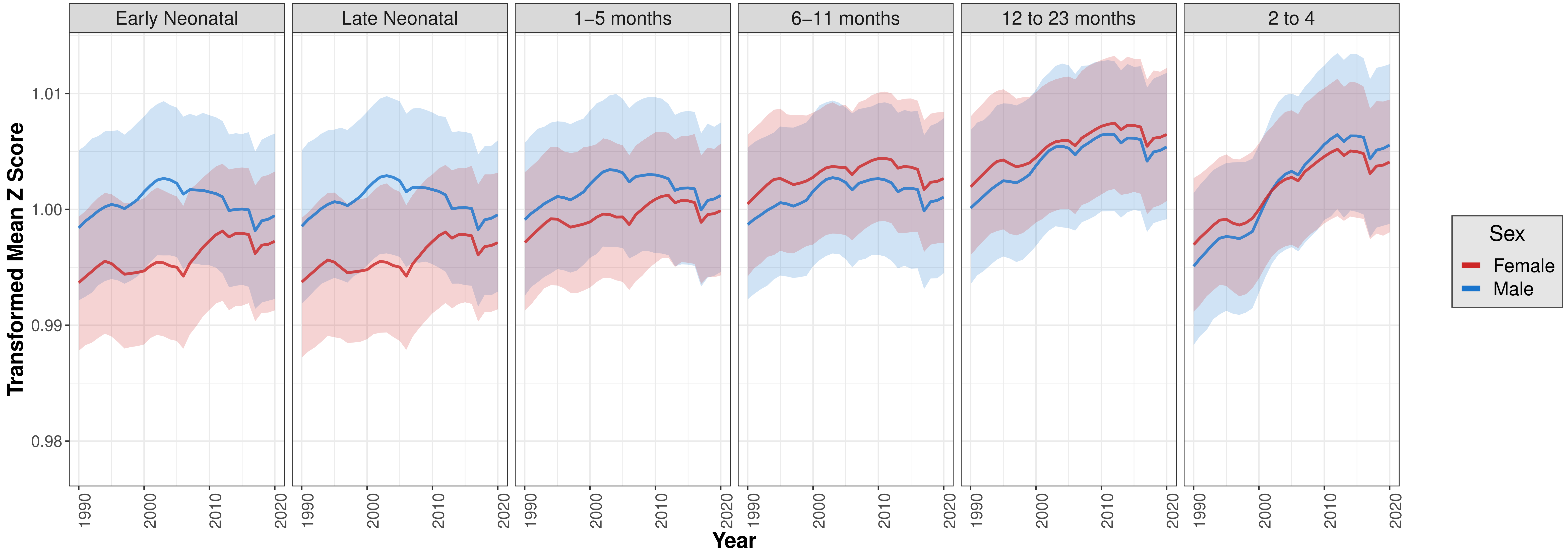

F

Source
No sources for this location

Dominica – Underweight (WAZ)

G: Overall and Severe Underweight Prevalence

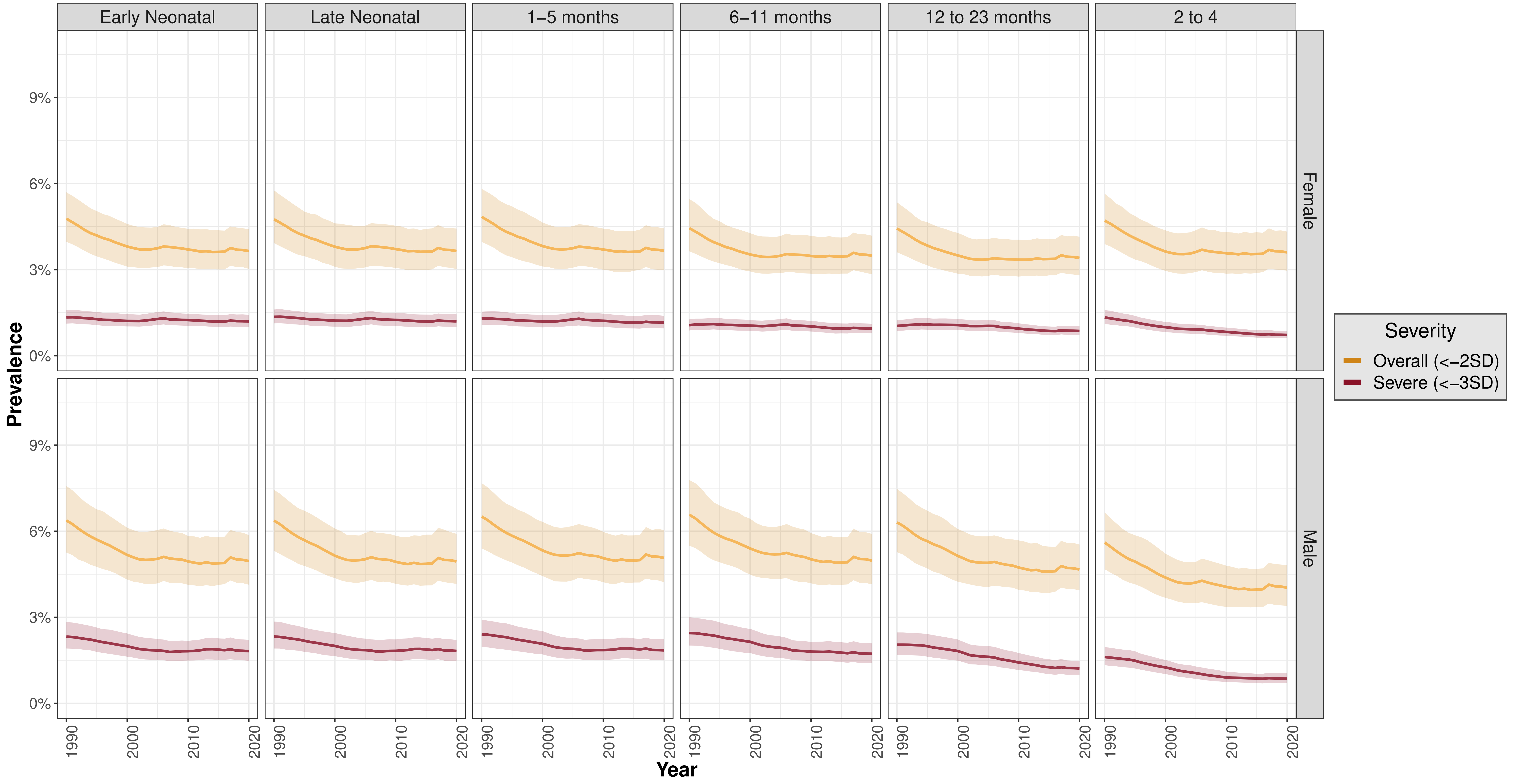

H: Transformed Mean Underweight Z Scores

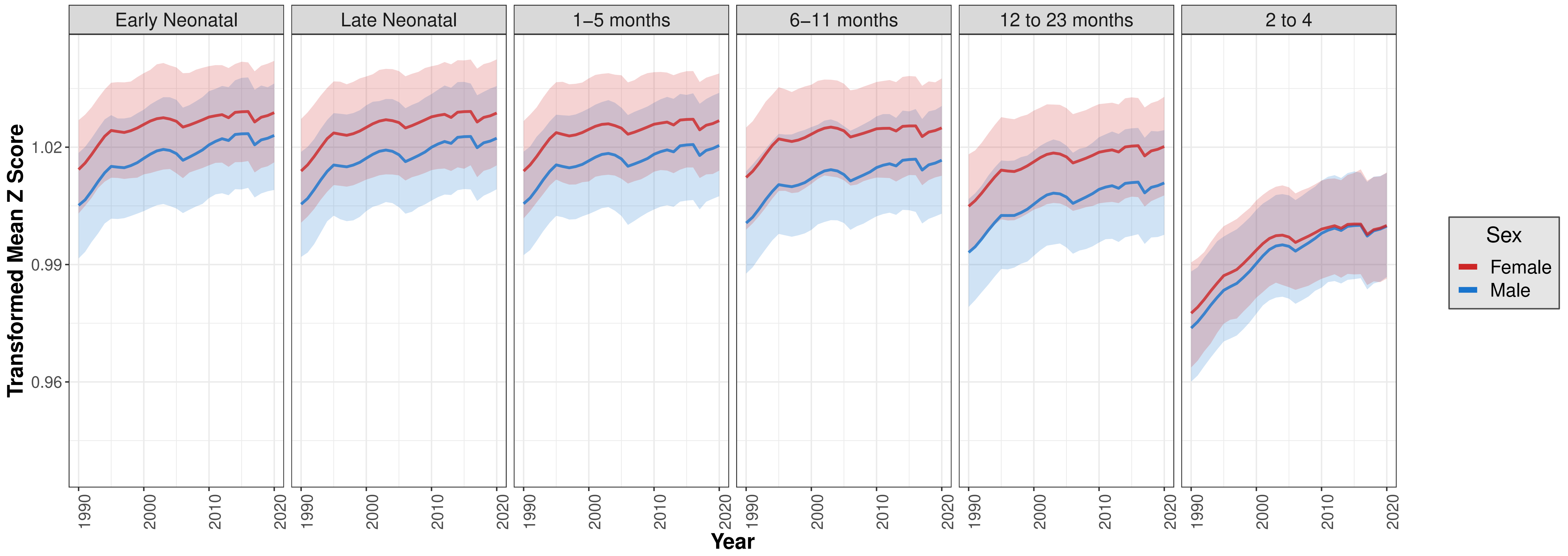

I

Source
No sources for this location

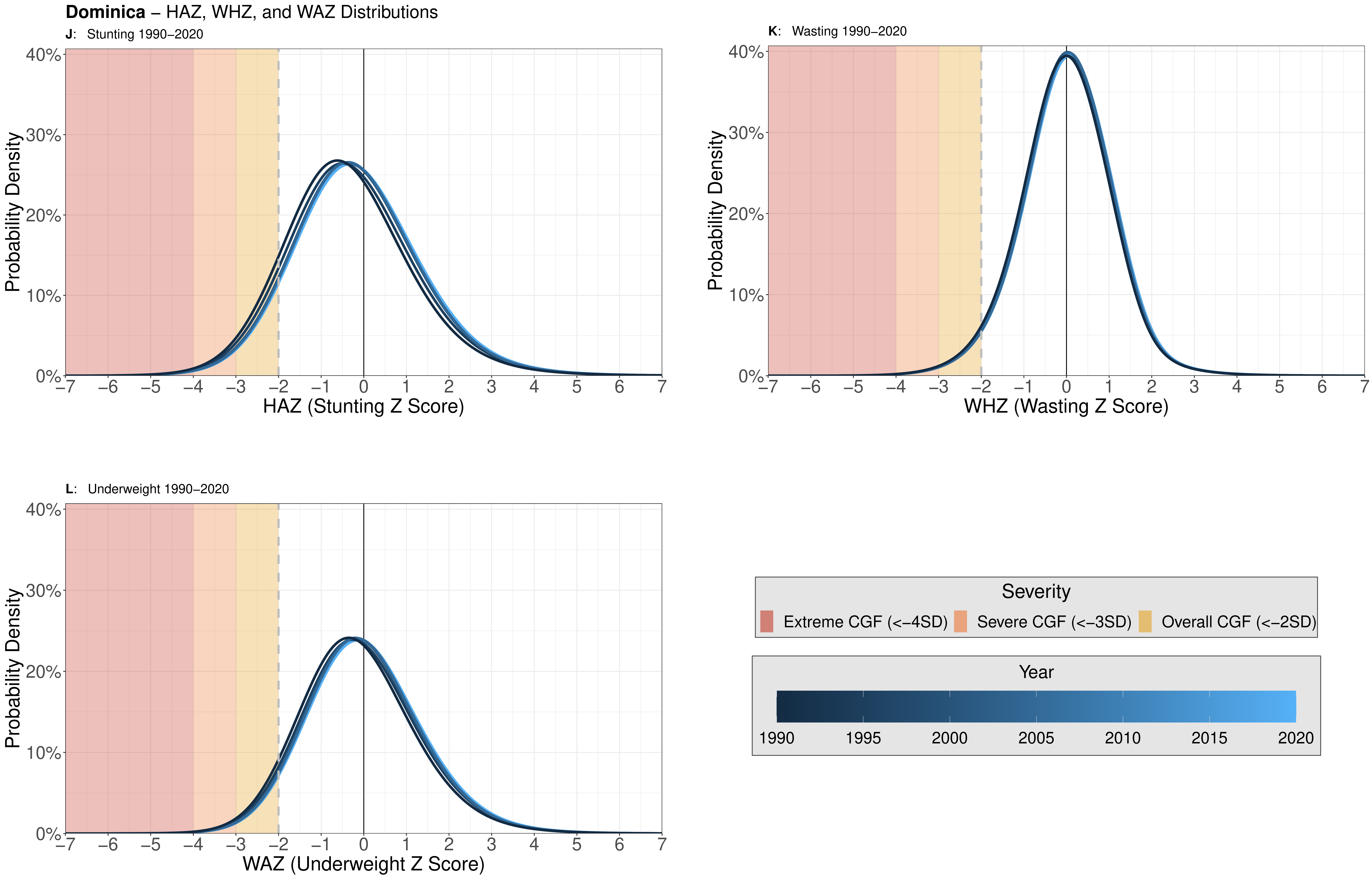

Dominica – HAZ, WHZ, and WAZ Distributions
J: Stunting 1990–2020
Probability Density
40%
30%
20%
10%
0%
−7 −6 −5 −4 −3 −2 −1 0 1 2 3 4 5 6 7
HAZ (Stunting Z Score)
K: Wasting 1990–2020
Probability Density
WHZ (Wasting Z Score)
L: Underweight 1990–2020
Probability Density
WAZ (Underweight Z Score)
Severity
Extreme CGF (<−4SD)
Severe CGF (<−3SD)
Overall CGF (<−2SD)
Year
1990
1995
2000
2005
2010
2015
2020

Dominican Republic – Stunting (HAZ)

A: Overall and Severe Stunting Prevalence

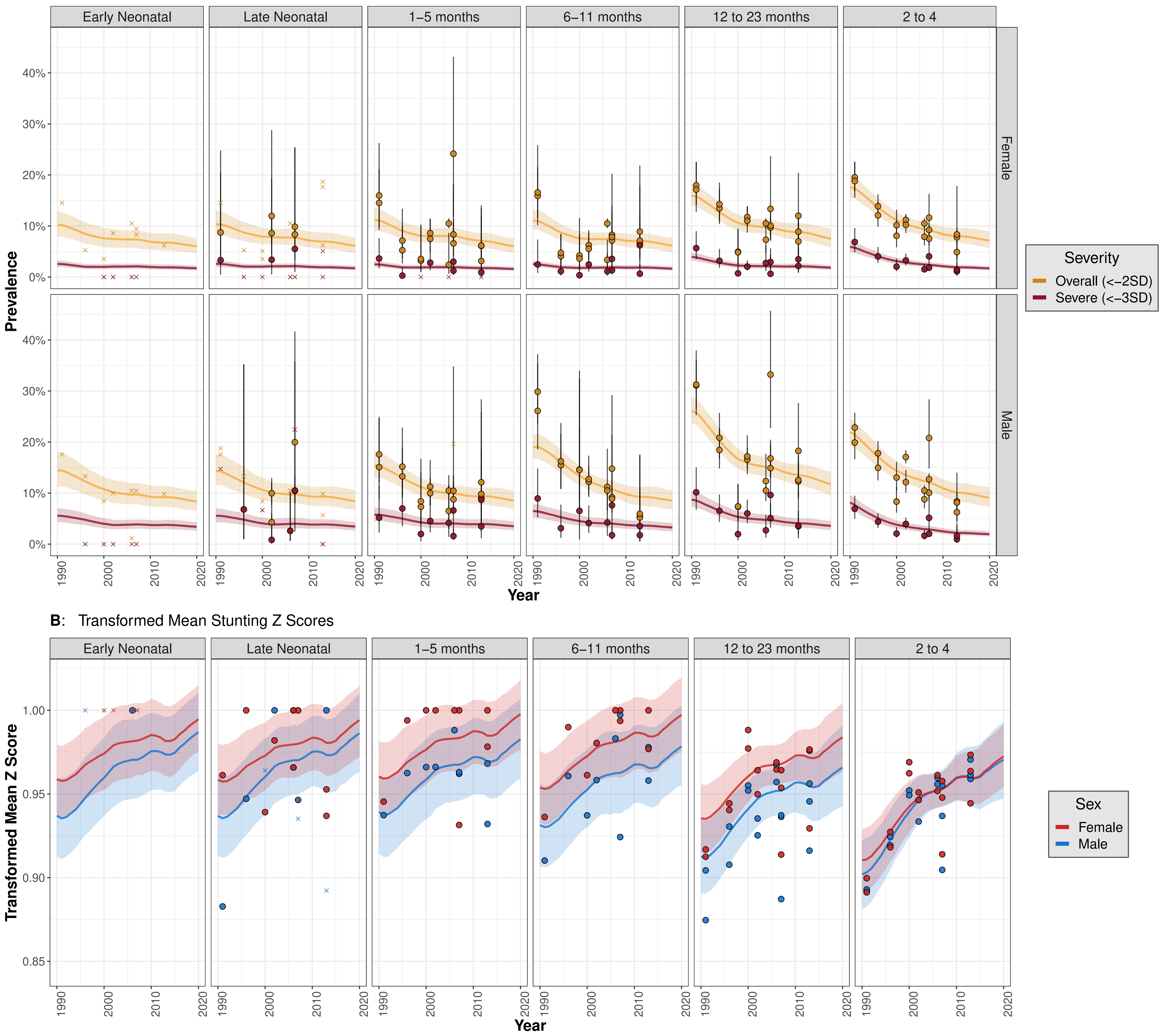

C

Year	Source
1986	DHS
1986	Experimental DHS
1986	WHO CGM Database
1991	DHS
1991	WHO CGM Database
1996	DHS
1996	WHO CGM Database
2000	MICS
2000	WHO CGM Database
2002	DHS
2002	WHO CGM Database
2006	National Multipurpose Household Survey
2006	WHO CGM Database
2007	DHS
2007	Special DHS
2007	WHO CGM Database
2013	DHS
2013	WHO CGM Database
2013	Special DHS

Dominican Republic – Wasting (WHZ)

D: Overall and Severe Wasting Prevalence

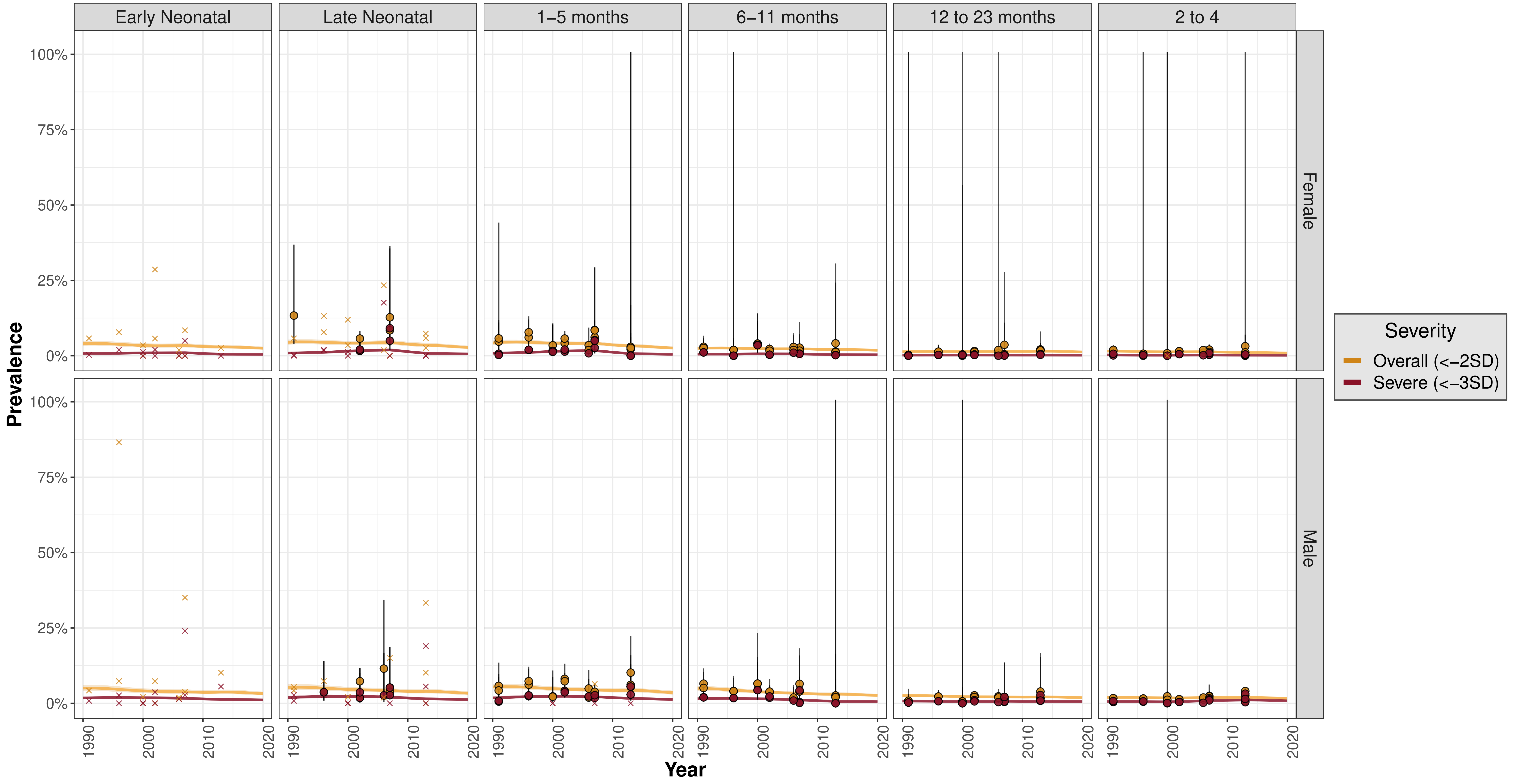

E: Transformed Mean Wasting Z Scores

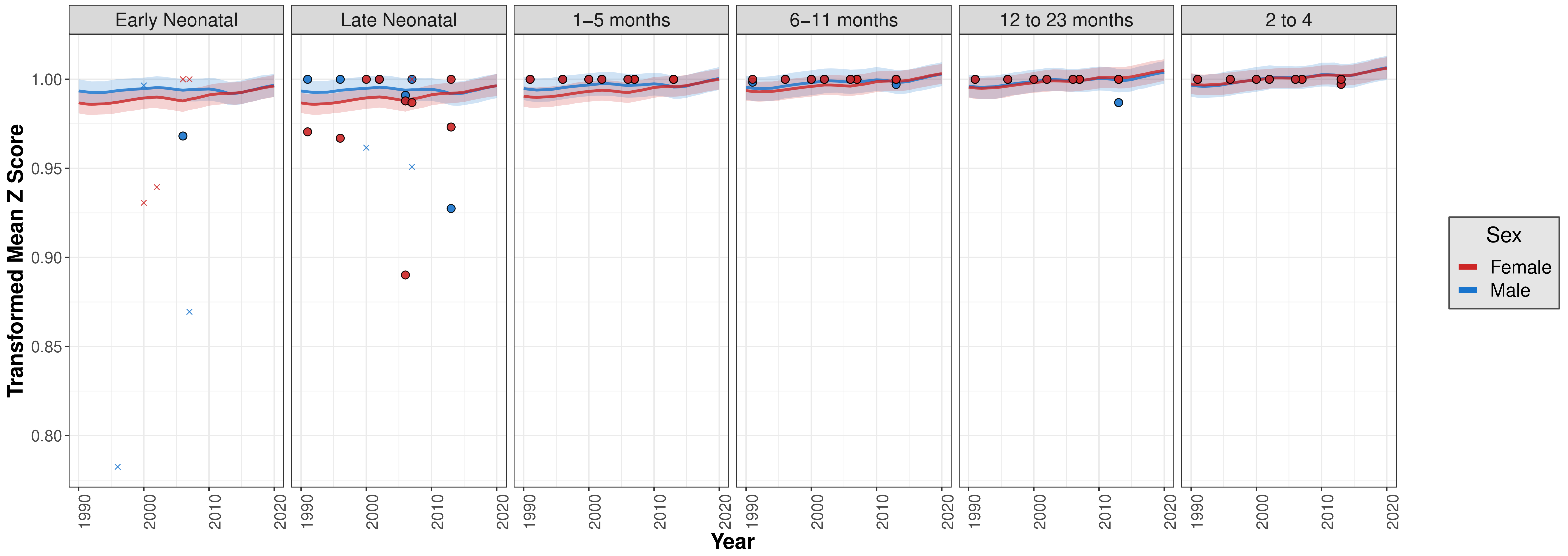

F

Year	Source
1986	DHS
1986	Experimental DHS
1986	WHO CGM Database
1991	DHS
1991	WHO CGM Database
1996	DHS
1996	WHO CGM Database
2000	MICS
2000	WHO CGM Database
2002	DHS
2002	WHO CGM Database
2006	National Multipurpose Household Survey
2006	WHO CGM Database
2007	DHS
2007	Special DHS
2007	WHO CGM Database
2013	DHS
2013	WHO CGM Database
2013	Special DHS

Dominican Republic – Underweight (WAZ)

G: Overall and Severe Underweight Prevalence

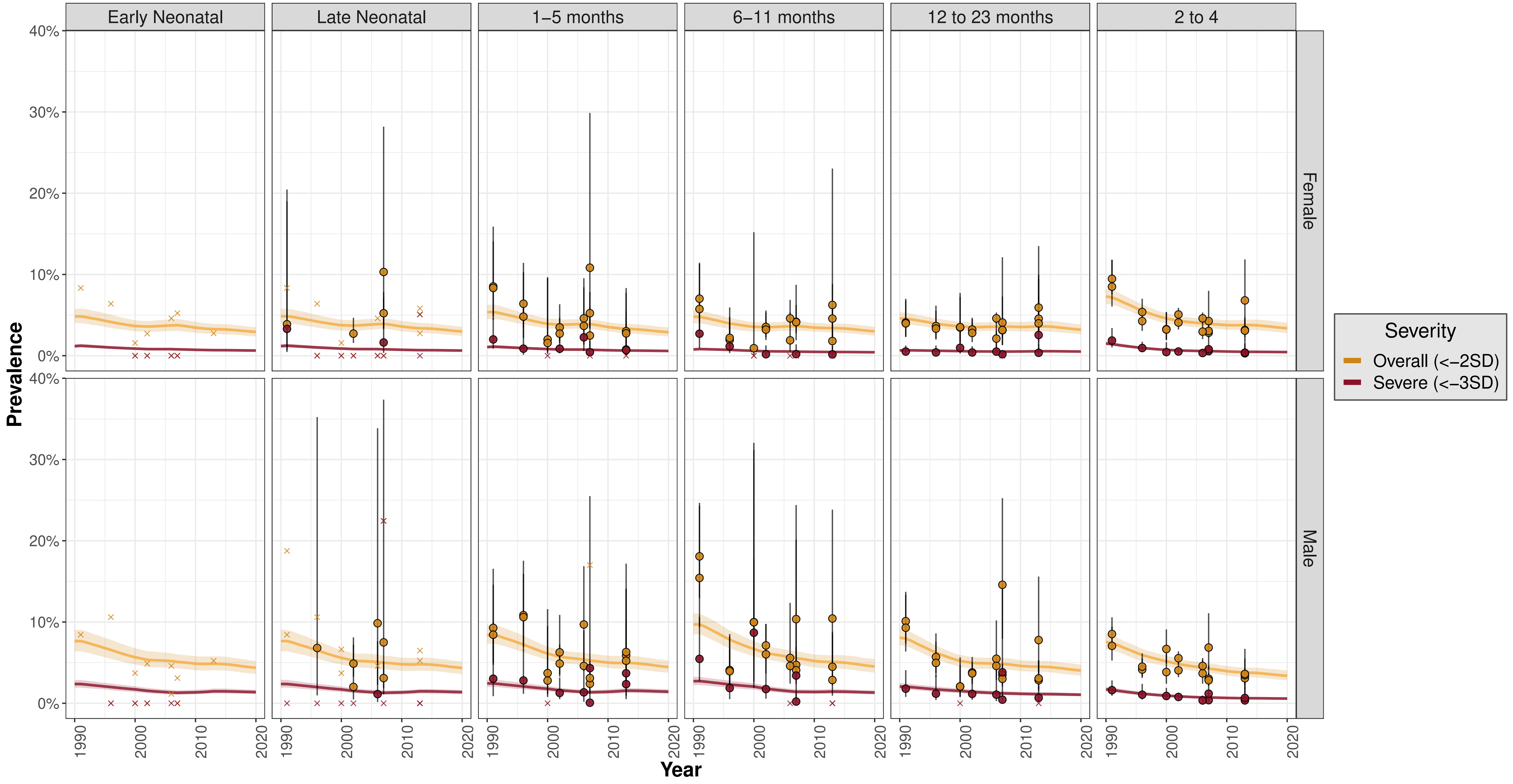

H: Transformed Mean Underweight Z Scores

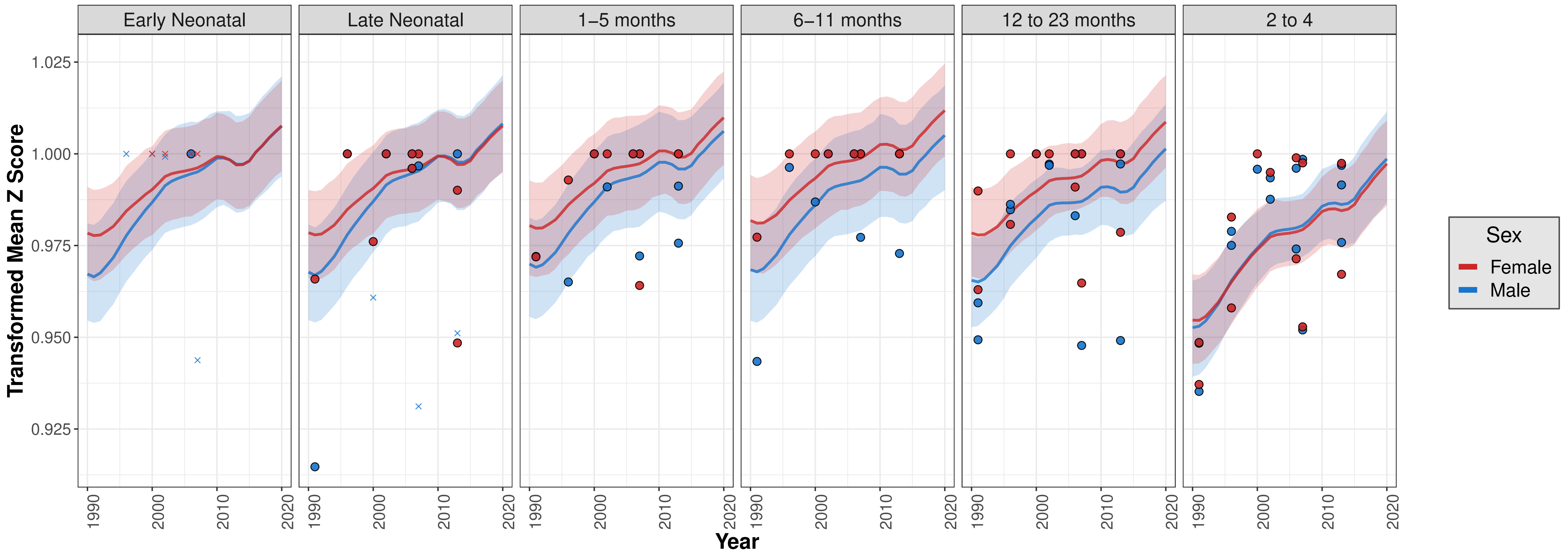

I

Year	Source
1986	DHS
1986	Experimental DHS
1986	WHO CGM Database
1991	DHS
1991	WHO CGM Database
1996	DHS
1996	WHO CGM Database
2000	MICS
2000	WHO CGM Database
2002	DHS
2002	WHO CGM Database
2006	National Multipurpose Household Survey
2006	WHO CGM Database
2007	DHS
2007	Special DHS
2007	WHO CGM Database
2013	DHS
2013	WHO CGM Database
2013	Special DHS

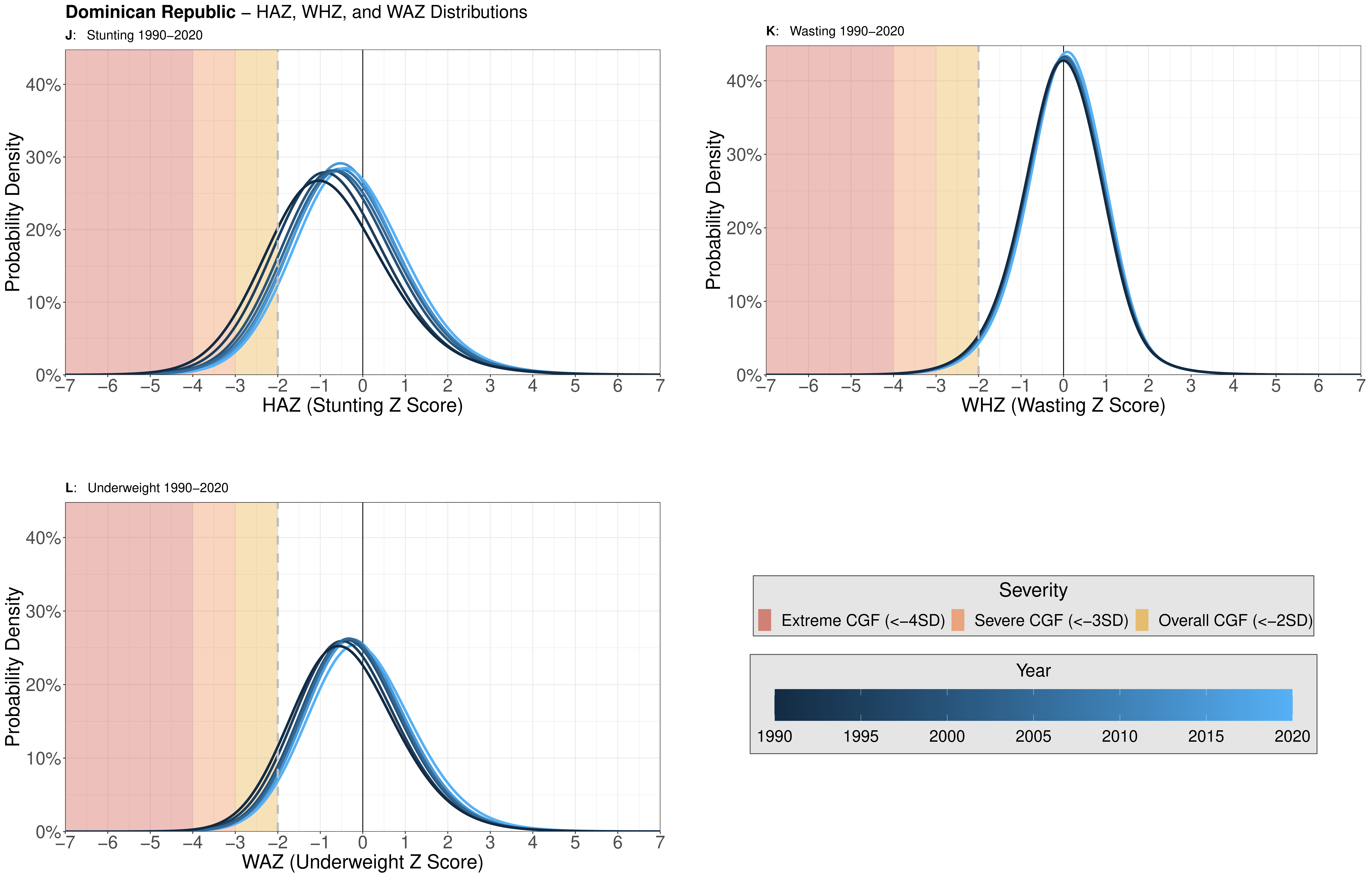

Dominican Republic – HAZ, WHZ, and WAZ Distributions
J: Stunting 1990–2020
Probability Density
40%
30%
20%
10%
0%
−7 −6 −5 −4 −3 −2 −1 0 1 2 3 4 5 6 7
HAZ (Stunting Z Score)
K: Wasting 1990–2020
Probability Density
40%
30%
20%
10%
0%
−7 −6 −5 −4 −3 −2 −1 0 1 2 3 4 5 6 7
WHZ (Wasting Z Score)
L: Underweight 1990–2020
Probability Density
40%
30%
20%
10%
0%
−7 −6 −5 −4 −3 −2 −1 0 1 2 3 4 5 6 7
WAZ (Underweight Z Score)
Severity
Extreme CGF (<−4SD)
Severe CGF (<−3SD)
Overall CGF (<−2SD)
Year
1990
1995
2000
2005
2010
2015
2020

Grenada – Stunting (HAZ)

A: Overall and Severe Stunting Prevalence

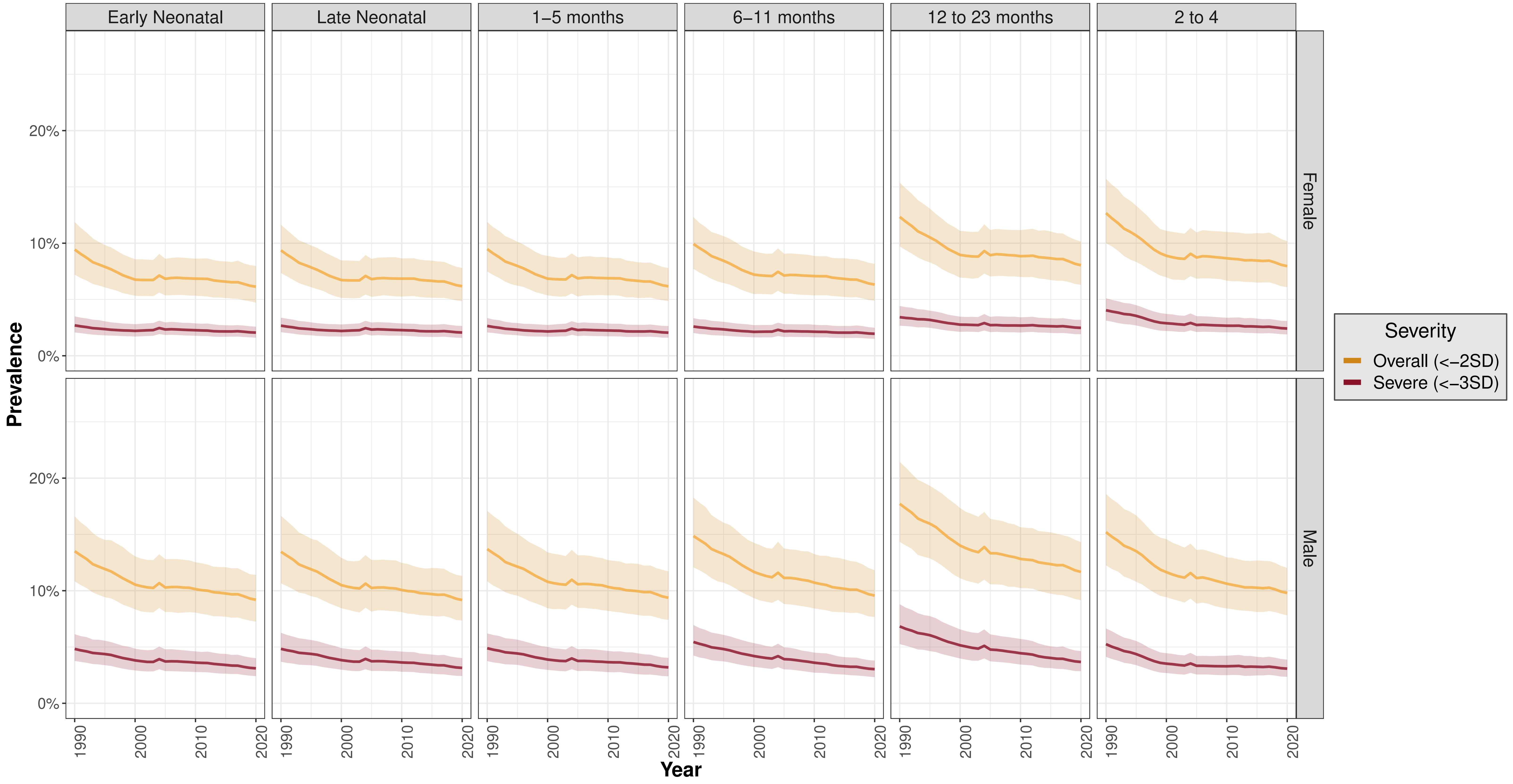

B: Transformed Mean Stunting Z Scores

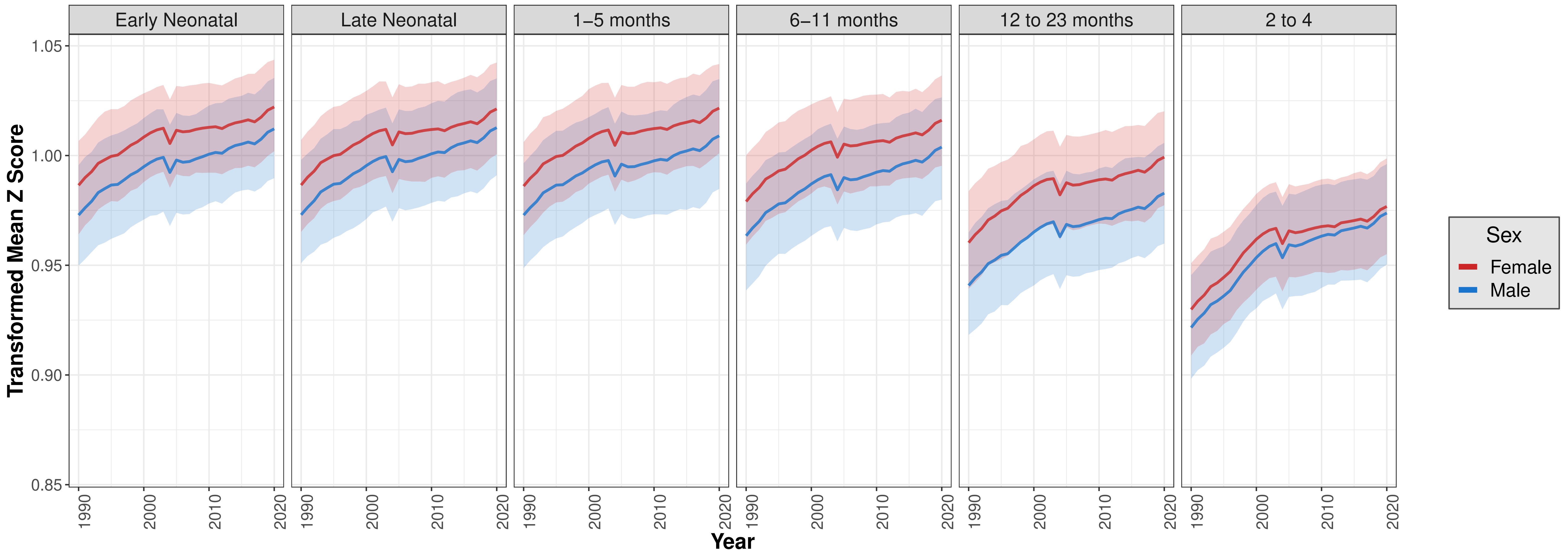

C

Source
No sources for this location

Grenada – Wasting (WHZ)

D: Overall and Severe Wasting Prevalence

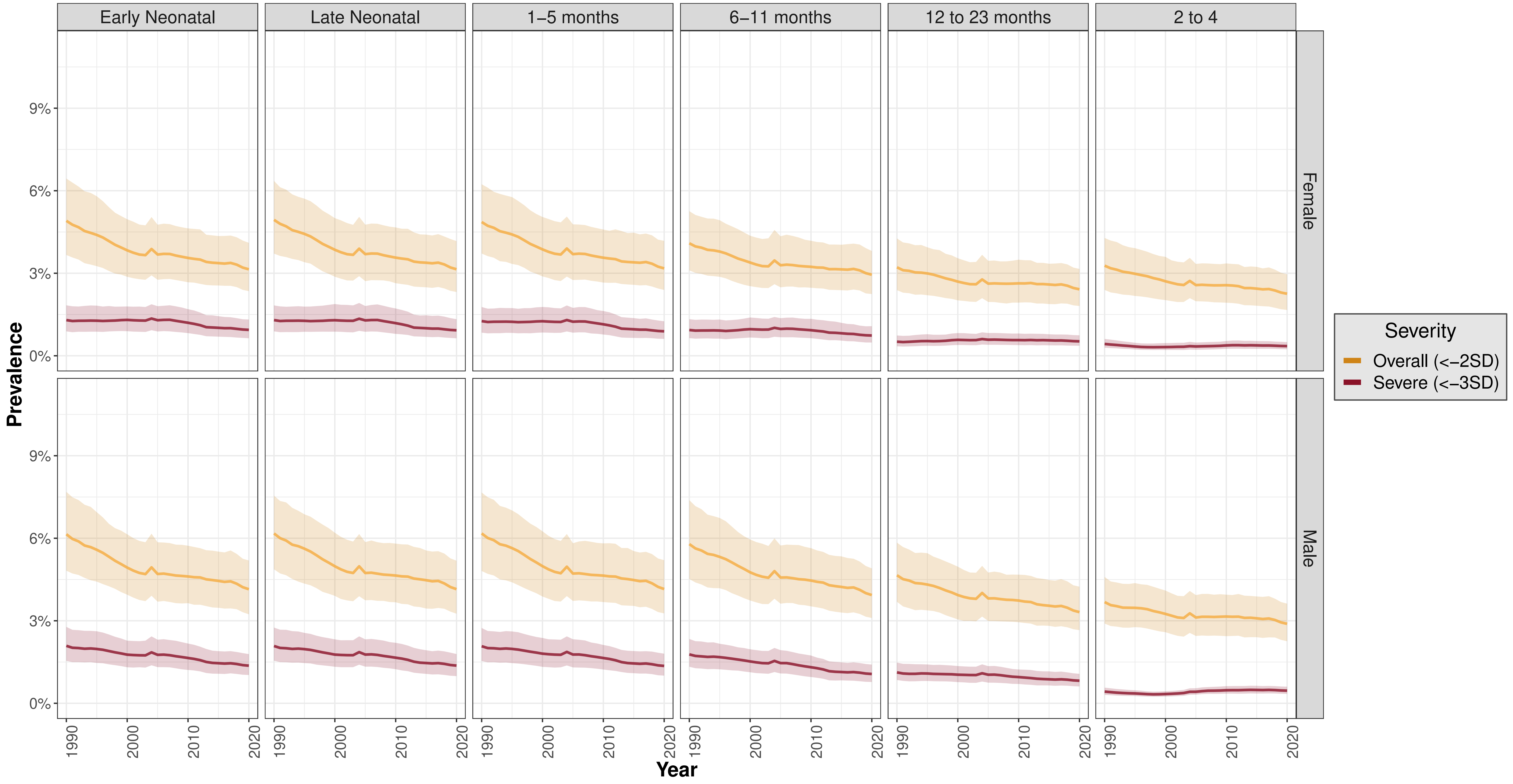

E: Transformed Mean Wasting Z Scores

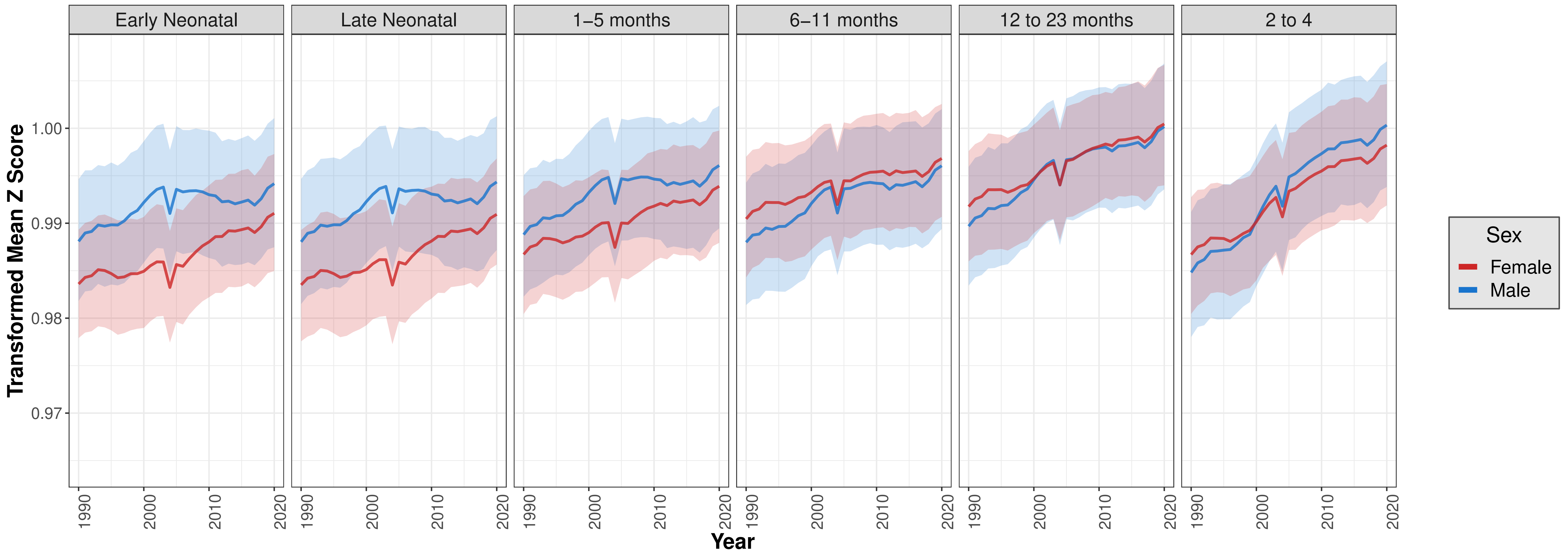

F

Source
No sources for this location

Grenada – Underweight (WAZ)

G: Overall and Severe Underweight Prevalence

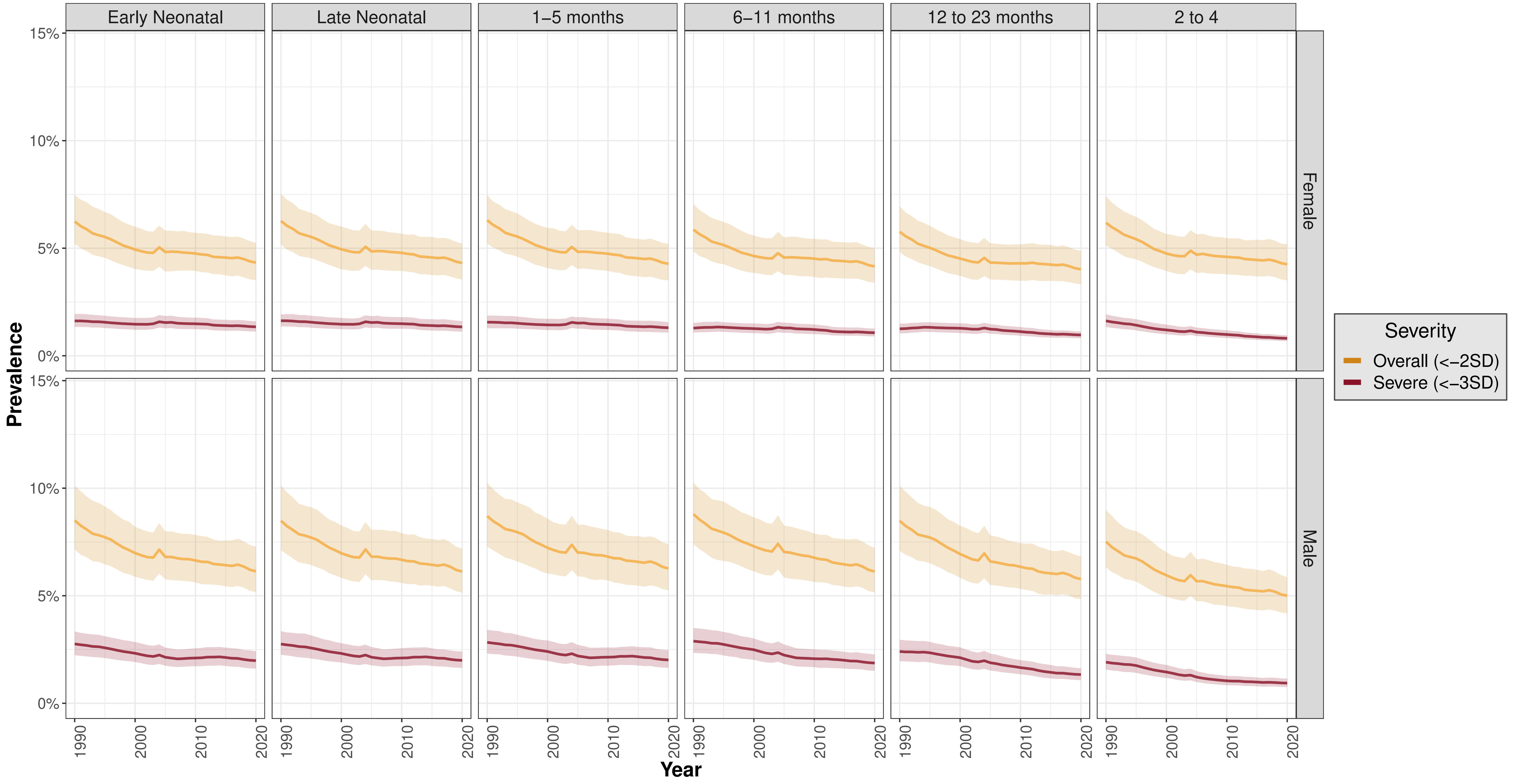

H: Transformed Mean Underweight Z Scores

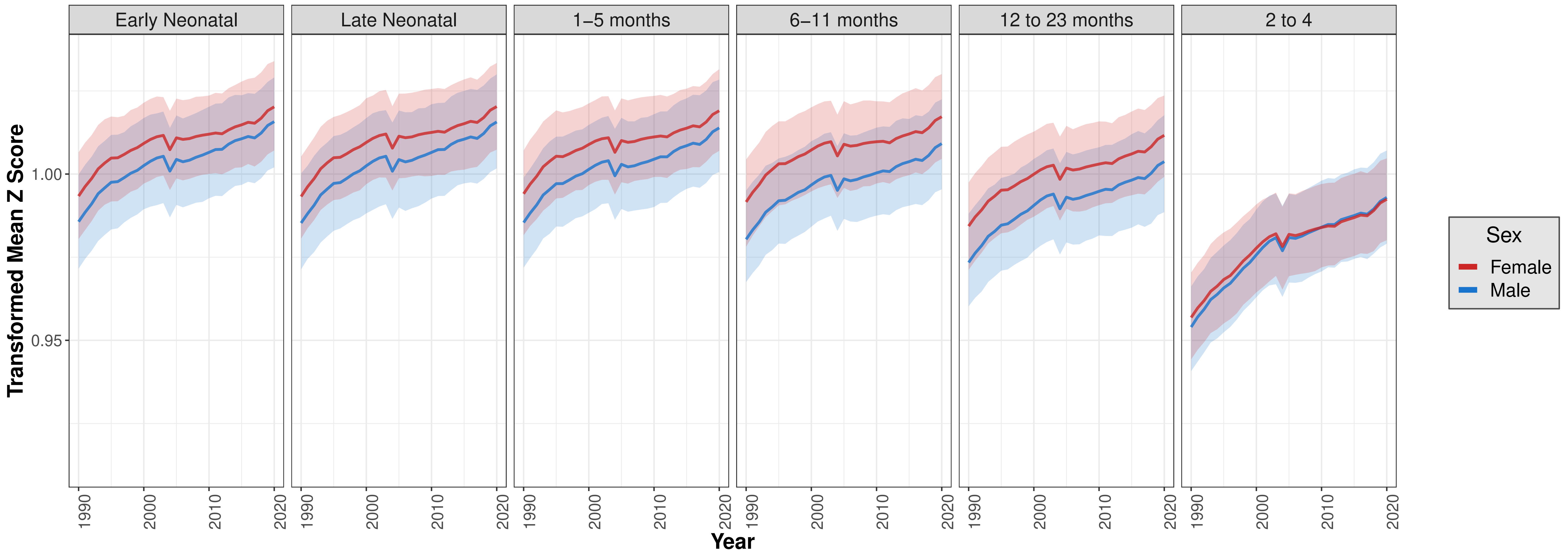

I

Source
No sources for this location

Grenada – HAZ, WHZ, and WAZ Distributions

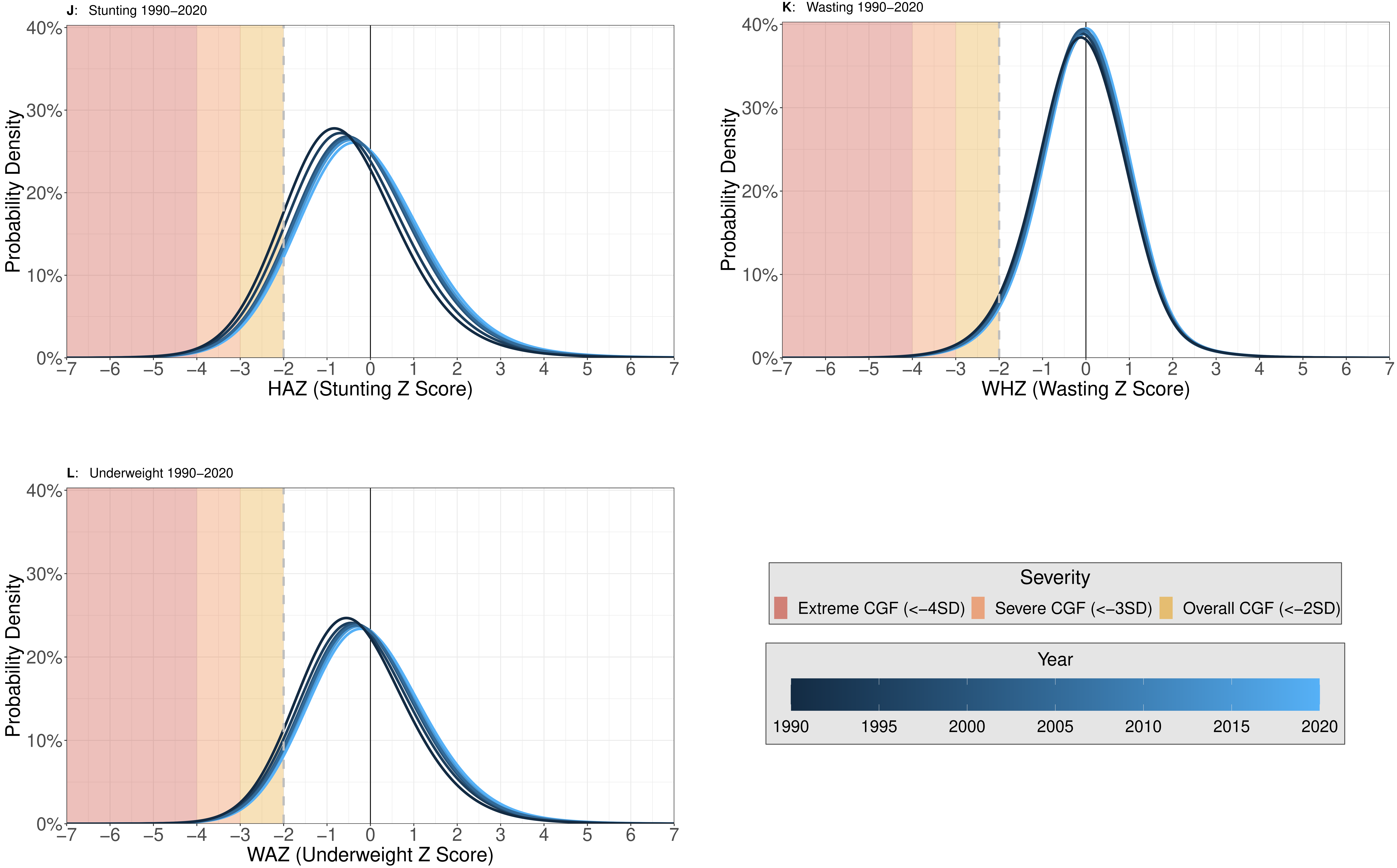

Guyana – Stunting (HAZ)

A: Overall and Severe Stunting Prevalence

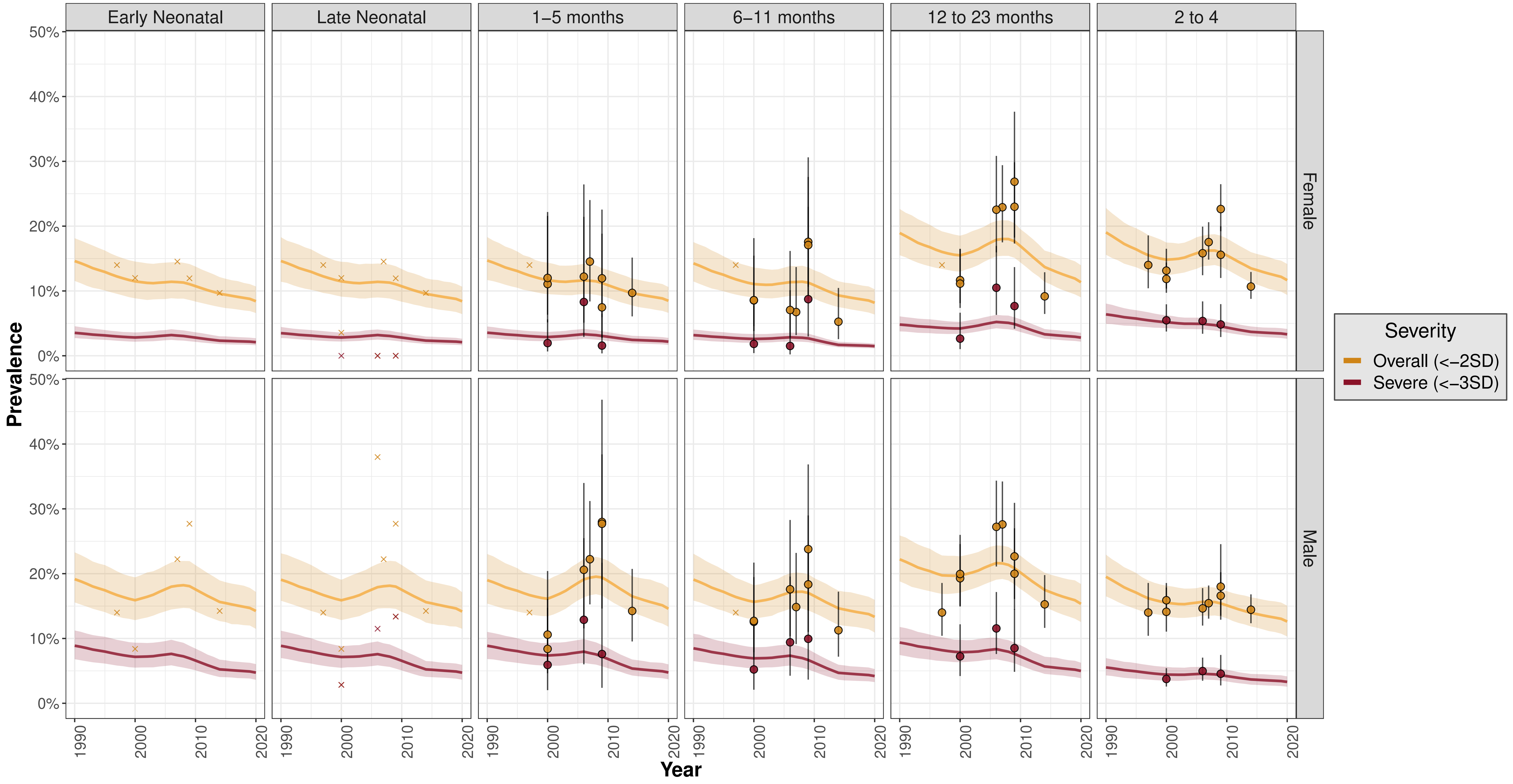

B: Transformed Mean Stunting Z Scores

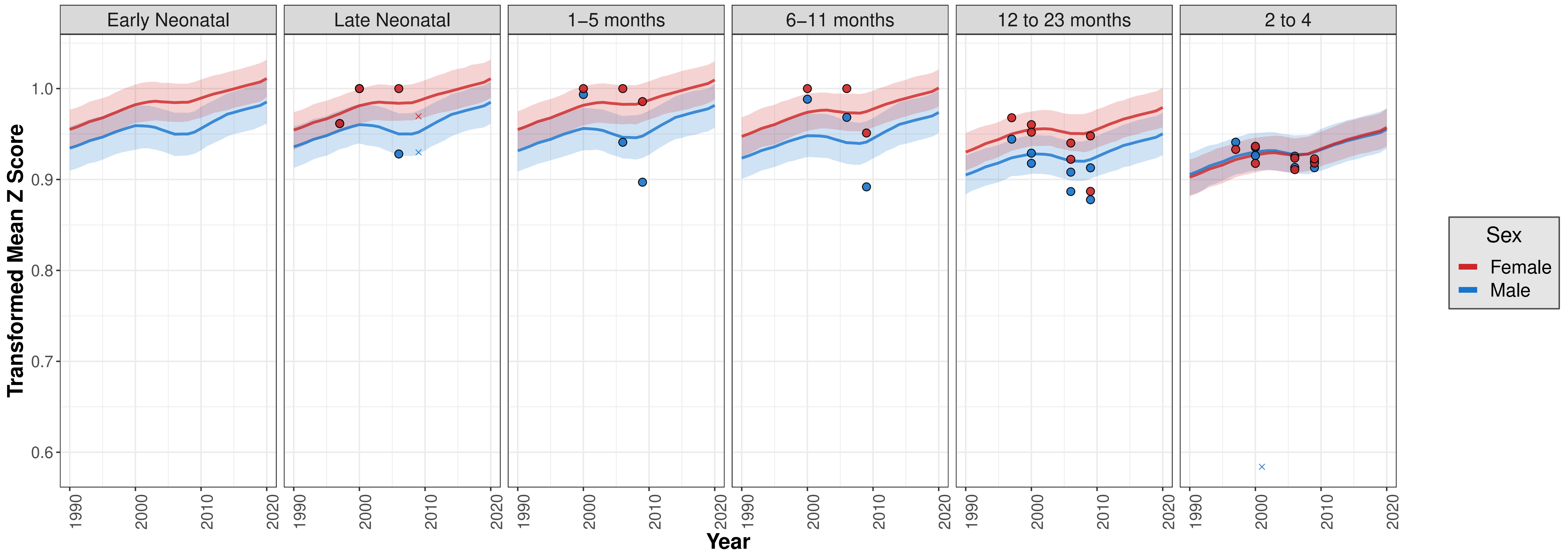

C

Year	Source
1971	WHO CGM Database
1981	WHO CGM Database
1997	WHO CGM Database
2000	MICS
2000	WHO CGM Database
2001	MICS
2006	MICS
2006	WHO CGM Database
2007	WHO CGM Database
2009	DHS
2009	WHO CGM Database
2014	WHO CGM Database
2014	MICS

Guyana – Wasting (WHZ)

D: Overall and Severe Wasting Prevalence

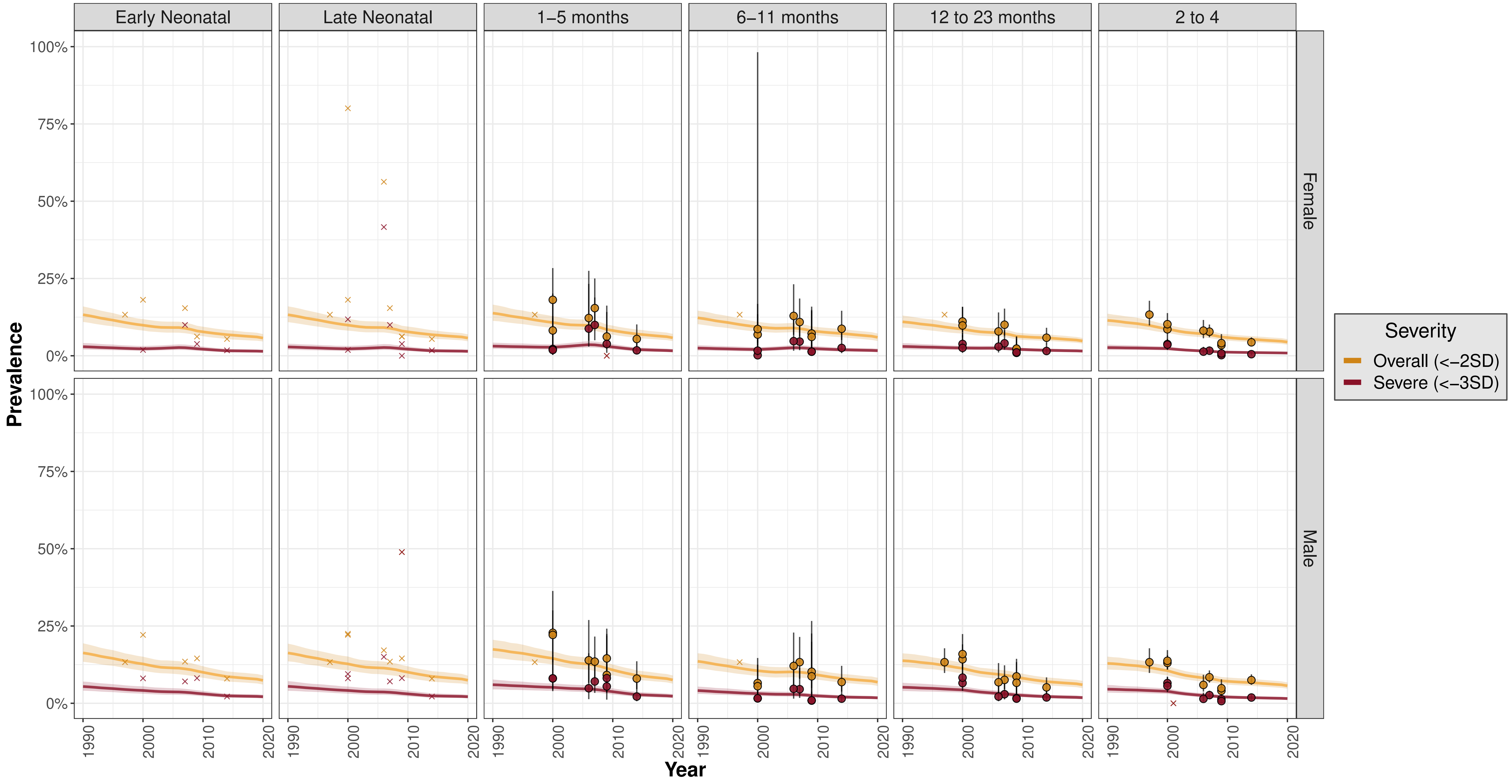

E: Transformed Mean Wasting Z Scores

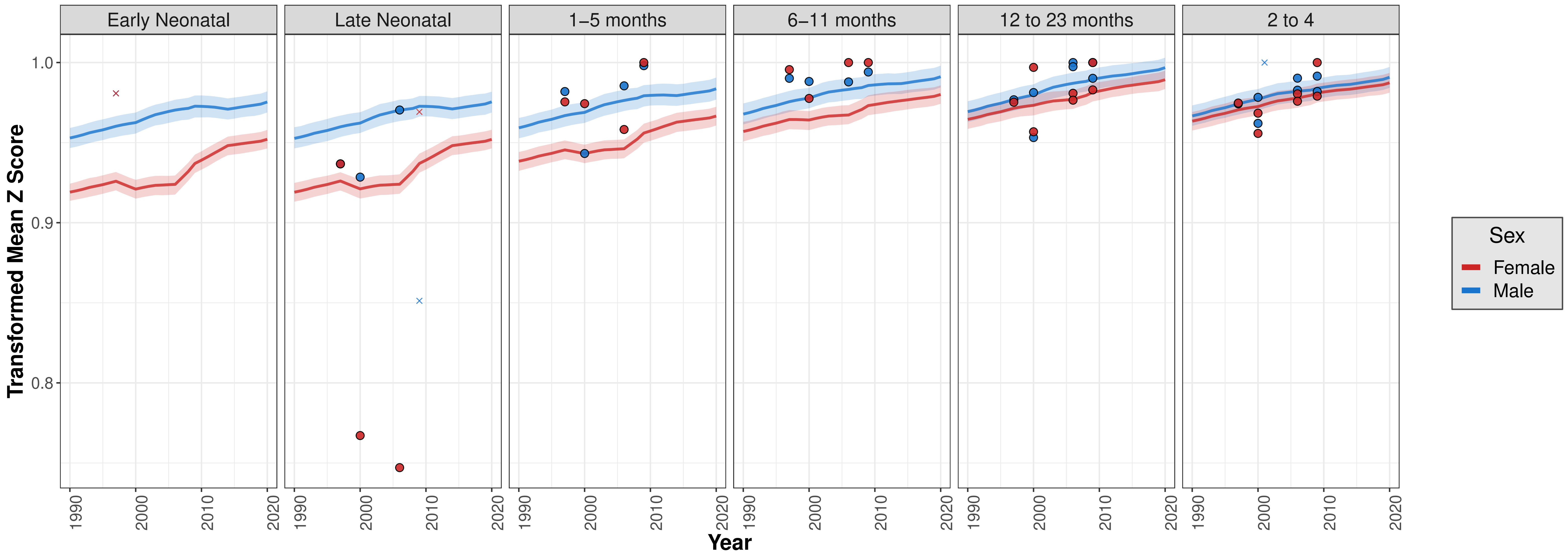

F

Year	Source
1981	WHO CGM Database
1997	WHO CGM Database
2000	MICS
2000	WHO CGM Database
2001	MICS
2006	MICS
2006	WHO CGM Database
2007	WHO CGM Database
2009	DHS
2009	WHO CGM Database
2014	WHO CGM Database
2014	MICS

Guyana – Underweight (WAZ)

G: Overall and Severe Underweight Prevalence

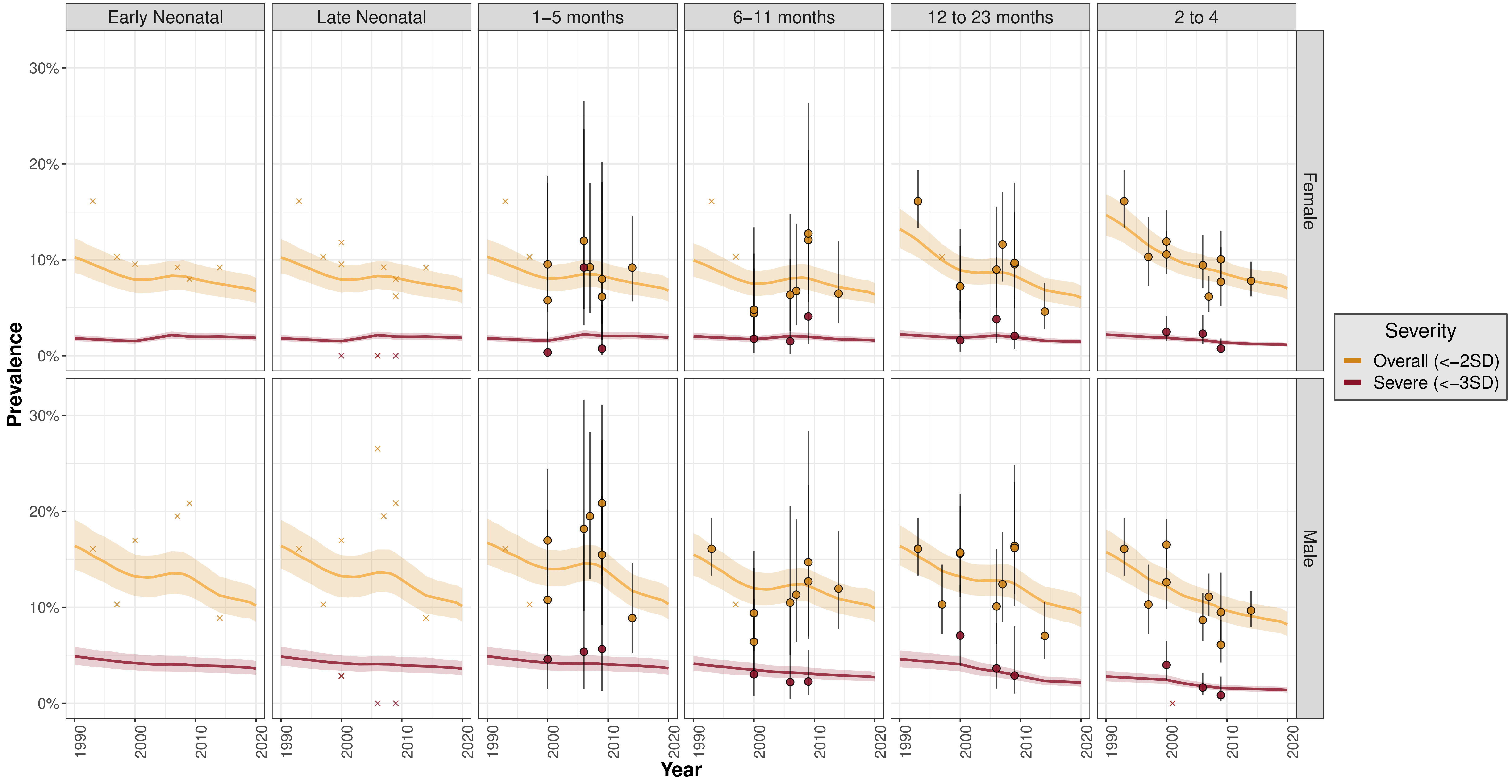

H: Transformed Mean Underweight Z Scores

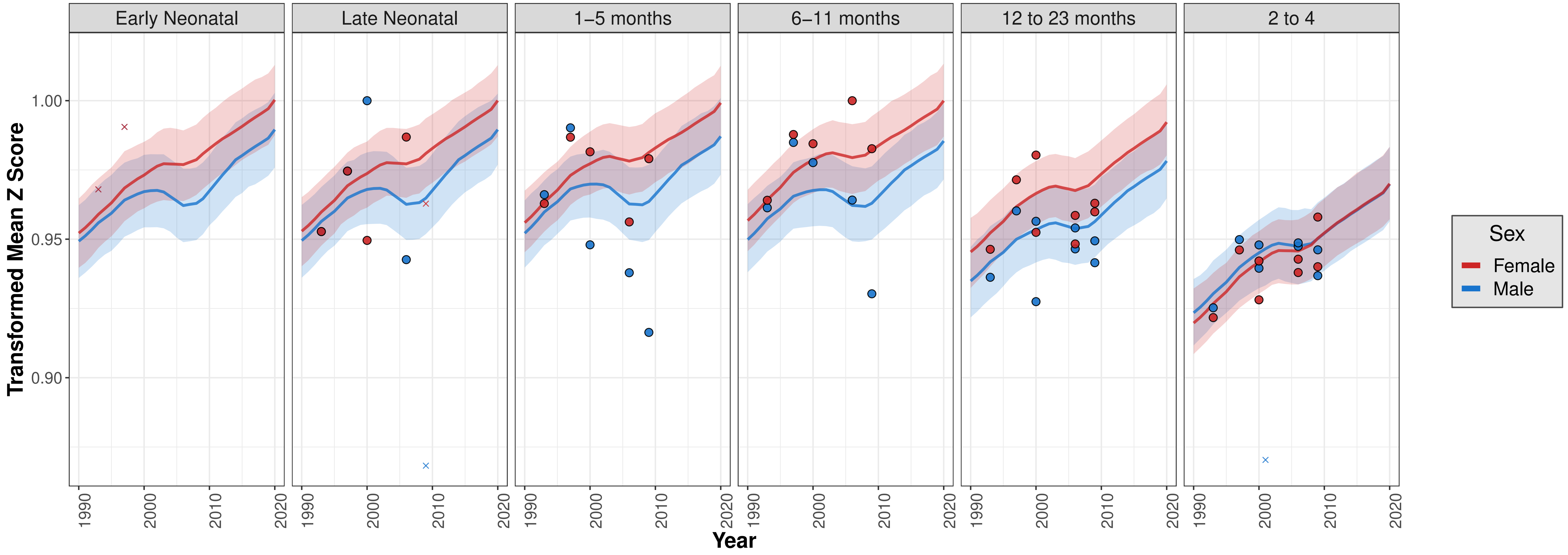

I

Year	Source
1971	WHO CGM Database
1981	WHO CGM Database
1993	WHO CGM Database
1997	WHO CGM Database
2000	MICS
2000	WHO CGM Database
2001	MICS
2006	MICS
2006	WHO CGM Database
2007	WHO CGM Database
2009	DHS
2009	WHO CGM Database
2014	WHO CGM Database
2014	MICS

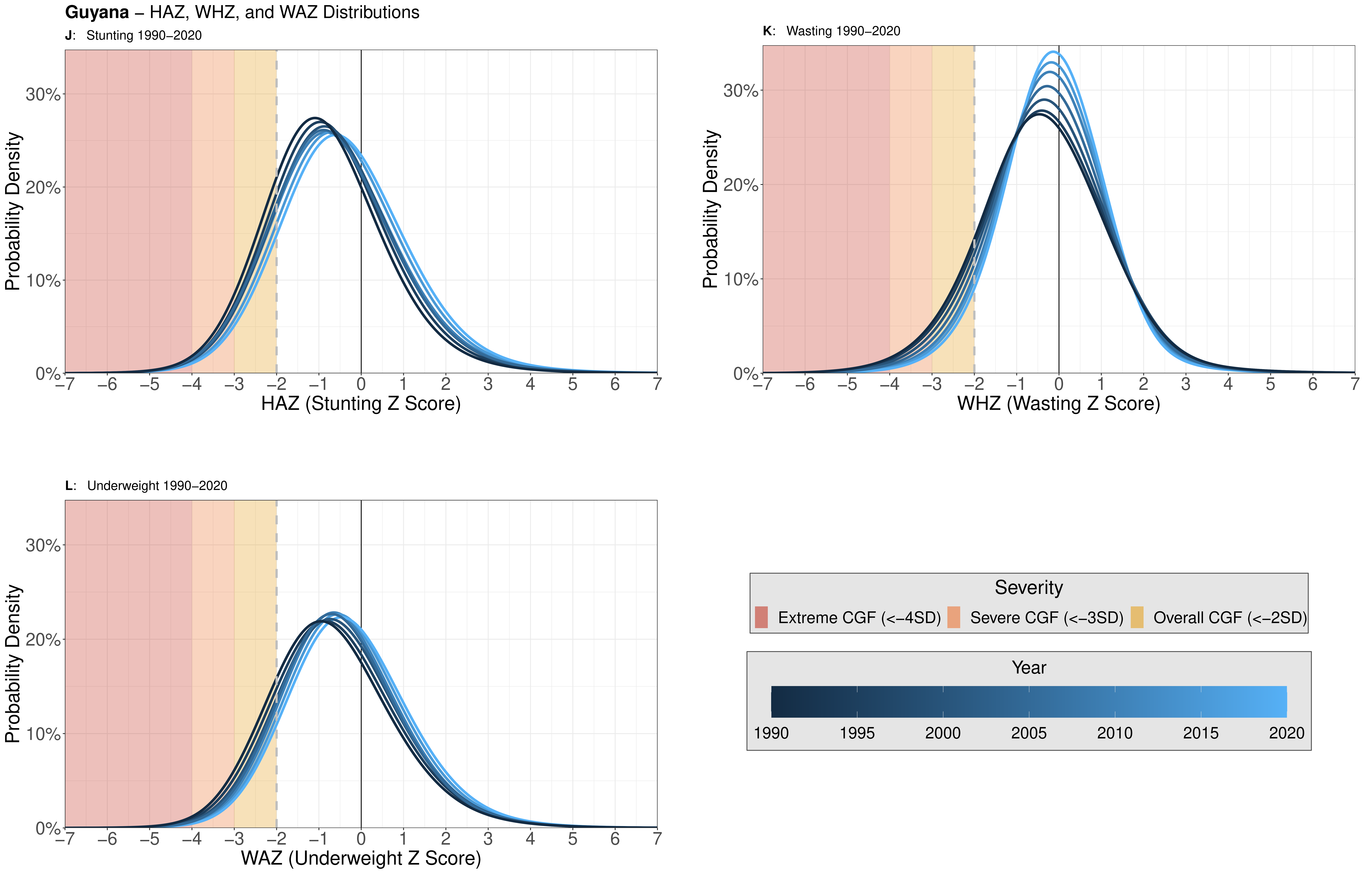

Guyana – HAZ, WHZ, and WAZ Distributions
J: Stunting 1990–2020
K: Wasting 1990–2020
L: Underweight 1990–2020
Probability Density
0%
10%
20%
30%
−7
−6
−5
−4
−3
−2
−1
0
1
2
3
4
5
6
7
HAZ (Stunting Z Score)
WHZ (Wasting Z Score)
WAZ (Underweight Z Score)
Severity
Extreme CGF (<−4SD)
Severe CGF (<−3SD)
Overall CGF (<−2SD)
Year
1990
1995
2000
2005
2010
2015
2020

Haiti – Stunting (HAZ)

A: Overall and Severe Stunting Prevalence

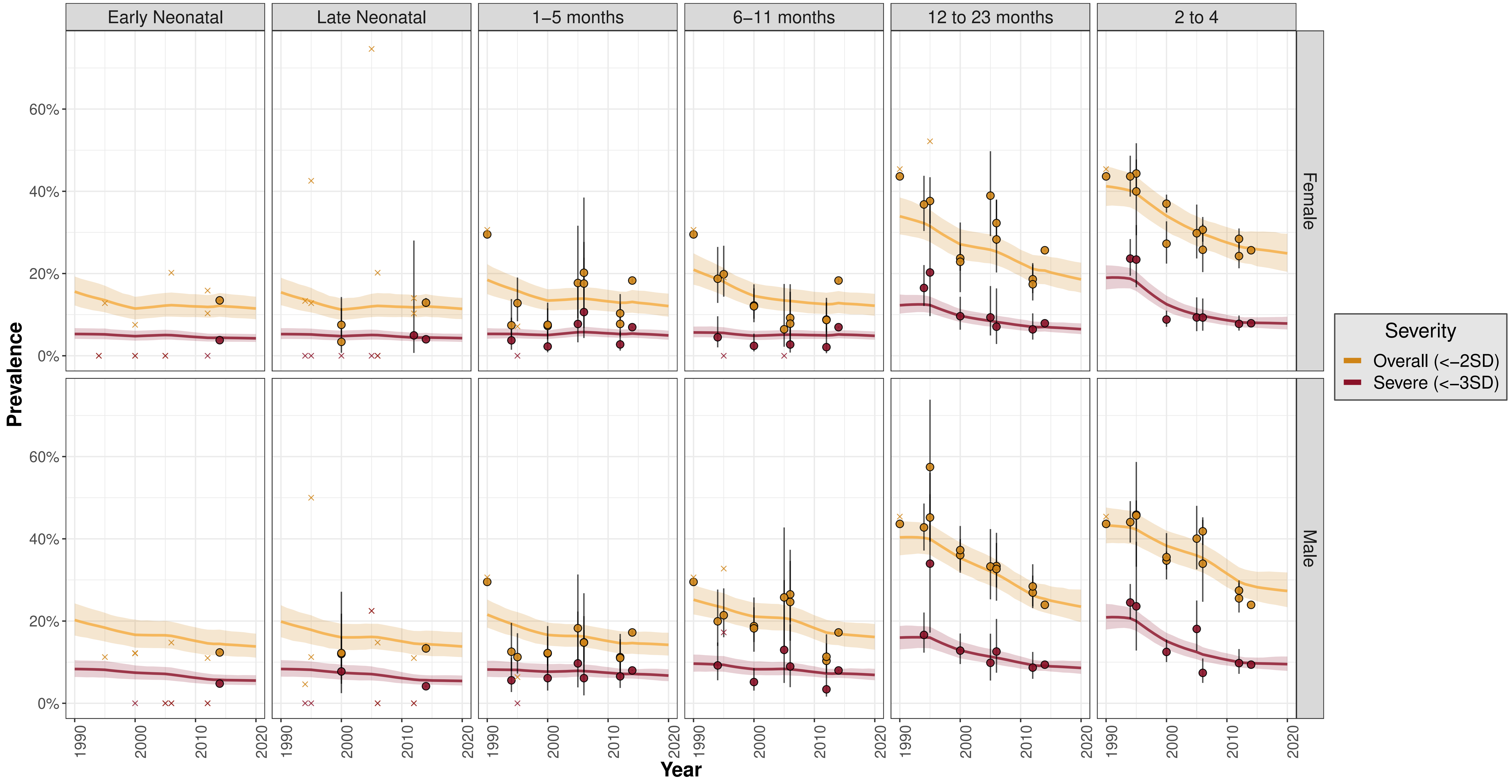

B: Transformed Mean Stunting Z Scores

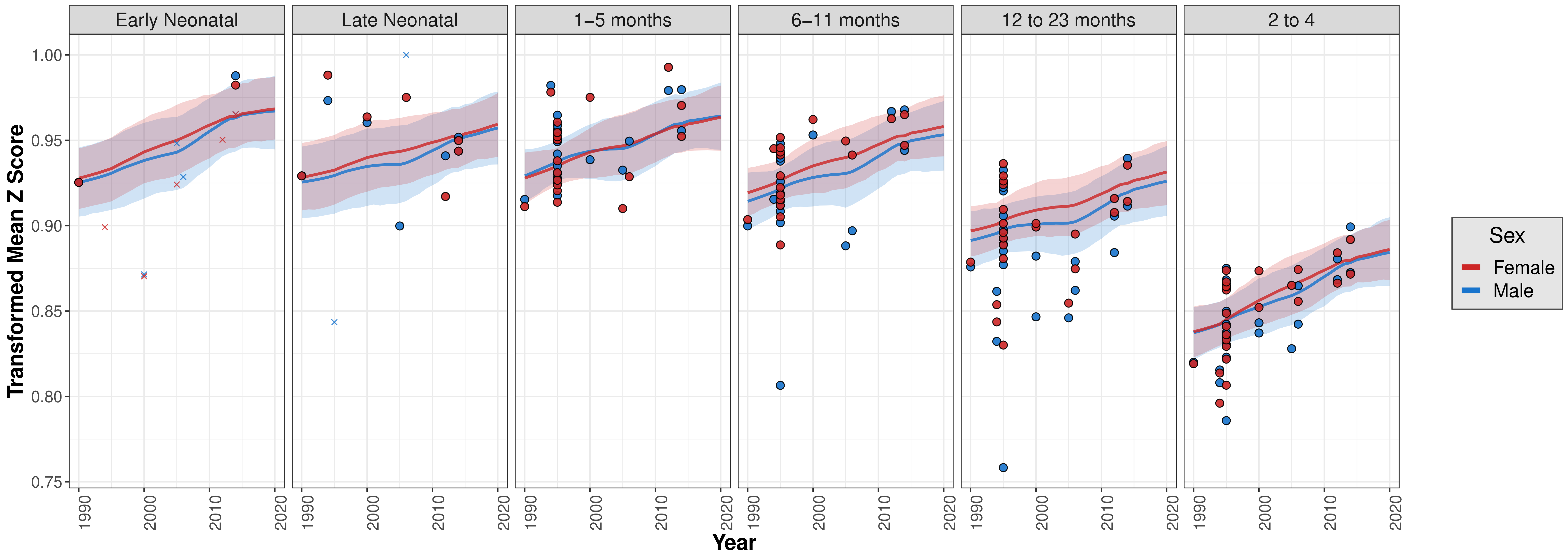

C

Year	Source
1990	WHO CGM Database
1994	DHS
1994	WHO CGM Database
1995	DHS
1995	WHO CGM Database
2000	DHS
2000	WHO CGM Database
2005	DHS
2006	DHS
2006	WHO CGM Database
2012	DHS
2012	WHO CGM Database
2014	Nutritional Survey Using SMART Methodology July–August

Haiti – Wasting (WHZ)

D: Overall and Severe Wasting Prevalence

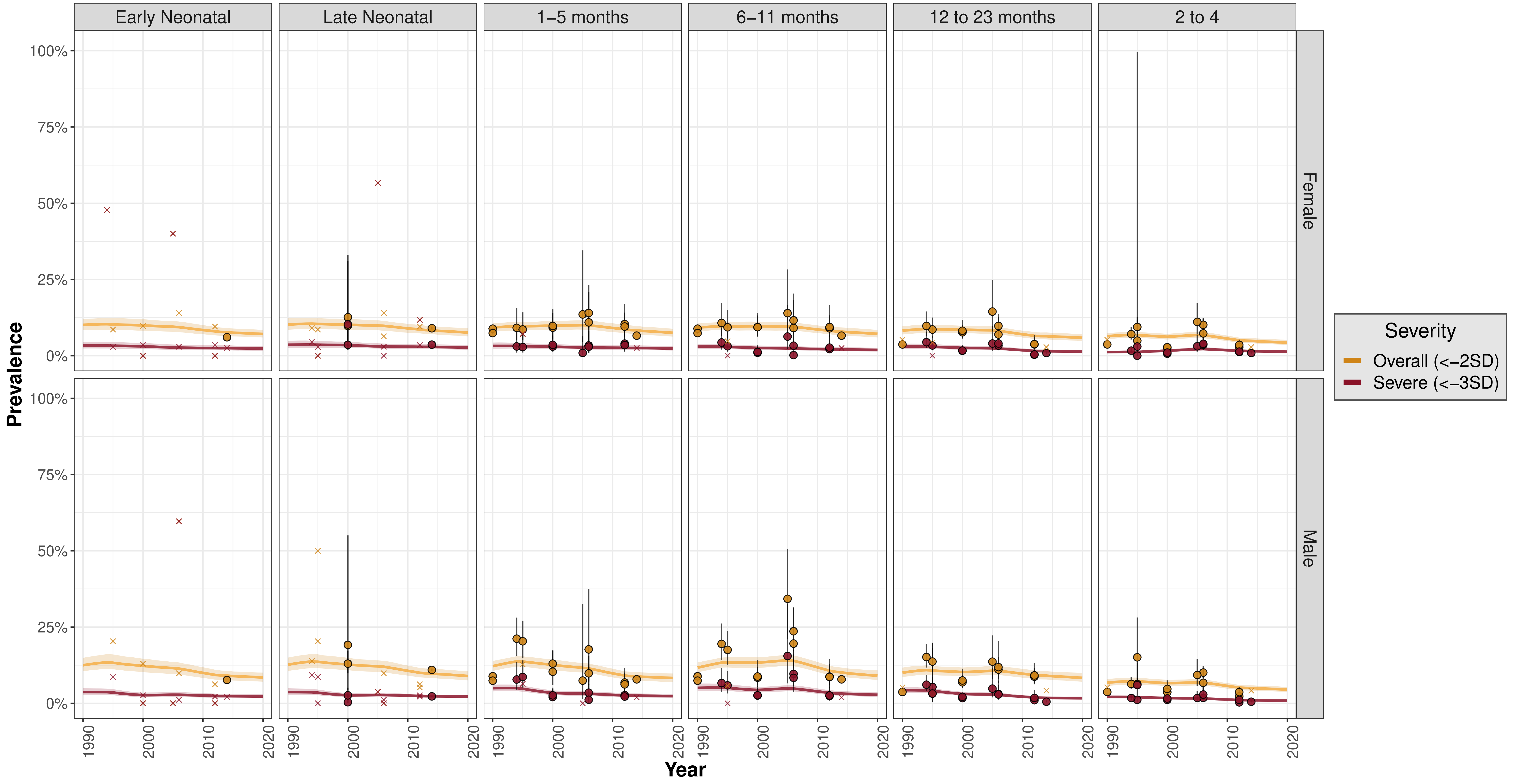

E: Transformed Mean Wasting Z Scores

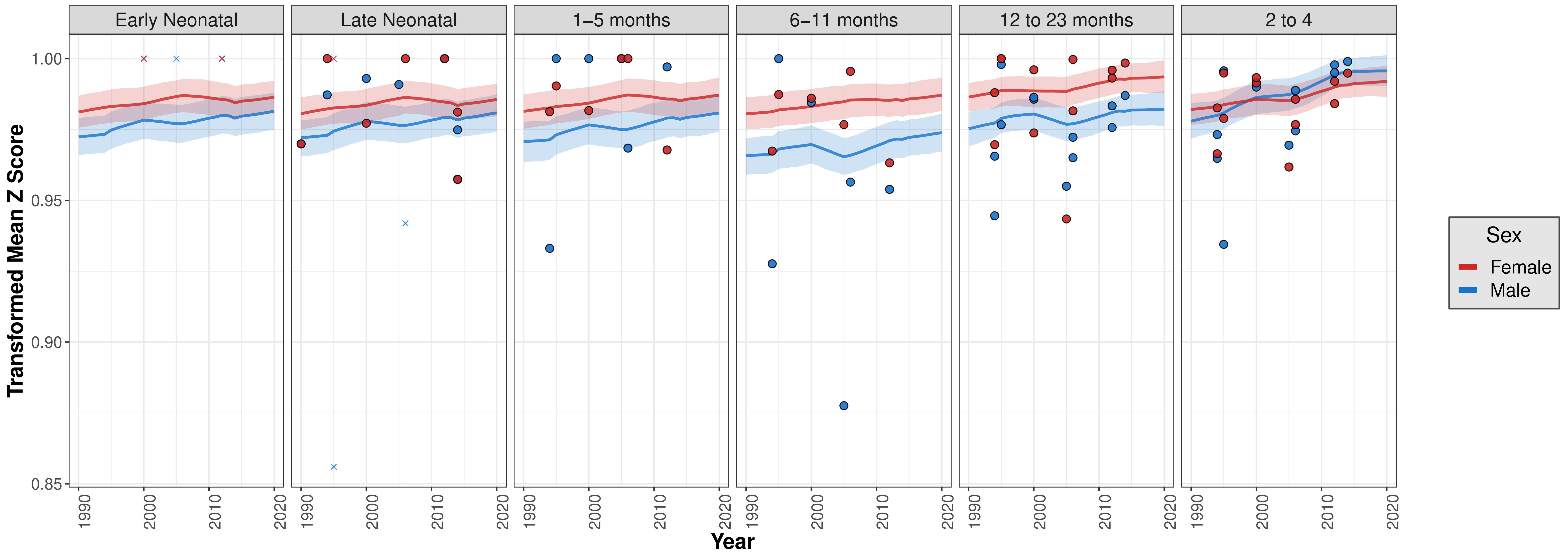

F

Year	Source
1990	WHO CGM Database
1994	DHS
1994	WHO CGM Database
1995	DHS
1995	WHO CGM Database
2000	DHS
2000	WHO CGM Database
2005	DHS
2006	DHS
2006	WHO CGM Database
2012	DHS
2012	WHO CGM Database
2014	Nutritional Survey Using SMART Methodology July–August

Haiti – Underweight (WAZ)

G: Overall and Severe Underweight Prevalence

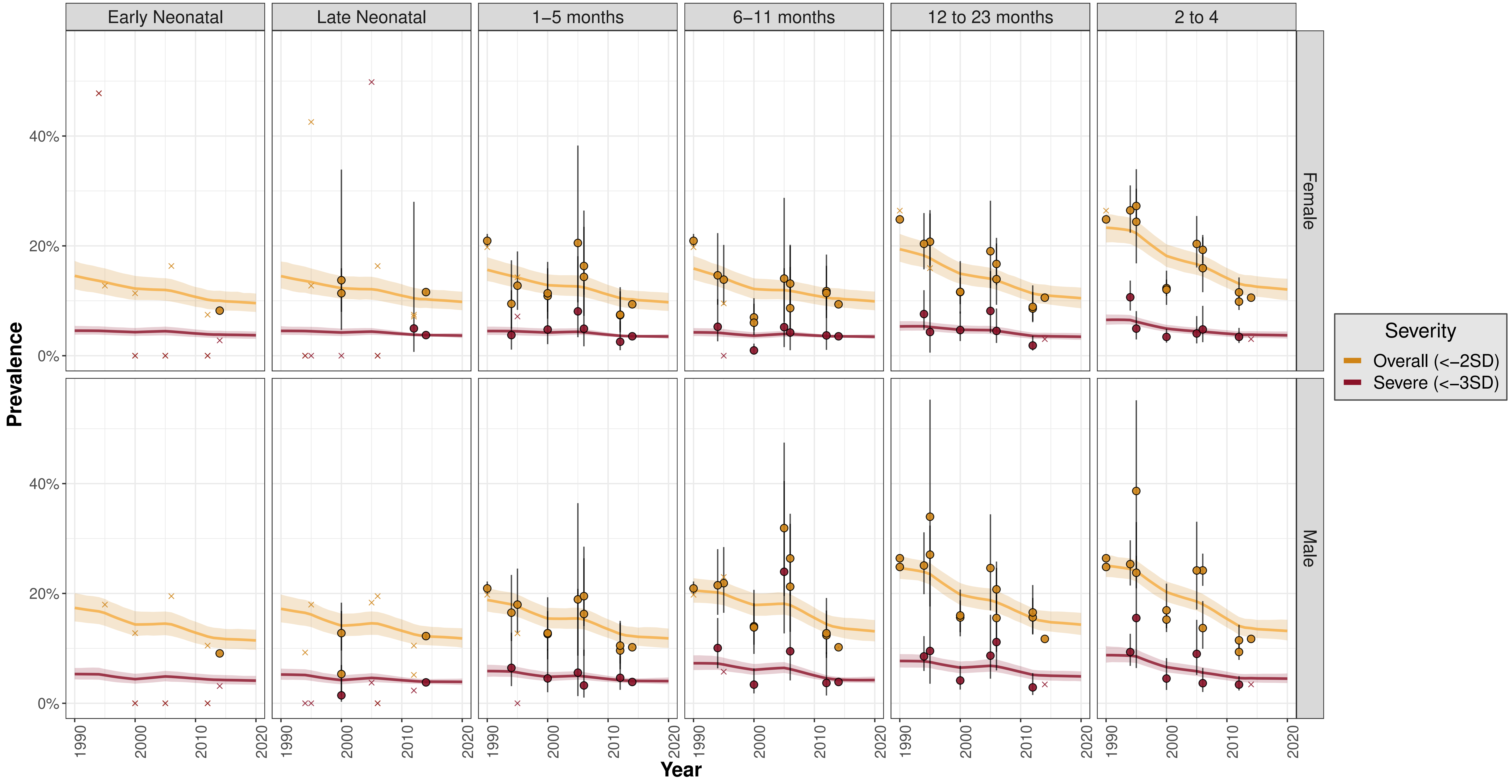

H: Transformed Mean Underweight Z Scores

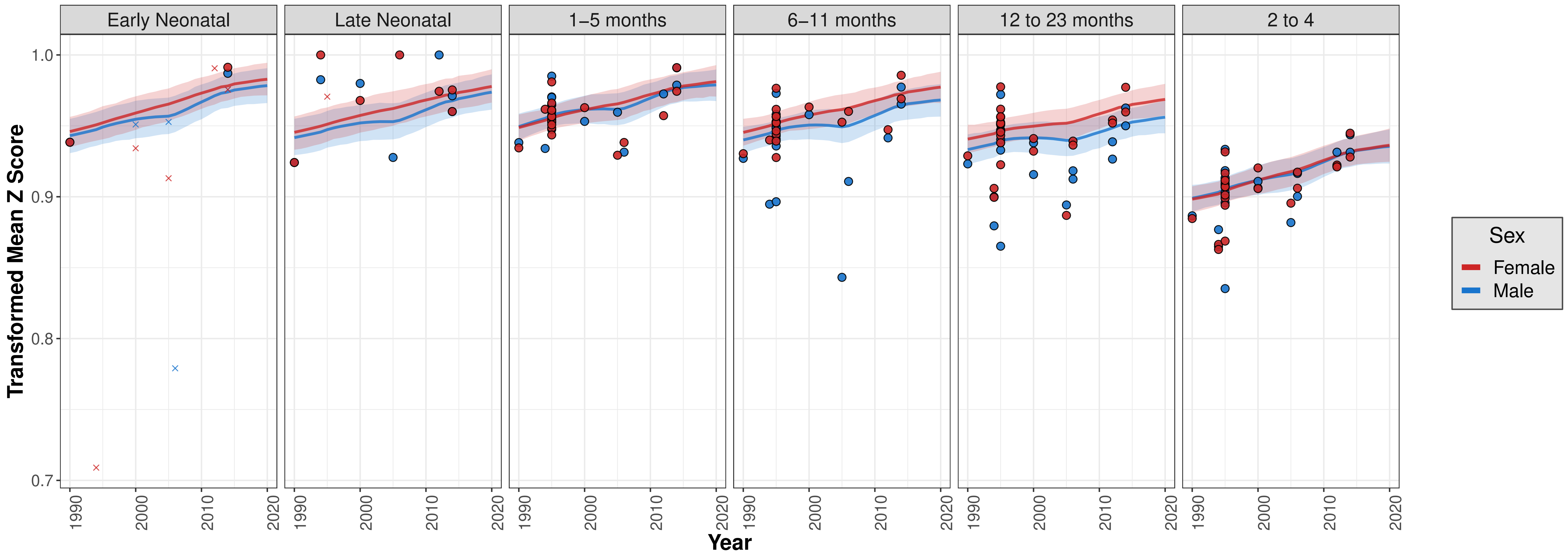

I

Year	Source
1990	WHO CGM Database
1994	DHS
1994	WHO CGM Database
1995	DHS
1995	WHO CGM Database
2000	DHS
2000	WHO CGM Database
2005	DHS
2006	DHS
2006	WHO CGM Database
2012	DHS
2012	WHO CGM Database
2014	Nutritional Survey Using SMART Methodology July–August

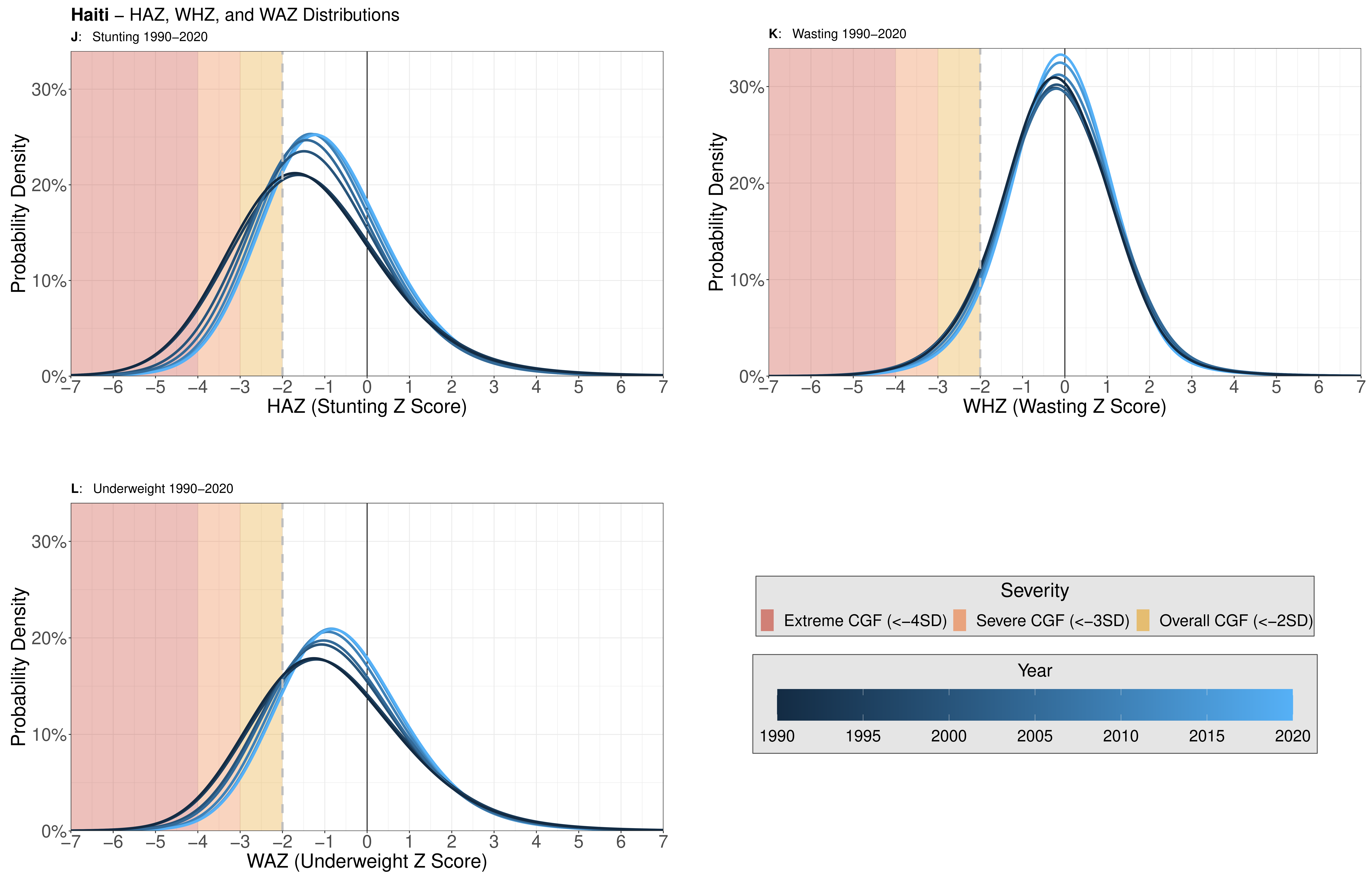
Haiti – HAZ, WHZ, and WAZ Distributions
J: Stunting 1990–2020
Probability Density
0%
10%
20%
30%
−7 −6 −5 −4 −3 −2 −1 0 1 2 3 4 5 6 7
HAZ (Stunting Z Score)
K: Wasting 1990–2020
Probability Density
0%
10%
20%
30%
−7 −6 −5 −4 −3 −2 −1 0 1 2 3 4 5 6 7
WHZ (Wasting Z Score)
L: Underweight 1990–2020
Probability Density
0%
10%
20%
30%
−7 −6 −5 −4 −3 −2 −1 0 1 2 3 4 5 6 7
WAZ (Underweight Z Score)
Severity
Extreme CGF (<−4SD)
Severe CGF (<−3SD)
Overall CGF (<−2SD)
Year
1990
1995
2000
2005
2010
2015
2020

Jamaica – Stunting (HAZ)

A: Overall and Severe Stunting Prevalence

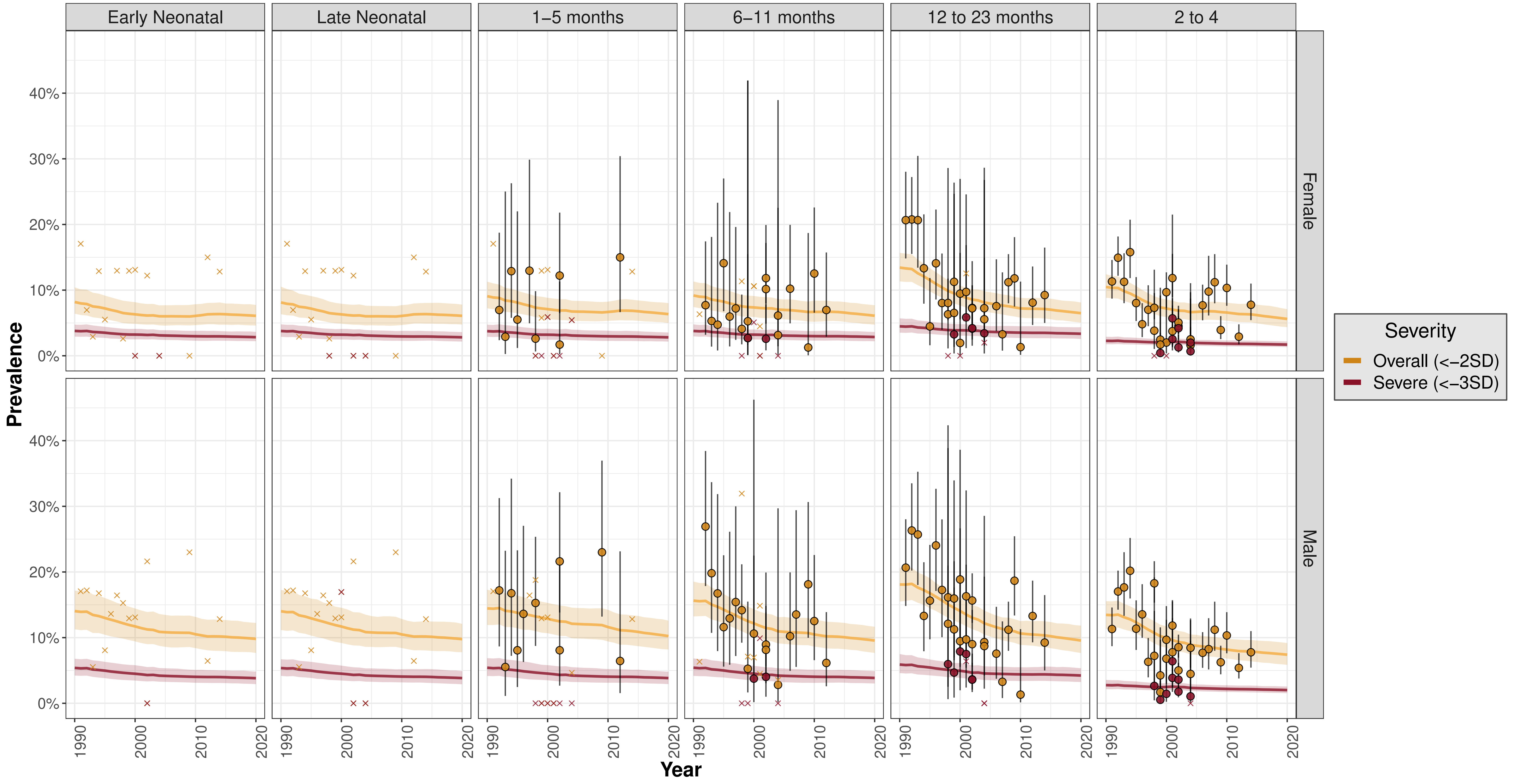

B: Transformed Mean Stunting Z Scores

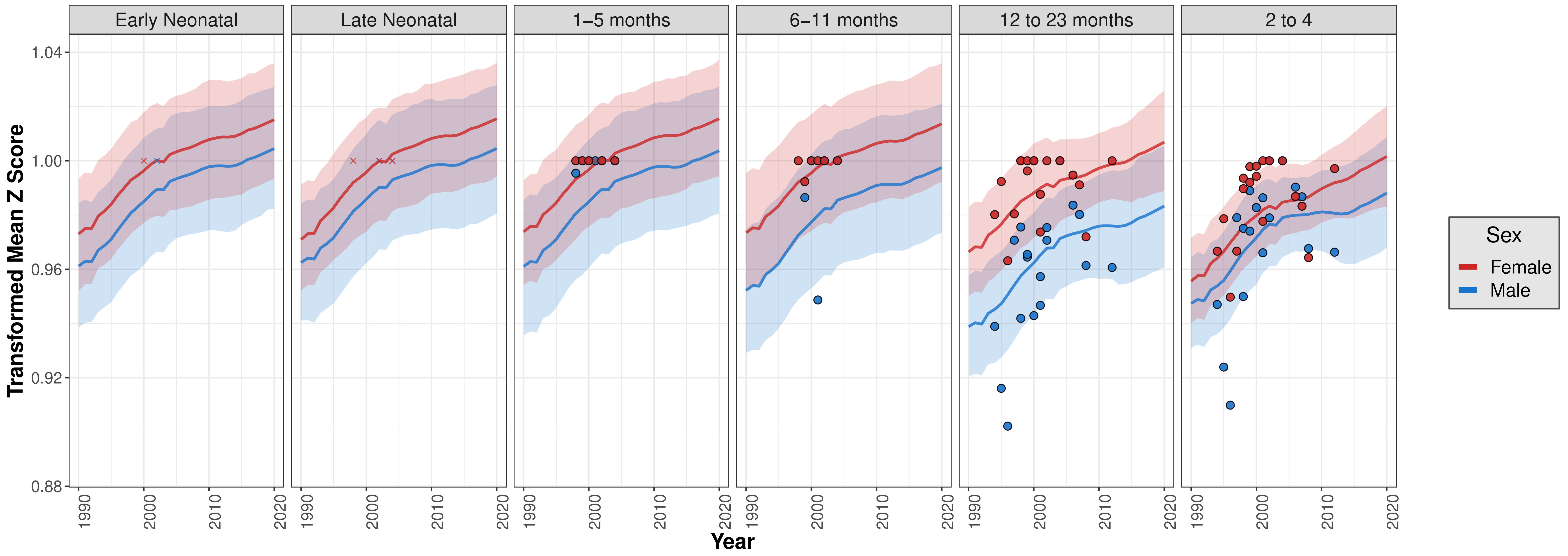

C

Year	Source
1978	WHO CGM Database
1989	WHO CGM Database
1991	WHO CGM Database
1992	WHO CGM Database
1993	WHO CGM Database
1994	WHO CGM Database
1995	WHO CGM Database
1996	WHO CGM Database
1997	WHO CGM Database
1998	Survey of Living Conditions
1998	WHO CGM Database
1999	Survey of Living Conditions
1999	WHO CGM Database
2000	Survey of Living Conditions
2000	WHO CGM Database
2001	Survey of Living Conditions
2001	WHO CGM Database
2002	Survey of Living Conditions
2002	WHO CGM Database
2004	WHO CGM Database
2004	Survey of Living Conditions
2006	WHO CGM Database
2007	WHO CGM Database
2008	WHO CGM Database
2009	WHO CGM Database
2010	WHO CGM Database
2012	WHO CGM Database
2014	WHO CGM Database

Jamaica – Wasting (WHZ)

D: Overall and Severe Wasting Prevalence

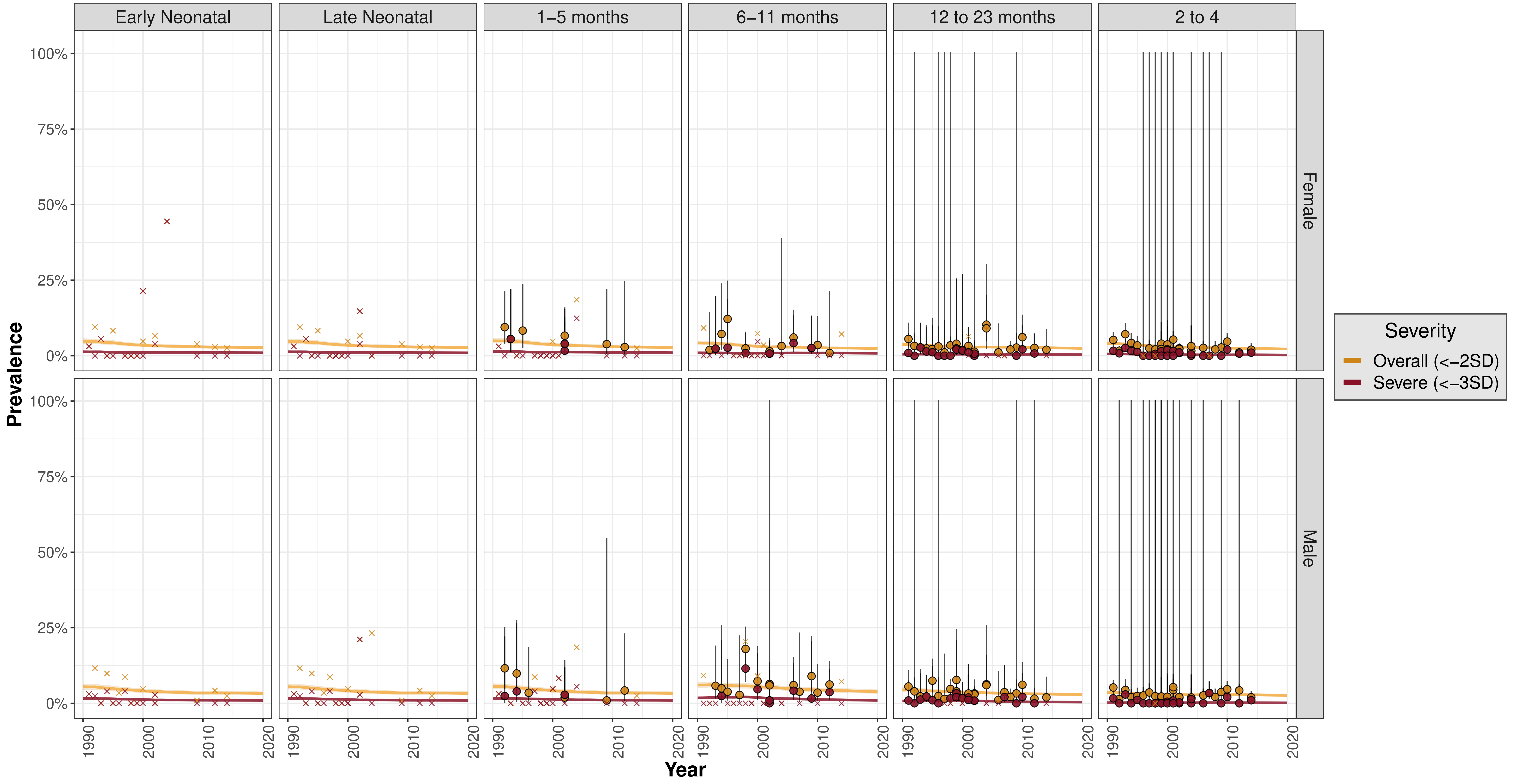

E: Transformed Mean Wasting Z Scores

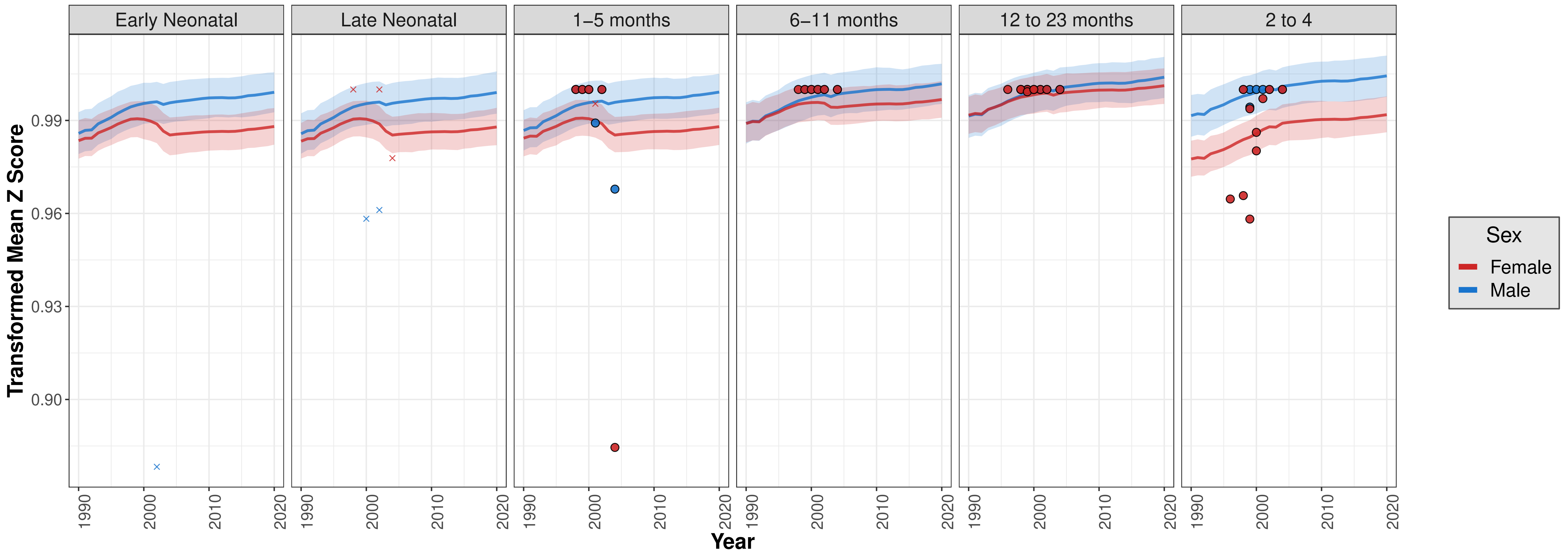

F

Year	Source
1978	WHO CGM Database
1989	WHO CGM Database
1991	WHO CGM Database
1992	WHO CGM Database
1993	WHO CGM Database
1994	WHO CGM Database
1995	WHO CGM Database
1996	WHO CGM Database
1997	WHO CGM Database
1998	Survey of Living Conditions
1998	WHO CGM Database
1999	Survey of Living Conditions
1999	WHO CGM Database
2000	Survey of Living Conditions
2000	WHO CGM Database
2001	Survey of Living Conditions
2001	WHO CGM Database
2002	Survey of Living Conditions
2002	WHO CGM Database
2004	WHO CGM Database
2004	Survey of Living Conditions
2006	WHO CGM Database
2007	WHO CGM Database
2008	WHO CGM Database
2009	WHO CGM Database
2010	WHO CGM Database
2012	WHO CGM Database
2014	WHO CGM Database

Jamaica – Underweight (WAZ)

G: Overall and Severe Underweight Prevalence

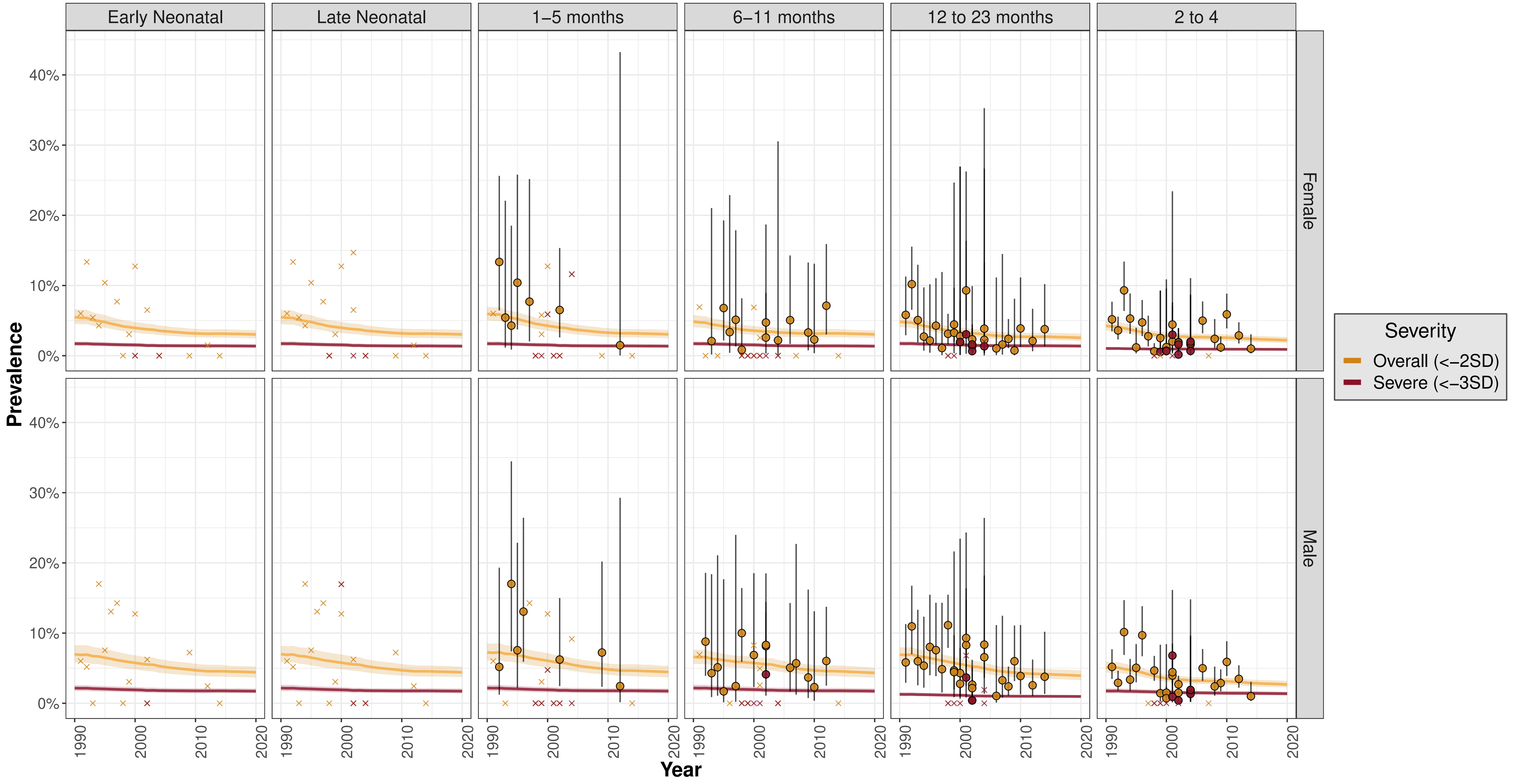

H: Transformed Mean Underweight Z Scores

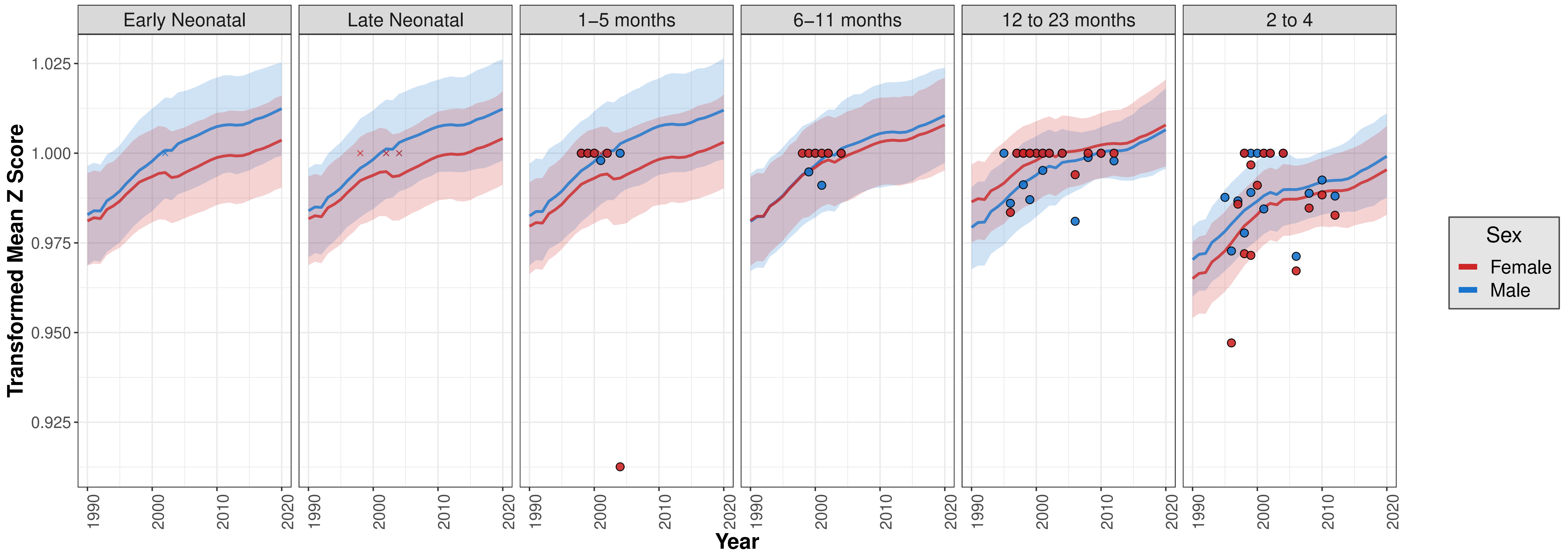

I

Year	Source
1978	WHO CGM Database
1989	WHO CGM Database
1991	WHO CGM Database
1992	WHO CGM Database
1993	WHO CGM Database
1994	WHO CGM Database
1995	WHO CGM Database
1996	WHO CGM Database
1997	WHO CGM Database
1998	Survey of Living Conditions
1998	WHO CGM Database
1999	Survey of Living Conditions
1999	WHO CGM Database
2000	Survey of Living Conditions
2000	WHO CGM Database
2001	Survey of Living Conditions
2001	WHO CGM Database
2002	Survey of Living Conditions
2002	WHO CGM Database
2004	WHO CGM Database
2004	Survey of Living Conditions
2006	WHO CGM Database
2007	WHO CGM Database
2008	WHO CGM Database
2009	WHO CGM Database
2010	WHO CGM Database
2012	WHO CGM Database
2014	WHO CGM Database

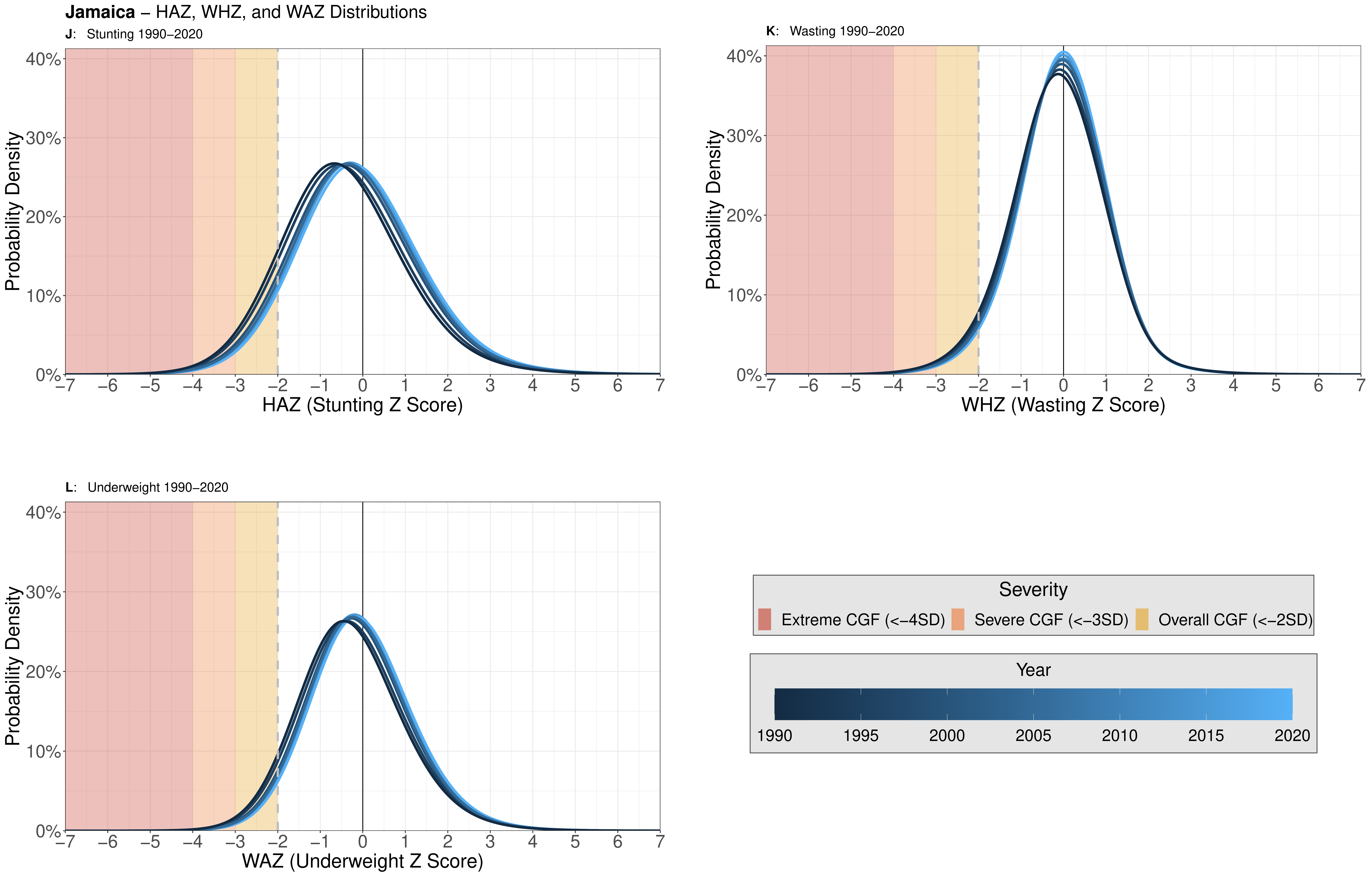

Jamaica – HAZ, WHZ, and WAZ Distributions
J: Stunting 1990–2020
K: Wasting 1990–2020
L: Underweight 1990–2020
Probability Density
0%
10%
20%
30%
40%
−7
−6
−5
−4
−3
−2
−1
0
1
2
3
4
5
6
7
HAZ (Stunting Z Score)
WHZ (Wasting Z Score)
WAZ (Underweight Z Score)
Severity
Extreme CGF (<−4SD)
Severe CGF (<−3SD)
Overall CGF (<−2SD)
Year
1990
1995
2000
2005
2010
2015
2020

Saint Lucia – Stunting (HAZ)

A: Overall and Severe Stunting Prevalence

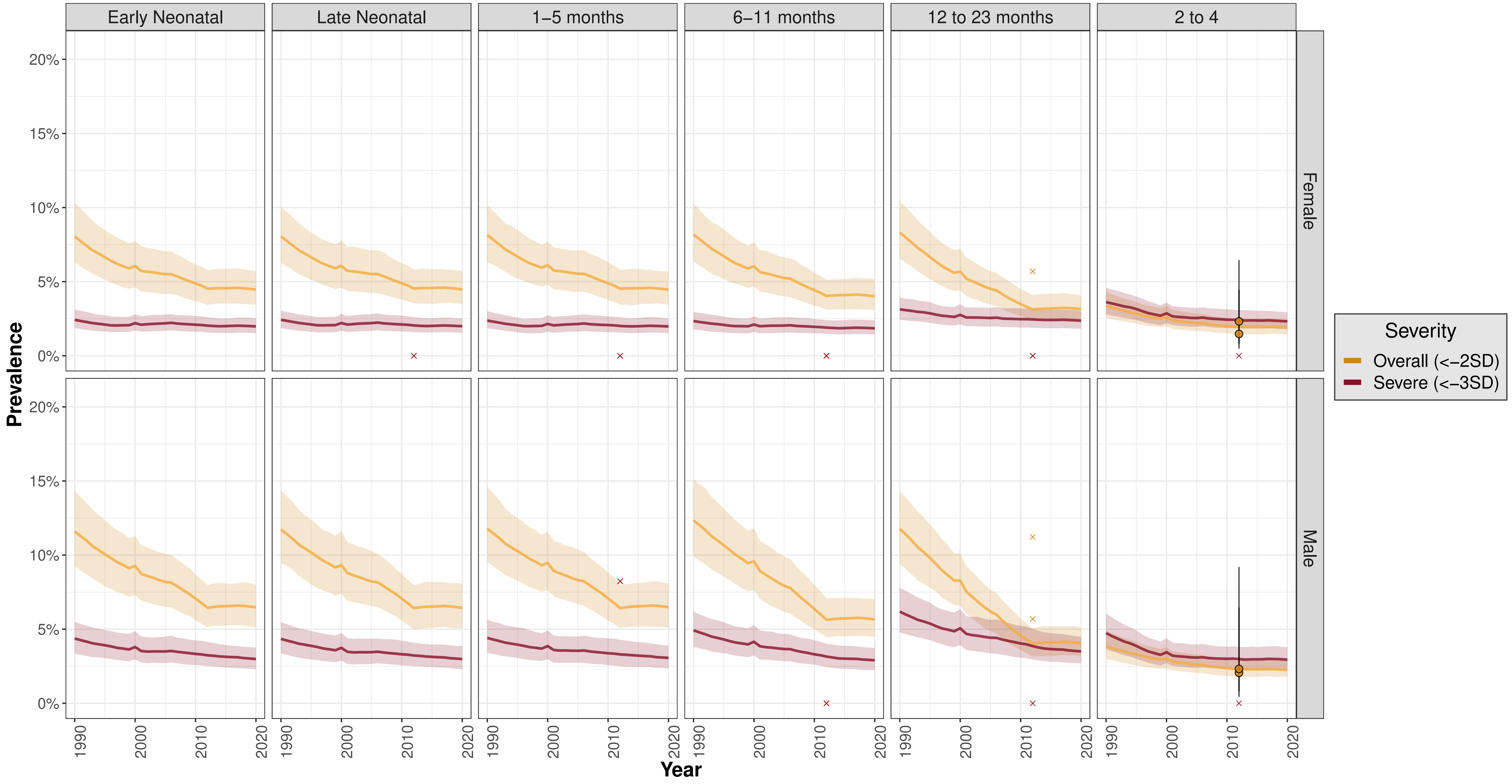

B: Transformed Mean Stunting Z Scores

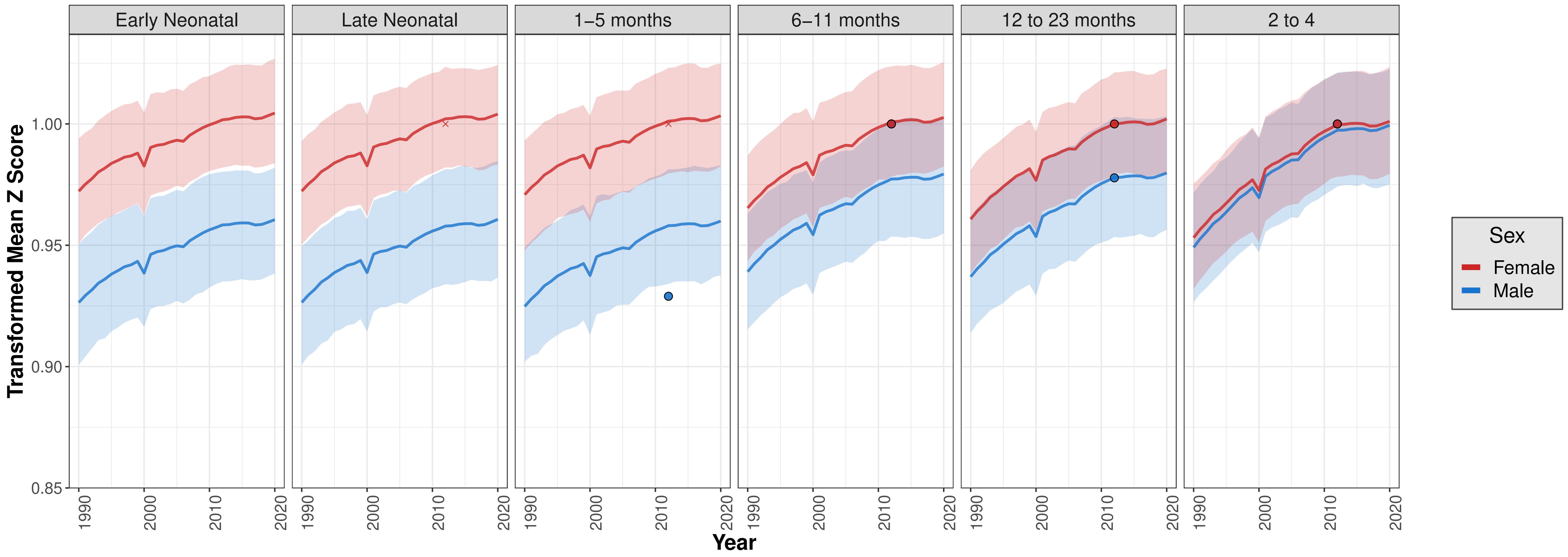

C

Year	Source
1976	WHO CGM Database
2012	WHO CGM Database
2012	MICS

Saint Lucia – Wasting (WHZ)

D: Overall and Severe Wasting Prevalence

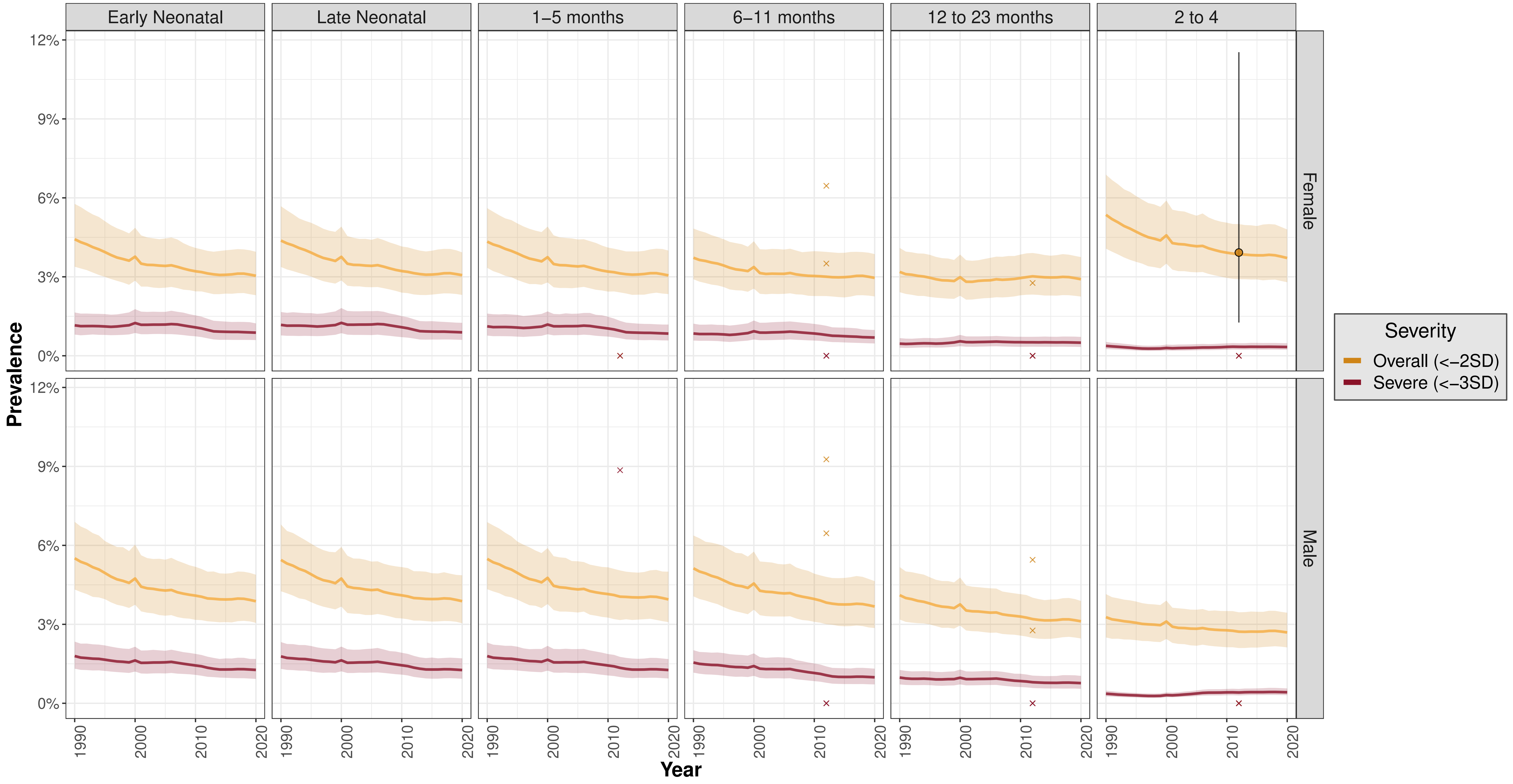

E: Transformed Mean Wasting Z Scores

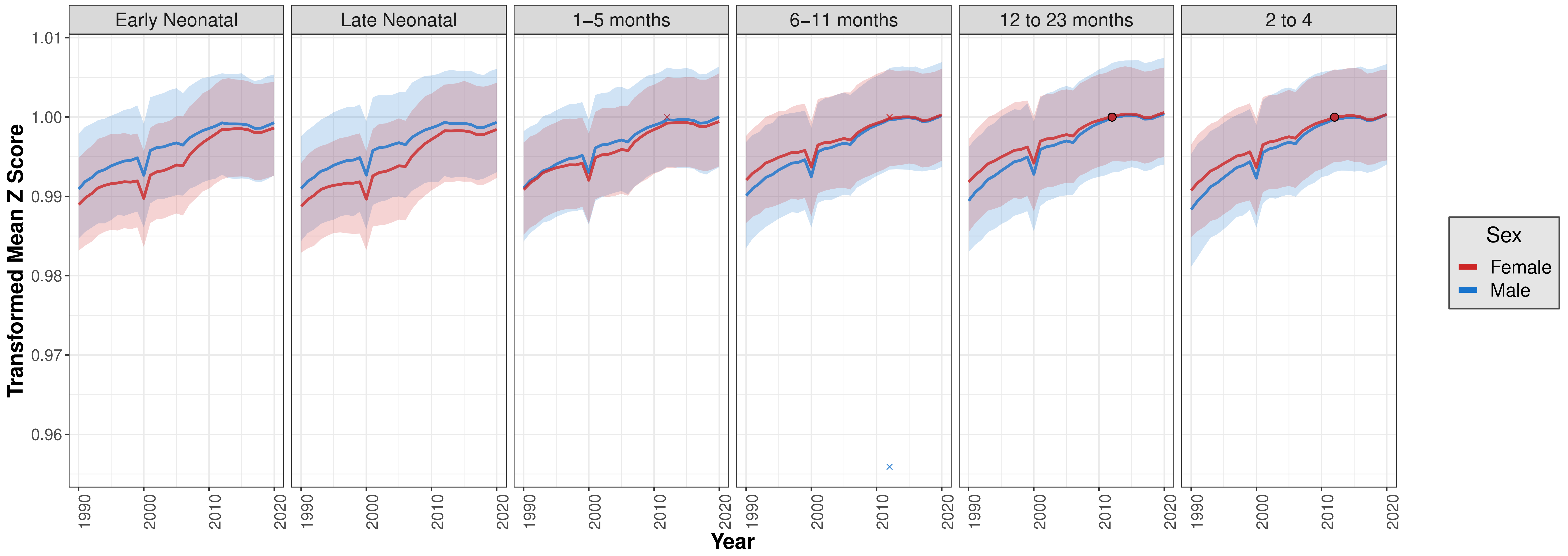

F

Year	Source
1976	WHO CGM Database
2012	WHO CGM Database
2012	MICS

Saint Lucia – Underweight (WAZ)

G: Overall and Severe Underweight Prevalence

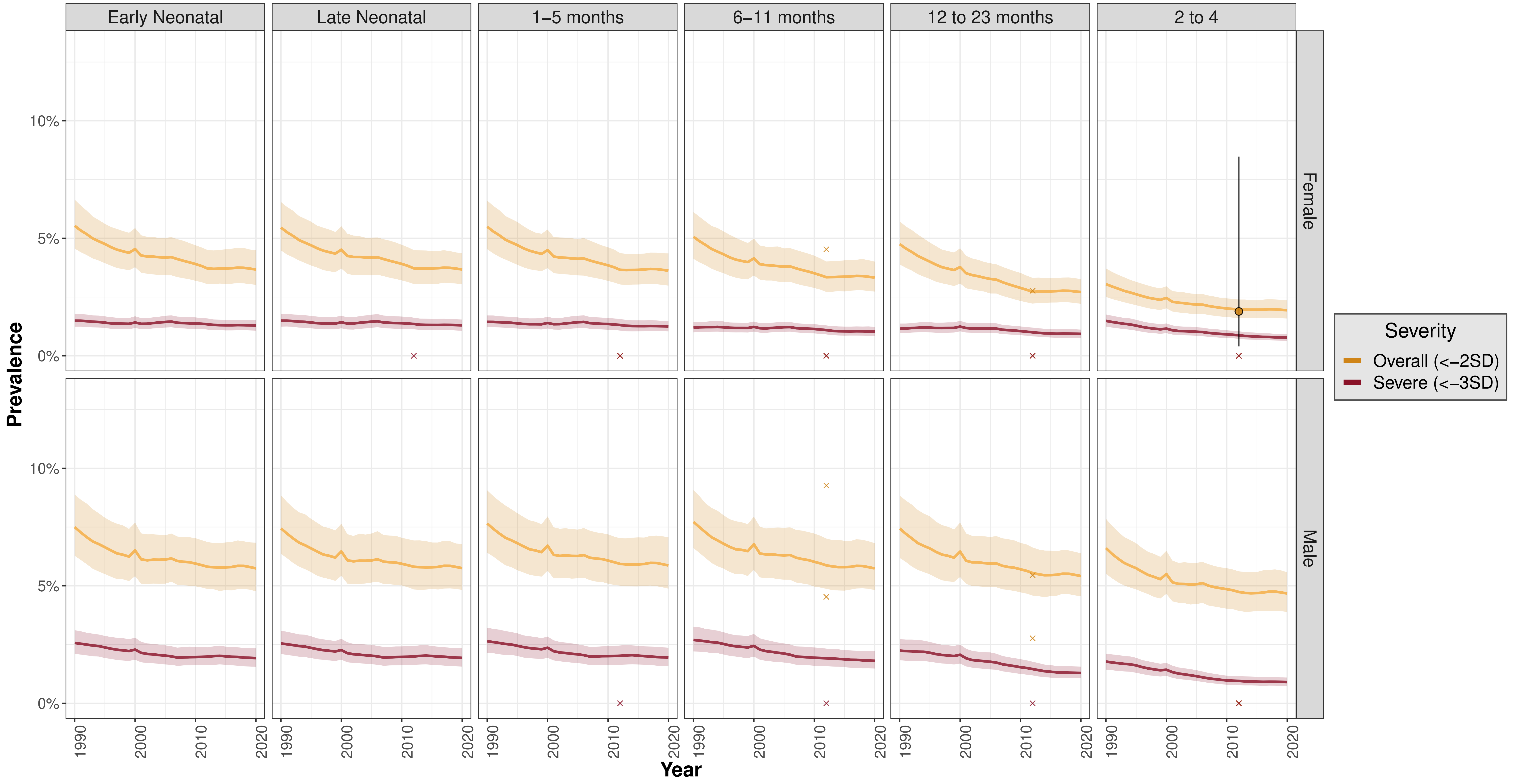

H: Transformed Mean Underweight Z Scores

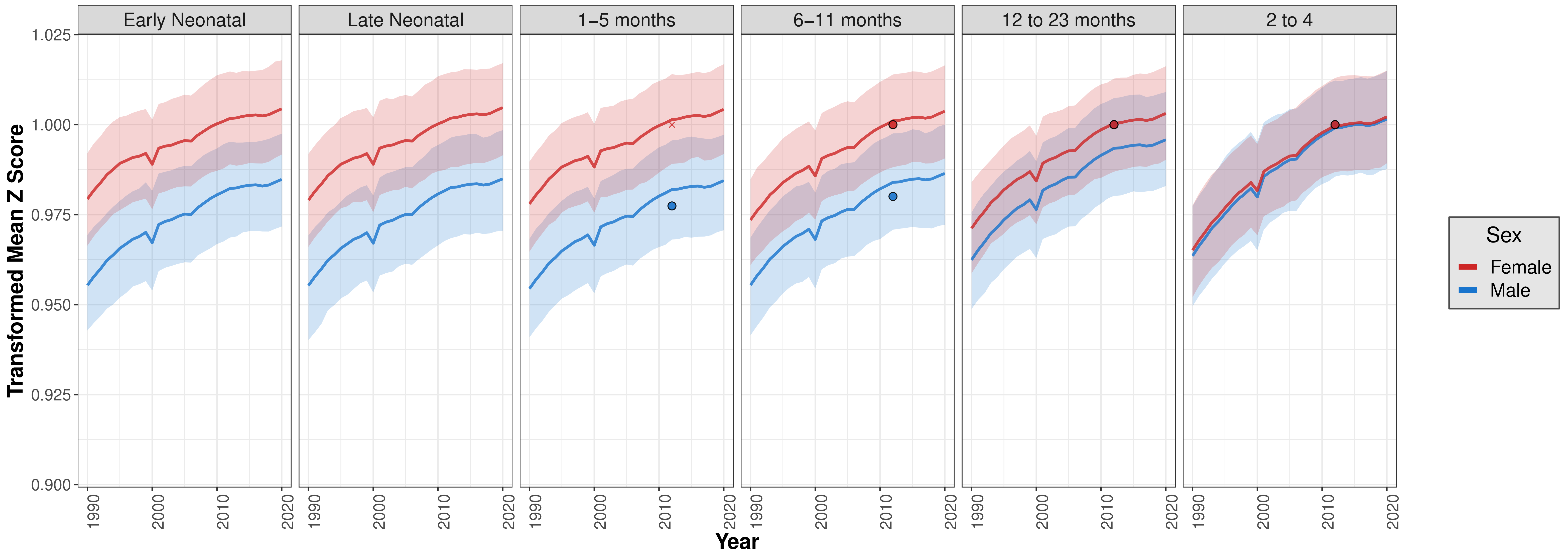

I

Year	Source
1976	WHO CGM Database
2012	WHO CGM Database
2012	MICS

Saint Lucia – HAZ, WHZ, and WAZ Distributions

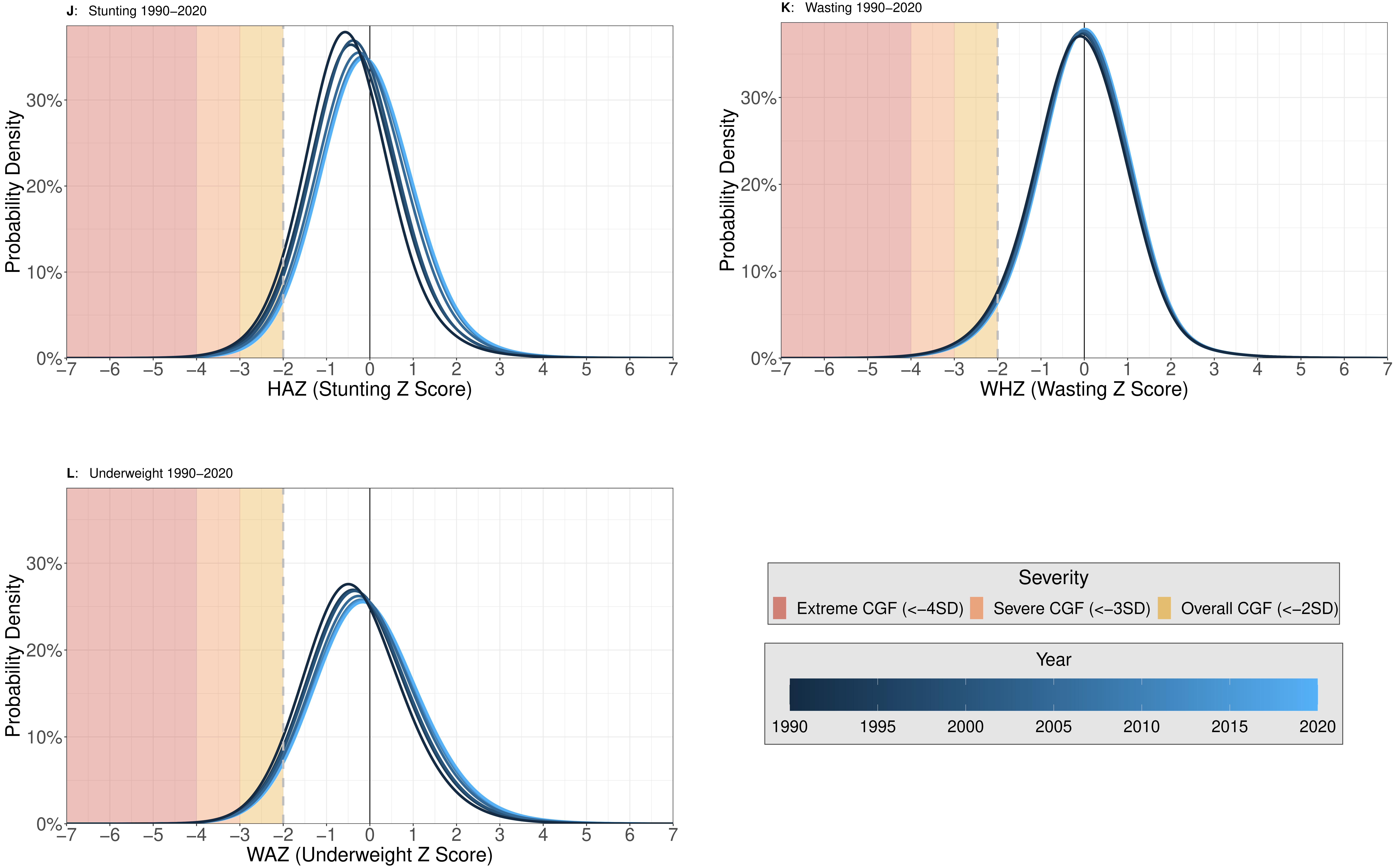

Saint Vincent and the Grenadines – Stunting (HAZ)

A: Overall and Severe Stunting Prevalence

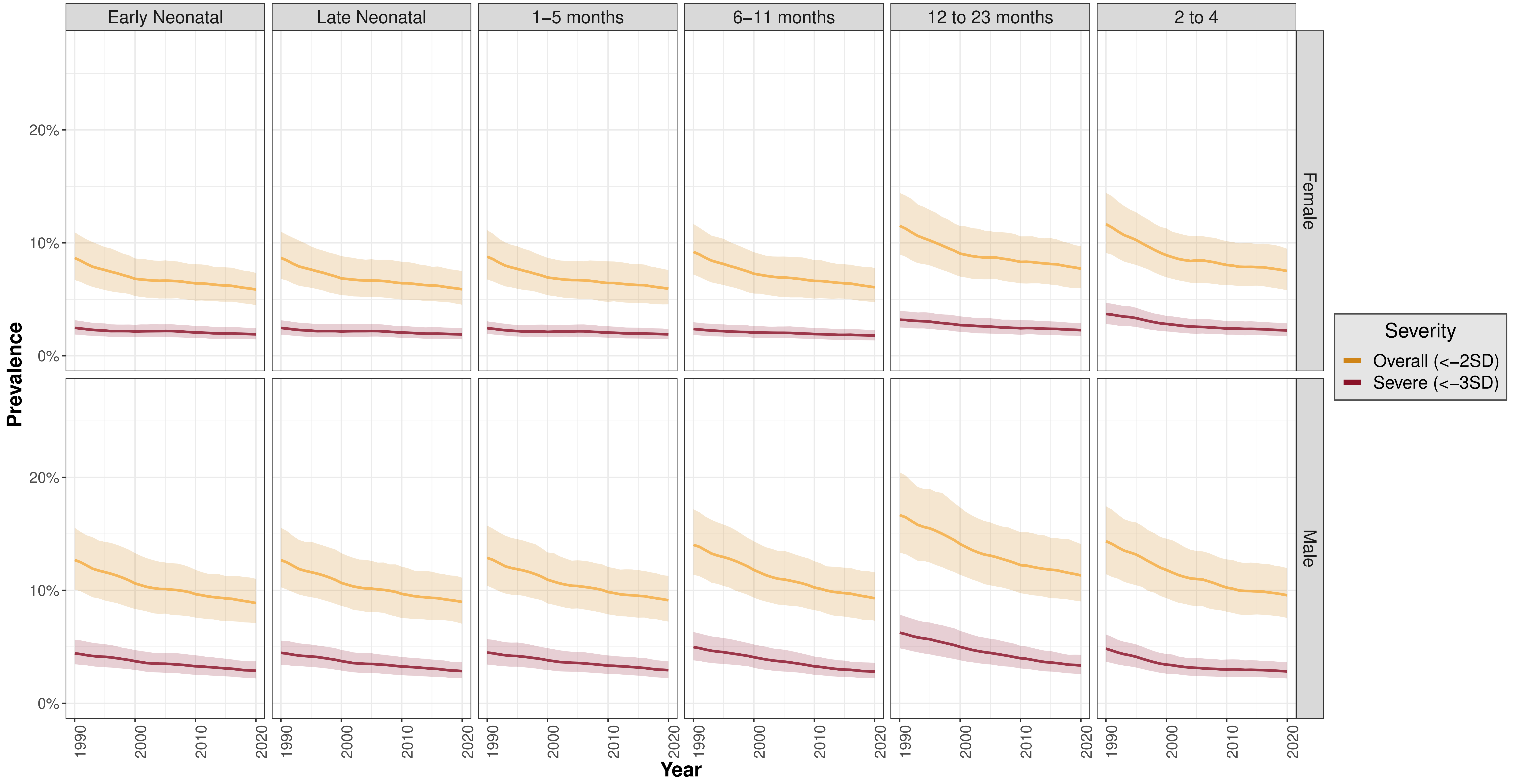

B: Transformed Mean Stunting Z Scores

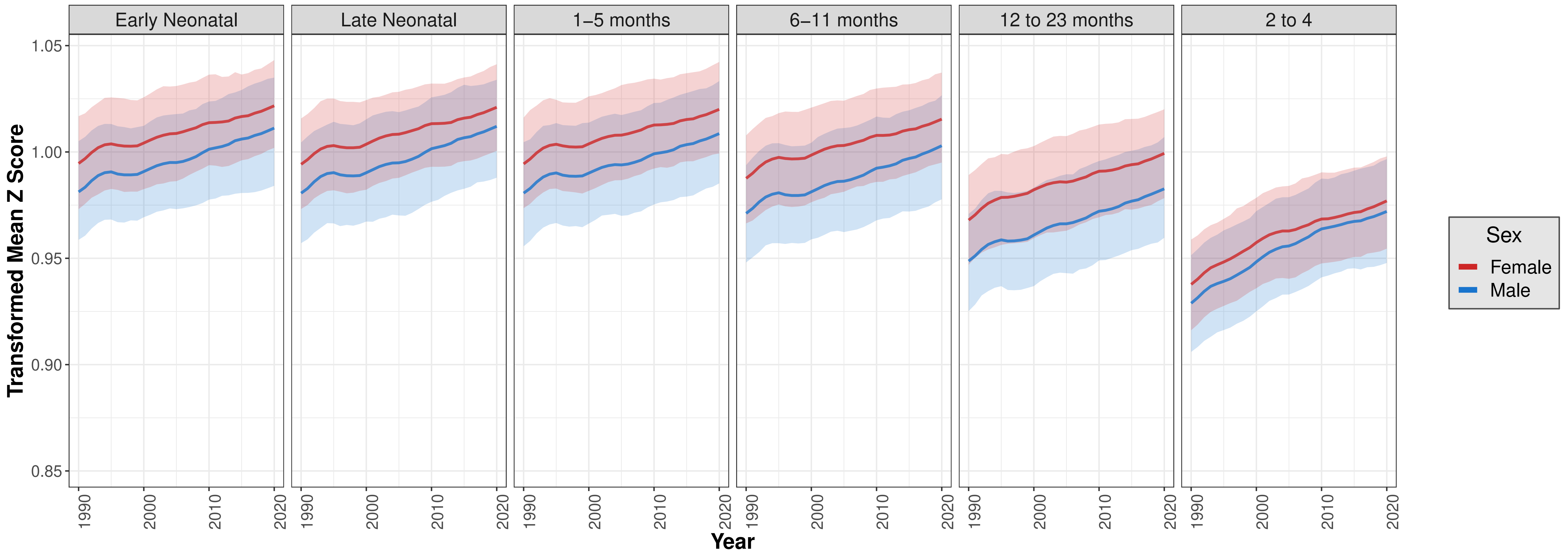

C

Source
No sources for this location

Saint Vincent and the Grenadines – Wasting (WHZ)

D: Overall and Severe Wasting Prevalence

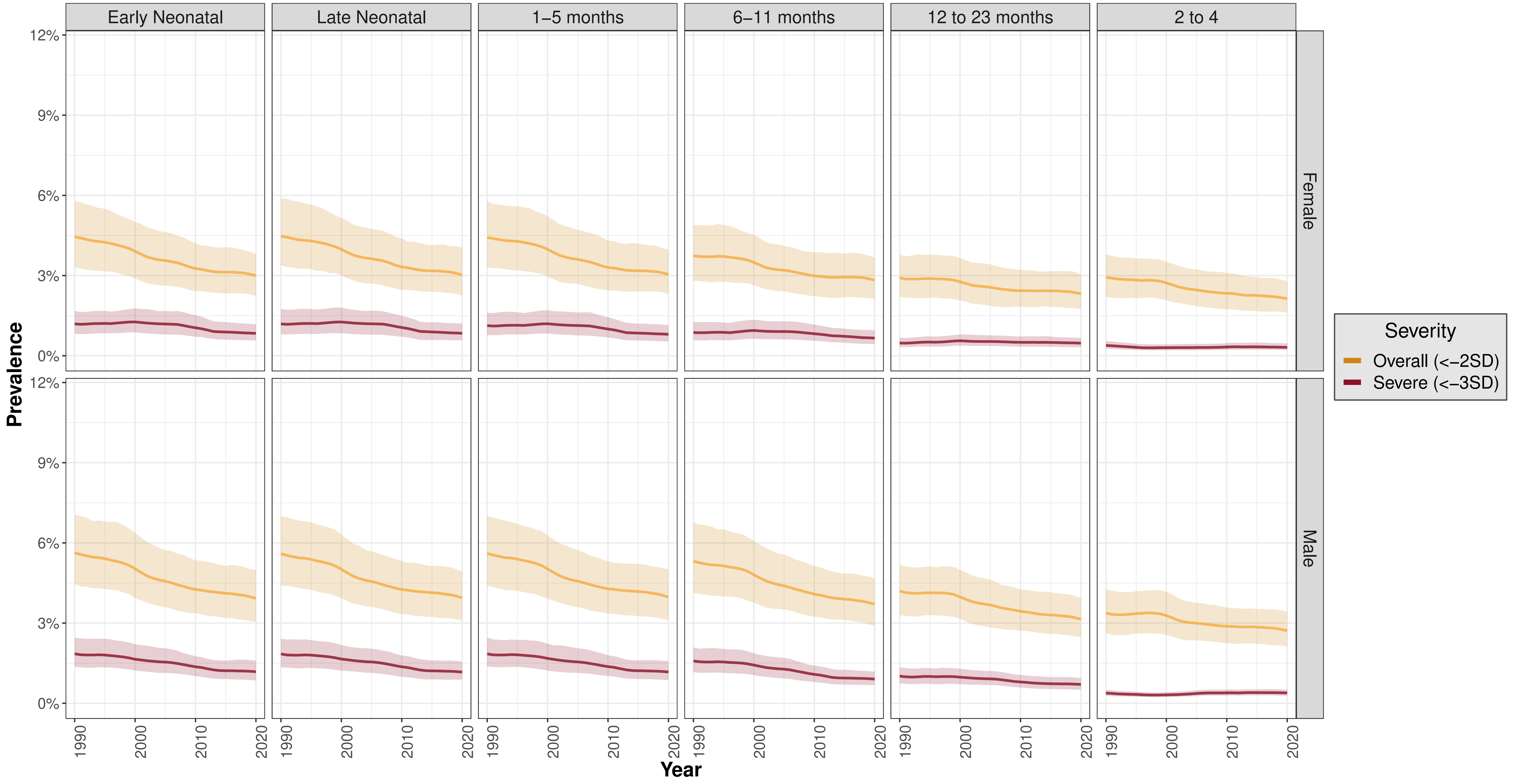

E: Transformed Mean Wasting Z Scores

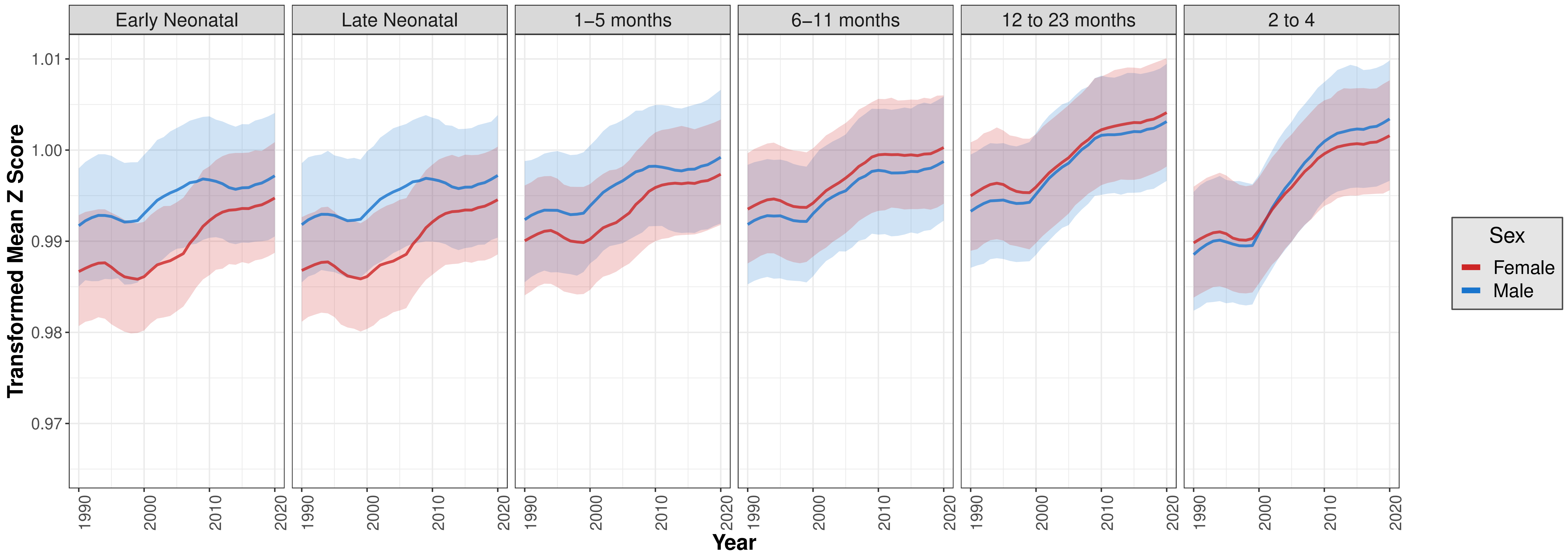

F

Source
No sources for this location

Saint Vincent and the Grenadines – Underweight (WAZ)

G: Overall and Severe Underweight Prevalence

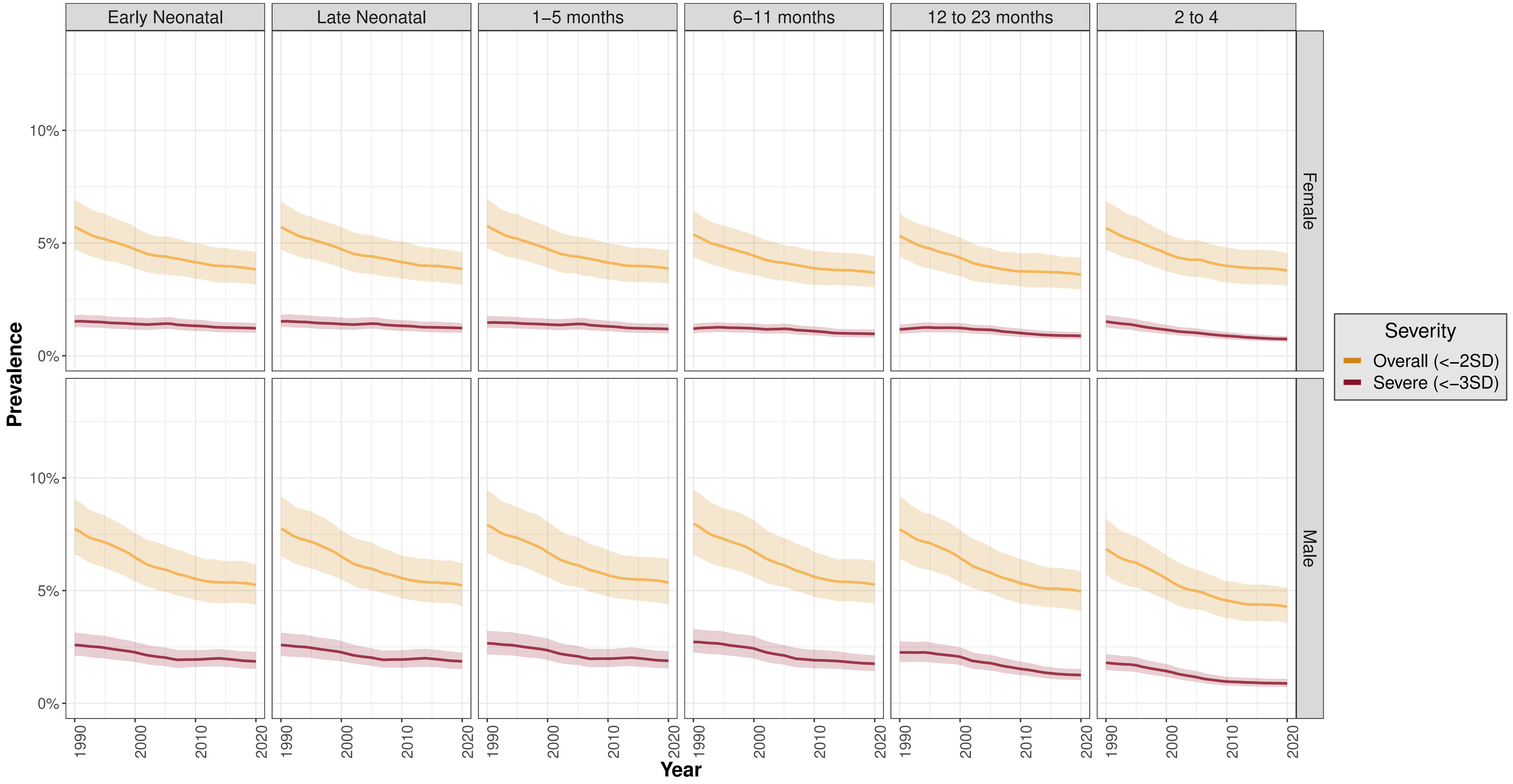

H: Transformed Mean Underweight Z Scores

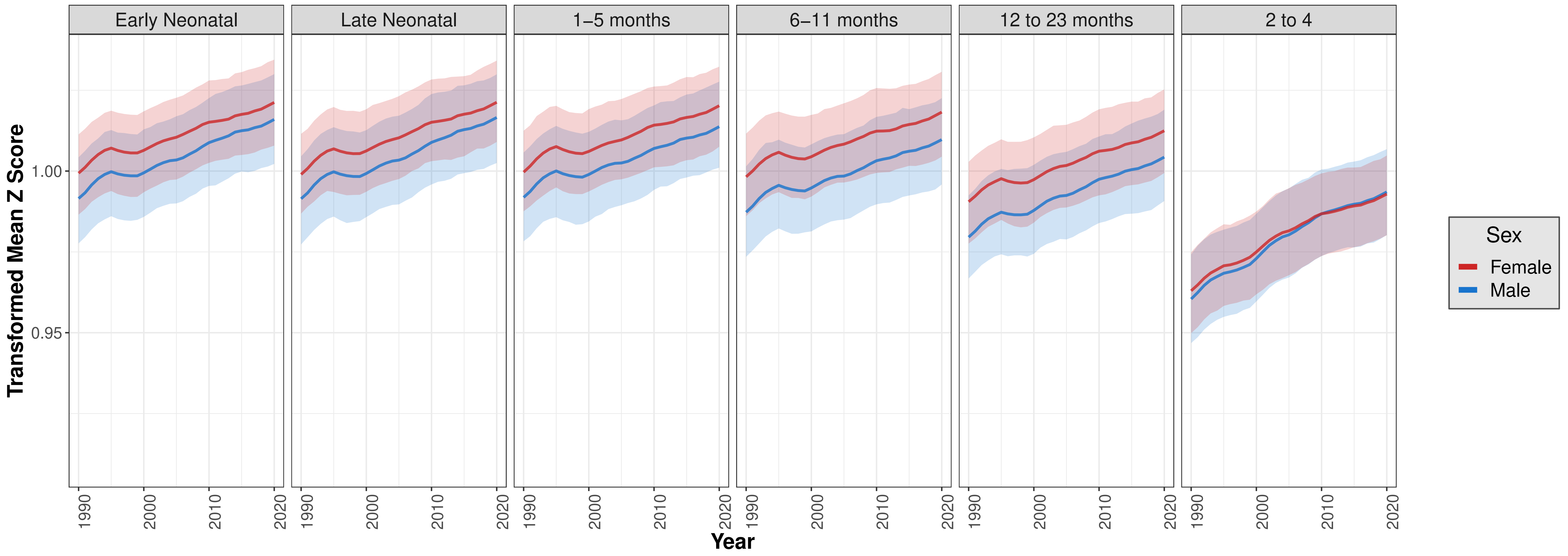

I

Source
No sources for this location

Saint Vincent and the Grenadines – HAZ, WHZ, and WAZ Distributions

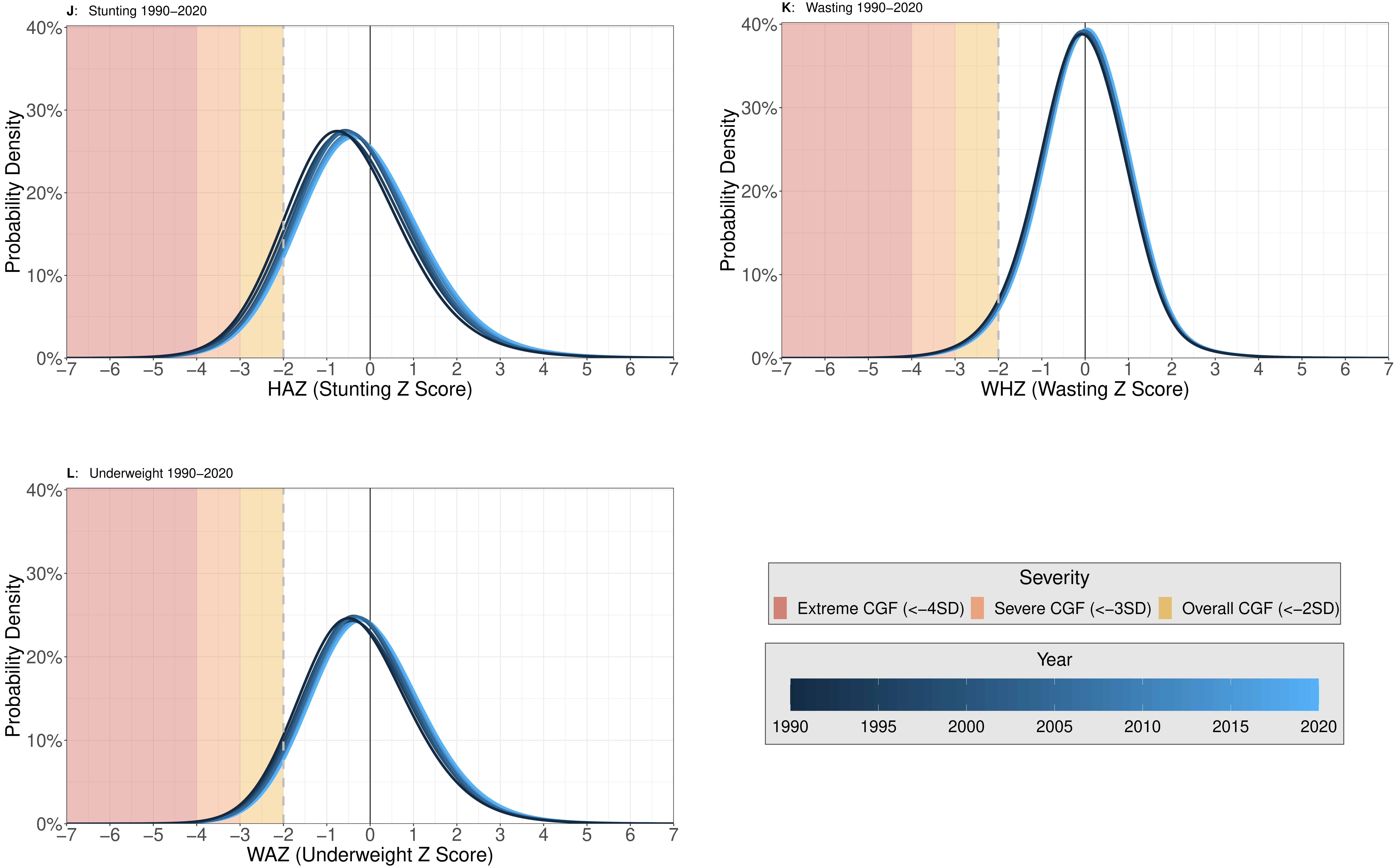

Suriname – Stunting (HAZ)

A: Overall and Severe Stunting Prevalence

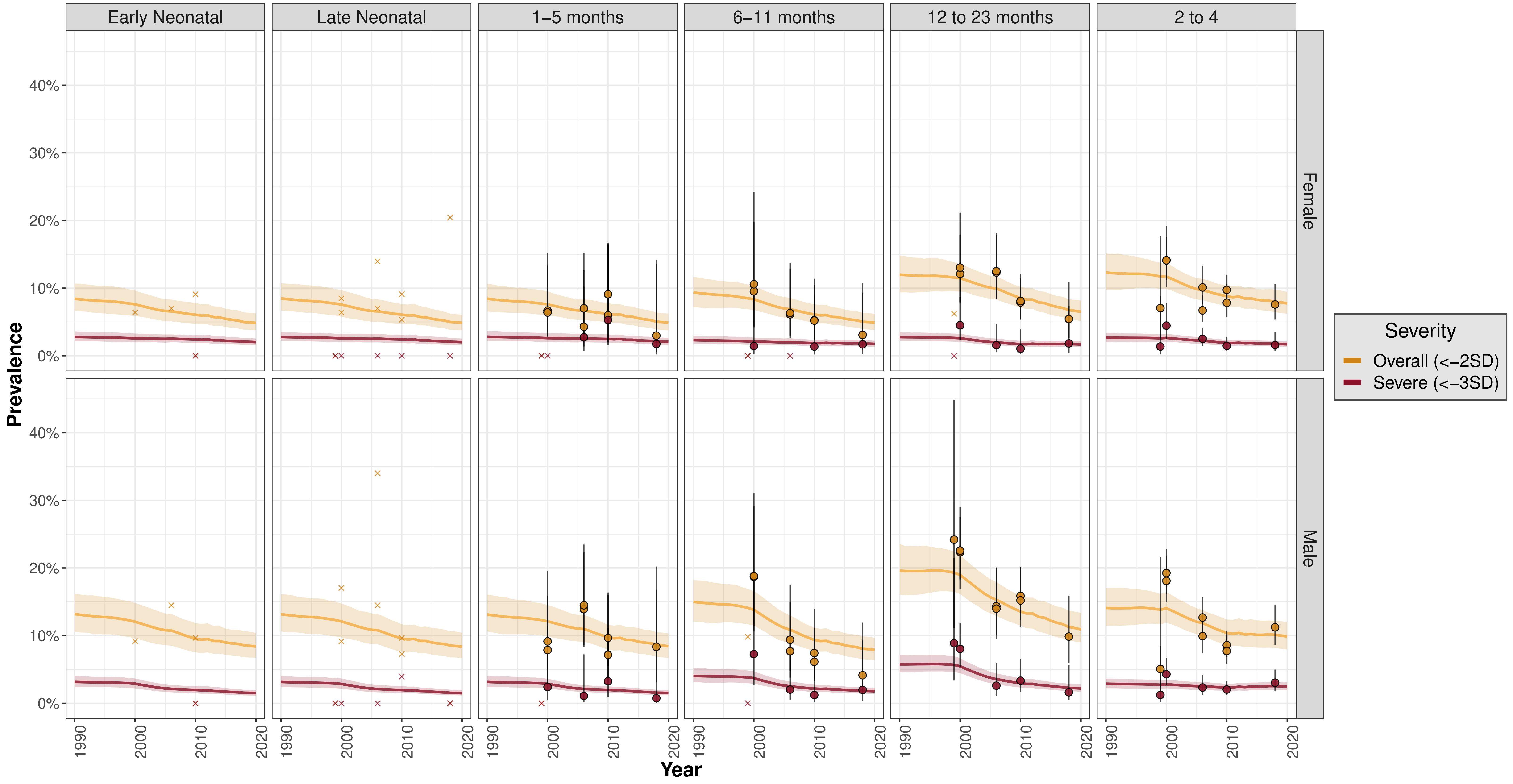

B: Transformed Mean Stunting Z Scores

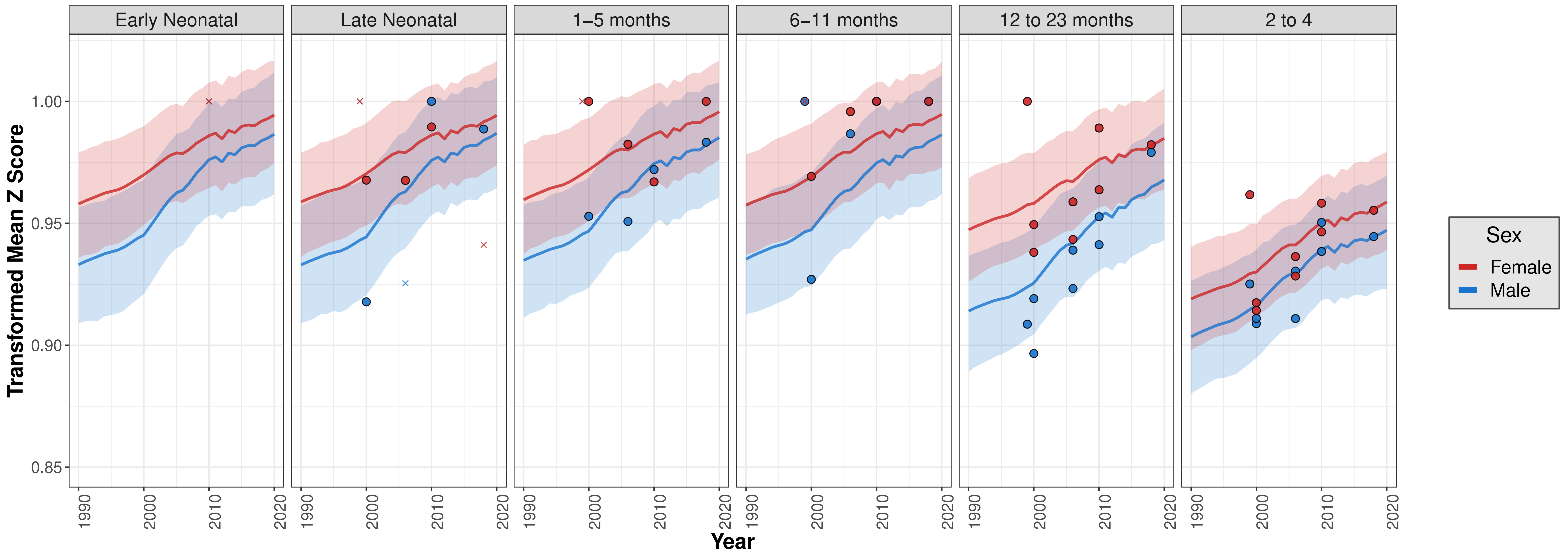

C

Year	Source
1999	MICS
2000	MICS
2000	WHO CGM Database
2006	MICS
2006	WHO CGM Database
2010	MICS
2010	WHO CGM Database
2018	MICS

Suriname – Wasting (WHZ)

D: Overall and Severe Wasting Prevalence

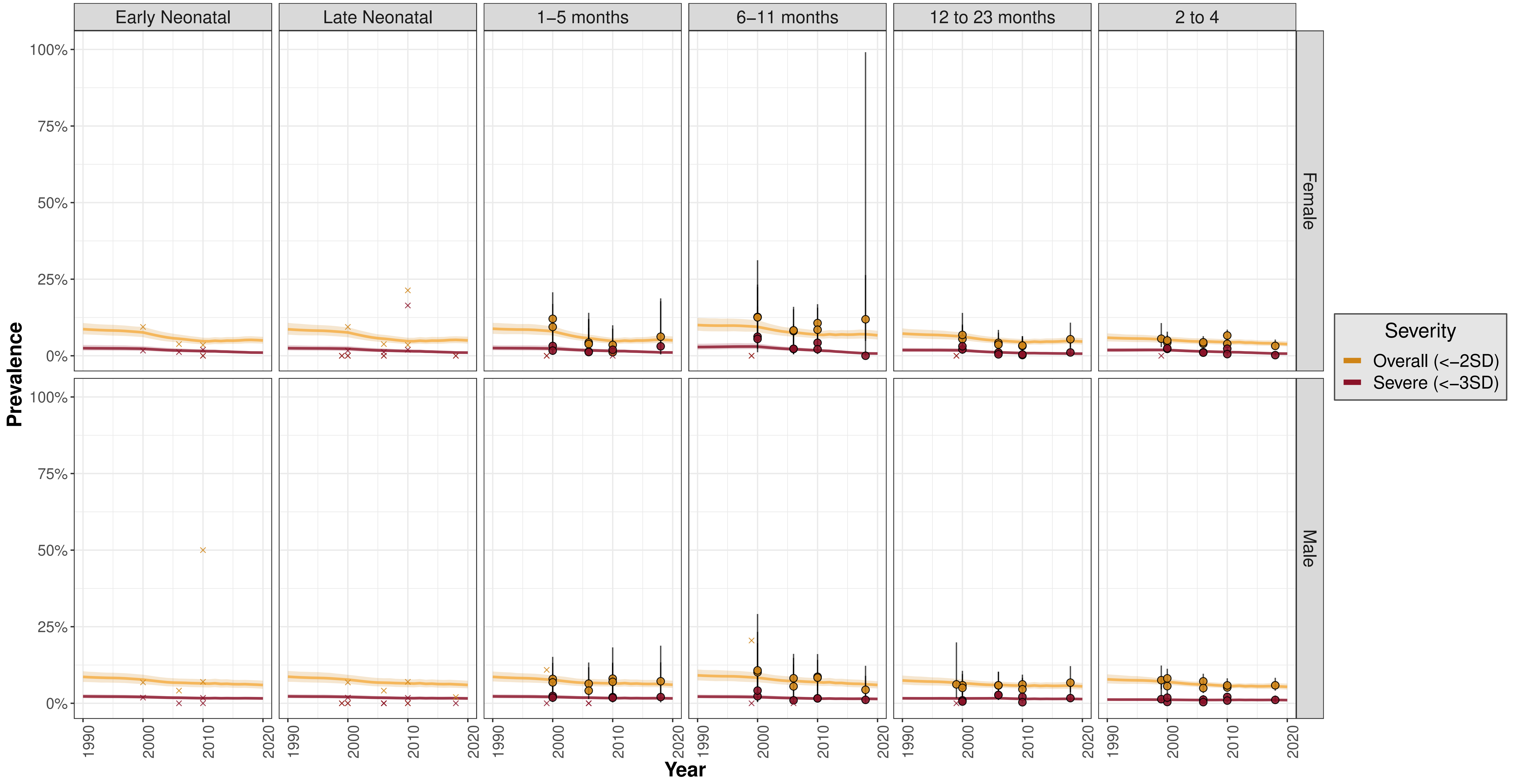

E: Transformed Mean Wasting Z Scores

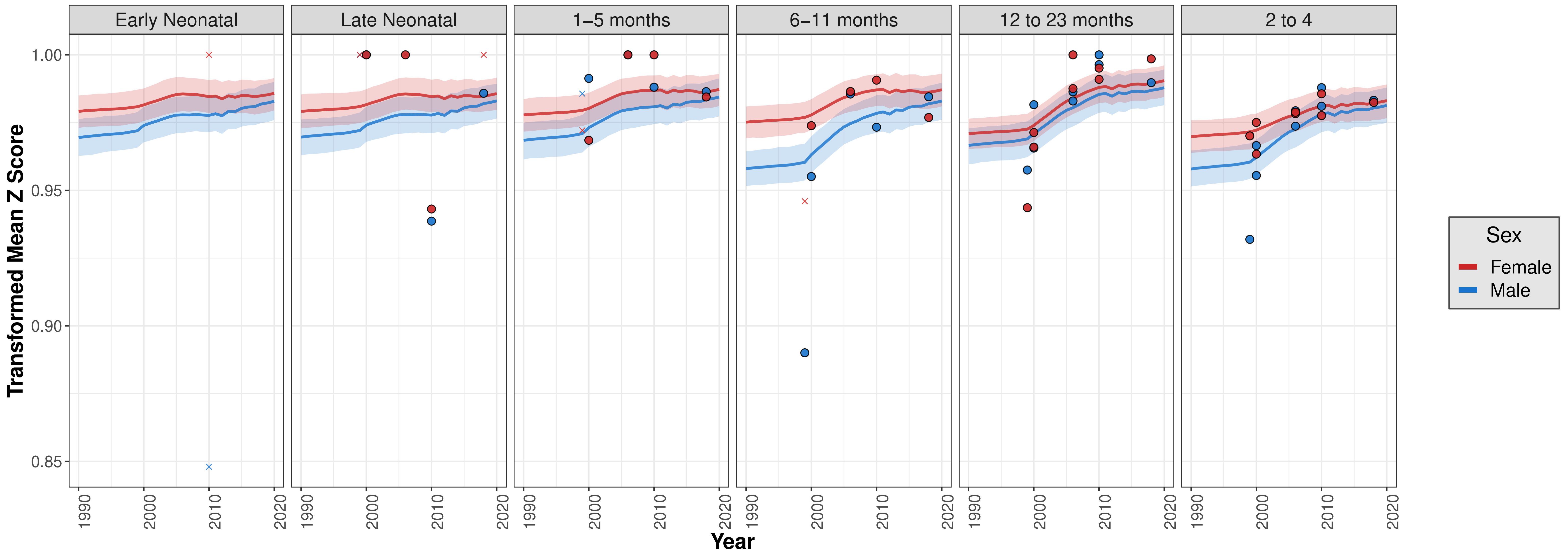

F

Year	Source
1999	MICS
2000	MICS
2000	WHO CGM Database
2006	MICS
2006	WHO CGM Database
2010	MICS
2010	WHO CGM Database
2018	MICS

Suriname – Underweight (WAZ)

G: Overall and Severe Underweight Prevalence

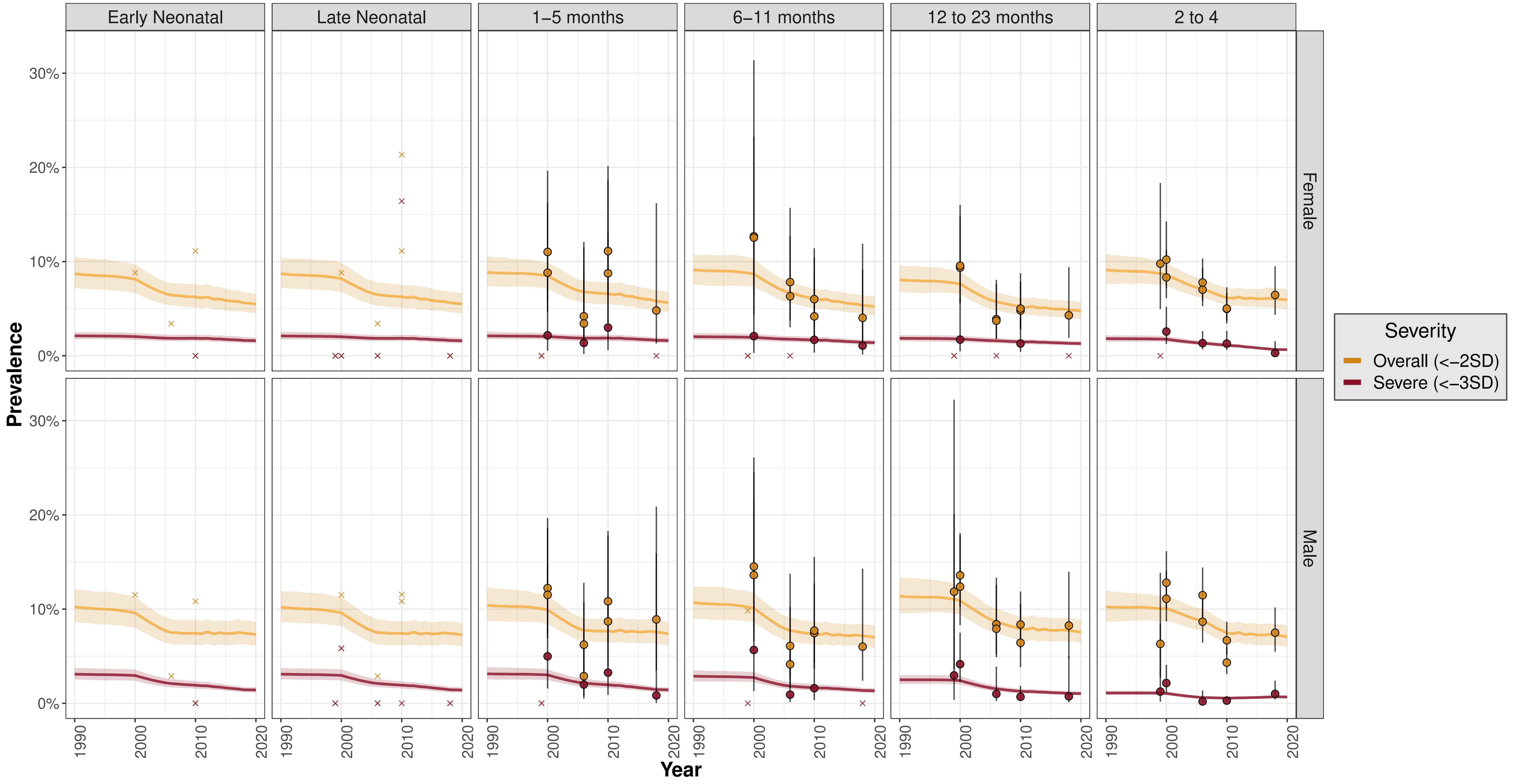

H: Transformed Mean Underweight Z Scores

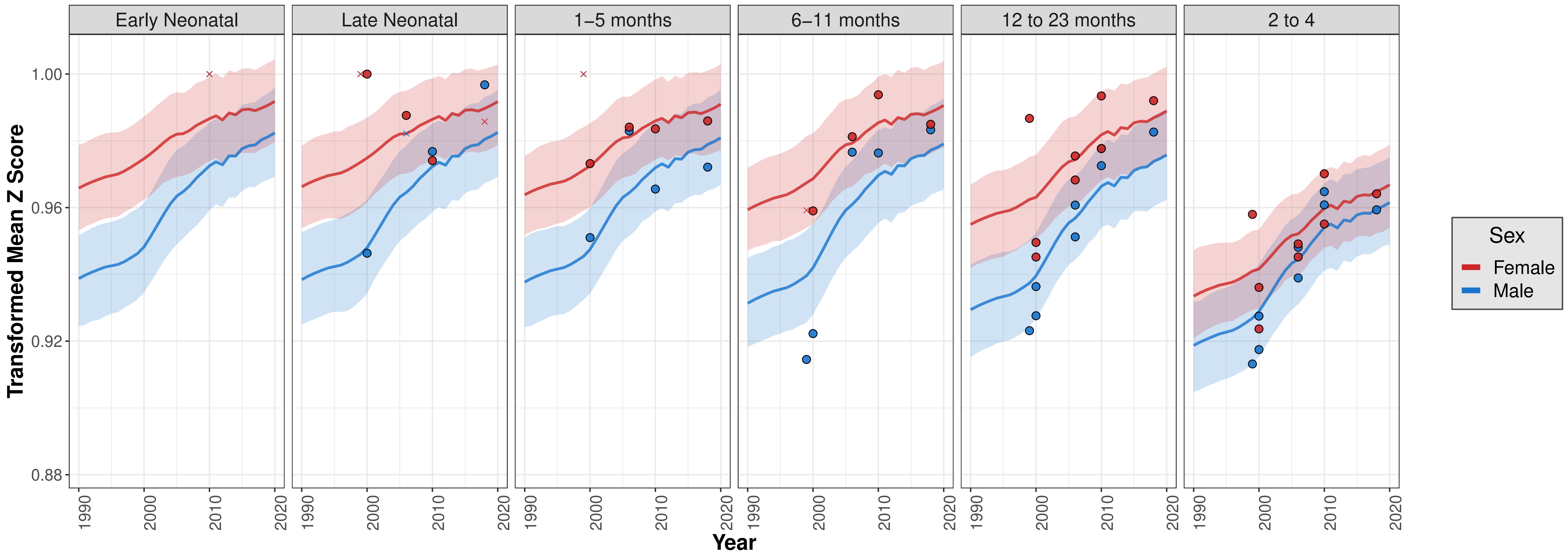

I

Year	Source
1999	MICS
2000	MICS
2000	WHO CGM Database
2006	MICS
2006	WHO CGM Database
2010	MICS
2010	WHO CGM Database
2018	MICS

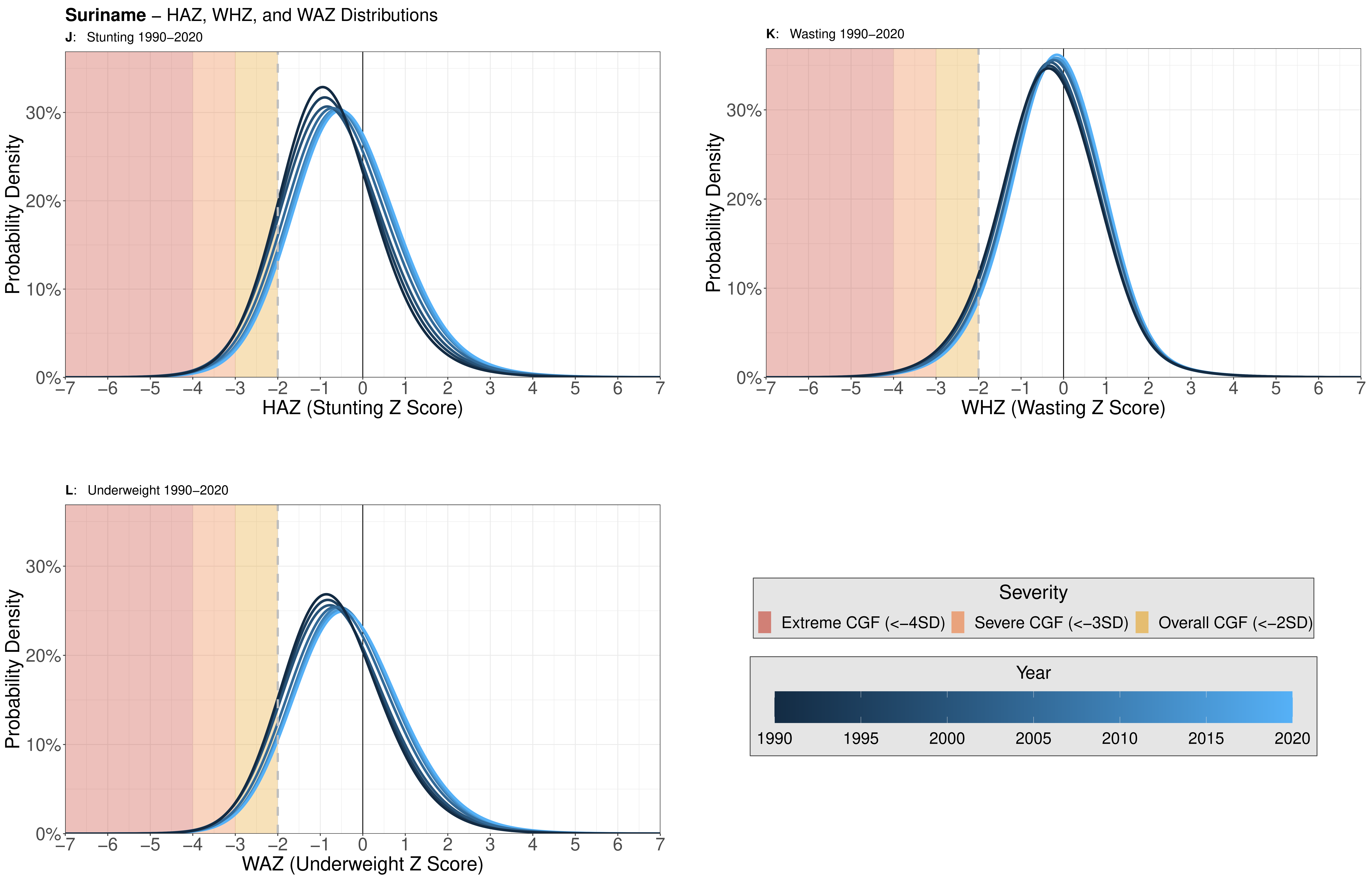
Suriname – HAZ, WHZ, and WAZ Distributions
J: Stunting 1990–2020
K: Wasting 1990–2020
L: Underweight 1990–2020
Probability Density
0%
10%
20%
30%
−7 −6 −5 −4 −3 −2 −1 0 1 2 3 4 5 6 7
HAZ (Stunting Z Score)
WHZ (Wasting Z Score)
WAZ (Underweight Z Score)
Severity
Extreme CGF (<−4SD)
Severe CGF (<−3SD)
Overall CGF (<−2SD)
Year
1990
1995
2000
2005
2010
2015
2020

Trinidad and Tobago – Stunting (HAZ)

A: Overall and Severe Stunting Prevalence

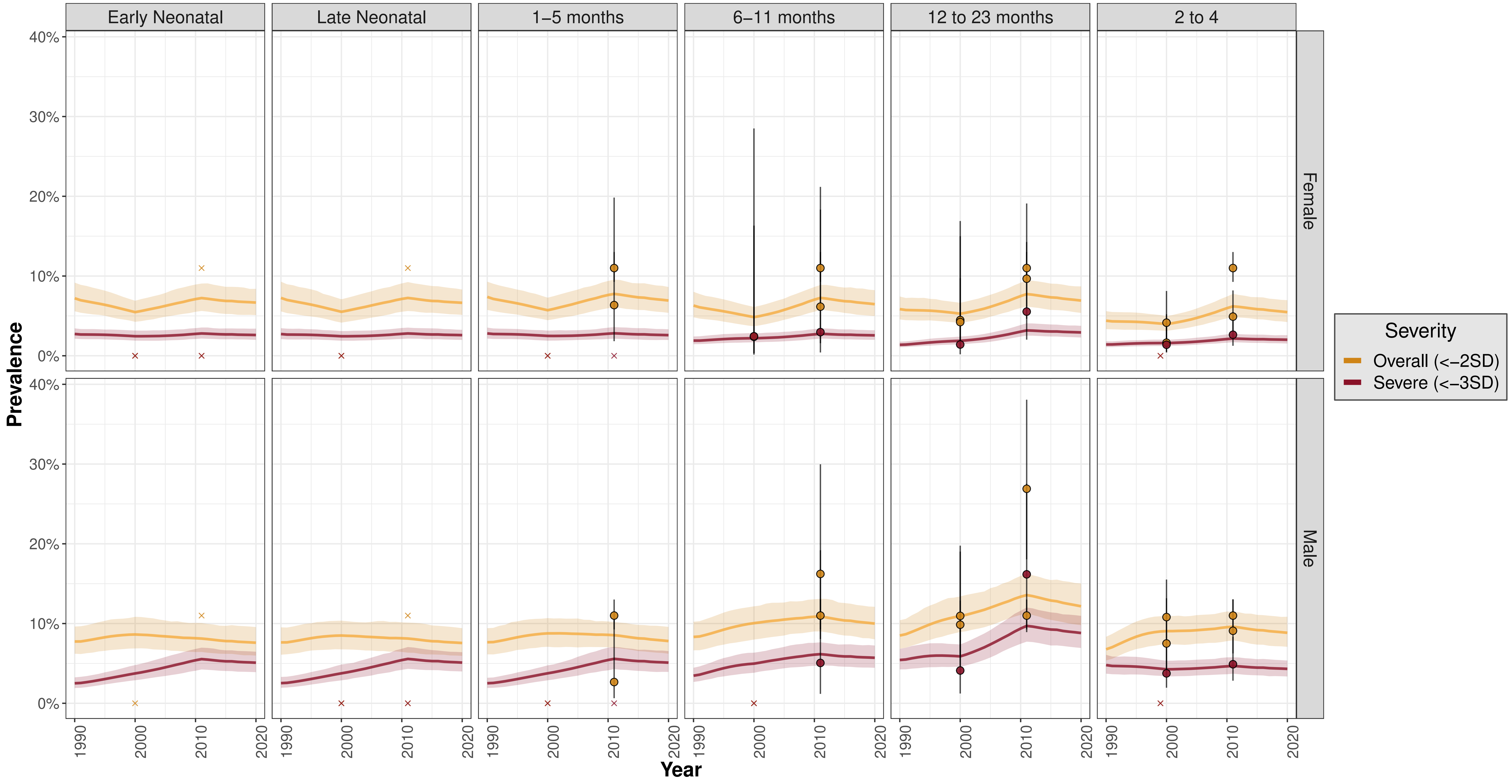

B: Transformed Mean Stunting Z Scores

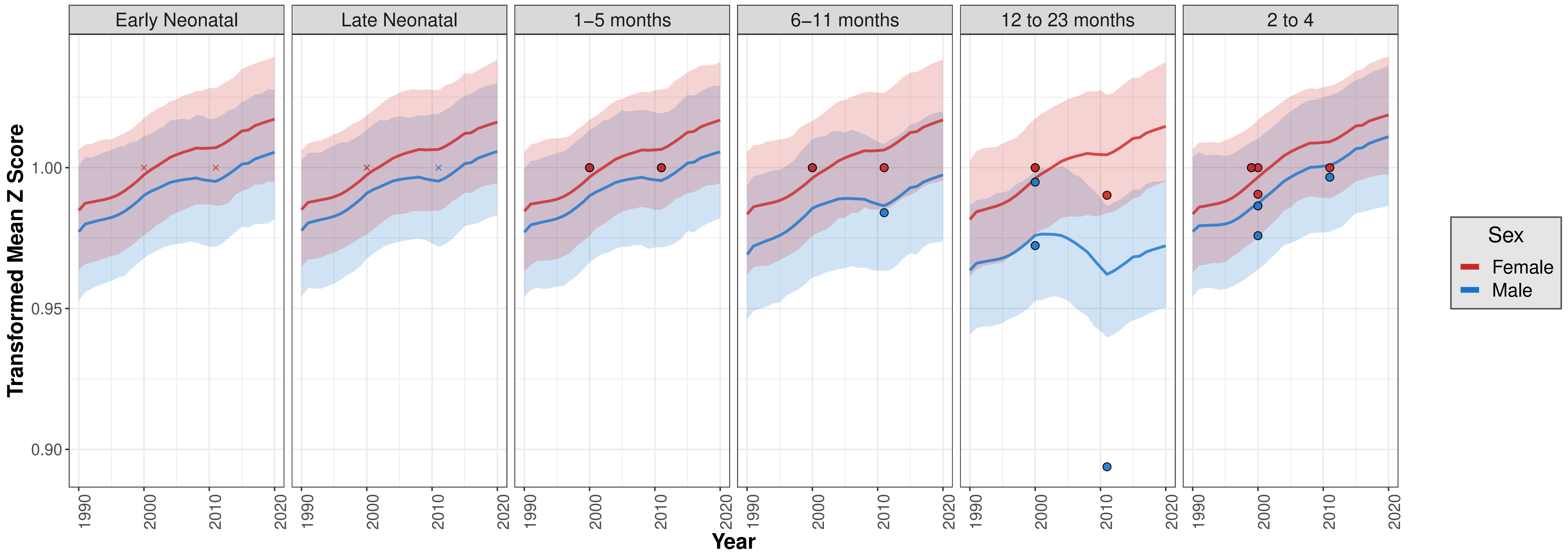

C

Year	Source
1976	WHO CGM Database
1987	DHS
1987	WHO CGM Database
1999	Child Health Survey
2000	MICS
2000	WHO CGM Database
2011	WHO CGM Database
2011	MICS

Trinidad and Tobago – Wasting (WHZ)

D: Overall and Severe Wasting Prevalence

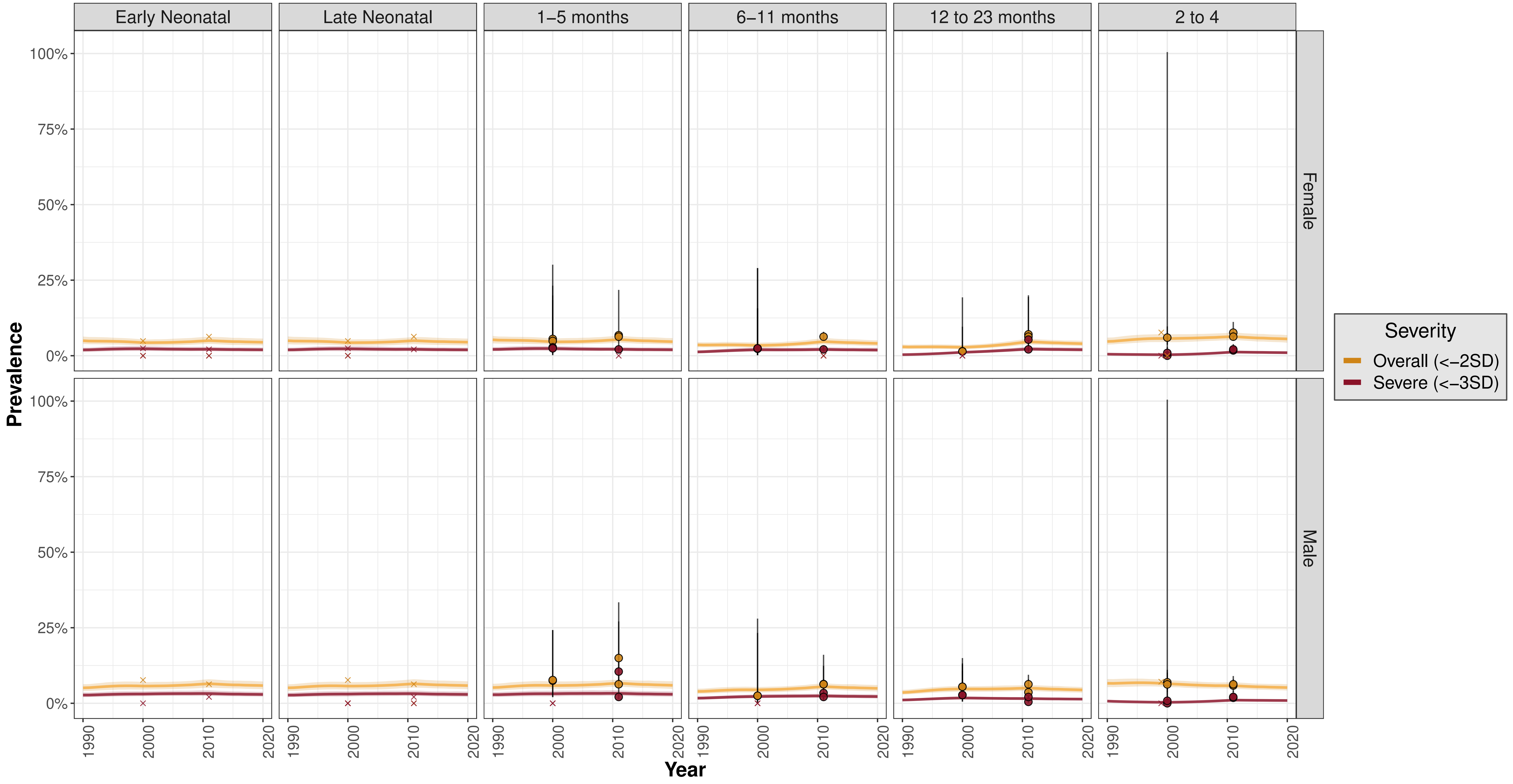

E: Transformed Mean Wasting Z Scores

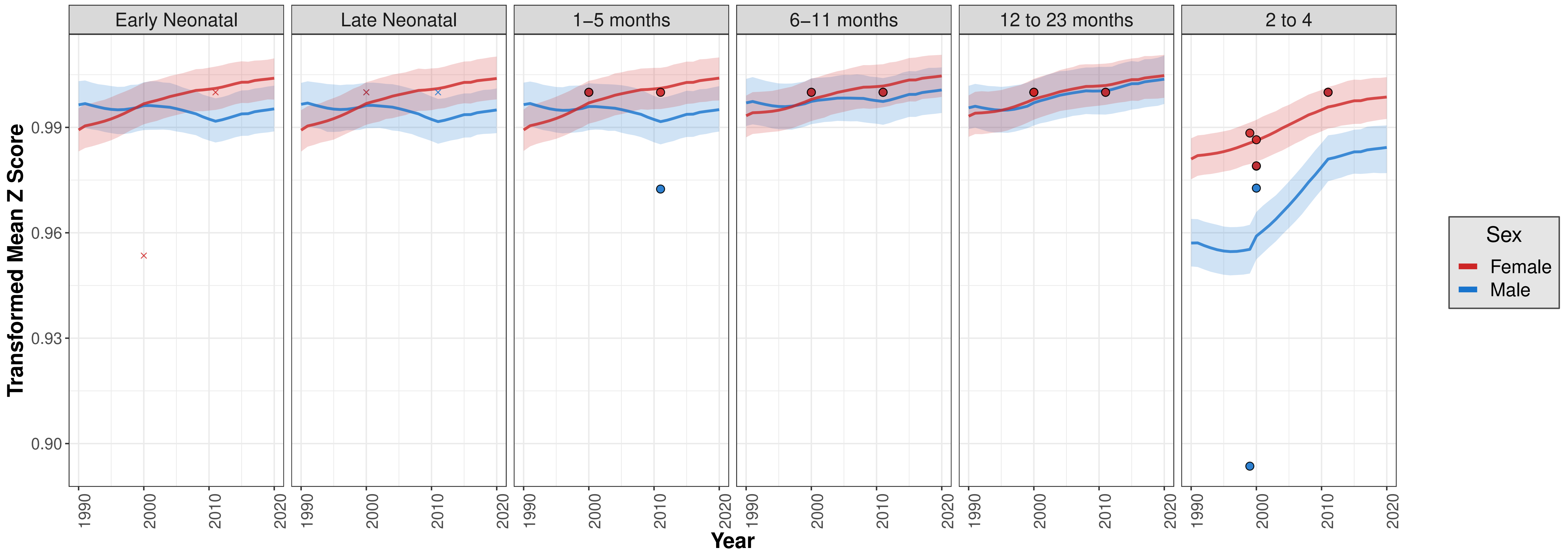

F

Year	Source
1976	WHO CGM Database
1987	DHS
1987	WHO CGM Database
1999	Child Health Survey
2000	MICS
2000	WHO CGM Database
2011	WHO CGM Database
2011	MICS

Trinidad and Tobago – Underweight (WAZ)

G: Overall and Severe Underweight Prevalence

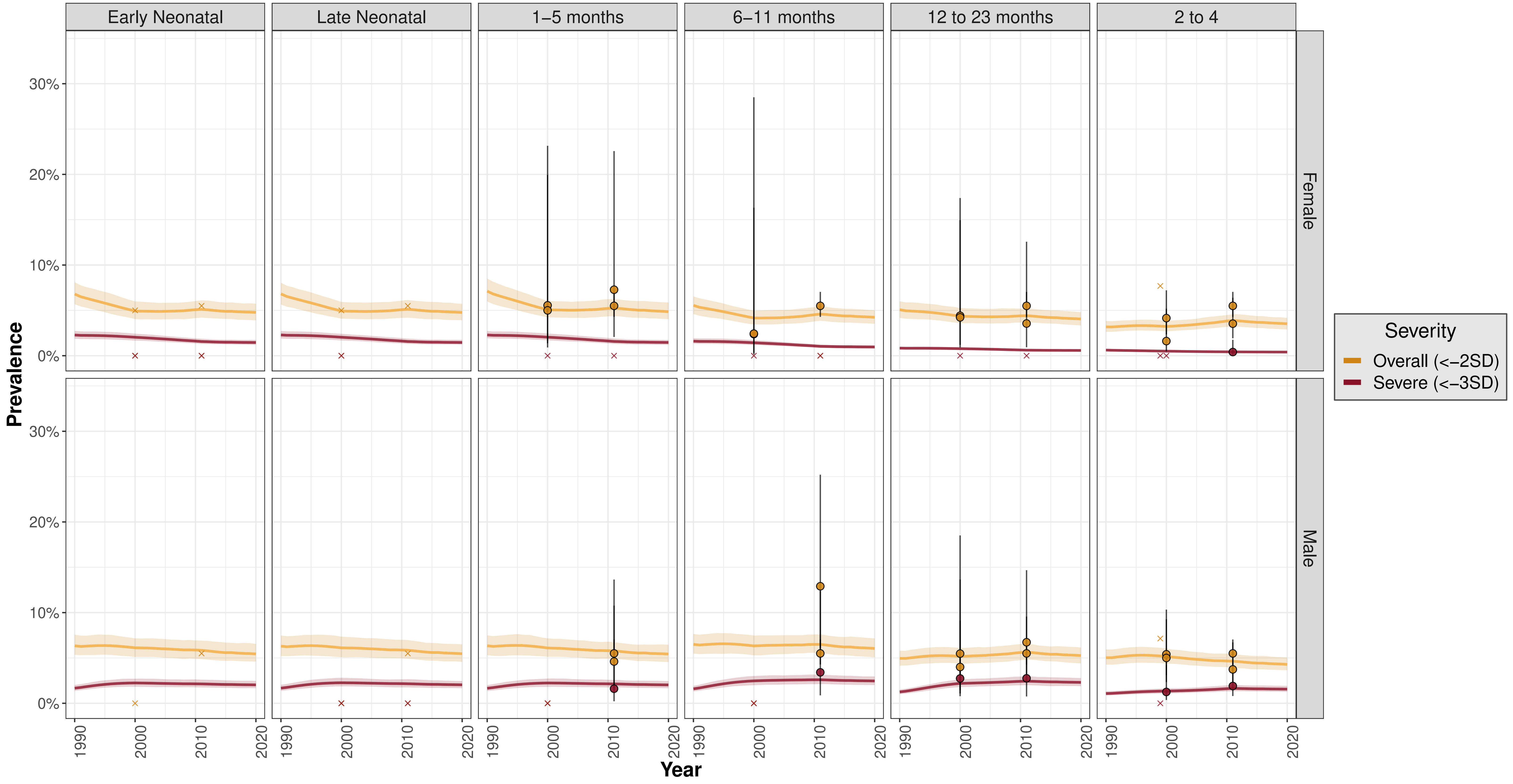

H: Transformed Mean Underweight Z Scores

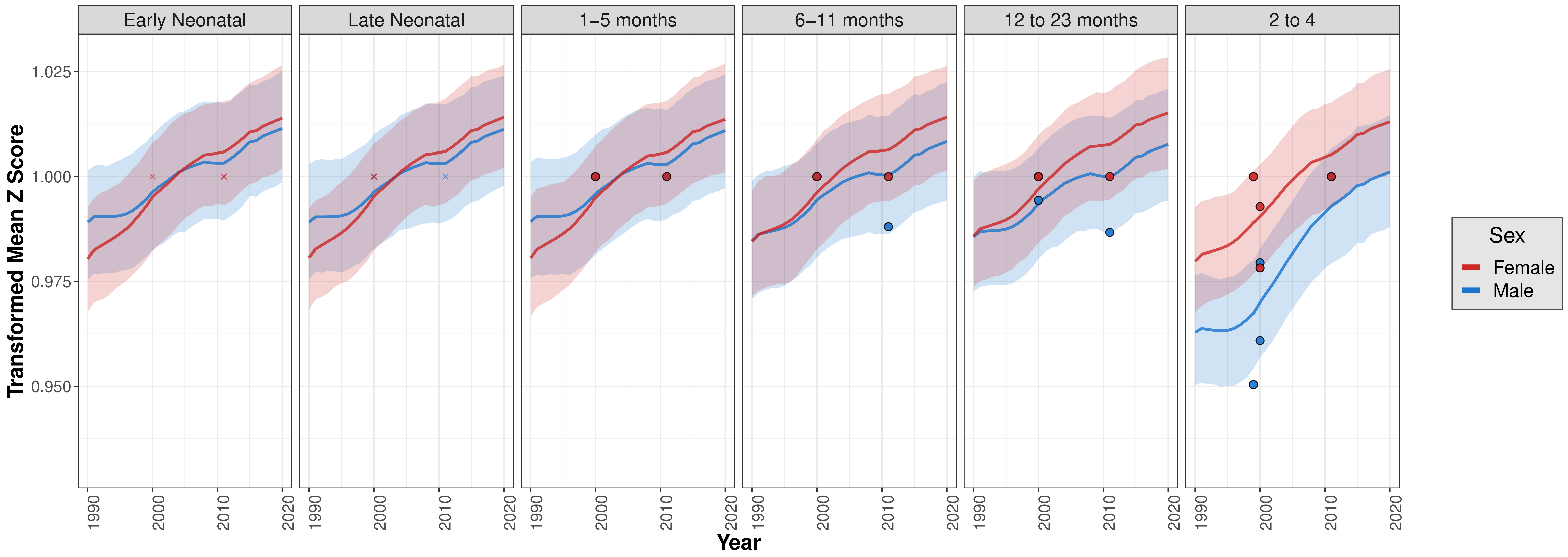

I

Year	Source
1976	WHO CGM Database
1987	DHS
1987	WHO CGM Database
1999	Child Health Survey
2000	MICS
2000	WHO CGM Database
2011	WHO CGM Database
2011	MICS

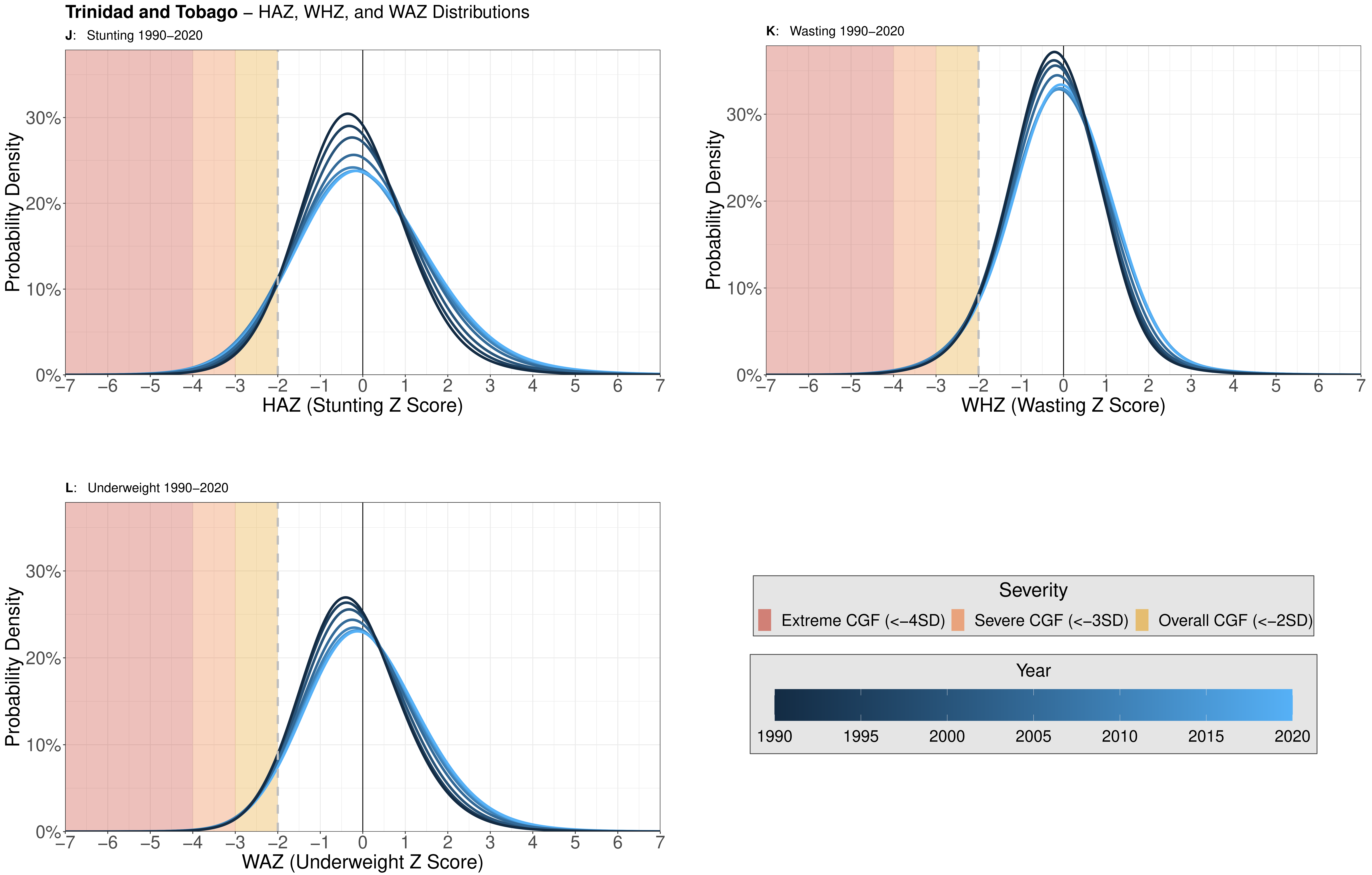

Trinidad and Tobago – HAZ, WHZ, and WAZ Distributions
J: Stunting 1990–2020
Probability Density
30%
20%
10%
0%
−7 −6 −5 −4 −3 −2 −1 0 1 2 3 4 5 6 7
HAZ (Stunting Z Score)
K: Wasting 1990–2020
Probability Density
30%
20%
10%
0%
−7 −6 −5 −4 −3 −2 −1 0 1 2 3 4 5 6 7
WHZ (Wasting Z Score)
L: Underweight 1990–2020
Probability Density
30%
20%
10%
0%
−7 −6 −5 −4 −3 −2 −1 0 1 2 3 4 5 6 7
WAZ (Underweight Z Score)
Severity
Extreme CGF (<−4SD)
Severe CGF (<−3SD)
Overall CGF (<−2SD)
Year
1990
1995
2000
2005
2010
2015
2020

Bermuda – Stunting (HAZ)

A: Overall and Severe Stunting Prevalence

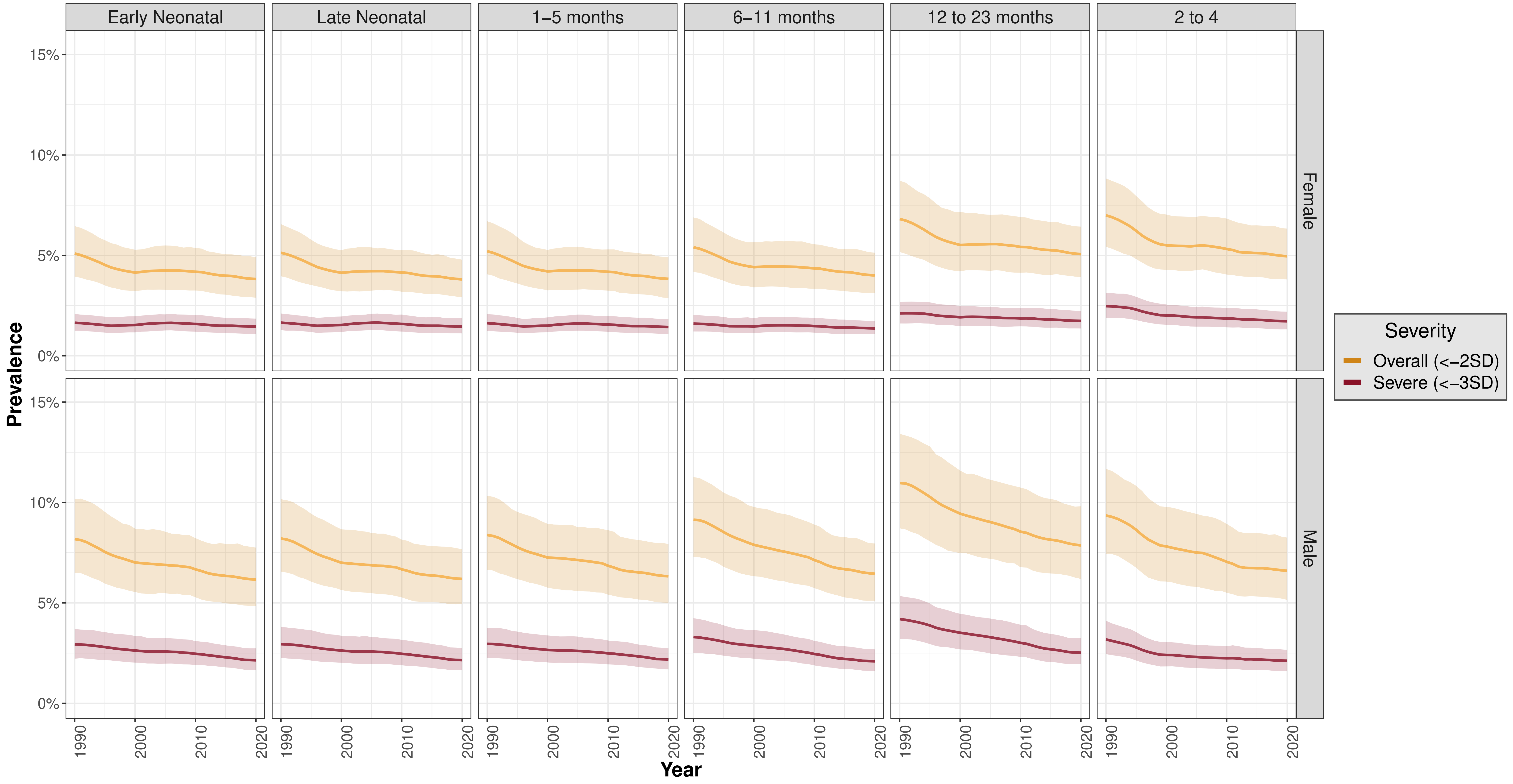

B: Transformed Mean Stunting Z Scores

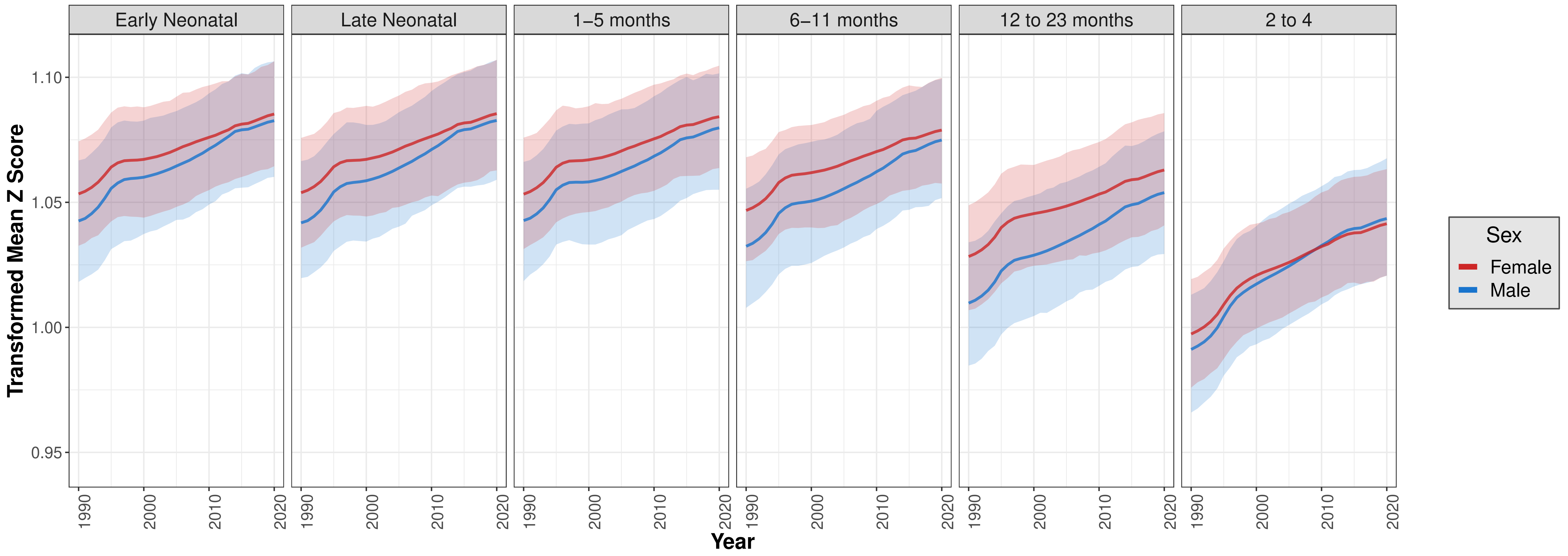

C

Source
No sources for this location

Bermuda – Wasting (WHZ)

D: Overall and Severe Wasting Prevalence

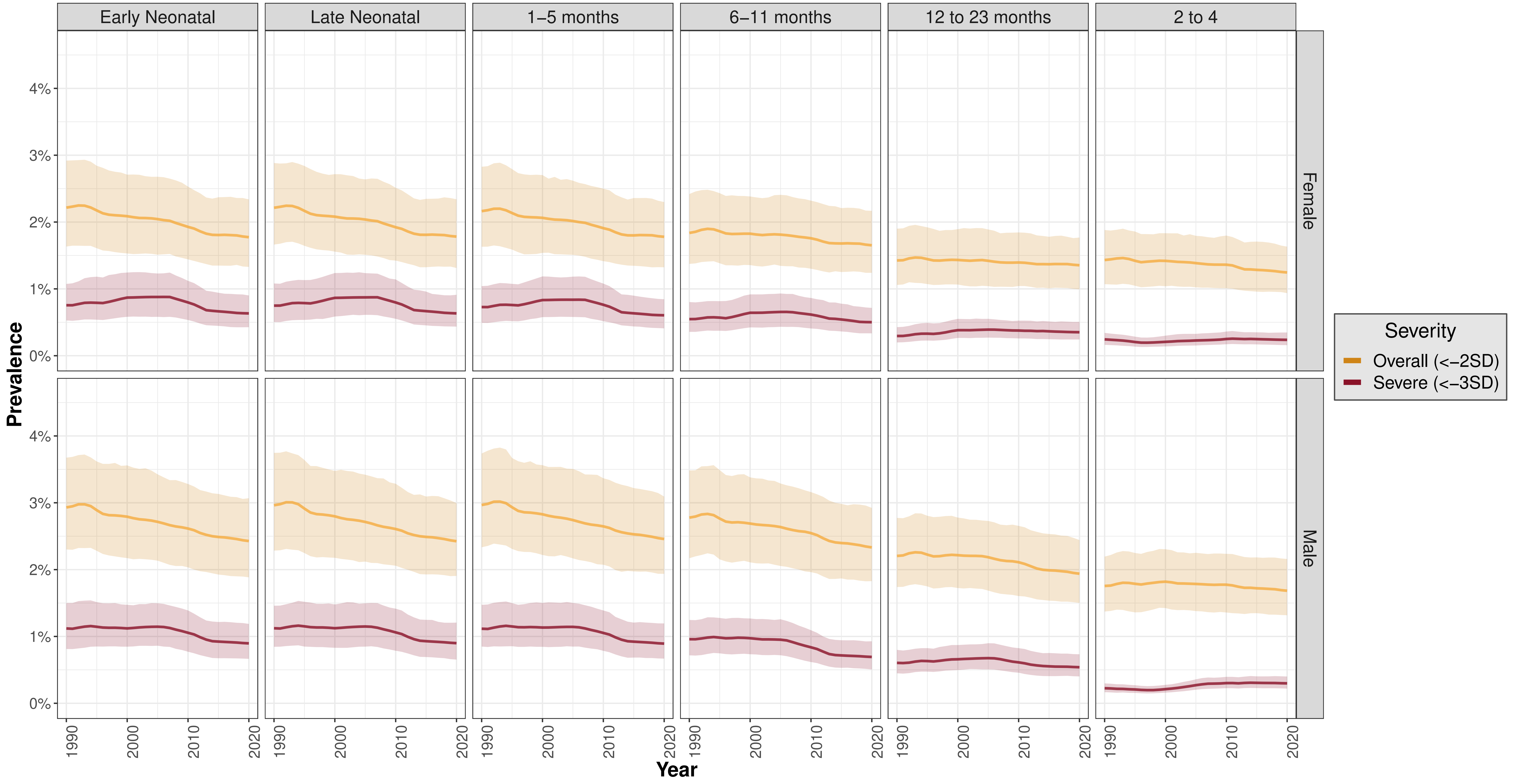

E: Transformed Mean Wasting Z Scores

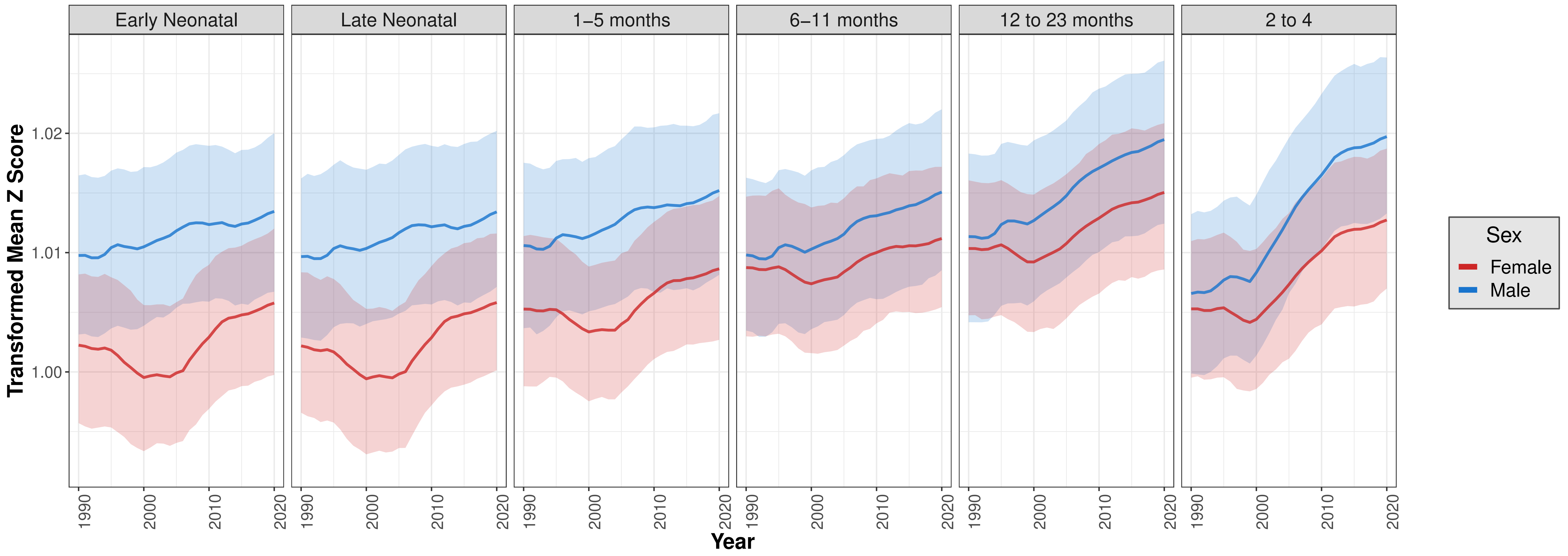

F

**Source**
No sources for this location

Bermuda – Underweight (WAZ)

G: Overall and Severe Underweight Prevalence

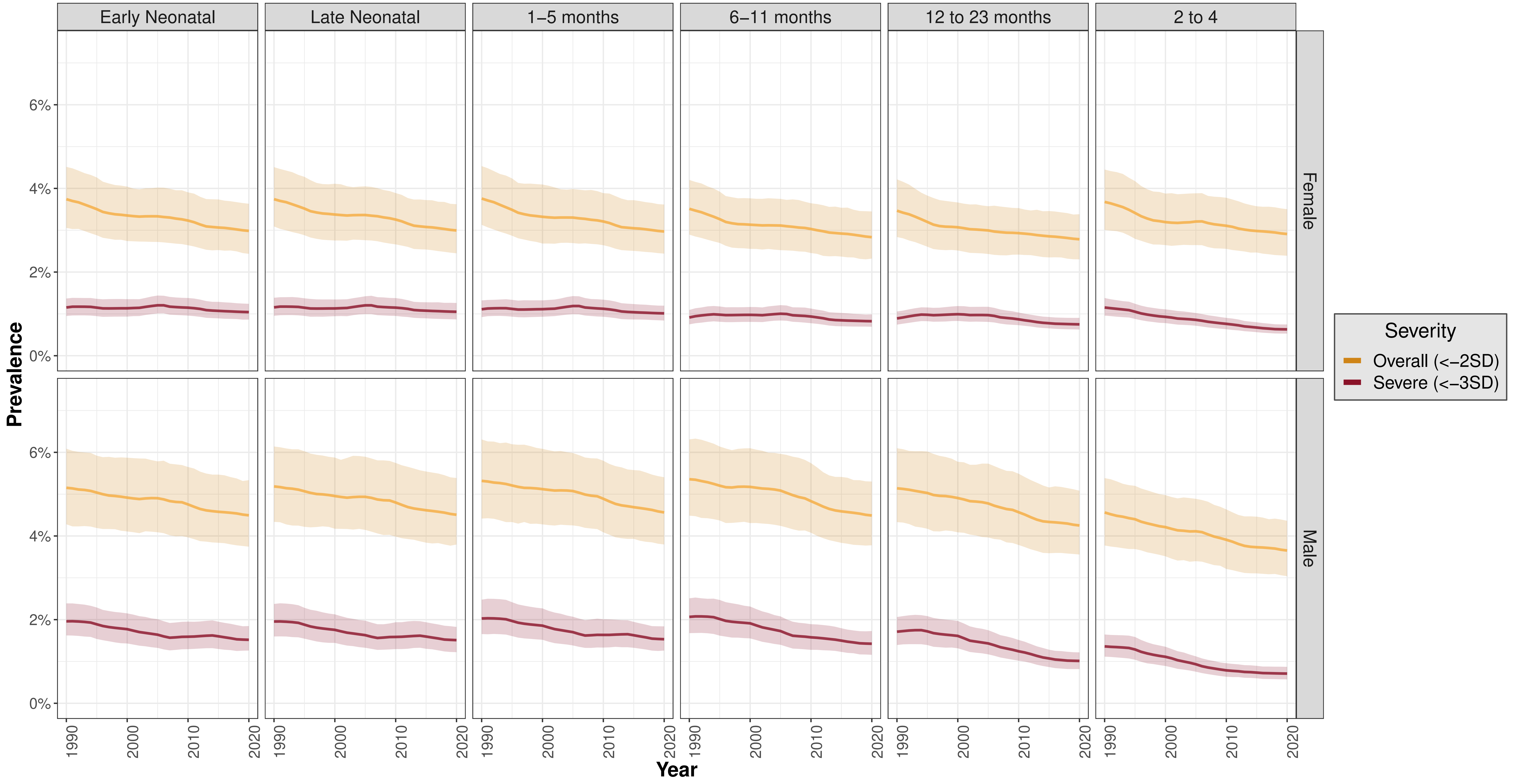

H: Transformed Mean Underweight Z Scores

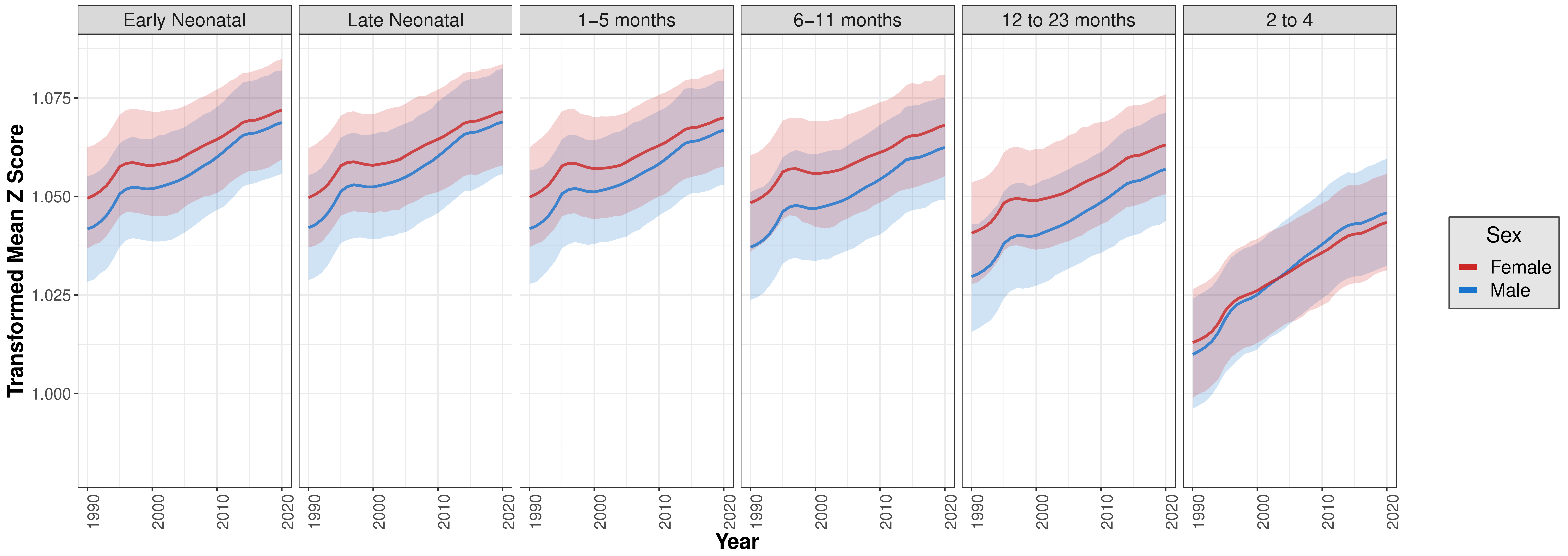

I

Source
No sources for this location

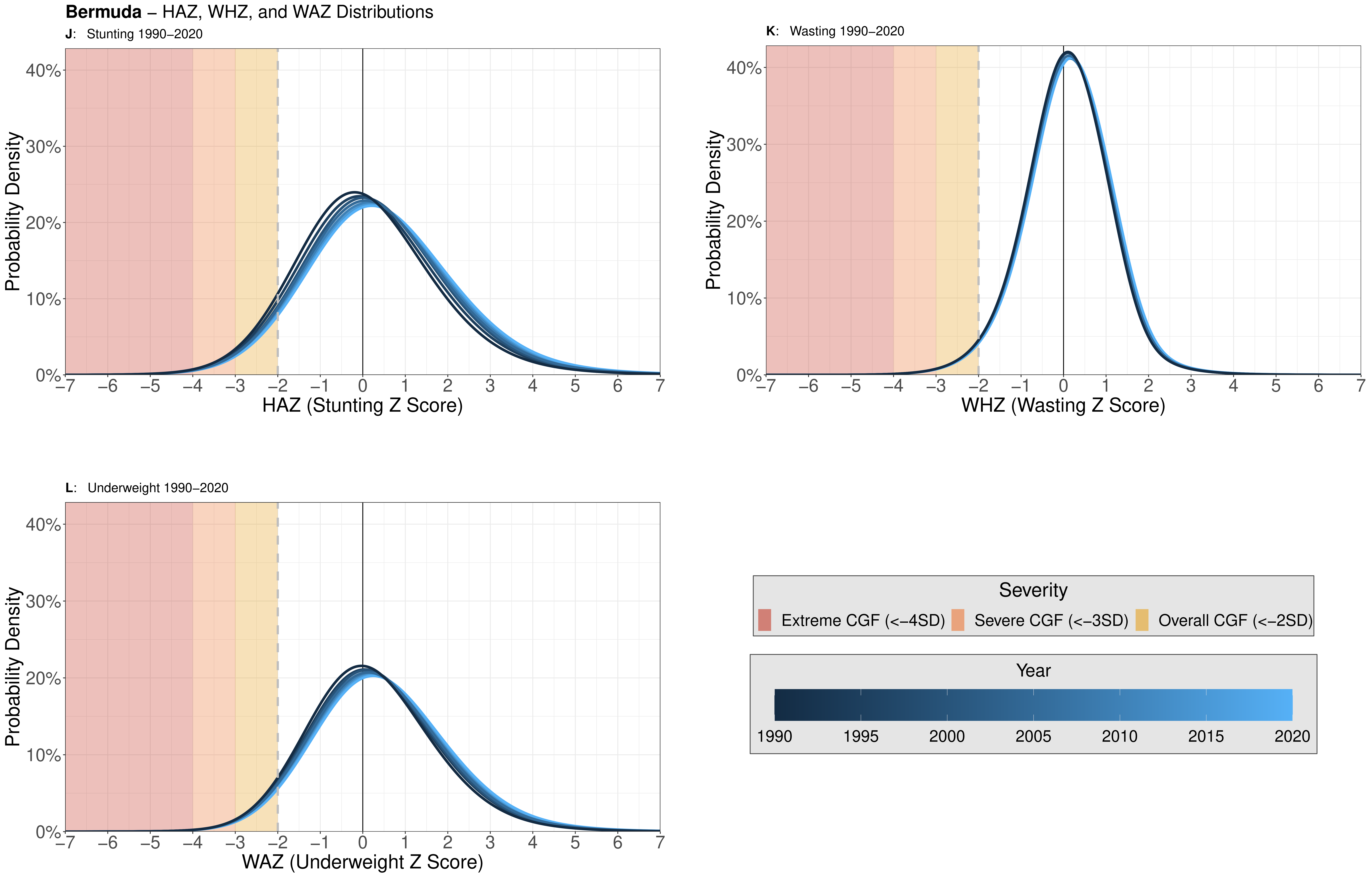
Bermuda – HAZ, WHZ, and WAZ Distributions
J: Stunting 1990–2020
Probability Density
40%
30%
20%
10%
0%
−7 −6 −5 −4 −3 −2 −1 0 1 2 3 4 5 6 7
HAZ (Stunting Z Score)
K: Wasting 1990–2020
Probability Density
40%
30%
20%
10%
0%
−7 −6 −5 −4 −3 −2 −1 0 1 2 3 4 5 6 7
WHZ (Wasting Z Score)
L: Underweight 1990–2020
Probability Density
40%
30%
20%
10%
0%
−7 −6 −5 −4 −3 −2 −1 0 1 2 3 4 5 6 7
WAZ (Underweight Z Score)
Severity
Extreme CGF (<−4SD)
Severe CGF (<−3SD)
Overall CGF (<−2SD)
Year
1990
1995
2000
2005
2010
2015
2020

Puerto Rico – Stunting (HAZ)

A: Overall and Severe Stunting Prevalence

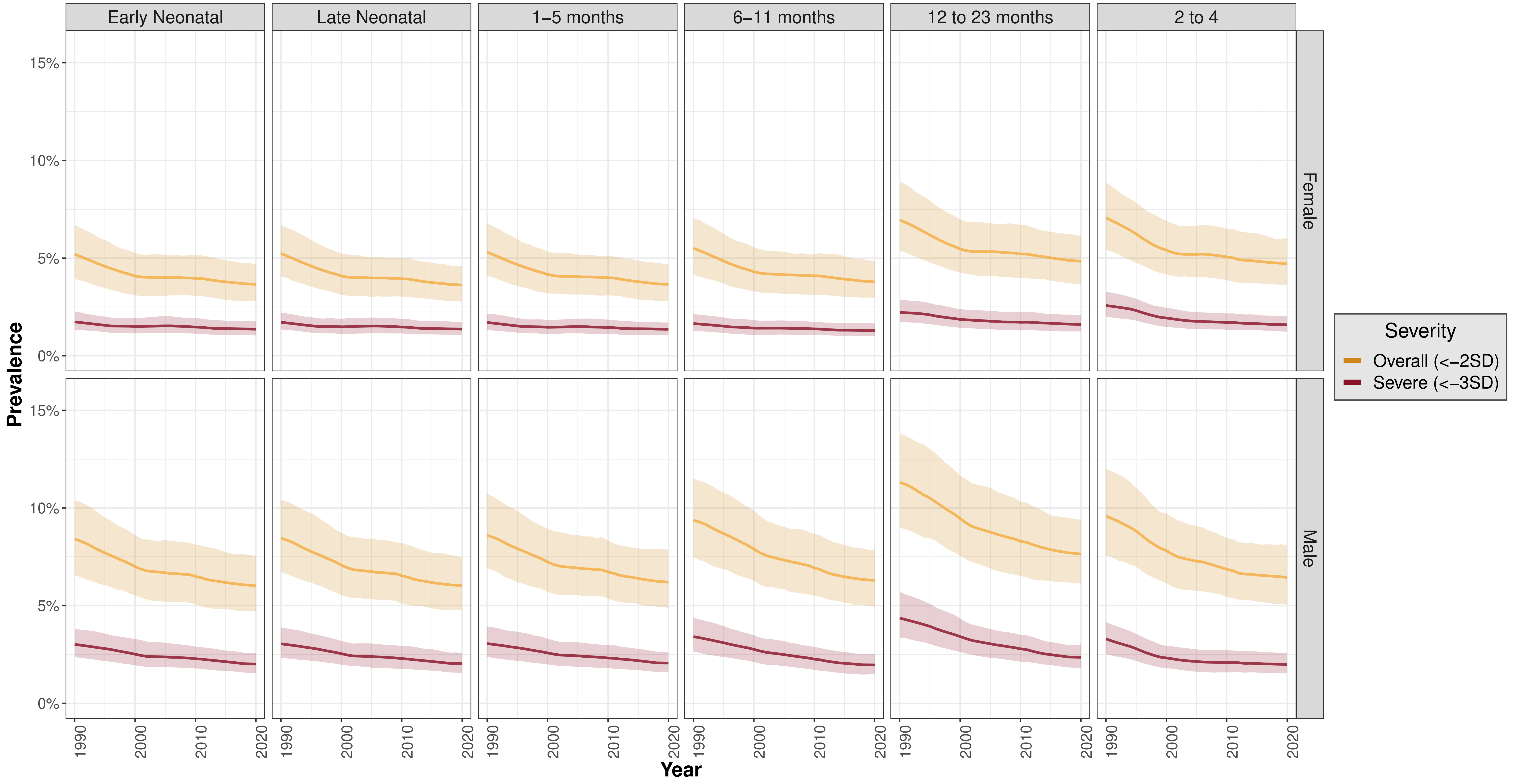

B: Transformed Mean Stunting Z Scores

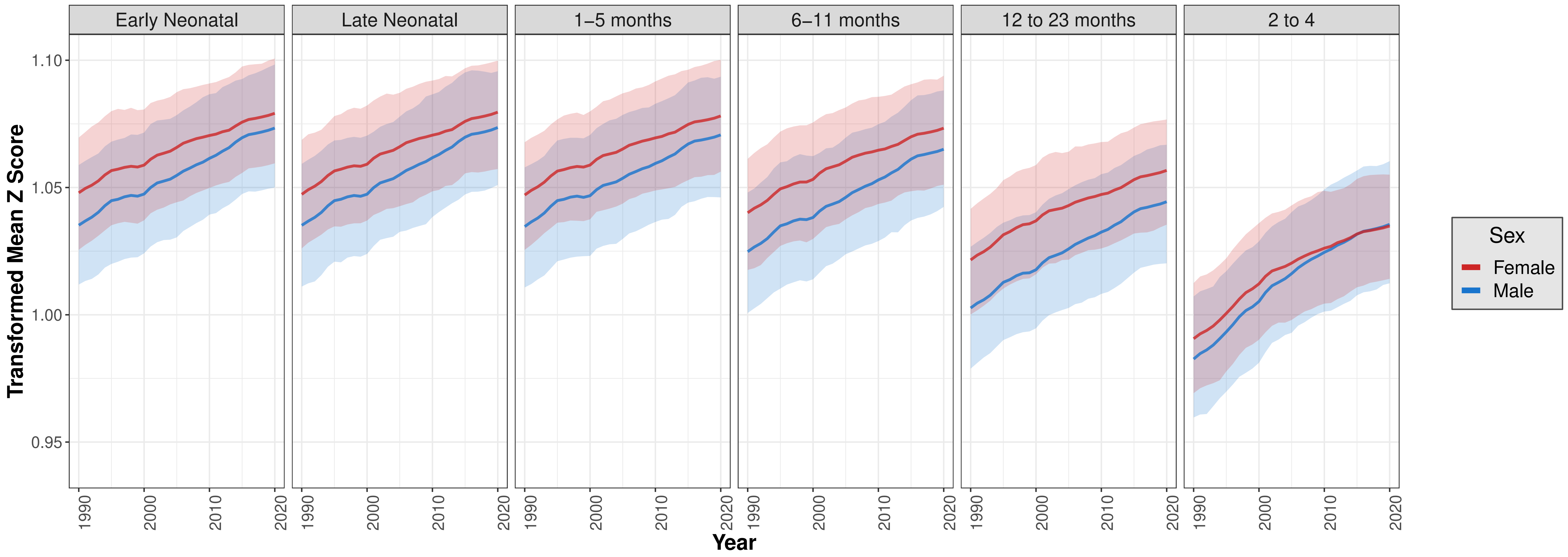

C

Source
No sources for this location

Puerto Rico – Wasting (WHZ)

D: Overall and Severe Wasting Prevalence

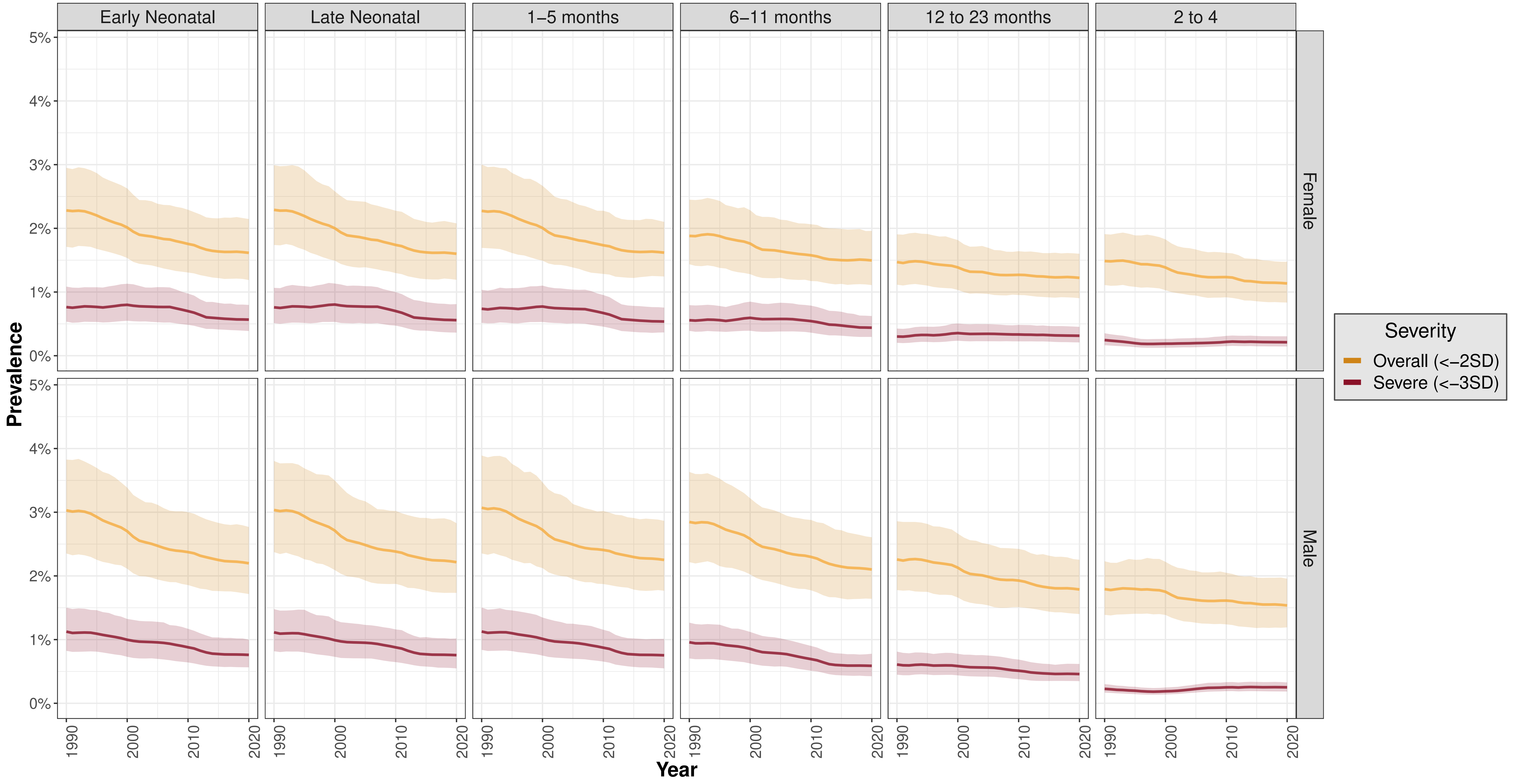

E: Transformed Mean Wasting Z Scores

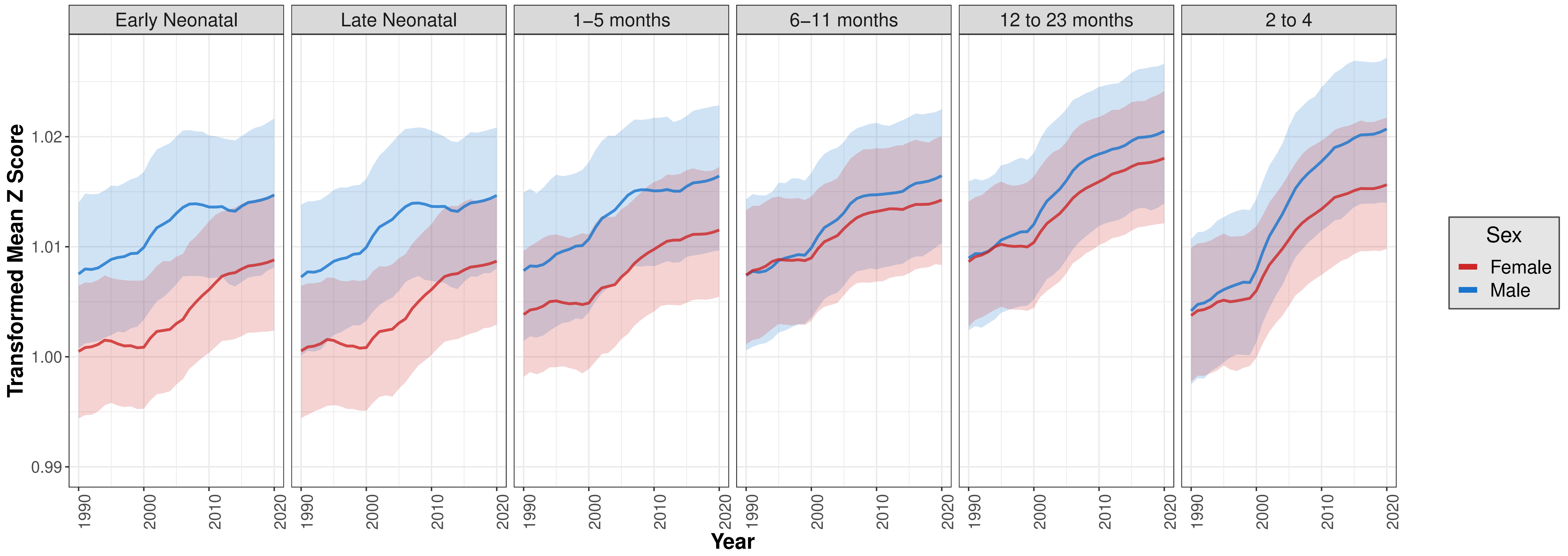

F

Source
No sources for this location

Puerto Rico – Underweight (WAZ)

G: Overall and Severe Underweight Prevalence

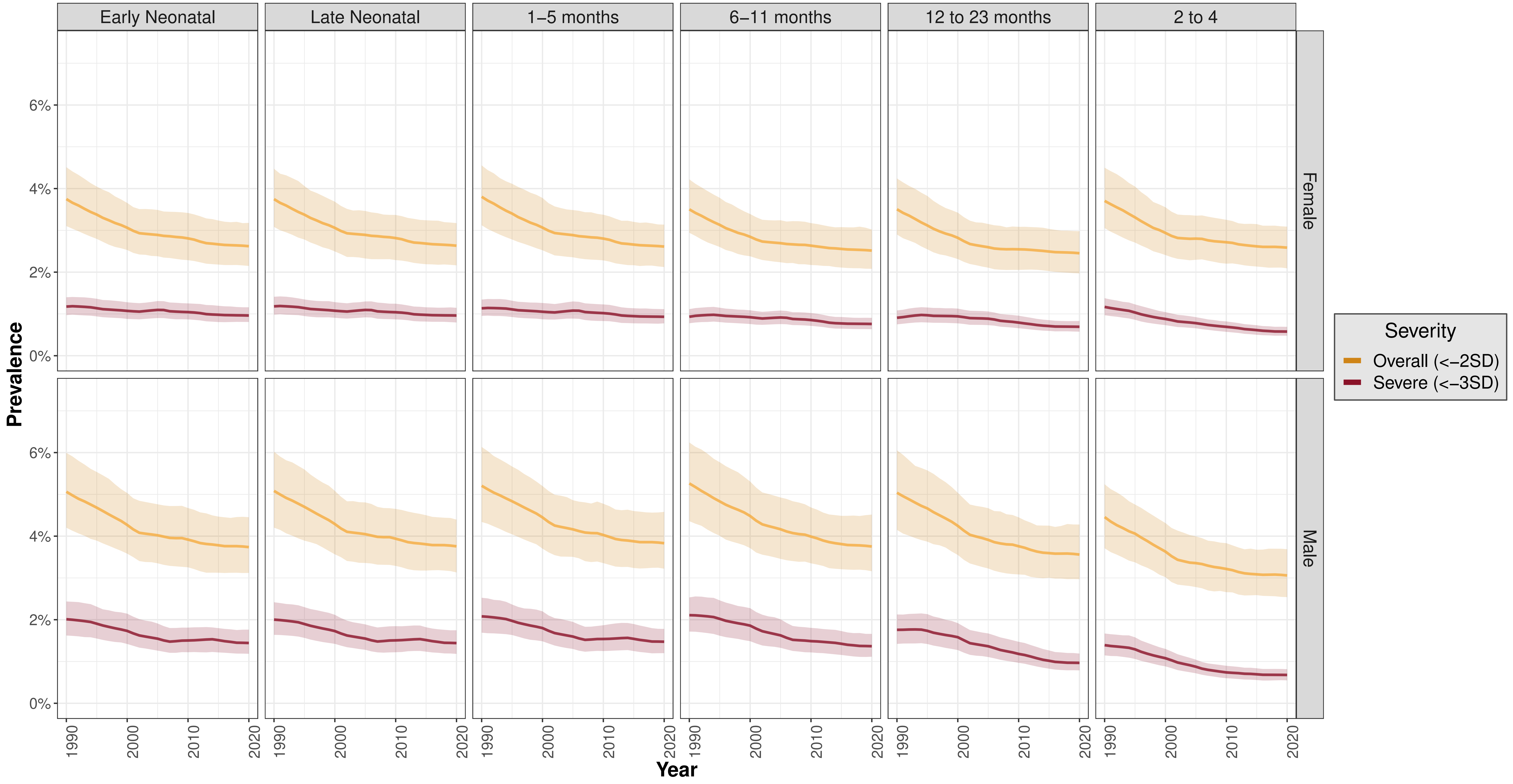

H: Transformed Mean Underweight Z Scores

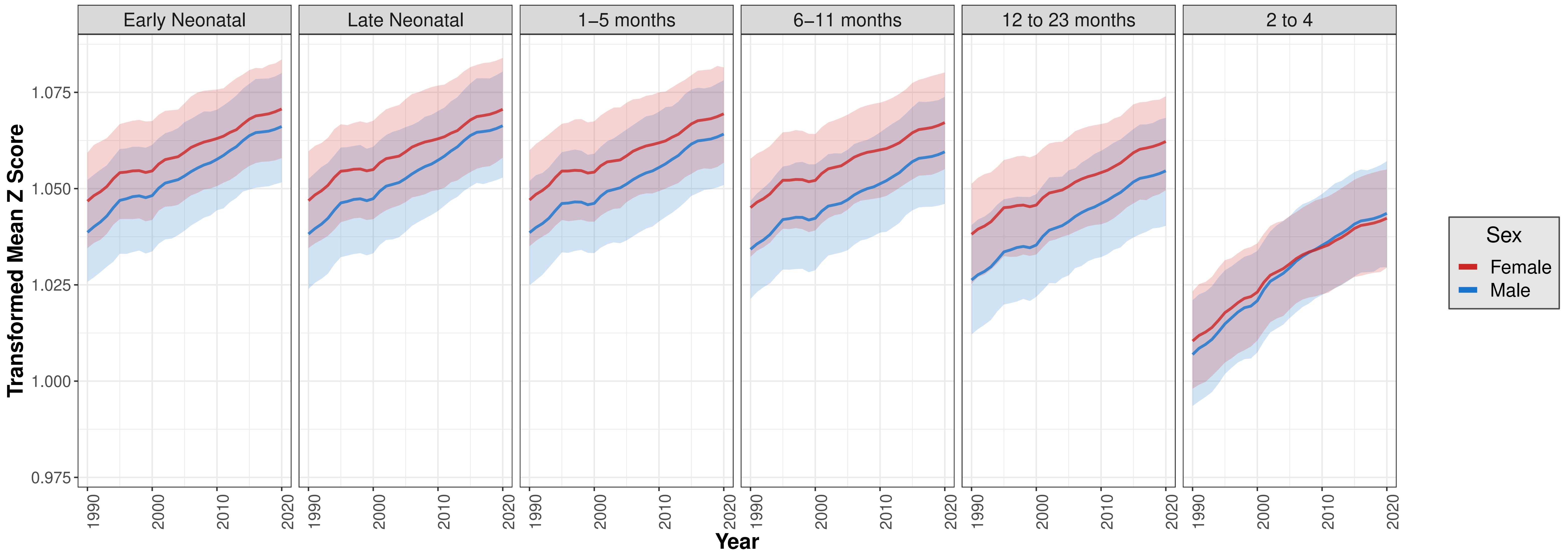

I

**Source**
No sources for this location

Puerto Rico – HAZ, WHZ, and WAZ Distributions

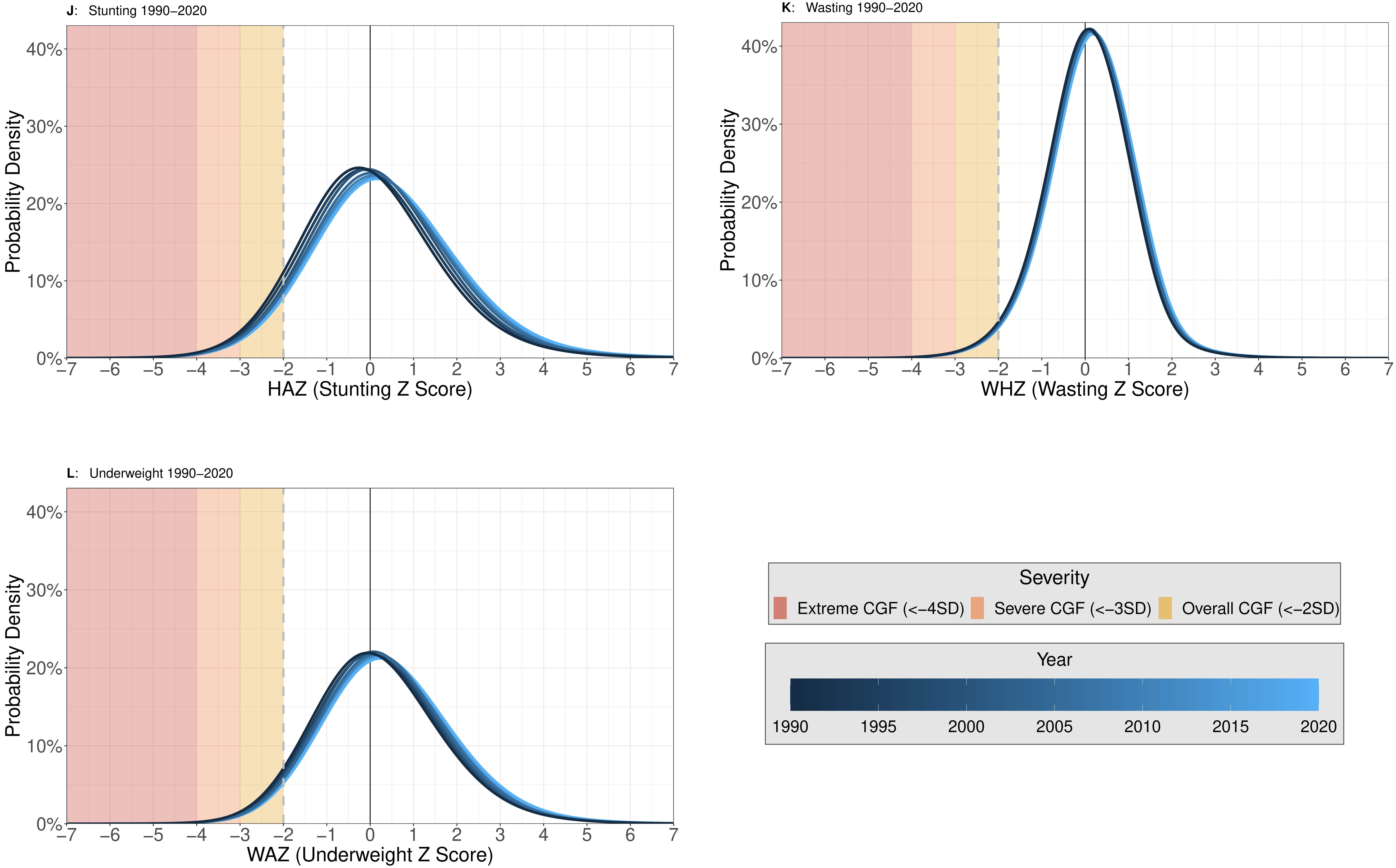

Saint Kitts and Nevis – Stunting (HAZ)

A: Overall and Severe Stunting Prevalence

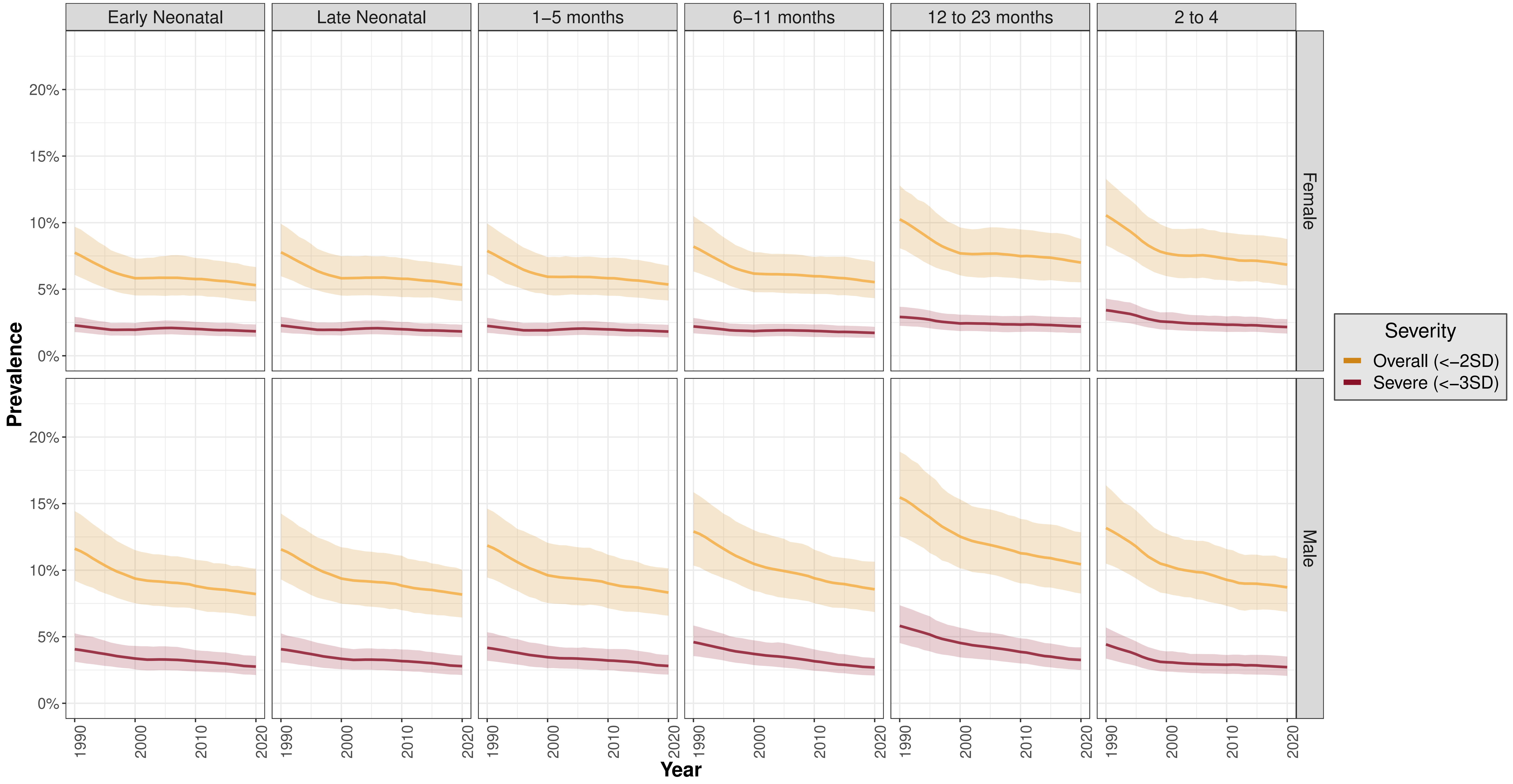

B: Transformed Mean Stunting Z Scores

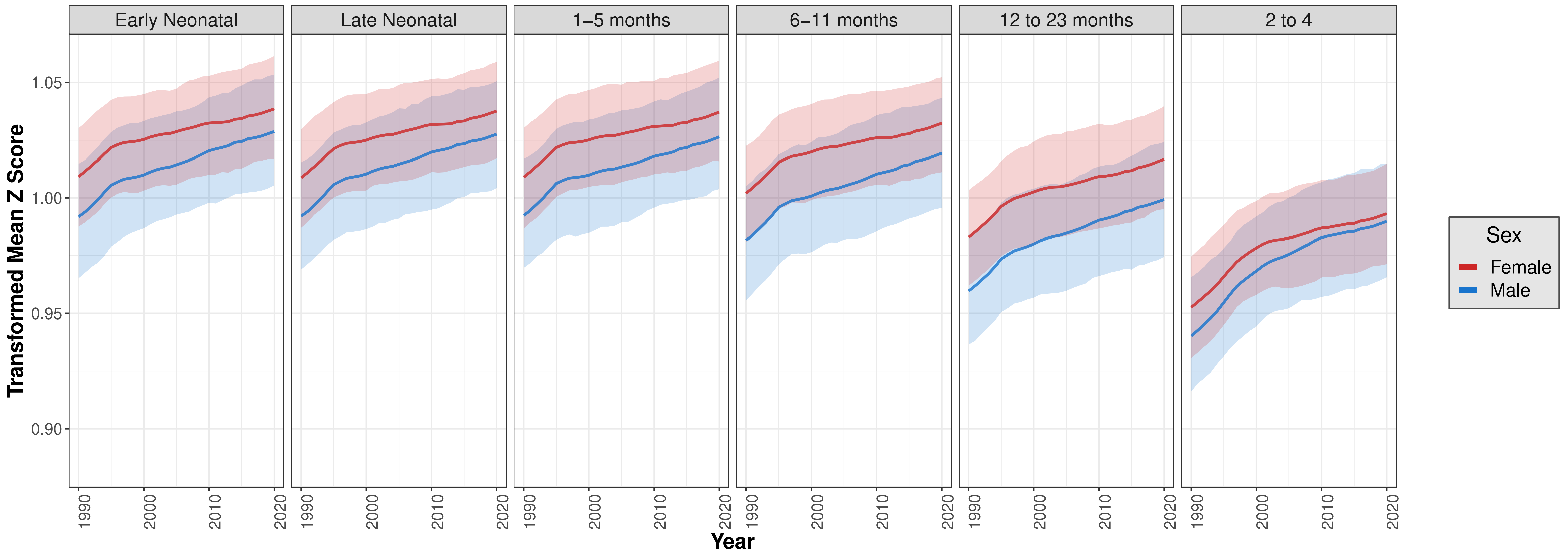

C

Source
No sources for this location

Saint Kitts and Nevis – Wasting (WHZ)

D: Overall and Severe Wasting Prevalence

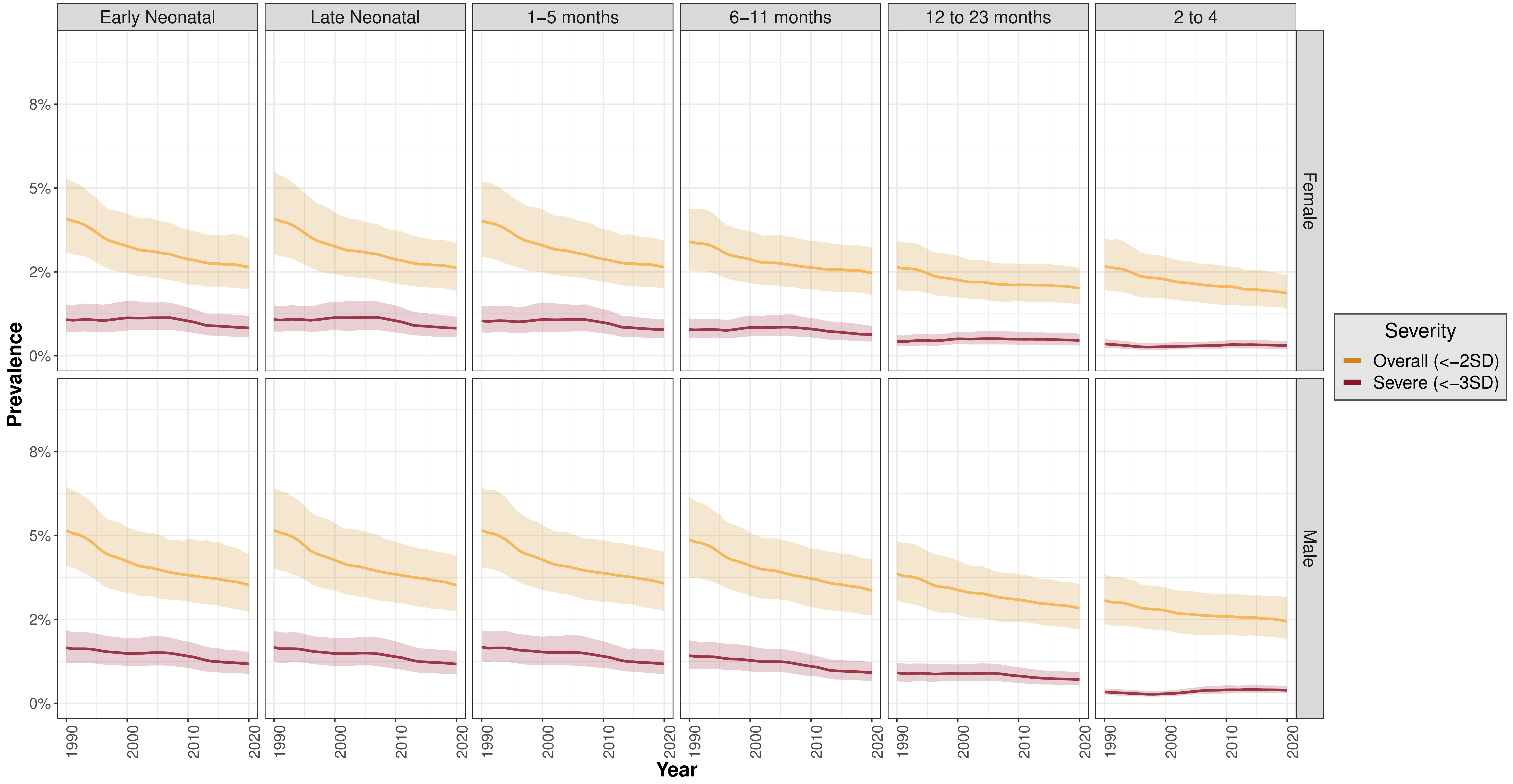

E: Transformed Mean Wasting Z Scores

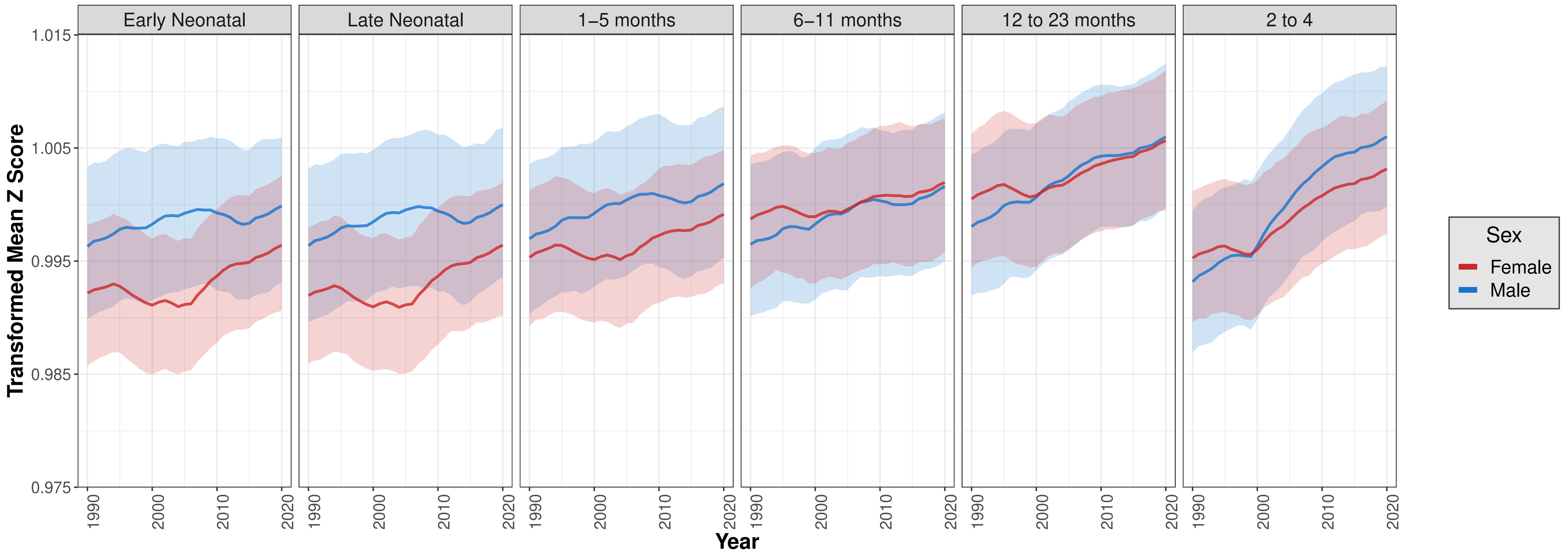

F

Source
No sources for this location

Saint Kitts and Nevis – Underweight (WAZ)

G: Overall and Severe Underweight Prevalence

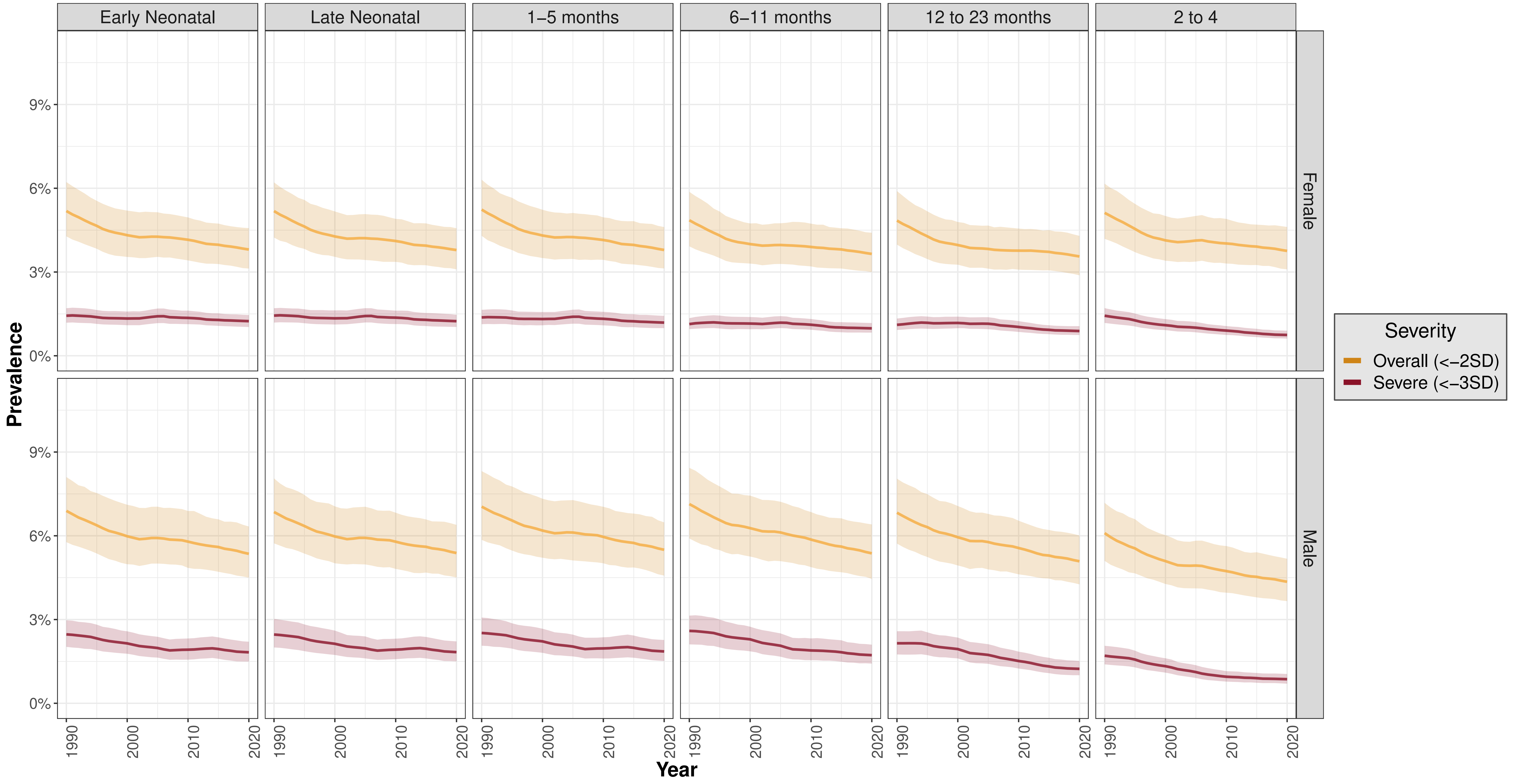

H: Transformed Mean Underweight Z Scores

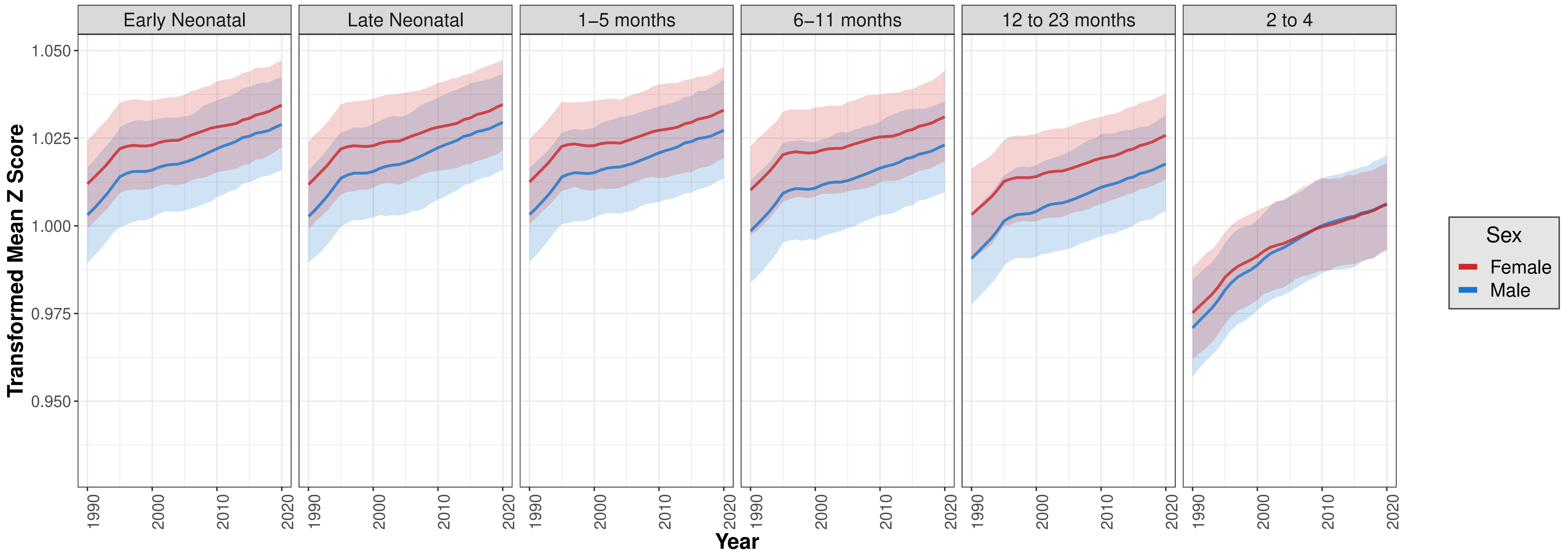

I

Source
No sources for this location

Saint Kitts and Nevis – HAZ, WHZ, and WAZ Distributions

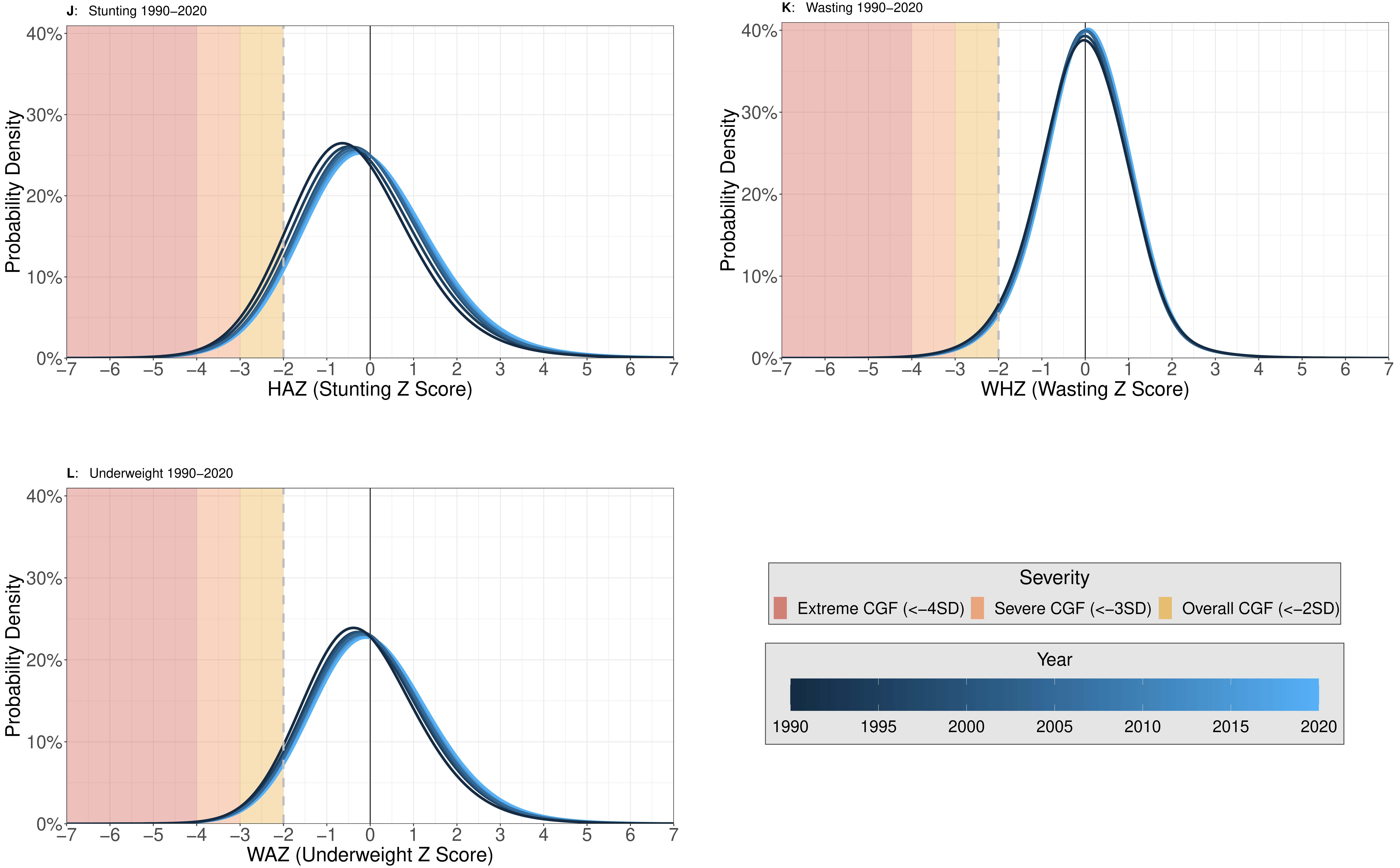

United States Virgin Islands – Stunting (HAZ)

A: Overall and Severe Stunting Prevalence

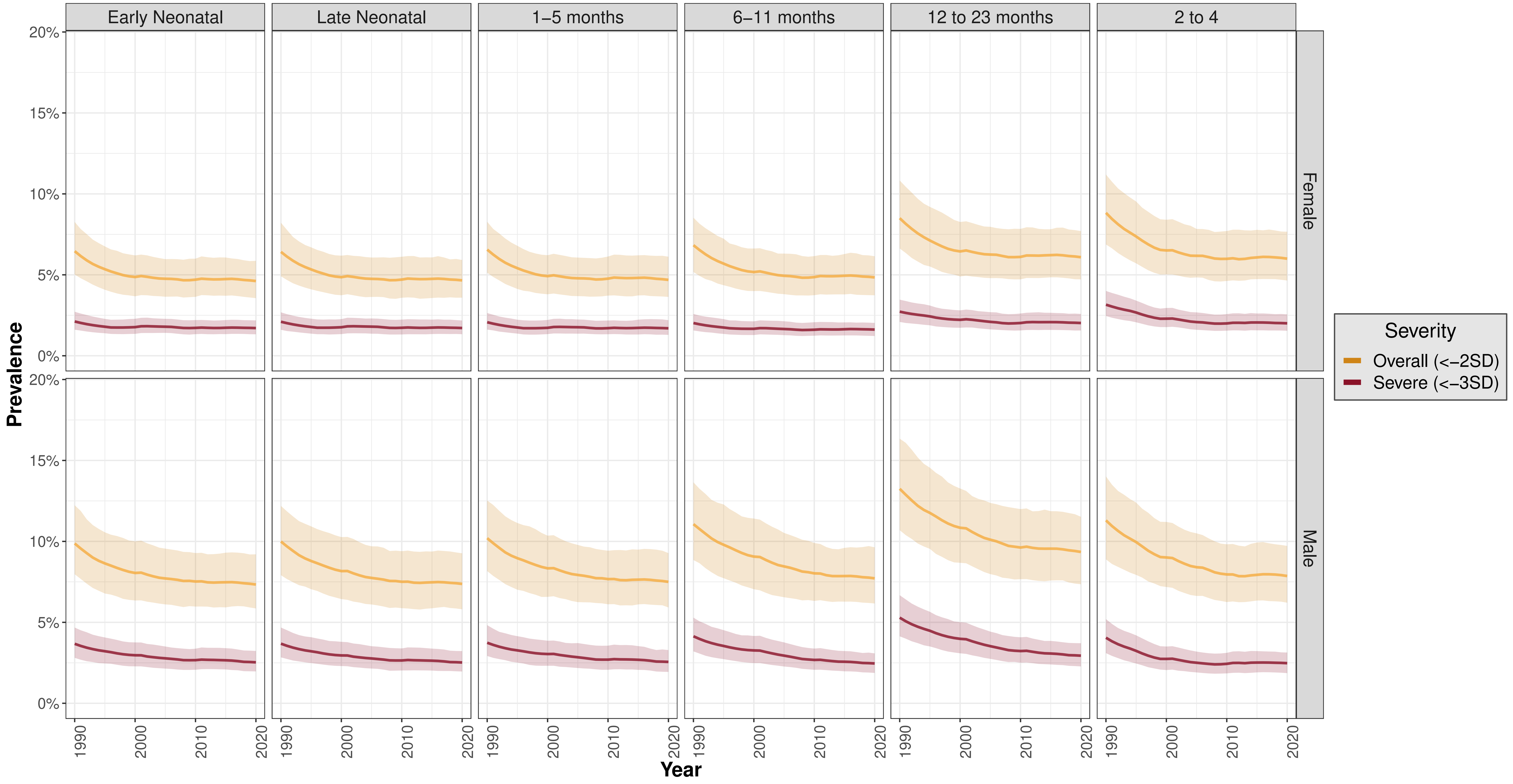

B: Transformed Mean Stunting Z Scores

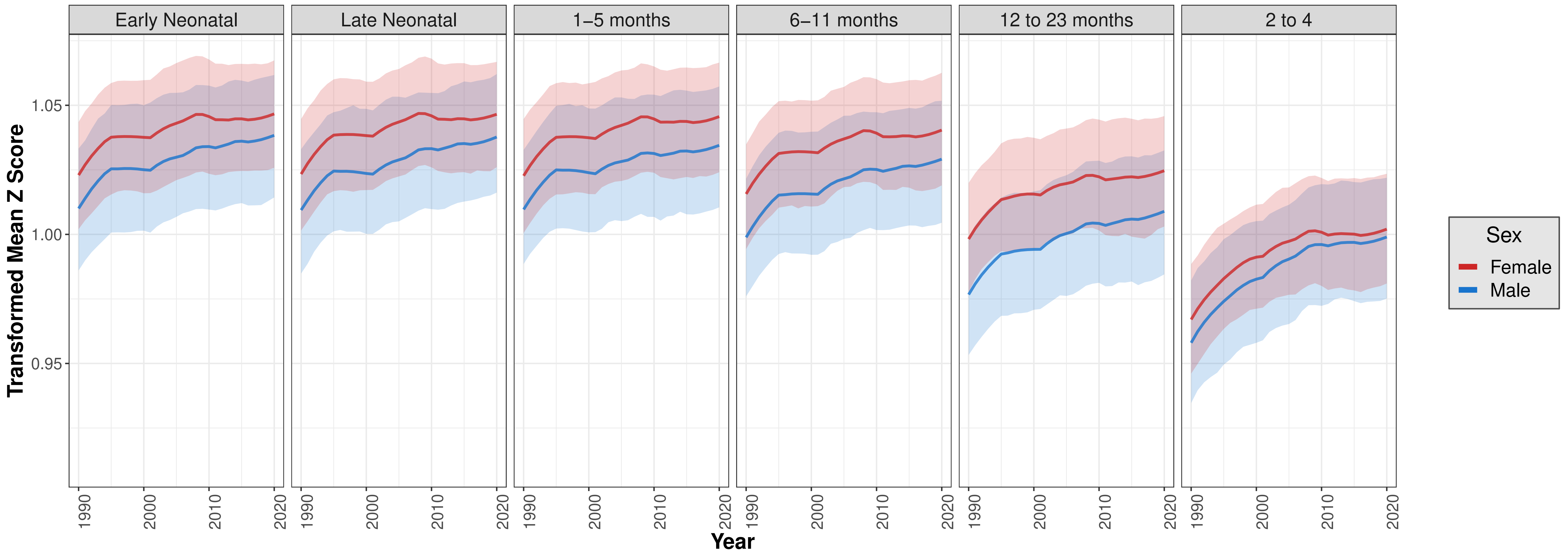

C

Source
No sources for this location

United States Virgin Islands – Wasting (WHZ)

D: Overall and Severe Wasting Prevalence

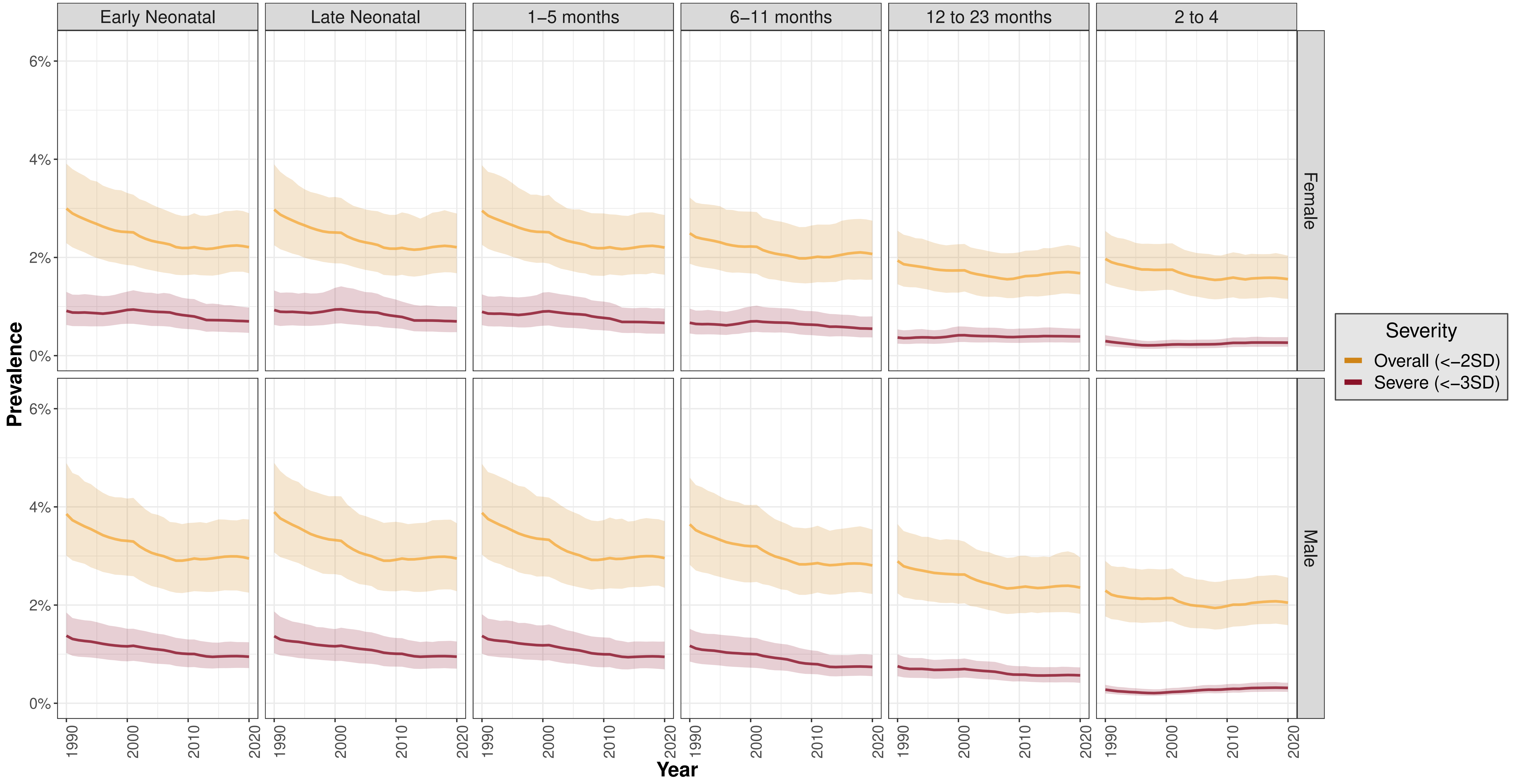

E: Transformed Mean Wasting Z Scores

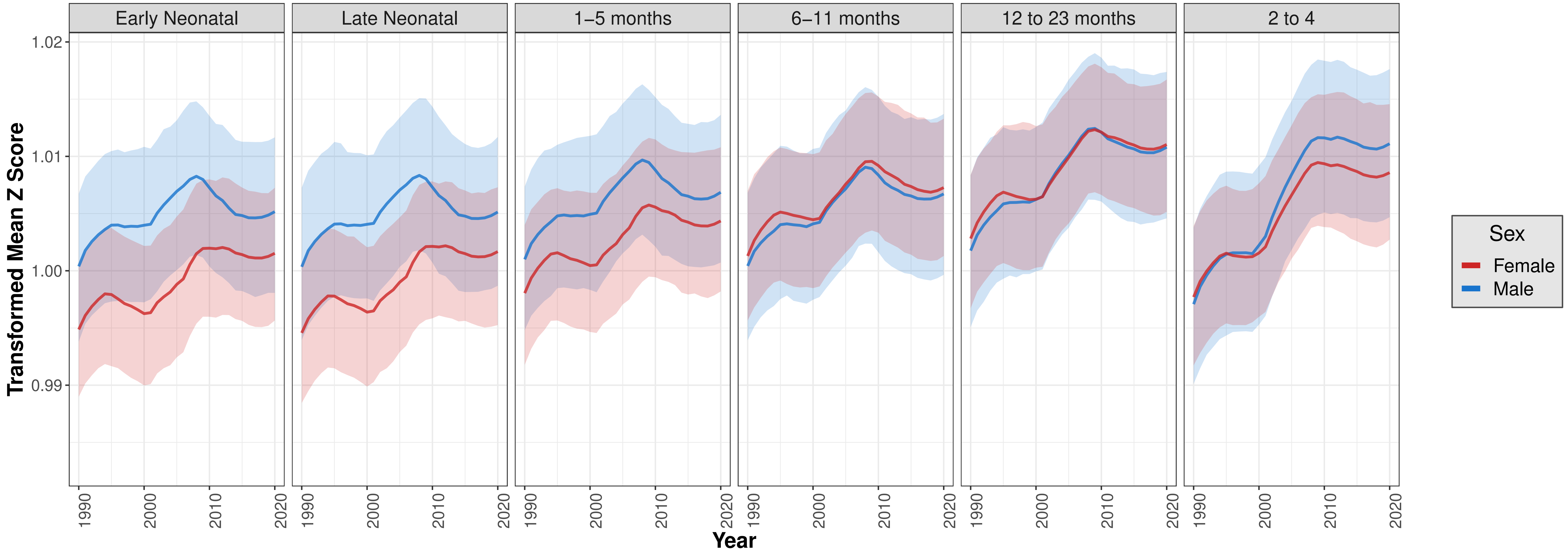

F

Source
No sources for this location

United States Virgin Islands – Underweight (WAZ)

G: Overall and Severe Underweight Prevalence

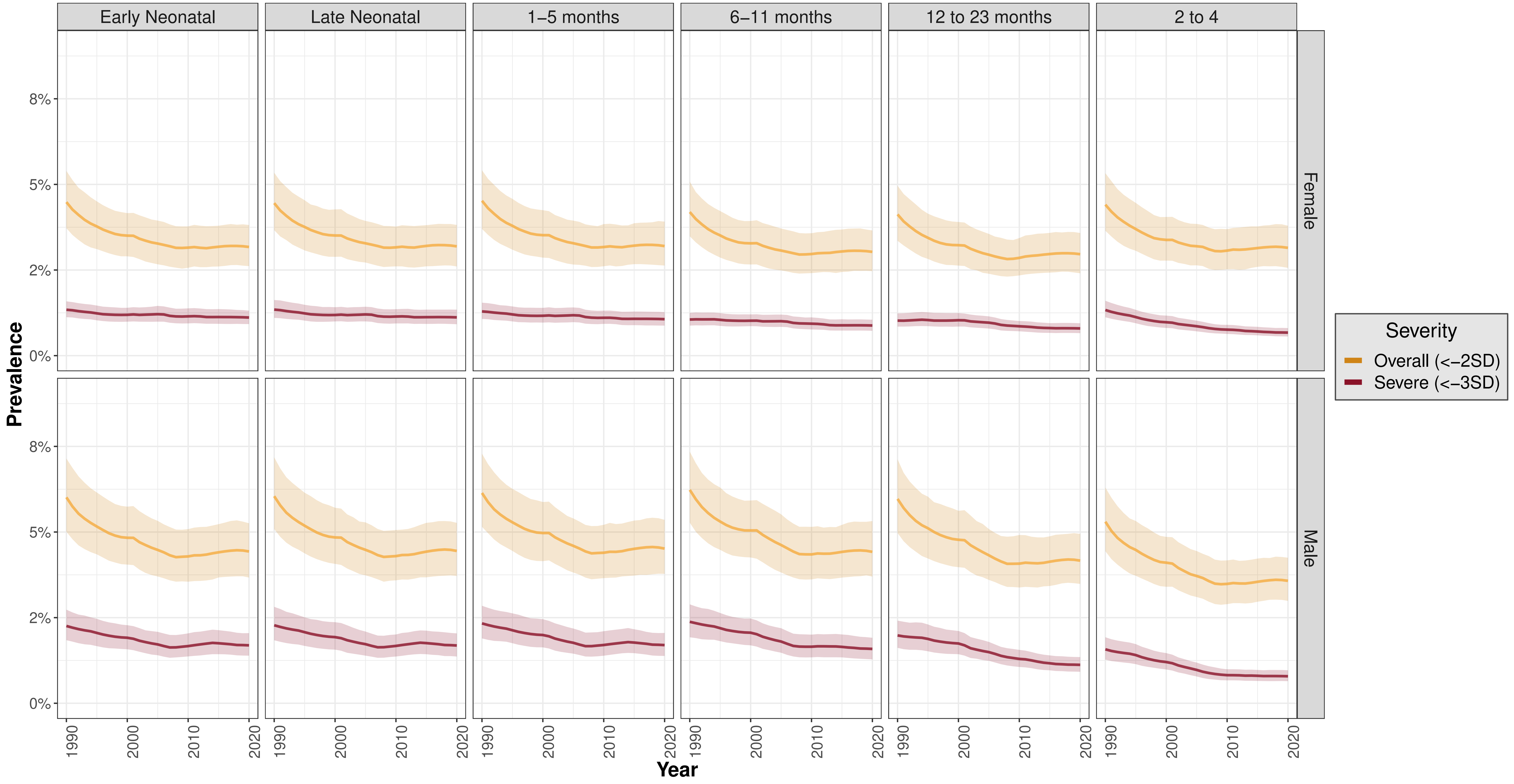

H: Transformed Mean Underweight Z Scores

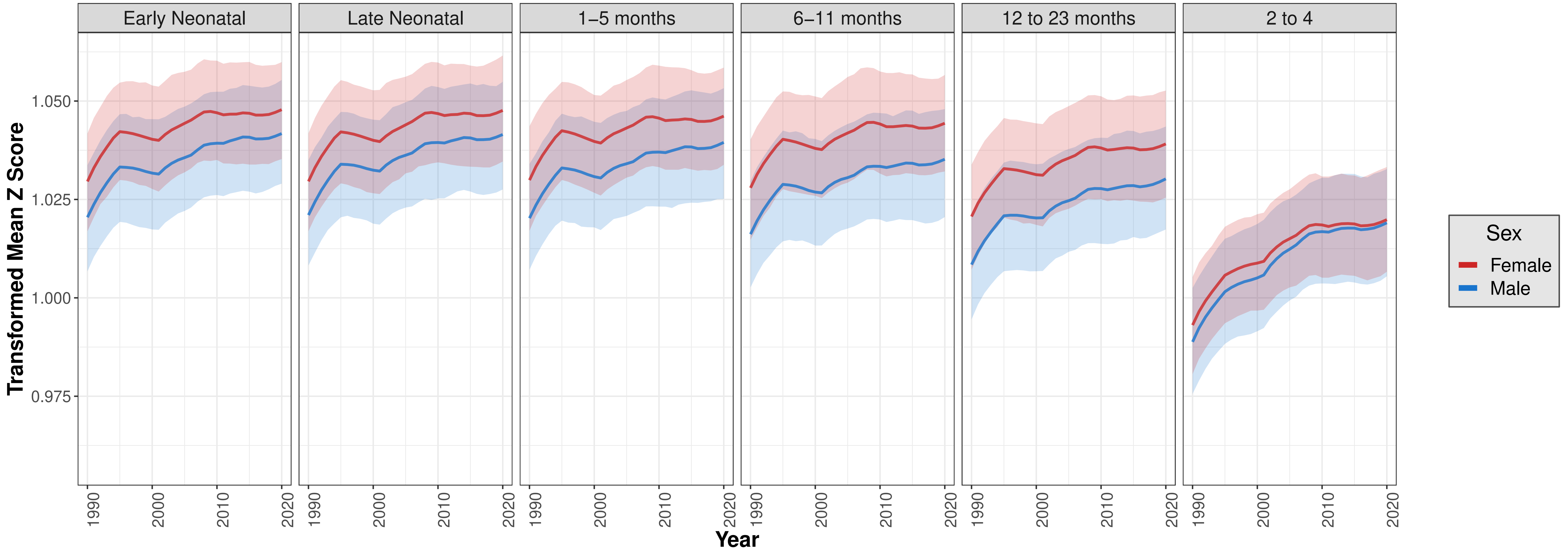

I

**Source**
No sources for this location

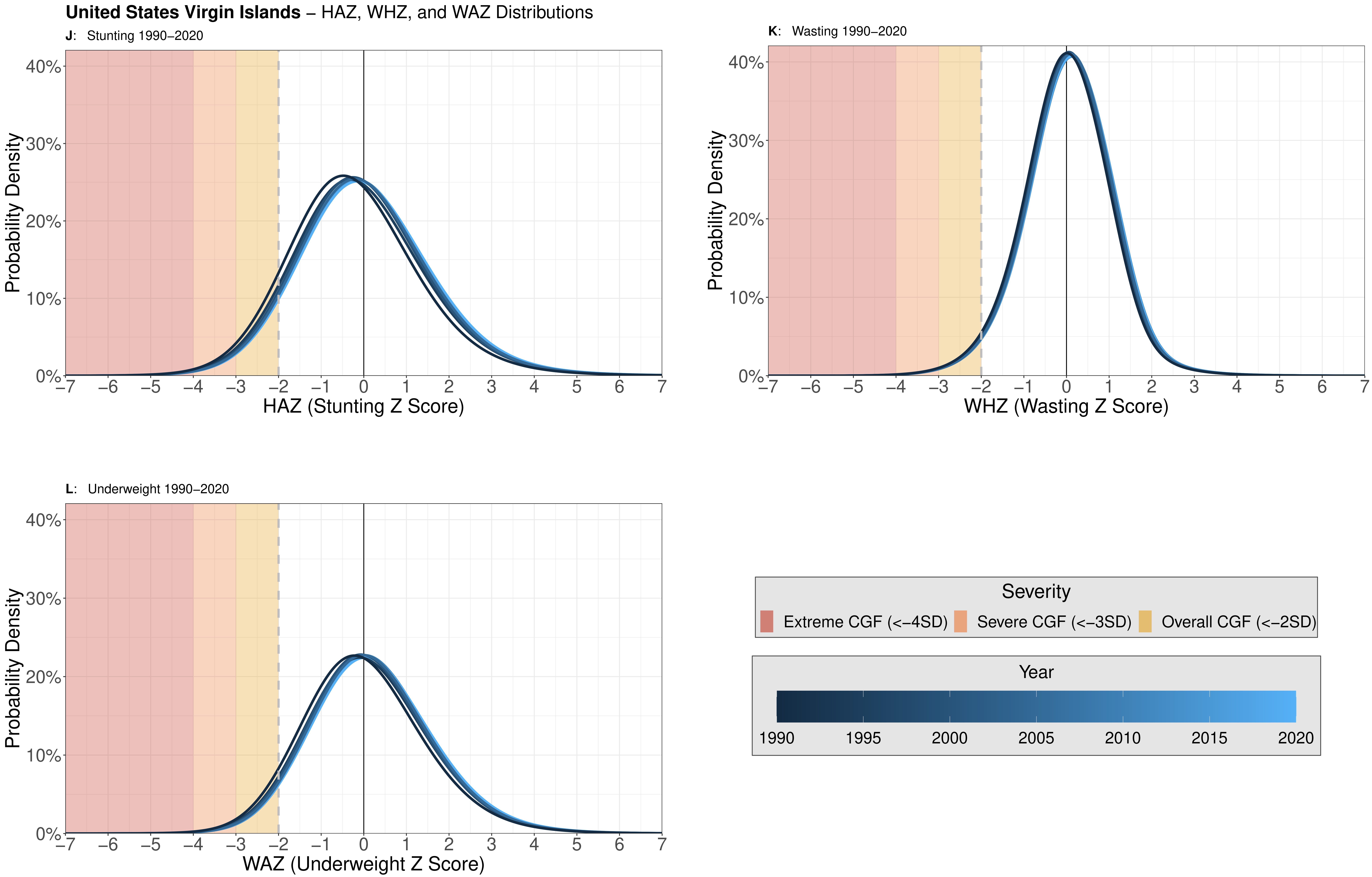
United States Virgin Islands – HAZ, WHZ, and WAZ Distributions
J: Stunting 1990–2020
K: Wasting 1990–2020
L: Underweight 1990–2020
Probability Density
0%
10%
20%
30%
40%
−7 −6 −5 −4 −3 −2 −1 0 1 2 3 4 5 6 7
HAZ (Stunting Z Score)
WHZ (Wasting Z Score)
WAZ (Underweight Z Score)
Severity
Extreme CGF (<−4SD)
Severe CGF (<−3SD)
Overall CGF (<−2SD)
Year
1990
1995
2000
2005
2010
2015
2020

Colombia – Stunting (HAZ)

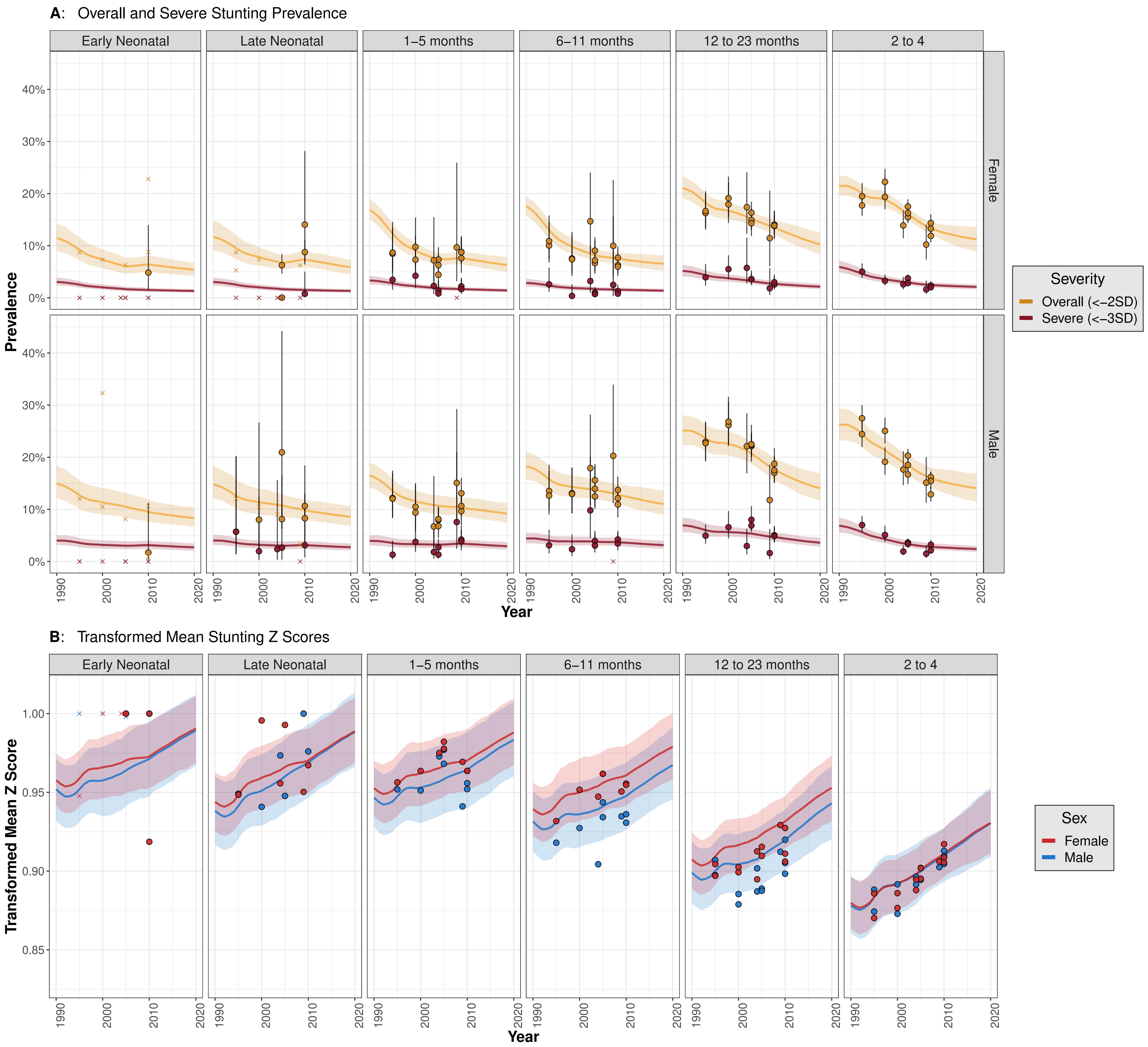

C

Year	Source
1966	WHO CGM Database
1979	WHO CGM Database
1986	DHS
1986	WHO CGM Database
1989	WHO CGM Database
1995	DHS
1995	WHO CGM Database
2000	DHS
2000	WHO CGM Database
2004	DHS
2004	WHO CGM Database
2005	DHS
2005	National Survey of the Nutritional Situation
2005	WHO CGM Database
2009	DHS
2010	DHS
2010	National Survey of the Nutritional Situation
2010	WHO CGM Database

Colombia – Wasting (WHZ)

D: Overall and Severe Wasting Prevalence

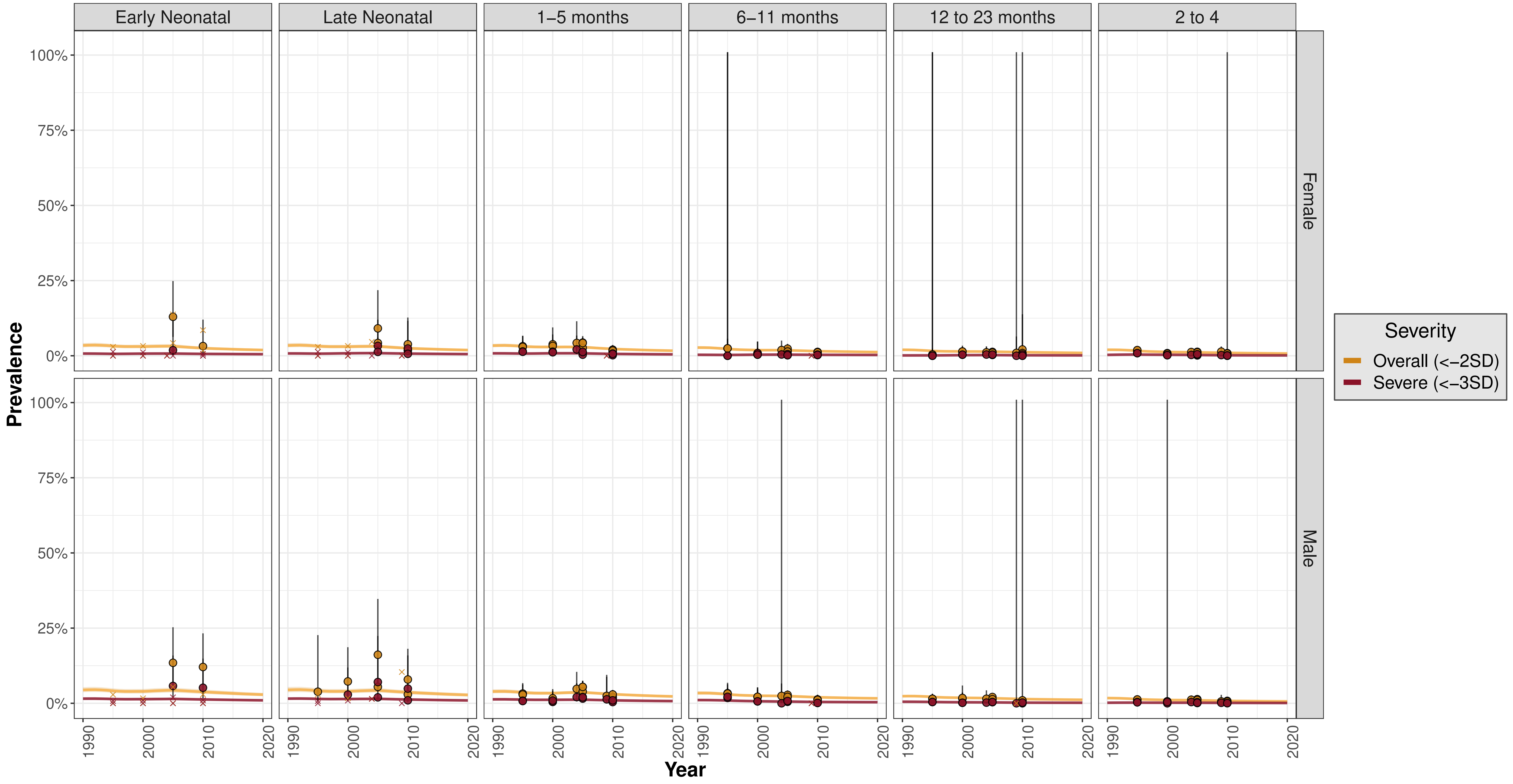

E: Transformed Mean Wasting Z Scores

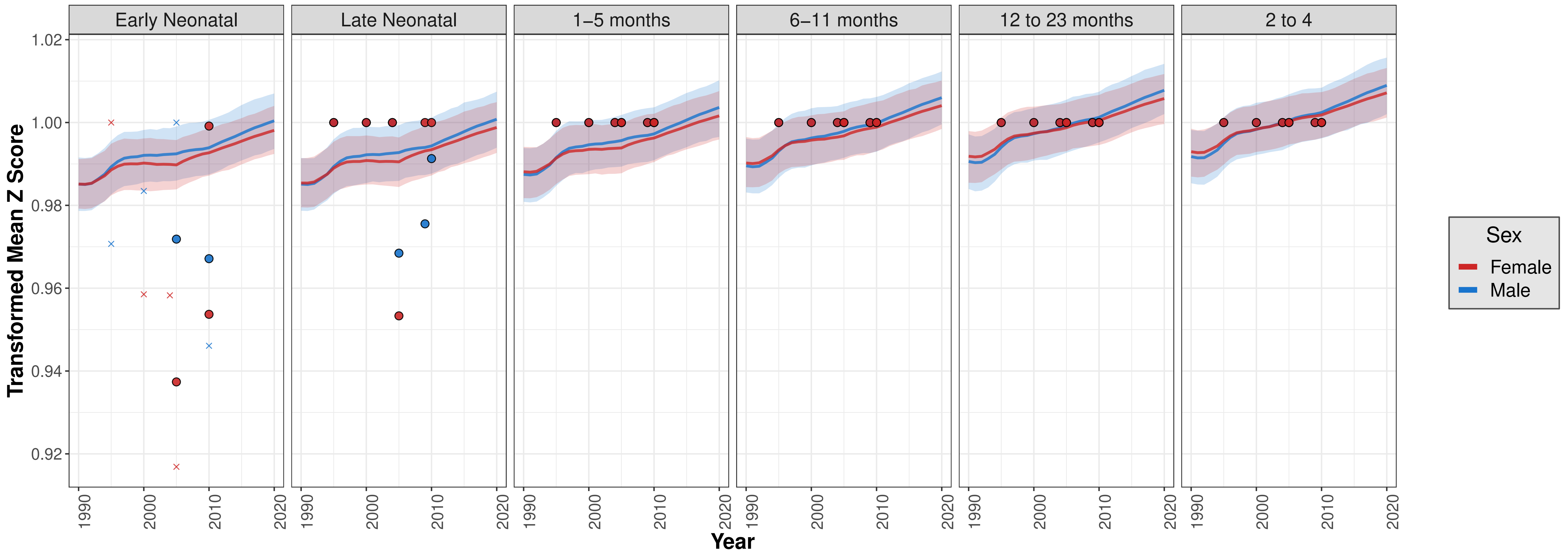

F

Year	Source
1966	WHO CGM Database
1979	WHO CGM Database
1986	DHS
1986	WHO CGM Database
1989	WHO CGM Database
1995	DHS
1995	WHO CGM Database
2000	DHS
2000	WHO CGM Database
2004	DHS
2005	DHS
2005	National Survey of the Nutritional Situation
2005	WHO CGM Database
2009	DHS
2010	DHS
2010	National Survey of the Nutritional Situation
2010	WHO CGM Database

Colombia – Underweight (WAZ)

G: Overall and Severe Underweight Prevalence

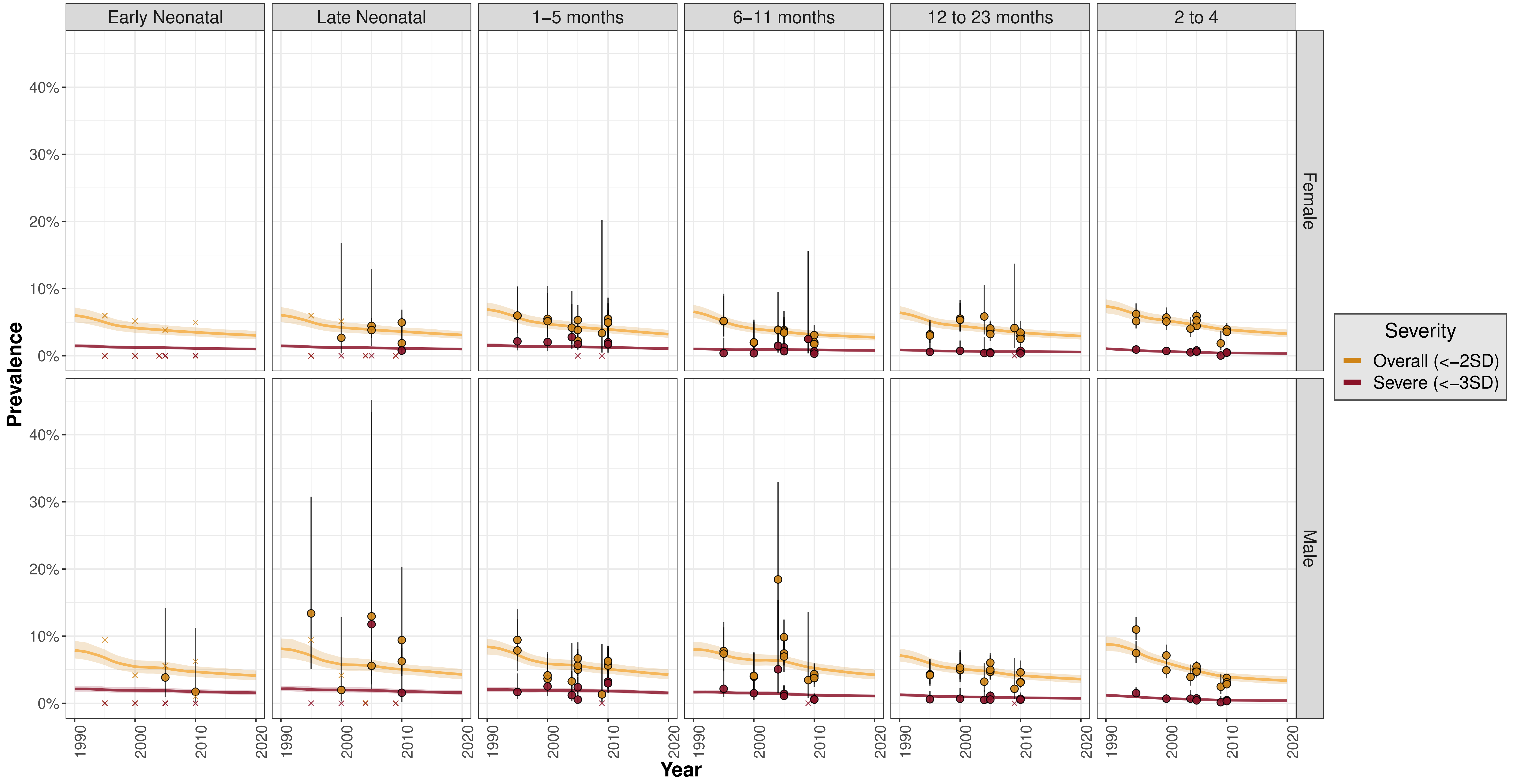

H: Transformed Mean Underweight Z Scores

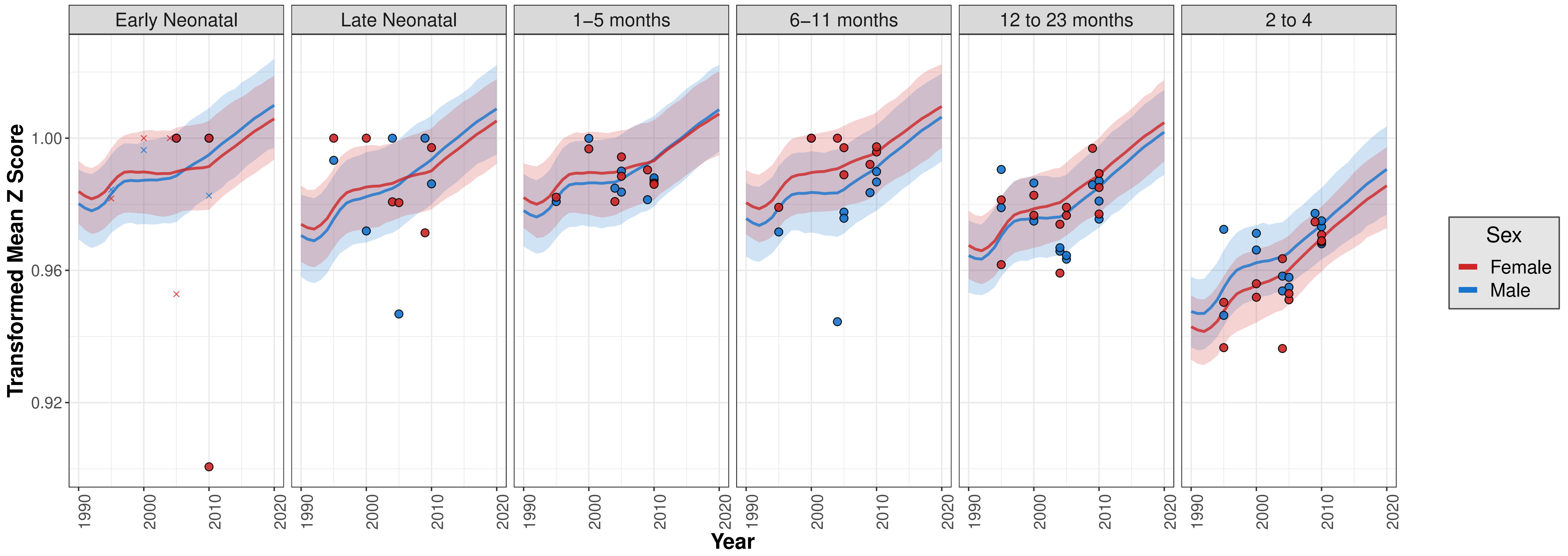

I

Year	Source
1966	WHO CGM Database
1979	WHO CGM Database
1986	DHS
1986	WHO CGM Database
1989	WHO CGM Database
1995	DHS
1995	WHO CGM Database
2000	DHS
2000	WHO CGM Database
2004	DHS
2004	WHO CGM Database
2005	DHS
2005	National Survey of the Nutritional Situation
2005	WHO CGM Database
2009	DHS
2010	DHS
2010	National Survey of the Nutritional Situation
2010	WHO CGM Database

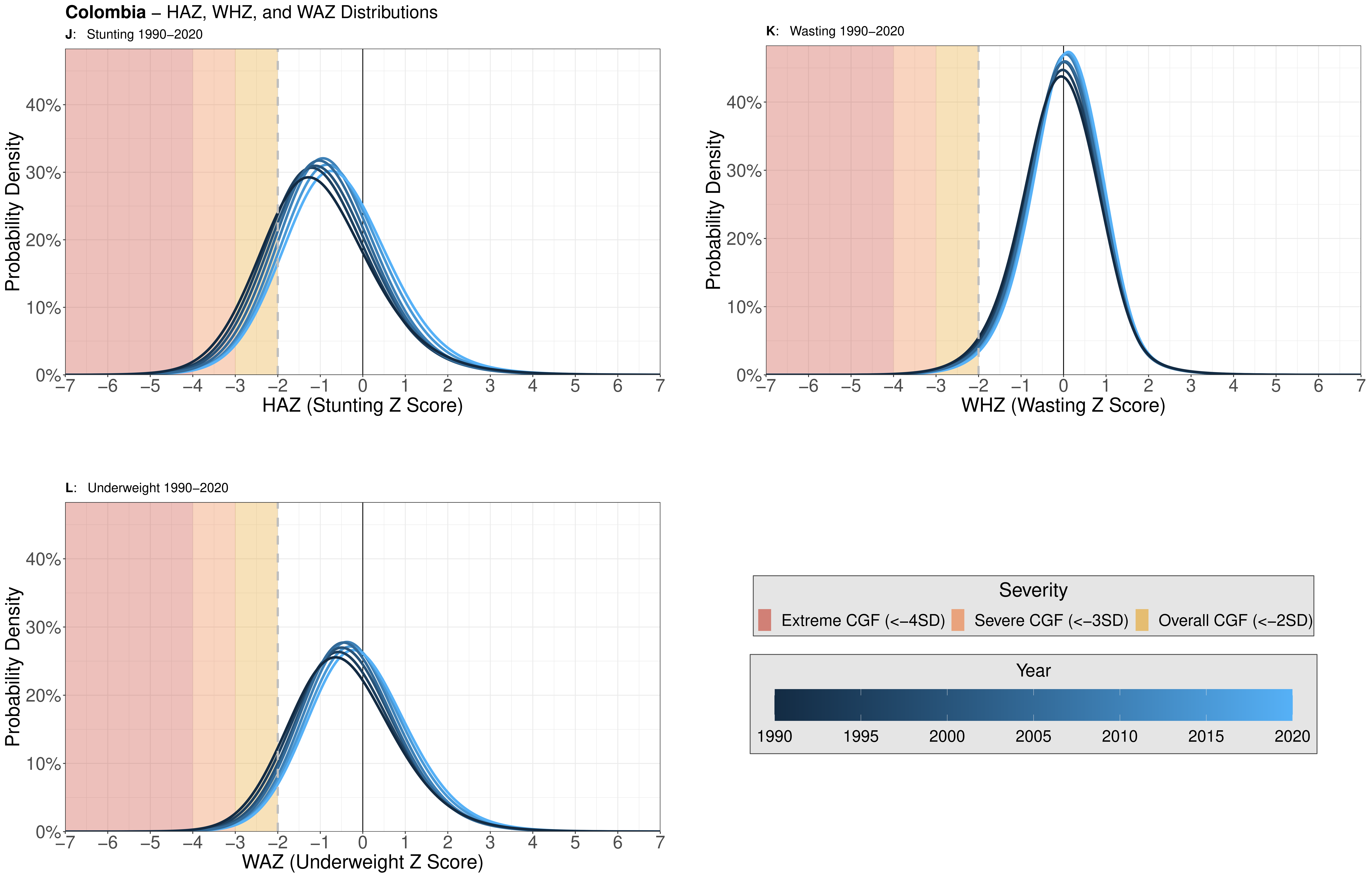

Colombia – HAZ, WHZ, and WAZ Distributions
J: Stunting 1990–2020
K: Wasting 1990–2020
L: Underweight 1990–2020
Probability Density
0%
10%
20%
30%
40%
−7 −6 −5 −4 −3 −2 −1 0 1 2 3 4 5 6 7
HAZ (Stunting Z Score)
WHZ (Wasting Z Score)
WAZ (Underweight Z Score)
Severity
Extreme CGF (<−4SD)
Severe CGF (<−3SD)
Overall CGF (<−2SD)
Year
1990
1995
2000
2005
2010
2015
2020

Costa Rica – Stunting (HAZ)

A: Overall and Severe Stunting Prevalence

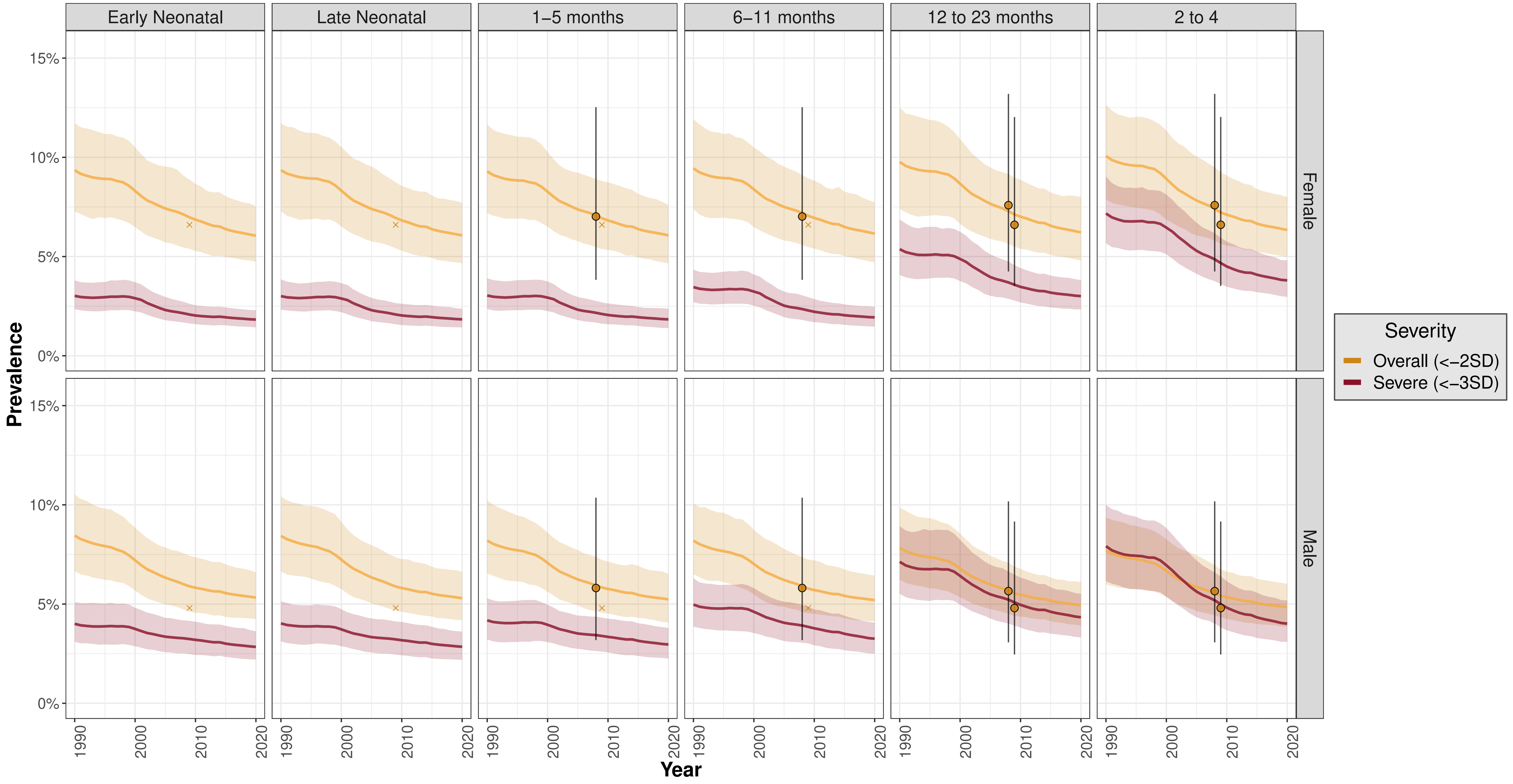

B: Transformed Mean Stunting Z Scores

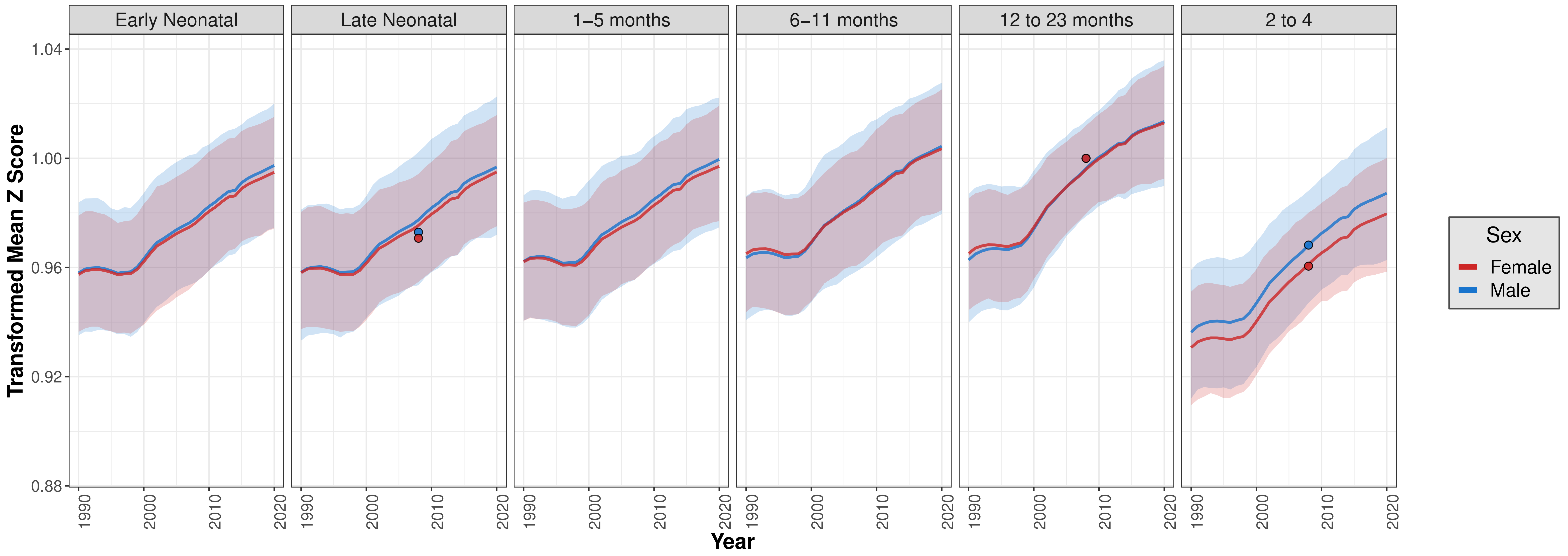

C

Year	Source
2008	WHO CGM Database
2009	WHO CGM Database

Costa Rica – Wasting (WHZ)

D: Overall and Severe Wasting Prevalence

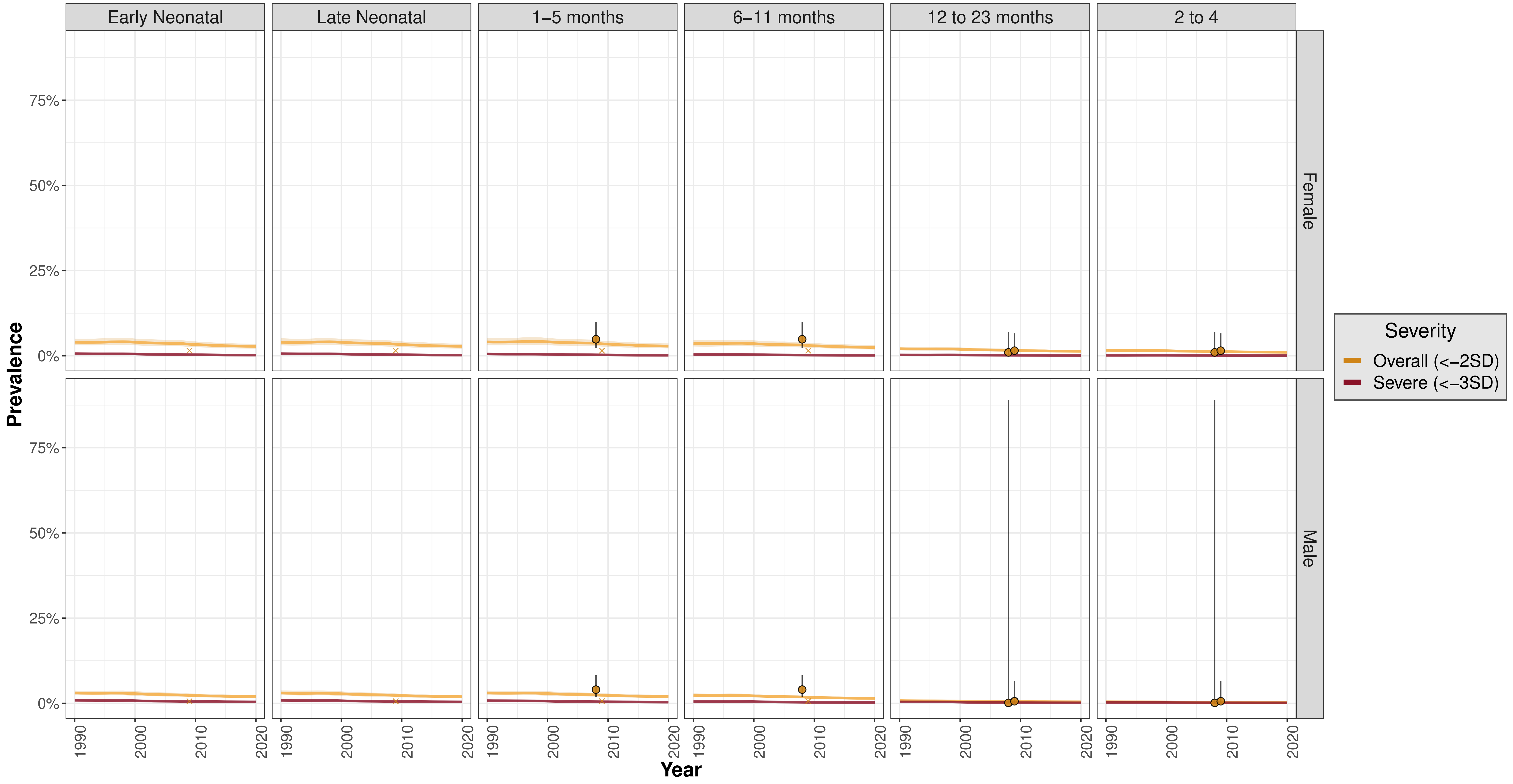

E: Transformed Mean Wasting Z Scores

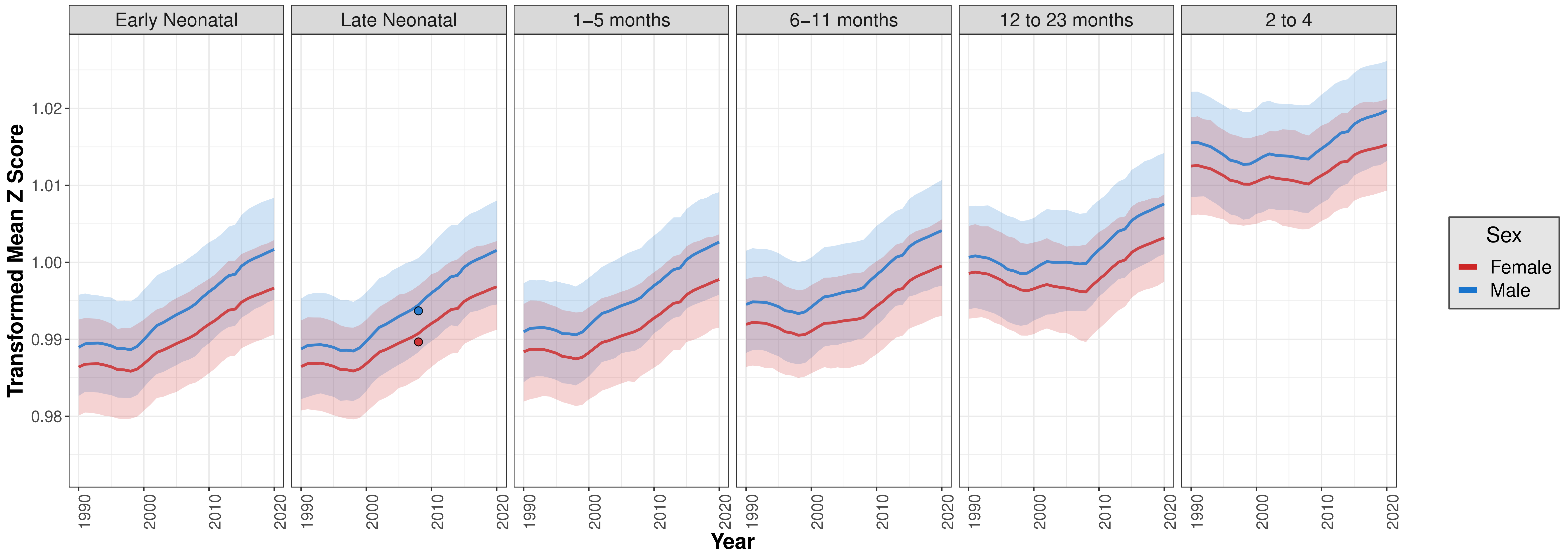

F

Year	Source
2008	WHO CGM Database
2009	WHO CGM Database

Costa Rica – Underweight (WAZ)

G: Overall and Severe Underweight Prevalence

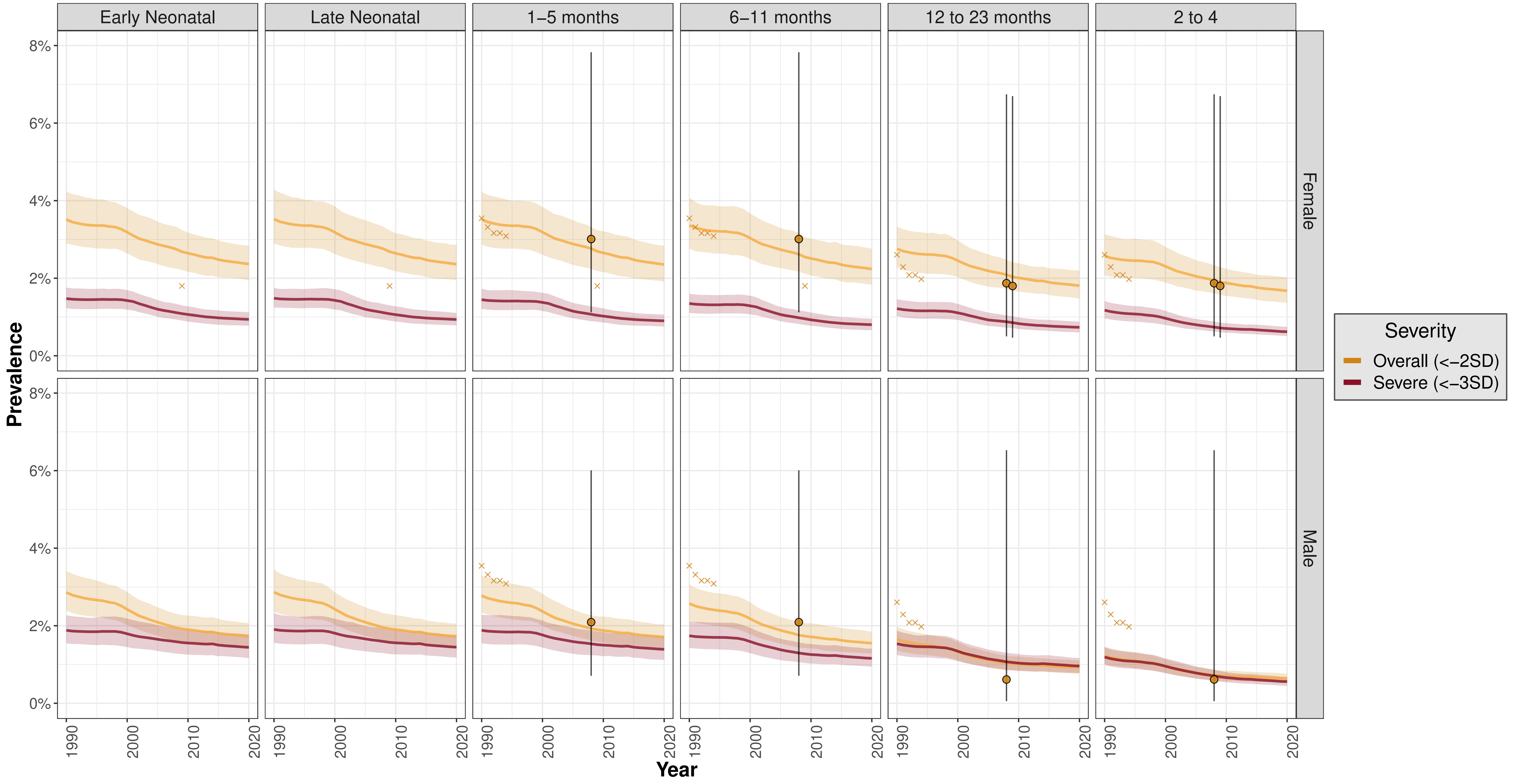

H: Transformed Mean Underweight Z Scores

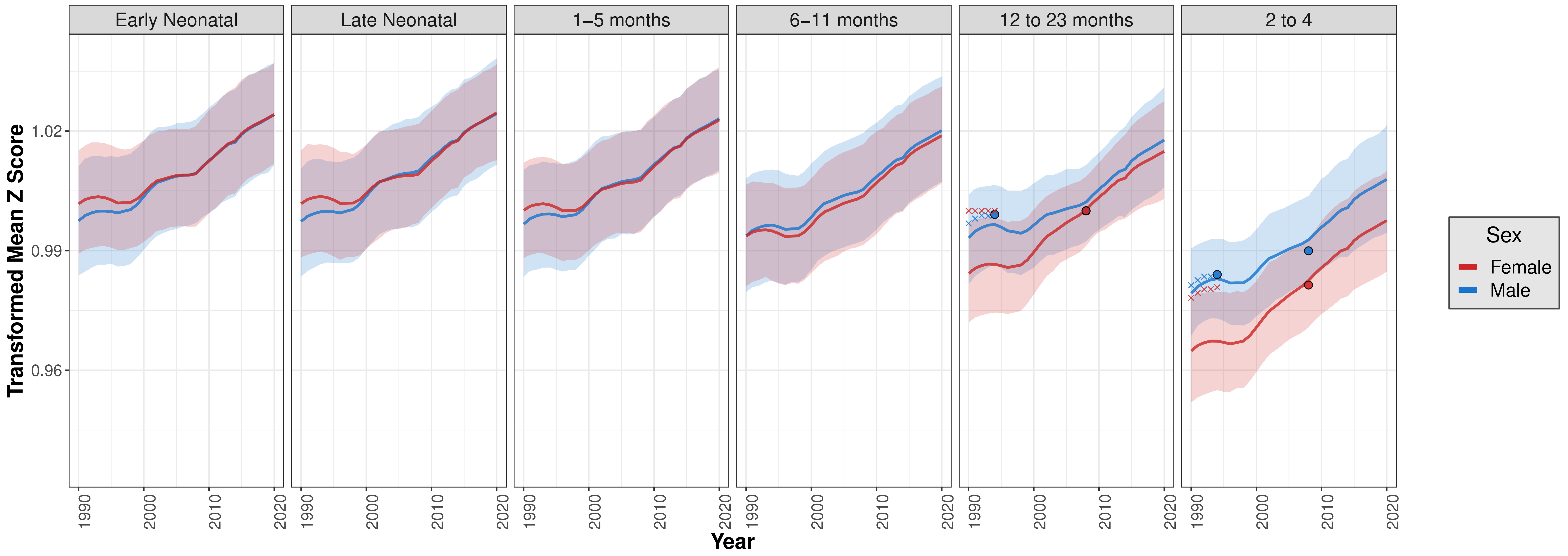

I

Year	Source
1989	WHO CGM Database
1990	WHO CGM Database
1991	WHO CGM Database
1992	WHO CGM Database
1993	WHO CGM Database
1994	WHO CGM Database
2008	WHO CGM Database
2009	WHO CGM Database

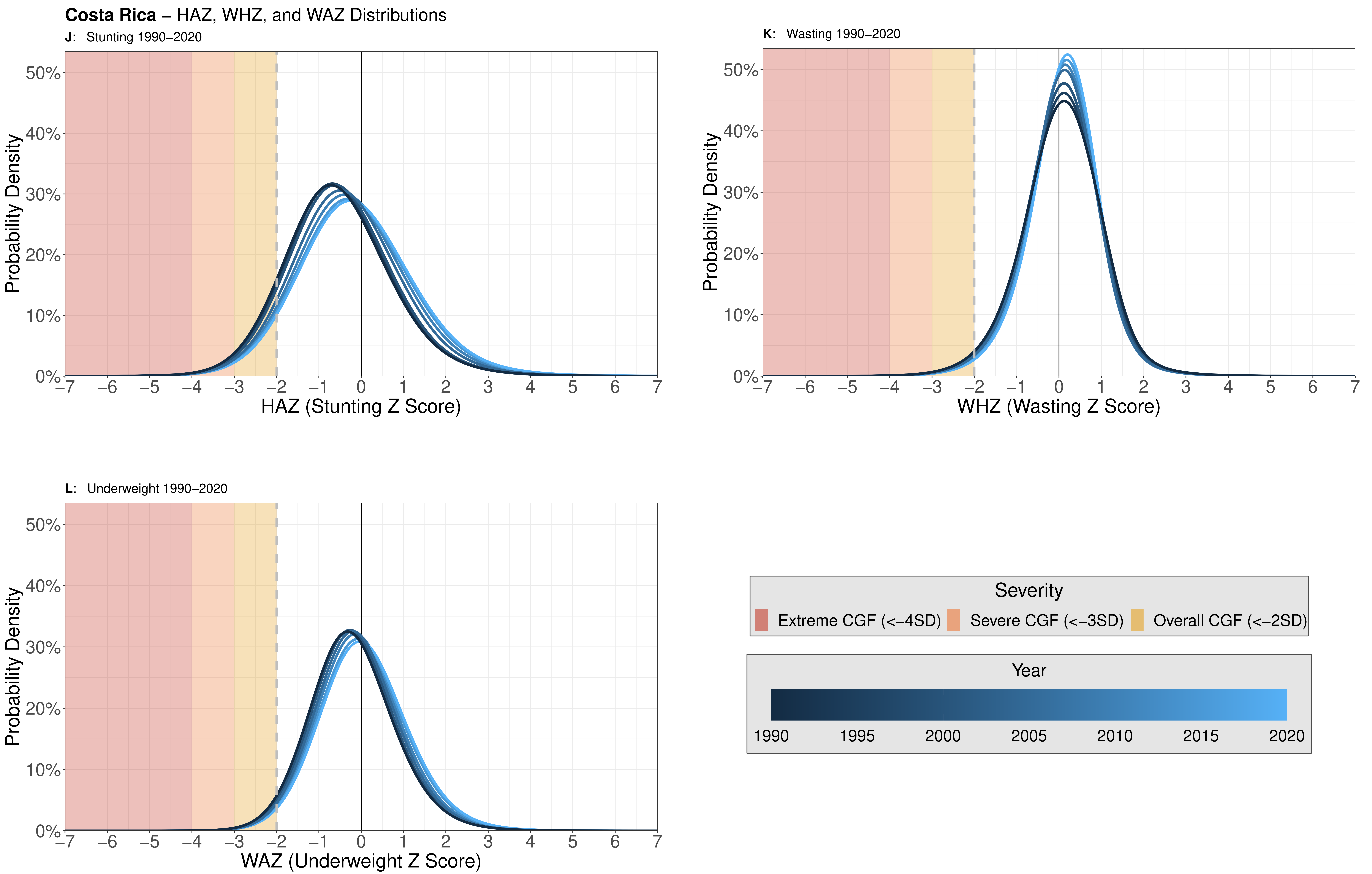

Costa Rica – HAZ, WHZ, and WAZ Distributions
J: Stunting 1990–2020
Probability Density
HAZ (Stunting Z Score)
K: Wasting 1990–2020
Probability Density
WHZ (Wasting Z Score)
L: Underweight 1990–2020
Probability Density
WAZ (Underweight Z Score)
Severity
Extreme CGF (<–4SD)
Severe CGF (<–3SD)
Overall CGF (<–2SD)
Year
1990
1995
2000
2005
2010
2015
2020

El Salvador – Stunting (HAZ)

A: Overall and Severe Stunting Prevalence

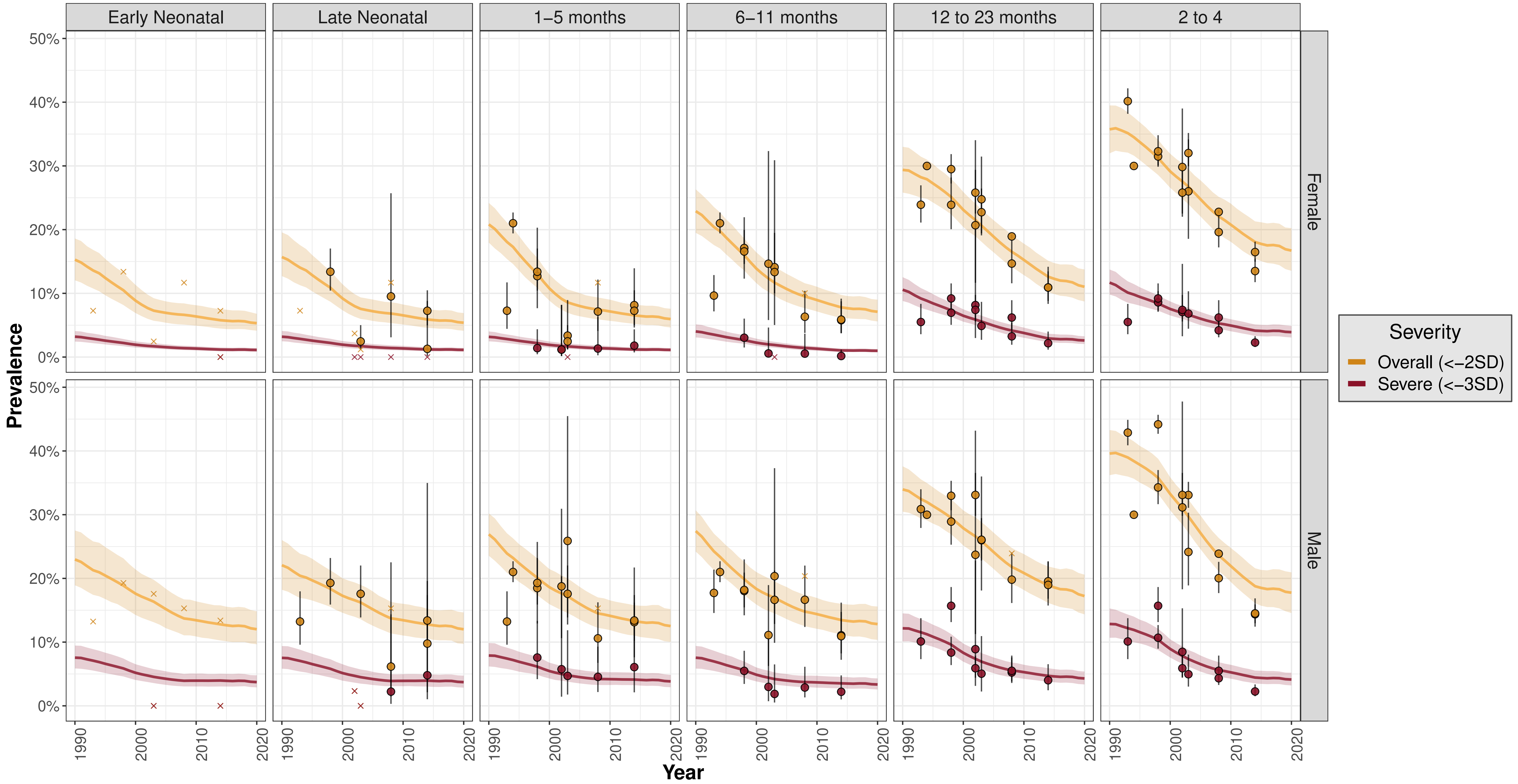

B: Transformed Mean Stunting Z Scores

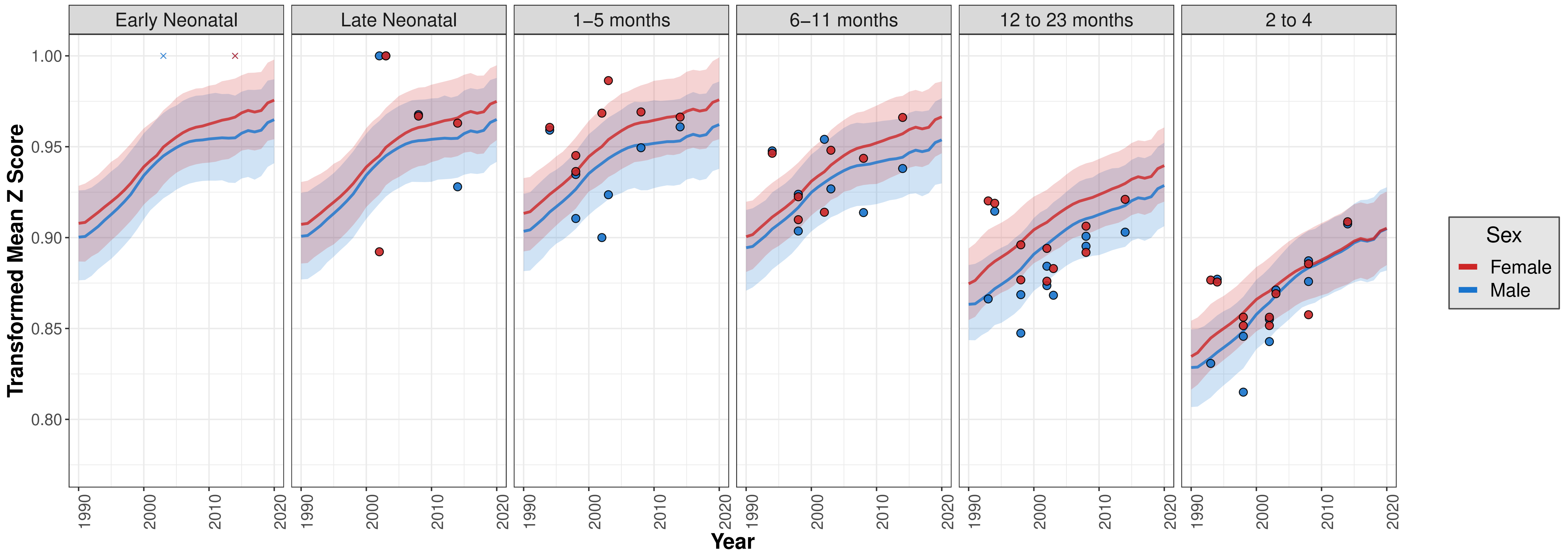

C

Year	Source
1988	WHO CGM Database
1993	WHO CGM Database
1994	WHO CGM Database
1998	Reproductive Health Survey
1998	WHO CGM Database
2002	Reproductive Health Survey
2002	WHO CGM Database
2003	Reproductive Health Survey
2003	WHO CGM Database
2008	Reproductive Health Survey
2008	WHO CGM Database
2014	WHO CGM Database
2014	MICS

El Salvador – Wasting (WHZ)

D: Overall and Severe Wasting Prevalence

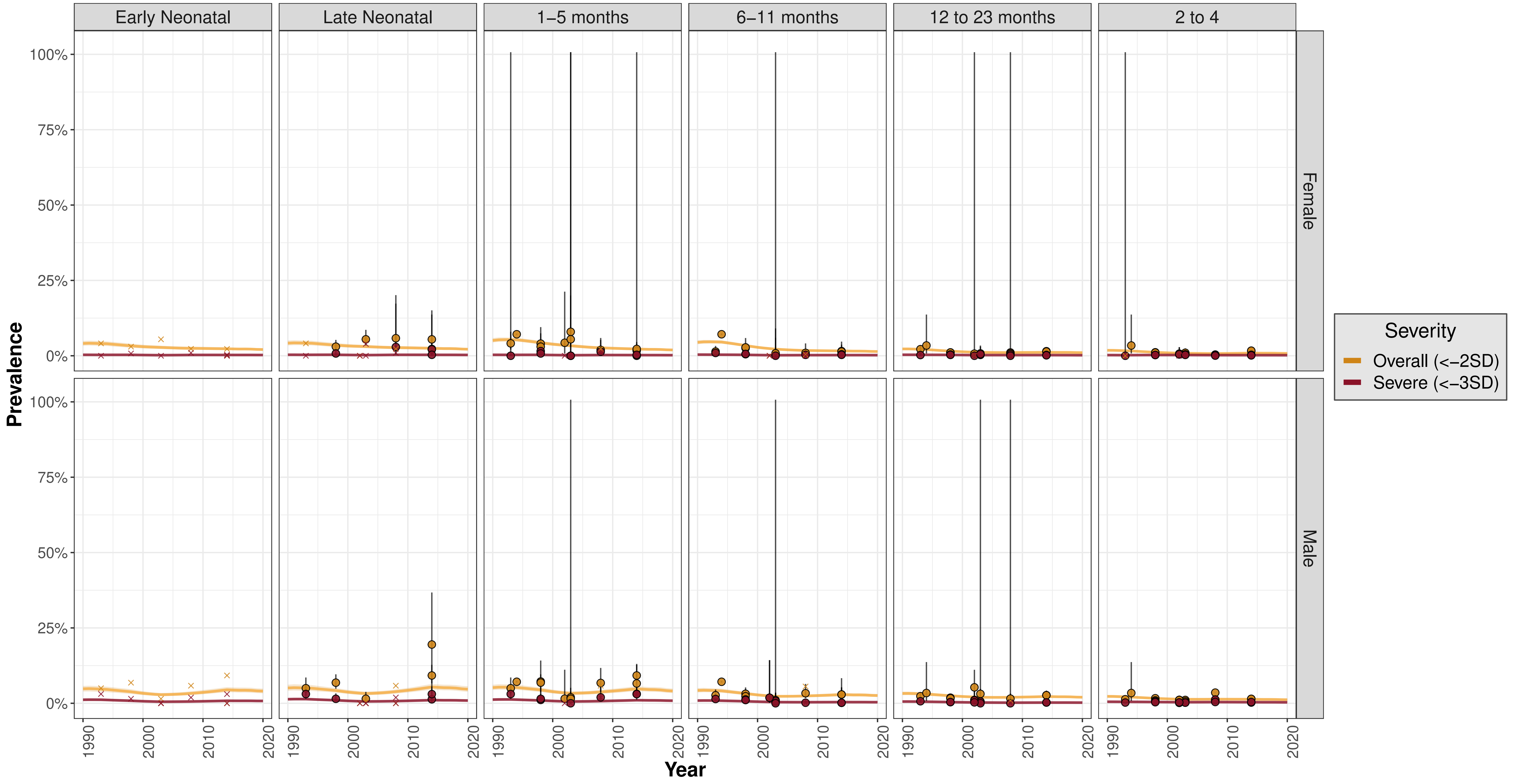

E: Transformed Mean Wasting Z Scores

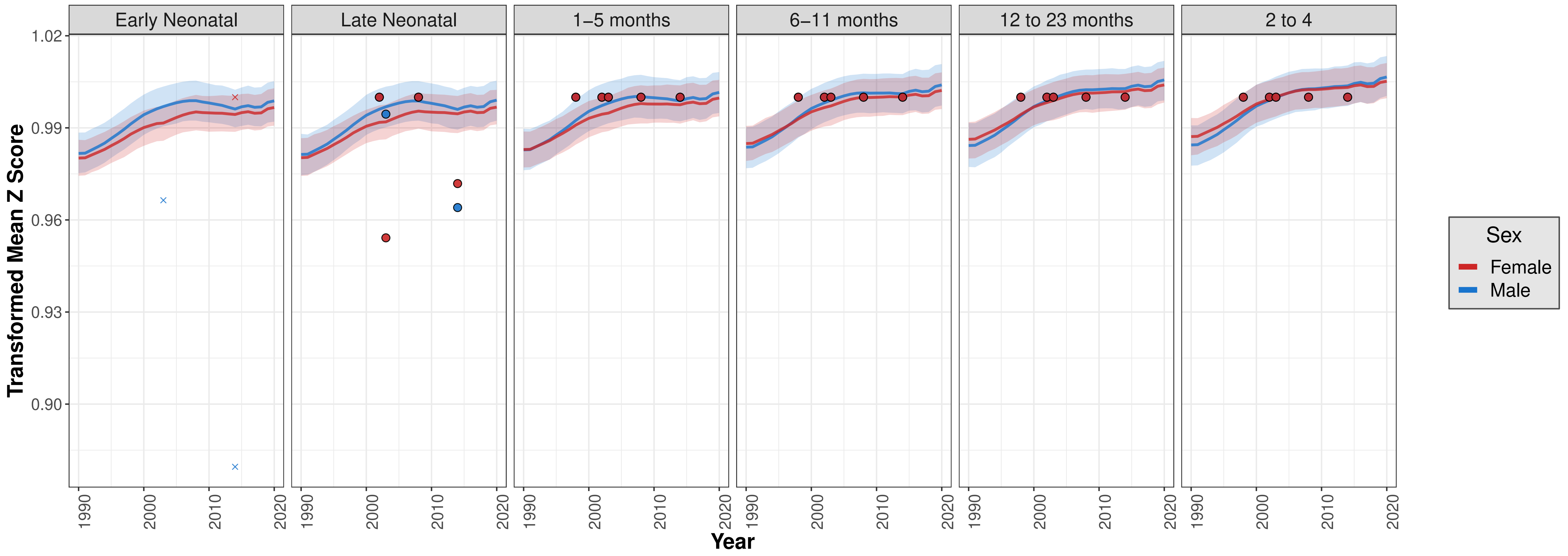

F

Year	Source
1988	WHO CGM Database
1993	WHO CGM Database
1994	WHO CGM Database
1998	Reproductive Health Survey
1998	WHO CGM Database
2002	Reproductive Health Survey
2002	WHO CGM Database
2003	Reproductive Health Survey
2003	WHO CGM Database
2008	Reproductive Health Survey
2008	WHO CGM Database
2014	WHO CGM Database
2014	MICS

El Salvador – Underweight (WAZ)

G: Overall and Severe Underweight Prevalence

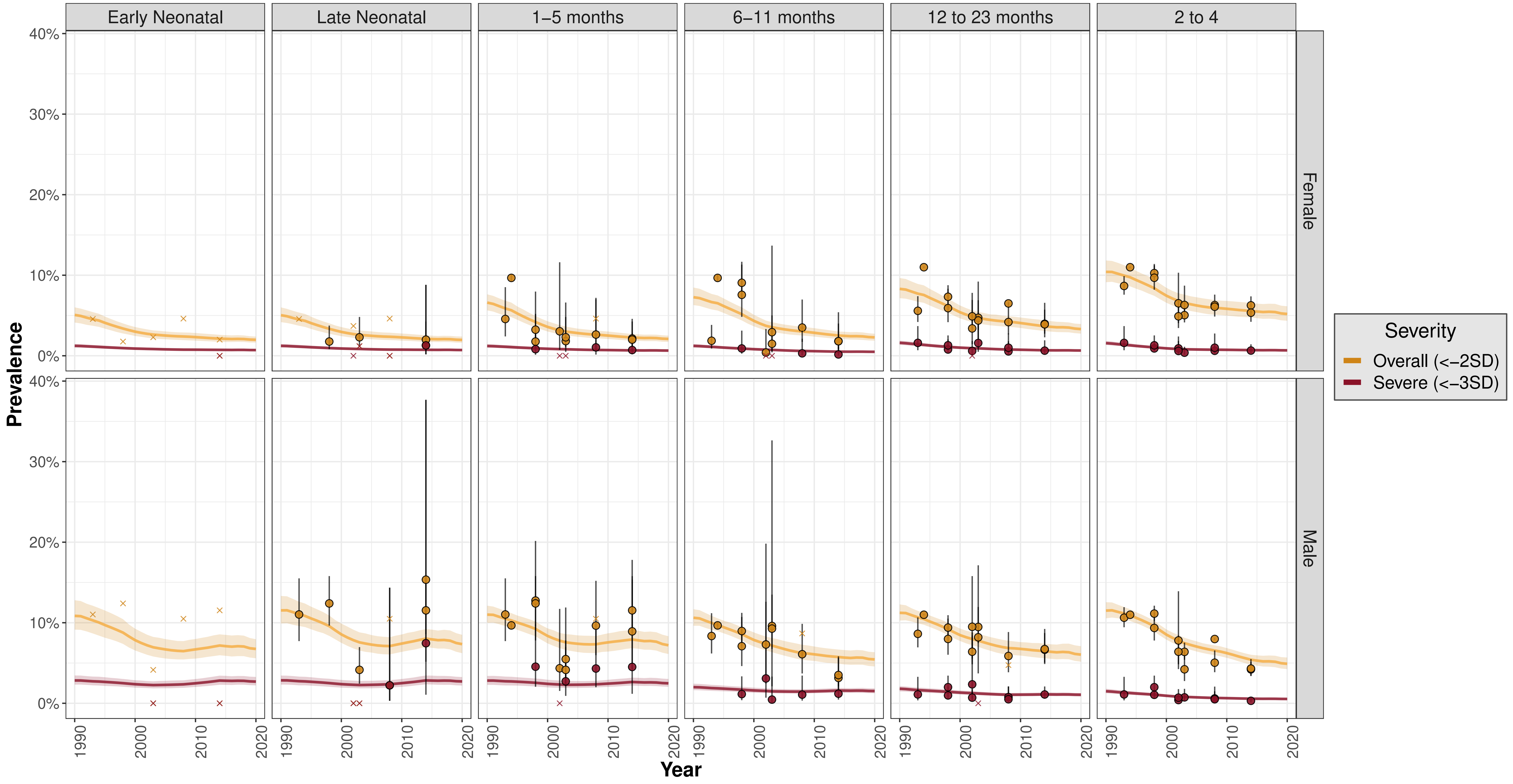

H: Transformed Mean Underweight Z Scores

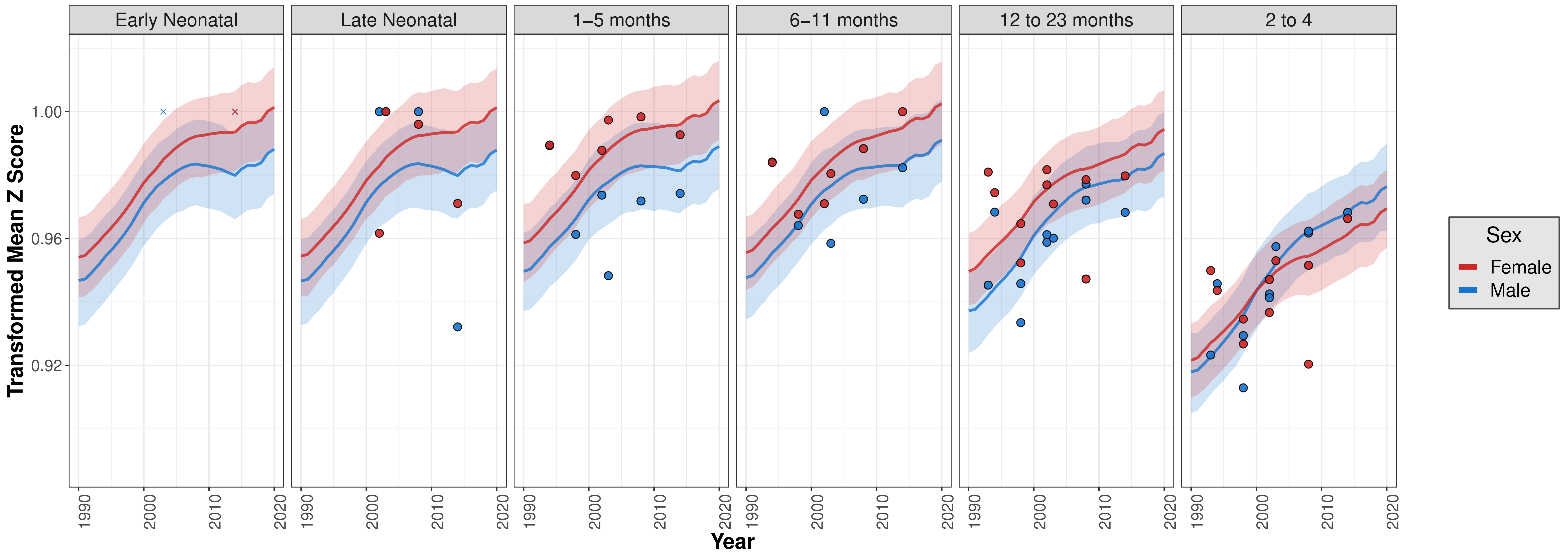

I

Year	Source
1988	WHO CGM Database
1993	WHO CGM Database
1994	WHO CGM Database
1998	Reproductive Health Survey
1998	WHO CGM Database
2002	Reproductive Health Survey
2002	WHO CGM Database
2003	Reproductive Health Survey
2003	WHO CGM Database
2008	Reproductive Health Survey
2008	WHO CGM Database
2014	WHO CGM Database
2014	MICS

El Salvador – HAZ, WHZ, and WAZ Distributions

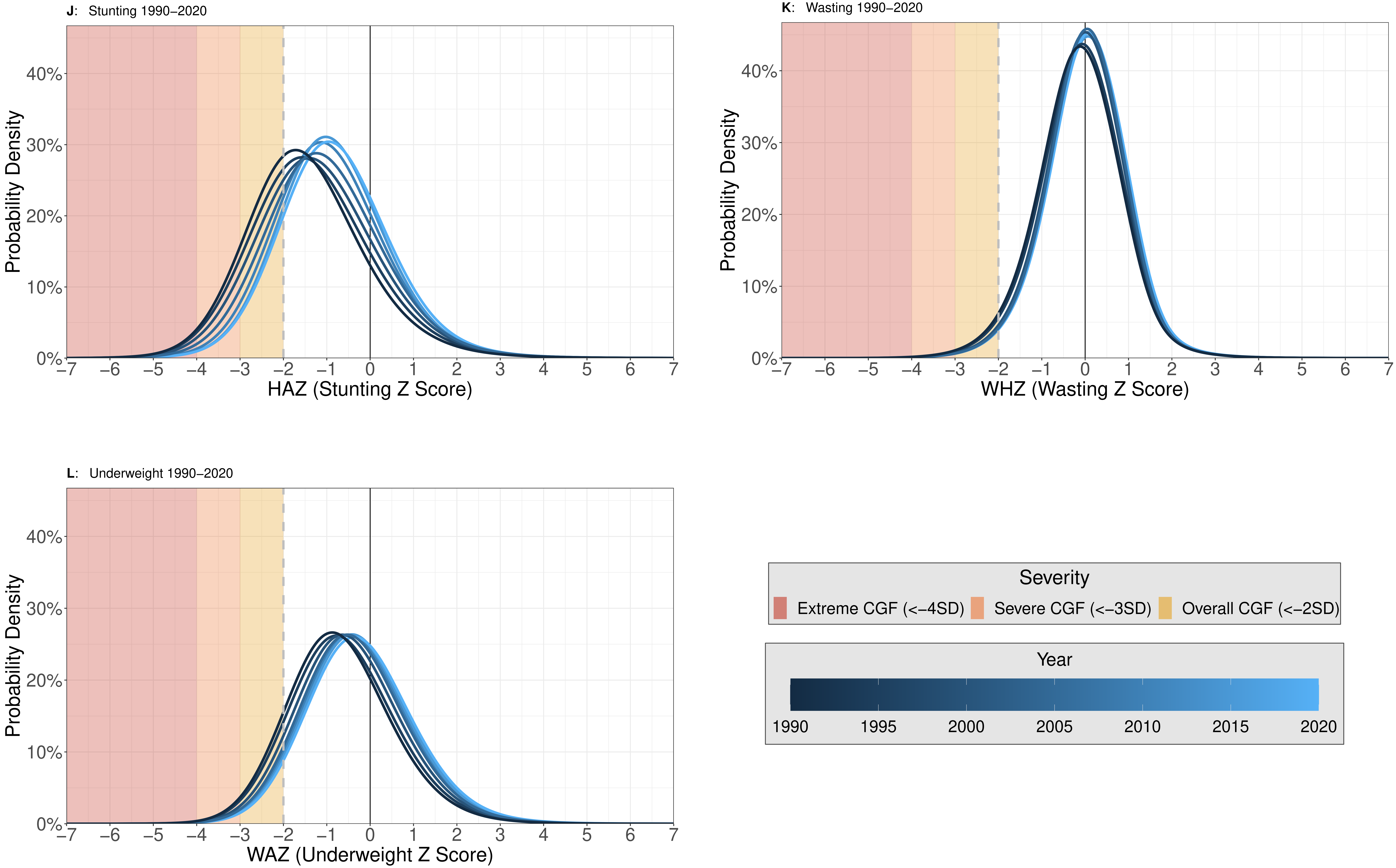

Guatemala – Stunting (HAZ)

A: Overall and Severe Stunting Prevalence

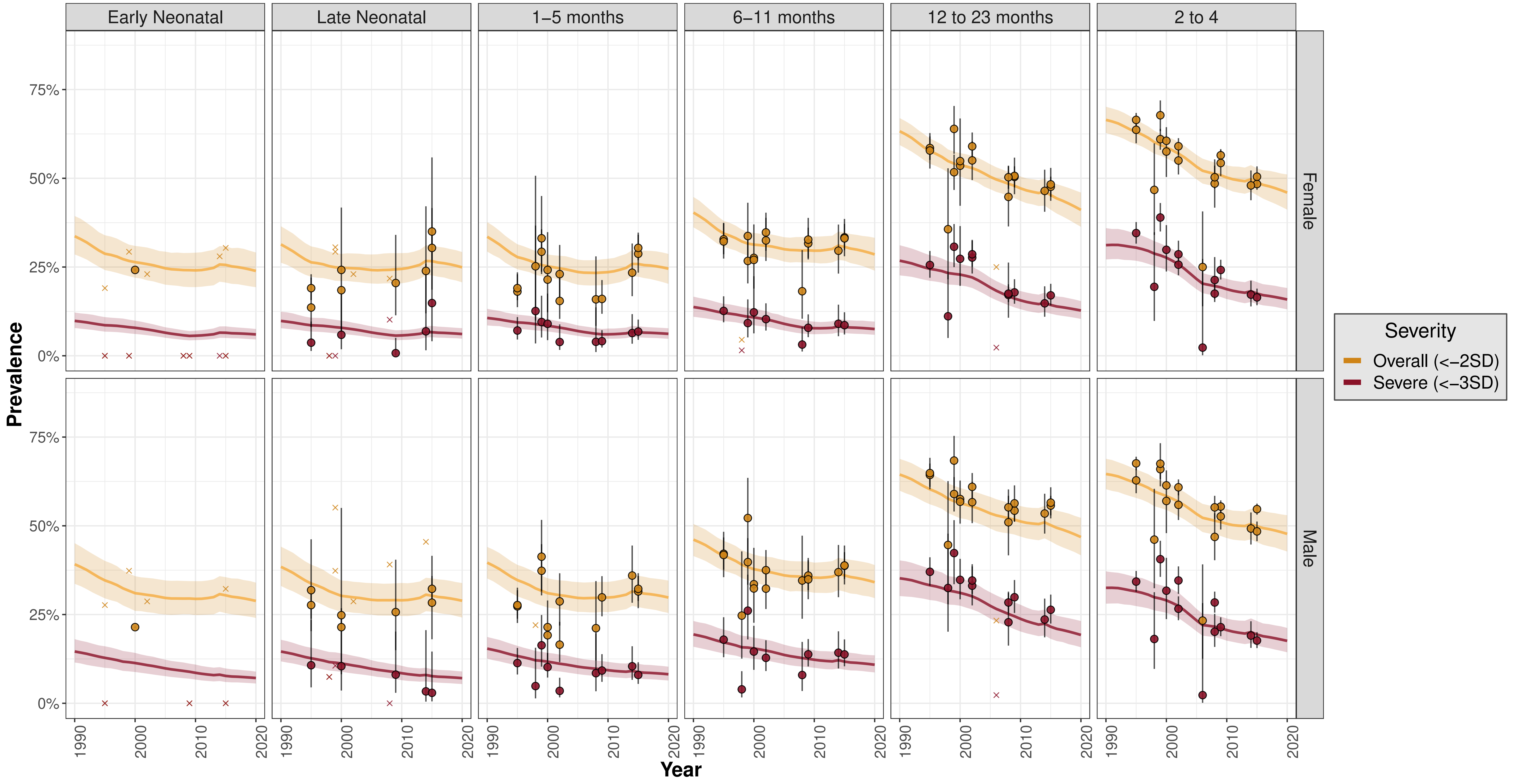

B: Transformed Mean Stunting Z Scores

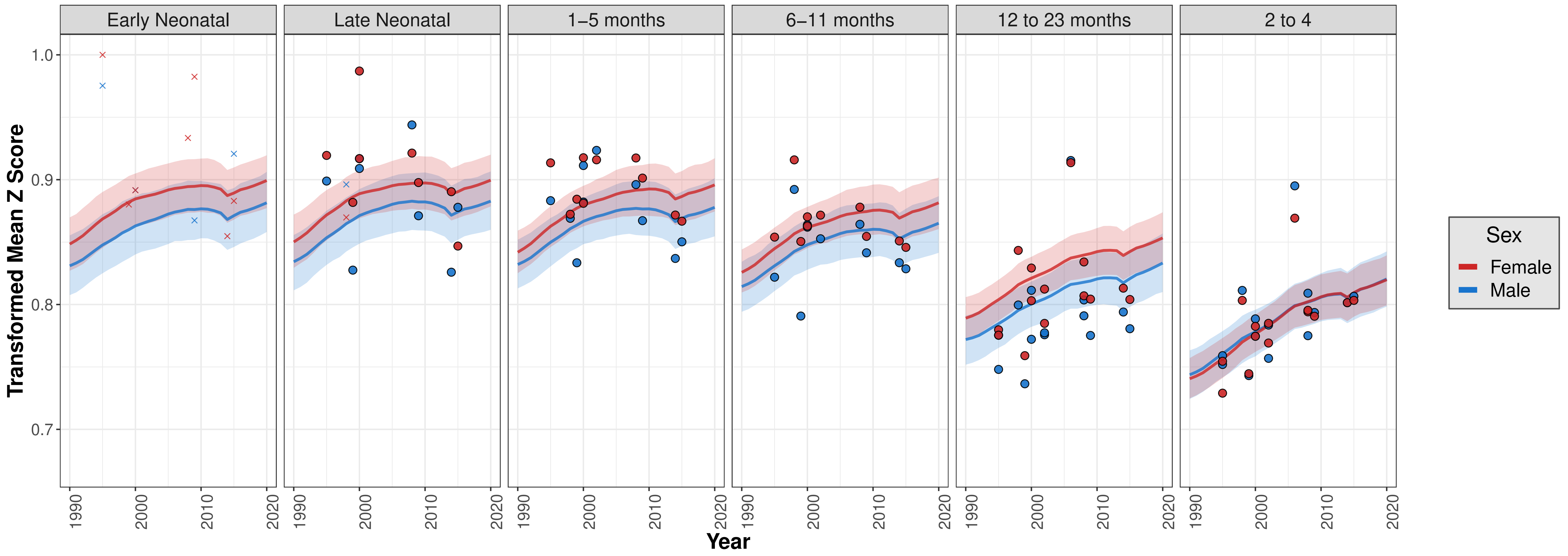

C

Year	Source
1985	WHO CGM Database
1987	DHS
1987	WHO CGM Database
1988	WHO CGM Database
1995	DHS
1995	WHO CGM Database
1998	Interim DHS
1999	Interim DHS
1999	WHO CGM Database
2000	Living Standards Measurement Survey
2000	WHO CGM Database
2002	Reproductive Health Survey
2002	WHO CGM Database
2006	WHO CGM Database
2008	Reproductive Health Survey
2008	WHO CGM Database
2009	Reproductive Health Survey
2009	WHO CGM Database
2014	DHS
2015	WHO CGM Database
2015	DHS

Guatemala – Wasting (WHZ)

D: Overall and Severe Wasting Prevalence

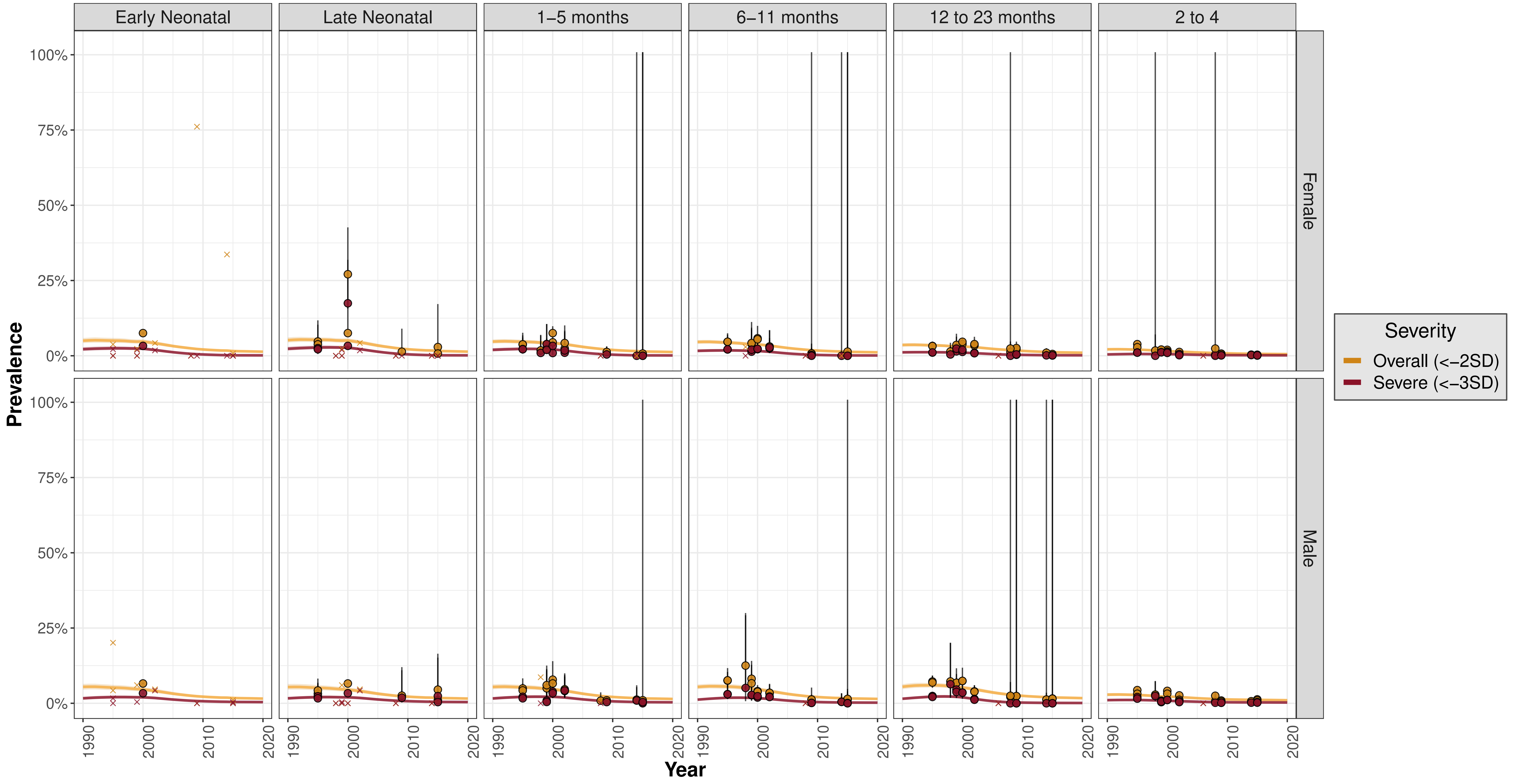

E: Transformed Mean Wasting Z Scores

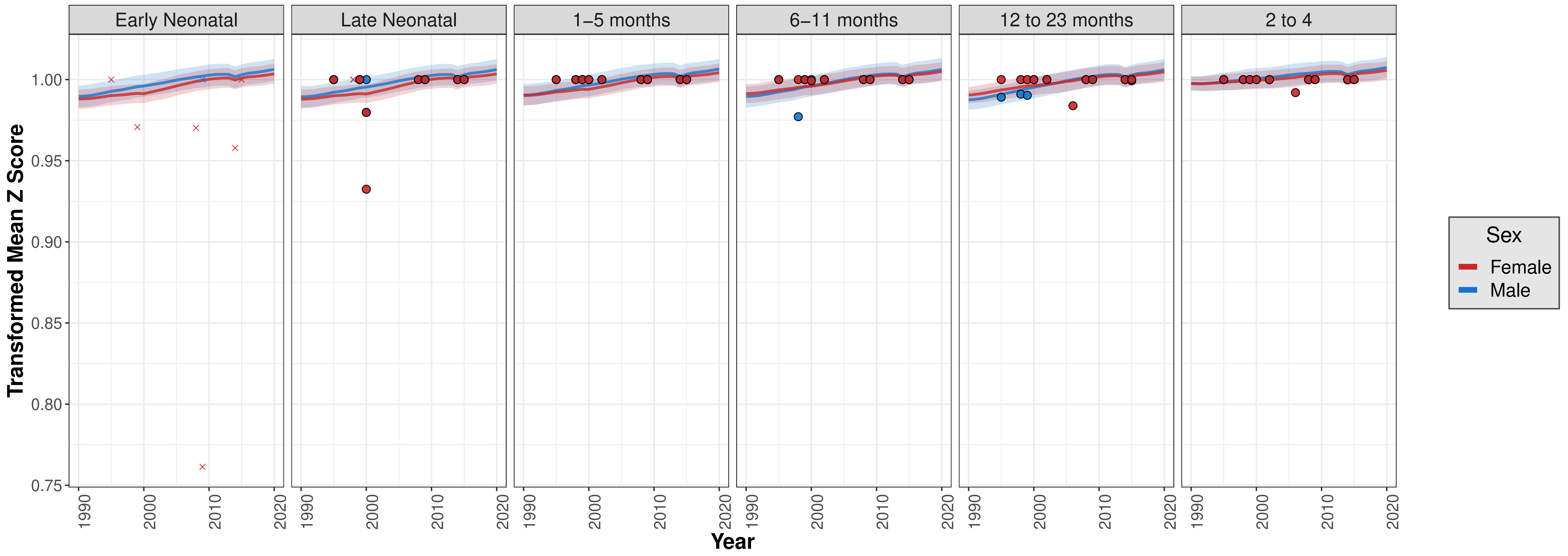

F

Year	Source
1985	WHO CGM Database
1987	DHS
1987	WHO CGM Database
1988	WHO CGM Database
1995	DHS
1995	WHO CGM Database
1998	Interim DHS
1999	Interim DHS
1999	WHO CGM Database
2000	Living Standards Measurement Survey
2000	WHO CGM Database
2002	Reproductive Health Survey
2002	WHO CGM Database
2006	WHO CGM Database
2008	Reproductive Health Survey
2008	WHO CGM Database
2009	Reproductive Health Survey
2009	WHO CGM Database
2014	DHS
2015	WHO CGM Database
2015	DHS

Guatemala – Underweight (WAZ)

G: Overall and Severe Underweight Prevalence

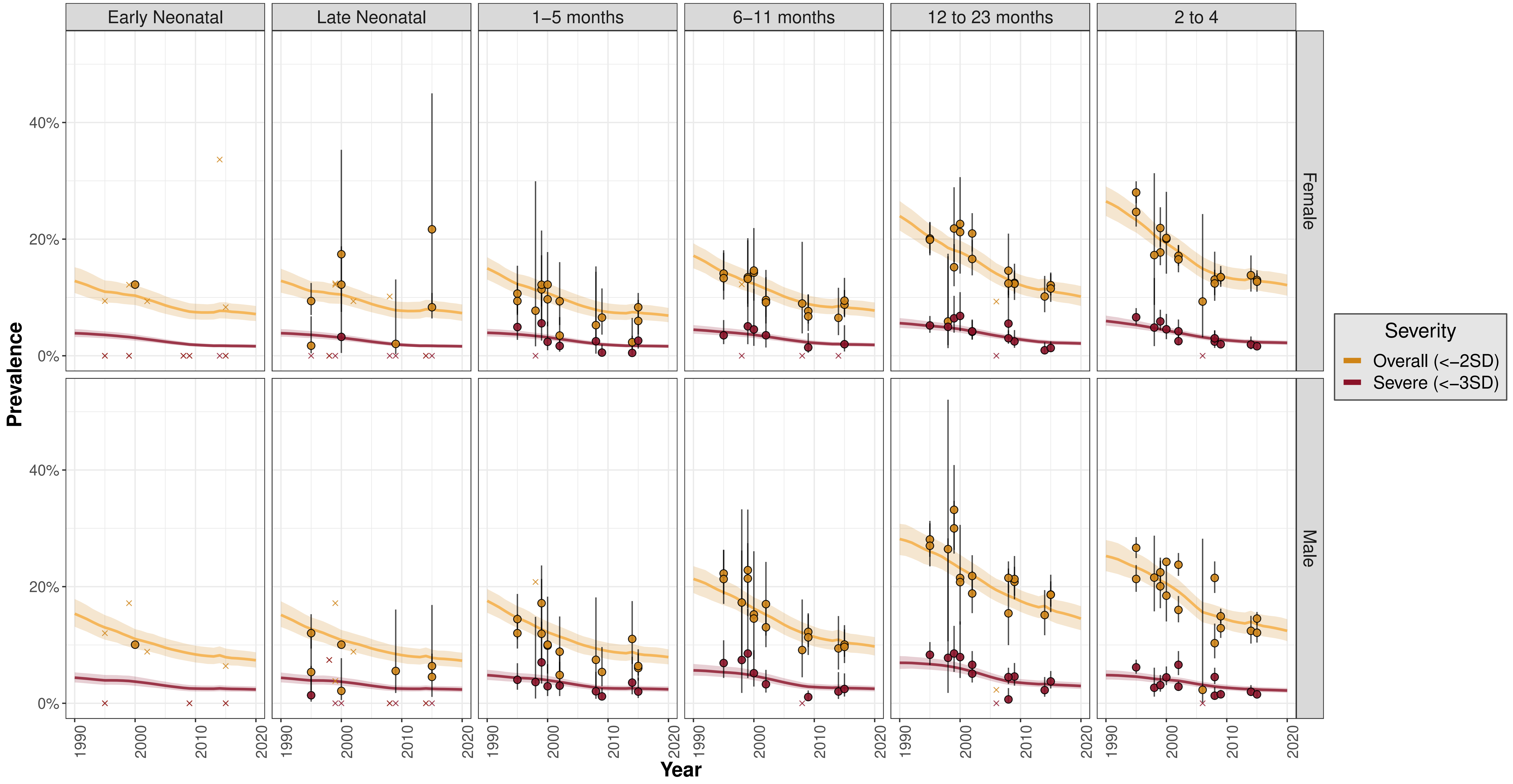

H: Transformed Mean Underweight Z Scores

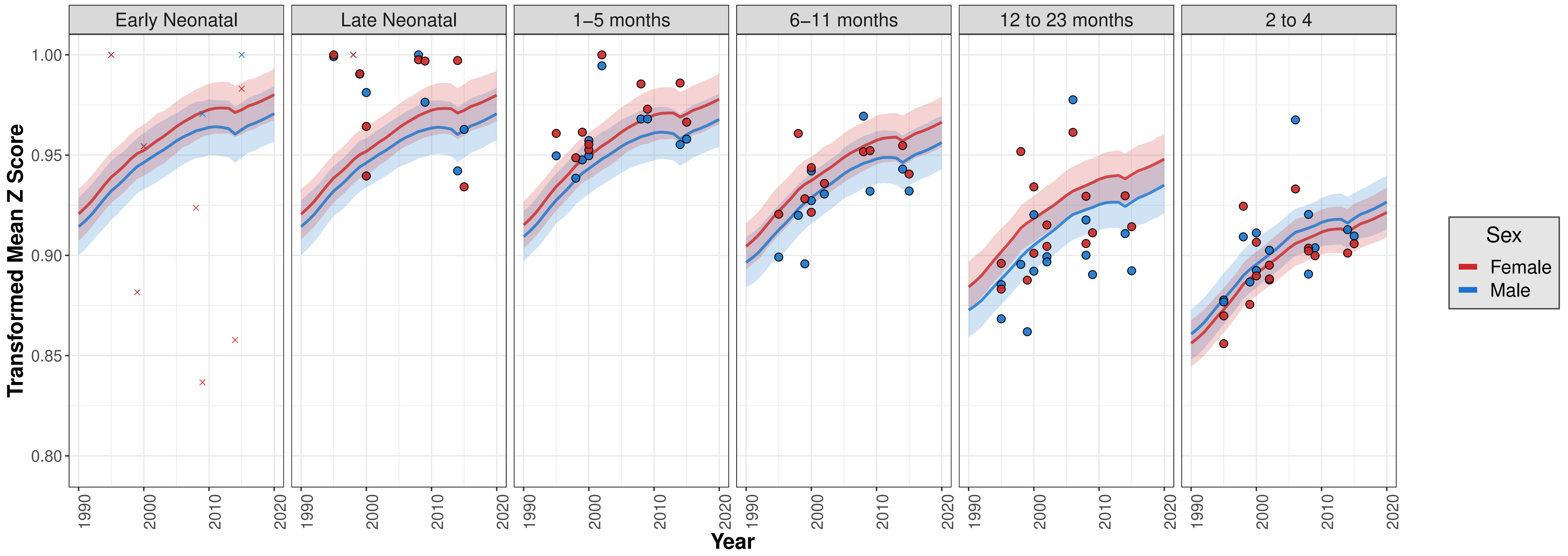

I

Year	Source
1985	WHO CGM Database
1987	DHS
1987	WHO CGM Database
1988	WHO CGM Database
1995	DHS
1995	WHO CGM Database
1998	Interim DHS
1999	Interim DHS
1999	WHO CGM Database
2000	Living Standards Measurement Survey
2000	WHO CGM Database
2002	Reproductive Health Survey
2002	WHO CGM Database
2006	WHO CGM Database
2008	Reproductive Health Survey
2008	WHO CGM Database
2009	Reproductive Health Survey
2009	WHO CGM Database
2014	DHS
2015	WHO CGM Database
2015	DHS

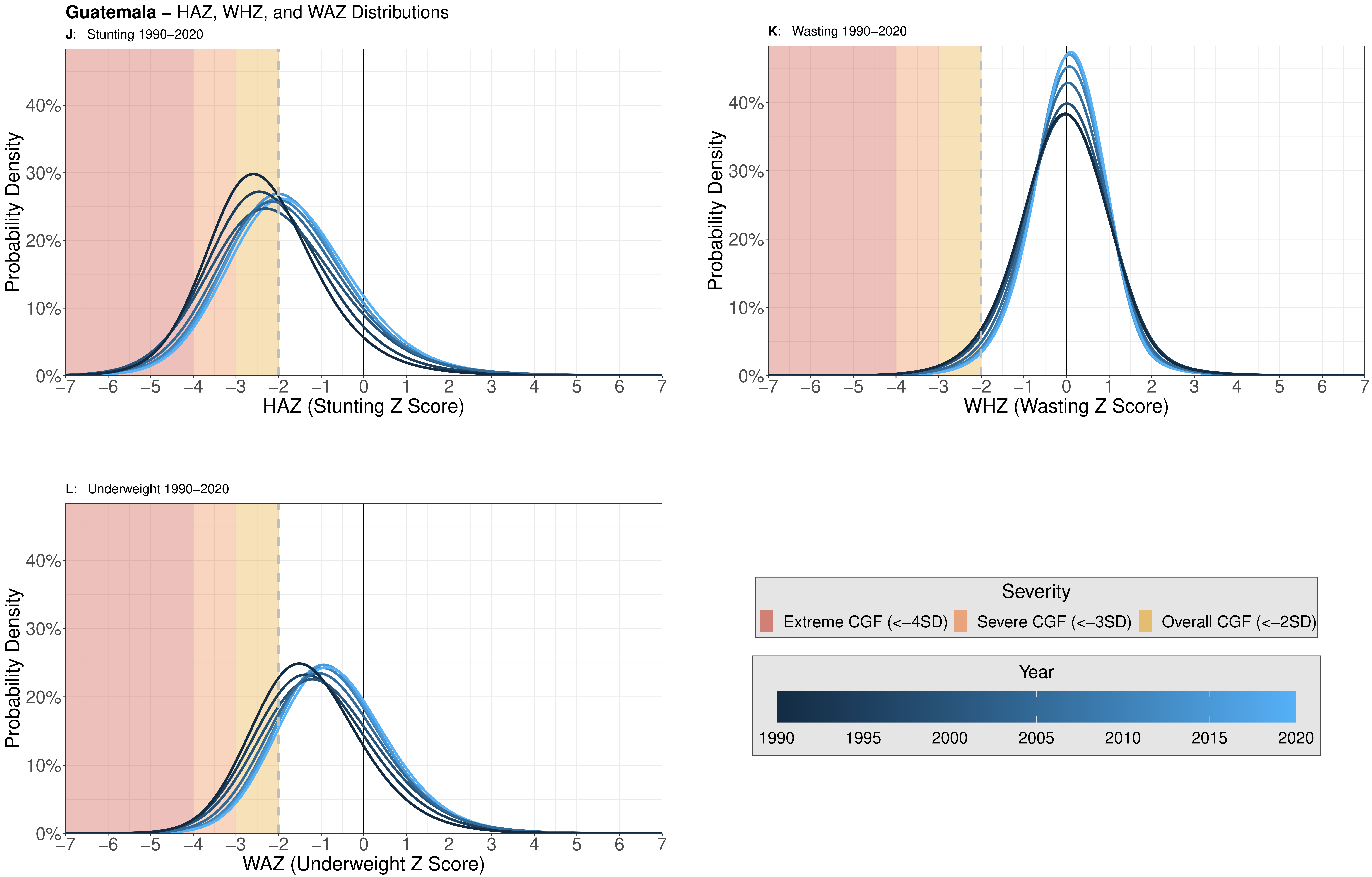
Guatemala – HAZ, WHZ, and WAZ Distributions
J: Stunting 1990–2020
Probability Density
0%
10%
20%
30%
40%
−7 −6 −5 −4 −3 −2 −1 0 1 2 3 4 5 6 7
HAZ (Stunting Z Score)
K: Wasting 1990–2020
Probability Density
WHZ (Wasting Z Score)
L: Underweight 1990–2020
Probability Density
WAZ (Underweight Z Score)
Severity
Extreme CGF (<−4SD)
Severe CGF (<−3SD)
Overall CGF (<−2SD)
Year
1990
1995
2000
2005
2010
2015
2020

Honduras – Stunting (HAZ)

A: Overall and Severe Stunting Prevalence

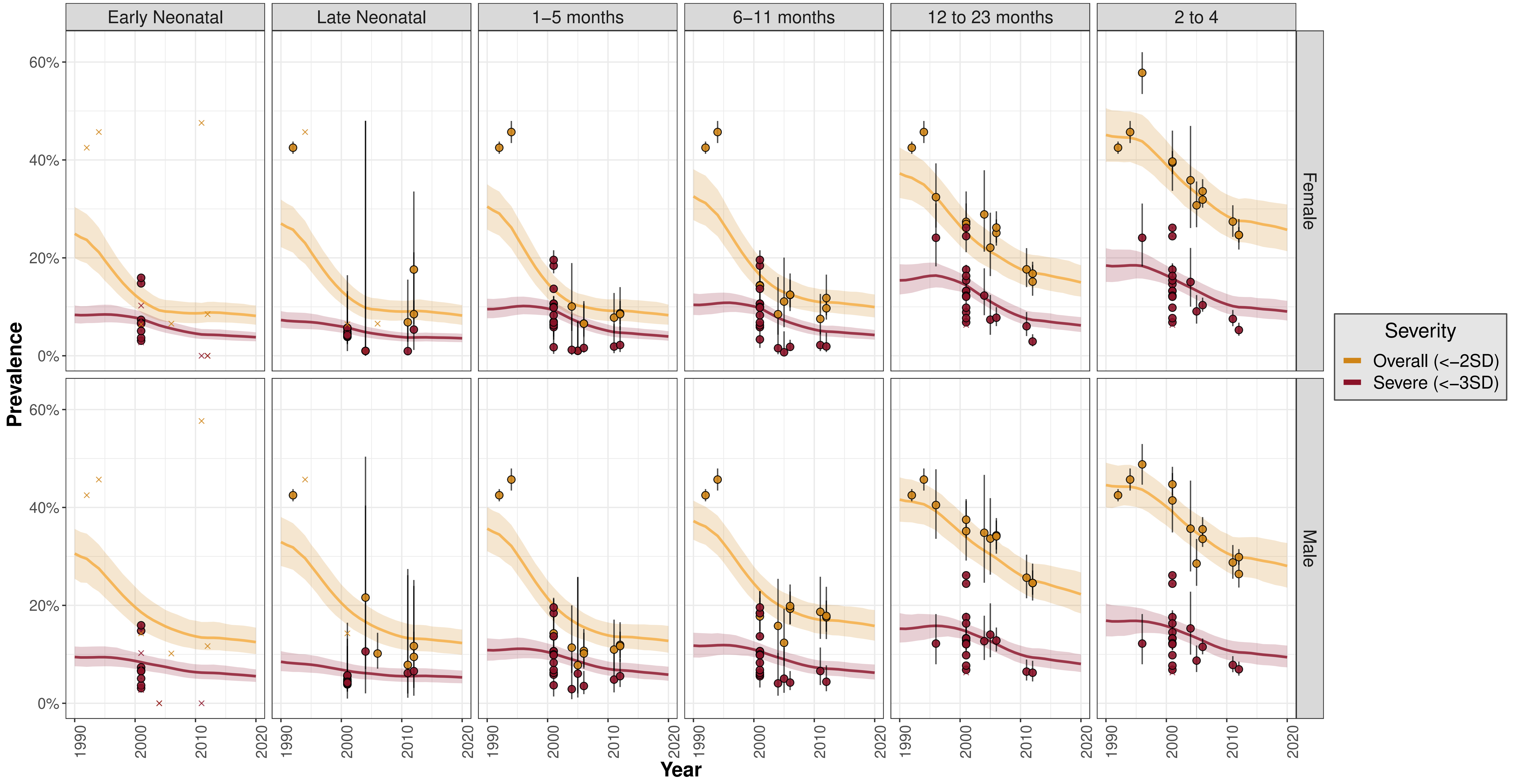

B: Transformed Mean Stunting Z Scores

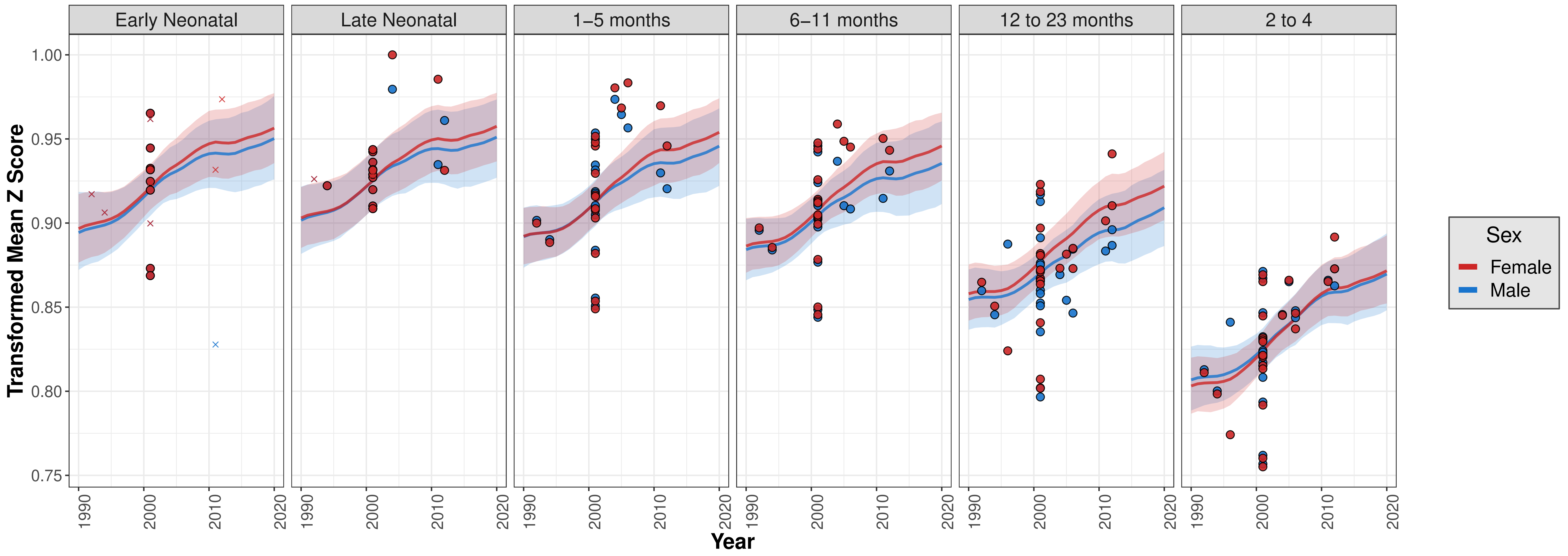

C

Year	Source
1987	WHO CGM Database
1992	WHO CGM Database
1994	WHO CGM Database
1996	WHO CGM Database
2001	Reproductive Health Survey
2001	WHO CGM Database
2004	Survey of Living Conditions
2005	DHS
2006	DHS
2006	WHO CGM Database
2011	DHS
2012	DHS
2012	WHO CGM Database

Honduras – Wasting (WHZ)

D: Overall and Severe Wasting Prevalence

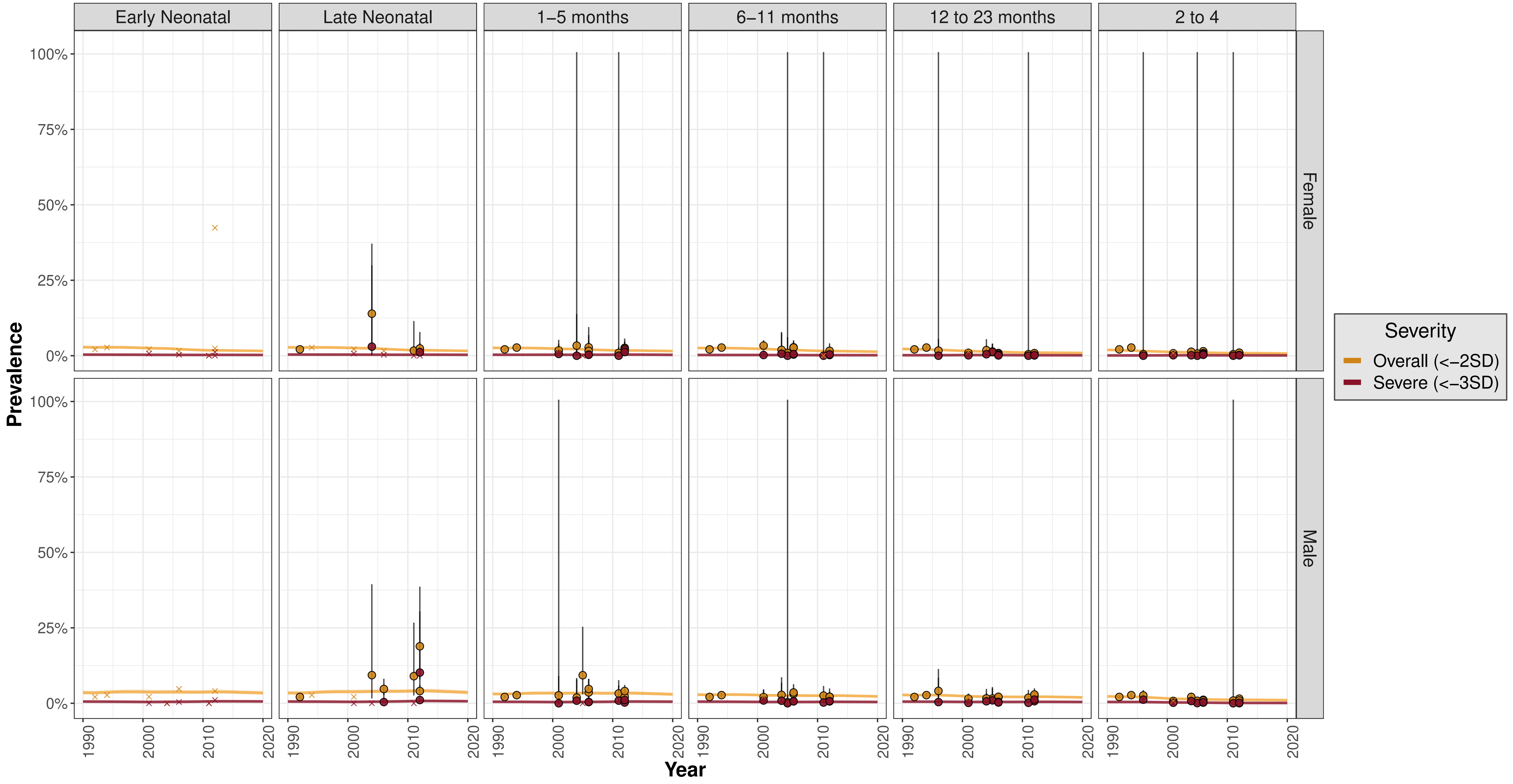

E: Transformed Mean Wasting Z Scores

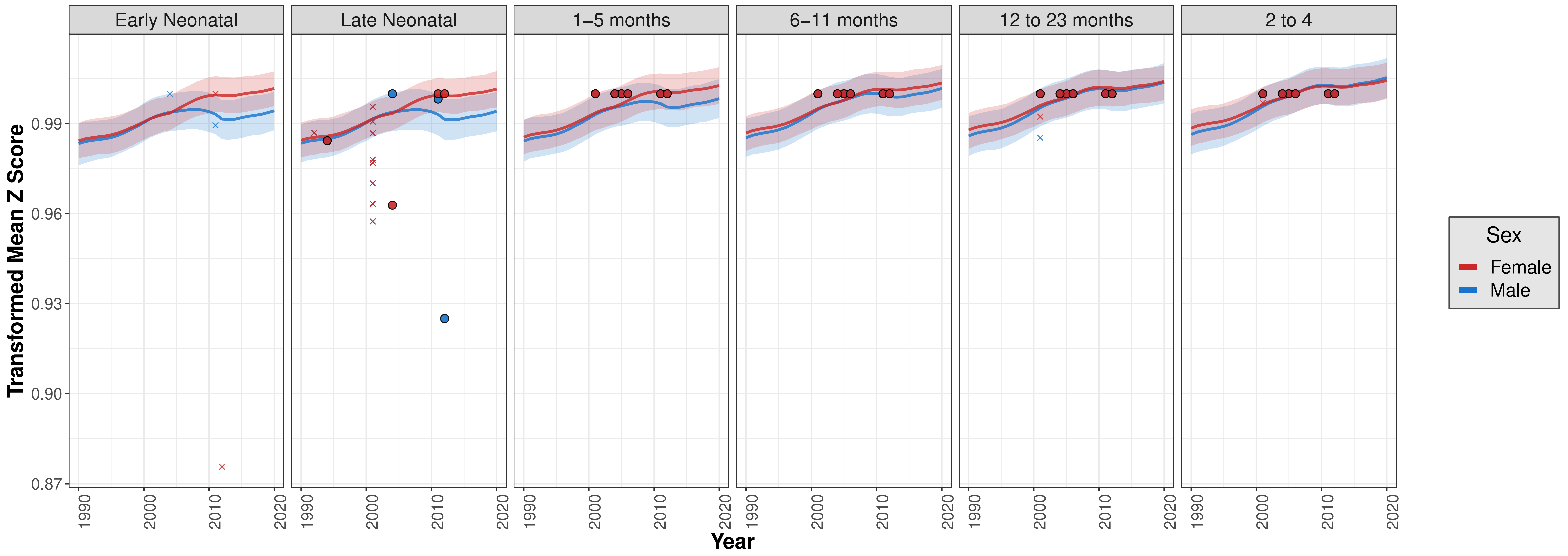

F

Year	Source
1987	WHO CGM Database
1992	WHO CGM Database
1994	WHO CGM Database
1996	WHO CGM Database
2001	Reproductive Health Survey
2001	WHO CGM Database
2004	Survey of Living Conditions
2005	DHS
2006	DHS
2006	WHO CGM Database
2011	DHS
2012	DHS
2012	WHO CGM Database

Honduras – Underweight (WAZ)

G: Overall and Severe Underweight Prevalence

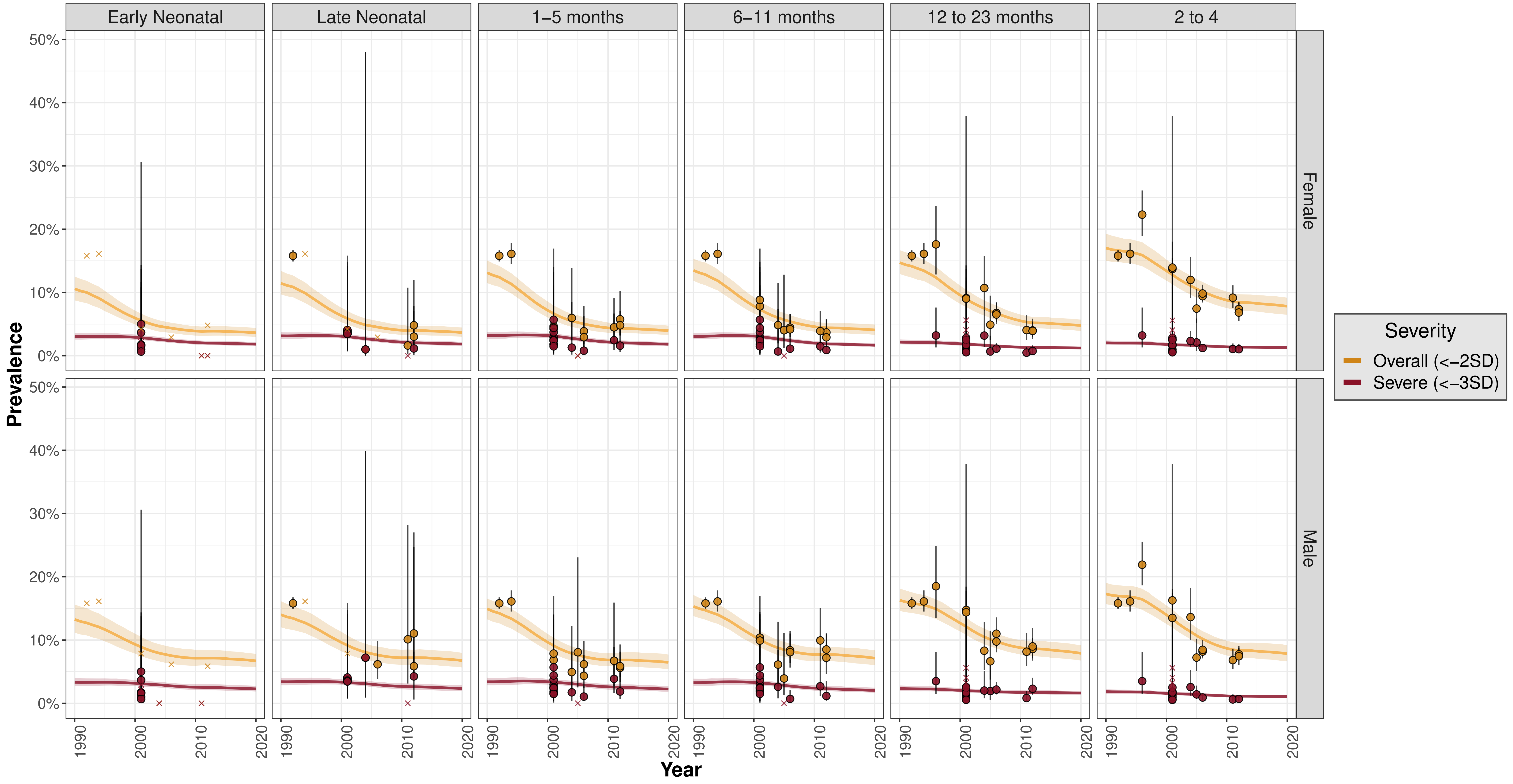

H: Transformed Mean Underweight Z Scores

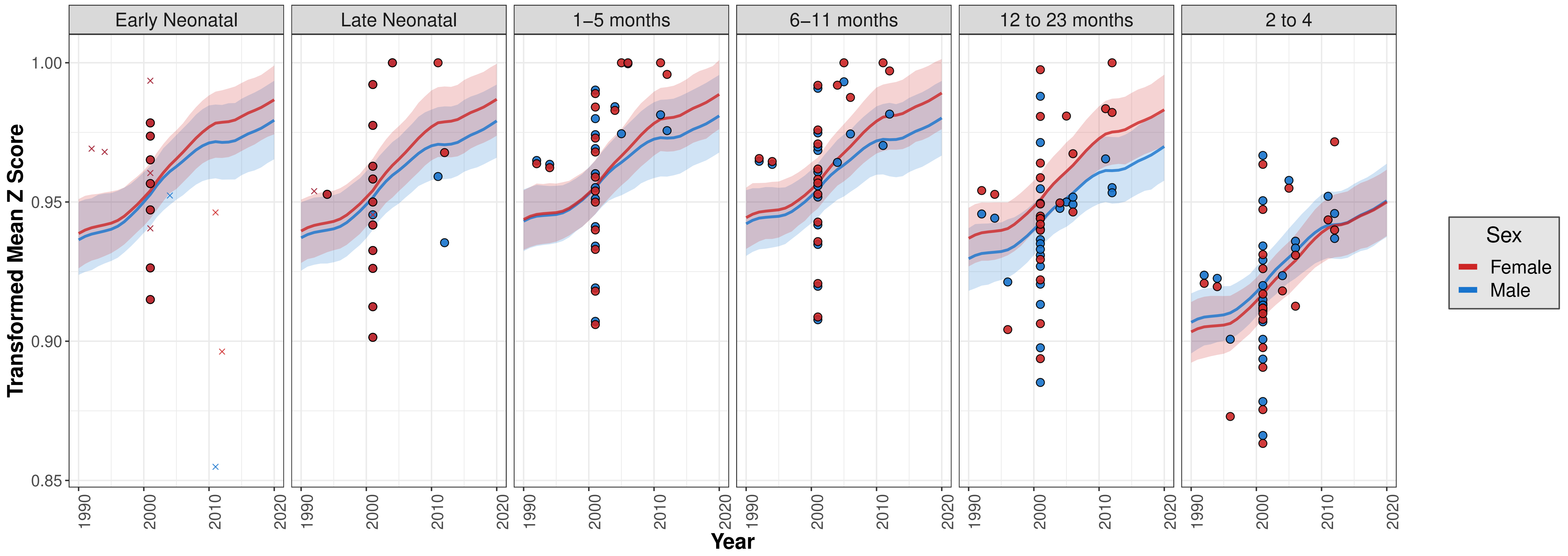

I

Year	Source
1987	WHO CGM Database
1992	WHO CGM Database
1994	WHO CGM Database
1996	WHO CGM Database
2001	Reproductive Health Survey
2001	WHO CGM Database
2004	Survey of Living Conditions
2005	DHS
2006	DHS
2006	WHO CGM Database
2011	DHS
2012	DHS
2012	WHO CGM Database

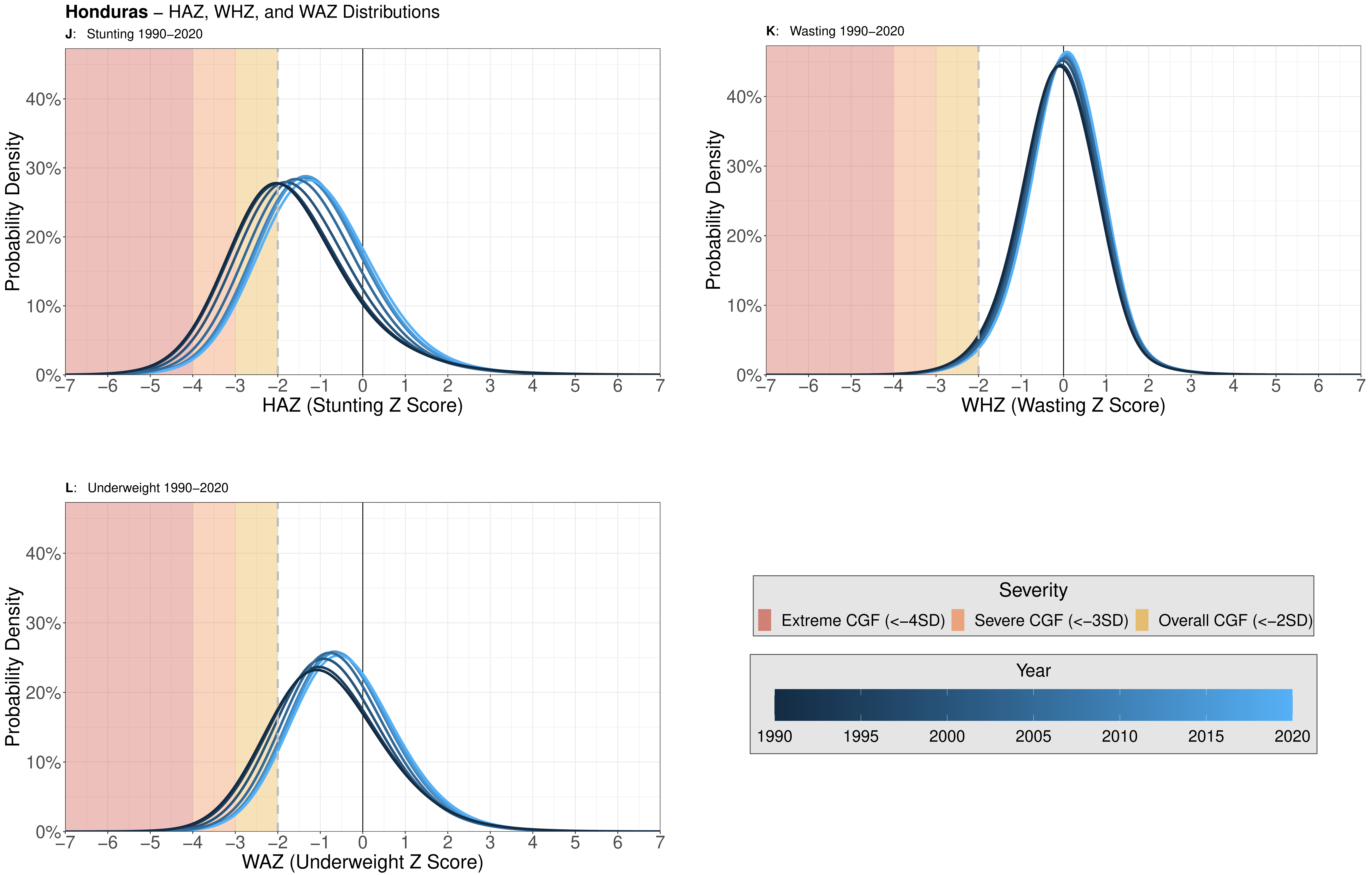

Honduras – HAZ, WHZ, and WAZ Distributions
J: Stunting 1990–2020
Probability Density
0%
10%
20%
30%
40%
−7 −6 −5 −4 −3 −2 −1 0 1 2 3 4 5 6 7
HAZ (Stunting Z Score)
K: Wasting 1990–2020
Probability Density
0%
10%
20%
30%
40%
−7 −6 −5 −4 −3 −2 −1 0 1 2 3 4 5 6 7
WHZ (Wasting Z Score)
L: Underweight 1990–2020
Probability Density
0%
10%
20%
30%
40%
−7 −6 −5 −4 −3 −2 −1 0 1 2 3 4 5 6 7
WAZ (Underweight Z Score)
Severity
Extreme CGF (<−4SD)
Severe CGF (<−3SD)
Overall CGF (<−2SD)
Year
1990
1995
2000
2005
2010
2015
2020

Mexico – Stunting (HAZ)

A: Overall and Severe Stunting Prevalence

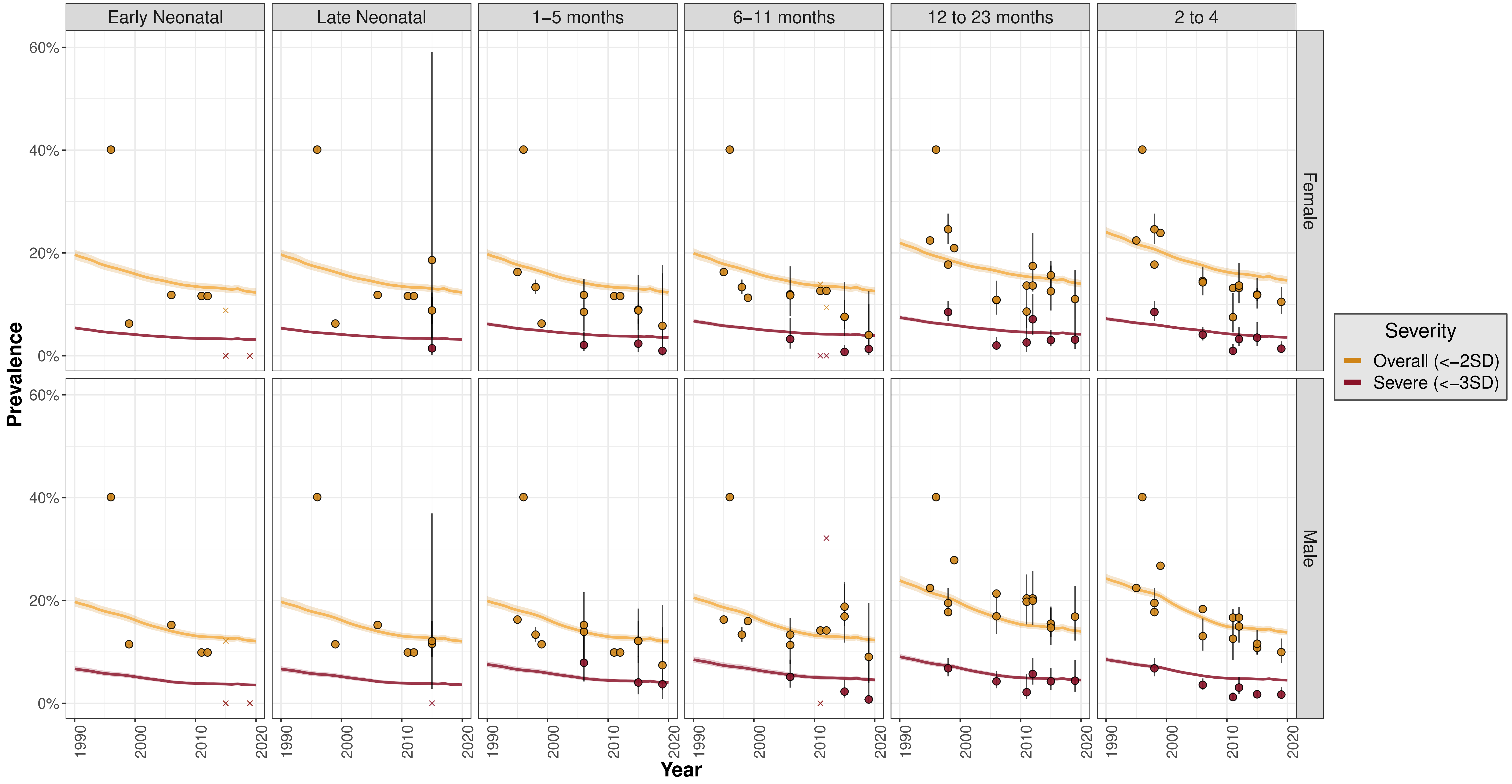

B: Transformed Mean Stunting Z Scores

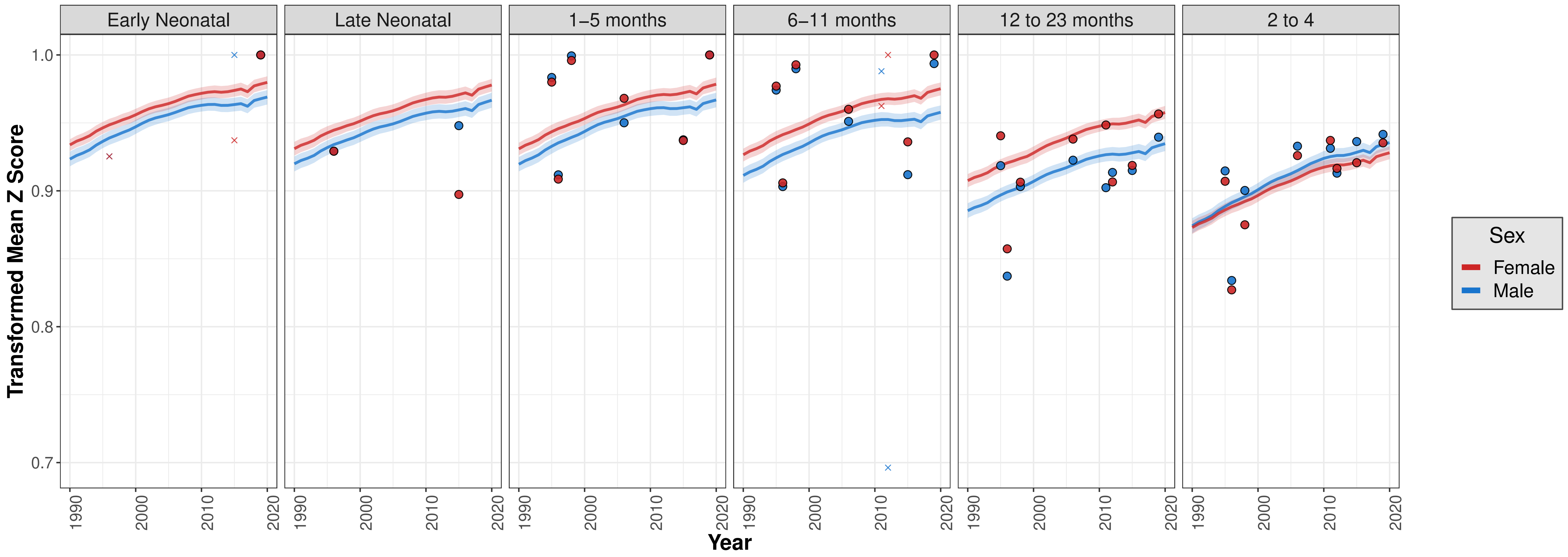

C

Year	Source	National	Subnational
1988	WHO CGM Database	X	
1989	WHO CGM Database	X	X
1990	WHO CGM Database		X
1994	WHO CGM Database		X
1995	WHO CGM Database	X	X
1996	WHO CGM Database	X	X
1997	WHO CGM Database		X
1998	National Nutrition Survey		X
1998	WHO CGM Database	X	X
1999	National Nutrition Survey		X
1999	WHO CGM Database	X	
2006	National Survey of Health and Nutrition	X	
2006	Family Life Survey		X
2006	WHO CGM Database	X	X
2008	WHO CGM Database		X
2009	Family Life Survey		X
2010	Family Life Survey		X
2011	National Survey of Health and Nutrition	X	
2011	WHO CGM Database	X	
2011	Family Life Survey		X
2012	National Survey of Health and Nutrition	X	
2012	WHO CGM Database	X	X
2012	Family Life Survey		X
2013	Family Life Survey		X
2015	WHO CGM Database	X	
2015	MICS	X	
2016	National Survey of Health and Nutrition Mid–way		X
2019	National Survey of Health and Nutrition (ENSANUT)	X	

Mexico – Wasting (WHZ)

D: Overall and Severe Wasting Prevalence

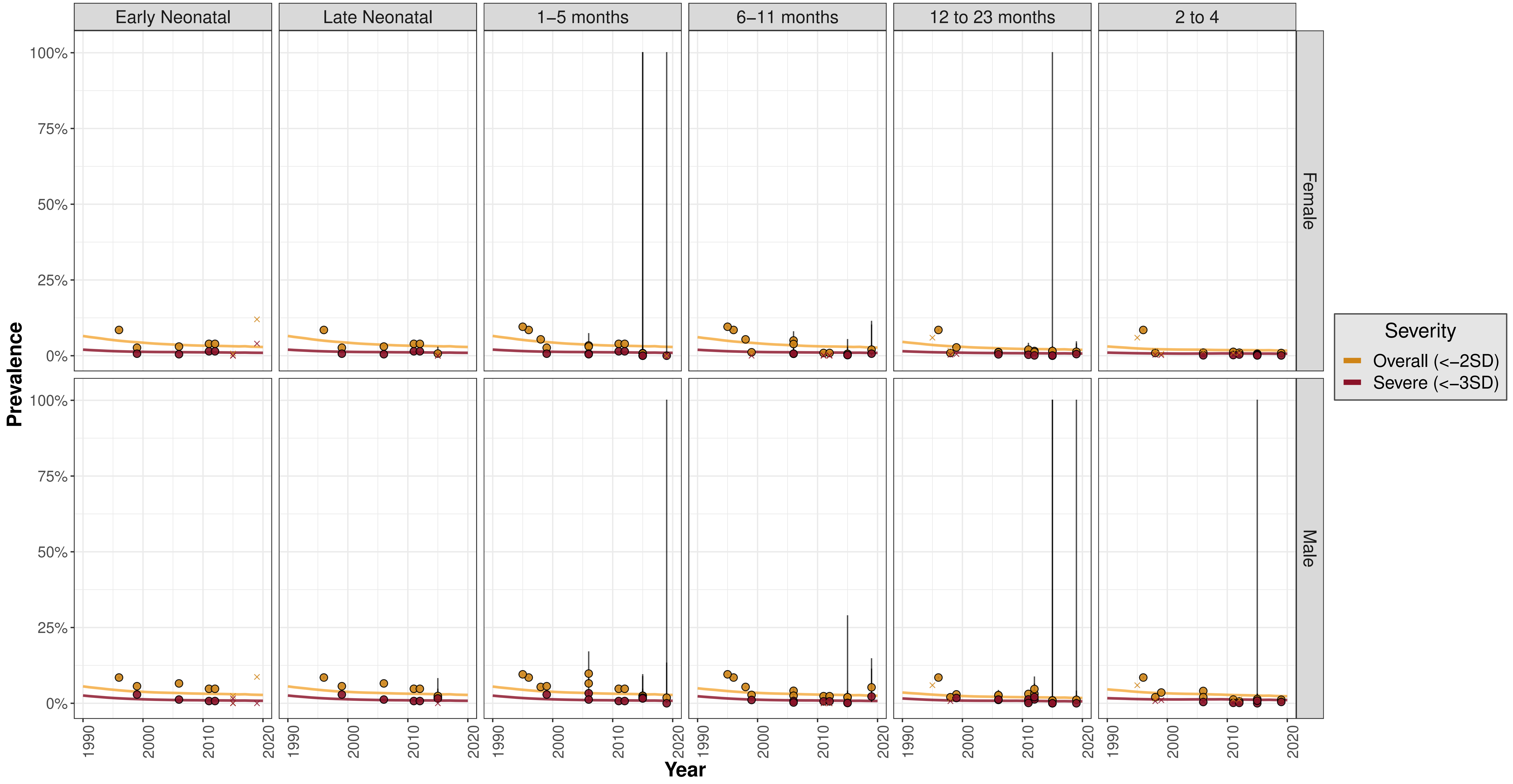

E: Transformed Mean Wasting Z Scores

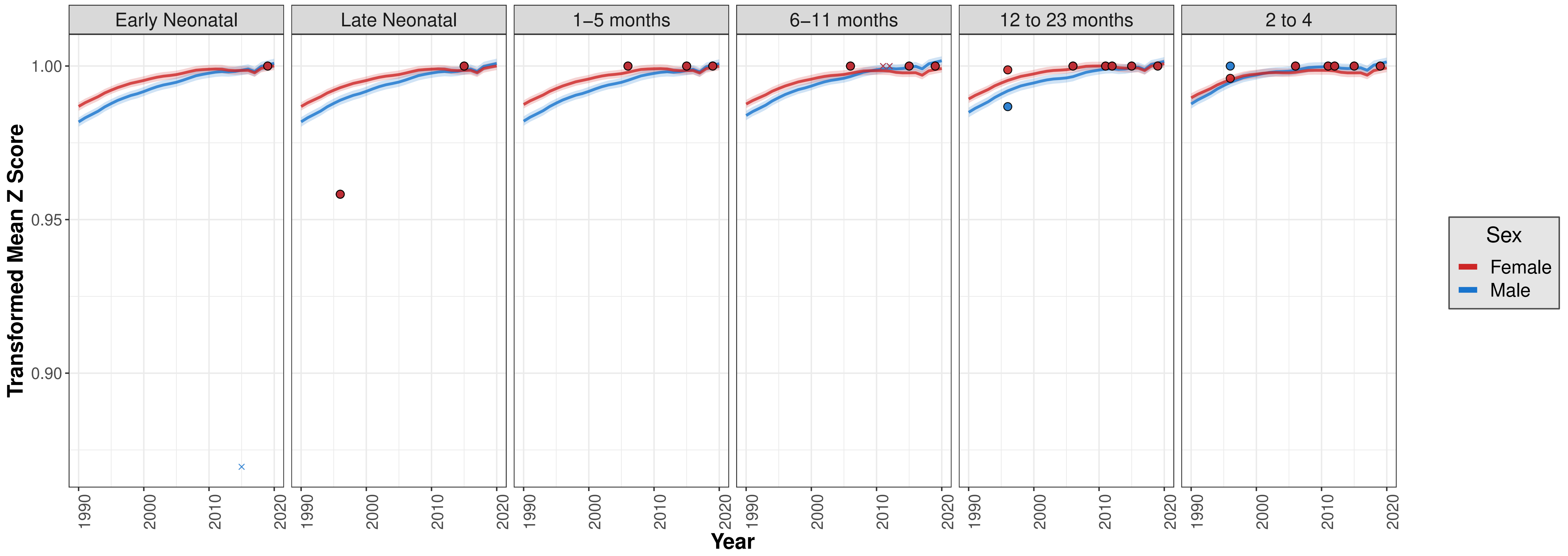

F

Year	Source	National	Subnational
1988	WHO CGM Database	X	
1989	WHO CGM Database	X	X
1994	WHO CGM Database		X
1995	WHO CGM Database	X	X
1996	WHO CGM Database	X	X
1997	WHO CGM Database		X
1998	National Nutrition Survey		X
1998	WHO CGM Database	X	X
1999	National Nutrition Survey		X
1999	WHO CGM Database	X	
2006	National Survey of Health and Nutrition	X	
2006	Family Life Survey		X
2006	WHO CGM Database	X	X
2008	WHO CGM Database		X
2009	Family Life Survey		X
2010	Family Life Survey		X
2011	National Survey of Health and Nutrition	X	
2011	WHO CGM Database	X	
2011	Family Life Survey		X
2012	National Survey of Health and Nutrition	X	
2012	WHO CGM Database	X	X
2012	Family Life Survey		X
2013	Family Life Survey		X
2015	WHO CGM Database	X	
2015	MICS	X	
2016	National Survey of Health and Nutrition Mid–way		X
2019	National Survey of Health and Nutrition (ENSANUT)	X	

Mexico – Underweight (WAZ)

G: Overall and Severe Underweight Prevalence

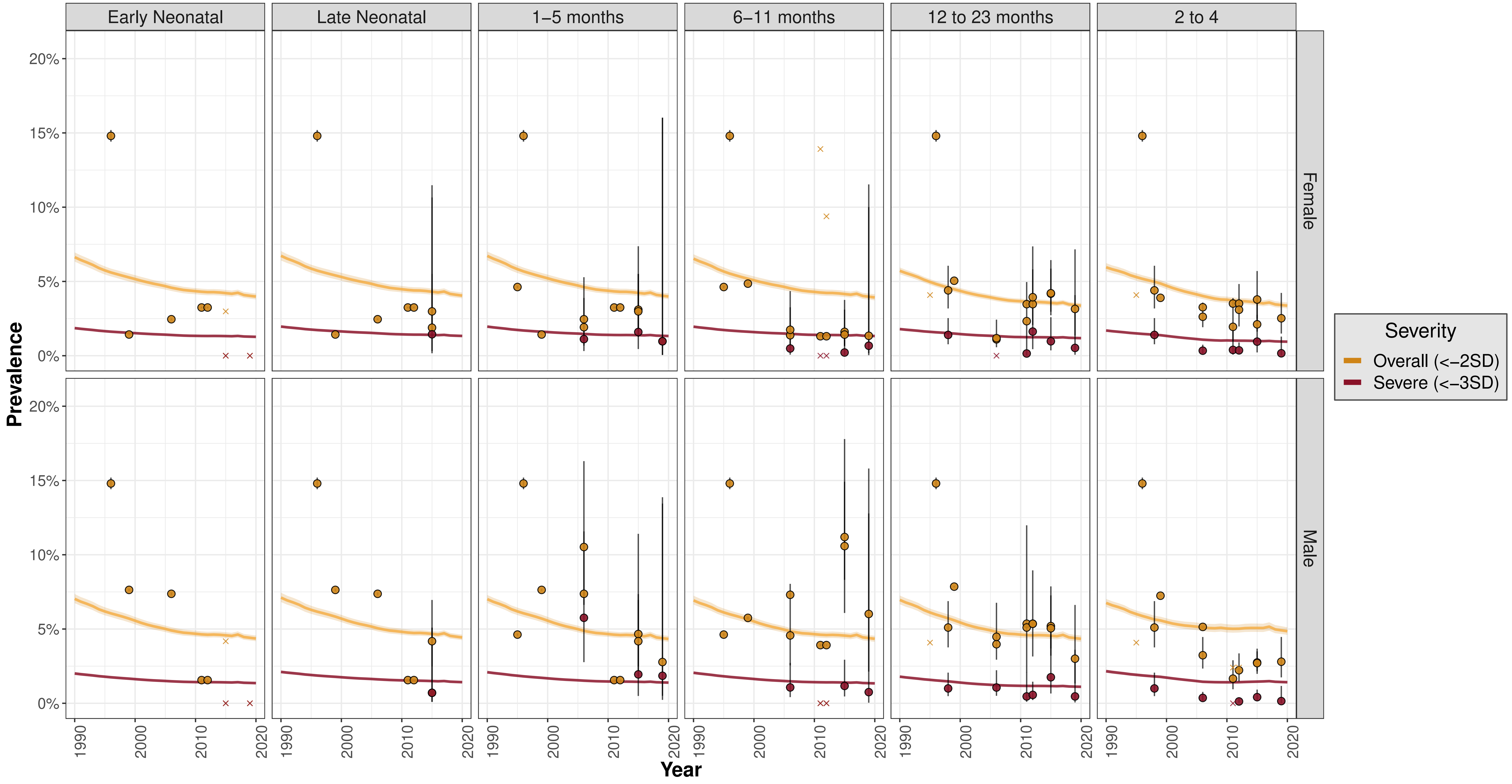

H: Transformed Mean Underweight Z Scores

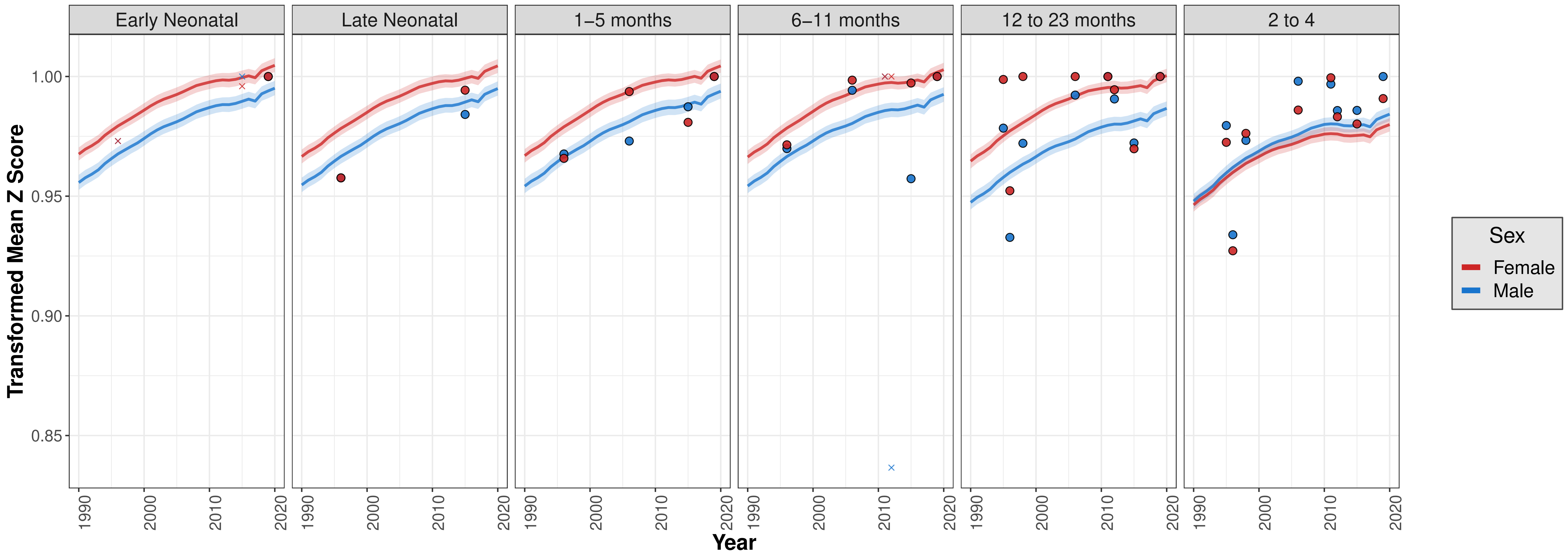

I

Year	Source	National	Subnational
1988	WHO CGM Database	X	
1989	WHO CGM Database	X	X
1990	WHO CGM Database		X
1991	WHO CGM Database		X
1994	WHO CGM Database		X
1995	WHO CGM Database	X	X
1996	WHO CGM Database	X	X
1997	WHO CGM Database		X
1998	National Nutrition Survey		X
1998	WHO CGM Database	X	X
1999	National Nutrition Survey		X
1999	WHO CGM Database	X	
2002	WHO CGM Database		X
2006	National Survey of Health and Nutrition	X	
2006	Family Life Survey		X
2006	Childhood Underweight		X
2006	WHO CGM Database	X	X
2008	WHO CGM Database		X
2009	Family Life Survey		X
2010	Family Life Survey		X
2011	National Survey of Health and Nutrition	X	
2011	WHO CGM Database	X	
2011	Family Life Survey		X
2012	National Survey of Health and Nutrition	X	
2012	Childhood Underweight		X
2012	WHO CGM Database	X	X
2012	Family Life Survey		X
2013	Family Life Survey		X
2015	WHO CGM Database	X	
2015	MICS	X	
2016	National Survey of Health and Nutrition Mid–way		X
2019	National Survey of Health and Nutrition (ENSANUT)	X	

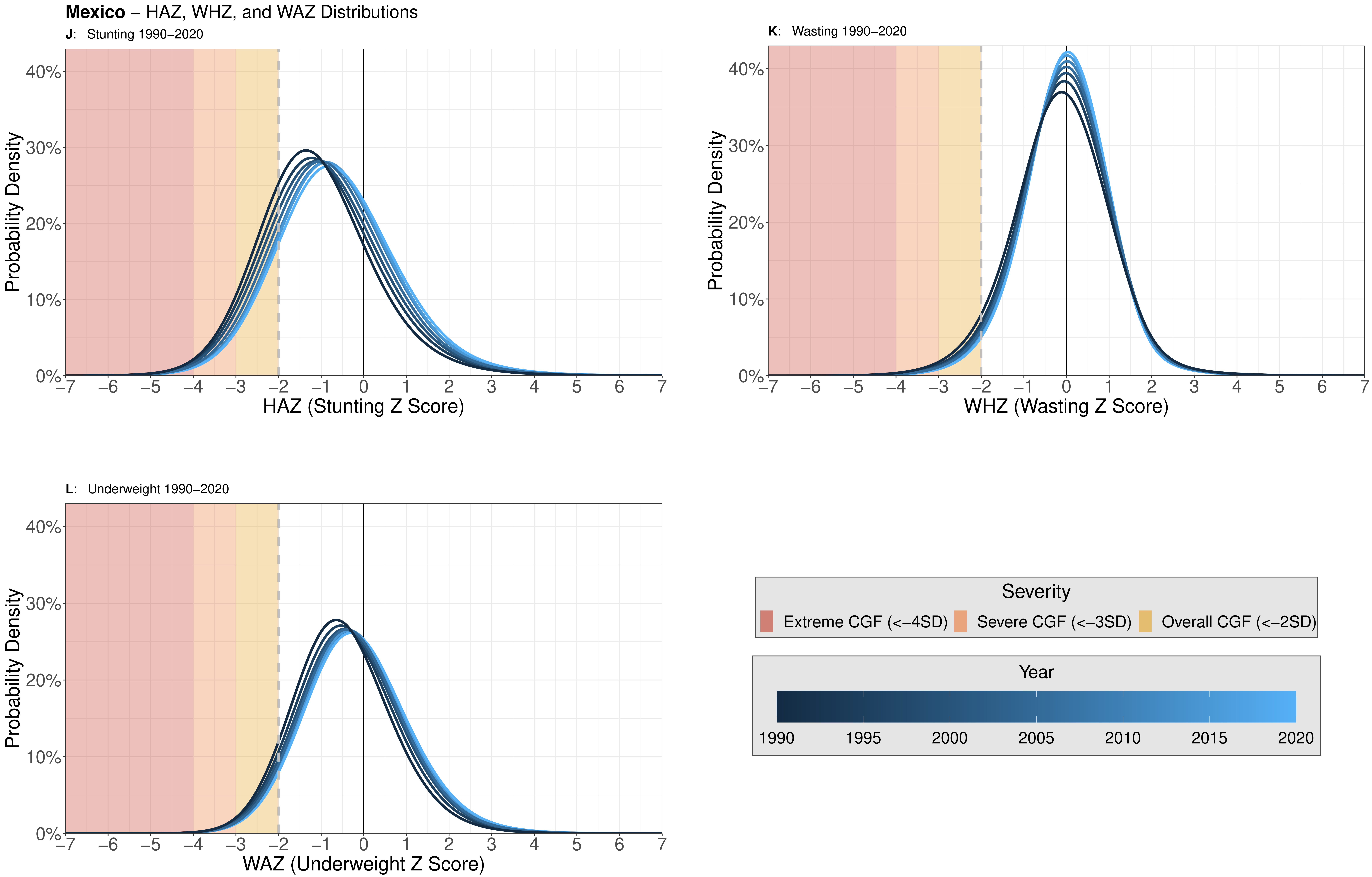

Mexico – HAZ, WHZ, and WAZ Distributions
J: Stunting 1990–2020
Probability Density
HAZ (Stunting Z Score)
K: Wasting 1990–2020
Probability Density
WHZ (Wasting Z Score)
L: Underweight 1990–2020
Probability Density
WAZ (Underweight Z Score)
0%
10%
20%
30%
40%
−7
−6
−5
−4
−3
−2
−1
0
1
2
3
4
5
6
7
Severity
Extreme CGF (<−4SD)
Severe CGF (<−3SD)
Overall CGF (<−2SD)
Year
1990
1995
2000
2005
2010
2015
2020

Nicaragua – Stunting (HAZ)

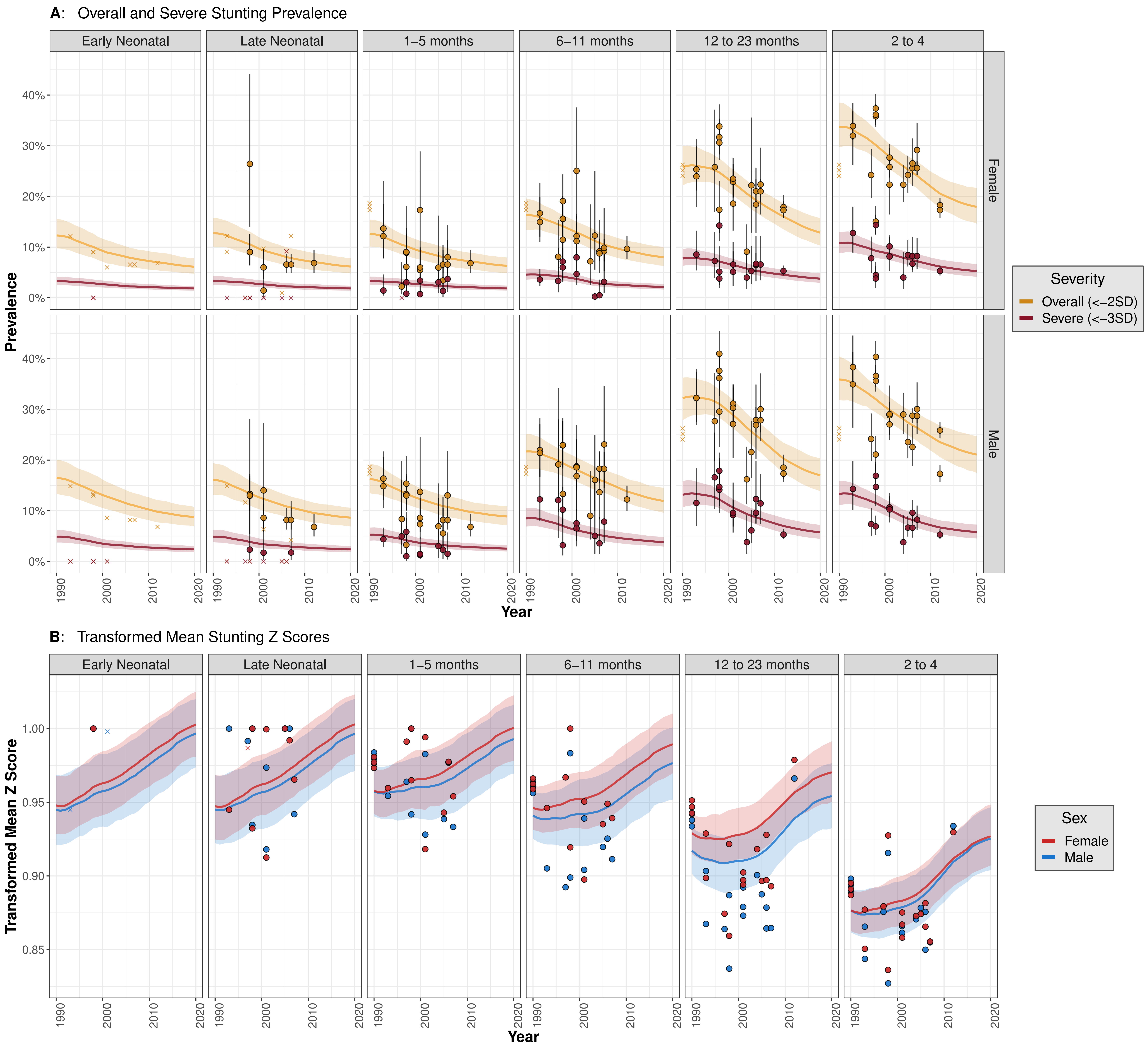

C

Year	Source
1988	WHO CGM Database
1990	WHO CGM Database
1993	Living Standards Measurement Survey
1993	WHO CGM Database
1997	DHS
1998	Living Standards Measurement Survey
1998	DHS
1998	WHO CGM Database
2001	Living Standards Measurement Survey
2001	DHS
2001	WHO CGM Database
2004	WHO CGM Database
2005	Living Standards Measurement Survey
2006	Reproductive Health Survey
2006	WHO CGM Database
2007	Reproductive Health Survey
2007	WHO CGM Database
2012	National DHS
2012	WHO CGM Database

Nicaragua – Wasting (WHZ)

D: Overall and Severe Wasting Prevalence

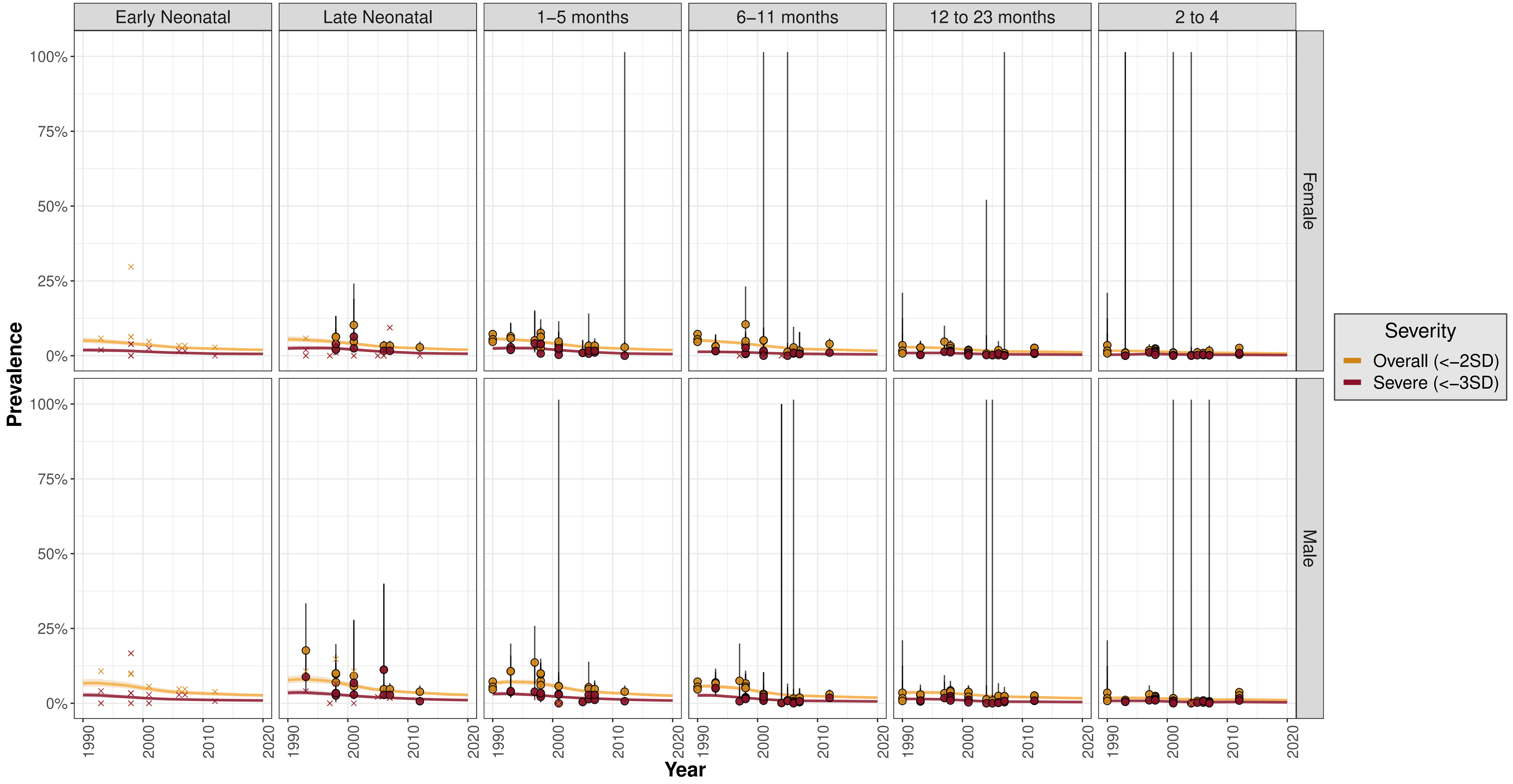

E: Transformed Mean Wasting Z Scores

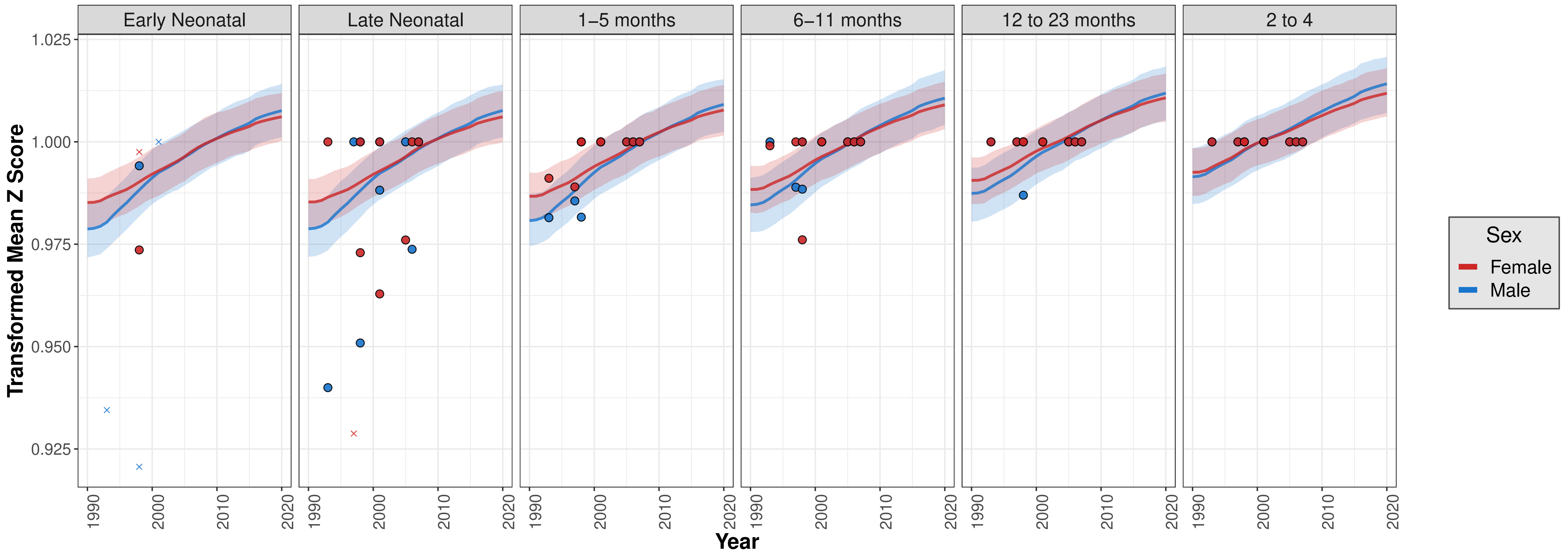

F

Year	Source
1988	WHO CGM Database
1990	WHO CGM Database
1993	Living Standards Measurement Survey
1993	WHO CGM Database
1997	DHS
1998	Living Standards Measurement Survey
1998	DHS
1998	WHO CGM Database
2001	Living Standards Measurement Survey
2001	DHS
2001	WHO CGM Database
2004	WHO CGM Database
2005	Living Standards Measurement Survey
2006	Reproductive Health Survey
2006	WHO CGM Database
2007	Reproductive Health Survey
2007	WHO CGM Database
2012	National DHS
2012	WHO CGM Database

Nicaragua – Underweight (WAZ)

G: Overall and Severe Underweight Prevalence

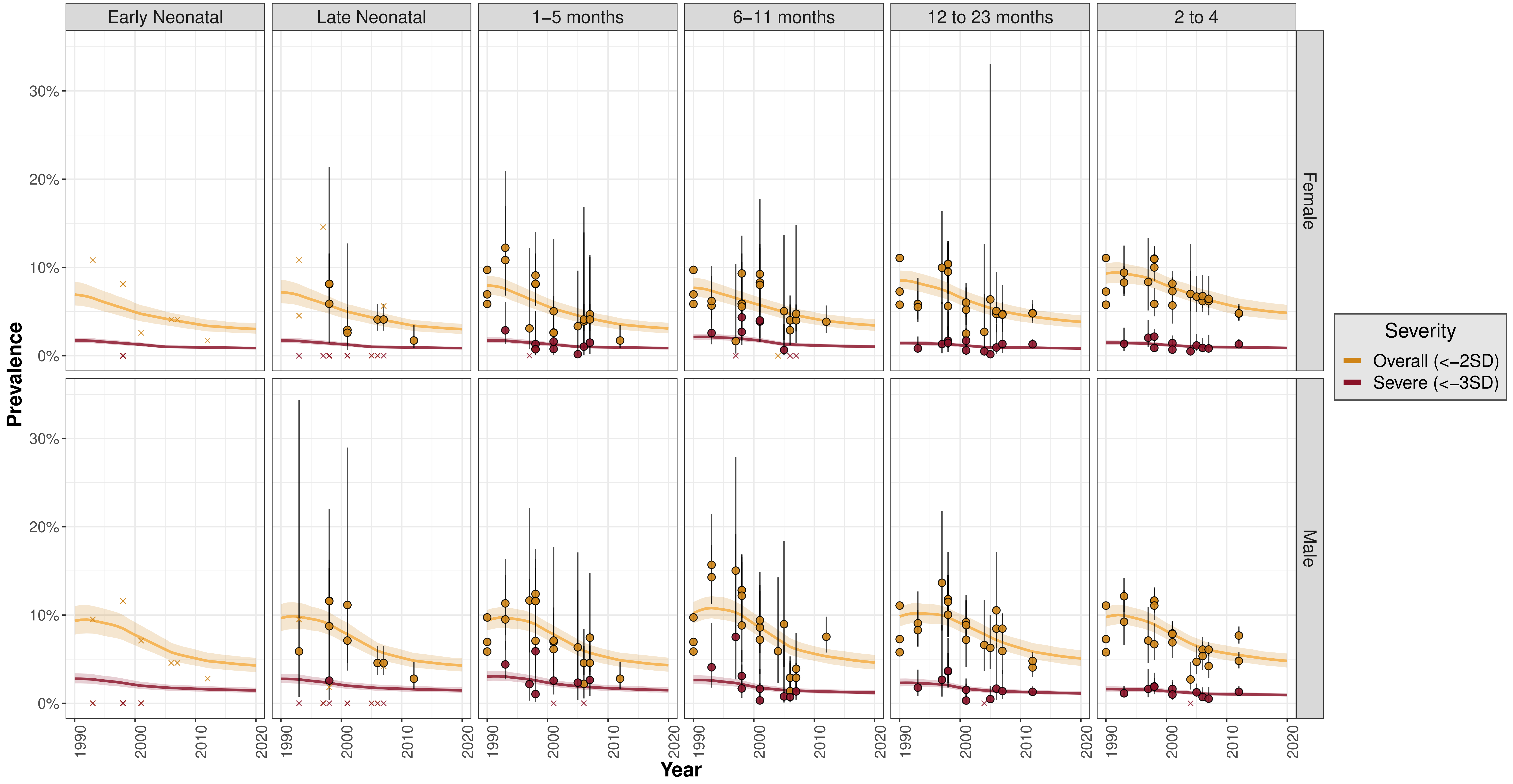

H: Transformed Mean Underweight Z Scores

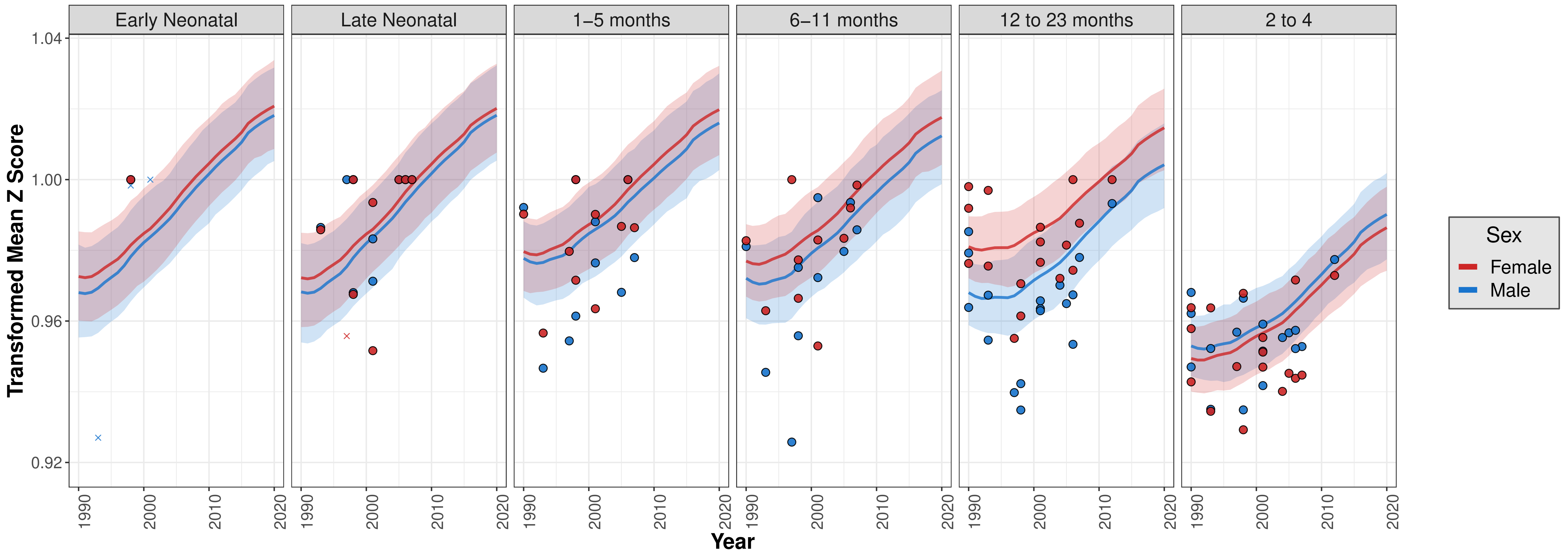

I

Year	Source
1988	WHO CGM Database
1990	WHO CGM Database
1993	Living Standards Measurement Survey
1993	WHO CGM Database
1997	DHS
1998	Living Standards Measurement Survey
1998	DHS
1998	WHO CGM Database
2001	Living Standards Measurement Survey
2001	DHS
2001	WHO CGM Database
2004	WHO CGM Database
2005	Living Standards Measurement Survey
2006	Reproductive Health Survey
2006	WHO CGM Database
2007	Reproductive Health Survey
2007	WHO CGM Database
2012	National DHS
2012	WHO CGM Database

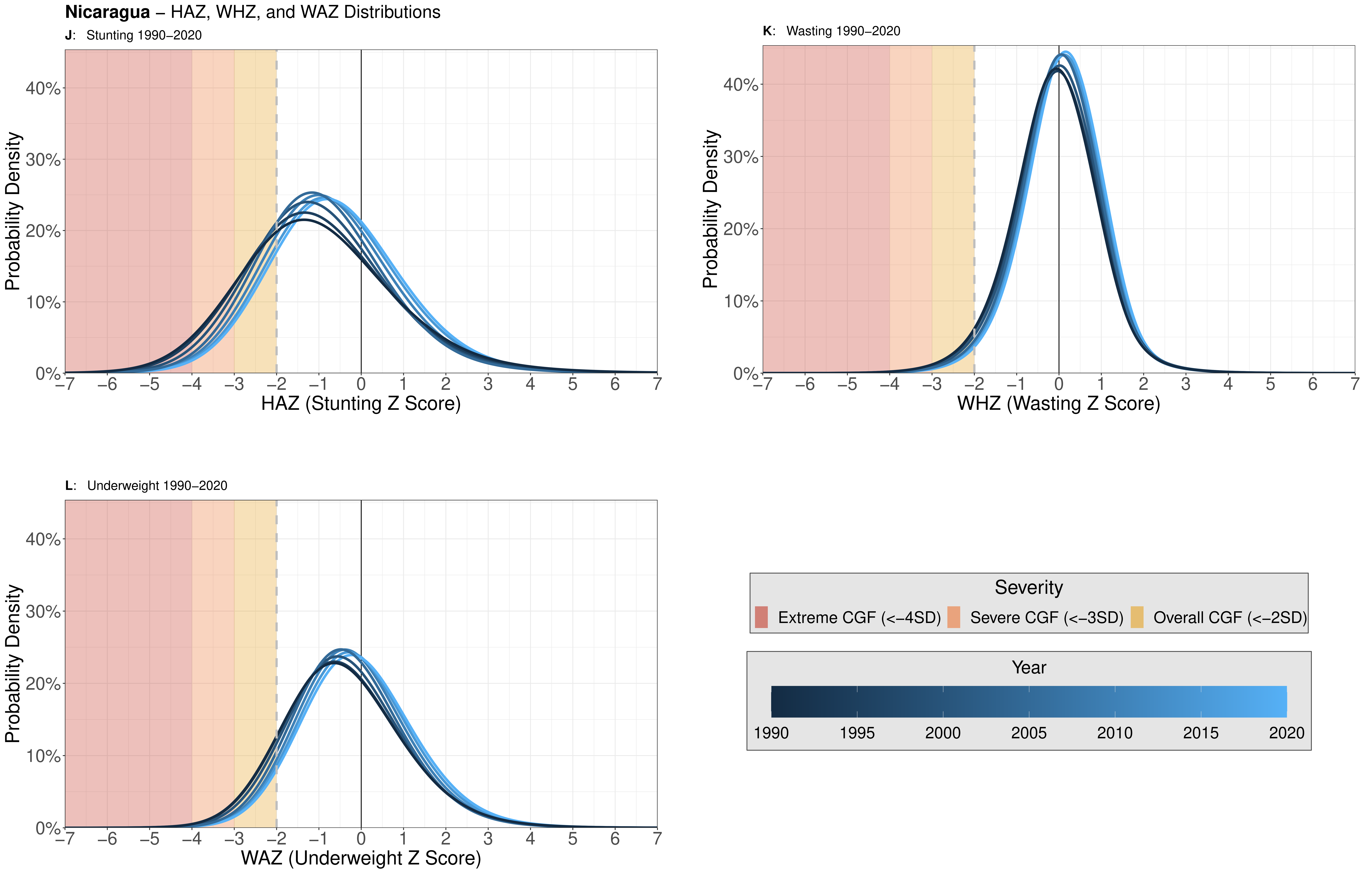

Nicaragua – HAZ, WHZ, and WAZ Distributions
J: Stunting 1990–2020
Probability Density
0%
10%
20%
30%
40%
−7 −6 −5 −4 −3 −2 −1 0 1 2 3 4 5 6 7
HAZ (Stunting Z Score)
K: Wasting 1990–2020
WHZ (Wasting Z Score)
L: Underweight 1990–2020
WAZ (Underweight Z Score)
Severity
Extreme CGF (<−4SD)
Severe CGF (<−3SD)
Overall CGF (<−2SD)
Year
1990
1995
2000
2005
2010
2015
2020

Panama – Stunting (HAZ)

A: Overall and Severe Stunting Prevalence

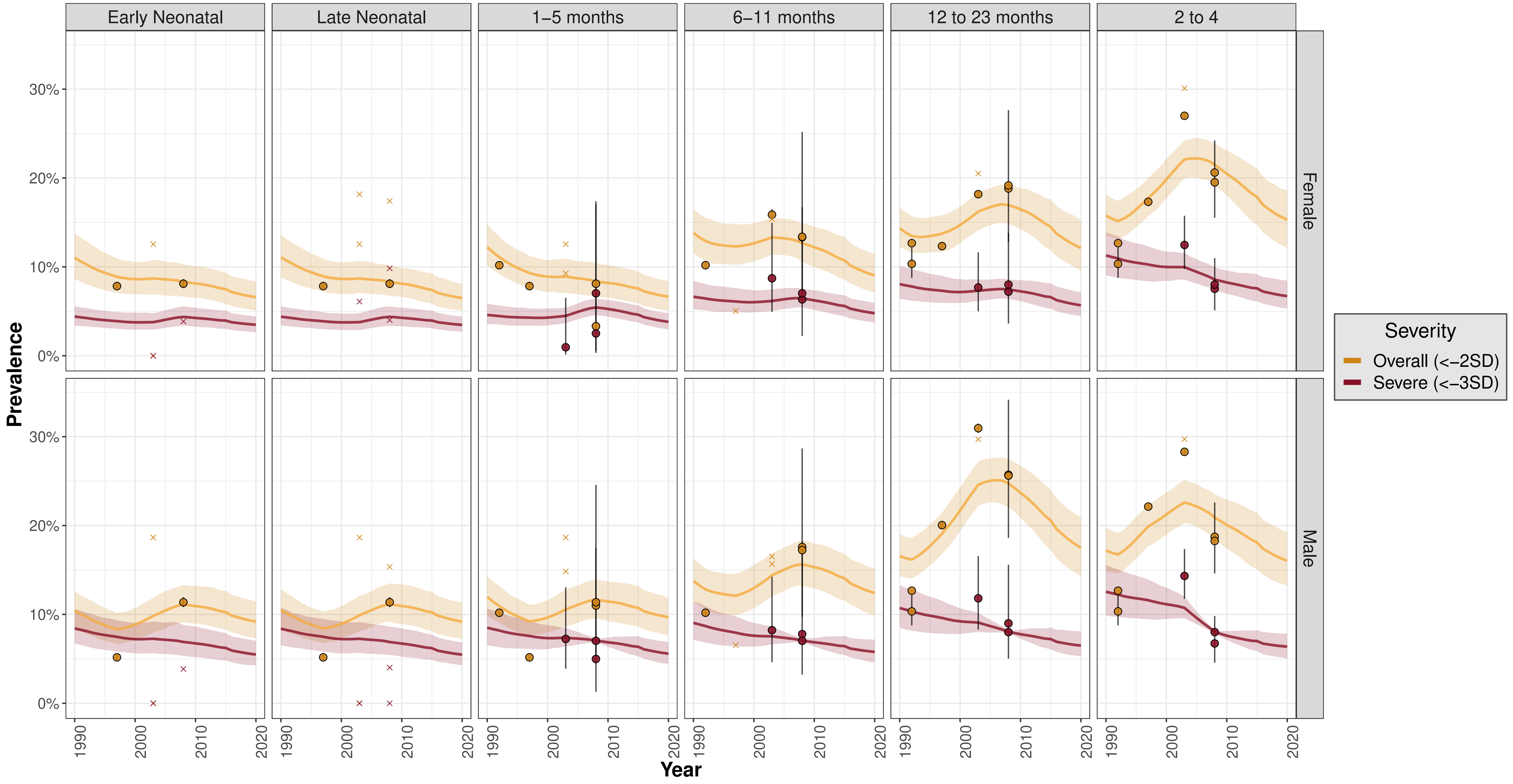

B: Transformed Mean Stunting Z Scores

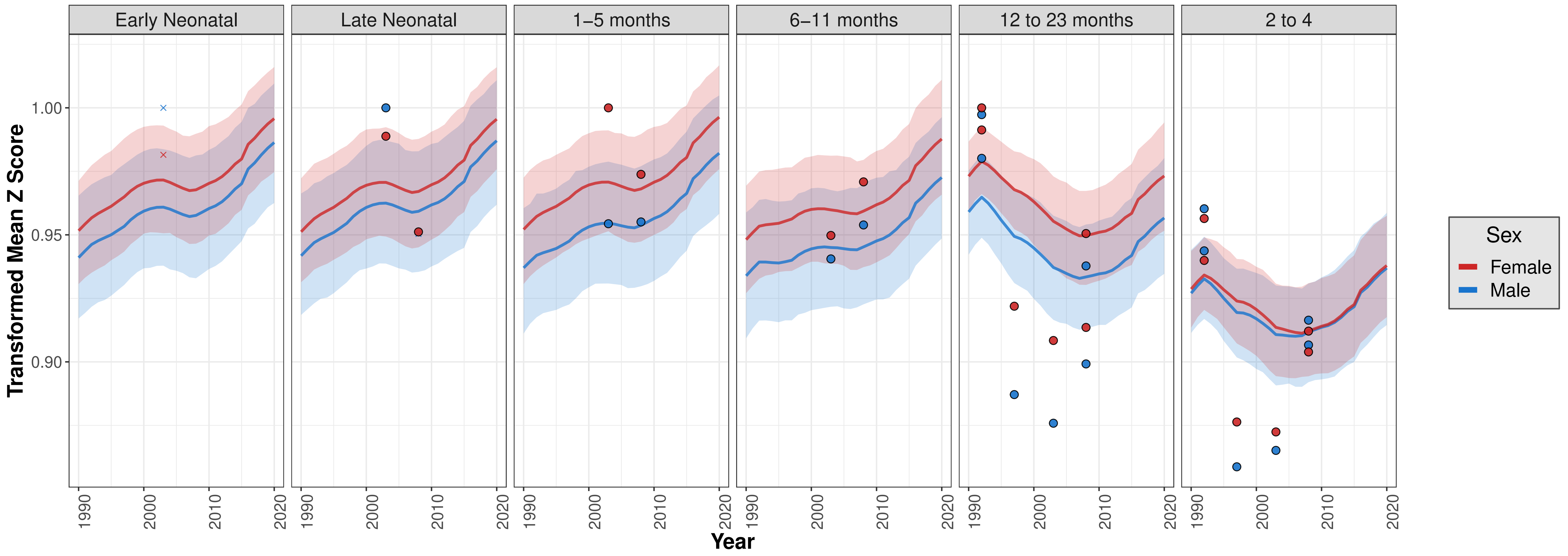

C

Year	Source
1980	WHO CGM Database
1992	WHO CGM Database
1997	WHO CGM Database
2003	Living Standard Measurement Survey
2003	WHO CGM Database
2008	Living Standard Measurement Survey
2008	WHO CGM Database

Panama – Wasting (WHZ)

D: Overall and Severe Wasting Prevalence

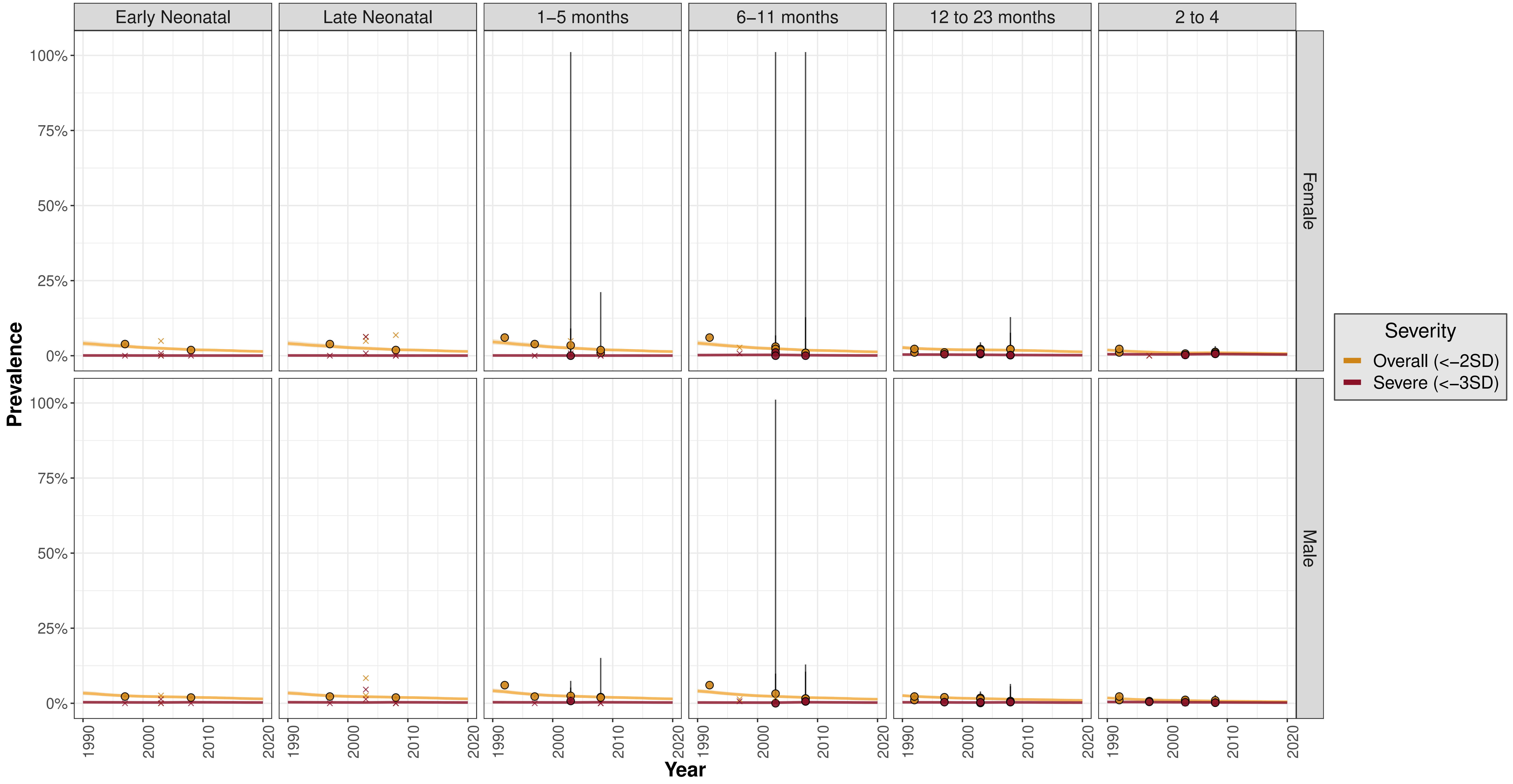

F

Year	Source
1980	WHO CGM Database
1992	WHO CGM Database
1997	WHO CGM Database
2003	Living Standard Measurement Survey
2003	WHO CGM Database
2008	Living Standard Measurement Survey
2008	WHO CGM Database

E: Transformed Mean Wasting Z Scores

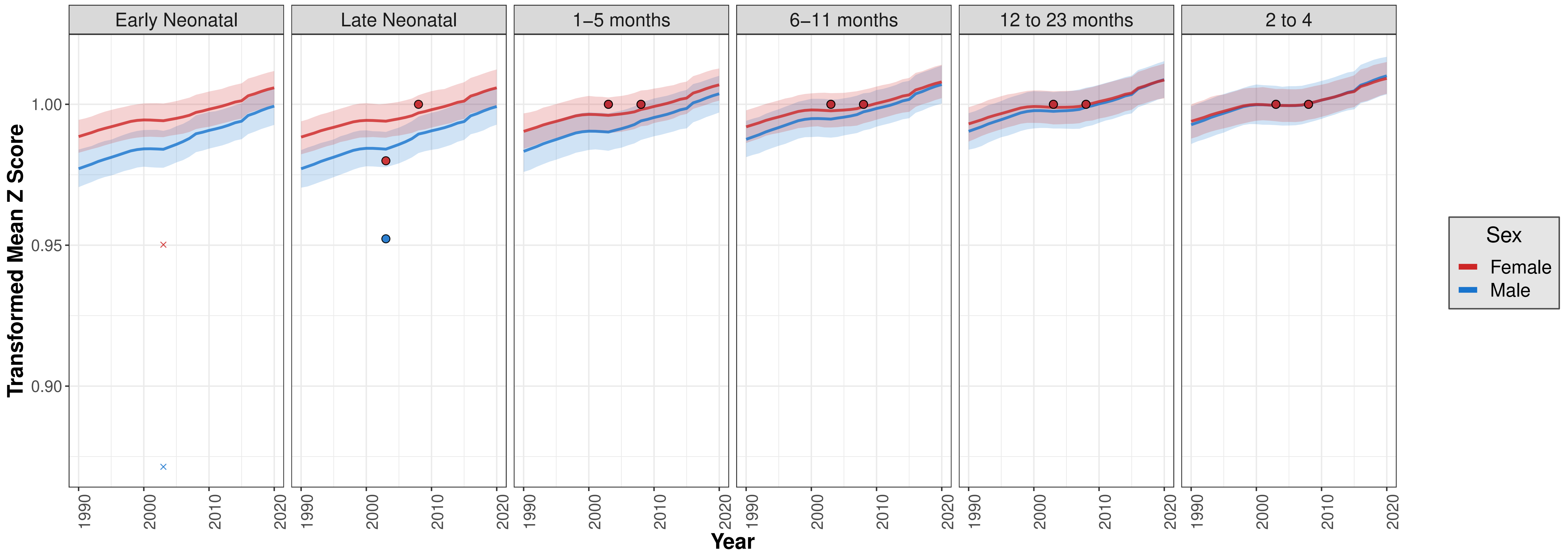

Panama – Underweight (WAZ)

G: Overall and Severe Underweight Prevalence

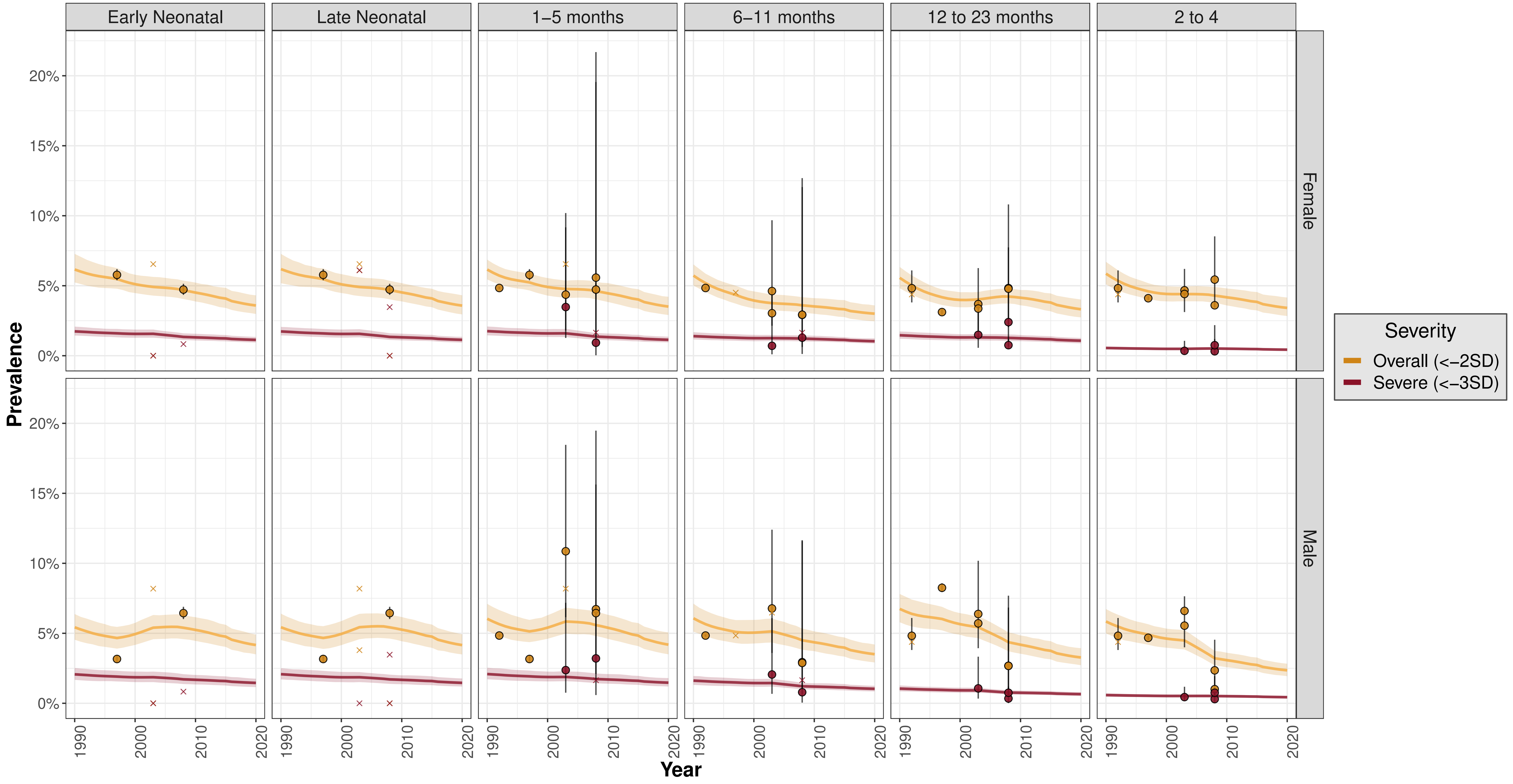

H: Transformed Mean Underweight Z Scores

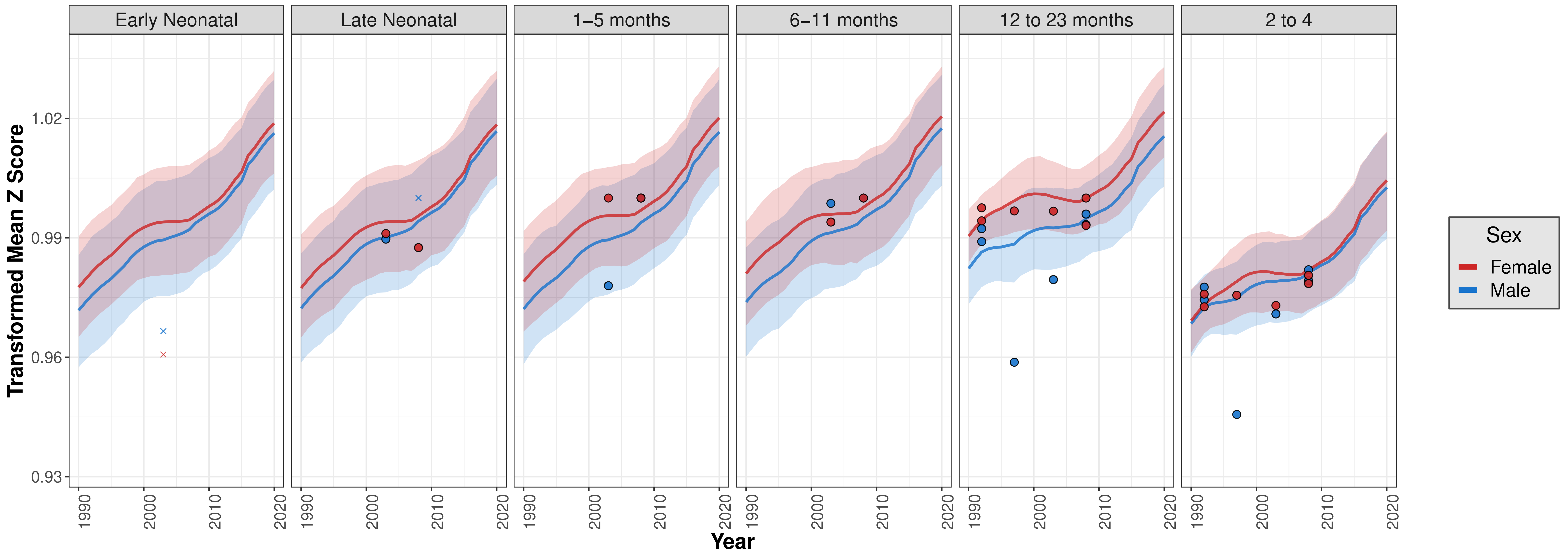

I

Year	Source
1980	WHO CGM Database
1992	WHO CGM Database
1997	WHO CGM Database
2003	Living Standard Measurement Survey
2003	WHO CGM Database
2008	Living Standard Measurement Survey
2008	WHO CGM Database

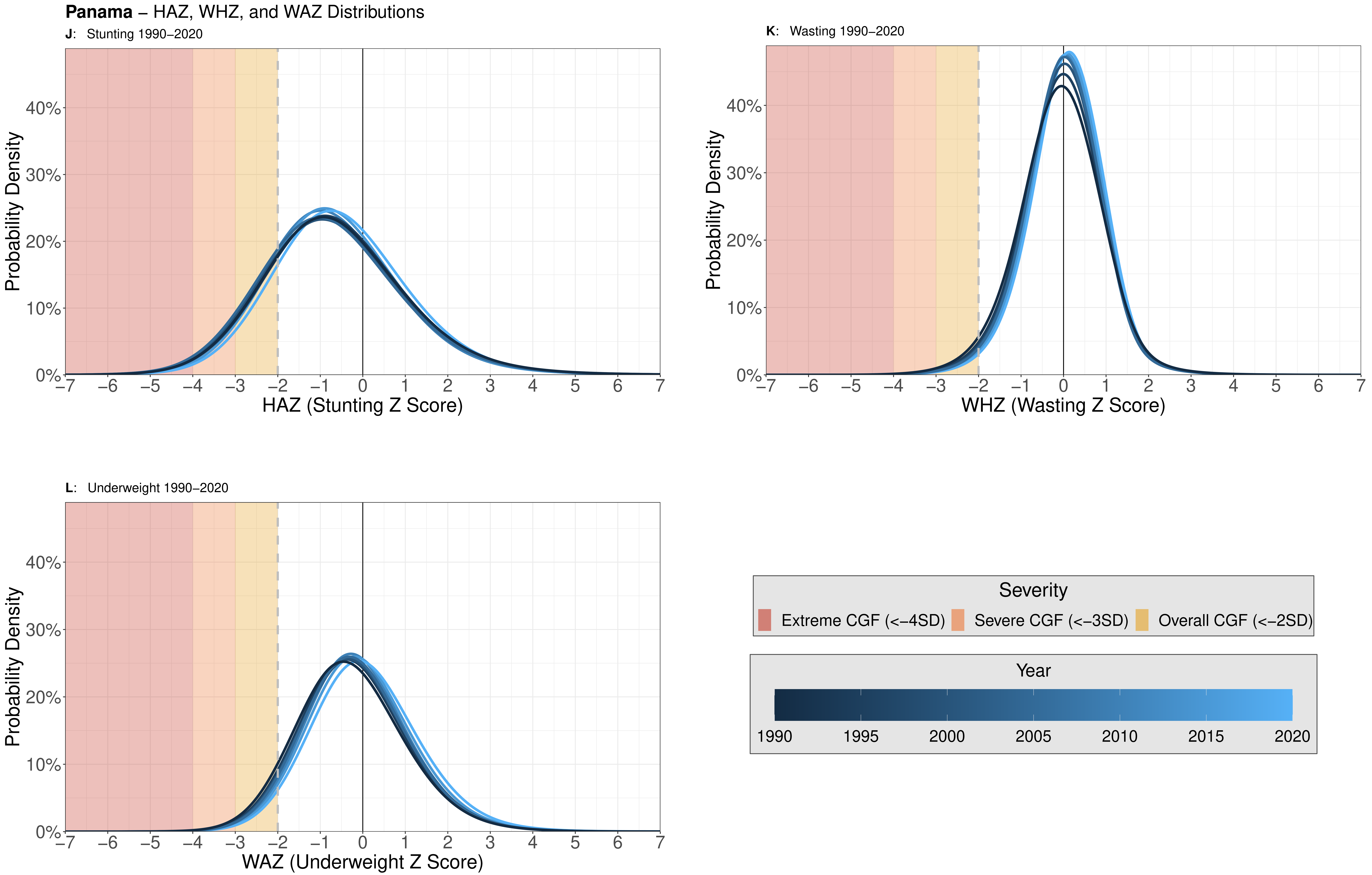
Panama – HAZ, WHZ, and WAZ Distributions
J: Stunting 1990–2020
K: Wasting 1990–2020
L: Underweight 1990–2020
Probability Density
0%
10%
20%
30%
40%
−7 −6 −5 −4 −3 −2 −1 0 1 2 3 4 5 6 7
HAZ (Stunting Z Score)
WHZ (Wasting Z Score)
WAZ (Underweight Z Score)
Severity
Extreme CGF (<−4SD)
Severe CGF (<−3SD)
Overall CGF (<−2SD)
Year
1990
1995
2000
2005
2010
2015
2020

Venezuela (Bolivarian Republic of) – Stunting (HAZ)

A: Overall and Severe Stunting Prevalence

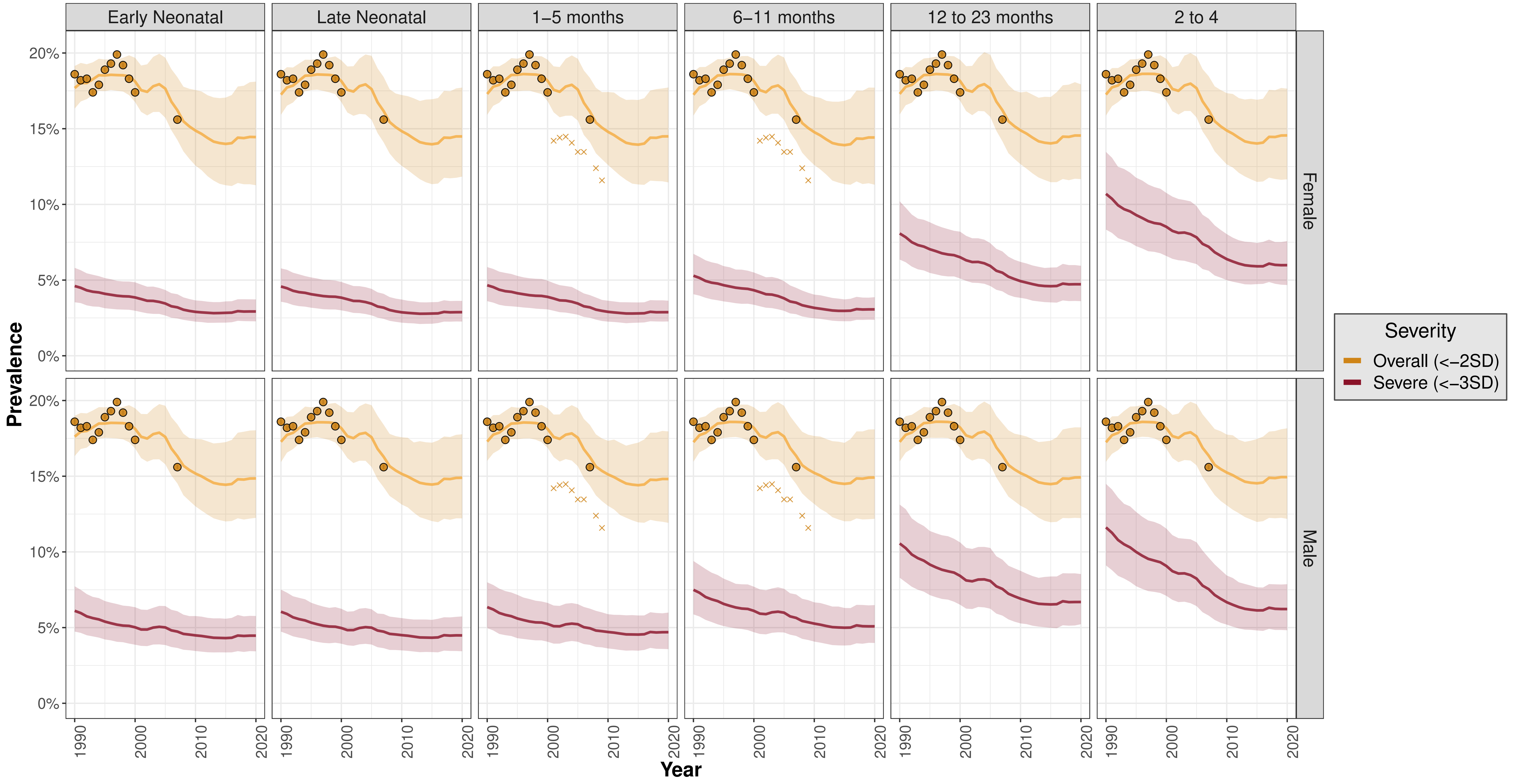

B: Transformed Mean Stunting Z Scores

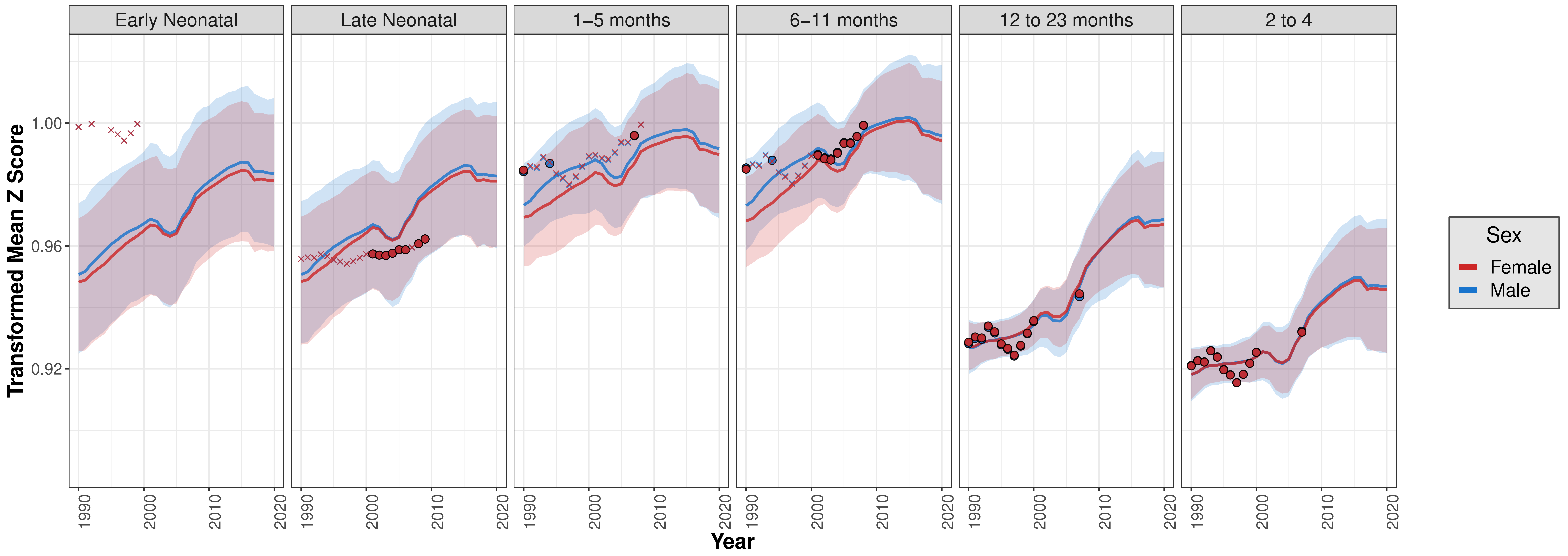

C

Year	Source
1982	WHO CGM Database
1987	WHO CGM Database
1990	WHO CGM Database
1991	WHO CGM Database
1992	WHO CGM Database
1993	WHO CGM Database
1994	WHO CGM Database
1995	WHO CGM Database
1996	WHO CGM Database
1997	WHO CGM Database
1998	WHO CGM Database
1999	WHO CGM Database
2000	WHO CGM Database
2001	WHO CGM Database
2002	WHO CGM Database
2003	WHO CGM Database
2004	WHO CGM Database
2005	WHO CGM Database
2006	WHO CGM Database
2007	WHO CGM Database
2008	WHO CGM Database
2009	WHO CGM Database

Venezuela (Bolivarian Republic of) – Wasting (WHZ)

D: Overall and Severe Wasting Prevalence

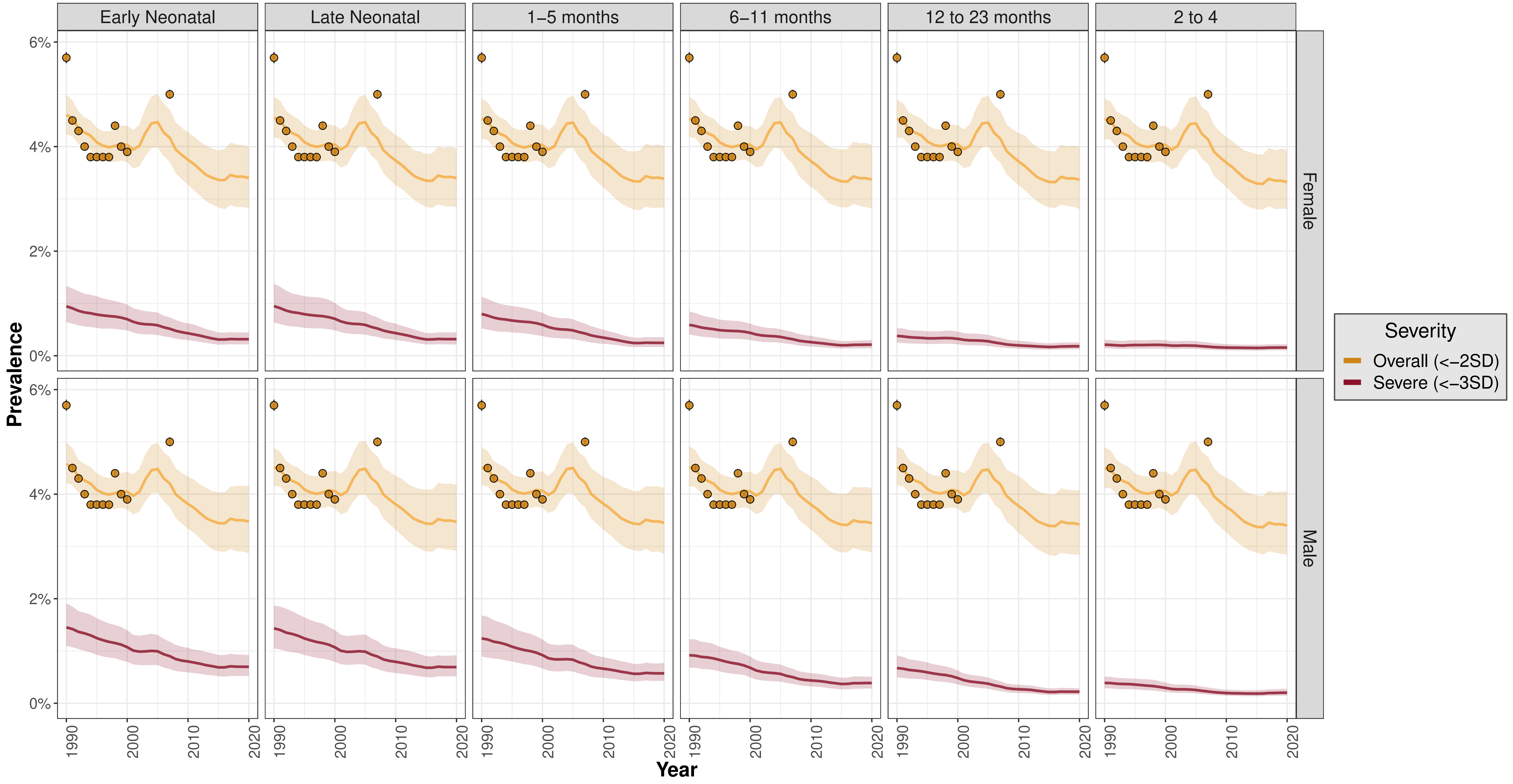

E: Transformed Mean Wasting Z Scores

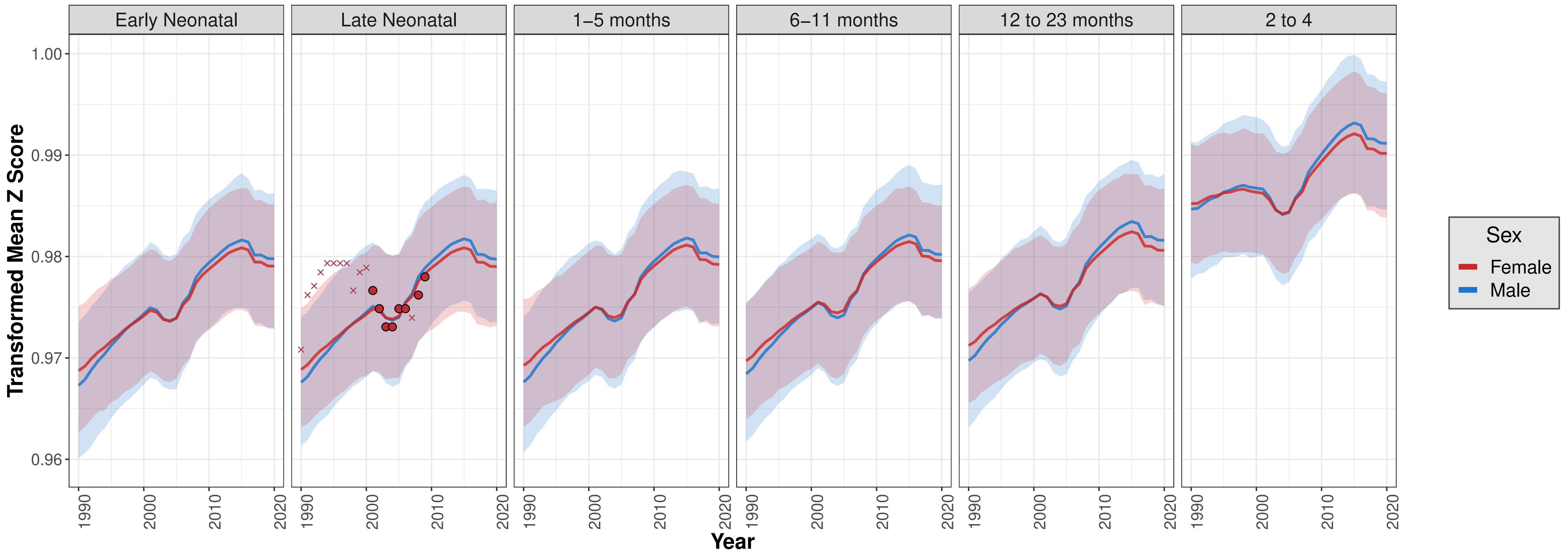

F

Year	Source
1982	WHO CGM Database
1987	WHO CGM Database
1990	WHO CGM Database
1991	WHO CGM Database
1992	WHO CGM Database
1993	WHO CGM Database
1994	WHO CGM Database
1995	WHO CGM Database
1996	WHO CGM Database
1997	WHO CGM Database
1998	WHO CGM Database
1999	WHO CGM Database
2000	WHO CGM Database
2001	WHO CGM Database
2002	WHO CGM Database
2003	WHO CGM Database
2004	WHO CGM Database
2005	WHO CGM Database
2006	WHO CGM Database
2007	WHO CGM Database
2008	WHO CGM Database
2009	WHO CGM Database

Venezuela (Bolivarian Republic of) – Underweight (WAZ)

G: Overall and Severe Underweight Prevalence

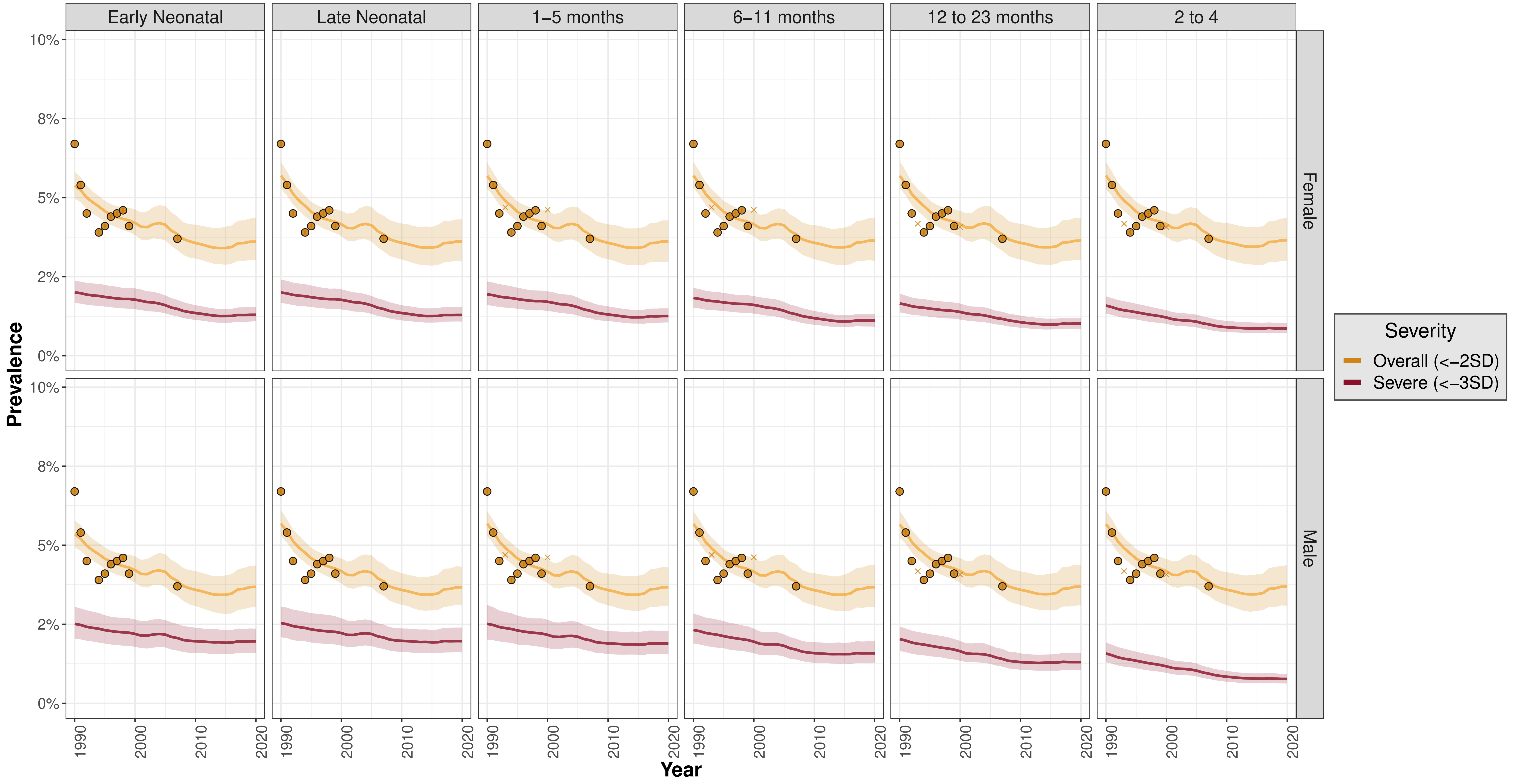

H: Transformed Mean Underweight Z Scores

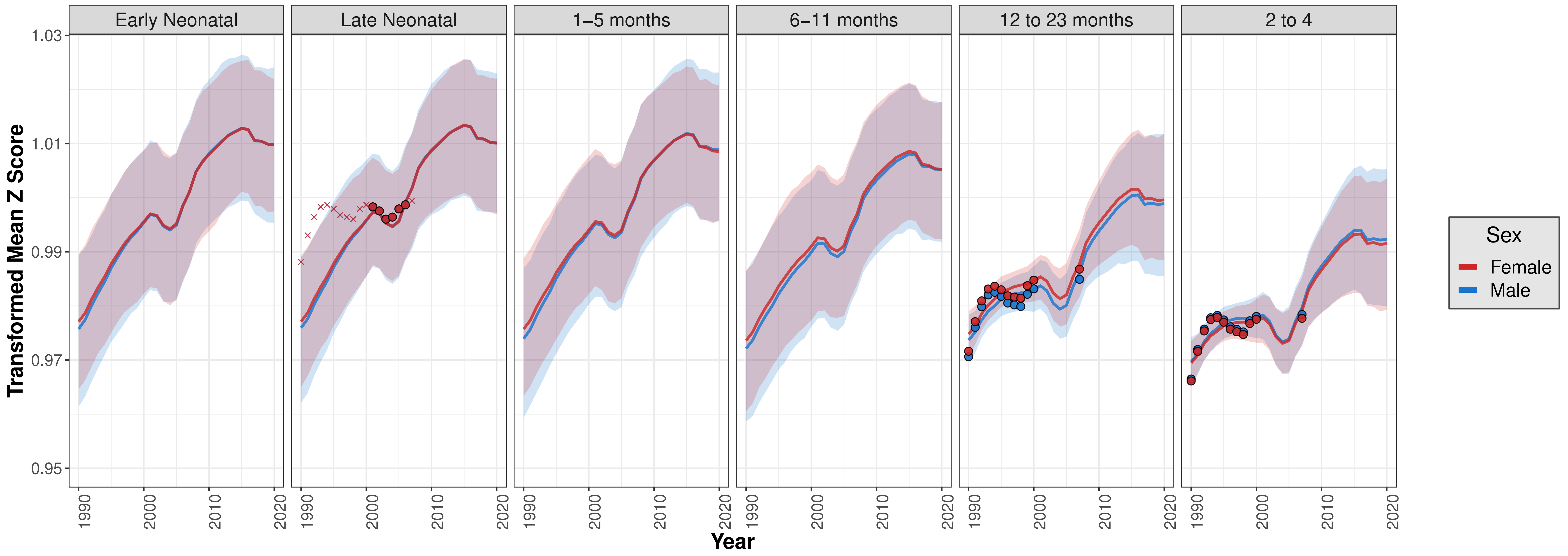

I

Year	Source
1982	WHO CGM Database
1987	WHO CGM Database
1990	WHO CGM Database
1991	WHO CGM Database
1992	WHO CGM Database
1993	WHO CGM Database
1994	WHO CGM Database
1995	WHO CGM Database
1996	WHO CGM Database
1997	WHO CGM Database
1998	WHO CGM Database
1999	WHO CGM Database
2000	WHO CGM Database
2001	WHO CGM Database
2002	WHO CGM Database
2003	WHO CGM Database
2004	WHO CGM Database
2005	WHO CGM Database
2006	WHO CGM Database
2007	WHO CGM Database

Venezuela (Bolivarian Republic of) – HAZ, WHZ, and WAZ Distributions

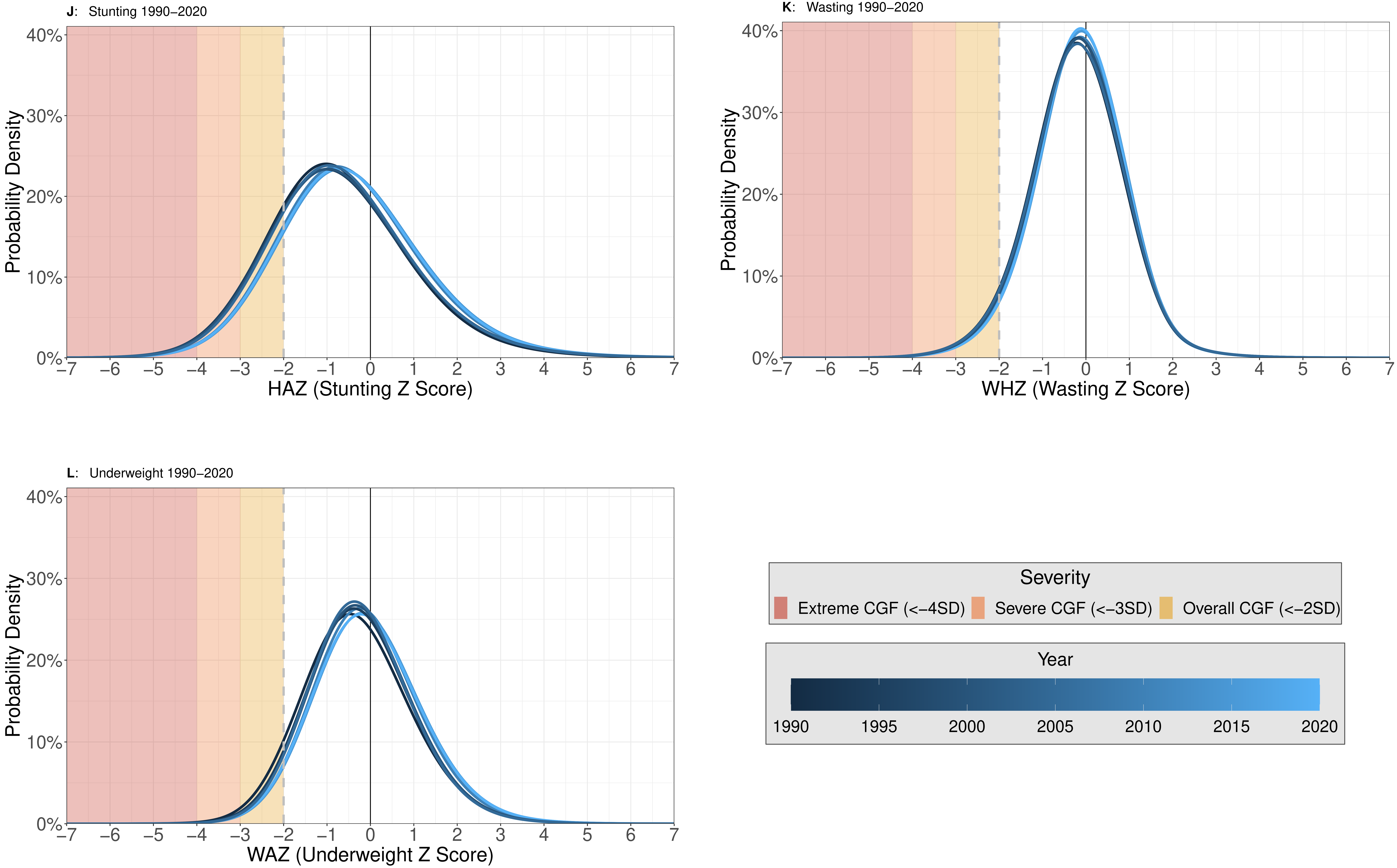

Brazil – Stunting (HAZ)

A: Overall and Severe Stunting Prevalence

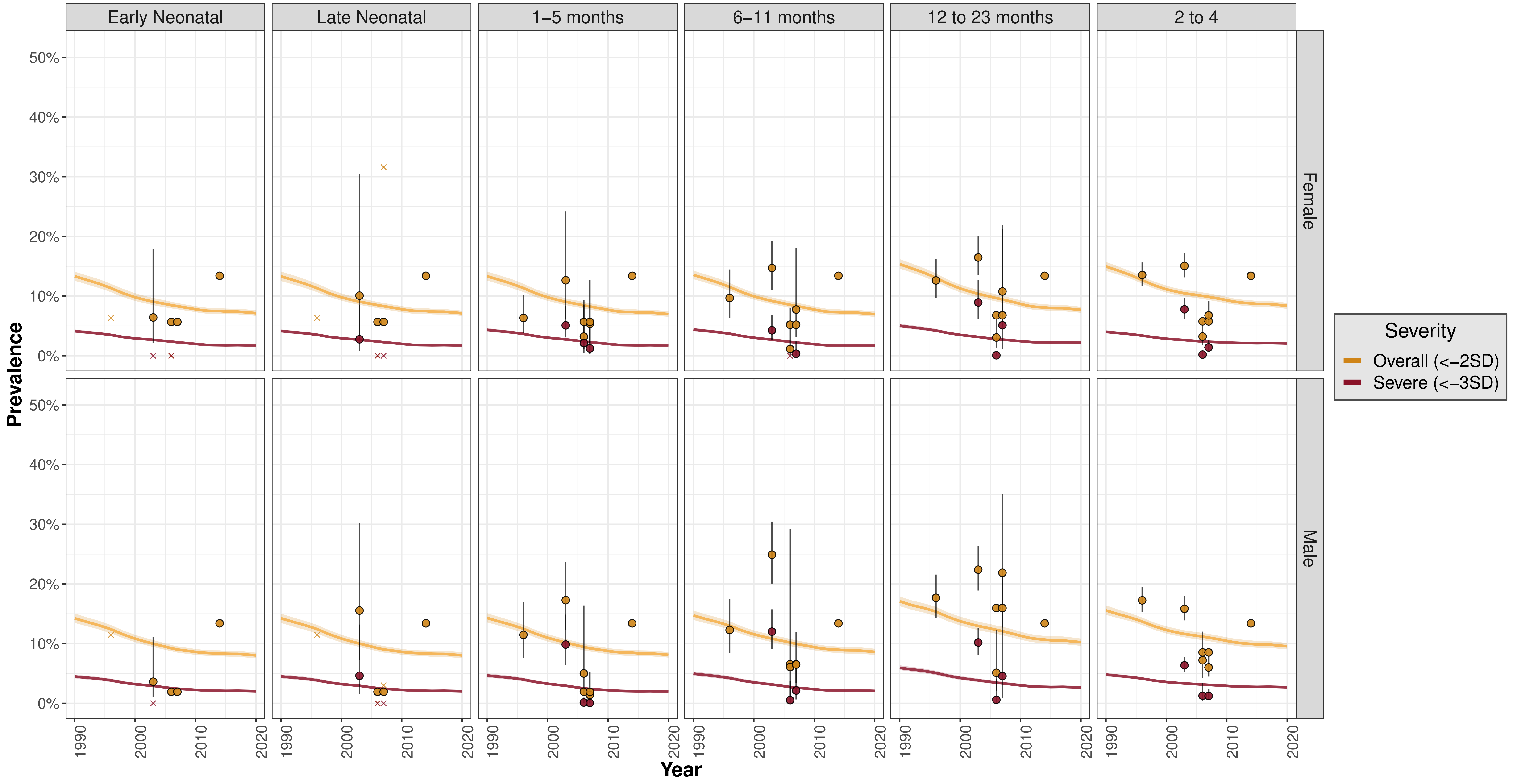

B: Transformed Mean Stunting Z Scores

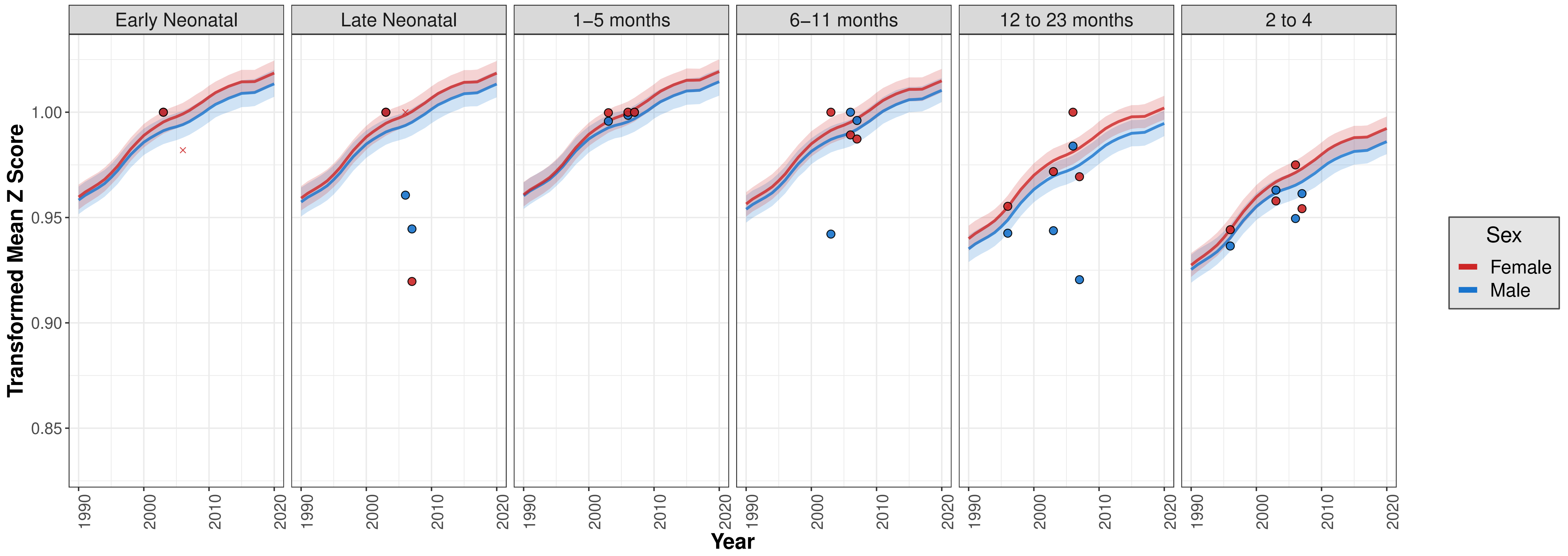

C

Year	Source	National	Subnational
1986	DHS	X	
1987	WHO CGM Database	X	
1989	WHO CGM Database	X	X
1991	WHO CGM Database		X
1992	WHO CGM Database		X
1995	WHO CGM Database		X
1996	DHS		X
1996	WHO CGM Database	X	X
1998	WHO CGM Database		X
2002	WHO CGM Database		X
2003	Consumer Expenditure Survey	X	
2006	WHO CGM Database	X	
2006	National DHS of Children and Women	X	
2007	WHO CGM Database	X	
2007	National DHS of Children and Women	X	
2008	WHO CGM Database		X
2012	Fortaleza Malnutrition and Enteric Disease Study		X
2014	WHO CGM Database	X	

Brazil – Wasting (WHZ)

D: Overall and Severe Wasting Prevalence

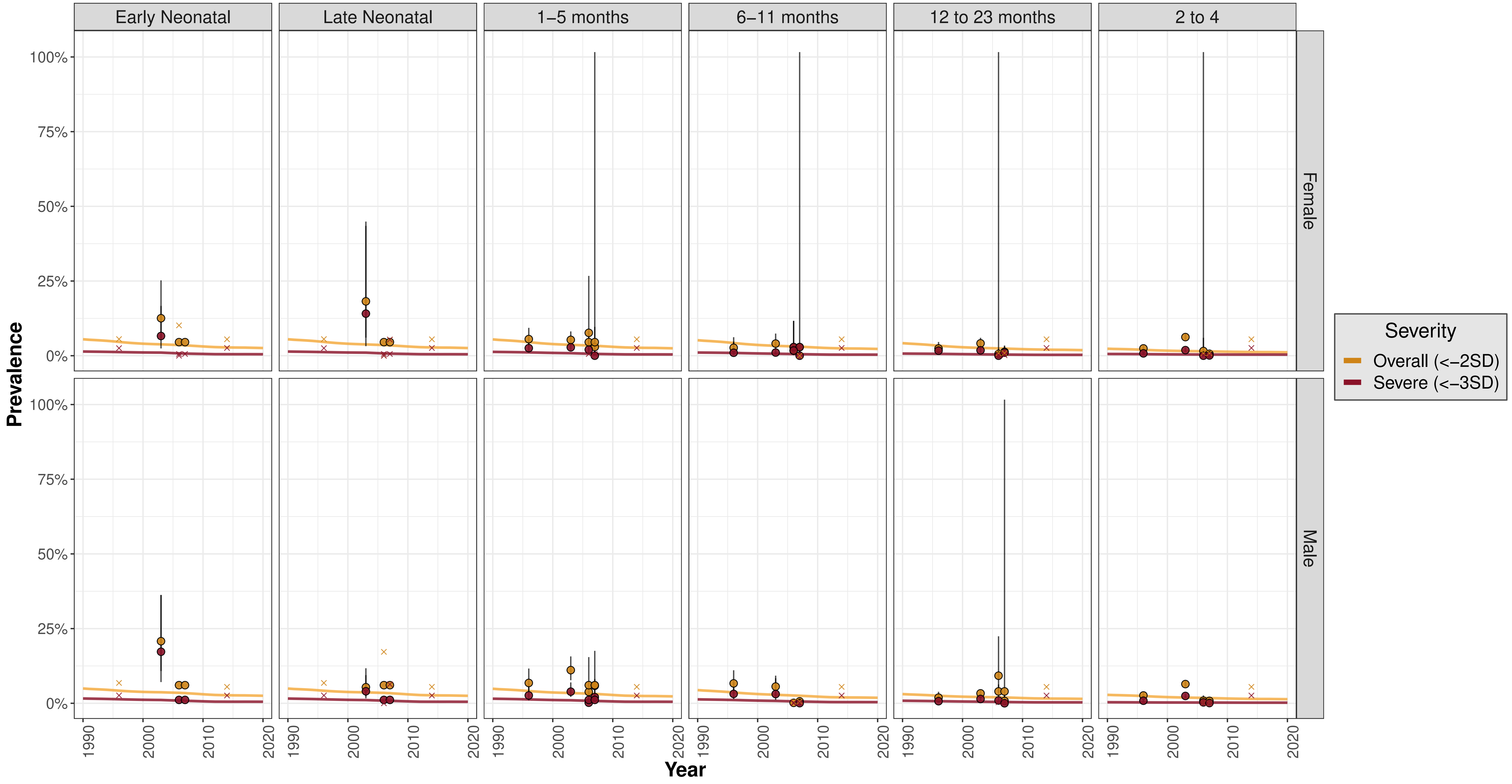

E: Transformed Mean Wasting Z Scores

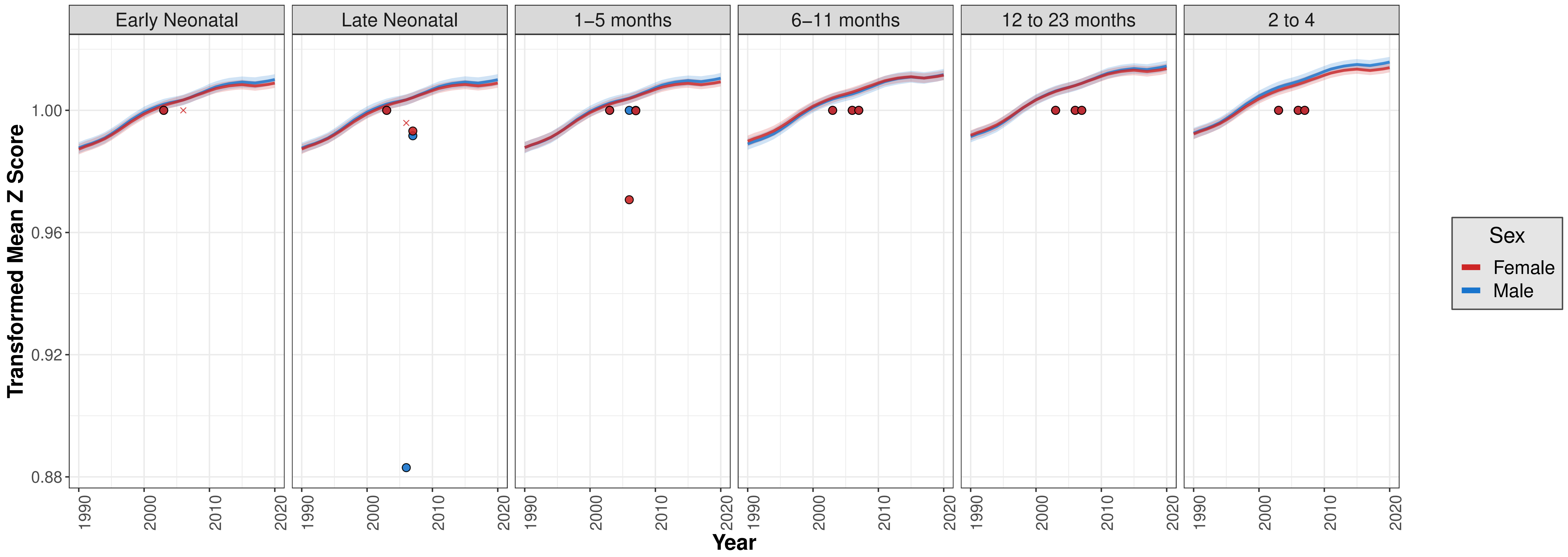

F

Year	Source	National	Subnational
1986	DHS	X	
1987	WHO CGM Database	X	
1989	WHO CGM Database	X	X
1991	WHO CGM Database		X
1995	WHO CGM Database		X
1996	DHS		X
1996	WHO CGM Database	X	X
1998	WHO CGM Database		X
2003	Consumer Expenditure Survey	X	
2006	WHO CGM Database	X	
2006	National DHS of Children and Women	X	
2007	WHO CGM Database	X	
2007	National DHS of Children and Women	X	
2008	WHO CGM Database		X
2012	Fortaleza Malnutrition and Enteric Disease Study		X
2014	WHO CGM Database	X	

Brazil – Underweight (WAZ)

G: Overall and Severe Underweight Prevalence

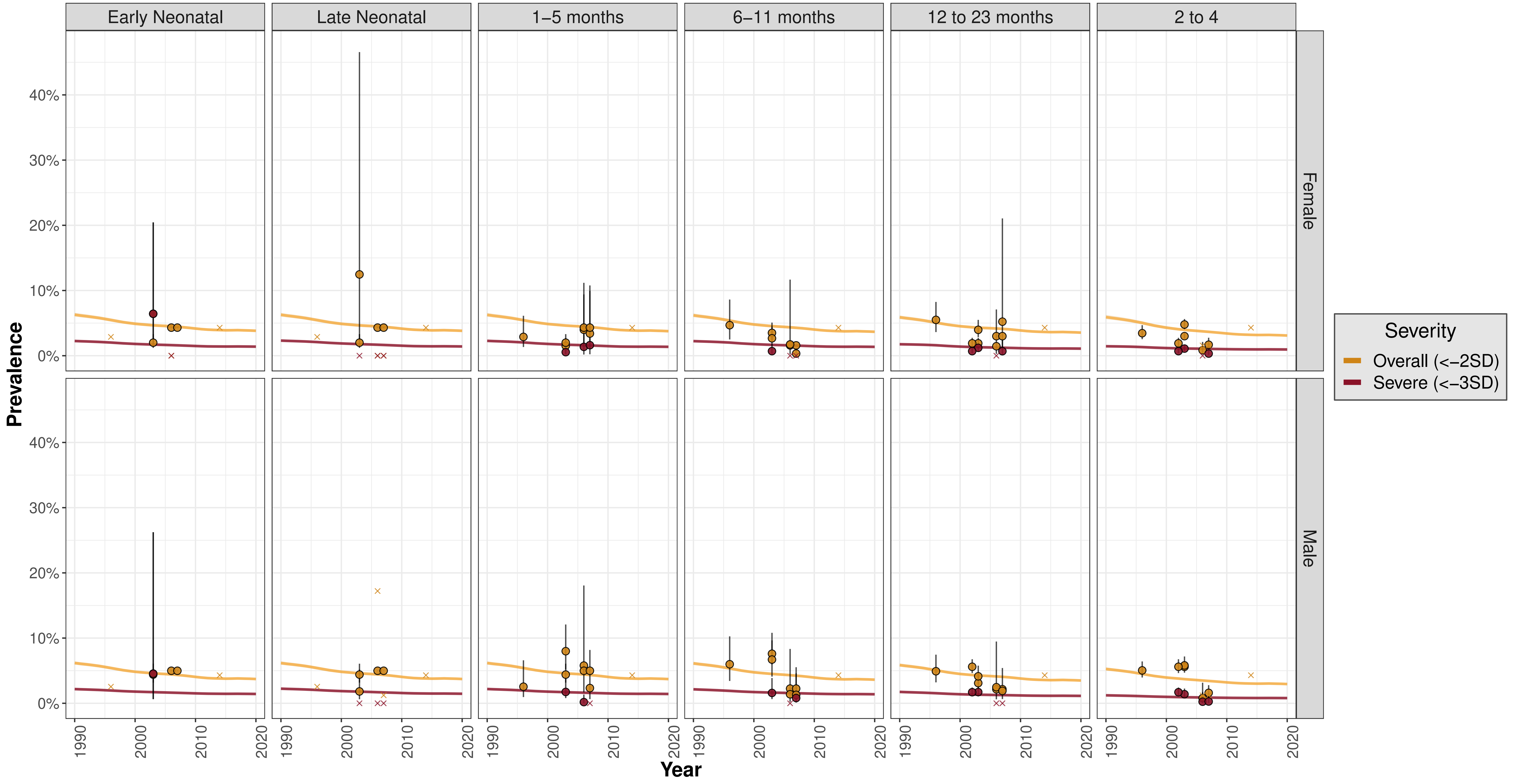

H: Transformed Mean Underweight Z Scores

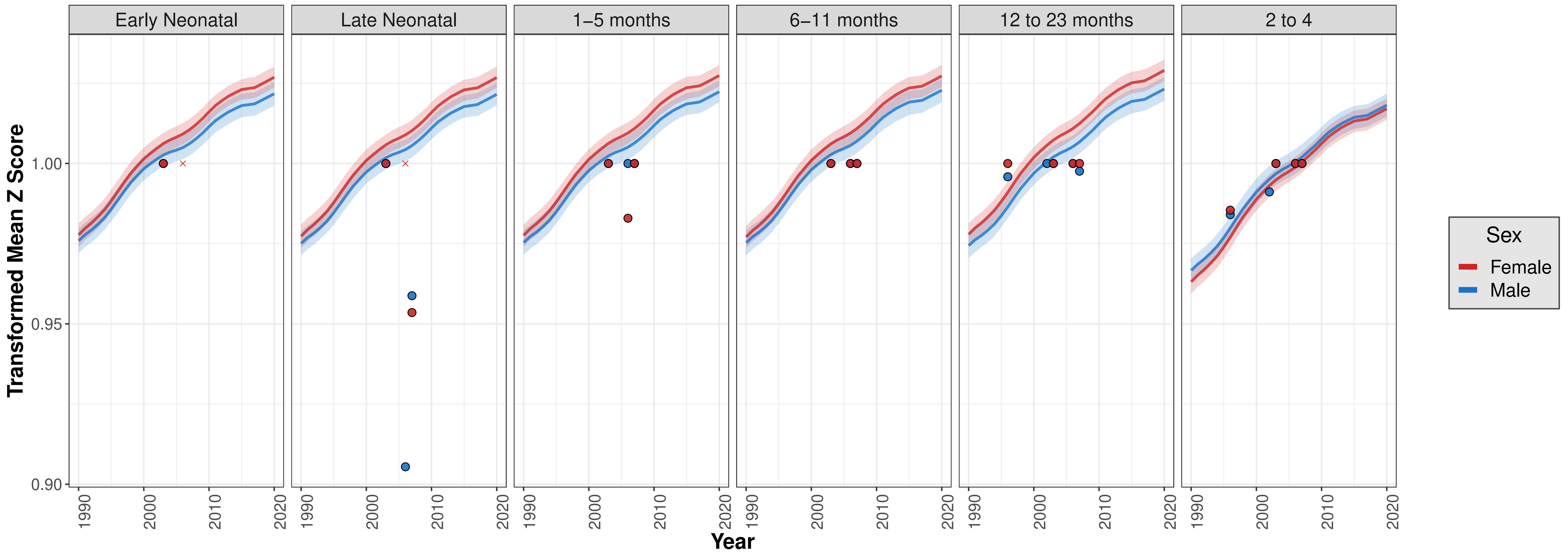

I

Year	Source	National	Subnational
1986	DHS	X	
1987	WHO CGM Database	X	
1989	WHO CGM Database	X	X
1991	WHO CGM Database		X
1995	WHO CGM Database		X
1996	DHS		X
1996	WHO CGM Database	X	X
1998	WHO CGM Database		X
2002	WHO CGM Database	X	X
2003	Consumer Expenditure Survey	X	
2003	WHO CGM Database	X	
2006	WHO CGM Database	X	
2006	National DHS of Children and Women	X	
2007	WHO CGM Database	X	
2007	National DHS of Children and Women	X	
2008	WHO CGM Database		X
2012	Fortaleza Malnutrition and Enteric Disease Study		X
2014	WHO CGM Database	X	

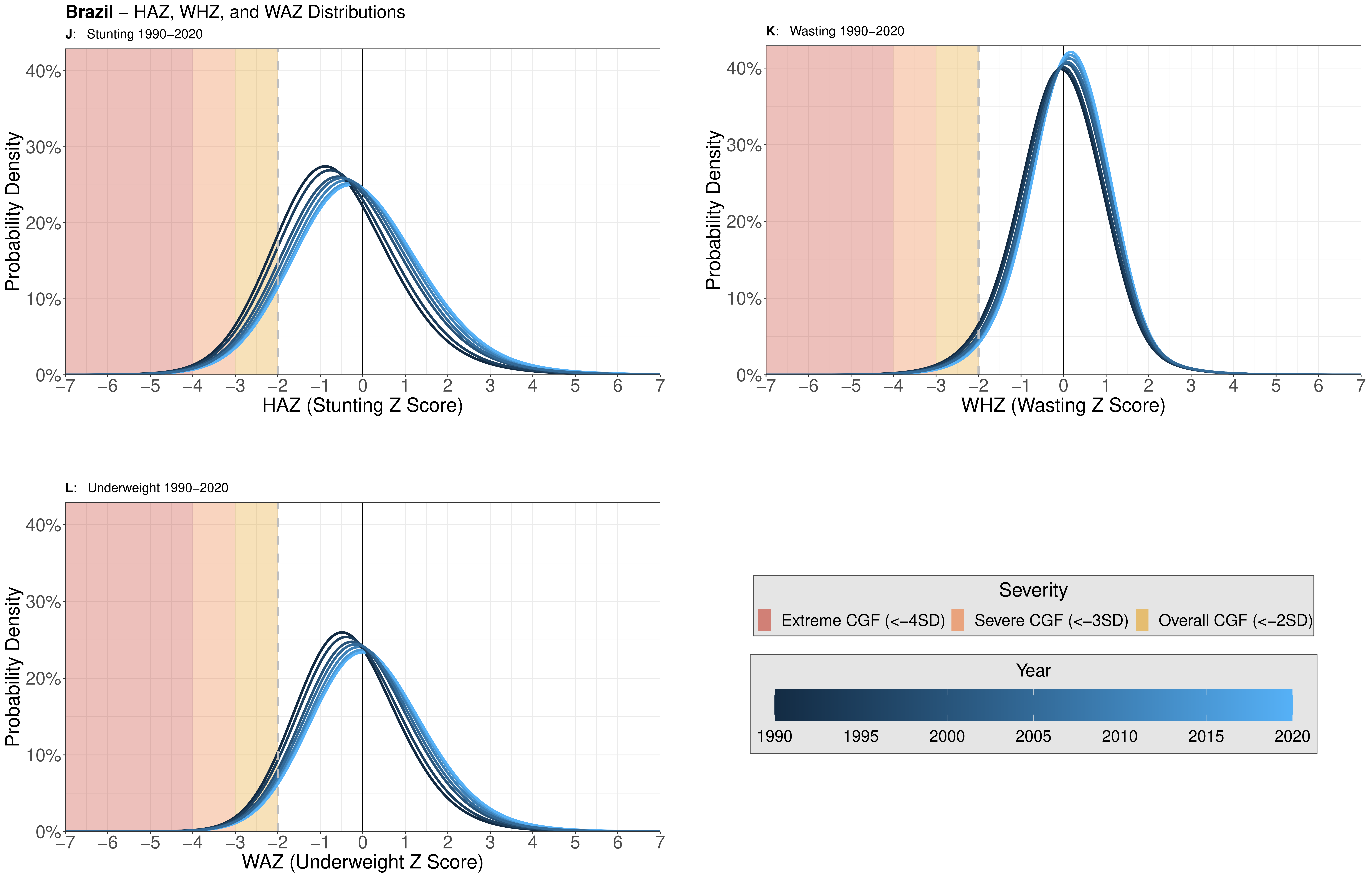

Brazil – HAZ, WHZ, and WAZ Distributions
J: Stunting 1990–2020
Probability Density
40%
30%
20%
10%
0%
−7 −6 −5 −4 −3 −2 −1 0 1 2 3 4 5 6 7
HAZ (Stunting Z Score)
K: Wasting 1990–2020
Probability Density
40%
30%
20%
10%
0%
−7 −6 −5 −4 −3 −2 −1 0 1 2 3 4 5 6 7
WHZ (Wasting Z Score)
L: Underweight 1990–2020
Probability Density
40%
30%
20%
10%
0%
−7 −6 −5 −4 −3 −2 −1 0 1 2 3 4 5 6 7
WAZ (Underweight Z Score)
Severity
Extreme CGF (<−4SD)
Severe CGF (<−3SD)
Overall CGF (<−2SD)
Year
1990
1995
2000
2005
2010
2015
2020

Paraguay – Stunting (HAZ)

A: Overall and Severe Stunting Prevalence

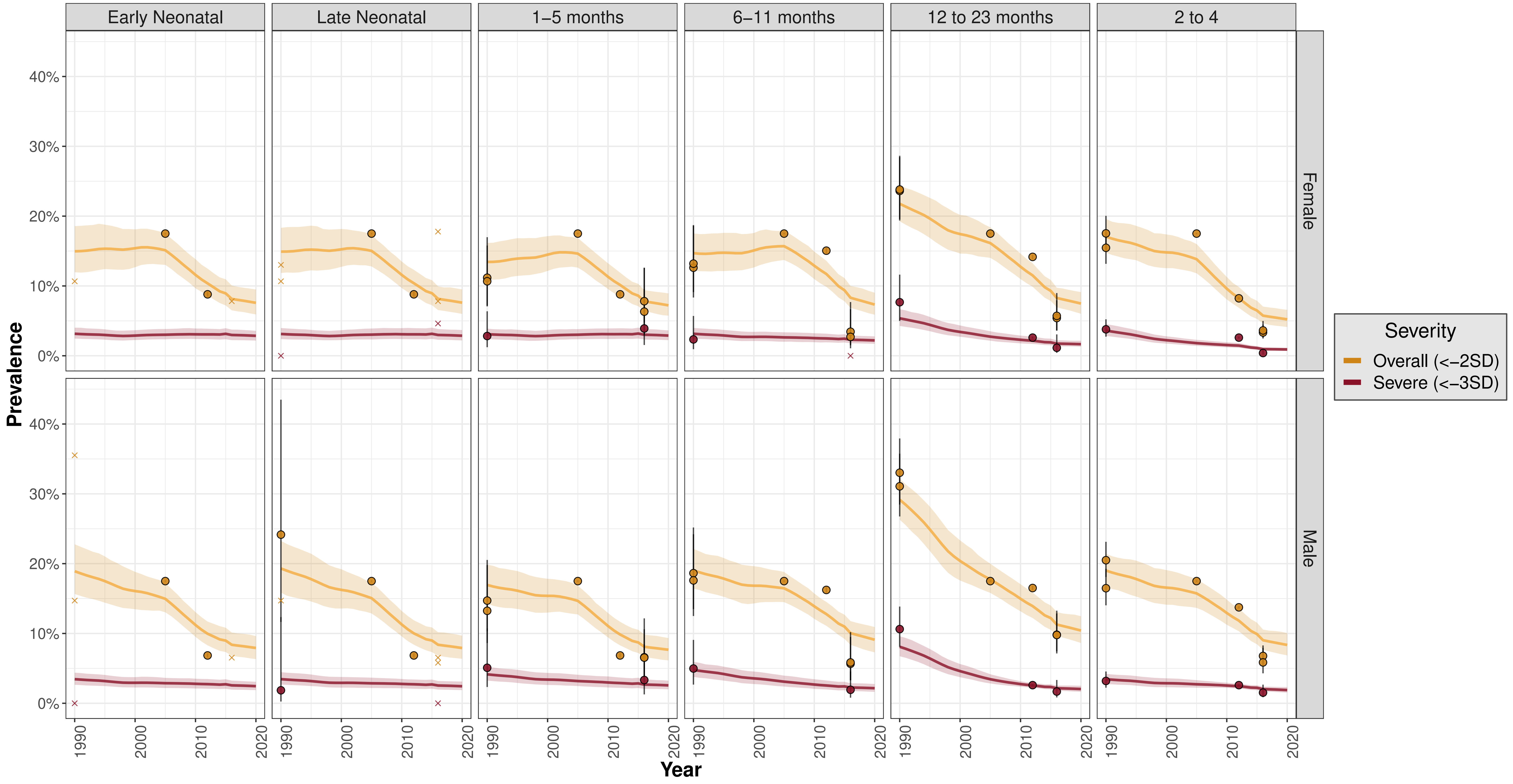

B: Transformed Mean Stunting Z Scores

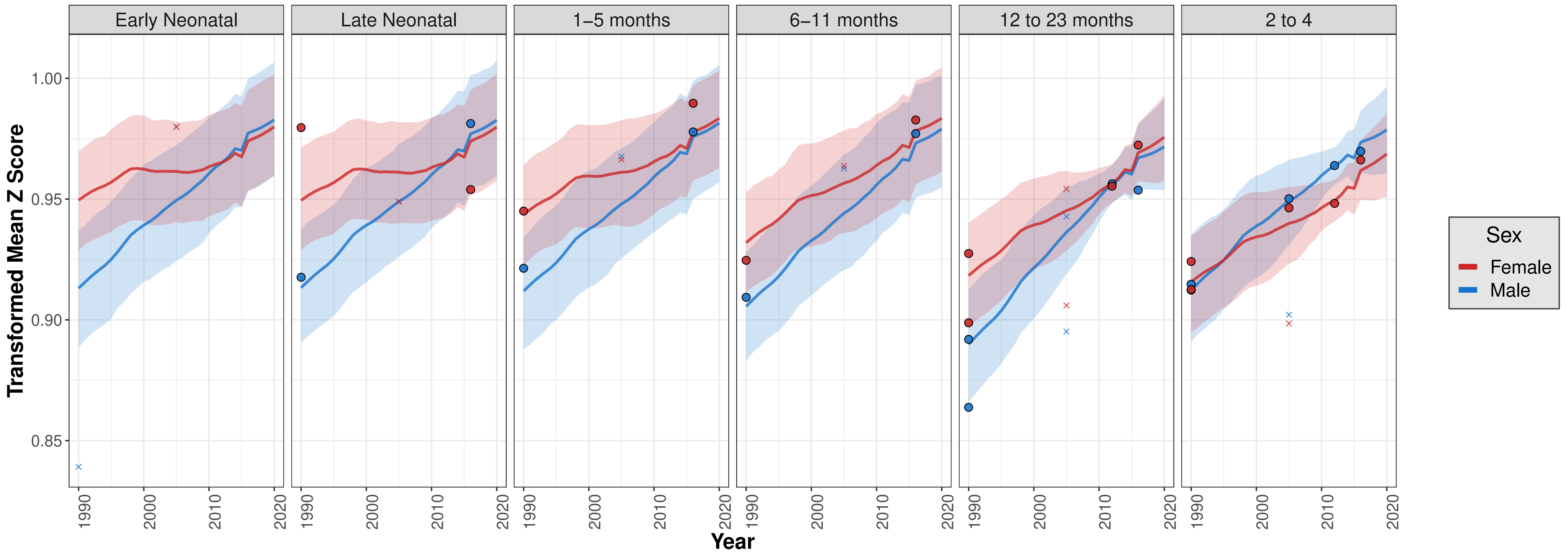

C

Year	Source
1990	DHS
1990	WHO CGM Database
2005	WHO CGM Database
2012	WHO CGM Database
2016	WHO CGM Database
2016	MICS

Paraguay – Wasting (WHZ)

D: Overall and Severe Wasting Prevalence

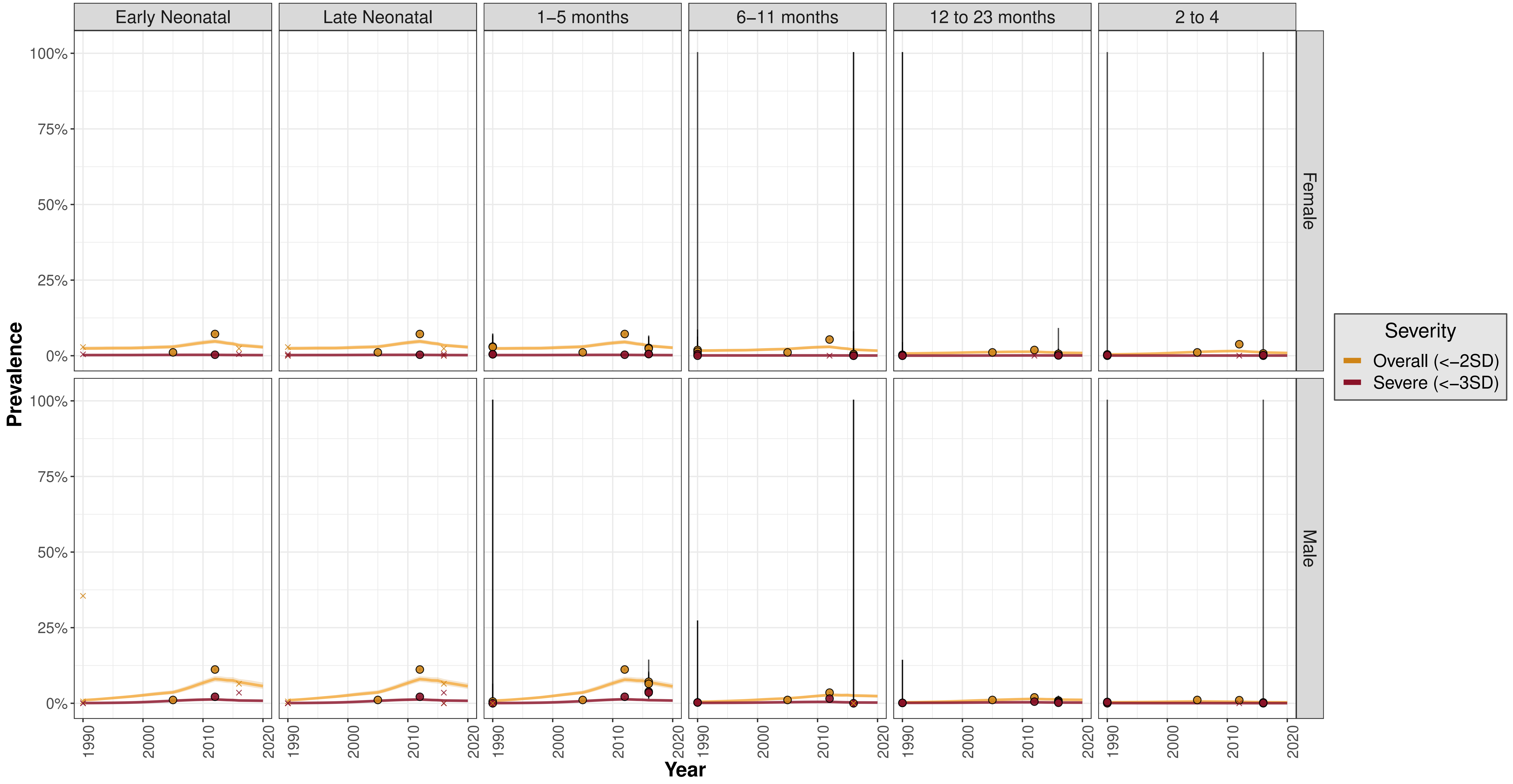

E: Transformed Mean Wasting Z Scores

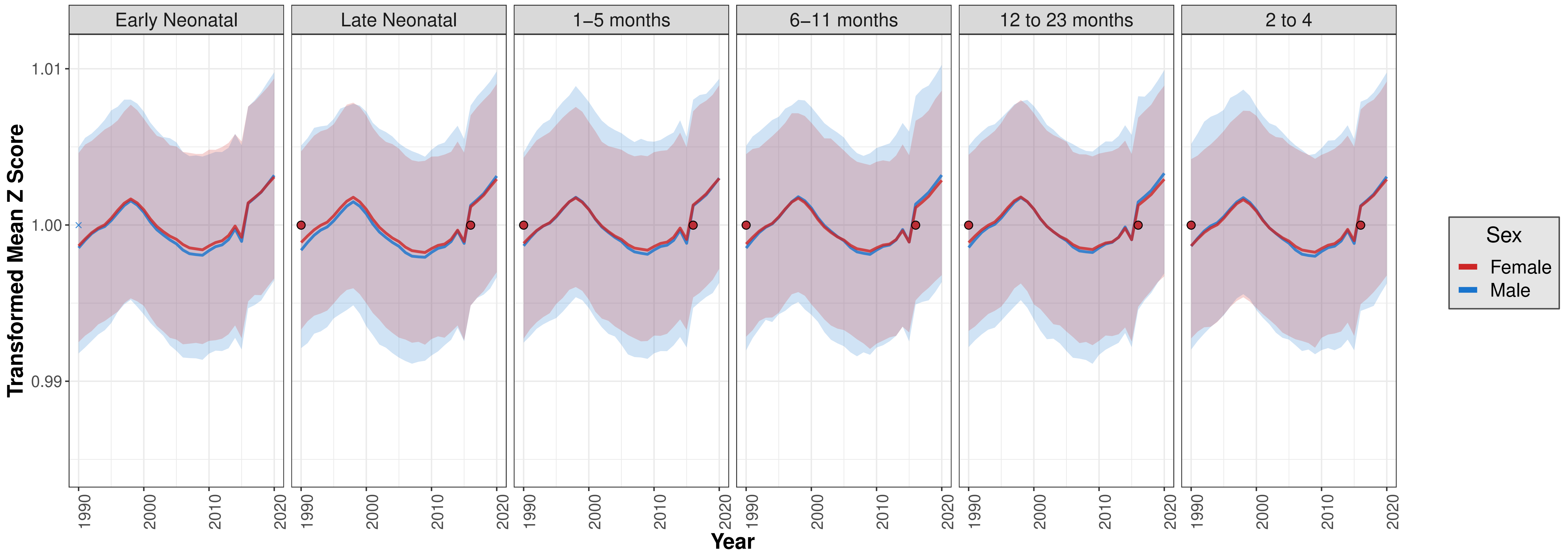

F

Year	Source
1990	DHS
1990	WHO CGM Database
2005	WHO CGM Database
2012	WHO CGM Database
2016	WHO CGM Database
2016	MICS

Paraguay – Underweight (WAZ)

G: Overall and Severe Underweight Prevalence

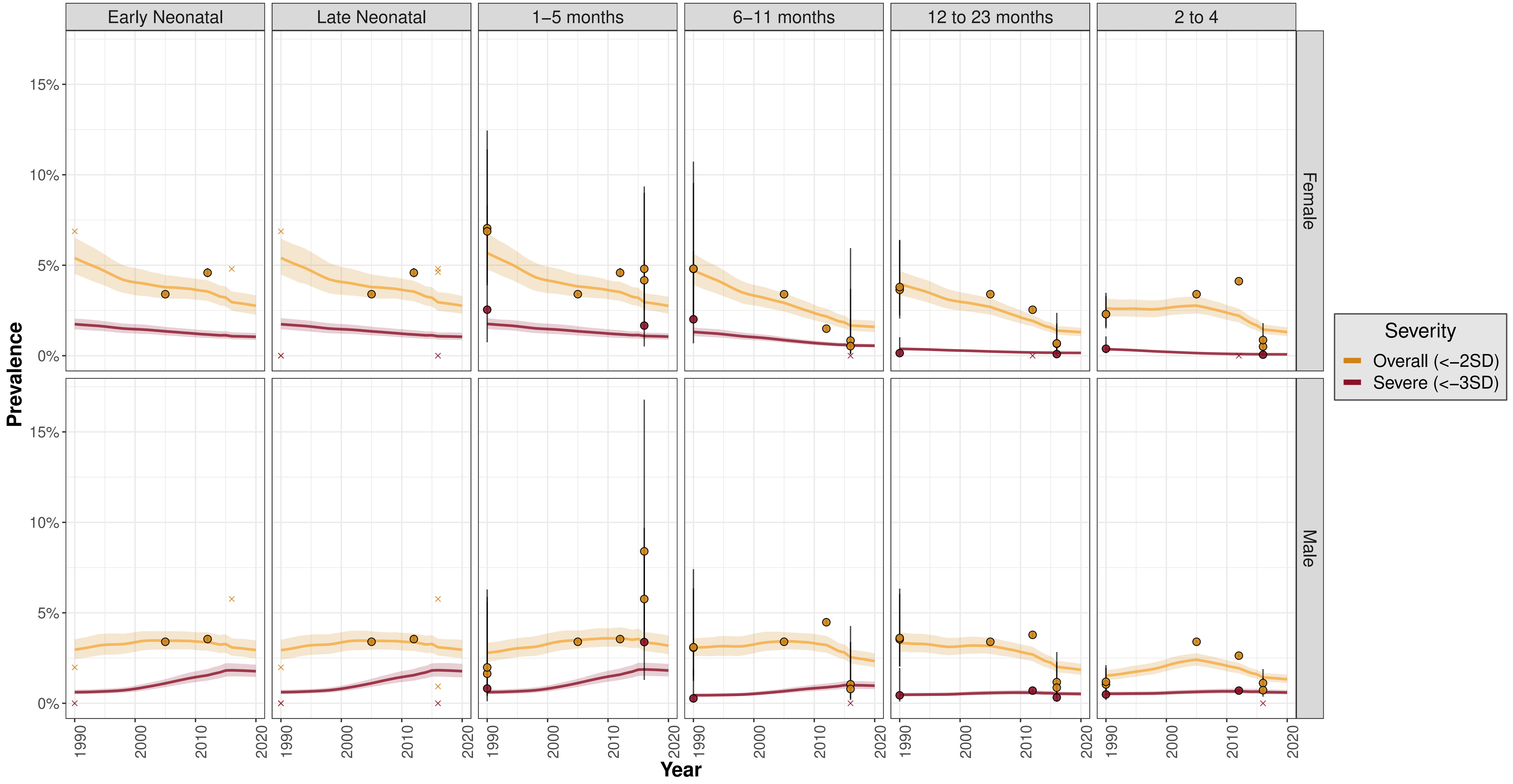

H: Transformed Mean Underweight Z Scores

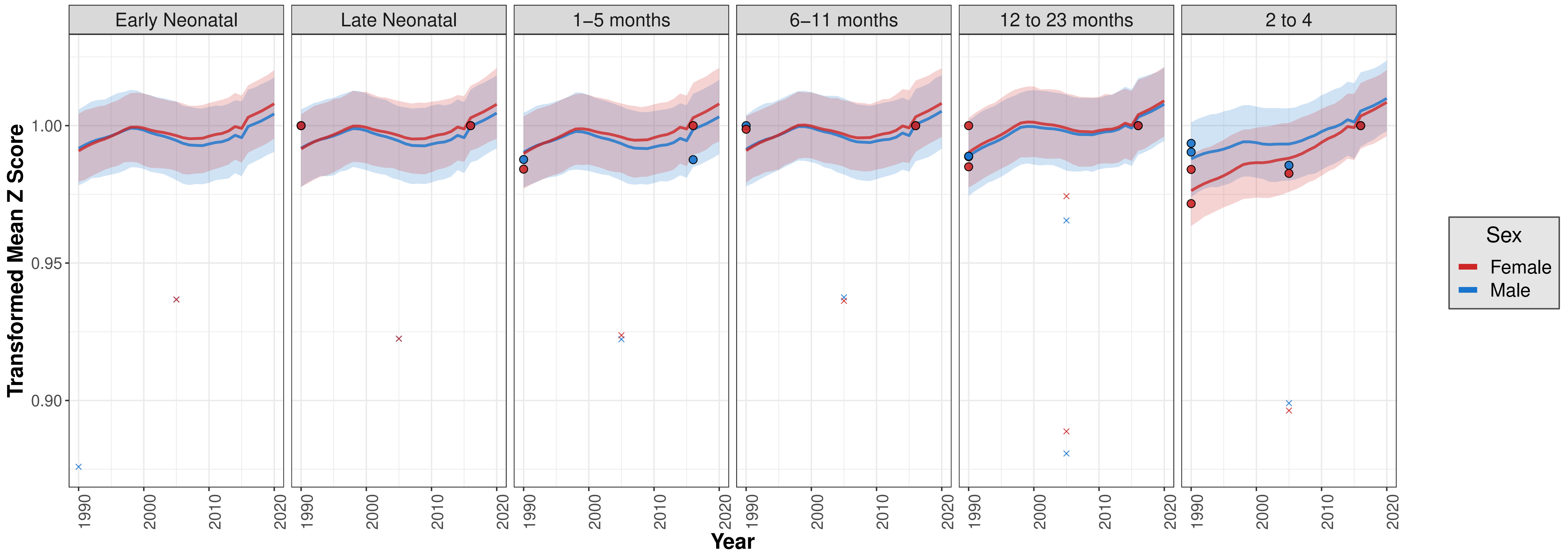

I

Year	Source
1990	DHS
1990	WHO CGM Database
2005	WHO CGM Database
2012	WHO CGM Database
2016	WHO CGM Database
2016	MICS

Paraguay – HAZ, WHZ, and WAZ Distributions

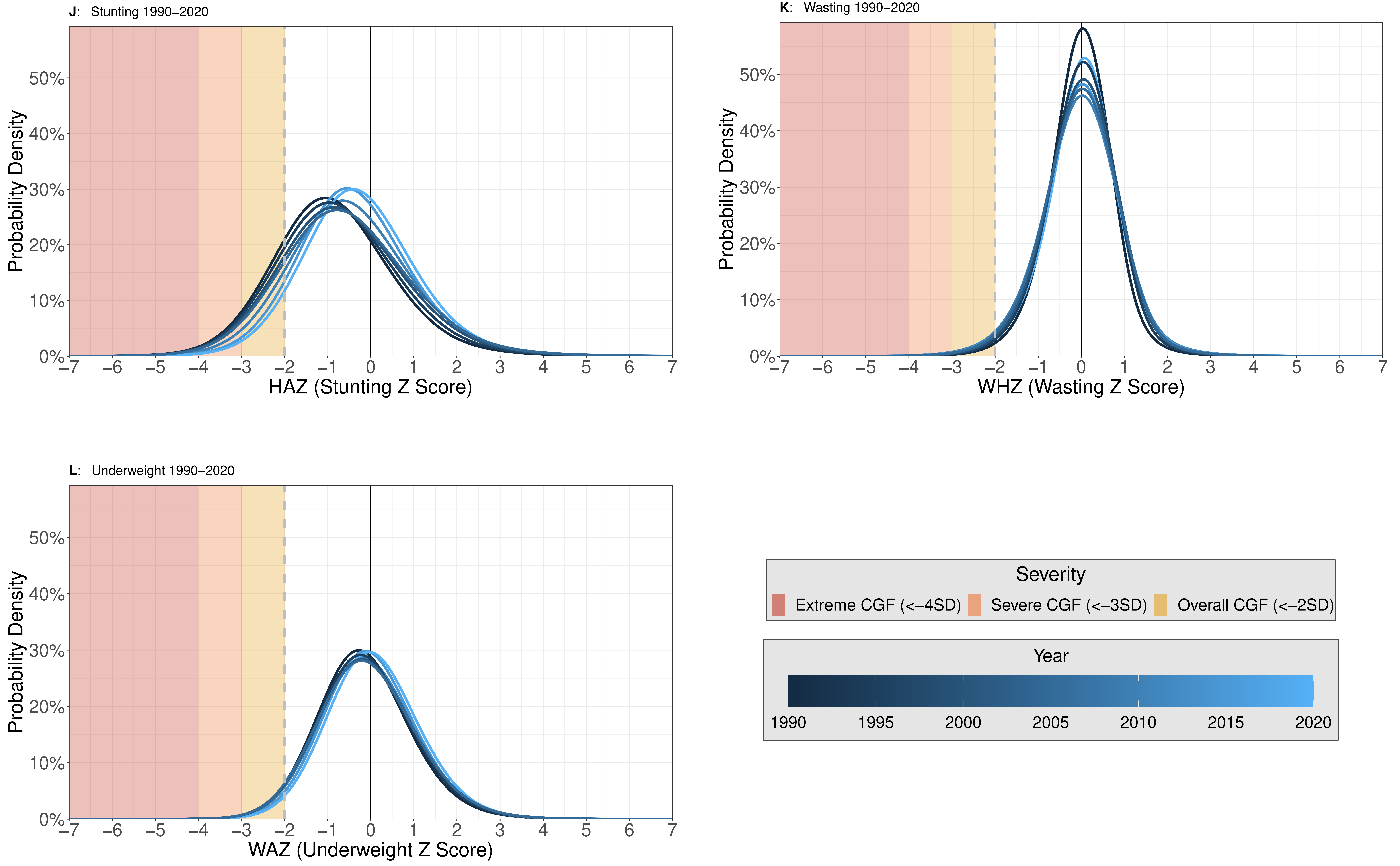

Supplement: Supplementary file 2 — Data S1 to S4 [file sciadv.abm8954_data_files_s1_to_s4.zip › sciadv.abm8954_data_file_s1c.pdf]
