# Supplementary material for: Current dichotomous metrics obscure trends in severe and extreme child growth failure
Source: Sci Adv. 2022 May 20;8(20):eabm8954. doi: 10.1126/sciadv.abm8954 (PMC9122330; doi:10.1126/sciadv.abm8954)
Supplement: Supplementary file 2 — Data S1 to S4 [file sciadv.abm8954_data_files_s1_to_s4.zip › sciadv.abm8954_data_file_s1d.pdf]

Data S1d. Spatio-temporal Gaussian Process Regression (ST-GPR) results for overall, severe, and mean CGF by location, including location-specific data sources; and distributions of stunting [HAZ], wasting [WHZ], and underweight [WAZ] for children under age five, both sexes, for every five years from 1990−2020. Country results are grouped by GBD super-region, including Central Europe, Eastern Europe, and Central Asia (S1a), High-income (S1b), Latin America and Caribbean (S1c), North Africa and Middle East (S1d), South Asia (S1e), Southeast Asia, East Asia, and Oceania (S1f), and Sub-Saharan Africa (S1g). Plots for each country include overall and severe stunting prevalence (A) and transformed mean stunting Z scores (B). A source list is shown which includes surveys included in the stunting models (C). Additional plots are shown for overall and severe wasting prevalence (D) and transformed mean wasting Z scores (E), followed by a source list with surveys included in the wasting models (F). Plots are then shown for overall and severe underweight prevalence (G), and transformed mean underweight Z scores (H), with a source list listing surveys included in the underweight models (I). Finally, distributions of stunting (J), wasting (K), and underweight (L) are shown for children under age five, both sexes, for every five years from 1990-2020. Surveys that were outliered are shown with X's on all plots. Surveys prior to 1990 may have been inputs to the models to inform trends, but estimates are only produced and shown for 1990−2020. For locations that are modeled nationally and subnationally, sources that are only included subnationally are not included in the plots of national level estimates. These sources were included in subnational models that influence national level models. Note that due to the transformation on mean Z scores, increasing values reflect improvements in mean Z score. Surveys conducted over a range of years were assigned to the midpoint year from that interval, which is the year reflected in the table and the plots. For the distributions of stunting, wasting, and underweight, the area under the curve reflects the estimated proportion of children experiencing that severity of CGF or worse. DHS is Demographic and Health Surveys. MICS is Multiple Indicator Cluster Survey. WHO CGM is the WHO Global Database on Child Growth and Malnutrition. SDNS is Survey of Diet and Nutritional Status.

This file contains the above for the following locations in the GBD super region of North Africa and the Middle East, in the following order:

Algeria, Bahrain, Egypt, Iran (Islamic Republic of), Iraq, Jordan, Kuwait, Lebanon, Libya, Morocco, Oman, Palestine, Qatar, Saudi Arabia, Syrian Arab Republic, Tunisia, Turkey, United Arab Emirates, Yemen, Afghanistan, Sudan

Algeria – Stunting (HAZ)

A: Overall and Severe Stunting Prevalence

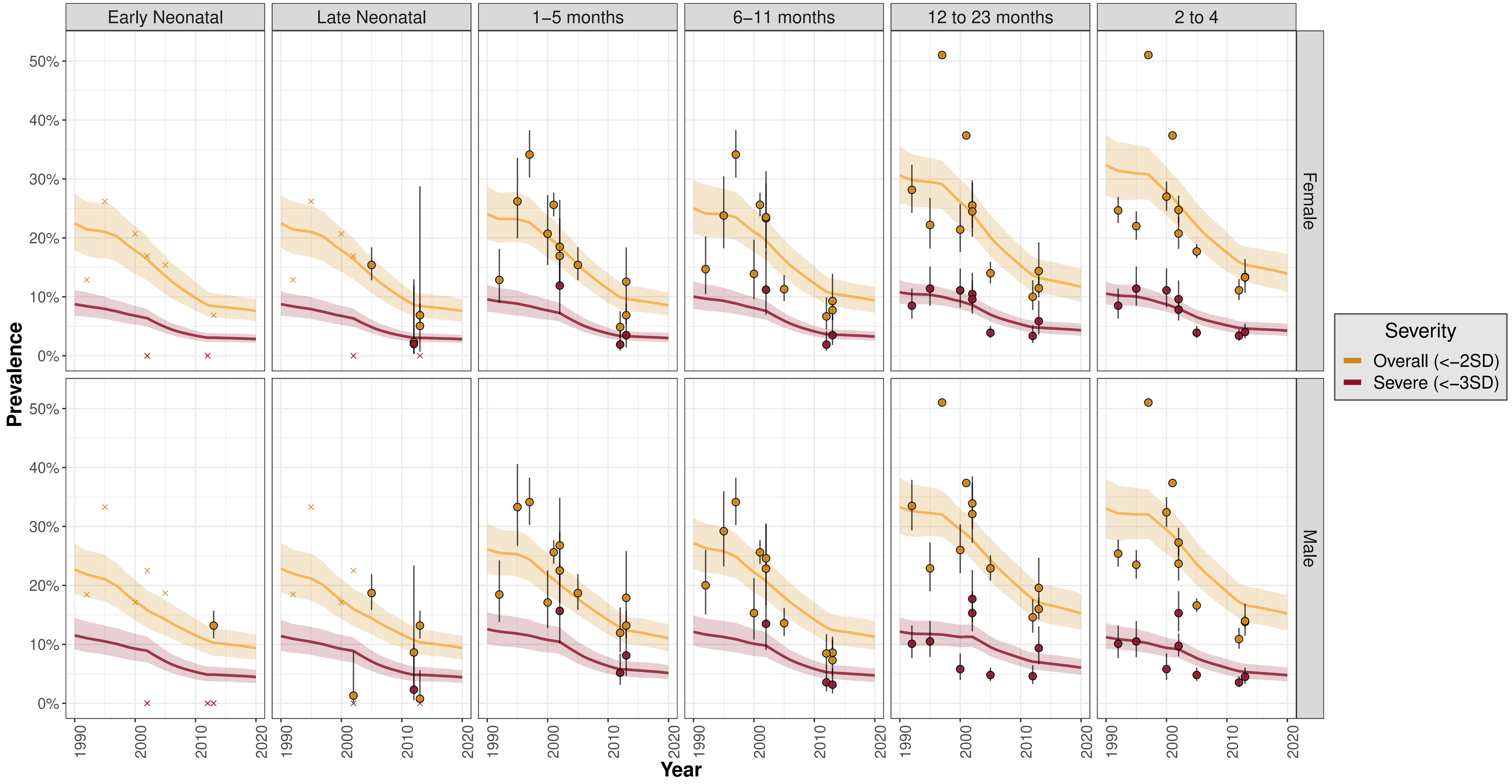

B: Transformed Mean Stunting Z Scores

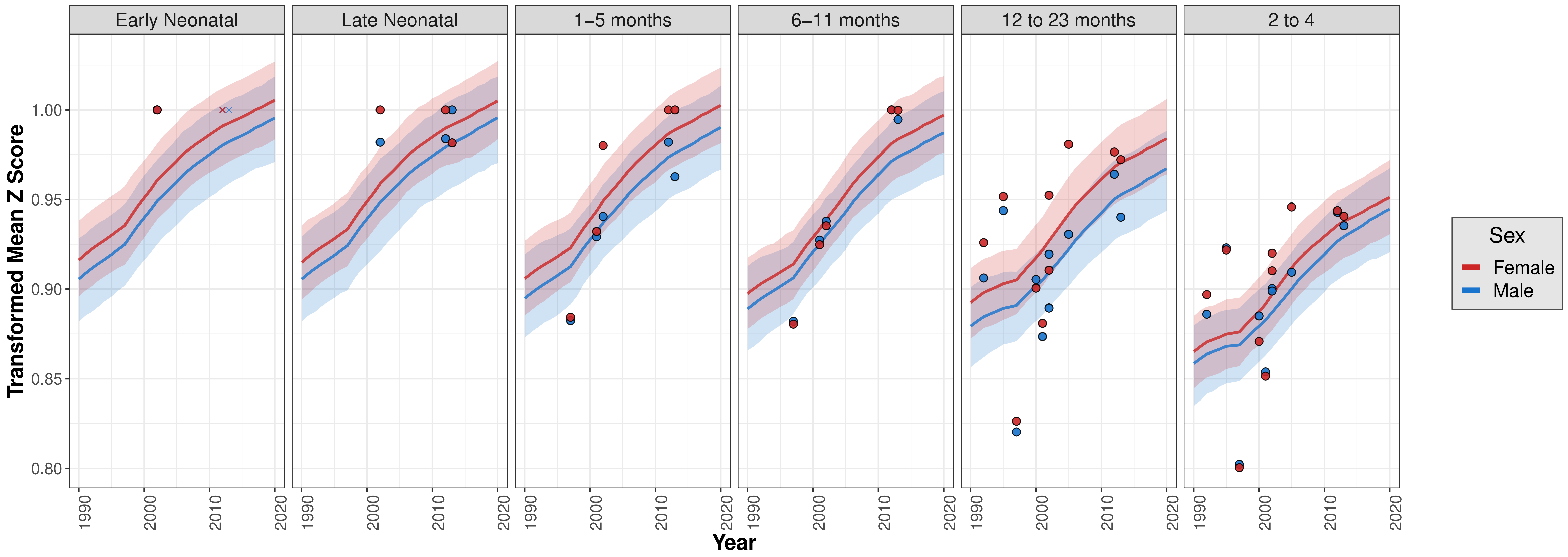

C

Year	Source
1987	WHO CGM Database
1992	WHO CGM Database
1995	WHO CGM Database
1997	WHO CGM Database
2000	WHO CGM Database
2001	WHO CGM Database
2002	Family Health Survey
2002	WHO CGM Database
2005	WHO CGM Database
2012	MICS
2013	WHO CGM Database
2013	MICS

Algeria – Wasting (WHZ)

D: Overall and Severe Wasting Prevalence

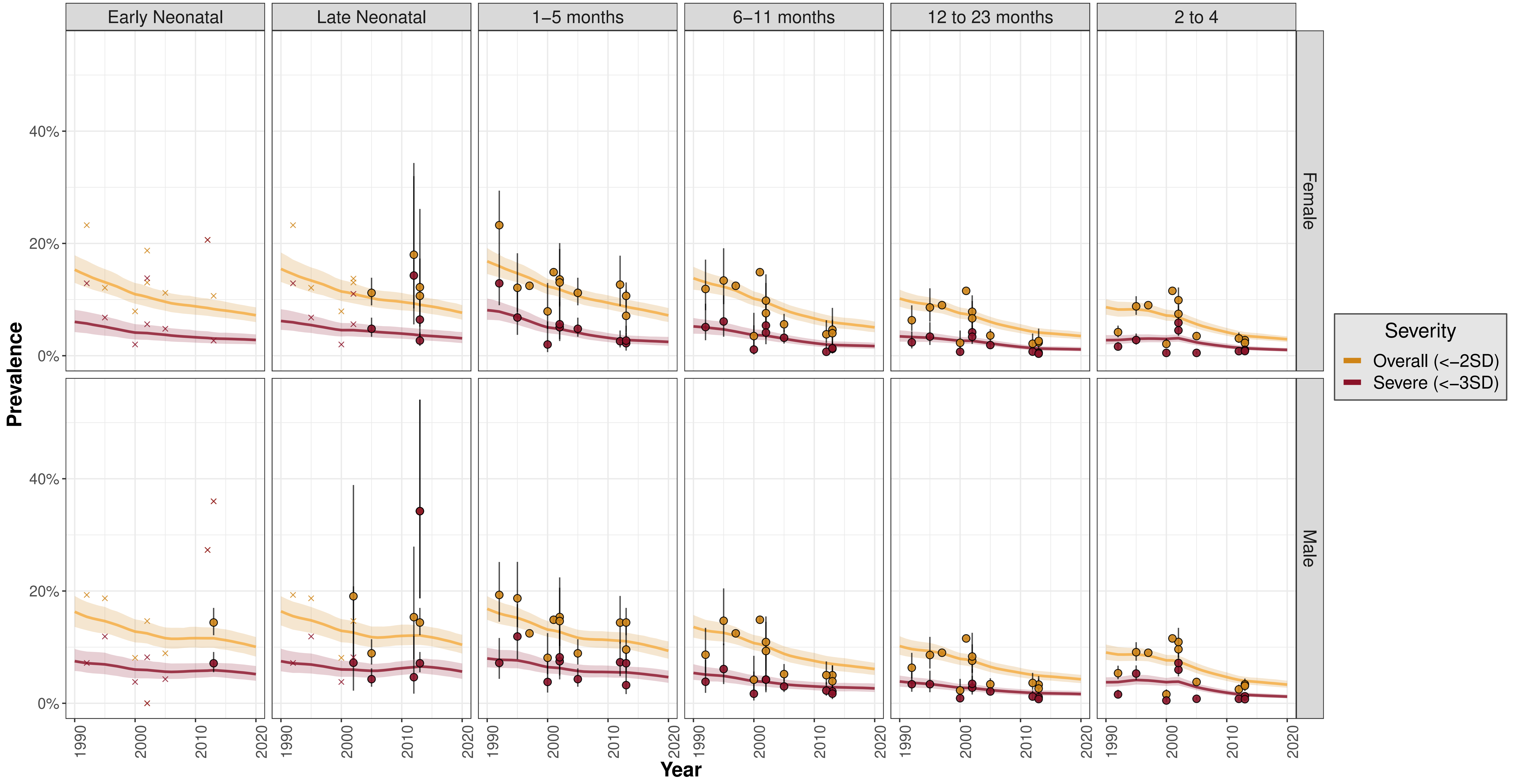

E: Transformed Mean Wasting Z Scores

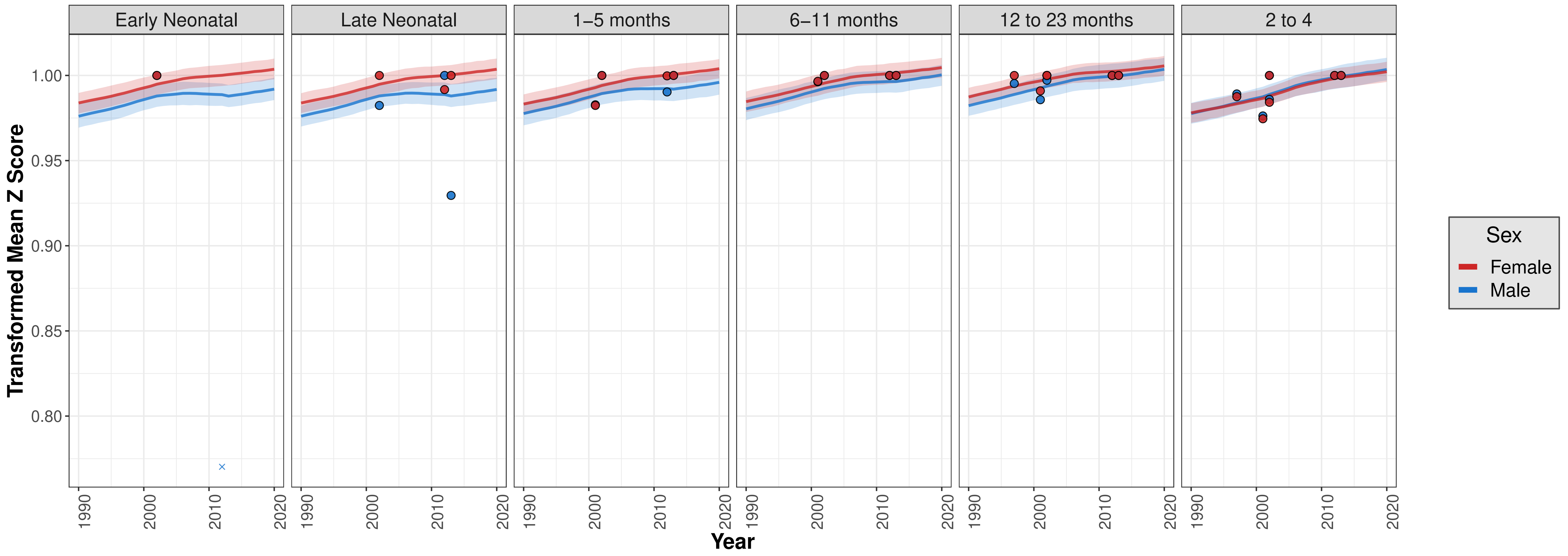

F

Year	Source
1987	WHO CGM Database
1992	WHO CGM Database
1995	WHO CGM Database
1997	WHO CGM Database
2000	WHO CGM Database
2001	WHO CGM Database
2002	Family Health Survey
2002	WHO CGM Database
2005	WHO CGM Database
2012	MICS
2013	WHO CGM Database
2013	MICS

Algeria – Underweight (WAZ)

G: Overall and Severe Underweight Prevalence

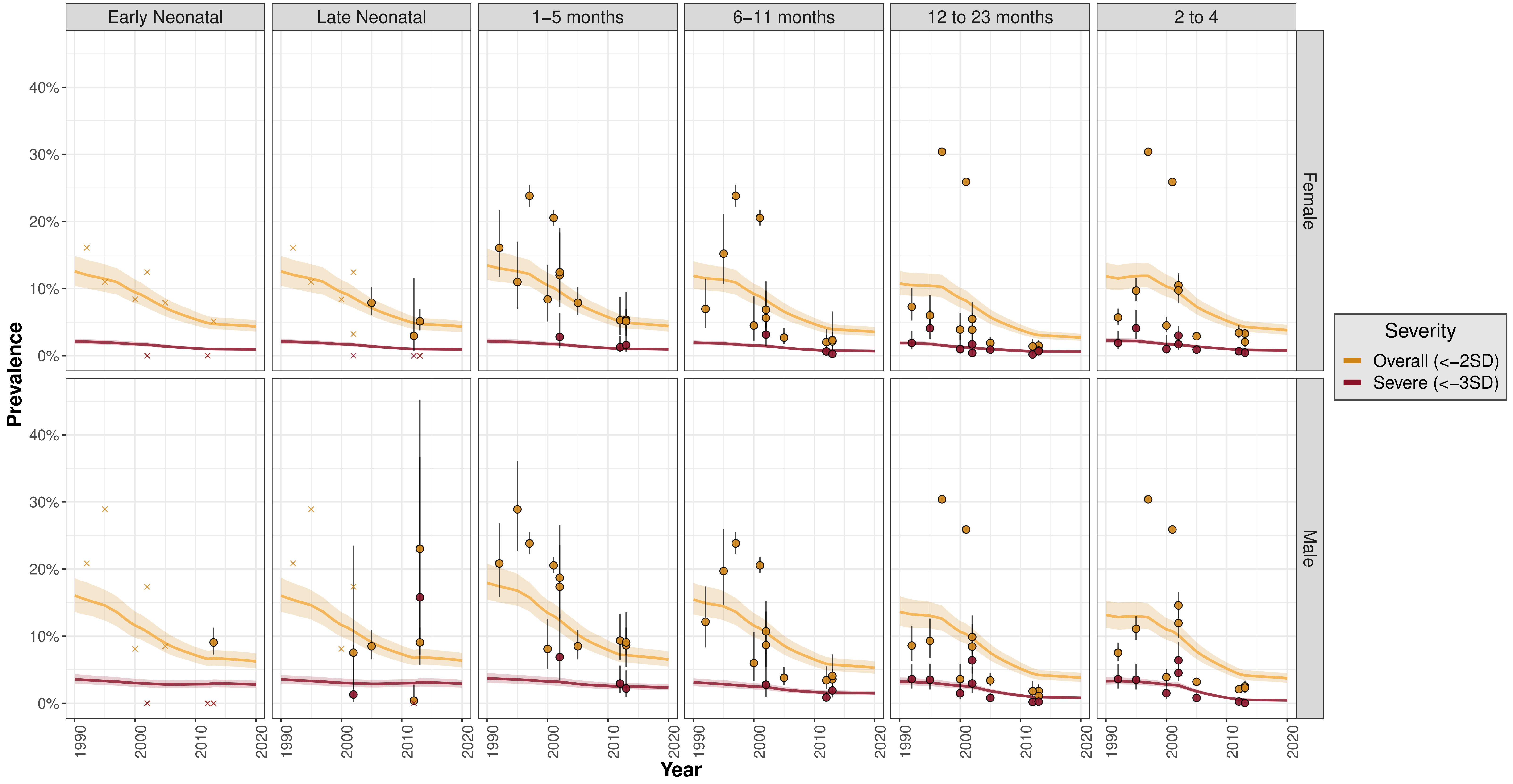

H: Transformed Mean Underweight Z Scores

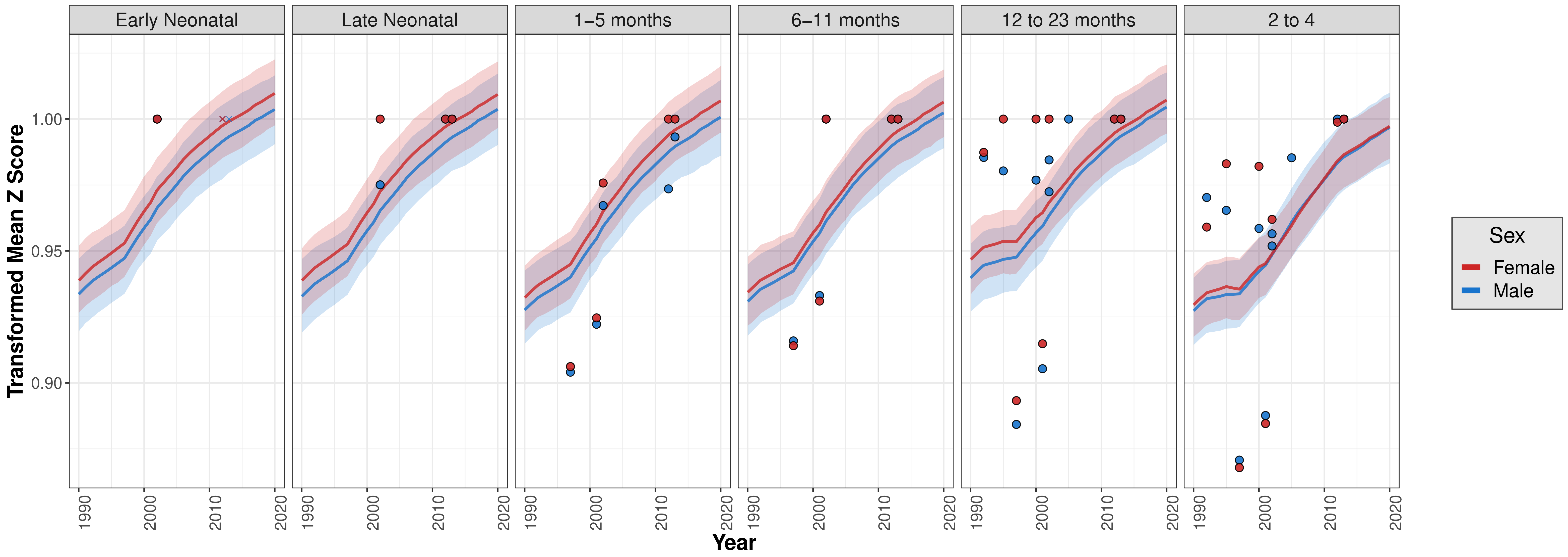

I

Year	Source
1987	WHO CGM Database
1992	WHO CGM Database
1995	WHO CGM Database
1997	WHO CGM Database
2000	WHO CGM Database
2001	WHO CGM Database
2002	Family Health Survey
2002	WHO CGM Database
2005	WHO CGM Database
2012	MICS
2013	WHO CGM Database
2013	MICS

Algeria – HAZ, WHZ, and WAZ Distributions

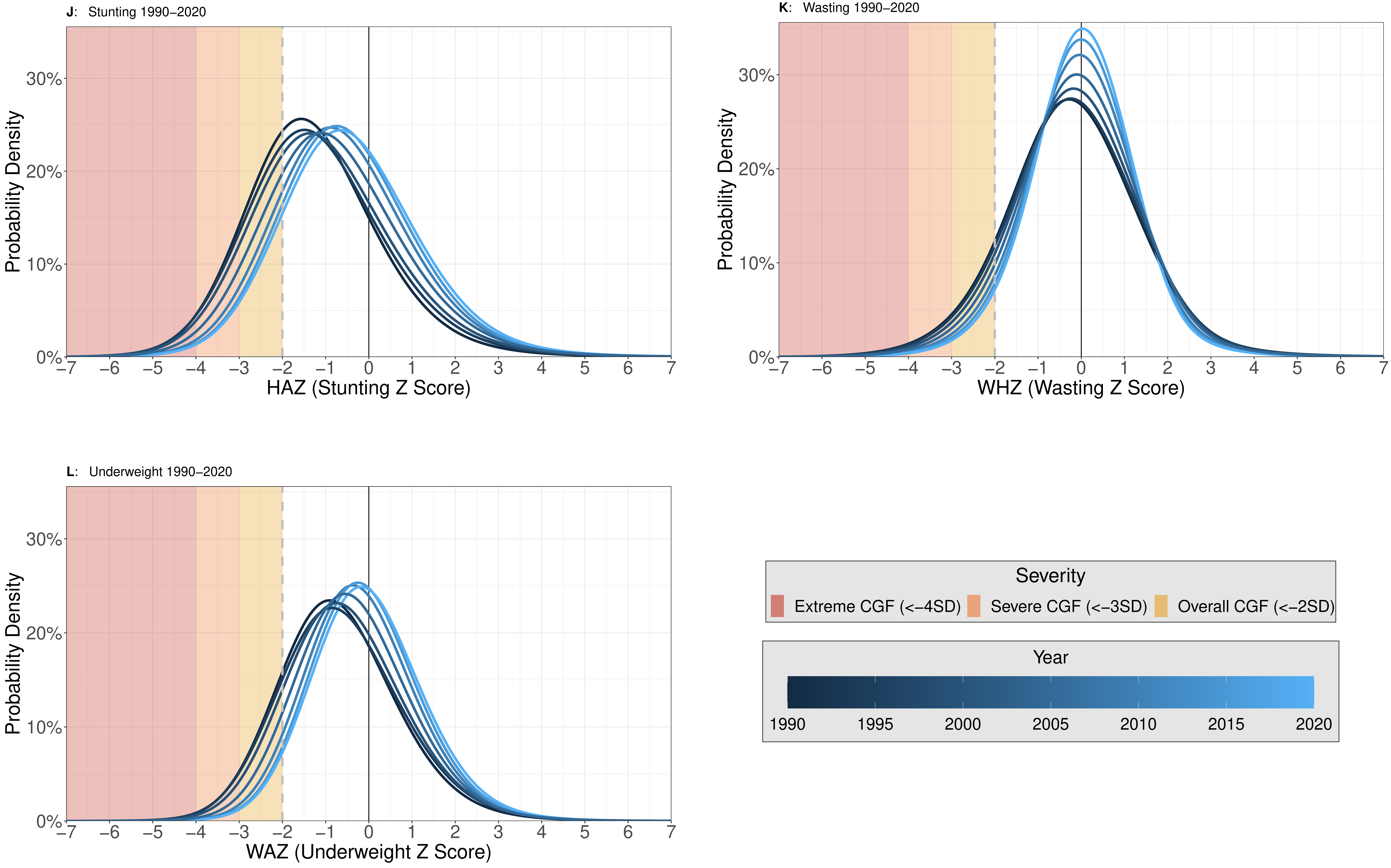

Bahrain – Stunting (HAZ)

A: Overall and Severe Stunting Prevalence

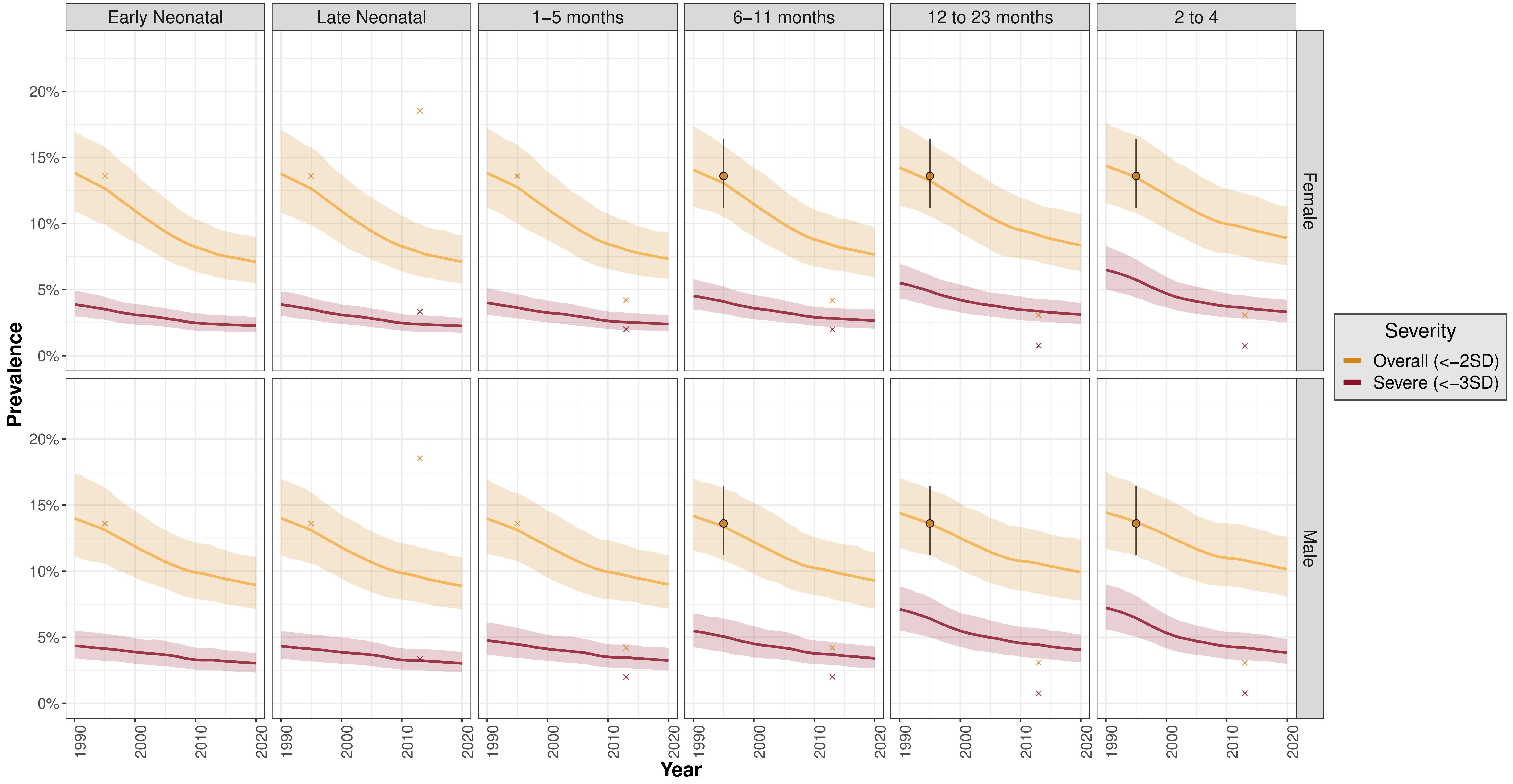

B: Transformed Mean Stunting Z Scores

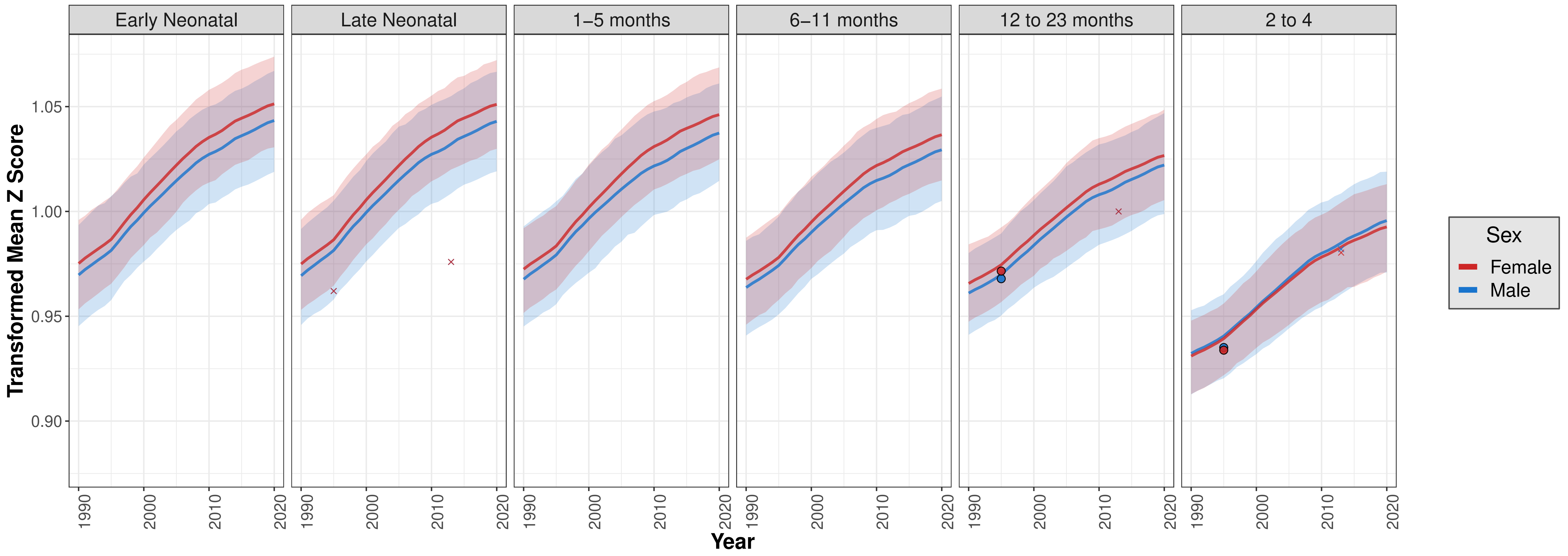

C

Year	Source
1989	WHO CGM Database
1995	WHO CGM Database
2013	Health Statistics

Bahrain – Wasting (WHZ)

D: Overall and Severe Wasting Prevalence

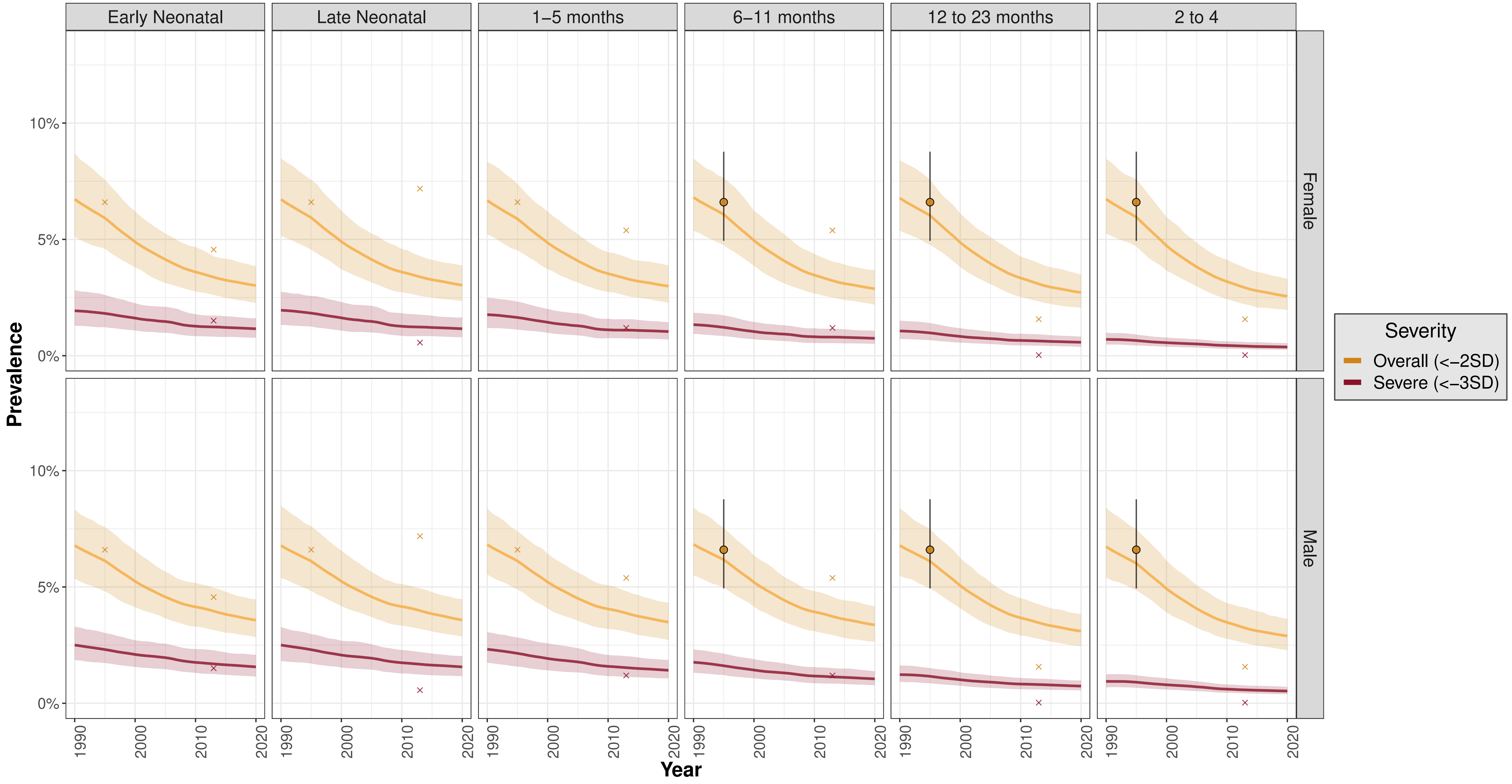

E: Transformed Mean Wasting Z Scores

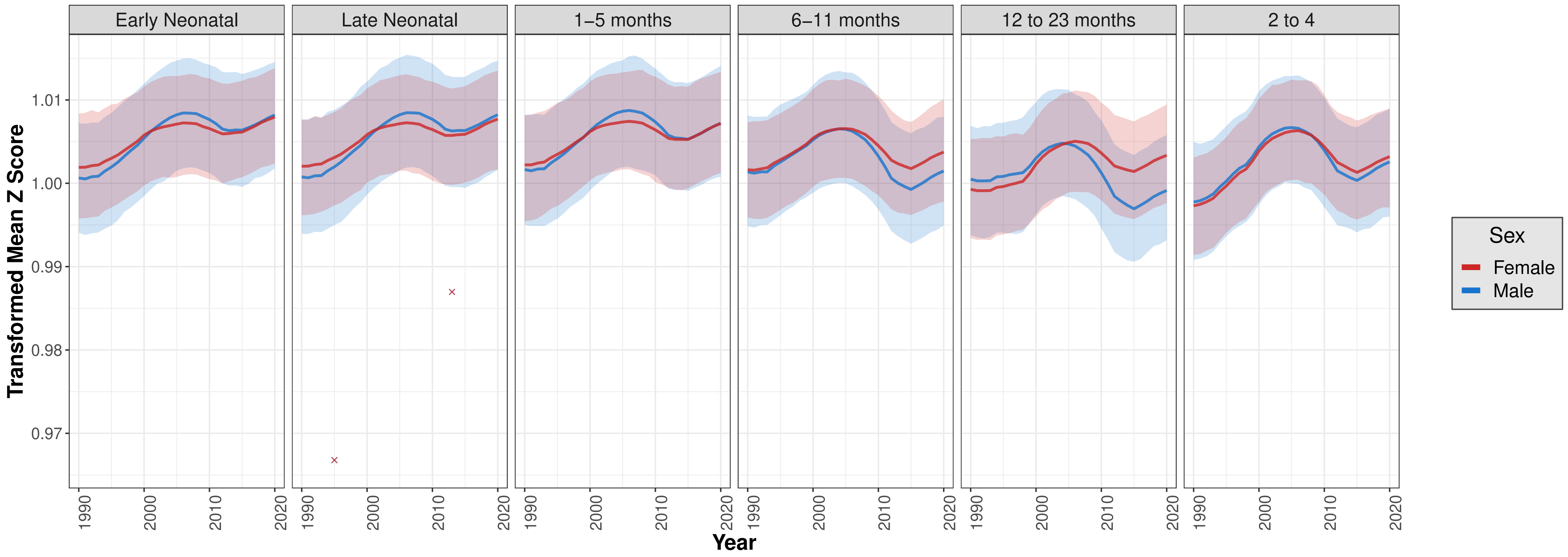

F

Year	Source
1989	WHO CGM Database
1995	WHO CGM Database
2013	Health Statistics

Bahrain – Underweight (WAZ)

G: Overall and Severe Underweight Prevalence

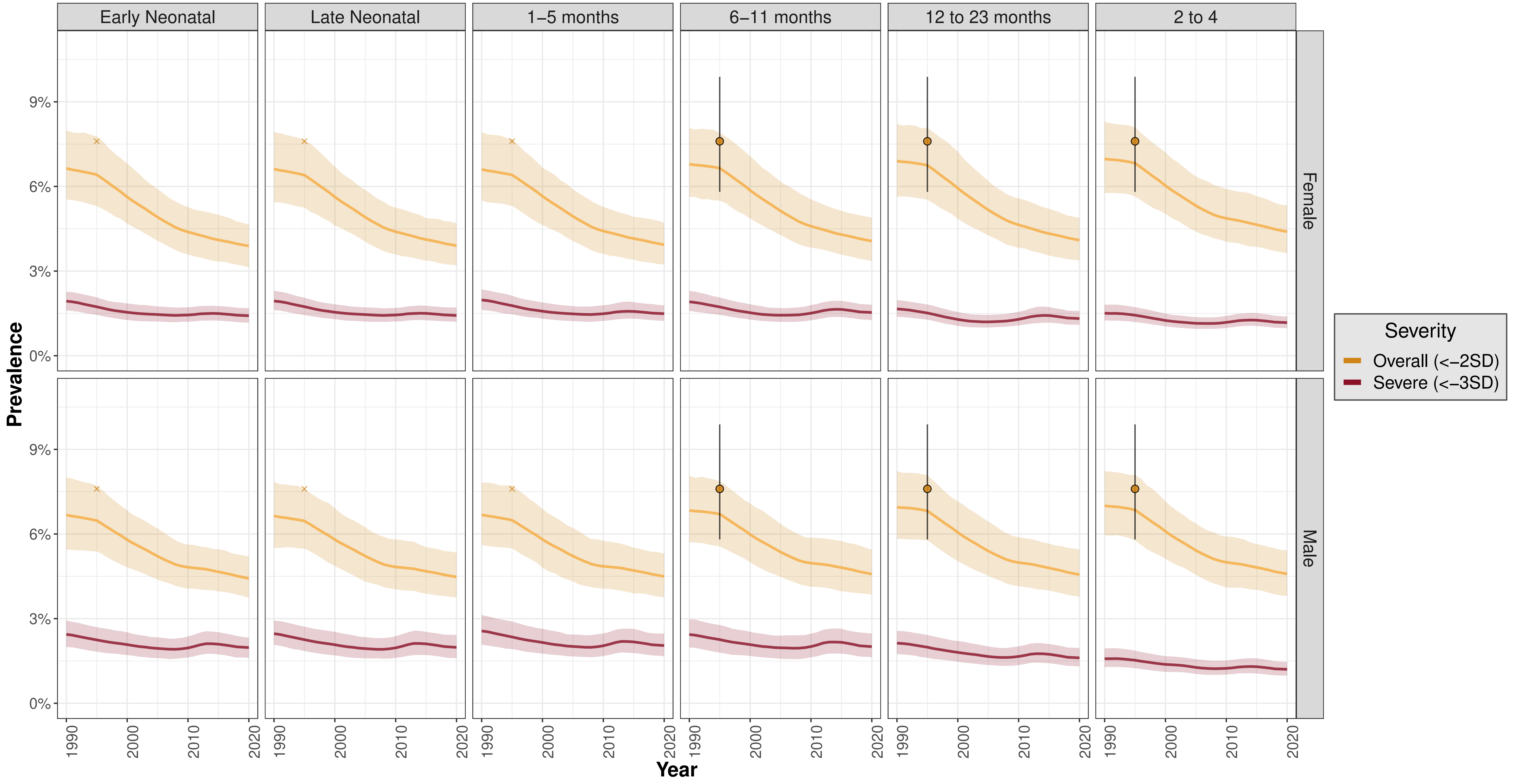

H: Transformed Mean Underweight Z Scores

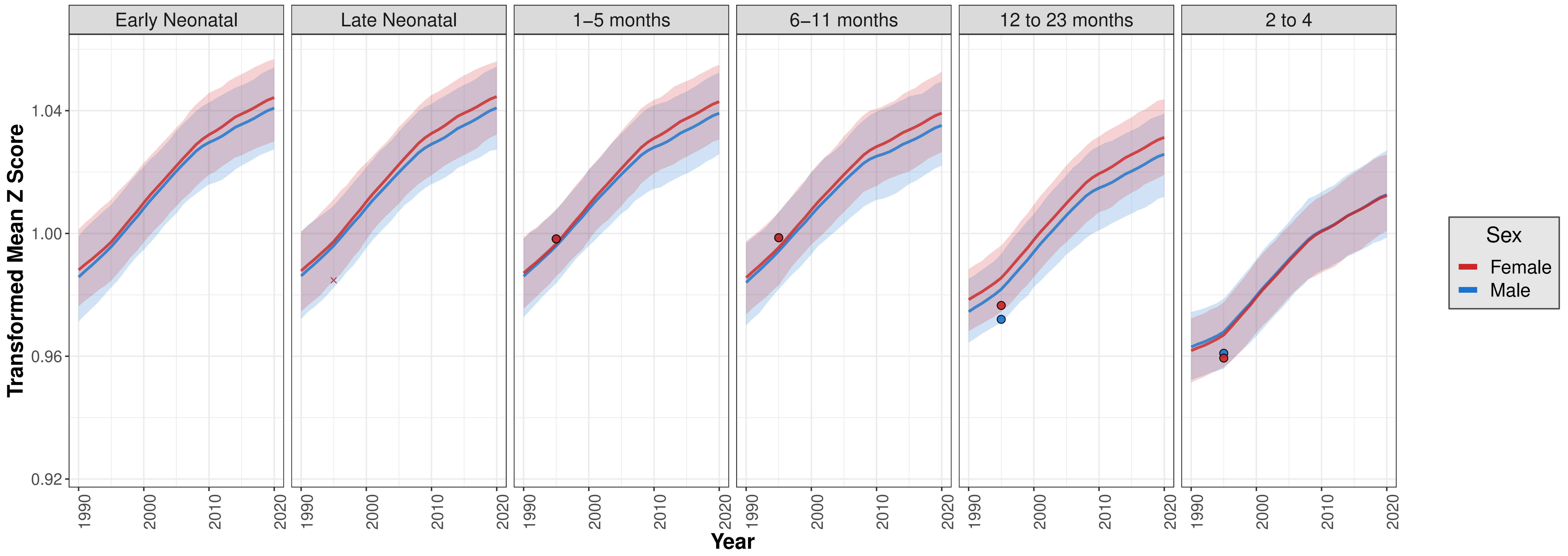

I

Year	Source
1989	WHO CGM Database
1995	WHO CGM Database

Bahrain – HAZ, WHZ, and WAZ Distributions

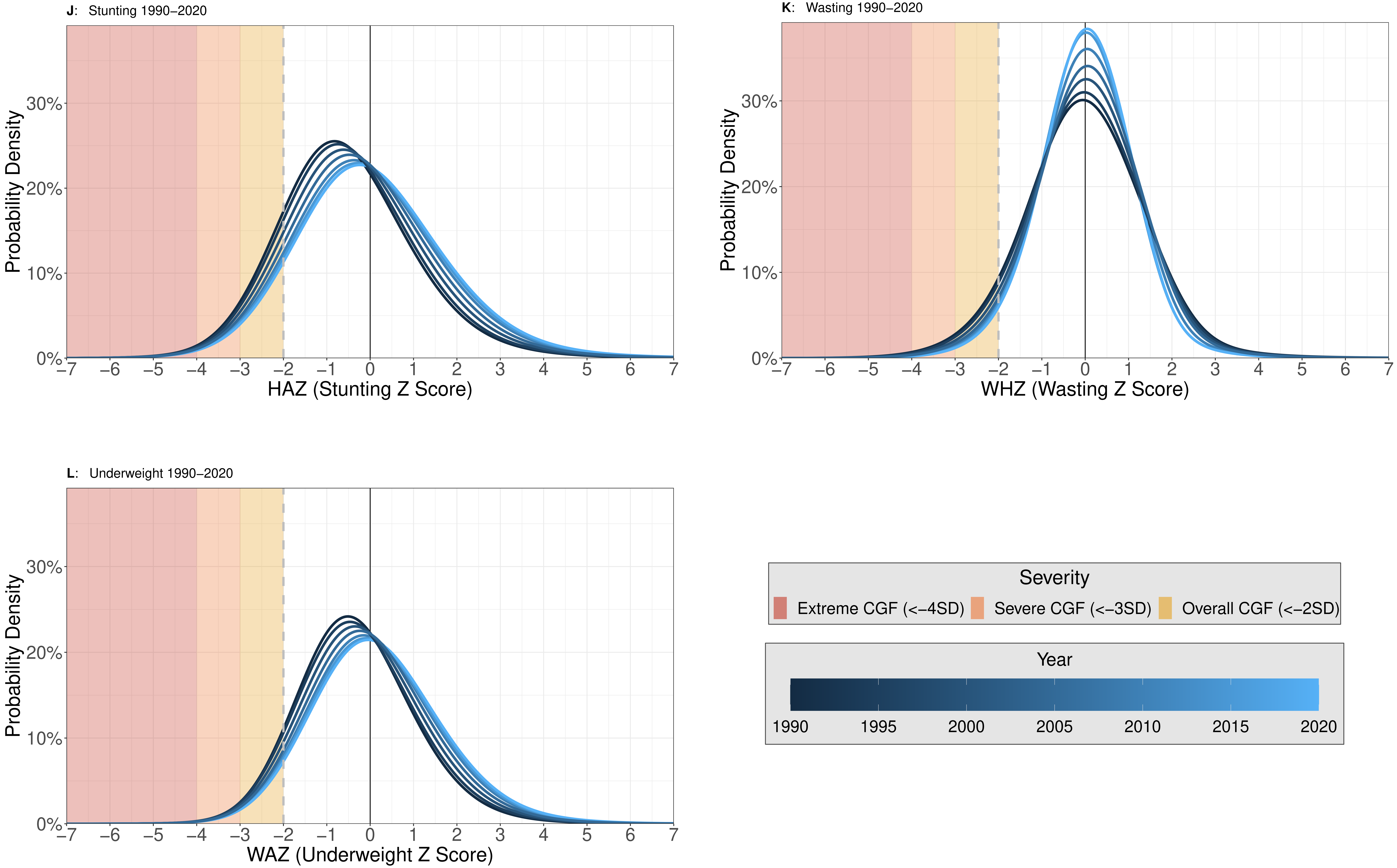

Egypt – Stunting (HAZ)

A: Overall and Severe Stunting Prevalence

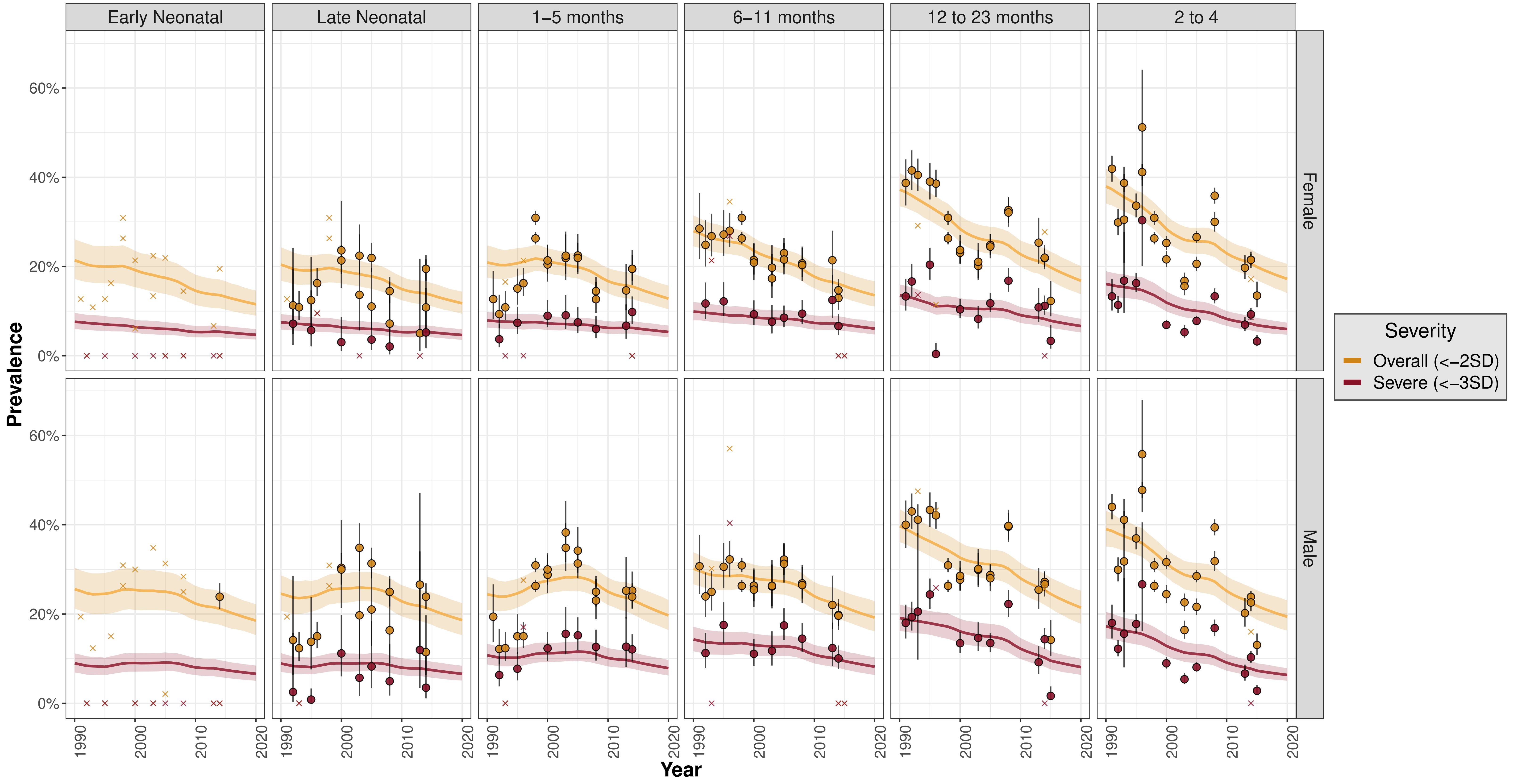

B: Transformed Mean Stunting Z Scores

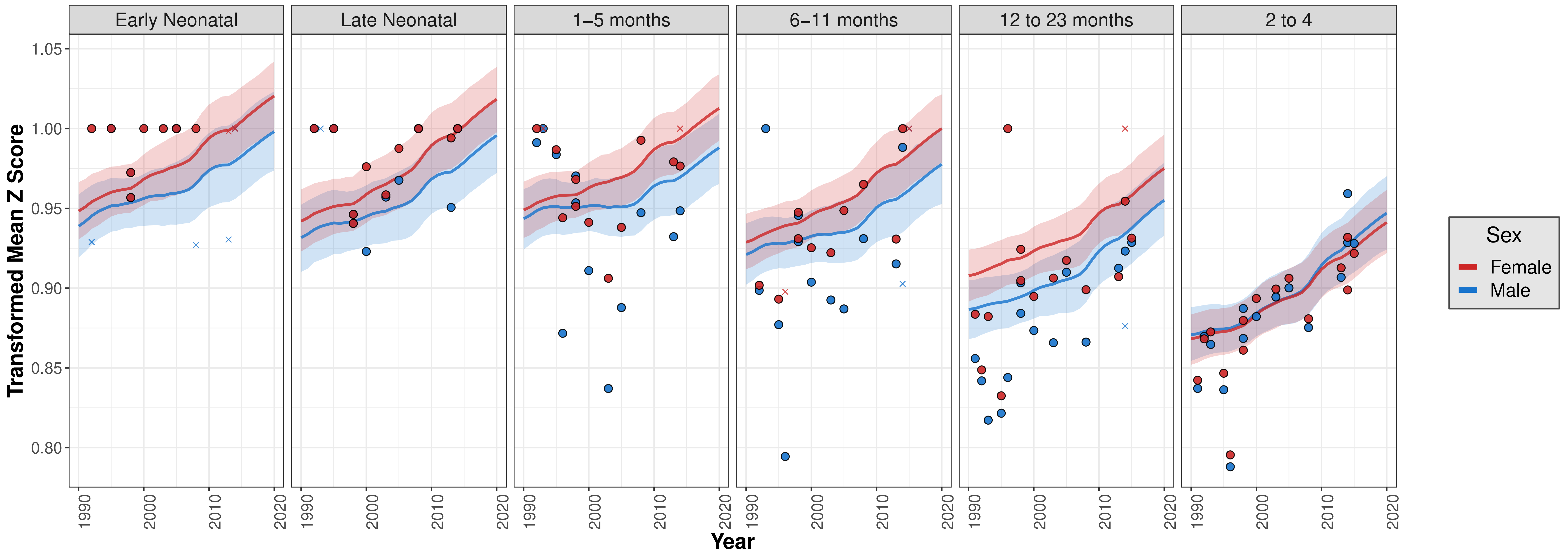

C

Year	Source
1978	WHO CGM Database
1988	DHS
1989	DHS
1989	WHO CGM Database
1991	WHO CGM Database
1992	DHS
1993	DHS
1993	WHO CGM Database
1995	DHS
1996	DHS
1996	WHO CGM Database
1998	WHO CGM Database
2000	DHS
2000	WHO CGM Database
2003	Interim DHS
2003	WHO CGM Database
2005	DHS
2005	WHO CGM Database
2008	DHS
2008	WHO CGM Database
2013	IPHN Rural Districts MICS
2014	WHO CGM Database
2014	DHS
2014	IPHN Rural Districts MICS
2015	Special DHS

Egypt – Wasting (WHZ)

D: Overall and Severe Wasting Prevalence

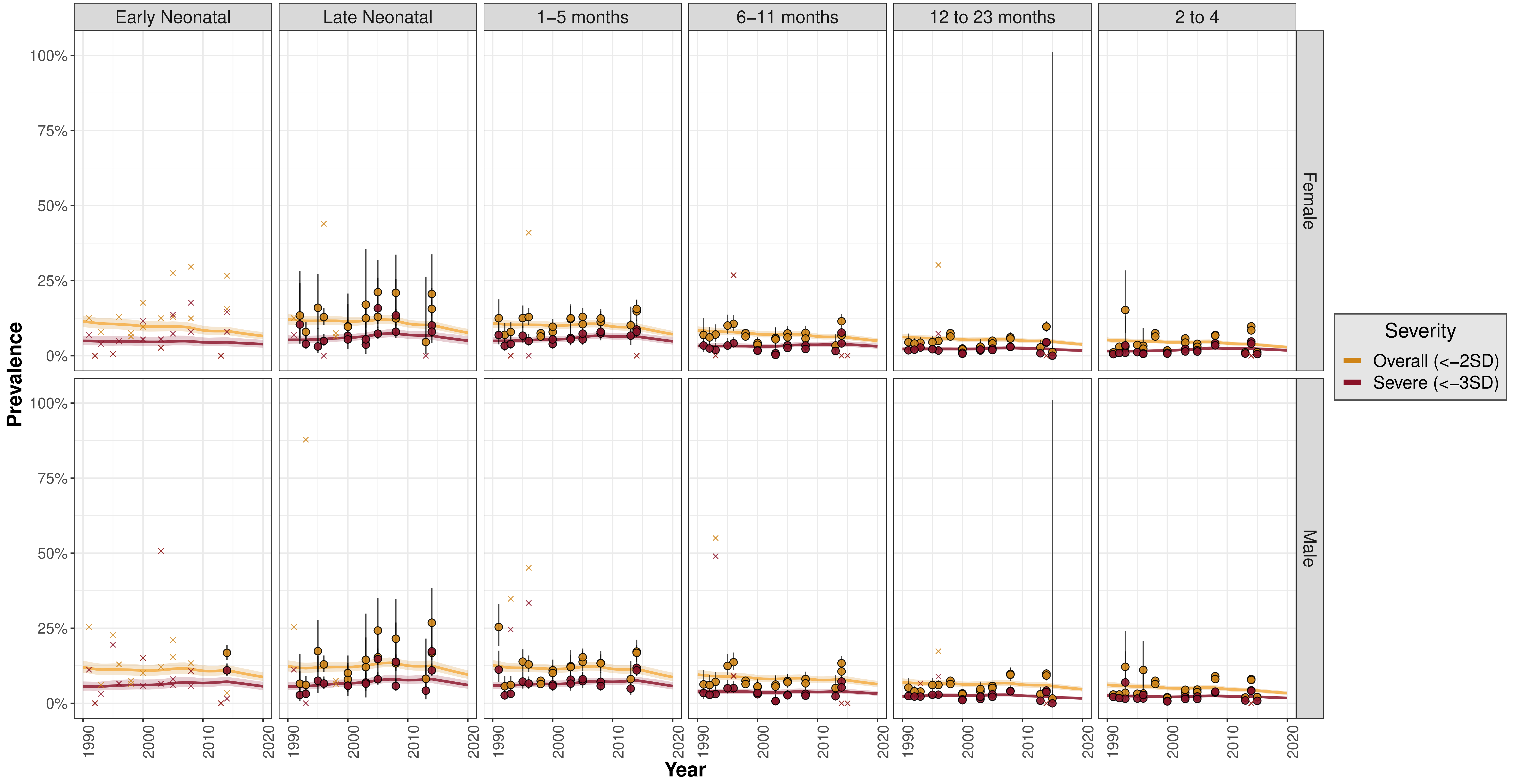

E: Transformed Mean Wasting Z Scores

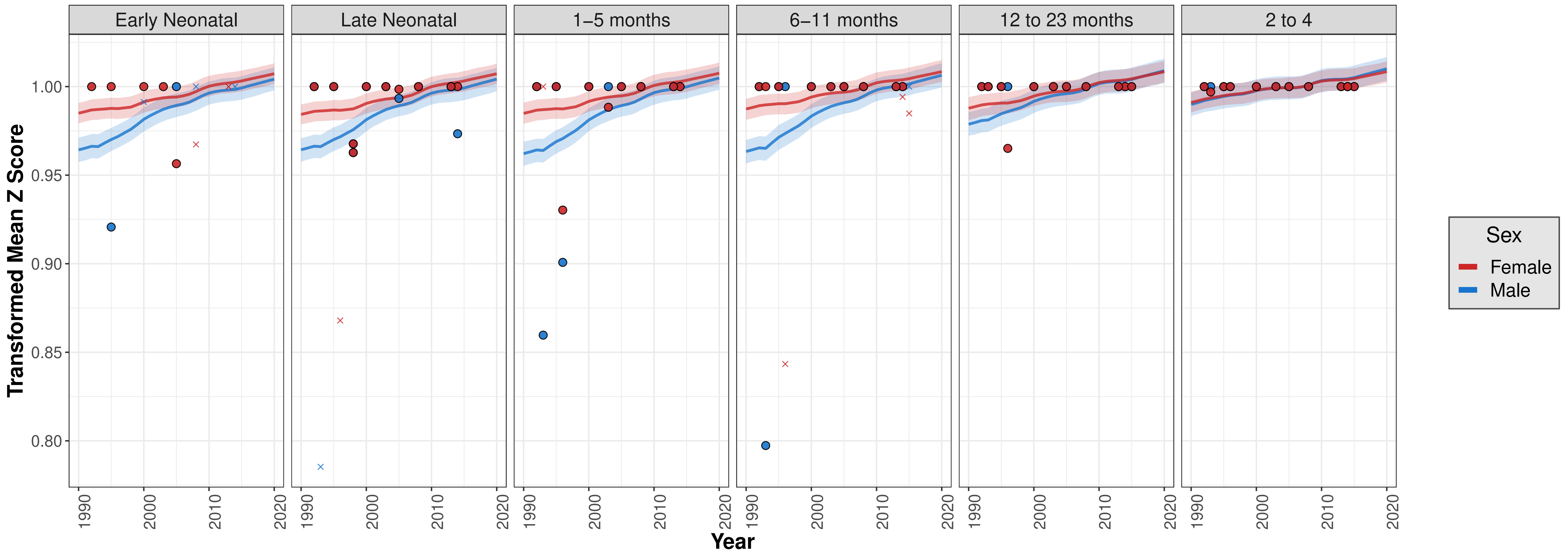

F

Year	Source
1978	WHO CGM Database
1988	DHS
1989	DHS
1989	WHO CGM Database
1991	WHO CGM Database
1992	DHS
1993	DHS
1993	WHO CGM Database
1995	DHS
1996	DHS
1996	WHO CGM Database
1998	WHO CGM Database
2000	DHS
2000	WHO CGM Database
2003	Interim DHS
2003	WHO CGM Database
2005	DHS
2005	WHO CGM Database
2008	DHS
2008	WHO CGM Database
2013	IPHN Rural Districts MICS
2014	WHO CGM Database
2014	DHS
2014	IPHN Rural Districts MICS
2015	Special DHS

Egypt – Underweight (WAZ)

G: Overall and Severe Underweight Prevalence

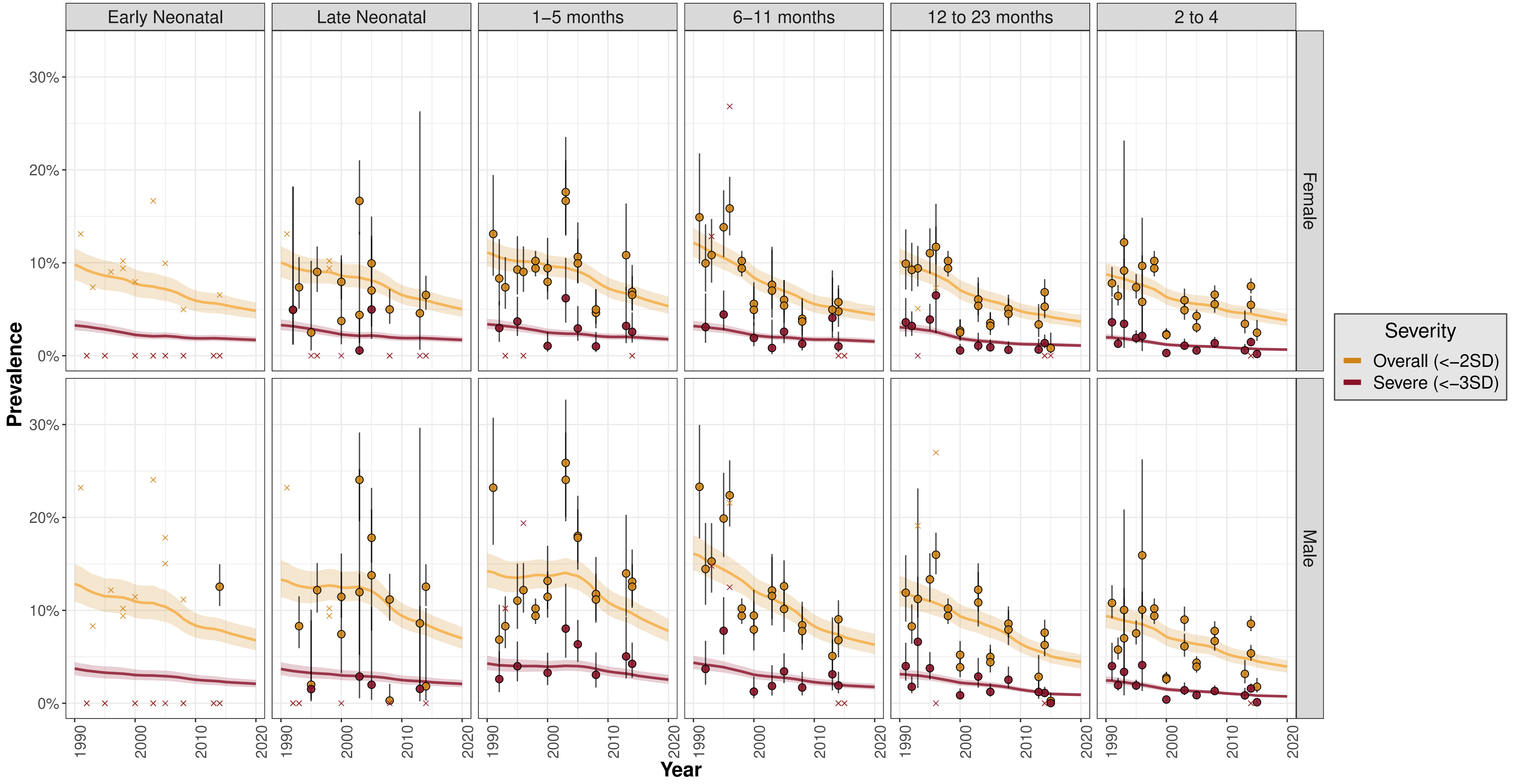

H: Transformed Mean Underweight Z Scores

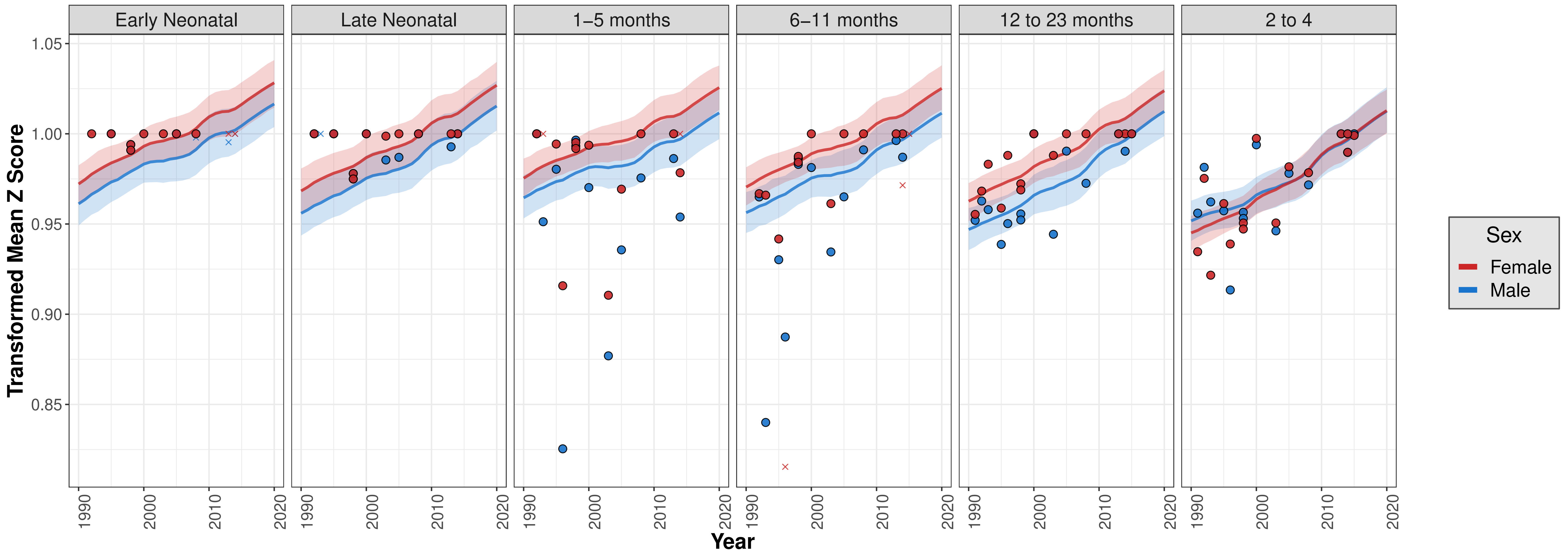

I

Year	Source
1978	WHO CGM Database
1988	DHS
1989	DHS
1989	WHO CGM Database
1991	WHO CGM Database
1992	DHS
1993	DHS
1993	WHO CGM Database
1995	DHS
1996	DHS
1996	WHO CGM Database
1998	WHO CGM Database
2000	DHS
2000	WHO CGM Database
2003	Interim DHS
2003	WHO CGM Database
2005	DHS
2005	WHO CGM Database
2008	DHS
2008	WHO CGM Database
2013	IPHN Rural Districts MICS
2014	WHO CGM Database
2014	DHS
2014	IPHN Rural Districts MICS
2015	Special DHS

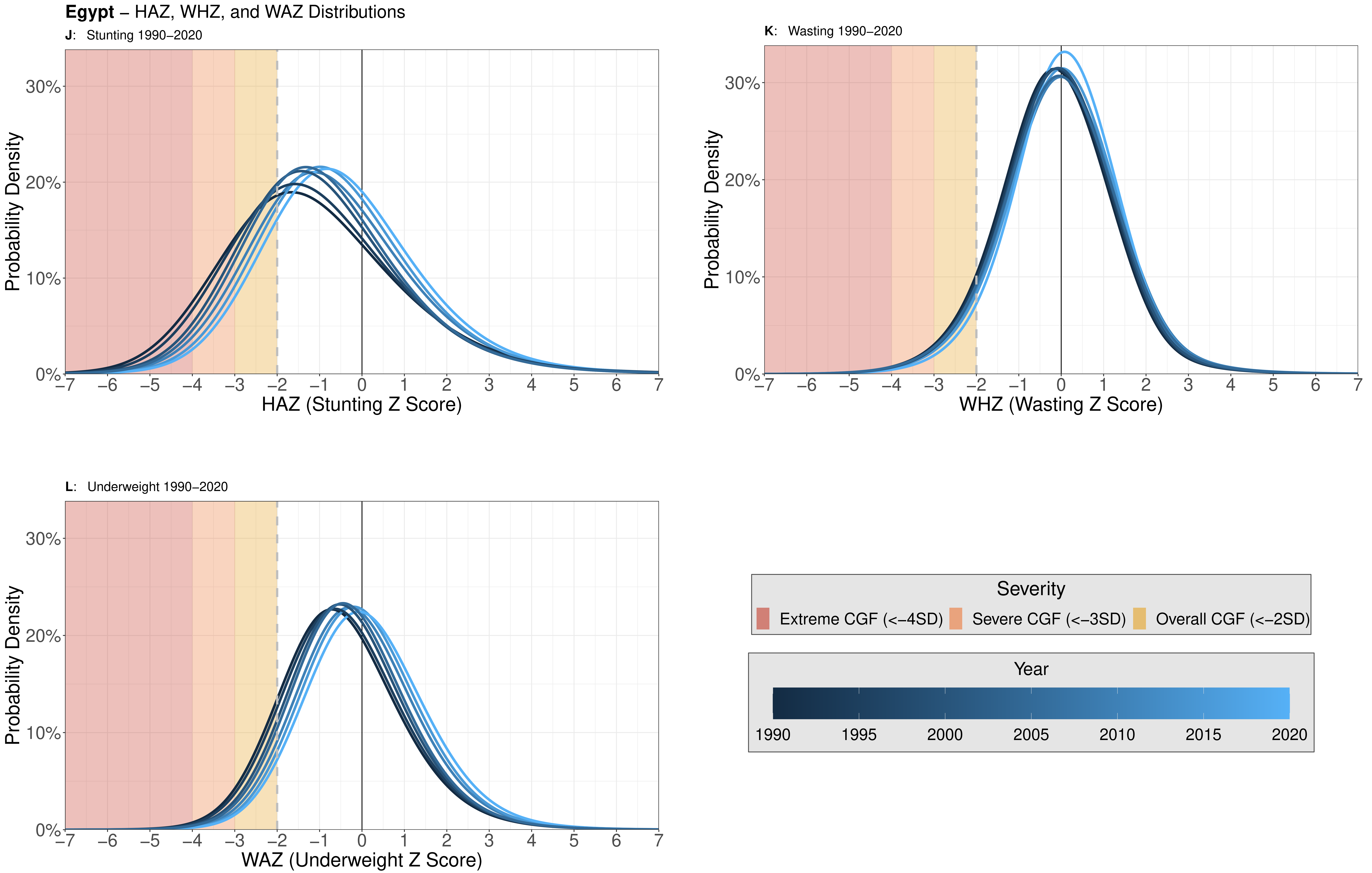
Egypt – HAZ, WHZ, and WAZ Distributions
J: Stunting 1990–2020
Probability Density
0%
10%
20%
30%
−7 −6 −5 −4 −3 −2 −1 0 1 2 3 4 5 6 7
HAZ (Stunting Z Score)
K: Wasting 1990–2020
Probability Density
0%
10%
20%
30%
−7 −6 −5 −4 −3 −2 −1 0 1 2 3 4 5 6 7
WHZ (Wasting Z Score)
L: Underweight 1990–2020
Probability Density
0%
10%
20%
30%
−7 −6 −5 −4 −3 −2 −1 0 1 2 3 4 5 6 7
WAZ (Underweight Z Score)
Severity
Extreme CGF (<−4SD)
Severe CGF (<−3SD)
Overall CGF (<−2SD)
Year
1990
1995
2000
2005
2010
2015
2020

Iran (Islamic Republic of) – Stunting (HAZ)

A: Overall and Severe Stunting Prevalence

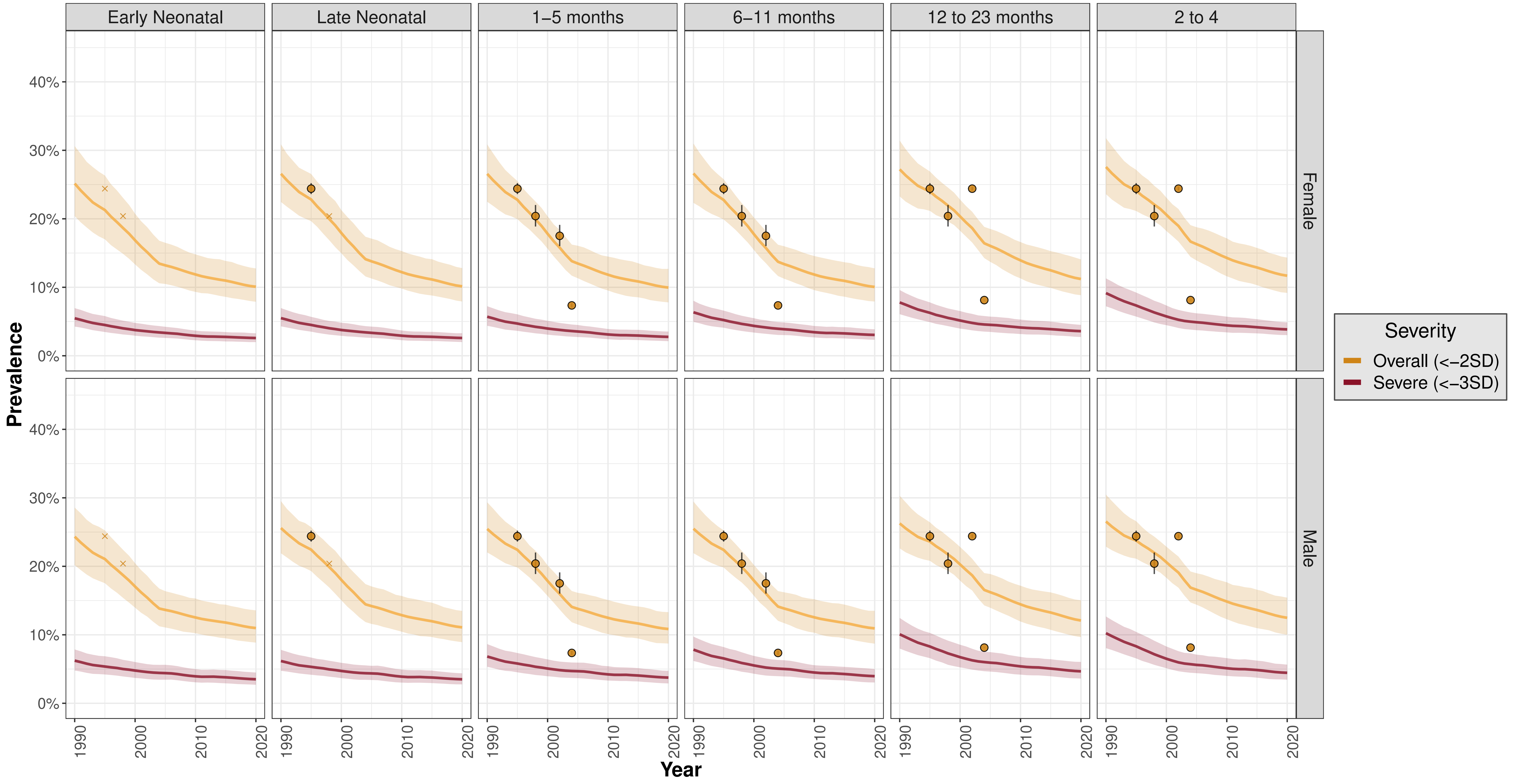

B: Transformed Mean Stunting Z Scores

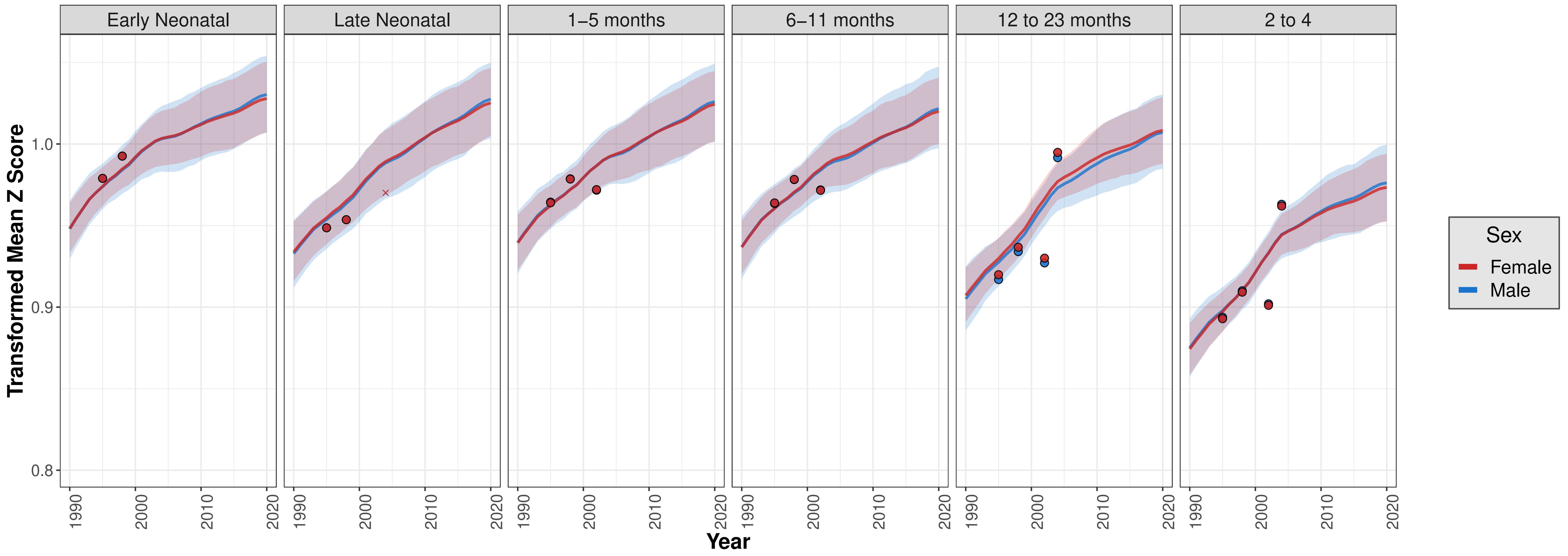

C

Year	Source	National	Subnational
1995	WHO CGM Database	X	
1998	WHO CGM Database	X	
1998	Iran Anthropometric Nutritional Indicators Survey		X
2002	WHO CGM Database	X	
2004	WHO CGM Database	X	
2004	Iran Anthropometric Nutritional Indicators Survey		X

Iran (Islamic Republic of) – Wasting (WHZ)

D: Overall and Severe Wasting Prevalence

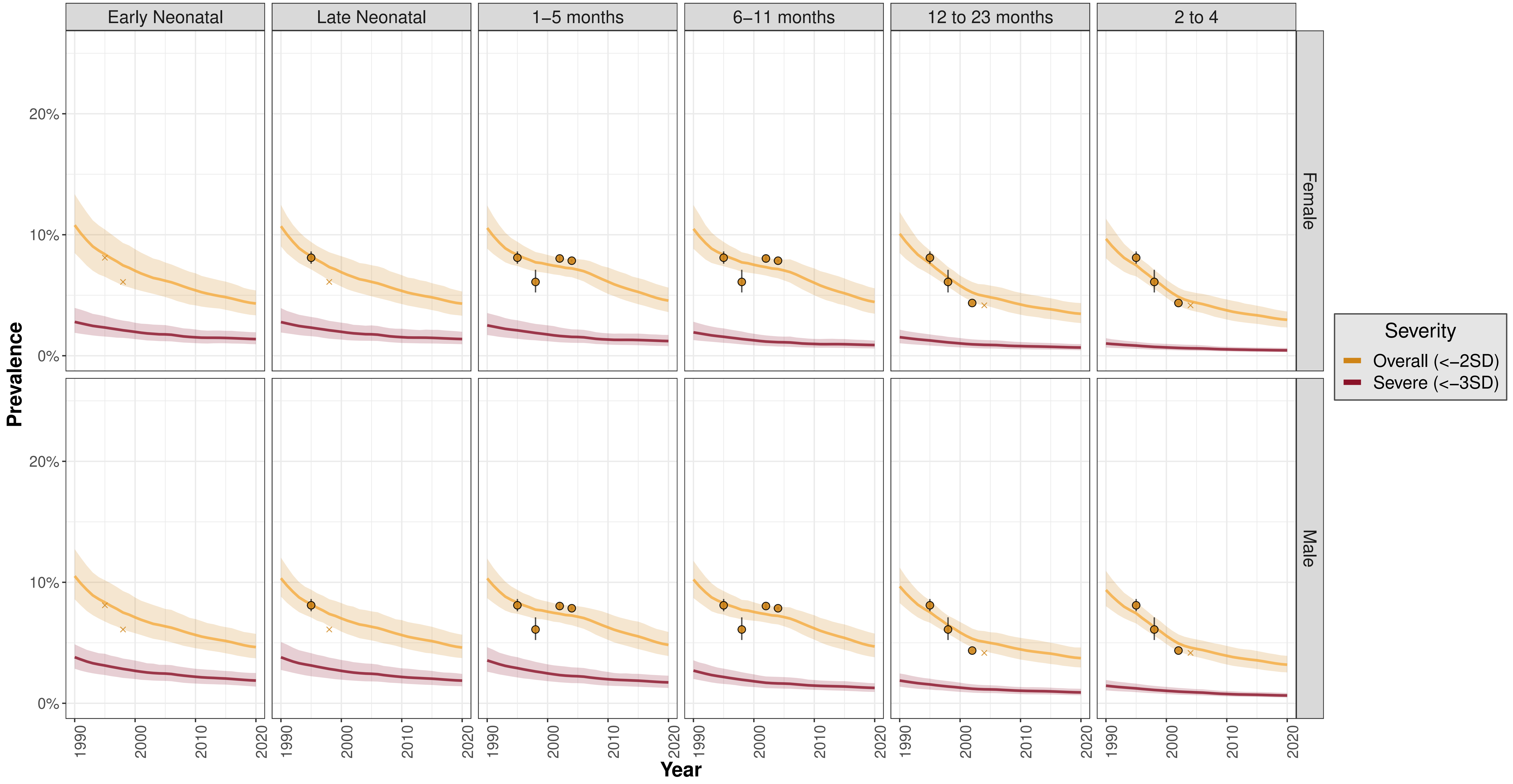

E: Transformed Mean Wasting Z Scores

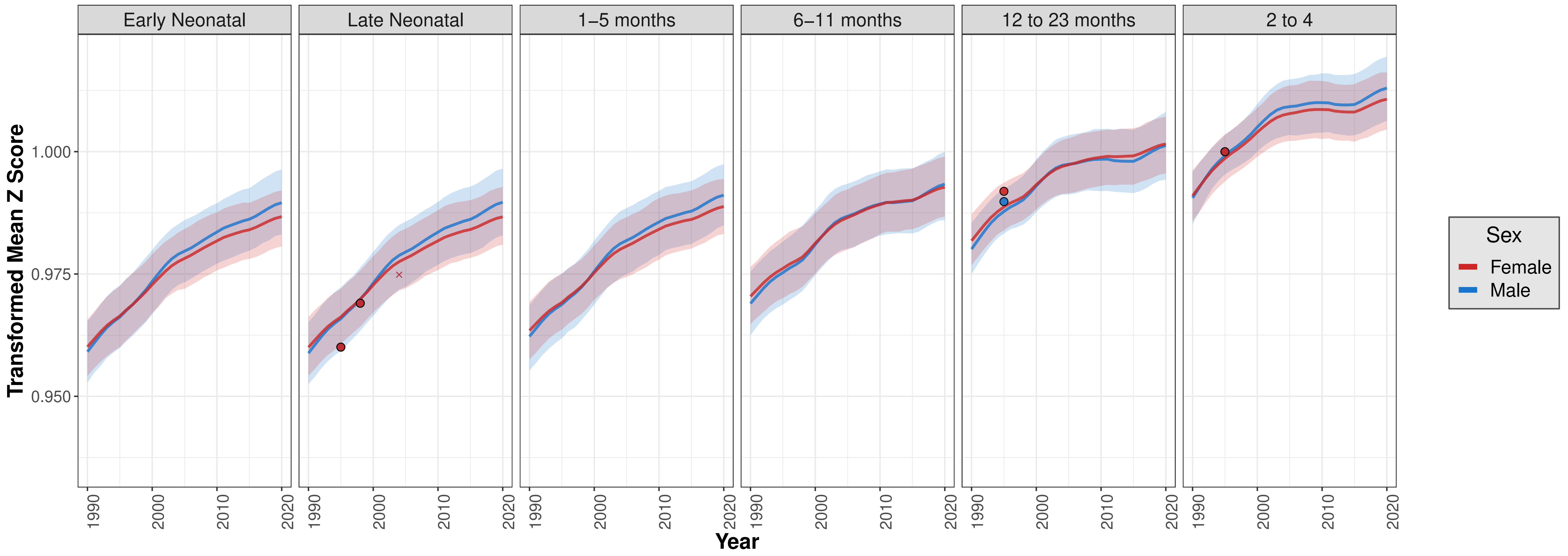

F

Year	Source	National	Subnational
1995	WHO CGM Database	X	
1998	WHO CGM Database	X	
1998	Iran Anthropometric Nutritional Indicators Survey		X
2002	WHO CGM Database	X	
2004	WHO CGM Database	X	
2004	Iran Anthropometric Nutritional Indicators Survey		X

Iran (Islamic Republic of) – Underweight (WAZ)

G: Overall and Severe Underweight Prevalence

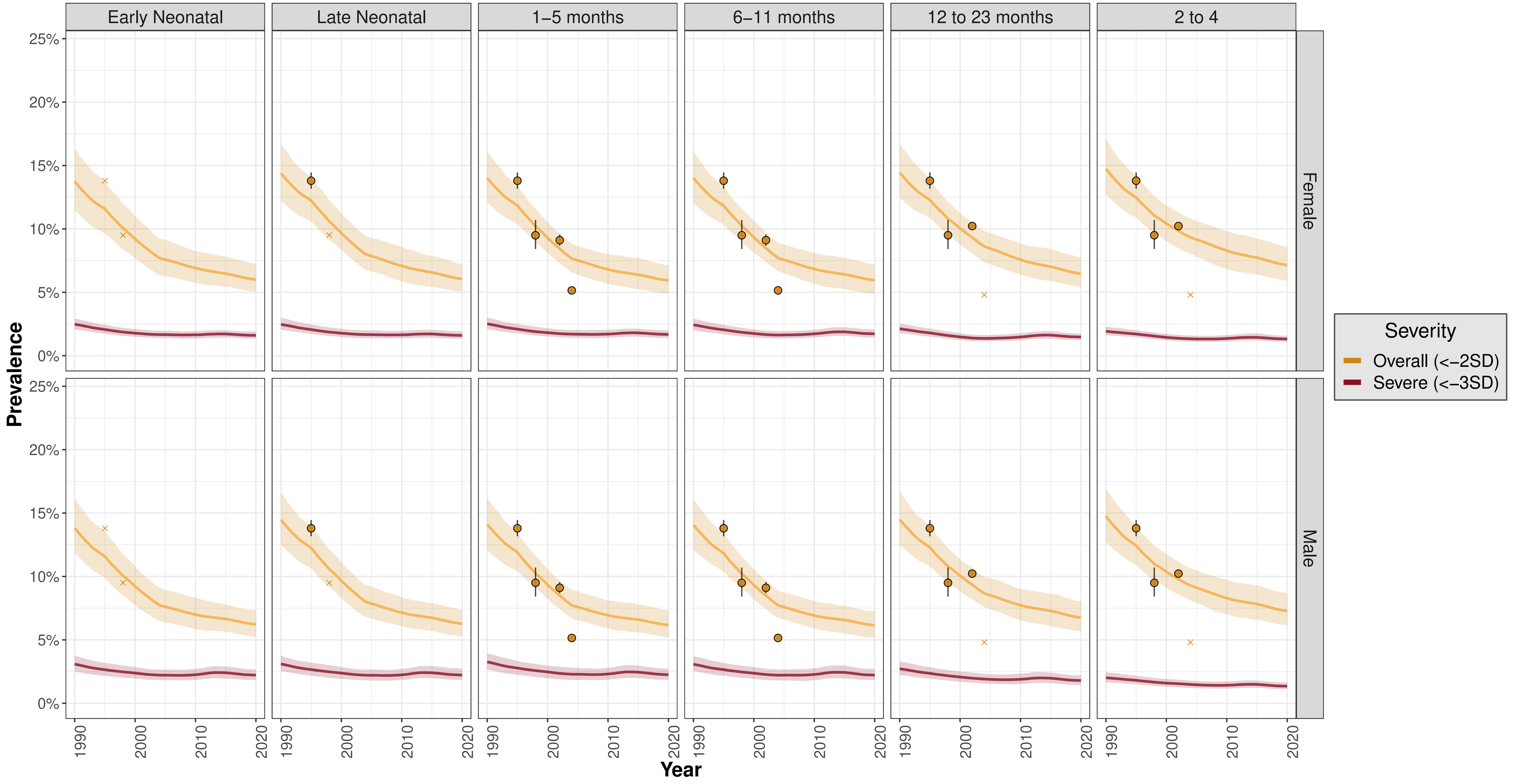

H: Transformed Mean Underweight Z Scores

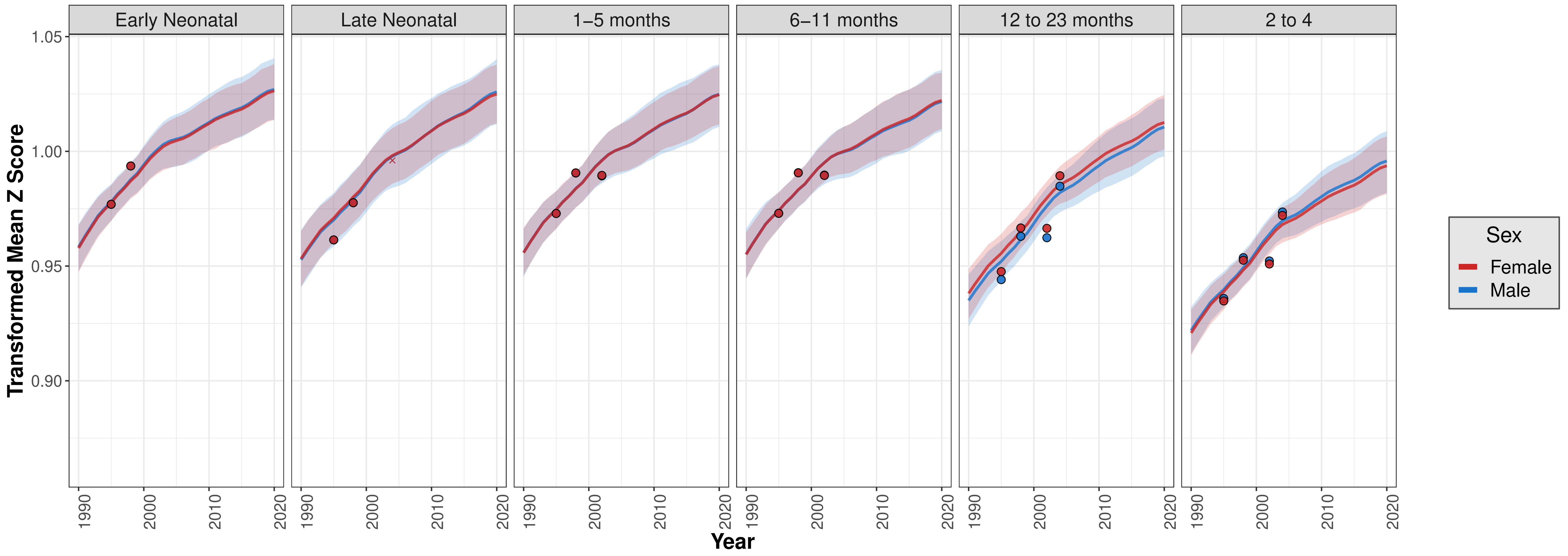

I

Year	Source	National	Subnational
1995	WHO CGM Database	X	
1998	WHO CGM Database	X	
1998	Iran Anthropometric Nutritional Indicators Survey		X
2002	WHO CGM Database	X	
2004	WHO CGM Database	X	
2004	Iran Anthropometric Nutritional Indicators Survey		X

Iran (Islamic Republic of) – HAZ, WHZ, and WAZ Distributions

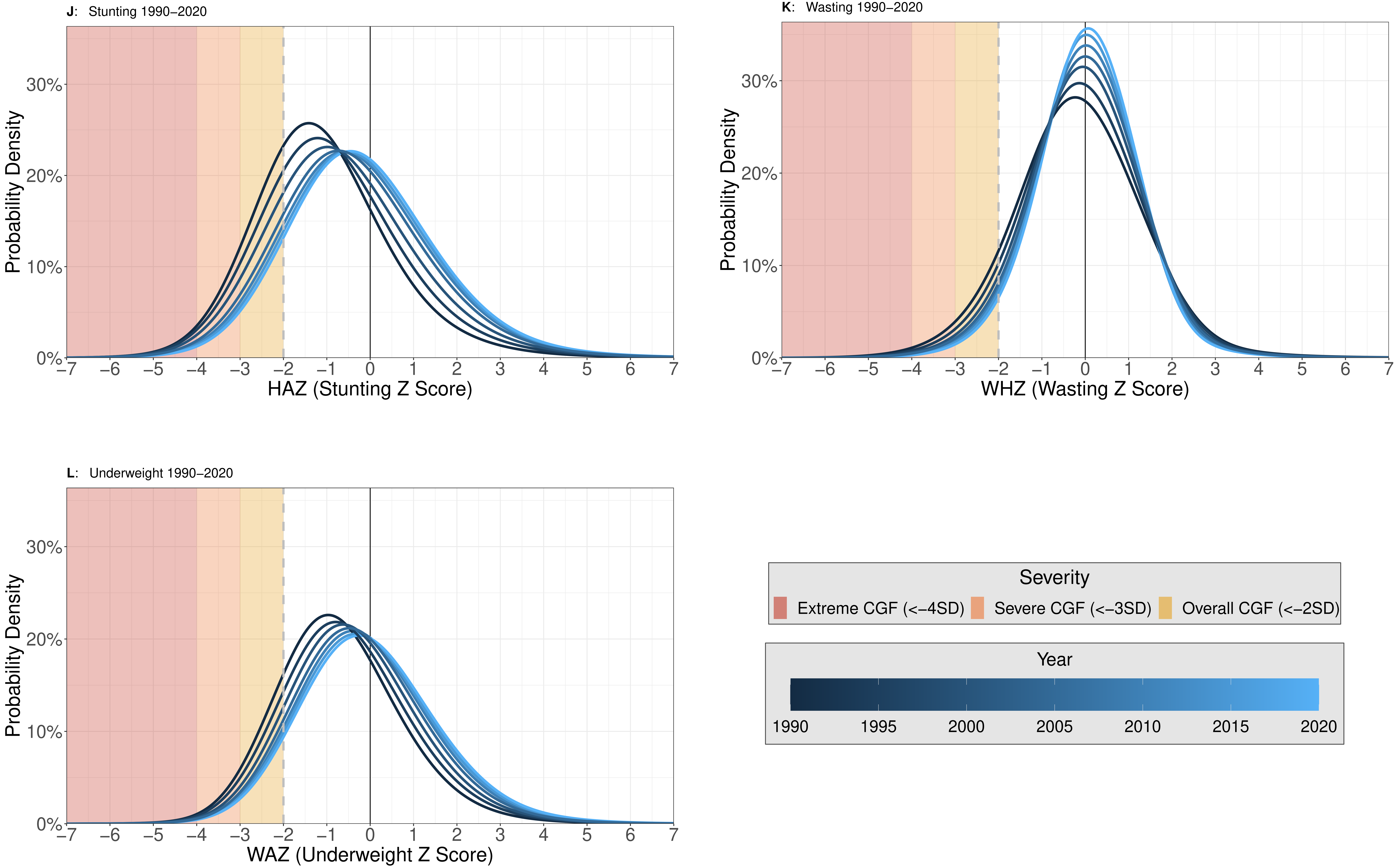

Iraq – Stunting (HAZ)

A: Overall and Severe Stunting Prevalence

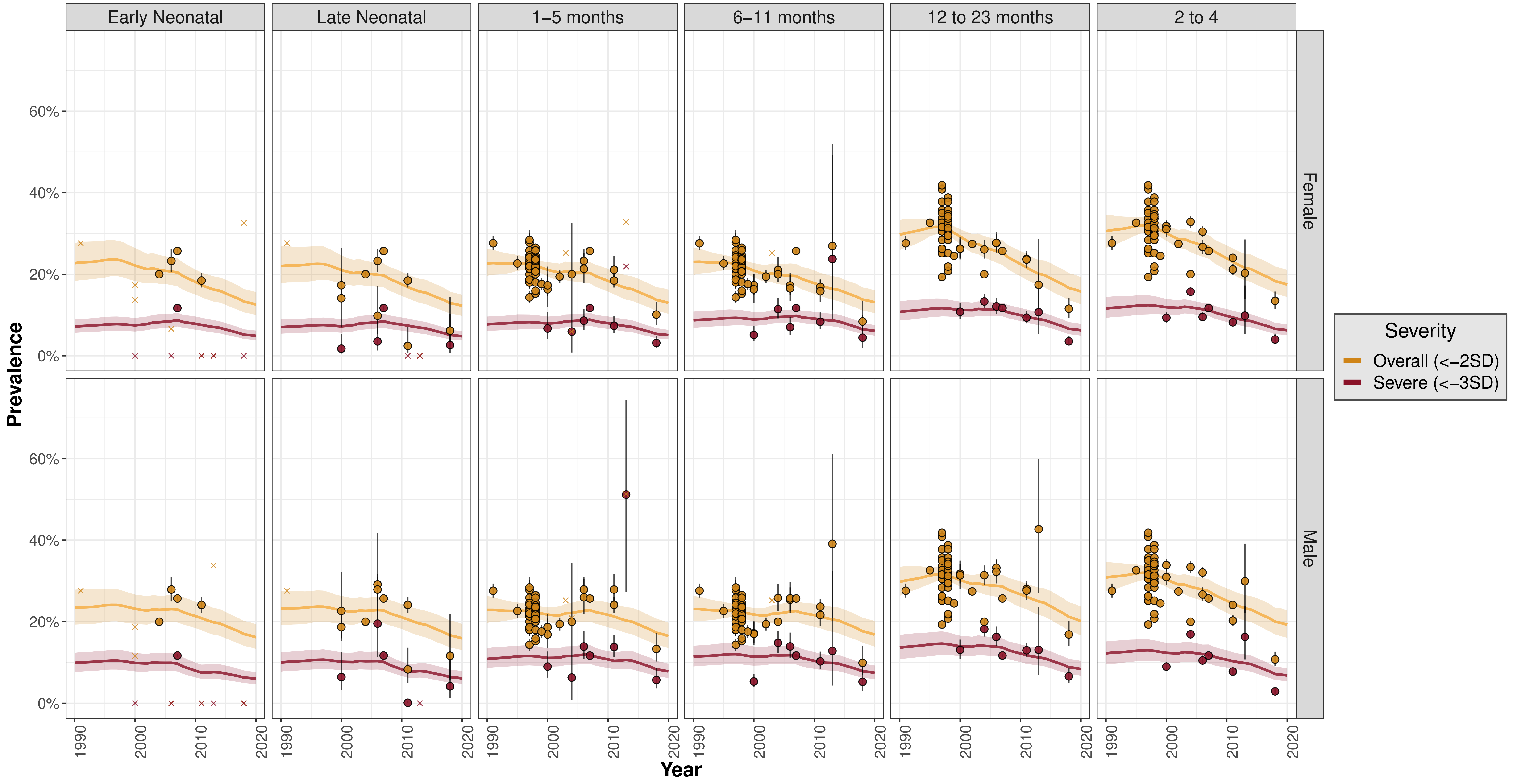

B: Transformed Mean Stunting Z Scores

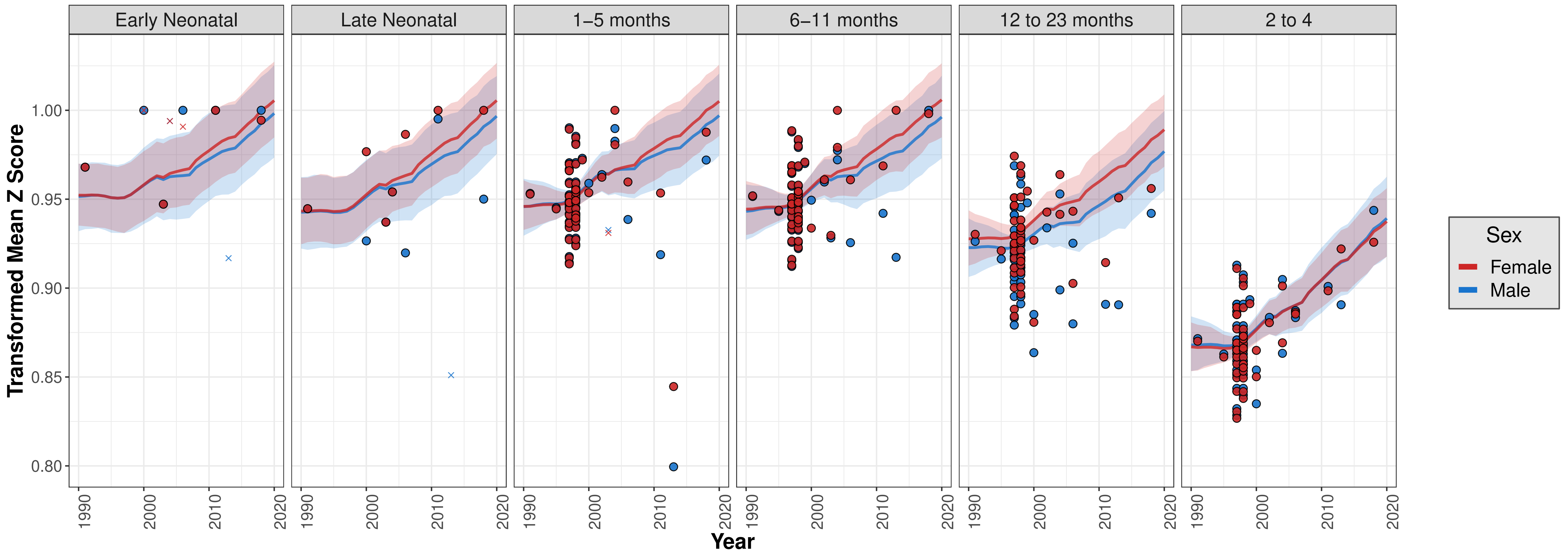

C

Year	Source
1991	WHO CGM Database
1995	WHO CGM Database
1997	WHO CGM Database
1998	WHO CGM Database
1999	WHO CGM Database
2000	MICS
2000	WHO CGM Database
2002	WHO CGM Database
2003	WHO CGM Database
2004	Multiple Indicator Rapid Assessment
2004	WHO CGM Database
2006	MICS
2006	WHO CGM Database
2007	Comprehensive Food Security and Vulnerability Analysis (CFSVA)
2011	MICS
2011	WHO CGM Database
2013	Household Socioeconomic Survey
2018	MICS

Iraq – Wasting (WHZ)

D: Overall and Severe Wasting Prevalence

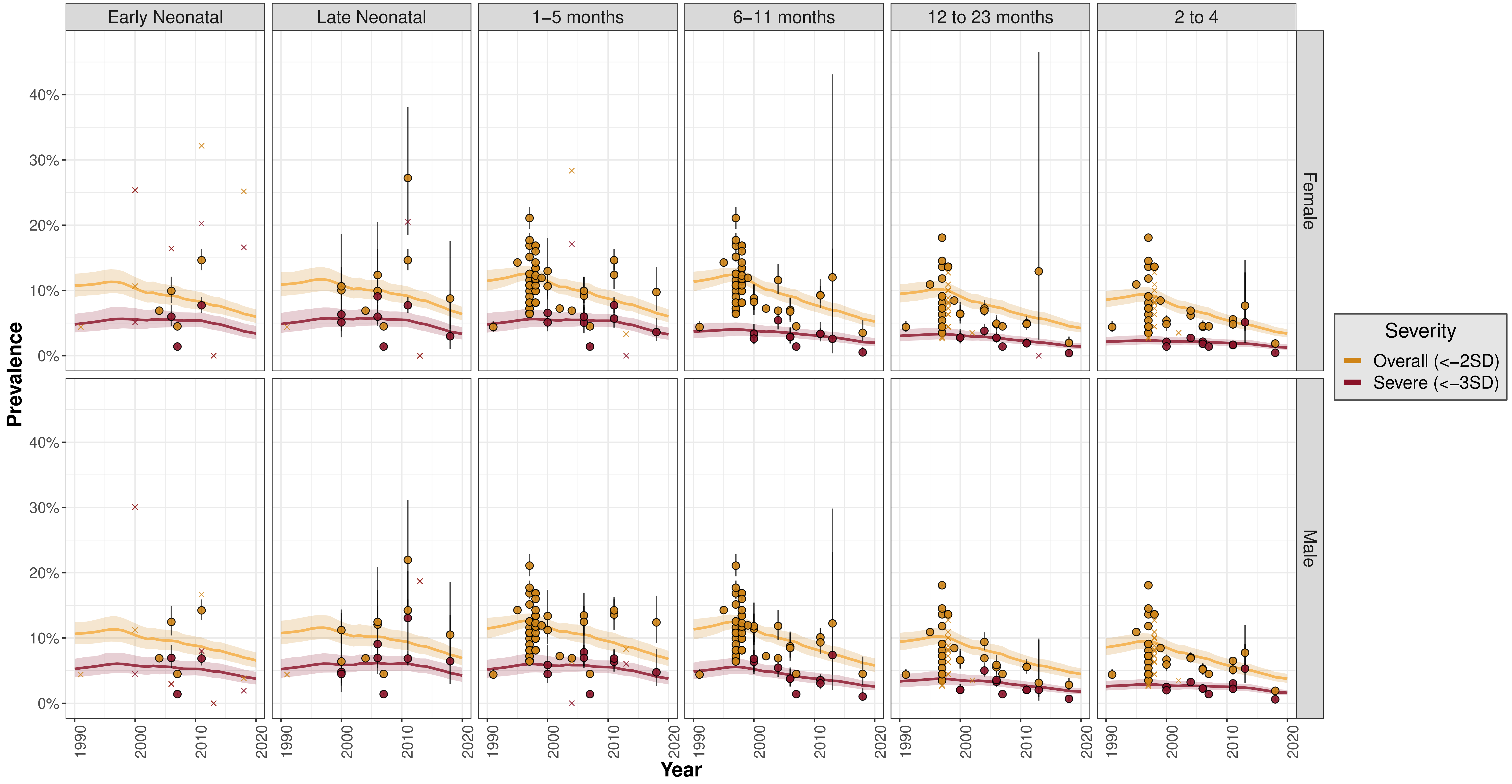

E: Transformed Mean Wasting Z Scores

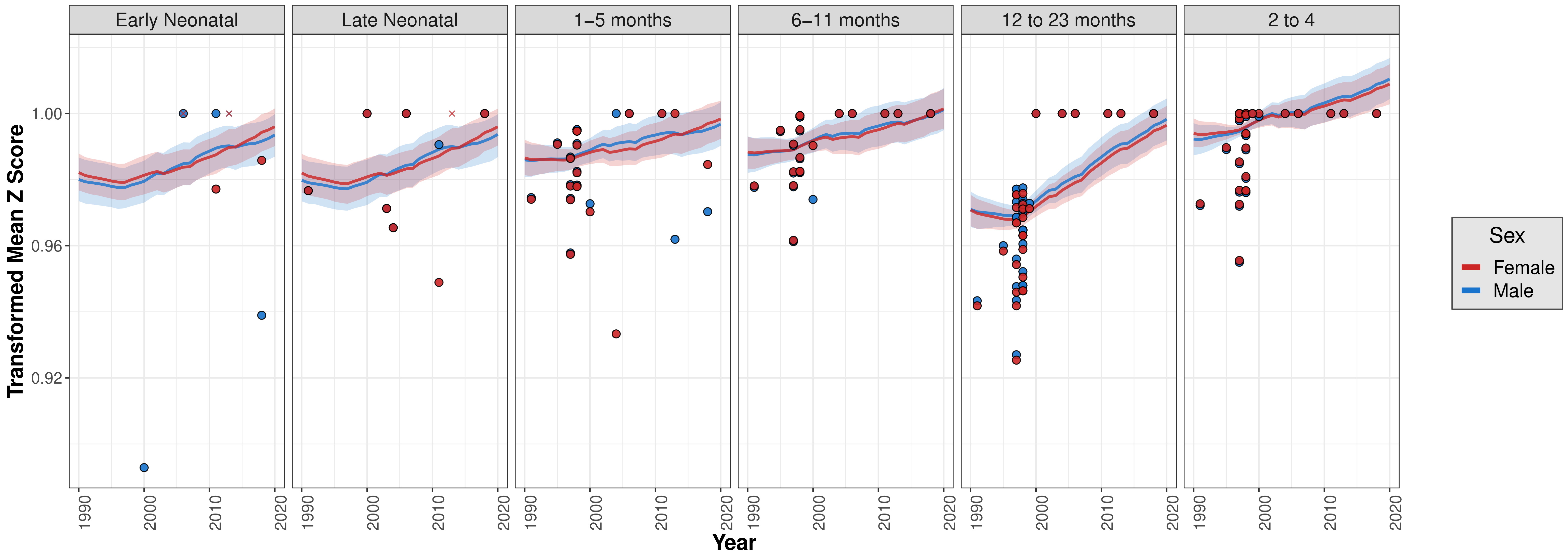

F

Year	Source
1991	WHO CGM Database
1995	WHO CGM Database
1997	WHO CGM Database
1998	WHO CGM Database
1999	WHO CGM Database
2000	MICS
2000	WHO CGM Database
2002	WHO CGM Database
2003	WHO CGM Database
2004	Multiple Indicator Rapid Assessment
2004	WHO CGM Database
2006	MICS
2006	WHO CGM Database
2007	Comprehensive Food Security and Vulnerability Analysis (CFSVA)
2011	MICS
2011	WHO CGM Database
2013	Household Socioeconomic Survey
2018	MICS

Iraq – Underweight (WAZ)

G: Overall and Severe Underweight Prevalence

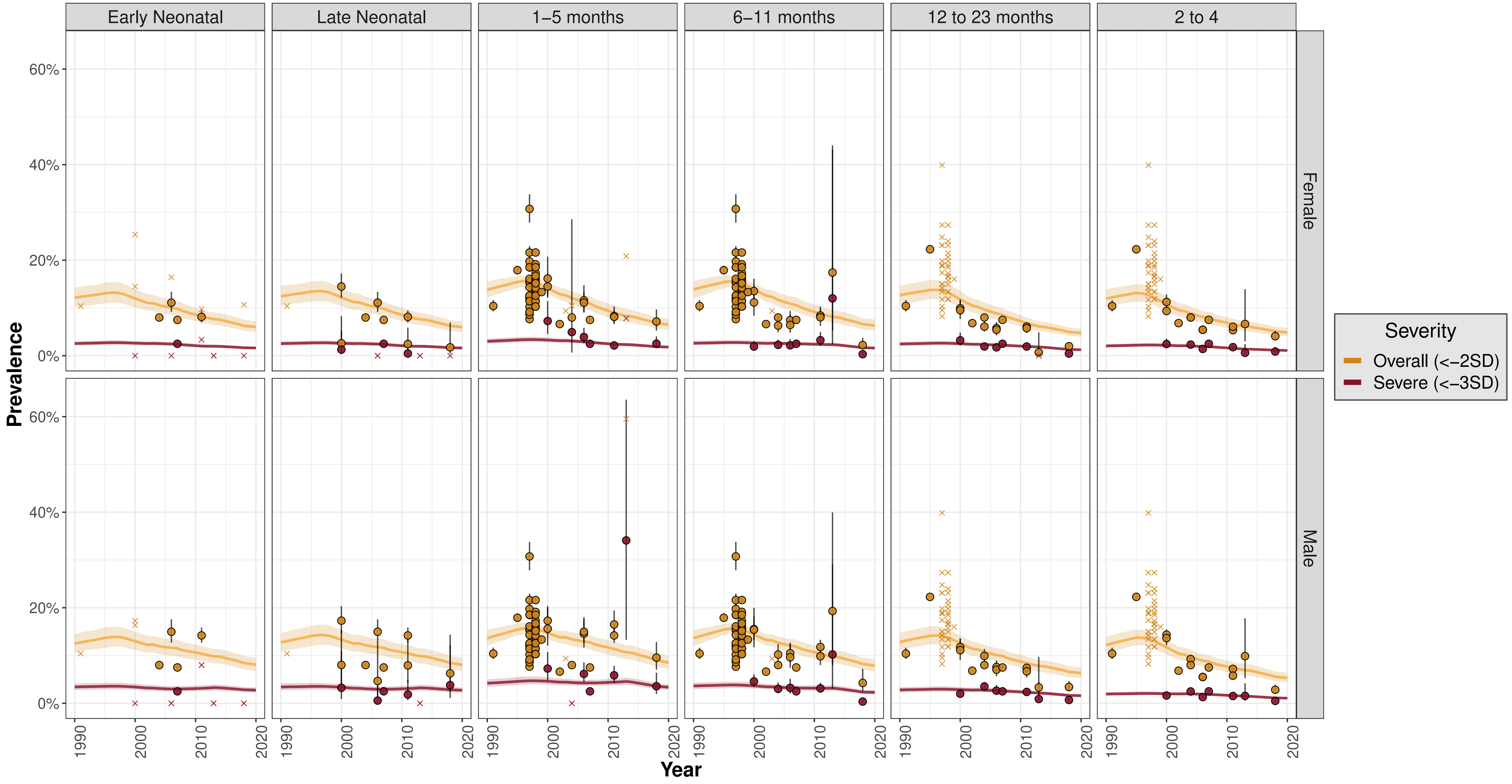

H: Transformed Mean Underweight Z Scores

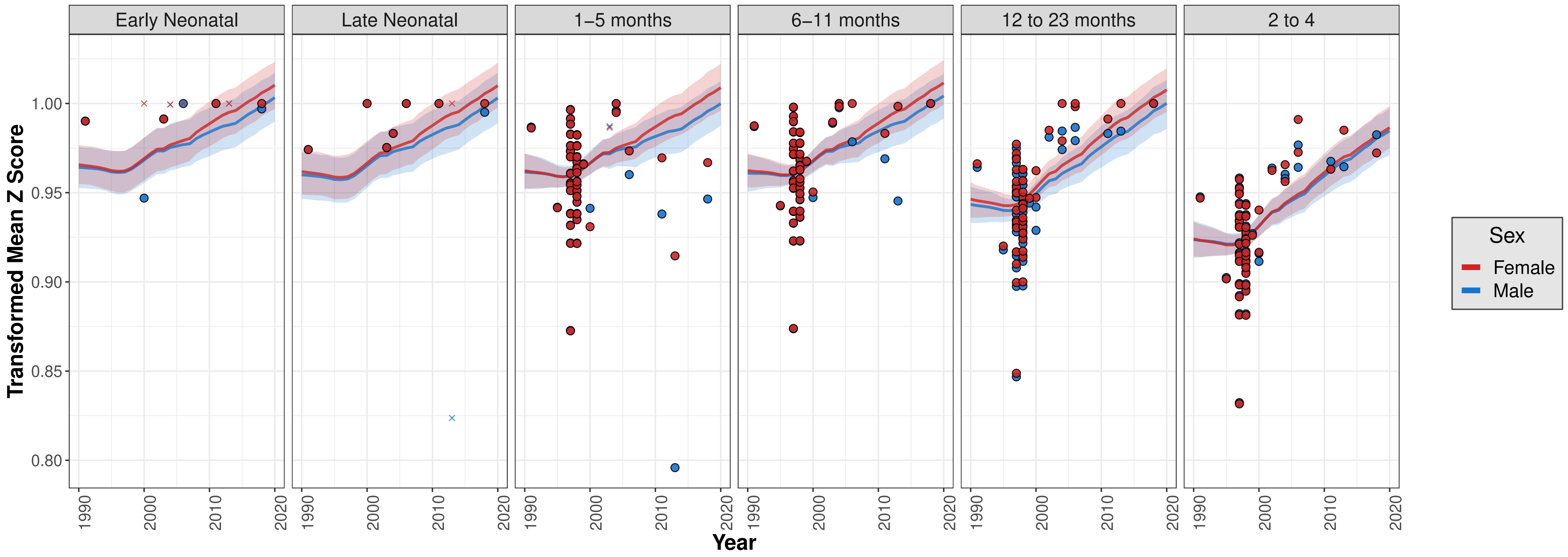

I

Year	Source
1991	WHO CGM Database
1995	WHO CGM Database
1997	WHO CGM Database
1998	WHO CGM Database
1999	WHO CGM Database
2000	MICS
2000	WHO CGM Database
2002	WHO CGM Database
2003	WHO CGM Database
2004	Multiple Indicator Rapid Assessment
2004	WHO CGM Database
2006	MICS
2006	WHO CGM Database
2007	Comprehensive Food Security and Vulnerability Analysis (CFSVA)
2011	MICS
2011	WHO CGM Database
2013	Household Socioeconomic Survey
2018	MICS

Iraq – HAZ, WHZ, and WAZ Distributions

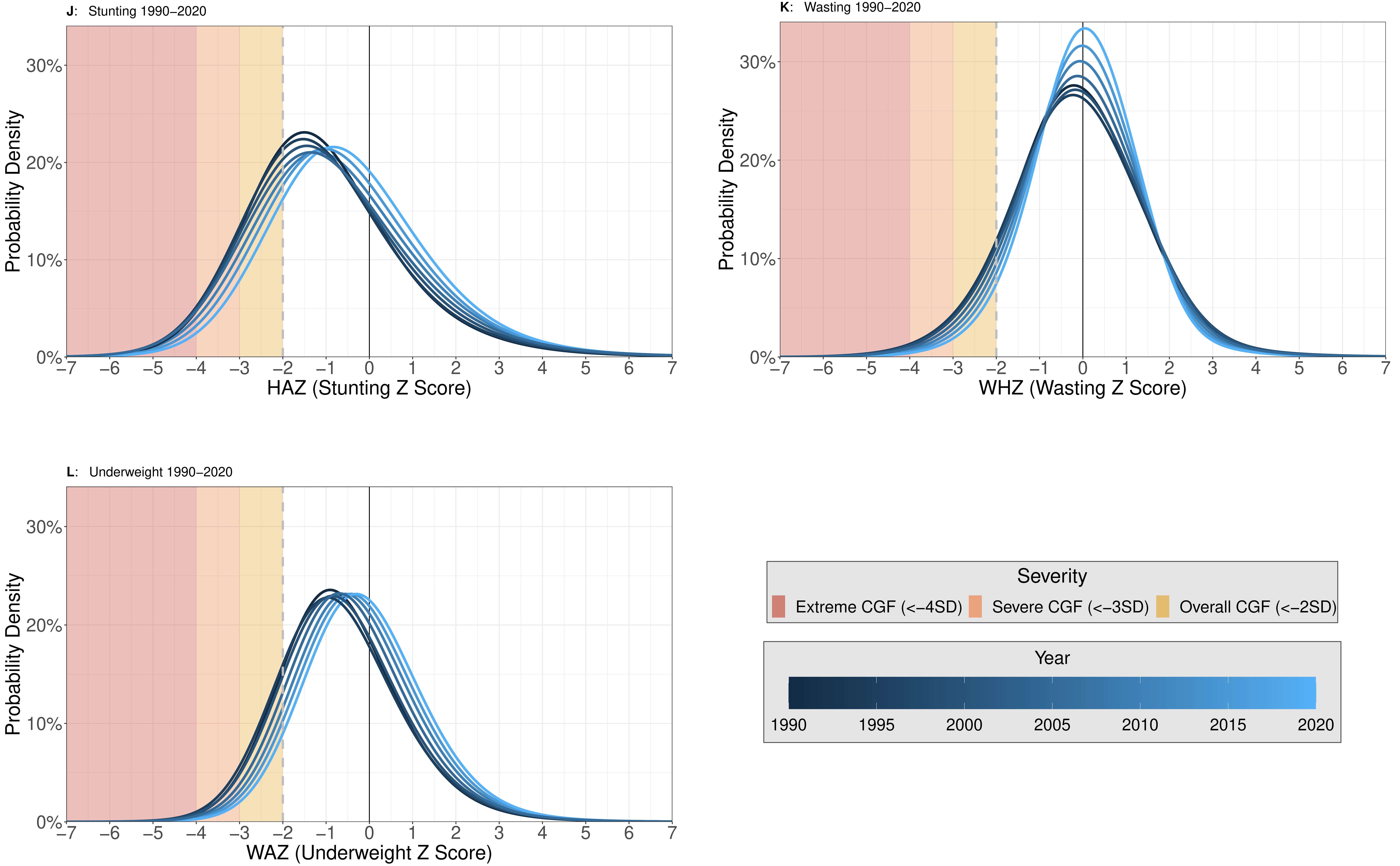

Jordan – Stunting (HAZ)

A: Overall and Severe Stunting Prevalence

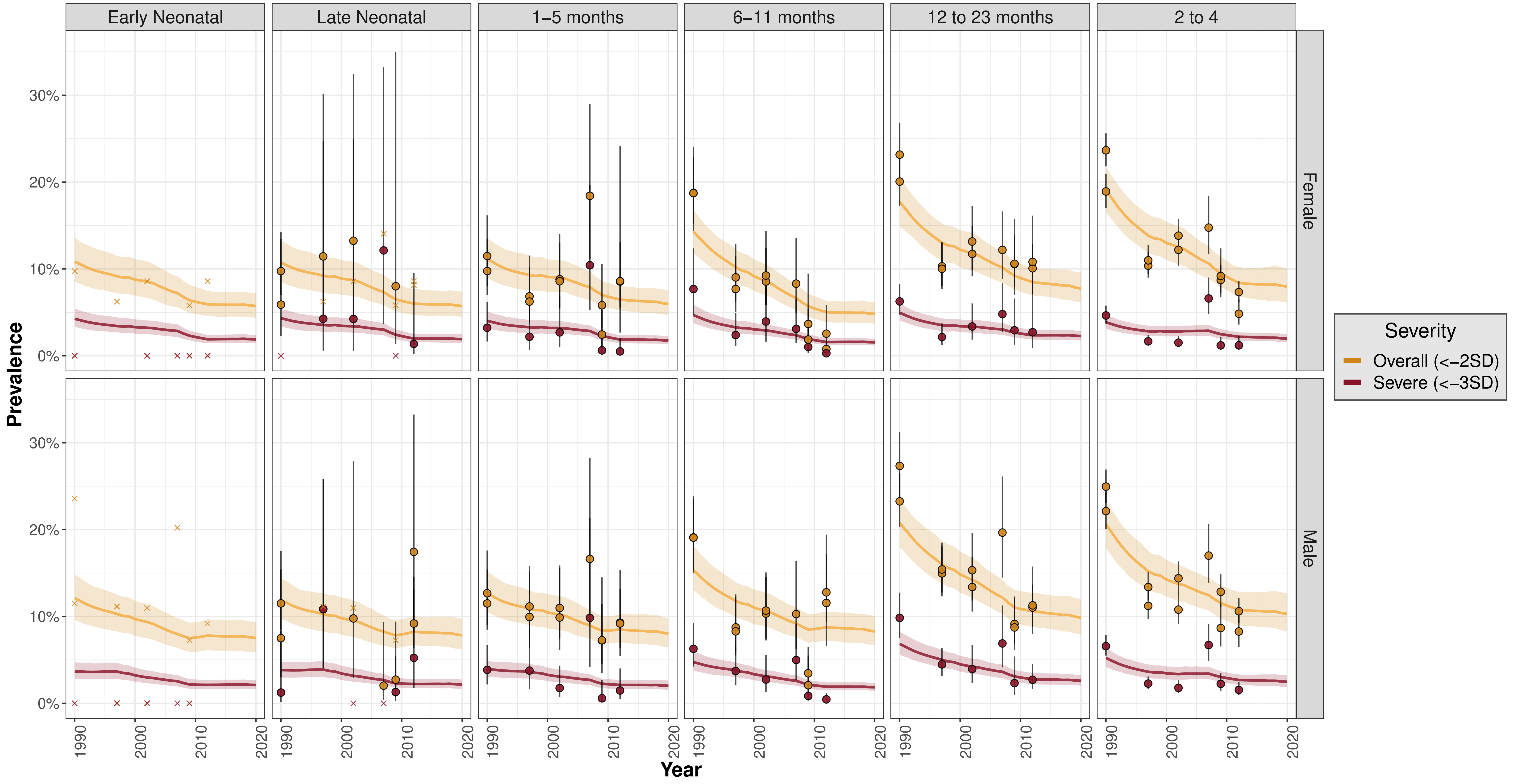

B: Transformed Mean Stunting Z Scores

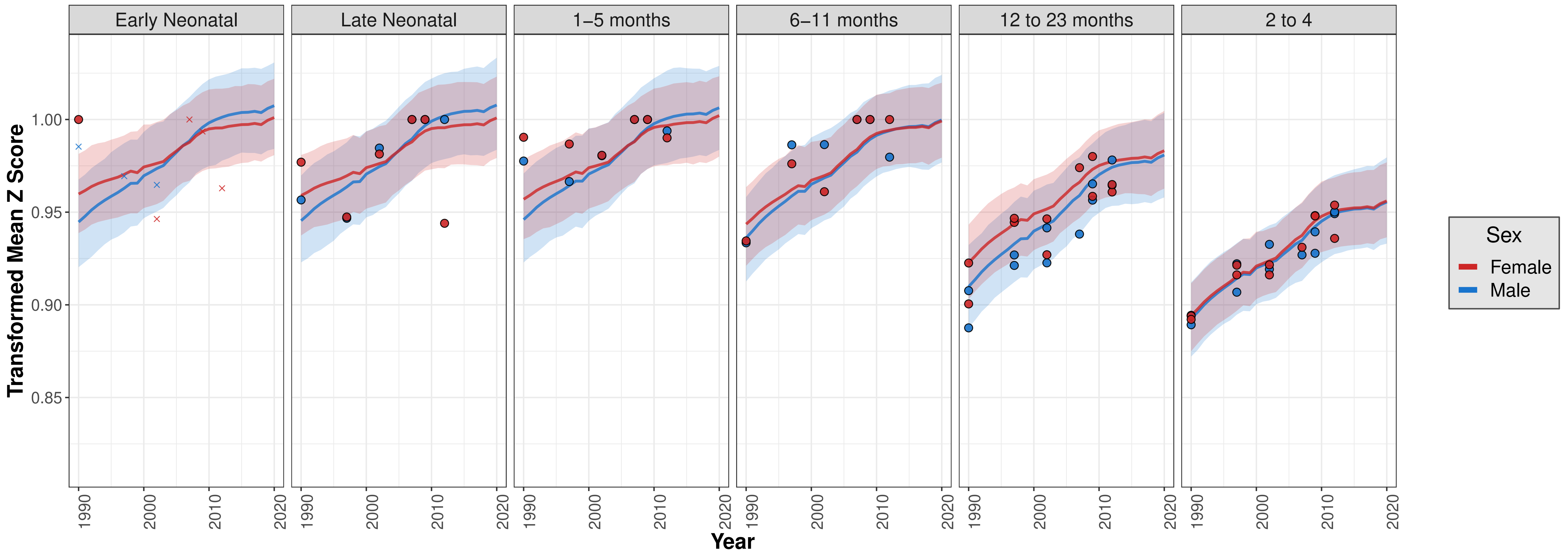

C

Year	Source
1990	DHS
1990	WHO CGM Database
1997	DHS
1997	WHO CGM Database
2002	DHS
2002	WHO CGM Database
2007	DHS
2009	Interim DHS
2009	WHO CGM Database
2012	DHS
2012	WHO CGM Database

Jordan – Wasting (WHZ)

D: Overall and Severe Wasting Prevalence

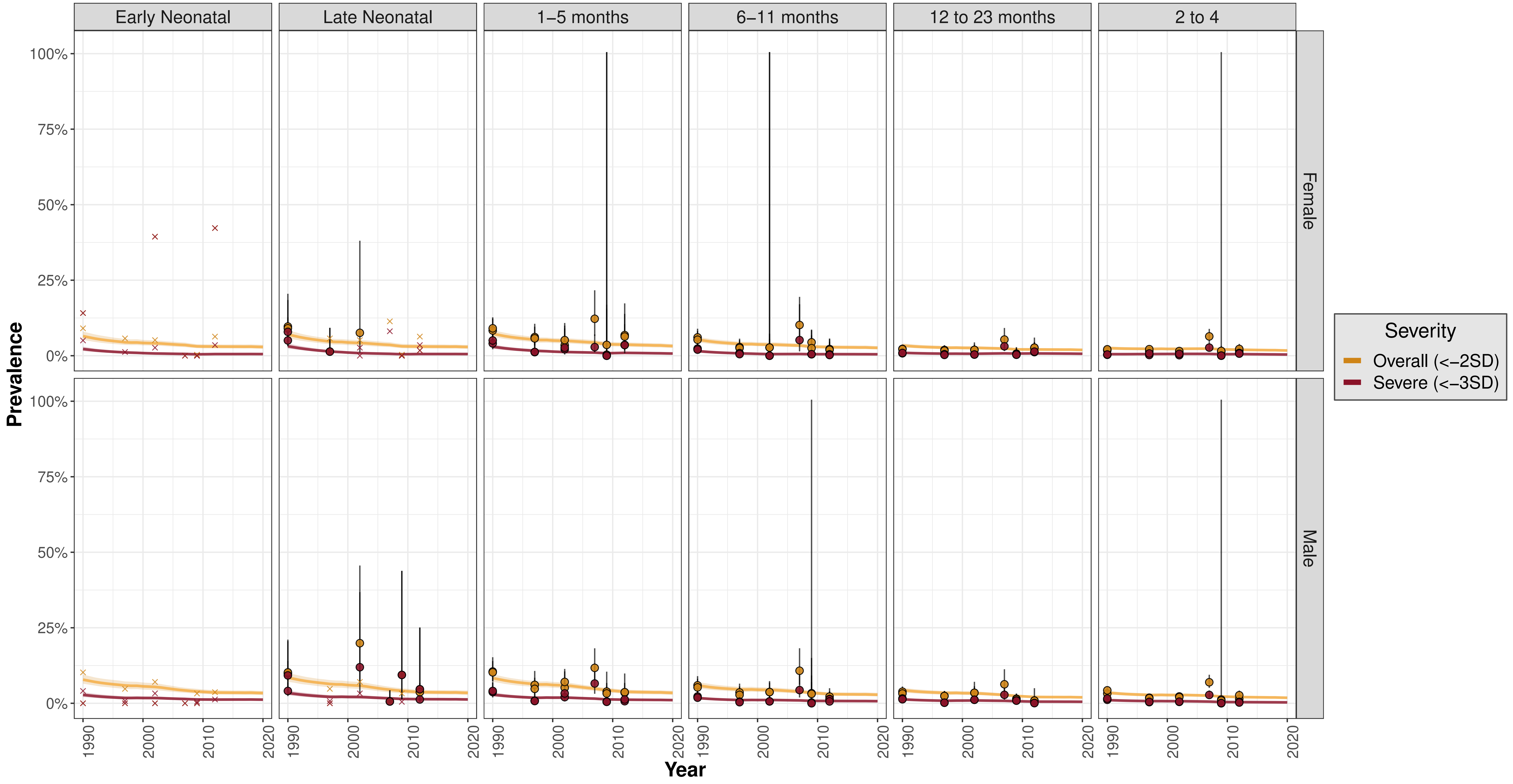

E: Transformed Mean Wasting Z Scores

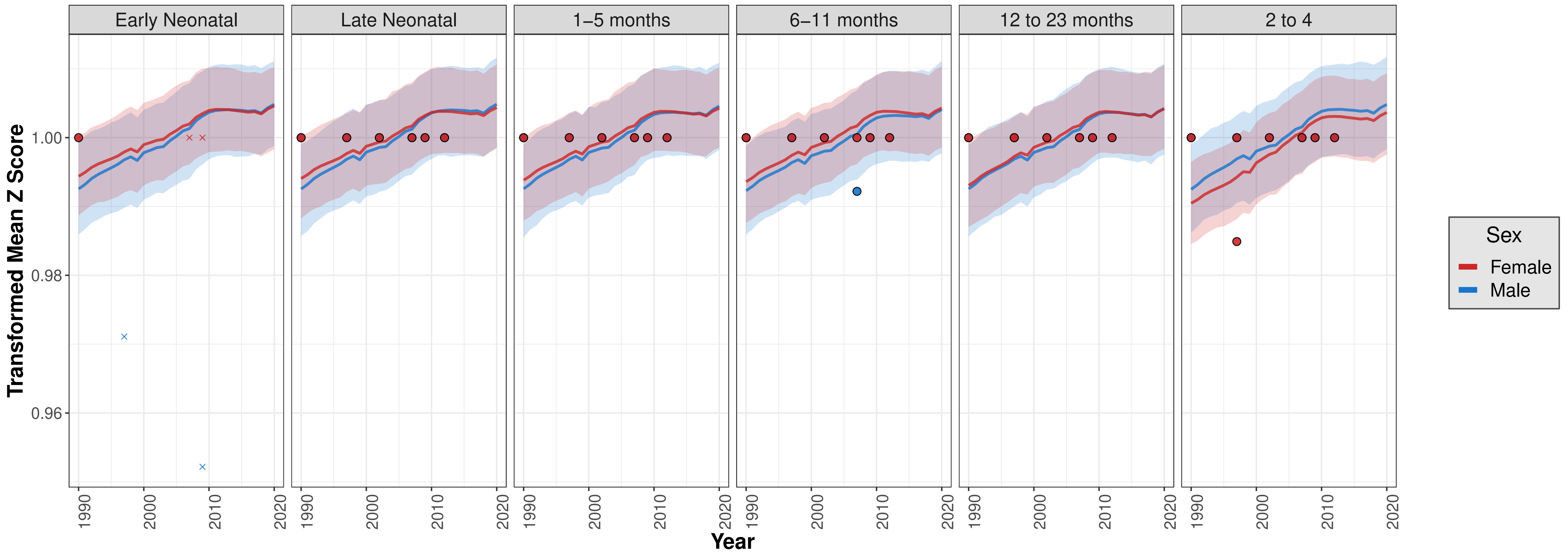

F

Year	Source
1990	DHS
1990	WHO CGM Database
1997	DHS
1997	WHO CGM Database
2002	DHS
2002	WHO CGM Database
2007	DHS
2009	Interim DHS
2009	WHO CGM Database
2012	DHS
2012	WHO CGM Database

Jordan – Underweight (WAZ)

G: Overall and Severe Underweight Prevalence

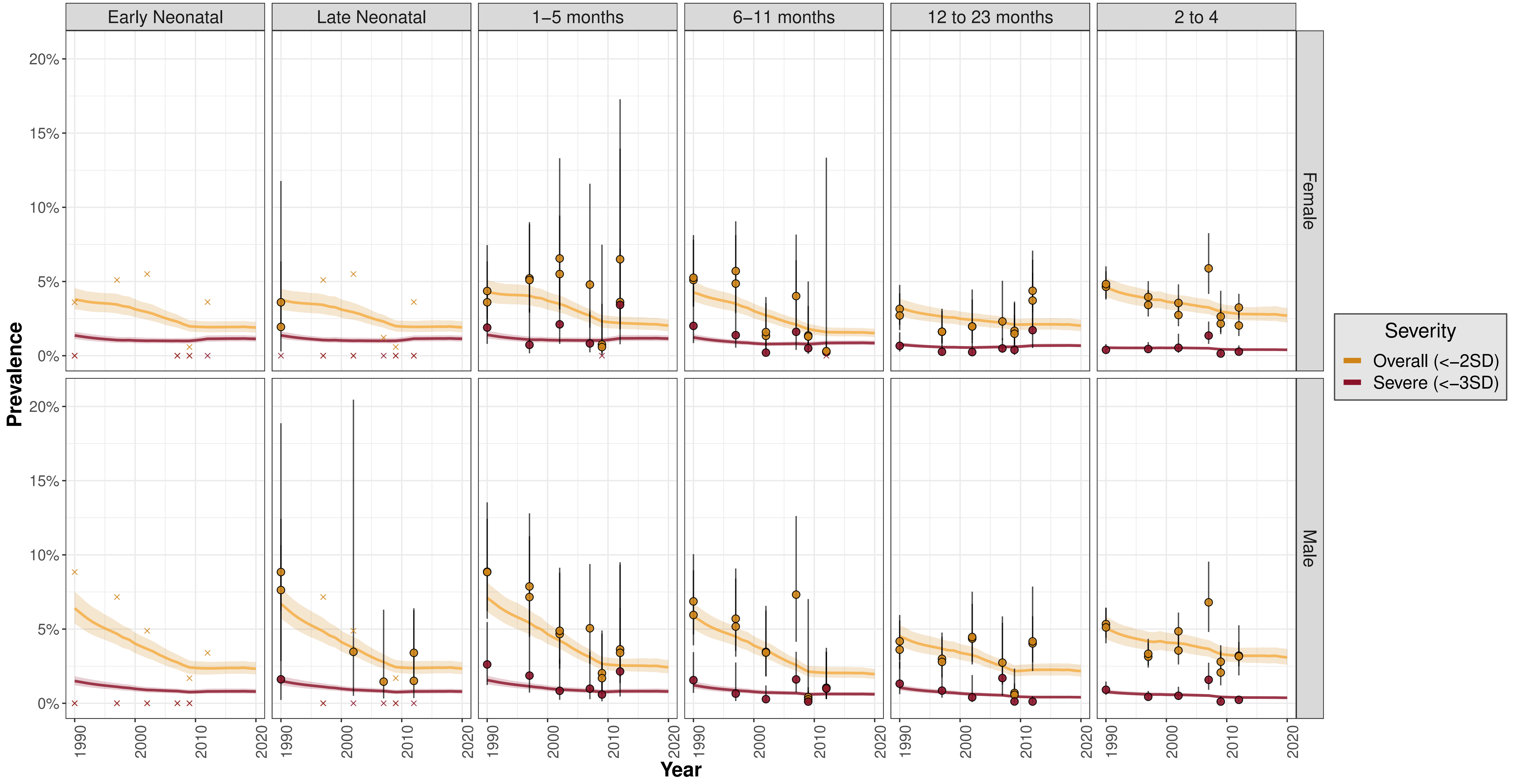

H: Transformed Mean Underweight Z Scores

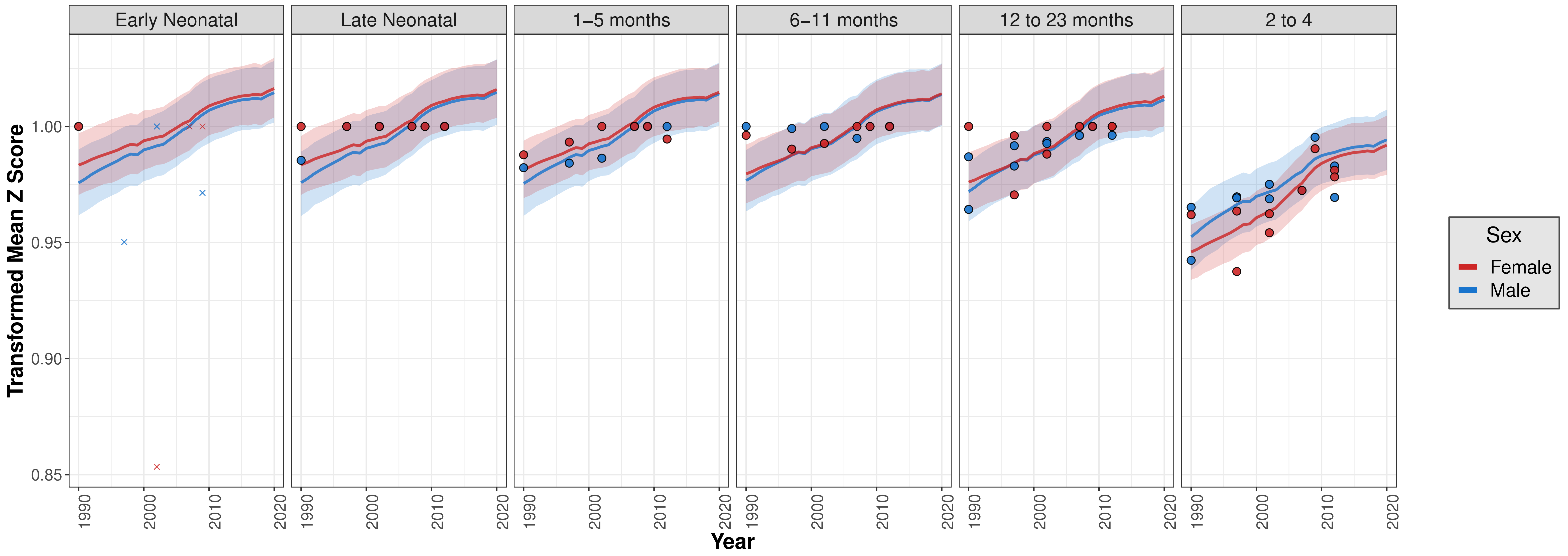

I

Year	Source
1990	DHS
1990	WHO CGM Database
1997	DHS
1997	WHO CGM Database
2002	DHS
2002	WHO CGM Database
2007	DHS
2009	Interim DHS
2009	WHO CGM Database
2012	DHS
2012	WHO CGM Database

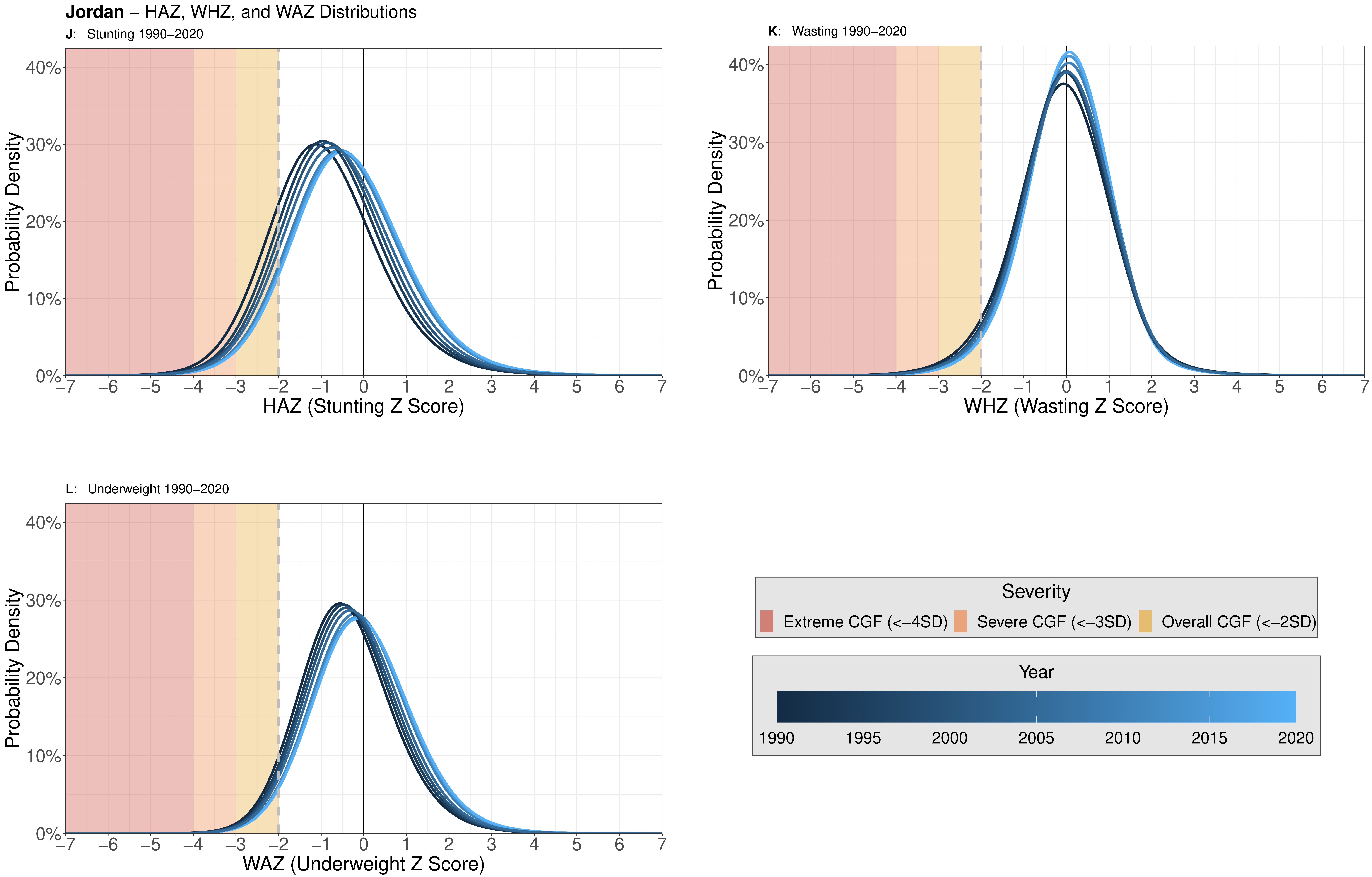
Jordan – HAZ, WHZ, and WAZ Distributions
J: Stunting 1990–2020
K: Wasting 1990–2020
L: Underweight 1990–2020
Probability Density
0%
10%
20%
30%
40%
−7
−6
−5
−4
−3
−2
−1
0
1
2
3
4
5
6
7
HAZ (Stunting Z Score)
WHZ (Wasting Z Score)
WAZ (Underweight Z Score)
Severity
Extreme CGF (<−4SD)
Severe CGF (<−3SD)
Overall CGF (<−2SD)
Year
1990
1995
2000
2005
2010
2015
2020

Kuwait – Stunting (HAZ)

A: Overall and Severe Stunting Prevalence

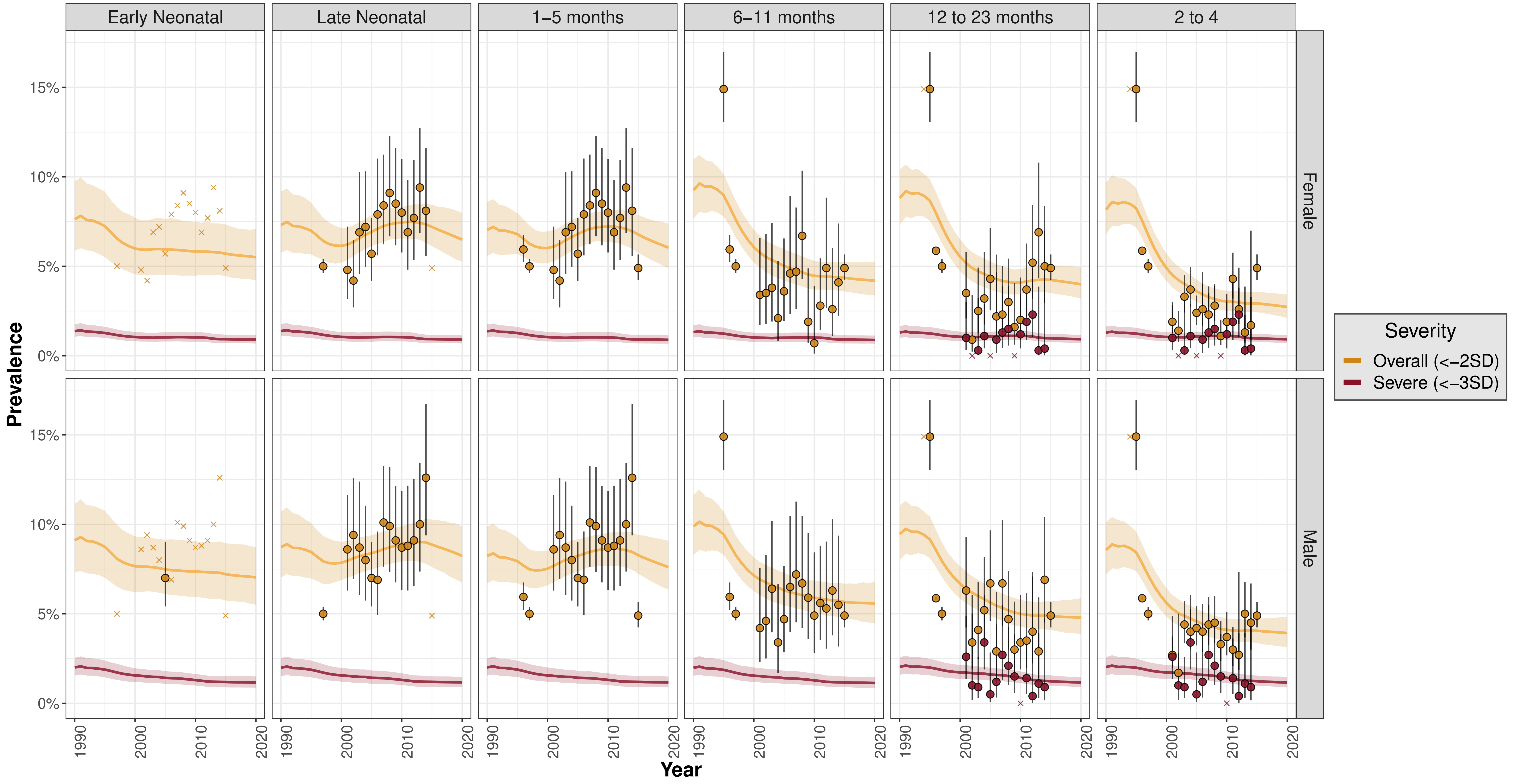

B: Transformed Mean Stunting Z Scores

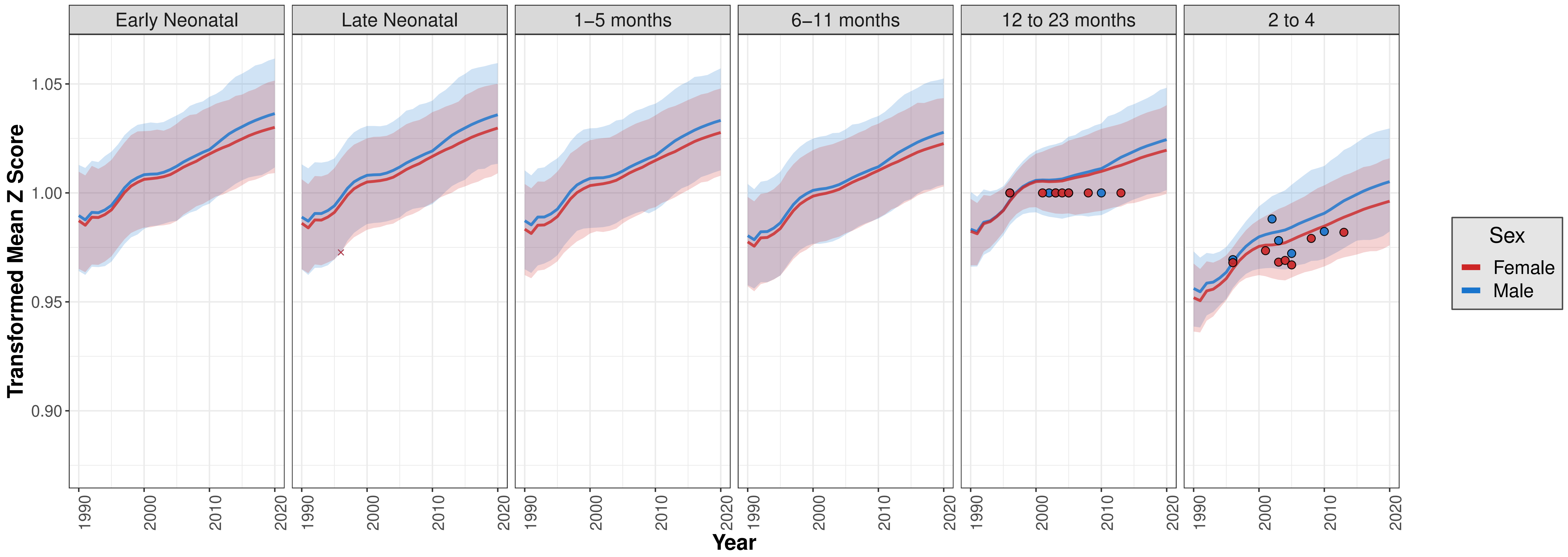

C

Year	Source
1994	WHO CGM Database
1995	WHO CGM Database
1996	WHO CGM Database
1997	WHO CGM Database
2001	WHO CGM Database
2002	WHO CGM Database
2003	WHO CGM Database
2004	WHO CGM Database
2005	WHO CGM Database
2006	WHO CGM Database
2007	WHO CGM Database
2008	WHO CGM Database
2009	WHO CGM Database
2010	WHO CGM Database
2011	WHO CGM Database
2012	WHO CGM Database
2013	WHO CGM Database
2014	WHO CGM Database
2015	WHO CGM Database

Kuwait – Wasting (WHZ)

D: Overall and Severe Wasting Prevalence

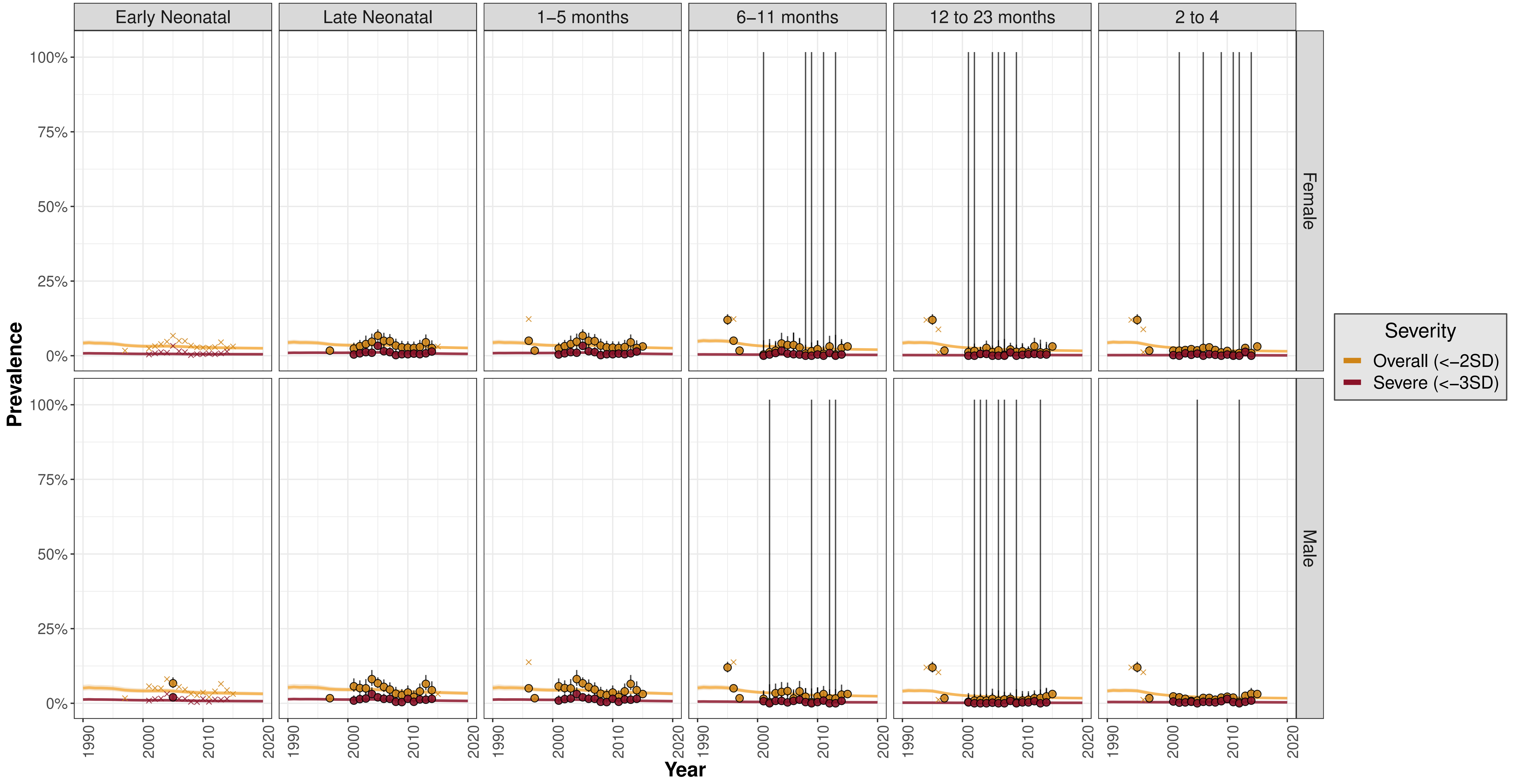

E: Transformed Mean Wasting Z Scores

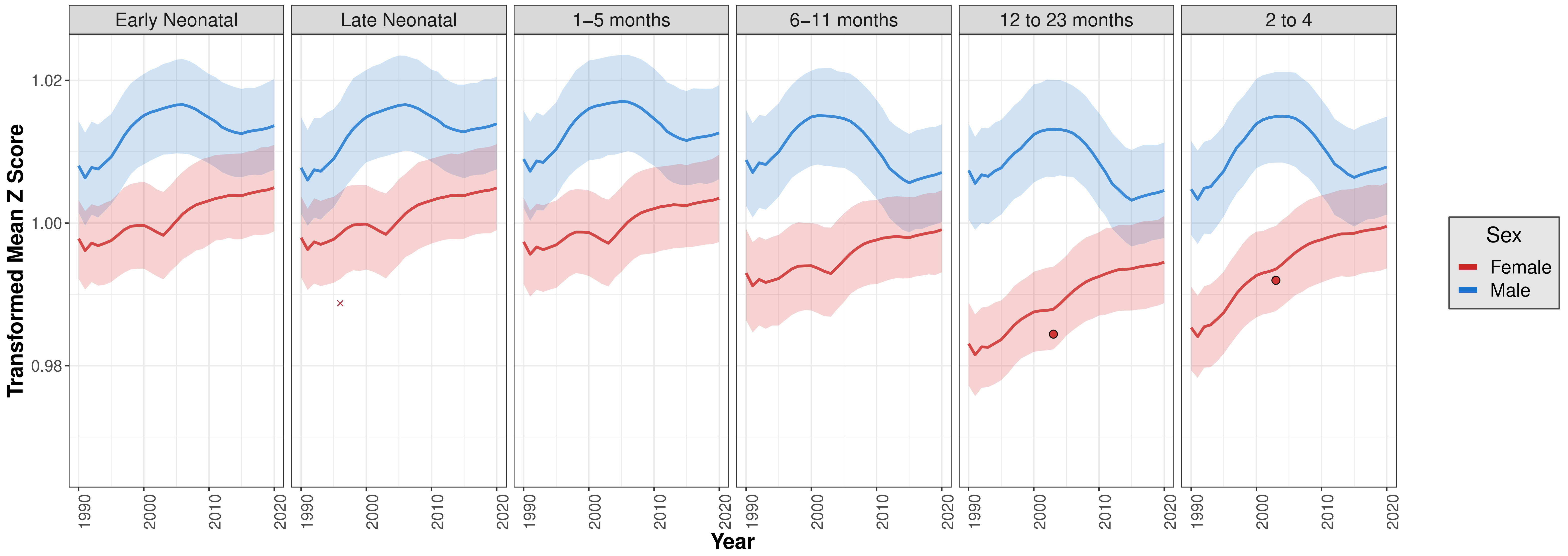

F

Year	Source
1994	WHO CGM Database
1995	WHO CGM Database
1996	WHO CGM Database
1997	WHO CGM Database
2001	WHO CGM Database
2002	WHO CGM Database
2003	WHO CGM Database
2004	WHO CGM Database
2005	WHO CGM Database
2006	WHO CGM Database
2007	WHO CGM Database
2008	WHO CGM Database
2009	WHO CGM Database
2010	WHO CGM Database
2011	WHO CGM Database
2012	WHO CGM Database
2013	WHO CGM Database
2014	WHO CGM Database
2015	WHO CGM Database

Kuwait – Underweight (WAZ)

G: Overall and Severe Underweight Prevalence

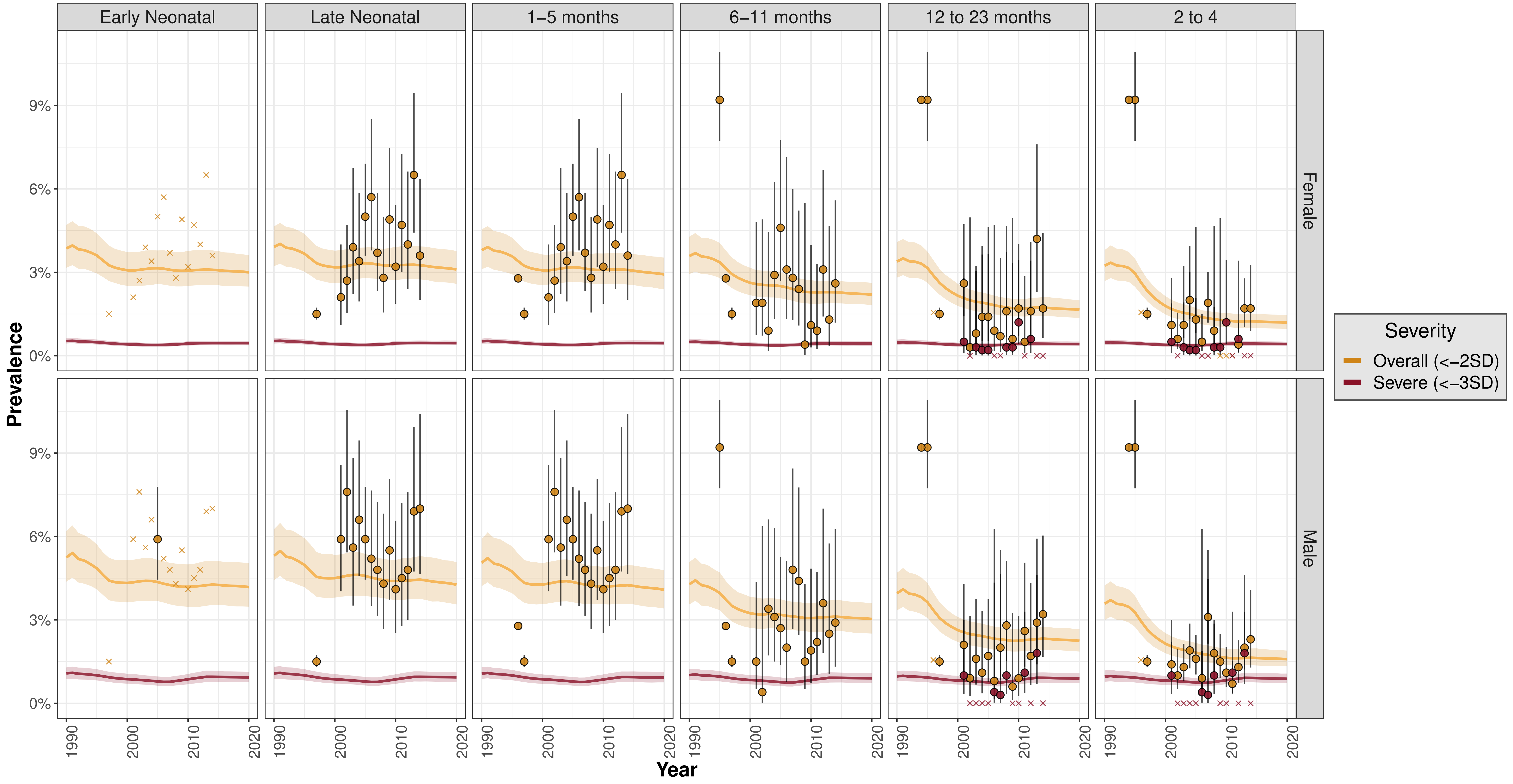

H: Transformed Mean Underweight Z Scores

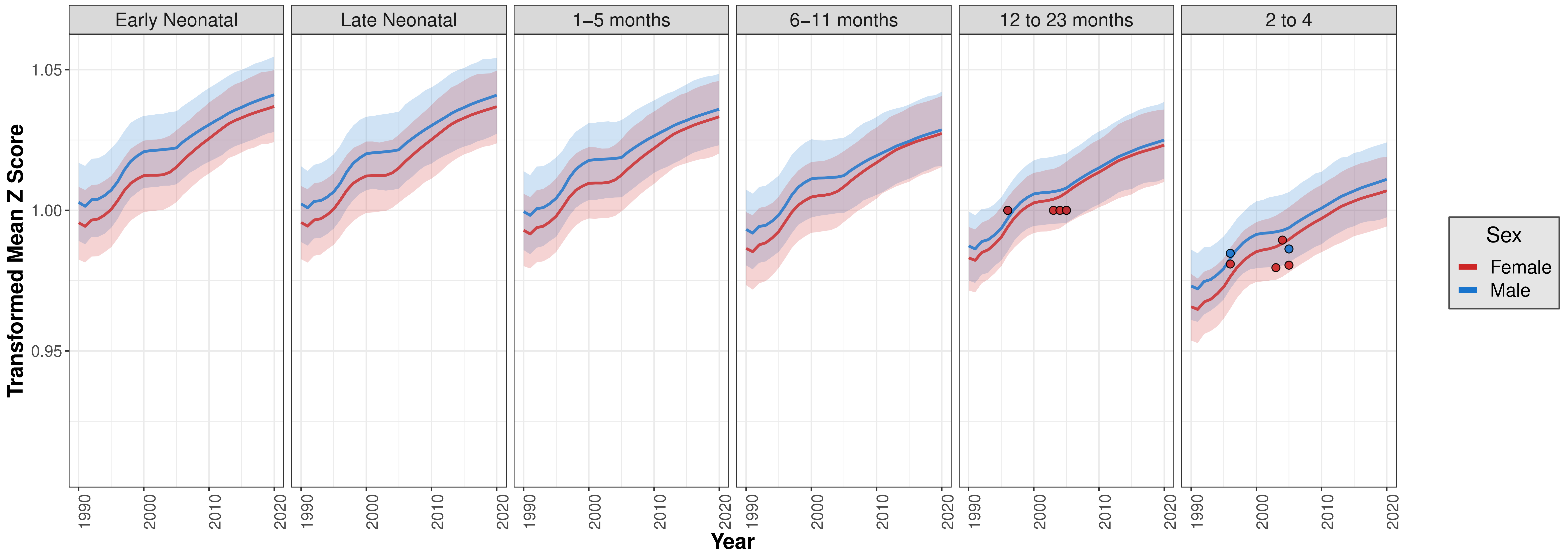

I

Year	Source
1994	WHO CGM Database
1995	WHO CGM Database
1996	WHO CGM Database
1997	WHO CGM Database
2001	WHO CGM Database
2002	WHO CGM Database
2003	WHO CGM Database
2004	WHO CGM Database
2005	WHO CGM Database
2006	WHO CGM Database
2007	WHO CGM Database
2008	WHO CGM Database
2009	WHO CGM Database
2010	WHO CGM Database
2011	WHO CGM Database
2012	WHO CGM Database
2013	WHO CGM Database
2014	WHO CGM Database

Kuwait – HAZ, WHZ, and WAZ Distributions

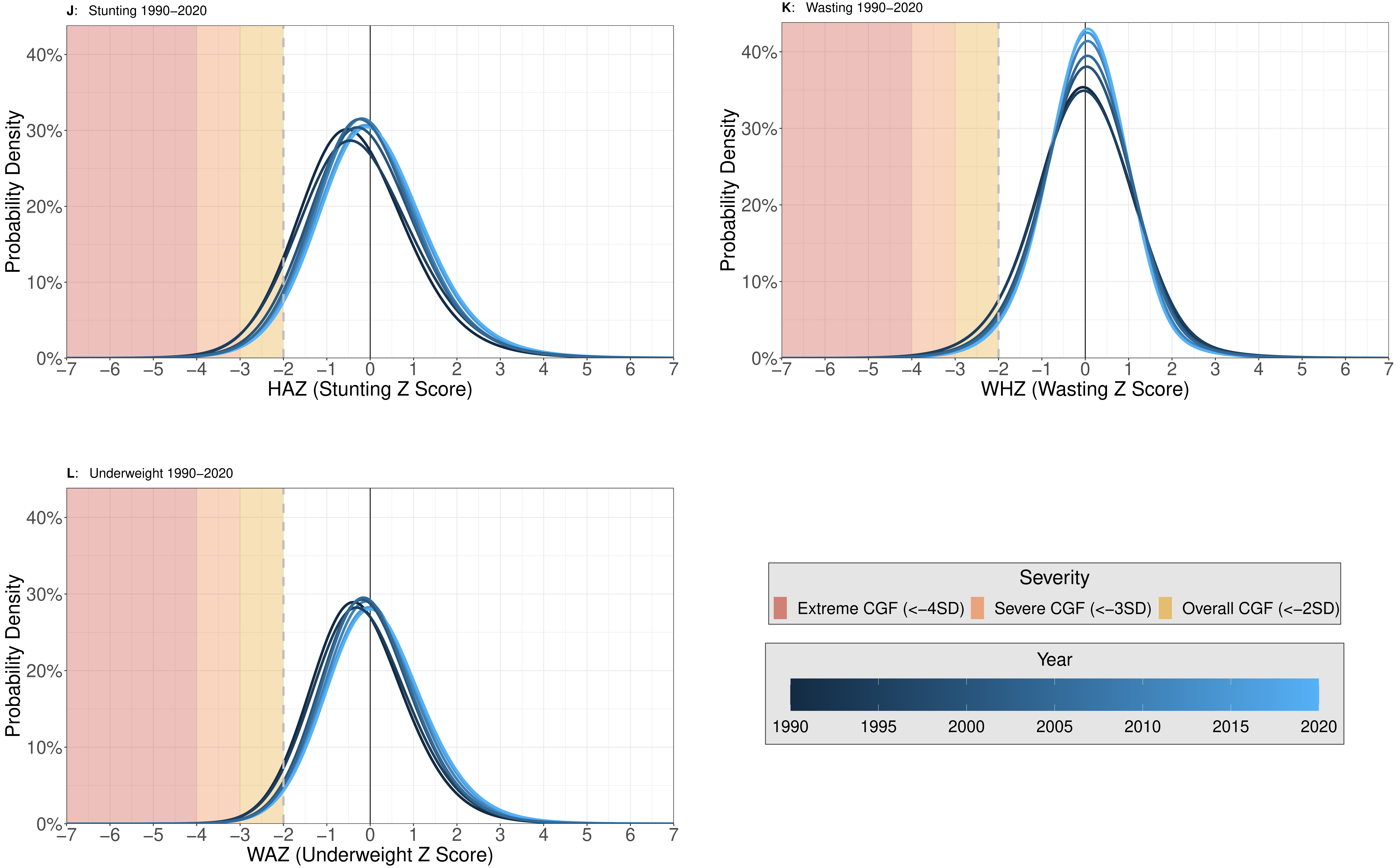

Lebanon – Stunting (HAZ)

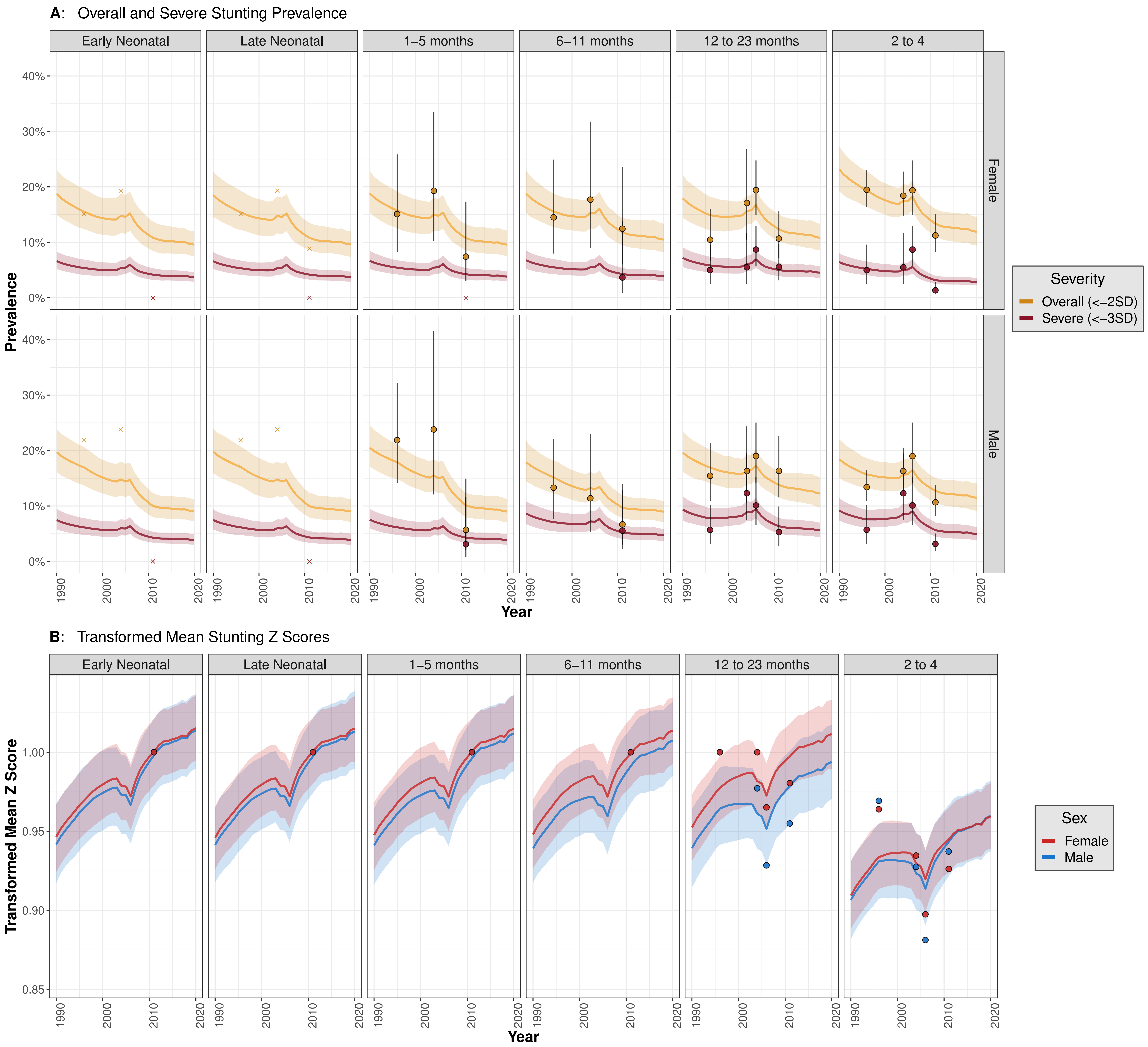

C

Year	Source
1996	WHO CGM Database
2004	WHO CGM Database
2006	WHO CGM Database
2011	Palestinians in MICS

Lebanon – Wasting (WHZ)

D: Overall and Severe Wasting Prevalence

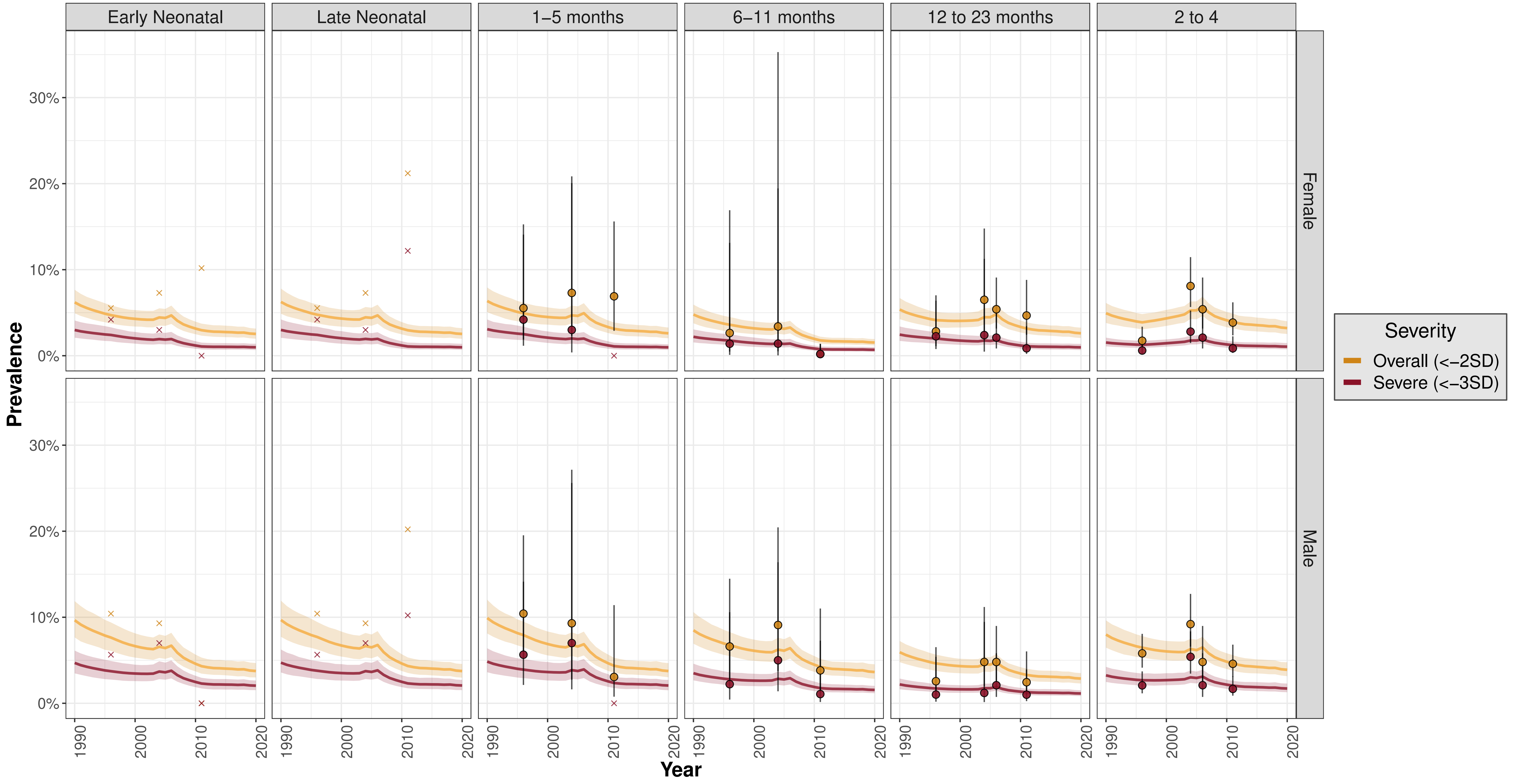

E: Transformed Mean Wasting Z Scores

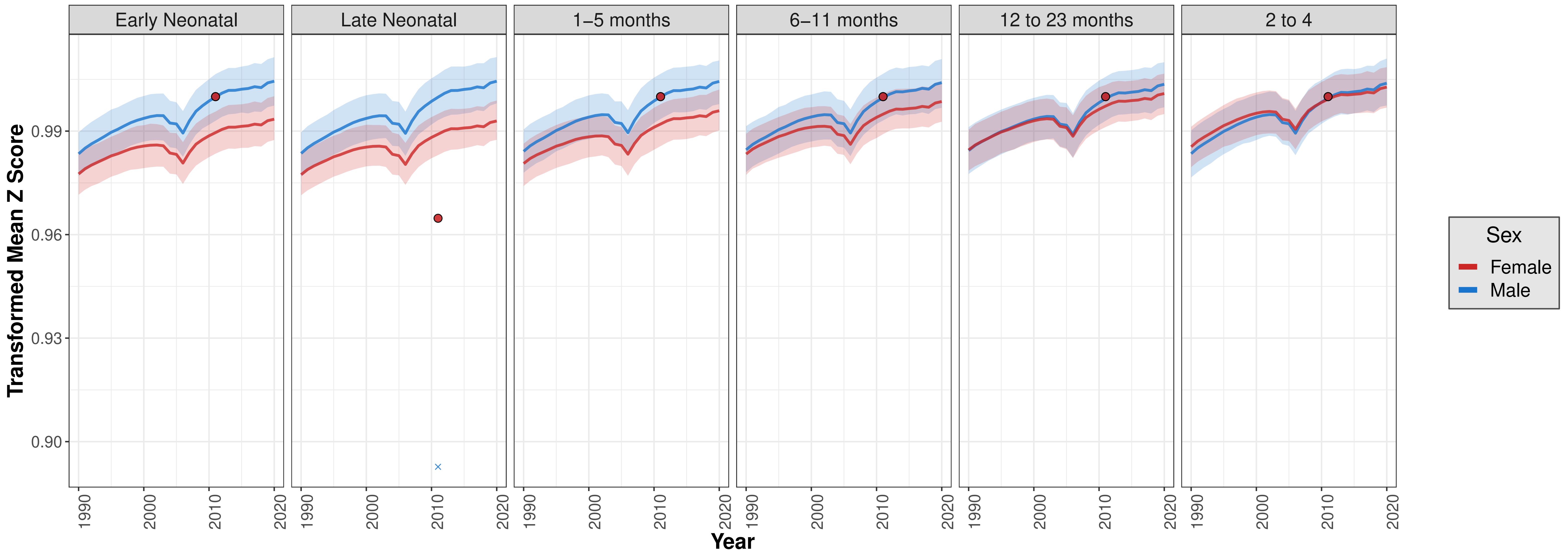

F

Year	Source
1996	WHO CGM Database
2004	WHO CGM Database
2006	WHO CGM Database
2011	Palestinians in MICS

Lebanon – Underweight (WAZ)

G: Overall and Severe Underweight Prevalence

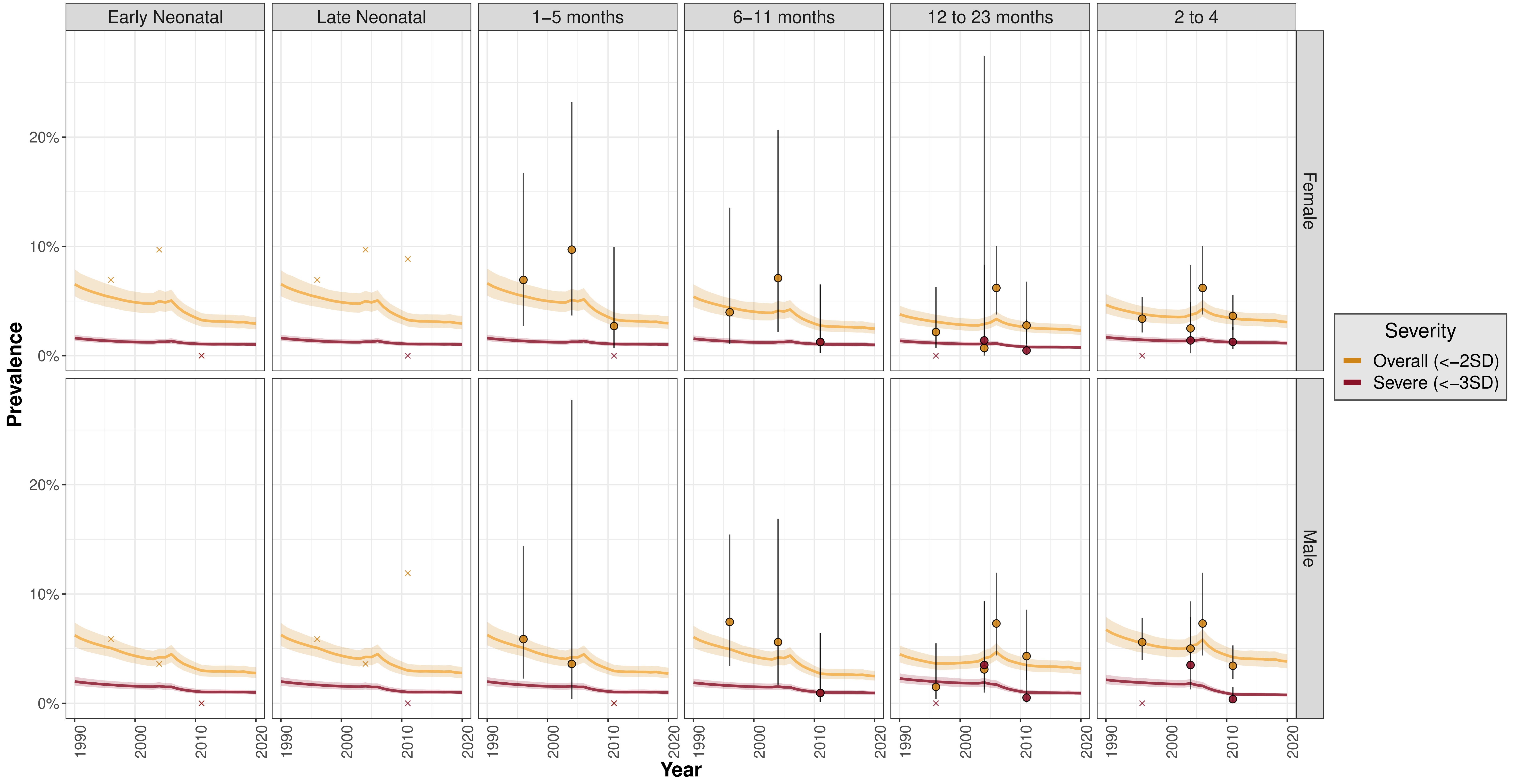

H: Transformed Mean Underweight Z Scores

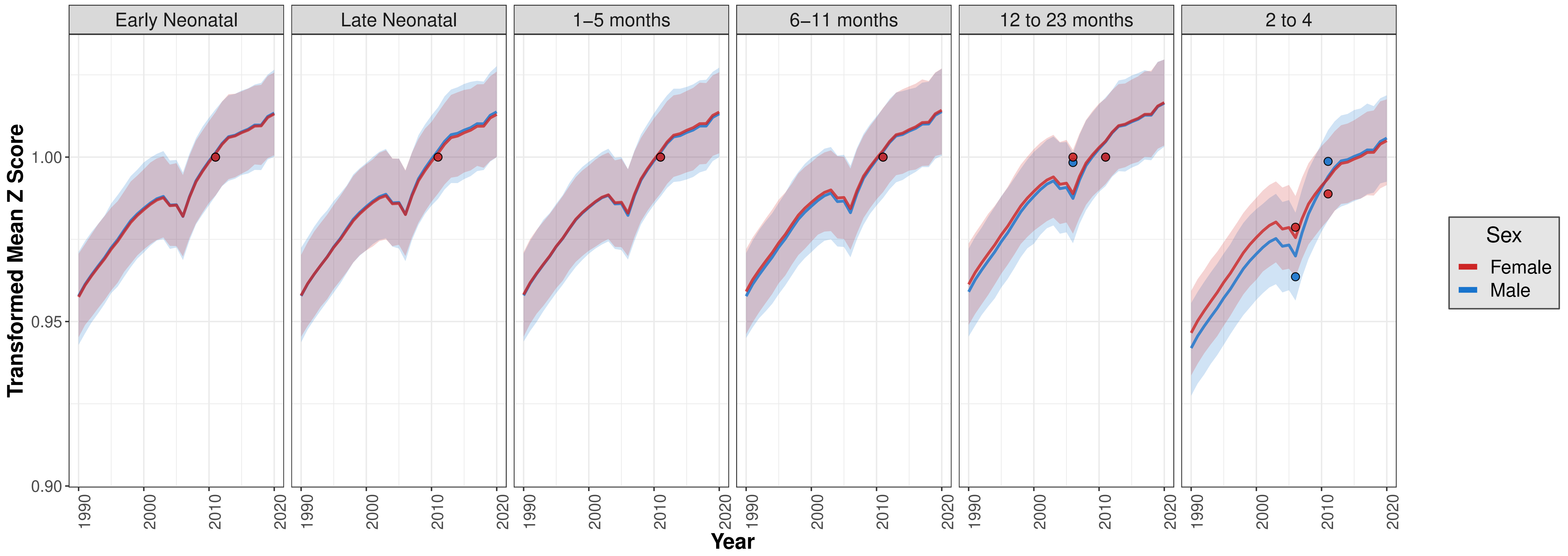

I

Year	Source
1996	WHO CGM Database
2004	WHO CGM Database
2006	WHO CGM Database
2011	Palestinians in MICS

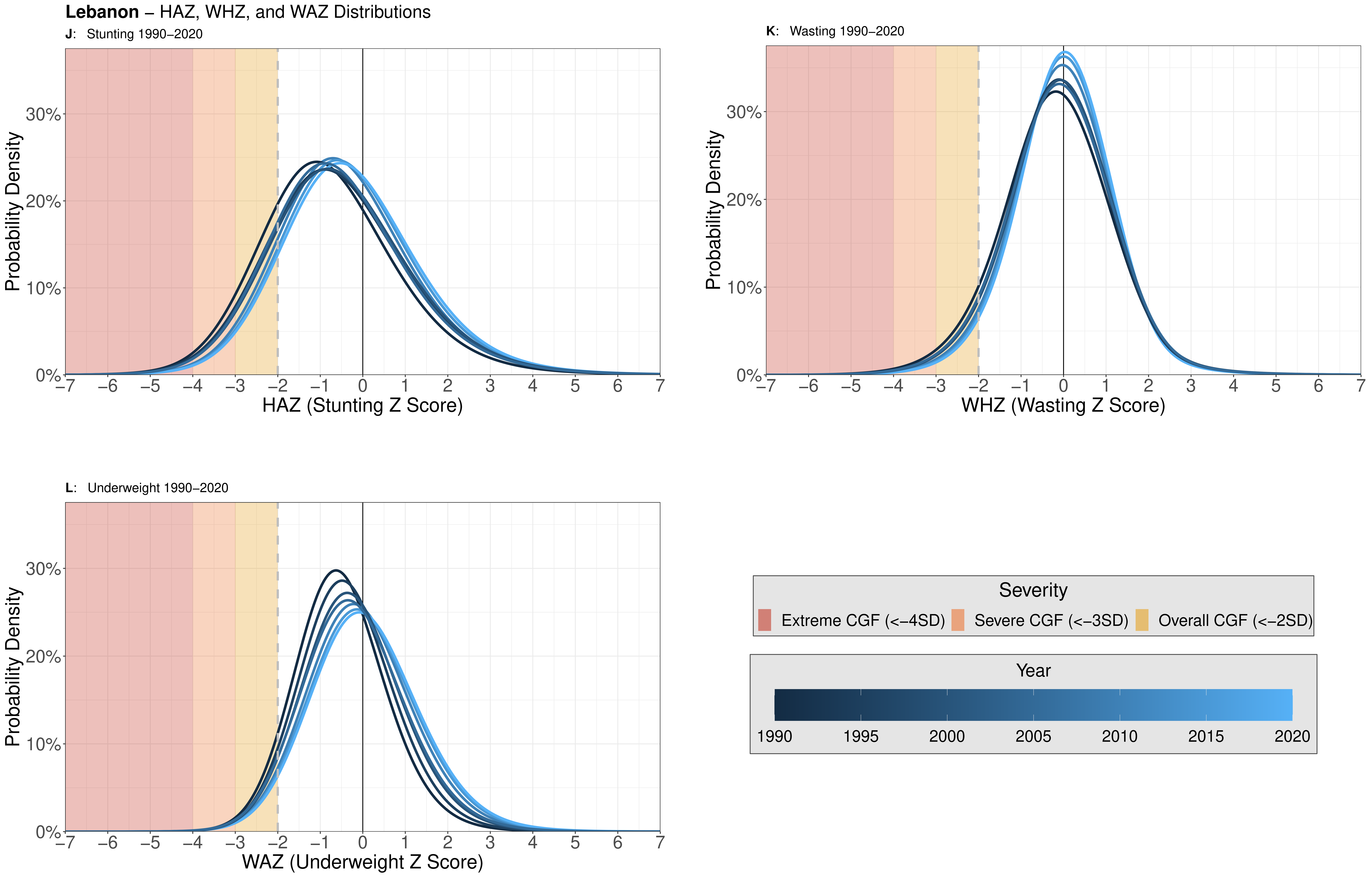

Lebanon – HAZ, WHZ, and WAZ Distributions
J: Stunting 1990–2020
K: Wasting 1990–2020
L: Underweight 1990–2020
Probability Density
0%
10%
20%
30%
−7 −6 −5 −4 −3 −2 −1 0 1 2 3 4 5 6 7
HAZ (Stunting Z Score)
WHZ (Wasting Z Score)
WAZ (Underweight Z Score)
Severity
Extreme CGF (<−4SD)
Severe CGF (<−3SD)
Overall CGF (<−2SD)
Year
1990
1995
2000
2005
2010
2015
2020

Libya – Stunting (HAZ)

A: Overall and Severe Stunting Prevalence

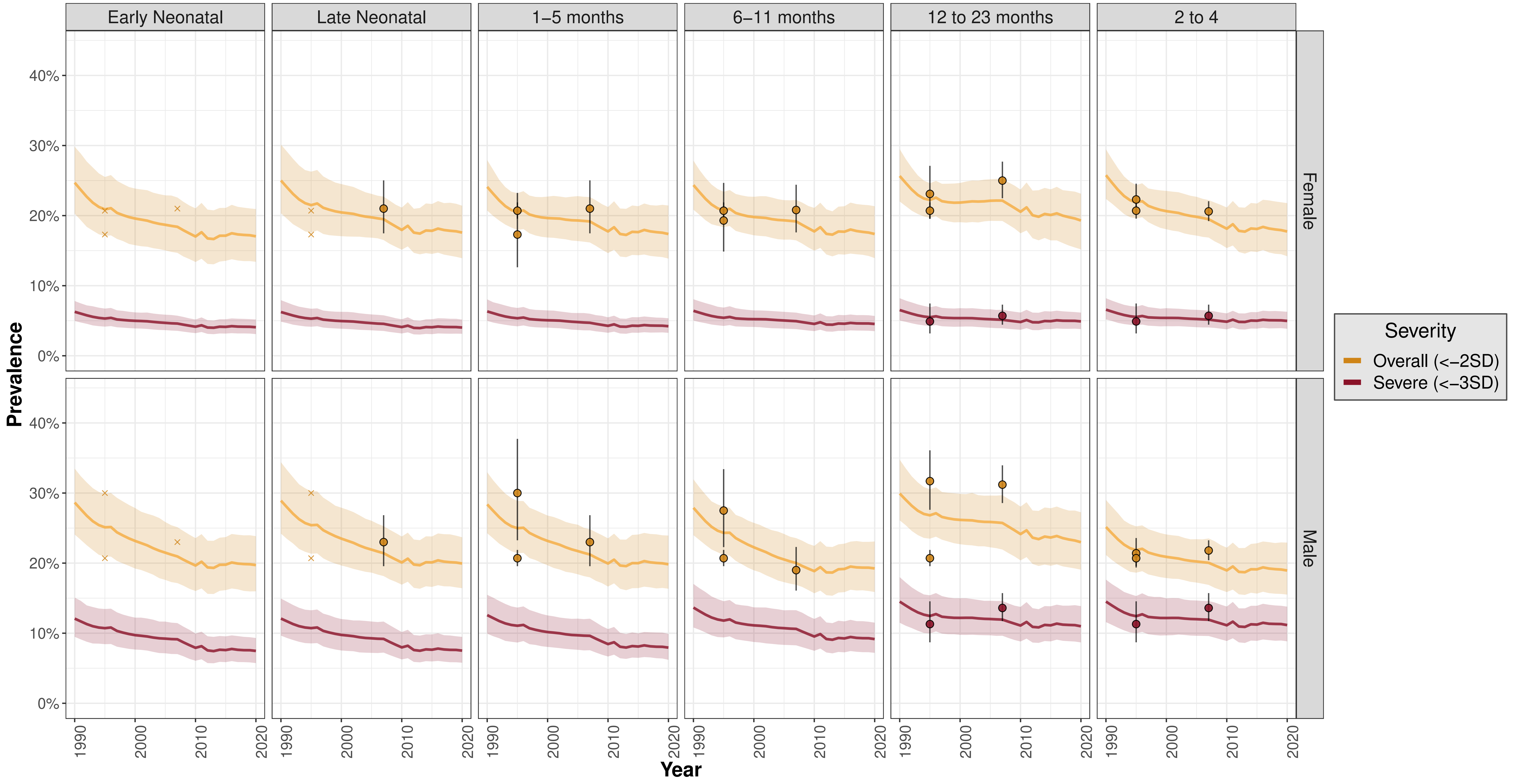

B: Transformed Mean Stunting Z Scores

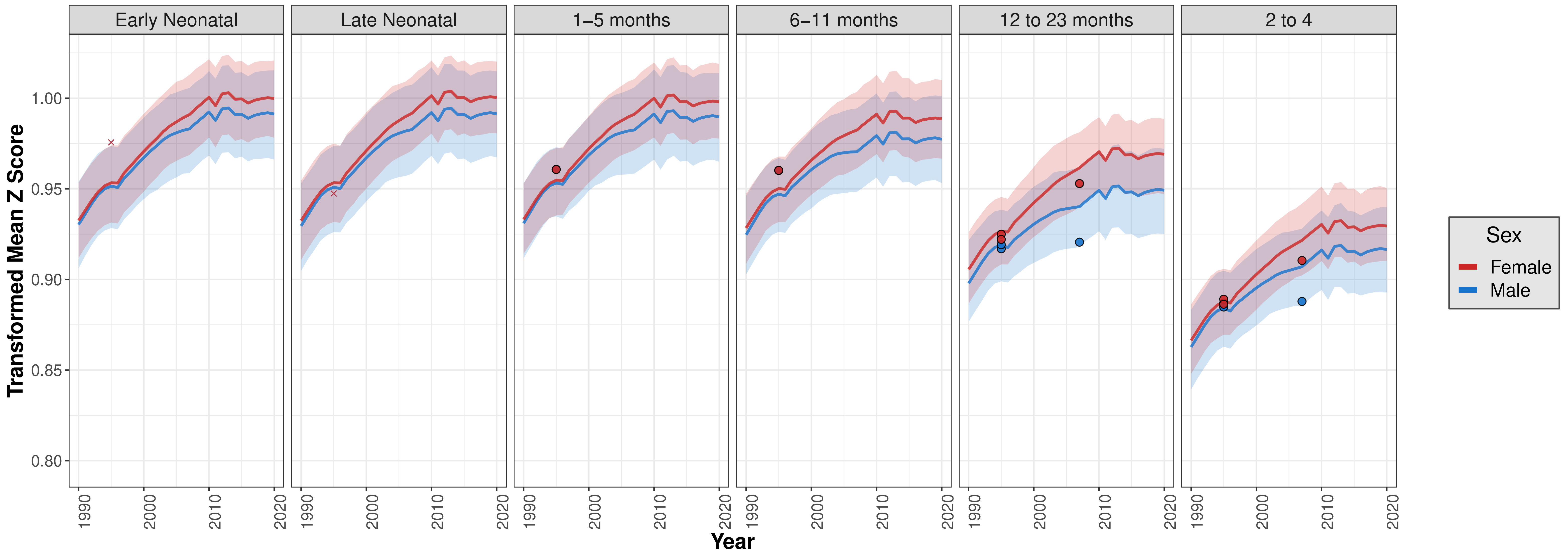

C

Year	Source
1995	WHO CGM Database
2007	WHO CGM Database

Libya – Wasting (WHZ)

D: Overall and Severe Wasting Prevalence

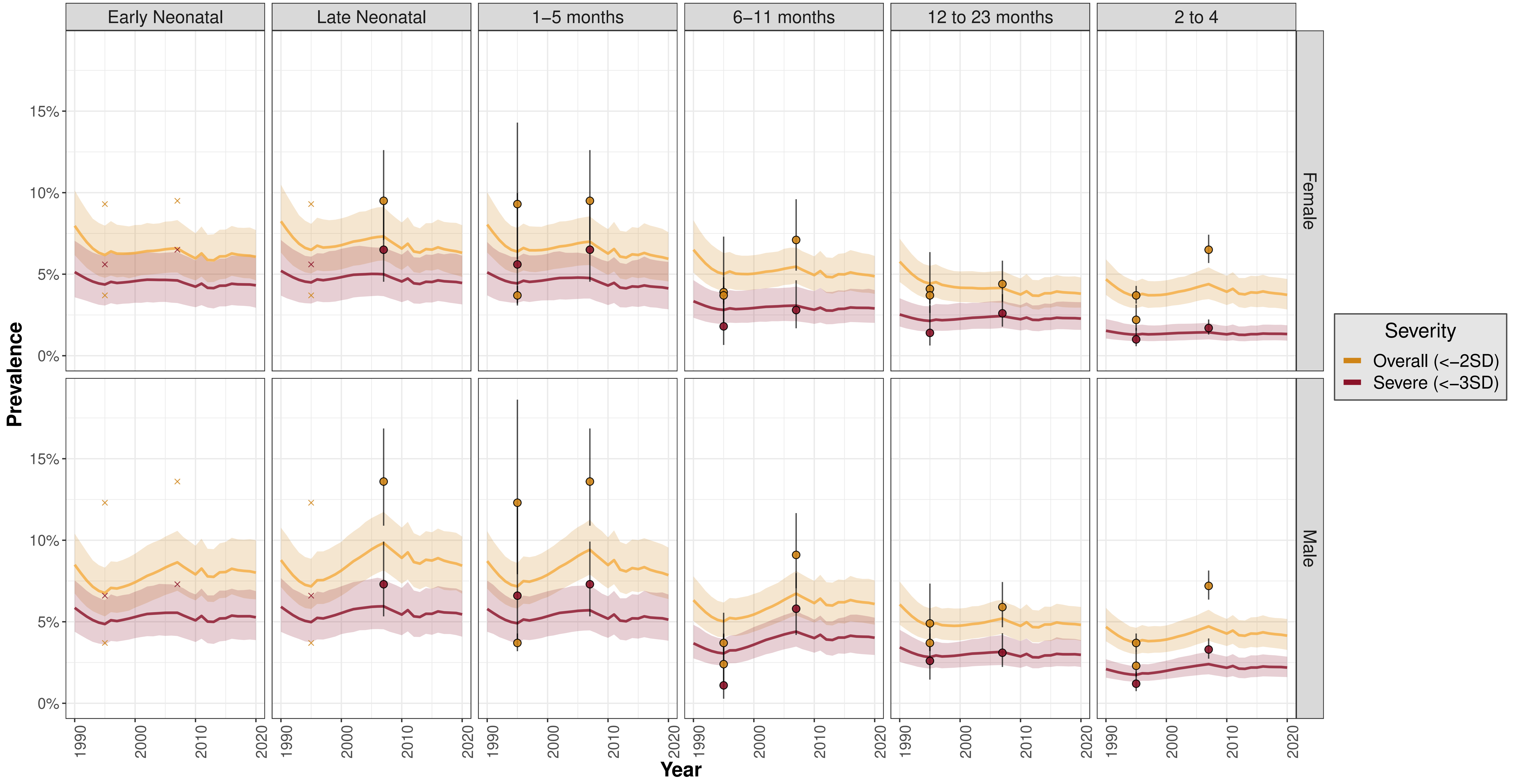

E: Transformed Mean Wasting Z Scores

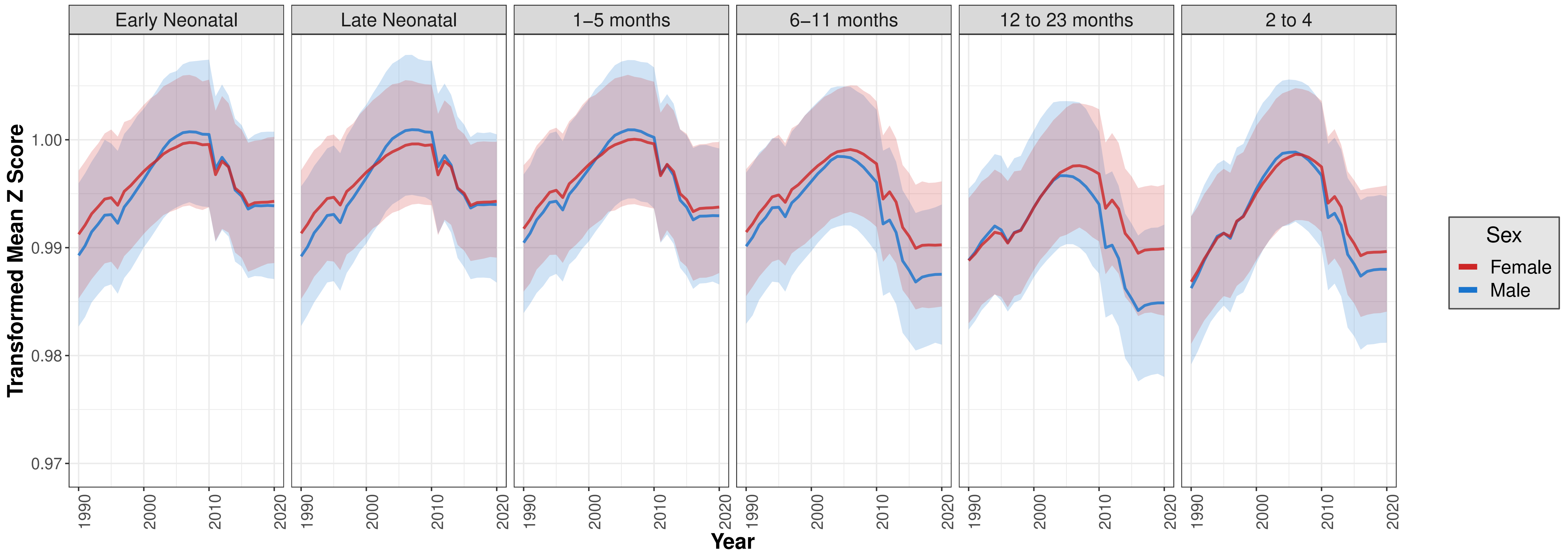

F

Year	Source
1995	WHO CGM Database
2007	WHO CGM Database

Libya – Underweight (WAZ)

G: Overall and Severe Underweight Prevalence

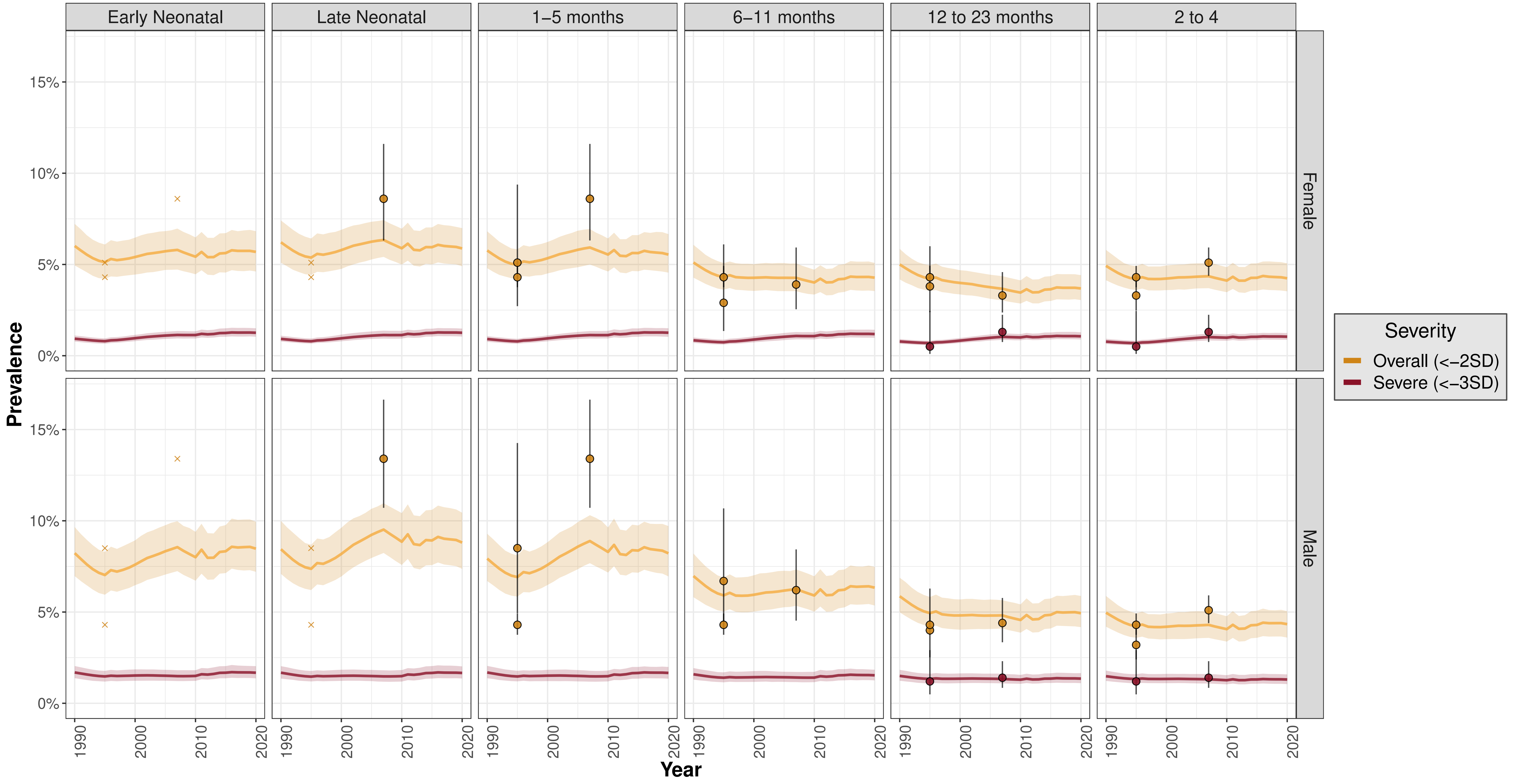

H: Transformed Mean Underweight Z Scores

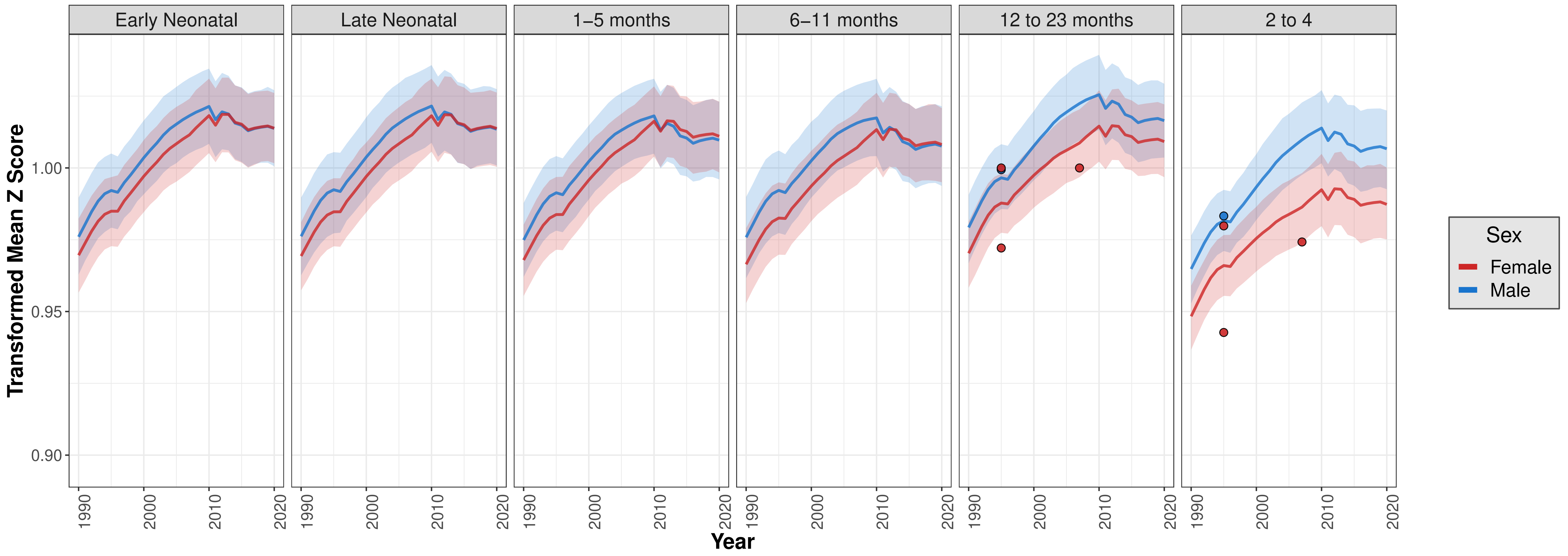

I

Year	Source
1995	WHO CGM Database
2007	WHO CGM Database

Libya – HAZ, WHZ, and WAZ Distributions

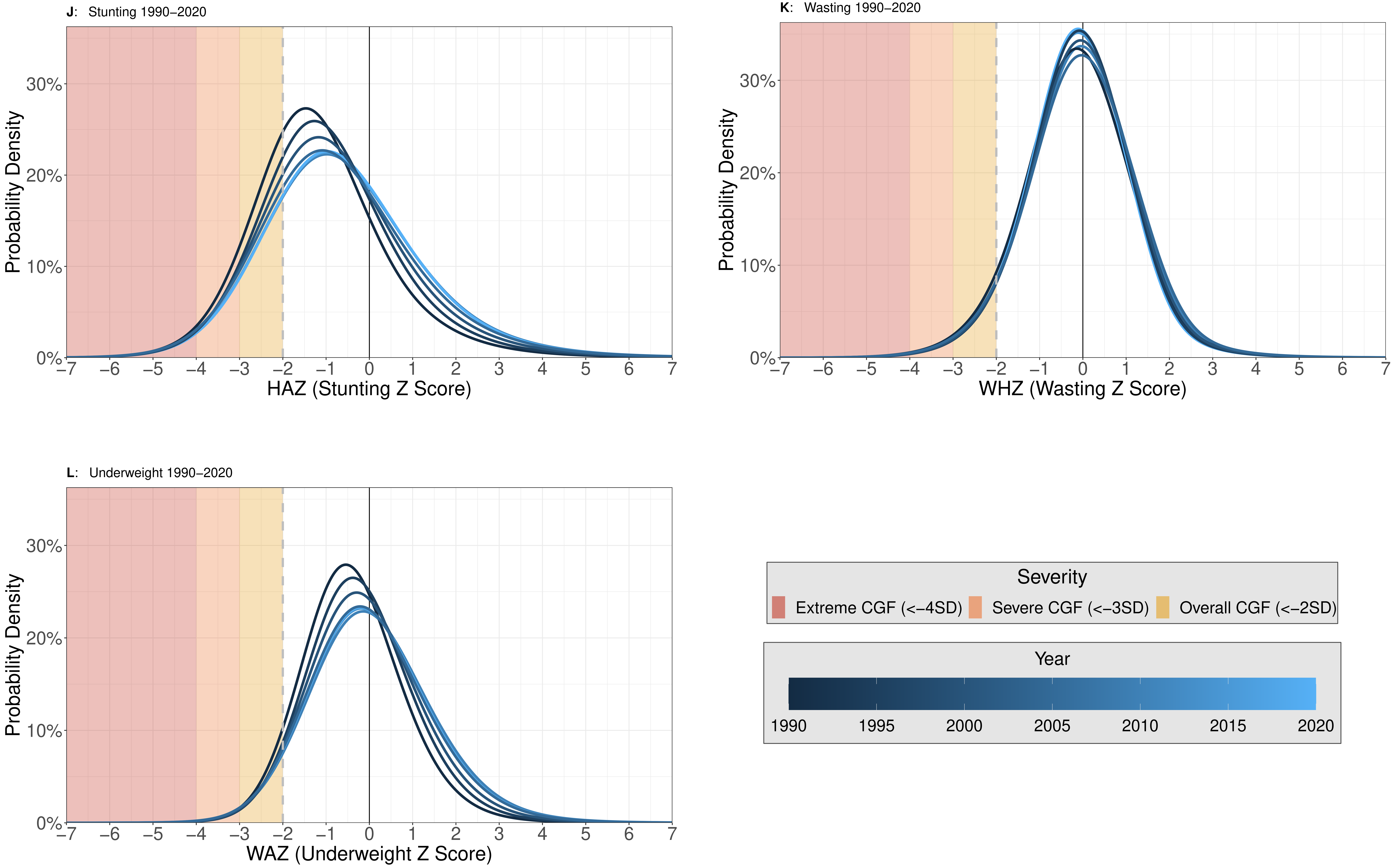

Morocco – Stunting (HAZ)

A: Overall and Severe Stunting Prevalence

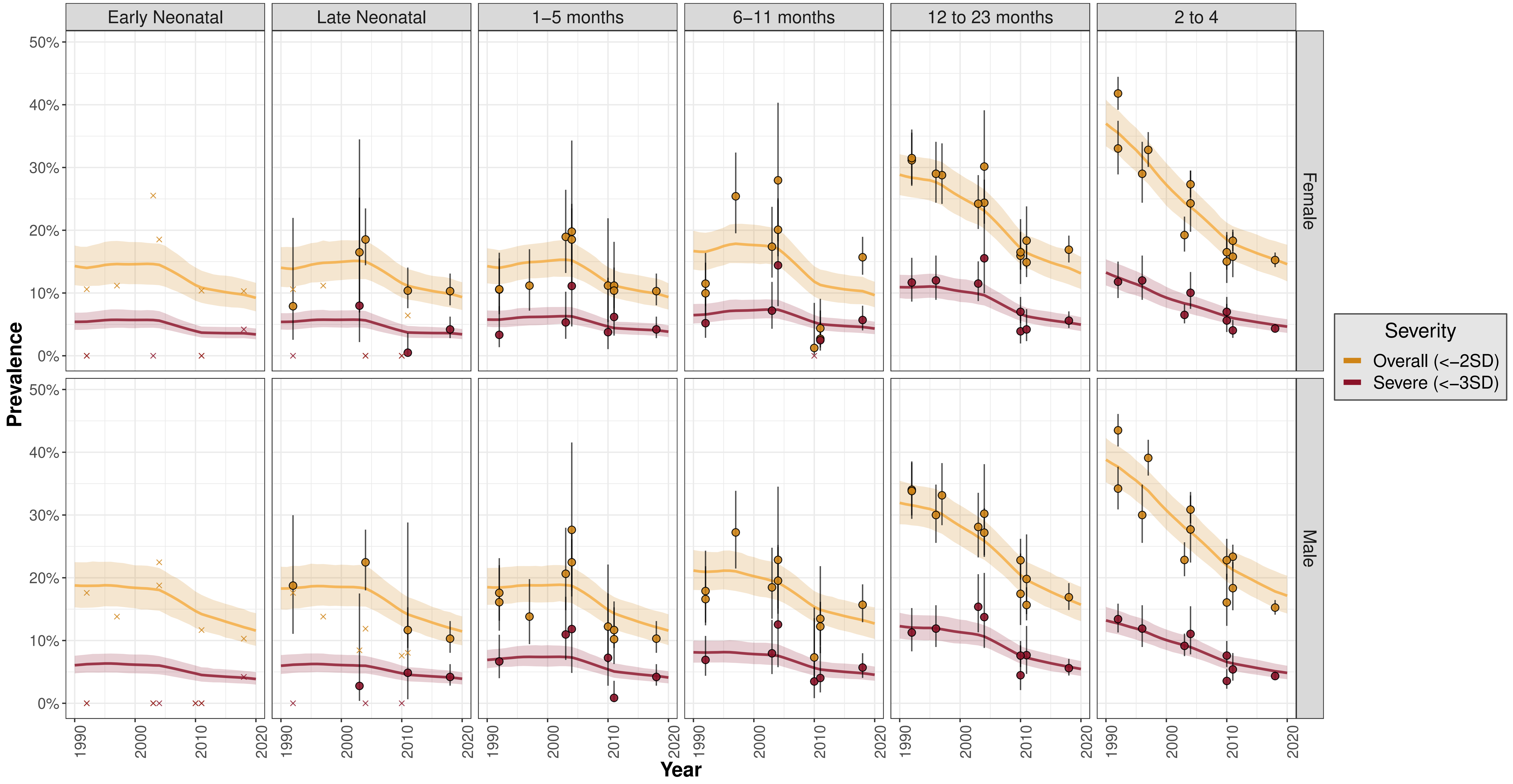

B: Transformed Mean Stunting Z Scores

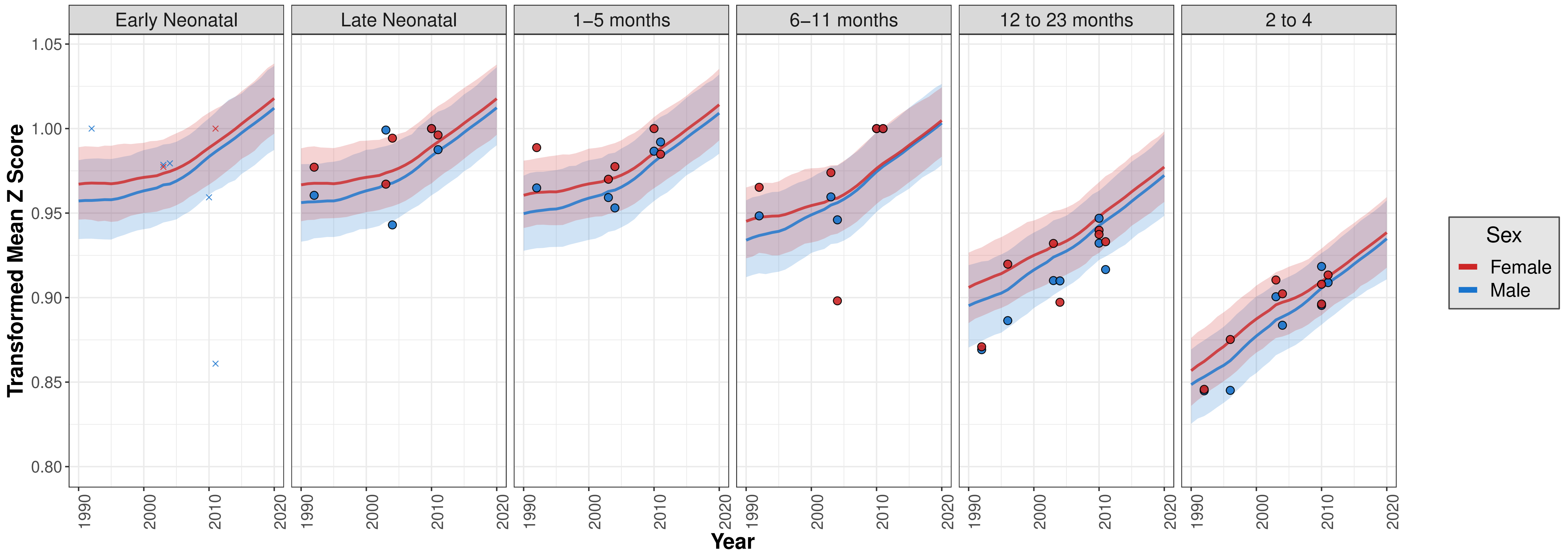

C

Year	Source
1987	DHS
1987	WHO CGM Database
1992	DHS
1992	WHO CGM Database
1996	WHO CGM Database
1997	WHO CGM Database
2003	DHS
2004	DHS
2004	WHO CGM Database
2010	National Survey on Population and Family Health
2010	WHO CGM Database
2011	National Survey on Population and Family Health
2011	WHO CGM Database
2018	National Survey on Population and Family Health

Morocco – Wasting (WHZ)

D: Overall and Severe Wasting Prevalence

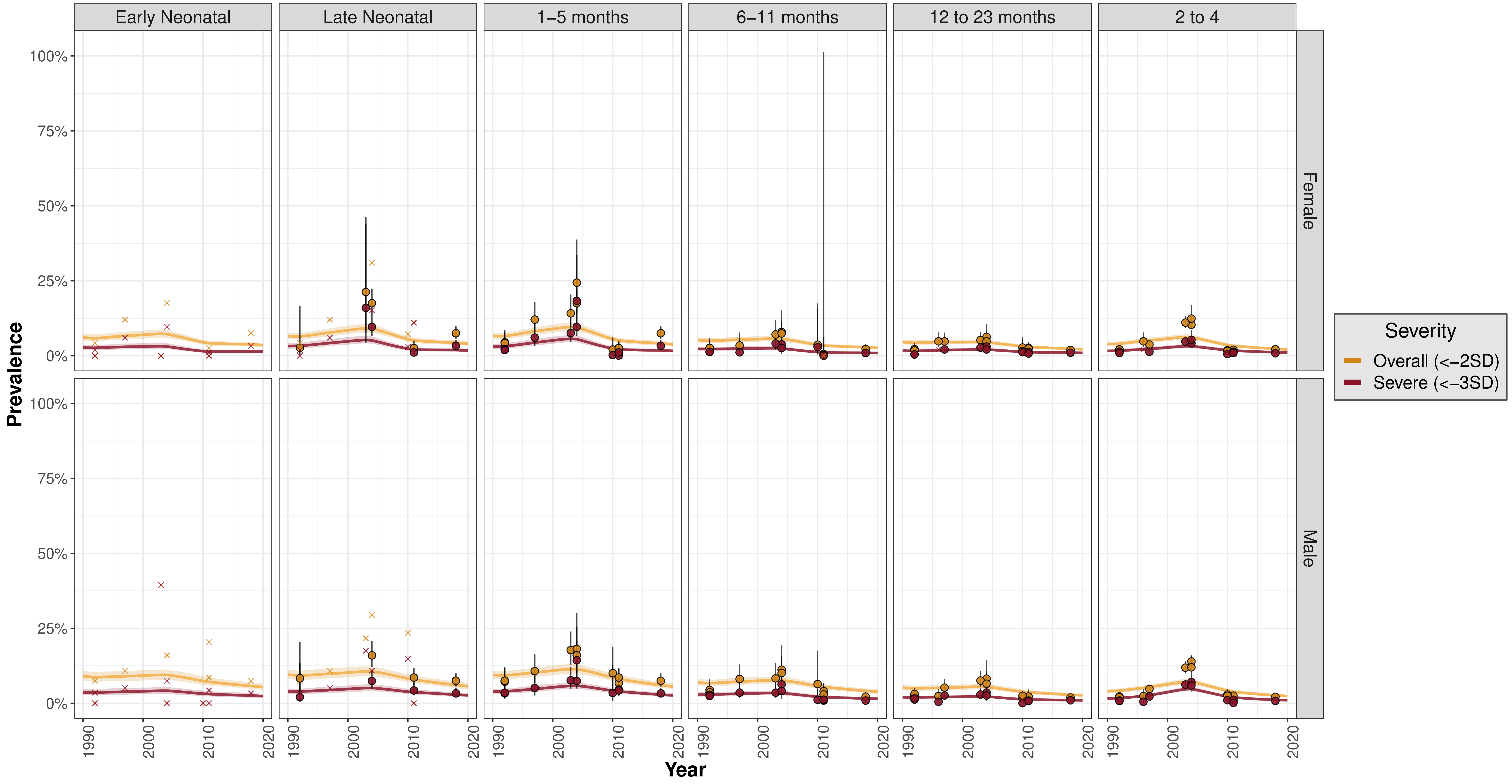

E: Transformed Mean Wasting Z Scores

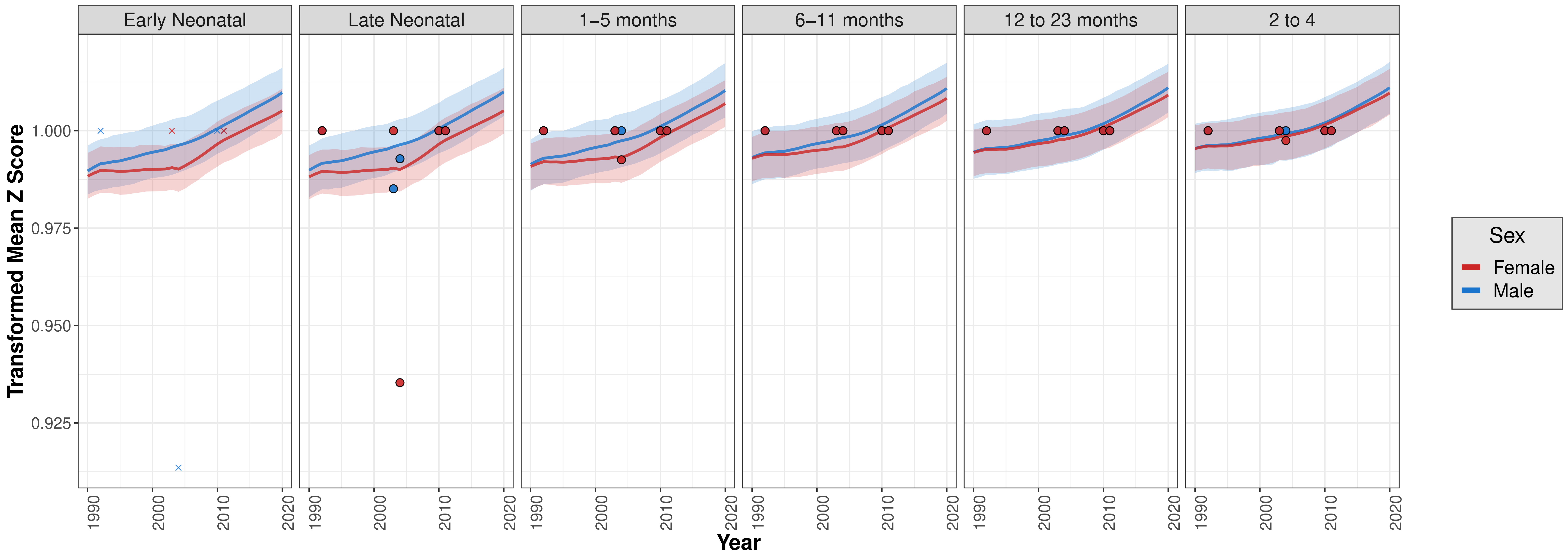

F

Year	Source
1987	DHS
1987	WHO CGM Database
1992	DHS
1992	WHO CGM Database
1996	WHO CGM Database
1997	WHO CGM Database
2003	DHS
2004	DHS
2004	WHO CGM Database
2010	National Survey on Population and Family Health
2010	WHO CGM Database
2011	National Survey on Population and Family Health
2011	WHO CGM Database
2018	National Survey on Population and Family Health

Morocco – Underweight (WAZ)

G: Overall and Severe Underweight Prevalence

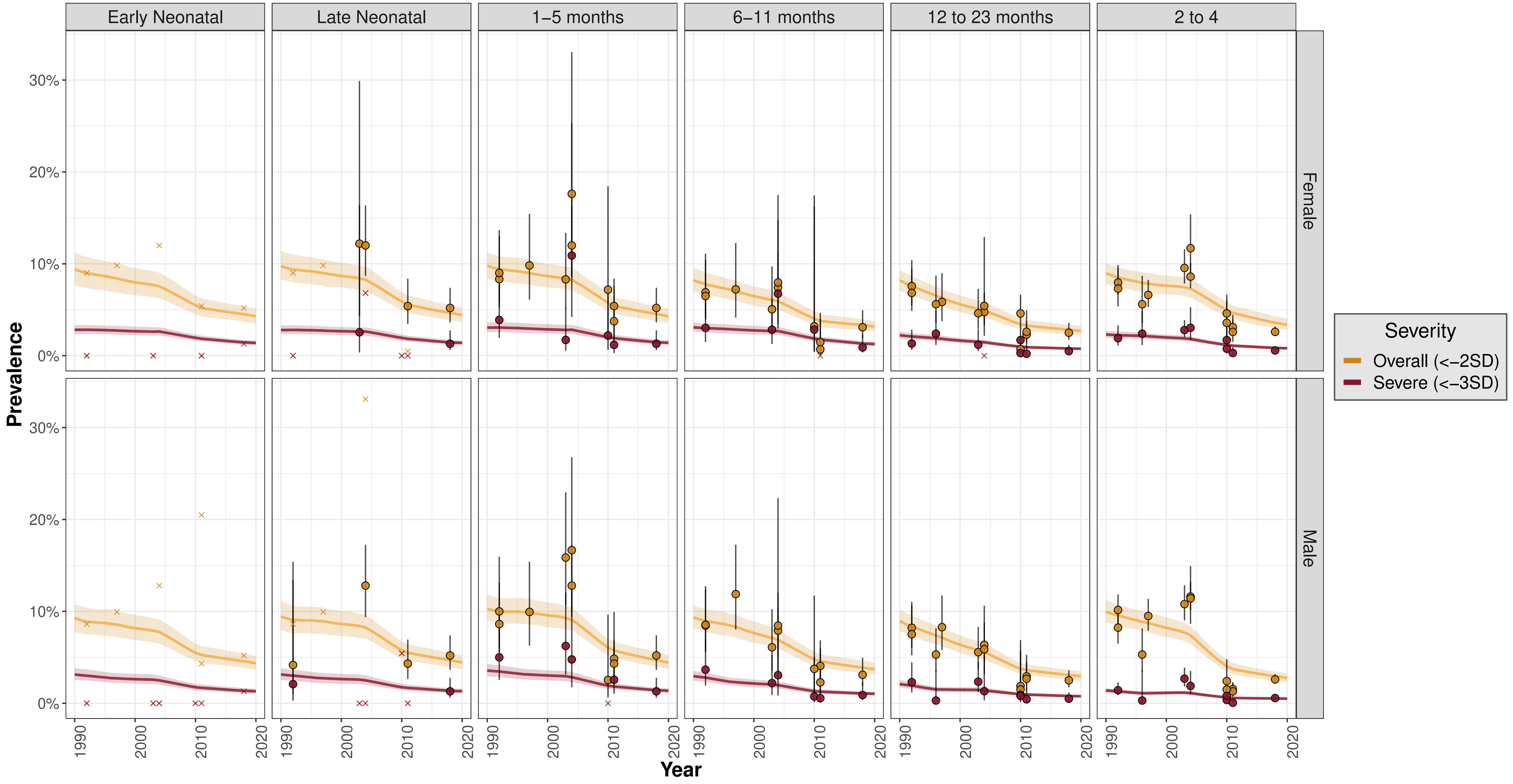

H: Transformed Mean Underweight Z Scores

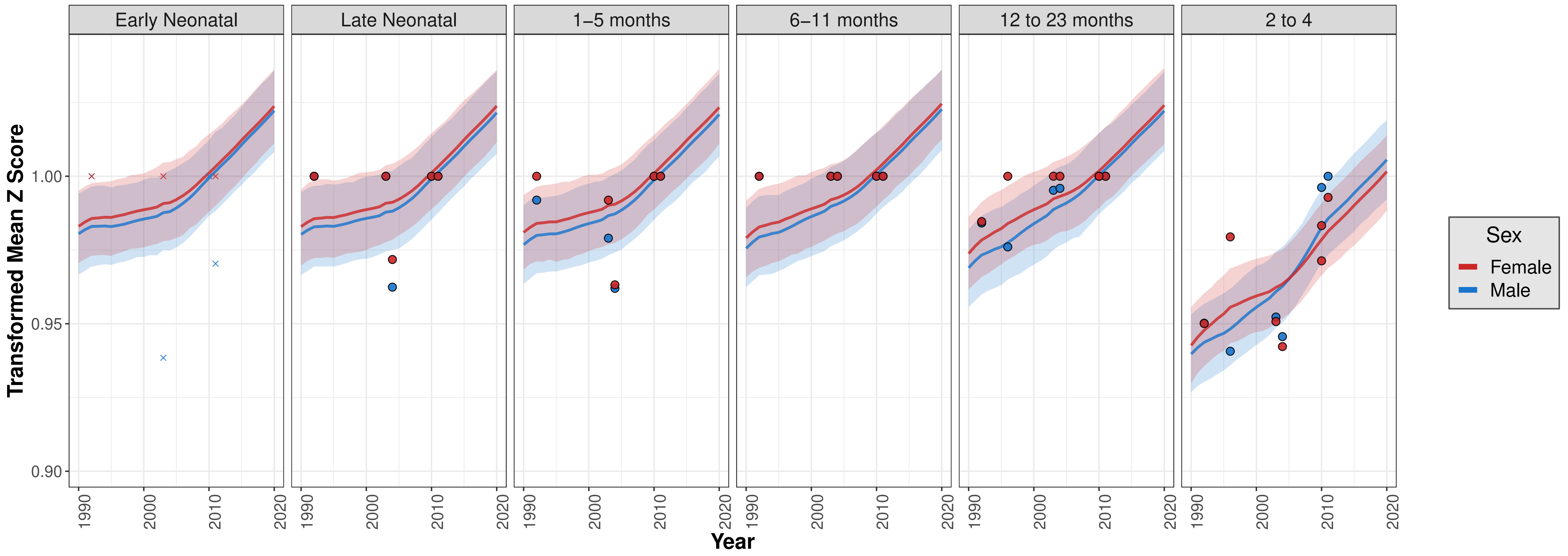

I

Year	Source
1987	DHS
1987	WHO CGM Database
1992	DHS
1992	WHO CGM Database
1996	WHO CGM Database
1997	WHO CGM Database
2003	DHS
2004	DHS
2004	WHO CGM Database
2010	National Survey on Population and Family Health
2010	WHO CGM Database
2011	National Survey on Population and Family Health
2011	WHO CGM Database
2018	National Survey on Population and Family Health

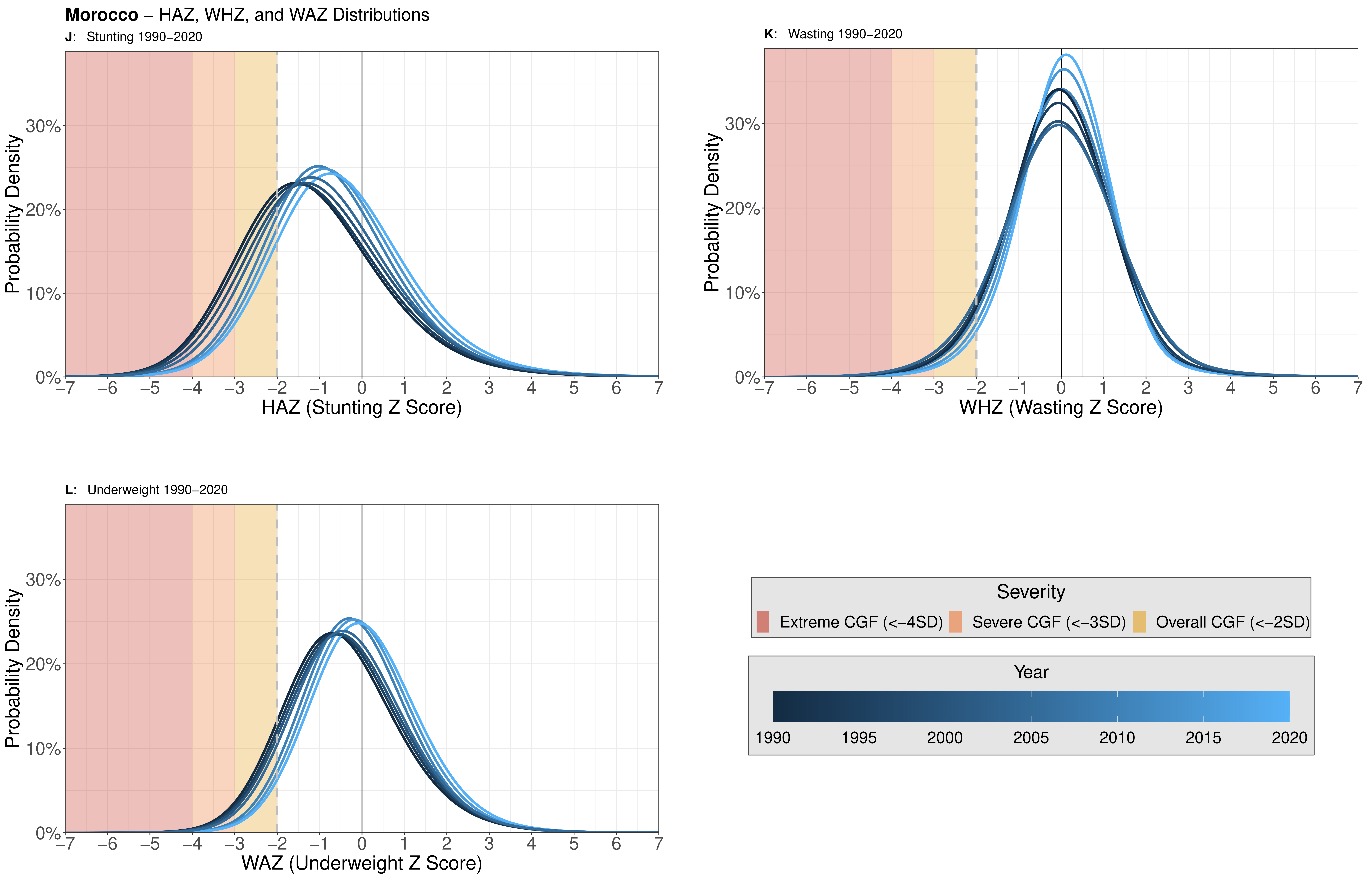

Morocco – HAZ, WHZ, and WAZ Distributions
J: Stunting 1990–2020
Probability Density
0%
10%
20%
30%
−7 −6 −5 −4 −3 −2 −1 0 1 2 3 4 5 6 7
HAZ (Stunting Z Score)
K: Wasting 1990–2020
Probability Density
0%
10%
20%
30%
−7 −6 −5 −4 −3 −2 −1 0 1 2 3 4 5 6 7
WHZ (Wasting Z Score)
L: Underweight 1990–2020
Probability Density
0%
10%
20%
30%
−7 −6 −5 −4 −3 −2 −1 0 1 2 3 4 5 6 7
WAZ (Underweight Z Score)
Severity
Extreme CGF (<−4SD)
Severe CGF (<−3SD)
Overall CGF (<−2SD)
Year
1990
1995
2000
2005
2010
2015
2020

Palestine – Stunting (HAZ)

A: Overall and Severe Stunting Prevalence

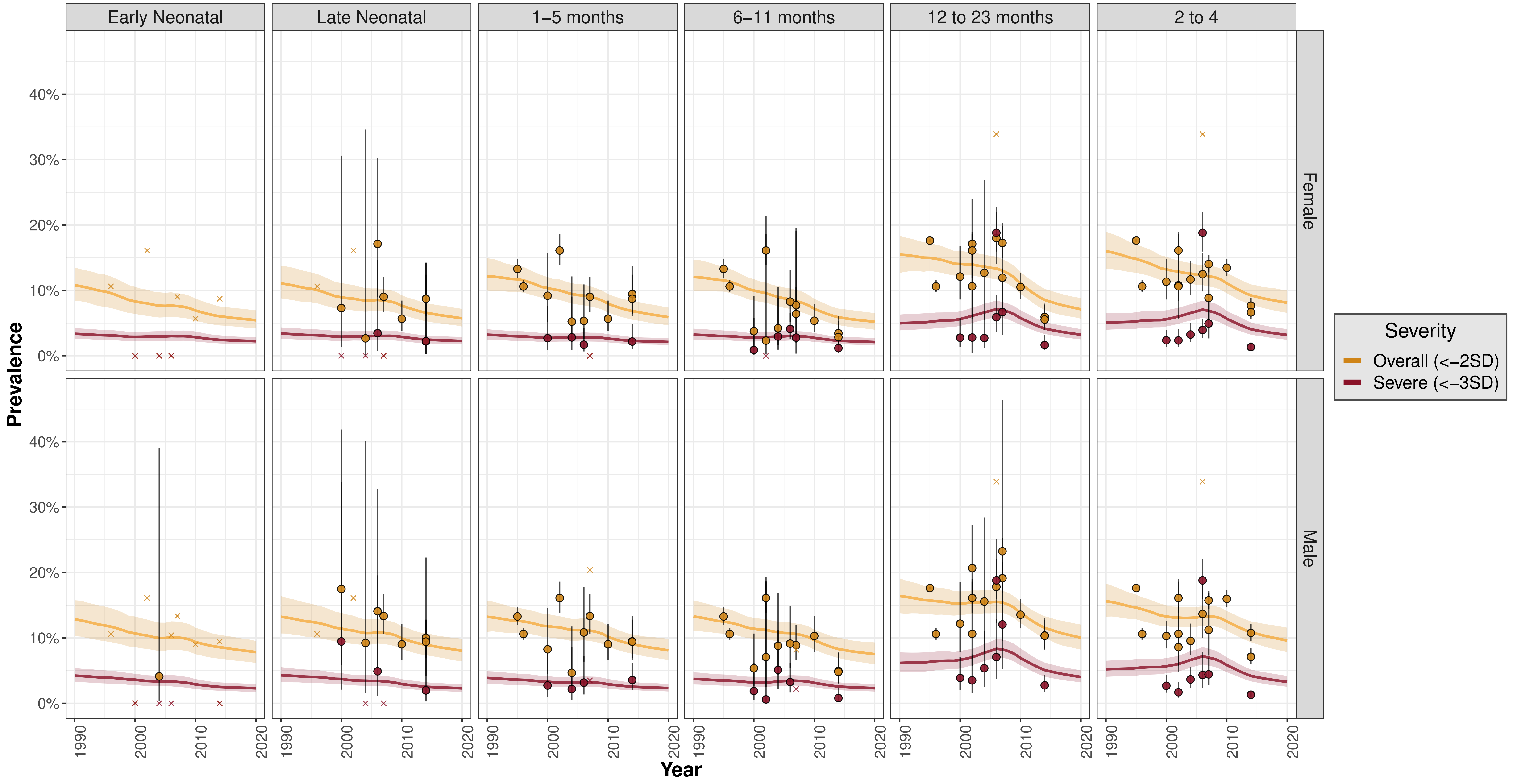

B: Transformed Mean Stunting Z Scores

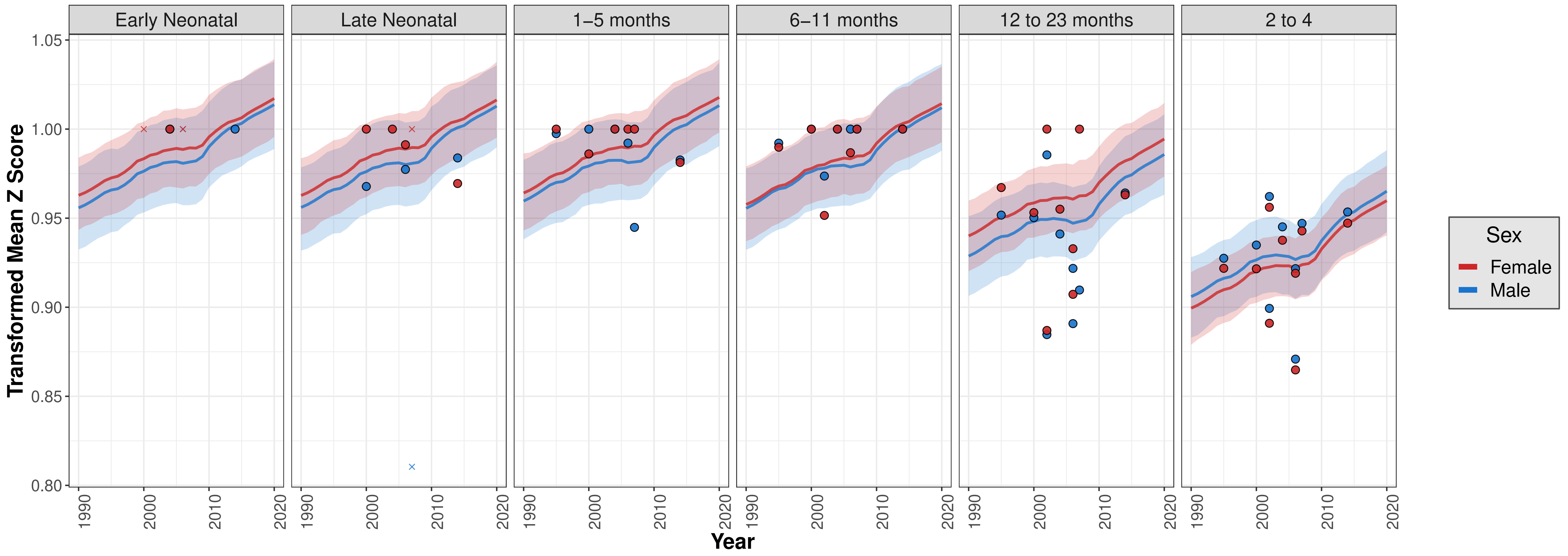

C

Year	Source
1995	WHO CGM Database
1996	WHO CGM Database
2000	West Bank and Gaza Strip MICS
2002	Nutrition Survey
2002	WHO CGM Database
2002	West Bank and Gaza Strip Rapid Nutritional Assessment
2004	DHS
2006	Family Health Survey
2006	Palestinians in Syria MICS
2007	Family Health Survey
2007	WHO CGM Database
2010	MICS
2010	WHO CGM Database
2014	WHO CGM Database
2014	MICS

Palestine – Wasting (WHZ)

D: Overall and Severe Wasting Prevalence

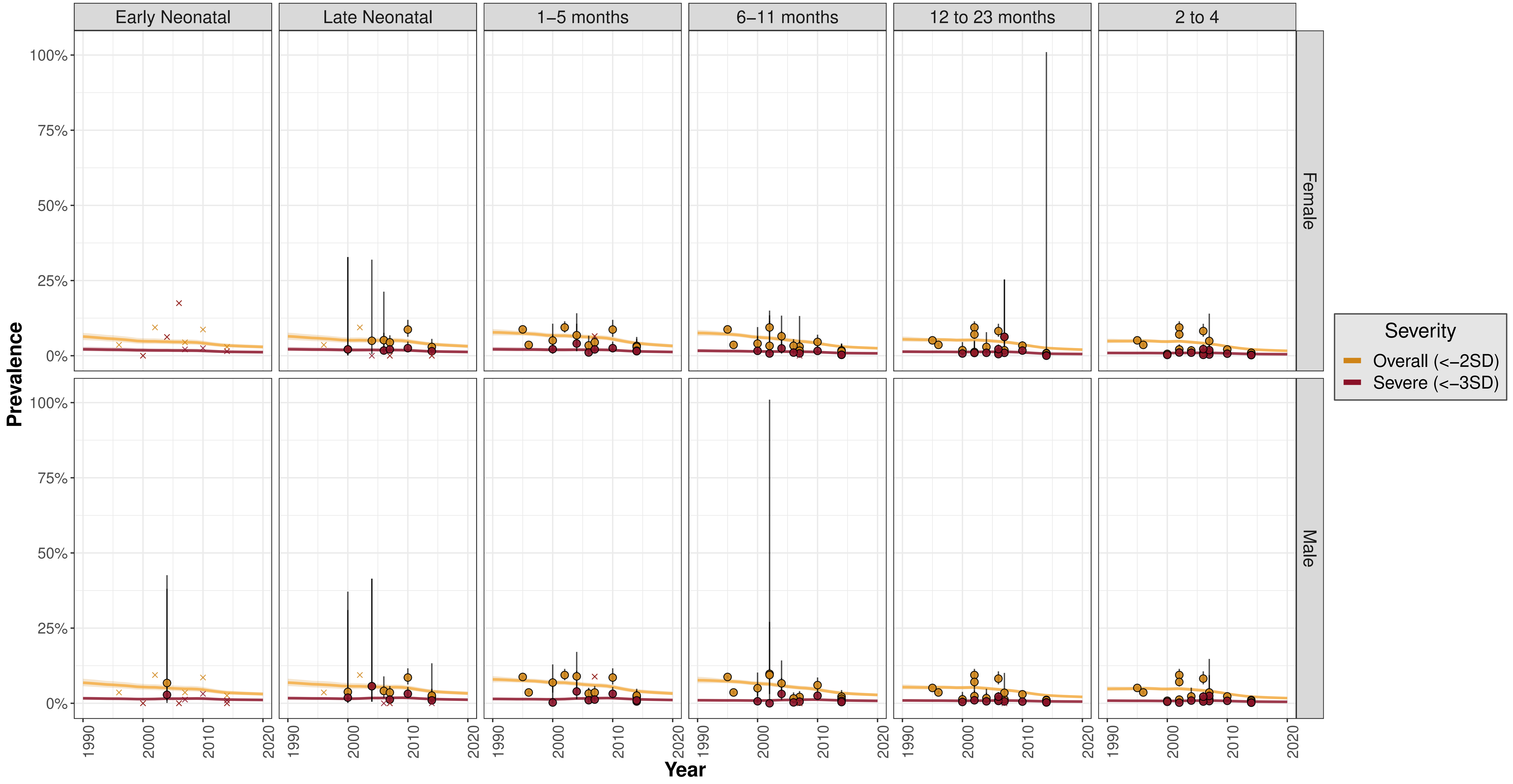

E: Transformed Mean Wasting Z Scores

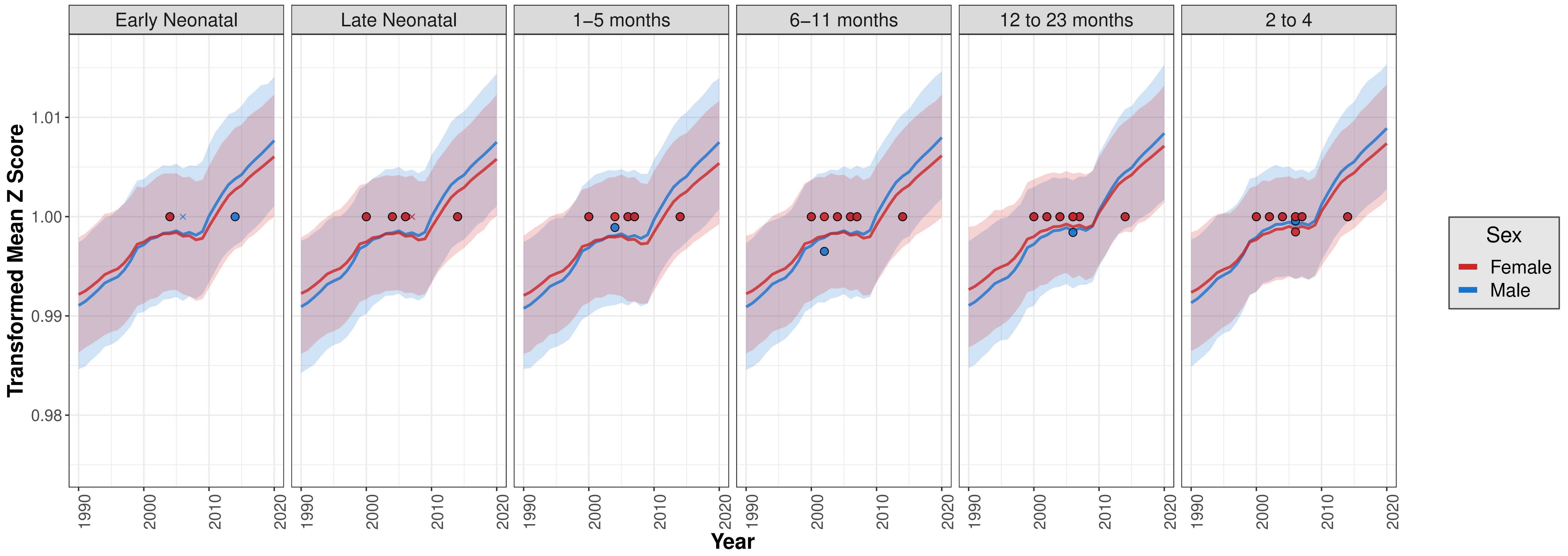

F

Year	Source
1995	WHO CGM Database
1996	WHO CGM Database
2000	West Bank and Gaza Strip MICS
2002	Nutrition Survey
2002	WHO CGM Database
2002	West Bank and Gaza Strip Rapid Nutritional Assessment
2004	DHS
2006	Family Health Survey
2006	Palestinians in Syria MICS
2007	Family Health Survey
2007	WHO CGM Database
2010	MICS
2010	WHO CGM Database
2014	WHO CGM Database
2014	MICS

Palestine – Underweight (WAZ)

G: Overall and Severe Underweight Prevalence

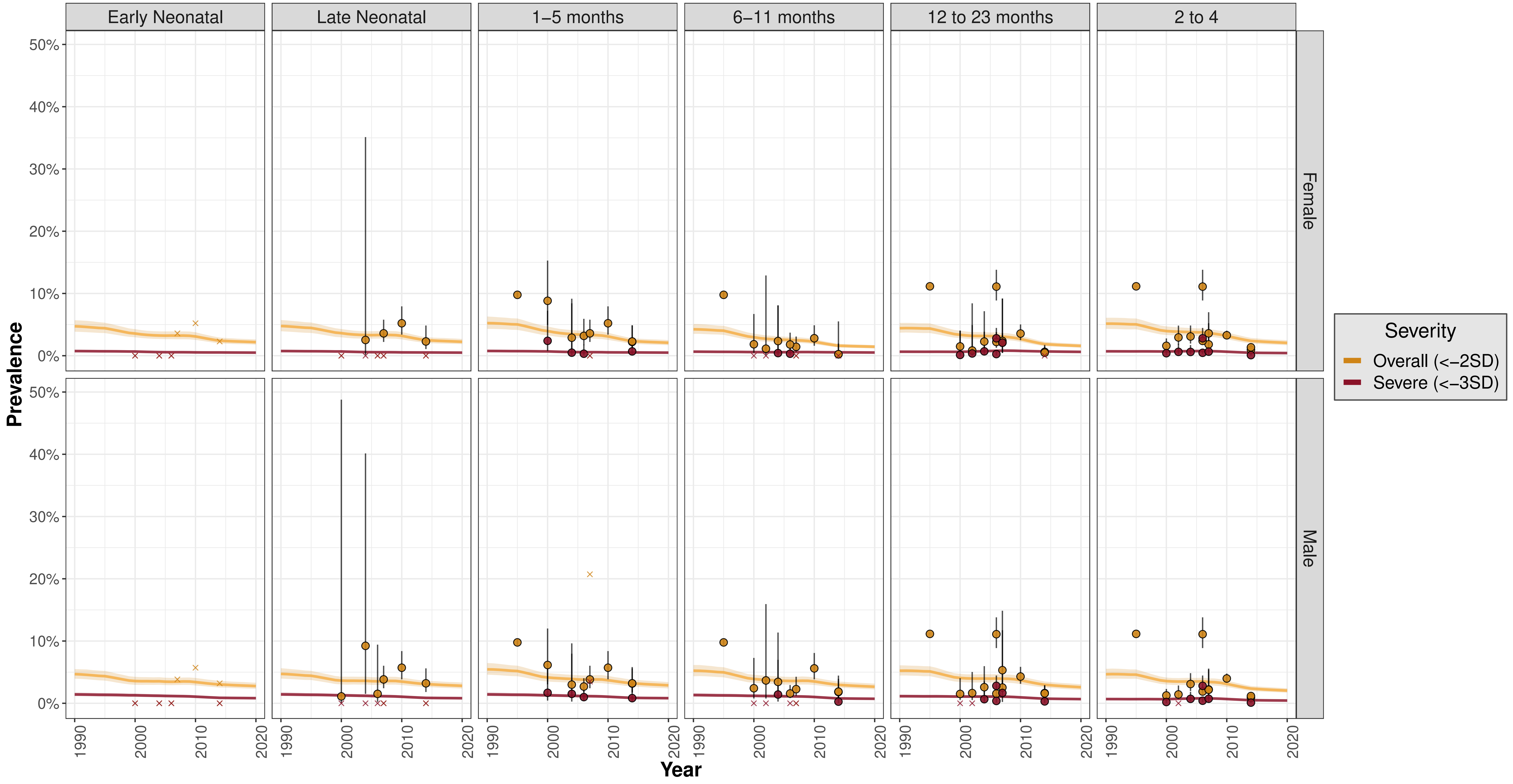

H: Transformed Mean Underweight Z Scores

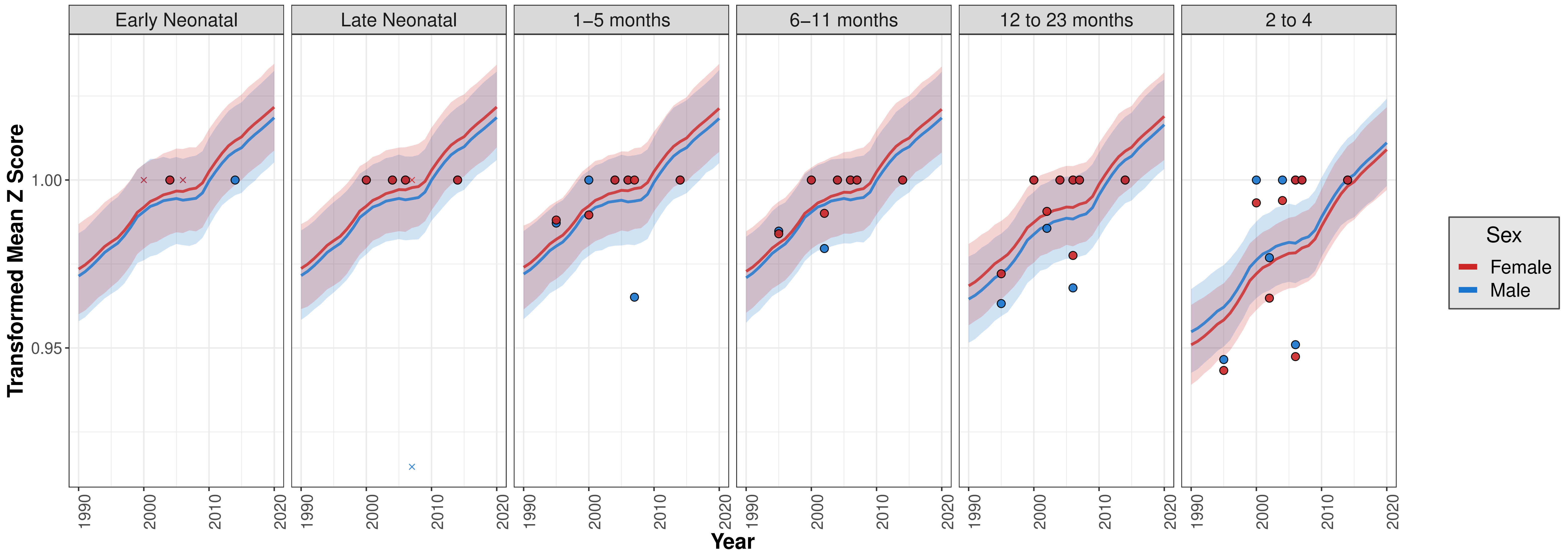

I

Year	Source
1995	WHO CGM Database
2000	West Bank and Gaza Strip MICS
2002	Nutrition Survey
2004	DHS
2006	Family Health Survey
2006	Palestinians in Syria MICS
2007	Family Health Survey
2007	WHO CGM Database
2010	MICS
2010	WHO CGM Database
2014	WHO CGM Database
2014	MICS

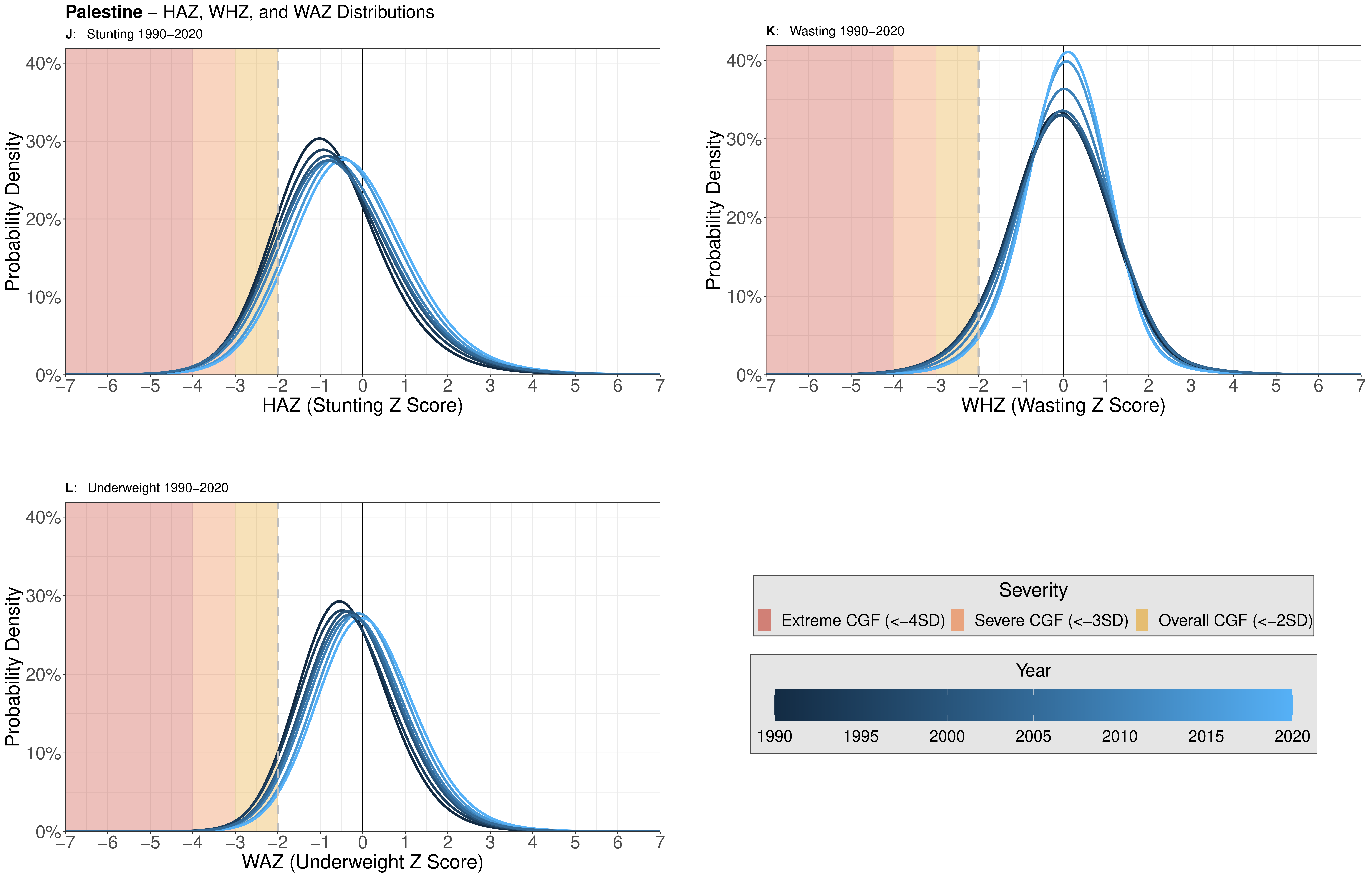
Palestine – HAZ, WHZ, and WAZ Distributions
J: Stunting 1990–2020
Probability Density
HAZ (Stunting Z Score)
K: Wasting 1990–2020
Probability Density
WHZ (Wasting Z Score)
L: Underweight 1990–2020
Probability Density
WAZ (Underweight Z Score)
0%
10%
20%
30%
40%
−7 −6 −5 −4 −3 −2 −1 0 1 2 3 4 5 6 7
Severity
Extreme CGF (<−4SD)
Severe CGF (<−3SD)
Overall CGF (<−2SD)
Year
1990
1995
2000
2005
2010
2015
2020

Oman – Stunting (HAZ)

A: Overall and Severe Stunting Prevalence

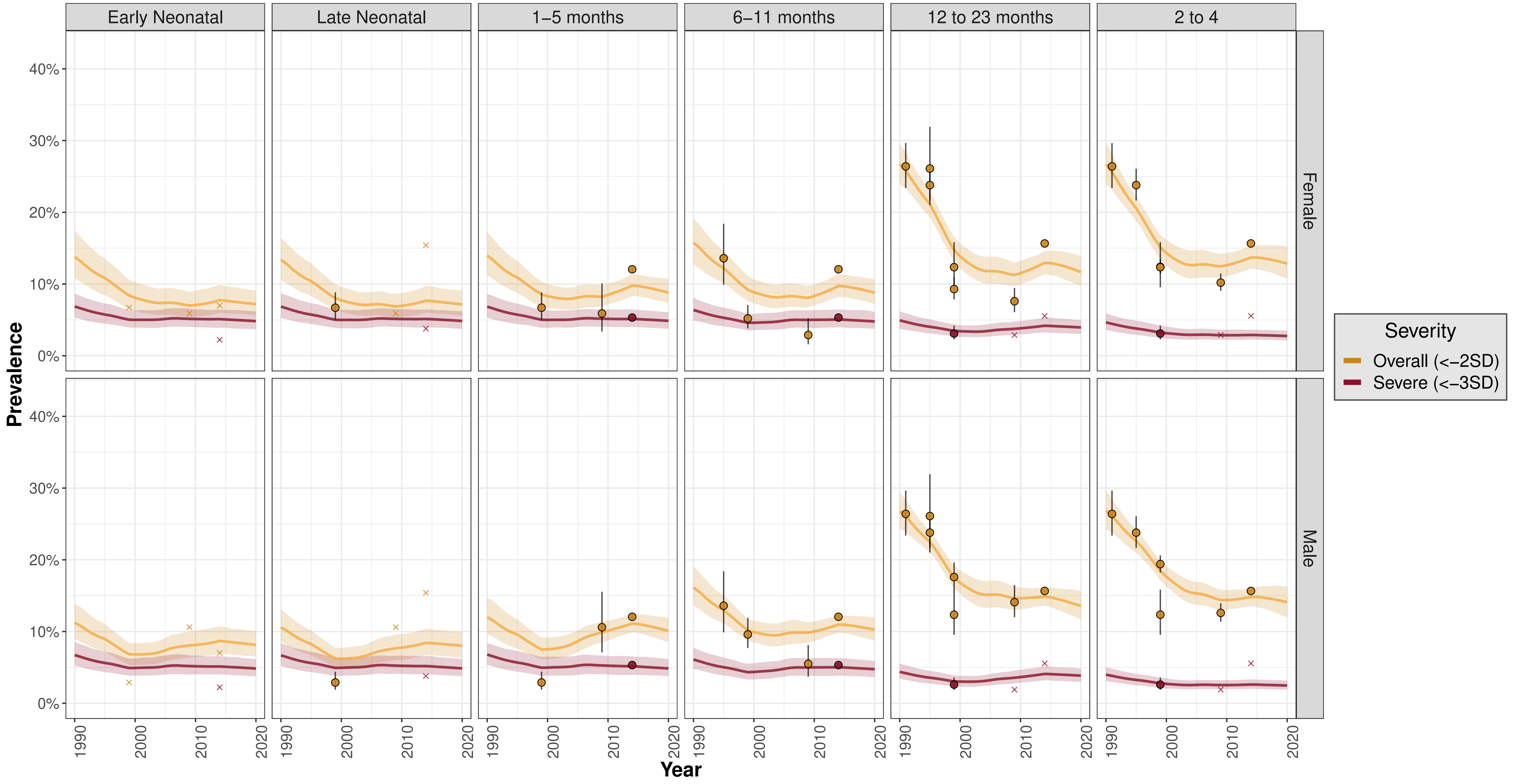

B: Transformed Mean Stunting Z Scores

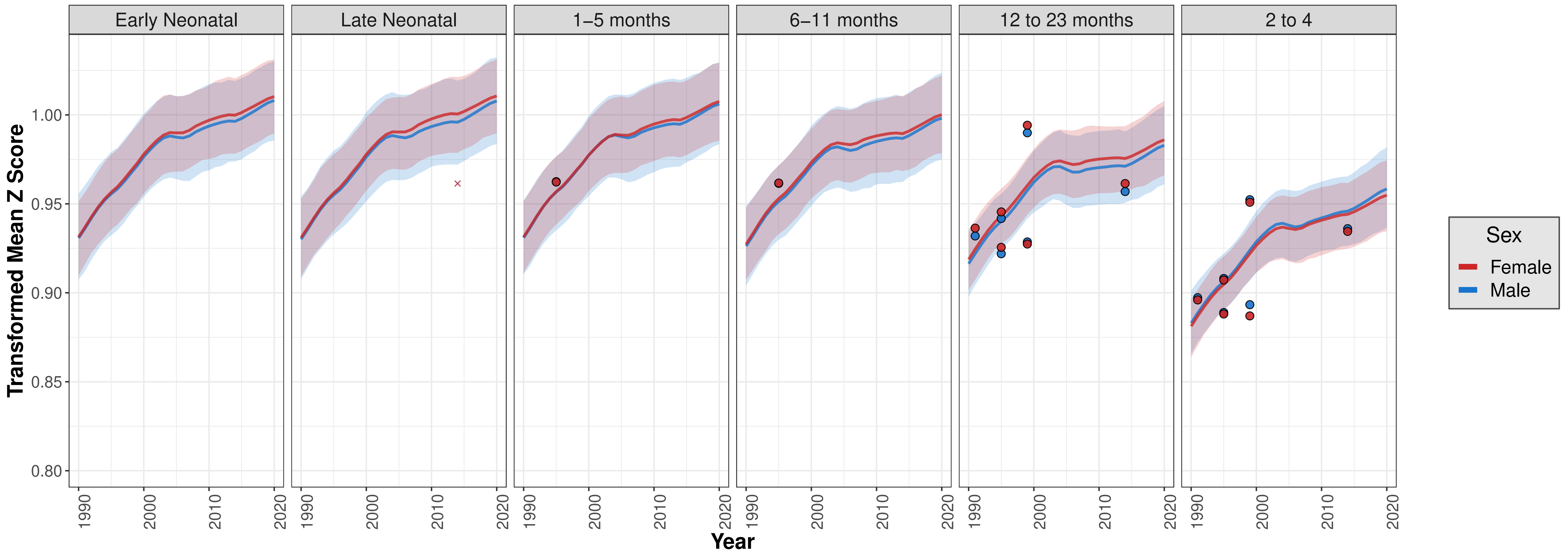

C

Year	Source
1991	WHO CGM Database
1995	WHO CGM Database
1995	Towards the Year Goals of the World Summit for Children: Report on Achievement of Mid–decade Goals in
1999	Protein–Energy Malnutrition Survey
1999	WHO CGM Database
2009	WHO CGM Database
2014	MICS

Oman – Wasting (WHZ)

D: Overall and Severe Wasting Prevalence

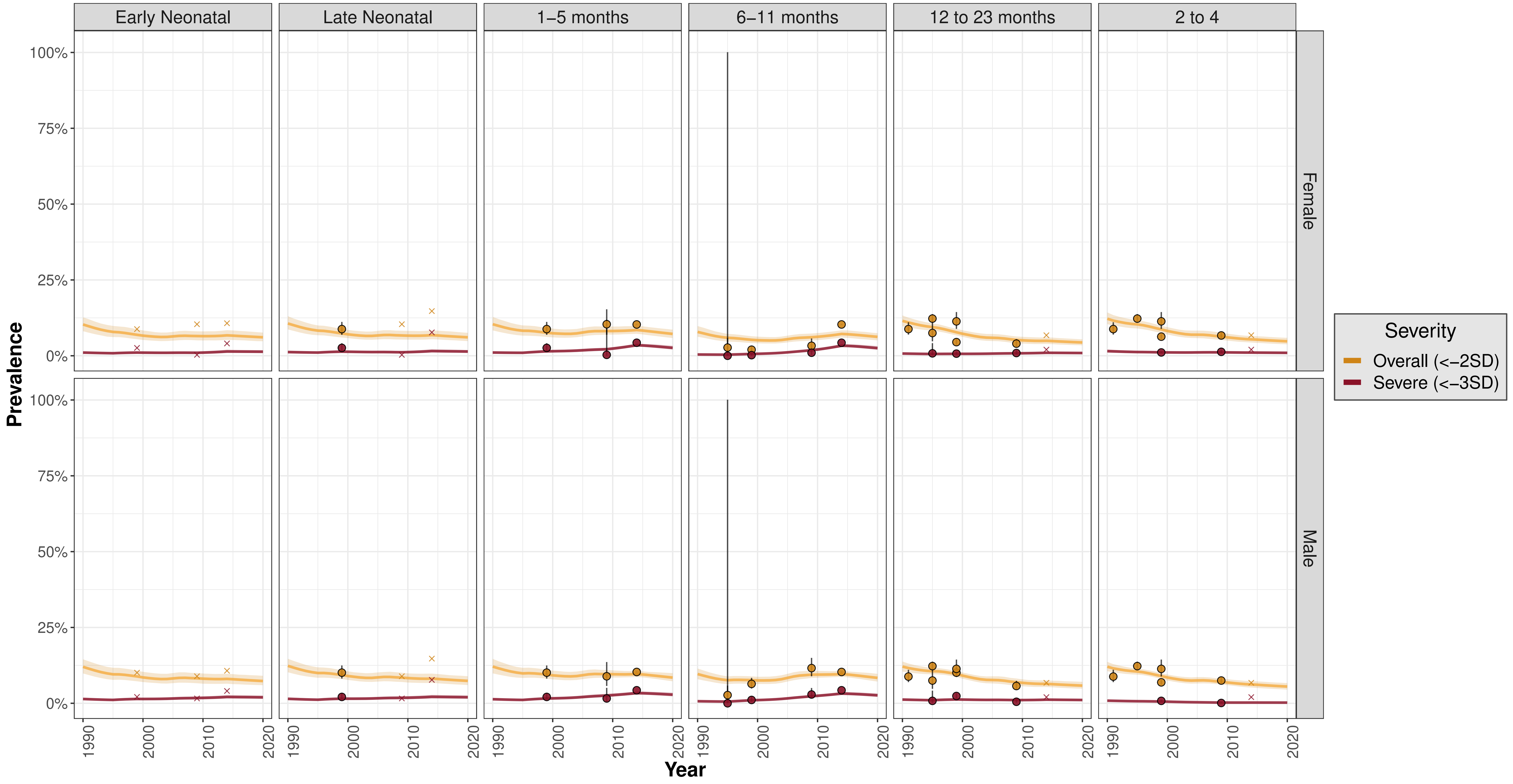

E: Transformed Mean Wasting Z Scores

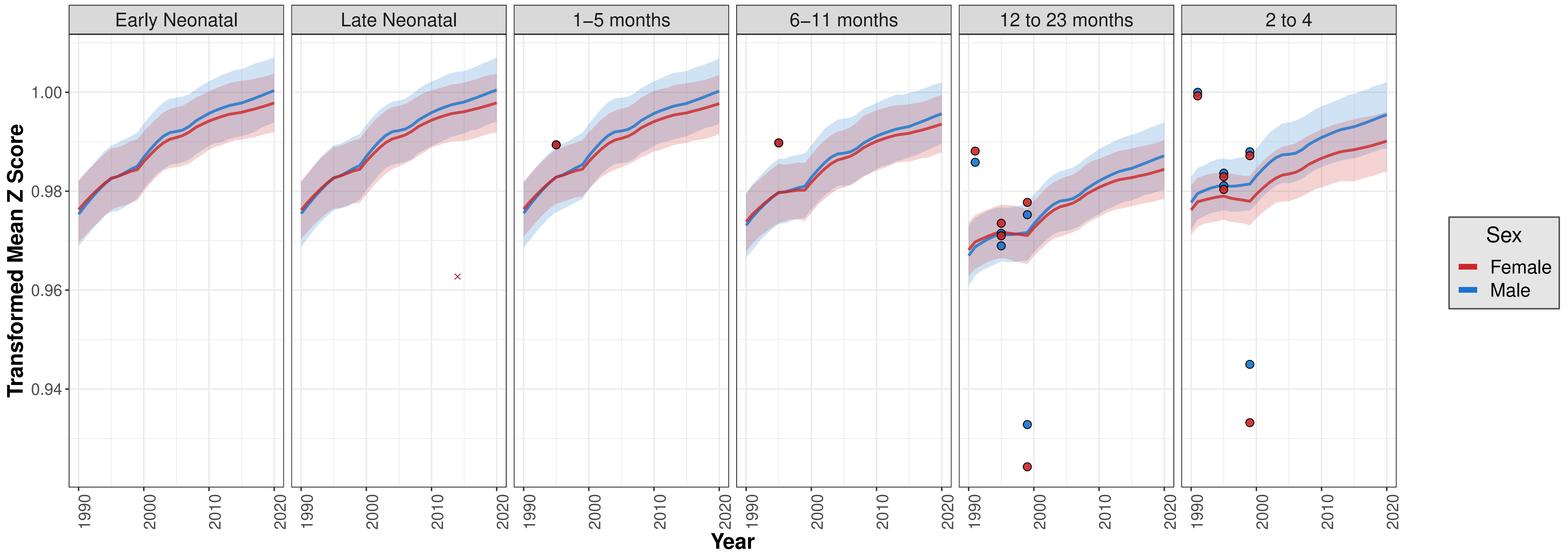

F

Year	Source
1991	WHO CGM Database
1995	WHO CGM Database
1995	Towards the Year Goals of the World Summit for Children: Report on Achievement of Mid–decade Goals in
1999	Protein–Energy Malnutrition Survey
1999	WHO CGM Database
2009	WHO CGM Database
2014	MICS

Oman – Underweight (WAZ)

G: Overall and Severe Underweight Prevalence

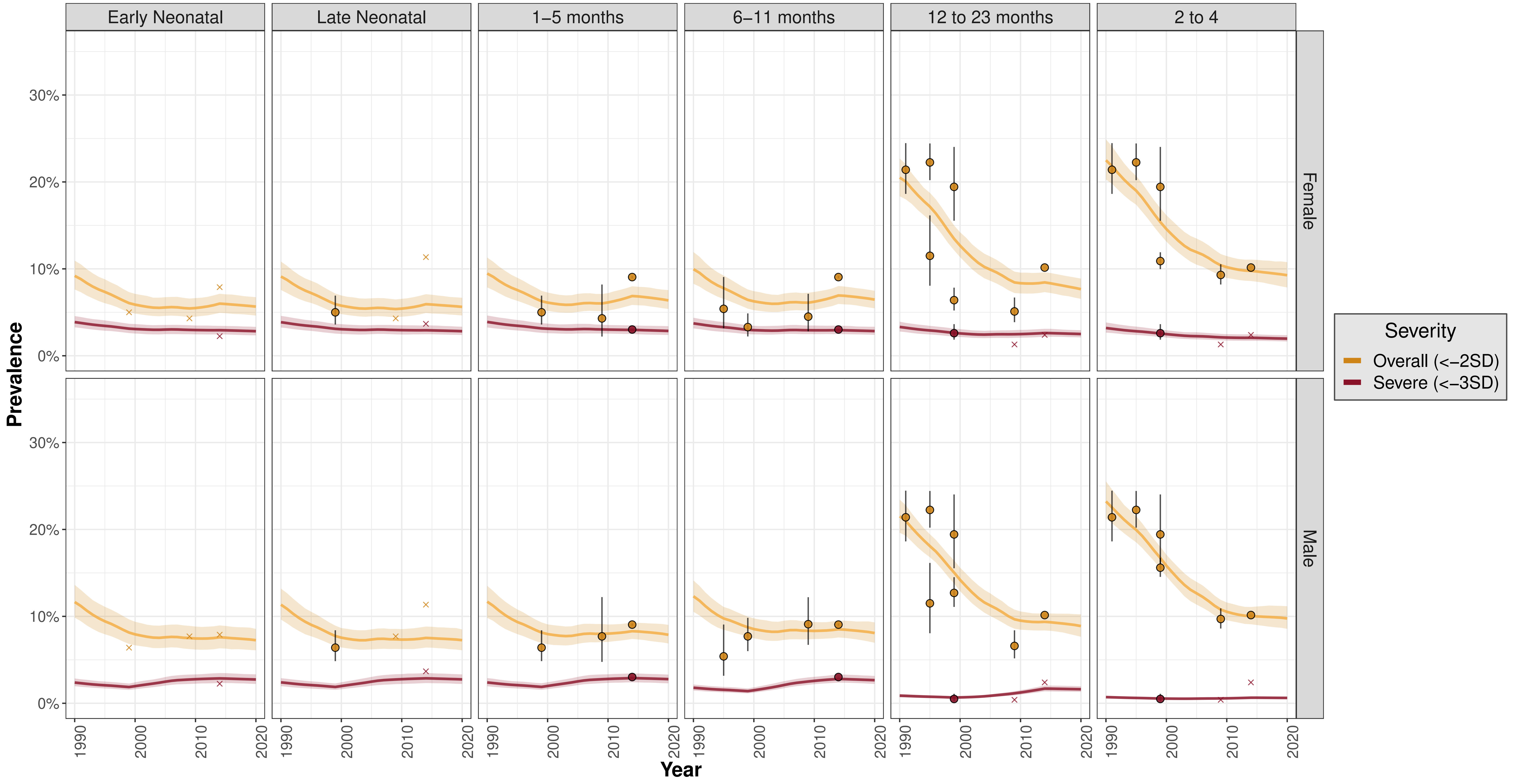

H: Transformed Mean Underweight Z Scores

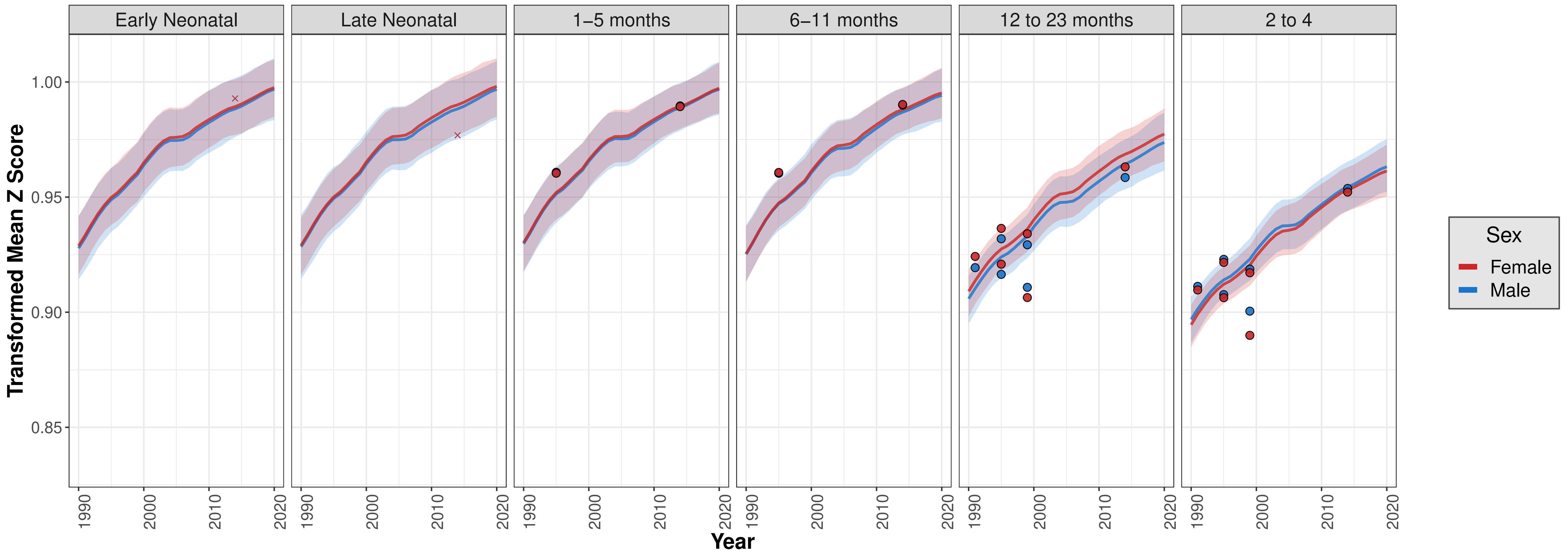

I

Year	Source
1991	WHO CGM Database
1995	WHO CGM Database
1995	Towards the Year Goals of the World Summit for Children: Report on Achievement of Mid–decade Goals in
1999	Protein–Energy Malnutrition Survey
1999	WHO CGM Database
2009	WHO CGM Database
2014	MICS

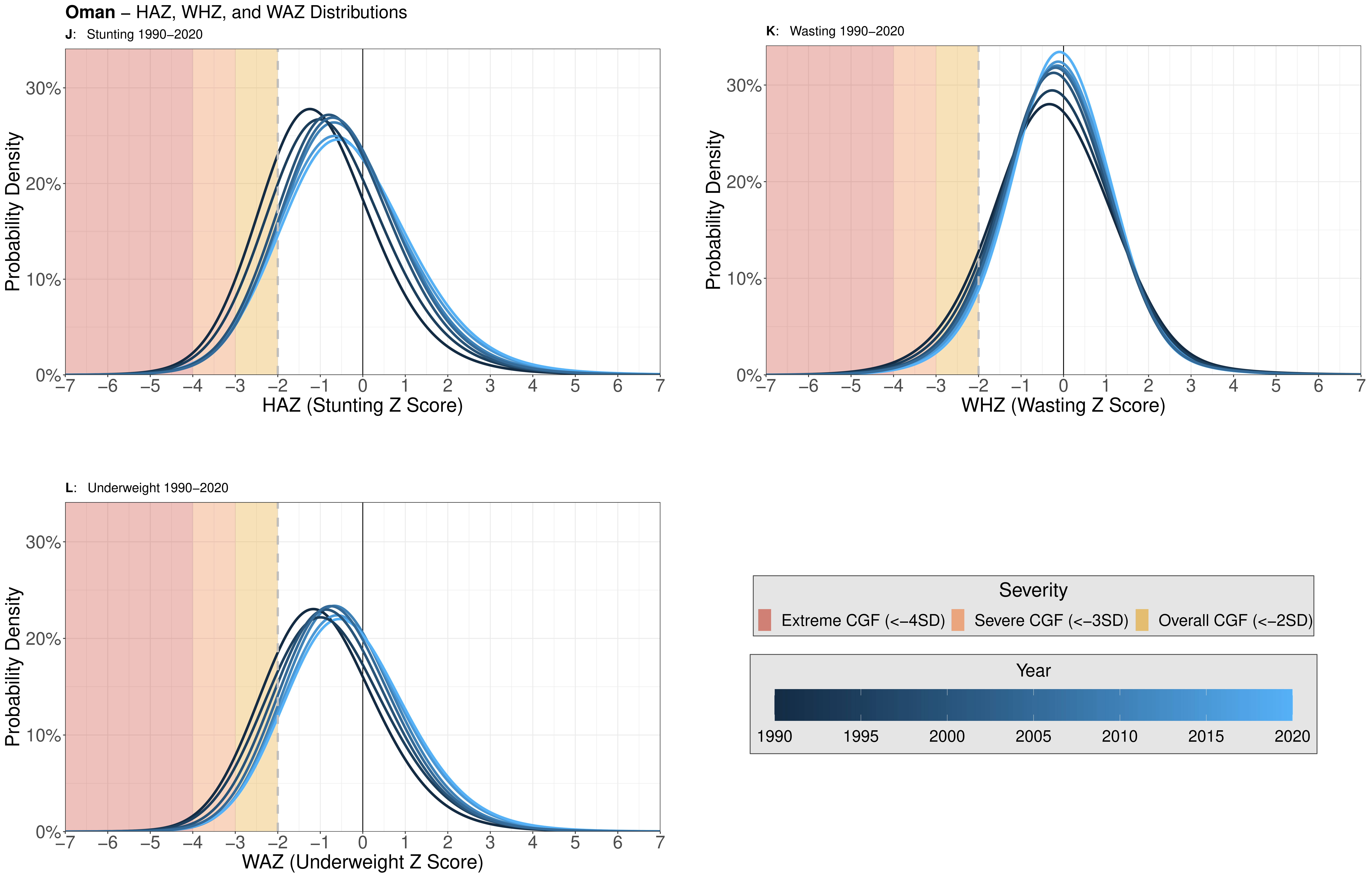

Oman – HAZ, WHZ, and WAZ Distributions
J: Stunting 1990–2020
Probability Density
30%
20%
10%
0%
−7 −6 −5 −4 −3 −2 −1 0 1 2 3 4 5 6 7
HAZ (Stunting Z Score)
K: Wasting 1990–2020
Probability Density
WHZ (Wasting Z Score)
L: Underweight 1990–2020
Probability Density
WAZ (Underweight Z Score)
Severity
Extreme CGF (<−4SD)
Severe CGF (<−3SD)
Overall CGF (<−2SD)
Year
1990
1995
2000
2005
2010
2015
2020

Qatar – Stunting (HAZ)

A: Overall and Severe Stunting Prevalence

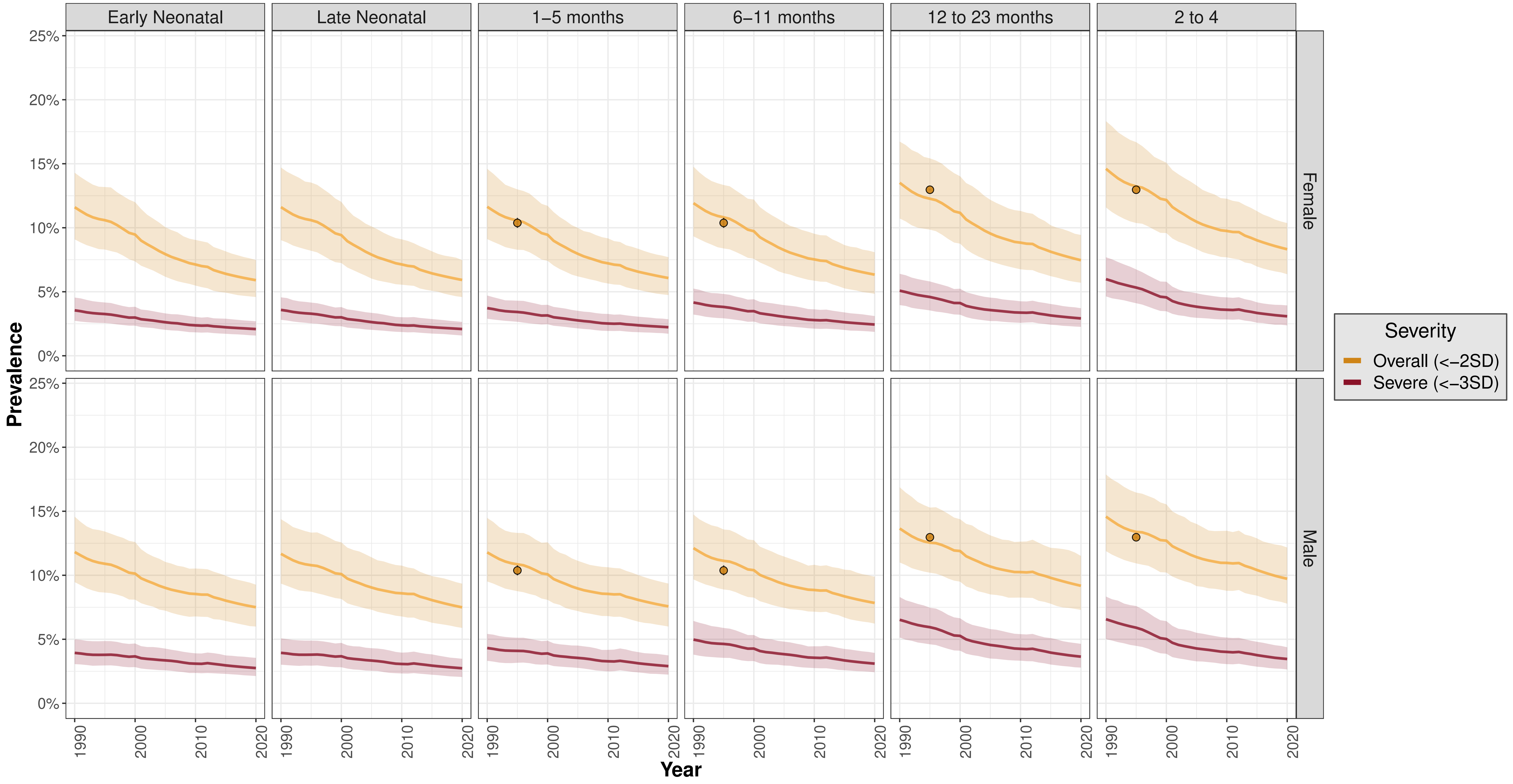

B: Transformed Mean Stunting Z Scores

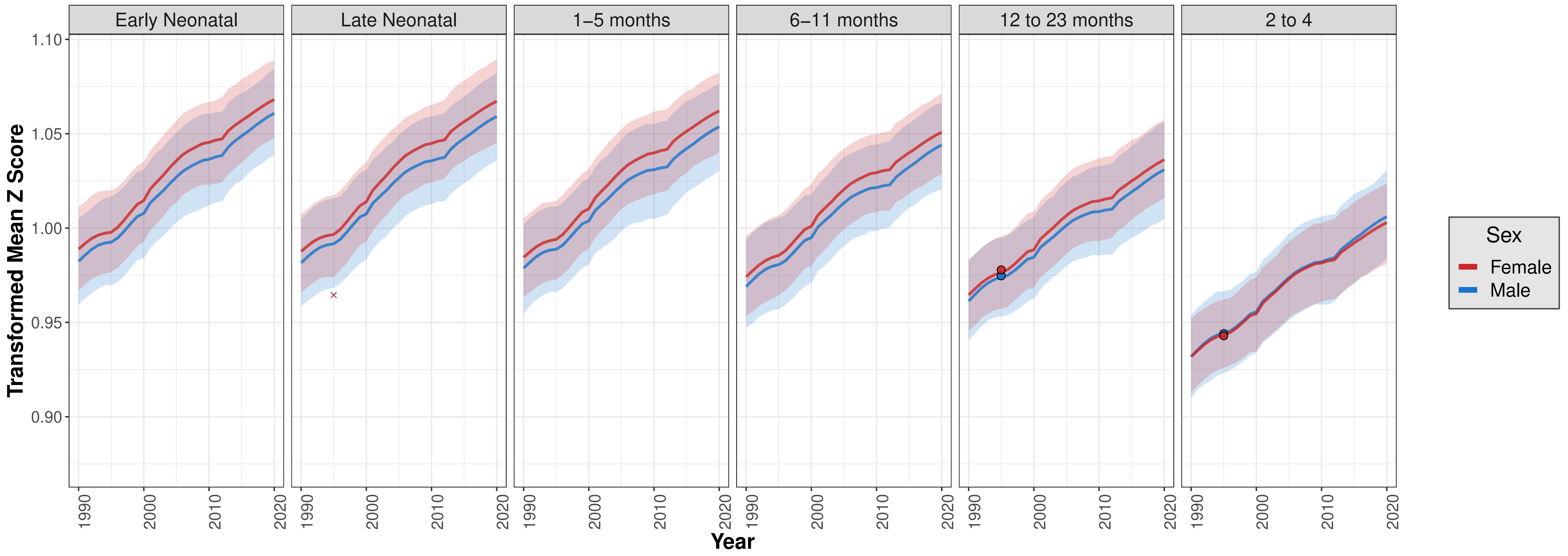

C

Year	Source
1995	WHO CGM Database

Qatar – Wasting (WHZ)

D: Overall and Severe Wasting Prevalence

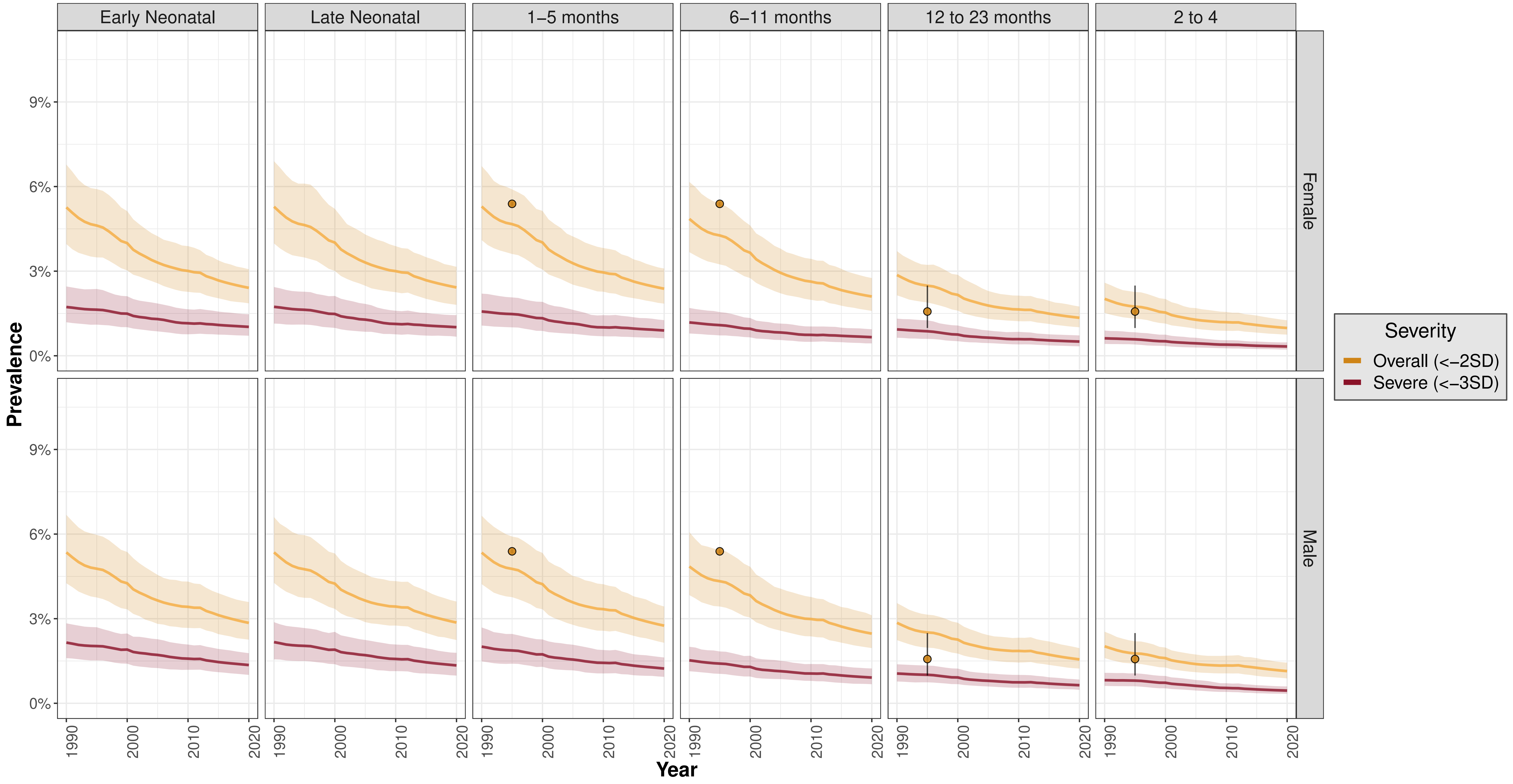

E: Transformed Mean Wasting Z Scores

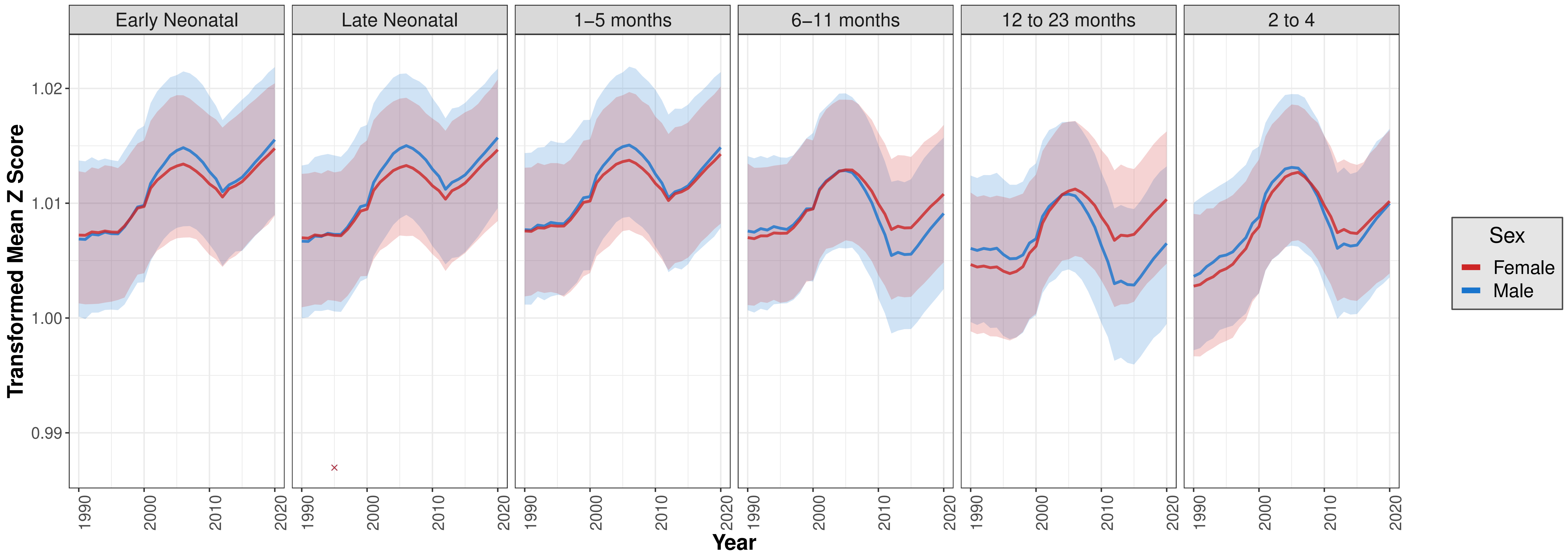

F

Year	Source
1995	WHO CGM Database

Qatar – Underweight (WAZ)

G: Overall and Severe Underweight Prevalence

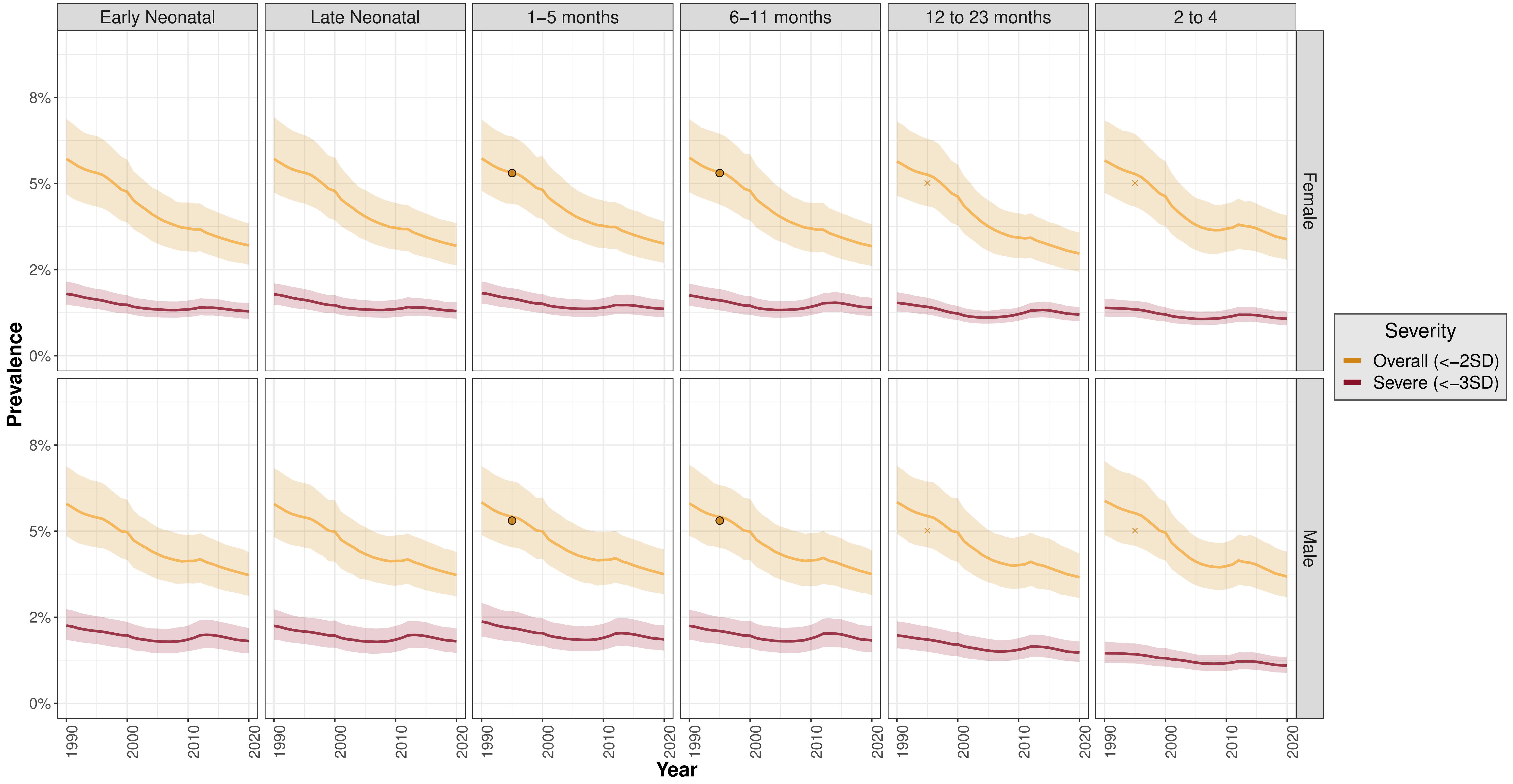

H: Transformed Mean Underweight Z Scores

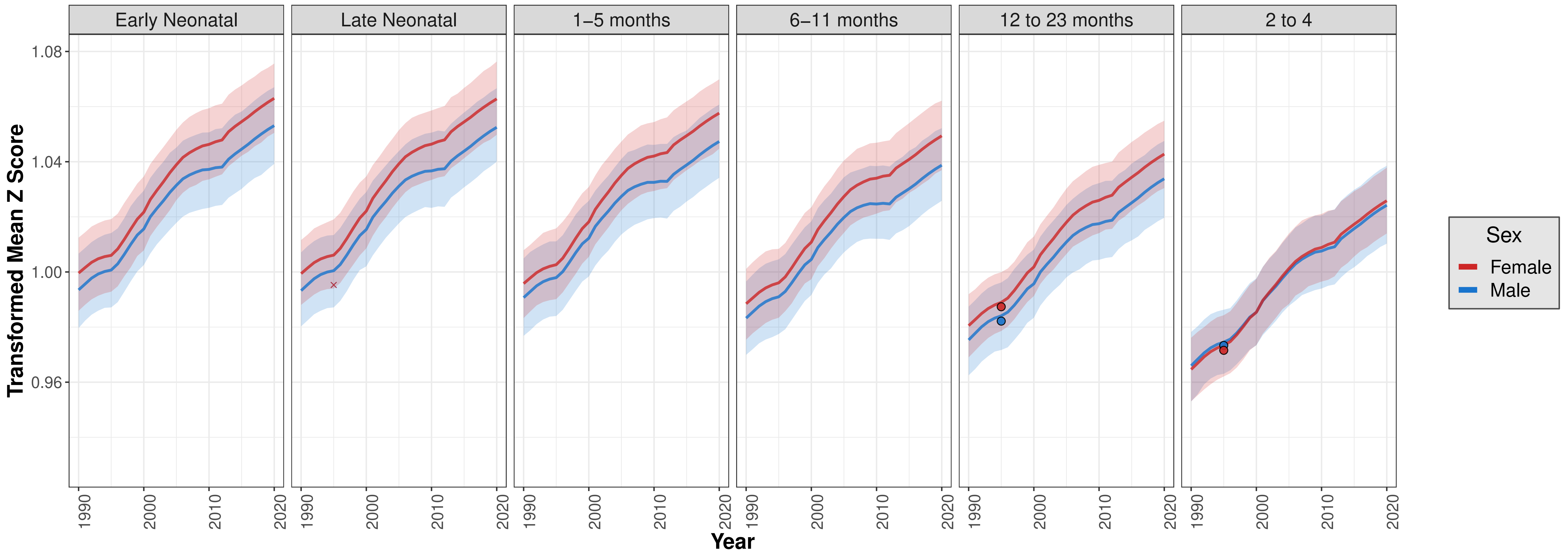

I

Year	Source
1995	WHO CGM Database

Qatar – HAZ, WHZ, and WAZ Distributions

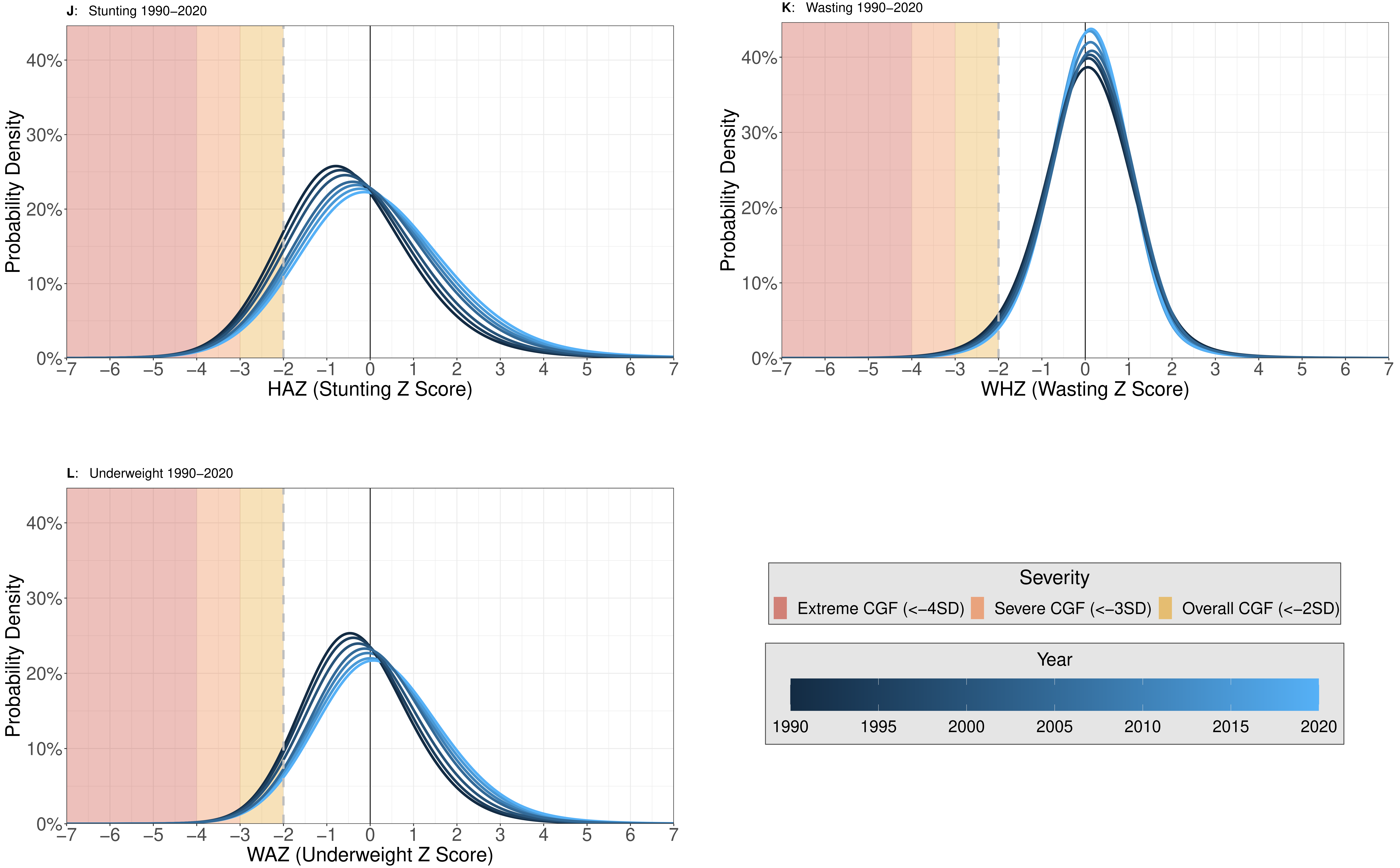

Saudi Arabia – Stunting (HAZ)

A: Overall and Severe Stunting Prevalence

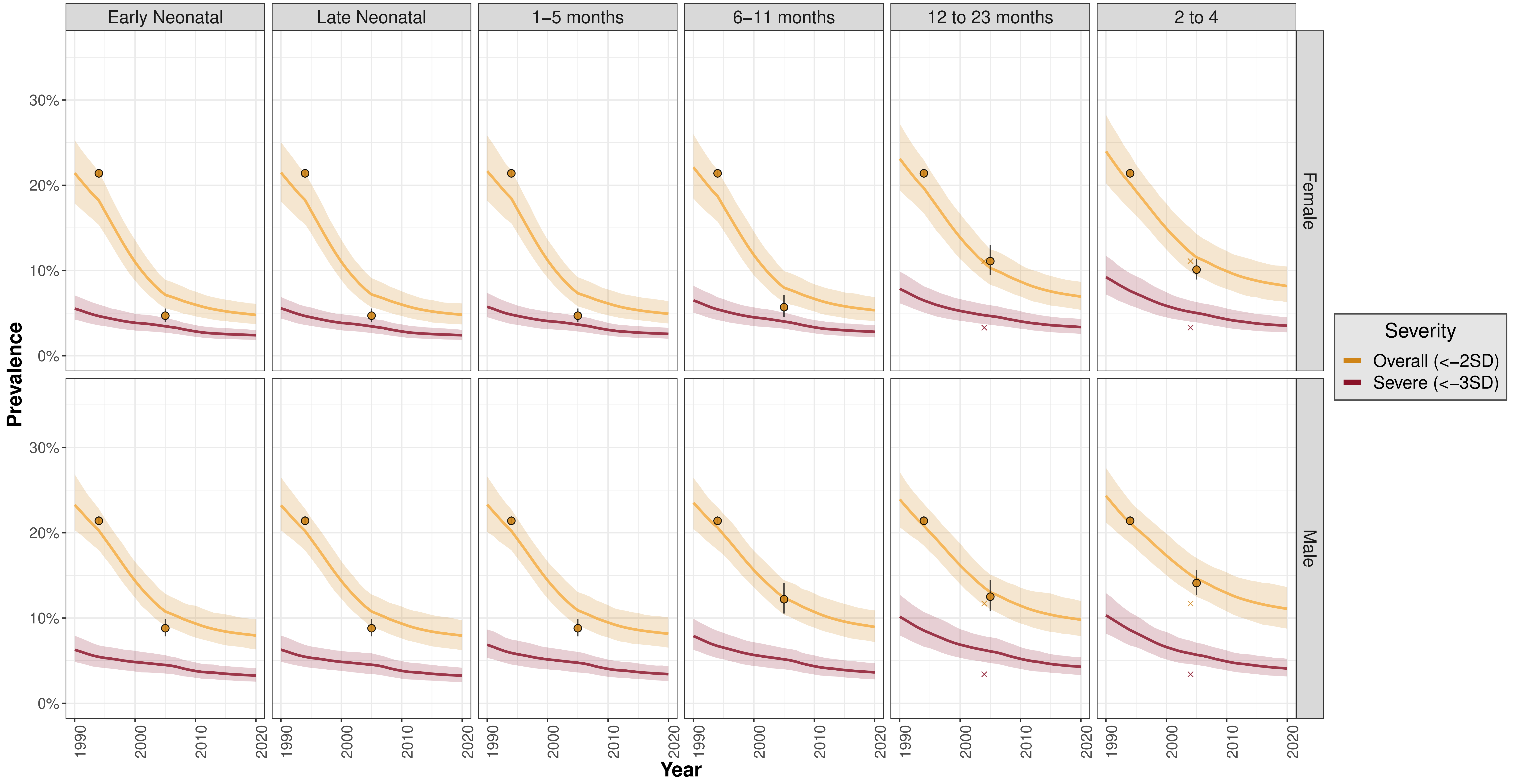

B: Transformed Mean Stunting Z Scores

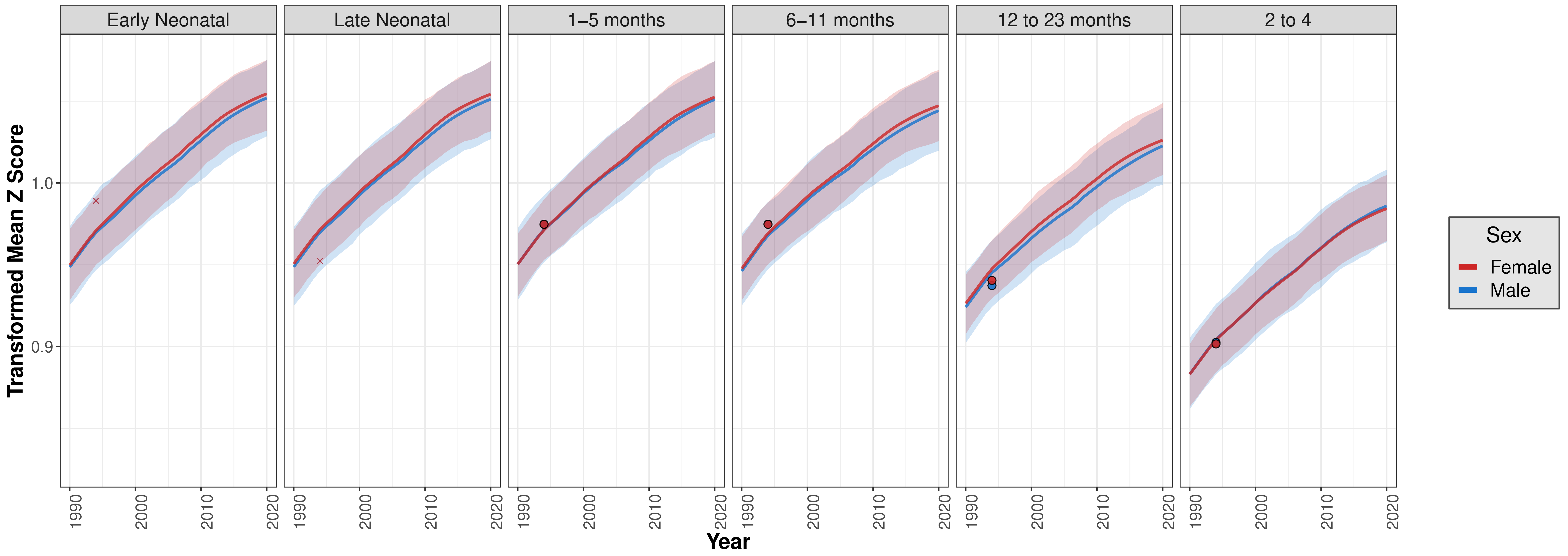

C

Year	Source
1994	WHO CGM Database
2004	WHO CGM Database
2005	WHO CGM Database

Saudi Arabia – Wasting (WHZ)

D: Overall and Severe Wasting Prevalence

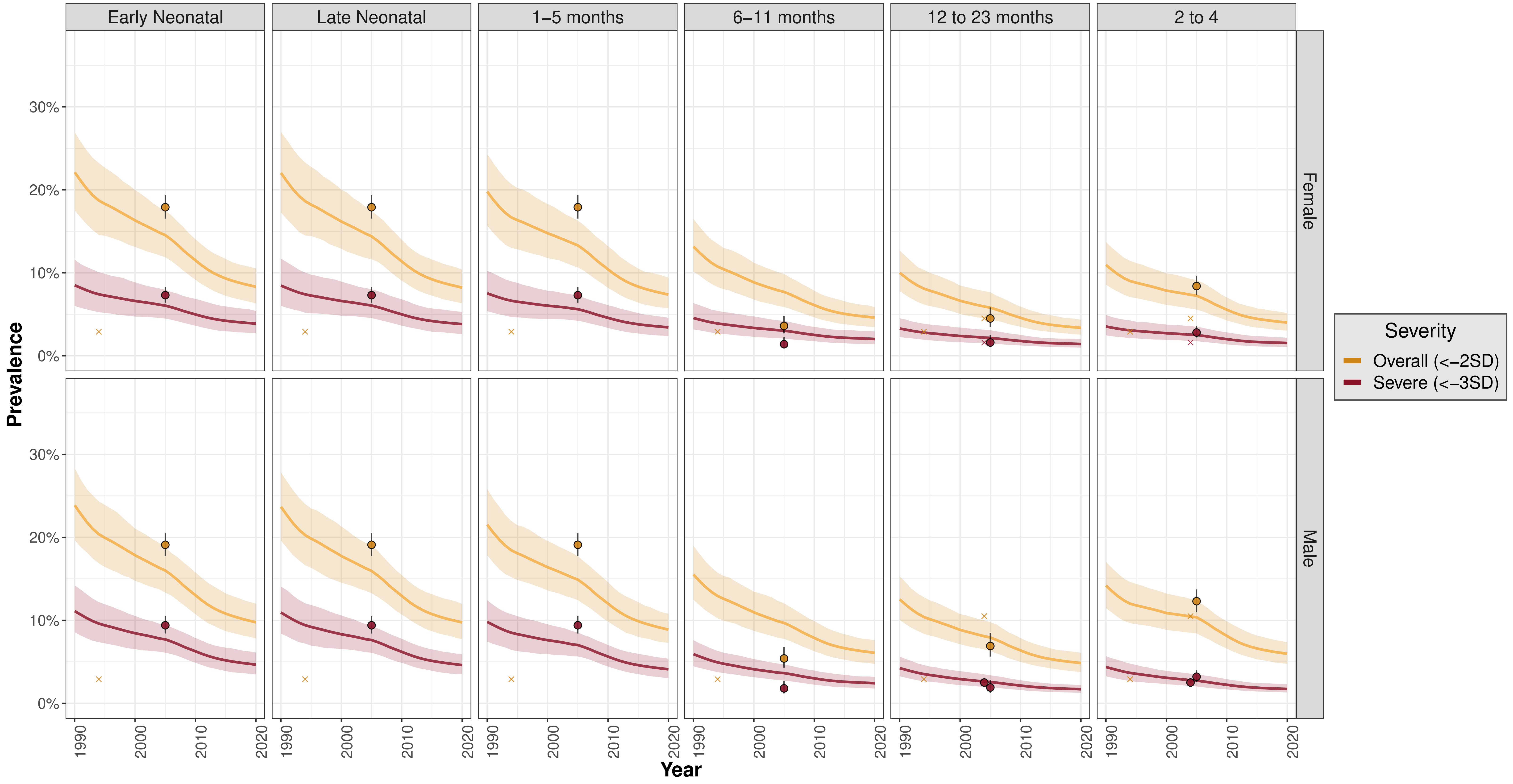

F

Year	Source
1994	WHO CGM Database
2004	WHO CGM Database
2005	WHO CGM Database

E: Transformed Mean Wasting Z Scores

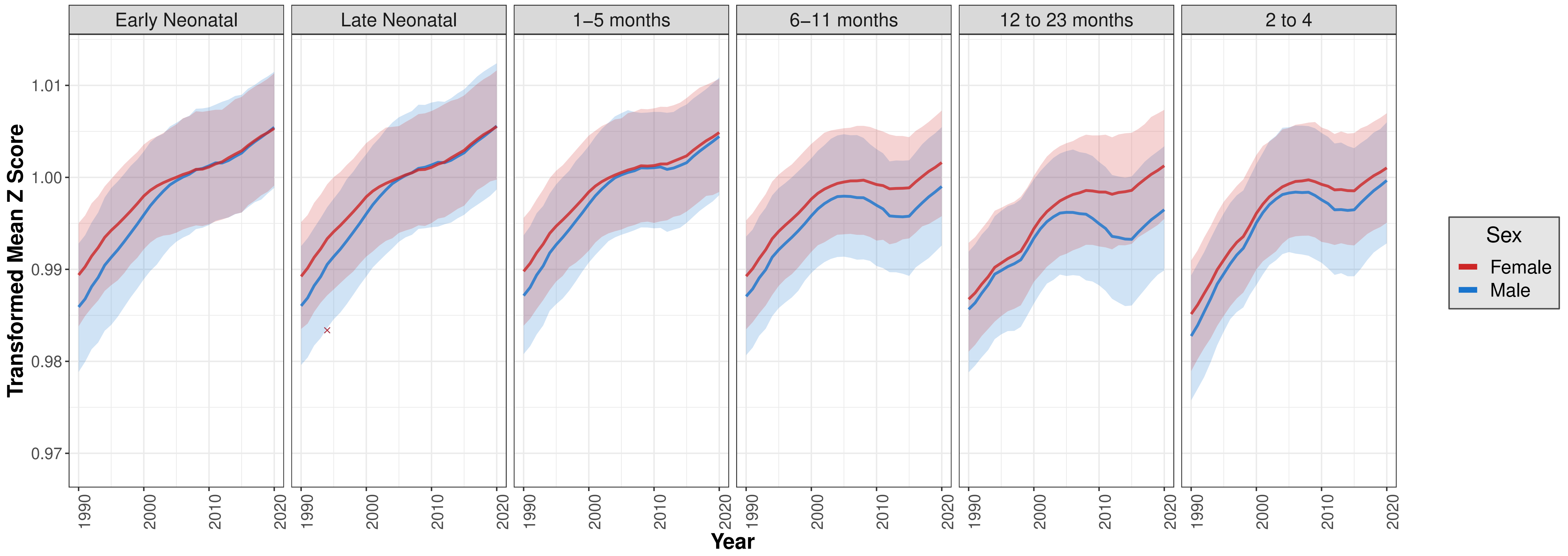

Saudi Arabia – Underweight (WAZ)

G: Overall and Severe Underweight Prevalence

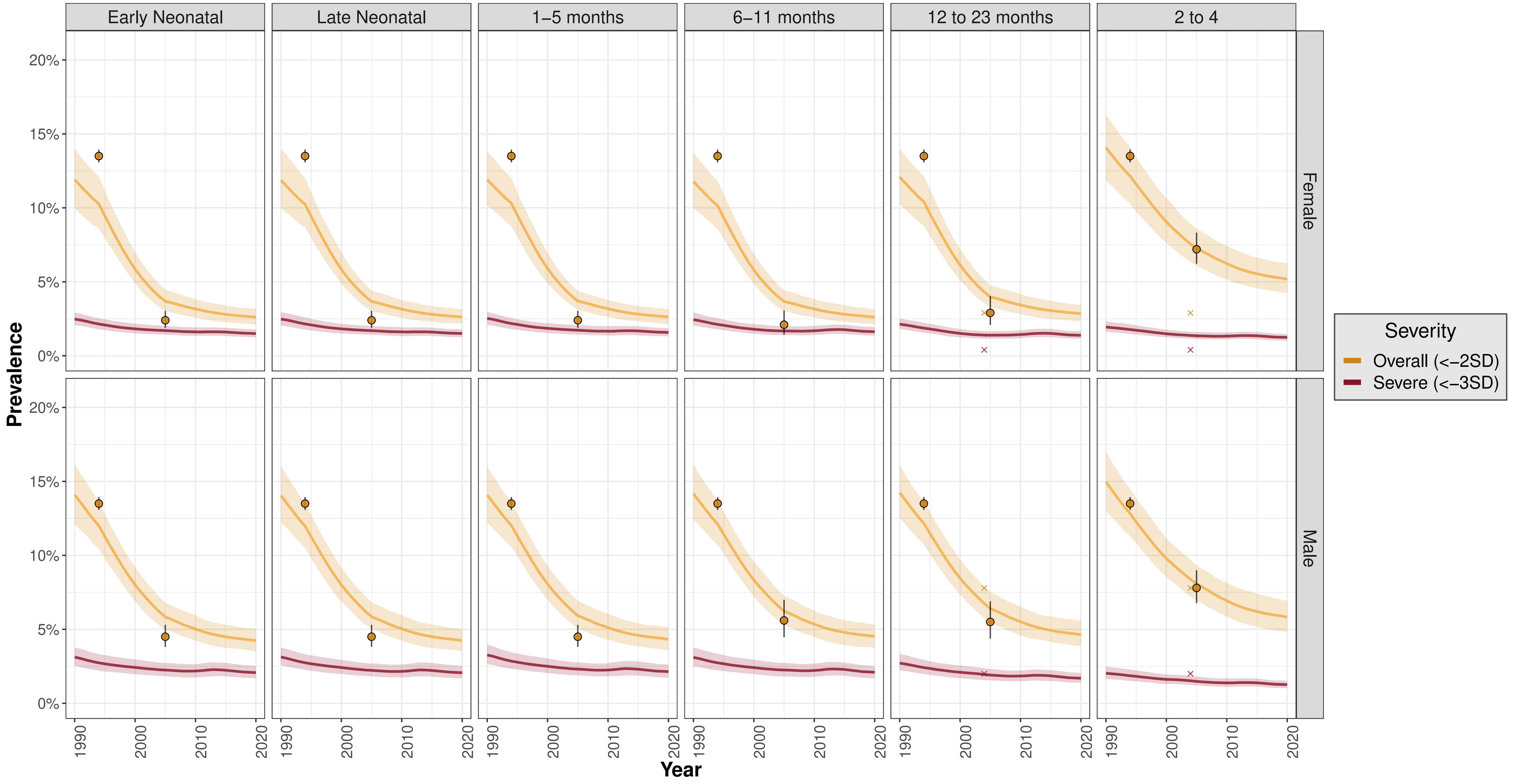

H: Transformed Mean Underweight Z Scores

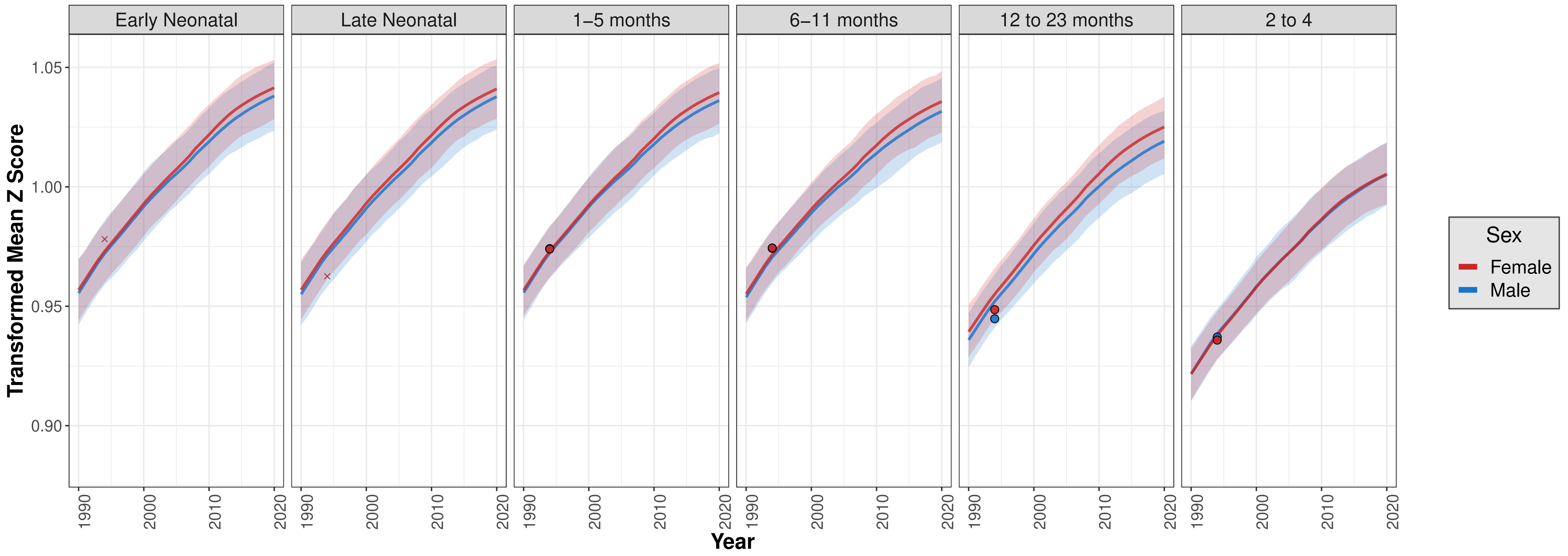

I

Year	Source
1994	WHO CGM Database
2004	WHO CGM Database
2005	WHO CGM Database

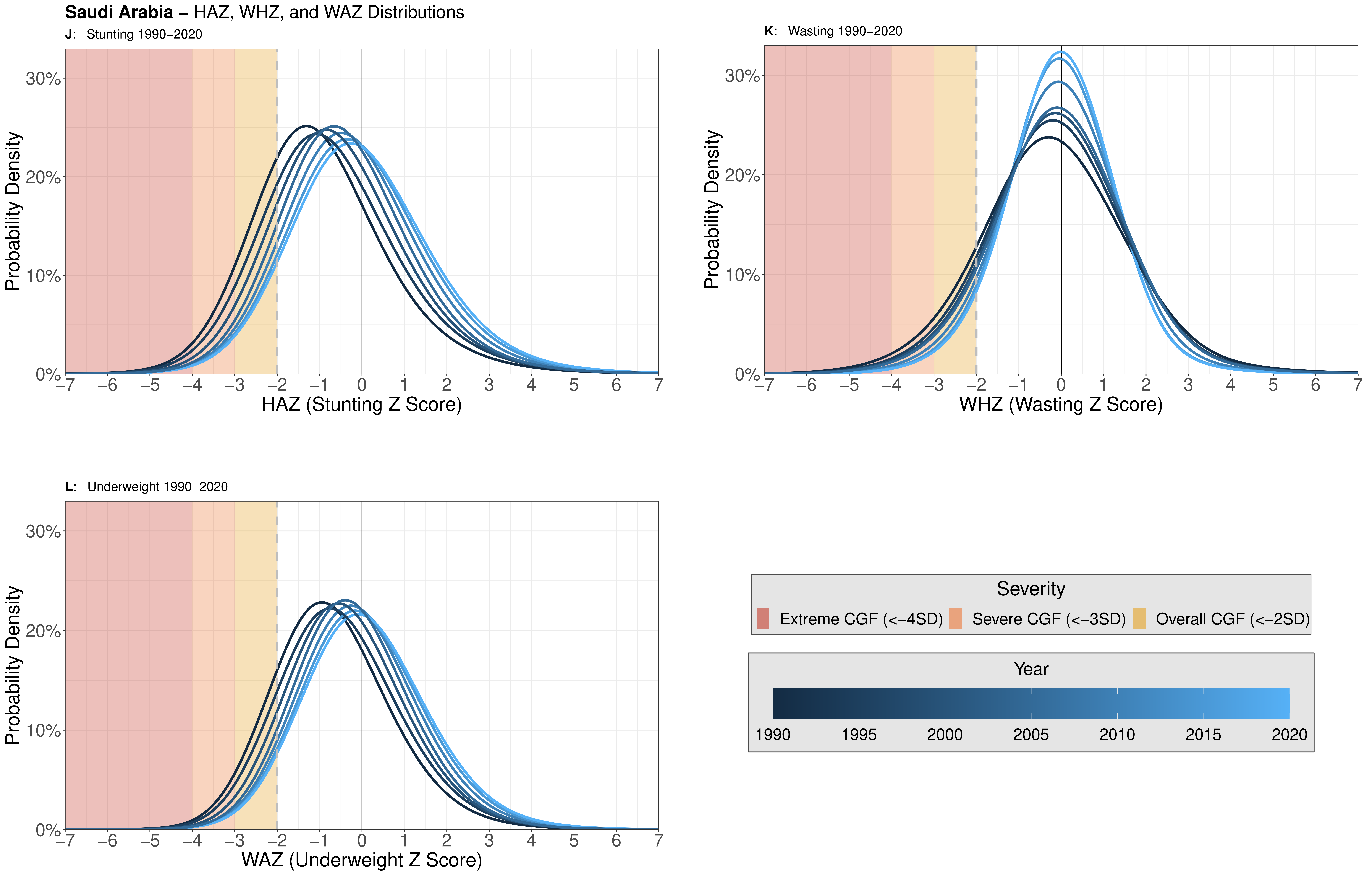

Saudi Arabia – HAZ, WHZ, and WAZ Distributions
J: Stunting 1990–2020
Probability Density
30%
20%
10%
0%
−7 −6 −5 −4 −3 −2 −1 0 1 2 3 4 5 6 7
HAZ (Stunting Z Score)
K: Wasting 1990–2020
Probability Density
30%
20%
10%
0%
−7 −6 −5 −4 −3 −2 −1 0 1 2 3 4 5 6 7
WHZ (Wasting Z Score)
L: Underweight 1990–2020
Probability Density
30%
20%
10%
0%
−7 −6 −5 −4 −3 −2 −1 0 1 2 3 4 5 6 7
WAZ (Underweight Z Score)
Severity
Extreme CGF (<−4SD)
Severe CGF (<−3SD)
Overall CGF (<−2SD)
Year
1990
1995
2000
2005
2010
2015
2020

Syrian Arab Republic – Stunting (HAZ)

A: Overall and Severe Stunting Prevalence

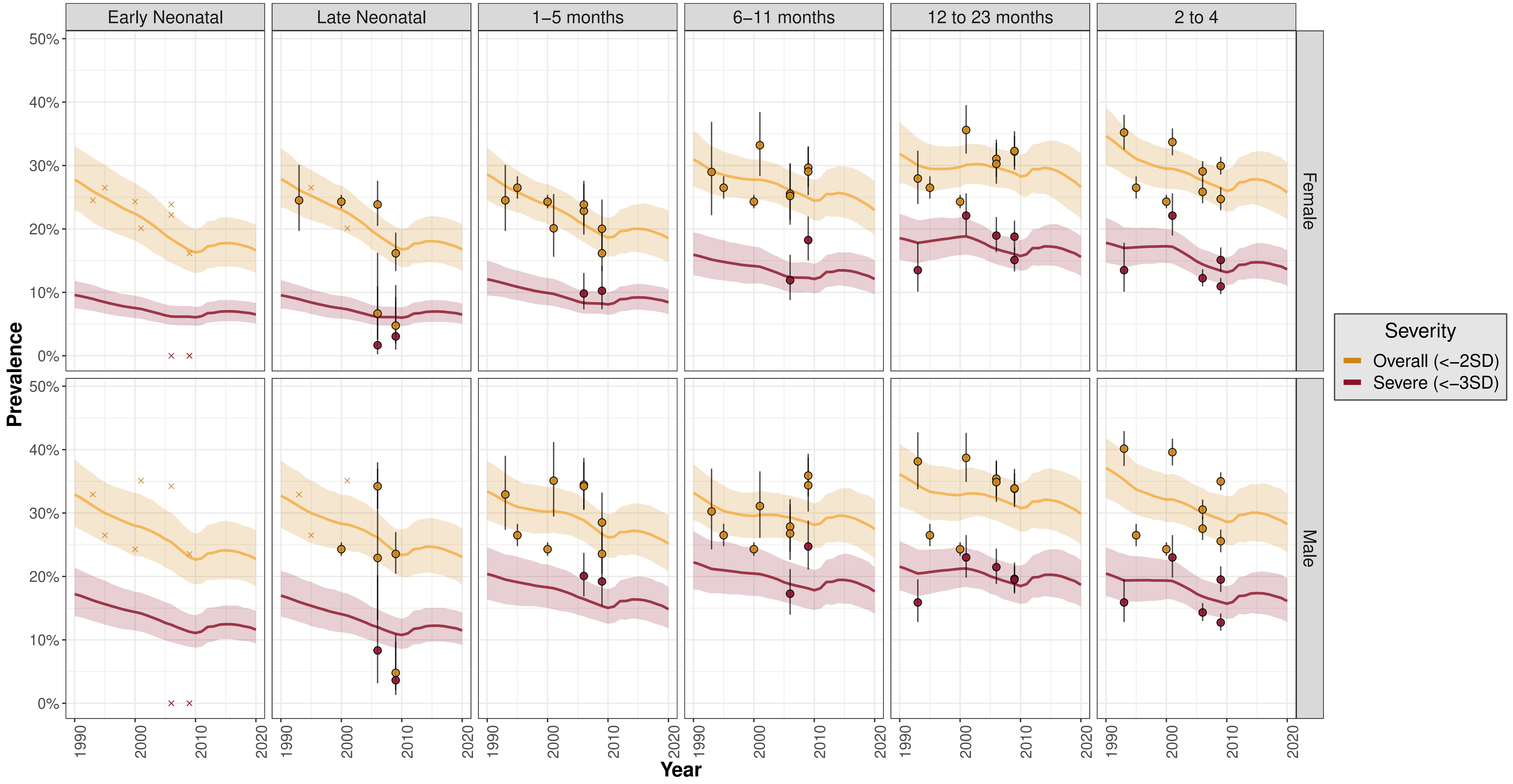

B: Transformed Mean Stunting Z Scores

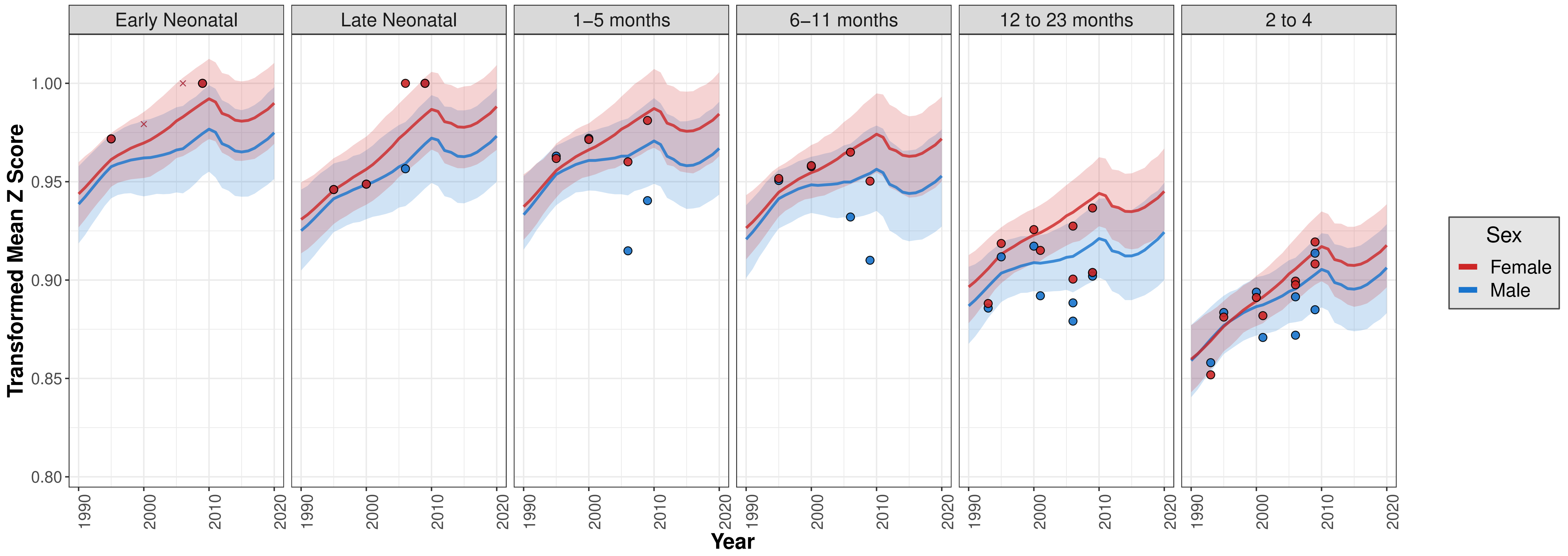

C

Year	Source
1993	WHO CGM Database
1995	WHO CGM Database
2000	WHO CGM Database
2001	WHO CGM Database
2006	Syria MICS
2006	WHO CGM Database
2009	Syria Family Health Survey
2009	WHO CGM Database

Syrian Arab Republic – Wasting (WHZ)

D: Overall and Severe Wasting Prevalence

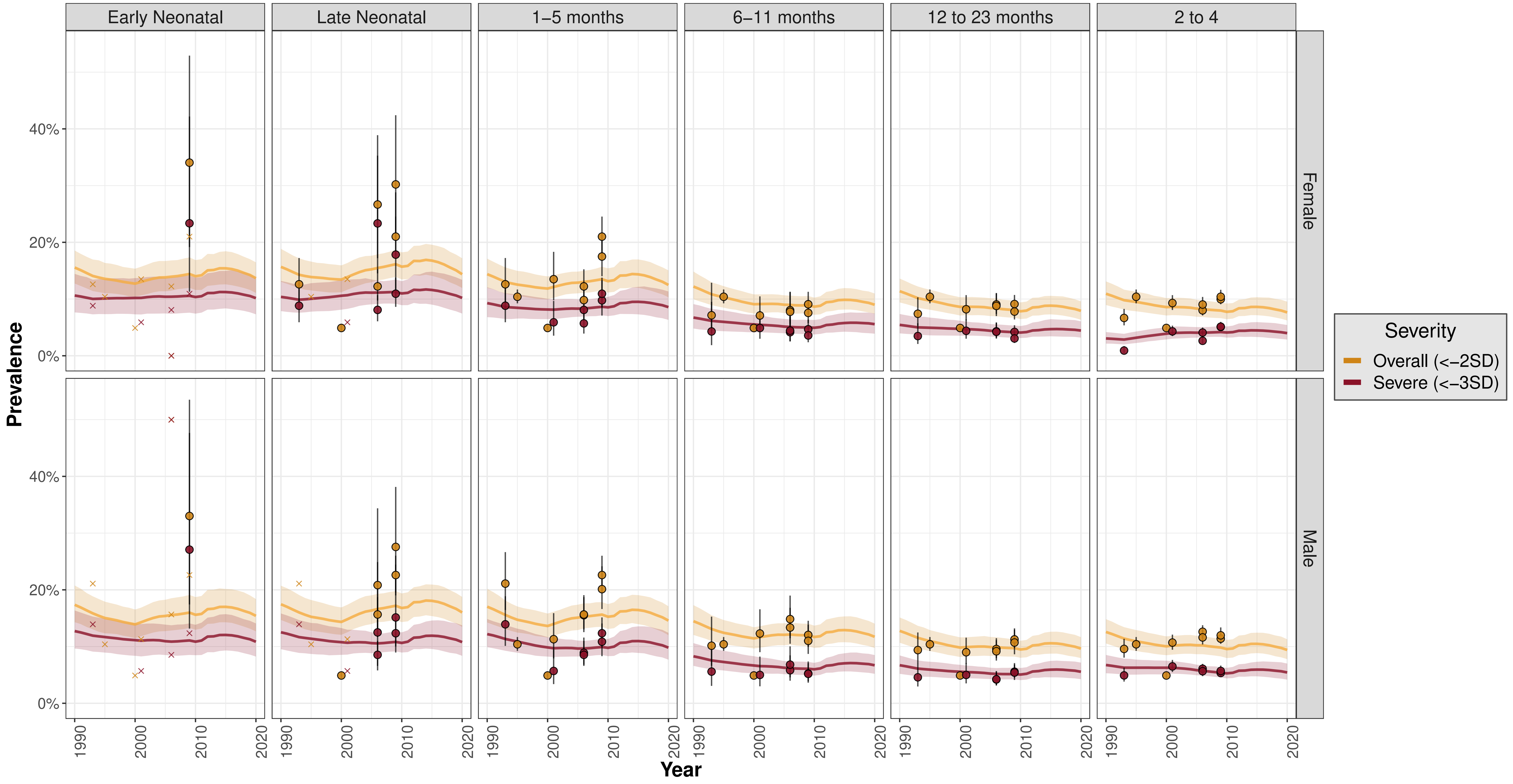

F

Year	Source
1993	WHO CGM Database
1995	WHO CGM Database
2000	WHO CGM Database
2001	WHO CGM Database
2006	Syria MICS
2006	WHO CGM Database
2009	Syria Family Health Survey
2009	WHO CGM Database

E: Transformed Mean Wasting Z Scores

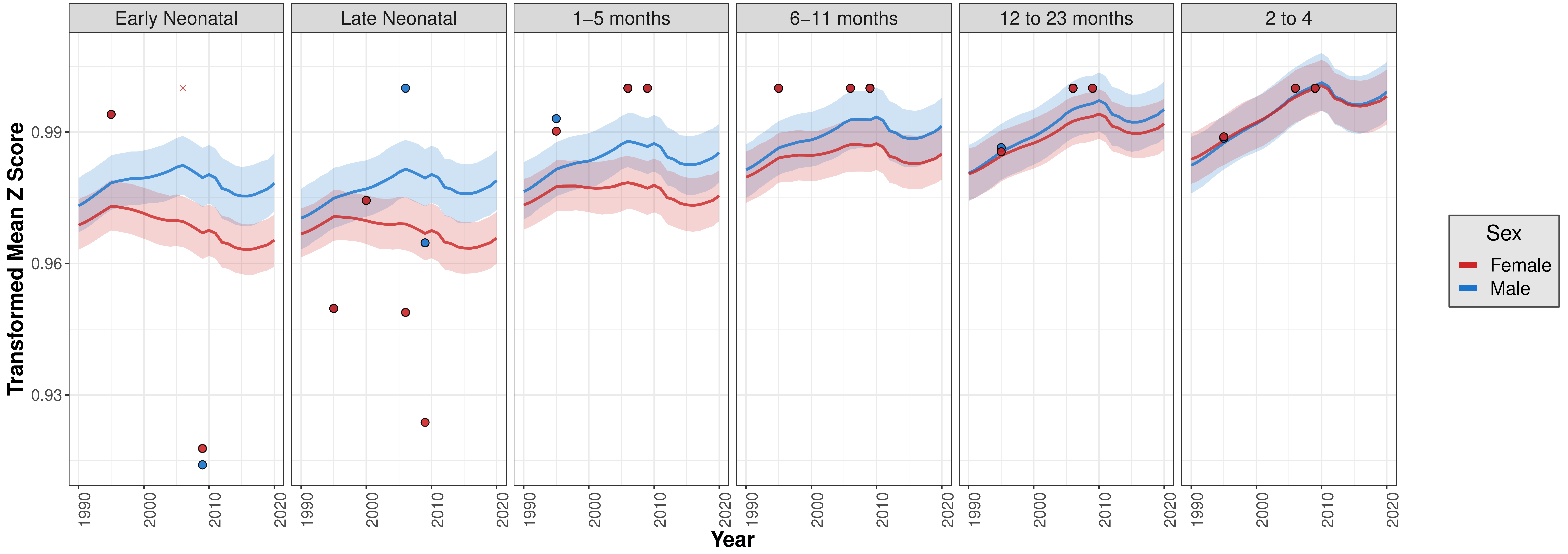

Syrian Arab Republic – Underweight (WAZ)

G: Overall and Severe Underweight Prevalence

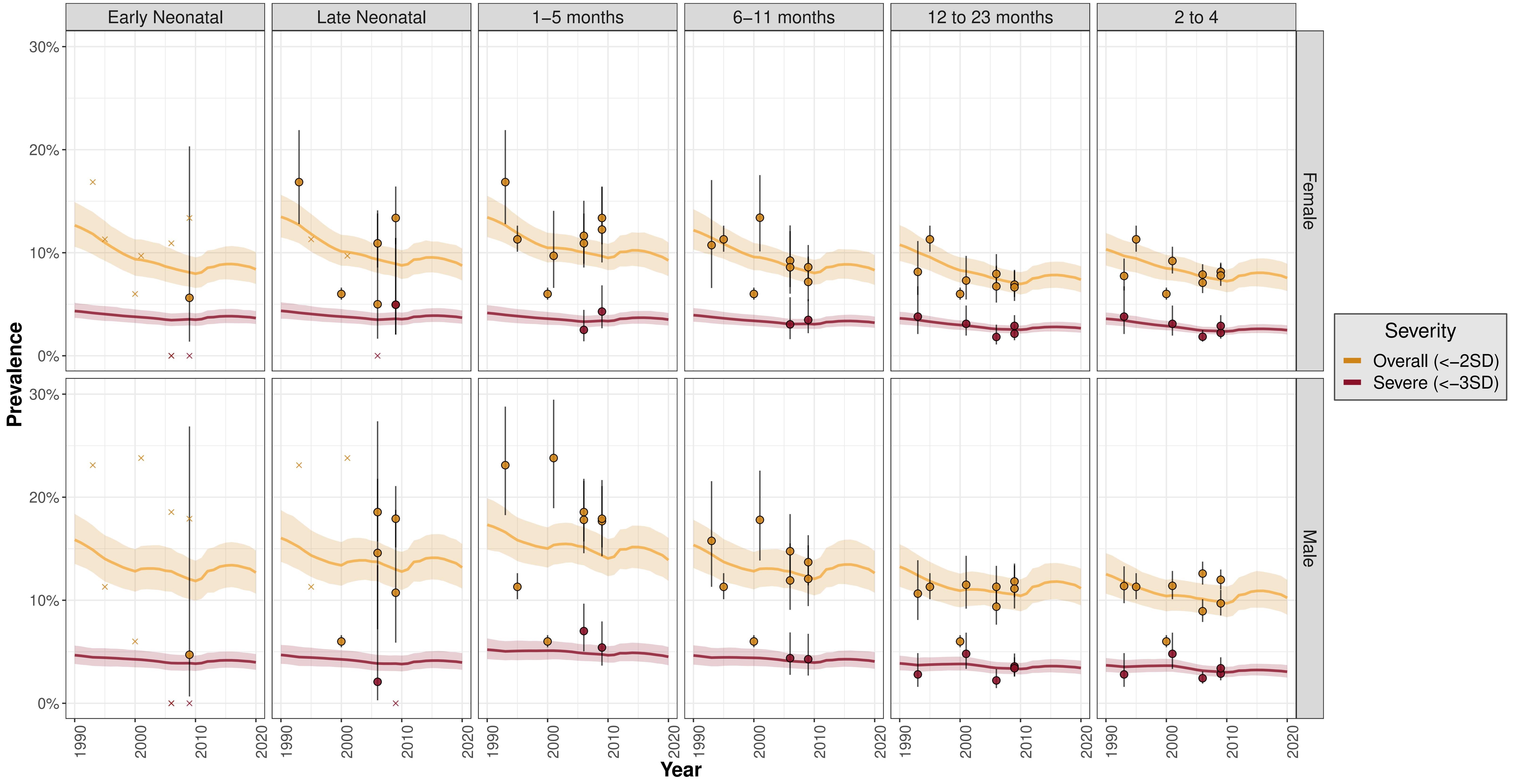

H: Transformed Mean Underweight Z Scores

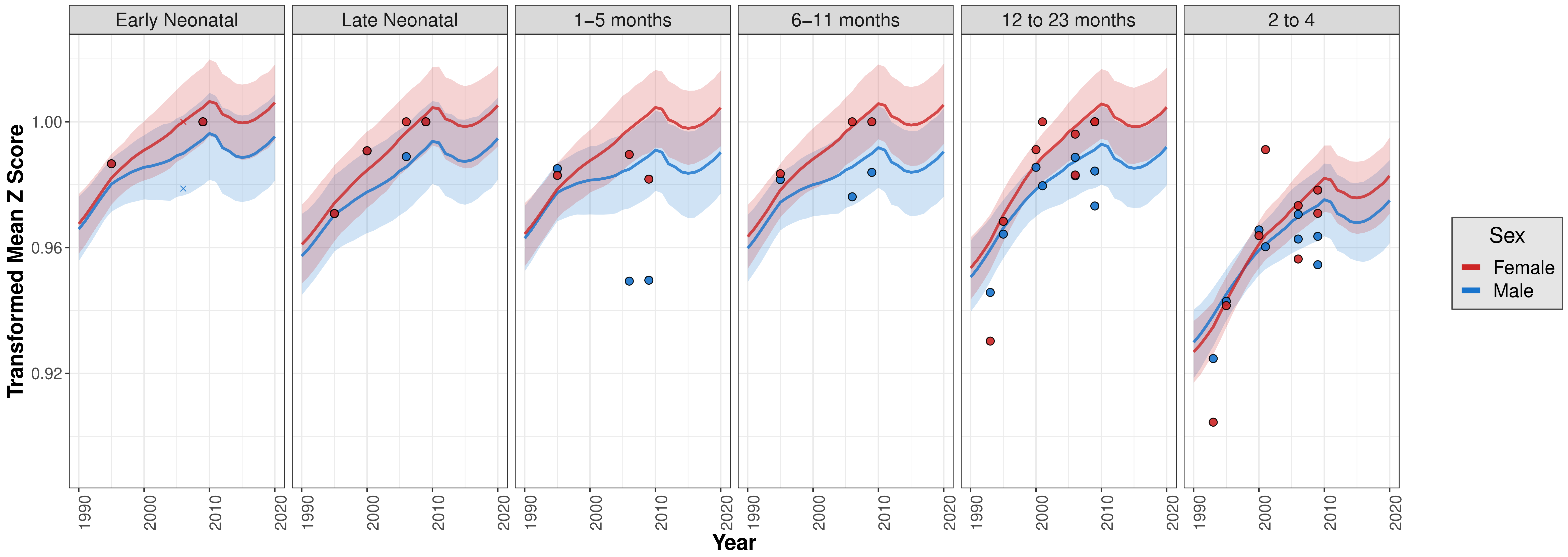

I

Year	Source
1993	WHO CGM Database
1995	WHO CGM Database
2000	WHO CGM Database
2001	WHO CGM Database
2006	Syria MICS
2006	WHO CGM Database
2009	Syria Family Health Survey
2009	WHO CGM Database

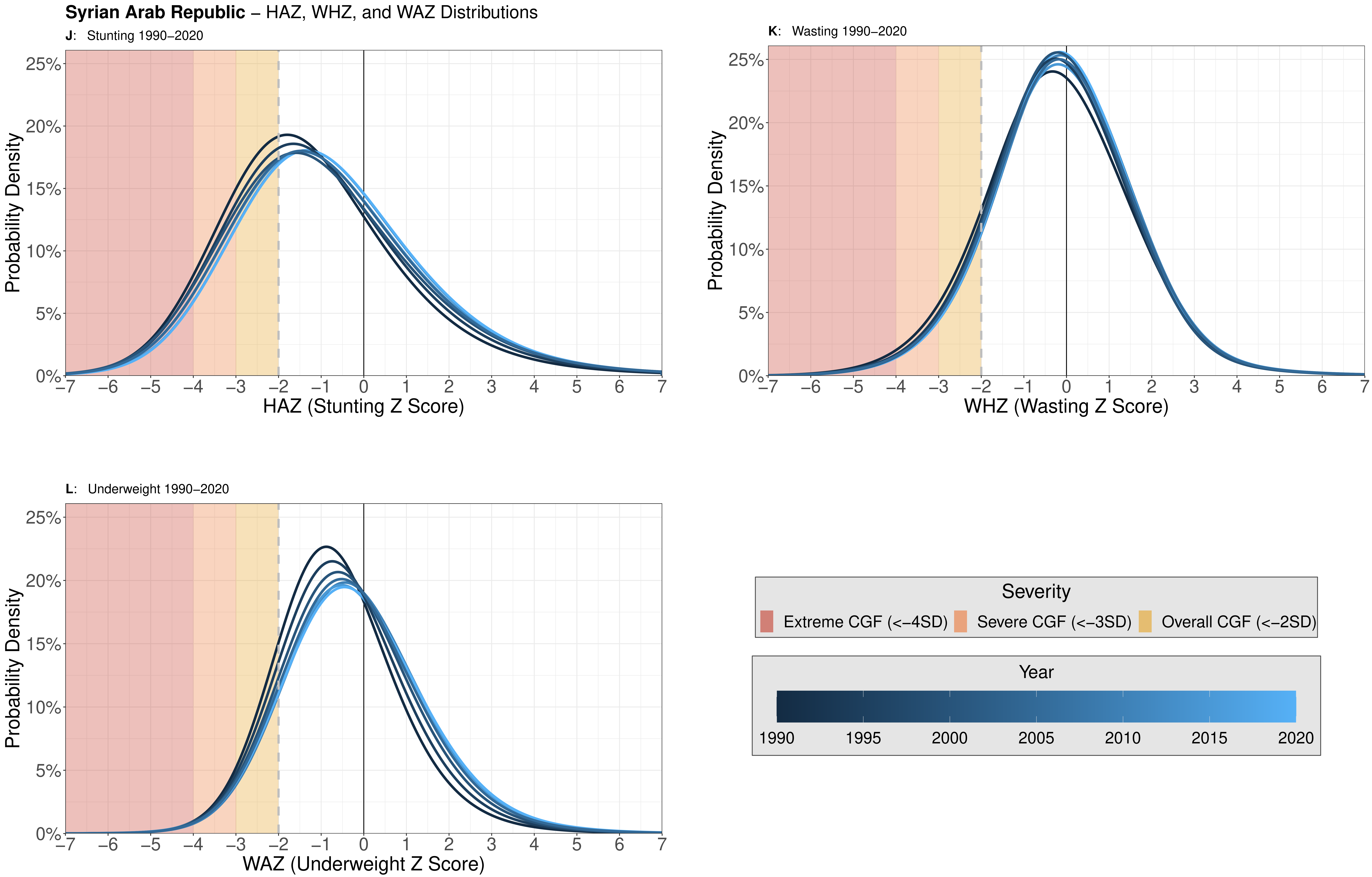
Syrian Arab Republic – HAZ, WHZ, and WAZ Distributions
J: Stunting 1990–2020
Probability Density
25%
20%
15%
10%
5%
0%
−7 −6 −5 −4 −3 −2 −1 0 1 2 3 4 5 6 7
HAZ (Stunting Z Score)
K: Wasting 1990–2020
Probability Density
WHZ (Wasting Z Score)
L: Underweight 1990–2020
Probability Density
WAZ (Underweight Z Score)
Severity
Extreme CGF (<−4SD)
Severe CGF (<−3SD)
Overall CGF (<−2SD)
Year
1990
1995
2000
2005
2010
2015
2020

Tunisia – Stunting (HAZ)

A: Overall and Severe Stunting Prevalence

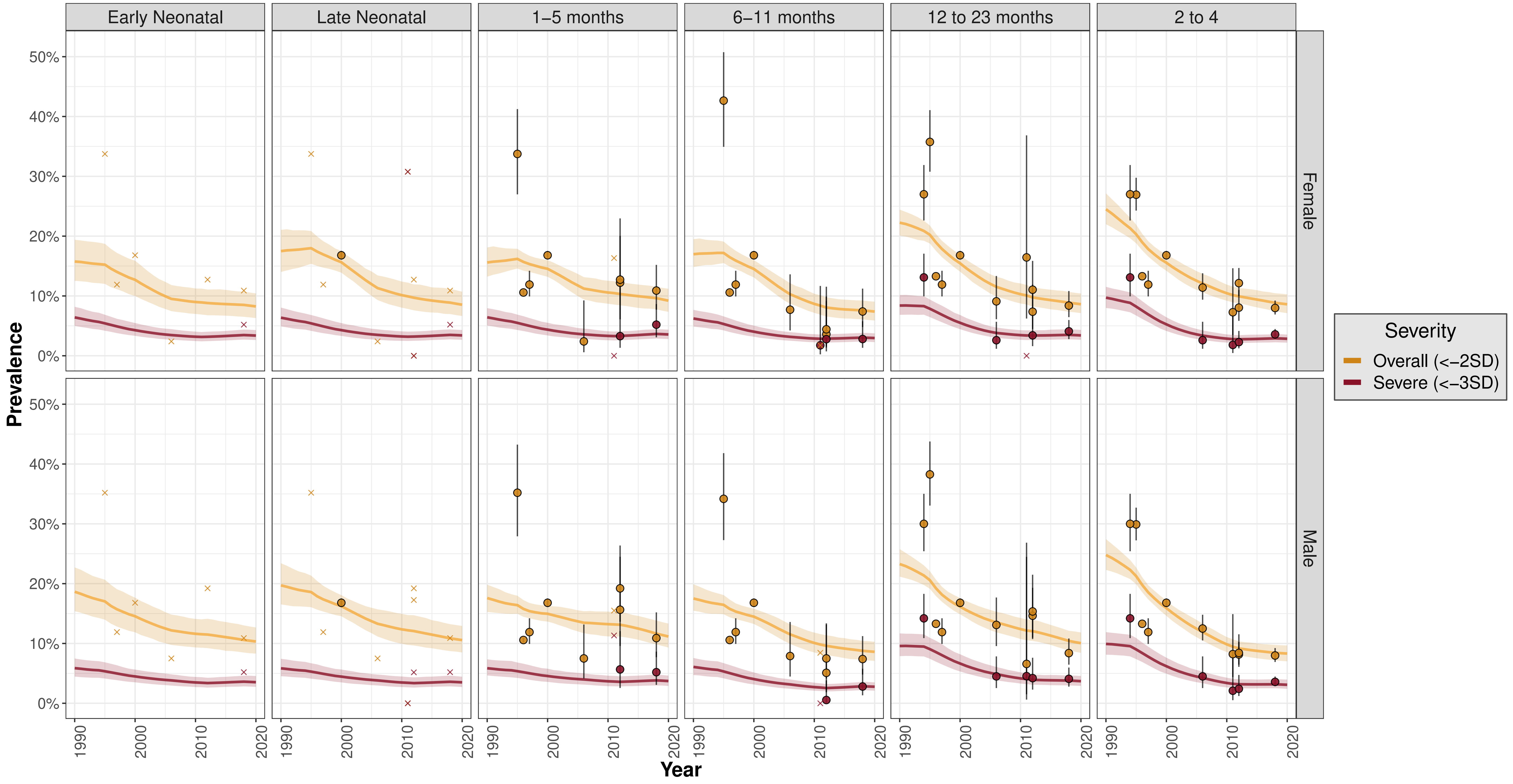

B: Transformed Mean Stunting Z Scores

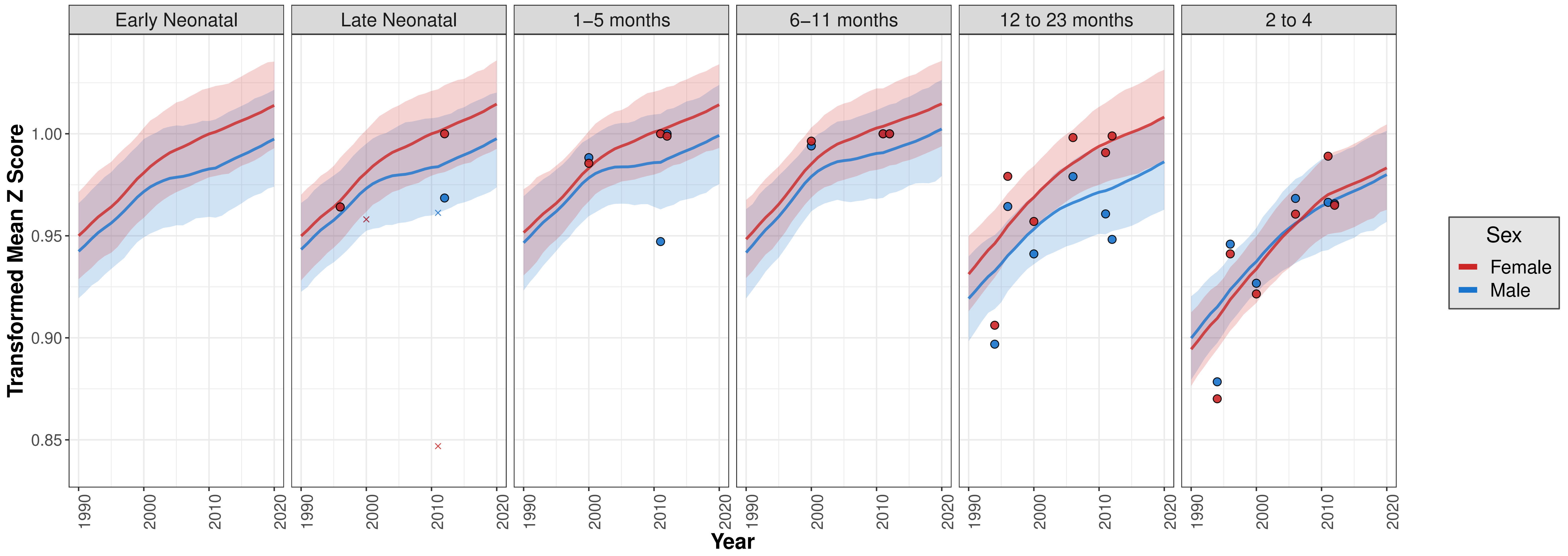

C

Year	Source
1974	WHO CGM Database
1988	DHS
1988	WHO CGM Database
1994	WHO CGM Database
1995	WHO CGM Database
1996	WHO CGM Database
1997	WHO CGM Database
2000	WHO CGM Database
2006	WHO CGM Database
2011	MICS
2012	MICS
2012	WHO CGM Database
2018	MICS

Tunisia – Wasting (WHZ)

D: Overall and Severe Wasting Prevalence

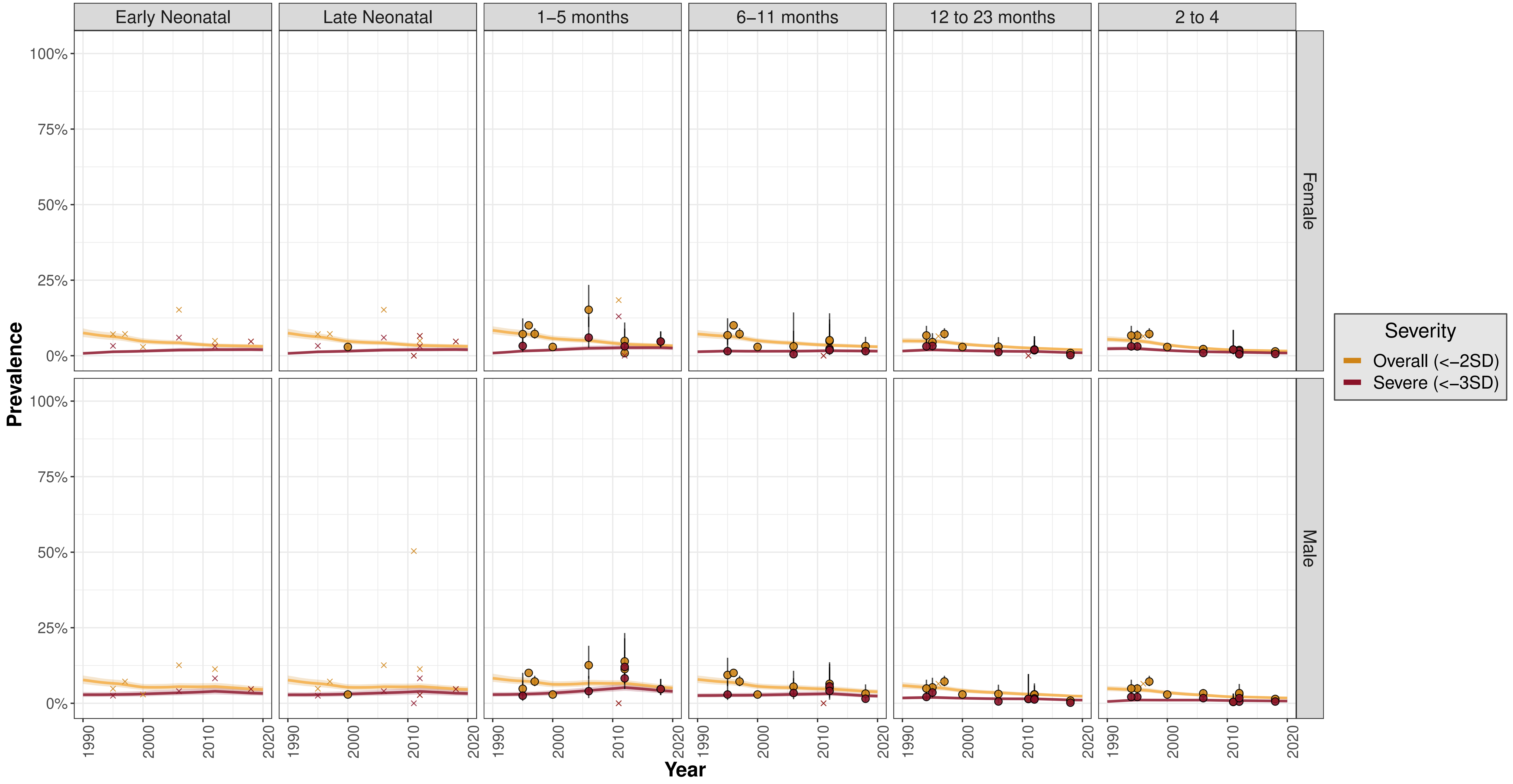

E: Transformed Mean Wasting Z Scores

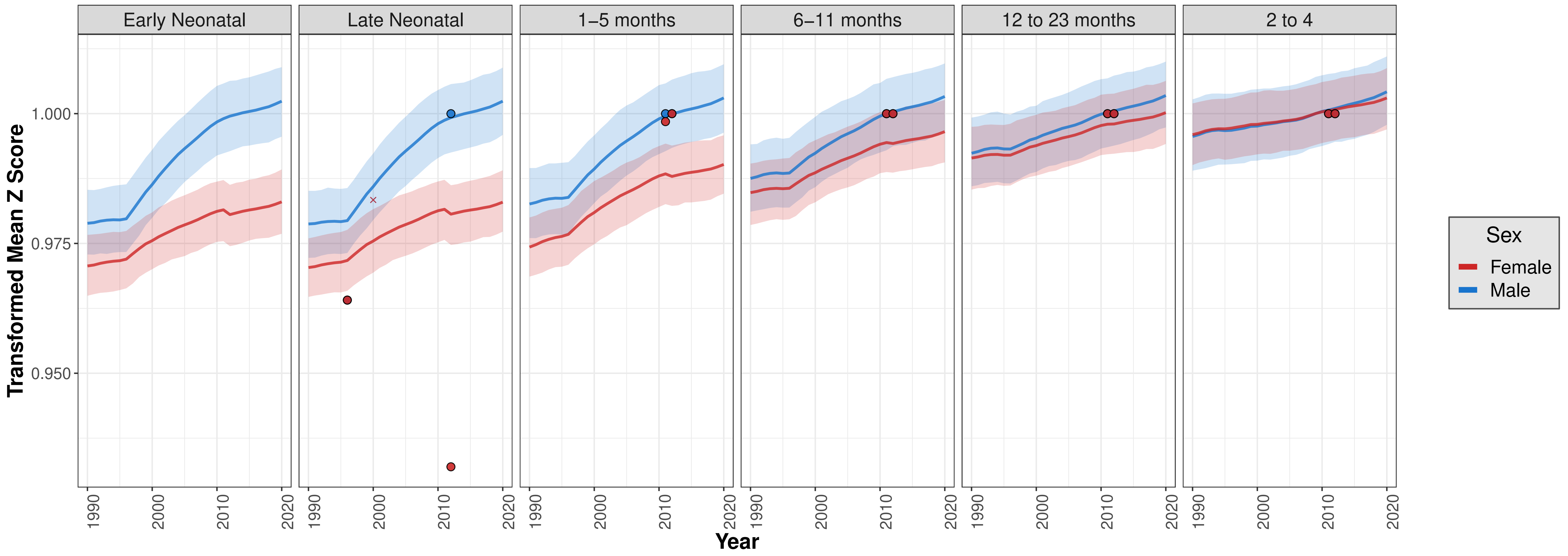

F

Year	Source
1974	WHO CGM Database
1988	DHS
1988	WHO CGM Database
1994	WHO CGM Database
1995	WHO CGM Database
1996	WHO CGM Database
1997	WHO CGM Database
2000	WHO CGM Database
2006	WHO CGM Database
2011	MICS
2012	MICS
2012	WHO CGM Database
2018	MICS

Tunisia – Underweight (WAZ)

G: Overall and Severe Underweight Prevalence

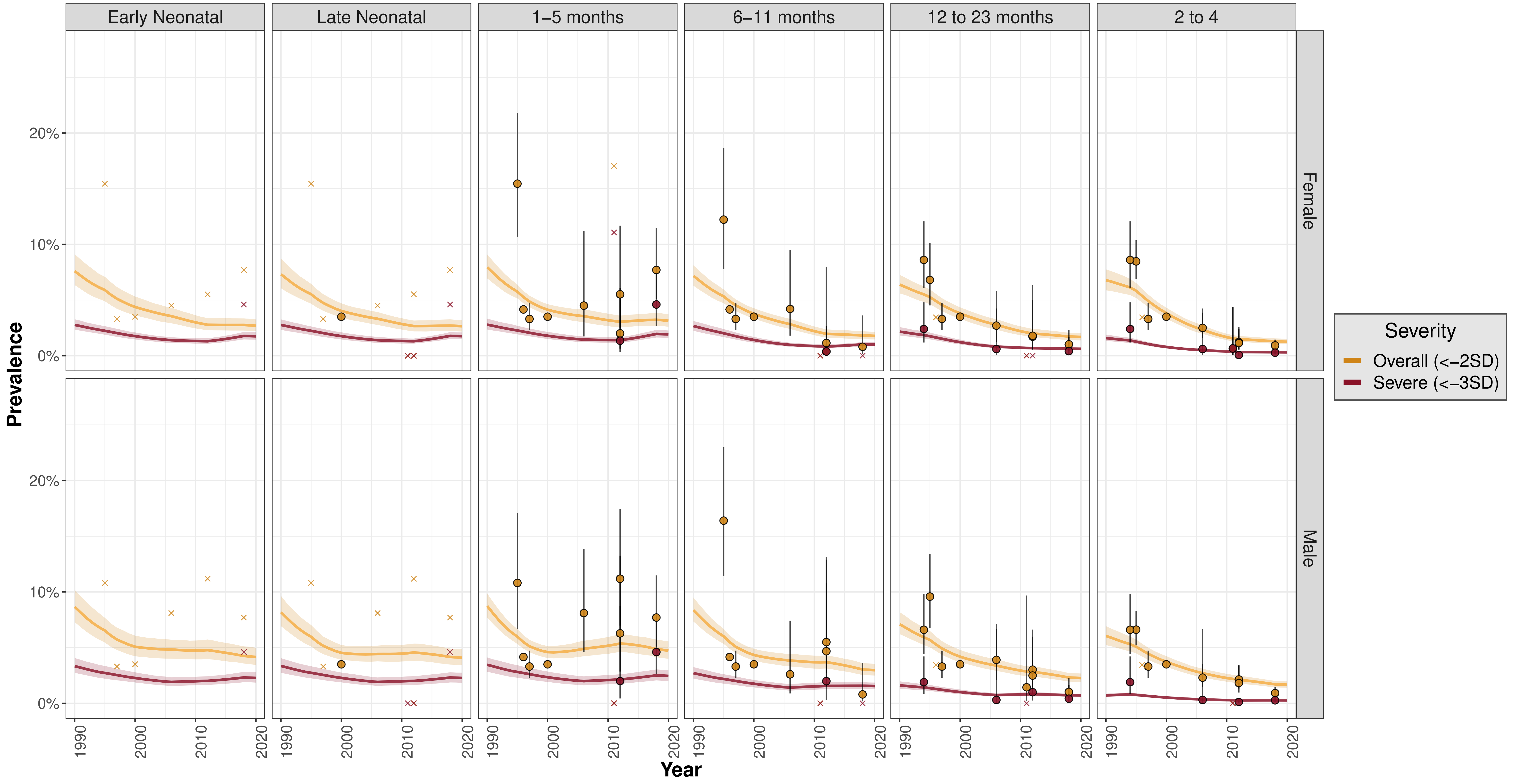

H: Transformed Mean Underweight Z Scores

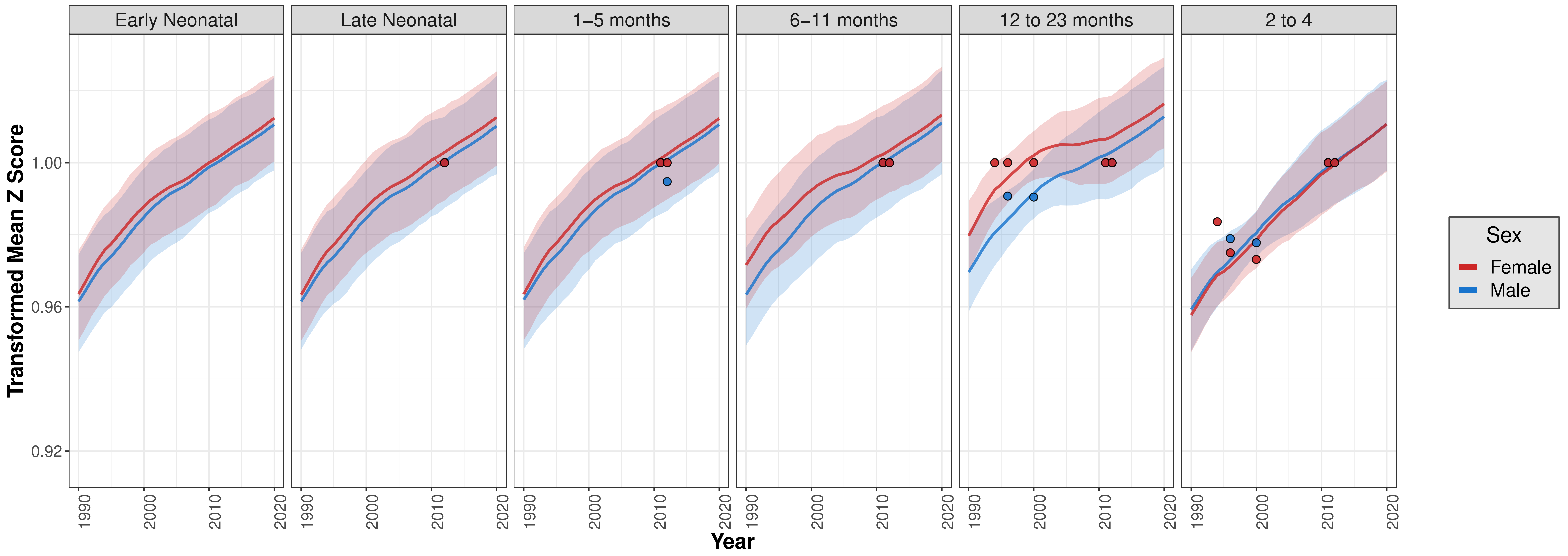

I

Year	Source
1974	WHO CGM Database
1988	DHS
1988	WHO CGM Database
1994	WHO CGM Database
1995	WHO CGM Database
1996	WHO CGM Database
1997	WHO CGM Database
2000	WHO CGM Database
2006	WHO CGM Database
2011	MICS
2012	MICS
2012	WHO CGM Database
2018	MICS

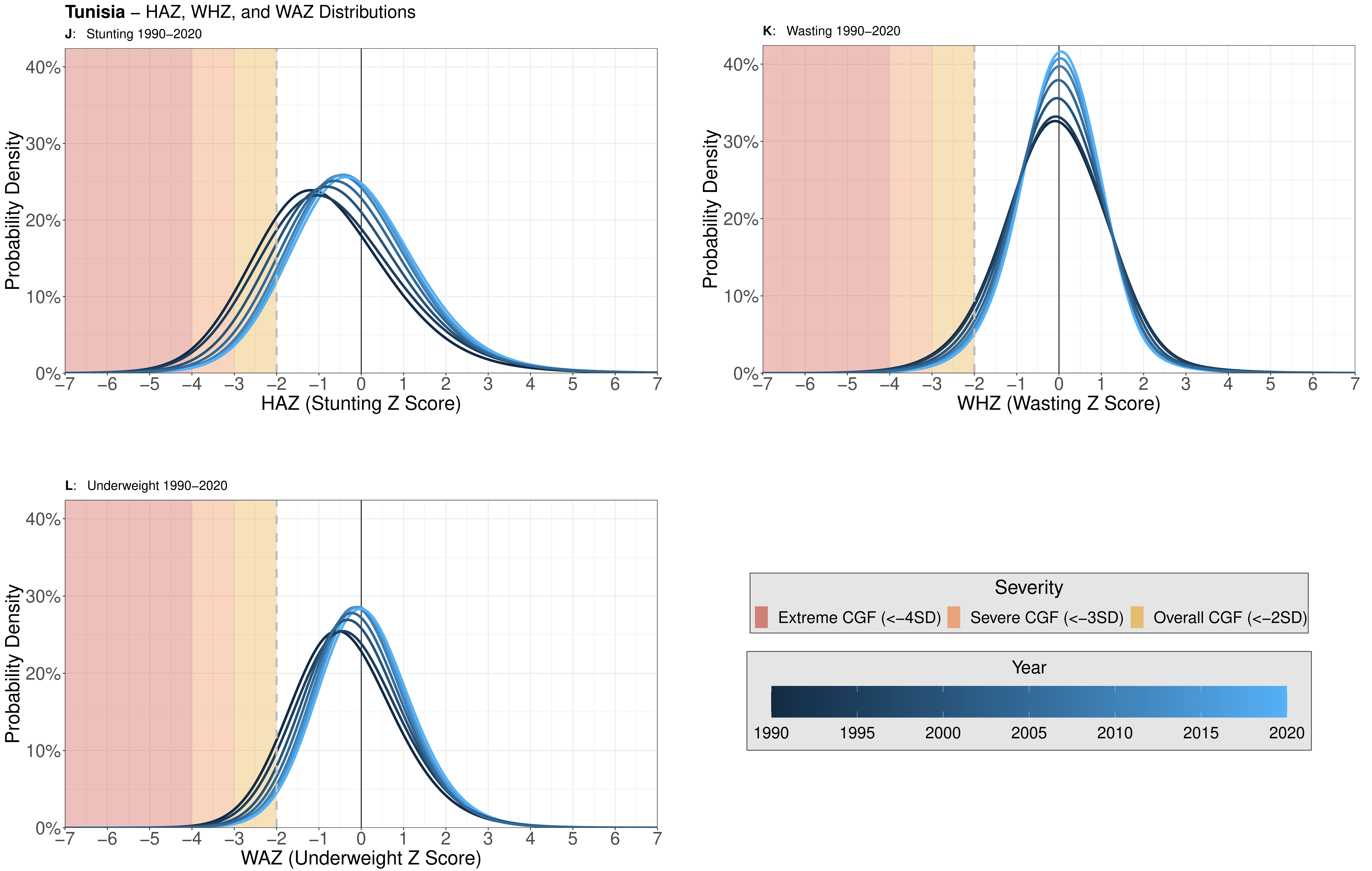

Tunisia – HAZ, WHZ, and WAZ Distributions
J: Stunting 1990–2020
K: Wasting 1990–2020
L: Underweight 1990–2020
Probability Density
0%
10%
20%
30%
40%
−7 −6 −5 −4 −3 −2 −1 0 1 2 3 4 5 6 7
HAZ (Stunting Z Score)
WHZ (Wasting Z Score)
WAZ (Underweight Z Score)
Severity
Extreme CGF (<−4SD)
Severe CGF (<−3SD)
Overall CGF (<−2SD)
Year
1990
1995
2000
2005
2010
2015
2020

Turkey – Stunting (HAZ)

A: Overall and Severe Stunting Prevalence

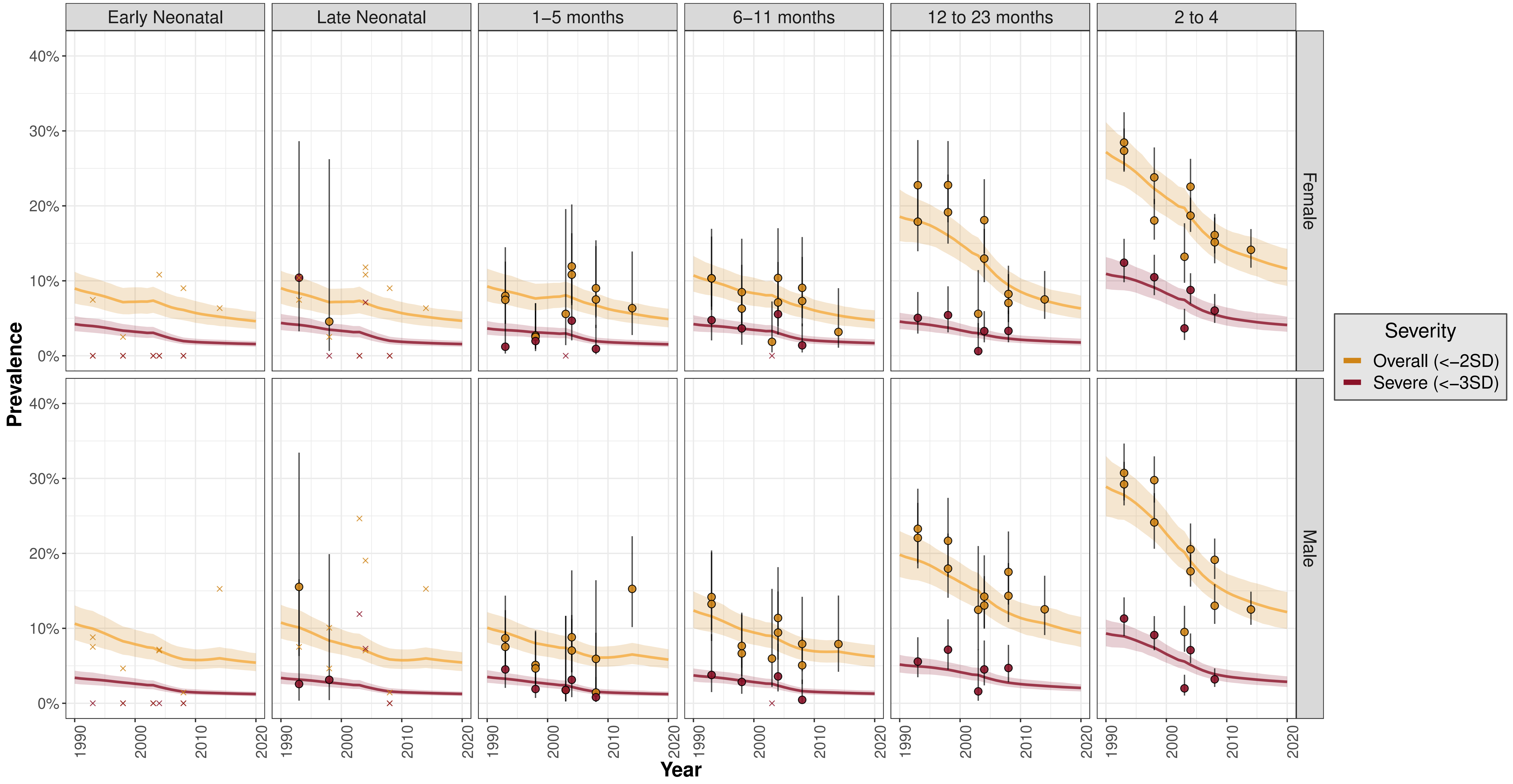

B: Transformed Mean Stunting Z Scores

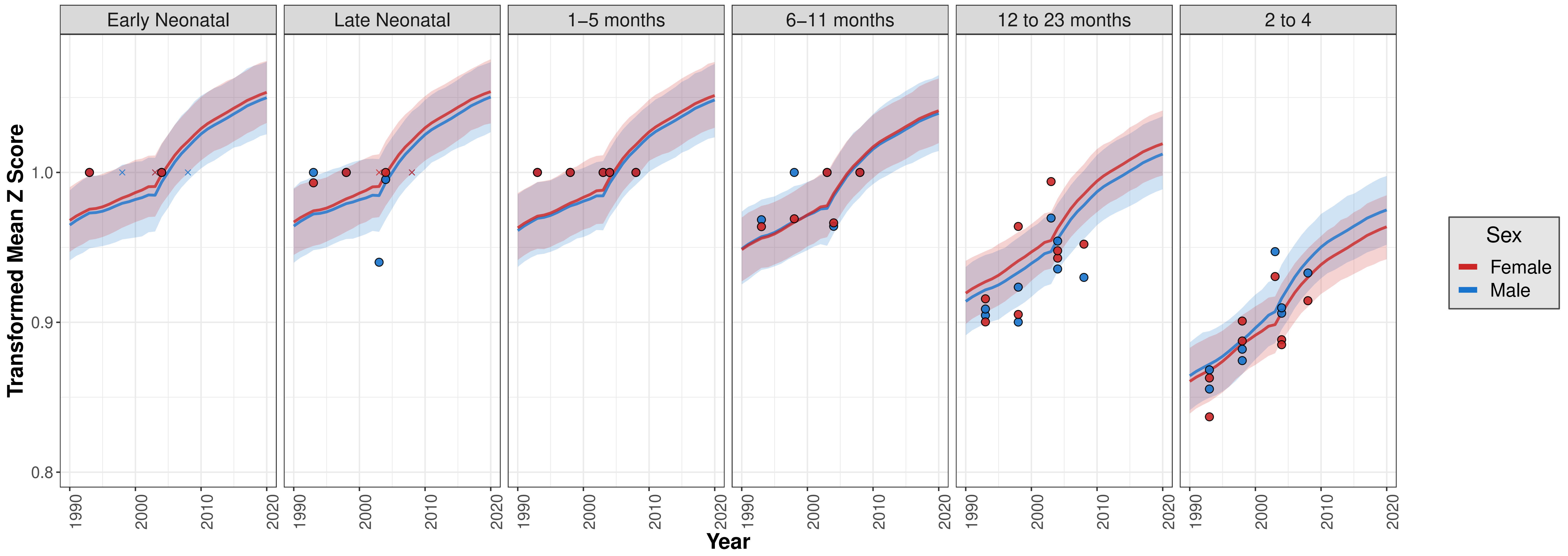

C

Year	Source
1993	DHS
1993	WHO CGM Database
1998	DHS
1998	WHO CGM Database
2003	DHS
2004	DHS
2004	WHO CGM Database
2008	DHS
2008	WHO CGM Database
2014	WHO CGM Database

Turkey – Wasting (WHZ)

D: Overall and Severe Wasting Prevalence

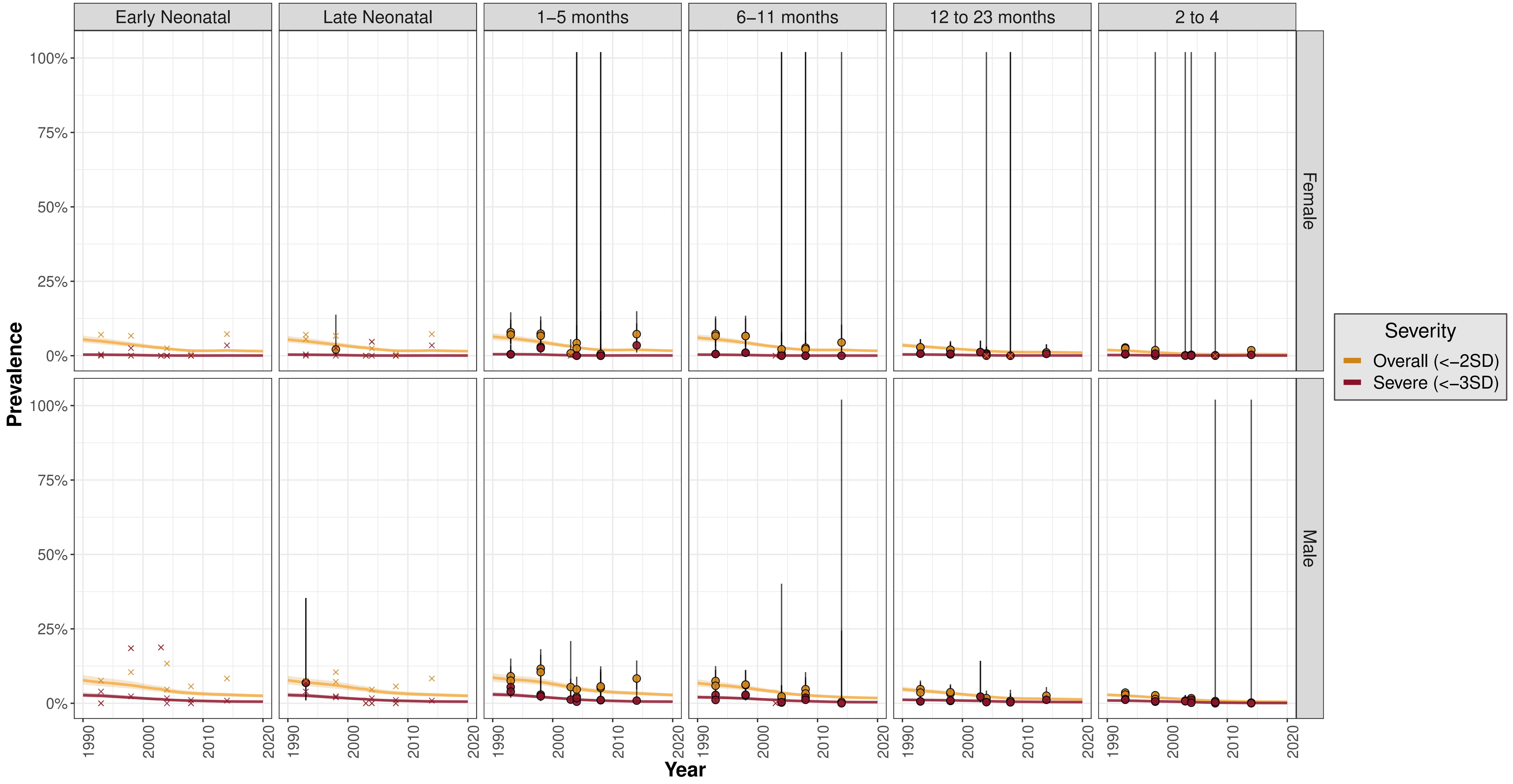

E: Transformed Mean Wasting Z Scores

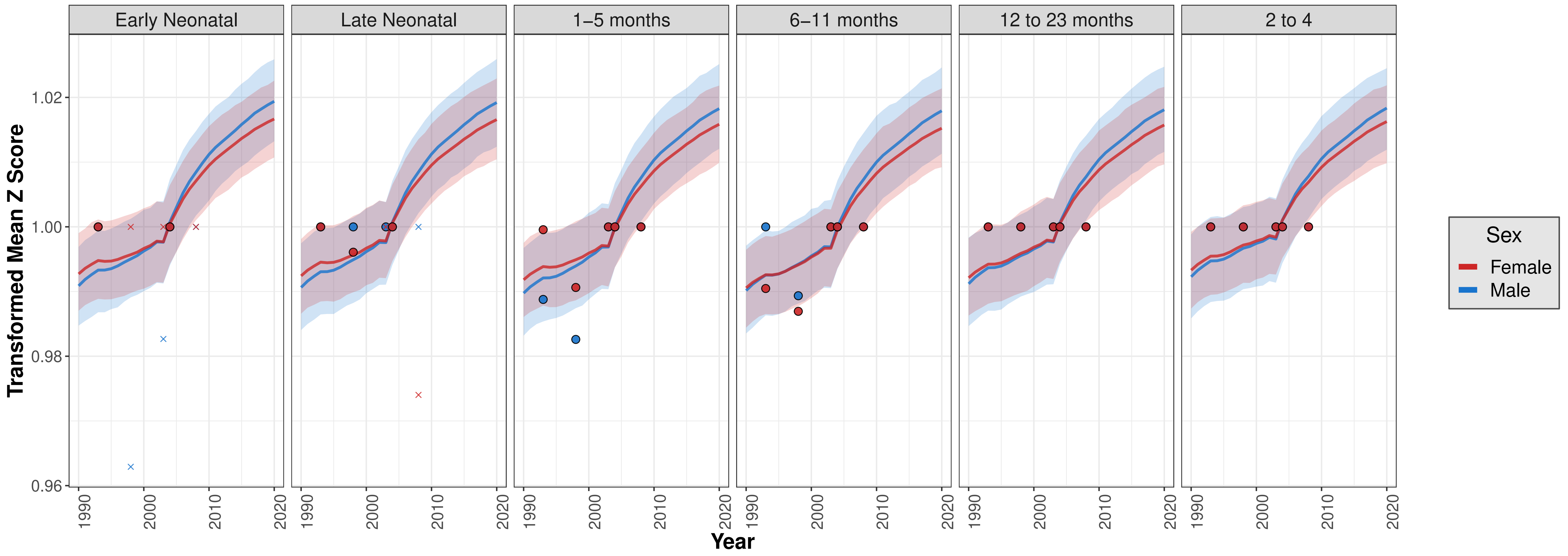

F

Year	Source
1993	DHS
1993	WHO CGM Database
1998	DHS
1998	WHO CGM Database
2003	DHS
2004	DHS
2004	WHO CGM Database
2008	DHS
2008	WHO CGM Database
2014	WHO CGM Database

Turkey – Underweight (WAZ)

G: Overall and Severe Underweight Prevalence

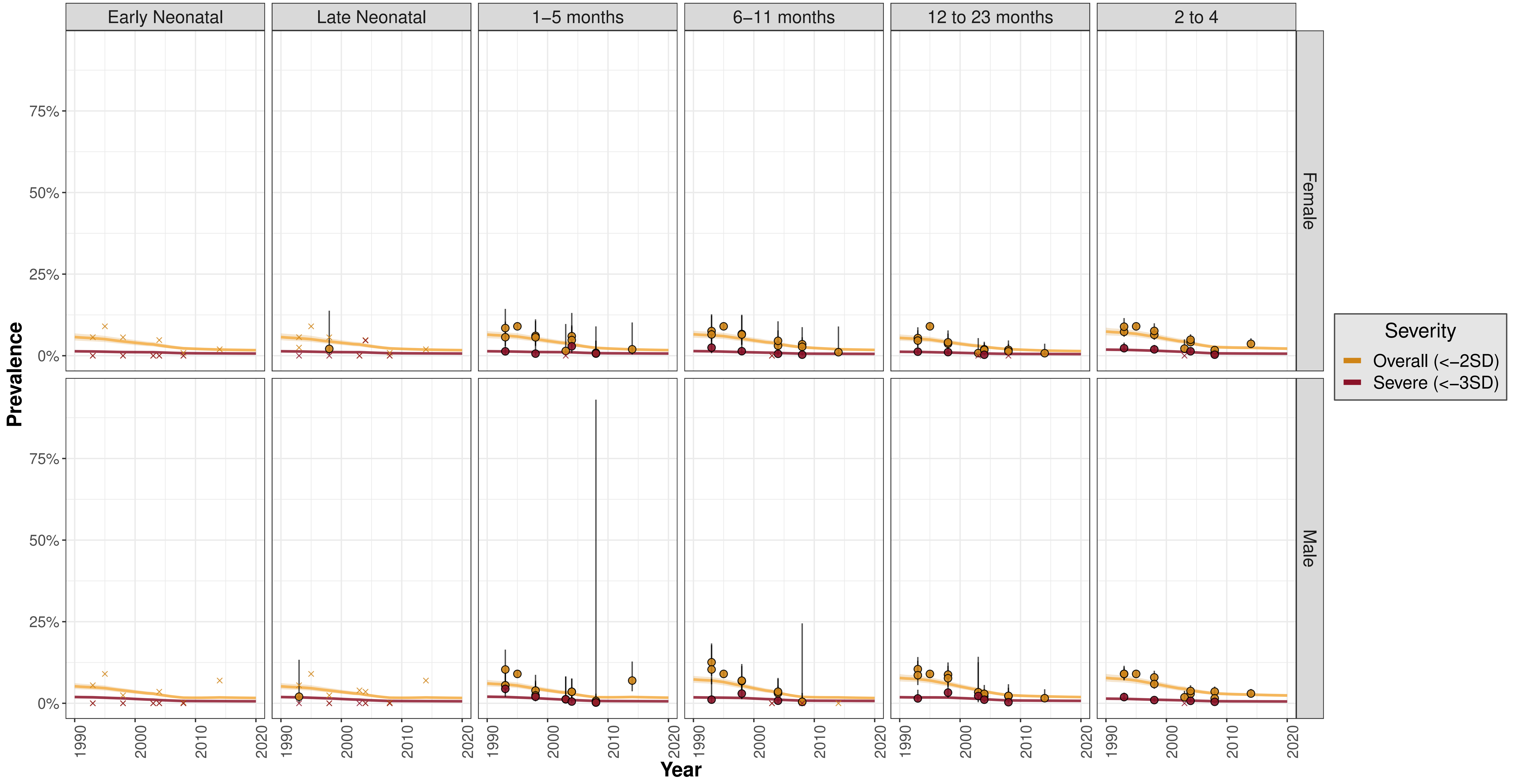

H: Transformed Mean Underweight Z Scores

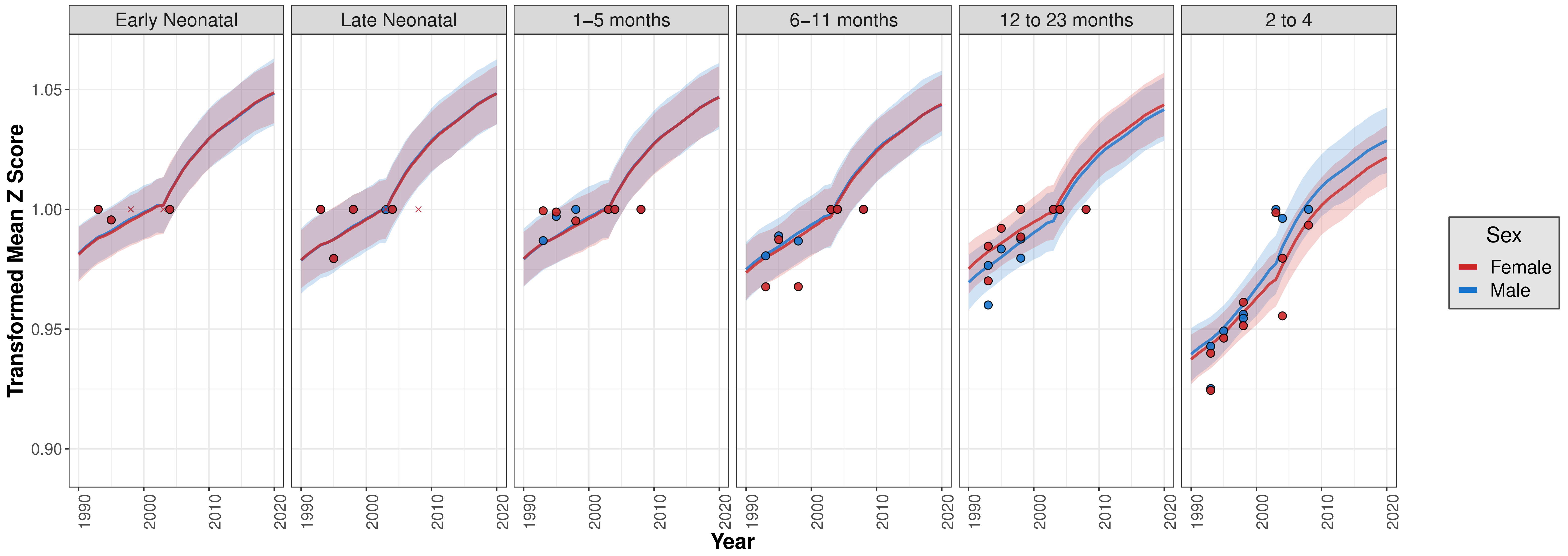

I

Year	Source
1993	DHS
1993	WHO CGM Database
1995	WHO CGM Database
1998	DHS
1998	WHO CGM Database
2003	DHS
2004	DHS
2004	WHO CGM Database
2008	DHS
2008	WHO CGM Database
2014	WHO CGM Database

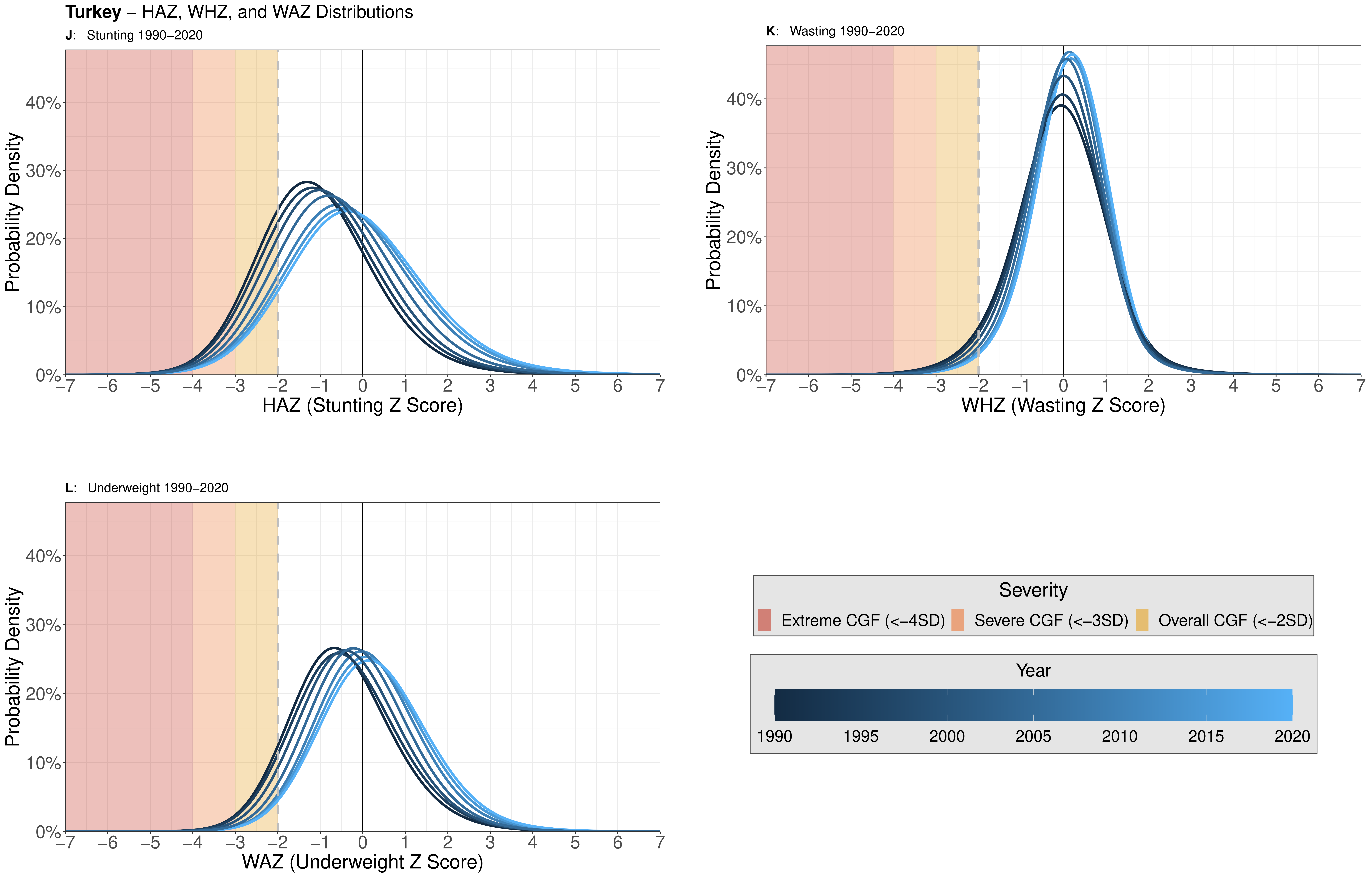

Turkey – HAZ, WHZ, and WAZ Distributions
J: Stunting 1990–2020
Probability Density
HAZ (Stunting Z Score)
K: Wasting 1990–2020
Probability Density
WHZ (Wasting Z Score)
L: Underweight 1990–2020
Probability Density
WAZ (Underweight Z Score)
0%
10%
20%
30%
40%
−7 −6 −5 −4 −3 −2 −1 0 1 2 3 4 5 6 7
Severity
Extreme CGF (<−4SD)
Severe CGF (<−3SD)
Overall CGF (<−2SD)
Year
1990
1995
2000
2005
2010
2015
2020

United Arab Emirates – Stunting (HAZ)

A: Overall and Severe Stunting Prevalence

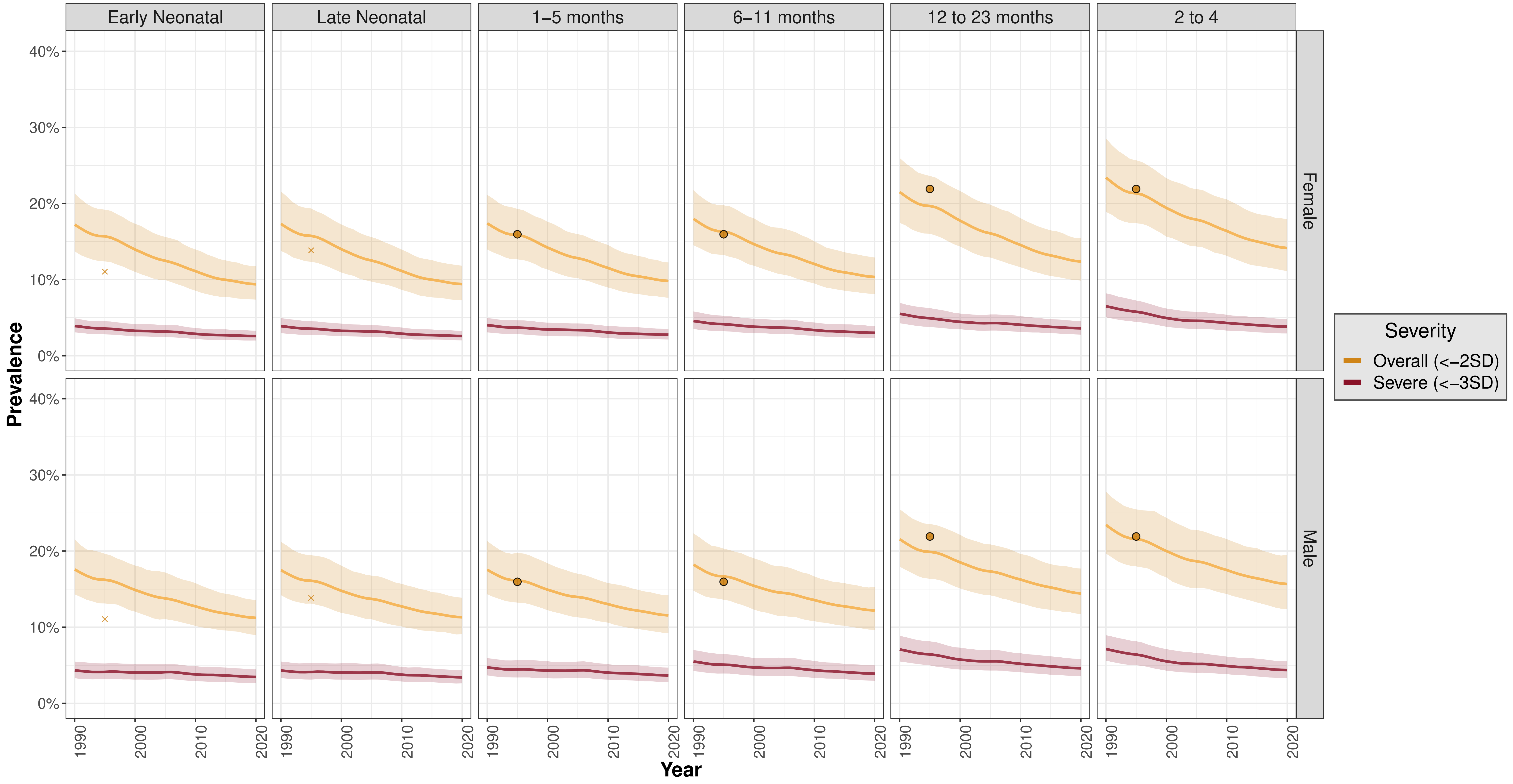

B: Transformed Mean Stunting Z Scores

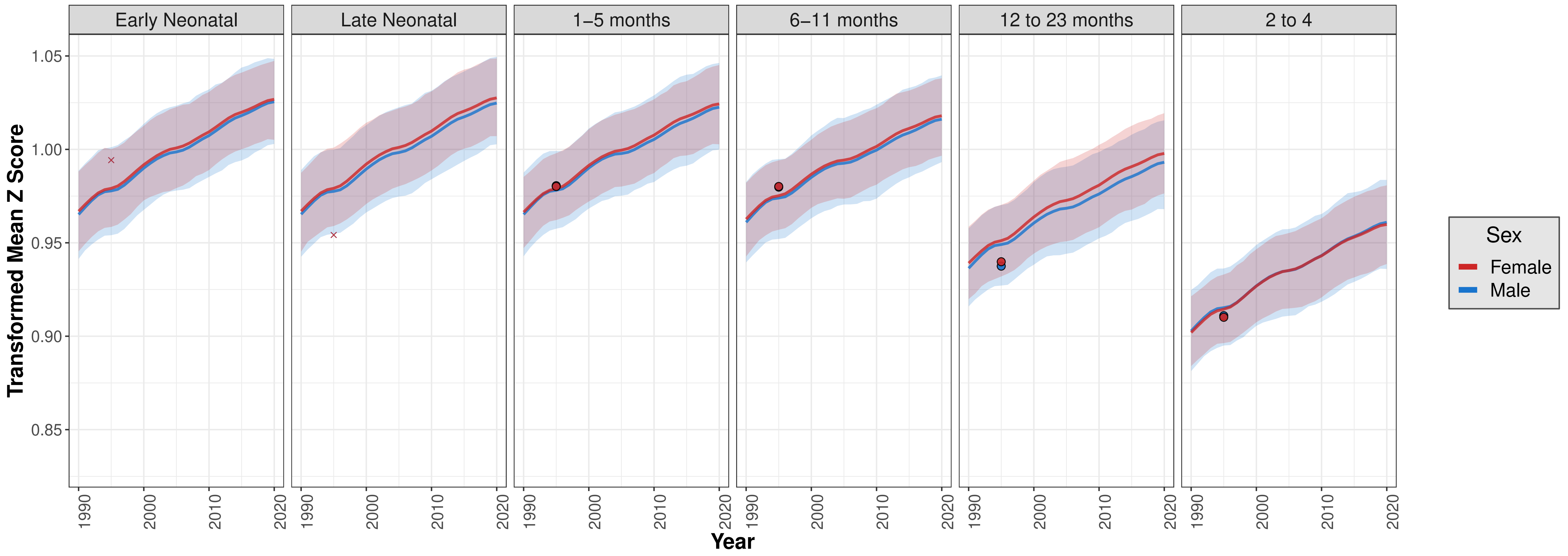

C

Year	Source
1995	Family Health Survey

United Arab Emirates – Wasting (WHZ)

D: Overall and Severe Wasting Prevalence

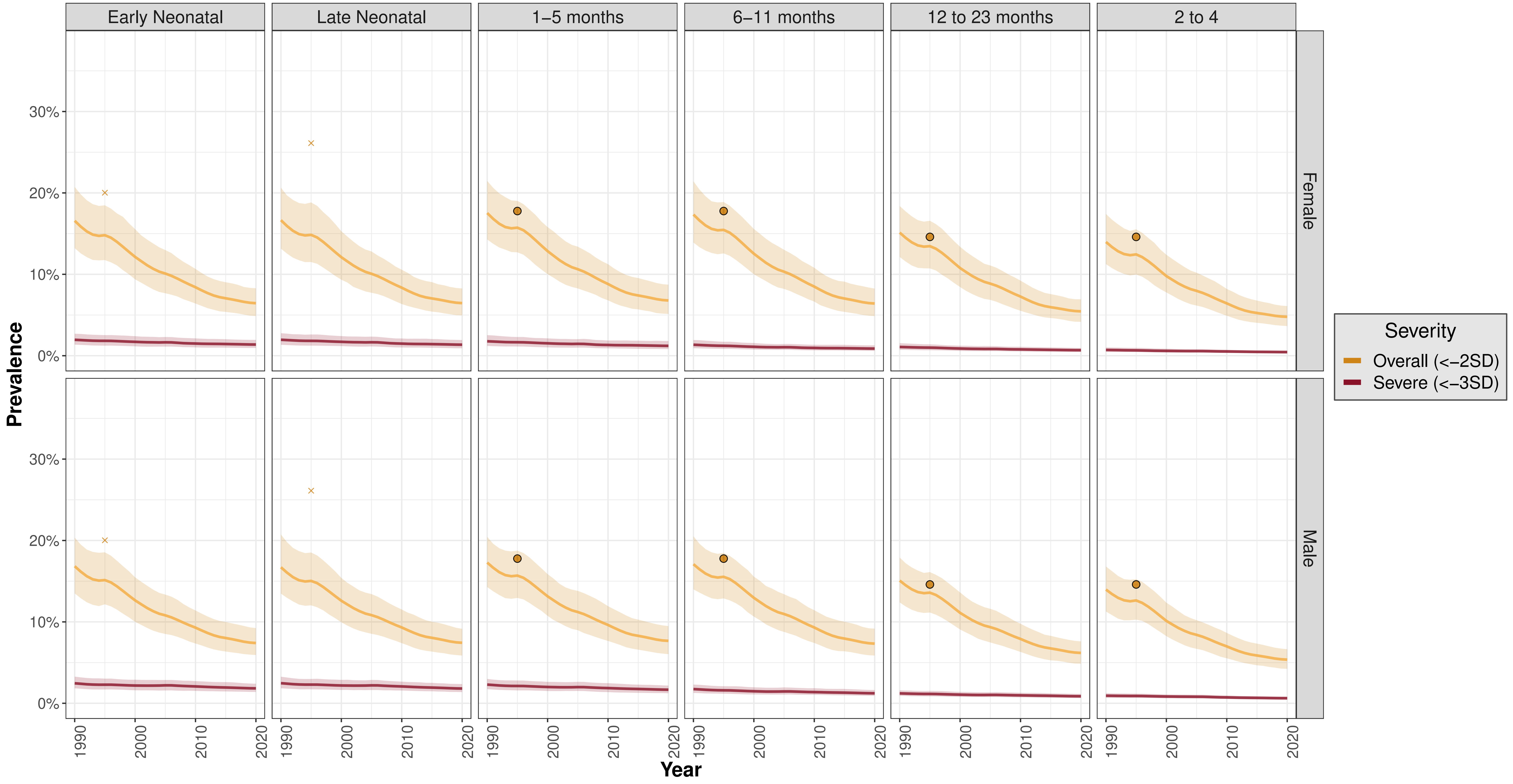

E: Transformed Mean Wasting Z Scores

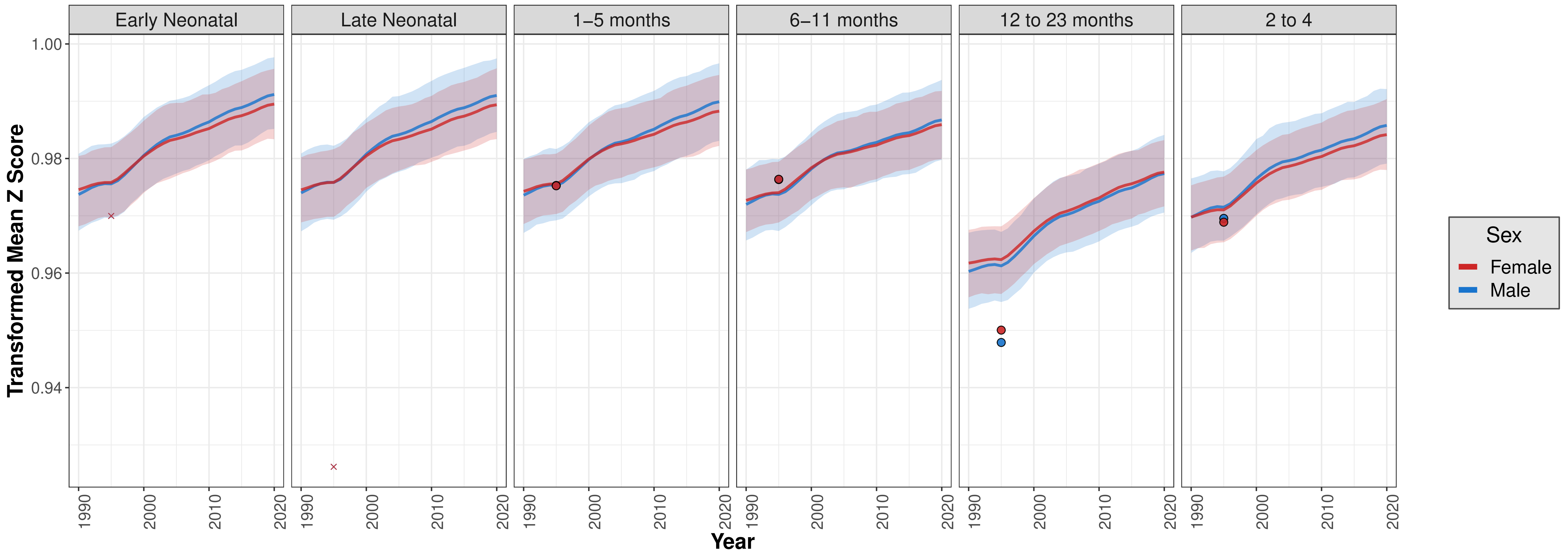

F

Year	Source
1995	Family Health Survey

United Arab Emirates – Underweight (WAZ)

G: Overall and Severe Underweight Prevalence

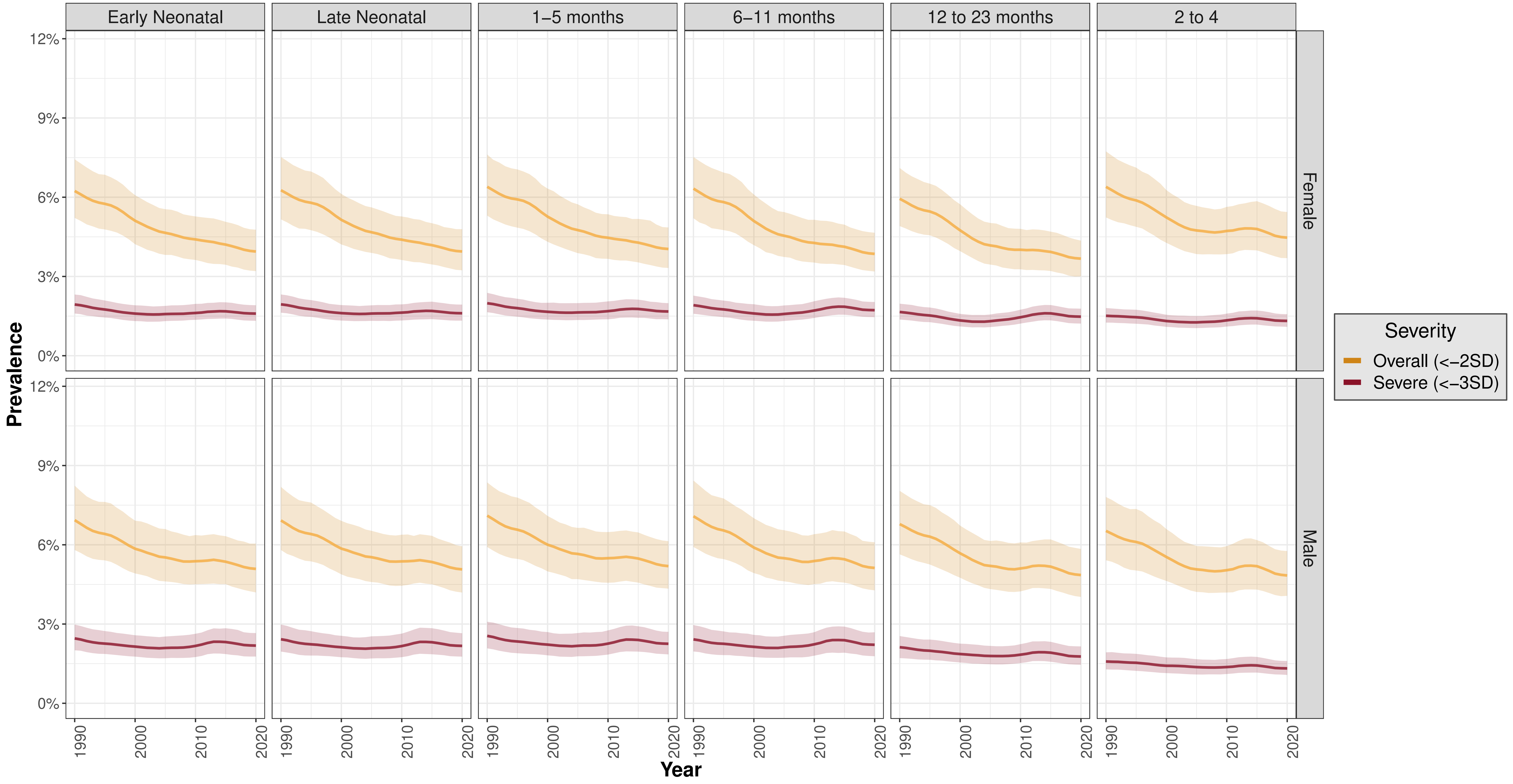

H: Transformed Mean Underweight Z Scores

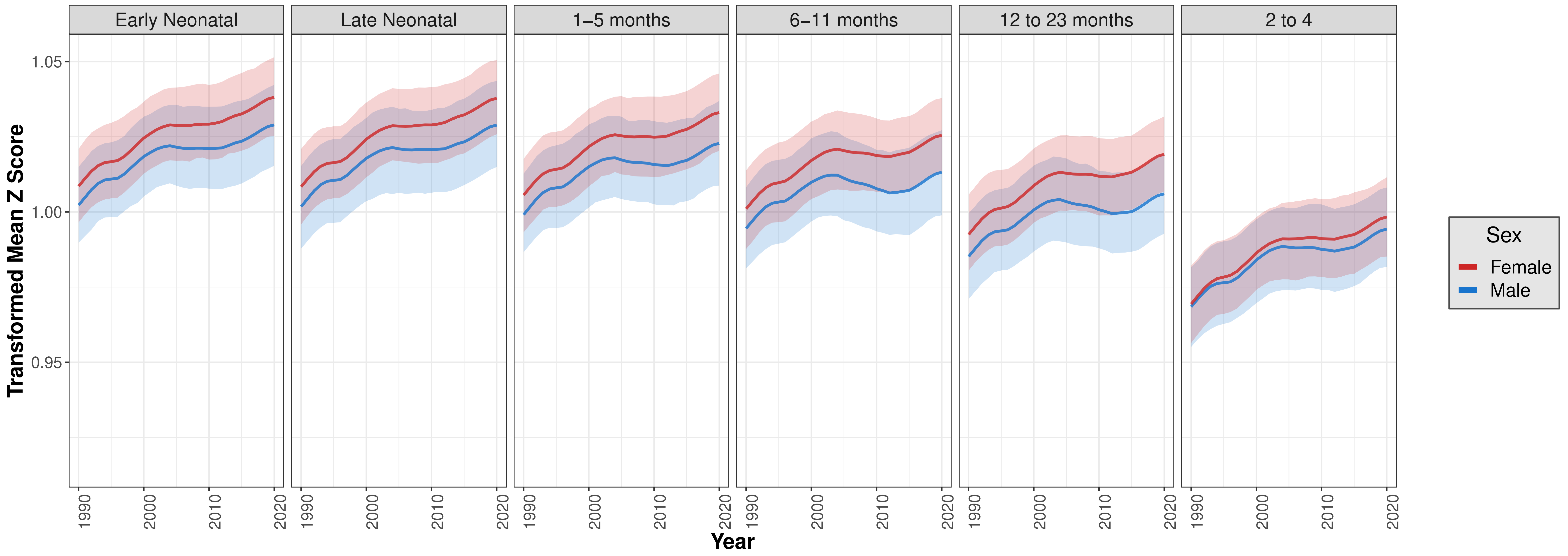

I

**Source**
No sources for this location

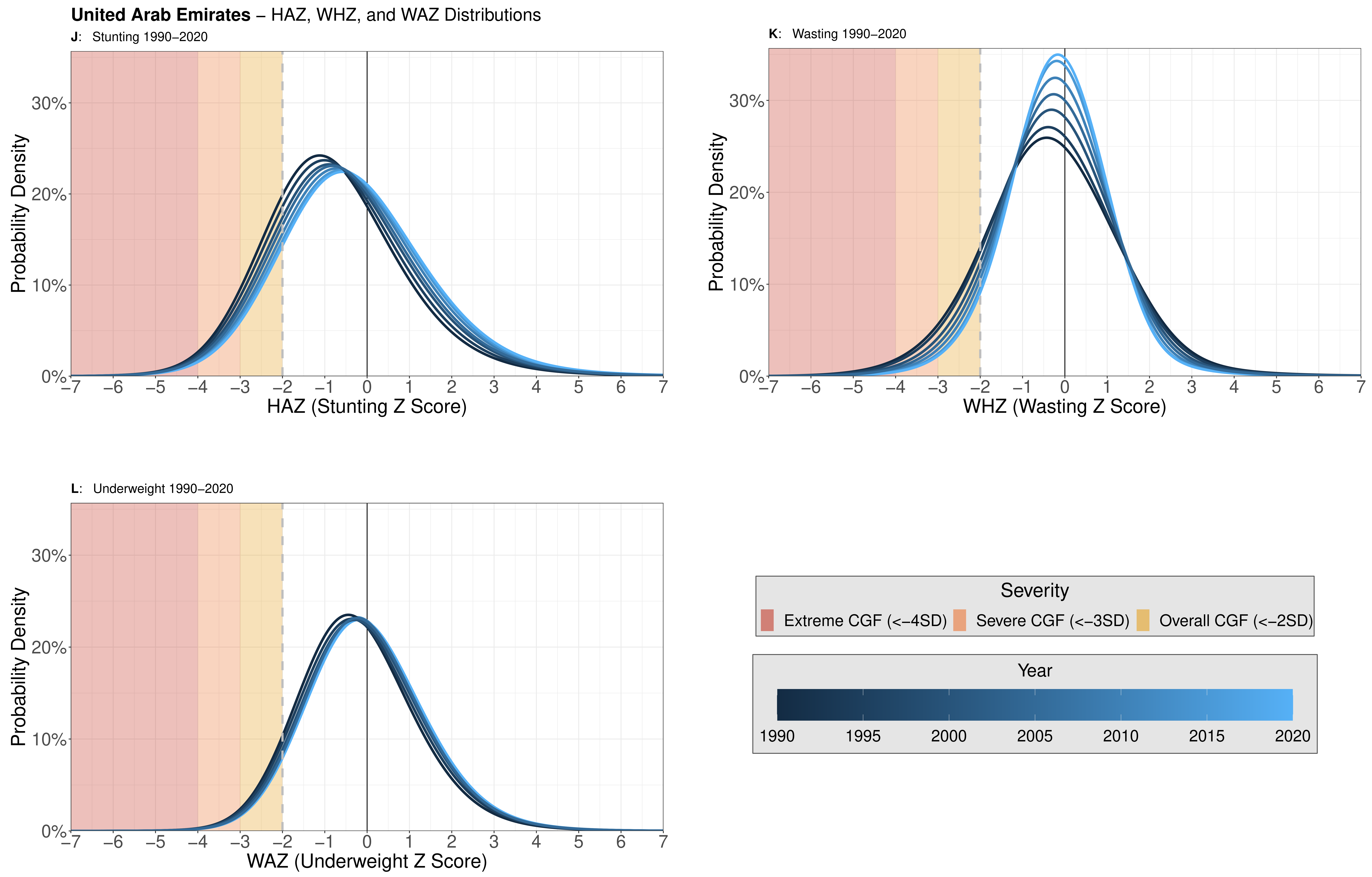
United Arab Emirates – HAZ, WHZ, and WAZ Distributions
J: Stunting 1990–2020
Probability Density
30%
20%
10%
0%
−7 −6 −5 −4 −3 −2 −1 0 1 2 3 4 5 6 7
HAZ (Stunting Z Score)
K: Wasting 1990–2020
Probability Density
30%
20%
10%
0%
−7 −6 −5 −4 −3 −2 −1 0 1 2 3 4 5 6 7
WHZ (Wasting Z Score)
L: Underweight 1990–2020
Probability Density
30%
20%
10%
0%
−7 −6 −5 −4 −3 −2 −1 0 1 2 3 4 5 6 7
WAZ (Underweight Z Score)
Severity
Extreme CGF (<−4SD)
Severe CGF (<−3SD)
Overall CGF (<−2SD)
Year
1990
1995
2000
2005
2010
2015
2020

Yemen – Stunting (HAZ)

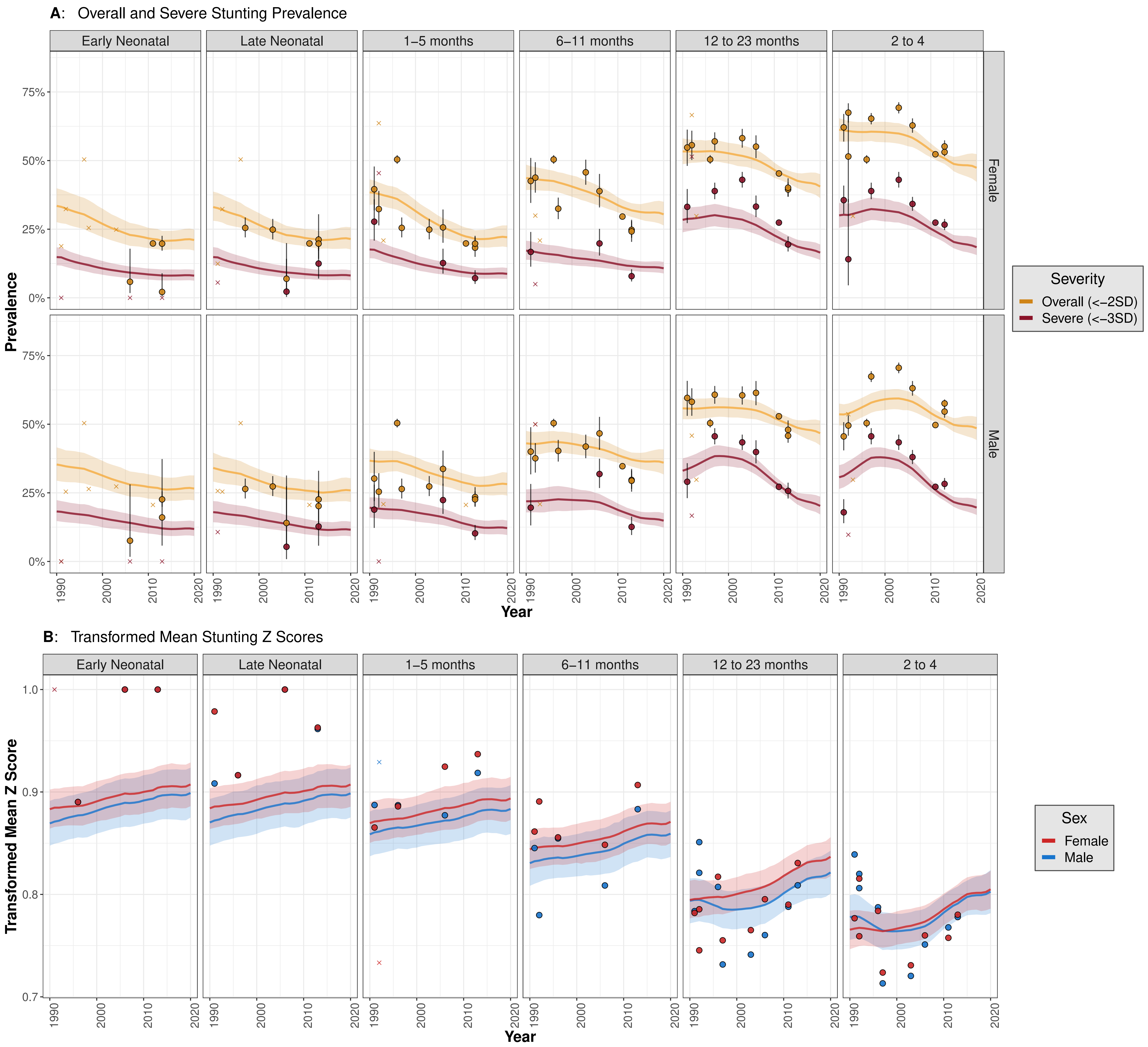

C

Year	Source
1991	DHS
1992	DHS
1992	WHO CGM Database
1993	WHO CGM Database
1996	WHO CGM Database
1997	WHO CGM Database
2003	WHO CGM Database
2006	Household Budget Survey
2011	WHO CGM Database
2012	National Social Protection Monitoring Survey
2013	DHS
2013	WHO CGM Database
2013	Dhamar Nutritional Status and Mortality Survey
2013	National Social Protection Monitoring Survey

Yemen – Wasting (WHZ)

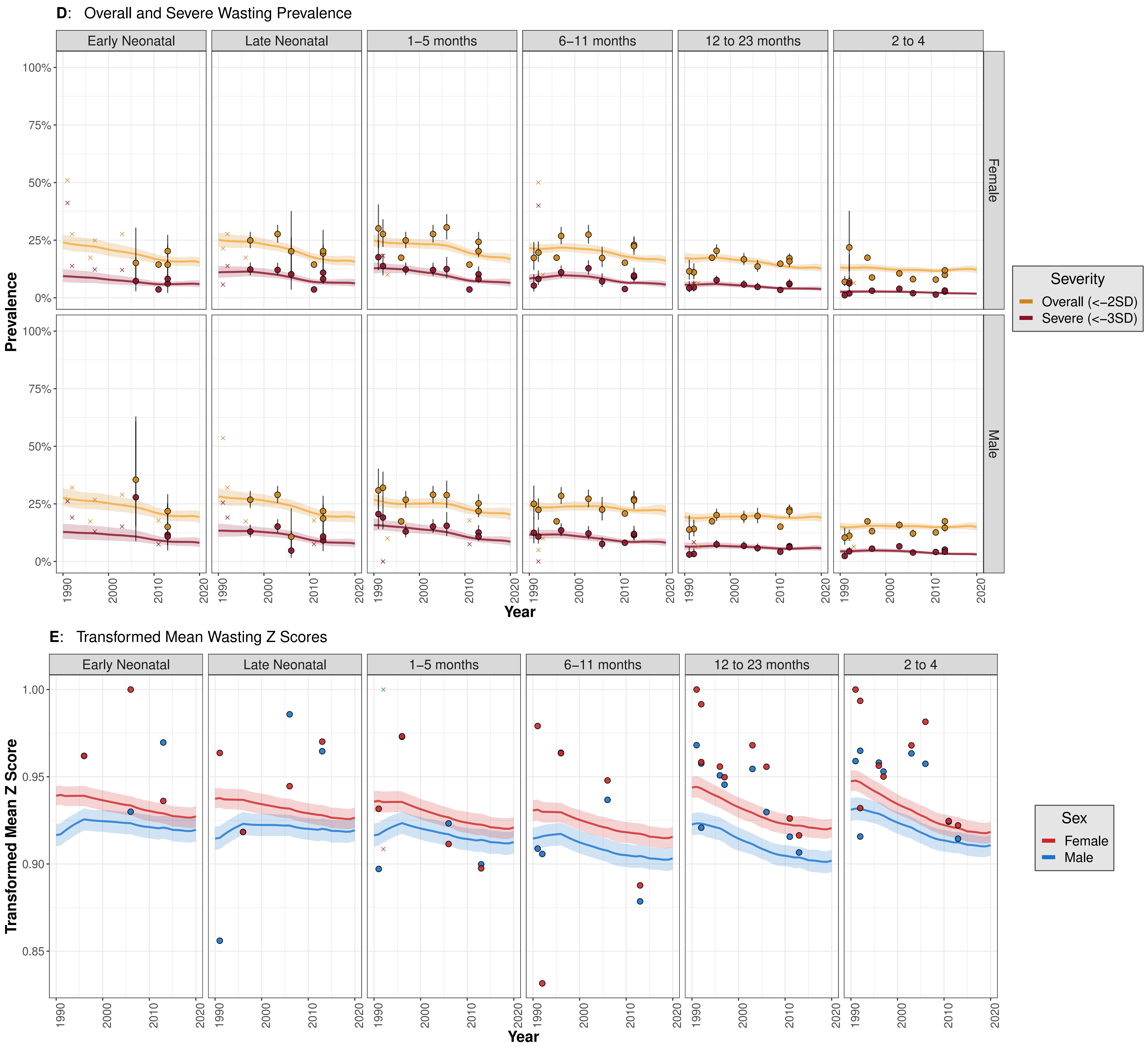

F

Year	Source
1991	DHS
1992	DHS
1992	WHO CGM Database
1993	WHO CGM Database
1996	WHO CGM Database
1997	WHO CGM Database
2003	WHO CGM Database
2006	Household Budget Survey
2011	WHO CGM Database
2012	National Social Protection Monitoring Survey
2013	DHS
2013	WHO CGM Database
2013	Dhamar Nutritional Status and Mortality Survey
2013	National Social Protection Monitoring Survey

Yemen – Underweight (WAZ)

G: Overall and Severe Underweight Prevalence

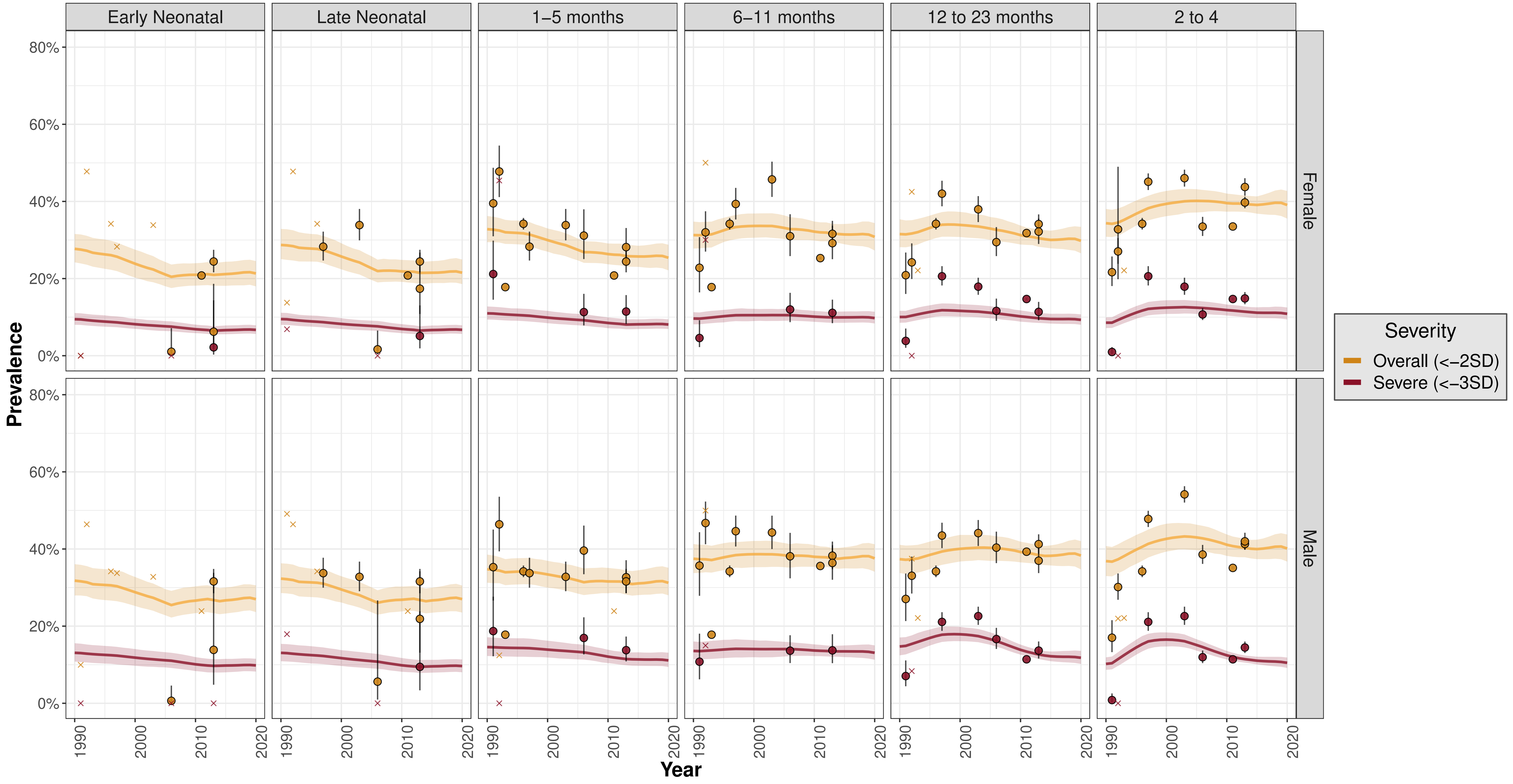

H: Transformed Mean Underweight Z Scores

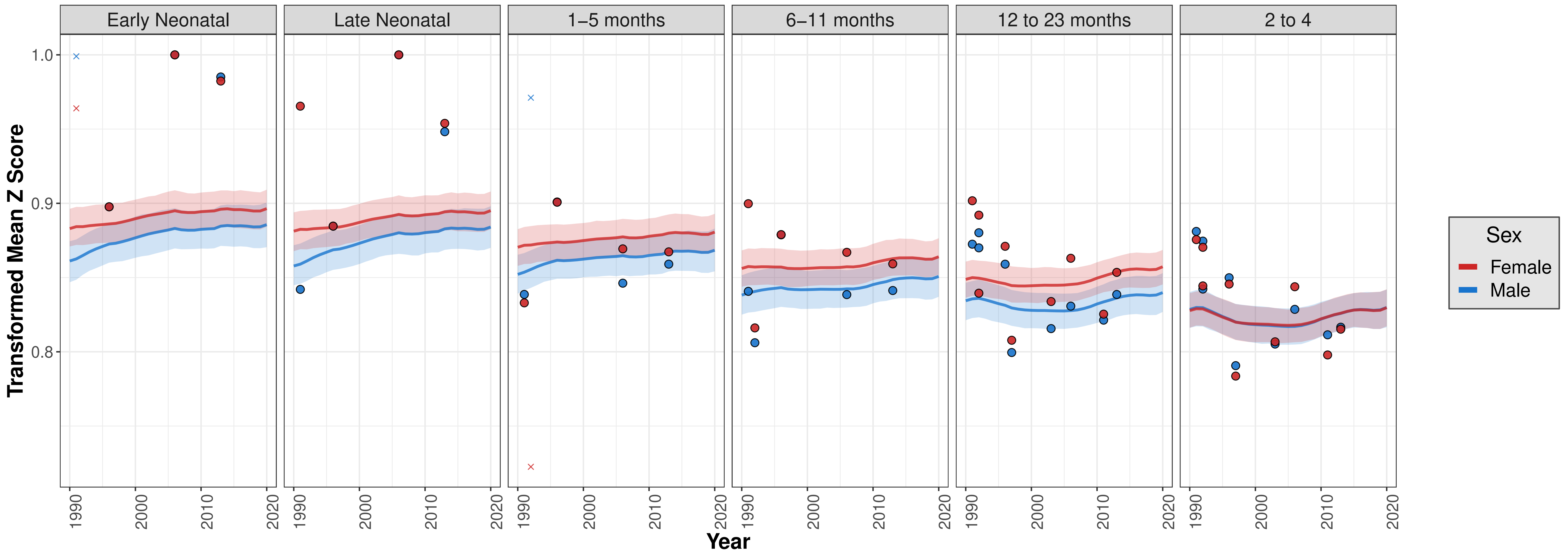

I

Year	Source
1991	DHS
1992	DHS
1992	WHO CGM Database
1993	WHO CGM Database
1996	WHO CGM Database
1997	WHO CGM Database
2003	WHO CGM Database
2006	Household Budget Survey
2011	WHO CGM Database
2012	National Social Protection Monitoring Survey
2013	DHS
2013	WHO CGM Database
2013	Dhamar Nutritional Status and Mortality Survey
2013	National Social Protection Monitoring Survey

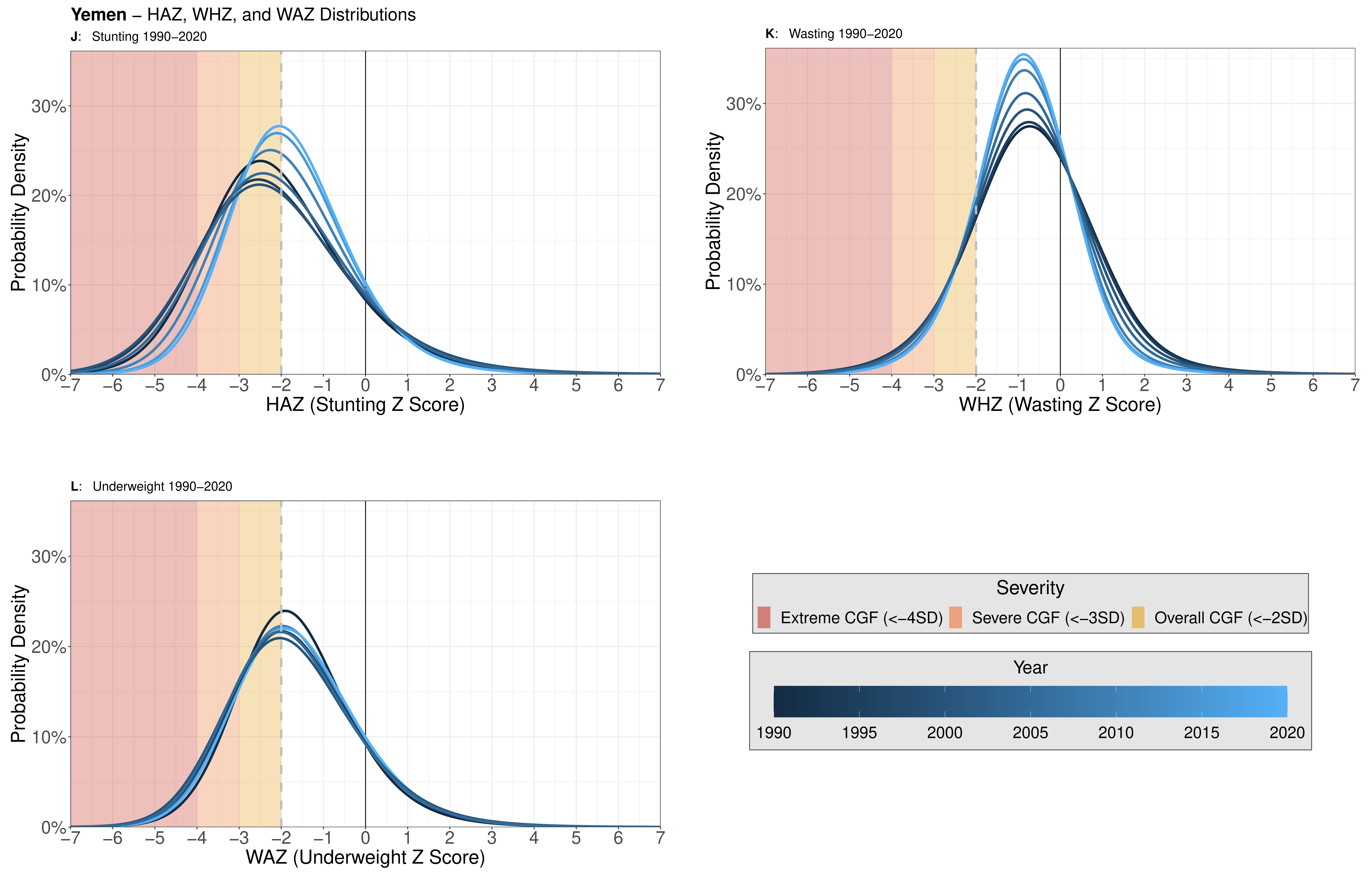

Yemen – HAZ, WHZ, and WAZ Distributions
J: Stunting 1990–2020
K: Wasting 1990–2020
L: Underweight 1990–2020
Probability Density
0%
10%
20%
30%
−7 −6 −5 −4 −3 −2 −1 0 1 2 3 4 5 6 7
HAZ (Stunting Z Score)
WHZ (Wasting Z Score)
WAZ (Underweight Z Score)
Severity
Extreme CGF (<−4SD)
Severe CGF (<−3SD)
Overall CGF (<−2SD)
Year
1990
1995
2000
2005
2010
2015
2020

Afghanistan – Stunting (HAZ)

A: Overall and Severe Stunting Prevalence

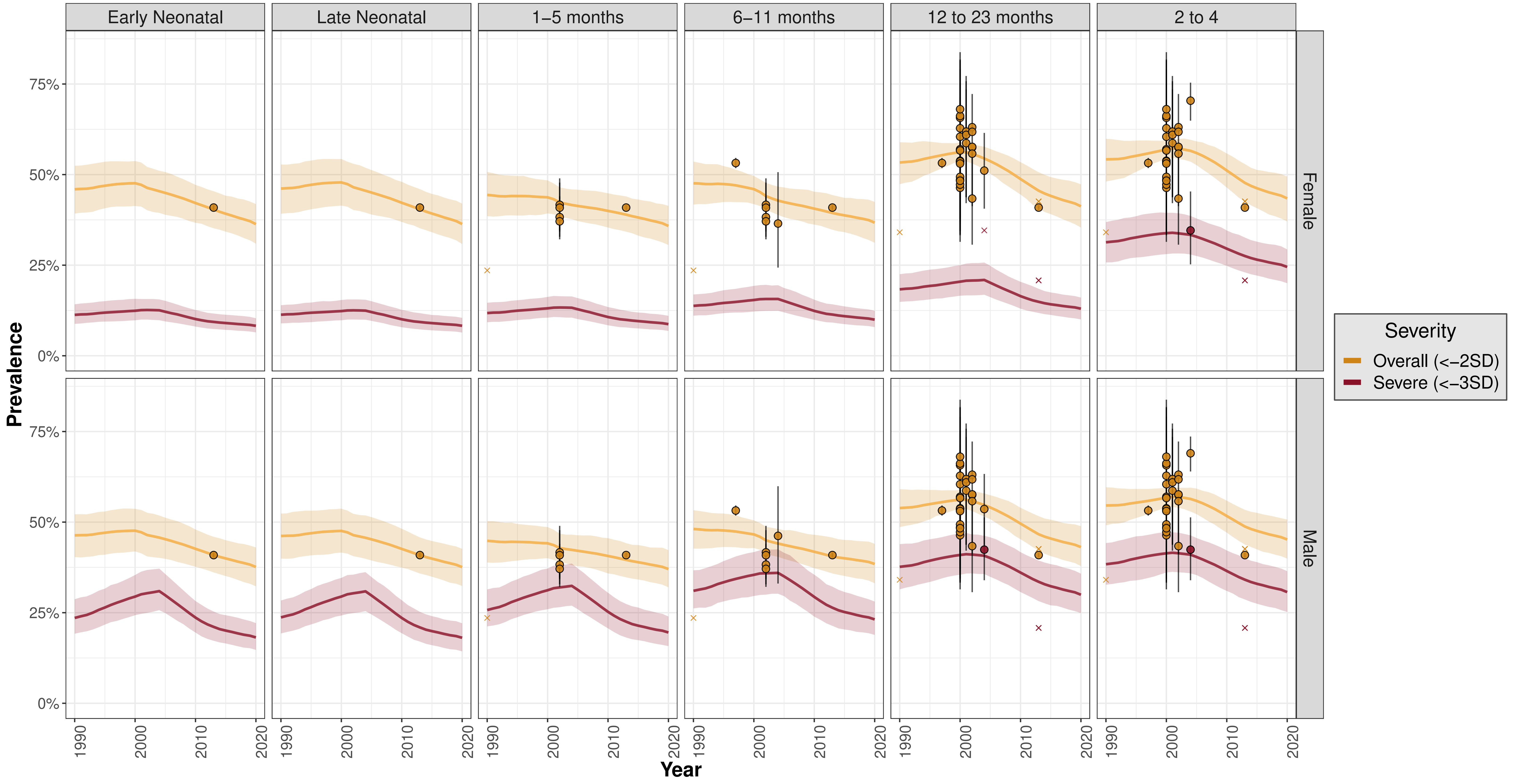

B: Transformed Mean Stunting Z Scores

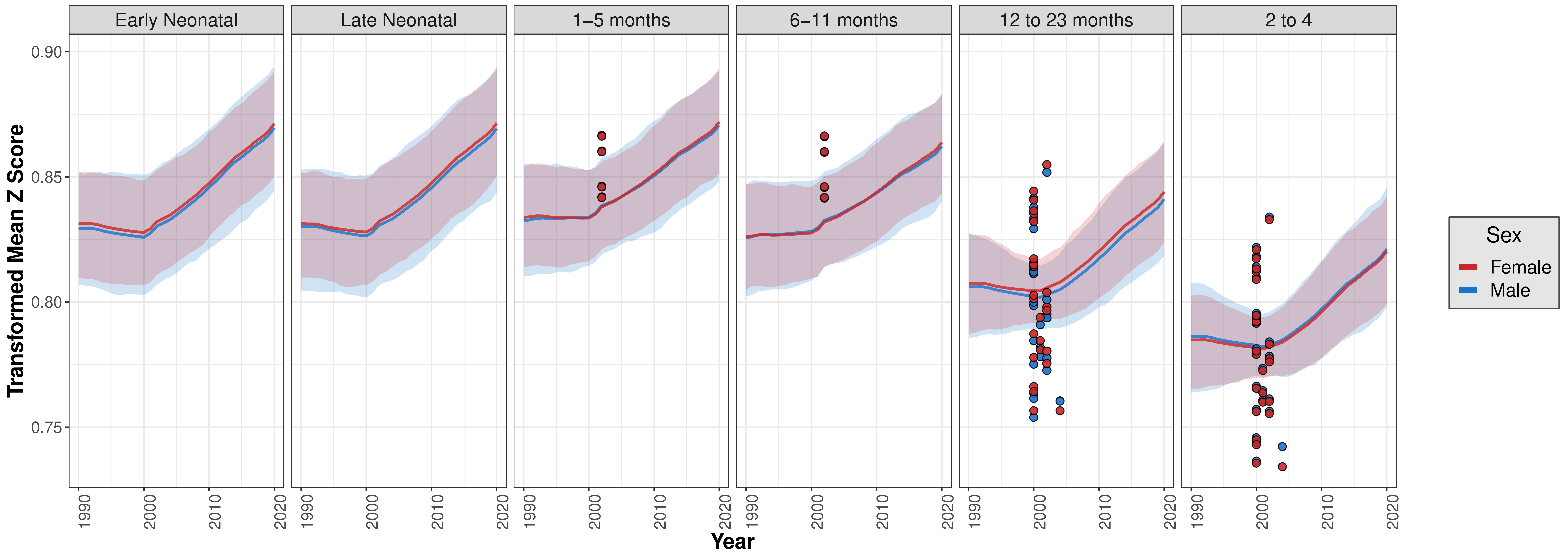

C

Year	Source
1990	WHO CGM Database
1997	WHO CGM Database
2000	WHO CGM Database
2001	WHO CGM Database
2002	WHO CGM Database
2004	WHO CGM Database
2013	WHO CGM Database
2013	National Nutrition Survey

Afghanistan – Wasting (WHZ)

D: Overall and Severe Wasting Prevalence

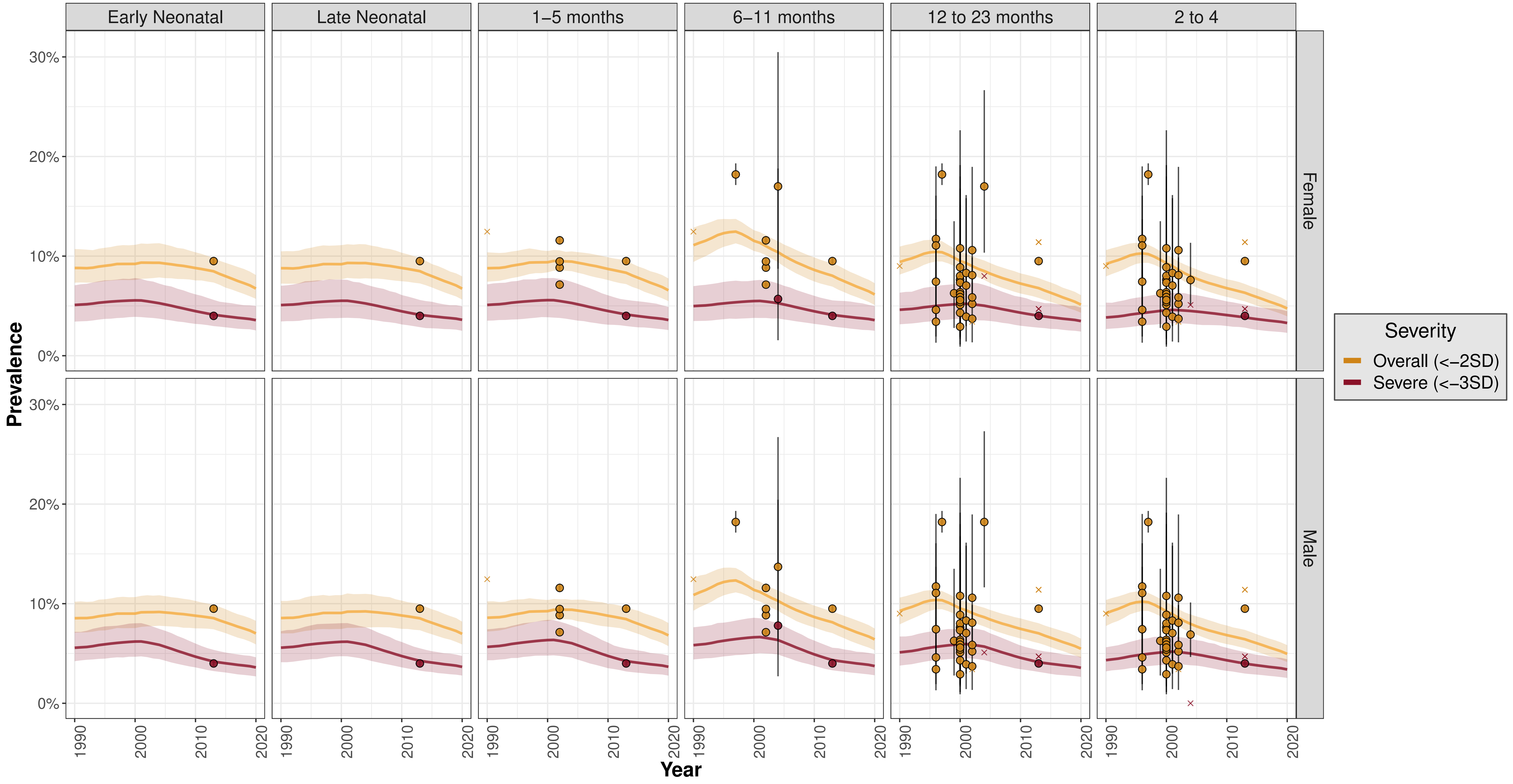

E: Transformed Mean Wasting Z Scores

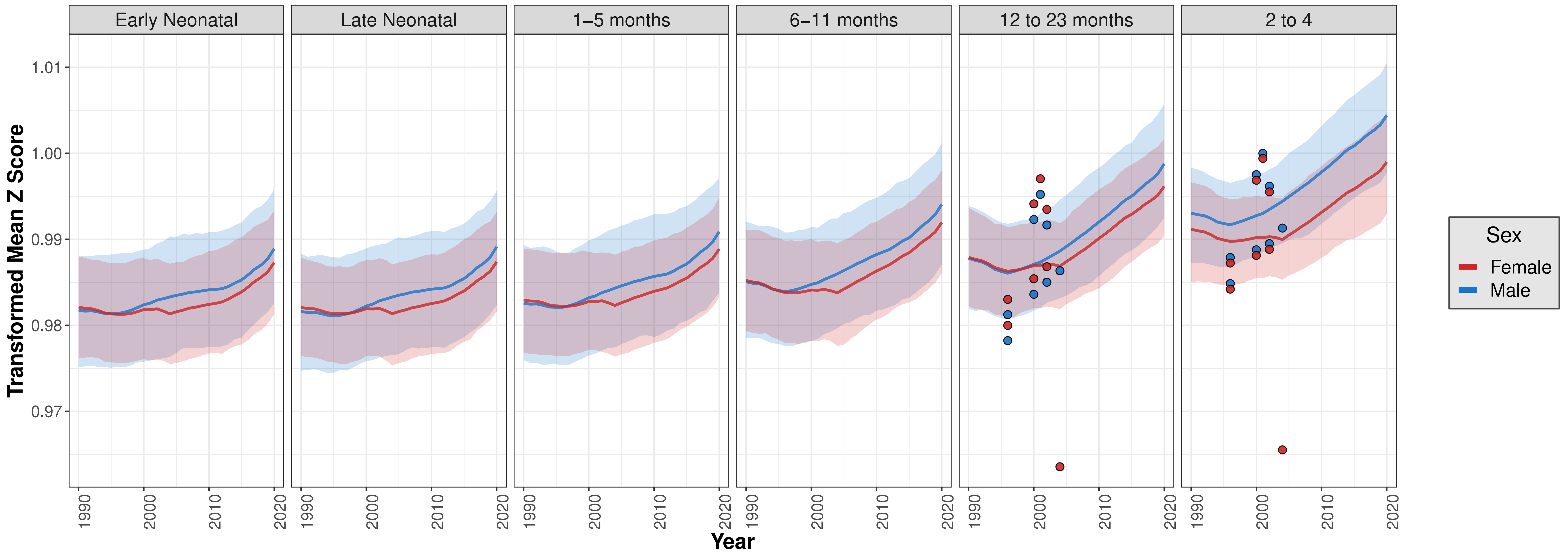

F

Year	Source
1990	WHO CGM Database
1996	WHO CGM Database
1997	WHO CGM Database
1999	WHO CGM Database
2000	WHO CGM Database
2001	WHO CGM Database
2002	WHO CGM Database
2004	WHO CGM Database
2013	WHO CGM Database
2013	National Nutrition Survey

Afghanistan – Underweight (WAZ)

G: Overall and Severe Underweight Prevalence

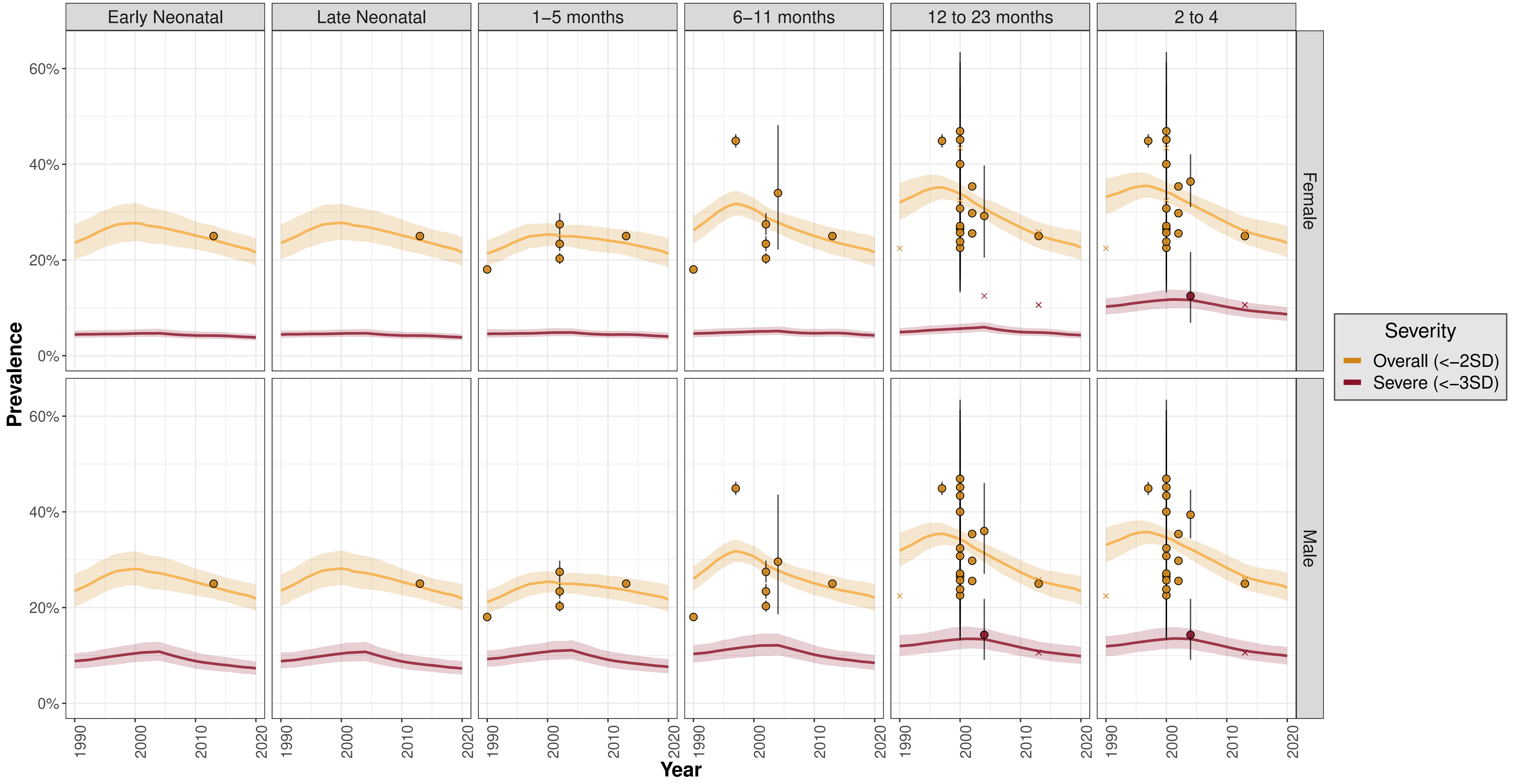

H: Transformed Mean Underweight Z Scores

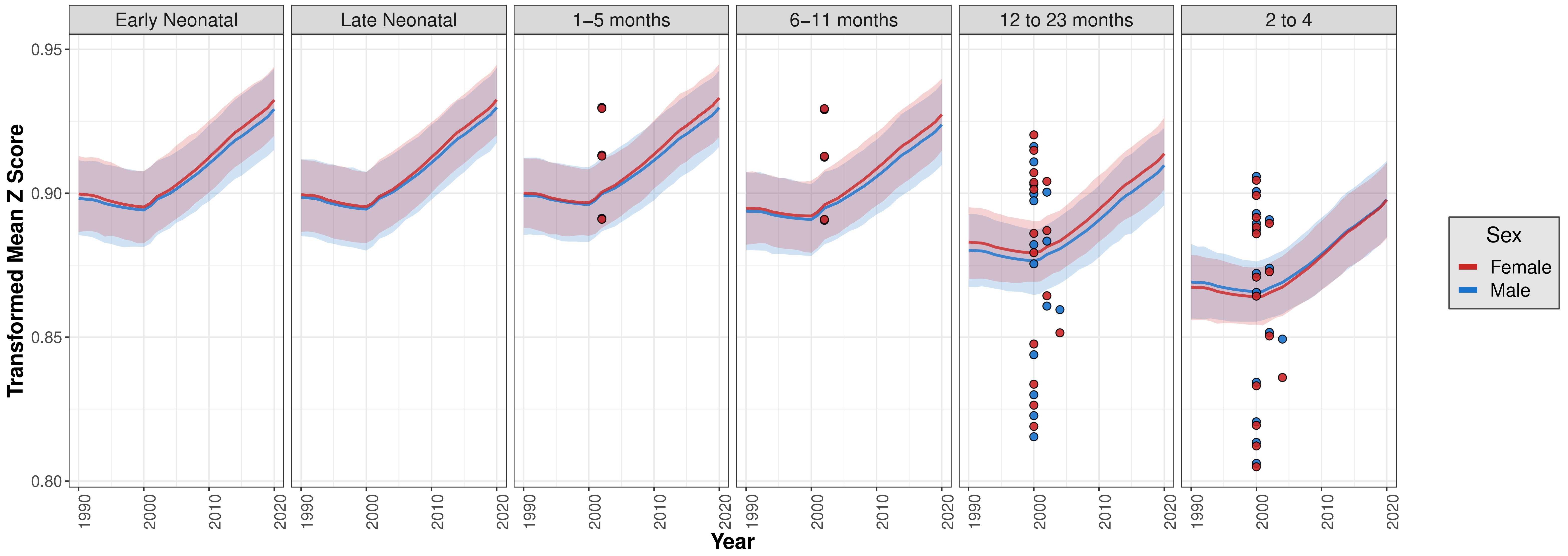

I

Year	Source
1990	WHO CGM Database
1997	WHO CGM Database
2000	WHO CGM Database
2002	WHO CGM Database
2004	WHO CGM Database
2013	WHO CGM Database
2013	National Nutrition Survey

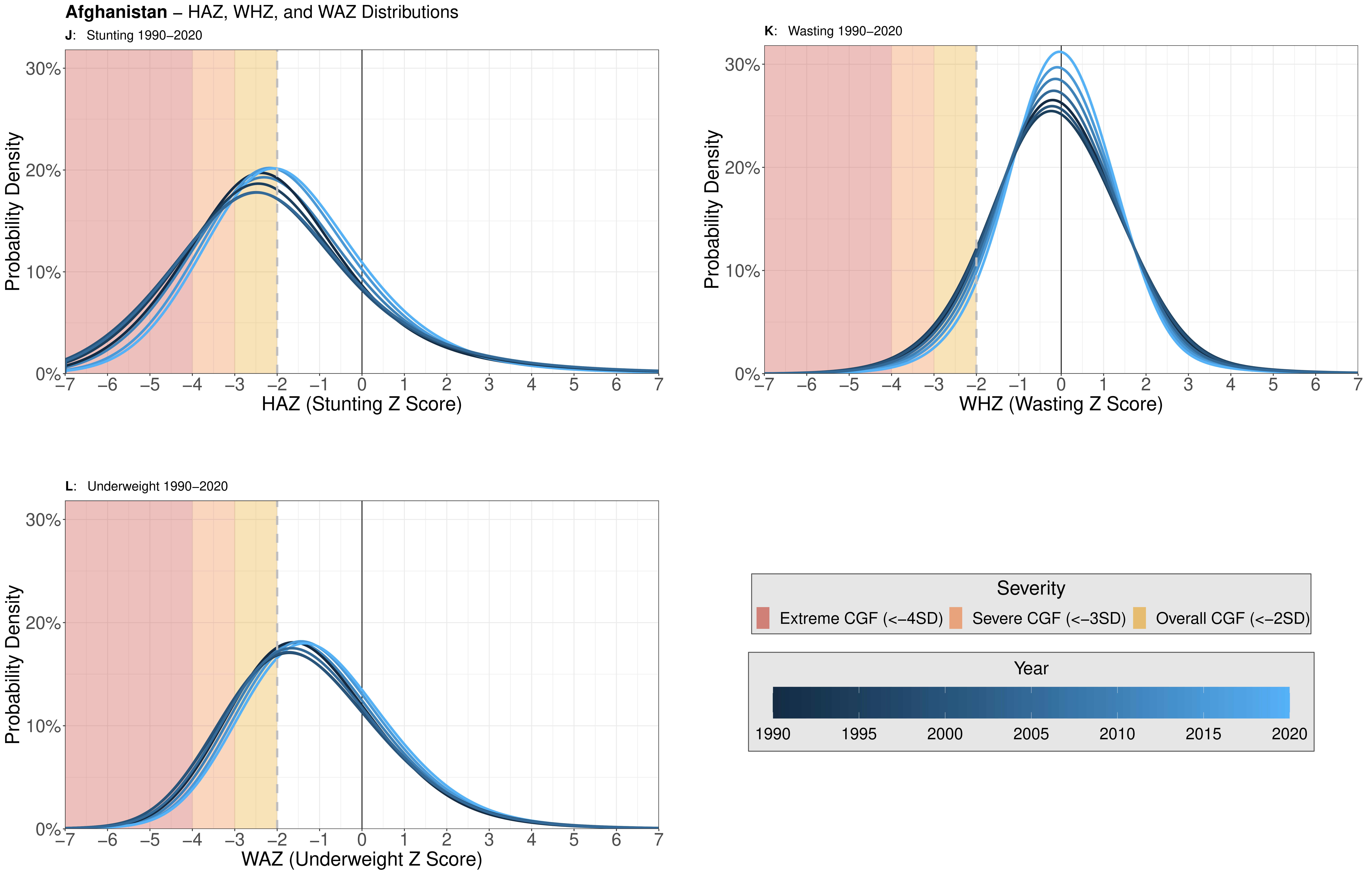
Afghanistan – HAZ, WHZ, and WAZ Distributions
J: Stunting 1990–2020
K: Wasting 1990–2020
L: Underweight 1990–2020
Probability Density
30%
20%
10%
0%
−7 −6 −5 −4 −3 −2 −1 0 1 2 3 4 5 6 7
HAZ (Stunting Z Score)
WHZ (Wasting Z Score)
WAZ (Underweight Z Score)
Severity
Extreme CGF (<−4SD)
Severe CGF (<−3SD)
Overall CGF (<−2SD)
Year
1990
1995
2000
2005
2010
2015
2020

Sudan – Stunting (HAZ)

A: Overall and Severe Stunting Prevalence

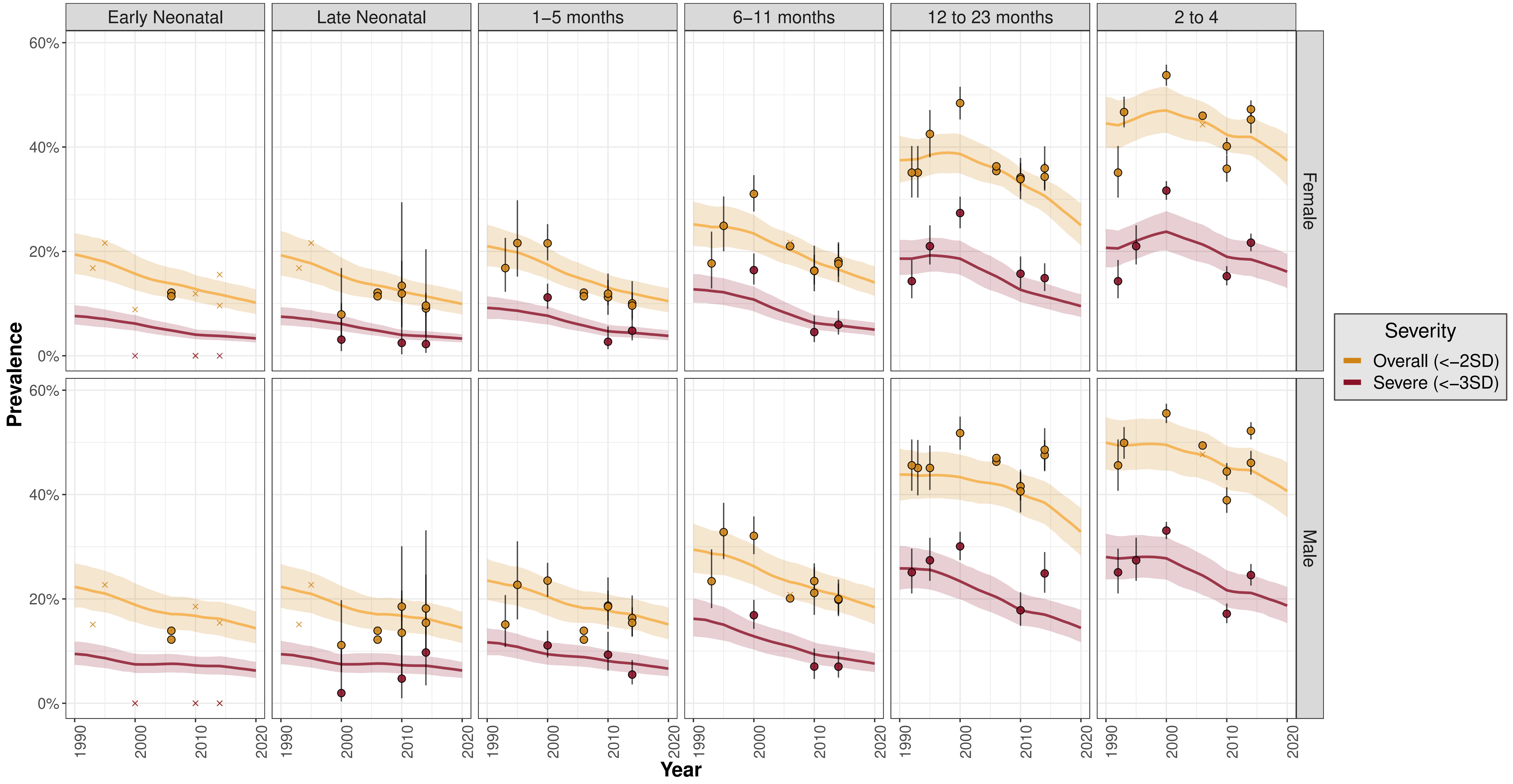

B: Transformed Mean Stunting Z Scores

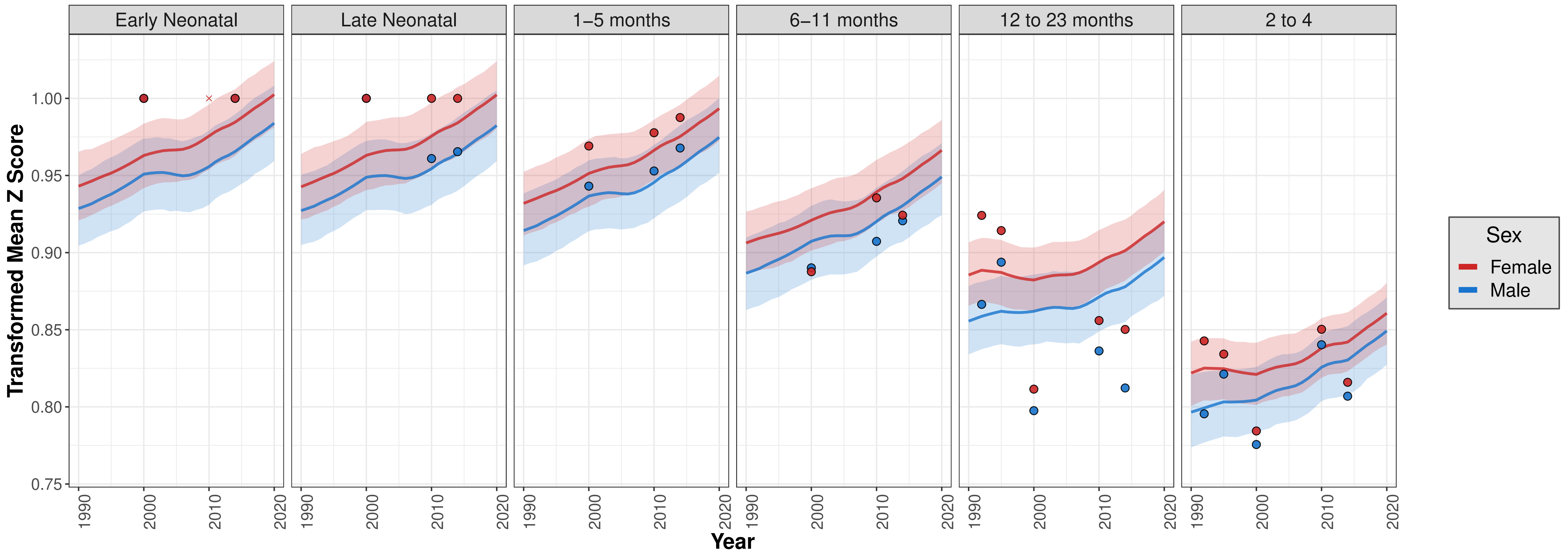

C

Year	Source
1992	WHO CGM Database
1993	WHO CGM Database
1995	WHO CGM Database
2000	MICS
2006	WHO CGM Database
2010	WHO CGM Database
2010	MICS
2014	WHO CGM Database
2014	MICS

Sudan – Wasting (WHZ)

D: Overall and Severe Wasting Prevalence

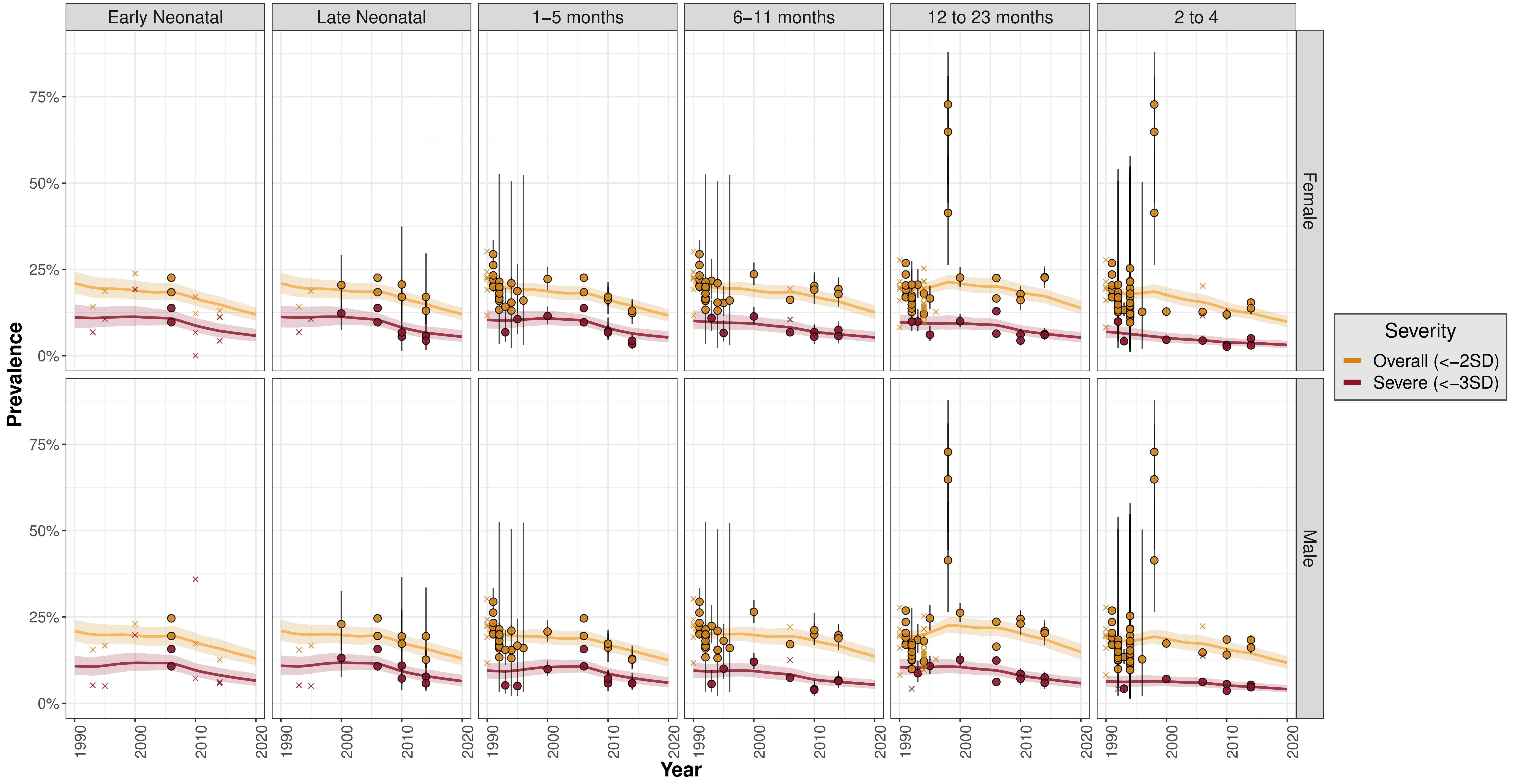

E: Transformed Mean Wasting Z Scores

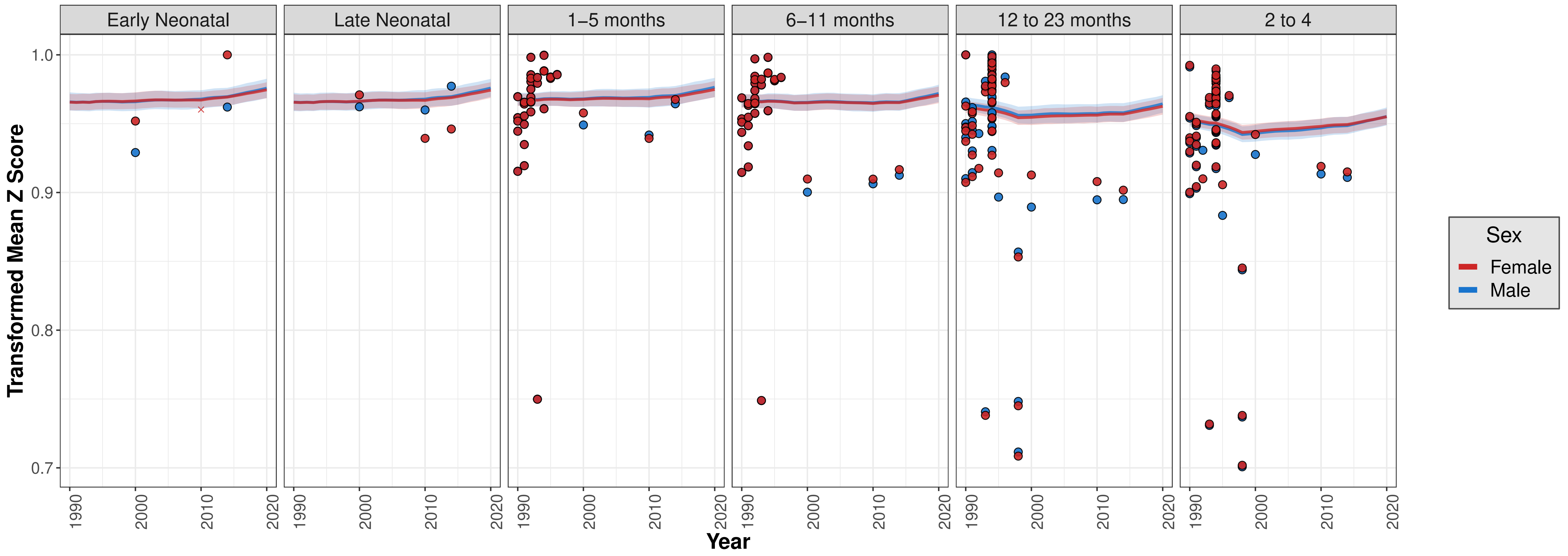

F

Year	Source
1986	WHO CGM Database
1987	WHO CGM Database
1988	WHO CGM Database
1990	WHO CGM Database
1991	WHO CGM Database
1992	WHO CGM Database
1993	WHO CGM Database
1994	WHO CGM Database
1995	WHO CGM Database
1996	WHO CGM Database
1998	WHO CGM Database
2000	MICS
2006	WHO CGM Database
2010	WHO CGM Database
2010	MICS
2014	WHO CGM Database
2014	MICS

Sudan – Underweight (WAZ)

G: Overall and Severe Underweight Prevalence

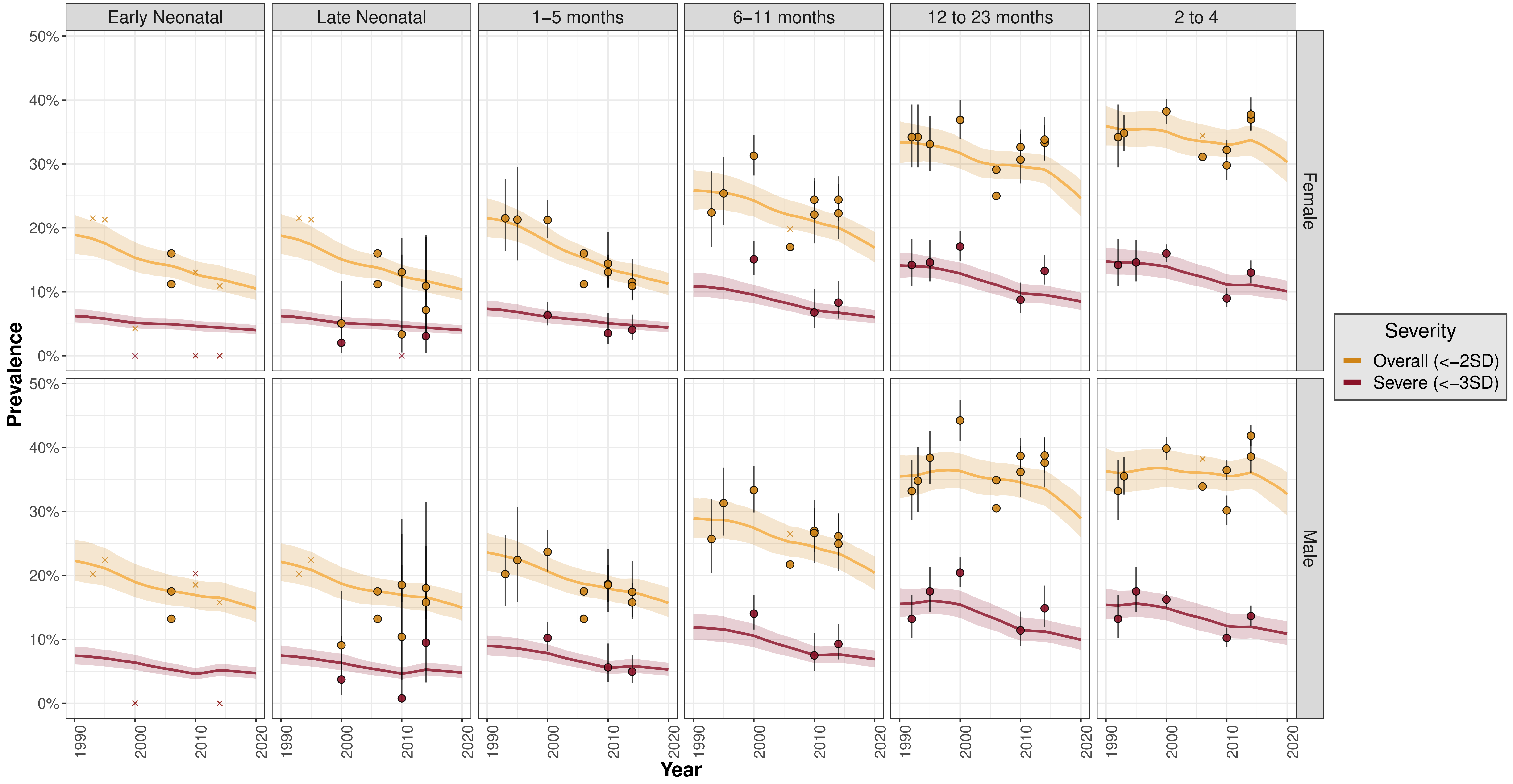

H: Transformed Mean Underweight Z Scores

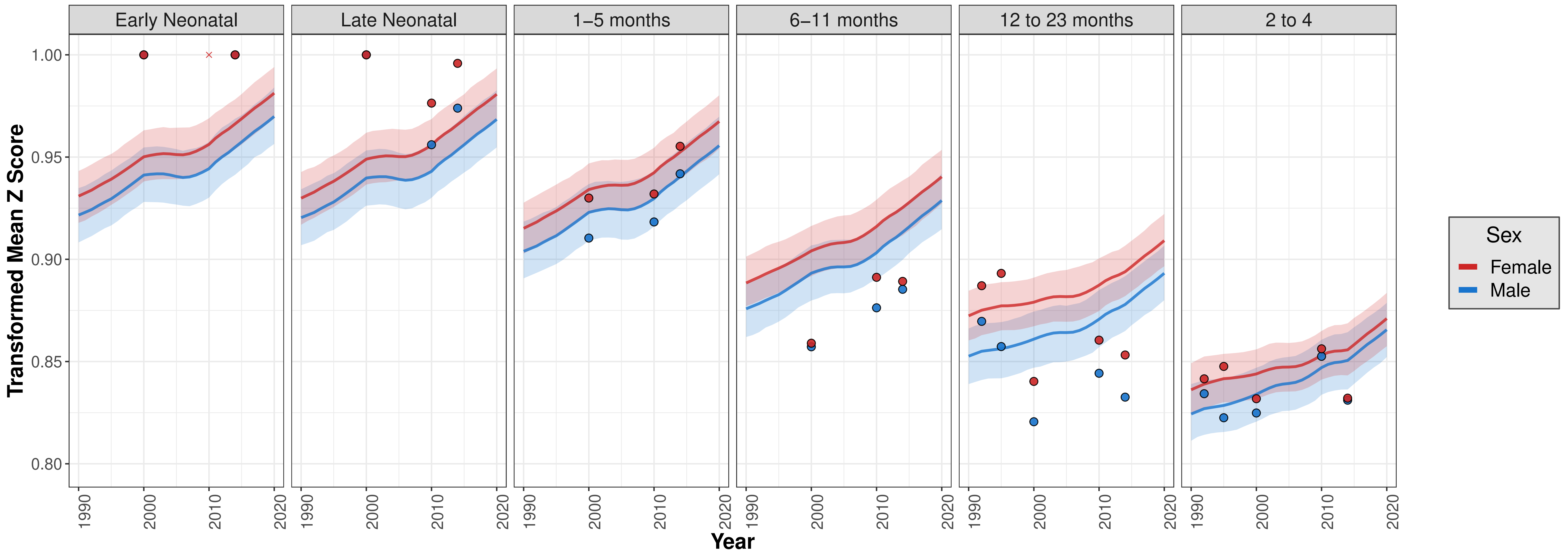

I

Year	Source
1992	WHO CGM Database
1993	WHO CGM Database
1995	WHO CGM Database
2000	MICS
2006	WHO CGM Database
2010	WHO CGM Database
2010	MICS
2014	WHO CGM Database
2014	MICS

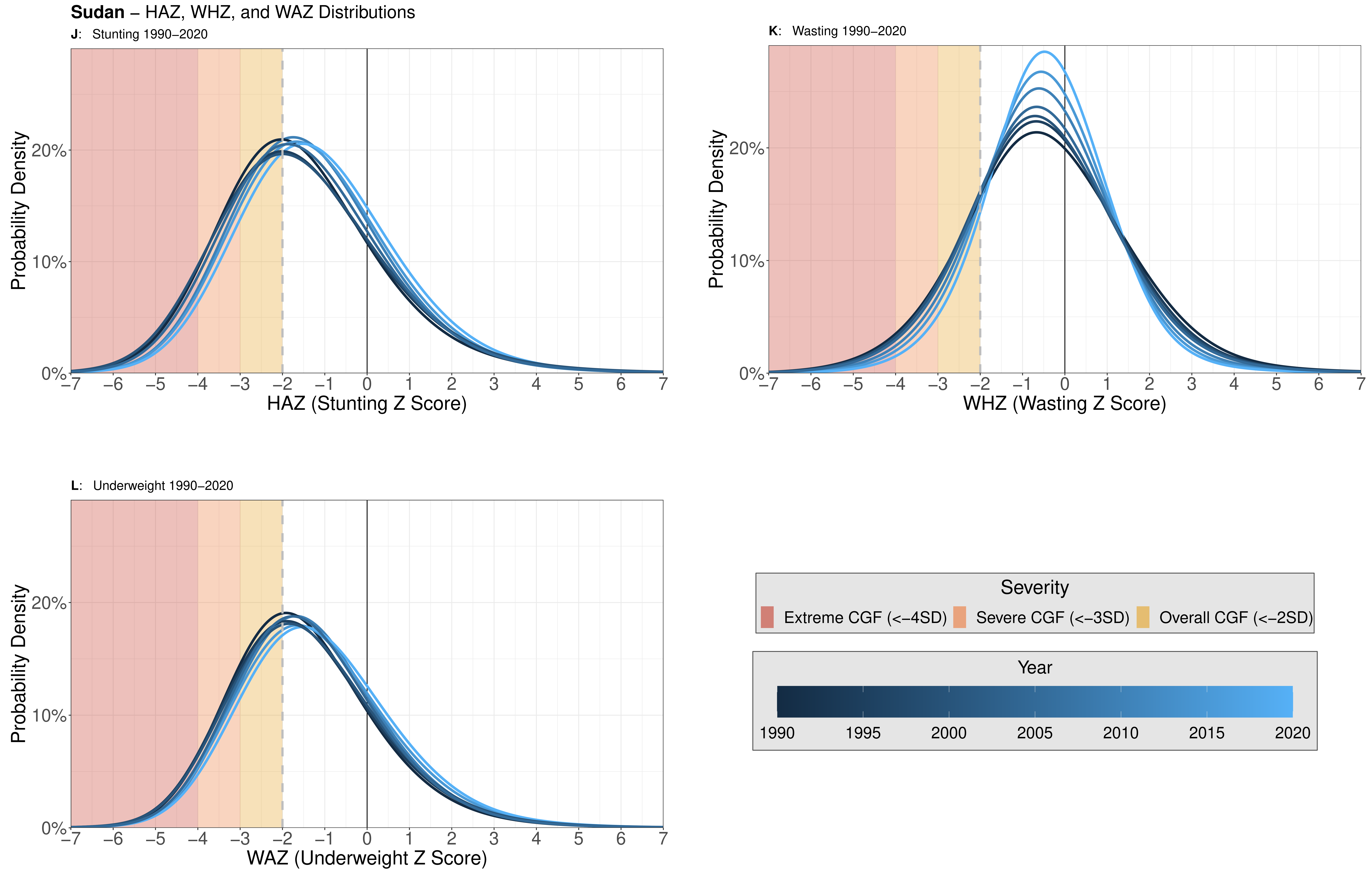

Sudan – HAZ, WHZ, and WAZ Distributions
J: Stunting 1990–2020
Probability Density
0%
10%
20%
−7 −6 −5 −4 −3 −2 −1 0 1 2 3 4 5 6 7
HAZ (Stunting Z Score)
K: Wasting 1990–2020
Probability Density
0%
10%
20%
−7 −6 −5 −4 −3 −2 −1 0 1 2 3 4 5 6 7
WHZ (Wasting Z Score)
L: Underweight 1990–2020
Probability Density
0%
10%
20%
−7 −6 −5 −4 −3 −2 −1 0 1 2 3 4 5 6 7
WAZ (Underweight Z Score)
Severity
Extreme CGF (<−4SD)
Severe CGF (<−3SD)
Overall CGF (<−2SD)
Year
1990
1995
2000
2005
2010
2015
2020
