# Supplementary material for: Current dichotomous metrics obscure trends in severe and extreme child growth failure
Source: Sci Adv. 2022 May 20;8(20):eabm8954. doi: 10.1126/sciadv.abm8954 (PMC9122330; doi:10.1126/sciadv.abm8954)
Supplement: Supplementary file 2 — Data S1 to S4 [file sciadv.abm8954_data_files_s1_to_s4.zip › sciadv.abm8954_data_file_s1e.pdf]

Data S1e. Spatio-temporal Gaussian Process Regression (ST-GPR) results for overall, severe, and mean CGF by location, including location-specific data sources; and distributions of stunting [HAZ], wasting [WHZ], and underweight [WAZ] for children under age five, both sexes, for every five years from 1990−2020. Country results are grouped by GBD super-region, including Central Europe, Eastern Europe, and Central Asia (S1a), High-income (S1b), Latin America and Caribbean (S1c), North Africa and Middle East (S1d), South Asia (S1e), Southeast Asia, East Asia, and Oceania (S1f), and Sub-Saharan Africa (S1g). Plots for each country include overall and severe stunting prevalence (A) and transformed mean stunting Z scores (B). A source list is shown which includes surveys included in the stunting models (C). Additional plots are shown for overall and severe wasting prevalence (D) and transformed mean wasting Z scores (E), followed by a source list with surveys included in the wasting models (F). Plots are then shown for overall and severe underweight prevalence (G), and transformed mean underweight Z scores (H), with a source list listing surveys included in the underweight models (I). Finally, distributions of stunting (J), wasting (K), and underweight (L) are shown for children under age five, both sexes, for every five years from 1990-2020. Surveys that were outliered are shown with X's on all plots. Surveys prior to 1990 may have been inputs to the models to inform trends, but estimates are only produced and shown for 1990−2020. For locations that are modeled nationally and subnationally, sources that are only included subnationally are not included in the plots of national level estimates. These sources were included in subnational models that influence national level models. Note that due to the transformation on mean Z scores, increasing values reflect improvements in mean Z score. Surveys conducted over a range of years were assigned to the midpoint year from that interval, which is the year reflected in the table and the plots. For the distributions of stunting, wasting, and underweight, the area under the curve reflects the estimated proportion of children experiencing that severity of CGF or worse. DHS is Demographic and Health Surveys. MICS is Multiple Indicator Cluster Survey. WHO CGM is the WHO Global Database on Child Growth and Malnutrition. SDNS is Survey of Diet and Nutritional Status.

This file contains the above for the following locations in the GBD super region of South Asia, in the following order:

Bangladesh, Bhutan, India, Nepal, Pakistan

Bangladesh – Stunting (HAZ)

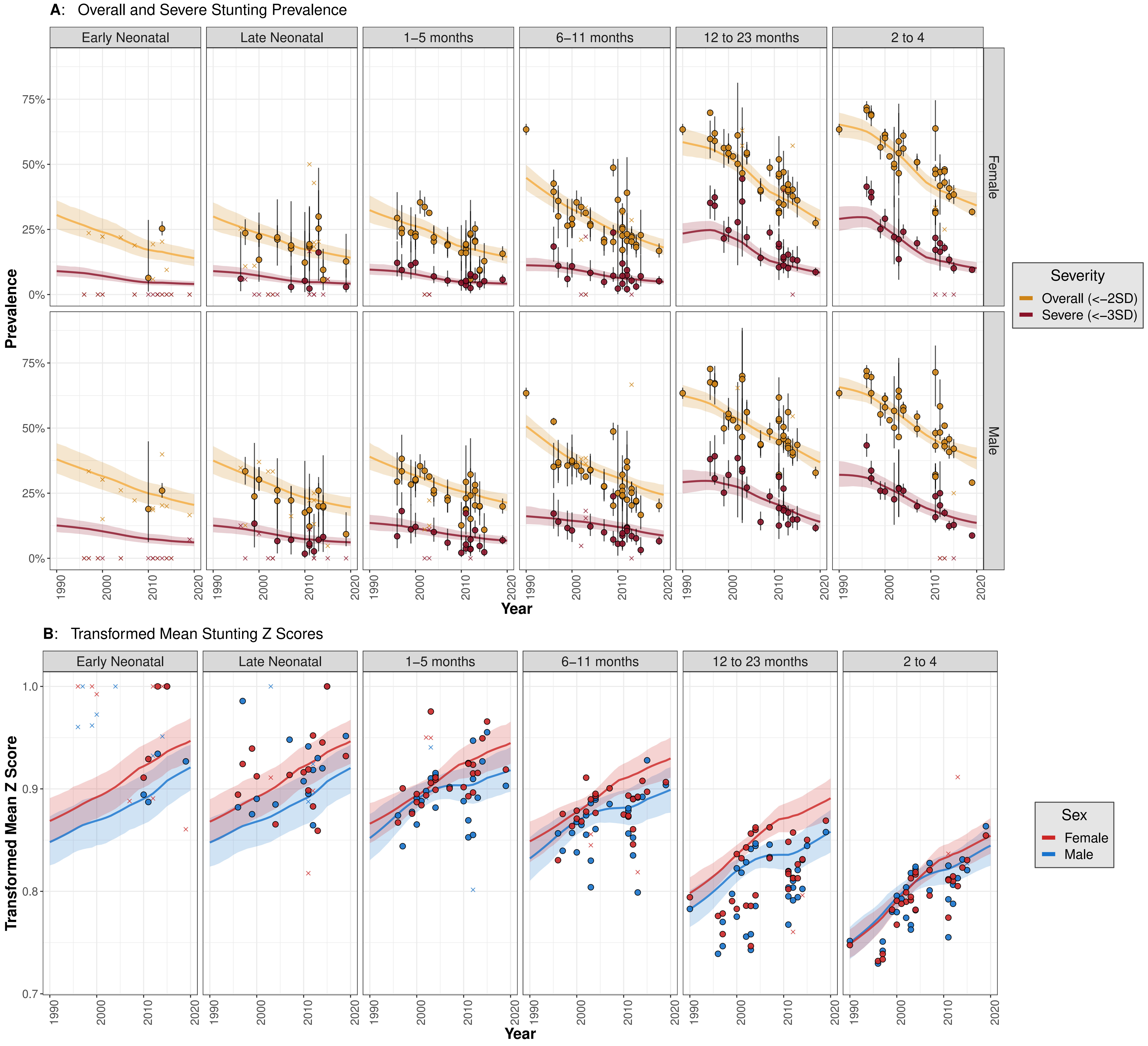

1986	WHO CGM Database
1990	WHO CGM Database
1996	DHS
1996	WHO CGM Database
1997	DHS
1997	WHO CGM Database
1999	DHS
2000	DHS
2000	WHO CGM Database
2001	WHO CGM Database
2002	WHO CGM Database
2002	Dinajpur Supporting Household Activities for Health, Assets, and Revenue Survey, Round 1
2003	WHO CGM Database
2003	Dinajpur Supporting Household Activities for Health, Assets, and Revenue Survey, Round 2
2003	Dinajpur Supporting Household Activities for Health, Assets, and Revenue Survey, Round 3
2004	DHS
2004	WHO CGM Database
2007	DHS
2007	WHO CGM Database
2009	Mizrapur Global Enteric Multicenter Study
2009	Household Food Security and Nutrition Assessment
2010	Dhaka Malnutrition and Enteric Disease Study
2011	DHS
2011	WHO CGM Database
2011	Integrated Household Survey
2011	Dhaka Cohort Study of Incidence of Amebiasis and Cryptosporidiosis in Children
2011	Dhaka Malnutrition and Enteric Disease Study
2011	National Micronutrients Status Survey
2012	MICS
2012	Integrated Household Survey
2012	Mizrapur Global Enteric Multicenter Study
2012	Dhaka Malnutrition and Enteric Disease Study
2013	WHO CGM Database
2013	MICS
2013	Dhaka Malnutrition and Enteric Disease Study
2014	WHO CGM Database
2014	DHS
2014	Dhaka Malnutrition and Enteric Disease Study
2015	Integrated Household Survey

Bangladesh – Wasting (WHZ)

D: Overall and Severe Wasting Prevalence

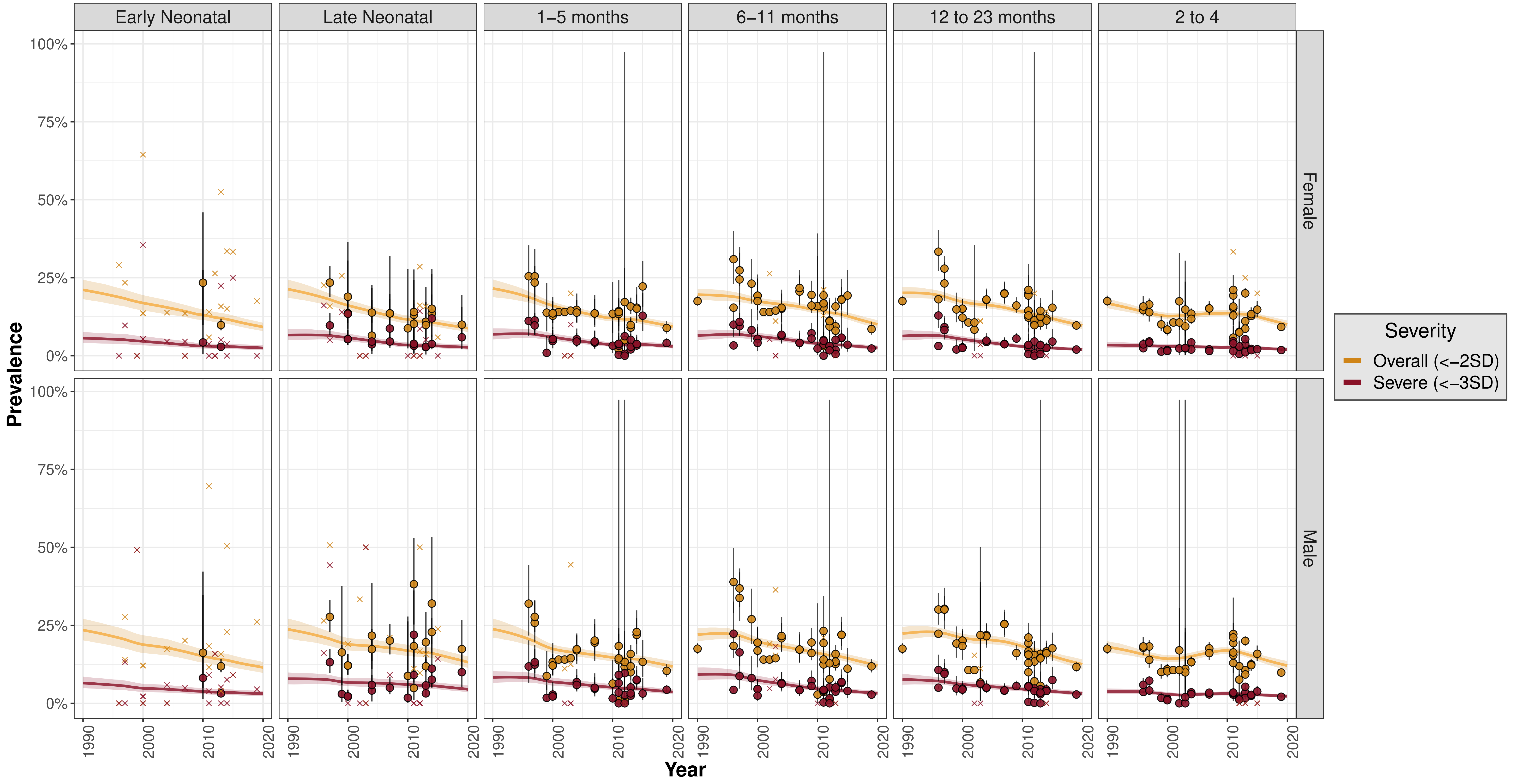

E: Transformed Mean Wasting Z Scores

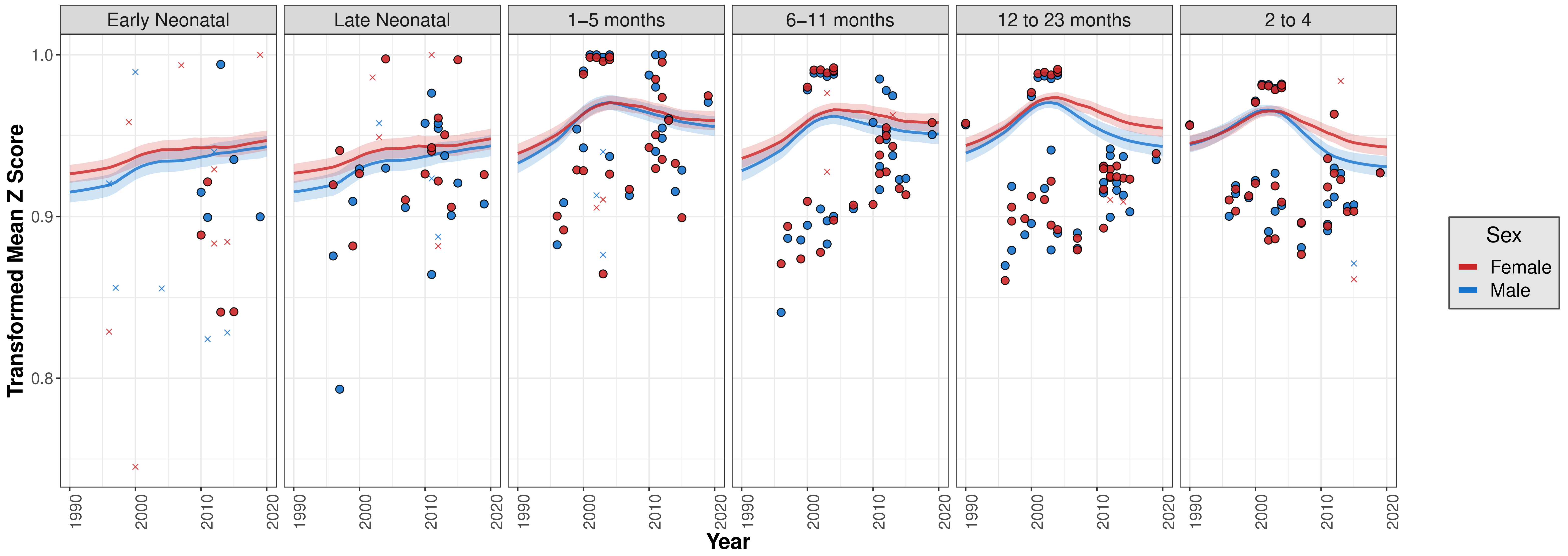

[illegible]	[illegible]
1986	WHO CGM Database
1990	WHO CGM Database
1996	DHS
1996	WHO CGM Database
1997	DHS
1997	WHO CGM Database
1999	DHS
2000	DHS
2000	WHO CGM Database
2001	WHO CGM Database
2002	WHO CGM Database
2002	Dinajpur Supporting Household Activities for Health, Assets, and Revenue Survey, Round 1
2003	WHO CGM Database
2003	Dinajpur Supporting Household Activities for Health, Assets, and Revenue Survey, Round 2
2003	Dinajpur Supporting Household Activities for Health, Assets, and Revenue Survey, Round 3
2004	DHS
2004	WHO CGM Database
2007	DHS
2007	WHO CGM Database
2009	Mizrapur Global Enteric Multicenter Study
2009	Household Food Security and Nutrition Assessment
2010	Dhaka Malnutrition and Enteric Disease Study
2011	DHS
2011	WHO CGM Database
2011	Integrated Household Survey
2011	Dhaka Cohort Study of Incidence of Amebiasis and Cryptosporidiosis in Children
2011	Dhaka Malnutrition and Enteric Disease Study
2011	National Micronutrients Status Survey
2012	MICS
2012	Integrated Household Survey
2012	Mizrapur Global Enteric Multicenter Study
2012	Dhaka Malnutrition and Enteric Disease Study
2013	WHO CGM Database
2013	MICS
2013	Dhaka Malnutrition and Enteric Disease Study
2014	WHO CGM Database
2014	DHS
2014	Dhaka Malnutrition and Enteric Disease Study
2015	Integrated Household Survey

Bangladesh – Underweight (WAZ)

G: Overall and Severe Underweight Prevalence

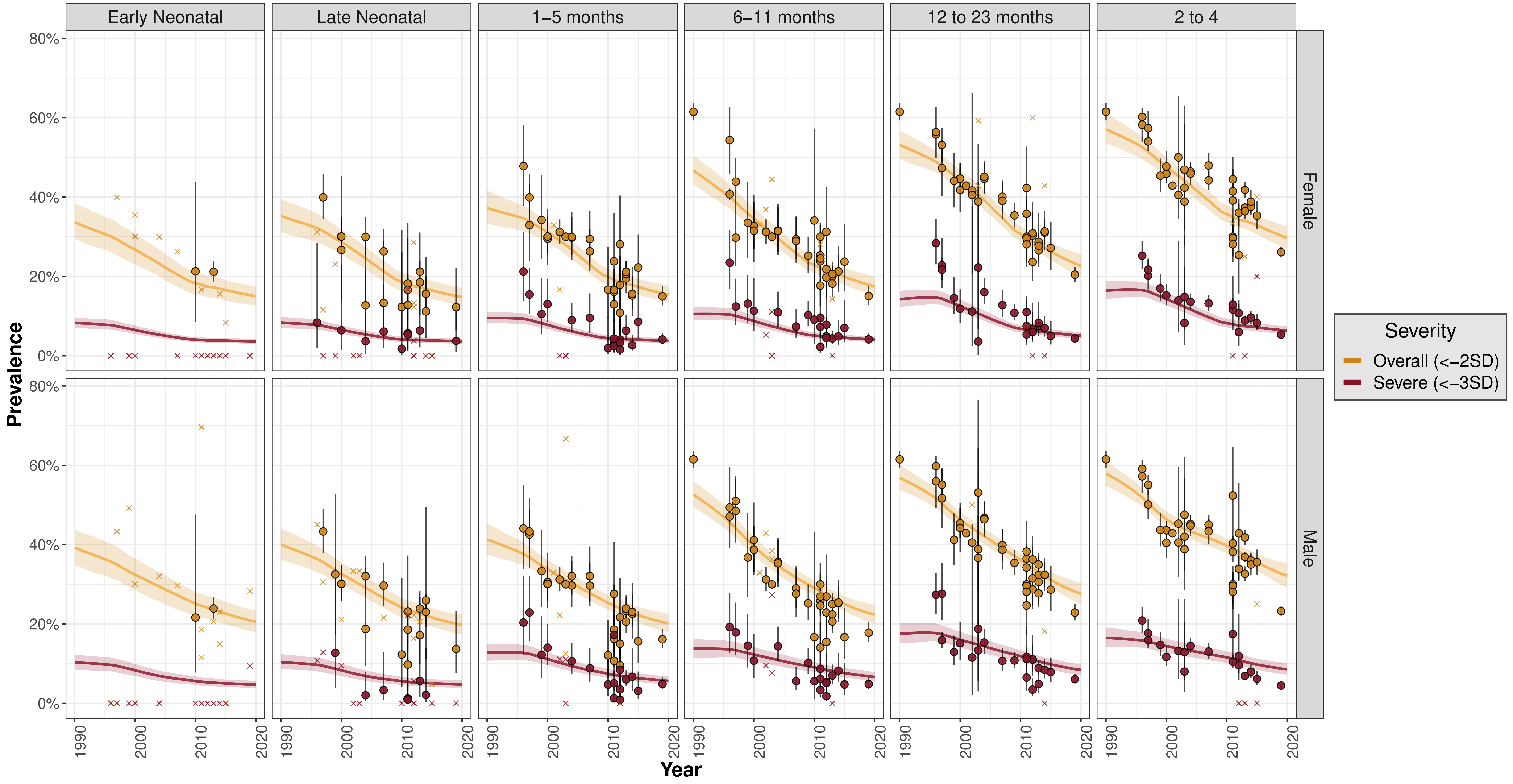

H: Transformed Mean Underweight Z Scores

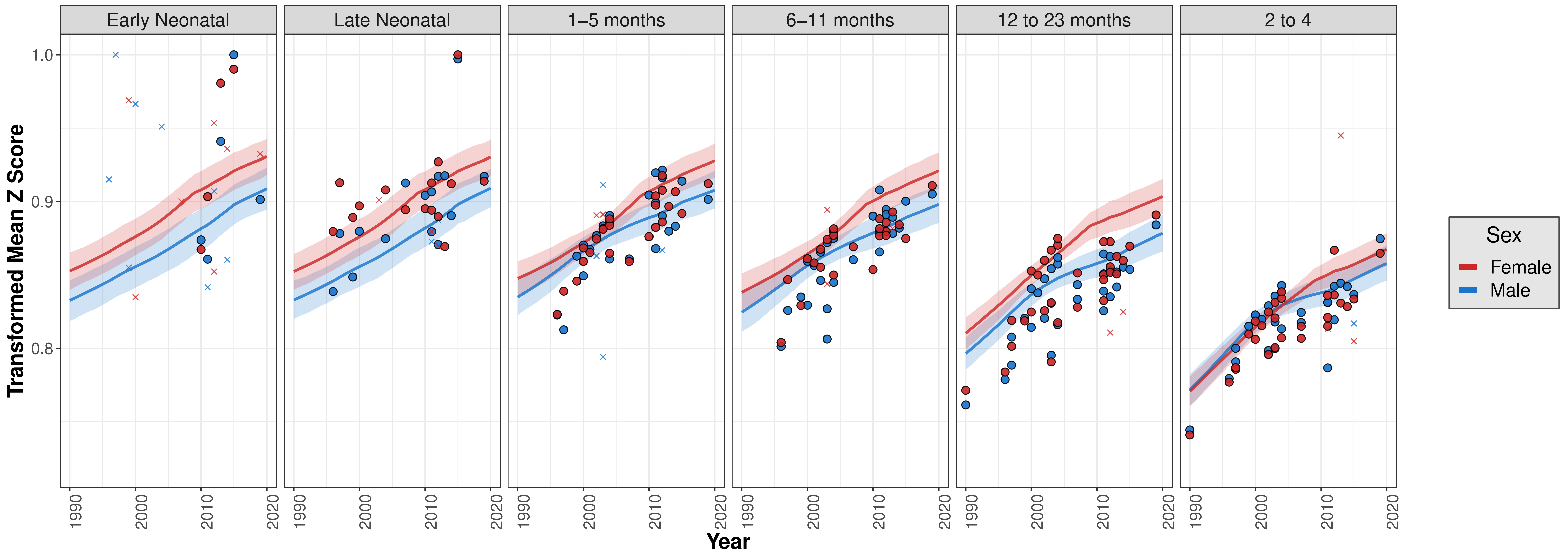

Year	Source
1986	WHO CGM Database
1990	WHO CGM Database
1996	DHS
1996	WHO CGM Database
1997	DHS
1997	WHO CGM Database
1999	DHS
2000	DHS
2000	WHO CGM Database
2001	WHO CGM Database
2002	WHO CGM Database
2002	Dinajpur Supporting Household Activities for Health, Assets, and Revenue Survey, Round 1
2003	WHO CGM Database
2003	Dinajpur Supporting Household Activities for Health, Assets, and Revenue Survey, Round 2
2003	Dinajpur Supporting Household Activities for Health, Assets, and Revenue Survey, Round 3
2004	DHS
2004	WHO CGM Database
2007	DHS
2007	WHO CGM Database
2009	Mizrapur Global Enteric Multicenter Study
2009	Household Food Security and Nutrition Assessment
2010	Dhaka Malnutrition and Enteric Disease Study
2011	DHS
2011	WHO CGM Database
2011	Integrated Household Survey
2011	Dhaka Cohort Study of Incidence of Amebiasis and Cryptosporidiosis in Children
2011	Dhaka Malnutrition and Enteric Disease Study
2011	National Micronutrients Status Survey
2012	MICS
2012	Integrated Household Survey
2012	Mizrapur Global Enteric Multicenter Study
2012	Dhaka Malnutrition and Enteric Disease Study
2013	WHO CGM Database
2013	MICS
2013	Dhaka Malnutrition and Enteric Disease Study
2014	WHO CGM Database
2014	DHS
2014	Dhaka Malnutrition and Enteric Disease Study
[illegible]	[illegible]

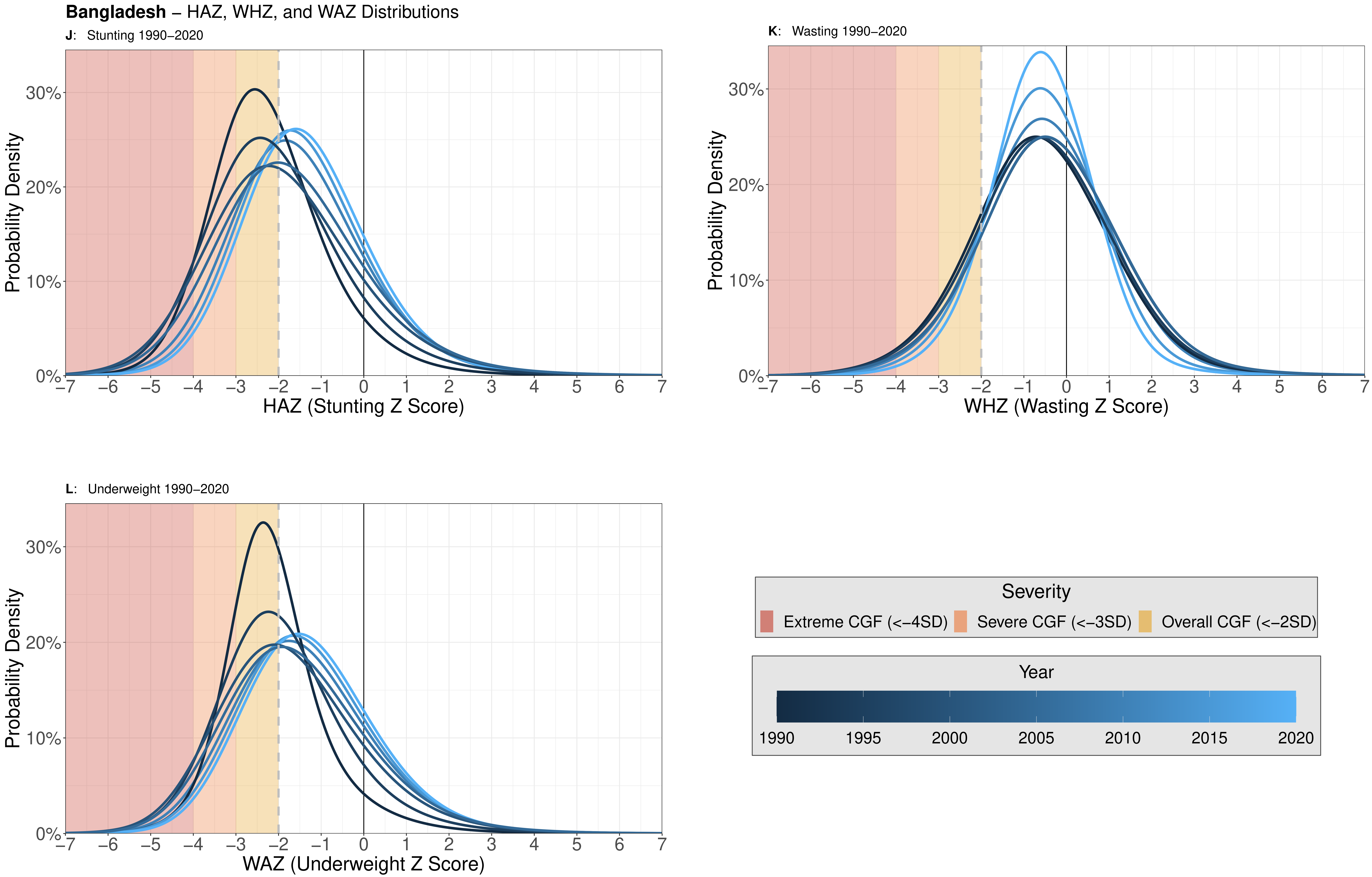
Bangladesh – HAZ, WHZ, and WAZ Distributions
J: Stunting 1990–2020
Probability Density
0%
10%
20%
30%
−7 −6 −5 −4 −3 −2 −1 0 1 2 3 4 5 6 7
HAZ (Stunting Z Score)
K: Wasting 1990–2020
Probability Density
0%
10%
20%
30%
−7 −6 −5 −4 −3 −2 −1 0 1 2 3 4 5 6 7
WHZ (Wasting Z Score)
L: Underweight 1990–2020
Probability Density
0%
10%
20%
30%
−7 −6 −5 −4 −3 −2 −1 0 1 2 3 4 5 6 7
WAZ (Underweight Z Score)
Severity
Extreme CGF (<−4SD)
Severe CGF (<−3SD)
Overall CGF (<−2SD)
Year
1990
1995
2000
2005
2010
2015
2020

Bhutan – Stunting (HAZ)

A: Overall and Severe Stunting Prevalence

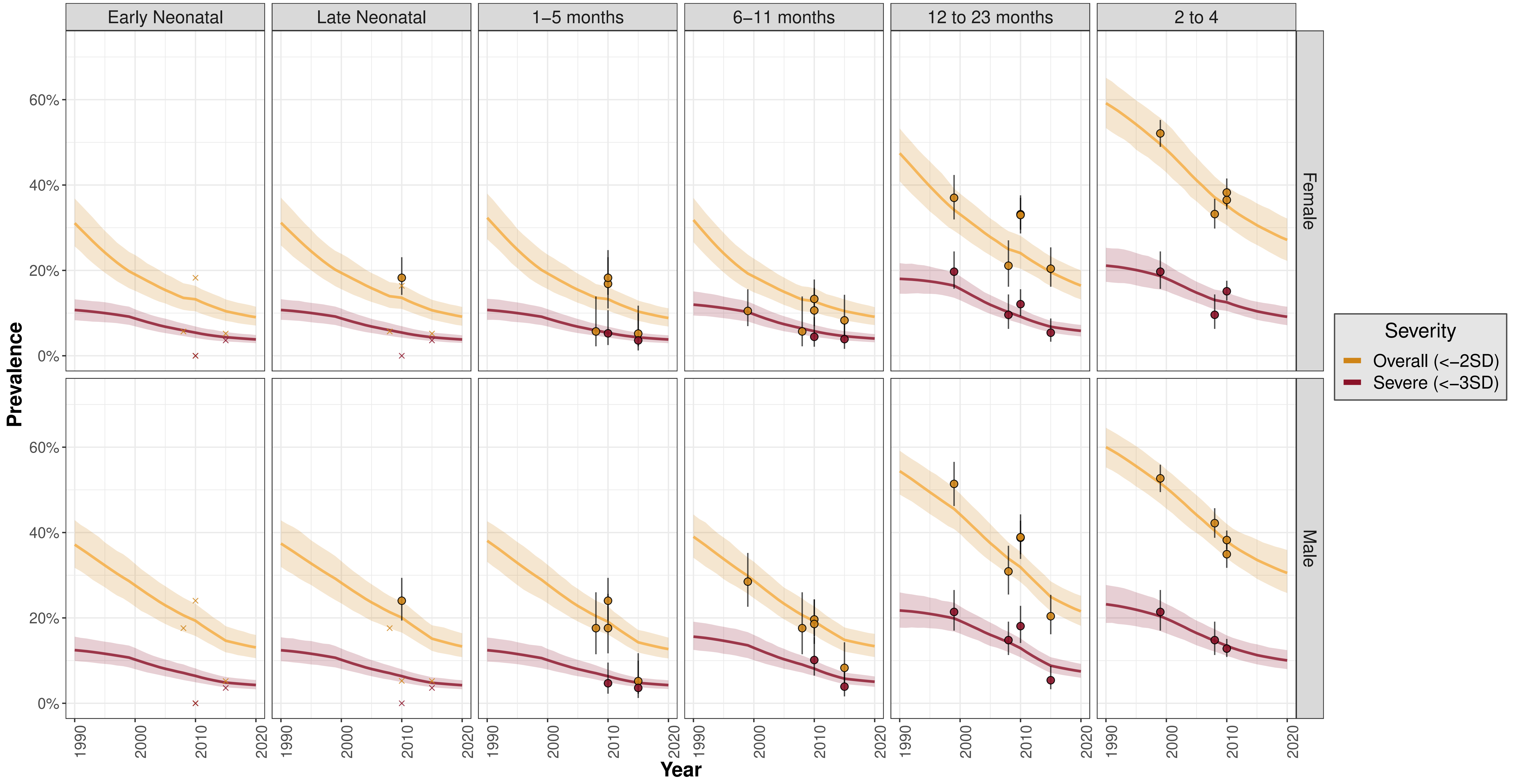

B: Transformed Mean Stunting Z Scores

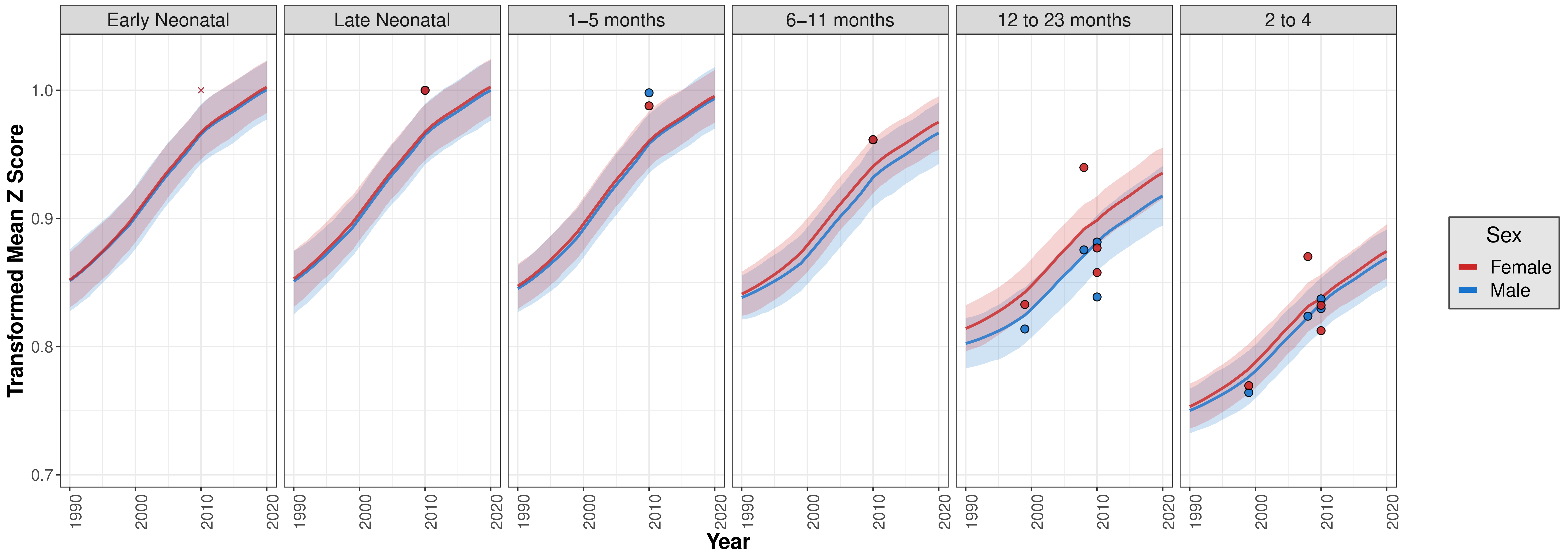

C

Year	Source
1987	WHO CGM Database
1999	WHO CGM Database
2008	WHO CGM Database
2010	MICS
2010	WHO CGM Database
2015	National Nutrition Survey

Bhutan – Wasting (WHZ)

D: Overall and Severe Wasting Prevalence

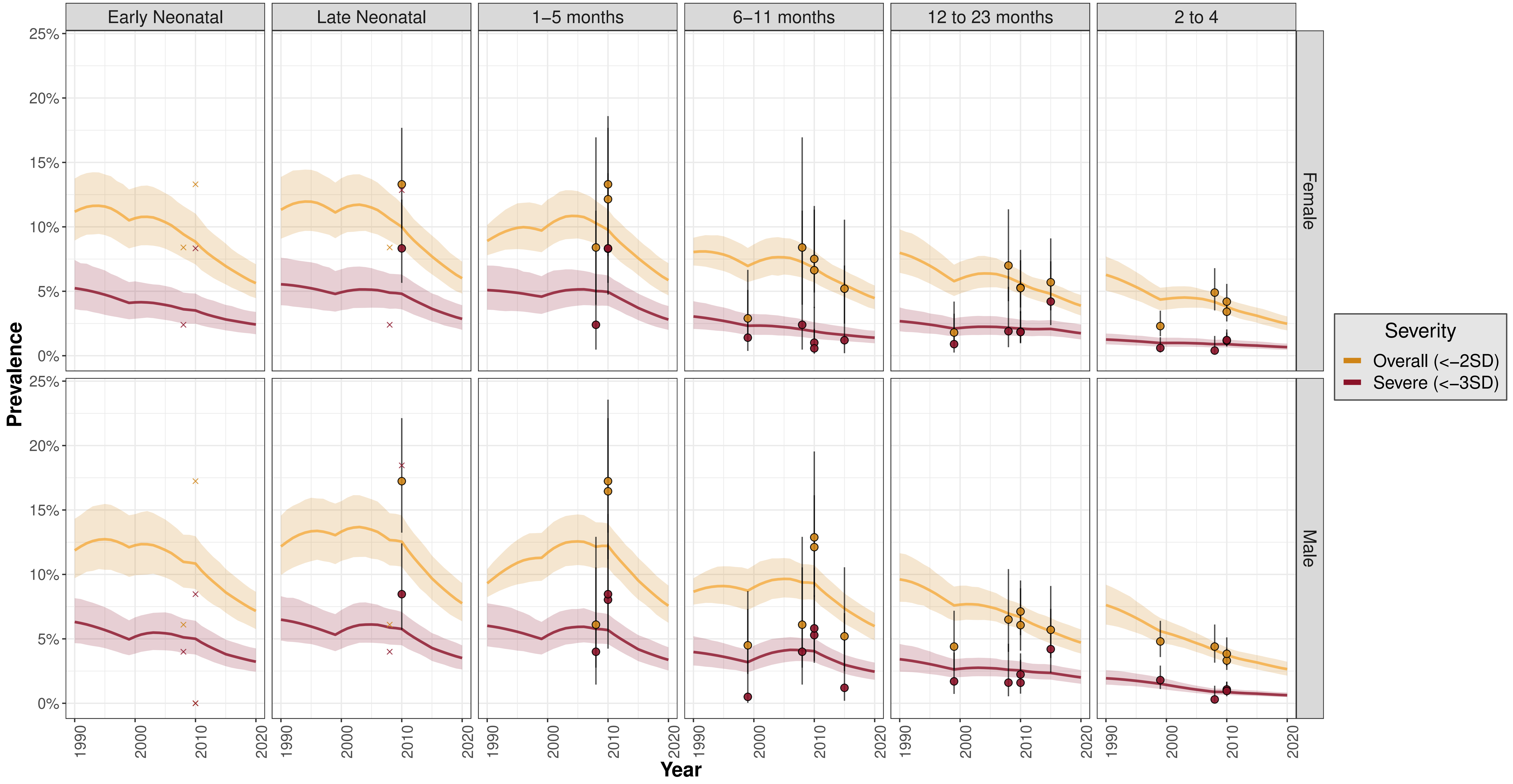

E: Transformed Mean Wasting Z Scores

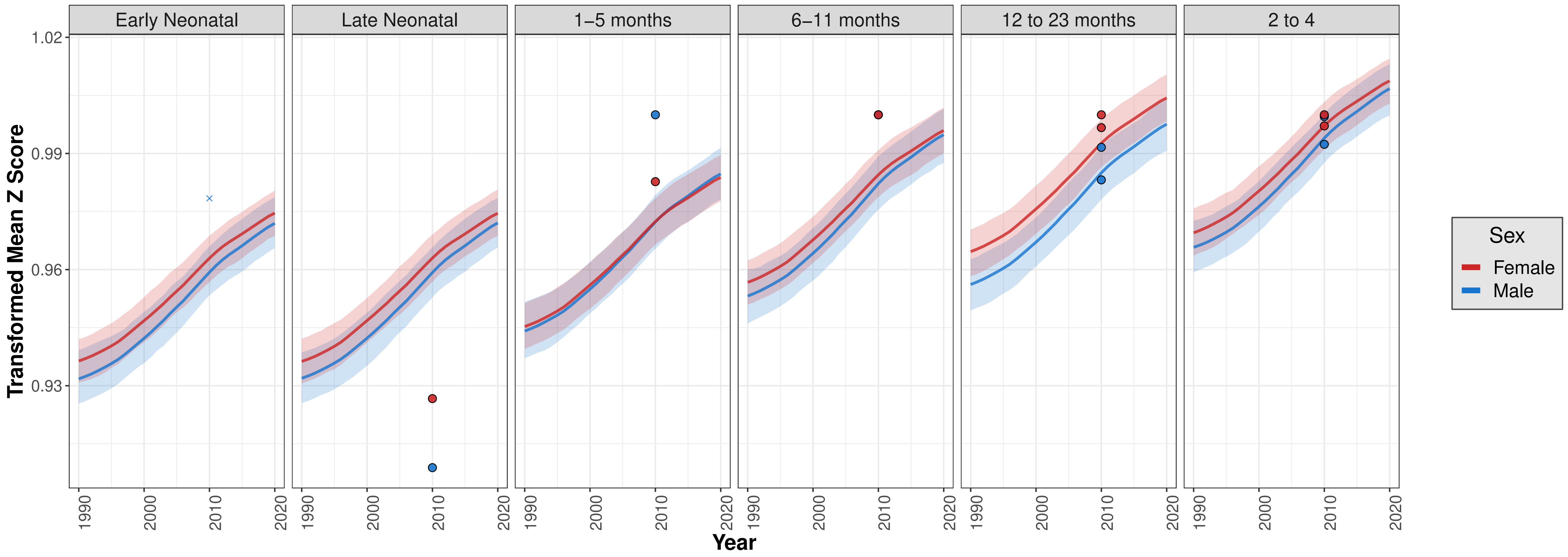

F

Year	Source
1987	WHO CGM Database
1999	WHO CGM Database
2008	WHO CGM Database
2010	MICS
2010	WHO CGM Database
2015	National Nutrition Survey

Bhutan – Underweight (WAZ)

G: Overall and Severe Underweight Prevalence

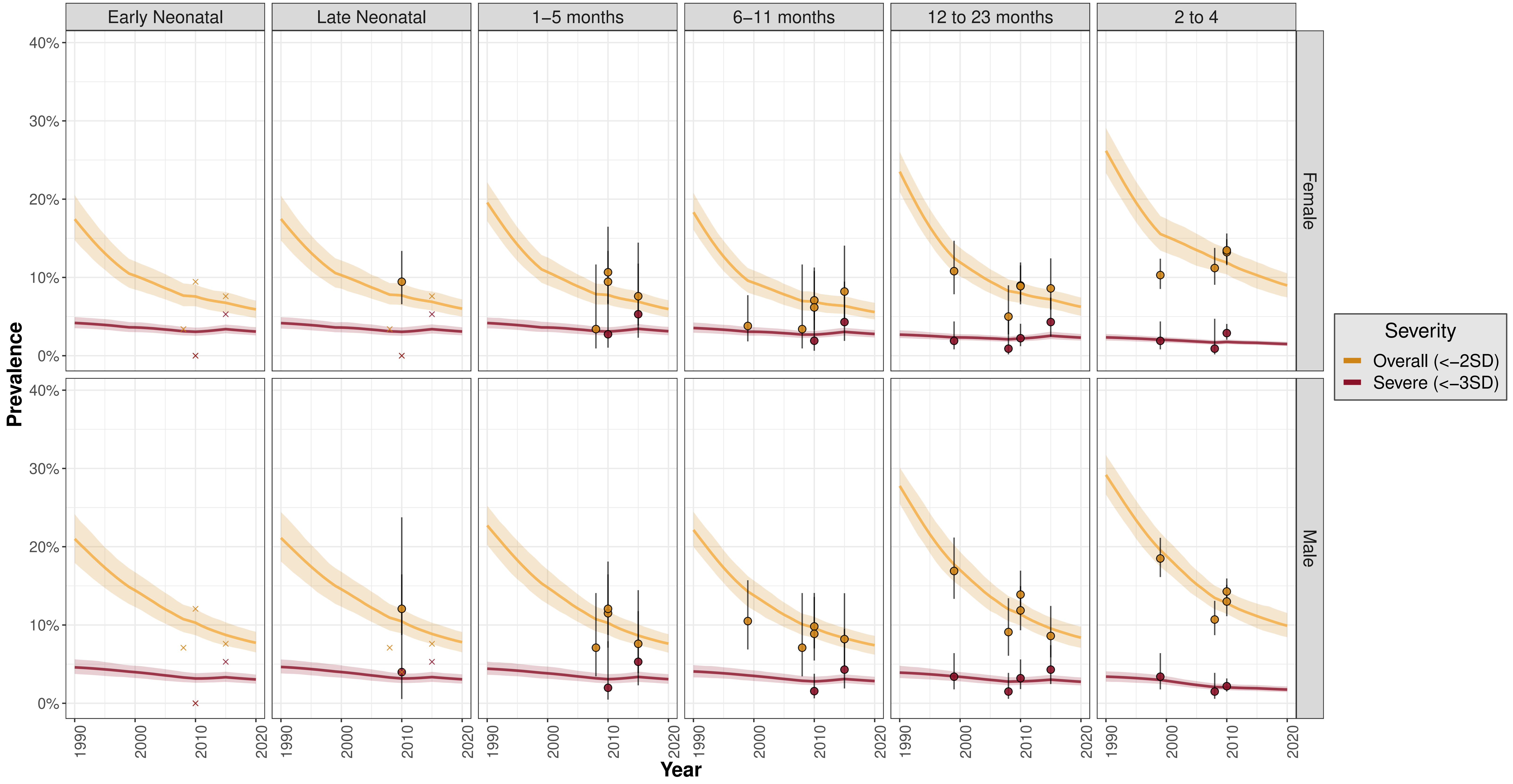

H: Transformed Mean Underweight Z Scores

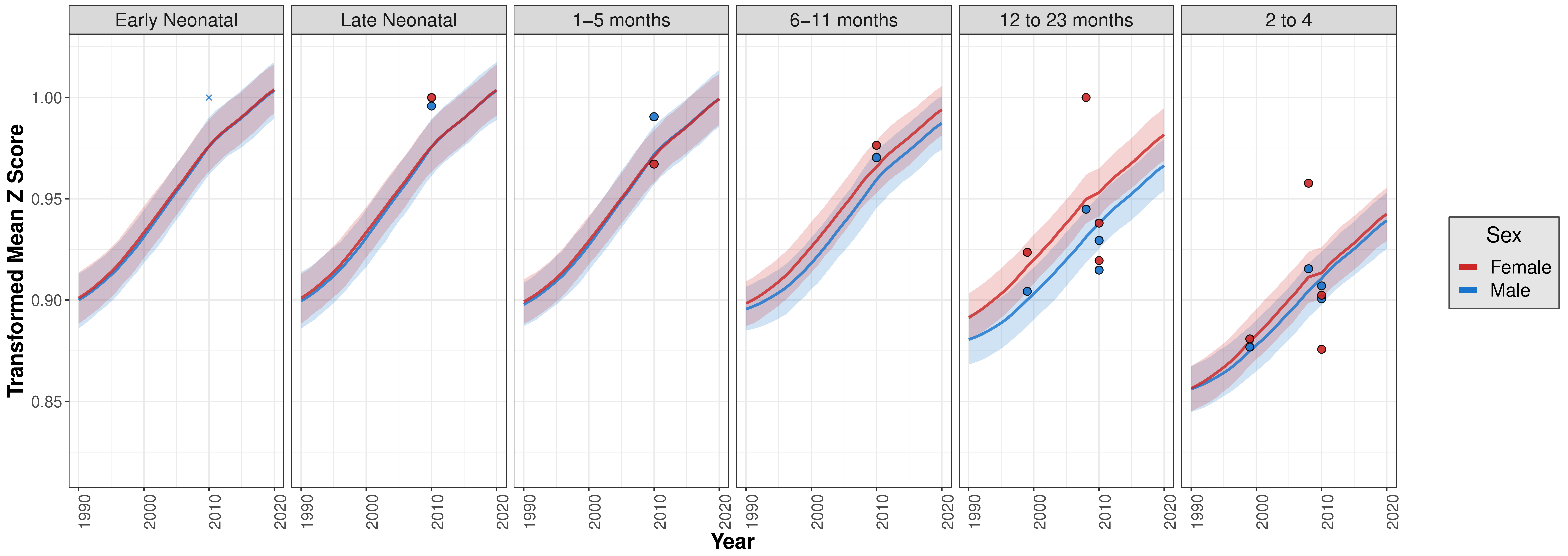

I

Year	Source
1987	WHO CGM Database
1999	WHO CGM Database
2008	WHO CGM Database
2010	MICS
2010	WHO CGM Database
2015	National Nutrition Survey

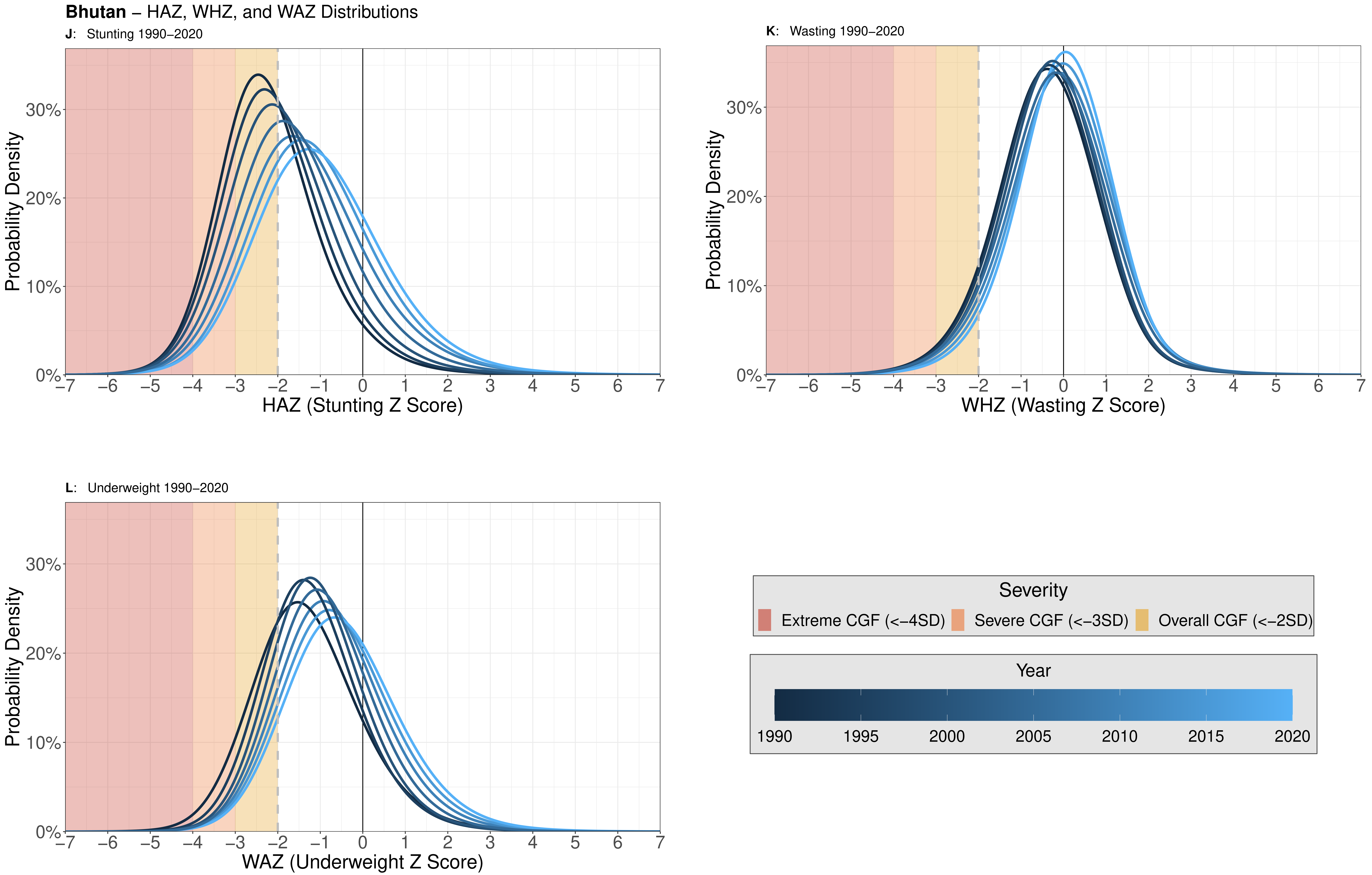
Bhutan – HAZ, WHZ, and WAZ Distributions
J: Stunting 1990–2020
Probability Density
0%
10%
20%
30%
−7 −6 −5 −4 −3 −2 −1 0 1 2 3 4 5 6 7
HAZ (Stunting Z Score)
K: Wasting 1990–2020
Probability Density
0%
10%
20%
30%
−7 −6 −5 −4 −3 −2 −1 0 1 2 3 4 5 6 7
WHZ (Wasting Z Score)
L: Underweight 1990–2020
Probability Density
0%
10%
20%
30%
−7 −6 −5 −4 −3 −2 −1 0 1 2 3 4 5 6 7
WAZ (Underweight Z Score)
Severity
Extreme CGF (<−4SD)
Severe CGF (<−3SD)
Overall CGF (<−2SD)
Year
1990
1995
2000
2005
2010
2015
2020

India – Stunting (HAZ)

A: Overall and Severe Stunting Prevalence

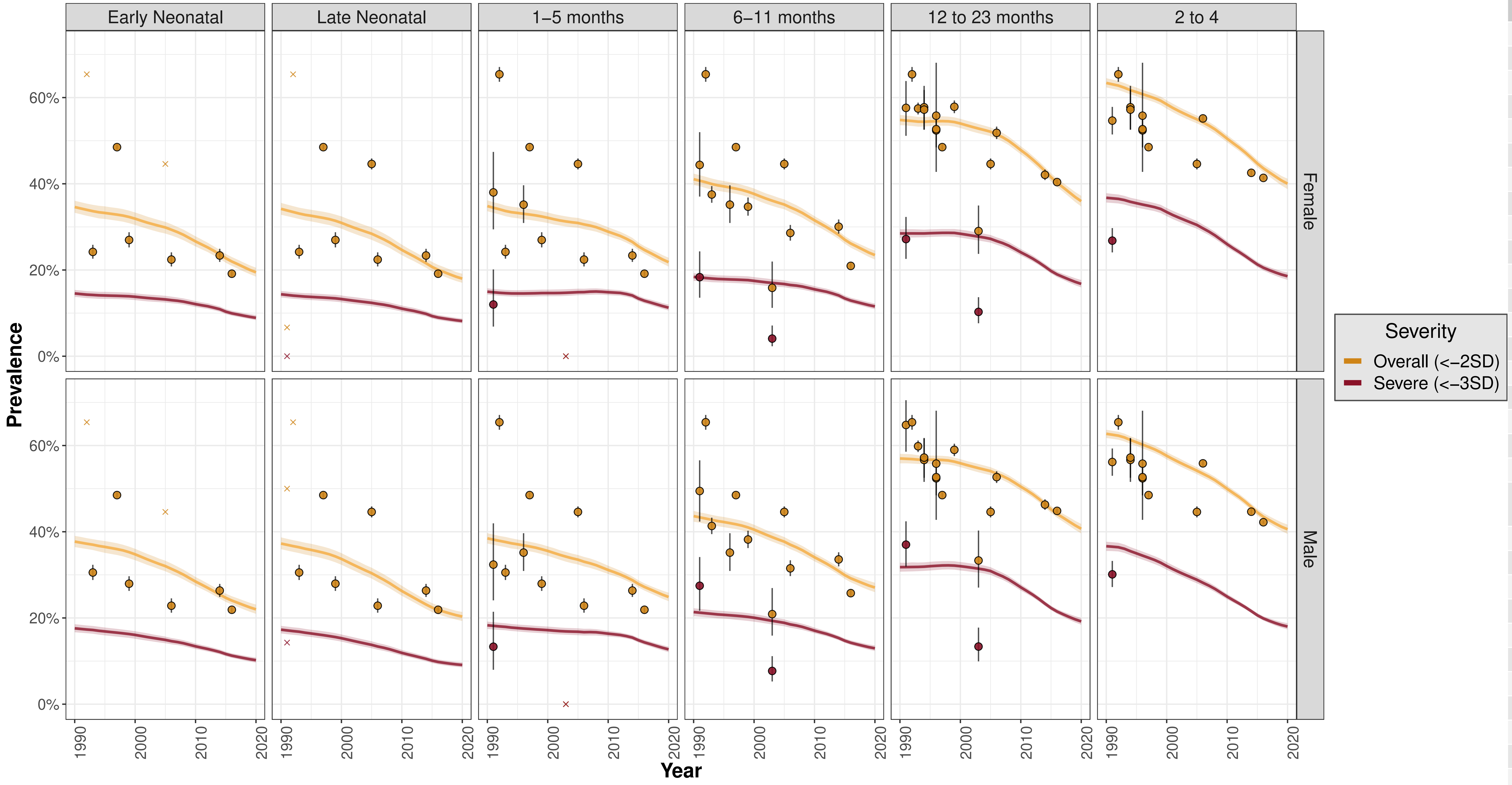

B: Transformed Mean Stunting Z Scores

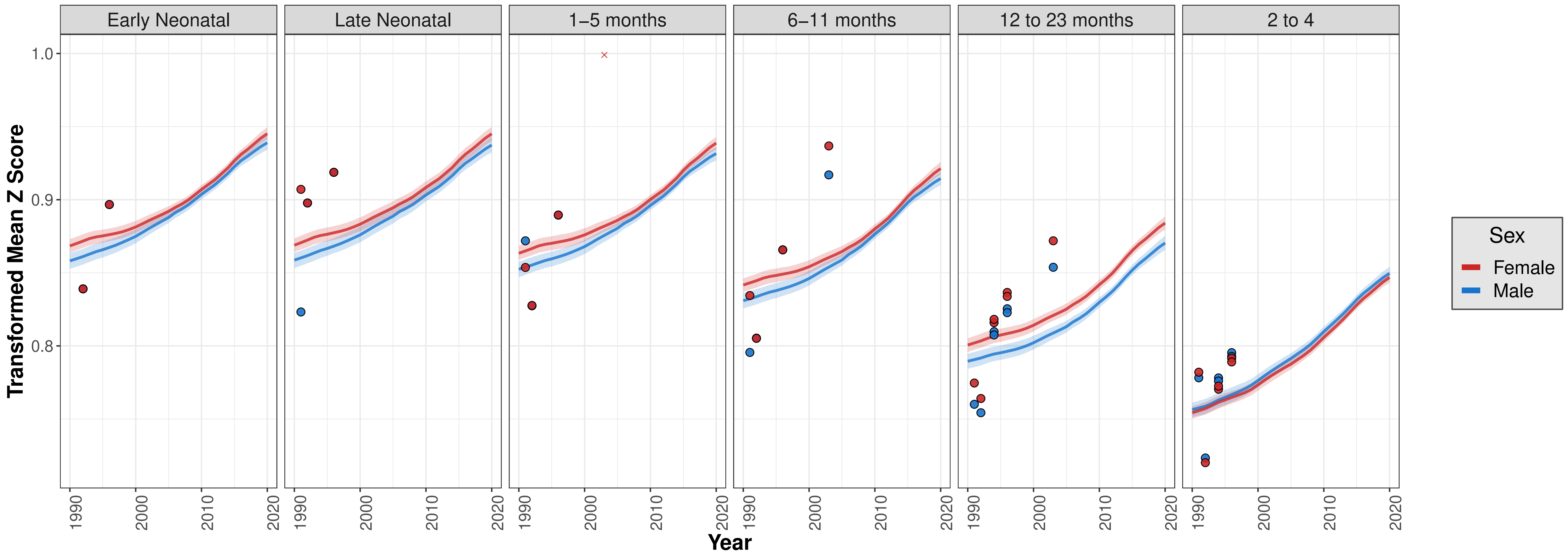

C

Year	Source	National	Subnational
1977	WHO CGM Database	X	
1989	WHO CGM Database	X	
1991	SDNS	X	
1992	DHS		X
1992	WHO CGM Database	X	
1993	DHS		X
1993	WHO CGM Database	X	
1994	Rural SDNS	X	
1995	WHO CGM Database		X
1996	Rural Second Repeat SDNS	X	
1996	WHO CGM Database	X	X
1997	WHO CGM Database	X	
1998	DHS		X
1998	Tribal First Repeat SDNS		X
1999	DHS		X
1999	WHO CGM Database	X	
2000	DHS		X
2000	Rural SDNS		X
2001	Rural SDNS		X
2003	Andhra Pradesh Young Lives: Household and Child Survey Round 1 – UK Data Service	X	X
2004	Human Development Survey		X
2005	DHS		X
2005	Human Development Survey		X
2005	Rural SDNS		X
2005	WHO CGM Database	X	
2006	DHS		X
2006	WHO CGM Database	X	X
2008	Tribal Second Repeat SDNS		X
2009	Kolkata Global Enteric Multicenter Study		X
2010	Vellore Malnutrition and Enteric Disease Study		X
2011	Rural Third Repeat SDNS		X
2011	Human Development Survey		X
2011	Vellore Malnutrition and Enteric Disease Study		X
2012	Rural Third Repeat SDNS		X
2012	District Level Household Survey		X
2012	Human Development Survey		X
2012	Kolkata Global Enteric Multicenter Study		X
2012	Vellore Malnutrition and Enteric Disease Study		X
2012	Maharashtra Comprehensive Nutrition Survey		X
2013	Rural SDNS		X
2013	District Level Household Survey		X
2013	Vellore Malnutrition and Enteric Disease Study		X
2014	WHO CGM Database	X	
2014	District Level Household Survey		X
2014	Clinical, Anthropometric and Bio–chemical Survey		X
2014	Vellore Malnutrition and Enteric Disease Study		X
2014	Rapid Survey on Children		X
2015	DHS		X
2015	Urban Nutrition Survey		X
2016	WHO CGM Database	X	
2016	DHS		X
2016	Urban Nutrition Survey		X

India – Wasting (WHZ)

D: Overall and Severe Wasting Prevalence

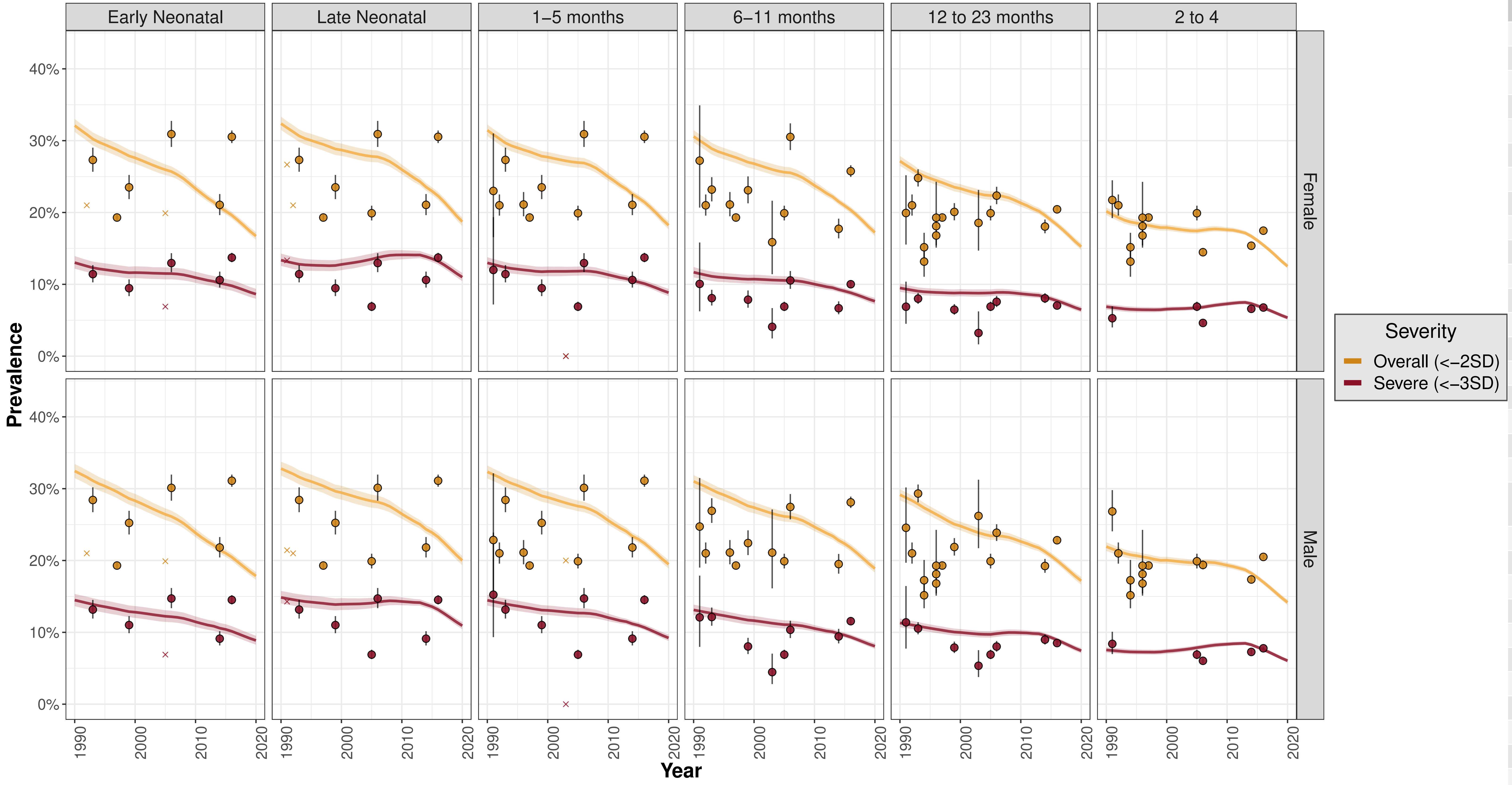

E: Transformed Mean Wasting Z Scores

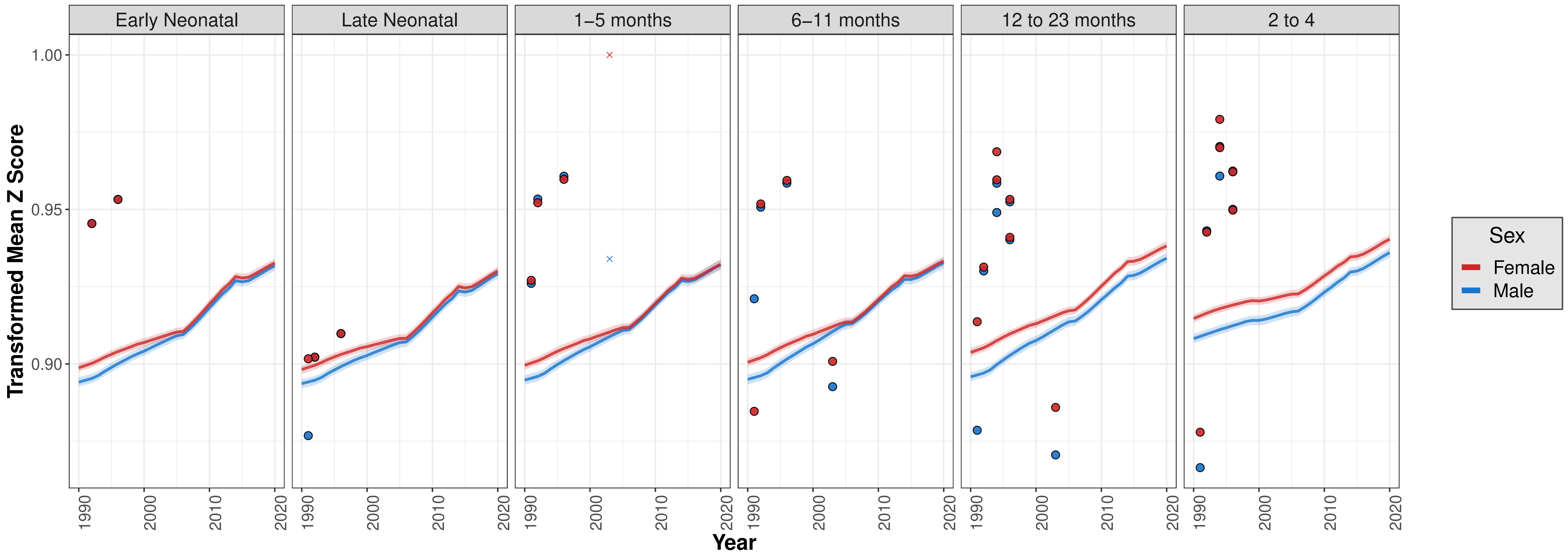

F

Year	Source	National	Subnational
1977	WHO CGM Database	X	
1989	WHO CGM Database	X	
1991	SDNS	X	
1992	DHS		X
1992	WHO CGM Database	X	
1993	DHS		X
1993	WHO CGM Database	X	
1994	Rural SDNS	X	
1995	WHO CGM Database		X
1996	Rural Second Repeat SDNS	X	
1996	WHO CGM Database	X	X
1997	WHO CGM Database	X	
1998	DHS		X
1998	Tribal First Repeat SDNS		X
1999	DHS		X
1999	WHO CGM Database	X	
2000	DHS		X
2000	Rural SDNS		X
2001	Rural SDNS		X
2003	Andhra Pradesh Young Lives: Household and Child Survey Round 1 – UK Data Service	X	X
2004	Human Development Survey		X
2005	DHS		X
2005	Human Development Survey		X
2005	Rural SDNS		X
2005	WHO CGM Database	X	
2006	DHS		X
2006	WHO CGM Database	X	X
2008	Tribal Second Repeat SDNS		X
2009	Kolkata Global Enteric Multicenter Study		X
2010	Vellore Malnutrition and Enteric Disease Study		X
2011	Rural Third Repeat SDNS		X
2011	Human Development Survey		X
2011	Vellore Malnutrition and Enteric Disease Study		X
2012	Rural Third Repeat SDNS		X
2012	District Level Household Survey		X
2012	Human Development Survey		X
2012	Kolkata Global Enteric Multicenter Study		X
2012	Vellore Malnutrition and Enteric Disease Study		X
2012	Maharashtra Comprehensive Nutrition Survey		X
2013	Rural SDNS		X
2013	District Level Household Survey		X
2013	Vellore Malnutrition and Enteric Disease Study		X
2014	WHO CGM Database	X	
2014	District Level Household Survey		X
2014	Clinical, Anthropometric and Bio–chemical Survey		X
2014	Vellore Malnutrition and Enteric Disease Study		X
2014	Rapid Survey on Children		X
2015	DHS		X
2015	Urban Nutrition Survey		X
2016	WHO CGM Database	X	
2016	DHS		X
2016	Urban Nutrition Survey		X

India – Underweight (WAZ)

G: Overall and Severe Underweight Prevalence

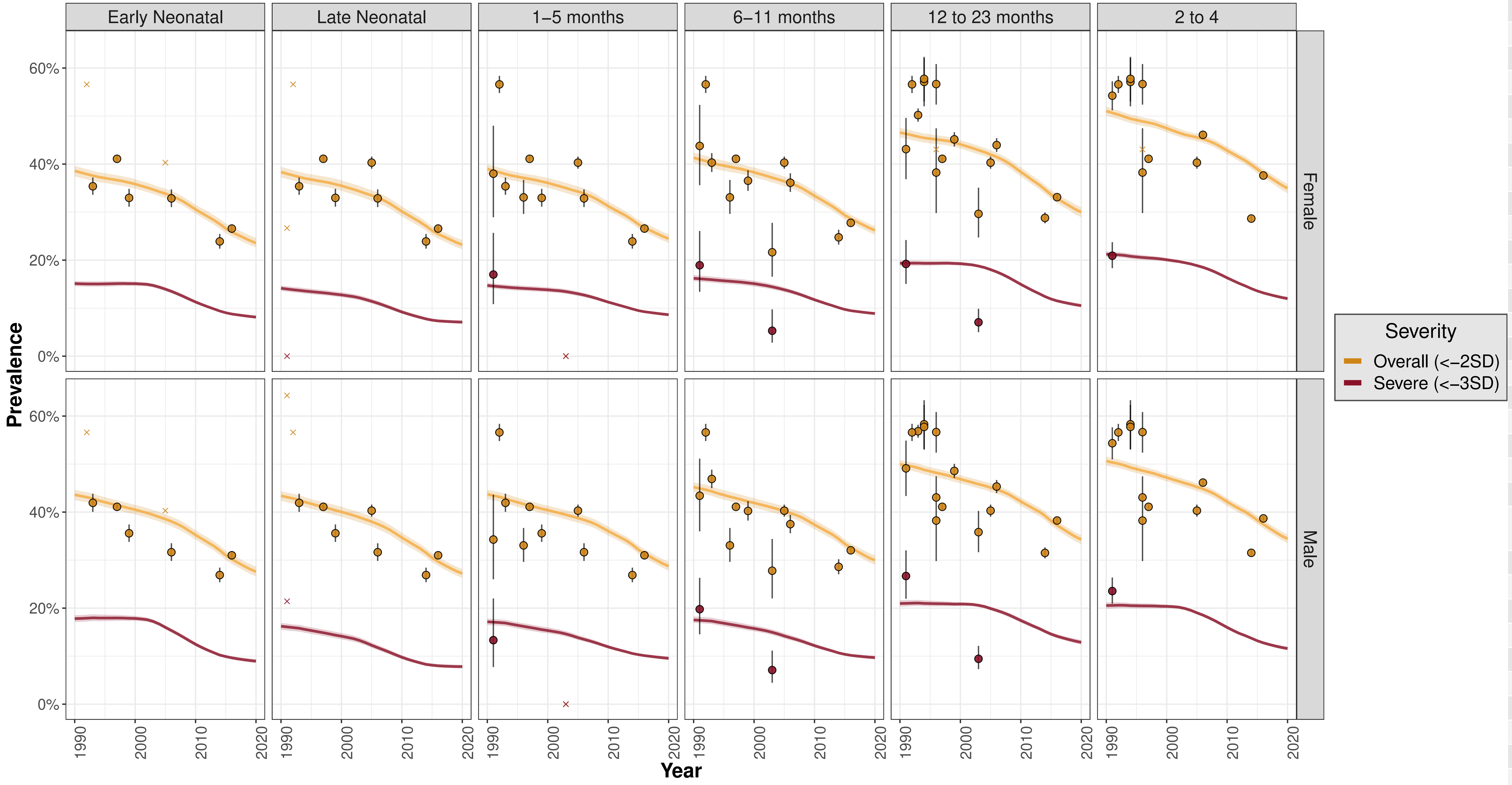

H: Transformed Mean Underweight Z Scores

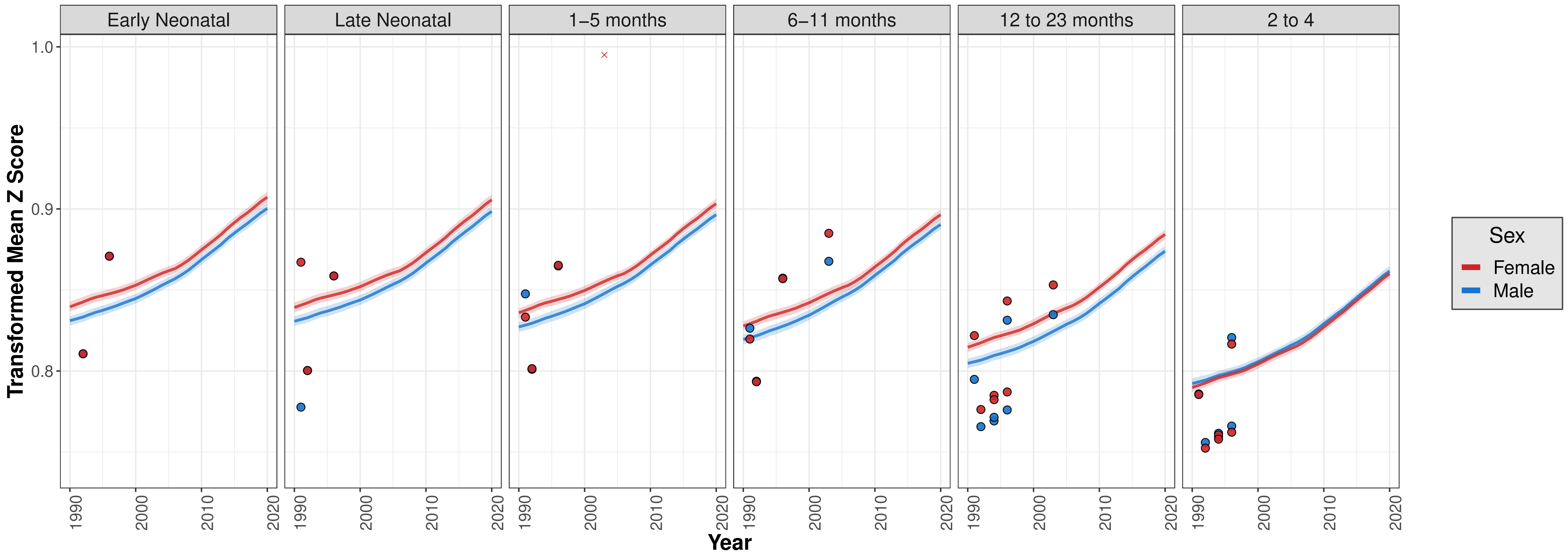

I

Year	Source	National	Subnational
1977	WHO CGM Database	X	
1989	WHO CGM Database	X	
1991	SDNS	X	
1992	DHS		X
1992	WHO CGM Database	X	
1993	DHS		X
1993	WHO CGM Database	X	
1994	Rural SDNS	X	
1994	Urban Slums SDNS		X
1995	WHO CGM Database		X
1996	Rural Second Repeat SDNS	X	
1996	WHO CGM Database	X	X
1997	WHO CGM Database	X	
1998	DHS		X
1998	Tribal First Repeat SDNS		X
1999	DHS		X
1999	WHO CGM Database	X	
2000	DHS		X
2000	Rural SDNS		X
2001	Rural SDNS		X
2002	District Level Household Survey		X
2003	District Level Household Survey		X
2003	Andhra Pradesh Young Lives: Household and Child Survey Round 1 – UK Data Service	X	X
2004	District Level Household Survey		X
2004	Human Development Survey		X
2005	DHS		X
2005	Human Development Survey		X
2005	Rural SDNS		X
2005	WHO CGM Database	X	
2006	DHS		X
2006	WHO CGM Database	X	X
2008	Tribal Second Repeat SDNS		X
2009	Kolkata Global Enteric Multicenter Study		X
2010	Vellore Malnutrition and Enteric Disease Study		X
2011	Rural Third Repeat SDNS		X
2011	Human Development Survey		X
2011	Vellore Malnutrition and Enteric Disease Study		X
2012	Rural Third Repeat SDNS		X
2012	District Level Household Survey		X
2012	Human Development Survey		X
2012	Kolkata Global Enteric Multicenter Study		X
2012	Vellore Malnutrition and Enteric Disease Study		X
2012	Maharashtra Comprehensive Nutrition Survey		X
2013	Rural SDNS		X
2013	District Level Household Survey		X
2013	Vellore Malnutrition and Enteric Disease Study		X
2014	WHO CGM Database	X	
2014	District Level Household Survey		X
2014	Clinical, Anthropometric and Bio–chemical Survey		X
2014	Vellore Malnutrition and Enteric Disease Study		X
2014	Rapid Survey on Children		X
2015	DHS		X
2015	Urban Nutrition Survey		X
2016	WHO CGM Database	X	
2016	DHS		X
2016	Urban Nutrition Survey		X

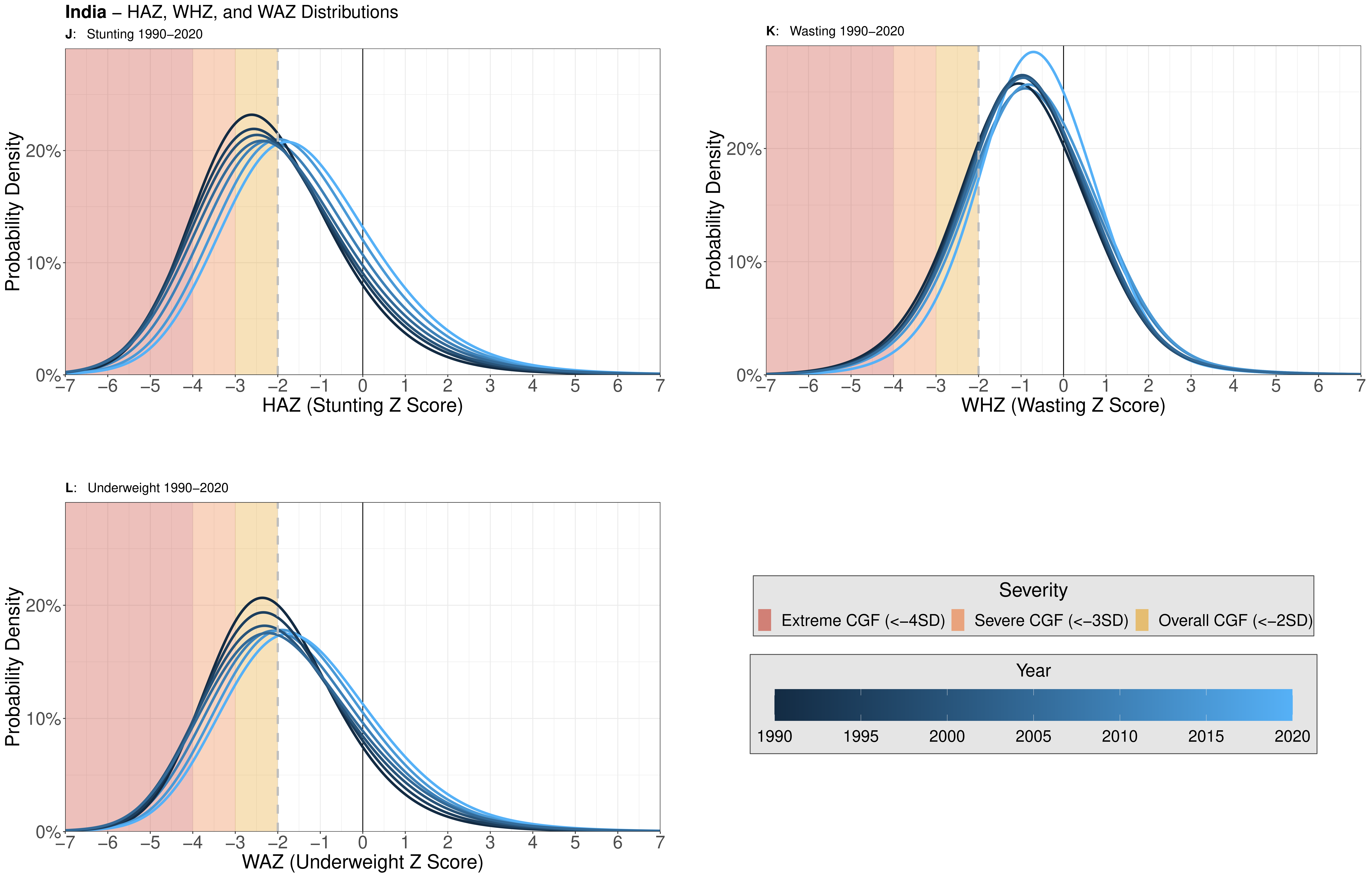
India – HAZ, WHZ, and WAZ Distributions
J: Stunting 1990–2020
K: Wasting 1990–2020
L: Underweight 1990–2020
Probability Density
0%
10%
20%
−7 −6 −5 −4 −3 −2 −1 0 1 2 3 4 5 6 7
HAZ (Stunting Z Score)
WHZ (Wasting Z Score)
WAZ (Underweight Z Score)
Severity
Extreme CGF (<−4SD)
Severe CGF (<−3SD)
Overall CGF (<−2SD)
Year
1990
1995
2000
2005
2010
2015
2020

Nepal – Stunting (HAZ)

A: Overall and Severe Stunting Prevalence

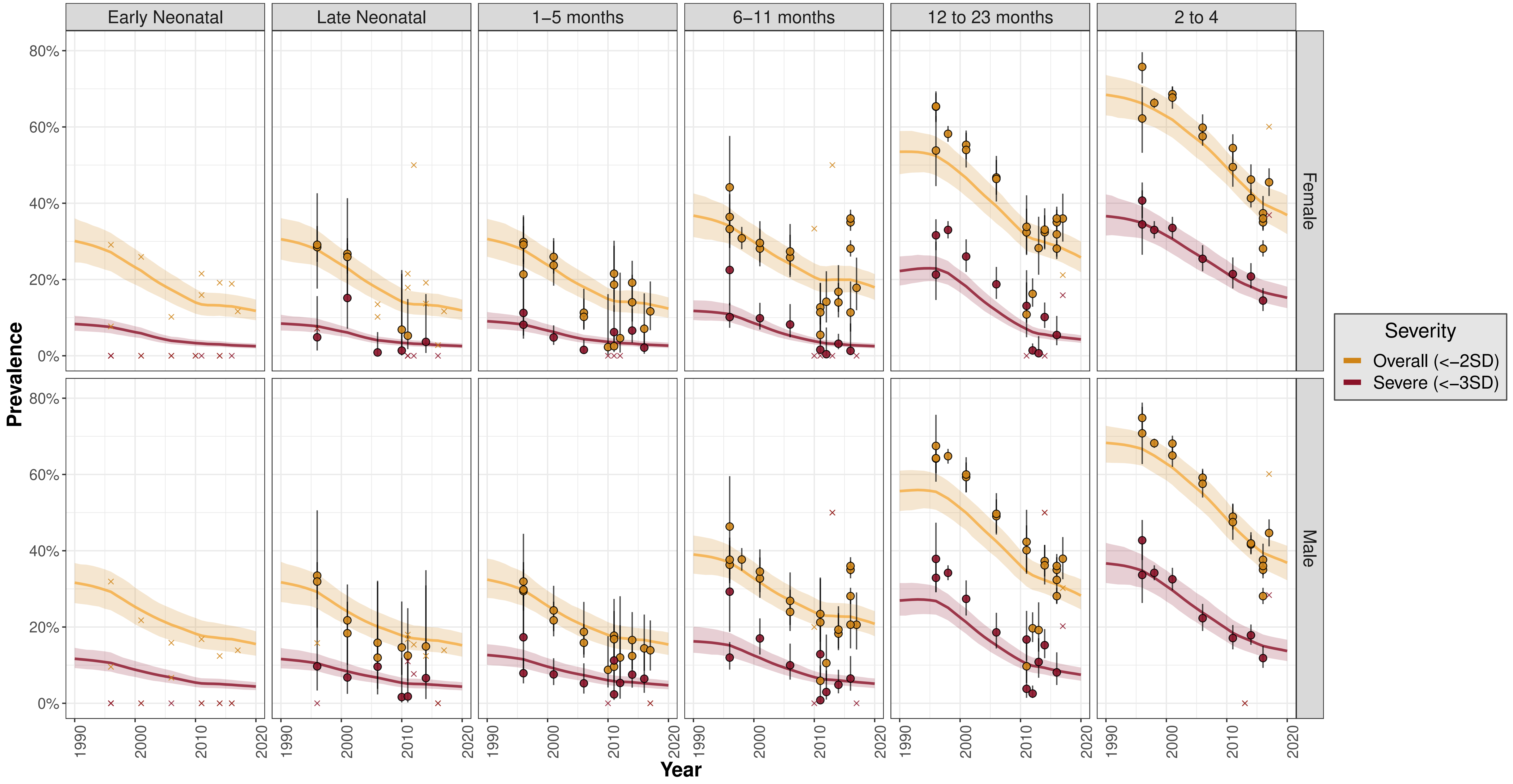

B: Transformed Mean Stunting Z Scores

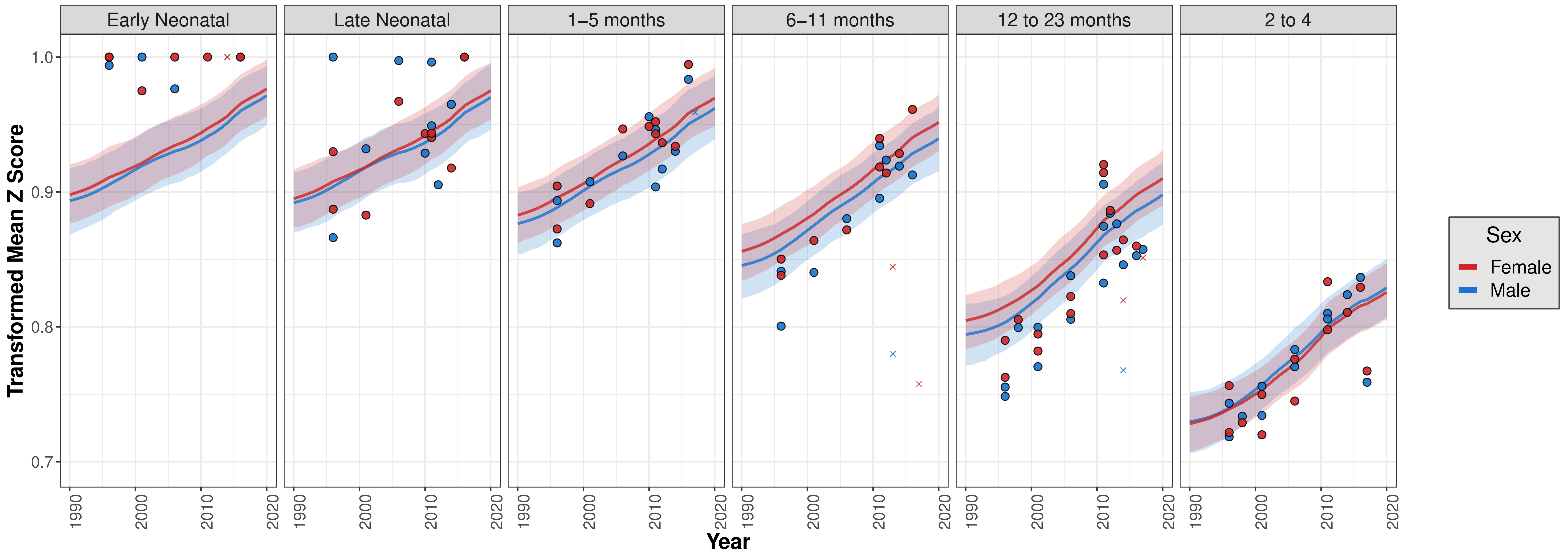

C

Year	Source
1975	WHO CGM Database
1996	Living Standards Survey
1996	DHS
1996	WHO CGM Database
1998	WHO CGM Database
2001	DHS
2001	WHO CGM Database
2006	DHS
2006	WHO CGM Database
2010	Bhaktapur Malnutrition and Enteric Disease Study
2011	DHS
2011	WHO CGM Database
2011	Bhaktapur Malnutrition and Enteric Disease Study
2012	Bhaktapur Malnutrition and Enteric Disease Study
2013	Bhaktapur Malnutrition and Enteric Disease Study
2014	WHO CGM Database
2014	MICS
2014	Bhaktapur Malnutrition and Enteric Disease Study
2016	DHS
2016	Micronutrient Status Survey
2017	WHO CGM Database
2017	DHS

Nepal – Wasting (WHZ)

D: Overall and Severe Wasting Prevalence

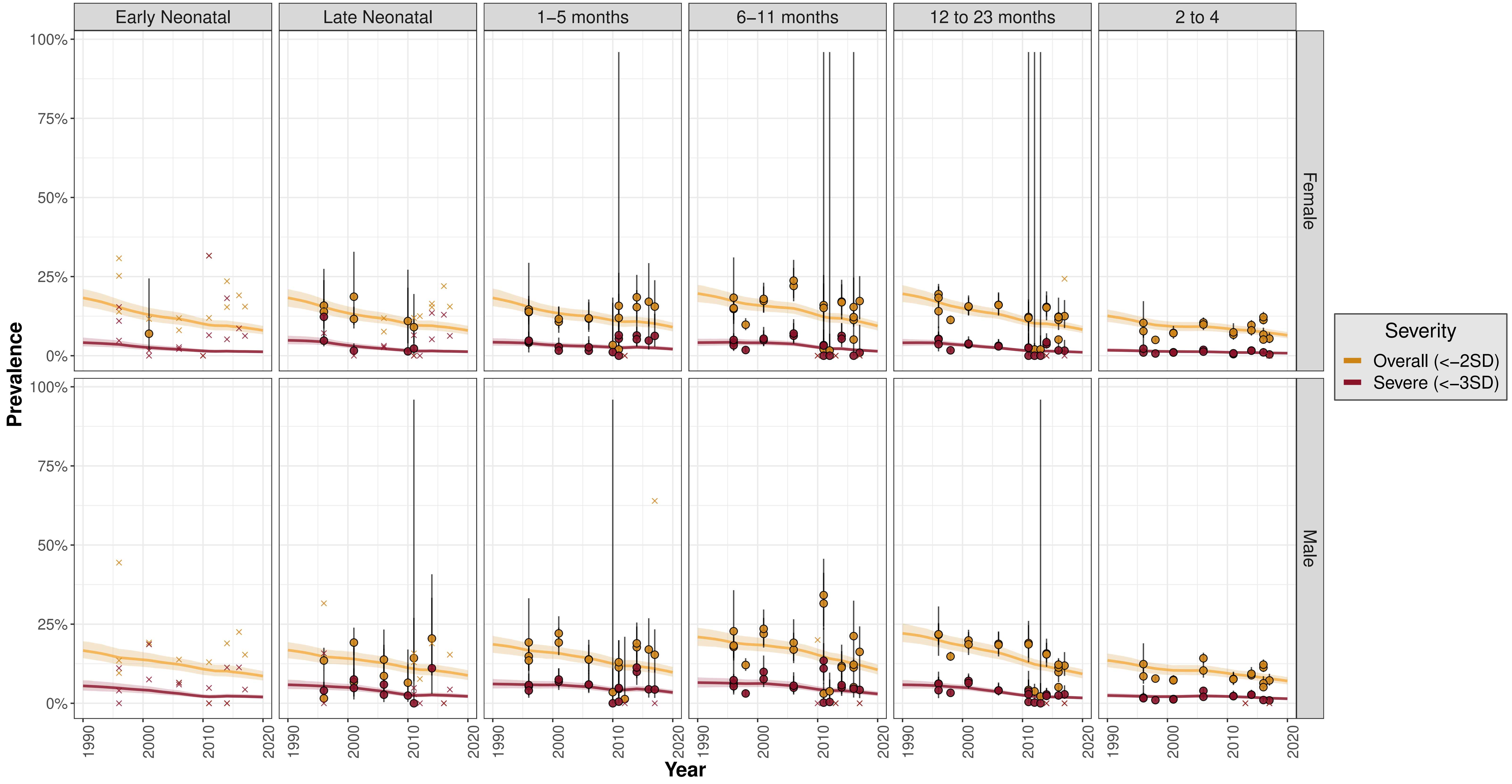

E: Transformed Mean Wasting Z Scores

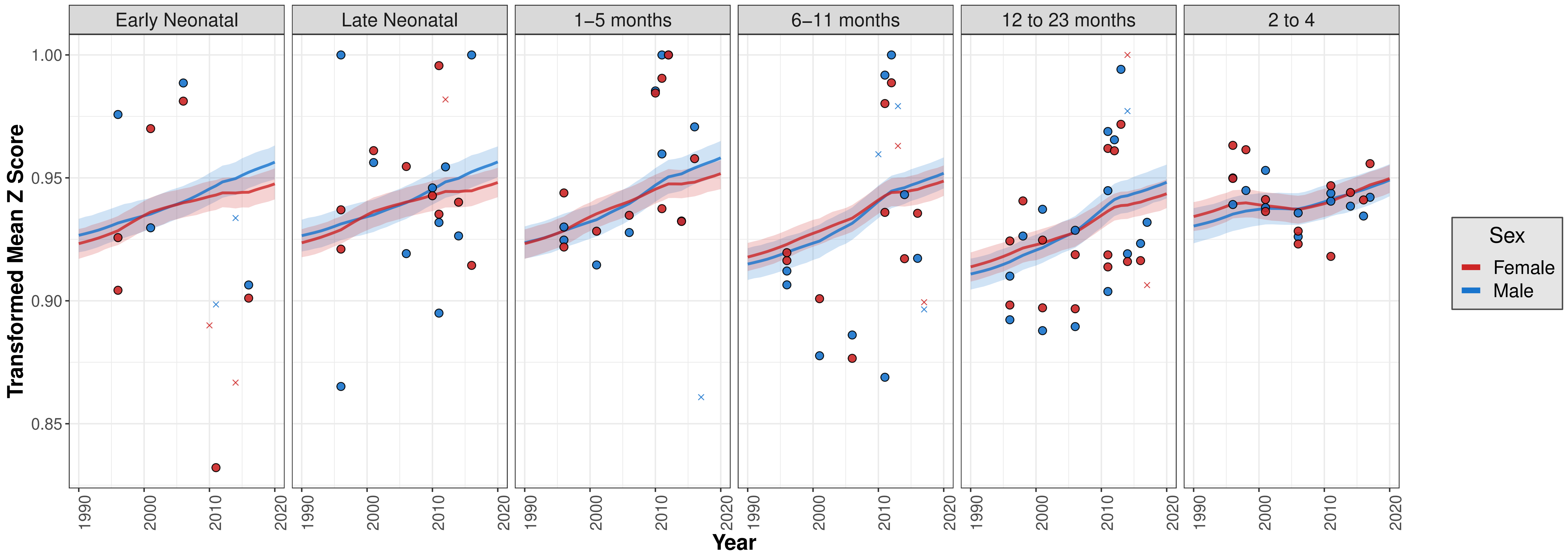

F

Year	Source
1975	WHO CGM Database
1996	Living Standards Survey
1996	DHS
1996	WHO CGM Database
1998	WHO CGM Database
2001	DHS
2001	WHO CGM Database
2006	DHS
2006	WHO CGM Database
2010	Bhaktapur Malnutrition and Enteric Disease Study
2011	DHS
2011	WHO CGM Database
2011	Bhaktapur Malnutrition and Enteric Disease Study
2012	Bhaktapur Malnutrition and Enteric Disease Study
2013	Bhaktapur Malnutrition and Enteric Disease Study
2014	WHO CGM Database
2014	MICS
2014	Bhaktapur Malnutrition and Enteric Disease Study
2016	DHS
2016	Micronutrient Status Survey
2017	WHO CGM Database
2017	DHS

Nepal – Underweight (WAZ)

G: Overall and Severe Underweight Prevalence

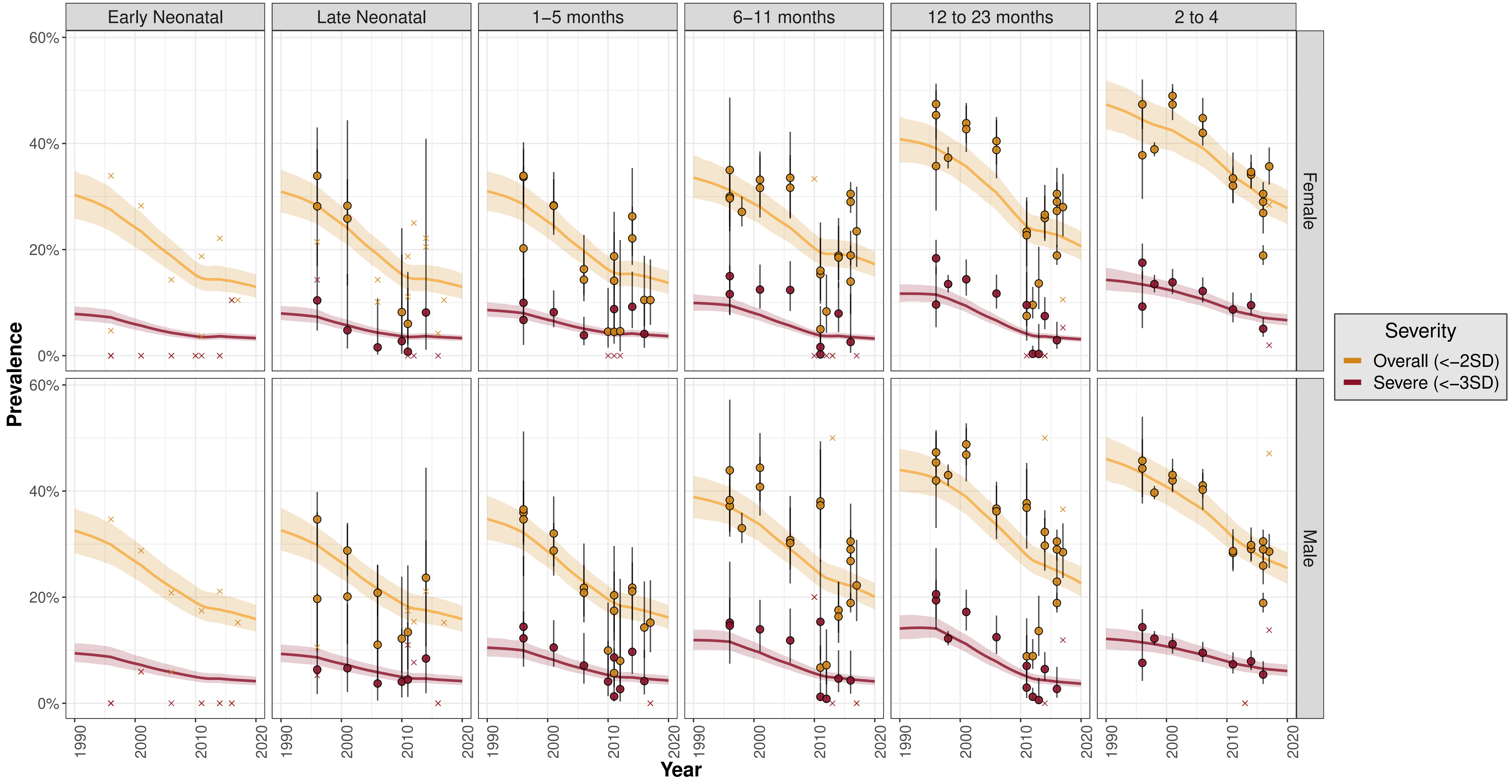

H: Transformed Mean Underweight Z Scores

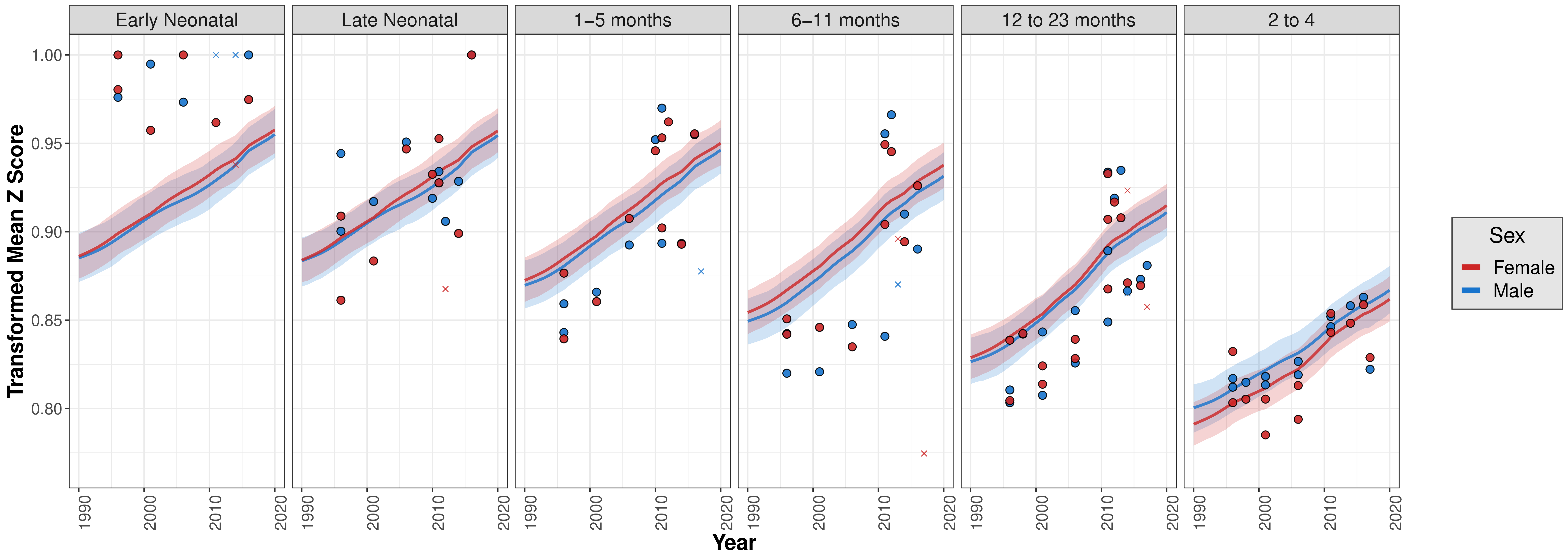

I

Year	Source
1975	WHO CGM Database
1996	Living Standards Survey
1996	DHS
1996	WHO CGM Database
1998	WHO CGM Database
2001	DHS
2001	WHO CGM Database
2006	DHS
2006	WHO CGM Database
2010	Bhaktapur Malnutrition and Enteric Disease Study
2011	DHS
2011	WHO CGM Database
2011	Bhaktapur Malnutrition and Enteric Disease Study
2012	Bhaktapur Malnutrition and Enteric Disease Study
2013	Bhaktapur Malnutrition and Enteric Disease Study
2014	WHO CGM Database
2014	MICS
2014	Bhaktapur Malnutrition and Enteric Disease Study
2016	DHS
2016	Micronutrient Status Survey
2017	WHO CGM Database
2017	DHS

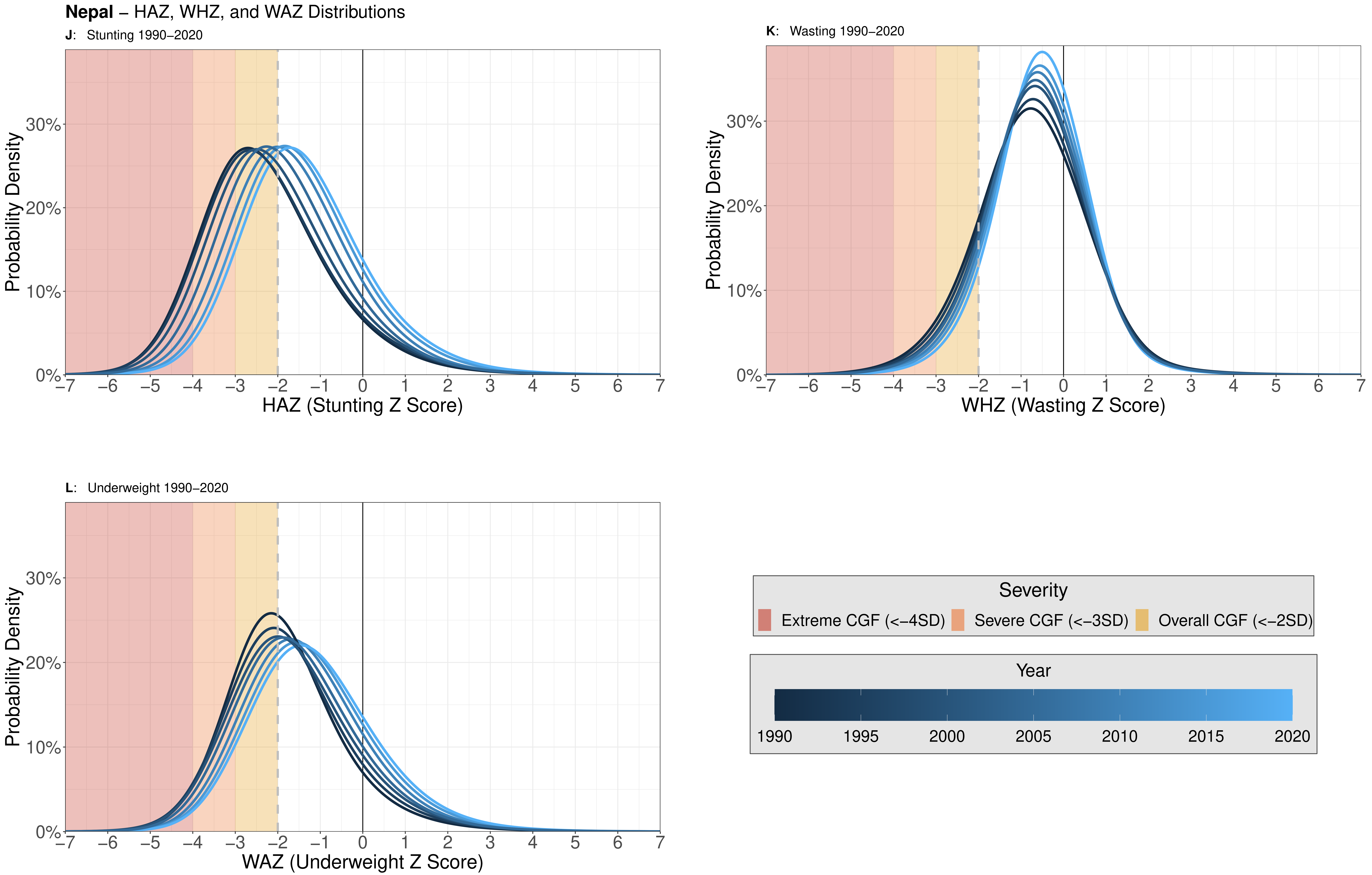

Nepal – HAZ, WHZ, and WAZ Distributions
J: Stunting 1990–2020
Probability Density
0%
10%
20%
30%
−7 −6 −5 −4 −3 −2 −1 0 1 2 3 4 5 6 7
HAZ (Stunting Z Score)
K: Wasting 1990–2020
Probability Density
0%
10%
20%
30%
−7 −6 −5 −4 −3 −2 −1 0 1 2 3 4 5 6 7
WHZ (Wasting Z Score)
L: Underweight 1990–2020
Probability Density
0%
10%
20%
30%
−7 −6 −5 −4 −3 −2 −1 0 1 2 3 4 5 6 7
WAZ (Underweight Z Score)
Severity
Extreme CGF (<−4SD)
Severe CGF (<−3SD)
Overall CGF (<−2SD)
Year
1990
1995
2000
2005
2010
2015
2020

Pakistan – Stunting (HAZ)

A: Overall and Severe Stunting Prevalence

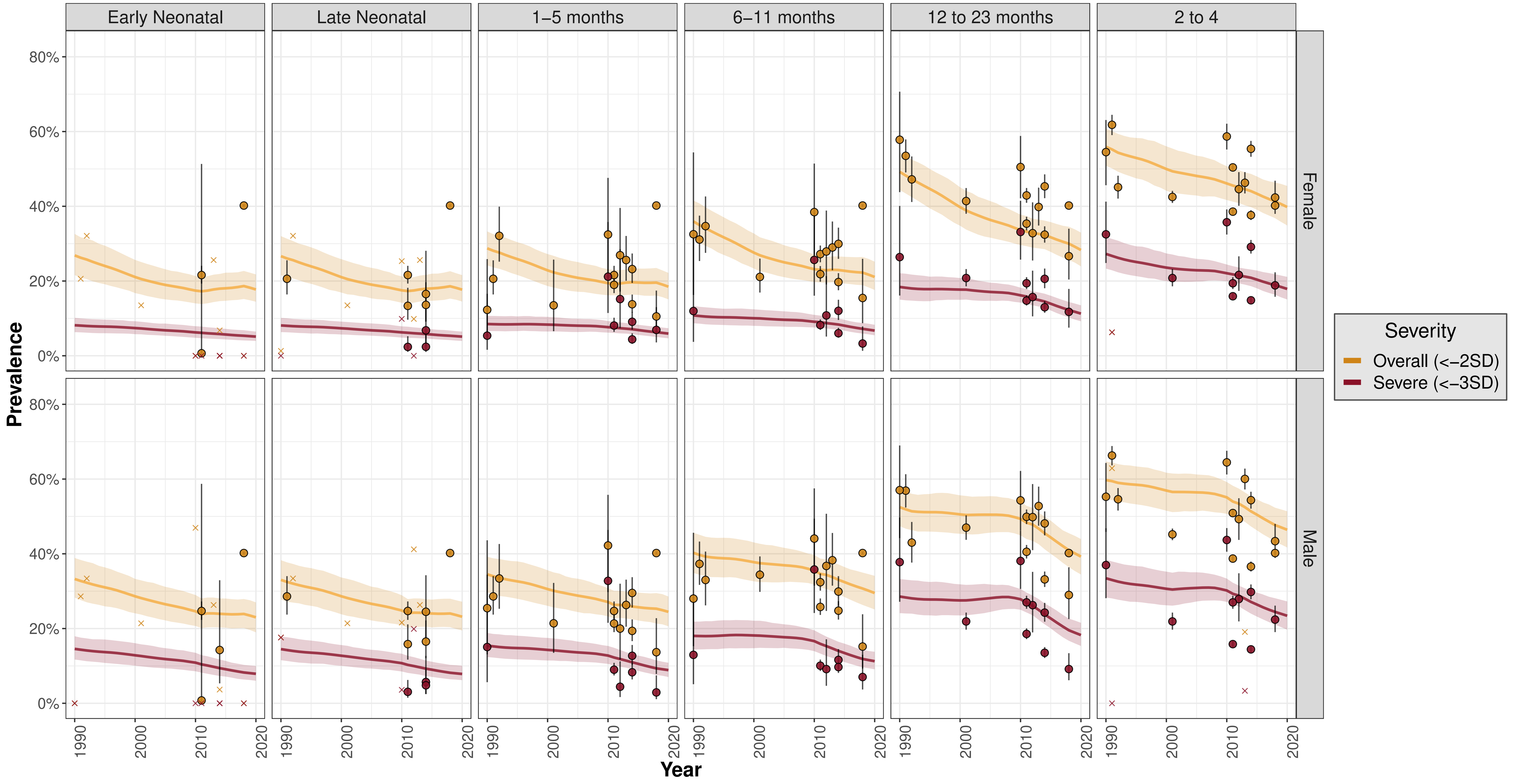

B: Transformed Mean Stunting Z Scores

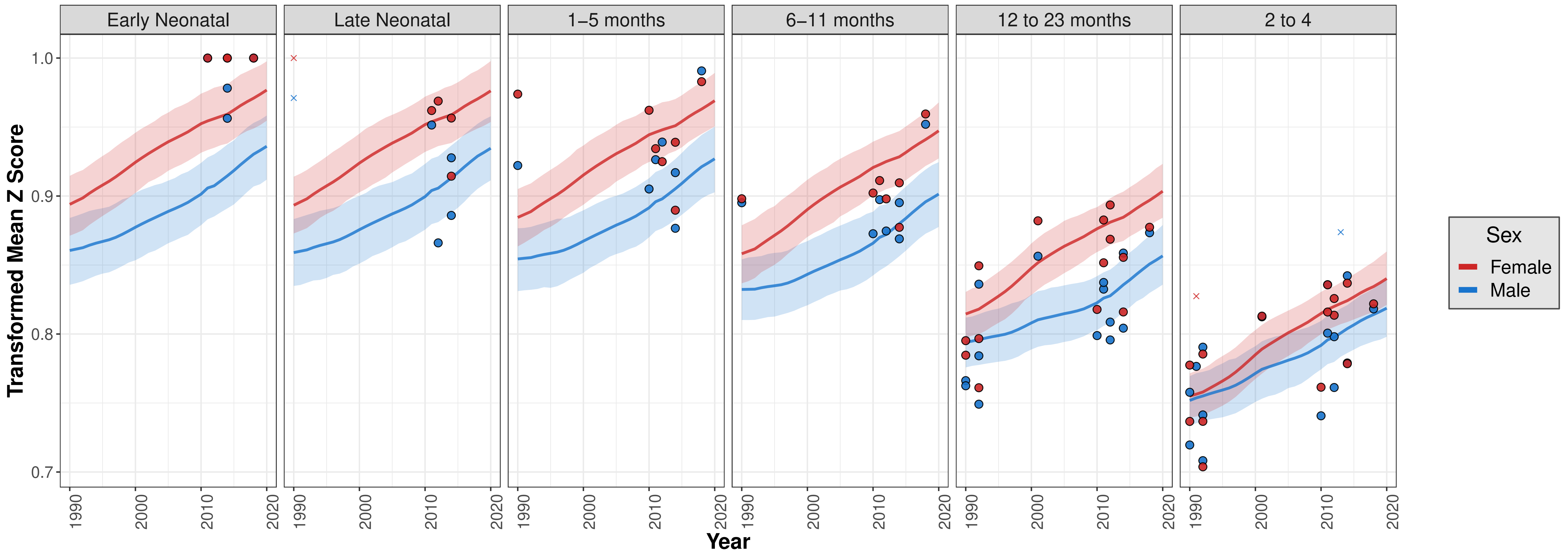

C

Year	Source	National	Subnational
1977	WHO CGM Database	X	
1986	WHO CGM Database	X	
1988	WHO CGM Database	X	
1990	DHS	X	
1990	WHO CGM Database	X	
1991	DHS	X	X
1991	WHO CGM Database	X	
1992	WHO CGM Database	X	
2001	WHO CGM Database	X	
2010	Balochistan MICS	X	
2010	Karachi Global Enteric Multicenter Study	X	
2011	Punjab MICS	X	
2011	WHO CGM Database	X	
2011	National Nutrition Survey	X	X
2012	DHS	X	
2012	WHO CGM Database	X	
2012	Karachi Global Enteric Multicenter Study	X	
2013	DHS	X	X
2013	WHO CGM Database	X	
2014	Sindh MICS	X	
2014	Punjab MICS	X	
2017	Gilgit–Baltistan MICS		X
2017	Khyber Pakhtunkhwa MICS		X
2018	DHS	X	X
2018	National Nutrition Survey	X	

Pakistan – Wasting (WHZ)

D: Overall and Severe Wasting Prevalence

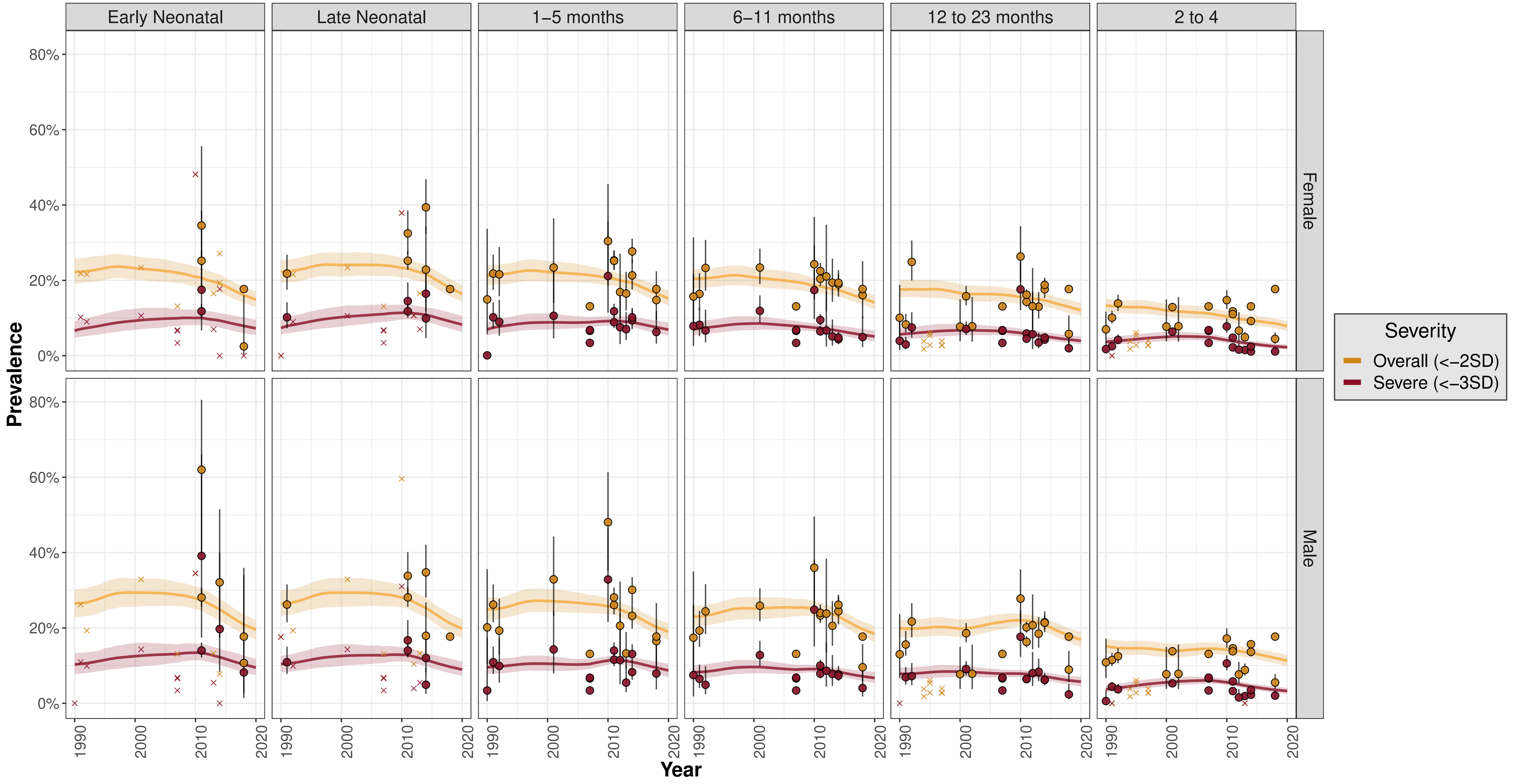

E: Transformed Mean Wasting Z Scores

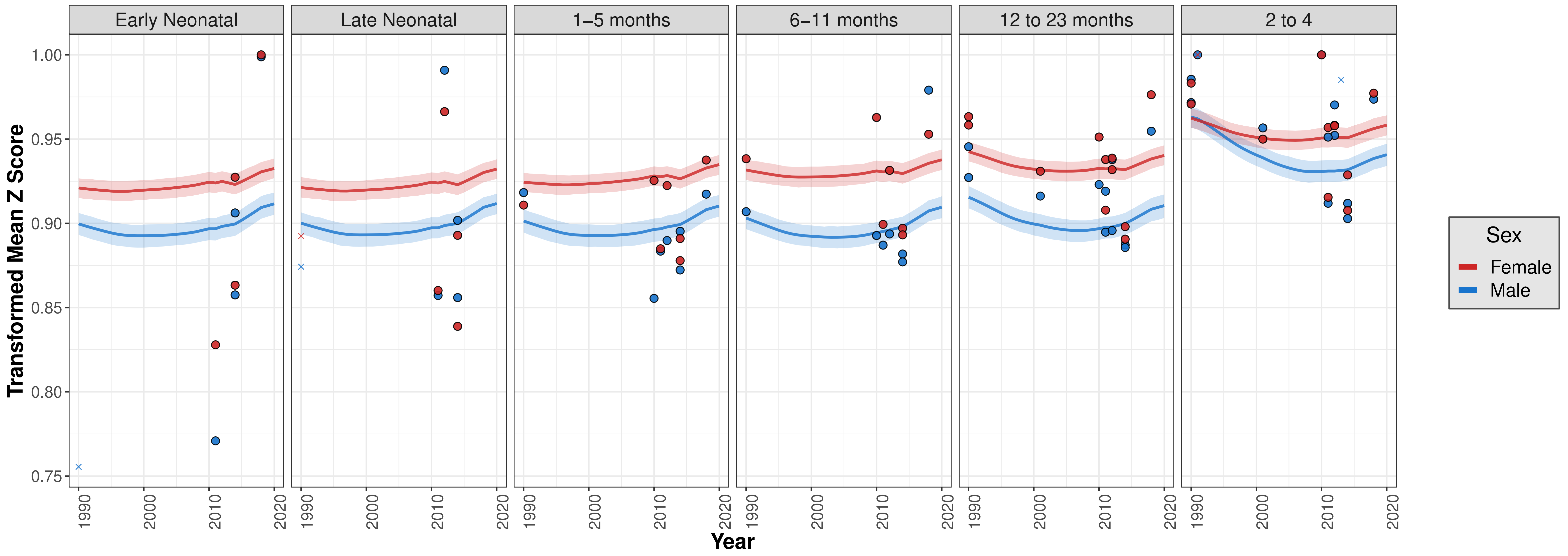

F

Year	Source	National	Subnational
1977	WHO CGM Database	X	
1986	WHO CGM Database	X	
1988	WHO CGM Database	X	
1990	DHS	X	
1990	WHO CGM Database	X	
1991	DHS	X	X
1991	WHO CGM Database	X	
1992	WHO CGM Database	X	
1994	WHO CGM Database	X	
1995	WHO CGM Database	X	
1997	WHO CGM Database	X	
2000	WHO CGM Database	X	
2001	WHO CGM Database	X	
2002	WHO CGM Database	X	
2007	Federally Administered Tribal Area MICS	X	
2010	Balochistan MICS	X	
2010	Karachi Global Enteric Multicenter Study	X	
2011	Punjab MICS	X	
2011	WHO CGM Database	X	
2011	National Nutrition Survey	X	X
2012	DHS	X	
2012	WHO CGM Database	X	
2012	Karachi Global Enteric Multicenter Study	X	
2013	DHS	X	X
2013	WHO CGM Database	X	
2014	Sindh MICS	X	
2014	Punjab MICS	X	
2017	Gilgit–Baltistan MICS		X
2017	Khyber Pakhtunkhwa MICS		X
2018	DHS	X	X
2018	National Nutrition Survey	X	

Pakistan – Underweight (WAZ)

G: Overall and Severe Underweight Prevalence

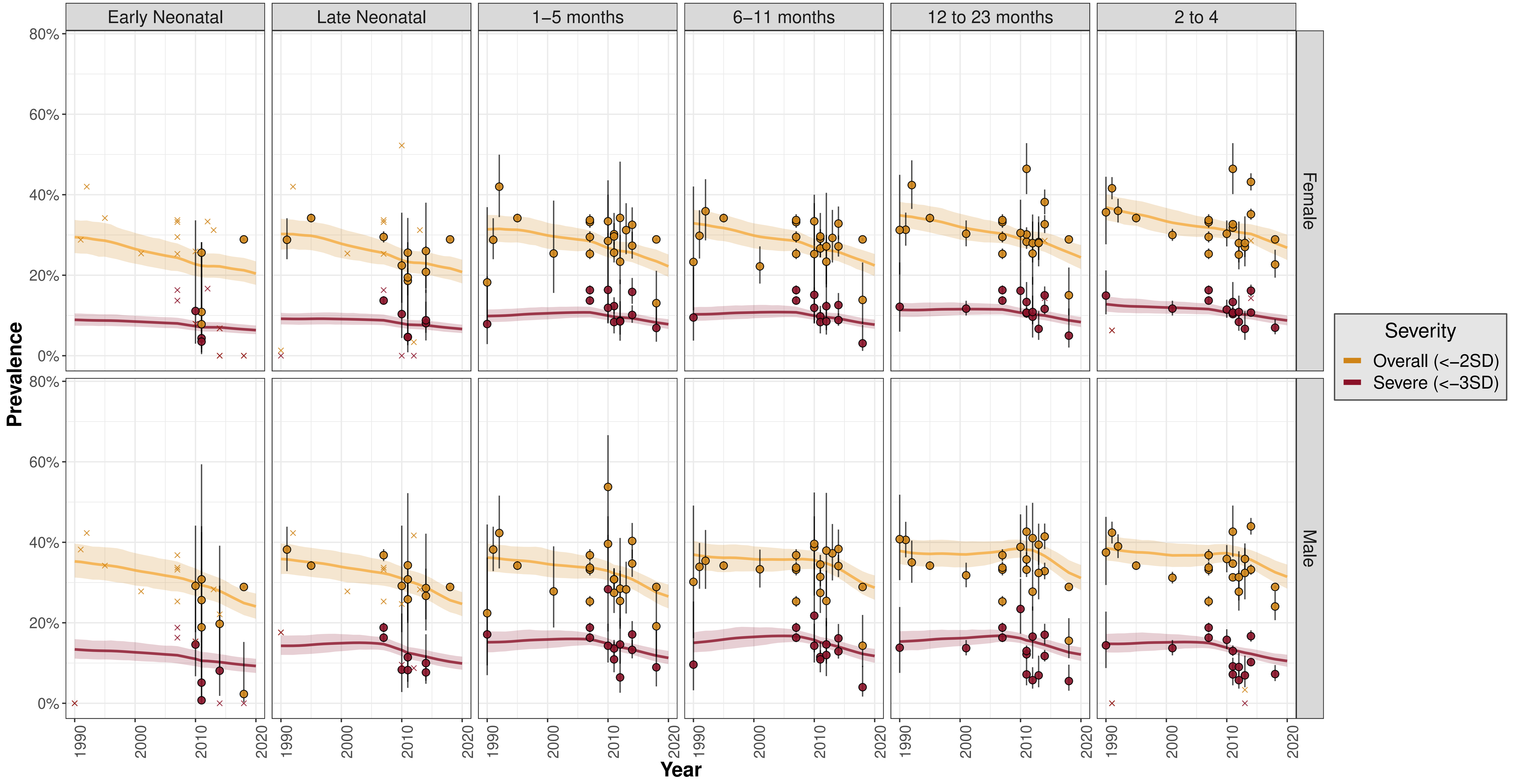

H: Transformed Mean Underweight Z Scores

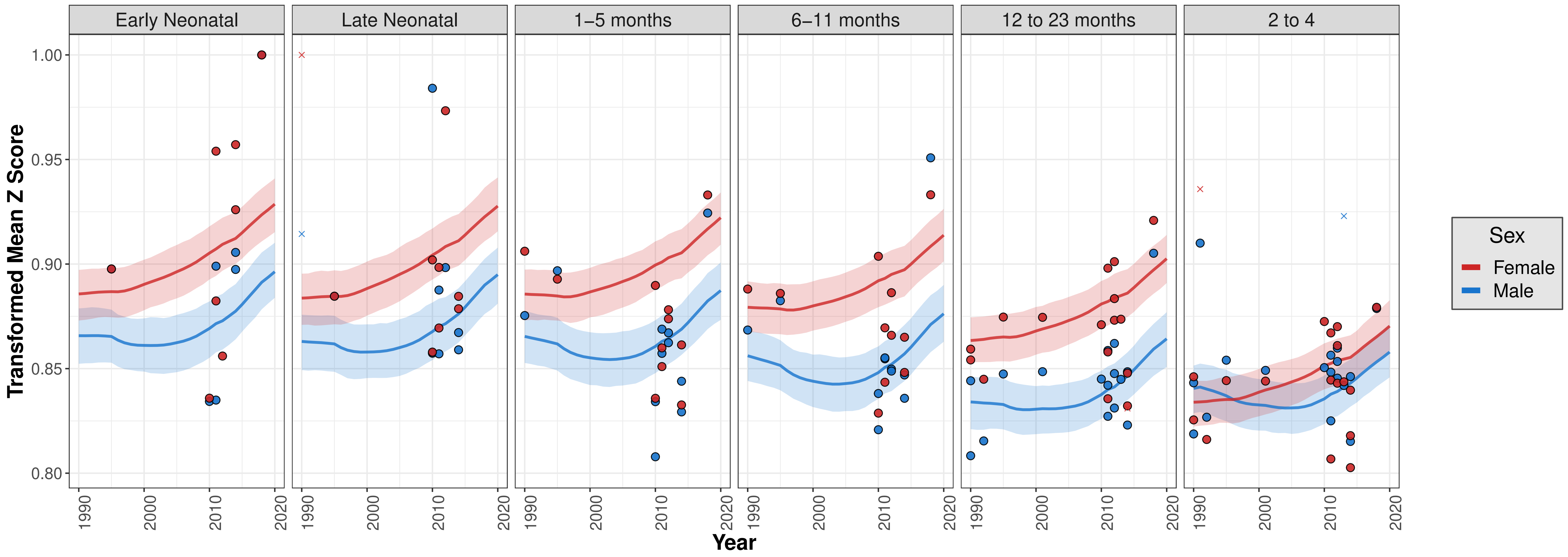

I

Year	Source	National	Subnational
1977	WHO CGM Database	X	
1986	WHO CGM Database	X	
1988	WHO CGM Database	X	
1990	DHS	X	
1990	WHO CGM Database	X	
1991	DHS	X	X
1991	WHO CGM Database	X	
1992	WHO CGM Database	X	
1995	WHO CGM Database	X	
2001	WHO CGM Database	X	
2004	Balochistan MICS		X
2004	Sindh MICS		X
2007	Federally Administered Tribal Area MICS	X	
2010	Balochistan MICS	X	
2010	Karachi Global Enteric Multicenter Study	X	
2010	Naushahro Feroze Malnutrition and Enteric Disease Study	X	
2011	Punjab MICS	X	
2011	WHO CGM Database	X	
2011	National Nutrition Survey	X	X
2011	Naushahro Feroze Malnutrition and Enteric Disease Study	X	
2012	DHS	X	
2012	WHO CGM Database	X	
2012	Karachi Global Enteric Multicenter Study	X	
2012	Naushahro Feroze Malnutrition and Enteric Disease Study	X	
2013	DHS	X	X
2013	WHO CGM Database	X	
2013	Naushahro Feroze Malnutrition and Enteric Disease Study	X	
2014	Sindh MICS	X	
2014	Punjab MICS	X	
2014	Naushahro Feroze Malnutrition and Enteric Disease Study	X	
2017	Gilgit–Baltistan MICS		X
2017	Khyber Pakhtunkhwa MICS		X
2018	DHS	X	X
2018	National Nutrition Survey	X	

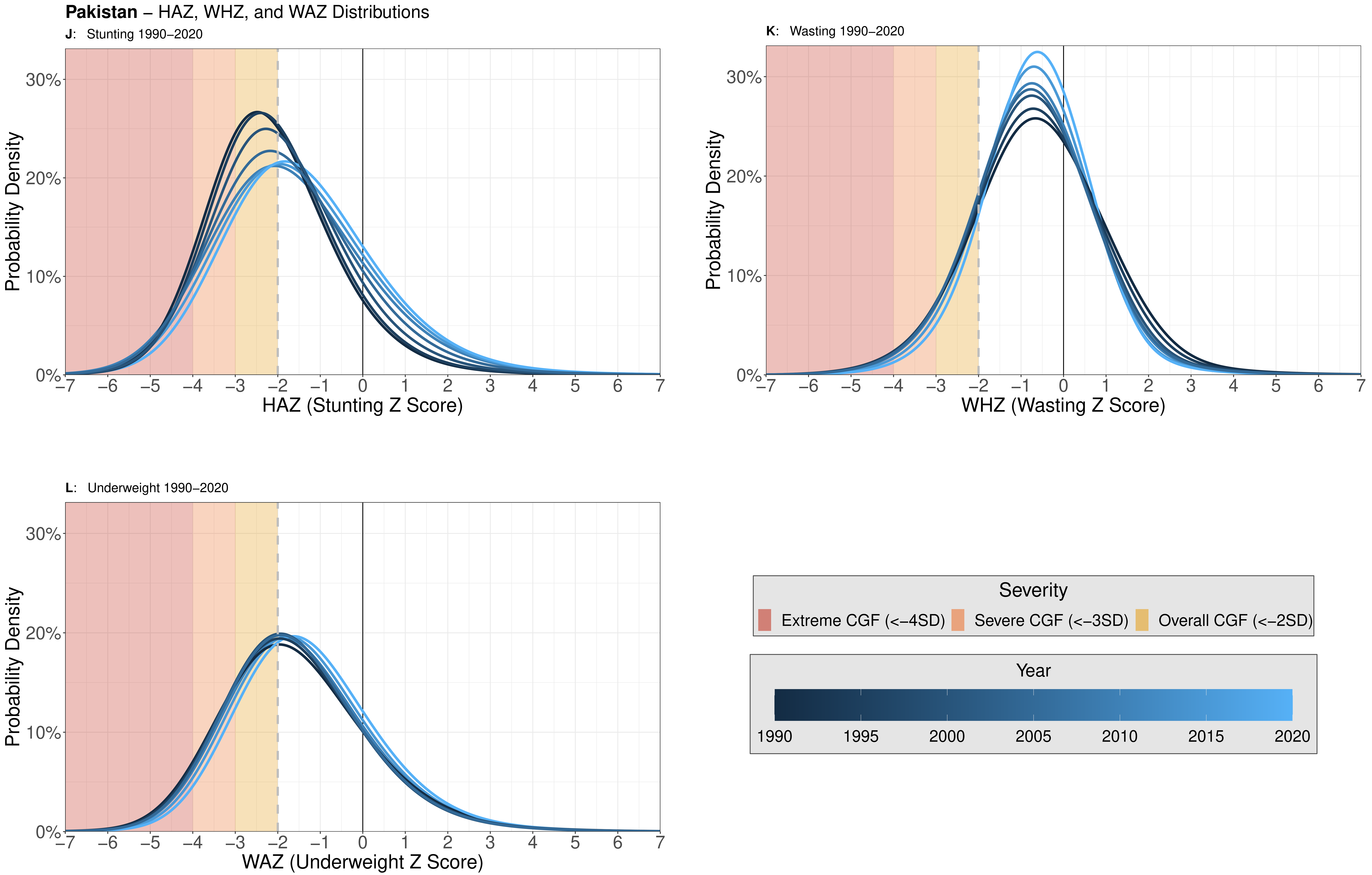
Pakistan – HAZ, WHZ, and WAZ Distributions
J: Stunting 1990–2020
K: Wasting 1990–2020
L: Underweight 1990–2020
Probability Density
30%
20%
10%
0%
−7 −6 −5 −4 −3 −2 −1 0 1 2 3 4 5 6 7
HAZ (Stunting Z Score)
WHZ (Wasting Z Score)
WAZ (Underweight Z Score)
Severity
Extreme CGF (<−4SD)
Severe CGF (<−3SD)
Overall CGF (<−2SD)
Year
1990
1995
2000
2005
2010
2015
2020
