# Supplementary material for: Current dichotomous metrics obscure trends in severe and extreme child growth failure
Source: Sci Adv. 2022 May 20;8(20):eabm8954. doi: 10.1126/sciadv.abm8954 (PMC9122330; doi:10.1126/sciadv.abm8954)
Supplement: Supplementary file 2 — Data S1 to S4 [file sciadv.abm8954_data_files_s1_to_s4.zip › sciadv.abm8954_data_file_s1f.pdf]

Data S1f. Spatio-temporal Gaussian Process Regression (ST-GPR) results for overall, severe, and mean CGF by location, including location-specific data sources; and distributions of stunting [HAZ], wasting [WHZ], and underweight [WAZ] for children under age five, both sexes, for every five years from 1990−2020. Country results are grouped by GBD super-region, including Central Europe, Eastern Europe, and Central Asia (S1a), High-income (S1b), Latin America and Caribbean (S1c), North Africa and Middle East (S1d), South Asia (S1e), Southeast Asia, East Asia, and Oceania (S1f), and Sub-Saharan Africa (S1g). Plots for each country include overall and severe stunting prevalence (A) and transformed mean stunting Z scores (B). A source list is shown which includes surveys included in the stunting models (C). Additional plots are shown for overall and severe wasting prevalence (D) and transformed mean wasting Z scores (E), followed by a source list with surveys included in the wasting models (F). Plots are then shown for overall and severe underweight prevalence (G), and transformed mean underweight Z scores (H), with a source list listing surveys included in the underweight models (I). Finally, distributions of stunting (J), wasting (K), and underweight (L) are shown for children under age five, both sexes, for every five years from 1990-2020. Surveys that were outliered are shown with X's on all plots. Surveys prior to 1990 may have been inputs to the models to inform trends, but estimates are only produced and shown for 1990−2020. For locations that are modeled nationally and subnationally, sources that are only included subnationally are not included in the plots of national level estimates. These sources were included in subnational models that influence national level models. Note that due to the transformation on mean Z scores, increasing values reflect improvements in mean Z score. Surveys conducted over a range of years were assigned to the midpoint year from that interval, which is the year reflected in the table and the plots. For the distributions of stunting, wasting, and underweight, the area under the curve reflects the estimated proportion of children experiencing that severity of CGF or worse. DHS is Demographic and Health Surveys. MICS is Multiple Indicator Cluster Survey. WHO CGM is the WHO Global Database on Child Growth and Malnutrition. SDNS is Survey of Diet and Nutritional Status.

This file contains the above for the following locations in the GBD super region of Southeast Asia, East Asia, and Oceania, in the following order:

East Asia: China, Democratic People's Republic of Korea, Taiwan (Province of China)

Oceania: Fiji, Kiribati, Marshall Islands, Micronesia (Federated States of), Nauru, Palau, Papua New Guinea, Samoa, Solomon Islands, Tonga, Tuvalu, Vanuatu

Southeast Asia: Cambodia, Indonesia, Lao People's Democratic Republic, Malaysia, Maldives, Mauritius, Myanmar, Philippines, Seychelles, Sri Lanka, Thailand, Timor-Leste, Viet Nam

China – Stunting (HAZ)

A: Overall and Severe Stunting Prevalence

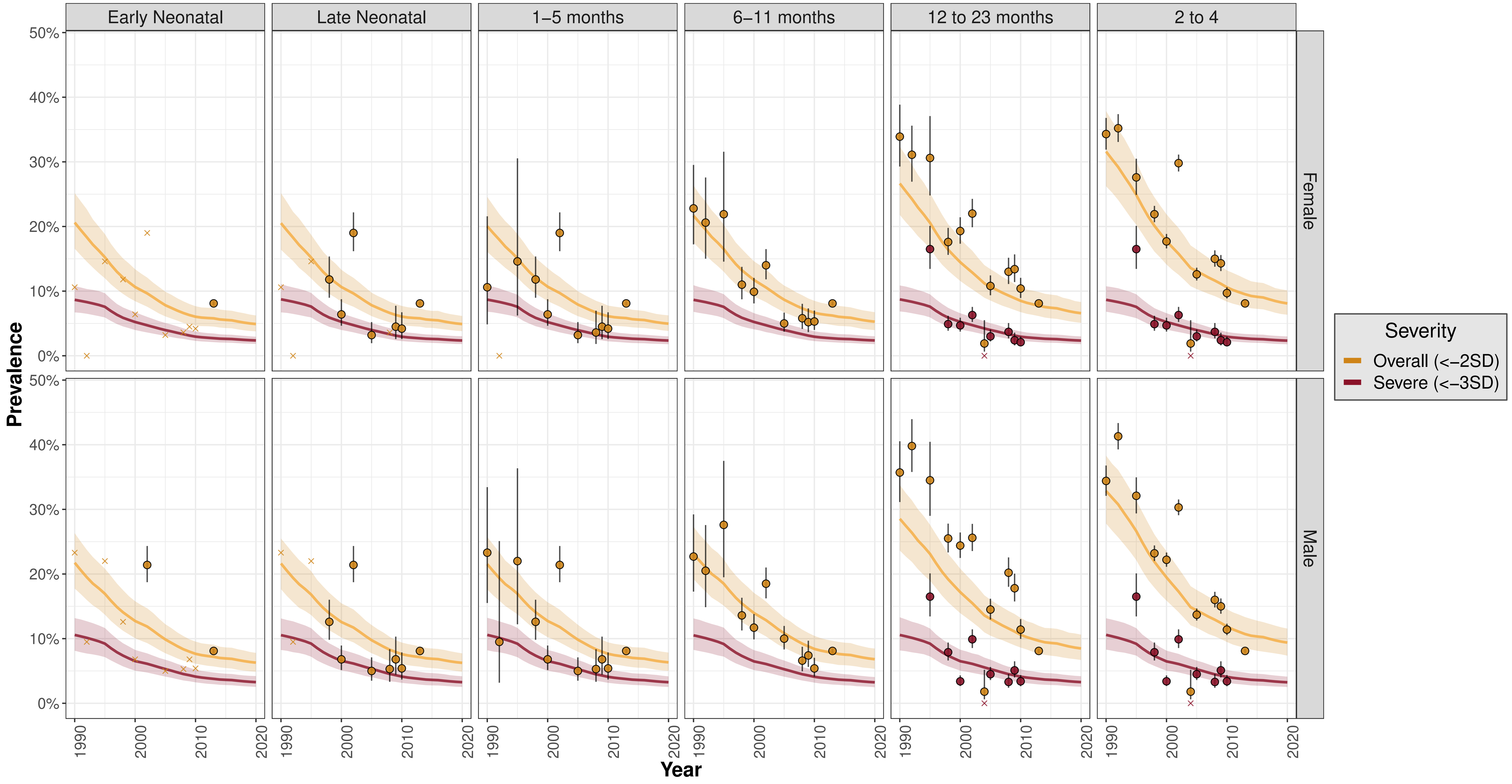

B: Transformed Mean Stunting Z Scores

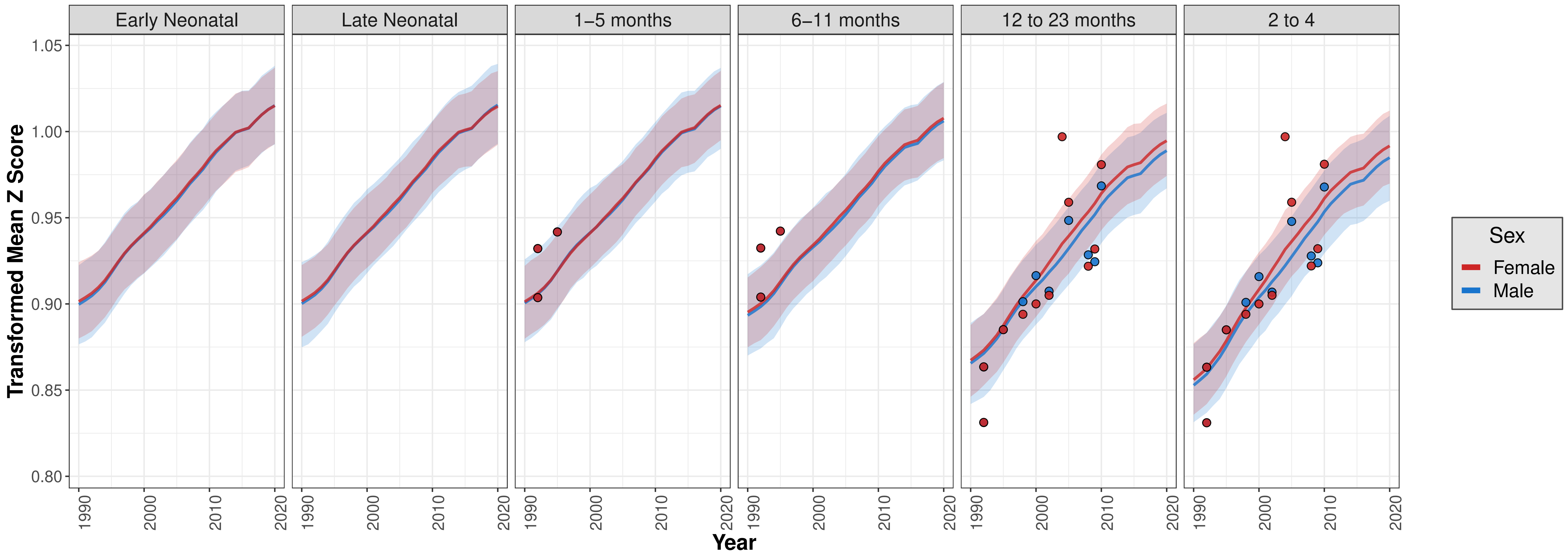

C

Year	Source	National	Subnational
1987	WHO CGM Database	X	
1989	WHO CGM Database	X	
1990	WHO CGM Database	X	
1991	Health and Nutrition Survey		X
1992	WHO CGM Database	X	X
1993	WHO CGM Database		X
1993	Health and Nutrition Survey		X
1995	WHO CGM Database	X	
1997	Health and Nutrition Survey		X
1998	WHO CGM Database	X	
2000	WHO CGM Database	X	
2000	Health and Nutrition Survey		X
2000	Shaanxi and Hubei Nutrition Assessment Baseline Survey		X
2002	WHO CGM Database	X	
2004	WHO CGM Database	X	
2004	Health and Nutrition Survey		X
2005	WHO CGM Database	X	
2006	Health and Nutrition Survey		X
2008	WHO CGM Database	X	
2009	WHO CGM Database	X	
2009	Health and Nutrition Survey		X
2010	WHO CGM Database	X	
2013	WHO CGM Database	X	

China – Wasting (WHZ)

D: Overall and Severe Wasting Prevalence

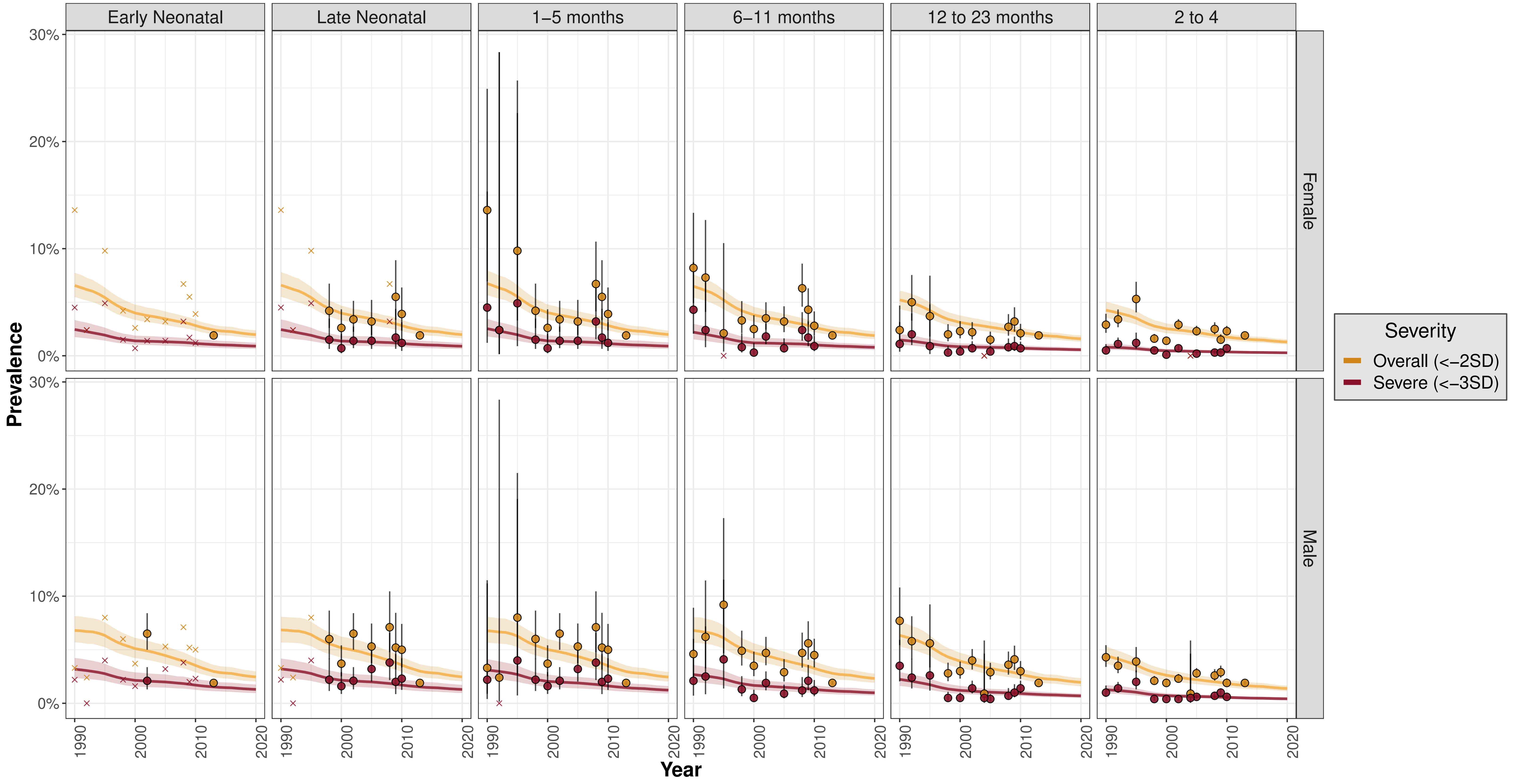

E: Transformed Mean Wasting Z Scores

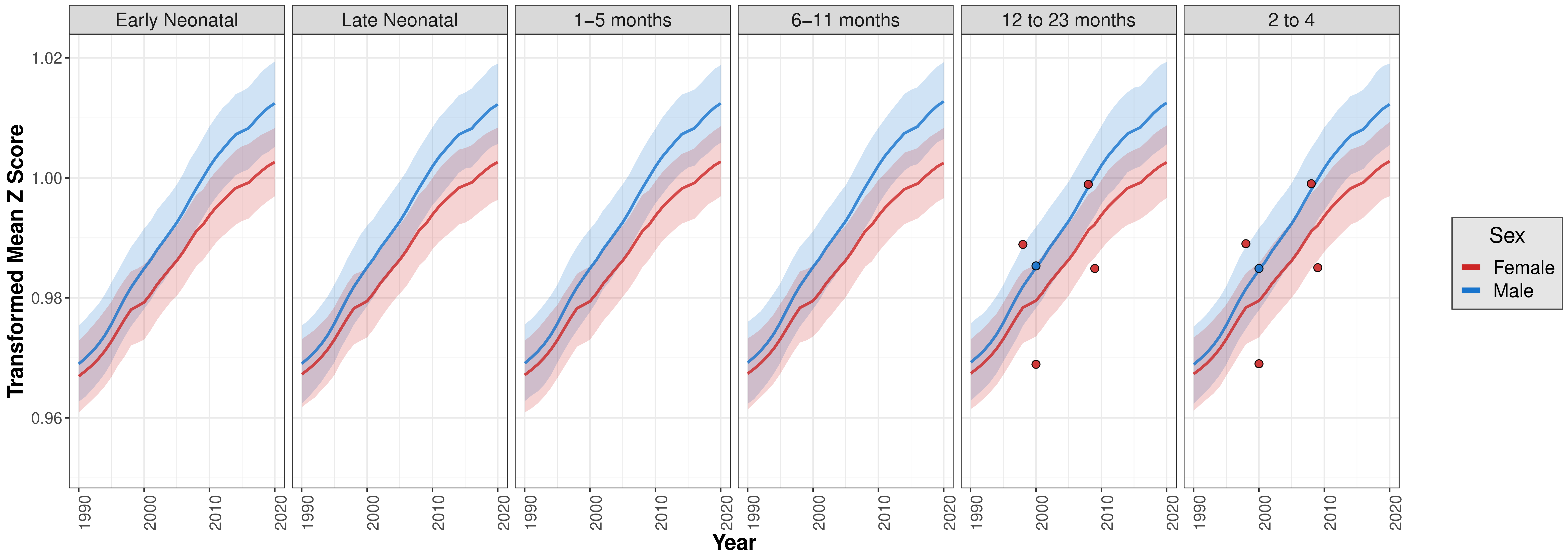

F

Year	Source	National	Subnational
1987	WHO CGM Database	X	
1989	WHO CGM Database	X	
1990	WHO CGM Database	X	
1991	Health and Nutrition Survey		X
1992	WHO CGM Database	X	X
1993	WHO CGM Database		X
1993	Health and Nutrition Survey		X
1995	WHO CGM Database	X	
1997	Health and Nutrition Survey		X
1998	WHO CGM Database	X	
2000	WHO CGM Database	X	
2000	Health and Nutrition Survey		X
2000	Shaanxi and Hubei Nutrition Assessment Baseline Survey		X
2002	WHO CGM Database	X	
2004	WHO CGM Database	X	
2004	Health and Nutrition Survey		X
2005	WHO CGM Database	X	
2006	Health and Nutrition Survey		X
2008	WHO CGM Database	X	
2009	WHO CGM Database	X	
2009	Health and Nutrition Survey		X
2010	WHO CGM Database	X	
2013	WHO CGM Database	X	

China – Underweight (WAZ)

G: Overall and Severe Underweight Prevalence

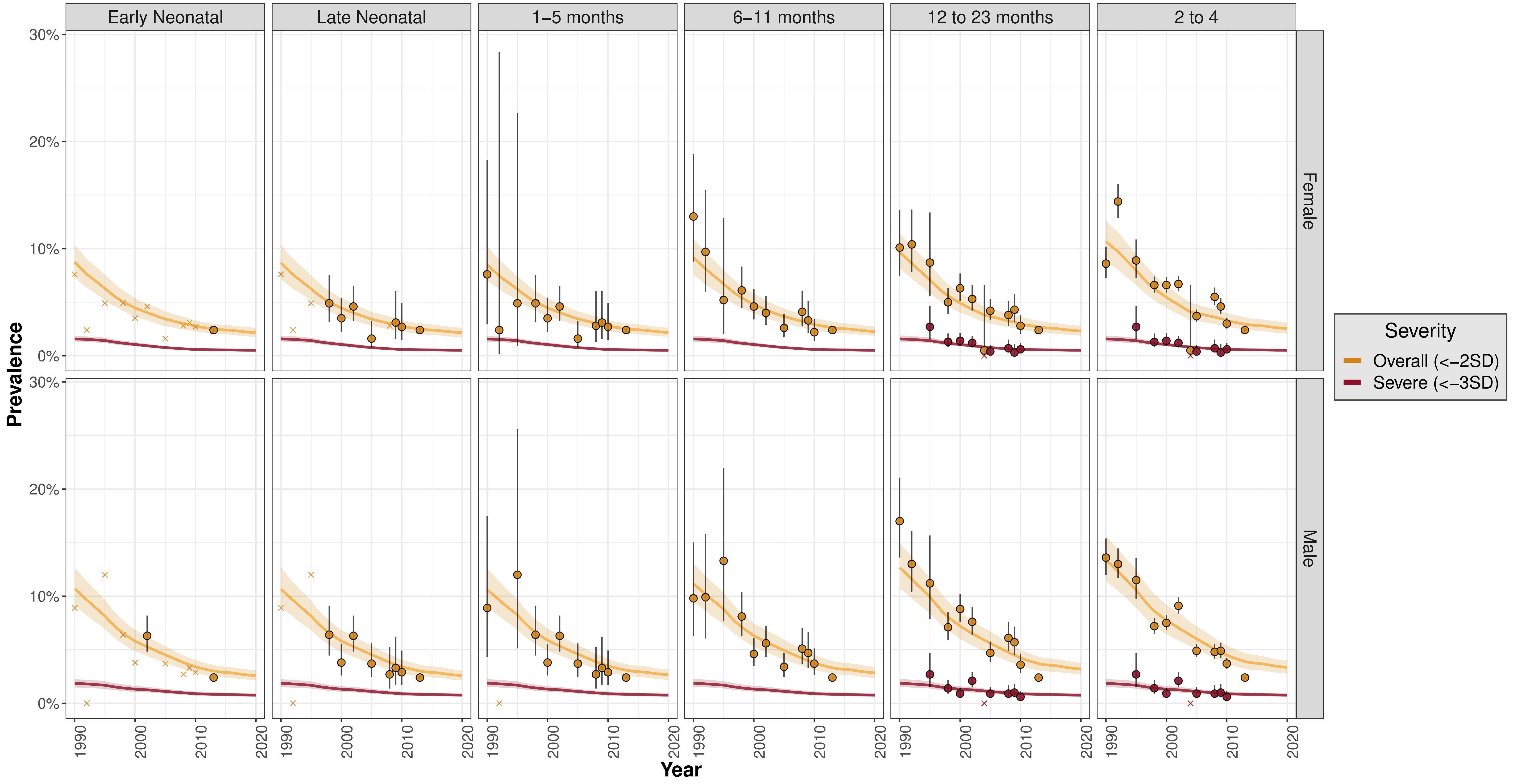

H: Transformed Mean Underweight Z Scores

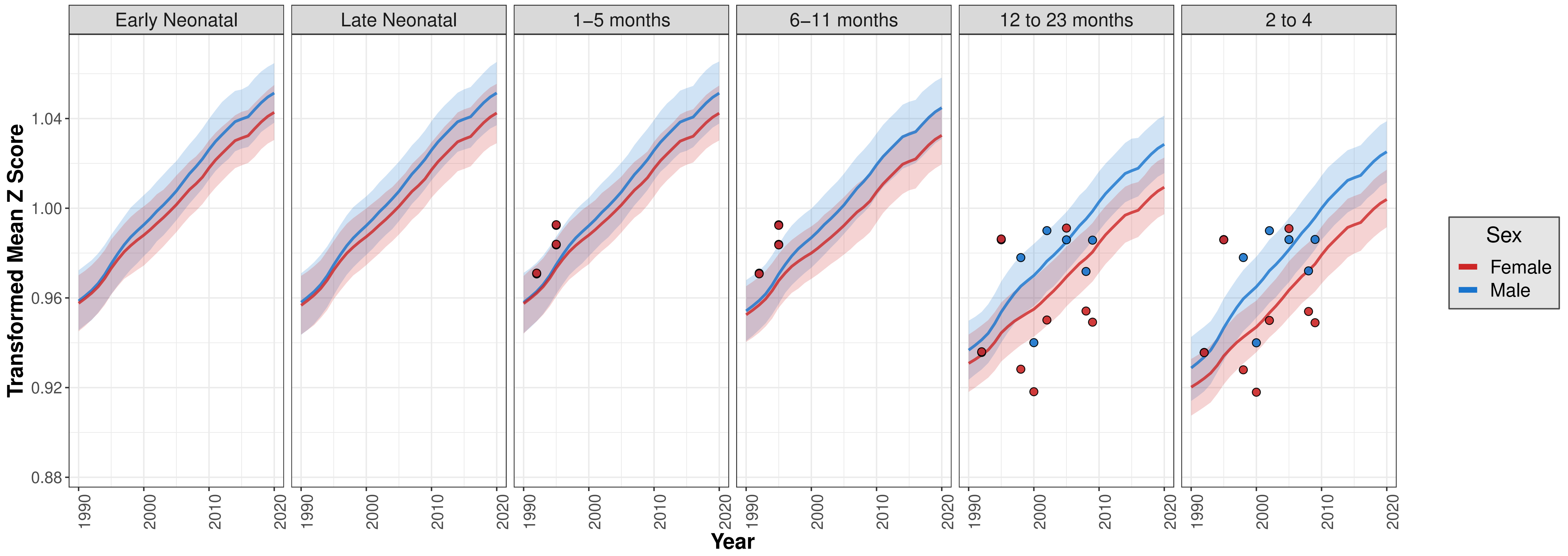

I

Year	Source	National	Subnational
1987	WHO CGM Database	X	
1989	WHO CGM Database	X	
1990	WHO CGM Database	X	
1991	Health and Nutrition Survey		X
1992	WHO CGM Database	X	X
1993	WHO CGM Database		X
1993	Health and Nutrition Survey		X
1995	WHO CGM Database	X	
1997	Health and Nutrition Survey		X
1998	WHO CGM Database	X	
2000	WHO CGM Database	X	
2000	Health and Nutrition Survey		X
2000	Shaanxi and Hubei Nutrition Assessment Baseline Survey		X
2002	WHO CGM Database	X	
2004	WHO CGM Database	X	
2004	Health and Nutrition Survey		X
2005	WHO CGM Database	X	
2006	Health and Nutrition Survey		X
2008	WHO CGM Database	X	
2009	WHO CGM Database	X	
2009	Health and Nutrition Survey		X
2010	WHO CGM Database	X	
2013	WHO CGM Database	X	

China – HAZ, WHZ, and WAZ Distributions

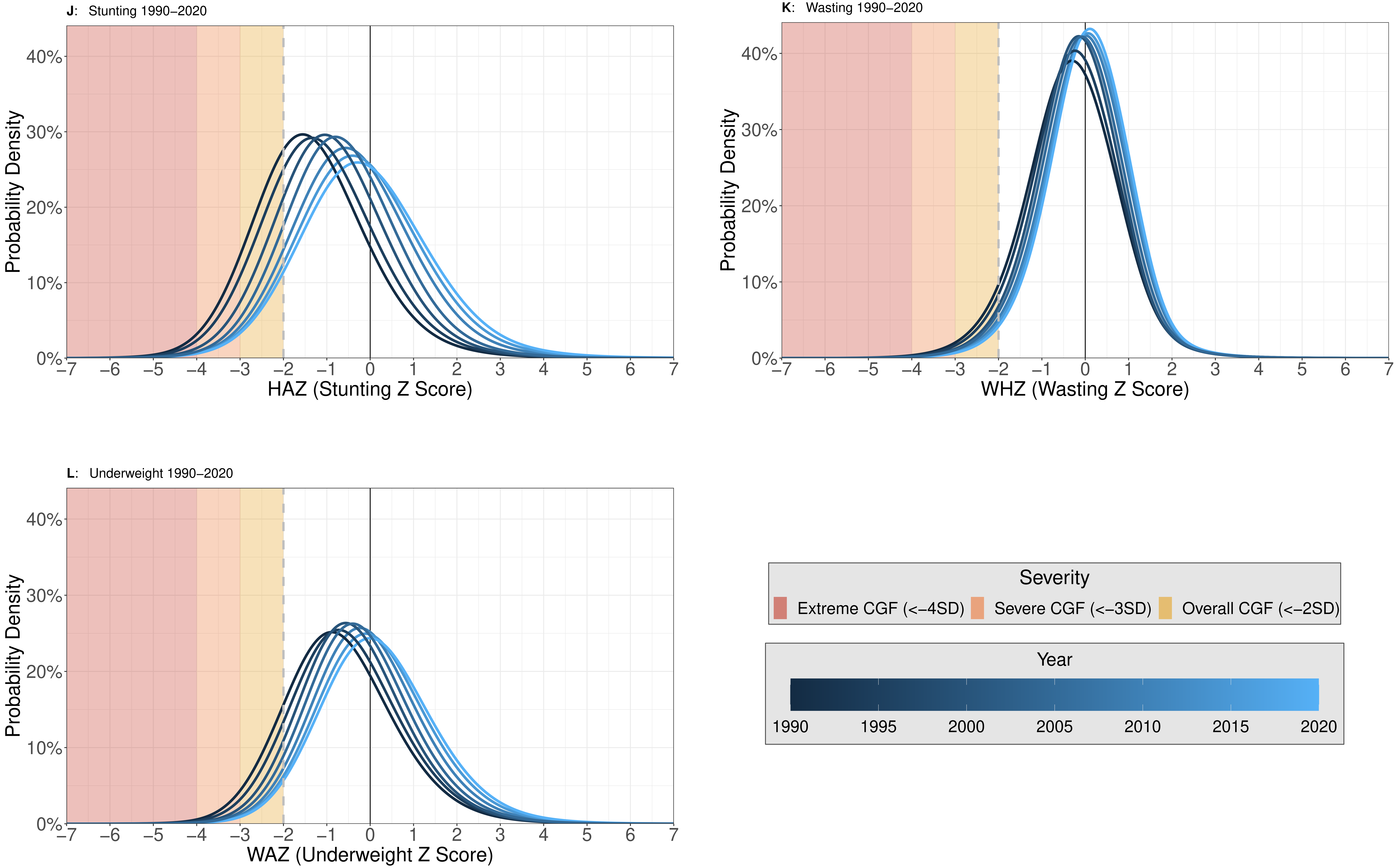

Democratic People's Republic of Korea – Stunting (HAZ)

A: Overall and Severe Stunting Prevalence

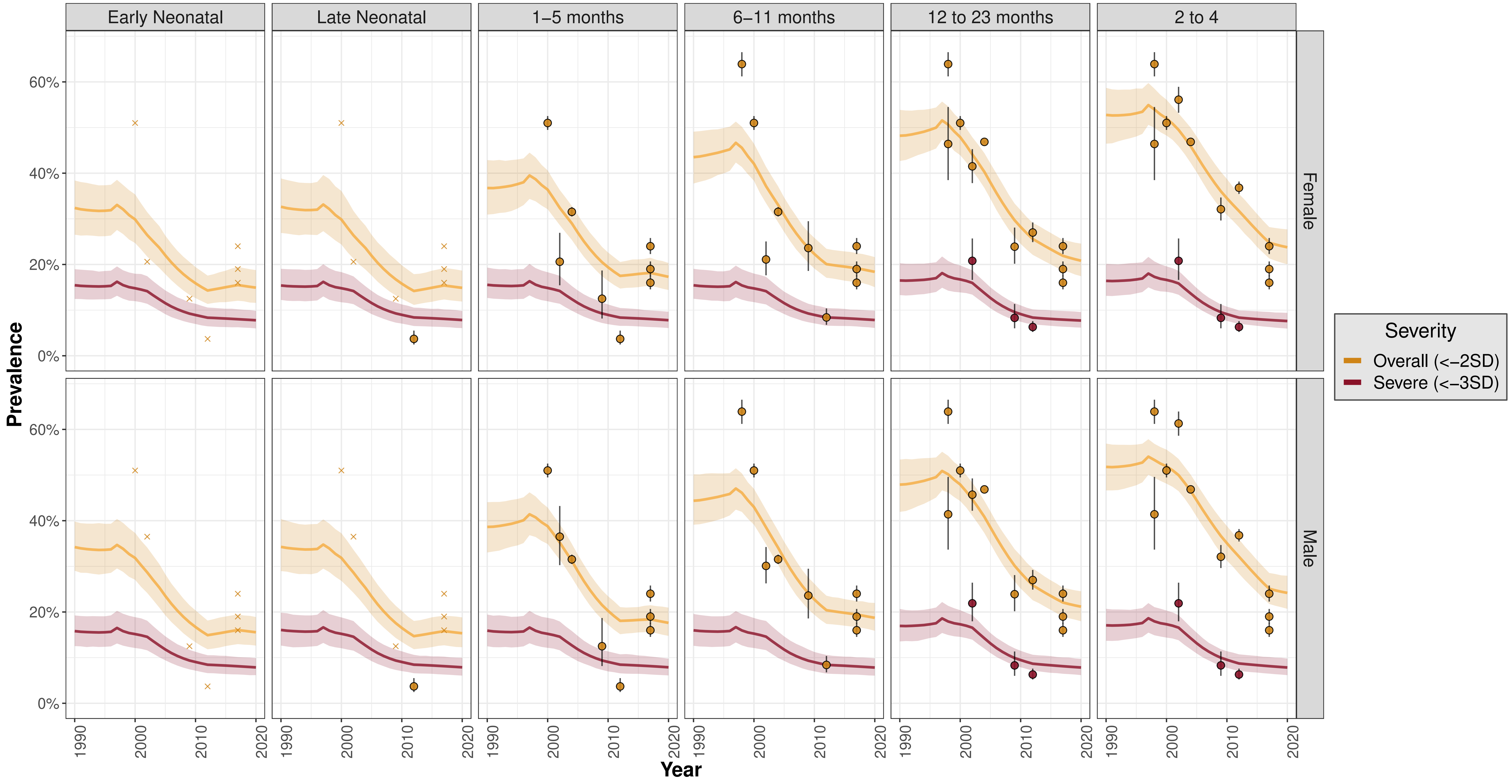

B: Transformed Mean Stunting Z Scores

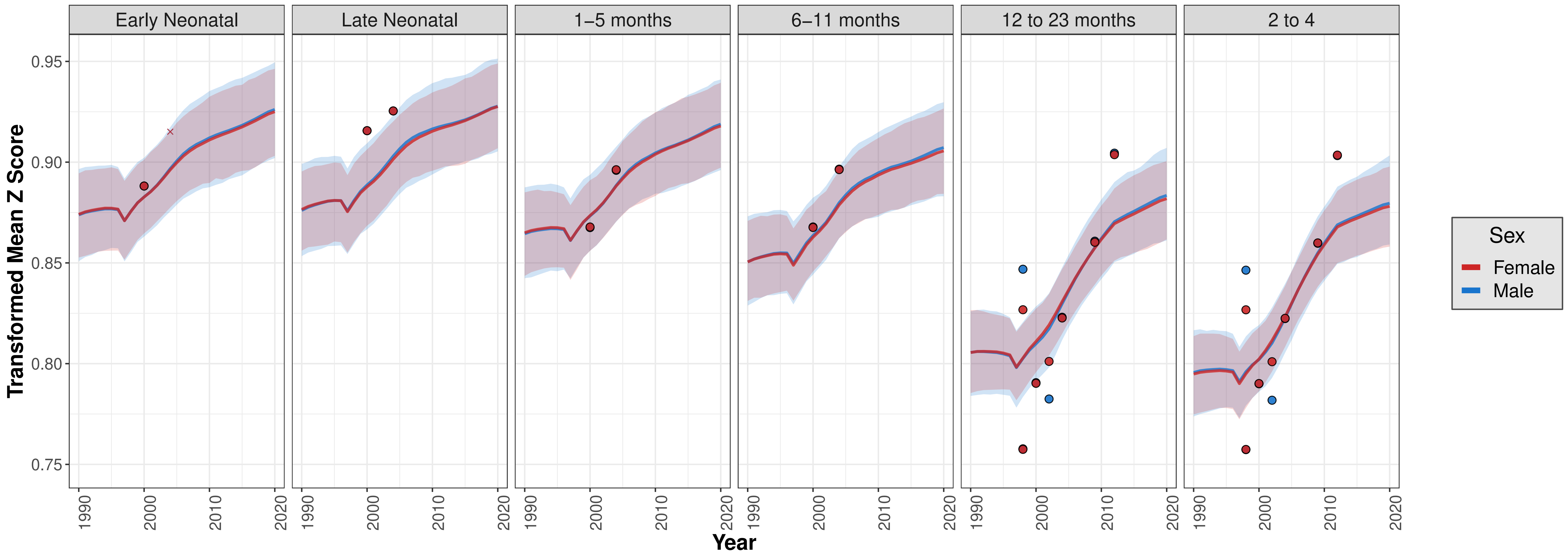

C

Year	Source
1998	MICS
1998	WHO CGM Database
2000	WHO CGM Database
2002	WHO CGM Database
2004	WHO CGM Database
2009	WHO CGM Database
2012	WHO CGM Database
2017	MICS

Democratic People's Republic of Korea – Wasting (WHZ)

D: Overall and Severe Wasting Prevalence

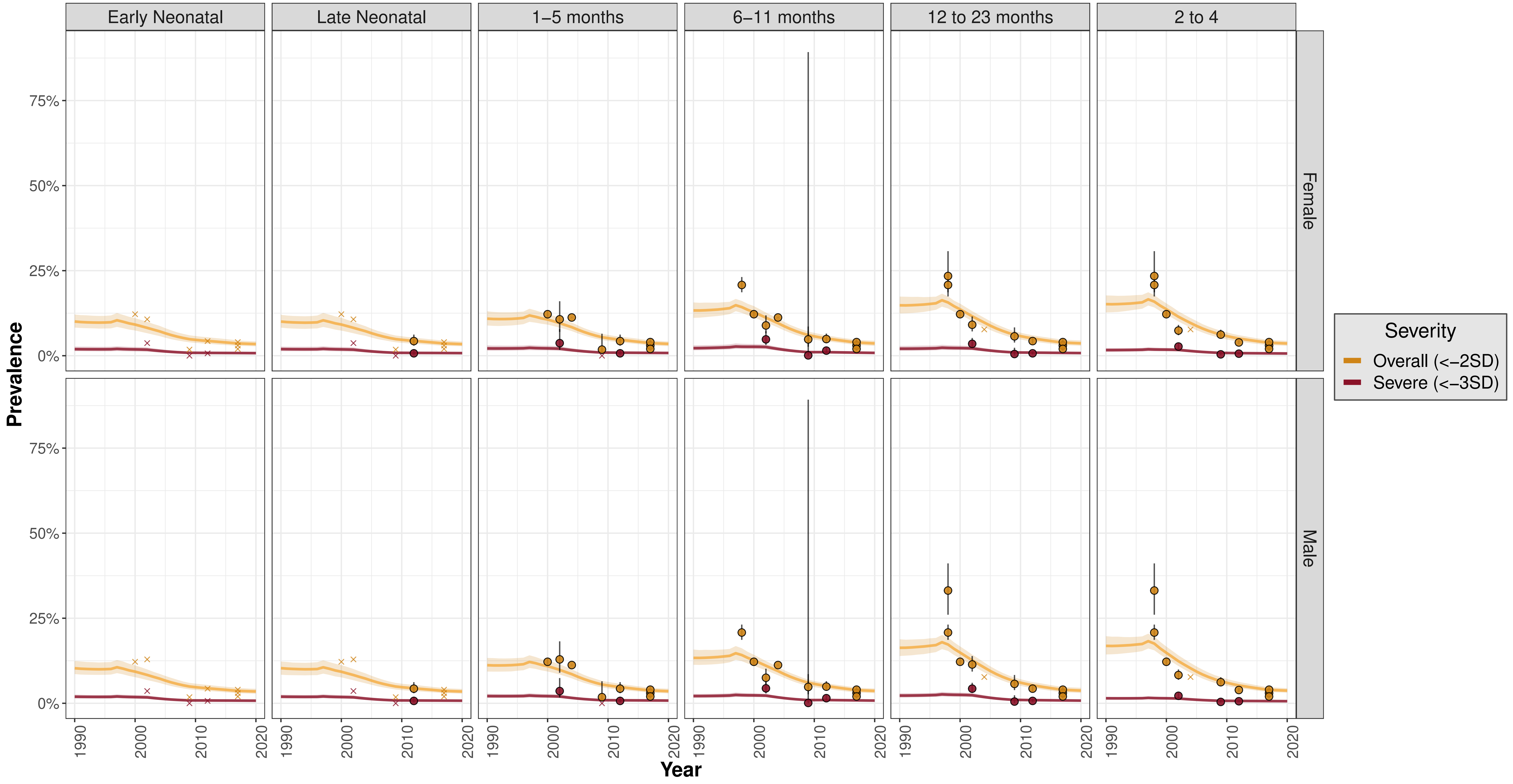

E: Transformed Mean Wasting Z Scores

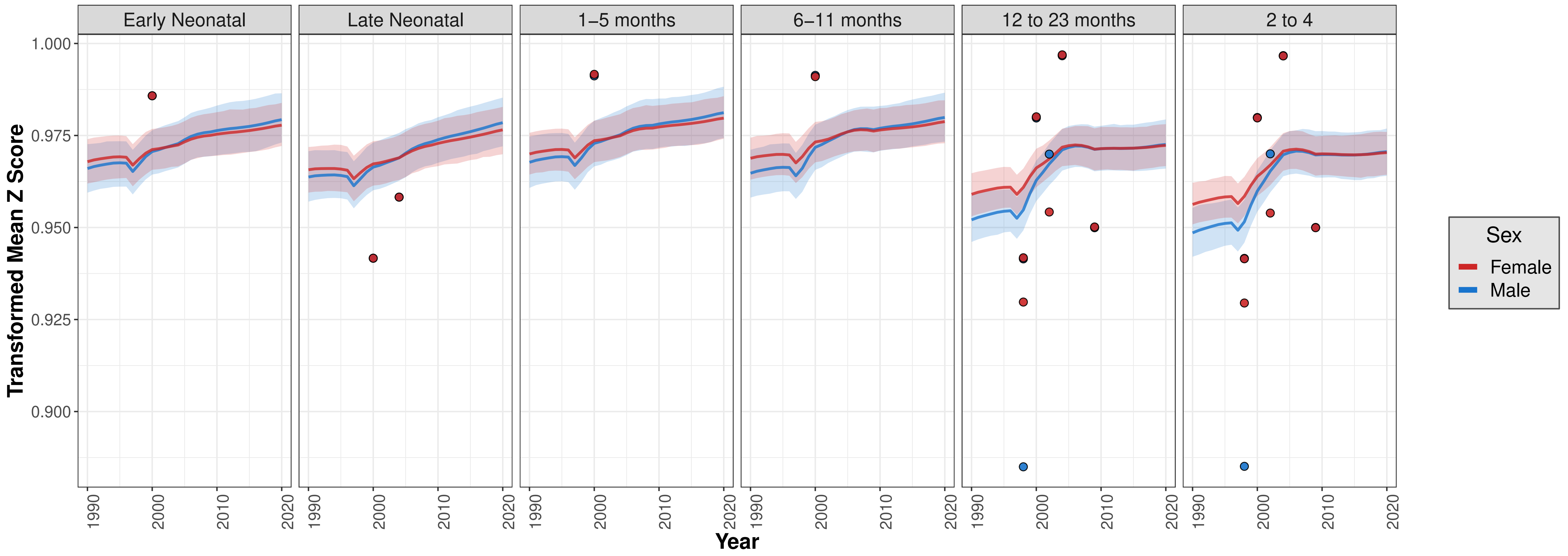

F

Year	Source
1998	MICS
1998	WHO CGM Database
2000	WHO CGM Database
2002	WHO CGM Database
2004	WHO CGM Database
2009	WHO CGM Database
2012	WHO CGM Database
2017	MICS

Democratic People's Republic of Korea – Underweight (WAZ)

G: Overall and Severe Underweight Prevalence

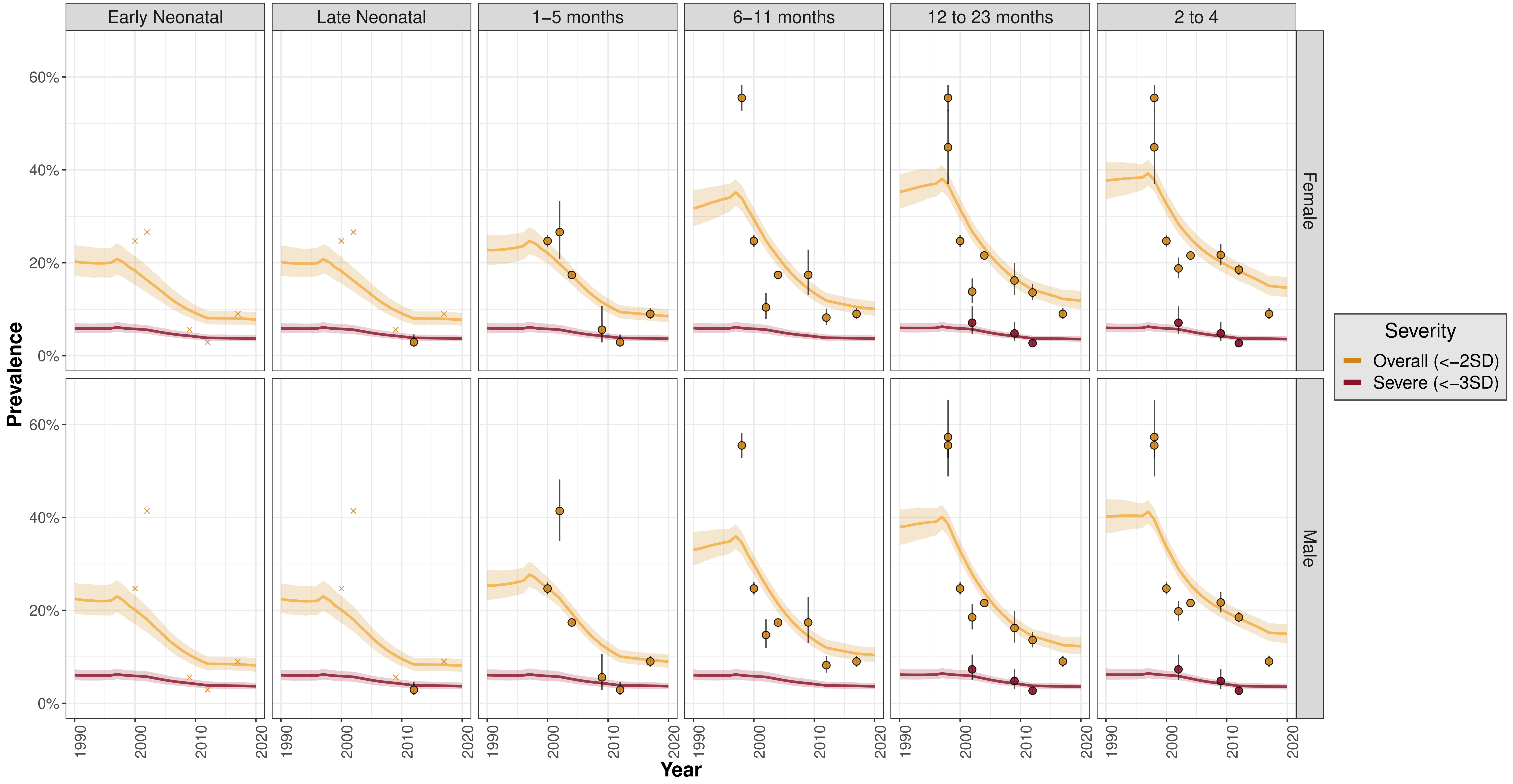

H: Transformed Mean Underweight Z Scores

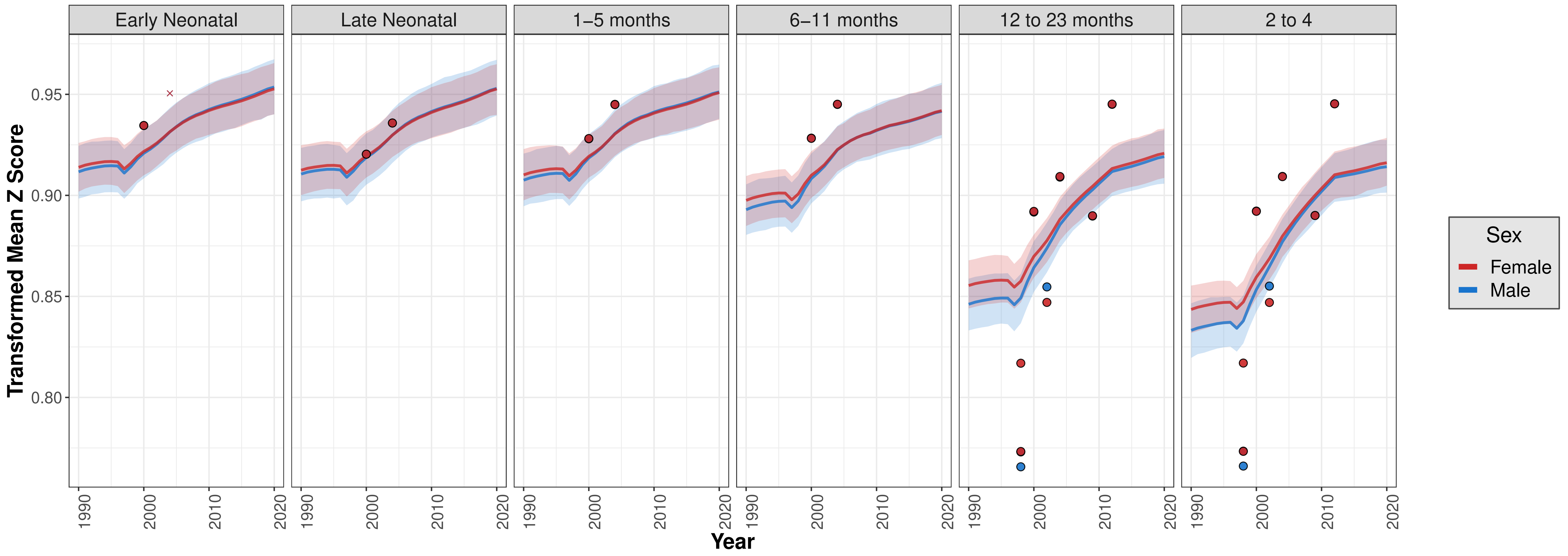

I

Year	Source
1998	MICS
1998	WHO CGM Database
2000	WHO CGM Database
2002	WHO CGM Database
2004	WHO CGM Database
2009	WHO CGM Database
2012	WHO CGM Database
2017	MICS

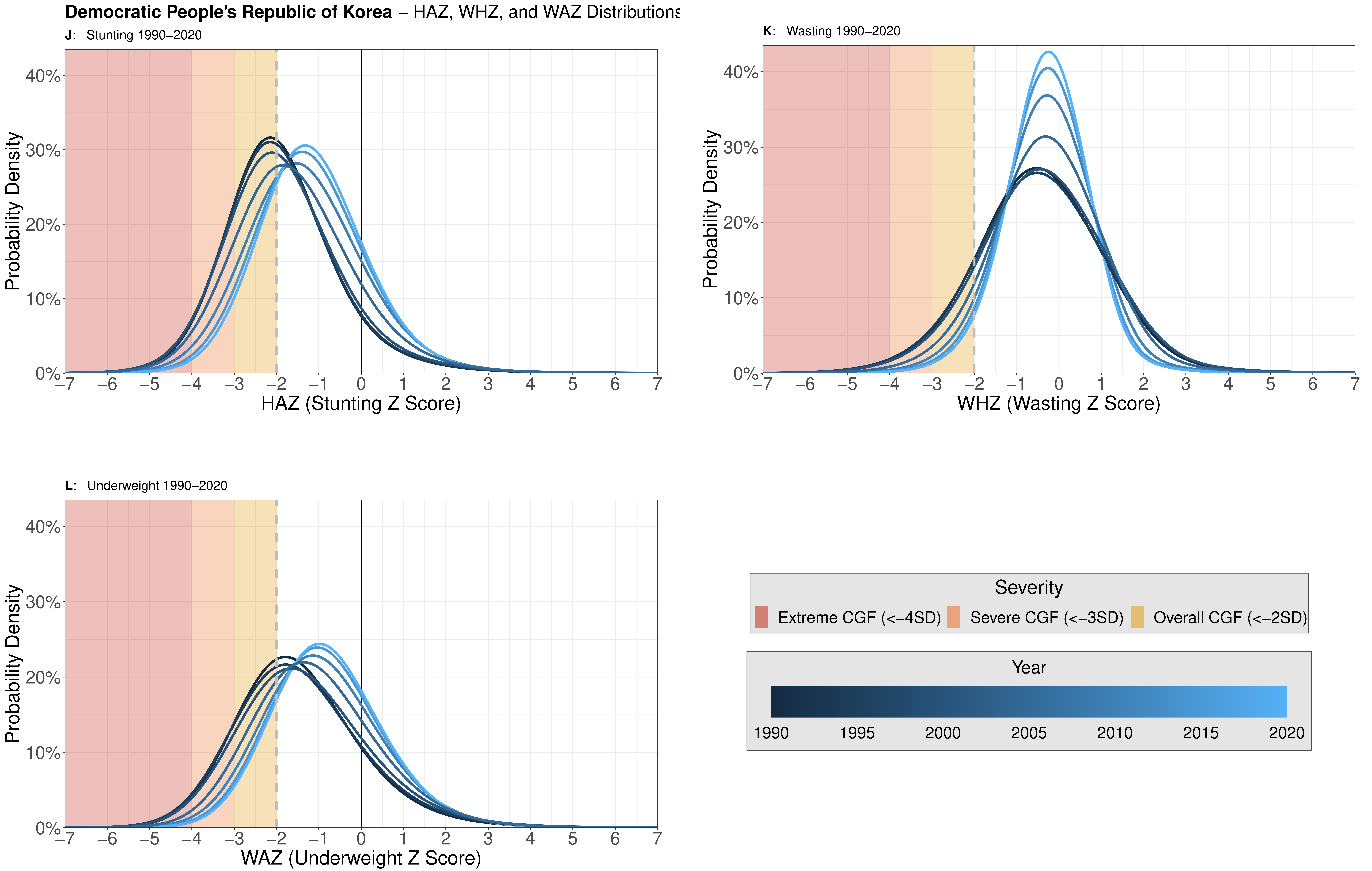
Democratic People's Republic of Korea – HAZ, WHZ, and WAZ Distributions
J: Stunting 1990–2020
Probability Density
HAZ (Stunting Z Score)
K: Wasting 1990–2020
WHZ (Wasting Z Score)
L: Underweight 1990–2020
WAZ (Underweight Z Score)
0%
10%
20%
30%
40%
−7 −6 −5 −4 −3 −2 −1 0 1 2 3 4 5 6 7
Severity
Extreme CGF (<−4SD)
Severe CGF (<−3SD)
Overall CGF (<−2SD)
Year
1990 1995 2000 2005 2010 2015 2020

Taiwan (Province of China) – Stunting (HAZ)

A: Overall and Severe Stunting Prevalence

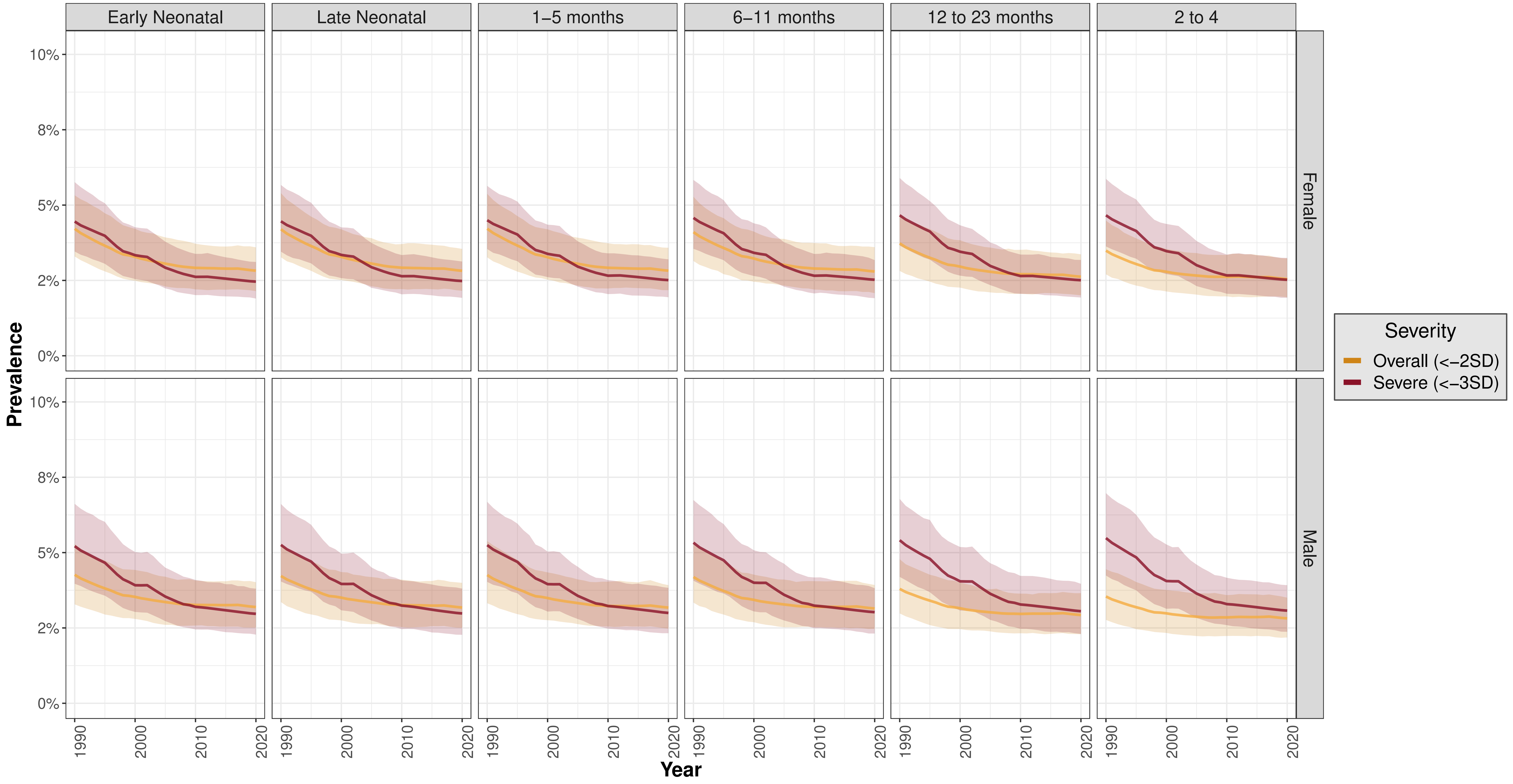

B: Transformed Mean Stunting Z Scores

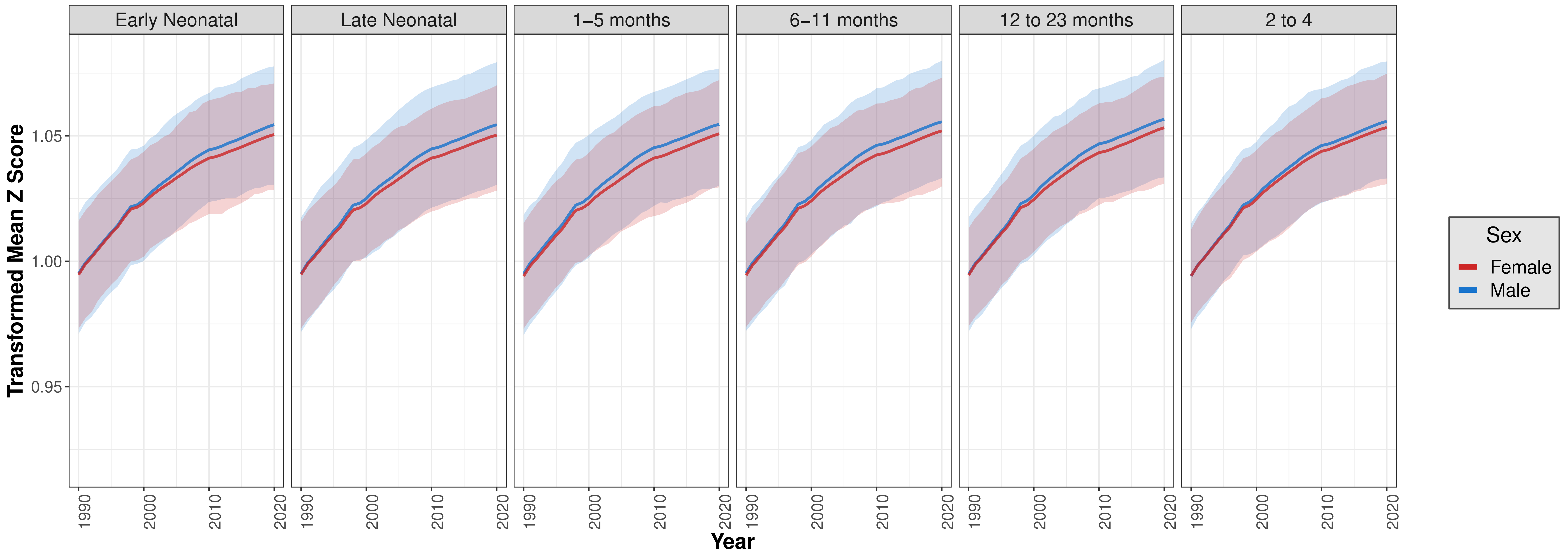

C

Year	Source
1988	WHO CGM Database

Taiwan (Province of China) – Wasting (WHZ)

D: Overall and Severe Wasting Prevalence

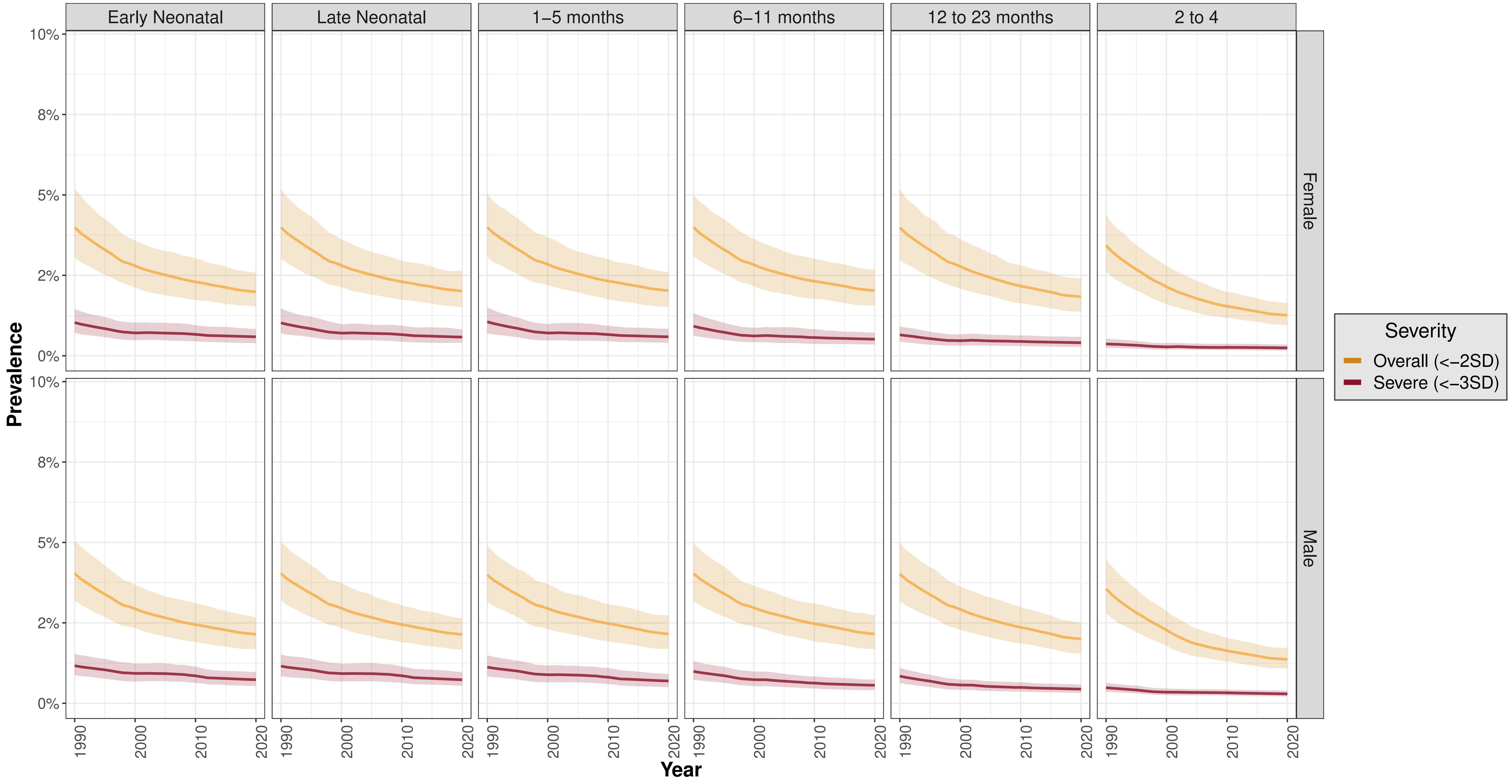

E: Transformed Mean Wasting Z Scores

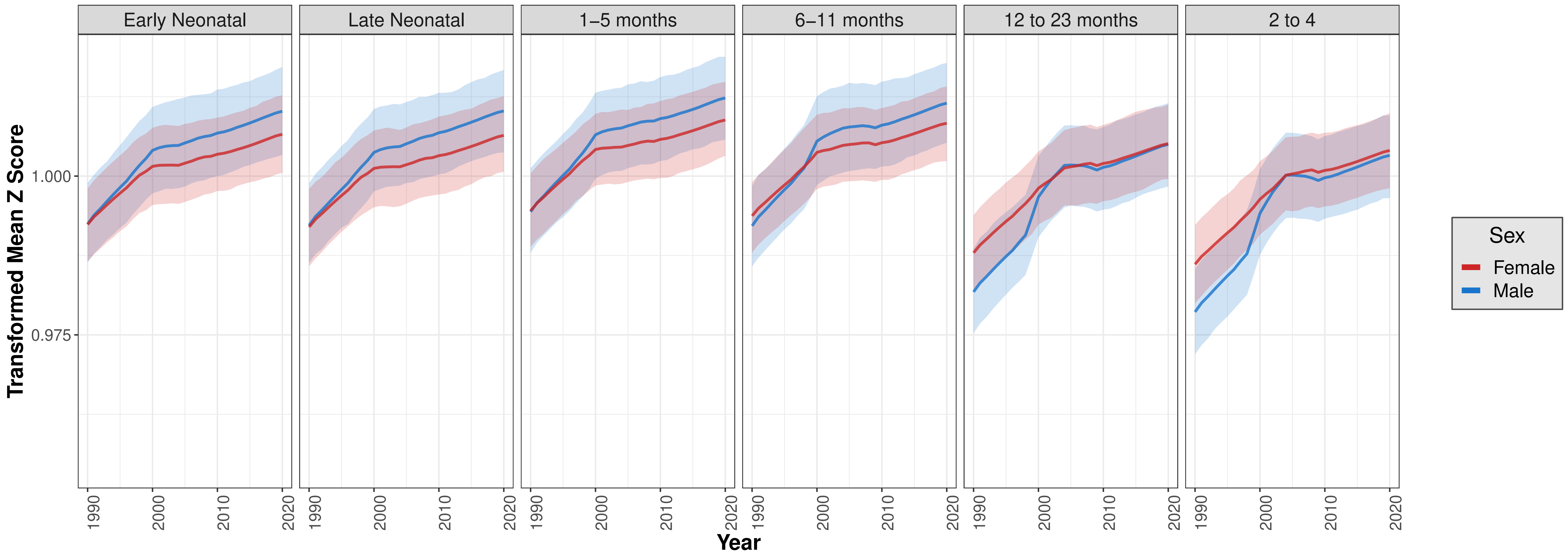

F

Year	Source
1988	WHO CGM Database

Taiwan (Province of China) – Underweight (WAZ)

G: Overall and Severe Underweight Prevalence

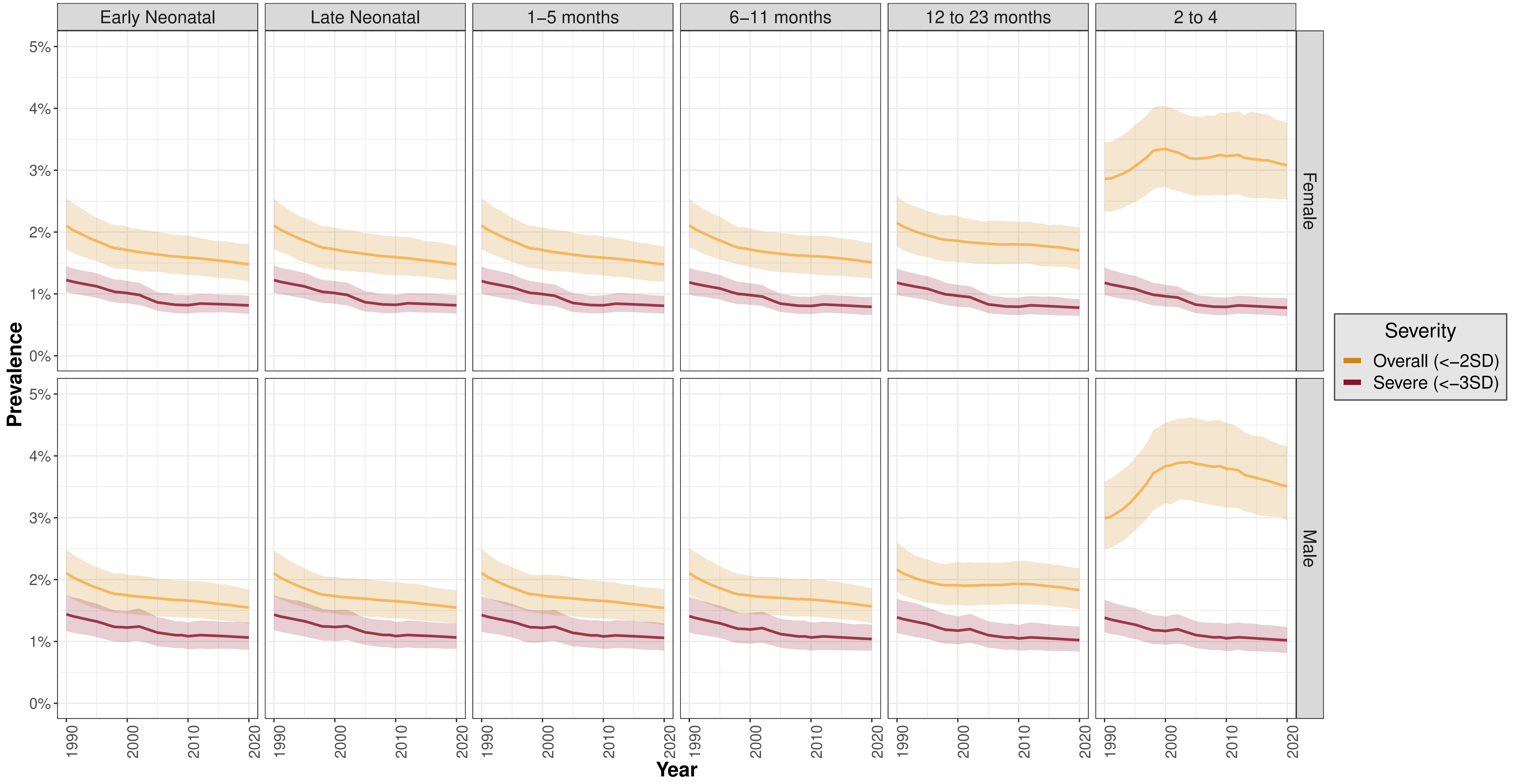

H: Transformed Mean Underweight Z Scores

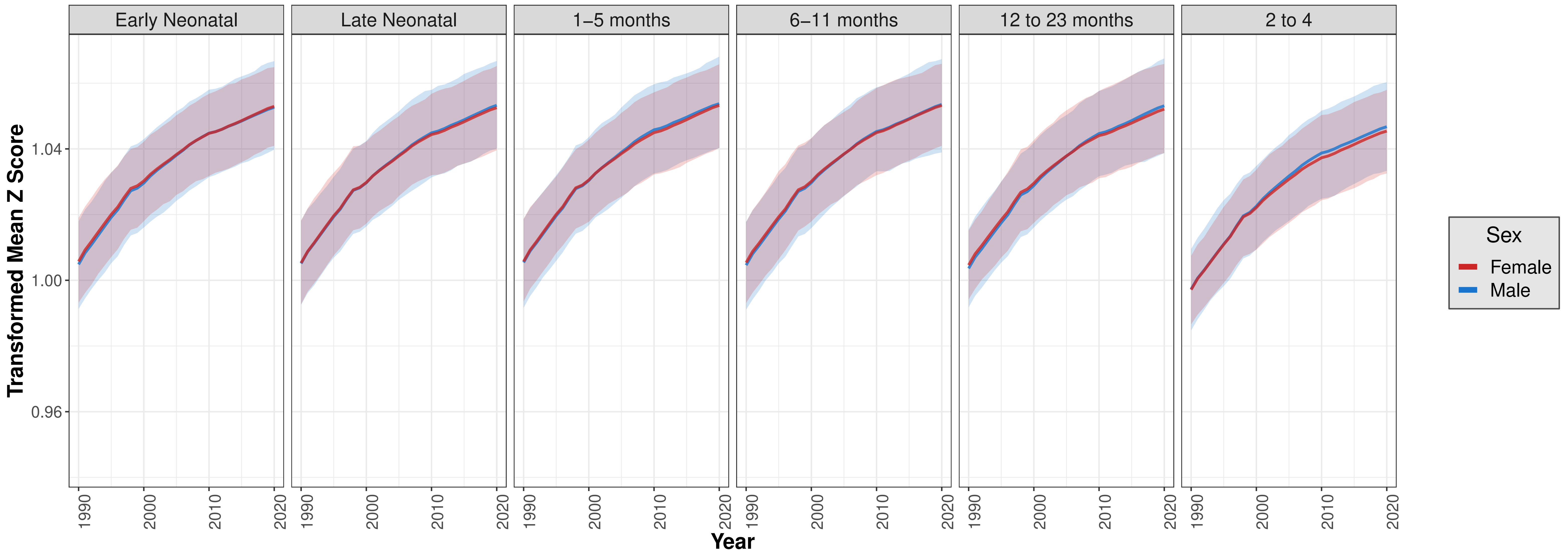

I

Year	Source
1988	WHO CGM Database

Taiwan (Province of China) – HAZ, WHZ, and WAZ Distributions

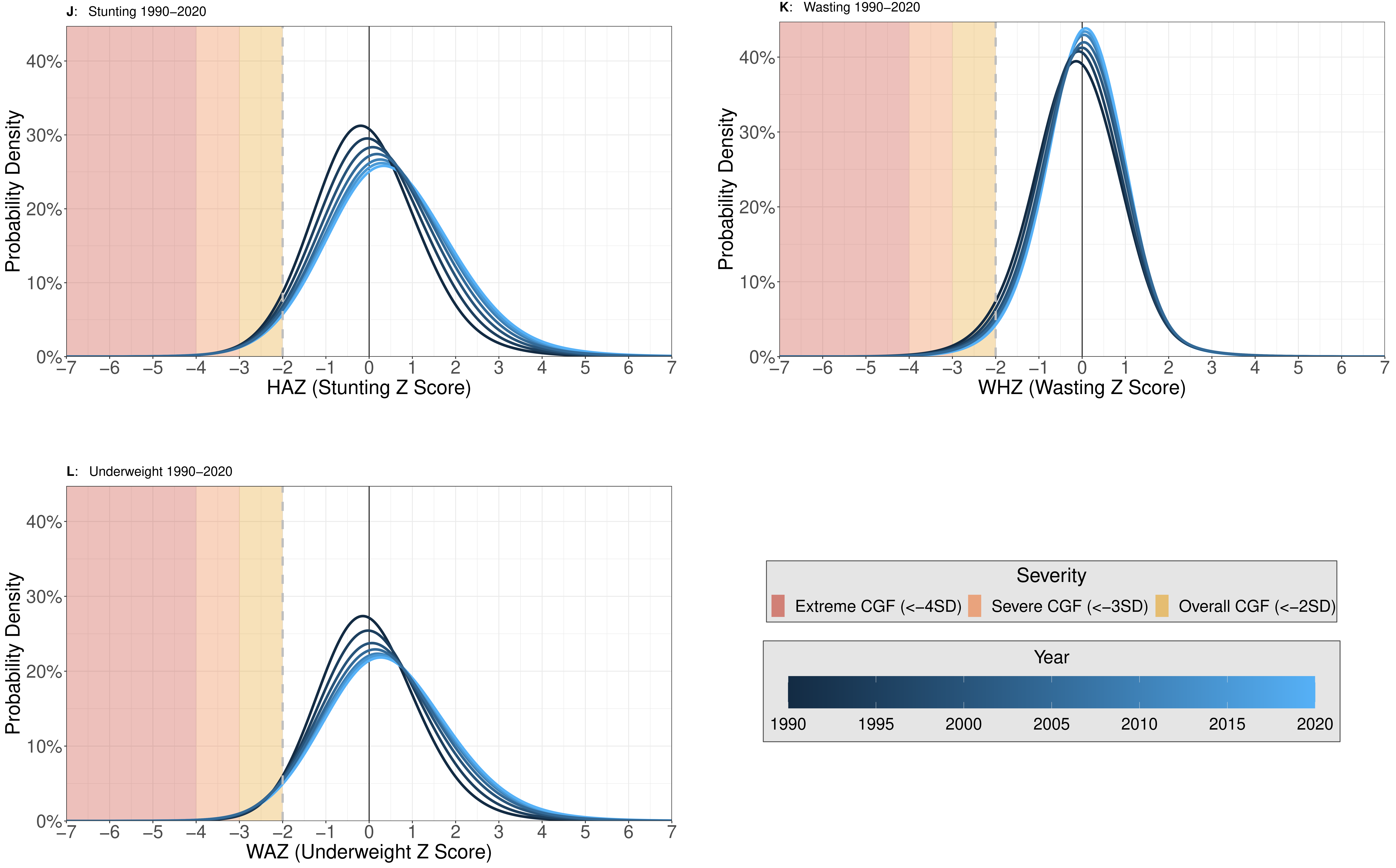

Fiji – Stunting (HAZ)

A: Overall and Severe Stunting Prevalence

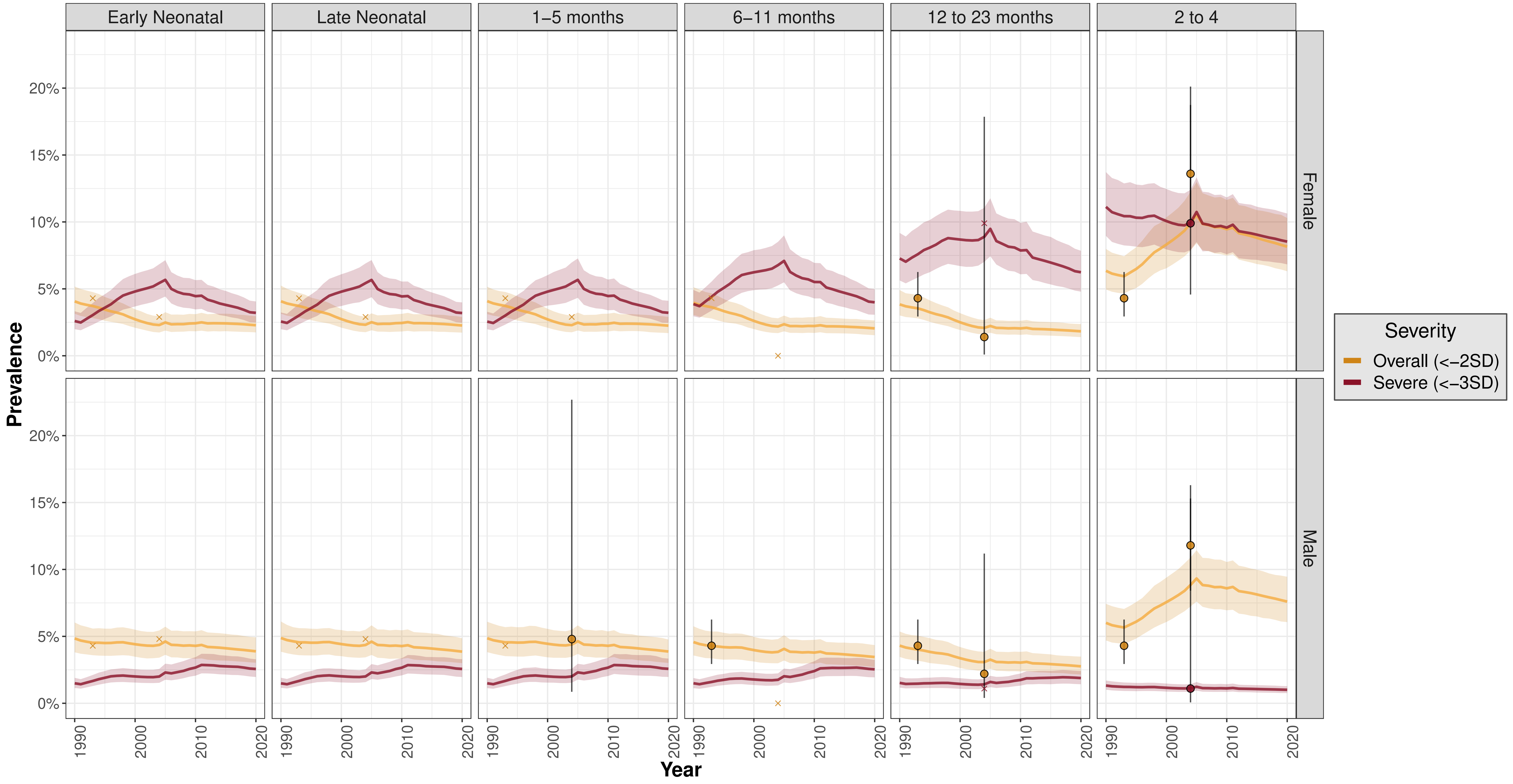

C

Year	Source
1993	WHO CGM Database
2004	WHO CGM Database

B: Transformed Mean Stunting Z Scores

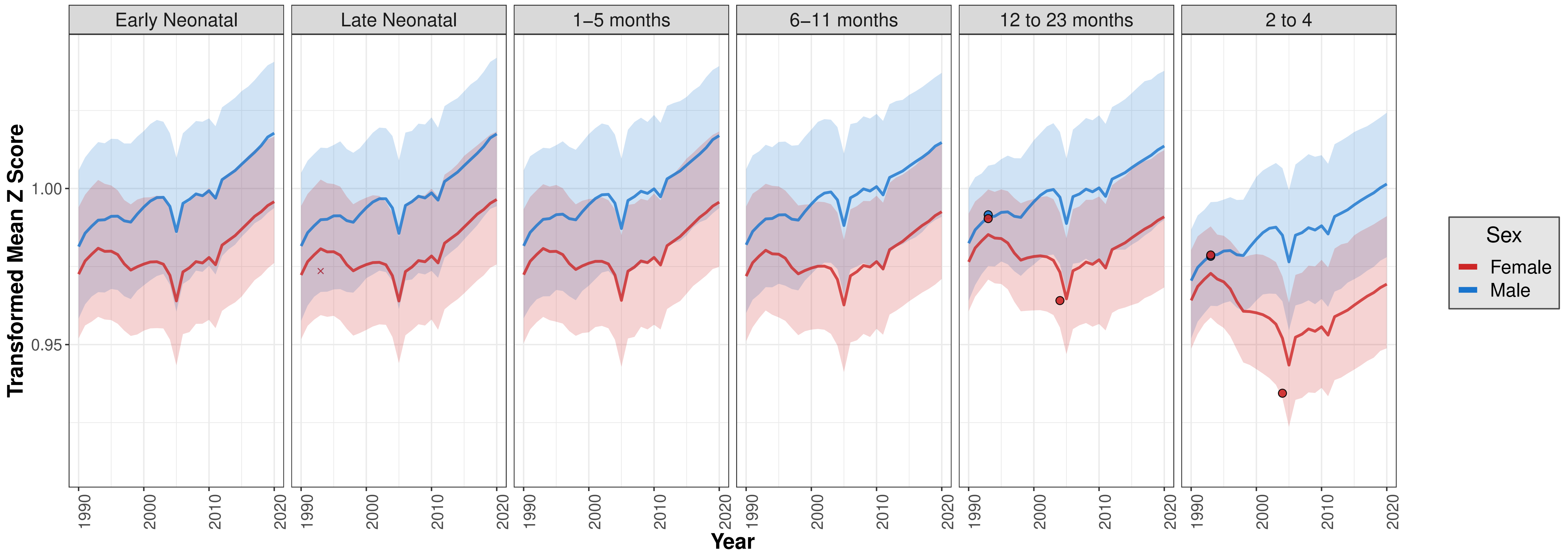

Fiji – Wasting (WHZ)

D: Overall and Severe Wasting Prevalence

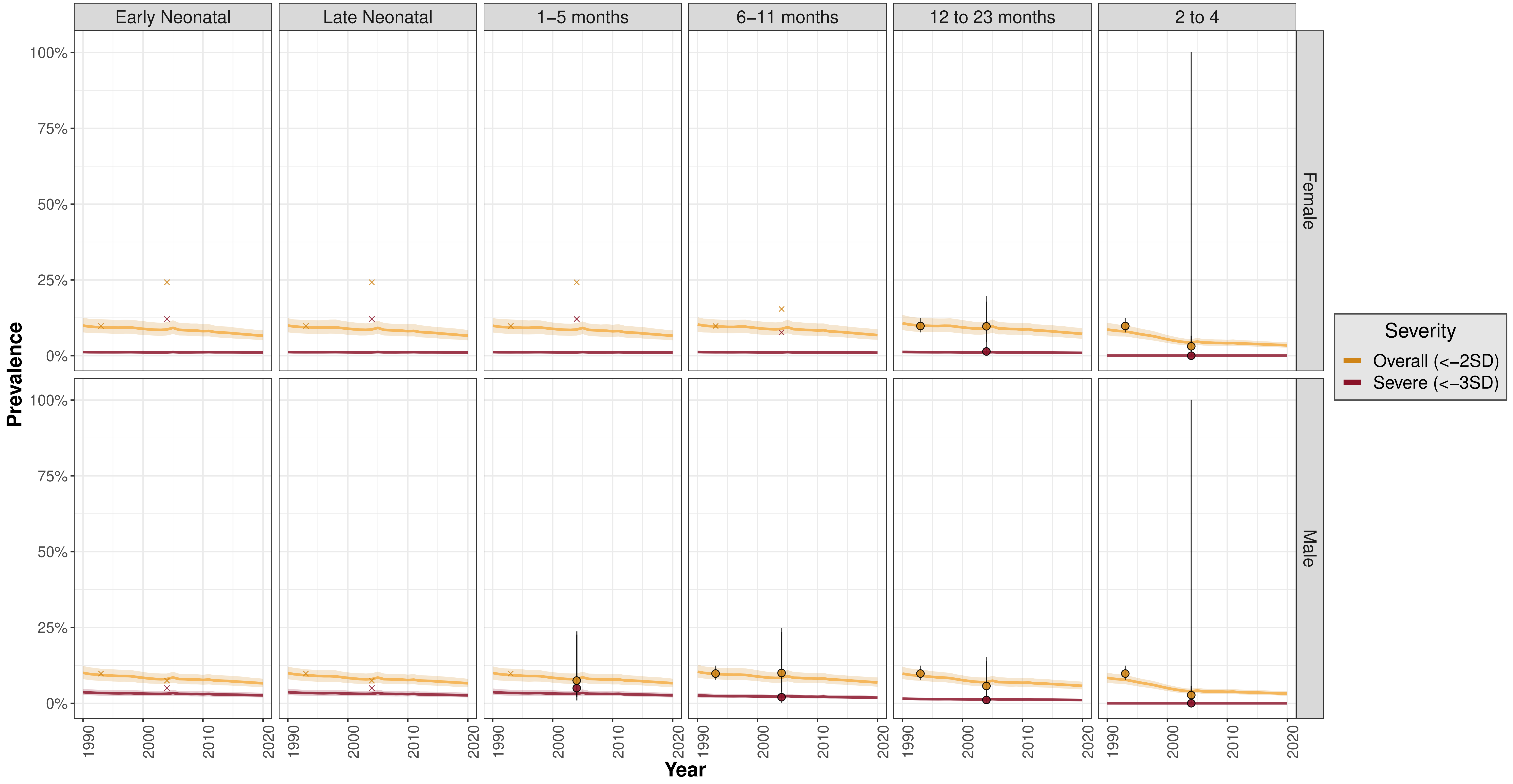

E: Transformed Mean Wasting Z Scores

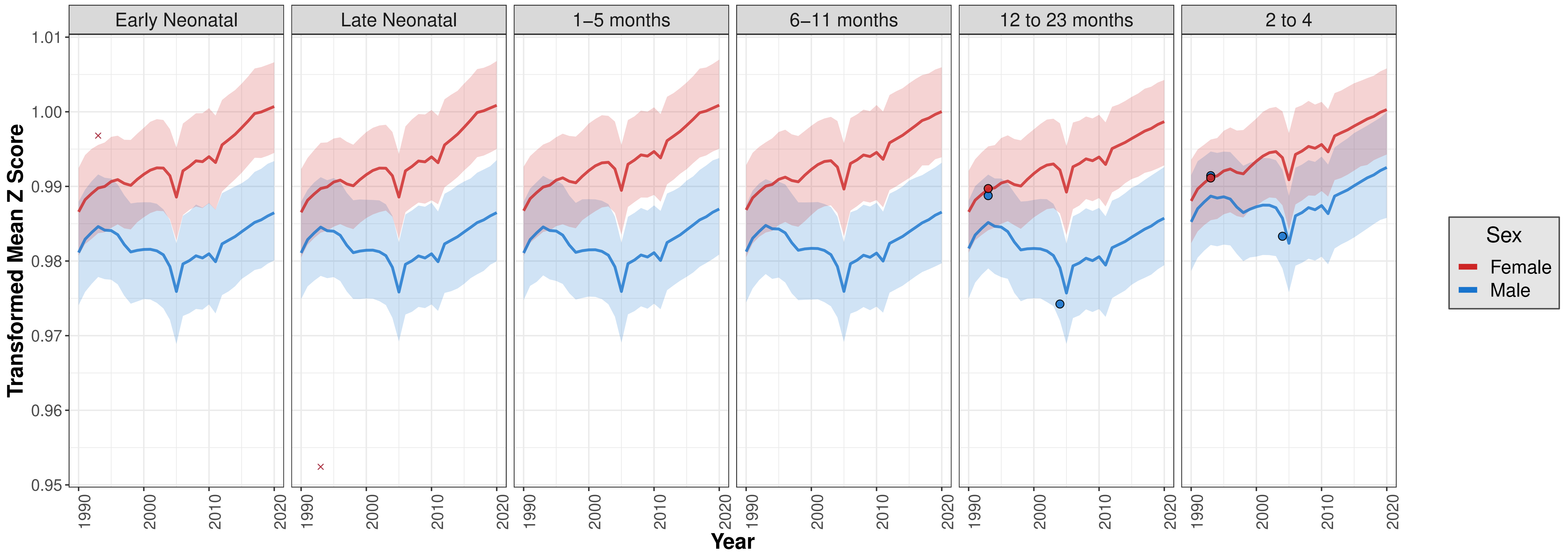

F

Year	Source
1993	WHO CGM Database
2004	WHO CGM Database

Fiji – Underweight (WAZ)

G: Overall and Severe Underweight Prevalence

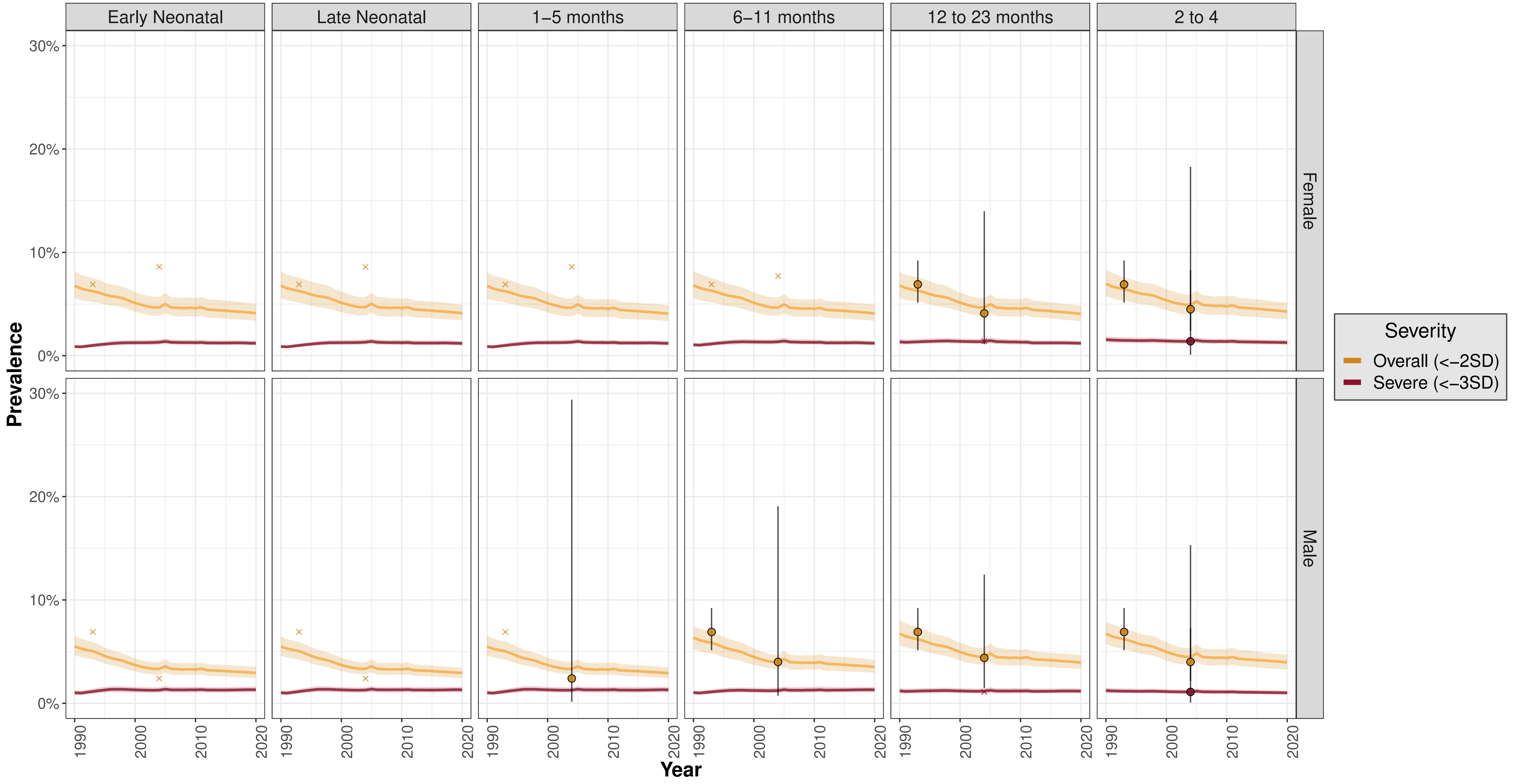

H: Transformed Mean Underweight Z Scores

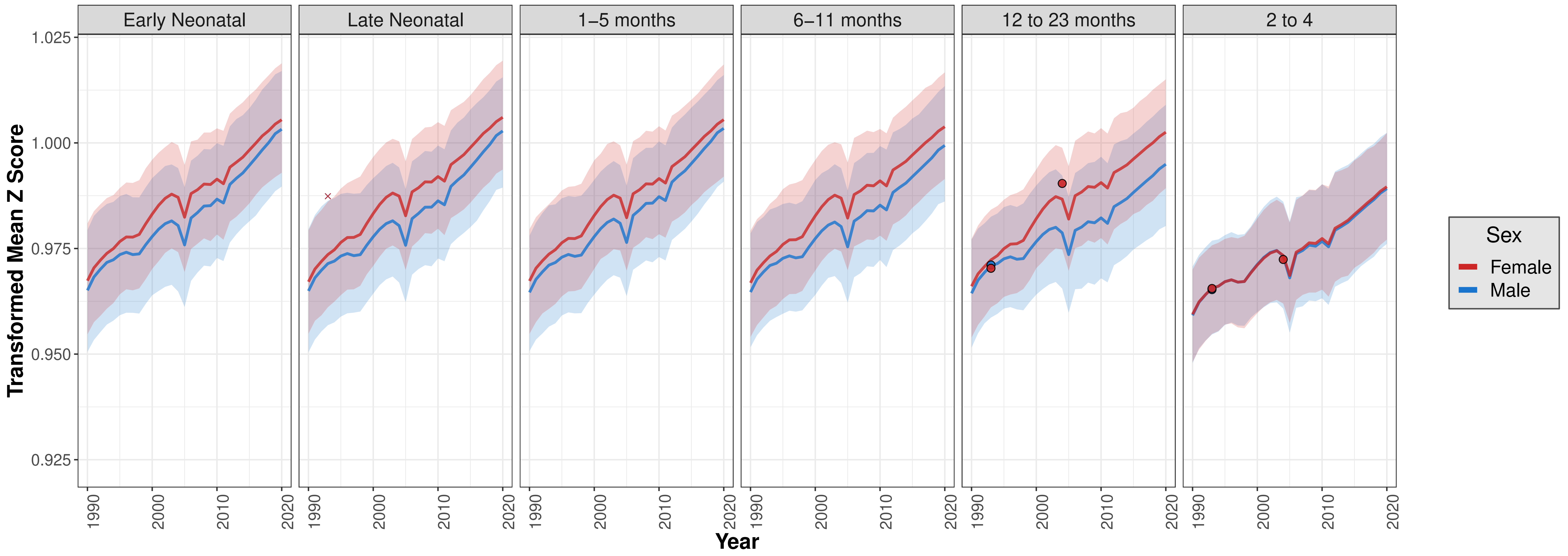

I

Year	Source
1993	WHO CGM Database
2004	WHO CGM Database

Fiji – HAZ, WHZ, and WAZ Distributions

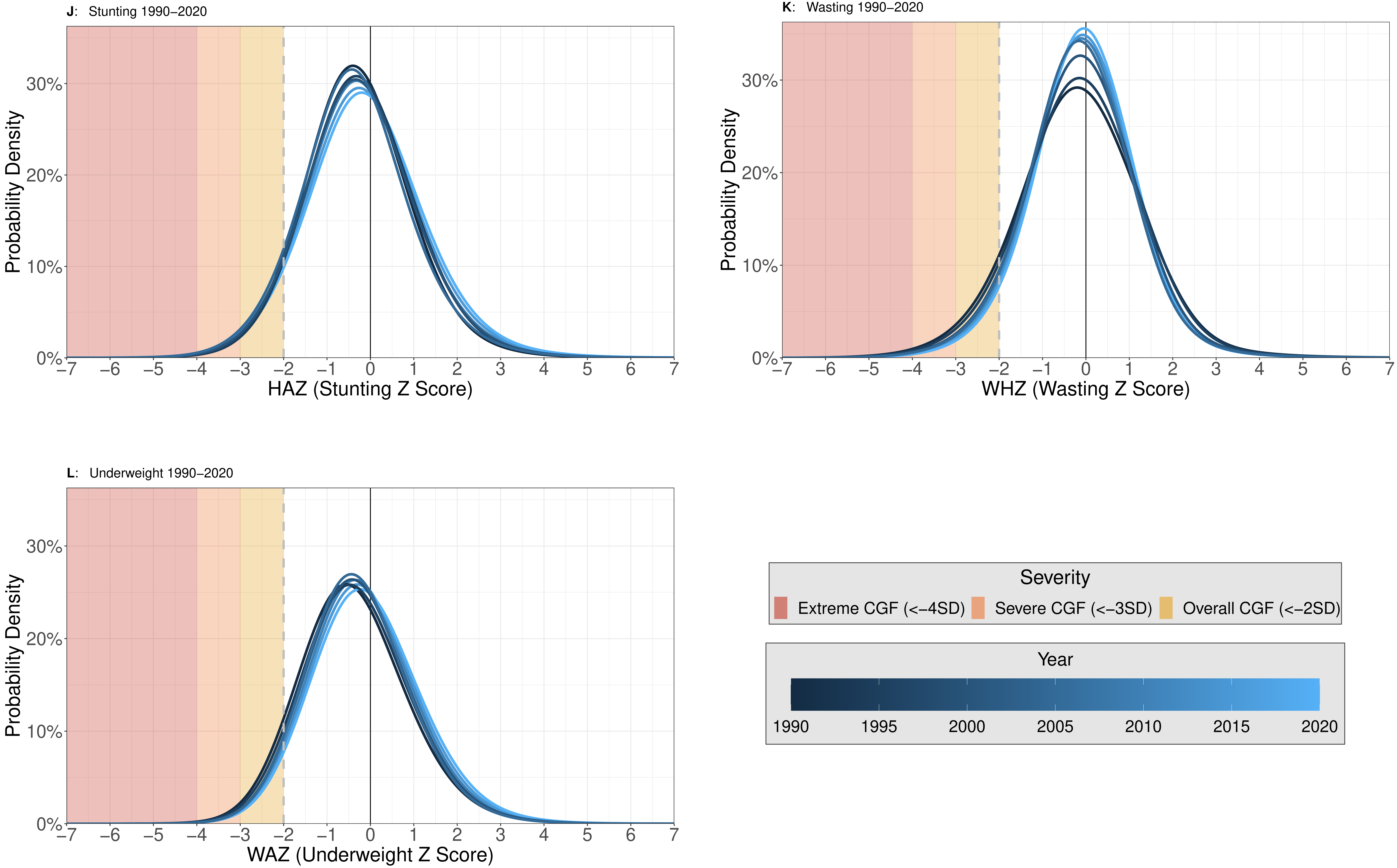

Kiribati – Stunting (HAZ)

A: Overall and Severe Stunting Prevalence

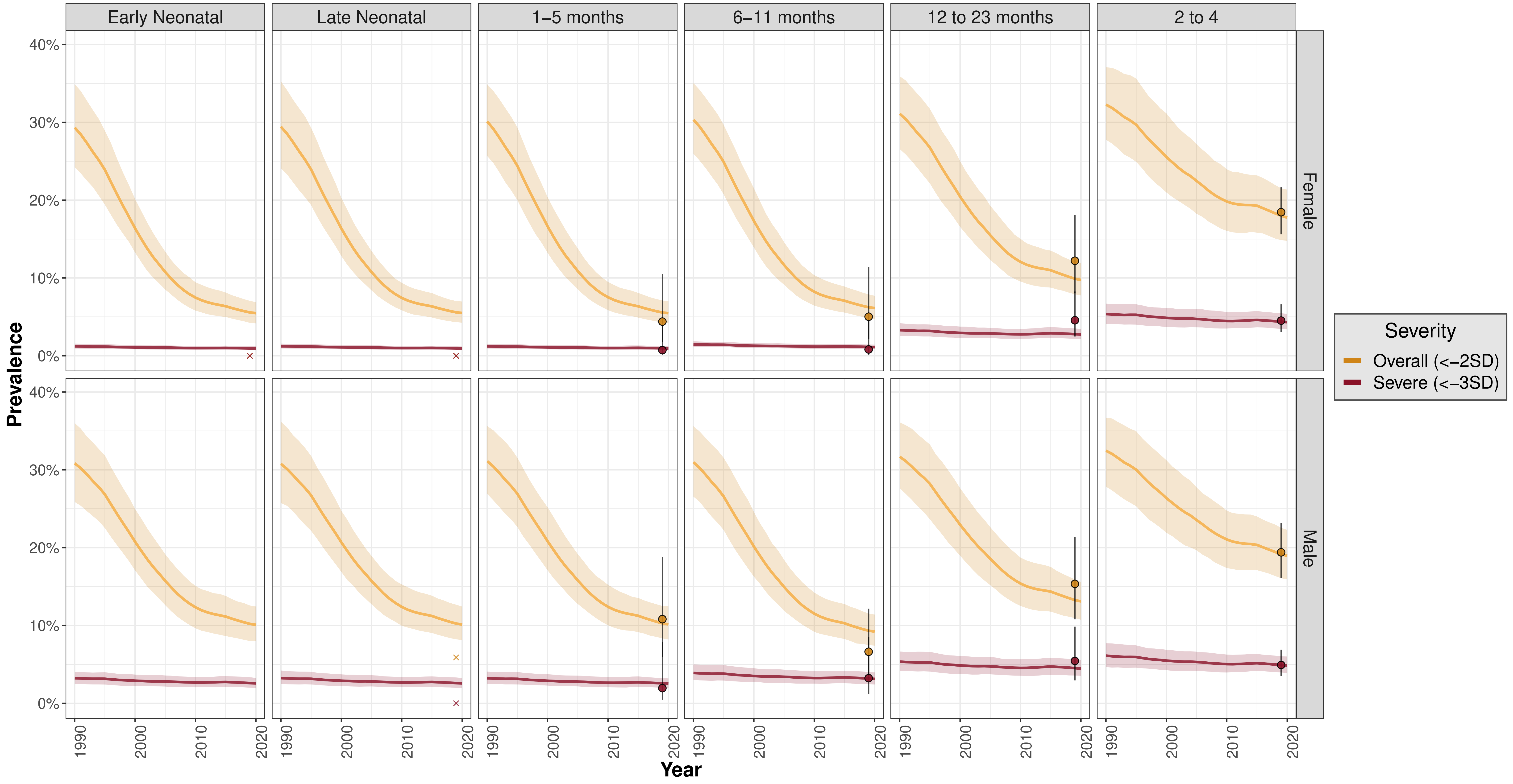

B: Transformed Mean Stunting Z Scores

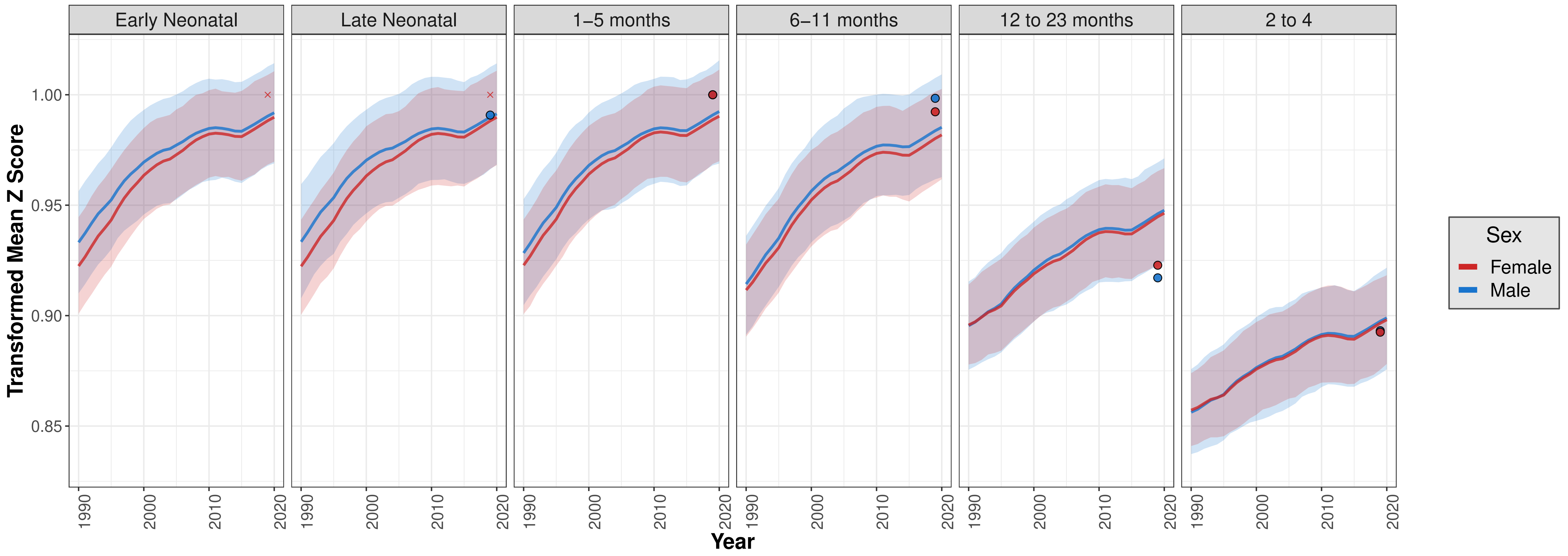

C

Year	Source
1985	WHO CGM Database
2019	MICS

Kiribati – Wasting (WHZ)

D: Overall and Severe Wasting Prevalence

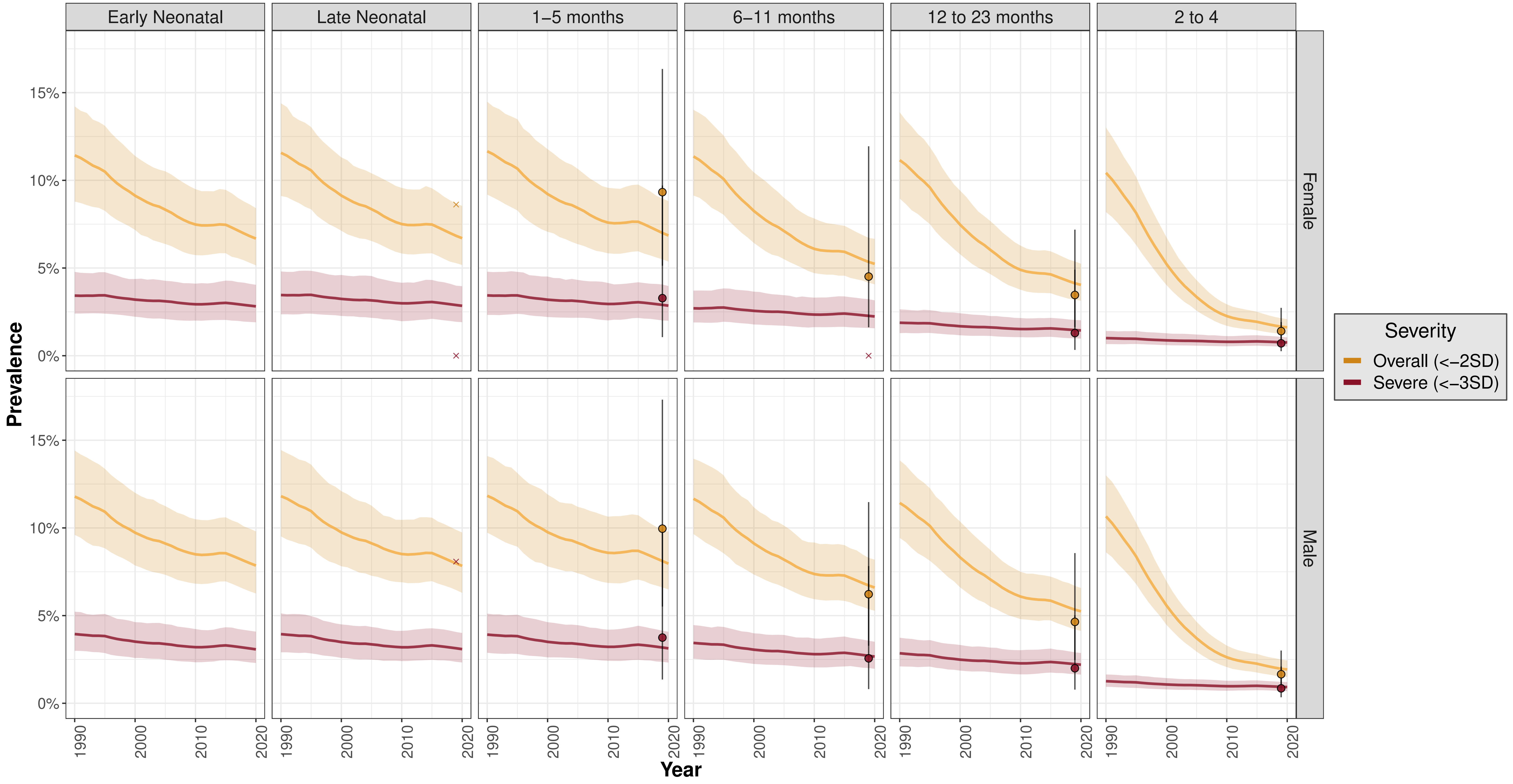

E: Transformed Mean Wasting Z Scores

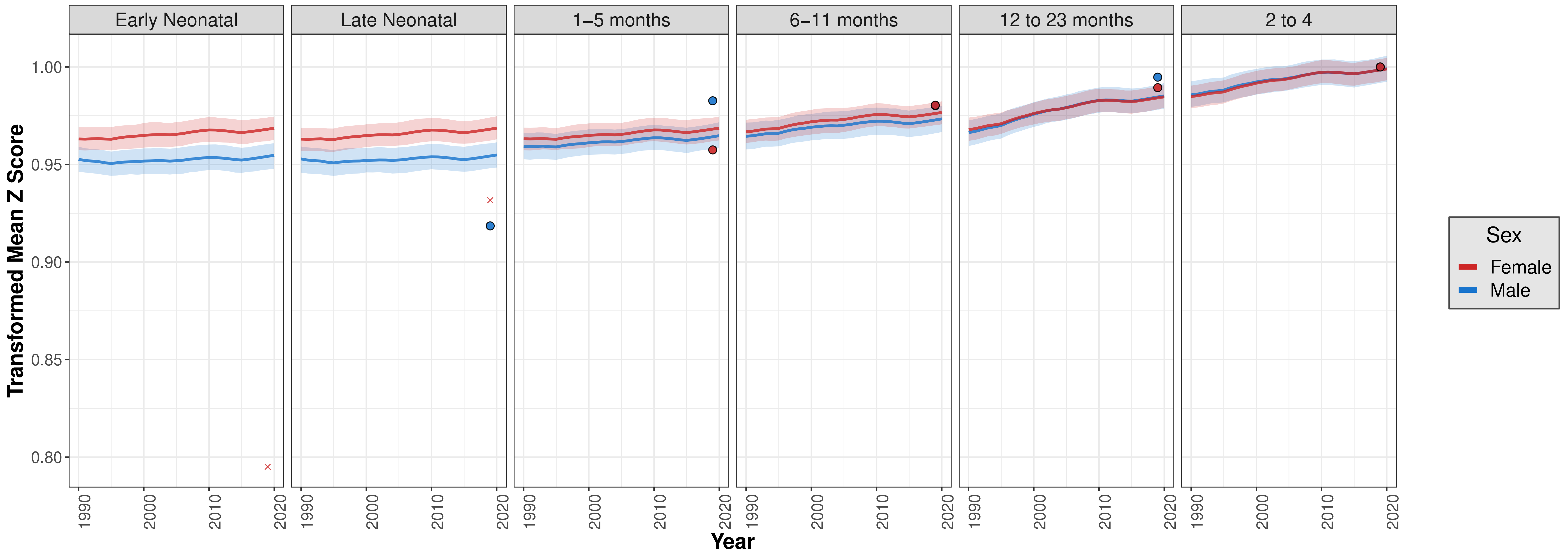

F

Year	Source
1985	WHO CGM Database
2019	MICS

Kiribati – Underweight (WAZ)

G: Overall and Severe Underweight Prevalence

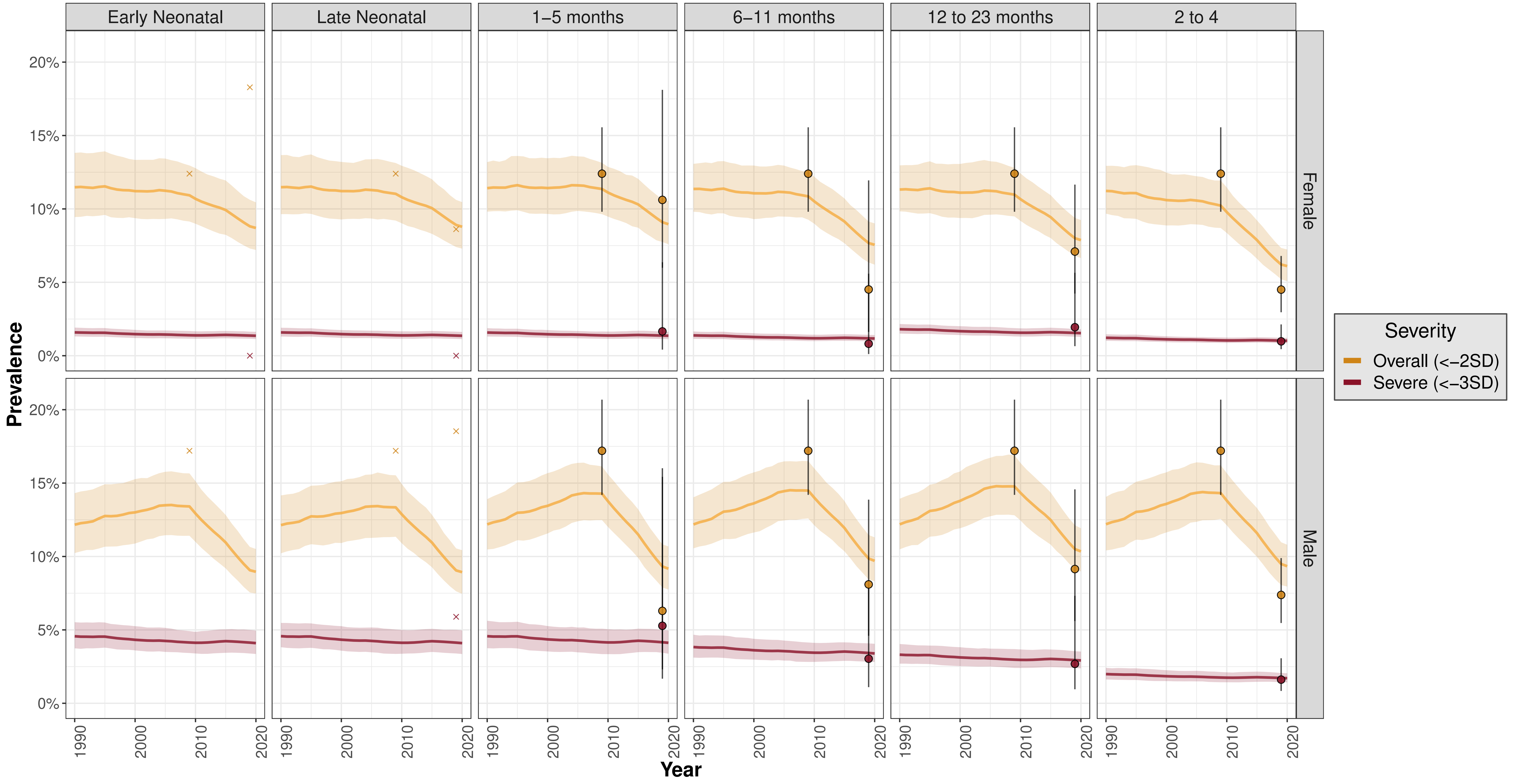

H: Transformed Mean Underweight Z Scores

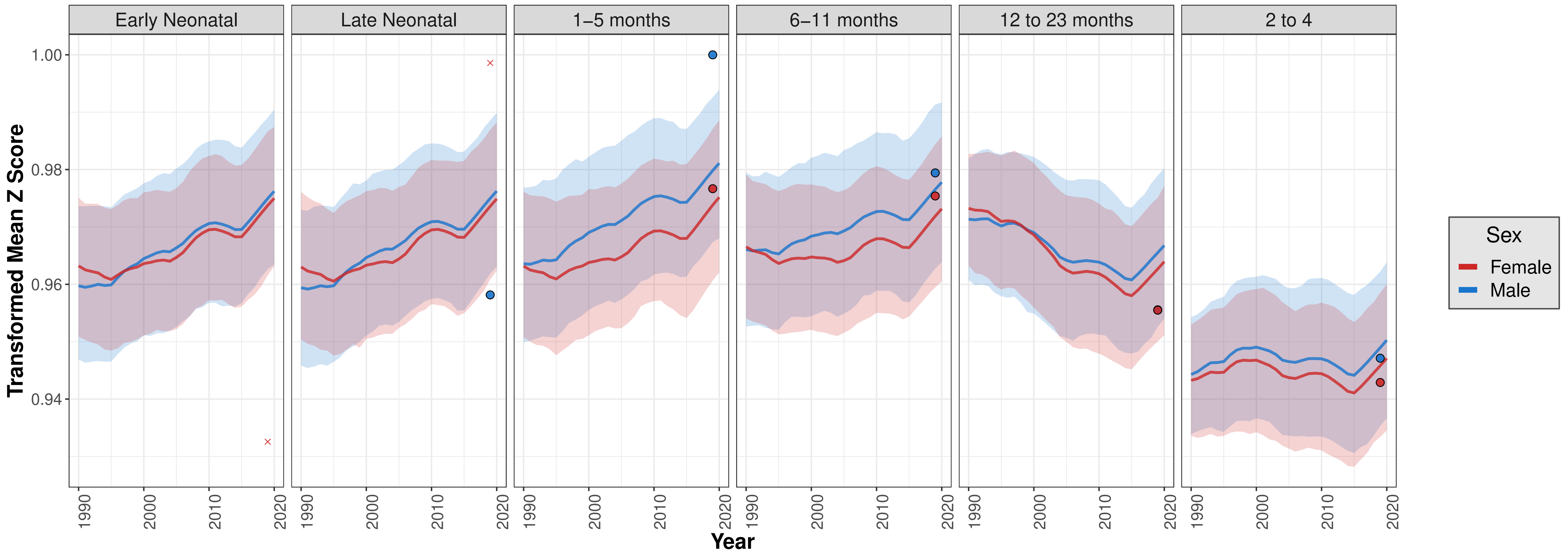

I

Year	Source
1985	WHO CGM Database
2009	WHO CGM Database
2019	MICS

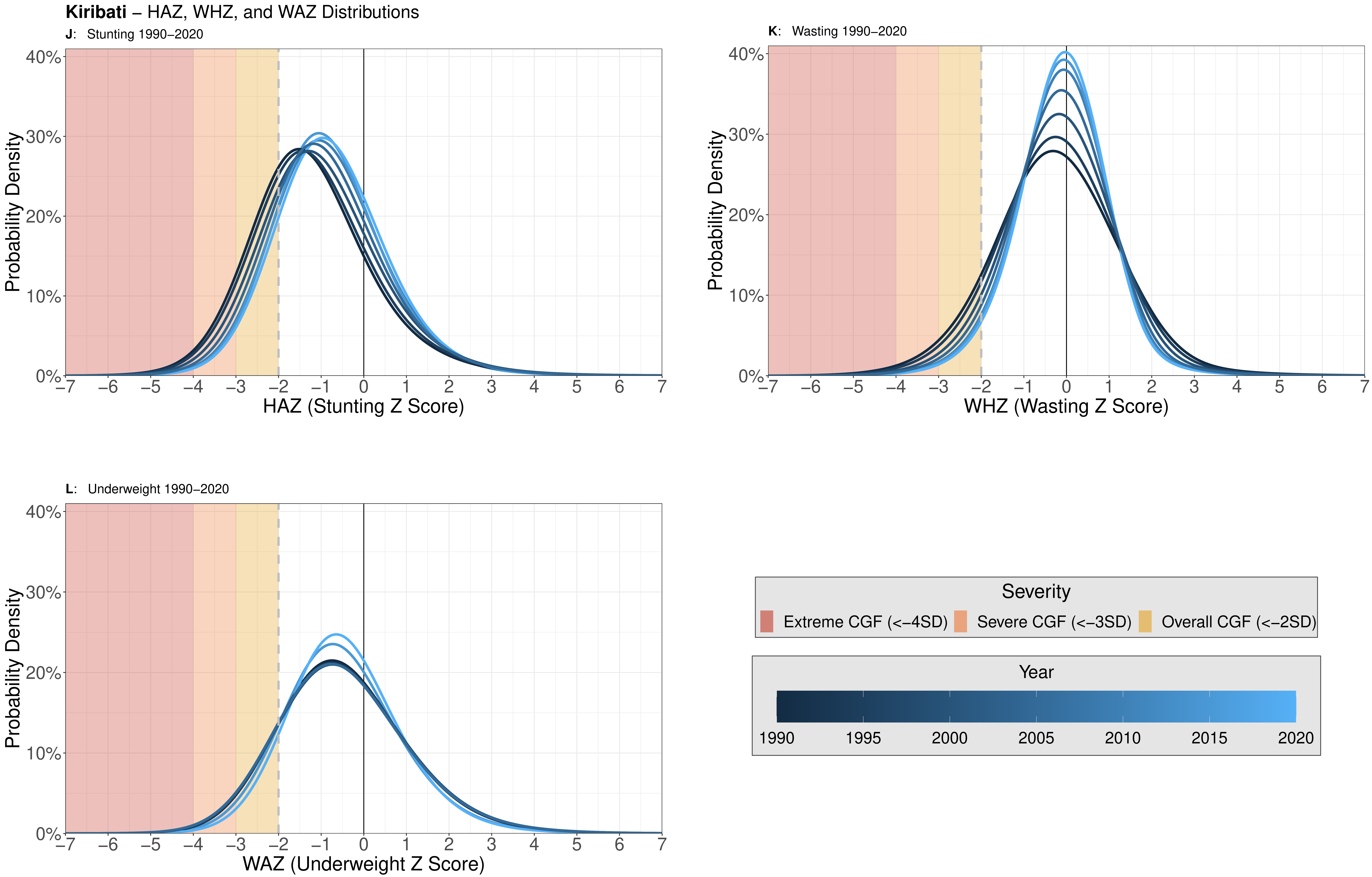

Kiribati – HAZ, WHZ, and WAZ Distributions
J: Stunting 1990–2020
Probability Density
40%
30%
20%
10%
0%
−7 −6 −5 −4 −3 −2 −1 0 1 2 3 4 5 6 7
HAZ (Stunting Z Score)
K: Wasting 1990–2020
Probability Density
40%
30%
20%
10%
0%
−7 −6 −5 −4 −3 −2 −1 0 1 2 3 4 5 6 7
WHZ (Wasting Z Score)
L: Underweight 1990–2020
Probability Density
40%
30%
20%
10%
0%
−7 −6 −5 −4 −3 −2 −1 0 1 2 3 4 5 6 7
WAZ (Underweight Z Score)
Severity
Extreme CGF (<−4SD)
Severe CGF (<−3SD)
Overall CGF (<−2SD)
Year
1990
1995
2000
2005
2010
2015
2020

Marshall Islands – Stunting (HAZ)

A: Overall and Severe Stunting Prevalence

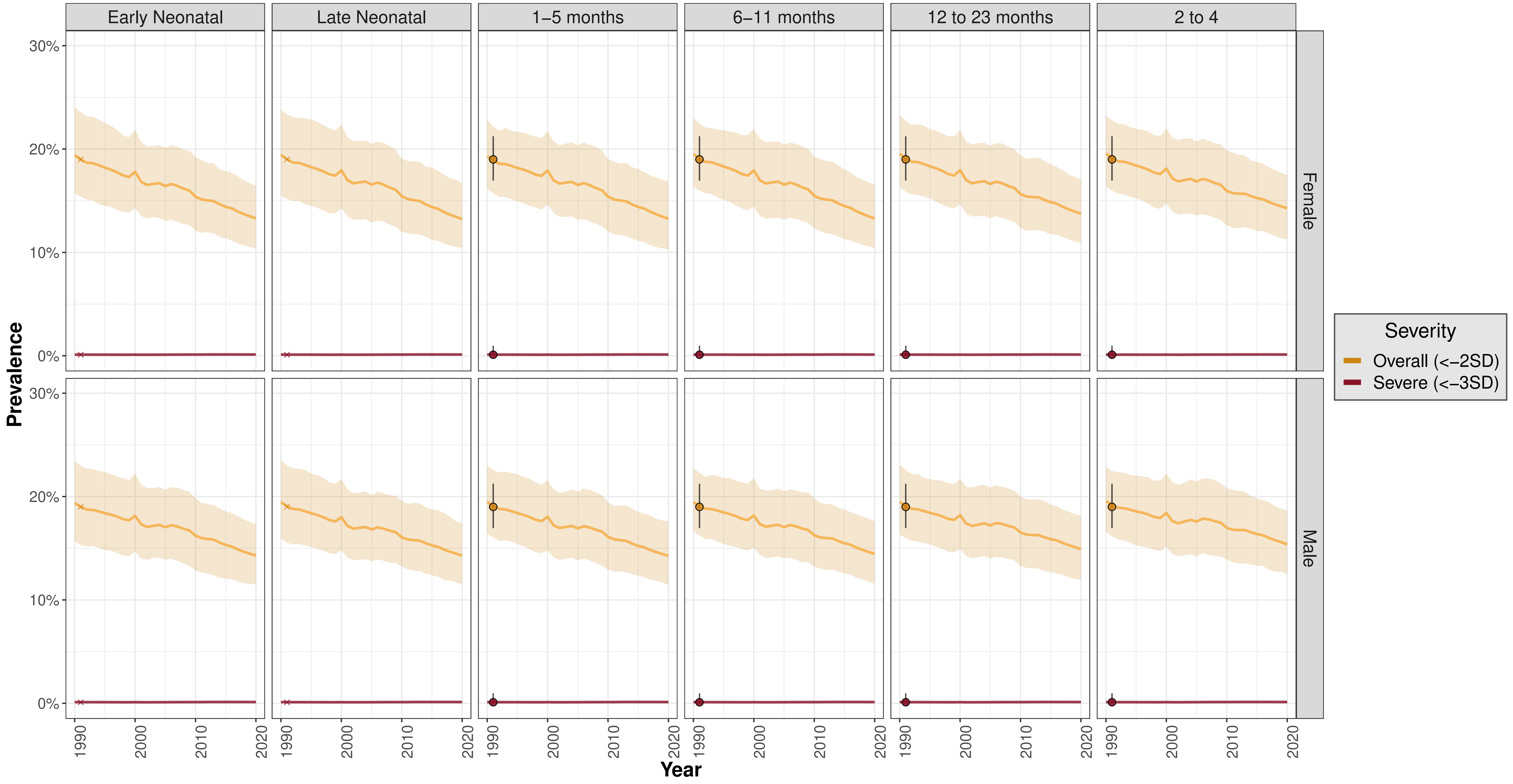

B: Transformed Mean Stunting Z Scores

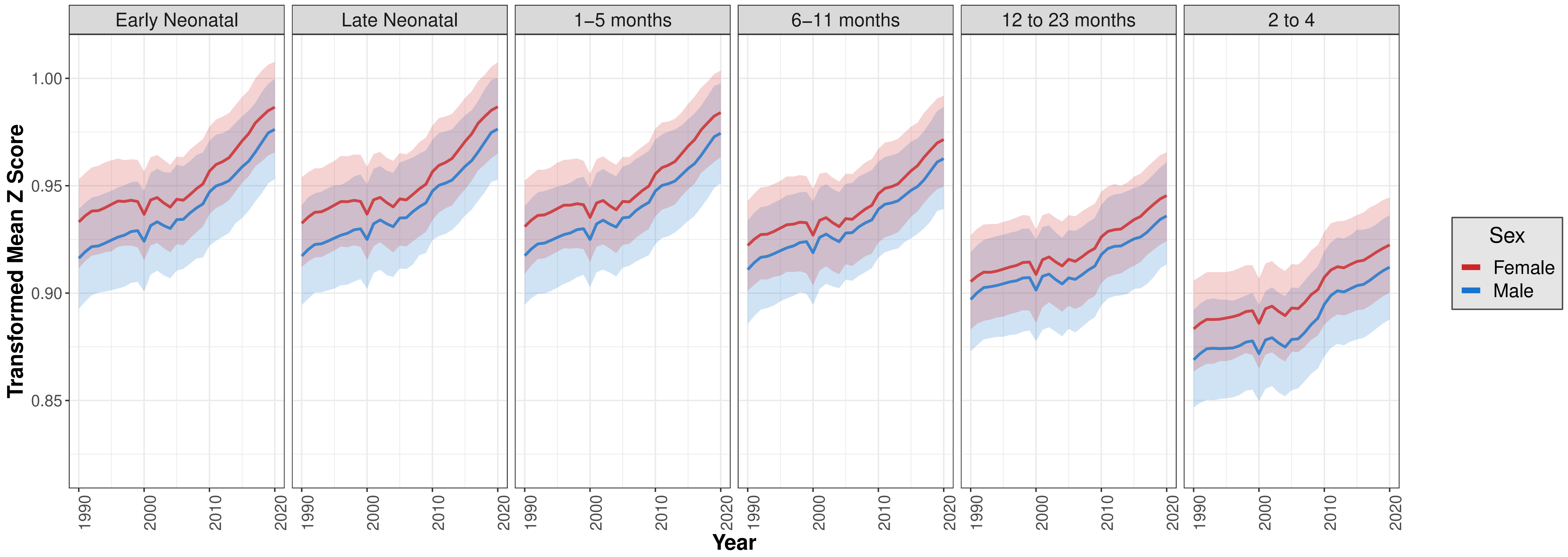

C

Year	Source
1991	National Nutrition Survey

Marshall Islands – Wasting (WHZ)

D: Overall and Severe Wasting Prevalence

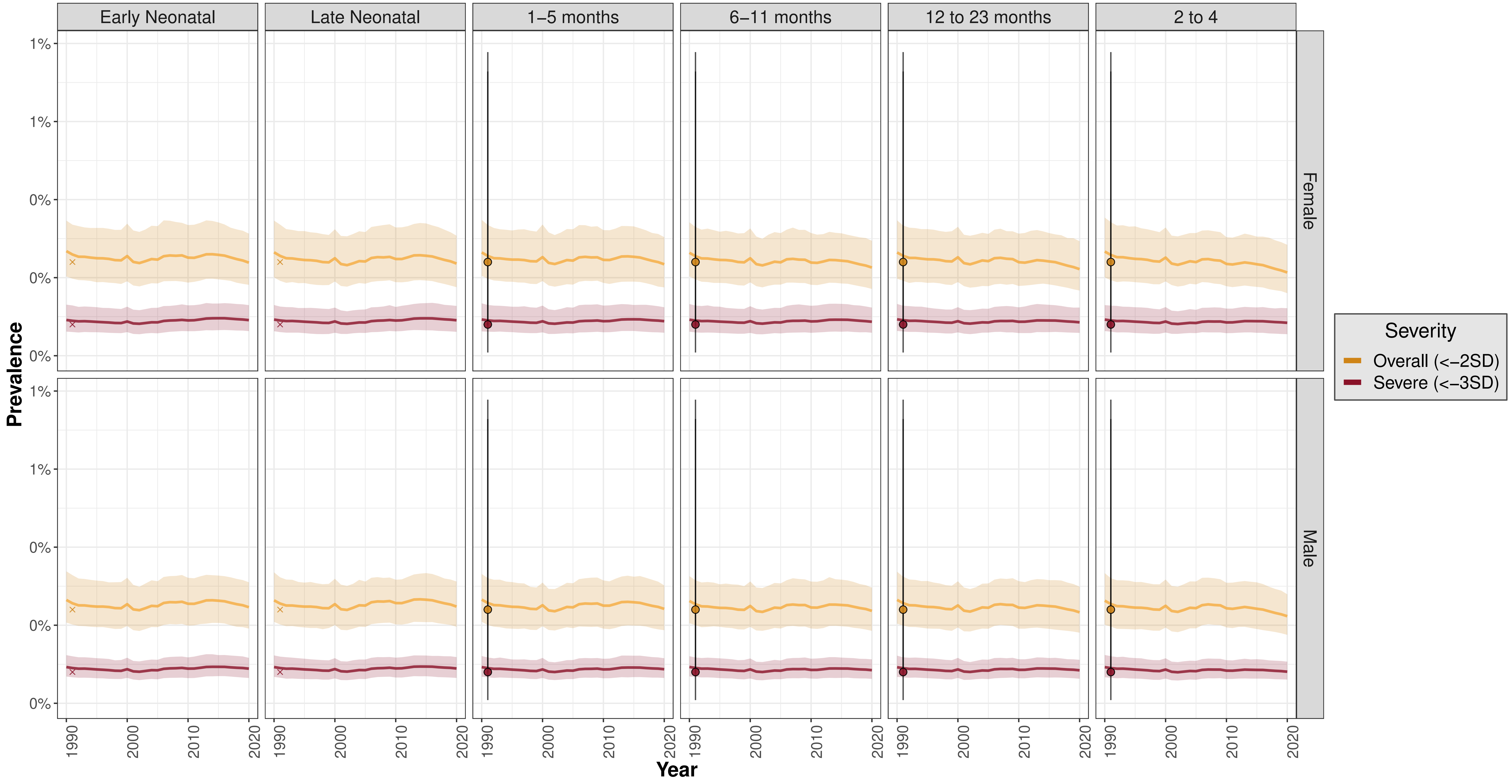

E: Transformed Mean Wasting Z Scores

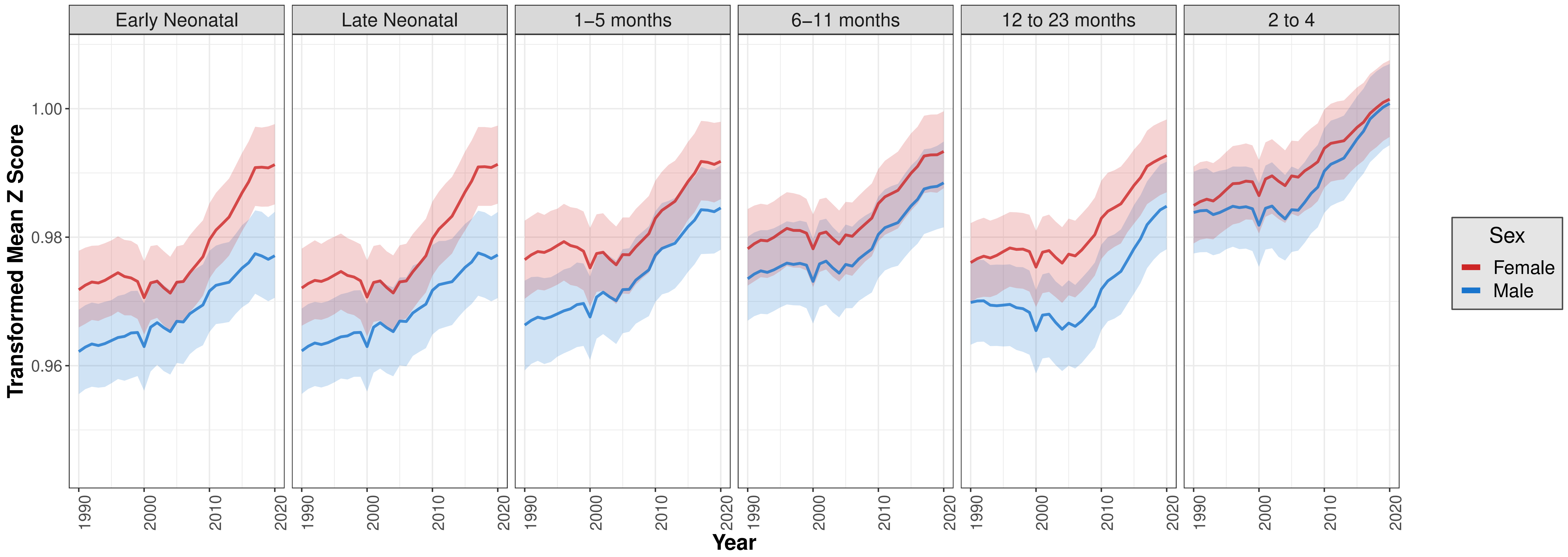

F

Year	Source
1991	National Nutrition Survey

Marshall Islands – Underweight (WAZ)

G: Overall and Severe Underweight Prevalence

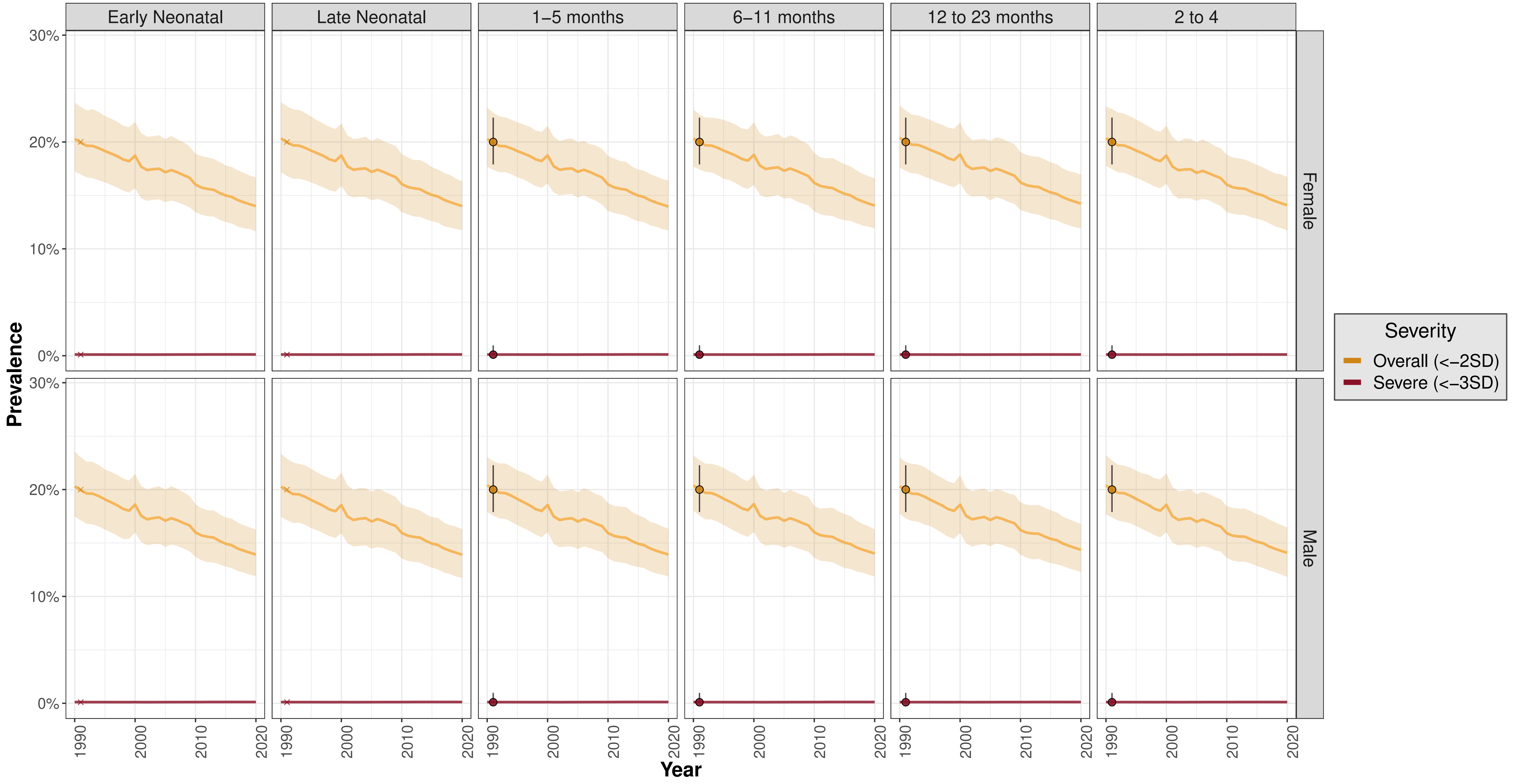

H: Transformed Mean Underweight Z Scores

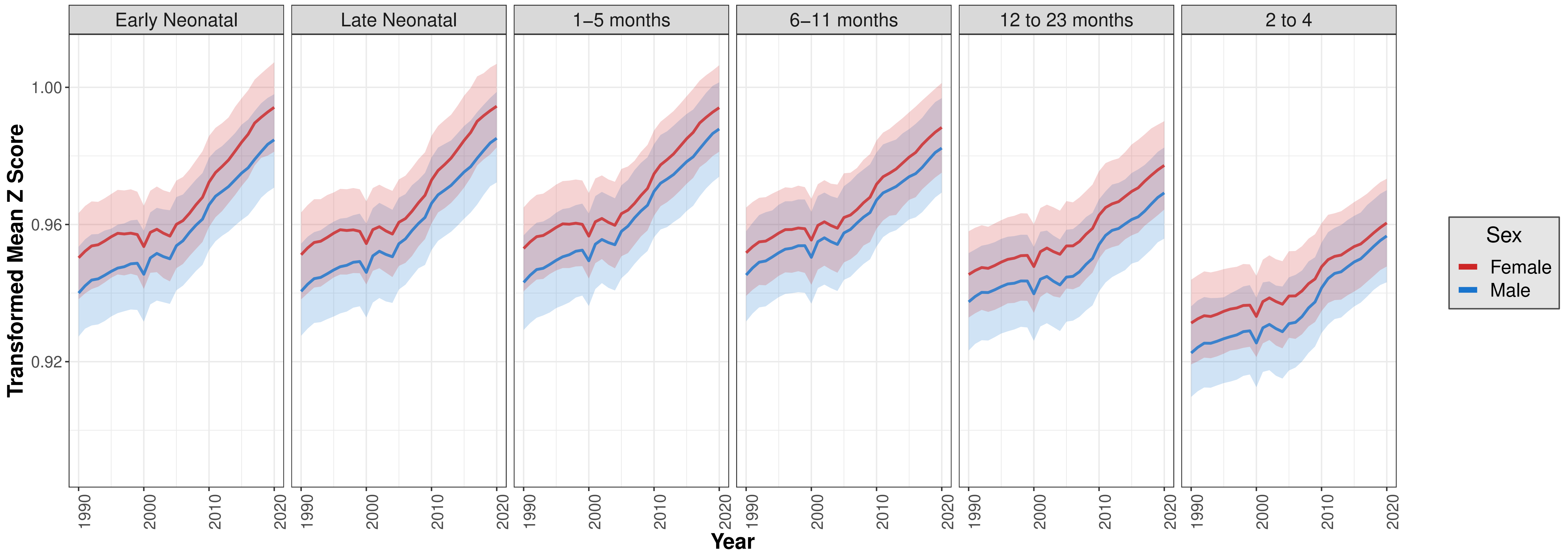

I

Year	Source
1991	National Nutrition Survey

Marshall Islands – HAZ, WHZ, and WAZ Distributions

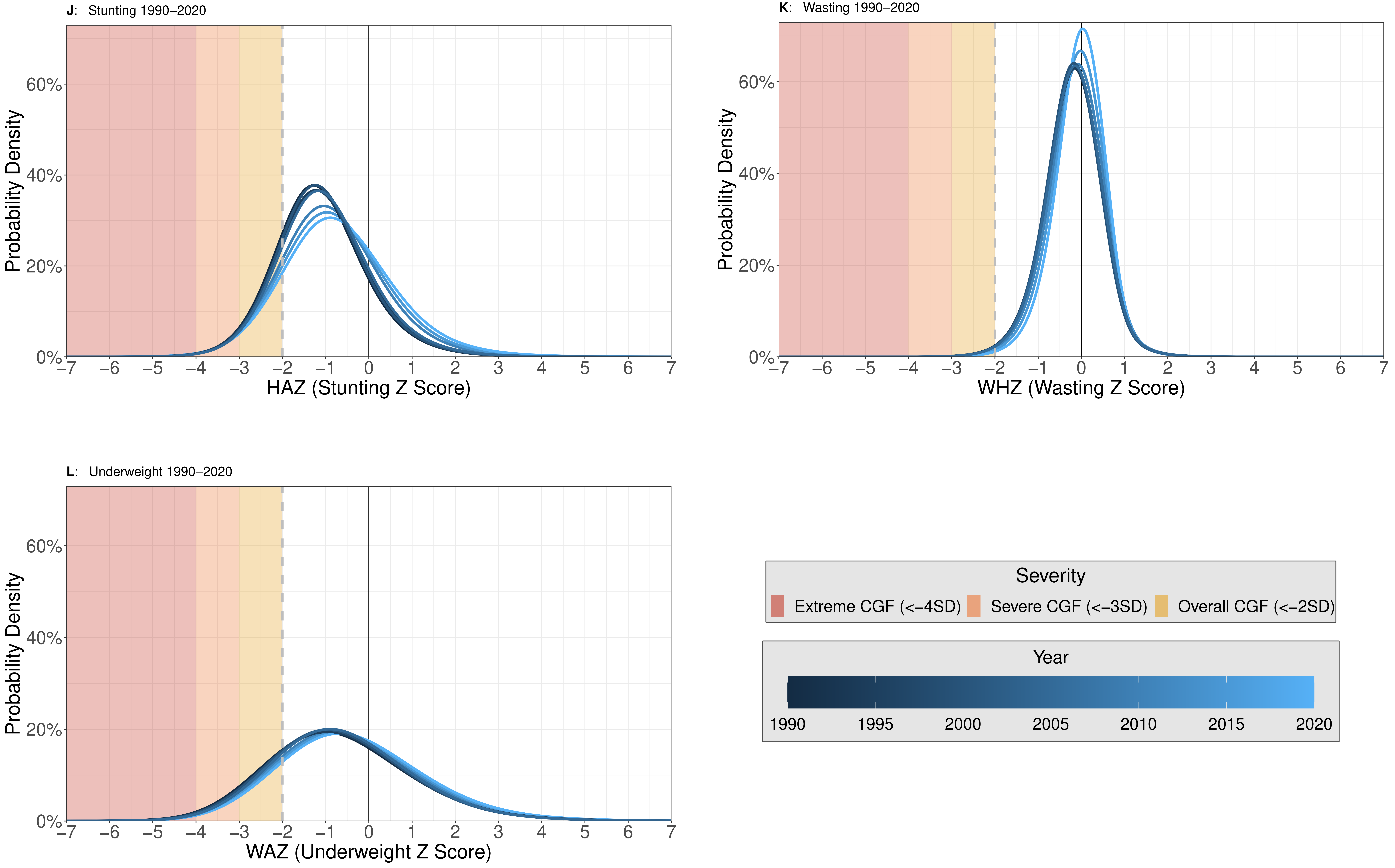

Micronesia (Federated States of) – Stunting (HAZ)

A: Overall and Severe Stunting Prevalence

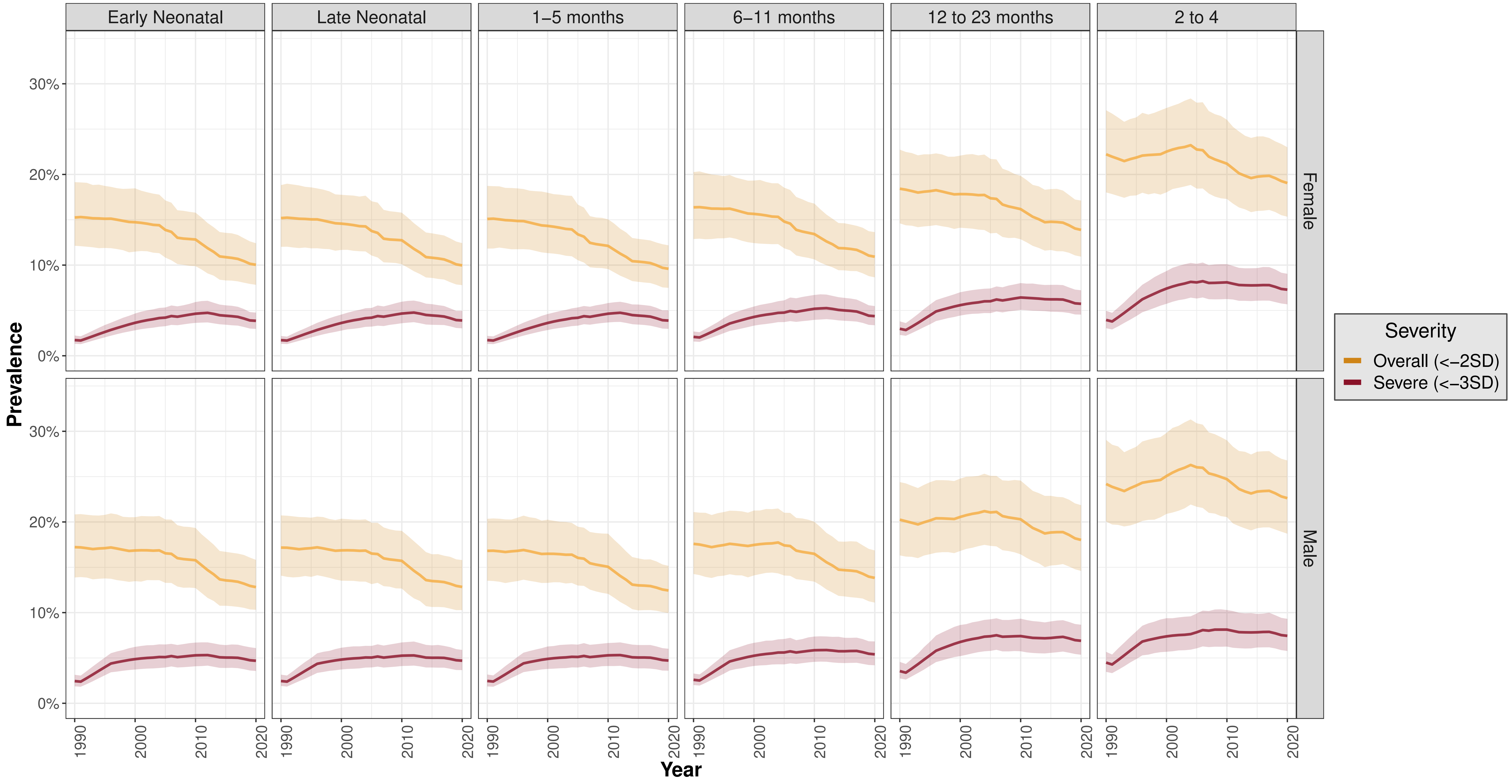

B: Transformed Mean Stunting Z Scores

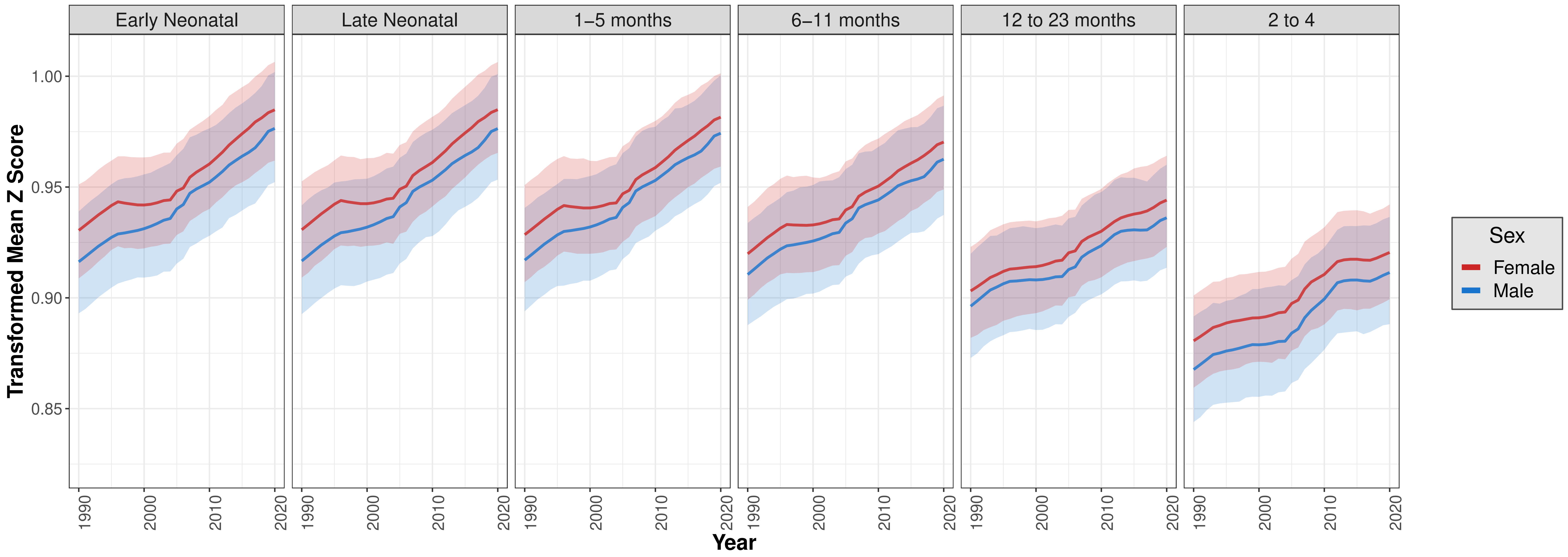

C

**Source**
No sources for this location

Micronesia (Federated States of) – Wasting (WHZ)

D: Overall and Severe Wasting Prevalence

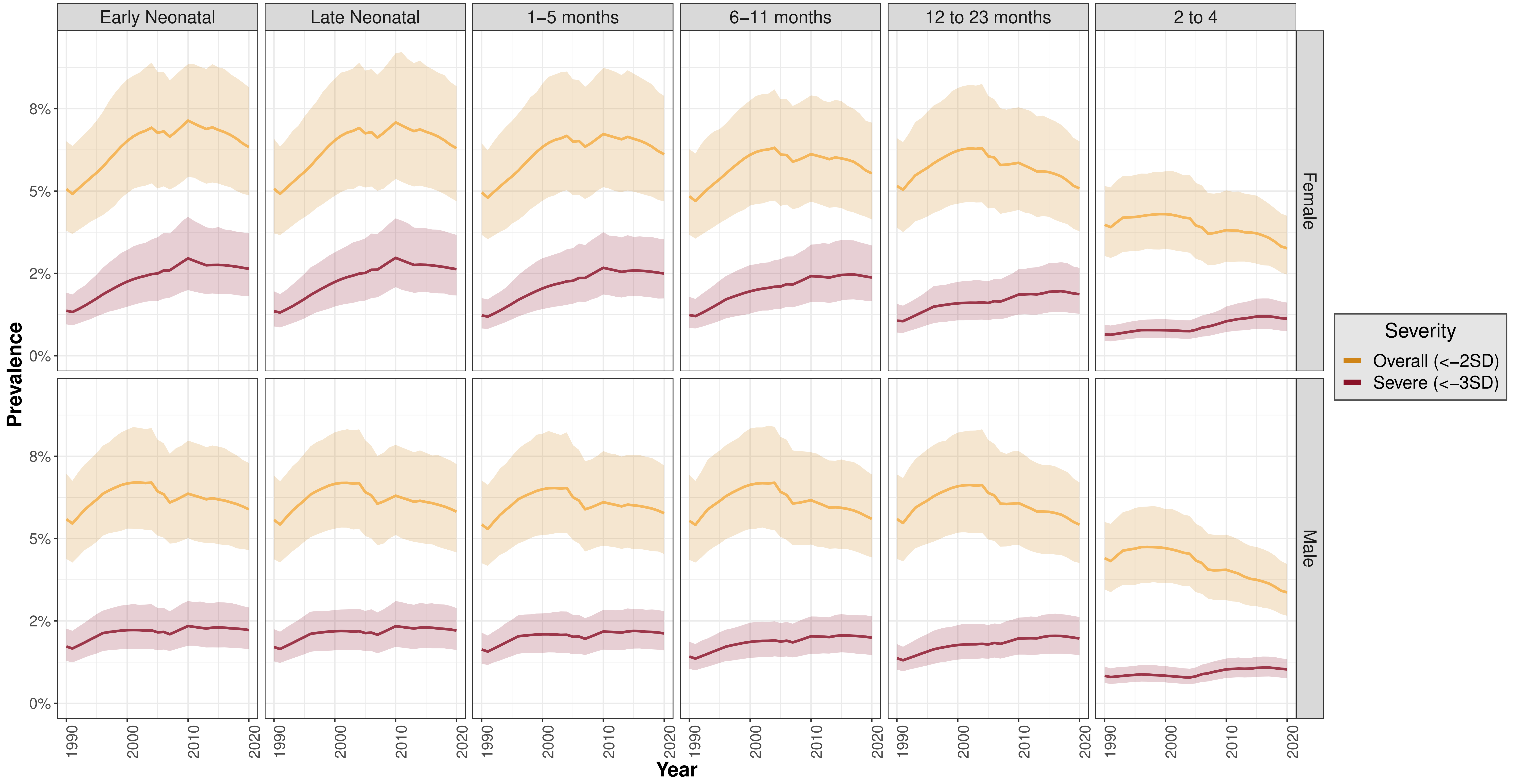

E: Transformed Mean Wasting Z Scores

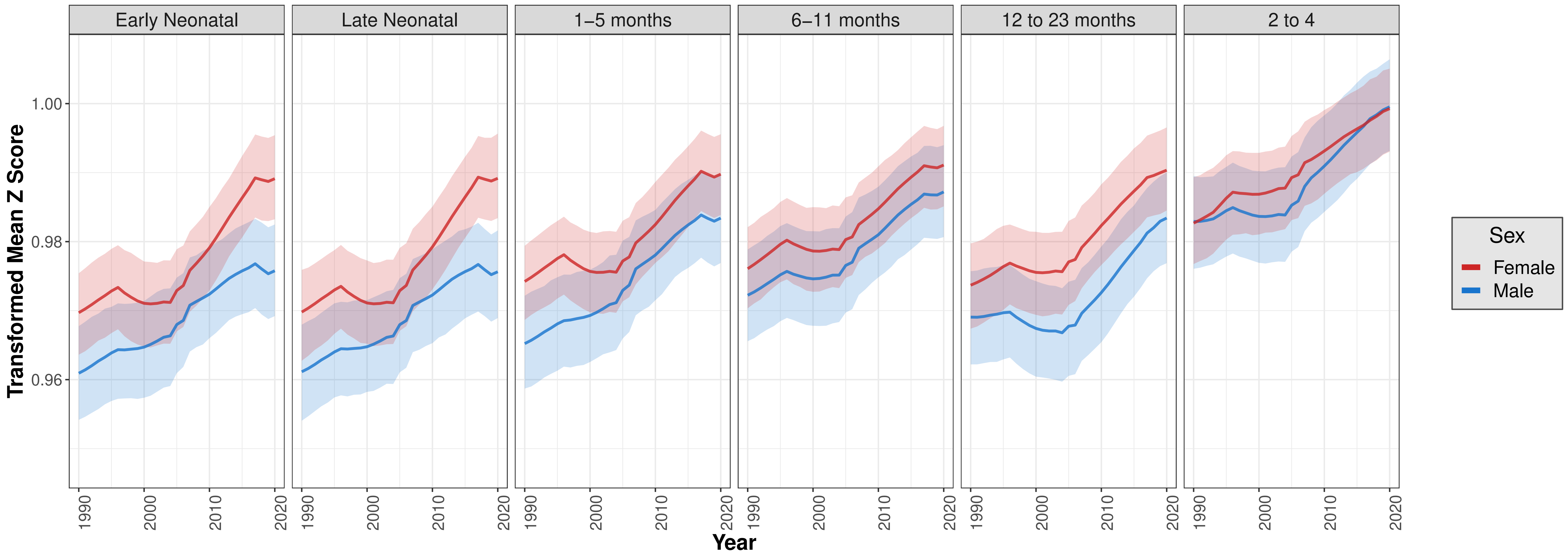

F

**Source**
No sources for this location

Micronesia (Federated States of) – Underweight (WAZ)

G: Overall and Severe Underweight Prevalence

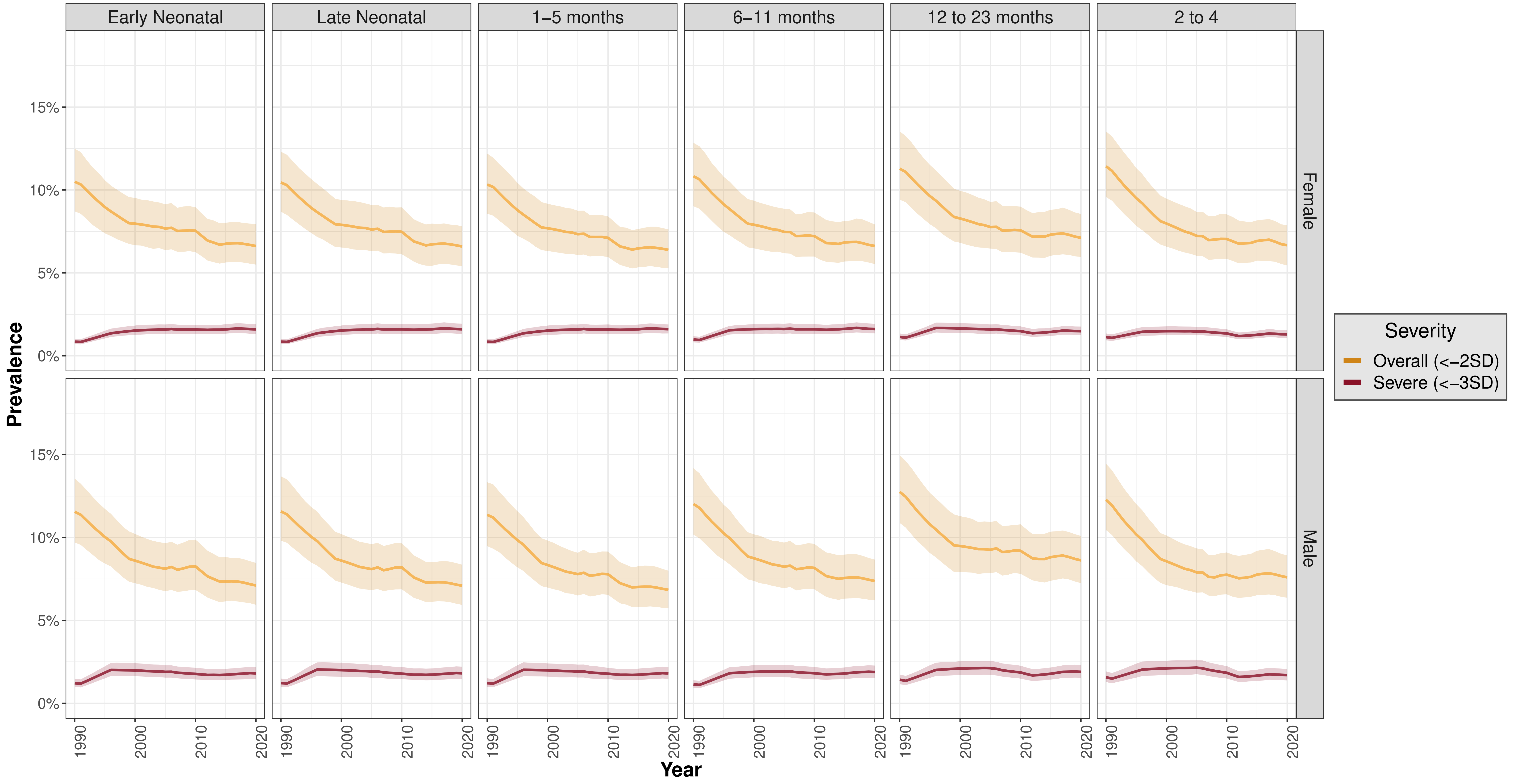

I

Source
No sources for this location

H: Transformed Mean Underweight Z Scores

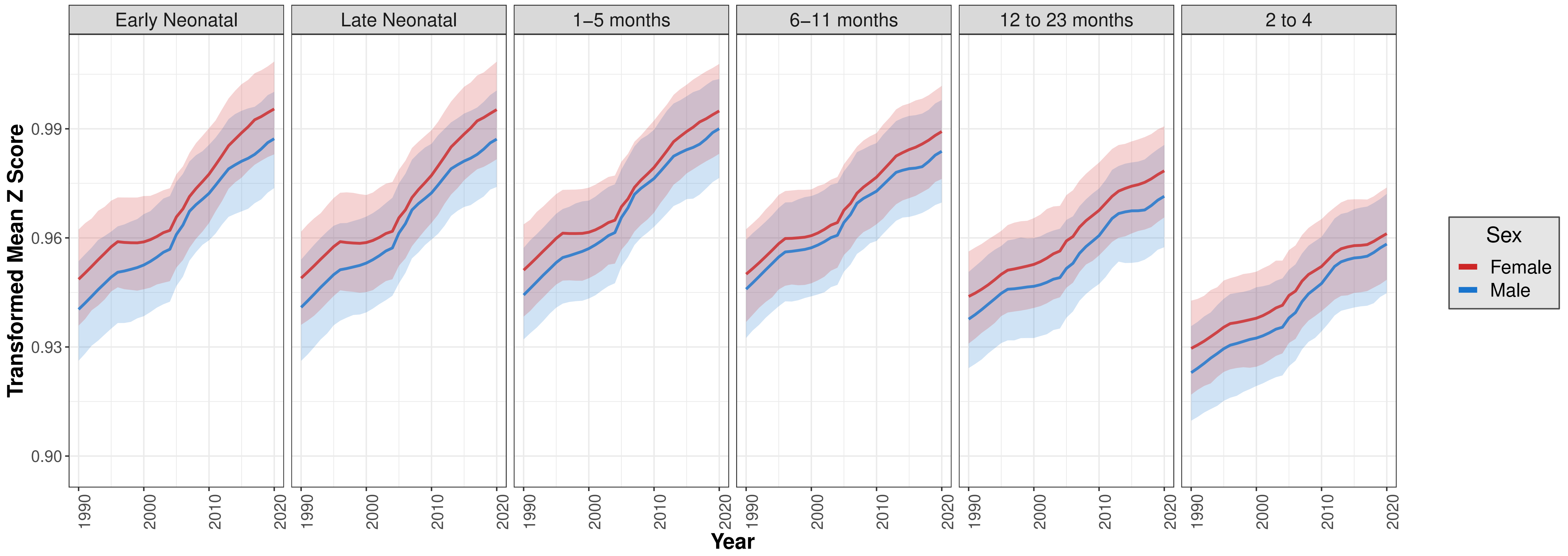

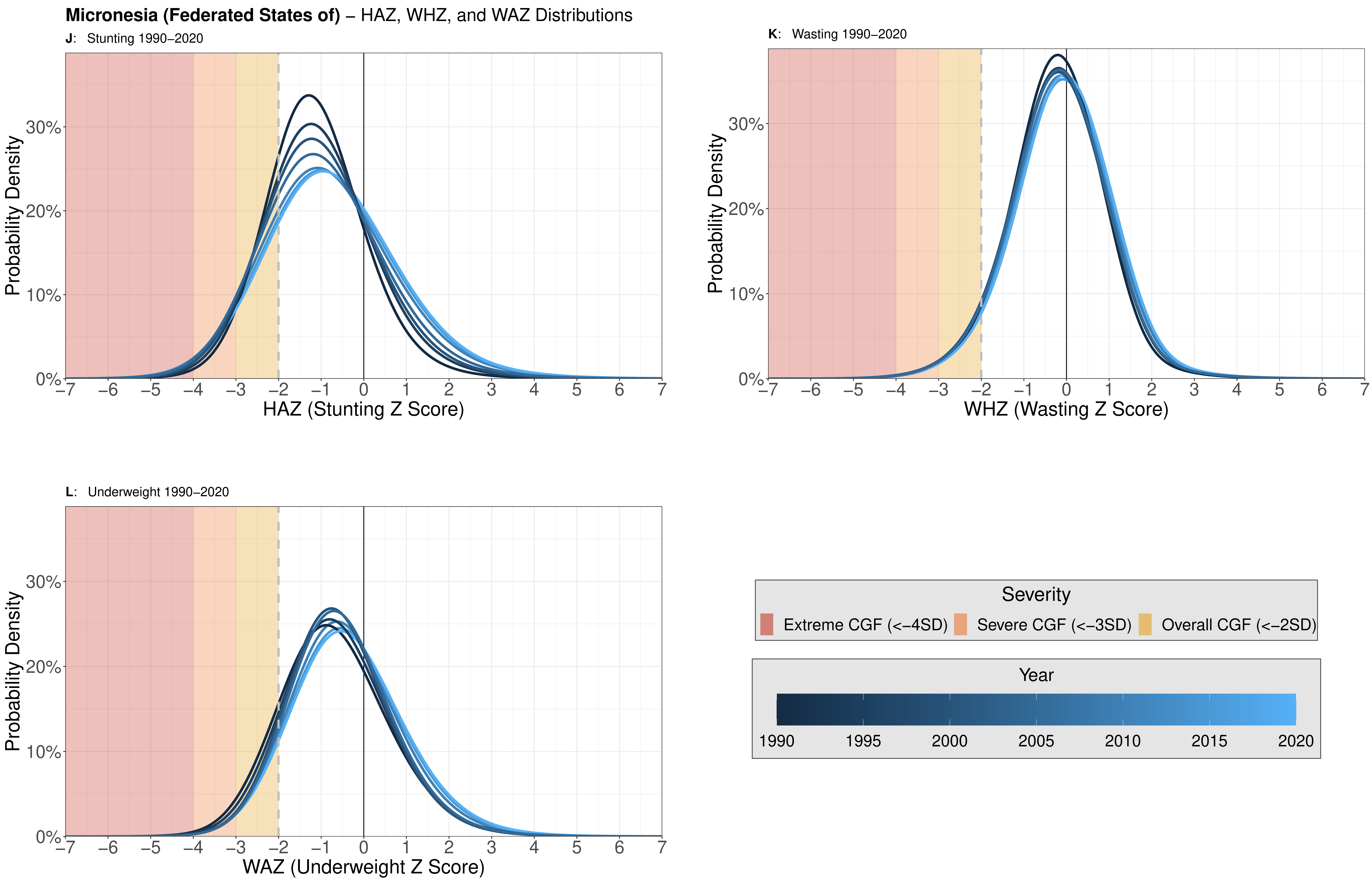

Micronesia (Federated States of) – HAZ, WHZ, and WAZ Distributions
J: Stunting 1990–2020
K: Wasting 1990–2020
L: Underweight 1990–2020
Probability Density
0%
10%
20%
30%
−7
−6
−5
−4
−3
−2
−1
0
1
2
3
4
5
6
7
HAZ (Stunting Z Score)
WHZ (Wasting Z Score)
WAZ (Underweight Z Score)
Severity
Extreme CGF (<−4SD)
Severe CGF (<−3SD)
Overall CGF (<−2SD)
Year
1990
1995
2000
2005
2010
2015
2020

Papua New Guinea – Stunting (HAZ)

A: Overall and Severe Stunting Prevalence

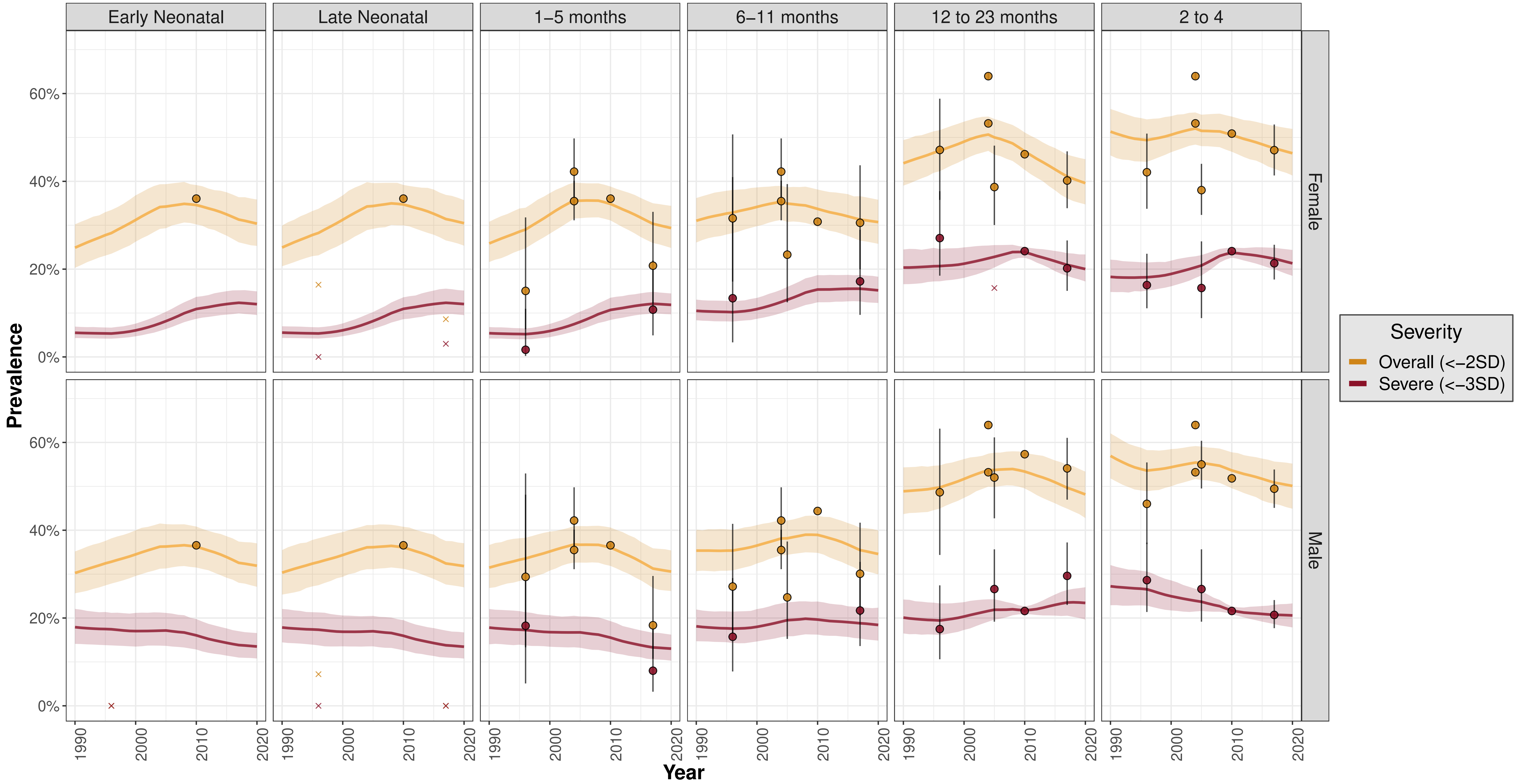

C

Year	Source
1983	WHO CGM Database
1986	WHO CGM Database
1996	Living Standards Measurement Survey
2004	WHO CGM Database
2005	WHO CGM Database
2010	WHO CGM Database
2017	DHS

B: Transformed Mean Stunting Z Scores

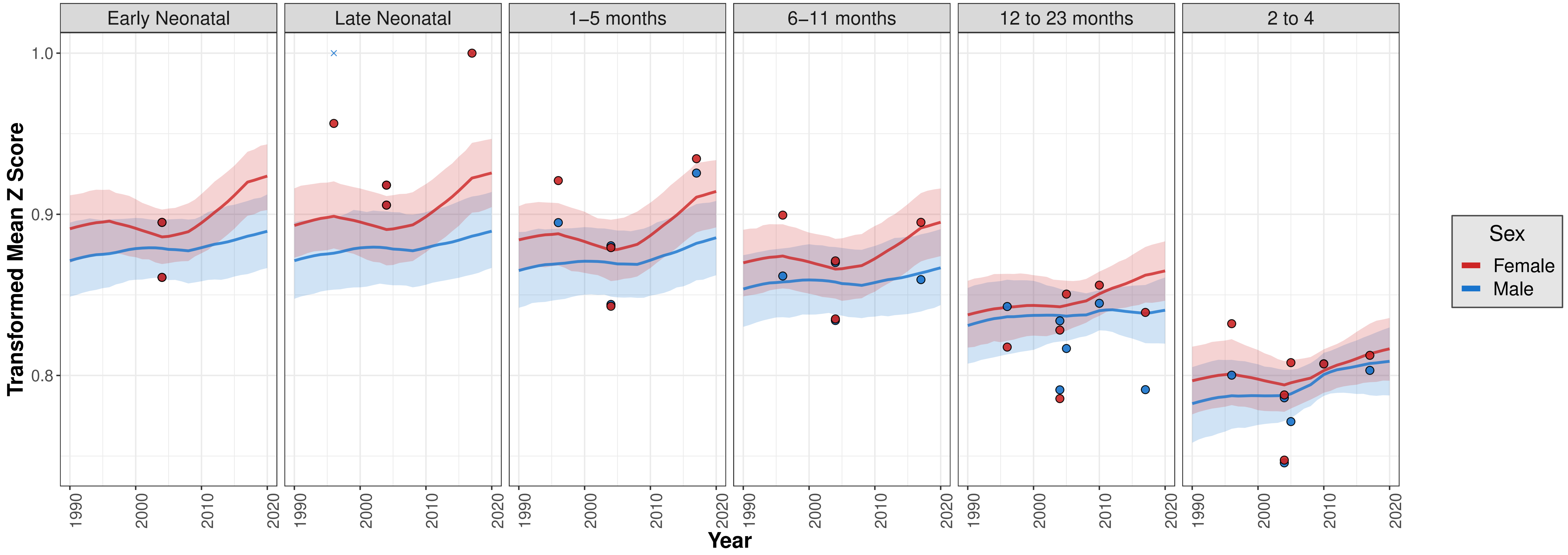

Papua New Guinea – Wasting (WHZ)

D: Overall and Severe Wasting Prevalence

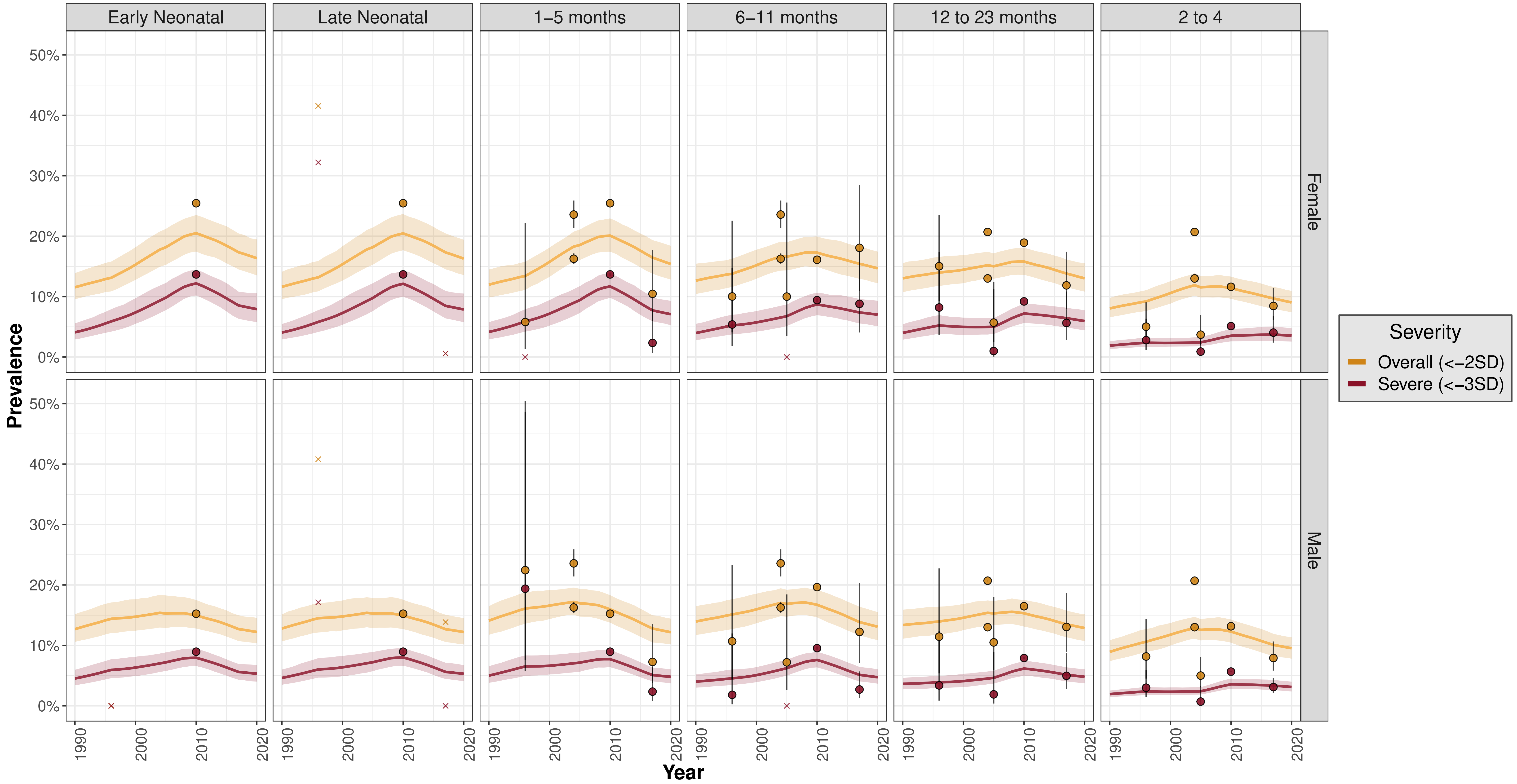

E: Transformed Mean Wasting Z Scores

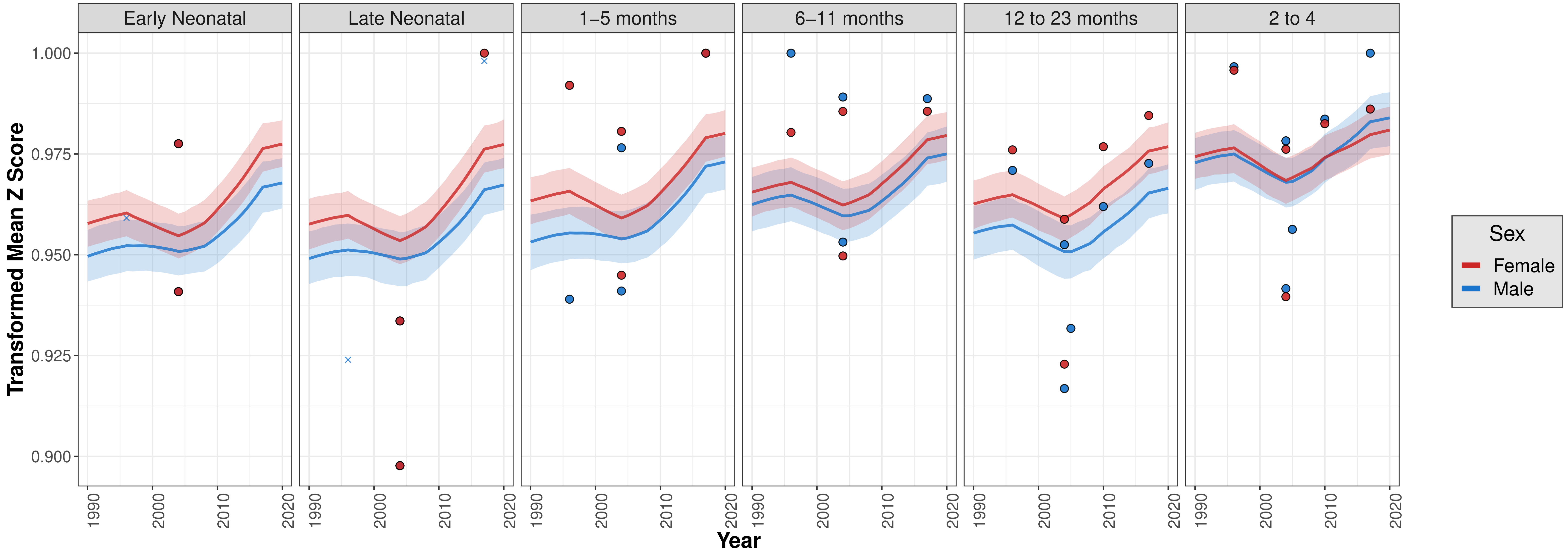

F

Year	Source
1983	WHO CGM Database
1986	WHO CGM Database
1996	Living Standards Measurement Survey
2004	WHO CGM Database
2005	WHO CGM Database
2010	WHO CGM Database
2017	DHS

Papua New Guinea – Underweight (WAZ)

G: Overall and Severe Underweight Prevalence

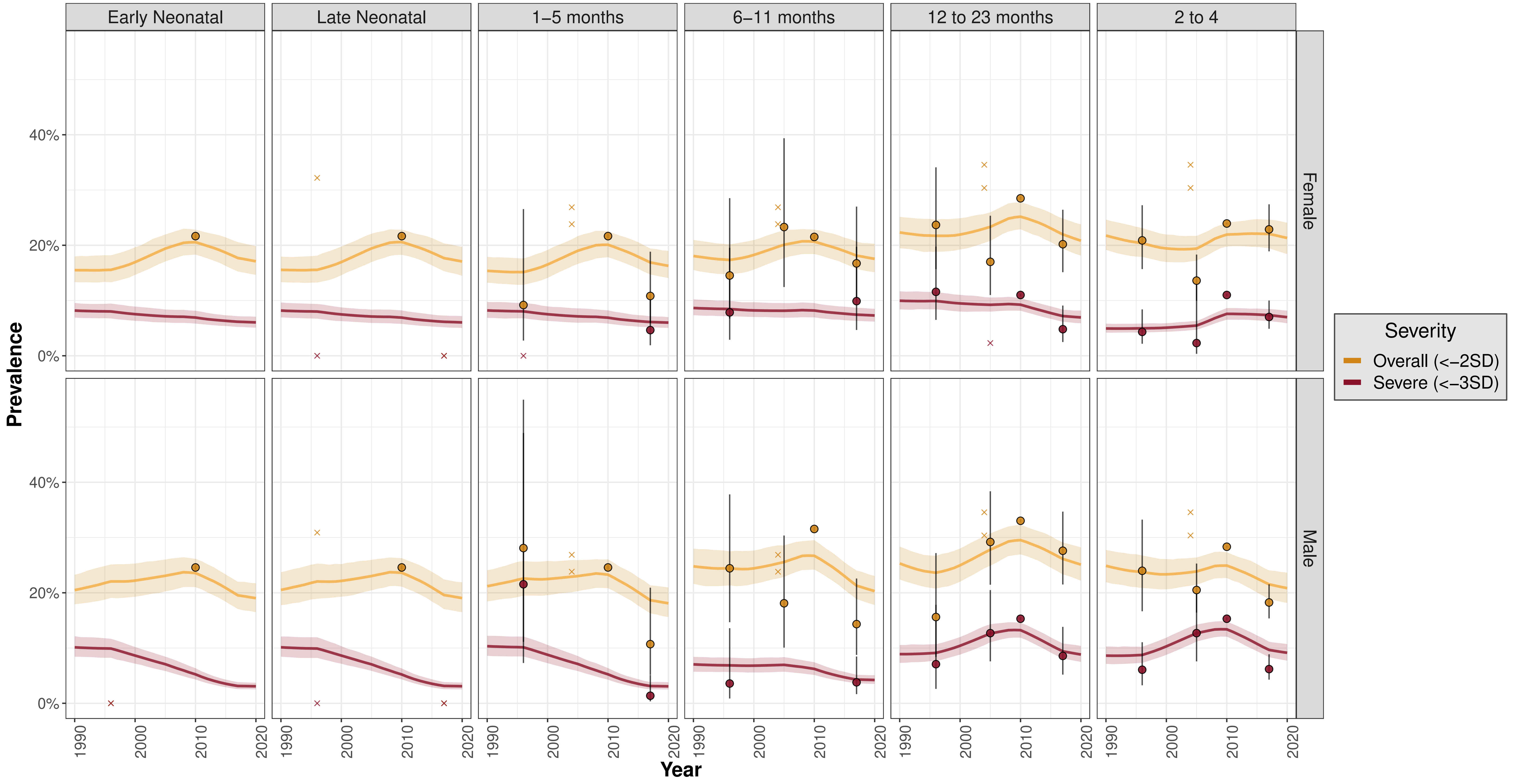

H: Transformed Mean Underweight Z Scores

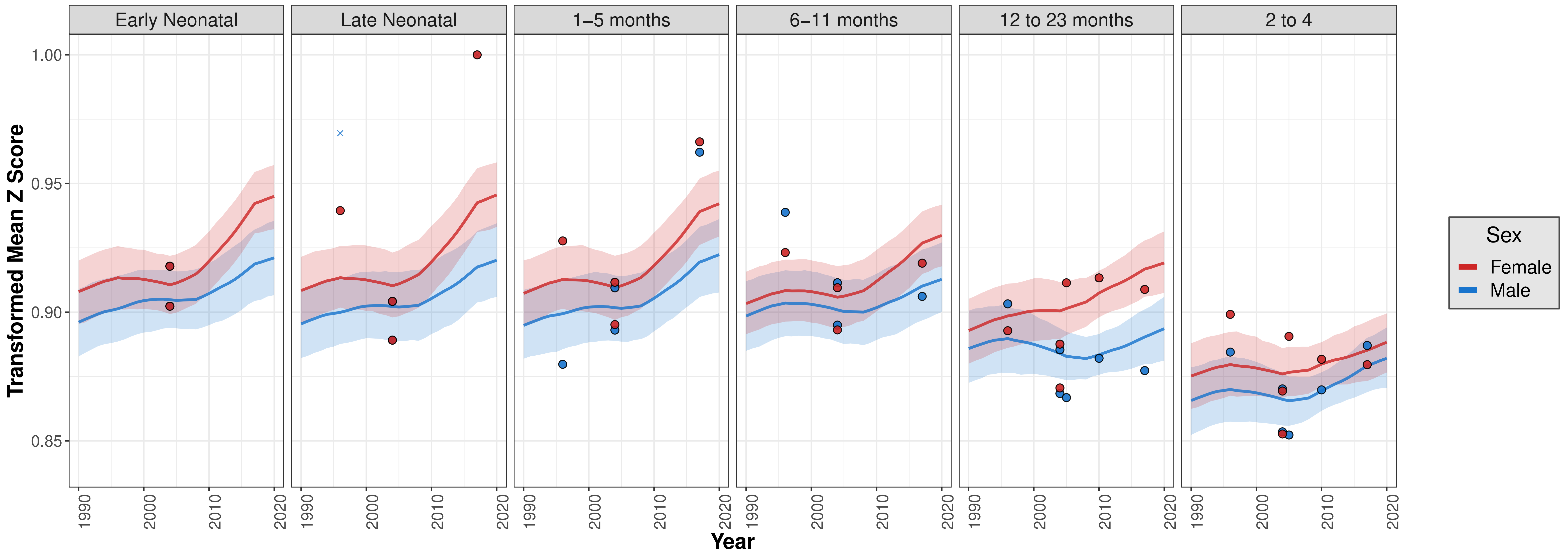

I

Year	Source
1983	WHO CGM Database
1986	WHO CGM Database
1996	Living Standards Measurement Survey
2004	WHO CGM Database
2005	WHO CGM Database
2010	WHO CGM Database
2017	DHS

Papua New Guinea – HAZ, WHZ, and WAZ Distributions

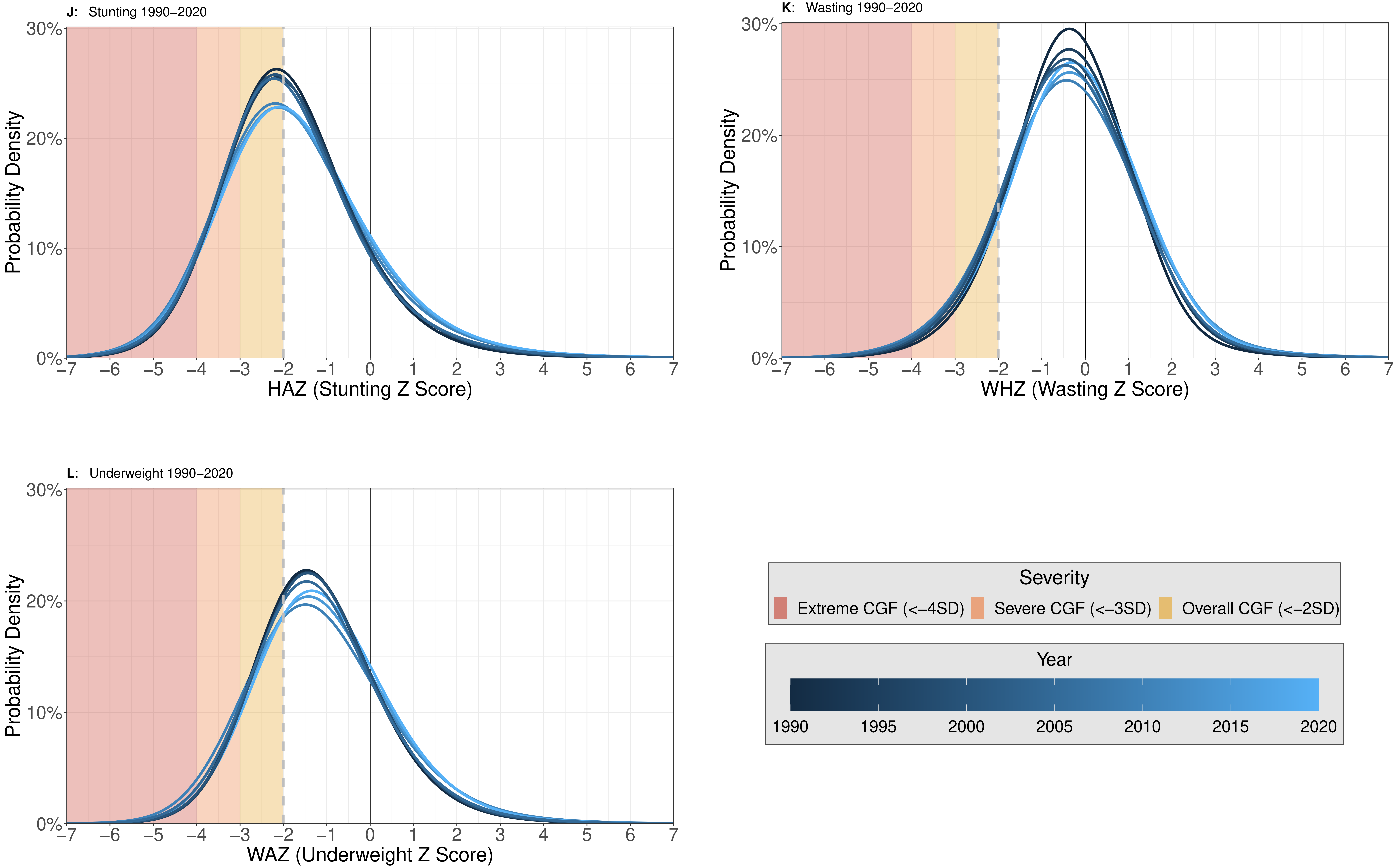

Samoa – Stunting (HAZ)

A: Overall and Severe Stunting Prevalence

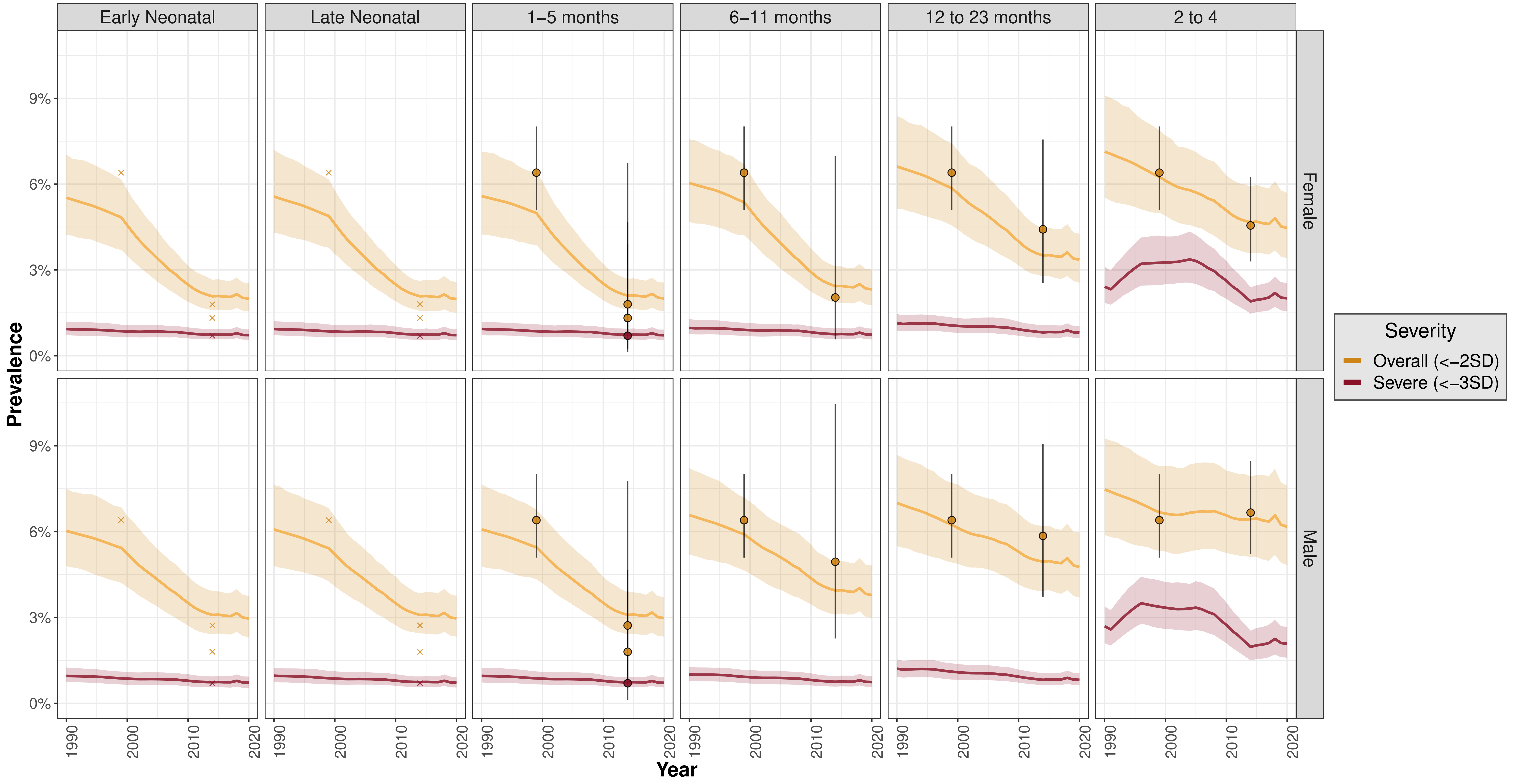

B: Transformed Mean Stunting Z Scores

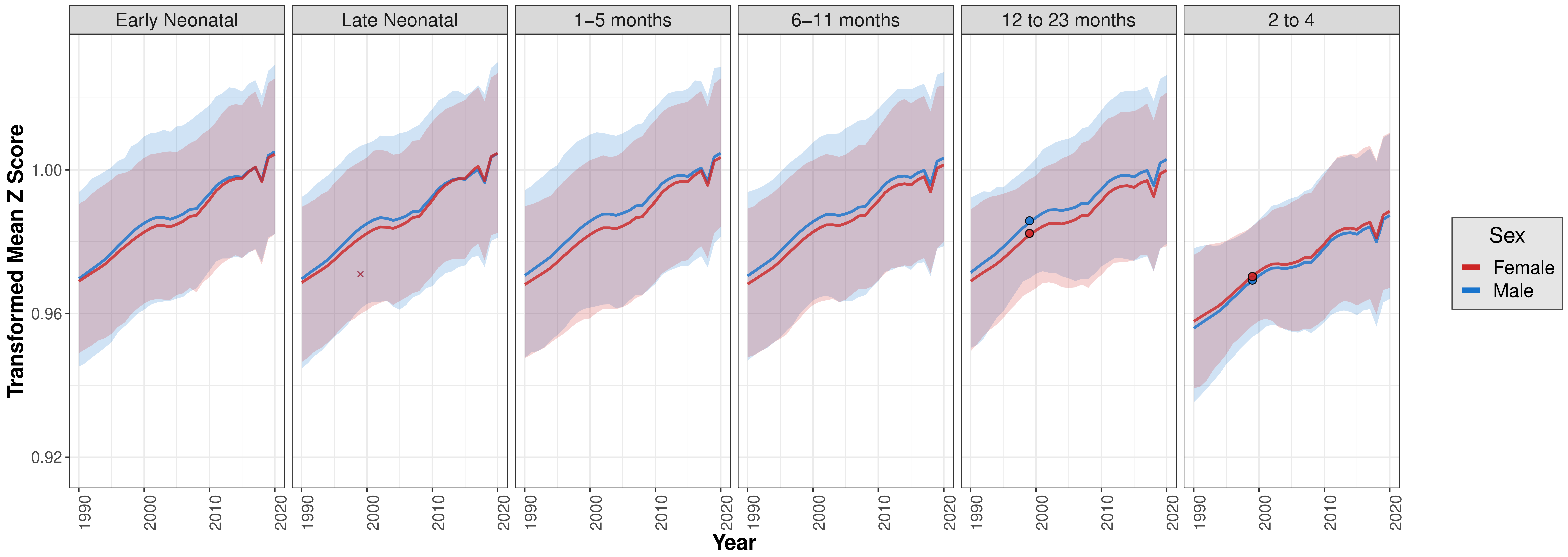

C

Year	Source
1999	WHO CGM Database
2014	WHO CGM Database
2014	DHS

Samoa – Wasting (WHZ)

D: Overall and Severe Wasting Prevalence

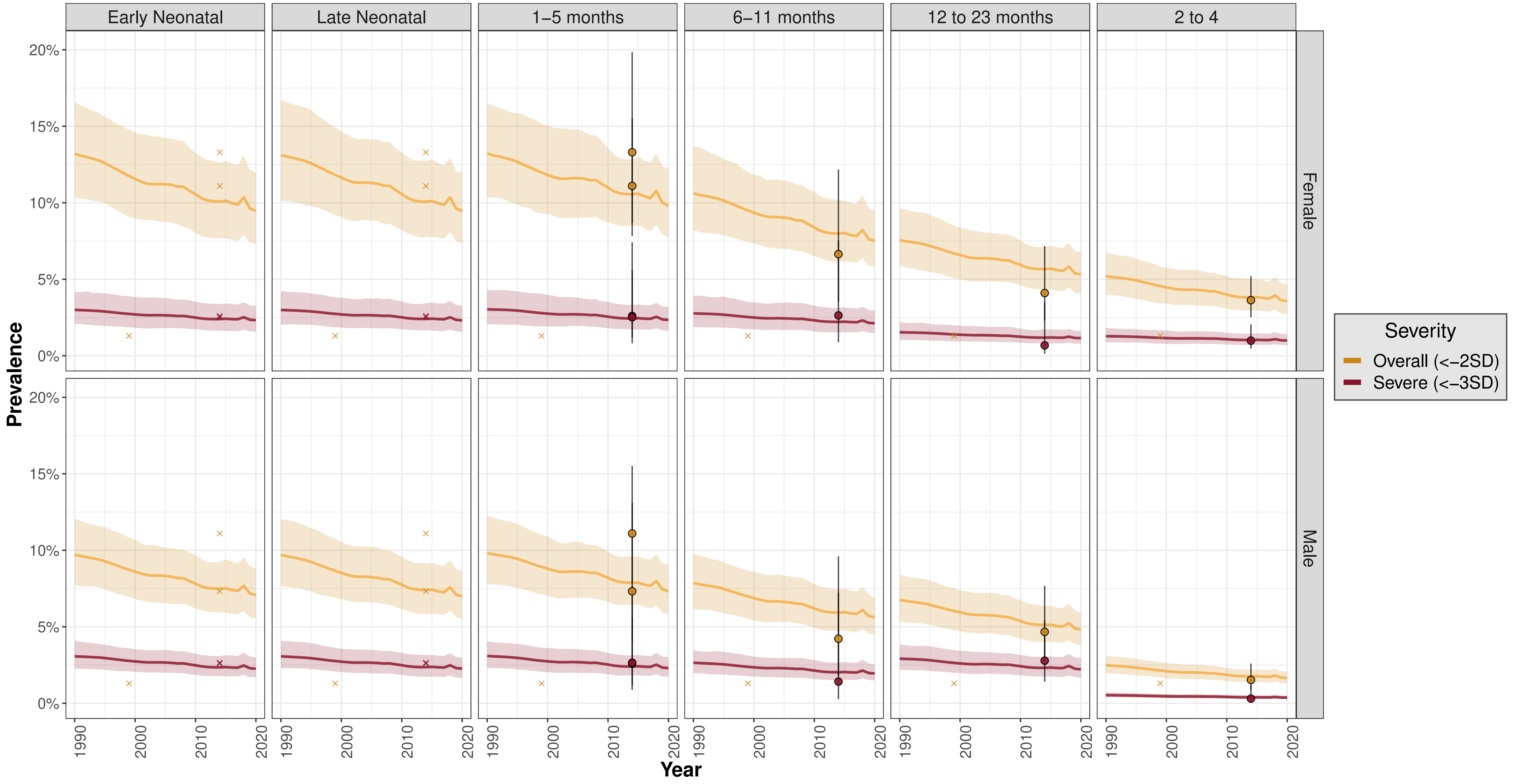

E: Transformed Mean Wasting Z Scores

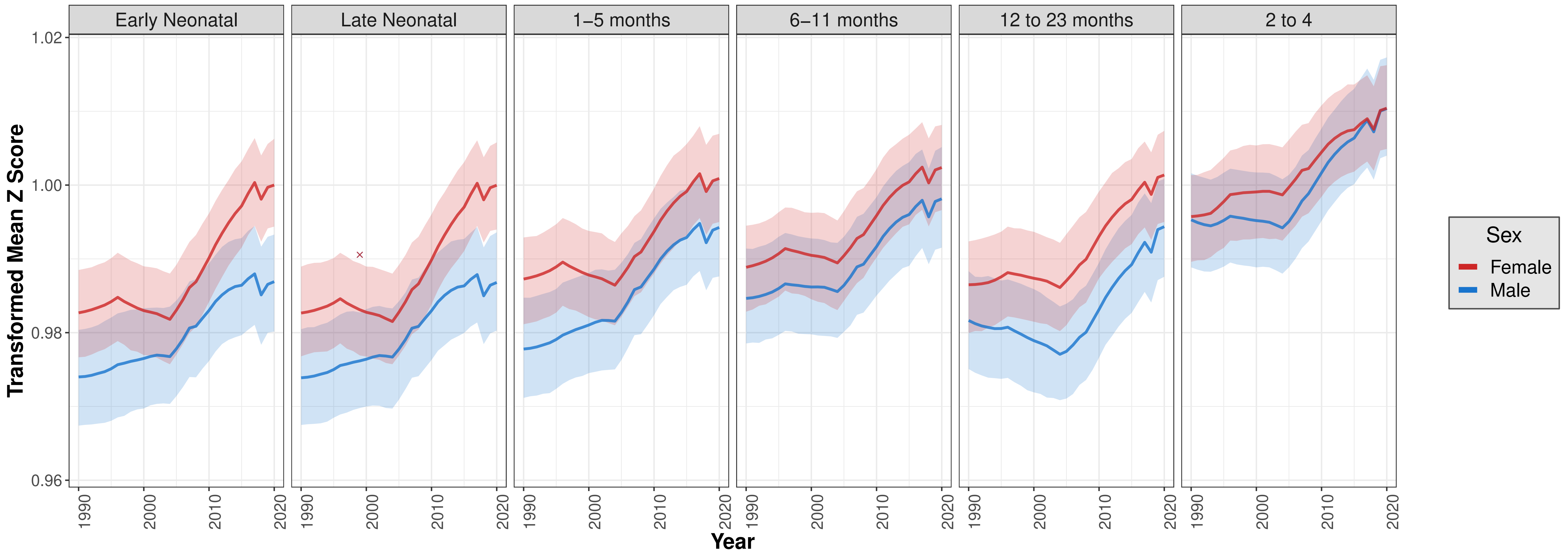

F

Year	Source
1999	WHO CGM Database
2014	WHO CGM Database
2014	DHS

Samoa – Underweight (WAZ)

G: Overall and Severe Underweight Prevalence

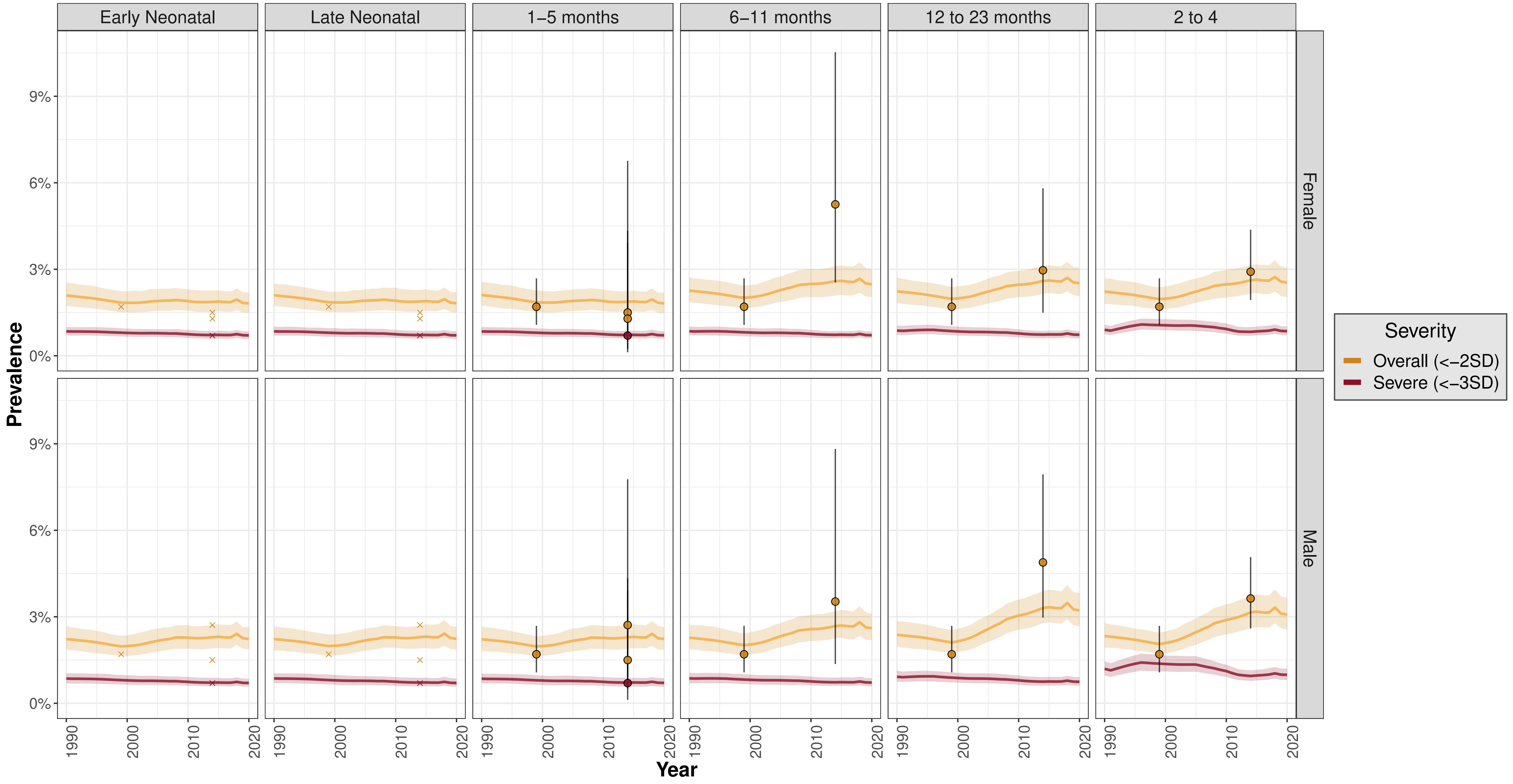

H: Transformed Mean Underweight Z Scores

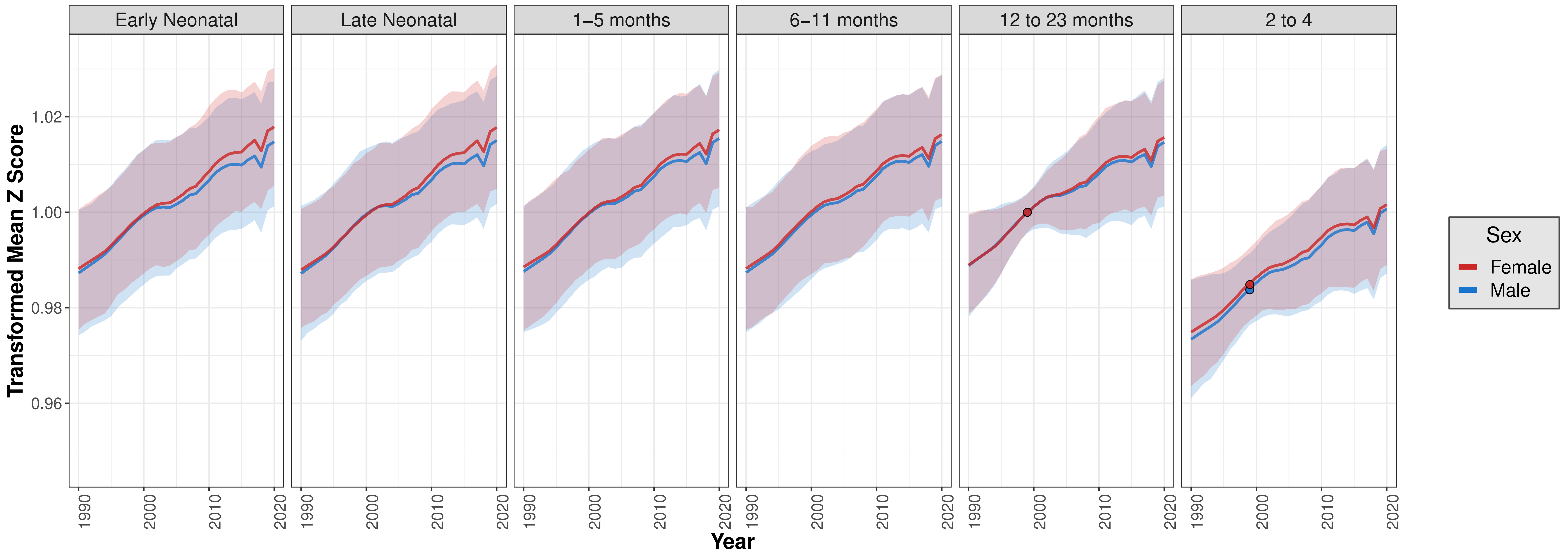

I

Year	Source
1999	WHO CGM Database
2014	WHO CGM Database
2014	DHS

Samoa – HAZ, WHZ, and WAZ Distributions

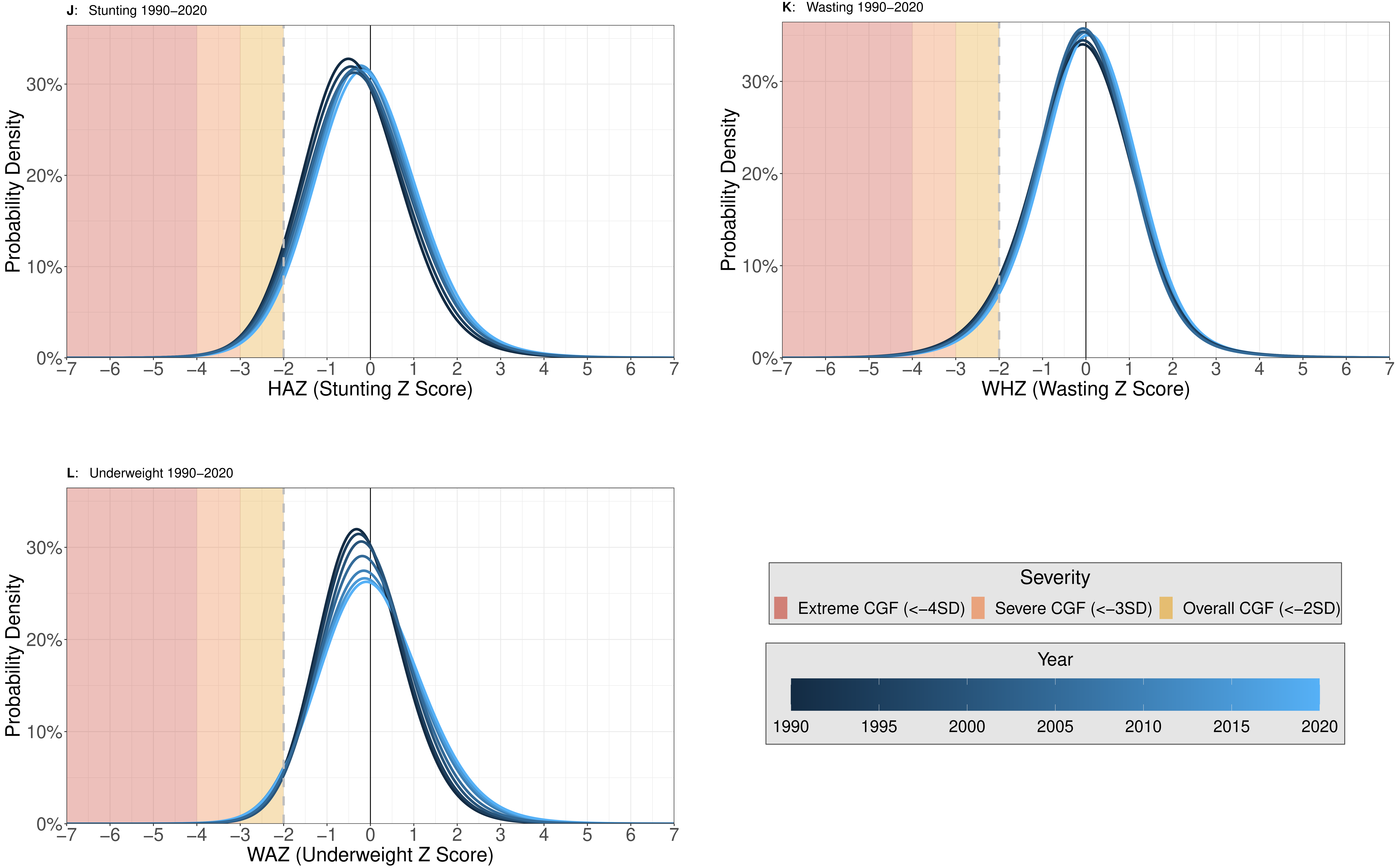

Solomon Islands – Stunting (HAZ)

A: Overall and Severe Stunting Prevalence

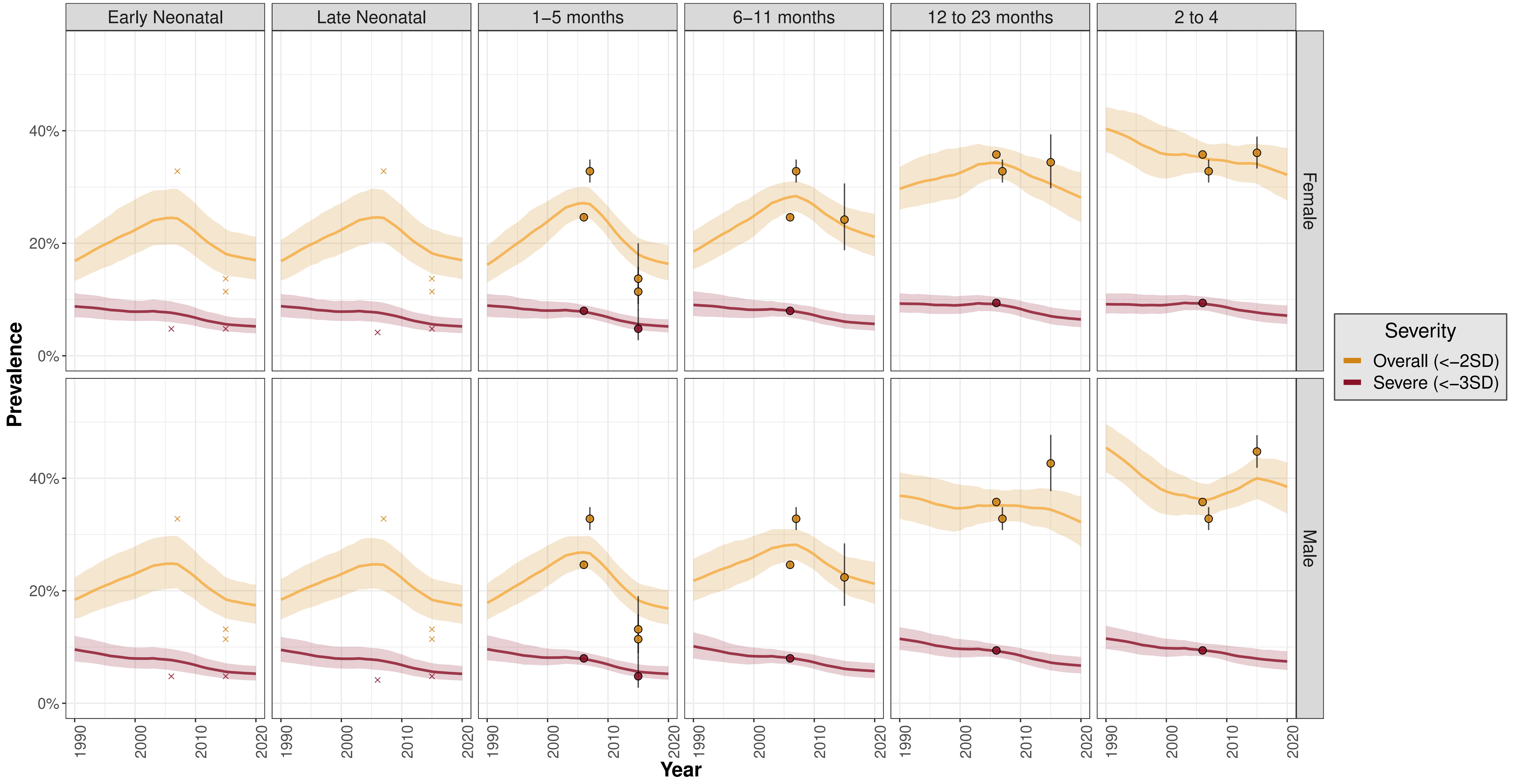

B: Transformed Mean Stunting Z Scores

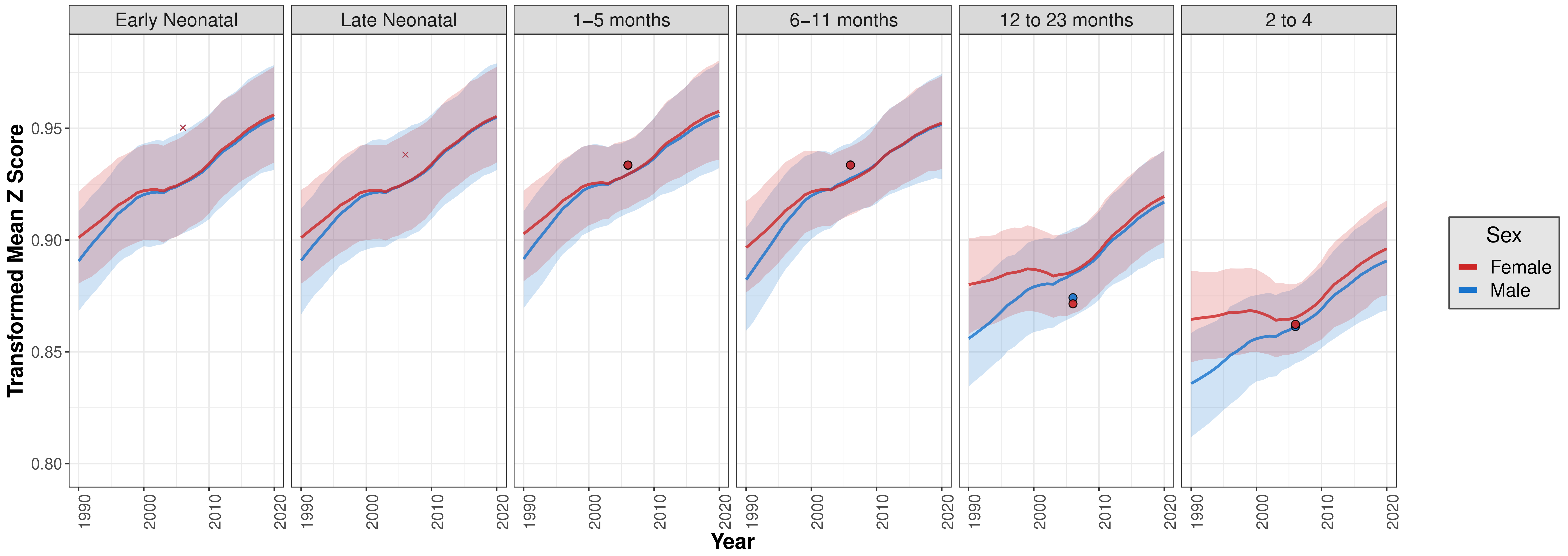

C

Year	Source
1970	WHO CGM Database
1989	WHO CGM Database
2006	WHO CGM Database
2007	WHO CGM Database
2015	WHO CGM Database
2015	DHS

Solomon Islands – Wasting (WHZ)

D: Overall and Severe Wasting Prevalence

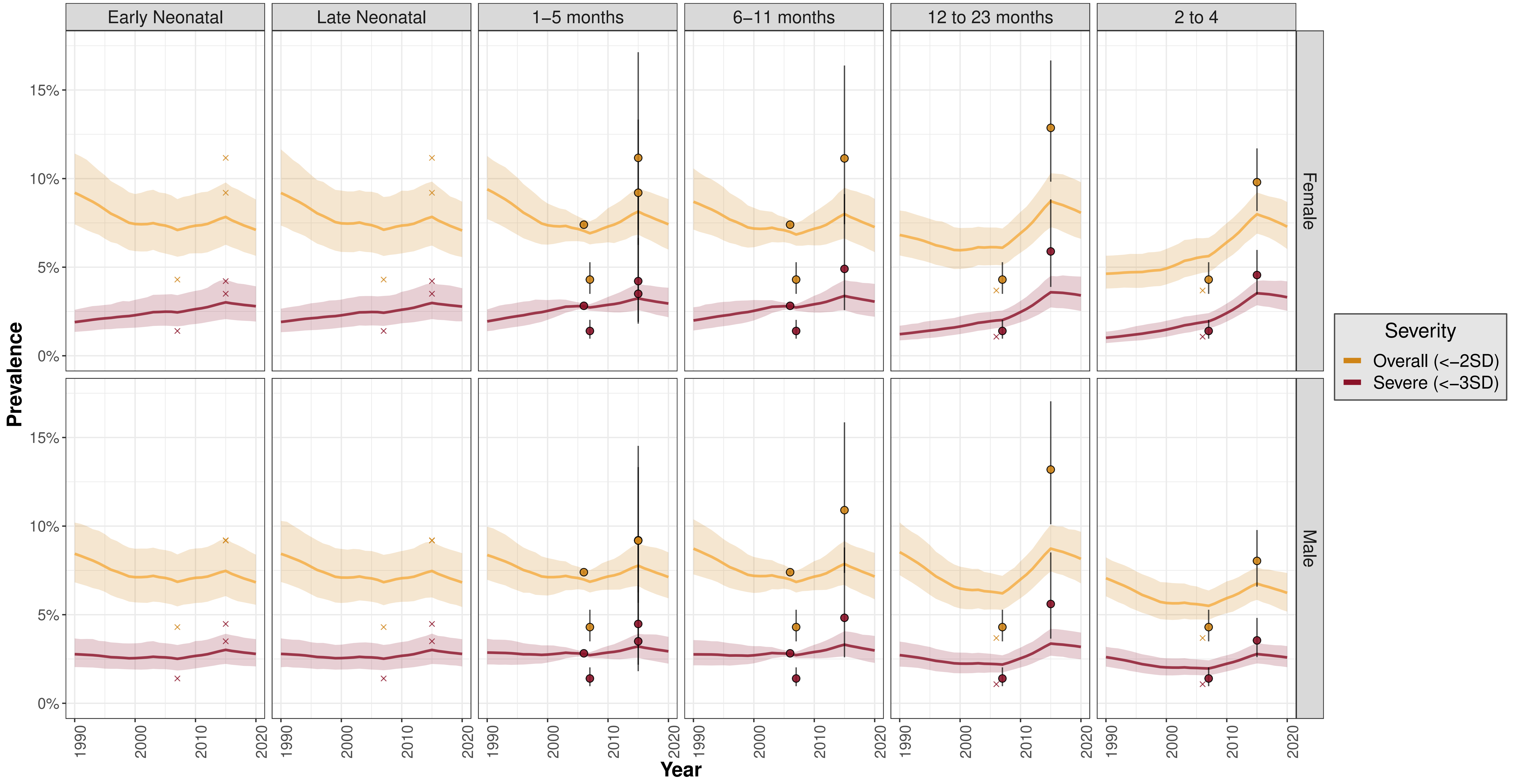

E: Transformed Mean Wasting Z Scores

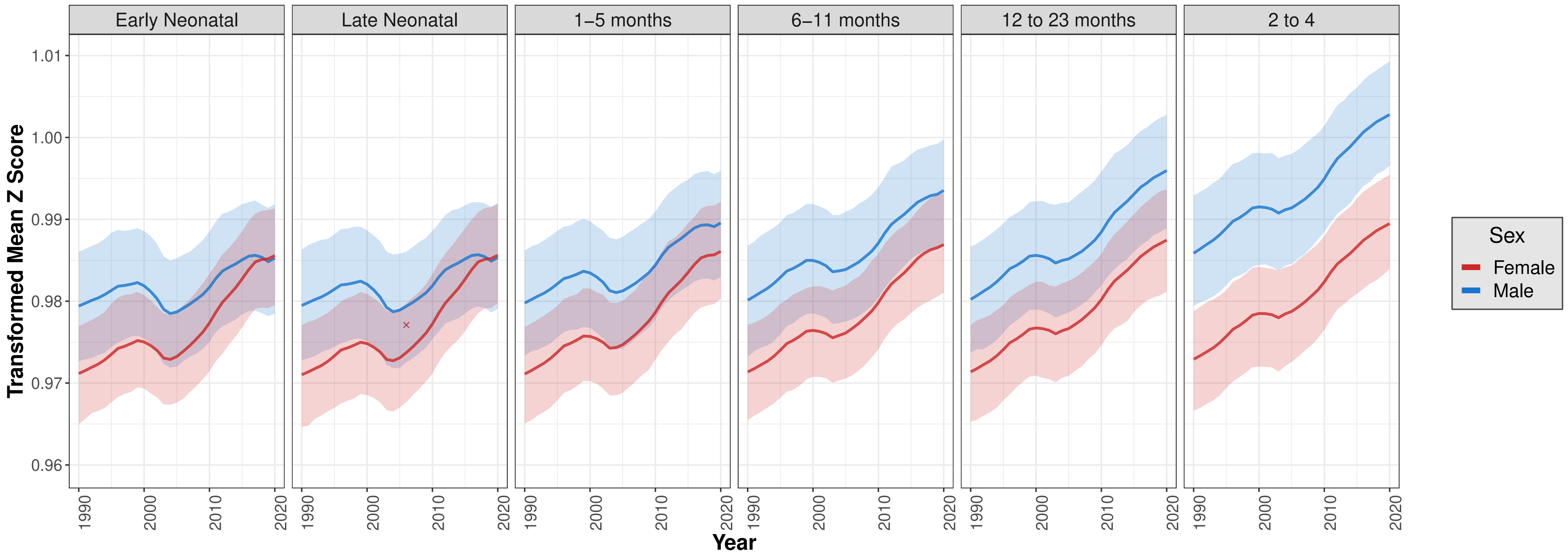

F

Year	Source
1970	WHO CGM Database
1989	WHO CGM Database
2006	WHO CGM Database
2007	WHO CGM Database
2015	WHO CGM Database
2015	DHS

Solomon Islands – Underweight (WAZ)

G: Overall and Severe Underweight Prevalence

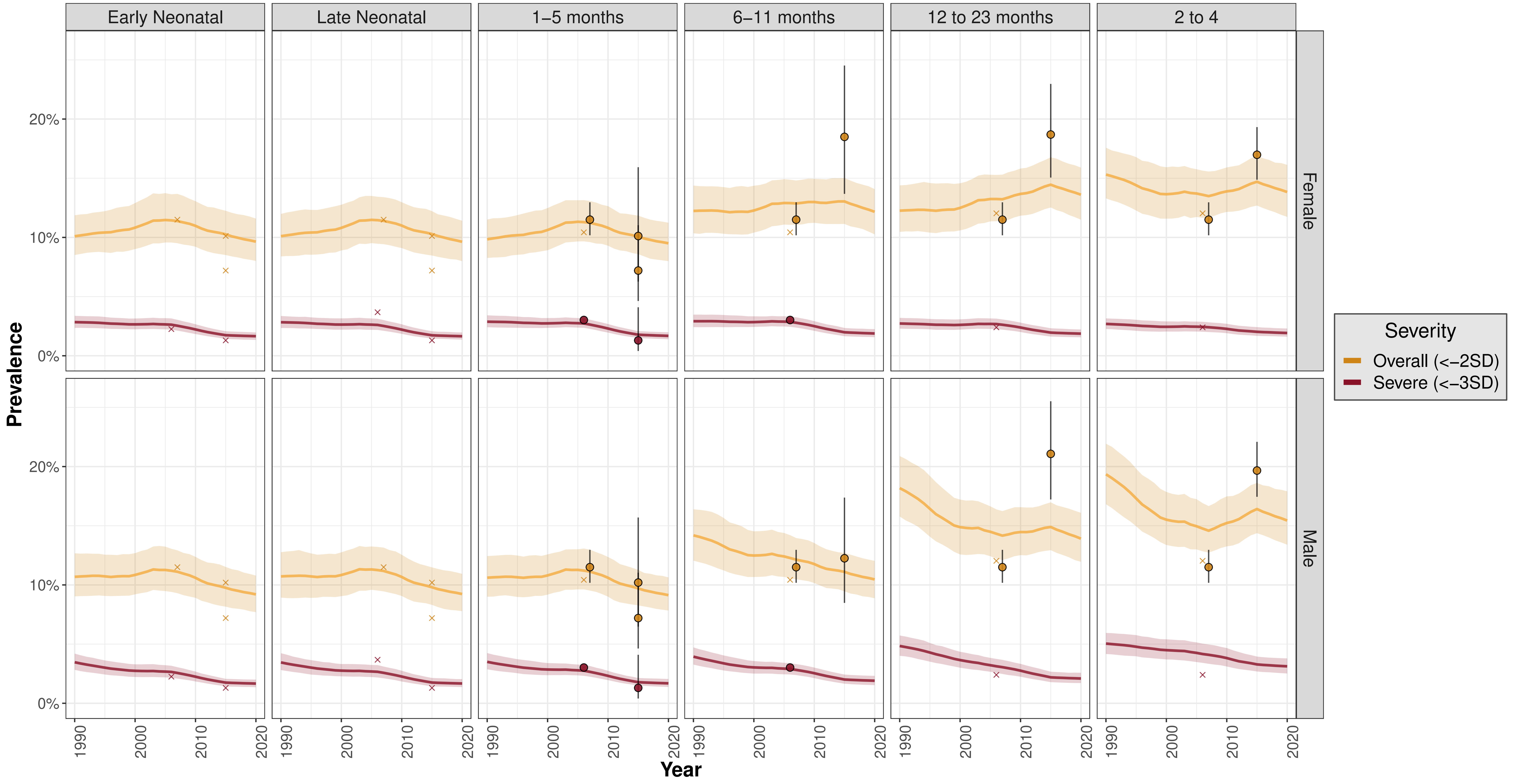

H: Transformed Mean Underweight Z Scores

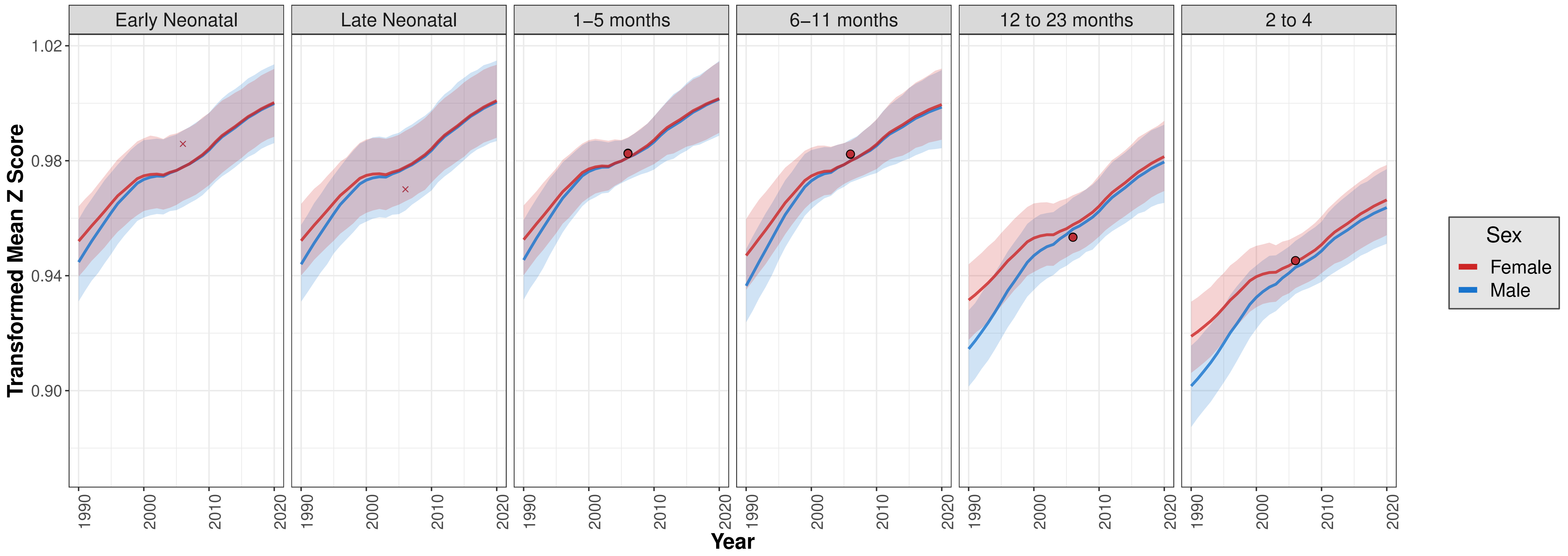

I

Year	Source
1970	WHO CGM Database
1989	WHO CGM Database
2006	WHO CGM Database
2007	WHO CGM Database
2015	WHO CGM Database
2015	DHS

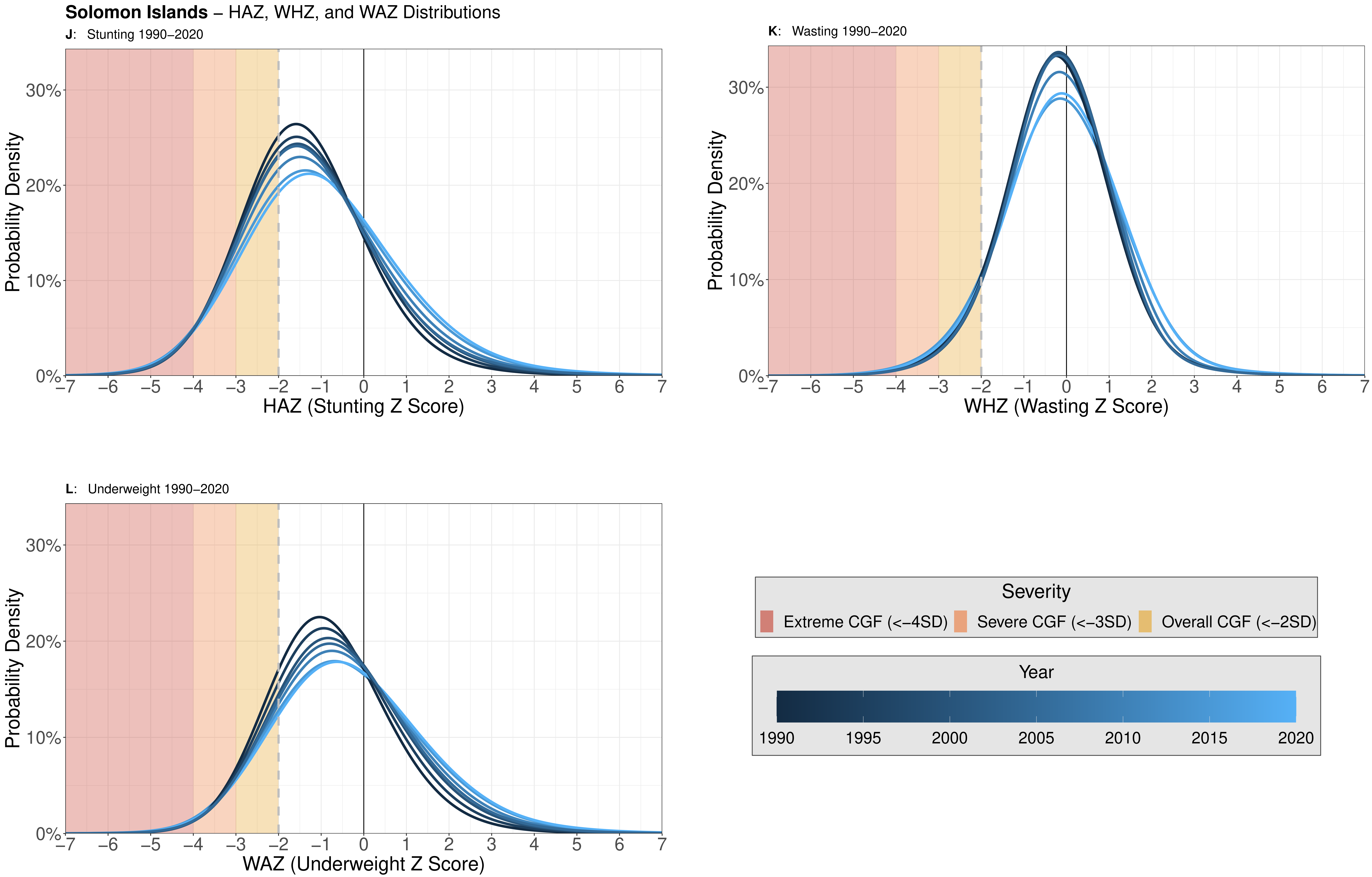

Solomon Islands – HAZ, WHZ, and WAZ Distributions
J: Stunting 1990–2020
Probability Density
30%
20%
10%
0%
−7 −6 −5 −4 −3 −2 −1 0 1 2 3 4 5 6 7
HAZ (Stunting Z Score)
K: Wasting 1990–2020
Probability Density
30%
20%
10%
0%
−7 −6 −5 −4 −3 −2 −1 0 1 2 3 4 5 6 7
WHZ (Wasting Z Score)
L: Underweight 1990–2020
Probability Density
30%
20%
10%
0%
−7 −6 −5 −4 −3 −2 −1 0 1 2 3 4 5 6 7
WAZ (Underweight Z Score)
Severity
Extreme CGF (<−4SD)
Severe CGF (<−3SD)
Overall CGF (<−2SD)
Year
1990 1995 2000 2005 2010 2015 2020

Tonga – Stunting (HAZ)

A: Overall and Severe Stunting Prevalence

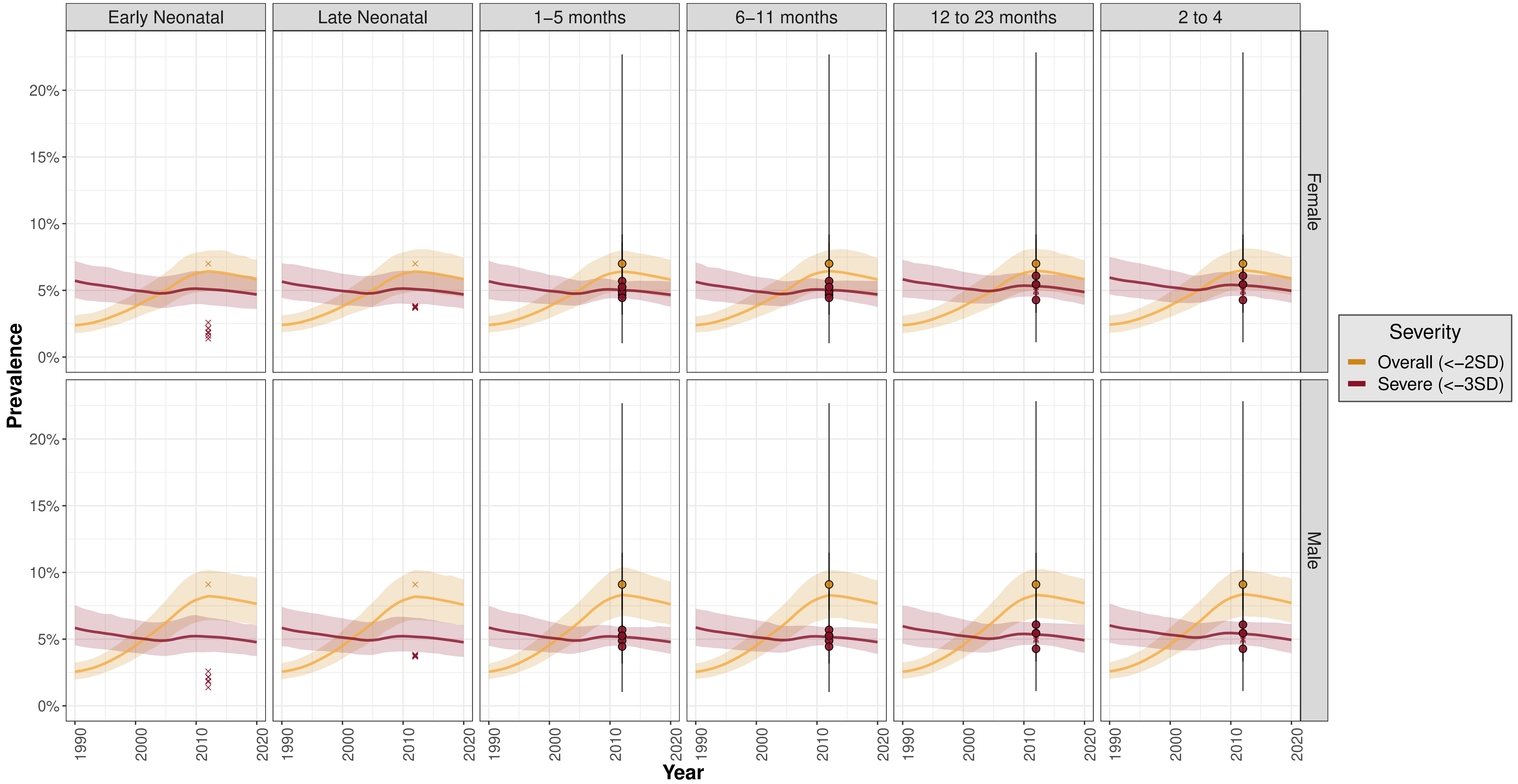

B: Transformed Mean Stunting Z Scores

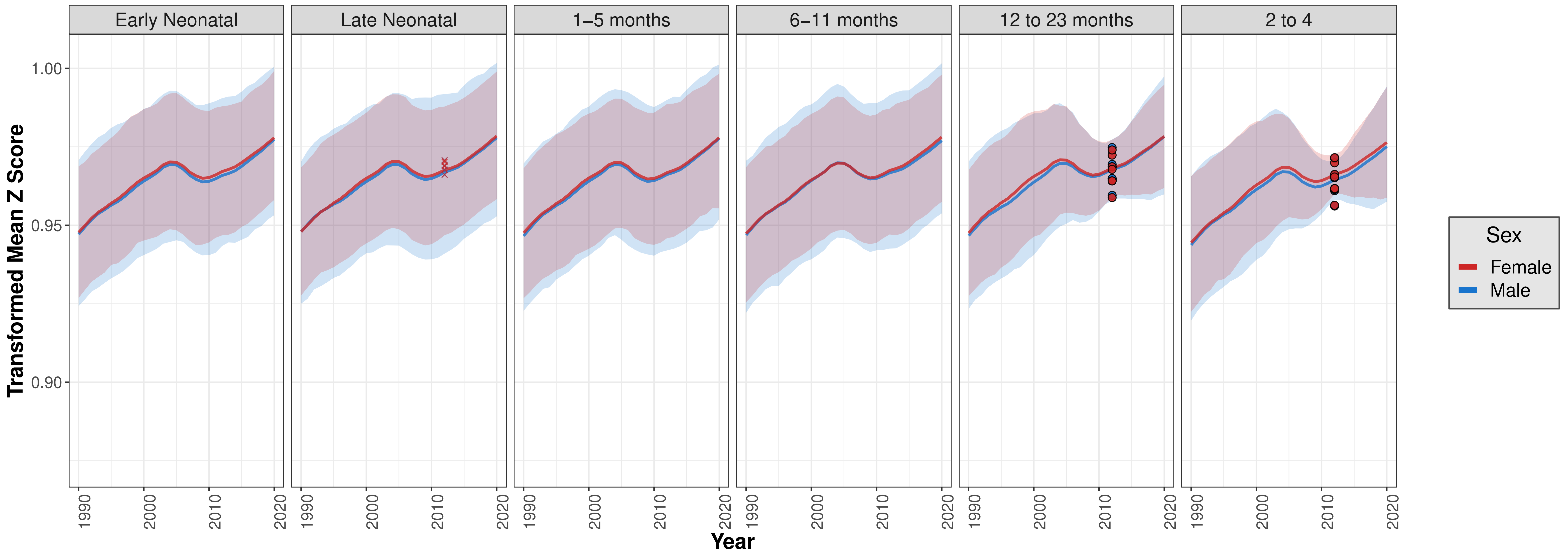

C

Year	Source
1986	WHO CGM Database
2012	WHO CGM Database

Tonga – Wasting (WHZ)

D: Overall and Severe Wasting Prevalence

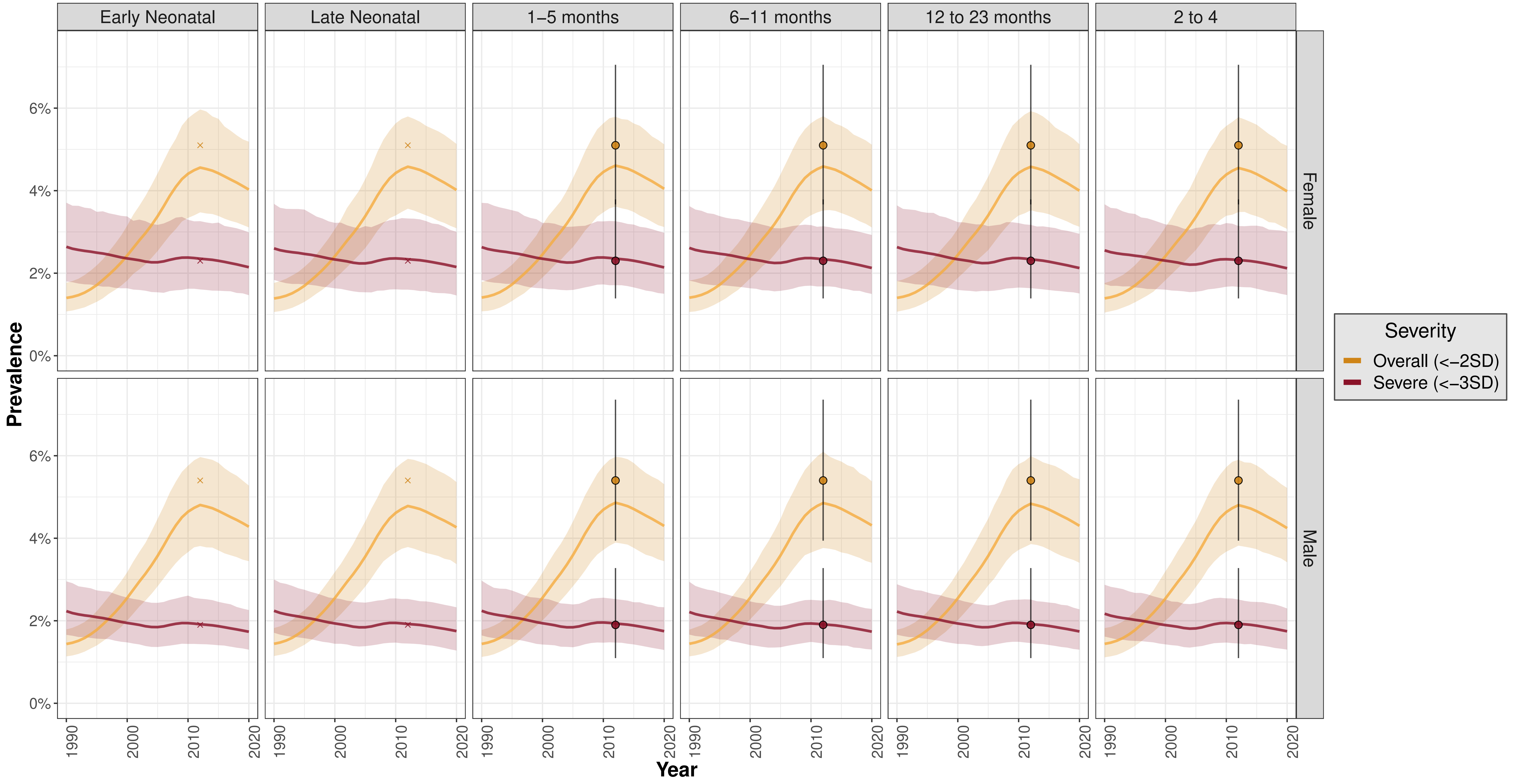

E: Transformed Mean Wasting Z Scores

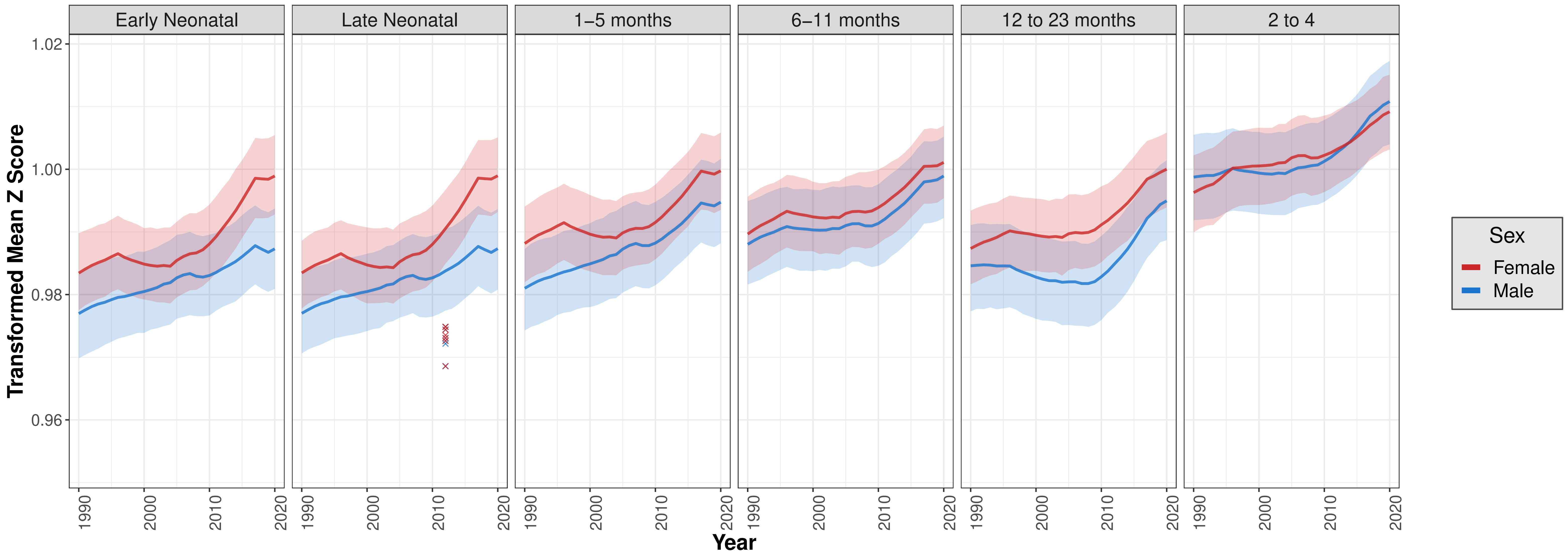

F

Year	Source
1986	WHO CGM Database
2012	WHO CGM Database

Tonga – Underweight (WAZ)

G: Overall and Severe Underweight Prevalence

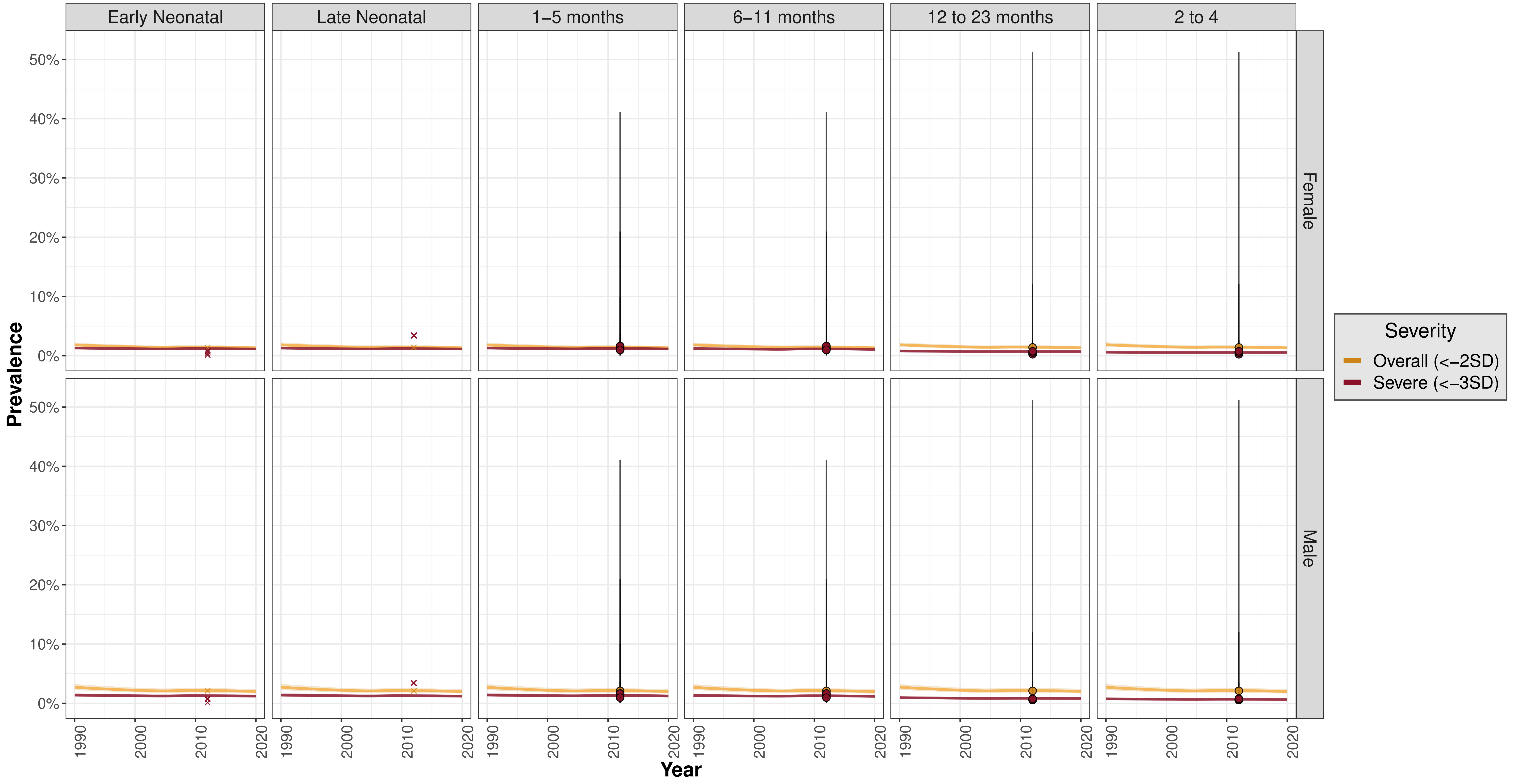

H: Transformed Mean Underweight Z Scores

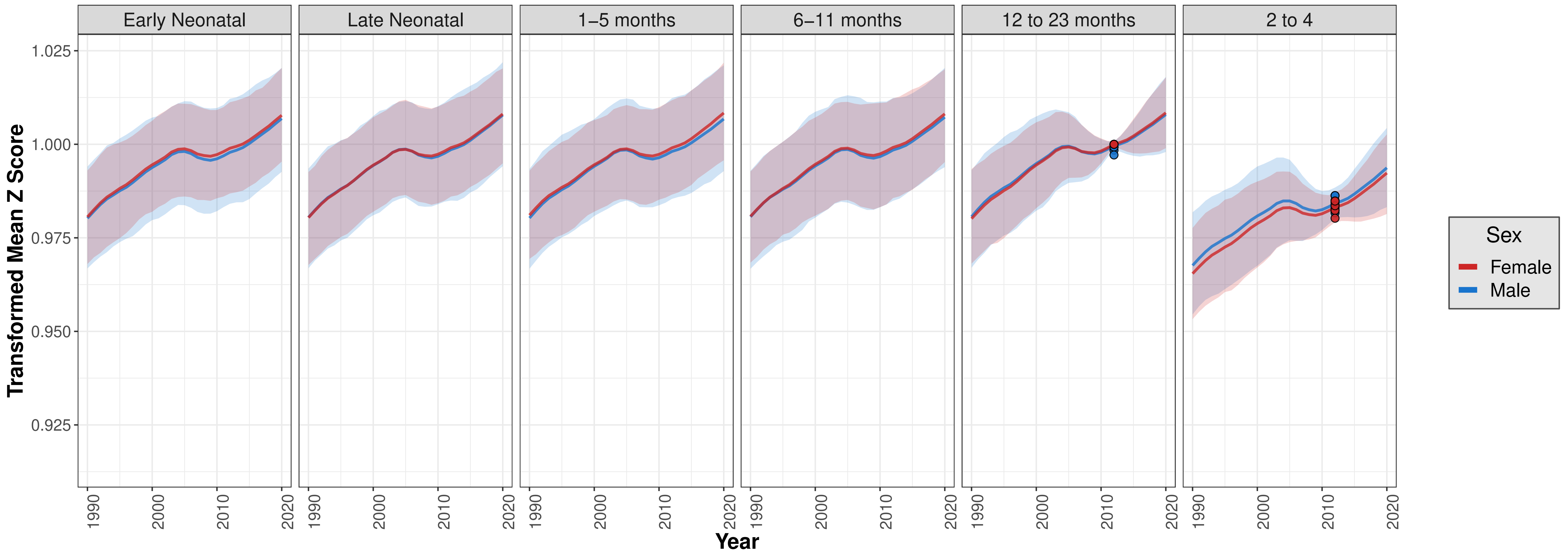

I

Year	Source
2012	WHO CGM Database

Tonga – HAZ, WHZ, and WAZ Distributions

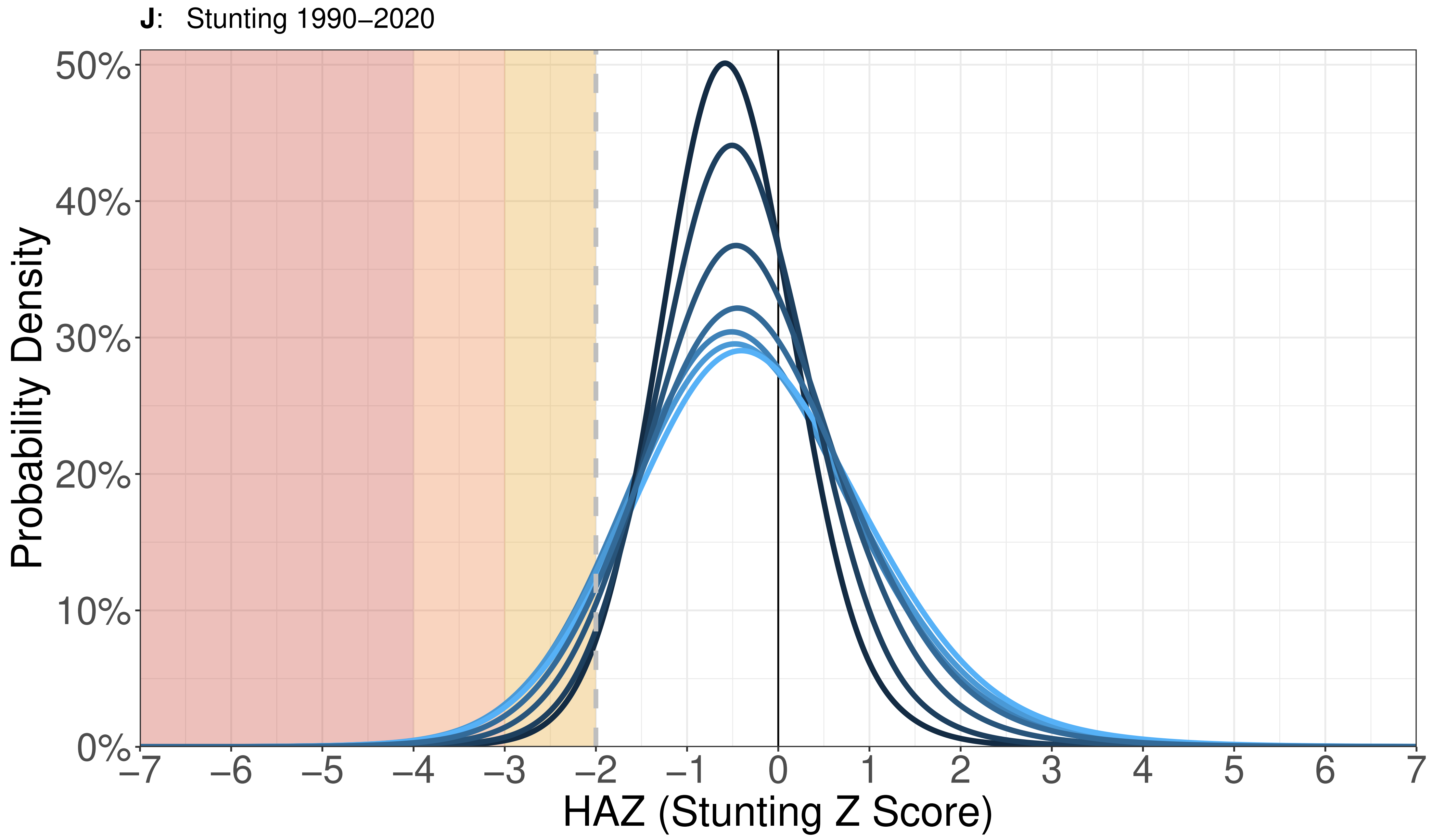

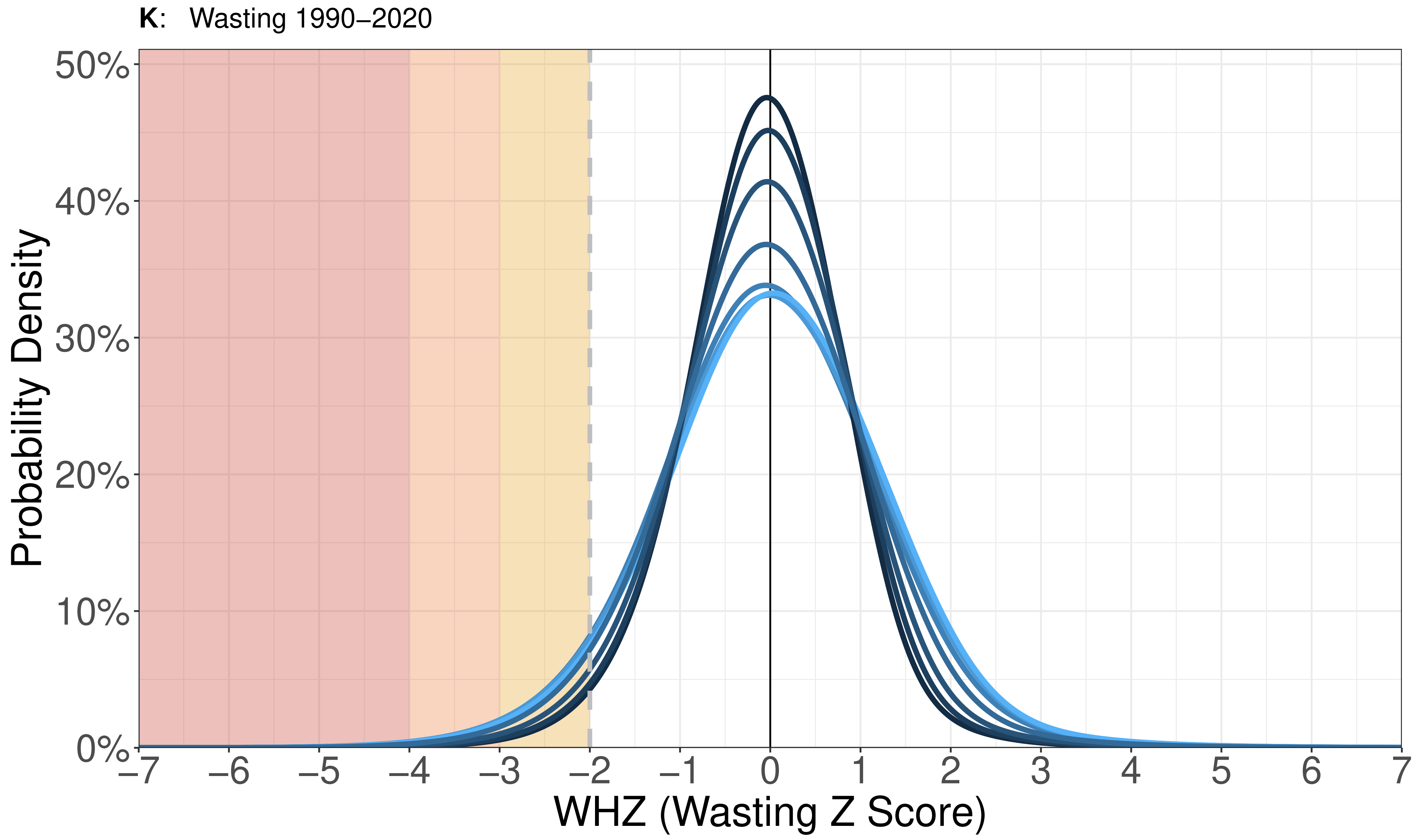

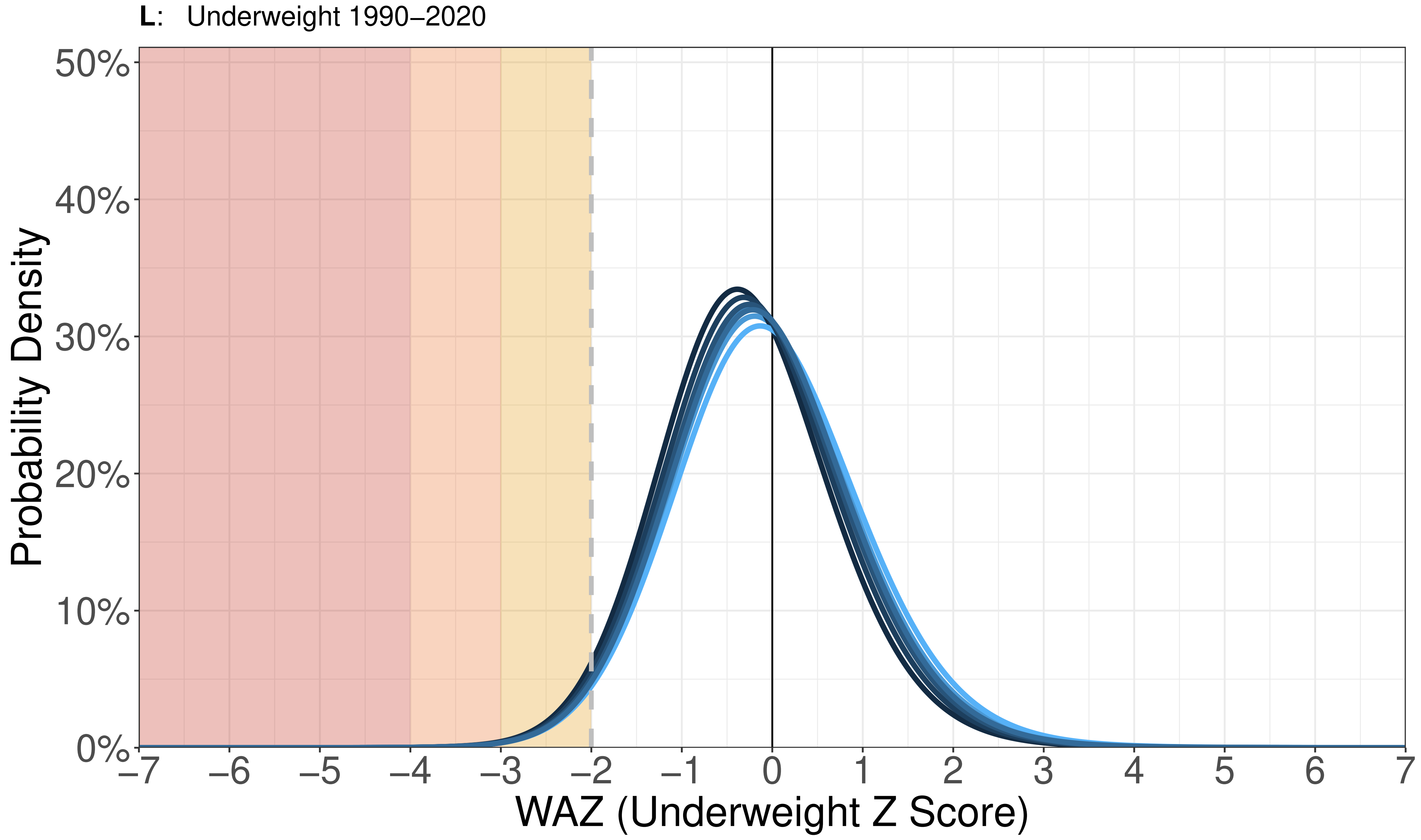

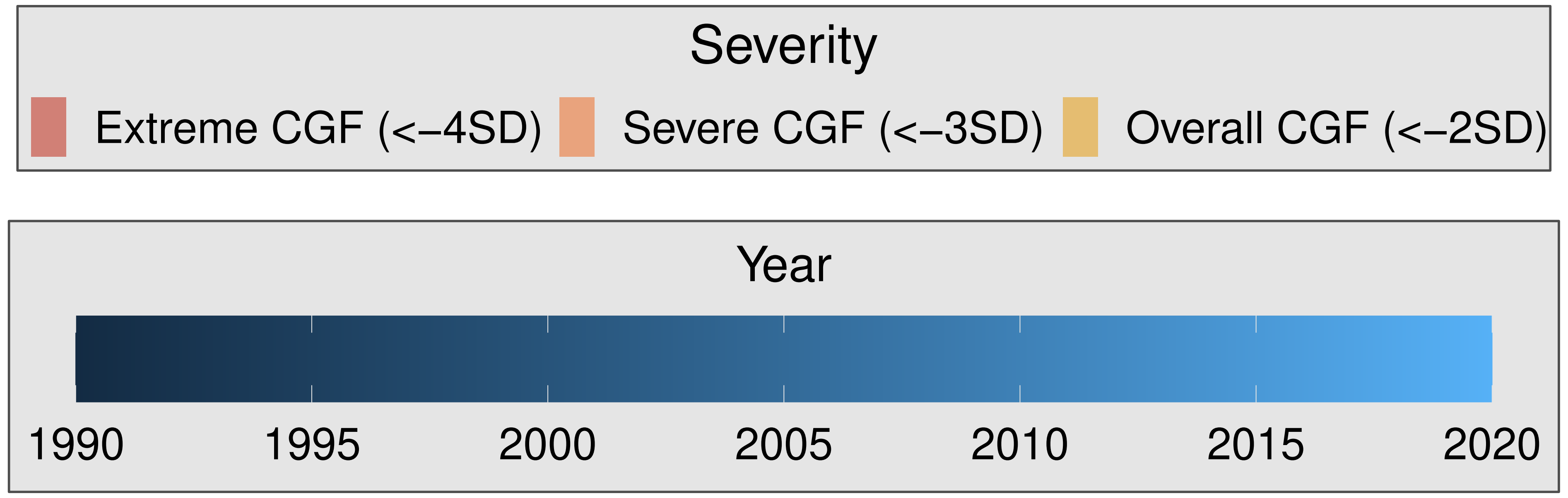

American Samoa – Stunting (HAZ)

A: Overall and Severe Stunting Prevalence

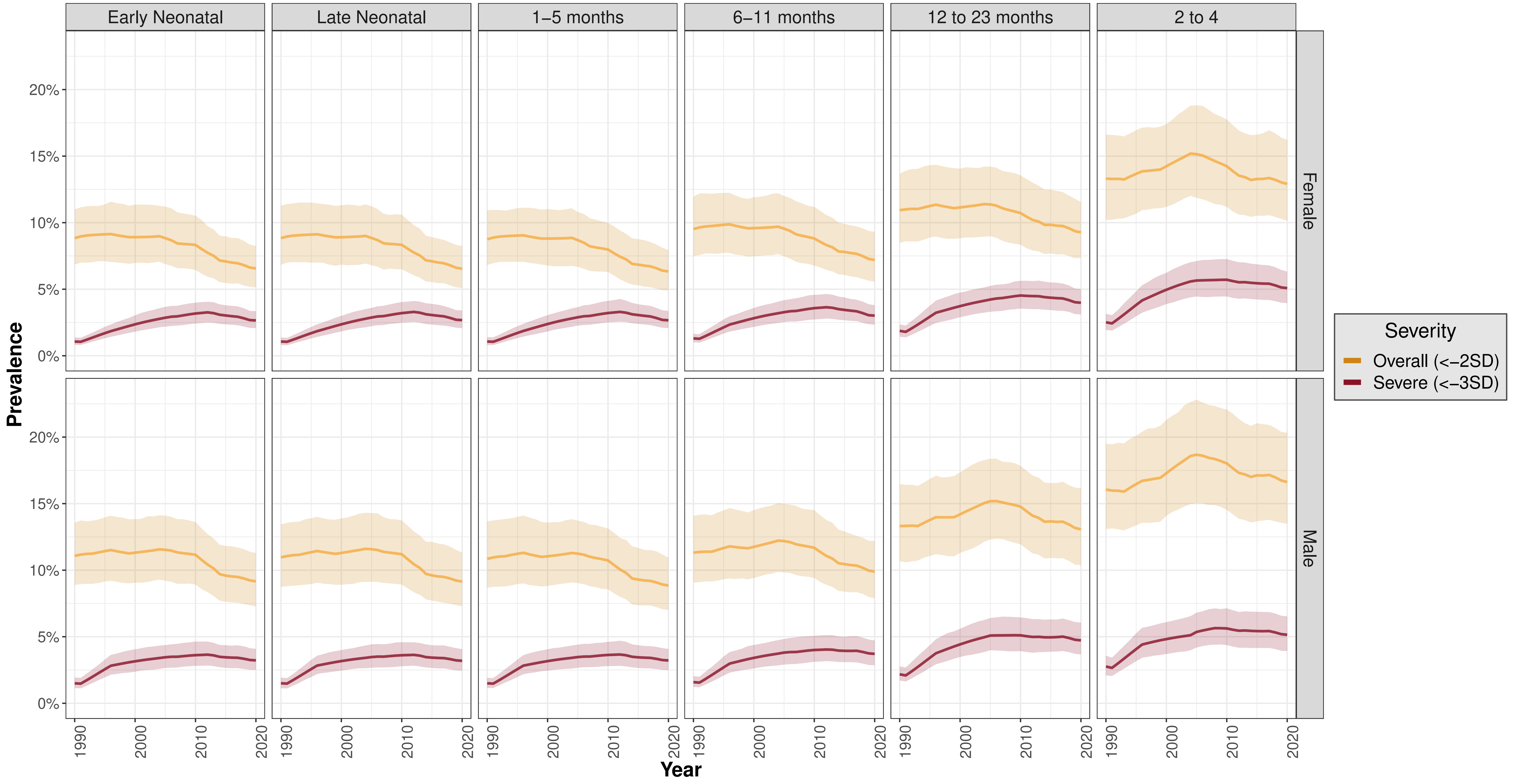

B: Transformed Mean Stunting Z Scores

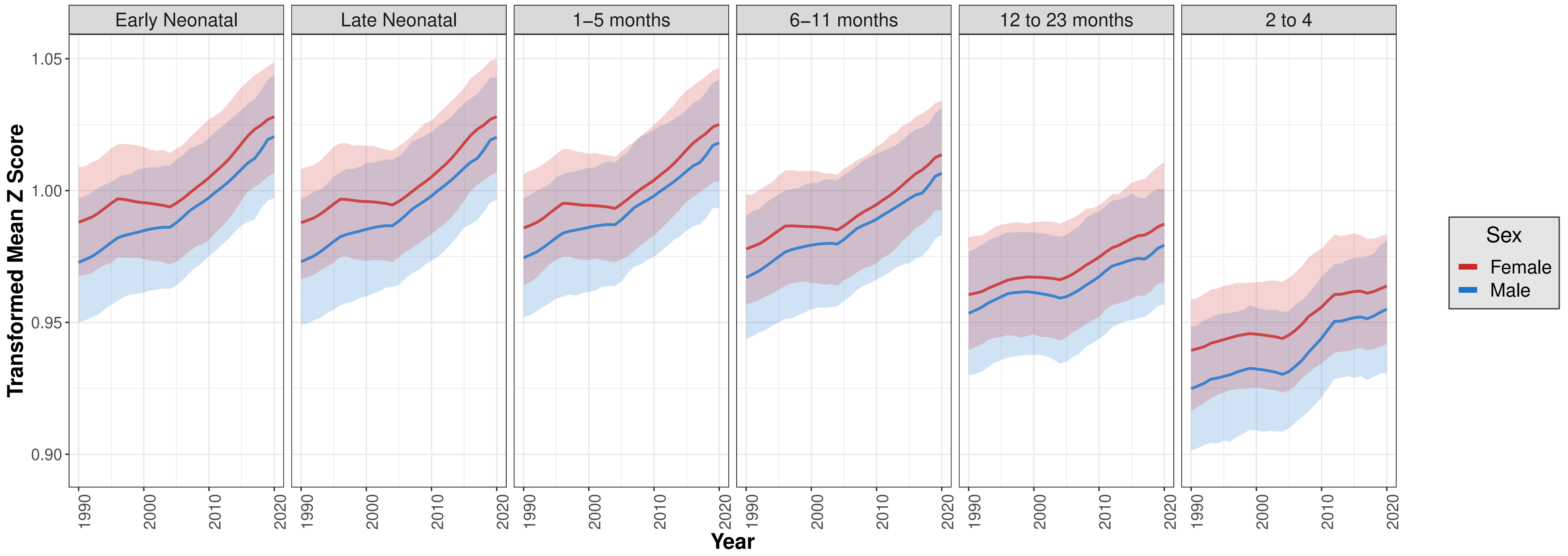

C

Source
No sources for this location

American Samoa – Wasting (WHZ)

D: Overall and Severe Wasting Prevalence

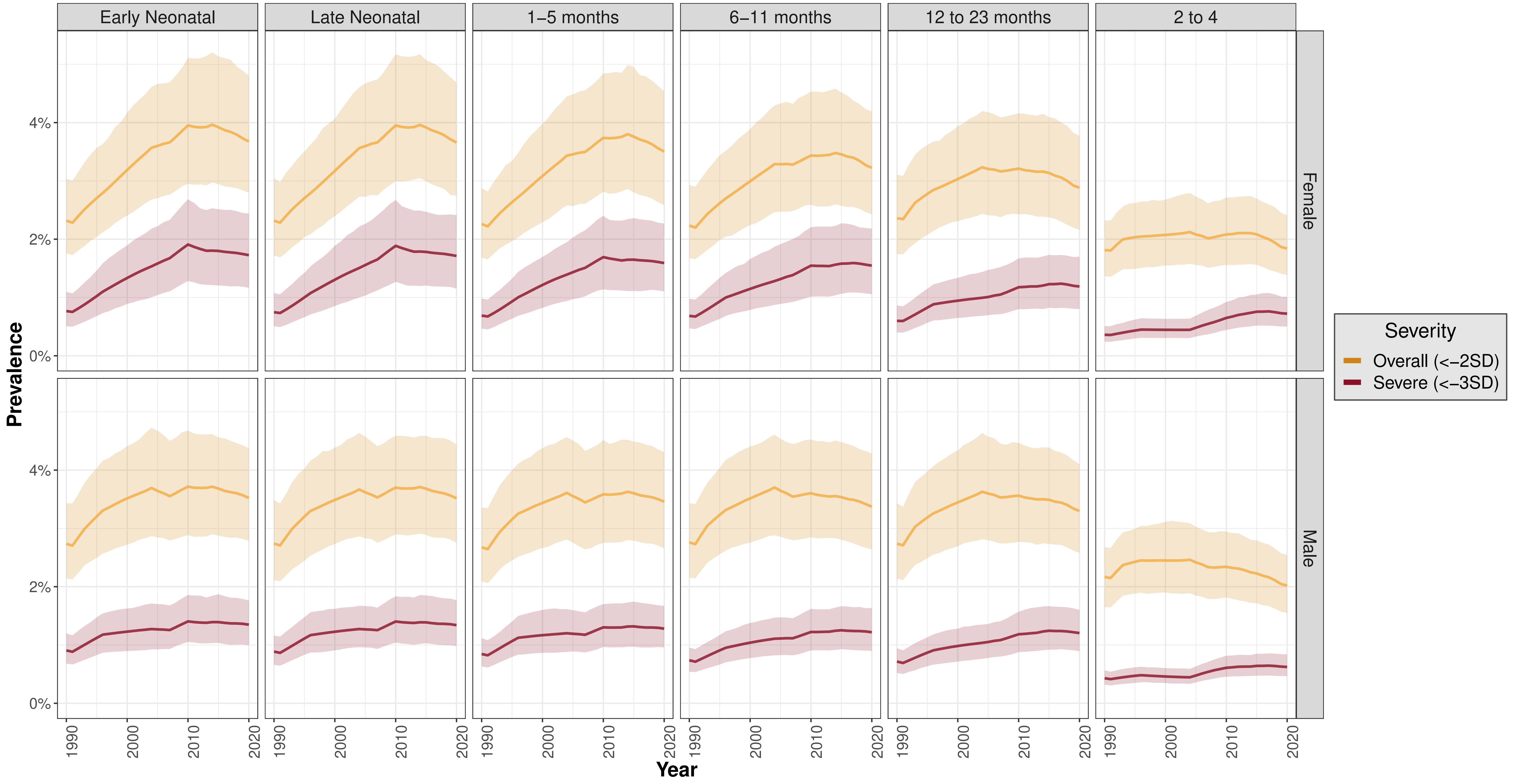

E: Transformed Mean Wasting Z Scores

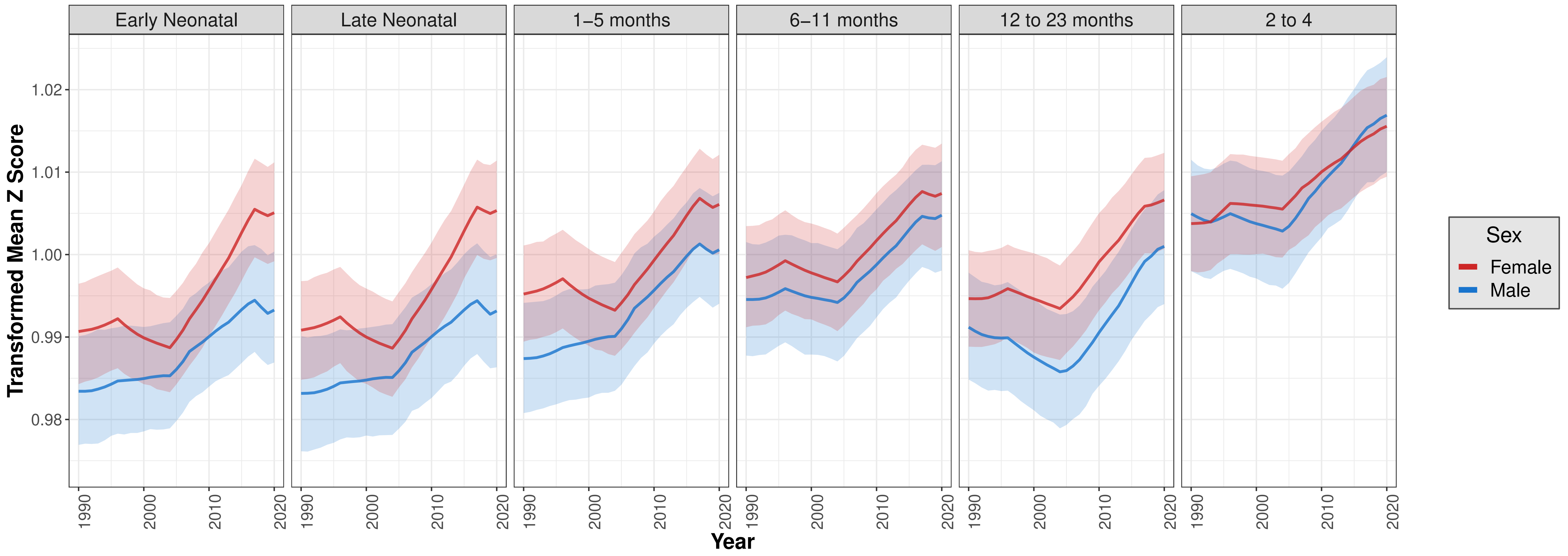

F

Source
No sources for this location

American Samoa – Underweight (WAZ)

G: Overall and Severe Underweight Prevalence

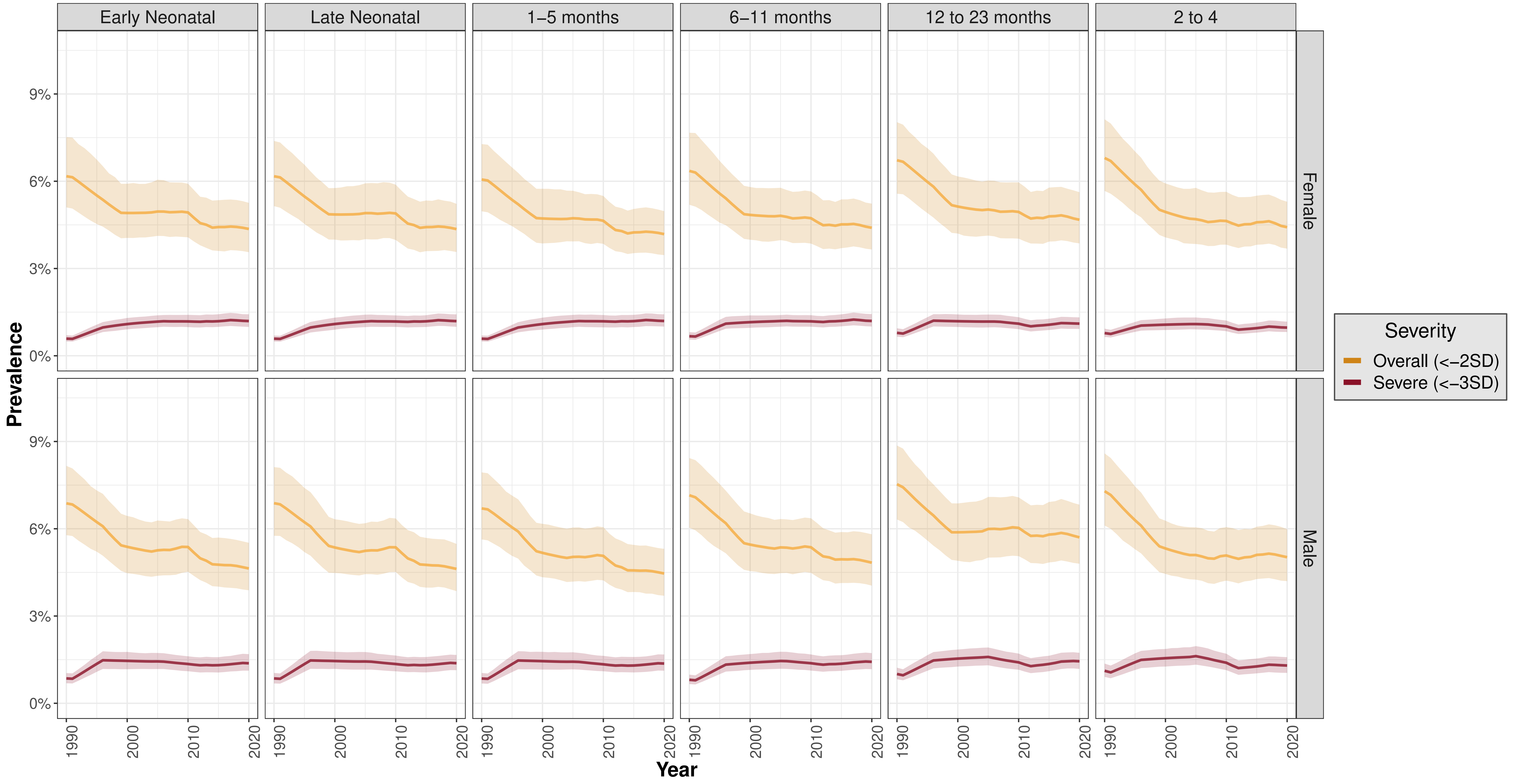

H: Transformed Mean Underweight Z Scores

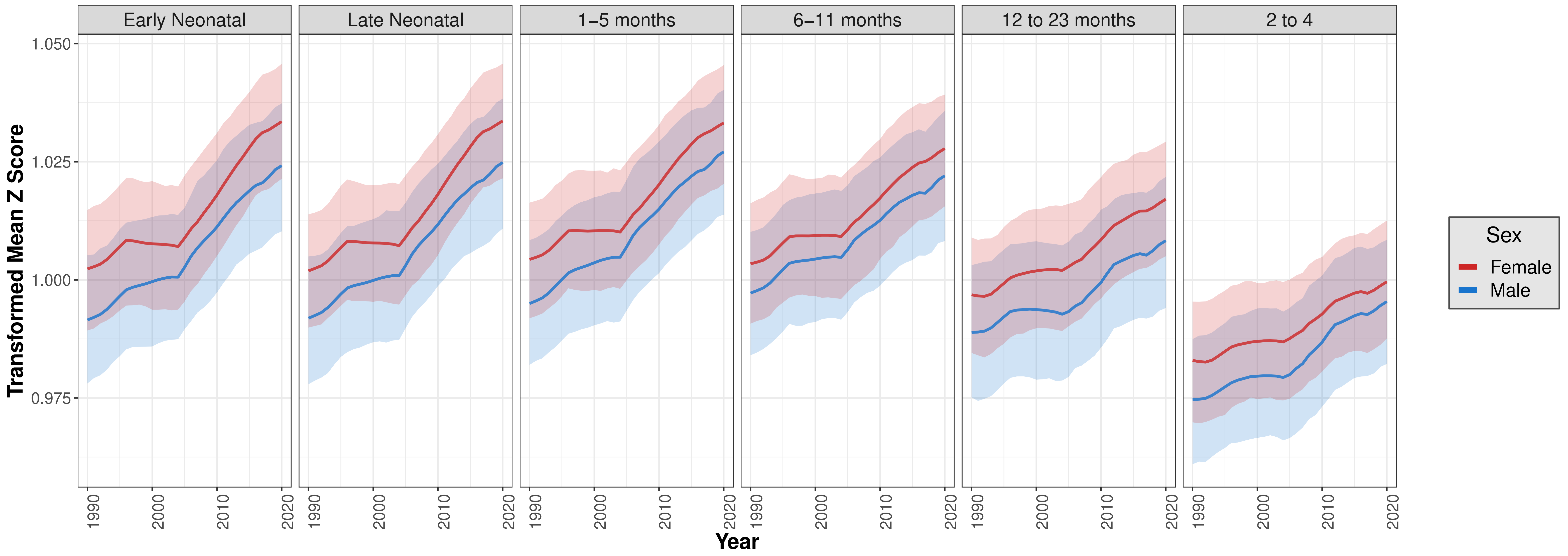

I

Source
No sources for this location

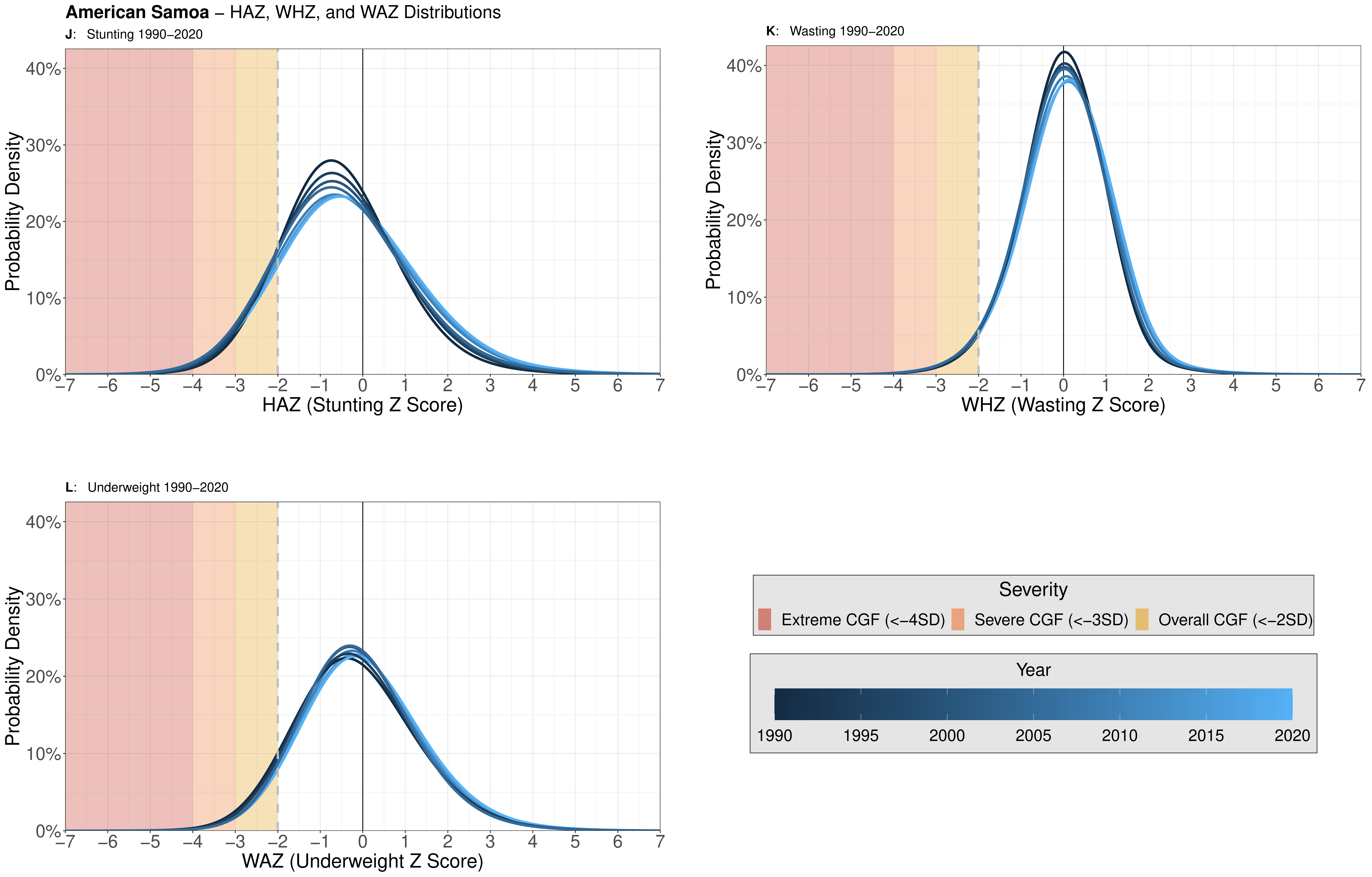
American Samoa – HAZ, WHZ, and WAZ Distributions
J: Stunting 1990–2020
K: Wasting 1990–2020
L: Underweight 1990–2020
Probability Density
0%
10%
20%
30%
40%
−7
−6
−5
−4
−3
−2
−1
0
1
2
3
4
5
6
7
HAZ (Stunting Z Score)
WHZ (Wasting Z Score)
WAZ (Underweight Z Score)
Severity
Extreme CGF (<−4SD)
Severe CGF (<−3SD)
Overall CGF (<−2SD)
Year
1990
1995
2000
2005
2010
2015
2020

Vanuatu – Stunting (HAZ)

A: Overall and Severe Stunting Prevalence

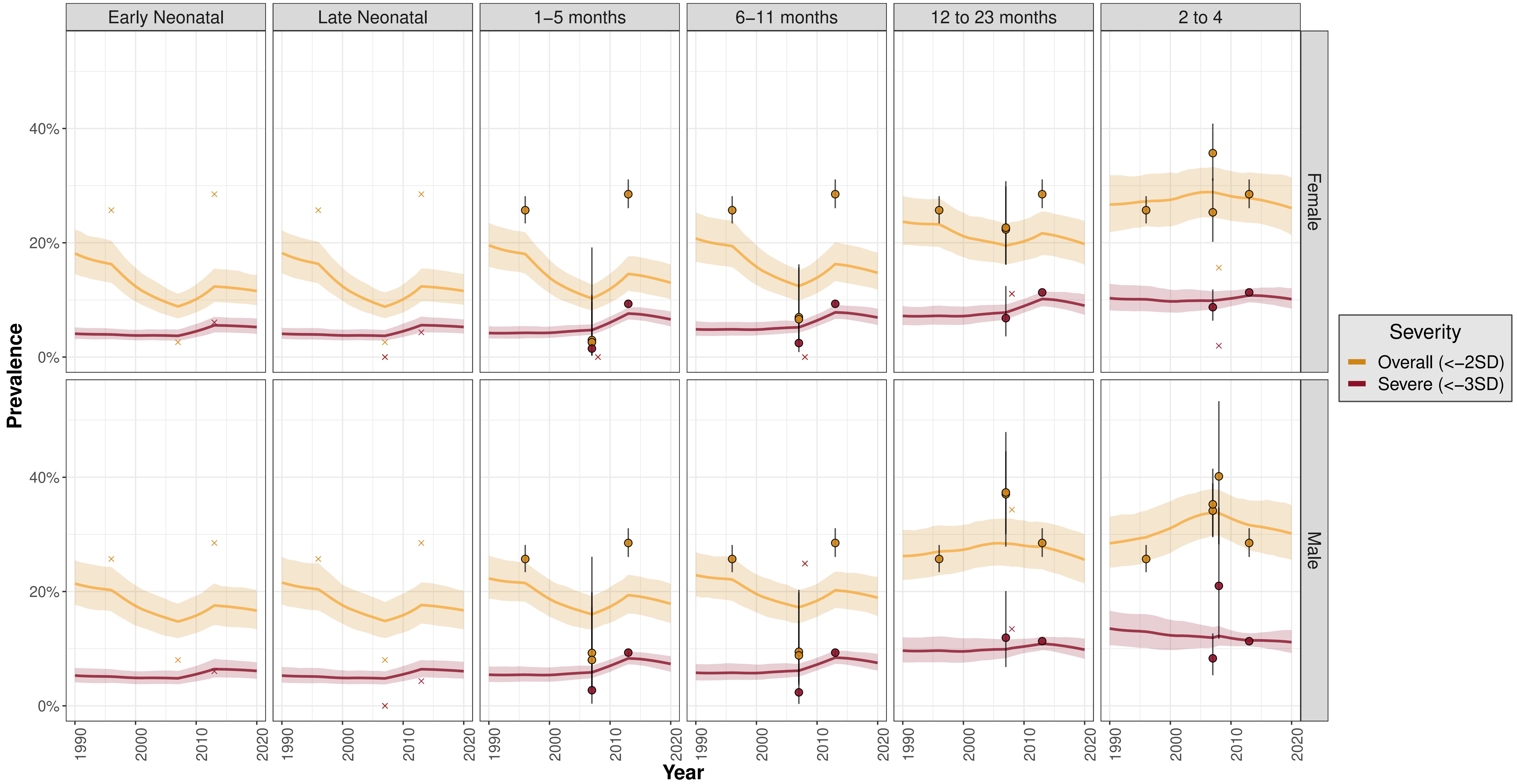

B: Transformed Mean Stunting Z Scores

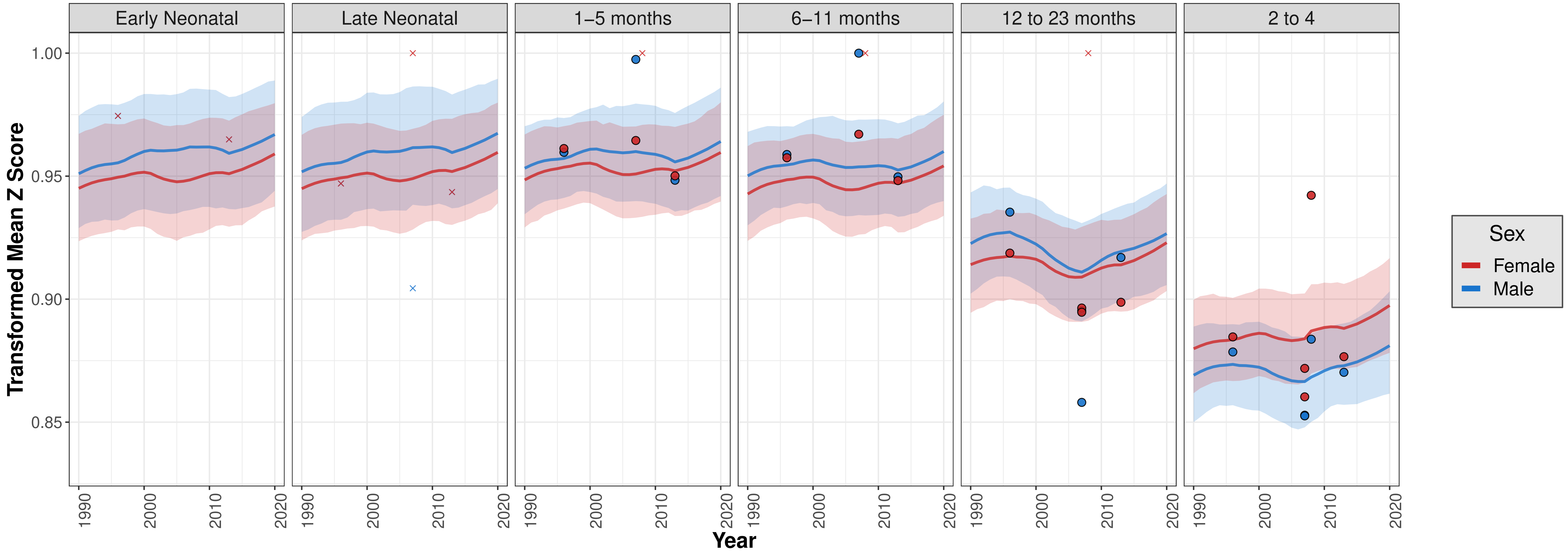

C

Year	Source
1983	WHO CGM Database
1996	WHO CGM Database
2007	MICS
2007	WHO CGM Database
2008	MICS
2013	WHO CGM Database

Vanuatu – Wasting (WHZ)

D: Overall and Severe Wasting Prevalence

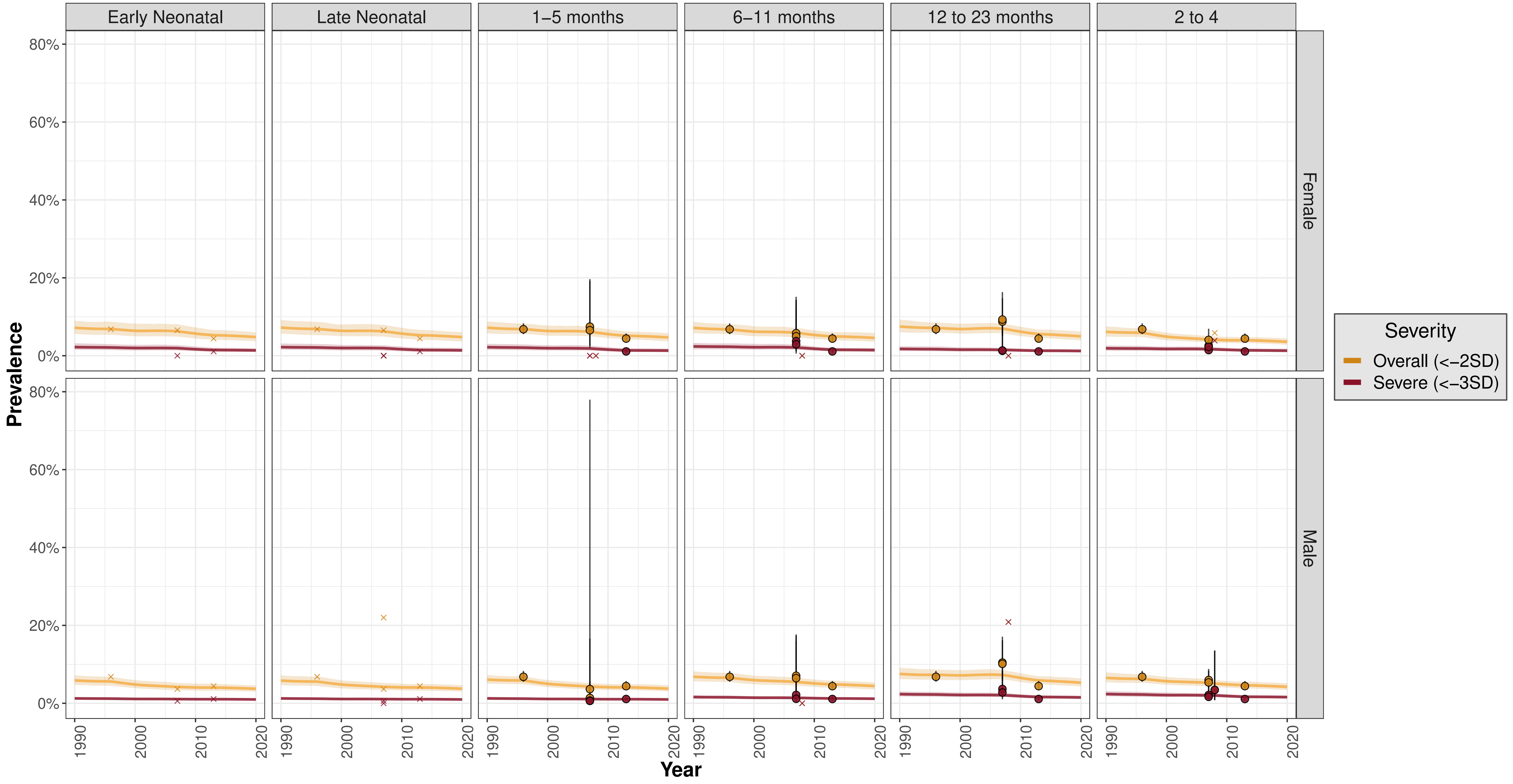

E: Transformed Mean Wasting Z Scores

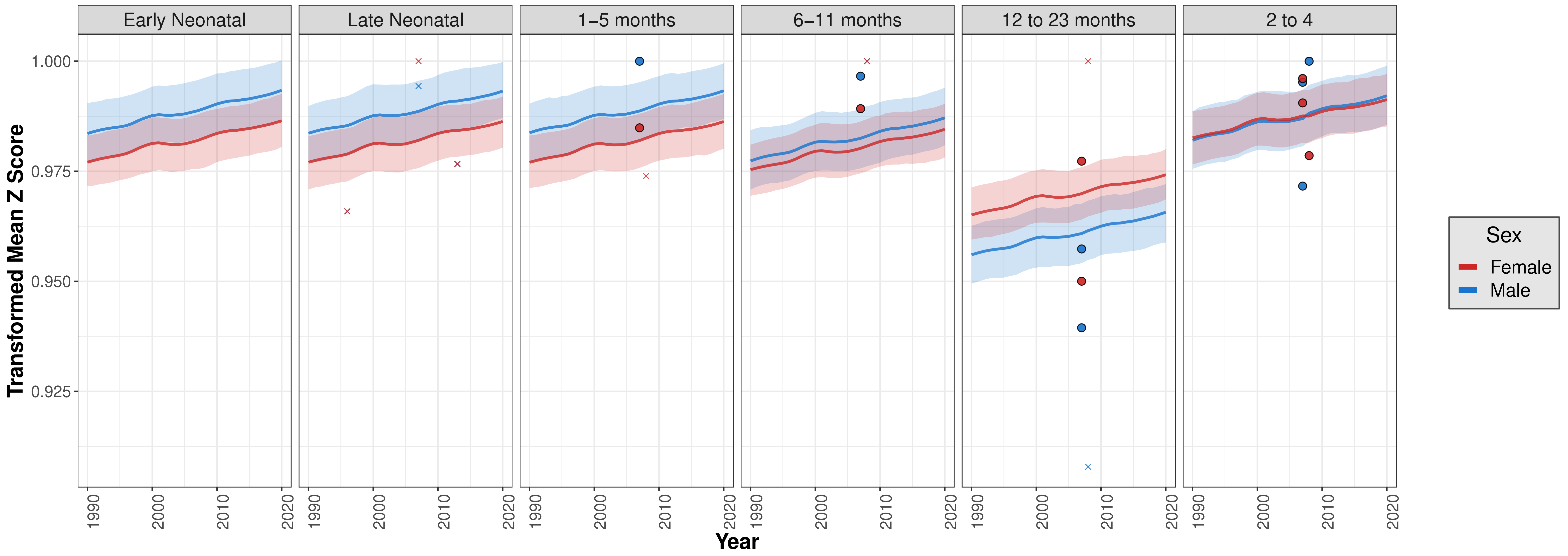

F

Year	Source
1996	WHO CGM Database
2007	MICS
2007	WHO CGM Database
2008	MICS
2013	WHO CGM Database

Vanuatu – Underweight (WAZ)

G: Overall and Severe Underweight Prevalence

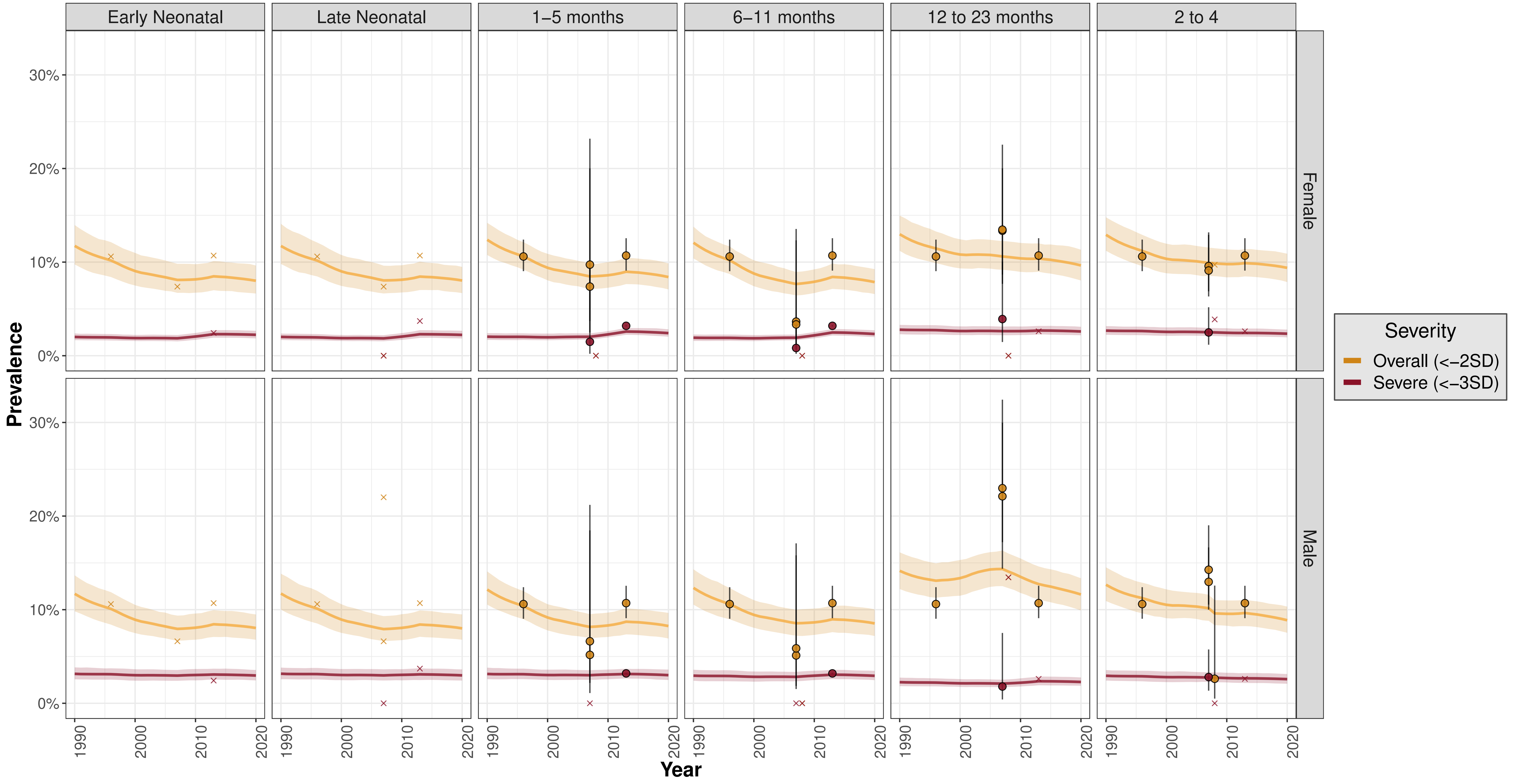

H: Transformed Mean Underweight Z Scores

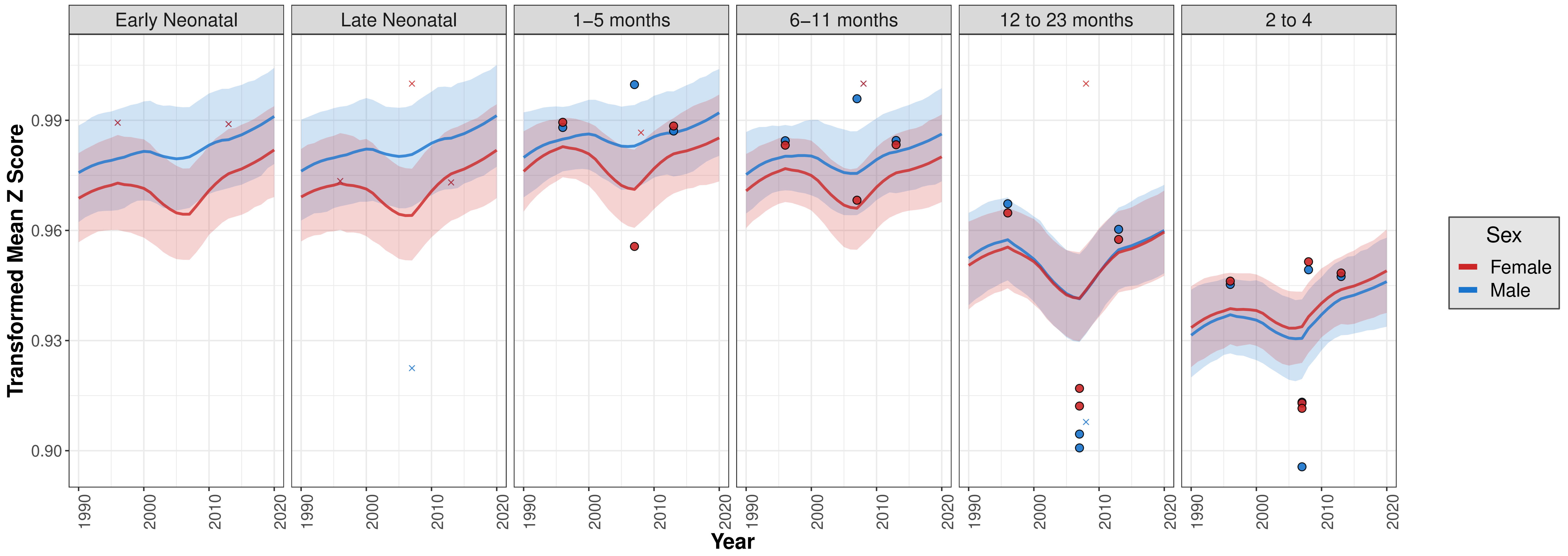

I

Year	Source
1983	WHO CGM Database
1996	WHO CGM Database
2007	MICS
2007	WHO CGM Database
2008	MICS
2013	WHO CGM Database

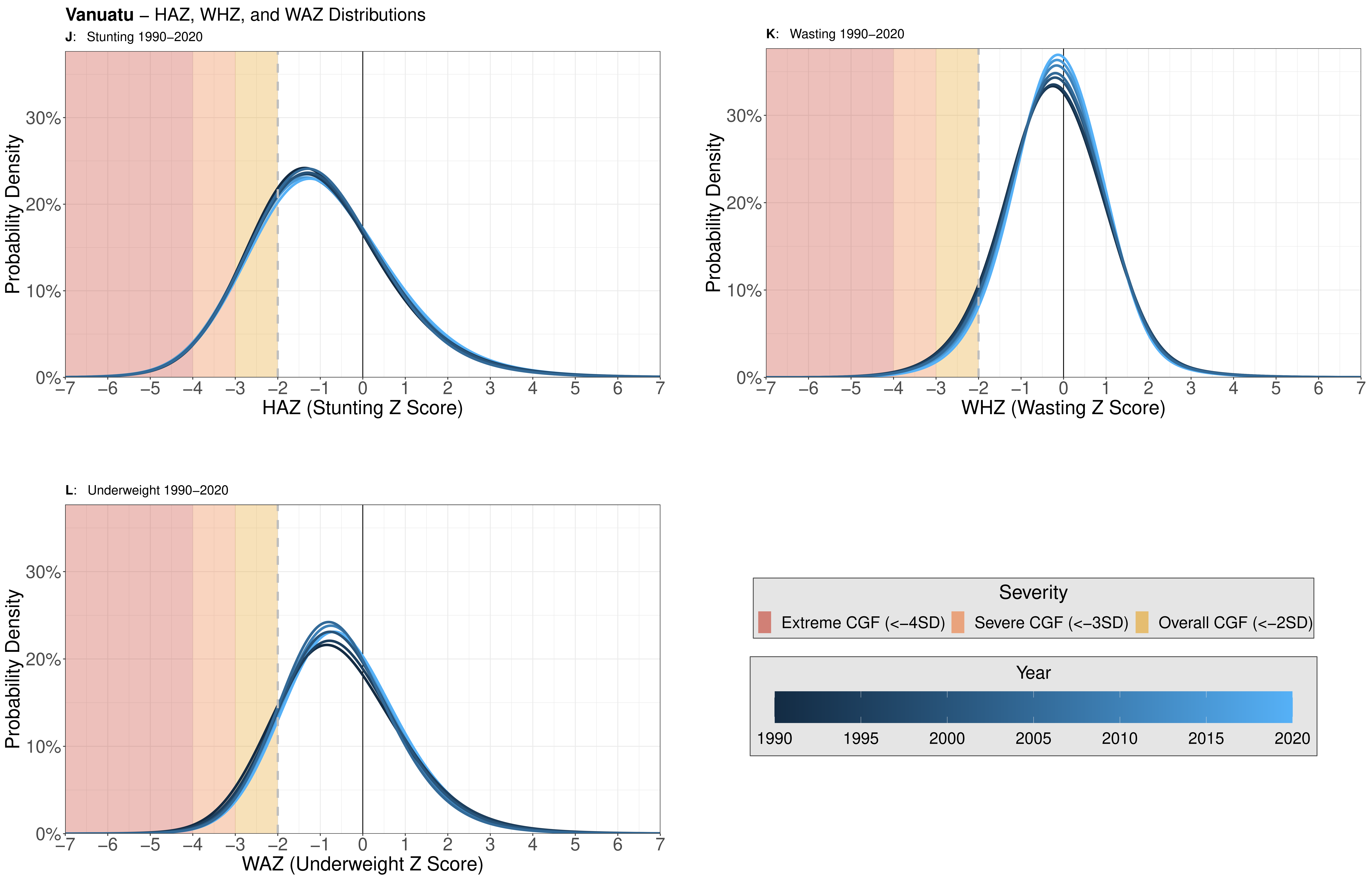
Vanuatu – HAZ, WHZ, and WAZ Distributions
J: Stunting 1990–2020
Probability Density
30%
20%
10%
0%
−7 −6 −5 −4 −3 −2 −1 0 1 2 3 4 5 6 7
HAZ (Stunting Z Score)
K: Wasting 1990–2020
Probability Density
30%
20%
10%
0%
−7 −6 −5 −4 −3 −2 −1 0 1 2 3 4 5 6 7
WHZ (Wasting Z Score)
L: Underweight 1990–2020
Probability Density
30%
20%
10%
0%
−7 −6 −5 −4 −3 −2 −1 0 1 2 3 4 5 6 7
WAZ (Underweight Z Score)
Severity
Extreme CGF (<−4SD)
Severe CGF (<−3SD)
Overall CGF (<−2SD)
Year
1990
1995
2000
2005
2010
2015
2020

Cook Islands – Stunting (HAZ)

A: Overall and Severe Stunting Prevalence

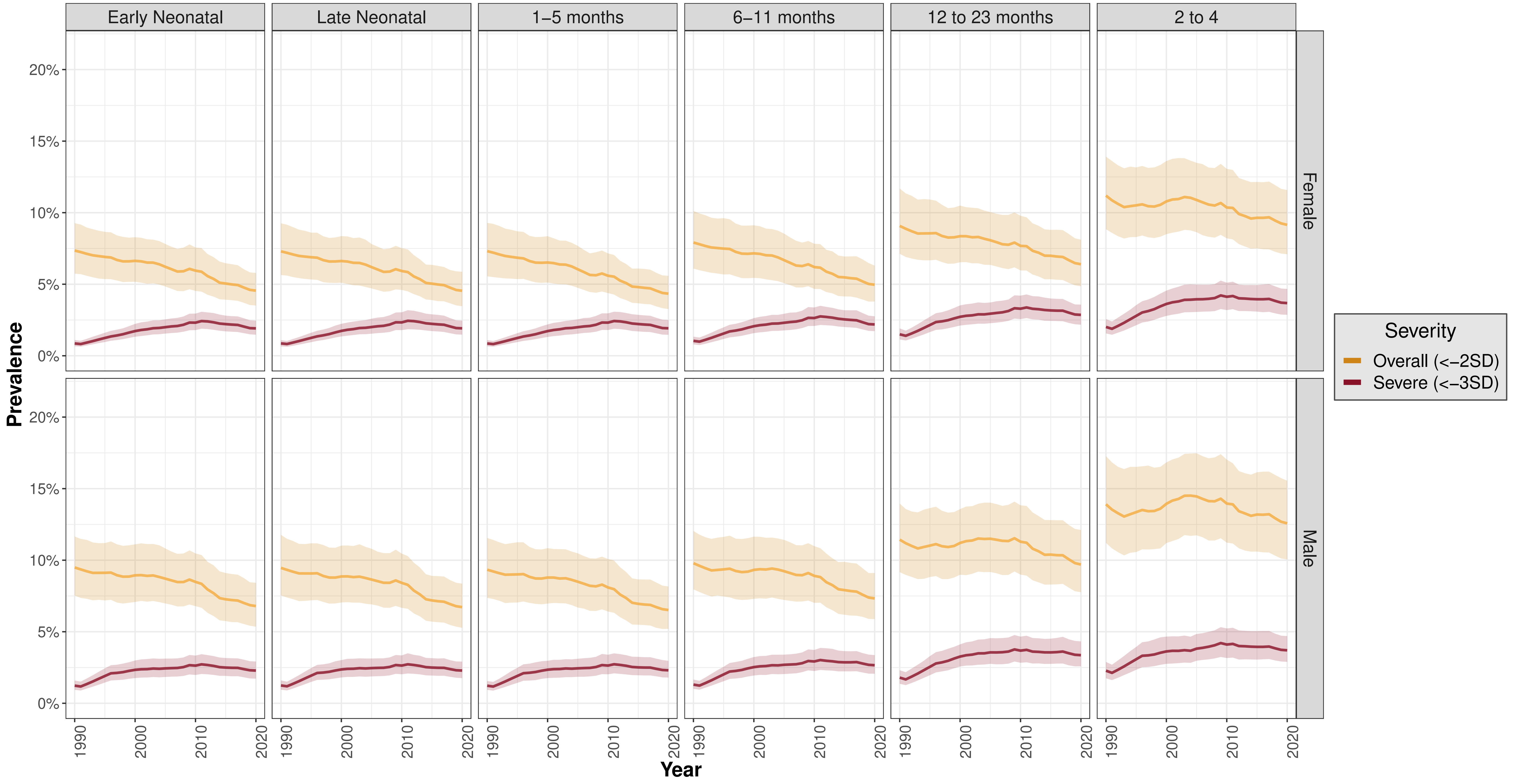

B: Transformed Mean Stunting Z Scores

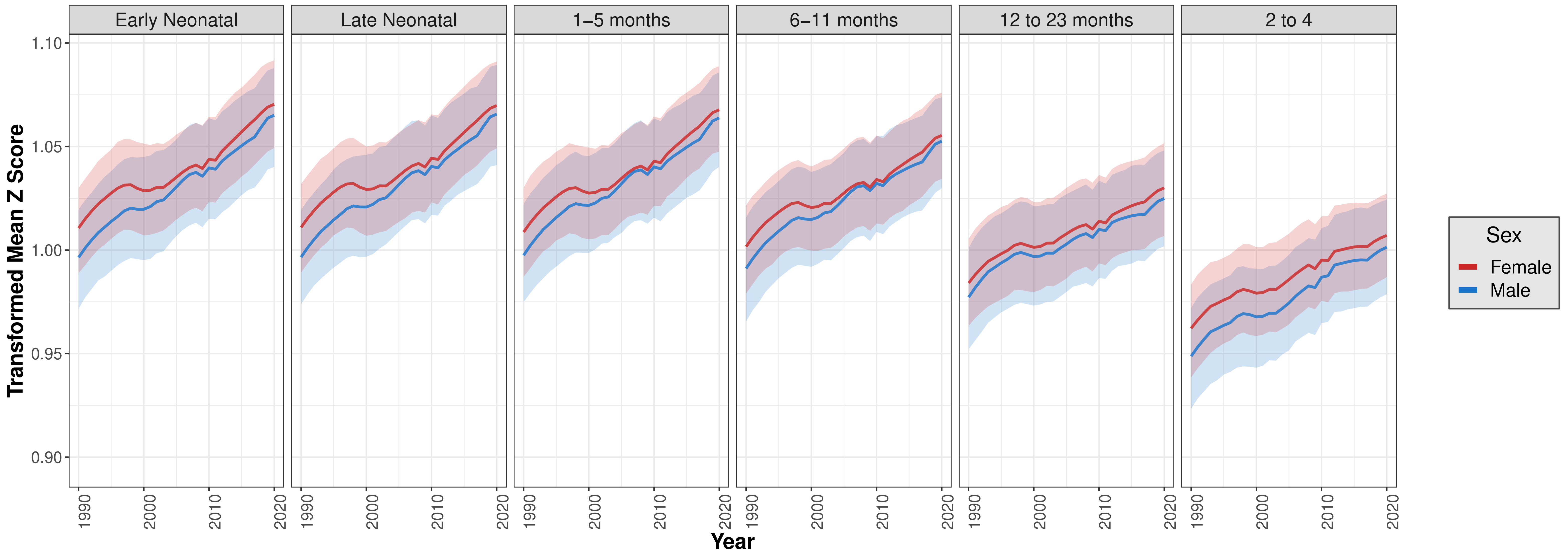

C

Source
No sources for this location

Cook Islands – Wasting (WHZ)

D: Overall and Severe Wasting Prevalence

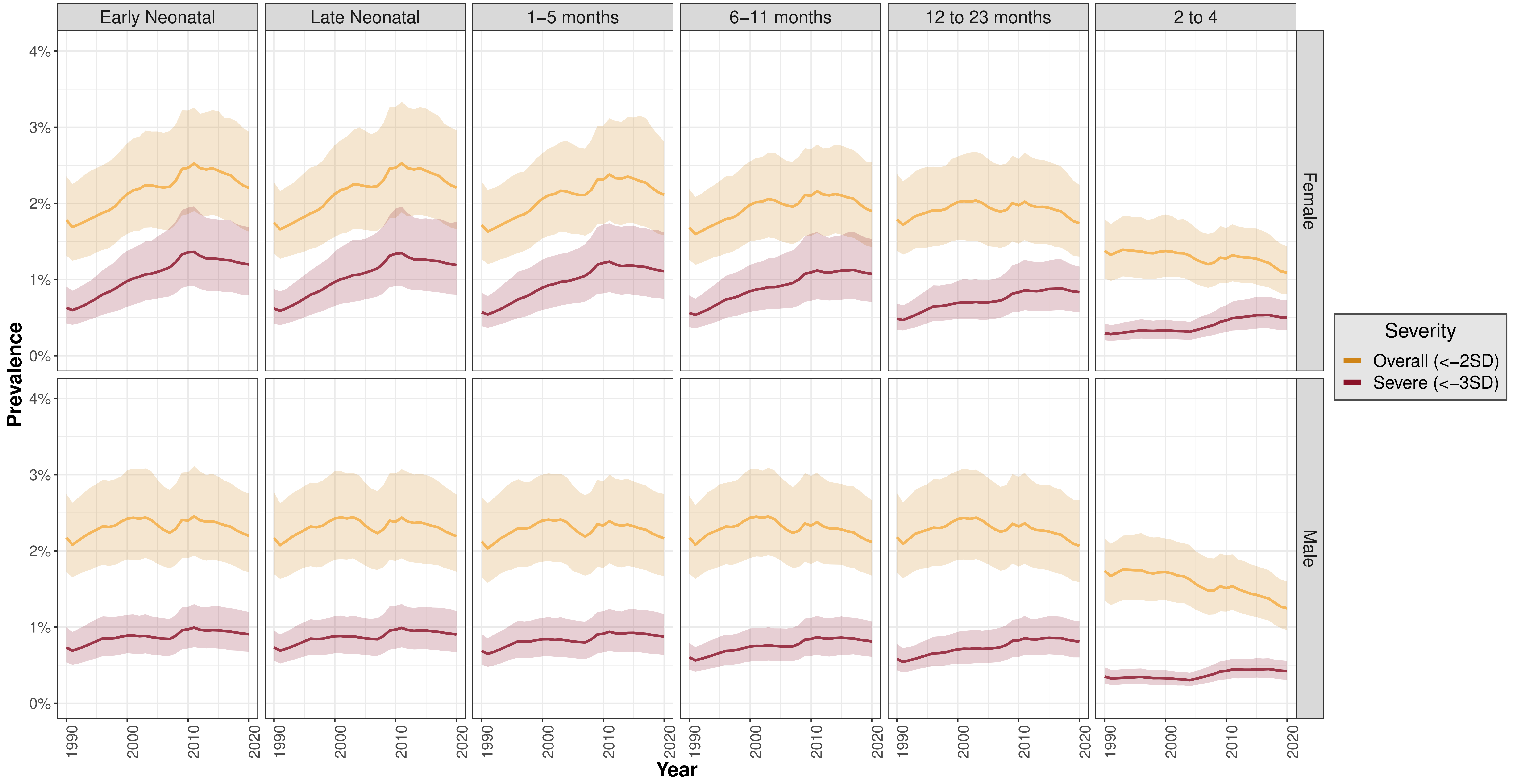

E: Transformed Mean Wasting Z Scores

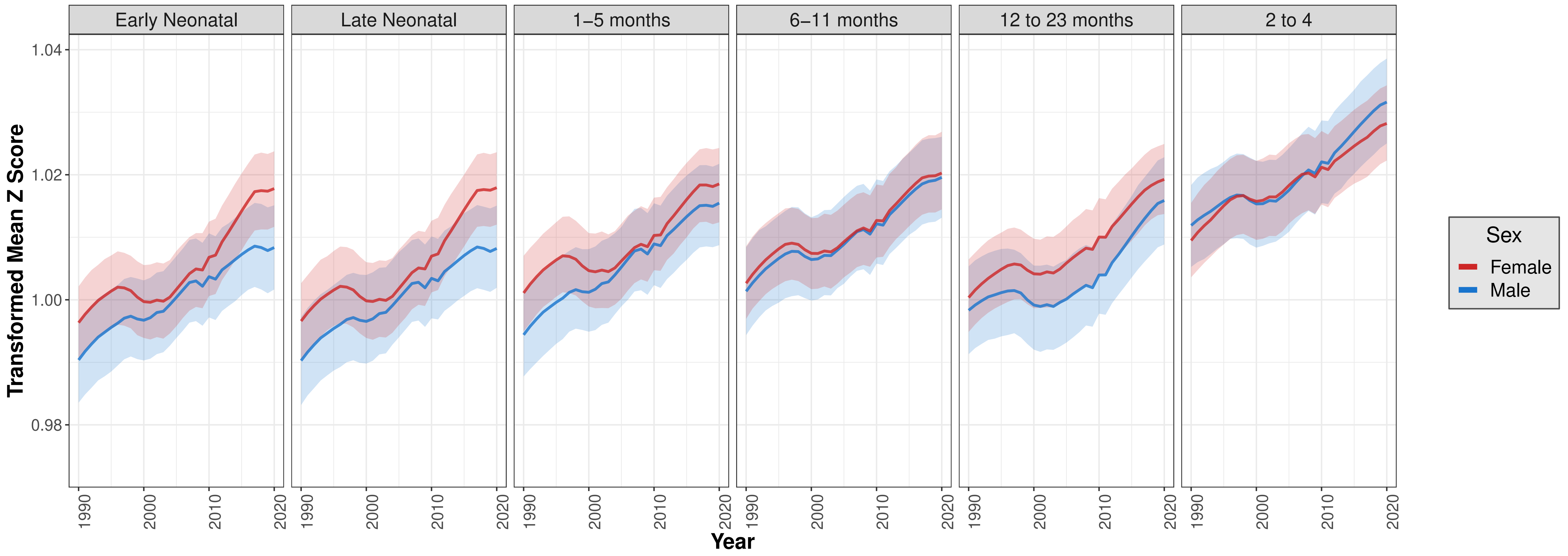

F

**Source**
No sources for this location

Cook Islands – Underweight (WAZ)

G: Overall and Severe Underweight Prevalence

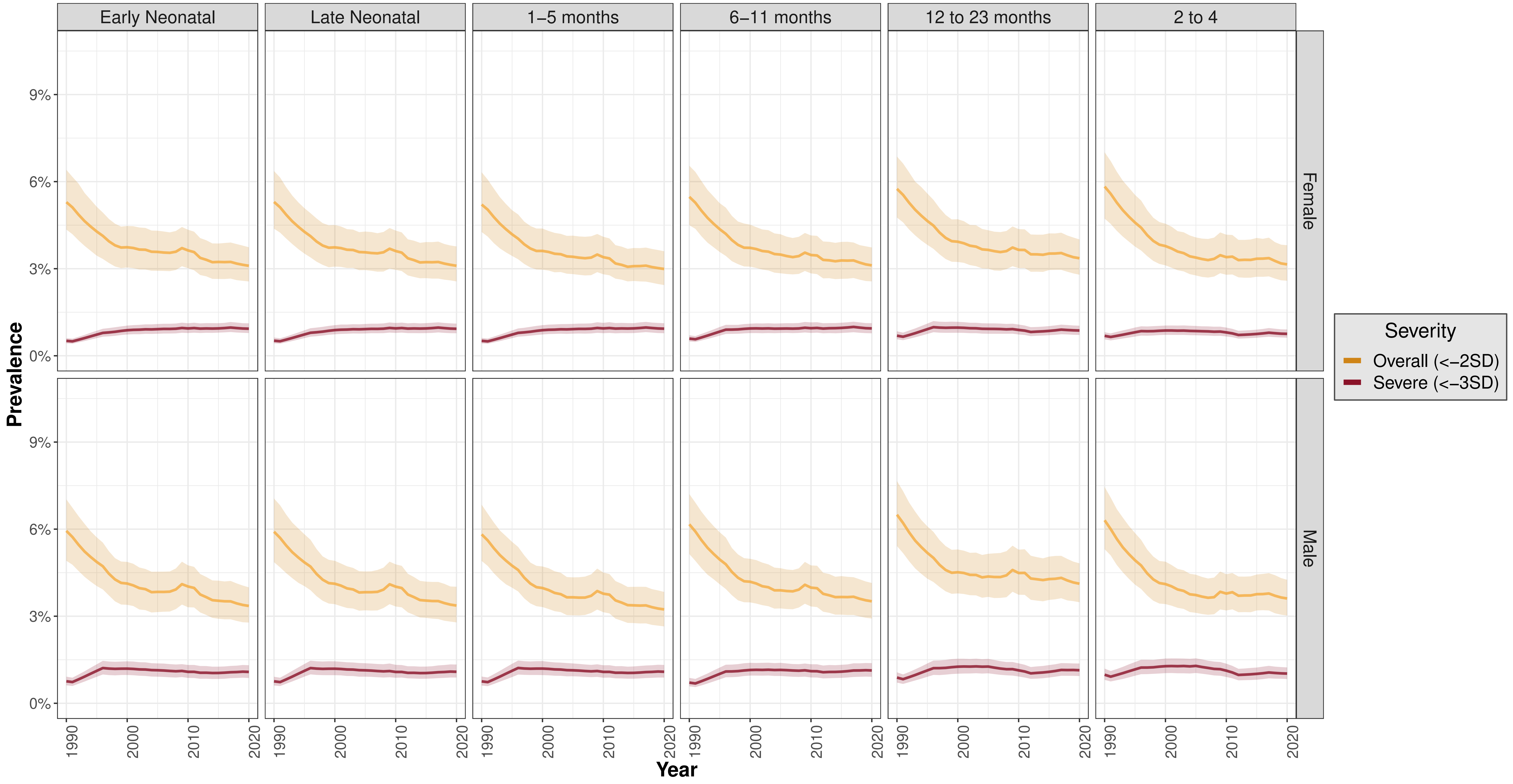

H: Transformed Mean Underweight Z Scores

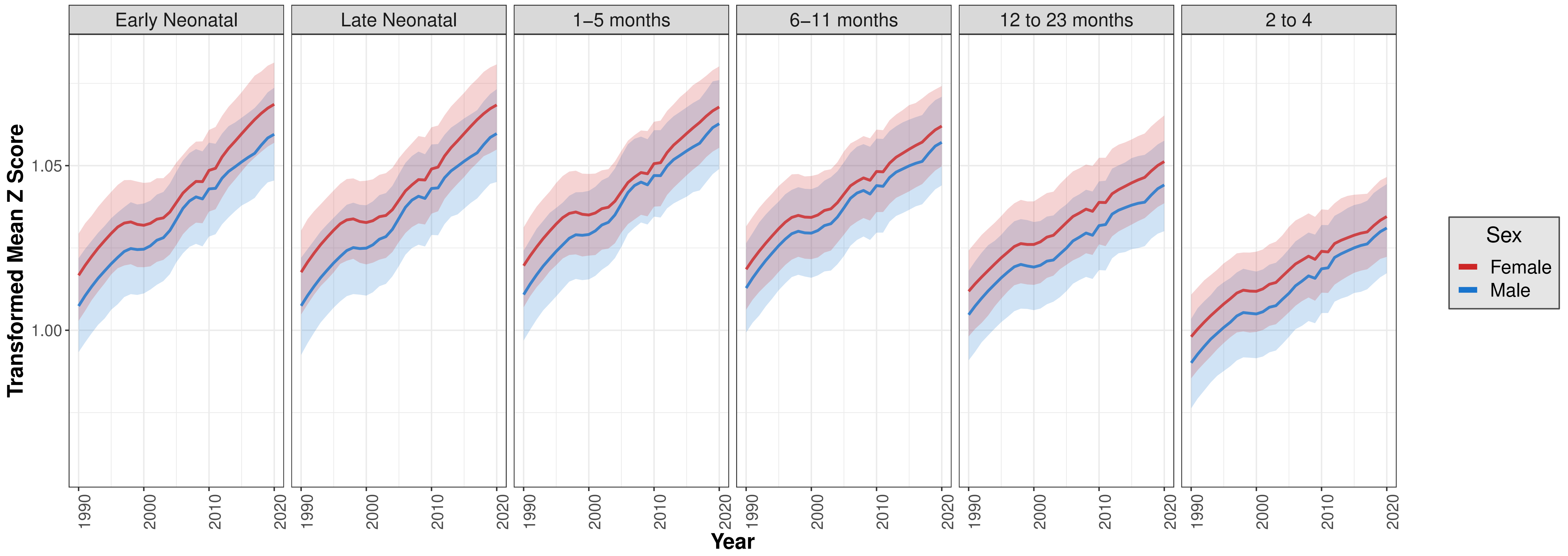

I

Source
No sources for this location

Cook Islands – HAZ, WHZ, and WAZ Distributions

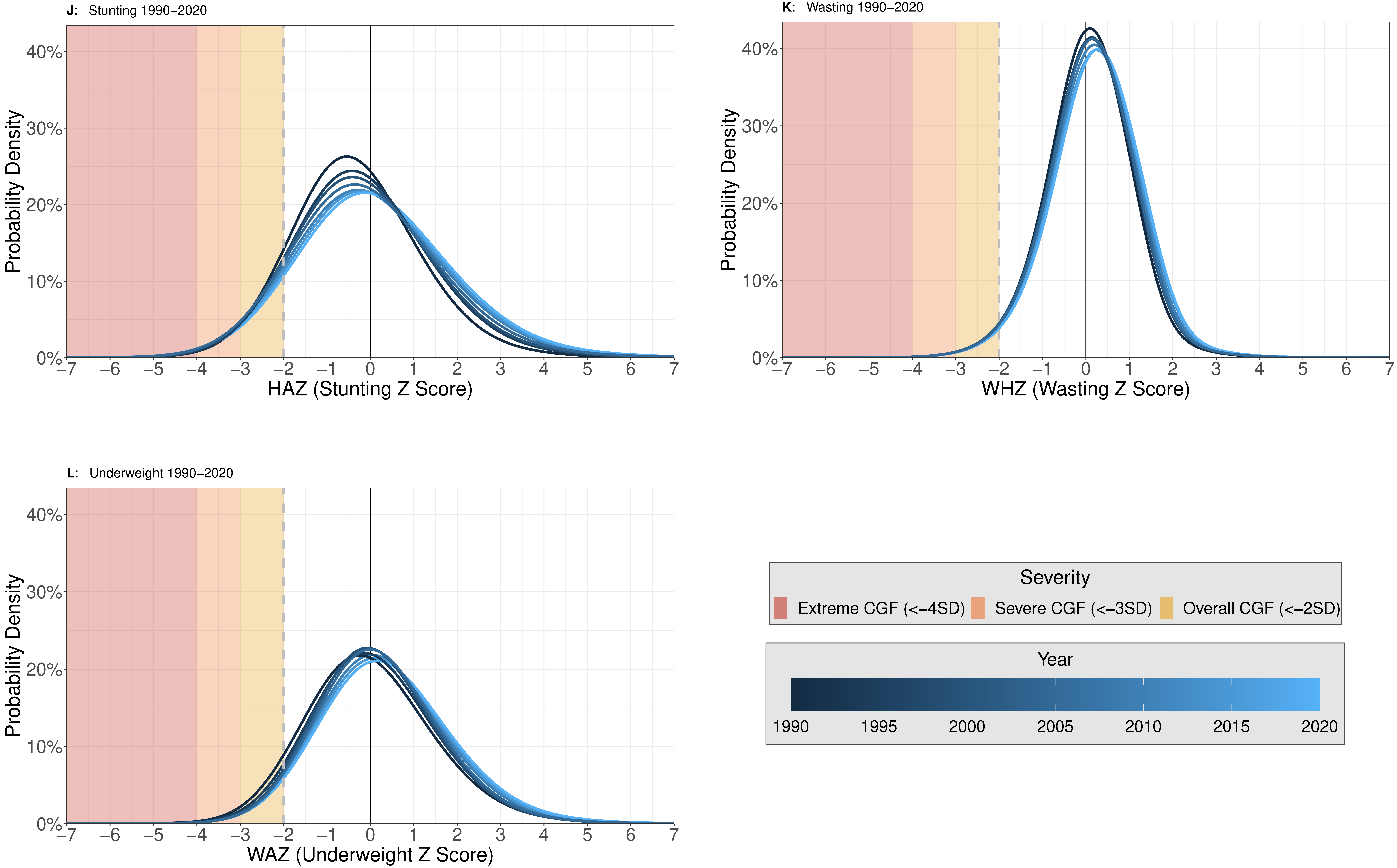

Guam – Stunting (HAZ)

A: Overall and Severe Stunting Prevalence

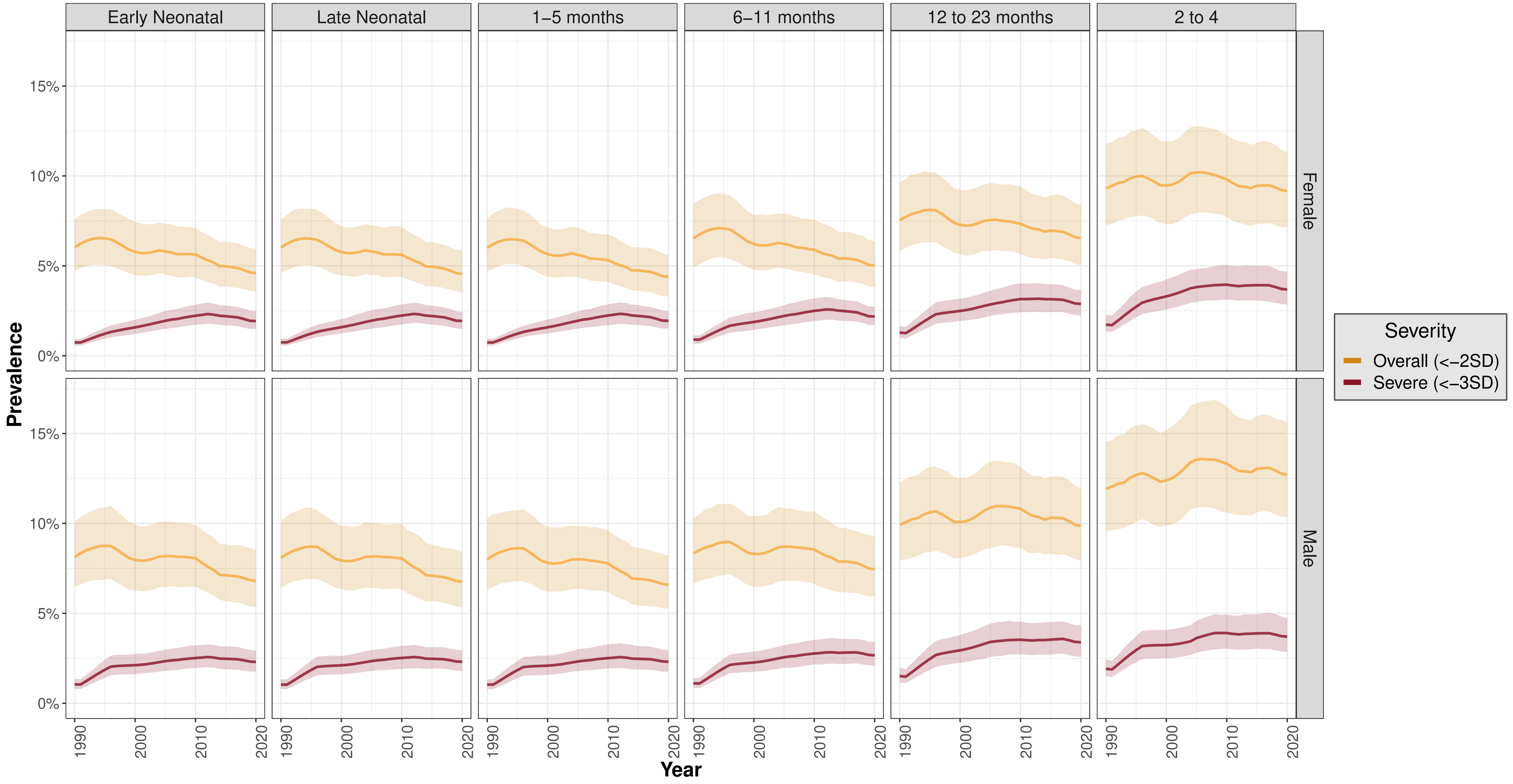

B: Transformed Mean Stunting Z Scores

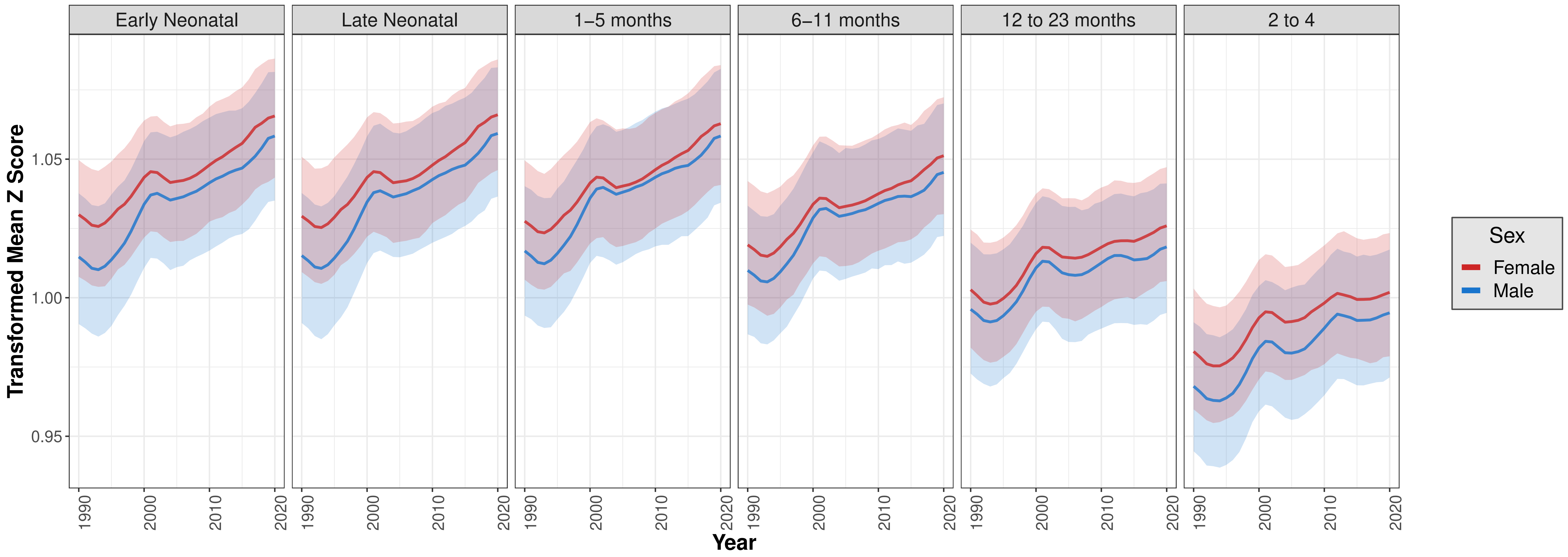

C

Source
No sources for this location

Guam – Wasting (WHZ)

D: Overall and Severe Wasting Prevalence

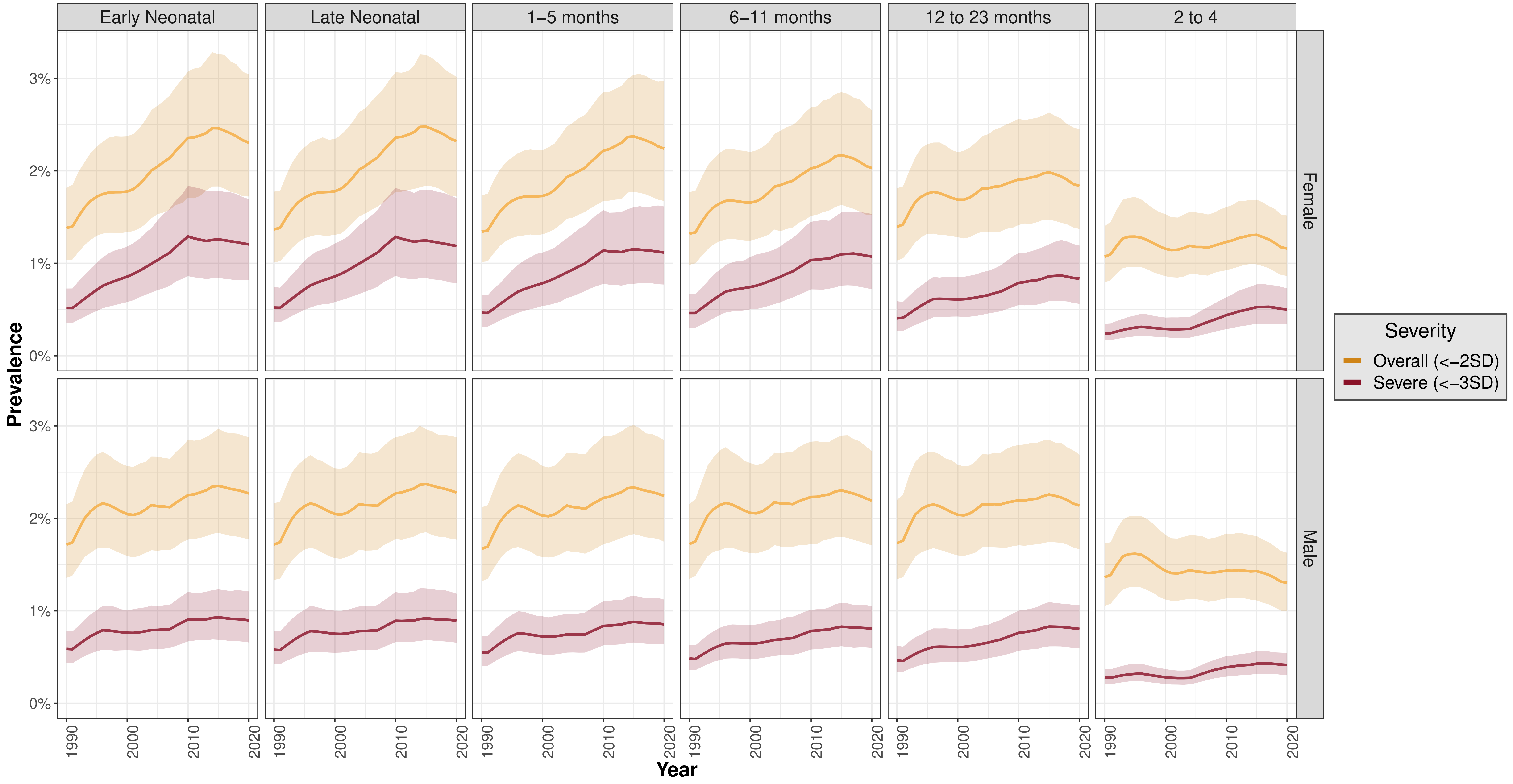

E: Transformed Mean Wasting Z Scores

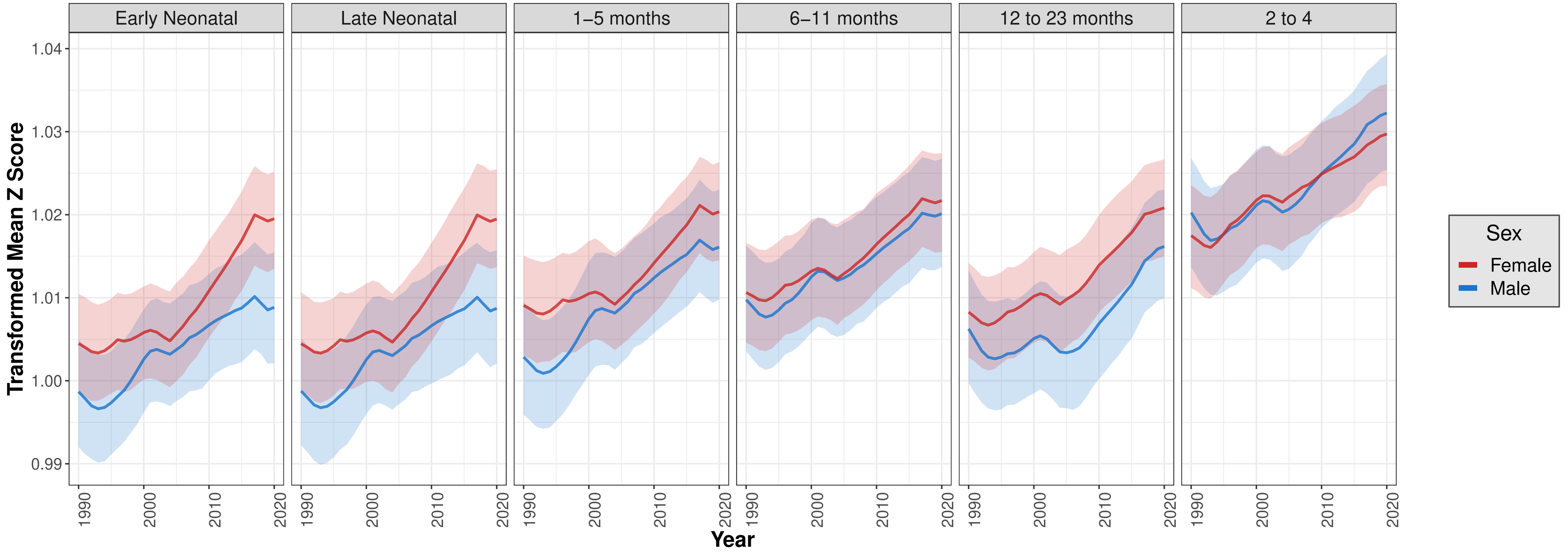

F

Source
No sources for this location

Guam – Underweight (WAZ)

G: Overall and Severe Underweight Prevalence

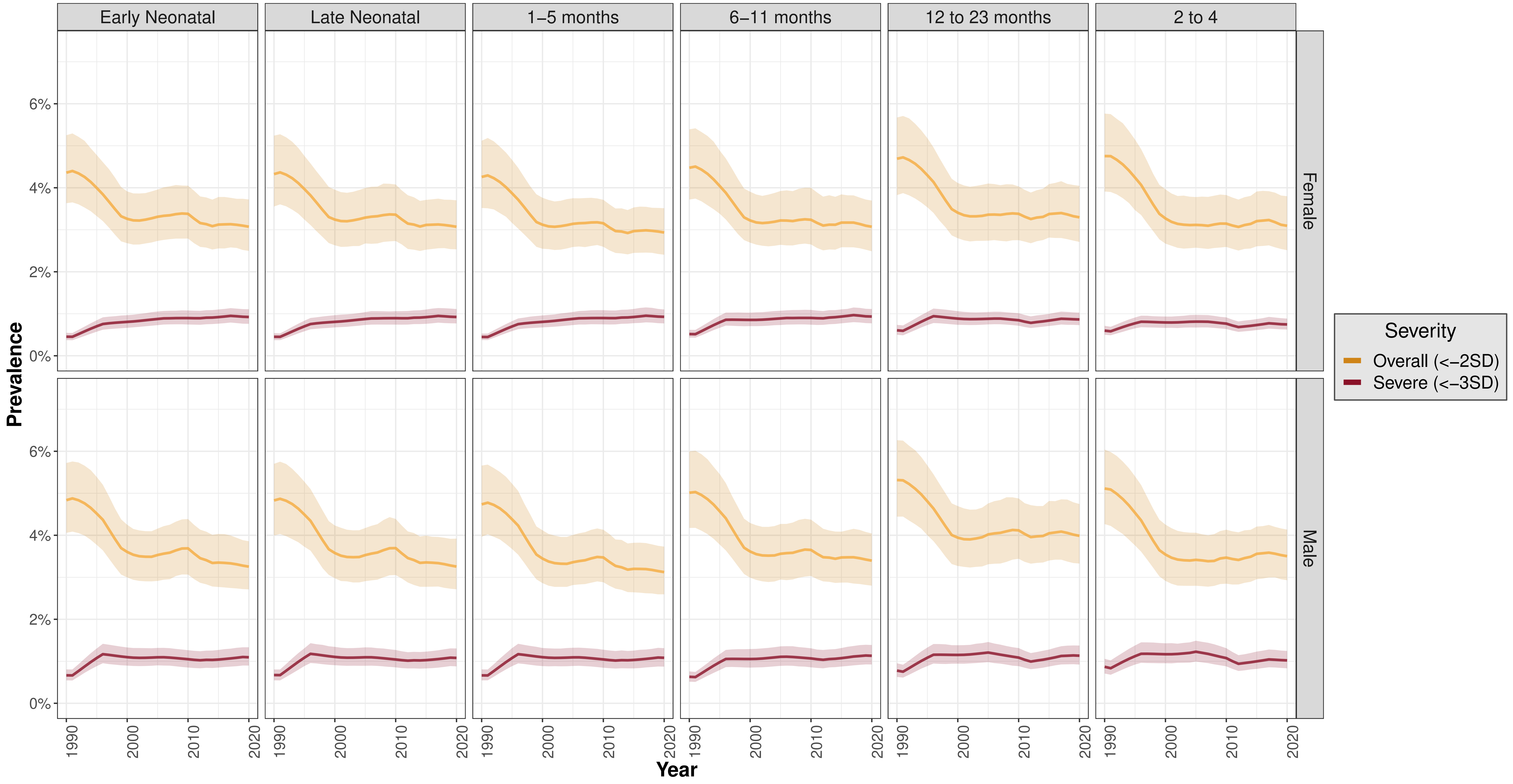

H: Transformed Mean Underweight Z Scores

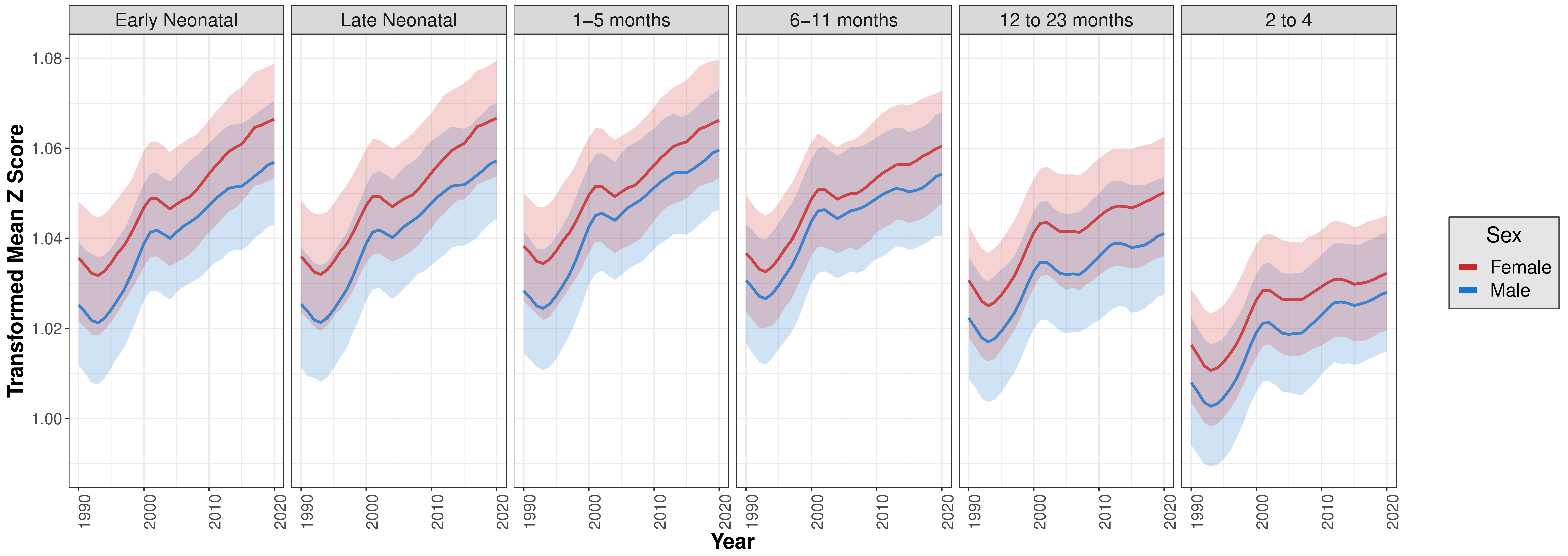

I

**Source**
No sources for this location

Guam – HAZ, WHZ, and WAZ Distributions

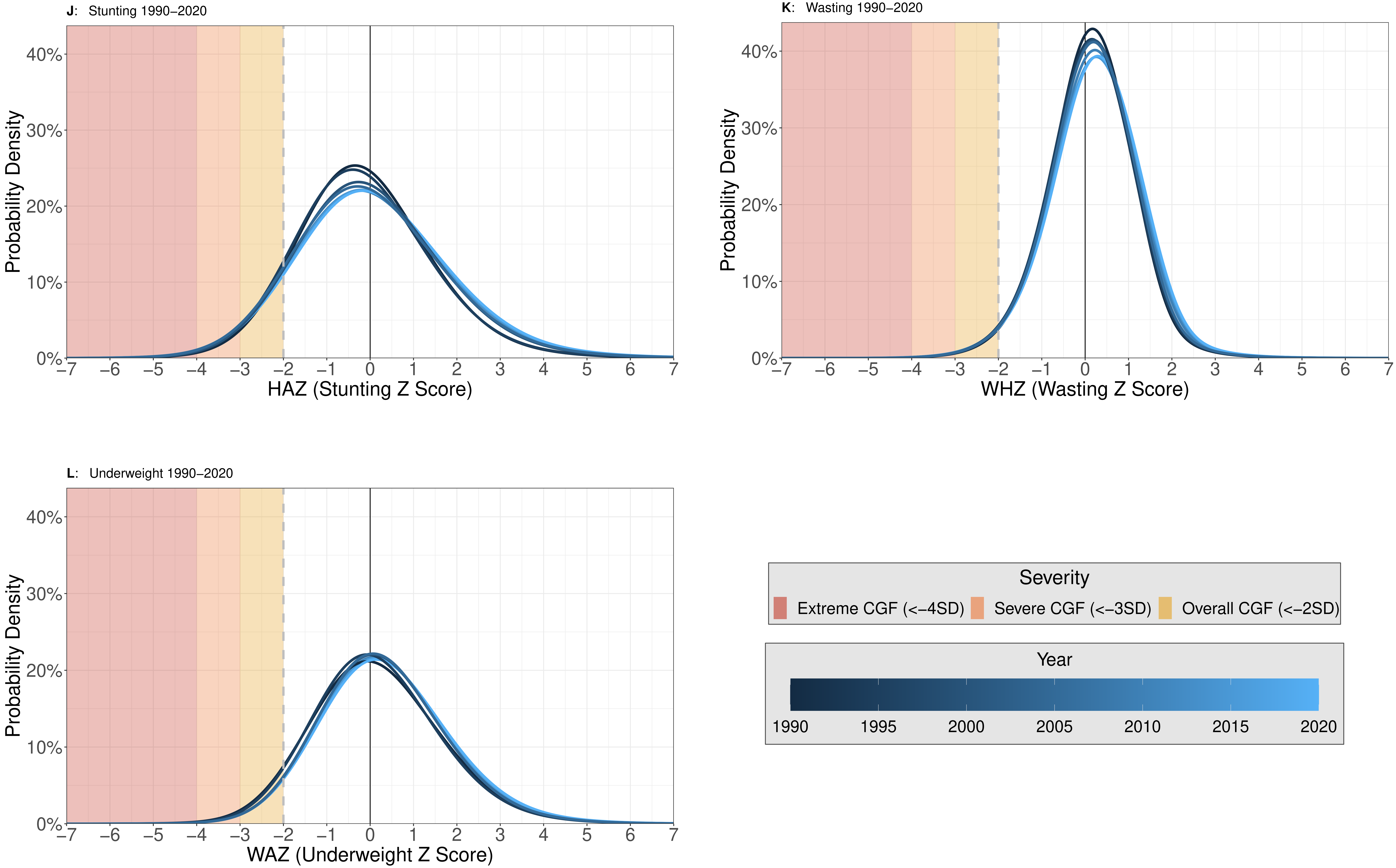

Nauru – Stunting (HAZ)

A: Overall and Severe Stunting Prevalence

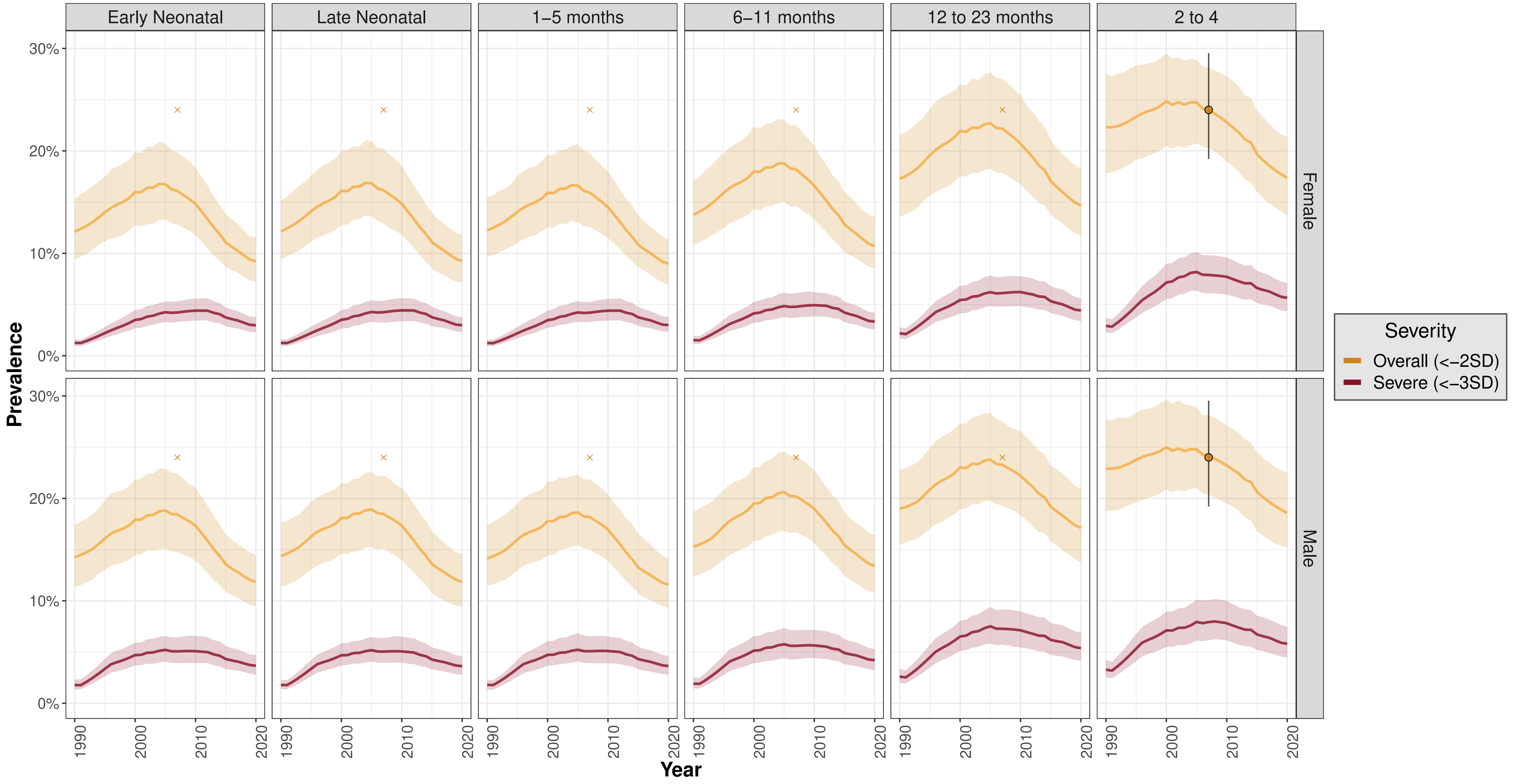

B: Transformed Mean Stunting Z Scores

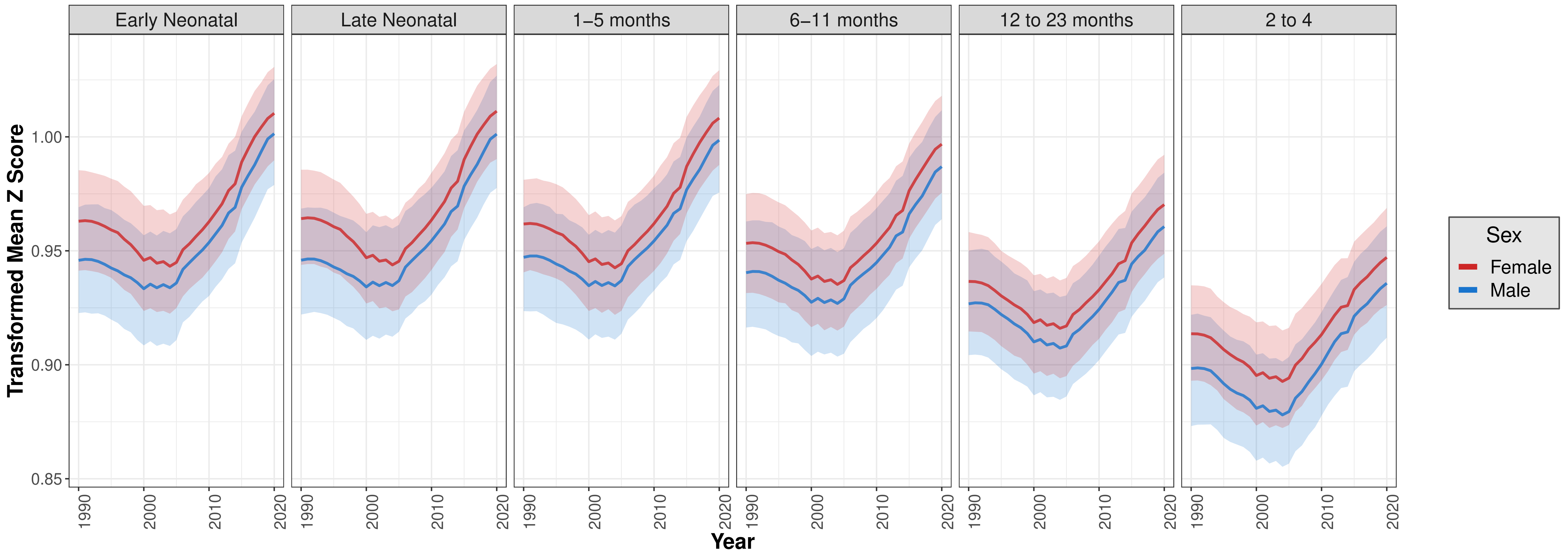

C

Year	Source
2007	WHO CGM Database

Nauru – Wasting (WHZ)

D: Overall and Severe Wasting Prevalence

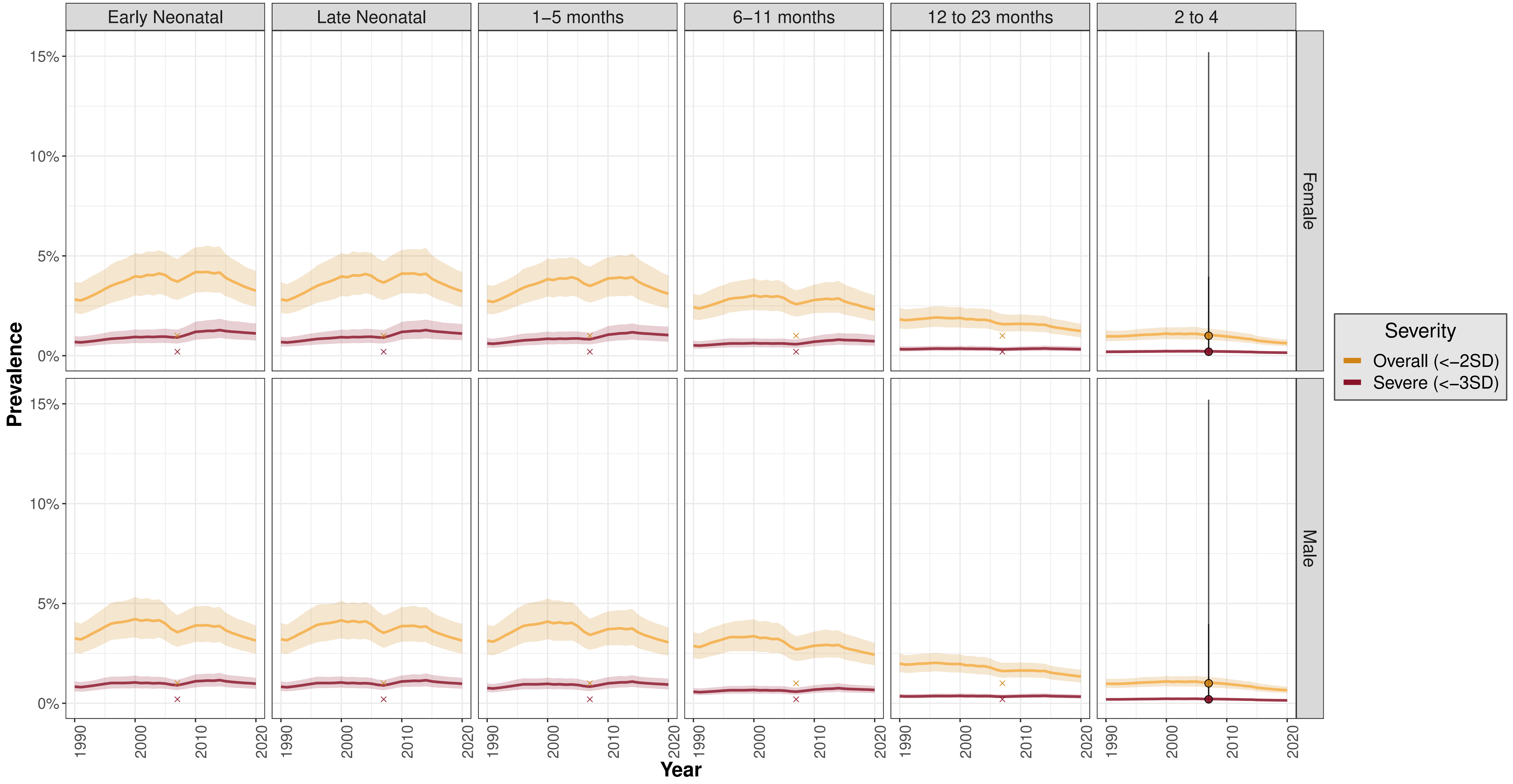

E: Transformed Mean Wasting Z Scores

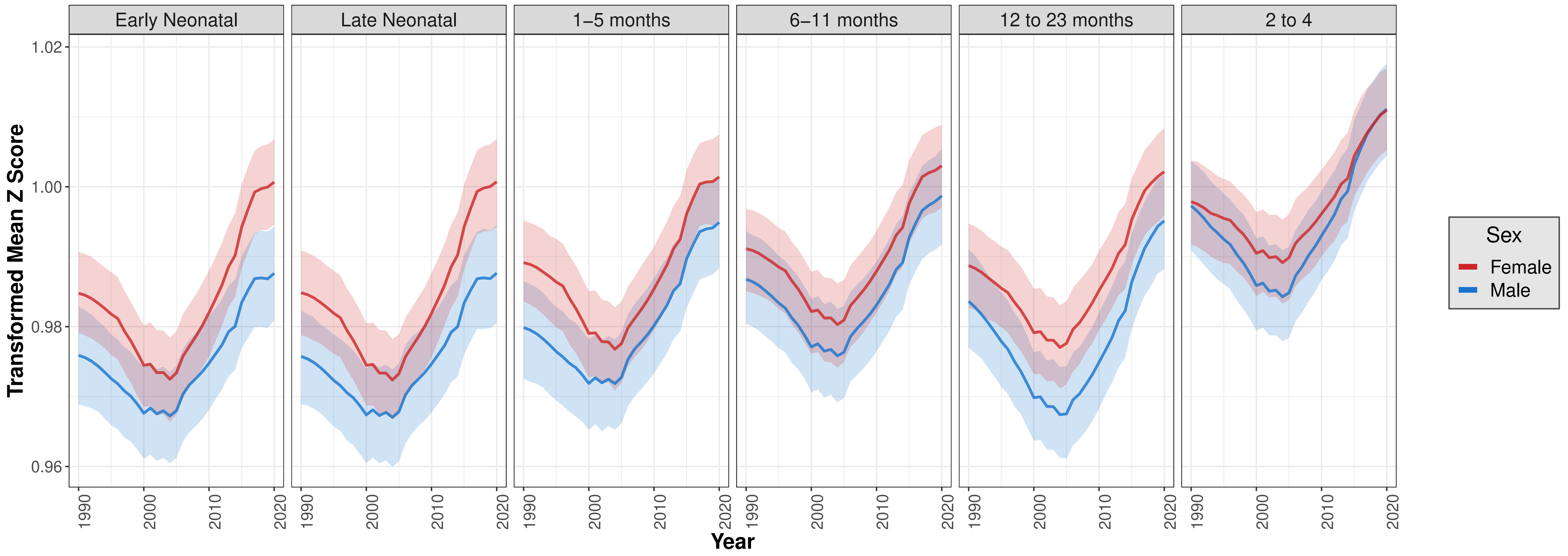

F

Year	Source
2007	WHO CGM Database

Nauru – Underweight (WAZ)

G: Overall and Severe Underweight Prevalence

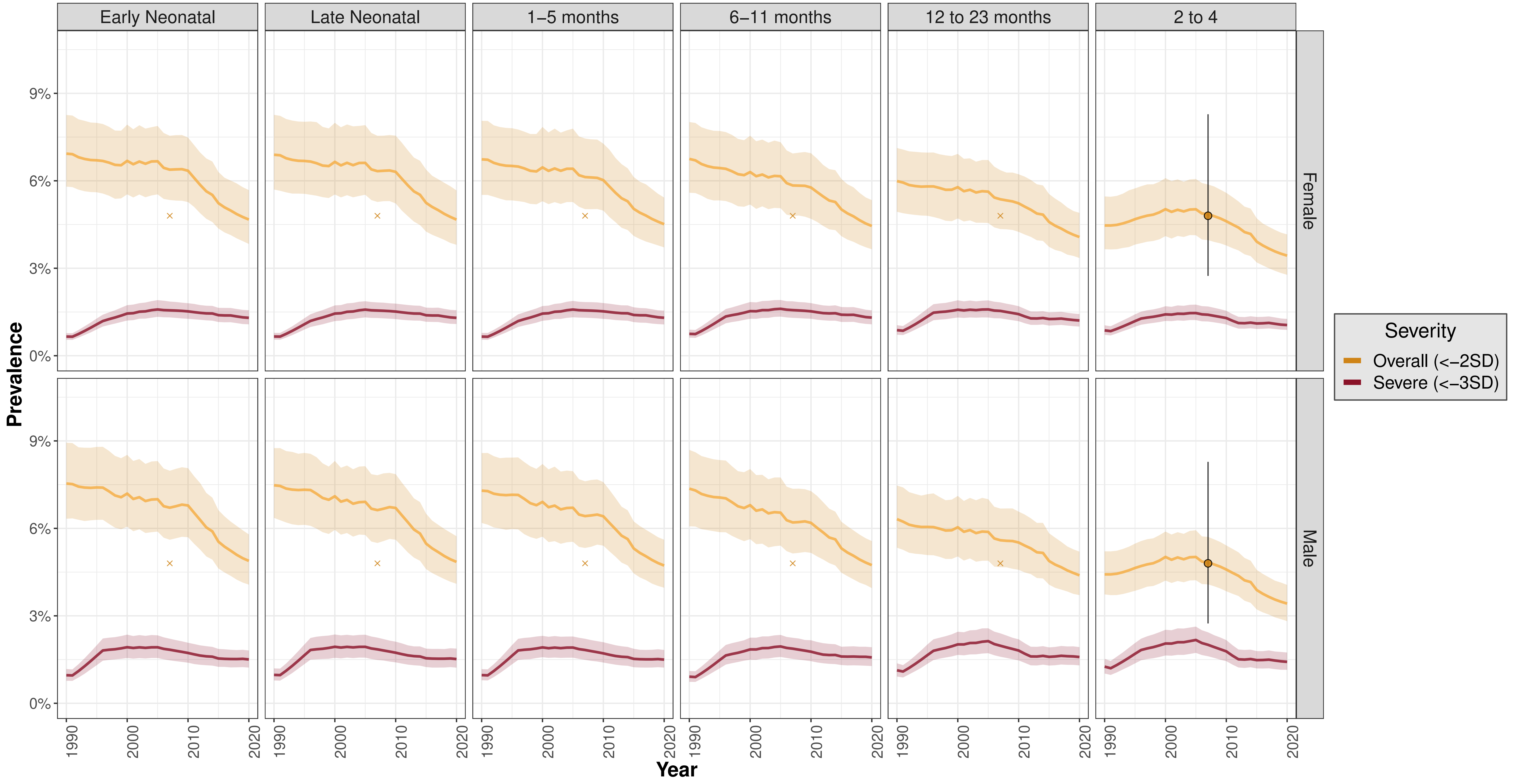

H: Transformed Mean Underweight Z Scores

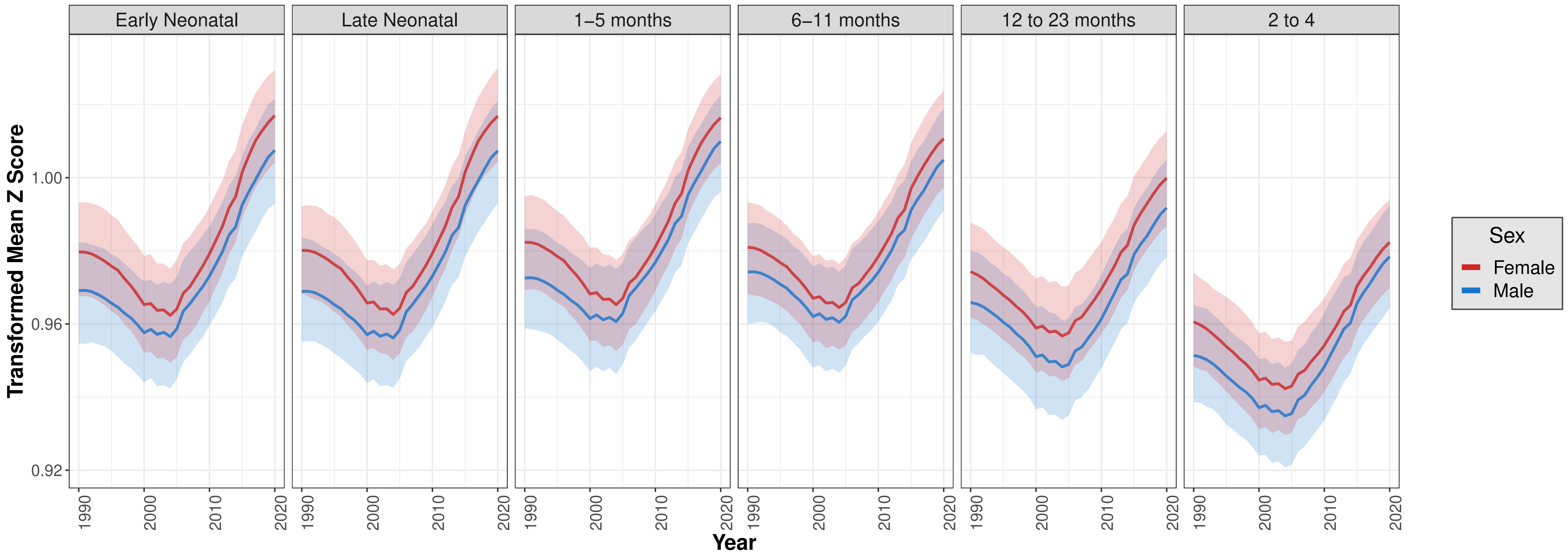

I

Year	Source
2007	WHO CGM Database

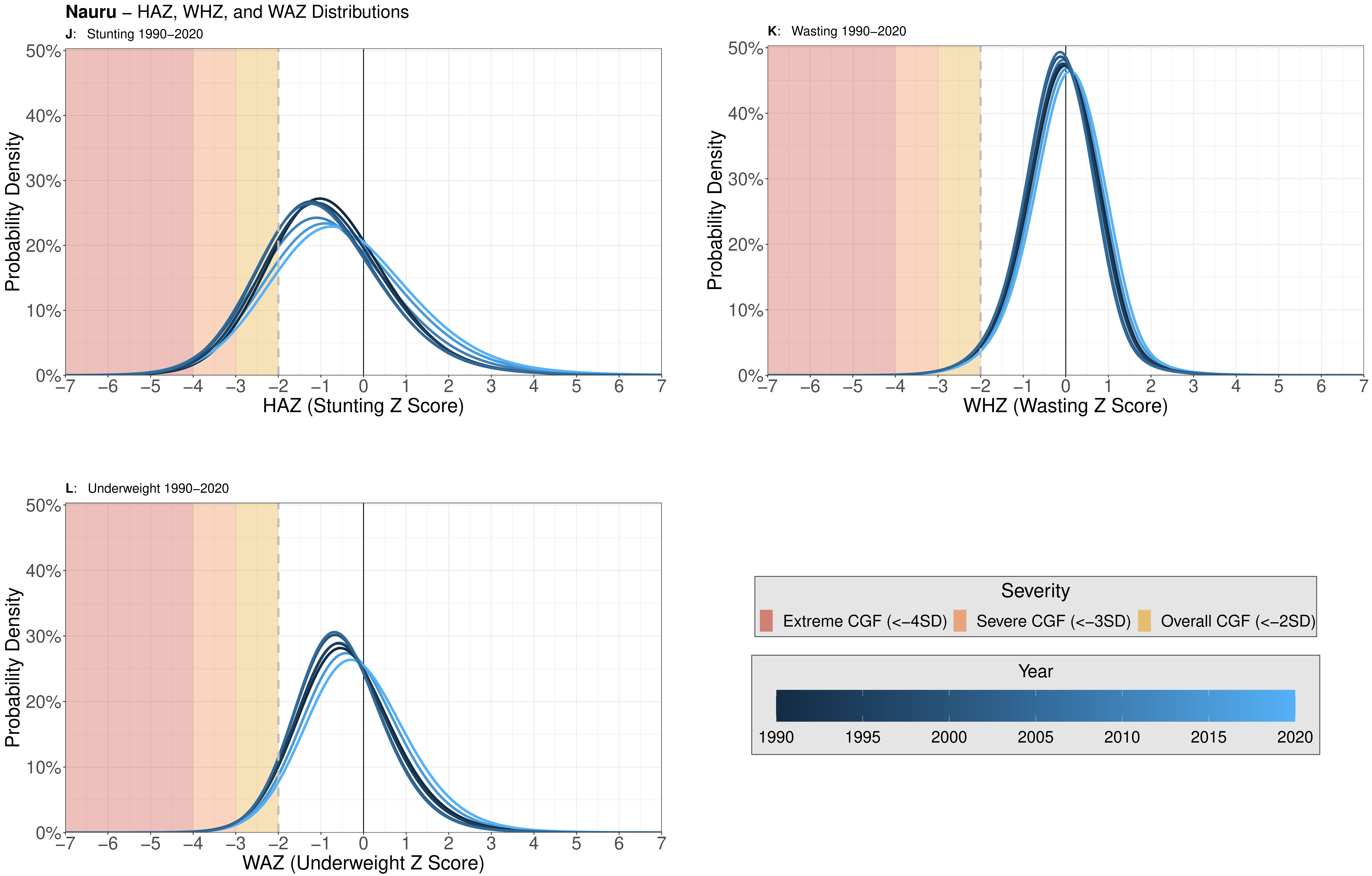
Nauru – HAZ, WHZ, and WAZ Distributions
J: Stunting 1990–2020
Probability Density
HAZ (Stunting Z Score)
K: Wasting 1990–2020
WHZ (Wasting Z Score)
L: Underweight 1990–2020
WAZ (Underweight Z Score)
0%
10%
20%
30%
40%
50%
−7 −6 −5 −4 −3 −2 −1 0 1 2 3 4 5 6 7
Severity
Extreme CGF (<−4SD)
Severe CGF (<−3SD)
Overall CGF (<−2SD)
Year
1990 1995 2000 2005 2010 2015 2020

Niue – Stunting (HAZ)

A: Overall and Severe Stunting Prevalence

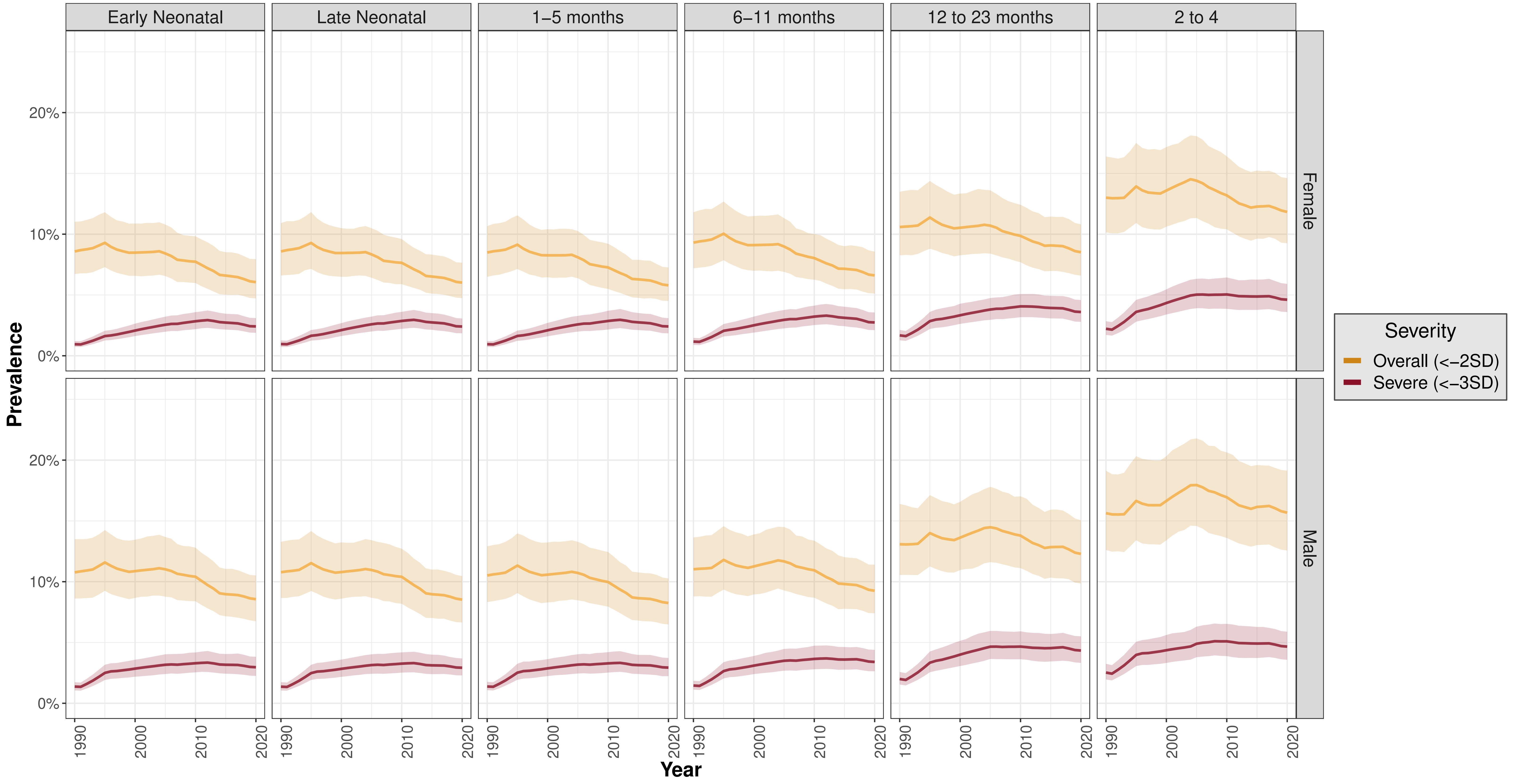

C

Source
No sources for this location

B: Transformed Mean Stunting Z Scores

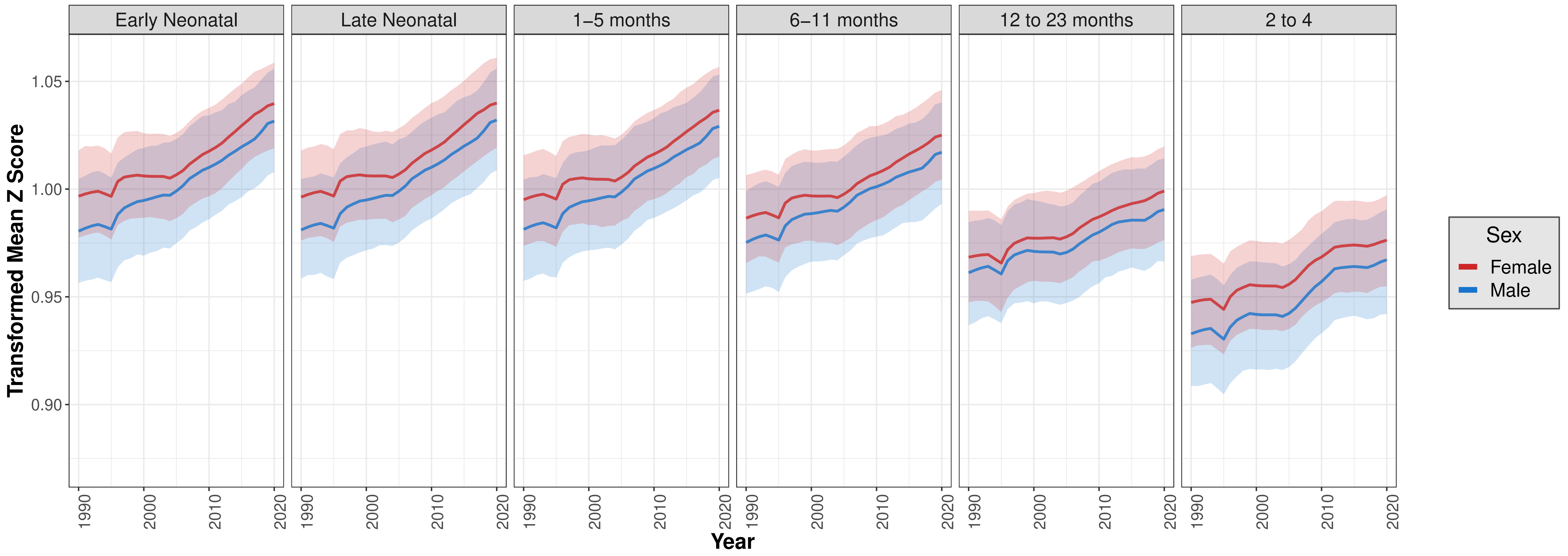

Niue – Wasting (WHZ)

D: Overall and Severe Wasting Prevalence

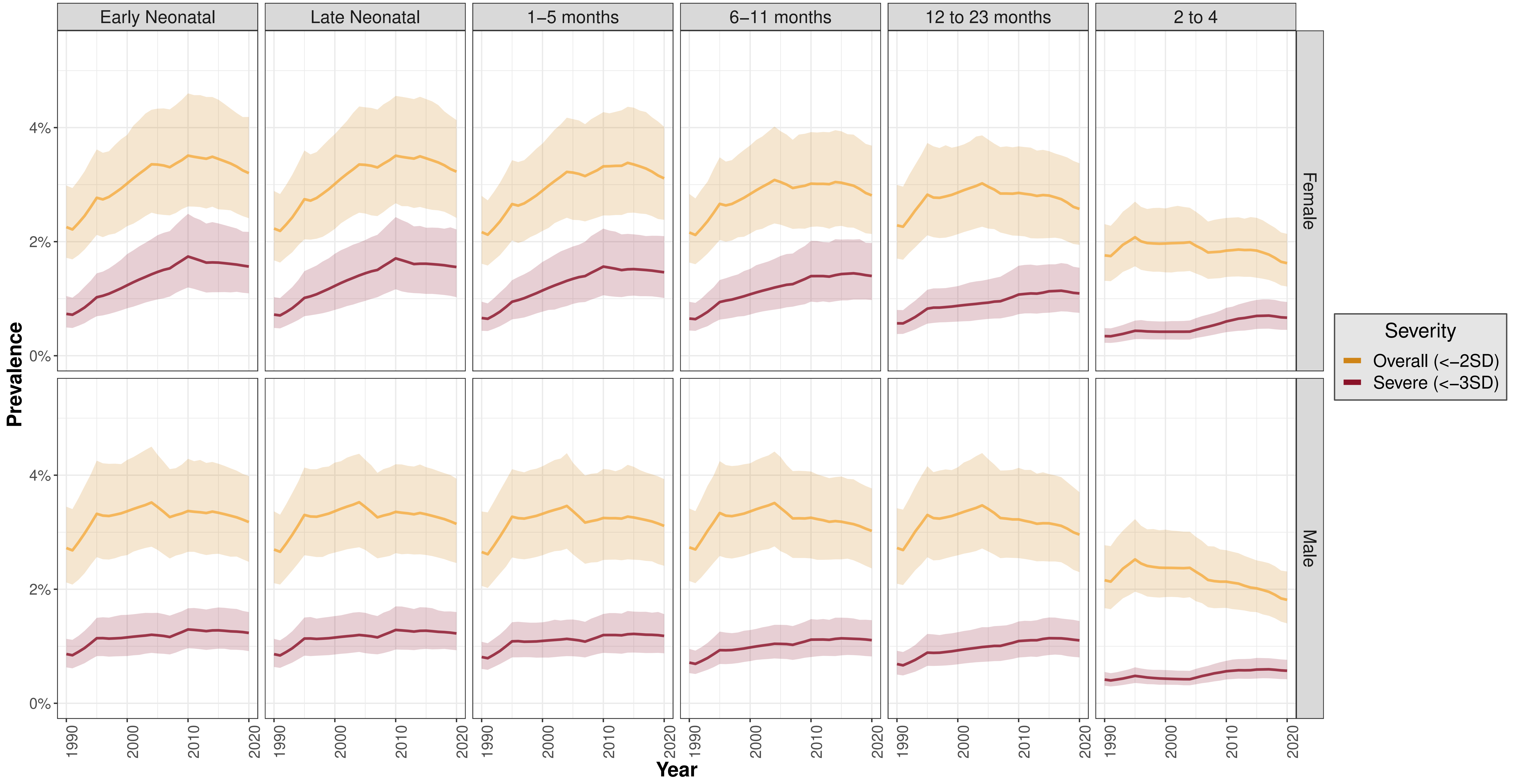

E: Transformed Mean Wasting Z Scores

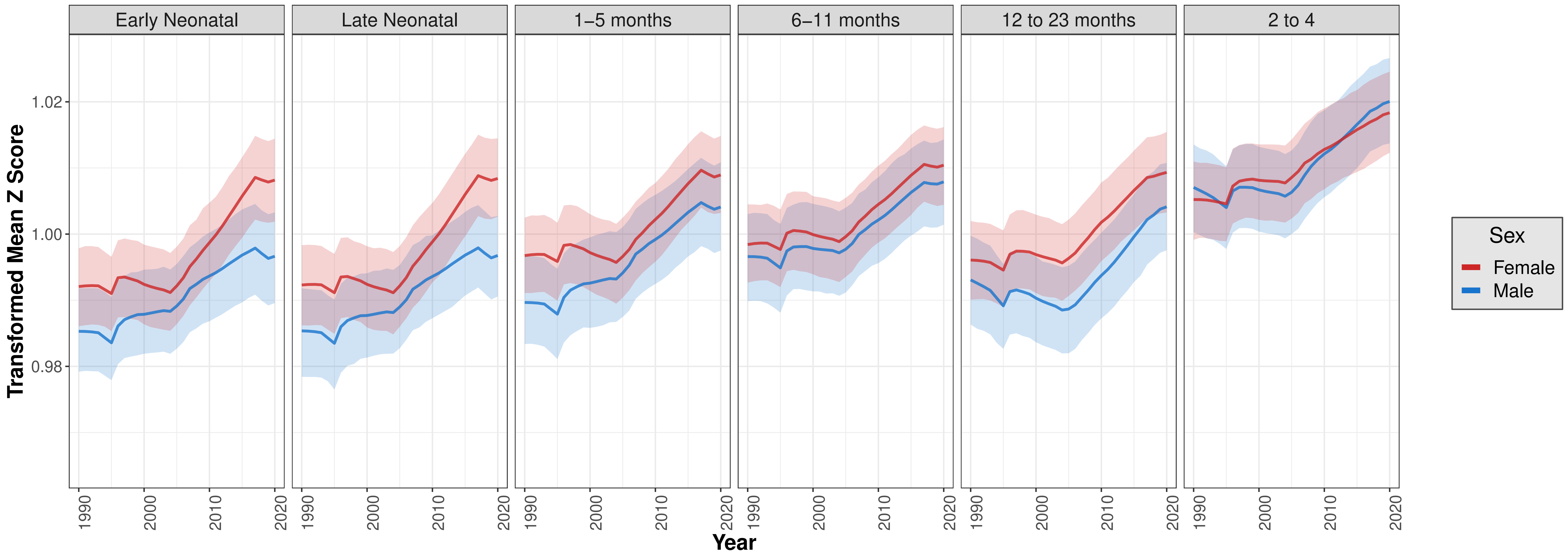

F

Source
No sources for this location

Niue – Underweight (WAZ)

G: Overall and Severe Underweight Prevalence

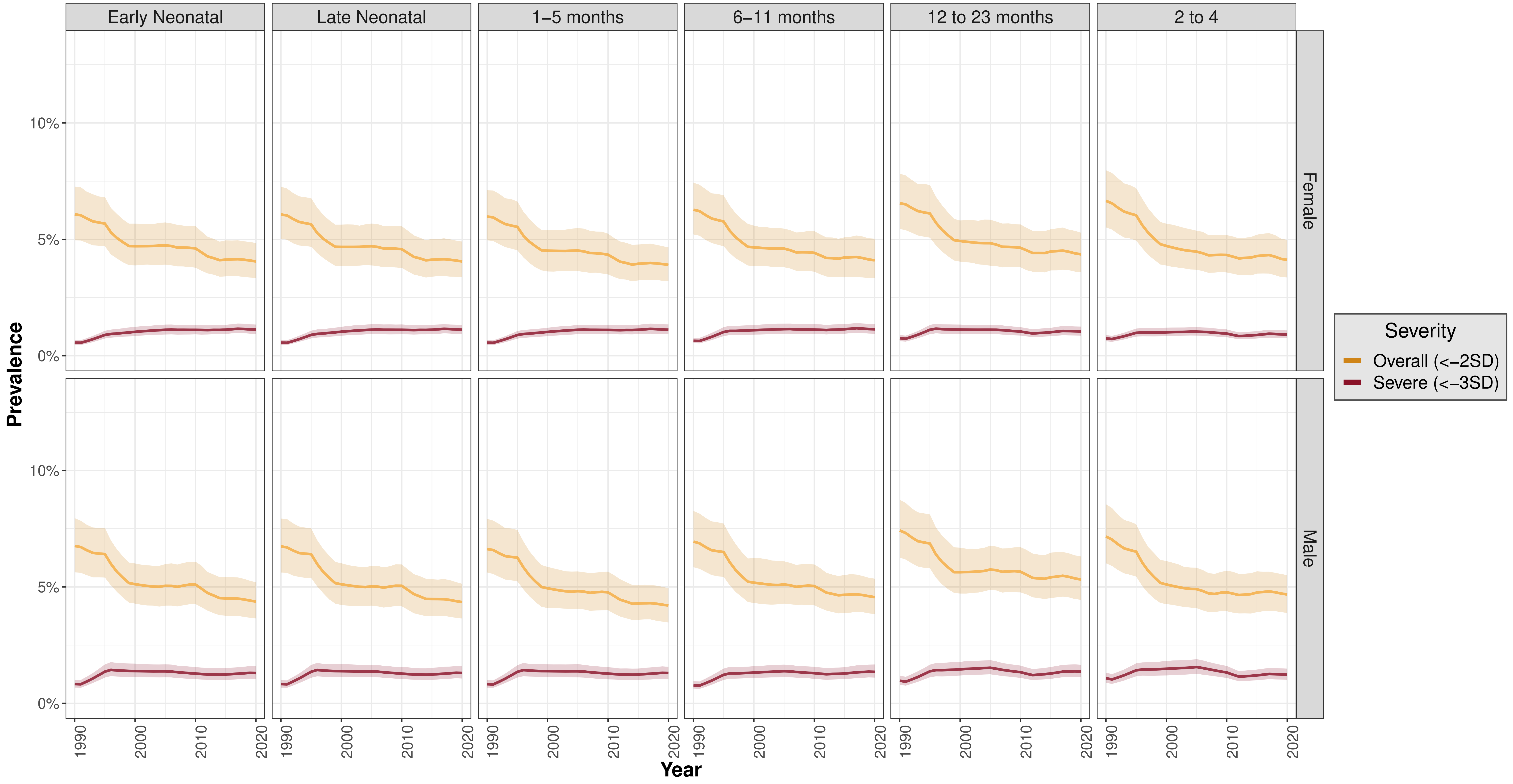

H: Transformed Mean Underweight Z Scores

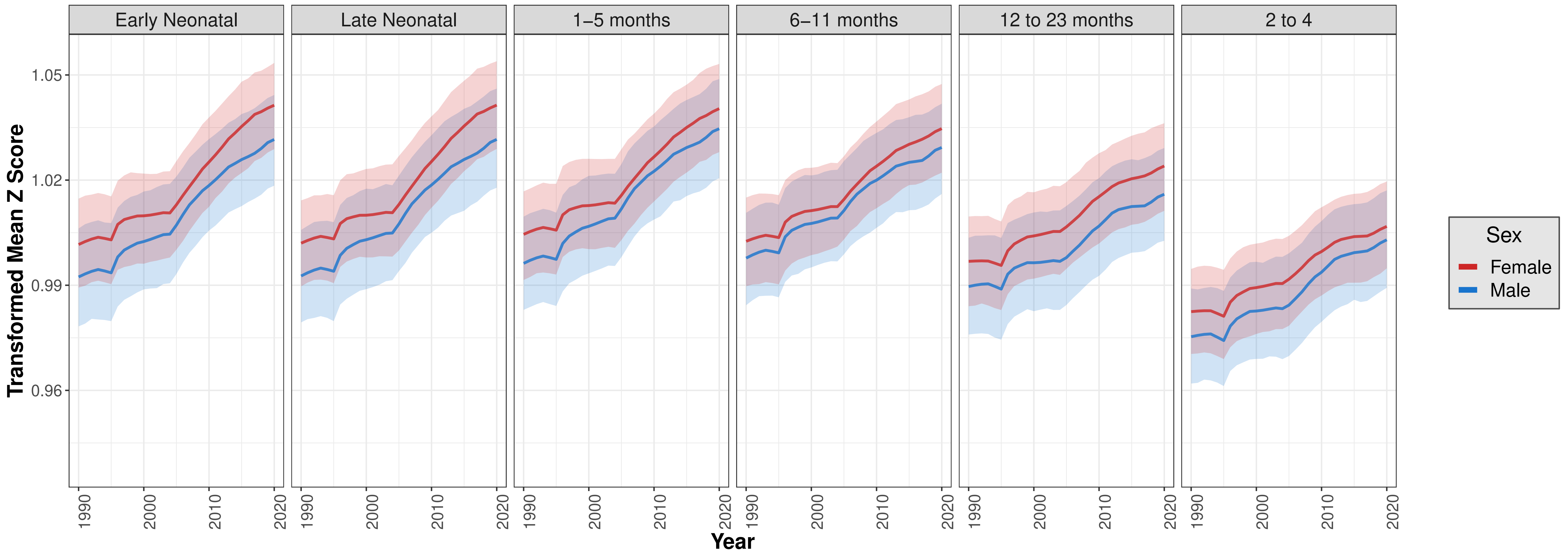

I

Source
No sources for this location

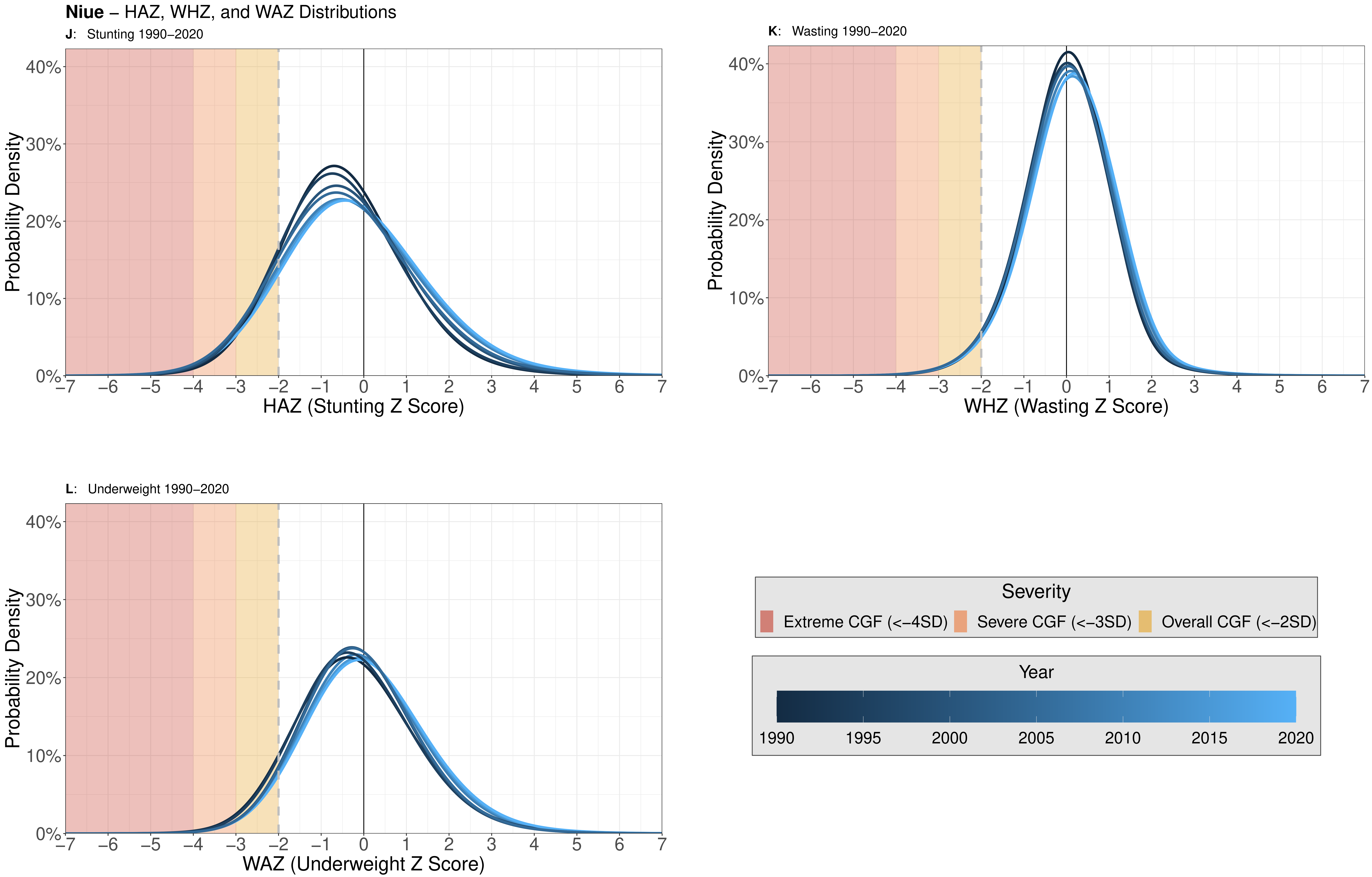

Niue – HAZ, WHZ, and WAZ Distributions
J: Stunting 1990–2020
Probability Density
0%
10%
20%
30%
40%
−7 −6 −5 −4 −3 −2 −1 0 1 2 3 4 5 6 7
HAZ (Stunting Z Score)
K: Wasting 1990–2020
Probability Density
WHZ (Wasting Z Score)
L: Underweight 1990–2020
Probability Density
WAZ (Underweight Z Score)
Severity
Extreme CGF (<−4SD)
Severe CGF (<−3SD)
Overall CGF (<−2SD)
Year
1990
1995
2000
2005
2010
2015
2020

Northern Mariana Islands – Stunting (HAZ)

A: Overall and Severe Stunting Prevalence

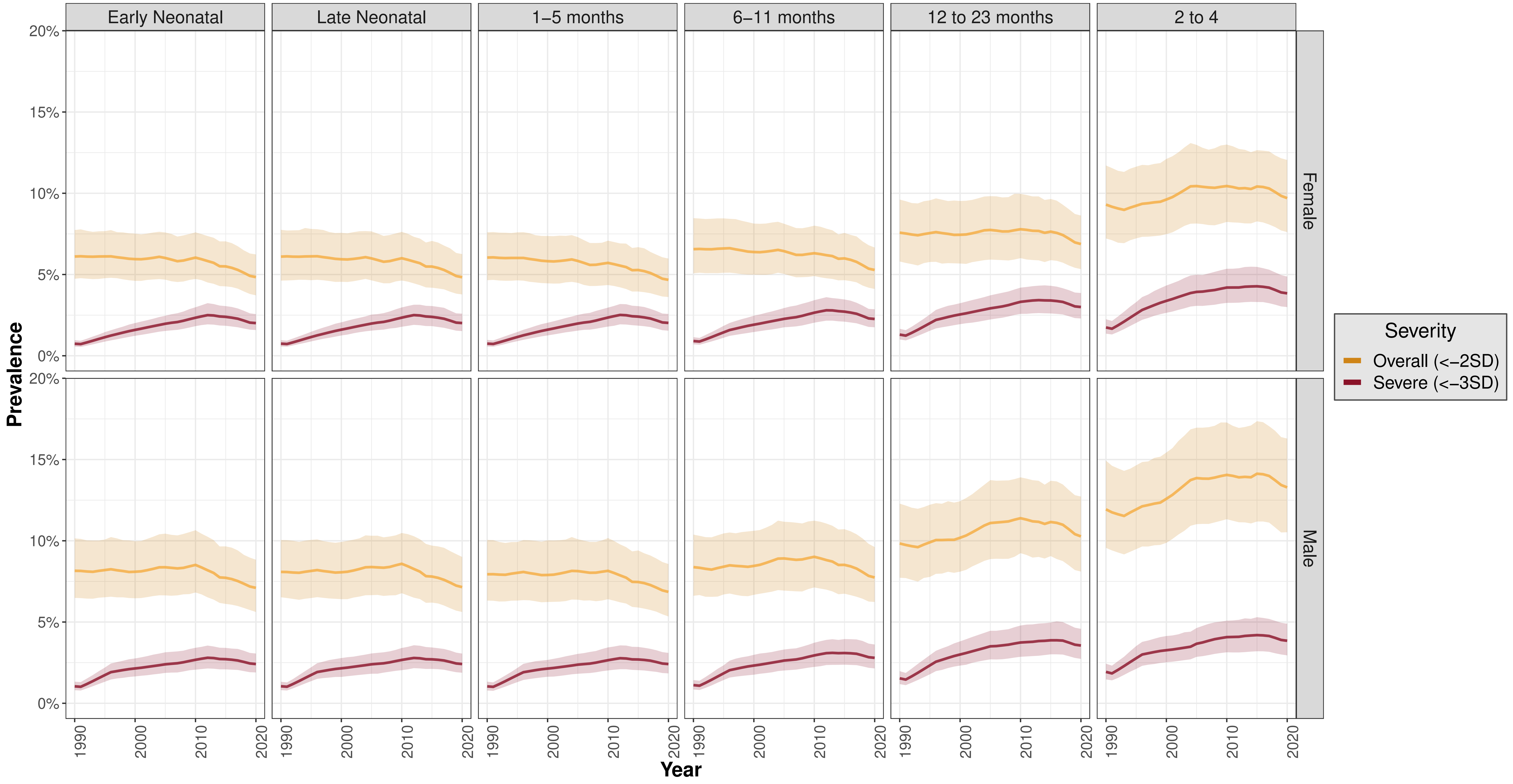

B: Transformed Mean Stunting Z Scores

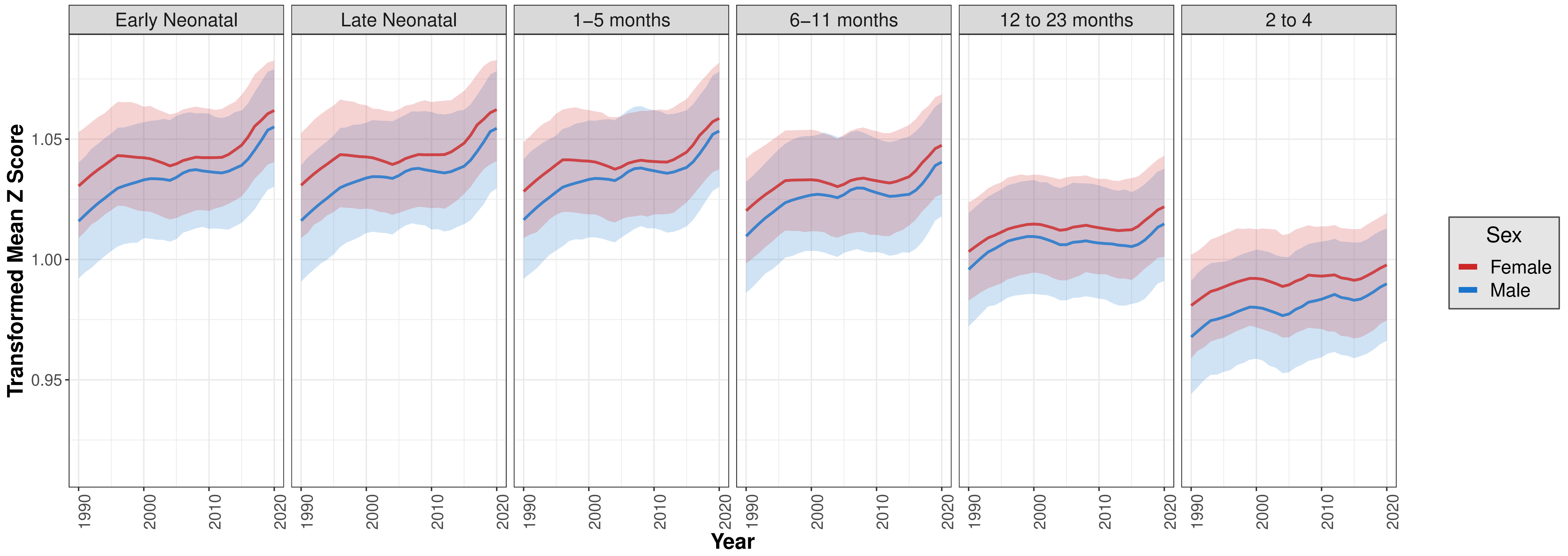

C

Source
No sources for this location

Northern Mariana Islands – Wasting (WHZ)

D: Overall and Severe Wasting Prevalence

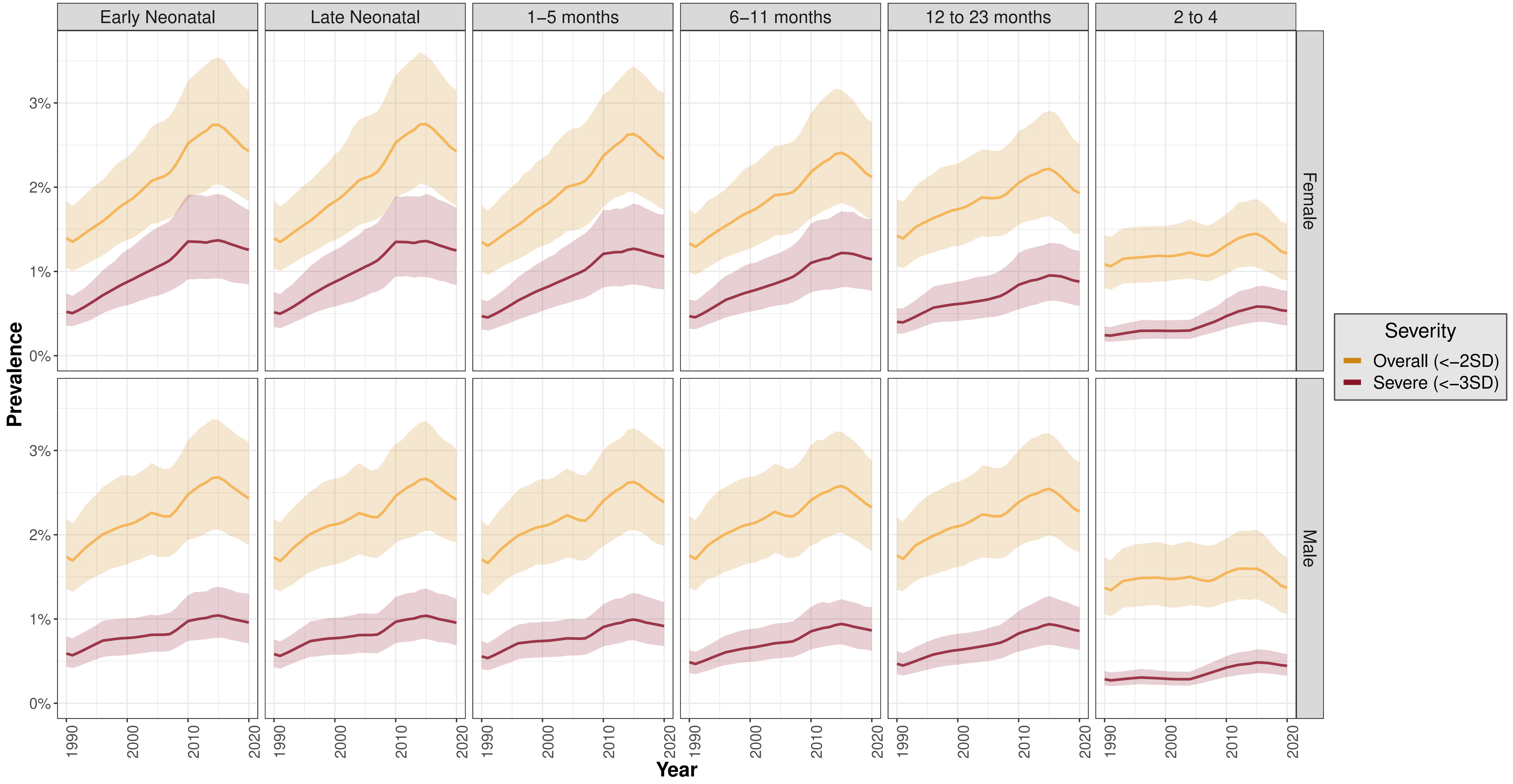

E: Transformed Mean Wasting Z Scores

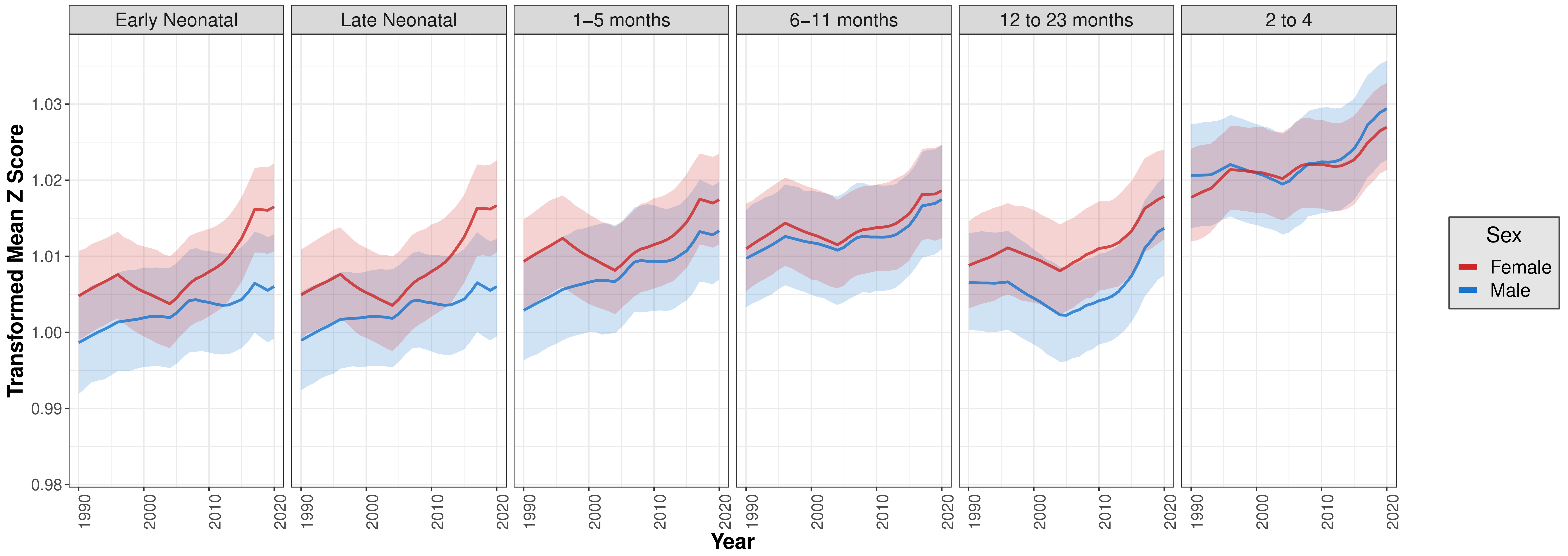

F

**Source**
No sources for this location

Northern Mariana Islands – Underweight (WAZ)

G: Overall and Severe Underweight Prevalence

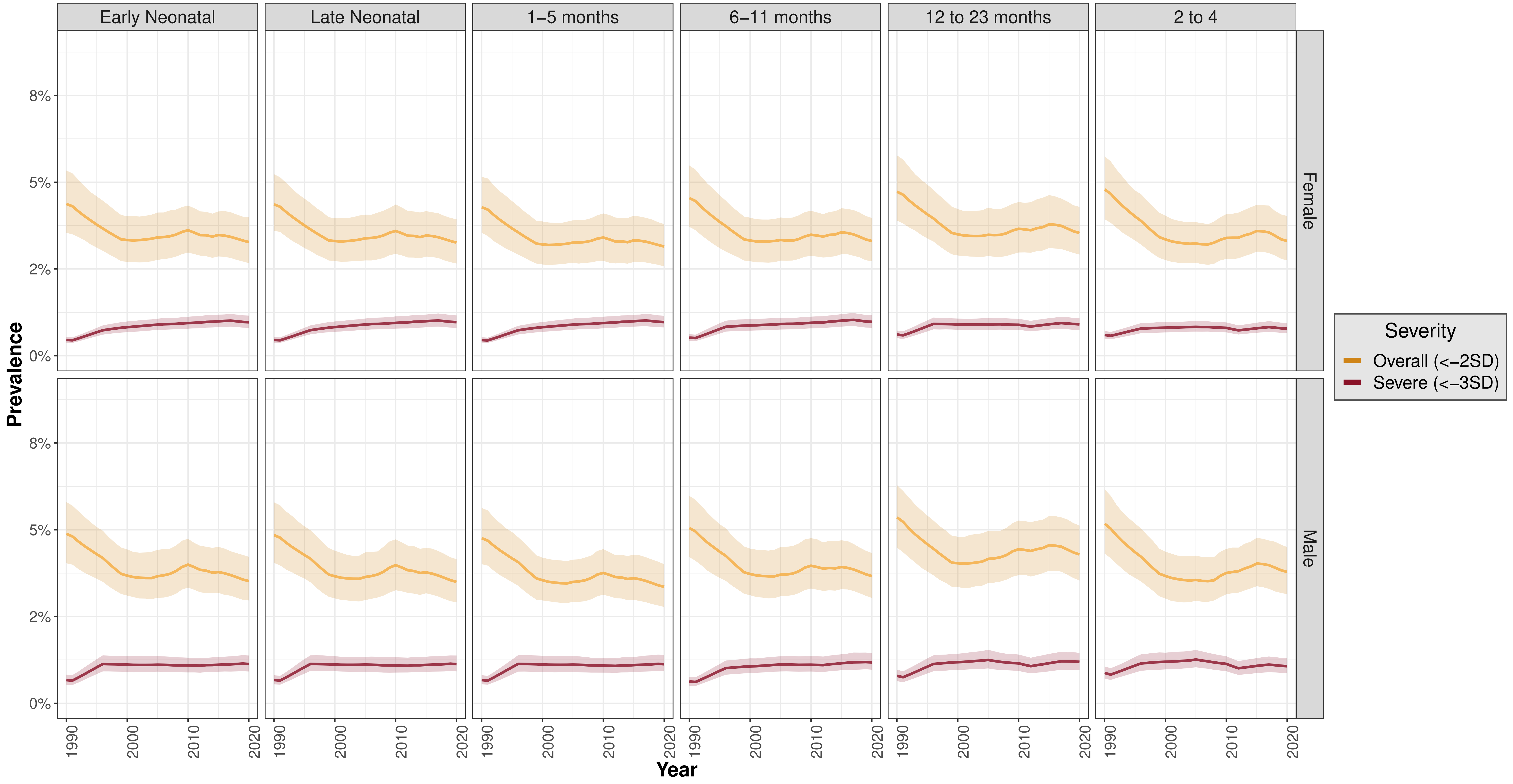

H: Transformed Mean Underweight Z Scores

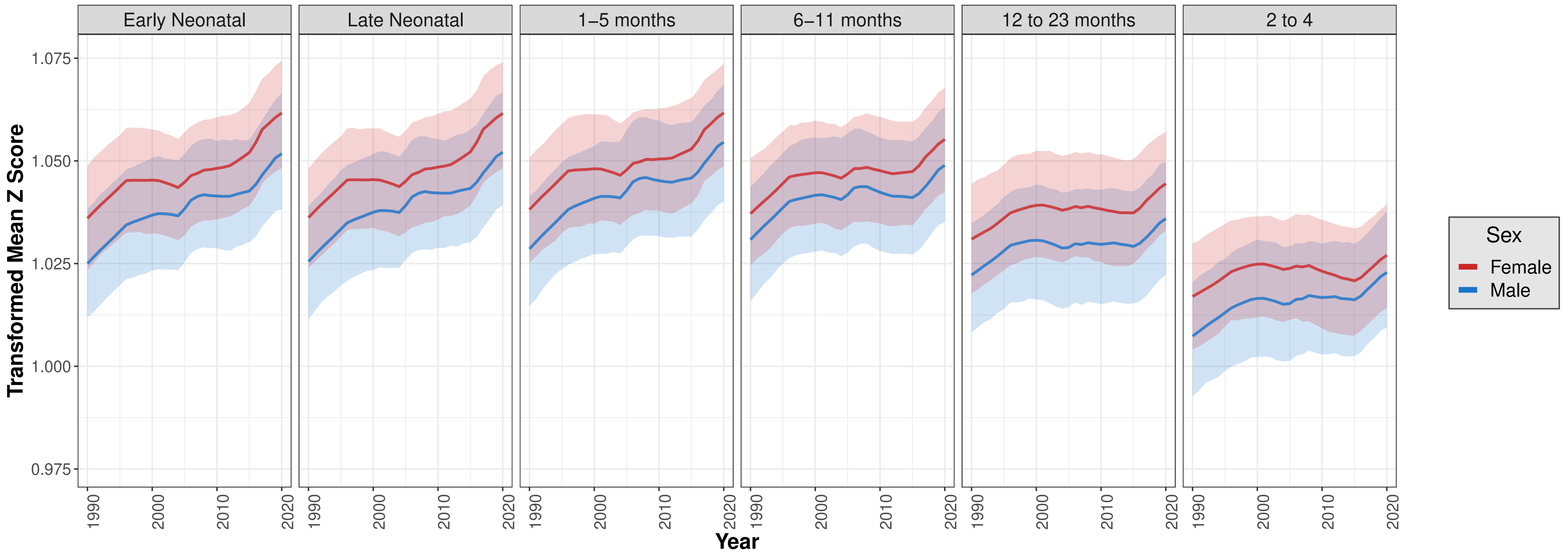

I

Source
No sources for this location

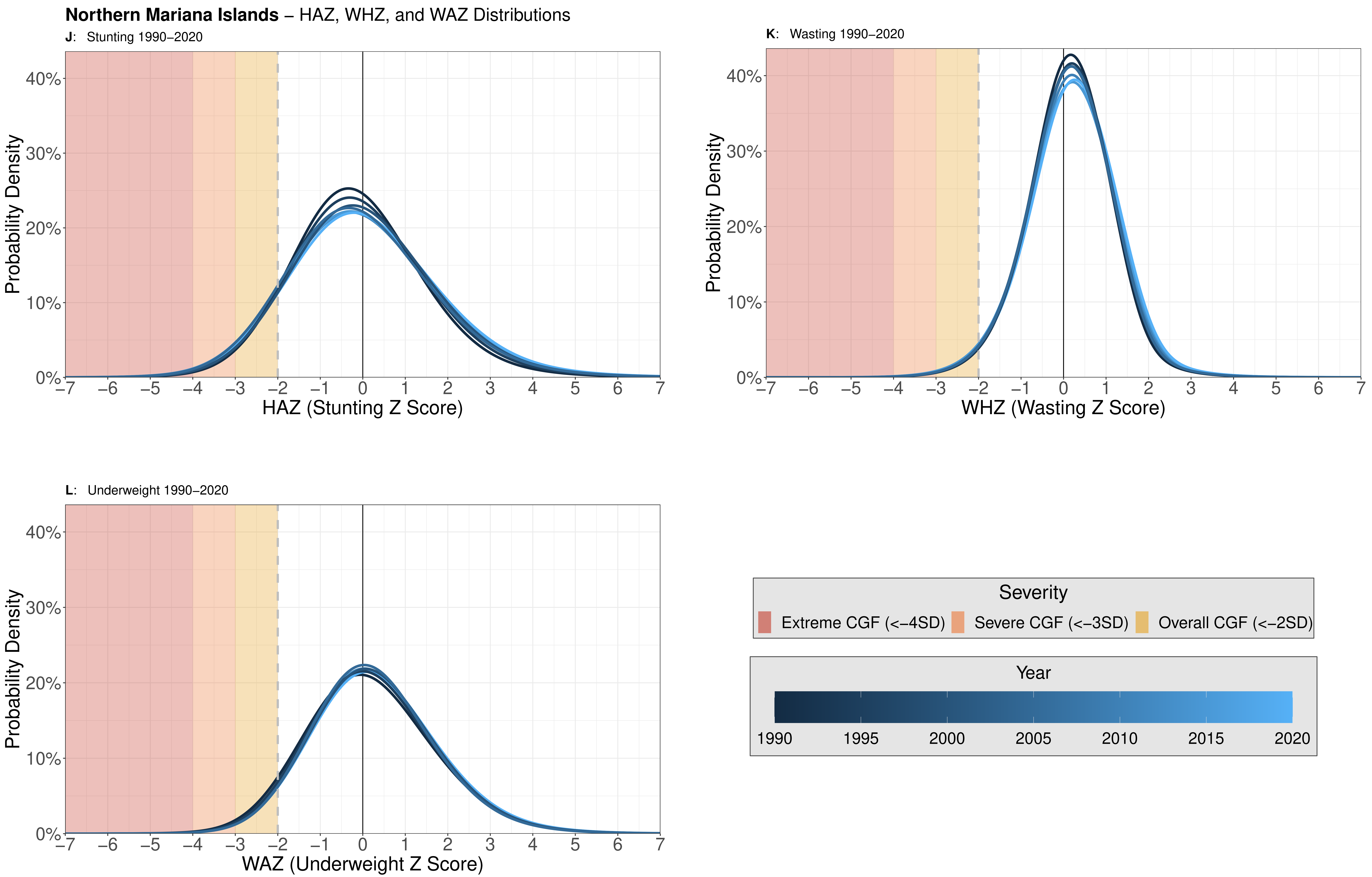
Northern Mariana Islands – HAZ, WHZ, and WAZ Distributions
J: Stunting 1990–2020
Probability Density
0%
10%
20%
30%
40%
−7 −6 −5 −4 −3 −2 −1 0 1 2 3 4 5 6 7
HAZ (Stunting Z Score)
K: Wasting 1990–2020
Probability Density
0%
10%
20%
30%
40%
−7 −6 −5 −4 −3 −2 −1 0 1 2 3 4 5 6 7
WHZ (Wasting Z Score)
L: Underweight 1990–2020
Probability Density
0%
10%
20%
30%
40%
−7 −6 −5 −4 −3 −2 −1 0 1 2 3 4 5 6 7
WAZ (Underweight Z Score)
Severity
Extreme CGF (<−4SD)
Severe CGF (<−3SD)
Overall CGF (<−2SD)
Year
1990
1995
2000
2005
2010
2015
2020

Palau – Stunting (HAZ)

A: Overall and Severe Stunting Prevalence

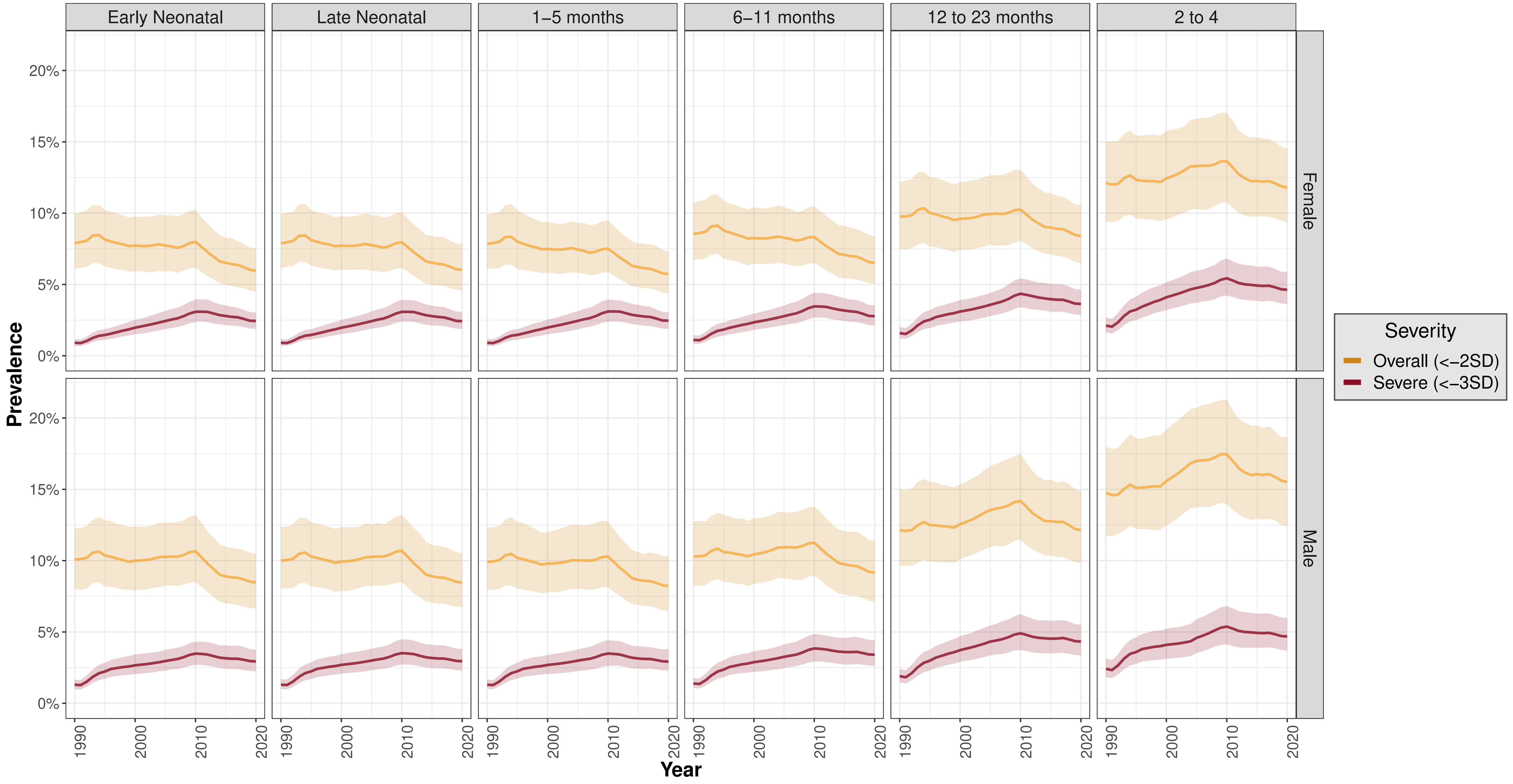

B: Transformed Mean Stunting Z Scores

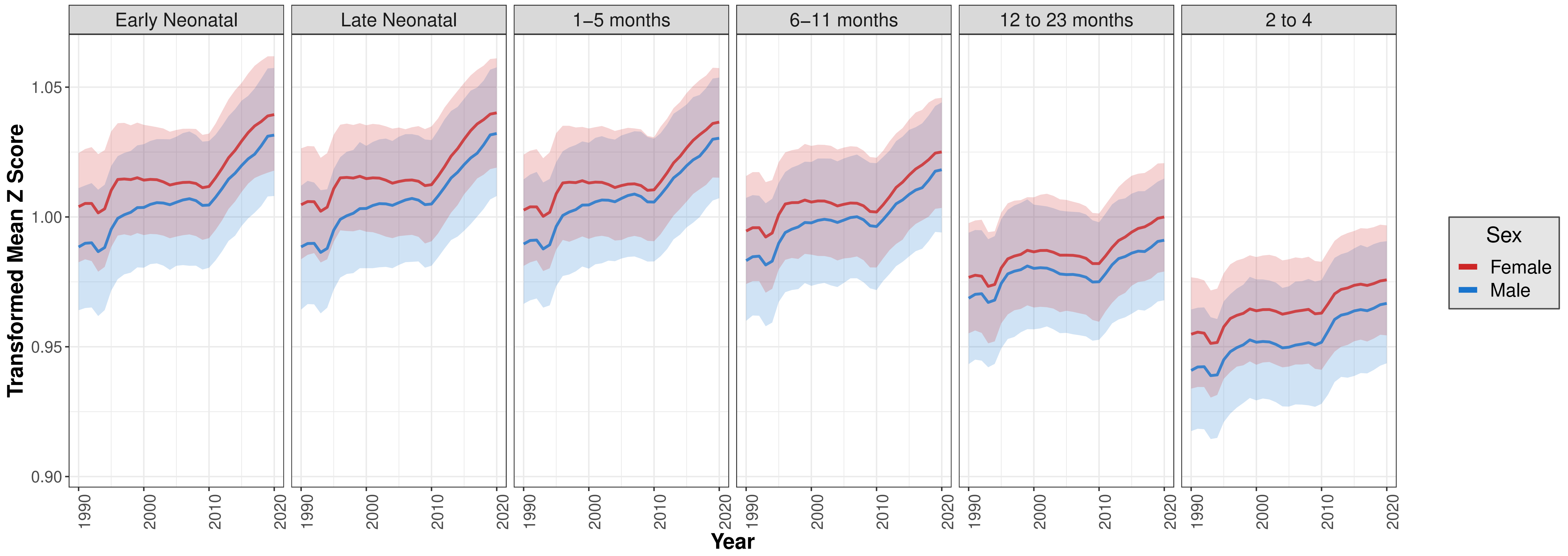

C

Source
No sources for this location

Palau – Wasting (WHZ)

D: Overall and Severe Wasting Prevalence

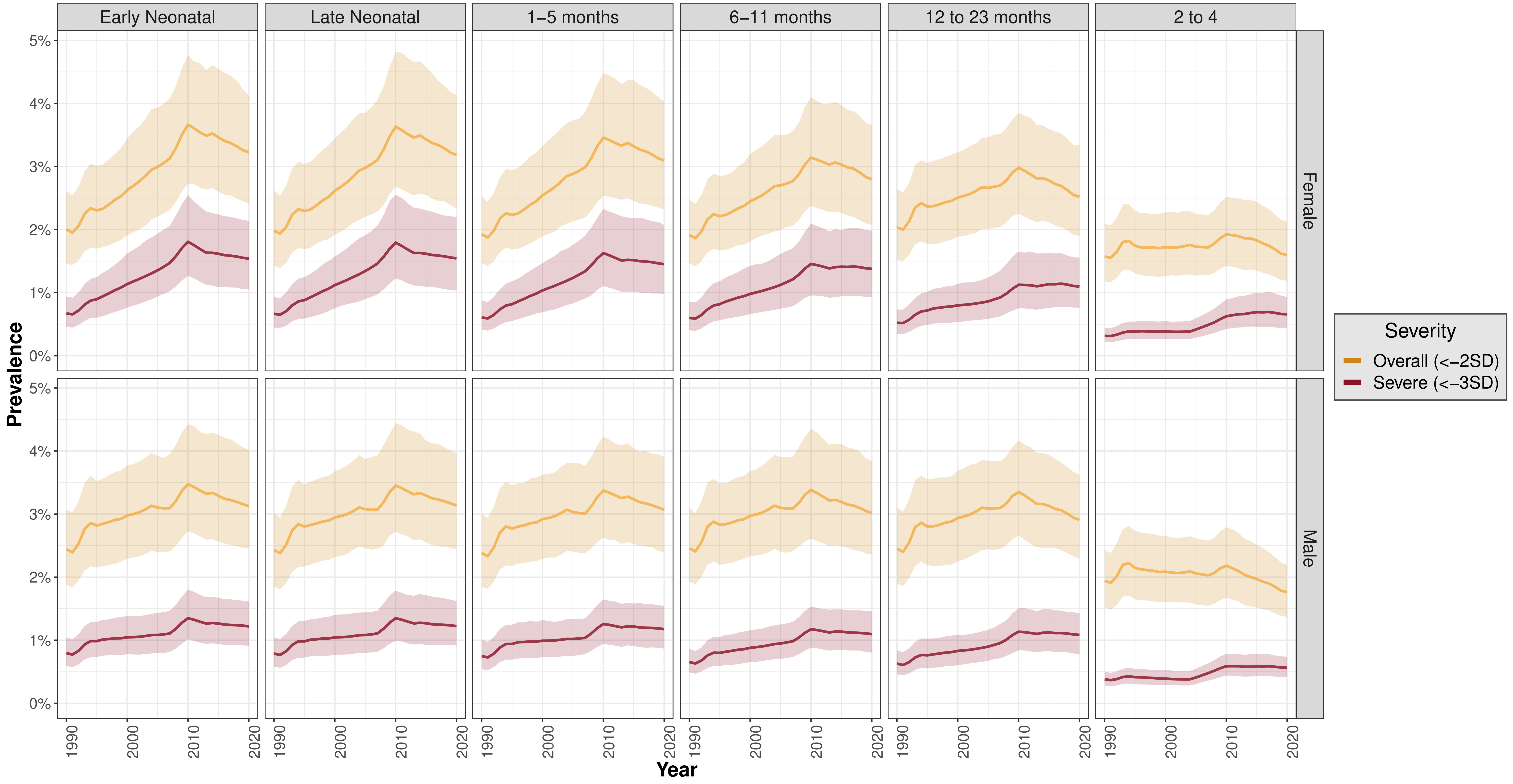

E: Transformed Mean Wasting Z Scores

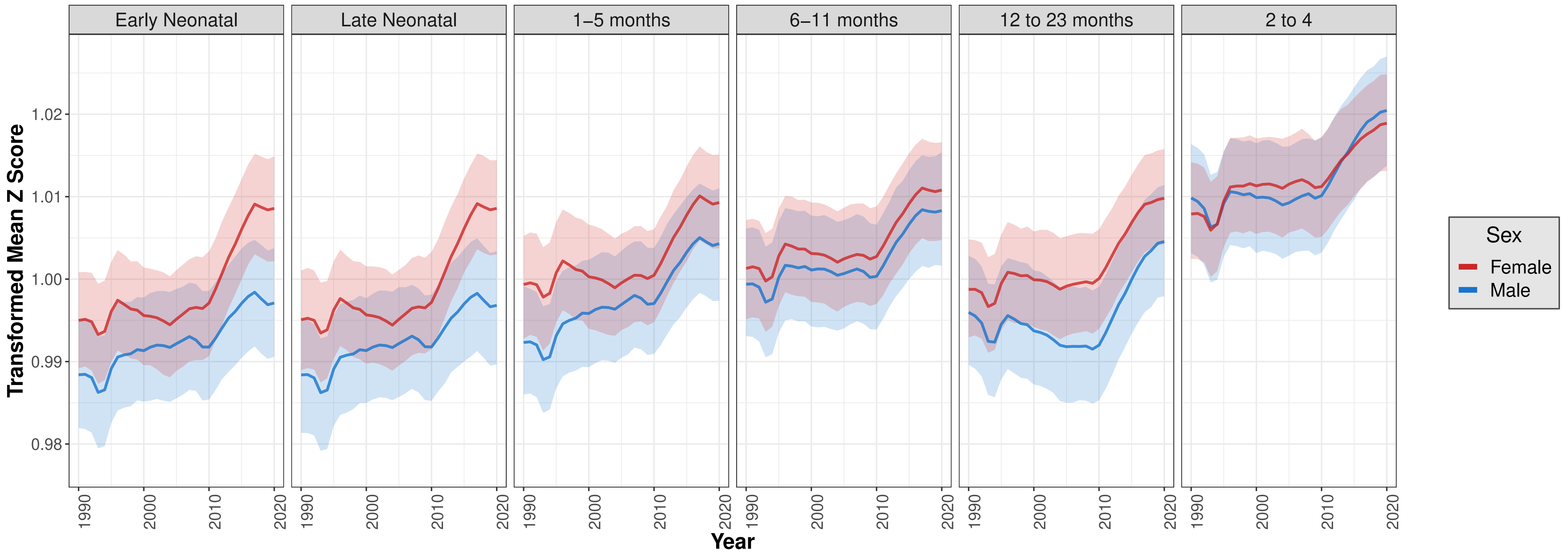

F

Source
No sources for this location

Palau – Underweight (WAZ)

G: Overall and Severe Underweight Prevalence

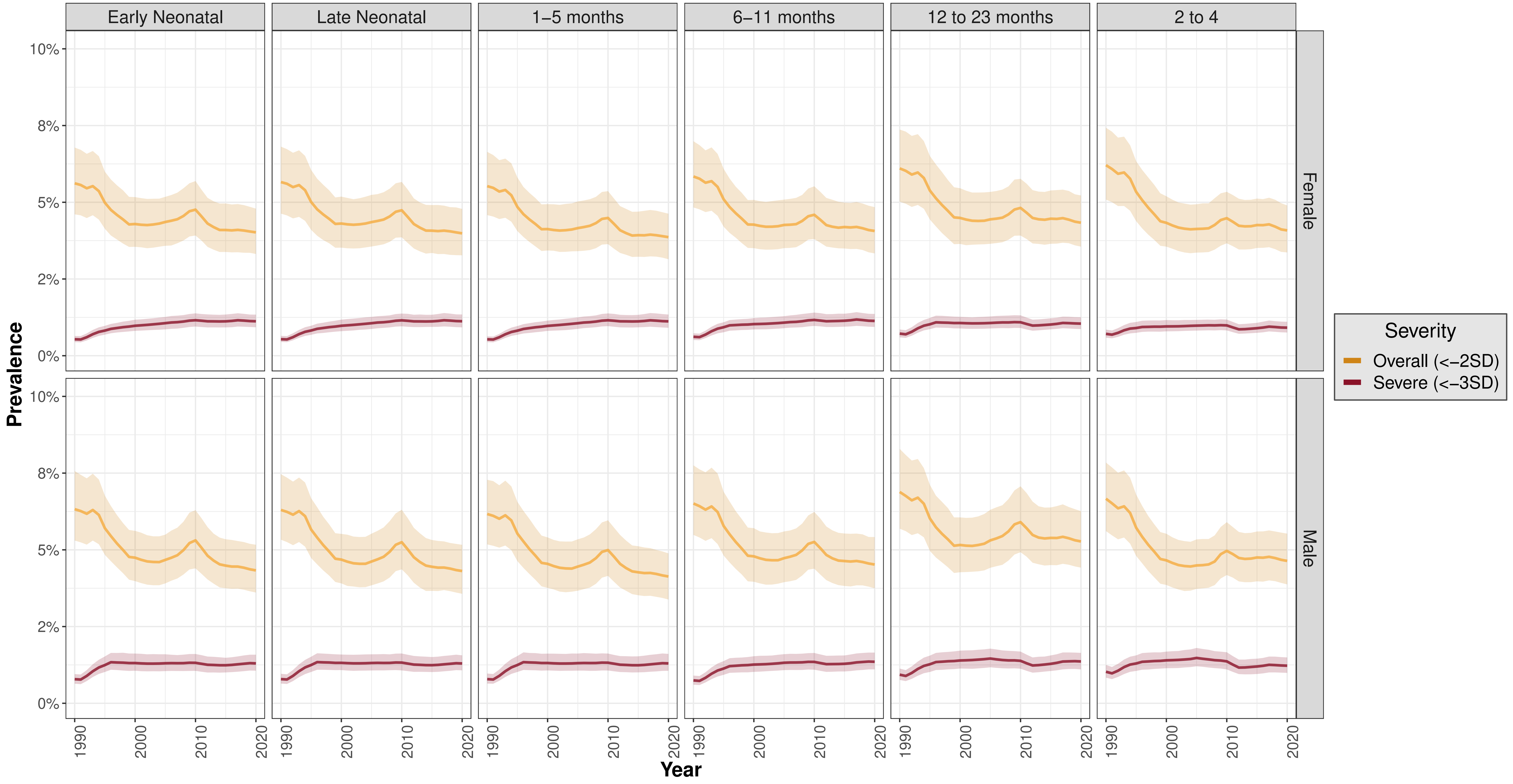

H: Transformed Mean Underweight Z Scores

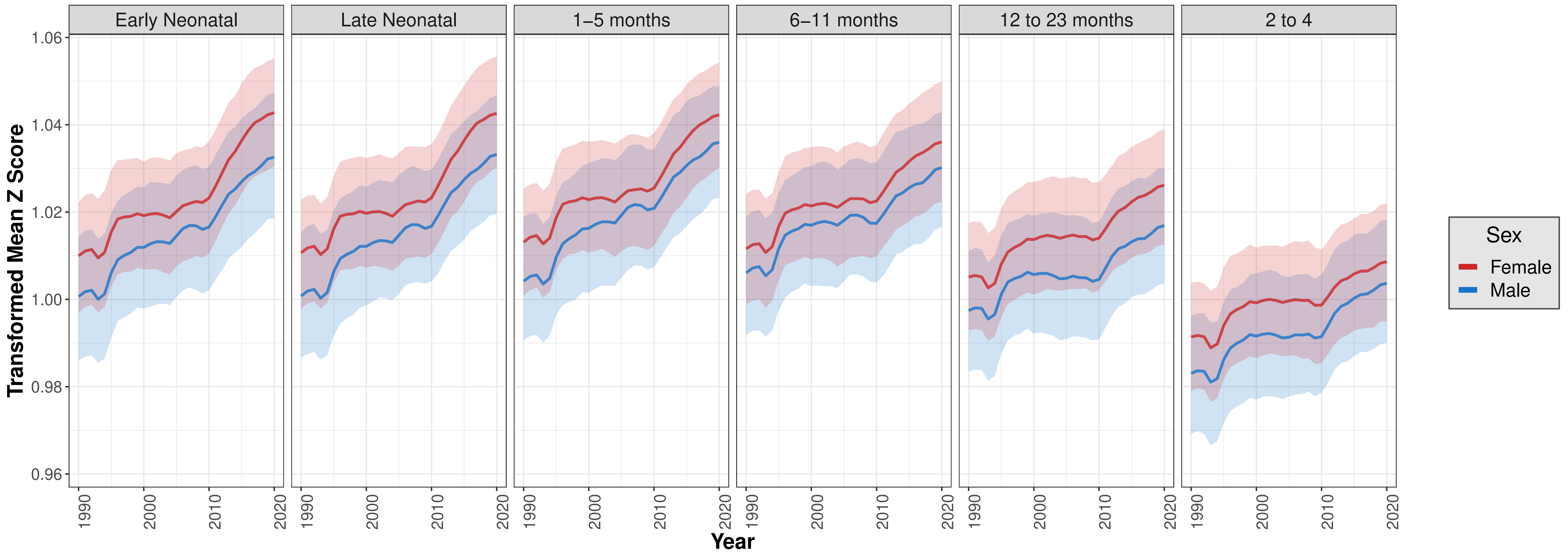

I

Source
No sources for this location

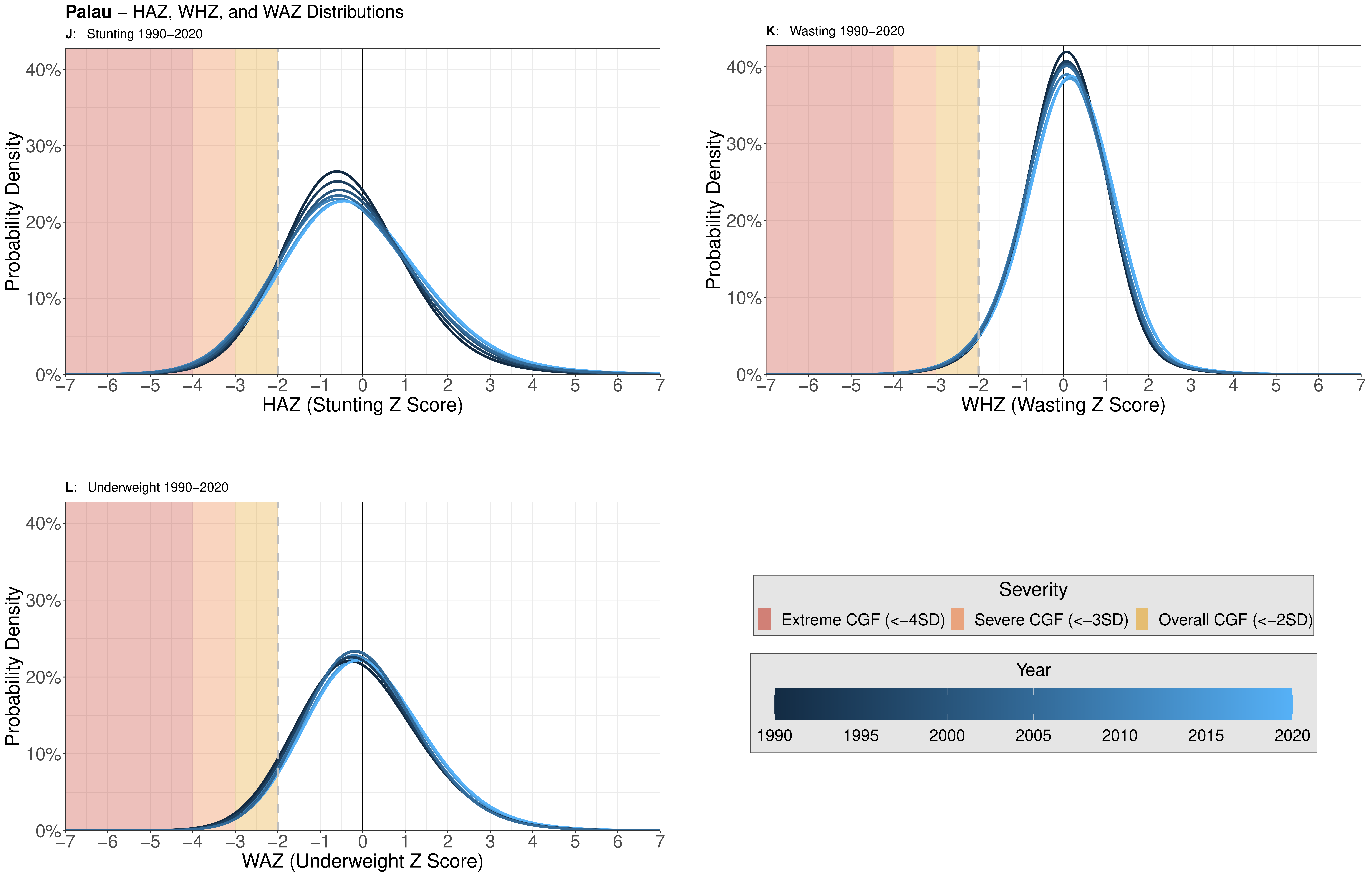

Palau – HAZ, WHZ, and WAZ Distributions
J: Stunting 1990–2020
Probability Density
40%
30%
20%
10%
0%
−7 −6 −5 −4 −3 −2 −1 0 1 2 3 4 5 6 7
HAZ (Stunting Z Score)
K: Wasting 1990–2020
Probability Density
WHZ (Wasting Z Score)
L: Underweight 1990–2020
Probability Density
WAZ (Underweight Z Score)
Severity
Extreme CGF (<−4SD)
Severe CGF (<−3SD)
Overall CGF (<−2SD)
Year
1990
1995
2000
2005
2010
2015
2020

Tokelau – Stunting (HAZ)

A: Overall and Severe Stunting Prevalence

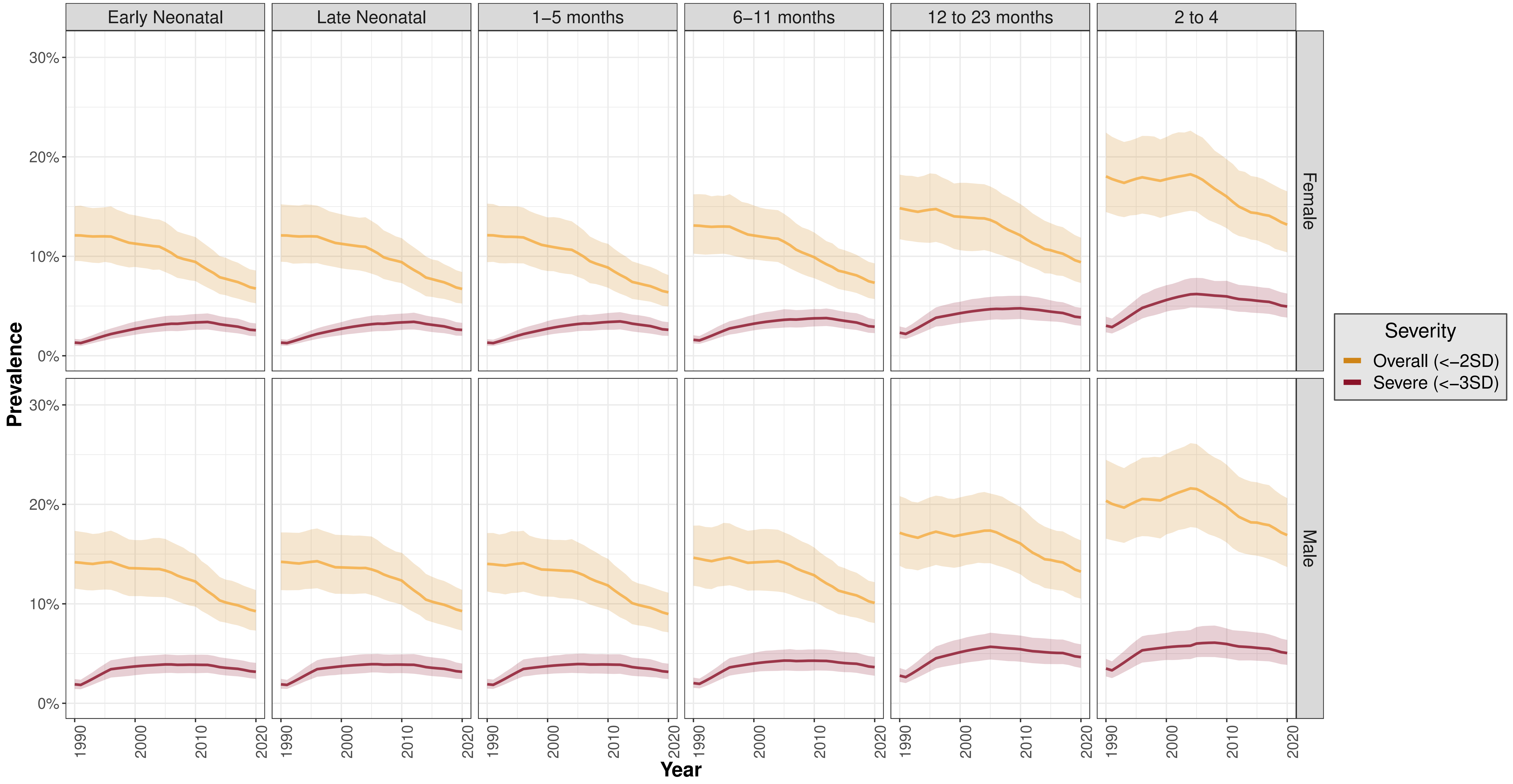

B: Transformed Mean Stunting Z Scores

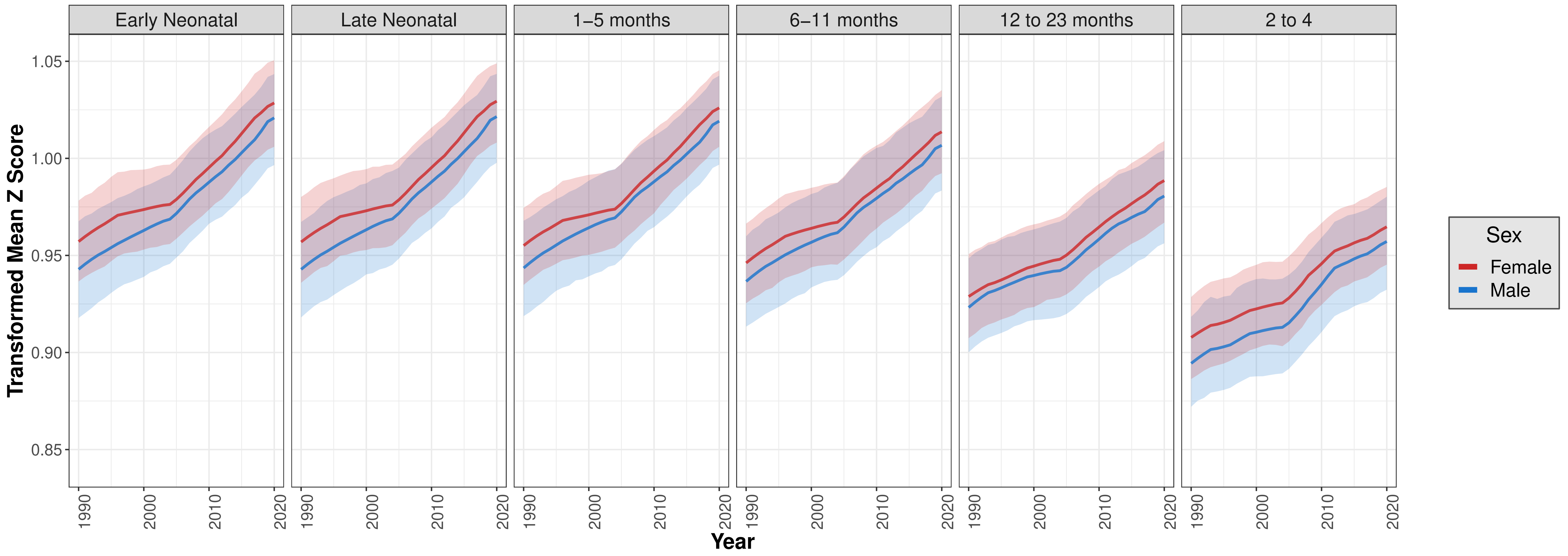

C

**Source**
No sources for this location

Tokelau – Wasting (WHZ)

D: Overall and Severe Wasting Prevalence

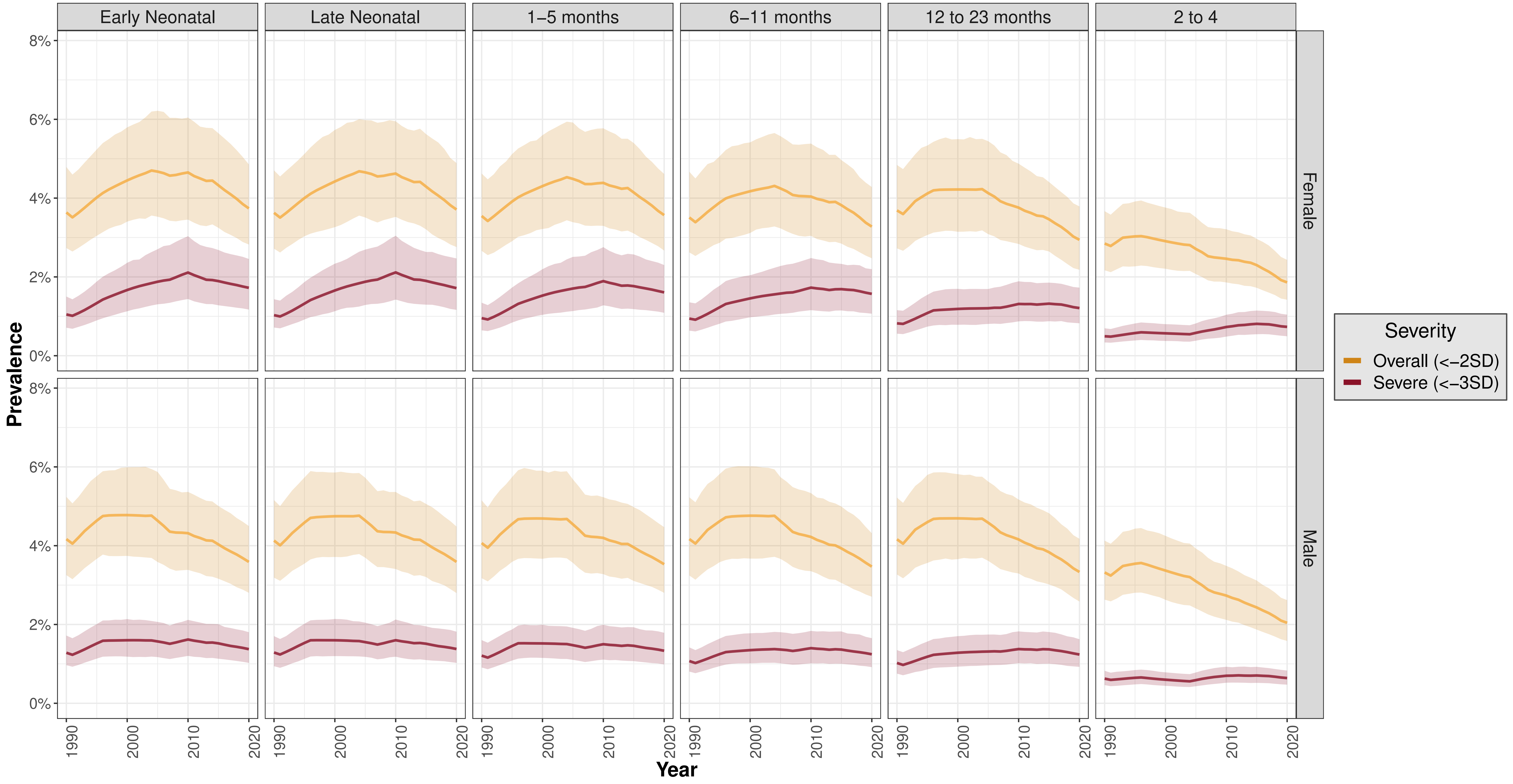

E: Transformed Mean Wasting Z Scores

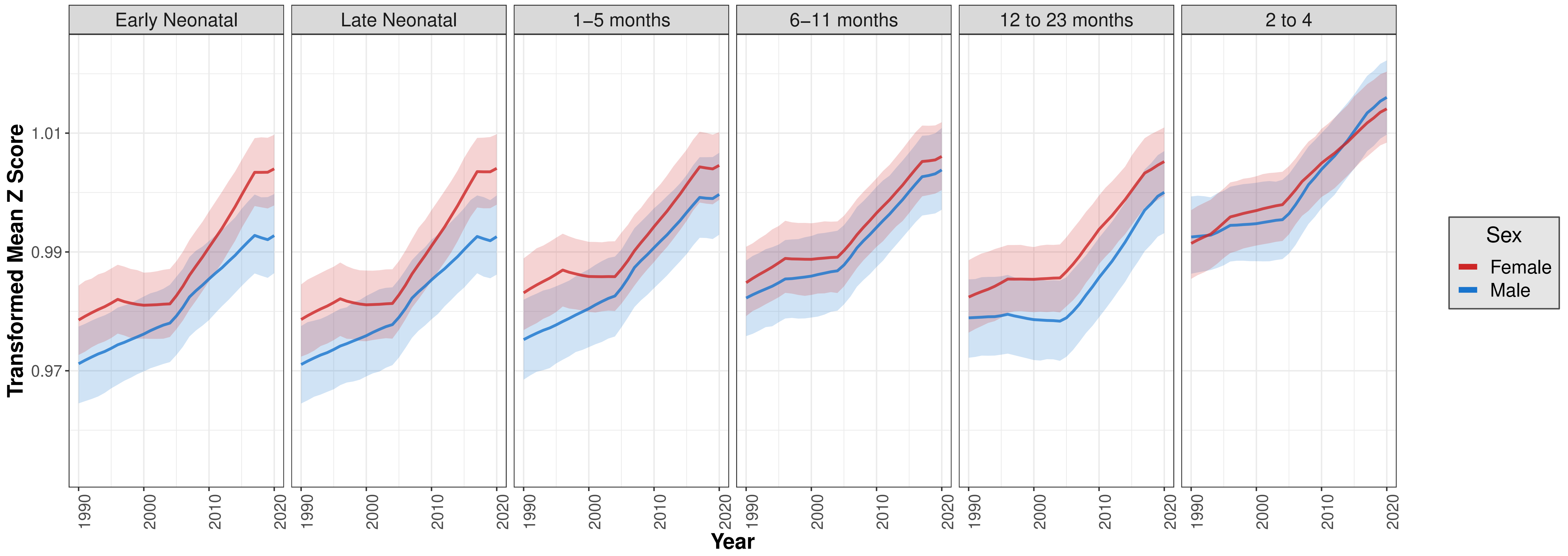

F

Source
No sources for this location

Tokelau – Underweight (WAZ)

G: Overall and Severe Underweight Prevalence

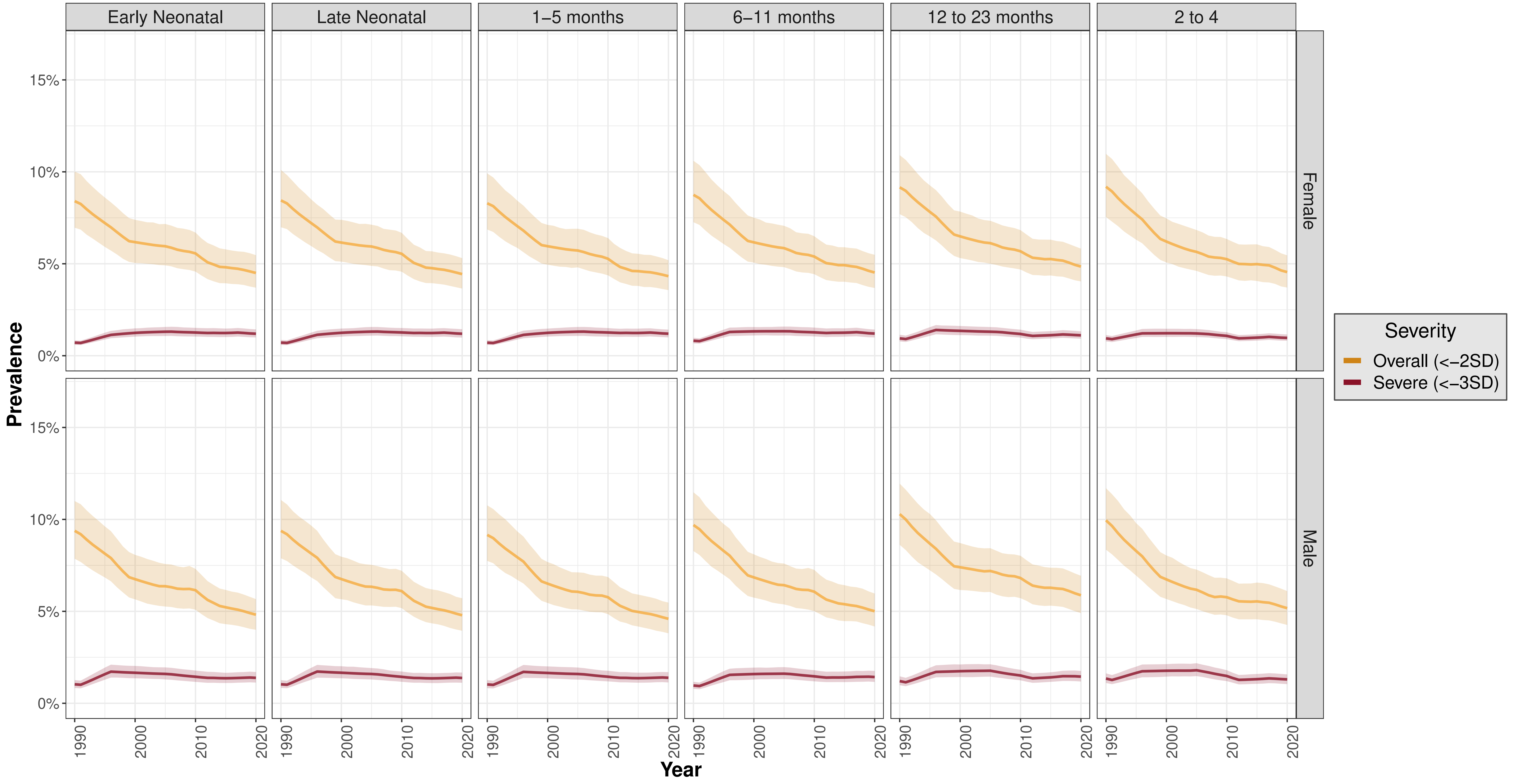

H: Transformed Mean Underweight Z Scores

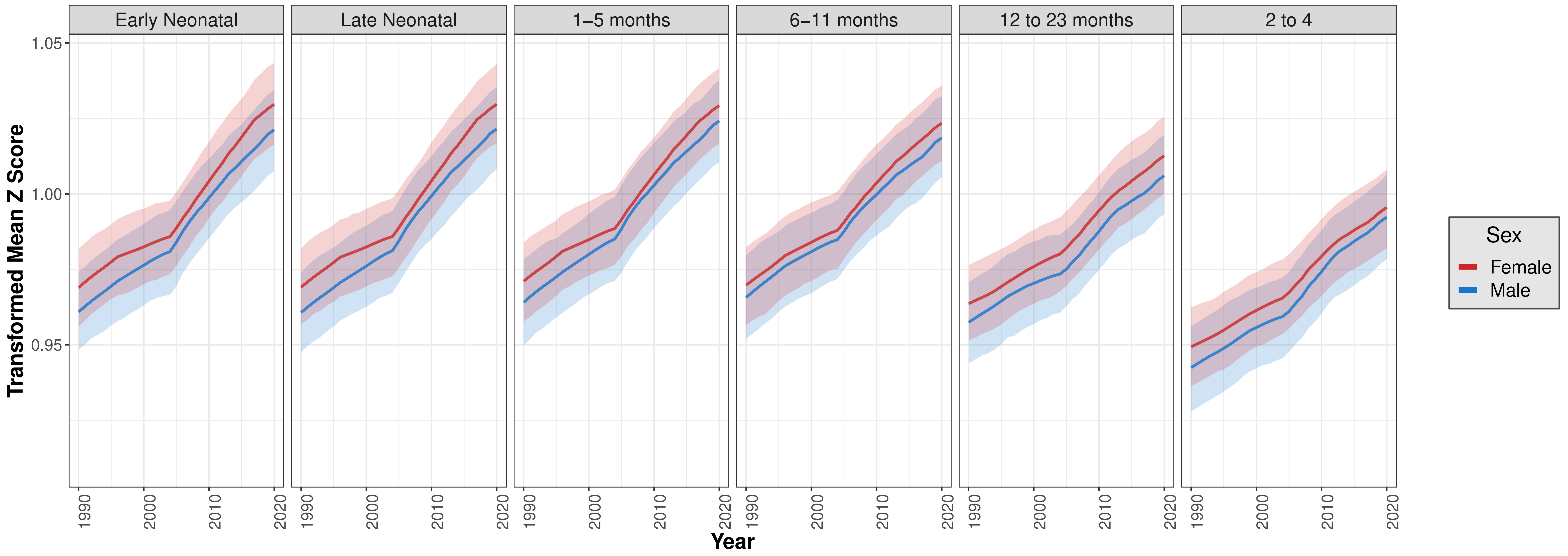

I

Source
No sources for this location

Tokelau – HAZ, WHZ, and WAZ Distributions

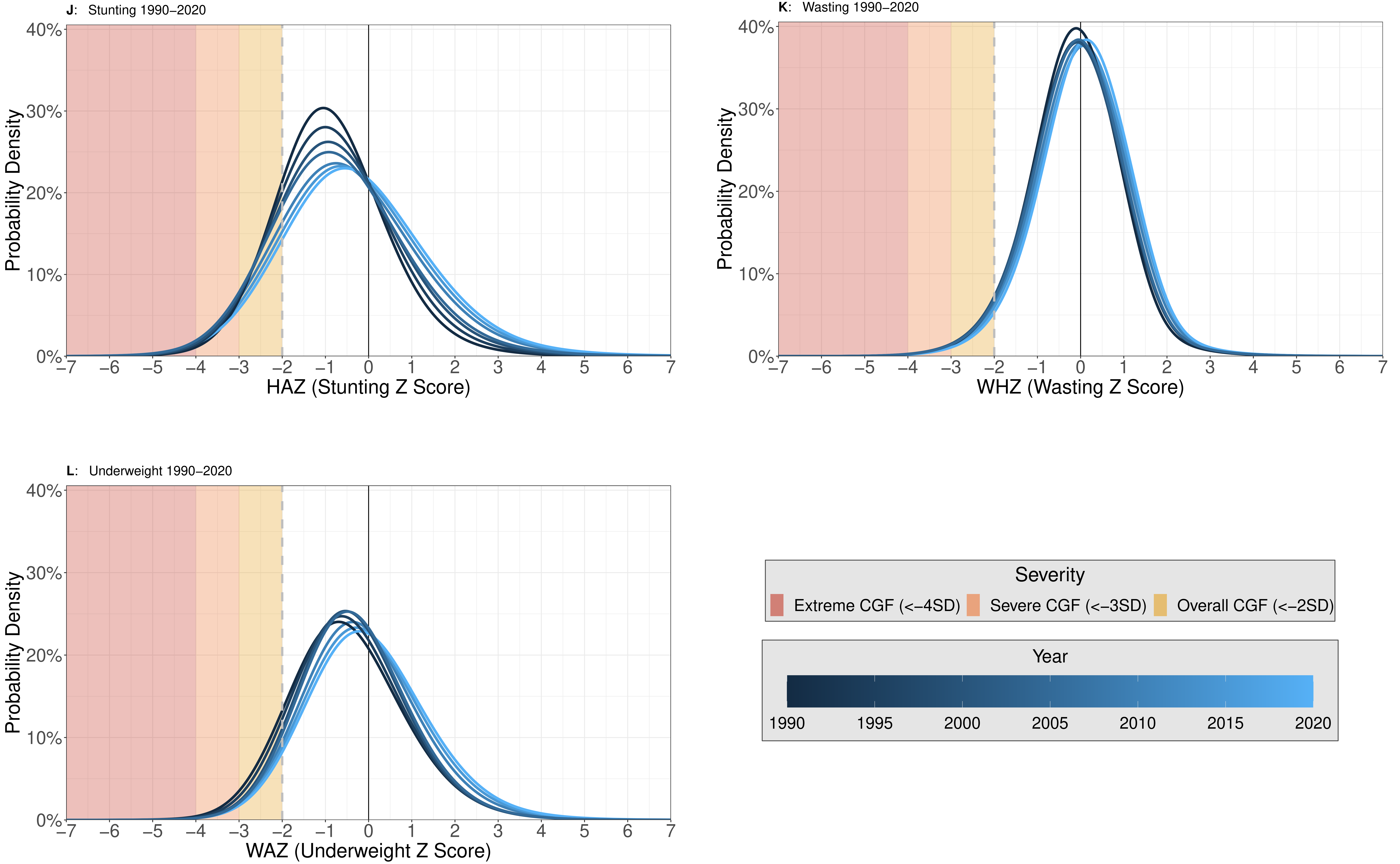

Tuvalu – Stunting (HAZ)

A: Overall and Severe Stunting Prevalence

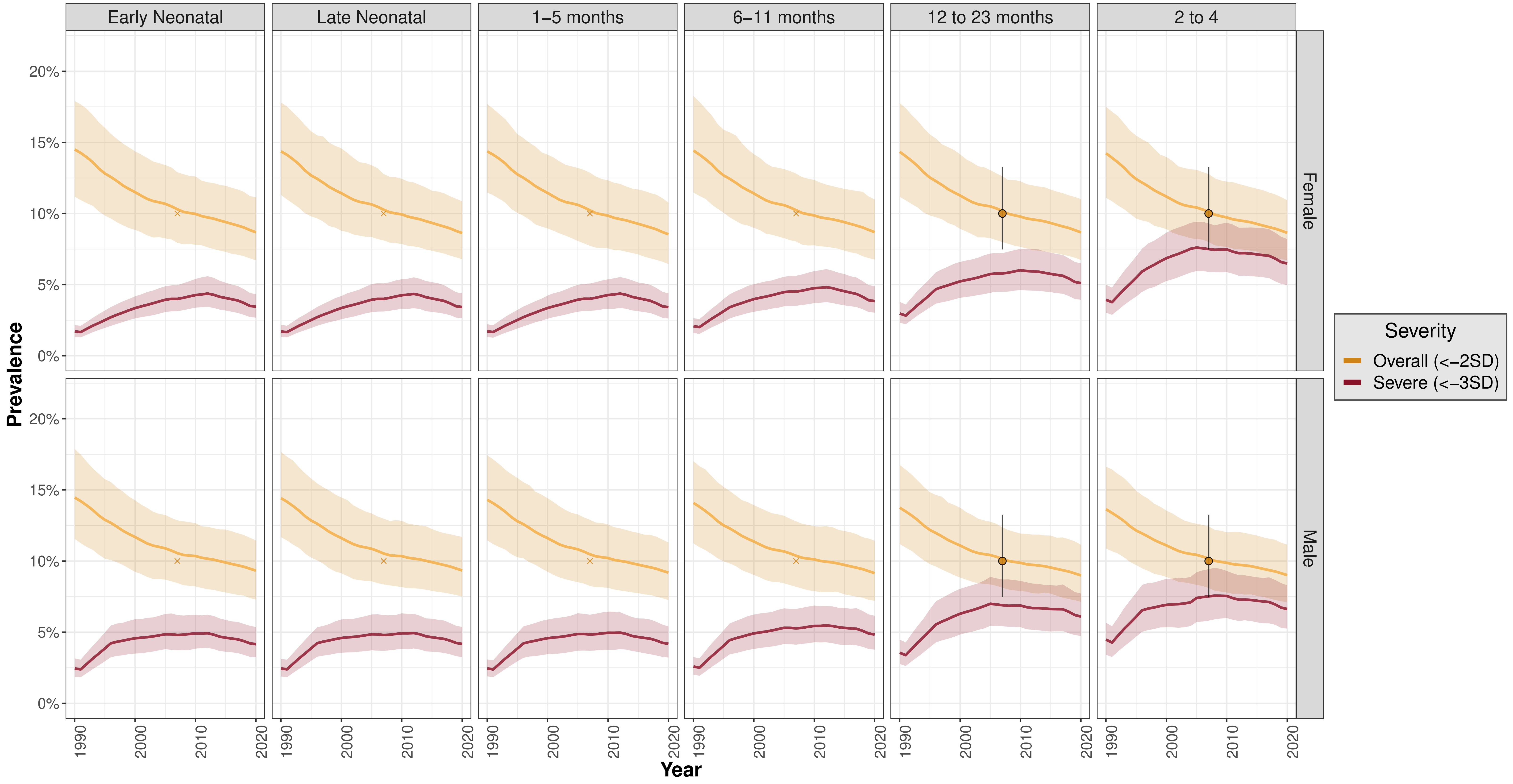

B: Transformed Mean Stunting Z Scores

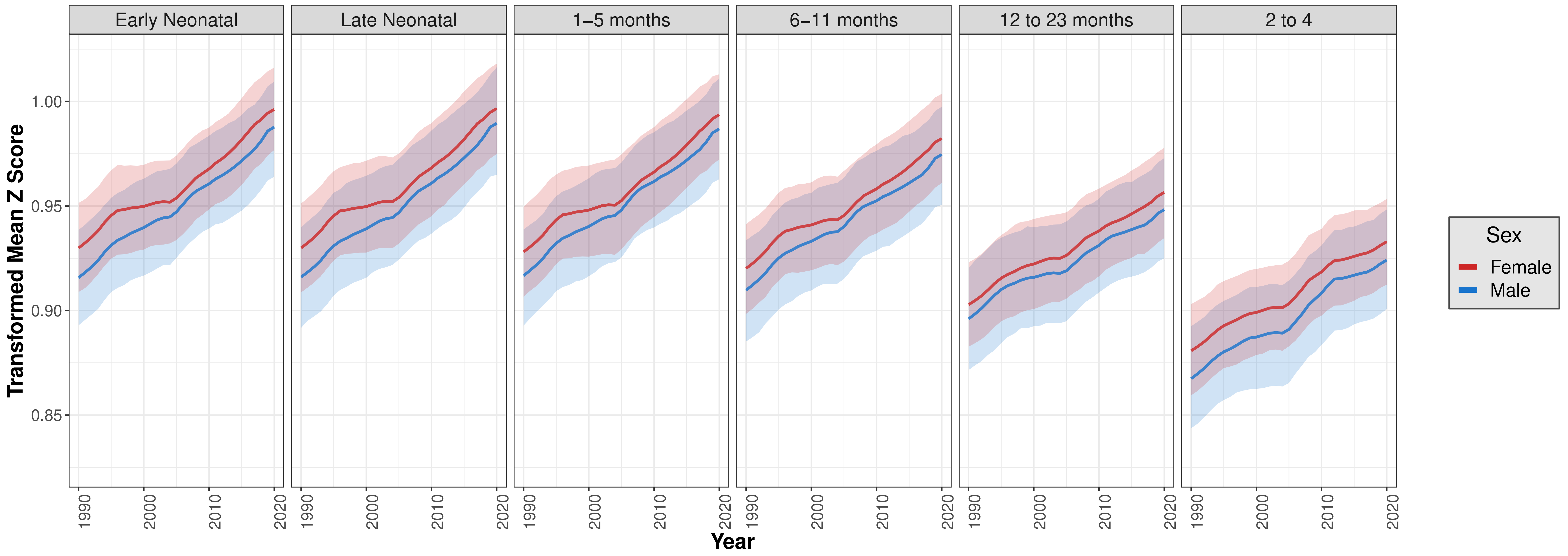

C

Year	Source
2007	WHO CGM Database

Tuvalu – Wasting (WHZ)

D: Overall and Severe Wasting Prevalence

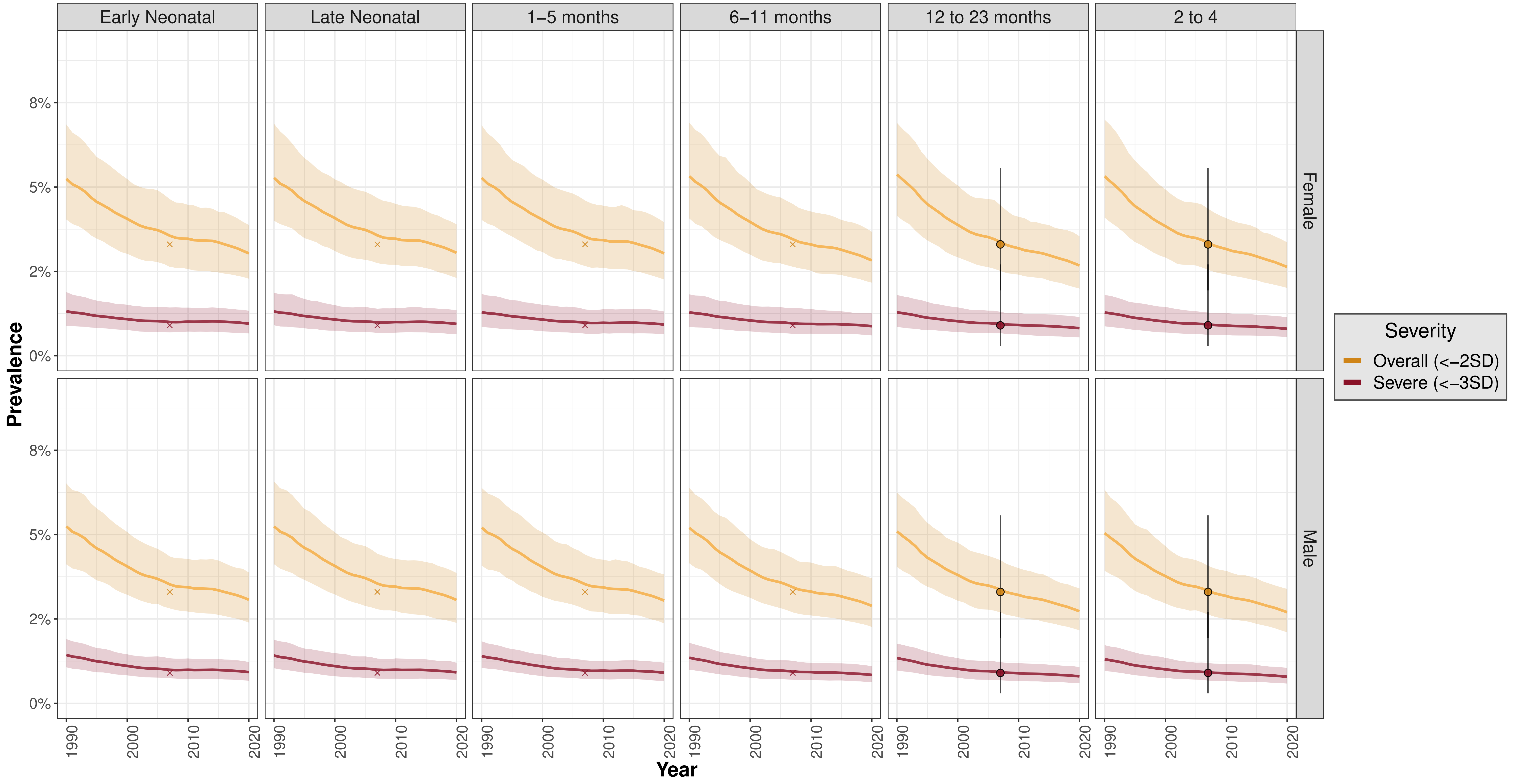

E: Transformed Mean Wasting Z Scores

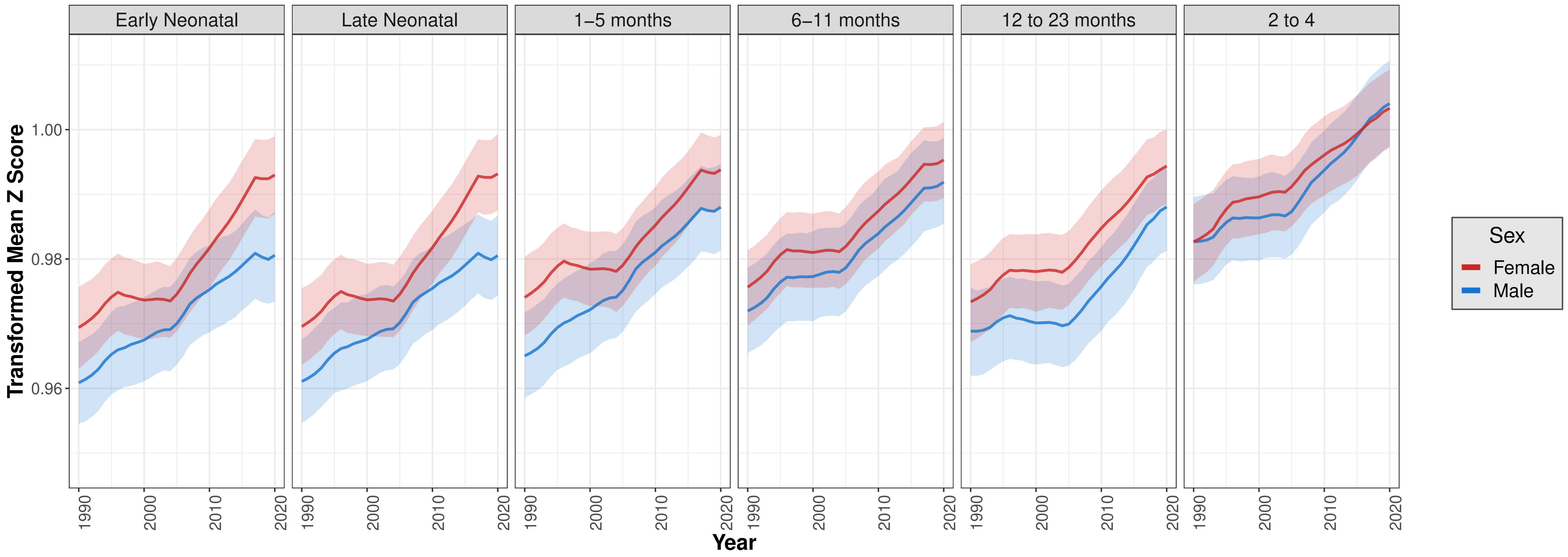

F

Year	Source
2007	WHO CGM Database

Tuvalu – Underweight (WAZ)

G: Overall and Severe Underweight Prevalence

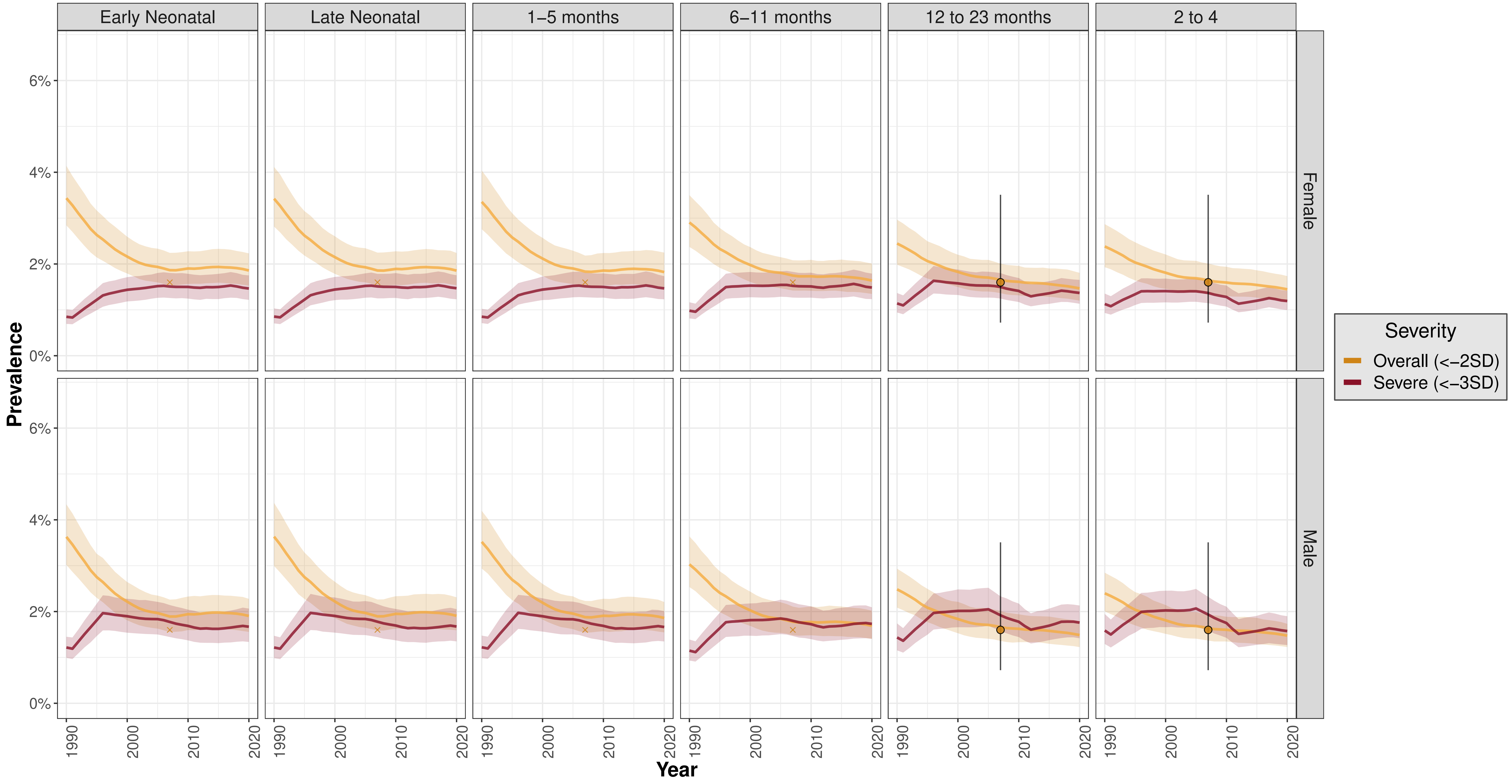

H: Transformed Mean Underweight Z Scores

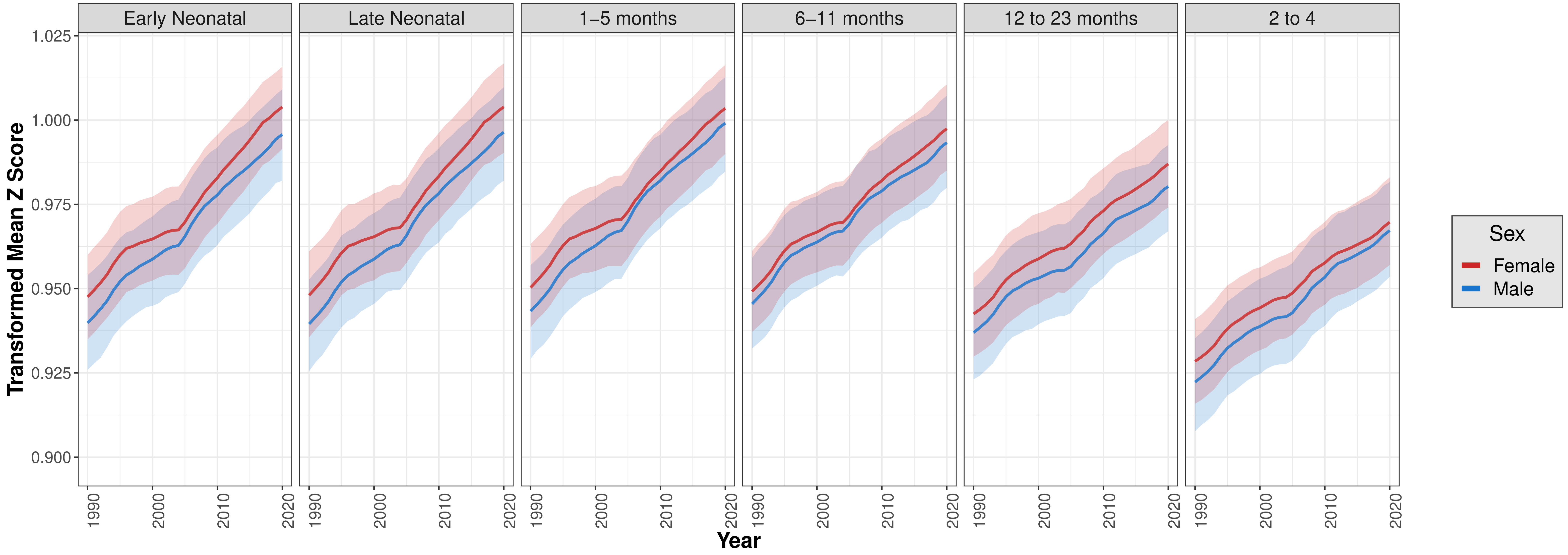

I

Year	Source
2007	WHO CGM Database

Tuvalu – HAZ, WHZ, and WAZ Distributions

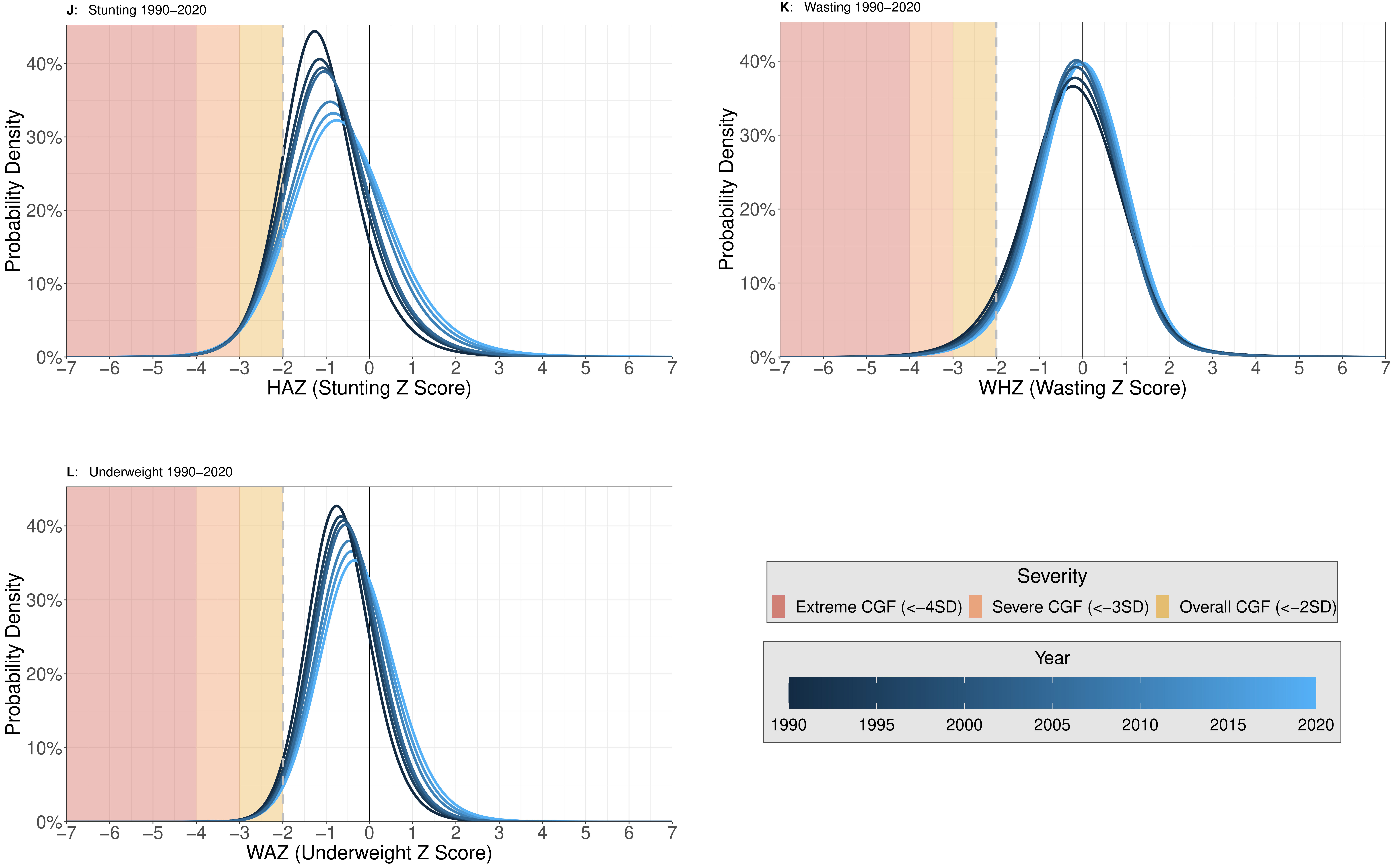

Cambodia – Stunting (HAZ)

A: Overall and Severe Stunting Prevalence

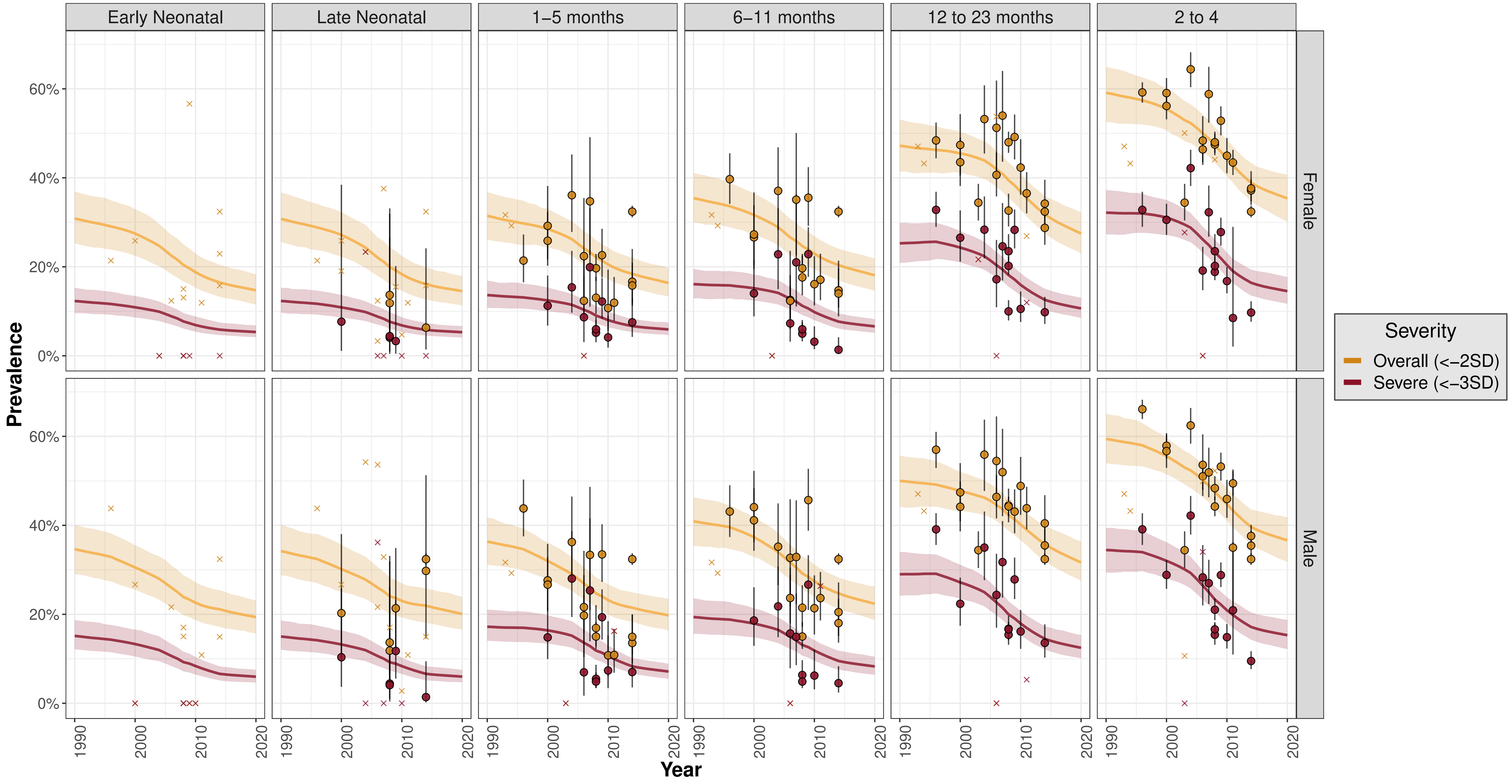

B: Transformed Mean Stunting Z Scores

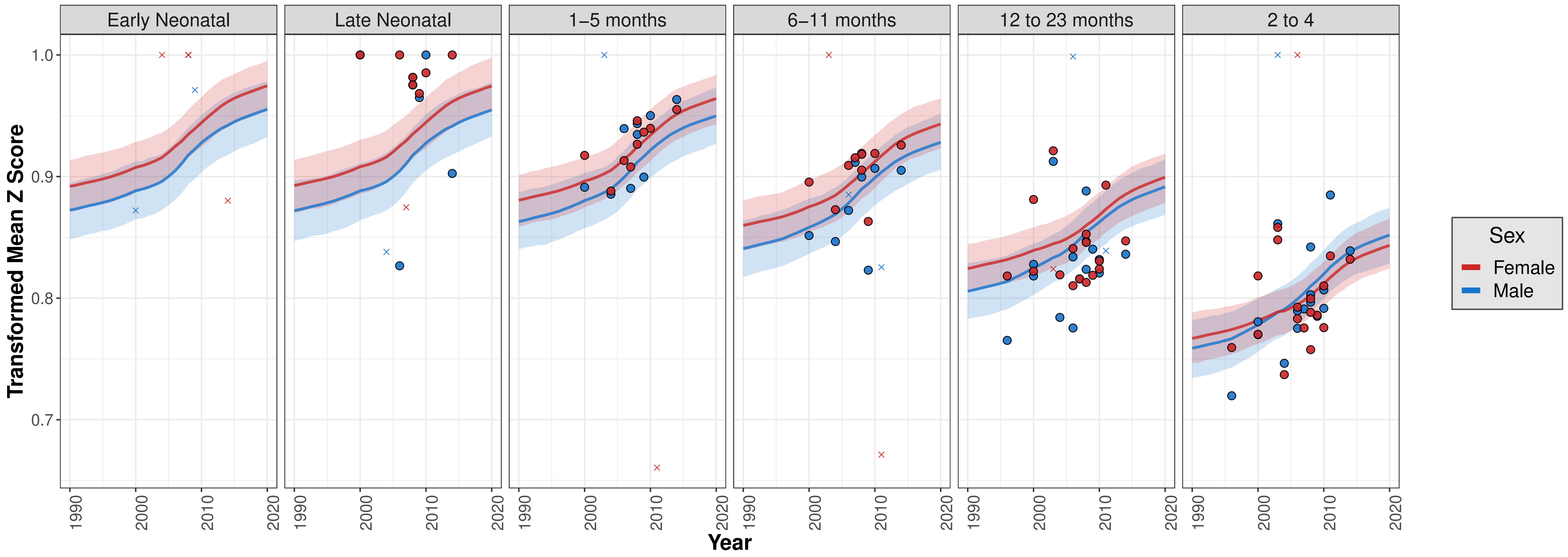

C

Year	Source
1993	WHO CGM Database
1994	WHO CGM Database
1996	WHO CGM Database
2000	DHS
2000	WHO CGM Database
2003	Socio–Economic Survey
2003	Seth Koma Follow–up Survey
2004	Socio–Economic Survey
2006	DHS
2006	Socio–Economic Survey
2006	WHO CGM Database
2007	Socio–Economic Survey
2008	Anthropometric Survey – National Institute of Statistics
2008	WHO CGM Database
2008	Anthropometrics Survey – CamNut
2009	Socio–Economic Survey
2010	DHS
2010	WHO CGM Database
2011	DHS
2011	WHO CGM Database
2014	WHO CGM Database
2014	DHS

Cambodia – Wasting (WHZ)

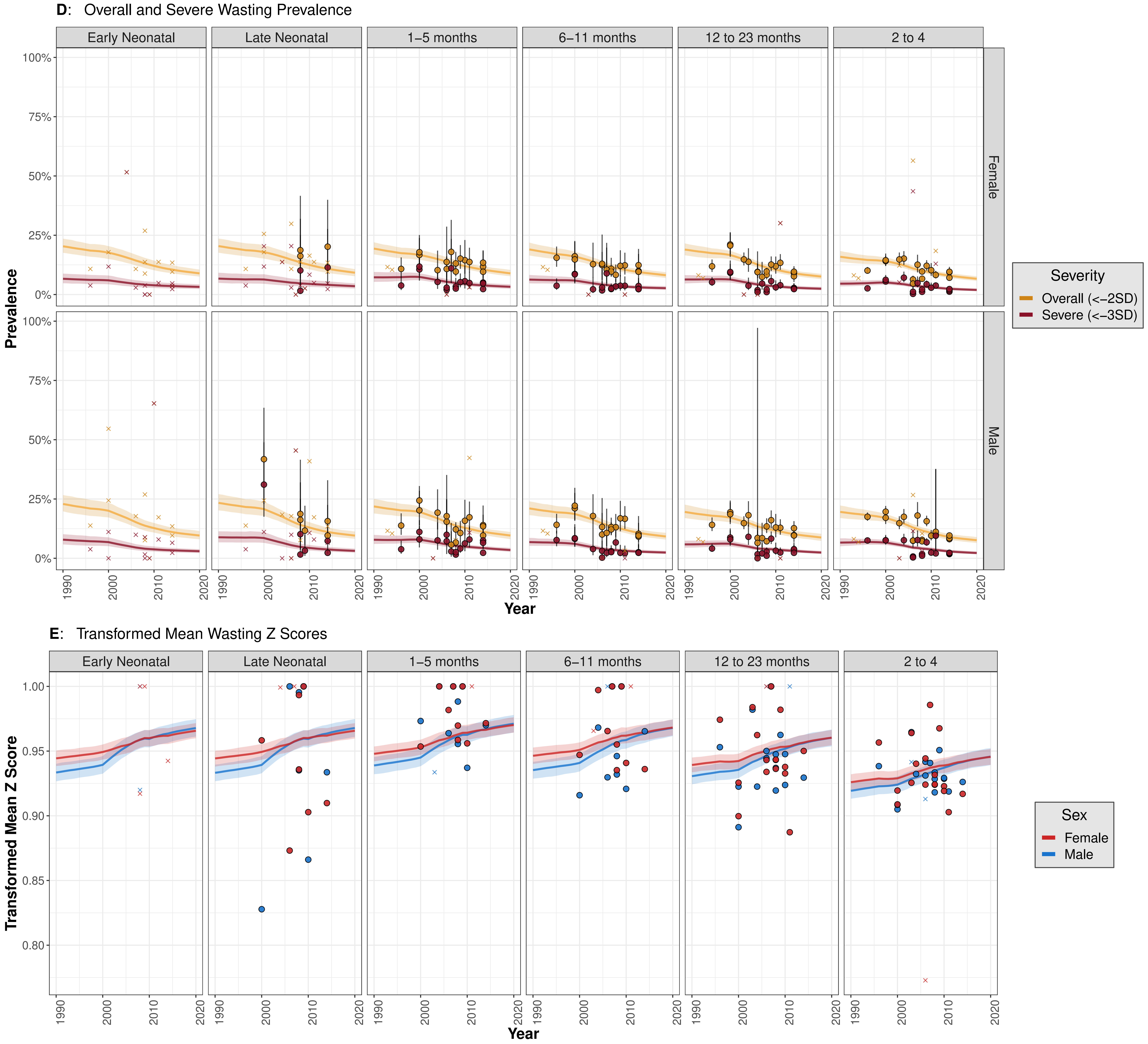

F

Year	Source
1993	WHO CGM Database
1994	WHO CGM Database
1996	WHO CGM Database
2000	DHS
2000	WHO CGM Database
2003	Socio–Economic Survey
2003	Seth Koma Follow–up Survey
2004	Socio–Economic Survey
2006	DHS
2006	Socio–Economic Survey
2006	WHO CGM Database
2007	Socio–Economic Survey
2008	Anthropometric Survey – National Institute of Statistics
2008	WHO CGM Database
2008	Anthropometrics Survey – CamNut
2009	Socio–Economic Survey
2010	DHS
2010	WHO CGM Database
2011	DHS
2011	WHO CGM Database
2014	WHO CGM Database
2014	DHS

Cambodia – Underweight (WAZ)

G: Overall and Severe Underweight Prevalence

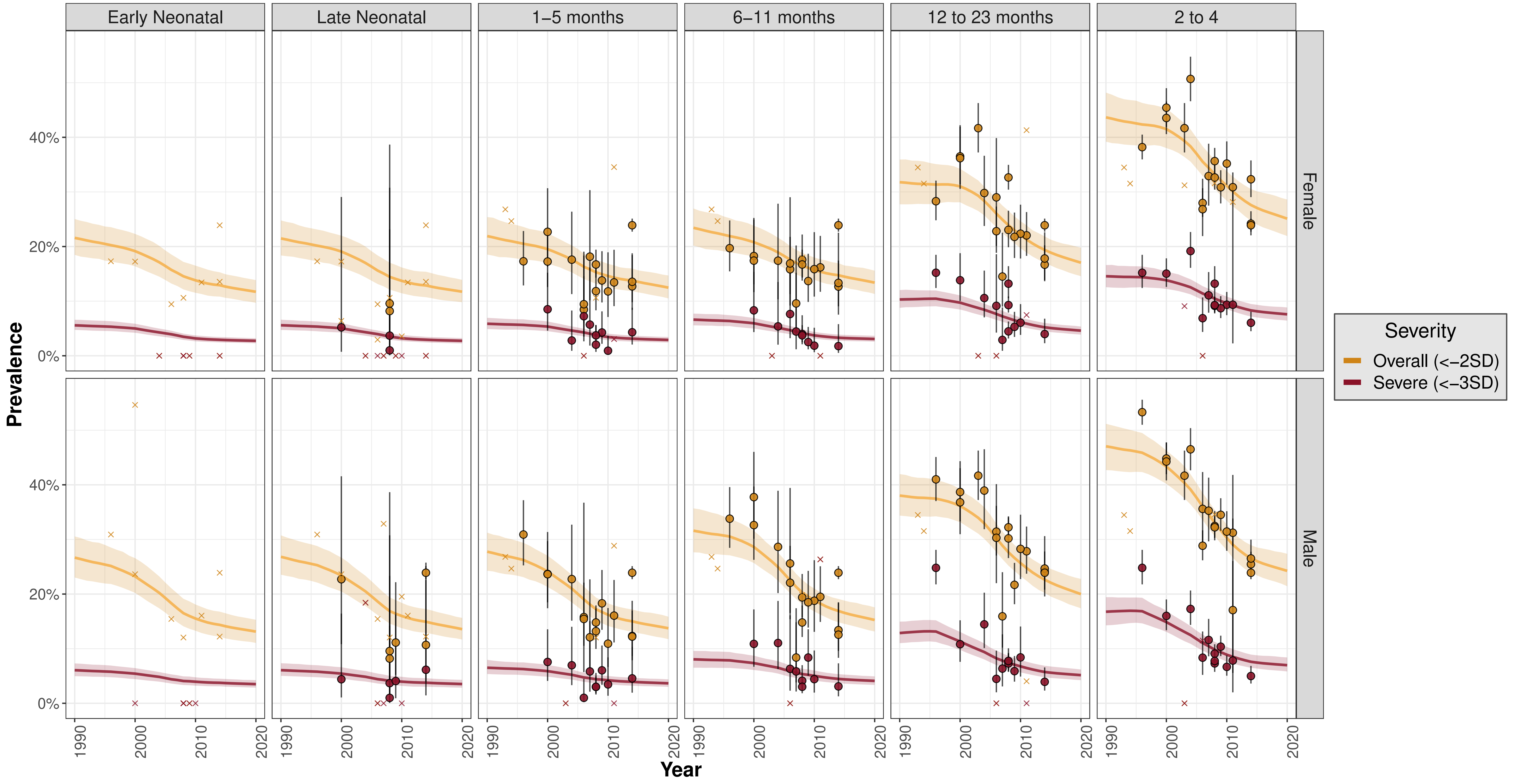

H: Transformed Mean Underweight Z Scores

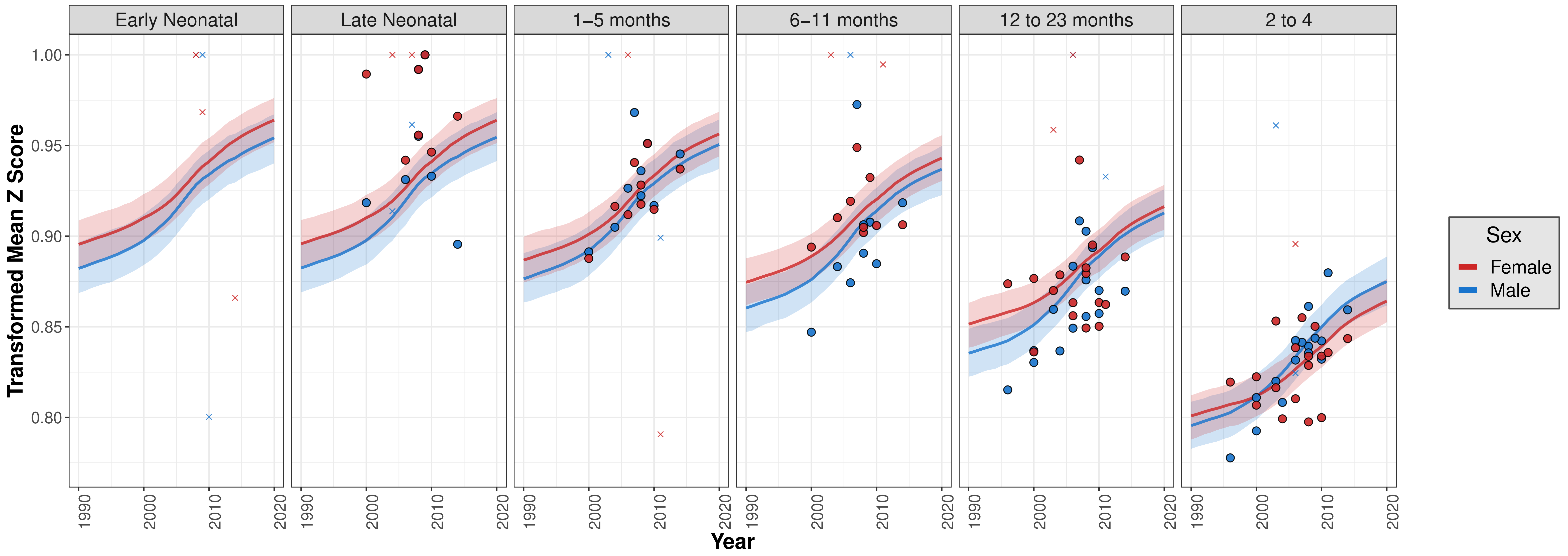

I

Year	Source
1993	WHO CGM Database
1994	WHO CGM Database
1996	WHO CGM Database
2000	DHS
2000	WHO CGM Database
2003	Socio–Economic Survey
2003	Seth Koma Follow–up Survey
2004	Socio–Economic Survey
2006	DHS
2006	Socio–Economic Survey
2006	WHO CGM Database
2007	Socio–Economic Survey
2008	Anthropometric Survey – National Institute of Statistics
2008	WHO CGM Database
2008	Anthropometrics Survey – CamNut
2009	Socio–Economic Survey
2010	DHS
2010	WHO CGM Database
2011	DHS
2011	WHO CGM Database
2014	WHO CGM Database
2014	DHS

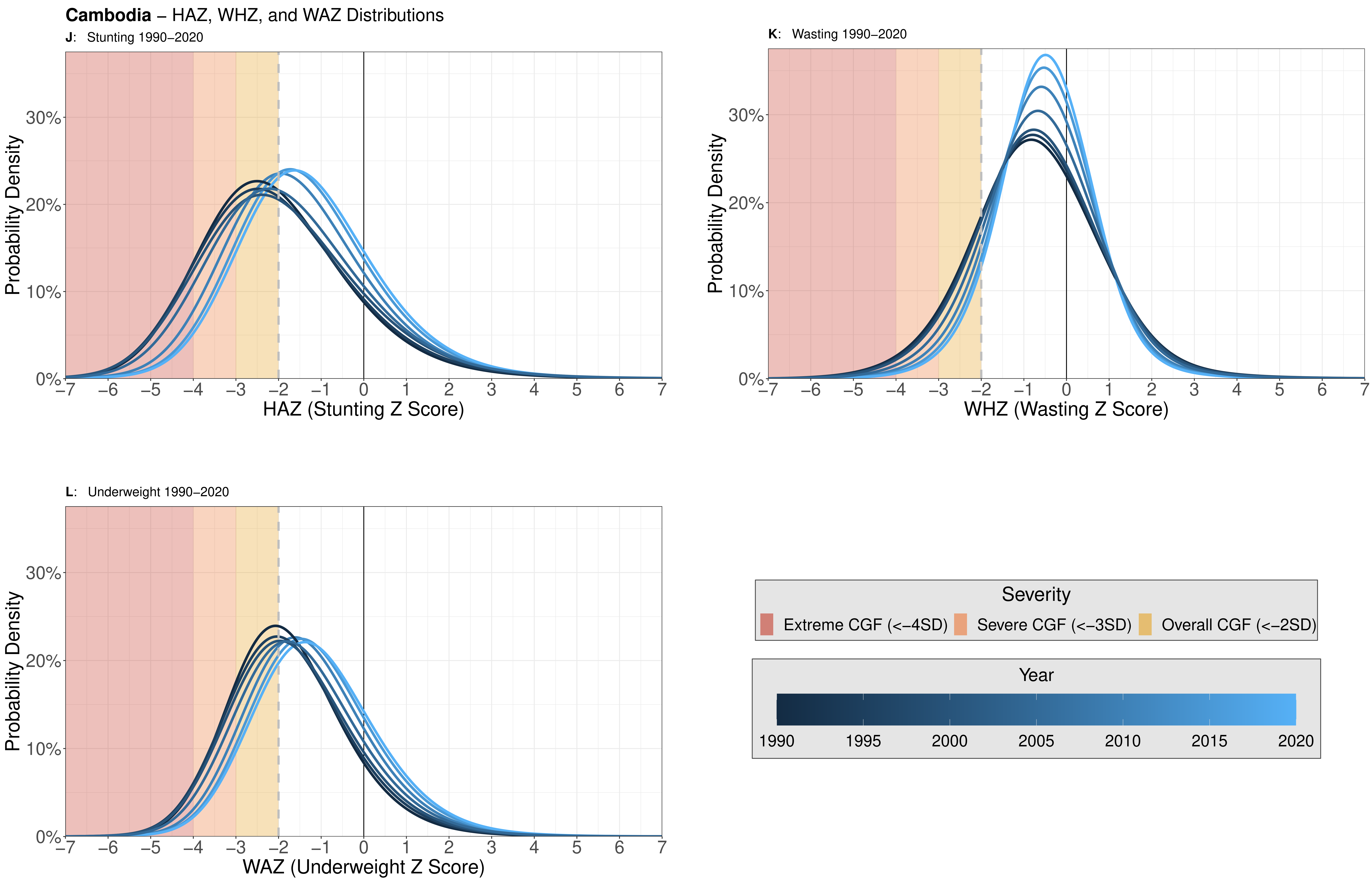

Cambodia – HAZ, WHZ, and WAZ Distributions
J: Stunting 1990–2020
K: Wasting 1990–2020
L: Underweight 1990–2020
Probability Density
0%
10%
20%
30%
−7 −6 −5 −4 −3 −2 −1 0 1 2 3 4 5 6 7
HAZ (Stunting Z Score)
WHZ (Wasting Z Score)
WAZ (Underweight Z Score)
Severity
Extreme CGF (<−4SD)
Severe CGF (<−3SD)
Overall CGF (<−2SD)
Year
1990
1995
2000
2005
2010
2015
2020

Indonesia – Stunting (HAZ)

A: Overall and Severe Stunting Prevalence

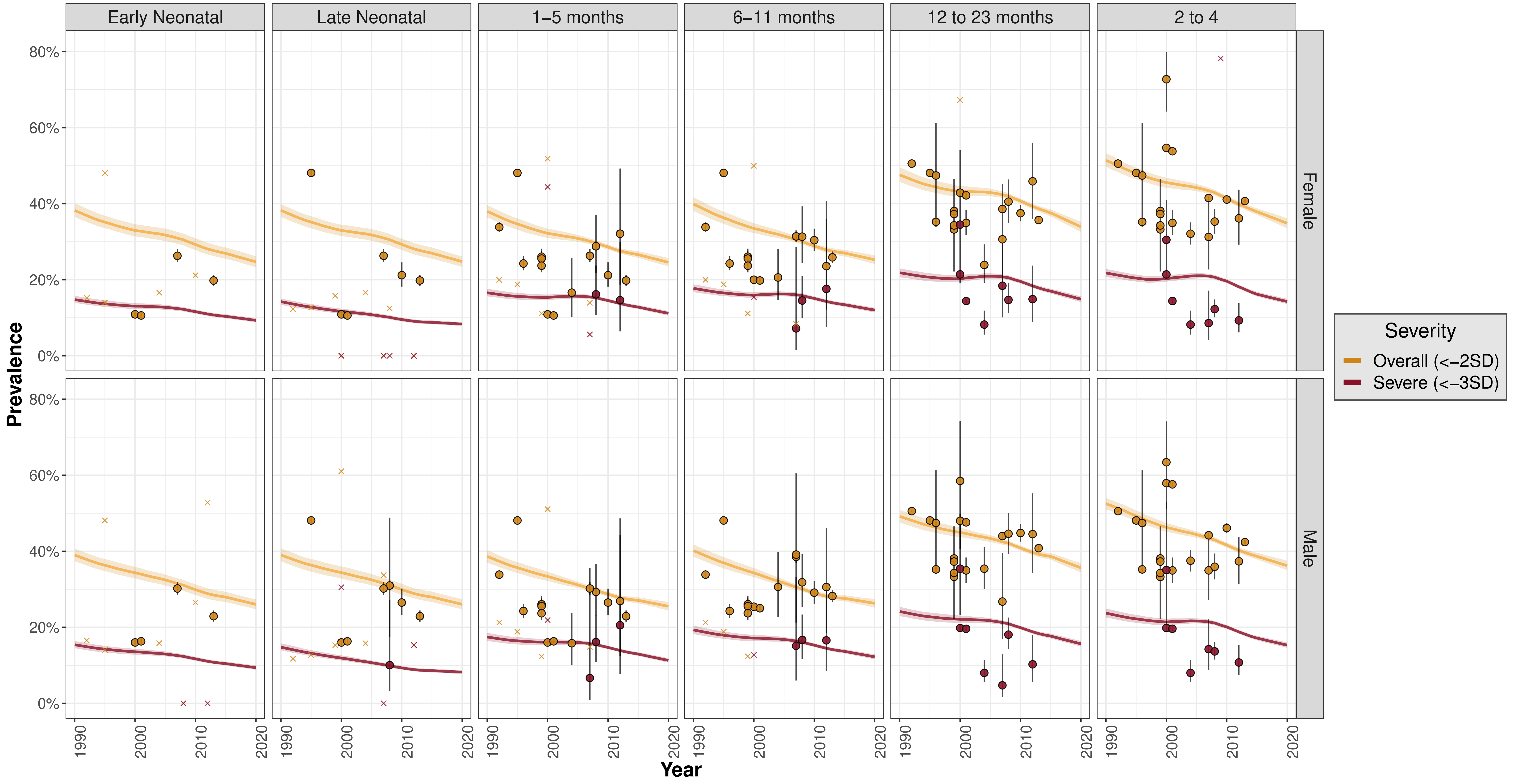

B: Transformed Mean Stunting Z Scores

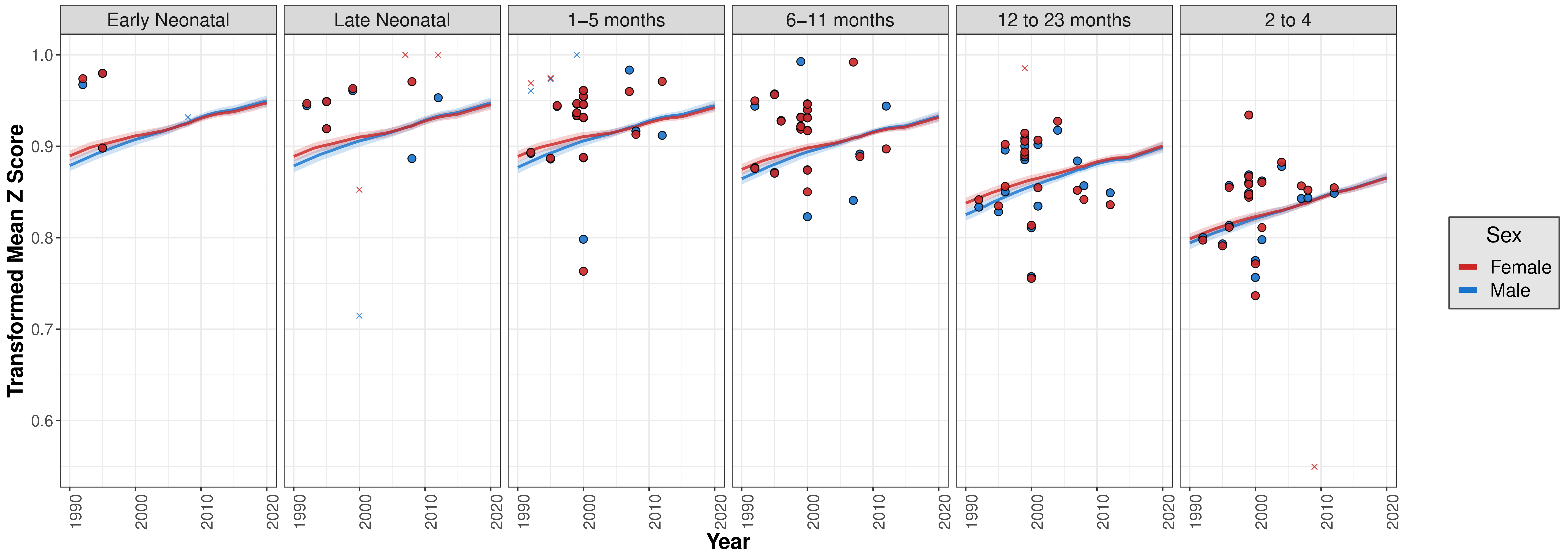

C

Year	Source	National	Subnational
1992	WHO CGM Database	X	
1992	End Decade Statistical Report: Data and Descriptive Analysis	X	
1993	Family Life Survey		X
1994	Family Life Survey		X
1995	WHO CGM Database	X	
1995	End Decade Statistical Report: Data and Descriptive Analysis	X	
1996	WHO CGM Database	X	
1997	Family Life Survey		X
1999	WHO CGM Database	X	
1999	End Decade Statistical Report: Data and Descriptive Analysis	X	
2000	Family Life Survey	X	X
2000	WHO CGM Database	X	
2001	National Health Survey – Round 1	X	
2001	WHO CGM Database	X	
2004	WHO CGM Database	X	
2007	Family Life Survey	X	
2007	WHO CGM Database	X	X
2008	Family Life Survey	X	
2009	Family Life Survey	X	
2010	WHO CGM Database	X	X
2012	Family Life Survey East	X	
2013	WHO CGM Database	X	X
2014	Family Life Survey		X
2015	Family Life Survey		X

Indonesia – Wasting (WHZ)

D: Overall and Severe Wasting Prevalence

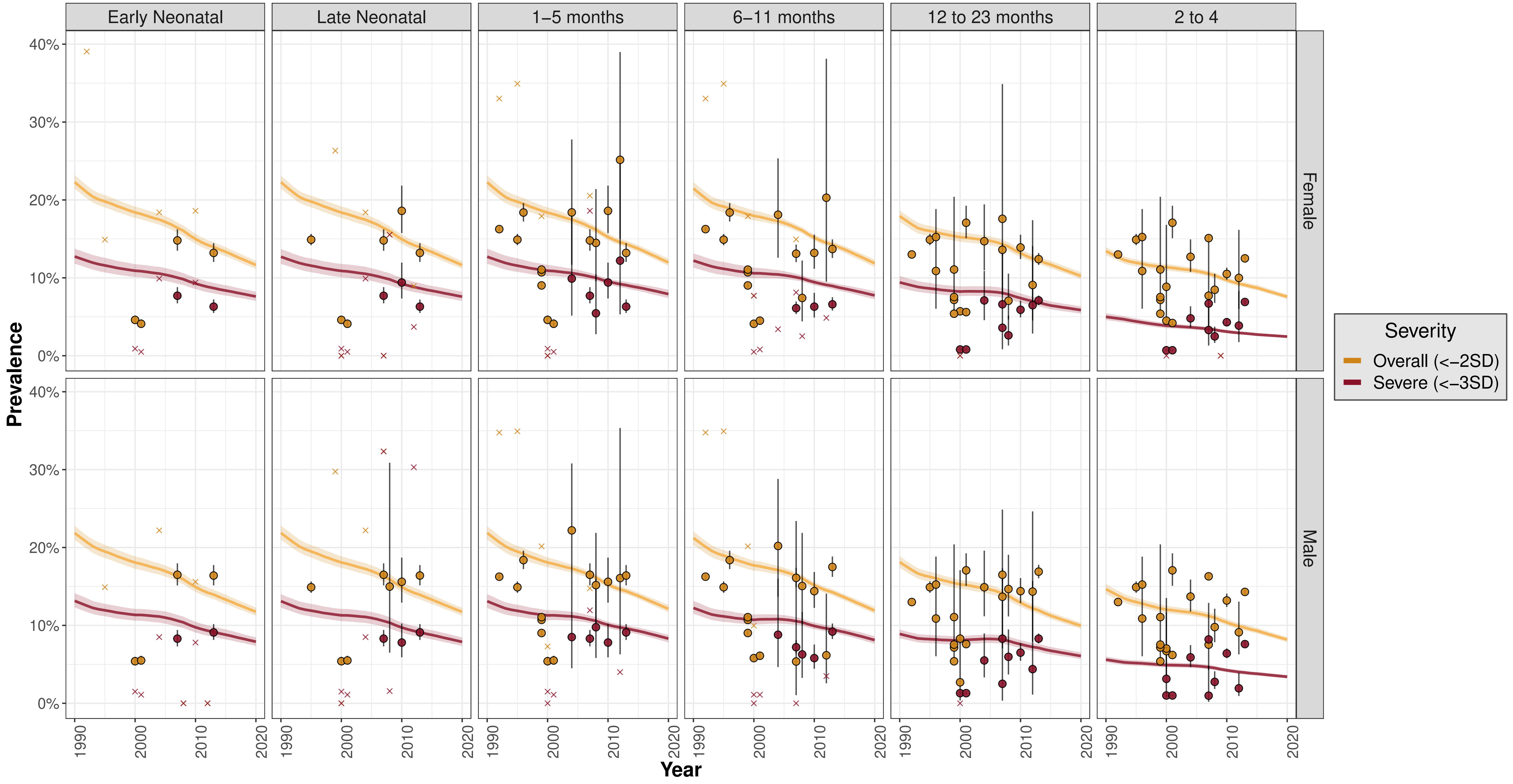

E: Transformed Mean Wasting Z Scores

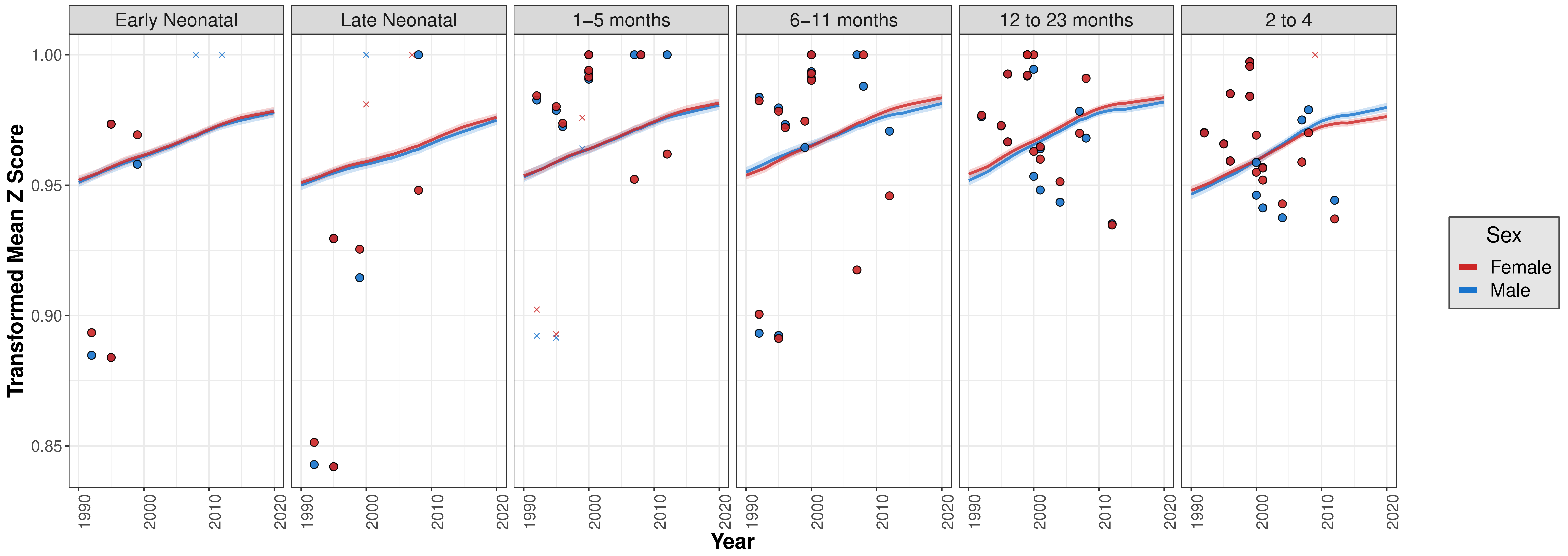

F

Year	Source	National	Subnational
1992	WHO CGM Database	X	
1992	End Decade Statistical Report: Data and Descriptive Analysis	X	
1993	Family Life Survey		X
1994	Family Life Survey		X
1995	WHO CGM Database	X	
1995	End Decade Statistical Report: Data and Descriptive Analysis	X	
1996	WHO CGM Database	X	
1997	Family Life Survey		X
1999	WHO CGM Database	X	
1999	End Decade Statistical Report: Data and Descriptive Analysis	X	
2000	Family Life Survey	X	X
2000	WHO CGM Database	X	
2001	National Health Survey – Round 1	X	
2001	WHO CGM Database	X	
2004	WHO CGM Database	X	
2007	Family Life Survey	X	
2007	WHO CGM Database	X	X
2008	Family Life Survey	X	
2009	Family Life Survey	X	
2010	WHO CGM Database	X	X
2012	Family Life Survey East	X	
2013	WHO CGM Database	X	X
2014	Family Life Survey		X
2015	Family Life Survey		X

Indonesia – Underweight (WAZ)

G: Overall and Severe Underweight Prevalence

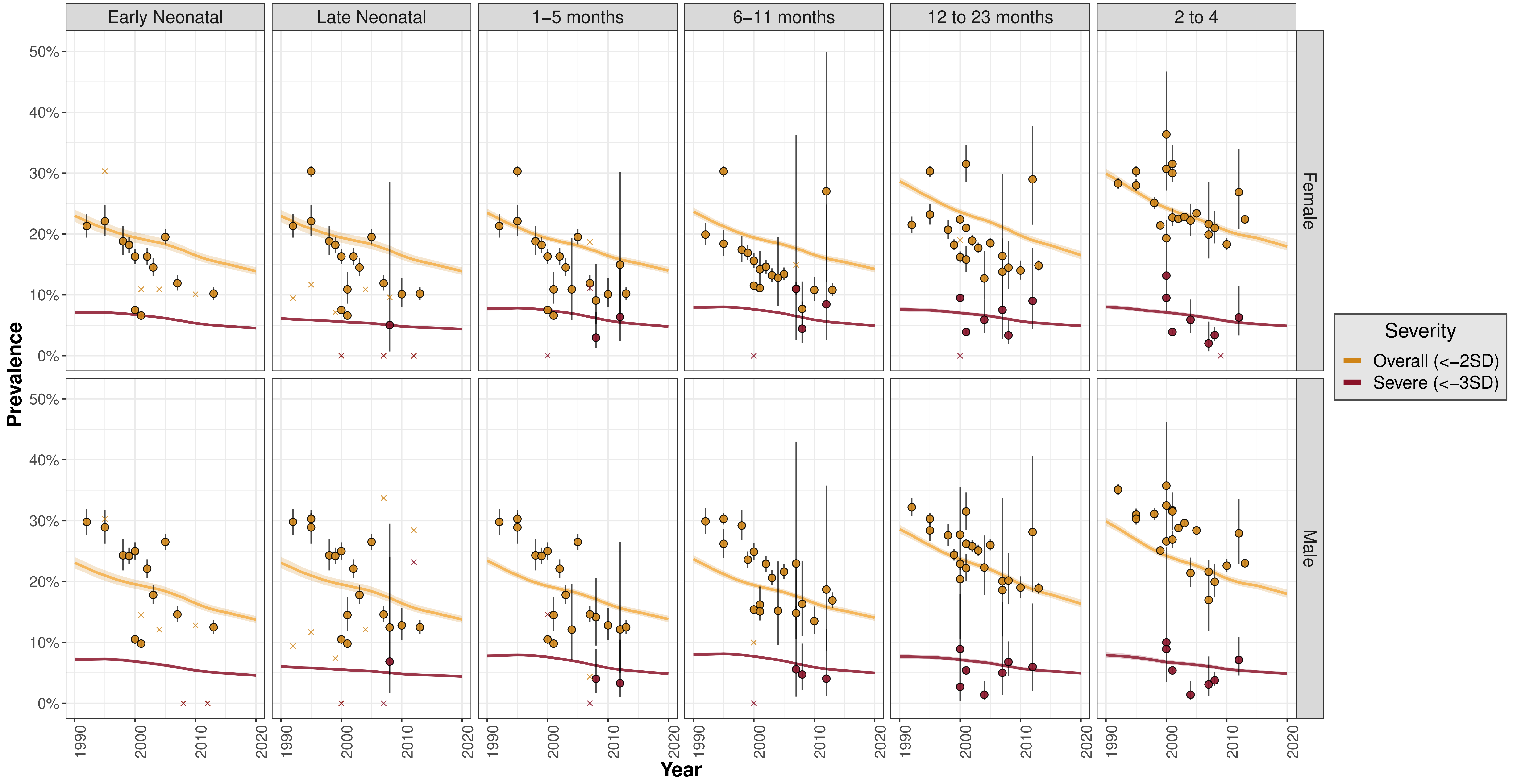

H: Transformed Mean Underweight Z Scores

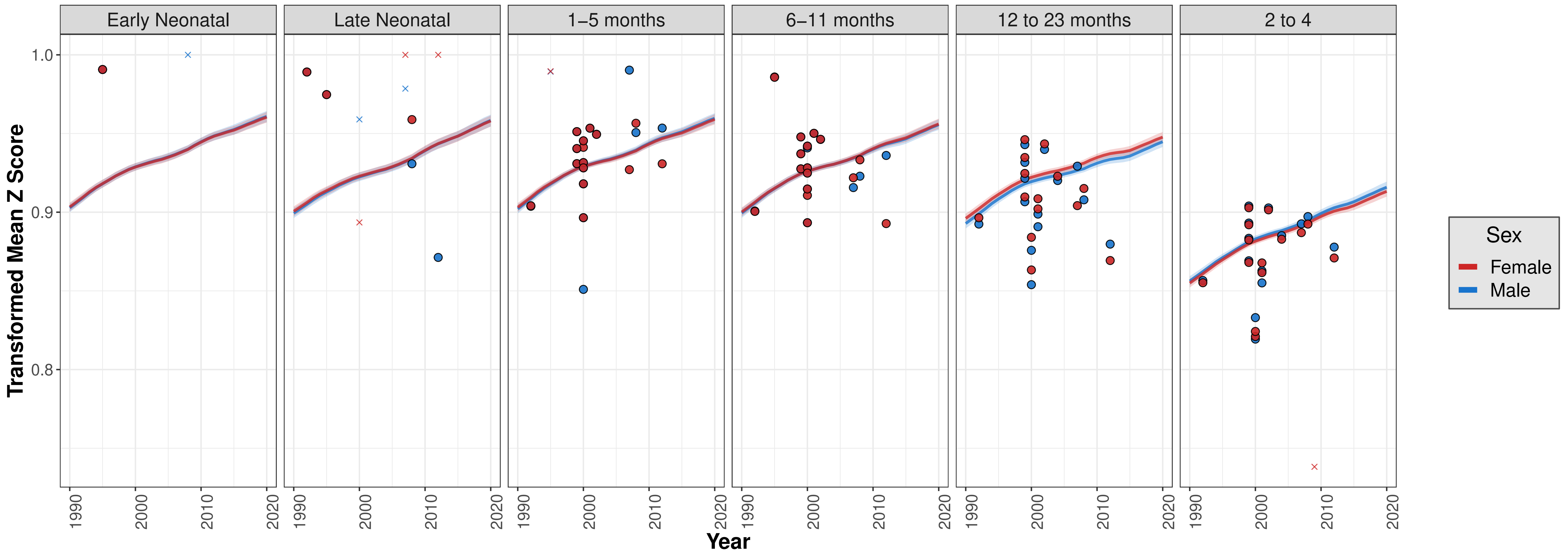

I

Year	Source	National	Subnational
1987	WHO CGM Database	X	
1989	WHO CGM Database	X	X
1992	WHO CGM Database	X	X
1992	End Decade Statistical Report: Data and Descriptive Analysis	X	
1993	Family Life Survey		X
1994	Family Life Survey		X
1995	WHO CGM Database	X	X
1995	End Decade Statistical Report: Data and Descriptive Analysis	X	
1997	Family Life Survey		X
1998	WHO CGM Database	X	X
1999	WHO CGM Database	X	X
1999	End Decade Statistical Report: Data and Descriptive Analysis	X	
2000	Family Life Survey	X	X
2000	WHO CGM Database	X	X
2001	National Health Survey – Round 1	X	
2001	WHO CGM Database	X	X
2002	WHO CGM Database	X	X
2003	WHO CGM Database	X	X
2004	WHO CGM Database	X	
2005	WHO CGM Database	X	X
2007	Family Life Survey	X	
2007	WHO CGM Database	X	X
2008	Family Life Survey	X	
2009	Family Life Survey	X	
2010	WHO CGM Database	X	X
2012	Family Life Survey East	X	
2013	WHO CGM Database	X	X
2014	Family Life Survey		X
2015	Family Life Survey		X

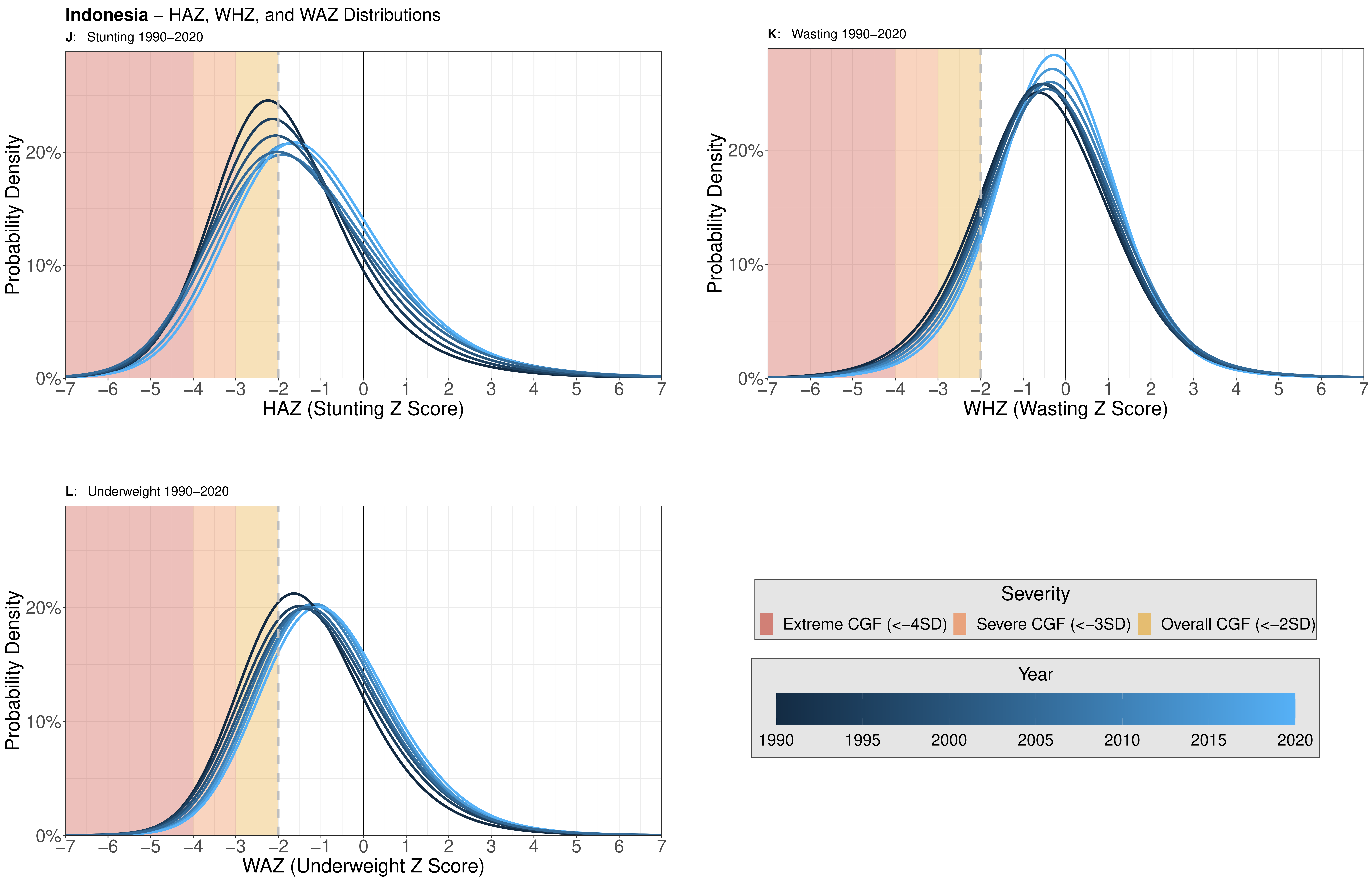
Indonesia – HAZ, WHZ, and WAZ Distributions
J: Stunting 1990–2020
Probability Density
0%
10%
20%
−7 −6 −5 −4 −3 −2 −1 0 1 2 3 4 5 6 7
HAZ (Stunting Z Score)
K: Wasting 1990–2020
Probability Density
0%
10%
20%
−7 −6 −5 −4 −3 −2 −1 0 1 2 3 4 5 6 7
WHZ (Wasting Z Score)
L: Underweight 1990–2020
Probability Density
0%
10%
20%
−7 −6 −5 −4 −3 −2 −1 0 1 2 3 4 5 6 7
WAZ (Underweight Z Score)
Severity
Extreme CGF (<−4SD)
Severe CGF (<−3SD)
Overall CGF (<−2SD)
Year
1990
1995
2000
2005
2010
2015
2020

Lao People's Democratic Republic – Stunting (HAZ)

A: Overall and Severe Stunting Prevalence

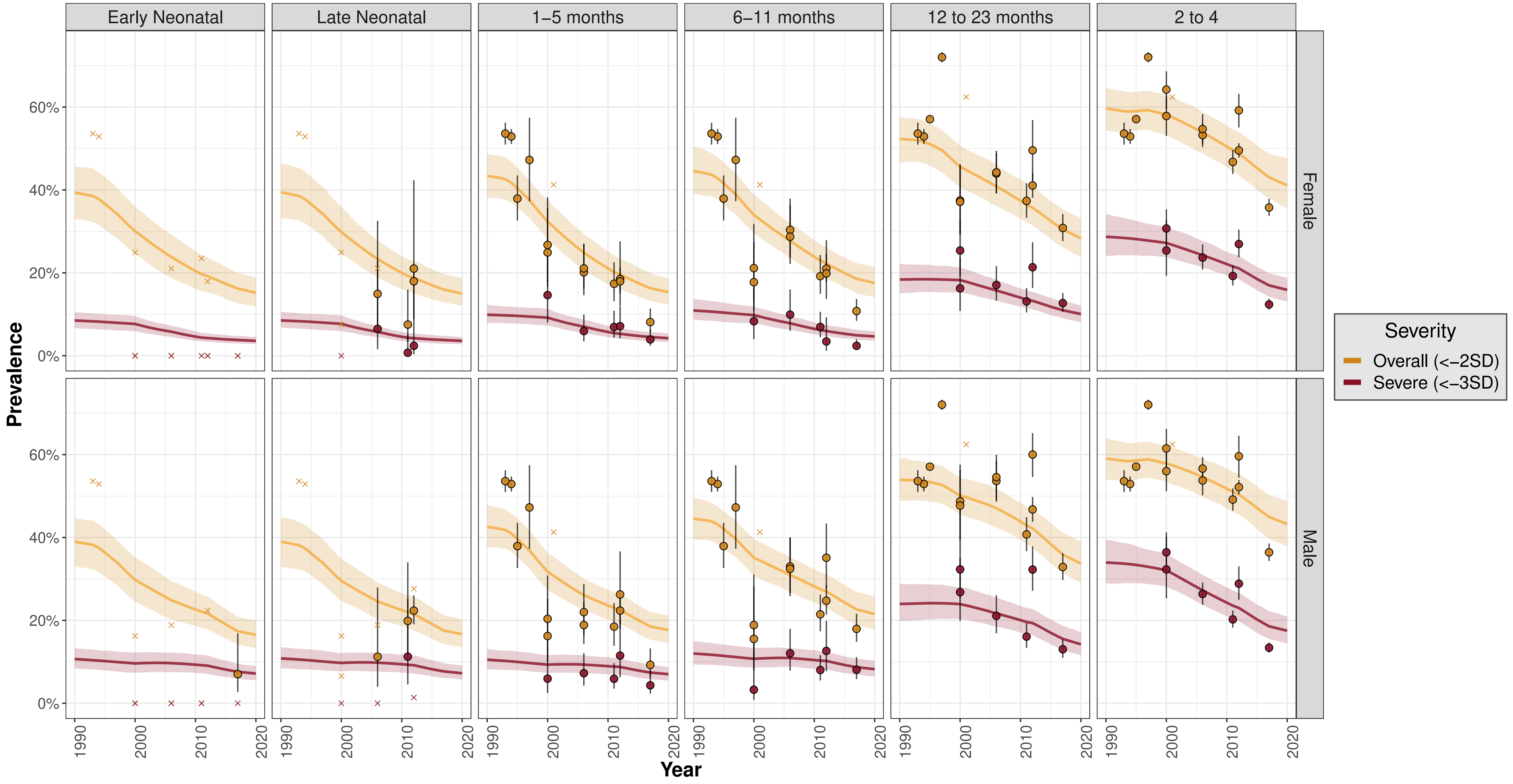

B: Transformed Mean Stunting Z Scores

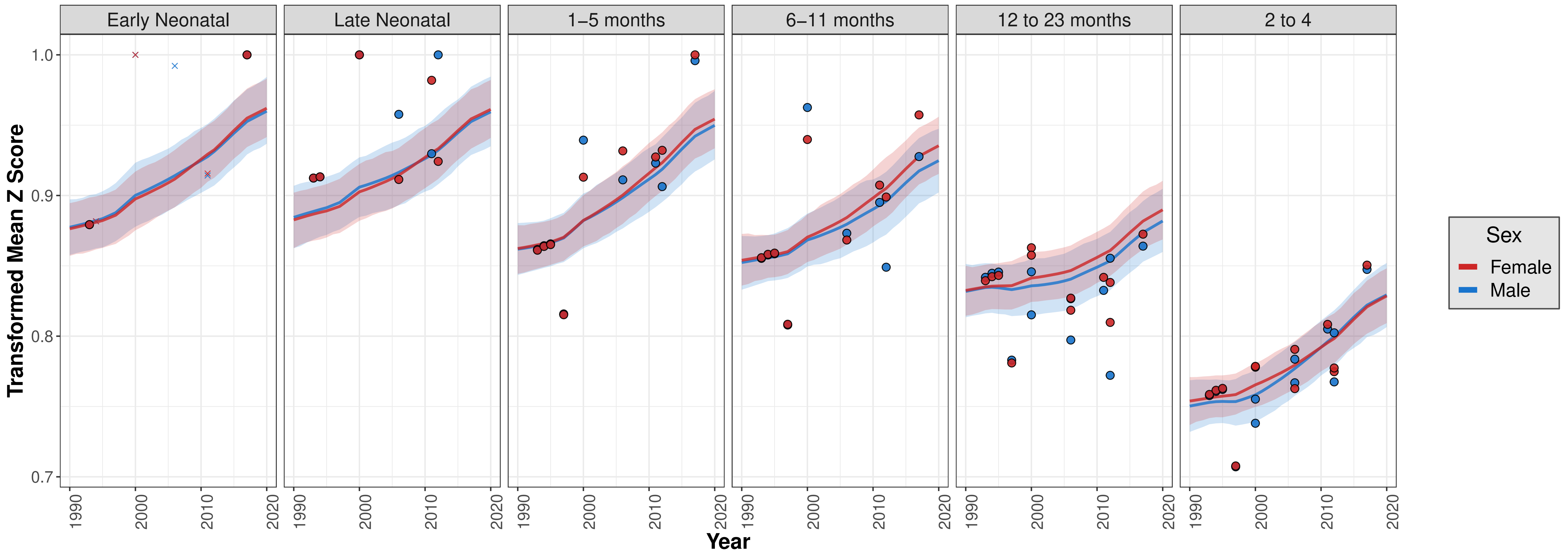

C

Year	Source
1986	WHO CGM Database
1993	WHO CGM Database
1994	WHO CGM Database
1995	WHO CGM Database
1997	WHO CGM Database
2000	MICS
2000	WHO CGM Database
2001	WHO CGM Database
2006	MICS
2006	WHO CGM Database
2011	MICS
2012	MICS
2012	WHO CGM Database
2017	MICS

Lao People's Democratic Republic – Wasting (WHZ)

D: Overall and Severe Wasting Prevalence

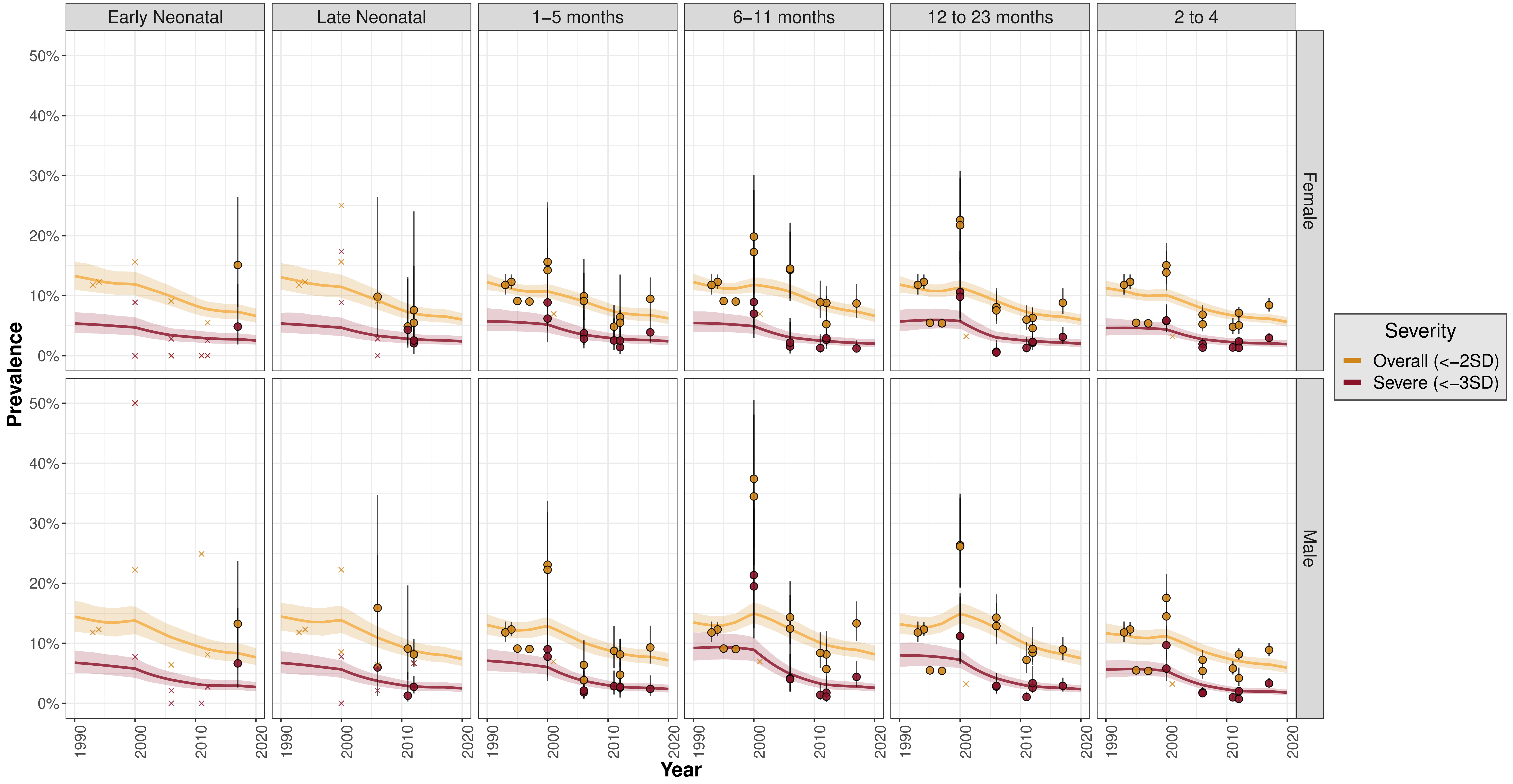

E: Transformed Mean Wasting Z Scores

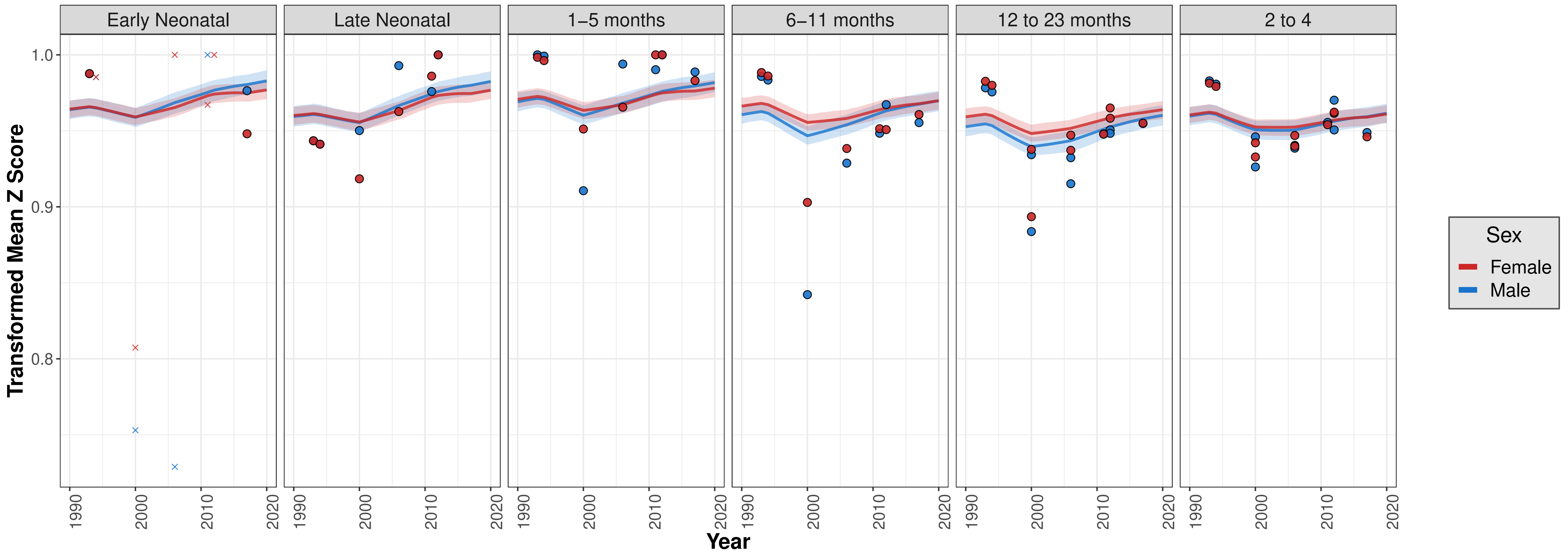

F

Year	Source
1993	WHO CGM Database
1994	WHO CGM Database
1995	WHO CGM Database
1997	WHO CGM Database
2000	MICS
2000	WHO CGM Database
2001	WHO CGM Database
2006	MICS
2006	WHO CGM Database
2011	MICS
2012	MICS
2012	WHO CGM Database
2017	MICS

Lao People's Democratic Republic – Underweight (WAZ)

G: Overall and Severe Underweight Prevalence

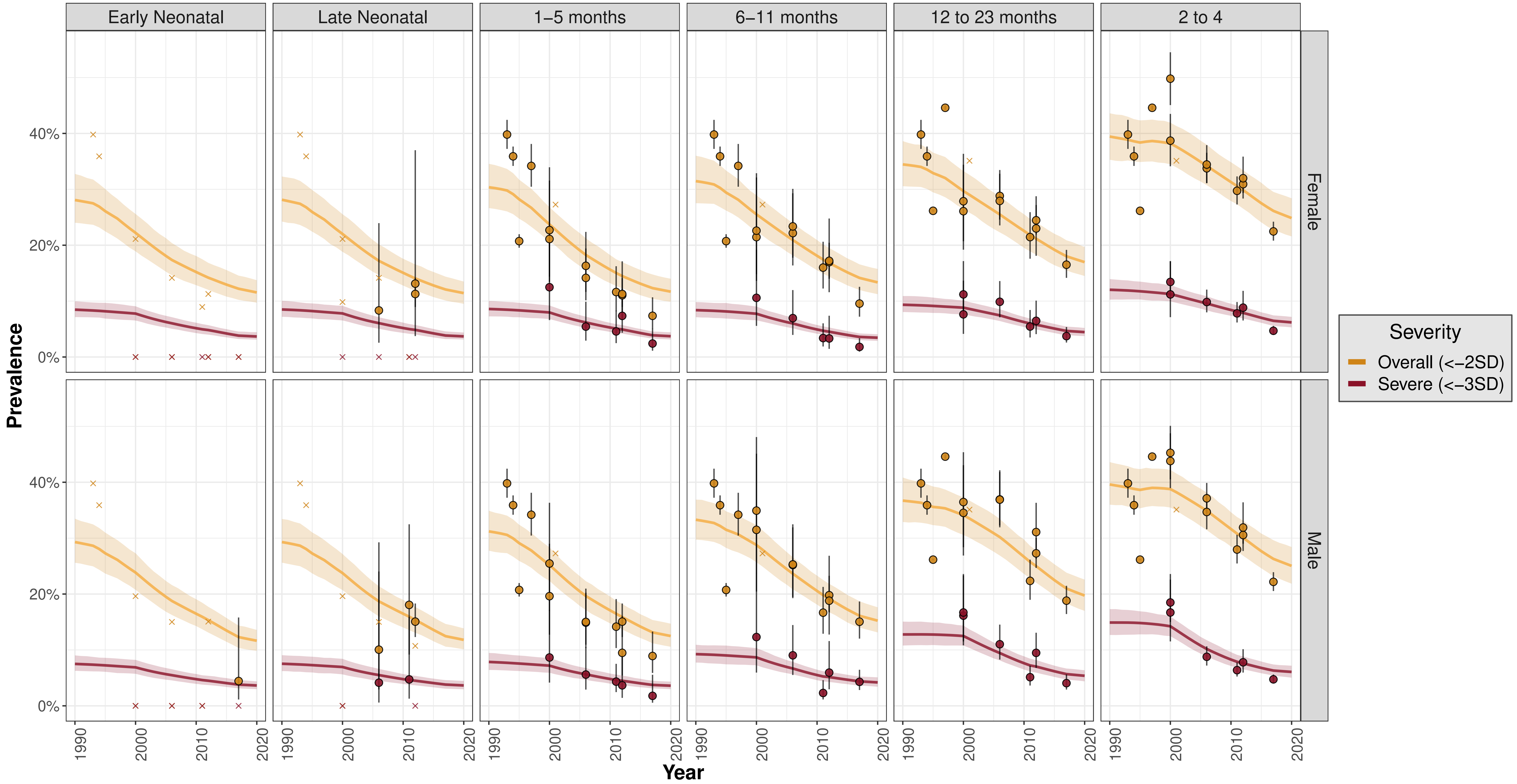

H: Transformed Mean Underweight Z Scores

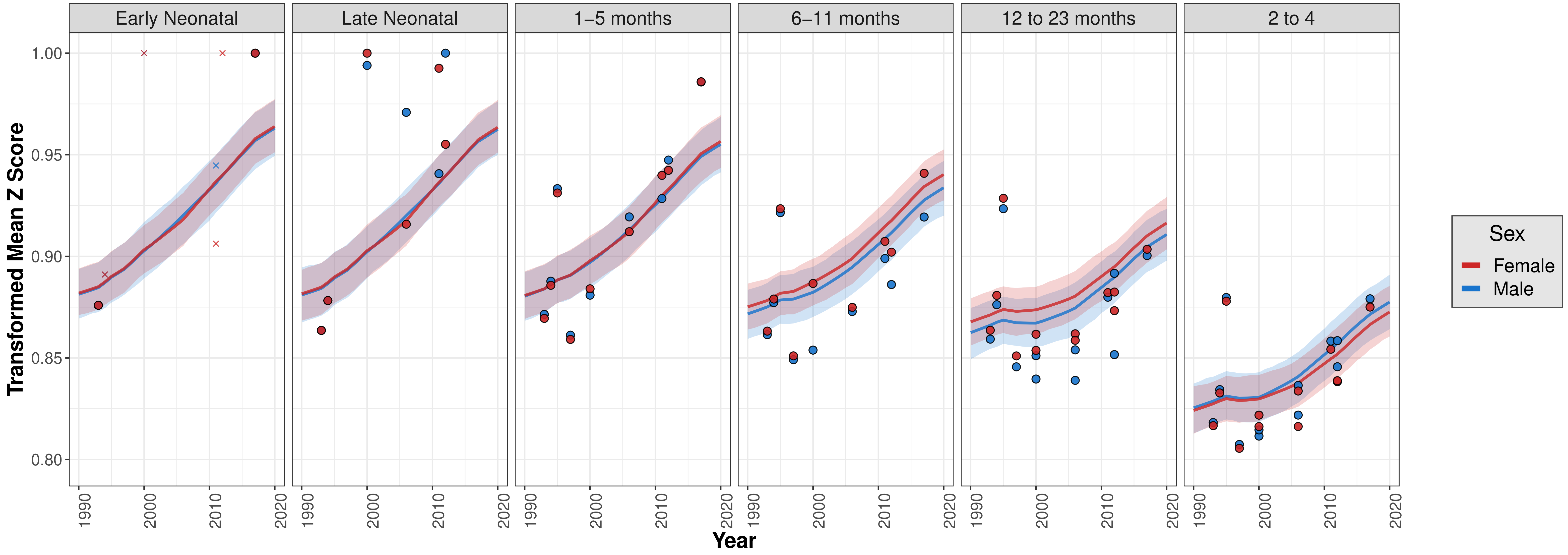

I

Year	Source
1993	WHO CGM Database
1994	WHO CGM Database
1995	WHO CGM Database
1997	WHO CGM Database
2000	MICS
2000	WHO CGM Database
2001	WHO CGM Database
2006	MICS
2006	WHO CGM Database
2011	MICS
2012	MICS
2012	WHO CGM Database
2017	MICS

Lao People's Democratic Republic – HAZ, WHZ, and WAZ Distributions

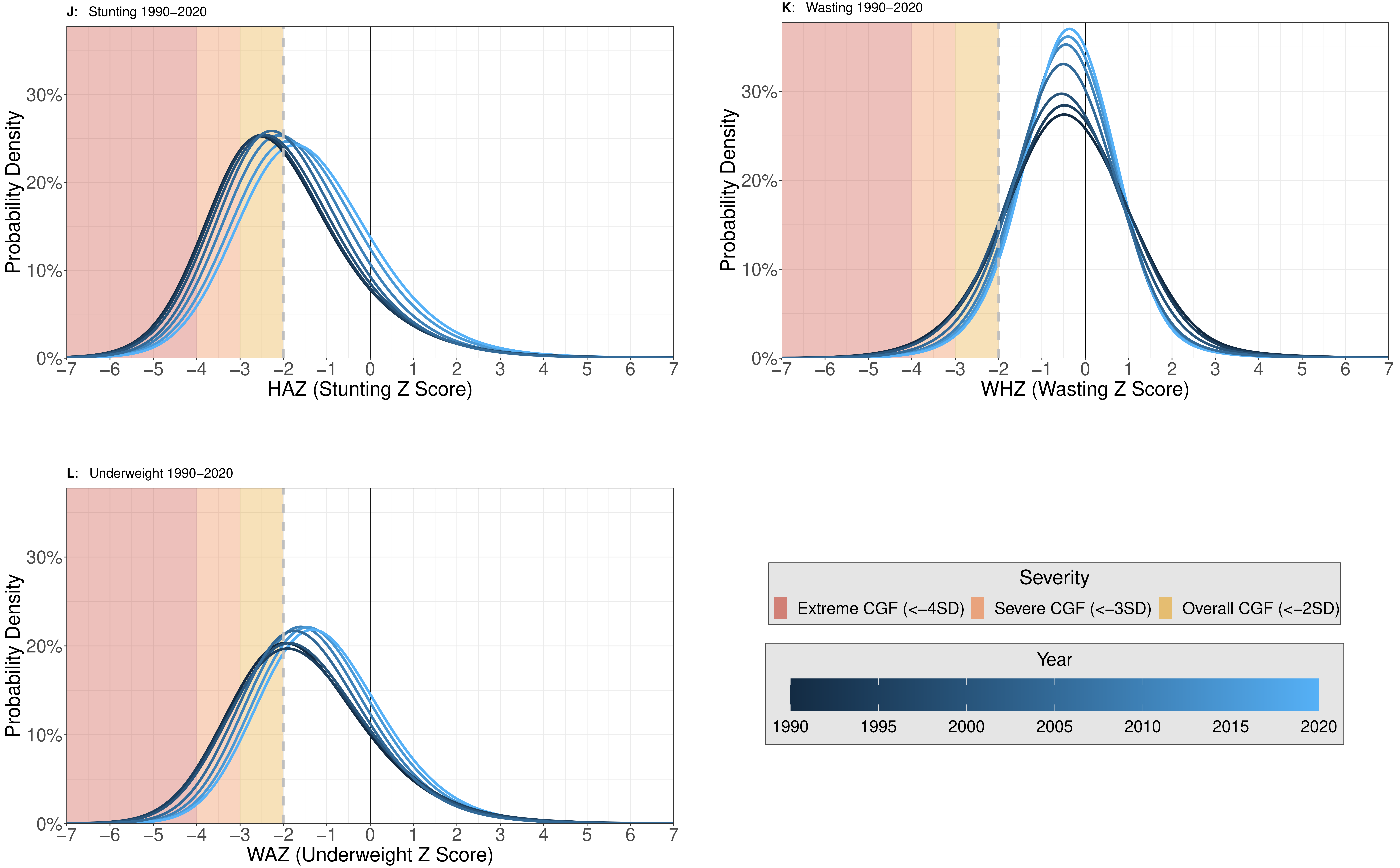

Malaysia – Stunting (HAZ)

A: Overall and Severe Stunting Prevalence

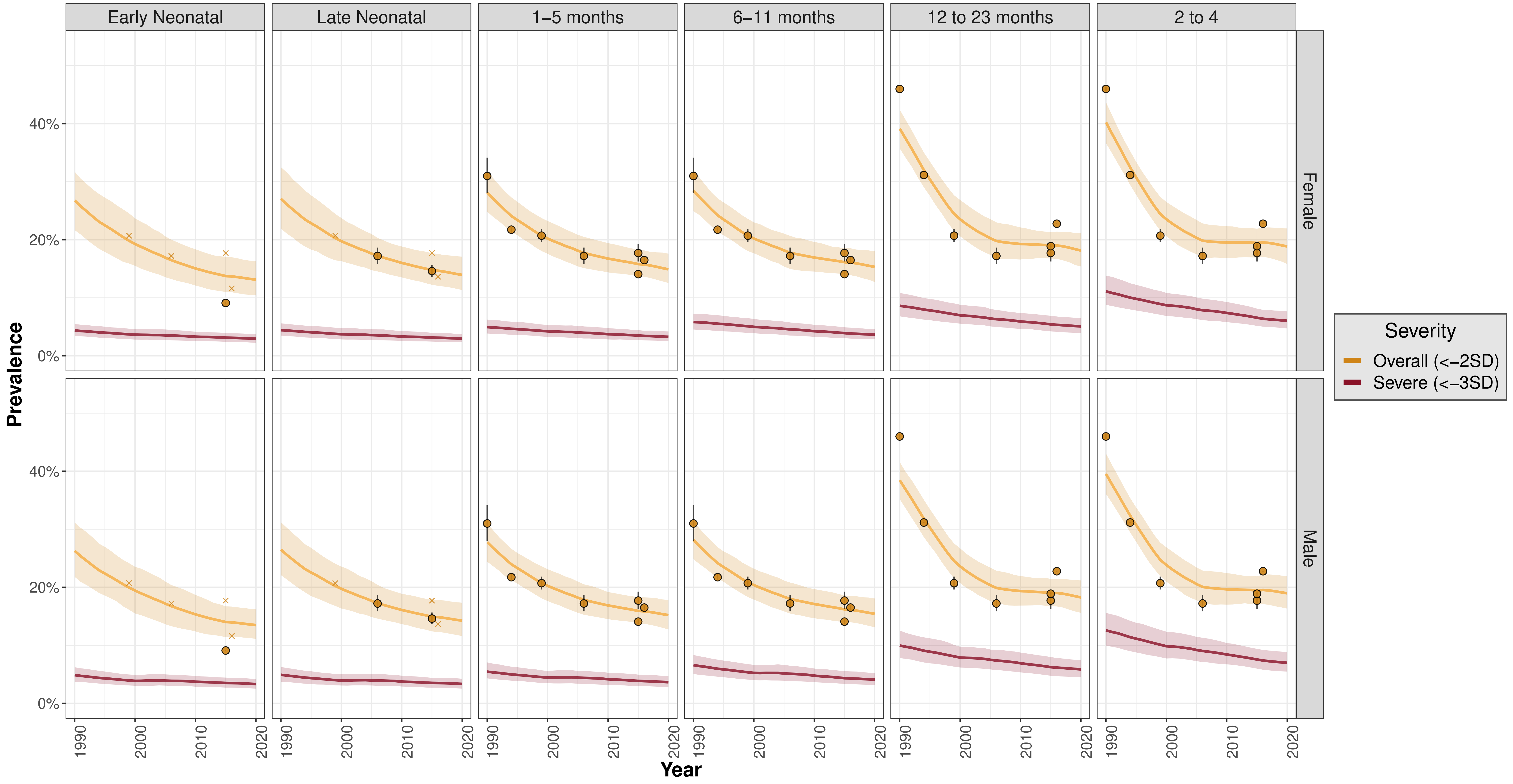

B: Transformed Mean Stunting Z Scores

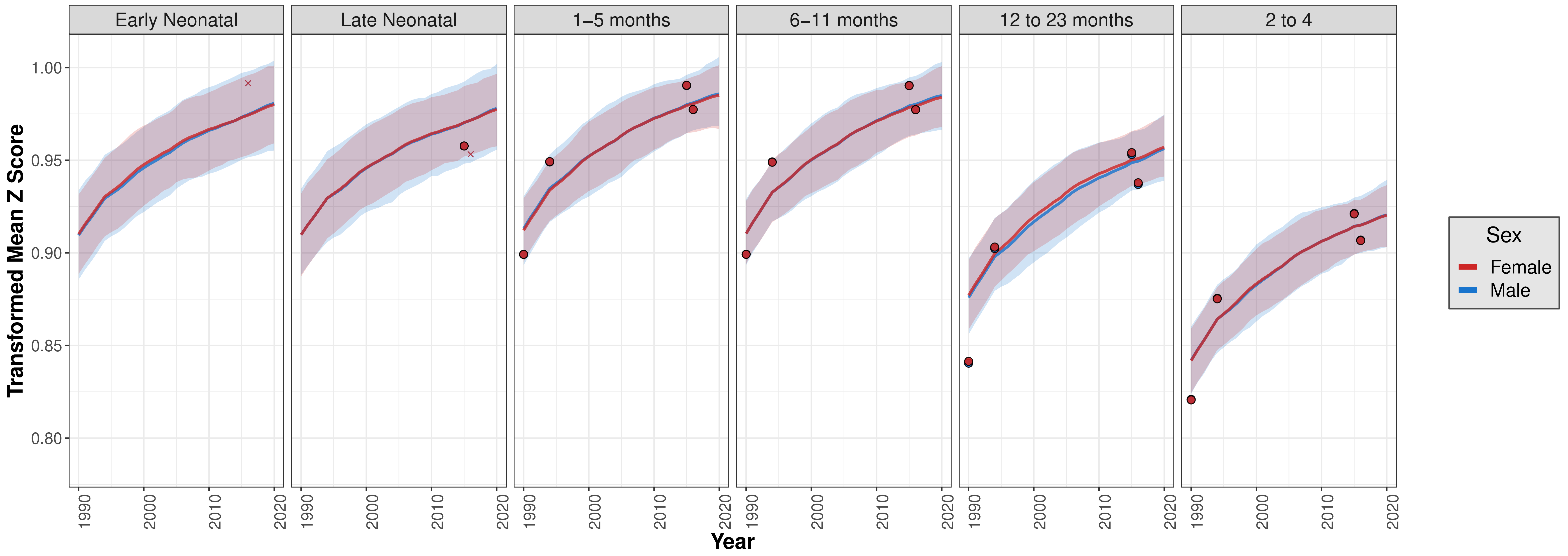

C

Year	Source
1990	WHO CGM Database
1994	WHO CGM Database
1999	WHO CGM Database
2006	WHO CGM Database
2015	WHO CGM Database
2015	National Health and Morbidity Survey
2016	National Health and Morbidity Survey

Malaysia – Wasting (WHZ)

D: Overall and Severe Wasting Prevalence

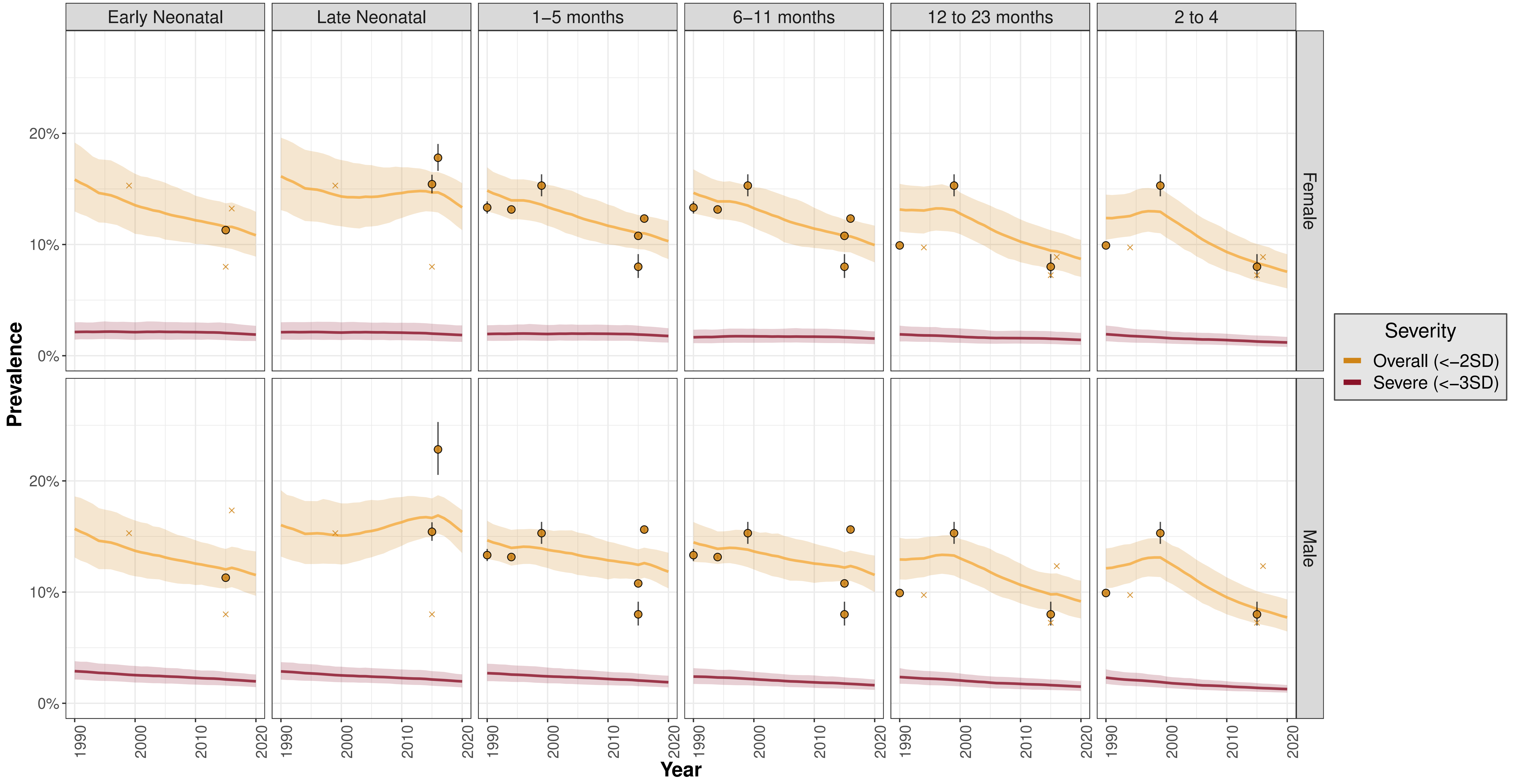

E: Transformed Mean Wasting Z Scores

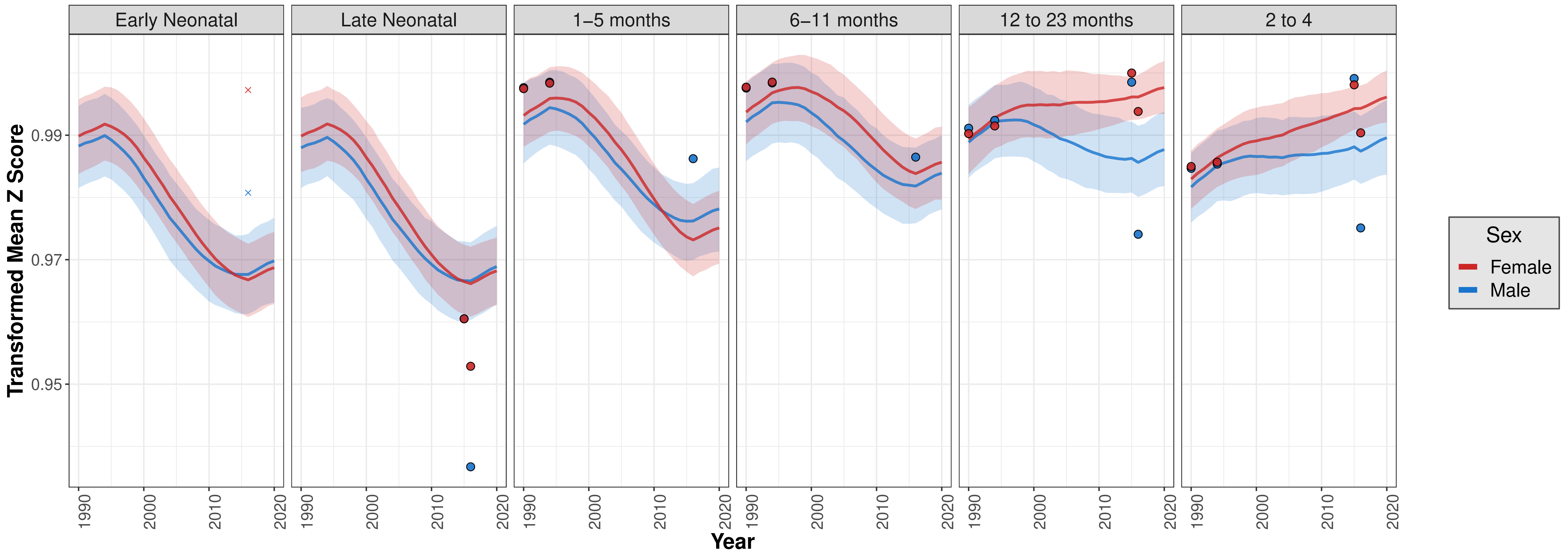

F

Year	Source
1990	WHO CGM Database
1994	WHO CGM Database
1999	WHO CGM Database
2015	WHO CGM Database
2015	National Health and Morbidity Survey
2016	National Health and Morbidity Survey

Malaysia – Underweight (WAZ)

G: Overall and Severe Underweight Prevalence

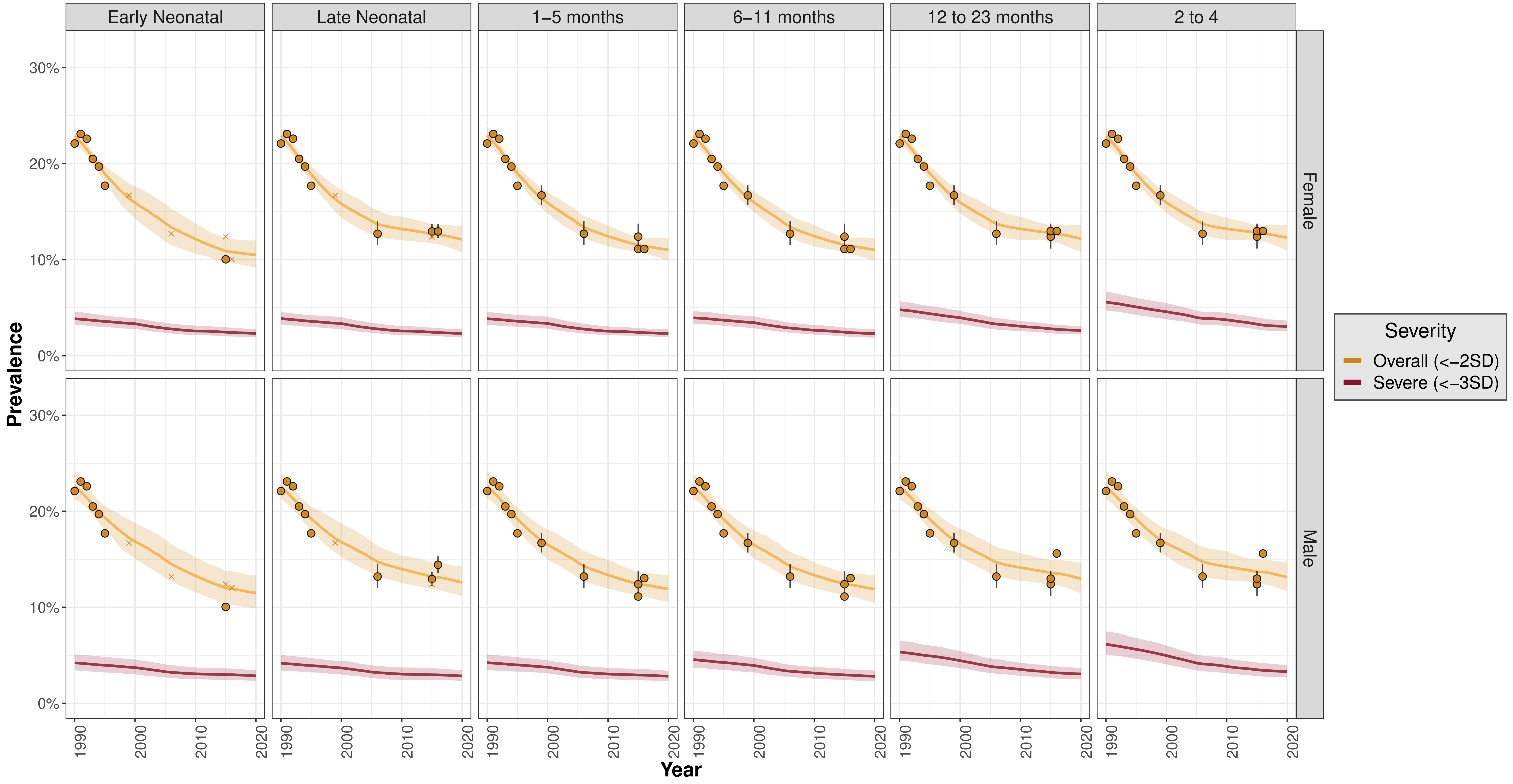

H: Transformed Mean Underweight Z Scores

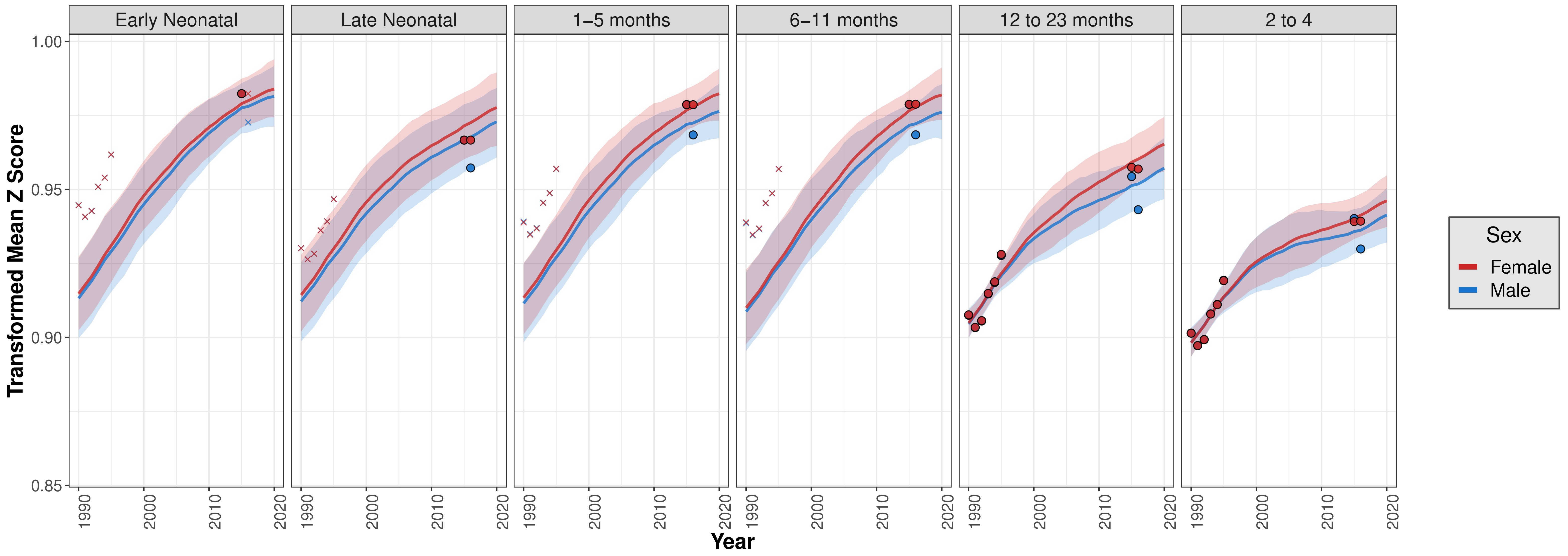

I

Year	Source
1990	WHO CGM Database
1991	WHO CGM Database
1992	WHO CGM Database
1993	WHO CGM Database
1994	WHO CGM Database
1995	WHO CGM Database
1999	WHO CGM Database
2006	WHO CGM Database
2015	WHO CGM Database
2015	National Health and Morbidity Survey
2016	National Health and Morbidity Survey

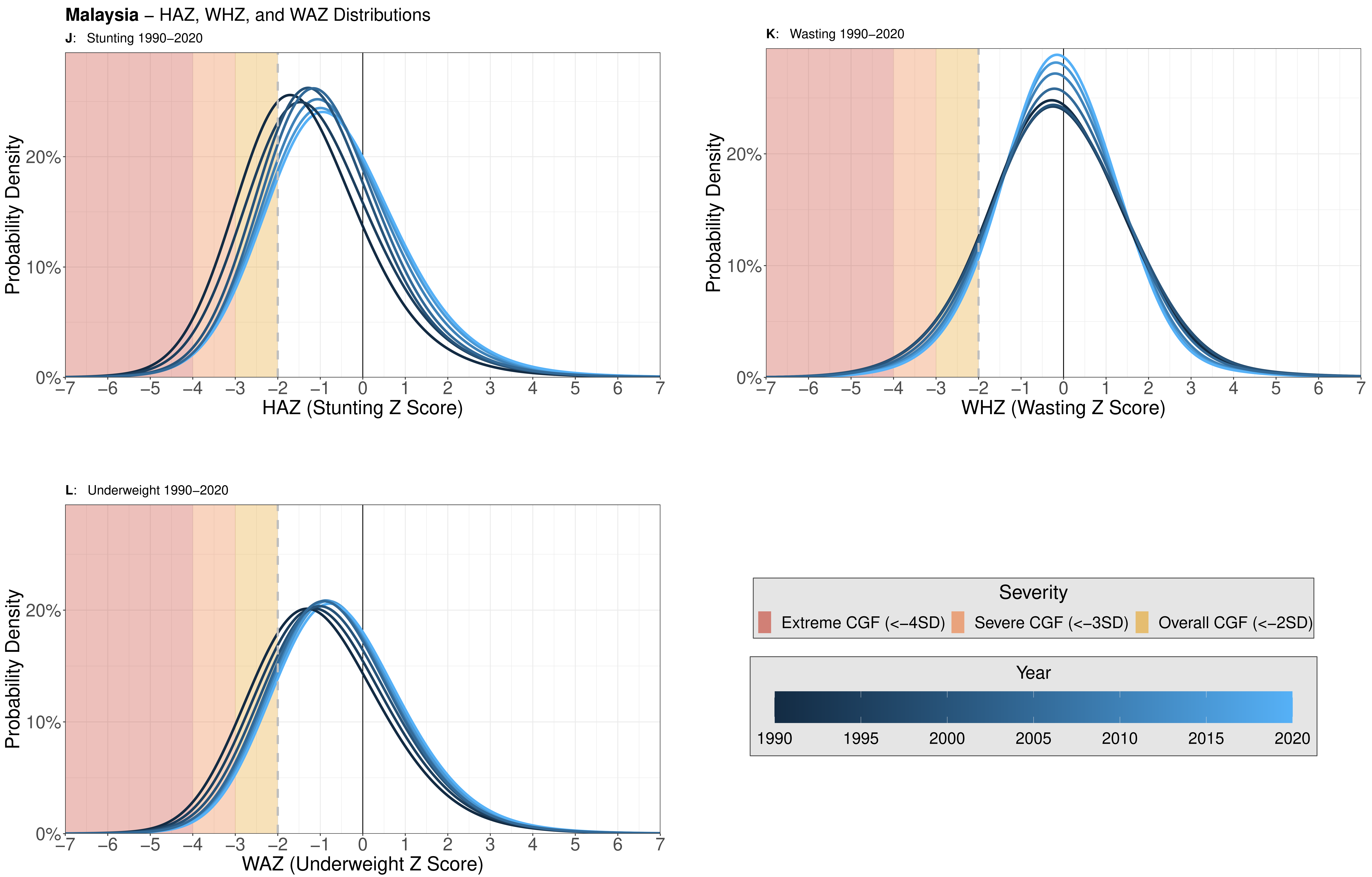
Malaysia – HAZ, WHZ, and WAZ Distributions
J: Stunting 1990–2020
Probability Density
20%
10%
0%
−7 −6 −5 −4 −3 −2 −1 0 1 2 3 4 5 6 7
HAZ (Stunting Z Score)
K: Wasting 1990–2020
Probability Density
20%
10%
0%
−7 −6 −5 −4 −3 −2 −1 0 1 2 3 4 5 6 7
WHZ (Wasting Z Score)
L: Underweight 1990–2020
Probability Density
20%
10%
0%
−7 −6 −5 −4 −3 −2 −1 0 1 2 3 4 5 6 7
WAZ (Underweight Z Score)
Severity
Extreme CGF (<−4SD)
Severe CGF (<−3SD)
Overall CGF (<−2SD)
Year
1990
1995
2000
2005
2010
2015
2020

Maldives – Stunting (HAZ)

A: Overall and Severe Stunting Prevalence

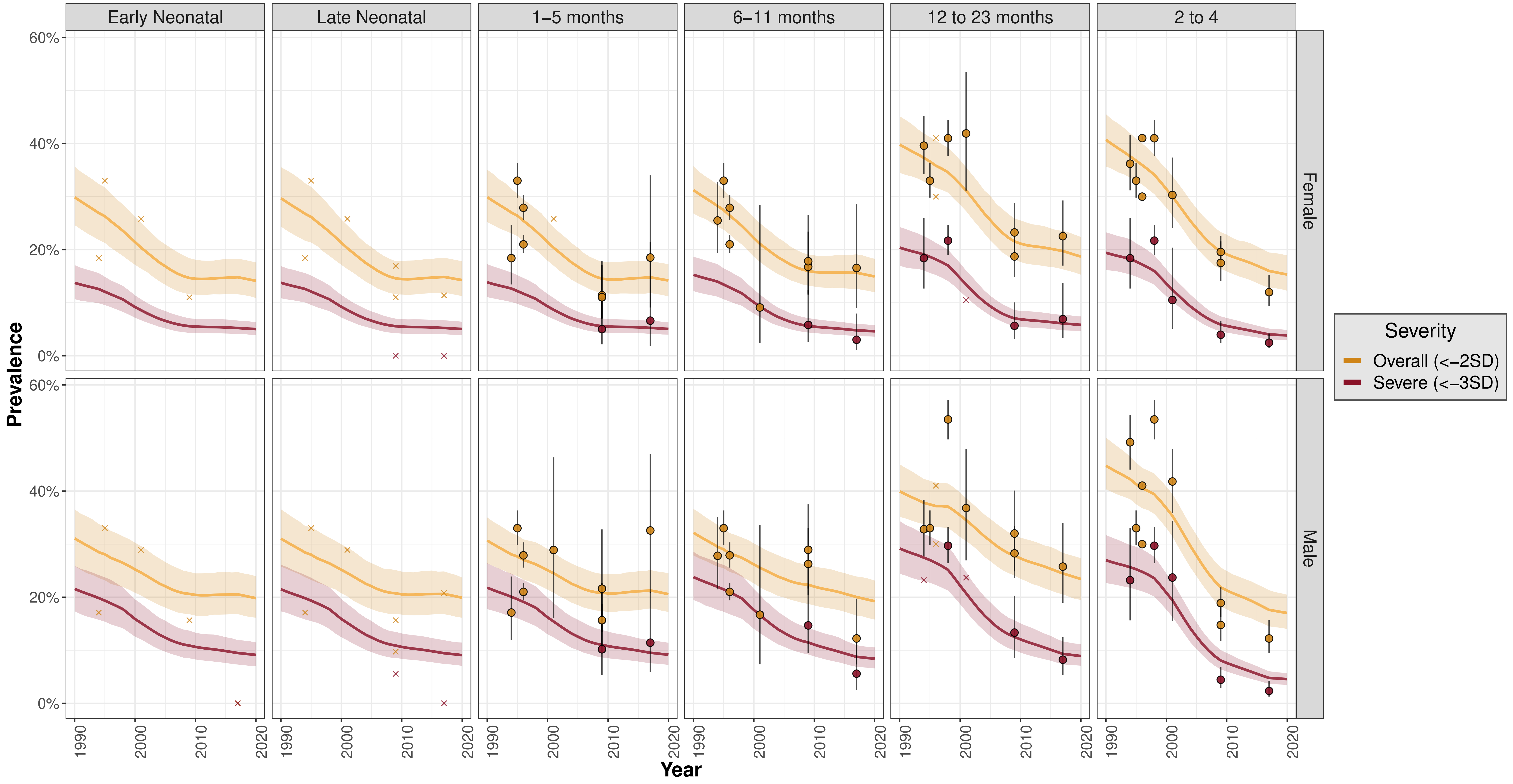

C

Year	Source
1994	WHO CGM Database
1995	WHO CGM Database
1996	WHO CGM Database
1998	WHO CGM Database
2001	WHO CGM Database
2009	DHS
2009	WHO CGM Database
2017	DHS

B: Transformed Mean Stunting Z Scores

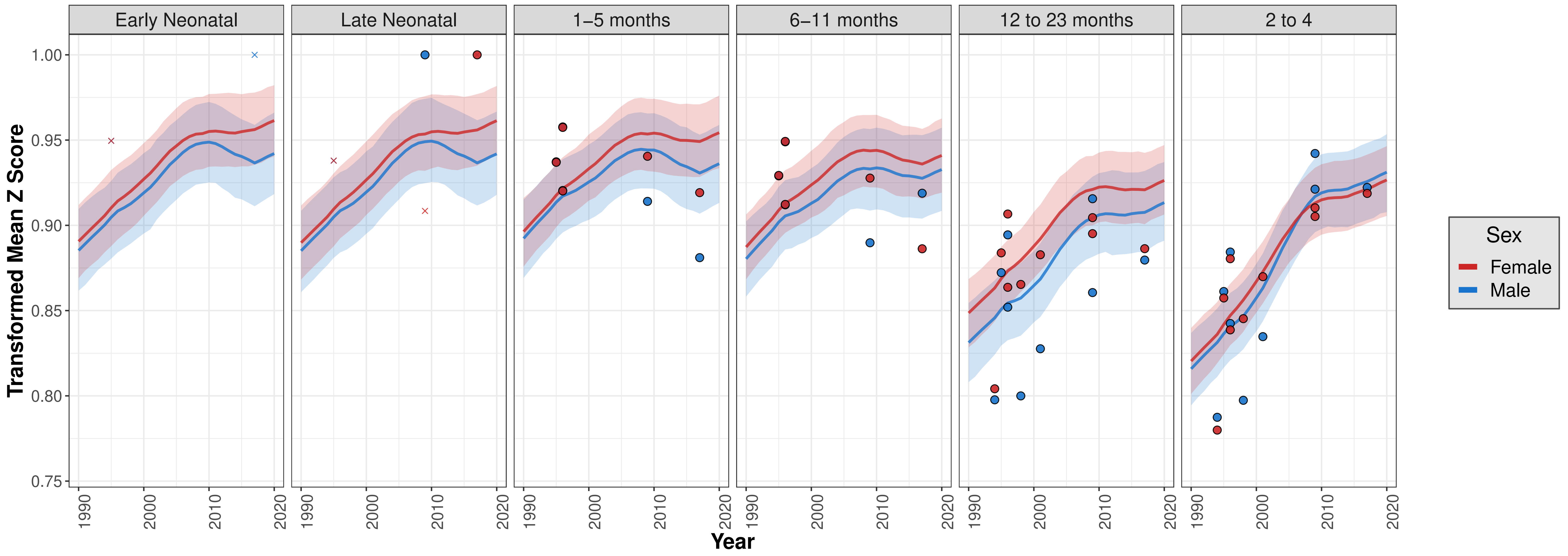

Maldives – Wasting (WHZ)

D: Overall and Severe Wasting Prevalence

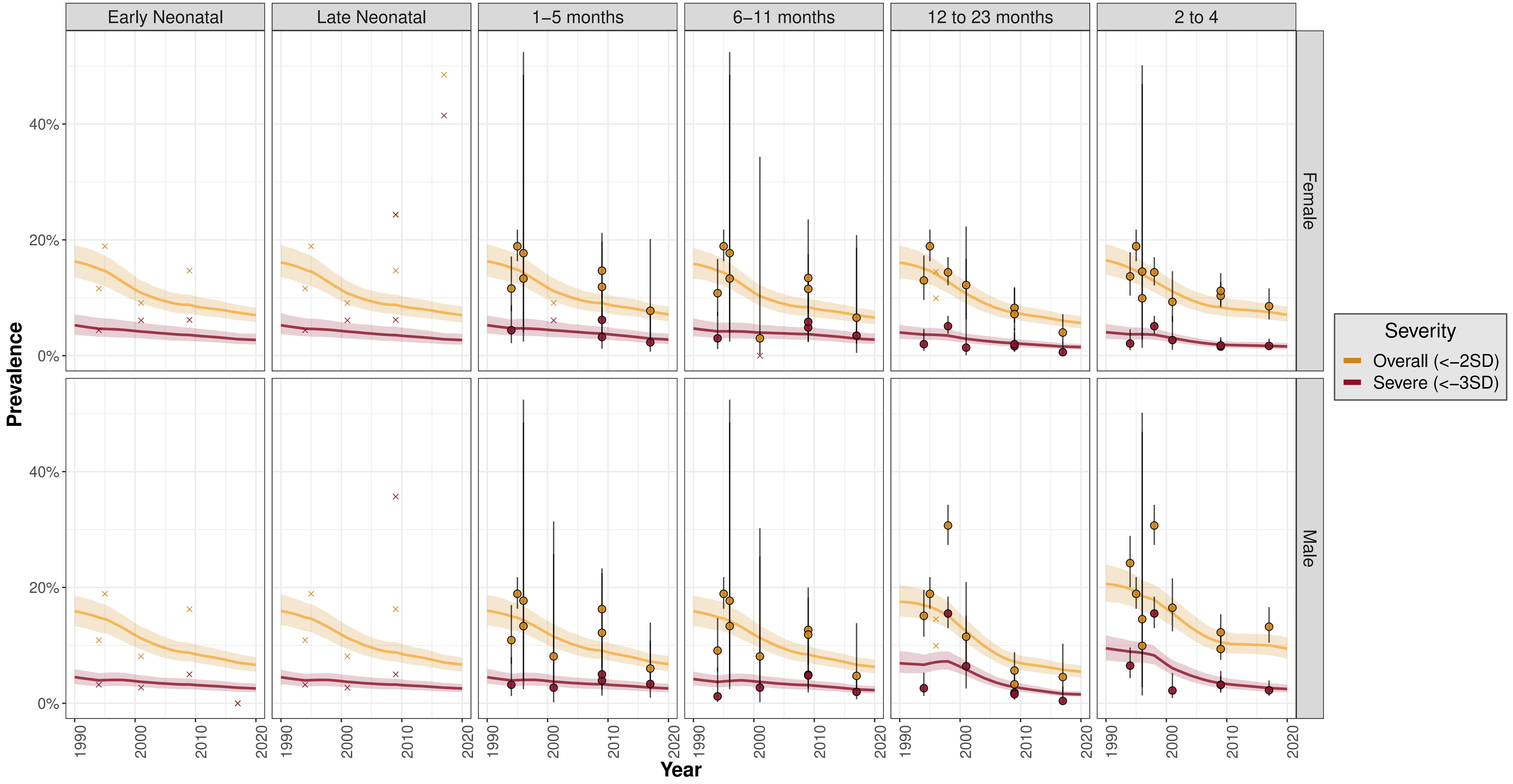

E: Transformed Mean Wasting Z Scores

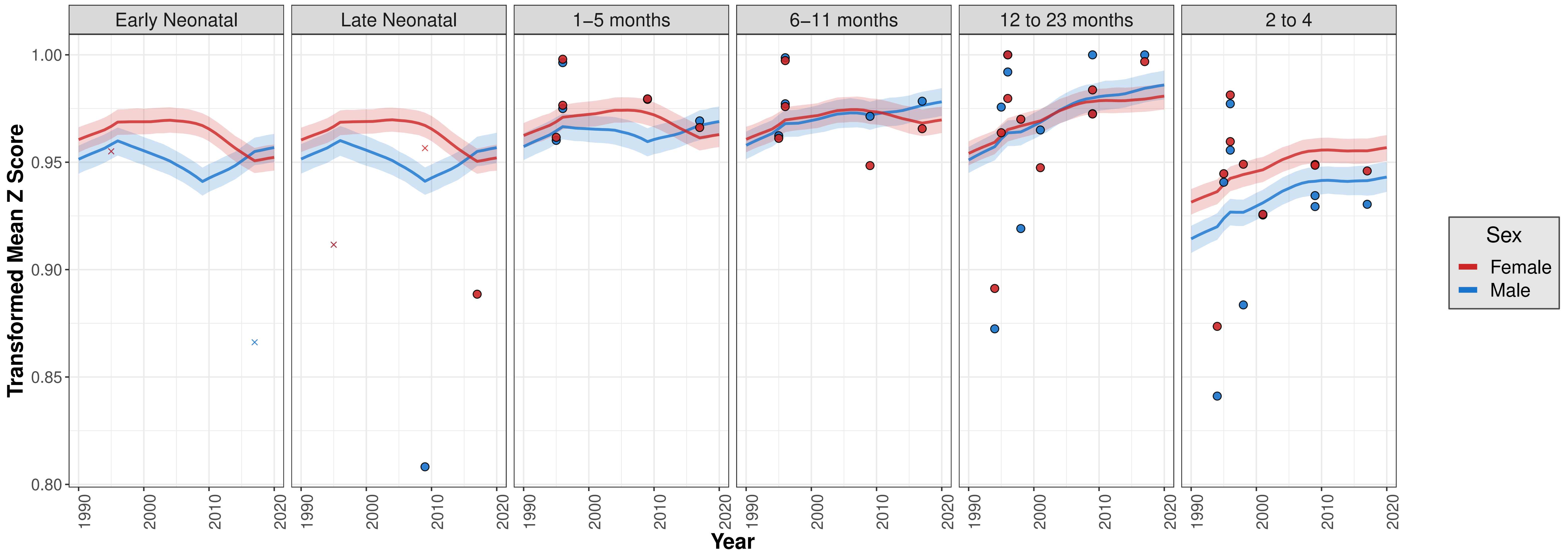

F

Year	Source
1983	WHO CGM Database
1994	WHO CGM Database
1995	WHO CGM Database
1996	WHO CGM Database
1998	WHO CGM Database
2001	WHO CGM Database
2009	DHS
2009	WHO CGM Database
2017	DHS

Maldives – Underweight (WAZ)

G: Overall and Severe Underweight Prevalence

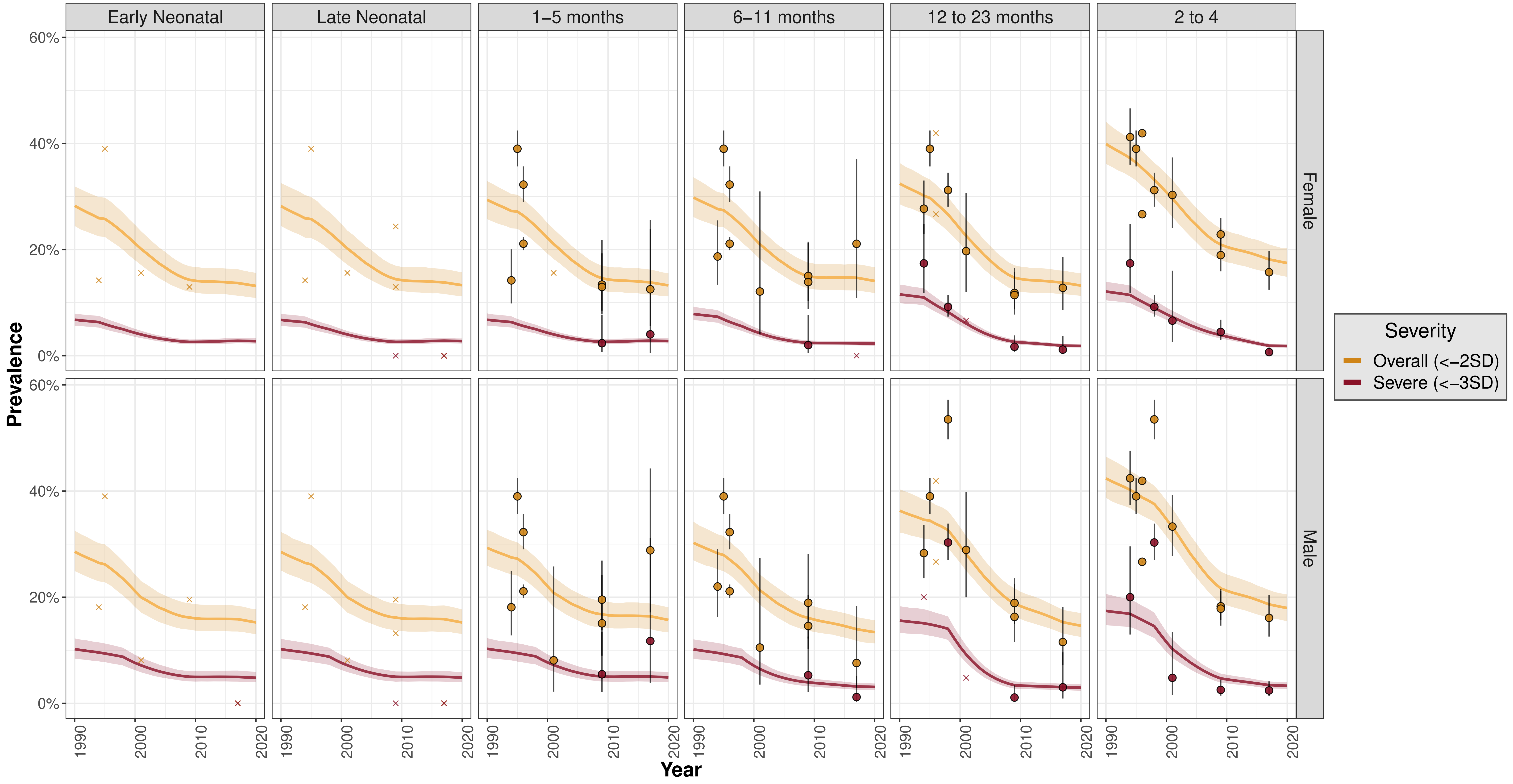

H: Transformed Mean Underweight Z Scores

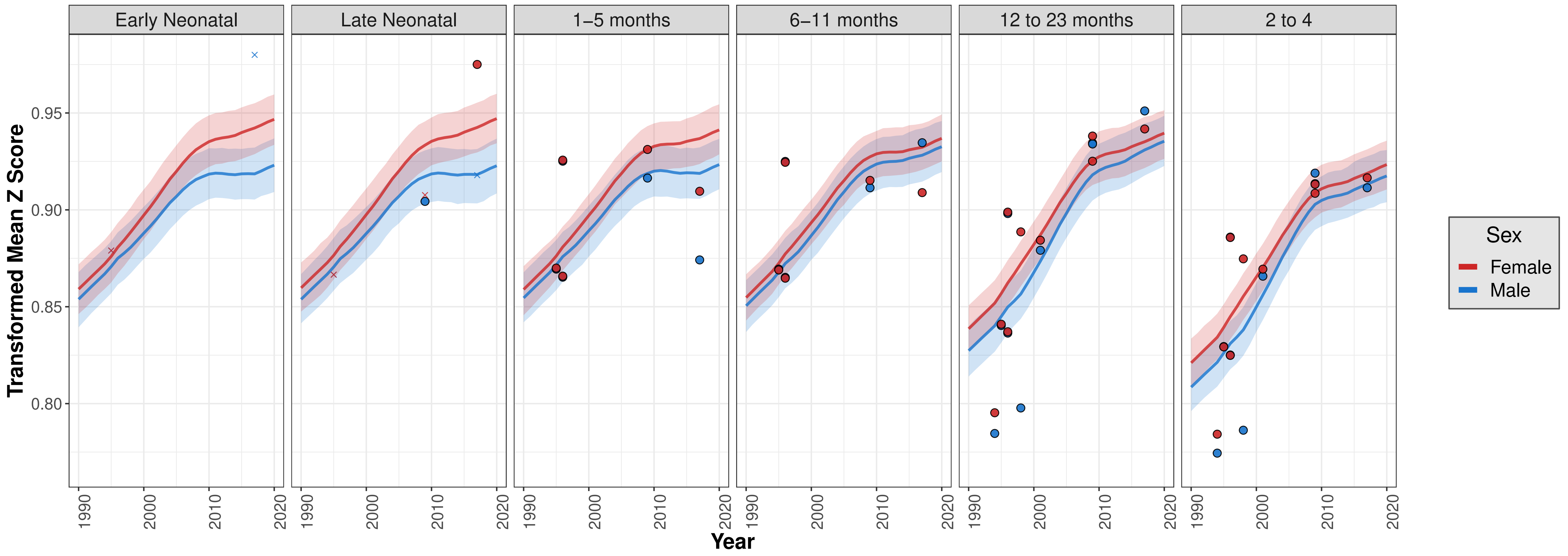

I

Year	Source
1994	WHO CGM Database
1995	WHO CGM Database
1996	WHO CGM Database
1998	WHO CGM Database
2001	WHO CGM Database
2009	DHS
2009	WHO CGM Database
2017	DHS

Maldives – HAZ, WHZ, and WAZ Distributions

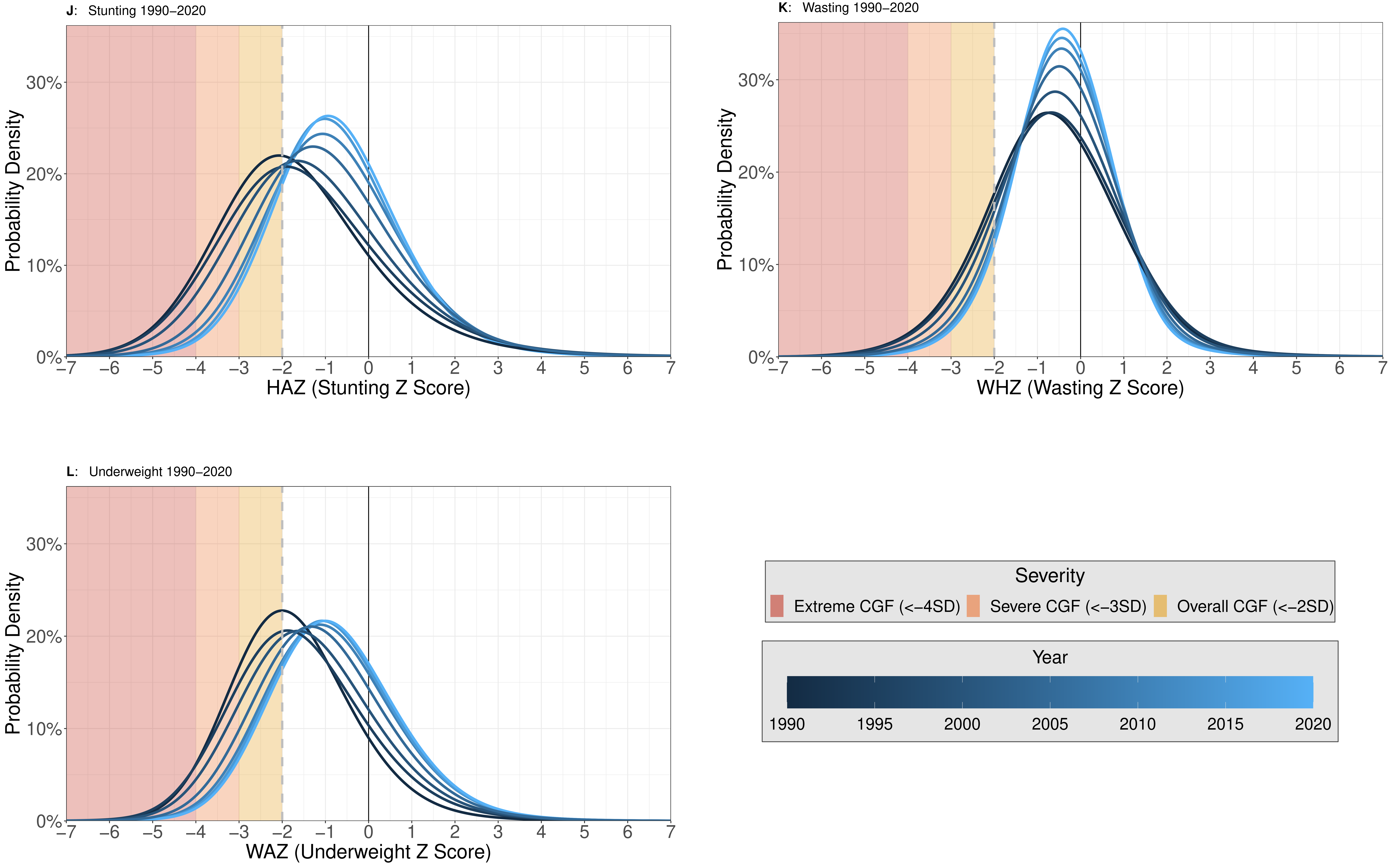

Myanmar – Stunting (HAZ)

A: Overall and Severe Stunting Prevalence

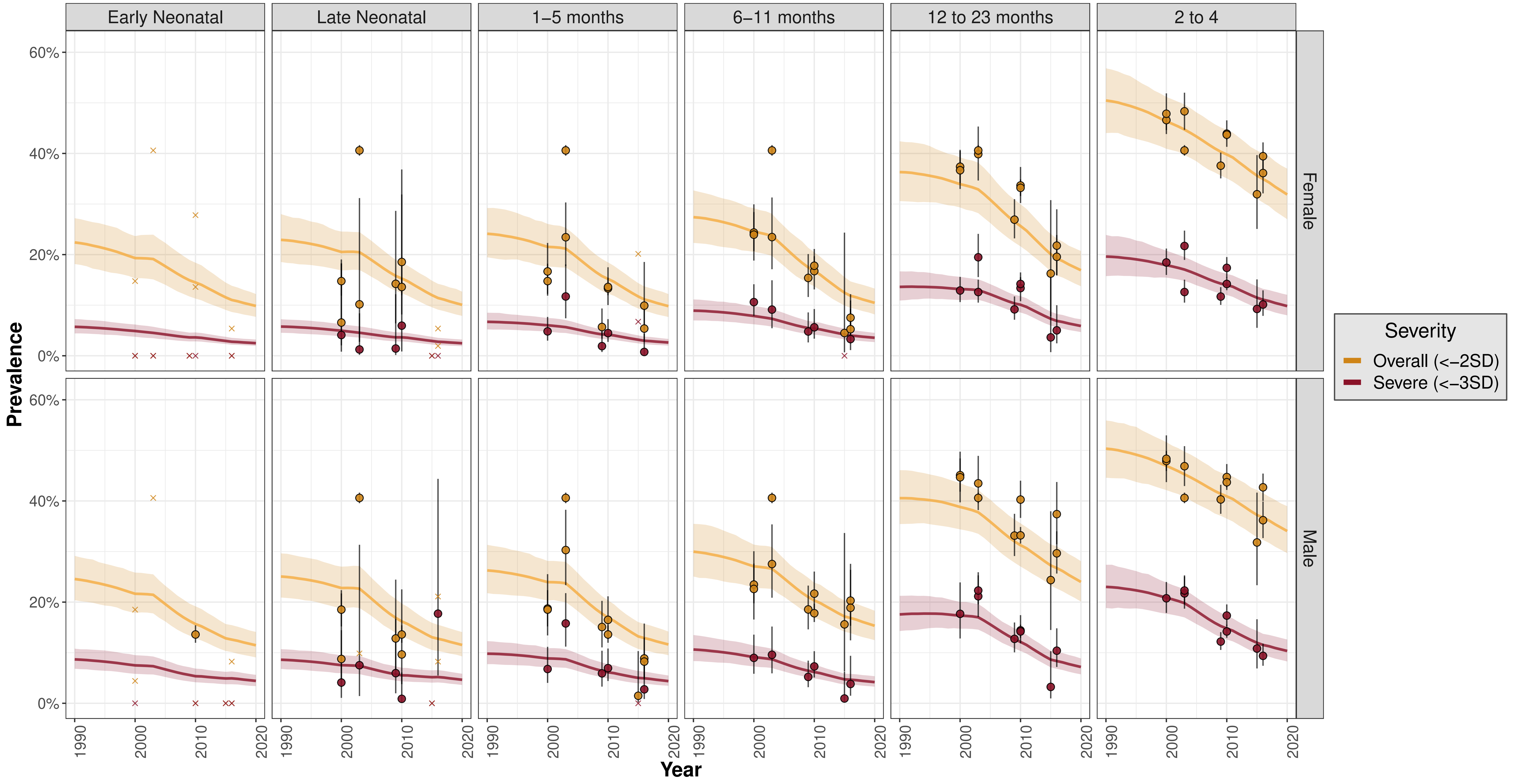

B: Transformed Mean Stunting Z Scores

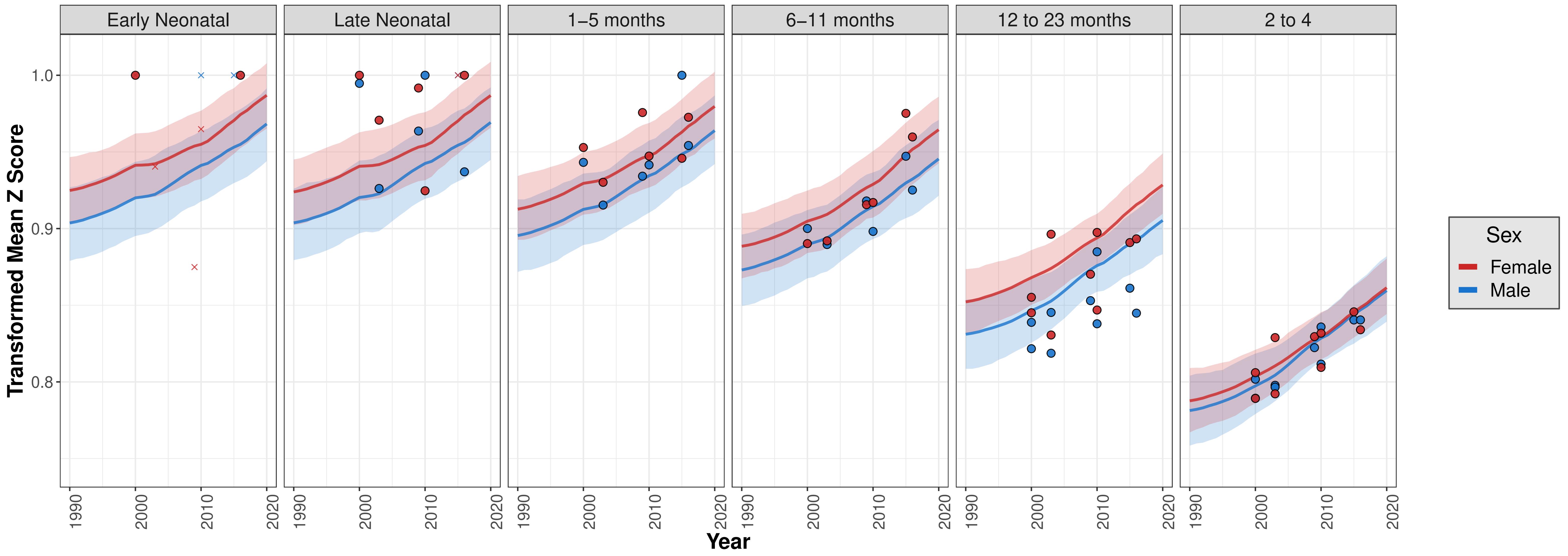

C

Year	Source
2000	MICS
2000	WHO CGM Database
2003	WHO CGM Database
2003	MICS
2009	MICS
2010	MICS
2010	WHO CGM Database
2015	DHS
2016	WHO CGM Database
2016	DHS

Myanmar – Wasting (WHZ)

D: Overall and Severe Wasting Prevalence

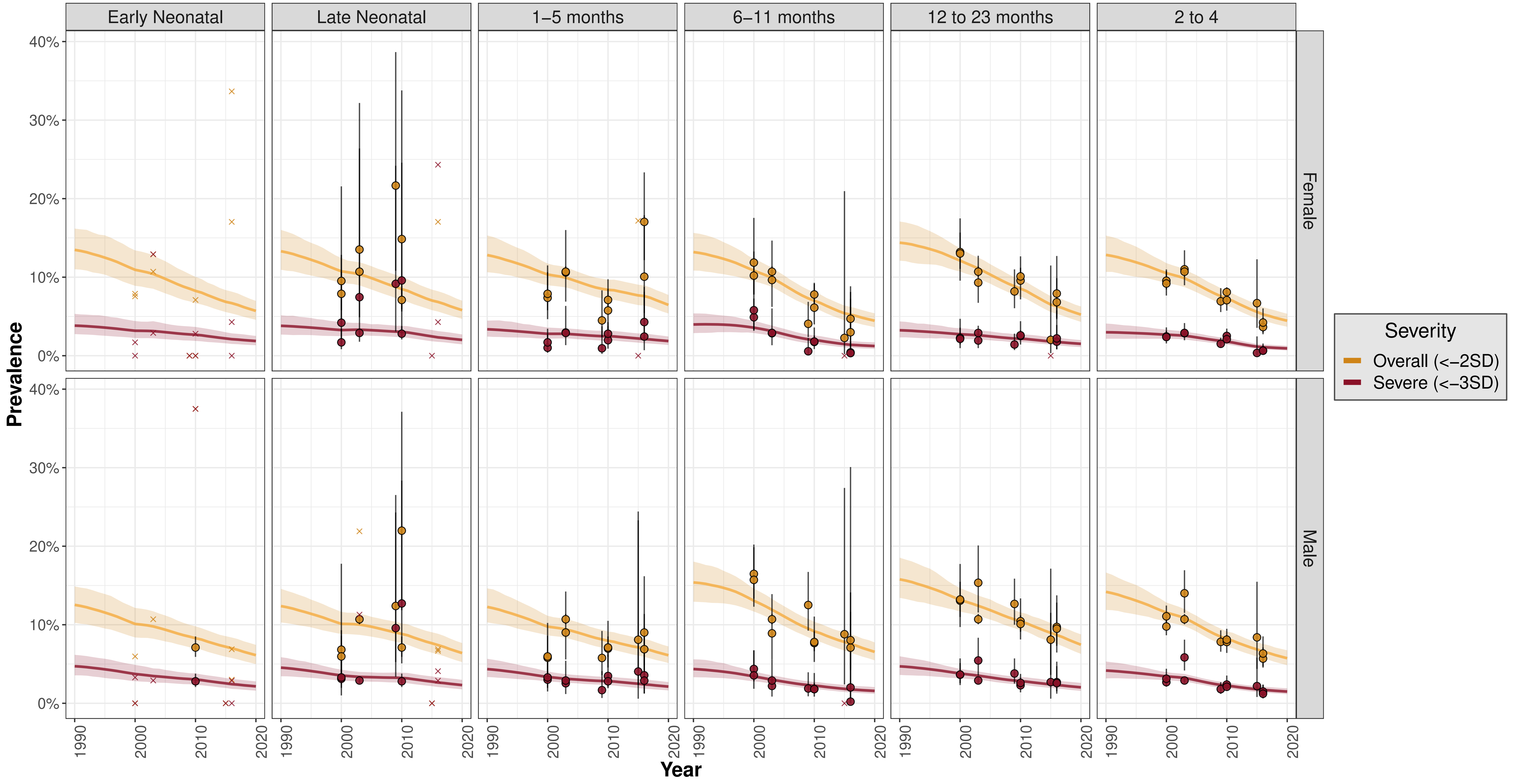

E: Transformed Mean Wasting Z Scores

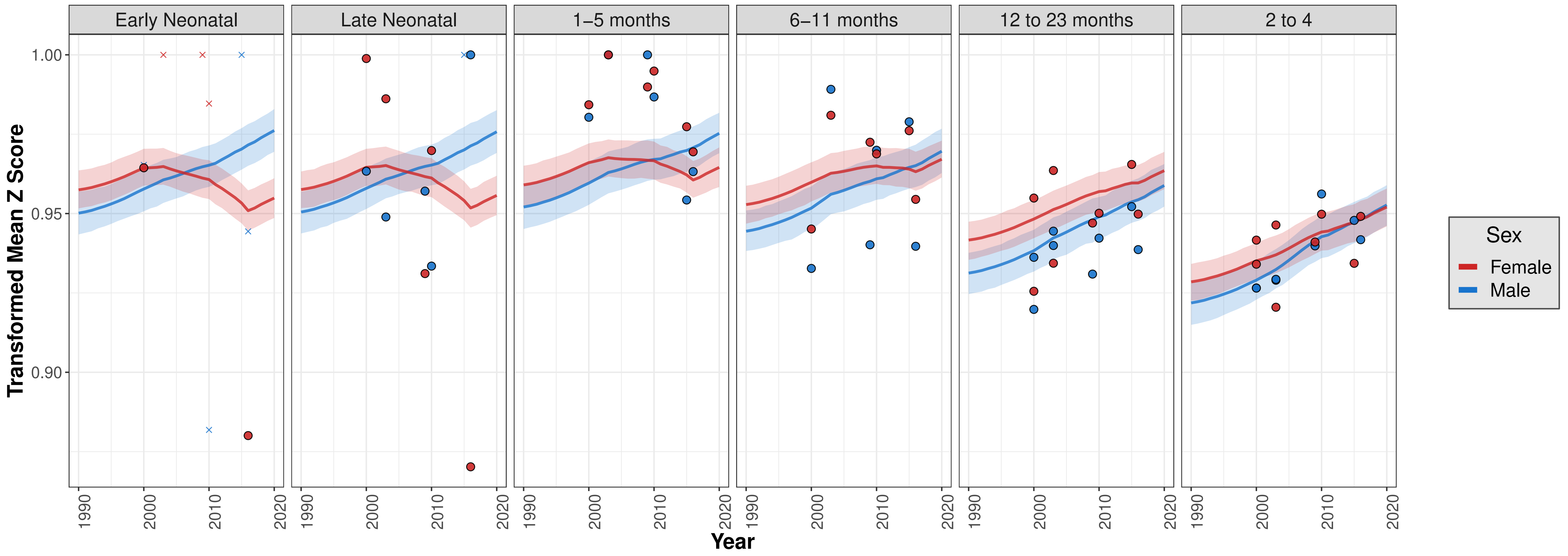

F

Year	Source
2000	MICS
2000	WHO CGM Database
2003	WHO CGM Database
2003	MICS
2009	MICS
2010	MICS
2010	WHO CGM Database
2015	DHS
2016	WHO CGM Database
2016	DHS

Myanmar – Underweight (WAZ)

G: Overall and Severe Underweight Prevalence

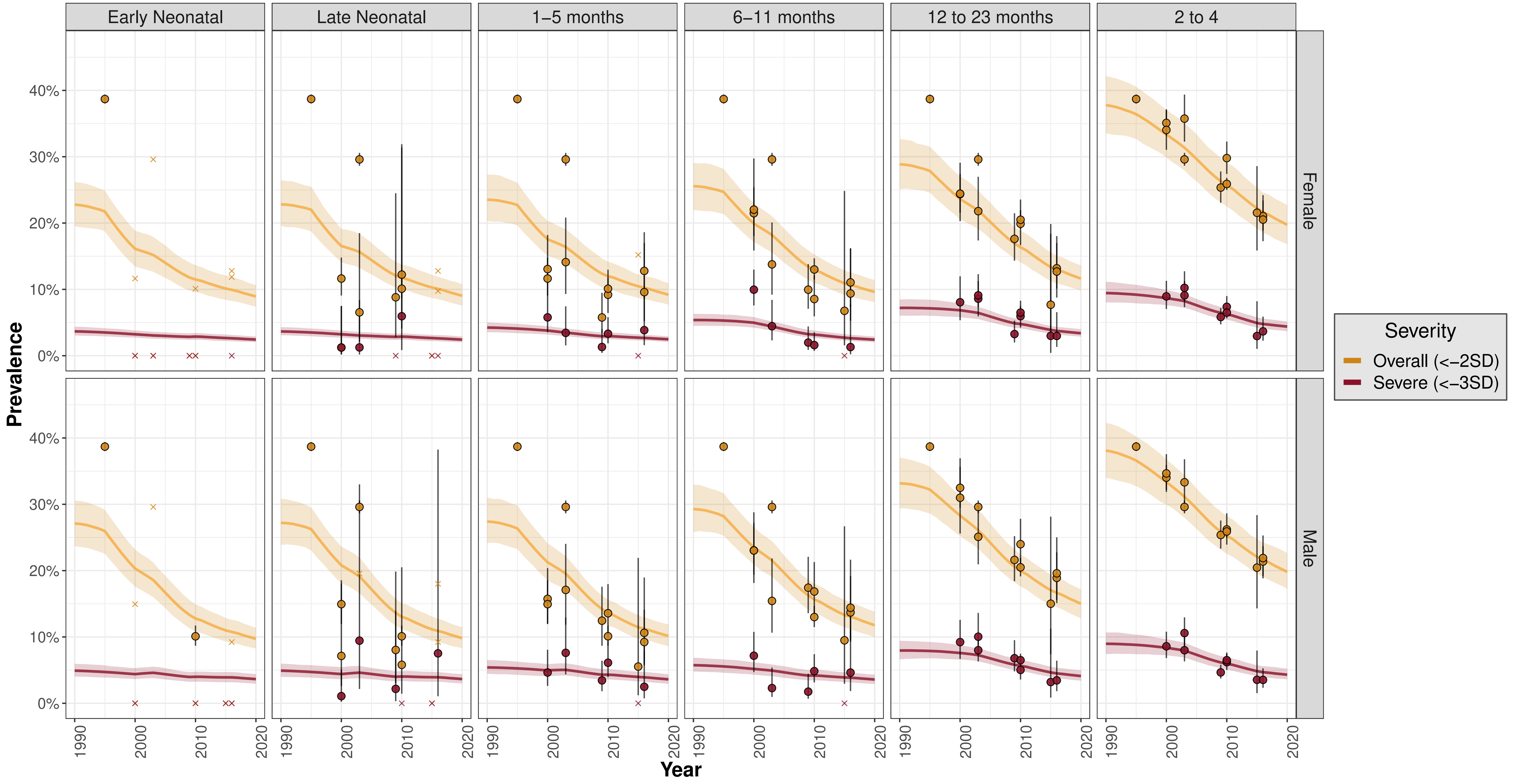

H: Transformed Mean Underweight Z Scores

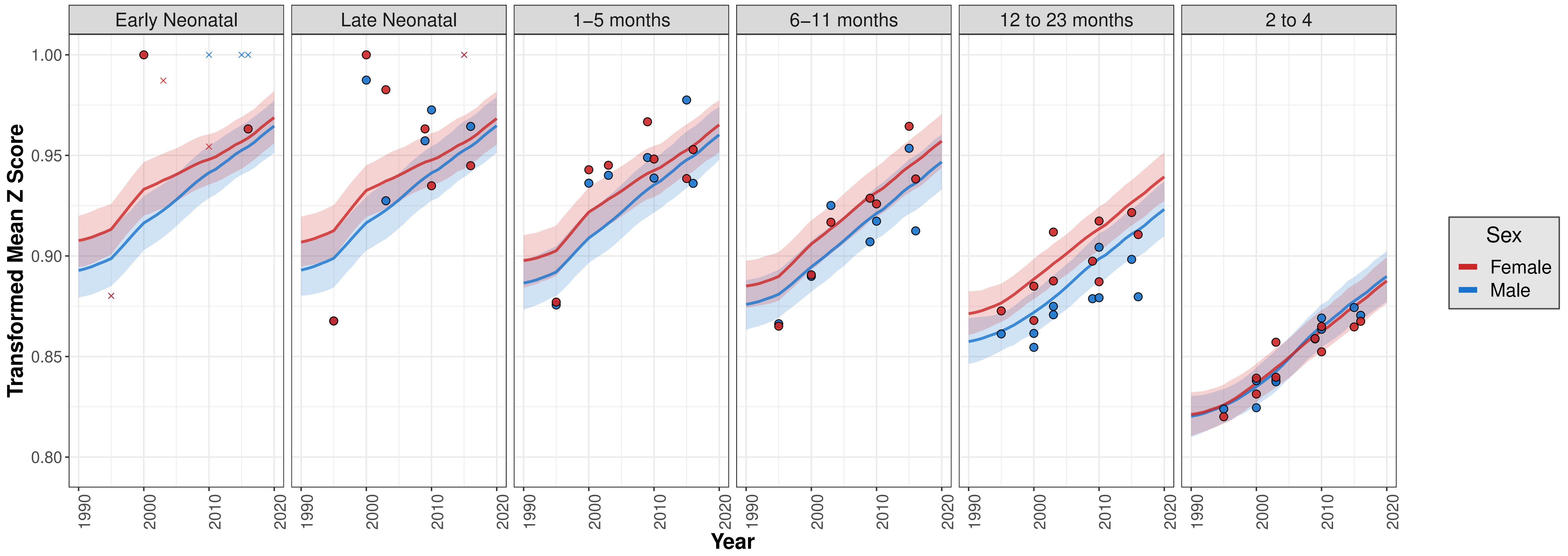

I

Year	Source
1995	WHO CGM Database
2000	MICS
2000	WHO CGM Database
2003	WHO CGM Database
2003	MICS
2009	MICS
2010	MICS
2010	WHO CGM Database
2015	DHS
2016	WHO CGM Database
2016	DHS

Myanmar – HAZ, WHZ, and WAZ Distributions

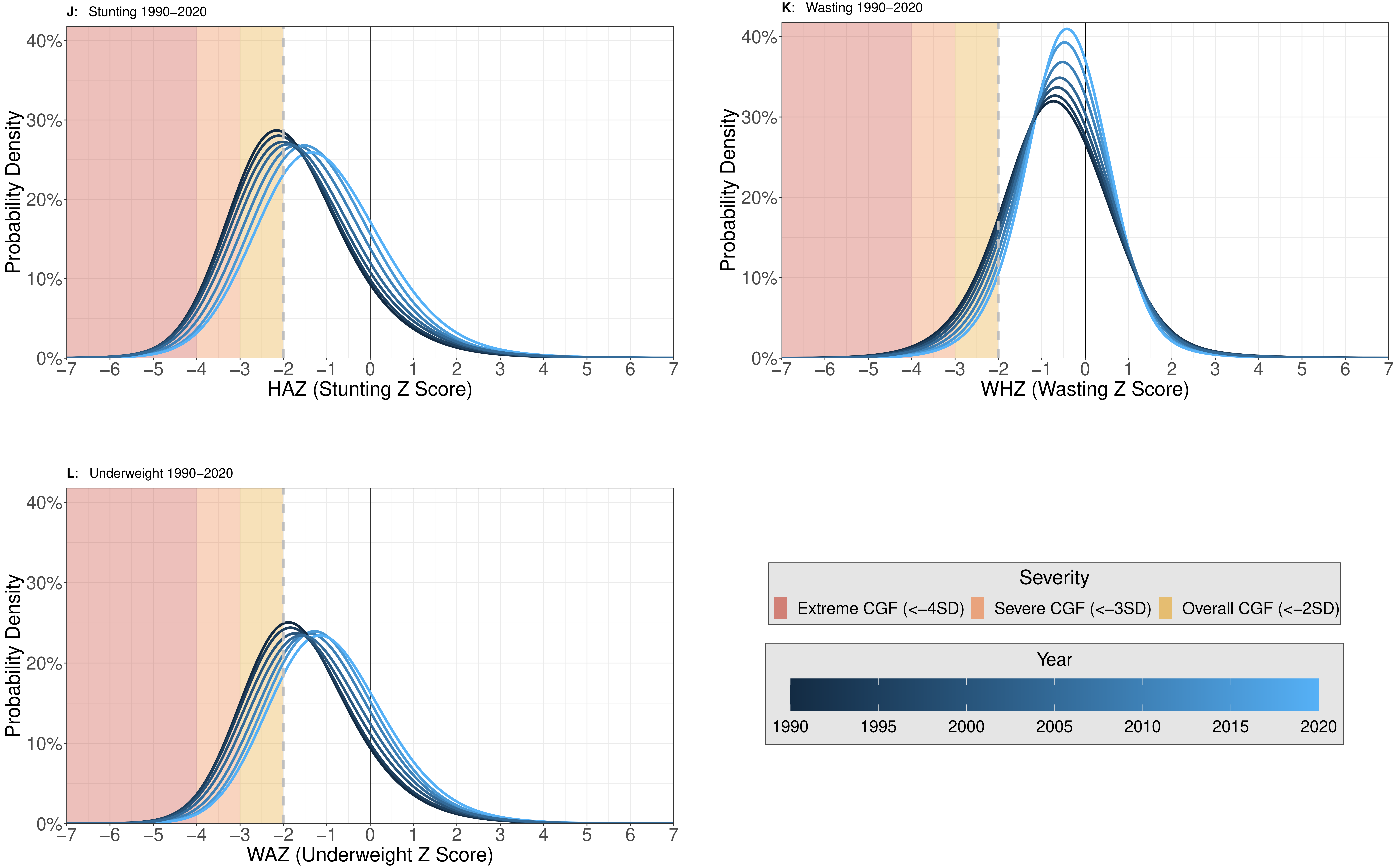

Philippines – Stunting (HAZ)

A: Overall and Severe Stunting Prevalence

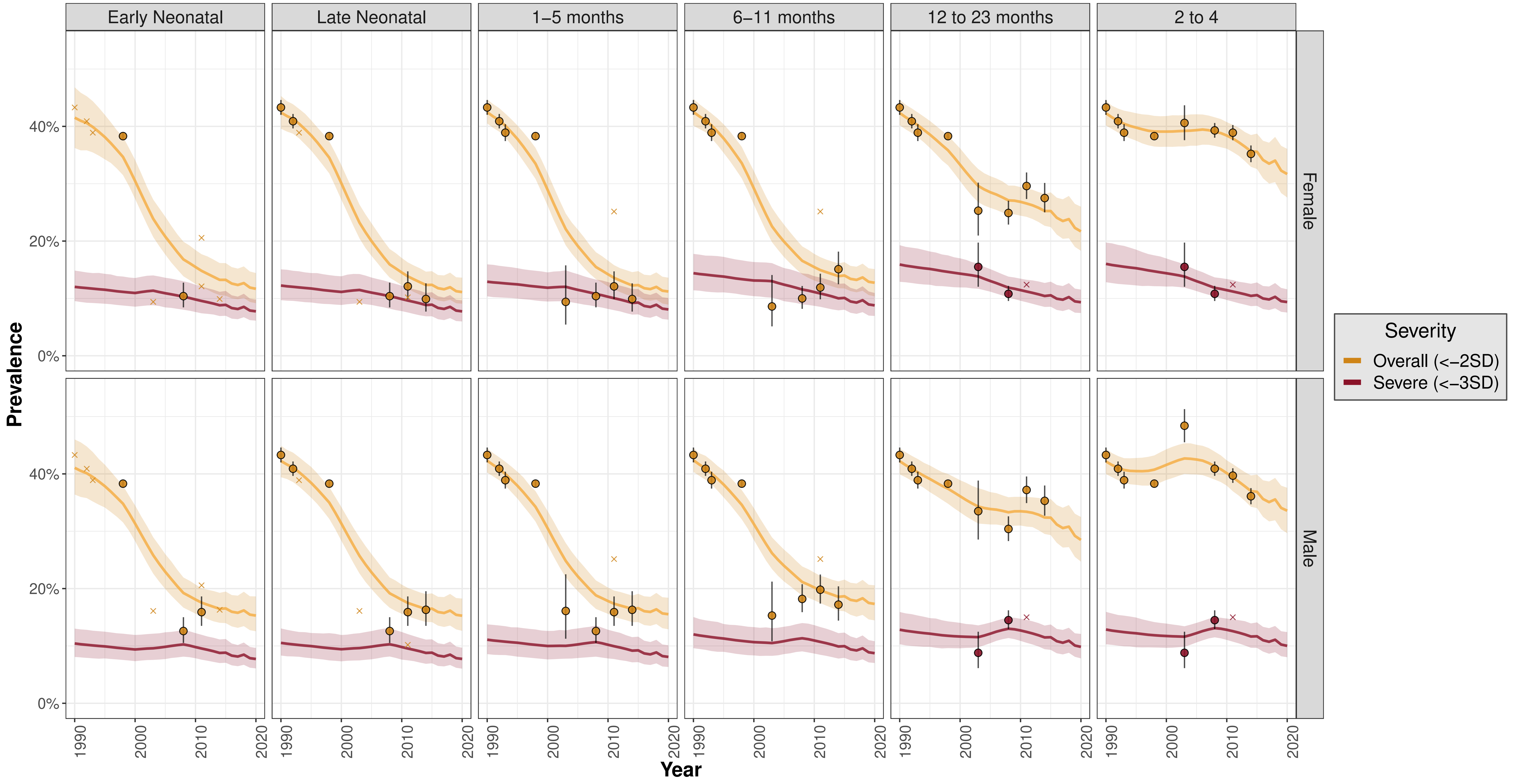

B: Transformed Mean Stunting Z Scores

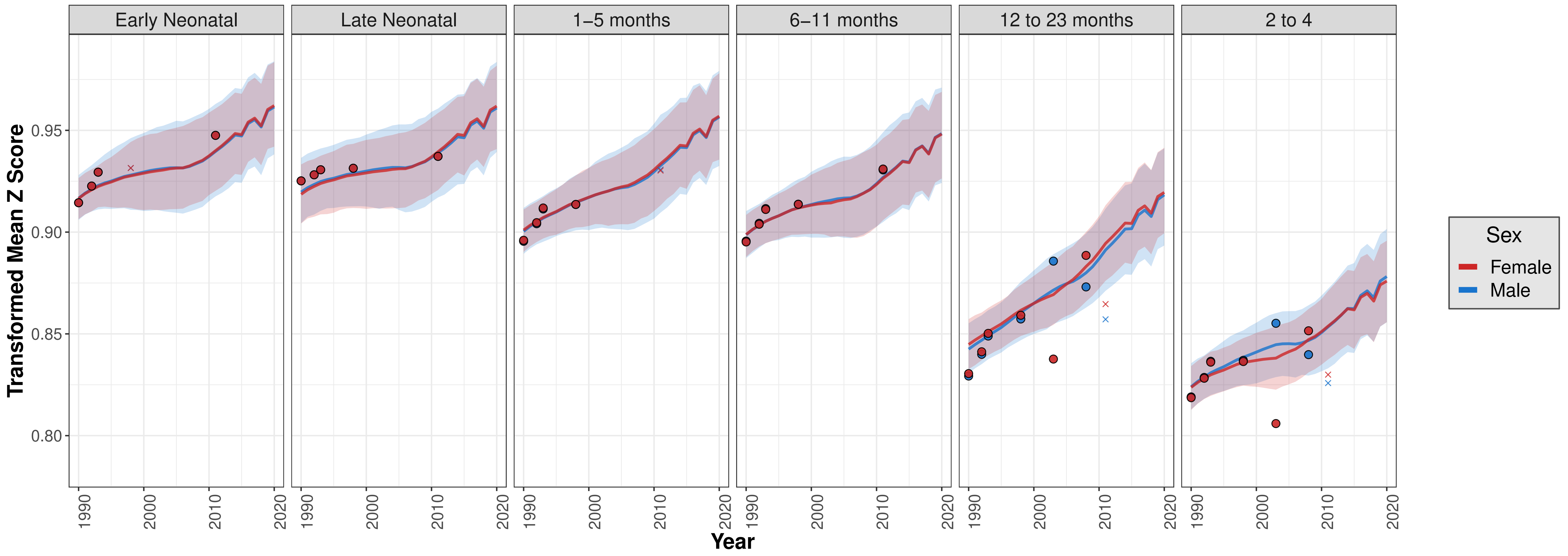

C

Year	Source	National	Subnational
1987	WHO CGM Database	X	
1990	WHO CGM Database	X	
1992	WHO CGM Database	X	
1993	WHO CGM Database	X	
1998	WHO CGM Database	X	
2003	WHO CGM Database	X	
2008	WHO CGM Database	X	
2011	WHO CGM Database	X	X
2011	Statistical Yearbook	X	
2014	WHO CGM Database	X	

Philippines – Wasting (WHZ)

D: Overall and Severe Wasting Prevalence

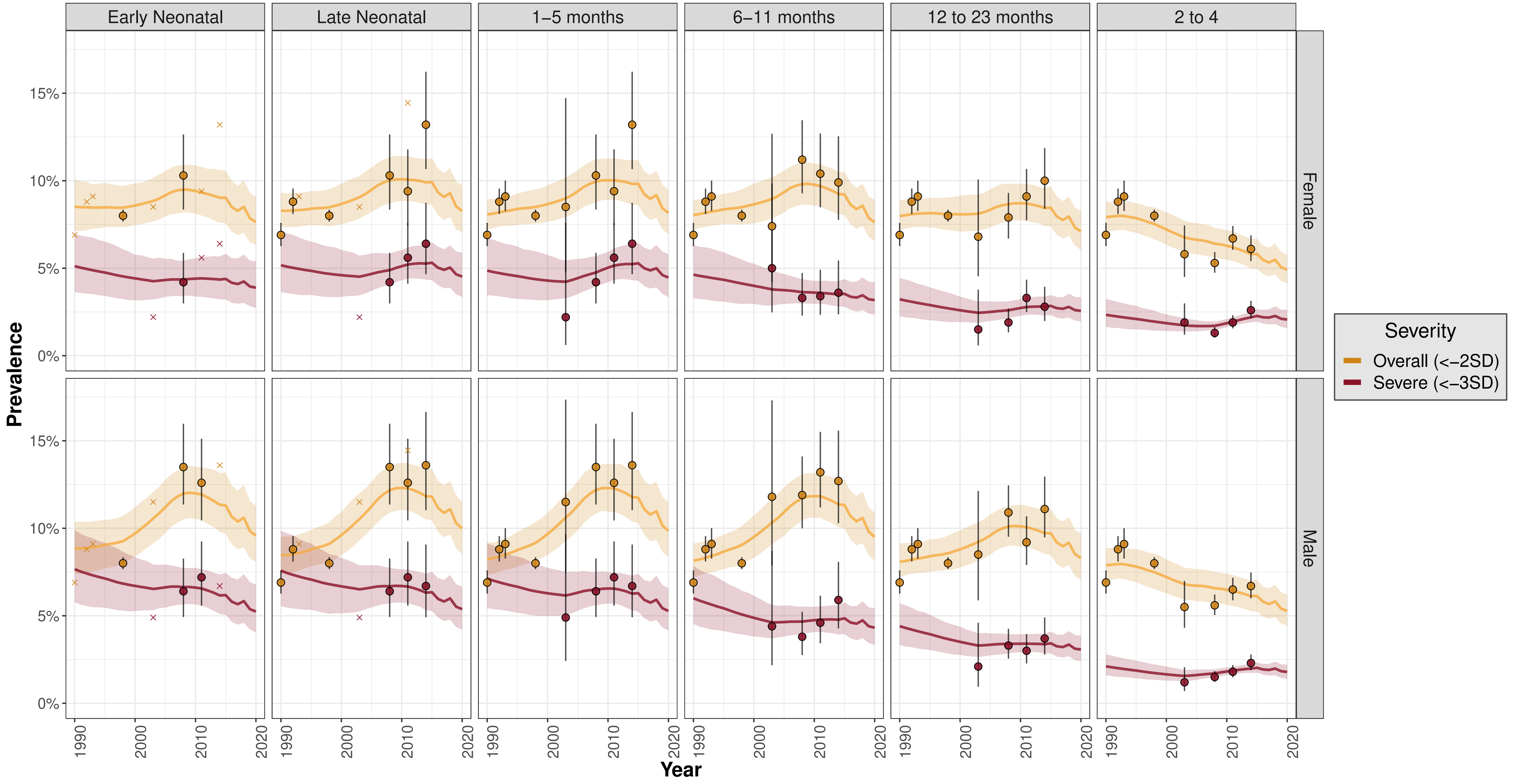

E: Transformed Mean Wasting Z Scores

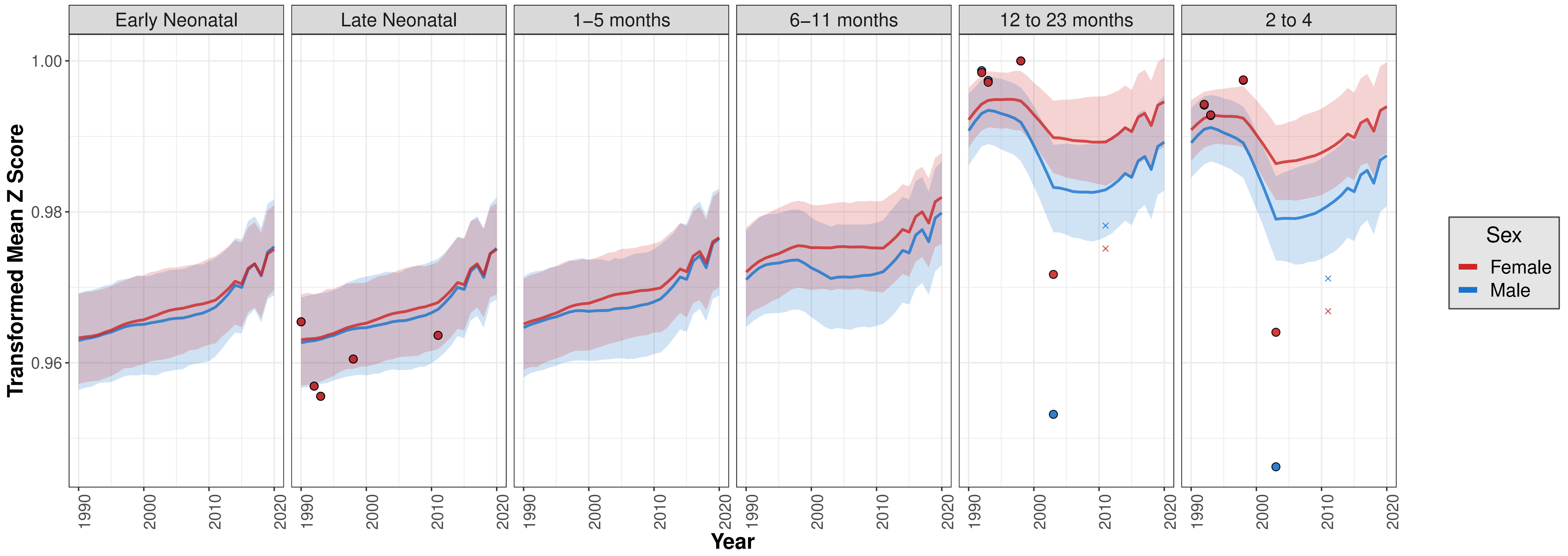

F

Year	Source	National	Subnational
1987	WHO CGM Database	X	
1990	WHO CGM Database	X	
1992	WHO CGM Database	X	
1993	WHO CGM Database	X	
1998	WHO CGM Database	X	
2003	WHO CGM Database	X	
2008	WHO CGM Database	X	
2011	WHO CGM Database	X	X
2011	Statistical Yearbook	X	
2014	WHO CGM Database	X	

Philippines – Underweight (WAZ)

G: Overall and Severe Underweight Prevalence

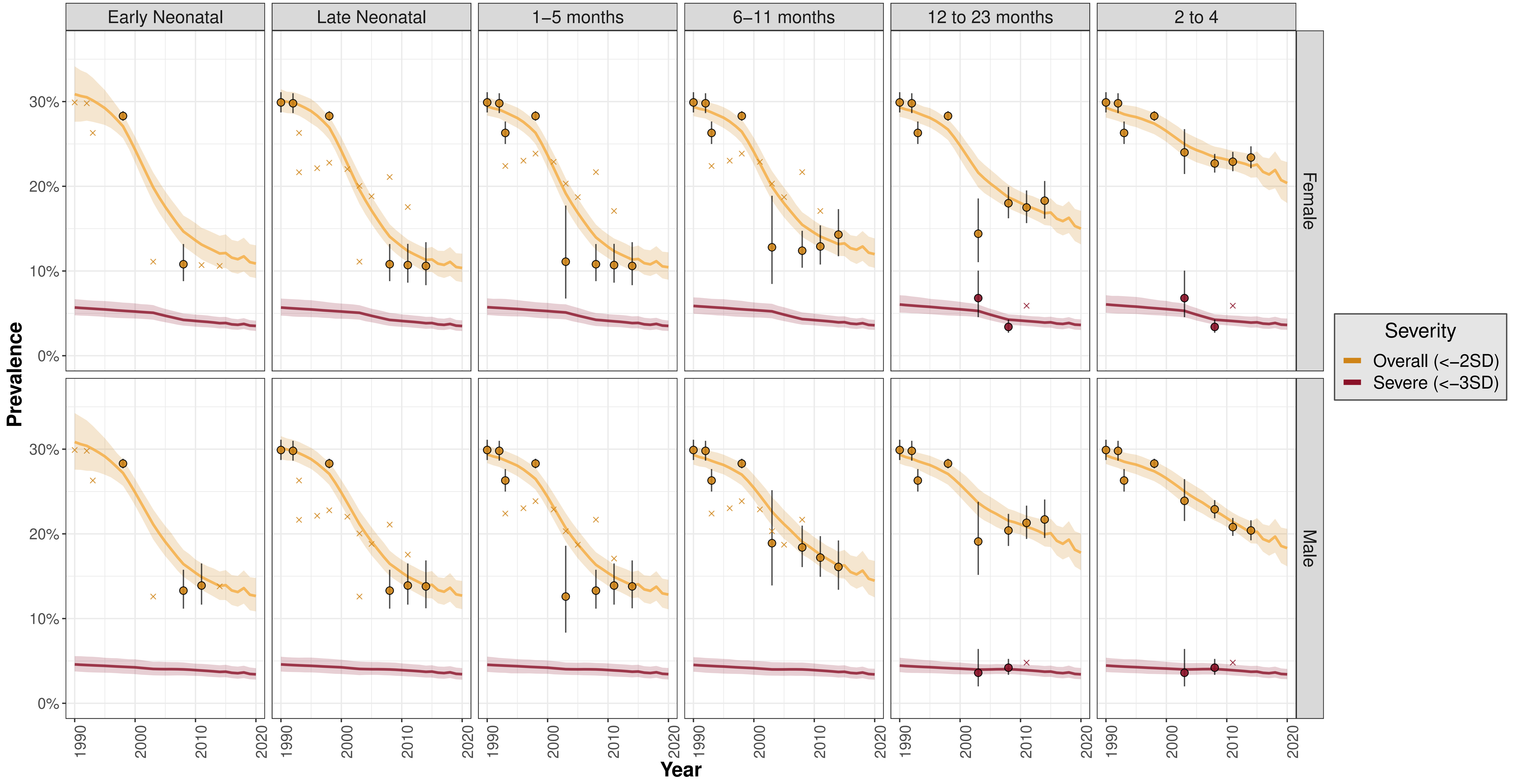

H: Transformed Mean Underweight Z Scores

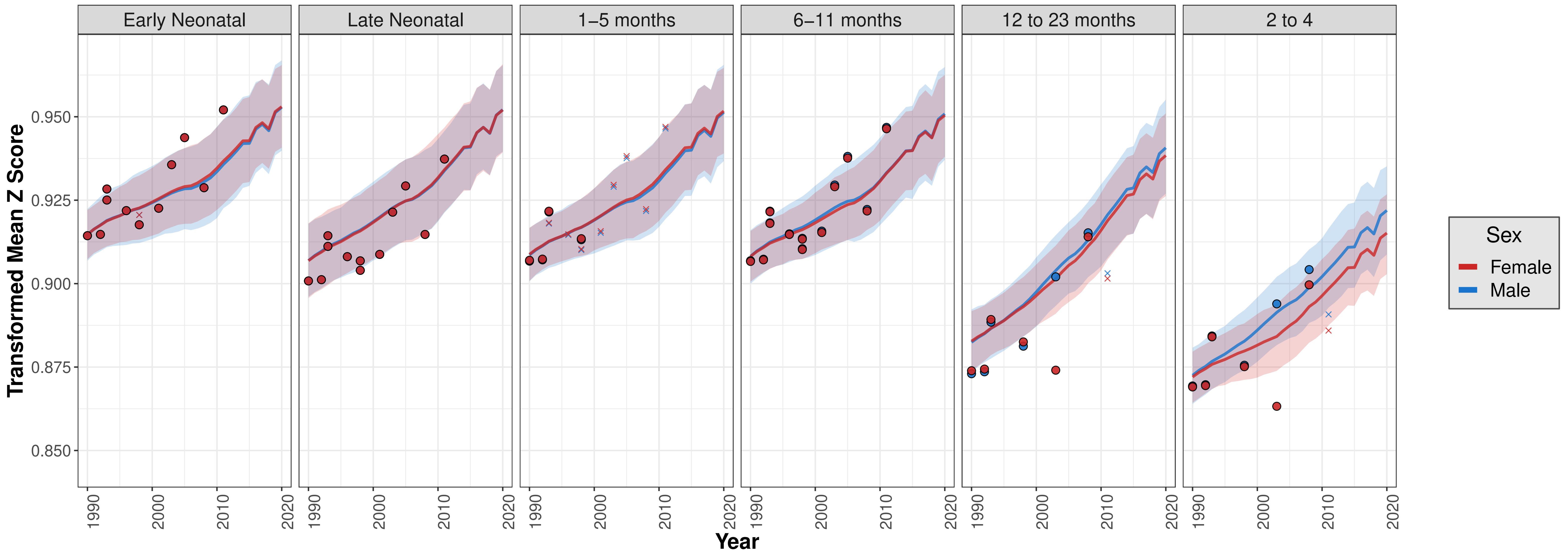

I

Year	Source	National	Subnational
1987	WHO CGM Database	X	
1990	WHO CGM Database	X	
1992	WHO CGM Database	X	
1993	WHO CGM Database	X	
1993	Statistical Yearbook	X	
1996	Statistical Yearbook	X	
1998	WHO CGM Database	X	
1998	Statistical Yearbook	X	
2001	Statistical Yearbook	X	
2003	WHO CGM Database	X	
2003	Statistical Yearbook	X	
2005	Statistical Yearbook	X	
2008	WHO CGM Database	X	
2008	Statistical Yearbook	X	
2011	WHO CGM Database	X	X
2011	Statistical Yearbook	X	
2014	WHO CGM Database	X	

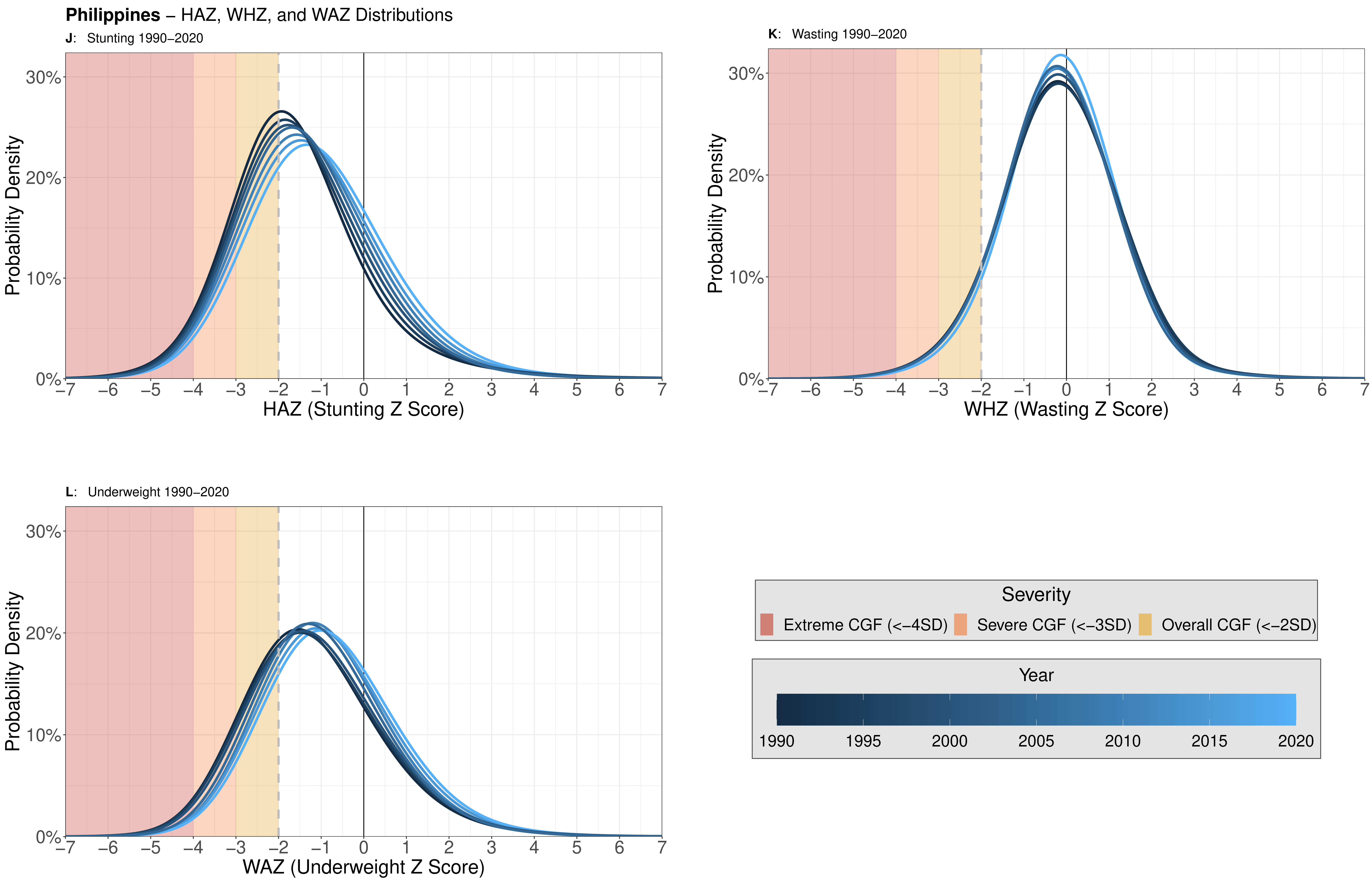
Philippines – HAZ, WHZ, and WAZ Distributions
J: Stunting 1990–2020
K: Wasting 1990–2020
L: Underweight 1990–2020
Probability Density
0%
10%
20%
30%
−7
−6
−5
−4
−3
−2
−1
0
1
2
3
4
5
6
7
HAZ (Stunting Z Score)
WHZ (Wasting Z Score)
WAZ (Underweight Z Score)
Severity
Extreme CGF (<−4SD)
Severe CGF (<−3SD)
Overall CGF (<−2SD)
Year
1990
1995
2000
2005
2010
2015
2020

Sri Lanka – Stunting (HAZ)

A: Overall and Severe Stunting Prevalence

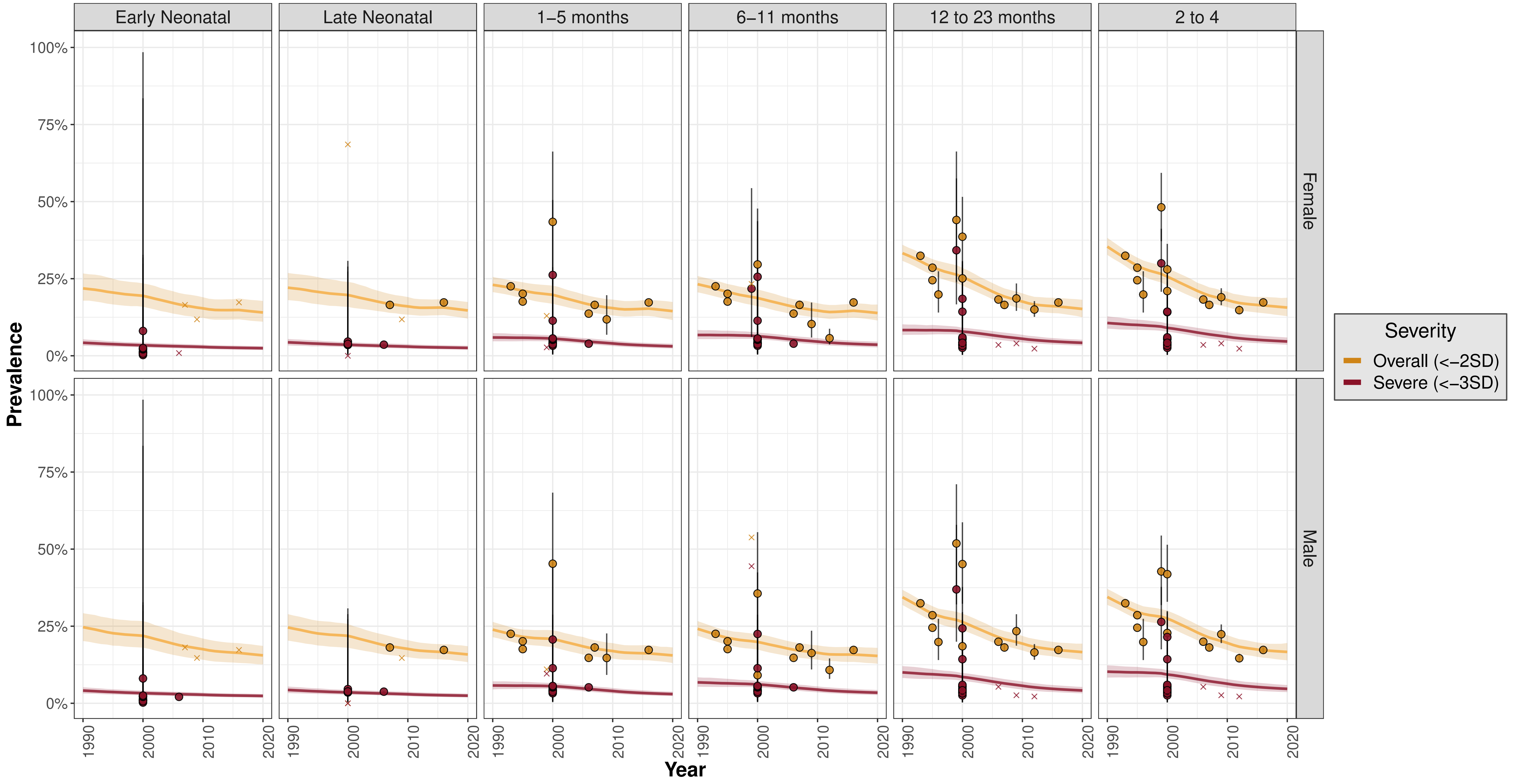

B: Transformed Mean Stunting Z Scores

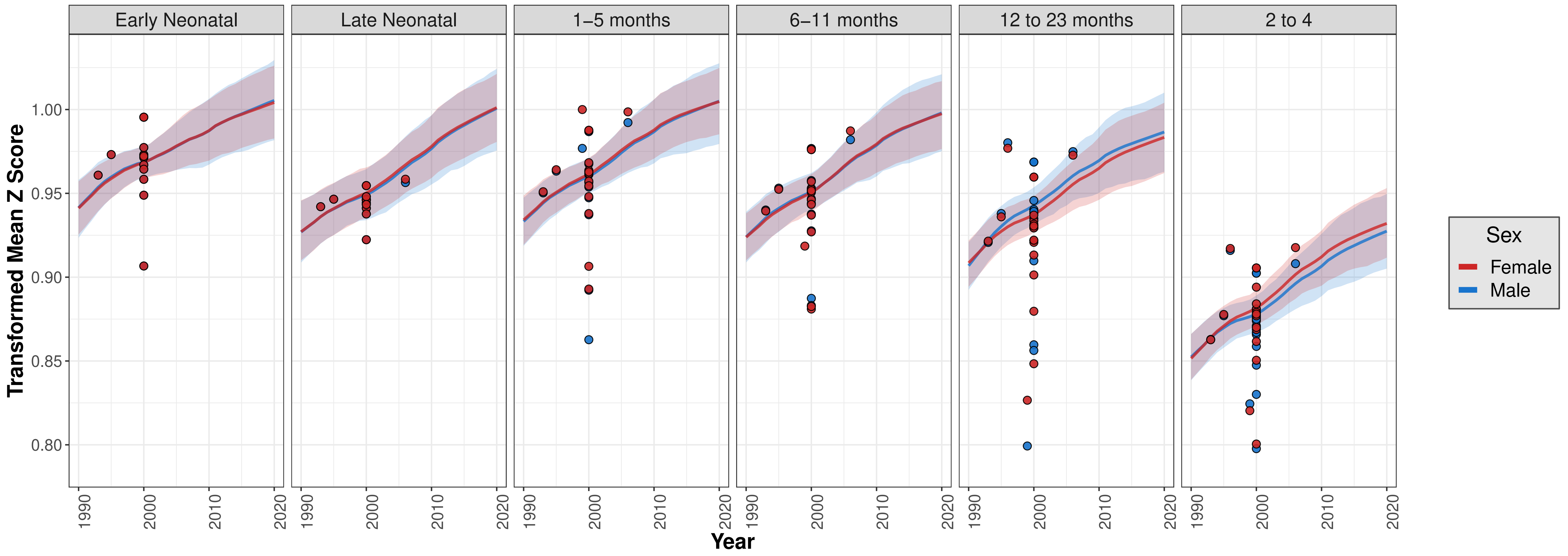

C

Year	Source
1978	WHO CGM Database
1987	DHS
1987	WHO CGM Database
1988	WHO CGM Database
1993	WHO CGM Database
1995	WHO CGM Database
1996	WHO CGM Database
1999	Integrated Survey
2000	Integrated Survey
2000	WHO CGM Database
2006	WHO CGM Database
2007	WHO CGM Database
2009	WHO CGM Database
2012	WHO CGM Database
2016	WHO CGM Database
2016	DHS

Sri Lanka – Wasting (WHZ)

D: Overall and Severe Wasting Prevalence

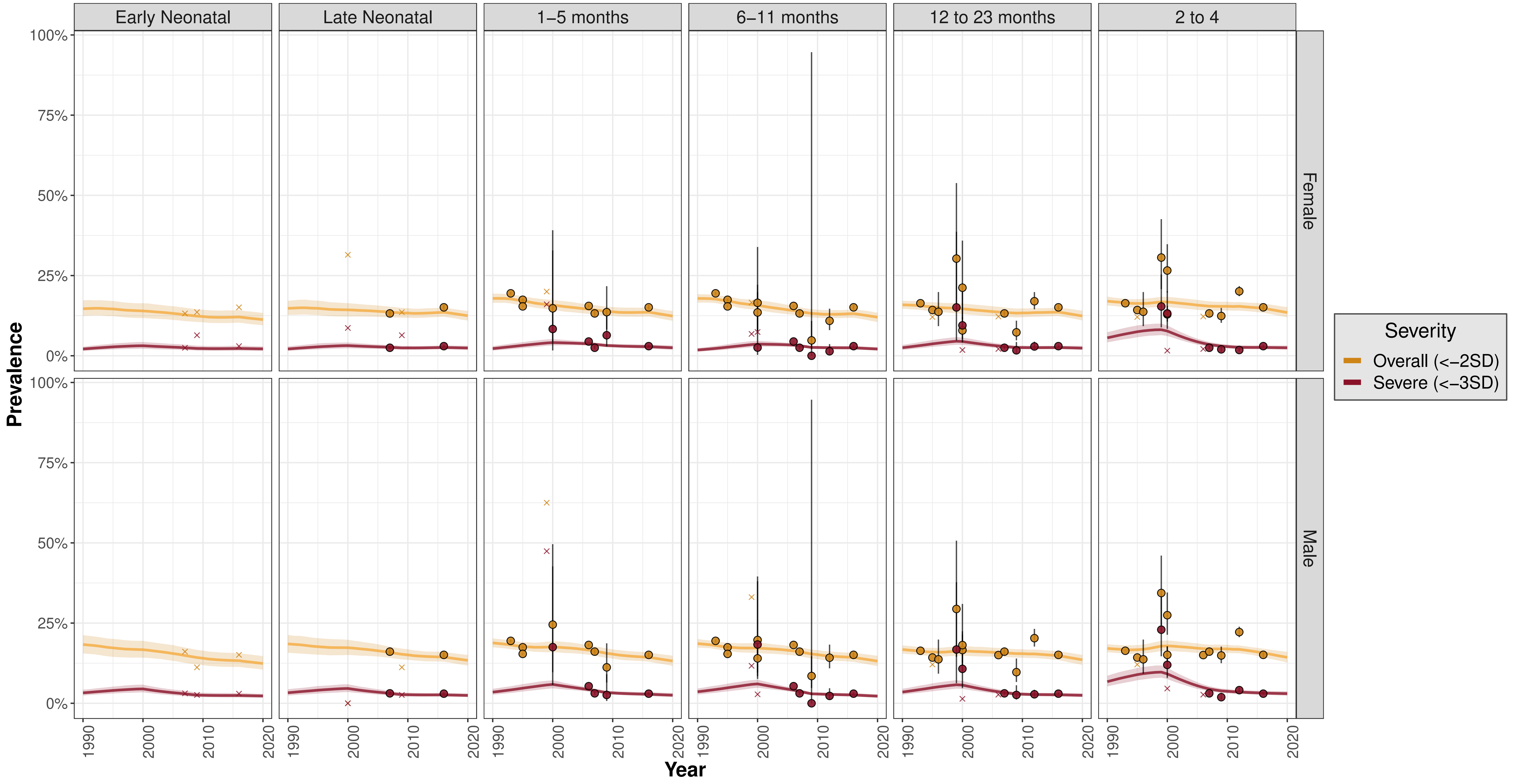

E: Transformed Mean Wasting Z Scores

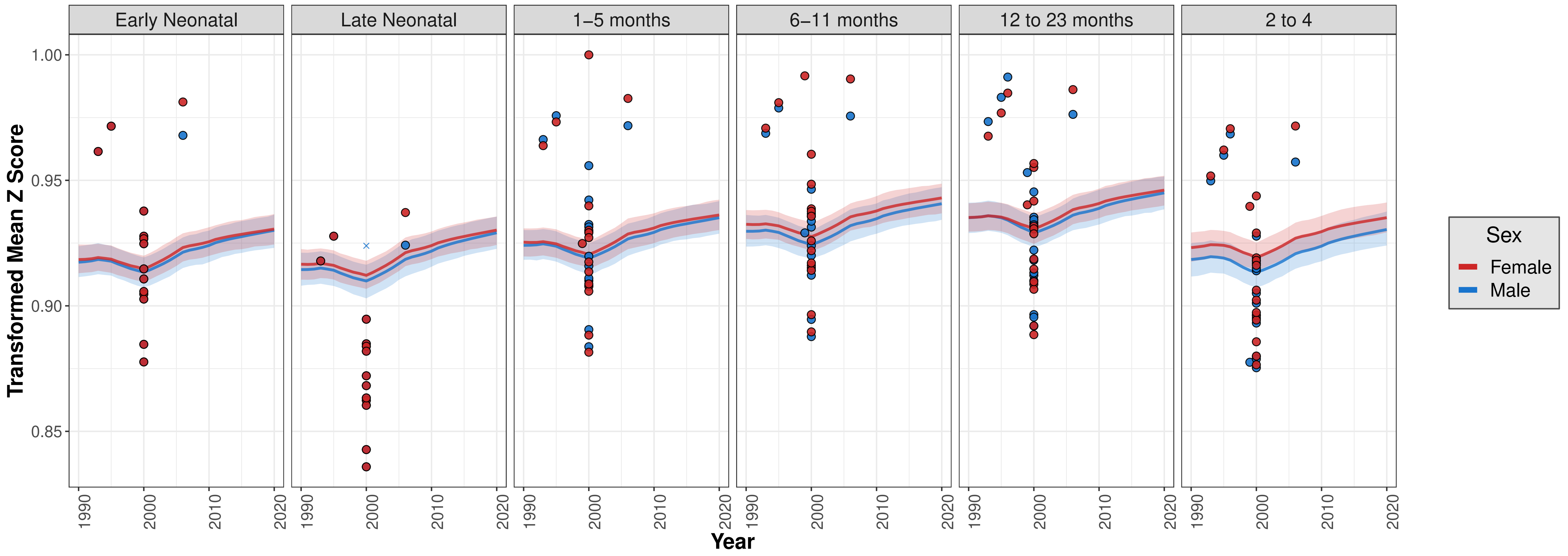

F

Year	Source
1978	WHO CGM Database
1987	DHS
1987	WHO CGM Database
1988	WHO CGM Database
1993	WHO CGM Database
1995	WHO CGM Database
1996	WHO CGM Database
1999	Integrated Survey
2000	Integrated Survey
2000	WHO CGM Database
2006	WHO CGM Database
2007	WHO CGM Database
2009	WHO CGM Database
2012	WHO CGM Database
2016	WHO CGM Database
2016	DHS

Sri Lanka – Underweight (WAZ)

G: Overall and Severe Underweight Prevalence

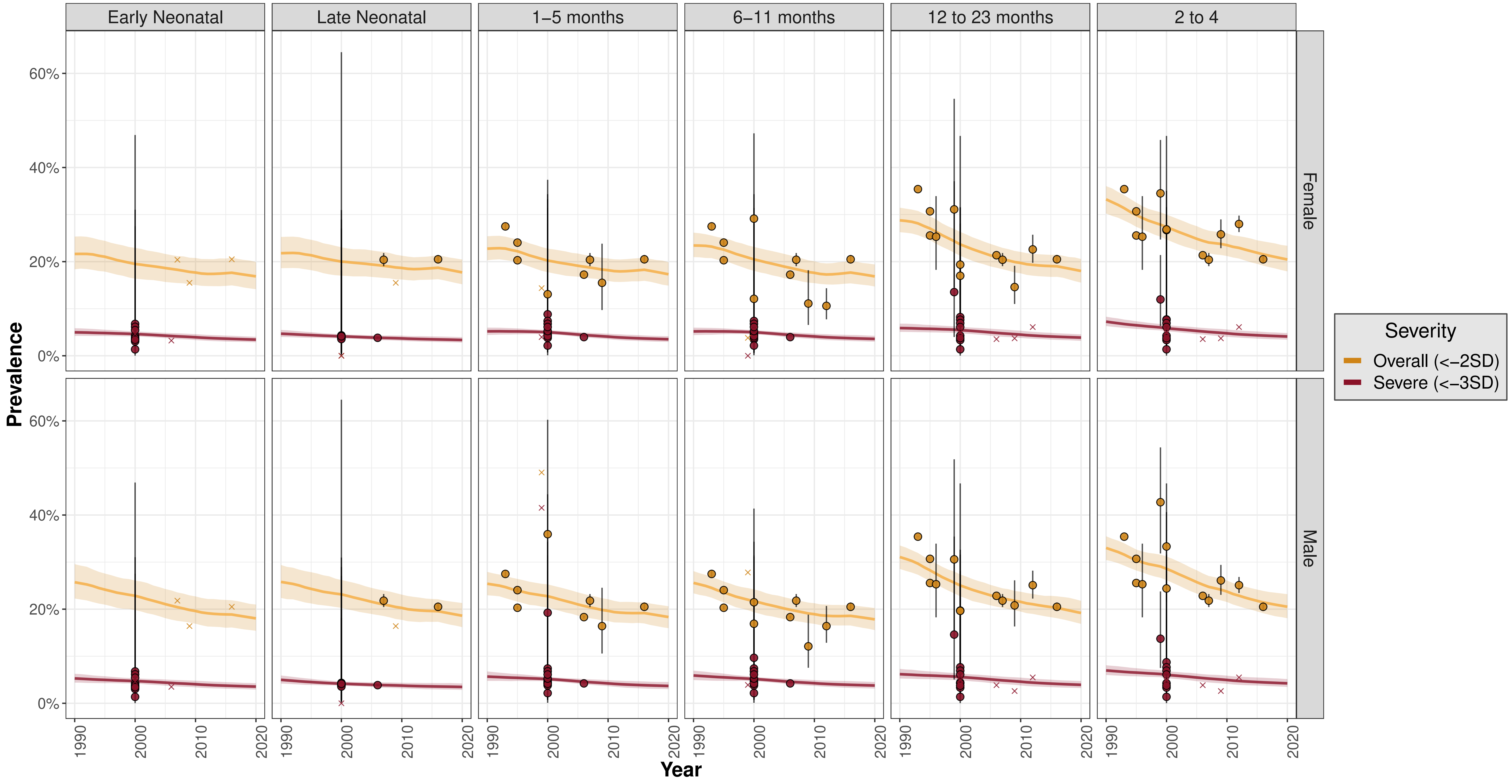

H: Transformed Mean Underweight Z Scores

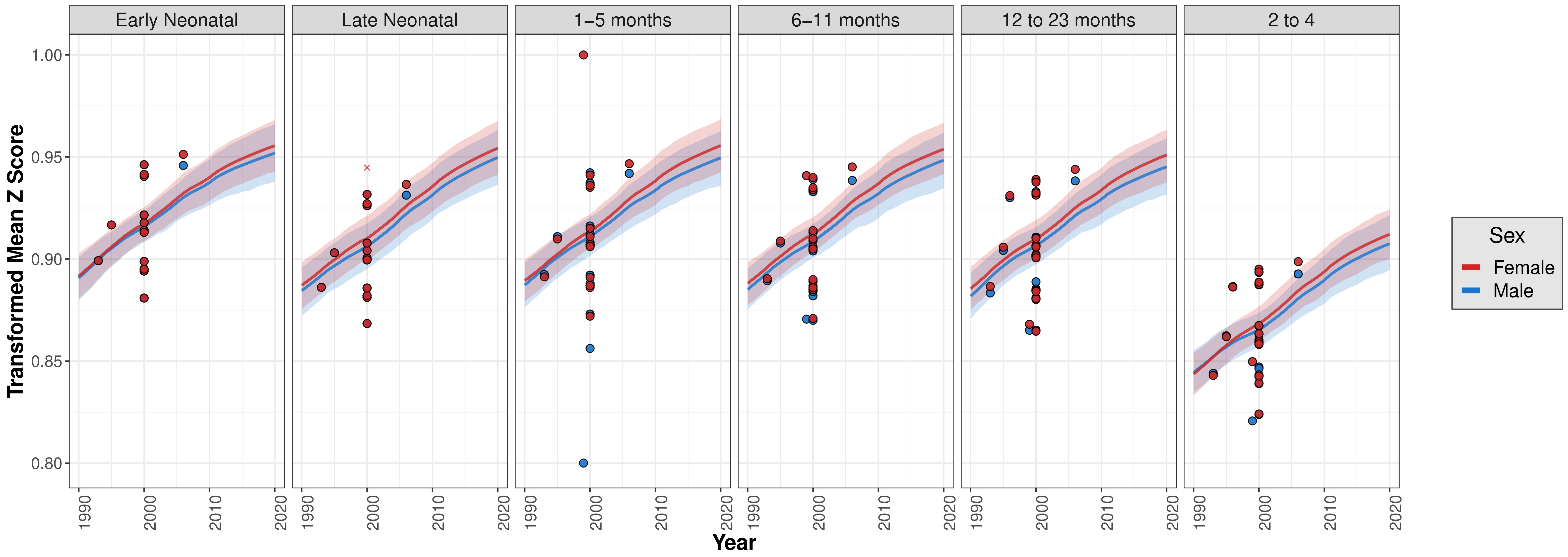

I

Year	Source
1978	WHO CGM Database
1987	DHS
1987	WHO CGM Database
1988	WHO CGM Database
1993	WHO CGM Database
1995	WHO CGM Database
1996	WHO CGM Database
1999	Integrated Survey
2000	Integrated Survey
2000	WHO CGM Database
2006	WHO CGM Database
2007	WHO CGM Database
2009	WHO CGM Database
2012	WHO CGM Database
2016	WHO CGM Database
2016	DHS

Sri Lanka – HAZ, WHZ, and WAZ Distributions

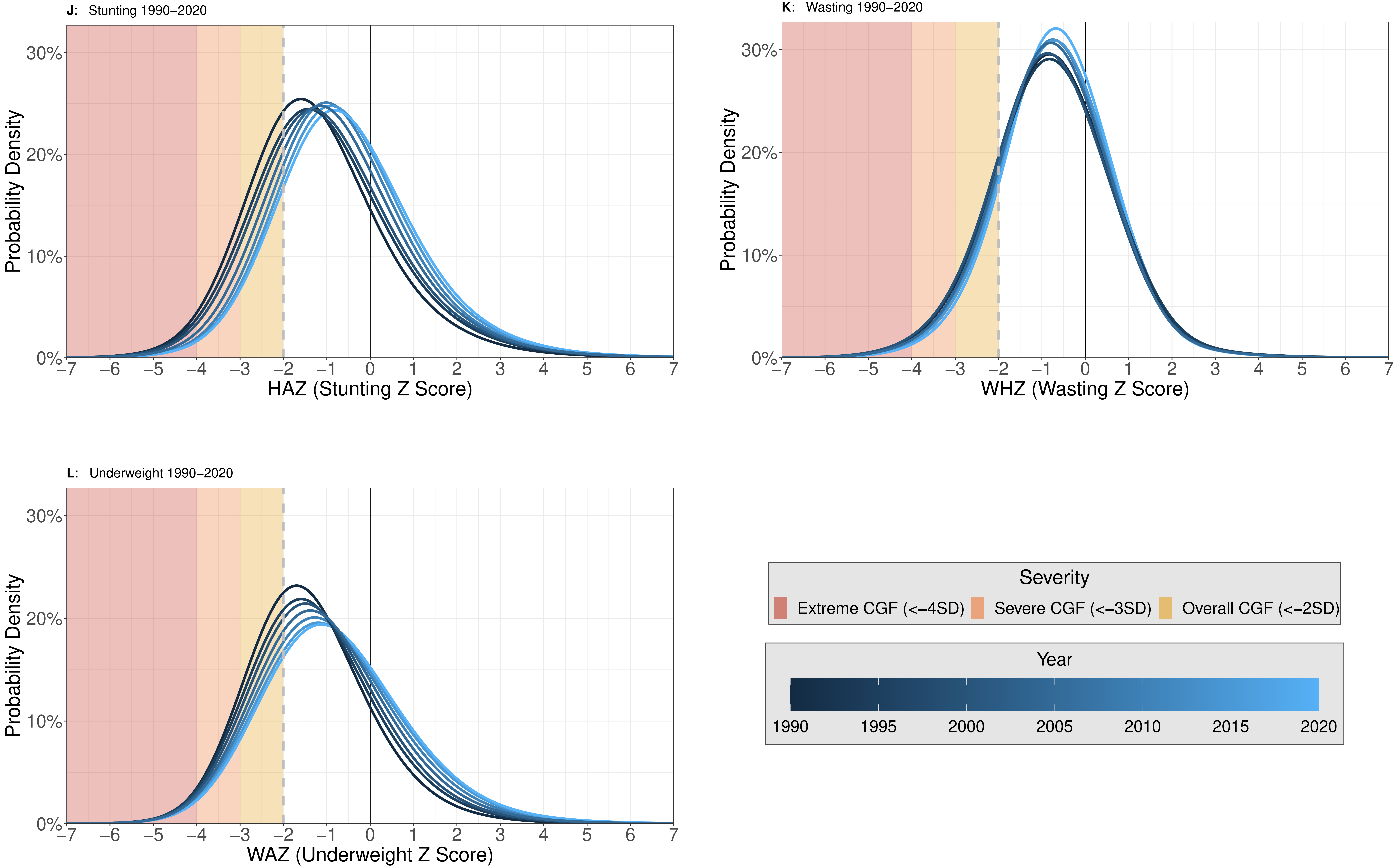

Thailand – Stunting (HAZ)

A: Overall and Severe Stunting Prevalence

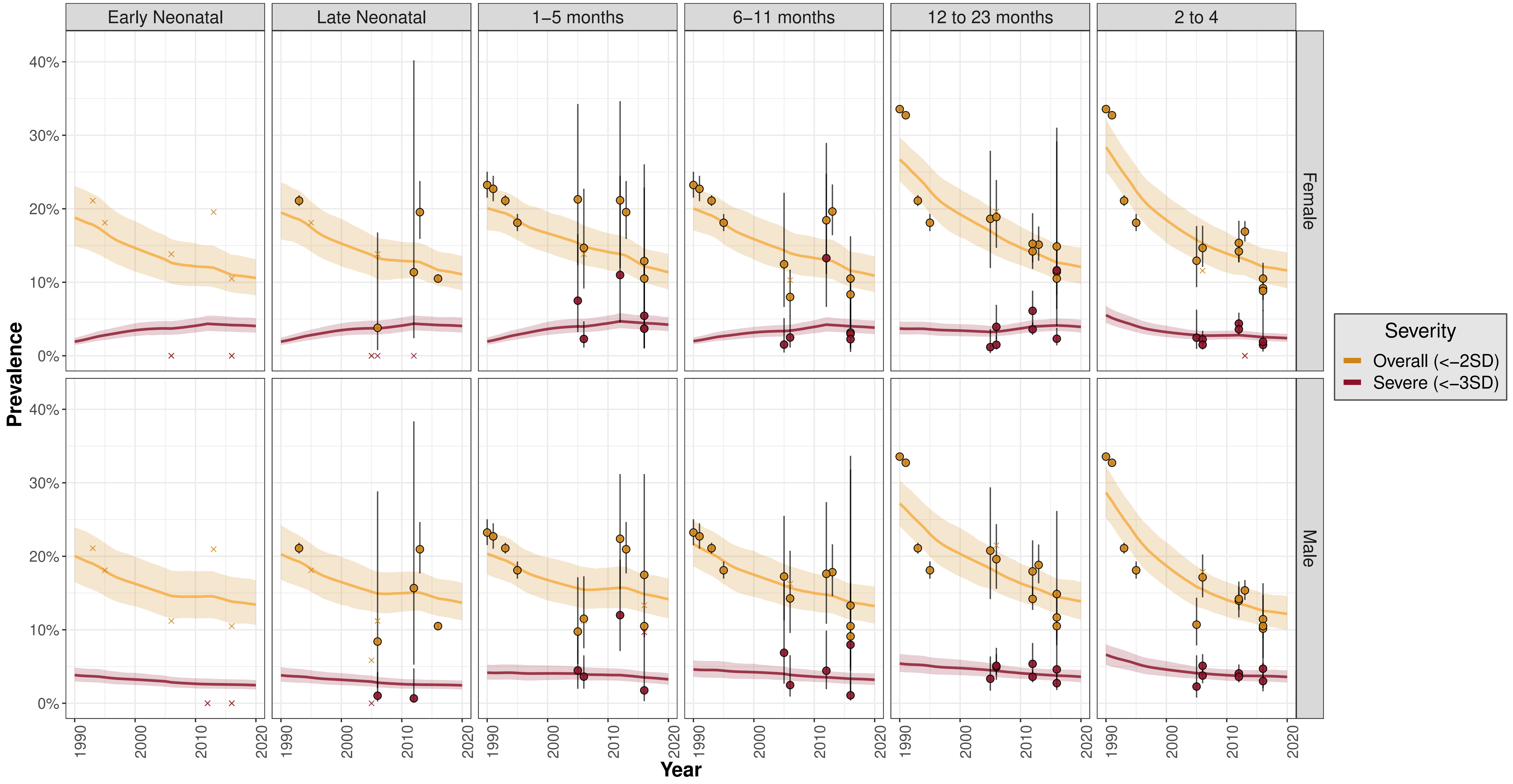

B: Transformed Mean Stunting Z Scores

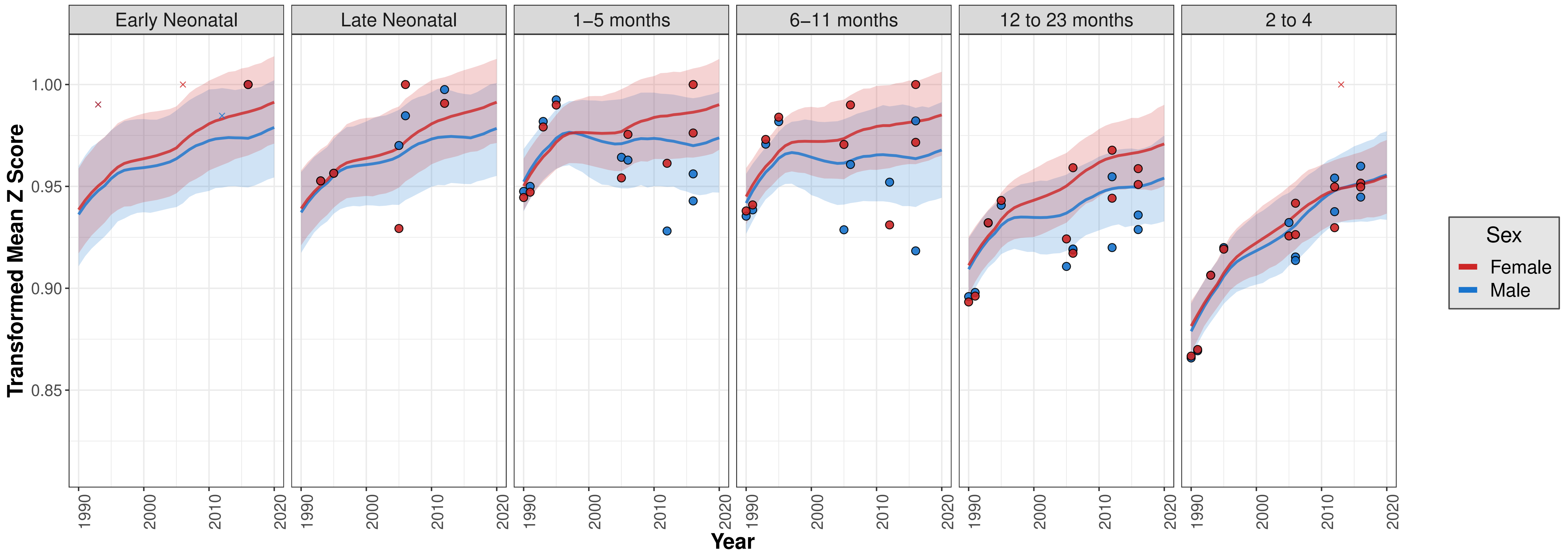

C

Year	Source
1987	DHS
1987	WHO CGM Database
1990	WHO CGM Database
1991	WHO CGM Database
1993	WHO CGM Database
1995	WHO CGM Database
2005	MICS
2006	MICS
2006	WHO CGM Database
2012	WHO CGM Database
2012	MICS
2013	WHO CGM Database
2013	MICS
2016	WHO CGM Database
2016	MICS
2016	Bangkok Small Community MICS

Thailand – Wasting (WHZ)

D: Overall and Severe Wasting Prevalence

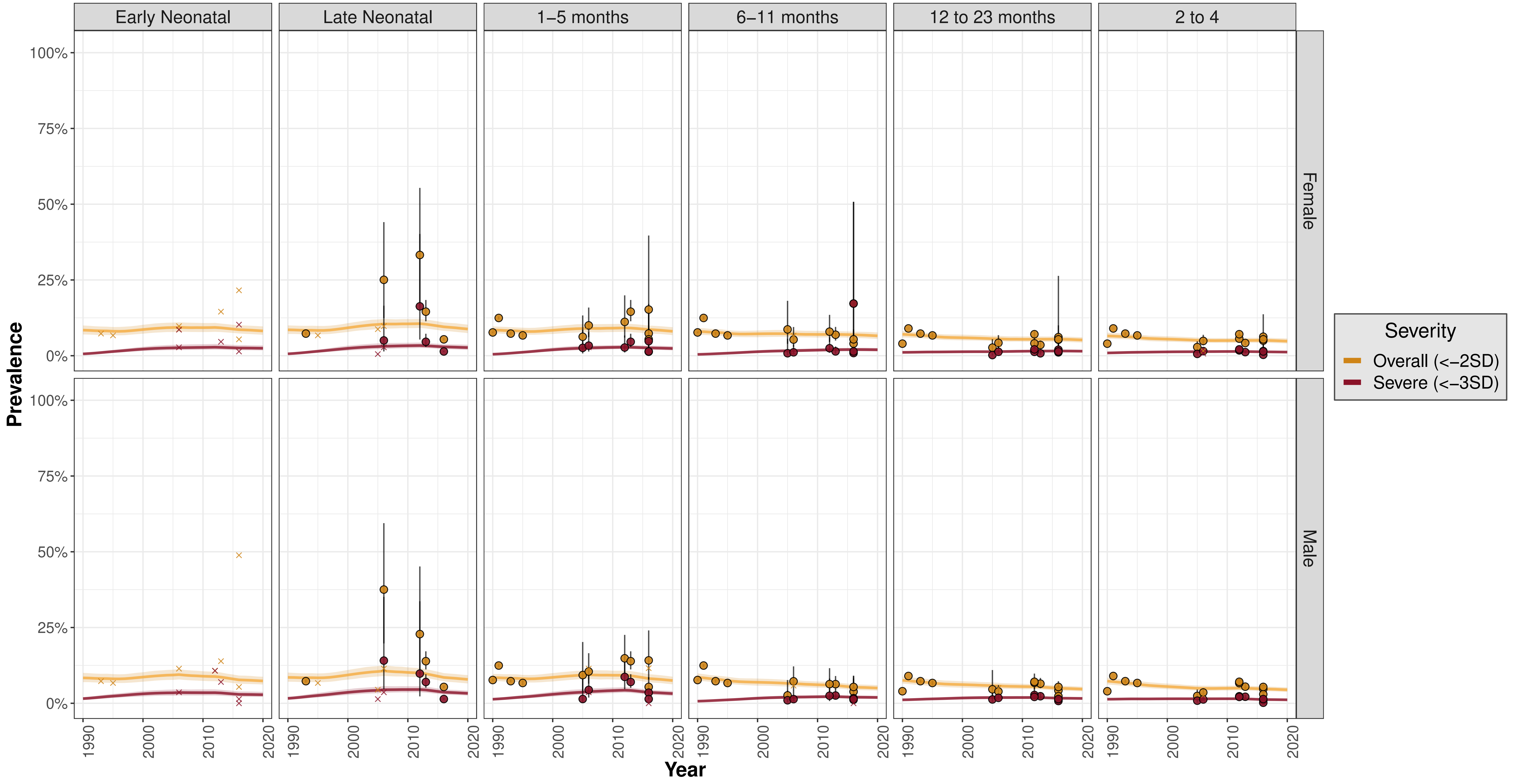

E: Transformed Mean Wasting Z Scores

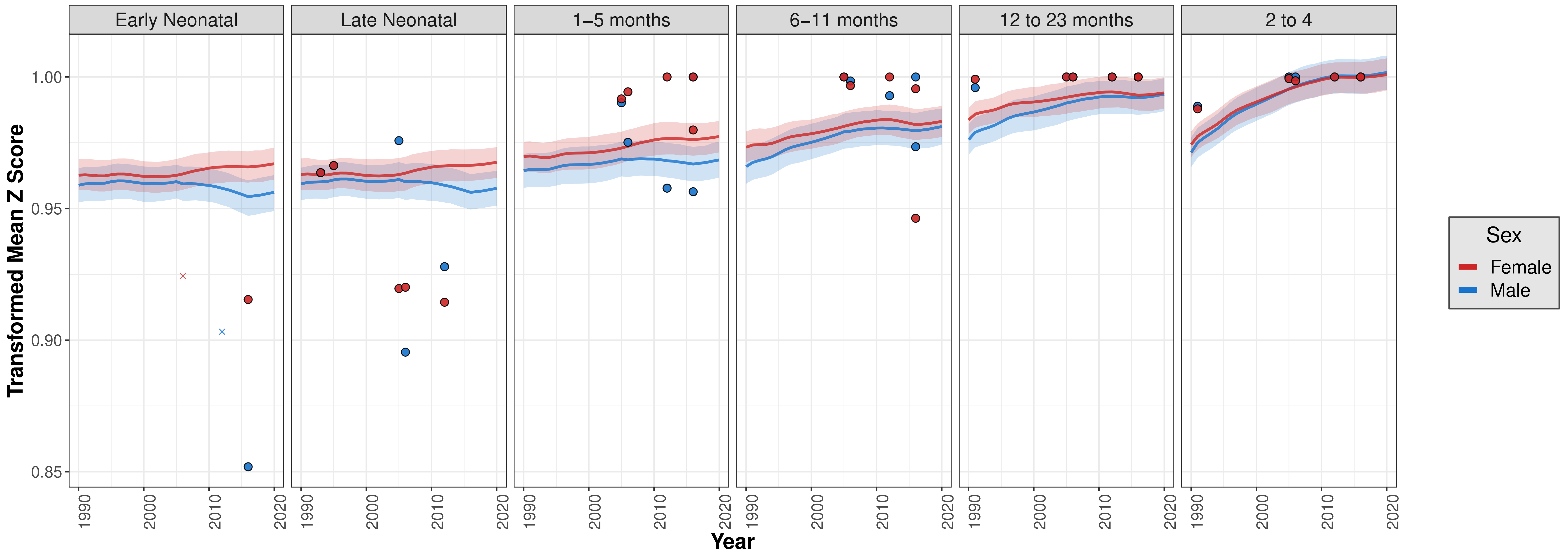

F

Year	Source
1987	DHS
1987	WHO CGM Database
1990	WHO CGM Database
1991	WHO CGM Database
1993	WHO CGM Database
1995	WHO CGM Database
2005	MICS
2006	MICS
2006	WHO CGM Database
2012	WHO CGM Database
2012	MICS
2013	WHO CGM Database
2016	WHO CGM Database
2016	MICS
2016	Bangkok Small Community MICS

Thailand – Underweight (WAZ)

G: Overall and Severe Underweight Prevalence

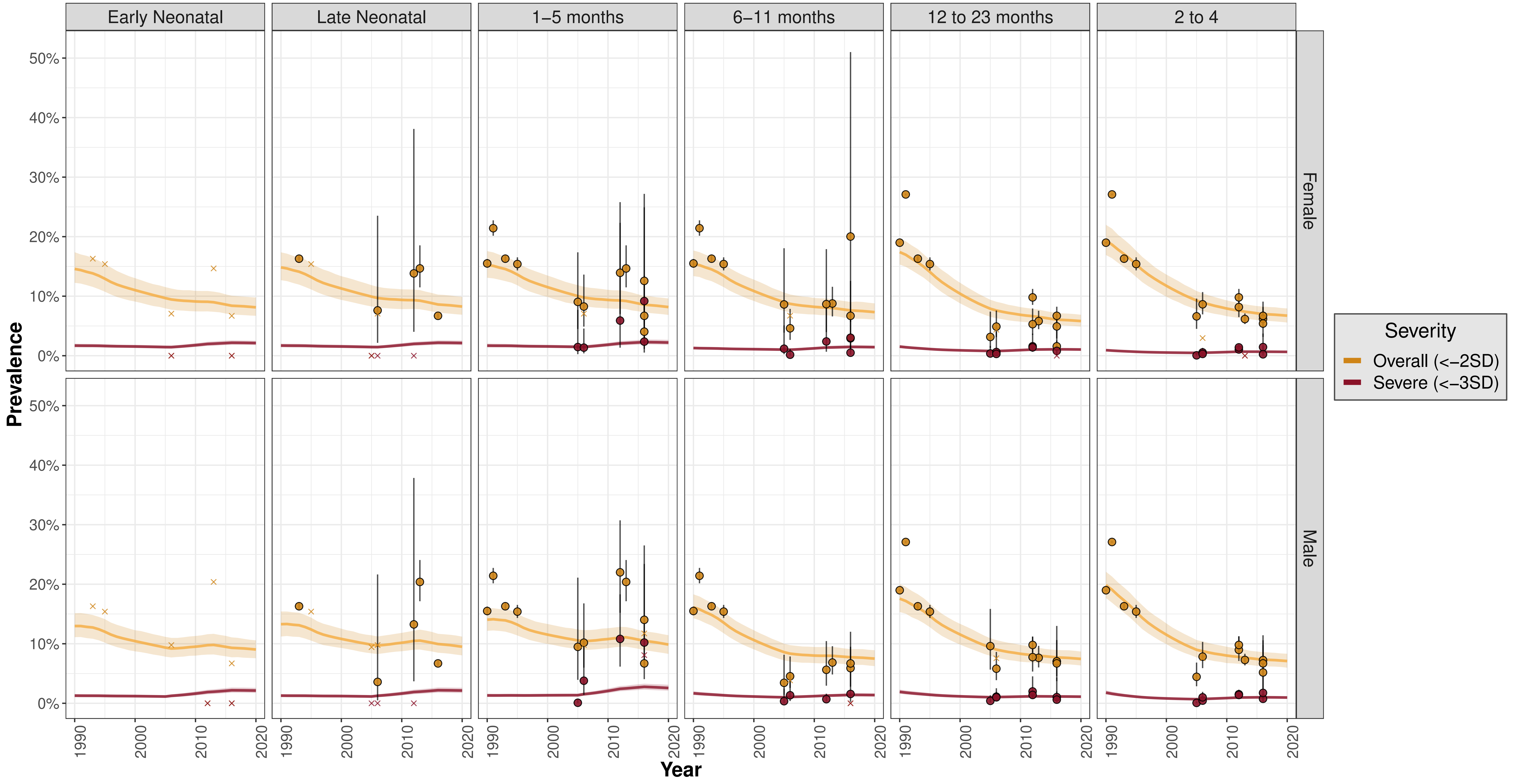

H: Transformed Mean Underweight Z Scores

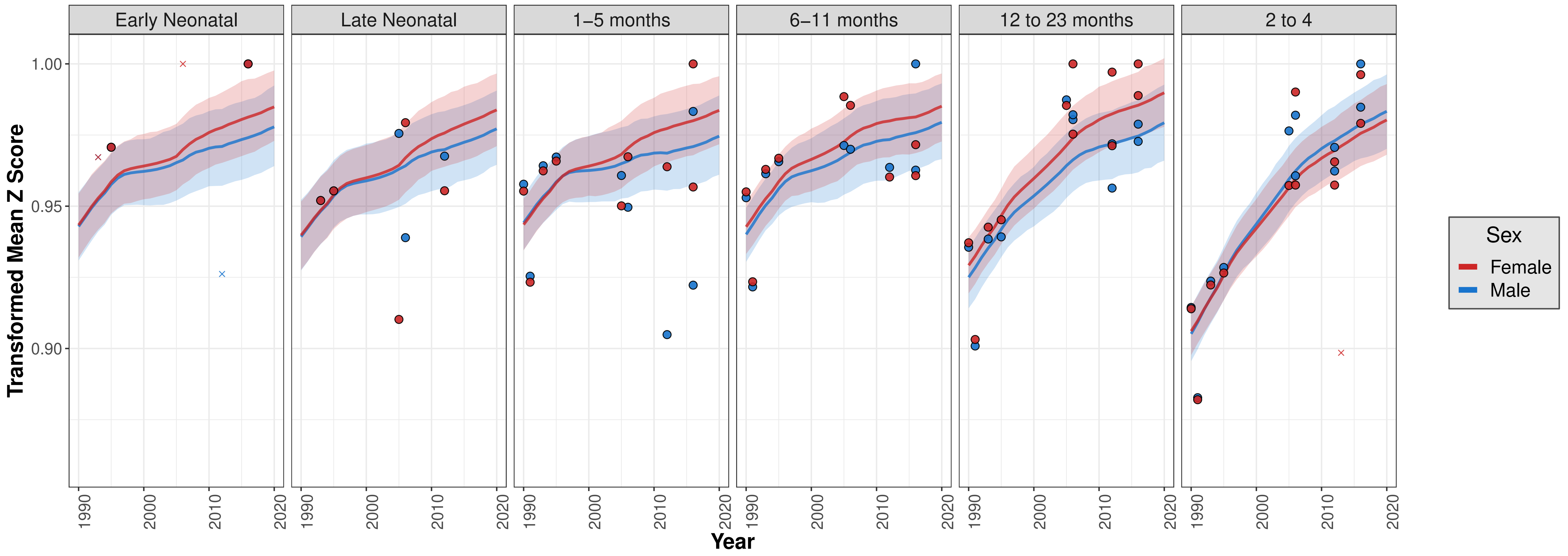

I

Year	Source
1987	DHS
1987	WHO CGM Database
1990	WHO CGM Database
1991	WHO CGM Database
1993	WHO CGM Database
1995	WHO CGM Database
2005	MICS
2006	MICS
2006	WHO CGM Database
2012	WHO CGM Database
2012	MICS
2013	WHO CGM Database
2013	MICS
2016	WHO CGM Database
2016	MICS
2016	Bangkok Small Community MICS

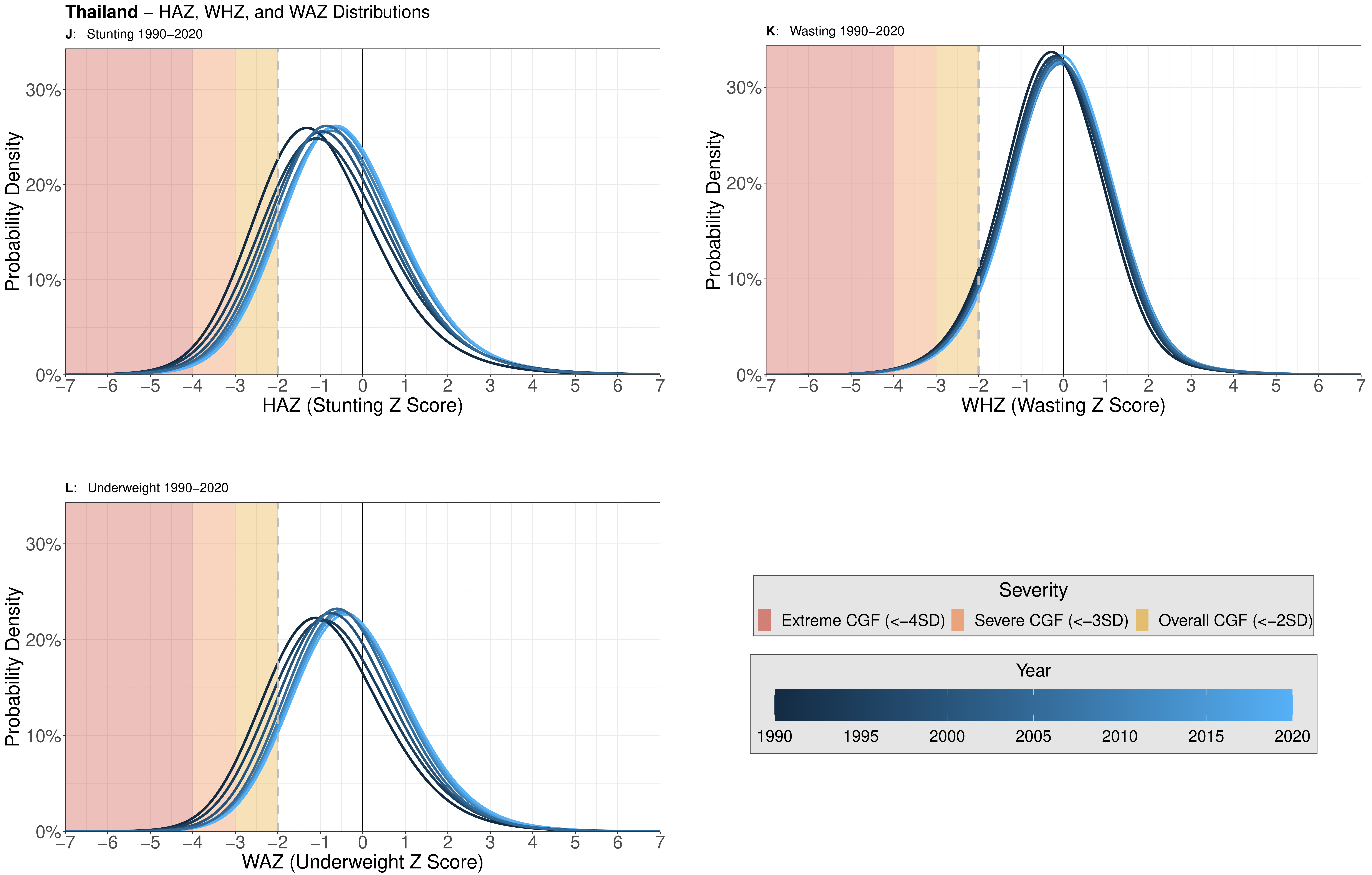

Thailand – HAZ, WHZ, and WAZ Distributions
J: Stunting 1990–2020
Probability Density
30%
20%
10%
0%
−7 −6 −5 −4 −3 −2 −1 0 1 2 3 4 5 6 7
HAZ (Stunting Z Score)
K: Wasting 1990–2020
Probability Density
WHZ (Wasting Z Score)
L: Underweight 1990–2020
Probability Density
WAZ (Underweight Z Score)
Severity
Extreme CGF (<−4SD)
Severe CGF (<−3SD)
Overall CGF (<−2SD)
Year
1990 1995 2000 2005 2010 2015 2020

Mauritius – Stunting (HAZ)

A: Overall and Severe Stunting Prevalence

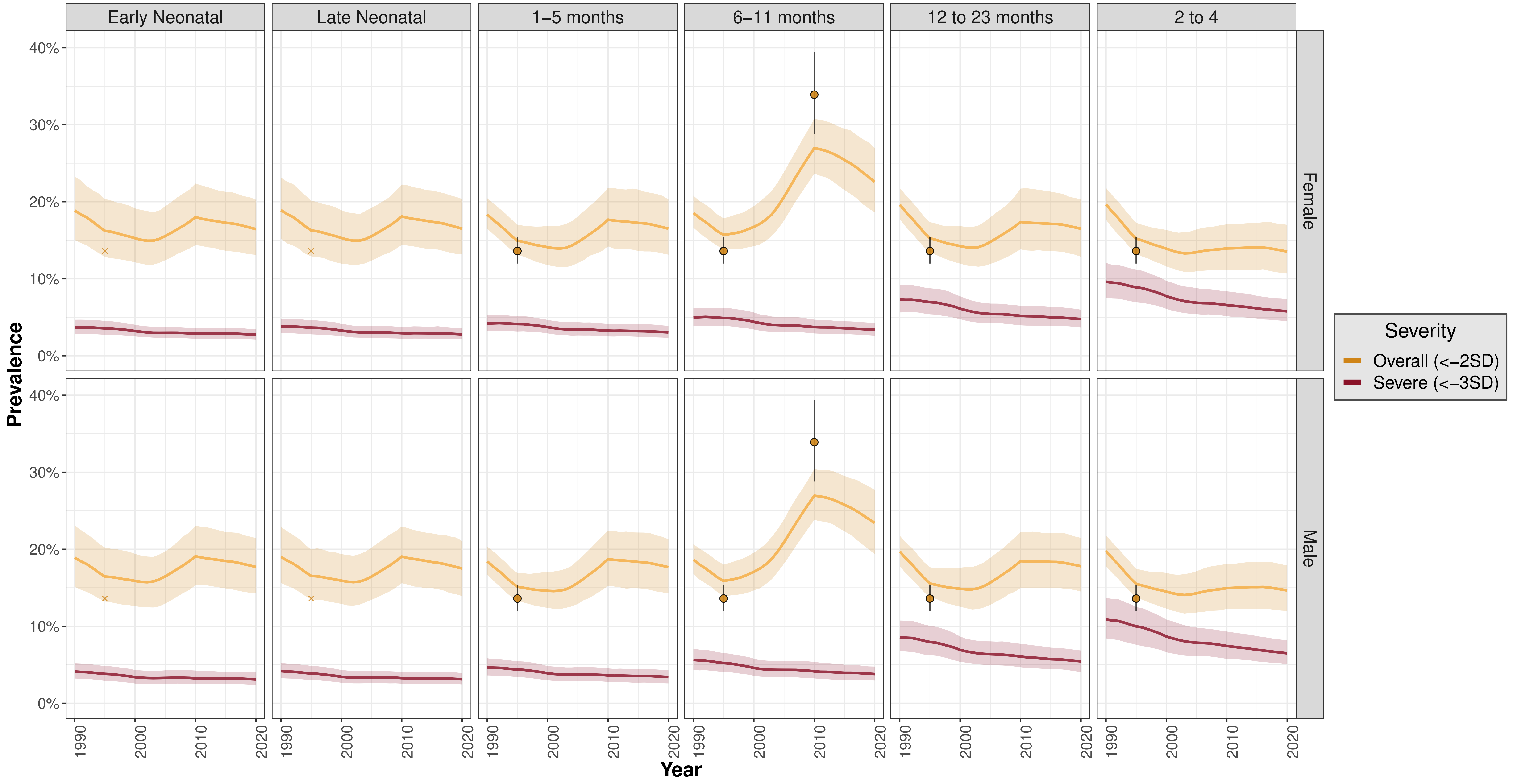

B: Transformed Mean Stunting Z Scores

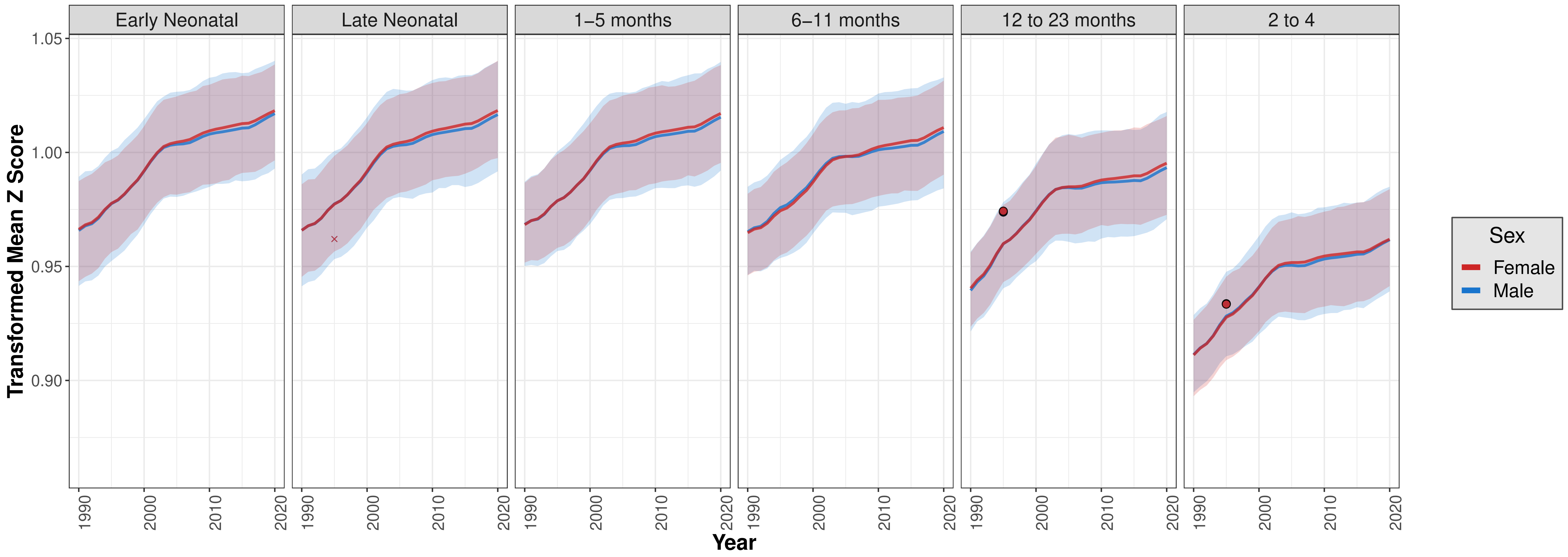

C

Year	Source
1985	WHO CGM Database
1985	Survey on the Nutritional Status of Preschool Children
1995	WHO CGM Database
2010	Comprehensive Food and Nutrition Security and Vulnerability Assessment

Mauritius – Wasting (WHZ)

D: Overall and Severe Wasting Prevalence

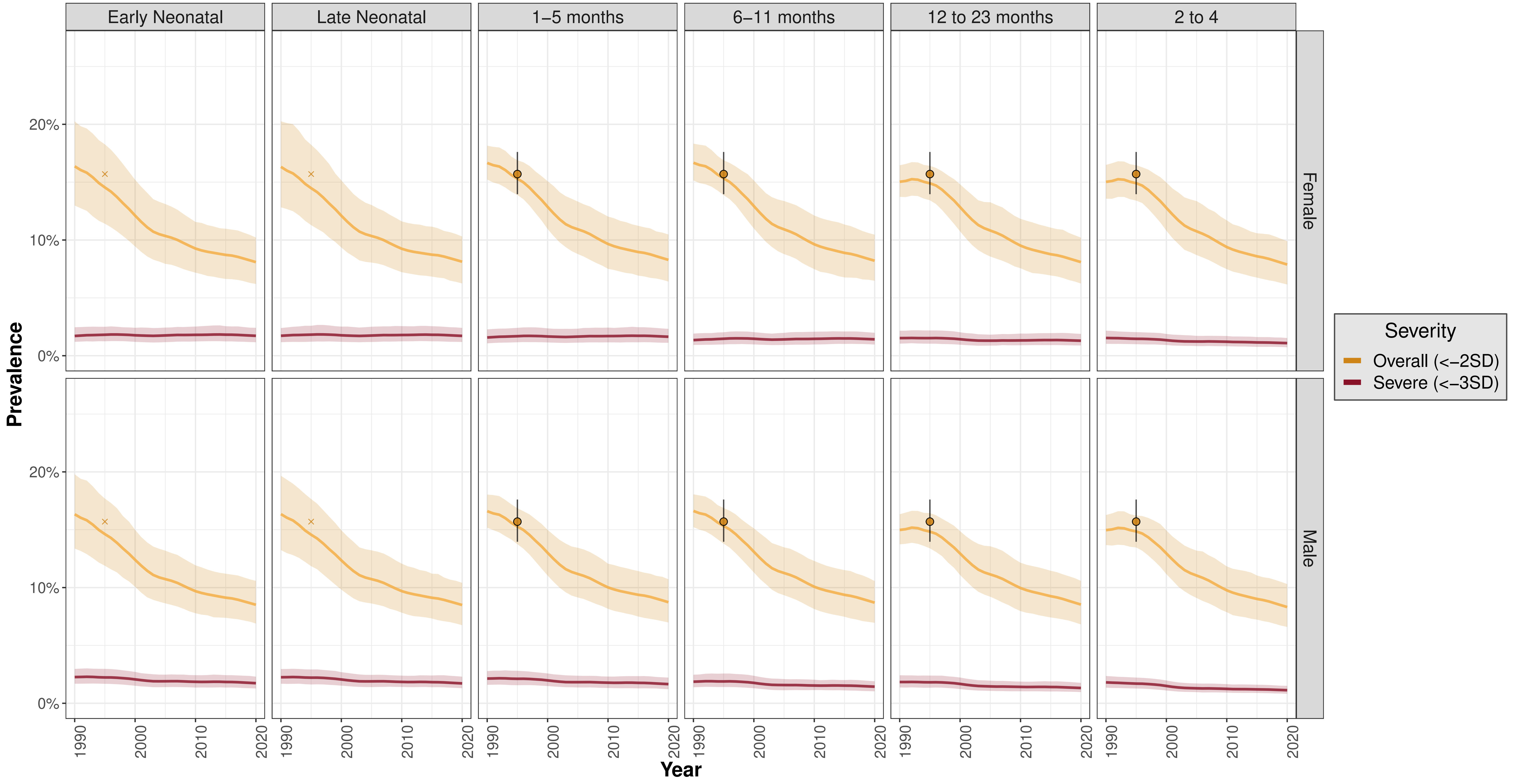

E: Transformed Mean Wasting Z Scores

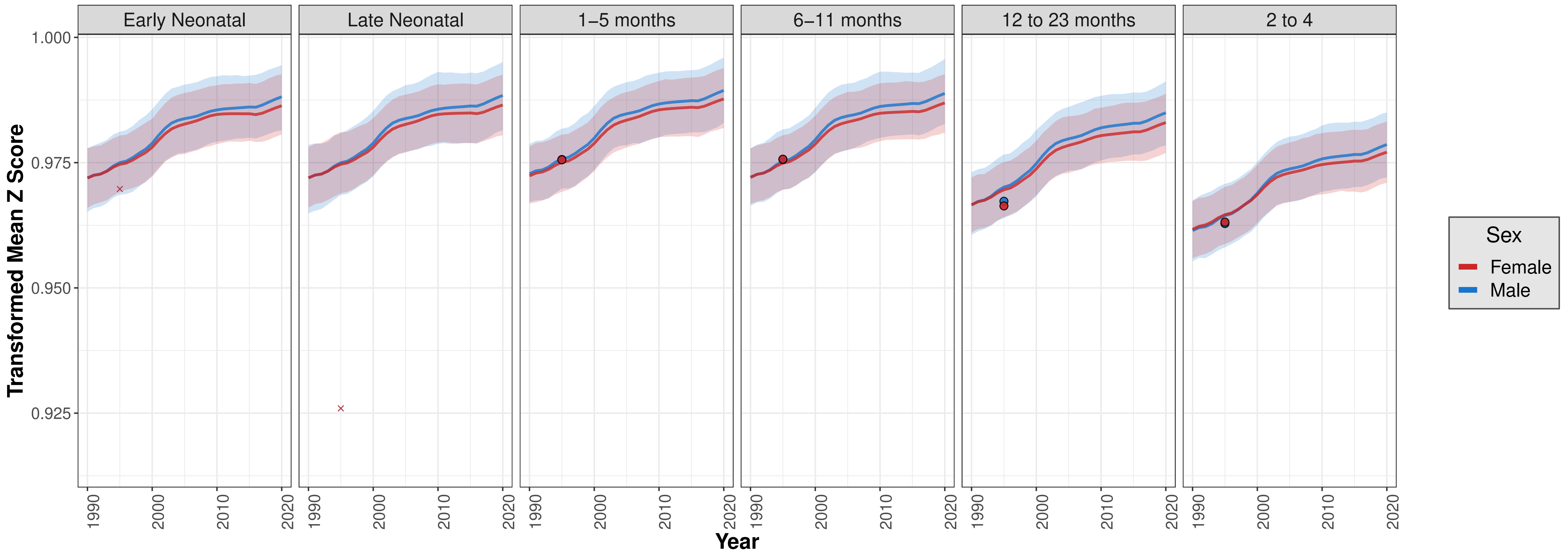

F

Year	Source
1985	WHO CGM Database
1985	Survey on the Nutritional Status of Preschool Children
1995	WHO CGM Database

Mauritius – Underweight (WAZ)

G: Overall and Severe Underweight Prevalence

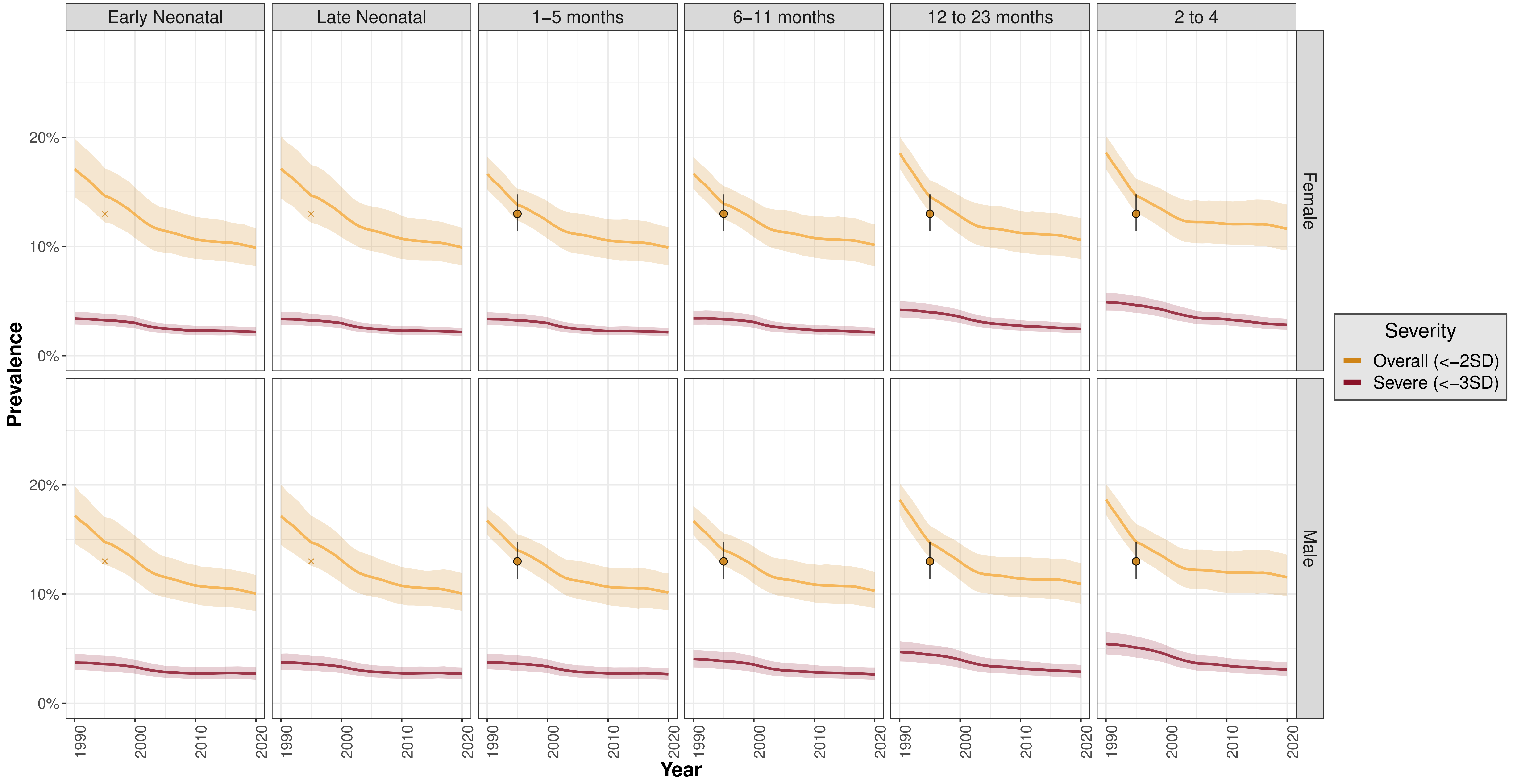

H: Transformed Mean Underweight Z Scores

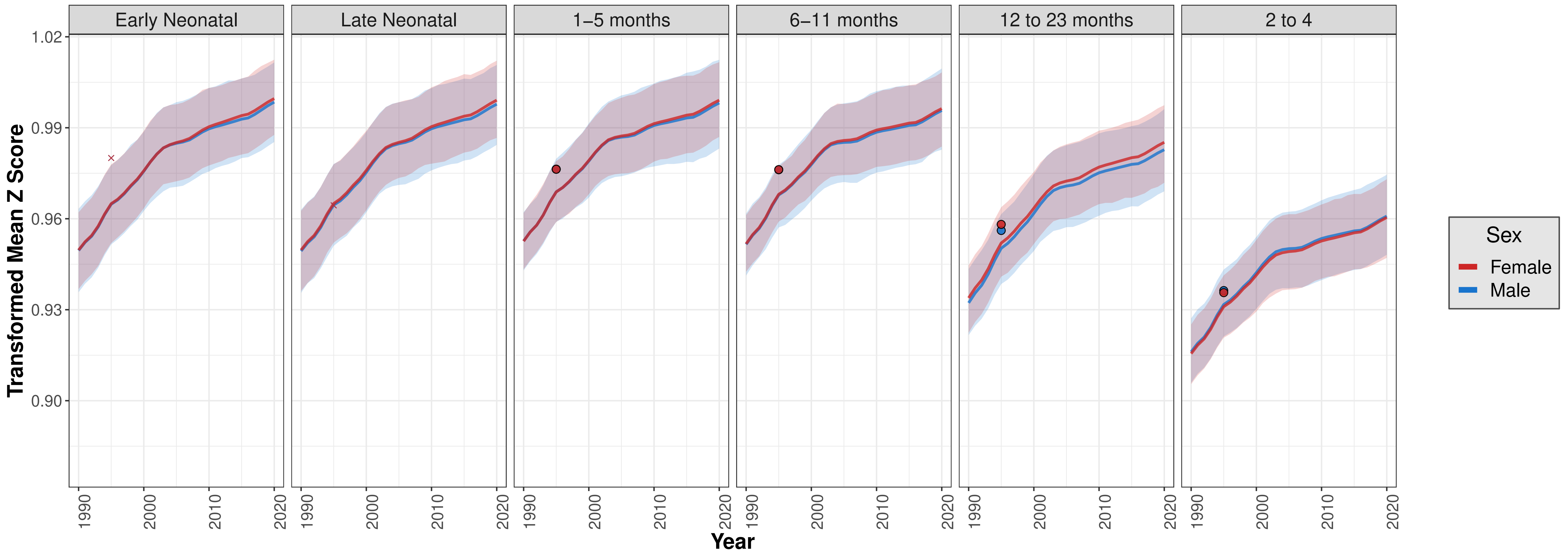

I

Year	Source
1985	WHO CGM Database
1985	Survey on the Nutritional Status of Preschool Children
1995	WHO CGM Database

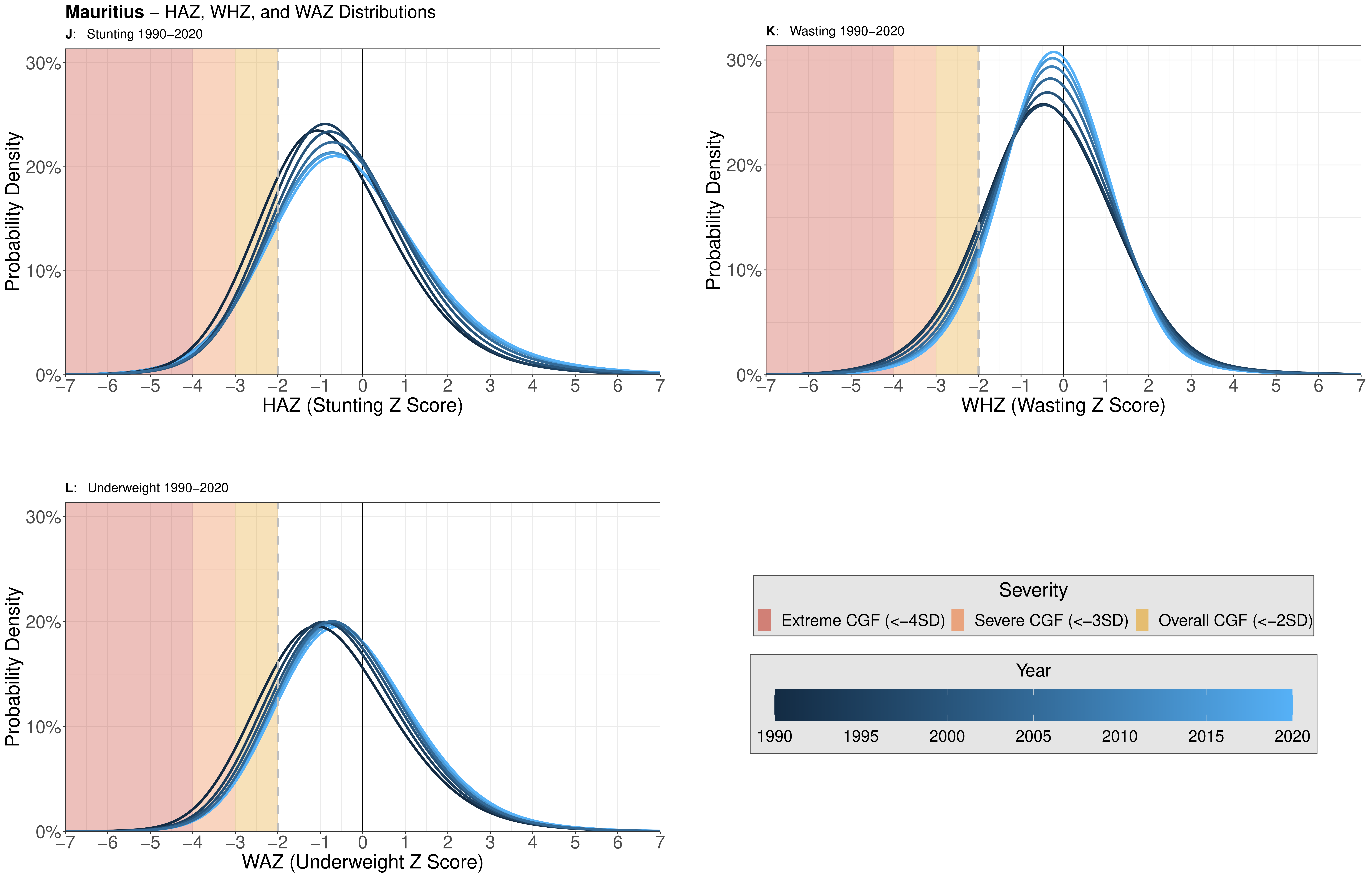
Mauritius – HAZ, WHZ, and WAZ Distributions
J: Stunting 1990–2020
Probability Density
30%
20%
10%
0%
−7 −6 −5 −4 −3 −2 −1 0 1 2 3 4 5 6 7
HAZ (Stunting Z Score)
K: Wasting 1990–2020
Probability Density
30%
20%
10%
0%
−7 −6 −5 −4 −3 −2 −1 0 1 2 3 4 5 6 7
WHZ (Wasting Z Score)
L: Underweight 1990–2020
Probability Density
30%
20%
10%
0%
−7 −6 −5 −4 −3 −2 −1 0 1 2 3 4 5 6 7
WAZ (Underweight Z Score)
Severity
Extreme CGF (<−4SD)
Severe CGF (<−3SD)
Overall CGF (<−2SD)
Year
1990
1995
2000
2005
2010
2015
2020

Seychelles – Stunting (HAZ)

A: Overall and Severe Stunting Prevalence

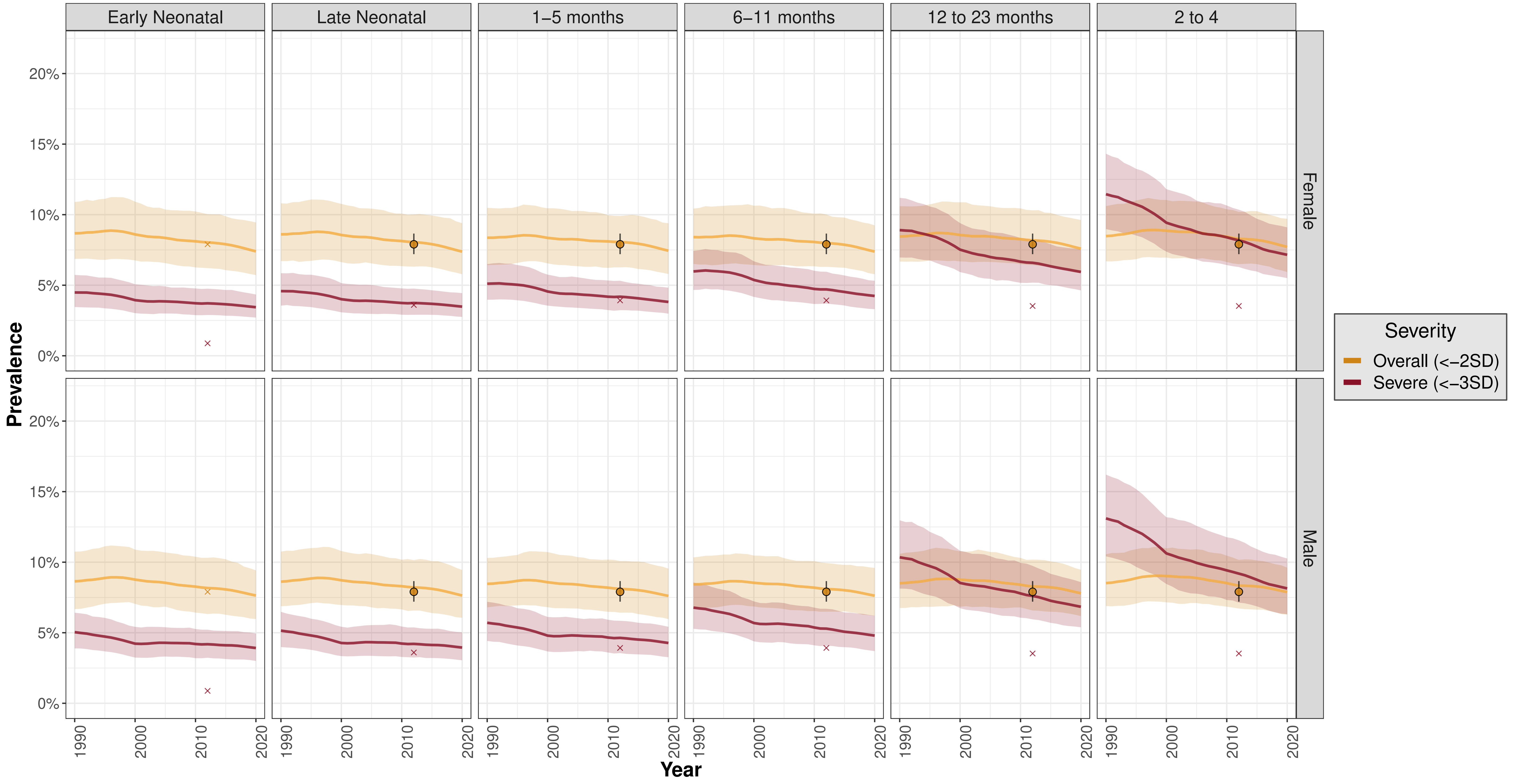

B: Transformed Mean Stunting Z Scores

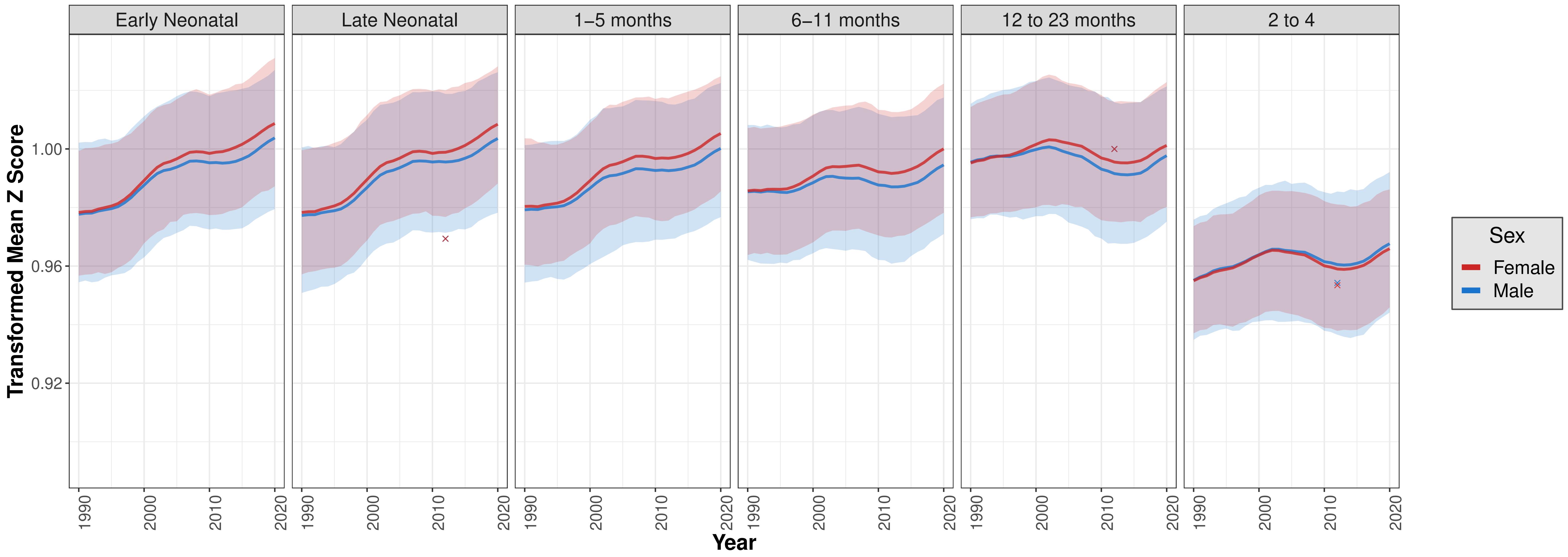

C

Year	Source
1988	WHO CGM Database
2012	WHO CGM Database

Seychelles – Wasting (WHZ)

D: Overall and Severe Wasting Prevalence

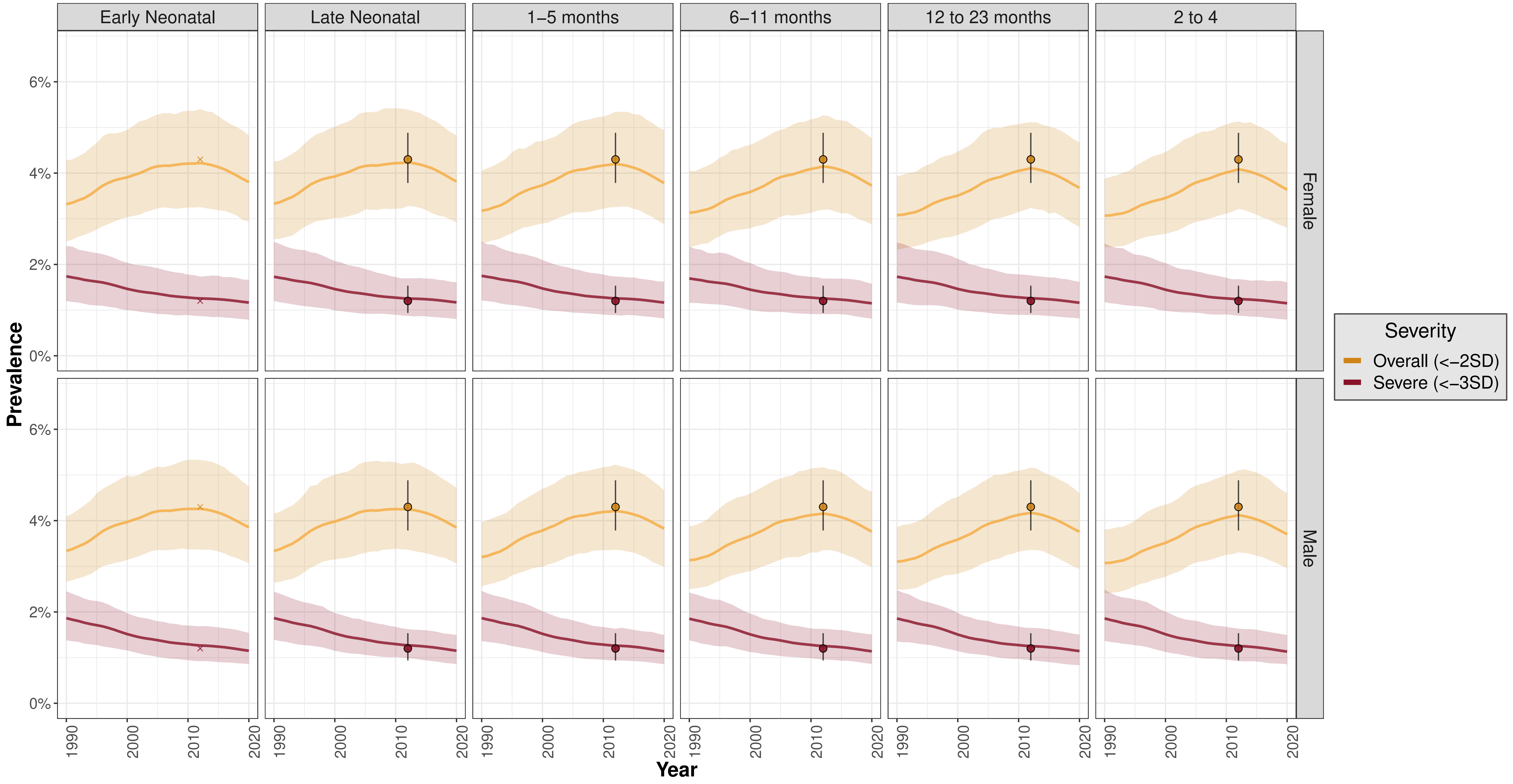

E: Transformed Mean Wasting Z Scores

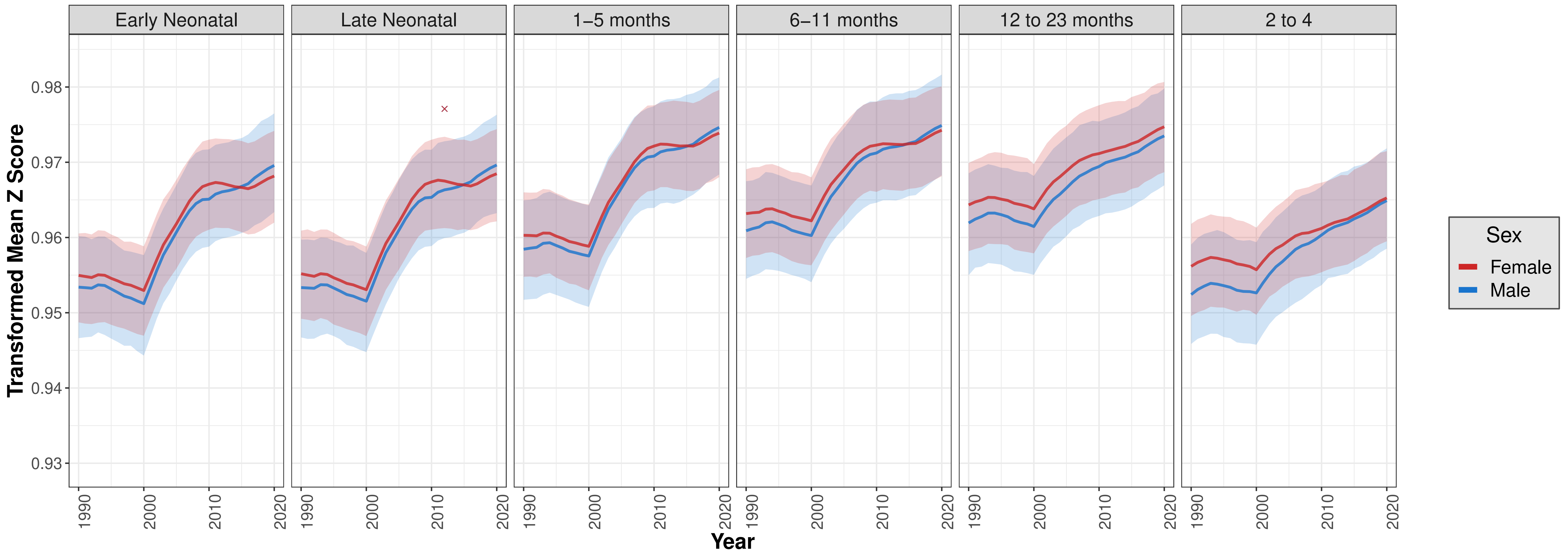

F

Year	Source
1988	WHO CGM Database
2012	WHO CGM Database

Seychelles – Underweight (WAZ)

G: Overall and Severe Underweight Prevalence

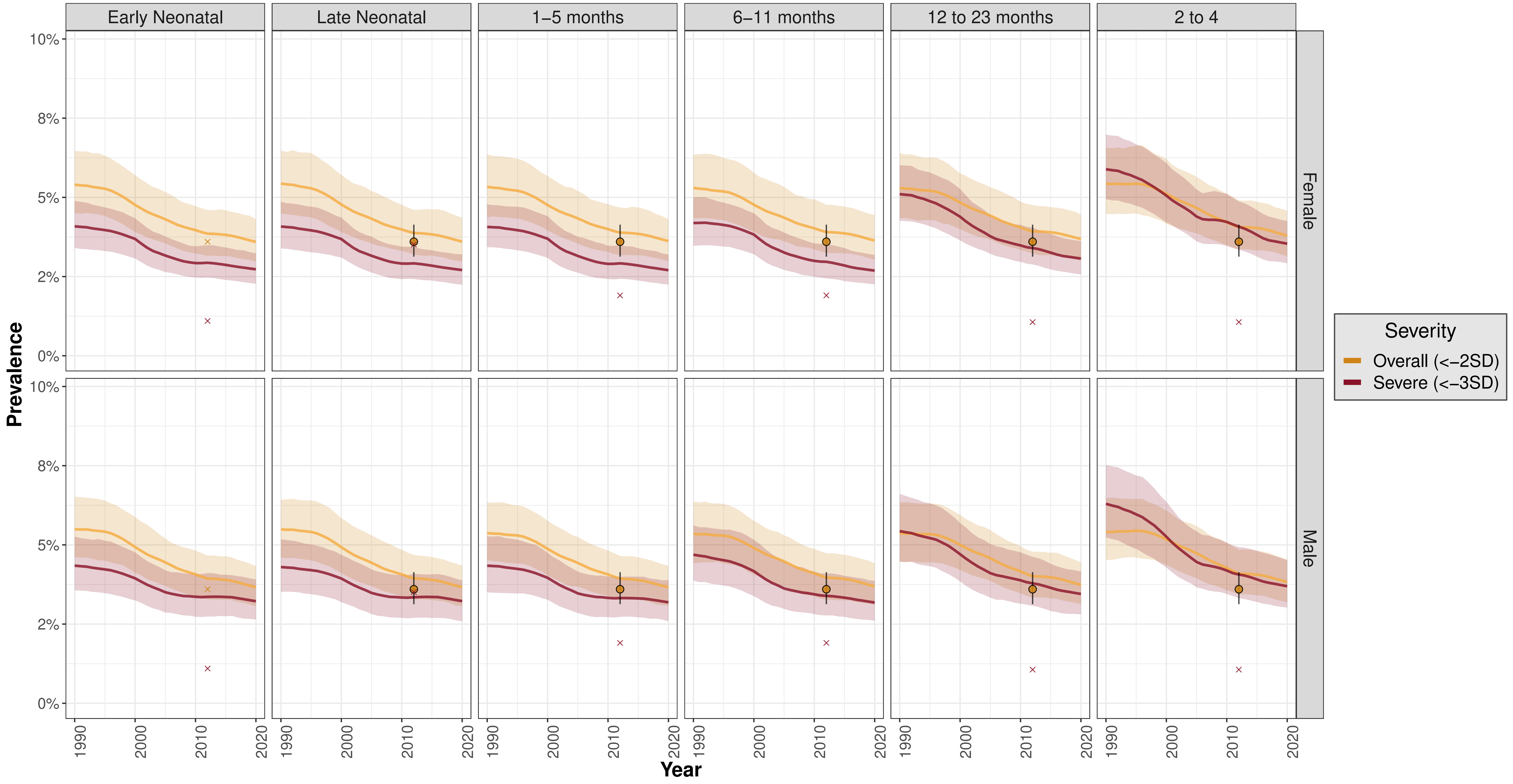

H: Transformed Mean Underweight Z Scores

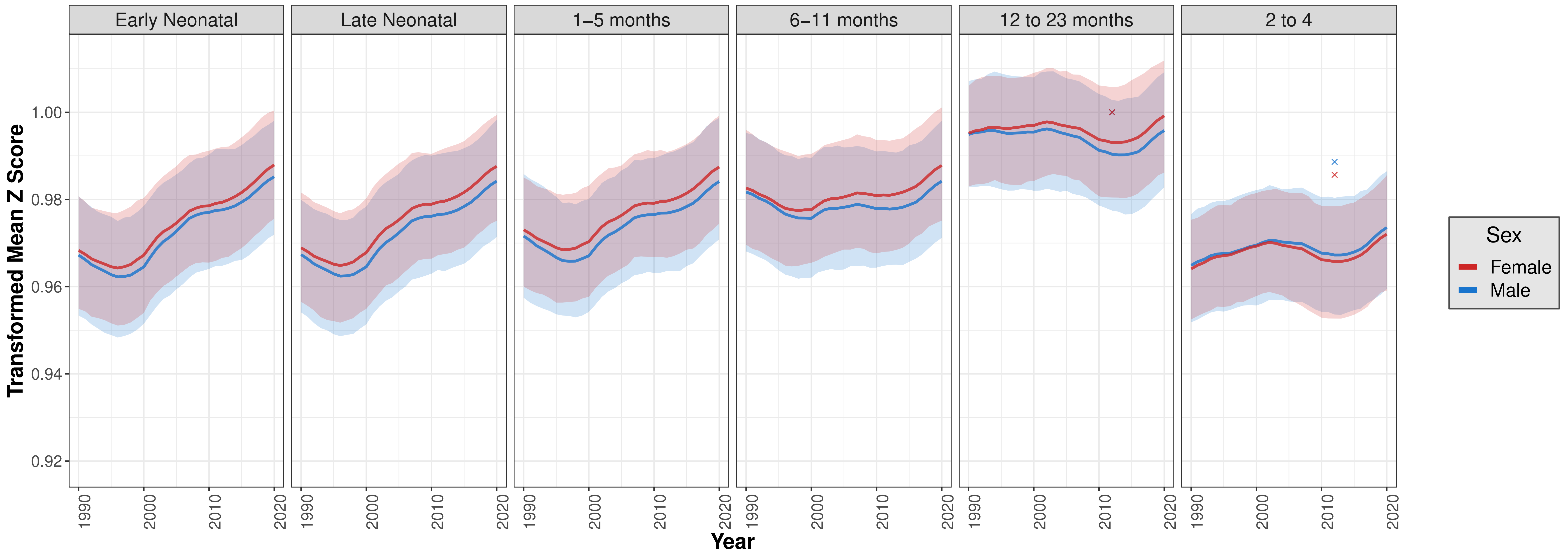

I

Year	Source
1988	WHO CGM Database
2012	WHO CGM Database

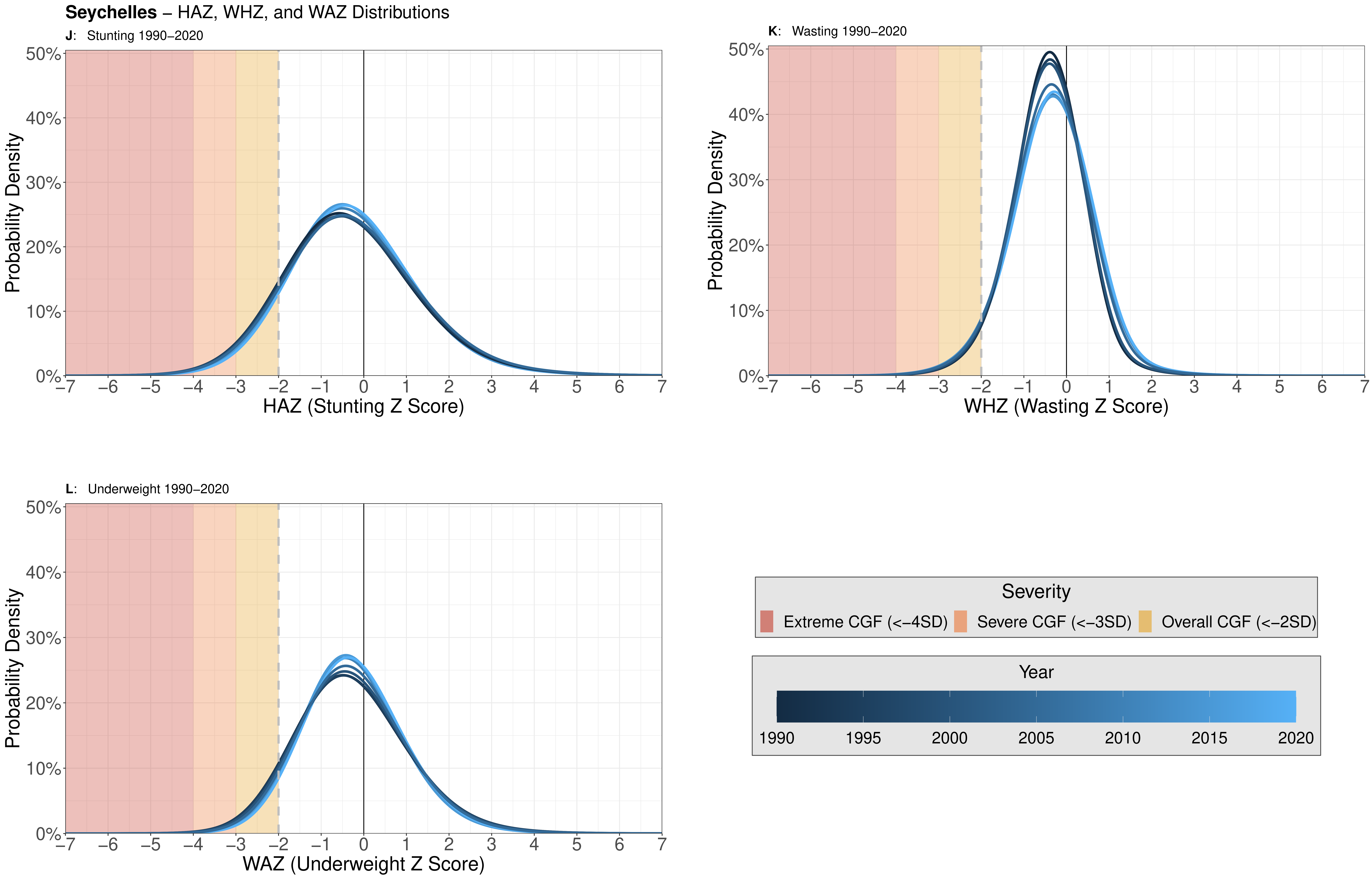
Seychelles – HAZ, WHZ, and WAZ Distributions
J: Stunting 1990–2020
K: Wasting 1990–2020
L: Underweight 1990–2020
Probability Density
0%
10%
20%
30%
40%
50%
−7 −6 −5 −4 −3 −2 −1 0 1 2 3 4 5 6 7
HAZ (Stunting Z Score)
WHZ (Wasting Z Score)
WAZ (Underweight Z Score)
Severity
Extreme CGF (<−4SD)
Severe CGF (<−3SD)
Overall CGF (<−2SD)
Year
1990
1995
2000
2005
2010
2015
2020

Timor–Leste

A: Overall and Severe Stunting Prevalence

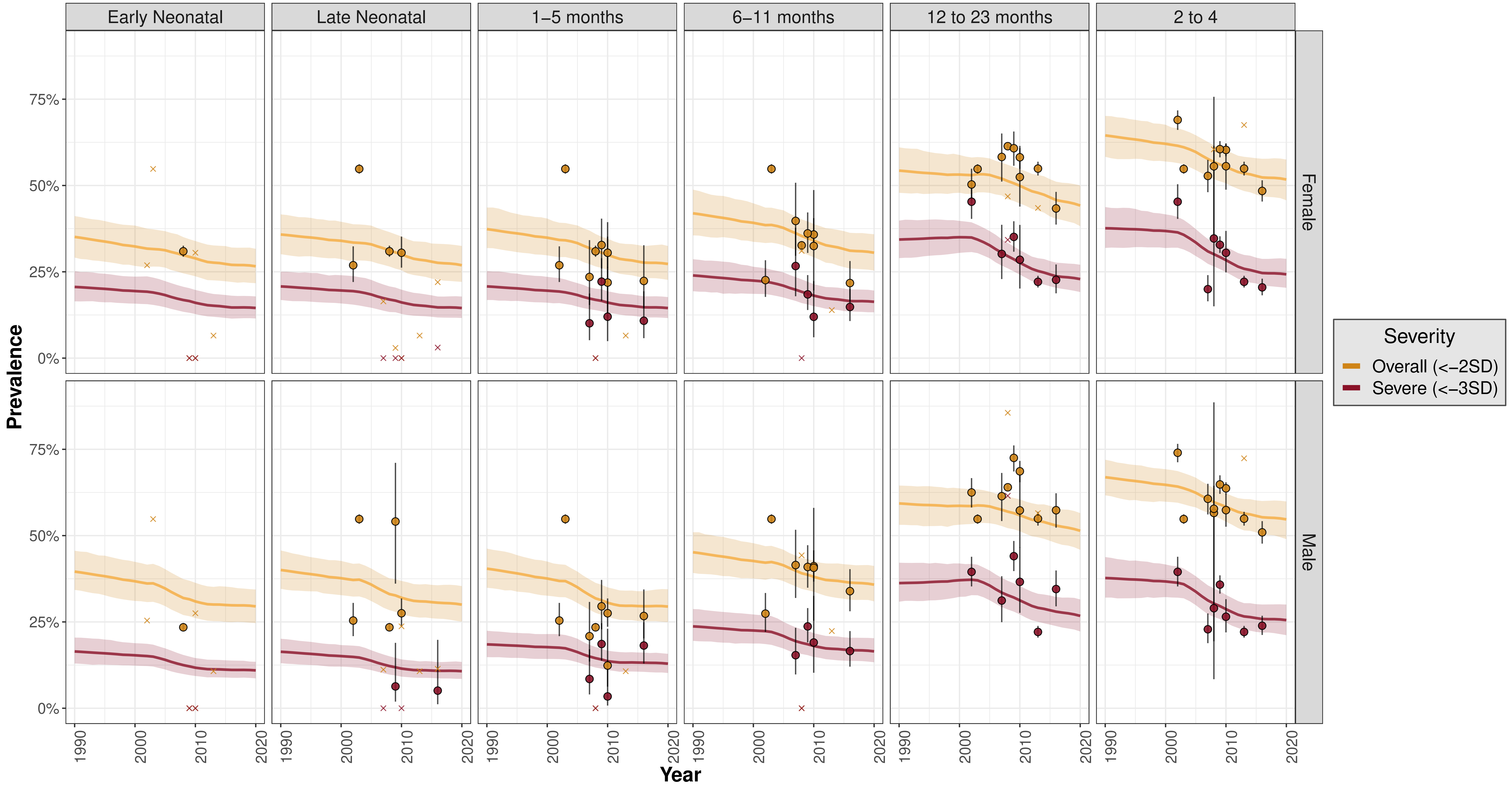

B: Transformed Mean Stunting Z Scores

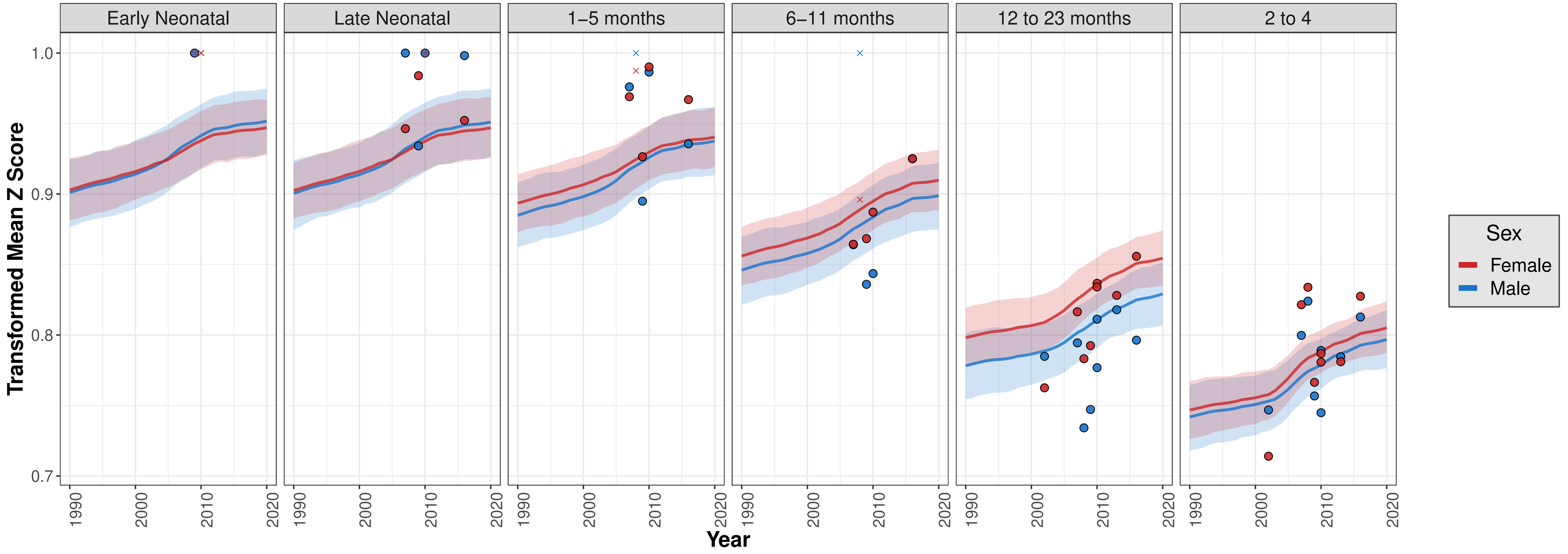

C

Year	Source
2002	WHO CGM Database
2003	DHS
2003	WHO CGM Database
2007	Living Standards and Measurement Survey
2008	Living Standards and Measurement Survey
2008	WHO CGM Database
2009	DHS
2010	DHS
2010	WHO CGM Database
2013	WHO CGM Database
2013	Food and Nutrition Survey
2016	DHS

Timor−Leste

D: Overall and Severe Wasting Prevalence

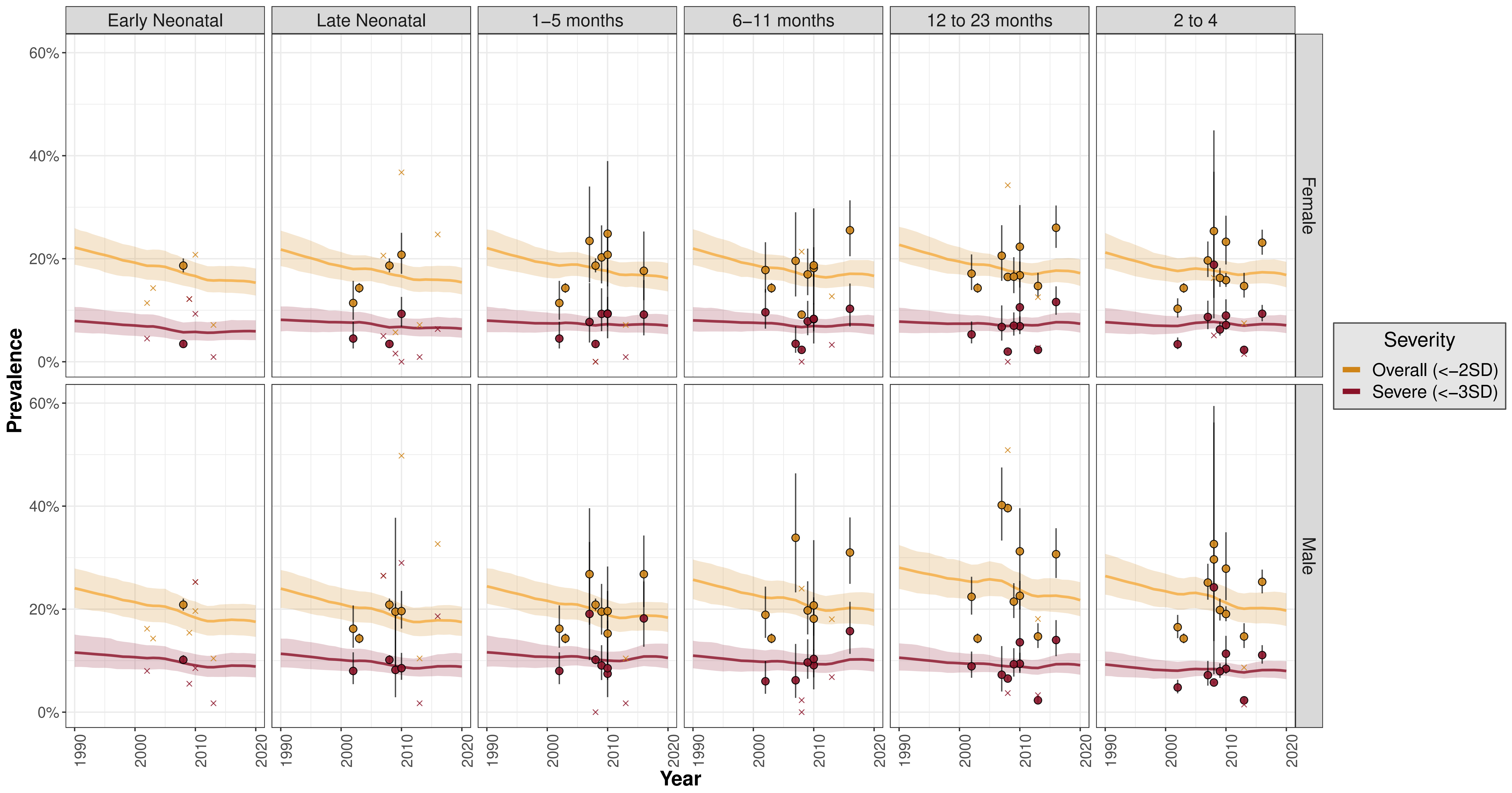

E: Transformed Mean Wasting Z Scores

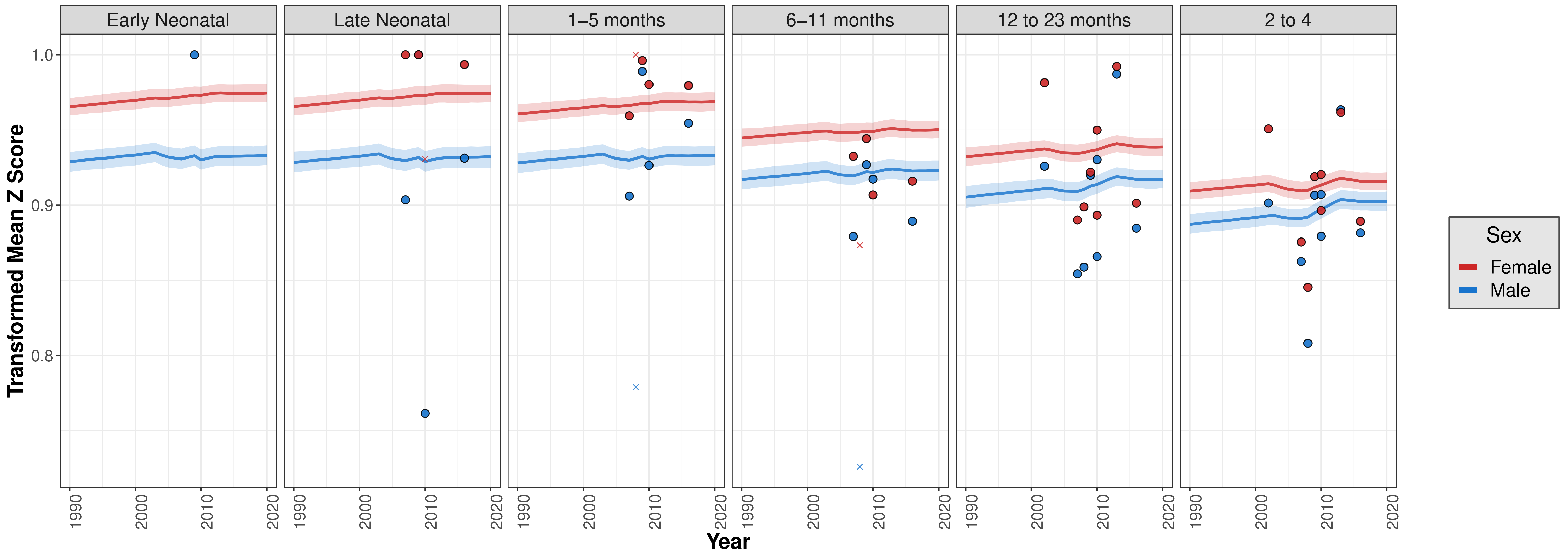

F

Year	Source
2002	WHO CGM Database
2003	DHS
2003	WHO CGM Database
2007	Living Standards and Measurement Survey
2008	Living Standards and Measurement Survey
2008	WHO CGM Database
2009	DHS
2010	DHS
2010	WHO CGM Database
2013	WHO CGM Database
2013	Food and Nutrition Survey
2016	DHS

Timor–Leste

G: Overall and Severe Underweight Prevalence

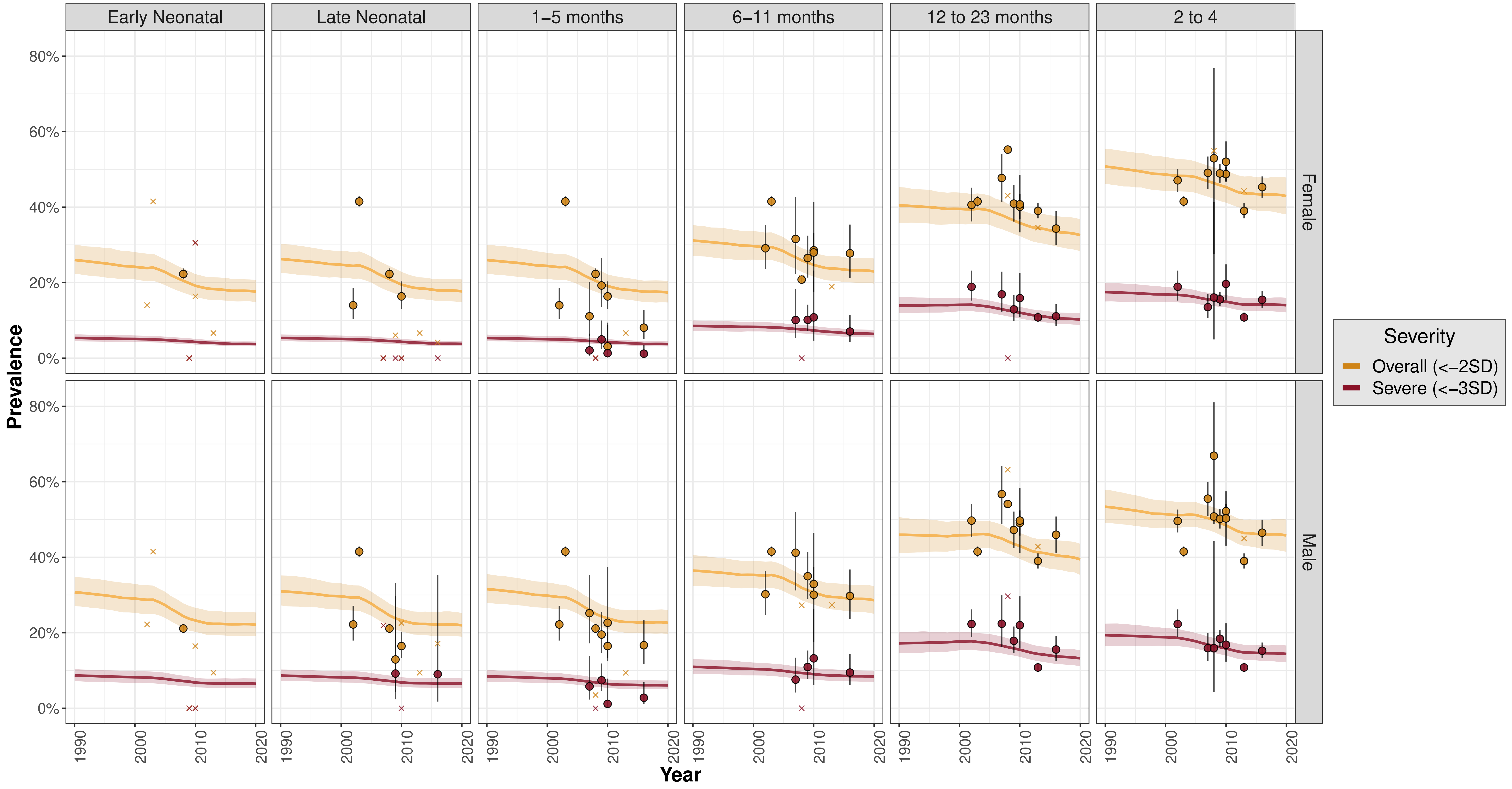

H: Transformed Mean Underweight Z Scores

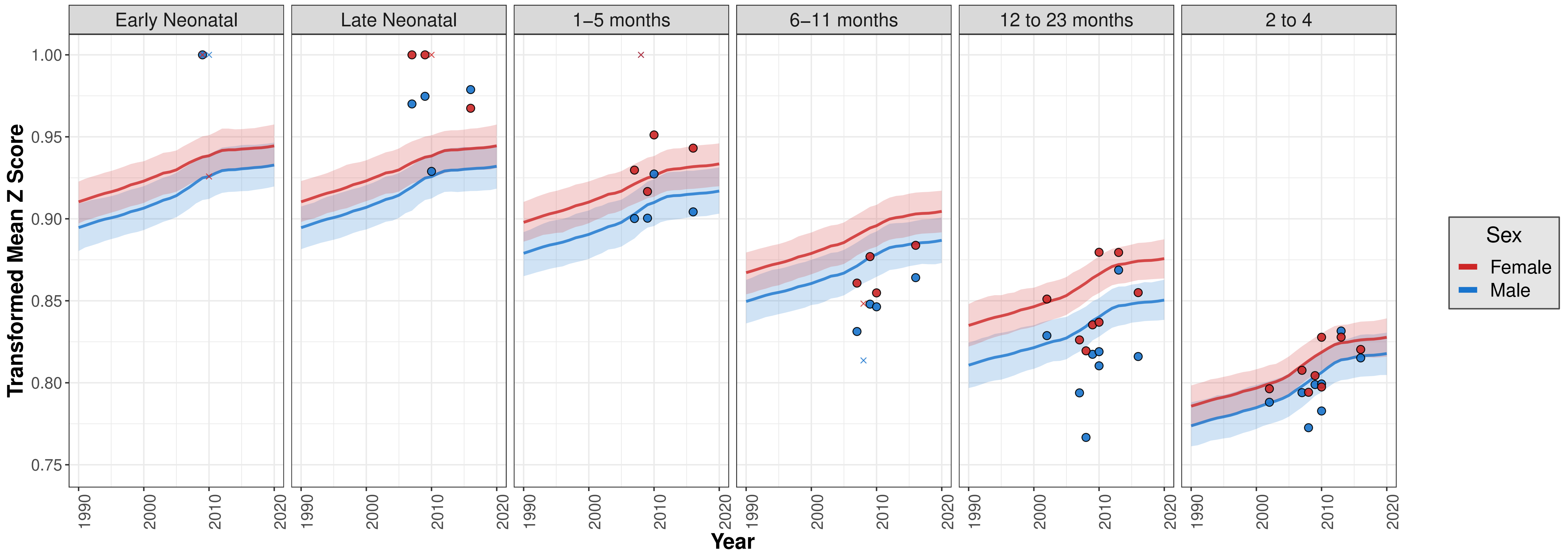

I

Year	Source
2002	WHO CGM Database
2003	DHS
2003	WHO CGM Database
2007	Living Standards and Measurement Survey
2008	Living Standards and Measurement Survey
2008	WHO CGM Database
2009	DHS
2010	DHS
2010	WHO CGM Database
2013	WHO CGM Database
2013	Food and Nutrition Survey
2016	DHS

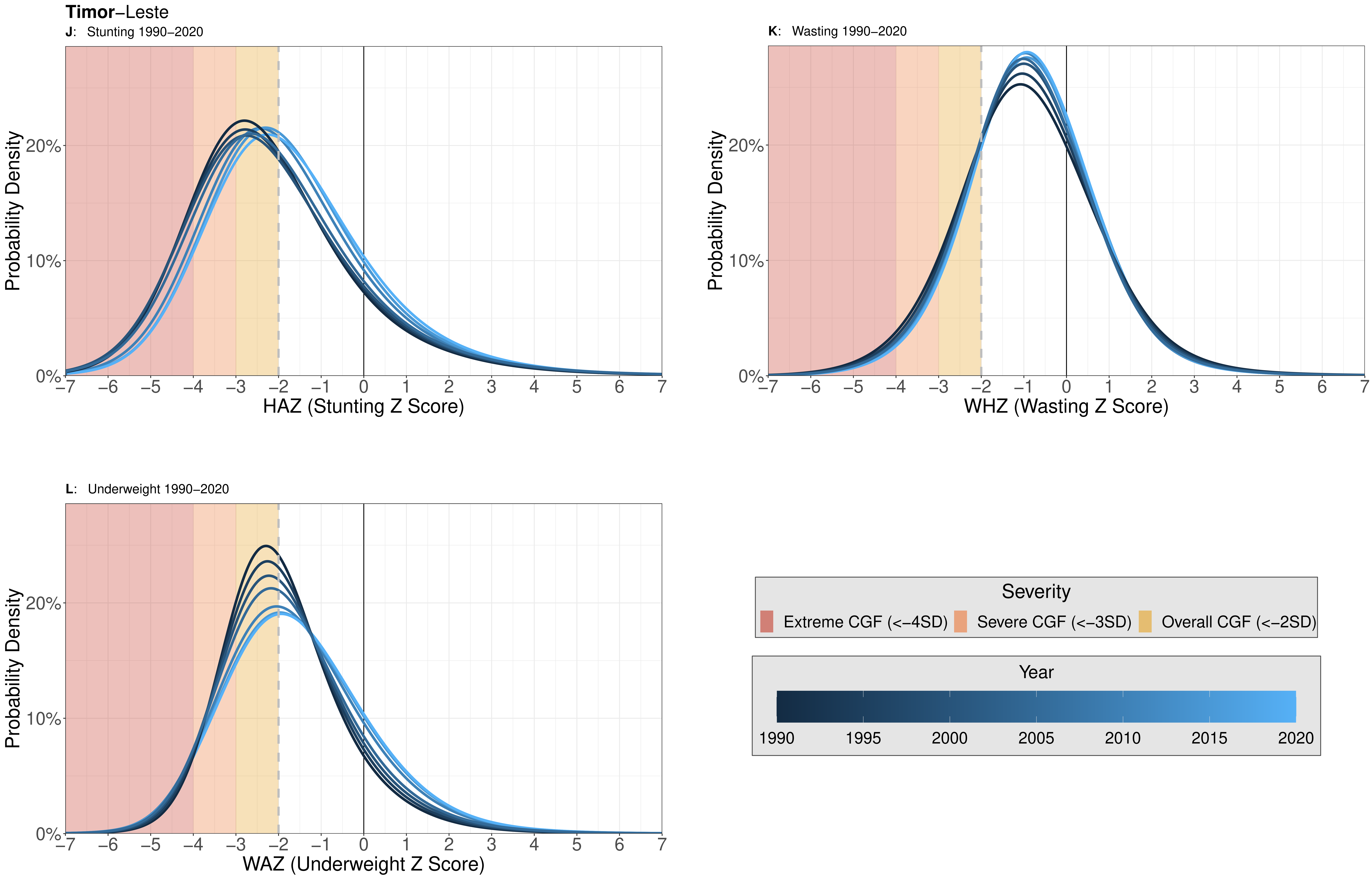

Timor–Leste
J: Stunting 1990–2020
Probability Density
0%
10%
20%
−7
−6
−5
−4
−3
−2
−1
0
1
2
3
4
5
6
7
HAZ (Stunting Z Score)
K: Wasting 1990–2020
Probability Density
0%
10%
20%
WHZ (Wasting Z Score)
L: Underweight 1990–2020
Probability Density
0%
10%
20%
WAZ (Underweight Z Score)
Severity
Extreme CGF (<−4SD)
Severe CGF (<−3SD)
Overall CGF (<−2SD)
Year
1990
1995
2000
2005
2010
2015
2020

Viet Nam – Stunting (HAZ)

A: Overall and Severe Stunting Prevalence

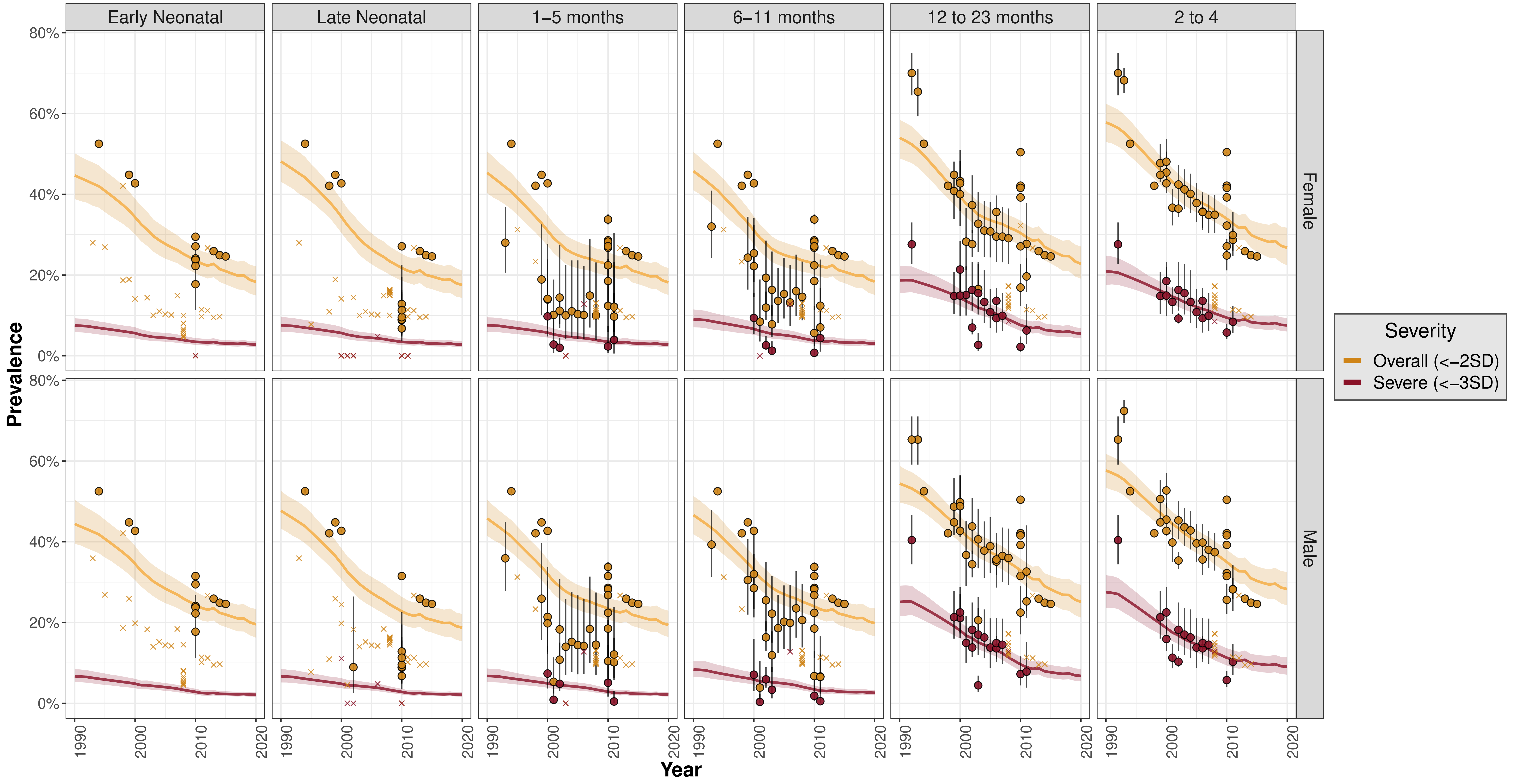

B: Transformed Mean Stunting Z Scores

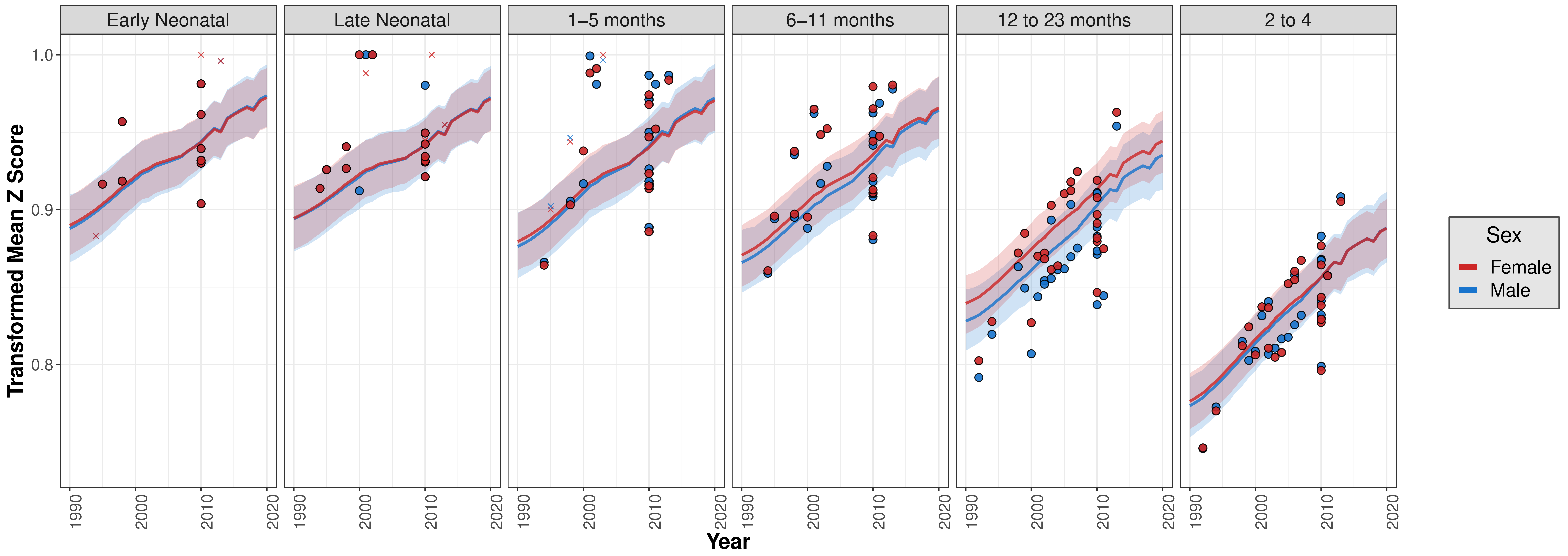

C

Year	Source
1984	WHO CGM Database
1986	Growing Healthy: A Review of 's Health Sector
1988	WHO CGM Database
1988	Growing Healthy: A Review of 's Health Sector
1992	WHO CGM Database
1993	WHO CGM Database
1994	WHO CGM Database
1995	Growing Healthy: A Review of 's Health Sector
1998	WHO CGM Database
1998	Growing Healthy: A Review of 's Health Sector
1999	WHO CGM Database
2000	MICS
2000	WHO CGM Database
2001	National Health Survey
2002	National Health Survey
2002	WHO CGM Database
2003	WHO CGM Database
2003	Young Lives: Household and Child Survey Round 1 – UK Data Service
2004	WHO CGM Database
2005	WHO CGM Database
2006	MICS
2006	WHO CGM Database
2007	WHO CGM Database
2008	Health Statistics Yearbook
2008	WHO CGM Database
2010	MICS
2010	WHO CGM Database
2010	Sanitation, Water Supply, and Child Nutrition Survey
2011	MICS
2011	WHO CGM Database
2011	Rate of Malnutrition in Children Under 5 years old by Type –
2012	Nutrition Surveillance
2012	Rate of Malnutrition in Children Under 5 years old by Type –
2013	WHO CGM Database
2013	Rate of Malnutrition in Children Under 5 years old by Type –
2014	WHO CGM Database
2014	Rate of Malnutrition in Children Under 5 years old by Type –
2015	WHO CGM Database

Viet Nam – Wasting (WHZ)

D: Overall and Severe Wasting Prevalence

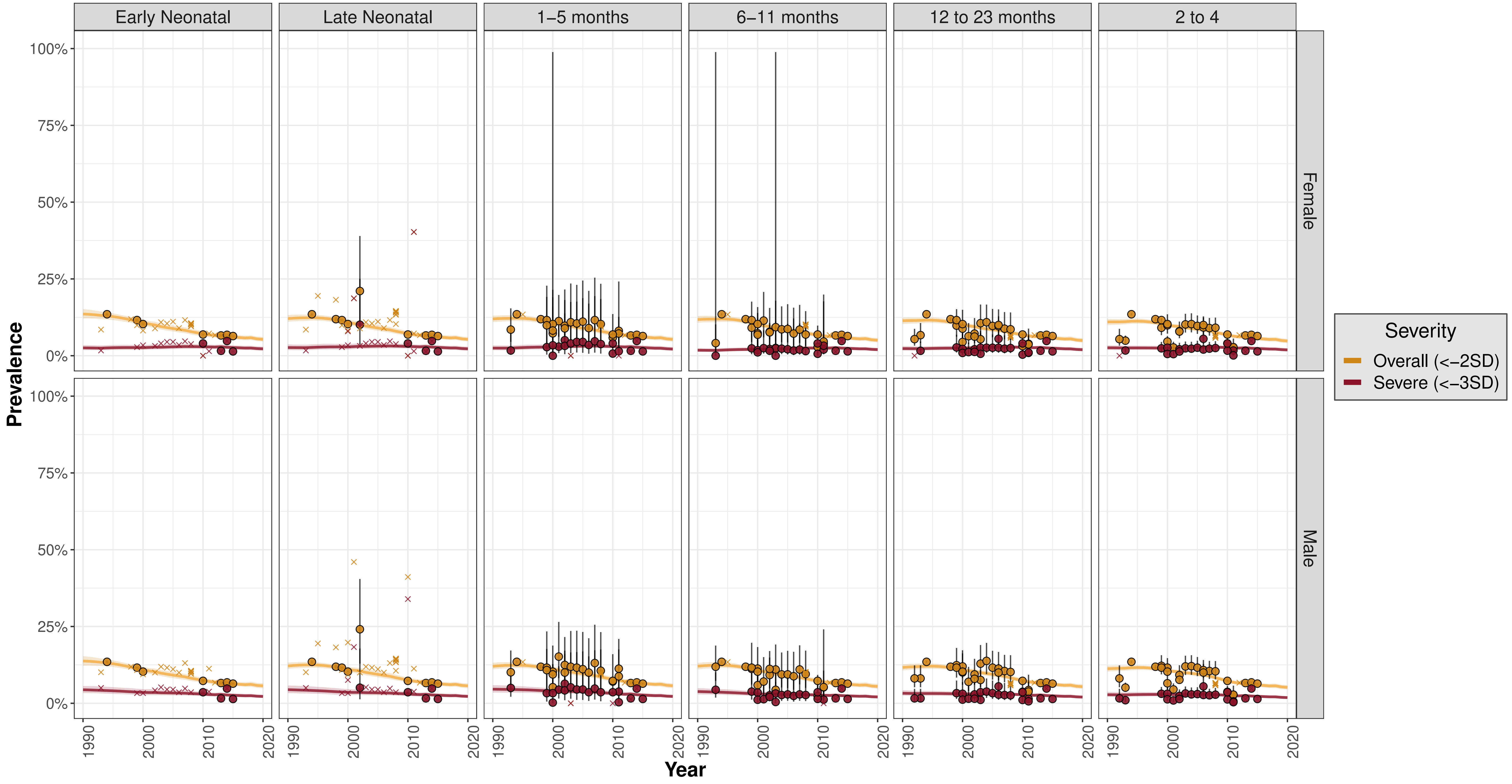

E: Transformed Mean Wasting Z Scores

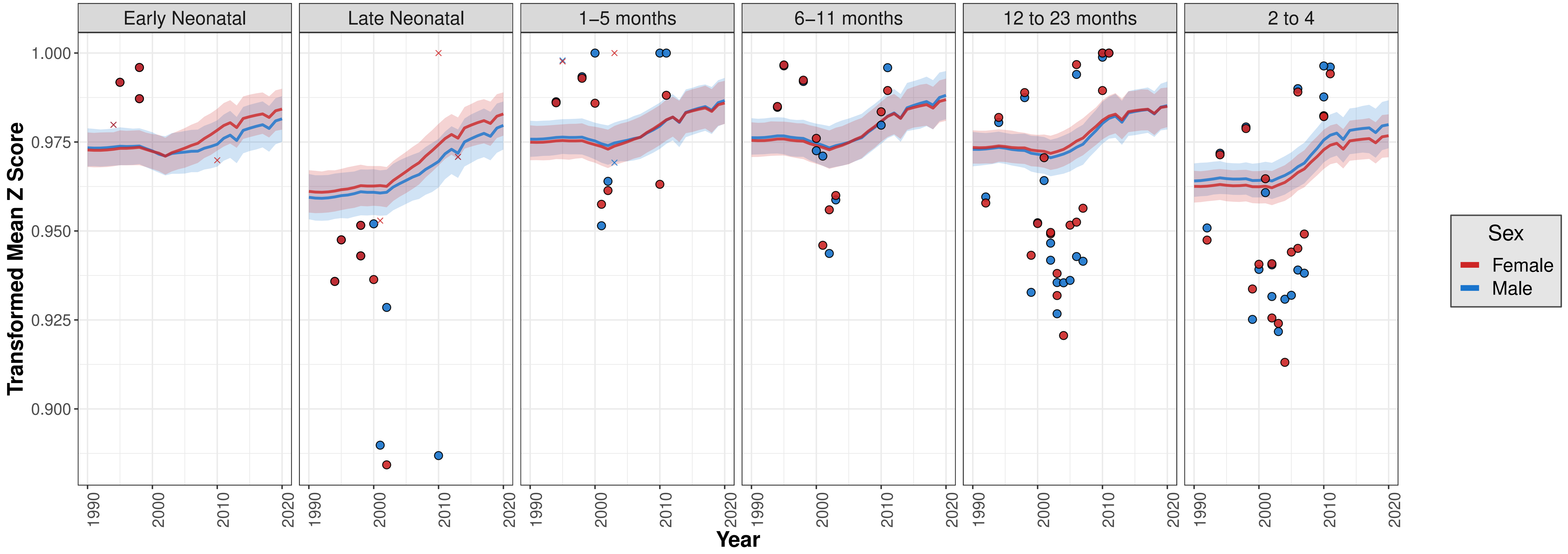

F

Year	Source
1984	WHO CGM Database
1986	Growing Healthy: A Review of 's Health Sector
1988	WHO CGM Database
1988	Growing Healthy: A Review of 's Health Sector
1992	WHO CGM Database
1993	WHO CGM Database
1994	WHO CGM Database
1995	Growing Healthy: A Review of 's Health Sector
1998	WHO CGM Database
1998	Growing Healthy: A Review of 's Health Sector
1999	WHO CGM Database
2000	MICS
2000	WHO CGM Database
2001	National Health Survey
2002	National Health Survey
2002	WHO CGM Database
2003	WHO CGM Database
2003	Young Lives: Household and Child Survey Round 1 – UK Data Service
2004	WHO CGM Database
2005	WHO CGM Database
2006	MICS
2006	WHO CGM Database
2007	WHO CGM Database
2008	Health Statistics Yearbook
2008	WHO CGM Database
2010	MICS
2010	WHO CGM Database
2011	MICS
2011	WHO CGM Database
2012	Nutrition Surveillance
2013	WHO CGM Database
2013	Rate of Malnutrition in Children Under 5 years old by Type –
2014	WHO CGM Database
2014	Rate of Malnutrition in Children Under 5 years old by Type –
2015	WHO CGM Database

Viet Nam – Underweight (WAZ)

G: Overall and Severe Underweight Prevalence

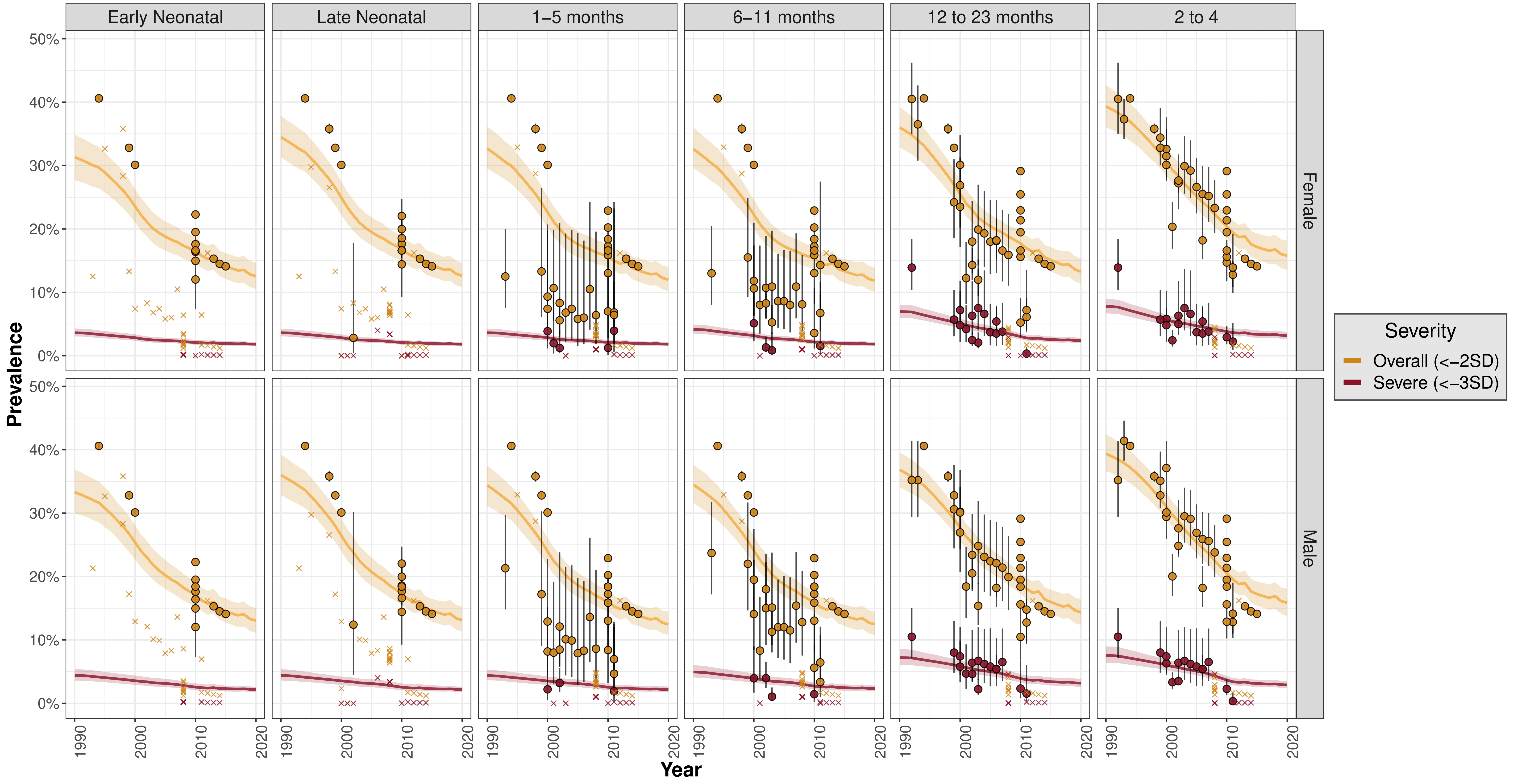

H: Transformed Mean Underweight Z Scores

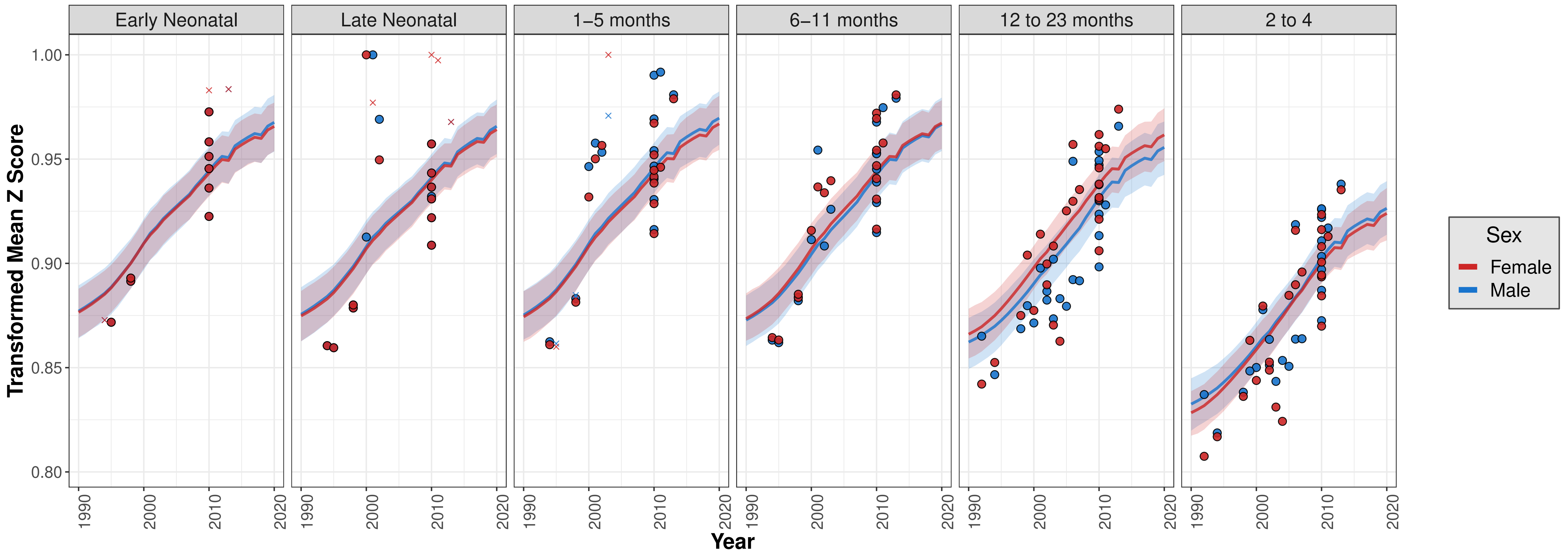

I

Year	Source
1984	WHO CGM Database
1986	Growing Healthy: A Review of 's Health Sector
1988	WHO CGM Database
1988	Growing Healthy: A Review of 's Health Sector
1992	WHO CGM Database
1993	WHO CGM Database
1994	WHO CGM Database
1995	Growing Healthy: A Review of 's Health Sector
1998	WHO CGM Database
1998	Growing Healthy: A Review of 's Health Sector
1999	WHO CGM Database
2000	MICS
2000	WHO CGM Database
2001	National Health Survey
2002	National Health Survey
2002	WHO CGM Database
2003	WHO CGM Database
2003	Young Lives: Household and Child Survey Round 1 – UK Data Service
2004	WHO CGM Database
2005	WHO CGM Database
2006	MICS
2006	WHO CGM Database
2007	WHO CGM Database
2008	Health Statistics Yearbook
2008	WHO CGM Database
2010	MICS
2010	WHO CGM Database
2010	Sanitation, Water Supply, and Child Nutrition Survey
2011	MICS
2011	WHO CGM Database
2011	Rate of Malnutrition in Children Under 5 years old by Type –
2012	Nutrition Surveillance
2012	Rate of Malnutrition in Children Under 5 years old by Type –
2013	WHO CGM Database
2013	Rate of Malnutrition in Children Under 5 years old by Type –
2014	WHO CGM Database
2014	Rate of Malnutrition in Children Under 5 years old by Type –
2015	WHO CGM Database

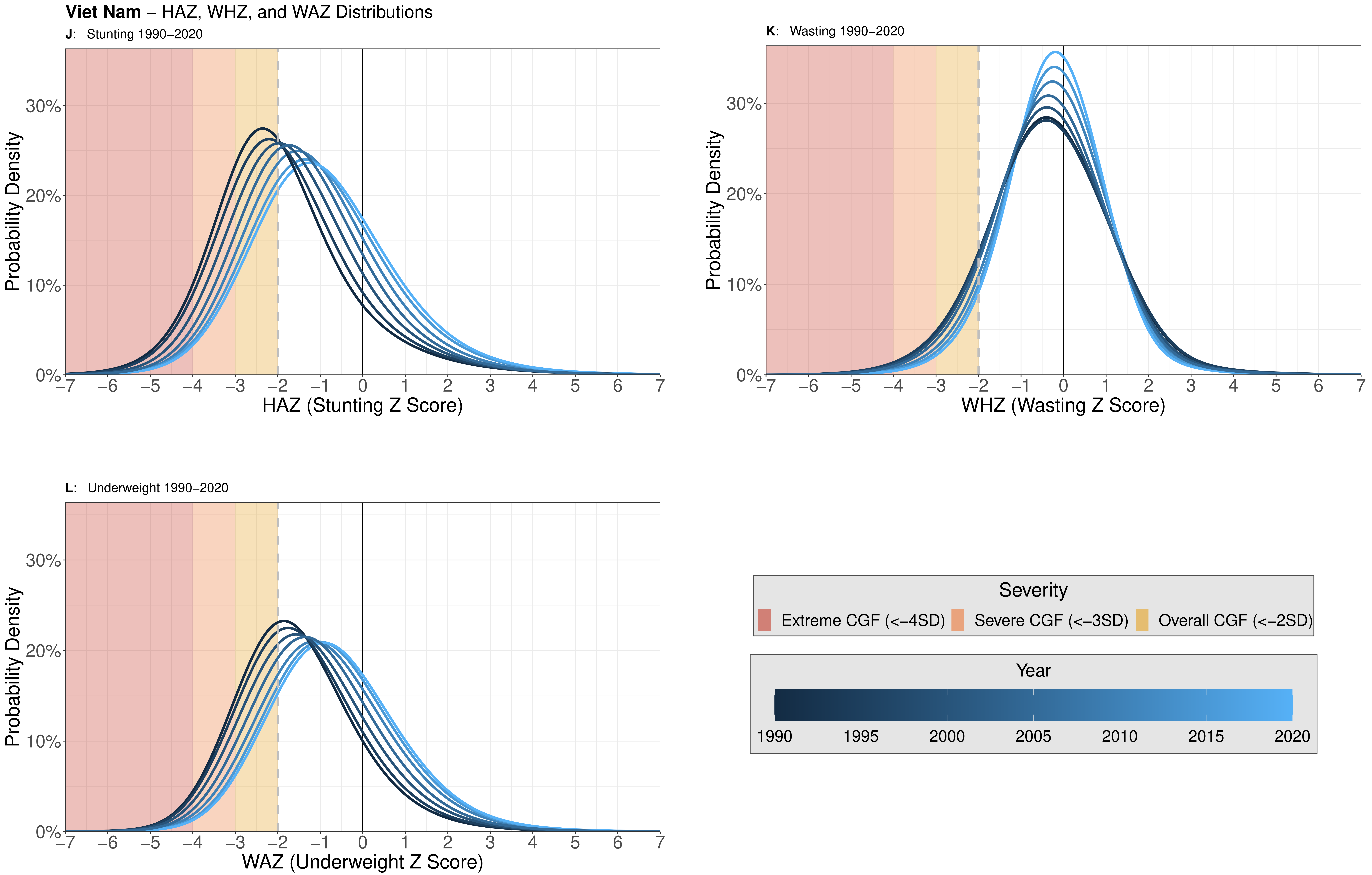
Viet Nam – HAZ, WHZ, and WAZ Distributions
J: Stunting 1990–2020
Probability Density
30%
20%
10%
0%
−7 −6 −5 −4 −3 −2 −1 0 1 2 3 4 5 6 7
HAZ (Stunting Z Score)
K: Wasting 1990–2020
Probability Density
30%
20%
10%
0%
−7 −6 −5 −4 −3 −2 −1 0 1 2 3 4 5 6 7
WHZ (Wasting Z Score)
L: Underweight 1990–2020
Probability Density
30%
20%
10%
0%
−7 −6 −5 −4 −3 −2 −1 0 1 2 3 4 5 6 7
WAZ (Underweight Z Score)
Severity
Extreme CGF (<−4SD)
Severe CGF (<−3SD)
Overall CGF (<−2SD)
Year
1990
1995
2000
2005
2010
2015
2020
