# Supplementary material for: Current dichotomous metrics obscure trends in severe and extreme child growth failure
Source: Sci Adv. 2022 May 20;8(20):eabm8954. doi: 10.1126/sciadv.abm8954 (PMC9122330; doi:10.1126/sciadv.abm8954)
Supplement: Supplementary file 2 — Data S1 to S4 [file sciadv.abm8954_data_files_s1_to_s4.zip › sciadv.abm8954_data_file_s1g.pdf]

Data S1g. Spatio-temporal Gaussian Process Regression (ST-GPR) results for overall, severe, and mean CGF by location, including location-specific data sources; and distributions of stunting [HAZ], wasting [WHZ], and underweight [WAZ] for children under age five, both sexes, for every five years from 1990−2020. Country results are grouped by GBD super-region, including Central Europe, Eastern Europe, and Central Asia (S1a), High-income (S1b), Latin America and Caribbean (S1c), North Africa and Middle East (S1d), South Asia (S1e), Southeast Asia, East Asia, and Oceania (S1f), and Sub-Saharan Africa (S1g). Plots for each country include overall and severe stunting prevalence (A) and transformed mean stunting Z scores (B). A source list is shown which includes surveys included in the stunting models (C). Additional plots are shown for overall and severe wasting prevalence (D) and transformed mean wasting Z scores (E), followed by a source list with surveys included in the wasting models (F). Plots are then shown for overall and severe underweight prevalence (G), and transformed mean underweight Z scores (H), with a source list listing surveys included in the underweight models (I). Finally, distributions of stunting (J), wasting (K), and underweight (L) are shown for children under age five, both sexes, for every five years from 1990-2020. Surveys that were outliered are shown with X's on all plots. Surveys prior to 1990 may have been inputs to the models to inform trends, but estimates are only produced and shown for 1990−2020. For locations that are modeled nationally and subnationally, sources that are only included subnationally are not included in the plots of national level estimates. These sources were included in subnational models that influence national level models. Note that due to the transformation on mean Z scores, increasing values reflect improvements in mean Z score. Surveys conducted over a range of years were assigned to the midpoint year from that interval, which is the year reflected in the table and the plots. For the distributions of stunting, wasting, and underweight, the area under the curve reflects the estimated proportion of children experiencing that severity of CGF or worse. DHS is Demographic and Health Surveys. MICS is Multiple Indicator Cluster Survey. WHO CGM is the WHO Global Database on Child Growth and Malnutrition. SDNS is Survey of Diet and Nutritional Status.

This file contains the above for the following locations in the GBD super region of sub-Saharan Africa, in the following order:

Central sub-Saharan Africa: Angola, Central African Republic, Congo, Democratic Republic of the Congo, Equatorial Guinea, Gabon

Eastern Sub-Saharan Africa: Burundi, Comoros, Djibouti, Eritrea, Ethiopia, Kenya, Madagascar, Malawi, Mozambique, Rwanda, Somalia, South Sudan, Uganda, United Republic of Tanzania, Zambia

Southern Sub-Saharan Africa: Botswana, Eswatini, Lesotho, Namibia, South Africa, Zimbabwe

Western Sub-Saharan Africa: Benin, Burkina Faso, Cabo Verde, Cameroon, Chad, Côte d'Ivoire, Gambia, Ghana, Guinea, Guinea-Bissau, Liberia, Mali, Mauritania, Niger, Nigeria, Sao Tome and Principe, Senegal, Sierra Leone, Togo

Angola – Stunting (HAZ)

A: Overall and Severe Stunting Prevalence

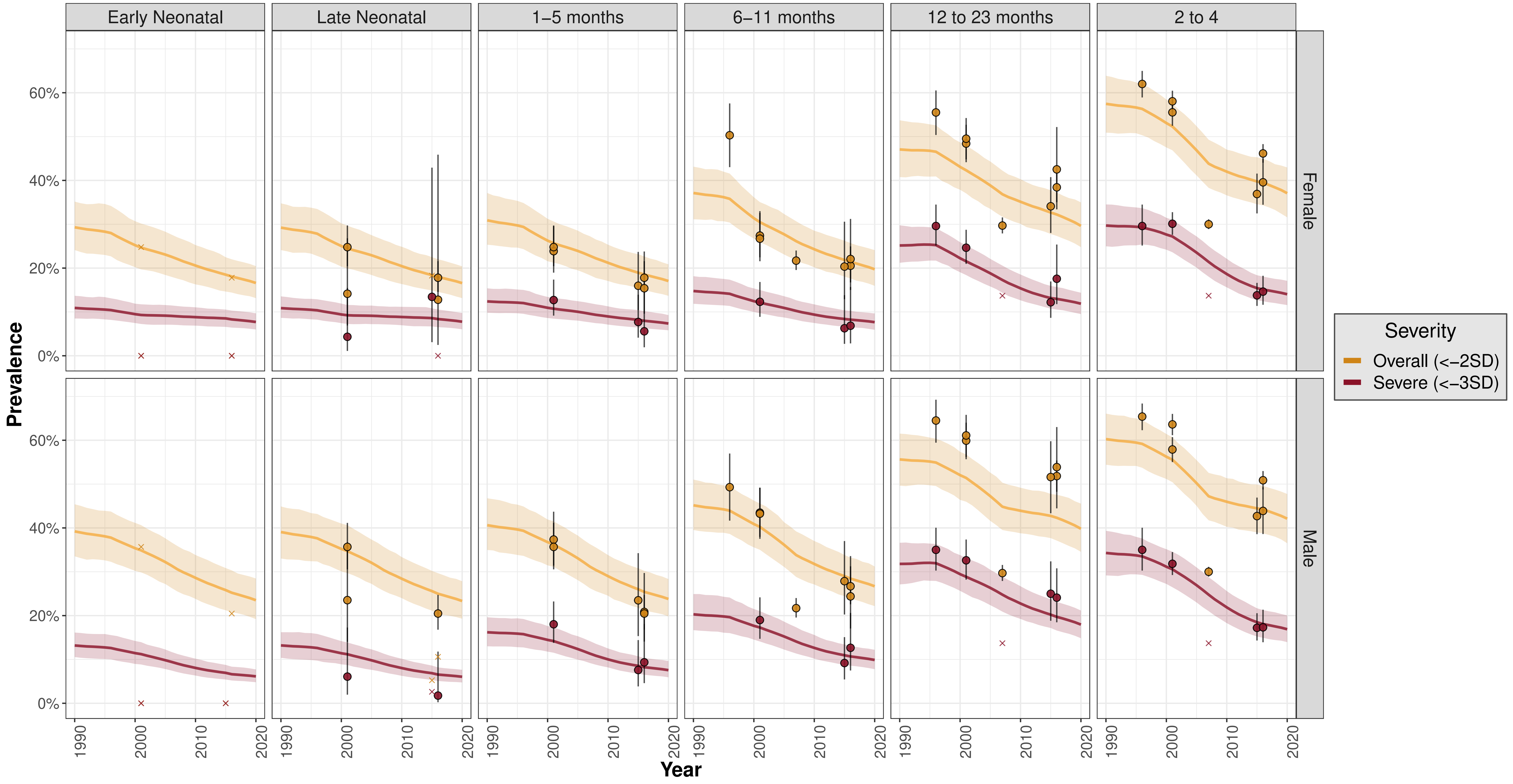

B: Transformed Mean Stunting Z Scores

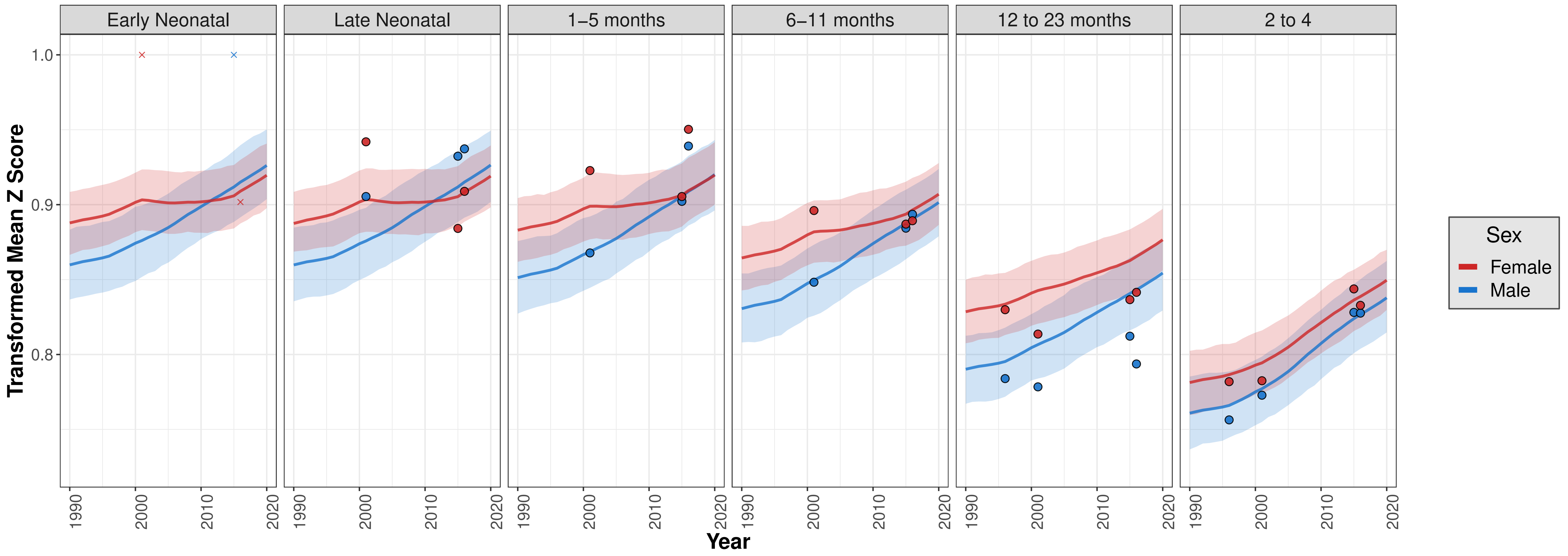

C

Year	Source
1996	WHO CGM Database
2001	MICS
2001	WHO CGM Database
2007	WHO CGM Database
2015	DHS
2016	WHO CGM Database
2016	DHS

Angola – Wasting (WHZ)

D: Overall and Severe Wasting Prevalence

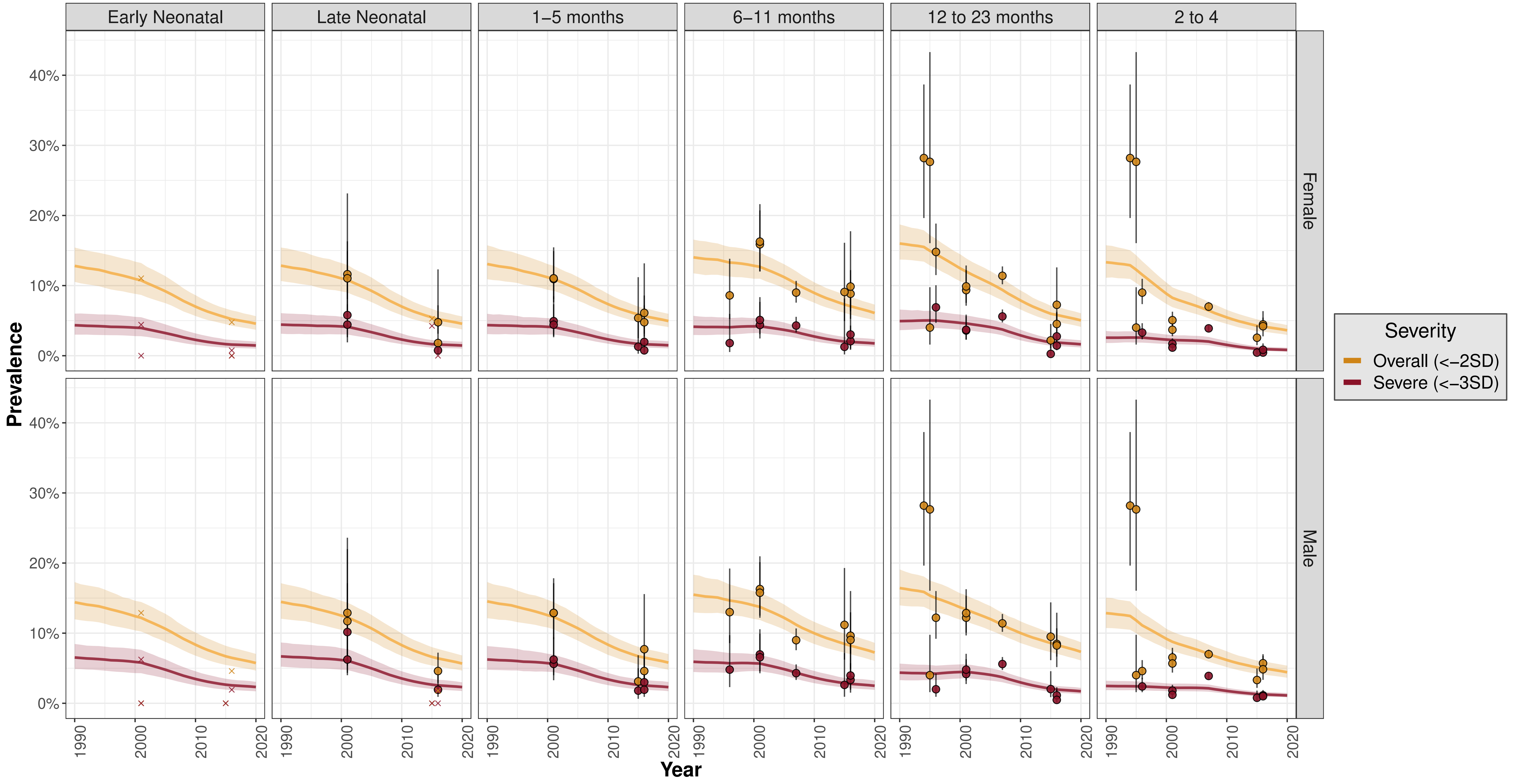

E: Transformed Mean Wasting Z Scores

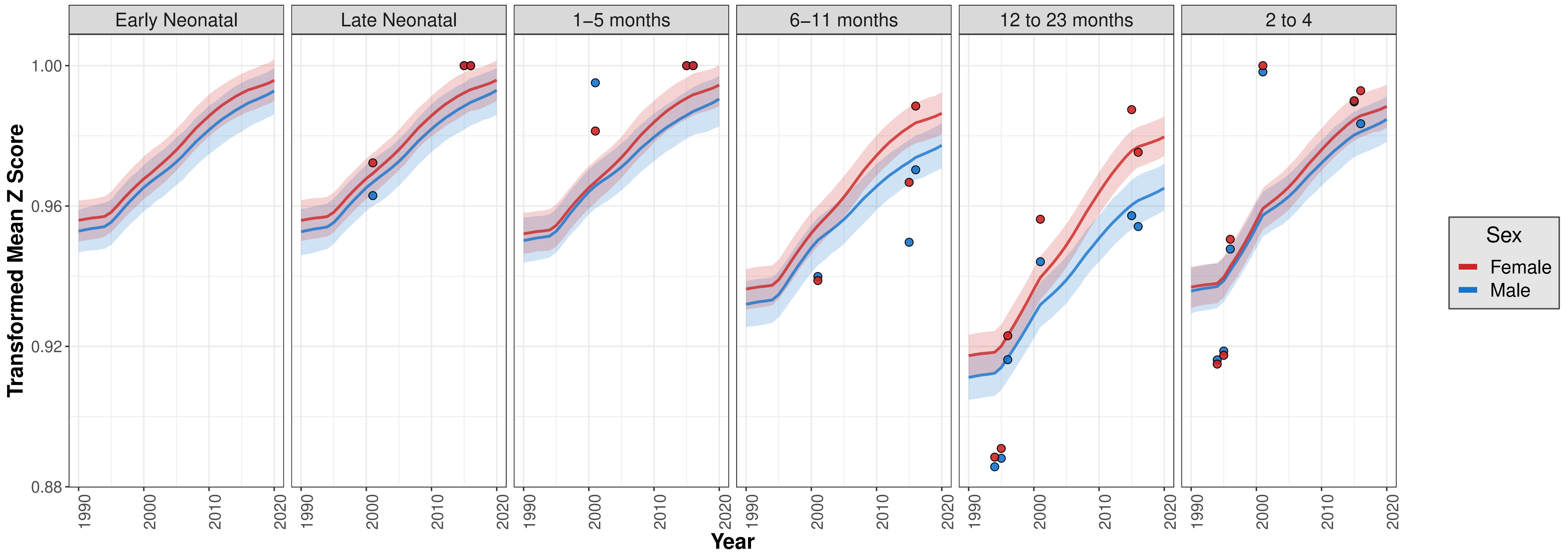

F

Year	Source
1994	WHO CGM Database
1995	WHO CGM Database
1996	WHO CGM Database
2001	MICS
2001	WHO CGM Database
2007	WHO CGM Database
2015	DHS
2016	WHO CGM Database
2016	DHS

Angola – Underweight (WAZ)

G: Overall and Severe Underweight Prevalence

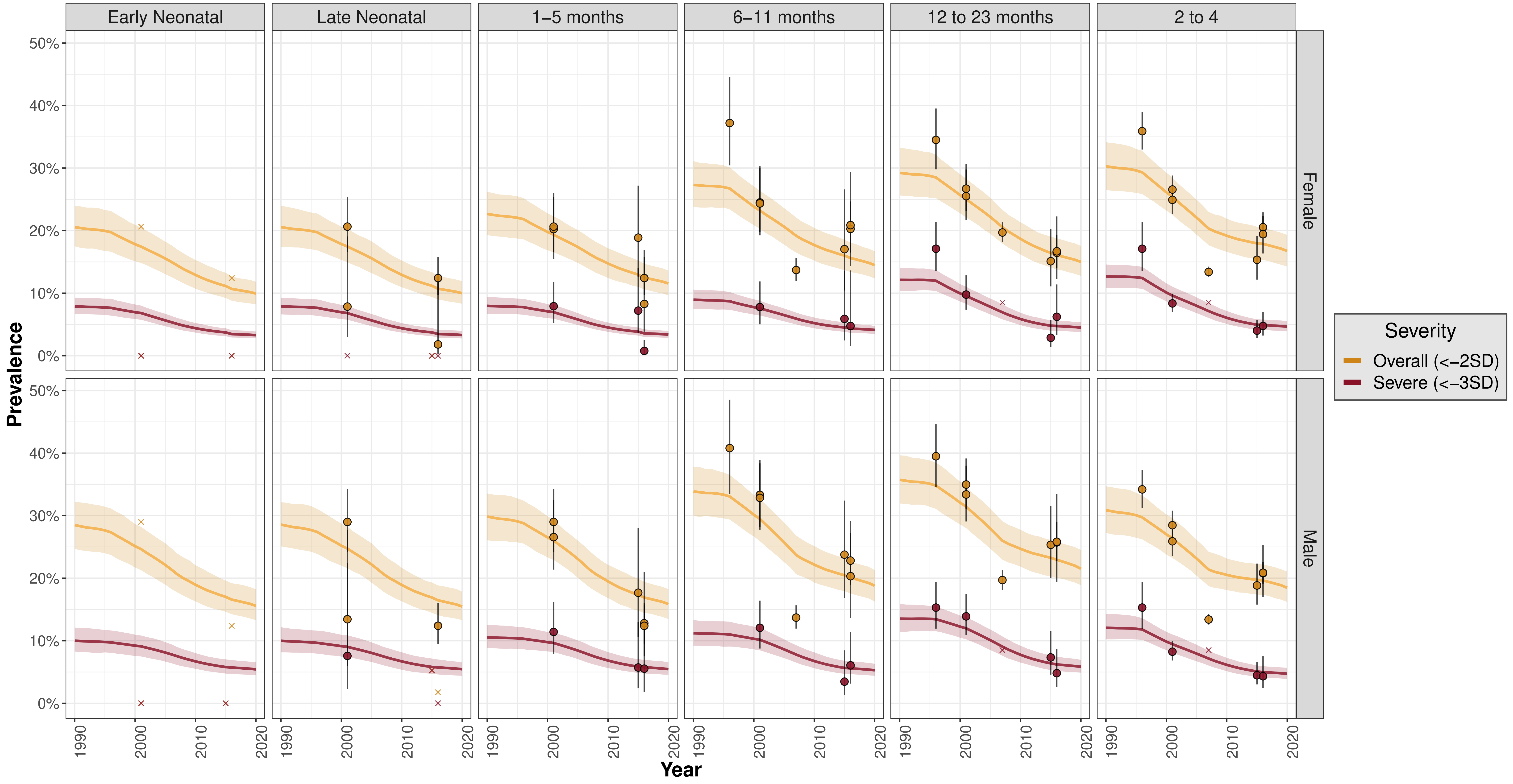

H: Transformed Mean Underweight Z Scores

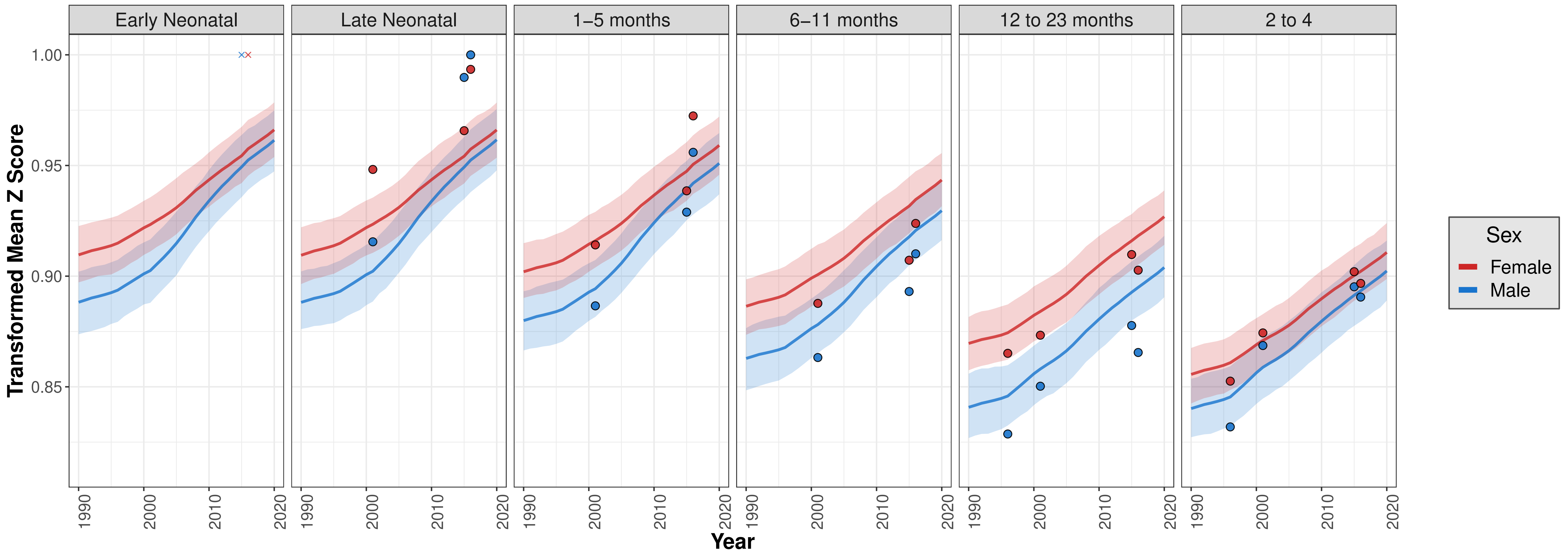

I

Year	Source
1996	WHO CGM Database
2001	MICS
2001	WHO CGM Database
2007	WHO CGM Database
2015	DHS
2016	WHO CGM Database
2016	DHS

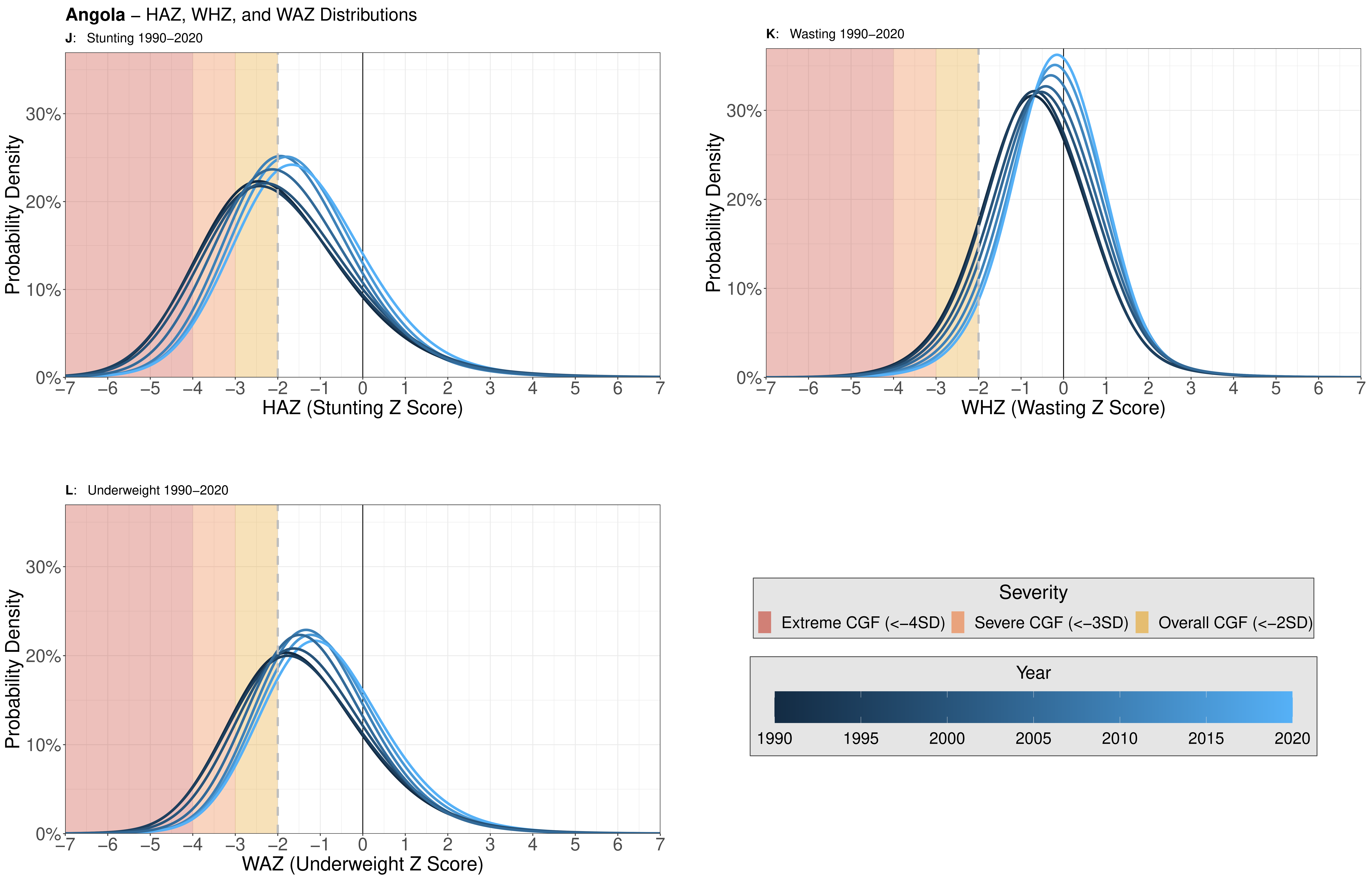

Angola – HAZ, WHZ, and WAZ Distributions
J: Stunting 1990–2020
K: Wasting 1990–2020
L: Underweight 1990–2020
Probability Density
0%
10%
20%
30%
−7 −6 −5 −4 −3 −2 −1 0 1 2 3 4 5 6 7
HAZ (Stunting Z Score)
WHZ (Wasting Z Score)
WAZ (Underweight Z Score)
Severity
Extreme CGF (<−4SD)
Severe CGF (<−3SD)
Overall CGF (<−2SD)
Year
1990
1995
2000
2005
2010
2015
2020

Central African Republic – Stunting (HAZ)

A: Overall and Severe Stunting Prevalence

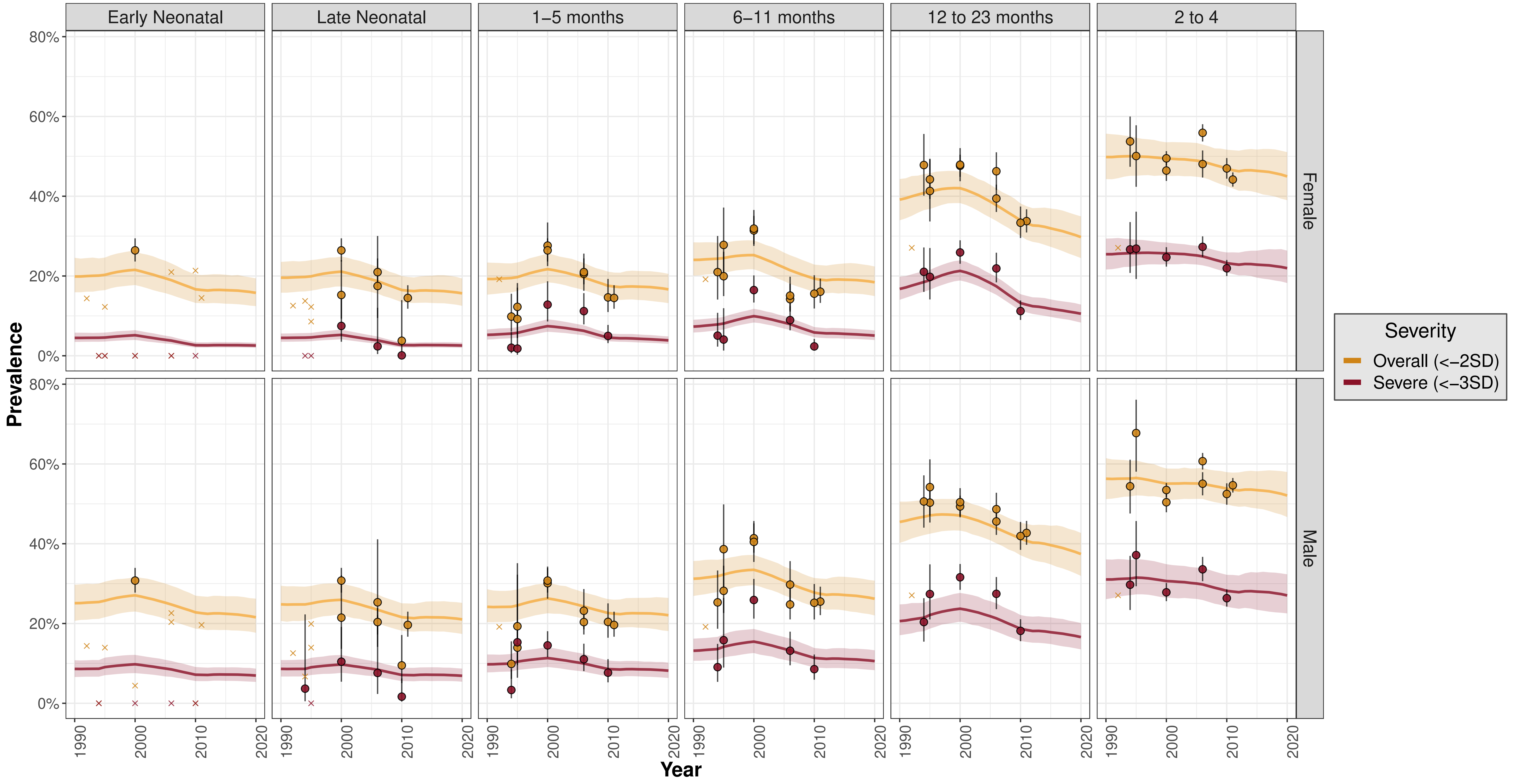

B: Transformed Mean Stunting Z Scores

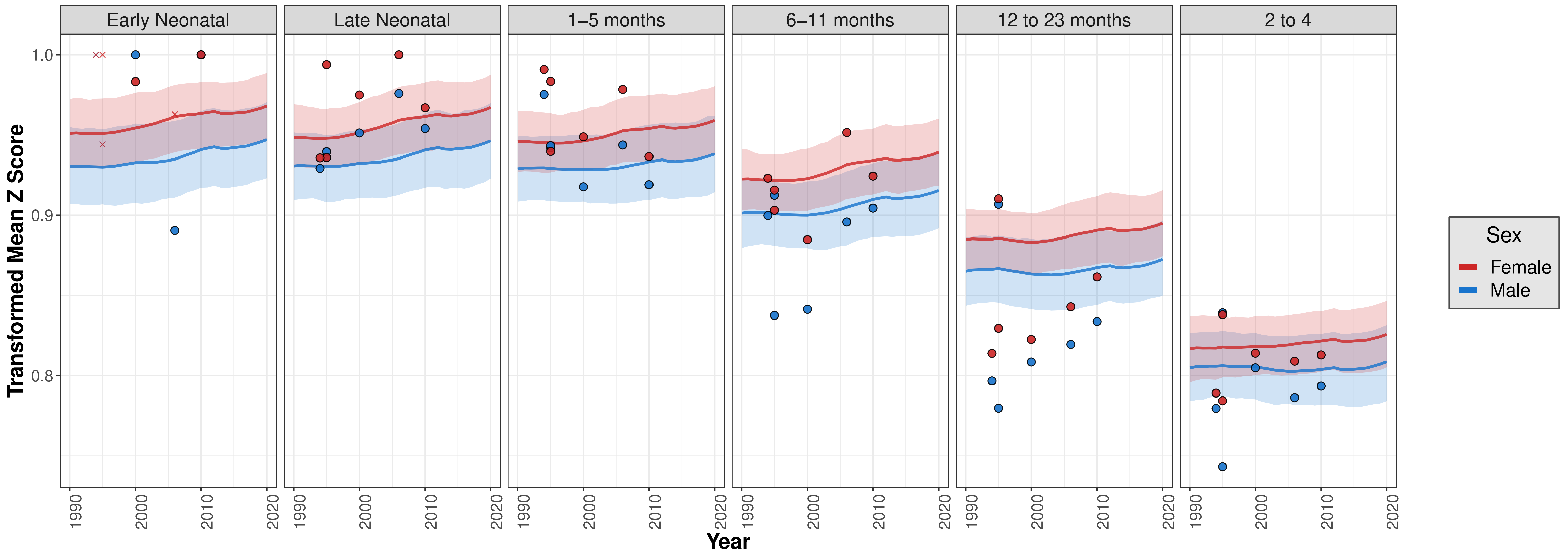

C

Year	Source
1992	Priority Survey on Household Living Conditions
1994	DHS
1995	DHS
1995	WHO CGM Database
2000	MICS
2000	WHO CGM Database
2006	MICS
2006	WHO CGM Database
2010	MICS
2011	WHO CGM Database

Central African Republic – Wasting (WHZ)

D: Overall and Severe Wasting Prevalence

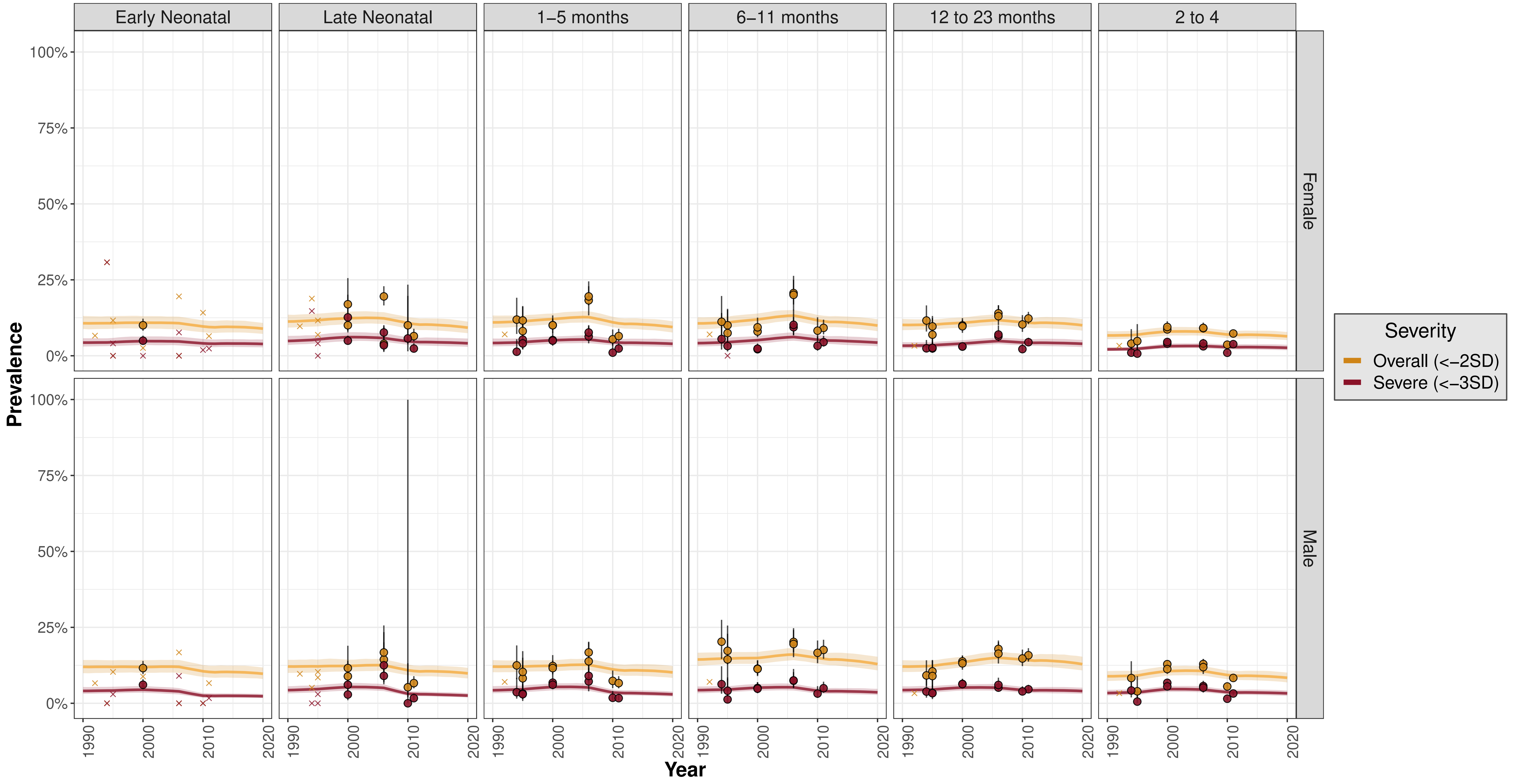

E: Transformed Mean Wasting Z Scores

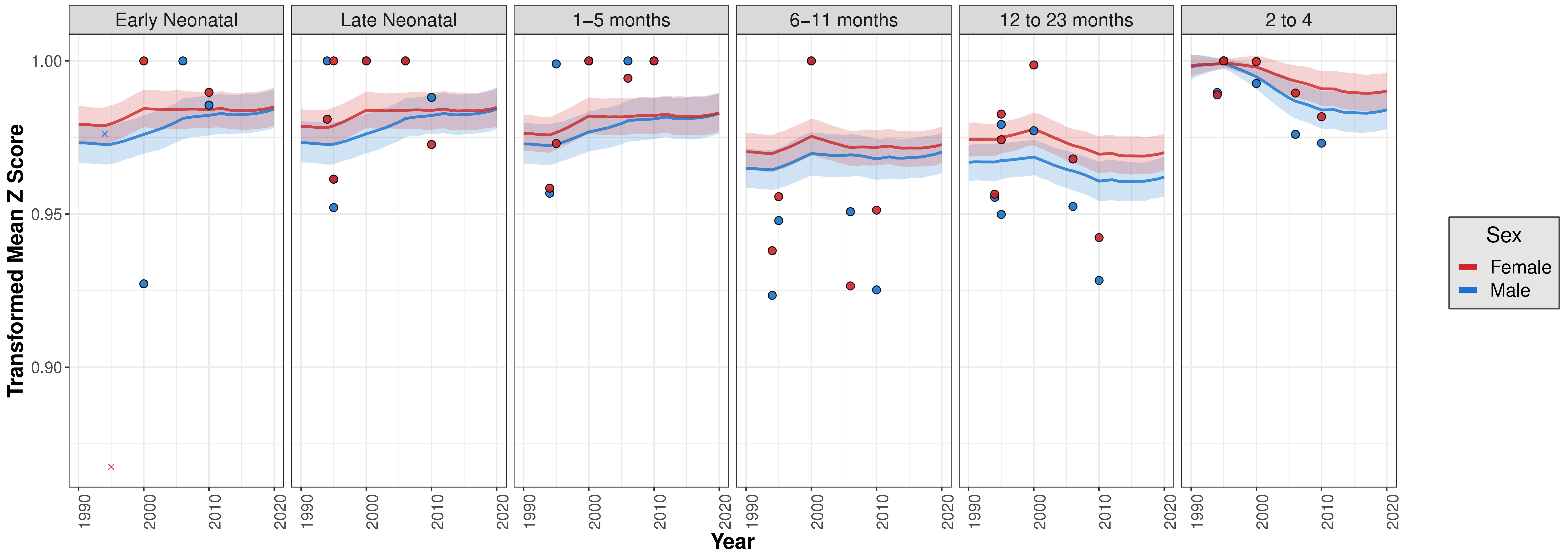

F

Year	Source
1992	Priority Survey on Household Living Conditions
1994	DHS
1995	DHS
1995	WHO CGM Database
2000	MICS
2000	WHO CGM Database
2006	MICS
2006	WHO CGM Database
2010	MICS
2011	WHO CGM Database

Central African Republic – Underweight (WAZ)

G: Overall and Severe Underweight Prevalence

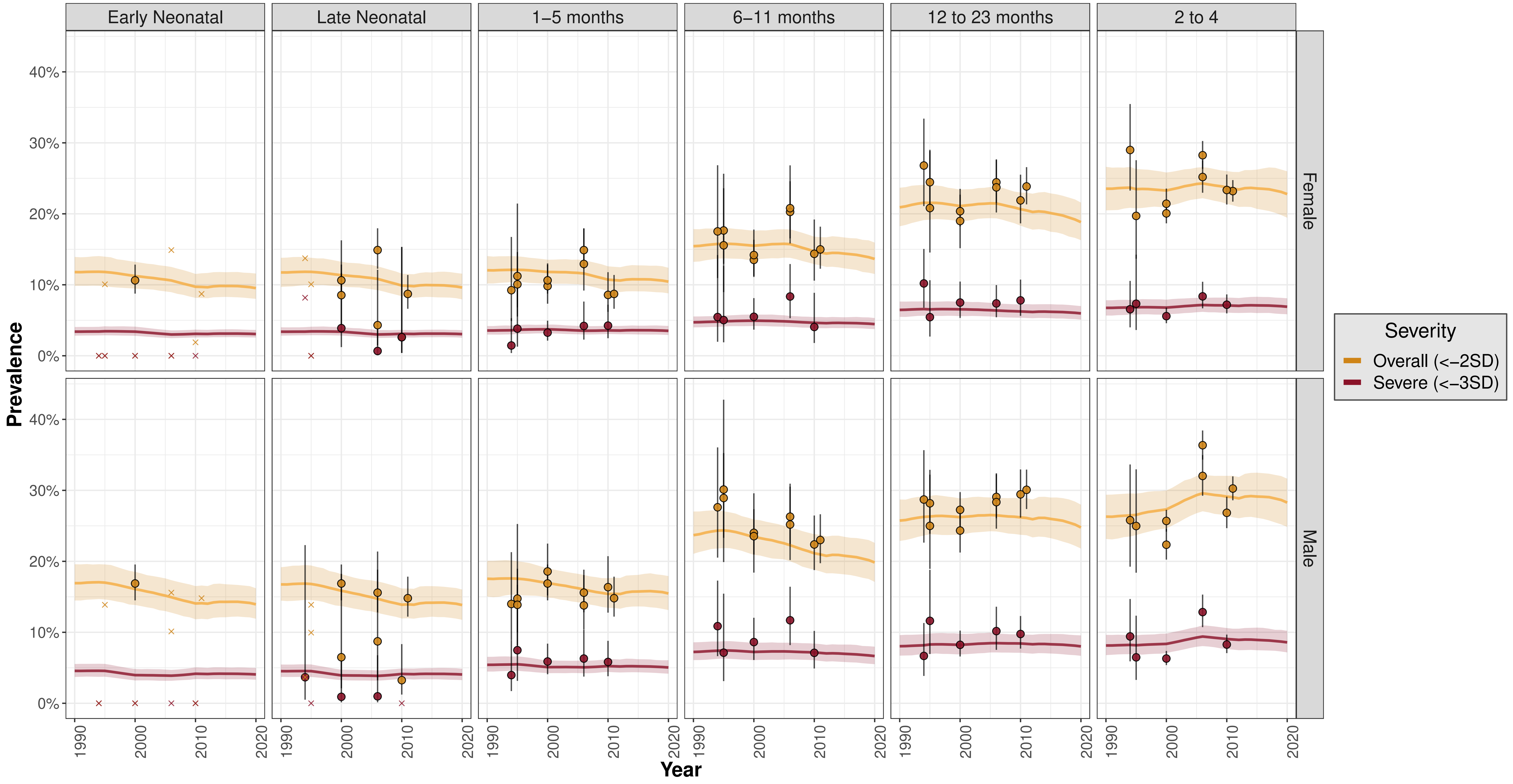

H: Transformed Mean Underweight Z Scores

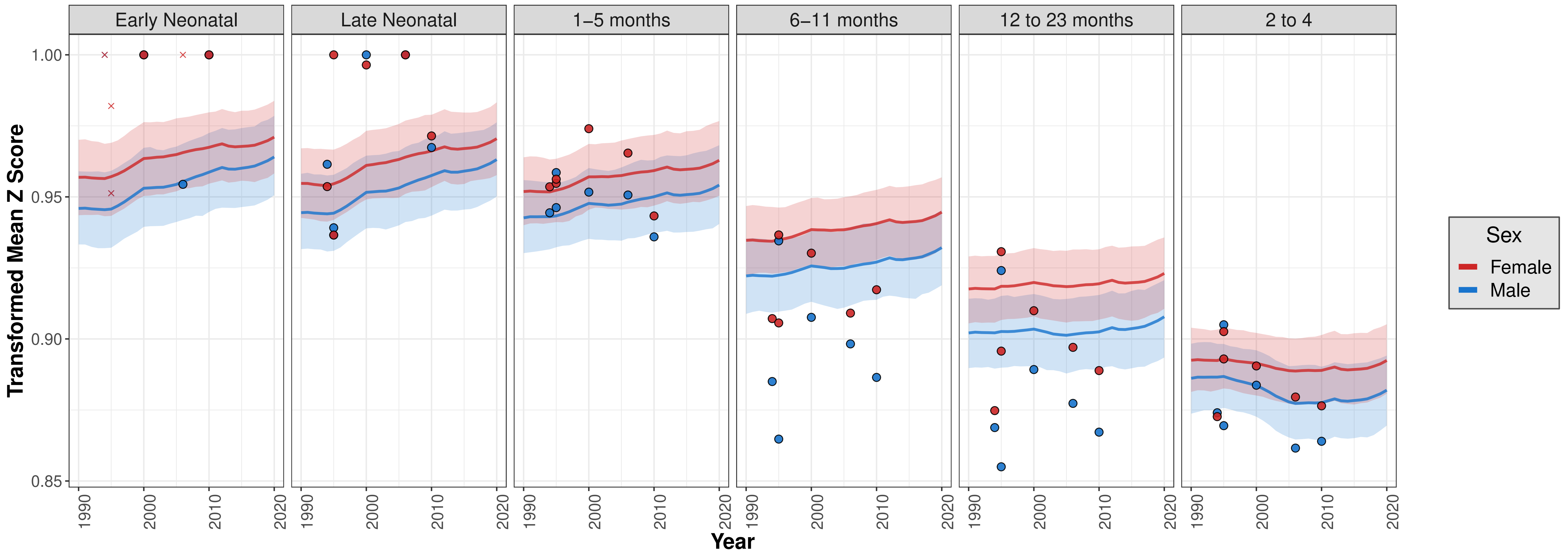

I

Year	Source
1994	DHS
1995	DHS
1995	WHO CGM Database
2000	MICS
2000	WHO CGM Database
2006	MICS
2006	WHO CGM Database
2010	MICS
2011	WHO CGM Database

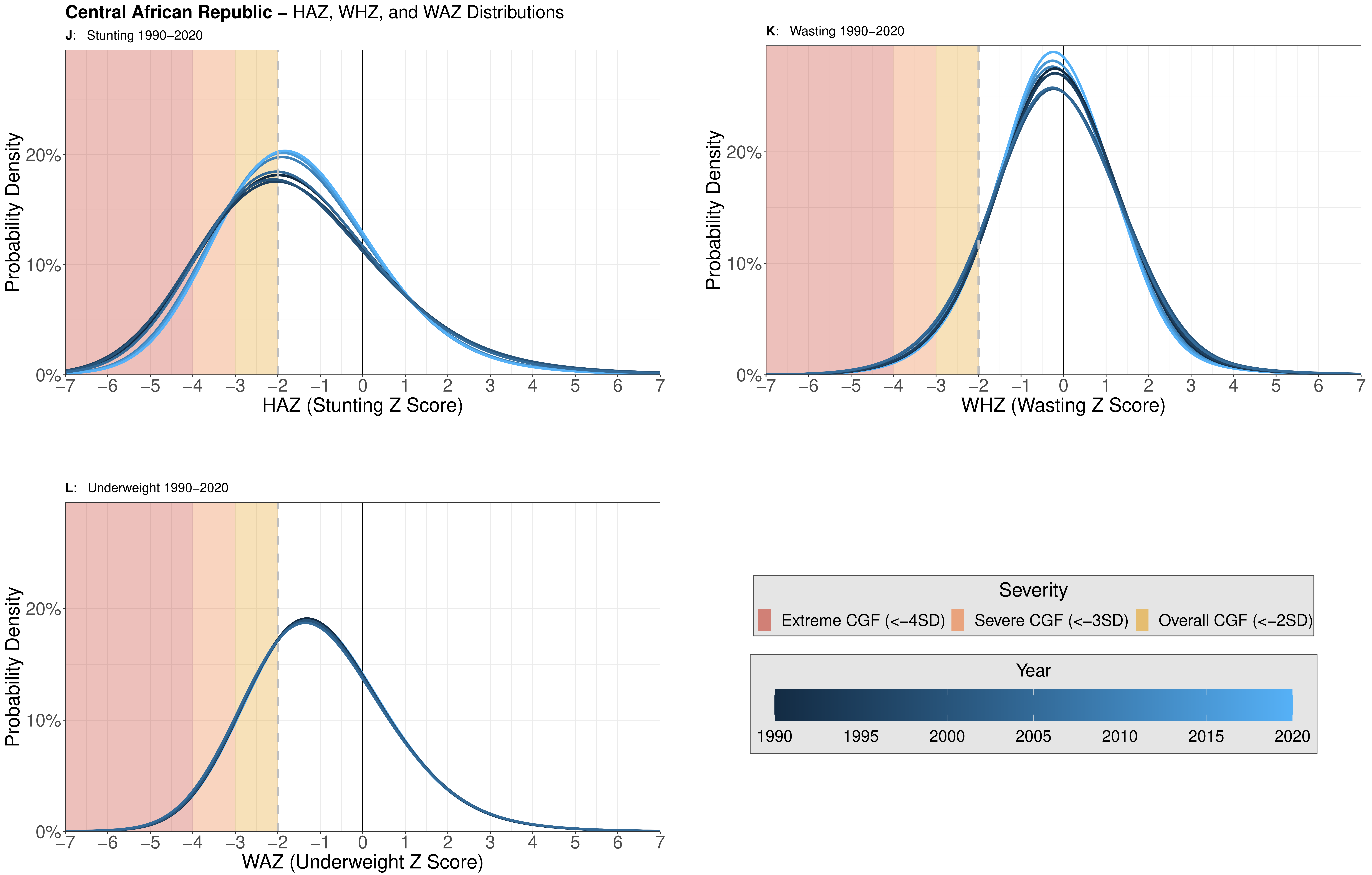
Central African Republic – HAZ, WHZ, and WAZ Distributions
J: Stunting 1990–2020
Probability Density
0%
10%
20%
−7 −6 −5 −4 −3 −2 −1 0 1 2 3 4 5 6 7
HAZ (Stunting Z Score)
K: Wasting 1990–2020
Probability Density
0%
10%
20%
−7 −6 −5 −4 −3 −2 −1 0 1 2 3 4 5 6 7
WHZ (Wasting Z Score)
L: Underweight 1990–2020
Probability Density
0%
10%
20%
−7 −6 −5 −4 −3 −2 −1 0 1 2 3 4 5 6 7
WAZ (Underweight Z Score)
Severity
Extreme CGF (<−4SD)
Severe CGF (<−3SD)
Overall CGF (<−2SD)
Year
1990
1995
2000
2005
2010
2015
2020

Congo – Stunting (HAZ)

A: Overall and Severe Stunting Prevalence

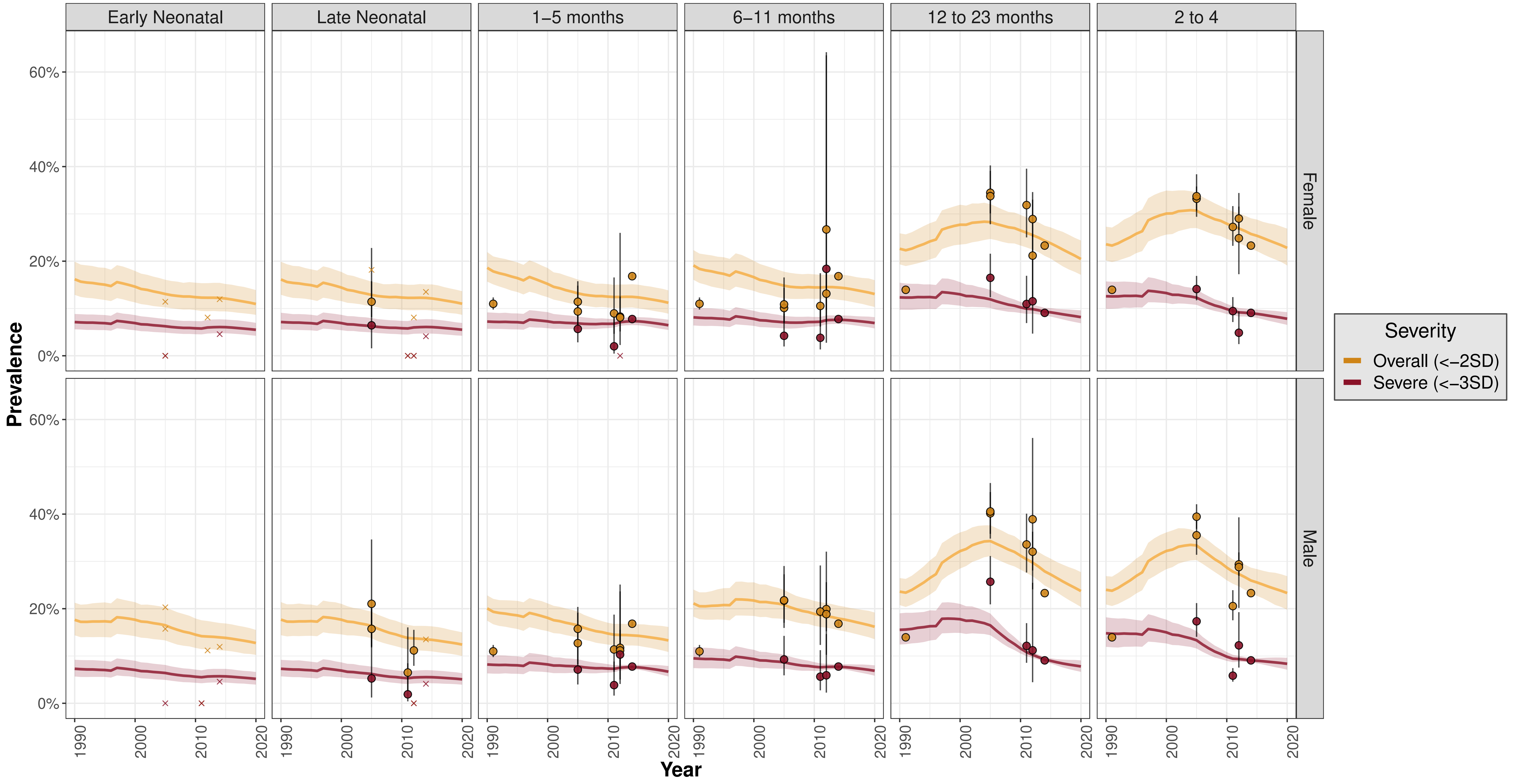

B: Transformed Mean Stunting Z Scores

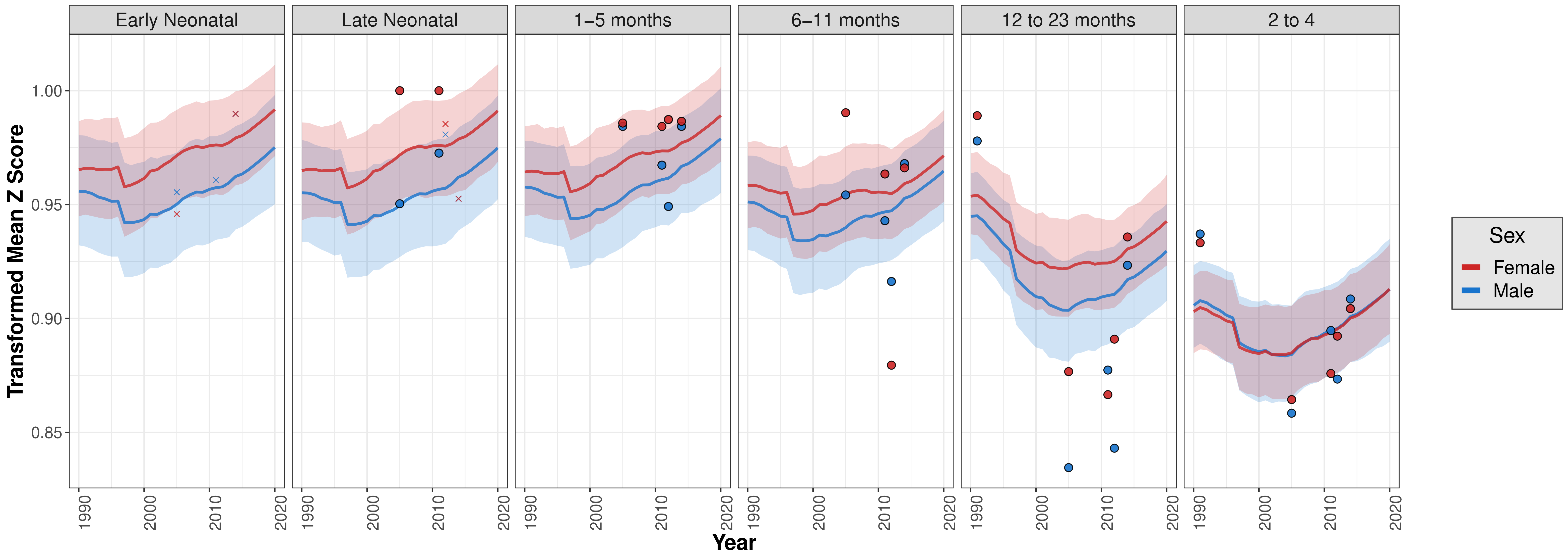

C

Year	Source
1986	WHO CGM Database
1987	WHO CGM Database
1991	WHO CGM Database
2005	DHS
2005	WHO CGM Database
2011	DHS
2012	DHS
2012	WHO CGM Database
2014	MICS

Congo – Wasting (WHZ)

D: Overall and Severe Wasting Prevalence

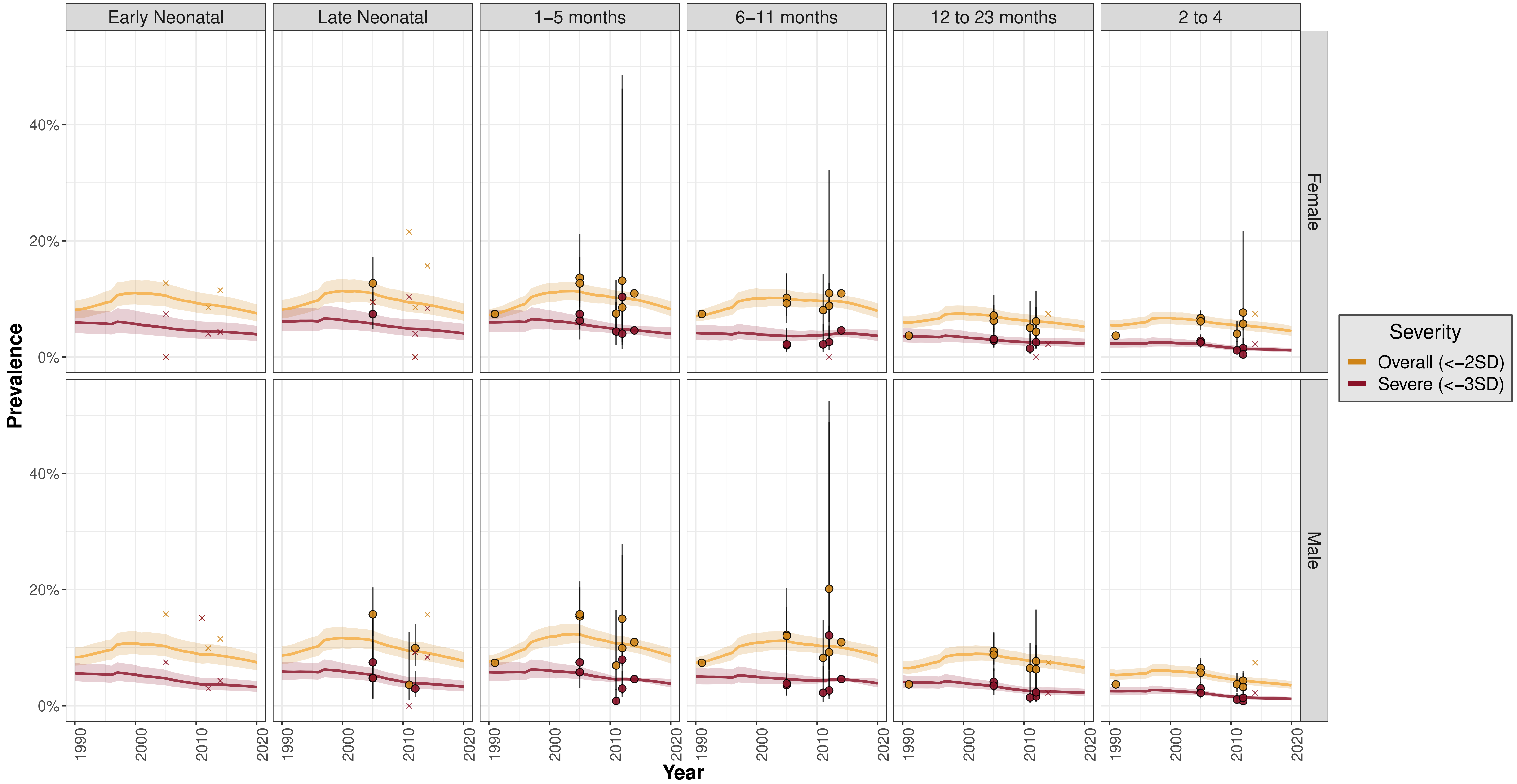

E: Transformed Mean Wasting Z Scores

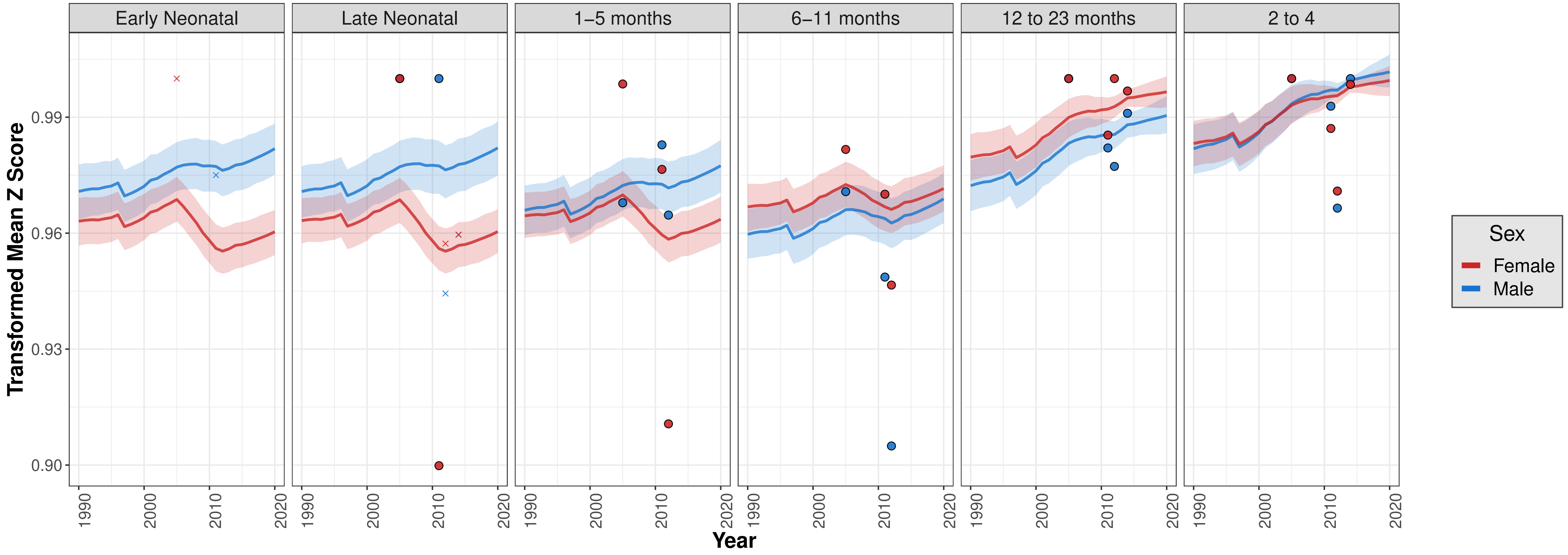

F

Year	Source
1986	WHO CGM Database
1987	WHO CGM Database
1991	WHO CGM Database
2005	DHS
2005	WHO CGM Database
2011	DHS
2012	DHS
2012	WHO CGM Database
2014	MICS

Congo – Underweight (WAZ)

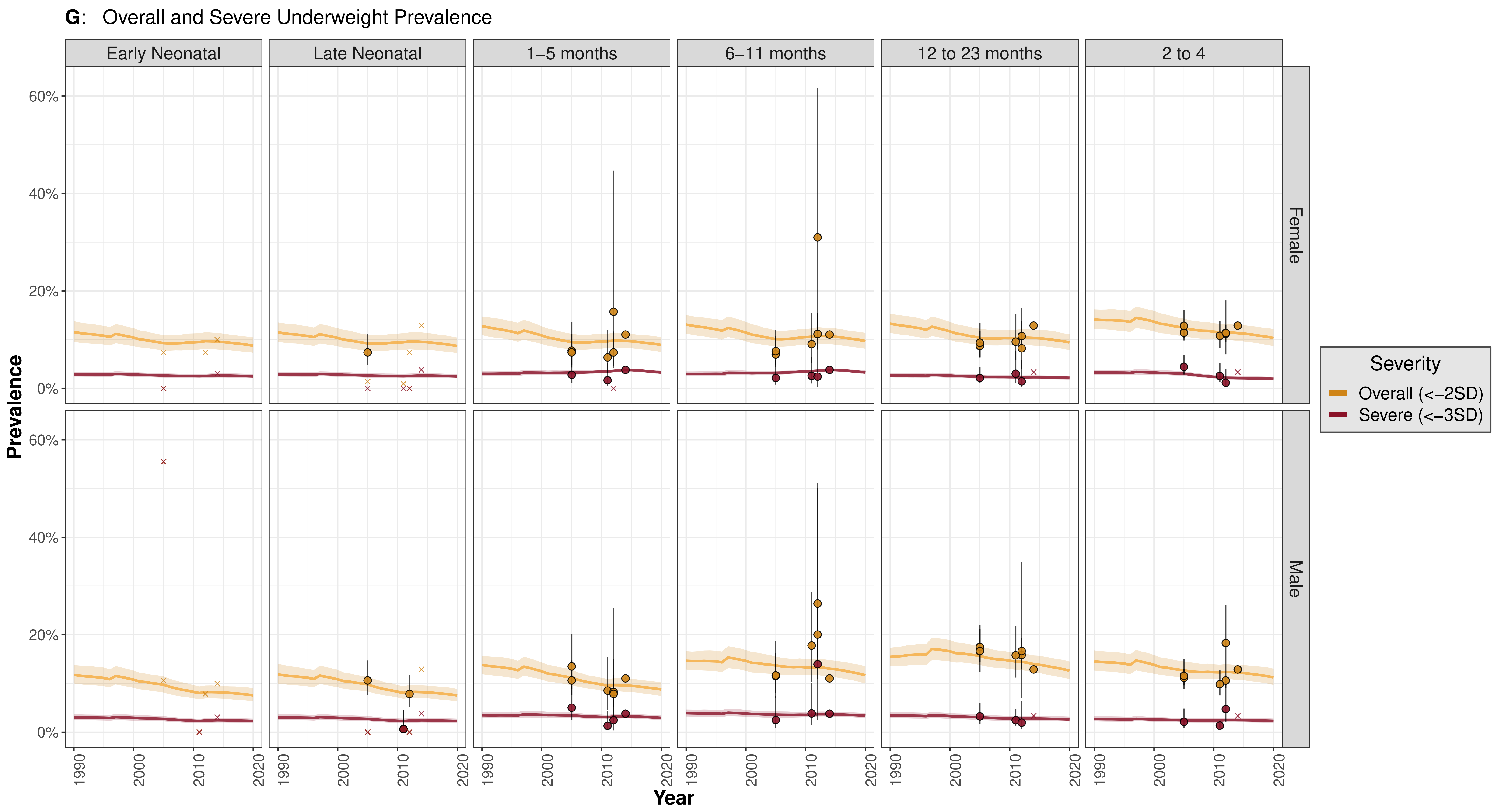

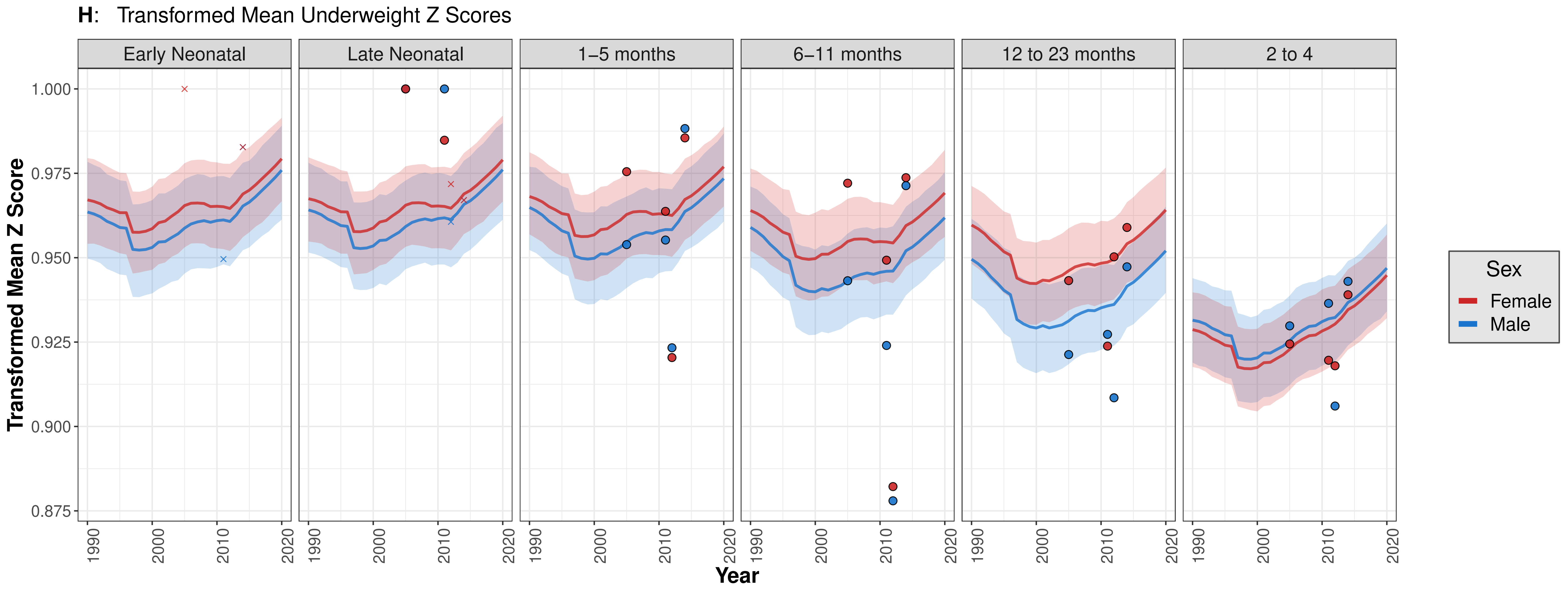

I

Year	Source
1986	WHO CGM Database
1987	WHO CGM Database
2005	DHS
2005	WHO CGM Database
2011	DHS
2012	DHS
2012	WHO CGM Database
2014	MICS

Congo – HAZ, WHZ, and WAZ Distributions

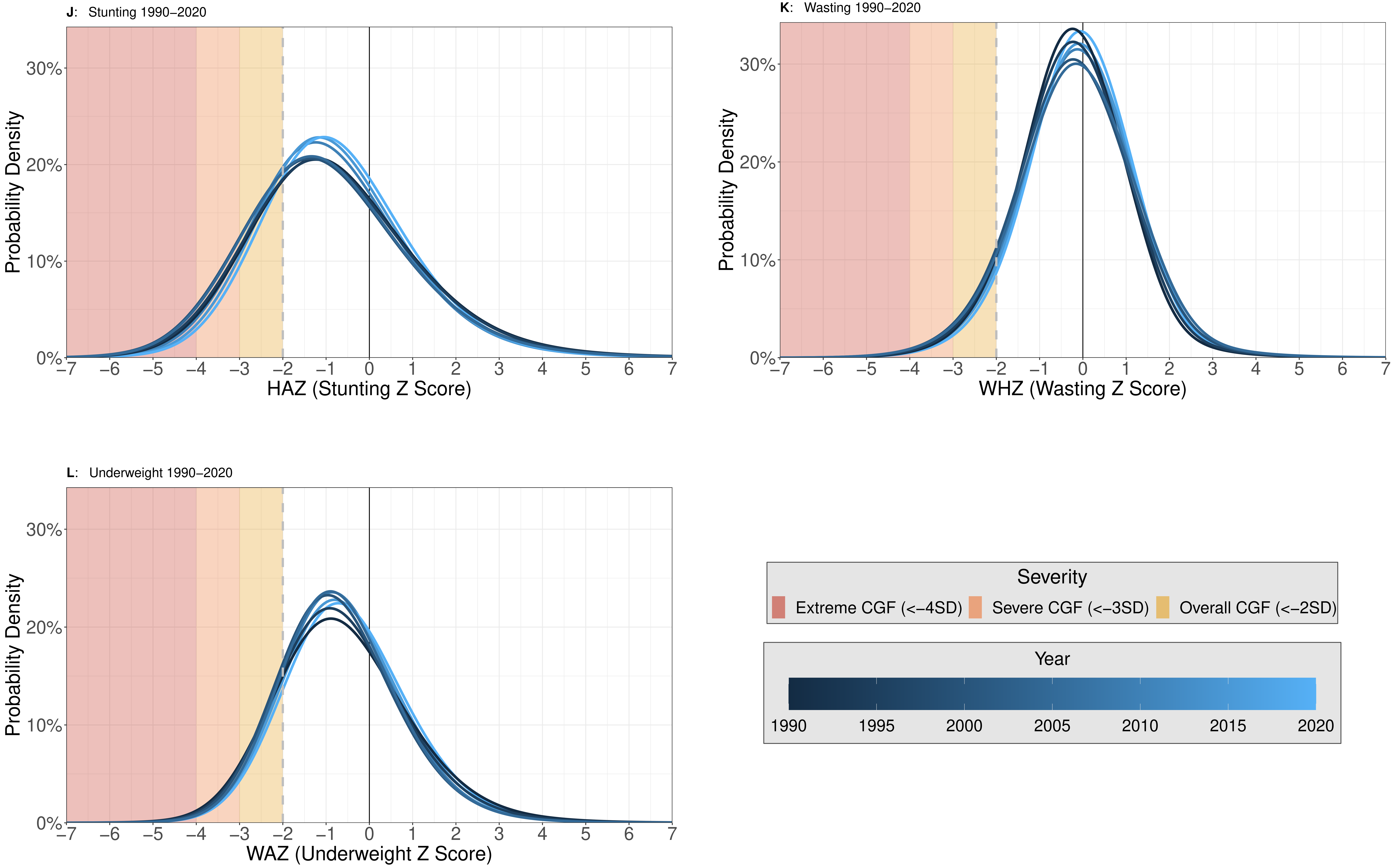

Democratic Republic of the Congo – Stunting (HAZ)

A: Overall and Severe Stunting Prevalence

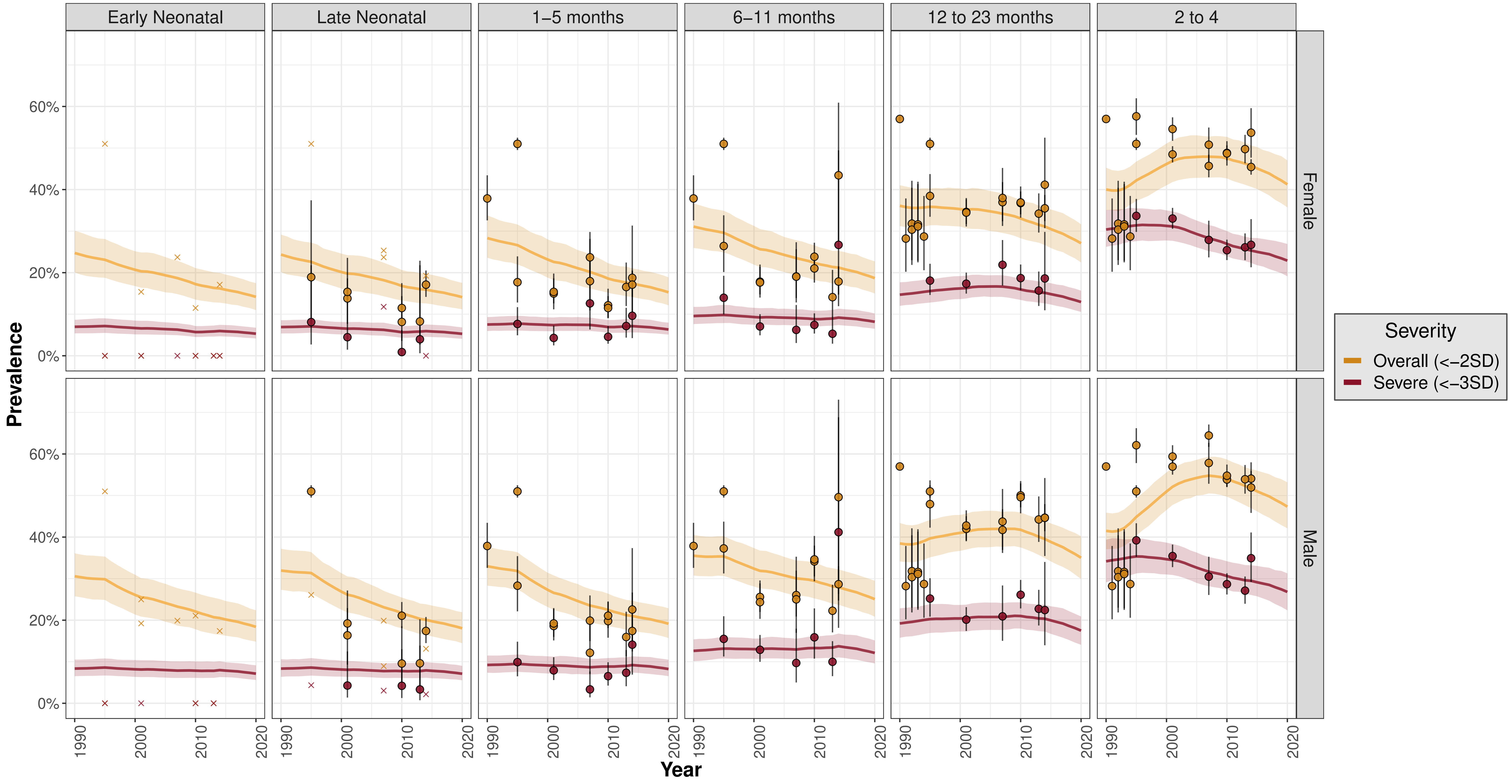

B: Transformed Mean Stunting Z Scores

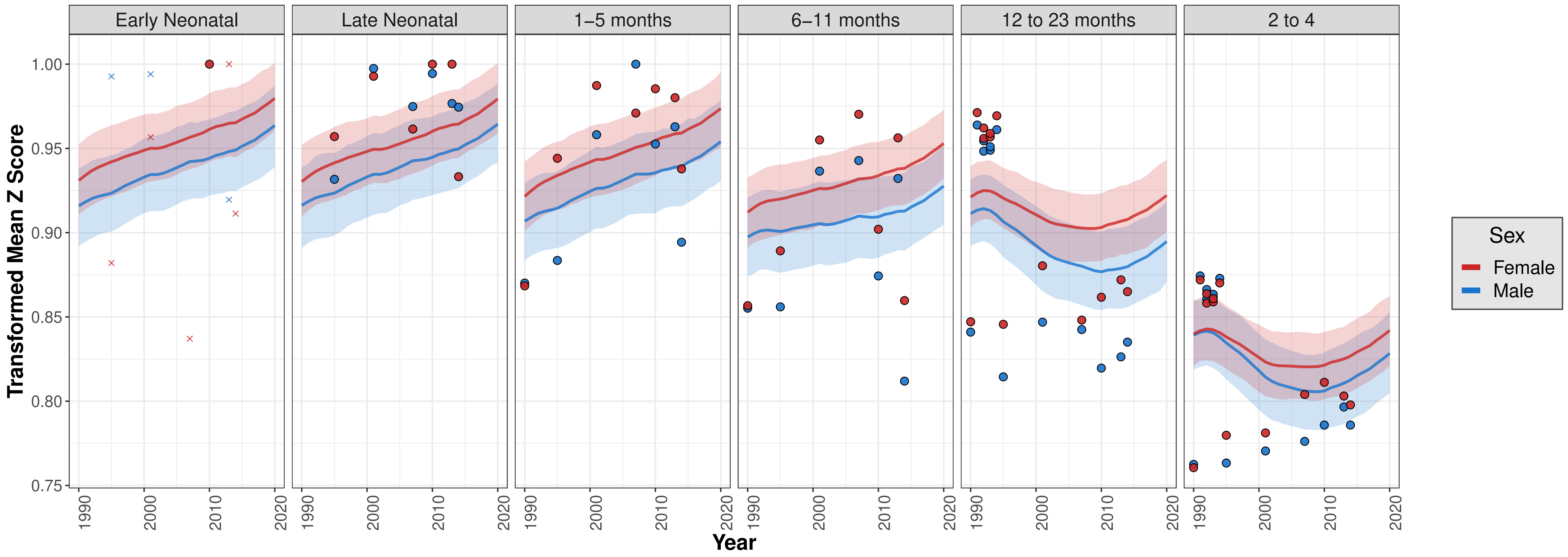

C

Year	Source
1985	WHO CGM Database
1986	WHO CGM Database
1990	WHO CGM Database
1991	WHO CGM Database
1992	WHO CGM Database
1993	WHO CGM Database
1994	WHO CGM Database
1995	MICS
1995	WHO CGM Database
2001	MICS
2001	WHO CGM Database
2007	DHS
2007	WHO CGM Database
2010	MICS
2010	WHO CGM Database
2013	DHS
2014	DHS
2014	WHO CGM Database

Democratic Republic of the Congo – Wasting (WHZ)

D: Overall and Severe Wasting Prevalence

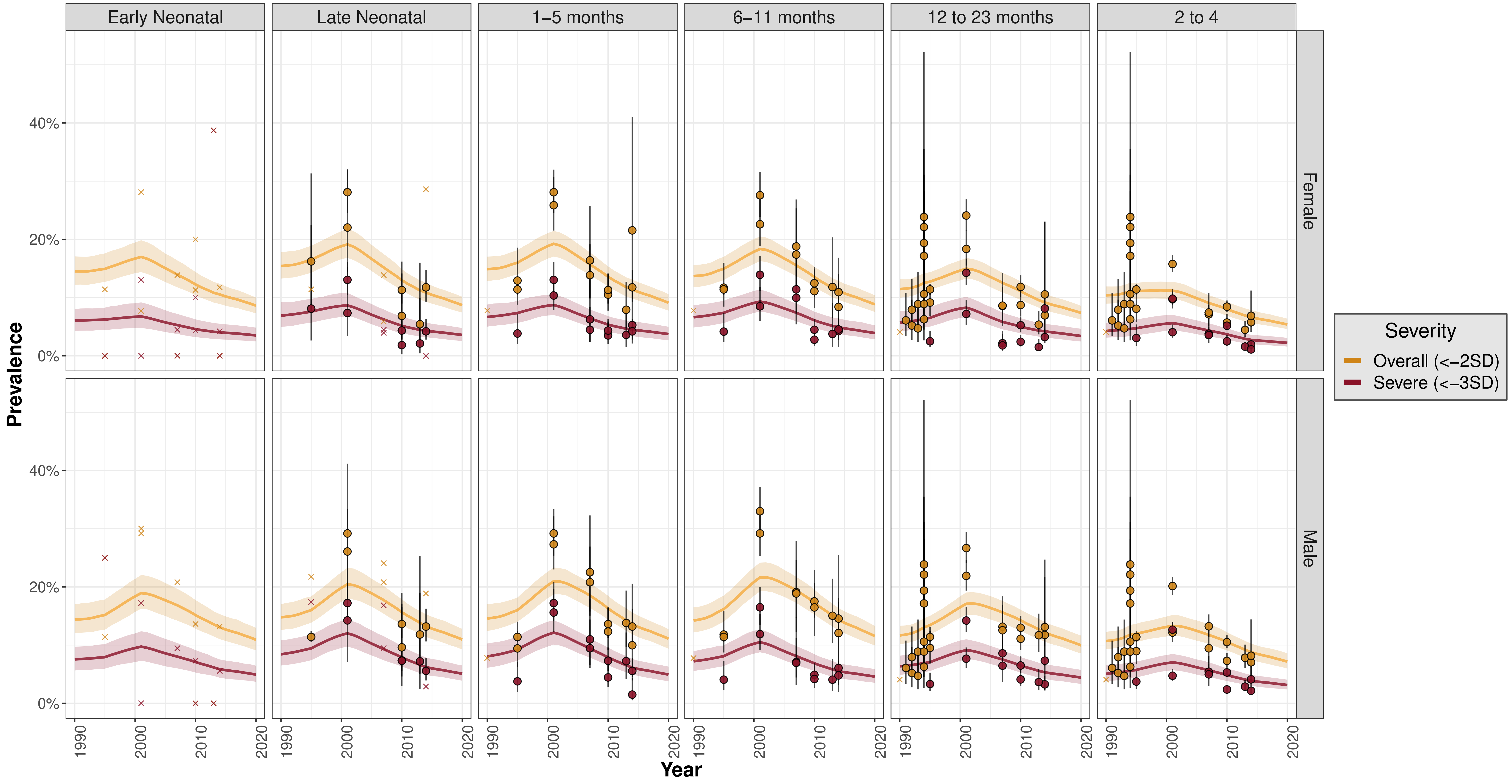

E: Transformed Mean Wasting Z Scores

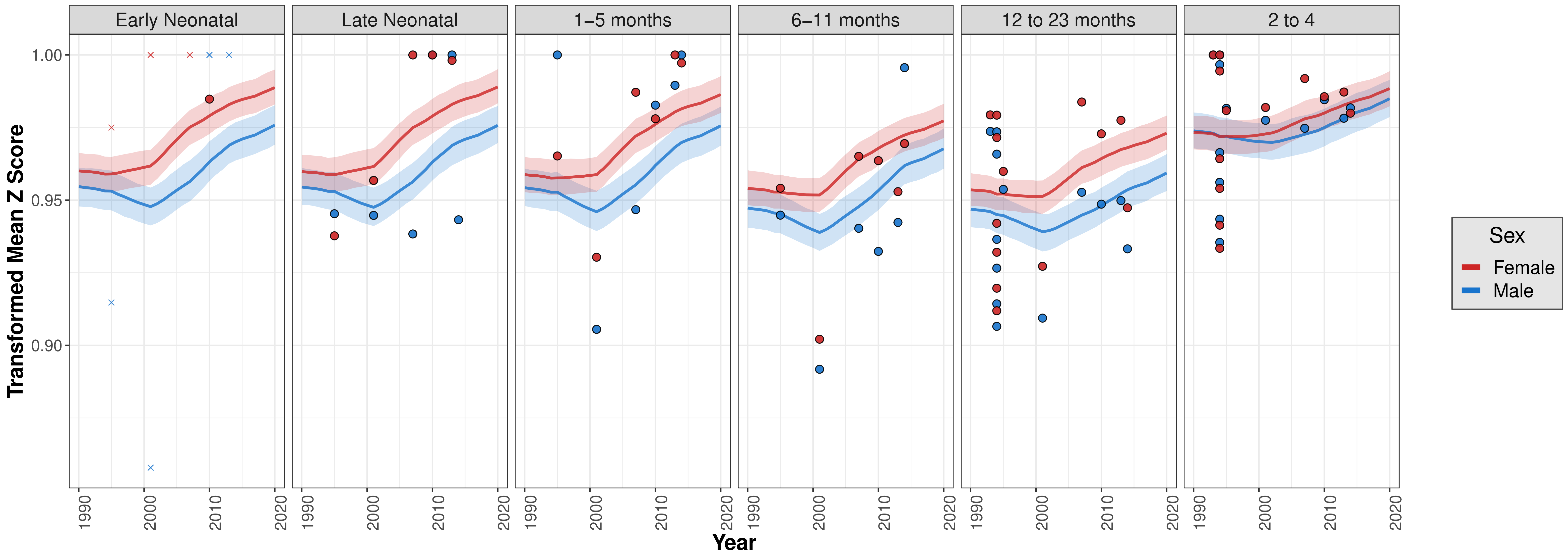

F

Year	Source
1985	WHO CGM Database
1986	WHO CGM Database
1990	WHO CGM Database
1991	WHO CGM Database
1992	WHO CGM Database
1993	WHO CGM Database
1994	WHO CGM Database
1995	MICS
1995	WHO CGM Database
2001	MICS
2001	WHO CGM Database
2007	DHS
2007	WHO CGM Database
2010	MICS
2010	WHO CGM Database
2013	DHS
2014	DHS
2014	WHO CGM Database

Democratic Republic of the Congo – Underweight (WAZ)

G: Overall and Severe Underweight Prevalence

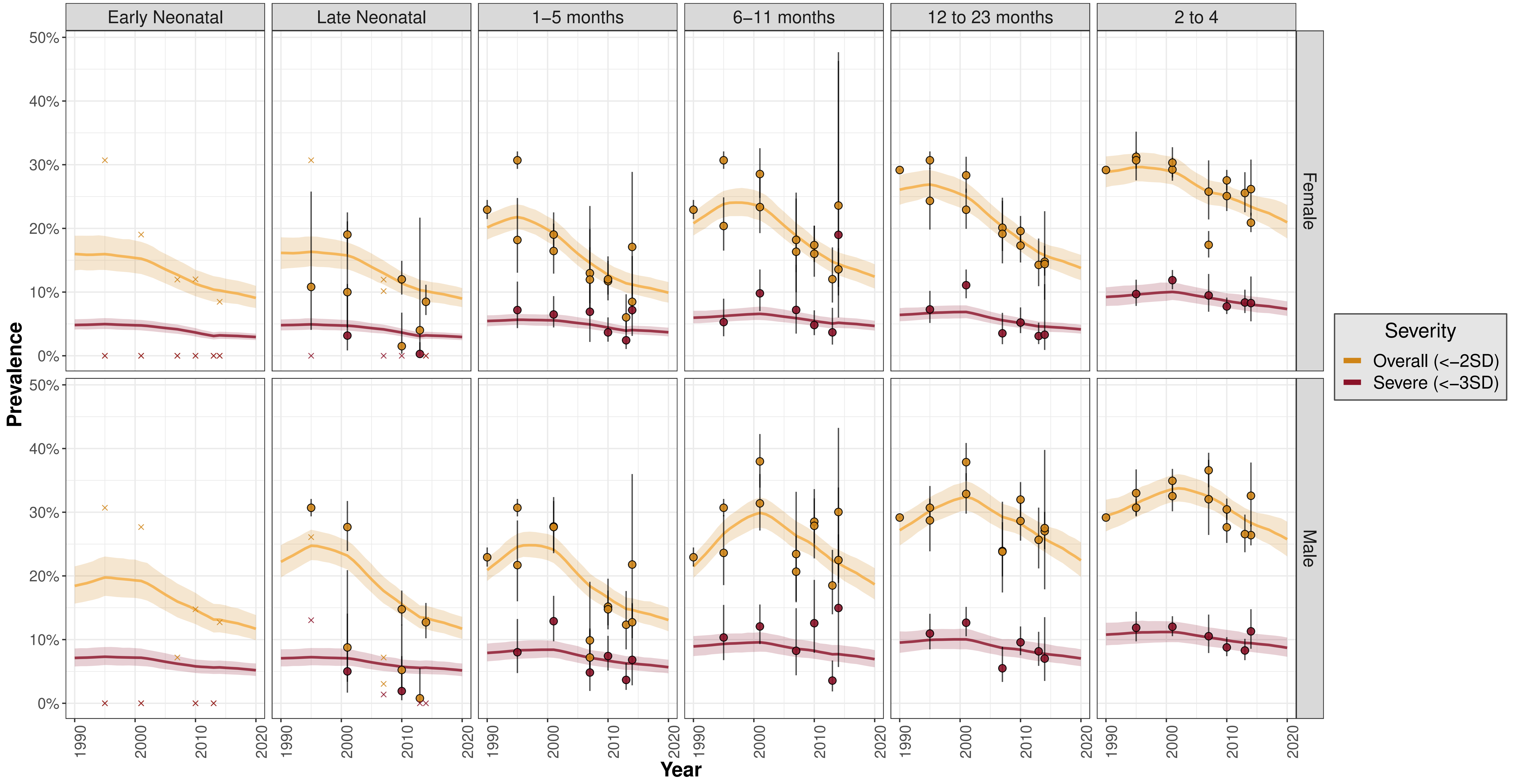

H: Transformed Mean Underweight Z Scores

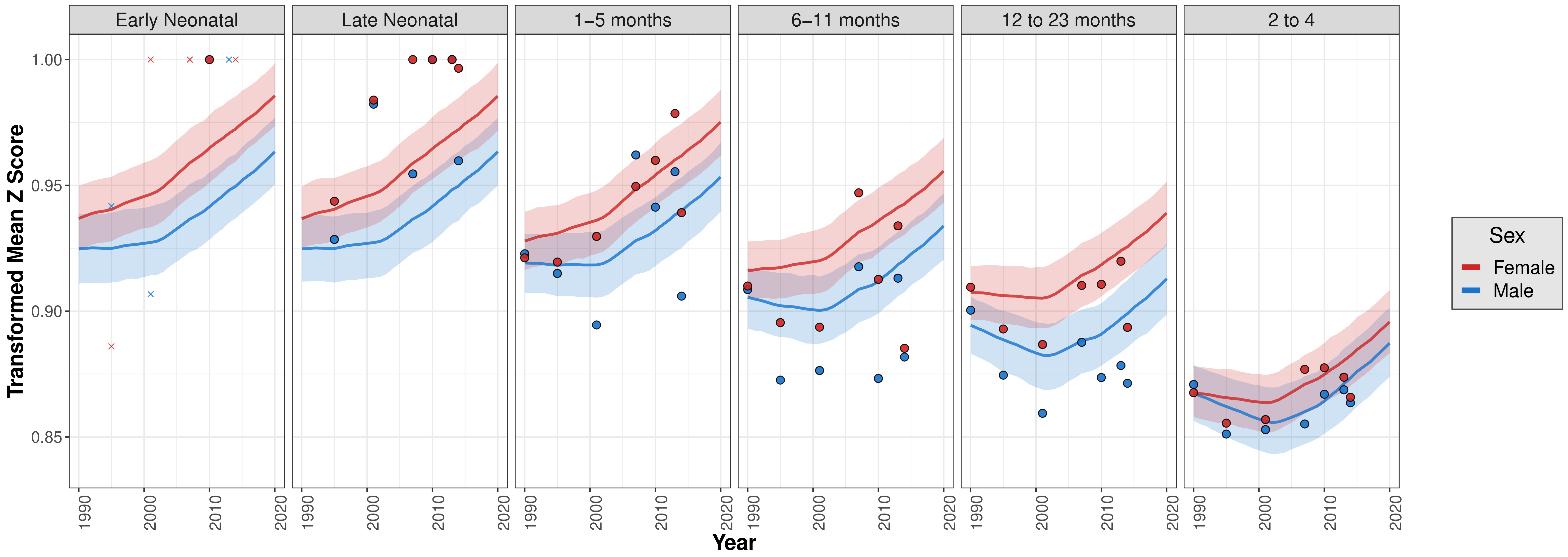

I

Year	Source
1985	WHO CGM Database
1986	WHO CGM Database
1990	WHO CGM Database
1995	MICS
1995	WHO CGM Database
2001	MICS
2001	WHO CGM Database
2007	DHS
2007	WHO CGM Database
2010	MICS
2010	WHO CGM Database
2013	DHS
2014	DHS
2014	WHO CGM Database

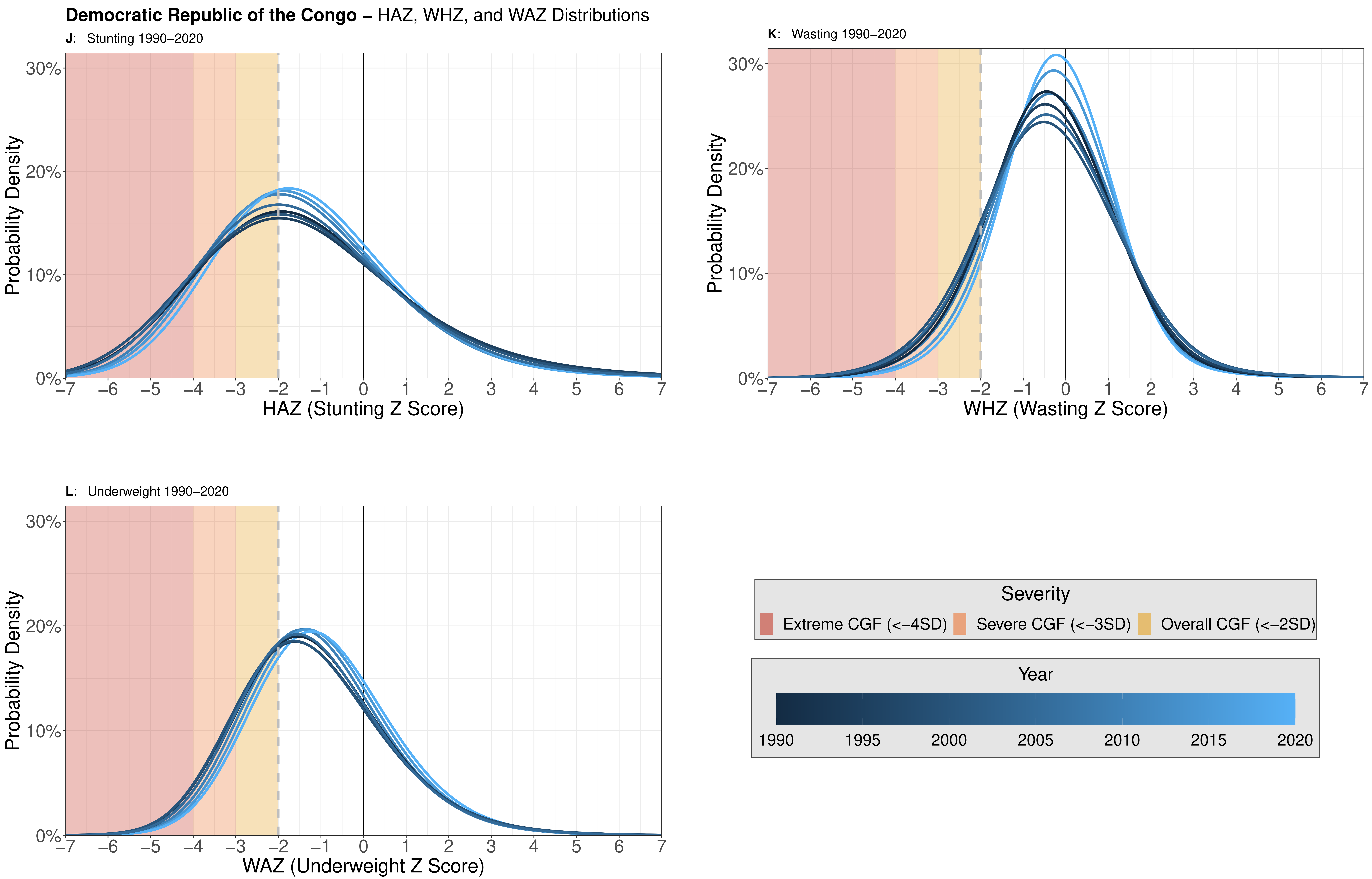
Democratic Republic of the Congo – HAZ, WHZ, and WAZ Distributions
J: Stunting 1990–2020
Probability Density
30%
20%
10%
0%
−7 −6 −5 −4 −3 −2 −1 0 1 2 3 4 5 6 7
HAZ (Stunting Z Score)
K: Wasting 1990–2020
Probability Density
30%
20%
10%
0%
−7 −6 −5 −4 −3 −2 −1 0 1 2 3 4 5 6 7
WHZ (Wasting Z Score)
L: Underweight 1990–2020
Probability Density
30%
20%
10%
0%
−7 −6 −5 −4 −3 −2 −1 0 1 2 3 4 5 6 7
WAZ (Underweight Z Score)
Severity
Extreme CGF (<−4SD)
Severe CGF (<−3SD)
Overall CGF (<−2SD)
Year
1990
1995
2000
2005
2010
2015
2020

Equatorial Guinea – Stunting (HAZ)

A: Overall and Severe Stunting Prevalence

B: Transformed Mean Stunting Z Scores

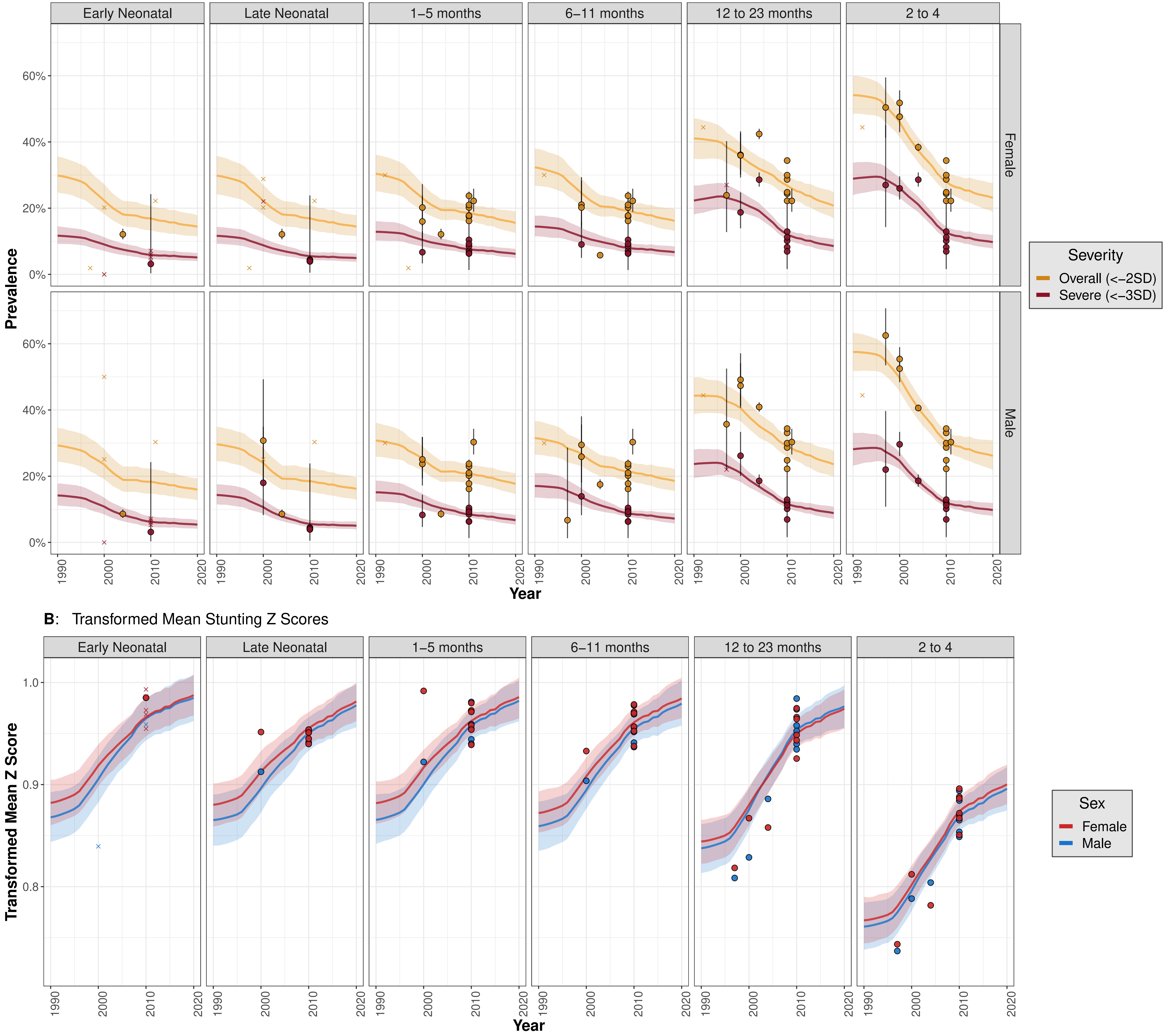

C

Year	Source
1992	WHO CGM Database
1997	WHO CGM Database
2000	MICS
2000	WHO CGM Database
2004	WHO CGM Database
2010	WHO CGM Database
2011	WHO CGM Database

Equatorial Guinea – Wasting (WHZ)

D: Overall and Severe Wasting Prevalence

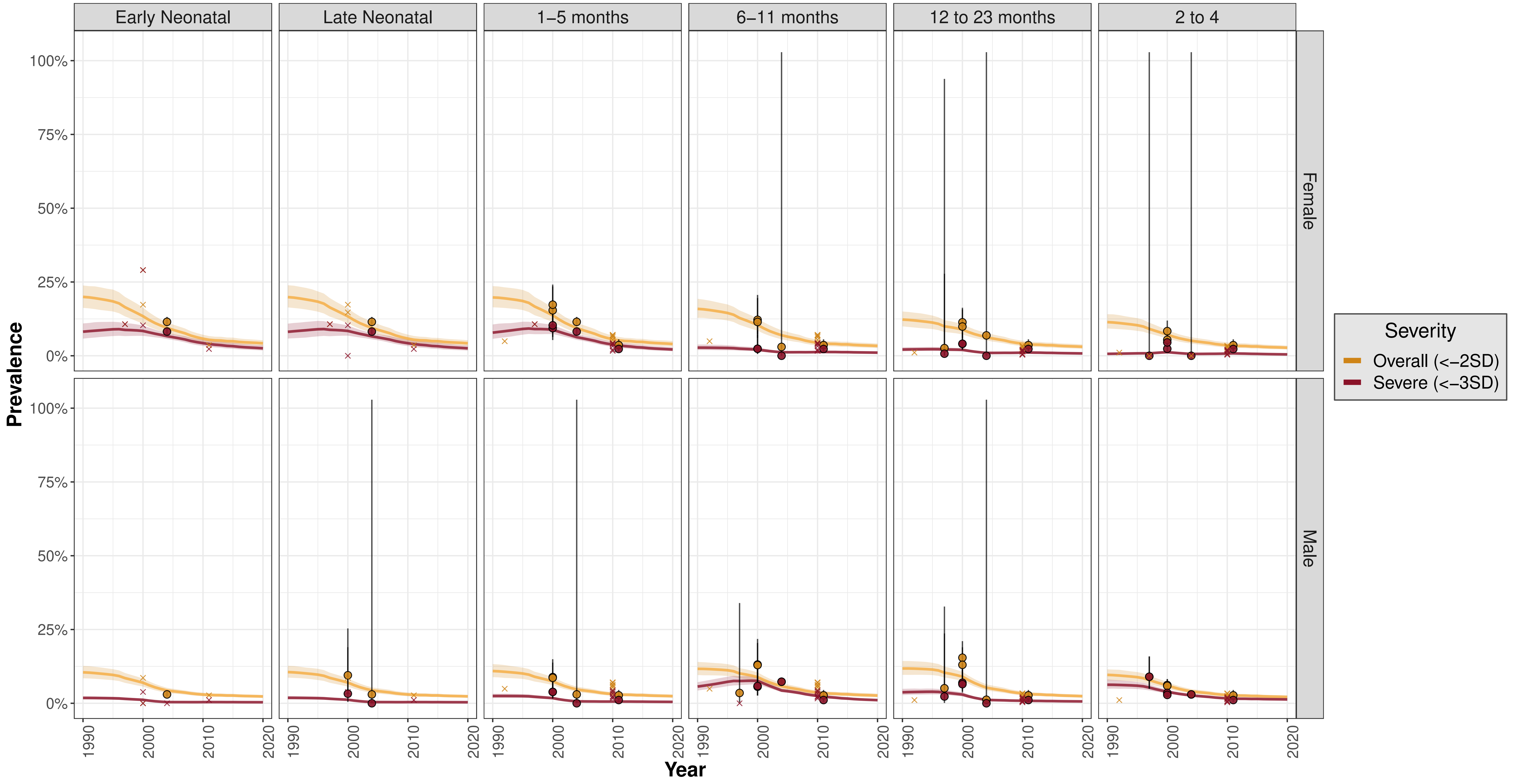

E: Transformed Mean Wasting Z Scores

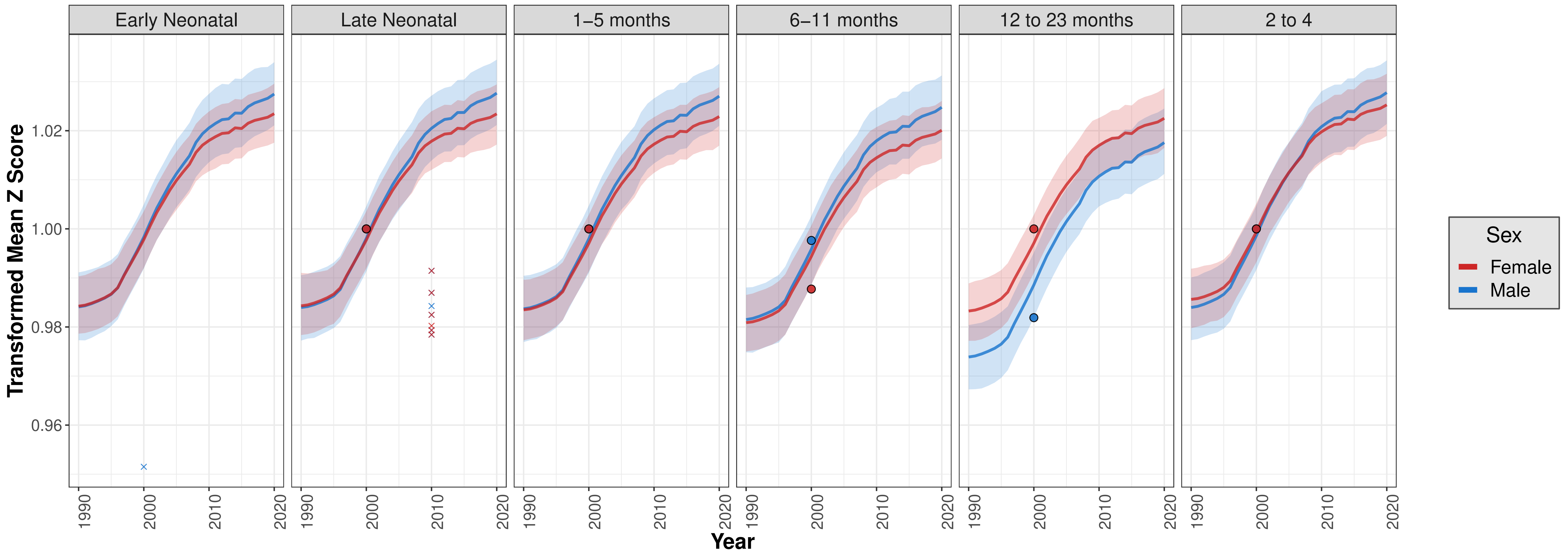

F

Year	Source
1992	WHO CGM Database
1997	WHO CGM Database
2000	MICS
2000	WHO CGM Database
2004	WHO CGM Database
2010	WHO CGM Database
2011	WHO CGM Database

Equatorial Guinea – Underweight (WAZ)

G: Overall and Severe Underweight Prevalence

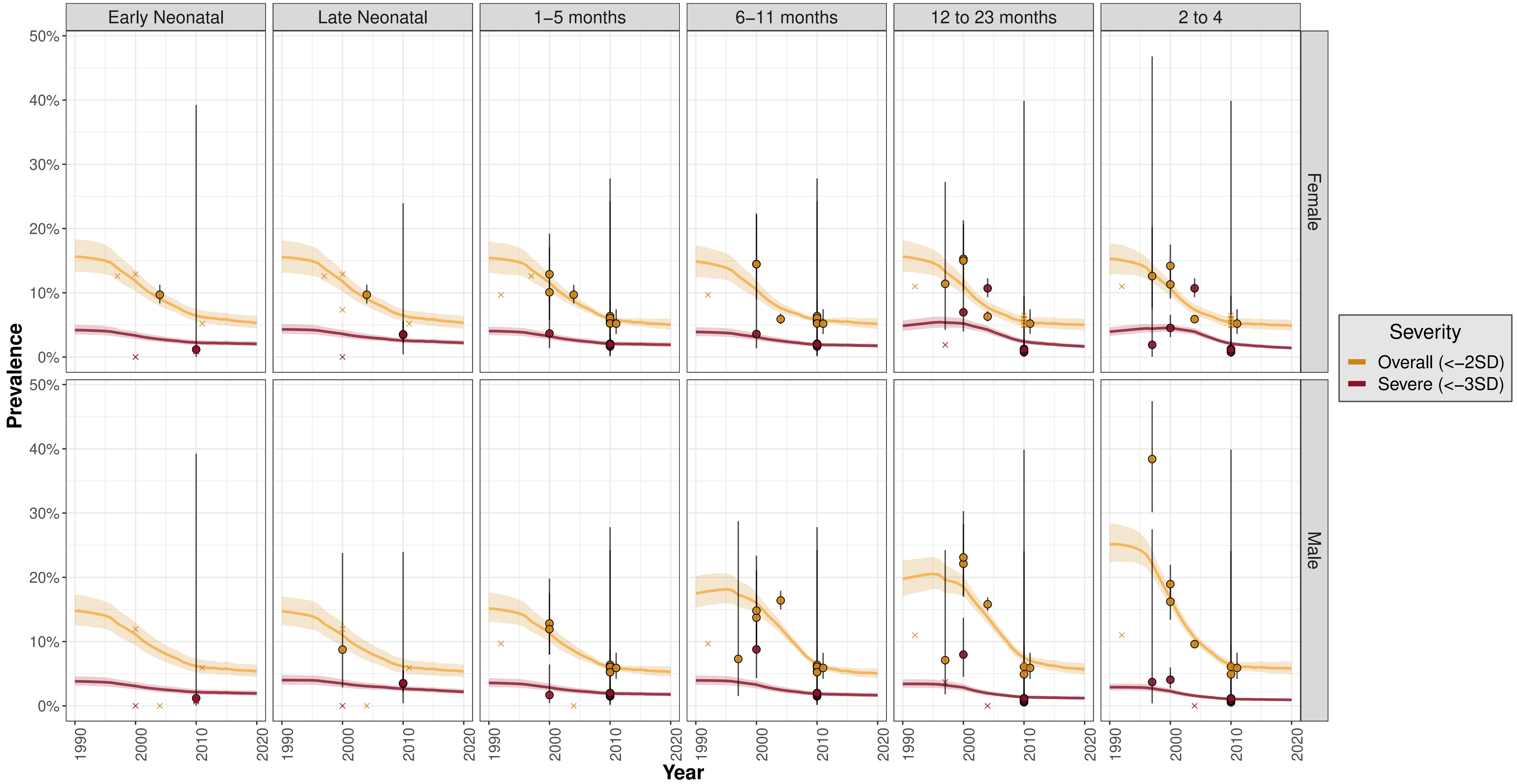

H: Transformed Mean Underweight Z Scores

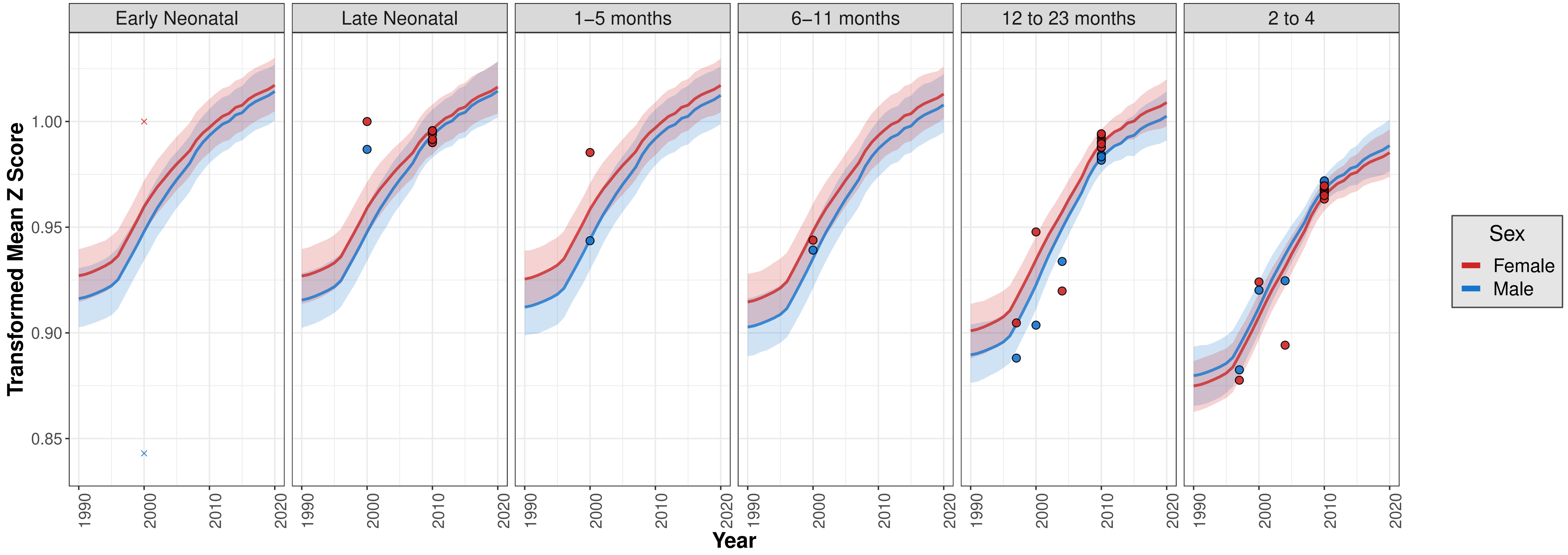

I

Year	Source
1992	WHO CGM Database
1997	WHO CGM Database
2000	MICS
2000	WHO CGM Database
2004	WHO CGM Database
2010	WHO CGM Database
2011	WHO CGM Database

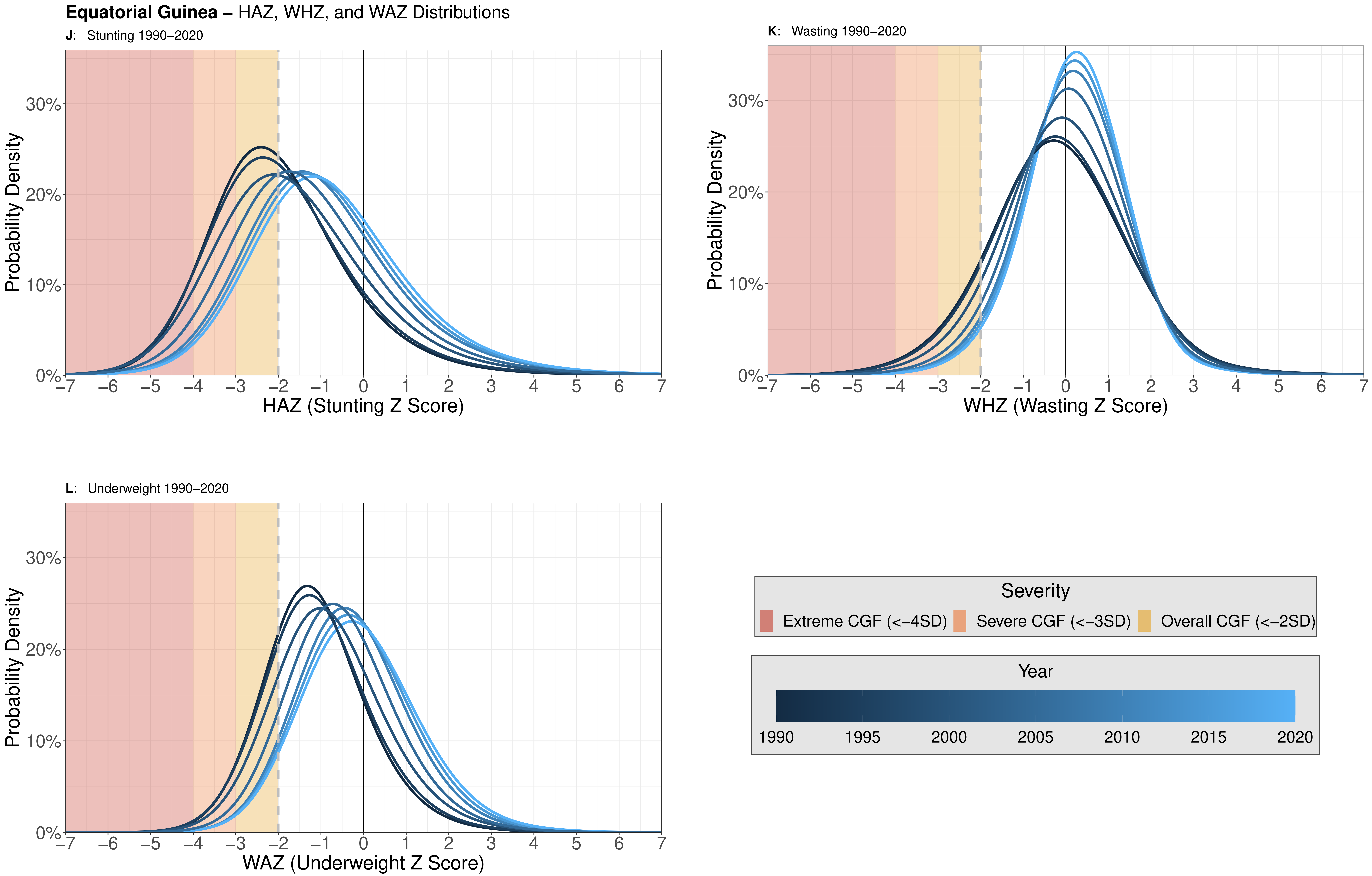
Equatorial Guinea – HAZ, WHZ, and WAZ Distributions
J: Stunting 1990–2020
Probability Density
30%
20%
10%
0%
−7
−6
−5
−4
−3
−2
−1
0
1
2
3
4
5
6
7
HAZ (Stunting Z Score)
K: Wasting 1990–2020
Probability Density
WHZ (Wasting Z Score)
L: Underweight 1990–2020
Probability Density
WAZ (Underweight Z Score)
Severity
Extreme CGF (<−4SD)
Severe CGF (<−3SD)
Overall CGF (<−2SD)
Year
1990
1995
2000
2005
2010
2015
2020

Gabon – Stunting (HAZ)

A: Overall and Severe Stunting Prevalence

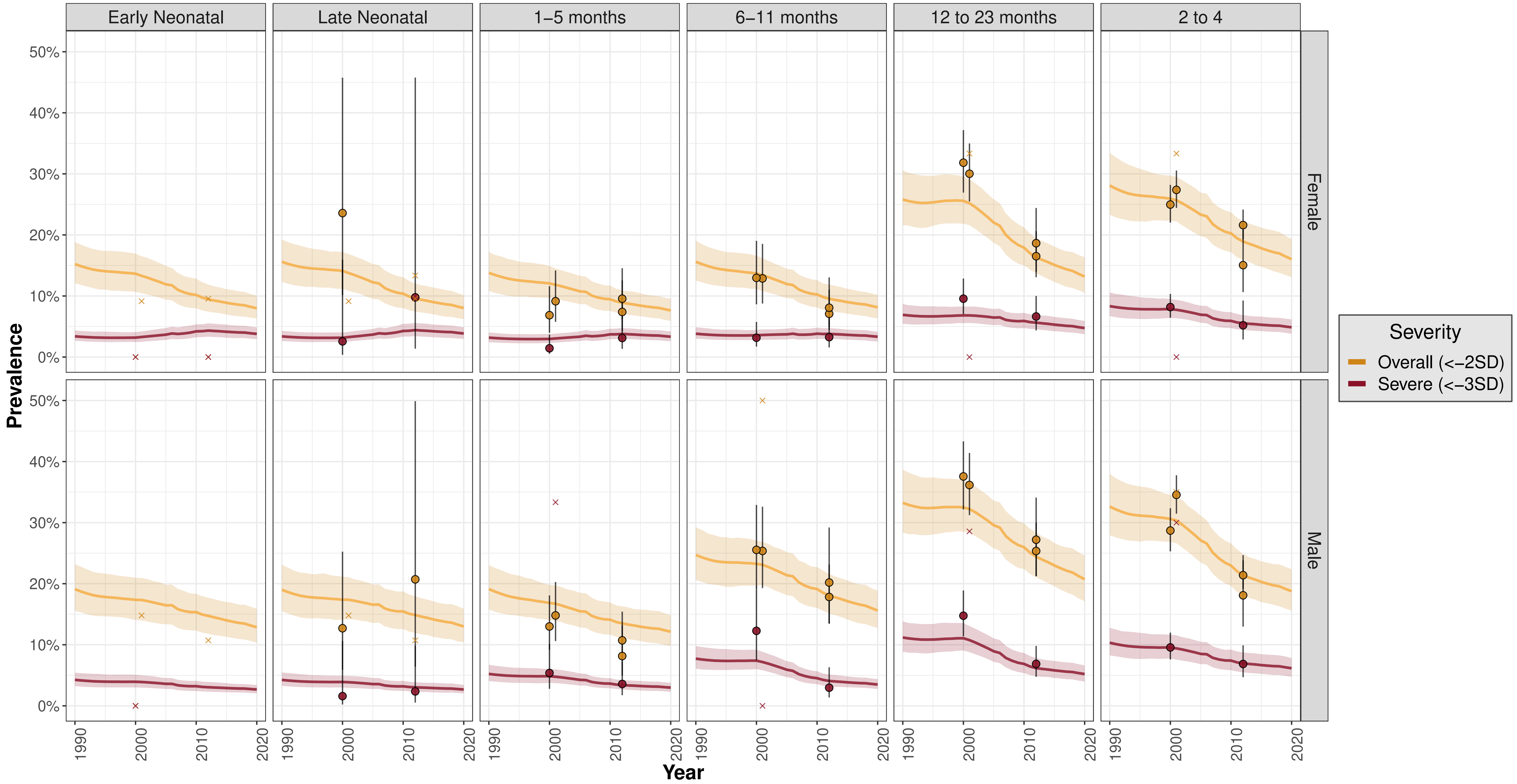

B: Transformed Mean Stunting Z Scores

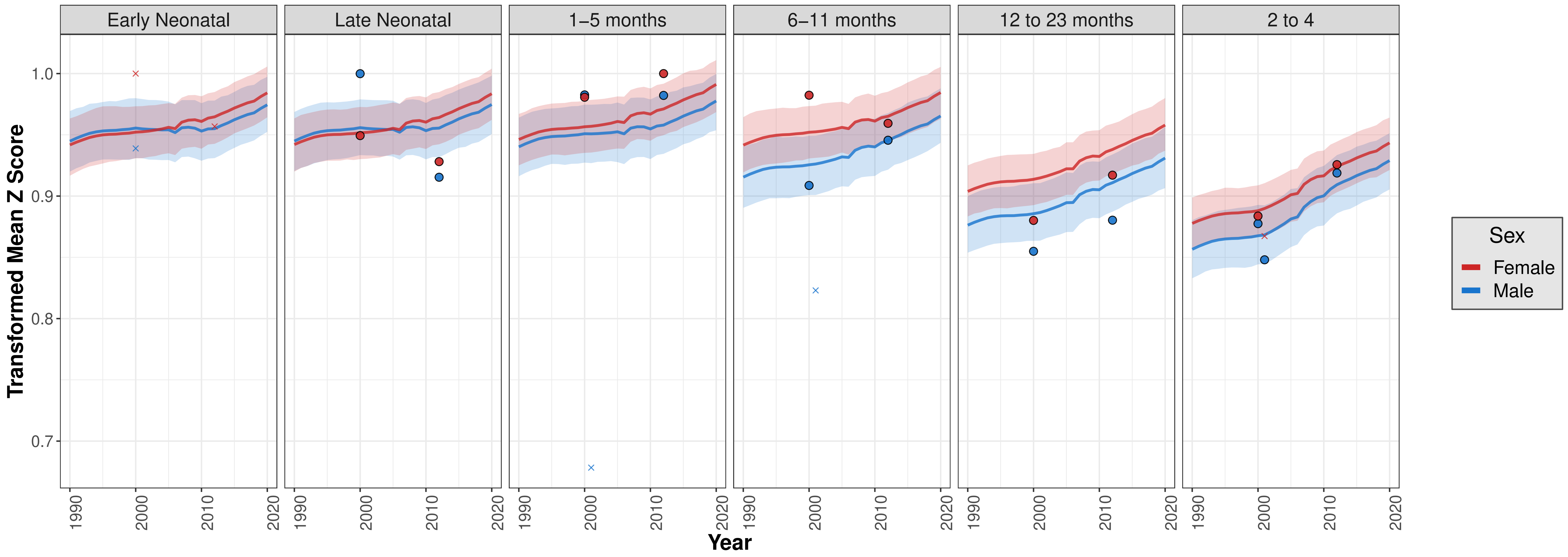

C

Year	Source
2000	DHS
2001	DHS
2001	WHO CGM Database
2012	DHS
2012	WHO CGM Database

Gabon – Wasting (WHZ)

D: Overall and Severe Wasting Prevalence

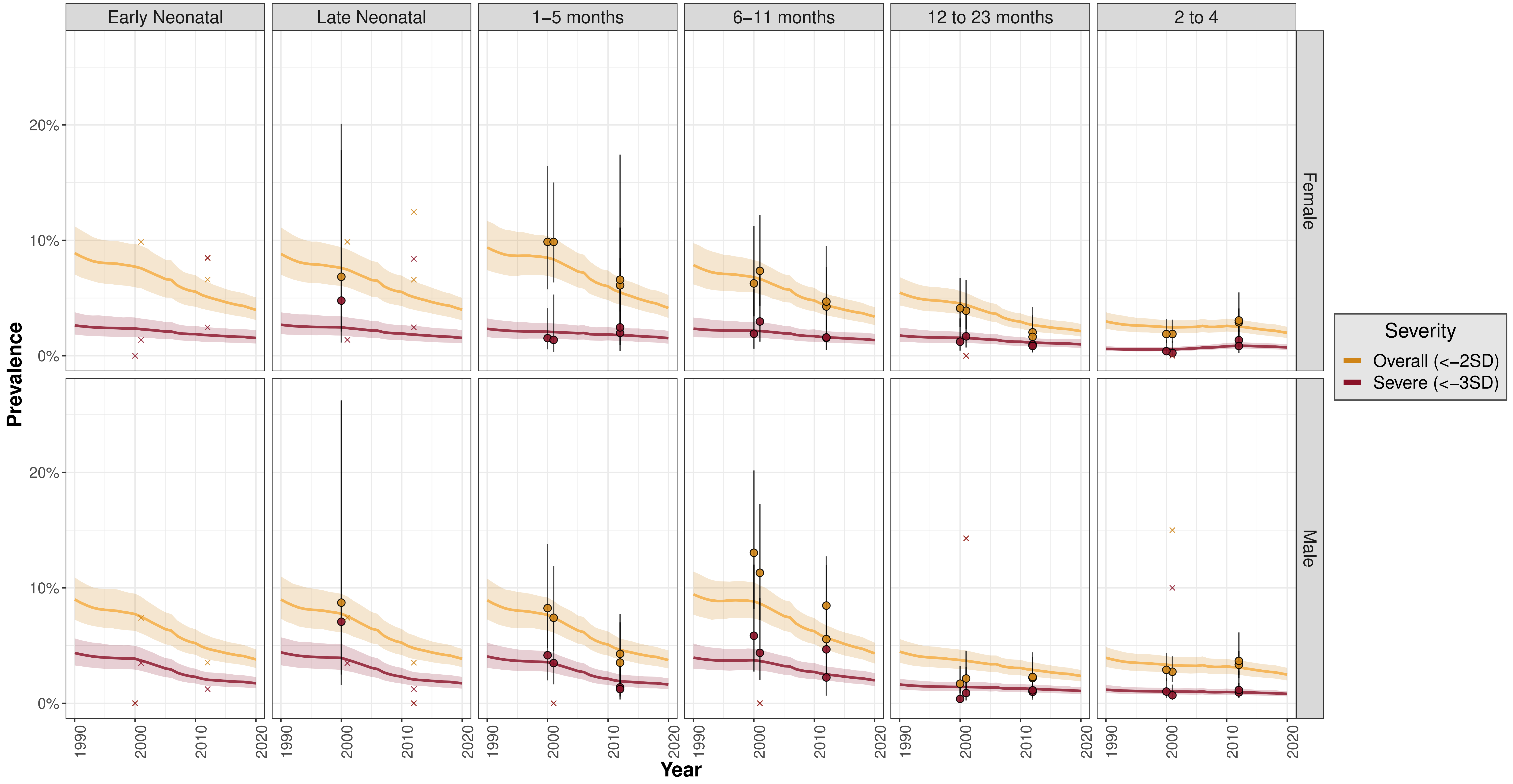

E: Transformed Mean Wasting Z Scores

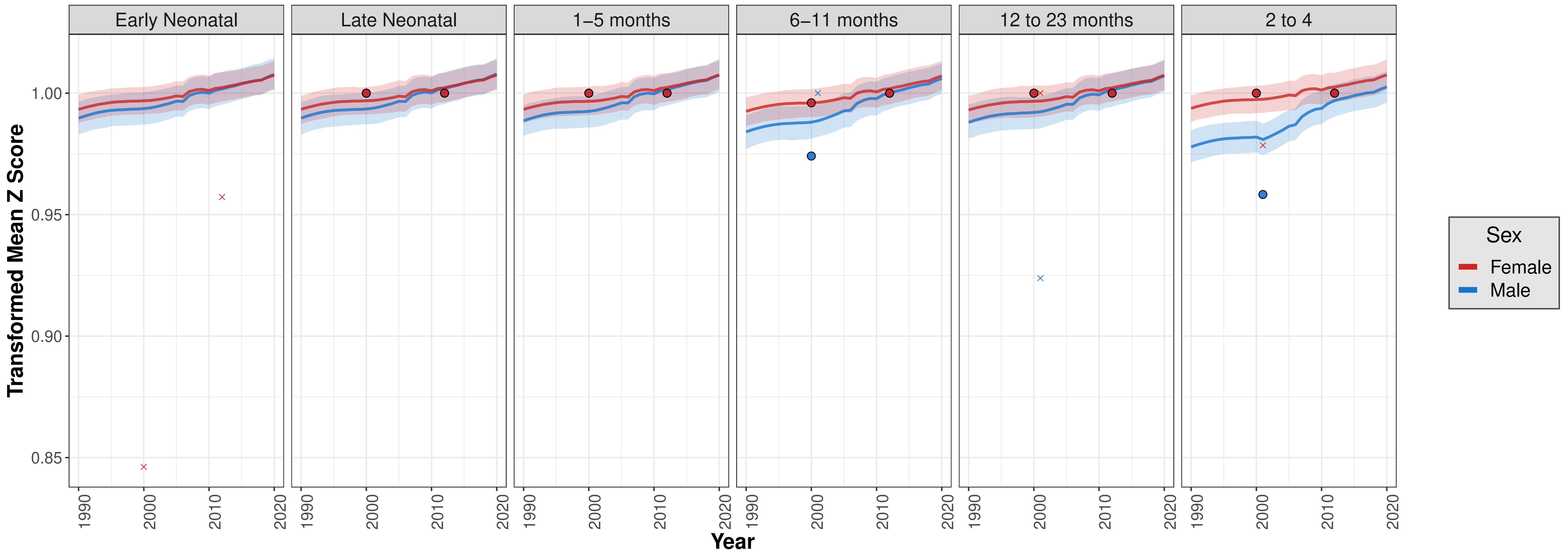

F

Year	Source
2000	DHS
2001	DHS
2001	WHO CGM Database
2012	DHS
2012	WHO CGM Database

Gabon – Underweight (WAZ)

G: Overall and Severe Underweight Prevalence

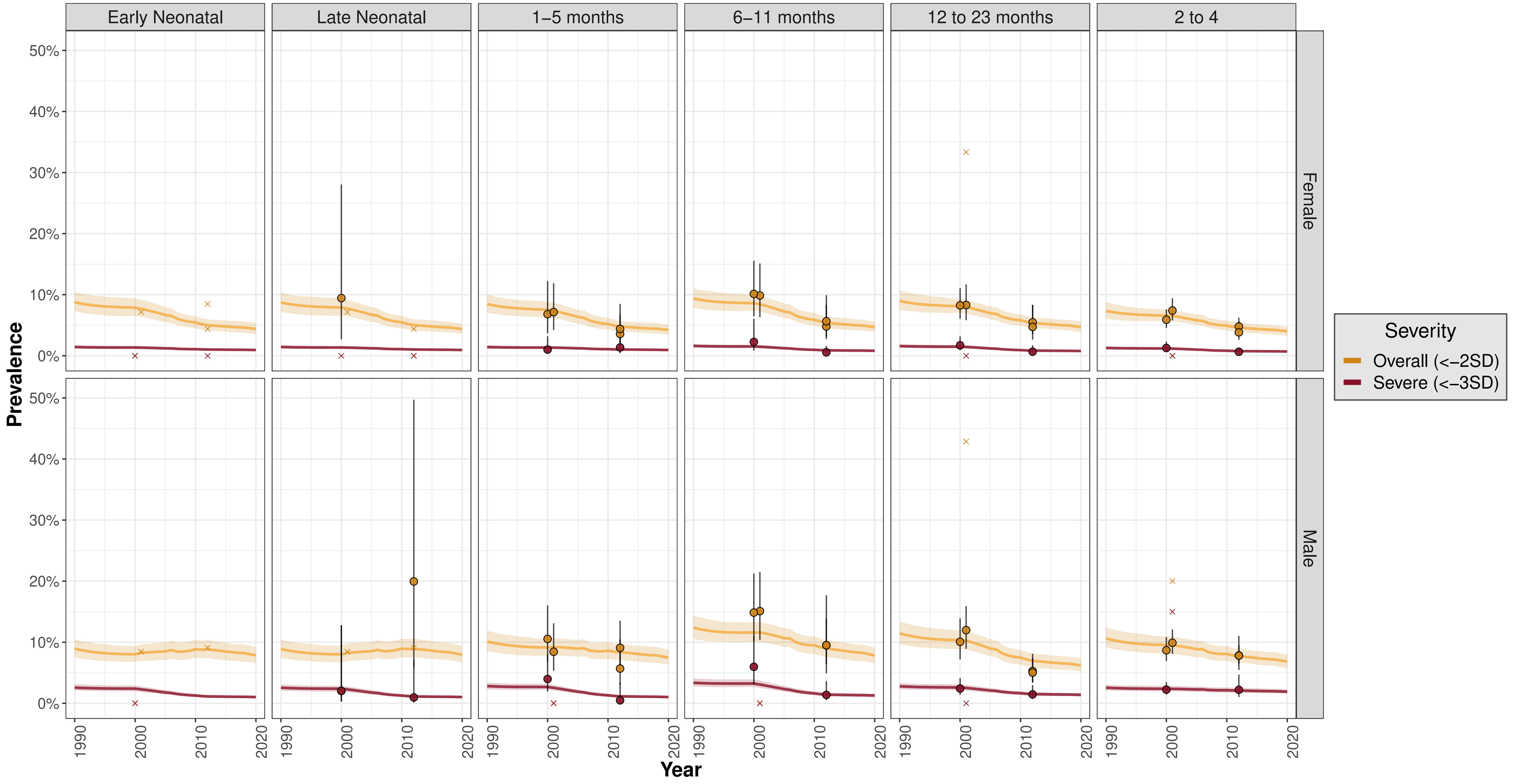

I

Year	Source
2000	DHS
2001	DHS
2001	WHO CGM Database
2012	DHS
2012	WHO CGM Database

H: Transformed Mean Underweight Z Scores

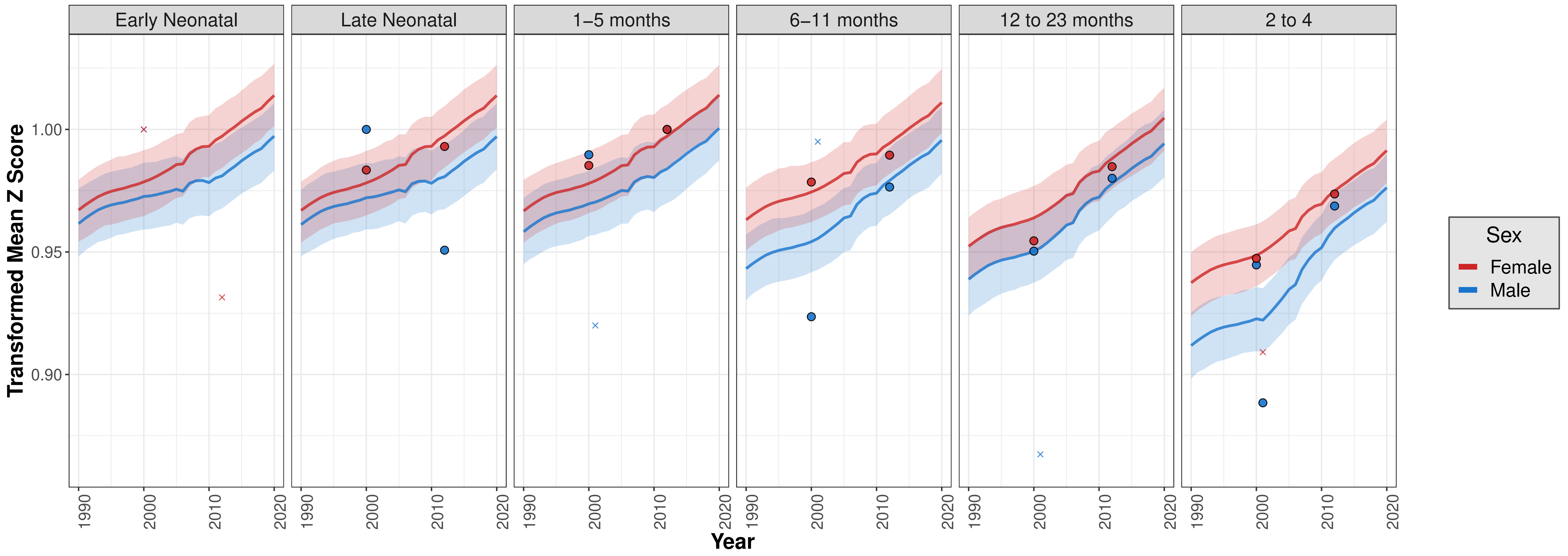

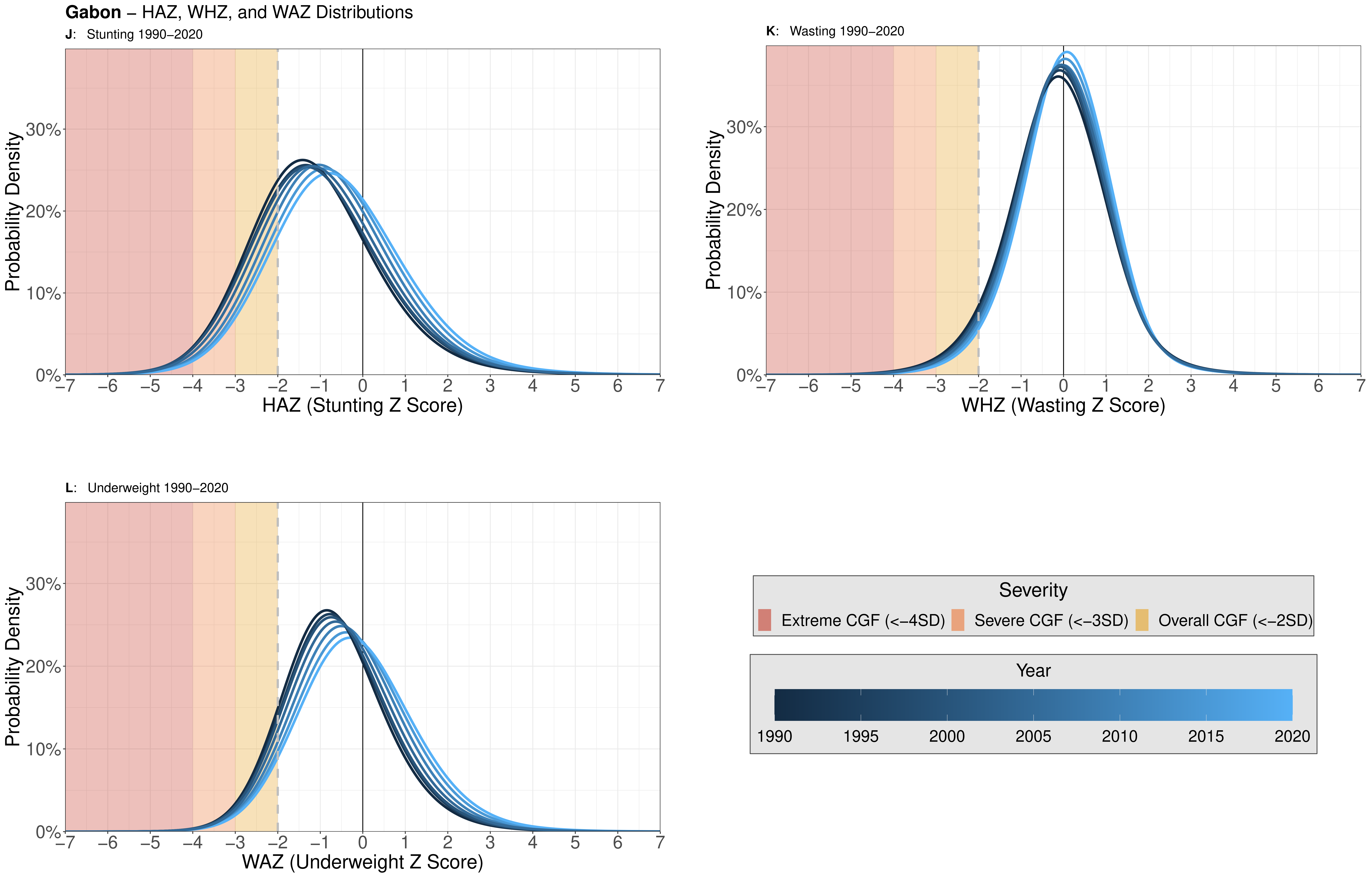
Gabon – HAZ, WHZ, and WAZ Distributions
J: Stunting 1990–2020
Probability Density
0%
10%
20%
30%
−7 −6 −5 −4 −3 −2 −1 0 1 2 3 4 5 6 7
HAZ (Stunting Z Score)
K: Wasting 1990–2020
Probability Density
0%
10%
20%
30%
−7 −6 −5 −4 −3 −2 −1 0 1 2 3 4 5 6 7
WHZ (Wasting Z Score)
L: Underweight 1990–2020
Probability Density
0%
10%
20%
30%
−7 −6 −5 −4 −3 −2 −1 0 1 2 3 4 5 6 7
WAZ (Underweight Z Score)
Severity
Extreme CGF (<−4SD)
Severe CGF (<−3SD)
Overall CGF (<−2SD)
Year
1990
1995
2000
2005
2010
2015
2020

Burundi – Stunting (HAZ)

A: Overall and Severe Stunting Prevalence

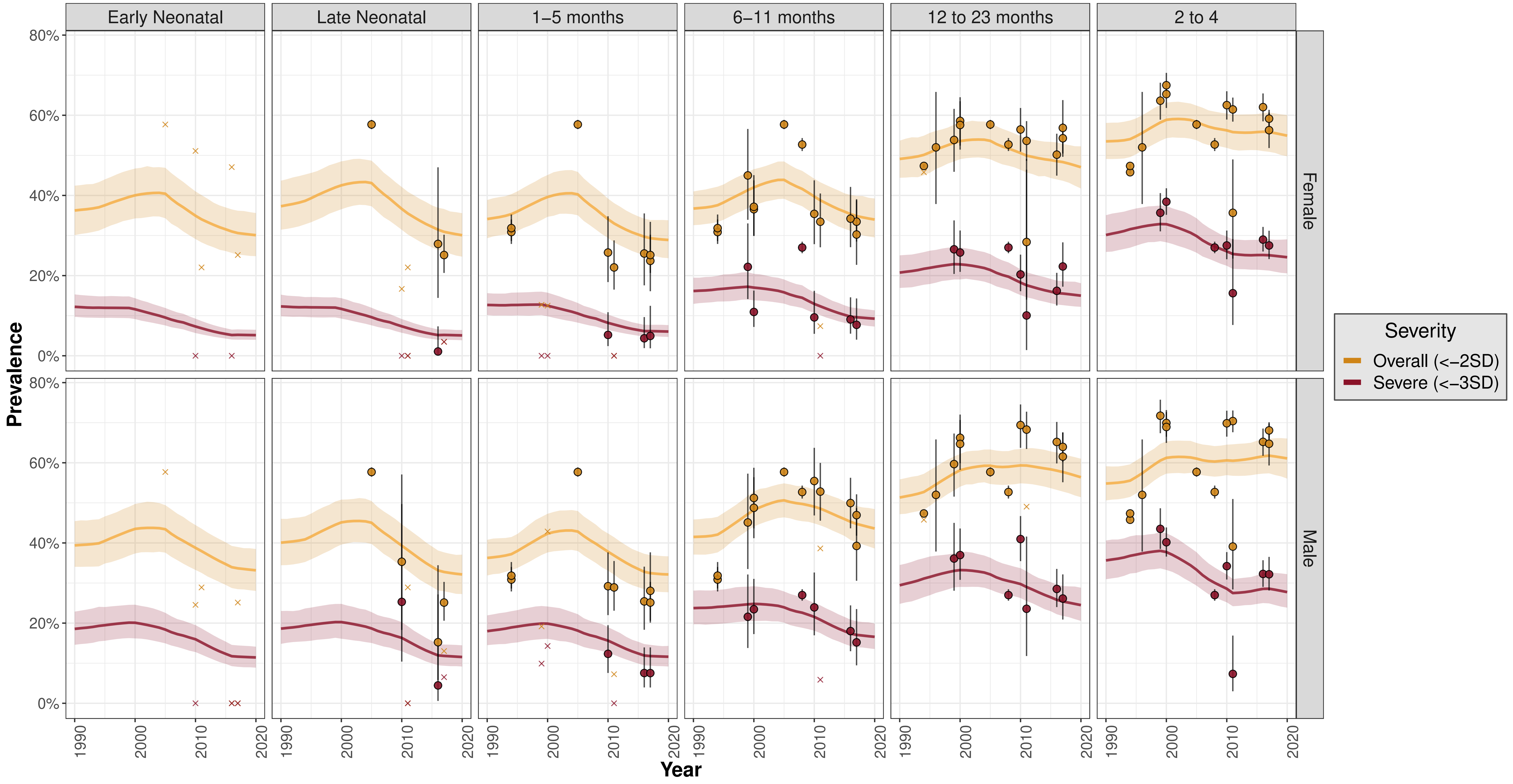

B: Transformed Mean Stunting Z Scores

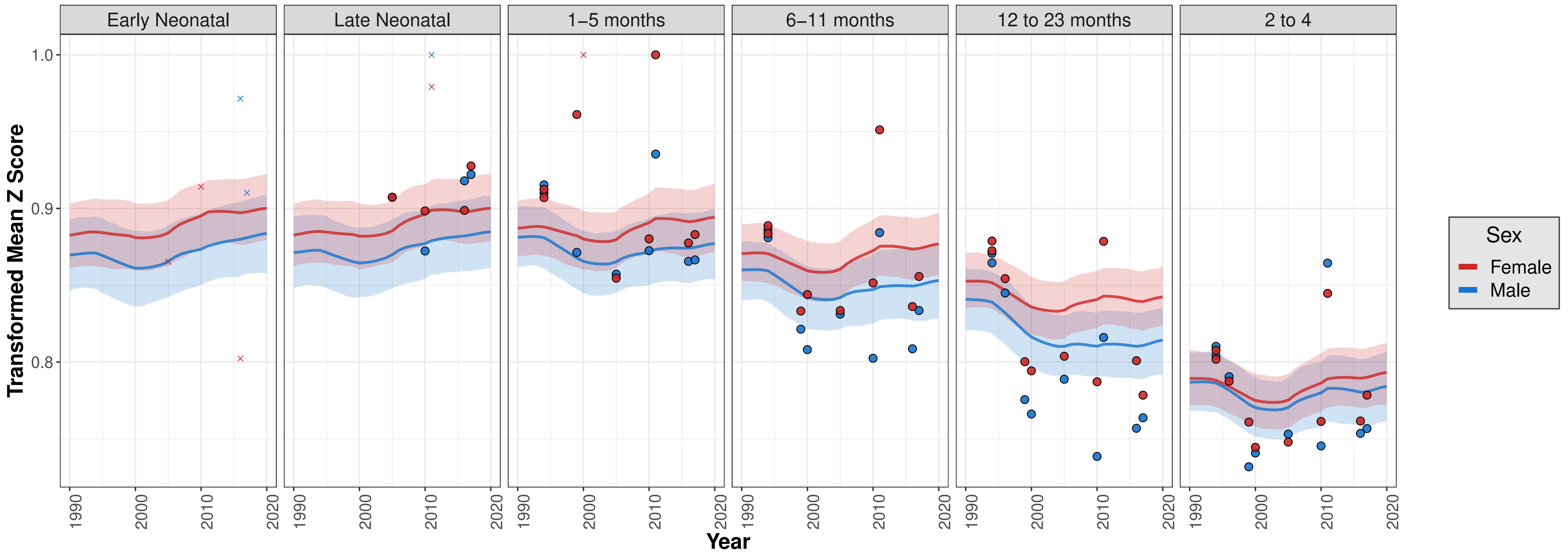

C

Year	Source
1985	WHO CGM Database
1986	WHO CGM Database
1987	DHS
1987	WHO CGM Database
1994	WHO CGM Database
1996	WHO CGM Database
1999	Priority Survey
2000	MICS
2000	WHO CGM Database
2005	WHO CGM Database
2008	Comprehensive Food Security and Vulnerability Analysis (CFSVA)
2010	DHS
2011	DHS
2011	WHO CGM Database
2016	DHS
2017	WHO CGM Database
2017	DHS

Burundi – Wasting (WHZ)

D: Overall and Severe Wasting Prevalence

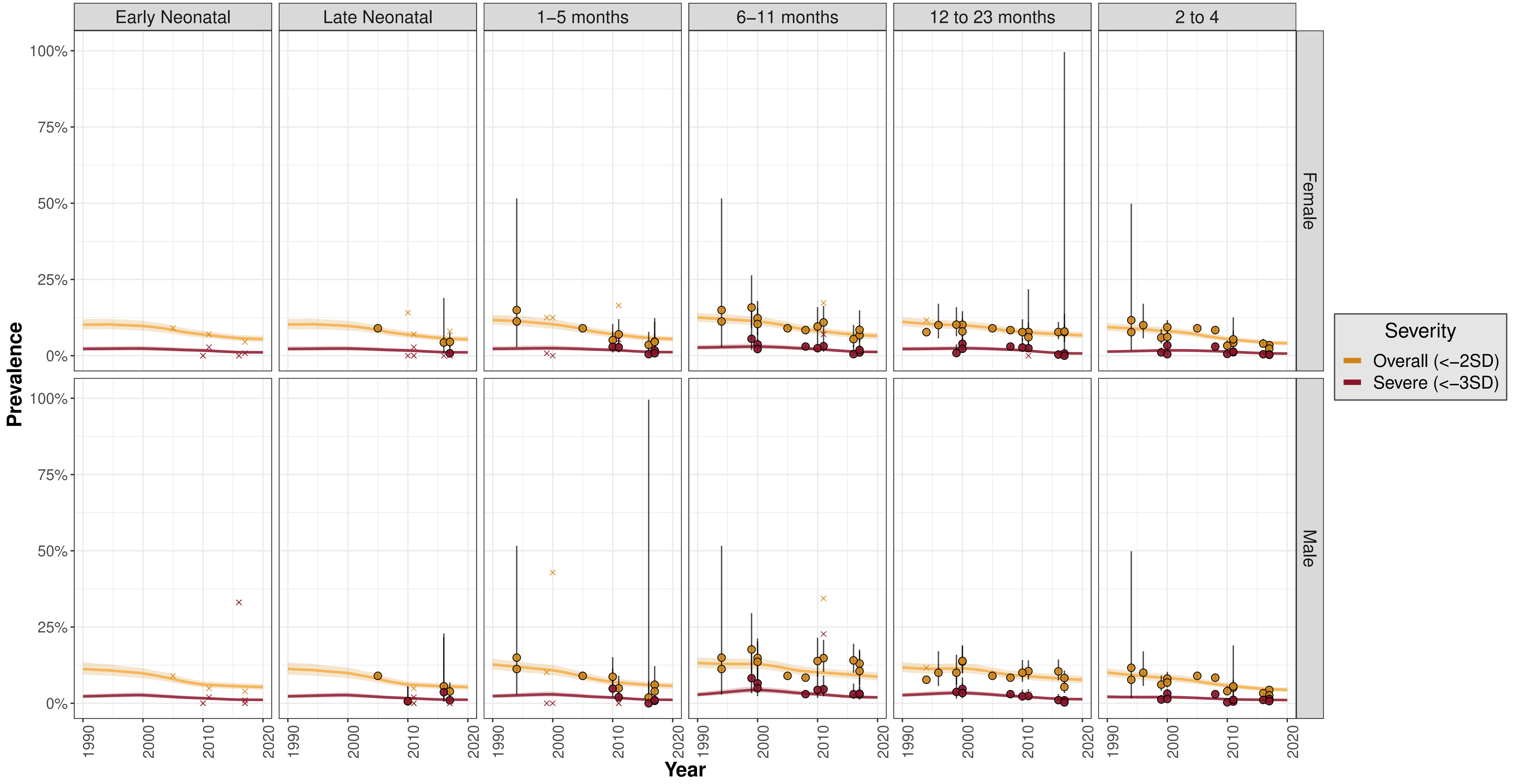

E: Transformed Mean Wasting Z Scores

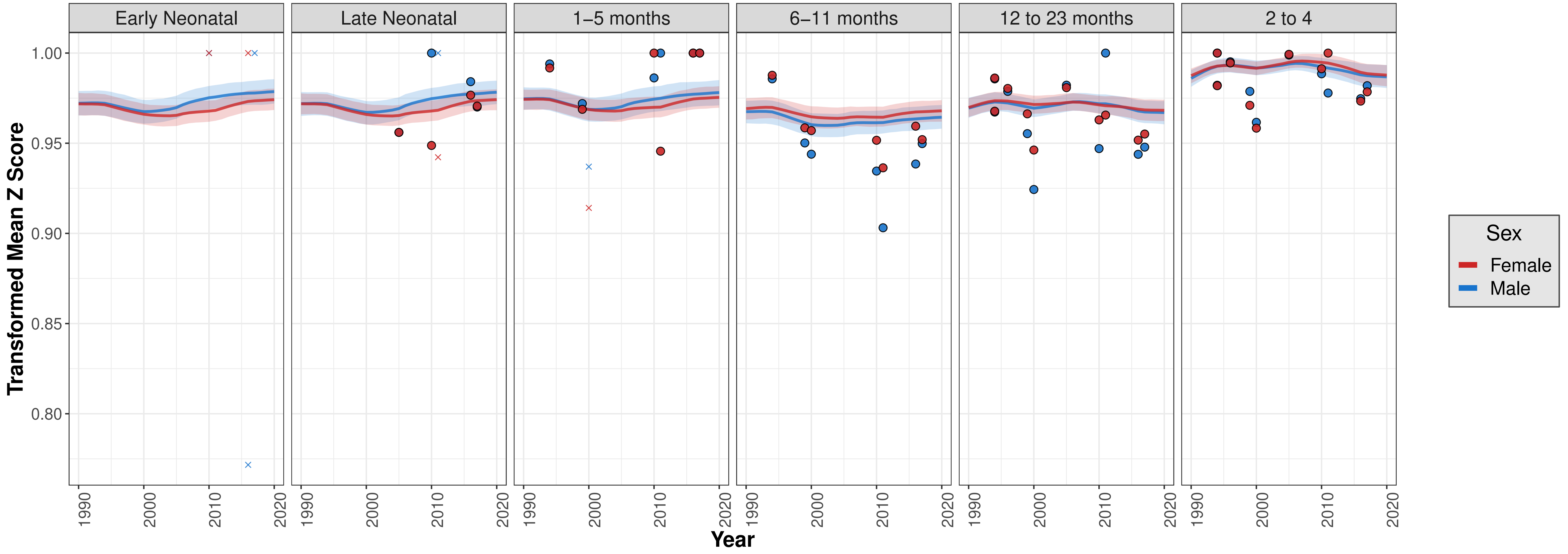

F

Year	Source
1985	WHO CGM Database
1986	WHO CGM Database
1987	DHS
1987	WHO CGM Database
1994	WHO CGM Database
1996	WHO CGM Database
1999	Priority Survey
2000	MICS
2000	WHO CGM Database
2005	WHO CGM Database
2008	Comprehensive Food Security and Vulnerability Analysis (CFSVA)
2010	DHS
2011	DHS
2011	WHO CGM Database
2016	DHS
2017	WHO CGM Database
2017	DHS

Burundi – Underweight (WAZ)

G: Overall and Severe Underweight Prevalence

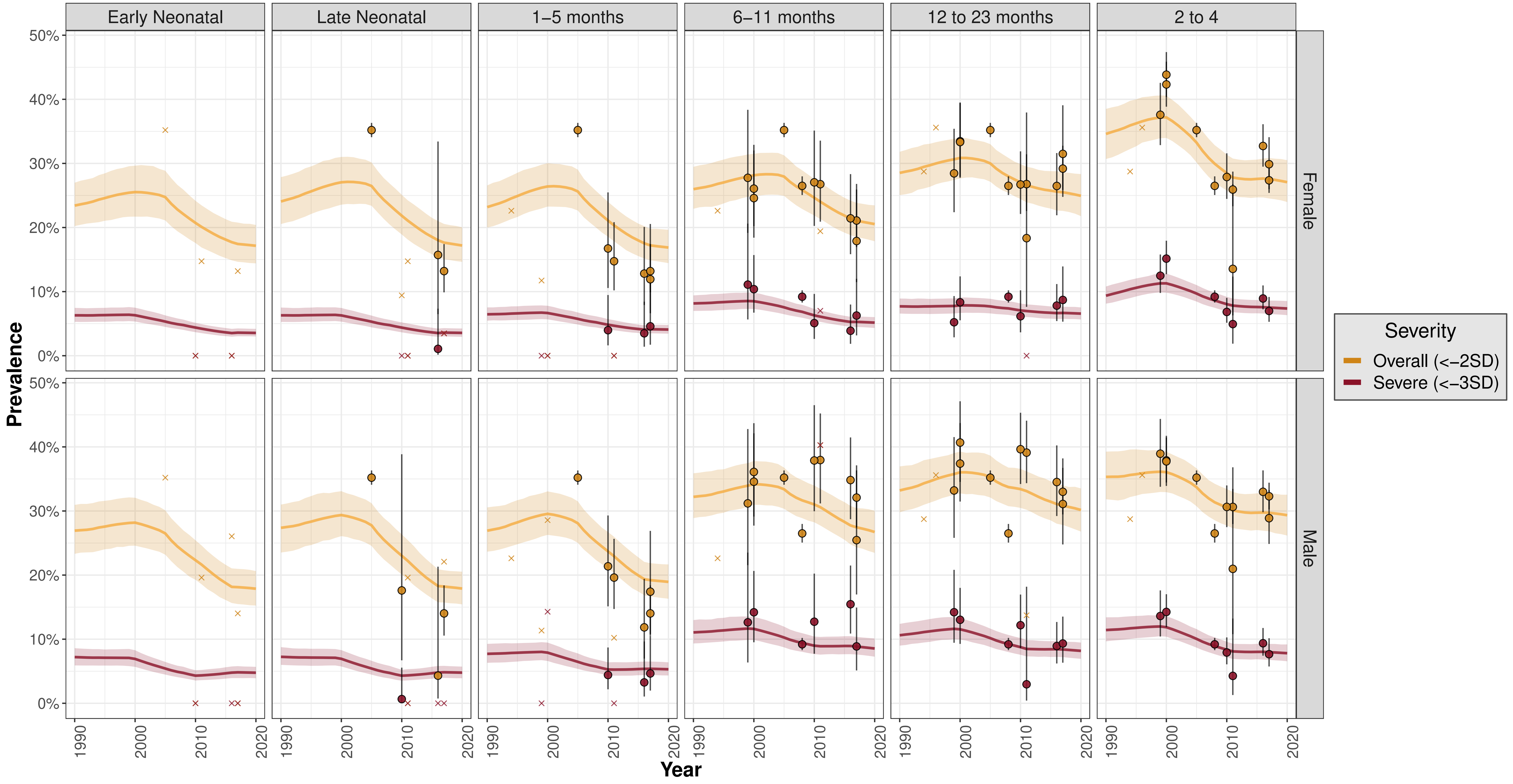

H: Transformed Mean Underweight Z Scores

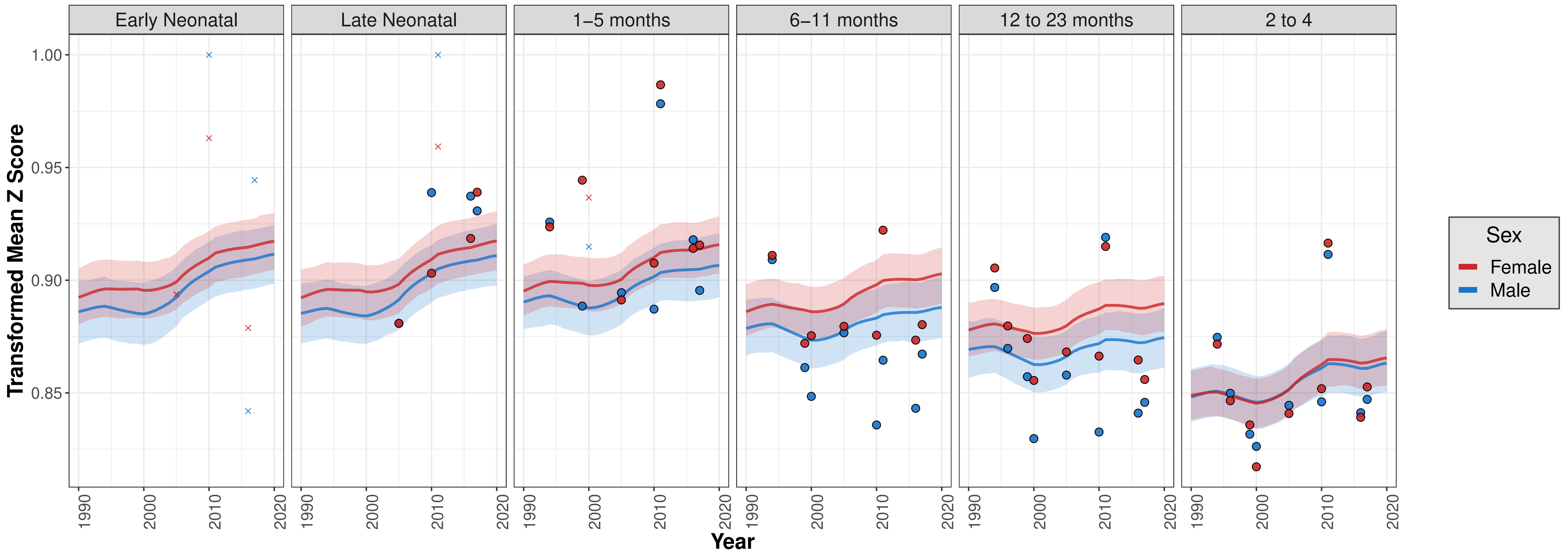

I

Year	Source
1985	WHO CGM Database
1986	WHO CGM Database
1987	DHS
1987	WHO CGM Database
1994	WHO CGM Database
1996	WHO CGM Database
1999	Priority Survey
2000	MICS
2000	WHO CGM Database
2005	WHO CGM Database
2008	Comprehensive Food Security and Vulnerability Analysis (CFSVA)
2010	DHS
2011	DHS
2011	WHO CGM Database
2016	DHS
2017	WHO CGM Database
2017	DHS

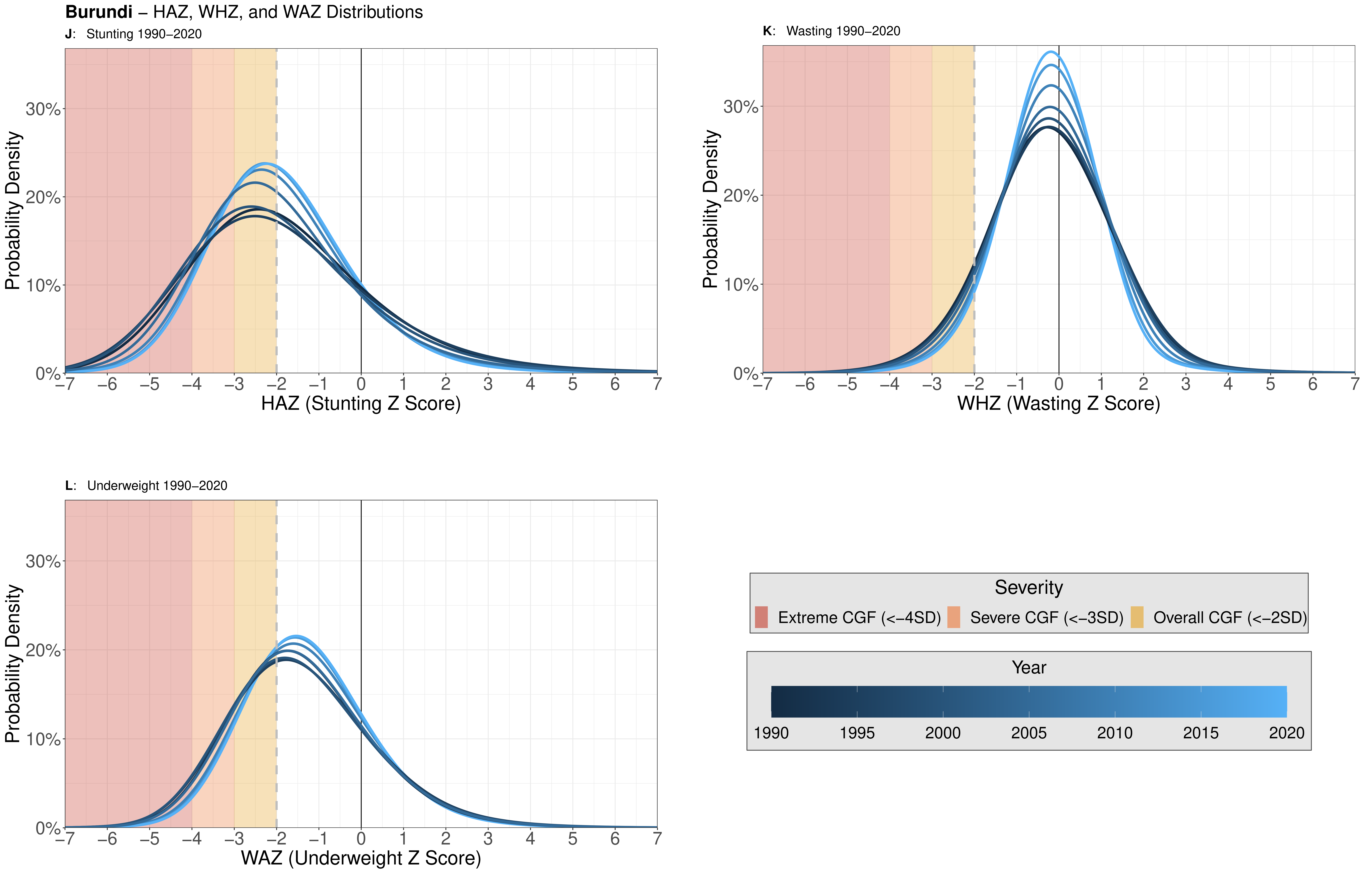
Burundi – HAZ, WHZ, and WAZ Distributions
J: Stunting 1990–2020
Probability Density
30%
20%
10%
0%
−7 −6 −5 −4 −3 −2 −1 0 1 2 3 4 5 6 7
HAZ (Stunting Z Score)
K: Wasting 1990–2020
Probability Density
30%
20%
10%
0%
−7 −6 −5 −4 −3 −2 −1 0 1 2 3 4 5 6 7
WHZ (Wasting Z Score)
L: Underweight 1990–2020
Probability Density
30%
20%
10%
0%
−7 −6 −5 −4 −3 −2 −1 0 1 2 3 4 5 6 7
WAZ (Underweight Z Score)
Severity
Extreme CGF (<−4SD)
Severe CGF (<−3SD)
Overall CGF (<−2SD)
Year
1990 1995 2000 2005 2010 2015 2020

Comoros – Stunting (HAZ)

A: Overall and Severe Stunting Prevalence

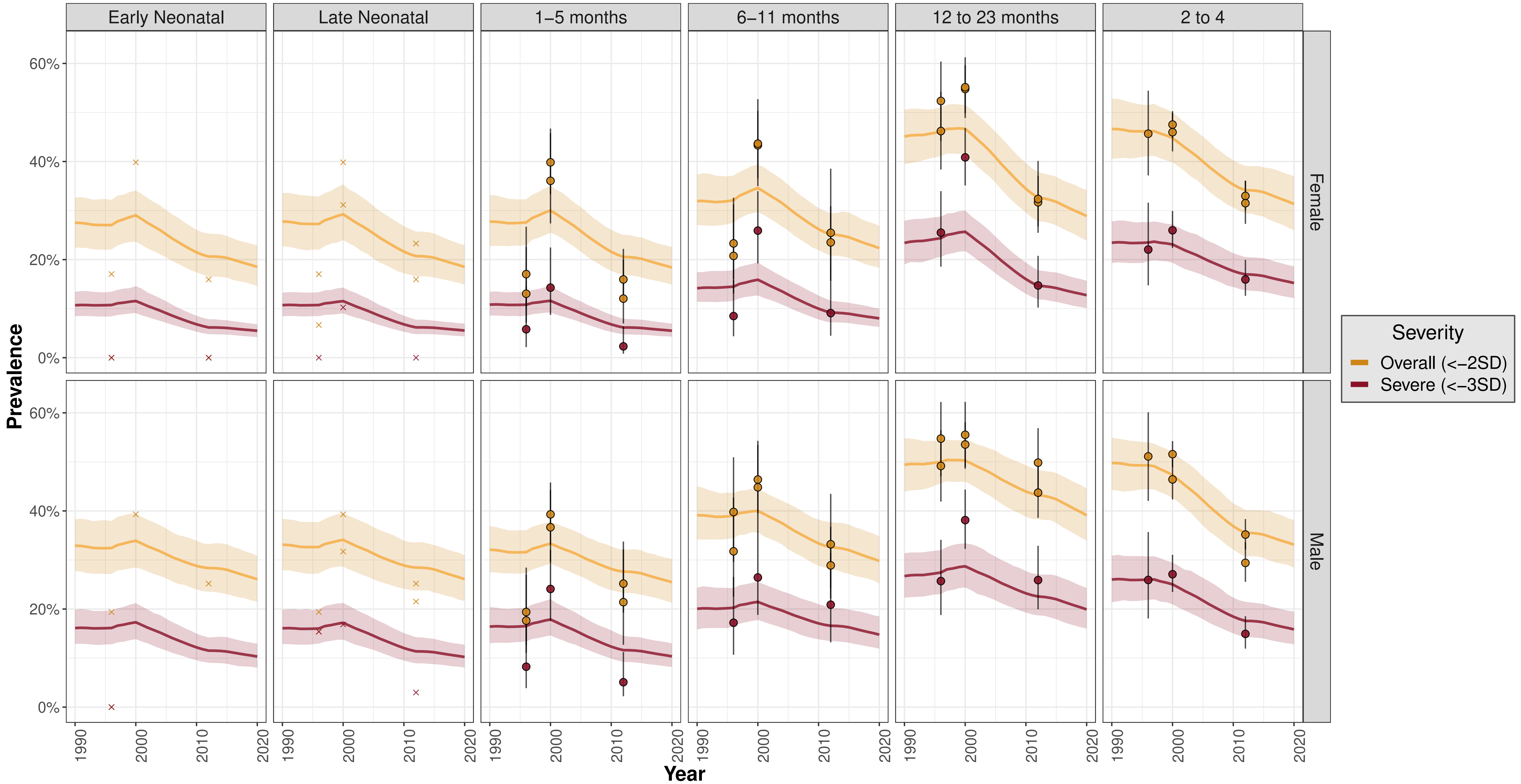

B: Transformed Mean Stunting Z Scores

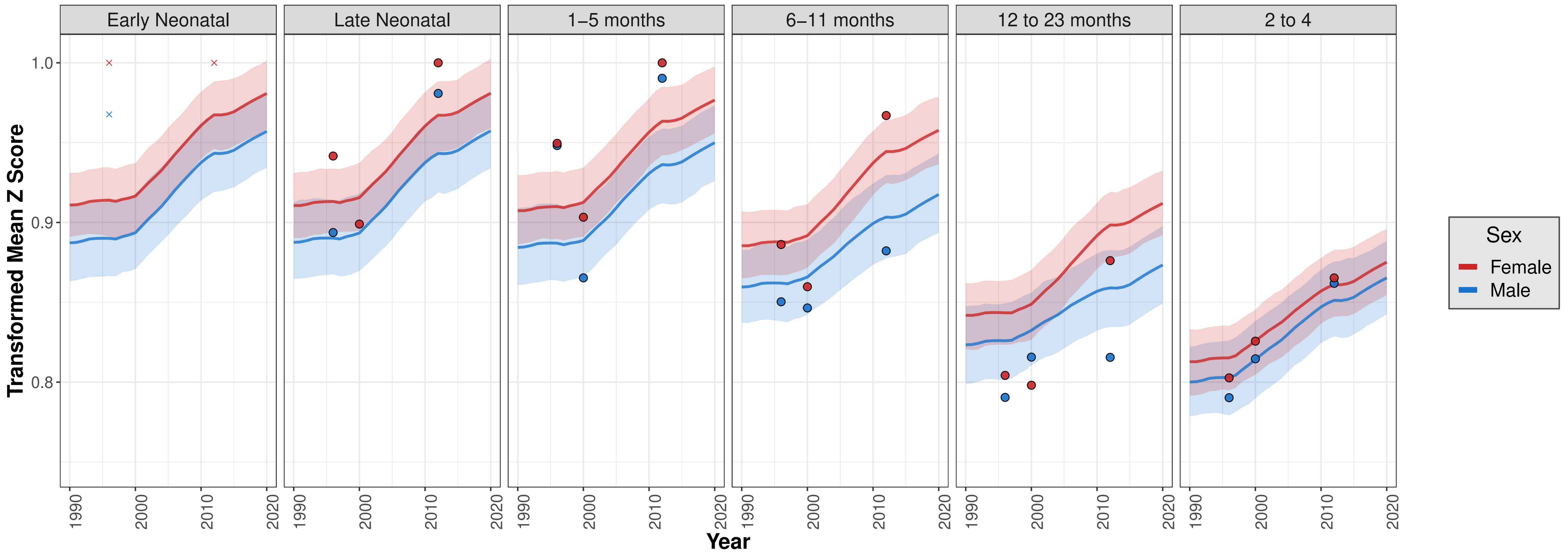

C

Year	Source
1996	DHS
1996	WHO CGM Database
2000	MICS
2000	WHO CGM Database
2012	DHS
2012	WHO CGM Database

Comoros – Wasting (WHZ)

D: Overall and Severe Wasting Prevalence

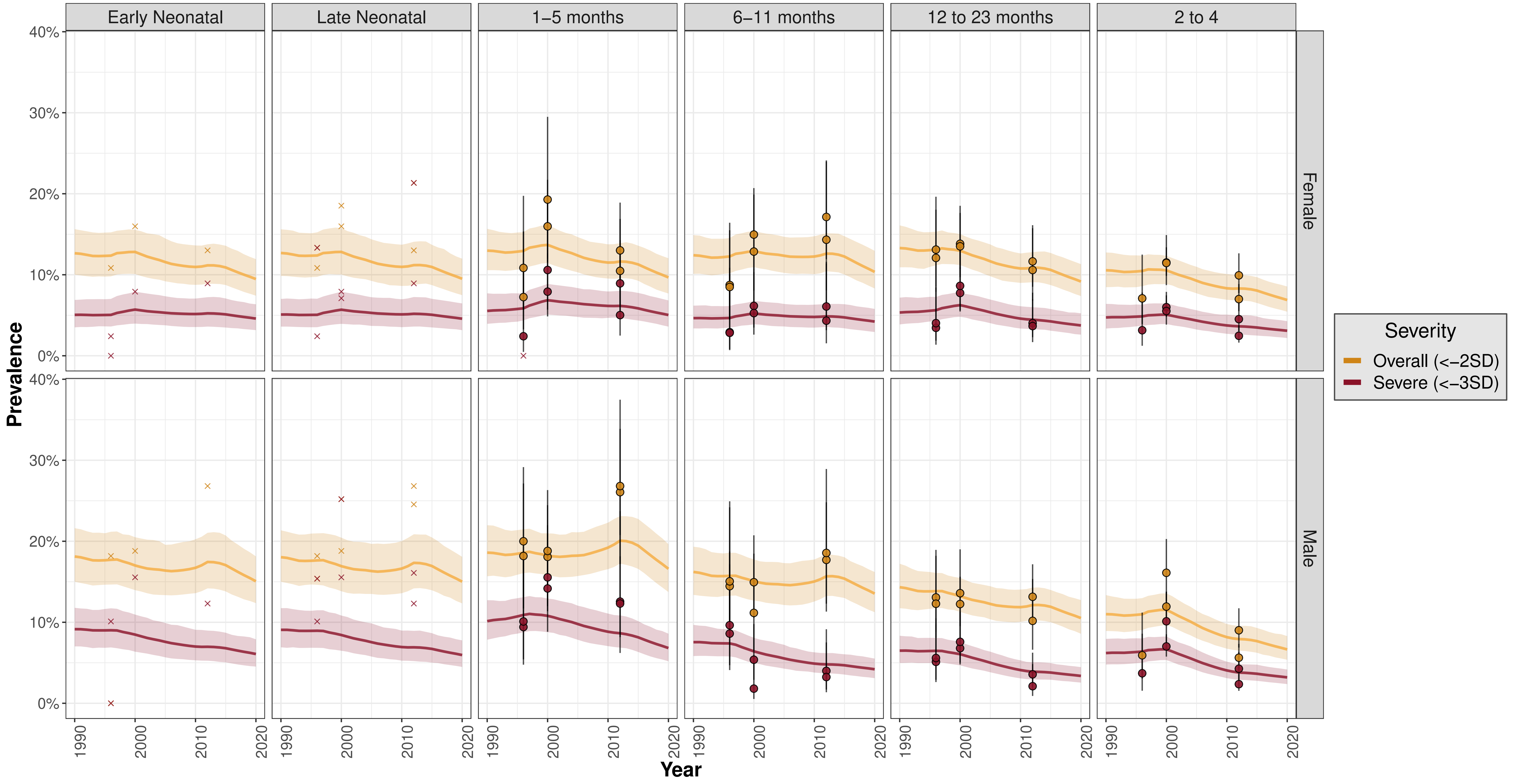

E: Transformed Mean Wasting Z Scores

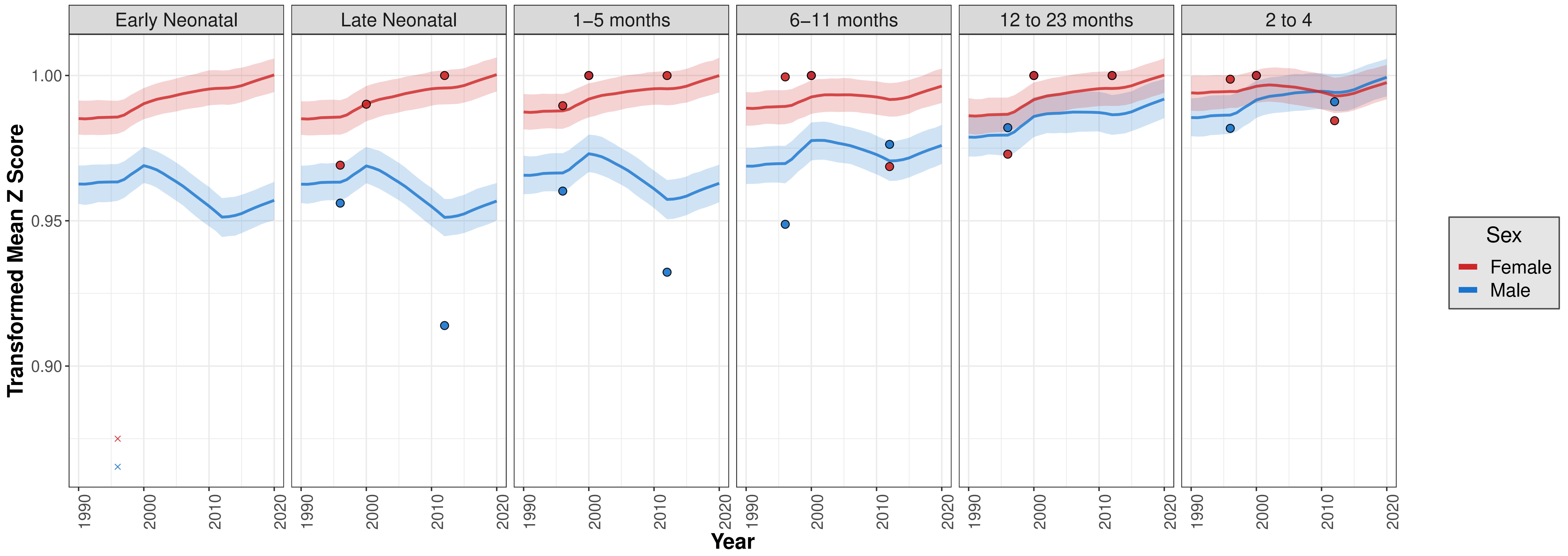

F

Year	Source
1996	DHS
1996	WHO CGM Database
2000	MICS
2000	WHO CGM Database
2012	DHS
2012	WHO CGM Database

Comoros – Underweight (WAZ)

G: Overall and Severe Underweight Prevalence

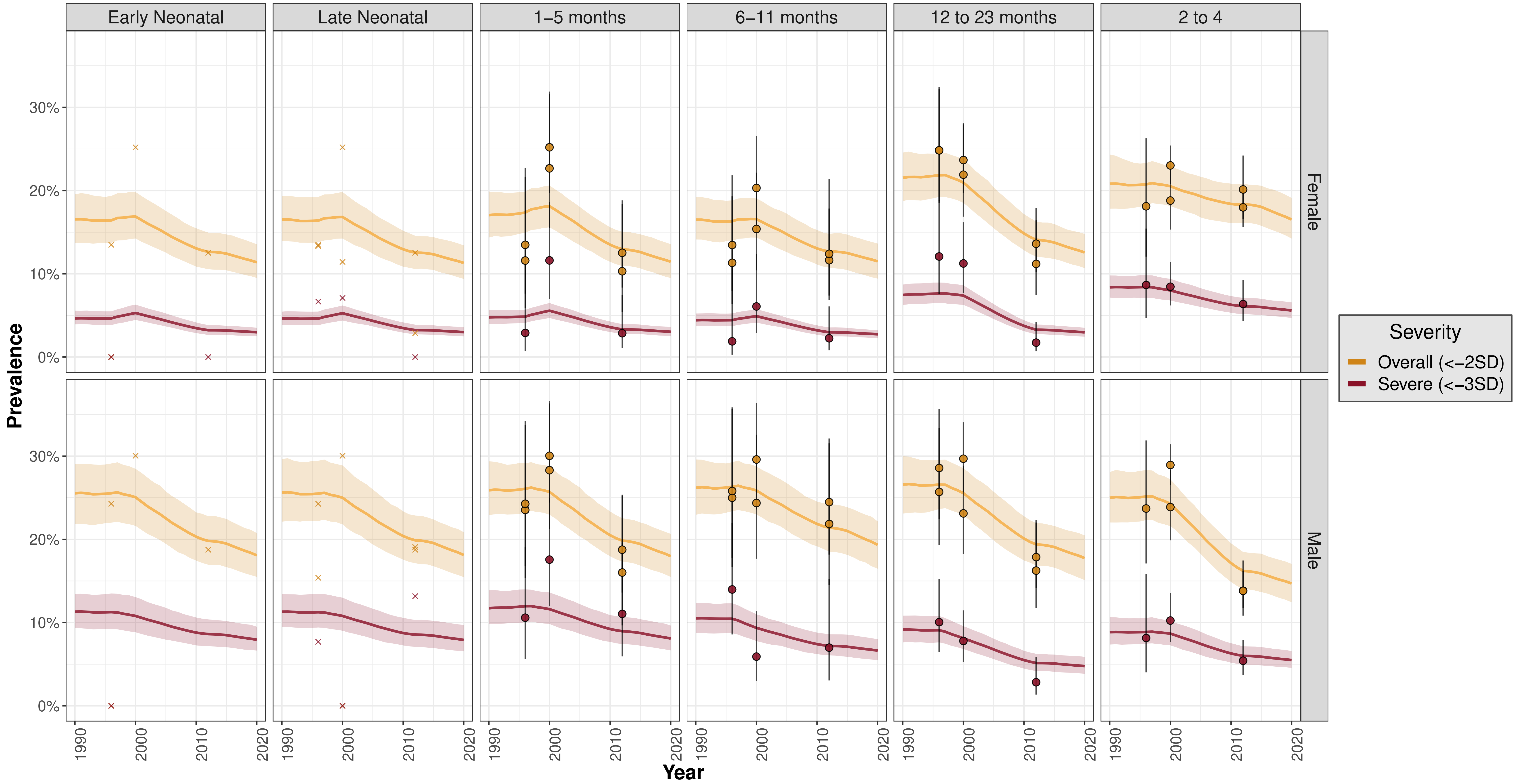

H: Transformed Mean Underweight Z Scores

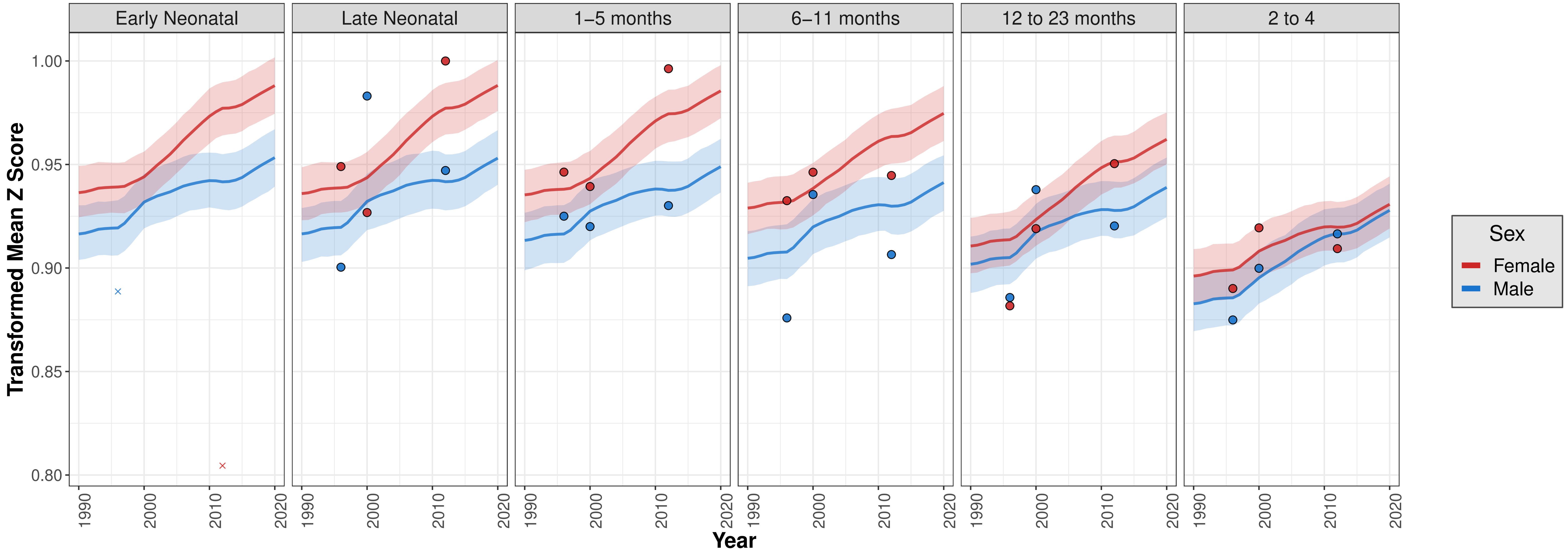

I

Year	Source
1996	DHS
1996	WHO CGM Database
2000	MICS
2000	WHO CGM Database
2012	DHS
2012	WHO CGM Database

Comoros – HAZ, WHZ, and WAZ Distributions

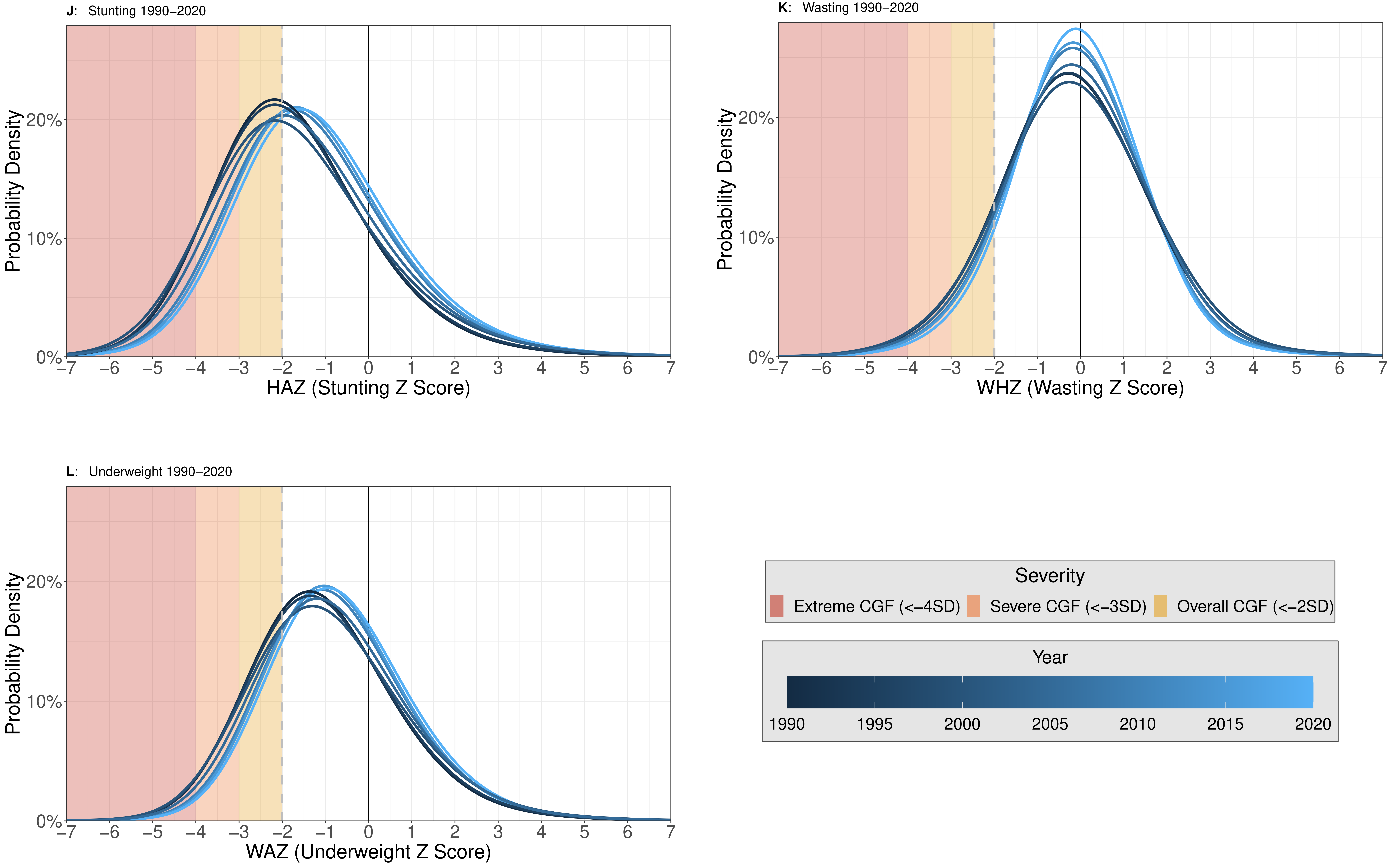

Djibouti – Stunting (HAZ)

A: Overall and Severe Stunting Prevalence

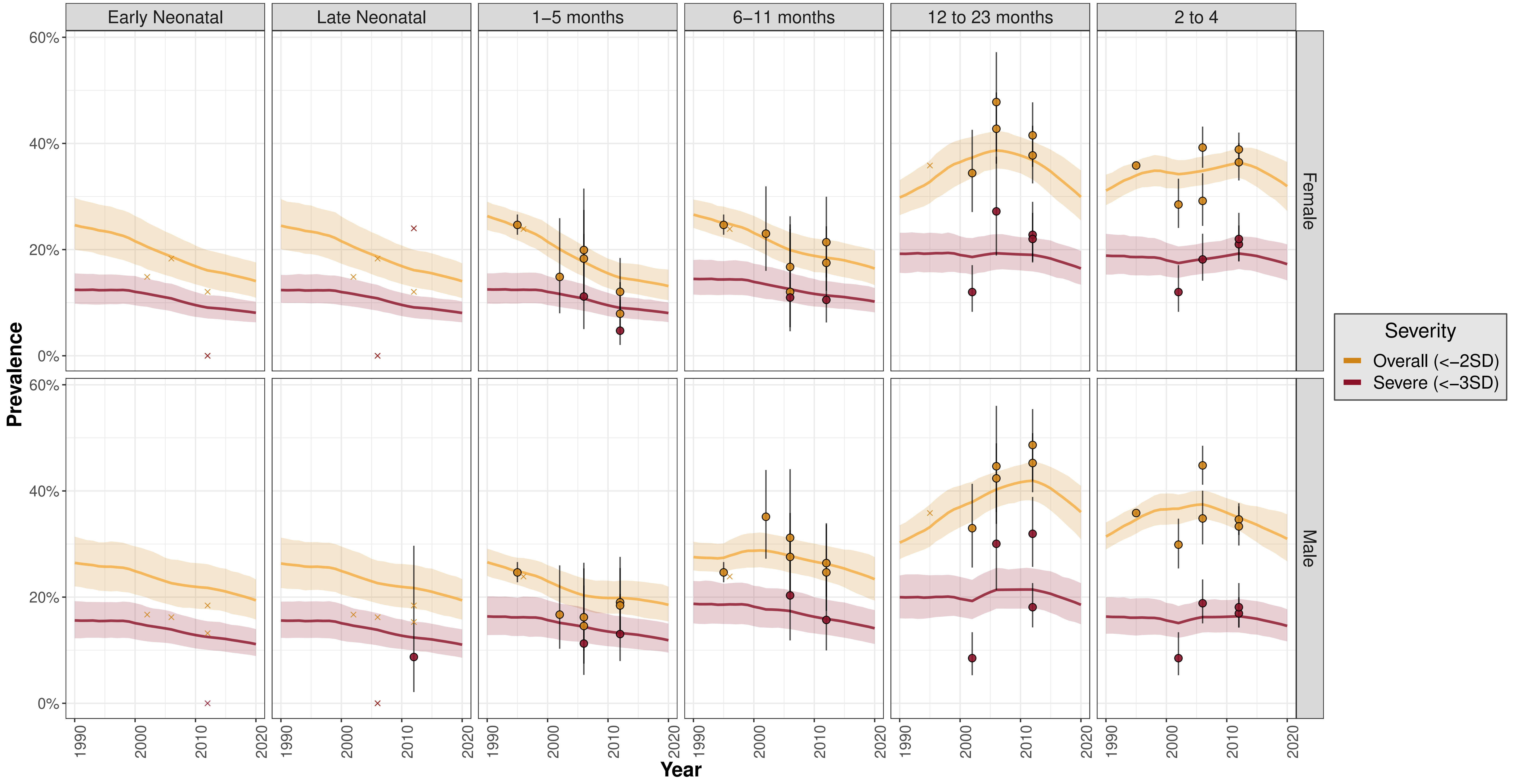

B: Transformed Mean Stunting Z Scores

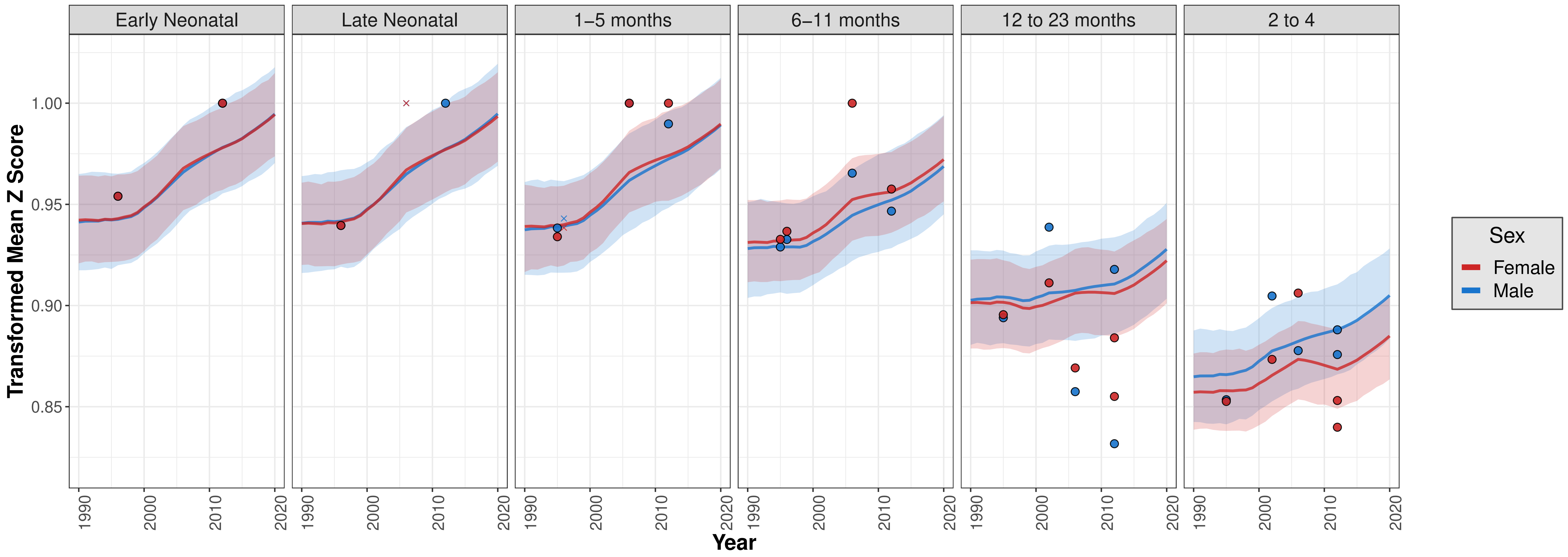

C

Year	Source
1989	WHO CGM Database
1995	WHO CGM Database
1996	WHO CGM Database
2002	WHO CGM Database
2006	MICS
2006	WHO CGM Database
2012	WHO CGM Database
2012	Family Health Survey

Djibouti – Wasting (WHZ)

D: Overall and Severe Wasting Prevalence

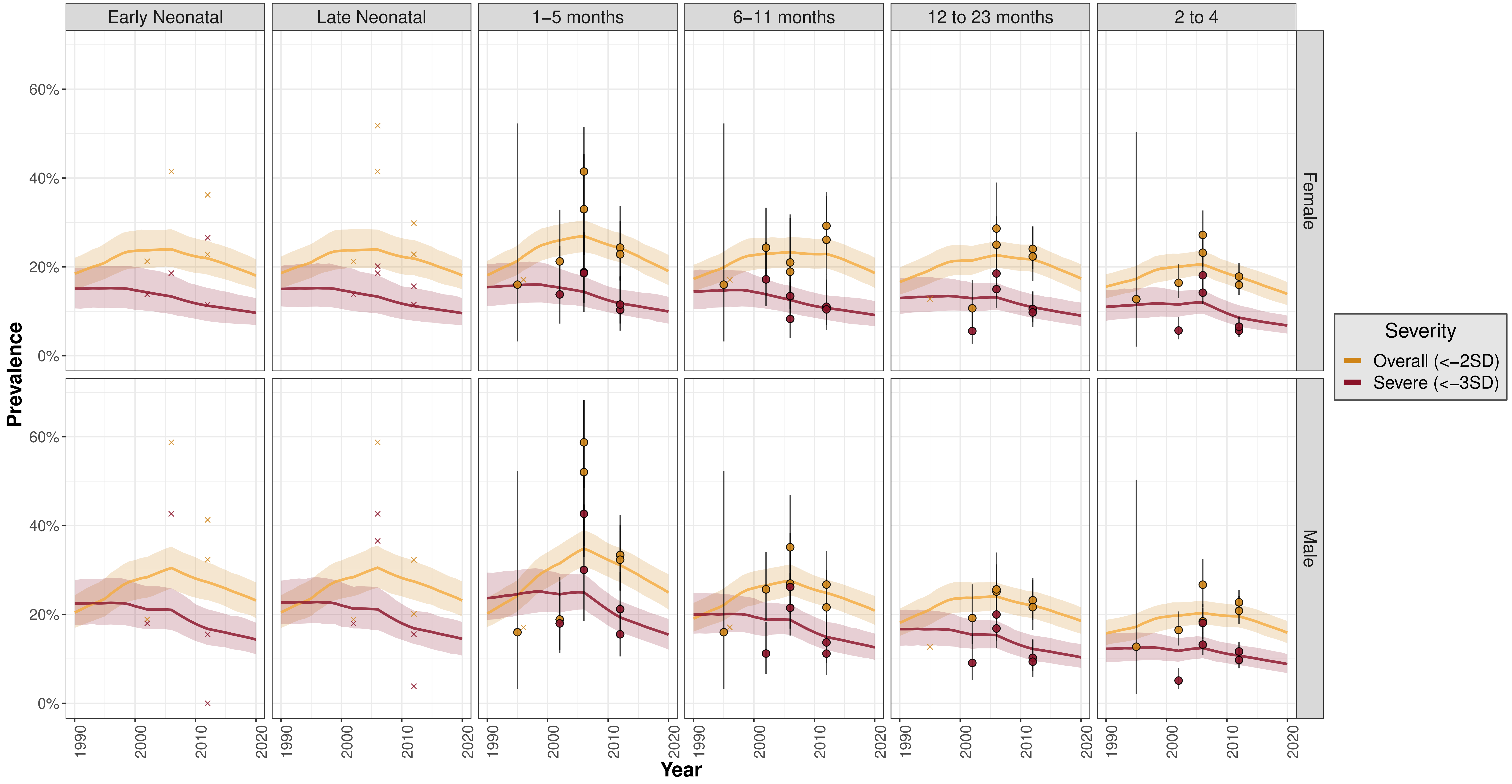

E: Transformed Mean Wasting Z Scores

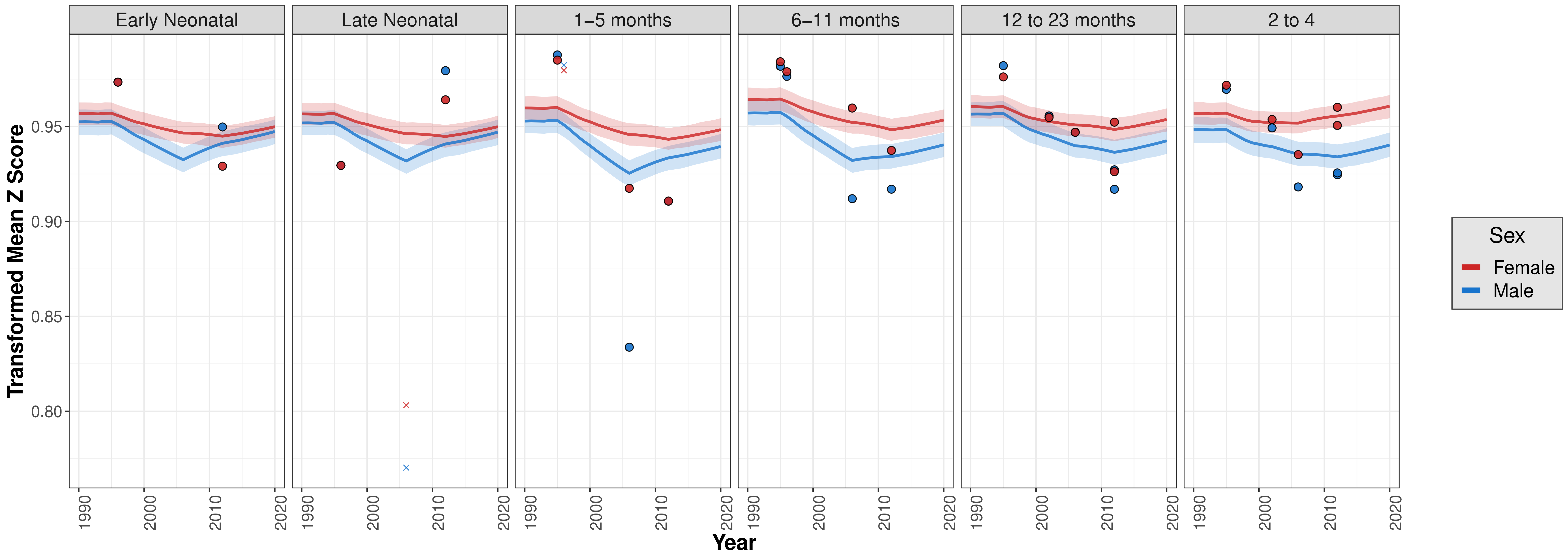

F

Year	Source
1989	WHO CGM Database
1995	WHO CGM Database
1996	WHO CGM Database
2002	WHO CGM Database
2006	MICS
2006	WHO CGM Database
2012	WHO CGM Database
2012	Family Health Survey

Djibouti – Underweight (WAZ)

G: Overall and Severe Underweight Prevalence

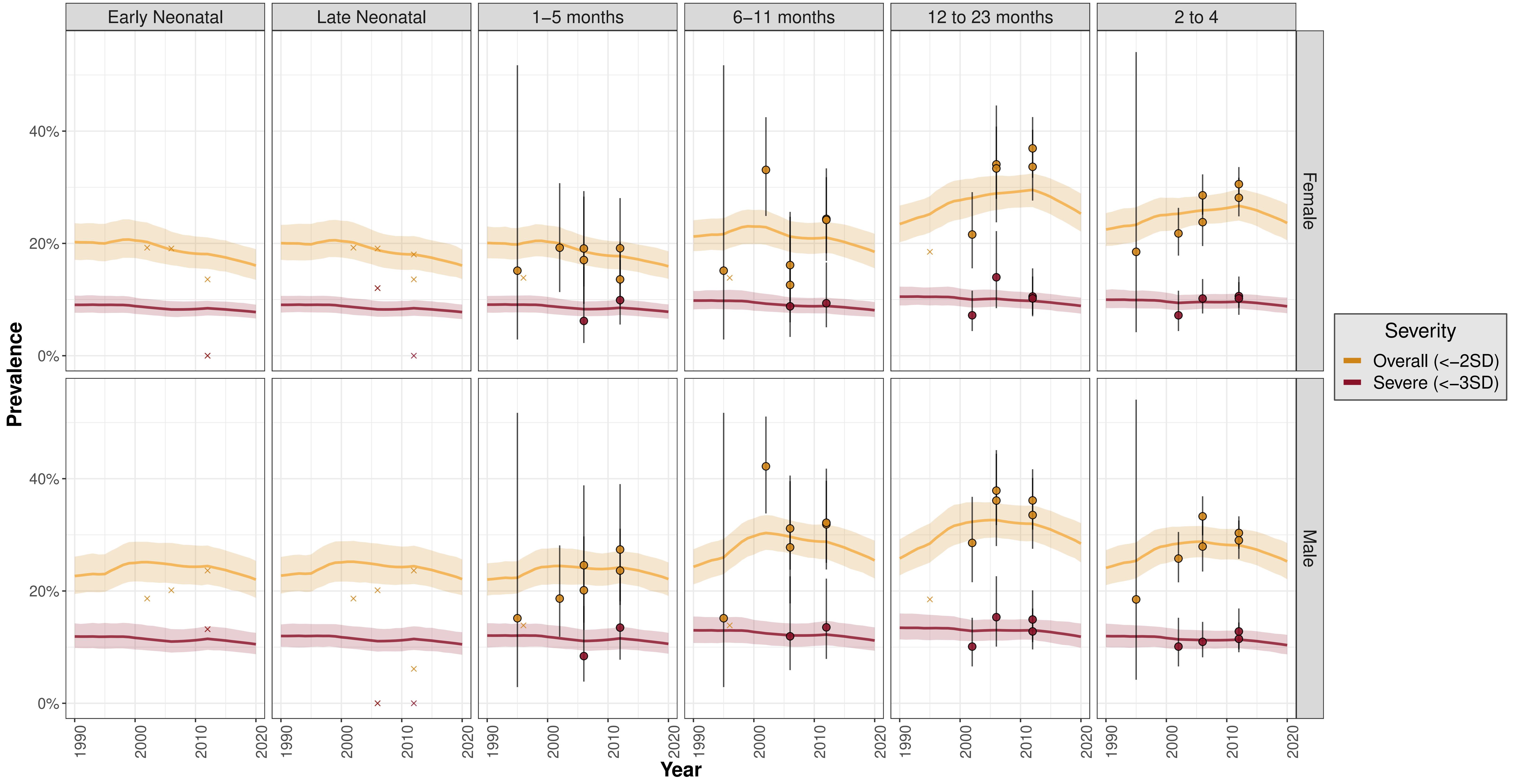

H: Transformed Mean Underweight Z Scores

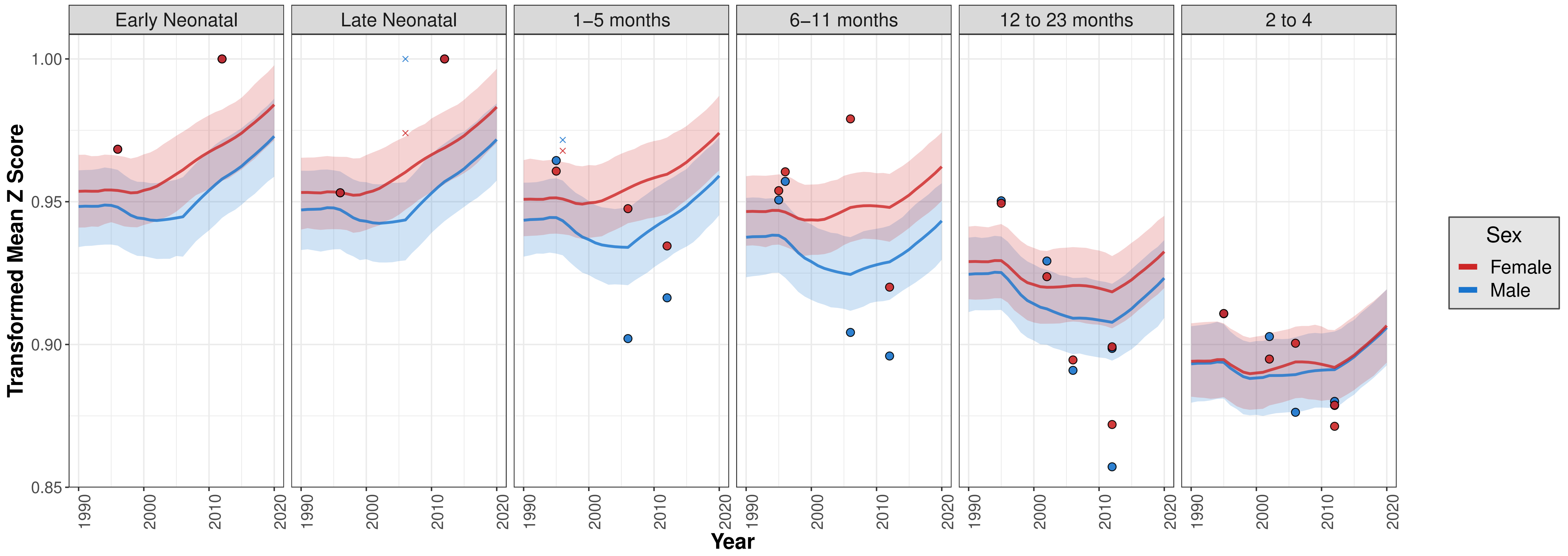

I

Year	Source
1989	WHO CGM Database
1995	WHO CGM Database
1996	WHO CGM Database
2002	WHO CGM Database
2006	MICS
2006	WHO CGM Database
2012	WHO CGM Database
2012	Family Health Survey

Djibouti – HAZ, WHZ, and WAZ Distributions

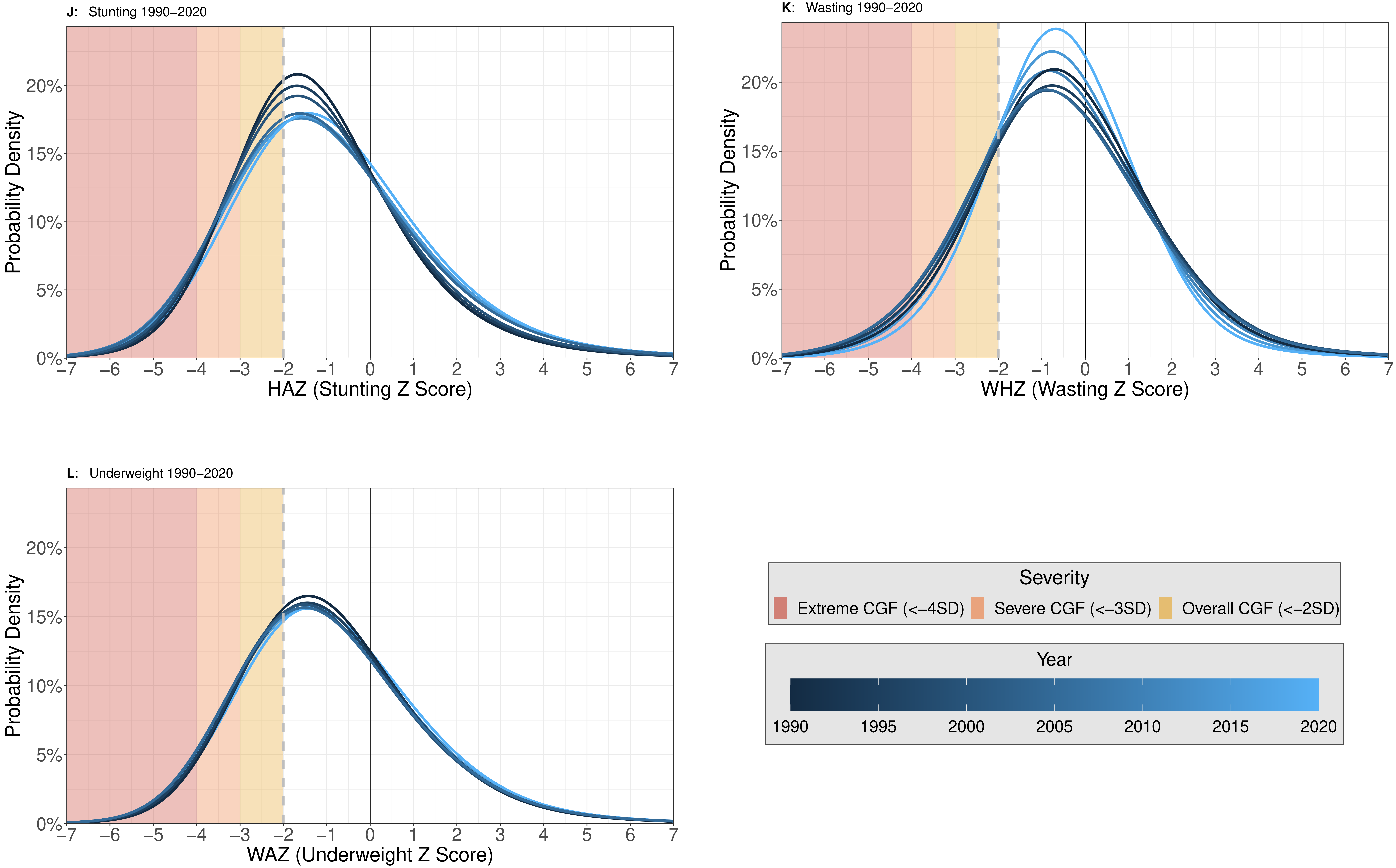

Eritrea – Stunting (HAZ)

A: Overall and Severe Stunting Prevalence

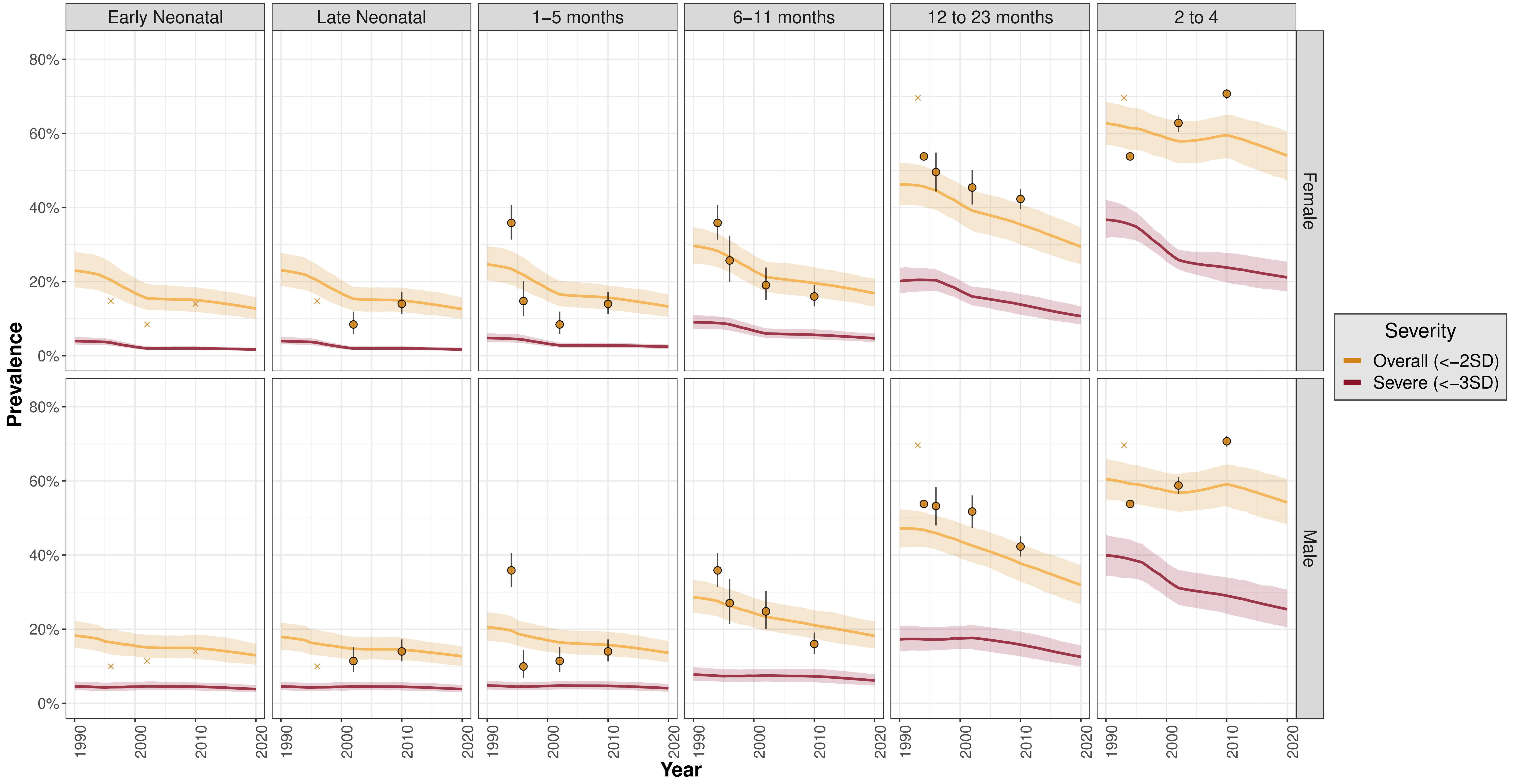

B: Transformed Mean Stunting Z Scores

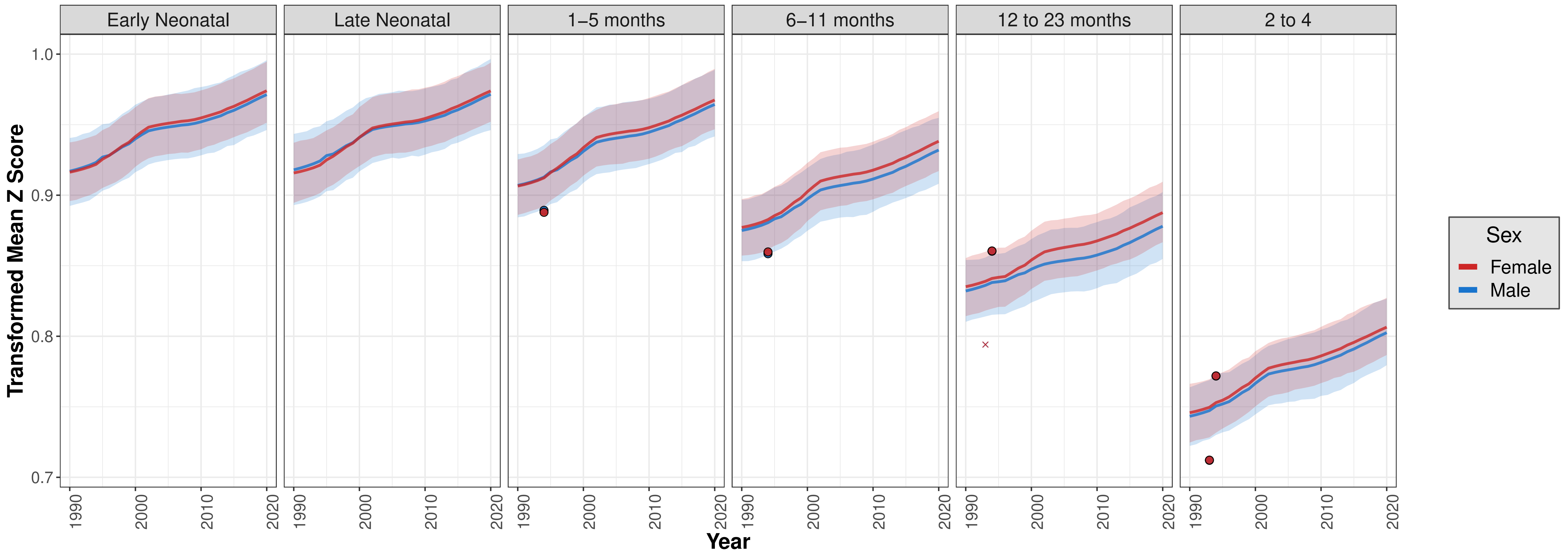

C

Year	Source
1993	WHO CGM Database
1994	WHO CGM Database
1995	DHS
1996	DHS
1996	WHO CGM Database
2002	DHS
2002	WHO CGM Database
2010	WHO CGM Database

Eritrea – Wasting (WHZ)

D: Overall and Severe Wasting Prevalence

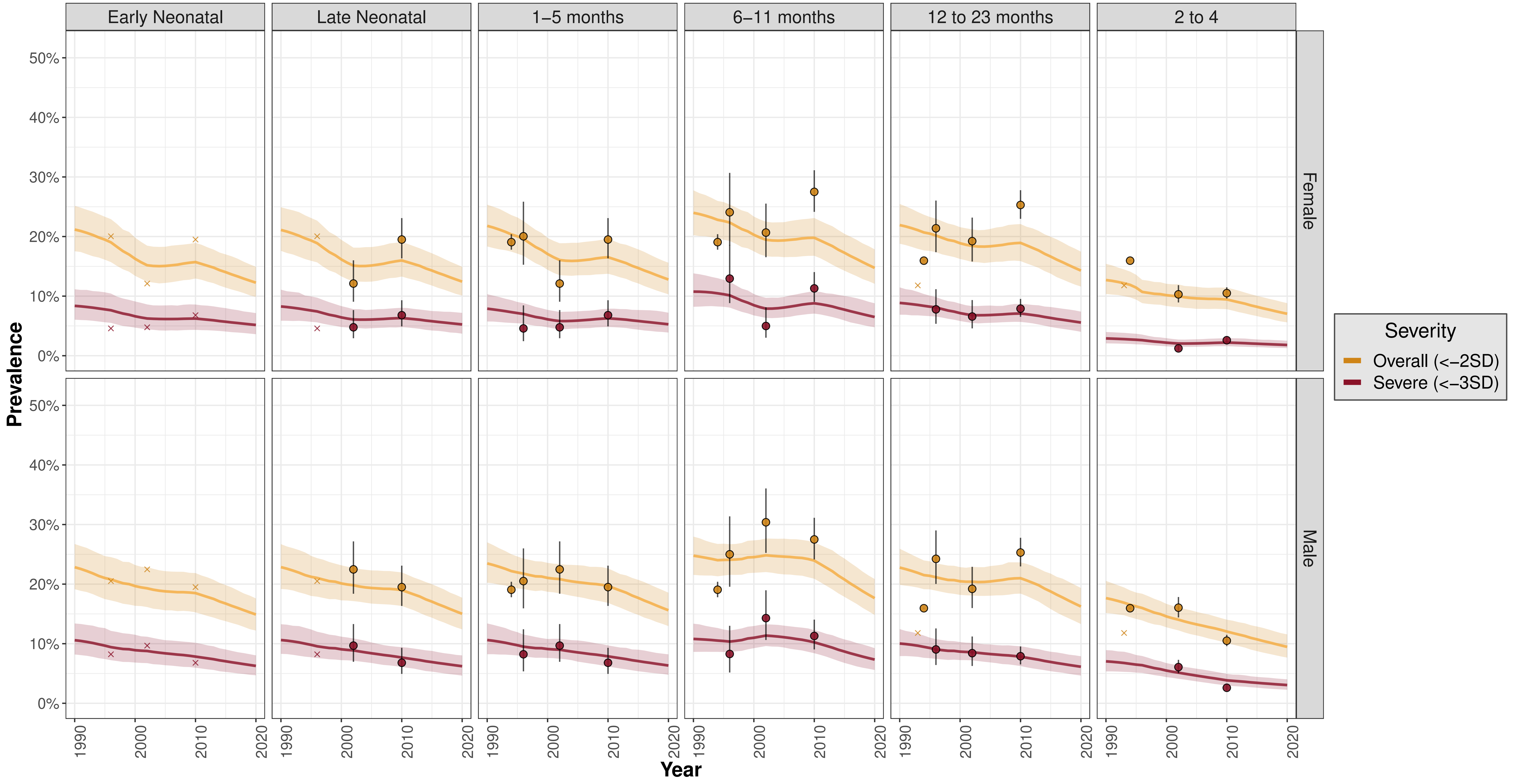

E: Transformed Mean Wasting Z Scores

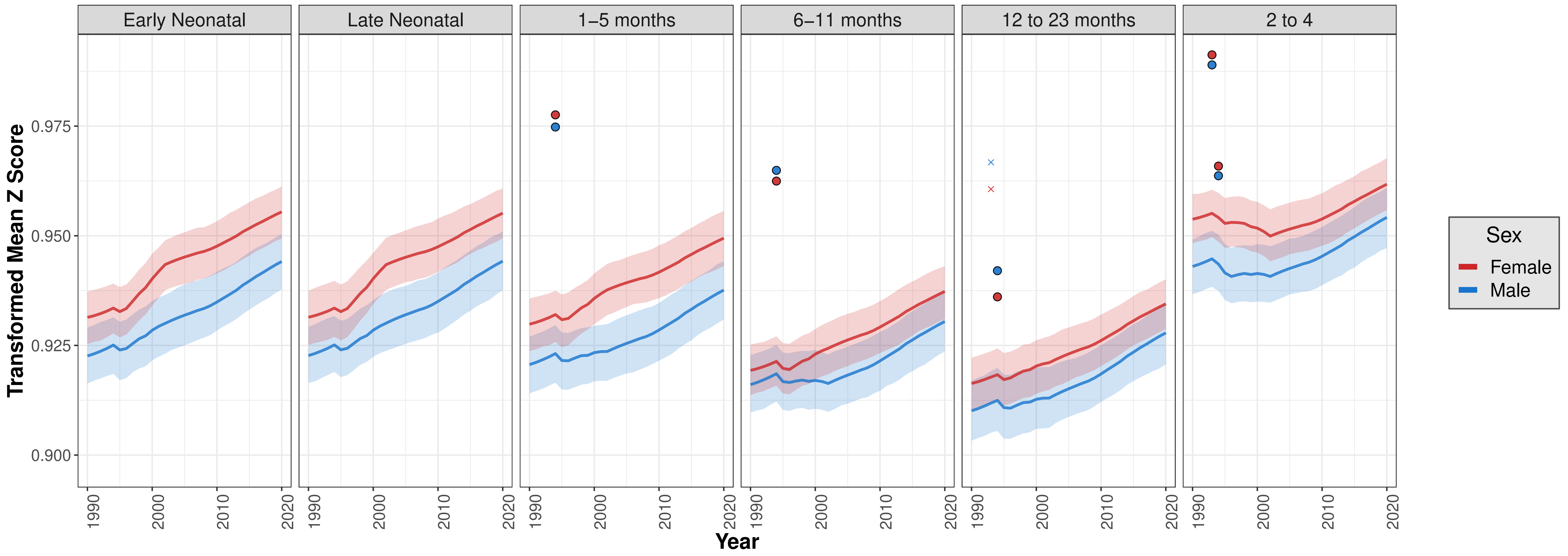

F

Year	Source
1993	WHO CGM Database
1994	WHO CGM Database
1995	DHS
1996	DHS
1996	WHO CGM Database
2002	DHS
2002	WHO CGM Database
2010	WHO CGM Database

Eritrea – Underweight (WAZ)

G: Overall and Severe Underweight Prevalence

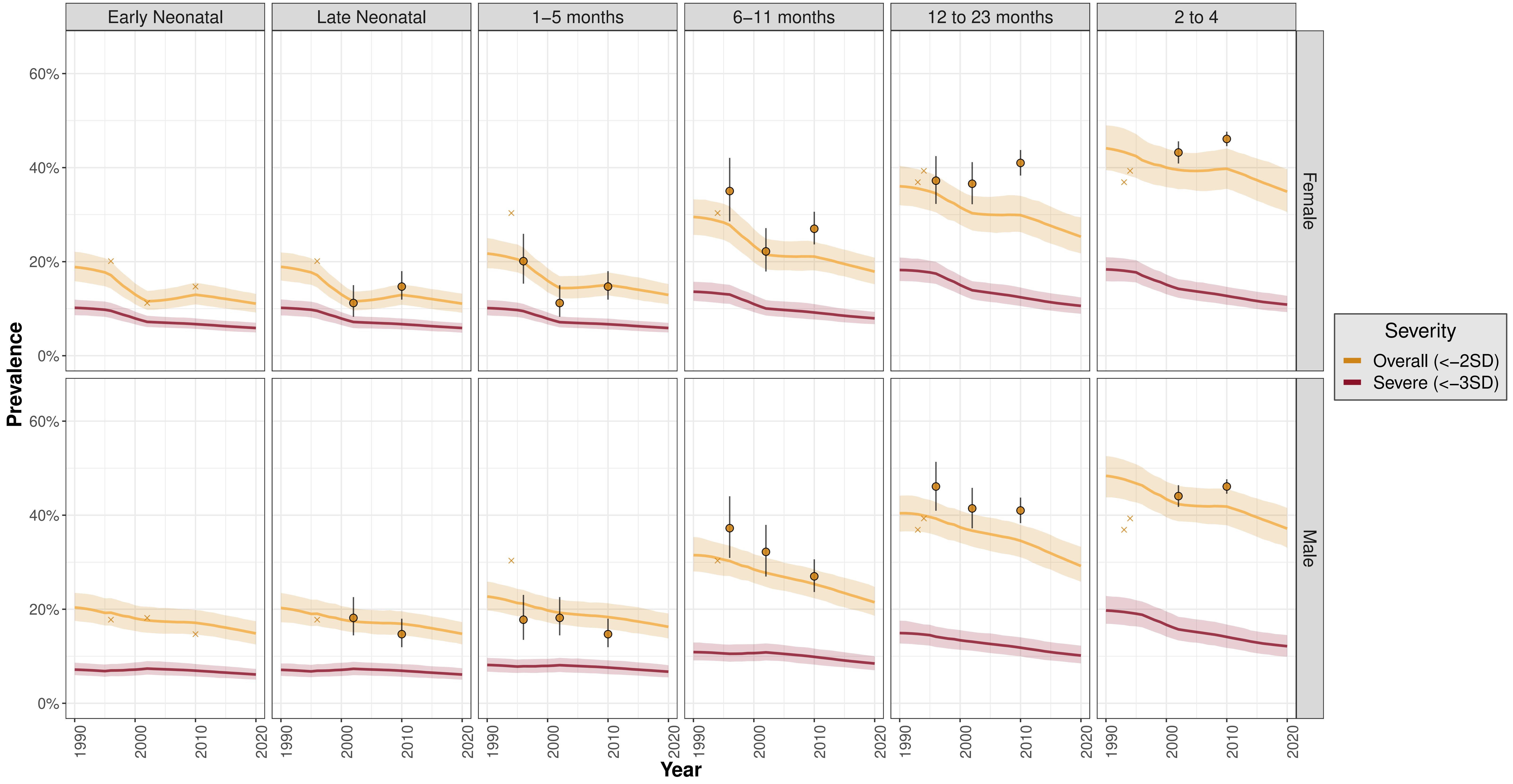

H: Transformed Mean Underweight Z Scores

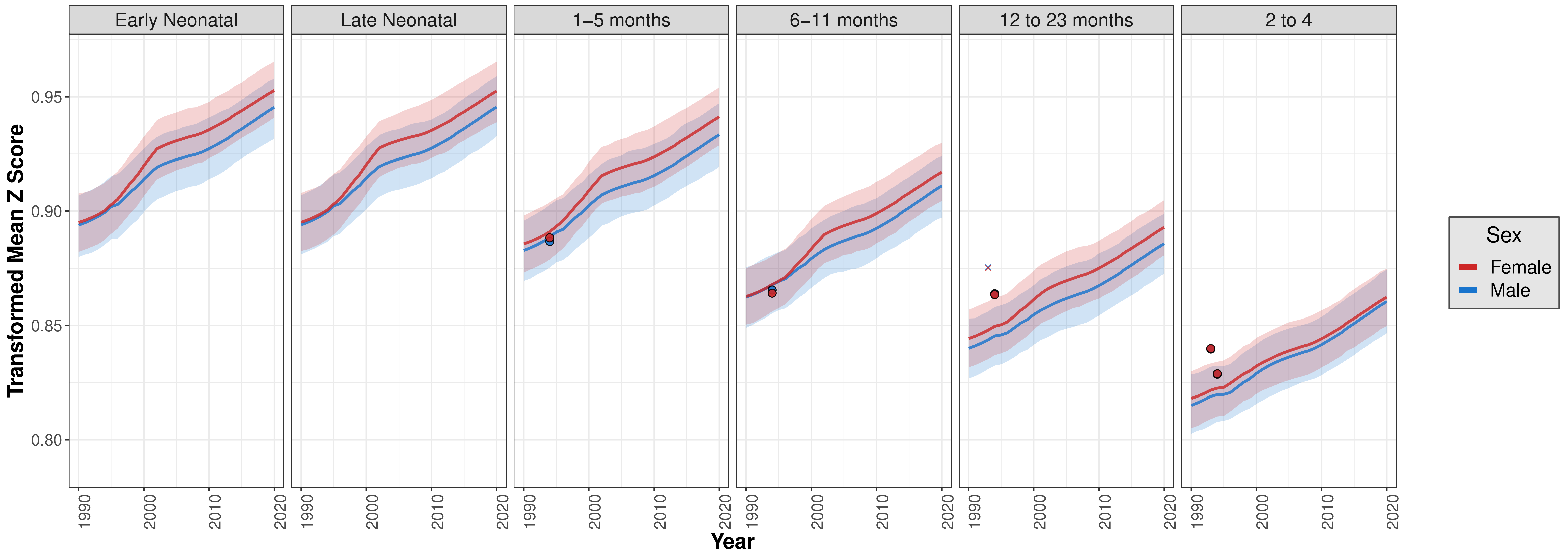

I

Year	Source
1993	WHO CGM Database
1994	WHO CGM Database
1995	DHS
1996	DHS
1996	WHO CGM Database
2002	DHS
2002	WHO CGM Database
2010	WHO CGM Database

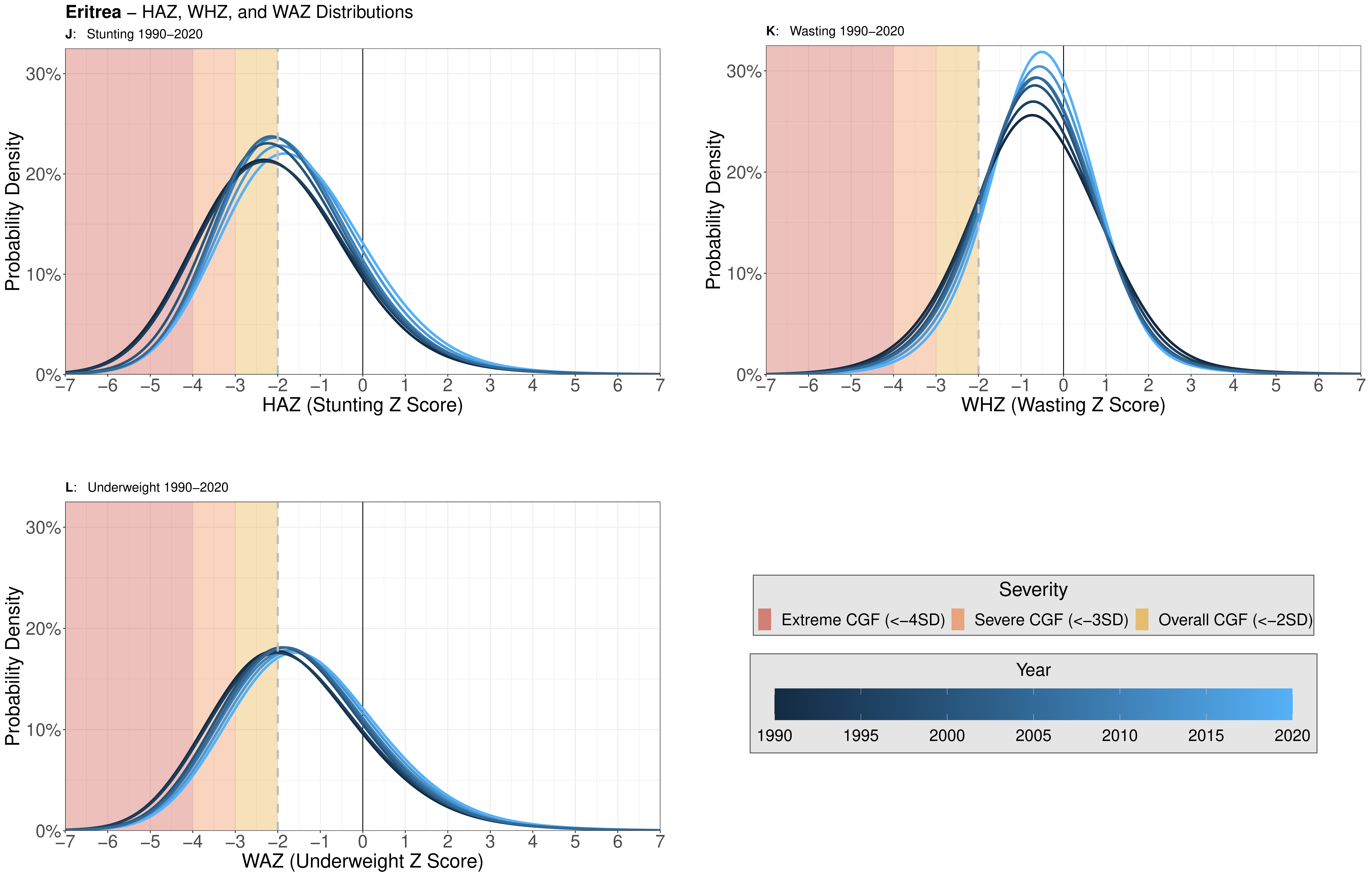

Eritrea – HAZ, WHZ, and WAZ Distributions
J: Stunting 1990–2020
K: Wasting 1990–2020
L: Underweight 1990–2020
Probability Density
30%
20%
10%
0%
−7 −6 −5 −4 −3 −2 −1 0 1 2 3 4 5 6 7
HAZ (Stunting Z Score)
WHZ (Wasting Z Score)
WAZ (Underweight Z Score)
Severity
Extreme CGF (<−4SD)
Severe CGF (<−3SD)
Overall CGF (<−2SD)
Year
1990
1995
2000
2005
2010
2015
2020

Ethiopia – Stunting (HAZ)

A: Overall and Severe Stunting Prevalence

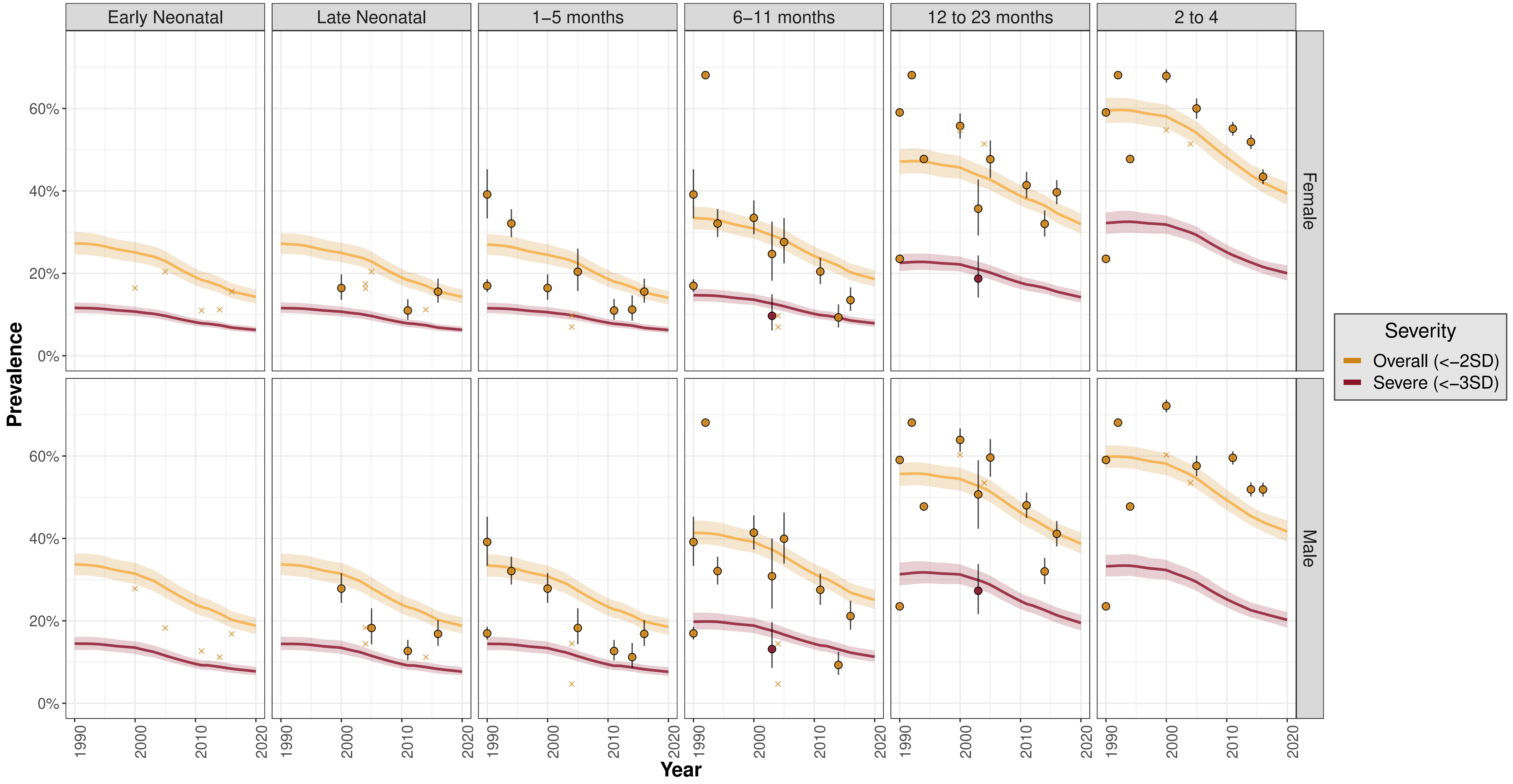

B: Transformed Mean Stunting Z Scores

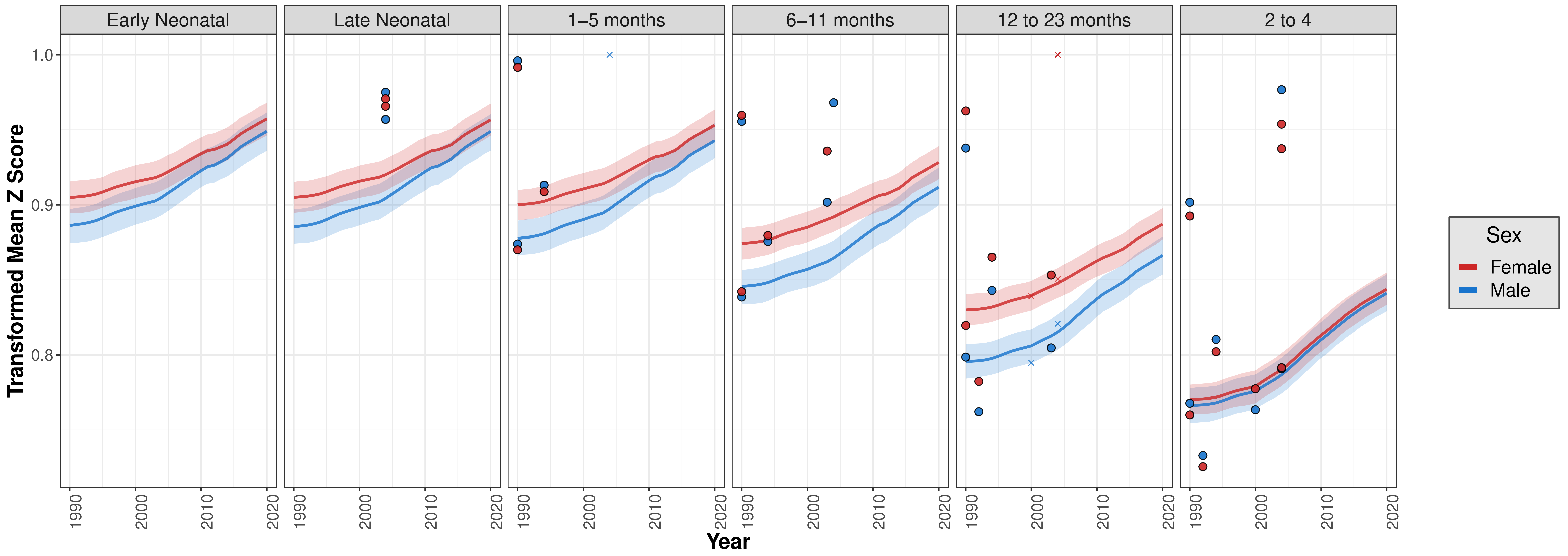

C

Year	Source	National	Subnational
1990	WHO CGM Database	X	
1992	WHO CGM Database	X	
1994	WHO CGM Database	X	
2000	DHS		X
2000	Welfare Monitoring Survey	X	
2000	WHO CGM Database	X	
2003	Young Lives: Household and Child Survey Round 1 – UK Data Service	X	X
2004	Welfare Monitoring Survey	X	
2005	DHS		X
2005	WHO CGM Database	X	
2011	DHS		X
2011	WHO CGM Database	X	
2012	Rural Socioeconomic Survey		X
2014	WHO CGM Database	X	
2014	Socioeconomic Survey		X
2016	WHO CGM Database	X	
2016	DHS		X
2016	Socioeconomic Survey		X

Ethiopia – Wasting (WHZ)

D: Overall and Severe Wasting Prevalence

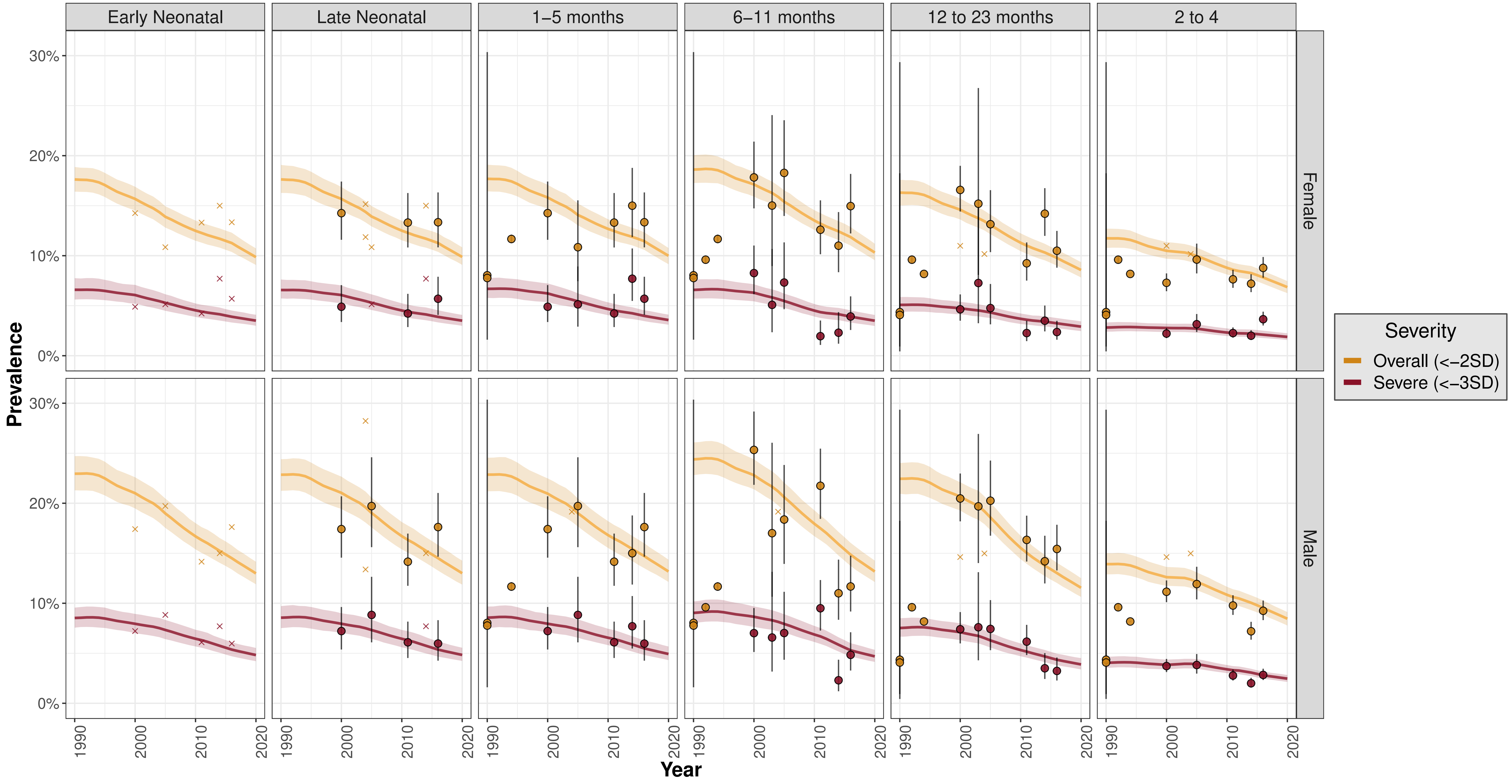

E: Transformed Mean Wasting Z Scores

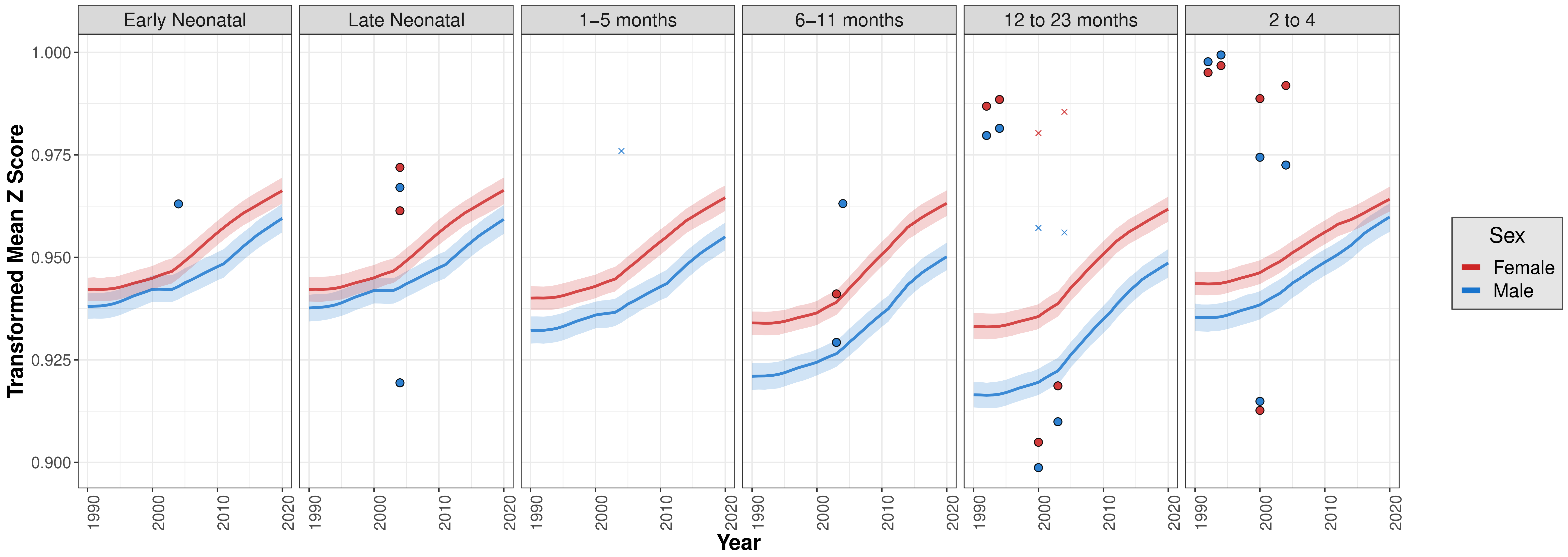

F

Year	Source	National	Subnational
1990	WHO CGM Database	X	
1992	WHO CGM Database	X	
1994	WHO CGM Database	X	
2000	DHS		X
2000	Welfare Monitoring Survey	X	
2000	WHO CGM Database	X	
2003	Young Lives: Household and Child Survey Round 1 – UK Data Service	X	X
2004	Welfare Monitoring Survey	X	
2005	DHS		X
2005	WHO CGM Database	X	
2011	DHS		X
2011	WHO CGM Database	X	
2012	Rural Socioeconomic Survey		X
2014	WHO CGM Database	X	
2014	Socioeconomic Survey		X
2016	WHO CGM Database	X	
2016	DHS		X
2016	Socioeconomic Survey		X

Ethiopia – Underweight (WAZ)

G: Overall and Severe Underweight Prevalence

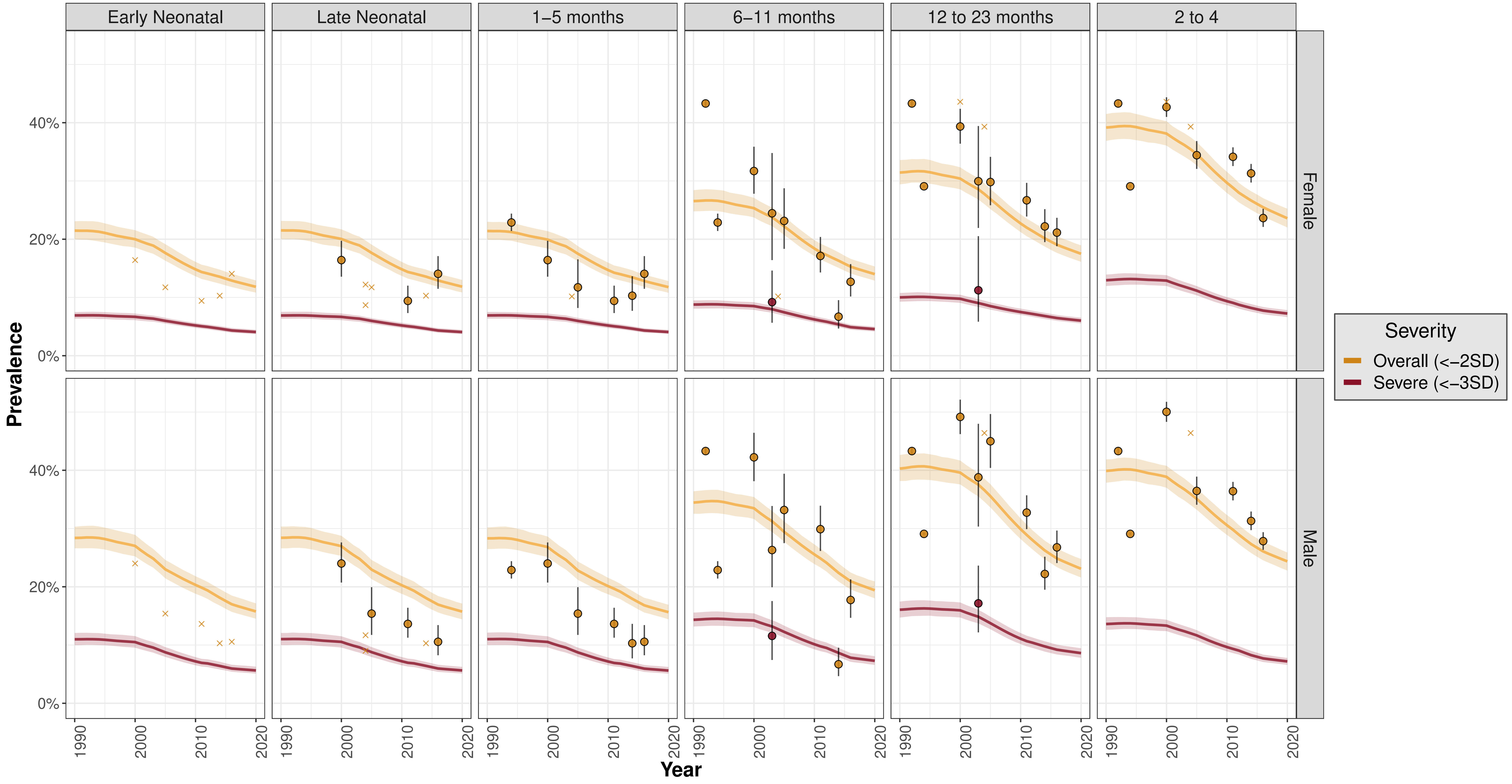

H: Transformed Mean Underweight Z Scores

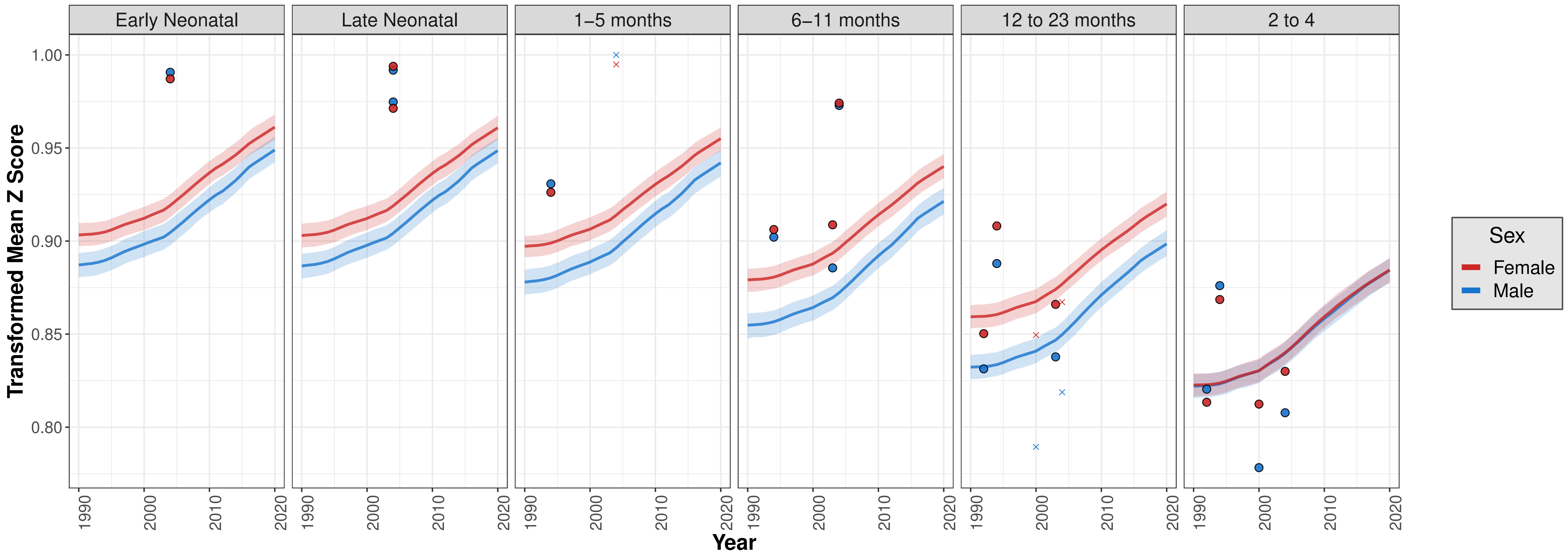

I

Year	Source	National	Subnational
1992	WHO CGM Database	X	
1994	WHO CGM Database	X	
2000	DHS		X
2000	Welfare Monitoring Survey	X	
2000	WHO CGM Database	X	
2003	Young Lives: Household and Child Survey Round 1 – UK Data Service	X	X
2004	Welfare Monitoring Survey	X	
2005	DHS		X
2005	WHO CGM Database	X	
2011	DHS		X
2011	WHO CGM Database	X	
2012	Rural Socioeconomic Survey		X
2014	WHO CGM Database	X	
2014	Socioeconomic Survey		X
2016	WHO CGM Database	X	
2016	DHS		X
2016	Socioeconomic Survey		X

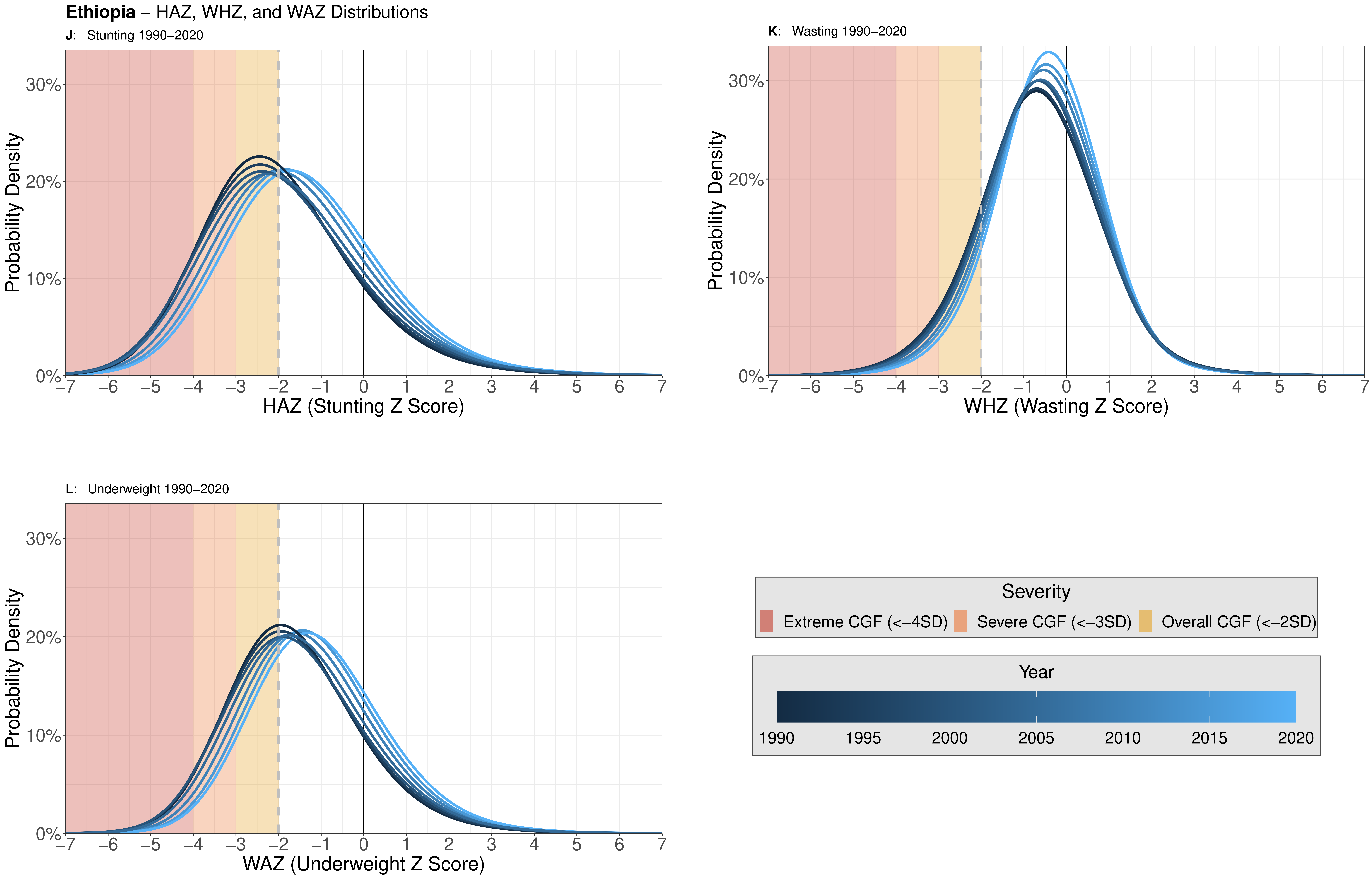

Ethiopia – HAZ, WHZ, and WAZ Distributions
J: Stunting 1990–2020
K: Wasting 1990–2020
L: Underweight 1990–2020
Probability Density
0%
10%
20%
30%
−7 −6 −5 −4 −3 −2 −1 0 1 2 3 4 5 6 7
HAZ (Stunting Z Score)
WHZ (Wasting Z Score)
WAZ (Underweight Z Score)
Severity
Extreme CGF (<−4SD)
Severe CGF (<−3SD)
Overall CGF (<−2SD)
Year
1990
1995
2000
2005
2010
2015
2020

Kenya – Stunting (HAZ)

A: Overall and Severe Stunting Prevalence

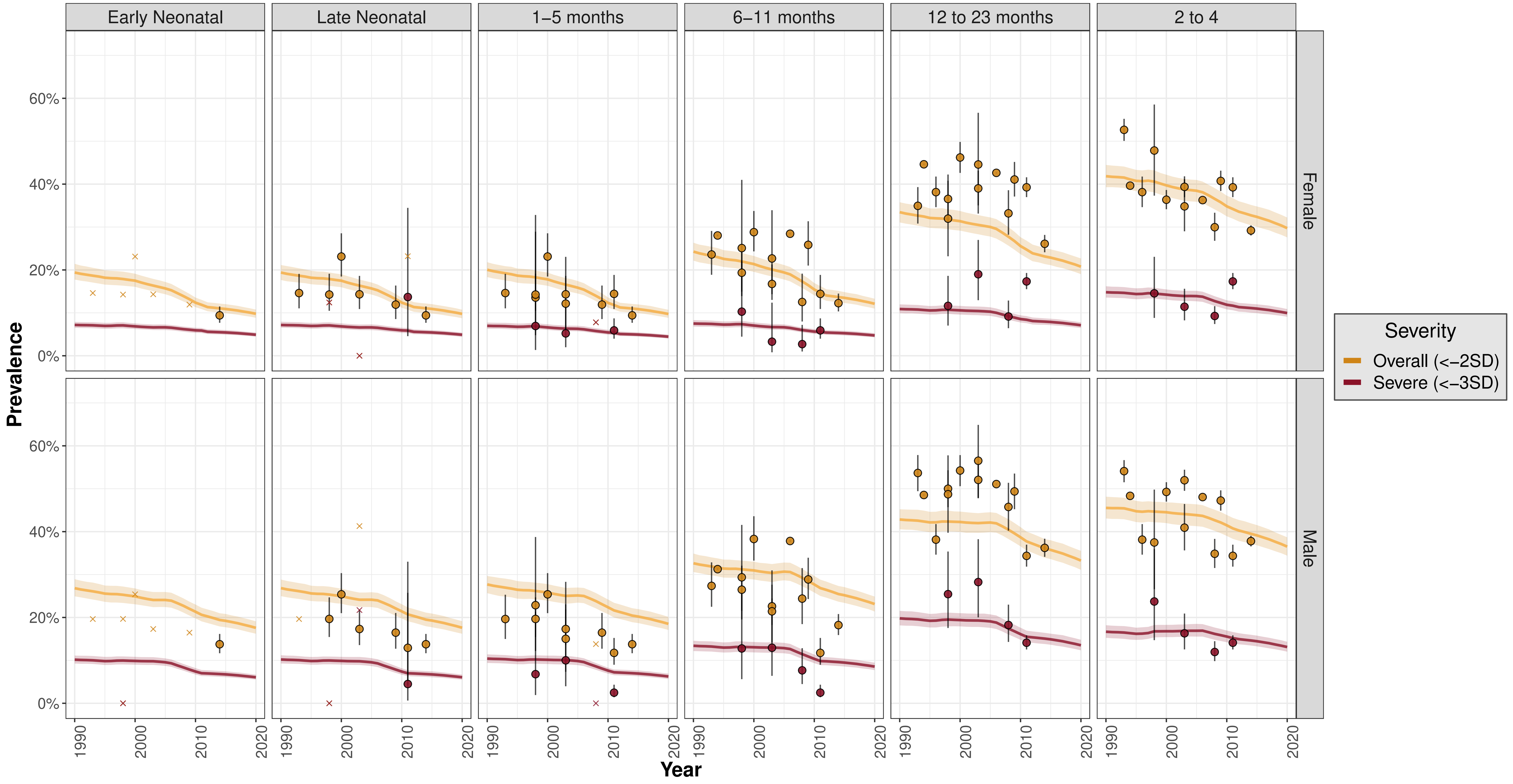

B: Transformed Mean Stunting Z Scores

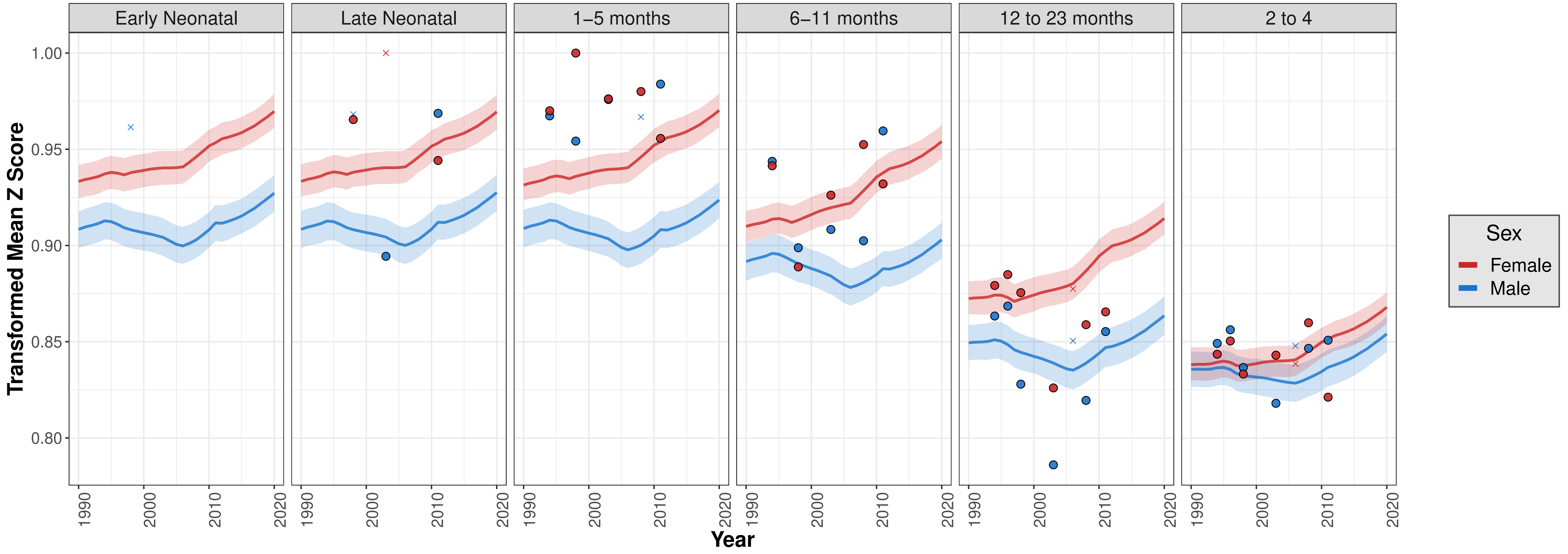

C

Year	Source	National	Subnational
1979	WHO CGM Database	X	
1987	WHO CGM Database	X	
1993	DHS		X
1993	WHO CGM Database	X	
1994	WHO CGM Database	X	
1996	MICS	X	
1997	Welfare Monitoring Survey III	X	X
1998	DHS	X	X
1998	WHO CGM Database	X	
2000	MICS		X
2000	WHO CGM Database	X	
2001	WHO CGM Database		X
2003	DHS	X	X
2003	WHO CGM Database	X	
2004	Greater Eldoret Health and Development Survey		X
2005	Integrated Household Budget Survey	X	X
2005	Greater Eldoret Health and Development Survey		X
2006	Integrated Household Budget Survey	X	X
2006	WHO CGM Database	X	
2006	Greater Eldoret Health and Development Survey		X
2007	North Eastern Province MICS		X
2008	Eastern Province MICS	X	X
2008	DHS		X
2009	DHS		X
2009	Coast MICS		X
2009	WHO CGM Database	X	
2010	Nyanza Global Enteric Multicenter Study	X	
2011	Nyanza Province MICS	X	
2012	Nyanza Global Enteric Multicenter Study	X	
2013	Bungoma County Multiple Indicator Survey		X
2013	Kakamega County Multiple Indicator Survey		X
2013	Turkana County Multiple Indicator Survey		X
2014	WHO CGM Database	X	
2014	DHS		X
2014	Bungoma County Multiple Indicator Survey		X
2014	Kakamega County Multiple Indicator Survey		X
2014	Turkana County Multiple Indicator Survey		X
2019	Marsabit Health and Nutrition SMART Survey July		X
2019	Samburu Health and Nutrition SMART Survey June		X
2019	Tana River Health and Nutrition SMART Survey February		X
2019	Turkana Health and Nutrition SMART Survey June		X
2019	Wajir Health and Nutrition SMART Survey June		X

Kenya – Wasting (WHZ)

D: Overall and Severe Wasting Prevalence

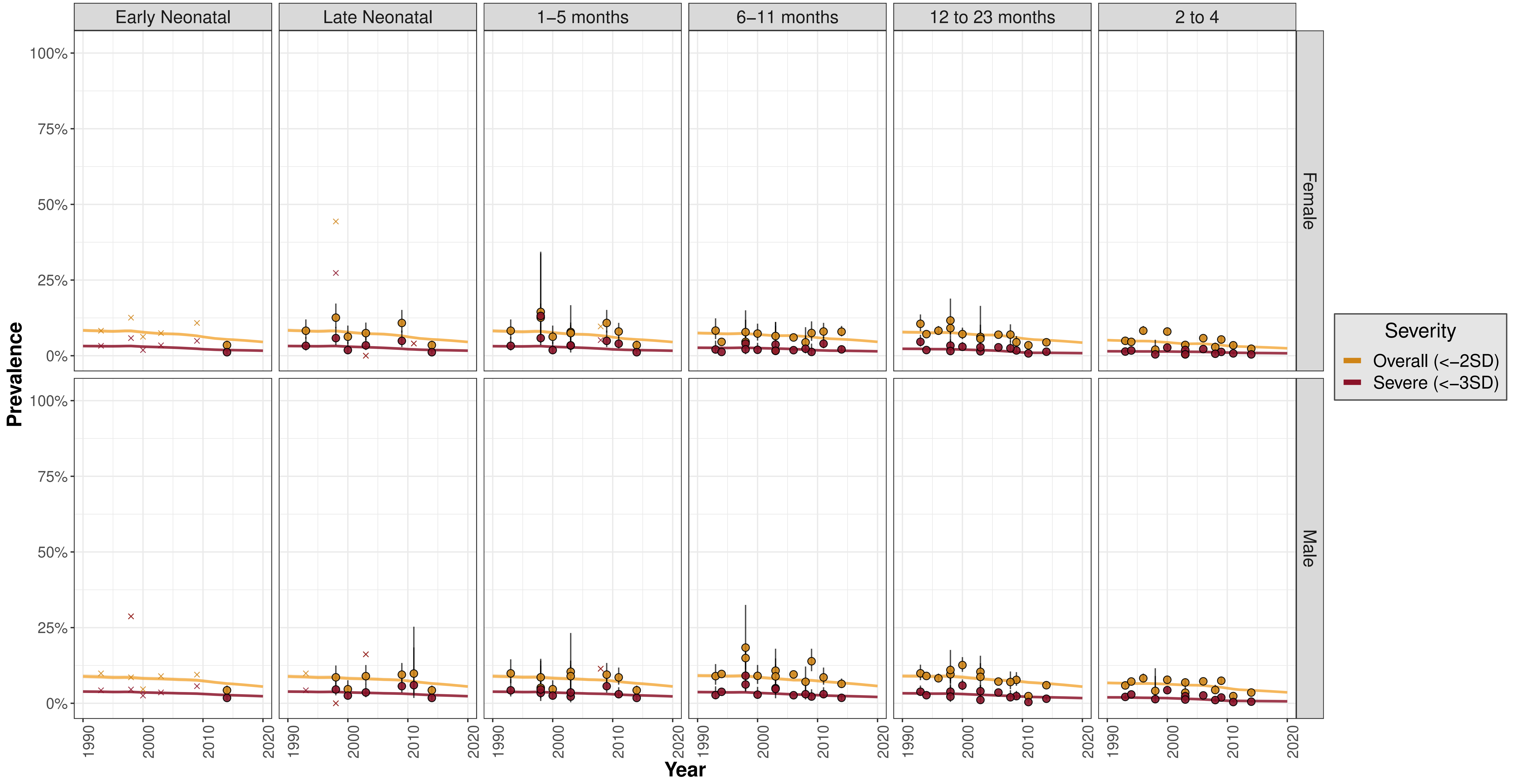

E: Transformed Mean Wasting Z Scores

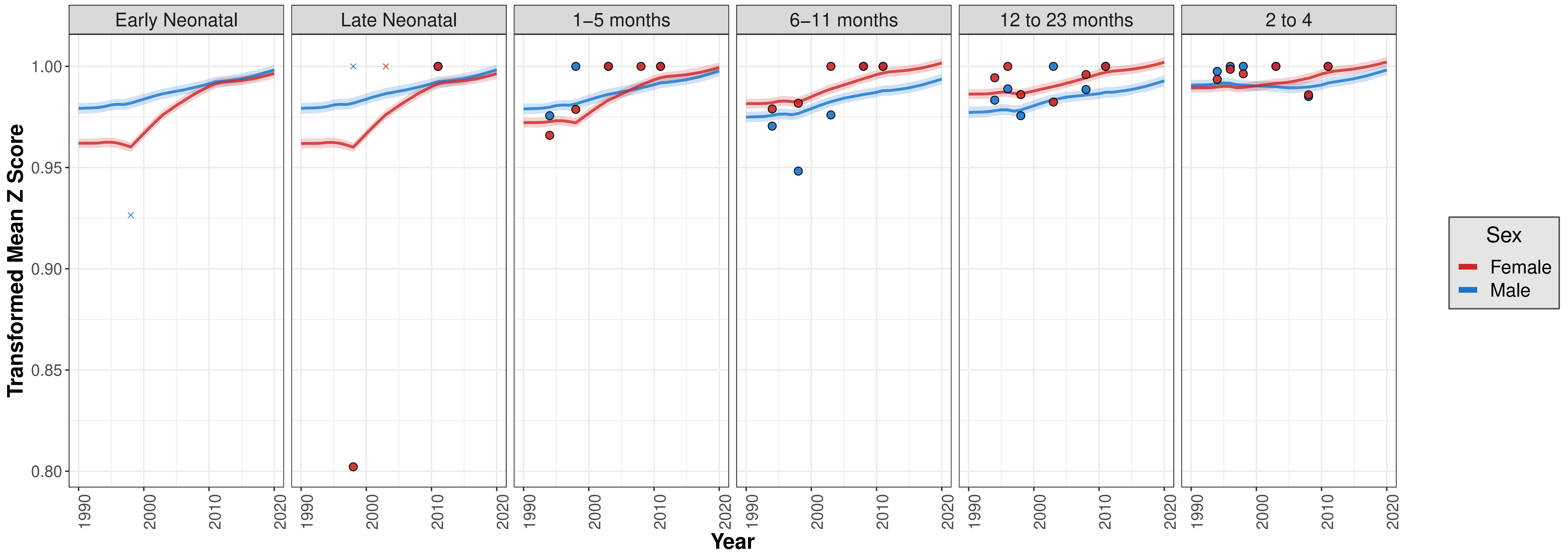

F

Year	Source	National	Subnational
1979	WHO CGM Database	X	
1987	WHO CGM Database	X	
1993	DHS		X
1993	WHO CGM Database	X	
1994	WHO CGM Database	X	
1996	MICS	X	
1997	Welfare Monitoring Survey III	X	X
1997	WHO CGM Database		X
1998	DHS	X	X
1998	WHO CGM Database	X	
2000	MICS		X
2000	WHO CGM Database	X	
2001	WHO CGM Database		X
2003	DHS	X	X
2003	WHO CGM Database	X	
2004	Greater Eldoret Health and Development Survey		X
2005	Integrated Household Budget Survey	X	X
2005	Greater Eldoret Health and Development Survey		X
2006	Integrated Household Budget Survey	X	X
2006	WHO CGM Database	X	
2006	Greater Eldoret Health and Development Survey		X
2007	North Eastern Province MICS		X
2008	Eastern Province MICS	X	X
2008	DHS		X
2009	DHS		X
2009	Coast MICS		X
2009	WHO CGM Database	X	
2010	Nyanza Global Enteric Multicenter Study	X	
2011	Nyanza Province MICS	X	
2012	Nyanza Global Enteric Multicenter Study	X	
2013	Bungoma County Multiple Indicator Survey		X
2013	Kakamega County Multiple Indicator Survey		X
2013	Turkana County Multiple Indicator Survey		X
2014	WHO CGM Database	X	
2014	DHS		X
2014	Bungoma County Multiple Indicator Survey		X
2014	Kakamega County Multiple Indicator Survey		X
2014	Turkana County Multiple Indicator Survey		X
2019	Marsabit Health and Nutrition SMART Survey July		X
2019	Samburu Health and Nutrition SMART Survey June		X
2019	Tana River Health and Nutrition SMART Survey February		X
2019	Turkana Health and Nutrition SMART Survey June		X
2019	Wajir Health and Nutrition SMART Survey June		X

Kenya – Underweight (WAZ)

G: Overall and Severe Underweight Prevalence

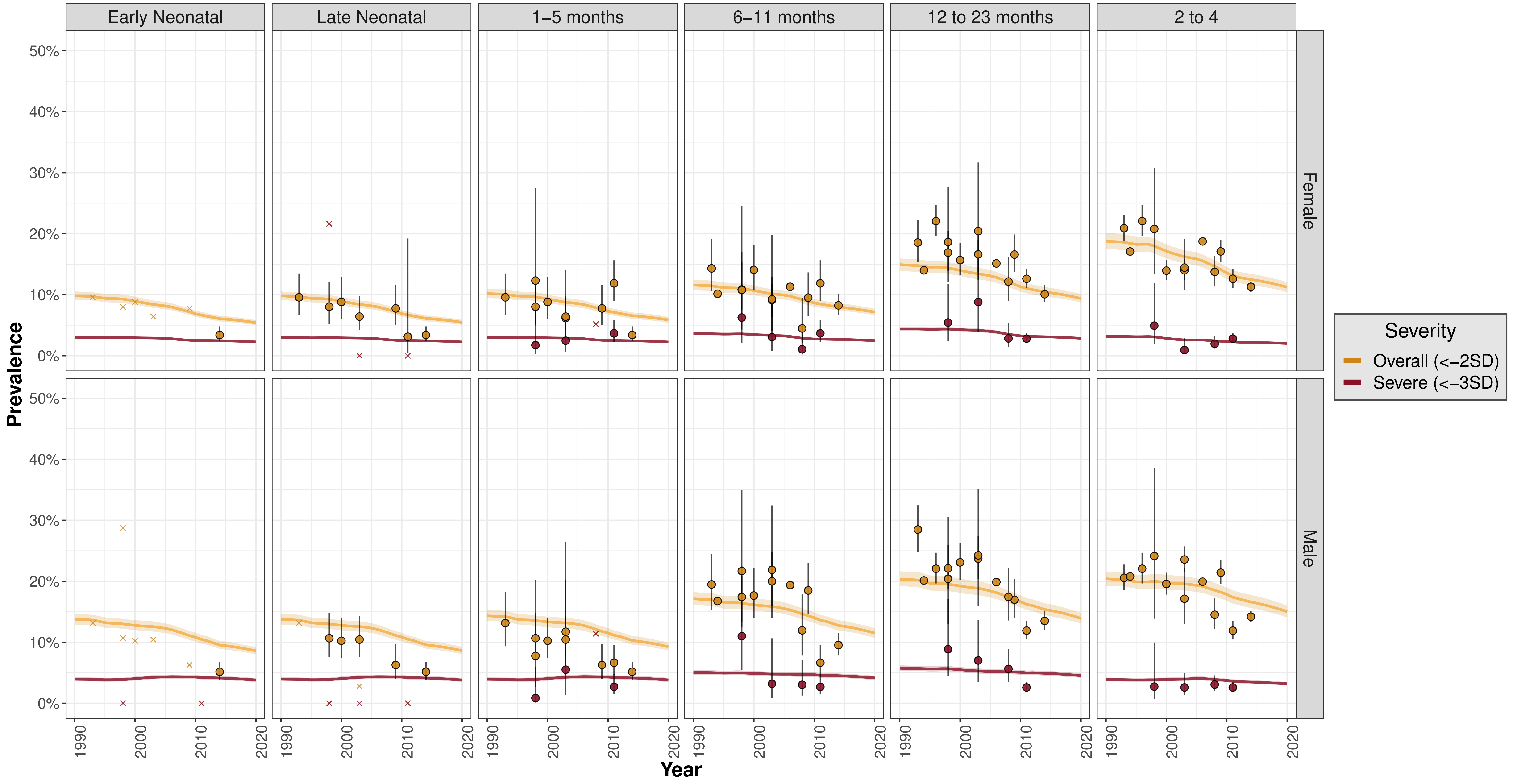

H: Transformed Mean Underweight Z Scores

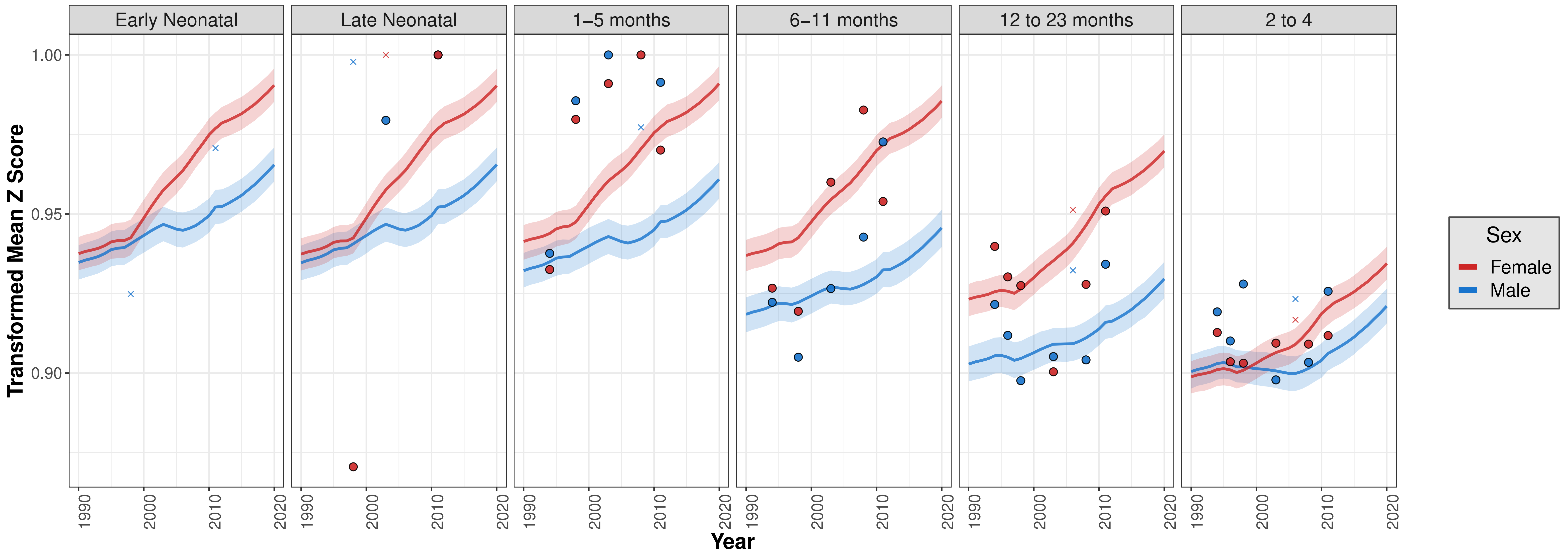

I

Year	Source	National	Subnational
1993	DHS		X
1993	WHO CGM Database	X	
1994	WHO CGM Database	X	
1996	MICS	X	
1997	Welfare Monitoring Survey III	X	X
1998	DHS	X	X
1998	WHO CGM Database	X	
2000	MICS		X
2000	WHO CGM Database	X	
2003	DHS	X	X
2003	WHO CGM Database	X	
2004	Greater Eldoret Health and Development Survey		X
2005	Integrated Household Budget Survey	X	X
2005	Greater Eldoret Health and Development Survey		X
2006	Integrated Household Budget Survey	X	X
2006	WHO CGM Database	X	
2006	Greater Eldoret Health and Development Survey		X
2007	North Eastern Province MICS		X
2008	Eastern Province MICS	X	X
2008	DHS		X
2009	DHS		X
2009	Coast MICS		X
2009	WHO CGM Database	X	
2010	Nyanza Global Enteric Multicenter Study	X	
2011	Nyanza Province MICS	X	
2012	Nyanza Global Enteric Multicenter Study	X	
2013	Bungoma County Multiple Indicator Survey		X
2013	Kakamega County Multiple Indicator Survey		X
2013	Turkana County Multiple Indicator Survey		X
2014	WHO CGM Database	X	
2014	DHS		X
2014	Bungoma County Multiple Indicator Survey		X
2014	Kakamega County Multiple Indicator Survey		X
2014	Turkana County Multiple Indicator Survey		X
2019	Marsabit Health and Nutrition SMART Survey July		X
2019	Samburu Health and Nutrition SMART Survey June		X
2019	Tana River Health and Nutrition SMART Survey February		X
2019	Turkana Health and Nutrition SMART Survey June		X
2019	Wajir Health and Nutrition SMART Survey June		X

Kenya – HAZ, WHZ, and WAZ Distributions

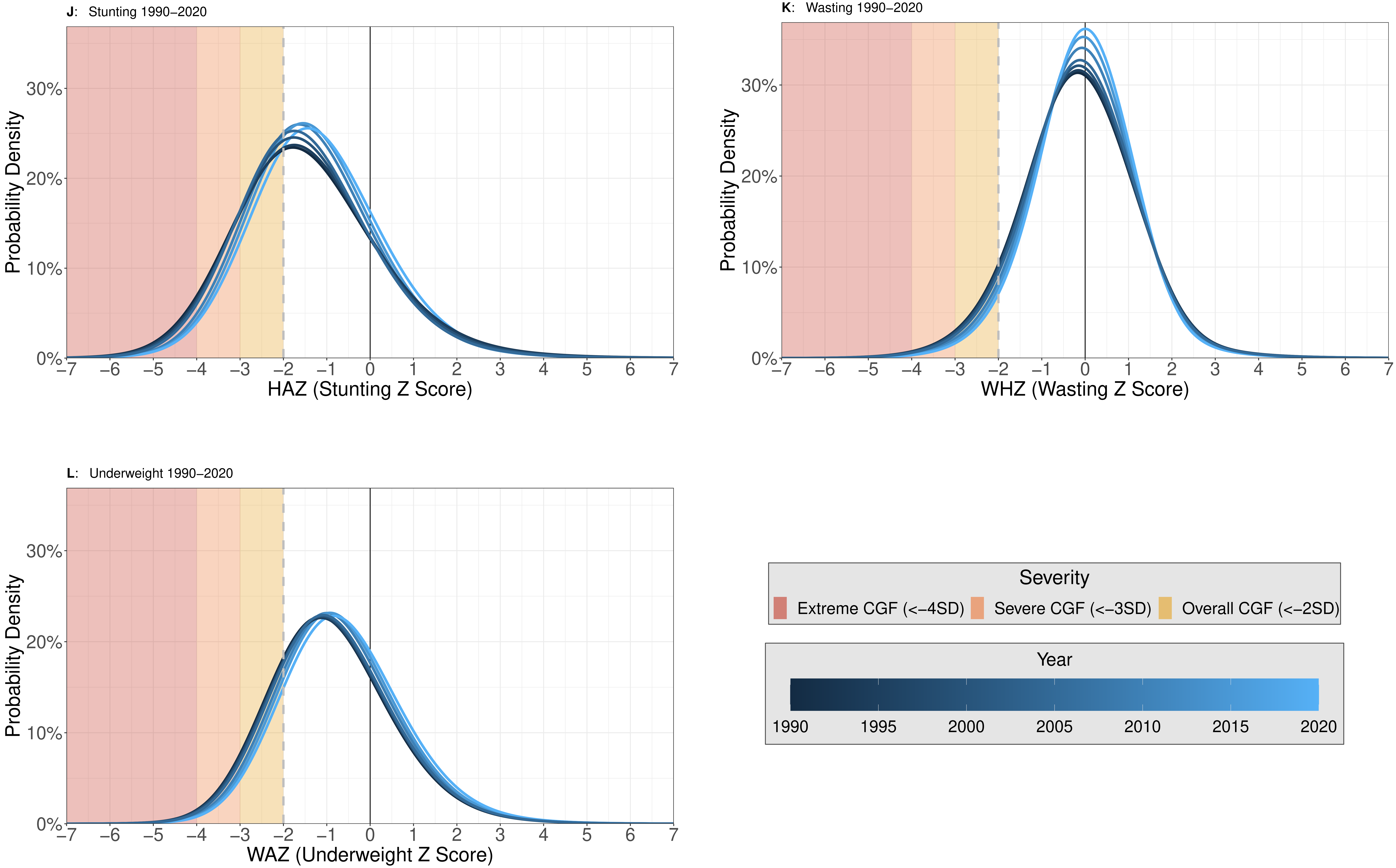

Madagascar – Stunting (HAZ)

A: Overall and Severe Stunting Prevalence

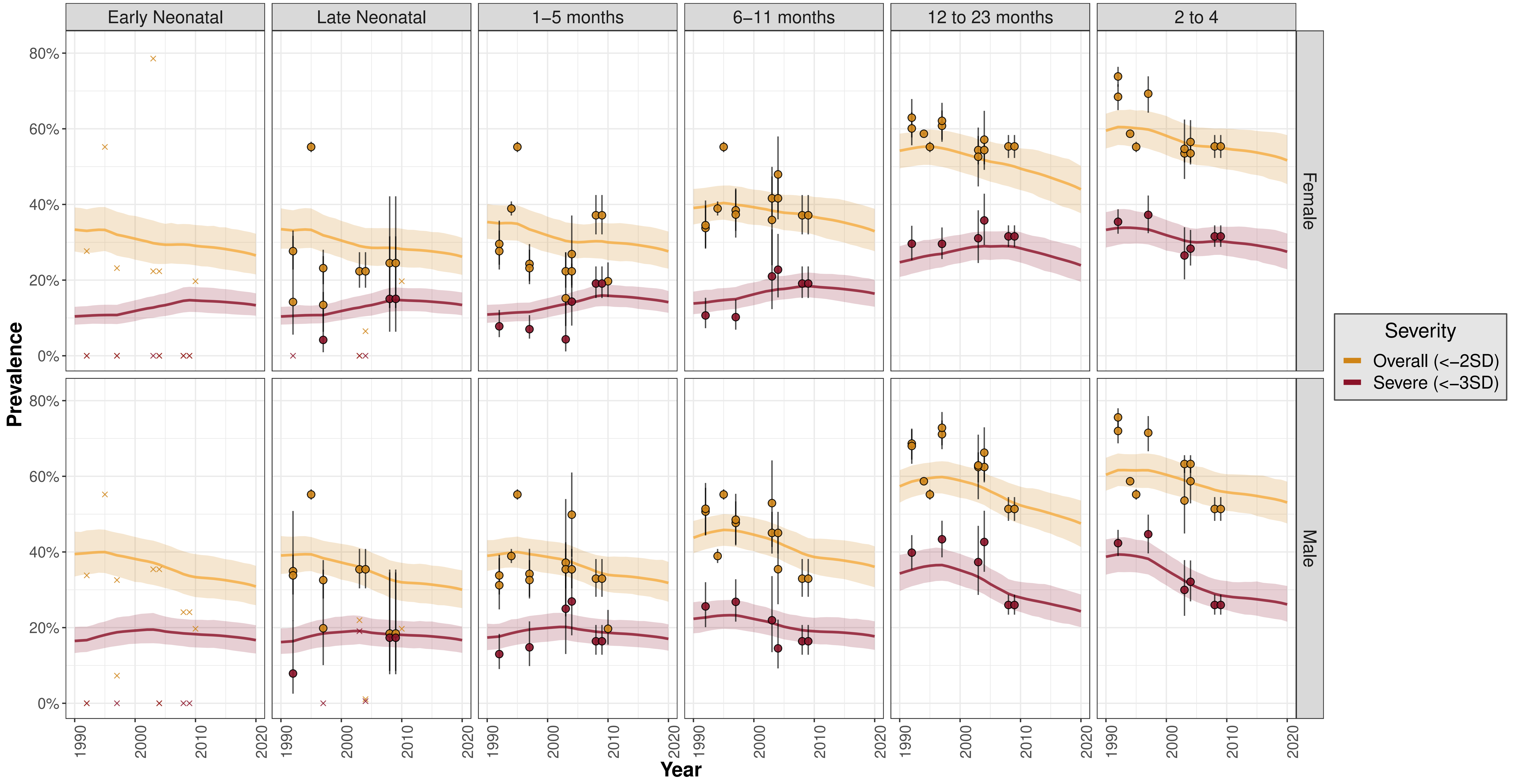

B: Transformed Mean Stunting Z Scores

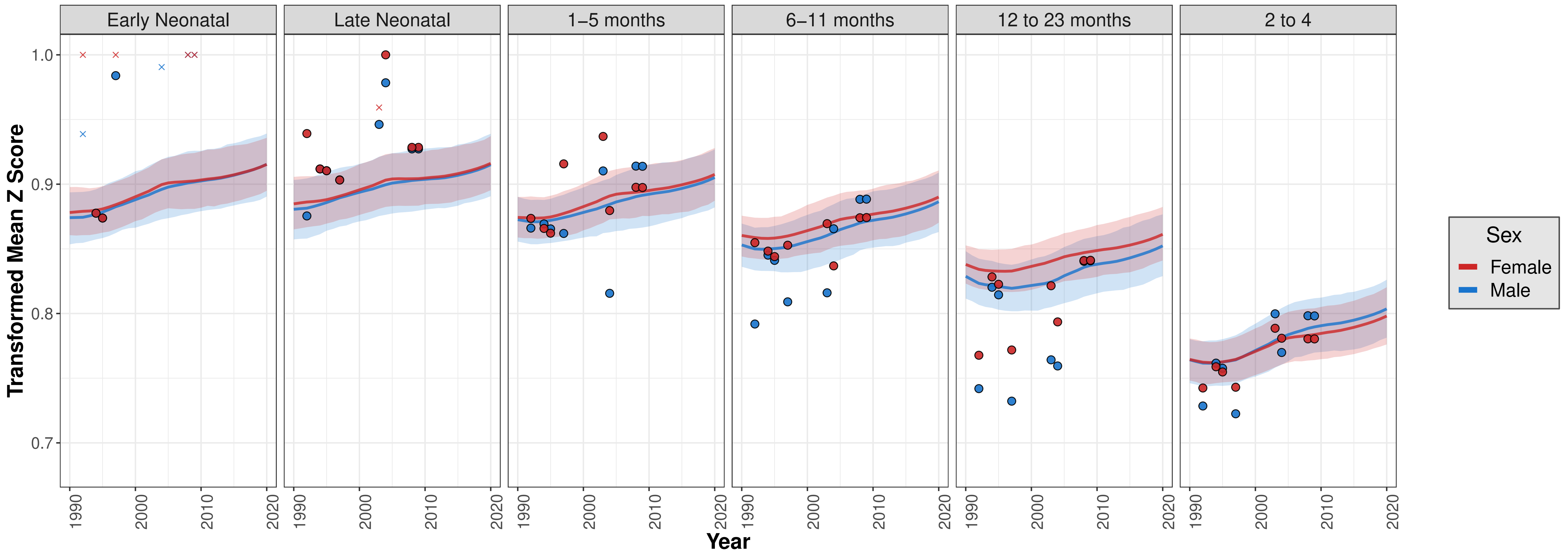

C

Year	Source
1985	WHO CGM Database
1986	WHO CGM Database
1988	WHO CGM Database
1992	DHS
1992	WHO CGM Database
1994	WHO CGM Database
1995	WHO CGM Database
1997	DHS
1997	WHO CGM Database
2003	DHS
2003	WHO CGM Database
2004	DHS
2004	WHO CGM Database
2008	DHS
2009	DHS
2010	Comprehensive Food and Nutrition Security and Vulnerability Assessment

Madagascar – Wasting (WHZ)

D: Overall and Severe Wasting Prevalence

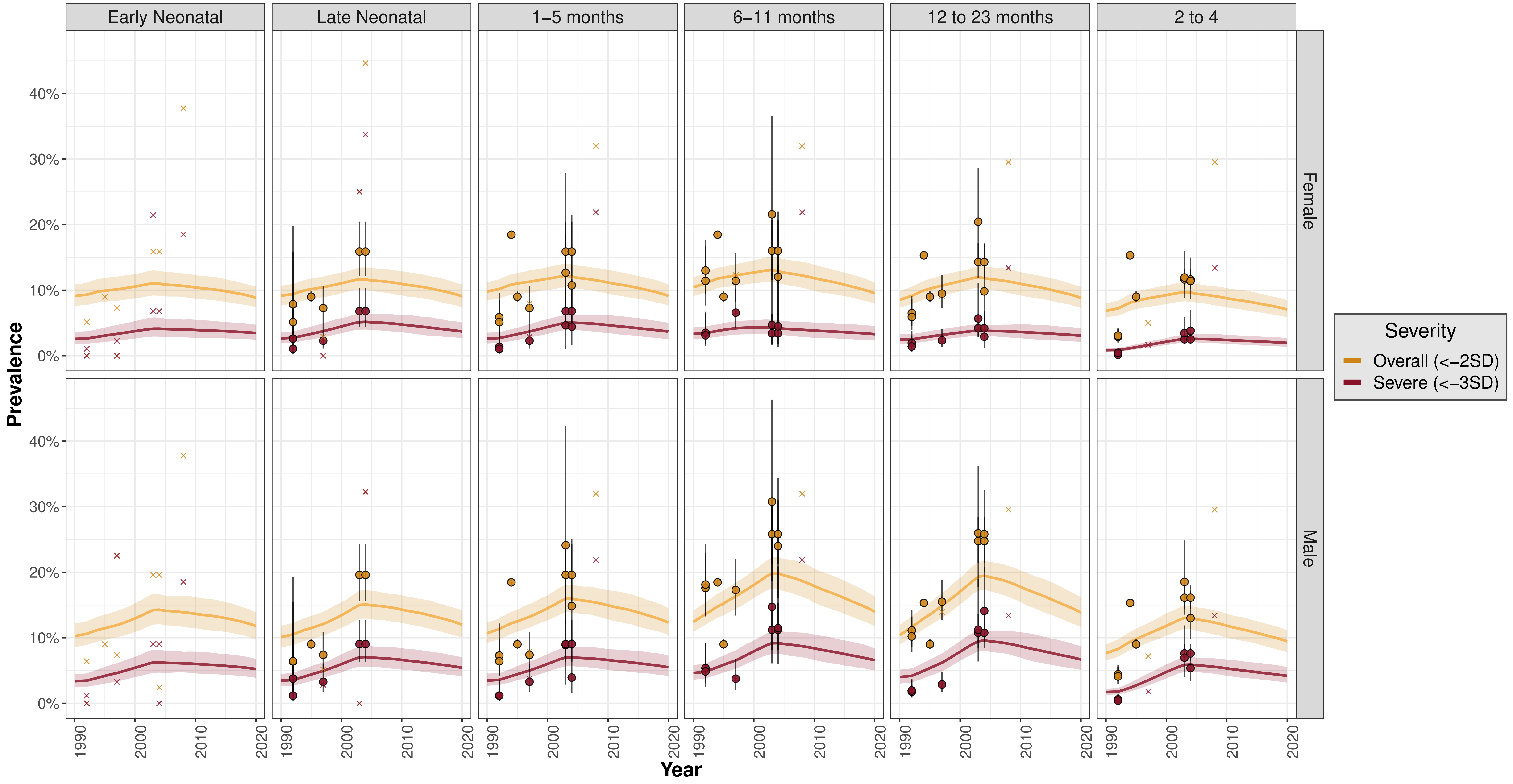

E: Transformed Mean Wasting Z Scores

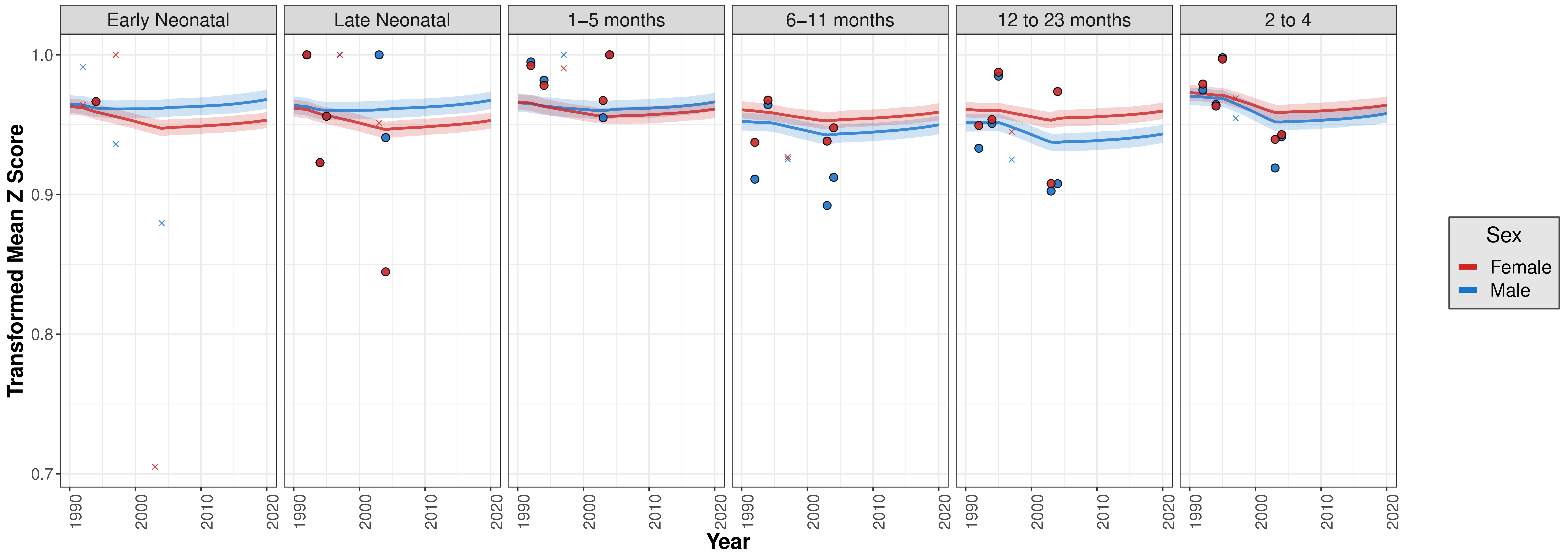

F

Year	Source
1985	WHO CGM Database
1986	WHO CGM Database
1988	WHO CGM Database
1992	DHS
1992	WHO CGM Database
1994	WHO CGM Database
1995	WHO CGM Database
1997	DHS
1997	WHO CGM Database
2003	DHS
2003	WHO CGM Database
2004	DHS
2004	WHO CGM Database
2008	DHS

Madagascar – Underweight (WAZ)

G: Overall and Severe Underweight Prevalence

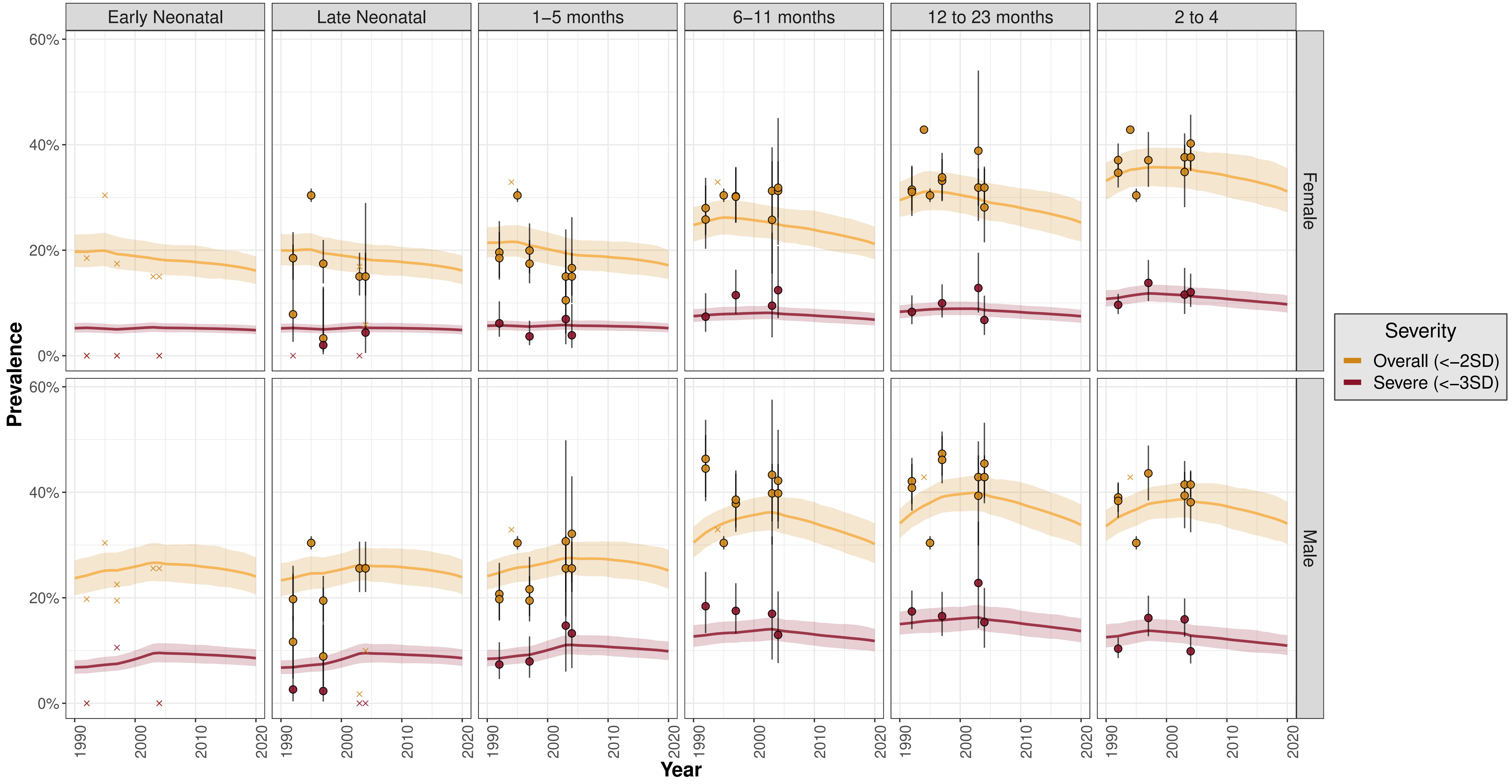

H: Transformed Mean Underweight Z Scores

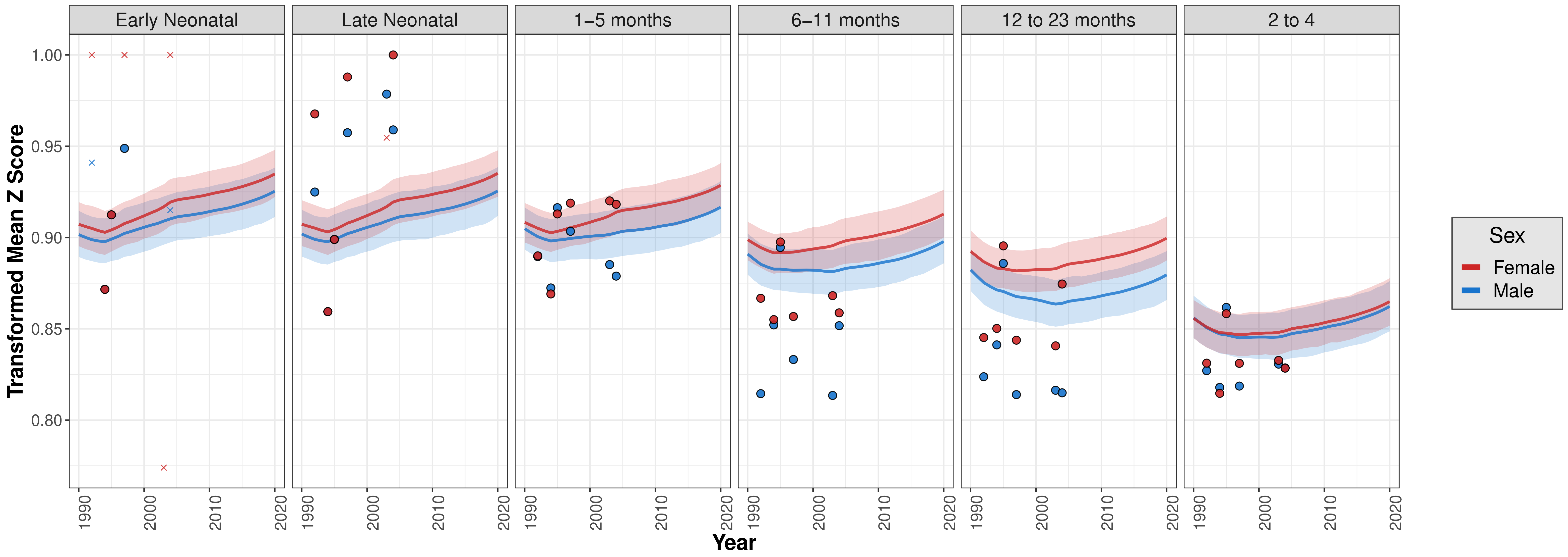

I

Year	Source
1985	WHO CGM Database
1986	WHO CGM Database
1988	WHO CGM Database
1992	DHS
1992	WHO CGM Database
1994	WHO CGM Database
1995	WHO CGM Database
1997	DHS
1997	WHO CGM Database
2003	DHS
2003	WHO CGM Database
2004	DHS
2004	WHO CGM Database

Madagascar – HAZ, WHZ, and WAZ Distributions

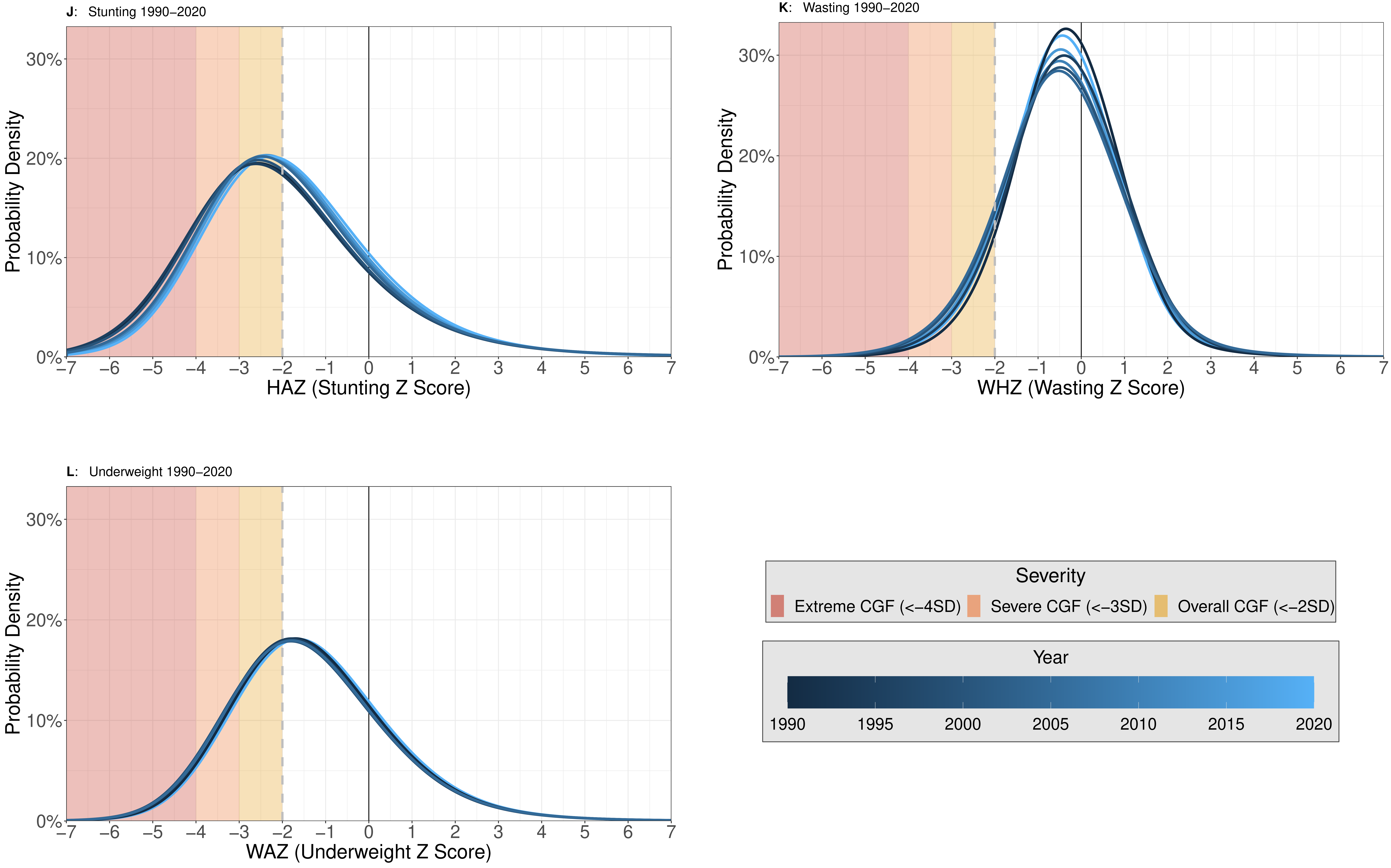

Malawi – Stunting (HAZ)

A: Overall and Severe Stunting Prevalence

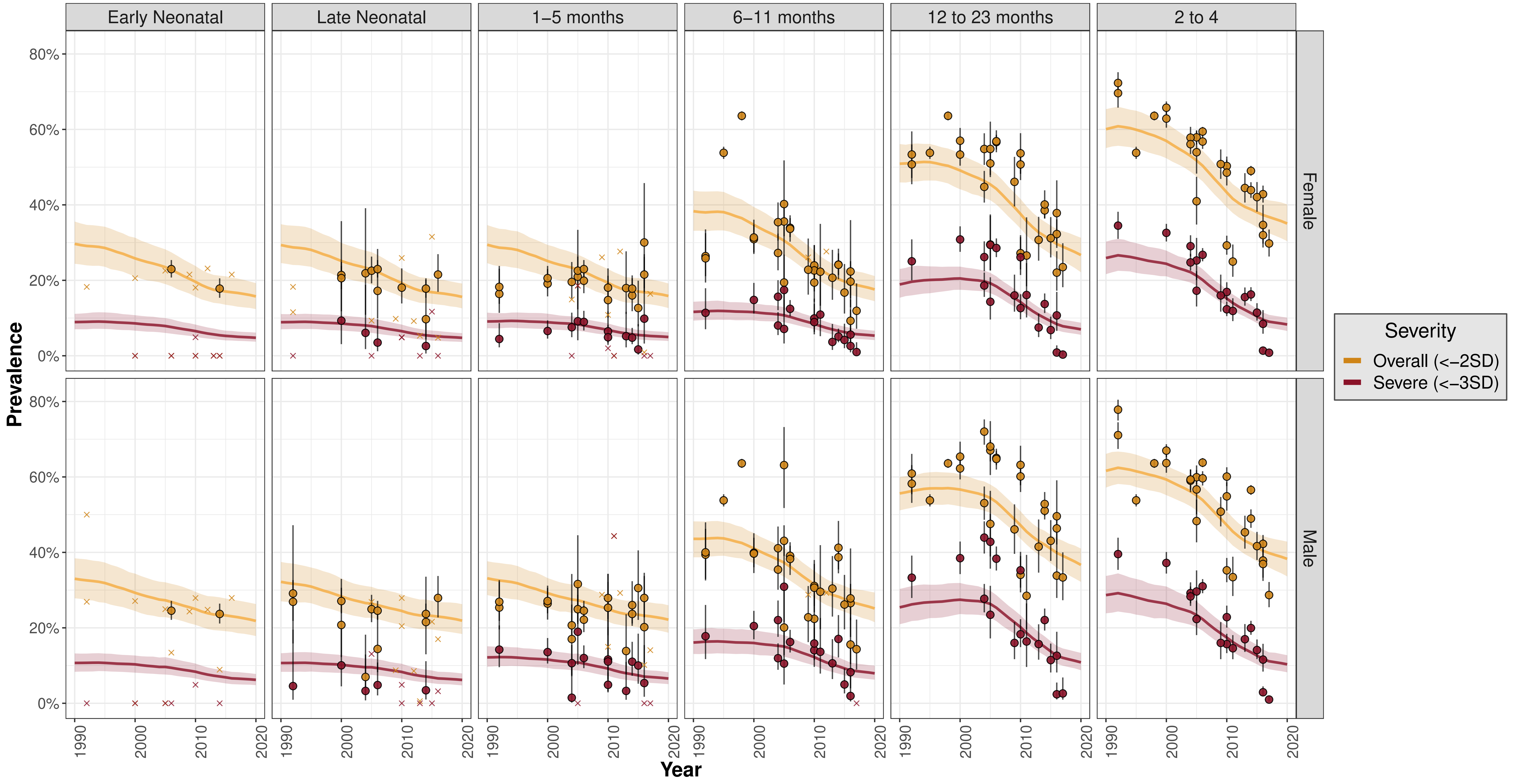

B: Transformed Mean Stunting Z Scores

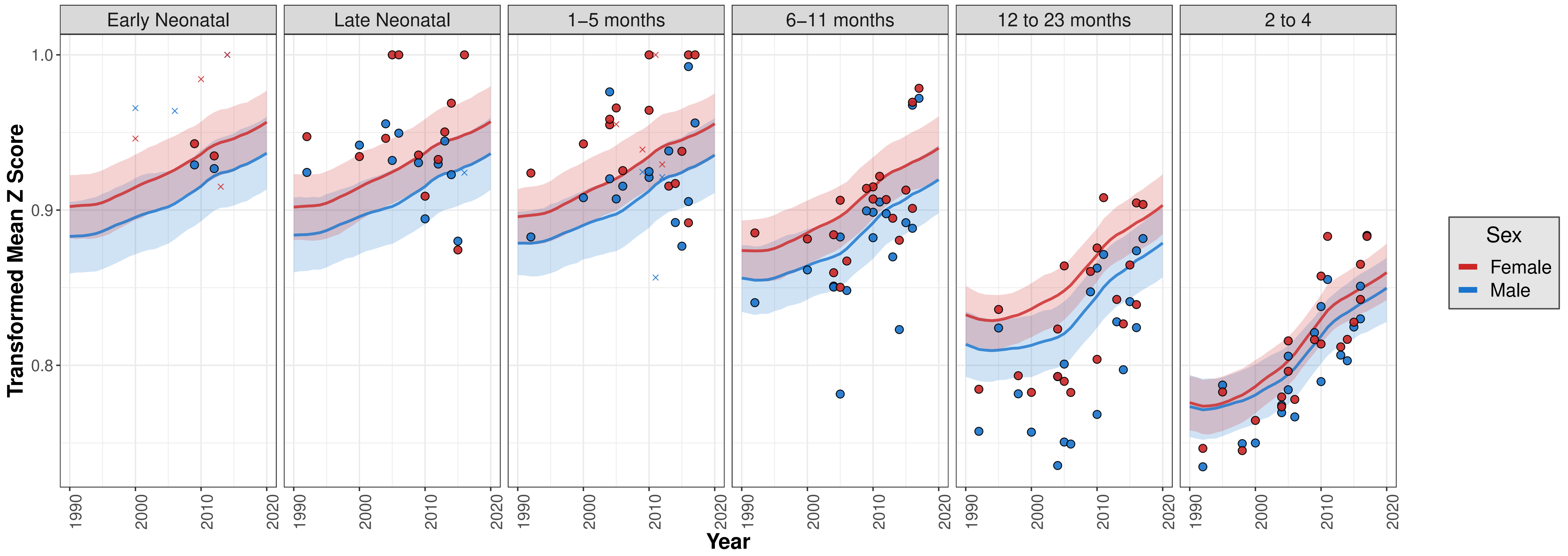

C

Year	Source
1981	WHO CGM Database
1982	WHO CGM Database
1985	WHO CGM Database
1992	DHS
1992	WHO CGM Database
1995	WHO CGM Database
1998	WHO CGM Database
2000	DHS
2000	WHO CGM Database
2004	DHS
2004	Living Standards Measurement Survey
2005	DHS
2005	Living Standards Measurement Survey
2005	WHO CGM Database
2006	MICS
2006	WHO CGM Database
2009	Welfare Monitoring Survey
2009	WHO CGM Database
2010	DHS
2010	Integrated Household Survey
2010	WHO CGM Database
2010	Integrated Household Panel Survey, Short–Term Panel,
2010	Comprehensive Food and Nutrition Security and Vulnerability Assessment
2011	Integrated Household Survey
2012	Welfare Monitoring Survey
2013	MICS
2013	Integrated Household Survey
2013	Integrated Household Panel Survey, Short–Term Panel,
2014	WHO CGM Database
2014	MICS
2015	DHS
2016	WHO CGM Database
2016	DHS
2016	Integrated Household Survey
2016	Integrated Household Panel Survey, Long–Term Panel,
2017	Integrated Household Survey

Malawi – Wasting (WHZ)

D: Overall and Severe Wasting Prevalence

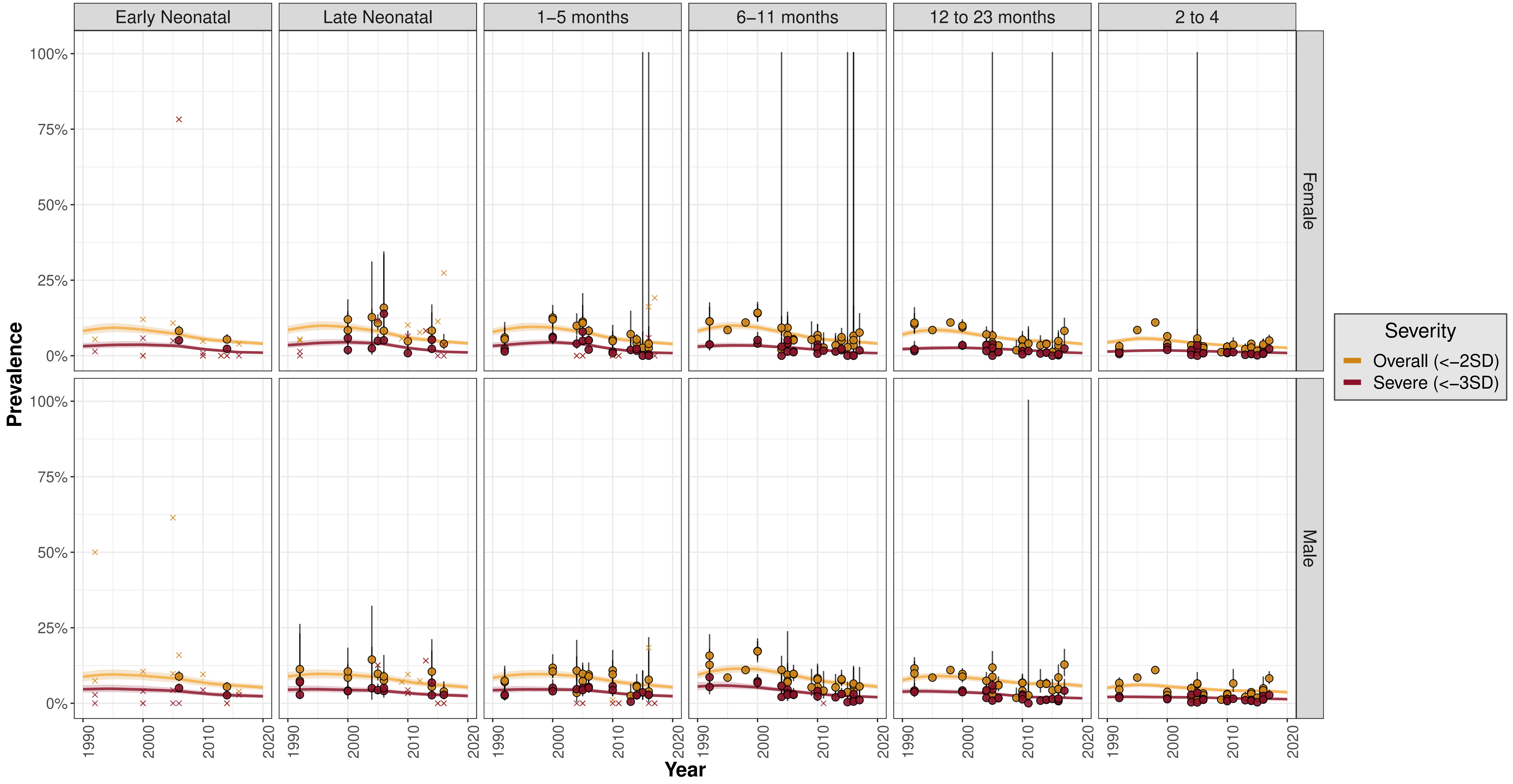

E: Transformed Mean Wasting Z Scores

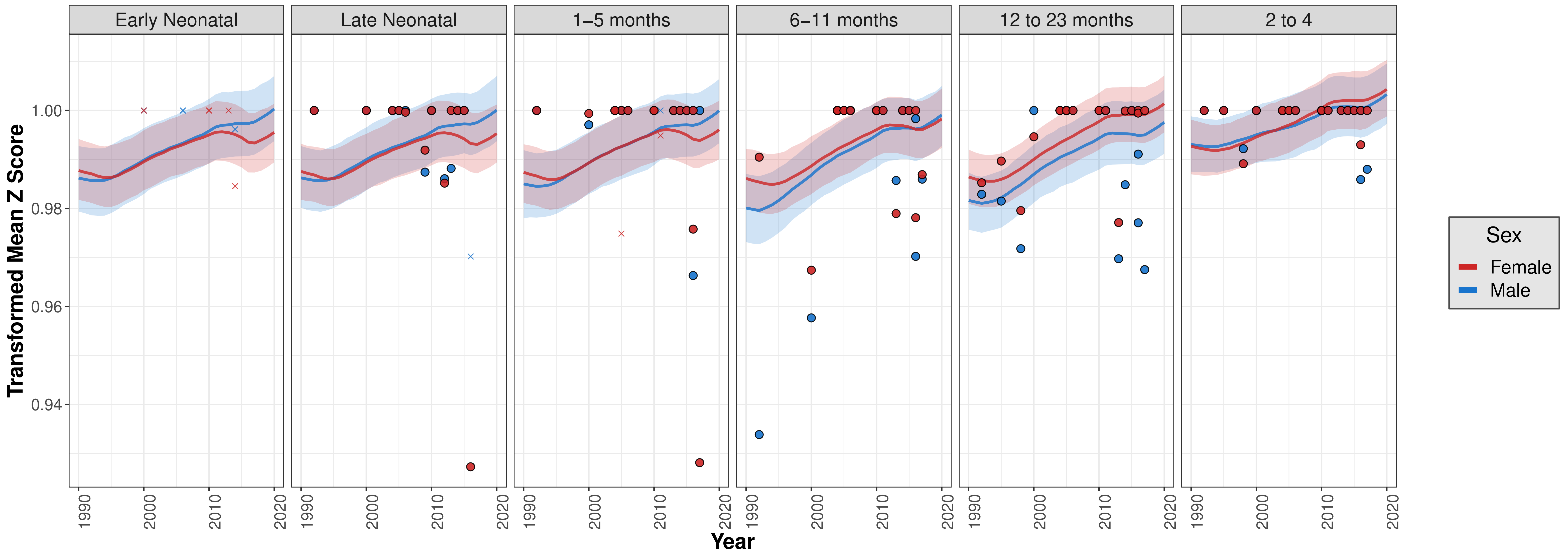

F

Year	Source
1981	WHO CGM Database
1982	WHO CGM Database
1985	WHO CGM Database
1992	DHS
1992	WHO CGM Database
1995	WHO CGM Database
1998	WHO CGM Database
2000	DHS
2000	WHO CGM Database
2004	DHS
2004	Living Standards Measurement Survey
2005	DHS
2005	Living Standards Measurement Survey
2005	WHO CGM Database
2006	MICS
2006	WHO CGM Database
2009	Welfare Monitoring Survey
2009	WHO CGM Database
2010	DHS
2010	Integrated Household Survey
2010	WHO CGM Database
2010	Integrated Household Panel Survey, Short−Term Panel,
2011	Integrated Household Survey
2012	Welfare Monitoring Survey
2013	MICS
2013	Integrated Household Survey
2013	Integrated Household Panel Survey, Short−Term Panel,
2014	WHO CGM Database
2014	MICS
2015	DHS
2016	WHO CGM Database
2016	DHS
2016	Integrated Household Survey
2016	Integrated Household Panel Survey, Long−Term Panel,
2017	Integrated Household Survey

Malawi – Underweight (WAZ)

G: Overall and Severe Underweight Prevalence

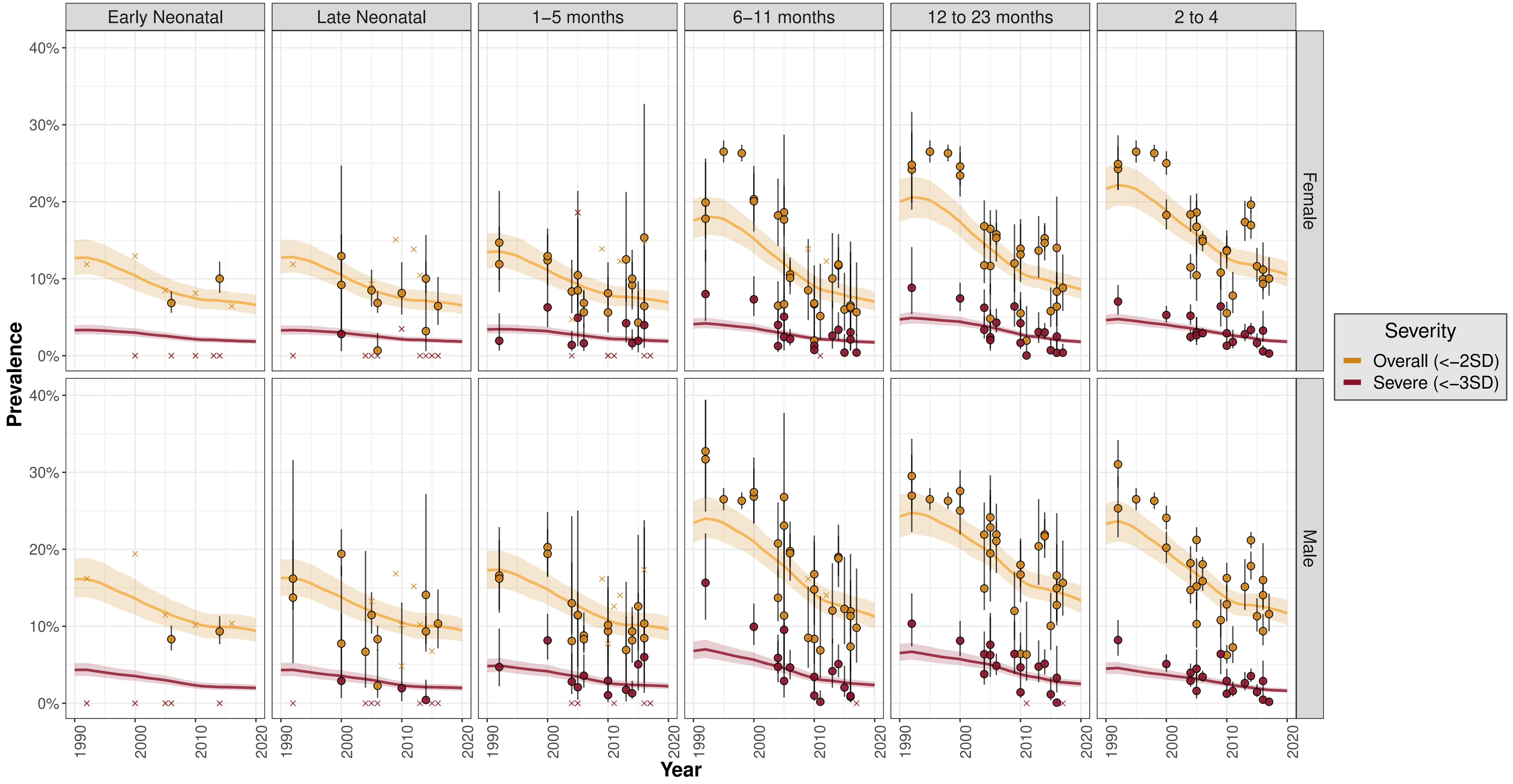

H: Transformed Mean Underweight Z Scores

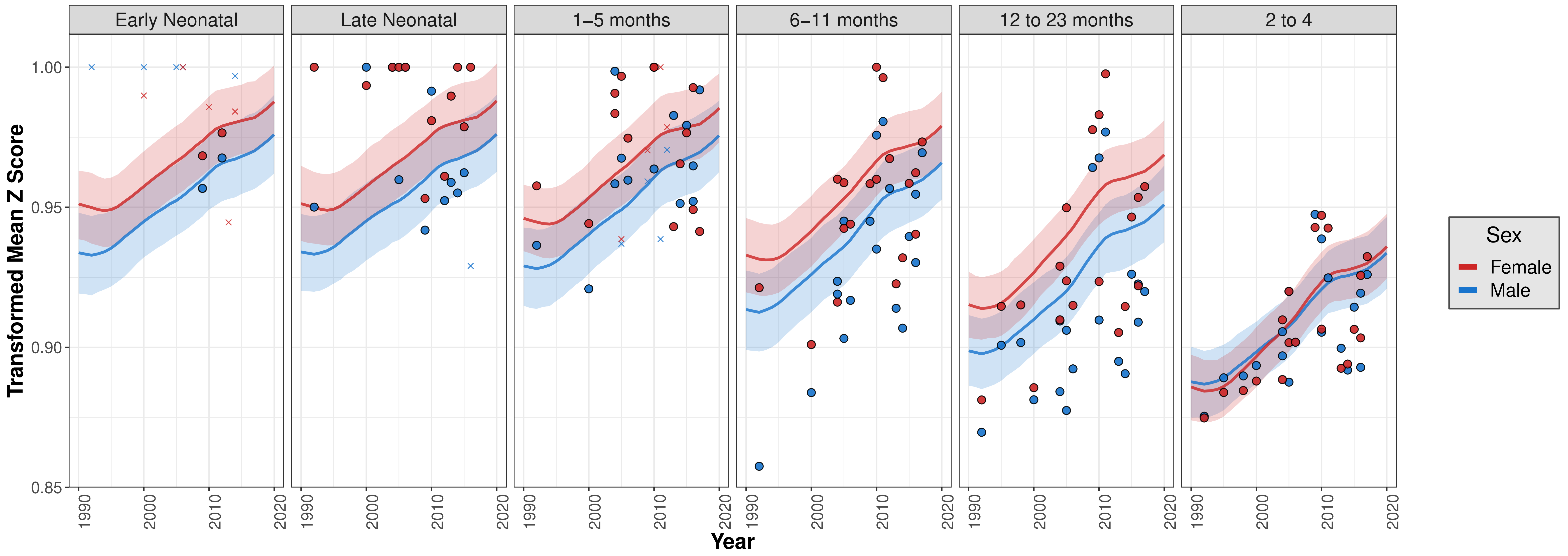

I

Year	Source
1981	WHO CGM Database
1992	DHS
1992	WHO CGM Database
1995	WHO CGM Database
1998	WHO CGM Database
2000	DHS
2000	WHO CGM Database
2004	DHS
2004	Living Standards Measurement Survey
2005	DHS
2005	Living Standards Measurement Survey
2005	WHO CGM Database
2006	MICS
2006	WHO CGM Database
2009	Welfare Monitoring Survey
2009	WHO CGM Database
2010	DHS
2010	Integrated Household Survey
2010	WHO CGM Database
2010	Integrated Household Panel Survey, Short–Term Panel,
2011	Integrated Household Survey
2012	Welfare Monitoring Survey
2013	MICS
2013	Integrated Household Survey
2013	Integrated Household Panel Survey, Short–Term Panel,
2014	WHO CGM Database
2014	MICS
2015	DHS
2016	WHO CGM Database
2016	DHS
2016	Integrated Household Survey
2016	Integrated Household Panel Survey, Long–Term Panel,
2017	Integrated Household Survey

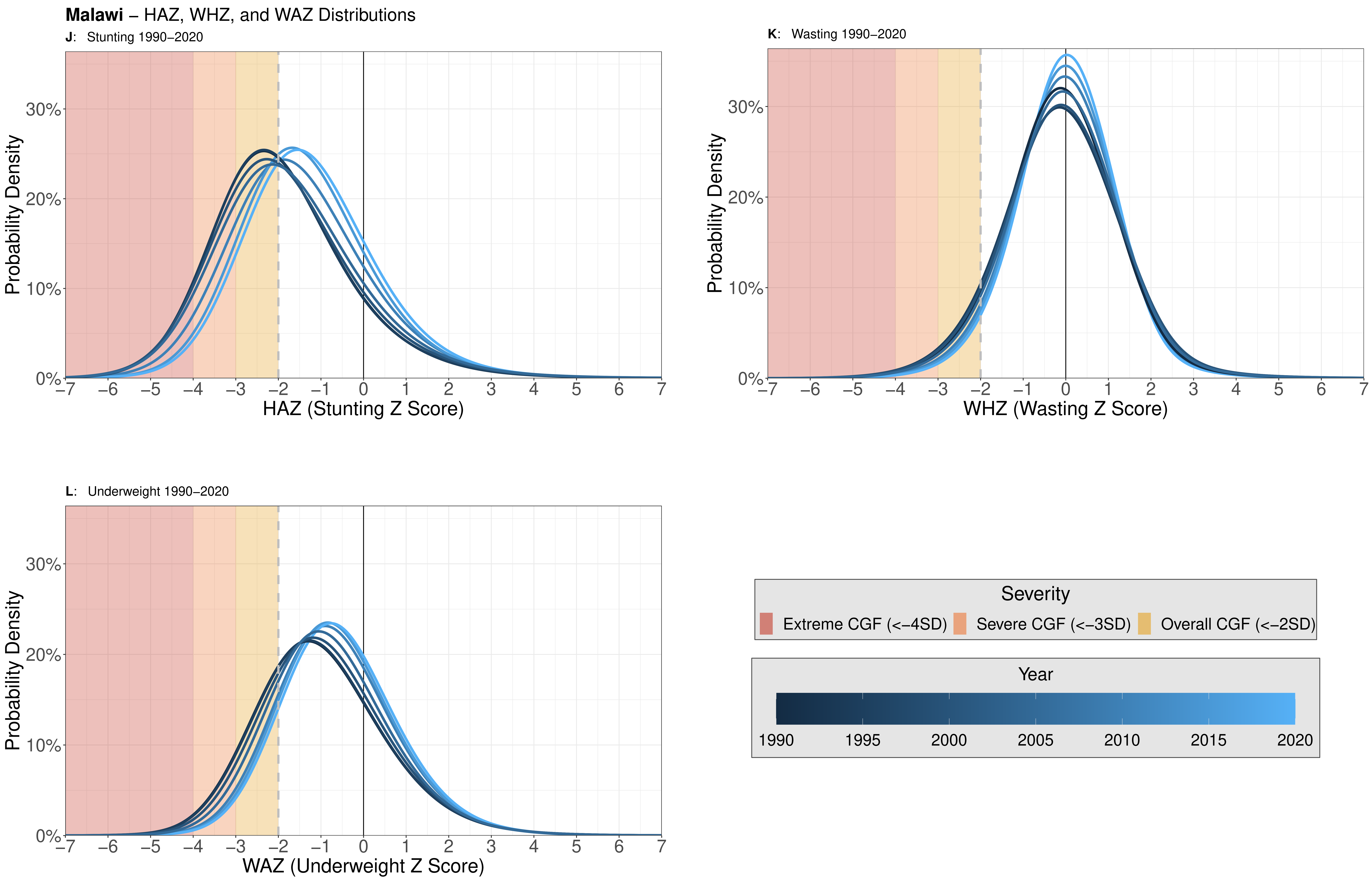

Malawi – HAZ, WHZ, and WAZ Distributions
J: Stunting 1990–2020
Probability Density
30%
20%
10%
0%
−7
−6
−5
−4
−3
−2
−1
0
1
2
3
4
5
6
7
HAZ (Stunting Z Score)
K: Wasting 1990–2020
WHZ (Wasting Z Score)
L: Underweight 1990–2020
WAZ (Underweight Z Score)
Severity
Extreme CGF (<−4SD)
Severe CGF (<−3SD)
Overall CGF (<−2SD)
Year
1990
1995
2000
2005
2010
2015
2020

Mozambique – Stunting (HAZ)

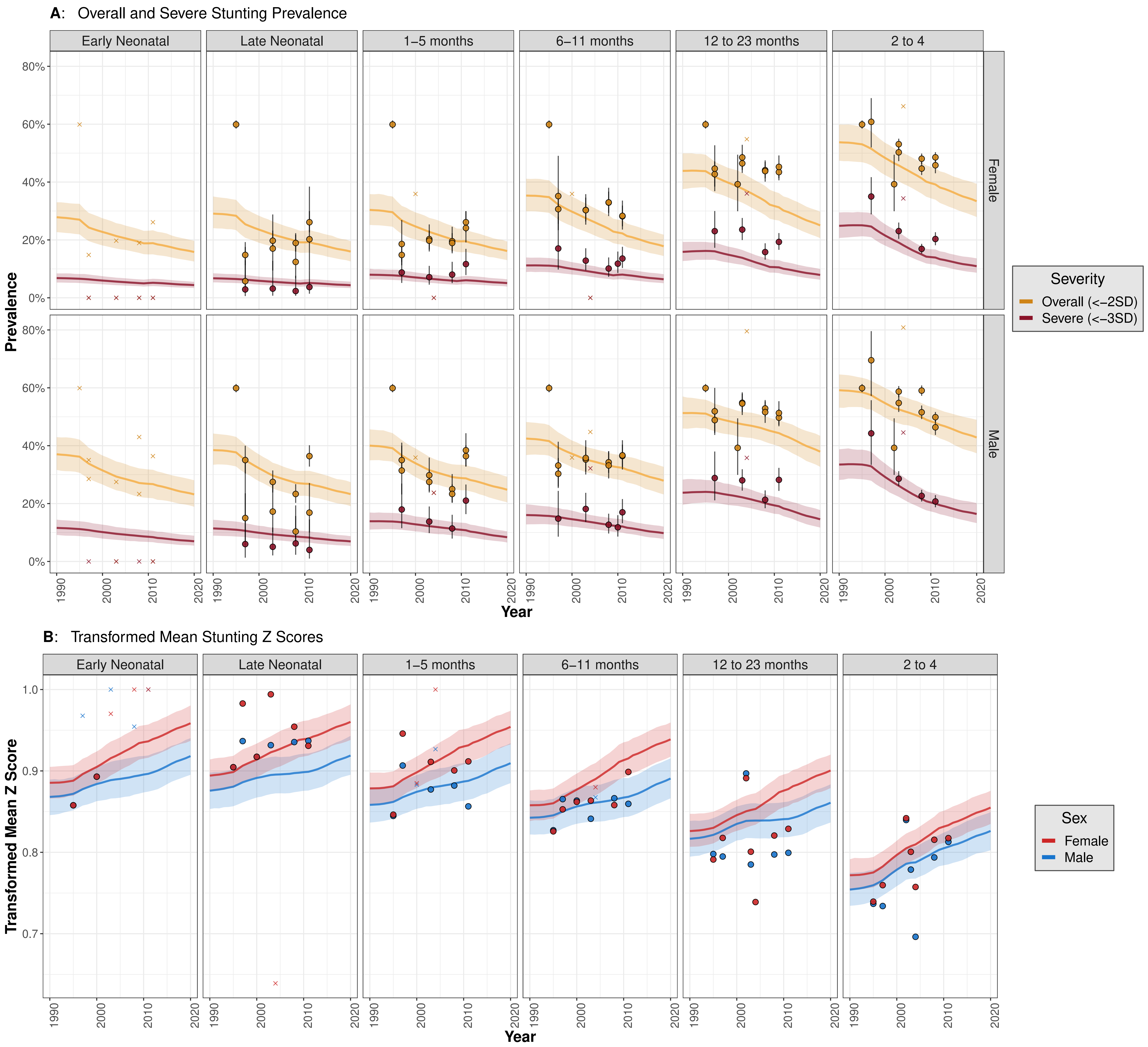

C

Year	Source
1989	WHO CGM Database
1995	WHO CGM Database
1997	DHS
1997	WHO CGM Database
2000	WHO CGM Database
2002	WHO CGM Database
2003	DHS
2003	WHO CGM Database
2004	DHS
2008	MICS
2008	WHO CGM Database
2009	Manhica Global Enteric Multicenter Study
2010	Comprehensive Food and Nutrition Security and Vulnerability Assessment
2011	DHS
2011	WHO CGM Database
2012	Manhica Global Enteric Multicenter Study

Mozambique – Wasting (WHZ)

D: Overall and Severe Wasting Prevalence

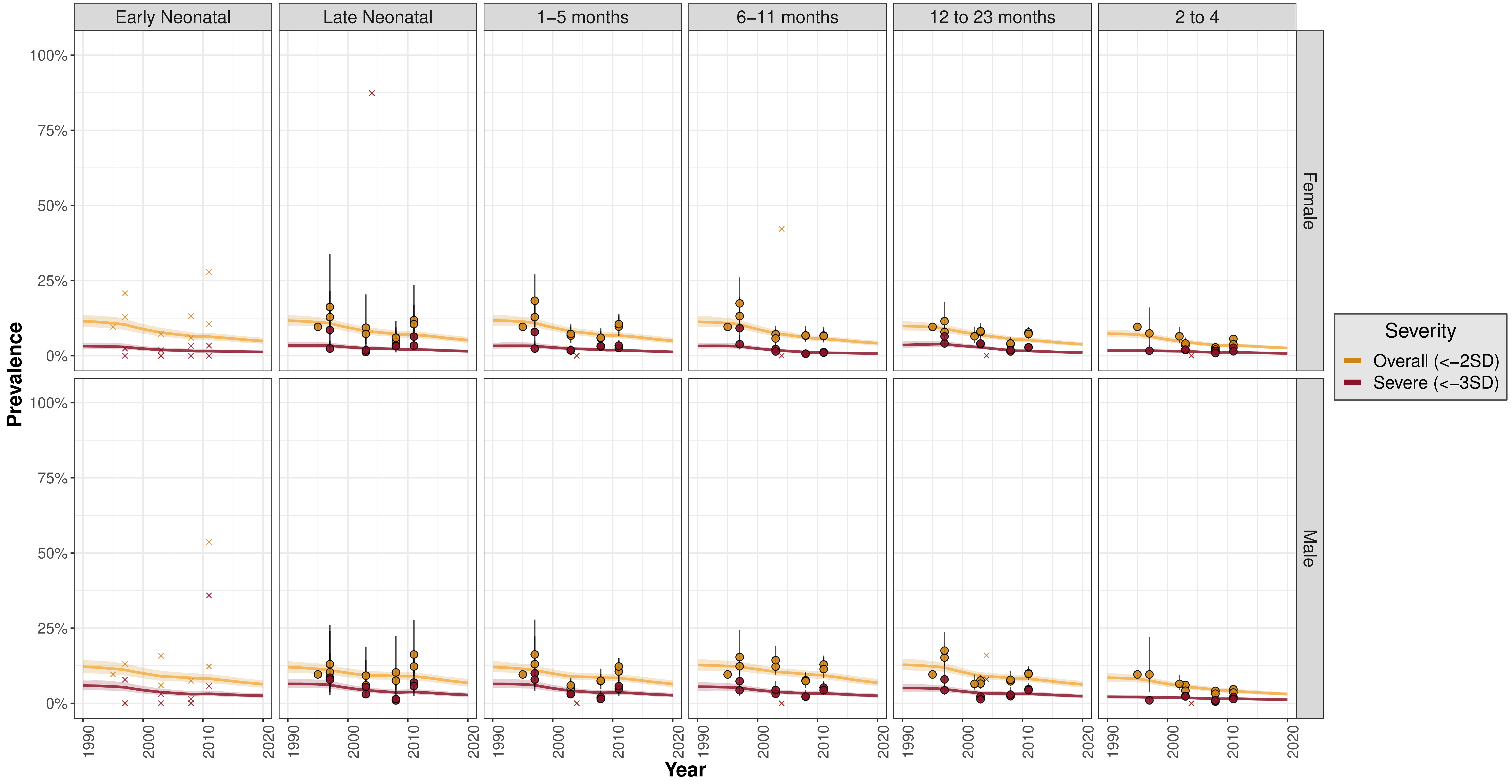

E: Transformed Mean Wasting Z Scores

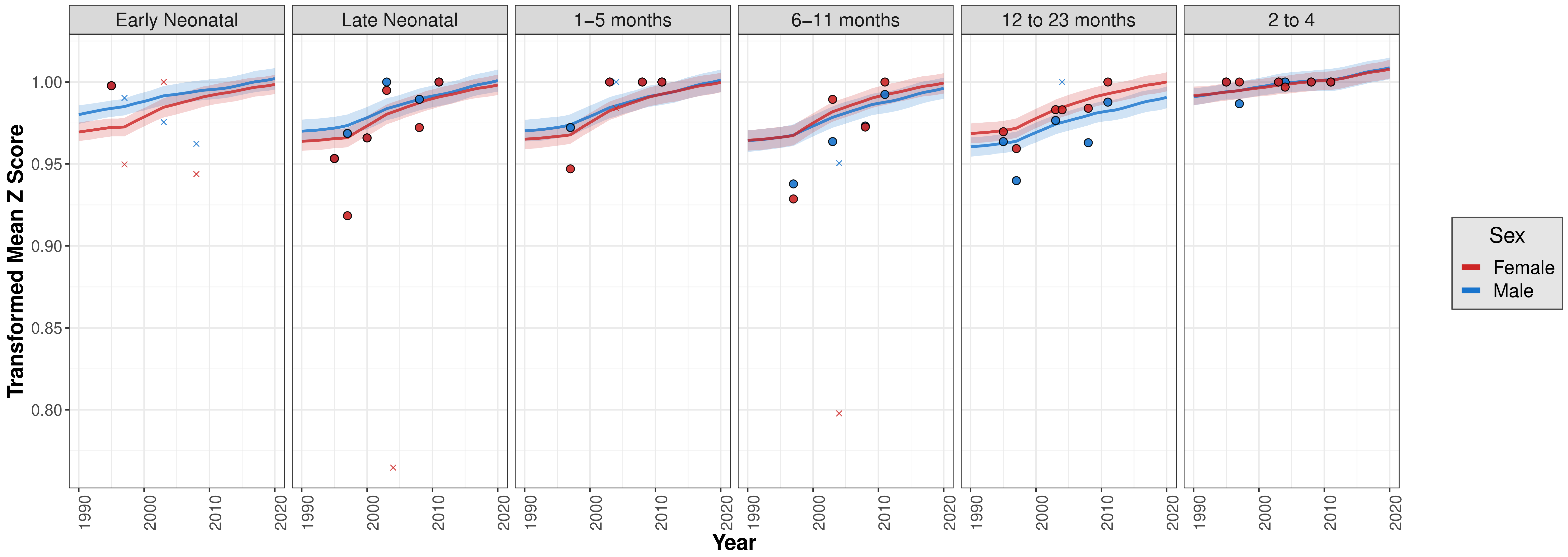

F

Year	Source
1989	WHO CGM Database
1995	WHO CGM Database
1997	DHS
1997	WHO CGM Database
2000	WHO CGM Database
2002	WHO CGM Database
2003	DHS
2003	WHO CGM Database
2004	DHS
2008	MICS
2008	WHO CGM Database
2009	Manhica Global Enteric Multicenter Study
2011	DHS
2011	WHO CGM Database
2012	Manhica Global Enteric Multicenter Study

Mozambique – Underweight (WAZ)

G: Overall and Severe Underweight Prevalence

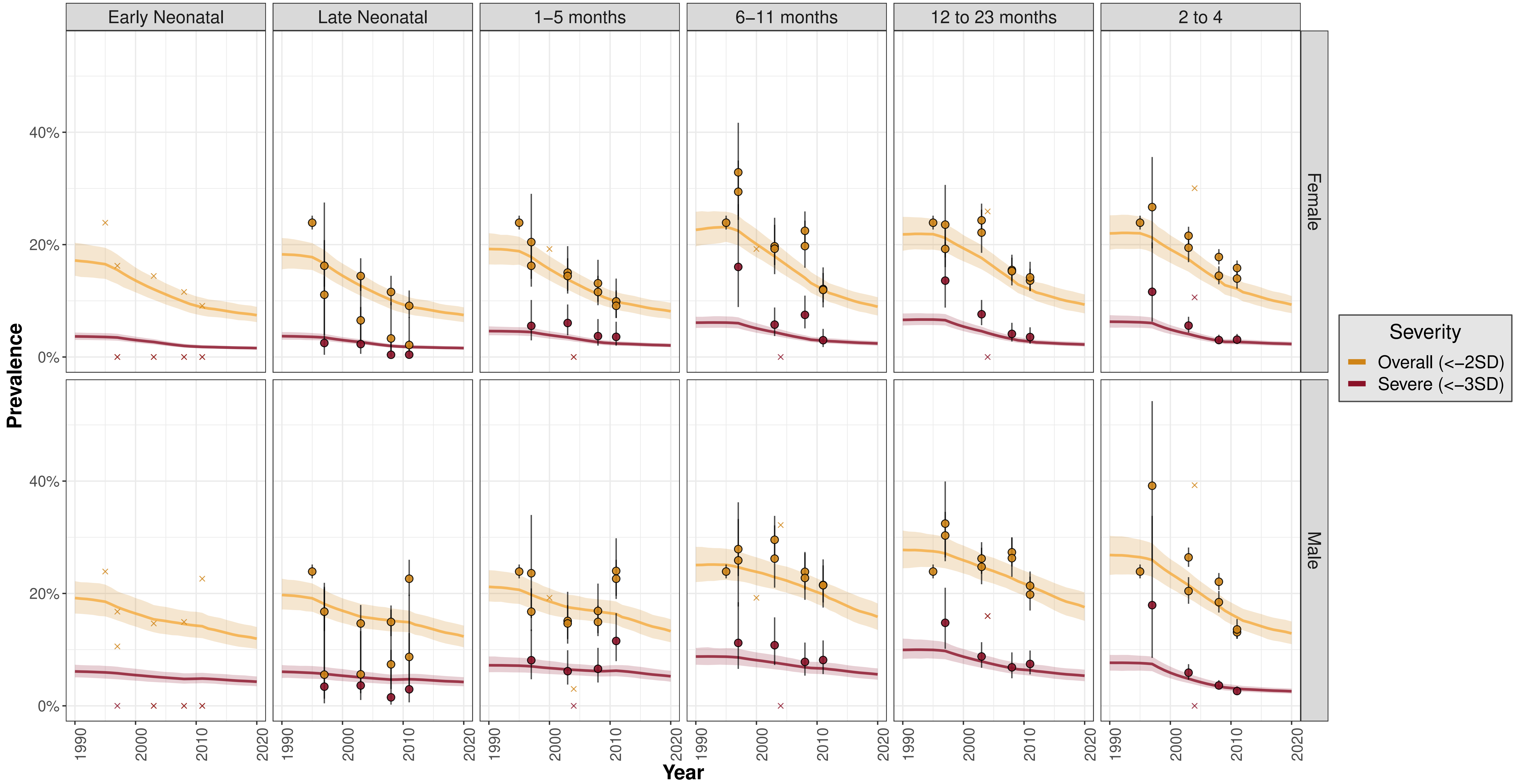

H: Transformed Mean Underweight Z Scores

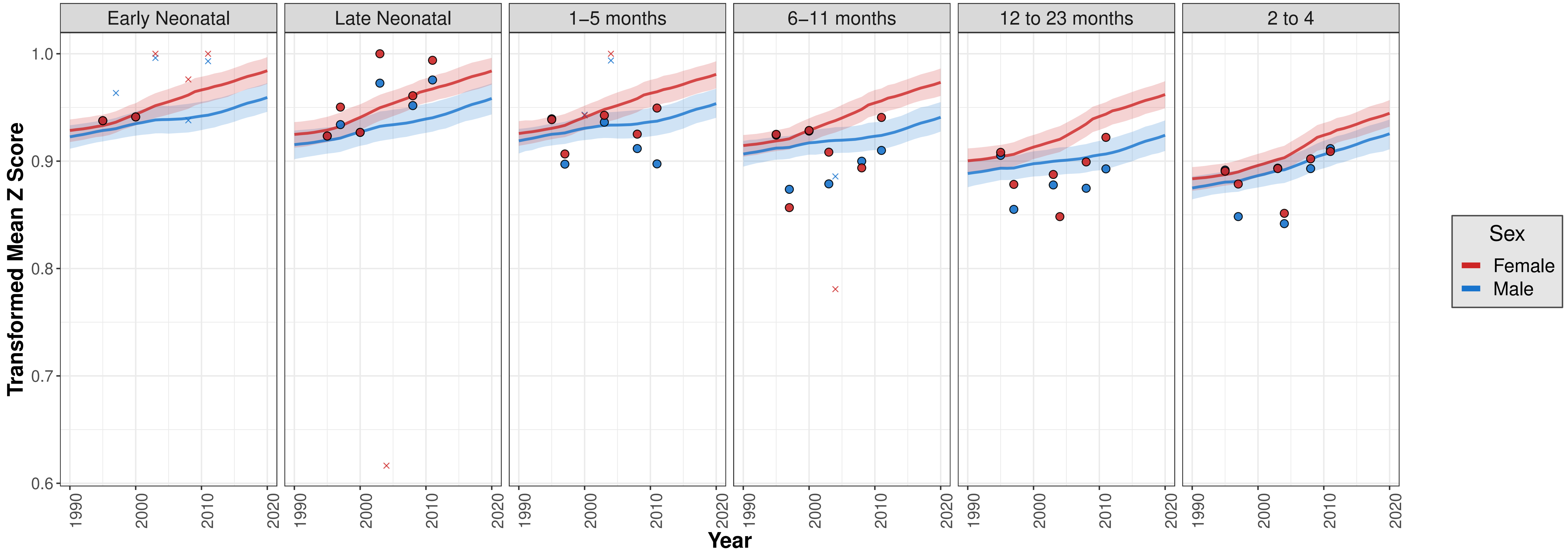

I

Year	Source
1989	WHO CGM Database
1995	WHO CGM Database
1997	DHS
1997	WHO CGM Database
2000	WHO CGM Database
2003	DHS
2003	WHO CGM Database
2004	DHS
2008	MICS
2008	WHO CGM Database
2009	Manhica Global Enteric Multicenter Study
2011	DHS
2011	WHO CGM Database
2012	Manhica Global Enteric Multicenter Study

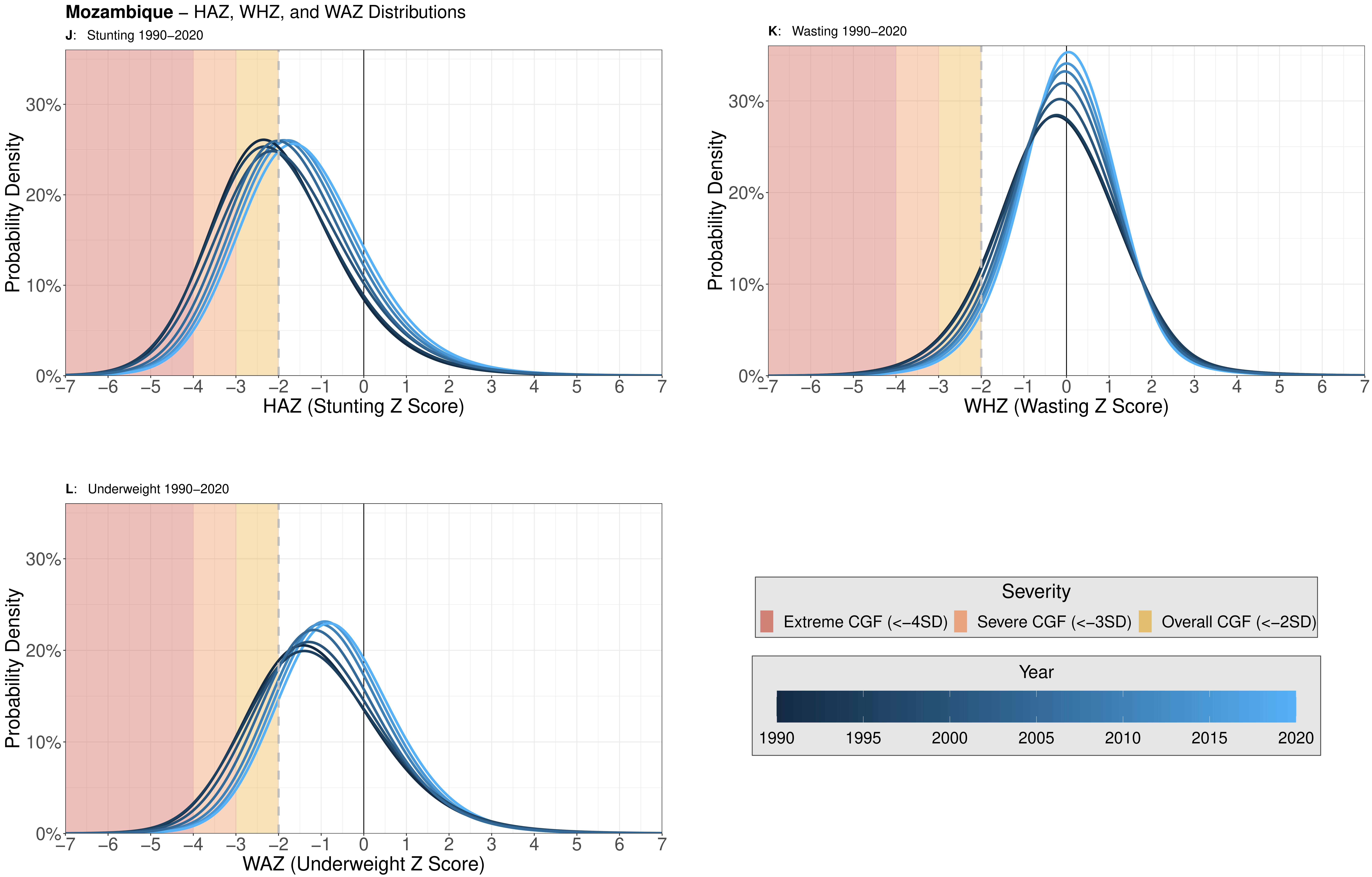

Mozambique – HAZ, WHZ, and WAZ Distributions
J: Stunting 1990–2020
Probability Density
0%
10%
20%
30%
−7 −6 −5 −4 −3 −2 −1 0 1 2 3 4 5 6 7
HAZ (Stunting Z Score)
K: Wasting 1990–2020
Probability Density
WHZ (Wasting Z Score)
L: Underweight 1990–2020
Probability Density
WAZ (Underweight Z Score)
Severity
Extreme CGF (<−4SD)
Severe CGF (<−3SD)
Overall CGF (<−2SD)
Year
1990
1995
2000
2005
2010
2015
2020

Rwanda – Stunting (HAZ)

A: Overall and Severe Stunting Prevalence

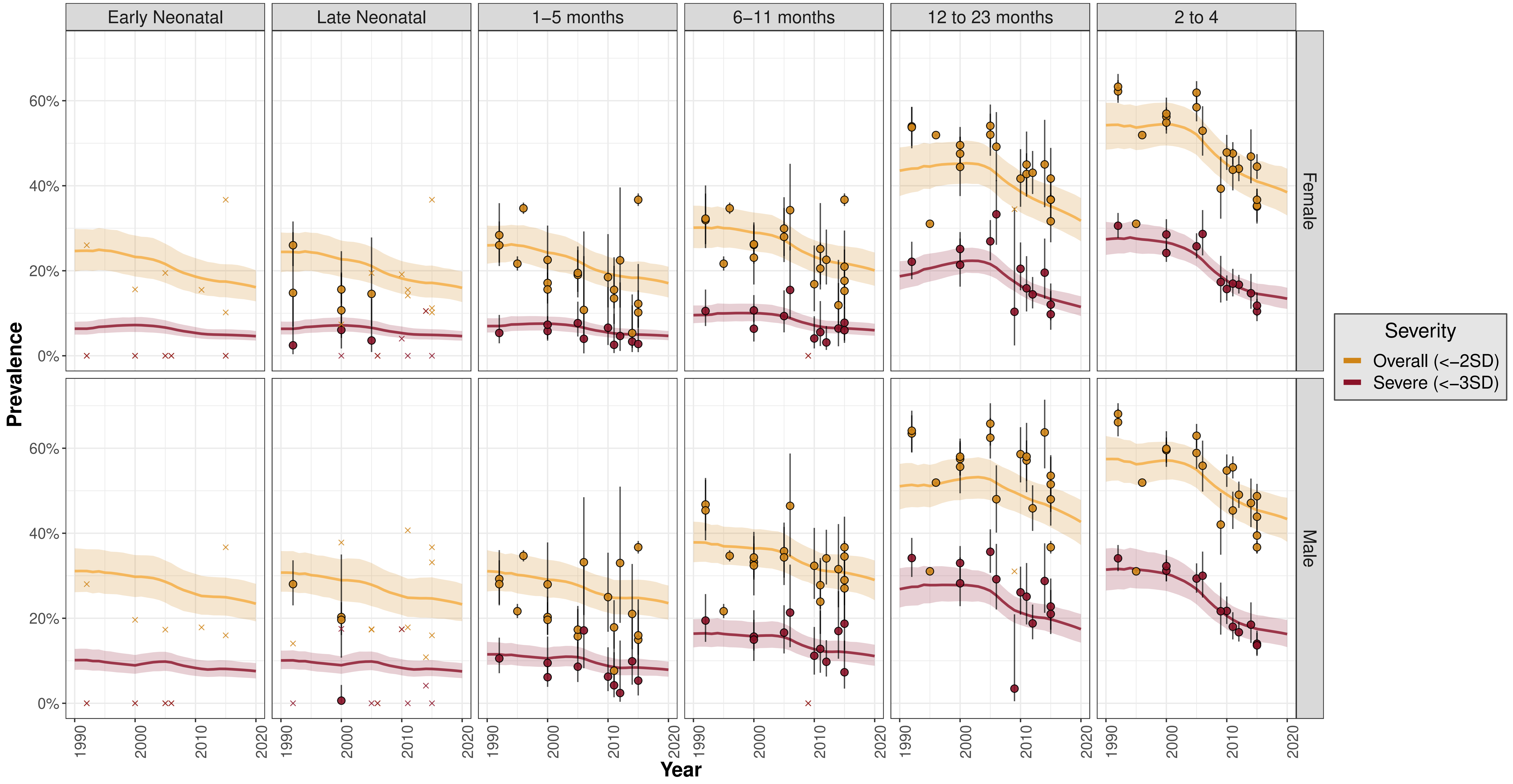

B: Transformed Mean Stunting Z Scores

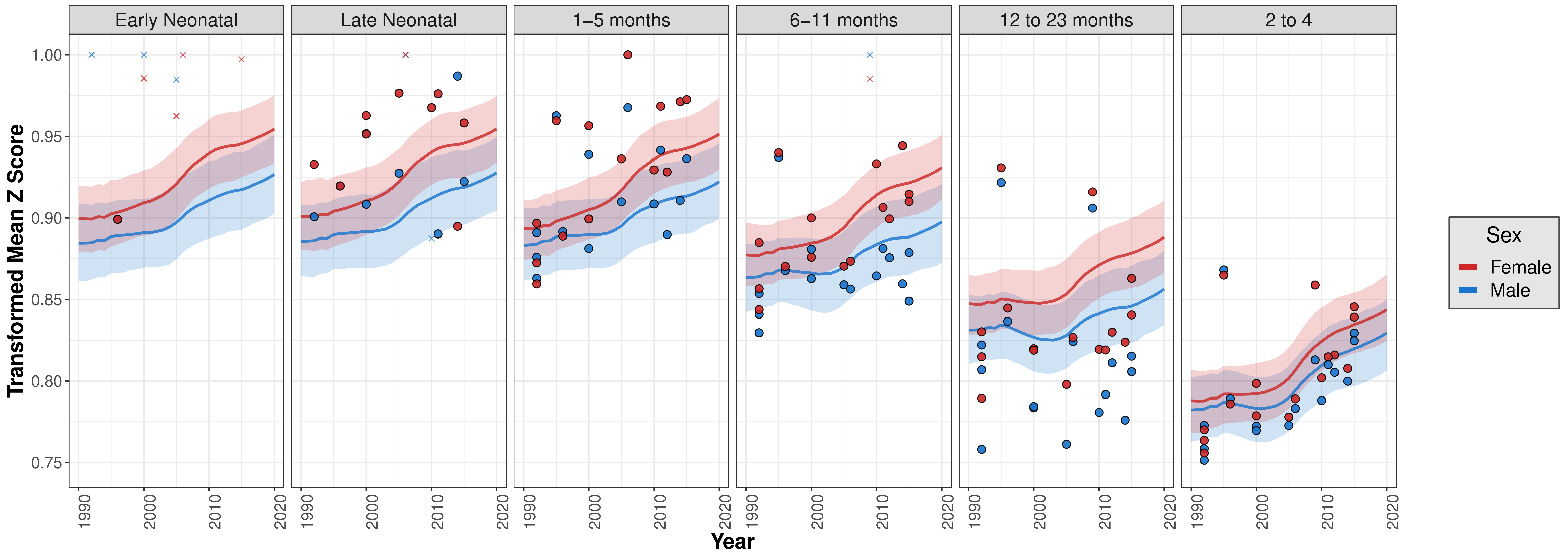

C

Year	Source
1992	DHS
1992	WHO CGM Database
1995	WHO CGM Database
1996	WHO CGM Database
2000	DHS
2000	MICS
2000	WHO CGM Database
2005	DHS
2005	WHO CGM Database
2006	Comprehensive Food Security and Vulnerability Analysis
2009	Comprehensive Food Security and Vulnerability Assessment and Nutrition Survey
2010	DHS
2011	DHS
2011	WHO CGM Database
2012	Comprehensive Food Security and Vulnerability Assessment and Nutrition Survey
2014	DHS
2015	WHO CGM Database
2015	DHS
2015	Comprehensive Food Security and Vulnerability Assessment and Nutrition Survey

Rwanda – Wasting (WHZ)

D: Overall and Severe Wasting Prevalence

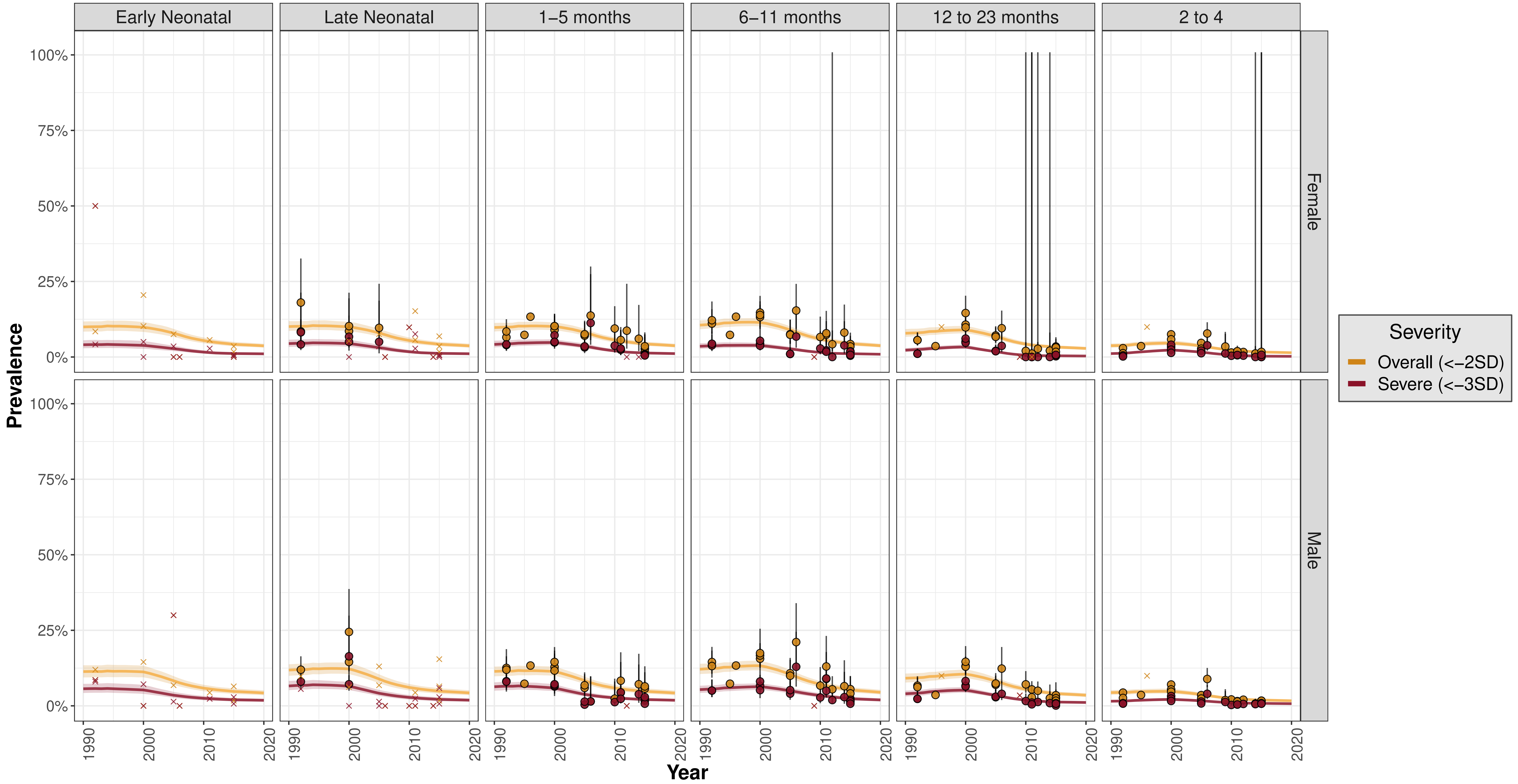

E: Transformed Mean Wasting Z Scores

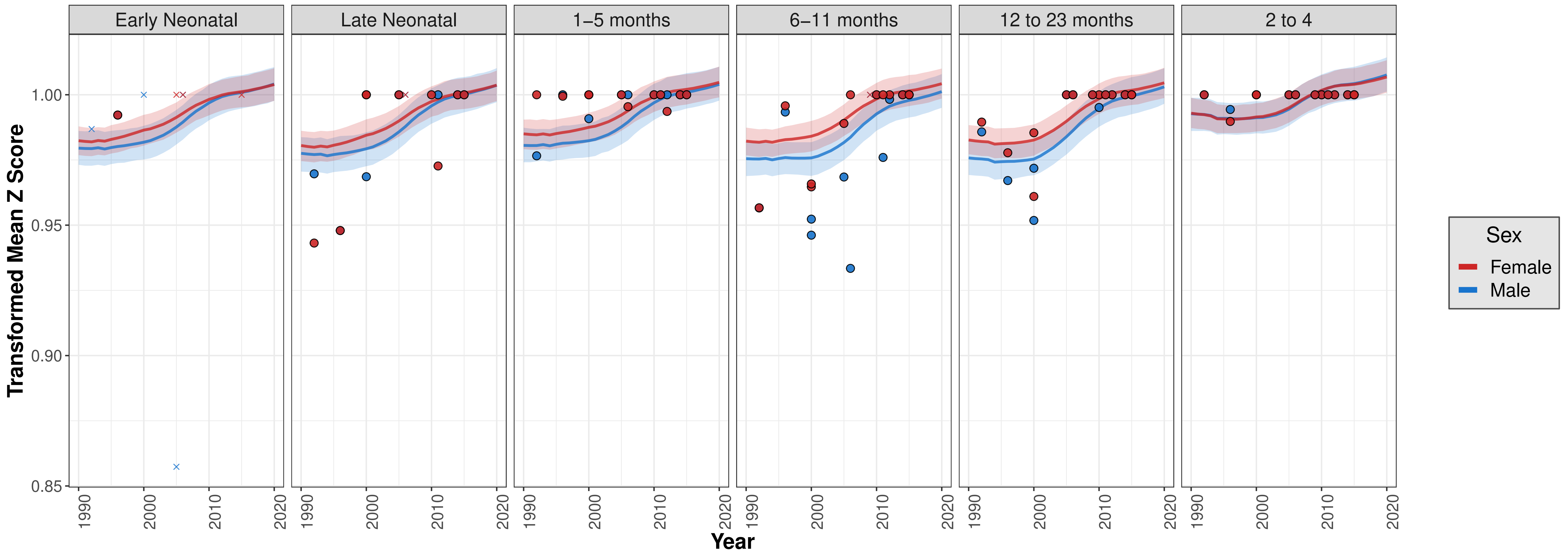

F

Year	Source
1992	DHS
1992	WHO CGM Database
1995	WHO CGM Database
1996	WHO CGM Database
2000	DHS
2000	MICS
2000	WHO CGM Database
2005	DHS
2005	WHO CGM Database
2006	Comprehensive Food Security and Vulnerability Analysis
2009	Comprehensive Food Security and Vulnerability Assessment and Nutrition Survey
2010	DHS
2011	DHS
2011	WHO CGM Database
2012	Comprehensive Food Security and Vulnerability Assessment and Nutrition Survey
2014	DHS
2015	WHO CGM Database
2015	DHS
2015	Comprehensive Food Security and Vulnerability Assessment and Nutrition Survey

Rwanda – Underweight (WAZ)

G: Overall and Severe Underweight Prevalence

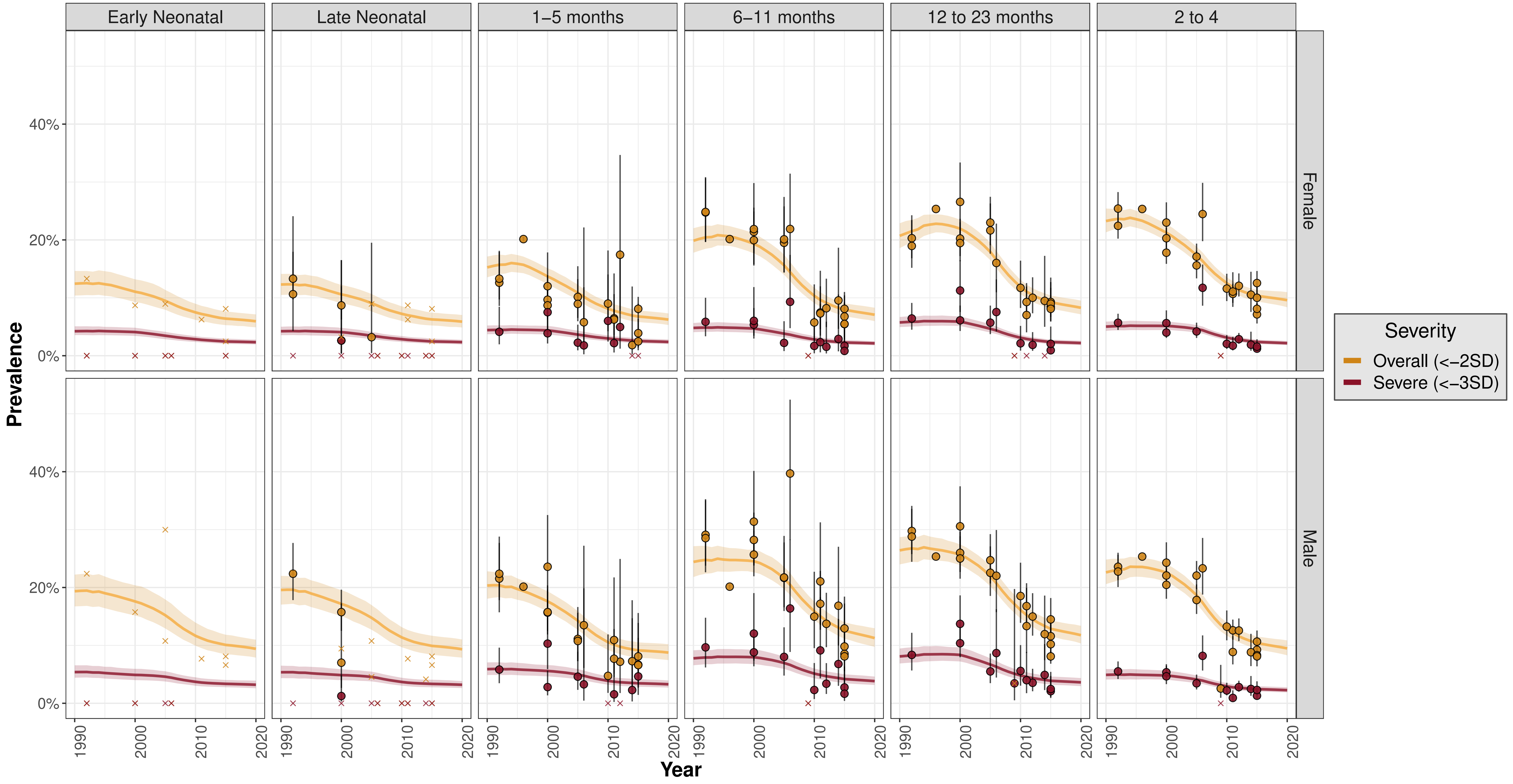

H: Transformed Mean Underweight Z Scores

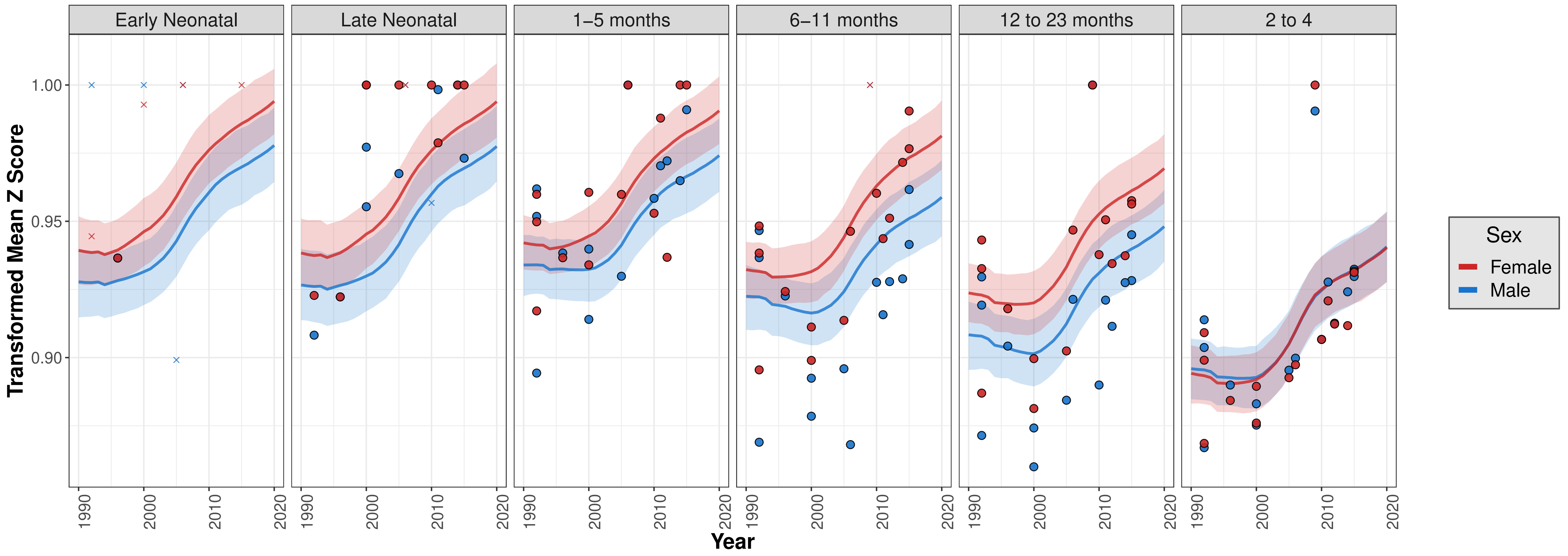

I

Year	Source
1992	DHS
1992	WHO CGM Database
1996	WHO CGM Database
2000	DHS
2000	MICS
2000	WHO CGM Database
2005	DHS
2005	WHO CGM Database
2006	Comprehensive Food Security and Vulnerability Analysis
2009	Comprehensive Food Security and Vulnerability Assessment and Nutrition Survey
2010	DHS
2011	DHS
2011	WHO CGM Database
2012	Comprehensive Food Security and Vulnerability Assessment and Nutrition Survey
2014	DHS
2015	WHO CGM Database
2015	DHS
2015	Comprehensive Food Security and Vulnerability Assessment and Nutrition Survey

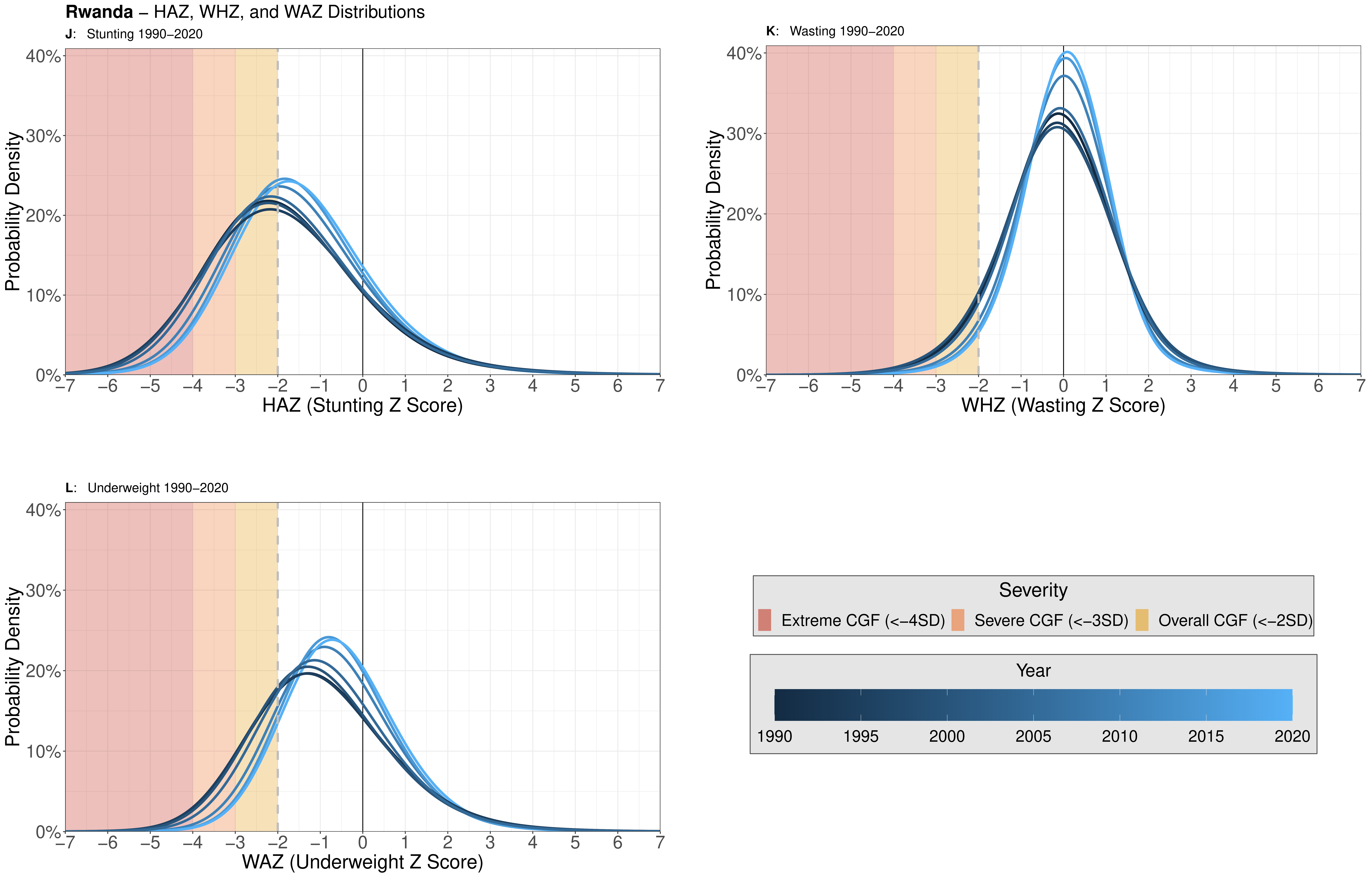

Rwanda – HAZ, WHZ, and WAZ Distributions
J: Stunting 1990–2020
Probability Density
40%
30%
20%
10%
0%
−7 −6 −5 −4 −3 −2 −1 0 1 2 3 4 5 6 7
HAZ (Stunting Z Score)
K: Wasting 1990–2020
WHZ (Wasting Z Score)
L: Underweight 1990–2020
WAZ (Underweight Z Score)
Severity
Extreme CGF (<−4SD)
Severe CGF (<−3SD)
Overall CGF (<−2SD)
Year
1990
1995
2000
2005
2010
2015
2020

Somalia – Stunting (HAZ)

A: Overall and Severe Stunting Prevalence

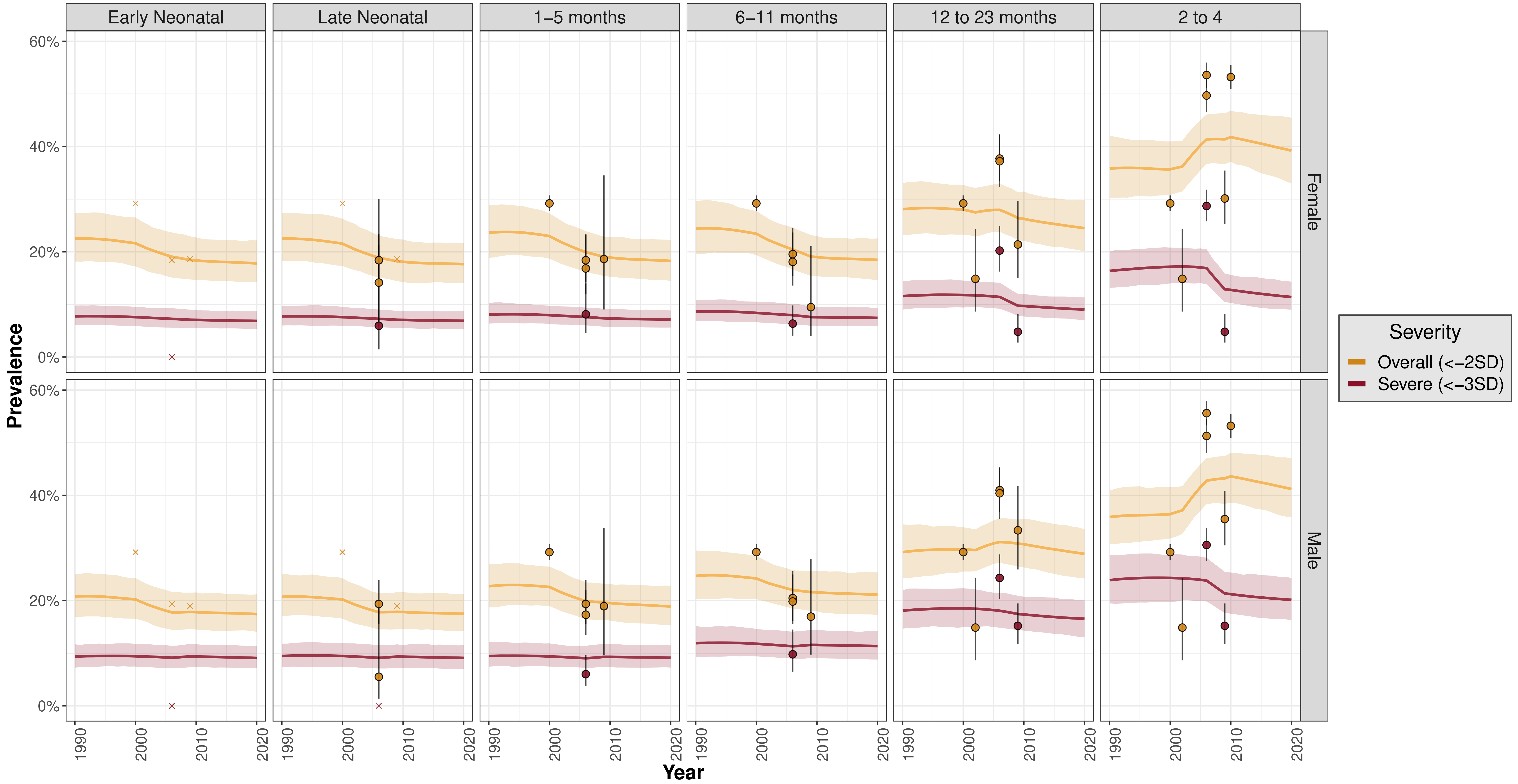

B: Transformed Mean Stunting Z Scores

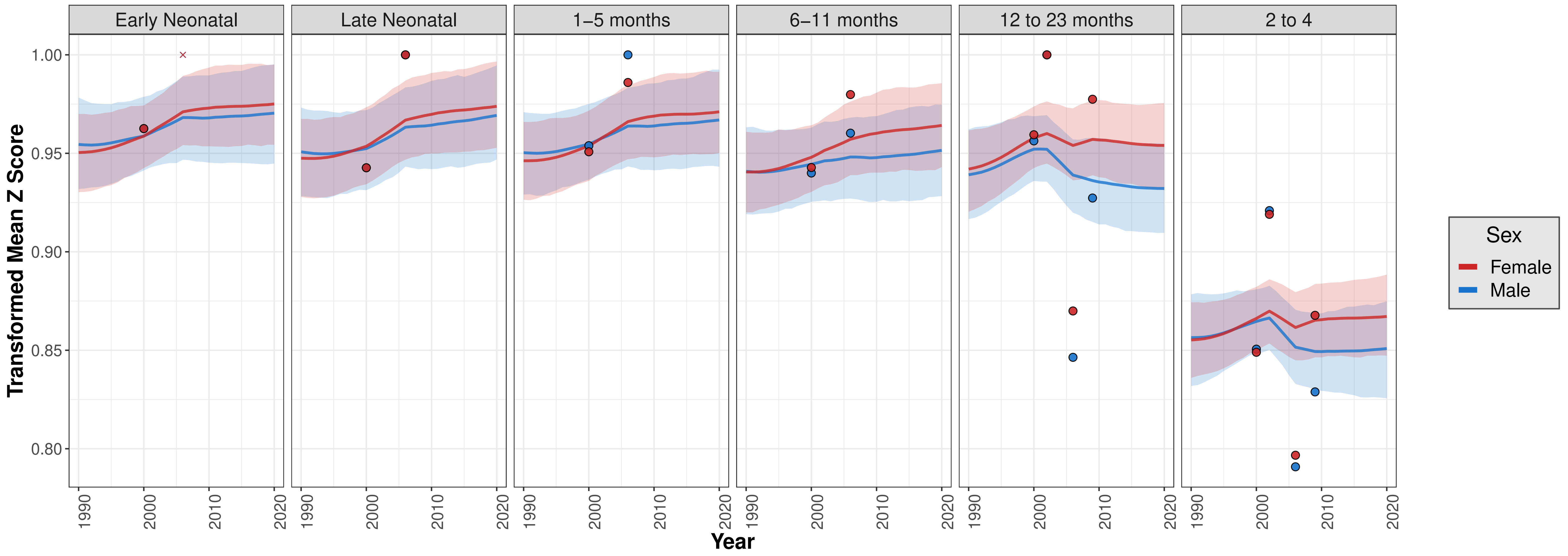

C

Year	Source
2000	WHO CGM Database
2002	WHO CGM Database
2006	MICS
2006	WHO CGM Database
2009	WHO CGM Database
2010	Comprehensive Food and Nutrition Security and Vulnerability Assessment

Somalia – Wasting (WHZ)

D: Overall and Severe Wasting Prevalence

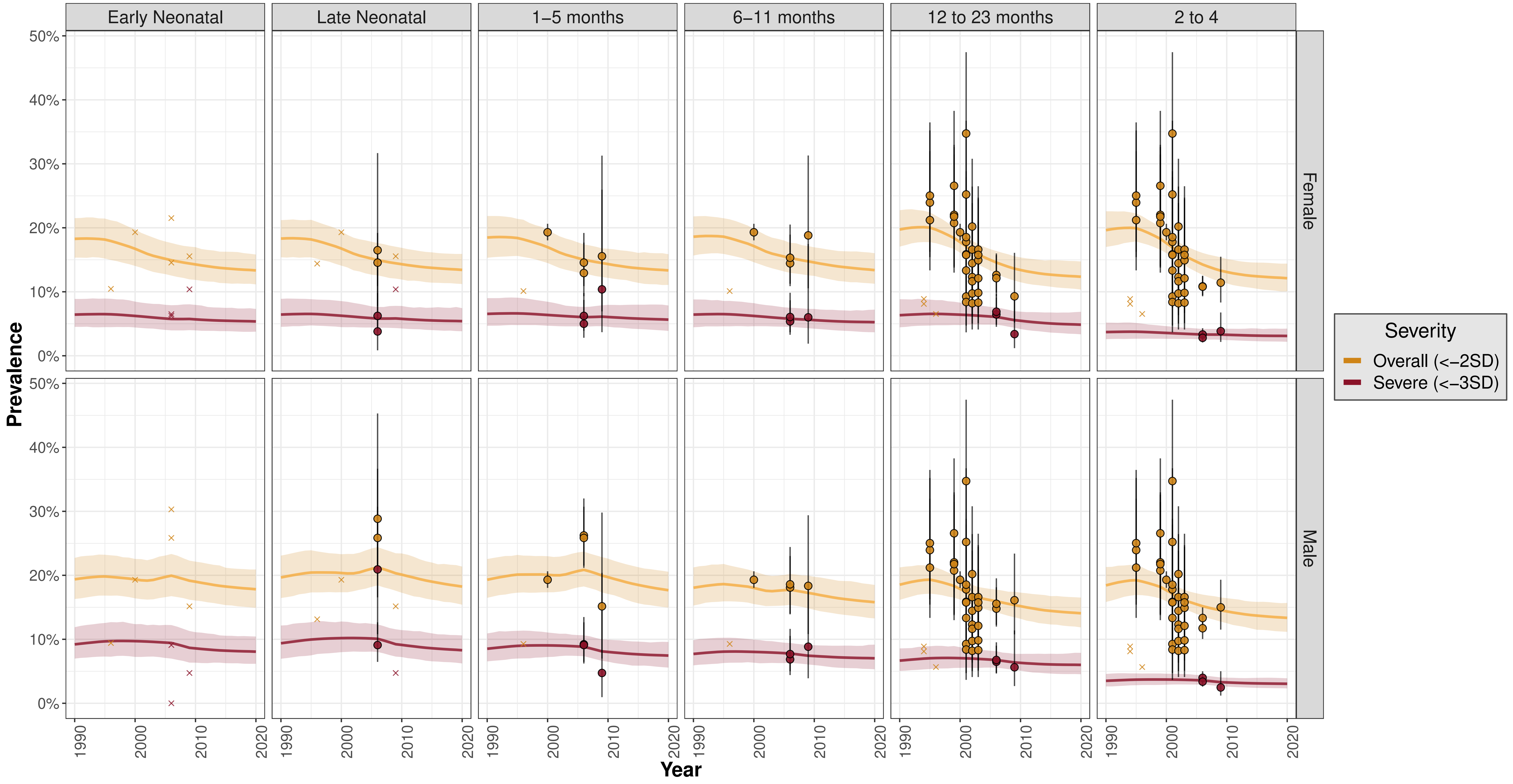

E: Transformed Mean Wasting Z Scores

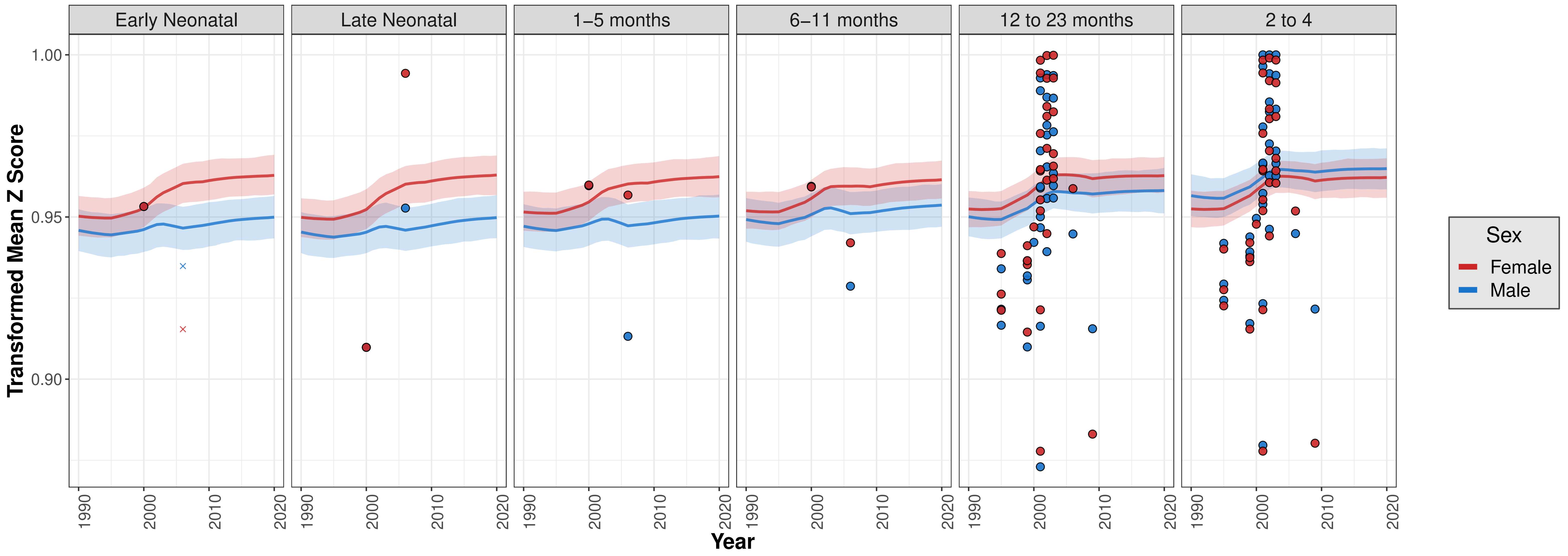

F

Year	Source
1994	WHO CGM Database
1995	WHO CGM Database
1996	MICS
1999	WHO CGM Database
2000	WHO CGM Database
2001	WHO CGM Database
2002	WHO CGM Database
2003	WHO CGM Database
2006	MICS
2006	WHO CGM Database
2009	WHO CGM Database

Somalia – Underweight (WAZ)

G: Overall and Severe Underweight Prevalence

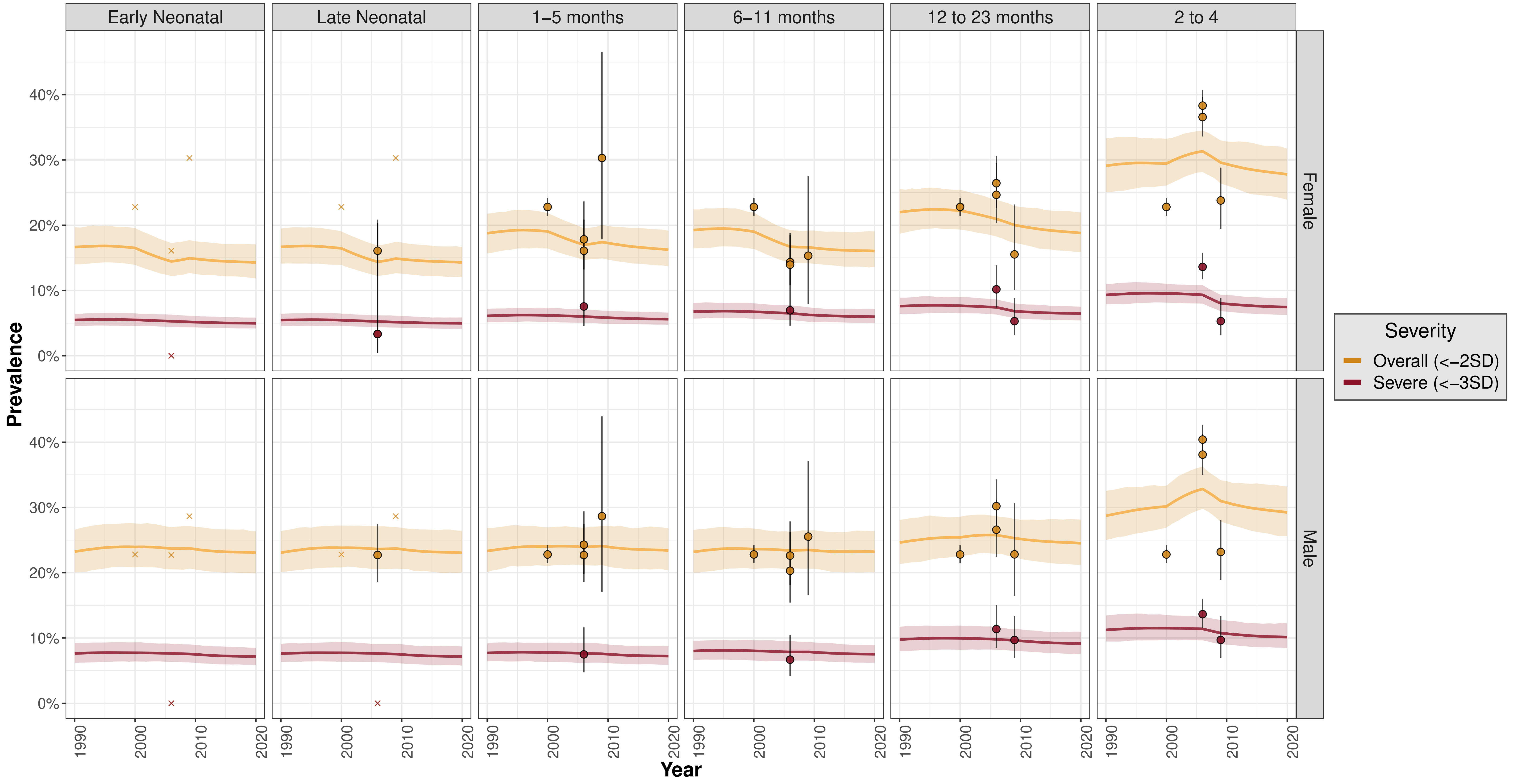

H: Transformed Mean Underweight Z Scores

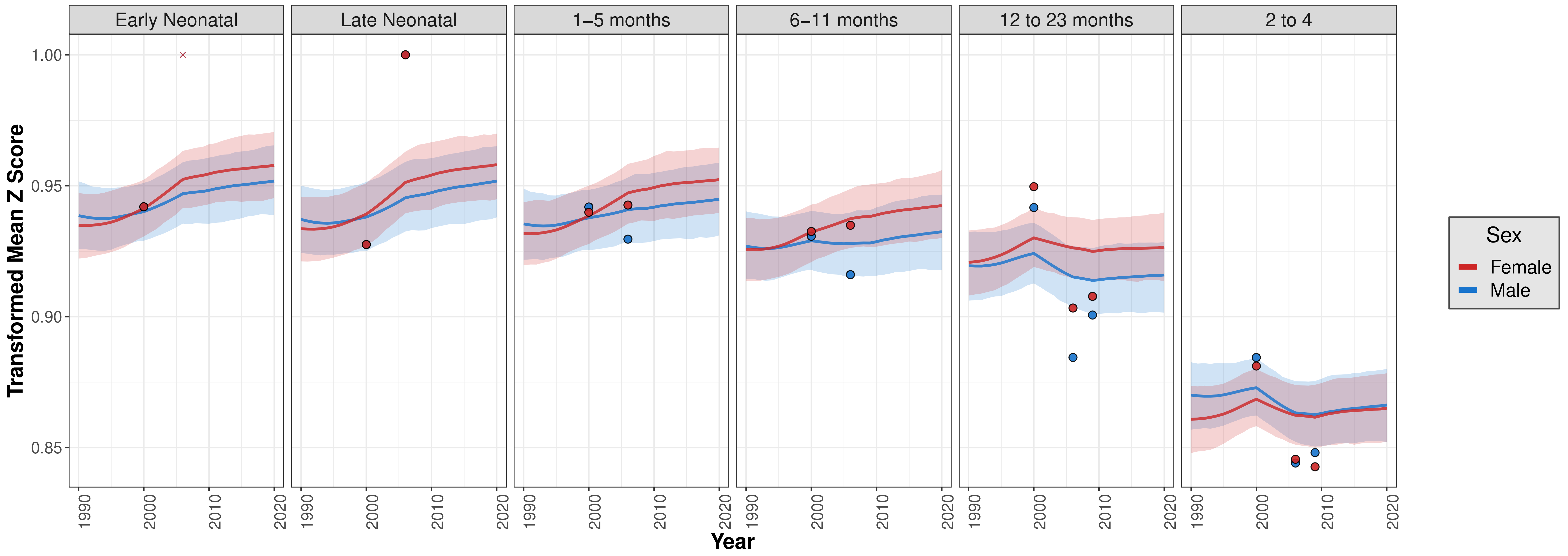

I

Year	Source
2000	WHO CGM Database
2006	MICS
2006	WHO CGM Database
2009	WHO CGM Database

Somalia – HAZ, WHZ, and WAZ Distributions

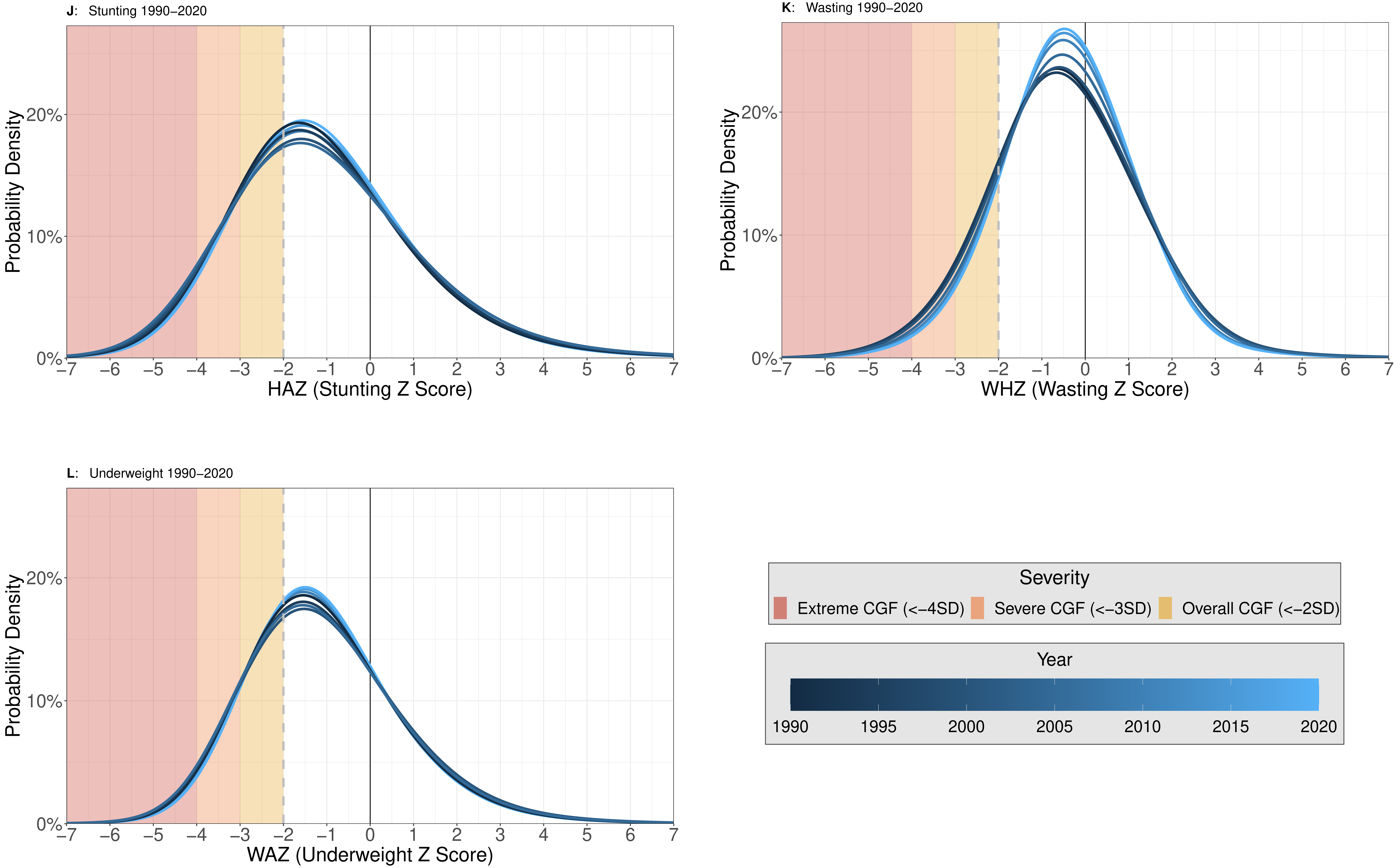

United Republic of Tanzania – Stunting (HAZ)

A: Overall and Severe Stunting Prevalence

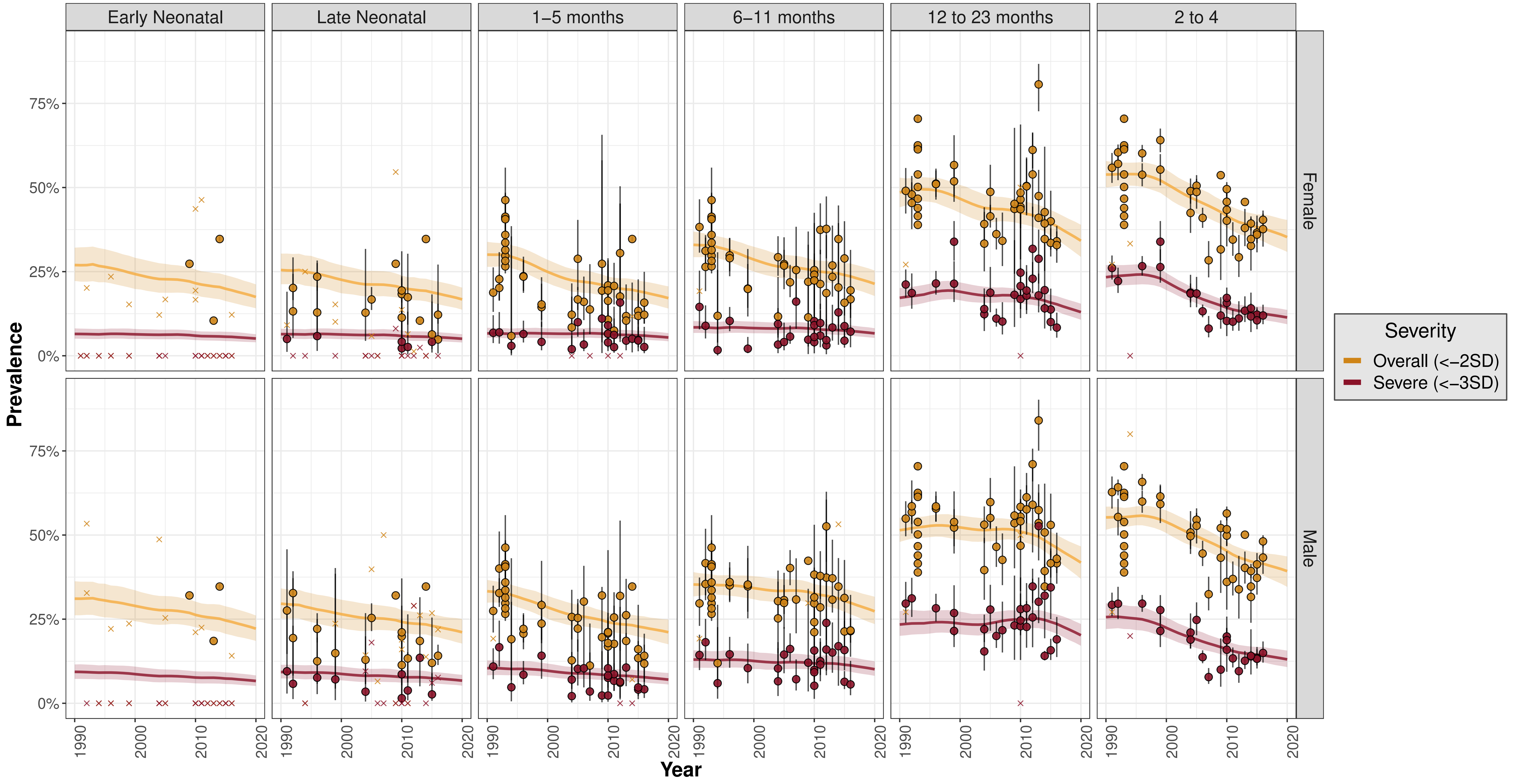

B: Transformed Mean Stunting Z Scores

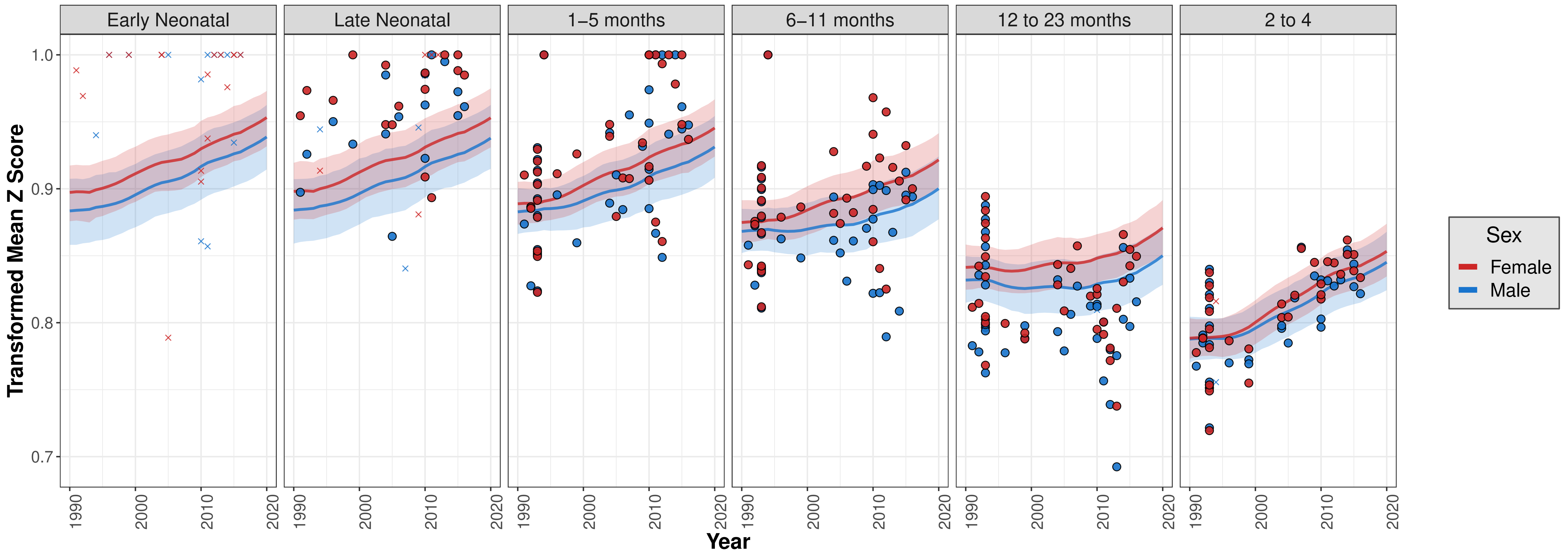

C

Year	Source
1985	WHO CGM Database
1991	DHS
1991	WHO CGM Database
1992	DHS
1992	WHO CGM Database
1993	WHO CGM Database
1994	Kagera Living Standards Measurement Study
1996	DHS
1996	WHO CGM Database
1999	DHS
1999	WHO CGM Database
2004	Kagera Living Standards Measurement Study
2004	DHS
2005	DHS
2005	WHO CGM Database
2006	Core Welfare Indicators Questionnaire Survey
2007	Core Welfare Indicators Questionnaire Survey
2009	DHS
2009	WHO CGM Database
2010	DHS
2010	National Panel Survey
2010	Kagera Living Standards Measurement Study
2010	WHO CGM Database
2010	Haydom Malnutrition and Enteric Disease Study
2011	National Panel Survey
2011	Haydom Malnutrition and Enteric Disease Study
2012	National Panel Survey
2012	Haydom Malnutrition and Enteric Disease Study
2013	WHO CGM Database
2013	National Panel Survey
2013	Haydom Malnutrition and Enteric Disease Study
2014	WHO CGM Database
2014	National Nutrition Survey
2014	National Panel Survey
2015	DHS
2015	National Panel Survey
2016	WHO CGM Database
2016	DHS

United Republic of Tanzania – Wasting (WHZ)

D: Overall and Severe Wasting Prevalence

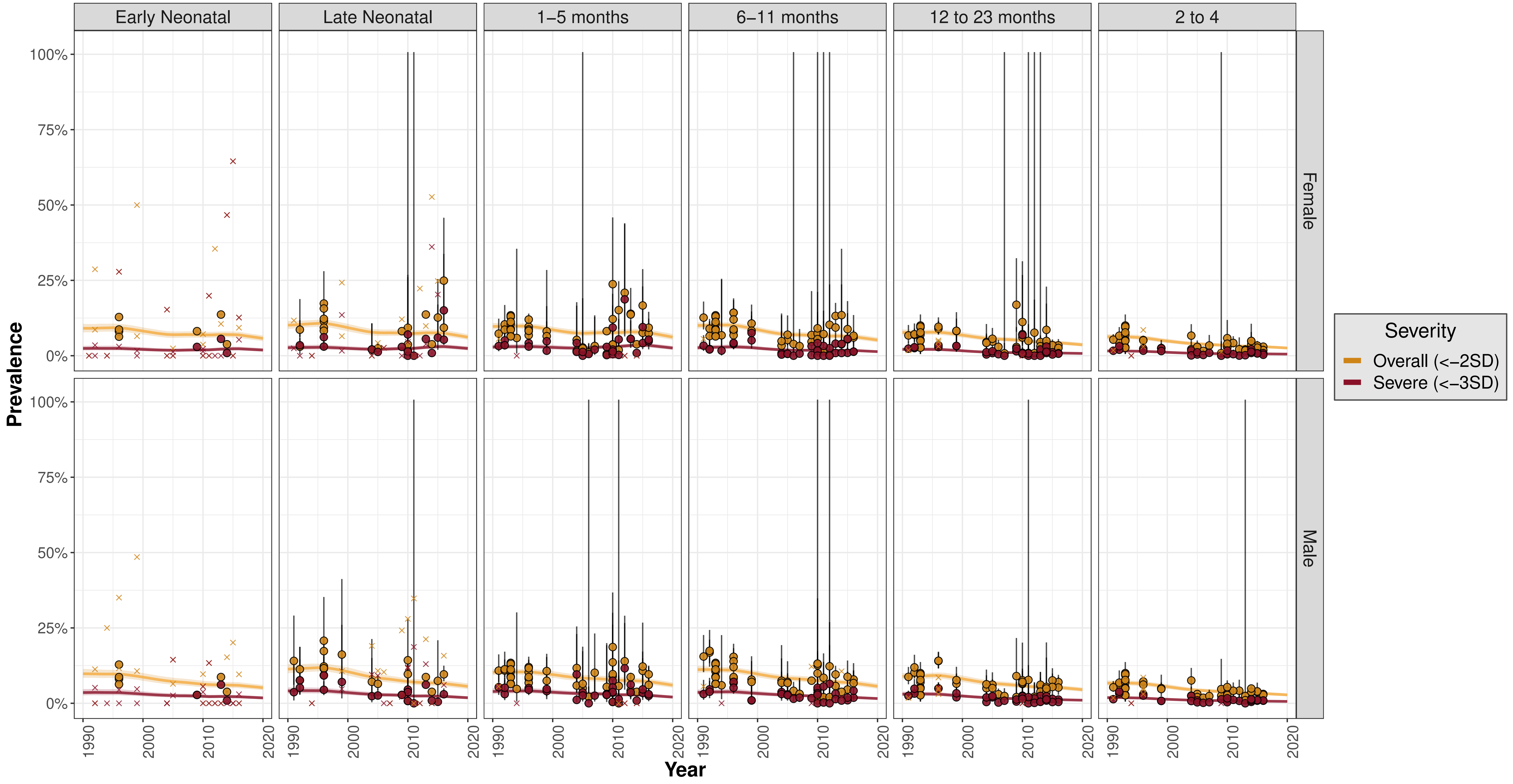

E: Transformed Mean Wasting Z Scores

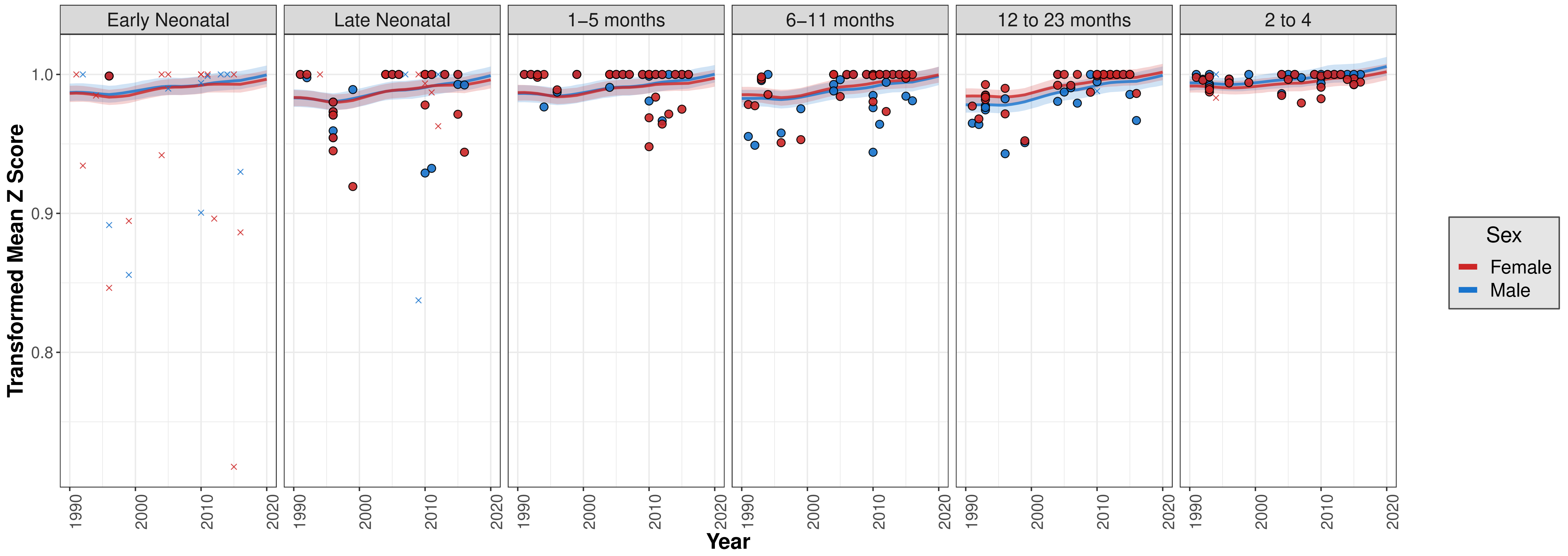

F

Year	Source
1985	WHO CGM Database
1991	DHS
1991	WHO CGM Database
1992	DHS
1992	WHO CGM Database
1993	WHO CGM Database
1994	Kagera Living Standards Measurement Study
1996	DHS
1996	WHO CGM Database
1996	MICS
1999	DHS
1999	WHO CGM Database
2004	Kagera Living Standards Measurement Study
2004	DHS
2005	DHS
2005	WHO CGM Database
2006	Core Welfare Indicators Questionnaire Survey
2007	Core Welfare Indicators Questionnaire Survey
2009	DHS
2009	WHO CGM Database
2010	DHS
2010	National Panel Survey
2010	Kagera Living Standards Measurement Study
2010	WHO CGM Database
2010	Haydom Malnutrition and Enteric Disease Study
2011	National Panel Survey
2011	Haydom Malnutrition and Enteric Disease Study
2012	National Panel Survey
2012	Haydom Malnutrition and Enteric Disease Study
2013	WHO CGM Database
2013	National Panel Survey
2013	Haydom Malnutrition and Enteric Disease Study
2014	WHO CGM Database
2014	National Nutrition Survey
2014	National Panel Survey
2015	DHS
2015	National Panel Survey
2016	WHO CGM Database
2016	DHS

United Republic of Tanzania – Underweight (WAZ)

G: Overall and Severe Underweight Prevalence

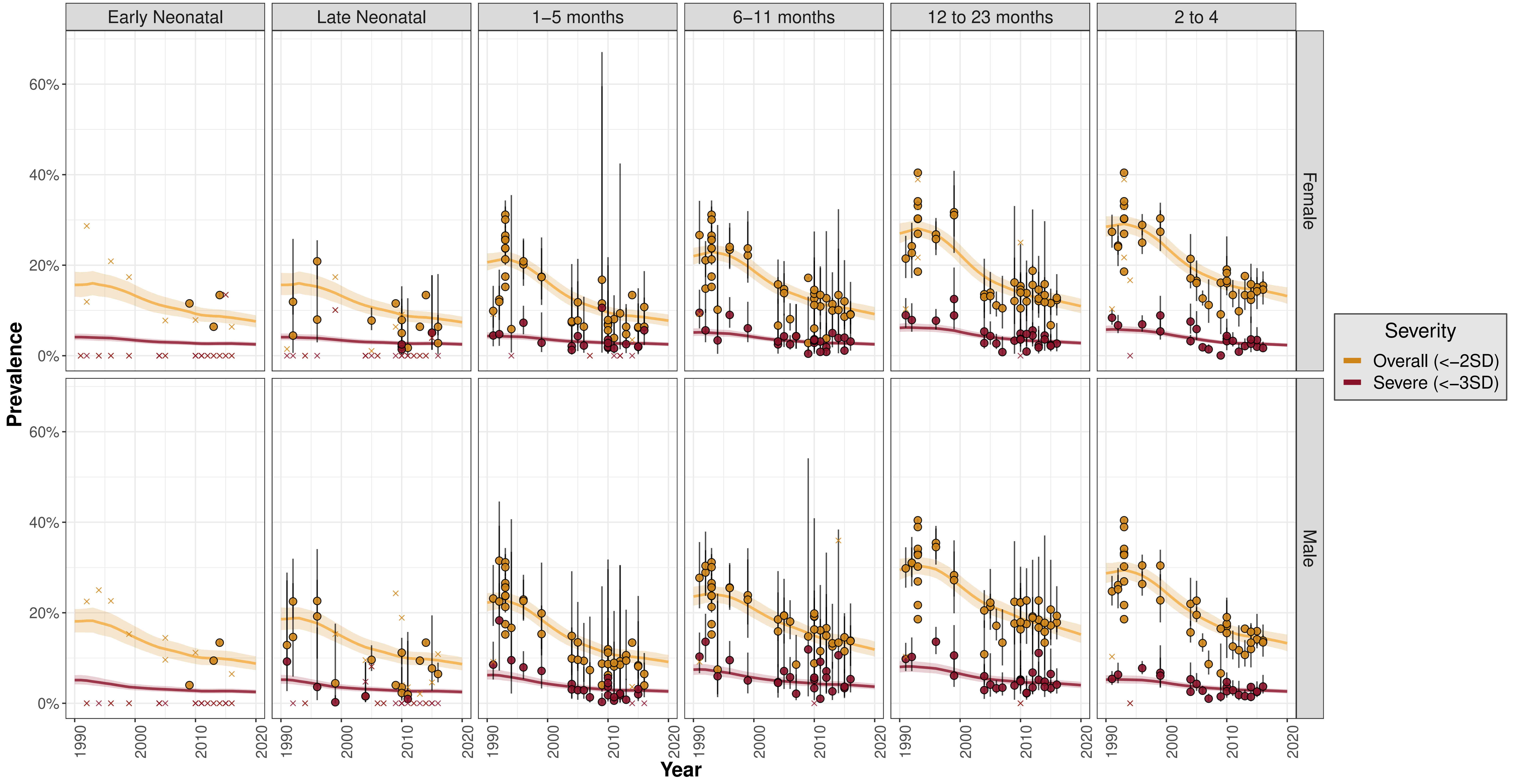

H: Transformed Mean Underweight Z Scores

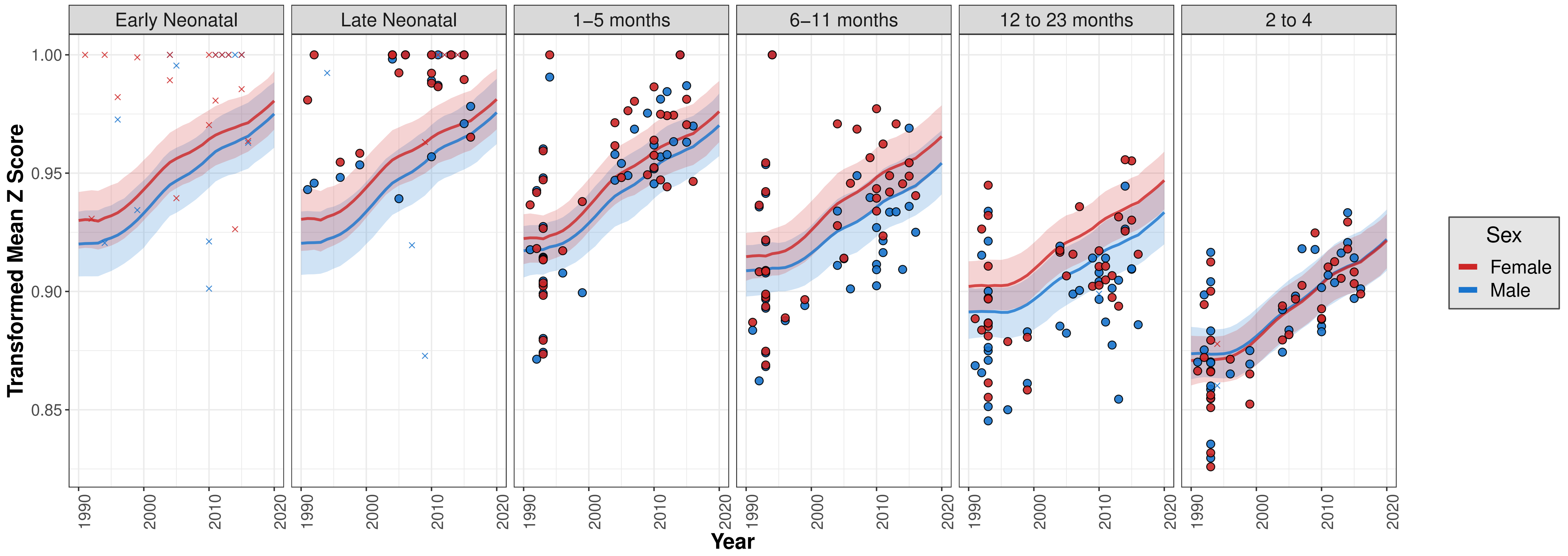

I

Year	Source
1985	WHO CGM Database
1991	DHS
1991	WHO CGM Database
1992	DHS
1992	WHO CGM Database
1993	WHO CGM Database
1994	Kagera Living Standards Measurement Study
1996	DHS
1996	WHO CGM Database
1999	DHS
1999	WHO CGM Database
2004	Kagera Living Standards Measurement Study
2004	DHS
2005	DHS
2005	WHO CGM Database
2006	Core Welfare Indicators Questionnaire Survey
2007	Core Welfare Indicators Questionnaire Survey
2009	DHS
2009	WHO CGM Database
2010	DHS
2010	National Panel Survey
2010	Kagera Living Standards Measurement Study
2010	WHO CGM Database
2010	Haydom Malnutrition and Enteric Disease Study
2011	National Panel Survey
2011	Haydom Malnutrition and Enteric Disease Study
2012	National Panel Survey
2012	Haydom Malnutrition and Enteric Disease Study
2013	WHO CGM Database
2013	National Panel Survey
2013	Haydom Malnutrition and Enteric Disease Study
2014	WHO CGM Database
2014	National Nutrition Survey
2014	National Panel Survey
2015	DHS
2015	National Panel Survey
2016	WHO CGM Database
2016	DHS

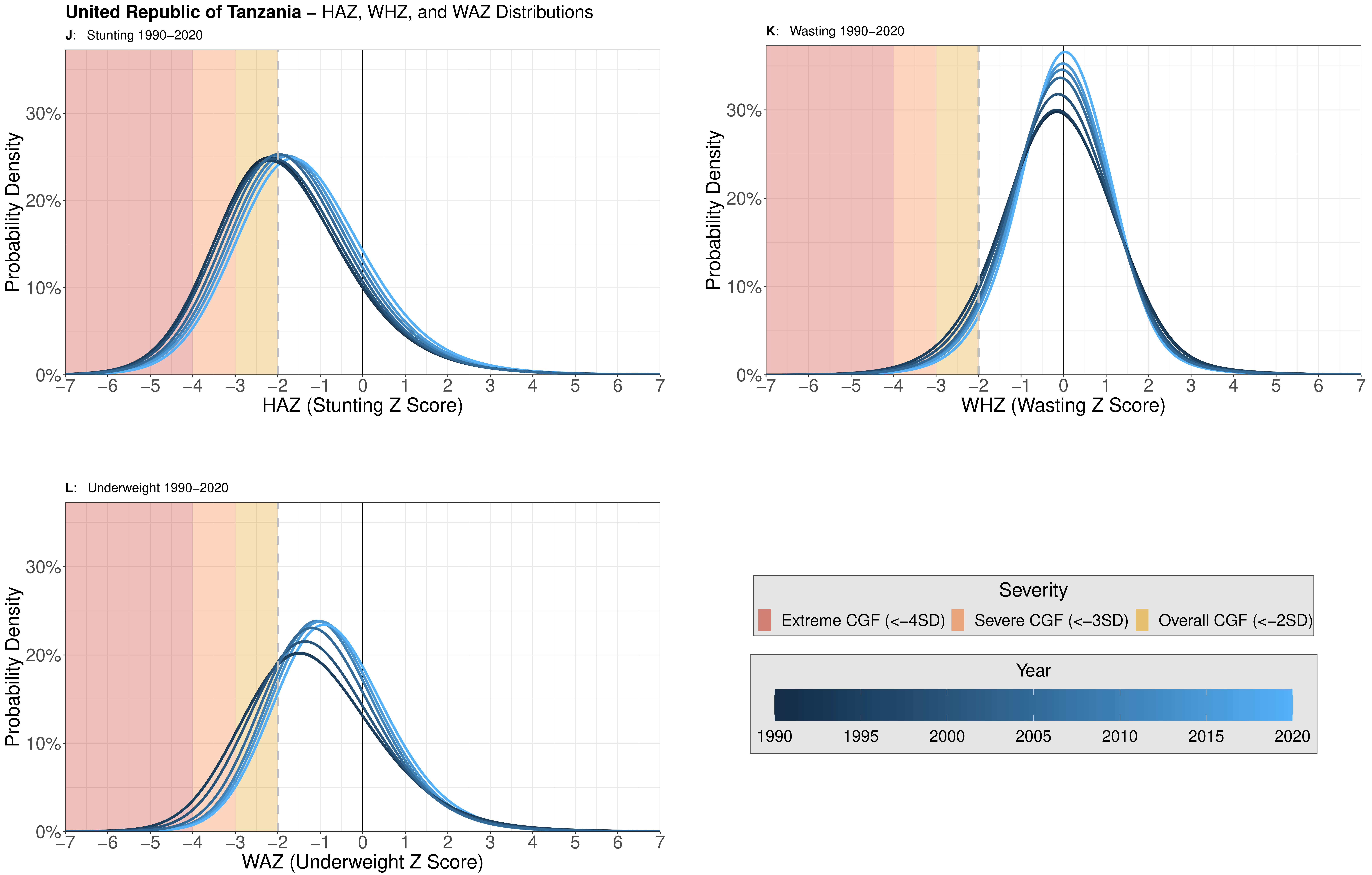
United Republic of Tanzania – HAZ, WHZ, and WAZ Distributions
J: Stunting 1990–2020
Probability Density
HAZ (Stunting Z Score)
K: Wasting 1990–2020
WHZ (Wasting Z Score)
L: Underweight 1990–2020
WAZ (Underweight Z Score)
0%
10%
20%
30%
−7 −6 −5 −4 −3 −2 −1 0 1 2 3 4 5 6 7
Severity
Extreme CGF (<−4SD)
Severe CGF (<−3SD)
Overall CGF (<−2SD)
Year
1990
1995
2000
2005
2010
2015
2020

Uganda – Stunting (HAZ)

A: Overall and Severe Stunting Prevalence

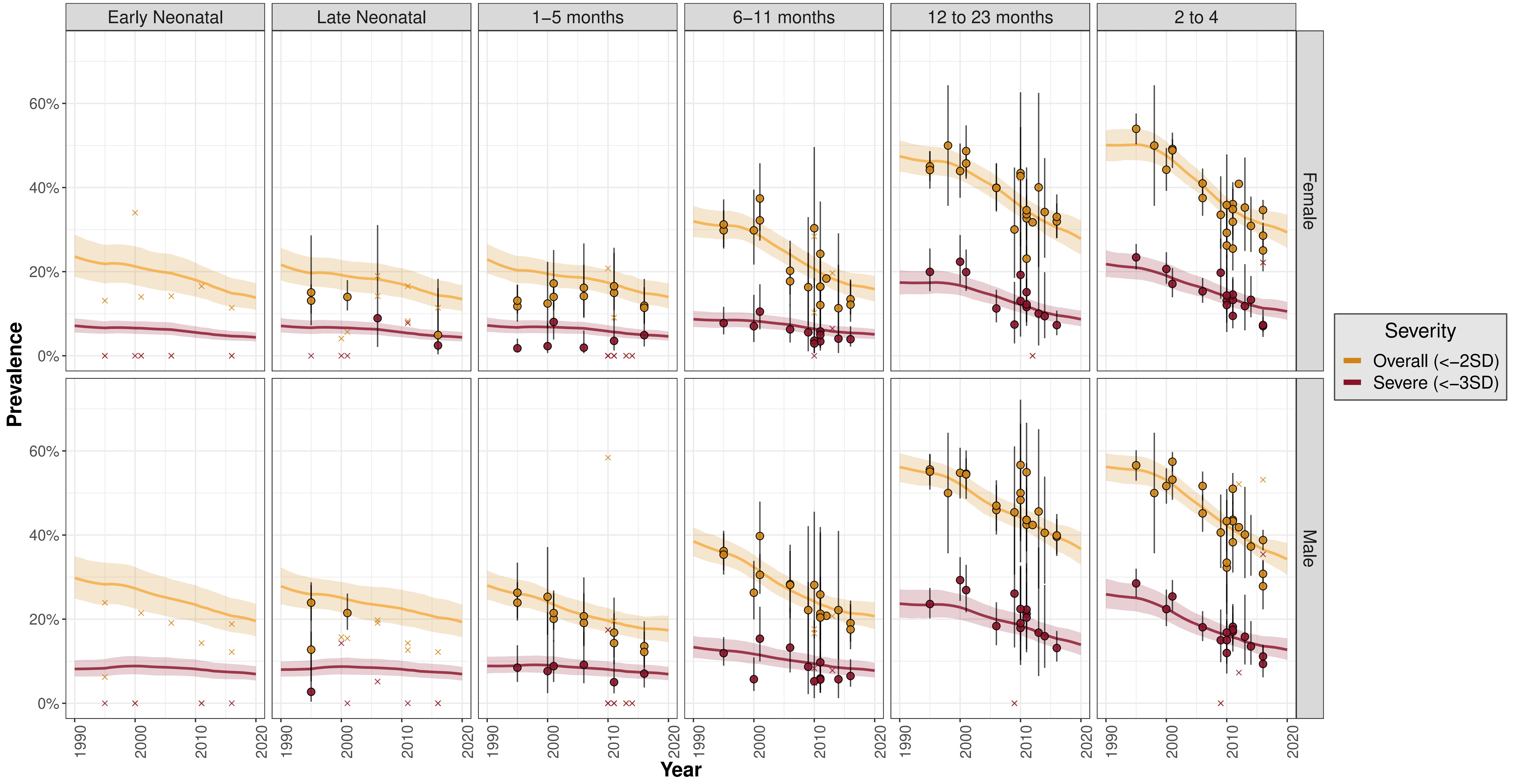

B: Transformed Mean Stunting Z Scores

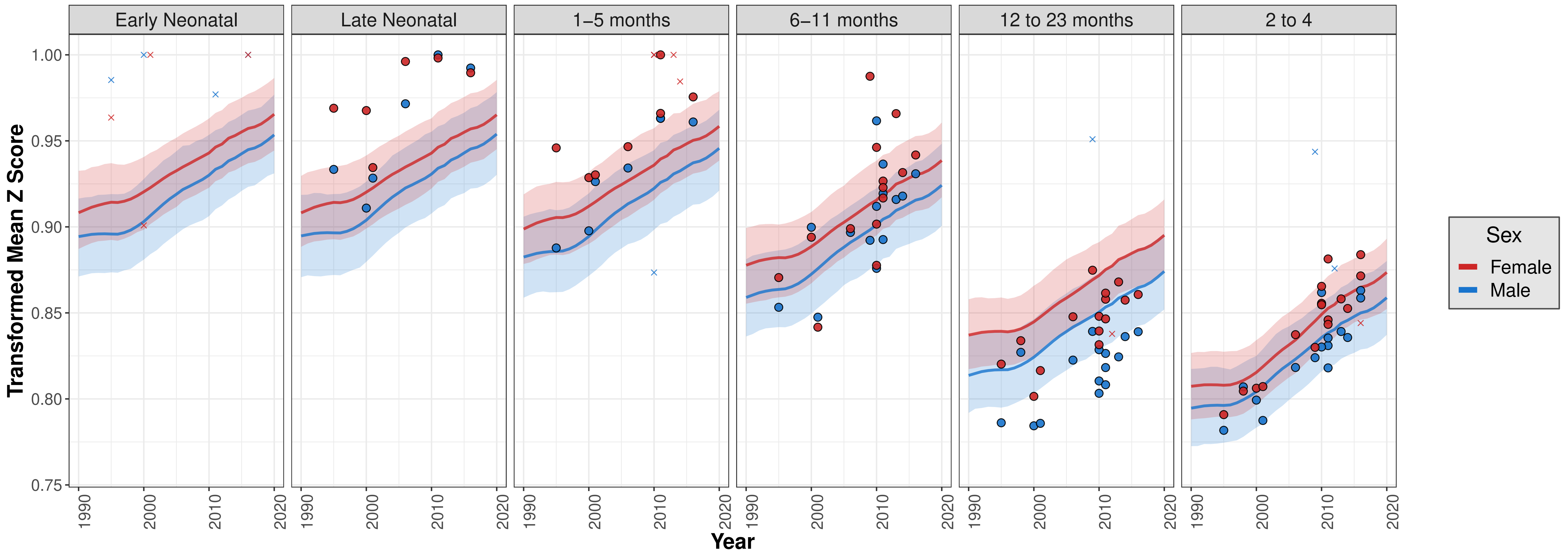

C

Year	Source
1985	WHO CGM Database
1987	Arua Child Mortality and Nutrition Survey
1987	WHO CGM Database
1988	DHS
1988	WHO CGM Database
1989	DHS
1989	WHO CGM Database
1995	DHS
1995	WHO CGM Database
1998	WHO CGM Database
2000	DHS
2001	DHS
2001	WHO CGM Database
2006	DHS
2006	Child Verbal Autopsy Study
2006	WHO CGM Database
2009	Living Standards Measurement Survey – Integrated Survey on Agriculture
2010	Living Standards Measurement Survey – Integrated Survey on Agriculture
2010	Living Standards Measurement Survey – Integrated Survey on Agriculture
2011	DHS
2011	WHO CGM Database
2011	Living Standards Measurement Survey – Integrated Survey on Agriculture
2011	Living Standards Measurement Survey – Integrated Survey on Agriculture
2012	WHO CGM Database
2012	Living Standards Measurement Survey – Integrated Survey on Agriculture
2013	Living Standards Measurement Survey – Integrated Survey on Agriculture
2014	Living Standards Measurement Survey – Integrated Survey on Agriculture
2016	WHO CGM Database
2016	DHS
2016	National Panel Survey (UNPS) , Wave 5

Uganda – Wasting (WHZ)

D: Overall and Severe Wasting Prevalence

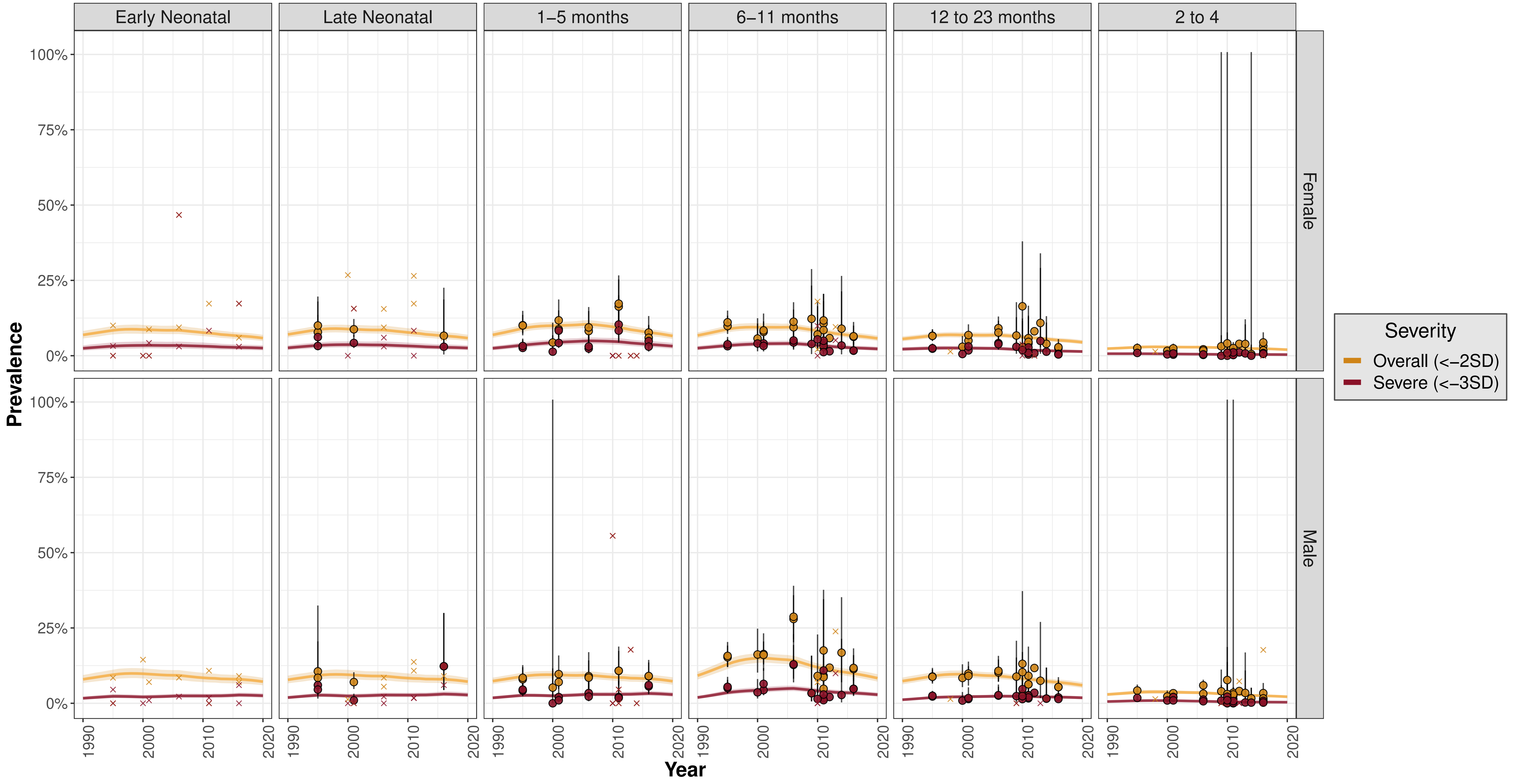

E: Transformed Mean Wasting Z Scores

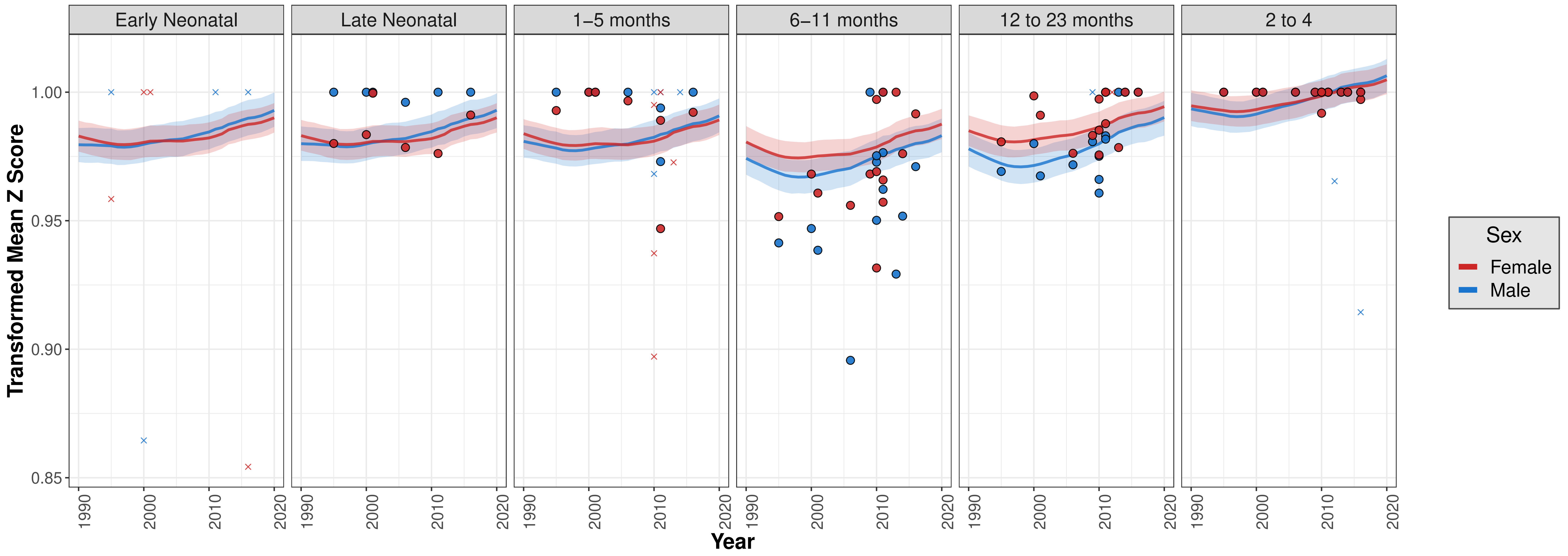

F

Year	Source
1985	WHO CGM Database
1987	Arua Child Mortality and Nutrition Survey
1987	WHO CGM Database
1988	DHS
1988	WHO CGM Database
1989	DHS
1989	WHO CGM Database
1995	DHS
1995	WHO CGM Database
1998	WHO CGM Database
2000	DHS
2001	DHS
2001	WHO CGM Database
2006	DHS
2006	Child Verbal Autopsy Study
2006	WHO CGM Database
2009	Living Standards Measurement Survey – Integrated Survey on Agriculture
2010	Living Standards Measurement Survey – Integrated Survey on Agriculture
2010	Living Standards Measurement Survey – Integrated Survey on Agriculture
2011	DHS
2011	WHO CGM Database
2011	Living Standards Measurement Survey – Integrated Survey on Agriculture
2011	Living Standards Measurement Survey – Integrated Survey on Agriculture
2012	WHO CGM Database
2012	Living Standards Measurement Survey – Integrated Survey on Agriculture
2013	Living Standards Measurement Survey – Integrated Survey on Agriculture
2014	Living Standards Measurement Survey – Integrated Survey on Agriculture
2016	WHO CGM Database
2016	DHS
2016	National Panel Survey (UNPS) , Wave 5

Uganda – Underweight (WAZ)

G: Overall and Severe Underweight Prevalence

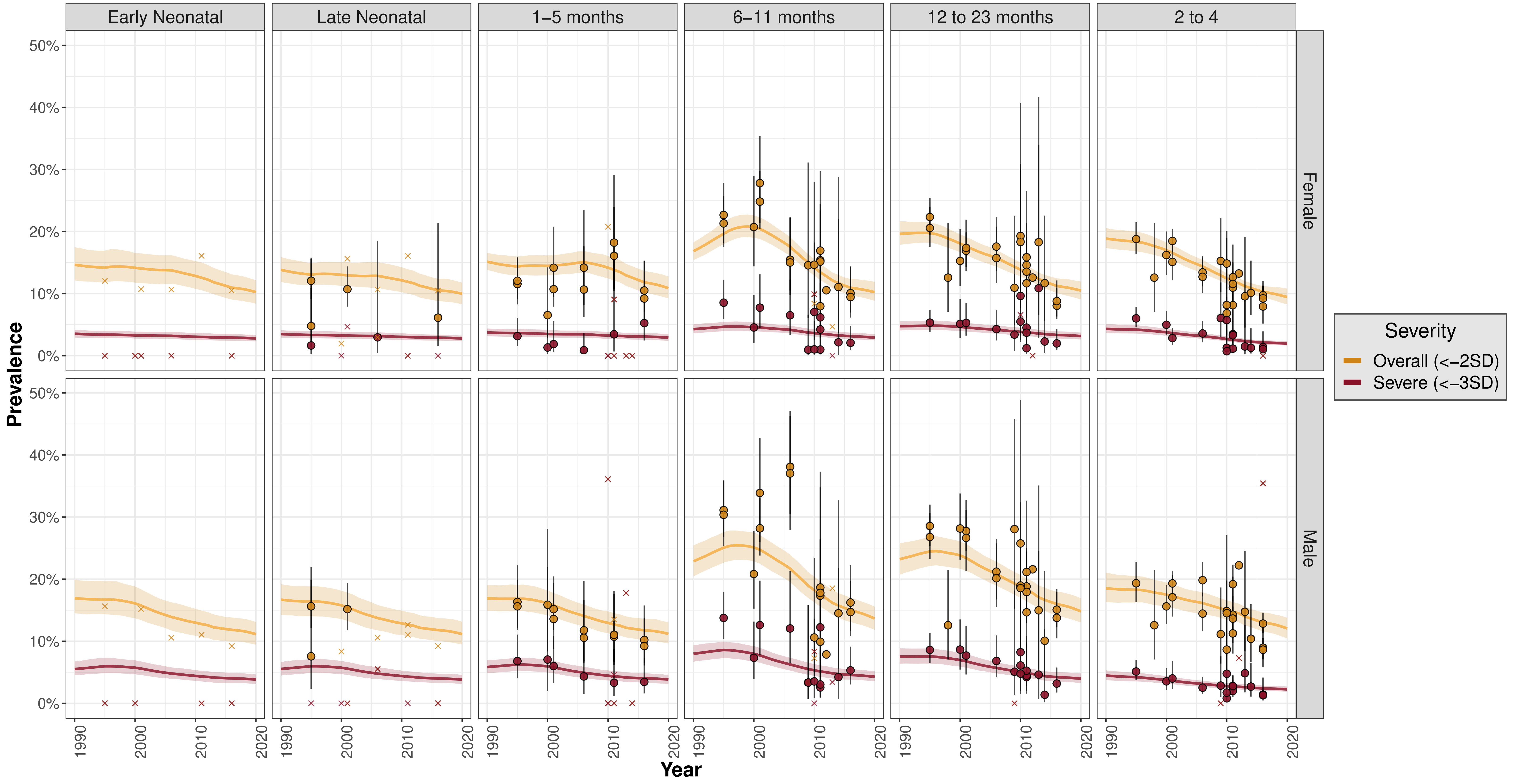

H: Transformed Mean Underweight Z Scores

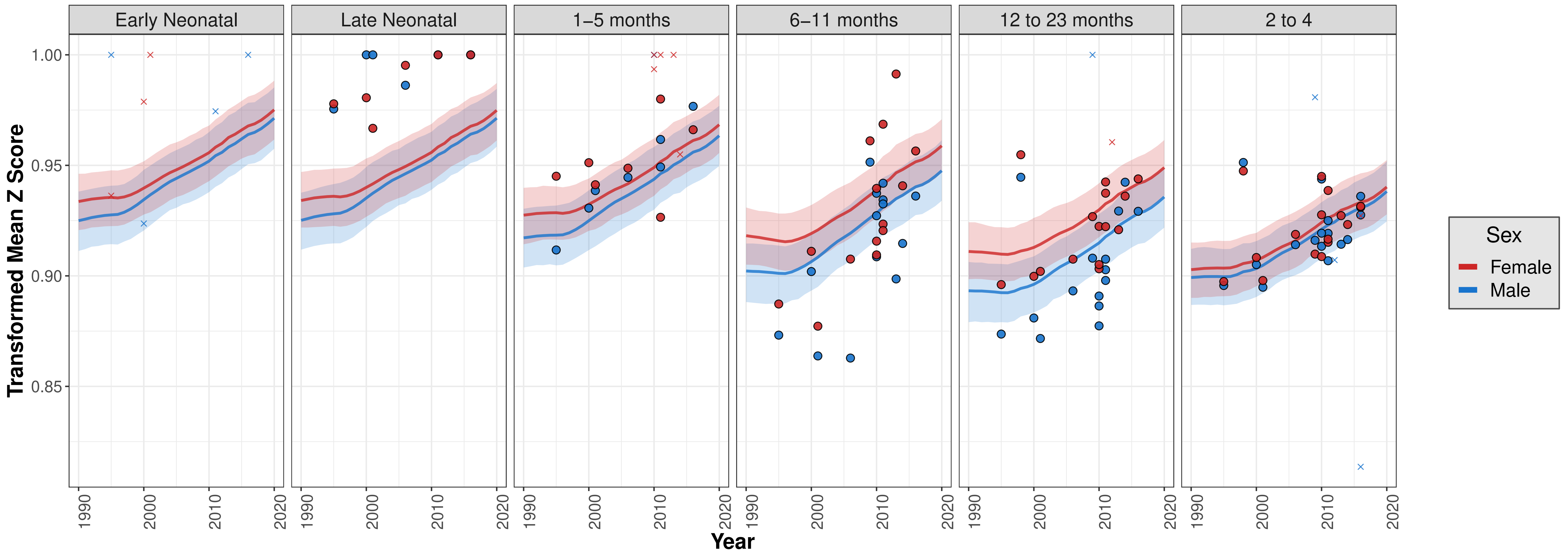

I

Year	Source
1985	WHO CGM Database
1987	Arua Child Mortality and Nutrition Survey
1987	WHO CGM Database
1988	DHS
1988	WHO CGM Database
1989	DHS
1989	WHO CGM Database
1995	DHS
1995	WHO CGM Database
1998	WHO CGM Database
2000	DHS
2001	DHS
2001	WHO CGM Database
2006	DHS
2006	Child Verbal Autopsy Study
2006	WHO CGM Database
2009	Living Standards Measurement Survey – Integrated Survey on Agriculture
2010	Living Standards Measurement Survey – Integrated Survey on Agriculture
2010	Living Standards Measurement Survey – Integrated Survey on Agriculture
2011	DHS
2011	WHO CGM Database
2011	Living Standards Measurement Survey – Integrated Survey on Agriculture
2011	Living Standards Measurement Survey – Integrated Survey on Agriculture
2012	WHO CGM Database
2012	Living Standards Measurement Survey – Integrated Survey on Agriculture
2013	Living Standards Measurement Survey – Integrated Survey on Agriculture
2014	Living Standards Measurement Survey – Integrated Survey on Agriculture
2016	WHO CGM Database
2016	DHS
2016	National Panel Survey (UNPS) , Wave 5

Uganda – HAZ, WHZ, and WAZ Distributions

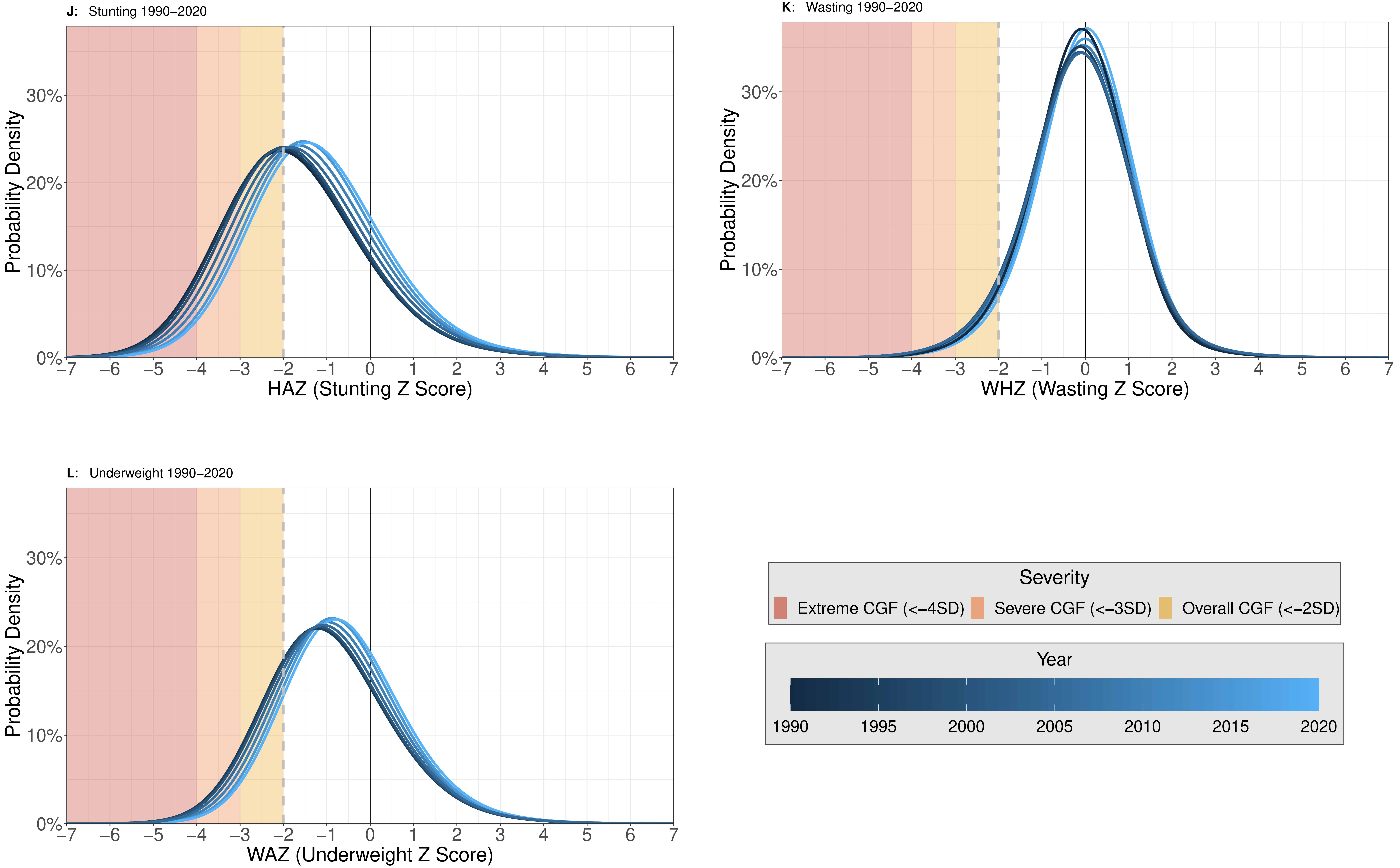

Zambia – Stunting (HAZ)

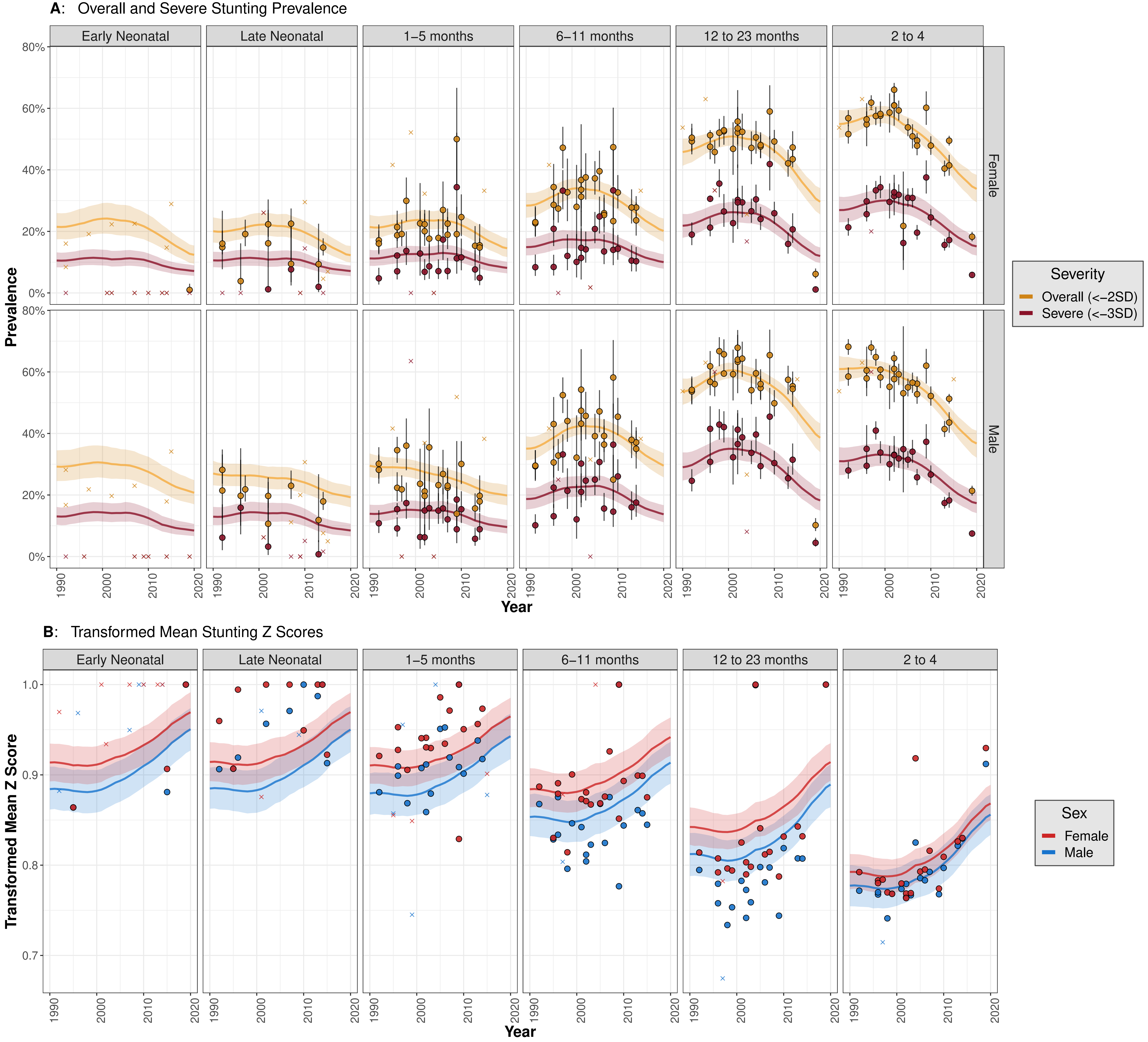

C

Year	Source
1990	WHO CGM Database
1992	DHS
1992	WHO CGM Database
1995	WHO CGM Database
1996	Living Conditions Monitoring Survey
1996	DHS
1997	DHS
1997	WHO CGM Database
1998	Living Conditions Monitoring Survey
1999	MICS
1999	WHO CGM Database
2001	DHS
2002	Living Conditions Monitoring Survey
2002	DHS
2002	WHO CGM Database
2003	Living Conditions Monitoring Survey
2004	Living Conditions Monitoring Survey
2005	Living Conditions Monitoring Survey
2006	Living Conditions Monitoring Survey
2007	DHS
2007	WHO CGM Database
2009	National Population Health Survey
2009	Access to ACT Initiative Survey
2010	Living Conditions Monitoring Survey
2013	DHS
2014	DHS
2014	WHO CGM Database
2015	Living Conditions Monitoring Survey
2019	DHS

Zambia – Wasting (WHZ)

D: Overall and Severe Wasting Prevalence

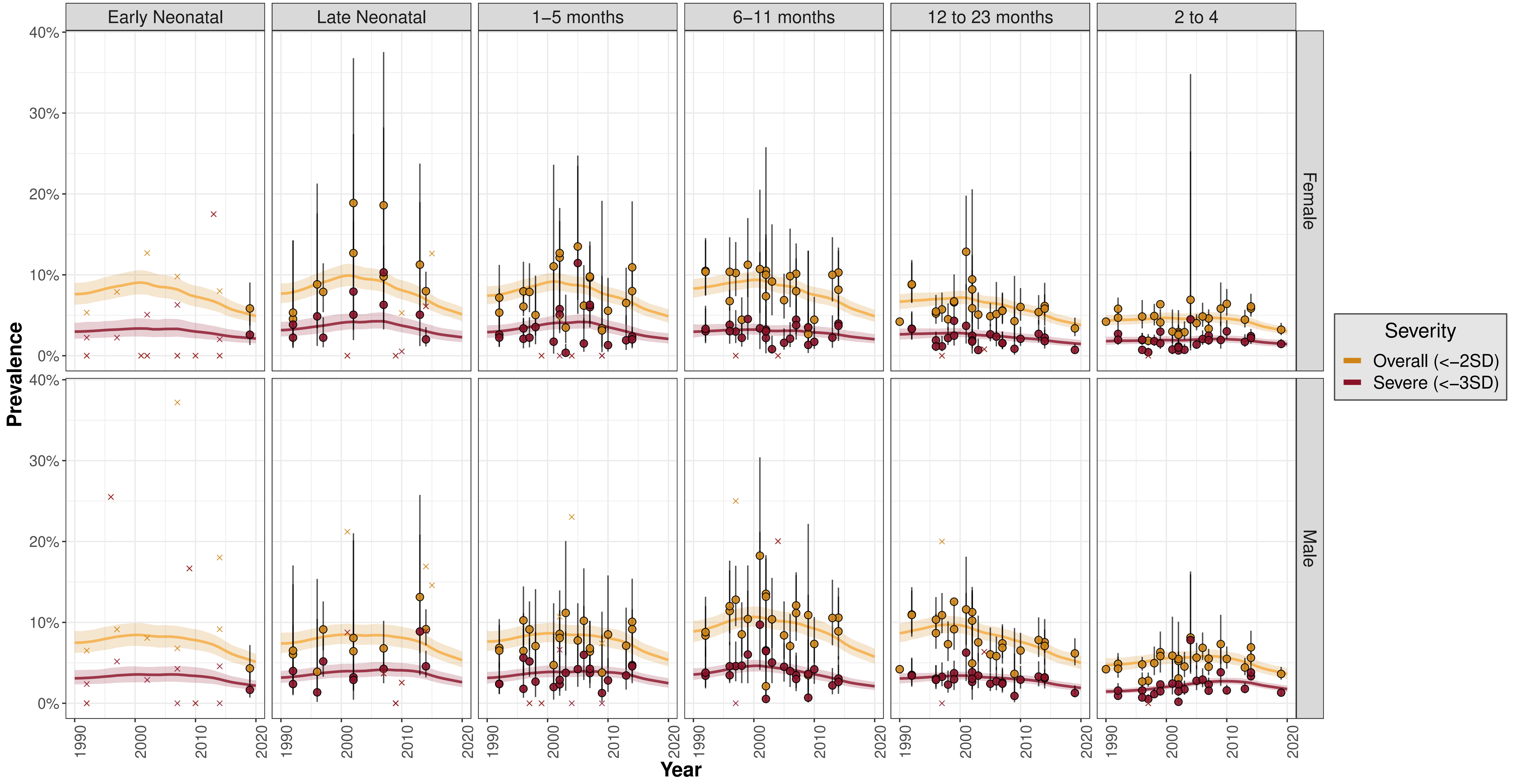

E: Transformed Mean Wasting Z Scores

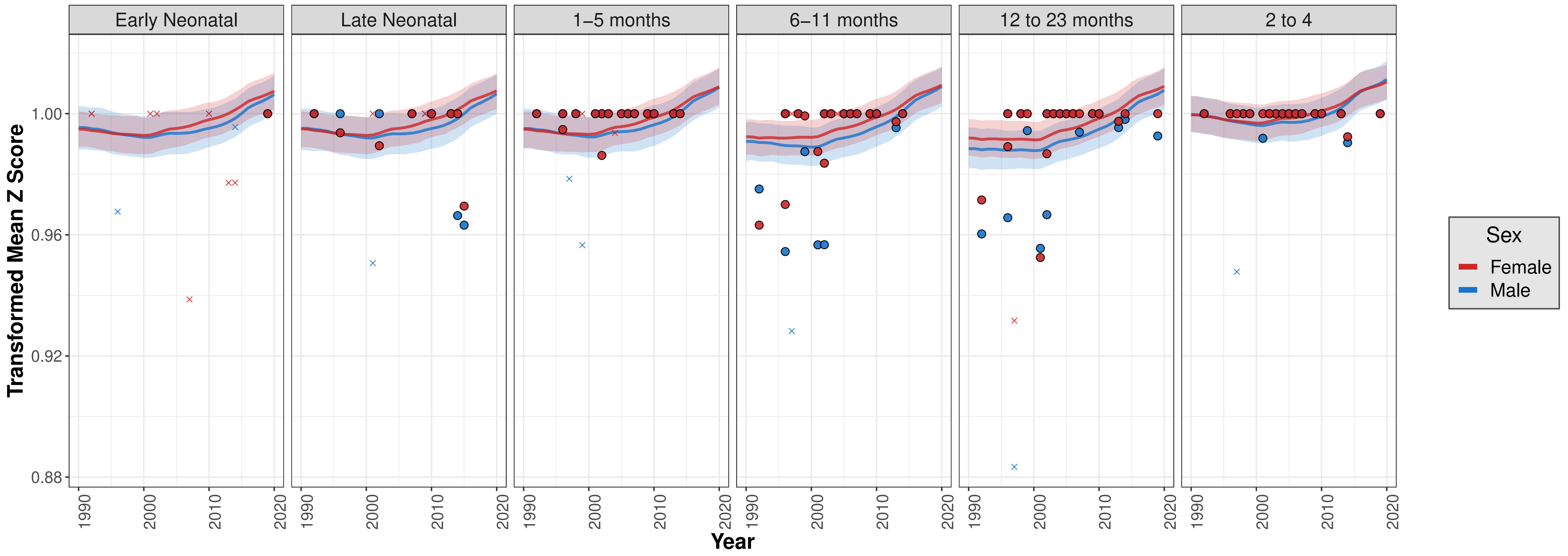

F

Year	Source
1990	WHO CGM Database
1992	DHS
1992	WHO CGM Database
1996	Living Conditions Monitoring Survey
1996	DHS
1997	DHS
1997	WHO CGM Database
1998	Living Conditions Monitoring Survey
1999	MICS
1999	WHO CGM Database
2001	DHS
2002	Living Conditions Monitoring Survey
2002	DHS
2002	WHO CGM Database
2003	Living Conditions Monitoring Survey
2004	Living Conditions Monitoring Survey
2005	Living Conditions Monitoring Survey
2006	Living Conditions Monitoring Survey
2007	DHS
2007	WHO CGM Database
2009	National Population Health Survey
2009	Access to ACT Initiative Survey
2010	Living Conditions Monitoring Survey
2013	DHS
2014	DHS
2014	WHO CGM Database
2015	Living Conditions Monitoring Survey
2019	DHS

Zambia – Underweight (WAZ)

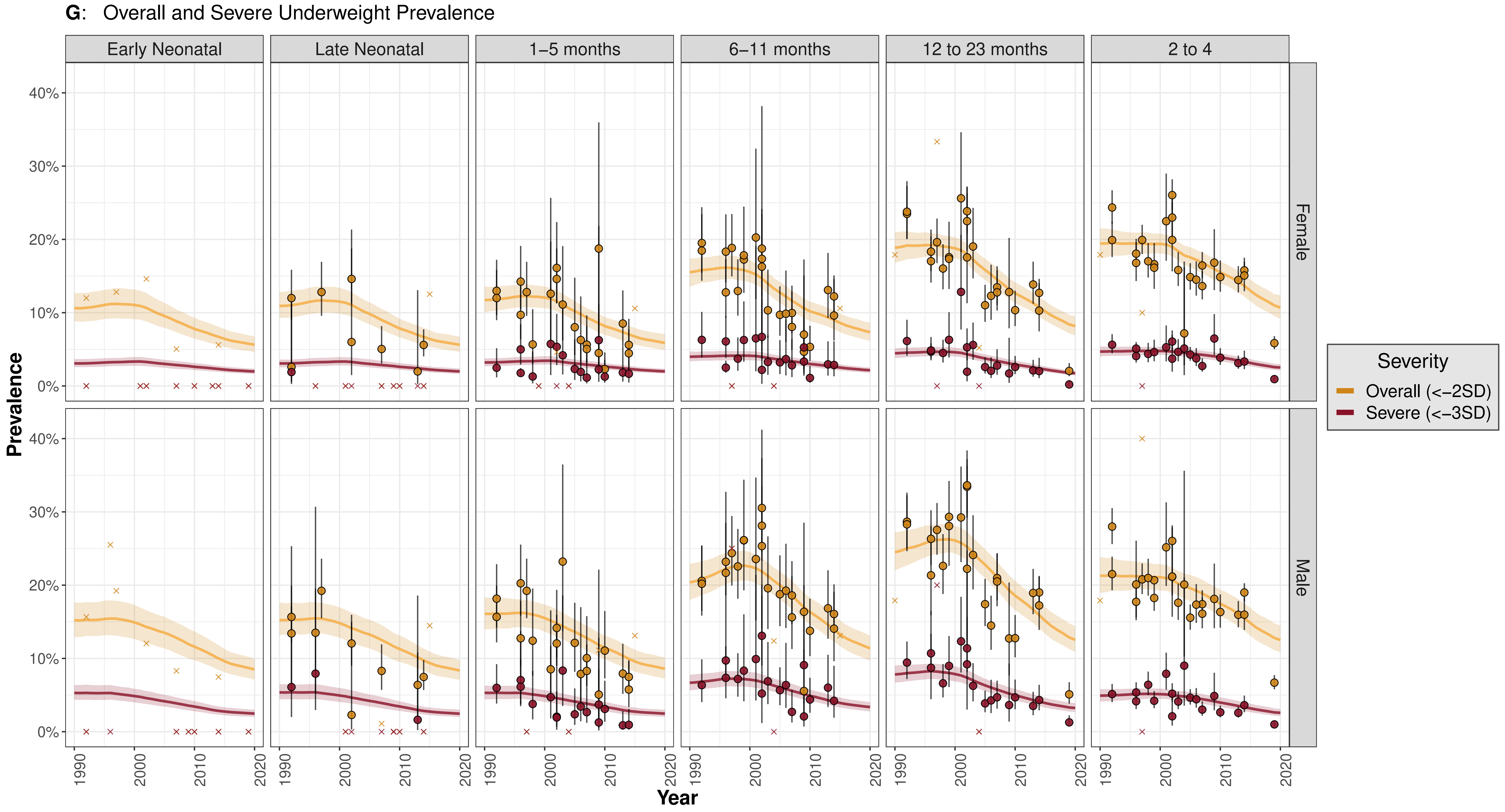

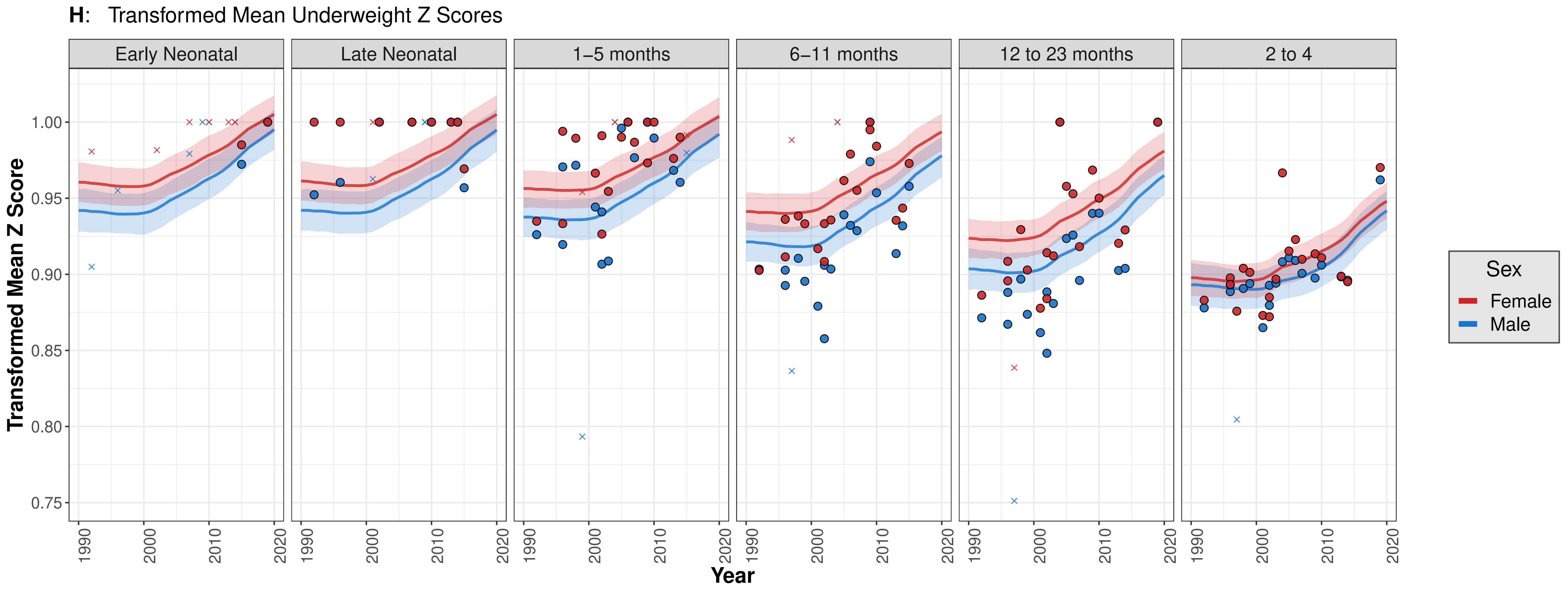

I

Year	Source
1990	WHO CGM Database
1992	DHS
1992	WHO CGM Database
1996	Living Conditions Monitoring Survey
1996	DHS
1997	DHS
1997	WHO CGM Database
1998	Living Conditions Monitoring Survey
1999	MICS
1999	WHO CGM Database
2001	DHS
2002	Living Conditions Monitoring Survey
2002	DHS
2002	WHO CGM Database
2003	Living Conditions Monitoring Survey
2004	Living Conditions Monitoring Survey
2005	Living Conditions Monitoring Survey
2006	Living Conditions Monitoring Survey
2007	DHS
2007	WHO CGM Database
2009	National Population Health Survey
2009	Access to ACT Initiative Survey
2010	Living Conditions Monitoring Survey
2013	DHS
2014	DHS
2014	WHO CGM Database
2015	Living Conditions Monitoring Survey
2019	DHS

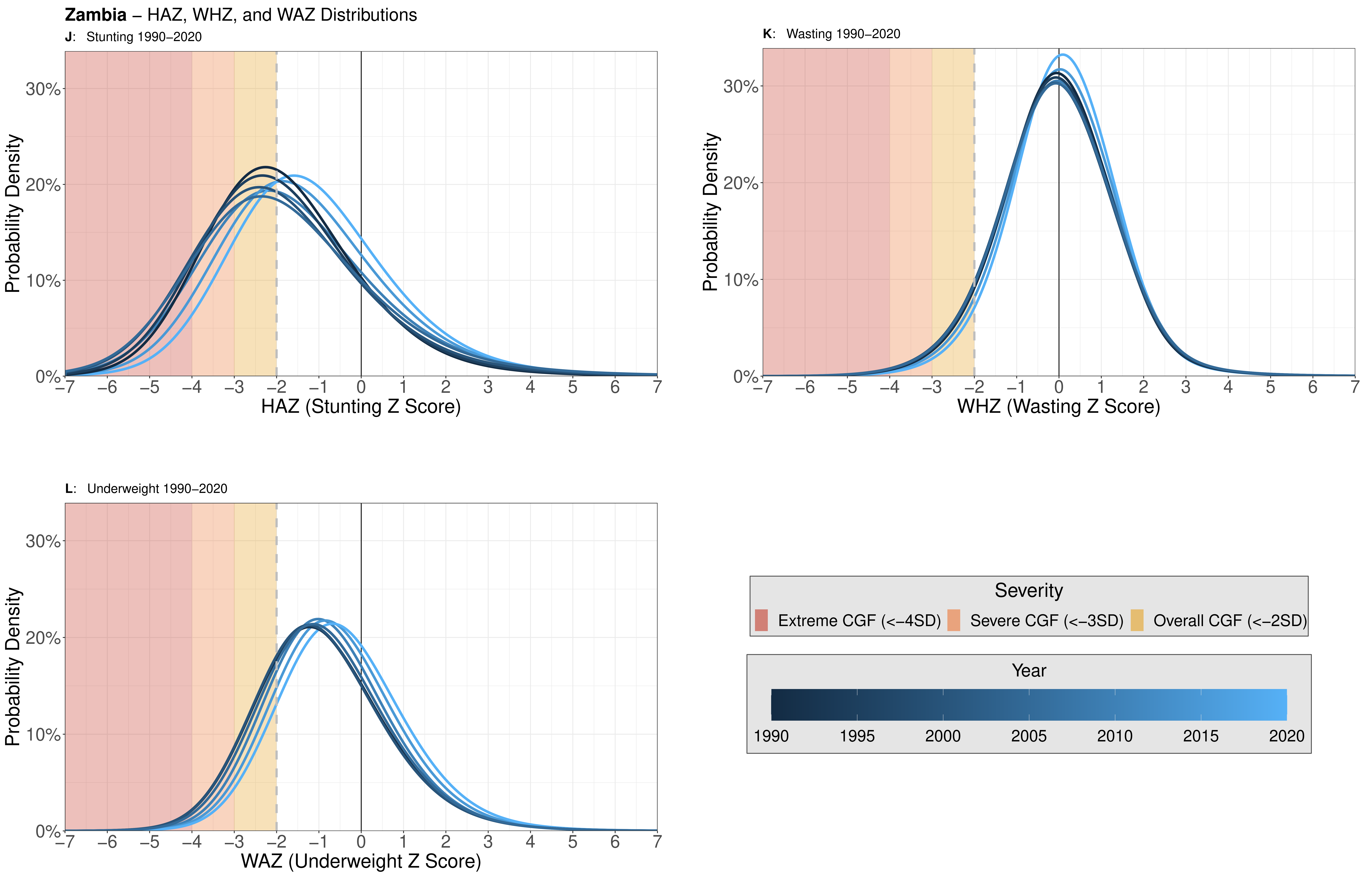
Zambia – HAZ, WHZ, and WAZ Distributions
J: Stunting 1990–2020
K: Wasting 1990–2020
L: Underweight 1990–2020
Probability Density
30%
20%
10%
0%
−7 −6 −5 −4 −3 −2 −1 0 1 2 3 4 5 6 7
HAZ (Stunting Z Score)
WHZ (Wasting Z Score)
WAZ (Underweight Z Score)
Severity
Extreme CGF (<−4SD)
Severe CGF (<−3SD)
Overall CGF (<−2SD)
Year
1990
1995
2000
2005
2010
2015
2020

South Sudan – Stunting (HAZ)

A: Overall and Severe Stunting Prevalence

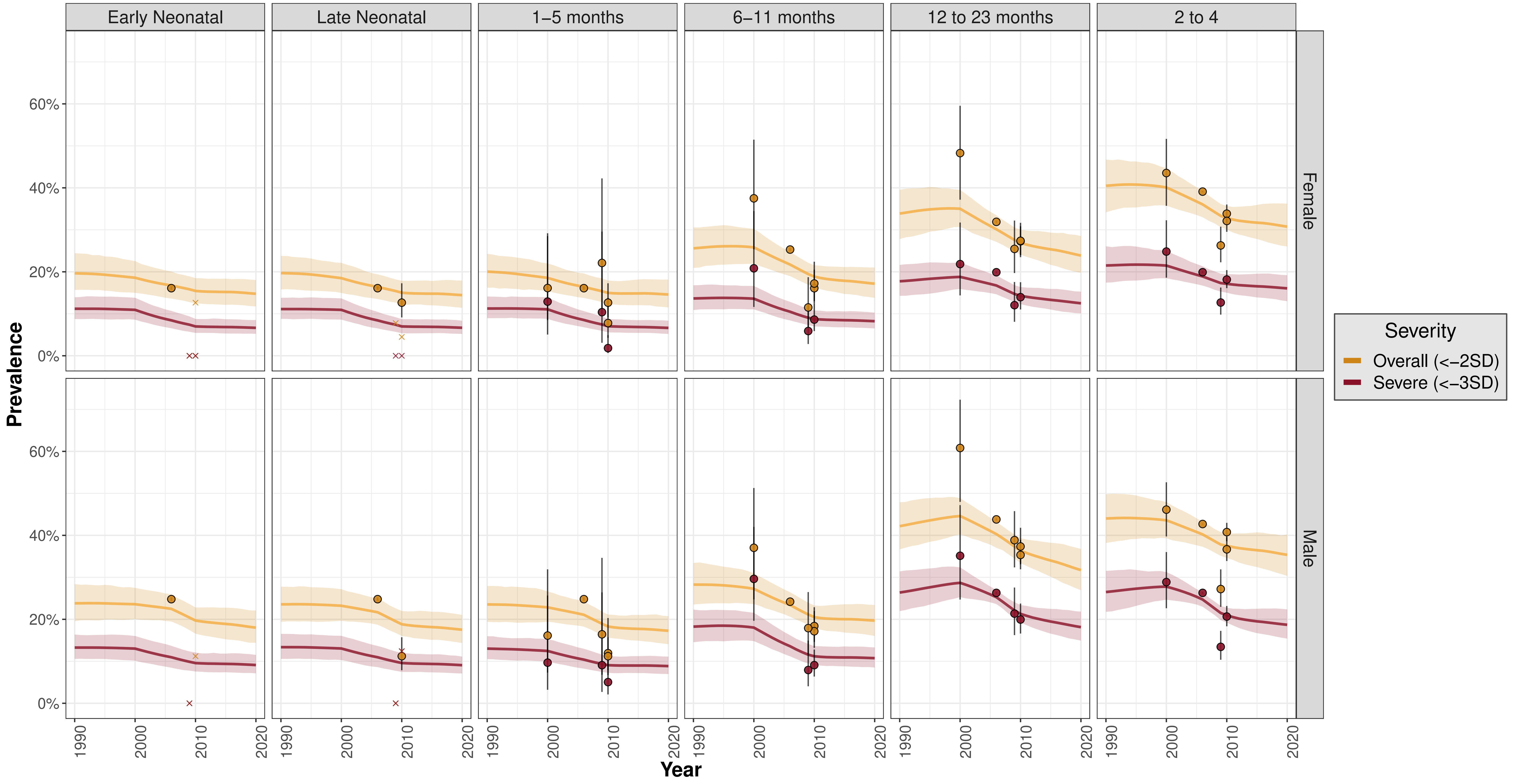

B: Transformed Mean Stunting Z Scores

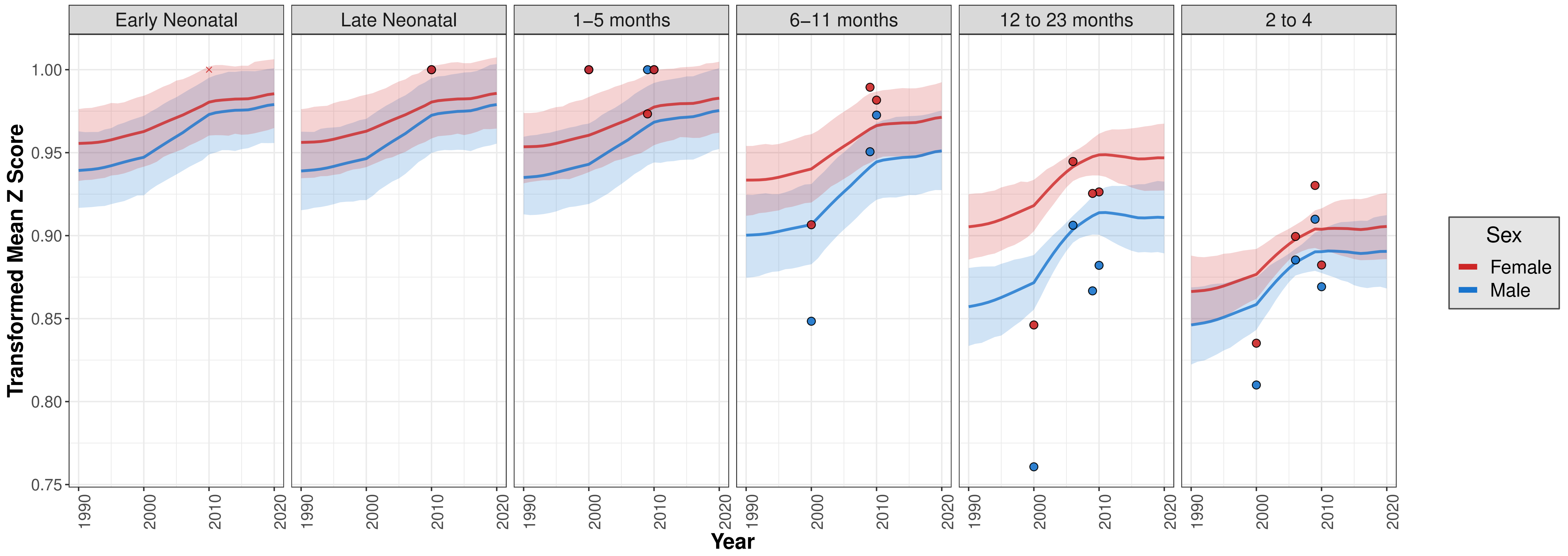

C

Year	Source
2000	MICS in the Southern Areas
2006	WHO CGM Database
2009	National Baseline Household Survey (NBHS)
2010	MICS
2010	WHO CGM Database

South Sudan – Wasting (WHZ)

D: Overall and Severe Wasting Prevalence

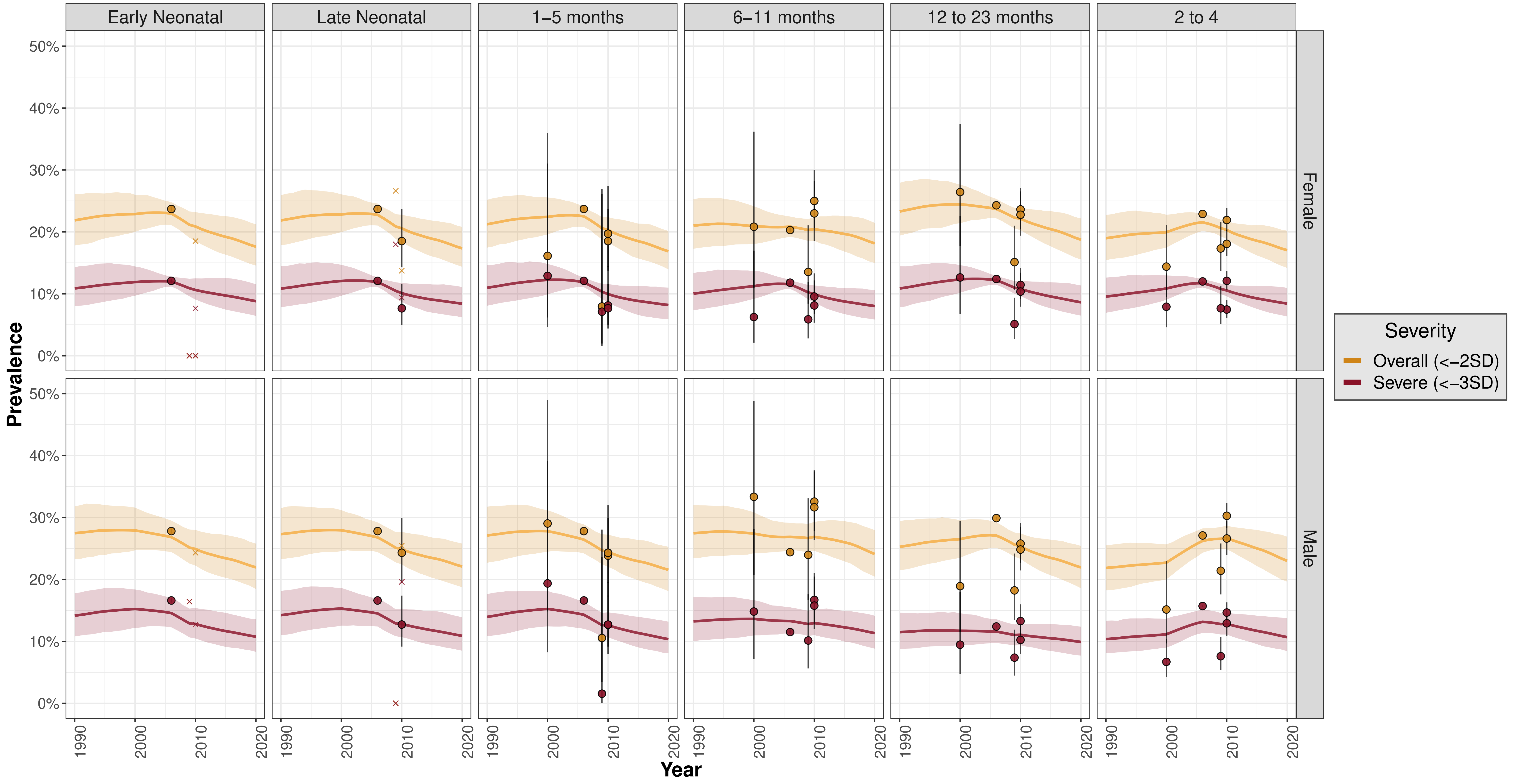

E: Transformed Mean Wasting Z Scores

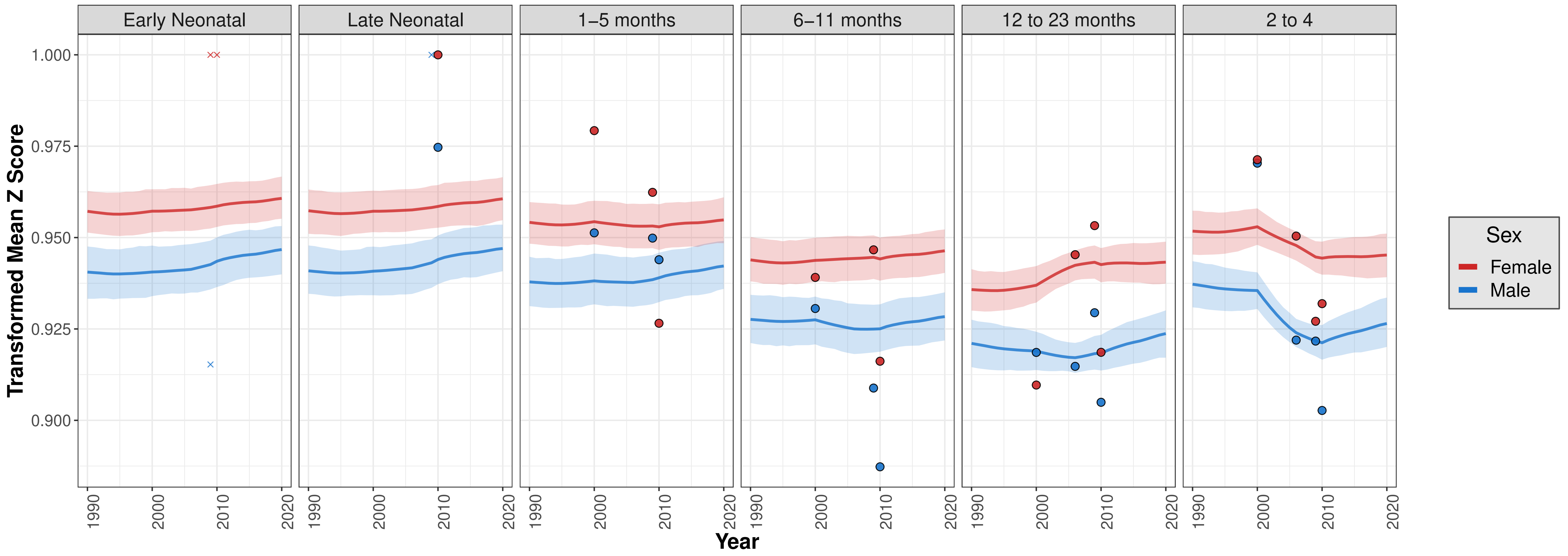

F

Year	Source
2000	MICS in the Southern Areas
2006	WHO CGM Database
2009	National Baseline Household Survey (NBHS)
2010	MICS
2010	WHO CGM Database

South Sudan – Underweight (WAZ)

G: Overall and Severe Underweight Prevalence

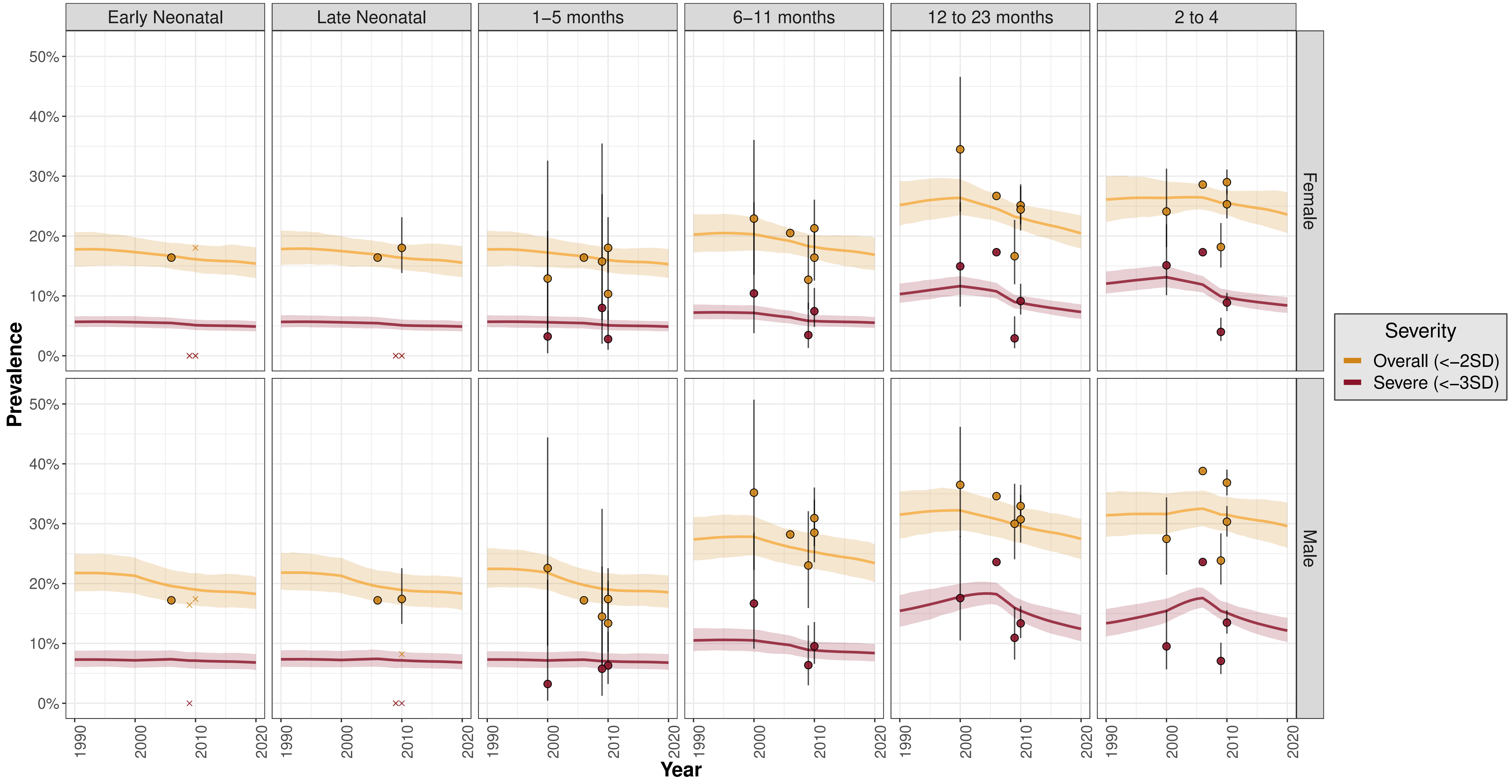

H: Transformed Mean Underweight Z Scores

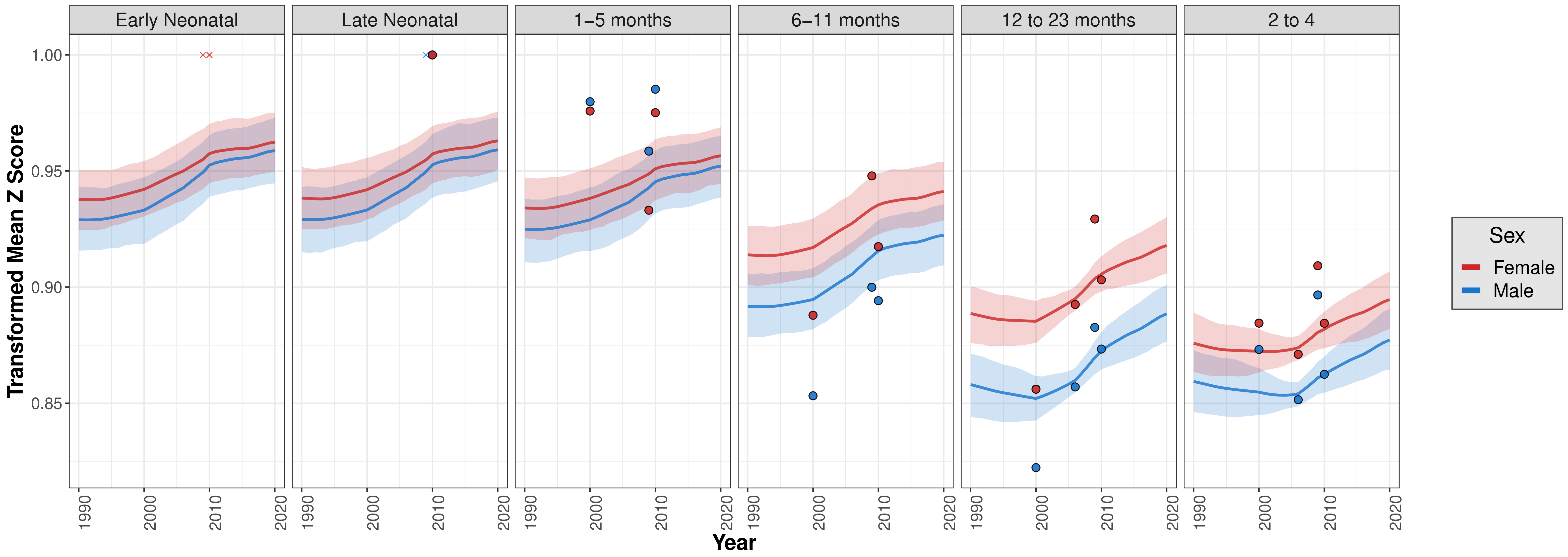

I

Year	Source
2000	MICS in the Southern Areas
2006	WHO CGM Database
2009	National Baseline Household Survey (NBHS)
2010	MICS
2010	WHO CGM Database

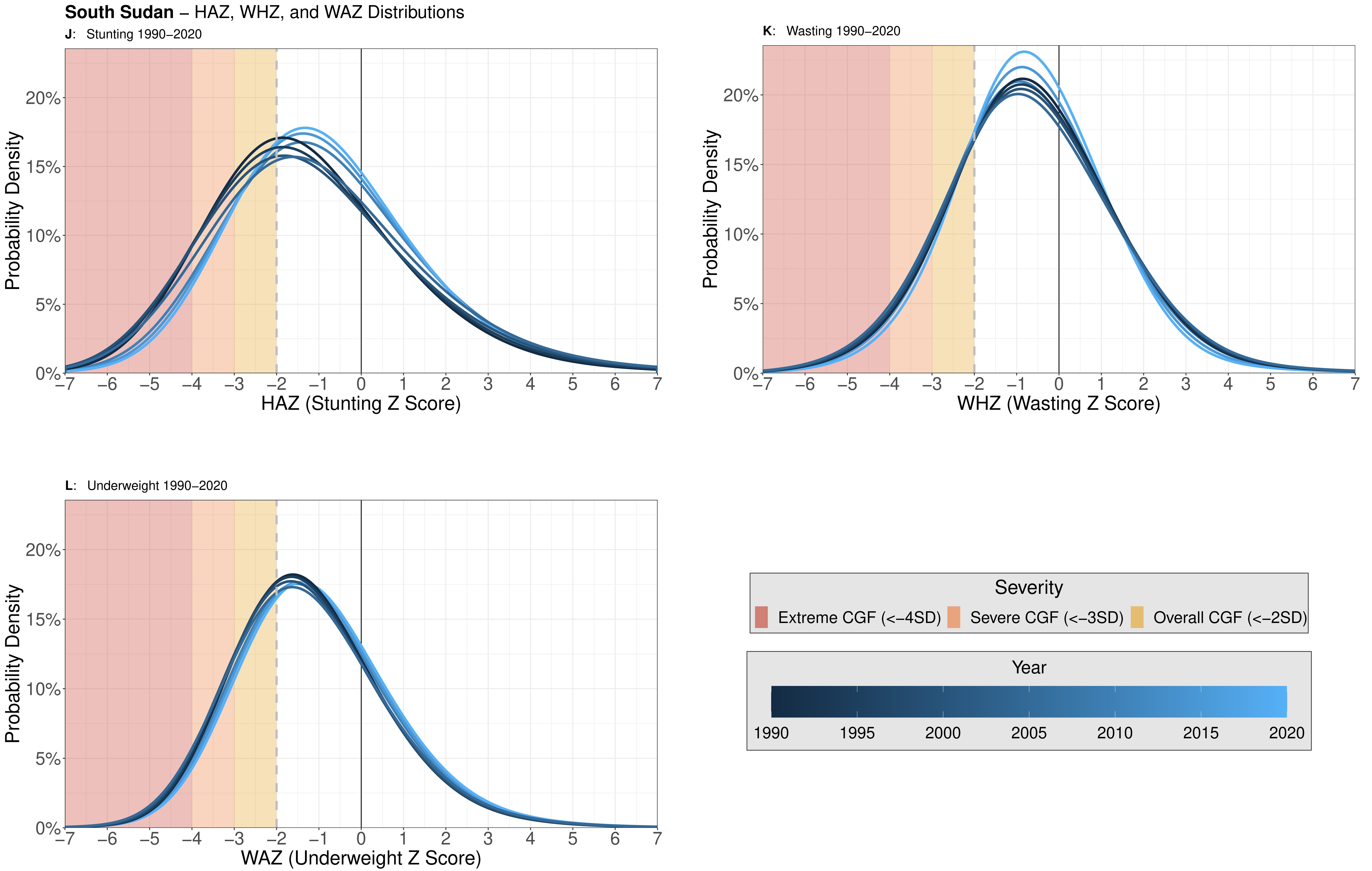
South Sudan – HAZ, WHZ, and WAZ Distributions
J: Stunting 1990–2020
K: Wasting 1990–2020
L: Underweight 1990–2020
Probability Density
0%
5%
10%
15%
20%
−7 −6 −5 −4 −3 −2 −1 0 1 2 3 4 5 6 7
HAZ (Stunting Z Score)
WHZ (Wasting Z Score)
WAZ (Underweight Z Score)
Severity
Extreme CGF (<−4SD)
Severe CGF (<−3SD)
Overall CGF (<−2SD)
Year
1990
1995
2000
2005
2010
2015
2020

Botswana – Stunting (HAZ)

A: Overall and Severe Stunting Prevalence

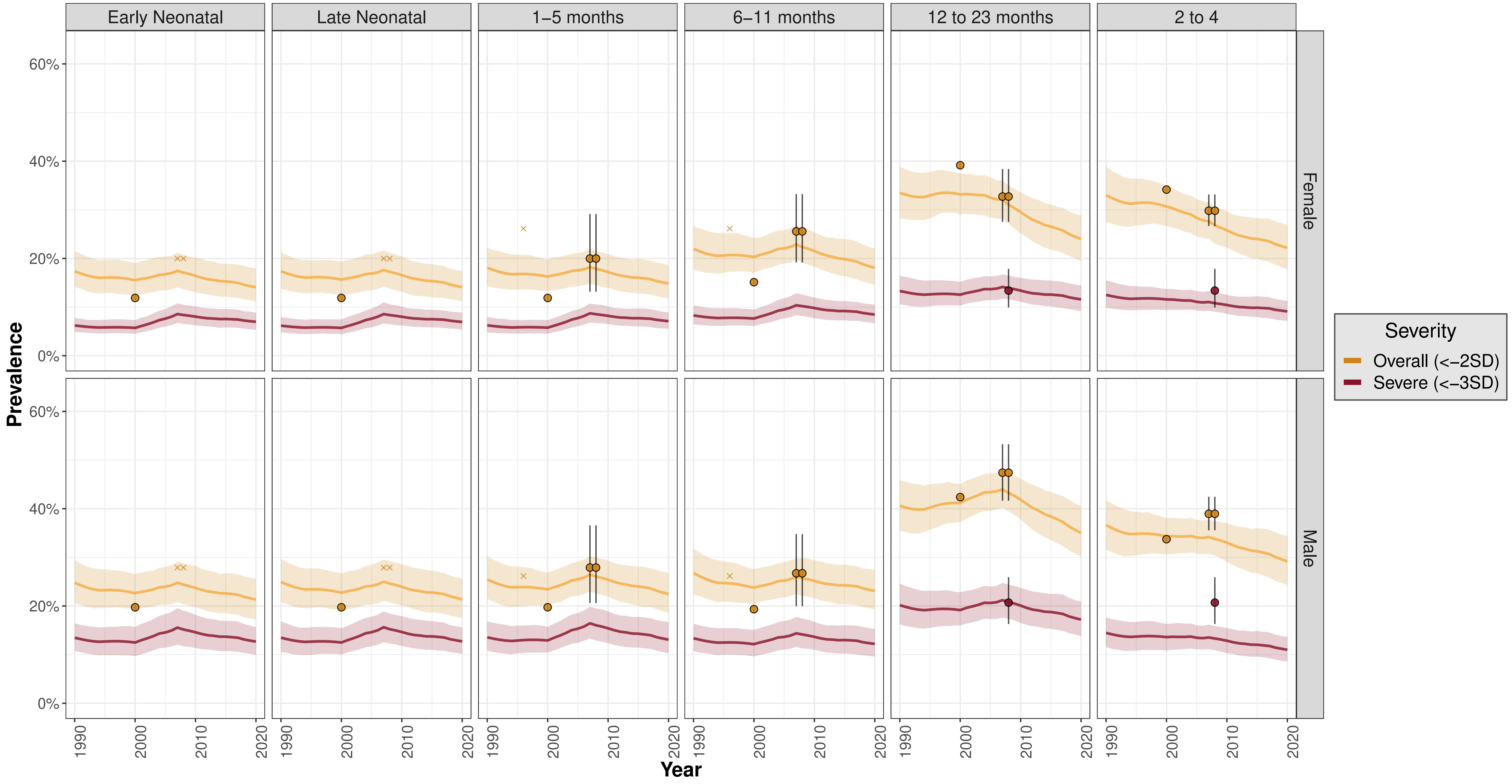

B: Transformed Mean Stunting Z Scores

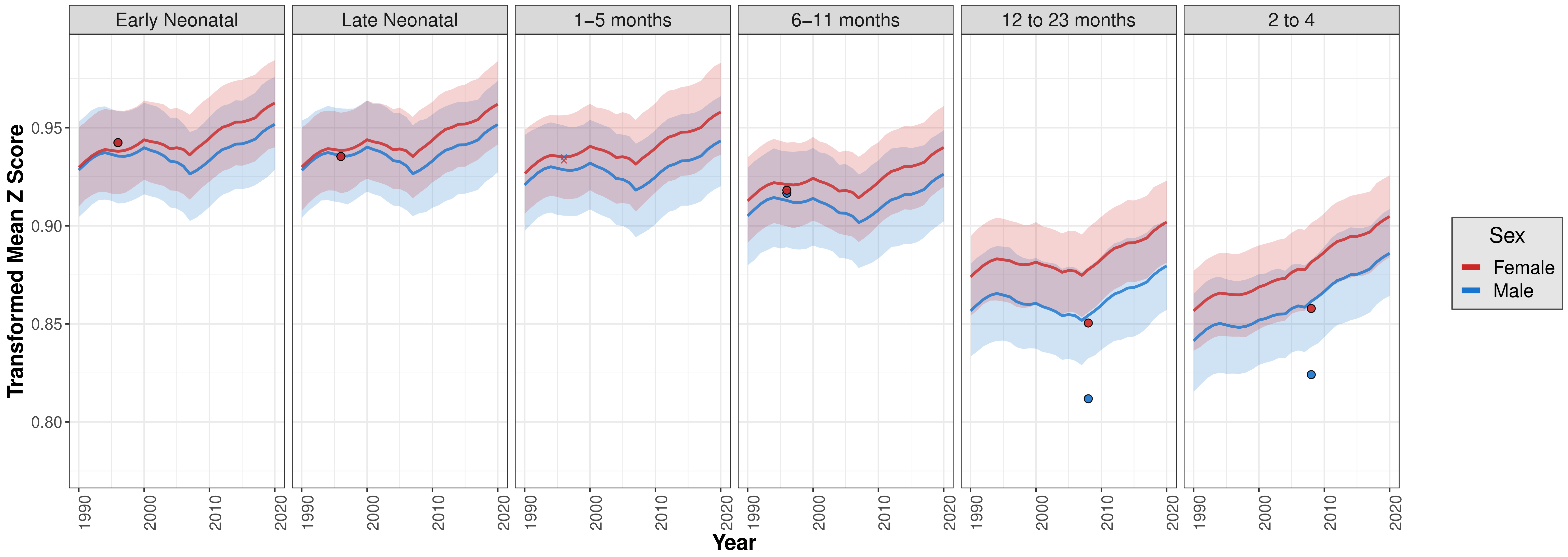

C

Year	Source
1985	WHO CGM Database
1996	WHO CGM Database
2000	MICS
2000	WHO CGM Database
2007	Family Health Survey
2007	WHO CGM Database
2008	Family Health Survey
2008	WHO CGM Database

Botswana – Wasting (WHZ)

D: Overall and Severe Wasting Prevalence

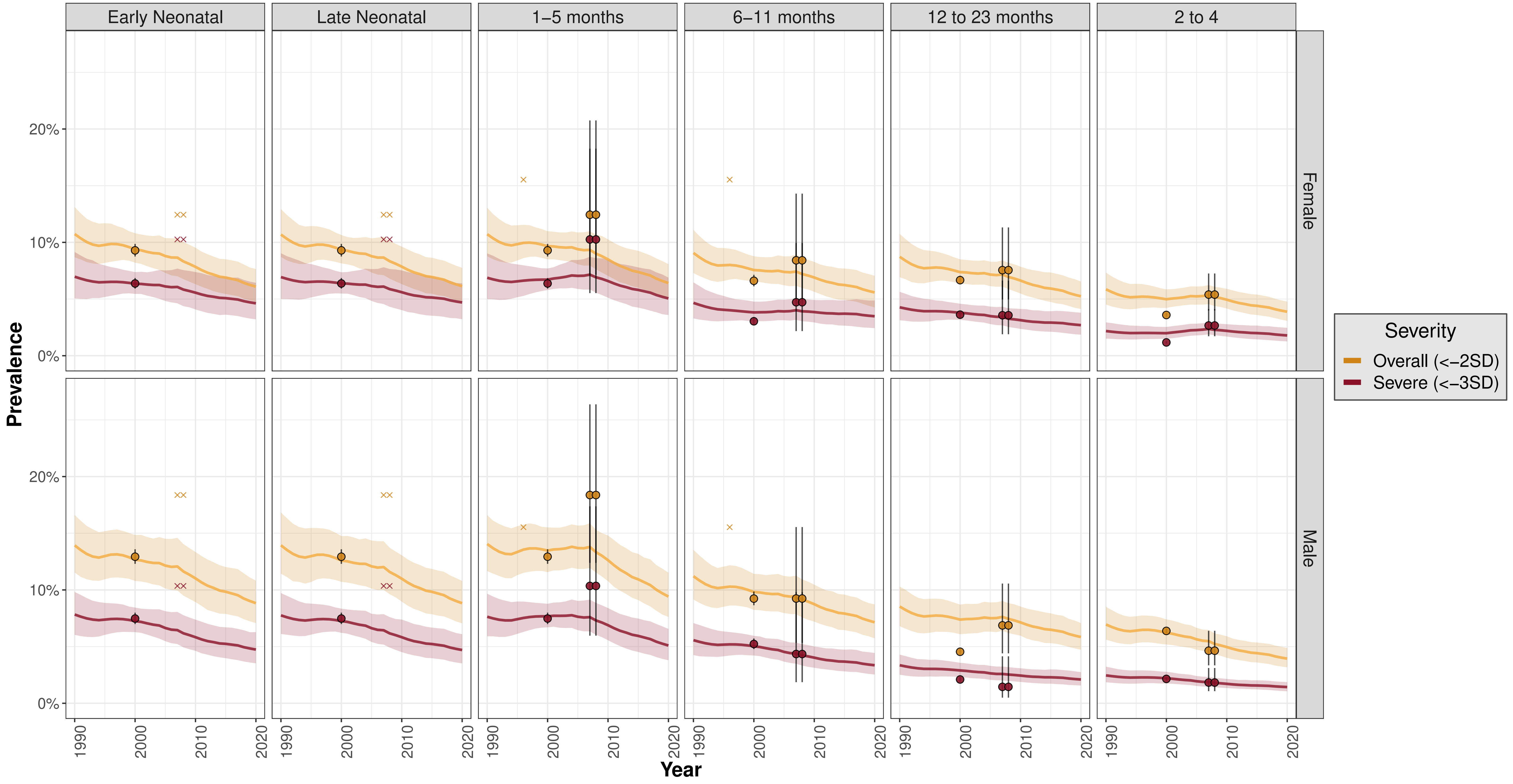

E: Transformed Mean Wasting Z Scores

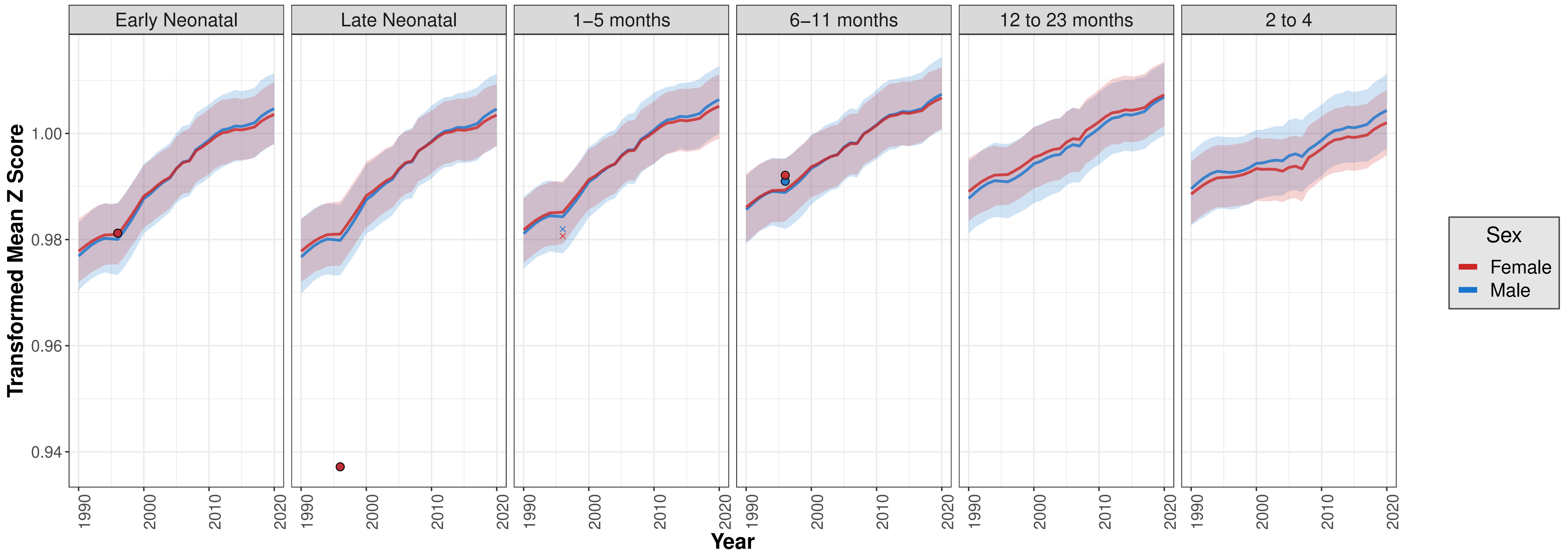

F

Year	Source
1985	WHO CGM Database
1996	WHO CGM Database
2000	MICS
2000	WHO CGM Database
2007	Family Health Survey
2007	WHO CGM Database
2008	Family Health Survey
2008	WHO CGM Database

Botswana – Underweight (WAZ)

G: Overall and Severe Underweight Prevalence

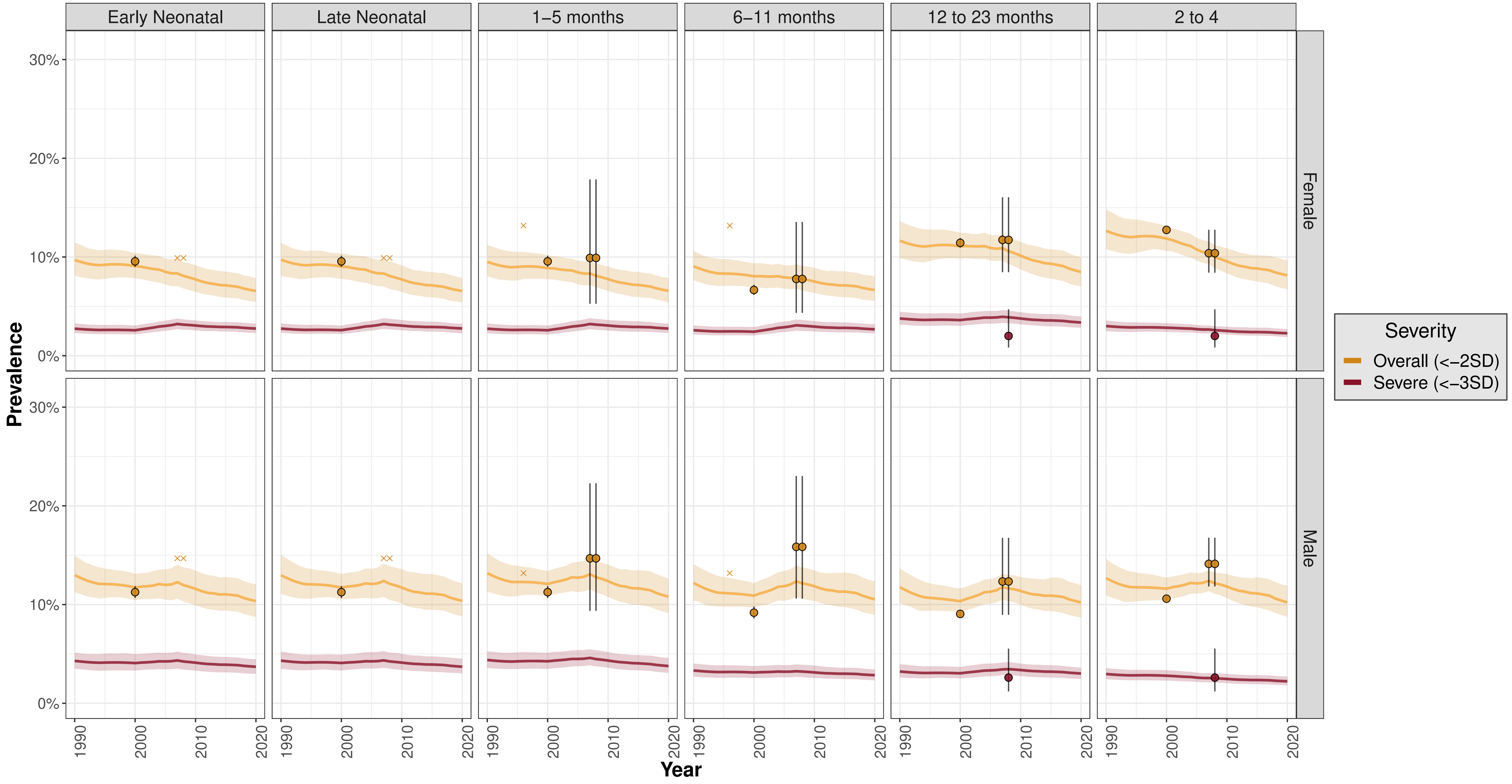

H: Transformed Mean Underweight Z Scores

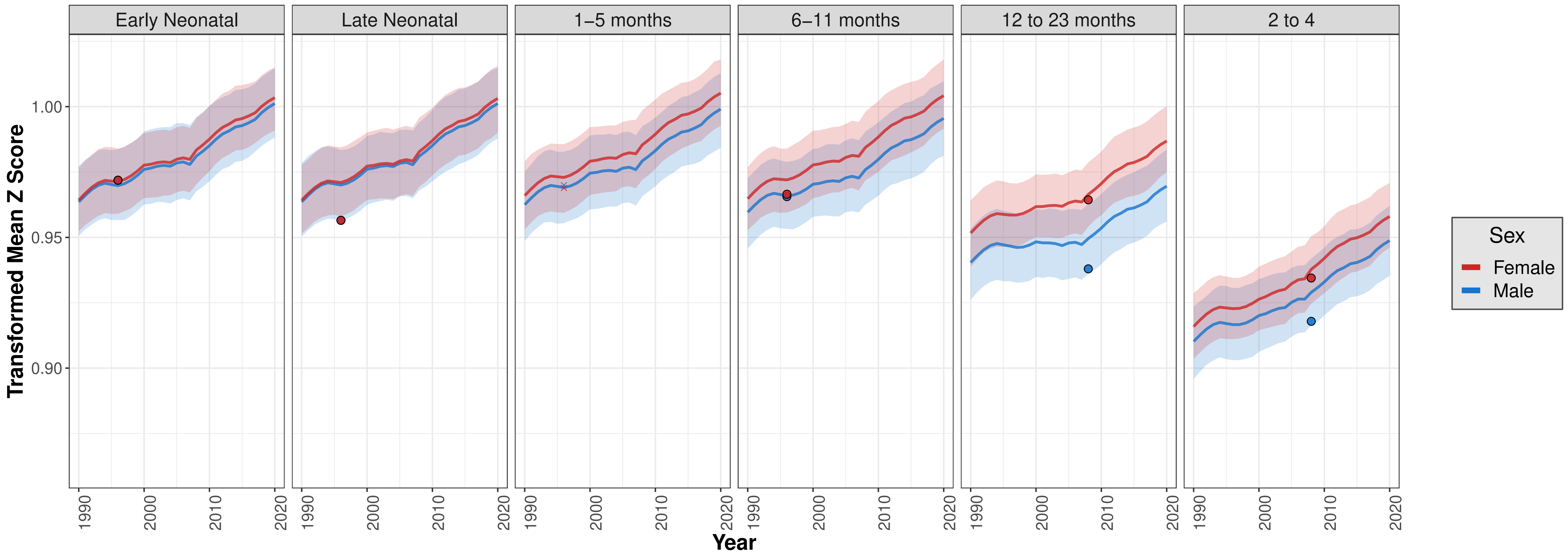

I

Year	Source
1985	WHO CGM Database
1996	WHO CGM Database
2000	MICS
2000	WHO CGM Database
2007	Family Health Survey
2007	WHO CGM Database
2008	Family Health Survey
2008	WHO CGM Database

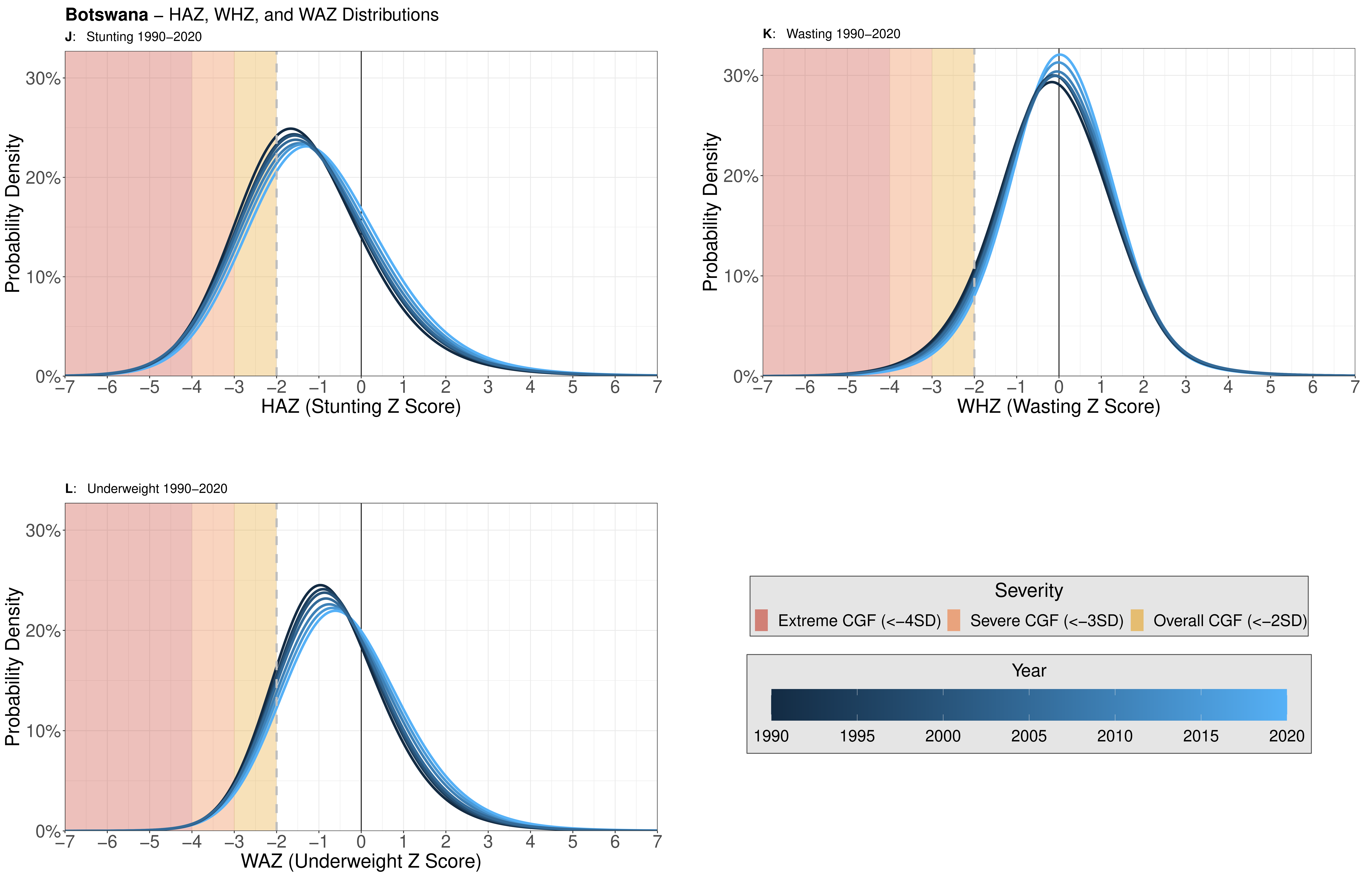
Botswana – HAZ, WHZ, and WAZ Distributions
J: Stunting 1990–2020
Probability Density
30%
20%
10%
0%
−7
−6
−5
−4
−3
−2
−1
0
1
2
3
4
5
6
7
HAZ (Stunting Z Score)
K: Wasting 1990–2020
Probability Density
WHZ (Wasting Z Score)
L: Underweight 1990–2020
Probability Density
WAZ (Underweight Z Score)
Severity
Extreme CGF (<−4SD)
Severe CGF (<−3SD)
Overall CGF (<−2SD)
Year
1990
1995
2000
2005
2010
2015
2020

Lesotho – Stunting (HAZ)

A: Overall and Severe Stunting Prevalence

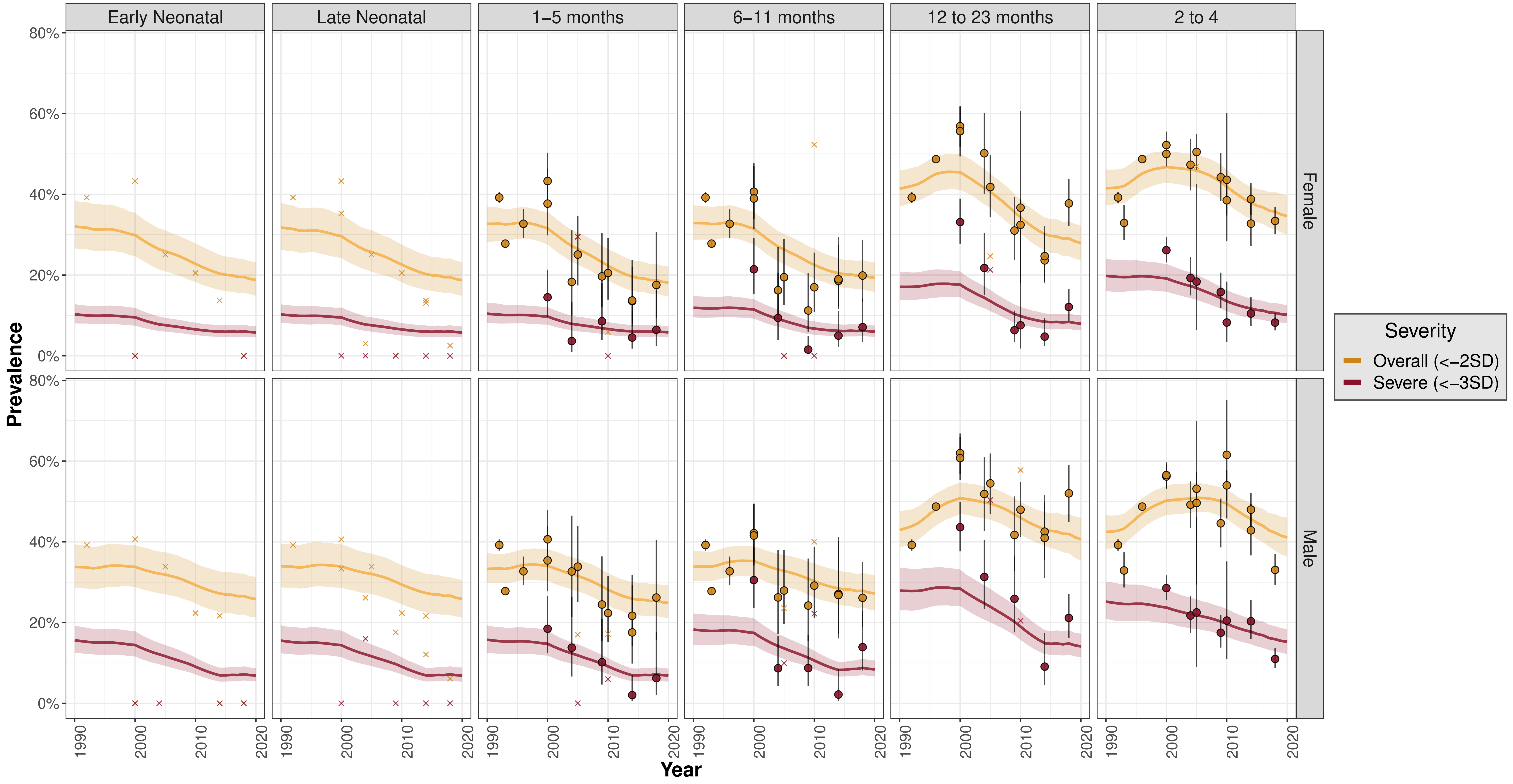

B: Transformed Mean Stunting Z Scores

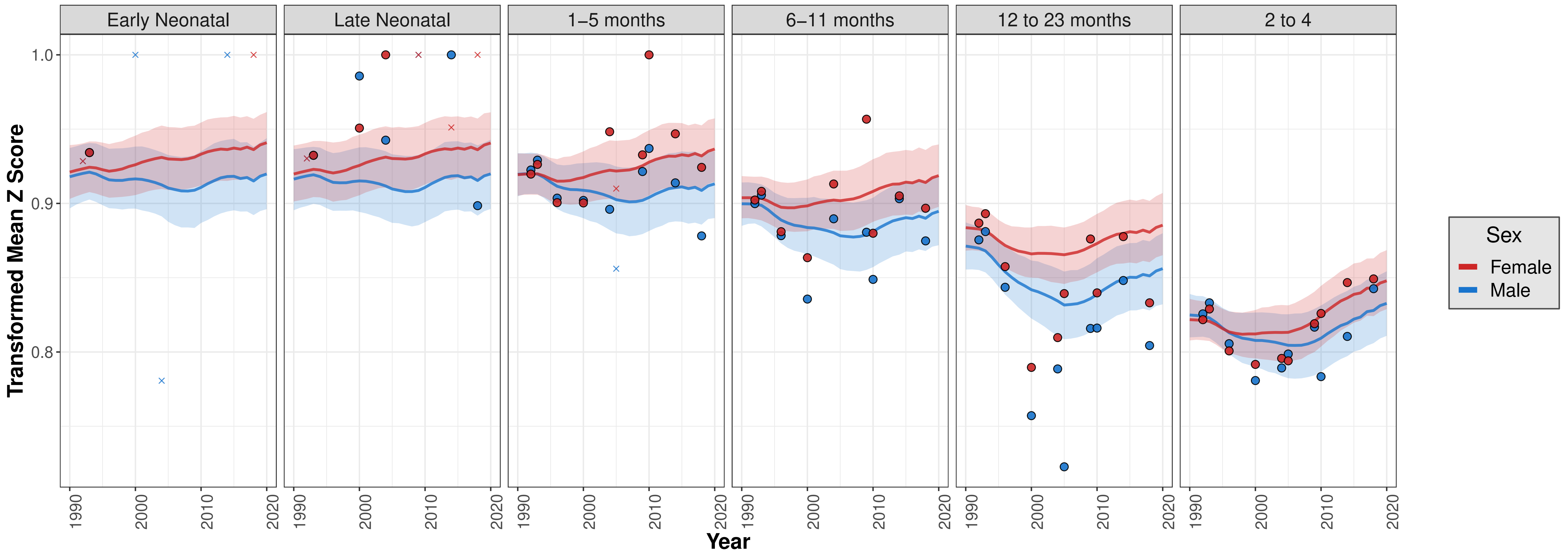

C

Year	Source
1976	WHO CGM Database
1988	WHO CGM Database
1992	WHO CGM Database
1993	WHO CGM Database
1996	WHO CGM Database
2000	MICS
2000	WHO CGM Database
2004	DHS
2005	DHS
2005	WHO CGM Database
2009	DHS
2010	DHS
2010	WHO CGM Database
2014	WHO CGM Database
2014	DHS
2018	MICS

Lesotho – Wasting (WHZ)

D: Overall and Severe Wasting Prevalence

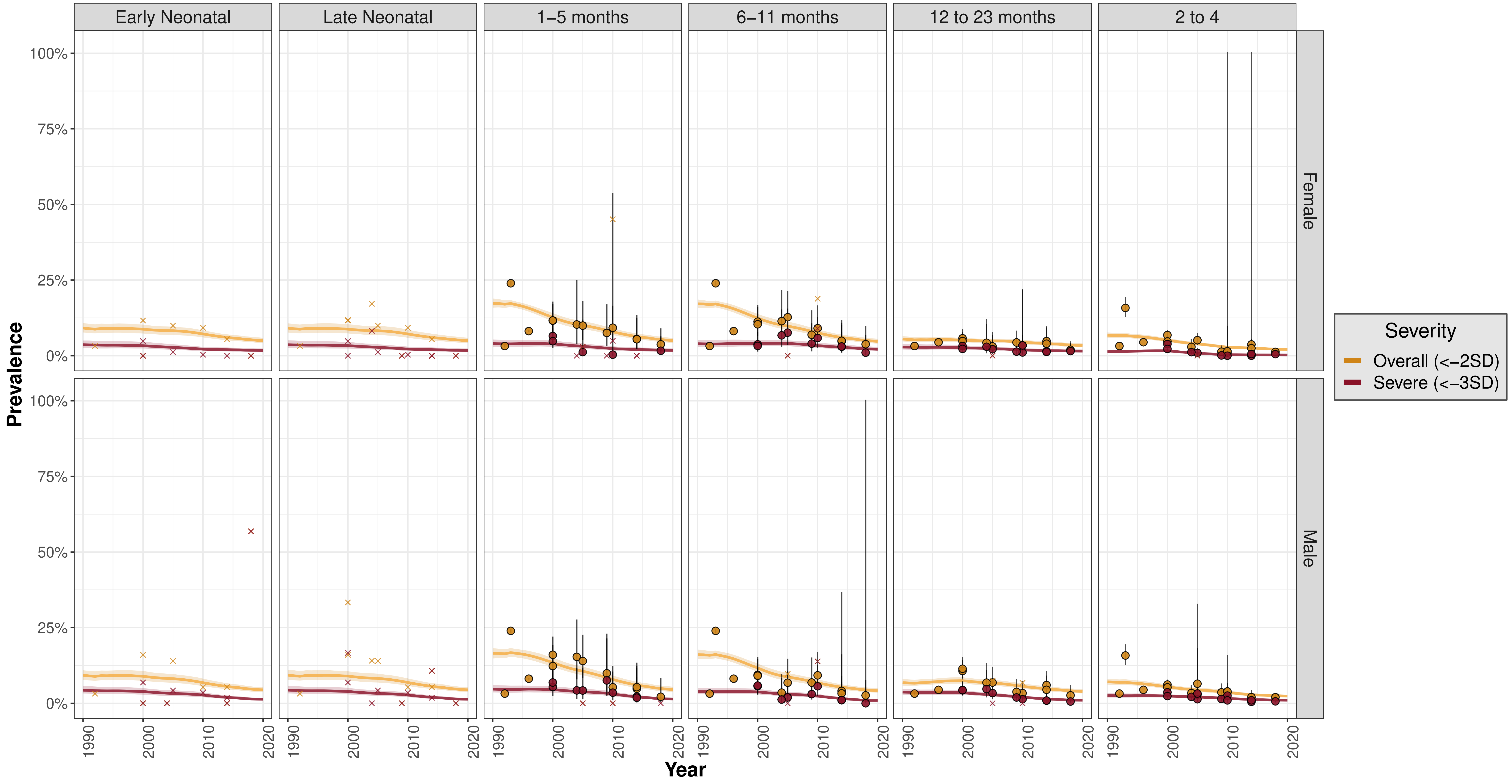

E: Transformed Mean Wasting Z Scores

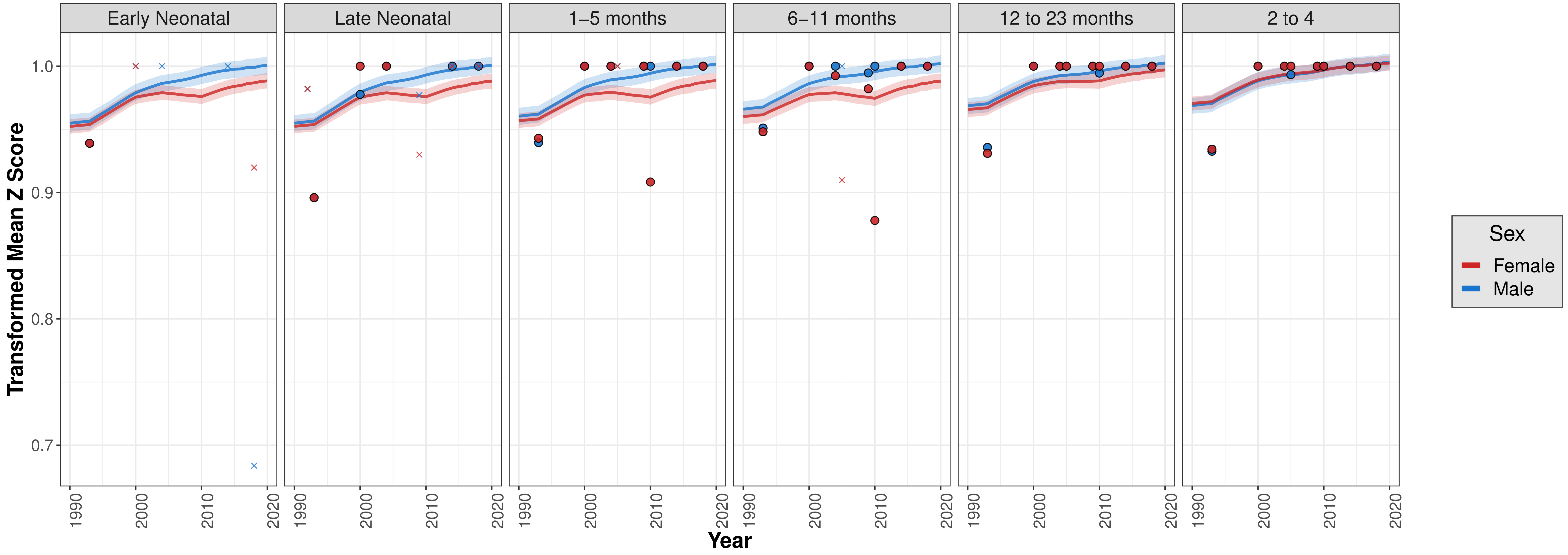

F

Year	Source
1992	WHO CGM Database
1993	WHO CGM Database
1996	WHO CGM Database
2000	MICS
2000	WHO CGM Database
2004	DHS
2005	DHS
2005	WHO CGM Database
2009	DHS
2010	DHS
2010	WHO CGM Database
2014	WHO CGM Database
2014	DHS
2018	MICS

Lesotho – Underweight (WAZ)

G: Overall and Severe Underweight Prevalence

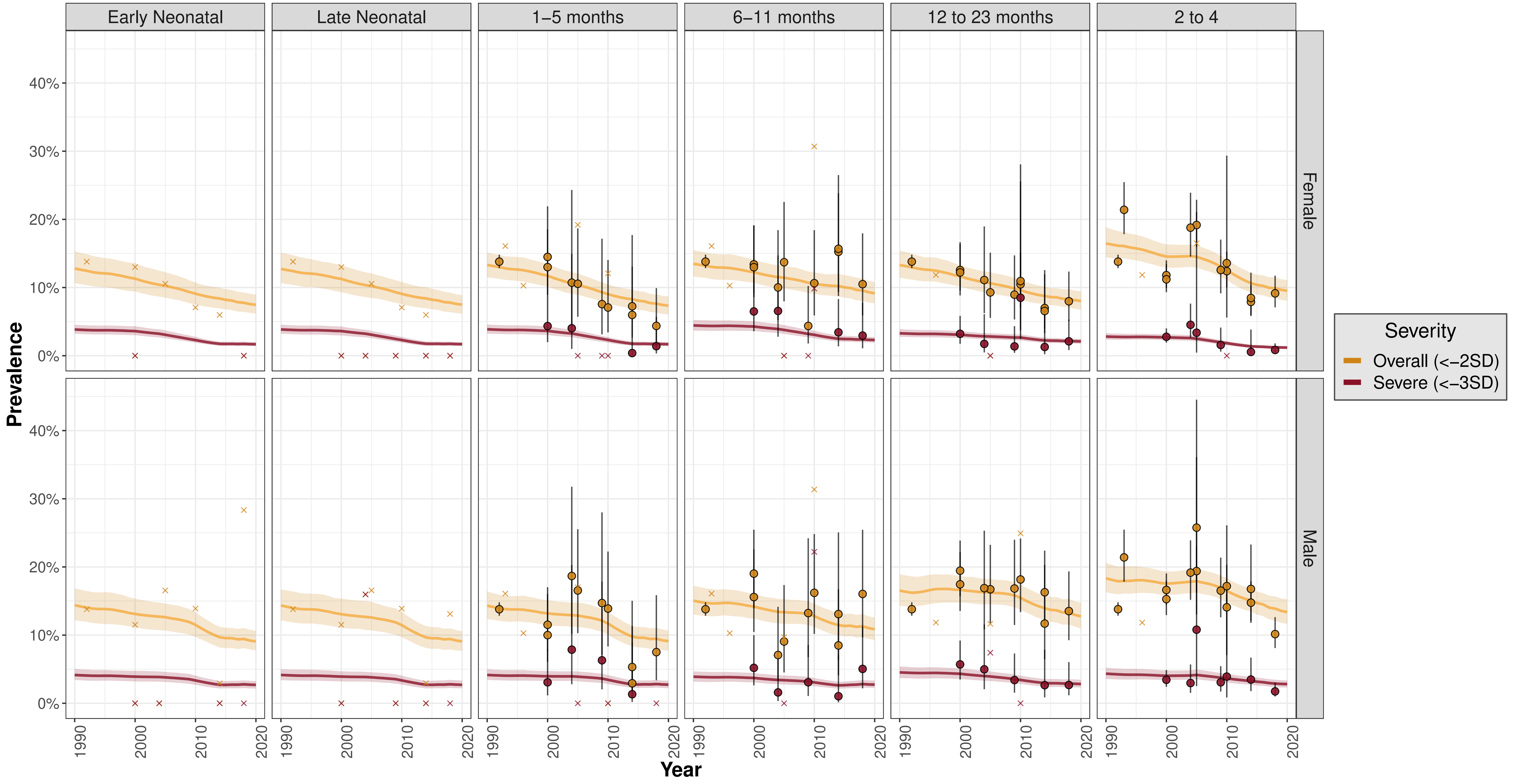

H: Transformed Mean Underweight Z Scores

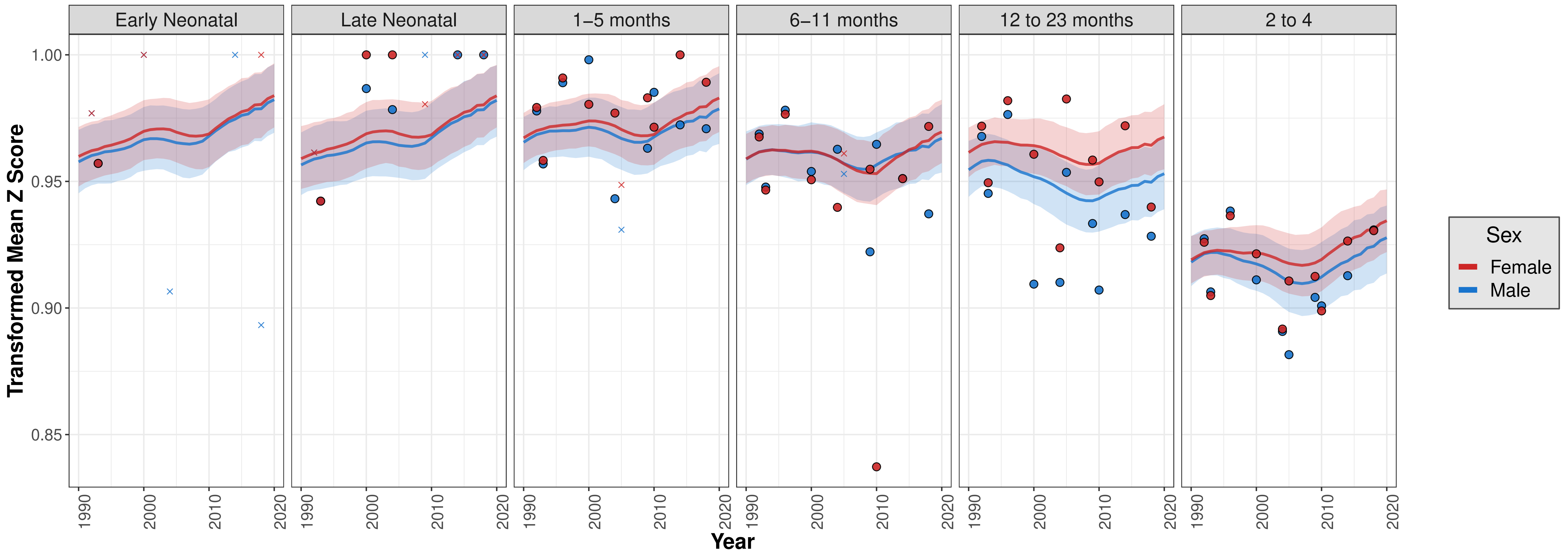

I

Year	Source
1976	WHO CGM Database
1992	WHO CGM Database
1993	WHO CGM Database
1996	WHO CGM Database
2000	MICS
2000	WHO CGM Database
2004	DHS
2005	DHS
2005	WHO CGM Database
2009	DHS
2010	DHS
2010	WHO CGM Database
2014	WHO CGM Database
2014	DHS
2018	MICS

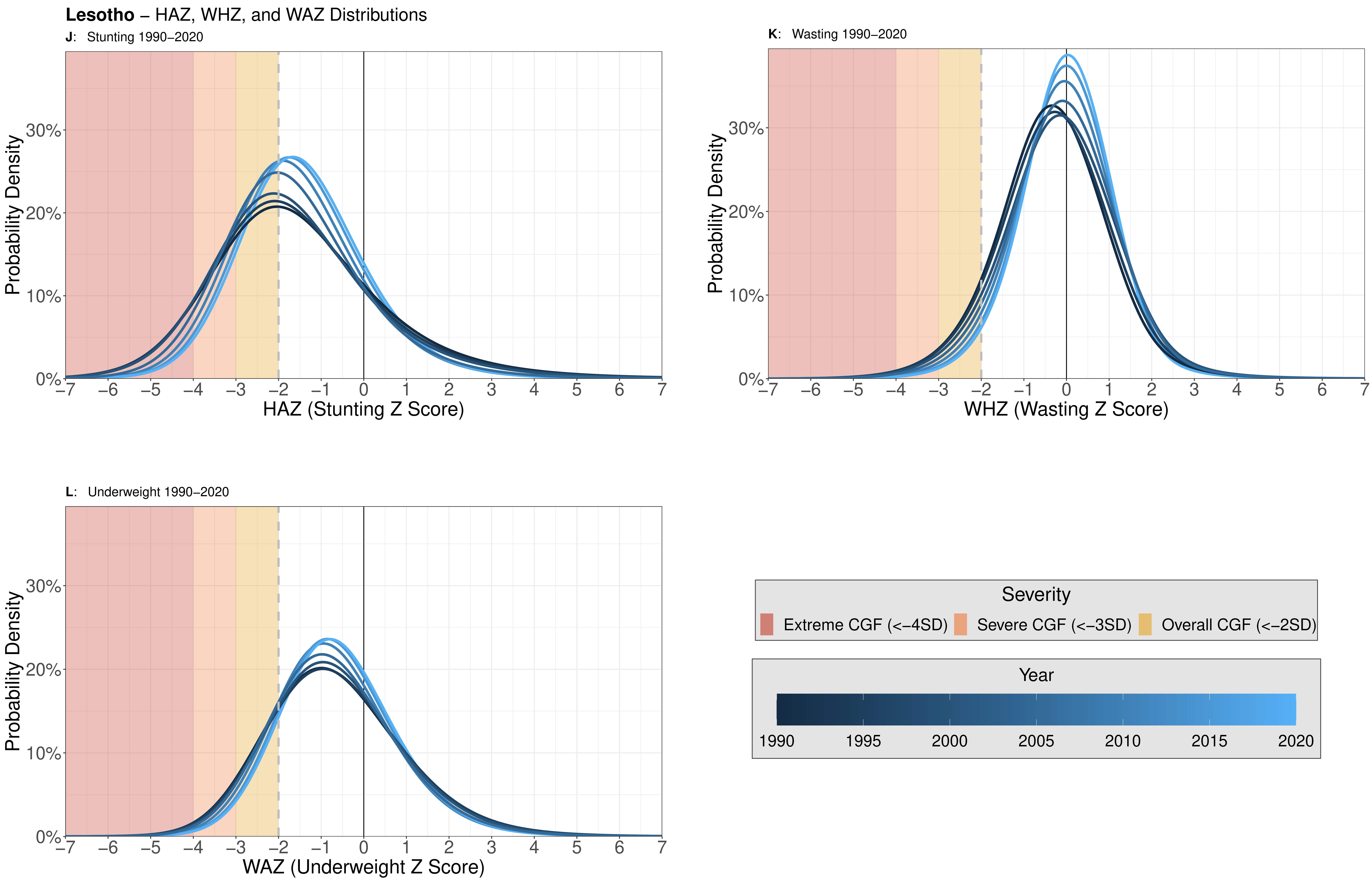

Lesotho – HAZ, WHZ, and WAZ Distributions
J: Stunting 1990–2020
Probability Density
0%
10%
20%
30%
−7 −6 −5 −4 −3 −2 −1 0 1 2 3 4 5 6 7
HAZ (Stunting Z Score)
K: Wasting 1990–2020
Probability Density
0%
10%
20%
30%
−7 −6 −5 −4 −3 −2 −1 0 1 2 3 4 5 6 7
WHZ (Wasting Z Score)
L: Underweight 1990–2020
Probability Density
0%
10%
20%
30%
−7 −6 −5 −4 −3 −2 −1 0 1 2 3 4 5 6 7
WAZ (Underweight Z Score)
Severity
Extreme CGF (<−4SD)
Severe CGF (<−3SD)
Overall CGF (<−2SD)
Year
1990
1995
2000
2005
2010
2015
2020

Namibia – Stunting (HAZ)

A: Overall and Severe Stunting Prevalence

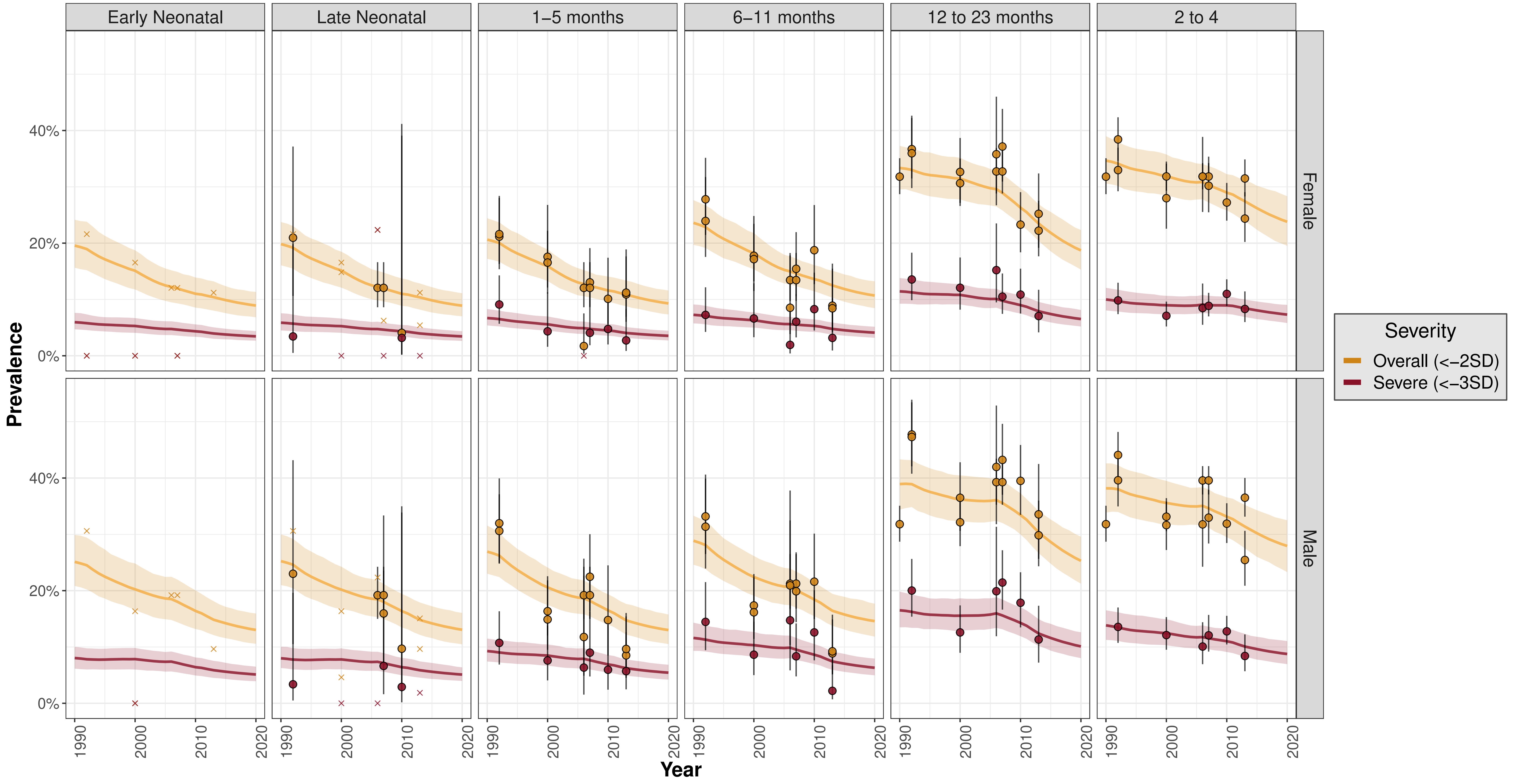

B: Transformed Mean Stunting Z Scores

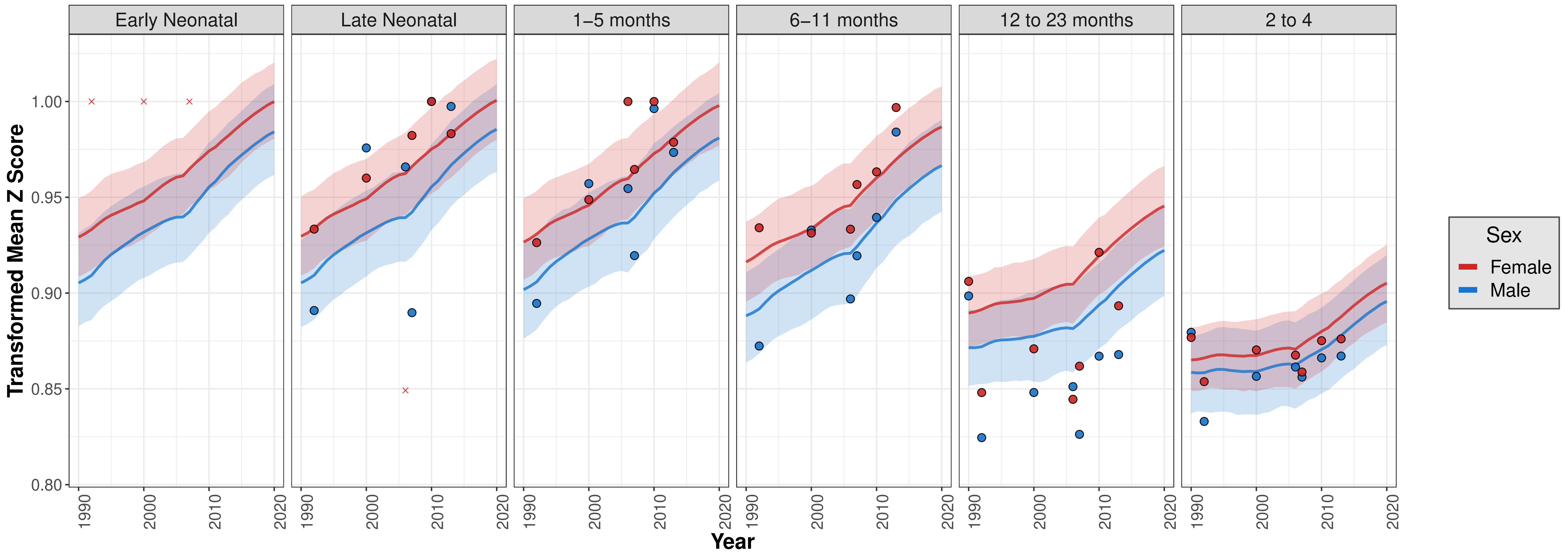

C

Year	Source
1990	WHO CGM Database
1992	DHS
1992	WHO CGM Database
2000	DHS
2000	WHO CGM Database
2006	DHS
2006	WHO CGM Database
2007	DHS
2007	WHO CGM Database
2010	Household Income and Expenditure Survey
2013	WHO CGM Database
2013	DHS

Namibia – Wasting (WHZ)

D: Overall and Severe Wasting Prevalence

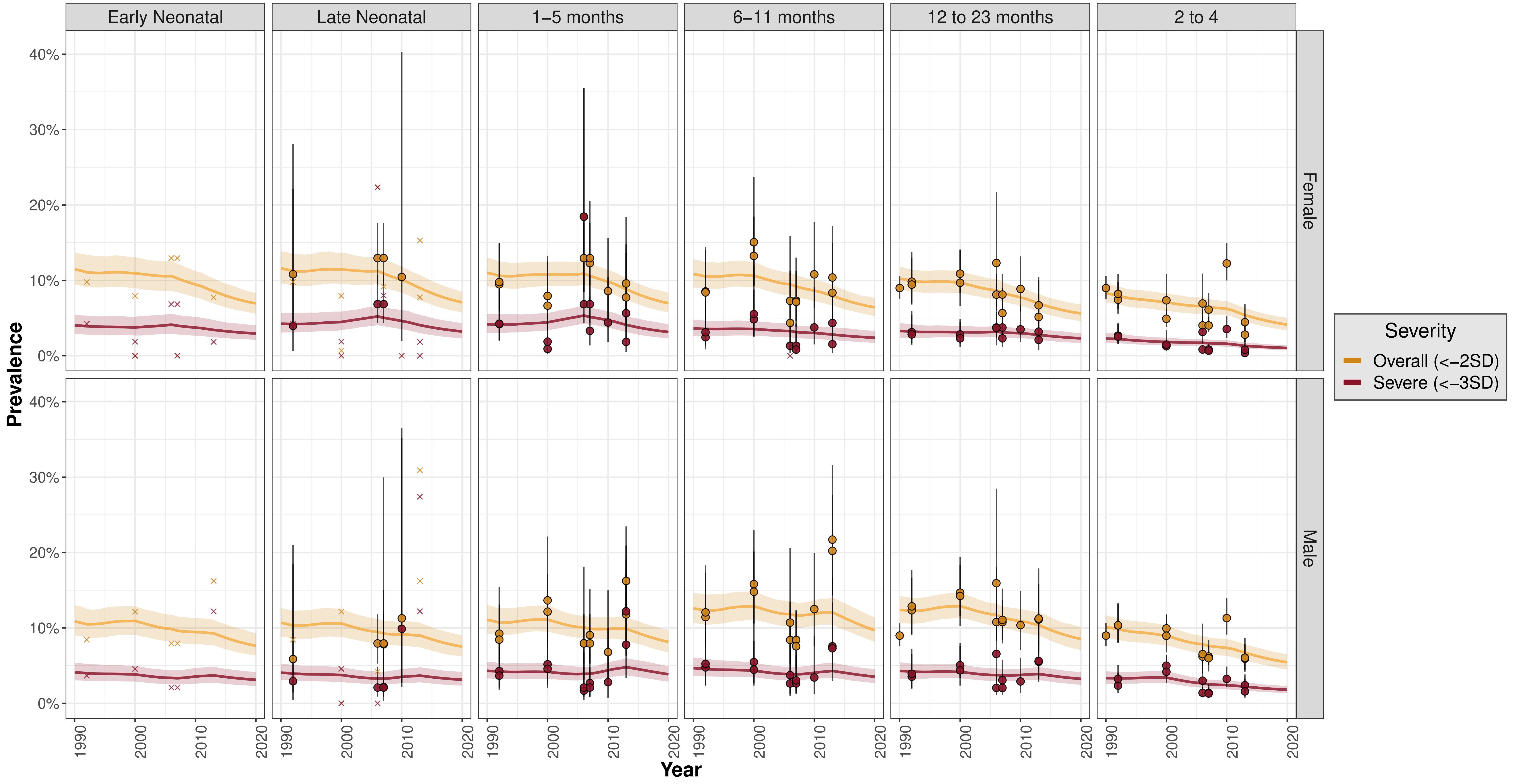

E: Transformed Mean Wasting Z Scores

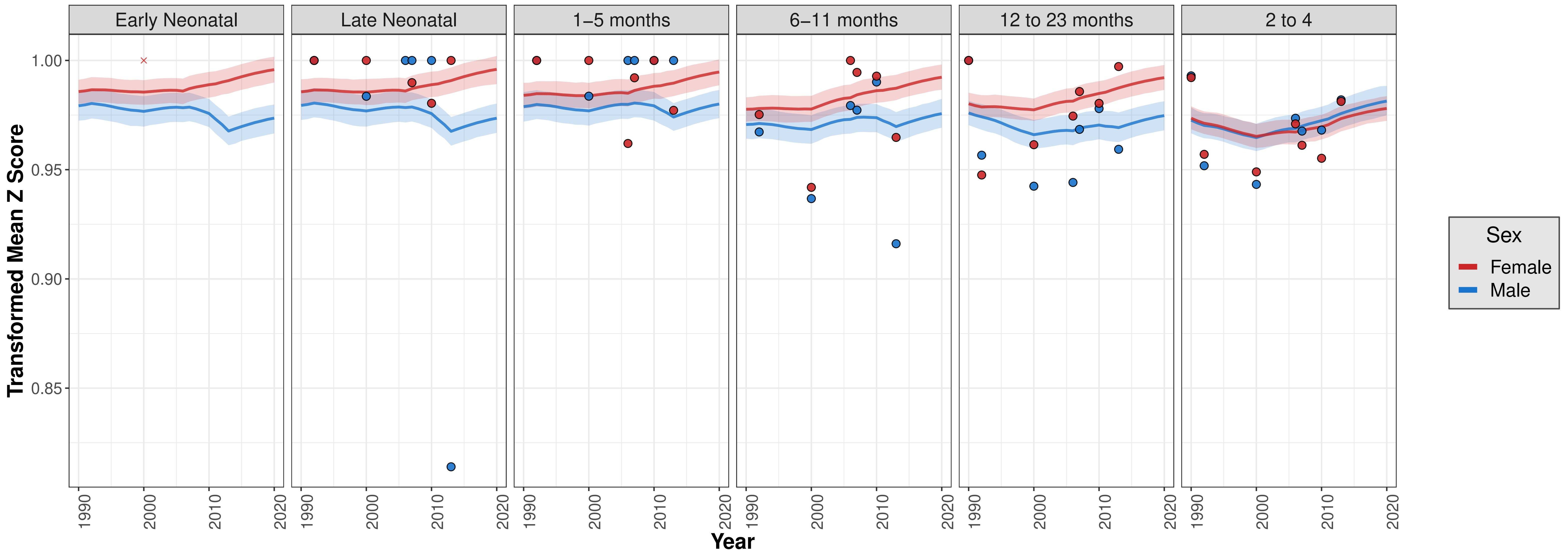

F

Year	Source
1990	WHO CGM Database
1992	DHS
1992	WHO CGM Database
2000	DHS
2000	WHO CGM Database
2006	DHS
2006	WHO CGM Database
2007	DHS
2007	WHO CGM Database
2010	Household Income and Expenditure Survey
2013	WHO CGM Database
2013	DHS

Namibia – Underweight (WAZ)

G: Overall and Severe Underweight Prevalence

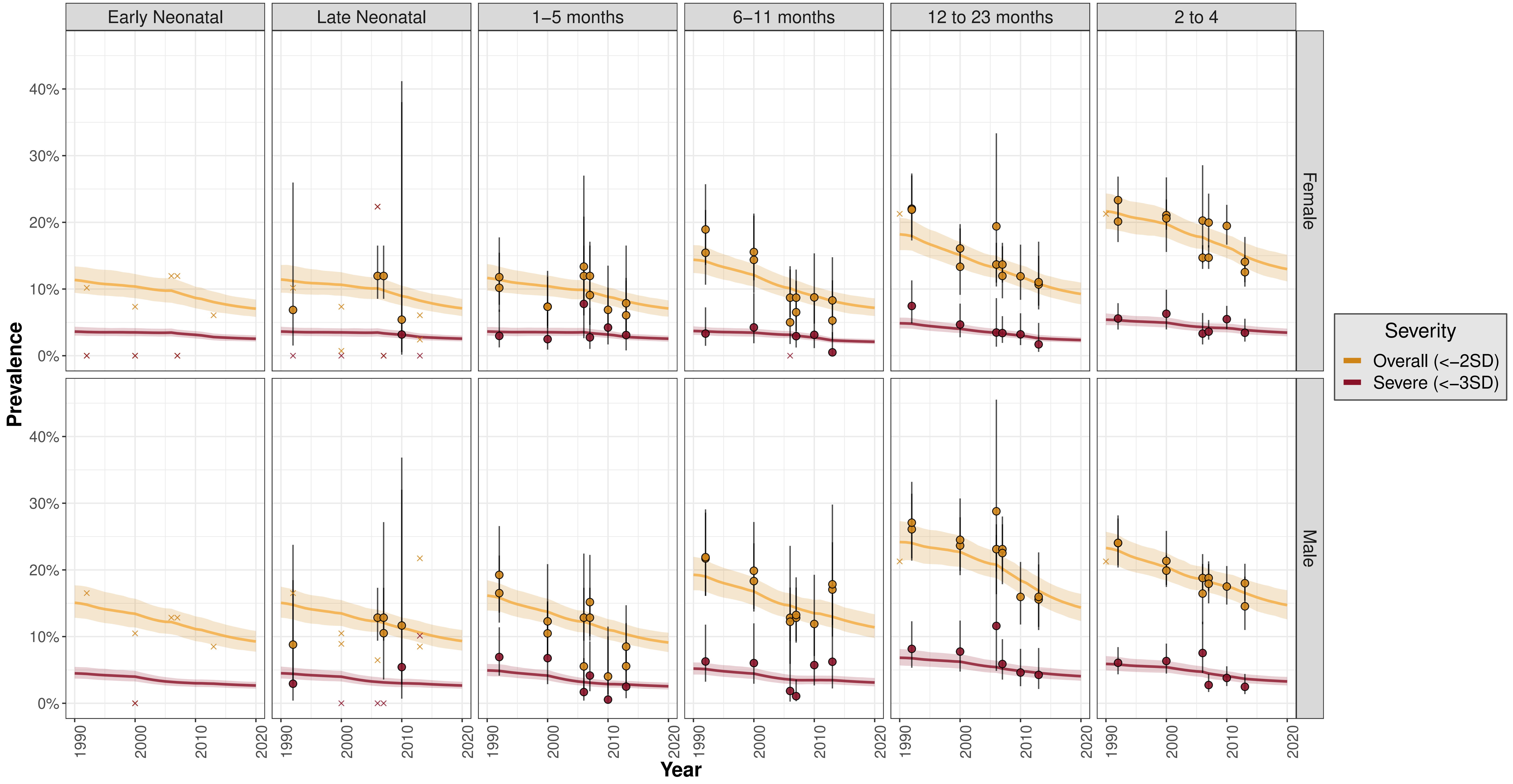

H: Transformed Mean Underweight Z Scores

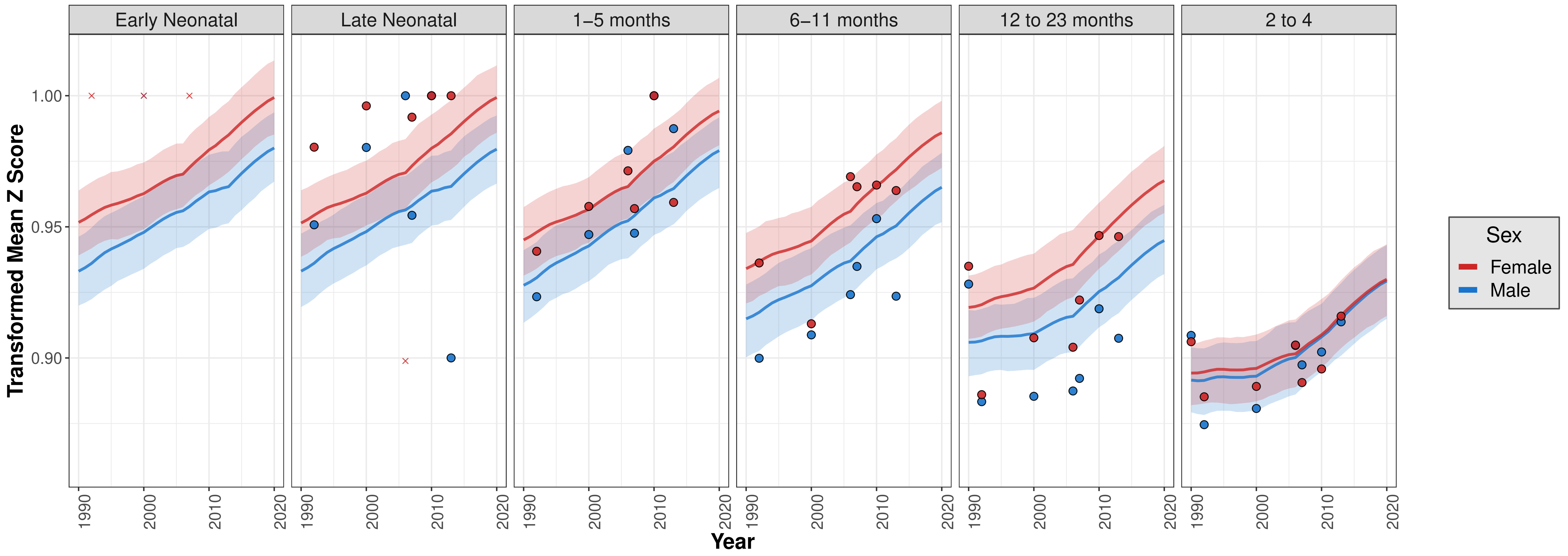

I

Year	Source
1990	WHO CGM Database
1992	DHS
1992	WHO CGM Database
2000	DHS
2000	WHO CGM Database
2006	DHS
2006	WHO CGM Database
2007	DHS
2007	WHO CGM Database
2010	Household Income and Expenditure Survey
2013	WHO CGM Database
2013	DHS

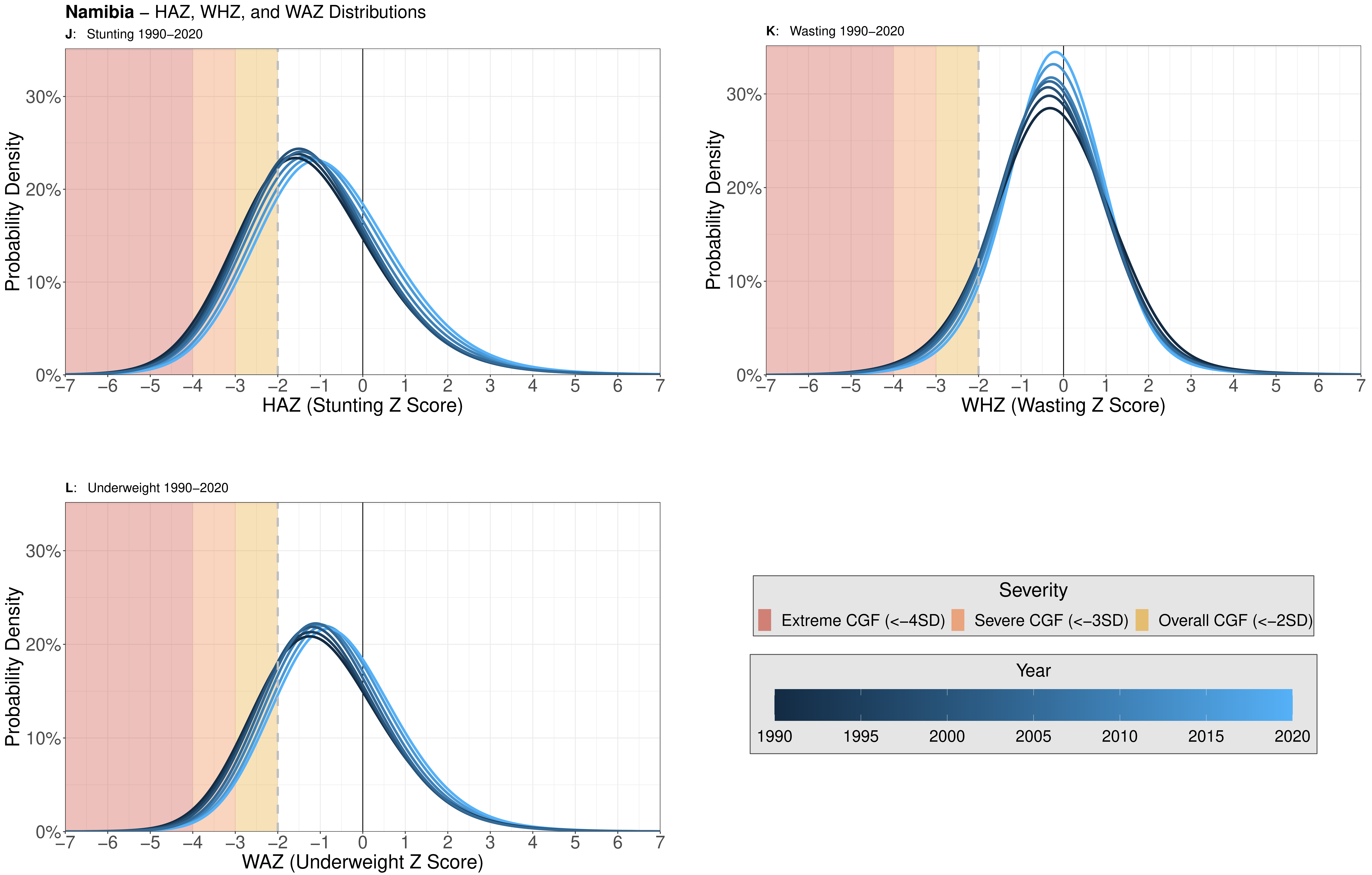

Namibia – HAZ, WHZ, and WAZ Distributions
J: Stunting 1990–2020
Probability Density
30%
20%
10%
0%
−7 −6 −5 −4 −3 −2 −1 0 1 2 3 4 5 6 7
HAZ (Stunting Z Score)
K: Wasting 1990–2020
WHZ (Wasting Z Score)
L: Underweight 1990–2020
WAZ (Underweight Z Score)
Severity
Extreme CGF (<−4SD)
Severe CGF (<−3SD)
Overall CGF (<−2SD)
Year
1990 1995 2000 2005 2010 2015 2020

South Africa – Stunting (HAZ)

A: Overall and Severe Stunting Prevalence

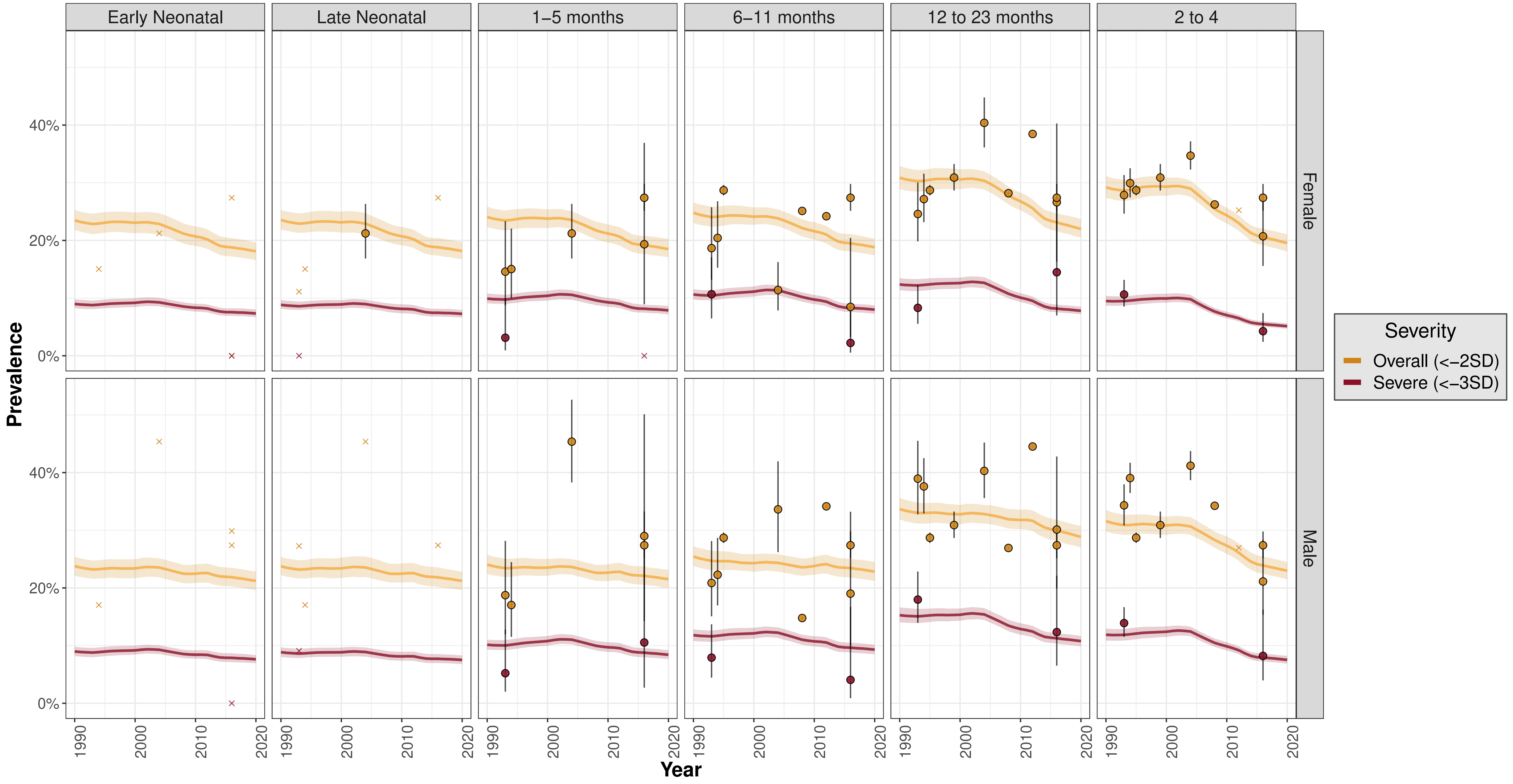

B: Transformed Mean Stunting Z Scores

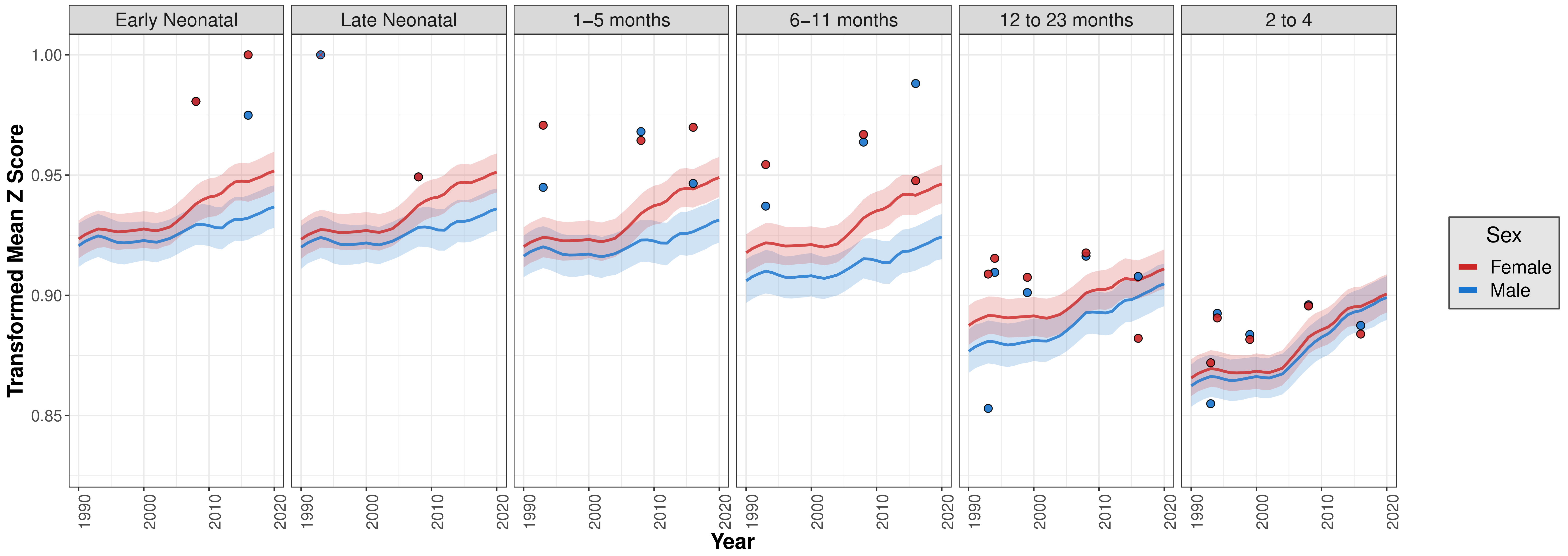

C

Year	Source	National	Subnational
1986	WHO CGM Database	X	X
1993	KwaZulu and Natal Income Dynamics Study		X
1993	Living Standards Measurement Study	X	X
1994	WHO CGM Database	X	
1995	WHO CGM Database	X	
1998	KwaZulu–Natal Income Dynamics Study		X
1999	Integrated Family Survey		X
1999	WHO CGM Database	X	
2002	Agincourt Integrated Family Survey		X
2004	KwaZulu–Natal Income Dynamics Study		X
2004	Agincourt Integrated Family Survey		X
2004	WHO CGM Database	X	X
2008	National Income Dynamics Study – Wave 1		X
2008	WHO CGM Database	X	
2010	National Income Dynamics Study – Wave 2		X
2011	National Income Dynamics Study – Wave 2		X
2012	National Income Dynamics Study – Wave 3		X
2012	WHO CGM Database	X	
2012	Venda Malnutrition and Enteric Disease Study		X
2014	National Income Dynamics Study – Wave 4		X
2015	National Income Dynamics Study – Wave 4		X
2016	WHO CGM Database	X	
2016	DHS	X	X

South Africa – Wasting (WHZ)

D: Overall and Severe Wasting Prevalence

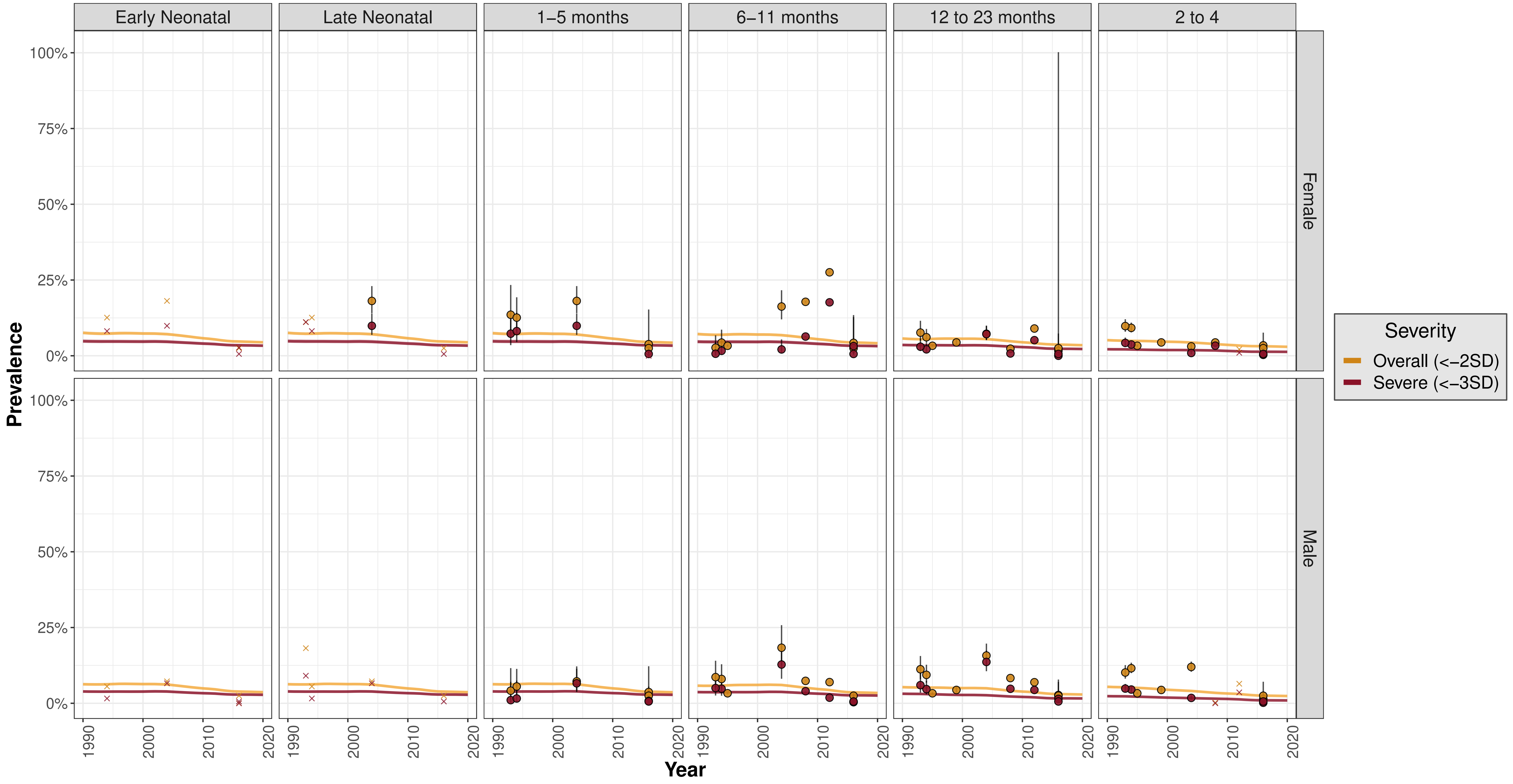

E: Transformed Mean Wasting Z Scores

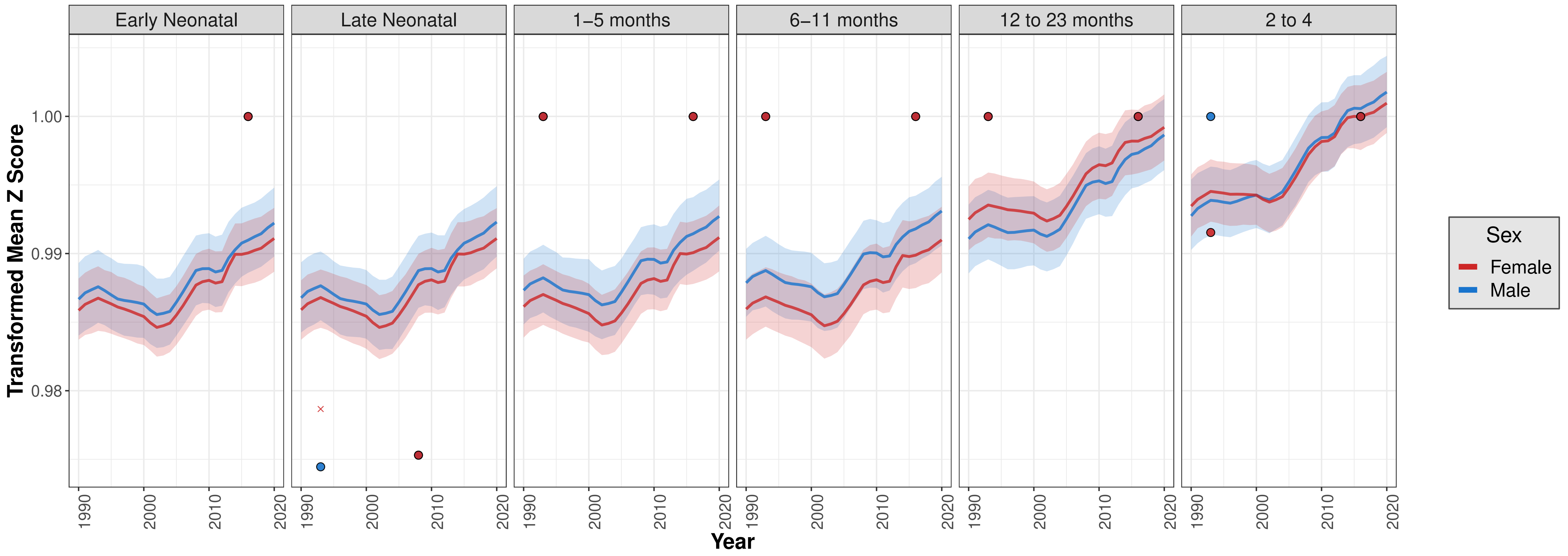

F

Year	Source	National	Subnational
1986	WHO CGM Database	X	X
1993	KwaZulu and Natal Income Dynamics Study		X
1993	Living Standards Measurement Study	X	X
1994	WHO CGM Database	X	
1995	WHO CGM Database	X	
1998	KwaZulu–Natal Income Dynamics Study		X
1999	Integrated Family Survey		X
1999	WHO CGM Database	X	
2002	Agincourt Integrated Family Survey		X
2004	KwaZulu–Natal Income Dynamics Study		X
2004	Agincourt Integrated Family Survey		X
2004	WHO CGM Database	X	X
2008	National Income Dynamics Study – Wave 1		X
2008	WHO CGM Database	X	
2010	National Income Dynamics Study – Wave 2		X
2011	National Income Dynamics Study – Wave 2		X
2012	National Income Dynamics Study – Wave 3		X
2012	WHO CGM Database	X	
2012	Venda Malnutrition and Enteric Disease Study		X
2014	National Income Dynamics Study – Wave 4		X
2015	National Income Dynamics Study – Wave 4		X
2016	WHO CGM Database	X	
2016	DHS	X	X

South Africa – Underweight (WAZ)

G: Overall and Severe Underweight Prevalence

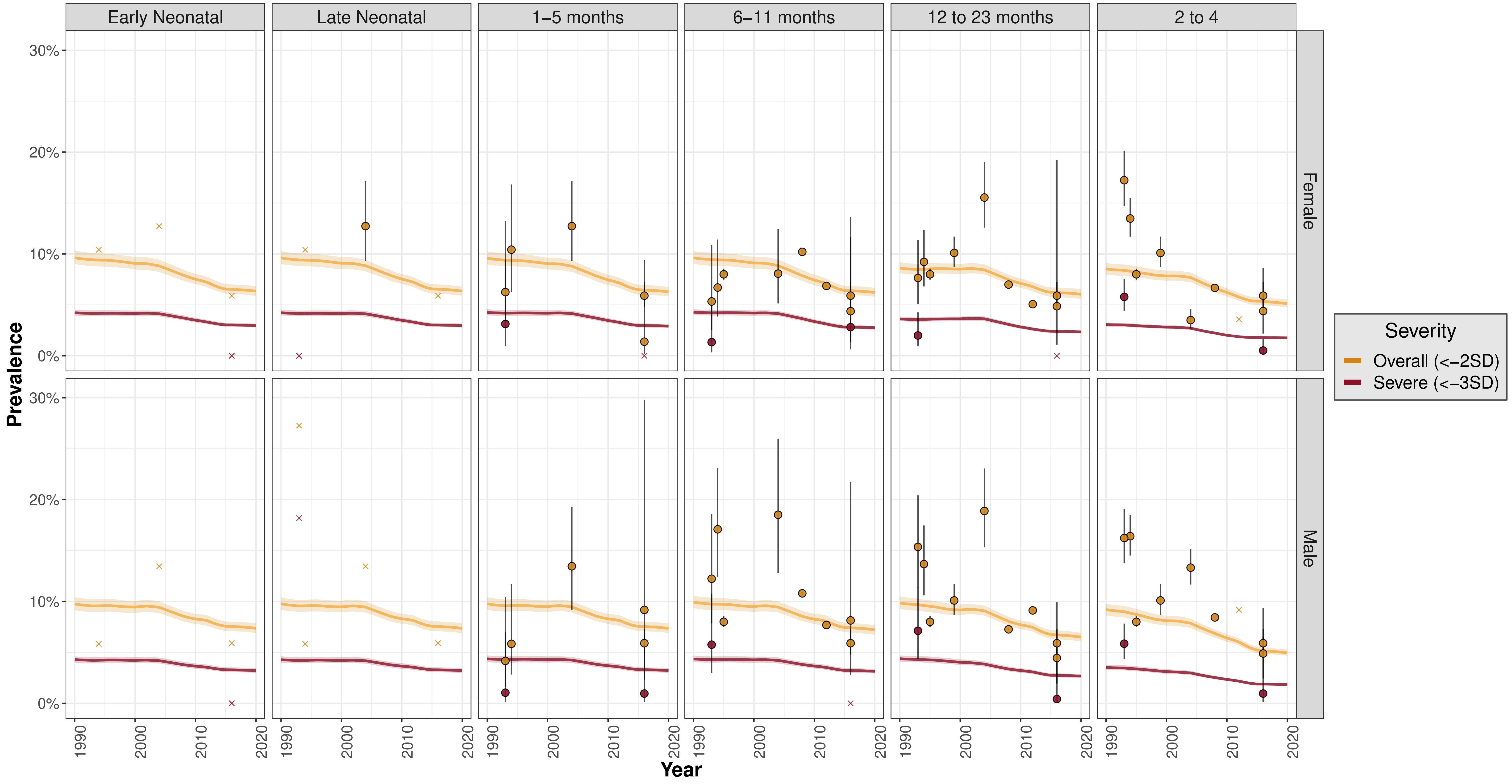

H: Transformed Mean Underweight Z Scores

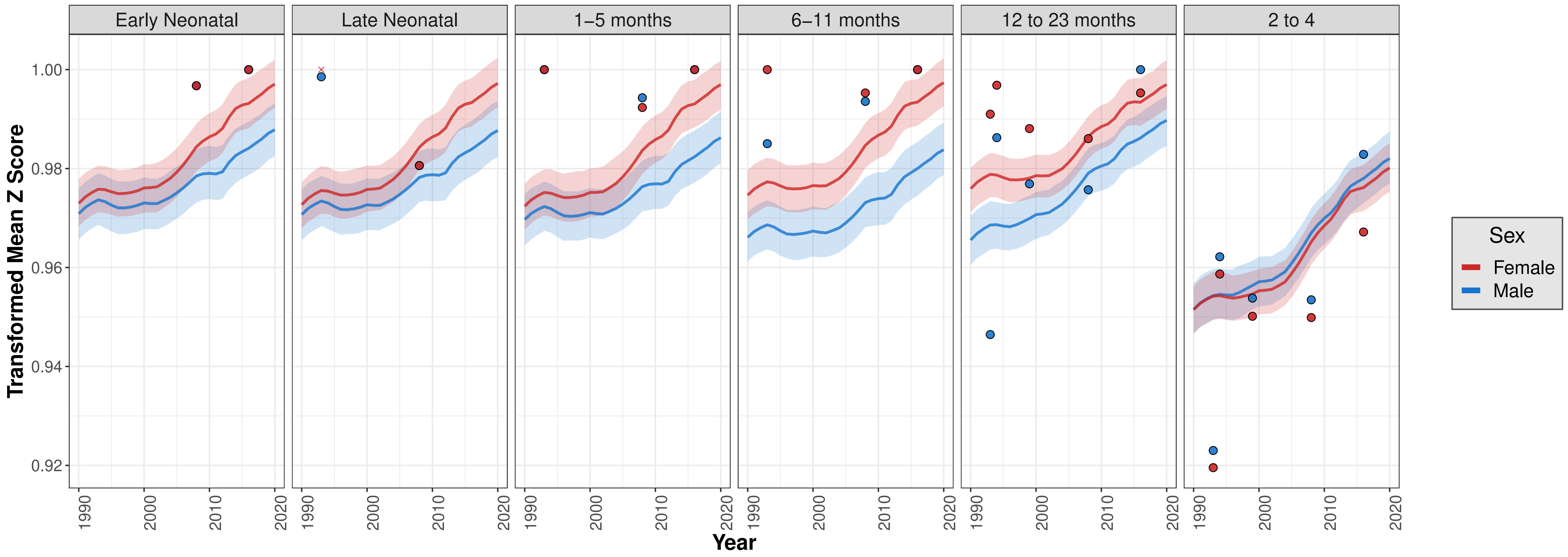

I

Year	Source	National	Subnational
1986	WHO CGM Database	X	X
1993	KwaZulu and Natal Income Dynamics Study		X
1993	Living Standards Measurement Study	X	X
1994	WHO CGM Database	X	
1995	WHO CGM Database	X	
1998	KwaZulu–Natal Income Dynamics Study		X
1999	Integrated Family Survey		X
1999	WHO CGM Database	X	
2002	Agincourt Integrated Family Survey		X
2004	KwaZulu–Natal Income Dynamics Study		X
2004	Agincourt Integrated Family Survey		X
2004	WHO CGM Database	X	X
2008	National Income Dynamics Study – Wave 1		X
2008	WHO CGM Database	X	
2010	National Income Dynamics Study – Wave 2		X
2011	National Income Dynamics Study – Wave 2		X
2012	National Income Dynamics Study – Wave 3		X
2012	WHO CGM Database	X	
2012	Venda Malnutrition and Enteric Disease Study		X
2014	National Income Dynamics Study – Wave 4		X
2015	National Income Dynamics Study – Wave 4		X
2016	WHO CGM Database	X	
2016	DHS	X	X

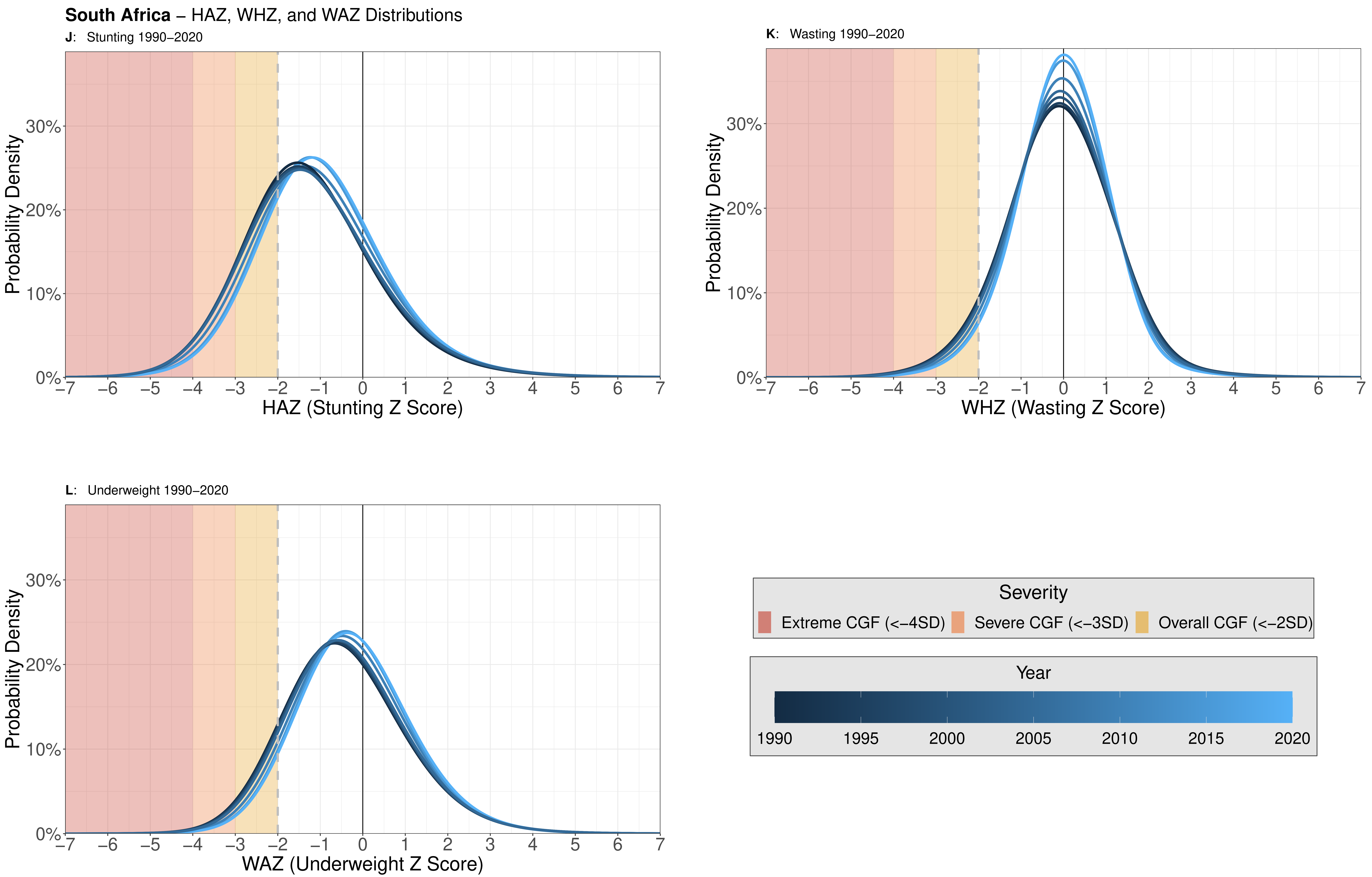
South Africa – HAZ, WHZ, and WAZ Distributions
J: Stunting 1990–2020
Probability Density
HAZ (Stunting Z Score)
K: Wasting 1990–2020
Probability Density
WHZ (Wasting Z Score)
L: Underweight 1990–2020
Probability Density
WAZ (Underweight Z Score)
0%
10%
20%
30%
−7 −6 −5 −4 −3 −2 −1 0 1 2 3 4 5 6 7
Severity
Extreme CGF (<−4SD)
Severe CGF (<−3SD)
Overall CGF (<−2SD)
Year
1990 1995 2000 2005 2010 2015 2020

Eswatini – Stunting (HAZ)

A: Overall and Severe Stunting Prevalence

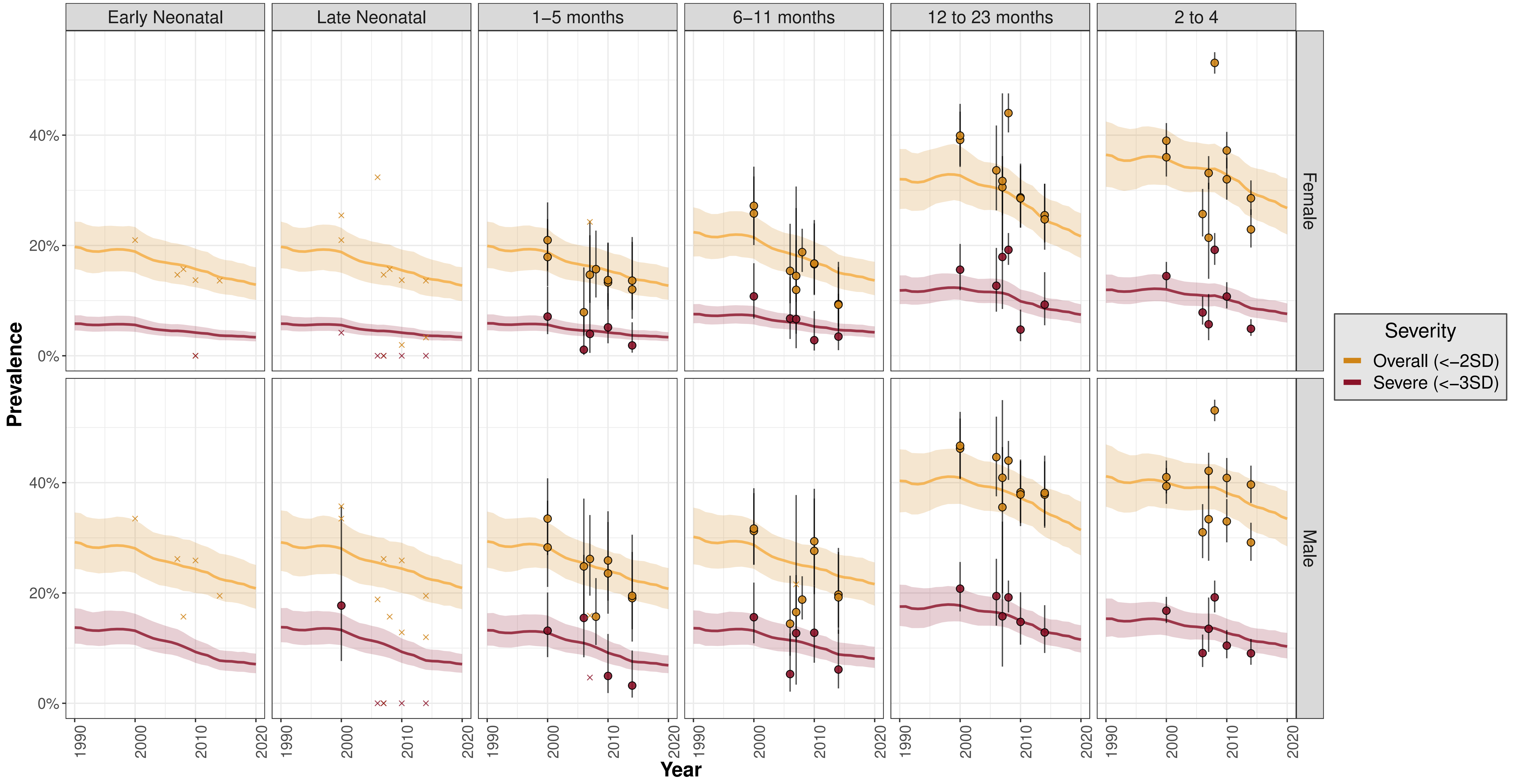

B: Transformed Mean Stunting Z Scores

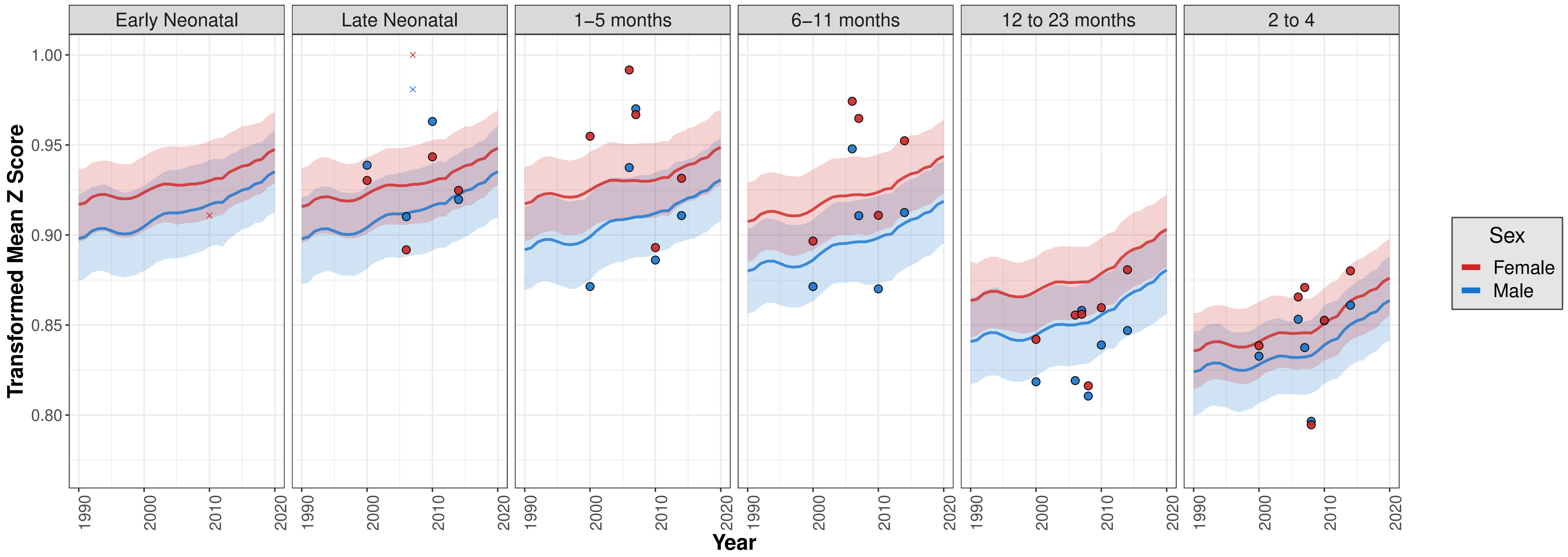

C

Year	Source
2000	MICS
2000	WHO CGM Database
2006	DHS
2007	DHS
2007	WHO CGM Database
2008	WHO CGM Database
2010	MICS
2010	WHO CGM Database
2014	WHO CGM Database
2014	MICS

Eswatini – Wasting (WHZ)

D: Overall and Severe Wasting Prevalence

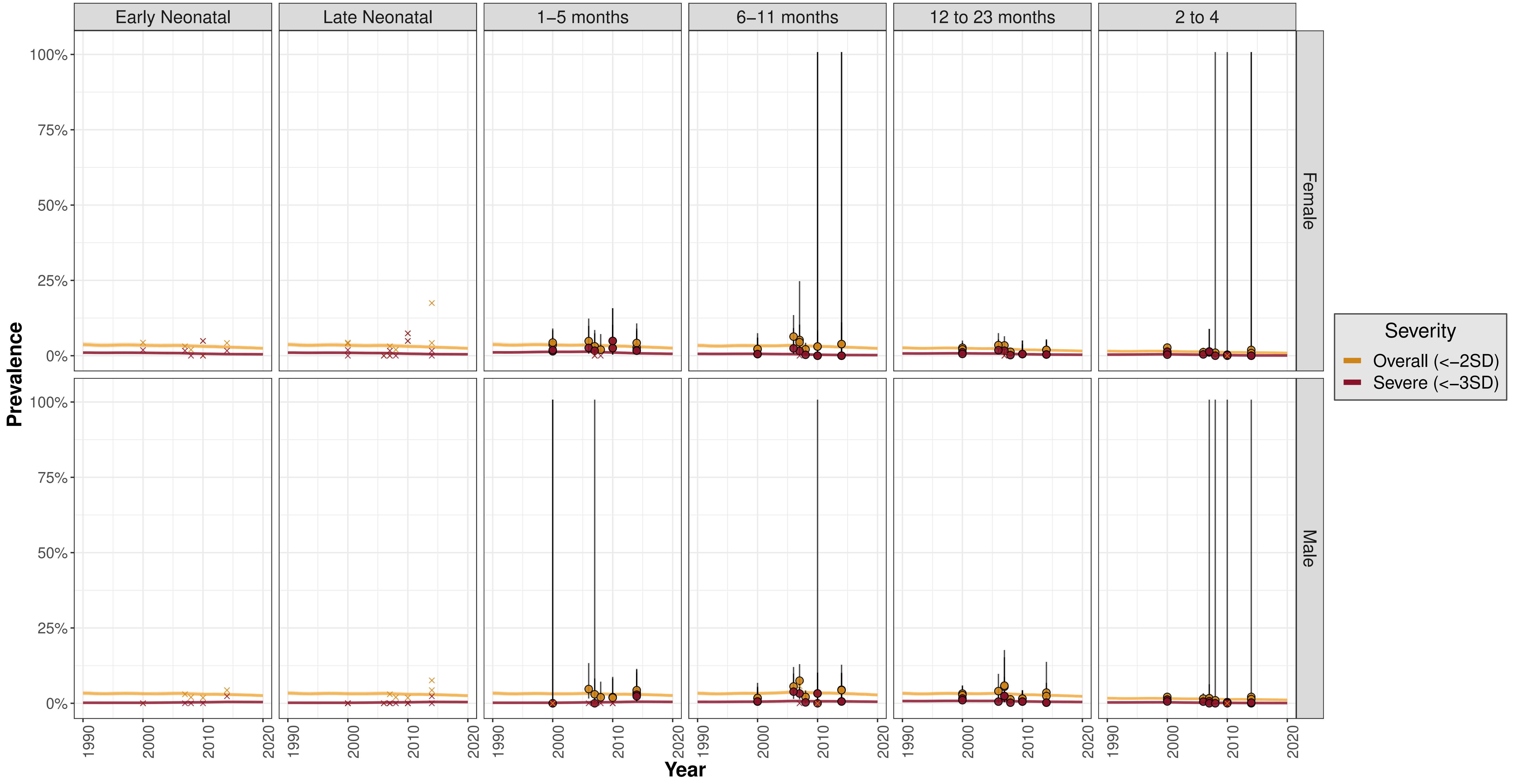

E: Transformed Mean Wasting Z Scores

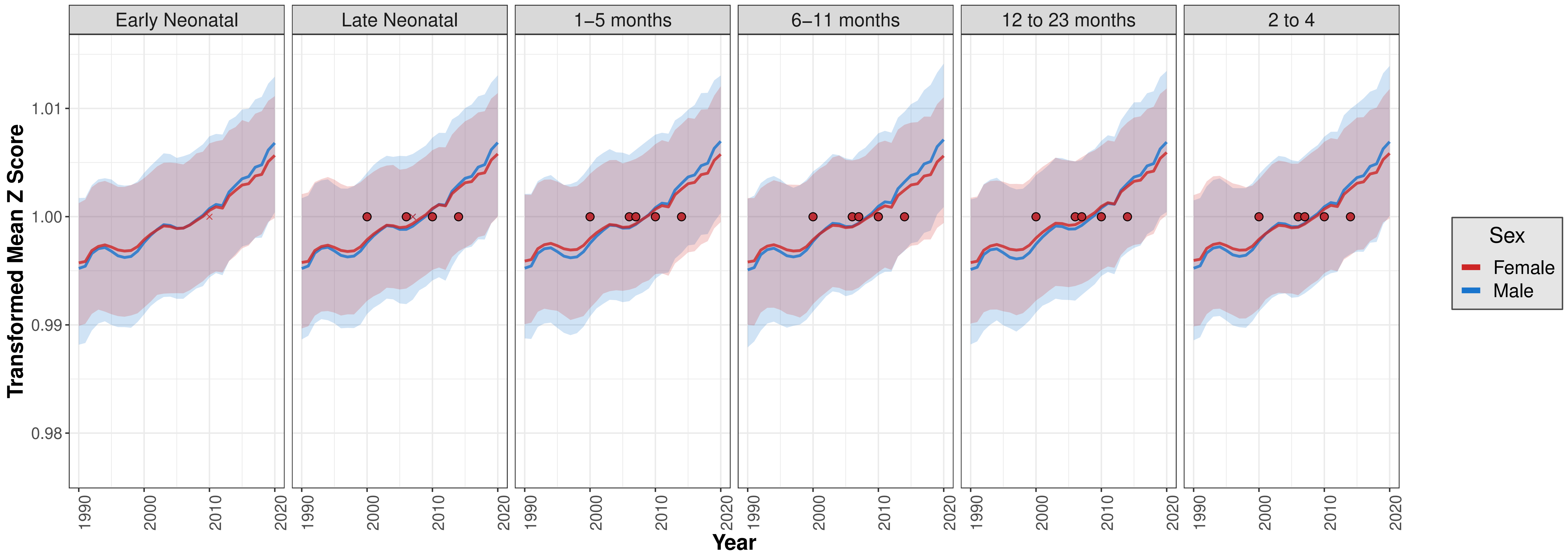

F

Year	Source
2000	MICS
2000	WHO CGM Database
2006	DHS
2007	DHS
2007	WHO CGM Database
2008	WHO CGM Database
2010	MICS
2010	WHO CGM Database
2014	WHO CGM Database
2014	MICS

Eswatini – Underweight (WAZ)

G: Overall and Severe Underweight Prevalence

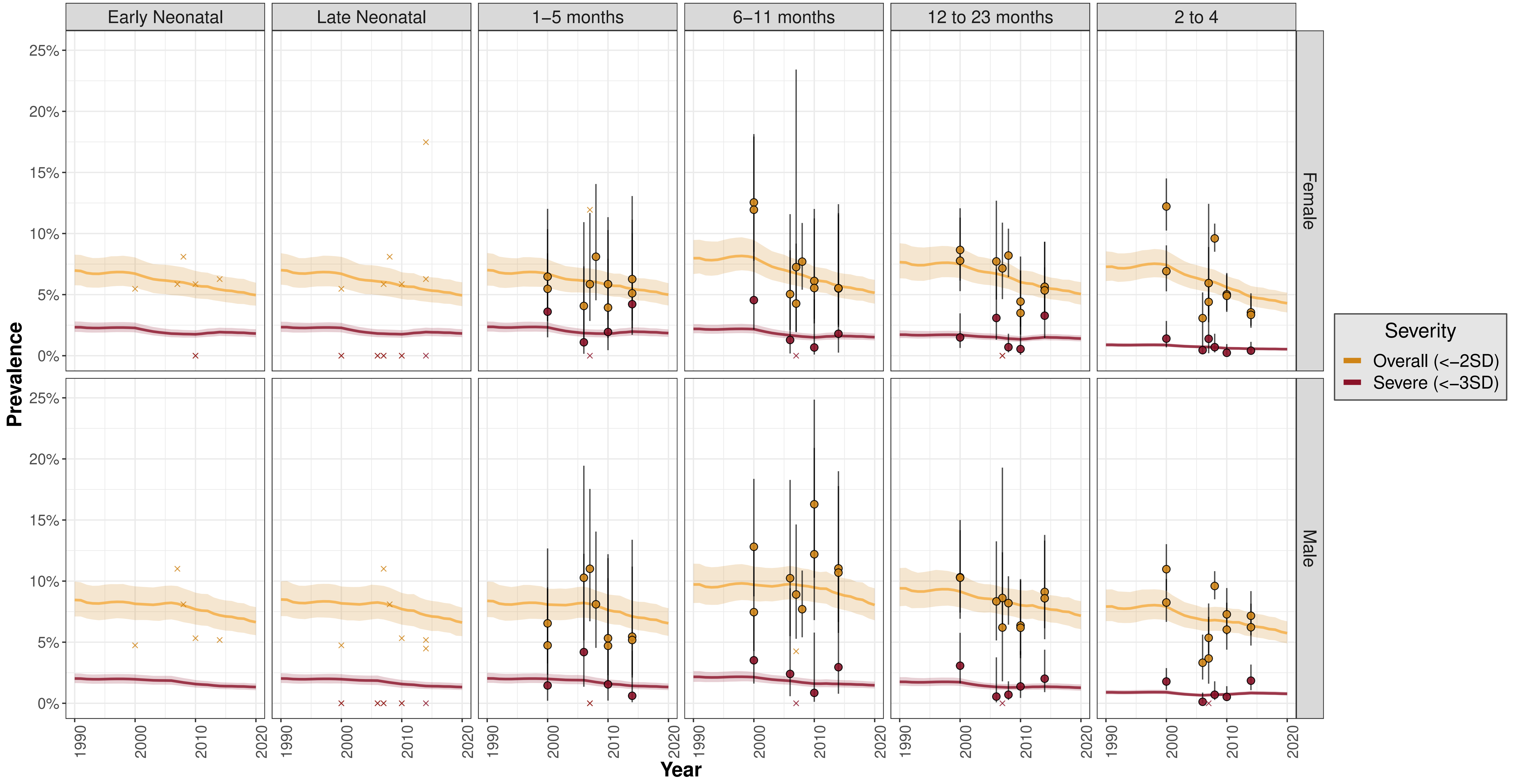

H: Transformed Mean Underweight Z Scores

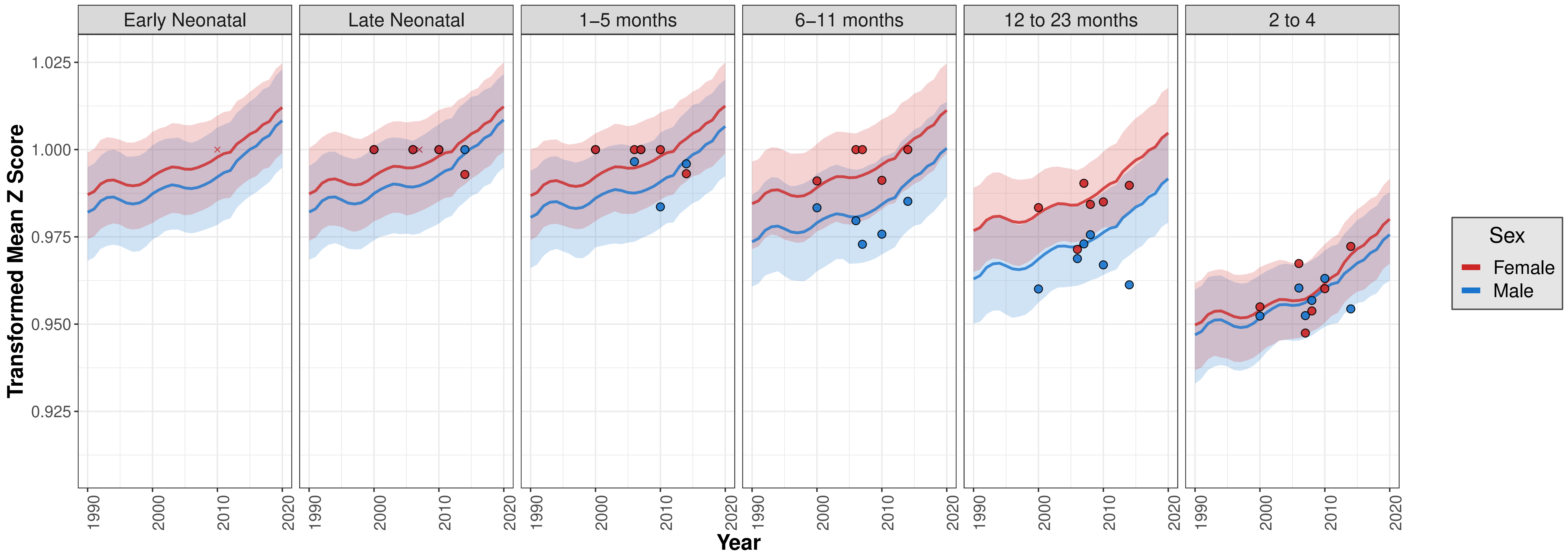

I

Year	Source
2000	MICS
2000	WHO CGM Database
2006	DHS
2007	DHS
2007	WHO CGM Database
2008	WHO CGM Database
2010	MICS
2010	WHO CGM Database
2014	WHO CGM Database
2014	MICS

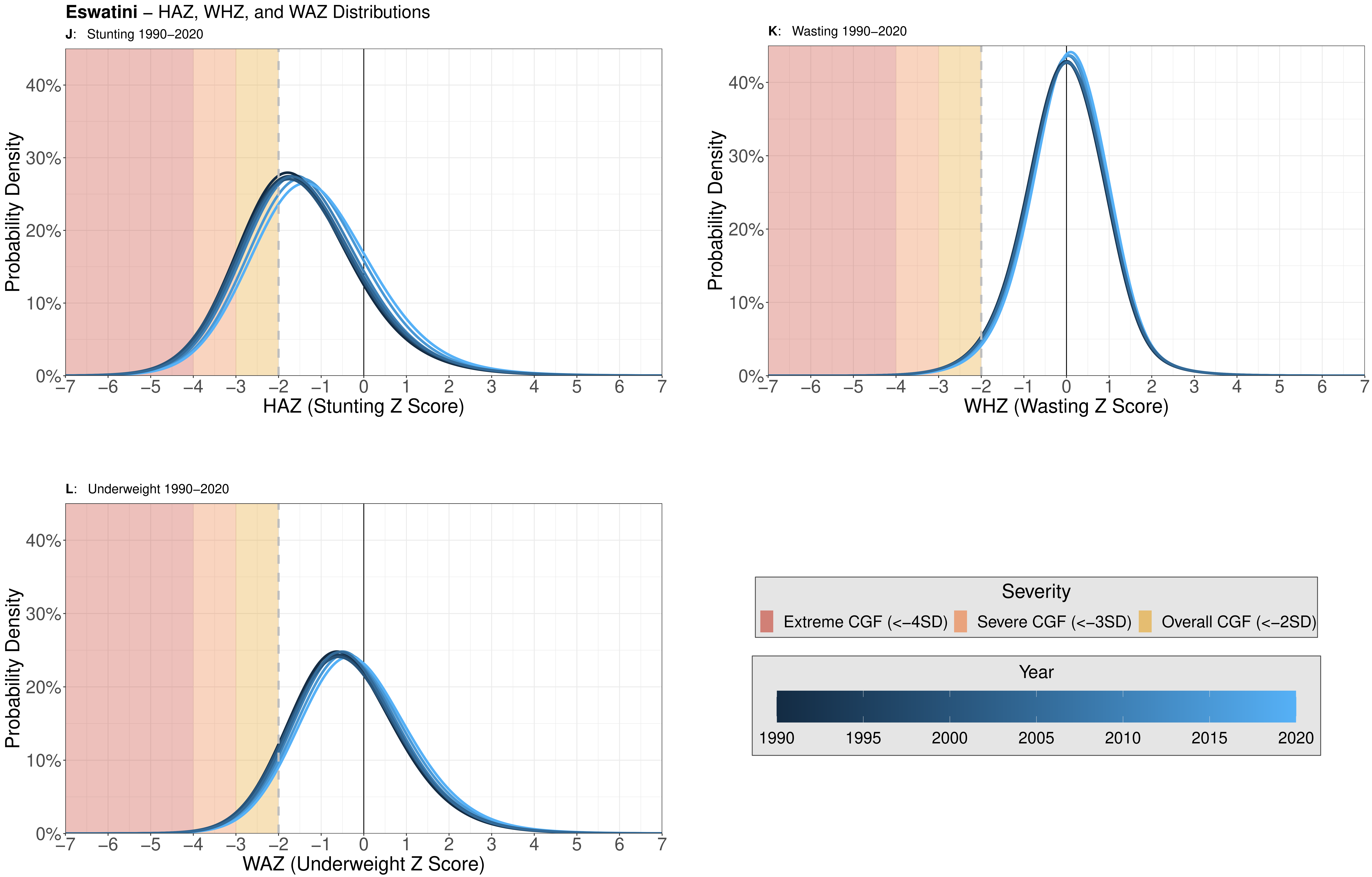

Eswatini – HAZ, WHZ, and WAZ Distributions
J: Stunting 1990–2020
Probability Density
40%
30%
20%
10%
0%
−7 −6 −5 −4 −3 −2 −1 0 1 2 3 4 5 6 7
HAZ (Stunting Z Score)
K: Wasting 1990–2020
Probability Density
40%
30%
20%
10%
0%
−7 −6 −5 −4 −3 −2 −1 0 1 2 3 4 5 6 7
WHZ (Wasting Z Score)
L: Underweight 1990–2020
Probability Density
40%
30%
20%
10%
0%
−7 −6 −5 −4 −3 −2 −1 0 1 2 3 4 5 6 7
WAZ (Underweight Z Score)
Severity
Extreme CGF (<−4SD)
Severe CGF (<−3SD)
Overall CGF (<−2SD)
Year
1990
1995
2000
2005
2010
2015
2020

Zimbabwe – Stunting (HAZ)

A: Overall and Severe Stunting Prevalence

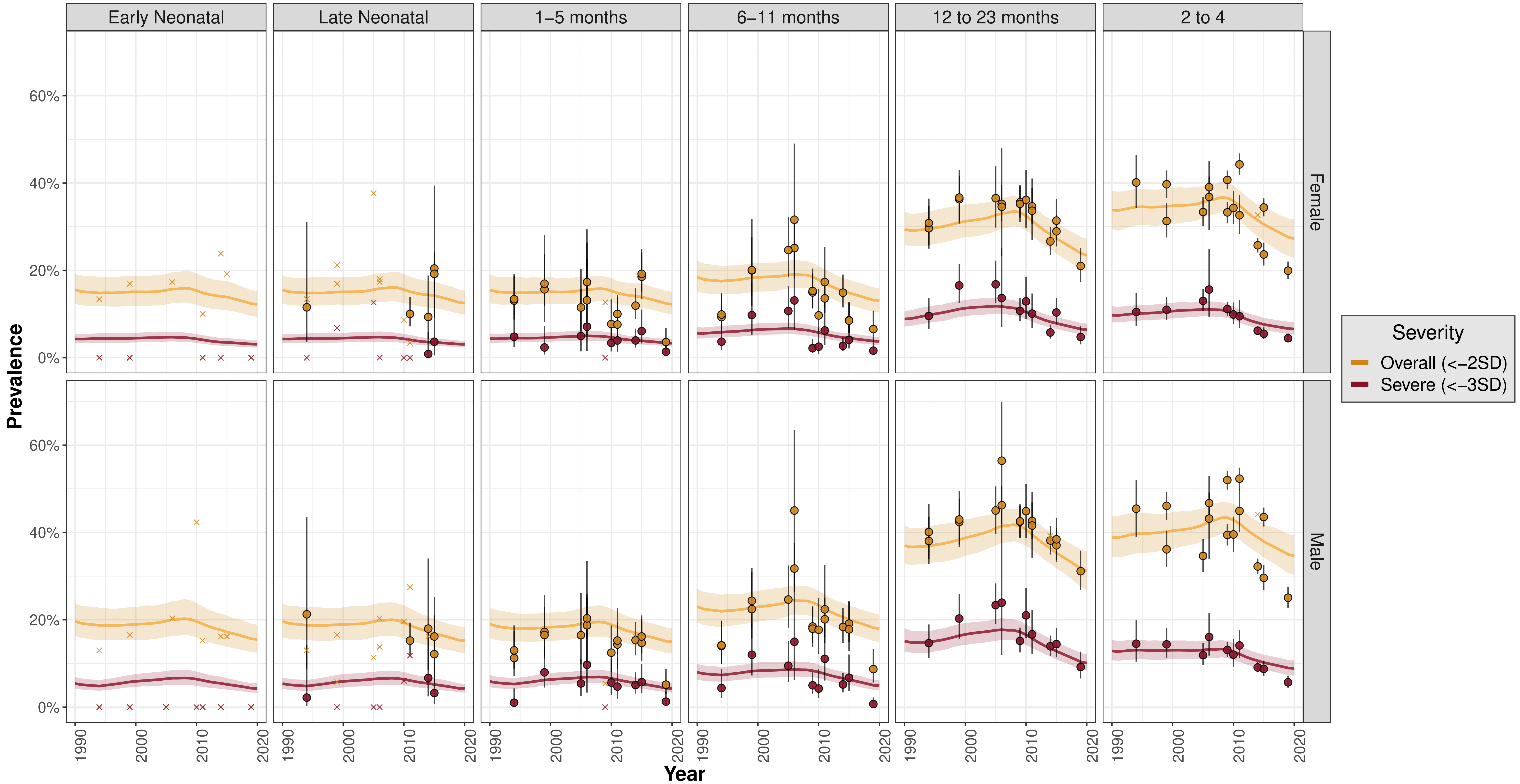

B: Transformed Mean Stunting Z Scores

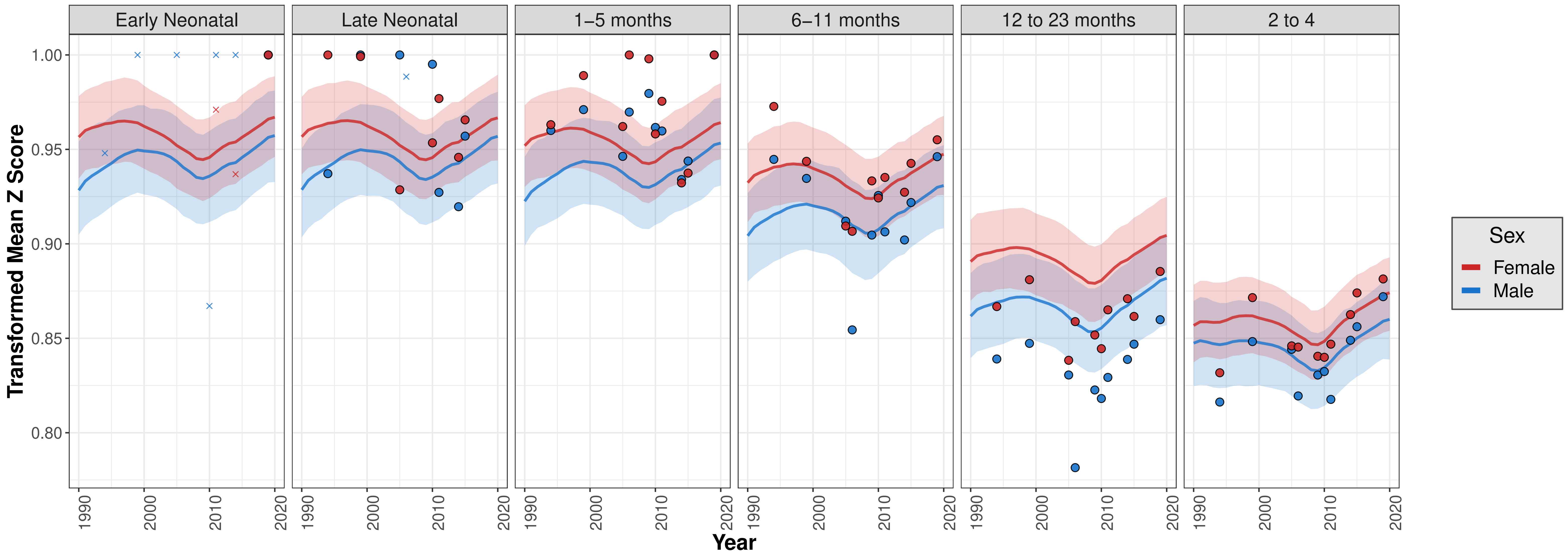

C

Year	Source
1988	DHS
1988	WHO CGM Database
1989	DHS
1994	DHS
1994	WHO CGM Database
1999	DHS
1999	WHO CGM Database
2005	DHS
2006	DHS
2006	WHO CGM Database
2009	Multiple Indicator Monitoring Survey
2009	WHO CGM Database
2010	DHS
2011	DHS
2011	WHO CGM Database
2014	WHO CGM Database
2014	MICS
2015	WHO CGM Database
2015	DHS
2019	MICS

Zimbabwe – Wasting (WHZ)

D: Overall and Severe Wasting Prevalence

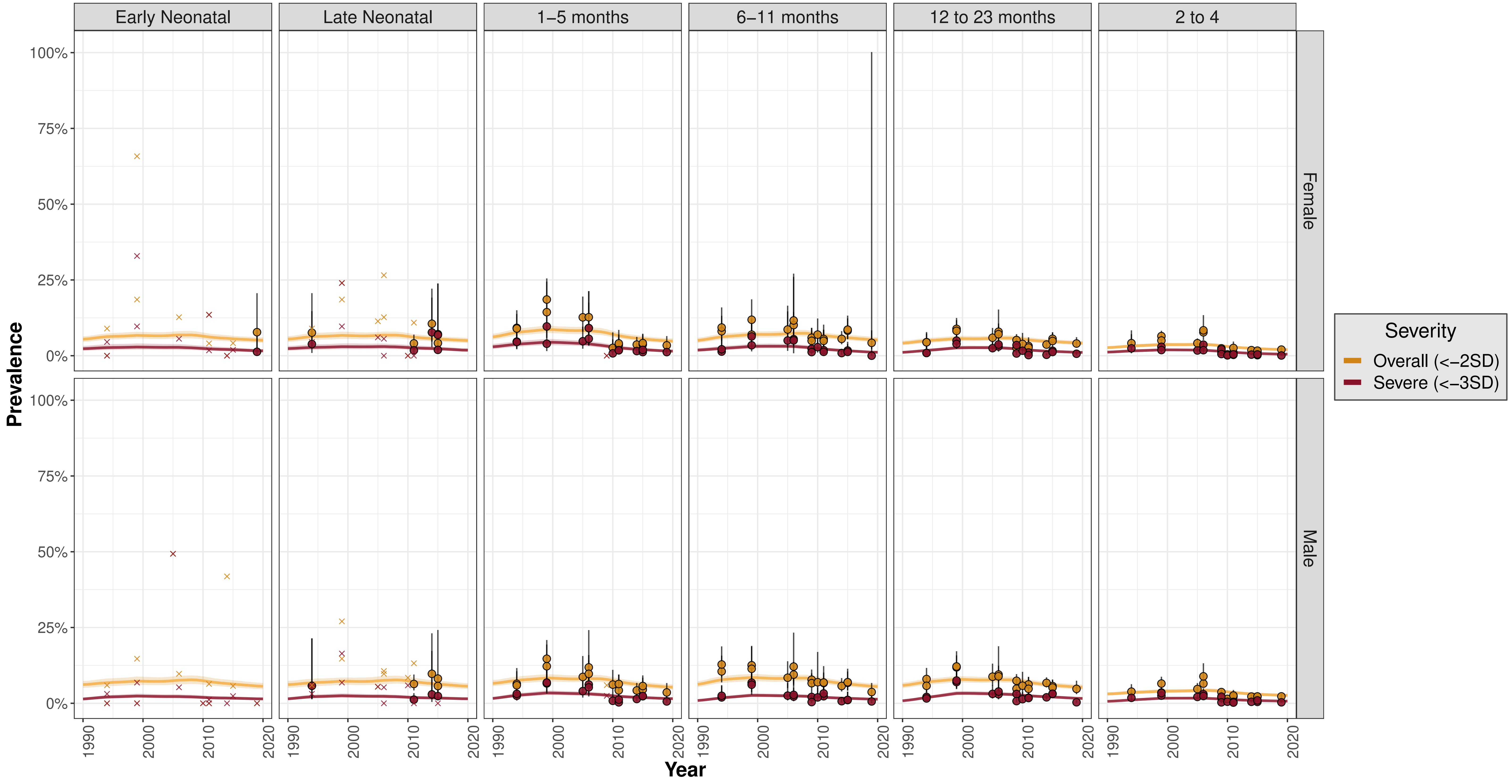

E: Transformed Mean Wasting Z Scores

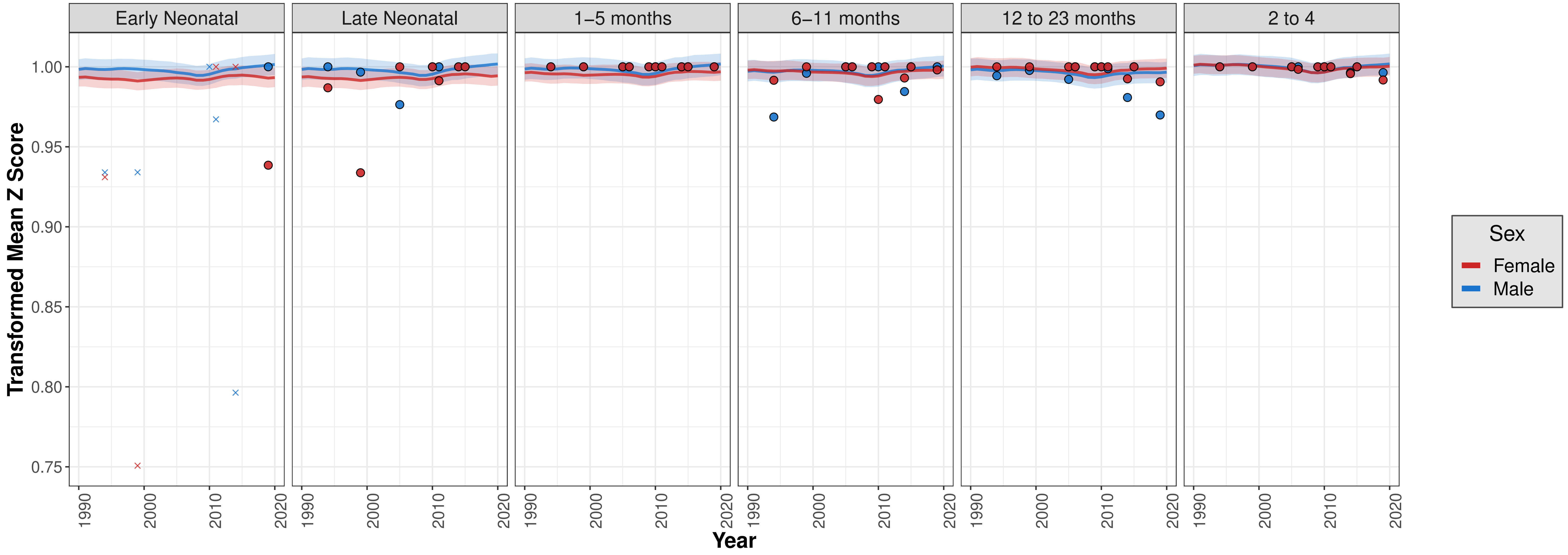

F

Year	Source
1988	DHS
1988	WHO CGM Database
1989	DHS
1994	DHS
1994	WHO CGM Database
1999	DHS
1999	WHO CGM Database
2005	DHS
2006	DHS
2006	WHO CGM Database
2009	Multiple Indicator Monitoring Survey
2009	WHO CGM Database
2010	DHS
2011	DHS
2011	WHO CGM Database
2014	MICS
2015	WHO CGM Database
2015	DHS
2019	MICS

Zimbabwe – Underweight (WAZ)

G: Overall and Severe Underweight Prevalence

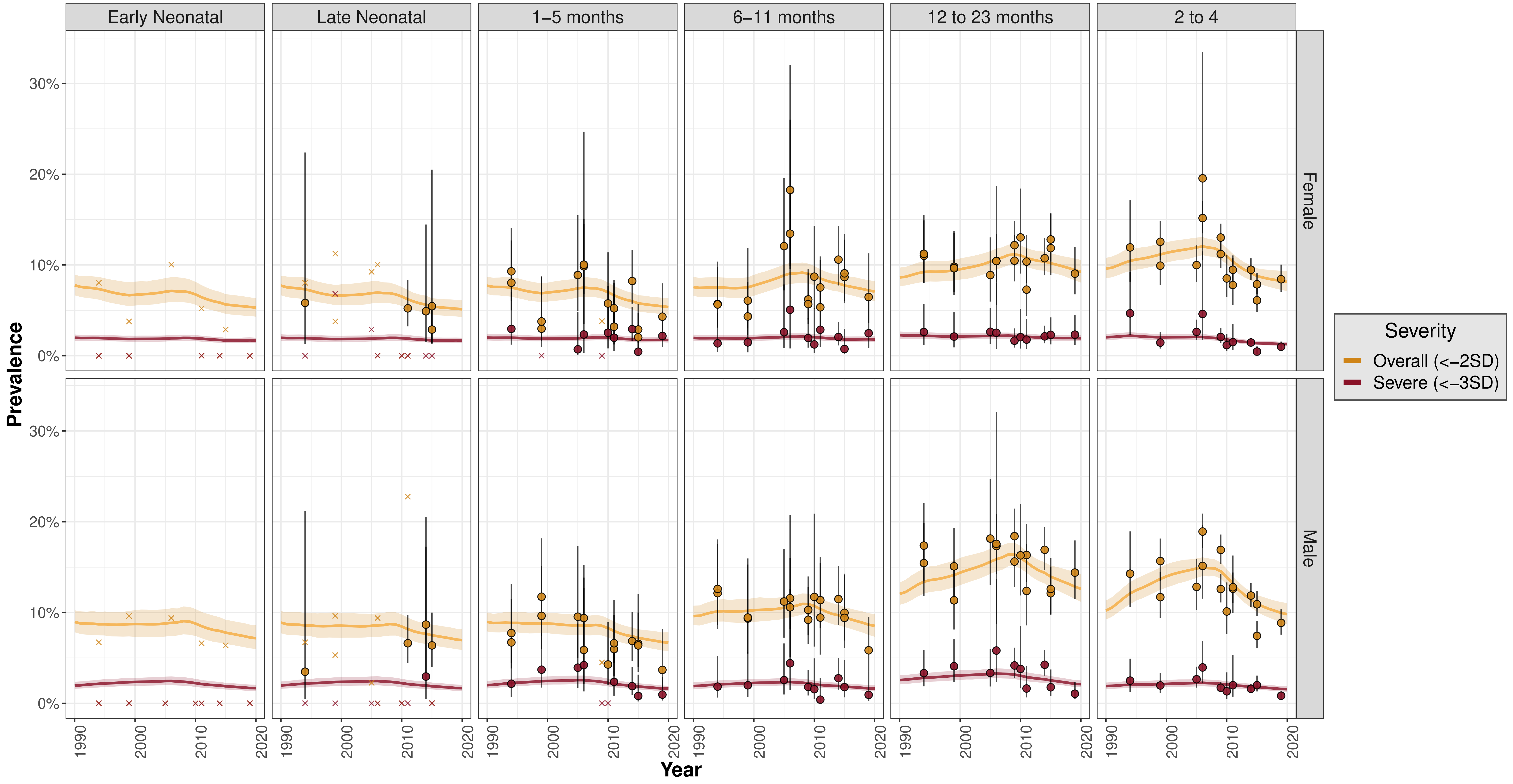

H: Transformed Mean Underweight Z Scores

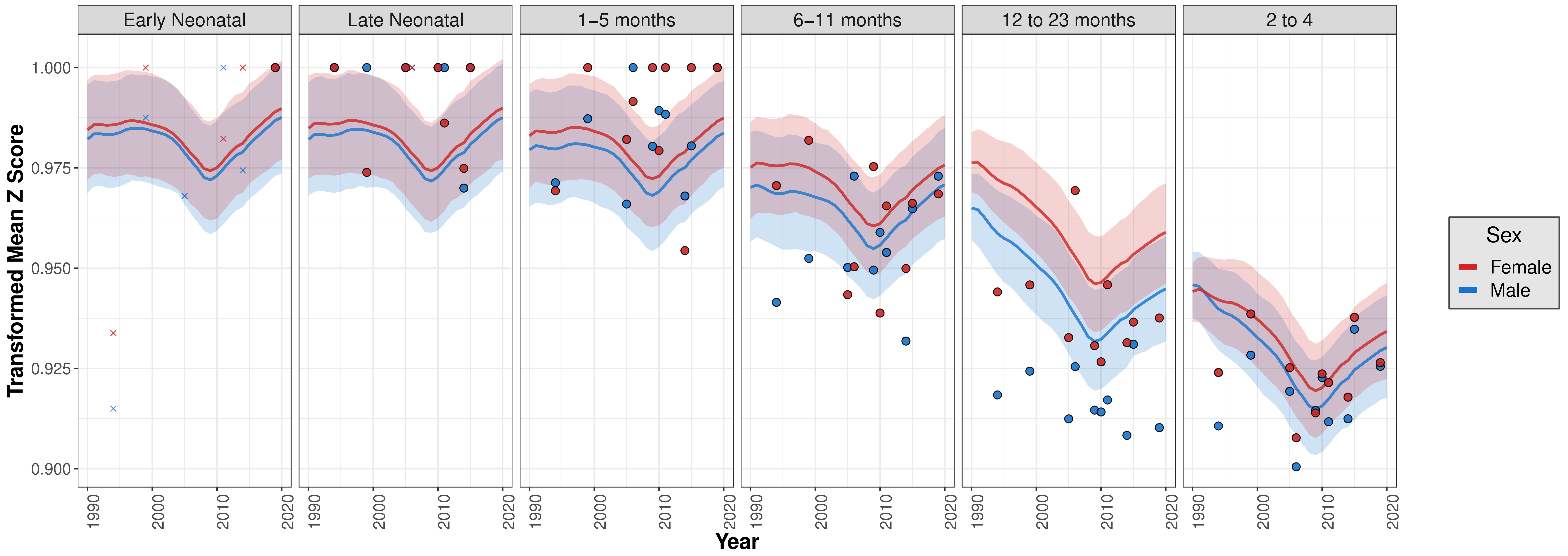

I

Year	Source
1987	WHO CGM Database
1988	DHS
1988	WHO CGM Database
1989	DHS
1994	DHS
1994	WHO CGM Database
1999	DHS
1999	WHO CGM Database
2005	DHS
2006	DHS
2006	WHO CGM Database
2009	Multiple Indicator Monitoring Survey
2009	WHO CGM Database
2010	DHS
2011	DHS
2011	WHO CGM Database
2014	MICS
2015	WHO CGM Database
2015	DHS
2019	MICS

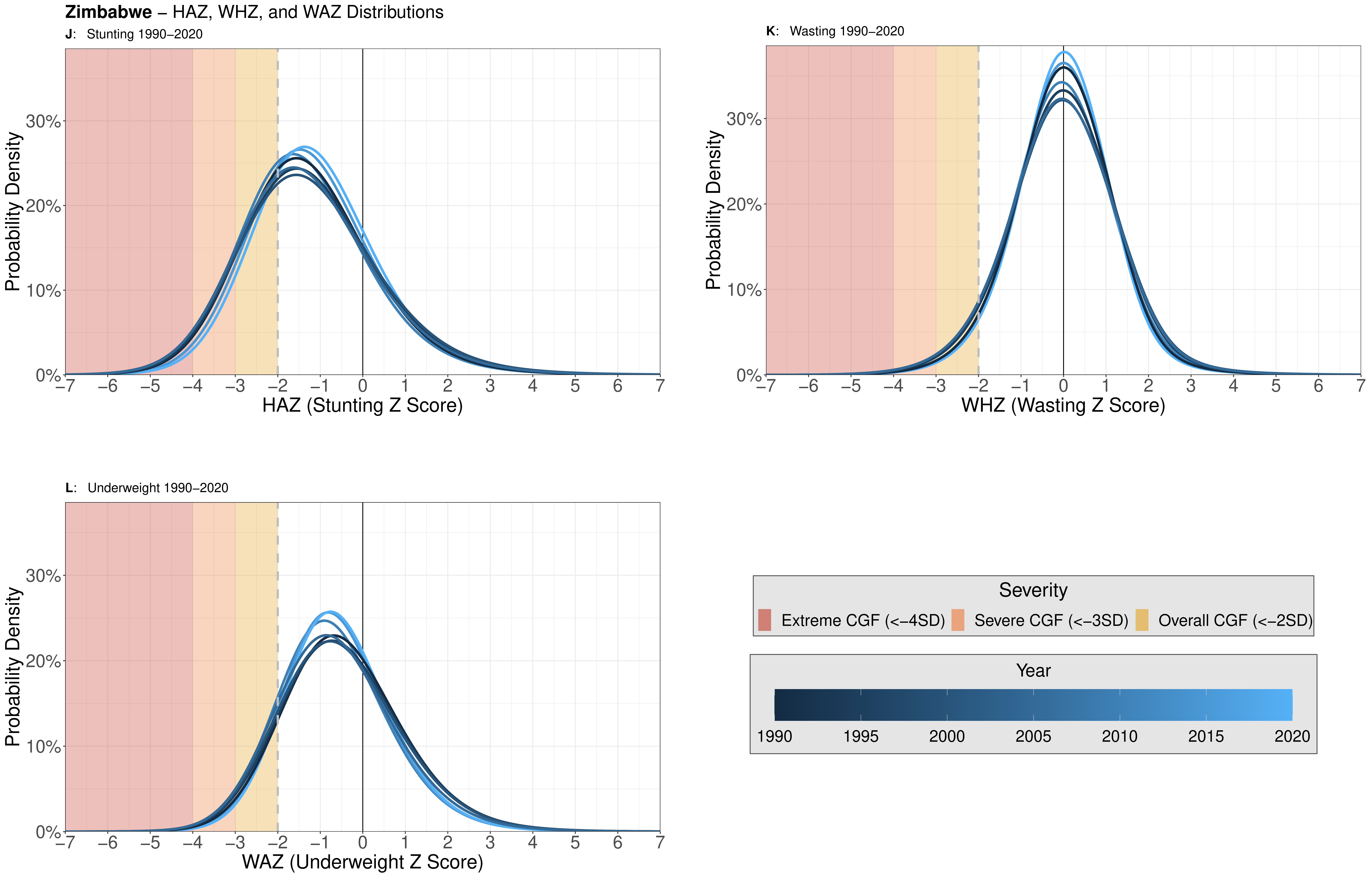

Zimbabwe – HAZ, WHZ, and WAZ Distributions
J: Stunting 1990–2020
Probability Density
HAZ (Stunting Z Score)
K: Wasting 1990–2020
Probability Density
WHZ (Wasting Z Score)
L: Underweight 1990–2020
Probability Density
WAZ (Underweight Z Score)
0%
10%
20%
30%
−7
−6
−5
−4
−3
−2
−1
0
1
2
3
4
5
6
7
Severity
Extreme CGF (<−4SD)
Severe CGF (<−3SD)
Overall CGF (<−2SD)
Year
1990
1995
2000
2005
2010
2015
2020

Benin – Stunting (HAZ)

A: Overall and Severe Stunting Prevalence

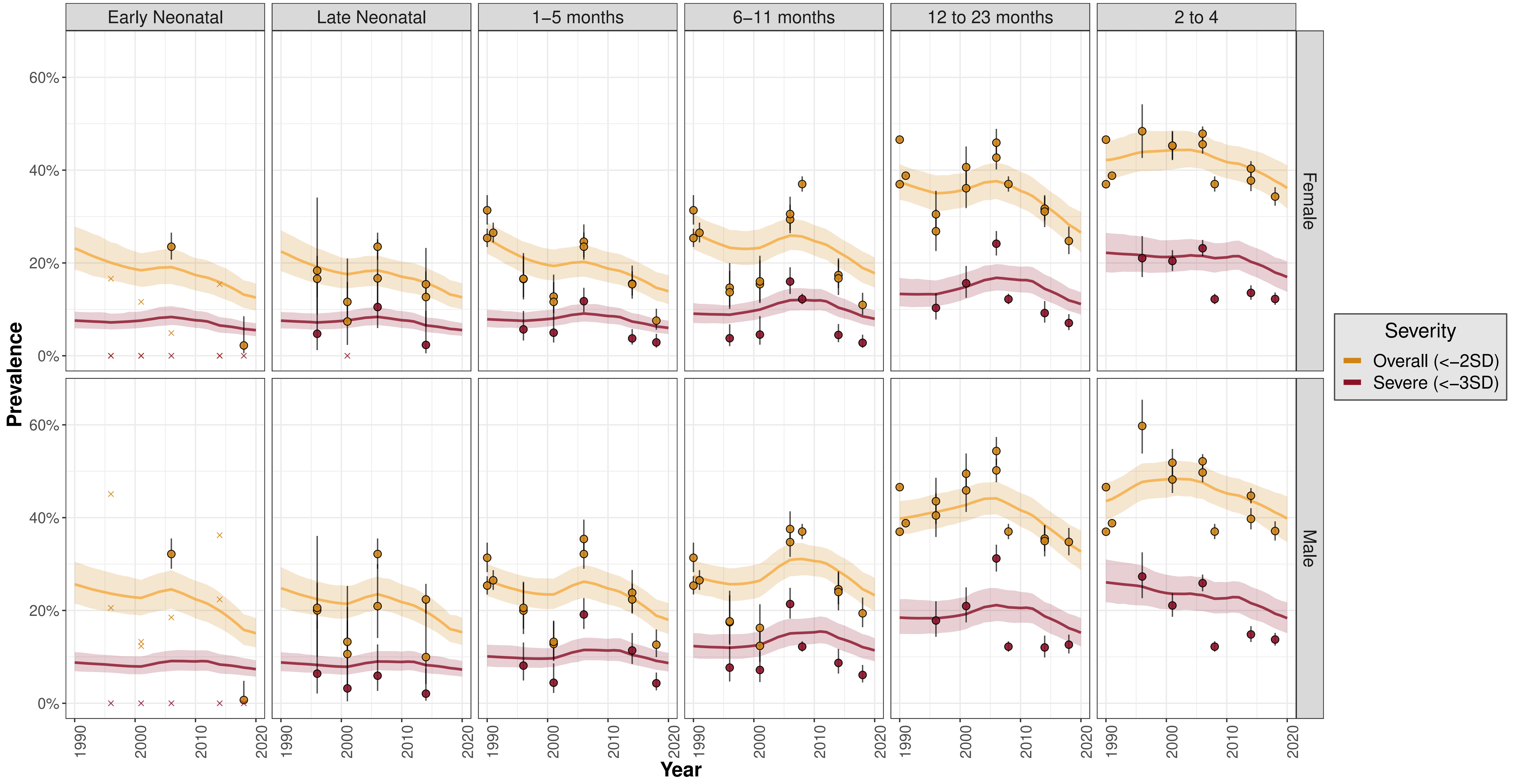

B: Transformed Mean Stunting Z Scores

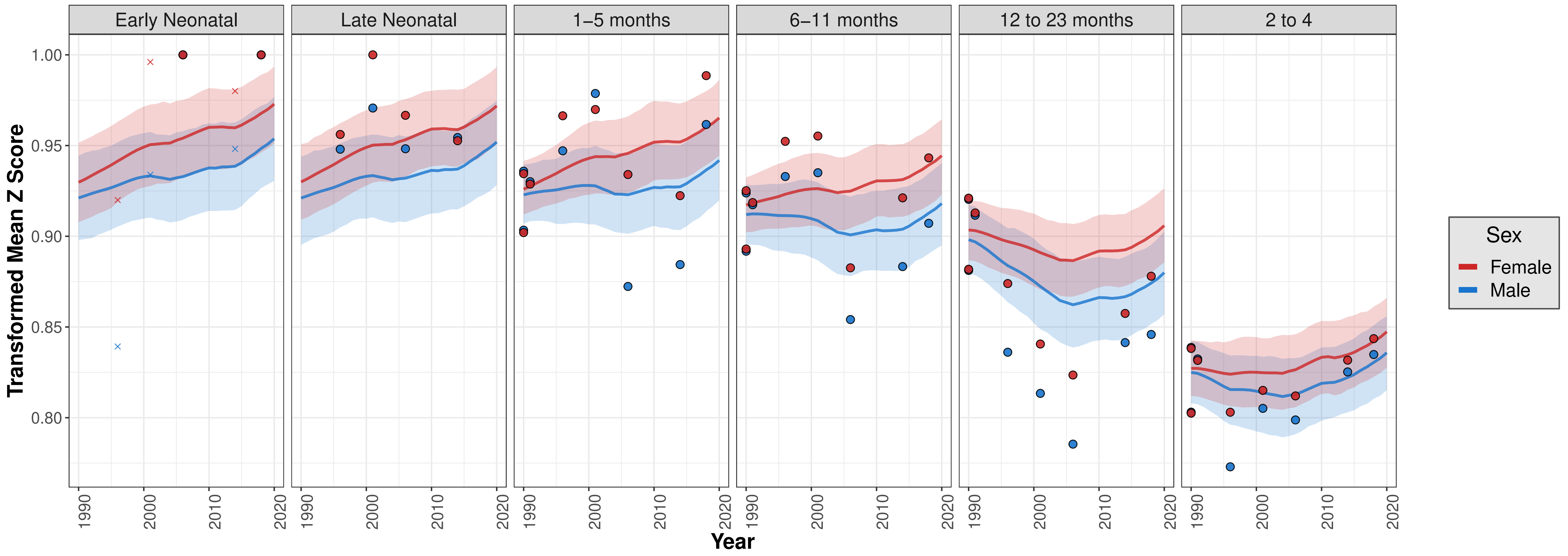

C

Year	Source
1989	WHO CGM Database
1990	WHO CGM Database
1991	WHO CGM Database
1996	DHS
1996	WHO CGM Database
2001	DHS
2001	WHO CGM Database
2006	DHS
2006	WHO CGM Database
2008	Comprehensive Food Security and Vulnerability Analysis
2011	DHS
2012	DHS
2014	WHO CGM Database
2014	MICS
2018	DHS

Benin – Wasting (WHZ)

D: Overall and Severe Wasting Prevalence

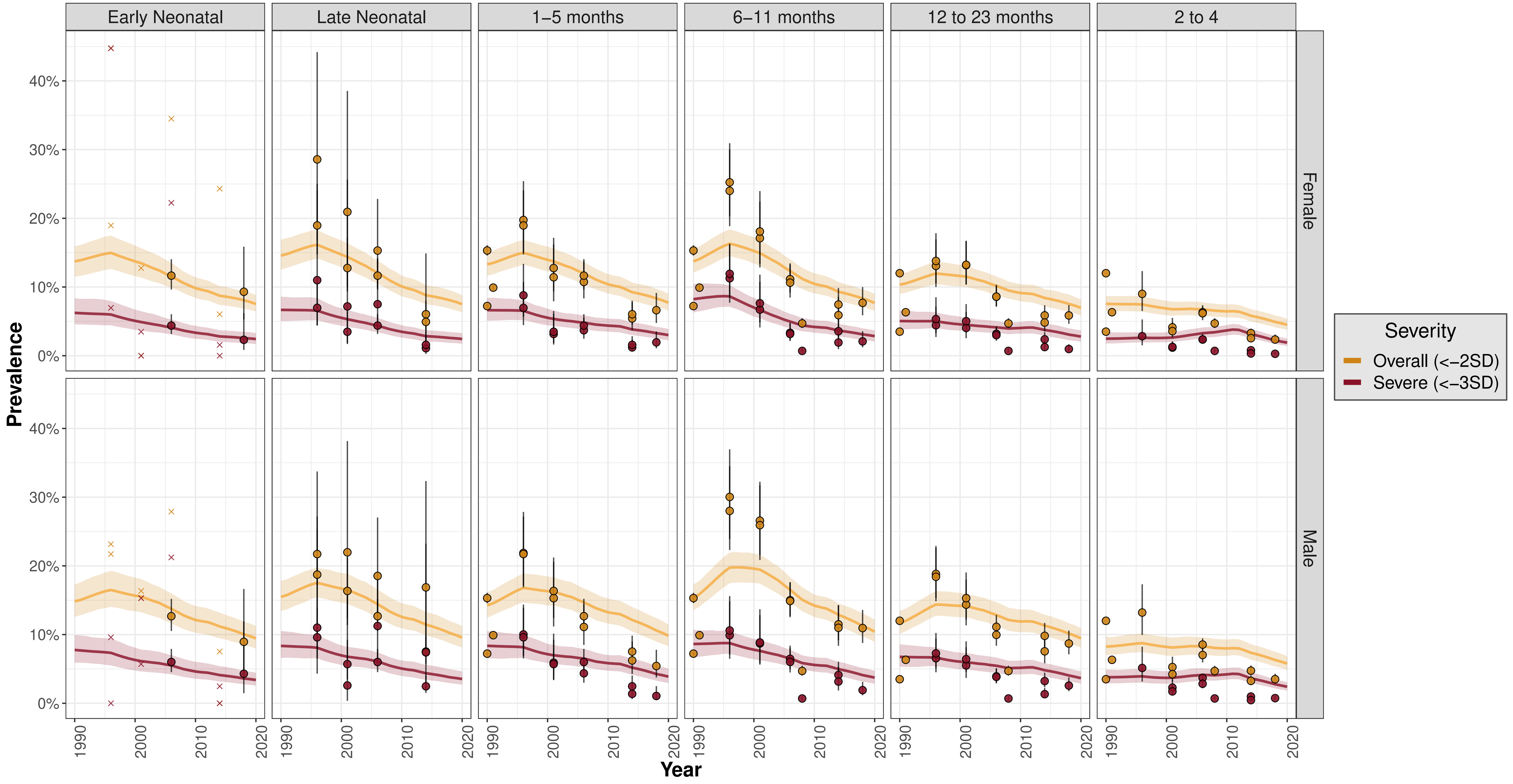

E: Transformed Mean Wasting Z Scores

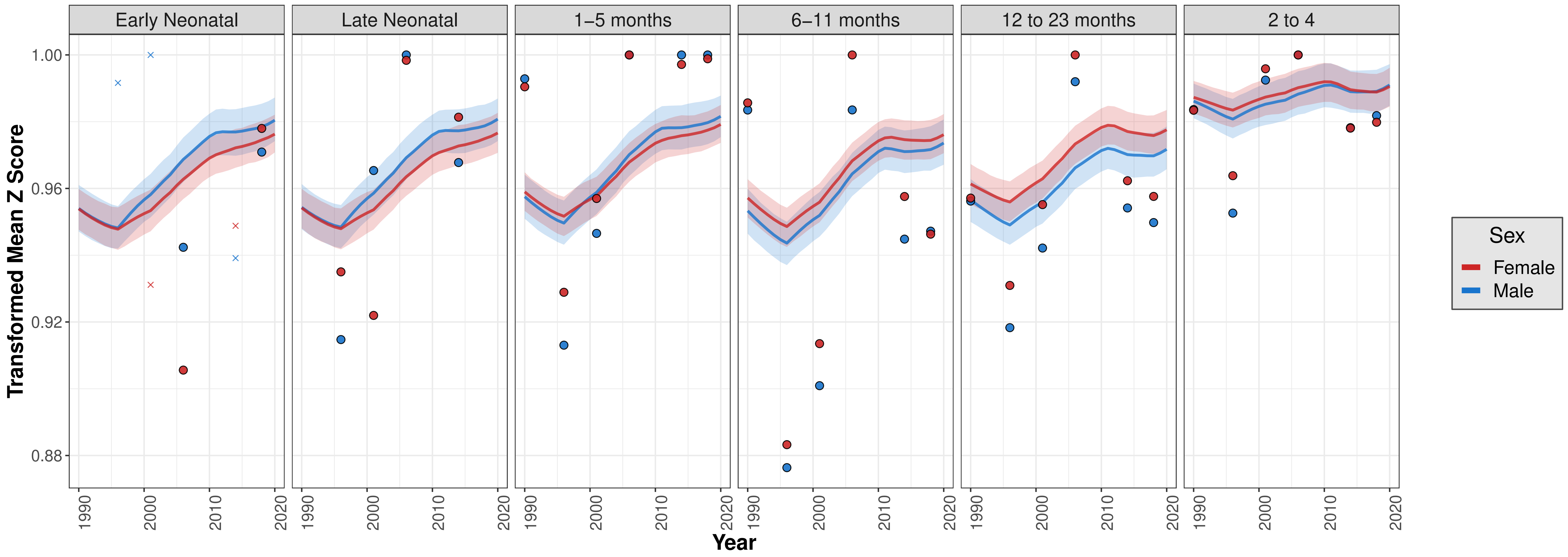

F

Year	Source
1989	WHO CGM Database
1990	WHO CGM Database
1991	WHO CGM Database
1996	DHS
1996	WHO CGM Database
2001	DHS
2001	WHO CGM Database
2006	DHS
2006	WHO CGM Database
2008	Comprehensive Food Security and Vulnerability Analysis
2011	DHS
2012	DHS
2014	WHO CGM Database
2014	MICS
2018	DHS

Benin – Underweight (WAZ)

G: Overall and Severe Underweight Prevalence

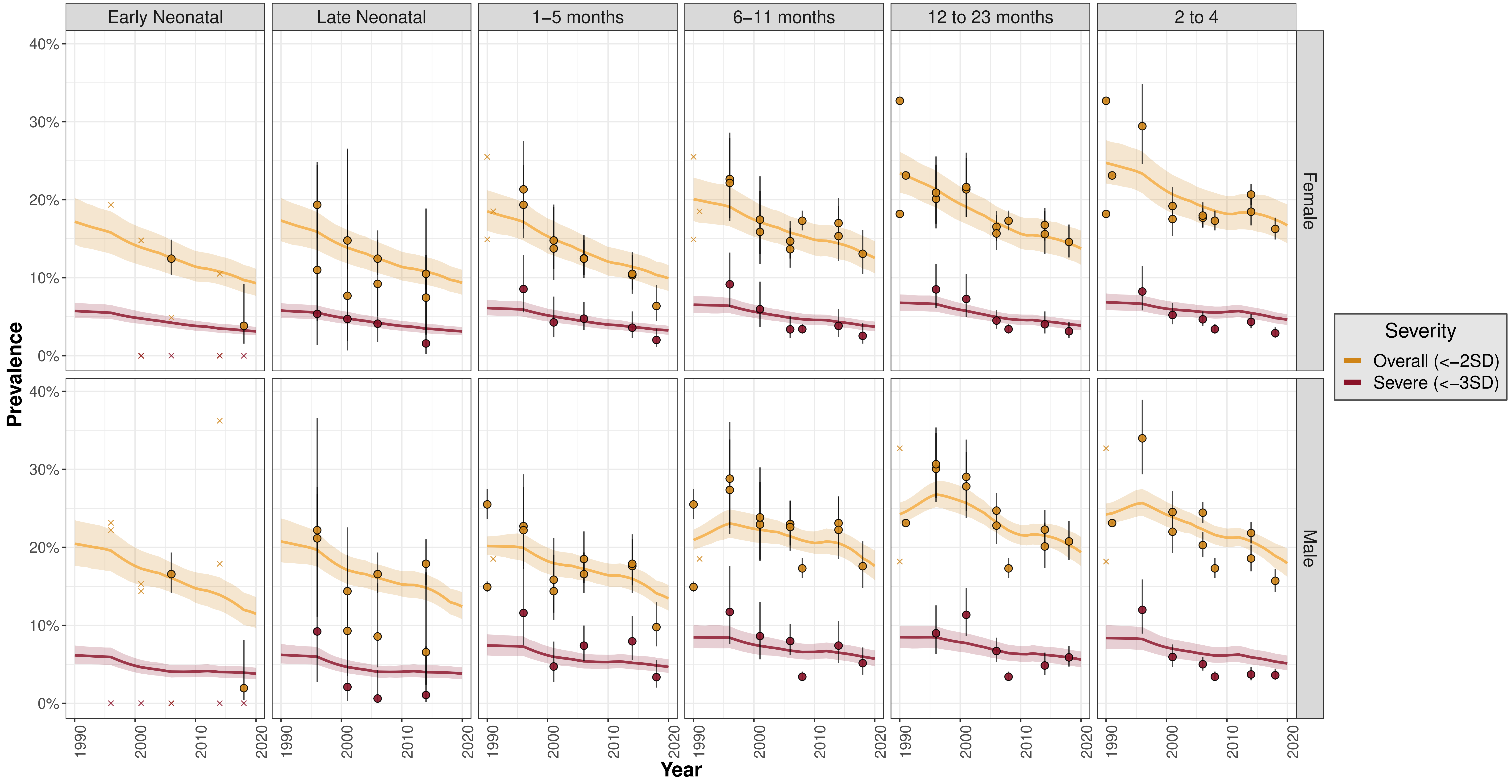

H: Transformed Mean Underweight Z Scores

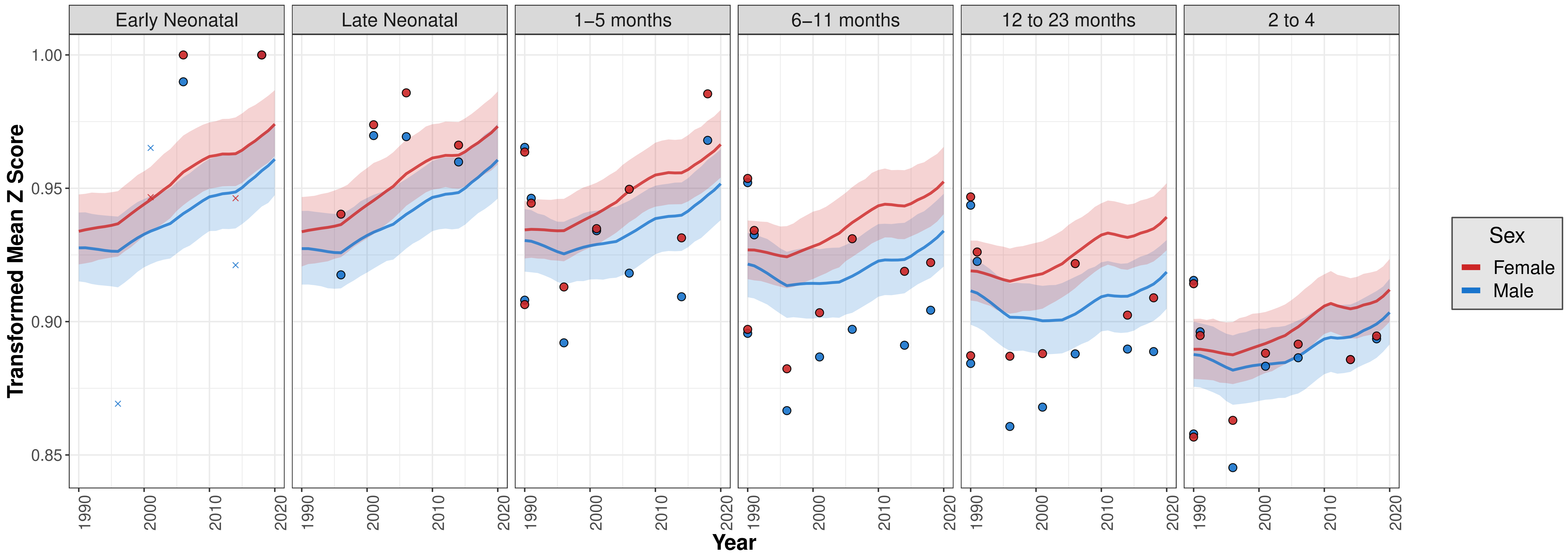

I

Year	Source
1989	WHO CGM Database
1990	WHO CGM Database
1991	WHO CGM Database
1996	DHS
1996	WHO CGM Database
2001	DHS
2001	WHO CGM Database
2006	DHS
2006	WHO CGM Database
2008	Comprehensive Food Security and Vulnerability Analysis
2011	DHS
2012	DHS
2014	WHO CGM Database
2014	MICS
2018	DHS

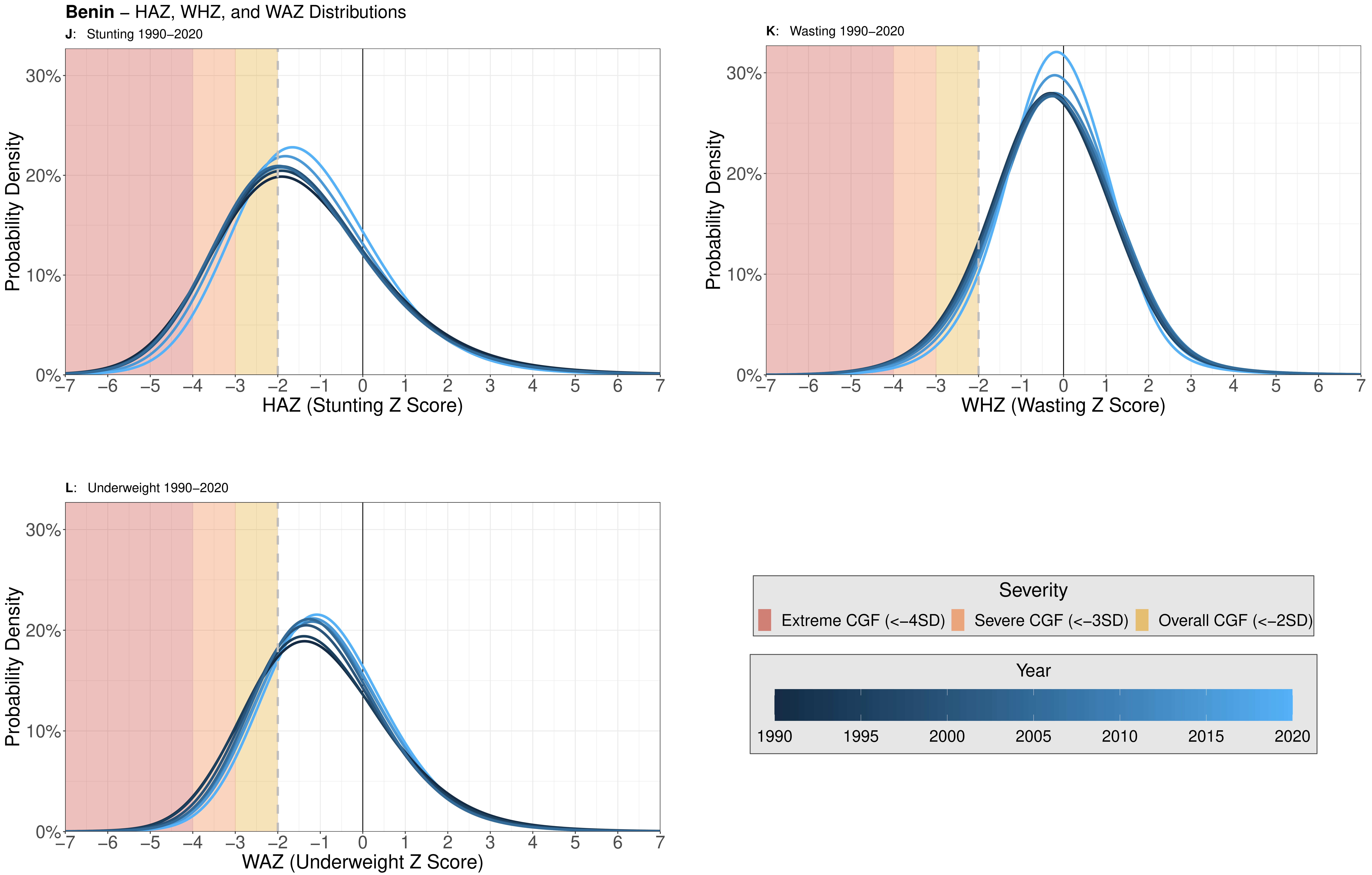
Benin – HAZ, WHZ, and WAZ Distributions
J: Stunting 1990–2020
K: Wasting 1990–2020
L: Underweight 1990–2020
Probability Density
0%
10%
20%
30%
−7 −6 −5 −4 −3 −2 −1 0 1 2 3 4 5 6 7
HAZ (Stunting Z Score)
WHZ (Wasting Z Score)
WAZ (Underweight Z Score)
Severity
Extreme CGF (<−4SD)
Severe CGF (<−3SD)
Overall CGF (<−2SD)
Year
1990
1995
2000
2005
2010
2015
2020

Burkina Faso – Stunting (HAZ)

A: Overall and Severe Stunting Prevalence

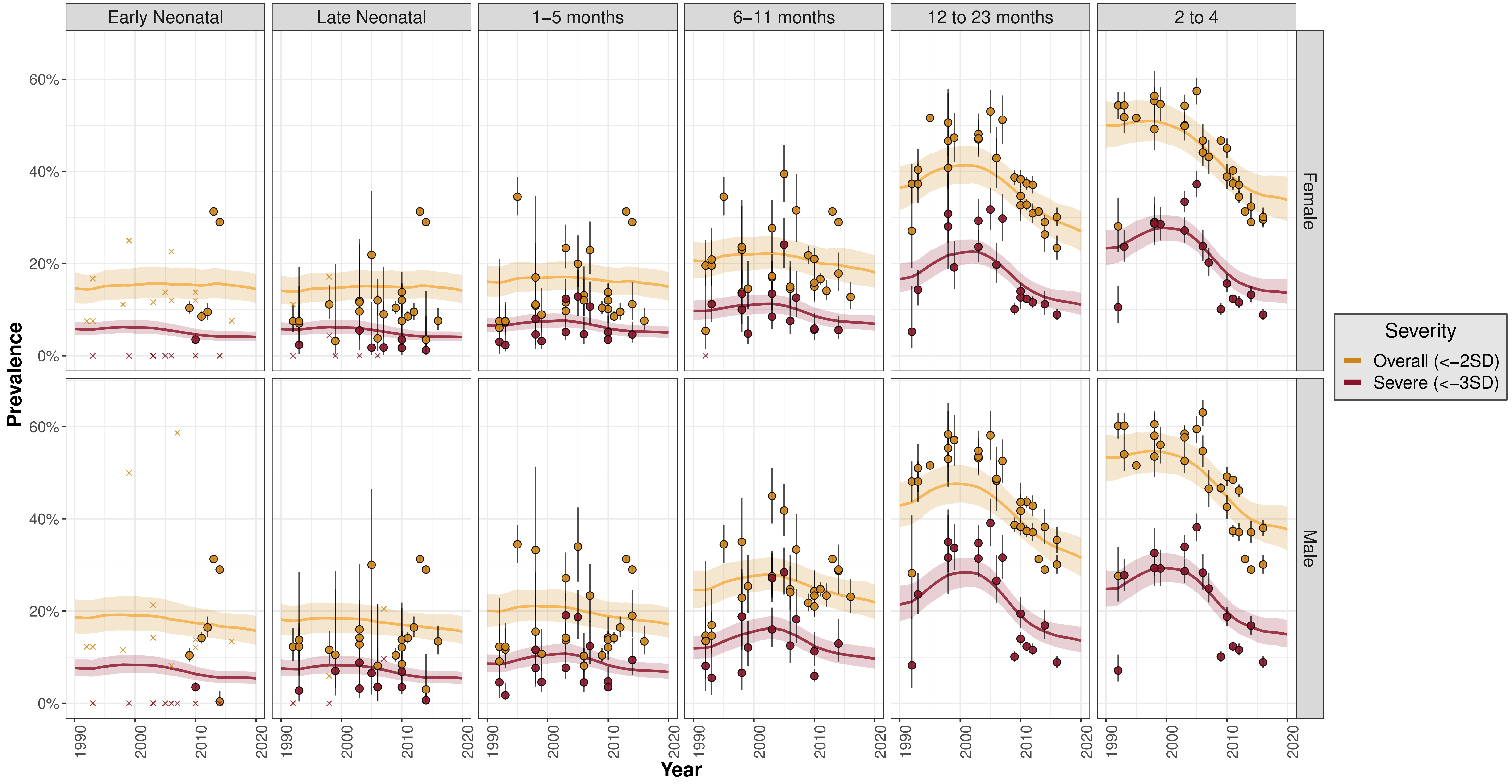

B: Transformed Mean Stunting Z Scores

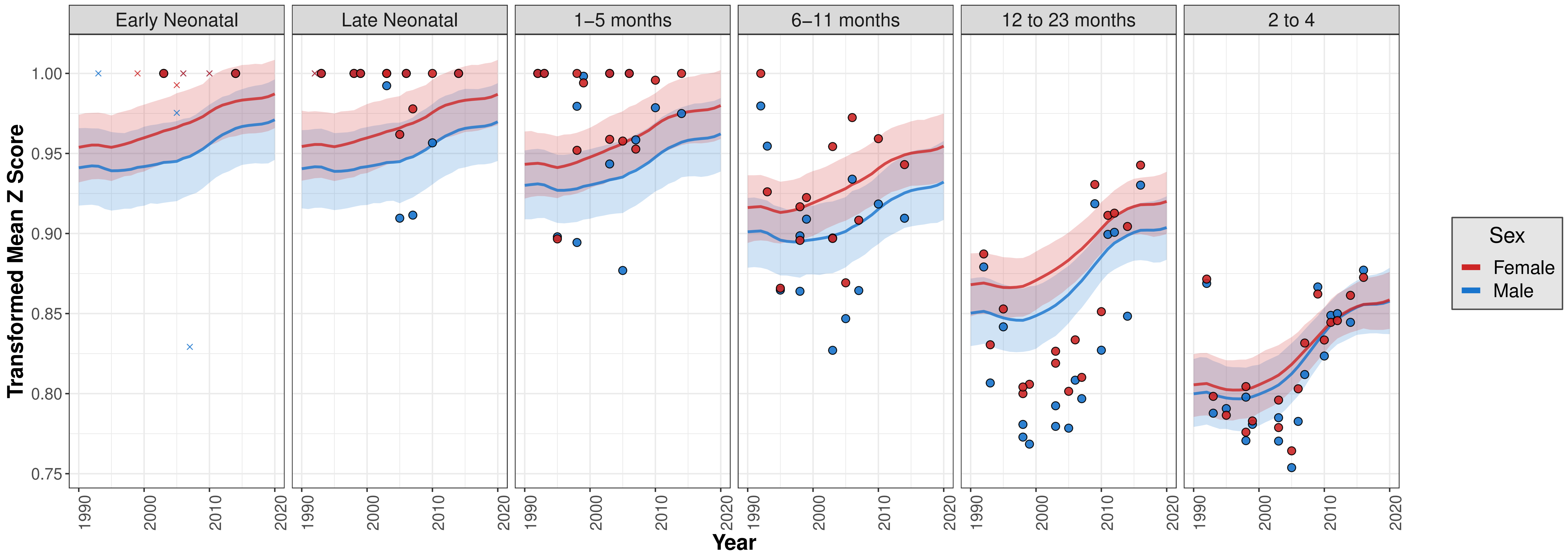

C

Year	Source
1992	DHS
1992	WHO CGM Database
1993	DHS
1993	WHO CGM Database
1995	WHO CGM Database
1998	Priority Survey
1998	DHS
1998	WHO CGM Database
1999	DHS
2003	Core Welfare Indicators Questionnaire Survey
2003	DHS
2003	WHO CGM Database
2005	Core Welfare Indicators Questionnaire Survey
2006	MICS
2006	WHO CGM Database
2007	Core Welfare Indicators Questionnaire Survey
2009	WHO CGM Database
2010	DHS
2010	National Nutrition Survey
2010	WHO CGM Database
2011	WHO CGM Database
2011	National Nutrition Survey
2012	WHO CGM Database
2012	National Nutrition Survey
2013	WHO CGM Database
2014	WHO CGM Database
2014	Continuous Multisectoral Survey
2016	WHO CGM Database
2016	National Nutrition Survey

Burkina Faso – Wasting (WHZ)

D: Overall and Severe Wasting Prevalence

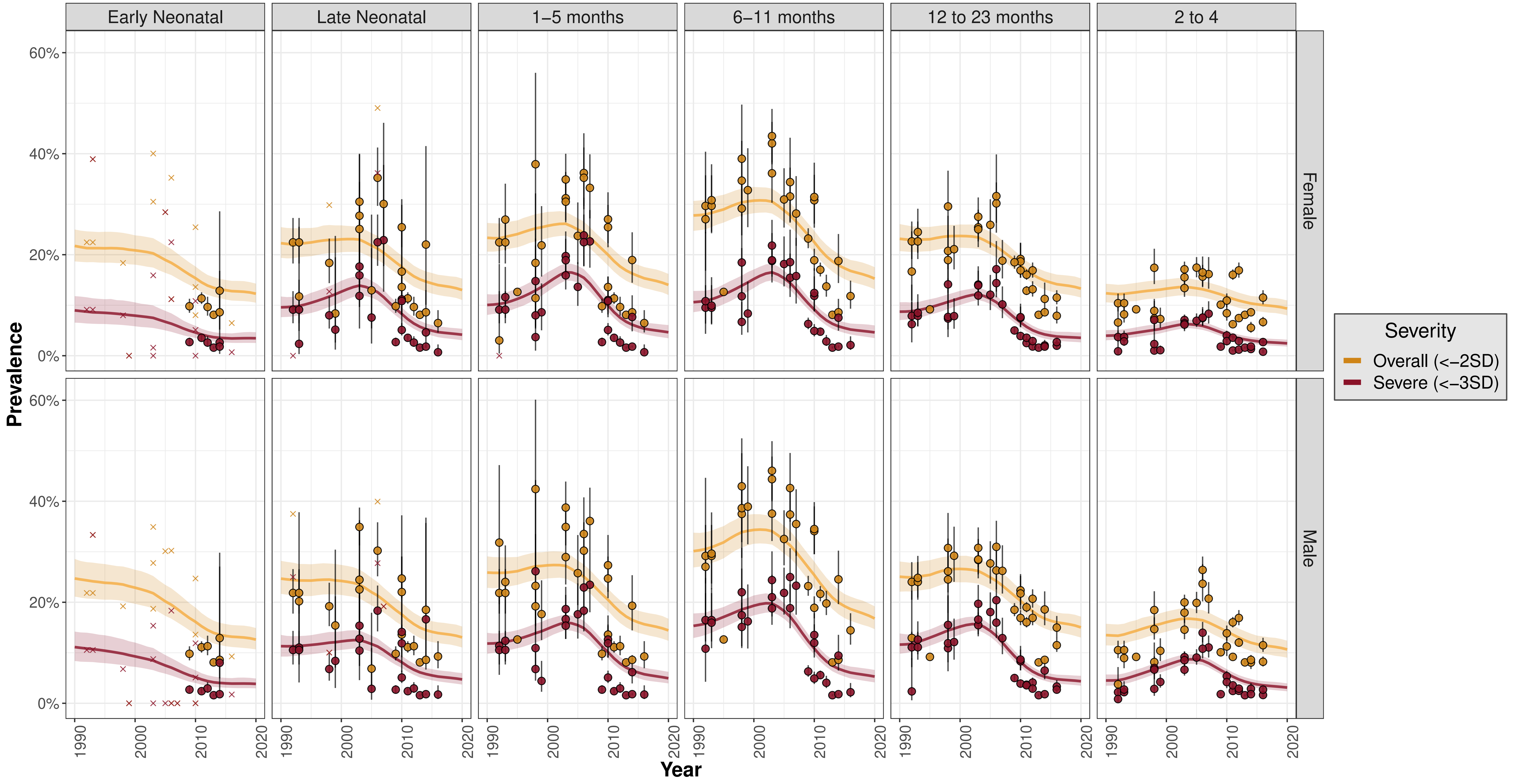

E: Transformed Mean Wasting Z Scores

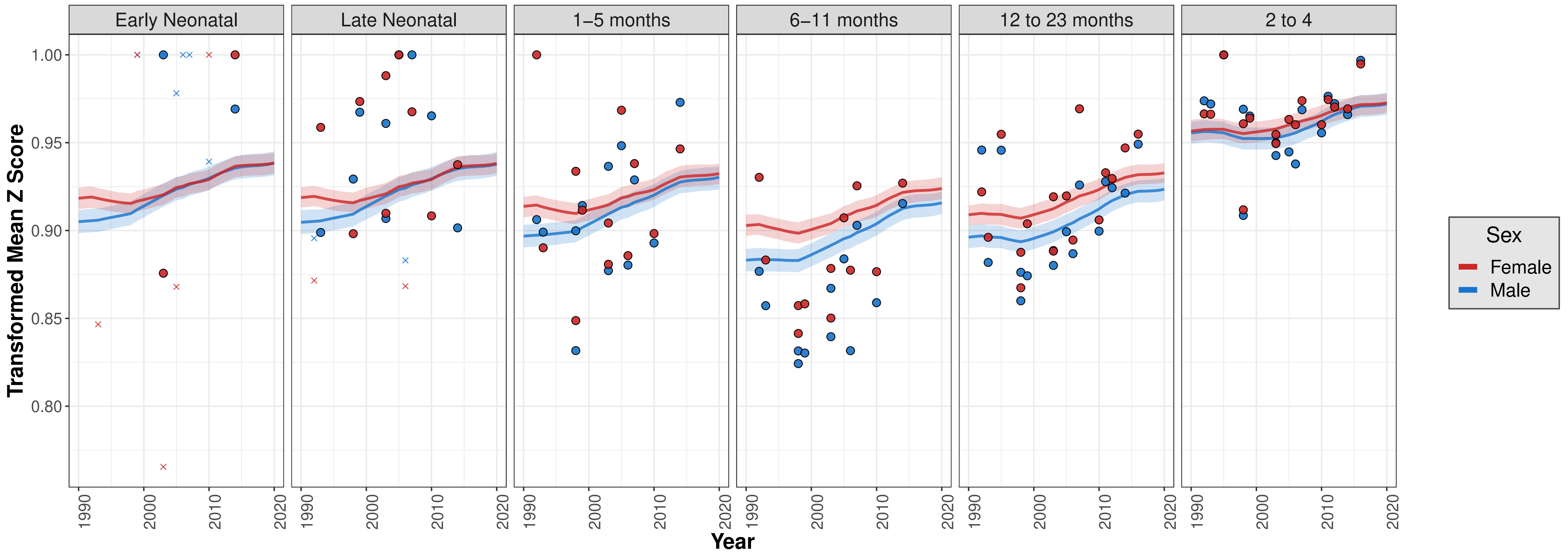

F

Year	Source
1992	DHS
1992	WHO CGM Database
1993	DHS
1993	WHO CGM Database
1995	WHO CGM Database
1998	Priority Survey
1998	DHS
1998	WHO CGM Database
1999	DHS
2003	Core Welfare Indicators Questionnaire Survey
2003	DHS
2003	WHO CGM Database
2005	Core Welfare Indicators Questionnaire Survey
2006	MICS
2006	WHO CGM Database
2007	Core Welfare Indicators Questionnaire Survey
2009	WHO CGM Database
2010	DHS
2010	National Nutrition Survey
2010	WHO CGM Database
2011	WHO CGM Database
2011	National Nutrition Survey
2012	WHO CGM Database
2012	National Nutrition Survey
2013	WHO CGM Database
2014	WHO CGM Database
2014	Continuous Multisectoral Survey
2016	WHO CGM Database
2016	National Nutrition Survey

Burkina Faso – Underweight (WAZ)

G: Overall and Severe Underweight Prevalence

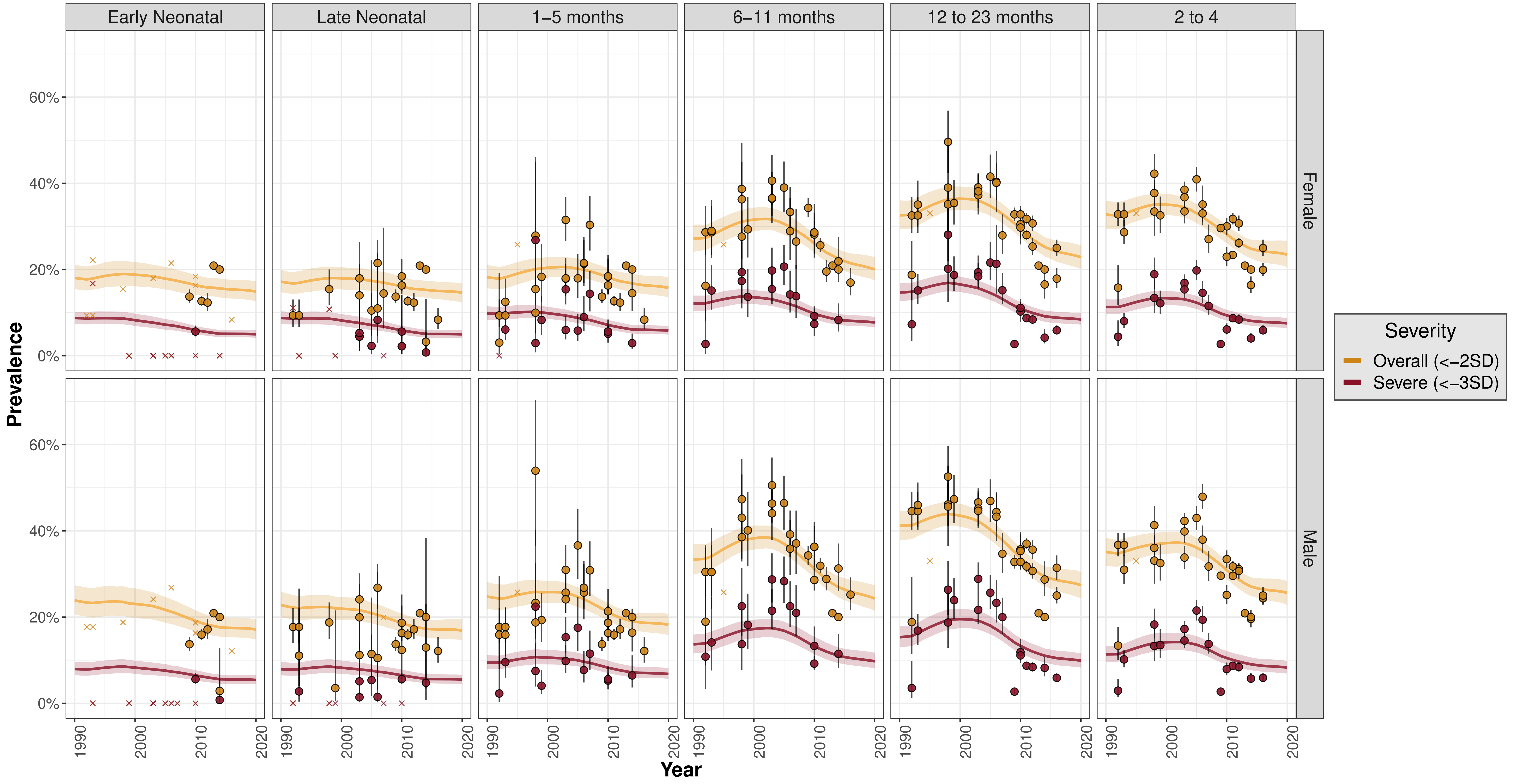

H: Transformed Mean Underweight Z Scores

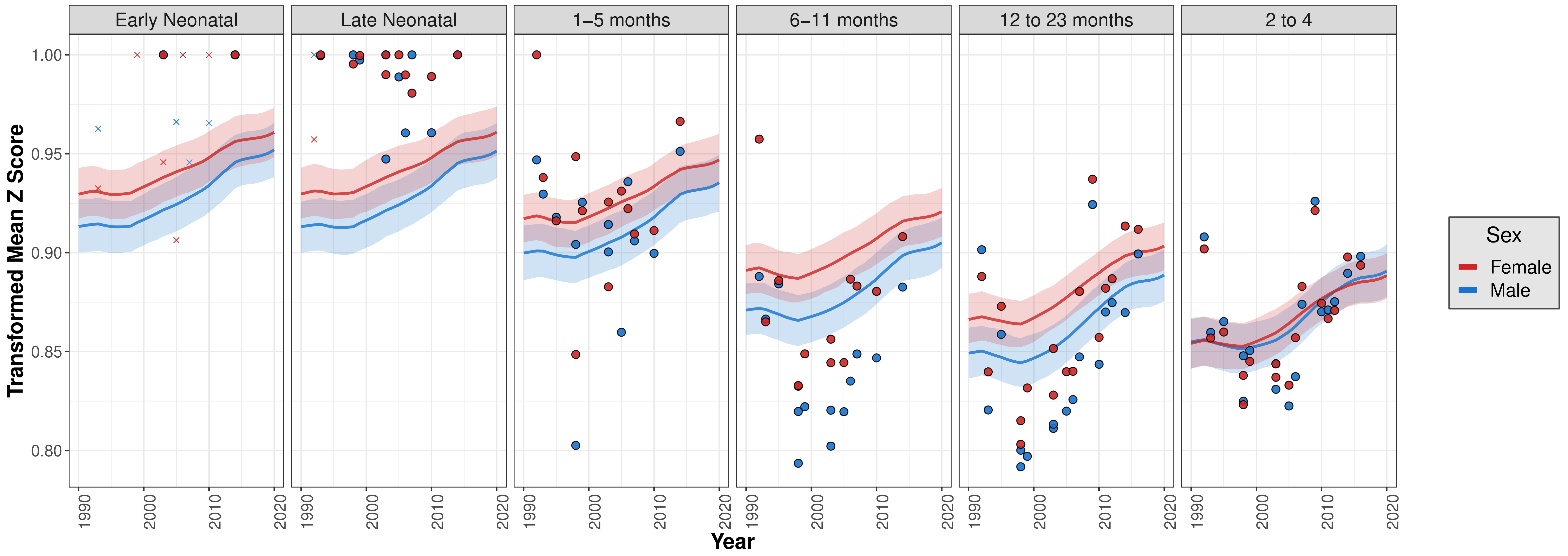

I

Year	Source
1992	DHS
1992	WHO CGM Database
1993	DHS
1993	WHO CGM Database
1995	WHO CGM Database
1998	Priority Survey
1998	DHS
1998	WHO CGM Database
1999	DHS
2003	Core Welfare Indicators Questionnaire Survey
2003	DHS
2003	WHO CGM Database
2005	Core Welfare Indicators Questionnaire Survey
2006	MICS
2006	WHO CGM Database
2007	Core Welfare Indicators Questionnaire Survey
2009	WHO CGM Database
2010	DHS
2010	National Nutrition Survey
2010	WHO CGM Database
2011	WHO CGM Database
2011	National Nutrition Survey
2012	WHO CGM Database
2012	National Nutrition Survey
2013	WHO CGM Database
2014	WHO CGM Database
2014	Continuous Multisectoral Survey
2016	WHO CGM Database
2016	National Nutrition Survey

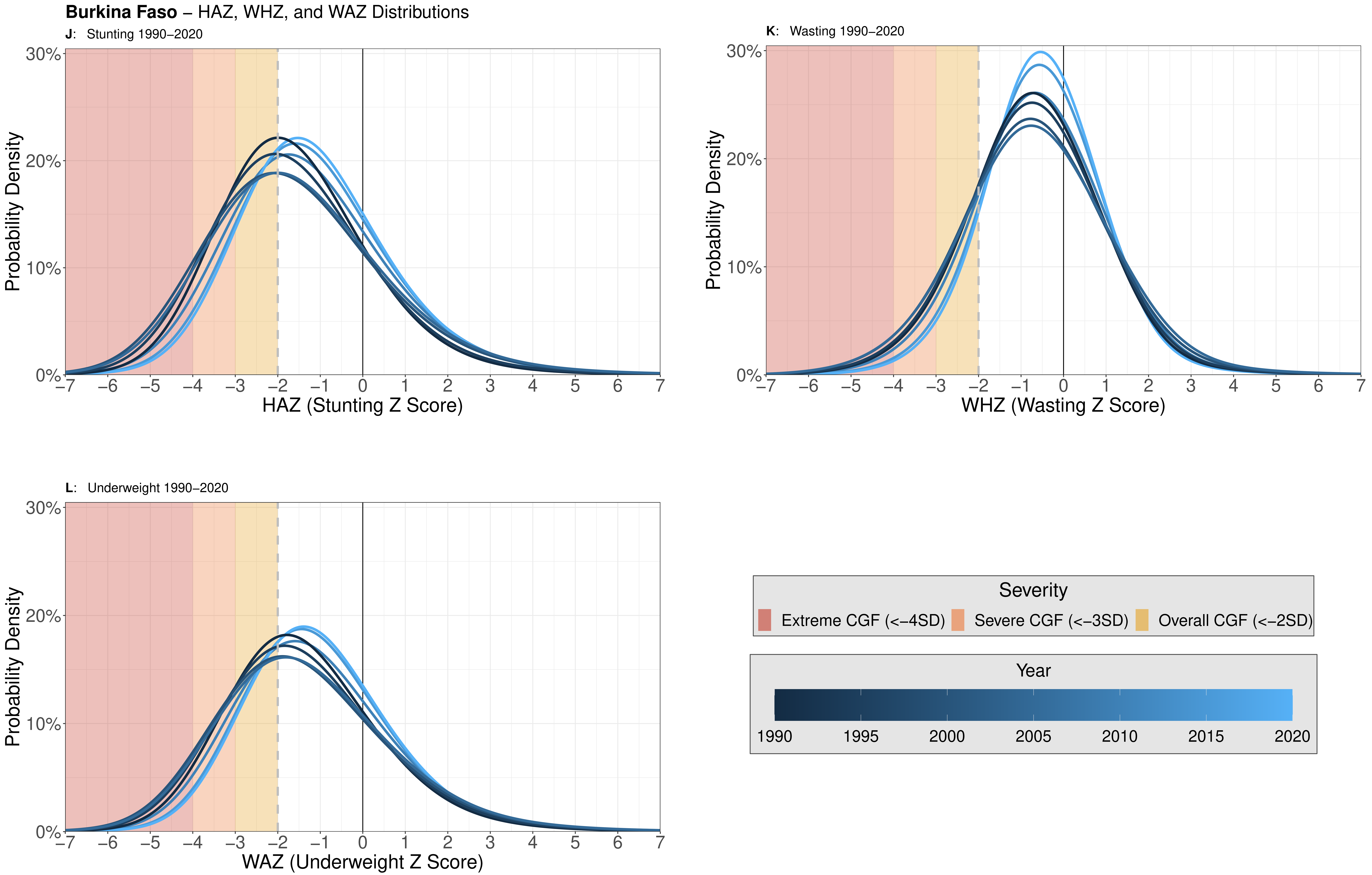
Burkina Faso – HAZ, WHZ, and WAZ Distributions
J: Stunting 1990–2020
K: Wasting 1990–2020
L: Underweight 1990–2020
Probability Density
30%
20%
10%
0%
−7 −6 −5 −4 −3 −2 −1 0 1 2 3 4 5 6 7
HAZ (Stunting Z Score)
WHZ (Wasting Z Score)
WAZ (Underweight Z Score)
Severity
Extreme CGF (<−4SD)
Severe CGF (<−3SD)
Overall CGF (<−2SD)
Year
1990
1995
2000
2005
2010
2015
2020

Cameroon – Stunting (HAZ)

A: Overall and Severe Stunting Prevalence

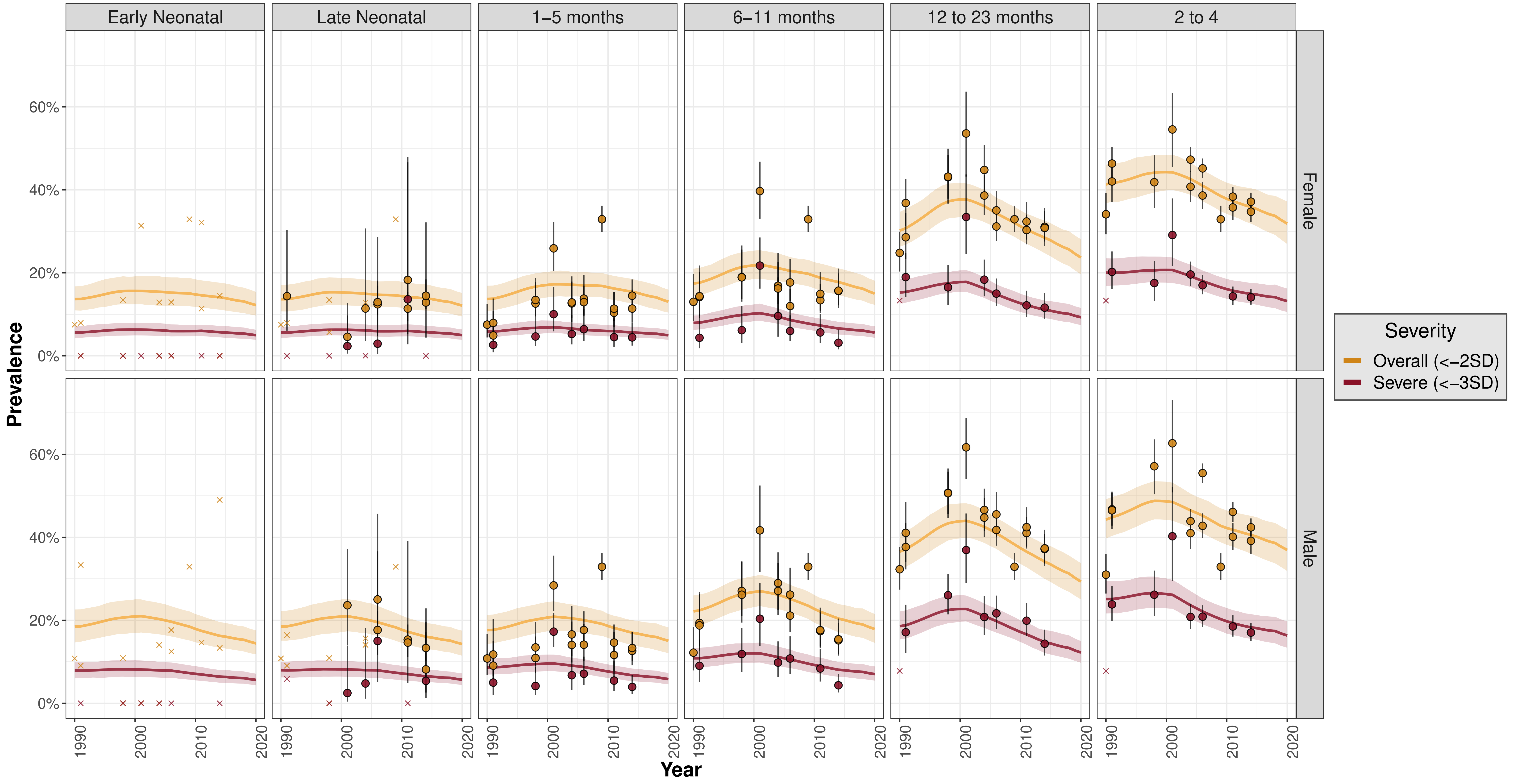

B: Transformed Mean Stunting Z Scores

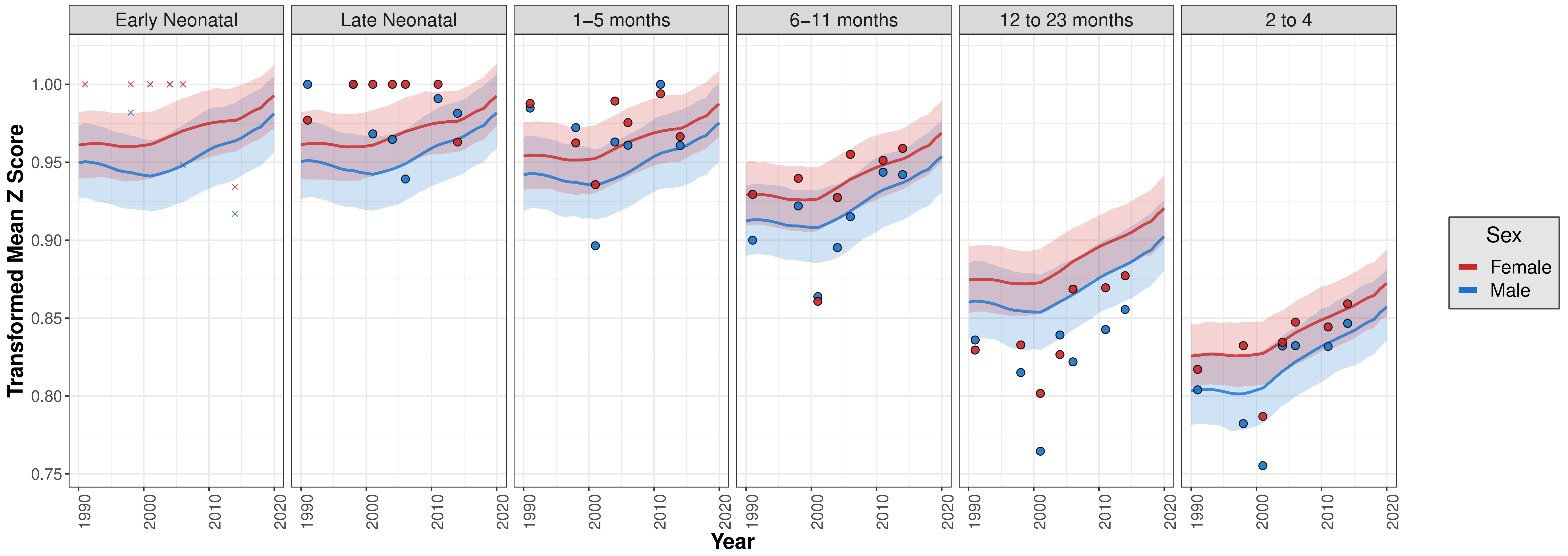

C

Year	Source
1990	WHO CGM Database
1991	DHS
1991	WHO CGM Database
1998	DHS
1998	WHO CGM Database
2001	Household Survey
2004	DHS
2004	WHO CGM Database
2006	MICS
2006	WHO CGM Database
2009	National Survey of Micronutrient Status and Consumption of Fortifiable Foods
2011	DHS
2011	WHO CGM Database
2014	WHO CGM Database
2014	MICS

Cameroon – Wasting (WHZ)

D: Overall and Severe Wasting Prevalence

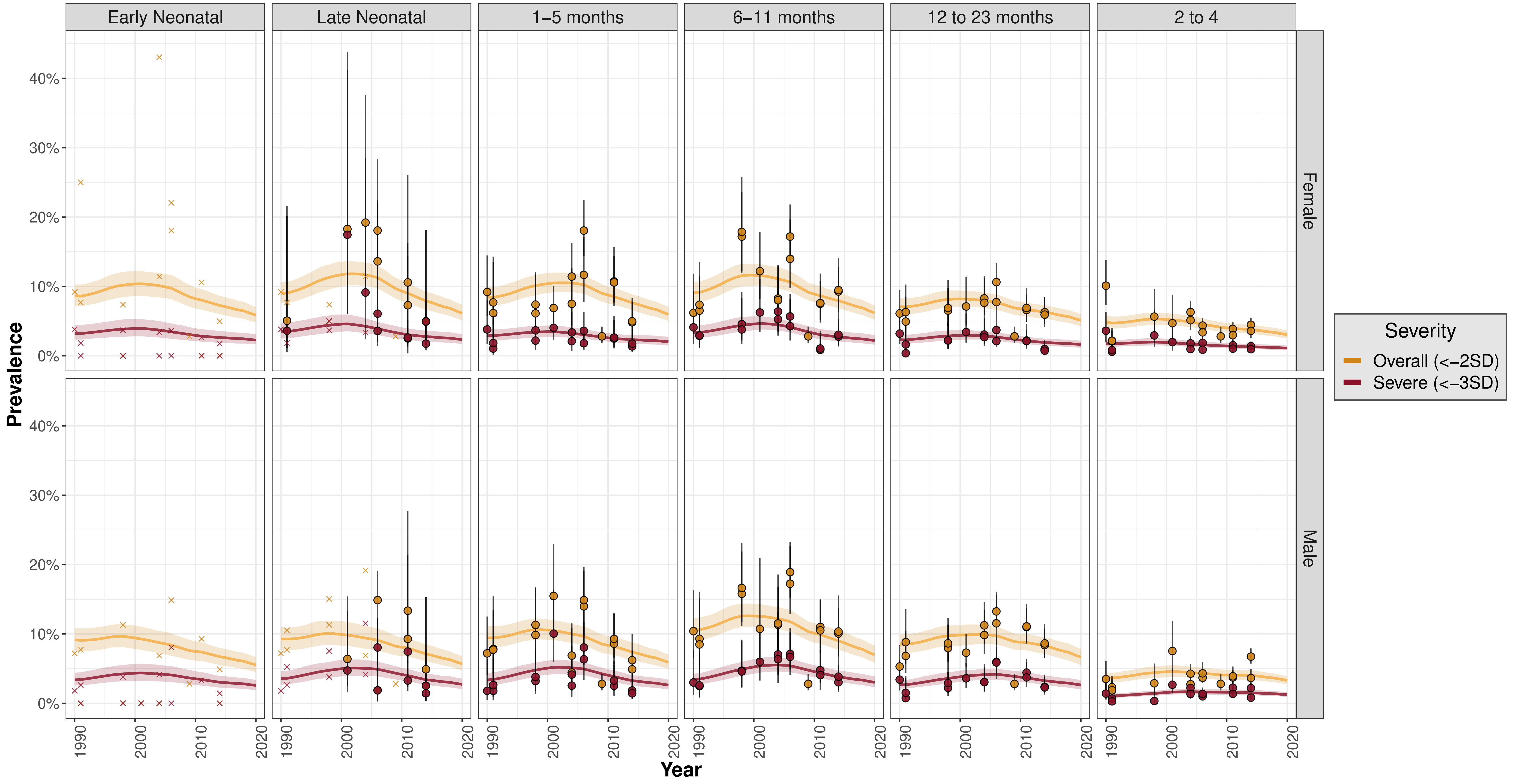

E: Transformed Mean Wasting Z Scores

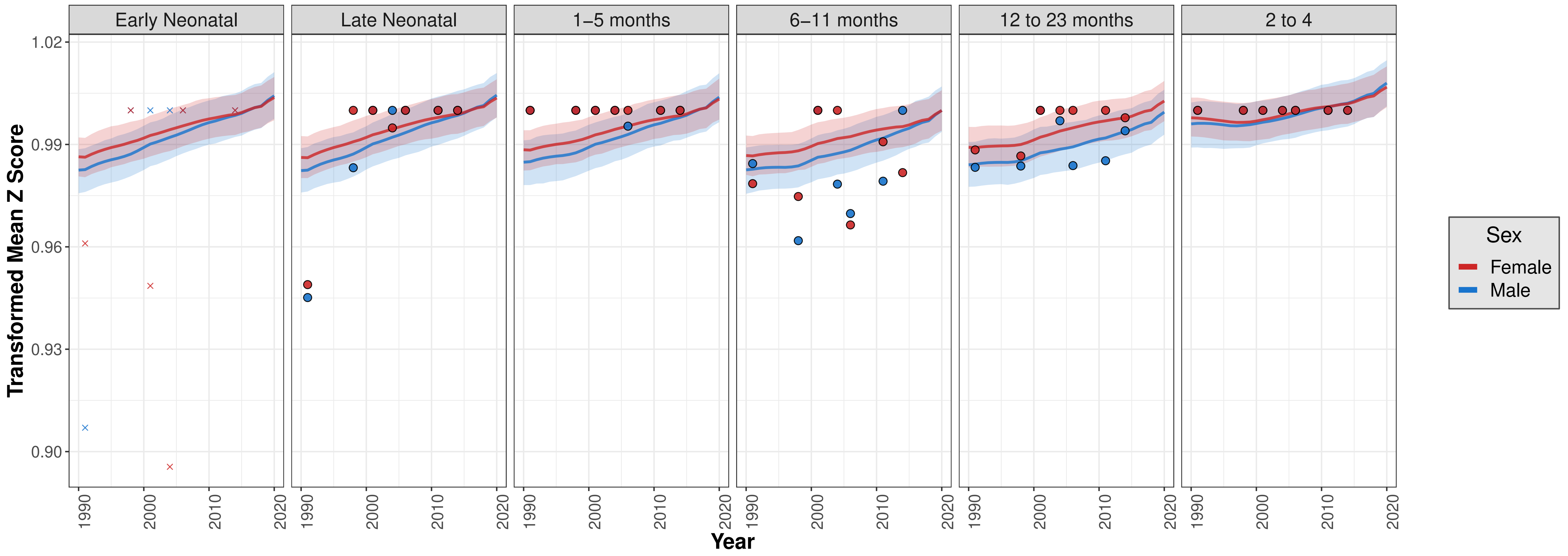

F

Year	Source
1990	WHO CGM Database
1991	DHS
1991	WHO CGM Database
1998	DHS
1998	WHO CGM Database
2001	Household Survey
2004	DHS
2004	WHO CGM Database
2006	MICS
2006	WHO CGM Database
2009	National Survey of Micronutrient Status and Consumption of Fortifiable Foods
2011	DHS
2011	WHO CGM Database
2014	WHO CGM Database
2014	MICS

Cameroon – Underweight (WAZ)

G: Overall and Severe Underweight Prevalence

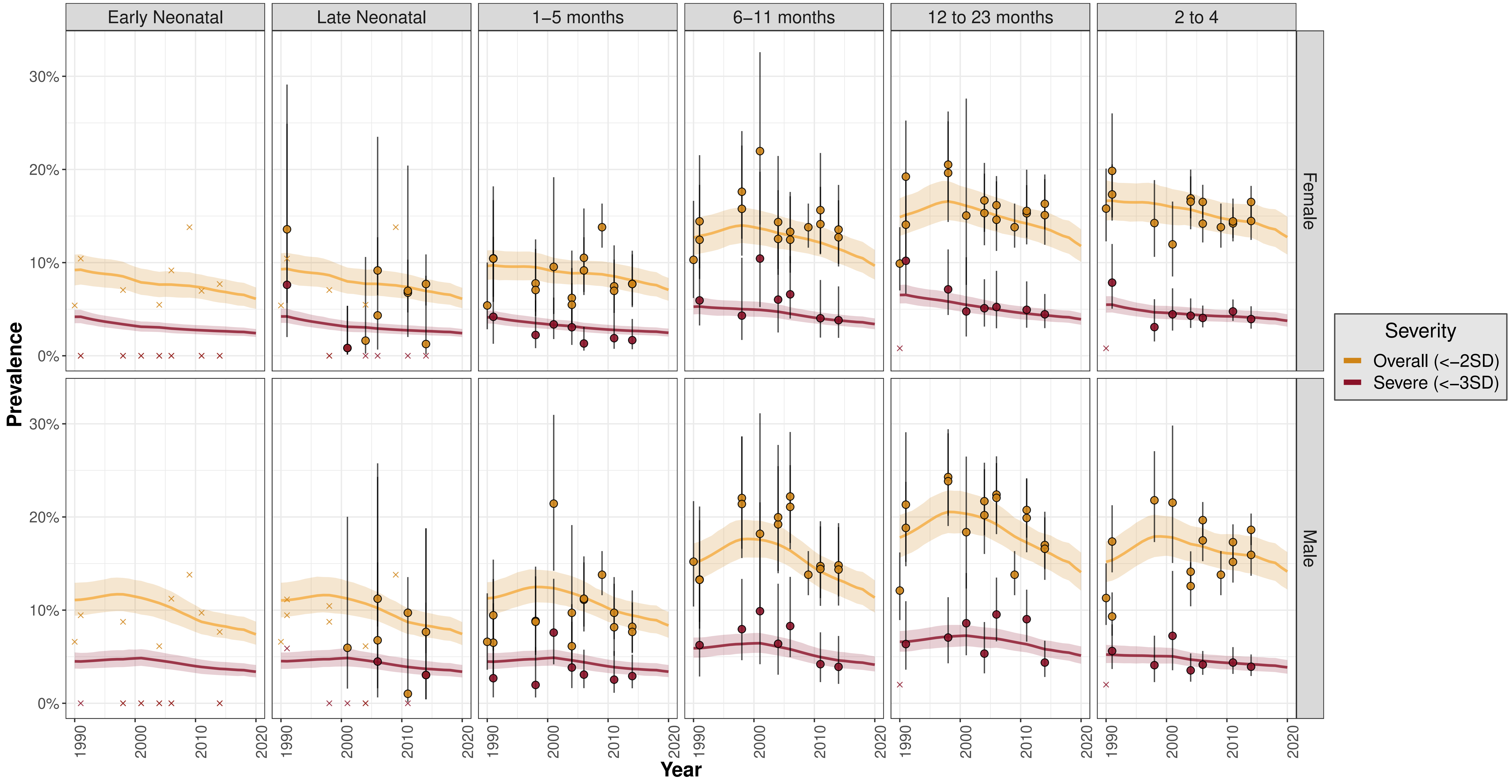

H: Transformed Mean Underweight Z Scores

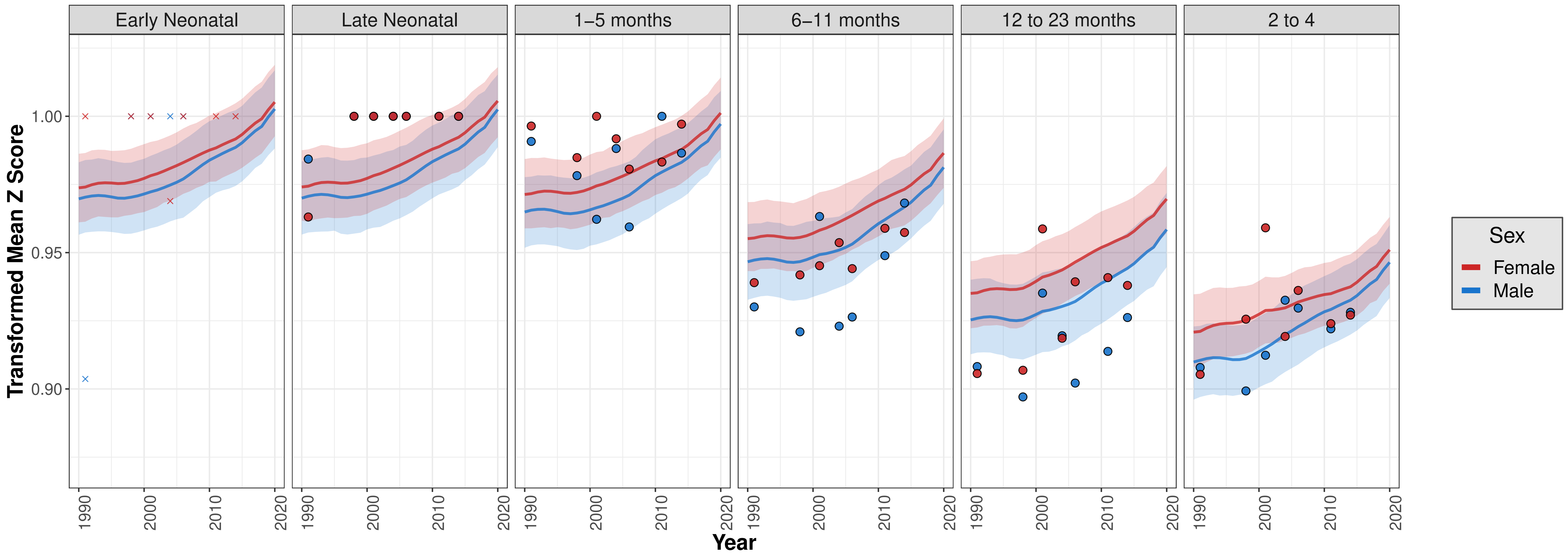

I

Year	Source
1990	WHO CGM Database
1991	DHS
1991	WHO CGM Database
1998	DHS
1998	WHO CGM Database
2001	Household Survey
2004	DHS
2004	WHO CGM Database
2006	MICS
2006	WHO CGM Database
2009	National Survey of Micronutrient Status and Consumption of Fortifiable Foods
2011	DHS
2011	WHO CGM Database
2014	WHO CGM Database
2014	MICS

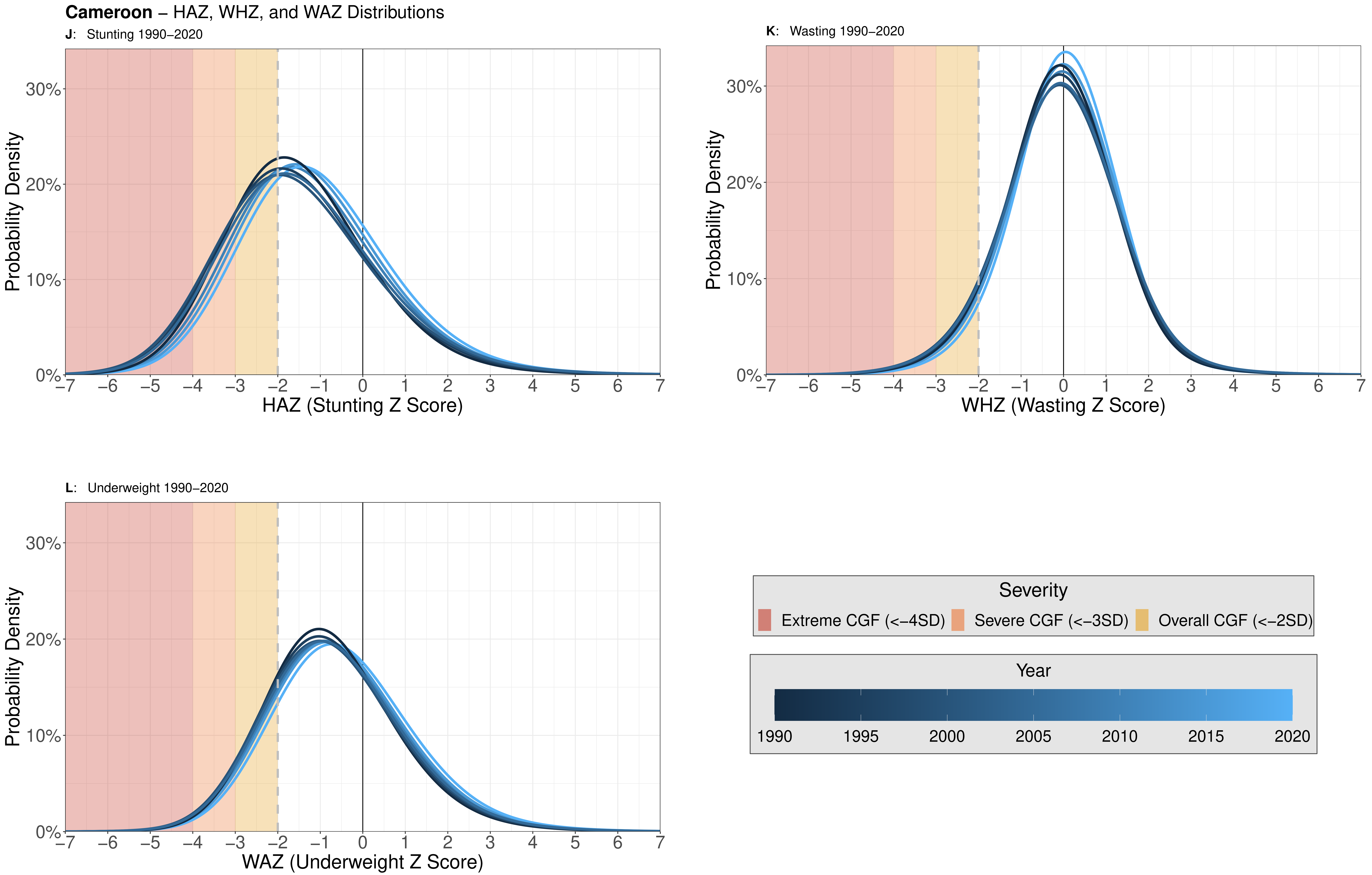
Cameroon – HAZ, WHZ, and WAZ Distributions
J: Stunting 1990–2020
Probability Density
HAZ (Stunting Z Score)
K: Wasting 1990–2020
Probability Density
WHZ (Wasting Z Score)
L: Underweight 1990–2020
Probability Density
WAZ (Underweight Z Score)
0%
10%
20%
30%
−7
−6
−5
−4
−3
−2
−1
0
1
2
3
4
5
6
7
Severity
Extreme CGF (<−4SD)
Severe CGF (<−3SD)
Overall CGF (<−2SD)
Year
1990
1995
2000
2005
2010
2015
2020

Cabo Verde – Stunting (HAZ)

A: Overall and Severe Stunting Prevalence

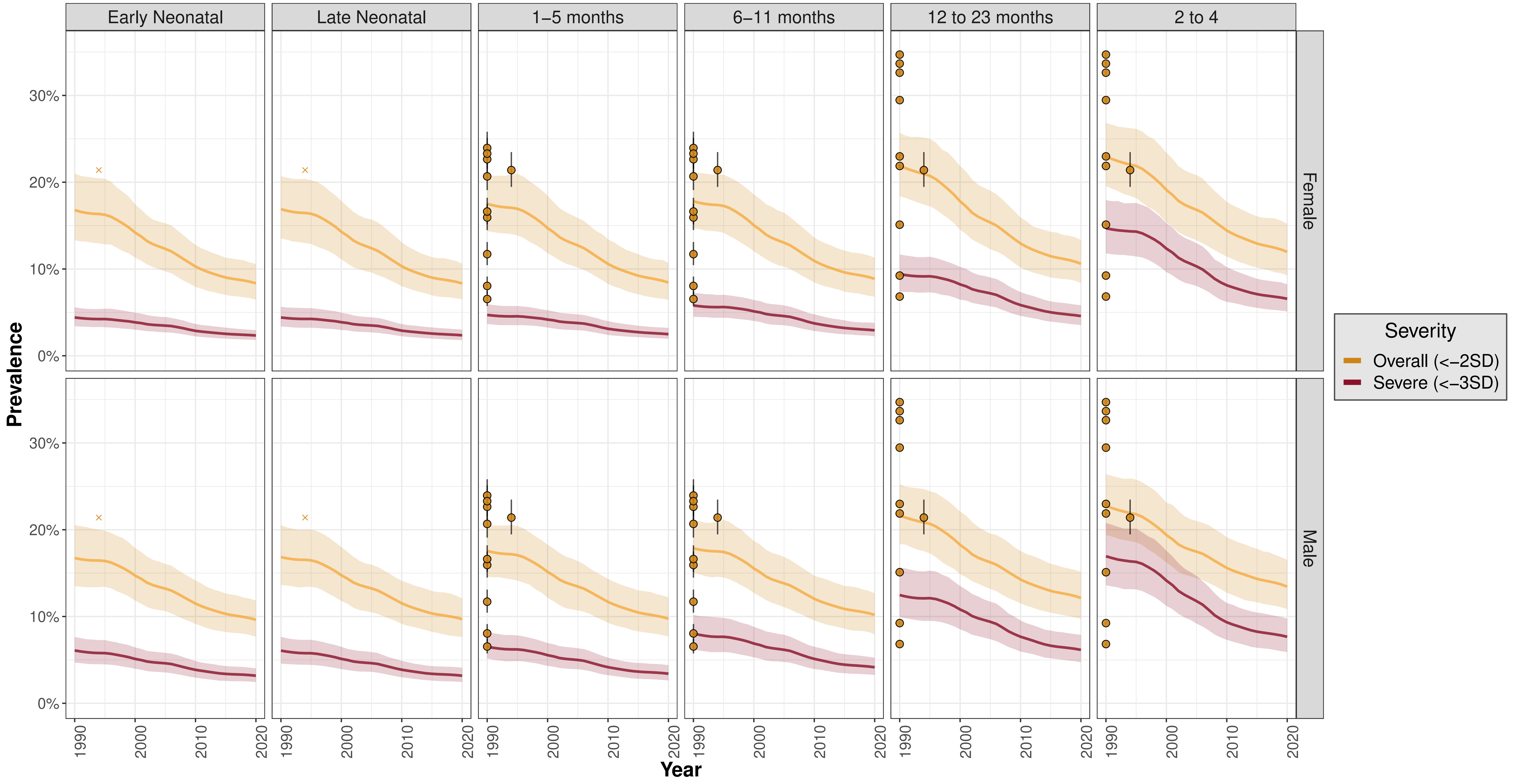

B: Transformed Mean Stunting Z Scores

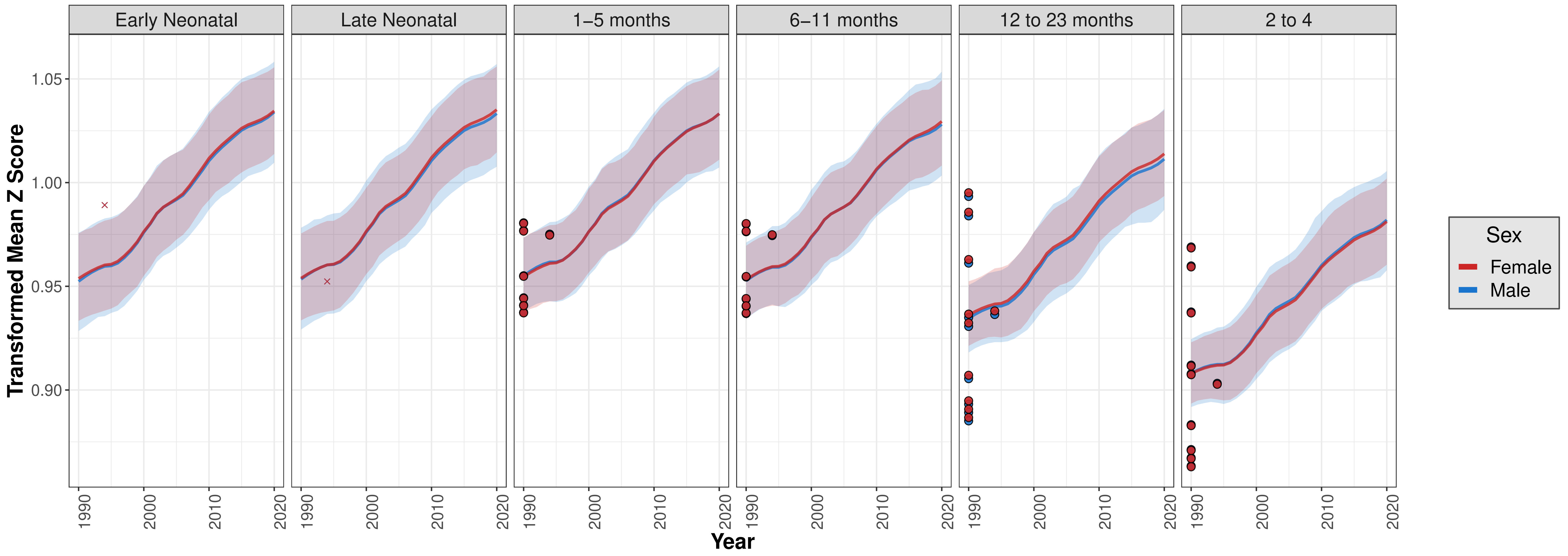

C

Year	Source
1983	WHO CGM Database
1990	WHO CGM Database
1994	WHO CGM Database

Cabo Verde – Wasting (WHZ)

D: Overall and Severe Wasting Prevalence

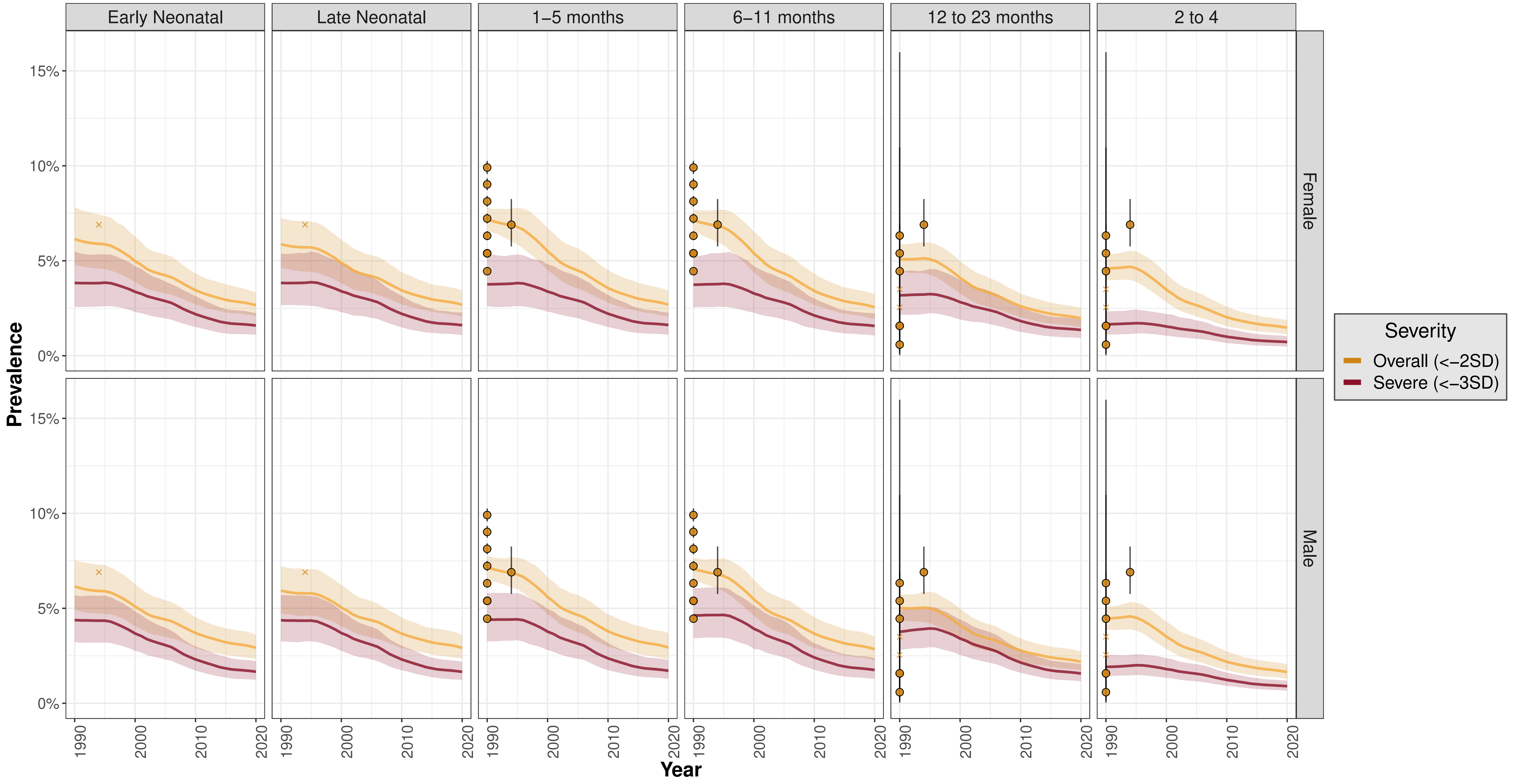

E: Transformed Mean Wasting Z Scores

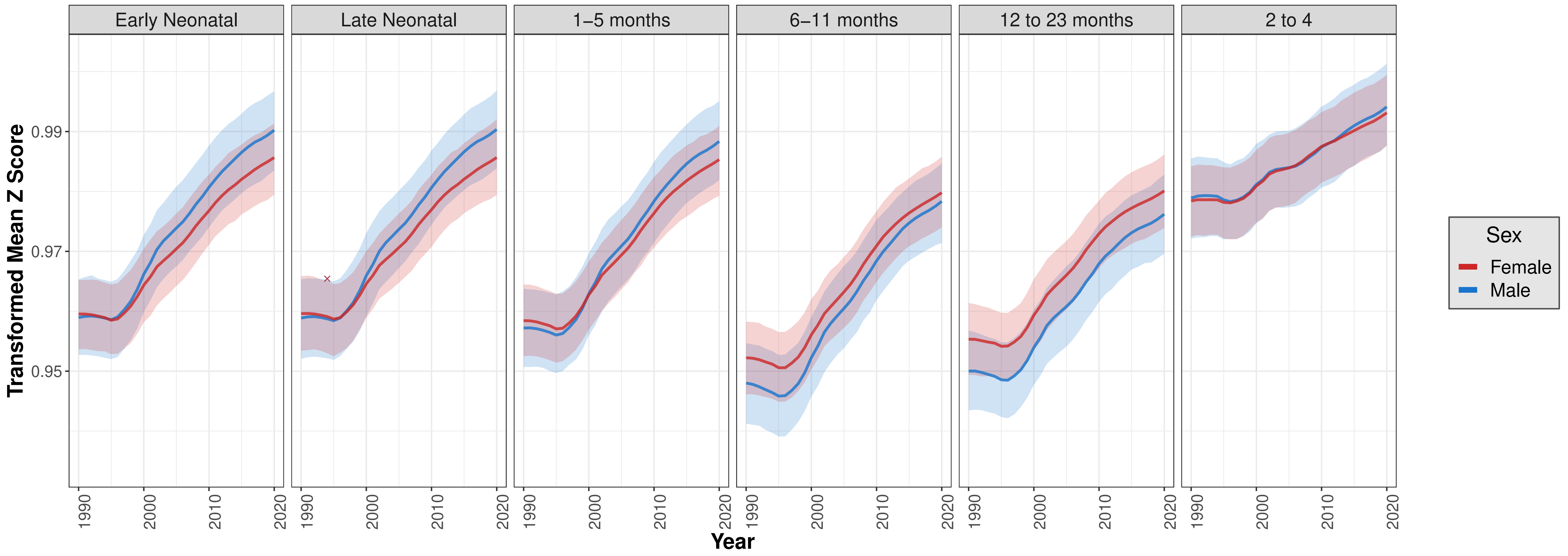

F

Year	Source
1983	WHO CGM Database
1990	WHO CGM Database
1994	WHO CGM Database

Cabo Verde – Underweight (WAZ)

G: Overall and Severe Underweight Prevalence

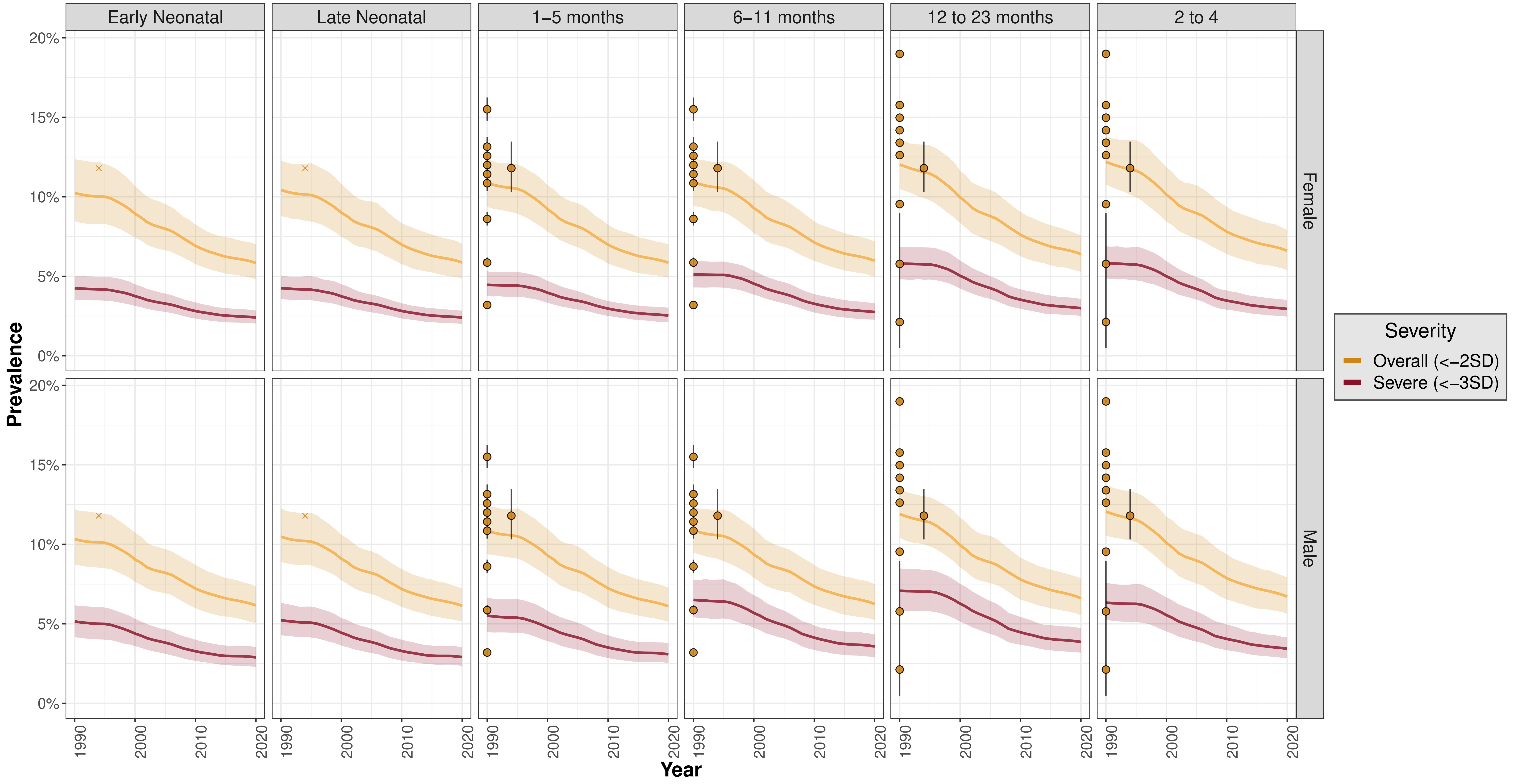

H: Transformed Mean Underweight Z Scores

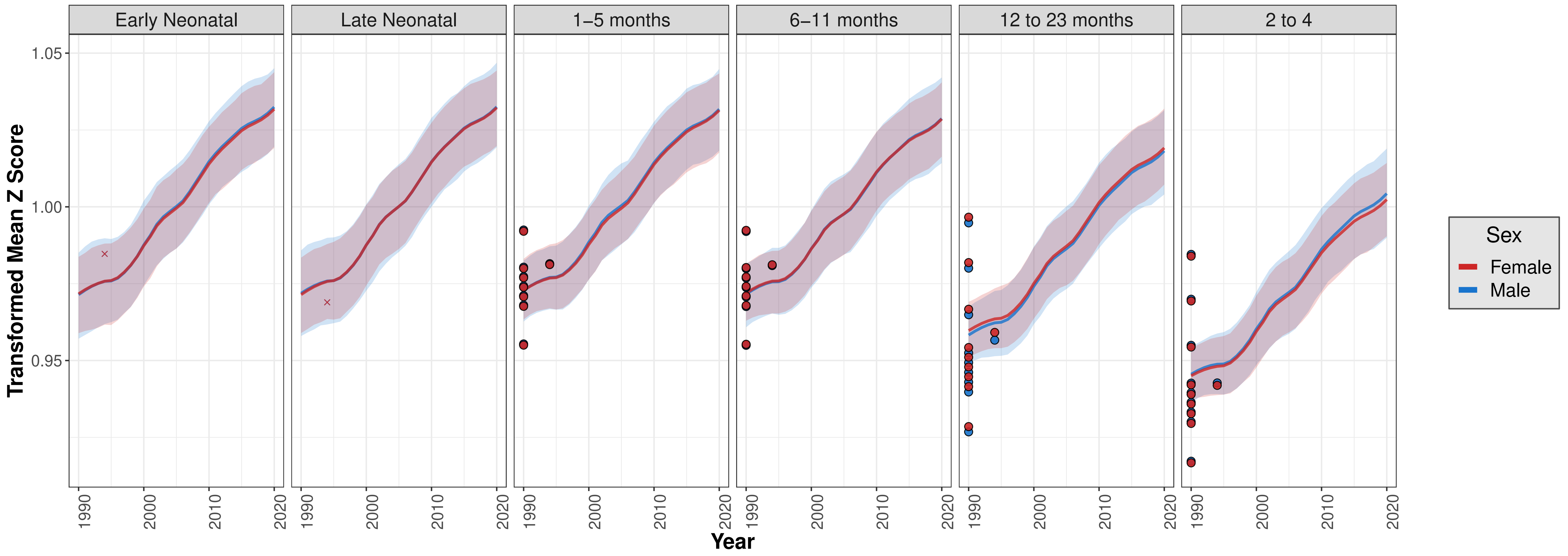

I

Year	Source
1983	WHO CGM Database
1990	WHO CGM Database
1994	WHO CGM Database

Cabo Verde – HAZ, WHZ, and WAZ Distributions

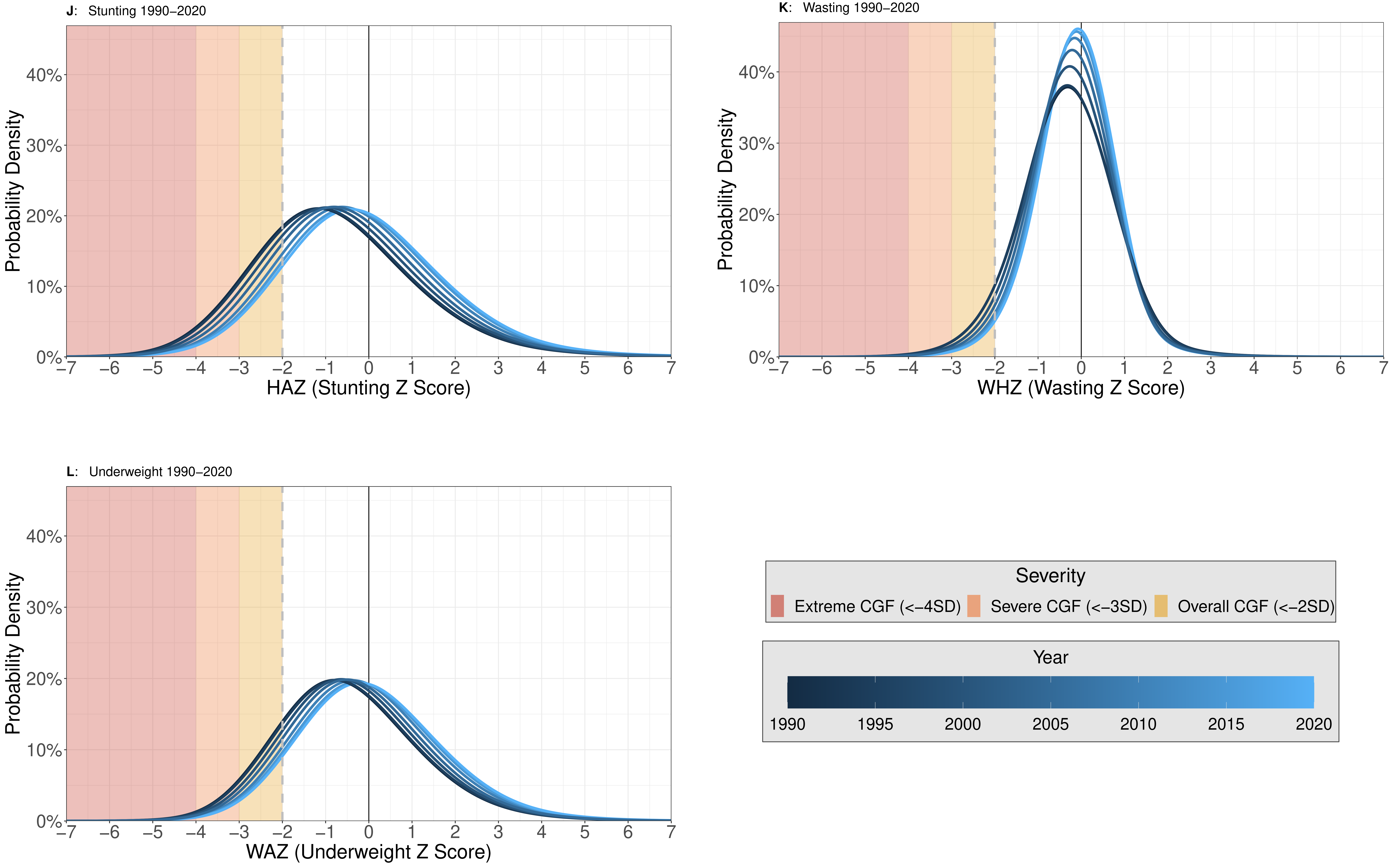

Chad – Stunting (HAZ)

A: Overall and Severe Stunting Prevalence

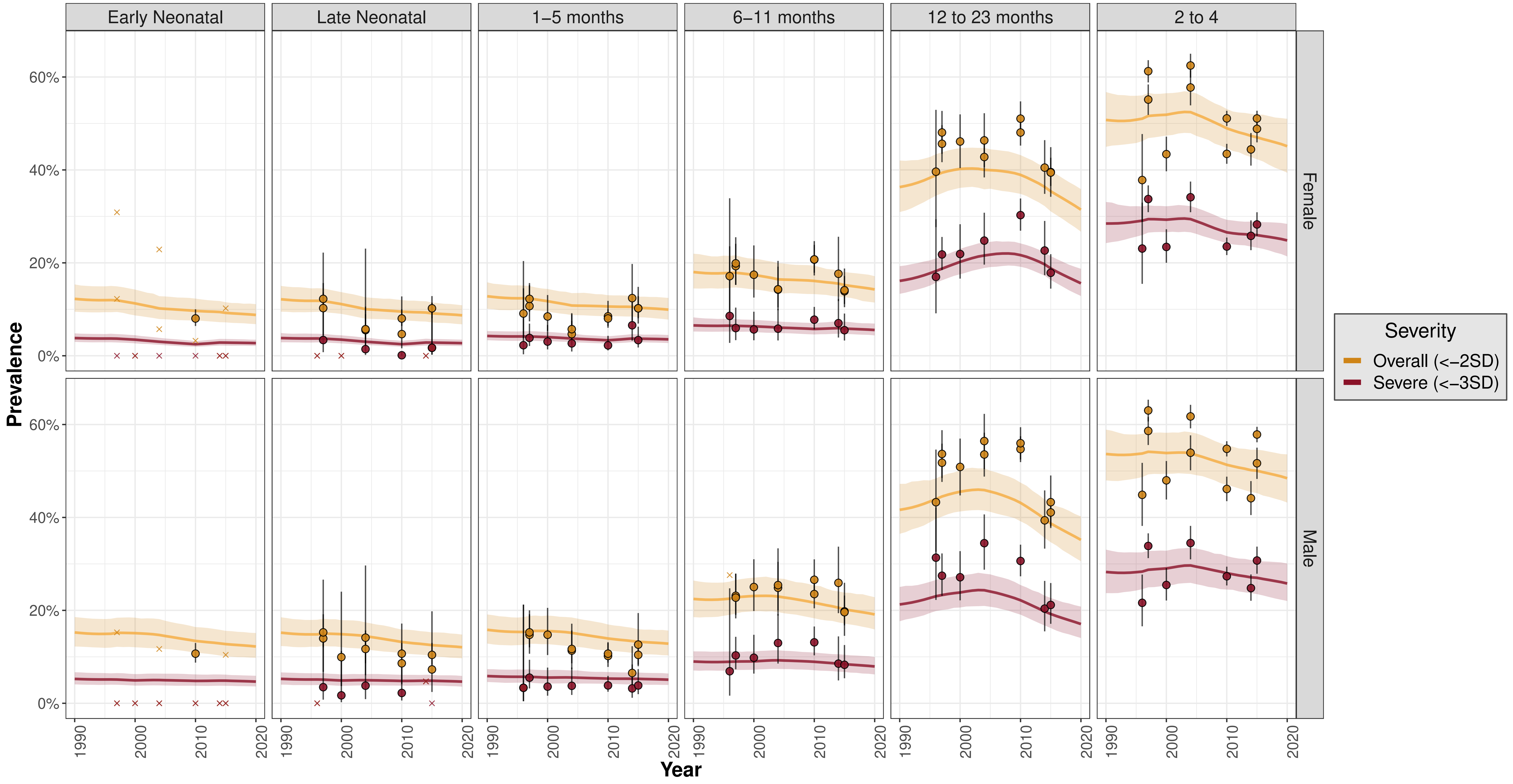

B: Transformed Mean Stunting Z Scores

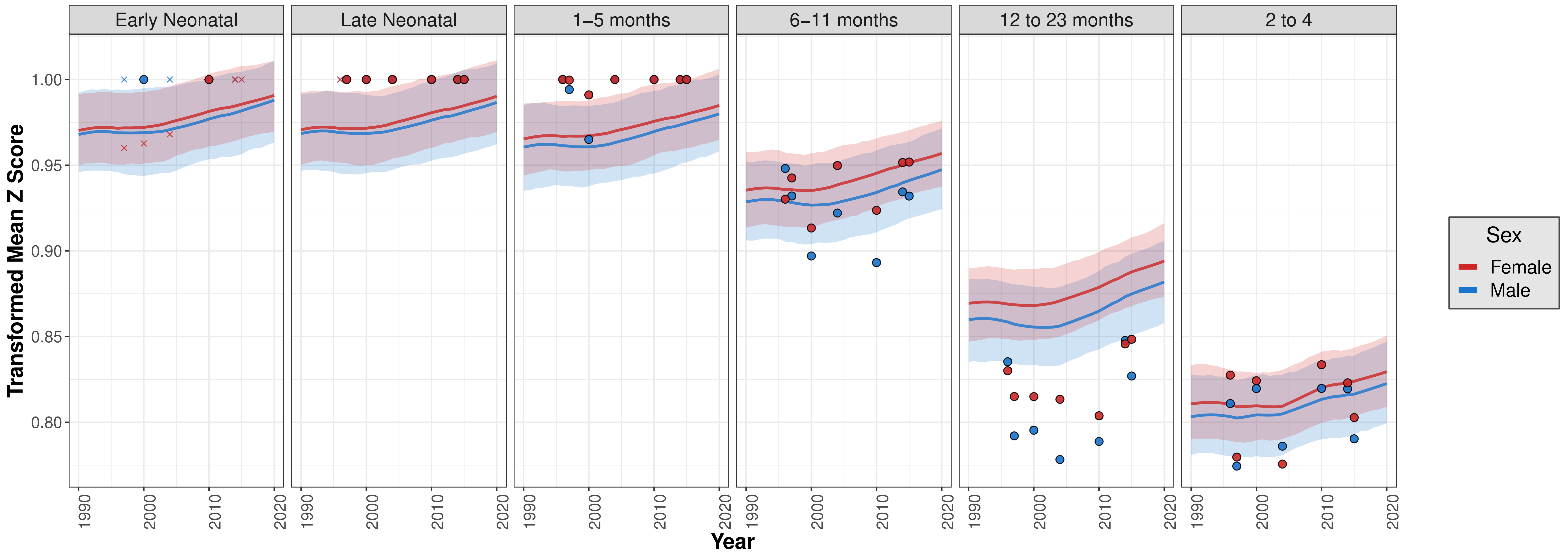

C

Year	Source
1996	DHS
1997	DHS
1997	WHO CGM Database
2000	MICS
2004	DHS
2004	WHO CGM Database
2010	MICS
2010	WHO CGM Database
2014	DHS
2015	WHO CGM Database
2015	DHS

Chad – Wasting (WHZ)

D: Overall and Severe Wasting Prevalence

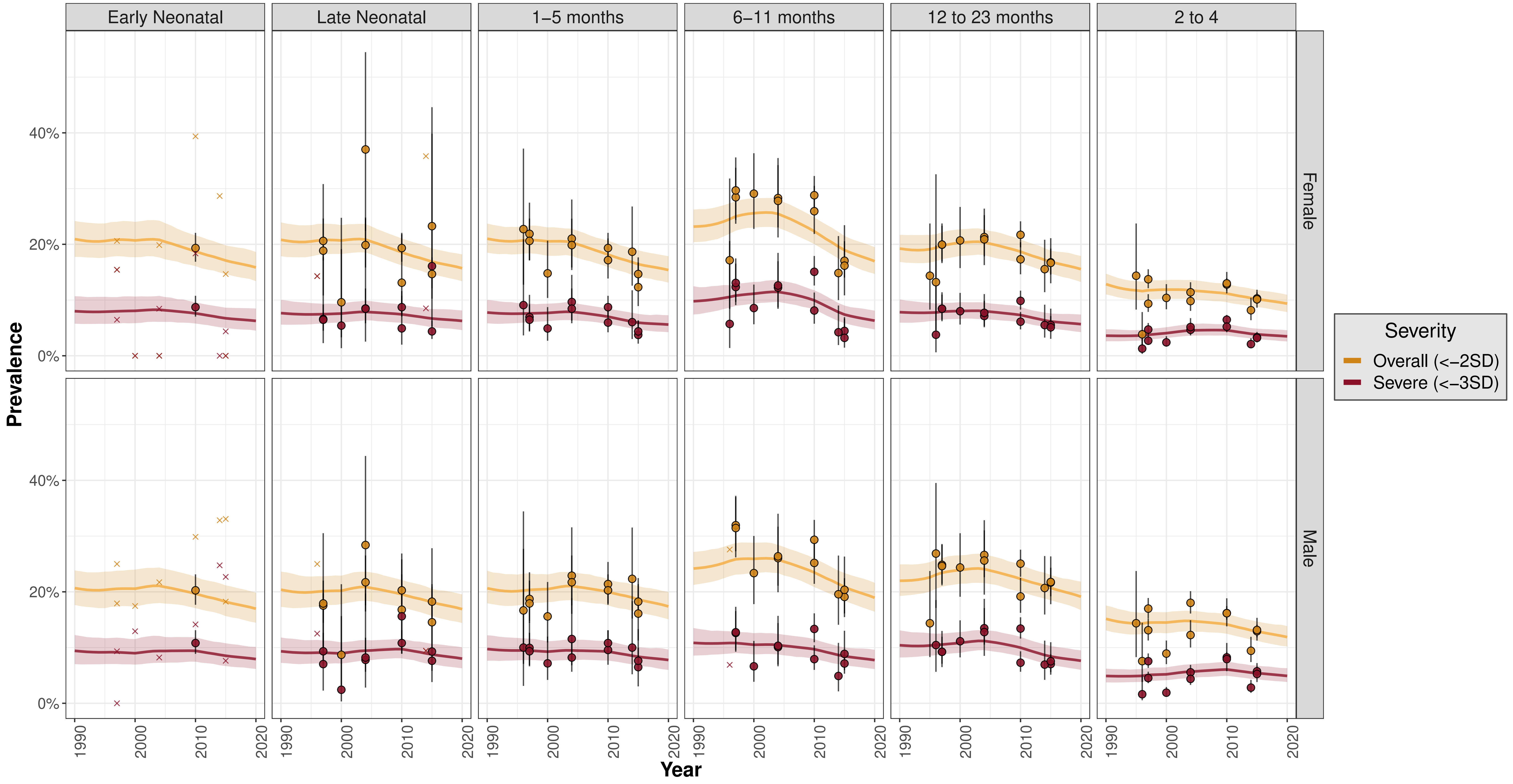

E: Transformed Mean Wasting Z Scores

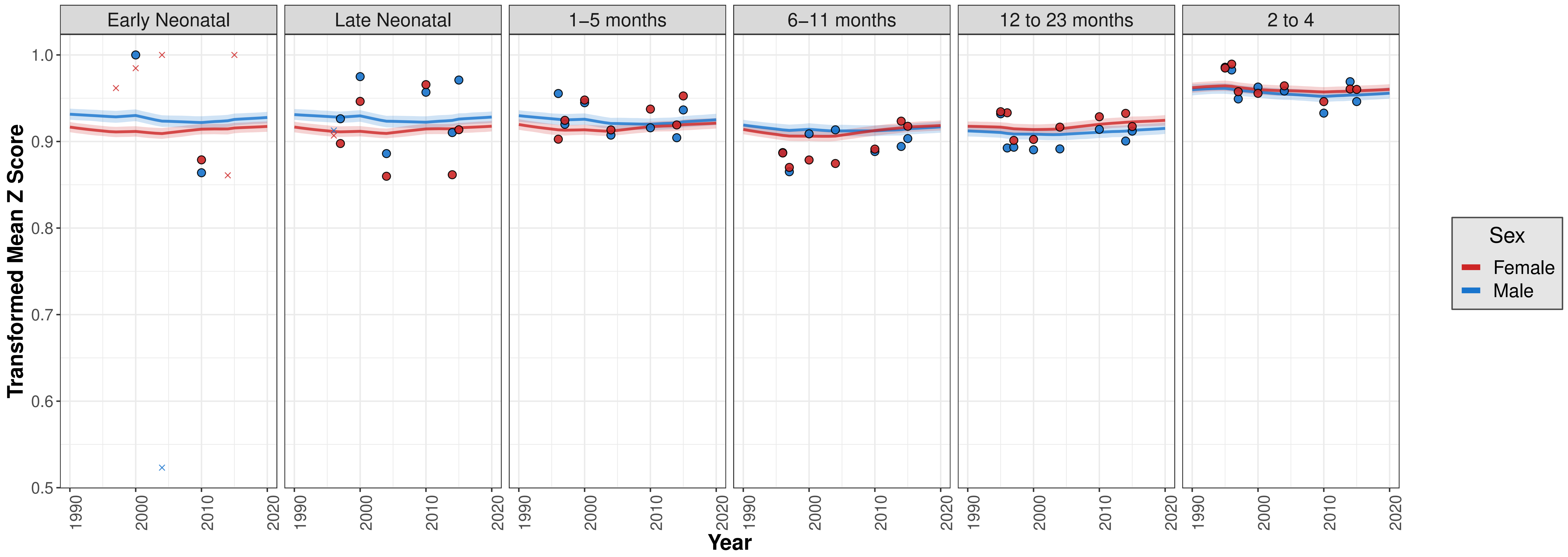

F

Year	Source
1988	WHO CGM Database
1995	WHO CGM Database
1996	DHS
1997	DHS
1997	WHO CGM Database
2000	MICS
2004	DHS
2004	WHO CGM Database
2010	MICS
2010	WHO CGM Database
2014	DHS
2015	WHO CGM Database
2015	DHS

Chad – Underweight (WAZ)

G: Overall and Severe Underweight Prevalence

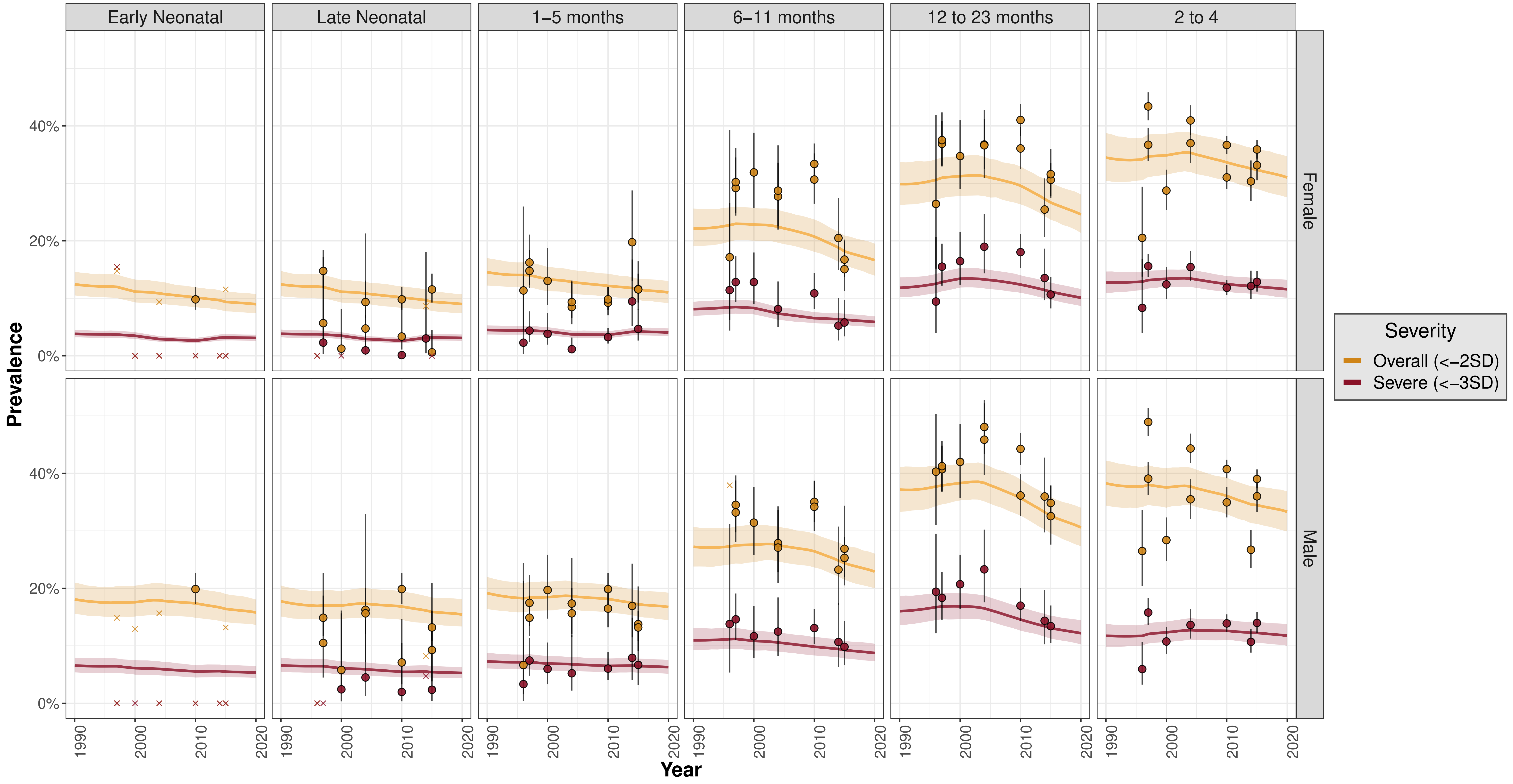

H: Transformed Mean Underweight Z Scores

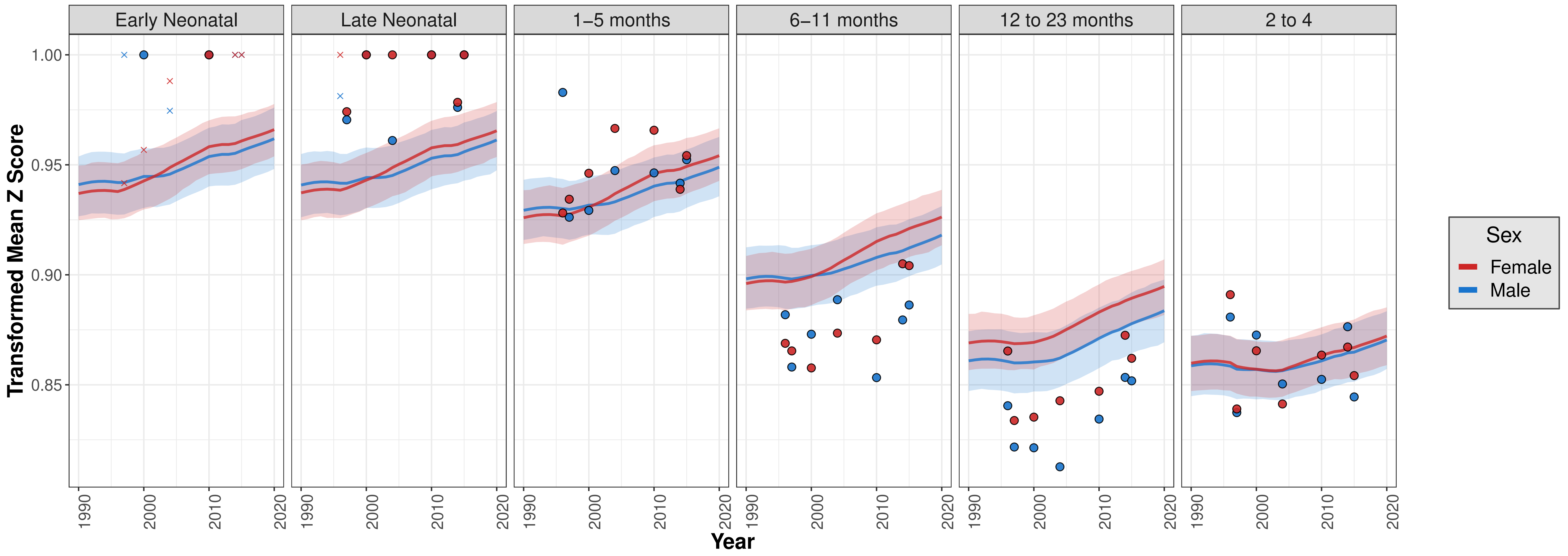

I

Year	Source
1996	DHS
1997	DHS
1997	WHO CGM Database
2000	MICS
2004	DHS
2004	WHO CGM Database
2010	MICS
2010	WHO CGM Database
2014	DHS
2015	WHO CGM Database
2015	DHS

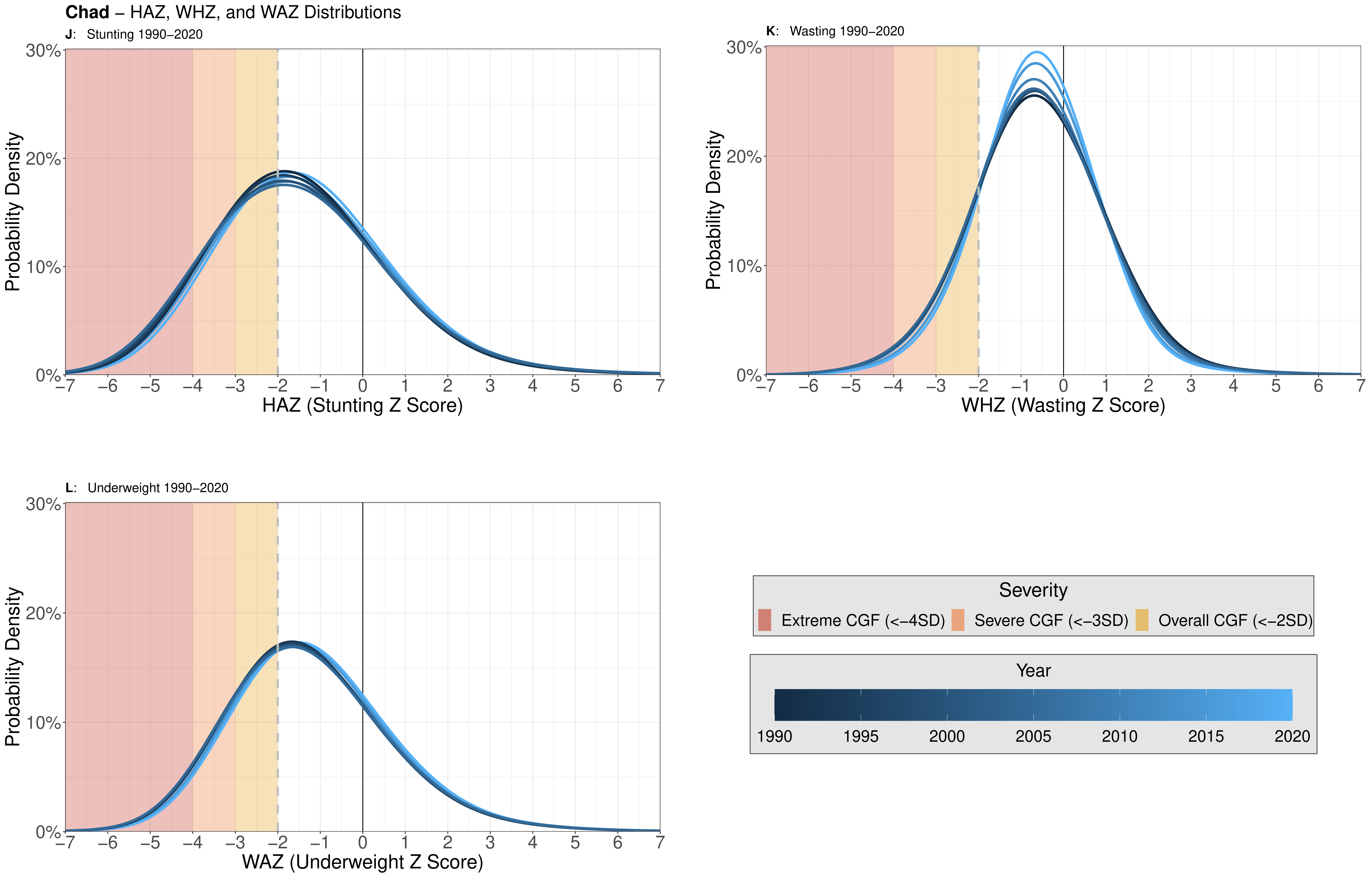

Chad – HAZ, WHZ, and WAZ Distributions
J: Stunting 1990–2020
K: Wasting 1990–2020
L: Underweight 1990–2020
Probability Density
30%
20%
10%
0%
−7 −6 −5 −4 −3 −2 −1 0 1 2 3 4 5 6 7
HAZ (Stunting Z Score)
WHZ (Wasting Z Score)
WAZ (Underweight Z Score)
Severity
Extreme CGF (<−4SD)
Severe CGF (<−3SD)
Overall CGF (<−2SD)
Year
1990
1995
2000
2005
2010
2015
2020

Côte d'Ivoire – Stunting (HAZ)

A: Overall and Severe Stunting Prevalence

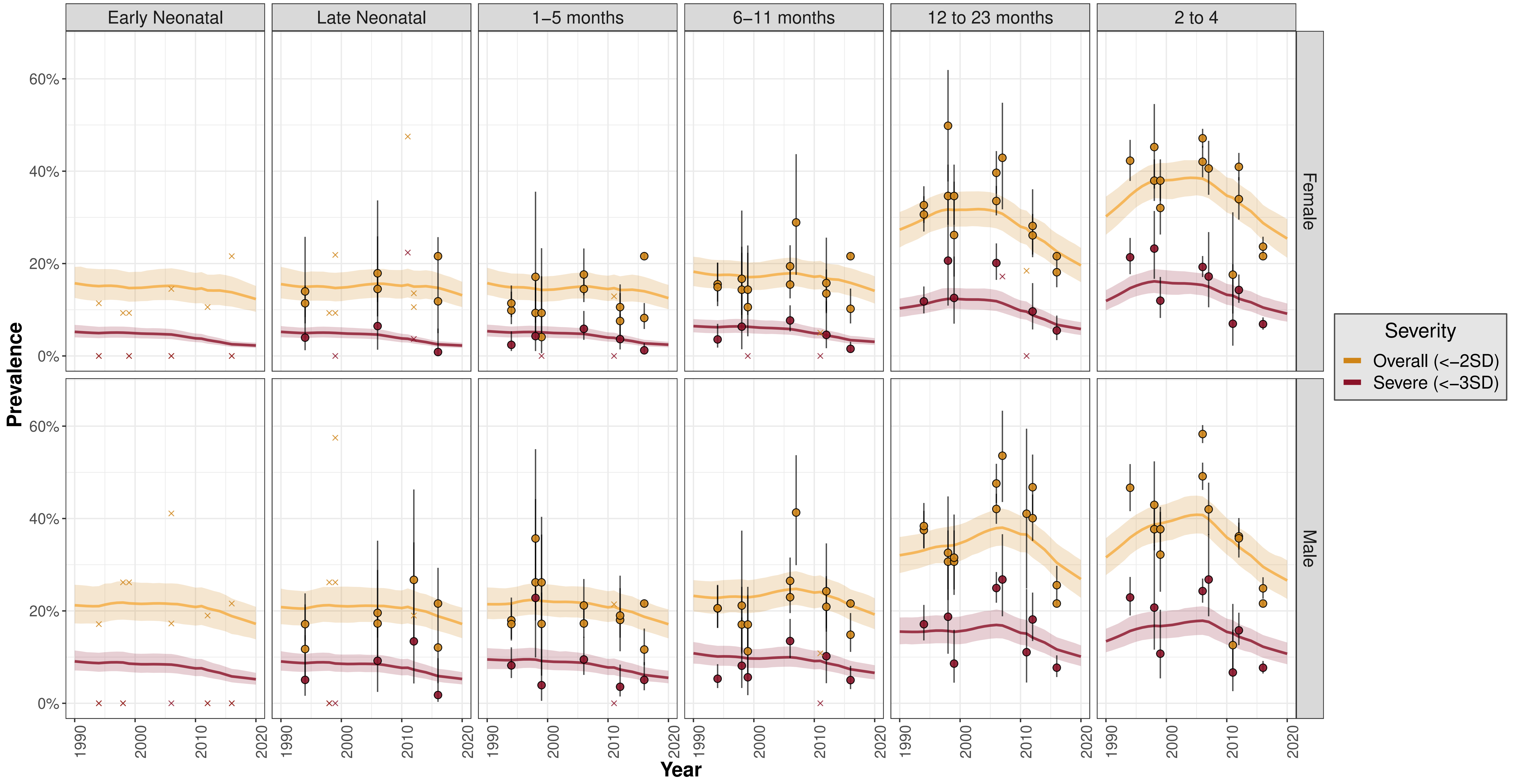

B: Transformed Mean Stunting Z Scores

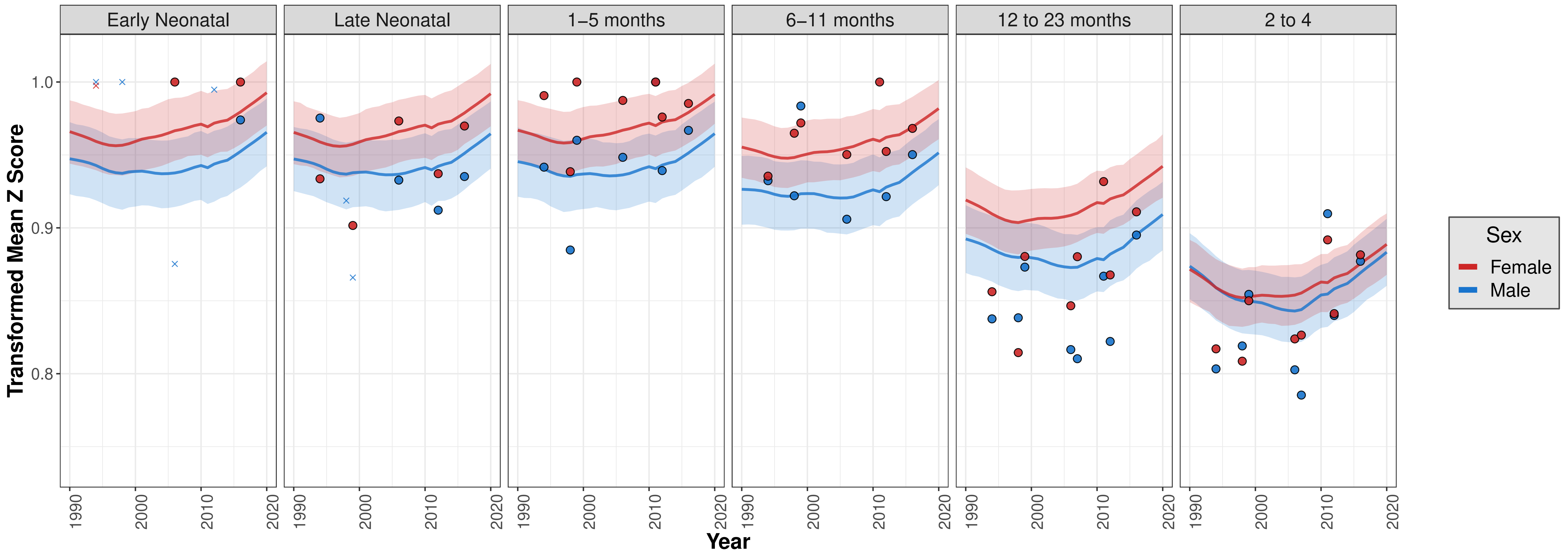

C

Year	Source
1986	Living Standards Measurement Survey
1986	Living Standards Measurement Survey
1986	WHO CGM Database
1987	Living Standards Measurement Survey
1987	Living Standards Measurement Survey
1988	Living Standards Measurement Survey
1988	Living Standards Measurement Survey
1989	Living Standards Measurement Survey
1994	DHS
1994	WHO CGM Database
1998	DHS
1998	WHO CGM Database
1999	DHS
1999	WHO CGM Database
2006	MICS
2006	WHO CGM Database
2007	WHO CGM Database
2011	DHS
2012	DHS
2012	WHO CGM Database
2016	WHO CGM Database
2016	Cote d'Ivoire MICS

Côte d'Ivoire – Wasting (WHZ)

D: Overall and Severe Wasting Prevalence

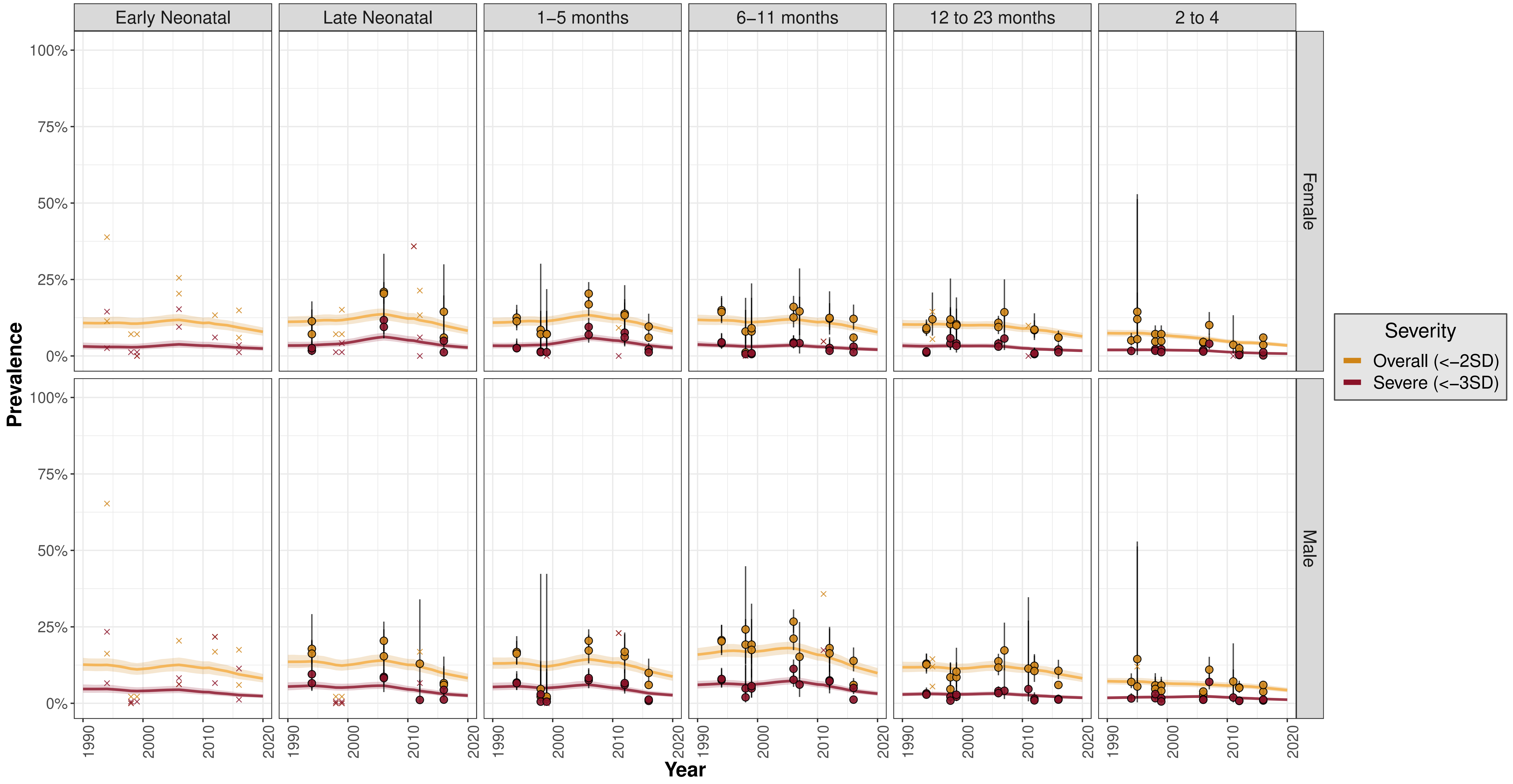

E: Transformed Mean Wasting Z Scores

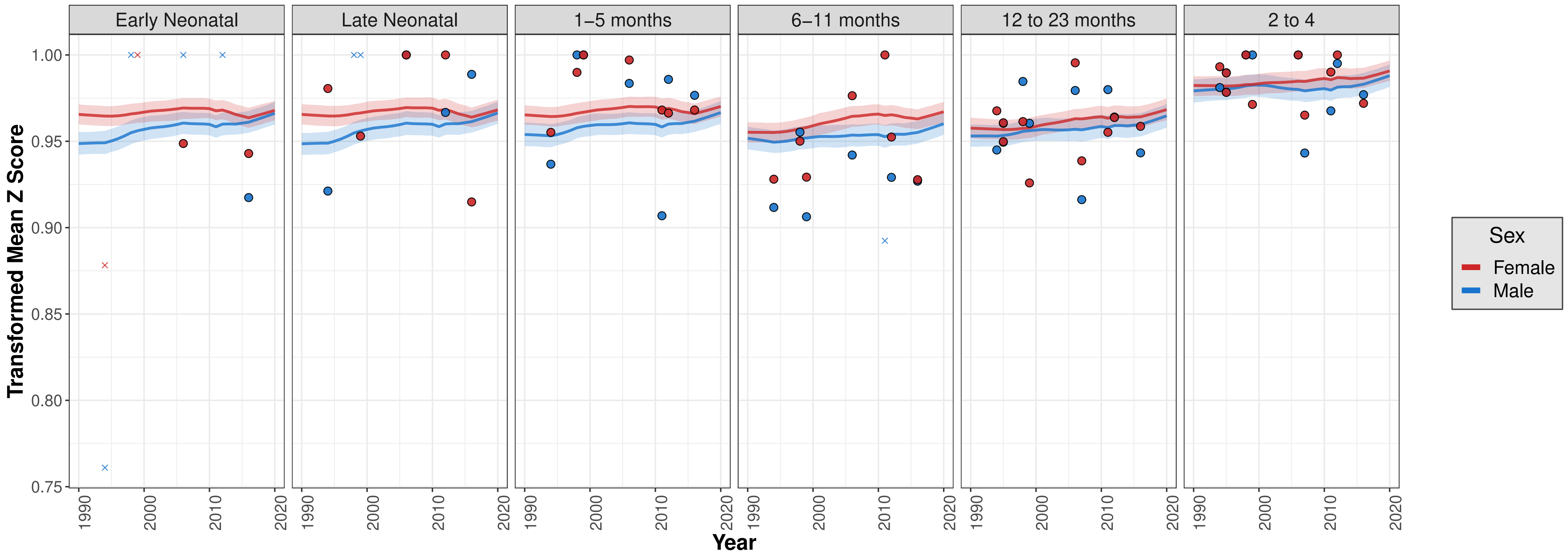

F

Year	Source
1986	Living Standards Measurement Survey
1986	Living Standards Measurement Survey
1986	WHO CGM Database
1987	Living Standards Measurement Survey
1987	Living Standards Measurement Survey
1988	Living Standards Measurement Survey
1988	Living Standards Measurement Survey
1989	Living Standards Measurement Survey
1994	DHS
1994	WHO CGM Database
1995	WHO CGM Database
1998	DHS
1998	WHO CGM Database
1999	DHS
1999	WHO CGM Database
2006	MICS
2006	WHO CGM Database
2007	WHO CGM Database
2011	DHS
2012	DHS
2012	WHO CGM Database
2016	WHO CGM Database
2016	Cote d'Ivoire MICS

Côte d'Ivoire – Underweight (WAZ)

G: Overall and Severe Underweight Prevalence

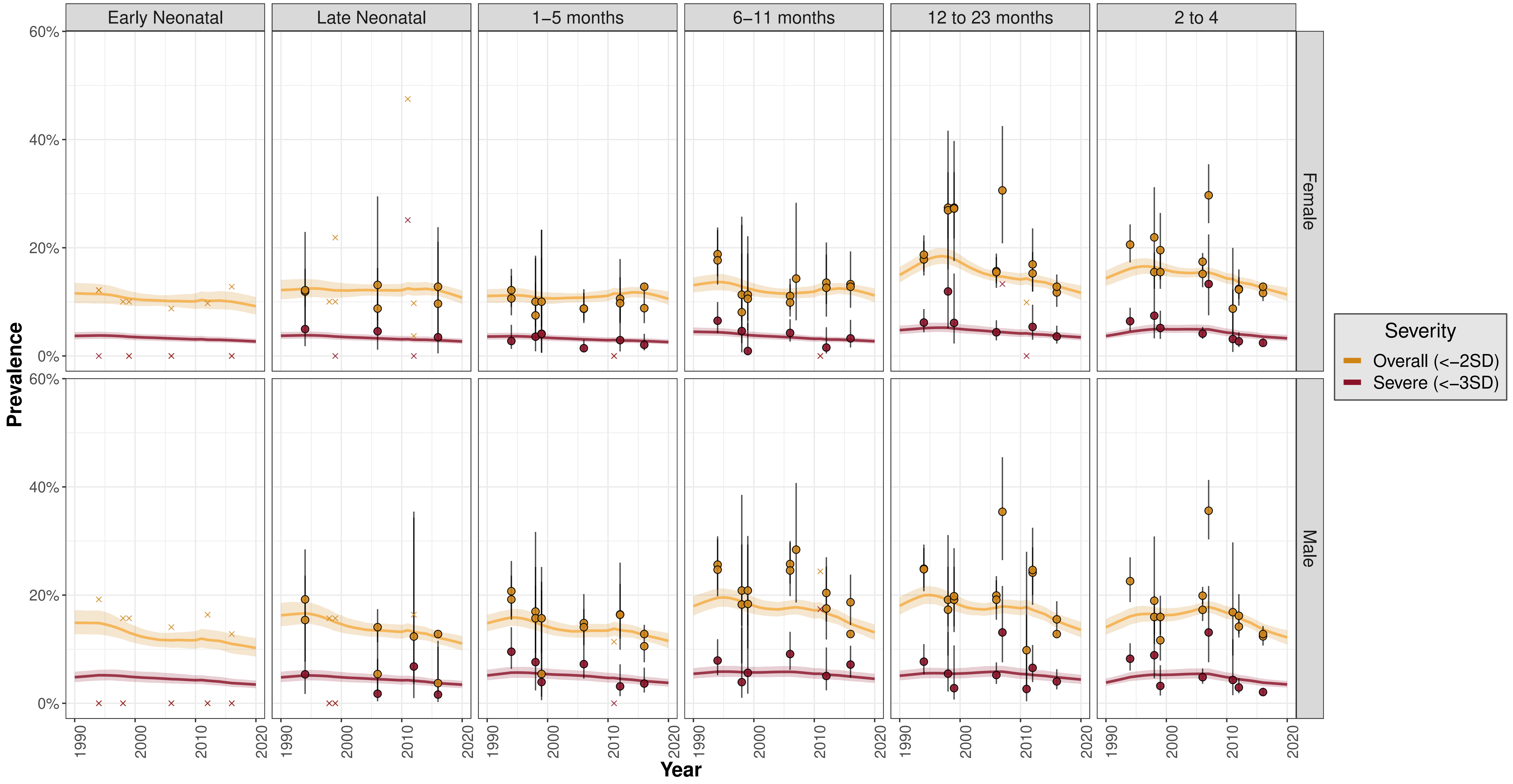

H: Transformed Mean Underweight Z Scores

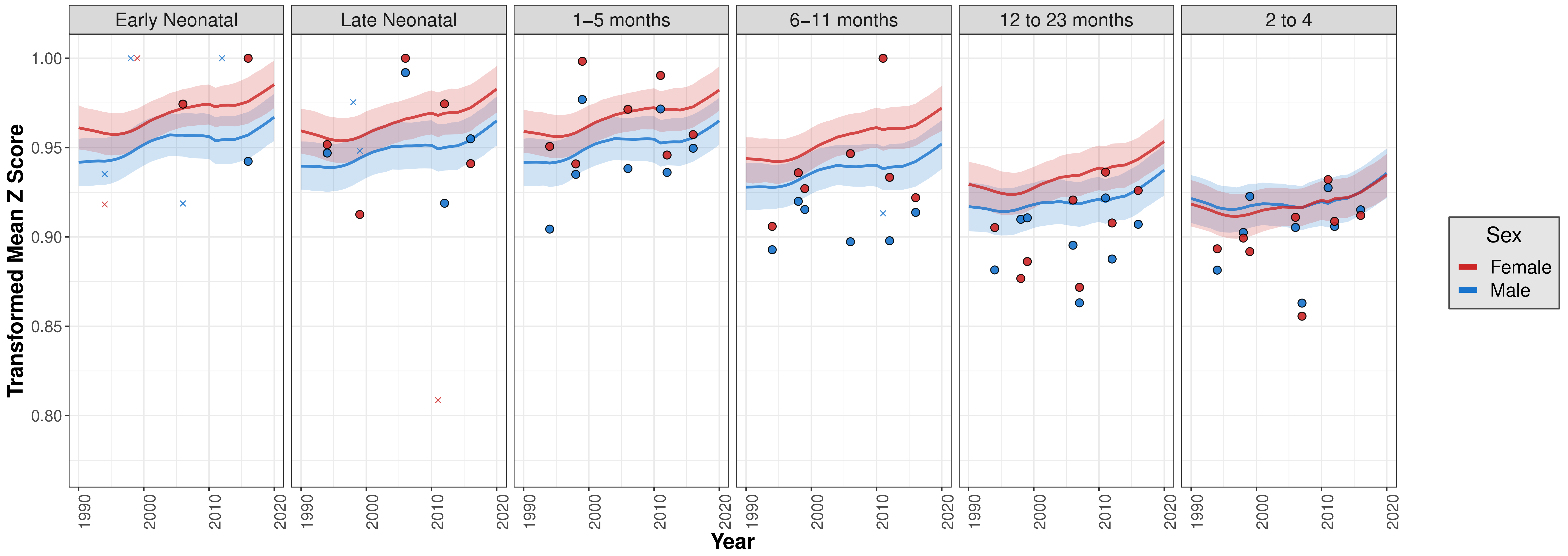

I

Year	Source
1986	Living Standards Measurement Survey
1986	Living Standards Measurement Survey
1986	WHO CGM Database
1987	Living Standards Measurement Survey
1987	Living Standards Measurement Survey
1988	Living Standards Measurement Survey
1988	Living Standards Measurement Survey
1989	Living Standards Measurement Survey
1994	DHS
1994	WHO CGM Database
1998	DHS
1998	WHO CGM Database
1999	DHS
1999	WHO CGM Database
2006	MICS
2006	WHO CGM Database
2007	WHO CGM Database
2011	DHS
2012	DHS
2012	WHO CGM Database
2016	WHO CGM Database
2016	Cote d'Ivoire MICS

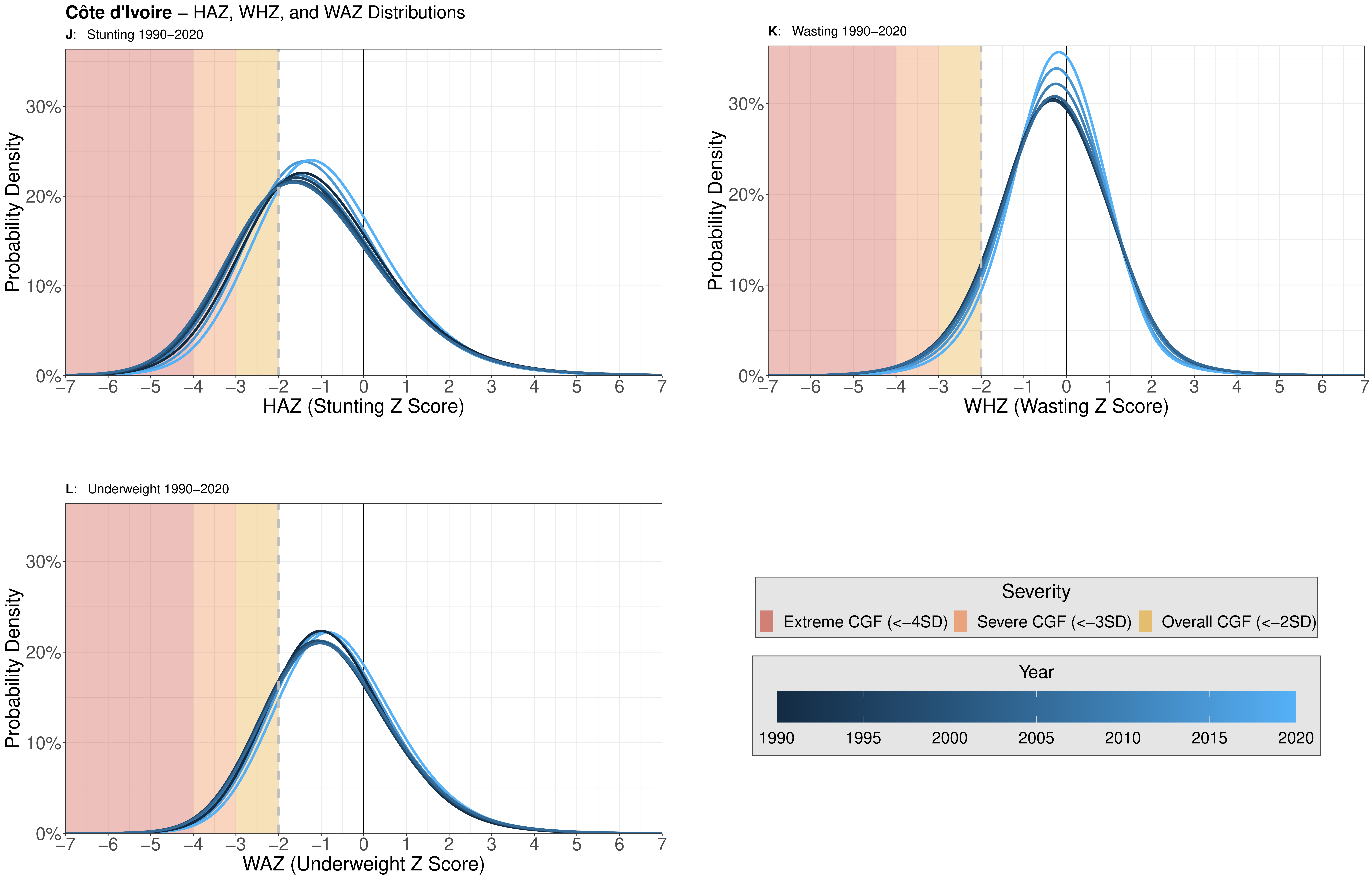

Côte d'Ivoire – HAZ, WHZ, and WAZ Distributions
J: Stunting 1990–2020
Probability Density
0%
10%
20%
30%
−7 −6 −5 −4 −3 −2 −1 0 1 2 3 4 5 6 7
HAZ (Stunting Z Score)
K: Wasting 1990–2020
Probability Density
WHZ (Wasting Z Score)
L: Underweight 1990–2020
Probability Density
WAZ (Underweight Z Score)
Severity
Extreme CGF (<−4SD)
Severe CGF (<−3SD)
Overall CGF (<−2SD)
Year
1990 1995 2000 2005 2010 2015 2020

Gambia – Stunting (HAZ)

A: Overall and Severe Stunting Prevalence

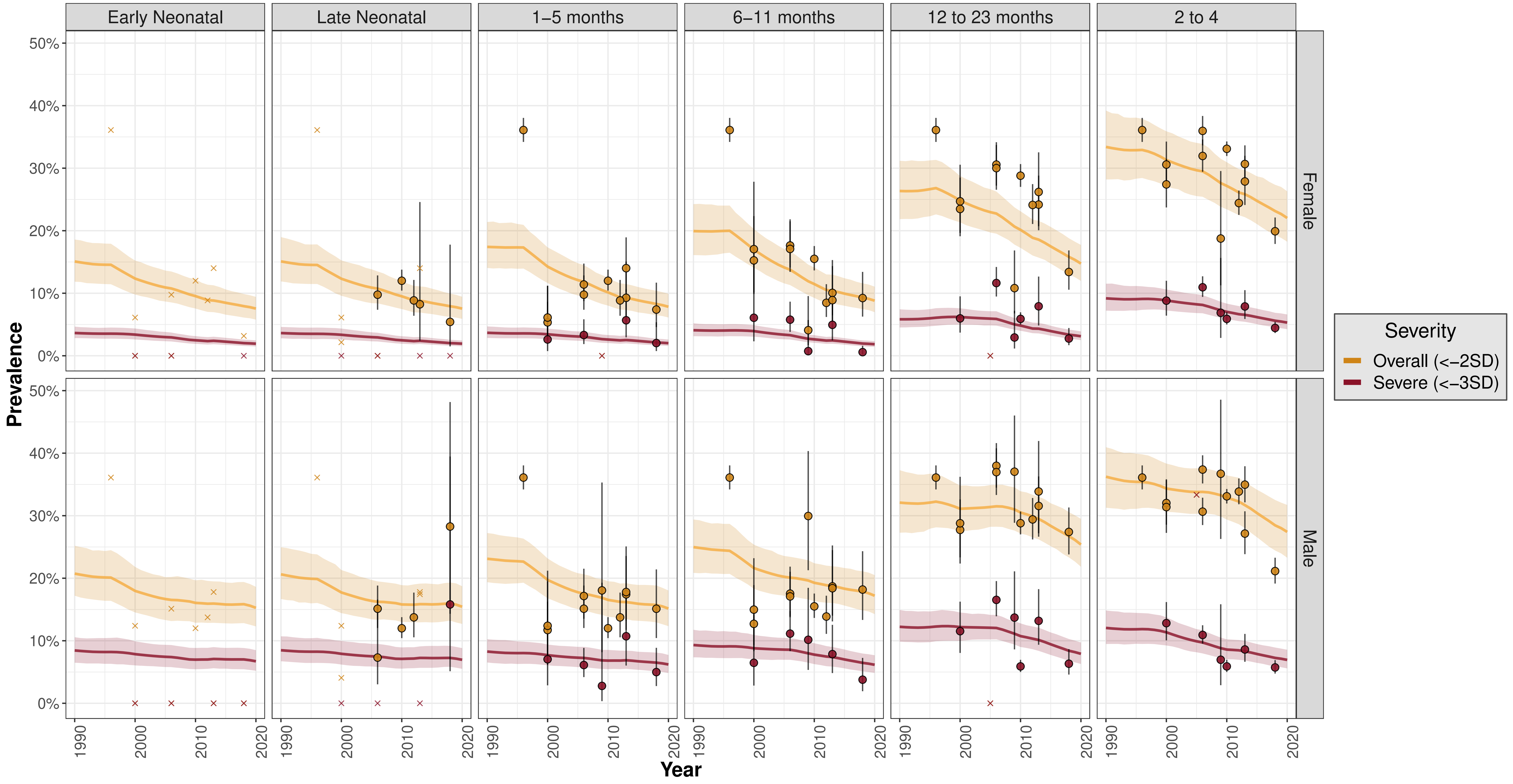

B: Transformed Mean Stunting Z Scores

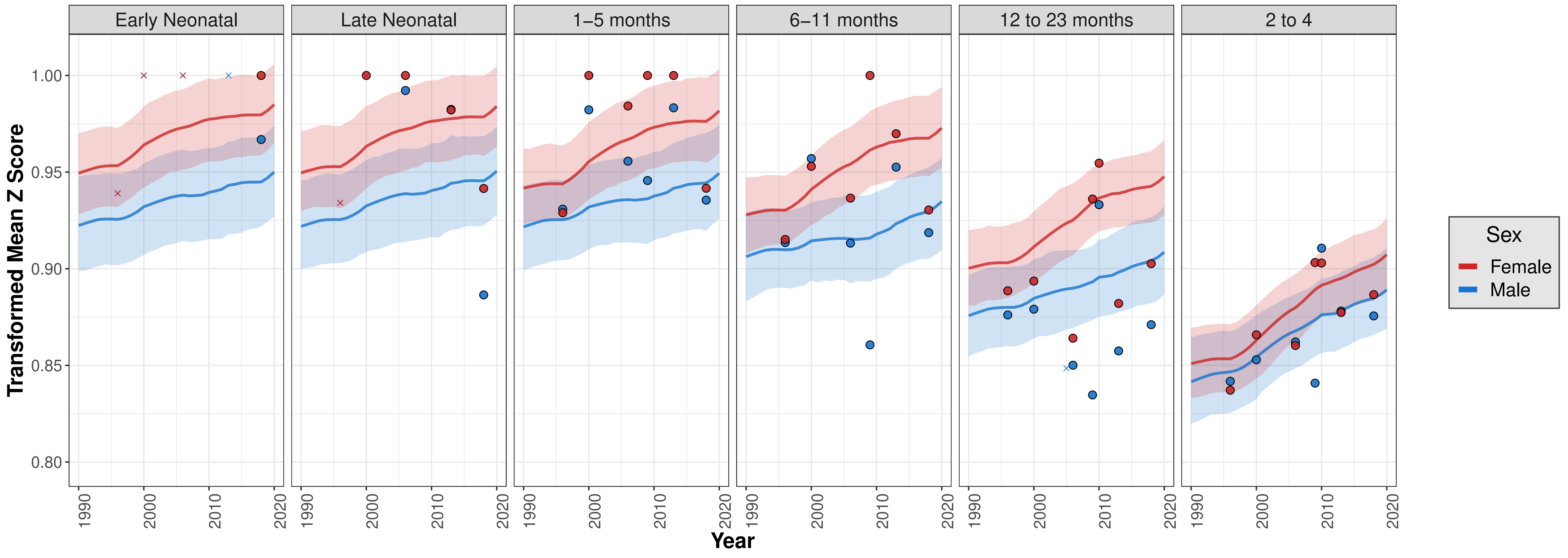

C

Year	Source
1996	WHO CGM Database
2000	MICS
2000	WHO CGM Database
2005	MICS
2006	MICS
2006	WHO CGM Database
2009	Basse Global Enteric Multicenter Study
2010	WHO CGM Database
2012	WHO CGM Database
2012	Basse Global Enteric Multicenter Study
2013	DHS
2013	WHO CGM Database
2018	MICS

Gambia – Wasting (WHZ)

D: Overall and Severe Wasting Prevalence

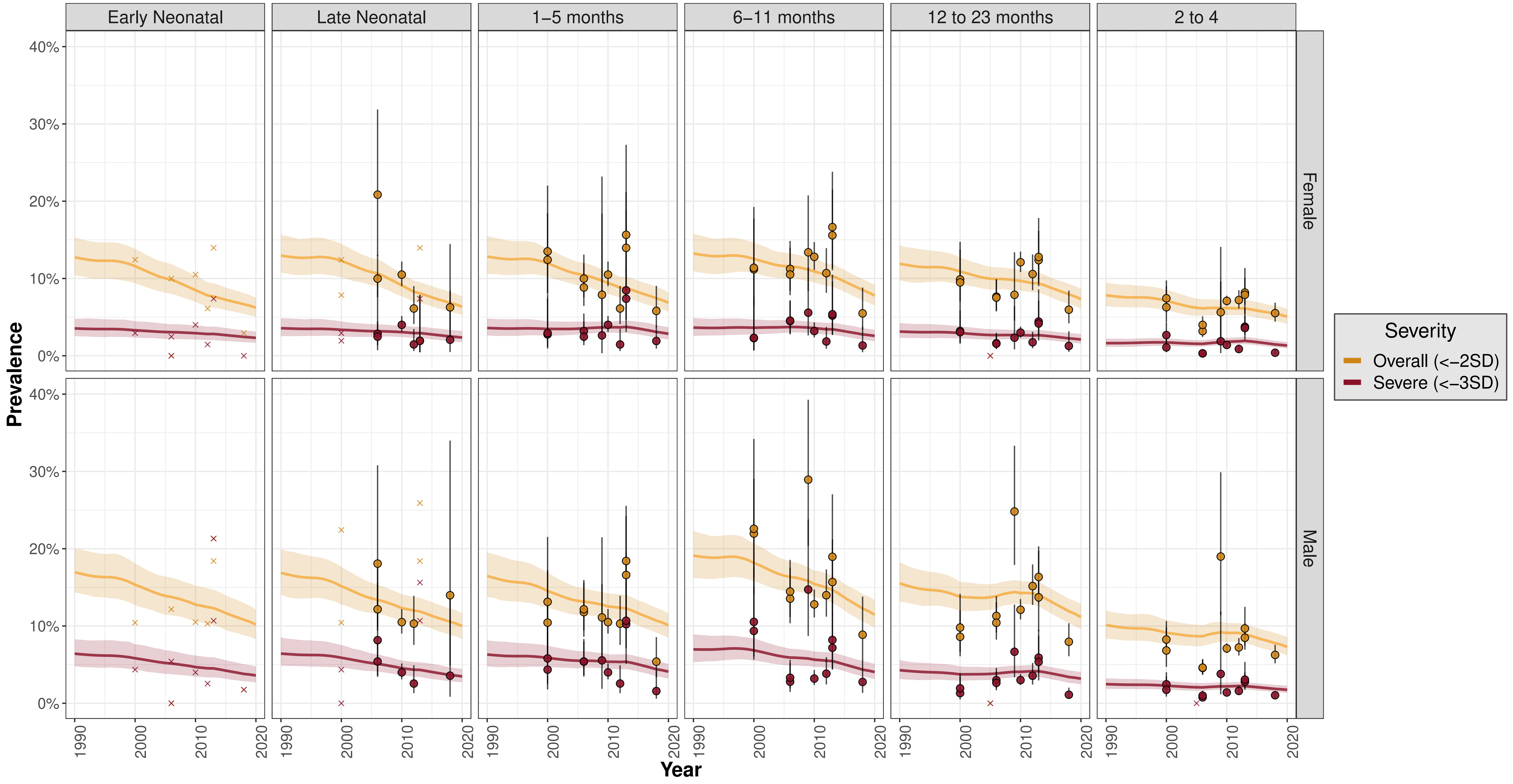

E: Transformed Mean Wasting Z Scores

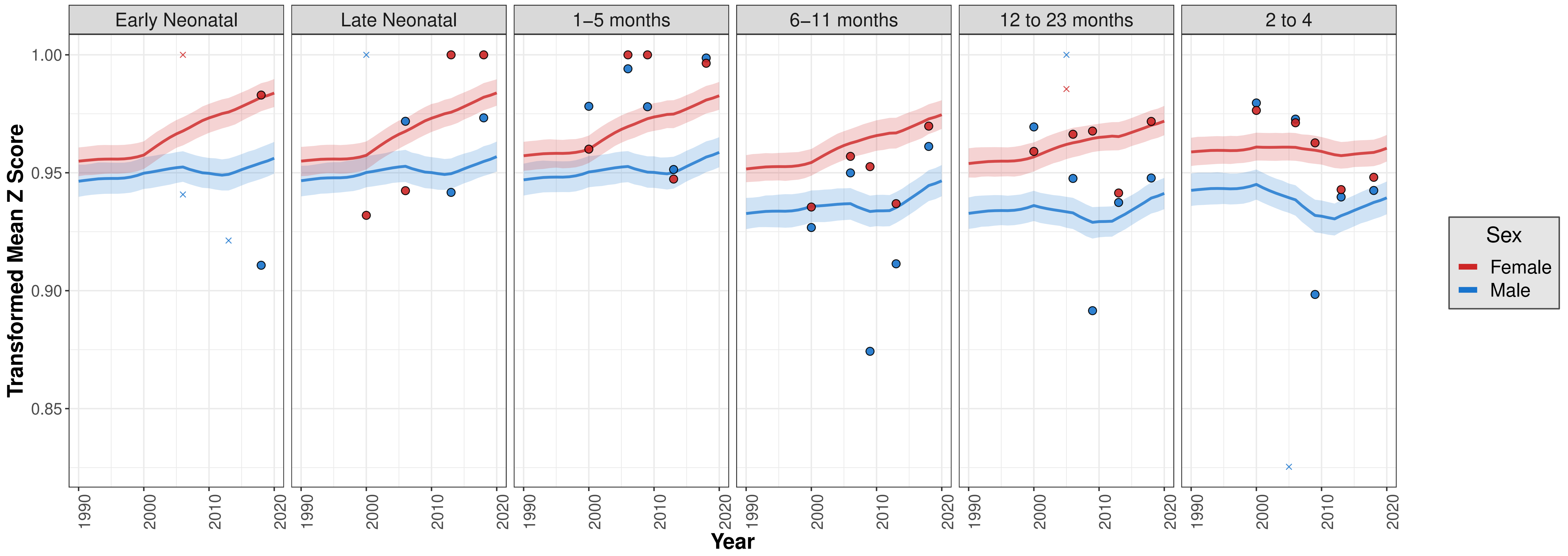

F

Year	Source
2000	MICS
2000	WHO CGM Database
2005	MICS
2006	MICS
2006	WHO CGM Database
2009	Basse Global Enteric Multicenter Study
2010	WHO CGM Database
2012	WHO CGM Database
2012	Basse Global Enteric Multicenter Study
2013	DHS
2013	WHO CGM Database
2018	MICS

Gambia – Underweight (WAZ)

G: Overall and Severe Underweight Prevalence

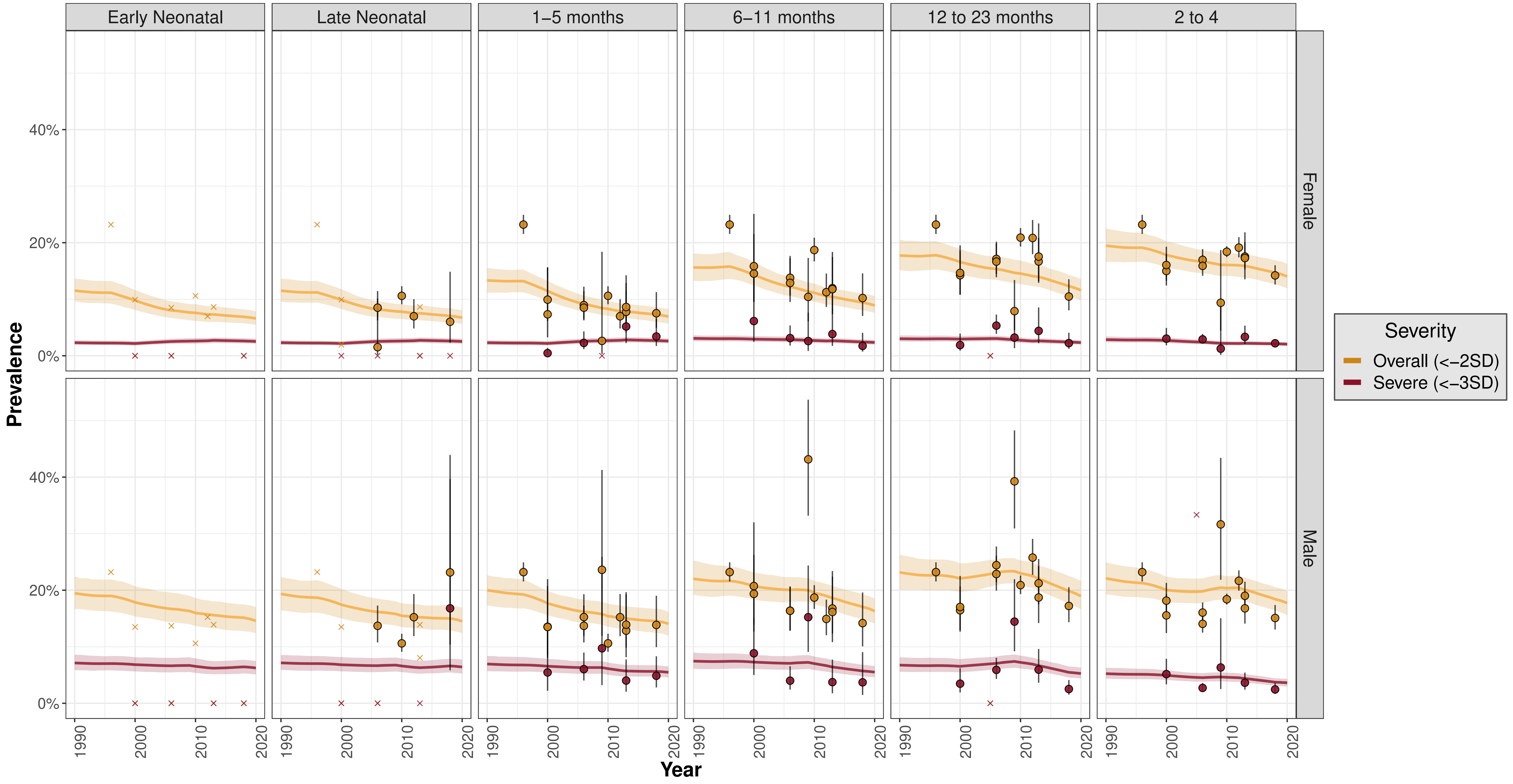

H: Transformed Mean Underweight Z Scores

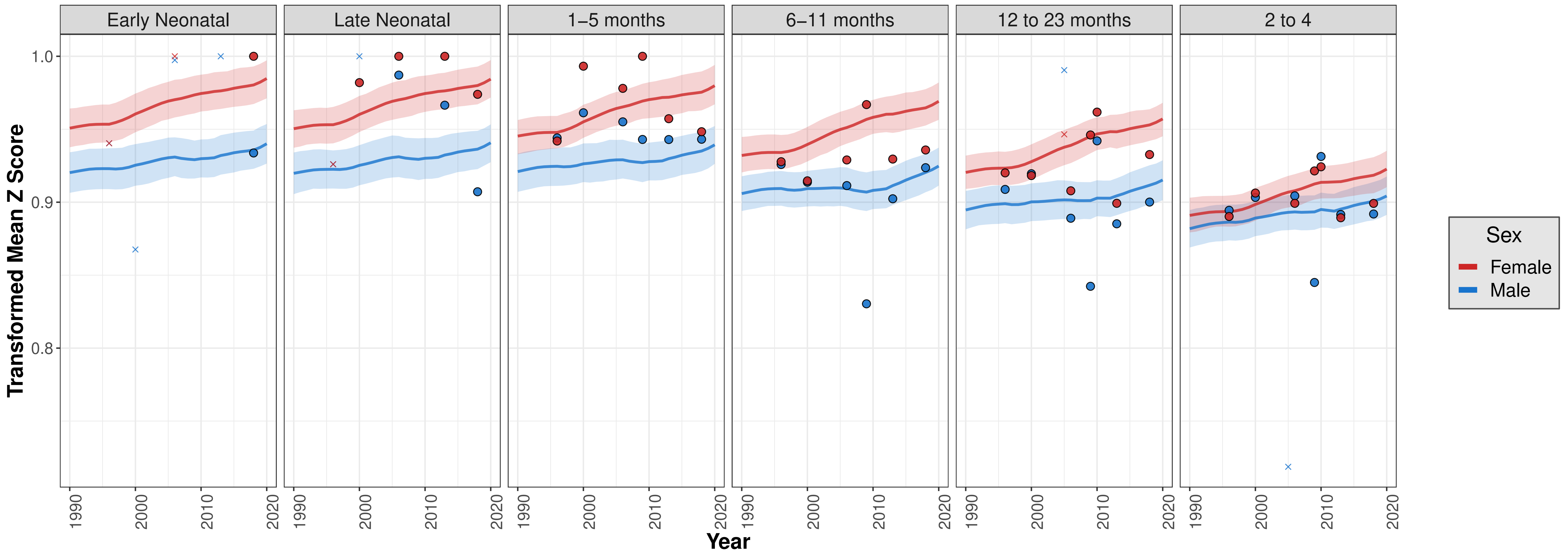

I

Year	Source
1996	WHO CGM Database
2000	MICS
2000	WHO CGM Database
2005	MICS
2006	MICS
2006	WHO CGM Database
2009	Basse Global Enteric Multicenter Study
2010	WHO CGM Database
2012	WHO CGM Database
2012	Basse Global Enteric Multicenter Study
2013	DHS
2013	WHO CGM Database
2018	MICS

Gambia – HAZ, WHZ, and WAZ Distributions

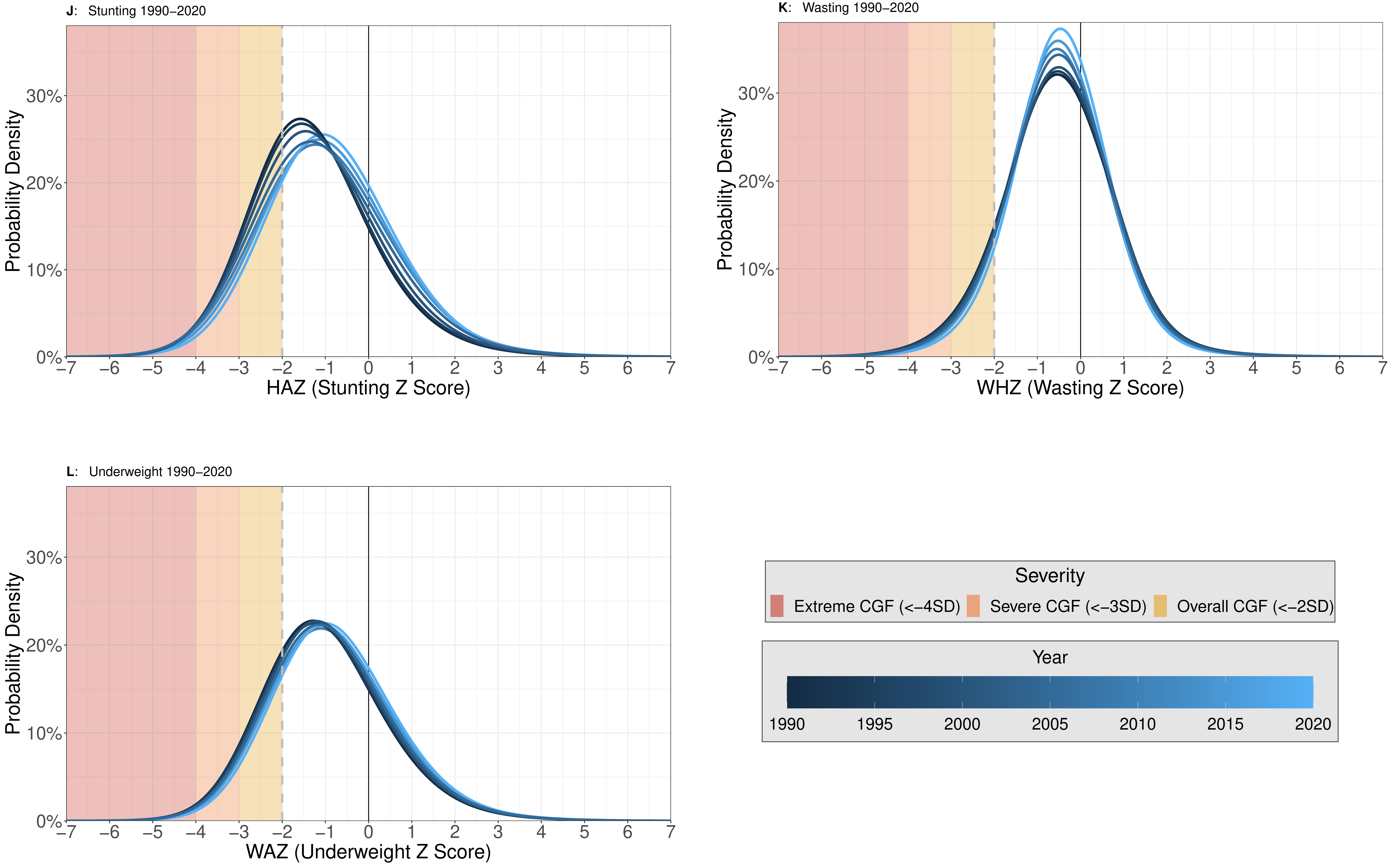

Ghana – Stunting (HAZ)

A: Overall and Severe Stunting Prevalence

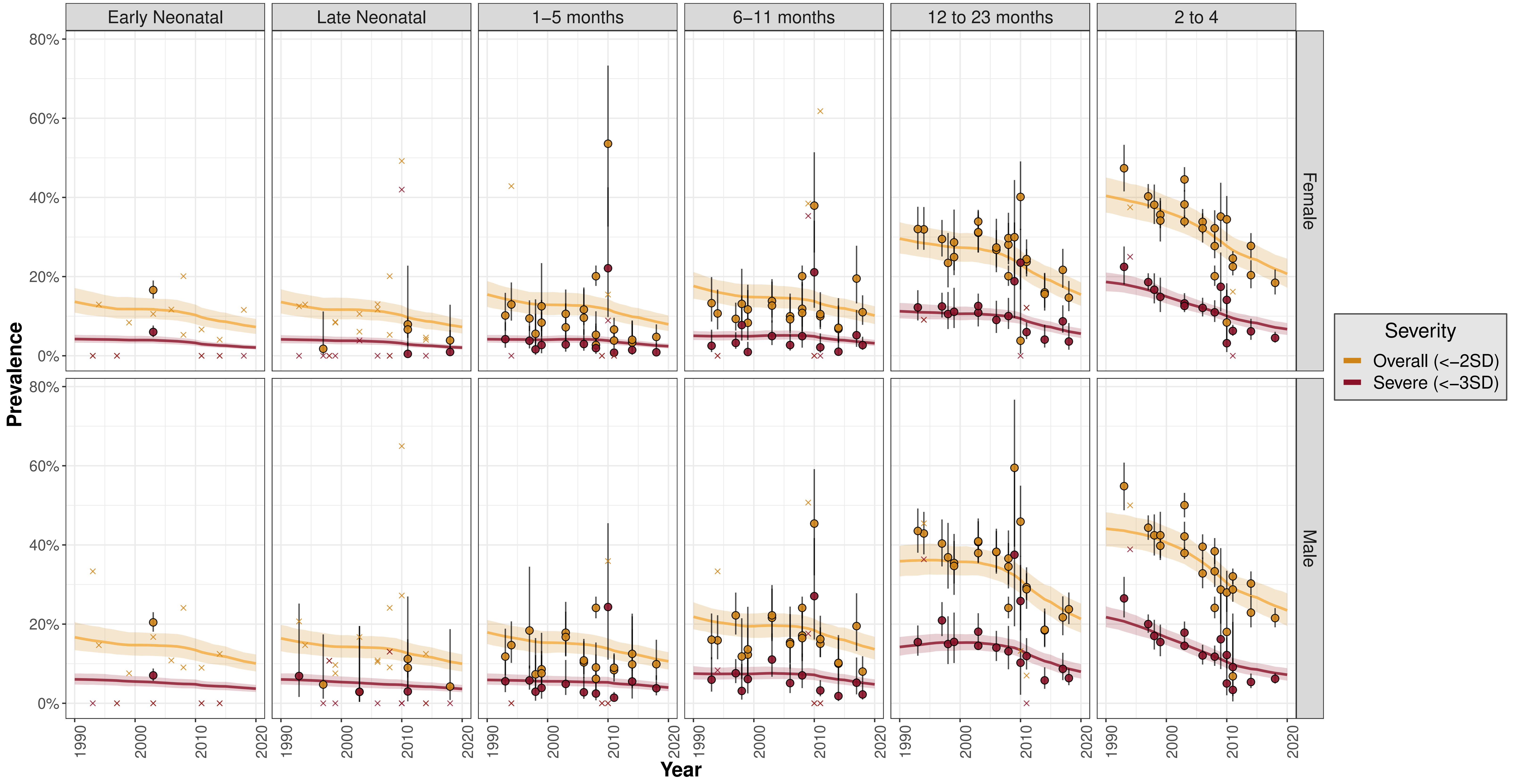

B: Transformed Mean Stunting Z Scores

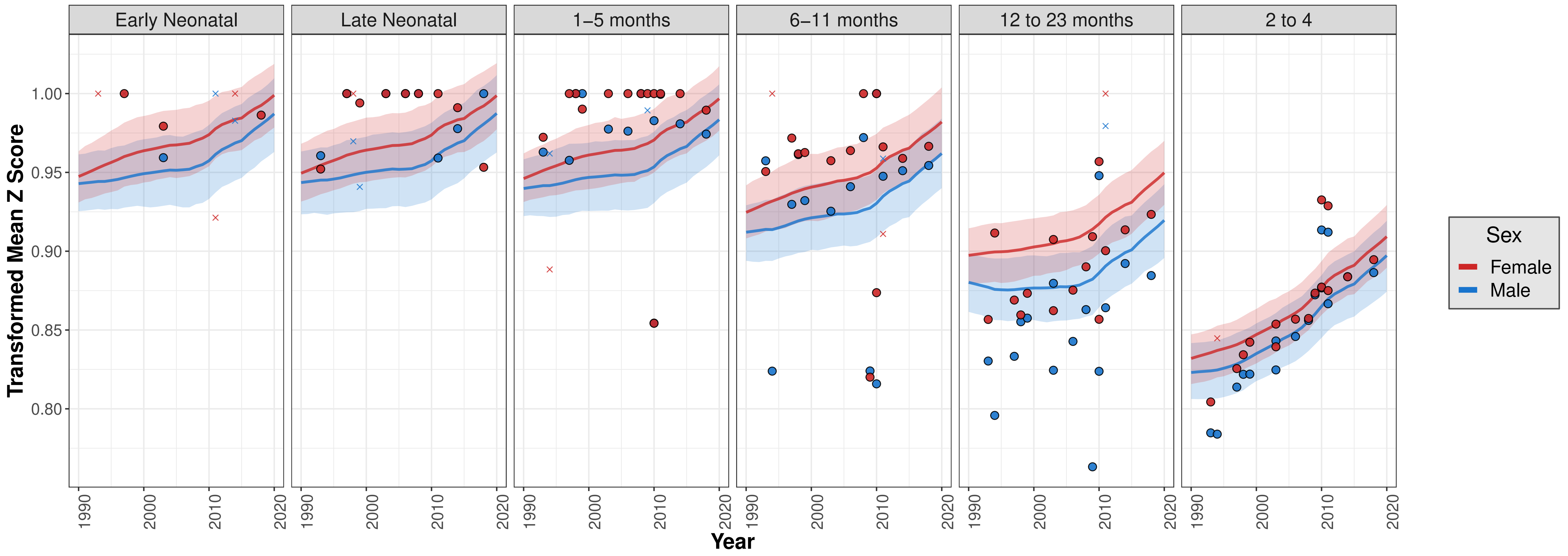

C

Year	Source
1988	Living Standards Measurement Survey
1988	Living Standards Measurement Survey
1988	DHS
1988	WHO CGM Database
1989	Living Standards Measurement Survey
1993	DHS
1994	DHS
1994	WHO CGM Database
1997	Core Welfare Indicators Survey
1998	DHS
1999	DHS
1999	WHO CGM Database
2003	DHS
2003	Core Welfare Indicators Questionnaire Survey
2003	WHO CGM Database
2006	MICS
2006	WHO CGM Database
2007	District MICS
2008	DHS
2008	WHO CGM Database
2008	District MICS
2008	Comprehensive Food Security and Vulnerability Analysis
2009	Socioeconomic Panel Survey
2010	Accra MICS
2010	Socioeconomic Panel Survey
2011	Accra MICS
2011	MICS
2011	WHO CGM Database
2014	WHO CGM Database
2014	DHS
2017	Micronutrient Survey
2018	MICS

Ghana – Wasting (WHZ)

D: Overall and Severe Wasting Prevalence

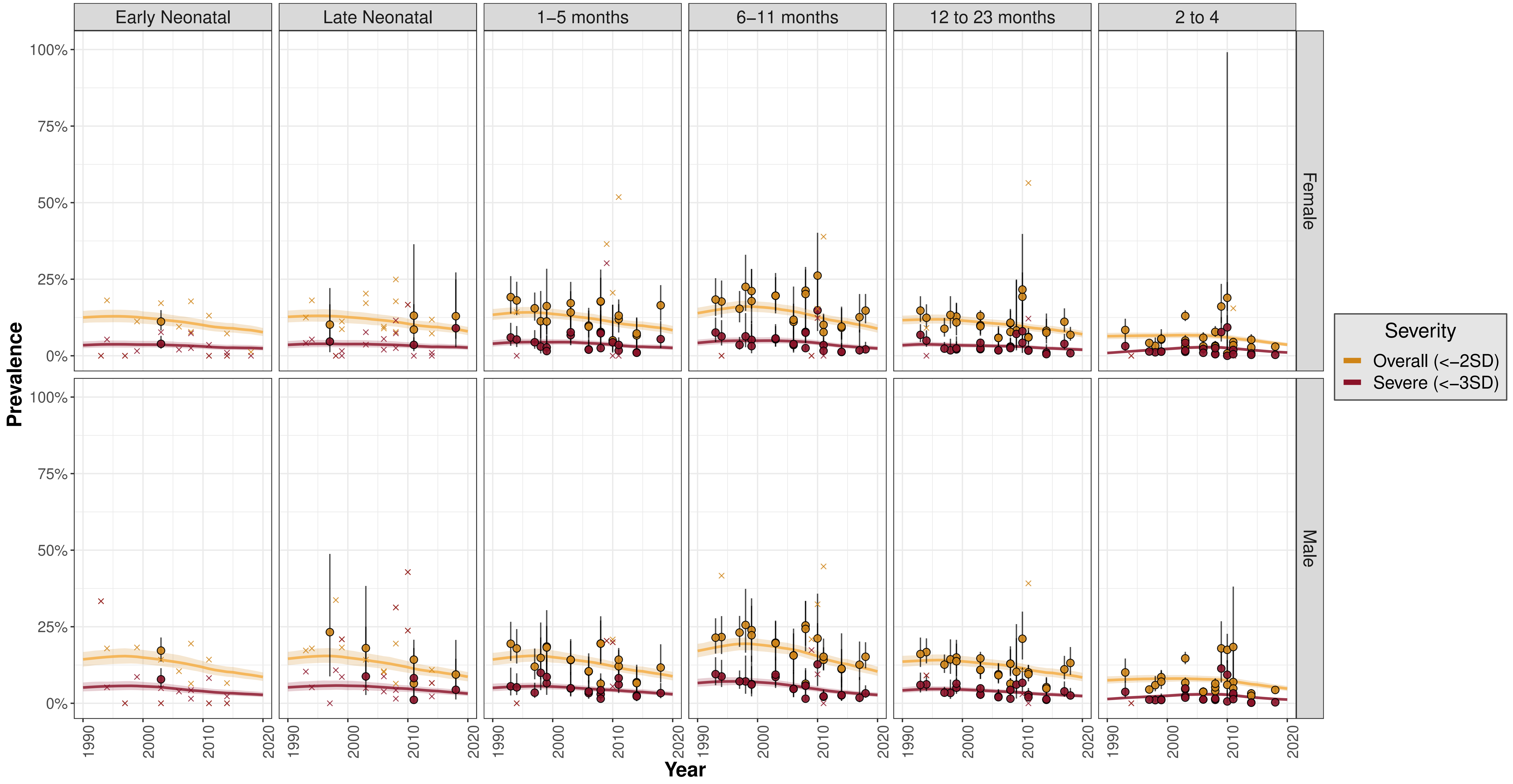

E: Transformed Mean Wasting Z Scores

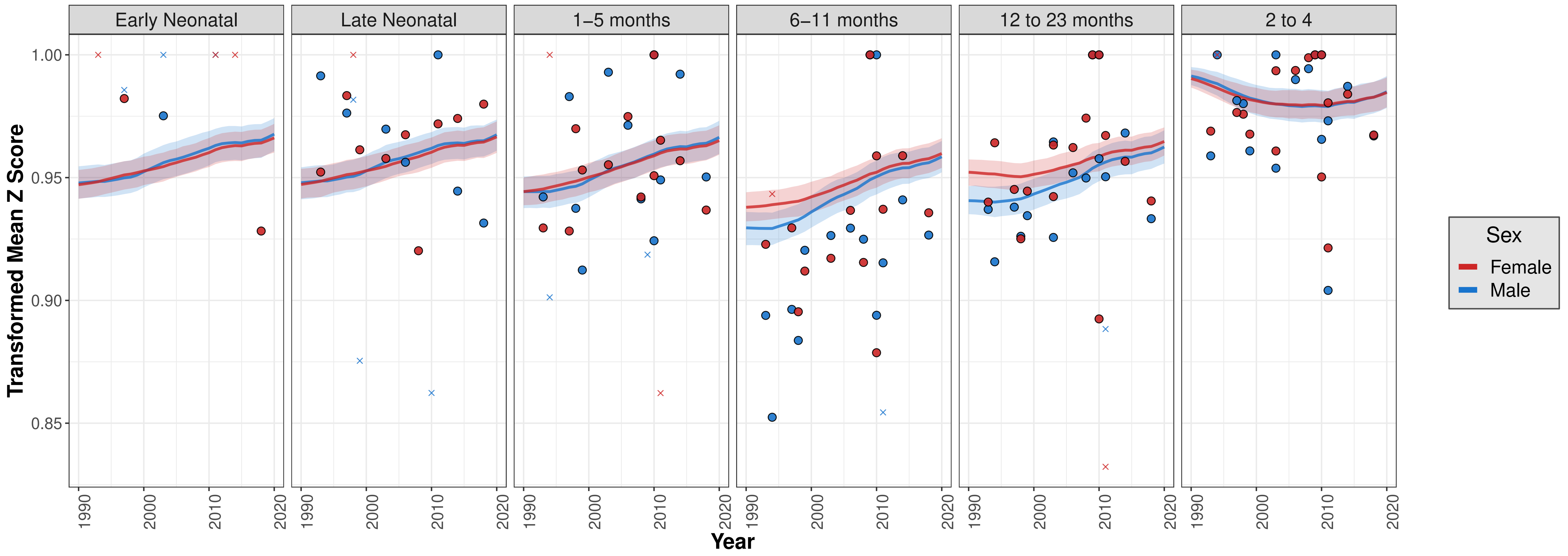

F

Year	Source
1988	Living Standards Measurement Survey
1988	Living Standards Measurement Survey
1988	DHS
1988	WHO CGM Database
1989	Living Standards Measurement Survey
1993	DHS
1994	DHS
1994	WHO CGM Database
1997	Core Welfare Indicators Survey
1998	DHS
1999	DHS
1999	WHO CGM Database
2003	DHS
2003	Core Welfare Indicators Questionnaire Survey
2003	WHO CGM Database
2006	MICS
2006	WHO CGM Database
2007	District MICS
2008	DHS
2008	WHO CGM Database
2008	District MICS
2008	Comprehensive Food Security and Vulnerability Analysis
2009	Socioeconomic Panel Survey
2010	Accra MICS
2010	Socioeconomic Panel Survey
2011	Accra MICS
2011	MICS
2011	WHO CGM Database
2014	WHO CGM Database
2014	DHS
2017	Micronutrient Survey
2018	MICS

Ghana – Underweight (WAZ)

G: Overall and Severe Underweight Prevalence

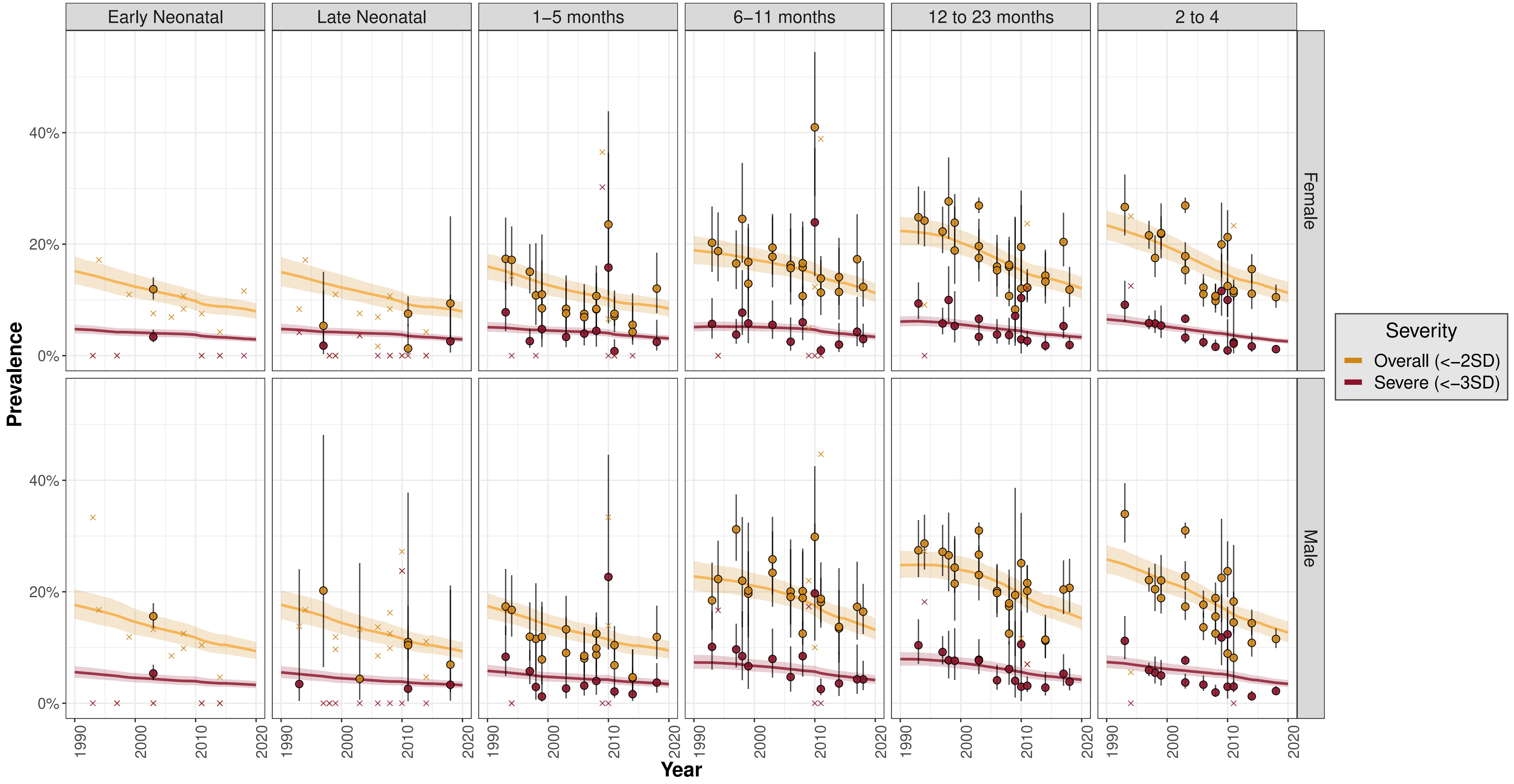

H: Transformed Mean Underweight Z Scores

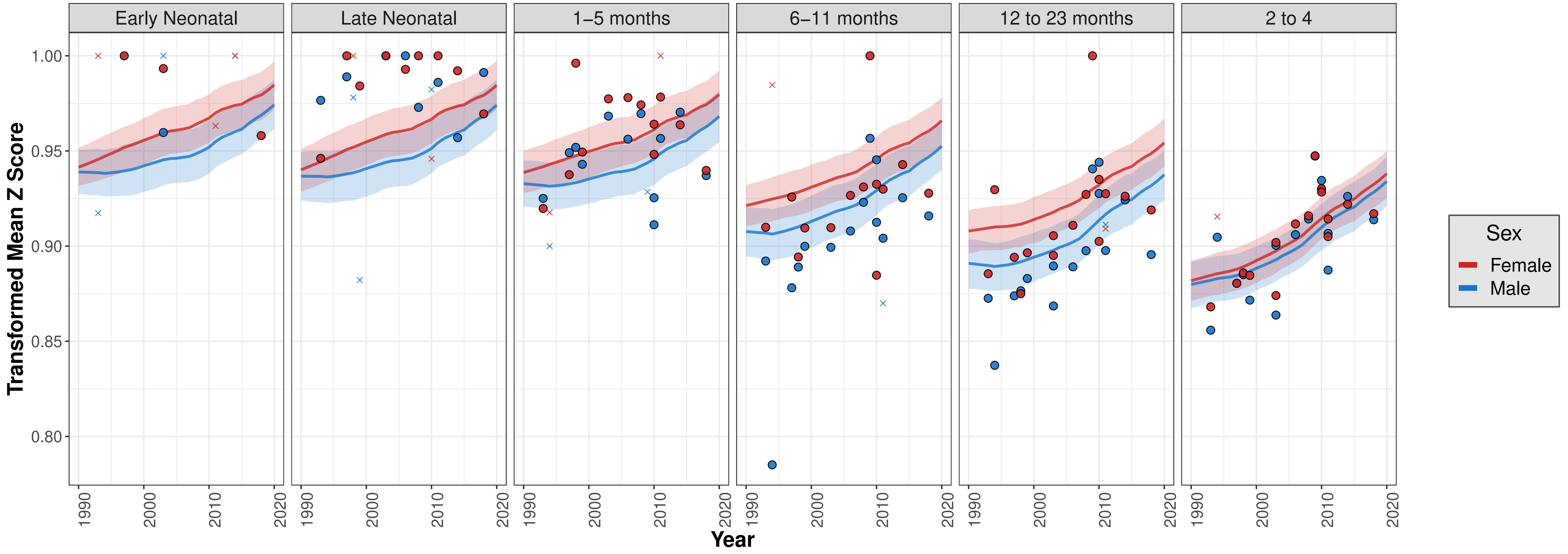

I

Year	Source
1988	Living Standards Measurement Survey
1988	Living Standards Measurement Survey
1988	DHS
1988	WHO CGM Database
1989	Living Standards Measurement Survey
1993	DHS
1994	DHS
1994	WHO CGM Database
1997	Core Welfare Indicators Survey
1998	DHS
1999	DHS
1999	WHO CGM Database
2003	DHS
2003	Core Welfare Indicators Questionnaire Survey
2003	WHO CGM Database
2006	MICS
2006	WHO CGM Database
2007	District MICS
2008	DHS
2008	WHO CGM Database
2008	District MICS
2008	Comprehensive Food Security and Vulnerability Analysis
2009	Socioeconomic Panel Survey
2010	Accra MICS
2010	Socioeconomic Panel Survey
2011	Accra MICS
2011	MICS
2011	WHO CGM Database
2014	WHO CGM Database
2014	DHS
2017	Micronutrient Survey
2018	MICS

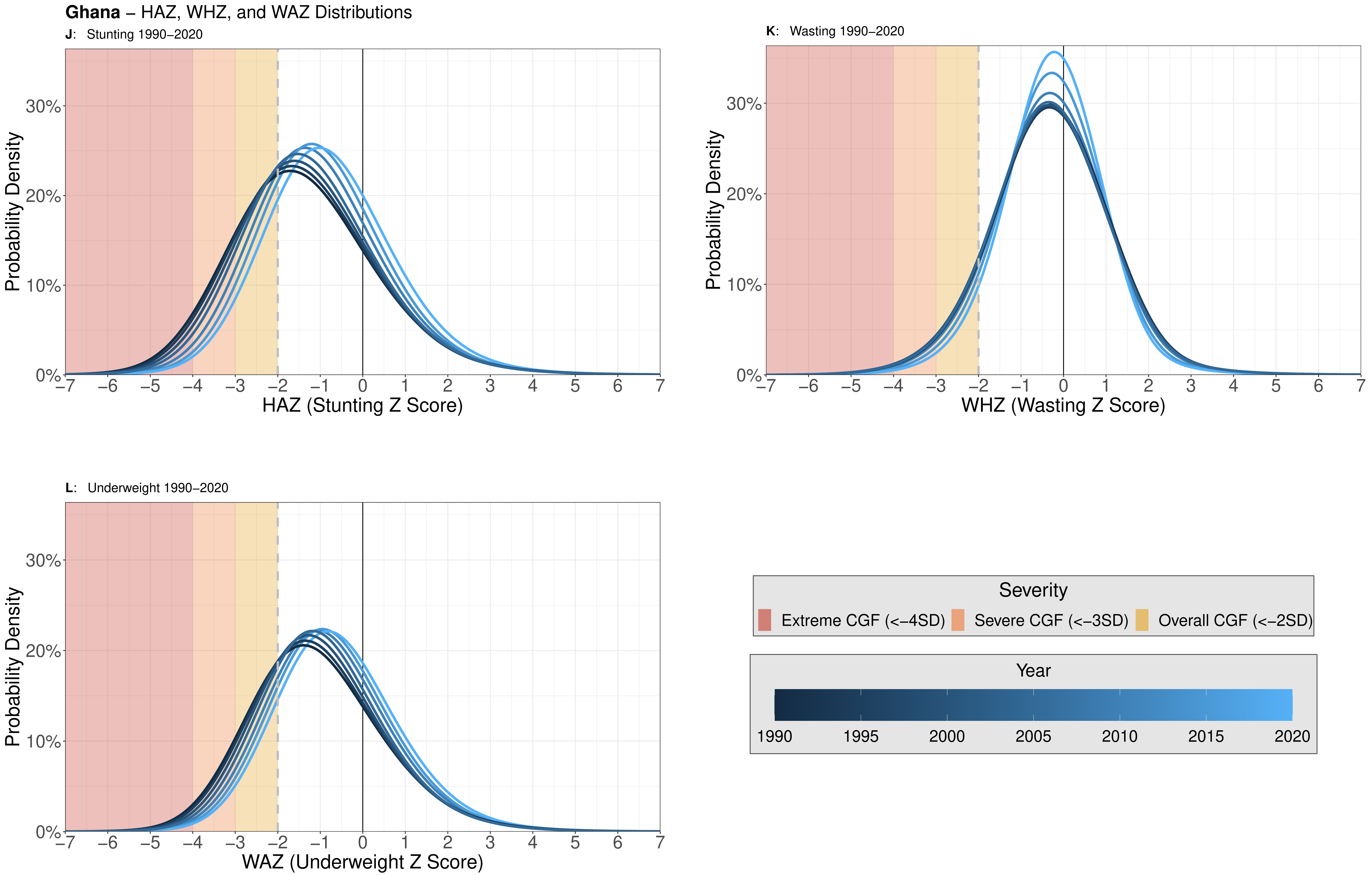

Ghana – HAZ, WHZ, and WAZ Distributions
J: Stunting 1990–2020
Probability Density
0%
10%
20%
30%
−7 −6 −5 −4 −3 −2 −1 0 1 2 3 4 5 6 7
HAZ (Stunting Z Score)
K: Wasting 1990–2020
Probability Density
WHZ (Wasting Z Score)
L: Underweight 1990–2020
Probability Density
WAZ (Underweight Z Score)
Severity
Extreme CGF (<−4SD)
Severe CGF (<−3SD)
Overall CGF (<−2SD)
Year
1990
1995
2000
2005
2010
2015
2020

Guinea – Stunting (HAZ)

A: Overall and Severe Stunting Prevalence

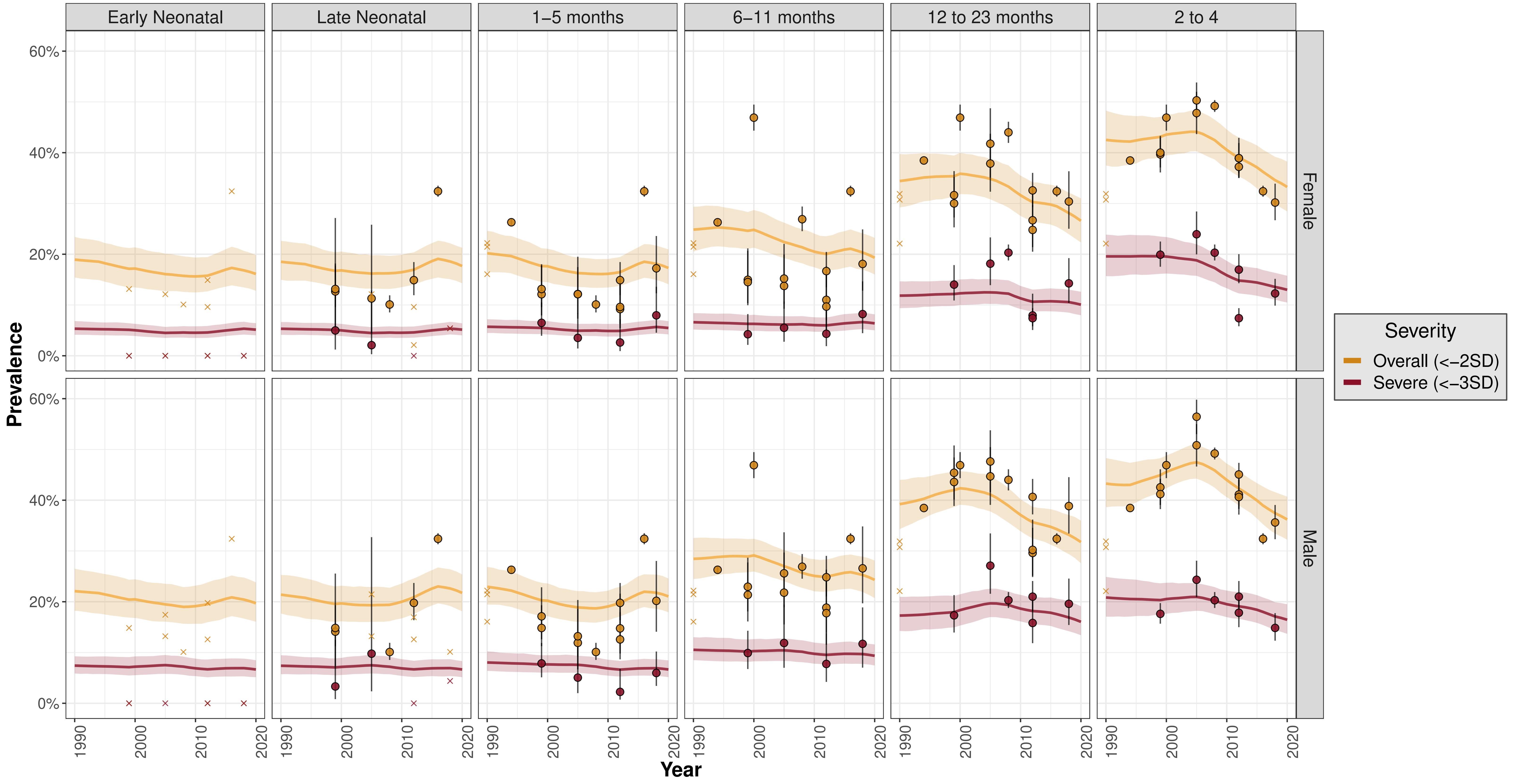

B: Transformed Mean Stunting Z Scores

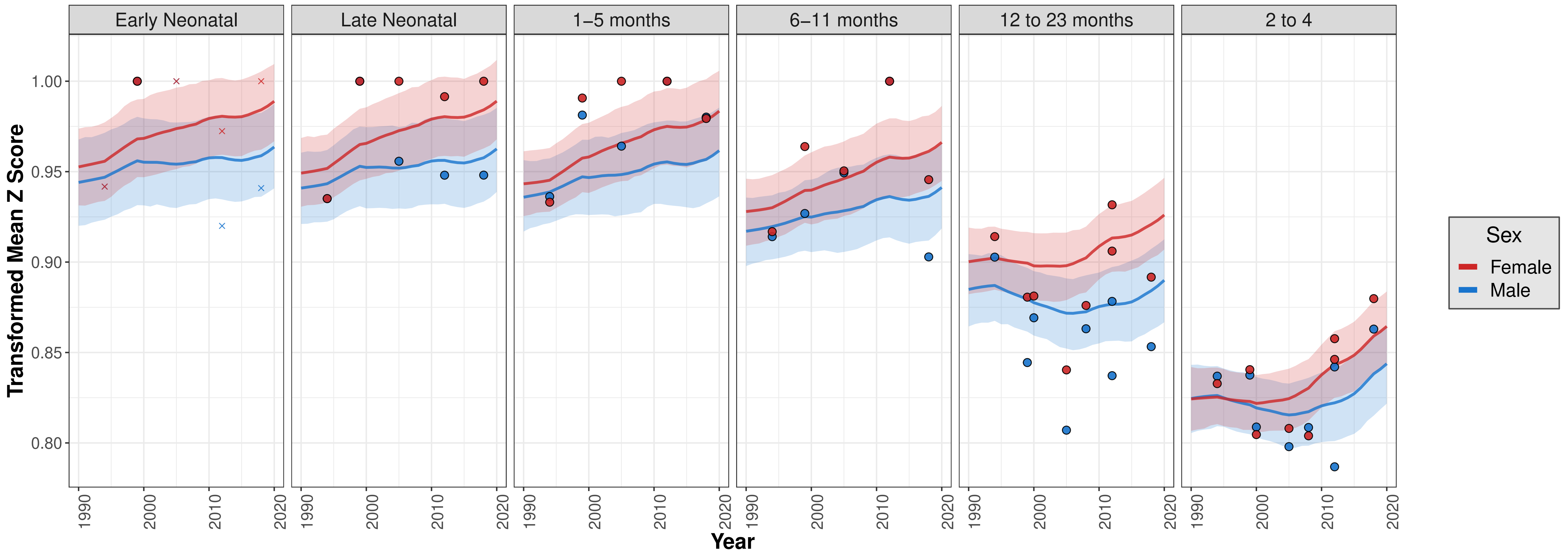

C

Year	Source
1990	WHO CGM Database
1994	WHO CGM Database
1999	DHS
1999	WHO CGM Database
2000	WHO CGM Database
2005	DHS
2005	WHO CGM Database
2008	WHO CGM Database
2012	DHS
2012	WHO CGM Database
2016	WHO CGM Database
2018	DHS

Guinea – Wasting (WHZ)

D: Overall and Severe Wasting Prevalence

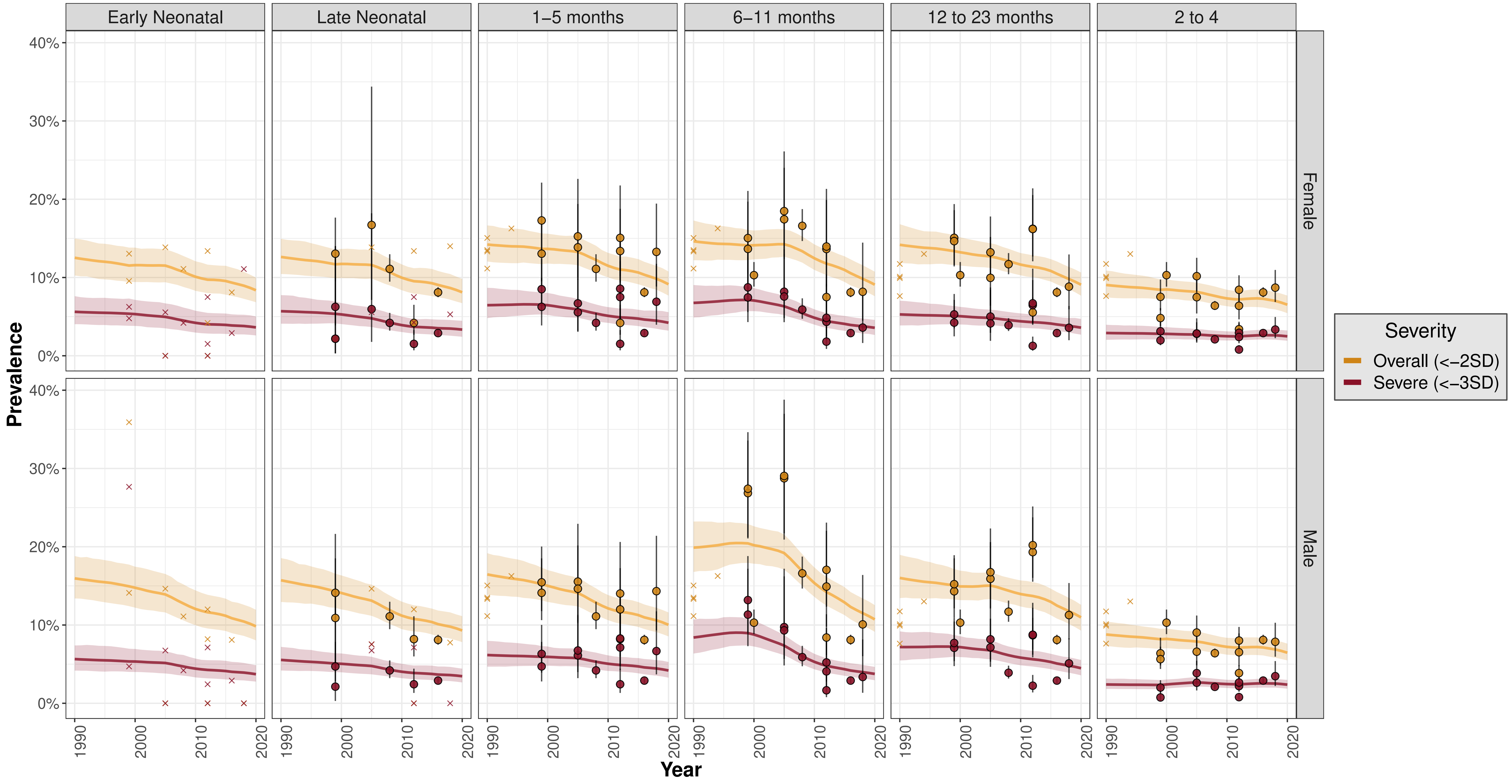

E: Transformed Mean Wasting Z Scores

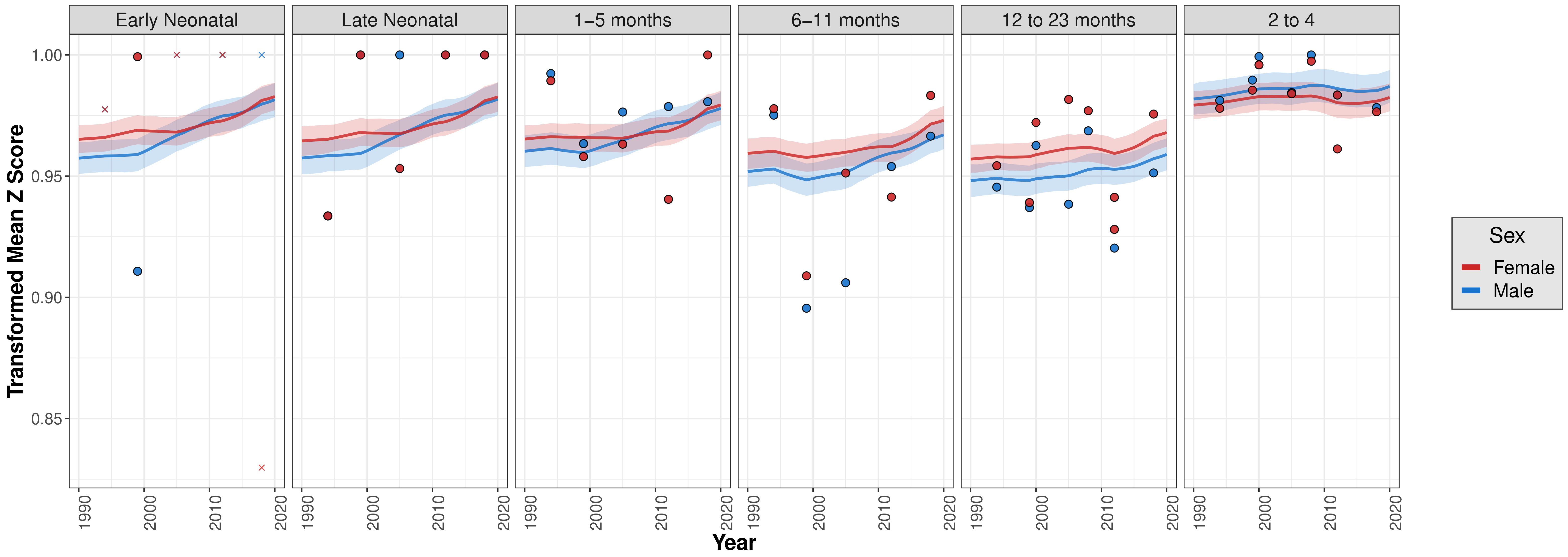

F

Year	Source
1990	WHO CGM Database
1994	WHO CGM Database
1999	DHS
1999	WHO CGM Database
2000	WHO CGM Database
2005	DHS
2005	WHO CGM Database
2008	WHO CGM Database
2012	DHS
2012	WHO CGM Database
2016	WHO CGM Database
2018	DHS

Guinea – Underweight (WAZ)

G: Overall and Severe Underweight Prevalence

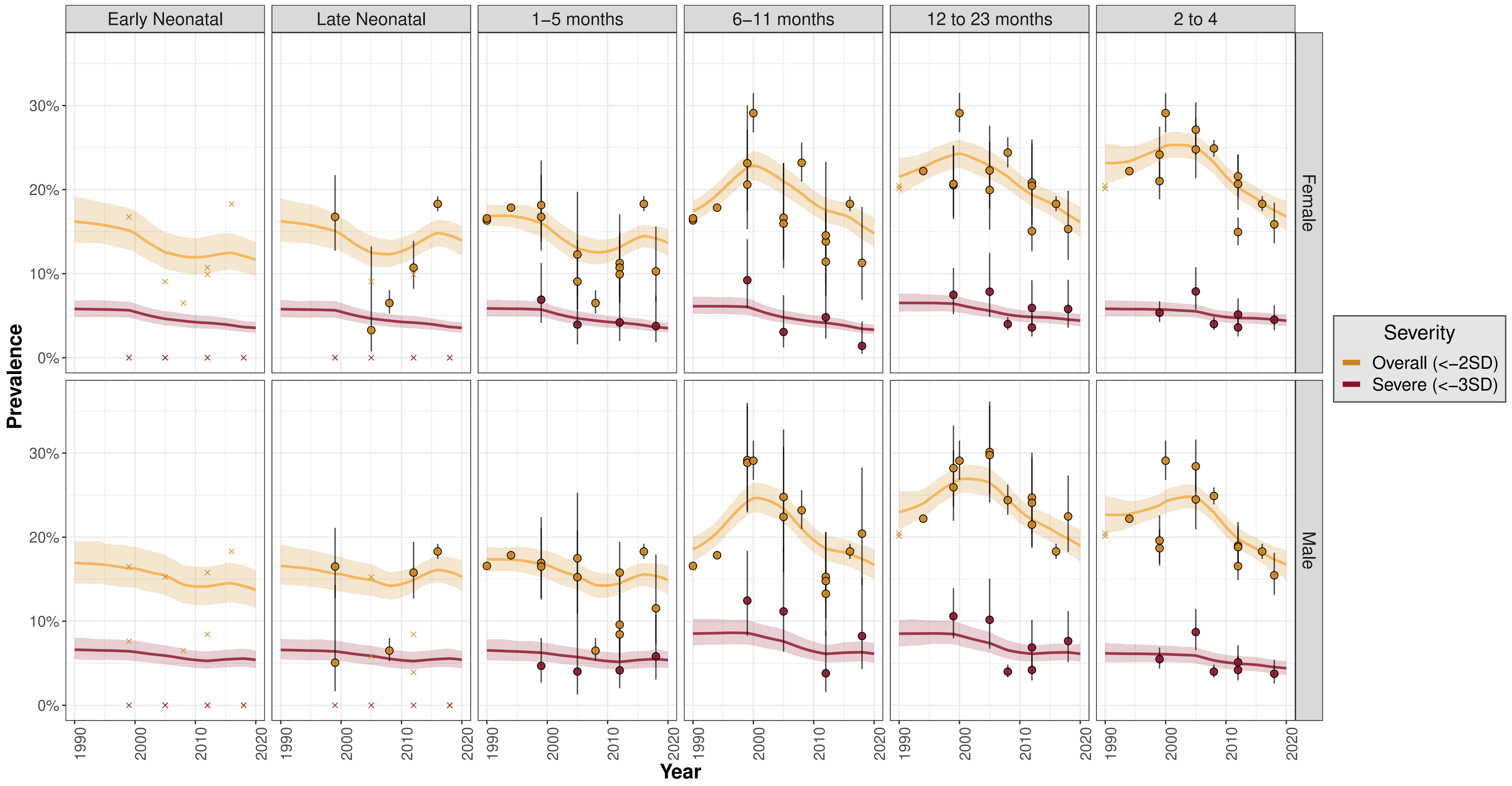

H: Transformed Mean Underweight Z Scores

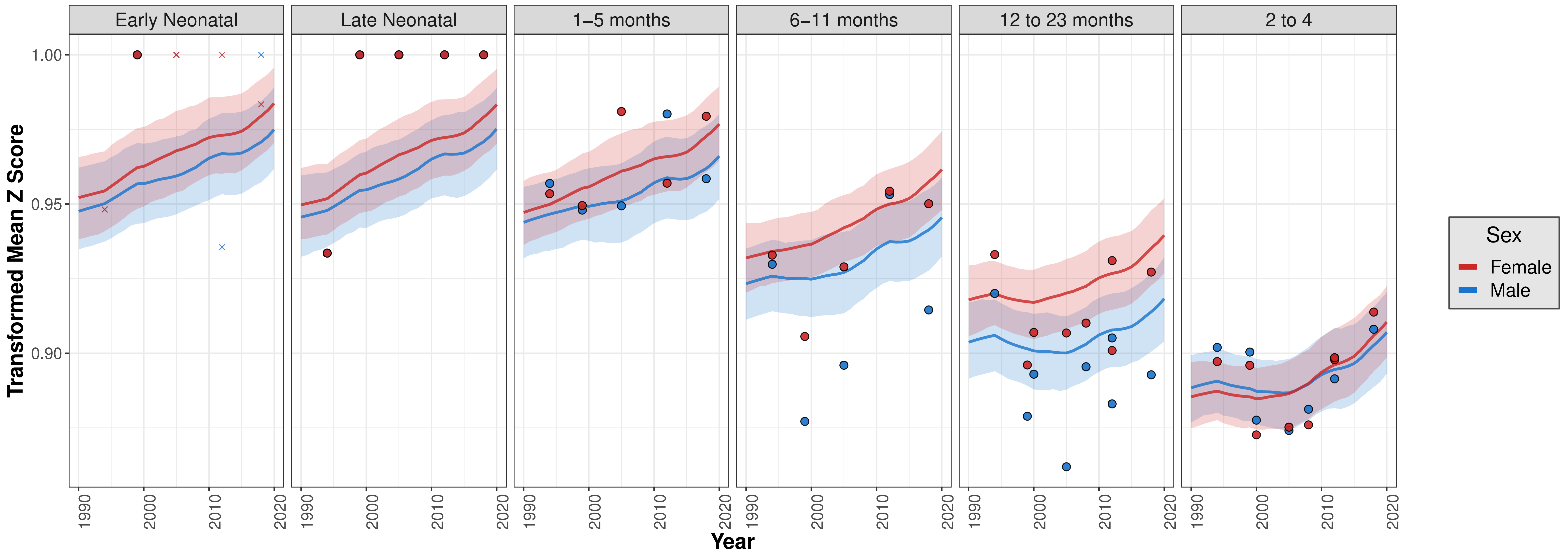

I

Year	Source
1990	WHO CGM Database
1994	WHO CGM Database
1999	DHS
1999	WHO CGM Database
2000	WHO CGM Database
2005	DHS
2005	WHO CGM Database
2008	WHO CGM Database
2012	DHS
2012	WHO CGM Database
2016	WHO CGM Database
2018	DHS

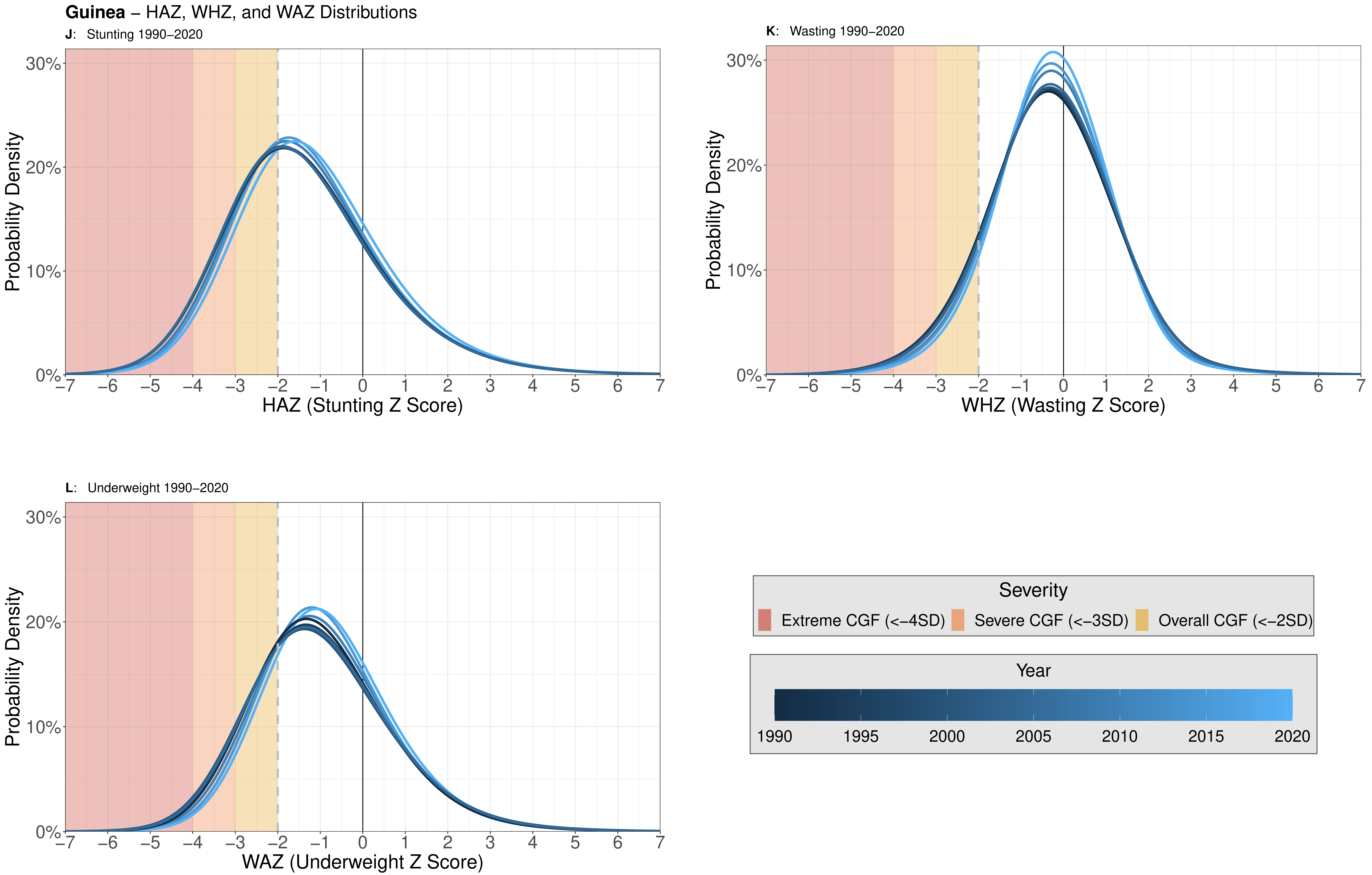

Guinea – HAZ, WHZ, and WAZ Distributions
J: Stunting 1990–2020
Probability Density
30%
20%
10%
0%
−7 −6 −5 −4 −3 −2 −1 0 1 2 3 4 5 6 7
HAZ (Stunting Z Score)
K: Wasting 1990–2020
Probability Density
WHZ (Wasting Z Score)
L: Underweight 1990–2020
Probability Density
WAZ (Underweight Z Score)
Severity
Extreme CGF (<−4SD)
Severe CGF (<−3SD)
Overall CGF (<−2SD)
Year
1990 1995 2000 2005 2010 2015 2020

Guinea–Bissau

A: Overall and Severe Stunting Prevalence

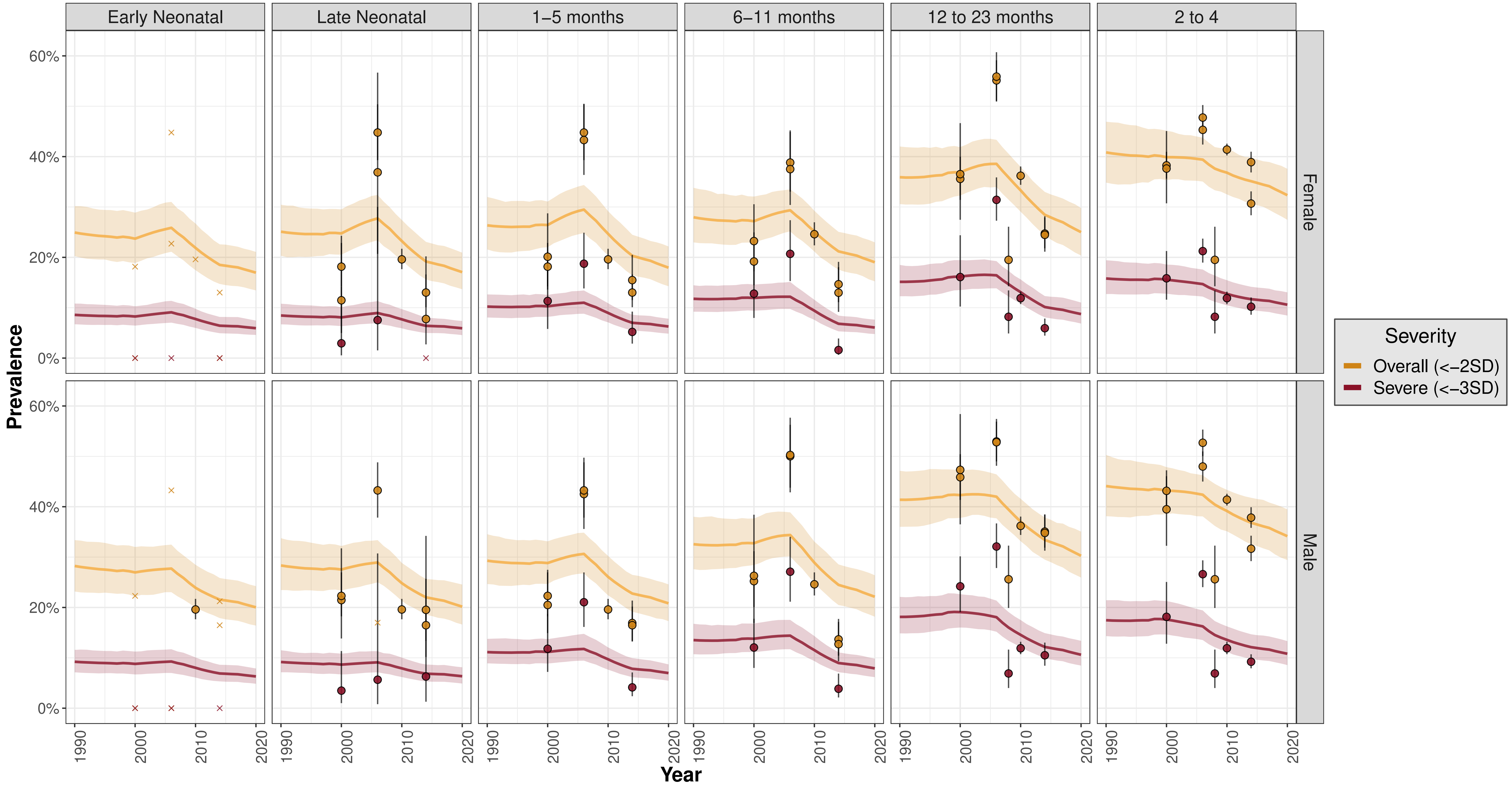

B: Transformed Mean Stunting Z Scores

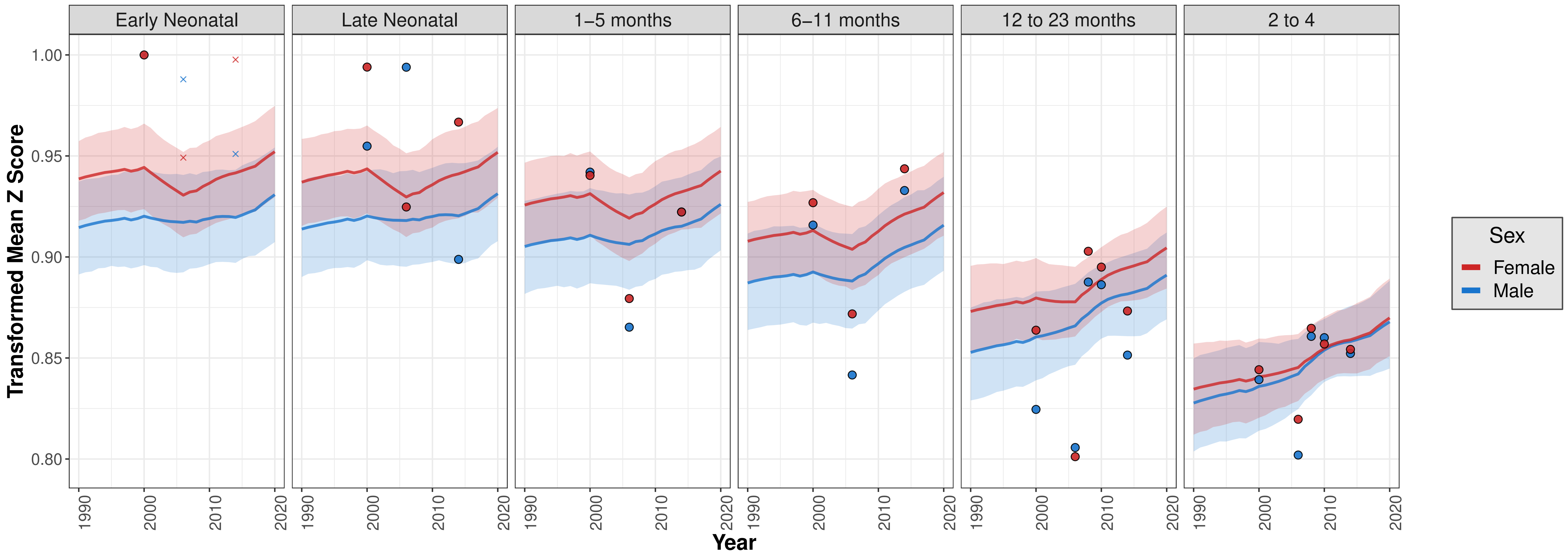

C

Year	Source
2000	MICS
2000	WHO CGM Database
2006	MICS
2006	WHO CGM Database
2008	WHO CGM Database
2010	WHO CGM Database
2014	WHO CGM Database
2014	MICS

Guinea–Bissau

D: Overall and Severe Wasting Prevalence

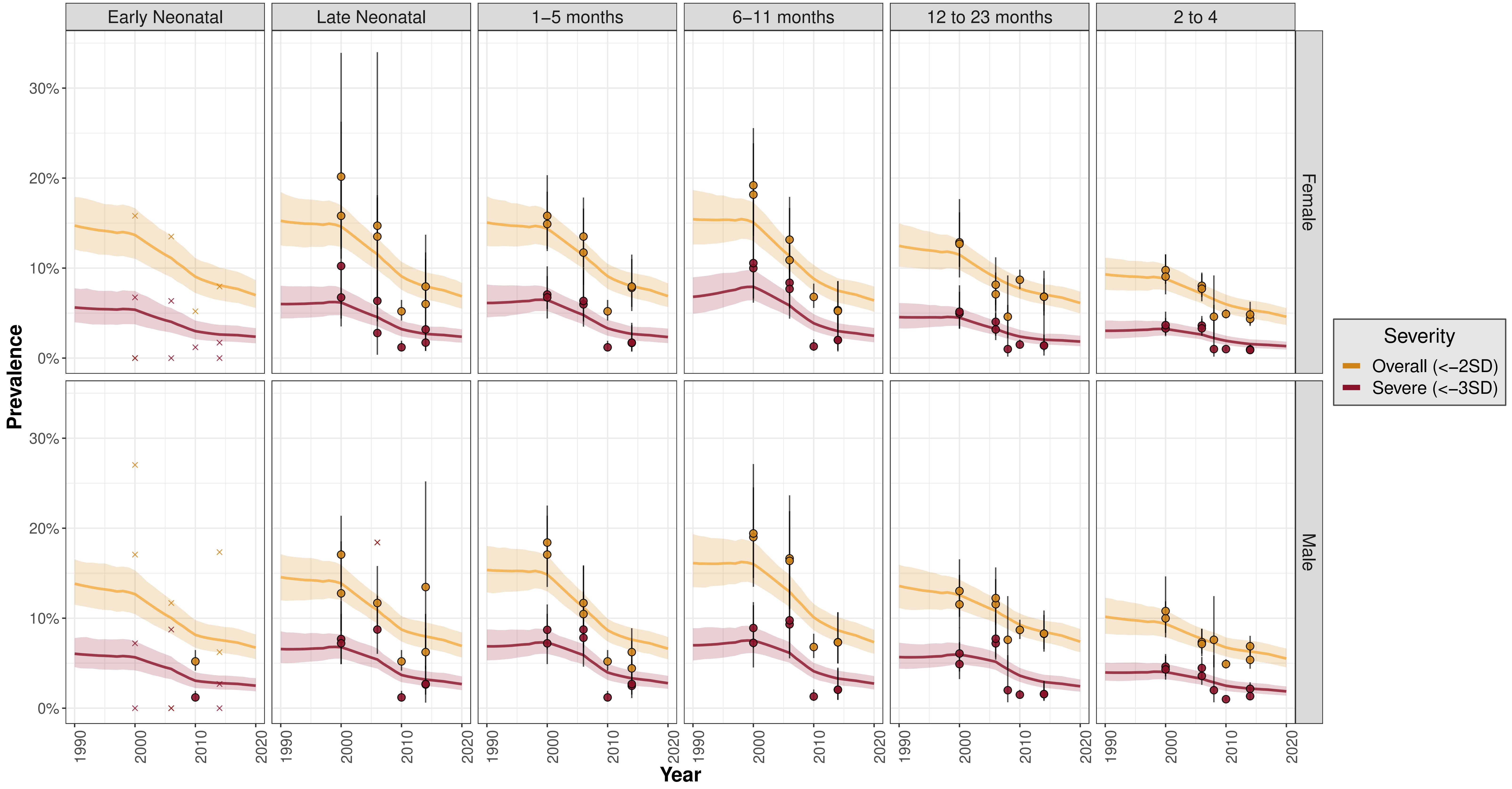

F

Year	Source
2000	MICS
2000	WHO CGM Database
2006	MICS
2006	WHO CGM Database
2008	WHO CGM Database
2010	WHO CGM Database
2014	WHO CGM Database
2014	MICS

E: Transformed Mean Wasting Z Scores

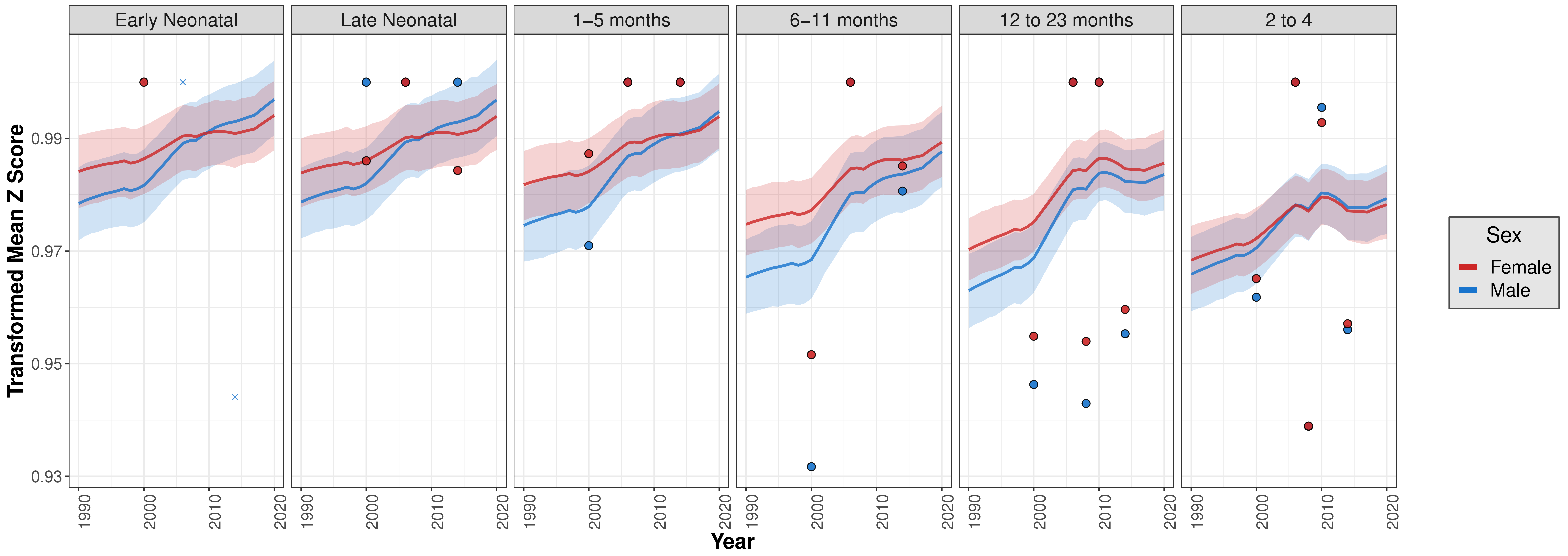

Guinea–Bissau

G: Overall and Severe Underweight Prevalence

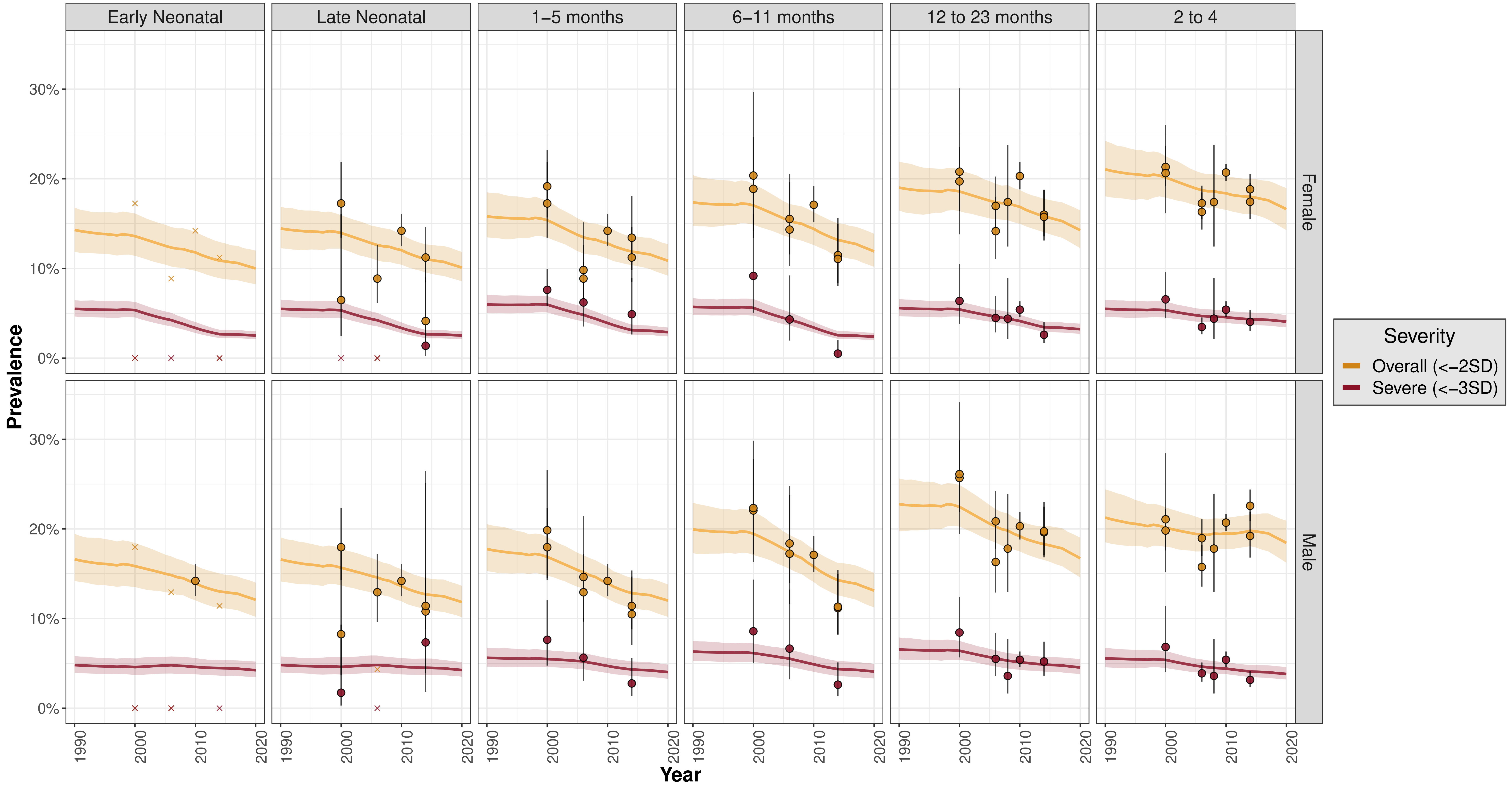

H: Transformed Mean Underweight Z Scores

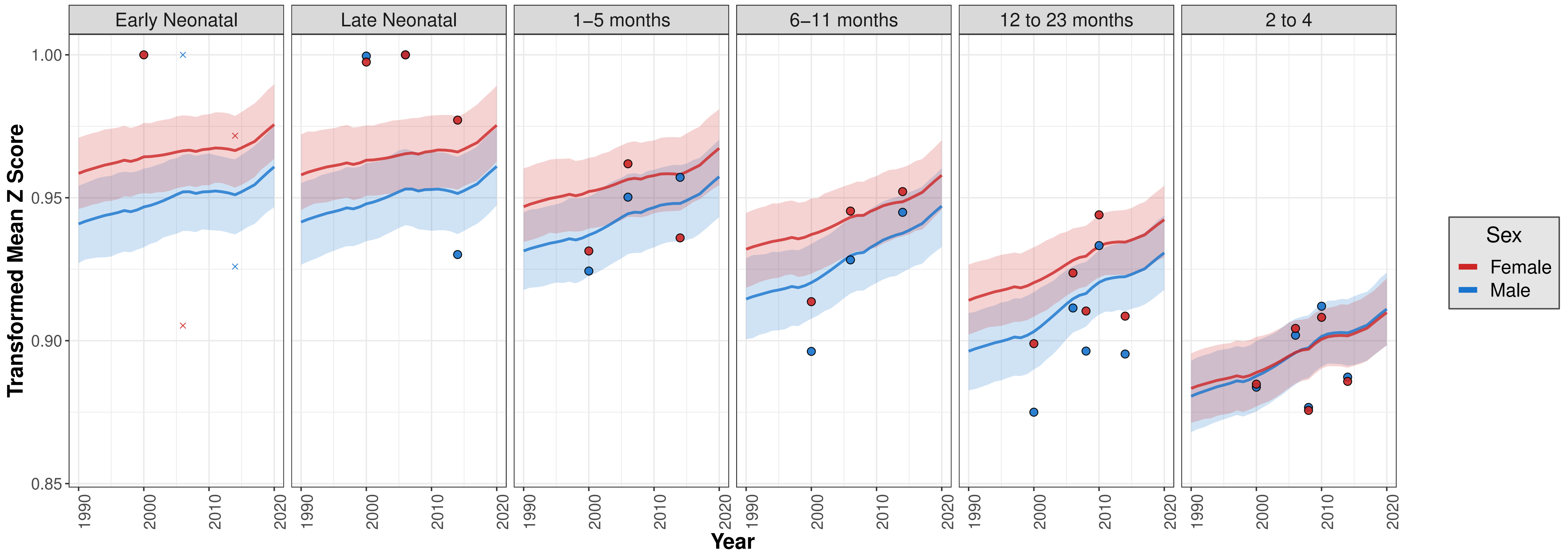

I

Year	Source
2000	MICS
2000	WHO CGM Database
2006	MICS
2006	WHO CGM Database
2008	WHO CGM Database
2010	WHO CGM Database
2014	WHO CGM Database
2014	MICS

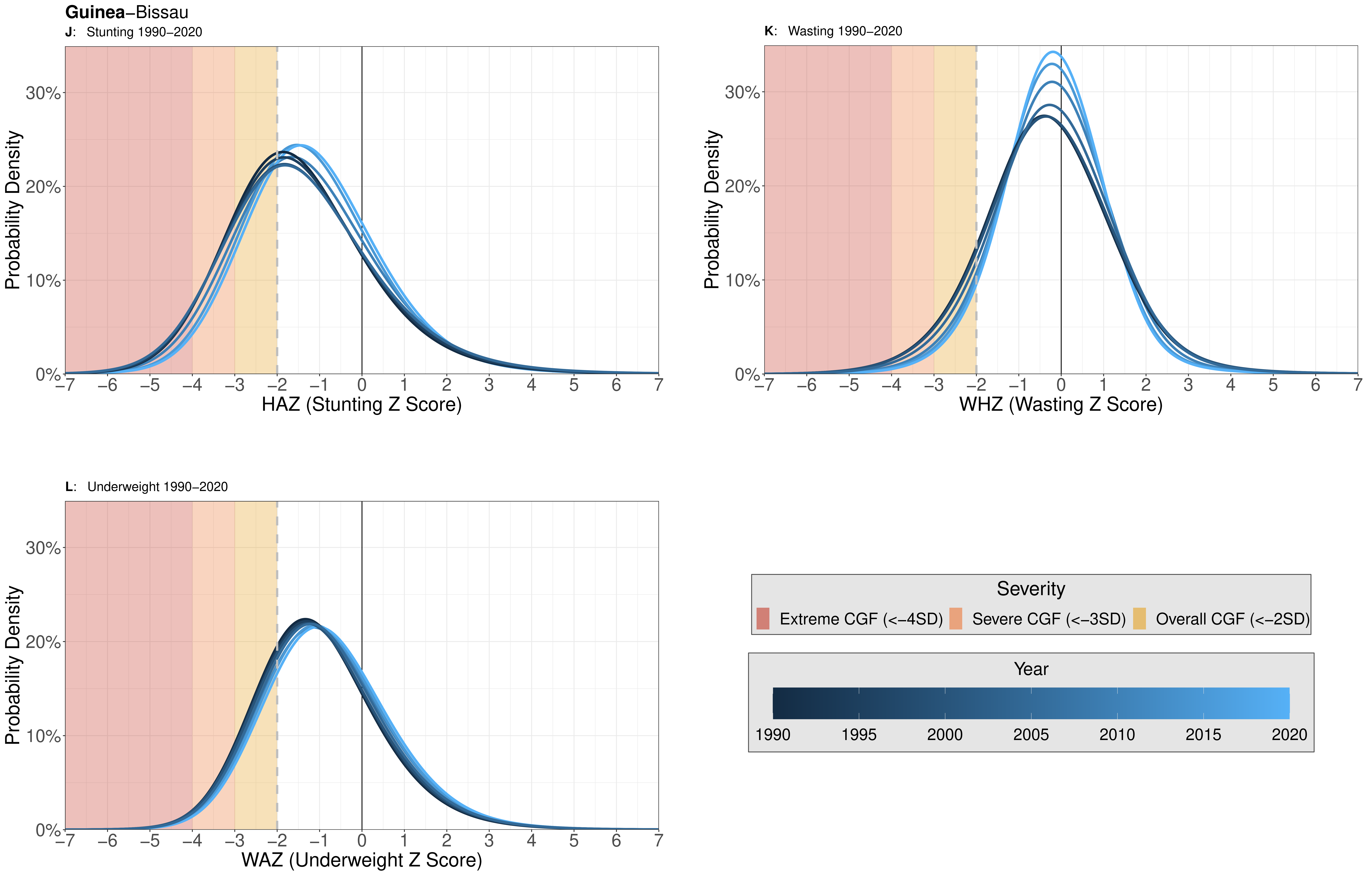
Guinea–Bissau
J: Stunting 1990–2020
K: Wasting 1990–2020
L: Underweight 1990–2020
Probability Density
0%
10%
20%
30%
−7 −6 −5 −4 −3 −2 −1 0 1 2 3 4 5 6 7
HAZ (Stunting Z Score)
WHZ (Wasting Z Score)
WAZ (Underweight Z Score)
Severity
Extreme CGF (<−4SD)
Severe CGF (<−3SD)
Overall CGF (<−2SD)
Year
1990
1995
2000
2005
2010
2015
2020

Liberia – Stunting (HAZ)

A: Overall and Severe Stunting Prevalence

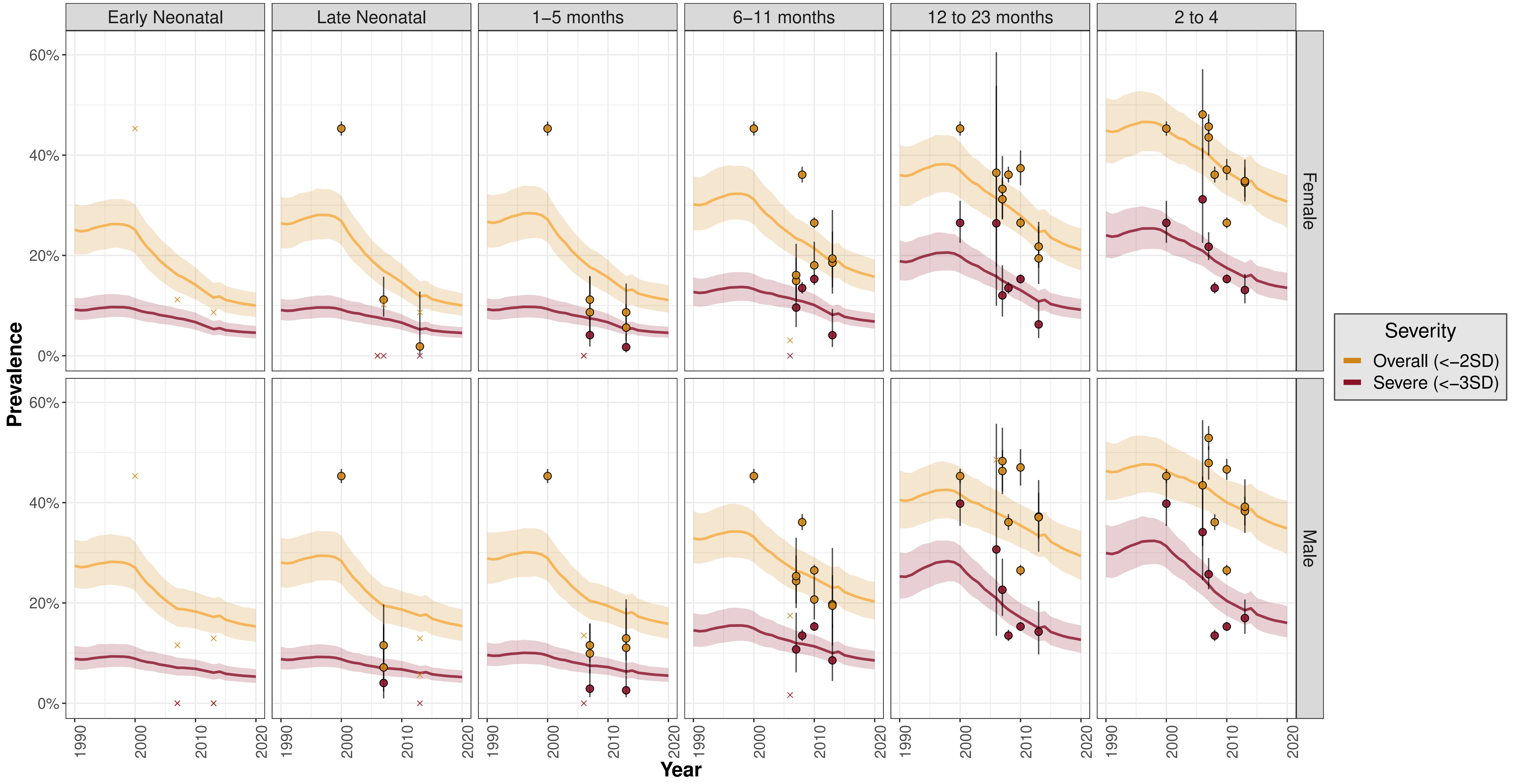

B: Transformed Mean Stunting Z Scores

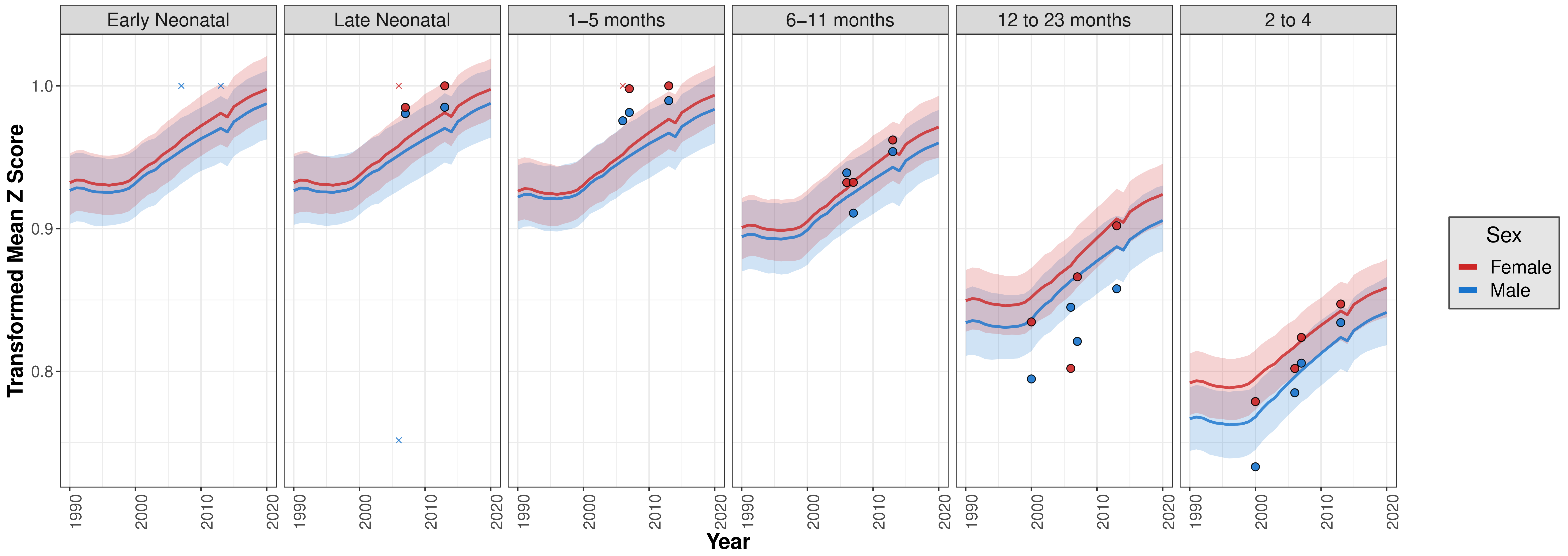

C

Year	Source
1976	WHO CGM Database
2000	WHO CGM Database
2006	DHS
2007	DHS
2007	WHO CGM Database
2008	Food Security and Nutrition Survey
2010	WHO CGM Database
2010	Comprehensive Food Security and Nutrition Survey
2013	DHS
2013	WHO CGM Database

Liberia – Wasting (WHZ)

D: Overall and Severe Wasting Prevalence

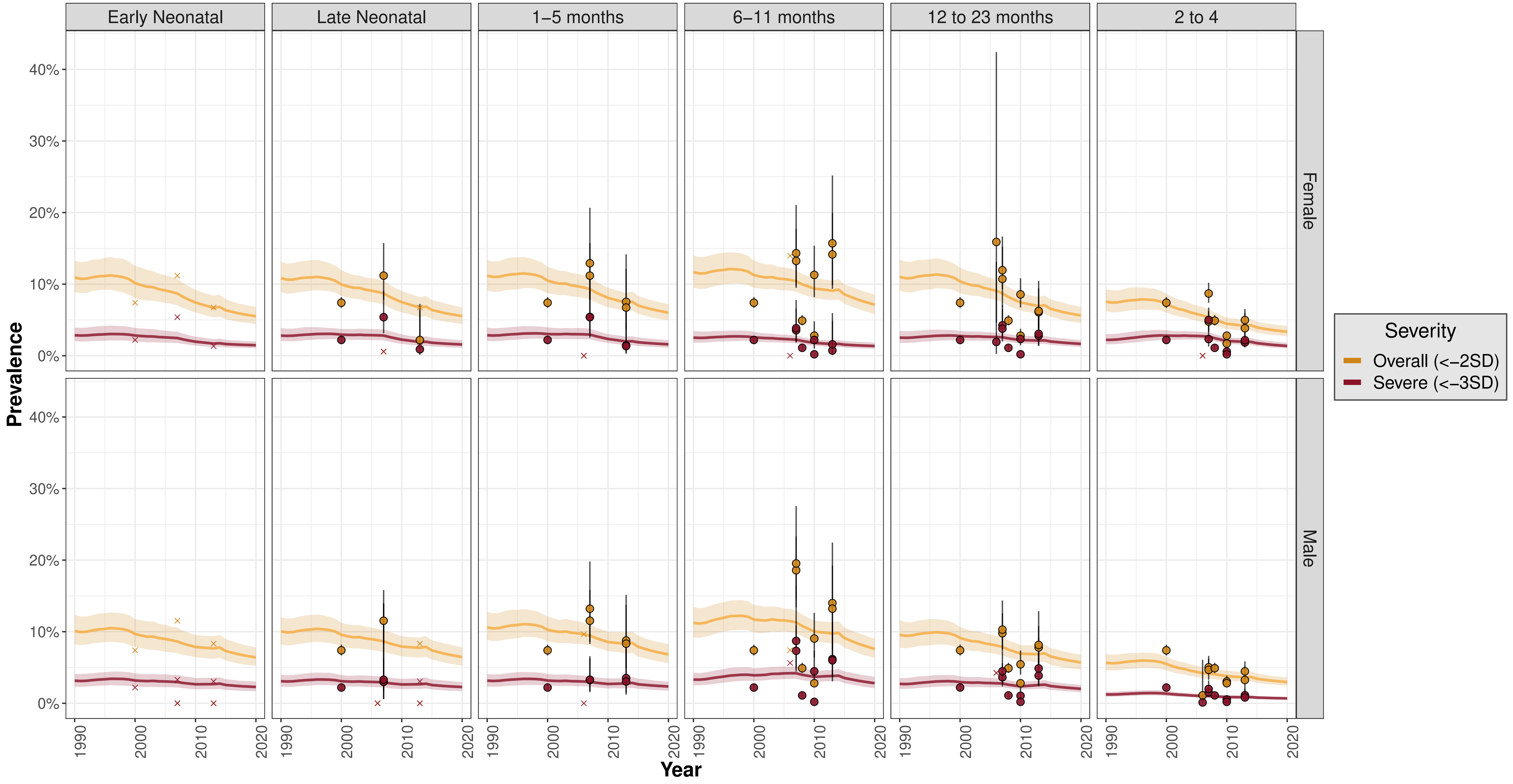

E: Transformed Mean Wasting Z Scores

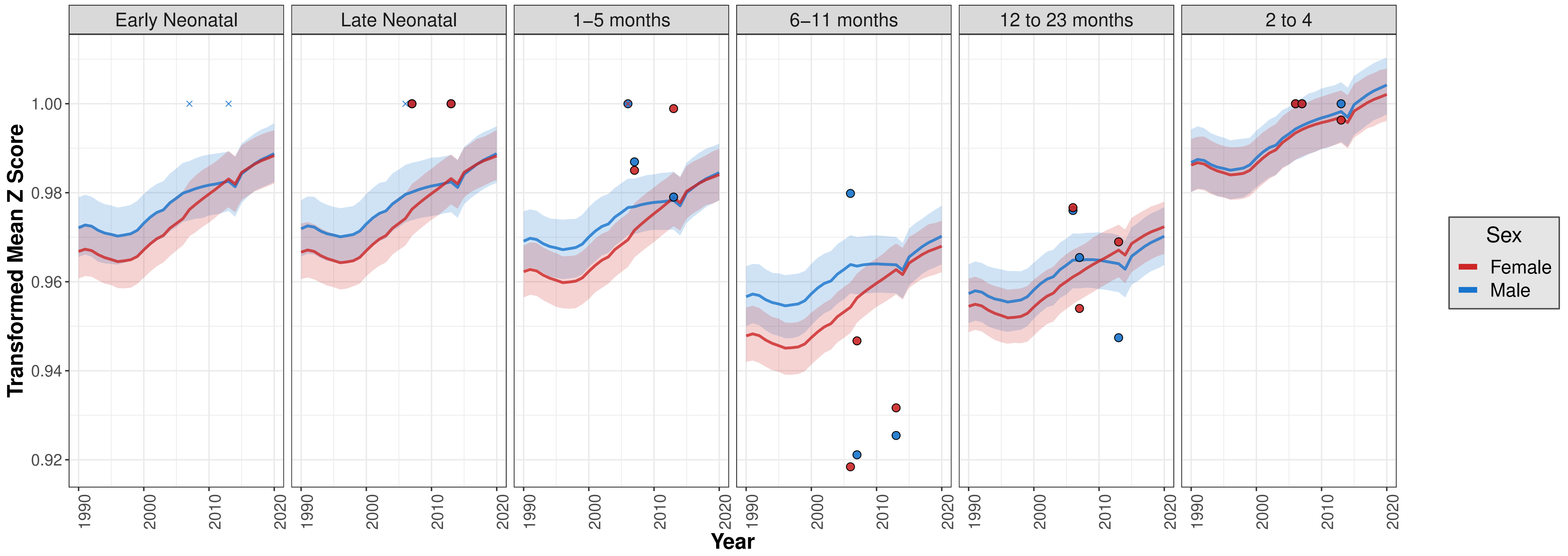

F

Year	Source
1976	WHO CGM Database
2000	WHO CGM Database
2006	DHS
2007	DHS
2007	WHO CGM Database
2008	Food Security and Nutrition Survey
2010	WHO CGM Database
2010	Comprehensive Food Security and Nutrition Survey
2013	DHS
2013	WHO CGM Database

Liberia – Underweight (WAZ)

G: Overall and Severe Underweight Prevalence

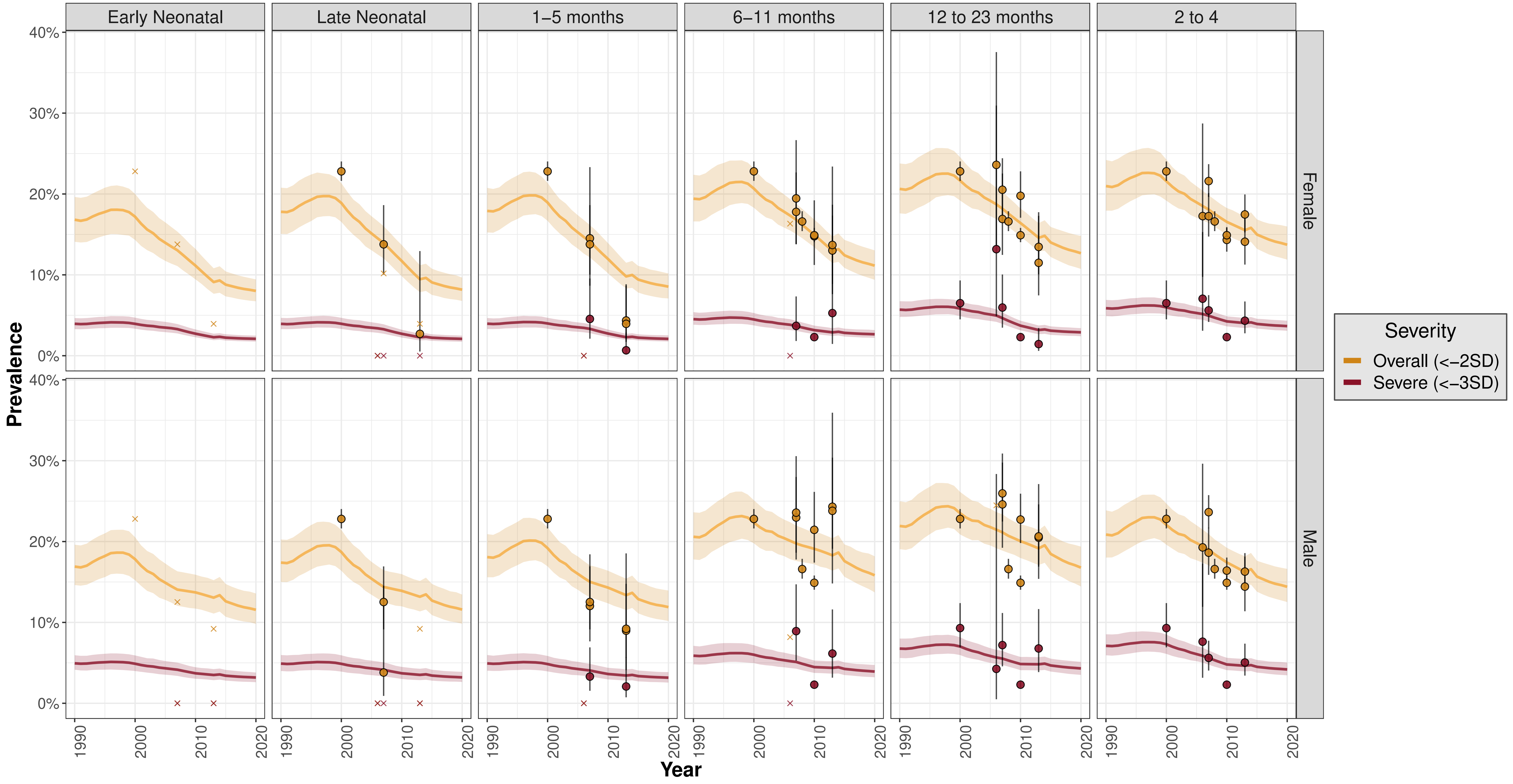

H: Transformed Mean Underweight Z Scores

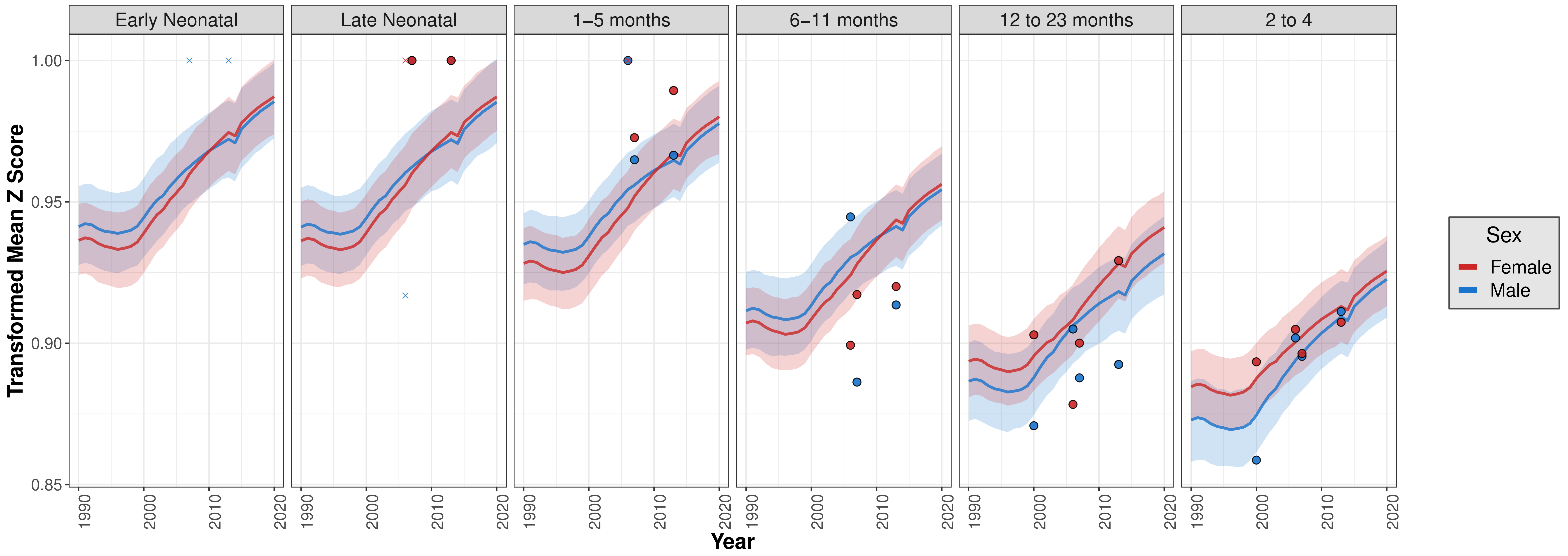

I

Year	Source
1976	WHO CGM Database
2000	WHO CGM Database
2006	DHS
2007	DHS
2007	WHO CGM Database
2008	Food Security and Nutrition Survey
2010	WHO CGM Database
2010	Comprehensive Food Security and Nutrition Survey
2013	DHS
2013	WHO CGM Database

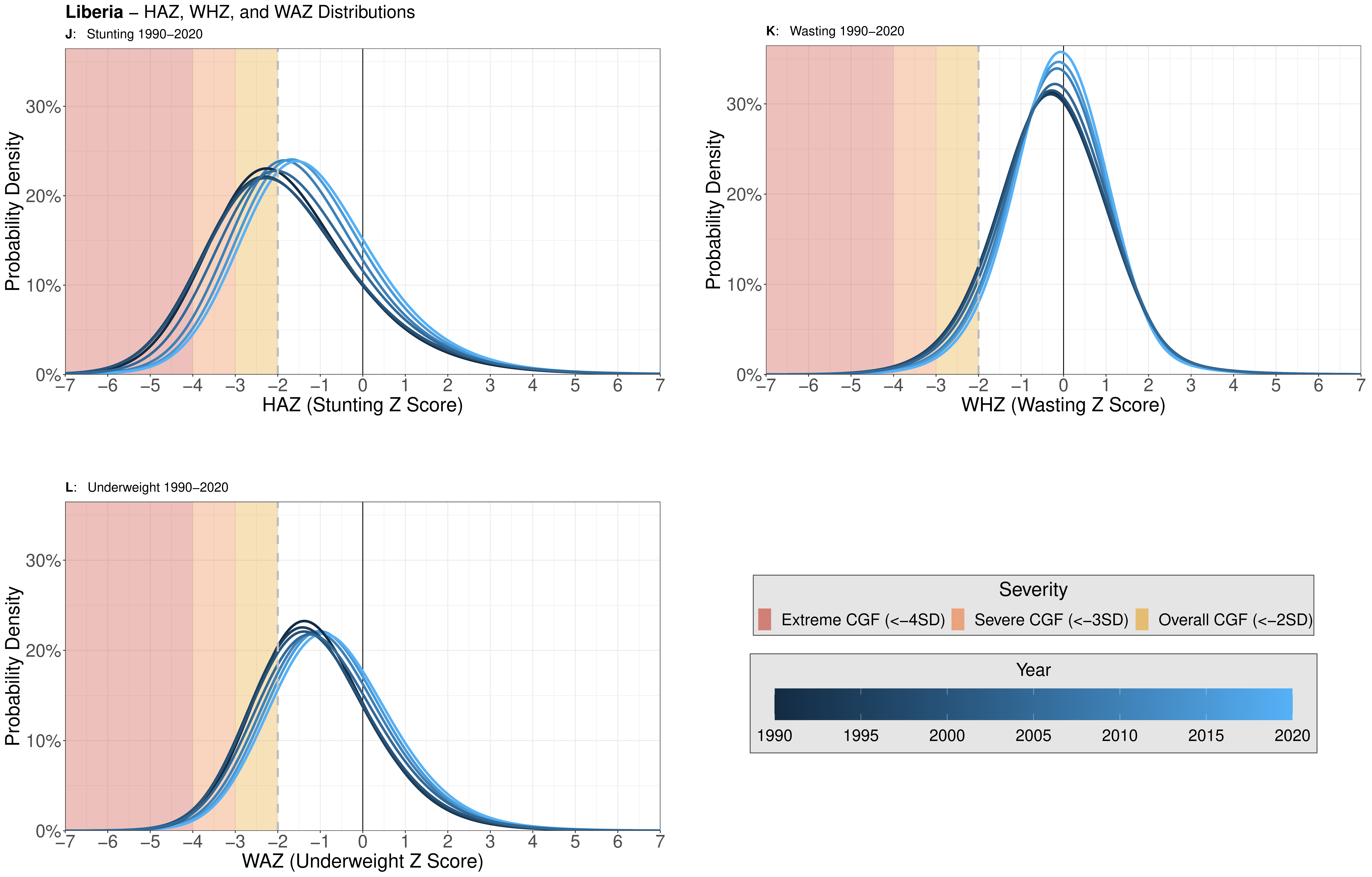
Liberia – HAZ, WHZ, and WAZ Distributions
J: Stunting 1990–2020
K: Wasting 1990–2020
L: Underweight 1990–2020
Probability Density
0%
10%
20%
30%
−7 −6 −5 −4 −3 −2 −1 0 1 2 3 4 5 6 7
HAZ (Stunting Z Score)
WHZ (Wasting Z Score)
WAZ (Underweight Z Score)
Severity
Extreme CGF (<−4SD)
Severe CGF (<−3SD)
Overall CGF (<−2SD)
Year
1990
1995
2000
2005
2010
2015
2020

Mali – Stunting (HAZ)

A: Overall and Severe Stunting Prevalence

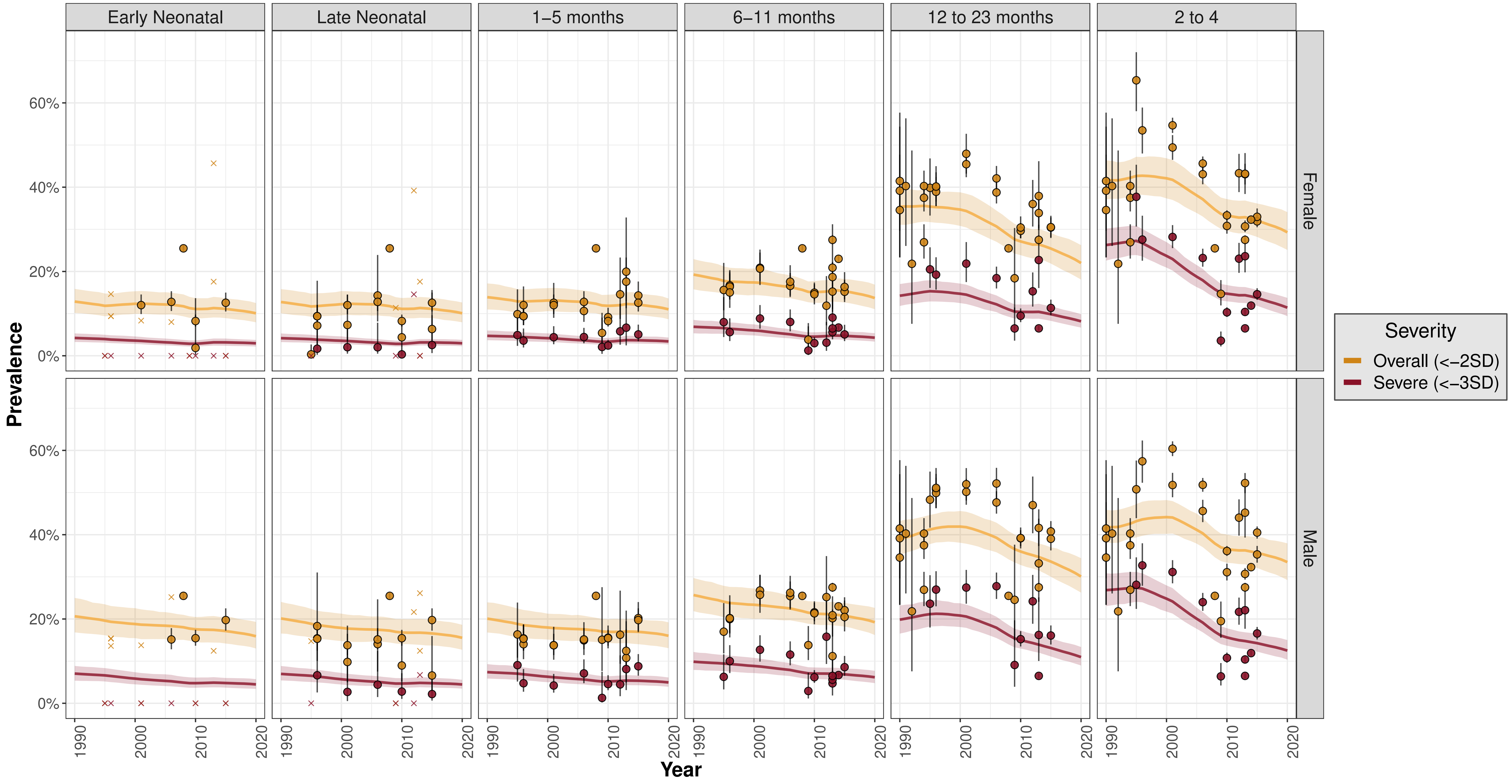

B: Transformed Mean Stunting Z Scores

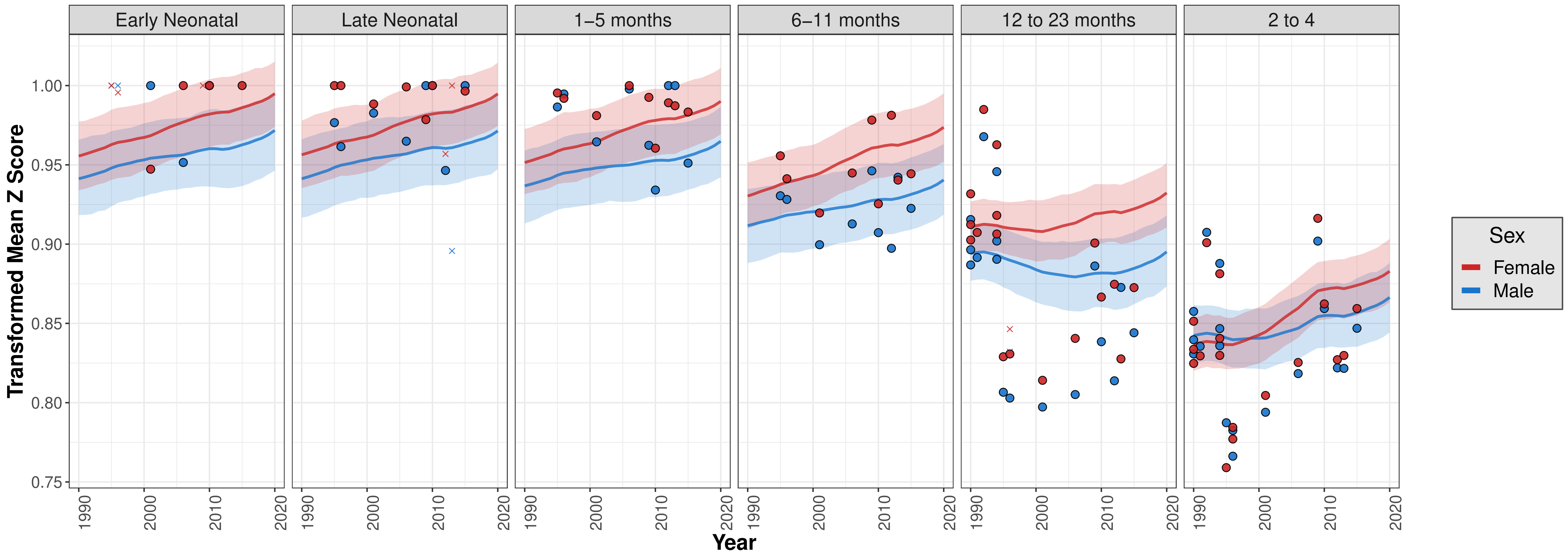

C

Year	Source
1987	DHS
1987	WHO CGM Database
1990	WHO CGM Database
1991	WHO CGM Database
1992	WHO CGM Database
1994	WHO CGM Database
1995	DHS
1996	DHS
1996	WHO CGM Database
2001	DHS
2001	WHO CGM Database
2006	DHS
2006	WHO CGM Database
2008	Food Security and Nutrition Study Baseline
2009	Bamako Global Enteric Multicenter Study
2009	MICS
2010	WHO CGM Database
2010	MICS
2012	DHS
2012	Bamako Global Enteric Multicenter Study
2013	DHS
2013	WHO CGM Database
2013	National Anthropometric Nutrition Survey and Mortality Retrospective July – August
2013	National Anthropometric Nutrition Survey and Mortality Retrospective June–August
2014	National Anthropometric Nutrition Survey and Mortality Retrospective June – August
2015	WHO CGM Database
2015	MICS

Mali – Wasting (WHZ)

D: Overall and Severe Wasting Prevalence

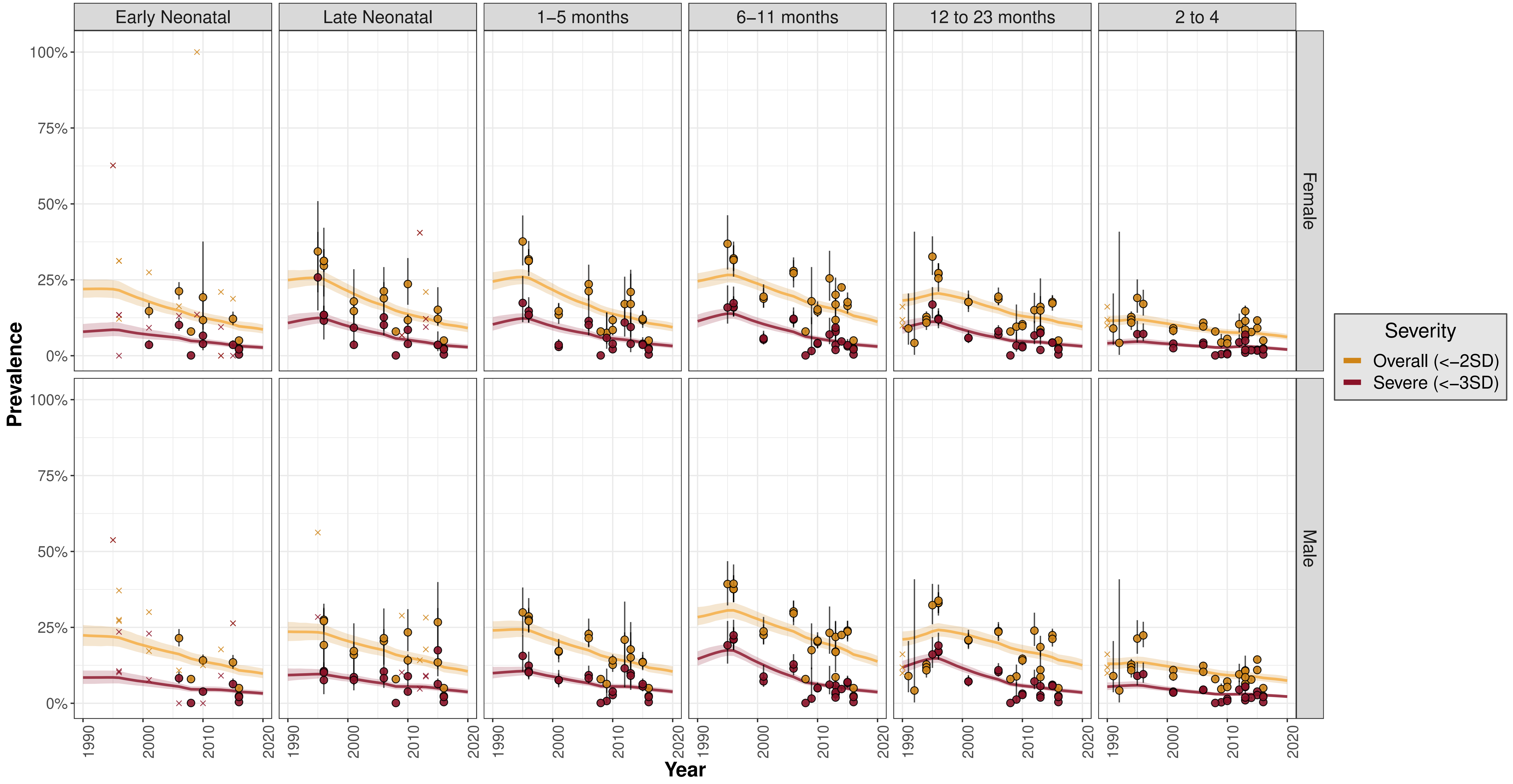

E: Transformed Mean Wasting Z Scores

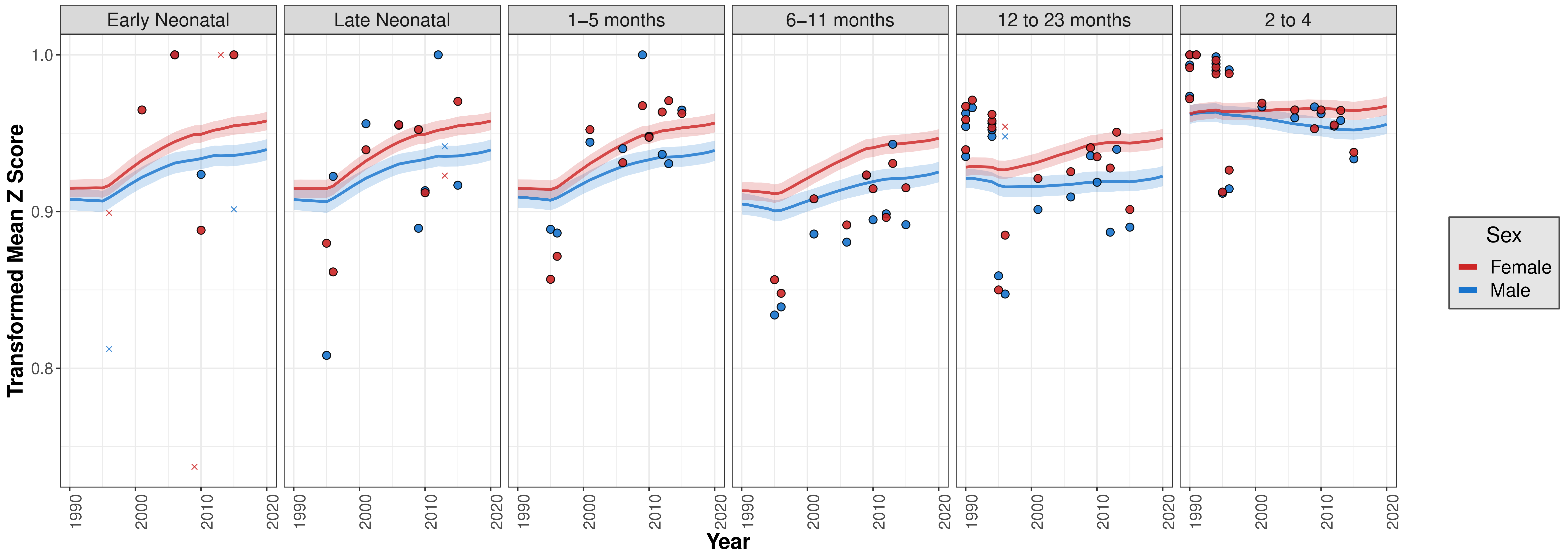

F

Year	Source
1985	WHO CGM Database
1987	DHS
1987	WHO CGM Database
1990	WHO CGM Database
1991	WHO CGM Database
1992	WHO CGM Database
1994	WHO CGM Database
1995	DHS
1996	DHS
1996	WHO CGM Database
2001	DHS
2001	WHO CGM Database
2006	DHS
2006	WHO CGM Database
2008	Food Security and Nutrition Study Baseline
2009	Bamako Global Enteric Multicenter Study
2009	MICS
2010	WHO CGM Database
2010	MICS
2012	DHS
2012	Bamako Global Enteric Multicenter Study
2013	DHS
2013	WHO CGM Database
2013	National Anthropometric Nutrition Survey and Mortality Retrospective July – August
2013	National Anthropometric Nutrition Survey and Mortality Retrospective June–August
2014	National Anthropometric Nutrition Survey and Mortality Retrospective June – August
2015	WHO CGM Database
2015	MICS
2016	Food and Nutritional Security Survey
2016	Food and Nutritional Security Survey, September

Mali – Underweight (WAZ)

G: Overall and Severe Underweight Prevalence

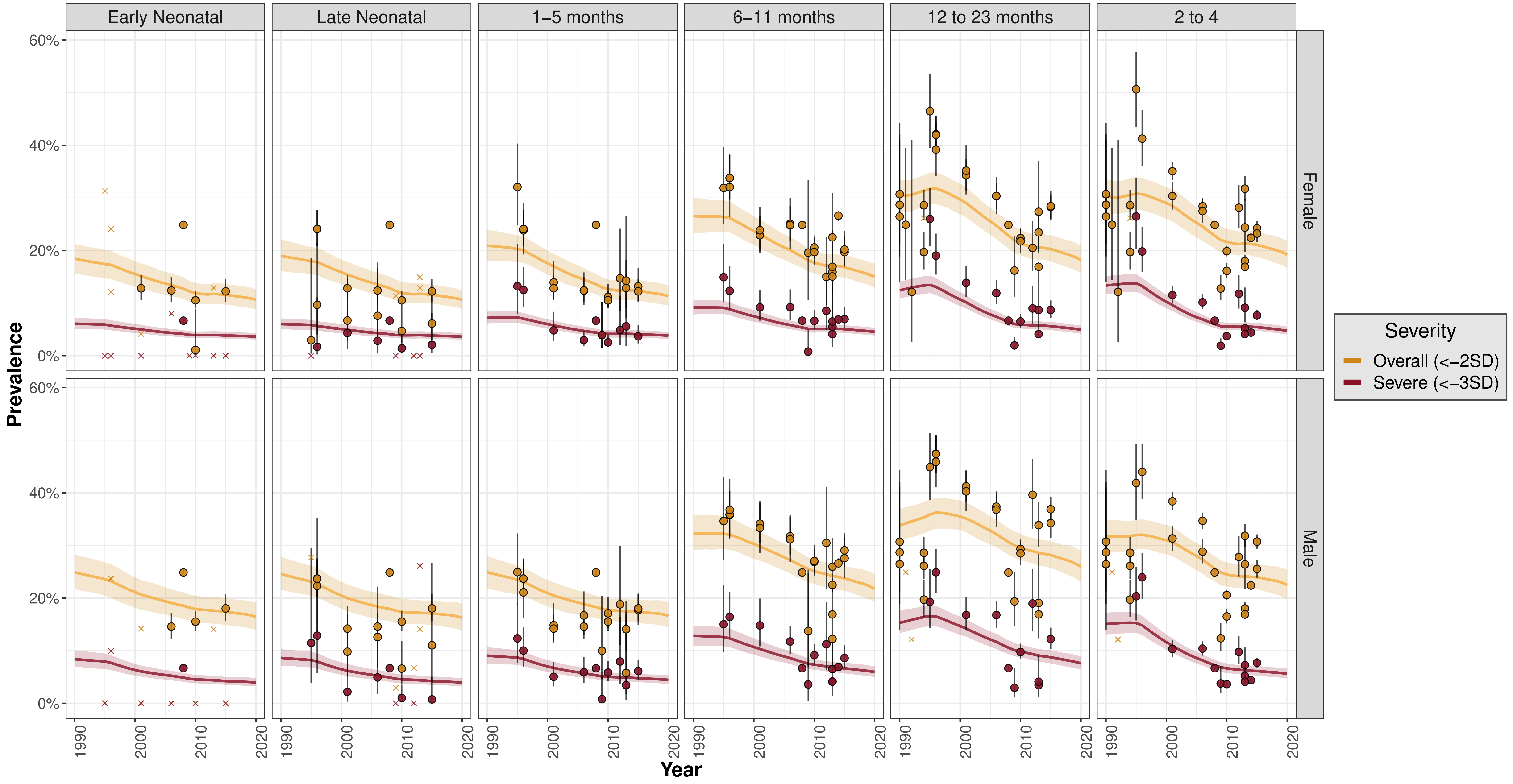

H: Transformed Mean Underweight Z Scores

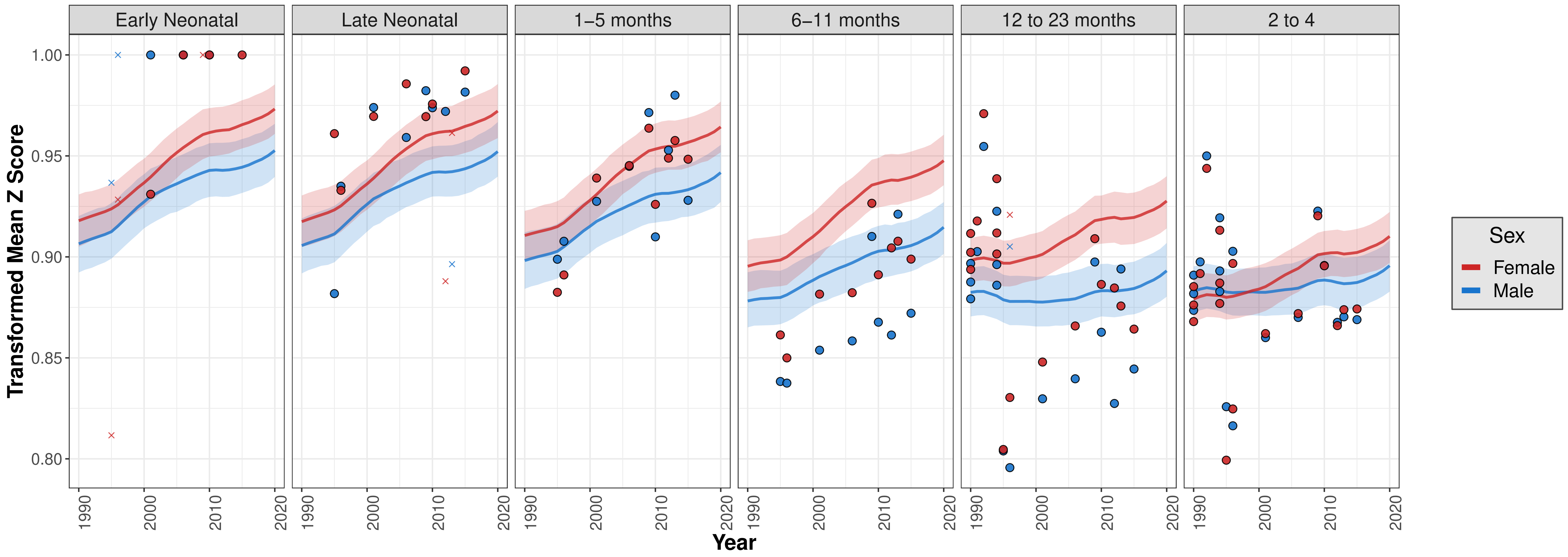

I

Year	Source
1987	DHS
1987	WHO CGM Database
1990	WHO CGM Database
1991	WHO CGM Database
1992	WHO CGM Database
1994	WHO CGM Database
1995	DHS
1996	DHS
1996	WHO CGM Database
2001	DHS
2001	WHO CGM Database
2006	DHS
2006	WHO CGM Database
2008	Food Security and Nutrition Study Baseline
2009	Bamako Global Enteric Multicenter Study
2009	MICS
2010	WHO CGM Database
2010	MICS
2012	DHS
2012	Bamako Global Enteric Multicenter Study
2013	DHS
2013	WHO CGM Database
2013	National Anthropometric Nutrition Survey and Mortality Retrospective July – August
2013	National Anthropometric Nutrition Survey and Mortality Retrospective June–August
2014	National Anthropometric Nutrition Survey and Mortality Retrospective June – August
2015	WHO CGM Database
2015	MICS

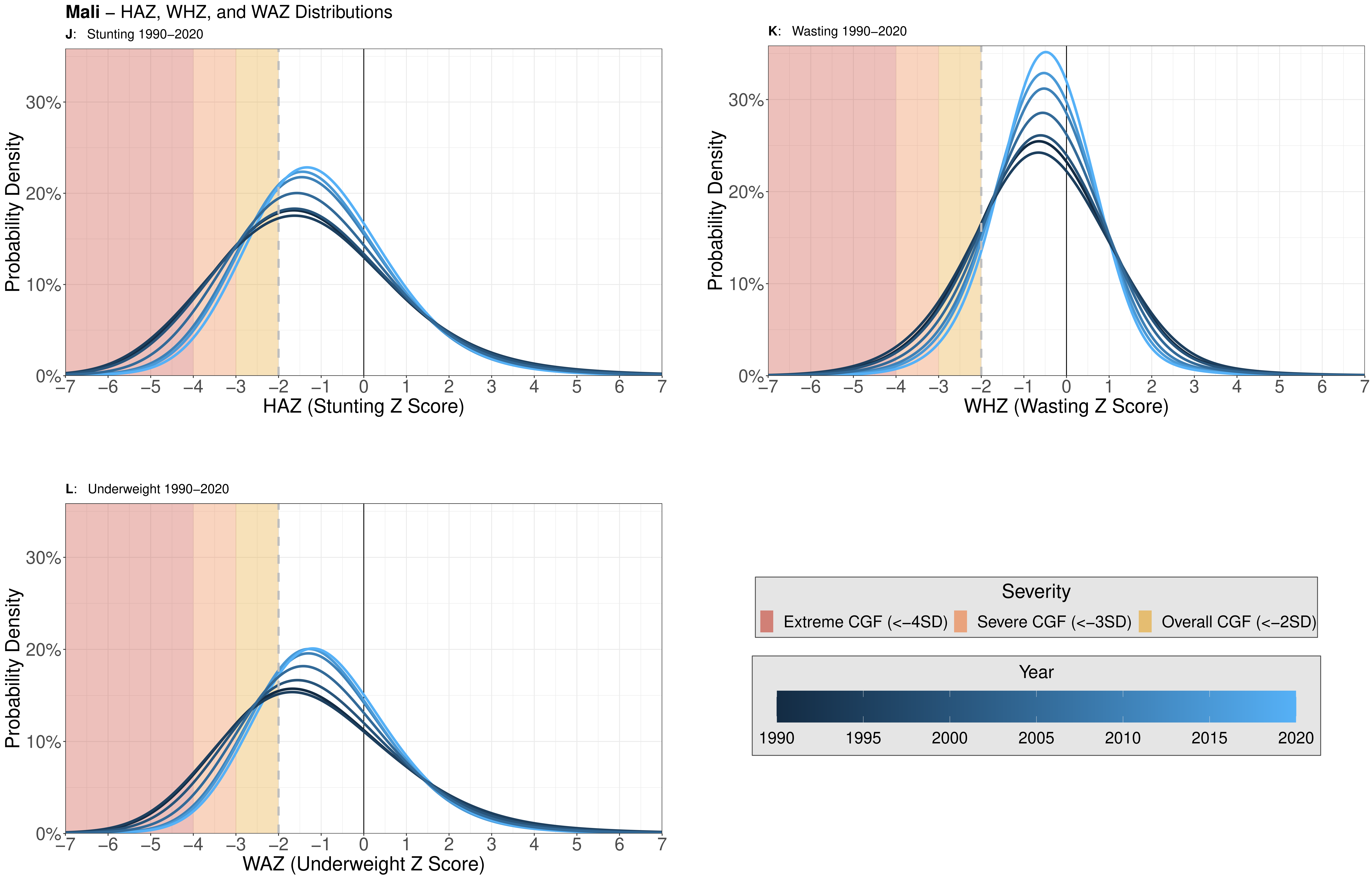
Mali – HAZ, WHZ, and WAZ Distributions
J: Stunting 1990–2020
K: Wasting 1990–2020
L: Underweight 1990–2020
Probability Density
0%
10%
20%
30%
−7 −6 −5 −4 −3 −2 −1 0 1 2 3 4 5 6 7
HAZ (Stunting Z Score)
WHZ (Wasting Z Score)
WAZ (Underweight Z Score)
Severity
Extreme CGF (<−4SD)
Severe CGF (<−3SD)
Overall CGF (<−2SD)
Year
1990
1995
2000
2005
2010
2015
2020

Mauritania – Stunting (HAZ)

A: Overall and Severe Stunting Prevalence

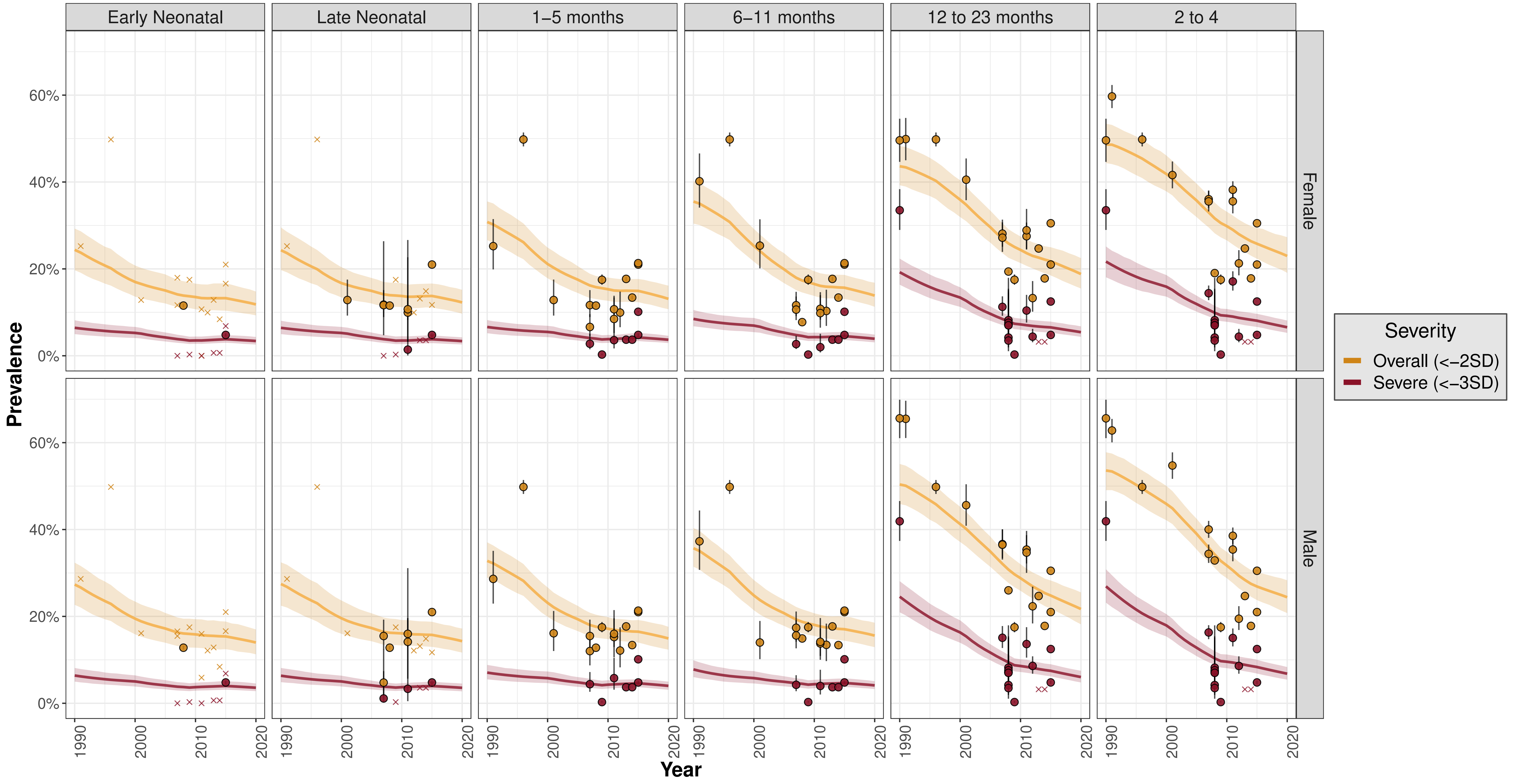

B: Transformed Mean Stunting Z Scores

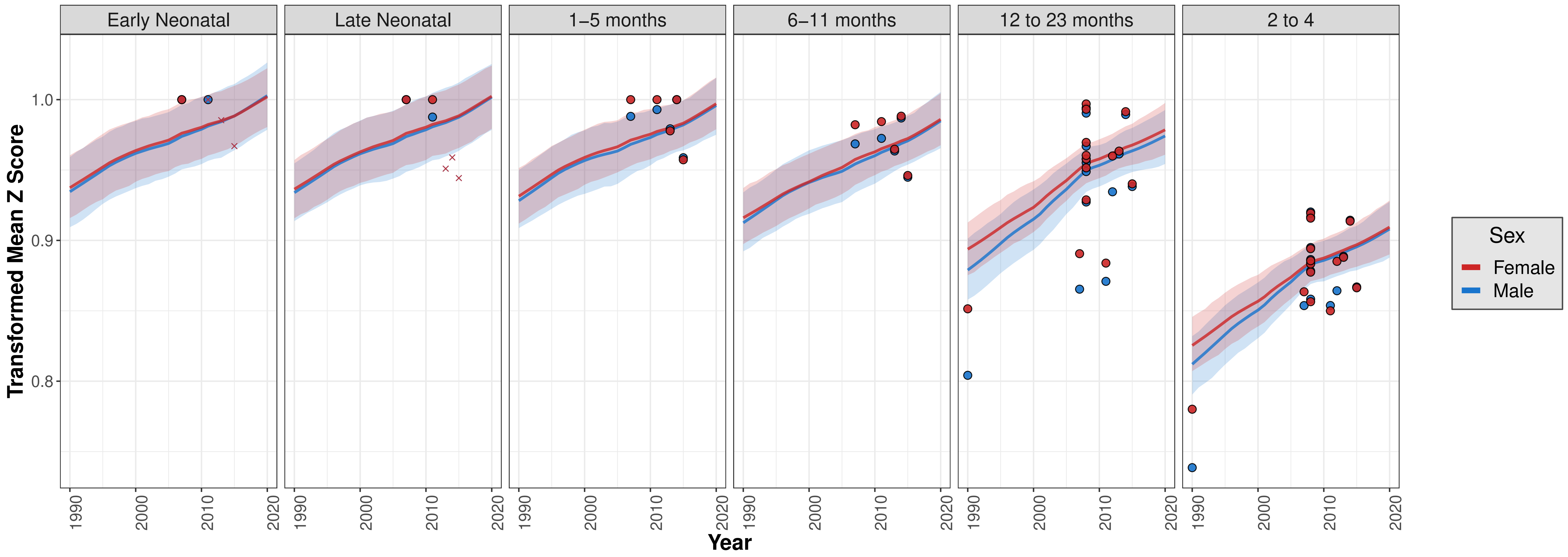

C

Year	Source
1988	WHO CGM Database
1990	WHO CGM Database
1991	WHO CGM Database
1996	WHO CGM Database
2000	DHS
2001	DHS
2001	WHO CGM Database
2007	MICS
2007	WHO CGM Database
2008	WHO CGM Database
2009	Household Food Security Survey
2011	WHO CGM Database
2011	MICS
2012	WHO CGM Database
2013	National Nutrition Survey July
2014	National Nutrition Survey Using the SMART Methodology August
2015	MICS
2015	National Nutrition Survey Using the SMART Methodology June

Mauritania – Wasting (WHZ)

D: Overall and Severe Wasting Prevalence

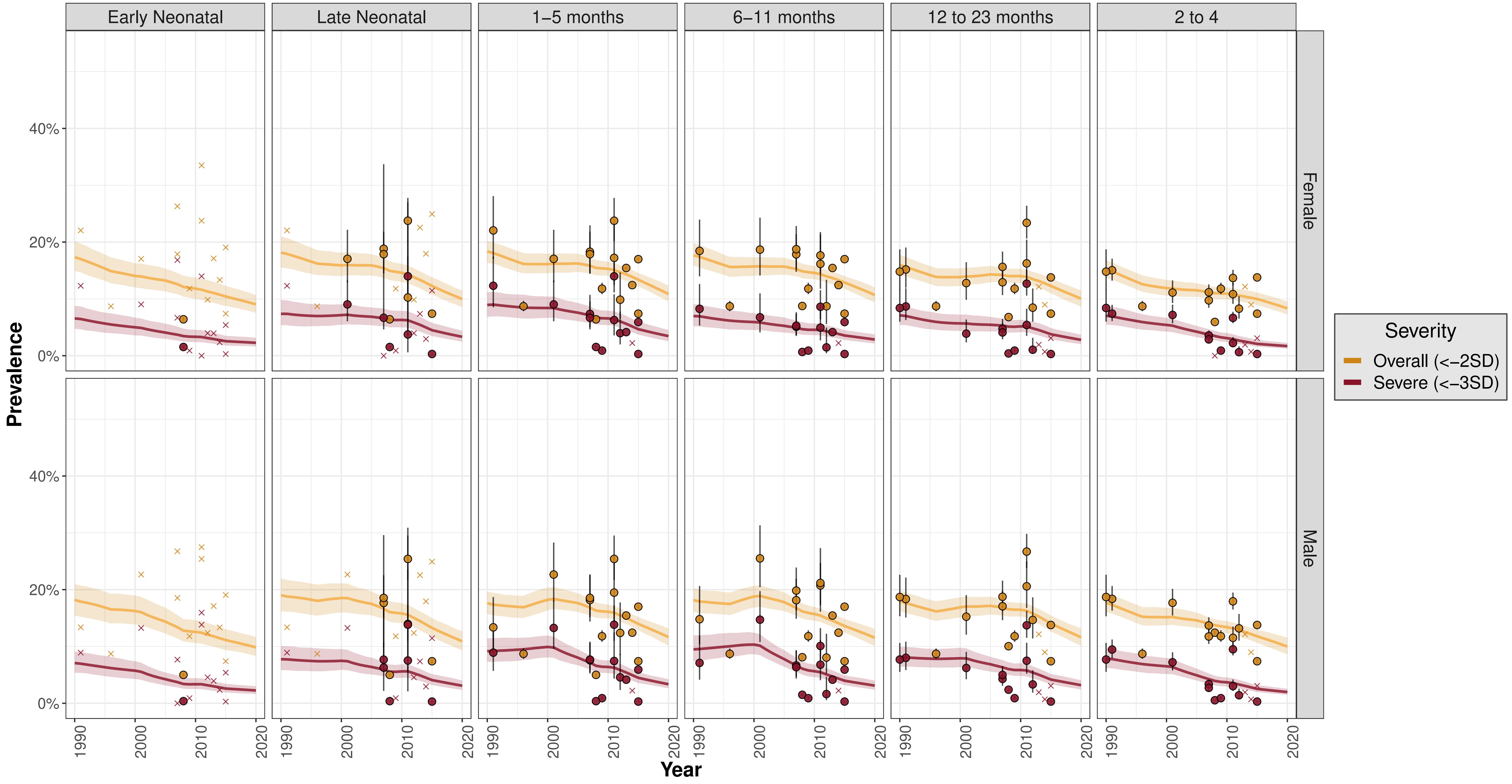

E: Transformed Mean Wasting Z Scores

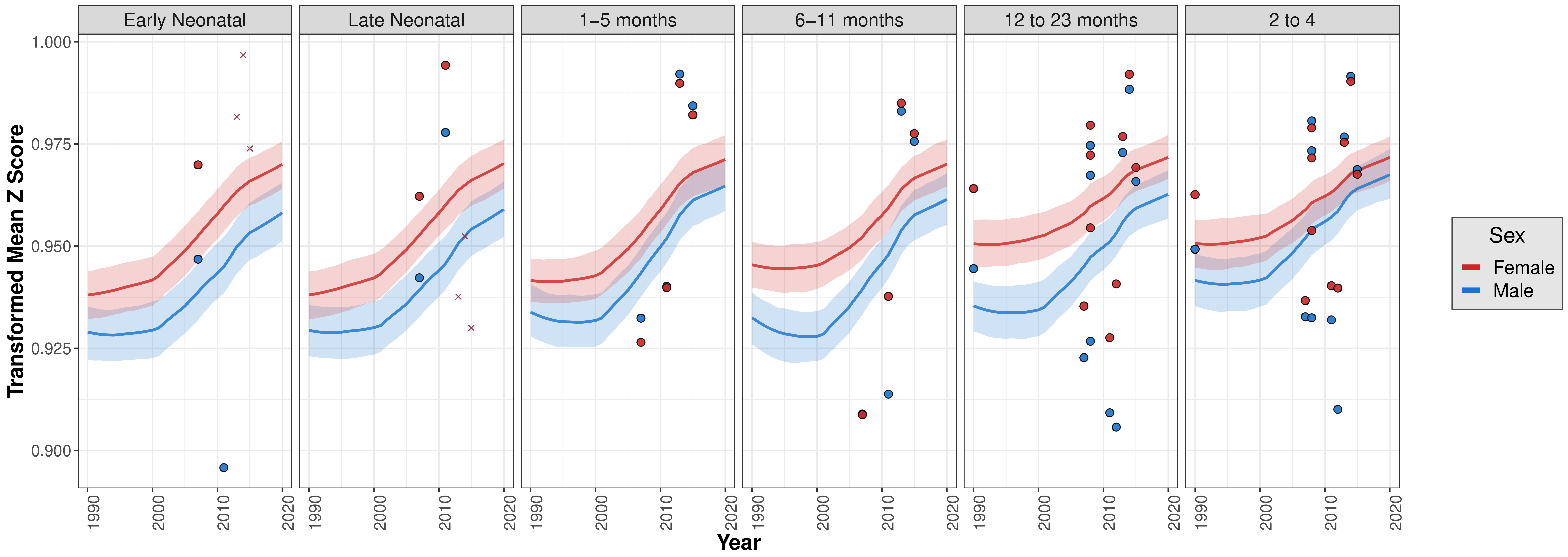

F

Year	Source
1988	WHO CGM Database
1990	WHO CGM Database
1991	WHO CGM Database
1996	WHO CGM Database
2000	DHS
2001	DHS
2001	WHO CGM Database
2007	MICS
2007	WHO CGM Database
2008	WHO CGM Database
2009	Household Food Security Survey
2011	WHO CGM Database
2011	MICS
2012	WHO CGM Database
2013	National Nutrition Survey July
2014	National Nutrition Survey Using the SMART Methodology August
2015	MICS
2015	National Nutrition Survey Using the SMART Methodology June

Mauritania – Underweight (WAZ)

G: Overall and Severe Underweight Prevalence

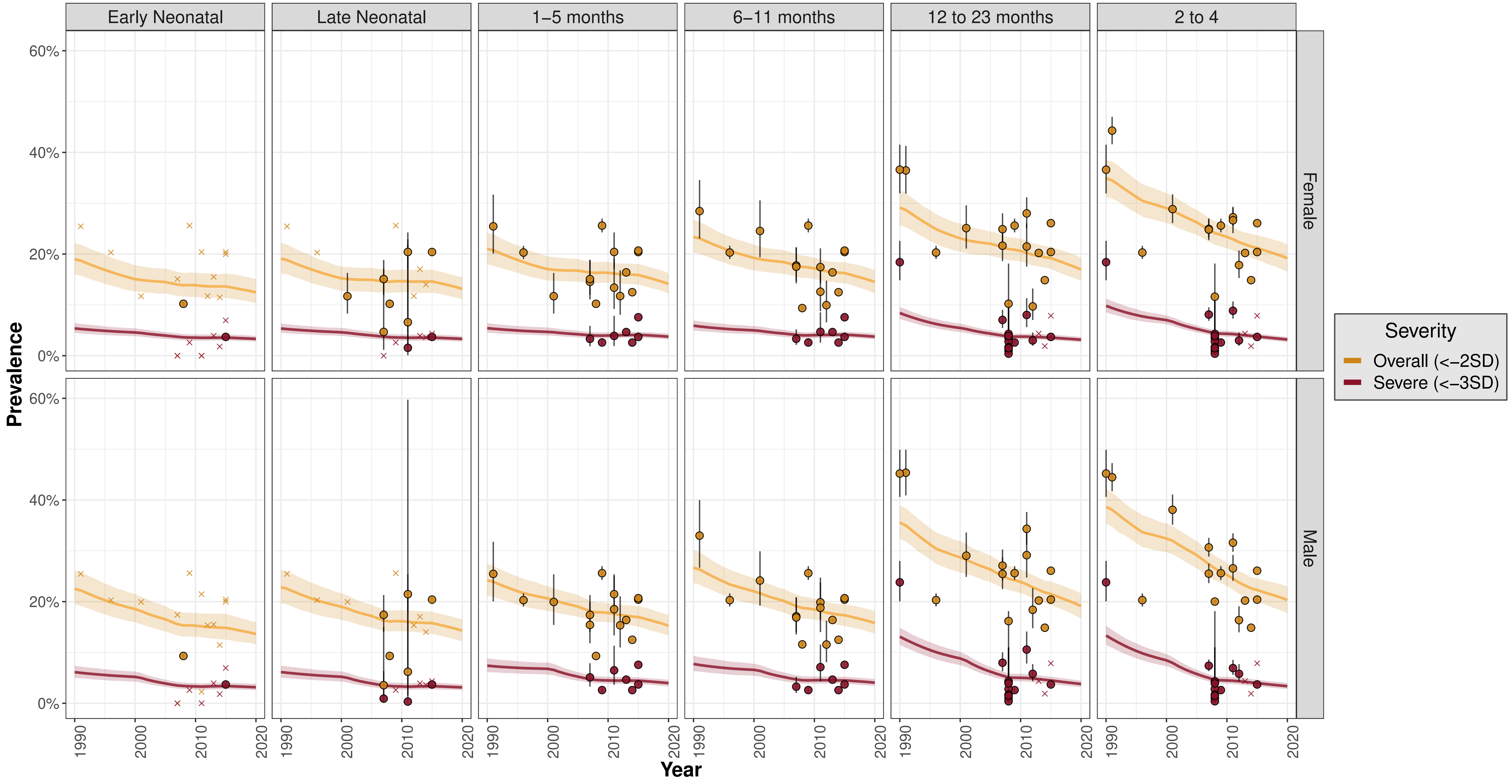

H: Transformed Mean Underweight Z Scores

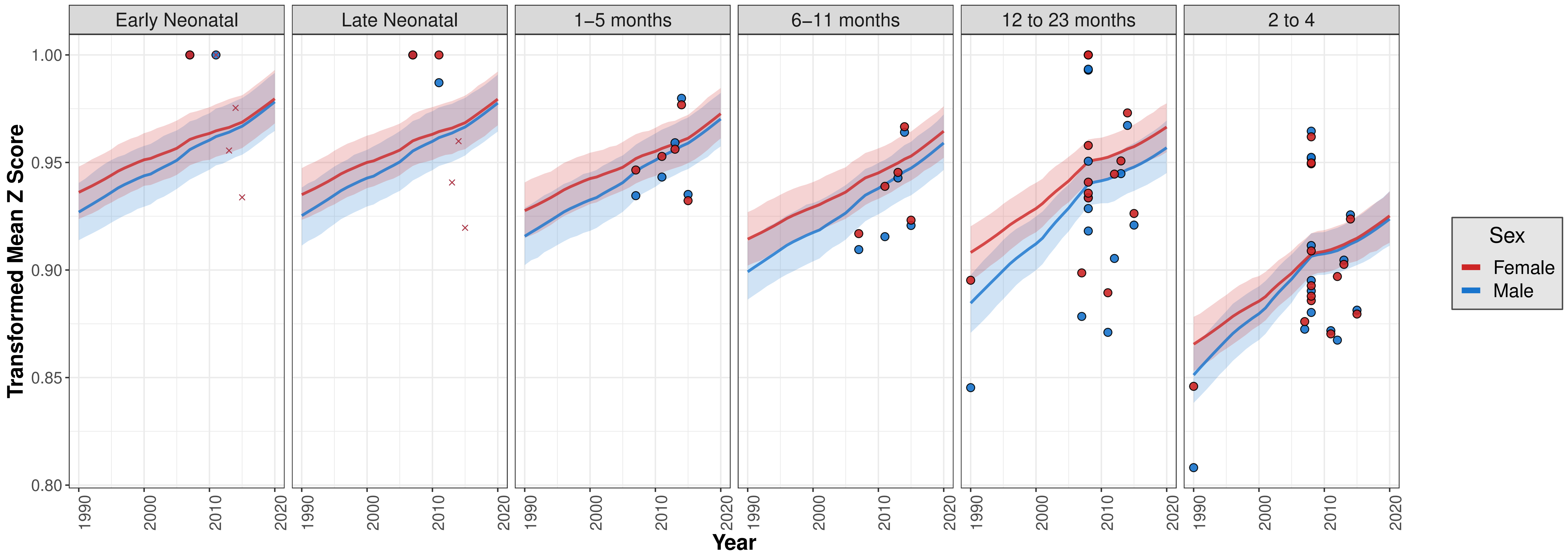

I

Year	Source
1990	WHO CGM Database
1991	WHO CGM Database
1996	WHO CGM Database
2000	DHS
2001	DHS
2001	WHO CGM Database
2007	MICS
2007	WHO CGM Database
2008	WHO CGM Database
2009	Household Food Security Survey
2011	WHO CGM Database
2011	MICS
2012	WHO CGM Database
2013	National Nutrition Survey July
2014	National Nutrition Survey Using the SMART Methodology August
2015	MICS
2015	National Nutrition Survey Using the SMART Methodology June

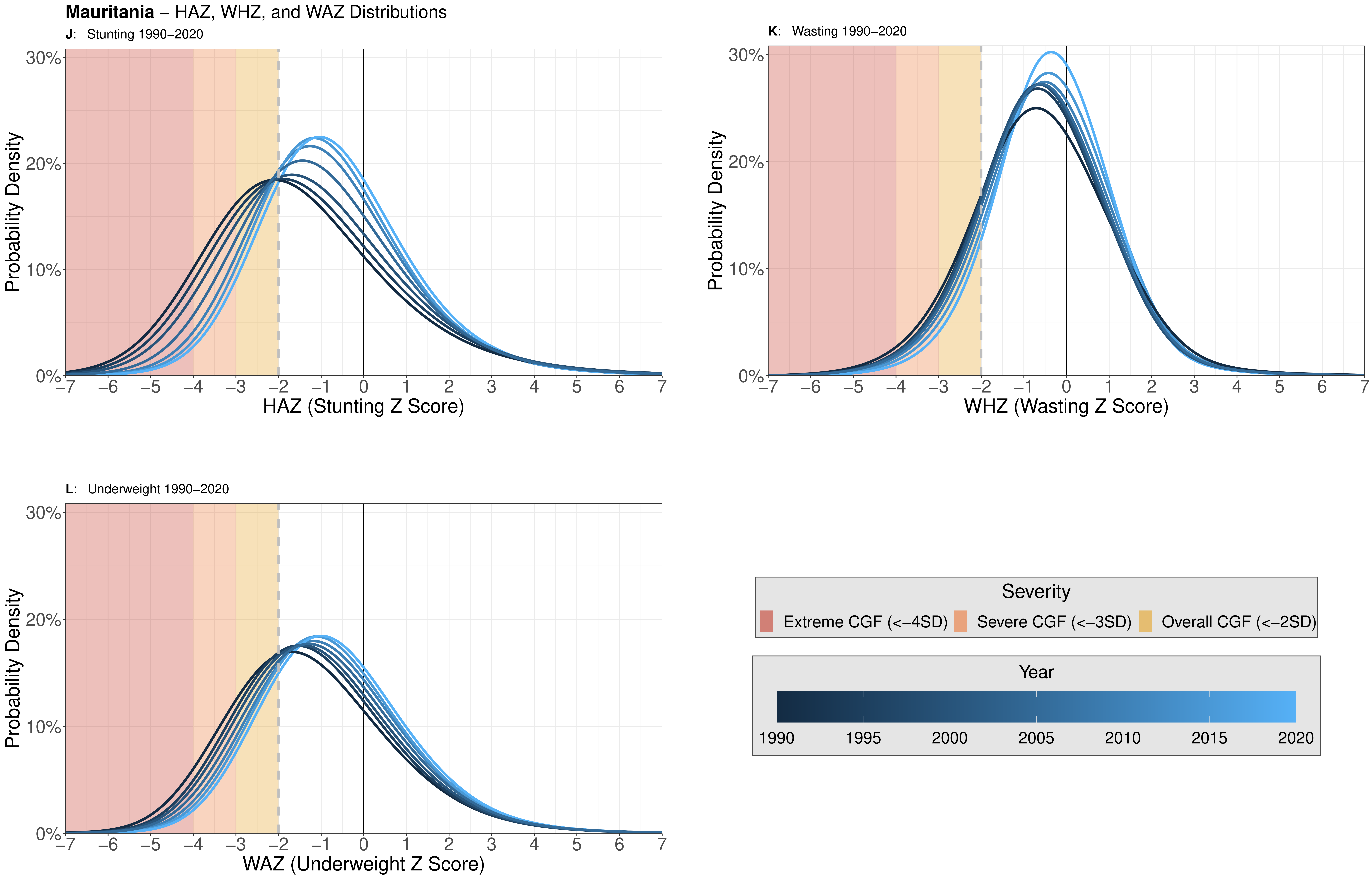
Mauritania – HAZ, WHZ, and WAZ Distributions
J: Stunting 1990–2020
Probability Density
0%
10%
20%
30%
−7 −6 −5 −4 −3 −2 −1 0 1 2 3 4 5 6 7
HAZ (Stunting Z Score)
K: Wasting 1990–2020
Probability Density
0%
10%
20%
30%
−7 −6 −5 −4 −3 −2 −1 0 1 2 3 4 5 6 7
WHZ (Wasting Z Score)
L: Underweight 1990–2020
Probability Density
0%
10%
20%
30%
−7 −6 −5 −4 −3 −2 −1 0 1 2 3 4 5 6 7
WAZ (Underweight Z Score)
Severity
Extreme CGF (<−4SD)
Severe CGF (<−3SD)
Overall CGF (<−2SD)
Year
1990
1995
2000
2005
2010
2015
2020

Niger – Stunting (HAZ)

A: Overall and Severe Stunting Prevalence

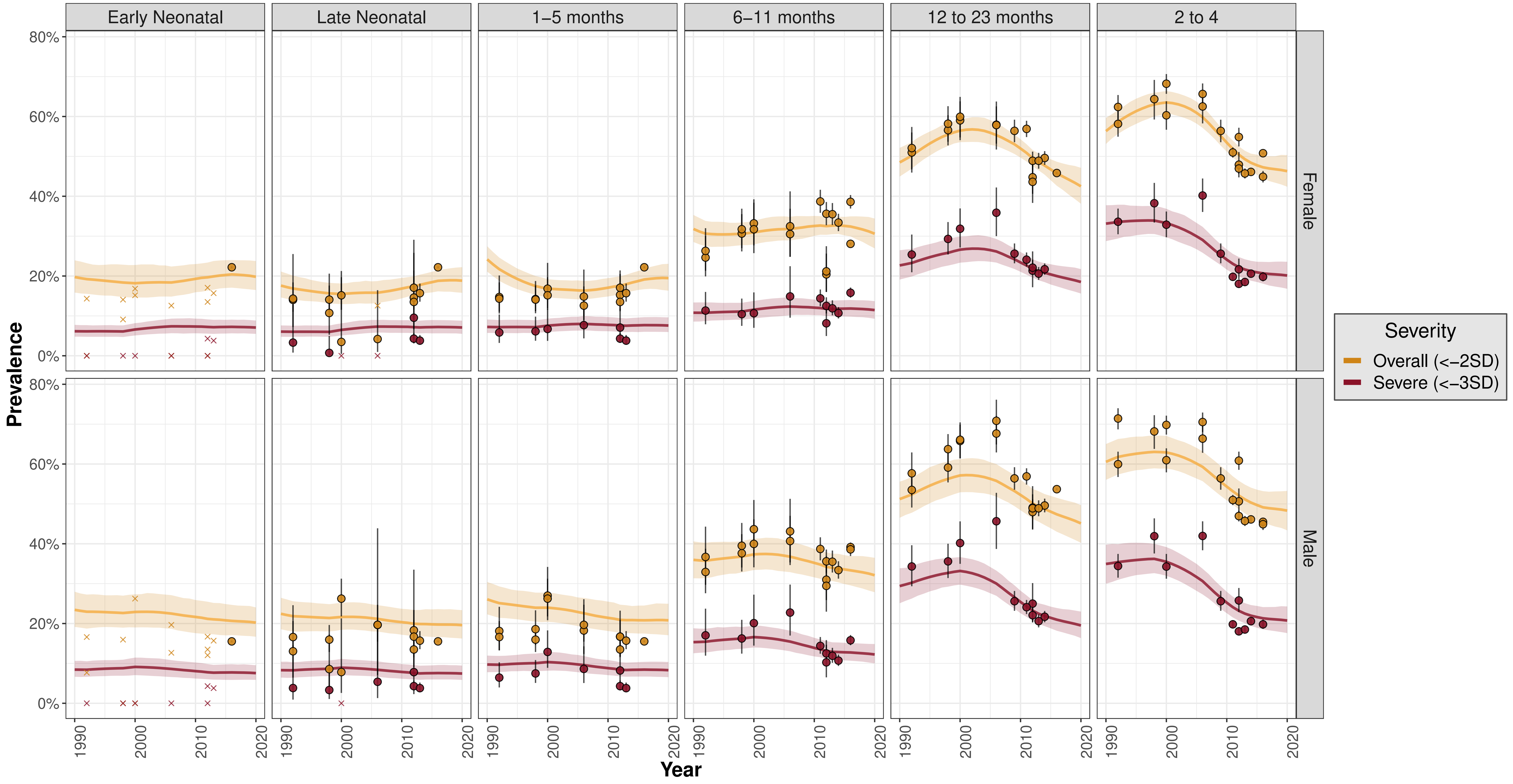

B: Transformed Mean Stunting Z Scores

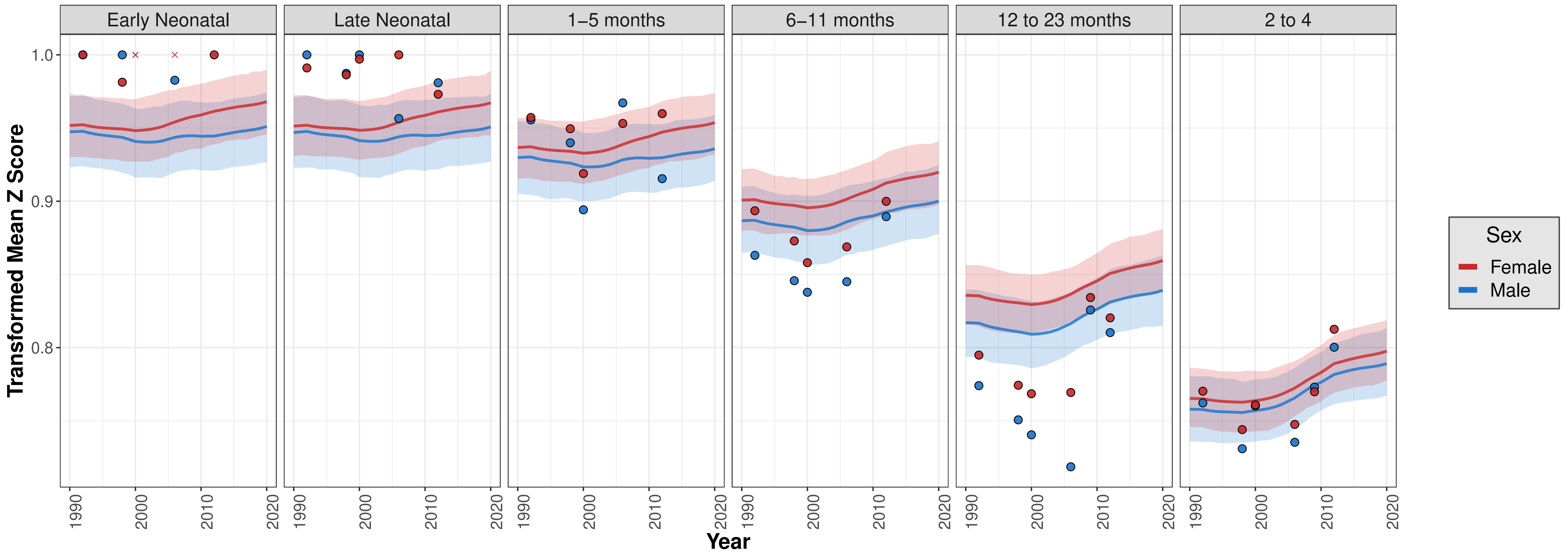

C

Year	Source
1985	WHO CGM Database
1987	WHO CGM Database
1992	DHS
1992	WHO CGM Database
1998	DHS
1998	WHO CGM Database
2000	MICS
2000	WHO CGM Database
2006	DHS
2006	WHO CGM Database
2009	Nutrition and Child Survival Survey
2011	Nutrition and Child Survival Survey
2012	DHS
2012	WHO CGM Database
2012	Nutrition and Child Survival Survey
2013	Nutrition and Child Survival Survey
2014	Nutrition and Child Survival Survey
2016	WHO CGM Database
2016	National Nutrition Survey Using SMART Methodology

Niger – Wasting (WHZ)

D: Overall and Severe Wasting Prevalence

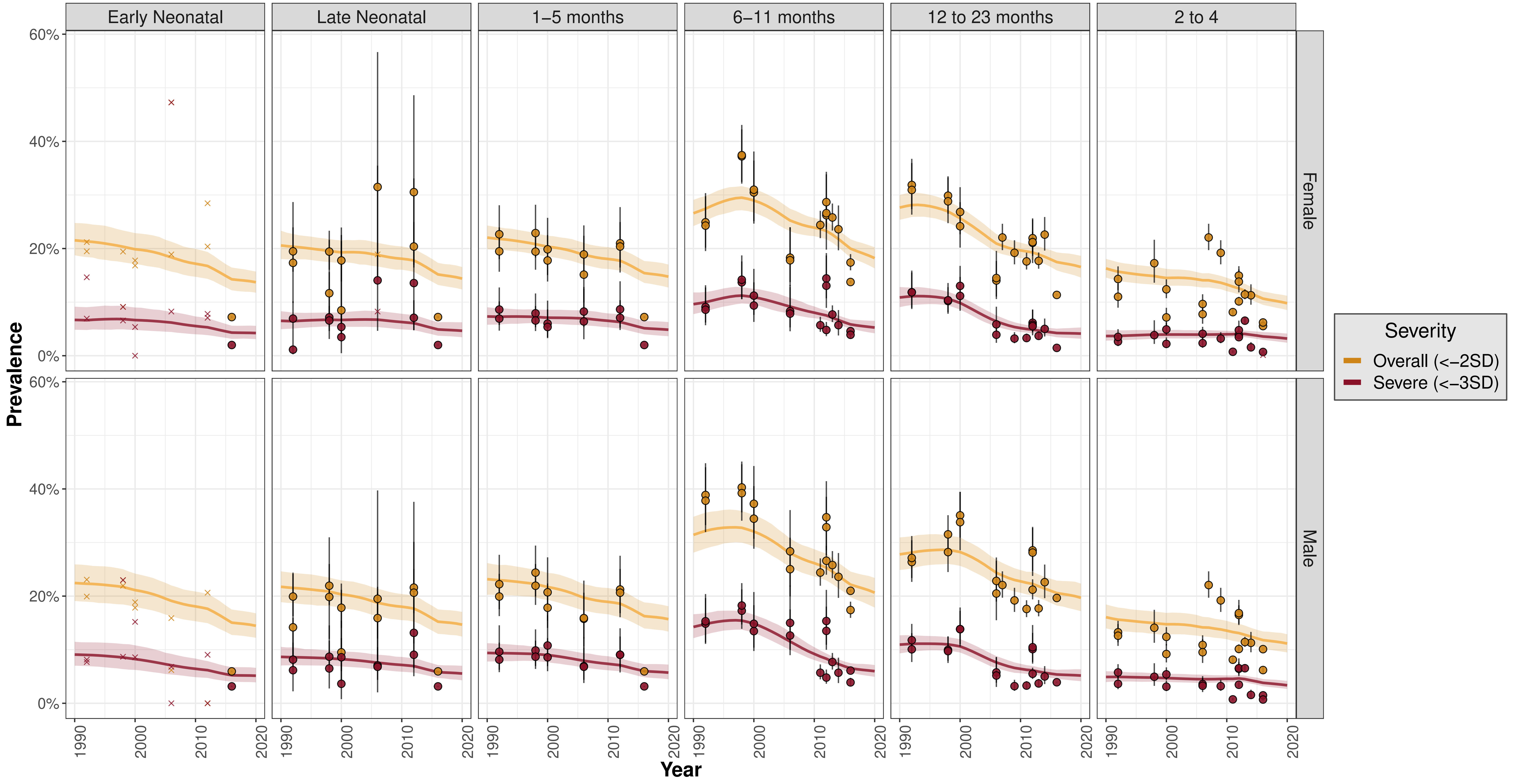

E: Transformed Mean Wasting Z Scores

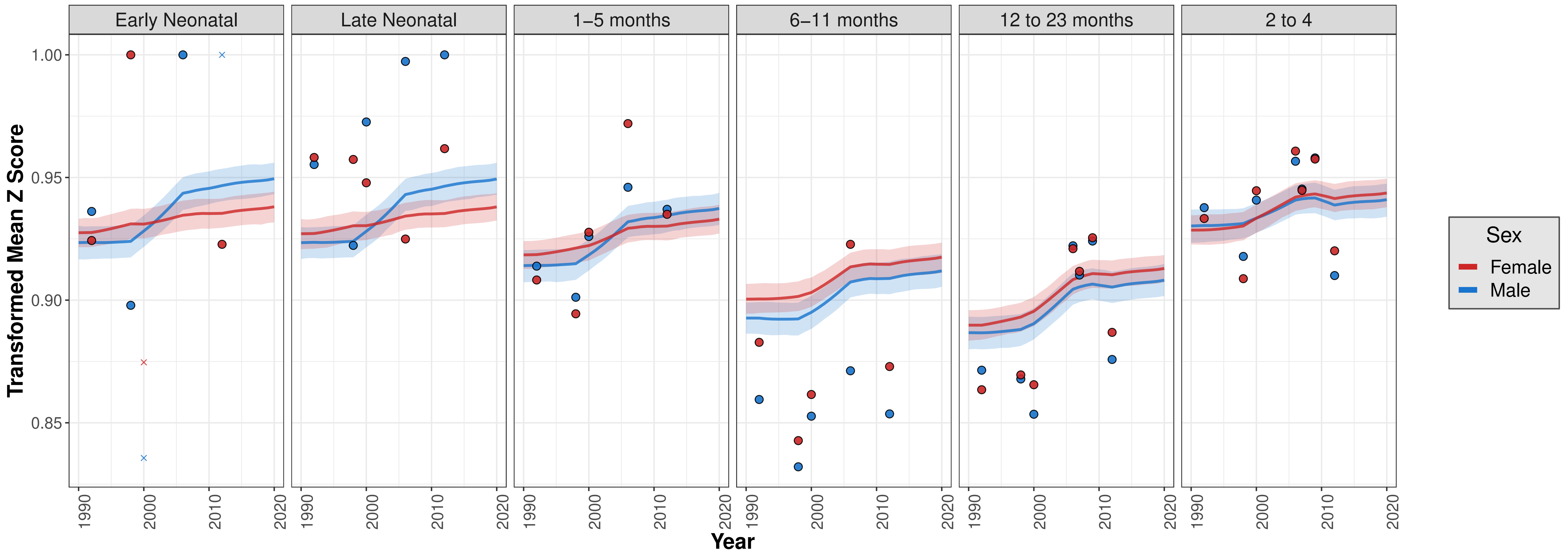

F

Year	Source
1985	WHO CGM Database
1987	WHO CGM Database
1992	DHS
1992	WHO CGM Database
1998	DHS
1998	WHO CGM Database
2000	MICS
2000	WHO CGM Database
2006	DHS
2006	WHO CGM Database
2007	Nutrition and Child Survival Survey
2009	Nutrition and Child Survival Survey
2011	Nutrition and Child Survival Survey
2012	DHS
2012	WHO CGM Database
2012	Nutrition and Child Survival Survey
2013	Nutrition and Child Survival Survey
2014	Nutrition and Child Survival Survey
2016	WHO CGM Database
2016	National Nutrition Survey Using SMART Methodology

Niger – Underweight (WAZ)

G: Overall and Severe Underweight Prevalence

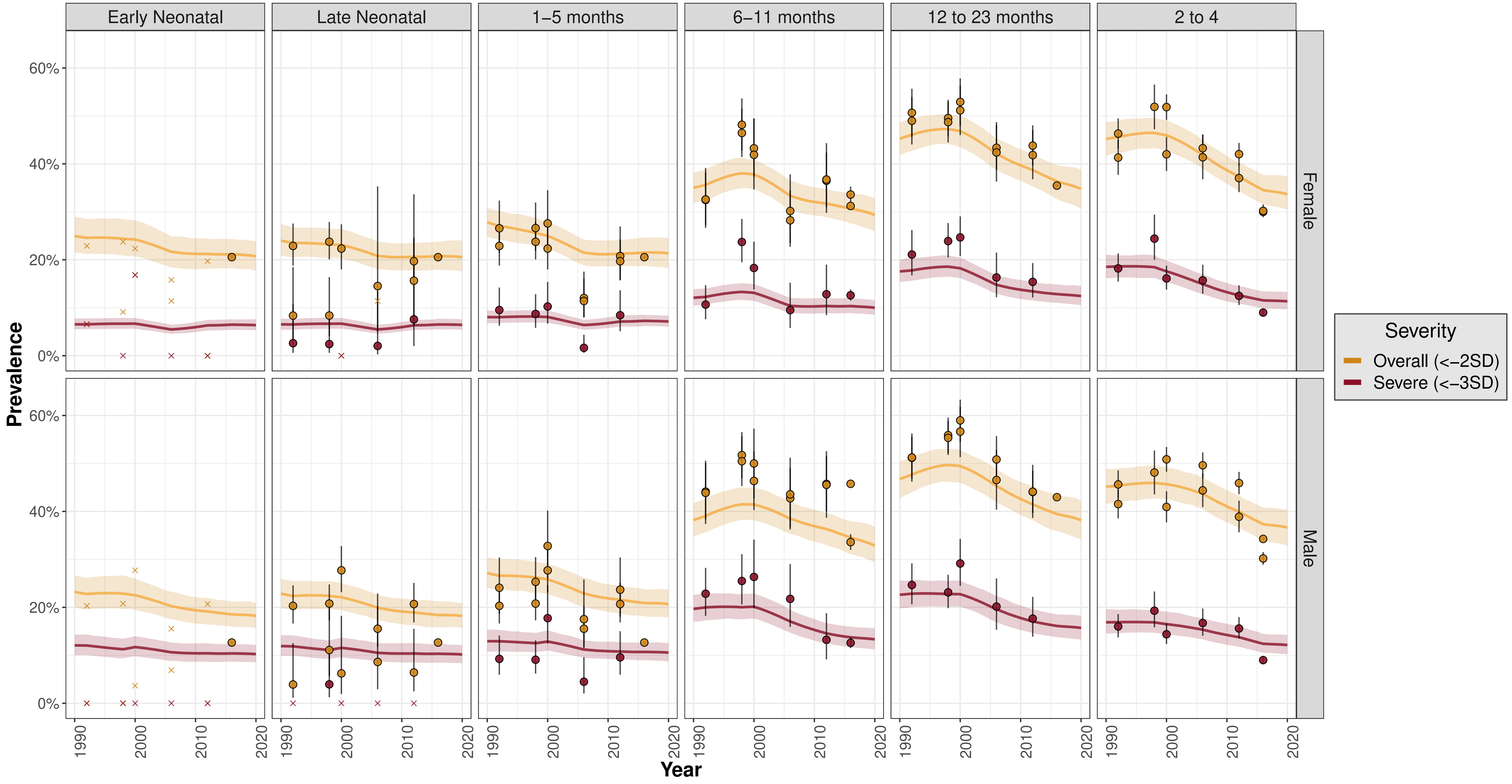

H: Transformed Mean Underweight Z Scores

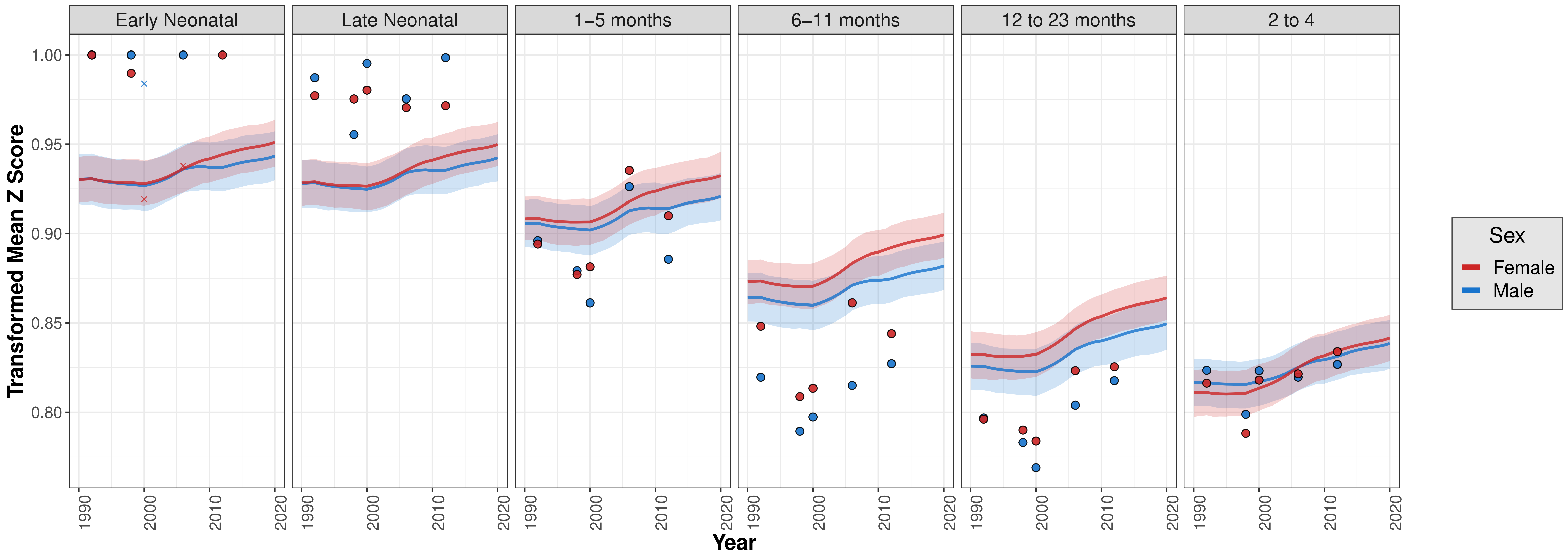

I

Year	Source
1985	WHO CGM Database
1987	WHO CGM Database
1992	DHS
1992	WHO CGM Database
1998	DHS
1998	WHO CGM Database
2000	MICS
2000	WHO CGM Database
2006	DHS
2006	WHO CGM Database
2012	DHS
2012	WHO CGM Database
2016	WHO CGM Database
2016	National Nutrition Survey Using SMART Methodology

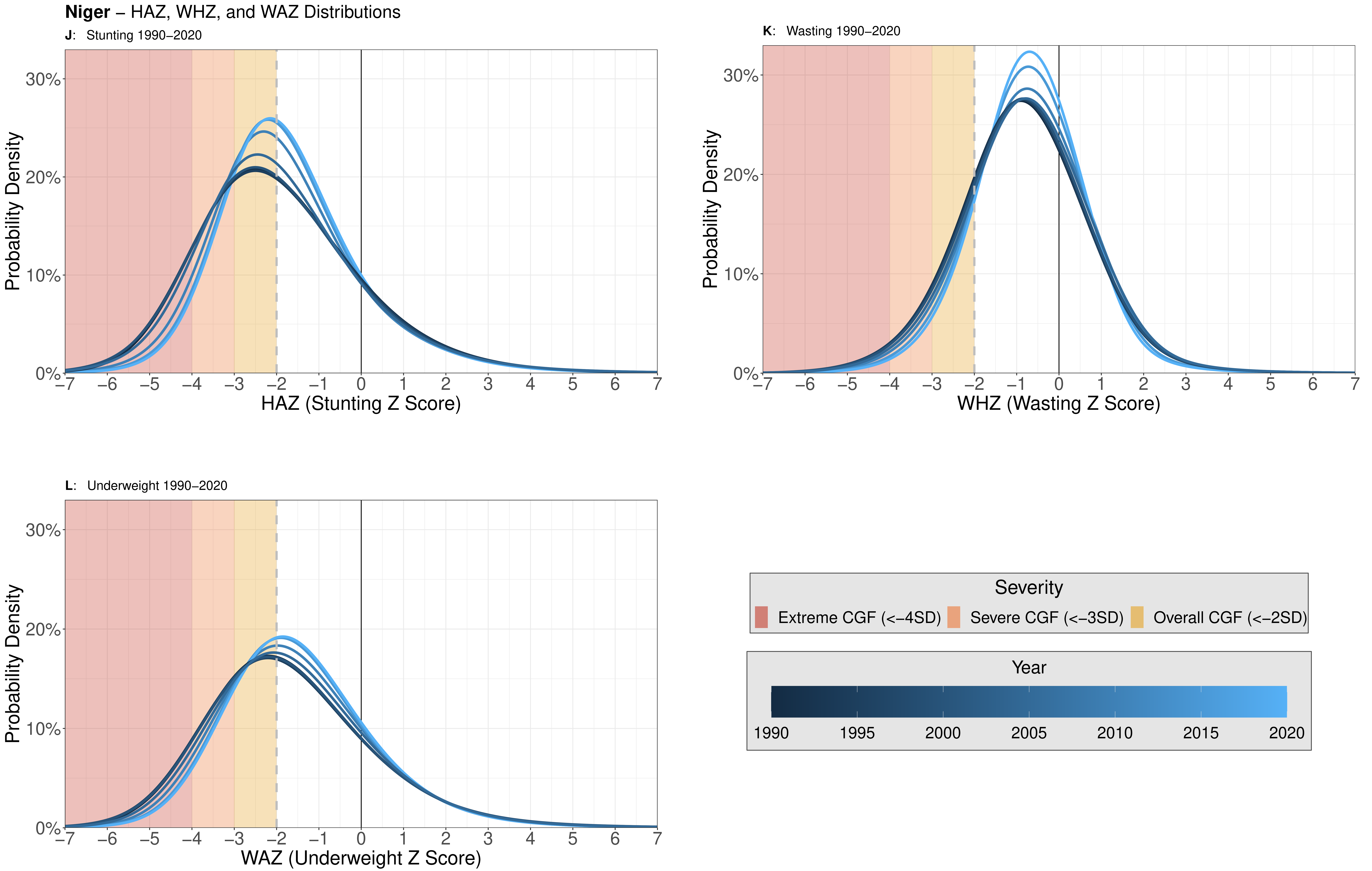

Niger – HAZ, WHZ, and WAZ Distributions
J: Stunting 1990–2020
Probability Density
HAZ (Stunting Z Score)
K: Wasting 1990–2020
WHZ (Wasting Z Score)
L: Underweight 1990–2020
WAZ (Underweight Z Score)
0%
10%
20%
30%
−7 −6 −5 −4 −3 −2 −1 0 1 2 3 4 5 6 7
Severity
Extreme CGF (<−4SD)
Severe CGF (<−3SD)
Overall CGF (<−2SD)
Year
1990
1995
2000
2005
2010
2015
2020

Nigeria – Stunting (HAZ)

A: Overall and Severe Stunting Prevalence

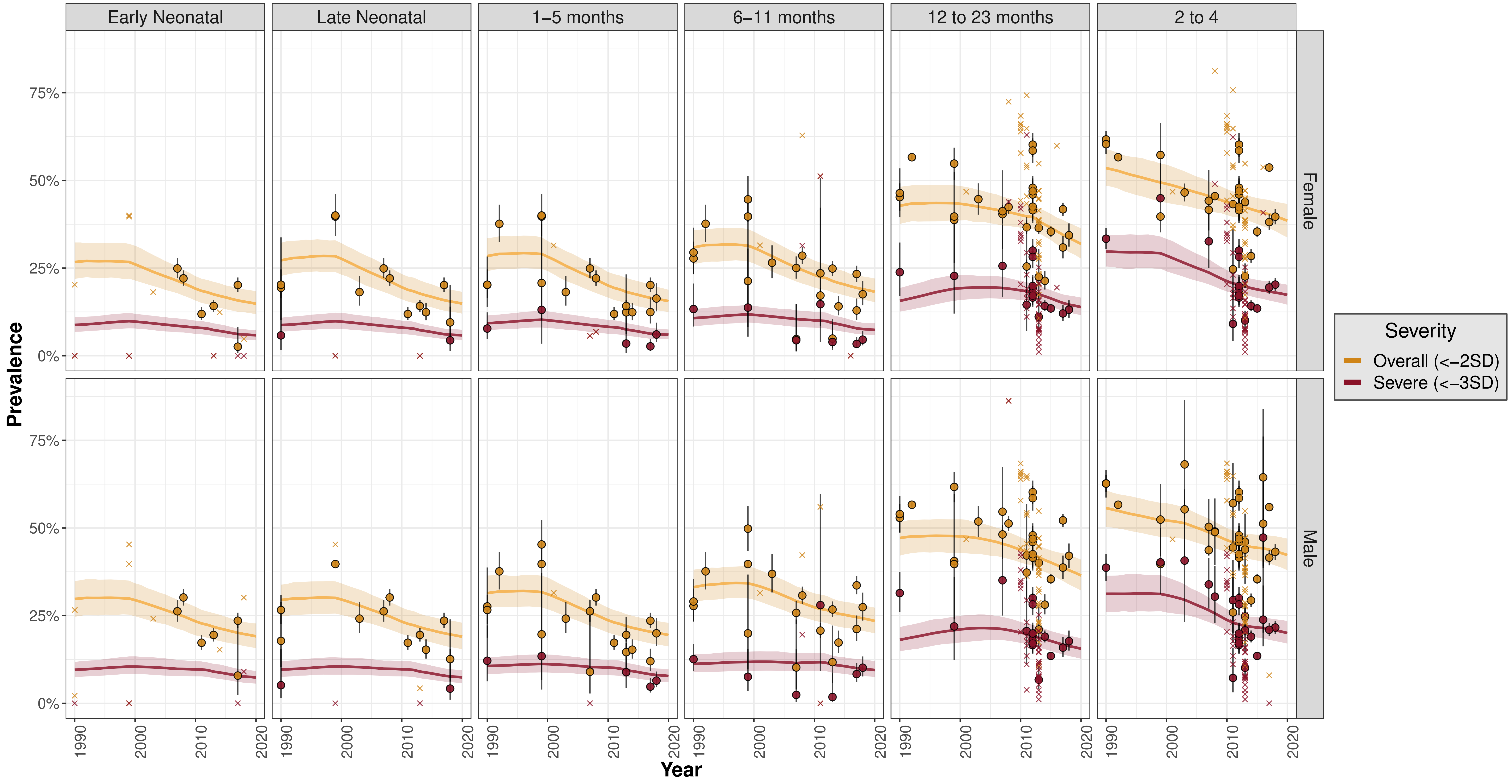

B: Transformed Mean Stunting Z Scores

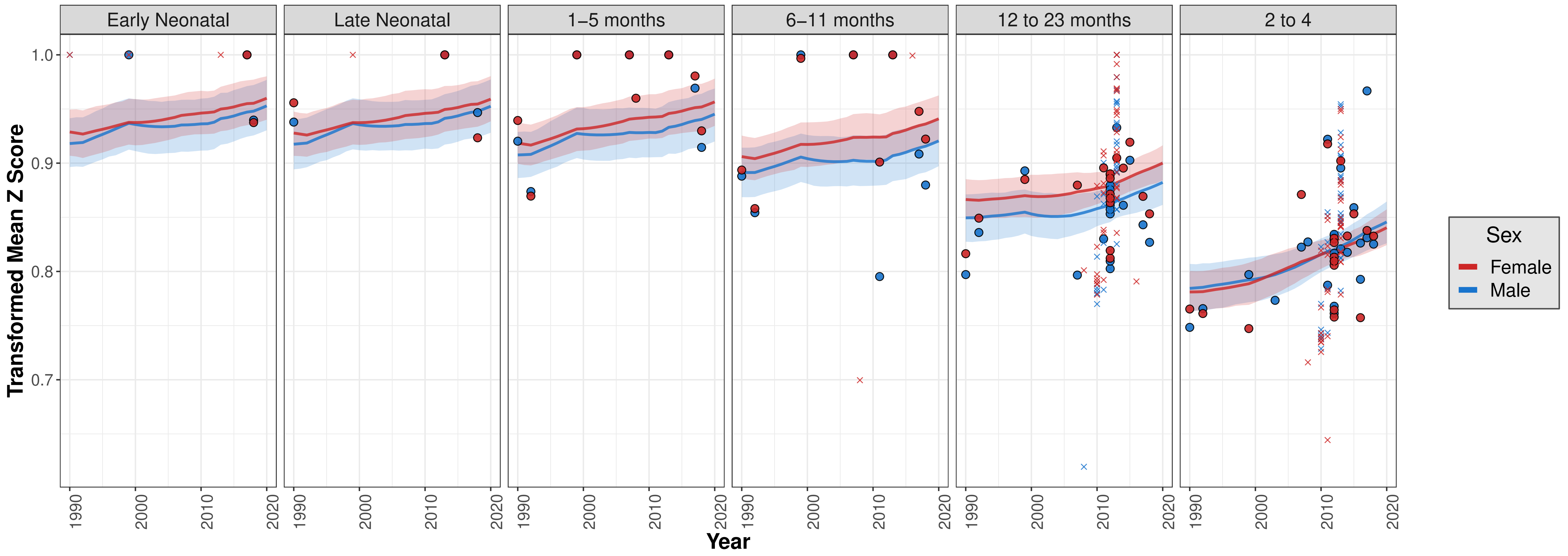

C

Year	Source	National	Subnational
1986	Ondo Special DHS	X	
1987	Ondo Special DHS	X	
1990	DHS	X	
1990	WHO CGM Database	X	
1992	WHO CGM Database	X	
1999	MICS	X	X
1999	WHO CGM Database	X	
2001	WHO CGM Database	X	
2003	DHS	X	X
2003	WHO CGM Database	X	
2007	MICS	X	X
2007	WHO CGM Database	X	
2008	DHS	X	X
2008	WHO CGM Database	X	
2010	Standardized Monitoring and Assessment of Relief and Transitions Survey, December	X	X
2011	MICS	X	X
2011	WHO CGM Database	X	
2011	Standardized Monitoring and Assessment of Relief and Transitions Survey, July–August	X	X
2011	Akwa Ibom Survey on Dietary Intakes, Vitamin A, and Iron Status of Women of Childbearing Age and Children 6–59 Months of Age	X	
2012	Standardized Monitoring and Assessment of Relief and Transitions Survey, February–March	X	X
2012	Standardized Monitoring and Assessment of Relief and Transitions Survey, September–October	X	
2013	DHS	X	X
2013	WHO CGM Database	X	
2013	Standardized Monitoring and Assessment of Relief and Transitions Survey, Round V	X	X
2013	General Household Survey, Panel , Wave 2	X	
2014	WHO CGM Database	X	
2015	National Nutrition and Health Survey	X	
2016	MICS with National Immunization Coverage Survey Supplement	X	X
2016	General Household Survey Panel Wave 3	X	X
2017	WHO CGM Database	X	
2017	MICS with National Immunization Coverage Survey Supplement	X	X
2017	MICS with National Immunization Coverage Survey Supplement – National Bureau of Statistics	X	
2018	DHS	X	X

Nigeria – Wasting (WHZ)

D: Overall and Severe Wasting Prevalence

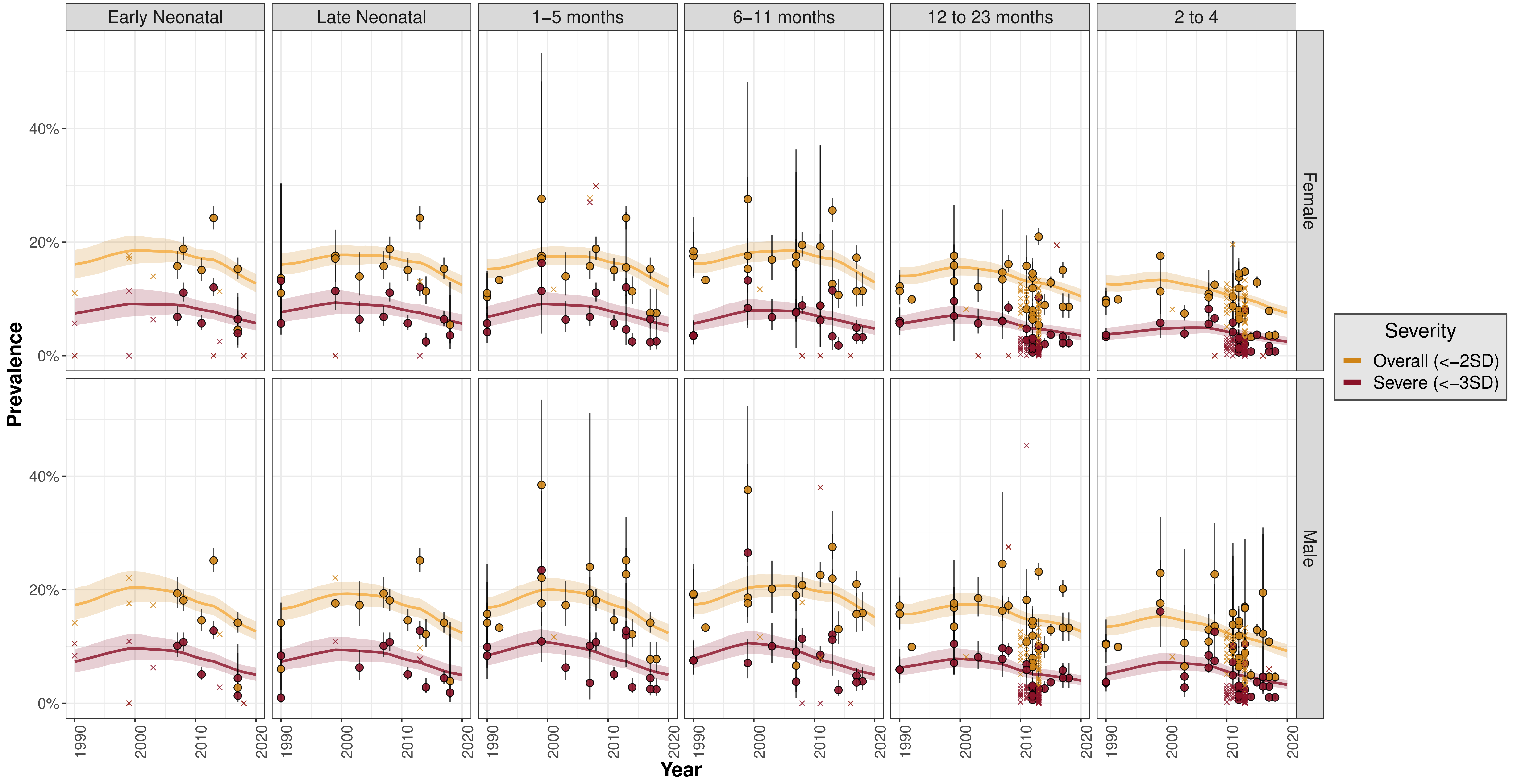

E: Transformed Mean Wasting Z Scores

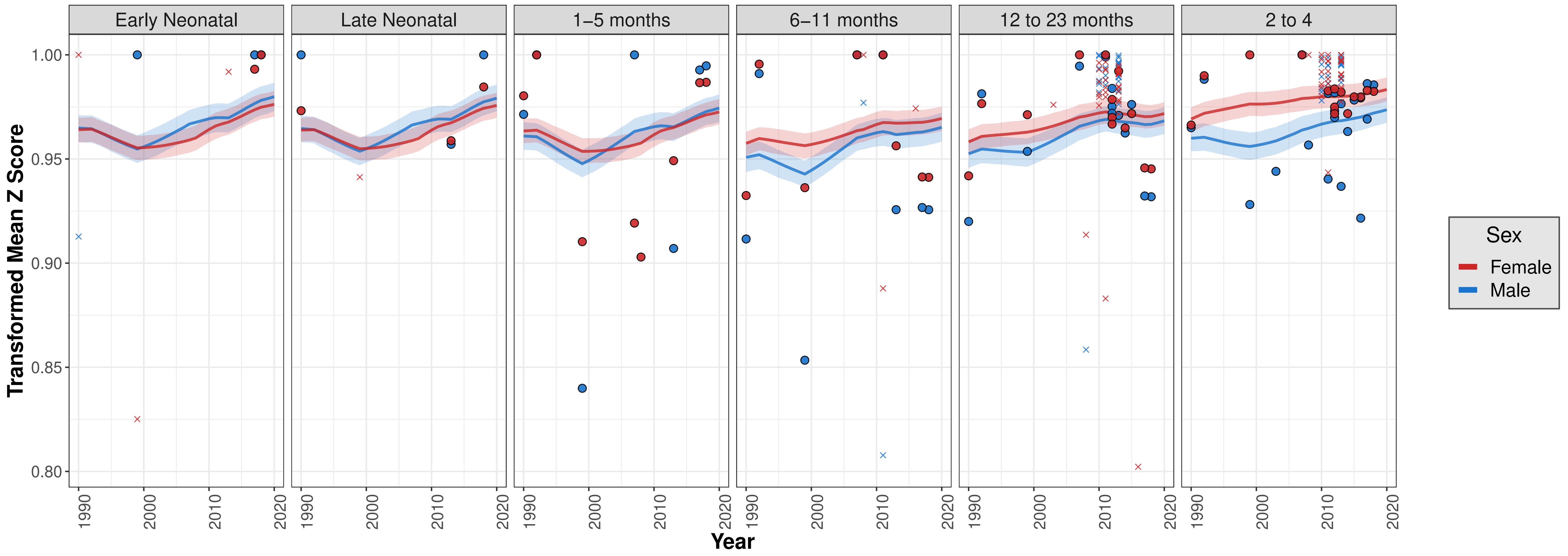

F

Year	Source	National	Subnational
1983	WHO CGM Database	X	
1986	Ondo Special DHS	X	
1987	Ondo Special DHS	X	
1990	DHS	X	
1990	WHO CGM Database	X	
1992	WHO CGM Database	X	
1999	MICS	X	X
1999	WHO CGM Database	X	
2001	WHO CGM Database	X	
2003	DHS	X	X
2003	WHO CGM Database	X	
2007	MICS	X	X
2007	WHO CGM Database	X	
2008	DHS	X	X
2008	WHO CGM Database	X	
2010	Standardized Monitoring and Assessment of Relief and Transitions Survey, December	X	X
2011	MICS	X	X
2011	WHO CGM Database	X	
2011	Standardized Monitoring and Assessment of Relief and Transitions Survey, July–August	X	X
2011	Akwa Ibom Survey on Dietary Intakes, Vitamin A, and Iron Status of Women of Childbearing Age and Children 6–59 Months of Age	X	
2012	Standardized Monitoring and Assessment of Relief and Transitions Survey, February–March	X	X
2012	Standardized Monitoring and Assessment of Relief and Transitions Survey, September–October	X	
2013	DHS	X	X
2013	WHO CGM Database	X	
2013	Standardized Monitoring and Assessment of Relief and Transitions Survey, Round V	X	X
2013	General Household Survey, Panel , Wave 2	X	
2014	WHO CGM Database	X	
2015	National Nutrition and Health Survey	X	
2016	MICS with National Immunization Coverage Survey Supplement	X	X
2016	General Household Survey Panel Wave 3	X	X
2017	WHO CGM Database	X	
2017	MICS with National Immunization Coverage Survey Supplement	X	X
2017	MICS with National Immunization Coverage Survey Supplement – National Bureau of Statistics	X	
2018	DHS	X	X

Nigeria – Underweight (WAZ)

G: Overall and Severe Underweight Prevalence

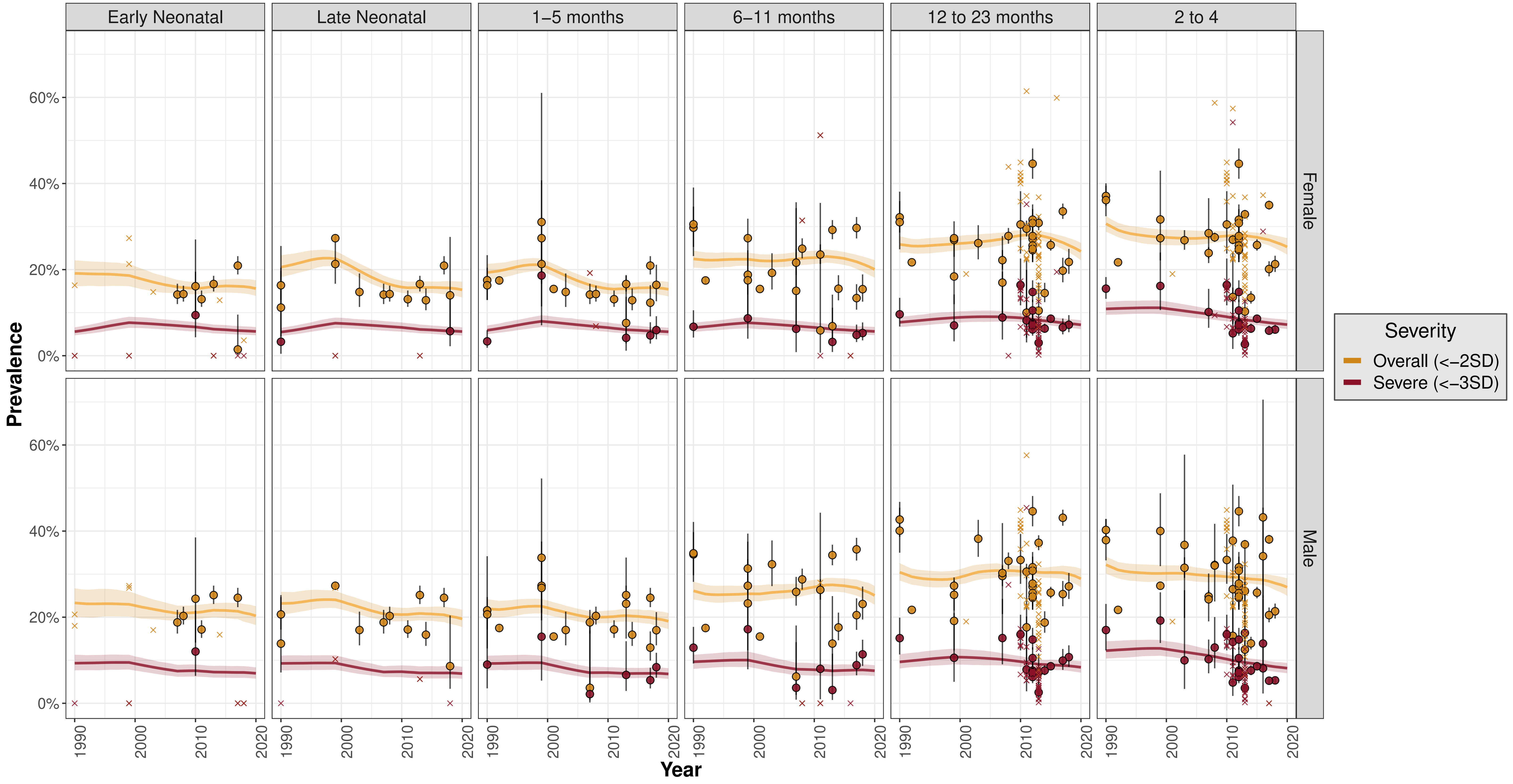

H: Transformed Mean Underweight Z Scores

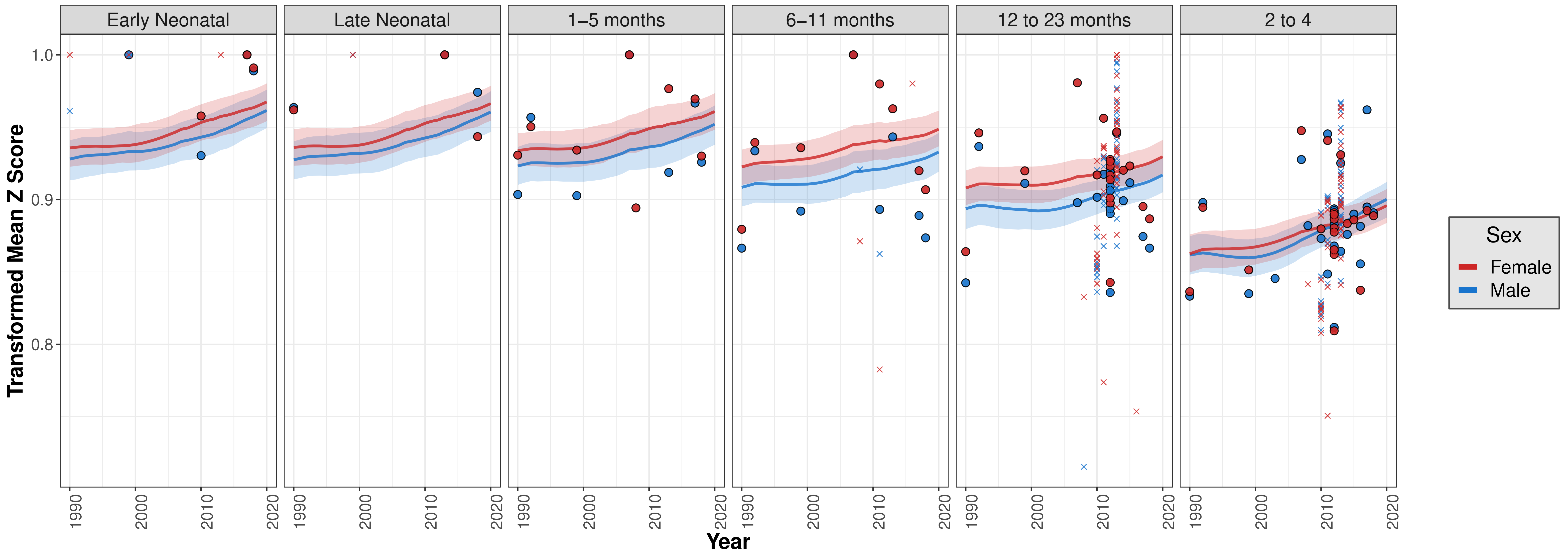

I

Year	Source	National	Subnational
1986	Ondo Special DHS	X	
1987	Ondo Special DHS	X	
1990	DHS	X	
1990	WHO CGM Database	X	
1992	WHO CGM Database	X	
1999	MICS	X	X
1999	WHO CGM Database	X	
2001	WHO CGM Database	X	
2003	DHS	X	X
2003	WHO CGM Database	X	
2007	MICS	X	X
2007	WHO CGM Database	X	
2008	DHS	X	X
2008	WHO CGM Database	X	
2010	Malaria Indicator Survey	X	
2010	Standardized Monitoring and Assessment of Relief and Transitions Survey, December	X	X
2011	MICS	X	X
2011	WHO CGM Database	X	
2011	Standardized Monitoring and Assessment of Relief and Transitions Survey, July–August	X	X
2011	Akwa Ibom Survey on Dietary Intakes, Vitamin A, and Iron Status of Women of Childbearing Age and Children 6–59 Months of Age	X	
2012	Standardized Monitoring and Assessment of Relief and Transitions Survey, February–March	X	X
2012	Standardized Monitoring and Assessment of Relief and Transitions Survey, September–October	X	
2013	DHS	X	X
2013	WHO CGM Database	X	
2013	Standardized Monitoring and Assessment of Relief and Transitions Survey, Round V	X	X
2013	General Household Survey, Panel , Wave 2	X	
2014	WHO CGM Database	X	
2015	National Nutrition and Health Survey	X	X
2016	MICS with National Immunization Coverage Survey Supplement	X	X
2016	General Household Survey Panel Wave 3	X	X
2017	WHO CGM Database	X	
2017	MICS with National Immunization Coverage Survey Supplement	X	X
2017	MICS with National Immunization Coverage Survey Supplement – National Bureau of Statistics	X	
2018	DHS	X	X

Nigeria – HAZ, WHZ, and WAZ Distributions

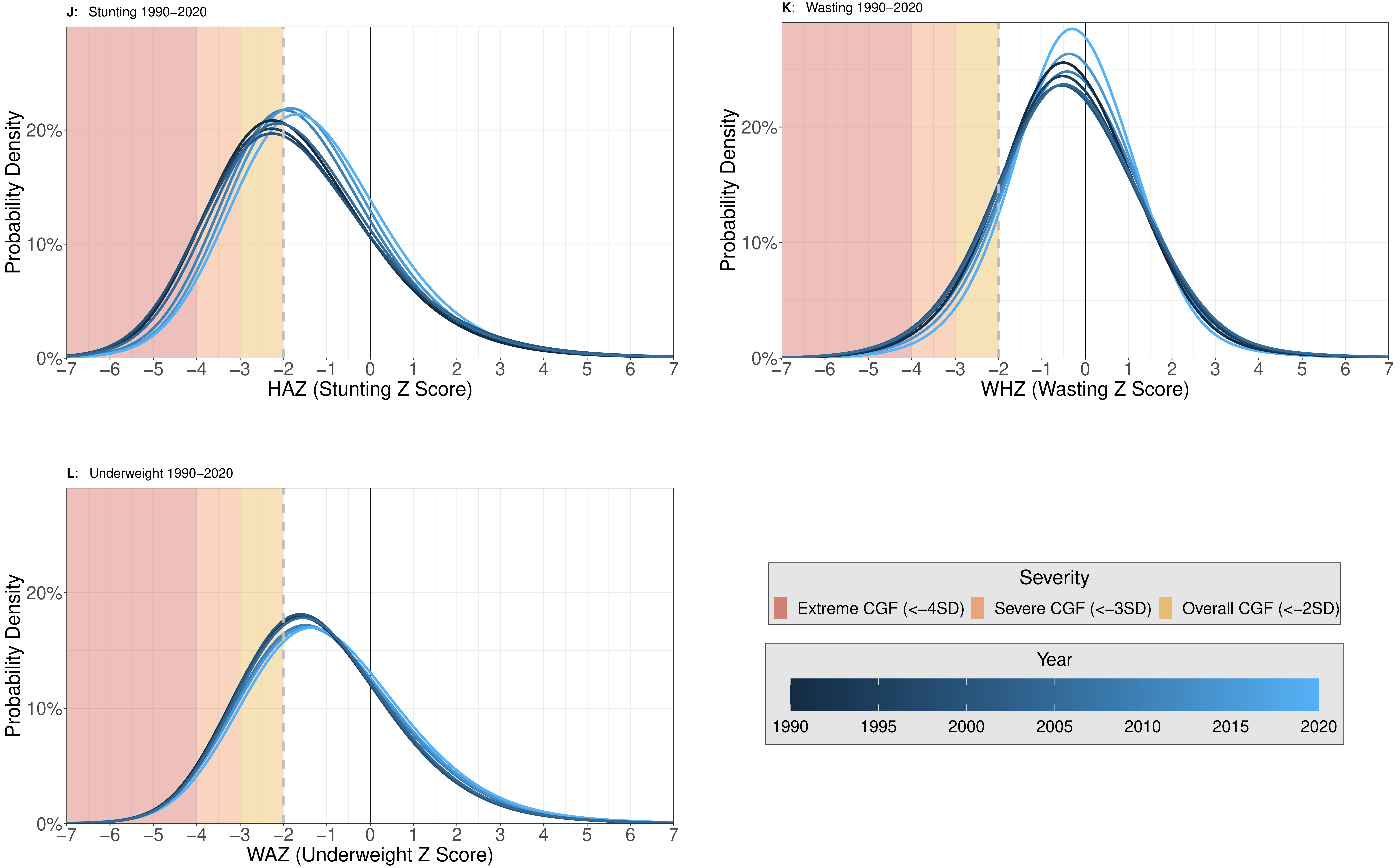

Sao Tome and Principe – Stunting (HAZ)

A: Overall and Severe Stunting Prevalence

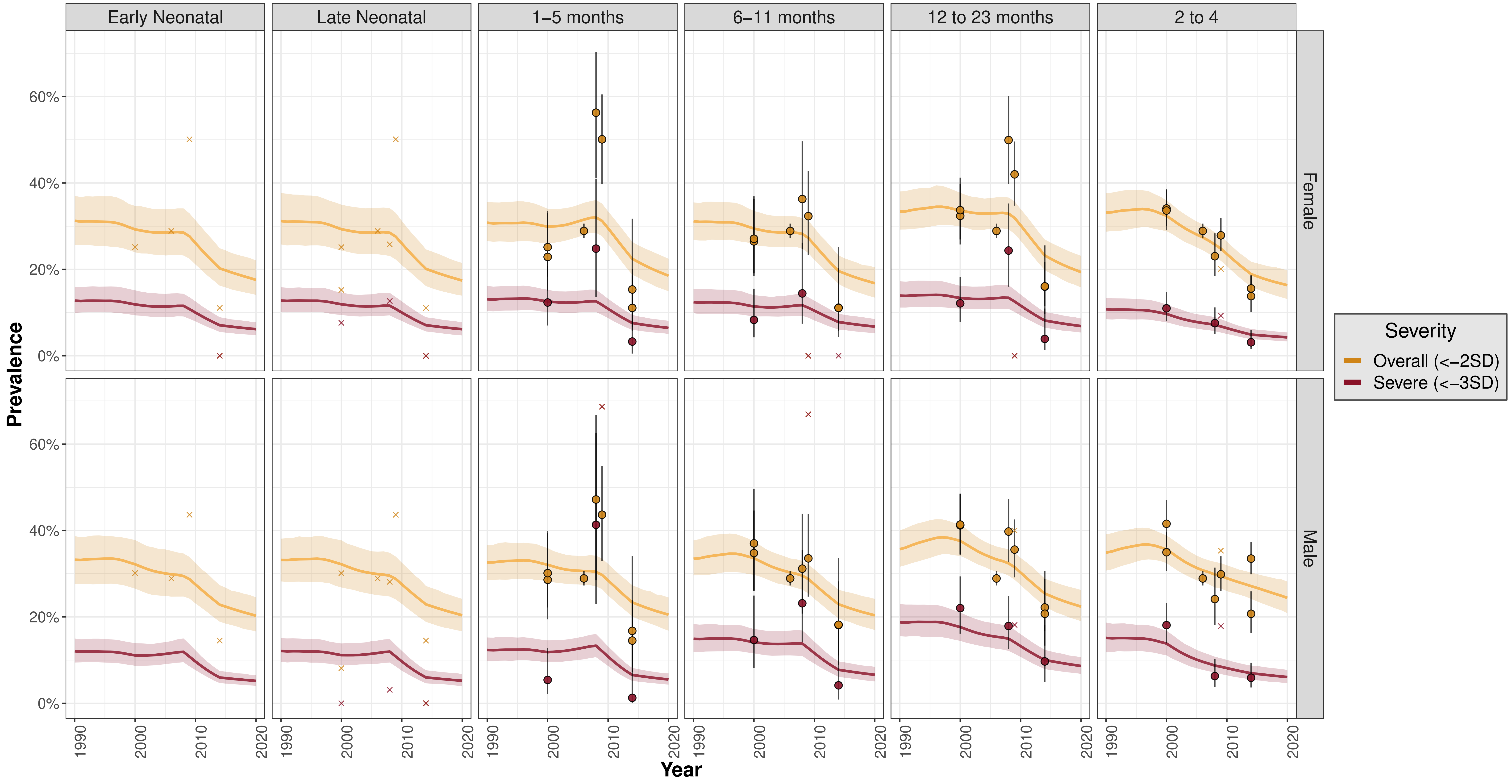

B: Transformed Mean Stunting Z Scores

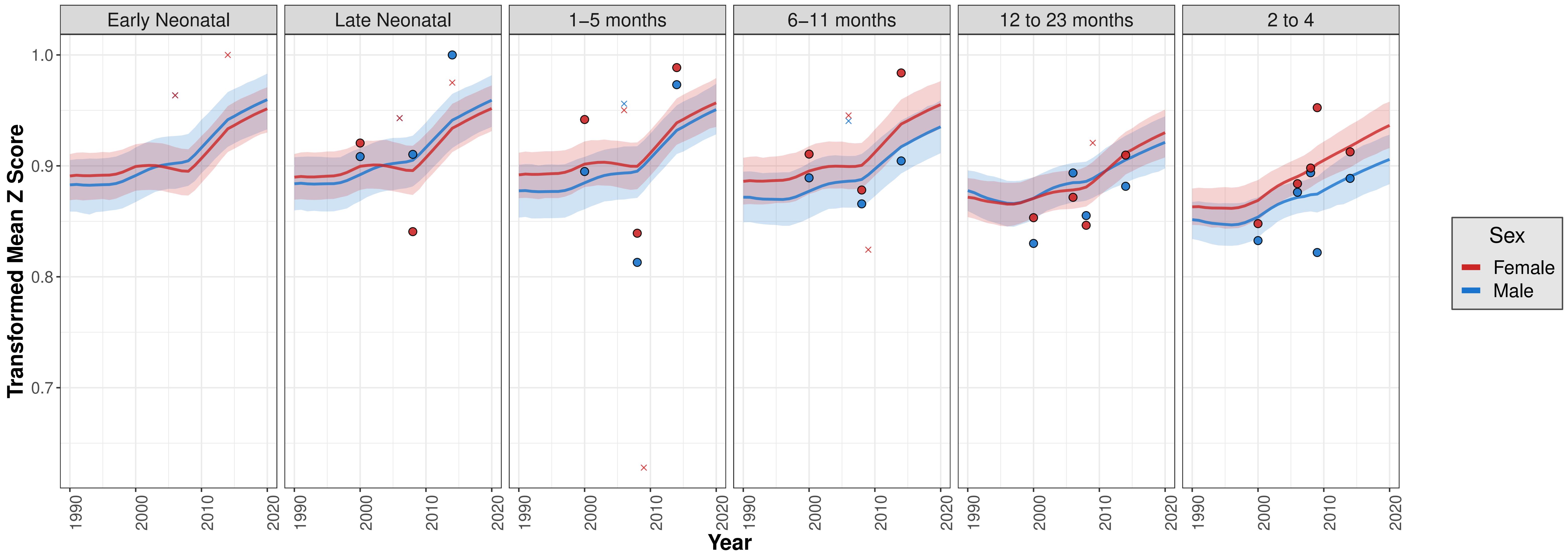

C

Year	Source
1986	WHO CGM Database
2000	MICS
2000	WHO CGM Database
2006	WHO CGM Database
2008	DHS
2009	DHS
2009	WHO CGM Database
2014	WHO CGM Database
2014	MICS

Sao Tome and Principe – Wasting (WHZ)

D: Overall and Severe Wasting Prevalence

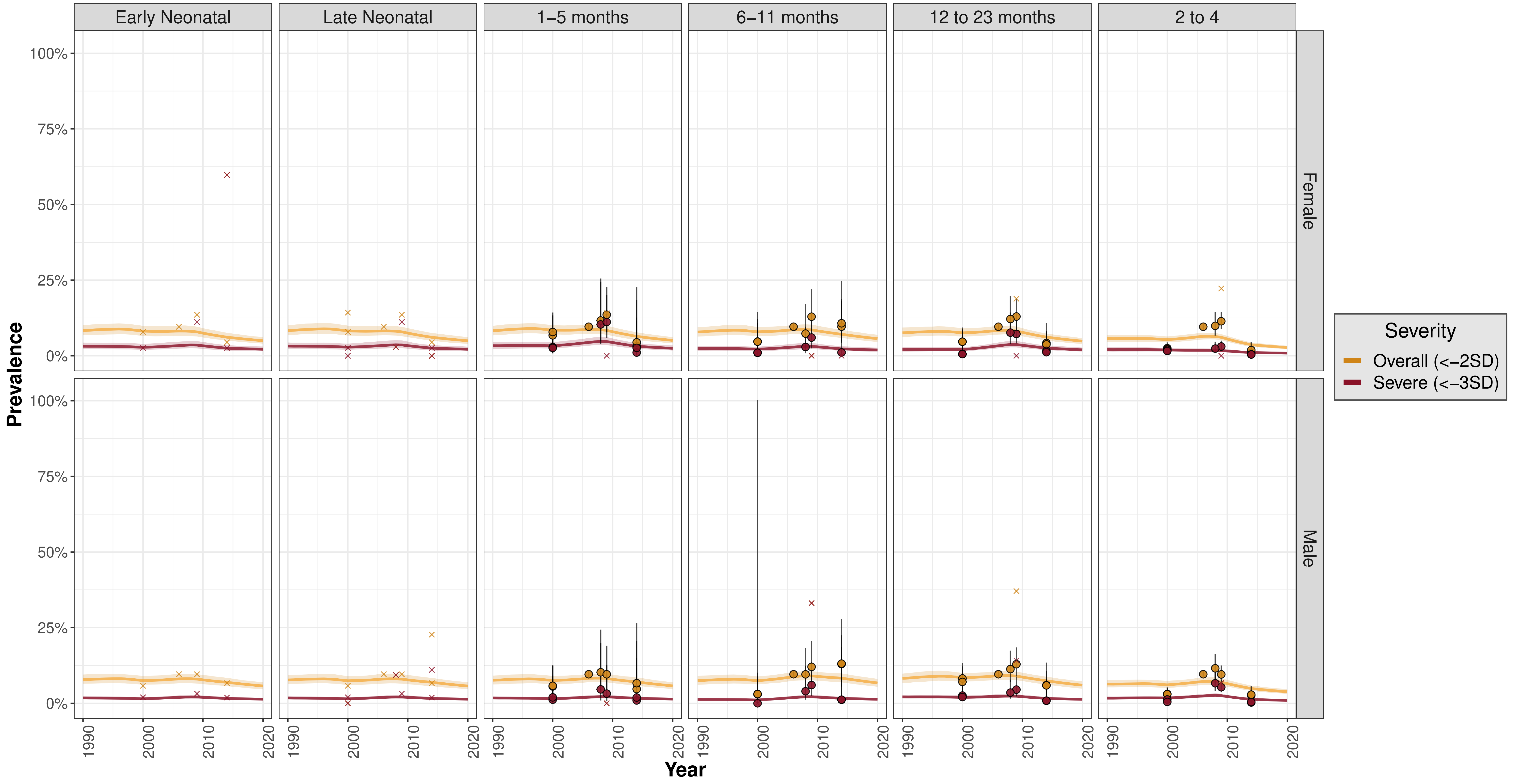

E: Transformed Mean Wasting Z Scores

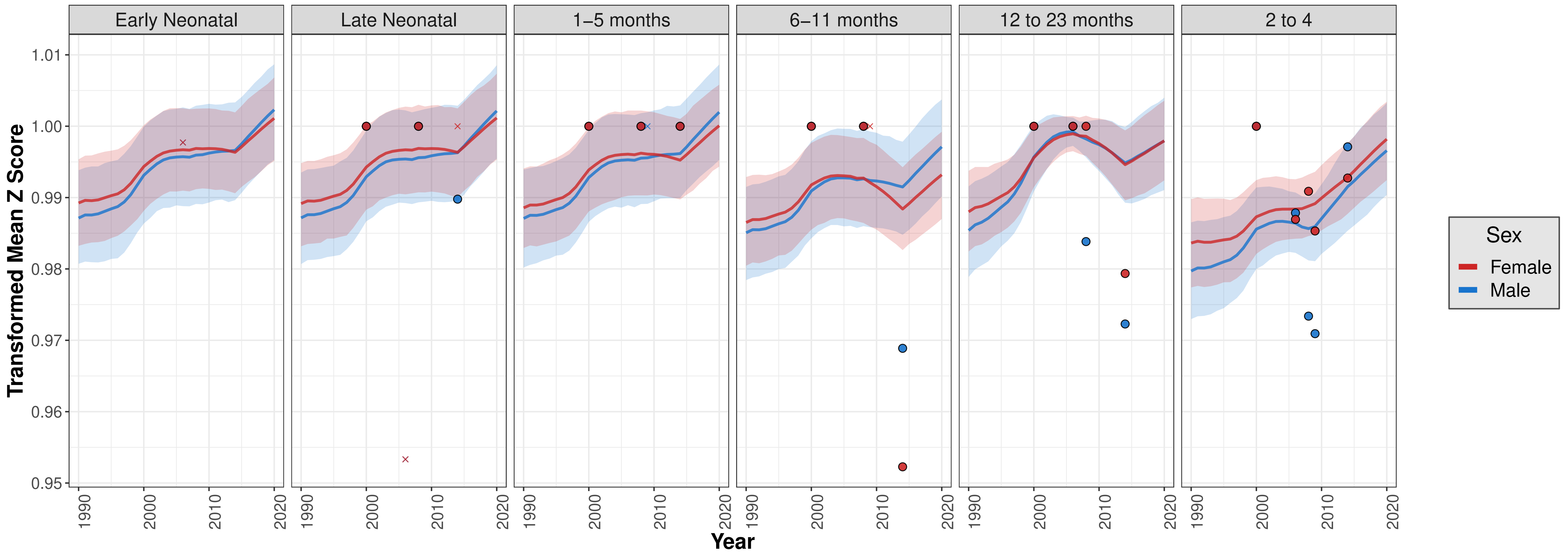

F

Year	Source
1986	WHO CGM Database
2000	MICS
2000	WHO CGM Database
2006	WHO CGM Database
2008	DHS
2009	DHS
2009	WHO CGM Database
2014	WHO CGM Database
2014	MICS

Sao Tome and Principe – Underweight (WAZ)

G: Overall and Severe Underweight Prevalence

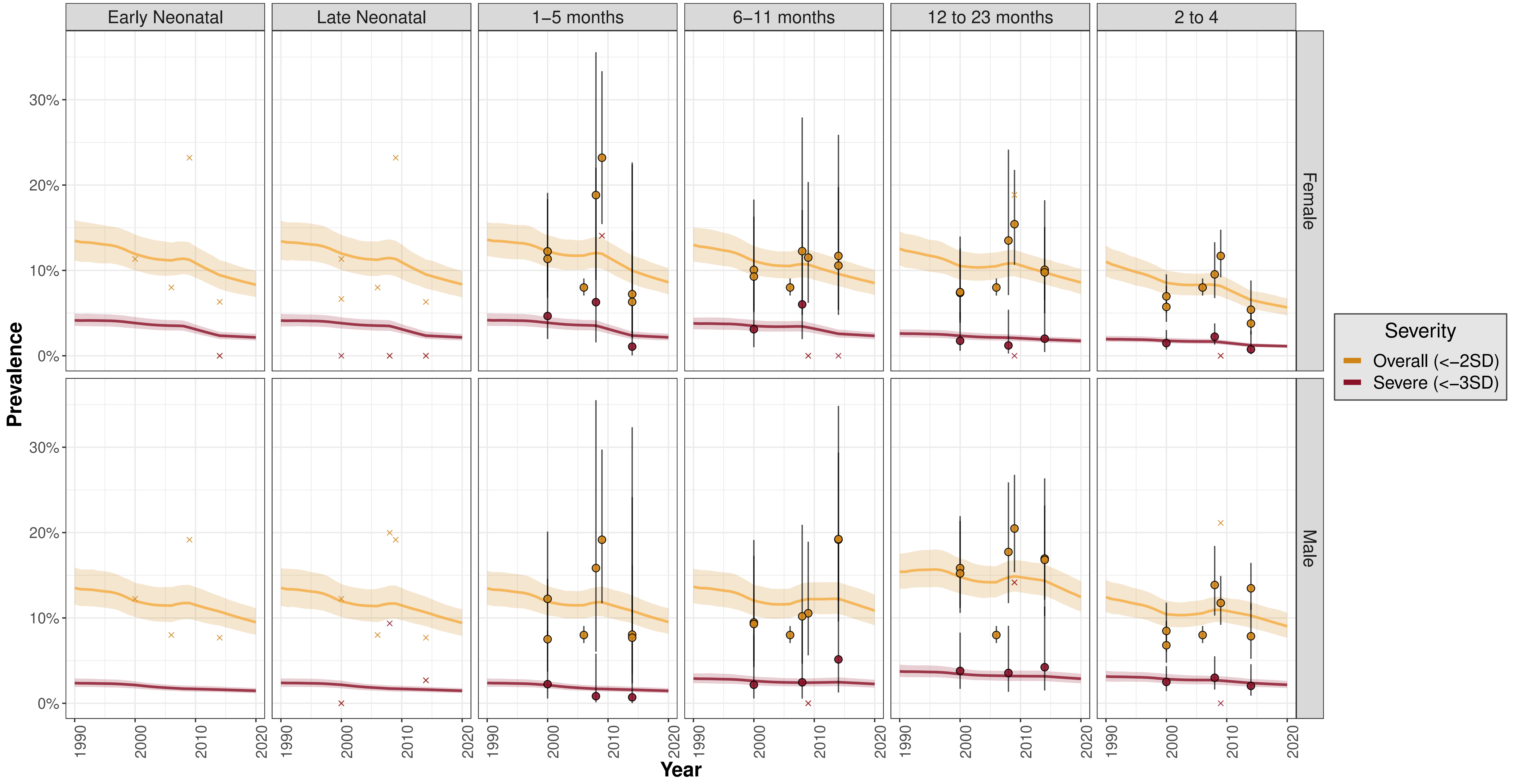

H: Transformed Mean Underweight Z Scores

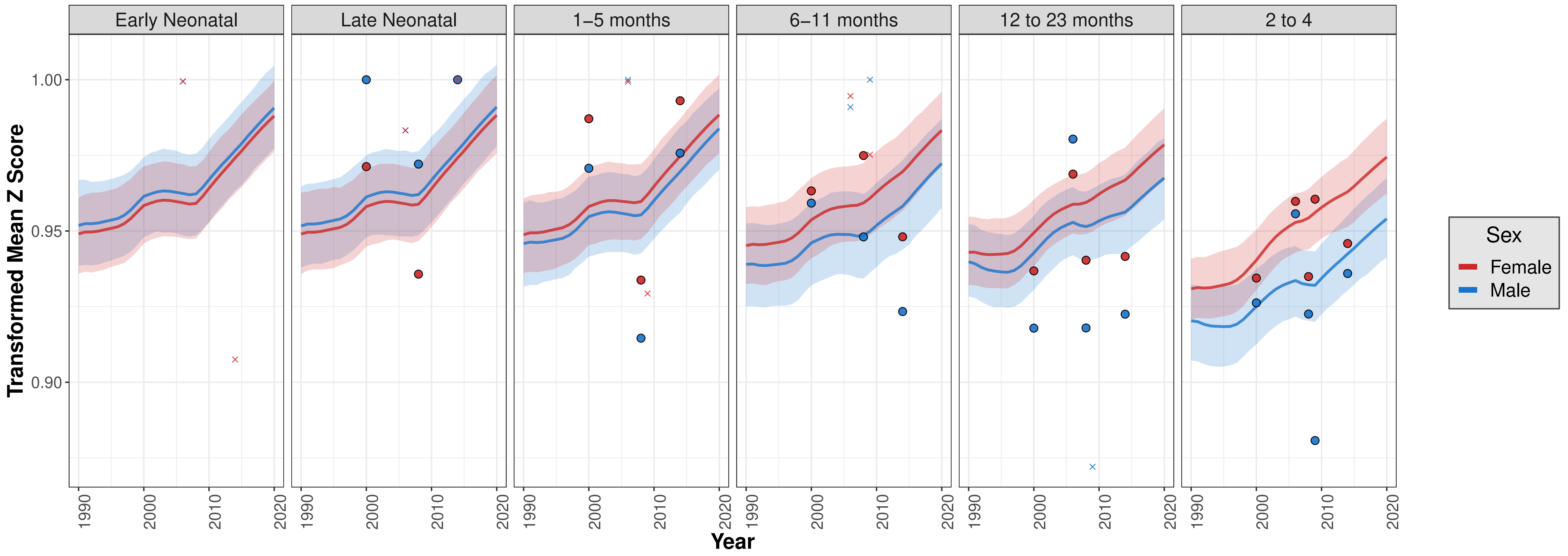

I

Year	Source
1986	WHO CGM Database
2000	MICS
2000	WHO CGM Database
2006	WHO CGM Database
2008	DHS
2009	DHS
2009	WHO CGM Database
2014	WHO CGM Database
2014	MICS

Sao Tome and Principe – HAZ, WHZ, and WAZ Distributions

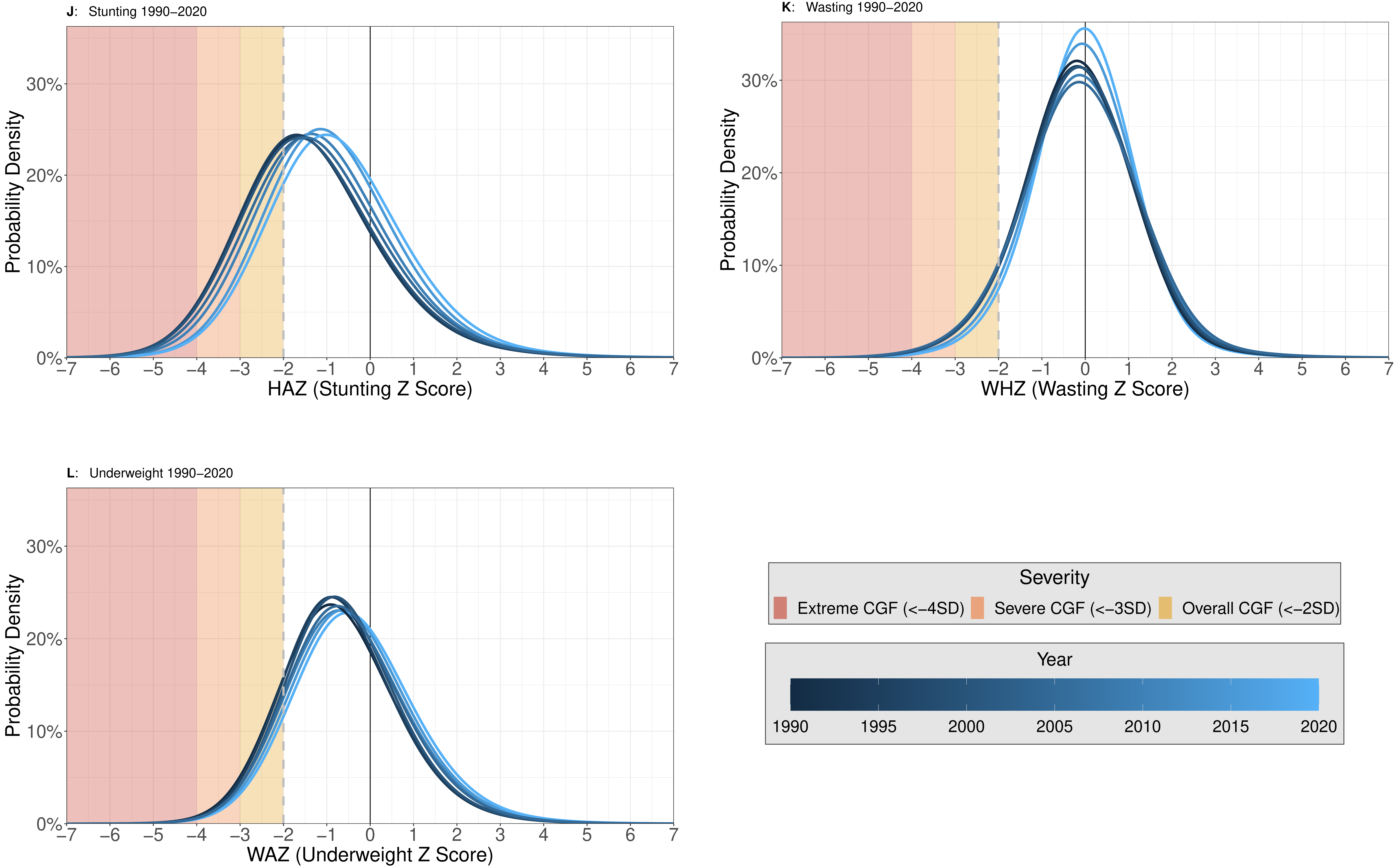

Senegal – Stunting (HAZ)

A: Overall and Severe Stunting Prevalence

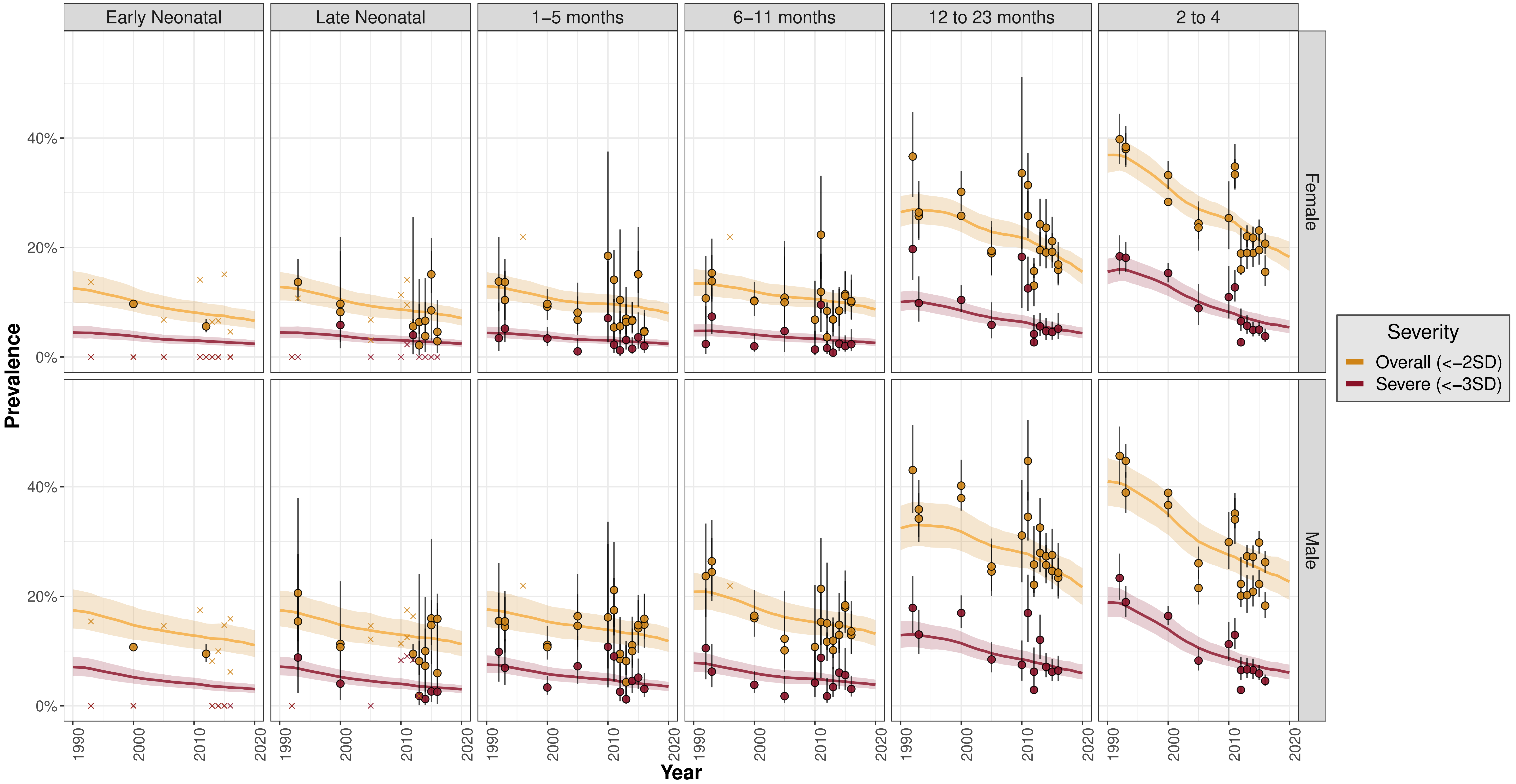

B: Transformed Mean Stunting Z Scores

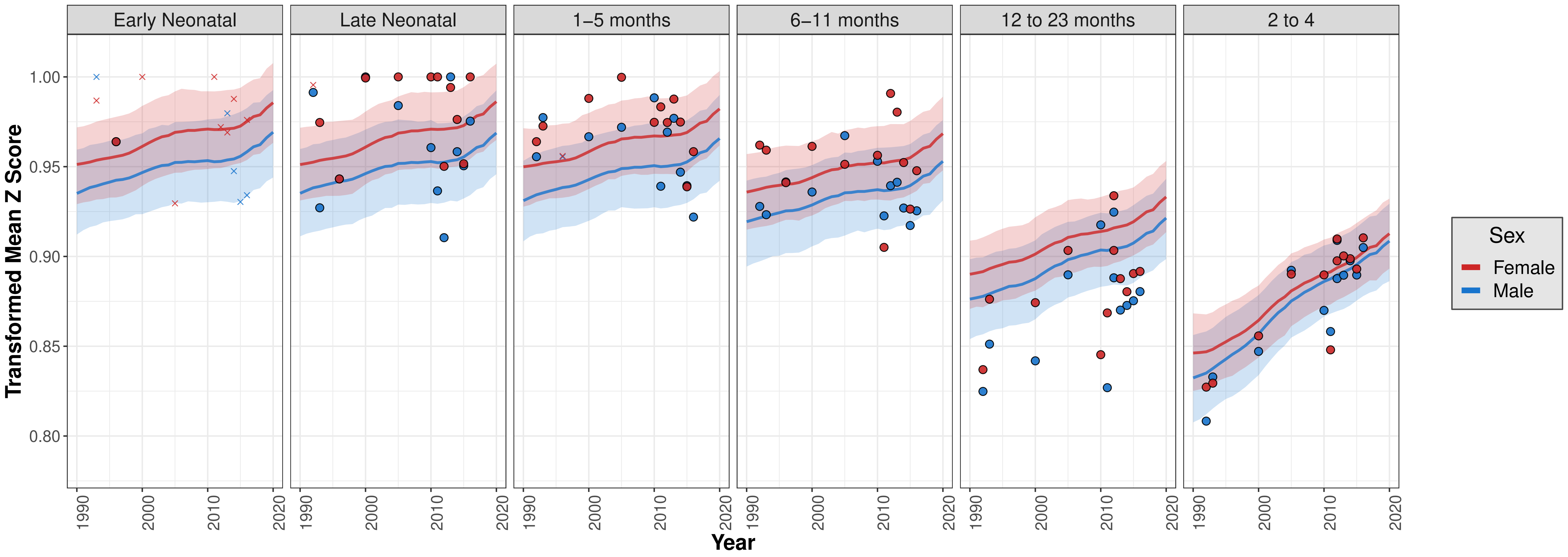

C

Year	Source
1986	DHS
1986	WHO CGM Database
1992	DHS
1993	DHS
1993	WHO CGM Database
1996	WHO CGM Database
2000	MICS
2000	WHO CGM Database
2005	DHS
2005	WHO CGM Database
2010	DHS
2011	DHS
2011	WHO CGM Database
2012	Continuous DHS
2012	WHO CGM Database
2013	Continuous DHS
2013	WHO CGM Database
2014	WHO CGM Database
2014	Continuous DHS
2015	WHO CGM Database
2015	Continuous DHS
2016	WHO CGM Database
2016	Continuous DHS

Senegal – Wasting (WHZ)

D: Overall and Severe Wasting Prevalence

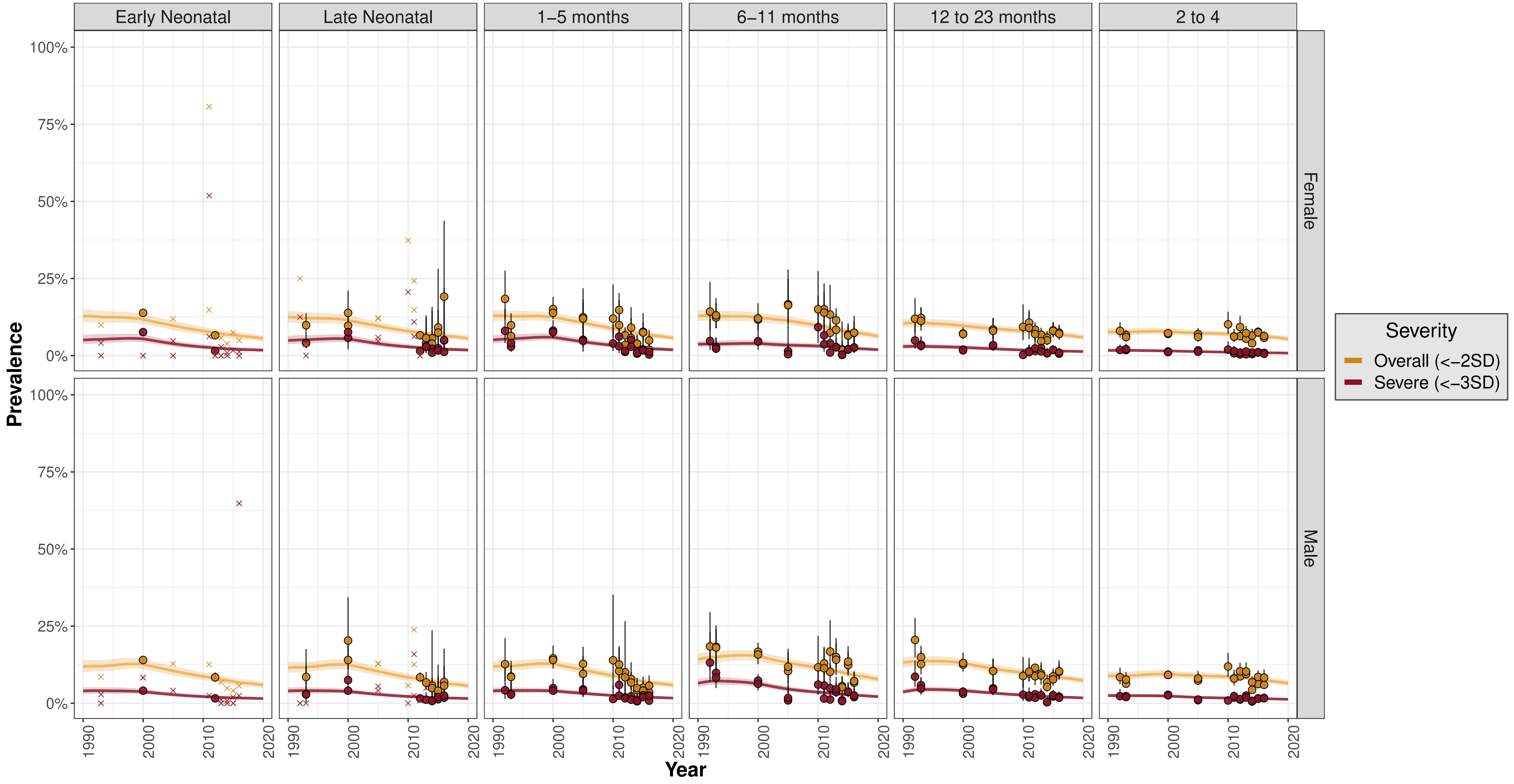

E: Transformed Mean Wasting Z Scores

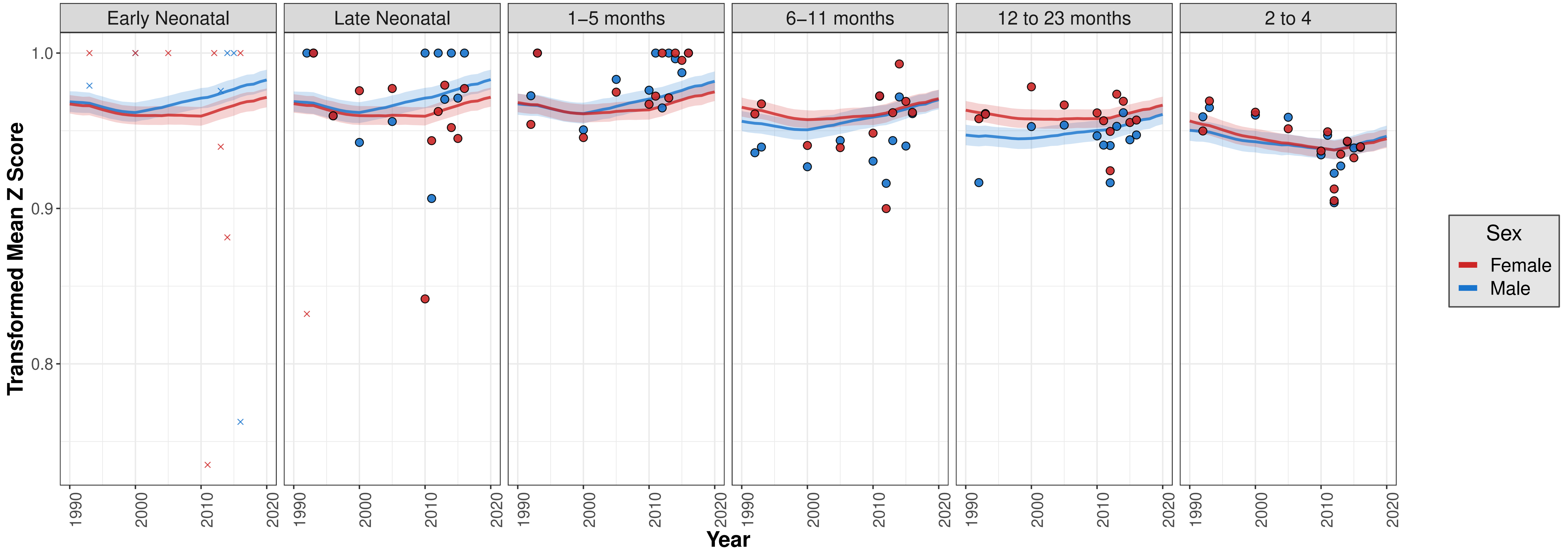

F

Year	Source
1986	DHS
1986	WHO CGM Database
1992	DHS
1993	DHS
1993	WHO CGM Database
1996	WHO CGM Database
2000	MICS
2000	WHO CGM Database
2005	DHS
2005	WHO CGM Database
2010	DHS
2011	DHS
2011	WHO CGM Database
2012	Continuous DHS
2012	WHO CGM Database
2013	Continuous DHS
2013	WHO CGM Database
2014	WHO CGM Database
2014	Continuous DHS
2015	WHO CGM Database
2015	Continuous DHS
2016	WHO CGM Database
2016	Continuous DHS

Senegal – Underweight (WAZ)

G: Overall and Severe Underweight Prevalence

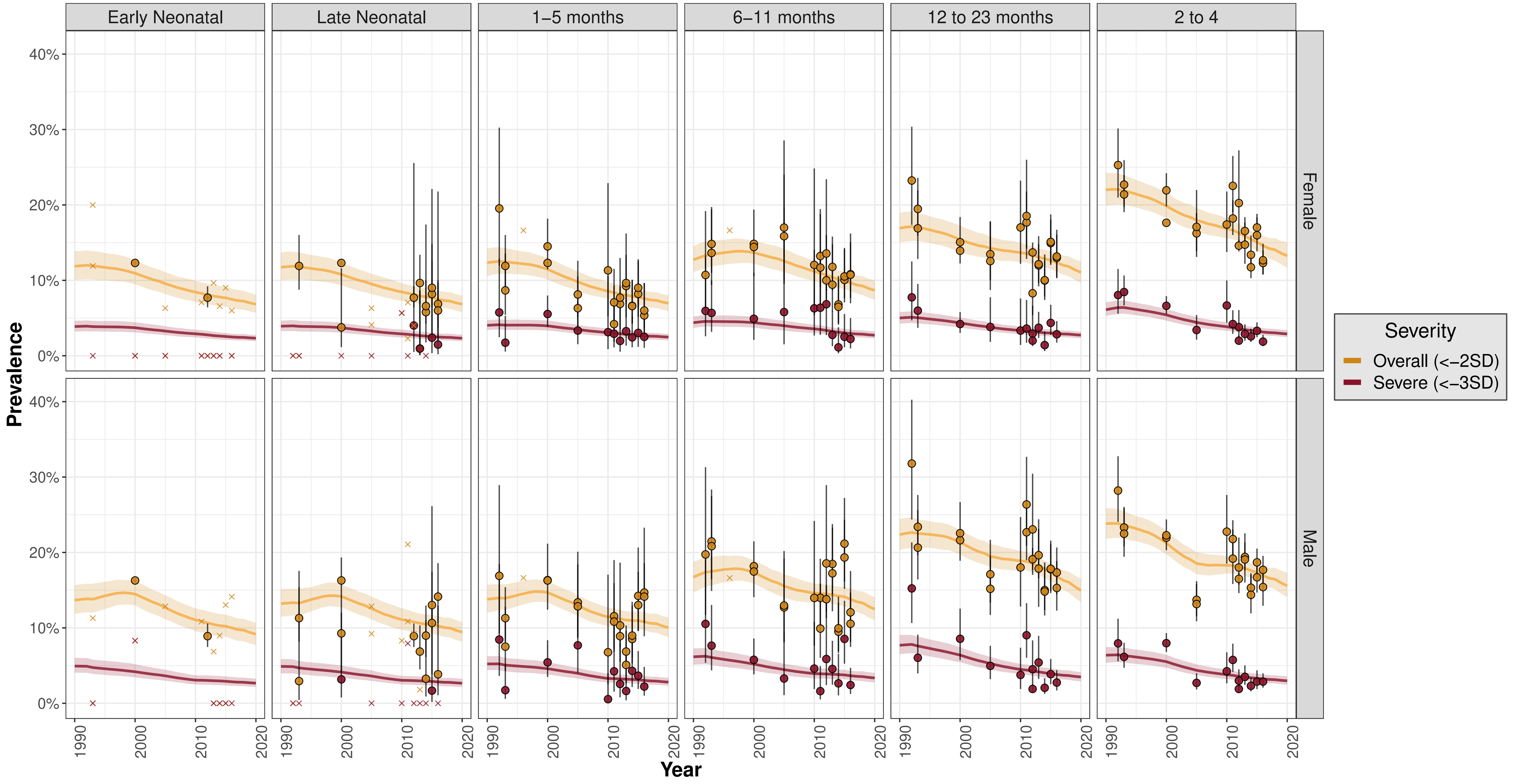

H: Transformed Mean Underweight Z Scores

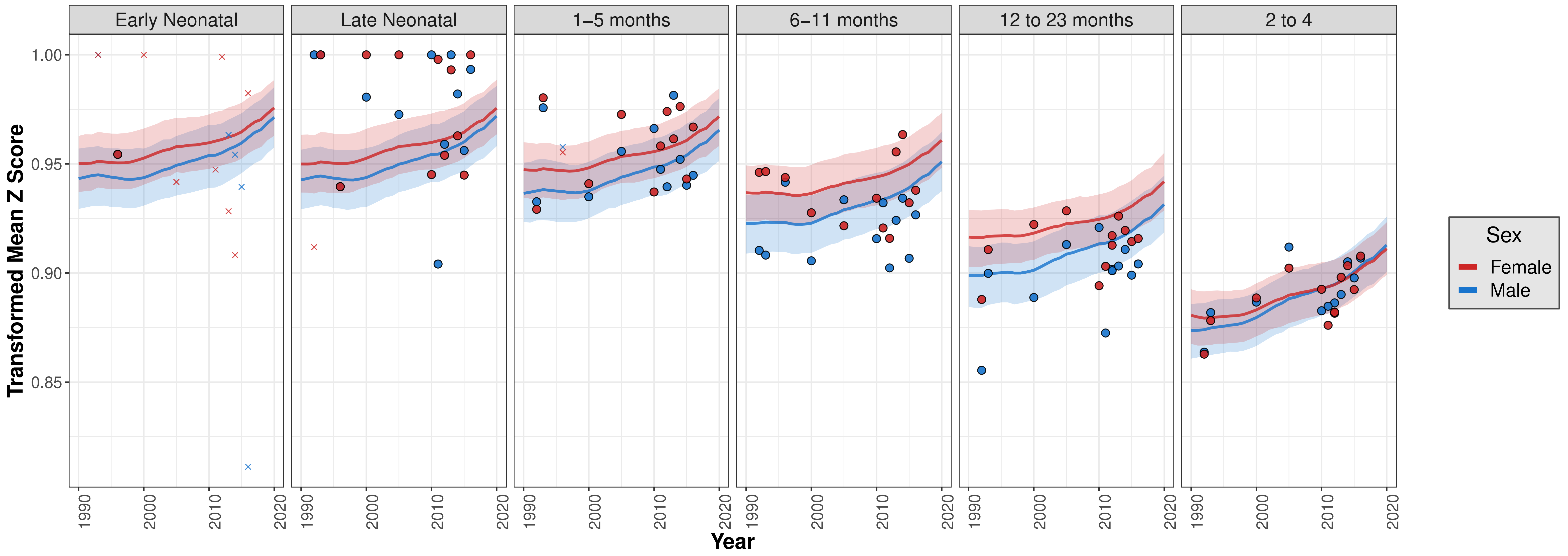

I

Year	Source
1986	DHS
1986	WHO CGM Database
1992	DHS
1993	DHS
1993	WHO CGM Database
1996	WHO CGM Database
2000	MICS
2000	WHO CGM Database
2005	DHS
2005	WHO CGM Database
2010	DHS
2011	DHS
2011	WHO CGM Database
2012	Continuous DHS
2012	WHO CGM Database
2013	Continuous DHS
2013	WHO CGM Database
2014	WHO CGM Database
2014	Continuous DHS
2015	WHO CGM Database
2015	Continuous DHS
2016	WHO CGM Database
2016	Continuous DHS

Senegal – HAZ, WHZ, and WAZ Distributions

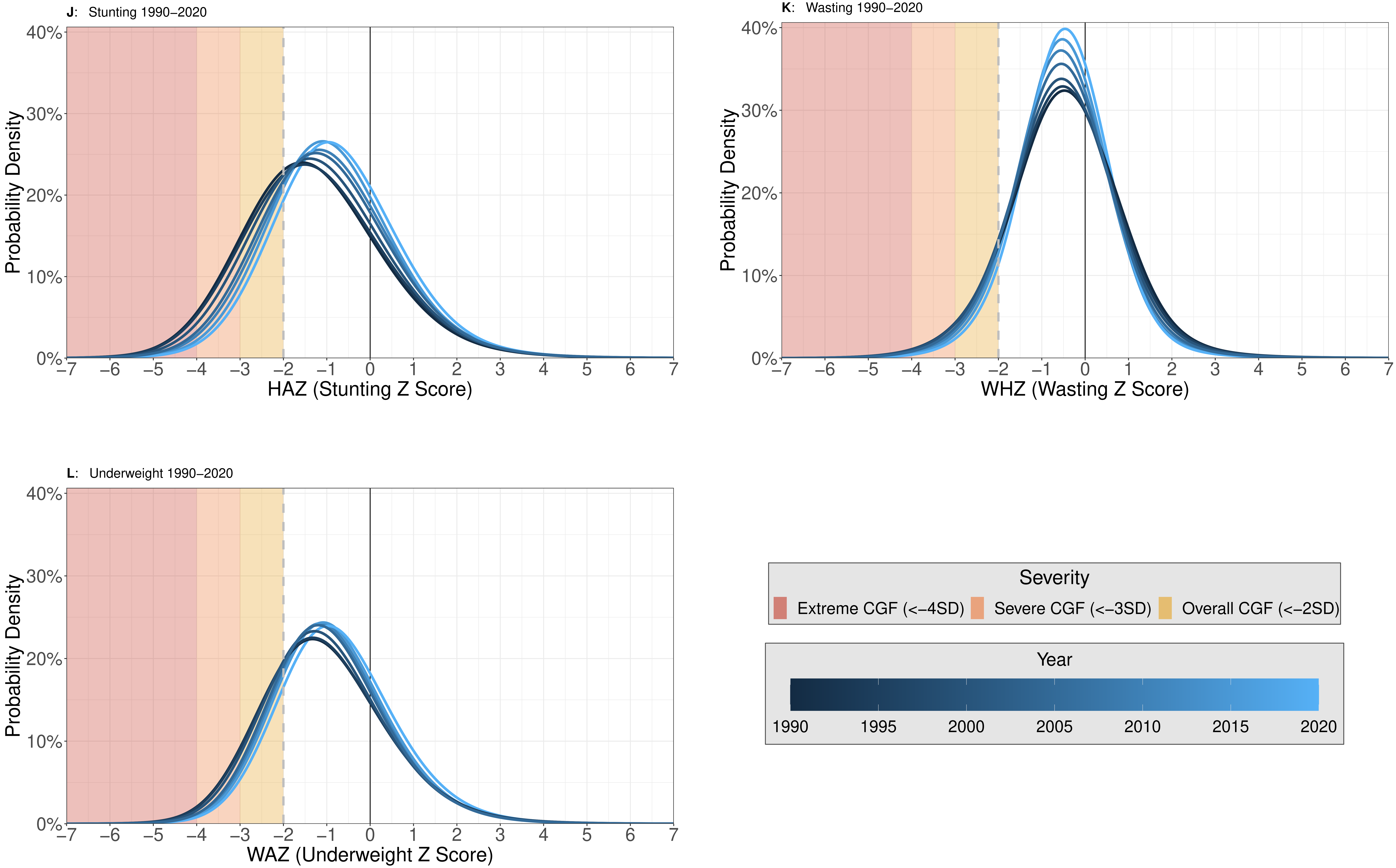

Sierra Leone – Stunting (HAZ)

A: Overall and Severe Stunting Prevalence

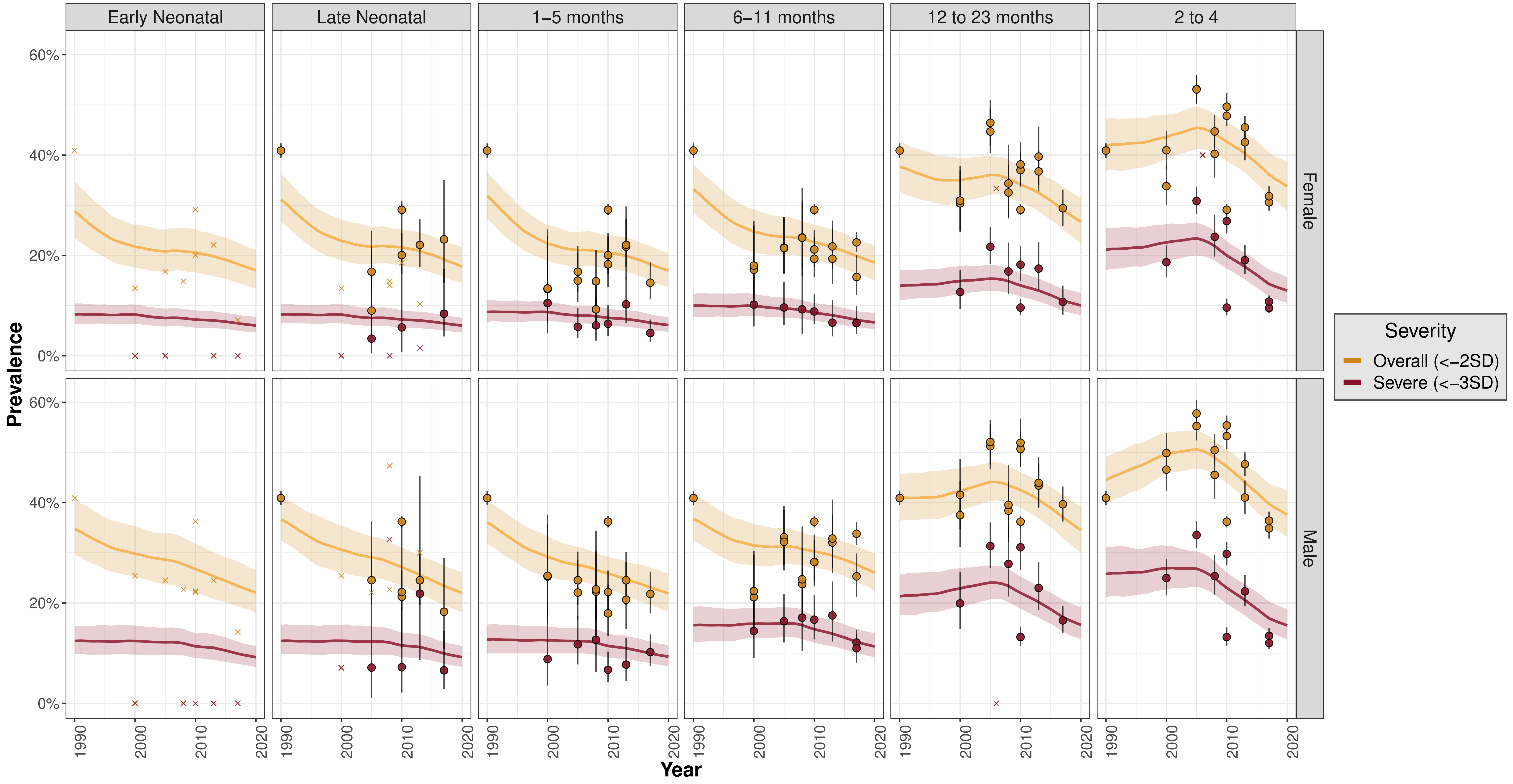

B: Transformed Mean Stunting Z Scores

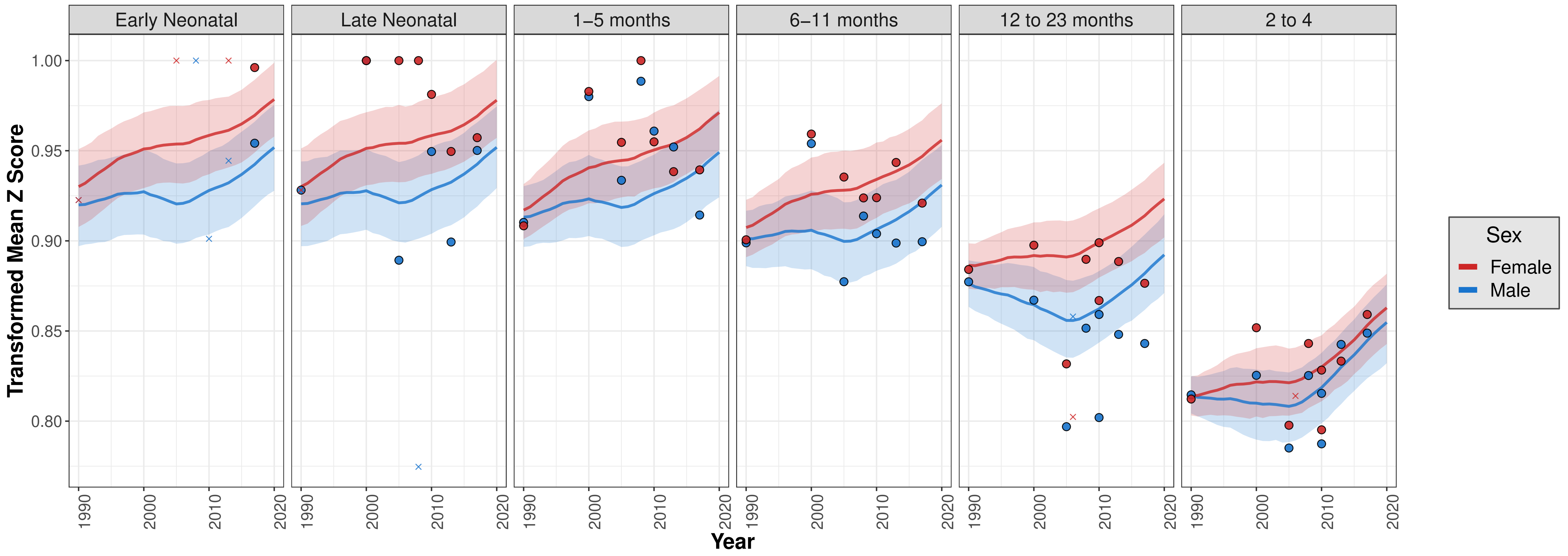

C

Year	Source
1989	WHO CGM Database
1990	WHO CGM Database
2000	MICS
2000	WHO CGM Database
2005	MICS
2005	WHO CGM Database
2006	MICS
2008	DHS
2008	WHO CGM Database
2010	MICS
2010	WHO CGM Database
2013	DHS
2013	WHO CGM Database
2017	MICS
2017	National Nutrition Survey

Sierra Leone – Wasting (WHZ)

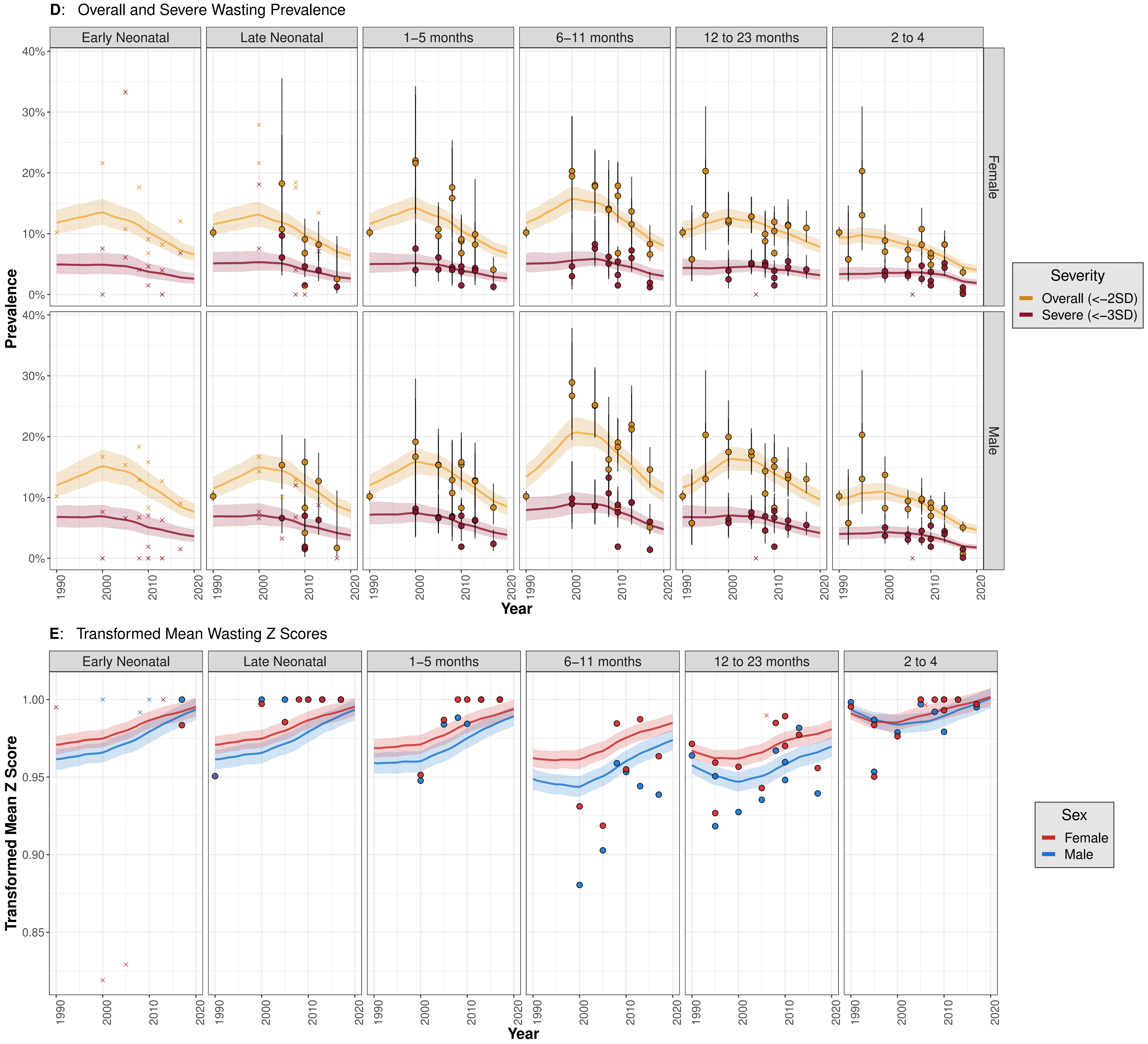

F

Year	Source
1989	WHO CGM Database
1990	WHO CGM Database
1992	WHO CGM Database
1995	WHO CGM Database
2000	MICS
2000	WHO CGM Database
2005	MICS
2005	WHO CGM Database
2006	MICS
2008	DHS
2008	WHO CGM Database
2010	MICS
2010	WHO CGM Database
2013	DHS
2013	WHO CGM Database
2017	MICS
2017	National Nutrition Survey

Sierra Leone – Underweight (WAZ)

G: Overall and Severe Underweight Prevalence

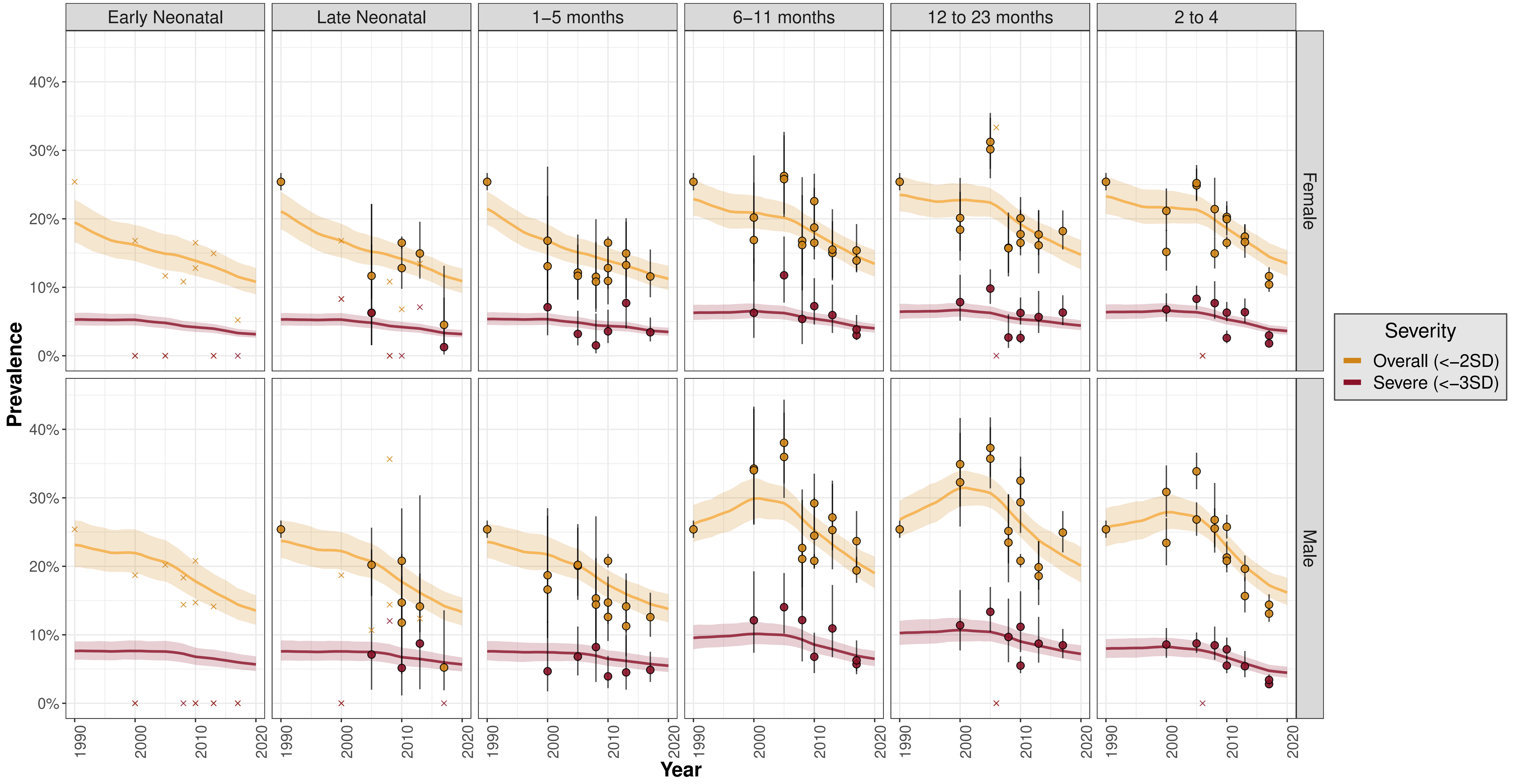

H: Transformed Mean Underweight Z Scores

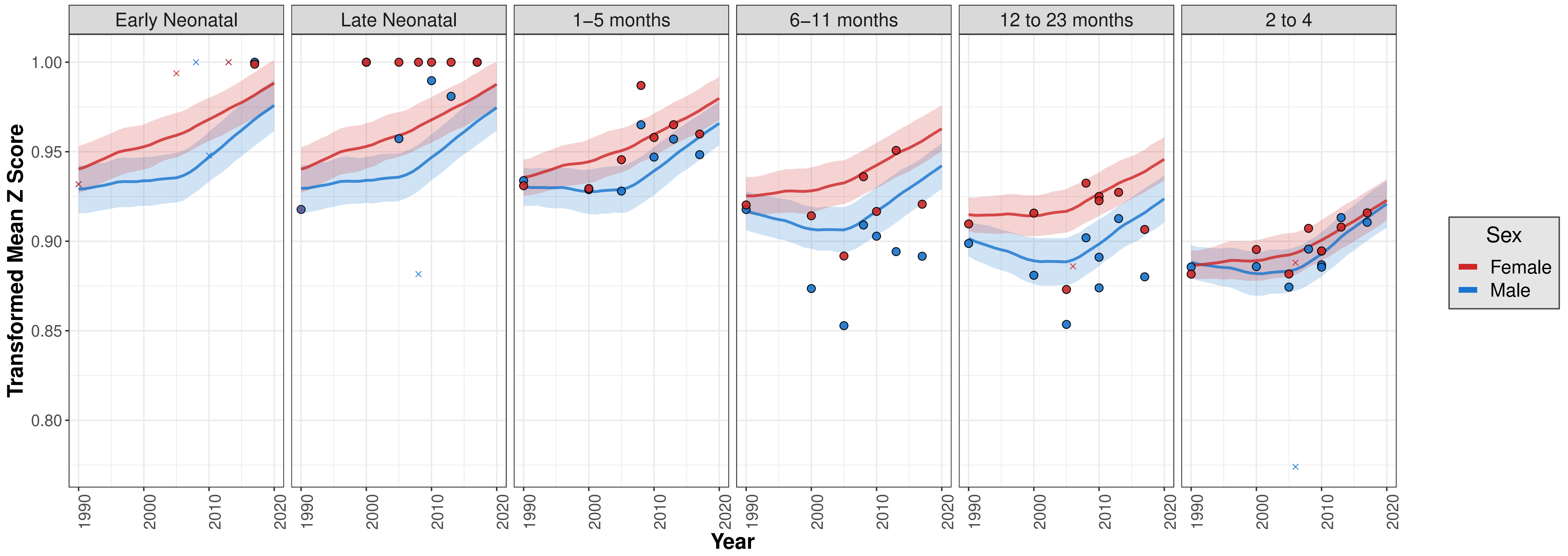

I

Year	Source
1989	WHO CGM Database
1990	WHO CGM Database
2000	MICS
2000	WHO CGM Database
2005	MICS
2005	WHO CGM Database
2006	MICS
2008	DHS
2008	WHO CGM Database
2010	MICS
2010	WHO CGM Database
2013	DHS
2013	WHO CGM Database
2017	MICS
2017	National Nutrition Survey

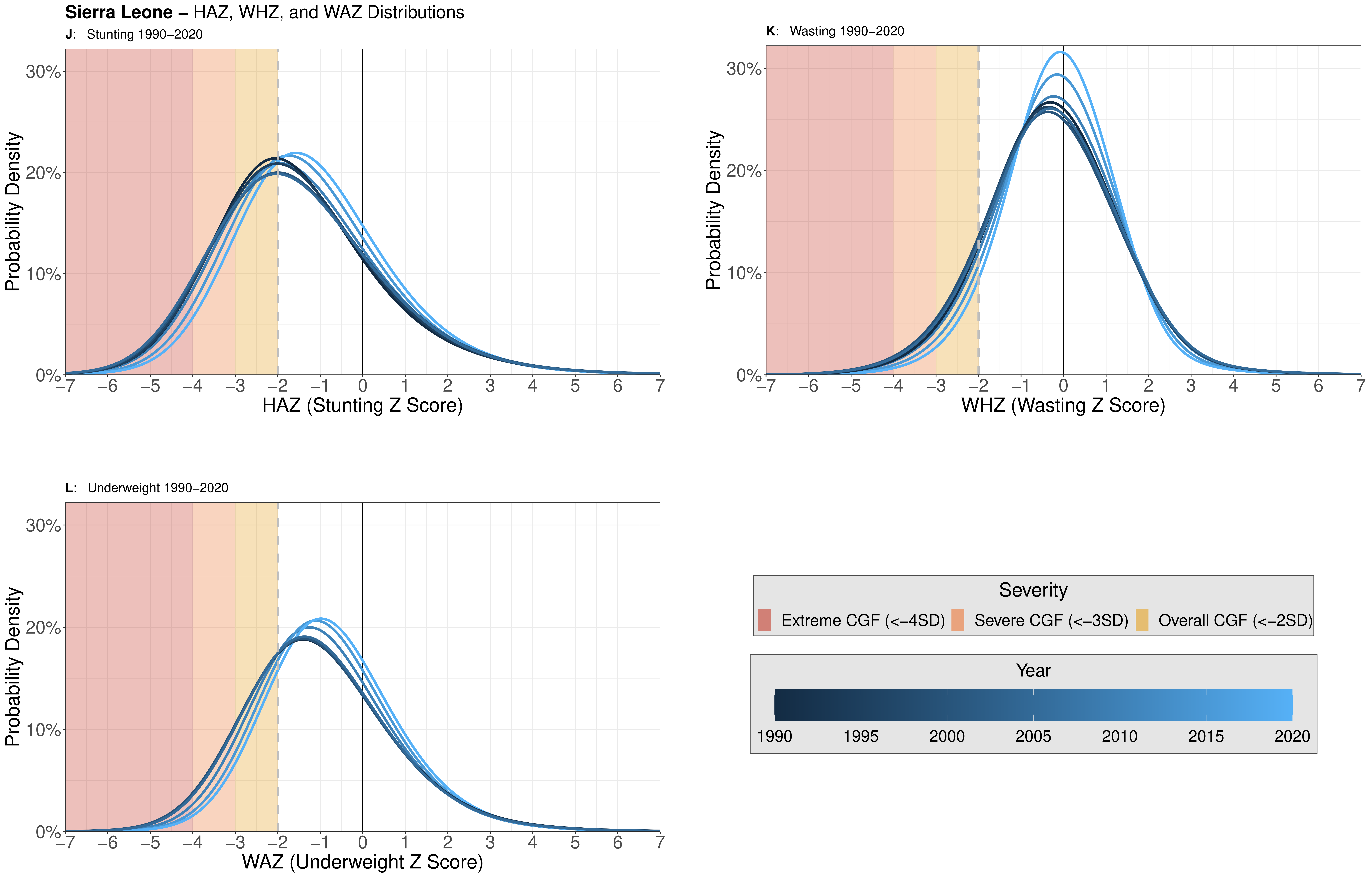
Sierra Leone – HAZ, WHZ, and WAZ Distributions
J: Stunting 1990–2020
Probability Density
0%
10%
20%
30%
HAZ (Stunting Z Score)
K: Wasting 1990–2020
WHZ (Wasting Z Score)
L: Underweight 1990–2020
WAZ (Underweight Z Score)
−7 −6 −5 −4 −3 −2 −1 0 1 2 3 4 5 6 7
Severity
Extreme CGF (<−4SD)
Severe CGF (<−3SD)
Overall CGF (<−2SD)
Year
1990
1995
2000
2005
2010
2015
2020

Togo – Stunting (HAZ)

A: Overall and Severe Stunting Prevalence

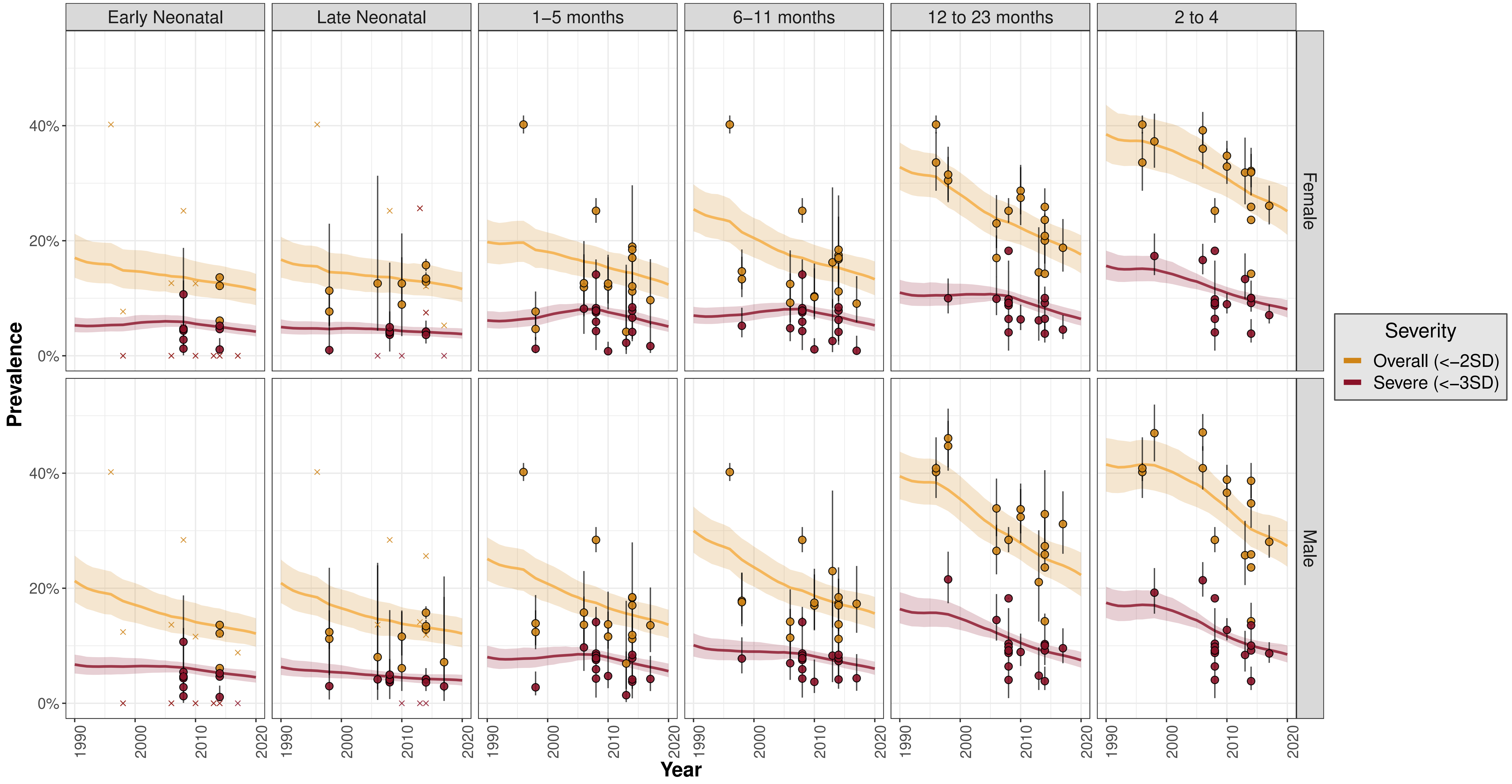

B: Transformed Mean Stunting Z Scores

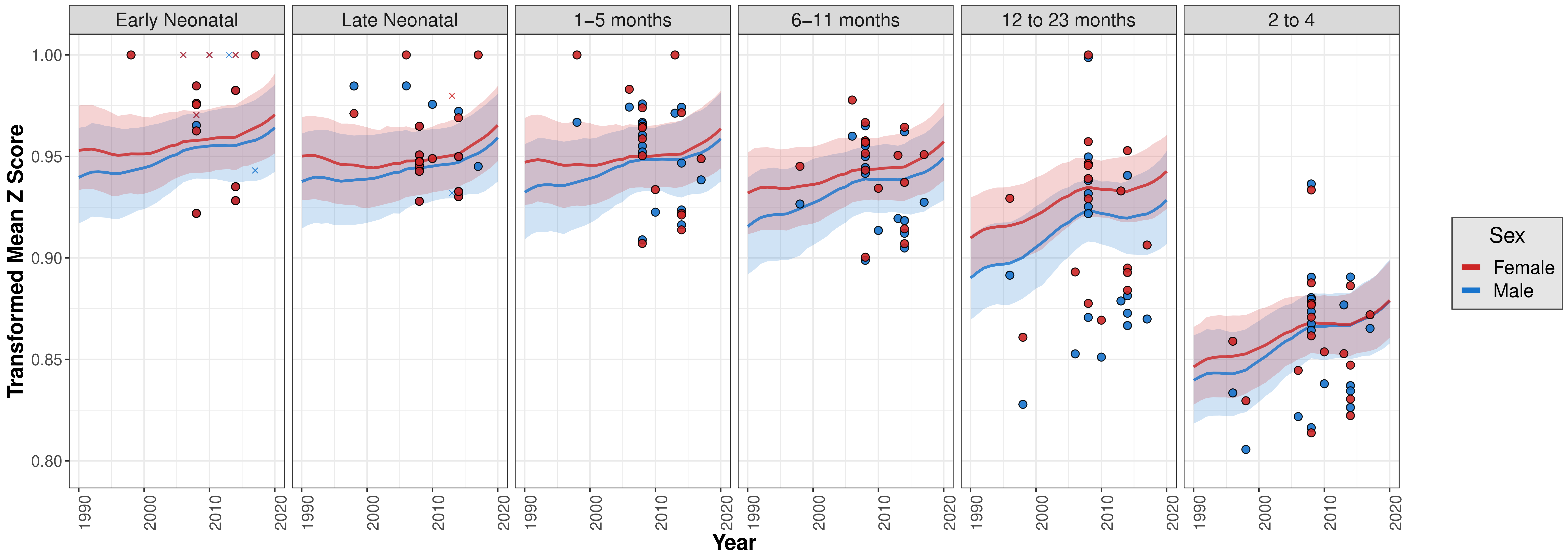

C

Year	Source
1977	WHO CGM Database
1988	DHS
1988	WHO CGM Database
1996	WHO CGM Database
1996	MICS
1998	DHS
1998	WHO CGM Database
2006	MICS
2006	WHO CGM Database
2008	WHO CGM Database
2010	MICS
2010	WHO CGM Database
2013	DHS
2014	DHS
2014	WHO CGM Database
2014	Nutritional Survey Using SMART Methodology July–August
2017	MICS

Togo – Wasting (WHZ)

D: Overall and Severe Wasting Prevalence

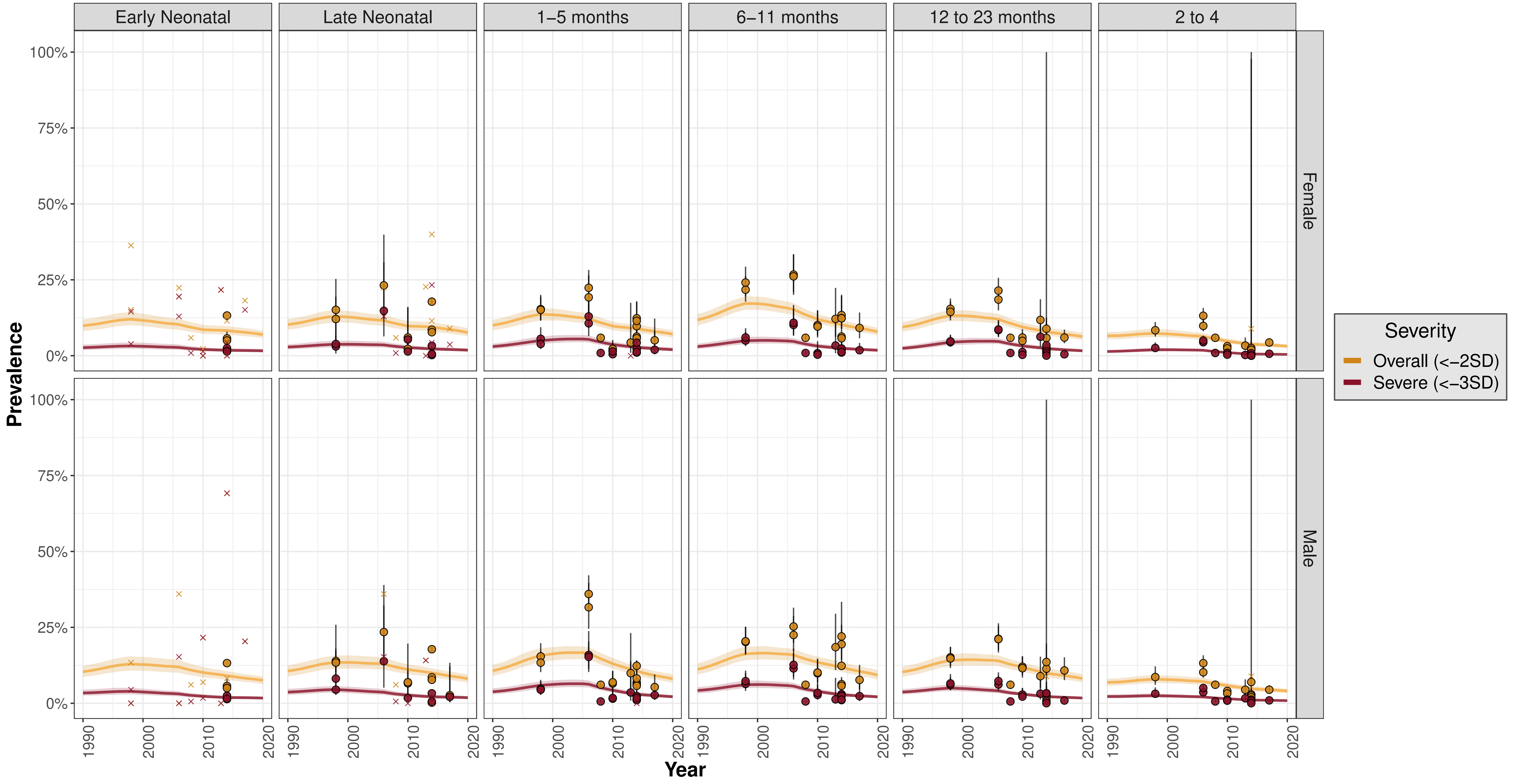

E: Transformed Mean Wasting Z Scores

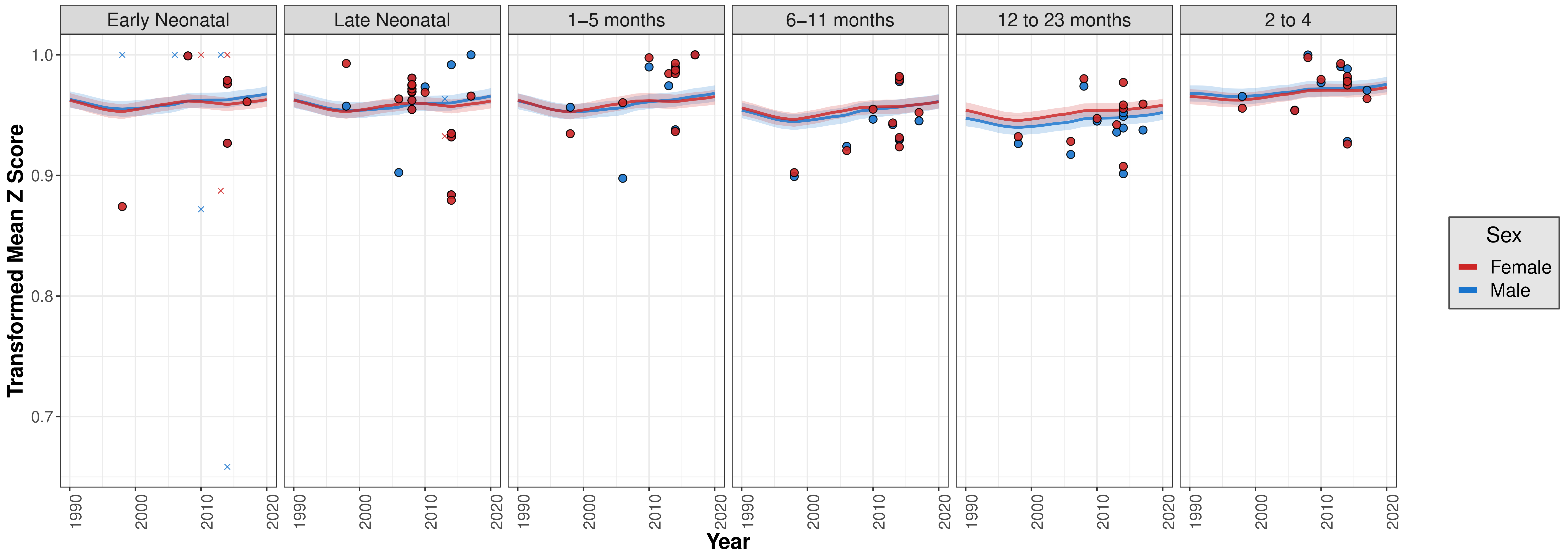

F

Year	Source
1977	WHO CGM Database
1988	DHS
1988	WHO CGM Database
1998	DHS
1998	WHO CGM Database
2006	MICS
2006	WHO CGM Database
2008	WHO CGM Database
2010	MICS
2010	WHO CGM Database
2013	DHS
2014	DHS
2014	WHO CGM Database
2014	Nutritional Survey Using SMART Methodology July–August
2017	MICS

Togo – Underweight (WAZ)

G: Overall and Severe Underweight Prevalence

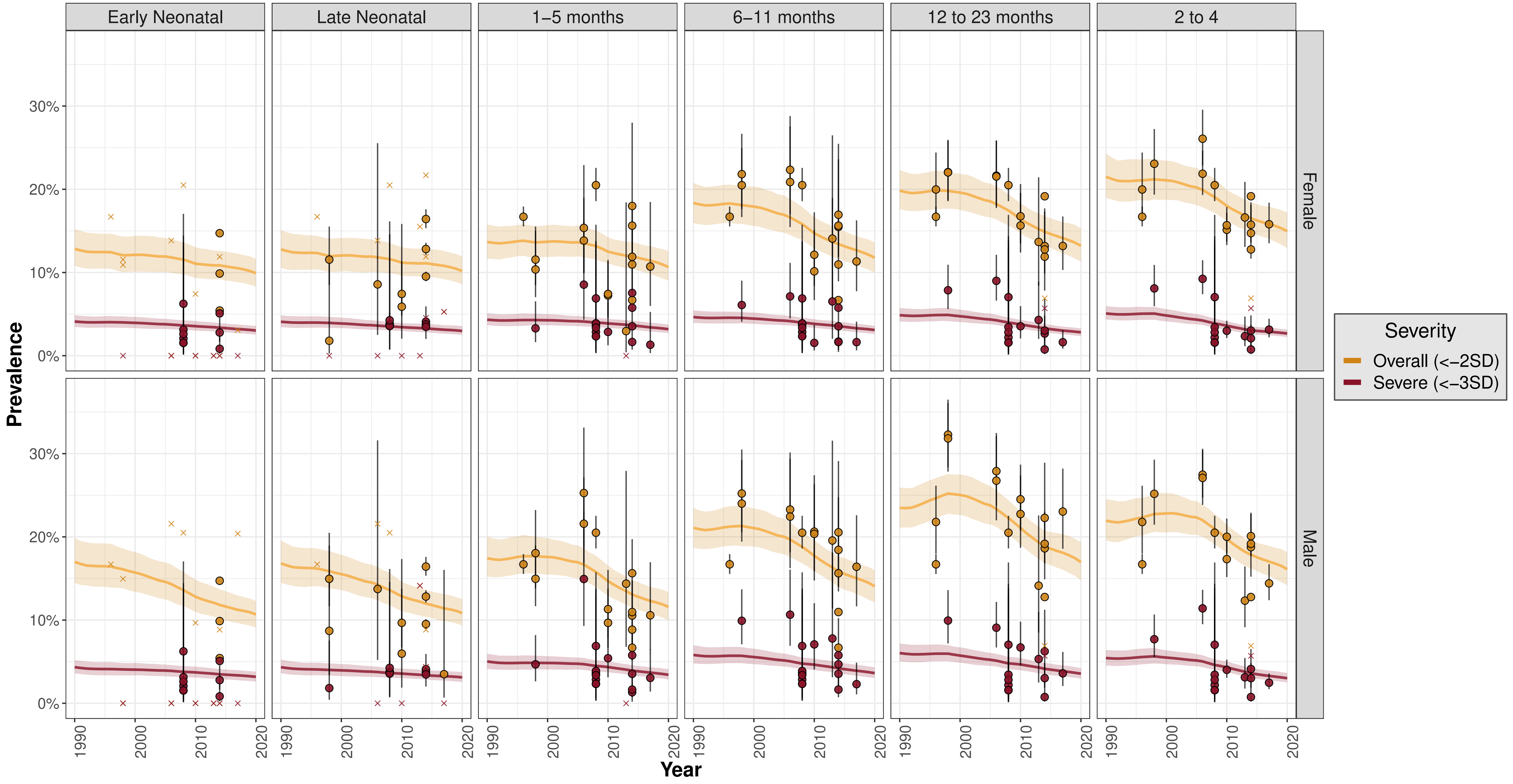

H: Transformed Mean Underweight Z Scores

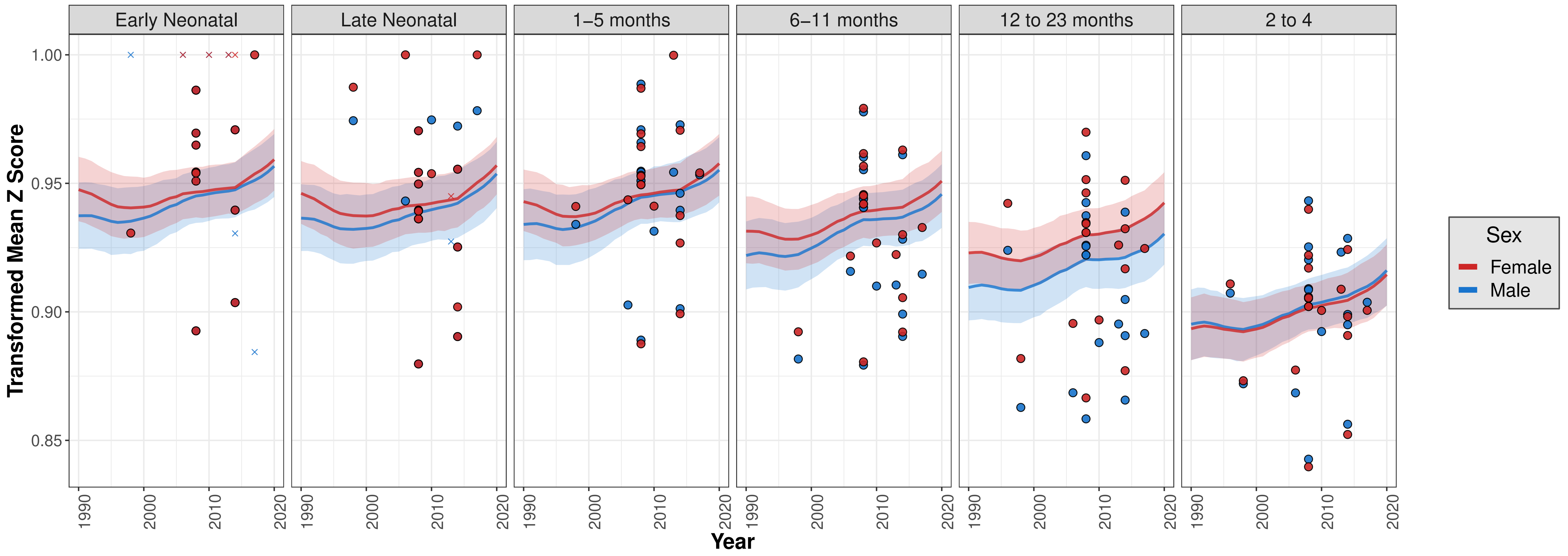

I

Year	Source
1977	WHO CGM Database
1988	DHS
1988	WHO CGM Database
1996	WHO CGM Database
1996	MICS
1998	DHS
1998	WHO CGM Database
2006	MICS
2006	WHO CGM Database
2008	WHO CGM Database
2010	MICS
2010	WHO CGM Database
2013	DHS
2014	DHS
2014	WHO CGM Database
2014	Nutritional Survey Using SMART Methodology July–August
2017	MICS

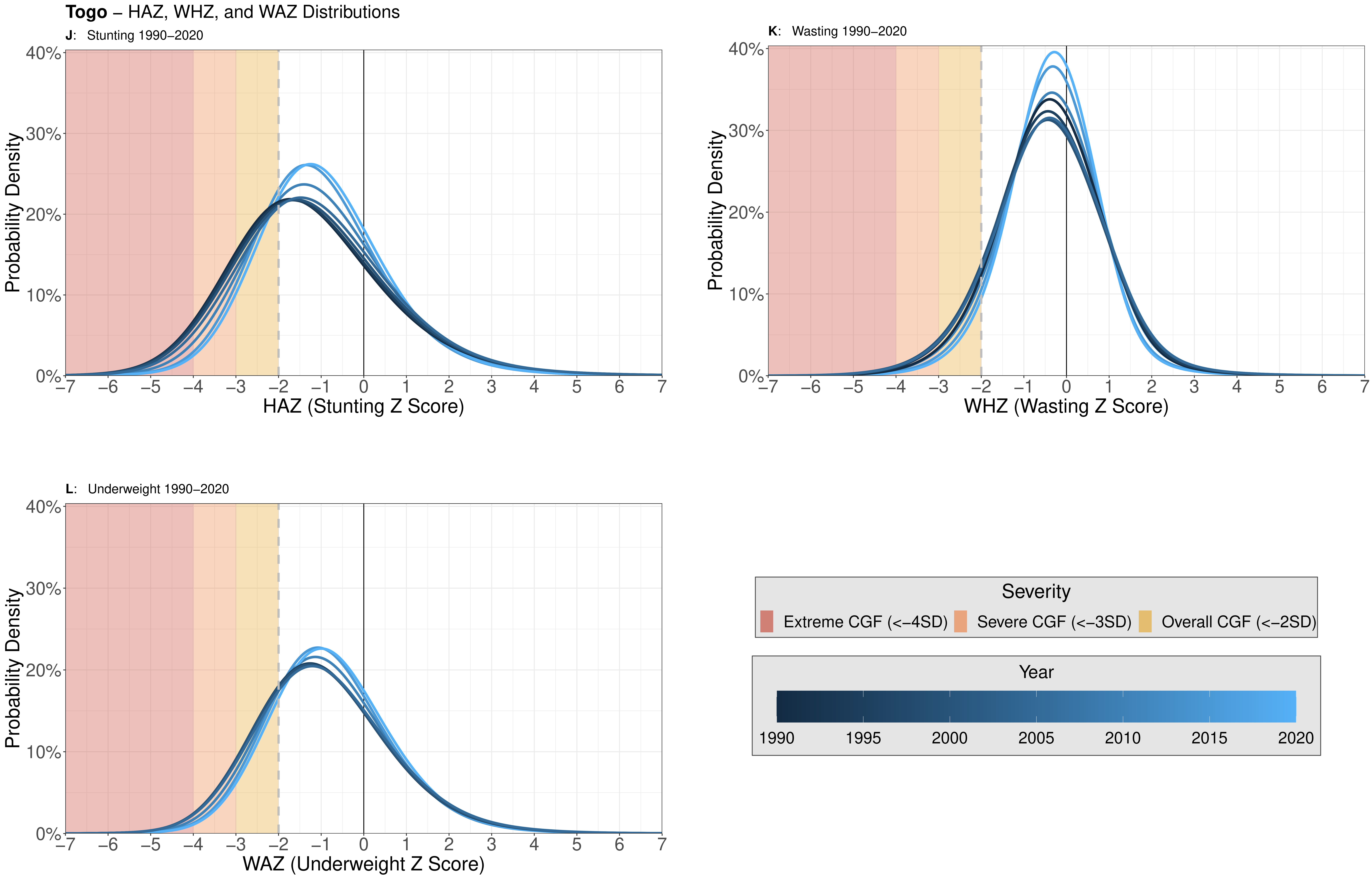

Togo – HAZ, WHZ, and WAZ Distributions
J: Stunting 1990–2020
Probability Density
0%
10%
20%
30%
40%
−7 −6 −5 −4 −3 −2 −1 0 1 2 3 4 5 6 7
HAZ (Stunting Z Score)
K: Wasting 1990–2020
Probability Density
0%
10%
20%
30%
40%
−7 −6 −5 −4 −3 −2 −1 0 1 2 3 4 5 6 7
WHZ (Wasting Z Score)
L: Underweight 1990–2020
Probability Density
0%
10%
20%
30%
40%
−7 −6 −5 −4 −3 −2 −1 0 1 2 3 4 5 6 7
WAZ (Underweight Z Score)
Severity
Extreme CGF (<−4SD)
Severe CGF (<−3SD)
Overall CGF (<−2SD)
Year
1990
1995
2000
2005
2010
2015
2020
