# Supplementary material for: Current dichotomous metrics obscure trends in severe and extreme child growth failure
Source: Sci Adv. 2022 May 20;8(20):eabm8954. doi: 10.1126/sciadv.abm8954 (PMC9122330; doi:10.1126/sciadv.abm8954)

Data S2. Age-sex specific fits of MR-BRT models for all forms and severities of CGF, 1990-2020. Prevalence models were fit in logit space, but are also shown in linear space for comparison. Figures shown include: overall stunting [HAZ < -2 standard deviations (SD)] prevalence shown in logit and linear space for males (A, B) and females (C, D); severe stunting [HAZ < -3 SD] for males (E, F) and females (G, H); extreme stunting [HAZ < -4 SD] for males (I, J) and females (K, L); overall wasting [WHZ < -2 SD] for males (M, N) and females (O, P); severe wasting [WHZ < -3 SD] for males (Q, R) and females (S, T); extreme wasting [WHZ < -4 SD] for males (U, V) and females (W, X); overall underweight [WAZ < -2 SD] for males (Y, Z) and females (AA, BB); severe underweight [WAZ < -3 SD] for males (CC, DD) and females (EE, FF); and extreme underweight [WAZ < -4 SD] for males (GG, HH) and females (II, JJ). Models were fit in logit space, but are also shown in linear space for comparison. All years were used as inputs to the models, but only 1990, 2000, 2010, and 2020 are shown here for simplicity.

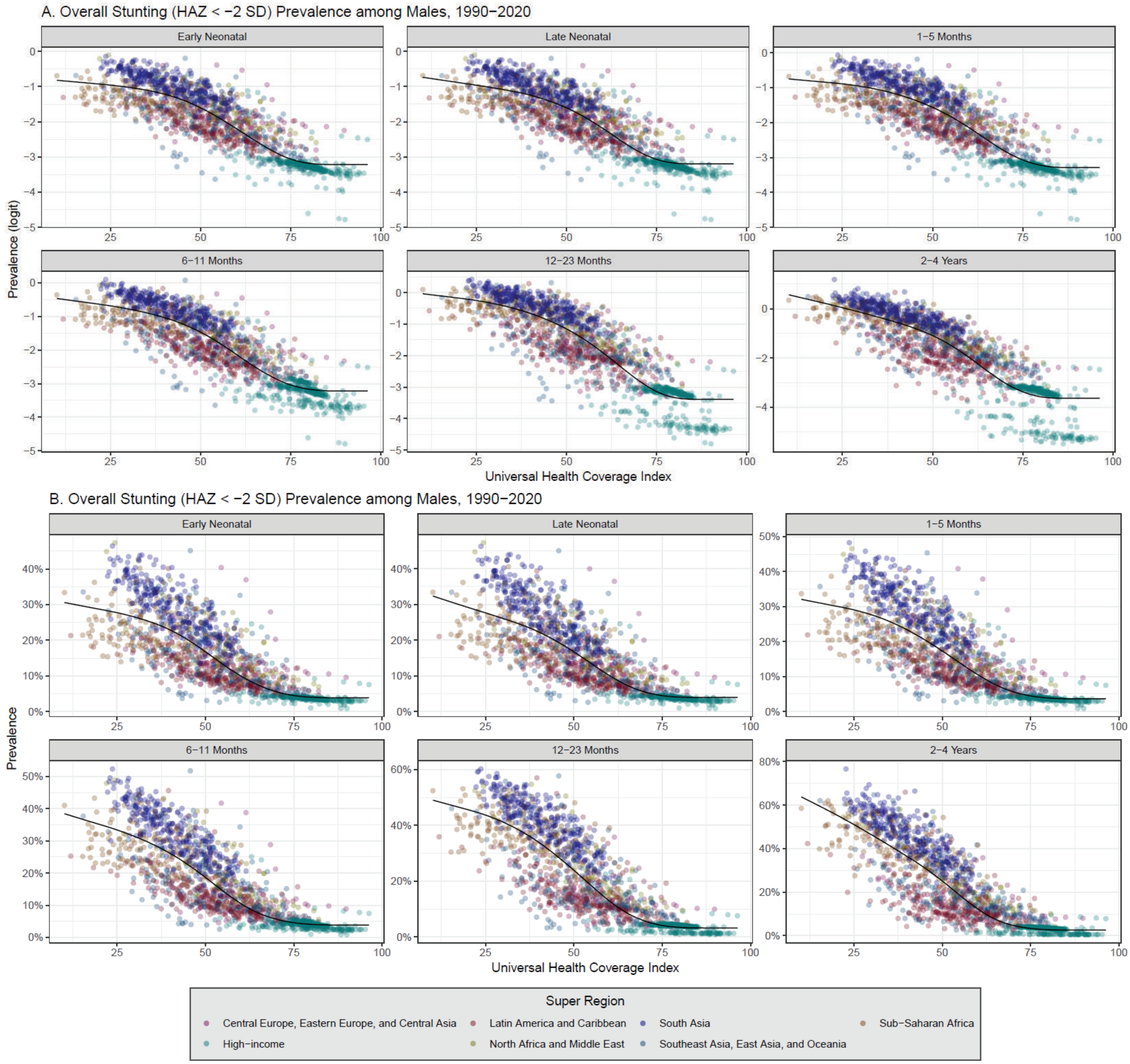

C. Overall Stunting (HAZ < −2 SD) Prevalence among Females, 1990−2020

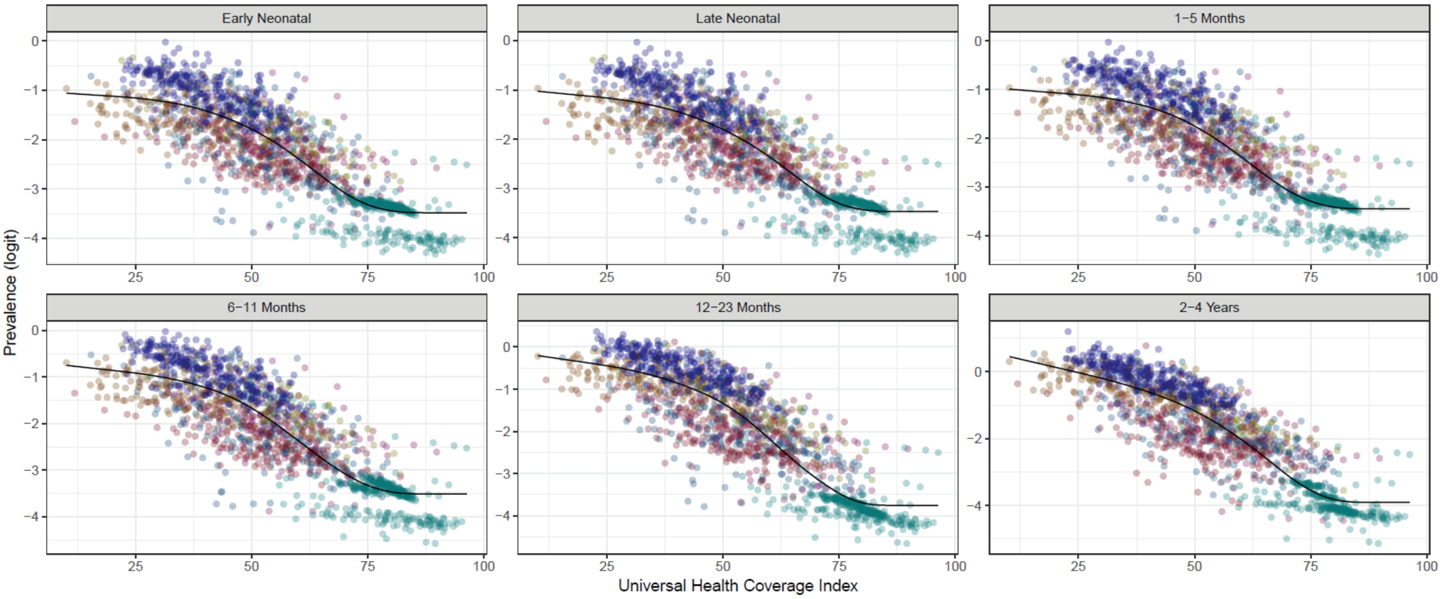

D. Overall Stunting (HAZ < −2 SD) Prevalence among Females, 1990−2020

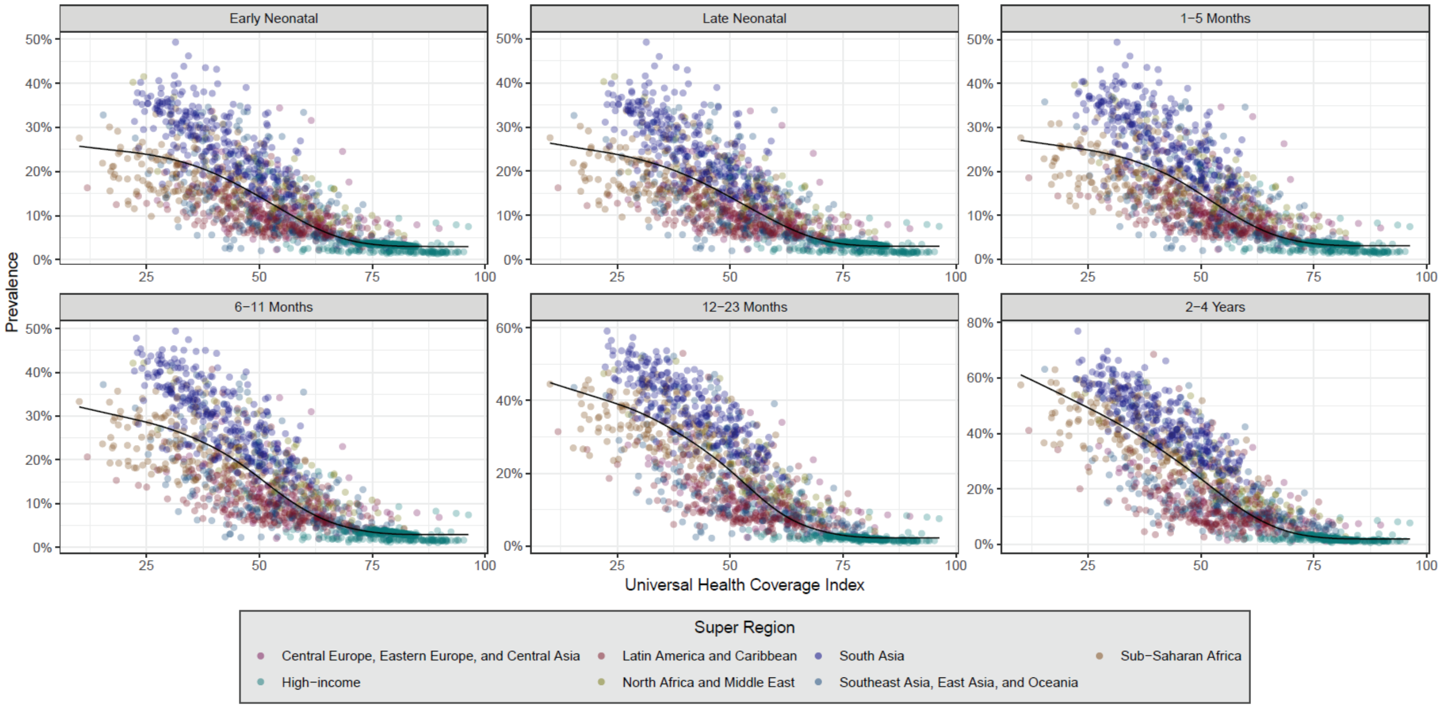

E. Severe Stunting (HAZ < −3 SD) Prevalence among Males, 1990−2020

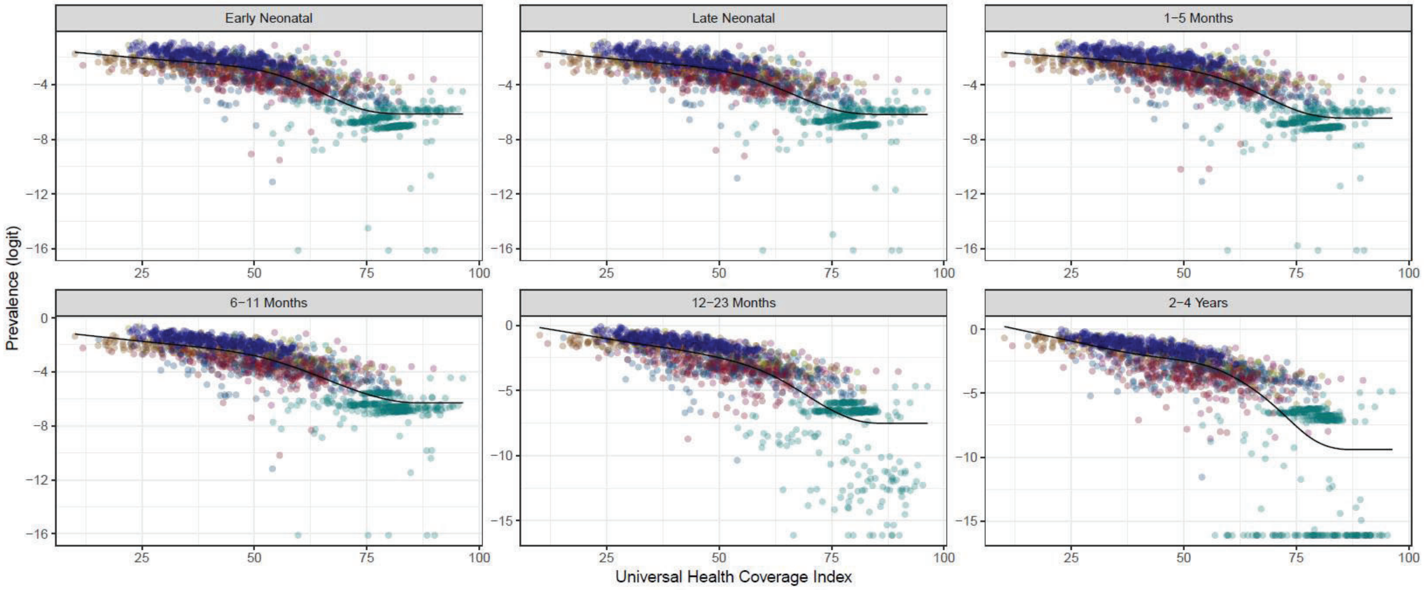

F. Severe Stunting (HAZ < −3 SD) Prevalence among Males, 1990−2020

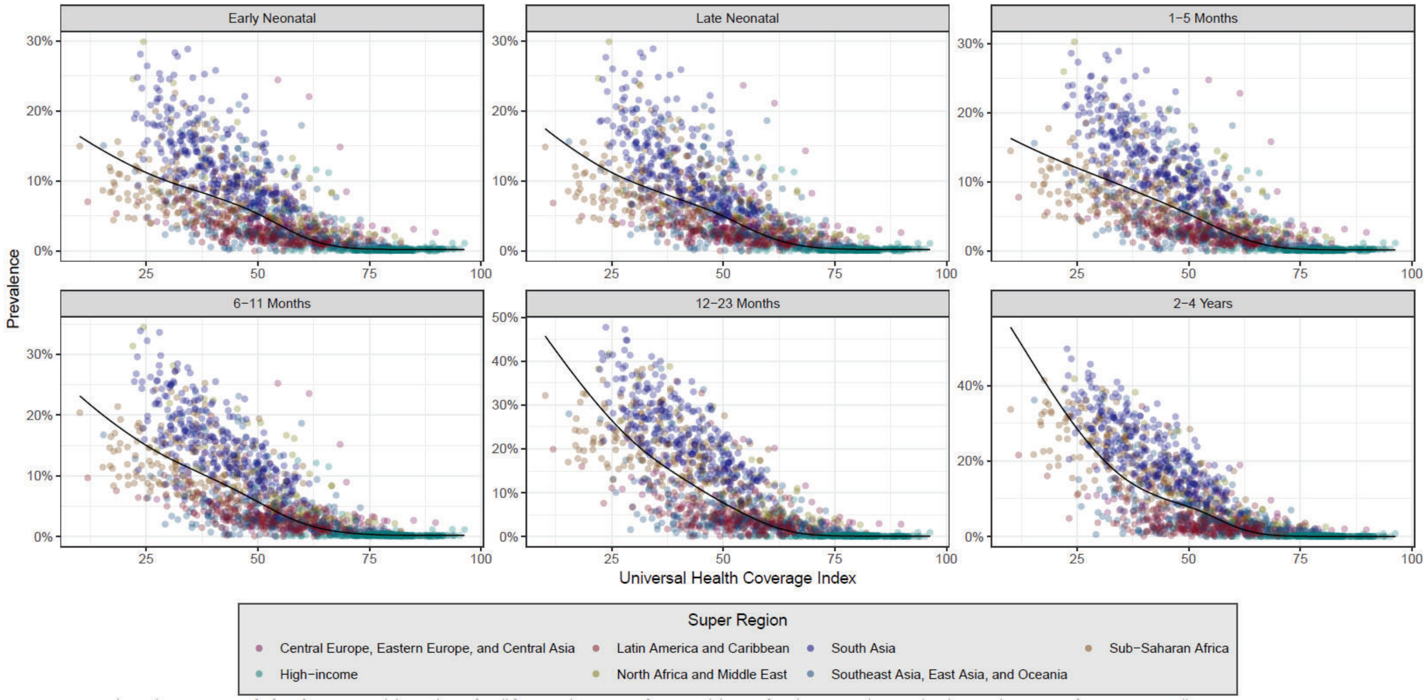

G. Severe Stunting (HAZ < −3 SD) Prevalence among Females, 1990−2020

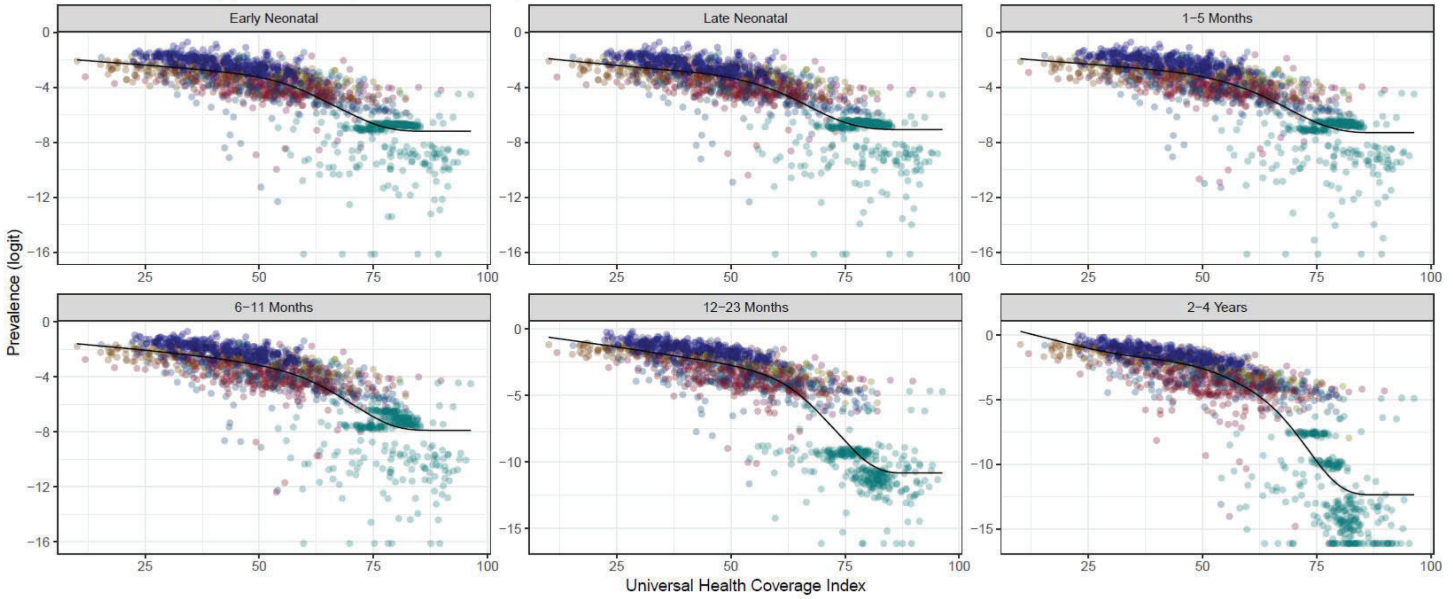

H. Severe Stunting (HAZ < −3 SD) Prevalence among Females, 1990−2020

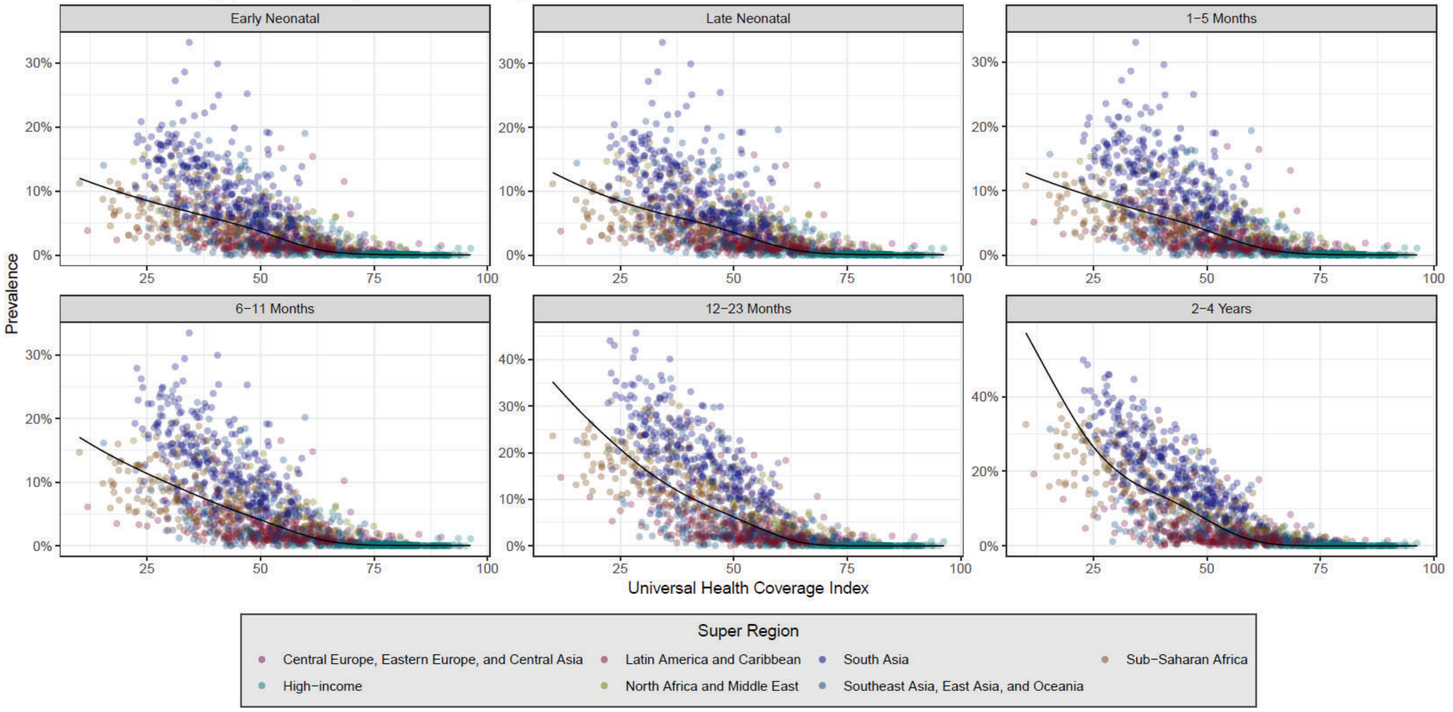

I. Extreme Stunting (HAZ < −4 SD) Prevalence among Males, 1990−2020

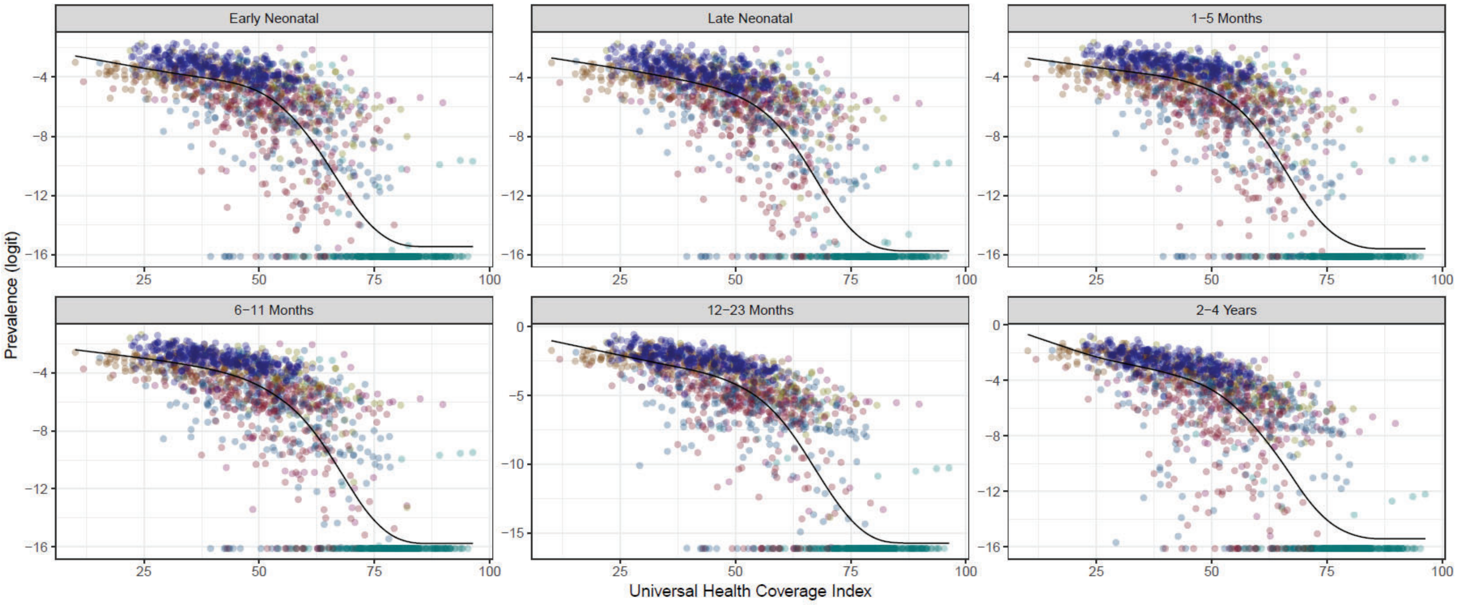

J. Extreme Stunting (HAZ < −4 SD) Prevalence among Males, 1990−2020

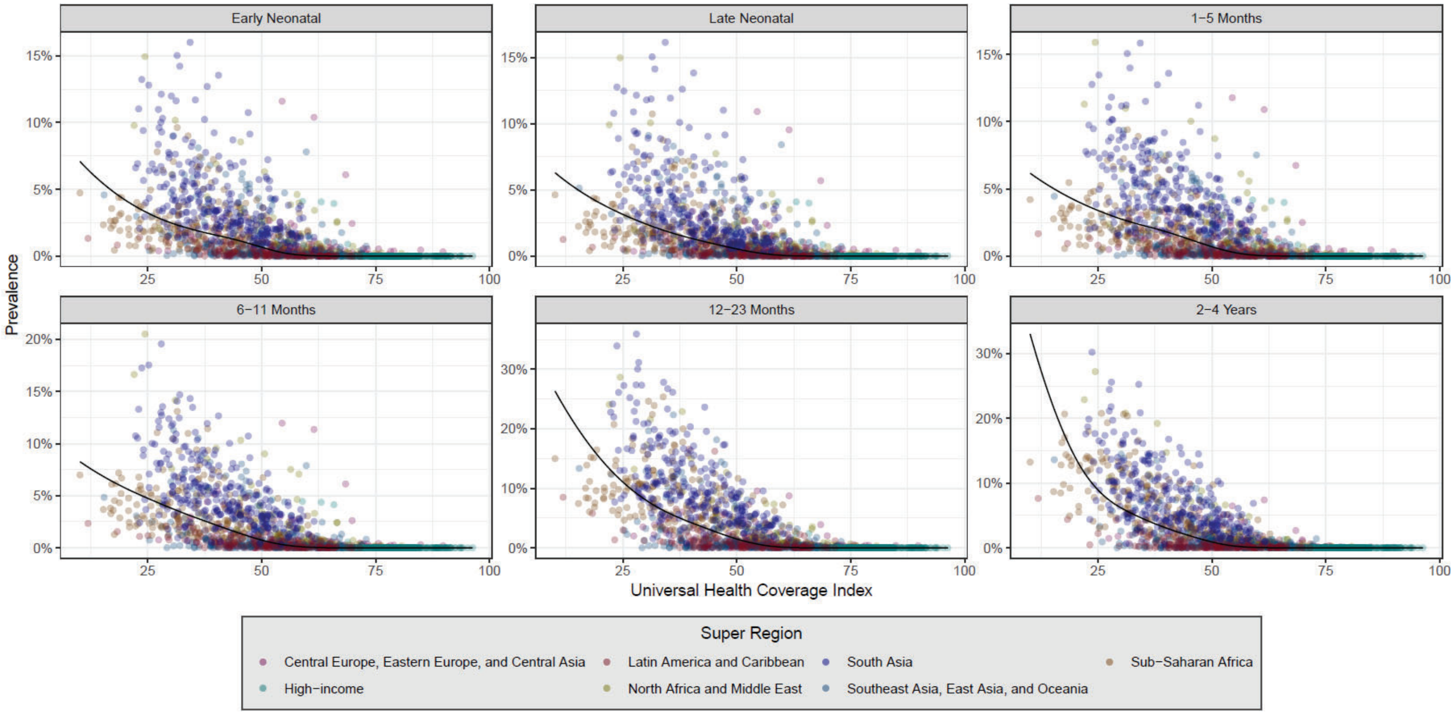

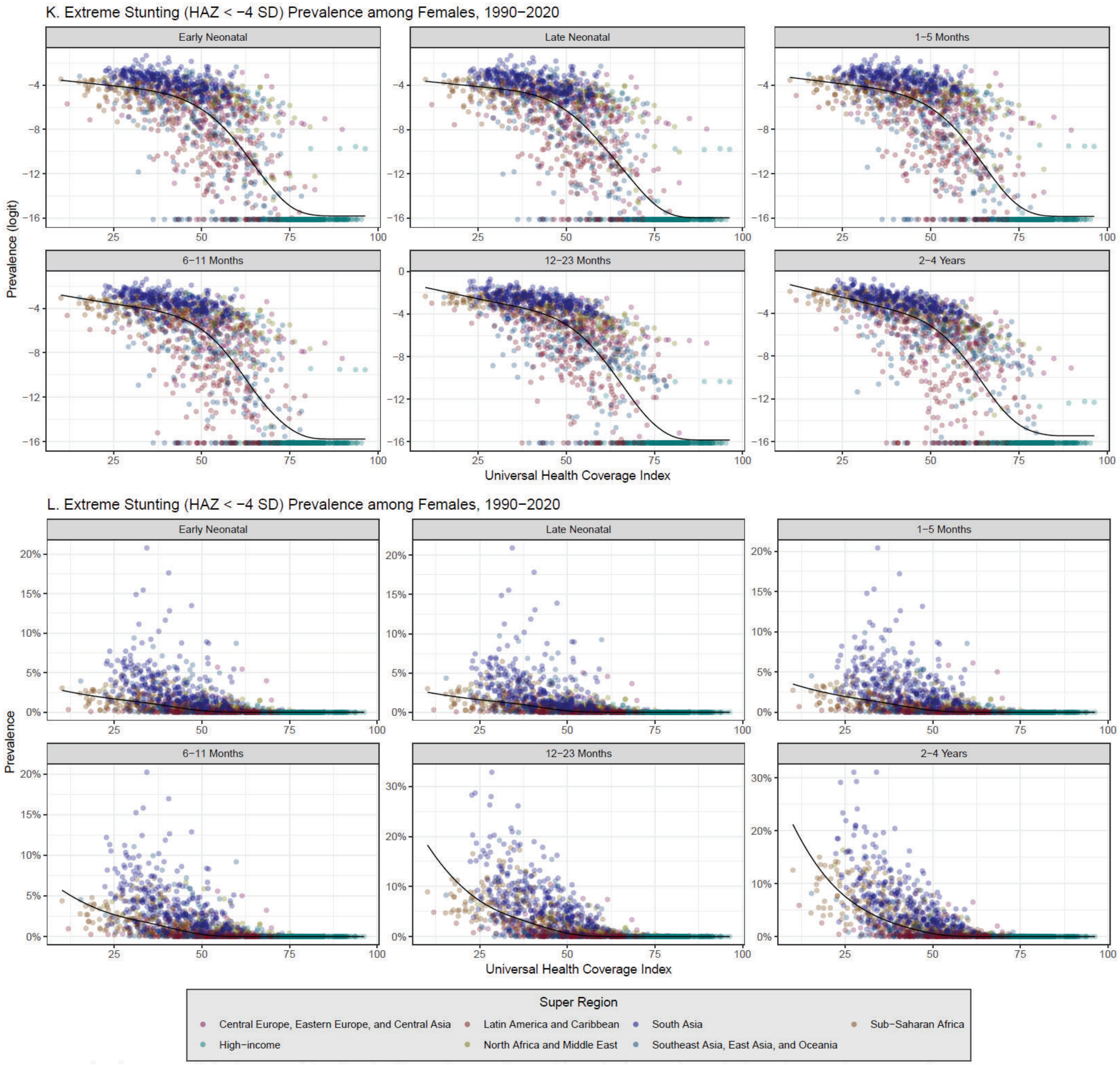

K. Extreme Stunting (HAZ < −4 SD) Prevalence among Females, 1990−2020
Early Neonatal
Late Neonatal
1−5 Months
6−11 Months
12−23 Months
2−4 Years
Prevalence (logit)
Universal Health Coverage Index
L. Extreme Stunting (HAZ < −4 SD) Prevalence among Females, 1990−2020
Early Neonatal
Late Neonatal
1−5 Months
6−11 Months
12−23 Months
2−4 Years
Prevalence
Universal Health Coverage Index
Super Region
Central Europe, Eastern Europe, and Central Asia
Latin America and Caribbean
South Asia
Sub−Saharan Africa
High−income
North Africa and Middle East
Southeast Asia, East Asia, and Oceania

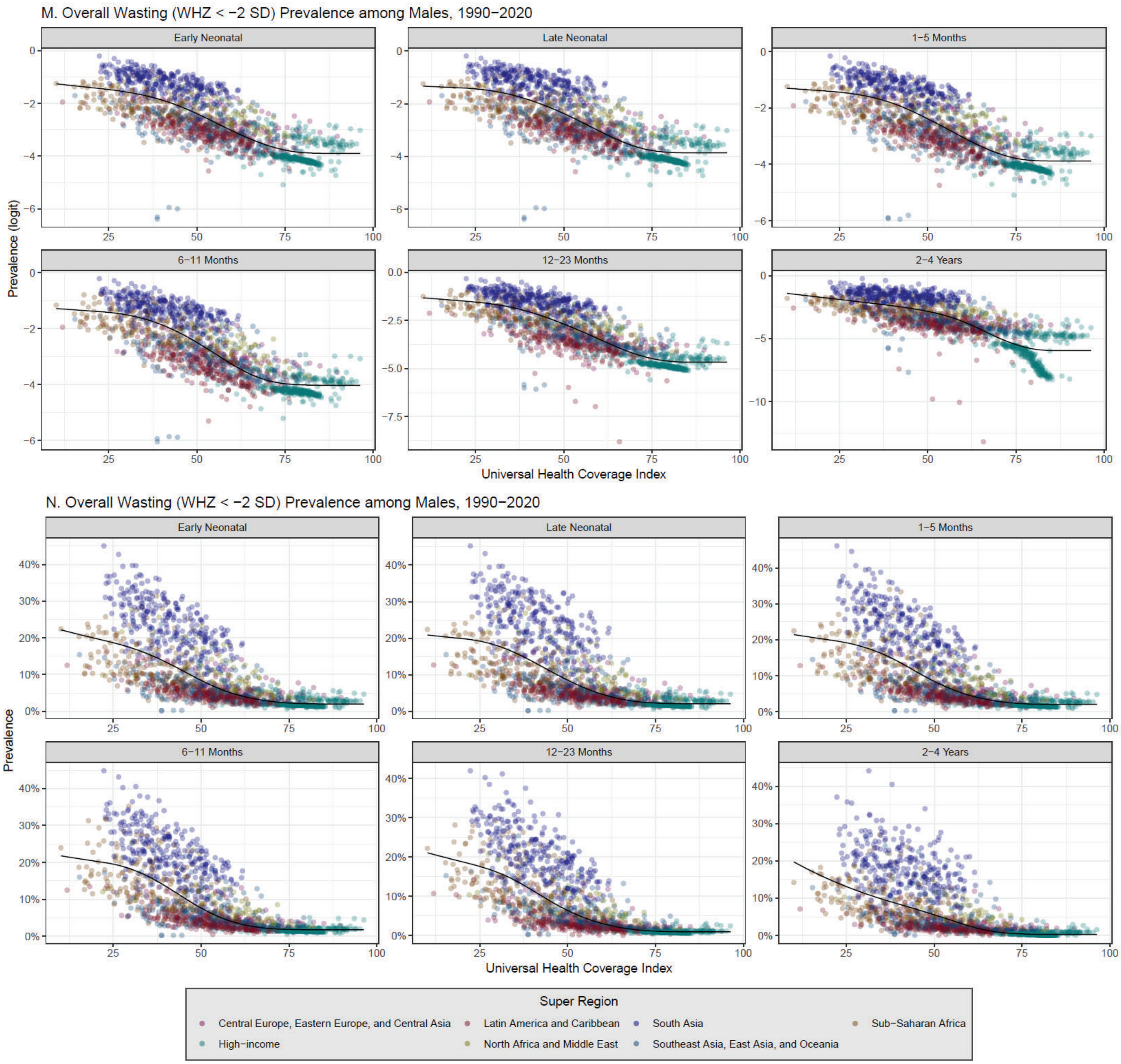

M. Overall Wasting (WHZ < −2 SD) Prevalence among Males, 1990−2020
Early Neonatal
Late Neonatal
1−5 Months
6−11 Months
12−23 Months
2−4 Years
Prevalence (logit)
Universal Health Coverage Index
N. Overall Wasting (WHZ < −2 SD) Prevalence among Males, 1990−2020
Early Neonatal
Late Neonatal
1−5 Months
6−11 Months
12−23 Months
2−4 Years
Prevalence
Universal Health Coverage Index
Super Region
Central Europe, Eastern Europe, and Central Asia
Latin America and Caribbean
South Asia
Sub−Saharan Africa
High−income
North Africa and Middle East
Southeast Asia, East Asia, and Oceania

O. Overall Wasting (WHZ < −2 SD) Prevalence among Females, 1990−2020

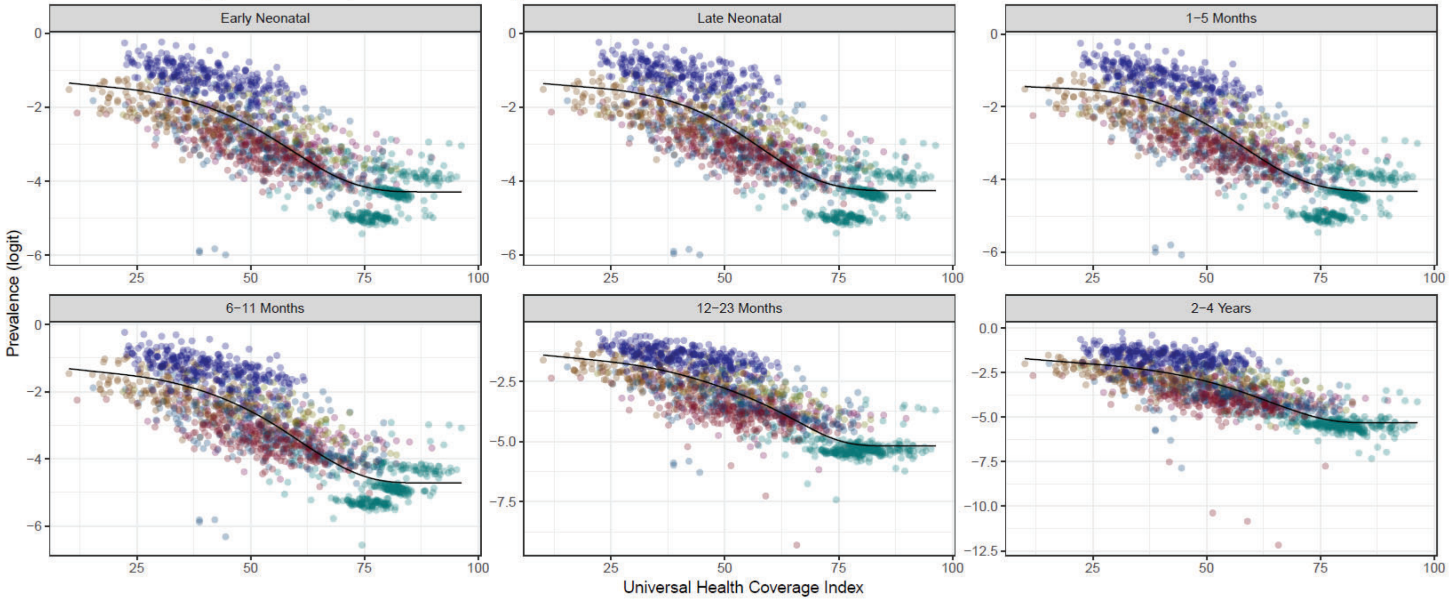

P. Overall Wasting (WHZ < −2 SD) Prevalence among Females, 1990−2020

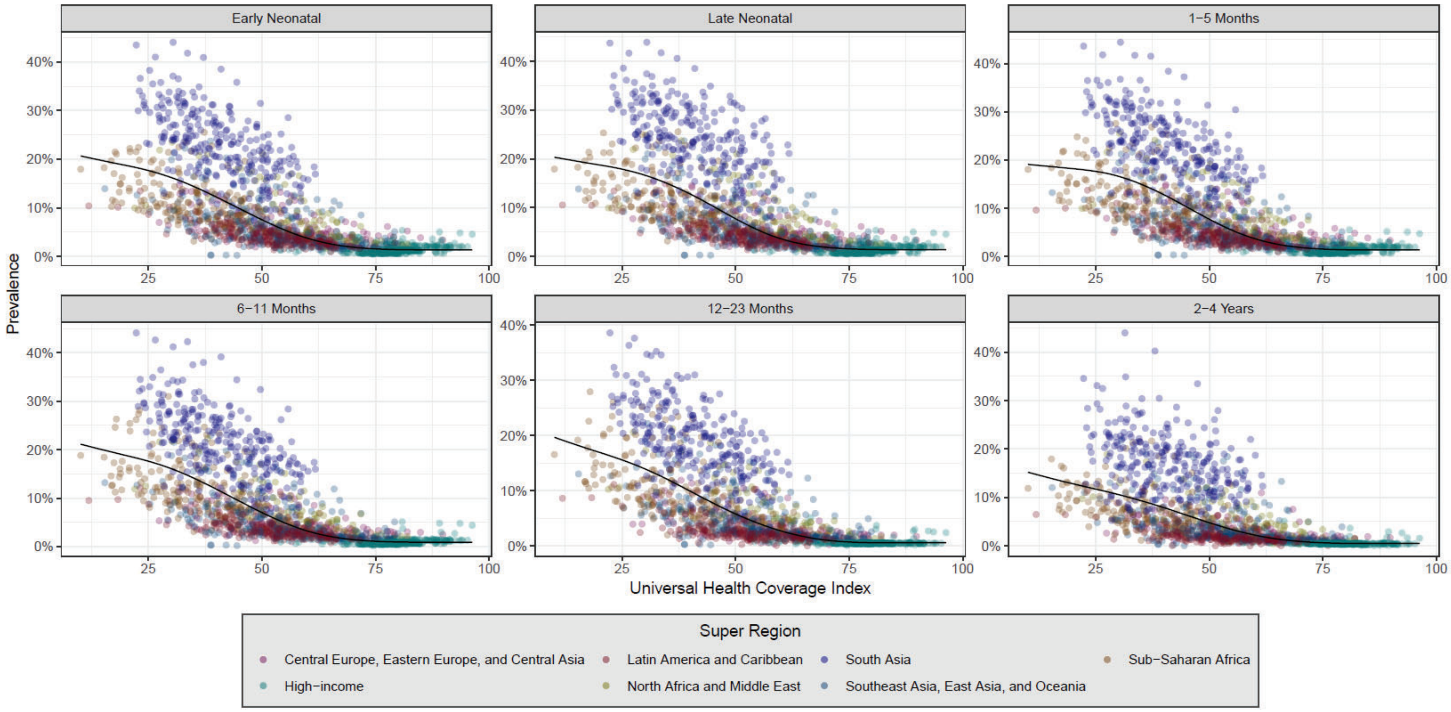

Q. Severe Wasting (WHZ < −3 SD) Prevalence among Males, 1990−2020

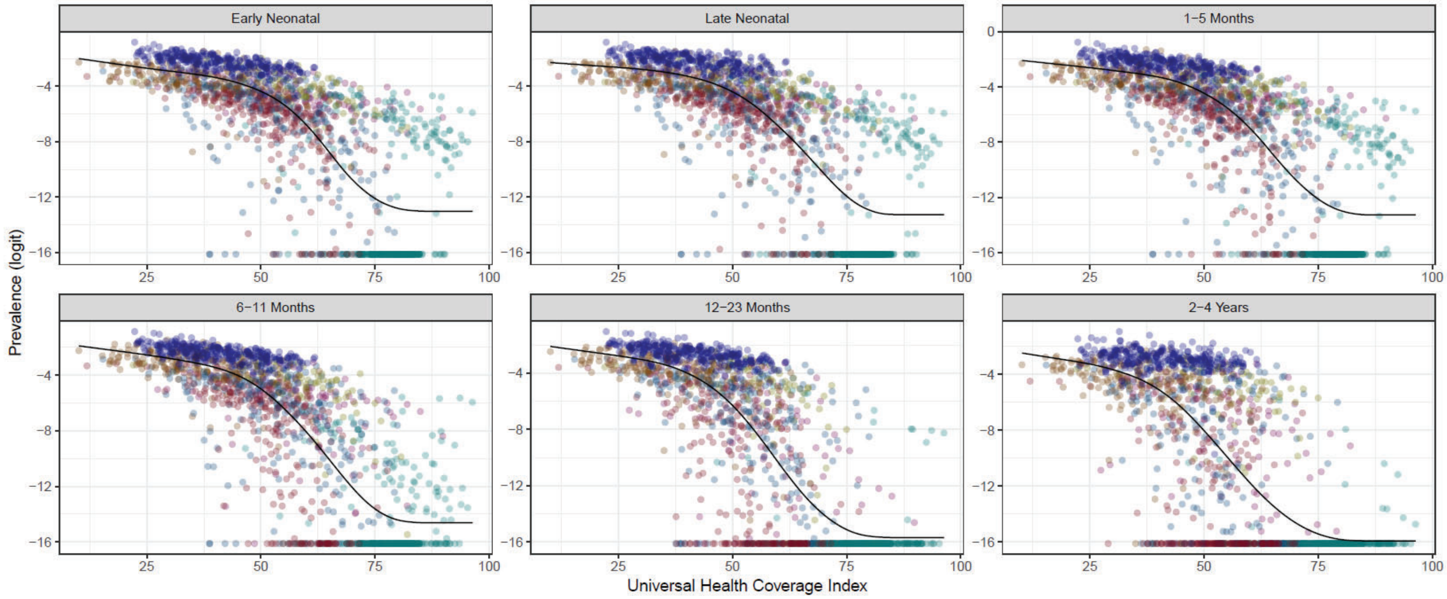

R. Severe Wasting (WHZ < −3 SD) Prevalence among Males, 1990−2020

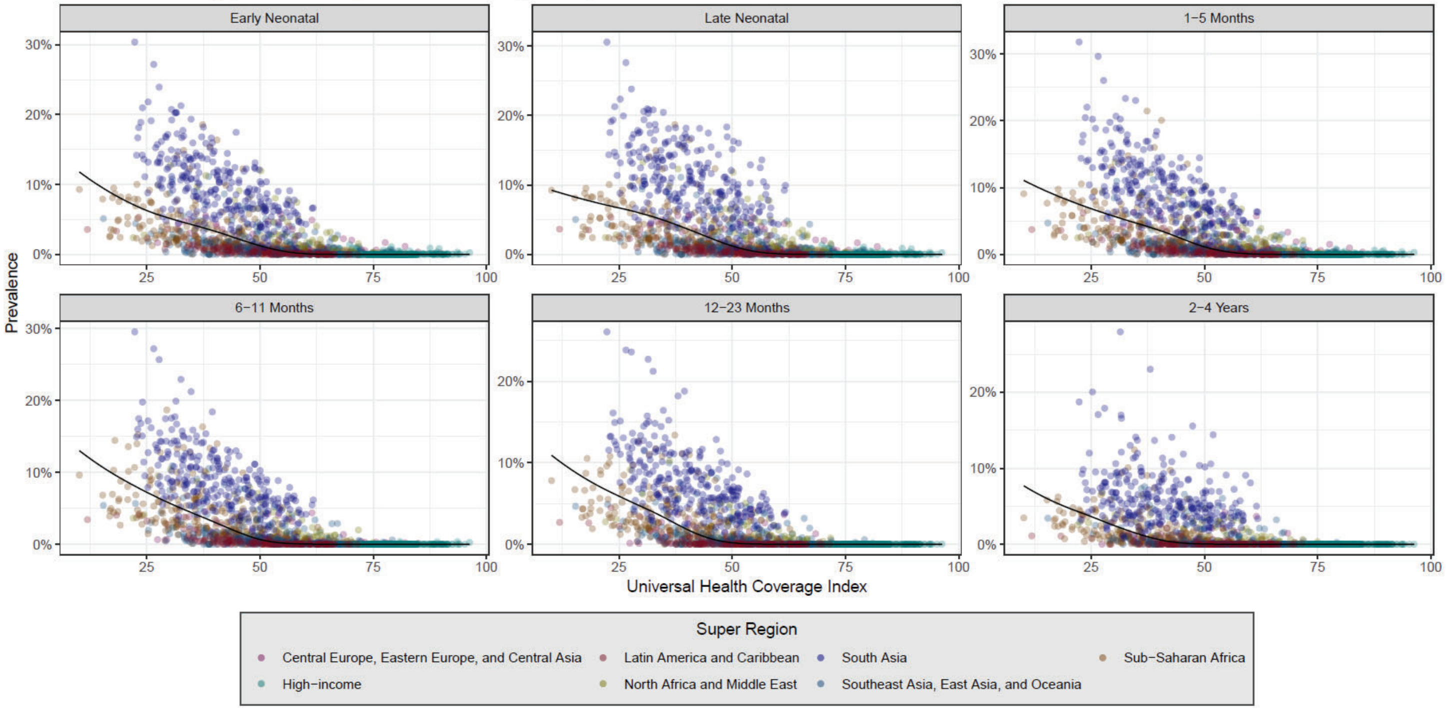

S. Severe Wasting (WHZ < −3 SD) Prevalence among Females, 1990−2020

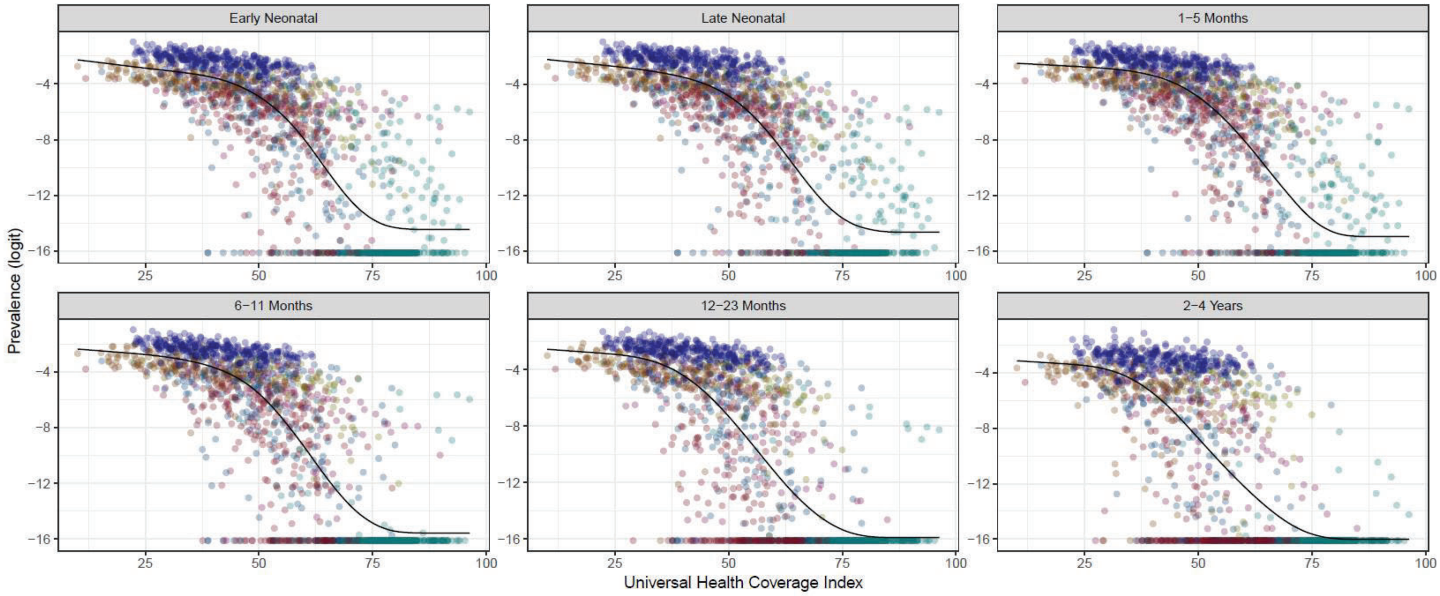

T. Severe Wasting (WHZ < −3 SD) Prevalence among Females, 1990−2020

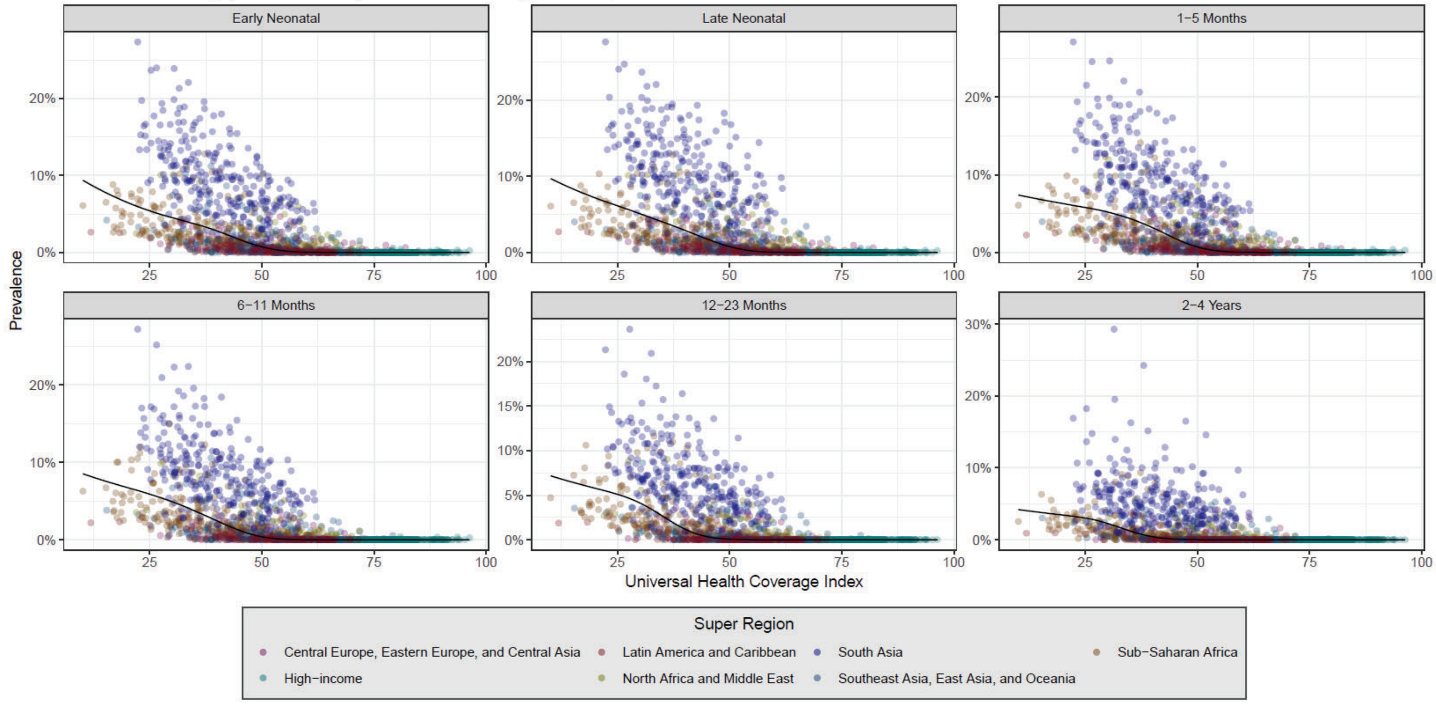

U. Extreme Wasting (WHZ < −4 SD) Prevalence among Males, 1990−2020

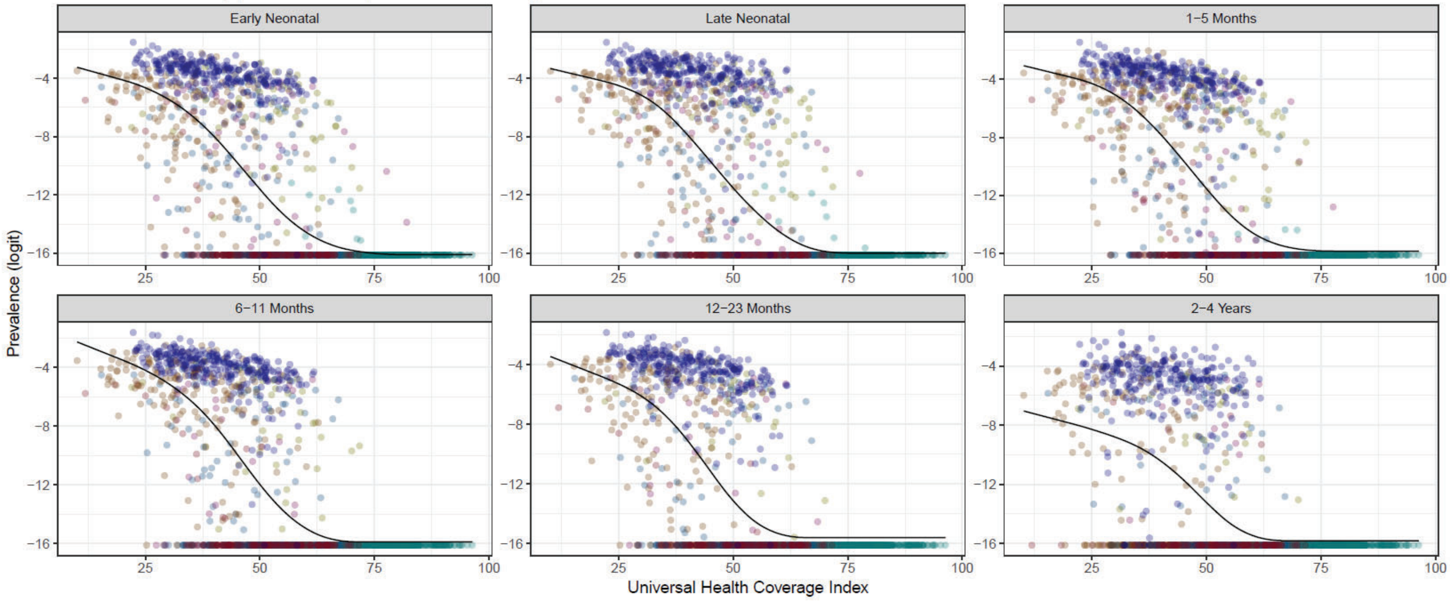

V. Extreme Wasting (WHZ < −4 SD) Prevalence among Males, 1990−2020

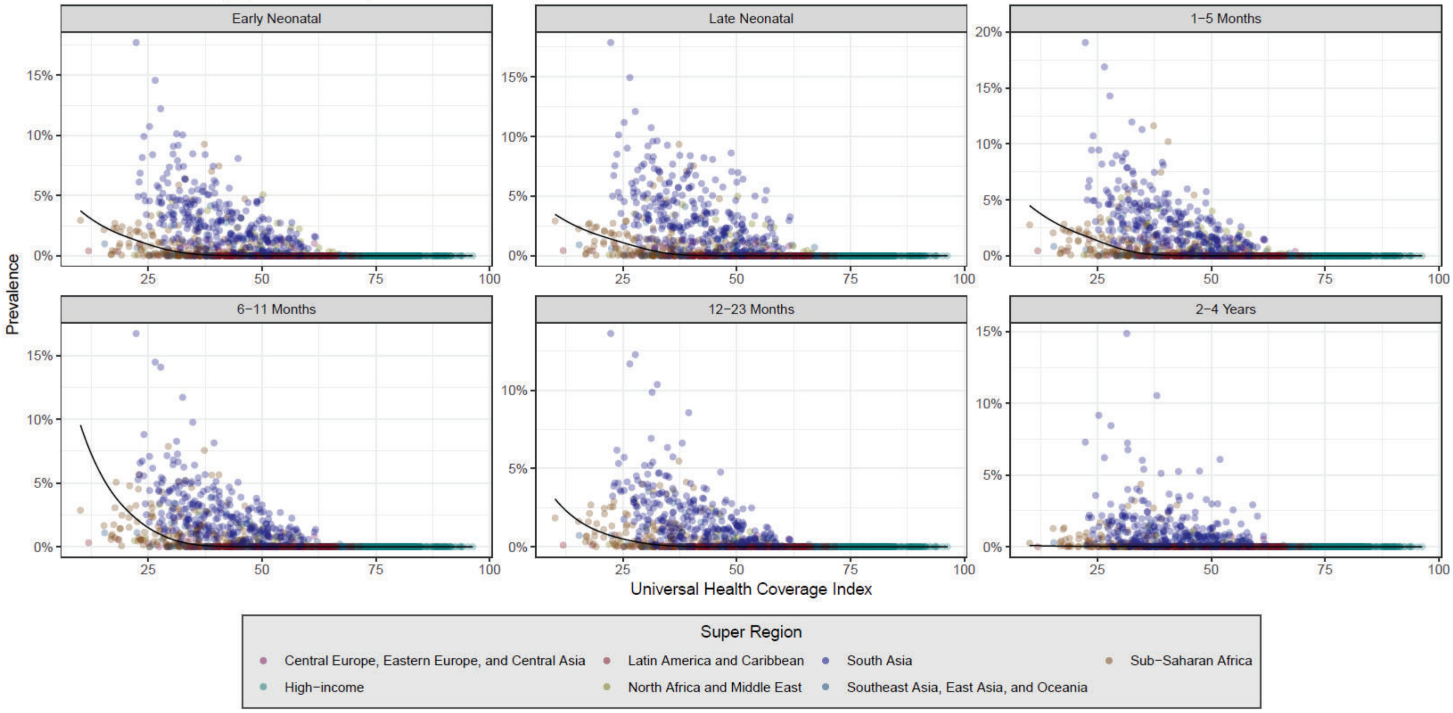

W. Extreme Wasting (WHZ < −4 SD) Prevalence among Females, 1990−2020

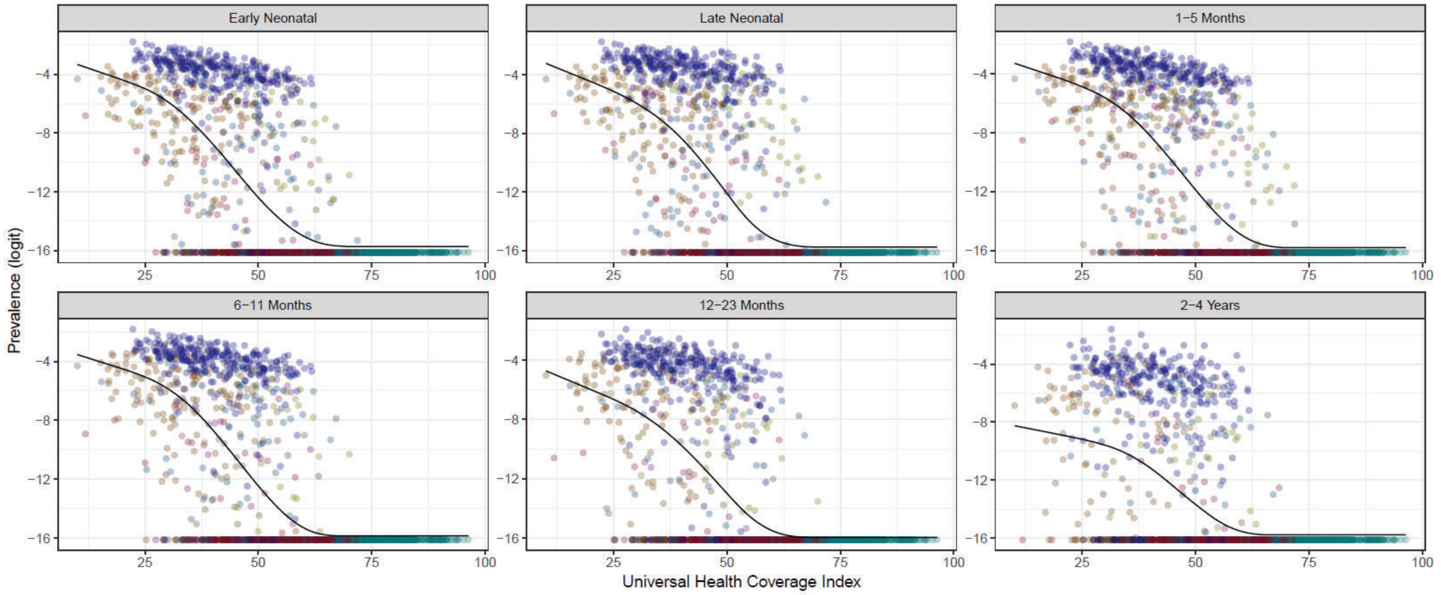

X. Extreme Wasting (WHZ < −4 SD) Prevalence among Females, 1990−2020

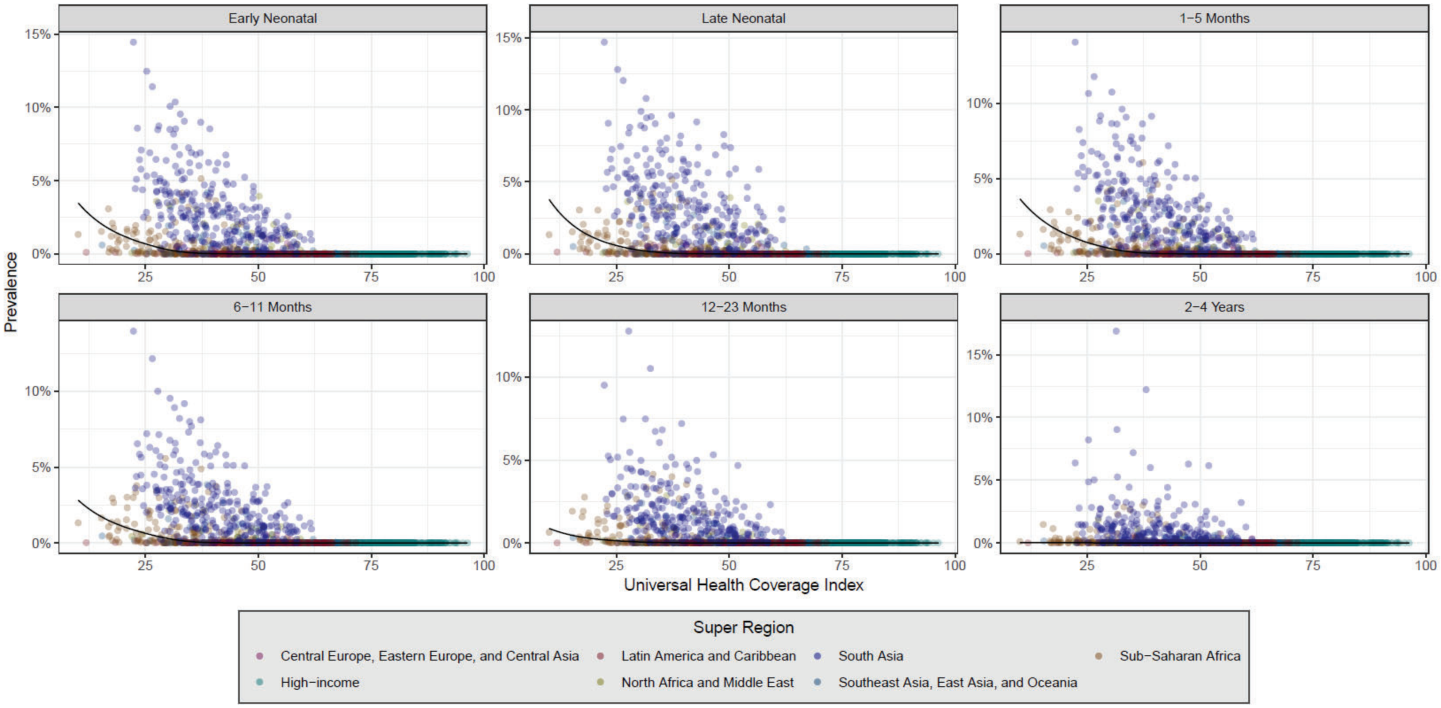

Y. Overall Underweight (WAZ < −2 SD) Prevalence among Males, 1990−2020

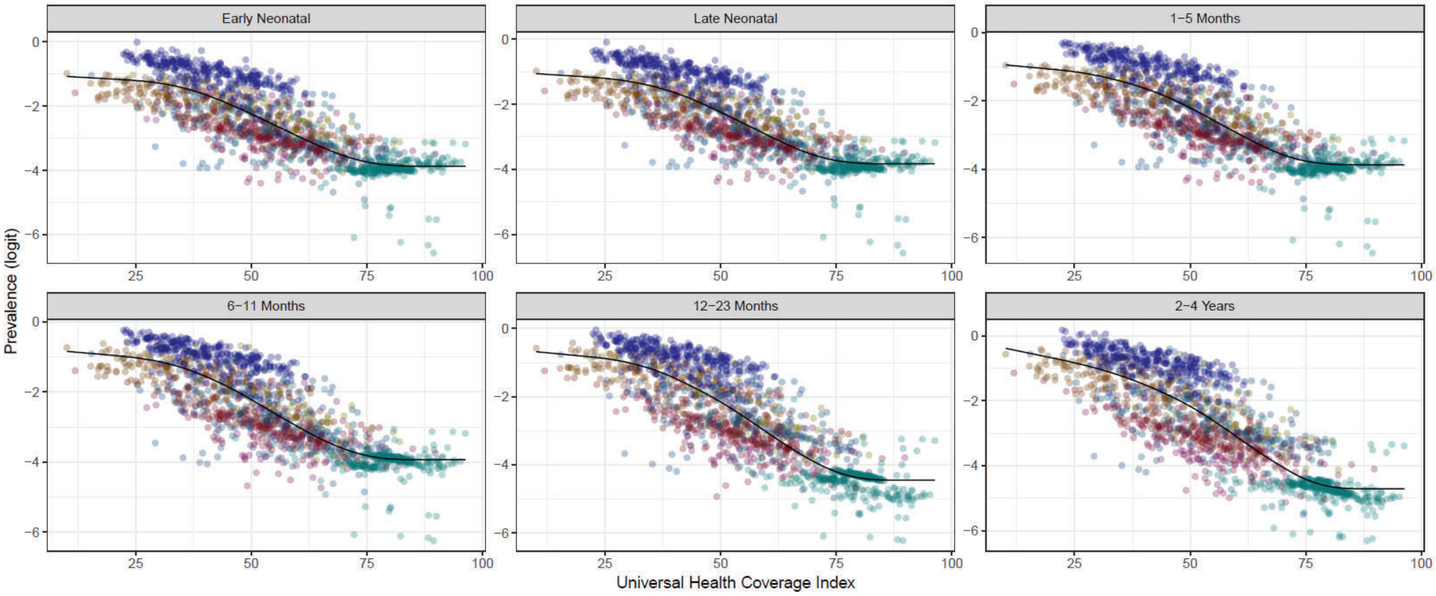

Z. Overall Underweight (WAZ < −2 SD) Prevalence among Males, 1990−2020

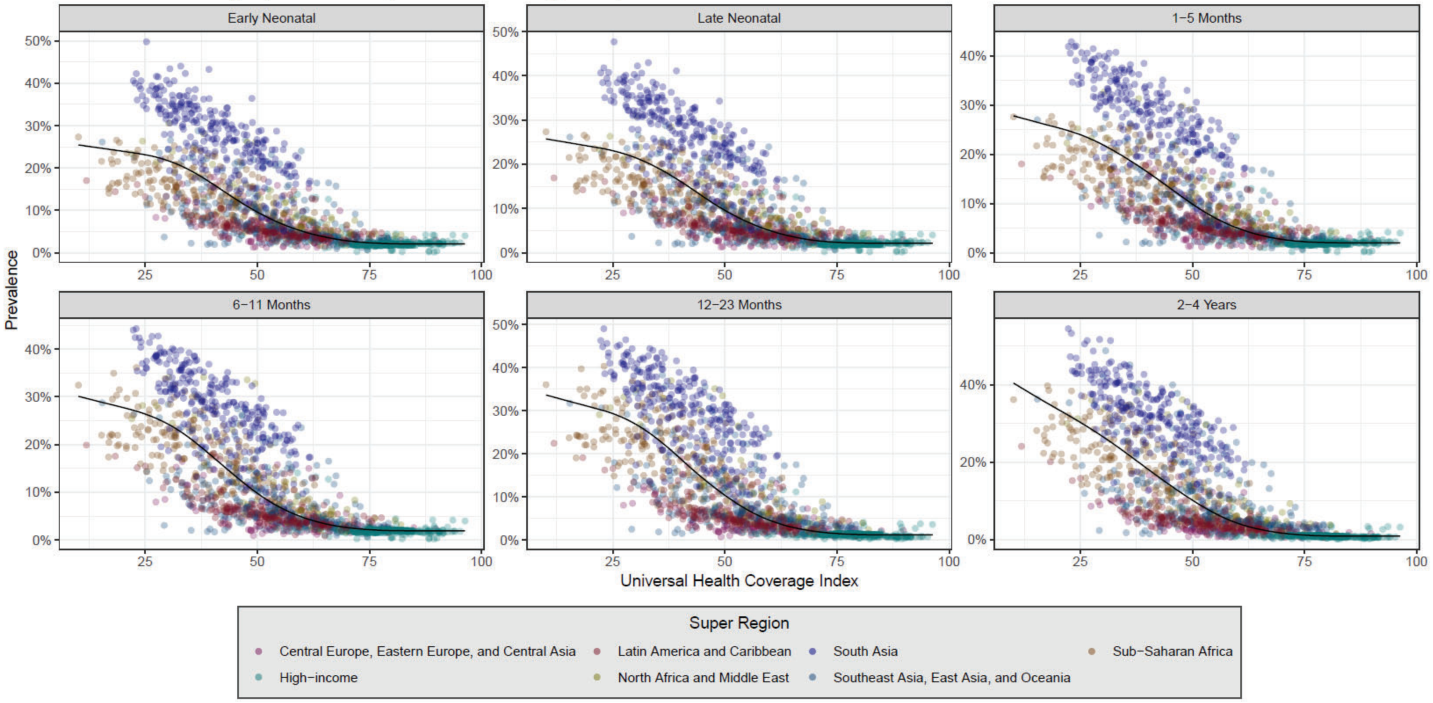

AA. Overall Underweight (WAZ < −2 SD) Prevalence among Females, 1990−2020

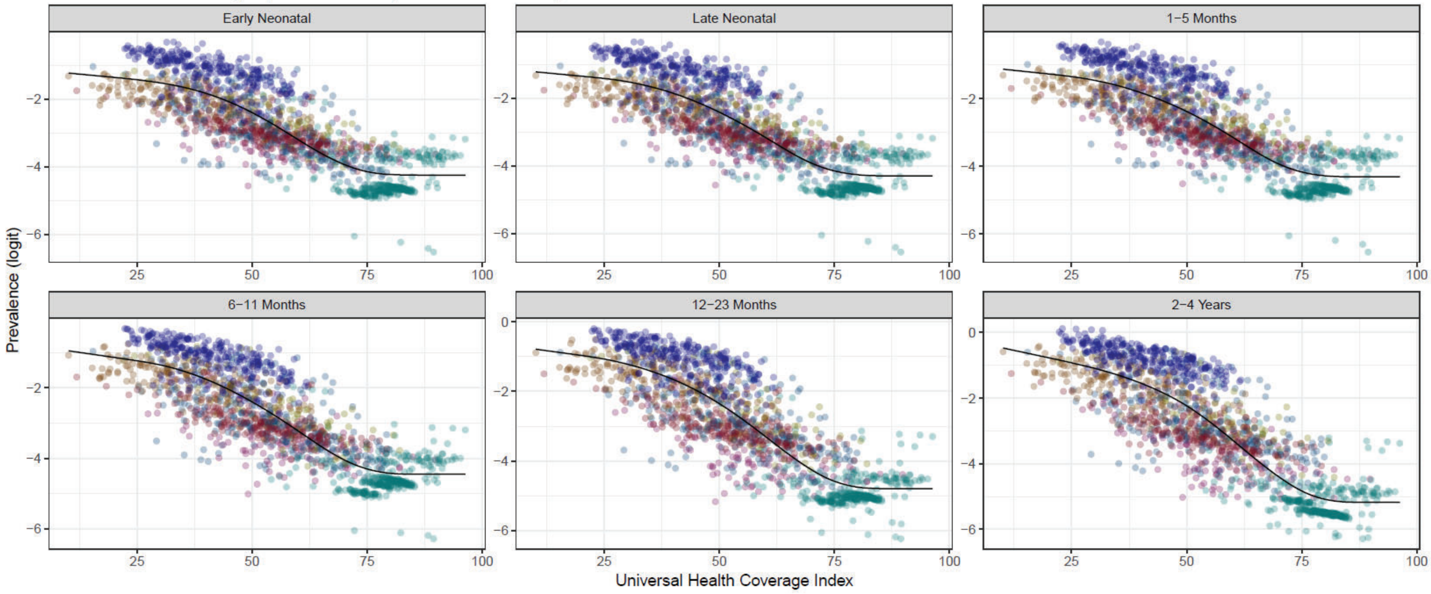

BB. Overall Underweight (WAZ < −2 SD) Prevalence among Females, 1990−2020

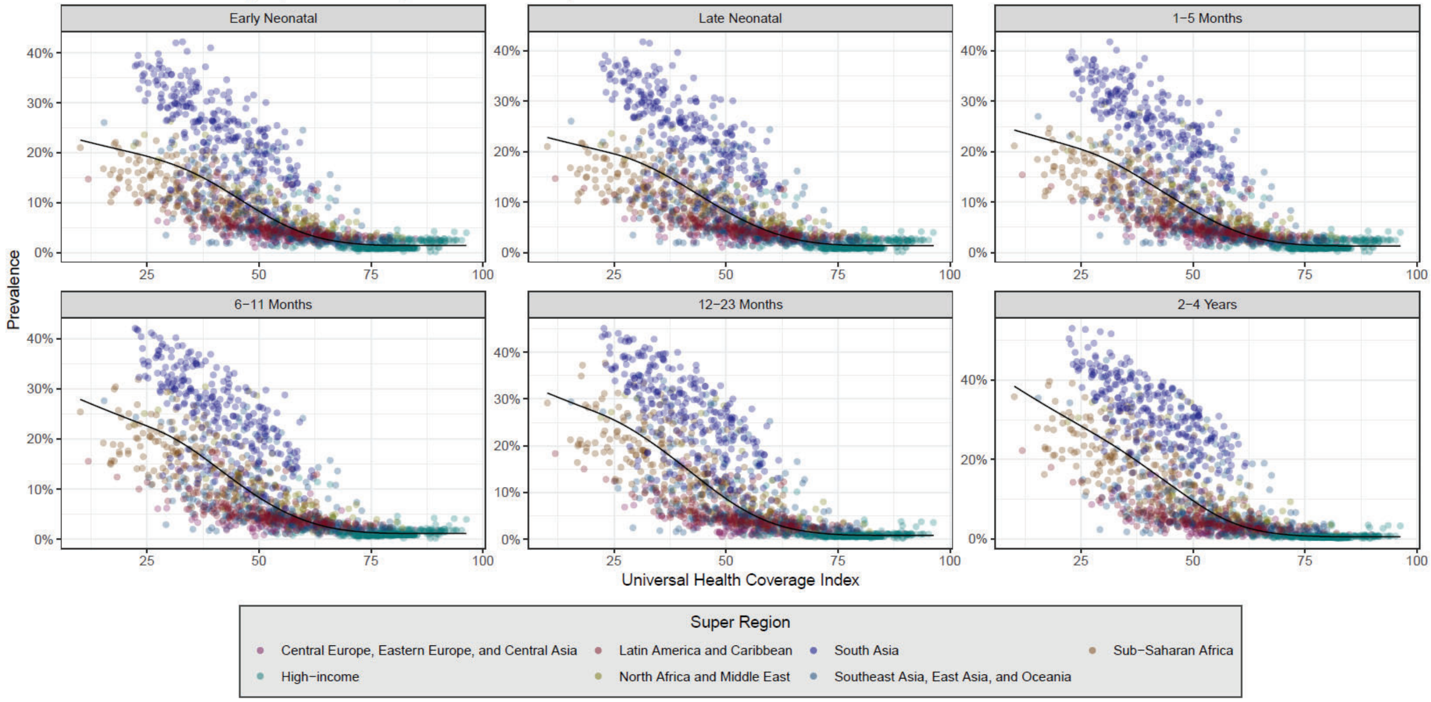

CC. Severe Underweight (WAZ < −3 SD) Prevalence among Males, 1990−2020

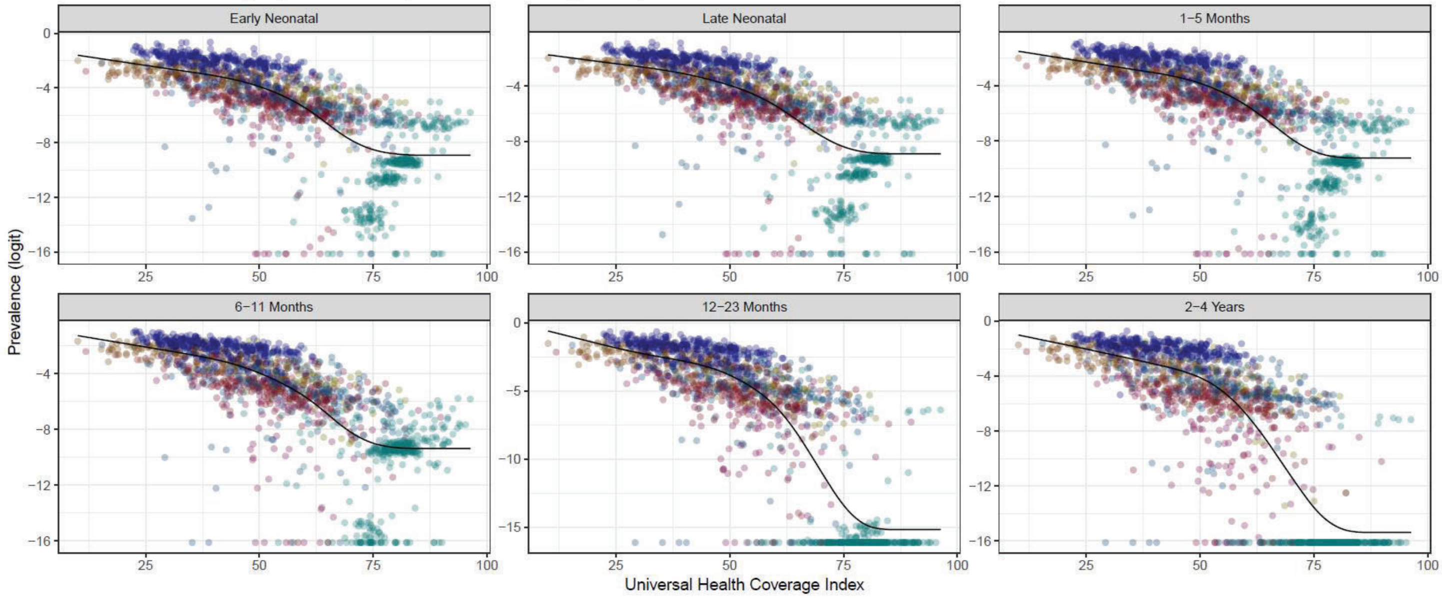

DD. Severe Underweight (WAZ < −3 SD) Prevalence among Males, 1990−2020

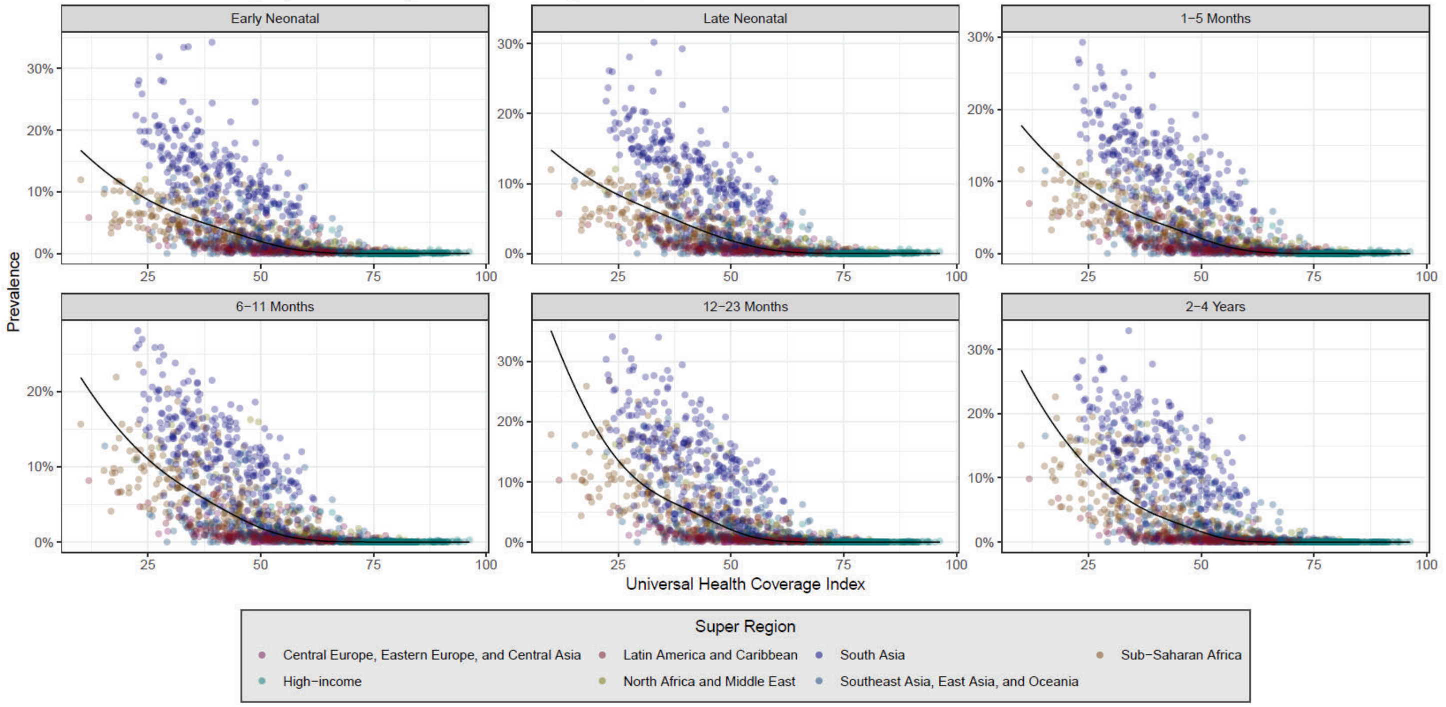

EE. Severe Underweight (WAZ < −3 SD) Prevalence among Females, 1990−2020

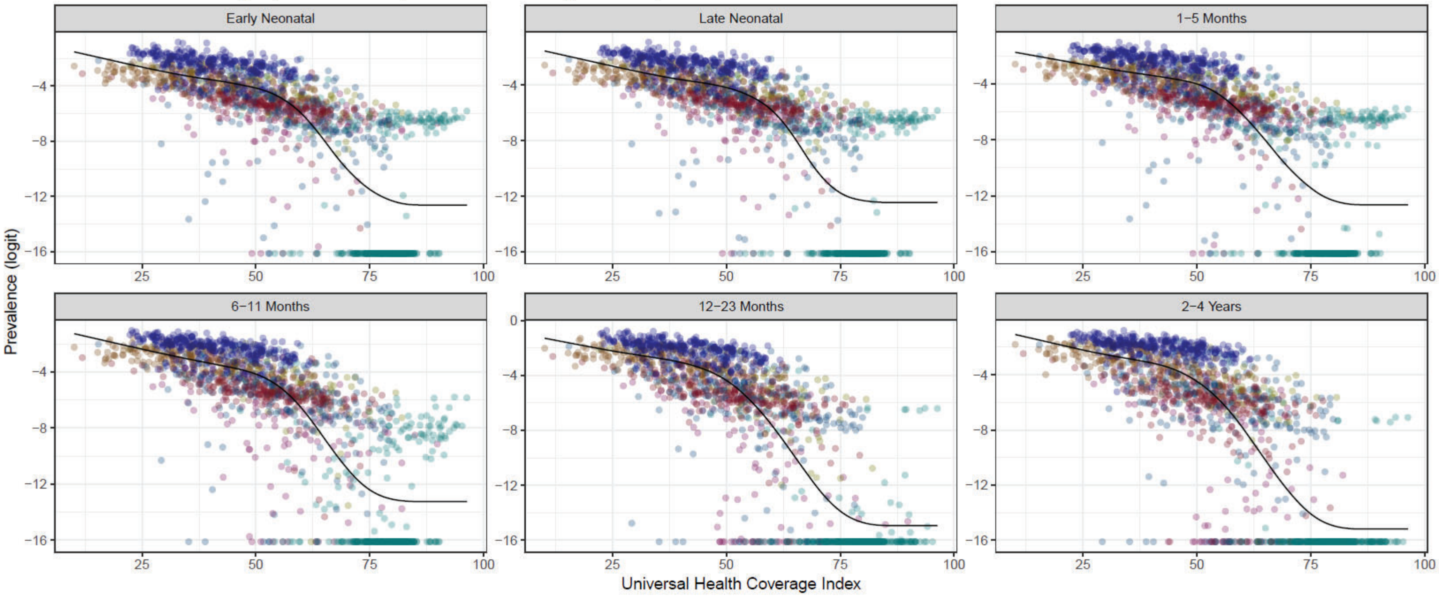

FF. Severe Underweight (WAZ < −3 SD) Prevalence among Females, 1990−2020

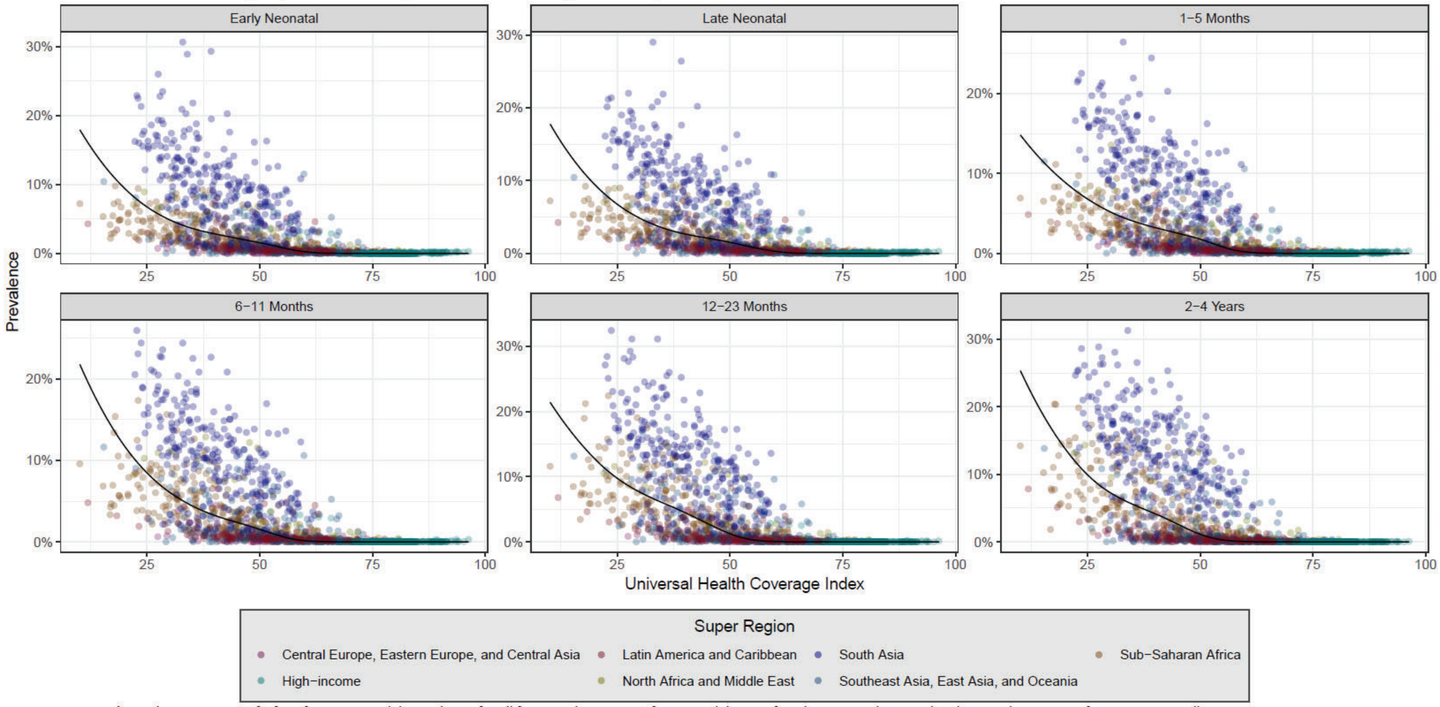

GG. Extreme Underweight (WAZ < −4 SD) Prevalence among Males, 1990−2020

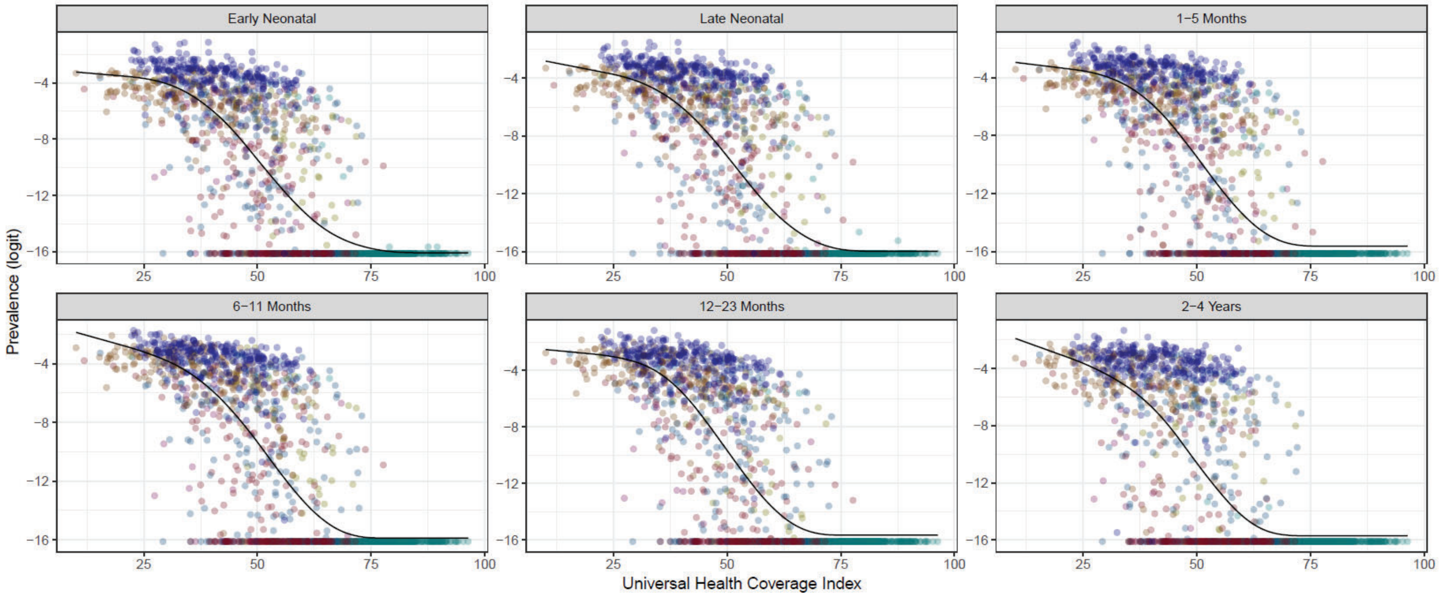

HH. Extreme Underweight (WAZ < −4 SD) Prevalence among Males, 1990−2020

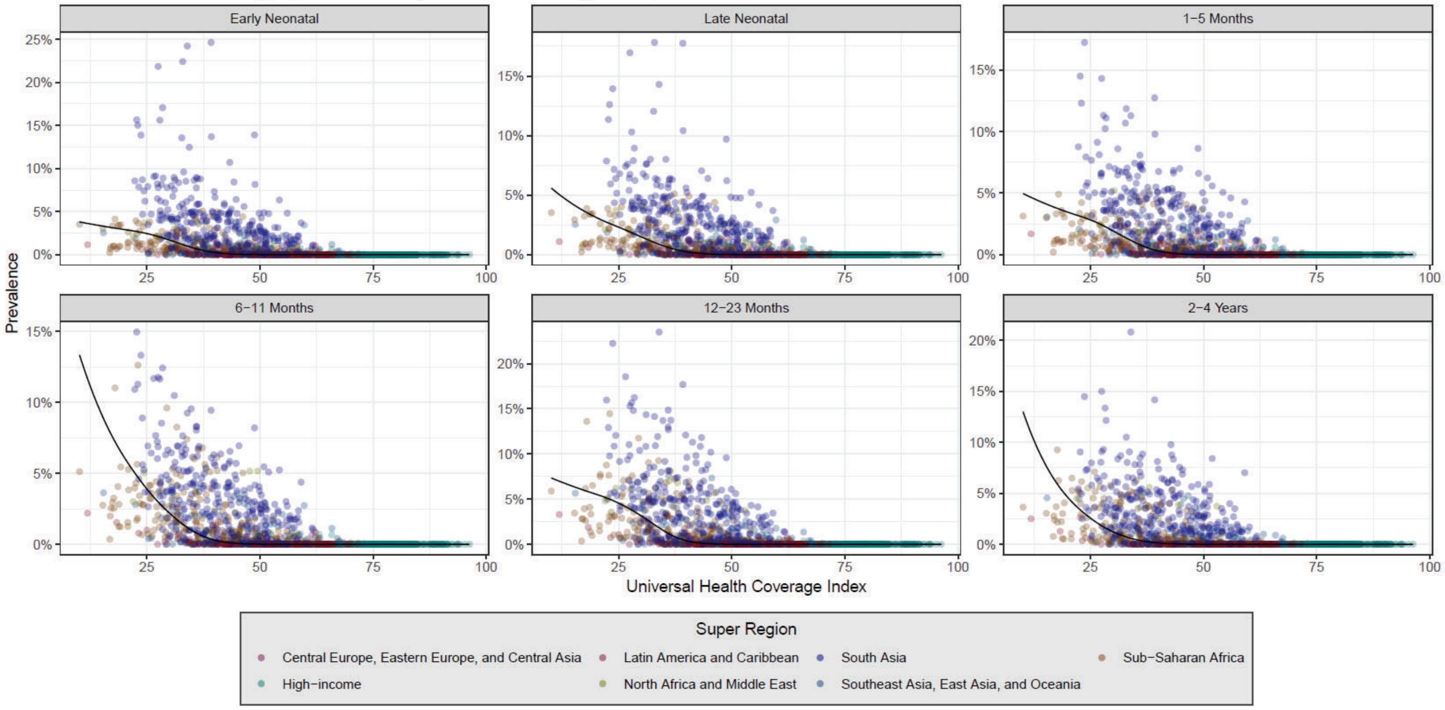

II. Extreme Underweight (WAZ < −4 SD) Prevalence among Females, 1990−2020

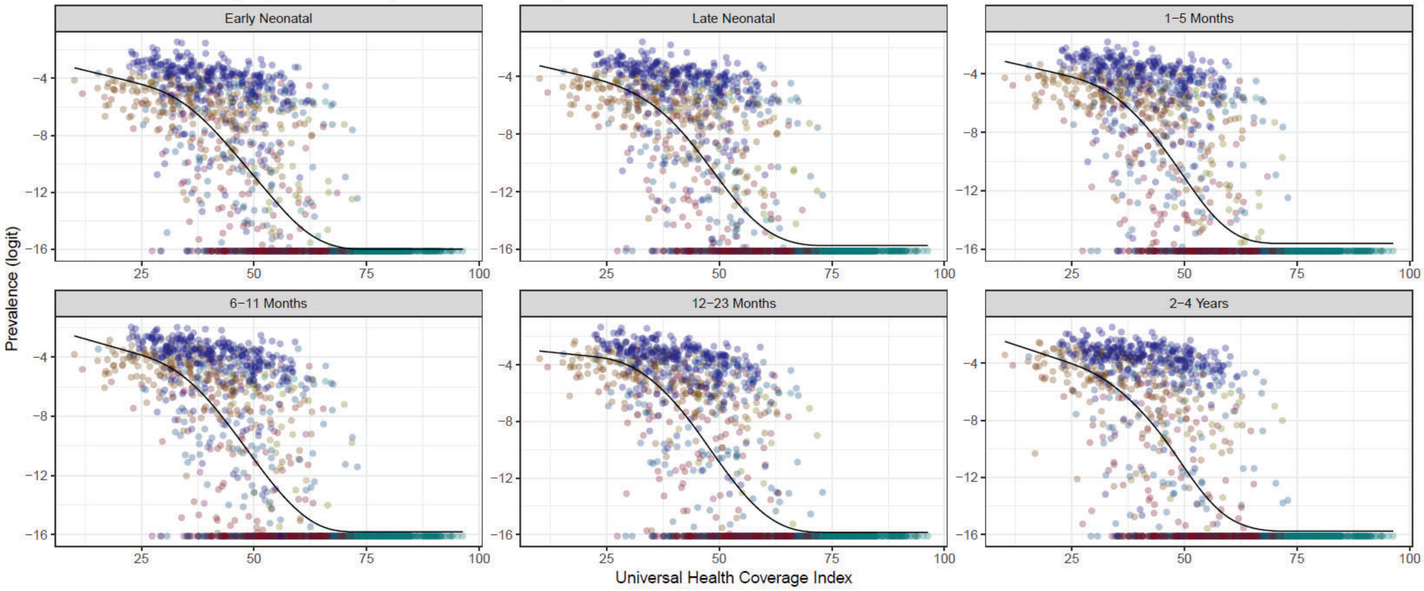

JJ. Extreme Underweight (WAZ < −4 SD) Prevalence among Females, 1990−2020

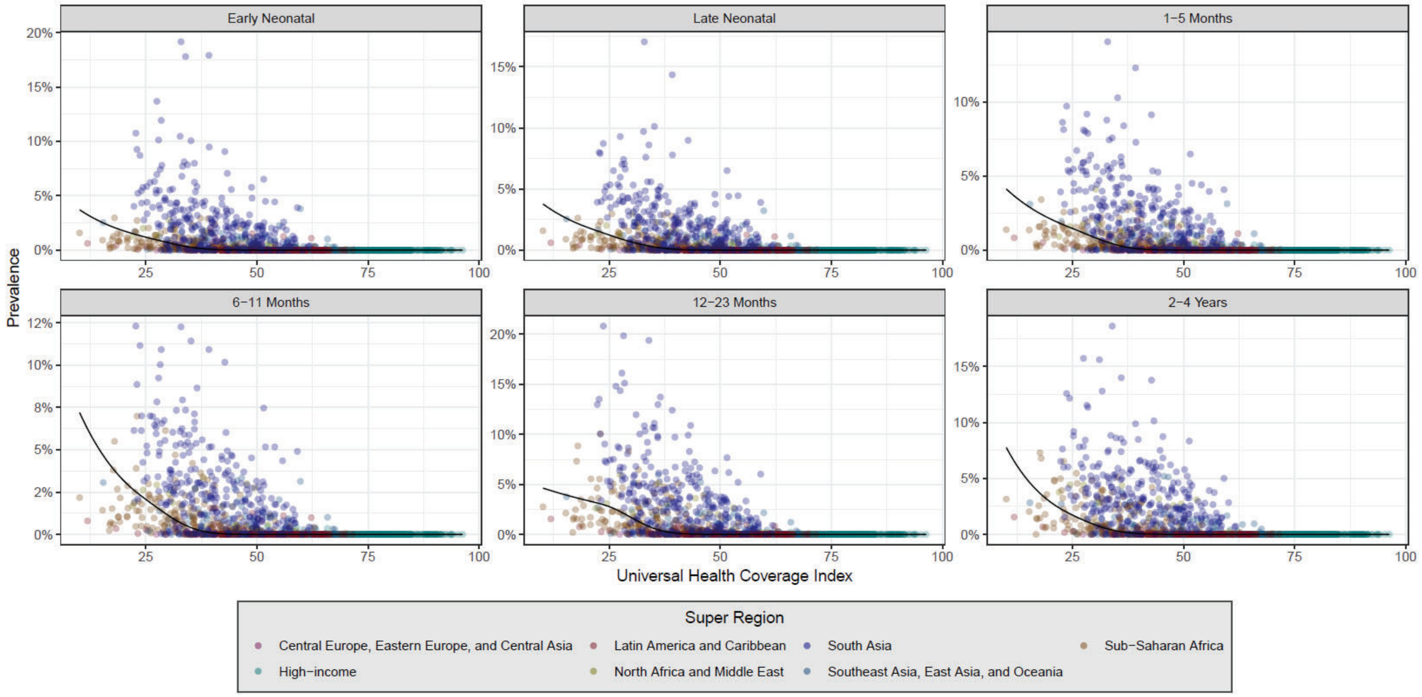

Supplement: Supplementary file 2 — Data S1 to S4 [file sciadv.abm8954_data_files_s1_to_s4.zip › sciadv.abm8954_data_file_s2.pdf]
