# Supplementary material for: Current dichotomous metrics obscure trends in severe and extreme child growth failure
Source: Sci Adv. 2022 May 20;8(20):eabm8954. doi: 10.1126/sciadv.abm8954 (PMC9122330; doi:10.1126/sciadv.abm8954)
Supplement: Supplementary file 2 — Data S1 to S4 [file sciadv.abm8954_data_files_s1_to_s4.zip › sciadv.abm8954_data_file_s3.pdf]

Data S3. Ensemble MR-BRT knot placements and weight given to each sub-model, for all forms and severities of CGF, by sex. Twenty MR−BRT models with different knot placements were fit to each age−sex, weighted based by their predictive performance. Knots 1 and 6 were always respectively placed at the lowest and highest estimated UHC index values input to the model. Knot placements for overall stunting in males and females are shown in (A, B), followed by severe stunting (C, D), extreme stunting (E, F), overall wasting (G, H), severe wasting (I, J), extreme wasting (K, L), overall underweight (M, N), severe underweight (O, P), and extreme underweight (Q, R)

A. Overall Stunting in Males: Ensemble MR−BRT Knot Placement

Early Neonatal

	Knot 1	Knot 2	Knot 3	Knot 4	Knot 5	Knot 6	Weight
1	0.091	0.263	0.463	0.651	0.757	0.966	5.50%
2	0.091	0.235	0.387	0.509	0.783	0.966	3.72%
3	0.091	0.124	0.232	0.773	0.880	0.966	7.04%
4	0.091	0.153	0.519	0.732	0.843	0.966	7.04%
5	0.091	0.150	0.270	0.440	0.775	0.966	1.80%
6	0.091	0.242	0.380	0.556	0.685	0.966	2.01%
7	0.091	0.220	0.490	0.708	0.814	0.966	6.66%
8	0.091	0.227	0.533	0.641	0.788	0.966	6.02%
9	0.091	0.143	0.496	0.707	0.880	0.966	17.11%
10	0.091	0.115	0.275	0.596	0.742	0.966	4.04%
11	0.091	0.234	0.336	0.503	0.733	0.966	2.16%
12	0.091	0.216	0.415	0.705	0.810	0.966	6.64%
13	0.091	0.327	0.429	0.760	0.885	0.966	6.84%
14	0.091	0.193	0.321	0.450	0.623	0.966	0.04%
15	0.091	0.178	0.423	0.544	0.736	0.966	3.32%
16	0.091	0.192	0.492	0.621	0.796	0.966	5.90%
17	0.091	0.167	0.305	0.437	0.892	0.966	4.45%
18	0.091	0.171	0.289	0.504	0.787	0.966	3.39%
19	0.091	0.426	0.543	0.682	0.850	0.966	6.33%
20	0.091	0.141	0.241	0.406	0.511	0.966	0.00%

Late Neonatal

	Knot 1	Knot 2	Knot 3	Knot 4	Knot 5	Knot 6	Weight
1	0.091	0.446	0.551	0.696	0.821	0.966	7.84%
2	0.091	0.179	0.434	0.560	0.769	0.966	3.77%
3	0.091	0.371	0.485	0.631	0.857	0.966	8.03%
4	0.091	0.274	0.453	0.612	0.774	0.966	5.50%
5	0.091	0.199	0.302	0.466	0.789	0.966	1.14%
6	0.091	0.221	0.333	0.530	0.881	0.966	6.38%
7	0.091	0.301	0.408	0.592	0.710	0.966	2.00%
8	0.091	0.214	0.424	0.564	0.682	0.966	0.60%
9	0.091	0.144	0.366	0.670	0.807	0.966	8.23%
10	0.091	0.129	0.274	0.443	0.817	0.966	1.27%
11	0.091	0.277	0.489	0.639	0.805	0.966	7.38%
12	0.091	0.120	0.319	0.515	0.714	0.966	0.28%
13	0.091	0.134	0.305	0.467	0.850	0.966	3.26%
14	0.091	0.137	0.242	0.552	0.741	0.966	1.45%
15	0.091	0.200	0.336	0.437	0.724	0.966	0.03%
16	0.091	0.278	0.616	0.751	0.863	0.966	20.08%
17	0.091	0.272	0.420	0.625	0.761	0.966	5.25%
18	0.091	0.246	0.405	0.685	0.828	0.966	9.62%
19	0.091	0.166	0.368	0.625	0.836	0.966	7.89%
20	0.091	0.163	0.341	0.493	0.594	0.966	0.00%

1–5 Months

	Knot 1	Knot 2	Knot 3	Knot 4	Knot 5	Knot 6	Weight
1	0.091	0.108	0.238	0.431	0.647	0.966	0.00%
2	0.091	0.134	0.353	0.594	0.697	0.966	1.52%
3	0.091	0.134	0.288	0.436	0.604	0.966	0.00%
4	0.091	0.161	0.280	0.657	0.779	0.966	5.31%
5	0.091	0.154	0.338	0.532	0.848	0.966	4.42%
6	0.091	0.132	0.268	0.610	0.884	0.966	6.35%
7	0.091	0.131	0.367	0.734	0.890	0.966	16.87%
8	0.091	0.232	0.524	0.682	0.870	0.966	6.75%
9	0.091	0.282	0.432	0.646	0.851	0.966	6.24%
10	0.091	0.144	0.375	0.773	0.885	0.966	10.84%
11	0.091	0.213	0.485	0.754	0.889	0.966	8.11%
12	0.091	0.114	0.576	0.676	0.789	0.966	6.14%
13	0.091	0.108	0.300	0.438	0.718	0.966	0.05%
14	0.091	0.423	0.540	0.748	0.891	0.966	6.35%
15	0.091	0.349	0.468	0.600	0.740	0.966	3.22%
16	0.091	0.352	0.459	0.656	0.845	0.966	6.11%
17	0.091	0.225	0.406	0.641	0.760	0.966	4.54%
18	0.091	0.106	0.240	0.427	0.619	0.966	0.00%
19	0.091	0.377	0.514	0.628	0.774	0.966	4.67%
20	0.091	0.266	0.415	0.579	0.737	0.966	2.52%

6–11 Months

	Knot 1	Knot 2	Knot 3	Knot 4	Knot 5	Knot 6	Weight
1	0.091	0.257	0.378	0.521	0.875	0.966	5.06%
2	0.091	0.154	0.279	0.403	0.887	0.966	3.24%
3	0.091	0.362	0.505	0.784	0.899	0.966	15.06%
4	0.091	0.132	0.347	0.700	0.835	0.966	6.09%
5	0.091	0.115	0.249	0.498	0.890	0.966	4.79%
6	0.091	0.176	0.331	0.579	0.815	0.966	4.75%
7	0.091	0.180	0.313	0.519	0.677	0.966	0.67%
8	0.091	0.170	0.331	0.572	0.803	0.966	4.44%
9	0.091	0.116	0.372	0.667	0.776	0.966	5.23%
10	0.091	0.287	0.421	0.587	0.788	0.966	4.42%
11	0.091	0.134	0.315	0.577	0.768	0.966	3.77%
12	0.091	0.352	0.588	0.732	0.895	0.966	6.06%
13	0.091	0.171	0.285	0.422	0.541	0.966	0.00%
14	0.091	0.125	0.435	0.598	0.893	0.966	6.02%
15	0.091	0.286	0.626	0.746	0.855	0.966	6.00%
16	0.091	0.136	0.428	0.603	0.763	0.966	4.26%
17	0.091	0.473	0.585	0.697	0.886	0.966	5.61%
18	0.091	0.251	0.427	0.552	0.758	0.966	3.34%
19	0.091	0.111	0.296	0.685	0.816	0.966	5.83%
20	0.091	0.144	0.465	0.644	0.799	0.966	5.35%

12–23 Months

	Knot 1	Knot 2	Knot 3	Knot 4	Knot 5	Knot 6	Weight
1	0.091	0.107	0.340	0.504	0.689	0.966	0.05%
2	0.091	0.114	0.445	0.557	0.784	0.966	3.51%
3	0.091	0.235	0.374	0.483	0.603	0.966	0.00%
4	0.091	0.151	0.358	0.478	0.761	0.966	0.75%
5	0.091	0.103	0.241	0.379	0.688	0.966	0.00%
6	0.091	0.101	0.233	0.718	0.825	0.966	8.03%
7	0.091	0.292	0.529	0.676	0.899	0.966	12.29%
8	0.091	0.103	0.359	0.610	0.861	0.966	6.98%
9	0.091	0.428	0.530	0.638	0.888	0.966	7.68%
10	0.091	0.118	0.421	0.726	0.854	0.966	10.83%
11	0.091	0.262	0.468	0.666	0.831	0.966	7.26%
12	0.091	0.150	0.380	0.742	0.855	0.966	20.07%
13	0.091	0.282	0.468	0.583	0.775	0.966	3.87%
14	0.091	0.152	0.500	0.607	0.794	0.966	5.38%
15	0.091	0.191	0.354	0.537	0.855	0.966	4.98%
16	0.091	0.275	0.432	0.657	0.782	0.966	5.72%
17	0.091	0.202	0.374	0.503	0.681	0.966	0.04%
18	0.091	0.111	0.211	0.567	0.765	0.966	2.15%
19	0.091	0.226	0.355	0.484	0.588	0.966	0.00%
20	0.091	0.232	0.334	0.447	0.765	0.966	0.41%

2–4 Years

	Knot 1	Knot 2	Knot 3	Knot 4	Knot 5	Knot 6	Weight
1	0.091	0.446	0.562	0.684	0.840	0.966	7.24%
2	0.091	0.123	0.229	0.346	0.494	0.966	0.00%
3	0.091	0.241	0.362	0.682	0.831	0.966	6.82%
4	0.091	0.164	0.333	0.470	0.604	0.966	0.11%
5	0.091	0.176	0.296	0.460	0.833	0.966	3.58%
6	0.091	0.284	0.428	0.532	0.723	0.966	2.79%
7	0.091	0.223	0.344	0.593	0.851	0.966	5.99%
8	0.091	0.102	0.296	0.776	0.885	0.966	17.48%
9	0.091	0.346	0.459	0.571	0.766	0.966	4.45%
10	0.091	0.350	0.520	0.639	0.804	0.966	6.26%
11	0.091	0.393	0.562	0.680	0.854	0.966	7.52%
12	0.091	0.110	0.521	0.639	0.849	0.966	6.99%
13	0.091	0.139	0.355	0.497	0.880	0.966	5.25%
14	0.091	0.134	0.373	0.522	0.666	0.966	1.19%
15	0.091	0.190	0.472	0.623	0.762	0.966	5.18%
16	0.091	0.135	0.373	0.493	0.710	0.966	1.68%
17	0.091	0.227	0.476	0.607	0.831	0.966	6.21%
18	0.091	0.223	0.417	0.587	0.799	0.966	5.15%
19	0.091	0.161	0.397	0.581	0.827	0.966	5.53%
20	0.091	0.119	0.228	0.502	0.657	0.966	0.59%

B. Overall Stunting in Females: Ensemble MR−BRT Knot Placement

Early Neonatal

	Knot 1	Knot 2	Knot 3	Knot 4	Knot 5	Knot 6	Weight
1	0.091	0.231	0.465	0.572	0.790	0.966	4.04%
2	0.091	0.155	0.528	0.639	0.812	0.966	5.48%
3	0.091	0.191	0.417	0.547	0.813	0.966	3.88%
4	0.091	0.134	0.359	0.695	0.870	0.966	6.58%
5	0.091	0.103	0.253	0.575	0.870	0.966	5.15%
6	0.091	0.195	0.454	0.654	0.892	0.966	6.52%
7	0.091	0.191	0.405	0.587	0.717	0.966	2.24%
8	0.091	0.187	0.426	0.659	0.863	0.966	5.99%
9	0.091	0.126	0.291	0.658	0.900	0.966	15.00%
10	0.091	0.264	0.413	0.699	0.861	0.966	6.00%
11	0.091	0.119	0.505	0.639	0.813	0.966	5.46%
12	0.091	0.313	0.455	0.567	0.825	0.966	4.48%
13	0.091	0.301	0.531	0.670	0.780	0.966	5.09%
14	0.091	0.103	0.216	0.321	0.671	0.966	0.00%
15	0.091	0.320	0.636	0.747	0.863	0.966	5.81%
16	0.091	0.111	0.252	0.493	0.892	0.966	3.98%
17	0.091	0.108	0.336	0.442	0.577	0.966	0.00%
18	0.091	0.131	0.272	0.539	0.789	0.966	2.74%
19	0.091	0.106	0.568	0.683	0.815	0.966	5.76%
20	0.091	0.135	0.428	0.660	0.839	0.966	5.80%

Late Neonatal

	Knot 1	Knot 2	Knot 3	Knot 4	Knot 5	Knot 6	Weight
1	0.091	0.167	0.348	0.622	0.777	0.966	4.47%
2	0.091	0.241	0.430	0.706	0.844	0.966	6.81%
3	0.091	0.183	0.338	0.489	0.780	0.966	1.13%
4	0.091	0.216	0.567	0.678	0.790	0.966	6.07%
5	0.091	0.192	0.533	0.649	0.771	0.966	5.50%
6	0.091	0.179	0.448	0.672	0.789	0.966	5.87%
7	0.091	0.101	0.476	0.611	0.783	0.966	4.90%
8	0.091	0.119	0.450	0.636	0.885	0.966	7.43%
9	0.091	0.336	0.501	0.673	0.785	0.966	5.48%
10	0.091	0.108	0.257	0.420	0.658	0.966	0.00%
11	0.091	0.147	0.500	0.779	0.887	0.966	17.09%
12	0.091	0.185	0.322	0.481	0.697	0.966	0.01%
13	0.091	0.102	0.405	0.537	0.645	0.966	0.01%
14	0.091	0.451	0.552	0.676	0.809	0.966	5.45%
15	0.091	0.300	0.442	0.753	0.855	0.966	6.83%
16	0.091	0.273	0.619	0.740	0.874	0.966	7.03%
17	0.091	0.132	0.300	0.696	0.812	0.966	6.51%
18	0.091	0.184	0.308	0.568	0.692	0.966	0.44%
19	0.091	0.284	0.428	0.612	0.817	0.966	5.29%
20	0.091	0.165	0.399	0.591	0.775	0.966	3.69%

1–5 Months

	Knot 1	Knot 2	Knot 3	Knot 4	Knot 5	Knot 6	Weight
1	0.091	0.133	0.241	0.662	0.865	0.966	5.60%
2	0.091	0.213	0.466	0.647	0.848	0.966	5.41%
3	0.091	0.299	0.530	0.743	0.846	0.966	5.42%
4	0.091	0.232	0.342	0.474	0.876	0.966	4.19%
5	0.091	0.259	0.378	0.608	0.895	0.966	5.49%
6	0.091	0.152	0.342	0.469	0.788	0.966	2.70%
7	0.091	0.336	0.451	0.639	0.759	0.966	4.54%
8	0.091	0.103	0.384	0.499	0.806	0.966	3.57%
9	0.091	0.213	0.476	0.593	0.830	0.966	5.04%
10	0.091	0.299	0.471	0.739	0.868	0.966	5.53%
11	0.091	0.153	0.377	0.581	0.793	0.966	4.38%
12	0.091	0.239	0.472	0.594	0.826	0.966	4.99%
13	0.091	0.188	0.465	0.703	0.830	0.966	5.46%
14	0.091	0.159	0.412	0.636	0.863	0.966	5.49%
15	0.091	0.232	0.383	0.601	0.757	0.966	4.07%
16	0.091	0.121	0.415	0.646	0.894	0.966	13.86%
17	0.091	0.163	0.496	0.707	0.846	0.966	5.54%
18	0.091	0.170	0.287	0.657	0.861	0.966	5.56%
19	0.091	0.123	0.251	0.594	0.728	0.966	3.16%
20	0.091	0.164	0.285	0.386	0.503	0.966	0.00%

6–11 Months

	Knot 1	Knot 2	Knot 3	Knot 4	Knot 5	Knot 6	Weight
1	0.091	0.107	0.416	0.539	0.692	0.966	1.68%
2	0.091	0.190	0.483	0.719	0.877	0.966	7.48%
3	0.091	0.351	0.454	0.665	0.786	0.966	5.85%
4	0.091	0.146	0.255	0.431	0.538	0.966	0.00%
5	0.091	0.235	0.350	0.507	0.870	0.966	5.24%
6	0.091	0.283	0.386	0.505	0.641	0.966	0.25%
7	0.091	0.173	0.418	0.711	0.871	0.966	7.59%
8	0.091	0.247	0.392	0.731	0.838	0.966	6.87%
9	0.091	0.255	0.407	0.592	0.797	0.966	5.25%
10	0.091	0.233	0.342	0.484	0.867	0.966	4.81%
11	0.091	0.153	0.603	0.770	0.885	0.966	17.40%
12	0.091	0.248	0.402	0.503	0.783	0.966	3.41%
13	0.091	0.140	0.580	0.700	0.852	0.966	6.93%
14	0.091	0.129	0.478	0.626	0.767	0.966	5.47%
15	0.091	0.110	0.518	0.712	0.849	0.966	6.96%
16	0.091	0.294	0.464	0.618	0.802	0.966	5.74%
17	0.091	0.144	0.278	0.425	0.712	0.966	0.26%
18	0.091	0.169	0.370	0.599	0.731	0.966	3.83%
19	0.091	0.150	0.336	0.442	0.821	0.966	3.07%
20	0.091	0.156	0.382	0.489	0.739	0.966	1.91%

12–23 Months

	Knot 1	Knot 2	Knot 3	Knot 4	Knot 5	Knot 6	Weight
1	0.091	0.211	0.332	0.442	0.658	0.966	0.02%
2	0.091	0.226	0.424	0.577	0.781	0.966	4.01%
3	0.091	0.143	0.422	0.546	0.734	0.966	2.27%
4	0.091	0.125	0.322	0.557	0.805	0.966	3.93%
5	0.091	0.154	0.388	0.607	0.816	0.966	5.20%
6	0.091	0.167	0.382	0.647	0.887	0.966	6.58%
7	0.091	0.217	0.357	0.546	0.713	0.966	1.50%
8	0.091	0.270	0.467	0.684	0.862	0.966	6.62%
9	0.091	0.182	0.376	0.587	0.830	0.966	5.12%
10	0.091	0.161	0.408	0.537	0.666	0.966	0.60%
11	0.091	0.174	0.471	0.691	0.855	0.966	6.75%
12	0.091	0.421	0.552	0.747	0.896	0.966	6.56%
13	0.091	0.386	0.513	0.647	0.864	0.966	6.26%
14	0.091	0.249	0.427	0.549	0.893	0.966	5.63%
15	0.091	0.115	0.500	0.752	0.856	0.966	16.56%
16	0.091	0.153	0.308	0.433	0.536	0.966	0.00%
17	0.091	0.193	0.304	0.747	0.848	0.966	15.16%
18	0.091	0.133	0.346	0.514	0.728	0.966	1.32%
19	0.091	0.158	0.426	0.600	0.707	0.966	2.57%
20	0.091	0.278	0.422	0.570	0.759	0.966	3.33%

2–4 Years

	Knot 1	Knot 2	Knot 3	Knot 4	Knot 5	Knot 6	Weight
1	0.091	0.237	0.436	0.650	0.815	0.966	5.93%
2	0.091	0.223	0.430	0.614	0.834	0.966	5.54%
3	0.091	0.292	0.402	0.665	0.851	0.966	7.28%
4	0.091	0.333	0.510	0.672	0.850	0.966	7.66%
5	0.091	0.212	0.324	0.499	0.826	0.966	1.68%
6	0.091	0.208	0.400	0.505	0.866	0.966	3.56%
7	0.091	0.109	0.299	0.462	0.826	0.966	0.90%
8	0.091	0.117	0.305	0.694	0.849	0.966	8.04%
9	0.091	0.167	0.495	0.665	0.828	0.966	7.12%
10	0.091	0.113	0.242	0.403	0.745	0.966	0.00%
11	0.091	0.189	0.488	0.728	0.878	0.966	13.25%
12	0.091	0.235	0.423	0.572	0.761	0.966	1.64%
13	0.091	0.137	0.264	0.565	0.716	0.966	0.19%
14	0.091	0.133	0.276	0.551	0.899	0.966	5.12%
15	0.091	0.190	0.596	0.762	0.892	0.966	19.15%
16	0.091	0.186	0.346	0.529	0.656	0.966	0.00%
17	0.091	0.161	0.285	0.456	0.659	0.966	0.00%
18	0.091	0.271	0.406	0.668	0.799	0.966	5.73%
19	0.091	0.277	0.485	0.683	0.823	0.966	7.20%
20	0.091	0.155	0.372	0.542	0.666	0.966	0.00%

C. Severe Stunting in Males: Ensemble MR–BRT Knot Placement

Early Neonatal

	Knot 1	Knot 2	Knot 3	Knot 4	Knot 5	Knot 6	Weight
1	0.091	0.120	0.302	0.588	0.887	0.966	6.03%
2	0.091	0.129	0.289	0.415	0.577	0.966	0.00%
3	0.091	0.103	0.340	0.479	0.625	0.966	0.00%
4	0.091	0.331	0.434	0.585	0.778	0.966	4.51%
5	0.091	0.192	0.428	0.597	0.722	0.966	2.67%
6	0.091	0.133	0.287	0.616	0.840	0.966	6.01%
7	0.091	0.223	0.374	0.559	0.740	0.966	1.90%
8	0.091	0.303	0.408	0.686	0.834	0.966	8.78%
9	0.091	0.169	0.510	0.699	0.839	0.966	19.46%
10	0.091	0.137	0.295	0.456	0.579	0.966	0.00%
11	0.091	0.166	0.289	0.628	0.826	0.966	6.05%
12	0.091	0.135	0.320	0.570	0.813	0.966	4.04%
13	0.091	0.126	0.276	0.439	0.699	0.966	0.01%
14	0.091	0.113	0.244	0.617	0.800	0.966	4.75%
15	0.091	0.262	0.395	0.676	0.806	0.966	7.58%
16	0.091	0.301	0.424	0.617	0.830	0.966	6.76%
17	0.091	0.130	0.469	0.622	0.877	0.966	7.79%
18	0.091	0.179	0.377	0.480	0.648	0.966	0.01%
19	0.091	0.188	0.400	0.589	0.772	0.966	3.93%
20	0.091	0.289	0.560	0.686	0.792	0.966	9.72%

Late Neonatal

	Knot 1	Knot 2	Knot 3	Knot 4	Knot 5	Knot 6	Weight
1	0.091	0.108	0.286	0.577	0.714	0.966	1.64%
2	0.091	0.232	0.382	0.752	0.877	0.966	6.92%
3	0.091	0.276	0.407	0.575	0.801	0.966	4.28%
4	0.091	0.240	0.397	0.568	0.736	0.966	2.53%
5	0.091	0.288	0.538	0.682	0.848	0.966	17.22%
6	0.091	0.244	0.451	0.616	0.802	0.966	5.32%
7	0.091	0.135	0.253	0.537	0.807	0.966	2.71%
8	0.091	0.223	0.445	0.671	0.791	0.966	6.05%
9	0.091	0.336	0.632	0.756	0.887	0.966	6.82%
10	0.091	0.117	0.230	0.570	0.843	0.966	4.01%
11	0.091	0.248	0.351	0.461	0.733	0.966	0.74%
12	0.091	0.428	0.646	0.750	0.880	0.966	6.89%
13	0.091	0.268	0.416	0.639	0.767	0.966	4.86%
14	0.091	0.163	0.293	0.554	0.865	0.966	4.29%
15	0.091	0.116	0.221	0.349	0.511	0.966	0.00%
16	0.091	0.238	0.405	0.529	0.846	0.966	4.22%
17	0.091	0.336	0.645	0.761	0.882	0.966	6.83%
18	0.091	0.216	0.395	0.729	0.846	0.966	7.66%
19	0.091	0.221	0.512	0.675	0.790	0.966	6.40%
20	0.091	0.116	0.242	0.358	0.829	0.966	0.59%

1–5 Months

	Knot 1	Knot 2	Knot 3	Knot 4	Knot 5	Knot 6	Weight
1	0.091	0.215	0.406	0.781	0.895	0.966	6.03%
2	0.091	0.269	0.455	0.756	0.876	0.966	6.46%
3	0.091	0.271	0.378	0.722	0.863	0.966	6.44%
4	0.091	0.115	0.397	0.650	0.887	0.966	5.60%
5	0.091	0.148	0.269	0.455	0.632	0.966	0.00%
6	0.091	0.132	0.495	0.646	0.770	0.966	4.26%
7	0.091	0.172	0.545	0.683	0.824	0.966	6.55%
8	0.091	0.278	0.426	0.576	0.688	0.966	0.29%
9	0.091	0.334	0.443	0.611	0.795	0.966	3.67%
10	0.091	0.151	0.412	0.728	0.844	0.966	6.62%
11	0.091	0.111	0.292	0.565	0.802	0.966	1.58%
12	0.091	0.104	0.408	0.683	0.836	0.966	5.92%
13	0.091	0.337	0.502	0.715	0.890	0.966	6.45%
14	0.091	0.121	0.245	0.493	0.806	0.966	0.34%
15	0.091	0.189	0.530	0.737	0.897	0.966	6.45%
16	0.091	0.118	0.240	0.605	0.865	0.966	3.67%
17	0.091	0.260	0.385	0.536	0.875	0.966	3.05%
18	0.091	0.192	0.507	0.613	0.769	0.966	3.47%
19	0.091	0.122	0.596	0.696	0.857	0.966	16.37%
20	0.091	0.184	0.469	0.720	0.858	0.966	6.78%

6–11 Months

	Knot 1	Knot 2	Knot 3	Knot 4	Knot 5	Knot 6	Weight
1	0.091	0.111	0.302	0.498	0.655	0.966	0.08%
2	0.091	0.140	0.278	0.464	0.668	0.966	0.03%
3	0.091	0.460	0.582	0.766	0.894	0.966	8.87%
4	0.091	0.155	0.268	0.380	0.546	0.966	0.00%
5	0.091	0.216	0.343	0.534	0.700	0.966	0.90%
6	0.091	0.138	0.375	0.497	0.636	0.966	0.06%
7	0.091	0.138	0.299	0.574	0.748	0.966	2.64%
8	0.091	0.360	0.475	0.657	0.768	0.966	6.45%
9	0.091	0.102	0.399	0.712	0.889	0.966	8.85%
10	0.091	0.122	0.226	0.461	0.718	0.966	0.17%
11	0.091	0.267	0.455	0.776	0.882	0.966	22.18%
12	0.091	0.241	0.609	0.790	0.895	0.966	13.66%
13	0.091	0.287	0.461	0.652	0.770	0.966	6.30%
14	0.091	0.122	0.384	0.521	0.729	0.966	1.47%
15	0.091	0.105	0.389	0.523	0.867	0.966	5.11%
16	0.091	0.158	0.348	0.595	0.786	0.966	4.54%
17	0.091	0.259	0.377	0.526	0.768	0.966	2.60%
18	0.091	0.148	0.272	0.616	0.780	0.966	4.61%
19	0.091	0.405	0.614	0.718	0.880	0.966	9.00%
20	0.091	0.165	0.273	0.431	0.884	0.966	2.47%

12–23 Months

	Knot 1	Knot 2	Knot 3	Knot 4	Knot 5	Knot 6	Weight
1	0.091	0.186	0.346	0.694	0.829	0.966	6.91%
2	0.091	0.104	0.291	0.486	0.609	0.966	0.00%
3	0.091	0.106	0.312	0.509	0.749	0.966	0.99%
4	0.091	0.115	0.259	0.554	0.719	0.966	0.91%
5	0.091	0.247	0.371	0.474	0.687	0.966	0.18%
6	0.091	0.220	0.380	0.519	0.745	0.966	1.41%
7	0.091	0.288	0.490	0.612	0.717	0.966	3.18%
8	0.091	0.322	0.488	0.650	0.848	0.966	7.23%
9	0.091	0.294	0.476	0.696	0.852	0.966	7.98%
10	0.091	0.419	0.576	0.714	0.828	0.966	8.00%
11	0.091	0.271	0.606	0.713	0.876	0.966	10.31%
12	0.091	0.144	0.316	0.541	0.877	0.966	4.47%
13	0.091	0.122	0.268	0.416	0.526	0.966	0.00%
14	0.091	0.133	0.528	0.672	0.838	0.966	7.56%
15	0.091	0.383	0.550	0.714	0.867	0.966	9.09%
16	0.091	0.264	0.534	0.641	0.827	0.966	6.85%
17	0.091	0.113	0.283	0.521	0.704	0.966	0.43%
18	0.091	0.180	0.311	0.629	0.776	0.966	4.05%
19	0.091	0.453	0.684	0.790	0.891	0.966	20.01%
20	0.091	0.161	0.352	0.545	0.672	0.966	0.44%

2–4 Years

	Knot 1	Knot 2	Knot 3	Knot 4	Knot 5	Knot 6	Weight
1	0.091	0.102	0.328	0.554	0.840	0.966	1.62%
2	0.091	0.113	0.334	0.512	0.727	0.966	0.05%
3	0.091	0.277	0.494	0.722	0.863	0.966	8.66%
4	0.091	0.158	0.450	0.559	0.719	0.966	0.42%
5	0.091	0.130	0.526	0.747	0.896	0.966	13.90%
6	0.091	0.181	0.343	0.479	0.759	0.966	0.08%
7	0.091	0.296	0.408	0.590	0.899	0.966	5.15%
8	0.091	0.260	0.439	0.614	0.836	0.966	4.06%
9	0.091	0.211	0.460	0.629	0.851	0.966	5.13%
10	0.091	0.288	0.472	0.684	0.886	0.966	8.36%
11	0.091	0.195	0.370	0.474	0.699	0.966	0.00%
12	0.091	0.176	0.310	0.420	0.600	0.966	0.00%
13	0.091	0.113	0.282	0.566	0.694	0.966	0.04%
14	0.091	0.109	0.647	0.767	0.892	0.966	21.66%
15	0.091	0.266	0.430	0.699	0.836	0.966	6.43%
16	0.091	0.342	0.610	0.729	0.842	0.966	9.02%
17	0.091	0.195	0.351	0.614	0.803	0.966	2.36%
18	0.091	0.189	0.344	0.496	0.803	0.966	0.43%
19	0.091	0.214	0.433	0.662	0.848	0.966	5.74%
20	0.091	0.237	0.485	0.641	0.880	0.966	6.89%

D. Severe Stunting in Females: Ensemble MR–BRT Knot Placement

Early Neonatal

	Knot 1	Knot 2	Knot 3	Knot 4	Knot 5	Knot 6	Weight
1	0.091	0.150	0.255	0.674	0.866	0.966	6.29%
2	0.091	0.114	0.428	0.592	0.826	0.966	4.31%
3	0.091	0.119	0.285	0.392	0.798	0.966	0.03%
4	0.091	0.246	0.502	0.706	0.840	0.966	7.03%
5	0.091	0.197	0.395	0.609	0.850	0.966	5.04%
6	0.091	0.277	0.429	0.715	0.838	0.966	6.87%
7	0.091	0.265	0.521	0.744	0.862	0.966	17.59%
8	0.091	0.138	0.483	0.614	0.897	0.966	6.86%
9	0.091	0.217	0.333	0.464	0.879	0.966	1.63%
10	0.091	0.225	0.360	0.460	0.578	0.966	0.00%
11	0.091	0.208	0.365	0.598	0.886	0.966	5.37%
12	0.091	0.248	0.391	0.574	0.825	0.966	3.51%
13	0.091	0.101	0.206	0.609	0.851	0.966	3.69%
14	0.091	0.113	0.627	0.730	0.846	0.966	7.89%
15	0.091	0.234	0.486	0.671	0.869	0.966	7.18%
16	0.091	0.292	0.467	0.626	0.771	0.966	4.13%
17	0.091	0.311	0.421	0.609	0.887	0.966	6.13%
18	0.091	0.108	0.416	0.632	0.837	0.966	5.45%
19	0.091	0.104	0.448	0.559	0.676	0.966	0.28%
20	0.091	0.101	0.267	0.602	0.715	0.966	0.73%

Late Neonatal

	Knot 1	Knot 2	Knot 3	Knot 4	Knot 5	Knot 6	Weight
1	0.091	0.183	0.406	0.534	0.705	0.966	1.06%
2	0.091	0.161	0.434	0.656	0.769	0.966	4.89%
3	0.091	0.123	0.283	0.566	0.871	0.966	4.20%
4	0.091	0.113	0.246	0.376	0.517	0.966	0.00%
5	0.091	0.105	0.264	0.423	0.828	0.966	0.89%
6	0.091	0.215	0.362	0.744	0.865	0.966	6.79%
7	0.091	0.339	0.552	0.689	0.843	0.966	6.53%
8	0.091	0.210	0.362	0.610	0.854	0.966	5.17%
9	0.091	0.167	0.467	0.604	0.817	0.966	5.05%
10	0.091	0.177	0.337	0.645	0.837	0.966	5.38%
11	0.091	0.154	0.559	0.667	0.836	0.966	6.45%
12	0.091	0.182	0.368	0.592	0.871	0.966	5.13%
13	0.091	0.270	0.403	0.601	0.749	0.966	3.25%
14	0.091	0.241	0.417	0.663	0.807	0.966	5.52%
15	0.091	0.207	0.564	0.672	0.839	0.966	6.51%
16	0.091	0.143	0.383	0.561	0.671	0.966	0.75%
17	0.091	0.328	0.513	0.652	0.881	0.966	6.68%
18	0.091	0.188	0.291	0.562	0.761	0.966	2.09%
19	0.091	0.348	0.566	0.715	0.894	0.966	16.70%
20	0.091	0.315	0.593	0.754	0.860	0.966	6.94%

1–5 Months

	Knot 1	Knot 2	Knot 3	Knot 4	Knot 5	Knot 6	Weight
1	0.091	0.150	0.390	0.698	0.806	0.966	5.37%
2	0.091	0.156	0.342	0.661	0.861	0.966	5.57%
3	0.091	0.156	0.413	0.632	0.889	0.966	5.95%
4	0.091	0.116	0.230	0.357	0.762	0.966	0.00%
5	0.091	0.130	0.589	0.695	0.848	0.966	6.52%
6	0.091	0.289	0.439	0.683	0.821	0.966	5.58%
7	0.091	0.252	0.508	0.686	0.872	0.966	6.65%
8	0.091	0.190	0.374	0.748	0.884	0.966	7.12%
9	0.091	0.113	0.286	0.678	0.881	0.966	6.07%
10	0.091	0.122	0.458	0.767	0.882	0.966	7.95%
11	0.091	0.139	0.397	0.509	0.835	0.966	1.97%
12	0.091	0.236	0.534	0.681	0.880	0.966	6.80%
13	0.091	0.275	0.401	0.700	0.821	0.966	5.69%
14	0.091	0.149	0.258	0.398	0.658	0.966	0.00%
15	0.091	0.217	0.354	0.508	0.674	0.966	0.00%
16	0.091	0.312	0.447	0.579	0.694	0.966	0.68%
17	0.091	0.173	0.352	0.511	0.752	0.966	0.32%
18	0.091	0.224	0.435	0.616	0.817	0.966	4.30%
19	0.091	0.364	0.647	0.773	0.900	0.966	17.00%
20	0.091	0.374	0.517	0.697	0.860	0.966	6.45%

6–11 Months

	Knot 1	Knot 2	Knot 3	Knot 4	Knot 5	Knot 6	Weight
1	0.091	0.103	0.578	0.745	0.856	0.966	8.88%
2	0.091	0.136	0.276	0.559	0.709	0.966	0.00%
3	0.091	0.106	0.247	0.412	0.829	0.966	0.00%
4	0.091	0.251	0.426	0.584	0.835	0.966	2.21%
5	0.091	0.172	0.275	0.519	0.860	0.966	0.14%
6	0.091	0.222	0.457	0.771	0.873	0.966	10.55%
7	0.091	0.189	0.439	0.554	0.709	0.966	0.00%
8	0.091	0.196	0.448	0.660	0.845	0.966	5.66%
9	0.091	0.248	0.518	0.684	0.803	0.966	5.30%
10	0.091	0.197	0.538	0.776	0.895	0.966	22.20%
11	0.091	0.122	0.475	0.607	0.892	0.966	6.32%
12	0.091	0.294	0.561	0.682	0.852	0.966	7.55%
13	0.091	0.116	0.256	0.379	0.860	0.966	0.00%
14	0.091	0.143	0.428	0.747	0.870	0.966	9.11%
15	0.091	0.336	0.468	0.638	0.770	0.966	2.21%
16	0.091	0.169	0.451	0.674	0.841	0.966	5.97%
17	0.091	0.270	0.525	0.729	0.835	0.966	7.38%
18	0.091	0.131	0.380	0.517	0.694	0.966	0.00%
19	0.091	0.263	0.371	0.706	0.843	0.966	6.29%
20	0.091	0.115	0.235	0.573	0.819	0.966	0.24%

12–23 Months

	Knot 1	Knot 2	Knot 3	Knot 4	Knot 5	Knot 6	Weight
1	0.091	0.171	0.449	0.571	0.817	0.966	2.11%
2	0.091	0.187	0.322	0.487	0.824	0.966	0.14%
3	0.091	0.251	0.437	0.791	0.895	0.966	10.33%
4	0.091	0.345	0.520	0.643	0.754	0.966	3.00%
5	0.091	0.137	0.335	0.533	0.877	0.966	1.24%
6	0.091	0.163	0.263	0.411	0.679	0.966	0.00%
7	0.091	0.108	0.566	0.763	0.878	0.966	11.16%
8	0.091	0.232	0.544	0.687	0.899	0.966	9.24%
9	0.091	0.246	0.593	0.769	0.873	0.966	12.35%
10	0.091	0.234	0.375	0.501	0.745	0.966	0.02%
11	0.091	0.195	0.397	0.689	0.807	0.966	5.03%
12	0.091	0.110	0.290	0.475	0.849	0.966	0.14%
13	0.091	0.218	0.430	0.708	0.859	0.966	7.70%
14	0.091	0.165	0.496	0.608	0.717	0.966	0.82%
15	0.091	0.119	0.583	0.774	0.887	0.966	25.82%
16	0.091	0.239	0.379	0.633	0.786	0.966	2.13%
17	0.091	0.243	0.353	0.472	0.808	0.966	0.09%
18	0.091	0.156	0.335	0.519	0.714	0.966	0.00%
19	0.091	0.220	0.338	0.547	0.652	0.966	0.00%
20	0.091	0.163	0.541	0.669	0.898	0.966	8.69%

2–4 Years

	Knot 1	Knot 2	Knot 3	Knot 4	Knot 5	Knot 6	Weight
1	0.091	0.291	0.574	0.691	0.857	0.966	10.68%
2	0.091	0.187	0.572	0.778	0.885	0.966	23.00%
3	0.091	0.134	0.324	0.674	0.886	0.966	6.09%
4	0.091	0.120	0.452	0.555	0.806	0.966	1.73%
5	0.091	0.190	0.446	0.620	0.759	0.966	1.91%
6	0.091	0.114	0.222	0.590	0.893	0.966	2.15%
7	0.091	0.138	0.305	0.466	0.573	0.966	0.00%
8	0.091	0.136	0.258	0.414	0.856	0.966	0.05%
9	0.091	0.107	0.294	0.643	0.895	0.966	4.86%
10	0.091	0.161	0.364	0.539	0.883	0.966	2.09%
11	0.091	0.155	0.334	0.634	0.862	0.966	4.09%
12	0.091	0.187	0.415	0.553	0.884	0.966	3.19%
13	0.091	0.382	0.498	0.611	0.751	0.966	2.08%
14	0.091	0.150	0.456	0.612	0.760	0.966	1.86%
15	0.091	0.178	0.304	0.463	0.693	0.966	0.00%
16	0.091	0.162	0.632	0.745	0.847	0.966	18.57%
17	0.091	0.137	0.343	0.735	0.888	0.966	9.20%
18	0.091	0.201	0.331	0.477	0.605	0.966	0.00%
19	0.091	0.129	0.395	0.508	0.872	0.966	1.54%
20	0.091	0.227	0.329	0.704	0.865	0.966	6.92%

E. Extreme Stunting in Males: Ensemble MR–BRT Knot Placement

Early Neonatal

	Knot 1	Knot 2	Knot 3	Knot 4	Knot 5	Knot 6	Weight
1	0.091	0.276	0.467	0.675	0.825	0.966	7.45%
2	0.091	0.143	0.358	0.778	0.882	0.966	7.58%
3	0.091	0.352	0.453	0.635	0.792	0.966	5.76%
4	0.091	0.102	0.253	0.663	0.866	0.966	6.19%
5	0.091	0.120	0.390	0.646	0.771	0.966	4.79%
6	0.091	0.322	0.452	0.561	0.772	0.966	2.85%
7	0.091	0.180	0.329	0.716	0.841	0.966	7.92%
8	0.091	0.268	0.427	0.643	0.871	0.966	7.19%
9	0.091	0.267	0.385	0.703	0.825	0.966	7.67%
10	0.091	0.183	0.286	0.456	0.857	0.966	0.43%
11	0.091	0.385	0.567	0.698	0.850	0.966	18.96%
12	0.091	0.177	0.303	0.546	0.677	0.966	0.01%
13	0.091	0.228	0.401	0.528	0.871	0.966	3.85%
14	0.091	0.243	0.478	0.630	0.823	0.966	6.55%
15	0.091	0.188	0.356	0.600	0.885	0.966	5.81%
16	0.091	0.169	0.290	0.471	0.859	0.966	0.68%
17	0.091	0.147	0.347	0.449	0.616	0.966	0.00%
18	0.091	0.291	0.459	0.626	0.755	0.966	4.24%
19	0.091	0.170	0.339	0.457	0.877	0.966	1.11%
20	0.091	0.170	0.327	0.517	0.802	0.966	0.95%

Late Neonatal

	Knot 1	Knot 2	Knot 3	Knot 4	Knot 5	Knot 6	Weight
1	0.091	0.192	0.319	0.473	0.834	0.966	0.01%
2	0.091	0.452	0.574	0.741	0.877	0.966	9.54%
3	0.091	0.181	0.305	0.567	0.787	0.966	0.30%
4	0.091	0.316	0.533	0.687	0.831	0.966	19.75%
5	0.091	0.247	0.387	0.622	0.894	0.966	6.39%
6	0.091	0.295	0.521	0.653	0.754	0.966	4.41%
7	0.091	0.111	0.350	0.491	0.869	0.966	0.42%
8	0.091	0.286	0.474	0.662	0.826	0.966	7.26%
9	0.091	0.153	0.338	0.469	0.722	0.966	0.00%
10	0.091	0.153	0.393	0.618	0.731	0.966	0.78%
11	0.091	0.245	0.396	0.556	0.810	0.966	1.42%
12	0.091	0.190	0.594	0.767	0.870	0.966	7.87%
13	0.091	0.114	0.564	0.733	0.899	0.966	7.90%
14	0.091	0.212	0.359	0.499	0.873	0.966	0.77%
15	0.091	0.215	0.426	0.570	0.811	0.966	2.42%
16	0.091	0.373	0.529	0.687	0.814	0.966	7.94%
17	0.091	0.107	0.467	0.579	0.763	0.966	1.43%
18	0.091	0.290	0.409	0.658	0.872	0.966	7.52%
19	0.091	0.129	0.491	0.624	0.895	0.966	7.38%
20	0.091	0.227	0.512	0.672	0.783	0.966	6.48%

1–5 Months

	Knot 1	Knot 2	Knot 3	Knot 4	Knot 5	Knot 6	Weight
1	0.091	0.218	0.343	0.676	0.820	0.966	7.47%
2	0.091	0.186	0.301	0.554	0.794	0.966	1.52%
3	0.091	0.237	0.524	0.673	0.896	0.966	8.69%
4	0.091	0.205	0.471	0.598	0.836	0.966	6.59%
5	0.091	0.166	0.502	0.691	0.880	0.966	21.52%
6	0.091	0.182	0.295	0.648	0.841	0.966	6.50%
7	0.091	0.120	0.364	0.618	0.787	0.966	4.45%
8	0.091	0.207	0.414	0.582	0.805	0.966	4.44%
9	0.091	0.100	0.236	0.451	0.847	0.966	0.15%
10	0.091	0.456	0.561	0.707	0.886	0.966	8.61%
11	0.091	0.148	0.288	0.394	0.871	0.966	0.07%
12	0.091	0.149	0.271	0.493	0.617	0.966	0.00%
13	0.091	0.297	0.518	0.668	0.880	0.966	8.61%
14	0.091	0.139	0.397	0.535	0.752	0.966	0.97%
15	0.091	0.175	0.355	0.556	0.705	0.966	0.24%
16	0.091	0.183	0.314	0.609	0.870	0.966	5.67%
17	0.091	0.127	0.380	0.521	0.704	0.966	0.08%
18	0.091	0.204	0.339	0.510	0.653	0.966	0.00%
19	0.091	0.322	0.439	0.631	0.856	0.966	7.60%
20	0.091	0.279	0.522	0.658	0.772	0.966	6.82%

6–11 Months

	Knot 1	Knot 2	Knot 3	Knot 4	Knot 5	Knot 6	Weight
1	0.091	0.210	0.531	0.678	0.873	0.966	6.42%
2	0.091	0.129	0.537	0.638	0.741	0.966	5.03%
3	0.091	0.180	0.578	0.715	0.894	0.966	6.32%
4	0.091	0.186	0.297	0.485	0.774	0.966	1.18%
5	0.091	0.104	0.237	0.347	0.726	0.966	0.01%
6	0.091	0.205	0.371	0.722	0.861	0.966	6.39%
7	0.091	0.206	0.445	0.623	0.832	0.966	5.73%
8	0.091	0.142	0.481	0.716	0.866	0.966	6.47%
9	0.091	0.109	0.323	0.694	0.875	0.966	6.25%
10	0.091	0.106	0.395	0.592	0.869	0.966	5.40%
11	0.091	0.144	0.593	0.695	0.855	0.966	6.69%
12	0.091	0.110	0.528	0.707	0.816	0.966	16.05%
13	0.091	0.391	0.523	0.657	0.845	0.966	6.29%
14	0.091	0.411	0.532	0.645	0.888	0.966	6.27%
15	0.091	0.107	0.275	0.379	0.829	0.966	0.52%
16	0.091	0.229	0.338	0.456	0.716	0.966	0.37%
17	0.091	0.131	0.501	0.767	0.872	0.966	6.20%
18	0.091	0.102	0.256	0.366	0.494	0.966	0.00%
19	0.091	0.123	0.410	0.581	0.838	0.966	5.01%
20	0.091	0.121	0.306	0.511	0.896	0.966	3.42%

12–23 Months

	Knot 1	Knot 2	Knot 3	Knot 4	Knot 5	Knot 6	Weight
1	0.091	0.171	0.276	0.497	0.787	0.966	0.22%
2	0.091	0.120	0.229	0.478	0.626	0.966	0.00%
3	0.091	0.112	0.496	0.599	0.843	0.966	6.62%
4	0.091	0.122	0.423	0.644	0.780	0.966	5.62%
5	0.091	0.135	0.348	0.462	0.708	0.966	0.00%
6	0.091	0.153	0.324	0.432	0.839	0.966	0.31%
7	0.091	0.154	0.568	0.747	0.848	0.966	9.22%
8	0.091	0.428	0.663	0.770	0.898	0.966	8.00%
9	0.091	0.243	0.353	0.600	0.759	0.966	2.57%
10	0.091	0.144	0.418	0.587	0.762	0.966	2.89%
11	0.091	0.236	0.338	0.498	0.731	0.966	0.06%
12	0.091	0.104	0.492	0.649	0.802	0.966	6.97%
13	0.091	0.349	0.572	0.691	0.868	0.966	20.00%
14	0.091	0.506	0.615	0.738	0.841	0.966	8.29%
15	0.091	0.214	0.497	0.785	0.898	0.966	7.49%
16	0.091	0.191	0.306	0.492	0.657	0.966	0.00%
17	0.091	0.172	0.297	0.692	0.820	0.966	7.18%
18	0.091	0.253	0.481	0.643	0.849	0.966	7.60%
19	0.091	0.101	0.328	0.438	0.642	0.966	0.00%
20	0.091	0.226	0.364	0.643	0.855	0.966	6.95%

2–4 Years

	Knot 1	Knot 2	Knot 3	Knot 4	Knot 5	Knot 6	Weight
1	0.091	0.119	0.259	0.669	0.802	0.966	6.50%
2	0.091	0.165	0.344	0.518	0.654	0.966	0.00%
3	0.091	0.152	0.343	0.522	0.687	0.966	0.00%
4	0.091	0.104	0.225	0.594	0.858	0.966	4.01%
5	0.091	0.106	0.277	0.404	0.753	0.966	0.00%
6	0.091	0.188	0.407	0.512	0.704	0.966	0.00%
7	0.091	0.123	0.388	0.563	0.867	0.966	5.63%
8	0.091	0.143	0.313	0.579	0.700	0.966	0.03%
9	0.091	0.206	0.373	0.569	0.801	0.966	2.85%
10	0.091	0.132	0.461	0.679	0.883	0.966	19.78%
11	0.091	0.154	0.432	0.542	0.848	0.966	4.93%
12	0.091	0.176	0.320	0.572	0.873	0.966	4.82%
13	0.091	0.228	0.581	0.693	0.884	0.966	20.43%
14	0.091	0.154	0.357	0.507	0.858	0.966	2.20%
15	0.091	0.258	0.423	0.609	0.828	0.966	7.35%
16	0.091	0.109	0.241	0.646	0.890	0.966	8.17%
17	0.091	0.291	0.545	0.669	0.783	0.966	10.57%
18	0.091	0.152	0.400	0.519	0.834	0.966	2.66%
19	0.091	0.133	0.332	0.462	0.780	0.966	0.01%
20	0.091	0.147	0.283	0.434	0.865	0.966	0.07%

F. Extreme Stunting in Females: Ensemble MR–BRT Knot Placement

Early Neonatal

	Knot 1	Knot 2	Knot 3	Knot 4	Knot 5	Knot 6	Weight
1	0.091	0.251	0.384	0.703	0.853	0.966	8.07%
2	0.091	0.225	0.536	0.712	0.815	0.966	19.33%
3	0.091	0.111	0.330	0.532	0.900	0.966	4.50%
4	0.091	0.258	0.394	0.527	0.788	0.966	2.06%
5	0.091	0.296	0.420	0.679	0.780	0.966	7.55%
6	0.091	0.130	0.270	0.411	0.885	0.966	0.16%
7	0.091	0.162	0.447	0.604	0.795	0.966	5.90%
8	0.091	0.359	0.590	0.712	0.851	0.966	7.73%
9	0.091	0.208	0.308	0.635	0.780	0.966	5.65%
10	0.091	0.137	0.248	0.430	0.782	0.966	0.00%
11	0.091	0.169	0.278	0.495	0.809	0.966	0.50%
12	0.091	0.173	0.322	0.597	0.706	0.966	0.84%
13	0.091	0.198	0.377	0.686	0.891	0.966	7.65%
14	0.091	0.191	0.301	0.748	0.897	0.966	6.68%
15	0.091	0.121	0.479	0.587	0.772	0.966	4.77%
16	0.091	0.369	0.489	0.608	0.710	0.966	2.84%
17	0.091	0.122	0.341	0.497	0.636	0.966	0.00%
18	0.091	0.194	0.398	0.538	0.758	0.966	1.38%
19	0.091	0.333	0.472	0.653	0.867	0.966	7.74%
20	0.091	0.104	0.332	0.620	0.834	0.966	6.64%

Late Neonatal

	Knot 1	Knot 2	Knot 3	Knot 4	Knot 5	Knot 6	Weight
1	0.091	0.163	0.385	0.550	0.899	0.966	5.43%
2	0.091	0.207	0.423	0.739	0.843	0.966	6.32%
3	0.091	0.143	0.319	0.563	0.837	0.966	4.84%
4	0.091	0.249	0.409	0.728	0.865	0.966	6.24%
5	0.091	0.207	0.311	0.587	0.834	0.966	5.30%
6	0.091	0.138	0.303	0.549	0.822	0.966	4.22%
7	0.091	0.292	0.620	0.767	0.881	0.966	6.15%
8	0.091	0.107	0.348	0.751	0.857	0.966	6.11%
9	0.091	0.398	0.509	0.665	0.873	0.966	6.23%
10	0.091	0.237	0.457	0.567	0.850	0.966	5.65%
11	0.091	0.333	0.451	0.701	0.869	0.966	6.30%
12	0.091	0.129	0.348	0.486	0.588	0.966	0.00%
13	0.091	0.389	0.537	0.737	0.892	0.966	6.01%
14	0.091	0.120	0.240	0.410	0.551	0.966	0.00%
15	0.091	0.194	0.400	0.547	0.700	0.966	1.77%
16	0.091	0.171	0.335	0.535	0.675	0.966	0.69%
17	0.091	0.284	0.419	0.732	0.876	0.966	6.16%
18	0.091	0.215	0.563	0.691	0.799	0.966	15.61%
19	0.091	0.148	0.322	0.552	0.672	0.966	0.80%
20	0.091	0.241	0.499	0.713	0.893	0.966	6.17%

1–5 Months

	Knot 1	Knot 2	Knot 3	Knot 4	Knot 5	Knot 6	Weight
1	0.091	0.184	0.338	0.573	0.729	0.966	2.11%
2	0.091	0.240	0.502	0.683	0.844	0.966	14.96%
3	0.091	0.236	0.431	0.615	0.734	0.966	3.98%
4	0.091	0.240	0.560	0.710	0.889	0.966	5.83%
5	0.091	0.294	0.410	0.595	0.785	0.966	4.64%
6	0.091	0.200	0.433	0.600	0.778	0.966	4.72%
7	0.091	0.250	0.405	0.575	0.845	0.966	5.13%
8	0.091	0.250	0.375	0.734	0.844	0.966	5.95%
9	0.091	0.106	0.257	0.370	0.644	0.966	0.00%
10	0.091	0.323	0.546	0.662	0.812	0.966	6.79%
11	0.091	0.226	0.346	0.452	0.793	0.966	1.08%
12	0.091	0.241	0.451	0.663	0.779	0.966	5.83%
13	0.091	0.314	0.514	0.629	0.845	0.966	5.98%
14	0.091	0.214	0.576	0.716	0.876	0.966	5.87%
15	0.091	0.258	0.520	0.711	0.866	0.966	5.95%
16	0.091	0.129	0.232	0.539	0.682	0.966	0.13%
17	0.091	0.373	0.527	0.696	0.832	0.966	6.13%
18	0.091	0.130	0.352	0.756	0.898	0.966	5.28%
19	0.091	0.435	0.536	0.664	0.807	0.966	5.88%
20	0.091	0.142	0.354	0.582	0.773	0.966	3.75%

6–11 Months

	Knot 1	Knot 2	Knot 3	Knot 4	Knot 5	Knot 6	Weight
1	0.091	0.200	0.307	0.538	0.850	0.966	4.07%
2	0.091	0.251	0.532	0.713	0.878	0.966	7.77%
3	0.091	0.261	0.436	0.581	0.746	0.966	3.17%
4	0.091	0.116	0.410	0.736	0.848	0.966	7.74%
5	0.091	0.109	0.220	0.405	0.800	0.966	0.00%
6	0.091	0.132	0.391	0.501	0.829	0.966	3.05%
7	0.091	0.239	0.506	0.620	0.854	0.966	19.38%
8	0.091	0.302	0.434	0.647	0.807	0.966	7.75%
9	0.091	0.114	0.296	0.577	0.759	0.966	2.23%
10	0.091	0.186	0.369	0.472	0.751	0.966	0.14%
11	0.091	0.224	0.364	0.539	0.785	0.966	2.55%
12	0.091	0.105	0.242	0.561	0.897	0.966	5.29%
13	0.091	0.194	0.408	0.625	0.845	0.966	7.72%
14	0.091	0.435	0.549	0.758	0.863	0.966	7.24%
15	0.091	0.284	0.487	0.622	0.833	0.966	7.82%
16	0.091	0.164	0.290	0.398	0.717	0.966	0.00%
17	0.091	0.167	0.391	0.495	0.685	0.966	0.00%
18	0.091	0.175	0.380	0.543	0.757	0.966	1.67%
19	0.091	0.158	0.341	0.543	0.869	0.966	5.12%
20	0.091	0.250	0.396	0.610	0.846	0.966	7.30%

12–23 Months

	Knot 1	Knot 2	Knot 3	Knot 4	Knot 5	Knot 6	Weight
1	0.091	0.306	0.437	0.724	0.891	0.966	9.29%
2	0.091	0.203	0.525	0.783	0.898	0.966	8.05%
3	0.091	0.292	0.522	0.684	0.840	0.966	22.58%
4	0.091	0.162	0.331	0.501	0.722	0.966	0.00%
5	0.091	0.145	0.393	0.543	0.706	0.966	0.02%
6	0.091	0.164	0.332	0.552	0.777	0.966	0.89%
7	0.091	0.162	0.347	0.566	0.746	0.966	0.59%
8	0.091	0.143	0.363	0.529	0.690	0.966	0.00%
9	0.091	0.130	0.407	0.516	0.792	0.966	1.19%
10	0.091	0.185	0.365	0.608	0.793	0.966	4.61%
11	0.091	0.184	0.371	0.605	0.714	0.966	0.76%
12	0.091	0.150	0.365	0.582	0.766	0.966	1.97%
13	0.091	0.111	0.211	0.445	0.764	0.966	0.00%
14	0.091	0.411	0.600	0.704	0.848	0.966	11.02%
15	0.091	0.322	0.425	0.620	0.871	0.966	8.35%
16	0.091	0.168	0.288	0.662	0.858	0.966	8.79%
17	0.091	0.251	0.432	0.551	0.788	0.966	2.63%
18	0.091	0.214	0.449	0.651	0.843	0.966	9.03%
19	0.091	0.433	0.606	0.794	0.899	0.966	8.56%
20	0.091	0.185	0.318	0.493	0.885	0.966	1.65%

2–4 Years

	Knot 1	Knot 2	Knot 3	Knot 4	Knot 5	Knot 6	Weight
1	0.091	0.269	0.542	0.646	0.782	0.966	8.73%
2	0.091	0.149	0.300	0.487	0.602	0.966	0.00%
3	0.091	0.267	0.412	0.544	0.741	0.966	1.76%
4	0.091	0.182	0.397	0.590	0.752	0.966	3.56%
5	0.091	0.116	0.333	0.617	0.741	0.966	3.67%
6	0.091	0.206	0.454	0.688	0.804	0.966	12.36%
7	0.091	0.192	0.325	0.572	0.765	0.966	2.77%
8	0.091	0.184	0.375	0.641	0.773	0.966	6.69%
9	0.091	0.186	0.400	0.536	0.674	0.966	0.10%
10	0.091	0.121	0.336	0.448	0.674	0.966	0.00%
11	0.091	0.128	0.468	0.609	0.757	0.966	5.29%
12	0.091	0.181	0.285	0.590	0.697	0.966	0.69%
13	0.091	0.238	0.346	0.481	0.811	0.966	1.85%
14	0.091	0.192	0.544	0.748	0.880	0.966	21.83%
15	0.091	0.142	0.246	0.533	0.756	0.966	0.67%
16	0.091	0.341	0.456	0.612	0.783	0.966	6.52%
17	0.091	0.168	0.399	0.668	0.891	0.966	17.23%
18	0.091	0.259	0.394	0.502	0.890	0.966	5.70%
19	0.091	0.152	0.286	0.436	0.827	0.966	0.55%
20	0.091	0.100	0.227	0.471	0.748	0.966	0.02%

G. Overall Wasting in Males: Ensemble MR–BRT Knot Placement

Early Neonatal

	Knot 1	Knot 2	Knot 3	Knot 4	Knot 5	Knot 6	Weight
1	0.091	0.355	0.537	0.676	0.824	0.966	5.17%
2	0.091	0.208	0.541	0.657	0.770	0.966	5.26%
3	0.091	0.118	0.245	0.379	0.677	0.966	0.26%
4	0.091	0.162	0.451	0.739	0.857	0.966	14.28%
5	0.091	0.326	0.442	0.585	0.815	0.966	5.17%
6	0.091	0.138	0.330	0.502	0.741	0.966	3.51%
7	0.091	0.137	0.258	0.425	0.852	0.966	4.83%
8	0.091	0.132	0.239	0.402	0.509	0.966	0.00%
9	0.091	0.245	0.393	0.712	0.860	0.966	6.42%
10	0.091	0.140	0.658	0.774	0.895	0.966	5.35%
11	0.091	0.370	0.546	0.762	0.899	0.966	5.00%
12	0.091	0.216	0.530	0.762	0.886	0.966	5.58%
13	0.091	0.208	0.455	0.598	0.807	0.966	5.43%
14	0.091	0.285	0.469	0.723	0.866	0.966	5.64%
15	0.091	0.254	0.559	0.758	0.882	0.966	5.41%
16	0.091	0.127	0.290	0.658	0.867	0.966	5.71%
17	0.091	0.387	0.583	0.688	0.812	0.966	4.93%
18	0.091	0.153	0.272	0.594	0.858	0.966	5.80%
19	0.091	0.266	0.524	0.731	0.883	0.966	5.49%
20	0.091	0.186	0.358	0.511	0.618	0.966	0.77%

Late Neonatal

	Knot 1	Knot 2	Knot 3	Knot 4	Knot 5	Knot 6	Weight
1	0.091	0.153	0.278	0.381	0.716	0.966	0.00%
2	0.091	0.223	0.332	0.538	0.811	0.966	4.47%
3	0.091	0.130	0.446	0.667	0.790	0.966	6.32%
4	0.091	0.295	0.429	0.560	0.773	0.966	2.46%
5	0.091	0.127	0.400	0.559	0.754	0.966	2.21%
6	0.091	0.117	0.246	0.632	0.732	0.966	2.95%
7	0.091	0.110	0.239	0.621	0.775	0.966	4.53%
8	0.091	0.126	0.299	0.696	0.817	0.966	8.16%
9	0.091	0.169	0.413	0.558	0.775	0.966	3.24%
10	0.091	0.103	0.387	0.642	0.762	0.966	4.57%
11	0.091	0.189	0.350	0.608	0.823	0.966	6.35%
12	0.091	0.248	0.601	0.706	0.815	0.966	4.58%
13	0.091	0.302	0.535	0.646	0.758	0.966	2.94%
14	0.091	0.110	0.568	0.757	0.874	0.966	5.74%
15	0.091	0.109	0.336	0.685	0.888	0.966	5.70%
16	0.091	0.303	0.450	0.565	0.771	0.966	2.37%
17	0.091	0.146	0.403	0.597	0.835	0.966	6.57%
18	0.091	0.154	0.492	0.716	0.894	0.966	15.88%
19	0.091	0.289	0.487	0.598	0.887	0.966	5.13%
20	0.091	0.130	0.571	0.693	0.830	0.966	5.82%

1–5 Months

	Knot 1	Knot 2	Knot 3	Knot 4	Knot 5	Knot 6	Weight
1	0.091	0.132	0.262	0.402	0.799	0.966	1.98%
2	0.091	0.193	0.398	0.631	0.754	0.966	5.52%
3	0.091	0.141	0.376	0.715	0.818	0.966	17.50%
4	0.091	0.136	0.257	0.489	0.656	0.966	0.01%
5	0.091	0.118	0.440	0.637	0.794	0.966	6.73%
6	0.091	0.247	0.570	0.741	0.897	0.966	5.89%
7	0.091	0.137	0.418	0.567	0.860	0.966	7.17%
8	0.091	0.357	0.554	0.660	0.814	0.966	5.04%
9	0.091	0.138	0.262	0.461	0.654	0.966	0.00%
10	0.091	0.109	0.252	0.427	0.773	0.966	1.44%
11	0.091	0.122	0.537	0.676	0.835	0.966	7.00%
12	0.091	0.120	0.579	0.788	0.891	0.966	6.47%
13	0.091	0.102	0.264	0.444	0.636	0.966	0.00%
14	0.091	0.313	0.456	0.660	0.798	0.966	5.98%
15	0.091	0.291	0.511	0.770	0.895	0.966	5.99%
16	0.091	0.246	0.538	0.721	0.862	0.966	6.44%
17	0.091	0.119	0.284	0.394	0.804	0.966	2.11%
18	0.091	0.125	0.274	0.390	0.652	0.966	0.00%
19	0.091	0.190	0.428	0.644	0.837	0.966	7.75%
20	0.091	0.174	0.408	0.548	0.855	0.966	6.97%

6–11 Months

	Knot 1	Knot 2	Knot 3	Knot 4	Knot 5	Knot 6	Weight
1	0.091	0.101	0.258	0.466	0.712	0.966	2.69%
2	0.091	0.144	0.375	0.646	0.758	0.966	5.35%
3	0.091	0.127	0.457	0.578	0.785	0.966	5.29%
4	0.091	0.264	0.406	0.518	0.792	0.966	5.01%
5	0.091	0.156	0.338	0.603	0.738	0.966	4.93%
6	0.091	0.180	0.309	0.543	0.745	0.966	4.54%
7	0.091	0.188	0.382	0.489	0.786	0.966	4.83%
8	0.091	0.308	0.459	0.560	0.761	0.966	4.75%
9	0.091	0.119	0.265	0.367	0.516	0.966	0.00%
10	0.091	0.260	0.361	0.538	0.716	0.966	3.90%
11	0.091	0.152	0.450	0.736	0.863	0.966	13.64%
12	0.091	0.169	0.361	0.579	0.732	0.966	4.65%
13	0.091	0.139	0.243	0.433	0.741	0.966	2.90%
14	0.091	0.152	0.548	0.678	0.883	0.966	5.46%
15	0.091	0.155	0.408	0.605	0.819	0.966	5.78%
16	0.091	0.148	0.412	0.587	0.837	0.966	6.22%
17	0.091	0.159	0.333	0.637	0.893	0.966	5.43%
18	0.091	0.142	0.497	0.615	0.742	0.966	4.99%
19	0.091	0.273	0.516	0.632	0.787	0.966	5.19%
20	0.091	0.111	0.269	0.555	0.733	0.966	4.47%

12–23 Months

	Knot 1	Knot 2	Knot 3	Knot 4	Knot 5	Knot 6	Weight
1	0.091	0.161	0.371	0.597	0.894	0.966	5.64%
2	0.091	0.323	0.485	0.615	0.854	0.966	5.20%
3	0.091	0.111	0.220	0.519	0.791	0.966	3.17%
4	0.091	0.138	0.252	0.405	0.861	0.966	2.78%
5	0.091	0.197	0.492	0.752	0.881	0.966	5.74%
6	0.091	0.125	0.560	0.747	0.875	0.966	5.71%
7	0.091	0.277	0.467	0.676	0.776	0.966	5.06%
8	0.091	0.128	0.320	0.667	0.808	0.966	5.74%
9	0.091	0.252	0.419	0.752	0.865	0.966	14.40%
10	0.091	0.122	0.449	0.608	0.735	0.966	3.41%
11	0.091	0.201	0.311	0.649	0.893	0.966	5.82%
12	0.091	0.235	0.347	0.613	0.754	0.966	3.86%
13	0.091	0.162	0.460	0.728	0.898	0.966	5.76%
14	0.091	0.182	0.306	0.416	0.606	0.966	0.00%
15	0.091	0.121	0.263	0.679	0.792	0.966	5.66%
16	0.091	0.173	0.343	0.461	0.662	0.966	0.01%
17	0.091	0.132	0.285	0.627	0.884	0.966	5.79%
18	0.091	0.268	0.605	0.737	0.893	0.966	5.25%
19	0.091	0.221	0.348	0.629	0.856	0.966	5.72%
20	0.091	0.154	0.492	0.624	0.812	0.966	5.27%

2–4 Years

	Knot 1	Knot 2	Knot 3	Knot 4	Knot 5	Knot 6	Weight
1	0.091	0.242	0.359	0.719	0.866	0.966	20.40%
2	0.091	0.197	0.341	0.642	0.764	0.966	2.13%
3	0.091	0.186	0.290	0.428	0.777	0.966	0.00%
4	0.091	0.154	0.367	0.572	0.772	0.966	0.41%
5	0.091	0.331	0.455	0.593	0.835	0.966	5.57%
6	0.091	0.181	0.310	0.612	0.750	0.966	0.44%
7	0.091	0.304	0.404	0.513	0.771	0.966	0.05%
8	0.091	0.360	0.489	0.631	0.758	0.966	2.49%
9	0.091	0.355	0.519	0.657	0.869	0.966	13.83%
10	0.091	0.192	0.350	0.536	0.818	0.966	0.91%
11	0.091	0.160	0.305	0.507	0.805	0.966	0.08%
12	0.091	0.163	0.364	0.508	0.826	0.966	0.60%
13	0.091	0.308	0.413	0.651	0.829	0.966	8.45%
14	0.091	0.297	0.612	0.782	0.883	0.966	22.43%
15	0.091	0.186	0.334	0.586	0.695	0.966	0.00%
16	0.091	0.165	0.365	0.466	0.854	0.966	0.54%
17	0.091	0.289	0.513	0.642	0.752	0.966	2.96%
18	0.091	0.213	0.469	0.596	0.818	0.966	4.78%
19	0.091	0.140	0.487	0.668	0.808	0.966	8.97%
20	0.091	0.256	0.466	0.602	0.817	0.966	4.95%

H. Overall Wasting in Females: Ensemble MR–BRT Knot Placement

Early Neonatal

	Knot 1	Knot 2	Knot 3	Knot 4	Knot 5	Knot 6	Weight
1	0.091	0.293	0.493	0.628	0.735	0.966	4.51%
2	0.091	0.357	0.520	0.679	0.897	0.966	4.75%
3	0.091	0.107	0.216	0.598	0.833	0.966	5.85%
4	0.091	0.229	0.444	0.560	0.723	0.966	2.89%
5	0.091	0.285	0.428	0.607	0.882	0.966	5.31%
6	0.091	0.147	0.268	0.755	0.889	0.966	4.58%
7	0.091	0.363	0.585	0.703	0.830	0.966	5.29%
8	0.091	0.267	0.425	0.556	0.805	0.966	4.85%
9	0.091	0.119	0.266	0.656	0.843	0.966	14.03%
10	0.091	0.222	0.443	0.665	0.772	0.966	7.27%
11	0.091	0.112	0.233	0.525	0.692	0.966	0.90%
12	0.091	0.139	0.303	0.518	0.865	0.966	5.07%
13	0.091	0.408	0.509	0.743	0.882	0.966	4.59%
14	0.091	0.217	0.390	0.687	0.885	0.966	5.61%
15	0.091	0.216	0.404	0.609	0.710	0.966	3.40%
16	0.091	0.131	0.266	0.446	0.572	0.966	0.00%
17	0.091	0.118	0.373	0.523	0.816	0.966	4.71%
18	0.091	0.243	0.643	0.768	0.883	0.966	5.60%
19	0.091	0.135	0.333	0.741	0.854	0.966	5.61%
20	0.091	0.155	0.328	0.528	0.859	0.966	5.16%

Late Neonatal

	Knot 1	Knot 2	Knot 3	Knot 4	Knot 5	Knot 6	Weight
1	0.091	0.153	0.407	0.715	0.875	0.966	7.25%
2	0.091	0.227	0.515	0.769	0.893	0.966	6.73%
3	0.091	0.105	0.267	0.383	0.558	0.966	0.00%
4	0.091	0.267	0.530	0.648	0.825	0.966	7.23%
5	0.091	0.178	0.351	0.549	0.705	0.966	3.42%
6	0.091	0.168	0.290	0.406	0.606	0.966	0.00%
7	0.091	0.145	0.301	0.443	0.639	0.966	0.11%
8	0.091	0.116	0.306	0.591	0.757	0.966	6.16%
9	0.091	0.126	0.272	0.601	0.837	0.966	18.07%
10	0.091	0.111	0.333	0.443	0.759	0.966	3.24%
11	0.091	0.404	0.548	0.675	0.802	0.966	6.70%
12	0.091	0.296	0.406	0.544	0.748	0.966	4.86%
13	0.091	0.137	0.530	0.743	0.858	0.966	7.26%
14	0.091	0.112	0.276	0.410	0.703	0.966	0.57%
15	0.091	0.198	0.304	0.550	0.830	0.966	6.85%
16	0.091	0.139	0.419	0.612	0.713	0.966	5.33%
17	0.091	0.148	0.371	0.556	0.662	0.966	2.08%
18	0.091	0.193	0.506	0.682	0.868	0.966	7.22%
19	0.091	0.324	0.512	0.753	0.873	0.966	6.61%
20	0.091	0.204	0.328	0.505	0.622	0.966	0.31%

1–5 Months

	Knot 1	Knot 2	Knot 3	Knot 4	Knot 5	Knot 6	Weight
1	0.091	0.106	0.621	0.739	0.895	0.966	6.66%
2	0.091	0.131	0.311	0.478	0.857	0.966	5.43%
3	0.091	0.148	0.322	0.635	0.789	0.966	7.37%
4	0.091	0.411	0.542	0.680	0.893	0.966	5.64%
5	0.091	0.244	0.436	0.595	0.772	0.966	5.91%
6	0.091	0.107	0.250	0.458	0.722	0.966	0.84%
7	0.091	0.179	0.410	0.718	0.836	0.966	17.01%
8	0.091	0.256	0.417	0.522	0.671	0.966	0.97%
9	0.091	0.108	0.343	0.503	0.775	0.966	4.00%
10	0.091	0.105	0.205	0.578	0.683	0.966	2.08%
11	0.091	0.174	0.452	0.595	0.769	0.966	6.01%
12	0.091	0.168	0.307	0.435	0.560	0.966	0.00%
13	0.091	0.155	0.335	0.446	0.721	0.966	0.96%
14	0.091	0.203	0.420	0.565	0.724	0.966	3.69%
15	0.091	0.180	0.316	0.528	0.858	0.966	6.15%
16	0.091	0.166	0.642	0.753	0.869	0.966	6.94%
17	0.091	0.128	0.478	0.596	0.899	0.966	6.56%
18	0.091	0.108	0.481	0.685	0.892	0.966	6.67%
19	0.091	0.186	0.321	0.433	0.695	0.966	0.29%
20	0.091	0.121	0.298	0.654	0.880	0.966	6.81%

6–11 Months

	Knot 1	Knot 2	Knot 3	Knot 4	Knot 5	Knot 6	Weight
1	0.091	0.330	0.553	0.731	0.867	0.966	6.46%
2	0.091	0.216	0.344	0.588	0.785	0.966	4.39%
3	0.091	0.115	0.396	0.543	0.739	0.966	1.24%
4	0.091	0.429	0.559	0.752	0.862	0.966	5.80%
5	0.091	0.203	0.304	0.538	0.830	0.966	4.33%
6	0.091	0.138	0.387	0.589	0.808	0.966	5.49%
7	0.091	0.134	0.356	0.490	0.727	0.966	0.13%
8	0.091	0.217	0.362	0.495	0.625	0.966	0.00%
9	0.091	0.153	0.363	0.727	0.892	0.966	6.98%
10	0.091	0.198	0.396	0.546	0.842	0.966	5.14%
11	0.091	0.196	0.352	0.580	0.792	0.966	4.48%
12	0.091	0.250	0.455	0.672	0.824	0.966	9.48%
13	0.091	0.170	0.276	0.555	0.839	0.966	5.10%
14	0.091	0.141	0.307	0.419	0.708	0.966	0.00%
15	0.091	0.212	0.460	0.621	0.810	0.966	6.48%
16	0.091	0.177	0.426	0.736	0.899	0.966	6.58%
17	0.091	0.183	0.489	0.607	0.885	0.966	6.57%
18	0.091	0.130	0.273	0.591	0.795	0.966	4.91%
19	0.091	0.118	0.275	0.684	0.858	0.966	16.44%
20	0.091	0.175	0.286	0.475	0.665	0.966	0.00%

12–23 Months

	Knot 1	Knot 2	Knot 3	Knot 4	Knot 5	Knot 6	Weight
1	0.091	0.309	0.465	0.636	0.856	0.966	5.60%
2	0.091	0.258	0.615	0.720	0.855	0.966	8.91%
3	0.091	0.268	0.482	0.675	0.796	0.966	5.29%
4	0.091	0.241	0.483	0.674	0.860	0.966	8.19%
5	0.091	0.175	0.531	0.715	0.842	0.966	14.12%
6	0.091	0.179	0.308	0.463	0.882	0.966	0.03%
7	0.091	0.176	0.286	0.638	0.895	0.966	7.62%
8	0.091	0.153	0.362	0.502	0.802	0.966	0.00%
9	0.091	0.117	0.363	0.704	0.840	0.966	20.48%
10	0.091	0.106	0.222	0.602	0.716	0.966	0.00%
11	0.091	0.292	0.416	0.538	0.840	0.966	0.53%
12	0.091	0.290	0.395	0.618	0.808	0.966	1.92%
13	0.091	0.114	0.276	0.698	0.802	0.966	8.10%
14	0.091	0.219	0.503	0.607	0.875	0.966	5.90%
15	0.091	0.160	0.356	0.531	0.828	0.966	0.10%
16	0.091	0.207	0.472	0.619	0.792	0.966	1.83%
17	0.091	0.198	0.501	0.632	0.815	0.966	4.54%
18	0.091	0.326	0.472	0.594	0.729	0.966	0.00%
19	0.091	0.176	0.368	0.680	0.787	0.966	5.04%
20	0.091	0.298	0.512	0.636	0.777	0.966	1.78%

2–4 Years

	Knot 1	Knot 2	Knot 3	Knot 4	Knot 5	Knot 6	Weight
1	0.091	0.150	0.344	0.467	0.695	0.966	0.00%
2	0.091	0.110	0.351	0.642	0.830	0.966	5.94%
3	0.091	0.199	0.401	0.633	0.756	0.966	2.48%
4	0.091	0.403	0.595	0.721	0.836	0.966	6.51%
5	0.091	0.179	0.455	0.619	0.802	0.966	4.24%
6	0.091	0.363	0.482	0.593	0.760	0.966	1.61%
7	0.091	0.126	0.323	0.435	0.886	0.966	1.44%
8	0.091	0.215	0.337	0.565	0.719	0.966	0.06%
9	0.091	0.326	0.487	0.647	0.810	0.966	5.10%
10	0.091	0.173	0.311	0.593	0.852	0.966	5.01%
11	0.091	0.298	0.406	0.587	0.876	0.966	5.49%
12	0.091	0.260	0.396	0.561	0.831	0.966	3.35%
13	0.091	0.182	0.362	0.519	0.768	0.966	0.28%
14	0.091	0.167	0.374	0.700	0.855	0.966	15.18%
15	0.091	0.111	0.306	0.439	0.899	0.966	1.67%
16	0.091	0.236	0.492	0.734	0.846	0.966	14.14%
17	0.091	0.166	0.297	0.491	0.686	0.966	0.00%
18	0.091	0.330	0.547	0.663	0.896	0.966	6.96%
19	0.091	0.344	0.463	0.573	0.814	0.966	3.16%
20	0.091	0.184	0.566	0.740	0.853	0.966	17.40%

I. Severe Wasting in Males: Ensemble MR–BRT Knot Placement

Early Neonatal

	Knot 1	Knot 2	Knot 3	Knot 4	Knot 5	Knot 6	Weight
1	0.091	0.205	0.412	0.776	0.890	0.966	7.06%
2	0.091	0.203	0.340	0.508	0.653	0.966	0.00%
3	0.091	0.130	0.353	0.453	0.736	0.966	0.00%
4	0.091	0.139	0.423	0.537	0.778	0.966	2.59%
5	0.091	0.228	0.457	0.662	0.847	0.966	26.26%
6	0.091	0.156	0.328	0.660	0.831	0.966	12.14%
7	0.091	0.222	0.325	0.534	0.739	0.966	0.08%
8	0.091	0.132	0.350	0.528	0.644	0.966	0.00%
9	0.091	0.218	0.492	0.646	0.779	0.966	12.12%
10	0.091	0.148	0.339	0.608	0.714	0.966	1.16%
11	0.091	0.170	0.310	0.468	0.839	0.966	0.33%
12	0.091	0.173	0.345	0.516	0.649	0.966	0.00%
13	0.091	0.167	0.424	0.569	0.746	0.966	2.43%
14	0.091	0.510	0.619	0.767	0.895	0.966	10.50%
15	0.091	0.163	0.294	0.415	0.875	0.966	0.04%
16	0.091	0.123	0.309	0.603	0.817	0.966	6.63%
17	0.091	0.216	0.375	0.745	0.883	0.966	9.32%
18	0.091	0.258	0.365	0.509	0.850	0.966	2.89%
19	0.091	0.146	0.276	0.604	0.819	0.966	5.54%
20	0.091	0.158	0.265	0.497	0.883	0.966	0.92%

Late Neonatal

	Knot 1	Knot 2	Knot 3	Knot 4	Knot 5	Knot 6	Weight
1	0.091	0.102	0.273	0.644	0.866	0.966	7.49%
2	0.091	0.184	0.593	0.695	0.858	0.966	21.13%
3	0.091	0.148	0.388	0.533	0.870	0.966	4.87%
4	0.091	0.176	0.339	0.543	0.724	0.966	0.30%
5	0.091	0.209	0.364	0.581	0.770	0.966	3.48%
6	0.091	0.170	0.462	0.565	0.888	0.966	7.16%
7	0.091	0.251	0.527	0.746	0.865	0.966	8.19%
8	0.091	0.181	0.355	0.559	0.784	0.966	2.98%
9	0.091	0.183	0.284	0.506	0.636	0.966	0.00%
10	0.091	0.183	0.612	0.716	0.855	0.966	10.16%
11	0.091	0.203	0.455	0.640	0.787	0.966	8.45%
12	0.091	0.187	0.289	0.391	0.812	0.966	0.00%
13	0.091	0.247	0.387	0.750	0.852	0.966	8.86%
14	0.091	0.105	0.206	0.551	0.752	0.966	0.17%
15	0.091	0.216	0.341	0.480	0.805	0.966	0.68%
16	0.091	0.143	0.244	0.379	0.848	0.966	0.00%
17	0.091	0.122	0.327	0.484	0.630	0.966	0.00%
18	0.091	0.156	0.426	0.746	0.889	0.966	7.08%
19	0.091	0.142	0.605	0.766	0.897	0.966	5.76%
20	0.091	0.234	0.368	0.497	0.890	0.966	3.23%

1–5 Months

	Knot 1	Knot 2	Knot 3	Knot 4	Knot 5	Knot 6	Weight
1	0.091	0.221	0.383	0.601	0.846	0.966	7.10%
2	0.091	0.148	0.346	0.521	0.822	0.966	3.58%
3	0.091	0.109	0.519	0.656	0.871	0.966	7.92%
4	0.091	0.188	0.363	0.768	0.886	0.966	6.22%
5	0.091	0.317	0.478	0.693	0.868	0.966	8.07%
6	0.091	0.133	0.367	0.541	0.666	0.966	0.27%
7	0.091	0.121	0.292	0.524	0.891	0.966	4.07%
8	0.091	0.227	0.517	0.670	0.890	0.966	7.39%
9	0.091	0.206	0.355	0.497	0.860	0.966	3.76%
10	0.091	0.103	0.358	0.480	0.680	0.966	0.04%
11	0.091	0.123	0.505	0.628	0.814	0.966	18.98%
12	0.091	0.193	0.341	0.747	0.880	0.966	6.93%
13	0.091	0.148	0.368	0.479	0.735	0.966	0.53%
14	0.091	0.332	0.439	0.748	0.892	0.966	6.57%
15	0.091	0.119	0.325	0.501	0.733	0.966	0.50%
16	0.091	0.165	0.281	0.570	0.728	0.966	1.44%
17	0.091	0.237	0.342	0.503	0.807	0.966	2.54%
18	0.091	0.272	0.435	0.728	0.868	0.966	7.59%
19	0.091	0.262	0.481	0.622	0.757	0.966	6.50%
20	0.091	0.143	0.274	0.389	0.633	0.966	0.00%

6–11 Months

	Knot 1	Knot 2	Knot 3	Knot 4	Knot 5	Knot 6	Weight
1	0.091	0.177	0.332	0.509	0.783	0.966	1.11%
2	0.091	0.101	0.306	0.502	0.644	0.966	0.00%
3	0.091	0.380	0.619	0.740	0.856	0.966	7.11%
4	0.091	0.107	0.275	0.708	0.825	0.966	17.94%
5	0.091	0.404	0.510	0.755	0.886	0.966	6.79%
6	0.091	0.300	0.521	0.652	0.830	0.966	7.17%
7	0.091	0.120	0.317	0.517	0.764	0.966	0.60%
8	0.091	0.364	0.558	0.689	0.835	0.966	8.25%
9	0.091	0.158	0.384	0.542	0.681	0.966	0.06%
10	0.091	0.109	0.246	0.475	0.860	0.966	0.86%
11	0.091	0.312	0.606	0.735	0.872	0.966	7.11%
12	0.091	0.175	0.529	0.651	0.896	0.966	6.55%
13	0.091	0.114	0.367	0.689	0.892	0.966	6.60%
14	0.091	0.316	0.521	0.630	0.858	0.966	7.02%
15	0.091	0.144	0.324	0.505	0.888	0.966	3.93%
16	0.091	0.112	0.243	0.524	0.775	0.966	0.45%
17	0.091	0.404	0.535	0.668	0.807	0.966	7.01%
18	0.091	0.359	0.490	0.672	0.812	0.966	7.30%
19	0.091	0.195	0.373	0.612	0.751	0.966	3.82%
20	0.091	0.104	0.204	0.516	0.799	0.966	0.30%

12–23 Months

	Knot 1	Knot 2	Knot 3	Knot 4	Knot 5	Knot 6	Weight
1	0.091	0.216	0.391	0.617	0.796	0.966	5.68%
2	0.091	0.151	0.287	0.581	0.836	0.966	5.69%
3	0.091	0.121	0.236	0.559	0.794	0.966	4.76%
4	0.091	0.126	0.297	0.416	0.646	0.966	0.00%
5	0.091	0.165	0.394	0.496	0.764	0.966	2.87%
6	0.091	0.277	0.402	0.531	0.900	0.966	5.41%
7	0.091	0.280	0.404	0.546	0.795	0.966	4.81%
8	0.091	0.174	0.385	0.502	0.862	0.966	5.14%
9	0.091	0.246	0.572	0.681	0.844	0.966	6.03%
10	0.091	0.339	0.456	0.558	0.709	0.966	2.47%
11	0.091	0.368	0.478	0.671	0.825	0.966	5.62%
12	0.091	0.246	0.474	0.627	0.801	0.966	14.23%
13	0.091	0.137	0.493	0.722	0.840	0.966	5.24%
14	0.091	0.273	0.578	0.729	0.834	0.966	5.75%
15	0.091	0.345	0.558	0.712	0.889	0.966	5.32%
16	0.091	0.143	0.620	0.729	0.855	0.966	5.58%
17	0.091	0.312	0.470	0.580	0.802	0.966	5.37%
18	0.091	0.292	0.420	0.594	0.796	0.966	5.46%
19	0.091	0.119	0.301	0.454	0.678	0.966	0.00%
20	0.091	0.129	0.492	0.758	0.887	0.966	4.55%

2–4 Years

	Knot 1	Knot 2	Knot 3	Knot 4	Knot 5	Knot 6	Weight
1	0.091	0.252	0.511	0.715	0.892	0.966	5.61%
2	0.091	0.128	0.241	0.576	0.833	0.966	5.62%
3	0.091	0.322	0.452	0.721	0.853	0.966	5.48%
4	0.091	0.145	0.294	0.567	0.672	0.966	1.24%
5	0.091	0.271	0.374	0.587	0.742	0.966	4.46%
6	0.091	0.182	0.305	0.466	0.607	0.966	0.00%
7	0.091	0.136	0.311	0.678	0.857	0.966	4.51%
8	0.091	0.283	0.527	0.688	0.840	0.966	5.35%
9	0.091	0.123	0.319	0.500	0.781	0.966	4.16%
10	0.091	0.141	0.262	0.559	0.848	0.966	5.73%
11	0.091	0.258	0.436	0.642	0.828	0.966	14.33%
12	0.091	0.281	0.519	0.672	0.838	0.966	5.43%
13	0.091	0.292	0.401	0.571	0.833	0.966	5.96%
14	0.091	0.324	0.440	0.733	0.850	0.966	5.52%
15	0.091	0.382	0.485	0.631	0.794	0.966	4.26%
16	0.091	0.118	0.522	0.776	0.893	0.966	5.71%
17	0.091	0.207	0.450	0.597	0.753	0.966	4.92%
18	0.091	0.164	0.418	0.792	0.899	0.966	5.01%
19	0.091	0.158	0.418	0.558	0.843	0.966	6.42%
20	0.091	0.130	0.292	0.434	0.718	0.966	0.27%

J. Severe Wasting in Females: Ensemble MR–BRT Knot Placement

Early Neonatal

	Knot 1	Knot 2	Knot 3	Knot 4	Knot 5	Knot 6	Weight
1	0.091	0.121	0.293	0.533	0.768	0.966	0.33%
2	0.091	0.175	0.344	0.489	0.822	0.966	1.05%
3	0.091	0.283	0.452	0.584	0.823	0.966	8.28%
4	0.091	0.155	0.309	0.483	0.756	0.966	0.00%
5	0.091	0.130	0.277	0.434	0.769	0.966	0.00%
6	0.091	0.200	0.362	0.600	0.711	0.966	1.34%
7	0.091	0.185	0.311	0.536	0.768	0.966	0.57%
8	0.091	0.119	0.337	0.485	0.689	0.966	0.00%
9	0.091	0.113	0.355	0.528	0.874	0.966	5.26%
10	0.091	0.120	0.373	0.561	0.687	0.966	0.02%
11	0.091	0.122	0.279	0.614	0.897	0.966	8.37%
12	0.091	0.309	0.525	0.670	0.801	0.966	22.01%
13	0.091	0.139	0.437	0.687	0.850	0.966	13.05%
14	0.091	0.217	0.396	0.709	0.878	0.966	8.80%
15	0.091	0.173	0.437	0.612	0.734	0.966	4.37%
16	0.091	0.166	0.307	0.419	0.756	0.966	0.00%
17	0.091	0.177	0.305	0.628	0.788	0.966	7.32%
18	0.091	0.112	0.374	0.587	0.793	0.966	5.94%
19	0.091	0.222	0.506	0.620	0.835	0.966	13.29%
20	0.091	0.217	0.336	0.549	0.680	0.966	0.00%

Late Neonatal

	Knot 1	Knot 2	Knot 3	Knot 4	Knot 5	Knot 6	Weight
1	0.091	0.362	0.542	0.676	0.808	0.966	7.47%
2	0.091	0.189	0.504	0.648	0.892	0.966	5.90%
3	0.091	0.228	0.340	0.557	0.872	0.966	4.96%
4	0.091	0.220	0.328	0.732	0.896	0.966	5.15%
5	0.091	0.173	0.467	0.595	0.736	0.966	3.72%
6	0.091	0.120	0.337	0.478	0.618	0.966	0.00%
7	0.091	0.157	0.422	0.700	0.800	0.966	15.09%
8	0.091	0.267	0.447	0.570	0.846	0.966	5.62%
9	0.091	0.293	0.468	0.681	0.881	0.966	5.96%
10	0.091	0.210	0.374	0.604	0.718	0.966	2.61%
11	0.091	0.158	0.382	0.646	0.783	0.966	5.86%
12	0.091	0.155	0.359	0.650	0.878	0.966	6.12%
13	0.091	0.188	0.294	0.597	0.765	0.966	3.39%
14	0.091	0.131	0.487	0.642	0.896	0.966	5.84%
15	0.091	0.406	0.609	0.745	0.881	0.966	5.92%
16	0.091	0.138	0.411	0.529	0.828	0.966	4.15%
17	0.091	0.203	0.578	0.772	0.893	0.966	5.08%
18	0.091	0.165	0.310	0.520	0.683	0.966	0.01%
19	0.091	0.109	0.304	0.444	0.875	0.966	1.08%
20	0.091	0.293	0.440	0.645	0.883	0.966	6.04%

1–5 Months

	Knot 1	Knot 2	Knot 3	Knot 4	Knot 5	Knot 6	Weight
1	0.091	0.212	0.313	0.720	0.880	0.966	7.33%
2	0.091	0.125	0.341	0.626	0.790	0.966	6.96%
3	0.091	0.126	0.277	0.411	0.817	0.966	0.00%
4	0.091	0.225	0.429	0.536	0.697	0.966	0.37%
5	0.091	0.212	0.323	0.443	0.820	0.966	0.22%
6	0.091	0.125	0.249	0.412	0.743	0.966	0.00%
7	0.091	0.115	0.380	0.783	0.889	0.966	5.29%
8	0.091	0.237	0.344	0.543	0.687	0.966	0.04%
9	0.091	0.269	0.521	0.622	0.847	0.966	9.90%
10	0.091	0.143	0.263	0.557	0.784	0.966	1.64%
11	0.091	0.108	0.327	0.477	0.793	0.966	0.40%
12	0.091	0.263	0.513	0.688	0.884	0.966	7.78%
13	0.091	0.132	0.393	0.551	0.872	0.966	6.73%
14	0.091	0.209	0.320	0.739	0.844	0.966	7.85%
15	0.091	0.186	0.368	0.718	0.879	0.966	7.38%
16	0.091	0.133	0.329	0.615	0.778	0.966	5.89%
17	0.091	0.170	0.301	0.707	0.831	0.966	19.52%
18	0.091	0.215	0.327	0.468	0.755	0.966	0.02%
19	0.091	0.140	0.344	0.712	0.866	0.966	7.81%
20	0.091	0.256	0.360	0.522	0.854	0.966	4.87%

6–11 Months

	Knot 1	Knot 2	Knot 3	Knot 4	Knot 5	Knot 6	Weight
1	0.091	0.283	0.385	0.515	0.864	0.966	5.61%
2	0.091	0.208	0.322	0.487	0.595	0.966	0.00%
3	0.091	0.128	0.297	0.716	0.896	0.966	4.78%
4	0.091	0.170	0.280	0.631	0.900	0.966	5.90%
5	0.091	0.153	0.400	0.613	0.791	0.966	6.23%
6	0.091	0.124	0.281	0.408	0.673	0.966	0.00%
7	0.091	0.364	0.588	0.742	0.869	0.966	6.23%
8	0.091	0.119	0.420	0.524	0.678	0.966	0.66%
9	0.091	0.257	0.386	0.597	0.732	0.966	4.51%
10	0.091	0.100	0.418	0.594	0.843	0.966	6.46%
11	0.091	0.232	0.472	0.597	0.716	0.966	4.22%
12	0.091	0.127	0.409	0.527	0.821	0.966	5.36%
13	0.091	0.108	0.218	0.557	0.718	0.966	0.80%
14	0.091	0.147	0.330	0.489	0.636	0.966	0.00%
15	0.091	0.210	0.319	0.484	0.801	0.966	2.44%
16	0.091	0.105	0.465	0.710	0.850	0.966	5.35%
17	0.091	0.181	0.315	0.484	0.871	0.966	4.17%
18	0.091	0.315	0.485	0.685	0.815	0.966	15.57%
19	0.091	0.244	0.390	0.680	0.833	0.966	6.15%
20	0.091	0.211	0.381	0.645	0.818	0.966	15.57%

12–23 Months

	Knot 1	Knot 2	Knot 3	Knot 4	Knot 5	Knot 6	Weight
1	0.091	0.139	0.392	0.496	0.671	0.966	0.05%
2	0.091	0.137	0.475	0.728	0.896	0.966	4.87%
3	0.091	0.330	0.535	0.708	0.842	0.966	5.59%
4	0.091	0.142	0.402	0.528	0.773	0.966	4.87%
5	0.091	0.203	0.343	0.613	0.849	0.966	6.11%
6	0.091	0.171	0.289	0.468	0.650	0.966	0.00%
7	0.091	0.319	0.444	0.583	0.706	0.966	3.15%
8	0.091	0.152	0.440	0.546	0.679	0.966	1.08%
9	0.091	0.295	0.458	0.713	0.871	0.966	5.74%
10	0.091	0.298	0.439	0.620	0.863	0.966	6.08%
11	0.091	0.166	0.497	0.701	0.879	0.966	5.79%
12	0.091	0.260	0.371	0.729	0.893	0.966	4.55%
13	0.091	0.229	0.566	0.696	0.797	0.966	7.56%
14	0.091	0.222	0.427	0.671	0.813	0.966	15.28%
15	0.091	0.178	0.458	0.735	0.847	0.966	5.61%
16	0.091	0.127	0.355	0.494	0.612	0.966	0.00%
17	0.091	0.170	0.604	0.721	0.860	0.966	6.56%
18	0.091	0.141	0.295	0.482	0.852	0.966	5.56%
19	0.091	0.206	0.354	0.587	0.760	0.966	5.81%
20	0.091	0.101	0.513	0.705	0.826	0.966	5.75%

2–4 Years

	Knot 1	Knot 2	Knot 3	Knot 4	Knot 5	Knot 6	Weight
1	0.091	0.116	0.479	0.625	0.833	0.966	7.83%
2	0.091	0.222	0.515	0.713	0.877	0.966	3.38%
3	0.091	0.289	0.500	0.663	0.868	0.966	1.41%
4	0.091	0.234	0.586	0.715	0.829	0.966	0.43%
5	0.091	0.270	0.543	0.754	0.856	0.966	0.61%
6	0.091	0.396	0.534	0.707	0.896	0.966	0.00%
7	0.091	0.250	0.412	0.631	0.740	0.966	2.73%
8	0.091	0.143	0.344	0.447	0.817	0.966	1.63%
9	0.091	0.305	0.507	0.662	0.789	0.966	0.56%
10	0.091	0.121	0.480	0.601	0.846	0.966	7.63%
11	0.091	0.181	0.325	0.639	0.844	0.966	4.12%
12	0.091	0.134	0.455	0.757	0.891	0.966	5.25%
13	0.091	0.182	0.322	0.460	0.895	0.966	7.45%
14	0.091	0.118	0.227	0.509	0.735	0.966	0.00%
15	0.091	0.245	0.402	0.698	0.816	0.966	19.07%
16	0.091	0.279	0.426	0.597	0.837	0.966	6.52%
17	0.091	0.173	0.417	0.668	0.775	0.966	15.37%
18	0.091	0.166	0.310	0.596	0.846	0.966	4.36%
19	0.091	0.187	0.410	0.526	0.811	0.966	5.98%
20	0.091	0.154	0.319	0.606	0.815	0.966	5.68%

K. Extreme Wasting in Males: Ensemble MR–BRT Knot Placement

Early Neonatal

	Knot 1	Knot 2	Knot 3	Knot 4	Knot 5	Knot 6	Weight
1	0.091	0.237	0.383	0.566	0.881	0.966	0.92%
2	0.091	0.126	0.486	0.626	0.797	0.966	8.30%
3	0.091	0.265	0.373	0.486	0.812	0.966	5.85%
4	0.091	0.108	0.302	0.467	0.678	0.966	6.26%
5	0.091	0.168	0.288	0.573	0.868	0.966	0.07%
6	0.091	0.106	0.247	0.663	0.885	0.966	0.84%
7	0.091	0.145	0.373	0.667	0.771	0.966	6.38%
8	0.091	0.113	0.252	0.409	0.860	0.966	5.70%
9	0.091	0.182	0.283	0.386	0.604	0.966	2.46%
10	0.091	0.112	0.273	0.612	0.801	0.966	5.74%
11	0.091	0.474	0.636	0.788	0.891	0.966	4.77%
12	0.091	0.164	0.356	0.483	0.897	0.966	0.00%
13	0.091	0.132	0.364	0.705	0.816	0.966	6.33%
14	0.091	0.346	0.490	0.686	0.812	0.966	5.94%
15	0.091	0.110	0.297	0.487	0.879	0.966	0.00%
16	0.091	0.212	0.379	0.506	0.850	0.966	5.91%
17	0.091	0.218	0.322	0.465	0.665	0.966	5.95%
18	0.091	0.233	0.344	0.525	0.712	0.966	6.49%
19	0.091	0.114	0.460	0.699	0.840	0.966	15.95%
20	0.091	0.234	0.477	0.581	0.858	0.966	6.14%

Late Neonatal

	Knot 1	Knot 2	Knot 3	Knot 4	Knot 5	Knot 6	Weight
1	0.091	0.223	0.406	0.508	0.853	0.966	0.00%
2	0.091	0.139	0.342	0.712	0.834	0.966	4.46%
3	0.091	0.209	0.312	0.551	0.692	0.966	17.29%
4	0.091	0.105	0.640	0.779	0.895	0.966	3.20%
5	0.091	0.220	0.386	0.518	0.751	0.966	6.67%
6	0.091	0.129	0.293	0.619	0.722	0.966	6.78%
7	0.091	0.132	0.286	0.544	0.882	0.966	0.00%
8	0.091	0.106	0.328	0.547	0.757	0.966	6.92%
9	0.091	0.155	0.392	0.624	0.864	0.966	2.02%
10	0.091	0.279	0.385	0.564	0.813	0.966	0.26%
11	0.091	0.129	0.278	0.416	0.628	0.966	3.41%
12	0.091	0.149	0.409	0.520	0.899	0.966	0.00%
13	0.091	0.255	0.431	0.659	0.880	0.966	5.10%
14	0.091	0.136	0.278	0.447	0.778	0.966	7.68%
15	0.091	0.146	0.344	0.557	0.778	0.966	6.87%
16	0.091	0.221	0.623	0.728	0.829	0.966	4.26%
17	0.091	0.198	0.322	0.542	0.764	0.966	7.19%
18	0.091	0.329	0.457	0.667	0.898	0.966	5.83%
19	0.091	0.275	0.463	0.709	0.843	0.966	6.36%
20	0.091	0.119	0.241	0.374	0.712	0.966	5.71%

1–5 Months

	Knot 1	Knot 2	Knot 3	Knot 4	Knot 5	Knot 6	Weight
1	0.091	0.259	0.395	0.497	0.601	0.966	4.86%
2	0.091	0.198	0.458	0.629	0.862	0.966	4.79%
3	0.091	0.108	0.274	0.376	0.578	0.966	2.77%
4	0.091	0.135	0.397	0.698	0.886	0.966	5.14%
5	0.091	0.117	0.235	0.358	0.864	0.966	0.00%
6	0.091	0.365	0.545	0.716	0.841	0.966	5.09%
7	0.091	0.392	0.496	0.667	0.811	0.966	5.15%
8	0.091	0.270	0.431	0.670	0.870	0.966	5.01%
9	0.091	0.191	0.512	0.644	0.855	0.966	5.26%
10	0.091	0.131	0.316	0.480	0.792	0.966	13.82%
11	0.091	0.127	0.286	0.407	0.768	0.966	5.54%
12	0.091	0.136	0.367	0.576	0.760	0.966	5.53%
13	0.091	0.152	0.373	0.624	0.880	0.966	3.57%
14	0.091	0.122	0.440	0.709	0.835	0.966	5.43%
15	0.091	0.131	0.280	0.669	0.867	0.966	3.14%
16	0.091	0.184	0.364	0.791	0.899	0.966	5.49%
17	0.091	0.502	0.636	0.739	0.852	0.966	4.88%
18	0.091	0.166	0.298	0.665	0.820	0.966	3.57%
19	0.091	0.101	0.250	0.422	0.762	0.966	5.53%
20	0.091	0.219	0.439	0.590	0.771	0.966	5.44%

6–11 Months

	Knot 1	Knot 2	Knot 3	Knot 4	Knot 5	Knot 6	Weight
1	0.091	0.113	0.286	0.401	0.701	0.966	7.47%
2	0.091	0.168	0.334	0.452	0.861	0.966	0.00%
3	0.091	0.320	0.433	0.556	0.728	0.966	4.02%
4	0.091	0.106	0.365	0.530	0.700	0.966	18.61%
5	0.091	0.133	0.568	0.685	0.832	0.966	7.04%
6	0.091	0.191	0.425	0.711	0.884	0.966	7.52%
7	0.091	0.260	0.389	0.613	0.778	0.966	5.03%
8	0.091	0.273	0.456	0.556	0.757	0.966	4.44%
9	0.091	0.197	0.305	0.542	0.798	0.966	0.81%
10	0.091	0.143	0.519	0.668	0.800	0.966	7.44%
11	0.091	0.285	0.394	0.594	0.712	0.966	7.22%
12	0.091	0.194	0.488	0.711	0.889	0.966	7.35%
13	0.091	0.194	0.328	0.502	0.836	0.966	0.23%
14	0.091	0.175	0.622	0.732	0.872	0.966	0.28%
15	0.091	0.127	0.292	0.397	0.721	0.966	0.00%
16	0.091	0.135	0.484	0.636	0.777	0.966	7.17%
17	0.091	0.273	0.434	0.563	0.710	0.966	7.25%
18	0.091	0.197	0.343	0.624	0.726	0.966	4.79%
19	0.091	0.165	0.396	0.526	0.784	0.966	1.54%
20	0.091	0.325	0.650	0.757	0.881	0.966	1.80%

12–23 Months

	Knot 1	Knot 2	Knot 3	Knot 4	Knot 5	Knot 6	Weight
1	0.091	0.298	0.418	0.522	0.764	0.966	3.61%
2	0.091	0.145	0.310	0.513	0.735	0.966	0.88%
3	0.091	0.161	0.281	0.511	0.828	0.966	0.63%
4	0.091	0.206	0.439	0.586	0.791	0.966	6.57%
5	0.091	0.128	0.286	0.581	0.757	0.966	2.91%
6	0.091	0.198	0.341	0.589	0.805	0.966	4.48%
7	0.091	0.113	0.370	0.645	0.774	0.966	7.09%
8	0.091	0.178	0.398	0.615	0.837	0.966	6.61%
9	0.091	0.197	0.320	0.423	0.782	0.966	0.02%
10	0.091	0.209	0.362	0.631	0.766	0.966	6.51%
11	0.091	0.367	0.495	0.638	0.759	0.966	19.92%
12	0.091	0.260	0.395	0.755	0.870	0.966	0.00%
13	0.091	0.250	0.359	0.547	0.751	0.966	3.01%
14	0.091	0.231	0.353	0.542	0.702	0.966	2.64%
15	0.091	0.253	0.419	0.548	0.777	0.966	4.55%
16	0.091	0.170	0.412	0.519	0.741	0.966	2.95%
17	0.091	0.136	0.281	0.415	0.664	0.966	8.22%
18	0.091	0.188	0.423	0.649	0.805	0.966	7.97%
19	0.091	0.232	0.429	0.659	0.838	0.966	8.24%
20	0.091	0.125	0.337	0.567	0.877	0.966	3.19%

2–4 Years

	Knot 1	Knot 2	Knot 3	Knot 4	Knot 5	Knot 6	Weight
1	0.091	0.146	0.558	0.760	0.878	0.966	0.07%
2	0.091	0.105	0.501	0.622	0.887	0.966	7.53%
3	0.091	0.184	0.295	0.436	0.552	0.966	4.23%
4	0.091	0.179	0.494	0.734	0.851	0.966	0.00%
5	0.091	0.101	0.256	0.443	0.675	0.966	7.93%
6	0.091	0.227	0.417	0.690	0.881	0.966	7.21%
7	0.091	0.101	0.482	0.635	0.780	0.966	7.39%
8	0.091	0.238	0.408	0.719	0.823	0.966	0.00%
9	0.091	0.335	0.443	0.718	0.869	0.966	0.00%
10	0.091	0.278	0.410	0.554	0.803	0.966	3.07%
11	0.091	0.109	0.293	0.602	0.768	0.966	1.63%
12	0.091	0.204	0.340	0.605	0.733	0.966	2.90%
13	0.091	0.236	0.435	0.611	0.802	0.966	5.77%
14	0.091	0.135	0.448	0.648	0.843	0.966	6.96%
15	0.091	0.192	0.387	0.548	0.774	0.966	1.79%
16	0.091	0.123	0.495	0.661	0.786	0.966	18.83%
17	0.091	0.156	0.257	0.455	0.785	0.966	0.00%
18	0.091	0.228	0.498	0.651	0.762	0.966	18.83%
19	0.091	0.351	0.547	0.766	0.884	0.966	0.32%
20	0.091	0.103	0.443	0.608	0.848	0.966	5.55%

L. Extreme Wasting in Females: Ensemble MR–BRT Knot Placement

Early Neonatal

	Knot 1	Knot 2	Knot 3	Knot 4	Knot 5	Knot 6	Weight
1	0.091	0.164	0.290	0.681	0.875	0.966	6.28%
2	0.091	0.233	0.536	0.645	0.802	0.966	7.98%
3	0.091	0.101	0.369	0.585	0.799	0.966	4.30%
4	0.091	0.280	0.387	0.521	0.807	0.966	3.10%
5	0.091	0.323	0.520	0.664	0.780	0.966	7.88%
6	0.091	0.162	0.271	0.557	0.825	0.966	1.87%
7	0.091	0.101	0.433	0.597	0.788	0.966	6.30%
8	0.091	0.160	0.321	0.436	0.898	0.966	0.12%
9	0.091	0.162	0.311	0.582	0.713	0.966	19.69%
10	0.091	0.371	0.548	0.687	0.802	0.966	7.68%
11	0.091	0.304	0.531	0.655	0.896	0.966	7.88%
12	0.091	0.384	0.570	0.728	0.883	0.966	2.05%
13	0.091	0.240	0.528	0.758	0.882	0.966	0.16%
14	0.091	0.173	0.403	0.554	0.739	0.966	4.30%
15	0.091	0.109	0.375	0.749	0.862	0.966	0.00%
16	0.091	0.107	0.346	0.523	0.773	0.966	1.67%
17	0.091	0.166	0.426	0.746	0.884	0.966	0.00%
18	0.091	0.111	0.445	0.677	0.799	0.966	7.89%
19	0.091	0.347	0.455	0.631	0.746	0.966	7.61%
20	0.091	0.148	0.381	0.543	0.753	0.966	3.23%

Late Neonatal

	Knot 1	Knot 2	Knot 3	Knot 4	Knot 5	Knot 6	Weight
1	0.091	0.309	0.475	0.600	0.776	0.966	6.53%
2	0.091	0.162	0.475	0.679	0.867	0.966	17.78%
3	0.091	0.345	0.487	0.622	0.761	0.966	7.11%
4	0.091	0.330	0.620	0.760	0.873	0.966	0.74%
5	0.091	0.235	0.381	0.572	0.767	0.966	2.76%
6	0.091	0.114	0.334	0.526	0.782	0.966	0.18%
7	0.091	0.283	0.450	0.561	0.780	0.966	4.61%
8	0.091	0.253	0.495	0.610	0.791	0.966	6.97%
9	0.091	0.103	0.341	0.693	0.856	0.966	5.62%
10	0.091	0.162	0.496	0.652	0.818	0.966	9.72%
11	0.091	0.214	0.371	0.639	0.896	0.966	5.05%
12	0.091	0.101	0.248	0.573	0.762	0.966	0.21%
13	0.091	0.120	0.256	0.437	0.818	0.966	0.00%
14	0.091	0.266	0.393	0.587	0.827	0.966	3.92%
15	0.091	0.355	0.543	0.657	0.853	0.966	7.23%
16	0.091	0.222	0.601	0.707	0.835	0.966	6.54%
17	0.091	0.250	0.375	0.478	0.582	0.966	6.41%
18	0.091	0.164	0.358	0.549	0.764	0.966	1.04%
19	0.091	0.174	0.308	0.553	0.836	0.966	0.53%
20	0.091	0.122	0.373	0.726	0.891	0.966	7.04%

1–5 Months

	Knot 1	Knot 2	Knot 3	Knot 4	Knot 5	Knot 6	Weight
1	0.091	0.318	0.607	0.723	0.843	0.966	0.06%
2	0.091	0.193	0.315	0.587	0.761	0.966	1.00%
3	0.091	0.151	0.322	0.537	0.813	0.966	0.03%
4	0.091	0.181	0.570	0.691	0.841	0.966	8.64%
5	0.091	0.172	0.370	0.508	0.609	0.966	8.58%
6	0.091	0.166	0.374	0.546	0.742	0.966	0.55%
7	0.091	0.105	0.241	0.714	0.889	0.966	3.67%
8	0.091	0.120	0.493	0.642	0.864	0.966	9.05%
9	0.091	0.239	0.524	0.694	0.826	0.966	9.34%
10	0.091	0.255	0.383	0.603	0.719	0.966	3.84%
11	0.091	0.259	0.367	0.521	0.870	0.966	0.23%
12	0.091	0.137	0.296	0.464	0.845	0.966	0.00%
13	0.091	0.162	0.330	0.462	0.651	0.966	8.66%
14	0.091	0.202	0.327	0.596	0.705	0.966	21.64%
15	0.091	0.282	0.438	0.555	0.830	0.966	3.56%
16	0.091	0.103	0.353	0.678	0.804	0.966	5.78%
17	0.091	0.538	0.651	0.781	0.896	0.966	6.42%
18	0.091	0.216	0.413	0.667	0.840	0.966	7.87%
19	0.091	0.108	0.425	0.534	0.765	0.966	1.07%
20	0.091	0.193	0.362	0.485	0.881	0.966	0.00%

6–11 Months

	Knot 1	Knot 2	Knot 3	Knot 4	Knot 5	Knot 6	Weight
1	0.091	0.113	0.581	0.705	0.838	0.966	0.00%
2	0.091	0.129	0.285	0.445	0.892	0.966	0.00%
3	0.091	0.211	0.498	0.618	0.882	0.966	8.10%
4	0.091	0.104	0.233	0.613	0.761	0.966	0.75%
5	0.091	0.193	0.345	0.542	0.655	0.966	10.45%
6	0.091	0.134	0.408	0.679	0.827	0.966	7.96%
7	0.091	0.122	0.341	0.546	0.815	0.966	0.48%
8	0.091	0.134	0.314	0.424	0.579	0.966	5.21%
9	0.091	0.106	0.389	0.571	0.785	0.966	2.50%
10	0.091	0.152	0.319	0.476	0.666	0.966	9.31%
11	0.091	0.317	0.447	0.629	0.872	0.966	7.54%
12	0.091	0.145	0.604	0.719	0.881	0.966	0.01%
13	0.091	0.125	0.345	0.658	0.760	0.966	5.80%
14	0.091	0.263	0.400	0.511	0.835	0.966	0.91%
15	0.091	0.182	0.311	0.569	0.674	0.966	20.25%
16	0.091	0.108	0.294	0.664	0.780	0.966	4.42%
17	0.091	0.399	0.505	0.617	0.764	0.966	7.97%
18	0.091	0.170	0.322	0.620	0.784	0.966	3.53%
19	0.091	0.258	0.364	0.617	0.801	0.966	4.76%
20	0.091	0.196	0.370	0.478	0.776	0.966	0.02%

12–23 Months

	Knot 1	Knot 2	Knot 3	Knot 4	Knot 5	Knot 6	Weight
1	0.091	0.129	0.282	0.442	0.887	0.966	0.00%
2	0.091	0.213	0.479	0.604	0.859	0.966	8.35%
3	0.091	0.185	0.497	0.672	0.878	0.966	11.07%
4	0.091	0.182	0.455	0.684	0.887	0.966	9.17%
5	0.091	0.152	0.311	0.651	0.842	0.966	4.56%
6	0.091	0.271	0.501	0.656	0.838	0.966	22.39%
7	0.091	0.202	0.358	0.500	0.700	0.966	8.95%
8	0.091	0.112	0.361	0.532	0.898	0.966	0.43%
9	0.091	0.199	0.442	0.578	0.801	0.966	5.78%
10	0.091	0.175	0.391	0.606	0.832	0.966	5.00%
11	0.091	0.137	0.316	0.439	0.736	0.966	0.00%
12	0.091	0.115	0.329	0.464	0.810	0.966	0.00%
13	0.091	0.281	0.388	0.491	0.706	0.966	0.31%
14	0.091	0.161	0.374	0.476	0.812	0.966	0.02%
15	0.091	0.434	0.558	0.723	0.884	0.966	1.09%
16	0.091	0.129	0.594	0.719	0.899	0.966	0.02%
17	0.091	0.159	0.389	0.608	0.853	0.966	4.98%
18	0.091	0.146	0.474	0.636	0.828	0.966	8.91%
19	0.091	0.120	0.464	0.631	0.792	0.966	8.63%
20	0.091	0.251	0.366	0.510	0.755	0.966	0.34%

2–4 Years

	Knot 1	Knot 2	Knot 3	Knot 4	Knot 5	Knot 6	Weight
1	0.091	0.357	0.533	0.648	0.815	0.966	11.95%
2	0.091	0.132	0.313	0.725	0.858	0.966	0.00%
3	0.091	0.157	0.333	0.641	0.808	0.966	5.91%
4	0.091	0.172	0.408	0.645	0.769	0.966	7.99%
5	0.091	0.216	0.416	0.517	0.851	0.966	3.93%
6	0.091	0.363	0.518	0.722	0.847	0.966	0.89%
7	0.091	0.352	0.504	0.644	0.807	0.966	22.44%
8	0.091	0.117	0.251	0.515	0.843	0.966	0.61%
9	0.091	0.137	0.241	0.355	0.565	0.966	4.37%
10	0.091	0.158	0.360	0.601	0.770	0.966	5.23%
11	0.091	0.179	0.368	0.572	0.734	0.966	4.44%
12	0.091	0.260	0.374	0.483	0.804	0.966	1.81%
13	0.091	0.177	0.411	0.544	0.829	0.966	4.65%
14	0.091	0.232	0.382	0.581	0.760	0.966	5.52%
15	0.091	0.194	0.297	0.401	0.870	0.966	0.02%
16	0.091	0.125	0.431	0.746	0.851	0.966	0.00%
17	0.091	0.273	0.525	0.743	0.869	0.966	0.42%
18	0.091	0.246	0.484	0.602	0.765	0.966	8.98%
19	0.091	0.107	0.491	0.648	0.850	0.966	10.19%
20	0.091	0.356	0.508	0.714	0.845	0.966	0.66%

M. Overall Underweight in Males: Ensemble MR–BRT Knot Placement

Early Neonatal

	Knot 1	Knot 2	Knot 3	Knot 4	Knot 5	Knot 6	Weight
1	0.091	0.156	0.592	0.739	0.849	0.966	5.08%
2	0.091	0.246	0.382	0.660	0.796	0.966	6.21%
3	0.091	0.232	0.448	0.623	0.758	0.966	2.70%
4	0.091	0.255	0.419	0.688	0.862	0.966	6.61%
5	0.091	0.222	0.330	0.525	0.864	0.966	5.74%
6	0.091	0.197	0.494	0.595	0.736	0.966	1.05%
7	0.091	0.196	0.384	0.492	0.838	0.966	3.75%
8	0.091	0.336	0.569	0.687	0.851	0.966	2.80%
9	0.091	0.209	0.617	0.741	0.883	0.966	4.04%
10	0.091	0.128	0.292	0.427	0.845	0.966	1.68%
11	0.091	0.338	0.576	0.740	0.886	0.966	2.56%
12	0.091	0.165	0.352	0.491	0.698	0.966	0.00%
13	0.091	0.147	0.382	0.676	0.815	0.966	16.92%
14	0.091	0.366	0.482	0.741	0.883	0.966	3.24%
15	0.091	0.193	0.373	0.557	0.734	0.966	0.25%
16	0.091	0.182	0.361	0.652	0.861	0.966	16.64%
17	0.091	0.160	0.349	0.468	0.786	0.966	0.44%
18	0.091	0.136	0.295	0.653	0.868	0.966	6.77%
19	0.091	0.116	0.472	0.679	0.900	0.966	7.02%
20	0.091	0.182	0.346	0.516	0.896	0.966	6.49%

Late Neonatal

	Knot 1	Knot 2	Knot 3	Knot 4	Knot 5	Knot 6	Weight
1	0.091	0.195	0.457	0.598	0.722	0.966	4.27%
2	0.091	0.137	0.407	0.652	0.820	0.966	16.18%
3	0.091	0.150	0.291	0.477	0.627	0.966	0.03%
4	0.091	0.360	0.514	0.659	0.765	0.966	5.09%
5	0.091	0.284	0.516	0.636	0.844	0.966	5.98%
6	0.091	0.257	0.407	0.515	0.615	0.966	0.11%
7	0.091	0.225	0.431	0.728	0.846	0.966	7.95%
8	0.091	0.168	0.308	0.502	0.880	0.966	6.47%
9	0.091	0.213	0.417	0.716	0.870	0.966	8.32%
10	0.091	0.126	0.285	0.556	0.689	0.966	2.18%
11	0.091	0.135	0.280	0.622	0.784	0.966	6.29%
12	0.091	0.150	0.273	0.654	0.771	0.966	6.44%
13	0.091	0.109	0.279	0.429	0.566	0.966	0.00%
14	0.091	0.172	0.336	0.495	0.600	0.966	0.01%
15	0.091	0.153	0.442	0.582	0.750	0.966	4.90%
16	0.091	0.189	0.310	0.539	0.674	0.966	1.34%
17	0.091	0.194	0.389	0.649	0.762	0.966	6.12%
18	0.091	0.282	0.391	0.706	0.891	0.966	6.45%
19	0.091	0.298	0.418	0.734	0.856	0.966	6.41%
20	0.091	0.103	0.232	0.728	0.887	0.966	5.45%

1–5 Months

	Knot 1	Knot 2	Knot 3	Knot 4	Knot 5	Knot 6	Weight
1	0.091	0.177	0.311	0.444	0.874	0.966	4.73%
2	0.091	0.340	0.465	0.694	0.891	0.966	5.32%
3	0.091	0.163	0.312	0.512	0.748	0.966	1.28%
4	0.091	0.352	0.455	0.659	0.859	0.966	5.33%
5	0.091	0.114	0.321	0.703	0.813	0.966	7.82%
6	0.091	0.221	0.328	0.466	0.642	0.966	0.00%
7	0.091	0.373	0.515	0.683	0.881	0.966	4.63%
8	0.091	0.272	0.512	0.631	0.786	0.966	4.86%
9	0.091	0.117	0.358	0.556	0.877	0.966	6.57%
10	0.091	0.302	0.474	0.625	0.772	0.966	4.26%
11	0.091	0.199	0.379	0.524	0.864	0.966	5.91%
12	0.091	0.145	0.451	0.682	0.841	0.966	16.14%
13	0.091	0.117	0.237	0.577	0.892	0.966	6.46%
14	0.091	0.346	0.491	0.623	0.773	0.966	3.84%
15	0.091	0.185	0.486	0.602	0.716	0.966	2.39%
16	0.091	0.119	0.299	0.505	0.694	0.966	0.04%
17	0.091	0.153	0.532	0.660	0.783	0.966	5.81%
18	0.091	0.139	0.486	0.603	0.782	0.966	5.12%
19	0.091	0.296	0.398	0.503	0.804	0.966	3.33%
20	0.091	0.110	0.215	0.670	0.830	0.966	6.16%

6–11 Months

	Knot 1	Knot 2	Knot 3	Knot 4	Knot 5	Knot 6	Weight
1	0.091	0.191	0.375	0.486	0.693	0.966	0.00%
2	0.091	0.144	0.301	0.507	0.812	0.966	4.22%
3	0.091	0.133	0.408	0.614	0.854	0.966	19.23%
4	0.091	0.265	0.421	0.539	0.750	0.966	0.85%
5	0.091	0.217	0.319	0.550	0.866	0.966	7.69%
6	0.091	0.142	0.337	0.478	0.697	0.966	0.00%
7	0.091	0.101	0.345	0.608	0.870	0.966	7.47%
8	0.091	0.253	0.460	0.711	0.831	0.966	6.87%
9	0.091	0.374	0.479	0.612	0.844	0.966	3.78%
10	0.091	0.339	0.474	0.651	0.830	0.966	4.63%
11	0.091	0.219	0.339	0.568	0.818	0.966	6.30%
12	0.091	0.177	0.292	0.606	0.812	0.966	7.46%
13	0.091	0.335	0.436	0.540	0.740	0.966	0.34%
14	0.091	0.102	0.499	0.701	0.831	0.966	7.77%
15	0.091	0.131	0.270	0.735	0.843	0.966	3.90%
16	0.091	0.285	0.435	0.625	0.743	0.966	2.61%
17	0.091	0.384	0.584	0.779	0.899	0.966	2.27%
18	0.091	0.263	0.437	0.647	0.887	0.966	6.86%
19	0.091	0.216	0.332	0.499	0.706	0.966	0.00%
20	0.091	0.189	0.457	0.689	0.818	0.966	7.75%

12–23 Months

	Knot 1	Knot 2	Knot 3	Knot 4	Knot 5	Knot 6	Weight
1	0.091	0.107	0.281	0.464	0.625	0.966	0.00%
2	0.091	0.158	0.279	0.391	0.656	0.966	0.00%
3	0.091	0.134	0.323	0.714	0.820	0.966	6.50%
4	0.091	0.234	0.578	0.743	0.890	0.966	6.51%
5	0.091	0.143	0.351	0.741	0.873	0.966	7.95%
6	0.091	0.194	0.461	0.601	0.788	0.966	5.23%
7	0.091	0.107	0.252	0.374	0.656	0.966	0.00%
8	0.091	0.120	0.271	0.396	0.606	0.966	0.00%
9	0.091	0.430	0.557	0.673	0.835	0.966	5.44%
10	0.091	0.113	0.428	0.617	0.792	0.966	5.50%
11	0.091	0.296	0.416	0.517	0.824	0.966	4.87%
12	0.091	0.165	0.455	0.618	0.753	0.966	4.75%
13	0.091	0.108	0.422	0.533	0.852	0.966	5.75%
14	0.091	0.246	0.359	0.709	0.854	0.966	6.67%
15	0.091	0.152	0.350	0.761	0.899	0.966	16.35%
16	0.091	0.136	0.421	0.534	0.899	0.966	6.36%
17	0.091	0.127	0.271	0.421	0.575	0.966	0.00%
18	0.091	0.179	0.460	0.656	0.848	0.966	6.45%
19	0.091	0.189	0.323	0.679	0.838	0.966	6.54%
20	0.091	0.148	0.309	0.479	0.864	0.966	5.14%

2–4 Years

	Knot 1	Knot 2	Knot 3	Knot 4	Knot 5	Knot 6	Weight
1	0.091	0.202	0.330	0.483	0.645	0.966	0.12%
2	0.091	0.250	0.384	0.534	0.844	0.966	4.60%
3	0.091	0.214	0.388	0.528	0.754	0.966	2.72%
4	0.091	0.135	0.527	0.687	0.885	0.966	5.79%
5	0.091	0.193	0.325	0.631	0.790	0.966	4.69%
6	0.091	0.194	0.434	0.563	0.838	0.966	4.84%
7	0.091	0.257	0.459	0.568	0.759	0.966	3.51%
8	0.091	0.226	0.371	0.712	0.885	0.966	9.59%
9	0.091	0.374	0.483	0.699	0.881	0.966	5.55%
10	0.091	0.277	0.565	0.720	0.868	0.966	5.61%
11	0.091	0.161	0.262	0.505	0.646	0.966	0.16%
12	0.091	0.123	0.545	0.741	0.881	0.966	14.48%
13	0.091	0.125	0.273	0.402	0.566	0.966	0.00%
14	0.091	0.363	0.563	0.718	0.849	0.966	5.38%
15	0.091	0.104	0.272	0.711	0.816	0.966	5.58%
16	0.091	0.443	0.577	0.699	0.865	0.966	5.18%
17	0.091	0.134	0.386	0.746	0.847	0.966	5.98%
18	0.091	0.387	0.496	0.714	0.860	0.966	5.46%
19	0.091	0.416	0.611	0.729	0.898	0.966	5.32%
20	0.091	0.162	0.294	0.645	0.837	0.966	5.43%

N. Overall Underweight in Females: Ensemble MR–BRT Knot Placement

Early Neonatal

	Knot 1	Knot 2	Knot 3	Knot 4	Knot 5	Knot 6	Weight
1	0.091	0.146	0.369	0.555	0.802	0.966	5.07%
2	0.091	0.225	0.514	0.693	0.875	0.966	5.63%
3	0.091	0.125	0.338	0.690	0.799	0.966	14.21%
4	0.091	0.242	0.435	0.599	0.883	0.966	5.52%
5	0.091	0.320	0.444	0.569	0.788	0.966	4.91%
6	0.091	0.238	0.466	0.737	0.841	0.966	5.87%
7	0.091	0.146	0.372	0.526	0.677	0.966	2.29%
8	0.091	0.177	0.508	0.639	0.808	0.966	5.69%
9	0.091	0.139	0.289	0.562	0.730	0.966	3.89%
10	0.091	0.237	0.475	0.598	0.759	0.966	4.93%
11	0.091	0.134	0.299	0.488	0.680	0.966	1.74%
12	0.091	0.100	0.217	0.320	0.497	0.966	0.00%
13	0.091	0.271	0.397	0.595	0.820	0.966	5.41%
14	0.091	0.220	0.329	0.486	0.679	0.966	1.74%
15	0.091	0.313	0.512	0.676	0.826	0.966	5.62%
16	0.091	0.116	0.405	0.519	0.892	0.966	5.35%
17	0.091	0.209	0.413	0.666	0.879	0.966	5.73%
18	0.091	0.229	0.462	0.596	0.796	0.966	5.31%
19	0.091	0.255	0.441	0.611	0.814	0.966	5.49%
20	0.091	0.148	0.422	0.637	0.786	0.966	5.59%

Late Neonatal

	Knot 1	Knot 2	Knot 3	Knot 4	Knot 5	Knot 6	Weight
1	0.091	0.364	0.614	0.758	0.868	0.966	5.32%
2	0.091	0.386	0.652	0.759	0.870	0.966	5.32%
3	0.091	0.224	0.454	0.558	0.693	0.966	1.56%
4	0.091	0.176	0.283	0.622	0.877	0.966	6.29%
5	0.091	0.209	0.433	0.649	0.779	0.966	5.92%
6	0.091	0.290	0.399	0.623	0.739	0.966	4.17%
7	0.091	0.174	0.381	0.730	0.847	0.966	15.73%
8	0.091	0.147	0.305	0.415	0.665	0.966	0.00%
9	0.091	0.165	0.366	0.609	0.798	0.966	5.69%
10	0.091	0.155	0.366	0.578	0.847	0.966	5.86%
11	0.091	0.376	0.592	0.761	0.874	0.966	5.18%
12	0.091	0.171	0.285	0.406	0.601	0.966	0.00%
13	0.091	0.121	0.573	0.698	0.894	0.966	5.88%
14	0.091	0.178	0.320	0.514	0.641	0.966	0.04%
15	0.091	0.329	0.561	0.702	0.876	0.966	5.50%
16	0.091	0.189	0.382	0.651	0.798	0.966	6.34%
17	0.091	0.305	0.406	0.637	0.740	0.966	4.51%
18	0.091	0.135	0.343	0.647	0.767	0.966	5.80%
19	0.091	0.115	0.482	0.656	0.871	0.966	6.31%
20	0.091	0.188	0.330	0.473	0.863	0.966	4.56%

1–5 Months

	Knot 1	Knot 2	Knot 3	Knot 4	Knot 5	Knot 6	Weight
1	0.091	0.273	0.547	0.705	0.895	0.966	5.10%
2	0.091	0.394	0.576	0.688	0.803	0.966	5.30%
3	0.091	0.198	0.370	0.624	0.742	0.966	3.82%
4	0.091	0.175	0.602	0.720	0.857	0.966	5.86%
5	0.091	0.171	0.309	0.634	0.877	0.966	6.34%
6	0.091	0.147	0.387	0.636	0.764	0.966	5.02%
7	0.091	0.179	0.492	0.789	0.896	0.966	5.11%
8	0.091	0.181	0.296	0.613	0.834	0.966	6.08%
9	0.091	0.101	0.526	0.673	0.852	0.966	14.65%
10	0.091	0.204	0.476	0.625	0.851	0.966	5.85%
11	0.091	0.225	0.408	0.578	0.831	0.966	5.16%
12	0.091	0.142	0.413	0.639	0.746	0.966	4.53%
13	0.091	0.433	0.588	0.710	0.890	0.966	4.20%
14	0.091	0.113	0.307	0.592	0.852	0.966	5.74%
15	0.091	0.263	0.490	0.646	0.808	0.966	5.73%
16	0.091	0.300	0.452	0.562	0.682	0.966	0.52%
17	0.091	0.198	0.309	0.468	0.655	0.966	0.00%
18	0.091	0.324	0.658	0.783	0.893	0.966	5.34%
19	0.091	0.108	0.265	0.609	0.814	0.966	5.65%
20	0.091	0.117	0.296	0.468	0.616	0.966	0.00%

6–11 Months

	Knot 1	Knot 2	Knot 3	Knot 4	Knot 5	Knot 6	Weight
1	0.091	0.190	0.313	0.668	0.879	0.966	8.73%
2	0.091	0.137	0.348	0.579	0.719	0.966	0.05%
3	0.091	0.203	0.378	0.643	0.834	0.966	18.25%
4	0.091	0.228	0.467	0.638	0.795	0.966	6.27%
5	0.091	0.172	0.578	0.681	0.810	0.966	8.65%
6	0.091	0.297	0.481	0.590	0.872	0.966	5.22%
7	0.091	0.112	0.606	0.720	0.852	0.966	7.24%
8	0.091	0.539	0.664	0.789	0.898	0.966	2.41%
9	0.091	0.117	0.337	0.475	0.827	0.966	1.21%
10	0.091	0.361	0.496	0.714	0.831	0.966	5.94%
11	0.091	0.497	0.631	0.765	0.896	0.966	2.13%
12	0.091	0.266	0.389	0.572	0.773	0.966	1.61%
13	0.091	0.145	0.575	0.789	0.889	0.966	6.17%
14	0.091	0.194	0.445	0.582	0.745	0.966	0.89%
15	0.091	0.183	0.381	0.610	0.866	0.966	7.30%
16	0.091	0.237	0.488	0.629	0.813	0.966	6.36%
17	0.091	0.149	0.289	0.545	0.876	0.966	5.81%
18	0.091	0.185	0.370	0.560	0.781	0.966	1.89%
19	0.091	0.158	0.283	0.456	0.742	0.966	0.00%
20	0.091	0.266	0.406	0.550	0.830	0.966	3.86%

12–23 Months

	Knot 1	Knot 2	Knot 3	Knot 4	Knot 5	Knot 6	Weight
1	0.091	0.118	0.239	0.606	0.767	0.966	3.73%
2	0.091	0.127	0.413	0.594	0.737	0.966	2.06%
3	0.091	0.115	0.325	0.480	0.860	0.966	3.61%
4	0.091	0.259	0.362	0.512	0.852	0.966	4.32%
5	0.091	0.161	0.558	0.699	0.831	0.966	17.11%
6	0.091	0.298	0.524	0.713	0.865	0.966	6.84%
7	0.091	0.208	0.432	0.754	0.890	0.966	6.79%
8	0.091	0.159	0.317	0.424	0.695	0.966	0.00%
9	0.091	0.385	0.491	0.603	0.760	0.966	3.15%
10	0.091	0.111	0.315	0.631	0.893	0.966	7.35%
11	0.091	0.259	0.425	0.632	0.811	0.966	6.18%
12	0.091	0.133	0.434	0.650	0.762	0.966	5.35%
13	0.091	0.106	0.220	0.696	0.895	0.966	6.72%
14	0.091	0.139	0.306	0.471	0.782	0.966	0.52%
15	0.091	0.134	0.388	0.558	0.740	0.966	1.13%
16	0.091	0.147	0.574	0.719	0.836	0.966	17.11%
17	0.091	0.110	0.335	0.606	0.849	0.966	6.71%
18	0.091	0.154	0.369	0.572	0.677	0.966	0.04%
19	0.091	0.136	0.295	0.397	0.779	0.966	0.01%
20	0.091	0.170	0.337	0.553	0.751	0.966	1.26%

2–4 Years

	Knot 1	Knot 2	Knot 3	Knot 4	Knot 5	Knot 6	Weight
1	0.091	0.242	0.354	0.587	0.896	0.966	6.01%
2	0.091	0.149	0.320	0.593	0.898	0.966	6.12%
3	0.091	0.381	0.648	0.797	0.898	0.966	6.20%
4	0.091	0.191	0.570	0.717	0.869	0.966	8.86%
5	0.091	0.215	0.446	0.726	0.871	0.966	16.29%
6	0.091	0.274	0.413	0.571	0.846	0.966	5.35%
7	0.091	0.112	0.252	0.495	0.627	0.966	0.00%
8	0.091	0.199	0.320	0.433	0.601	0.966	0.00%
9	0.091	0.107	0.446	0.614	0.879	0.966	6.33%
10	0.091	0.235	0.403	0.510	0.885	0.966	5.14%
11	0.091	0.255	0.418	0.542	0.708	0.966	1.04%
12	0.091	0.305	0.572	0.697	0.894	0.966	6.51%
13	0.091	0.144	0.277	0.427	0.718	0.966	0.02%
14	0.091	0.254	0.458	0.598	0.802	0.966	4.93%
15	0.091	0.251	0.578	0.732	0.839	0.966	9.80%
16	0.091	0.124	0.338	0.623	0.822	0.966	5.71%
17	0.091	0.187	0.322	0.485	0.716	0.966	0.30%
18	0.091	0.129	0.345	0.526	0.661	0.966	0.08%
19	0.091	0.260	0.405	0.509	0.891	0.966	5.19%
20	0.091	0.133	0.307	0.678	0.793	0.966	6.11%

O. Severe Underweight in Males: Ensemble MR–BRT Knot Placement

Early Neonatal

	Knot 1	Knot 2	Knot 3	Knot 4	Knot 5	Knot 6	Weight
1	0.091	0.207	0.522	0.630	0.871	0.966	7.09%
2	0.091	0.288	0.399	0.522	0.799	0.966	2.99%
3	0.091	0.276	0.377	0.541	0.797	0.966	3.22%
4	0.091	0.173	0.302	0.663	0.797	0.966	6.94%
5	0.091	0.174	0.414	0.555	0.662	0.966	0.19%
6	0.091	0.263	0.411	0.594	0.713	0.966	2.02%
7	0.091	0.543	0.648	0.788	0.899	0.966	17.72%
8	0.091	0.166	0.307	0.624	0.871	0.966	6.08%
9	0.091	0.101	0.210	0.524	0.798	0.966	1.47%
10	0.091	0.167	0.267	0.654	0.850	0.966	6.76%
11	0.091	0.168	0.301	0.613	0.749	0.966	3.37%
12	0.091	0.150	0.497	0.697	0.868	0.966	7.40%
13	0.091	0.161	0.311	0.603	0.880	0.966	5.60%
14	0.091	0.192	0.530	0.635	0.774	0.966	7.57%
15	0.091	0.277	0.377	0.658	0.867	0.966	6.92%
16	0.091	0.104	0.223	0.364	0.668	0.966	0.00%
17	0.091	0.161	0.272	0.574	0.747	0.966	1.80%
18	0.091	0.143	0.432	0.537	0.742	0.966	1.76%
19	0.091	0.103	0.444	0.563	0.871	0.966	5.46%
20	0.091	0.304	0.572	0.773	0.886	0.966	5.65%

Late Neonatal

	Knot 1	Knot 2	Knot 3	Knot 4	Knot 5	Knot 6	Weight
1	0.091	0.115	0.239	0.633	0.842	0.966	5.77%
2	0.091	0.162	0.447	0.582	0.780	0.966	4.65%
3	0.091	0.247	0.457	0.627	0.826	0.966	6.34%
4	0.091	0.123	0.274	0.403	0.570	0.966	0.00%
5	0.091	0.107	0.283	0.662	0.790	0.966	6.15%
6	0.091	0.146	0.483	0.782	0.886	0.966	5.13%
7	0.091	0.158	0.553	0.707	0.809	0.966	15.66%
8	0.091	0.137	0.327	0.664	0.875	0.966	6.20%
9	0.091	0.172	0.470	0.741	0.885	0.966	5.81%
10	0.091	0.103	0.379	0.518	0.891	0.966	4.23%
11	0.091	0.178	0.321	0.507	0.654	0.966	0.07%
12	0.091	0.140	0.241	0.426	0.831	0.966	1.12%
13	0.091	0.302	0.528	0.635	0.757	0.966	6.26%
14	0.091	0.468	0.581	0.782	0.895	0.966	5.54%
15	0.091	0.155	0.264	0.623	0.732	0.966	3.26%
16	0.091	0.222	0.329	0.450	0.584	0.966	0.00%
17	0.091	0.264	0.628	0.739	0.870	0.966	6.09%
18	0.091	0.408	0.513	0.649	0.824	0.966	7.36%
19	0.091	0.234	0.452	0.782	0.894	0.966	4.98%
20	0.091	0.172	0.471	0.594	0.791	0.966	5.38%

1–5 Months

	Knot 1	Knot 2	Knot 3	Knot 4	Knot 5	Knot 6	Weight
1	0.091	0.146	0.530	0.697	0.860	0.966	6.51%
2	0.091	0.130	0.254	0.478	0.669	0.966	0.00%
3	0.091	0.430	0.568	0.697	0.867	0.966	6.18%
4	0.091	0.100	0.386	0.762	0.865	0.966	5.26%
5	0.091	0.238	0.338	0.486	0.890	0.966	2.63%
6	0.091	0.139	0.537	0.678	0.800	0.966	16.70%
7	0.091	0.111	0.331	0.484	0.876	0.966	2.35%
8	0.091	0.147	0.419	0.665	0.789	0.966	6.68%
9	0.091	0.324	0.572	0.751	0.867	0.966	5.43%
10	0.091	0.164	0.362	0.494	0.649	0.966	0.00%
11	0.091	0.269	0.412	0.513	0.865	0.966	3.57%
12	0.091	0.157	0.307	0.606	0.840	0.966	4.78%
13	0.091	0.208	0.321	0.666	0.860	0.966	6.00%
14	0.091	0.212	0.494	0.636	0.820	0.966	6.62%
15	0.091	0.128	0.288	0.415	0.611	0.966	0.00%
16	0.091	0.102	0.344	0.630	0.761	0.966	4.29%
17	0.091	0.362	0.469	0.648	0.796	0.966	6.72%
18	0.091	0.187	0.388	0.602	0.838	0.966	5.13%
19	0.091	0.184	0.327	0.688	0.814	0.966	6.76%
20	0.091	0.274	0.394	0.580	0.813	0.966	4.39%

6–11 Months

	Knot 1	Knot 2	Knot 3	Knot 4	Knot 5	Knot 6	Weight
1	0.091	0.432	0.537	0.641	0.807	0.966	7.14%
2	0.091	0.352	0.497	0.735	0.874	0.966	5.73%
3	0.091	0.171	0.339	0.536	0.858	0.966	4.08%
4	0.091	0.212	0.374	0.697	0.811	0.966	7.29%
5	0.091	0.144	0.254	0.582	0.828	0.966	4.58%
6	0.091	0.223	0.545	0.695	0.850	0.966	6.78%
7	0.091	0.152	0.319	0.466	0.575	0.966	0.00%
8	0.091	0.285	0.409	0.598	0.712	0.966	2.61%
9	0.091	0.160	0.553	0.653	0.770	0.966	17.76%
10	0.091	0.142	0.455	0.748	0.887	0.966	5.38%
11	0.091	0.183	0.352	0.512	0.758	0.966	1.44%
12	0.091	0.182	0.306	0.638	0.772	0.966	5.58%
13	0.091	0.177	0.352	0.500	0.726	0.966	0.53%
14	0.091	0.144	0.267	0.509	0.844	0.966	2.75%
15	0.091	0.147	0.288	0.417	0.724	0.966	0.01%
16	0.091	0.222	0.338	0.668	0.877	0.966	6.28%
17	0.091	0.409	0.536	0.639	0.809	0.966	7.10%
18	0.091	0.146	0.498	0.611	0.735	0.966	4.92%
19	0.091	0.122	0.308	0.533	0.823	0.966	3.30%
20	0.091	0.137	0.359	0.681	0.846	0.966	6.74%

12–23 Months

	Knot 1	Knot 2	Knot 3	Knot 4	Knot 5	Knot 6	Weight
1	0.091	0.236	0.343	0.593	0.850	0.966	3.30%
2	0.091	0.158	0.272	0.453	0.722	0.966	0.00%
3	0.091	0.173	0.377	0.575	0.784	0.966	1.05%
4	0.091	0.279	0.540	0.648	0.876	0.966	8.89%
5	0.091	0.119	0.404	0.539	0.828	0.966	1.48%
6	0.091	0.240	0.409	0.728	0.846	0.966	8.83%
7	0.091	0.141	0.299	0.570	0.810	0.966	0.84%
8	0.091	0.103	0.219	0.672	0.777	0.966	2.24%
9	0.091	0.156	0.513	0.636	0.828	0.966	7.60%
10	0.091	0.338	0.544	0.688	0.818	0.966	9.67%
11	0.091	0.205	0.353	0.658	0.899	0.966	7.13%
12	0.091	0.187	0.309	0.485	0.727	0.966	0.00%
13	0.091	0.122	0.315	0.676	0.878	0.966	6.90%
14	0.091	0.122	0.274	0.616	0.795	0.966	1.40%
15	0.091	0.259	0.571	0.723	0.836	0.966	22.07%
16	0.091	0.328	0.431	0.606	0.746	0.966	1.51%
17	0.091	0.135	0.292	0.495	0.688	0.966	0.00%
18	0.091	0.155	0.320	0.758	0.871	0.966	8.18%
19	0.091	0.432	0.533	0.753	0.861	0.966	8.78%
20	0.091	0.333	0.453	0.562	0.713	0.966	0.15%

2–4 Years

	Knot 1	Knot 2	Knot 3	Knot 4	Knot 5	Knot 6	Weight
1	0.091	0.152	0.365	0.495	0.771	0.966	0.91%
2	0.091	0.238	0.396	0.561	0.685	0.966	0.55%
3	0.091	0.261	0.409	0.689	0.889	0.966	7.69%
4	0.091	0.208	0.312	0.436	0.570	0.966	0.00%
5	0.091	0.211	0.381	0.647	0.798	0.966	5.72%
6	0.091	0.115	0.478	0.734	0.845	0.966	19.38%
7	0.091	0.147	0.255	0.691	0.825	0.966	6.35%
8	0.091	0.150	0.282	0.480	0.591	0.966	0.00%
9	0.091	0.114	0.276	0.426	0.576	0.966	0.00%
10	0.091	0.323	0.434	0.576	0.750	0.966	2.73%
11	0.091	0.380	0.543	0.654	0.896	0.966	7.73%
12	0.091	0.124	0.439	0.735	0.848	0.966	8.77%
13	0.091	0.263	0.589	0.735	0.870	0.966	8.03%
14	0.091	0.357	0.610	0.752	0.877	0.966	7.75%
15	0.091	0.139	0.247	0.452	0.727	0.966	0.02%
16	0.091	0.435	0.596	0.772	0.891	0.966	7.57%
17	0.091	0.299	0.450	0.654	0.790	0.966	6.27%
18	0.091	0.207	0.313	0.421	0.717	0.966	0.01%
19	0.091	0.263	0.368	0.583	0.854	0.966	4.94%
20	0.091	0.196	0.471	0.595	0.818	0.966	5.58%

P. Severe Underweight in Females: Ensemble MR–BRT Knot Placement

Early Neonatal

	Knot 1	Knot 2	Knot 3	Knot 4	Knot 5	Knot 6	Weight
1	0.091	0.123	0.236	0.569	0.700	0.966	0.10%
2	0.091	0.135	0.343	0.536	0.864	0.966	3.56%
3	0.091	0.211	0.321	0.505	0.851	0.966	1.94%
4	0.091	0.119	0.443	0.602	0.844	0.966	8.04%
5	0.091	0.391	0.541	0.650	0.877	0.966	23.21%
6	0.091	0.285	0.469	0.607	0.782	0.966	6.91%
7	0.091	0.152	0.294	0.481	0.584	0.966	0.00%
8	0.091	0.143	0.322	0.525	0.713	0.966	0.09%
9	0.091	0.472	0.616	0.730	0.898	0.966	9.37%
10	0.091	0.151	0.333	0.673	0.866	0.966	9.28%
11	0.091	0.210	0.390	0.536	0.639	0.966	0.00%
12	0.091	0.255	0.398	0.539	0.663	0.966	0.04%
13	0.091	0.136	0.330	0.612	0.719	0.966	1.52%
14	0.091	0.138	0.256	0.450	0.639	0.966	0.00%
15	0.091	0.167	0.311	0.507	0.744	0.966	0.18%
16	0.091	0.245	0.420	0.624	0.768	0.966	6.03%
17	0.091	0.112	0.285	0.431	0.674	0.966	0.00%
18	0.091	0.282	0.453	0.567	0.673	0.966	0.41%
19	0.091	0.232	0.335	0.593	0.883	0.966	6.11%
20	0.091	0.336	0.485	0.653	0.858	0.966	23.21%

Late Neonatal

	Knot 1	Knot 2	Knot 3	Knot 4	Knot 5	Knot 6	Weight
1	0.091	0.142	0.290	0.477	0.787	0.966	0.11%
2	0.091	0.430	0.531	0.632	0.758	0.966	8.54%
3	0.091	0.151	0.497	0.635	0.818	0.966	10.32%
4	0.091	0.257	0.396	0.628	0.869	0.966	8.58%
5	0.091	0.287	0.568	0.692	0.882	0.966	11.25%
6	0.091	0.194	0.355	0.556	0.798	0.966	2.40%
7	0.091	0.110	0.218	0.585	0.751	0.966	0.49%
8	0.091	0.197	0.299	0.412	0.725	0.966	0.00%
9	0.091	0.211	0.318	0.440	0.865	0.966	0.48%
10	0.091	0.179	0.389	0.525	0.800	0.966	1.83%
11	0.091	0.292	0.406	0.578	0.895	0.966	6.59%
12	0.091	0.125	0.267	0.623	0.727	0.966	1.10%
13	0.091	0.277	0.459	0.662	0.787	0.966	10.08%
14	0.091	0.158	0.445	0.646	0.861	0.966	9.96%
15	0.091	0.179	0.318	0.480	0.613	0.966	0.00%
16	0.091	0.135	0.252	0.433	0.835	0.966	0.08%
17	0.091	0.219	0.321	0.590	0.722	0.966	0.63%
18	0.091	0.197	0.450	0.559	0.670	0.966	0.12%
19	0.091	0.212	0.371	0.532	0.817	0.966	2.24%
20	0.091	0.246	0.556	0.665	0.789	0.966	25.20%

1–5 Months

	Knot 1	Knot 2	Knot 3	Knot 4	Knot 5	Knot 6	Weight
1	0.091	0.146	0.289	0.443	0.599	0.966	0.00%
2	0.091	0.146	0.284	0.451	0.737	0.966	0.01%
3	0.091	0.233	0.447	0.663	0.801	0.966	20.72%
4	0.091	0.139	0.365	0.662	0.782	0.966	7.26%
5	0.091	0.212	0.423	0.554	0.864	0.966	5.48%
6	0.091	0.120	0.275	0.654	0.843	0.966	6.87%
7	0.091	0.261	0.675	0.783	0.894	0.966	7.43%
8	0.091	0.237	0.338	0.763	0.878	0.966	8.29%
9	0.091	0.101	0.422	0.547	0.660	0.966	0.09%
10	0.091	0.296	0.419	0.529	0.798	0.966	2.83%
11	0.091	0.262	0.371	0.508	0.767	0.966	0.82%
12	0.091	0.141	0.555	0.758	0.868	0.966	9.46%
13	0.091	0.286	0.438	0.551	0.891	0.966	5.90%
14	0.091	0.131	0.490	0.623	0.856	0.966	10.23%
15	0.091	0.207	0.335	0.490	0.706	0.966	0.03%
16	0.091	0.101	0.214	0.516	0.680	0.966	0.00%
17	0.091	0.109	0.504	0.777	0.885	0.966	7.53%
18	0.091	0.238	0.471	0.587	0.823	0.966	7.01%
19	0.091	0.260	0.382	0.546	0.652	0.966	0.03%
20	0.091	0.177	0.284	0.394	0.640	0.966	0.00%

6–11 Months

	Knot 1	Knot 2	Knot 3	Knot 4	Knot 5	Knot 6	Weight
1	0.091	0.312	0.424	0.532	0.719	0.966	1.01%
2	0.091	0.348	0.488	0.607	0.882	0.966	5.63%
3	0.091	0.189	0.385	0.657	0.874	0.966	5.79%
4	0.091	0.210	0.438	0.664	0.871	0.966	6.04%
5	0.091	0.112	0.540	0.662	0.776	0.966	6.45%
6	0.091	0.202	0.450	0.628	0.786	0.966	5.12%
7	0.091	0.435	0.541	0.703	0.844	0.966	7.83%
8	0.091	0.207	0.498	0.620	0.890	0.966	5.73%
9	0.091	0.111	0.242	0.613	0.851	0.966	4.13%
10	0.091	0.162	0.462	0.709	0.822	0.966	15.09%
11	0.091	0.290	0.439	0.723	0.887	0.966	5.76%
12	0.091	0.147	0.342	0.535	0.850	0.966	3.01%
13	0.091	0.276	0.398	0.576	0.808	0.966	3.93%
14	0.091	0.253	0.452	0.620	0.772	0.966	4.65%
15	0.091	0.149	0.328	0.607	0.797	0.966	3.77%
16	0.091	0.315	0.430	0.587	0.784	0.966	3.96%
17	0.091	0.213	0.387	0.553	0.794	0.966	2.86%
18	0.091	0.292	0.411	0.597	0.812	0.966	4.67%
19	0.091	0.364	0.495	0.632	0.743	0.966	4.57%
20	0.091	0.202	0.320	0.421	0.560	0.966	0.00%

12–23 Months

	Knot 1	Knot 2	Knot 3	Knot 4	Knot 5	Knot 6	Weight
1	0.091	0.104	0.317	0.504	0.683	0.966	0.41%
2	0.091	0.232	0.588	0.693	0.840	0.966	20.84%
3	0.091	0.119	0.334	0.495	0.859	0.966	4.29%
4	0.091	0.133	0.369	0.751	0.893	0.966	8.40%
5	0.091	0.256	0.368	0.479	0.647	0.966	0.12%
6	0.091	0.127	0.311	0.601	0.851	0.966	6.86%
7	0.091	0.189	0.409	0.635	0.813	0.966	7.58%
8	0.091	0.136	0.473	0.584	0.748	0.966	5.05%
9	0.091	0.101	0.290	0.725	0.871	0.966	8.90%
10	0.091	0.168	0.432	0.533	0.643	0.966	0.63%
11	0.091	0.108	0.379	0.510	0.729	0.966	1.80%
12	0.091	0.146	0.268	0.370	0.711	0.966	0.01%
13	0.091	0.121	0.362	0.511	0.778	0.966	3.10%
14	0.091	0.116	0.446	0.575	0.900	0.966	7.62%
15	0.091	0.261	0.408	0.613	0.779	0.966	6.34%
16	0.091	0.120	0.273	0.419	0.567	0.966	0.00%
17	0.091	0.171	0.398	0.579	0.710	0.966	3.03%
18	0.091	0.129	0.285	0.629	0.811	0.966	6.68%
19	0.091	0.171	0.544	0.673	0.788	0.966	8.34%
20	0.091	0.148	0.273	0.375	0.536	0.966	0.00%

2–4 Years

	Knot 1	Knot 2	Knot 3	Knot 4	Knot 5	Knot 6	Weight
1	0.091	0.265	0.375	0.527	0.848	0.966	4.15%
2	0.091	0.339	0.515	0.645	0.773	0.966	5.51%
3	0.091	0.116	0.346	0.722	0.869	0.966	16.40%
4	0.091	0.179	0.366	0.585	0.884	0.966	5.63%
5	0.091	0.202	0.399	0.659	0.787	0.966	5.74%
6	0.091	0.138	0.426	0.527	0.695	0.966	0.89%
7	0.091	0.212	0.487	0.590	0.818	0.966	5.28%
8	0.091	0.154	0.401	0.572	0.789	0.966	3.96%
9	0.091	0.114	0.267	0.607	0.844	0.966	4.90%
10	0.091	0.163	0.284	0.577	0.821	0.966	3.94%
11	0.091	0.209	0.336	0.518	0.676	0.966	0.20%
12	0.091	0.128	0.326	0.628	0.805	0.966	5.18%
13	0.091	0.149	0.470	0.583	0.836	0.966	5.42%
14	0.091	0.265	0.395	0.750	0.884	0.966	6.61%
15	0.091	0.299	0.433	0.717	0.881	0.966	7.40%
16	0.091	0.176	0.341	0.583	0.843	0.966	4.98%
17	0.091	0.405	0.520	0.683	0.820	0.966	6.56%
18	0.091	0.137	0.260	0.365	0.611	0.966	0.00%
19	0.091	0.120	0.234	0.481	0.809	0.966	1.06%
20	0.091	0.159	0.344	0.660	0.825	0.966	6.18%

Q. Extreme Underweight in Males: Ensemble MR–BRT Knot Placement

Early Neonatal

	Knot 1	Knot 2	Knot 3	Knot 4	Knot 5	Knot 6	Weight
1	0.091	0.216	0.343	0.590	0.729	0.966	5.07%
2	0.091	0.112	0.507	0.705	0.832	0.966	5.10%
3	0.091	0.345	0.453	0.708	0.897	0.966	4.66%
4	0.091	0.150	0.409	0.625	0.829	0.966	5.10%
5	0.091	0.130	0.254	0.723	0.847	0.966	4.51%
6	0.091	0.153	0.373	0.519	0.654	0.966	4.51%
7	0.091	0.270	0.416	0.582	0.884	0.966	5.11%
8	0.091	0.184	0.291	0.525	0.867	0.966	5.02%
9	0.091	0.252	0.486	0.717	0.878	0.966	5.08%
10	0.091	0.216	0.423	0.611	0.773	0.966	5.17%
11	0.091	0.138	0.394	0.626	0.819	0.966	5.07%
12	0.091	0.165	0.313	0.546	0.663	0.966	4.71%
13	0.091	0.168	0.585	0.692	0.802	0.966	4.95%
14	0.091	0.217	0.603	0.727	0.889	0.966	4.86%
15	0.091	0.114	0.227	0.336	0.899	0.966	0.00%
16	0.091	0.108	0.339	0.596	0.864	0.966	4.89%
17	0.091	0.235	0.336	0.691	0.892	0.966	4.99%
18	0.091	0.149	0.330	0.433	0.843	0.966	12.78%
19	0.091	0.132	0.346	0.450	0.601	0.966	3.30%
20	0.091	0.203	0.402	0.755	0.881	0.966	5.11%

Late Neonatal

	Knot 1	Knot 2	Knot 3	Knot 4	Knot 5	Knot 6	Weight
1	0.091	0.266	0.492	0.789	0.891	0.966	5.44%
2	0.091	0.301	0.515	0.702	0.882	0.966	4.74%
3	0.091	0.255	0.665	0.770	0.884	0.966	4.13%
4	0.091	0.325	0.563	0.754	0.854	0.966	4.79%
5	0.091	0.145	0.646	0.755	0.881	0.966	4.53%
6	0.091	0.245	0.437	0.643	0.850	0.966	5.75%
7	0.091	0.174	0.292	0.553	0.800	0.966	5.49%
8	0.091	0.132	0.359	0.471	0.607	0.966	2.17%
9	0.091	0.156	0.488	0.659	0.781	0.966	5.61%
10	0.091	0.161	0.526	0.648	0.867	0.966	5.52%
11	0.091	0.163	0.306	0.449	0.693	0.966	4.13%
12	0.091	0.169	0.410	0.528	0.808	0.966	6.03%
13	0.091	0.145	0.420	0.666	0.886	0.966	1.82%
14	0.091	0.390	0.586	0.784	0.899	0.966	4.27%
15	0.091	0.295	0.413	0.630	0.849	0.966	5.60%
16	0.091	0.200	0.369	0.526	0.823	0.966	14.02%
17	0.091	0.201	0.354	0.513	0.694	0.966	4.79%
18	0.091	0.157	0.434	0.568	0.771	0.966	5.61%
19	0.091	0.152	0.401	0.517	0.771	0.966	5.57%
20	0.091	0.156	0.259	0.361	0.465	0.966	0.00%

1–5 Months

	Knot 1	Knot 2	Knot 3	Knot 4	Knot 5	Knot 6	Weight
1	0.091	0.142	0.273	0.395	0.747	0.966	6.45%
2	0.091	0.143	0.407	0.607	0.763	0.966	17.98%
3	0.091	0.347	0.510	0.638	0.820	0.966	5.11%
4	0.091	0.211	0.334	0.737	0.863	0.966	3.69%
5	0.091	0.166	0.340	0.701	0.878	0.966	2.08%
6	0.091	0.154	0.320	0.636	0.806	0.966	0.27%
7	0.091	0.173	0.514	0.713	0.877	0.966	7.01%
8	0.091	0.149	0.336	0.540	0.767	0.966	11.11%
9	0.091	0.325	0.496	0.599	0.706	0.966	6.83%
10	0.091	0.177	0.461	0.581	0.698	0.966	7.14%
11	0.091	0.243	0.412	0.550	0.795	0.966	0.26%
12	0.091	0.178	0.425	0.759	0.899	0.966	6.54%
13	0.091	0.355	0.472	0.573	0.825	0.966	2.28%
14	0.091	0.141	0.576	0.735	0.895	0.966	7.19%
15	0.091	0.164	0.276	0.504	0.886	0.966	0.00%
16	0.091	0.158	0.350	0.612	0.841	0.966	0.23%
17	0.091	0.129	0.406	0.560	0.751	0.966	8.41%
18	0.091	0.111	0.227	0.359	0.502	0.966	0.00%
19	0.091	0.121	0.248	0.391	0.690	0.966	4.34%
20	0.091	0.326	0.465	0.601	0.826	0.966	3.06%

6–11 Months

	Knot 1	Knot 2	Knot 3	Knot 4	Knot 5	Knot 6	Weight
1	0.091	0.134	0.241	0.451	0.610	0.966	1.97%
2	0.091	0.127	0.499	0.646	0.855	0.966	4.21%
3	0.091	0.171	0.591	0.729	0.851	0.966	6.68%
4	0.091	0.221	0.387	0.500	0.774	0.966	18.49%
5	0.091	0.351	0.592	0.692	0.847	0.966	5.95%
6	0.091	0.278	0.564	0.700	0.844	0.966	6.64%
7	0.091	0.104	0.386	0.525	0.762	0.966	7.39%
8	0.091	0.317	0.652	0.775	0.898	0.966	4.82%
9	0.091	0.176	0.293	0.592	0.890	0.966	0.00%
10	0.091	0.194	0.349	0.521	0.835	0.966	0.00%
11	0.091	0.121	0.475	0.607	0.877	0.966	1.48%
12	0.091	0.117	0.291	0.668	0.787	0.966	0.03%
13	0.091	0.197	0.315	0.705	0.813	0.966	1.14%
14	0.091	0.172	0.299	0.463	0.596	0.966	1.88%
15	0.091	0.269	0.655	0.765	0.887	0.966	5.03%
16	0.091	0.229	0.376	0.541	0.694	0.966	6.90%
17	0.091	0.132	0.448	0.637	0.742	0.966	7.43%
18	0.091	0.152	0.284	0.462	0.757	0.966	7.31%
19	0.091	0.447	0.574	0.764	0.877	0.966	5.18%
20	0.091	0.118	0.285	0.481	0.764	0.966	7.48%

12–23 Months

	Knot 1	Knot 2	Knot 3	Knot 4	Knot 5	Knot 6	Weight
1	0.091	0.107	0.443	0.700	0.858	0.966	4.18%
2	0.091	0.213	0.455	0.605	0.809	0.966	1.17%
3	0.091	0.177	0.410	0.528	0.678	0.966	6.61%
4	0.091	0.164	0.490	0.609	0.852	0.966	2.16%
5	0.091	0.124	0.357	0.606	0.731	0.966	17.55%
6	0.091	0.153	0.331	0.669	0.838	0.966	0.12%
7	0.091	0.168	0.277	0.386	0.653	0.966	1.94%
8	0.091	0.125	0.244	0.399	0.708	0.966	4.89%
9	0.091	0.150	0.252	0.390	0.751	0.966	6.49%
10	0.091	0.102	0.391	0.553	0.838	0.966	0.00%
11	0.091	0.106	0.387	0.510	0.622	0.966	4.21%
12	0.091	0.160	0.278	0.602	0.784	0.966	0.00%
13	0.091	0.189	0.336	0.477	0.584	0.966	1.28%
14	0.091	0.156	0.541	0.693	0.823	0.966	7.02%
15	0.091	0.154	0.303	0.609	0.722	0.966	7.49%
16	0.091	0.307	0.478	0.613	0.728	0.966	6.98%
17	0.091	0.229	0.376	0.724	0.886	0.966	4.47%
18	0.091	0.205	0.421	0.773	0.895	0.966	15.07%
19	0.091	0.112	0.217	0.540	0.658	0.966	6.22%
20	0.091	0.113	0.429	0.672	0.886	0.966	2.17%

2–4 Years

	Knot 1	Knot 2	Knot 3	Knot 4	Knot 5	Knot 6	Weight
1	0.091	0.347	0.543	0.705	0.824	0.966	6.47%
2	0.091	0.136	0.421	0.554	0.709	0.966	15.18%
3	0.091	0.180	0.470	0.778	0.892	0.966	0.00%
4	0.091	0.327	0.447	0.563	0.895	0.966	3.16%
5	0.091	0.100	0.231	0.406	0.548	0.966	1.59%
6	0.091	0.189	0.308	0.581	0.806	0.966	0.82%
7	0.091	0.141	0.277	0.534	0.807	0.966	0.04%
8	0.091	0.111	0.352	0.467	0.771	0.966	0.00%
9	0.091	0.266	0.407	0.634	0.744	0.966	6.90%
10	0.091	0.144	0.381	0.638	0.785	0.966	3.46%
11	0.091	0.347	0.511	0.649	0.795	0.966	6.19%
12	0.091	0.315	0.548	0.681	0.891	0.966	6.51%
13	0.091	0.145	0.407	0.599	0.731	0.966	16.38%
14	0.091	0.160	0.291	0.493	0.884	0.966	0.00%
15	0.091	0.217	0.404	0.527	0.648	0.966	6.43%
16	0.091	0.279	0.567	0.670	0.832	0.966	6.55%
17	0.091	0.275	0.443	0.652	0.880	0.966	5.50%
18	0.091	0.285	0.429	0.598	0.819	0.966	3.78%
19	0.091	0.108	0.410	0.681	0.807	0.966	5.20%
20	0.091	0.280	0.437	0.673	0.837	0.966	5.85%

R. Extreme Underweight in Females: Ensemble MR–BRT Knot Placement

Early Neonatal

	Knot 1	Knot 2	Knot 3	Knot 4	Knot 5	Knot 6	Weight
1	0.091	0.181	0.288	0.412	0.569	0.966	0.00%
2	0.091	0.120	0.331	0.440	0.576	0.966	0.04%
3	0.091	0.203	0.626	0.759	0.891	0.966	1.89%
4	0.091	0.368	0.565	0.698	0.844	0.966	4.01%
5	0.091	0.336	0.534	0.647	0.862	0.966	4.72%
6	0.091	0.161	0.508	0.635	0.803	0.966	5.22%
7	0.091	0.126	0.285	0.661	0.878	0.966	0.00%
8	0.091	0.192	0.324	0.542	0.746	0.966	20.49%
9	0.091	0.404	0.574	0.736	0.895	0.966	2.90%
10	0.091	0.215	0.456	0.564	0.707	0.966	8.20%
11	0.091	0.202	0.422	0.615	0.893	0.966	0.27%
12	0.091	0.133	0.394	0.596	0.792	0.966	0.00%
13	0.091	0.287	0.453	0.570	0.710	0.966	7.12%
14	0.091	0.168	0.350	0.636	0.869	0.966	0.00%
15	0.091	0.111	0.416	0.724	0.897	0.966	6.29%
16	0.091	0.140	0.337	0.544	0.708	0.966	10.85%
17	0.091	0.230	0.519	0.781	0.892	0.966	5.05%
18	0.091	0.149	0.250	0.377	0.732	0.966	6.85%
19	0.091	0.201	0.383	0.582	0.735	0.966	10.26%
20	0.091	0.270	0.534	0.656	0.813	0.966	5.85%

Late Neonatal

	Knot 1	Knot 2	Knot 3	Knot 4	Knot 5	Knot 6	Weight
1	0.091	0.131	0.306	0.502	0.647	0.966	5.62%
2	0.091	0.411	0.653	0.762	0.862	0.966	2.12%
3	0.091	0.147	0.295	0.687	0.826	0.966	3.33%
4	0.091	0.292	0.464	0.604	0.725	0.966	5.76%
5	0.091	0.102	0.221	0.401	0.757	0.966	0.00%
6	0.091	0.156	0.627	0.732	0.877	0.966	5.07%
7	0.091	0.279	0.544	0.662	0.823	0.966	5.62%
8	0.091	0.154	0.346	0.608	0.803	0.966	2.29%
9	0.091	0.229	0.438	0.636	0.804	0.966	4.93%
10	0.091	0.323	0.435	0.571	0.863	0.966	3.62%
11	0.091	0.368	0.545	0.705	0.866	0.966	5.40%
12	0.091	0.221	0.413	0.696	0.797	0.966	5.57%
13	0.091	0.123	0.267	0.405	0.739	0.966	5.87%
14	0.091	0.283	0.482	0.611	0.785	0.966	4.96%
15	0.091	0.173	0.406	0.585	0.898	0.966	2.94%
16	0.091	0.240	0.377	0.690	0.872	0.966	5.23%
17	0.091	0.107	0.336	0.478	0.629	0.966	5.27%
18	0.091	0.214	0.502	0.715	0.849	0.966	5.84%
19	0.091	0.106	0.363	0.583	0.699	0.966	5.98%
20	0.091	0.132	0.343	0.490	0.733	0.966	14.59%

1–5 Months

	Knot 1	Knot 2	Knot 3	Knot 4	Knot 5	Knot 6	Weight
1	0.091	0.177	0.374	0.557	0.843	0.966	1.79%
2	0.091	0.128	0.238	0.368	0.898	0.966	0.00%
3	0.091	0.127	0.400	0.623	0.797	0.966	3.88%
4	0.091	0.272	0.509	0.696	0.894	0.966	6.04%
5	0.091	0.122	0.266	0.426	0.589	0.966	4.06%
6	0.091	0.160	0.318	0.518	0.662	0.966	6.12%
7	0.091	0.242	0.439	0.611	0.835	0.966	4.77%
8	0.091	0.130	0.250	0.403	0.680	0.966	5.60%
9	0.091	0.167	0.357	0.489	0.674	0.966	6.08%
10	0.091	0.395	0.508	0.616	0.844	0.966	5.47%
11	0.091	0.221	0.472	0.699	0.808	0.966	15.20%
12	0.091	0.280	0.578	0.712	0.825	0.966	5.73%
13	0.091	0.165	0.355	0.462	0.626	0.966	5.44%
14	0.091	0.181	0.311	0.709	0.877	0.966	4.66%
15	0.091	0.164	0.450	0.686	0.791	0.966	6.01%
16	0.091	0.195	0.349	0.668	0.890	0.966	4.50%
17	0.091	0.101	0.310	0.654	0.828	0.966	2.75%
18	0.091	0.312	0.523	0.645	0.750	0.966	5.80%
19	0.091	0.251	0.428	0.756	0.895	0.966	0.00%
20	0.091	0.160	0.361	0.505	0.669	0.966	6.10%

6–11 Months

	Knot 1	Knot 2	Knot 3	Knot 4	Knot 5	Knot 6	Weight
1	0.091	0.114	0.293	0.505	0.883	0.966	0.00%
2	0.091	0.161	0.415	0.534	0.756	0.966	0.86%
3	0.091	0.269	0.569	0.679	0.879	0.966	6.61%
4	0.091	0.151	0.394	0.574	0.892	0.966	1.60%
5	0.091	0.158	0.267	0.732	0.835	0.966	4.12%
6	0.091	0.251	0.375	0.587	0.723	0.966	7.30%
7	0.091	0.212	0.375	0.540	0.733	0.966	7.98%
8	0.091	0.200	0.308	0.536	0.673	0.966	7.32%
9	0.091	0.206	0.319	0.601	0.813	0.966	1.34%
10	0.091	0.256	0.464	0.709	0.817	0.966	7.12%
11	0.091	0.218	0.510	0.709	0.879	0.966	7.08%
12	0.091	0.164	0.358	0.599	0.716	0.966	18.25%
13	0.091	0.156	0.487	0.643	0.785	0.966	6.59%
14	0.091	0.119	0.286	0.495	0.778	0.966	0.00%
15	0.091	0.211	0.322	0.453	0.636	0.966	5.95%
16	0.091	0.270	0.411	0.560	0.810	0.966	2.15%
17	0.091	0.369	0.521	0.672	0.868	0.966	6.47%
18	0.091	0.104	0.263	0.450	0.553	0.966	2.83%
19	0.091	0.349	0.549	0.685	0.827	0.966	6.44%
20	0.091	0.120	0.295	0.451	0.868	0.966	0.00%

12–23 Months

	Knot 1	Knot 2	Knot 3	Knot 4	Knot 5	Knot 6	Weight
1	0.091	0.181	0.299	0.634	0.772	0.966	0.19%
2	0.091	0.117	0.410	0.606	0.847	0.966	1.05%
3	0.091	0.122	0.367	0.517	0.808	0.966	0.00%
4	0.091	0.161	0.263	0.454	0.606	0.966	3.71%
5	0.091	0.114	0.337	0.546	0.742	0.966	8.77%
6	0.091	0.205	0.427	0.693	0.838	0.966	7.28%
7	0.091	0.234	0.446	0.652	0.753	0.966	6.01%
8	0.091	0.151	0.257	0.517	0.739	0.966	9.49%
9	0.091	0.262	0.440	0.551	0.785	0.966	0.60%
10	0.091	0.107	0.325	0.610	0.870	0.966	0.01%
11	0.091	0.245	0.407	0.686	0.817	0.966	6.45%
12	0.091	0.134	0.259	0.513	0.659	0.966	7.65%
13	0.091	0.162	0.376	0.725	0.850	0.966	6.66%
14	0.091	0.248	0.416	0.685	0.786	0.966	6.64%
15	0.091	0.168	0.269	0.491	0.709	0.966	19.13%
16	0.091	0.146	0.247	0.718	0.899	0.966	1.07%
17	0.091	0.157	0.535	0.670	0.842	0.966	7.56%
18	0.091	0.214	0.377	0.573	0.804	0.966	0.11%
19	0.091	0.232	0.420	0.711	0.812	0.966	7.61%
20	0.091	0.132	0.278	0.631	0.786	0.966	0.01%

2–4 Years

	Knot 1	Knot 2	Knot 3	Knot 4	Knot 5	Knot 6	Weight
1	0.091	0.226	0.464	0.614	0.825	0.966	6.15%
2	0.091	0.308	0.517	0.784	0.889	0.966	0.00%
3	0.091	0.142	0.285	0.419	0.555	0.966	1.84%
4	0.091	0.343	0.445	0.578	0.823	0.966	4.58%
5	0.091	0.180	0.472	0.603	0.842	0.966	5.97%
6	0.091	0.264	0.438	0.603	0.851	0.966	4.79%
7	0.091	0.120	0.302	0.570	0.777	0.966	0.05%
8	0.091	0.162	0.404	0.593	0.841	0.966	2.64%
9	0.091	0.363	0.509	0.729	0.891	0.966	7.56%
10	0.091	0.201	0.347	0.696	0.831	0.966	6.04%
11	0.091	0.158	0.506	0.709	0.854	0.966	20.36%
12	0.091	0.180	0.400	0.506	0.796	0.966	0.06%
13	0.091	0.200	0.317	0.642	0.865	0.966	2.72%
14	0.091	0.348	0.470	0.603	0.825	0.966	6.49%
15	0.091	0.176	0.453	0.568	0.811	0.966	3.39%
16	0.091	0.119	0.292	0.517	0.870	0.966	0.00%
17	0.091	0.157	0.557	0.670	0.788	0.966	8.14%
18	0.091	0.170	0.541	0.660	0.784	0.966	8.50%
19	0.091	0.147	0.264	0.442	0.721	0.966	9.82%
20	0.091	0.140	0.343	0.596	0.773	0.966	0.89%
