# Supplementary material for: Current dichotomous metrics obscure trends in severe and extreme child growth failure
Source: Sci Adv. 2022 May 20;8(20):eabm8954. doi: 10.1126/sciadv.abm8954 (PMC9122330; doi:10.1126/sciadv.abm8954)

Data S4. Age-sex specific fits of MR-BRT models for all forms and severities of CGF, 1990-2020, with incorporation of SDI as a linear covariate. Prevalence models were fit in logit space, but are also shown in linear space for comparison. Figures shown include: overall stunting [HAZ < -2 standard deviations (SD)] prevalence shown in logit and linear space for males (A, B) and females (C, D); severe stunting [HAZ < -3 SD] for males (E, F) and females (G, H); extreme stunting [HAZ < -4 SD] for males (I, J) and females (K, L); overall wasting [WHZ < -2 SD] for males (M, N) and females (O, P); severe wasting [WHZ < -3 SD] for males (Q, R) and females (S, T); extreme wasting [WHZ < -4 SD] for males (U, V) and females (W, X); overall underweight [WAZ < -2 SD] for males (Y, Z) and females (AA, BB); severe underweight [WAZ < -3 SD] for males (CC, DD) and females (EE, FF); and extreme underweight [WAZ < -4 SD] for males (GG, HH) and females (II, JJ). Models were fit in logit space, but are also shown in linear space for comparison. All years were used as inputs to the models, but only 1990, 2000, 2010, and 2020 are shown here for simplicity. In these MR-BRT models, a linear covariate of Socio-demographic index (SDI) was incorporated, unlike the MR-BRT models shown in Fig. S6. The four black lines represent the expected values of CGF prevalence for a given location with SDI values of 20, 40, 60, and 80, with the highest SDI value having the lowest predicted CGF prevalence.

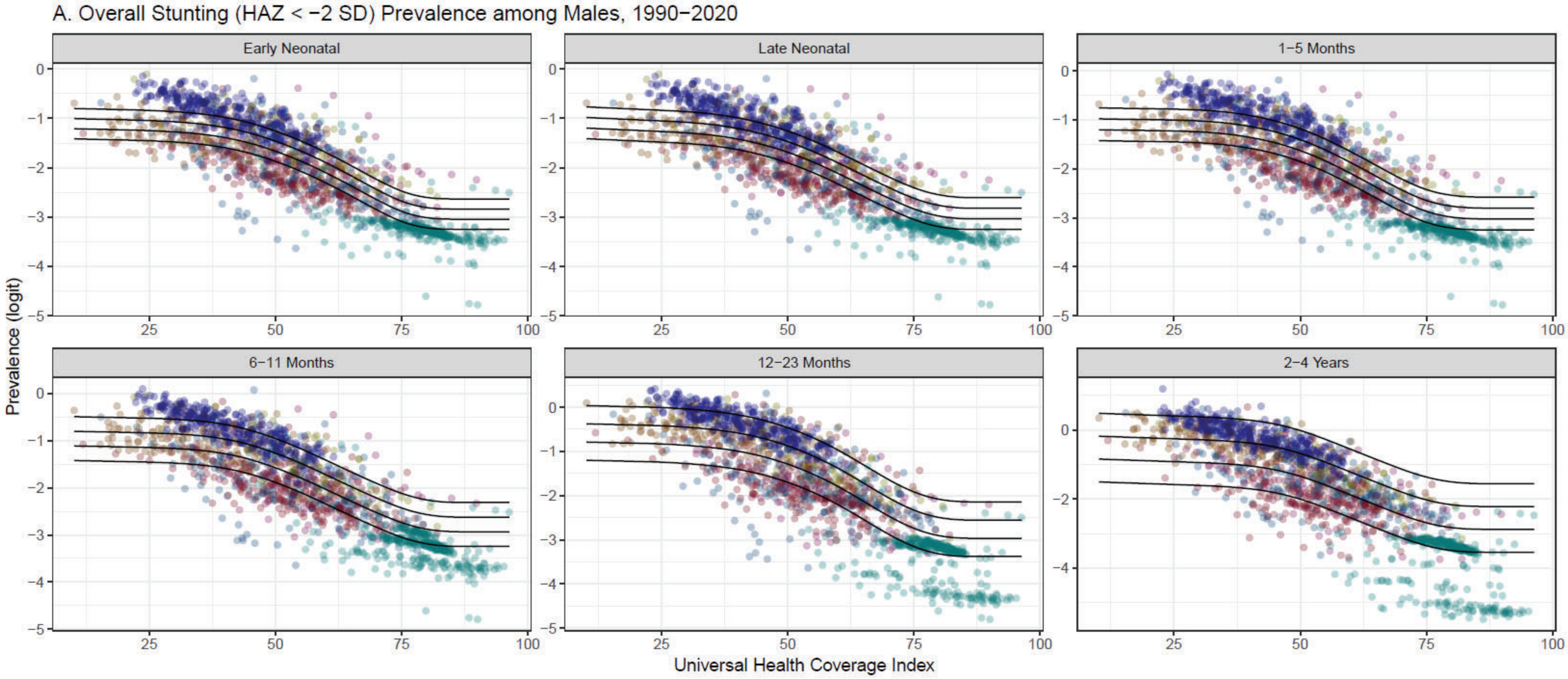

B. Overall Stunting (HAZ < −2 SD) Prevalence among Males, 1990−2020

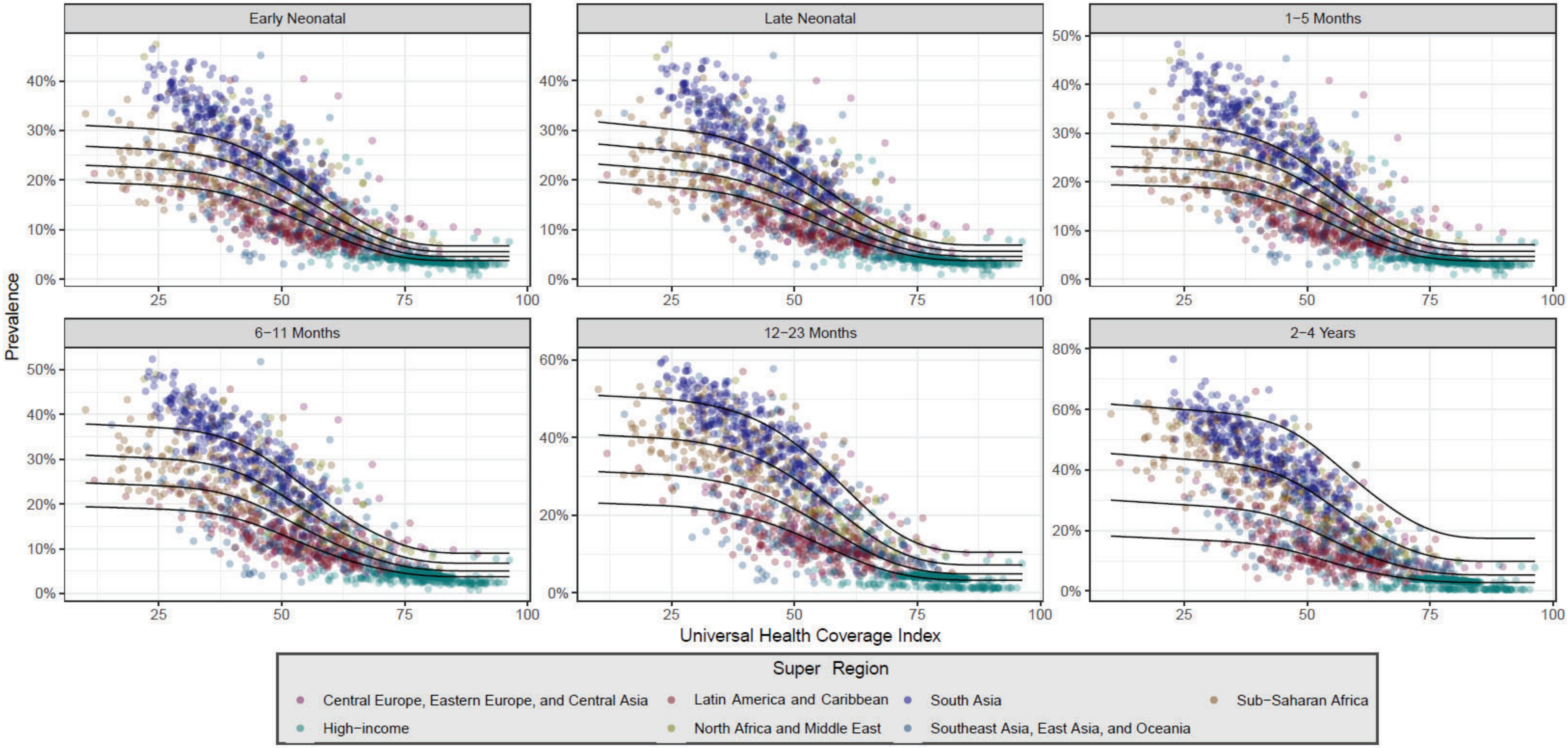

C. Overall Stunting (HAZ < −2 SD) Prevalence among Females, 1990−2020

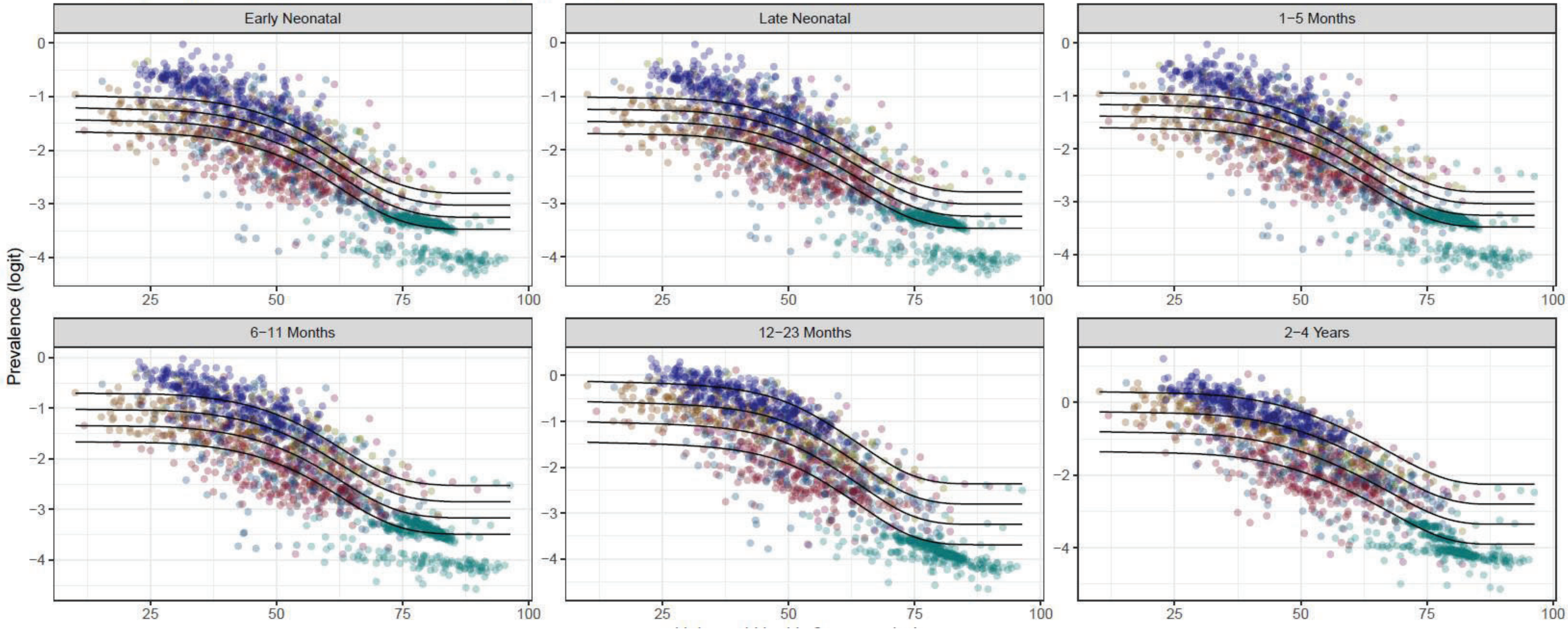

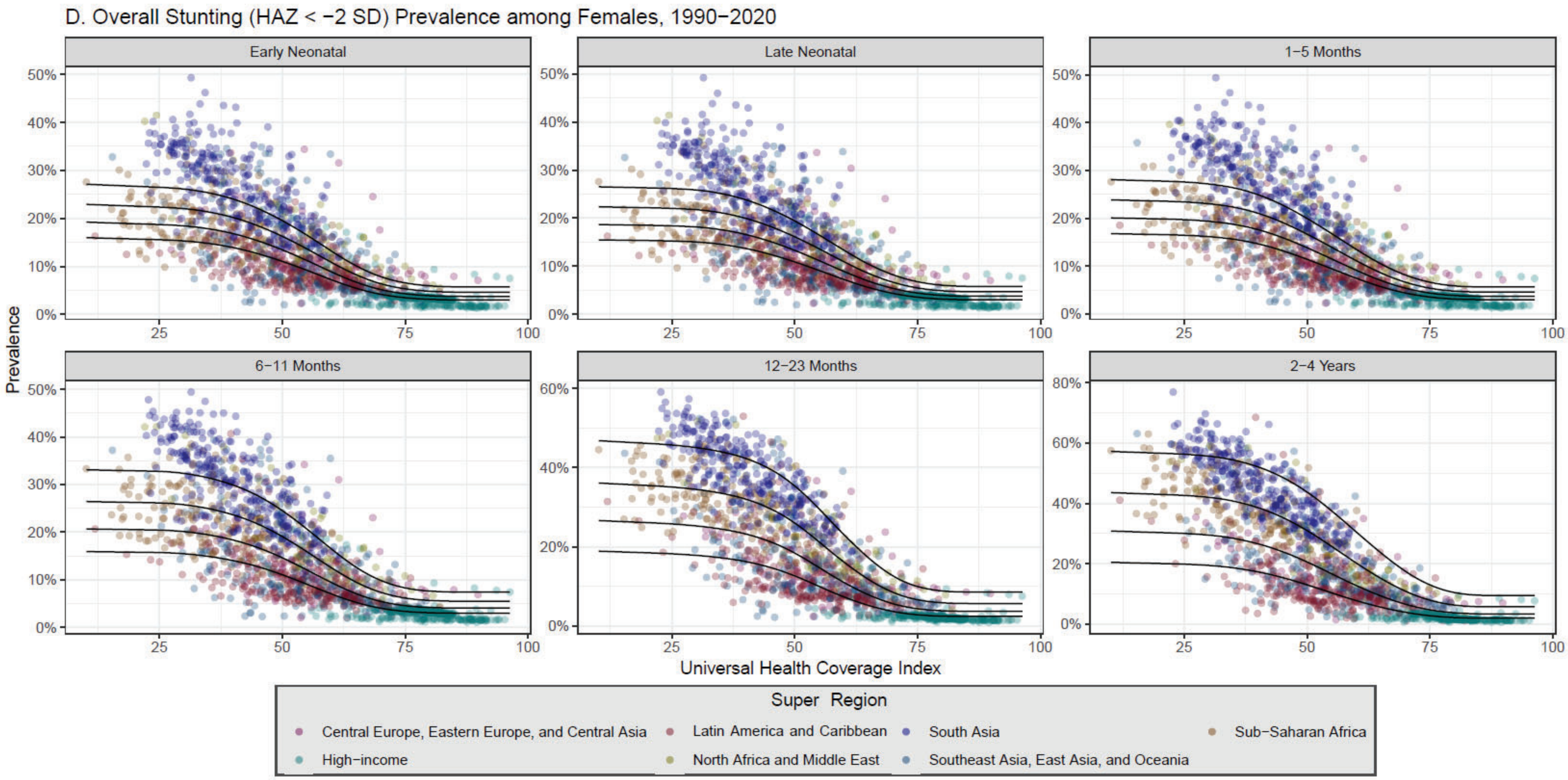

D. Overall Stunting (HAZ < −2 SD) Prevalence among Females, 1990−2020
Early Neonatal
Late Neonatal
1−5 Months
6−11 Months
12−23 Months
2−4 Years
Prevalence
Universal Health Coverage Index
Super Region
Central Europe, Eastern Europe, and Central Asia
Latin America and Caribbean
South Asia
Sub−Saharan Africa
High−income
North Africa and Middle East
Southeast Asia, East Asia, and Oceania

E. Severe Stunting (HAZ < −3 SD) Prevalence among Males, 1990−2020

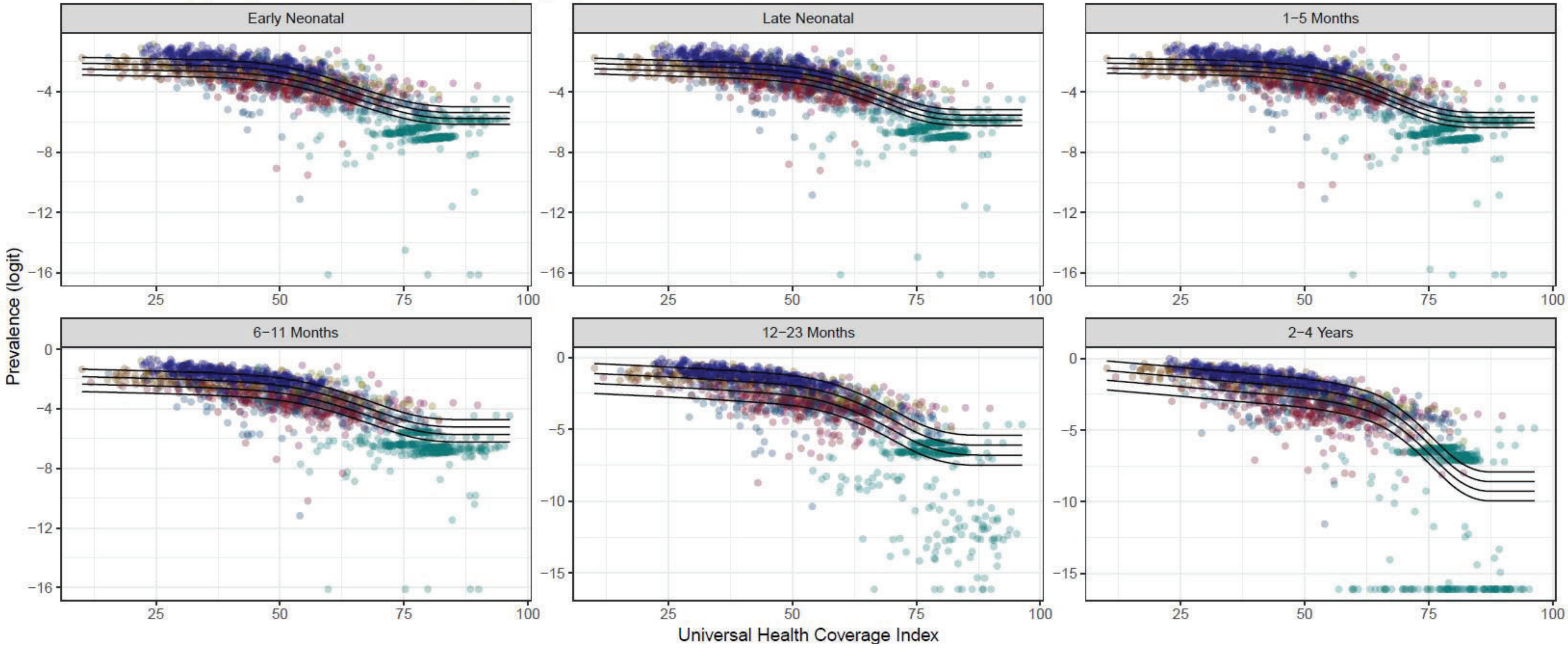

F. Severe Stunting (HAZ < −3 SD) Prevalence among Males, 1990−2020

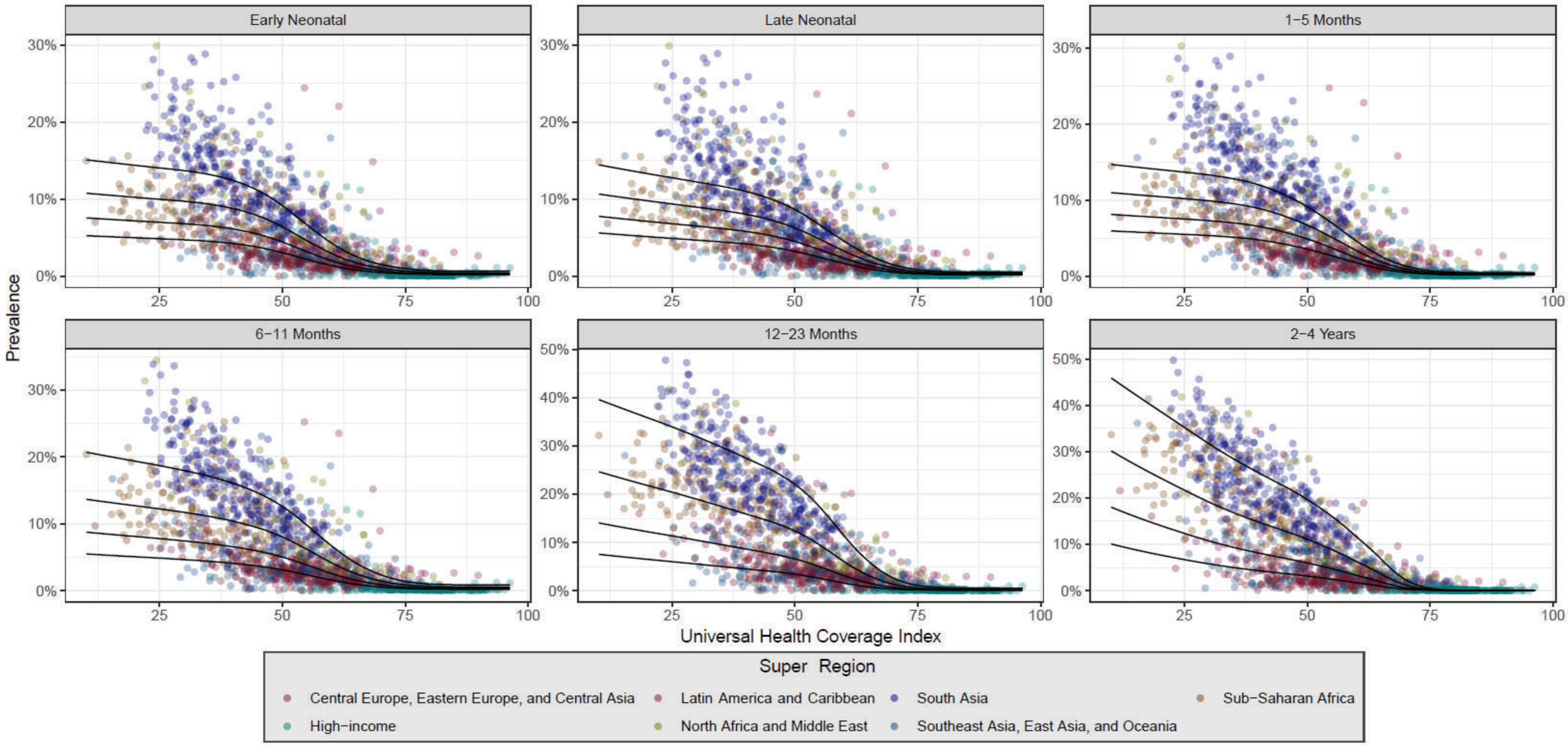

G. Severe Stunting (HAZ < −3 SD) Prevalence among Females, 1990−2020

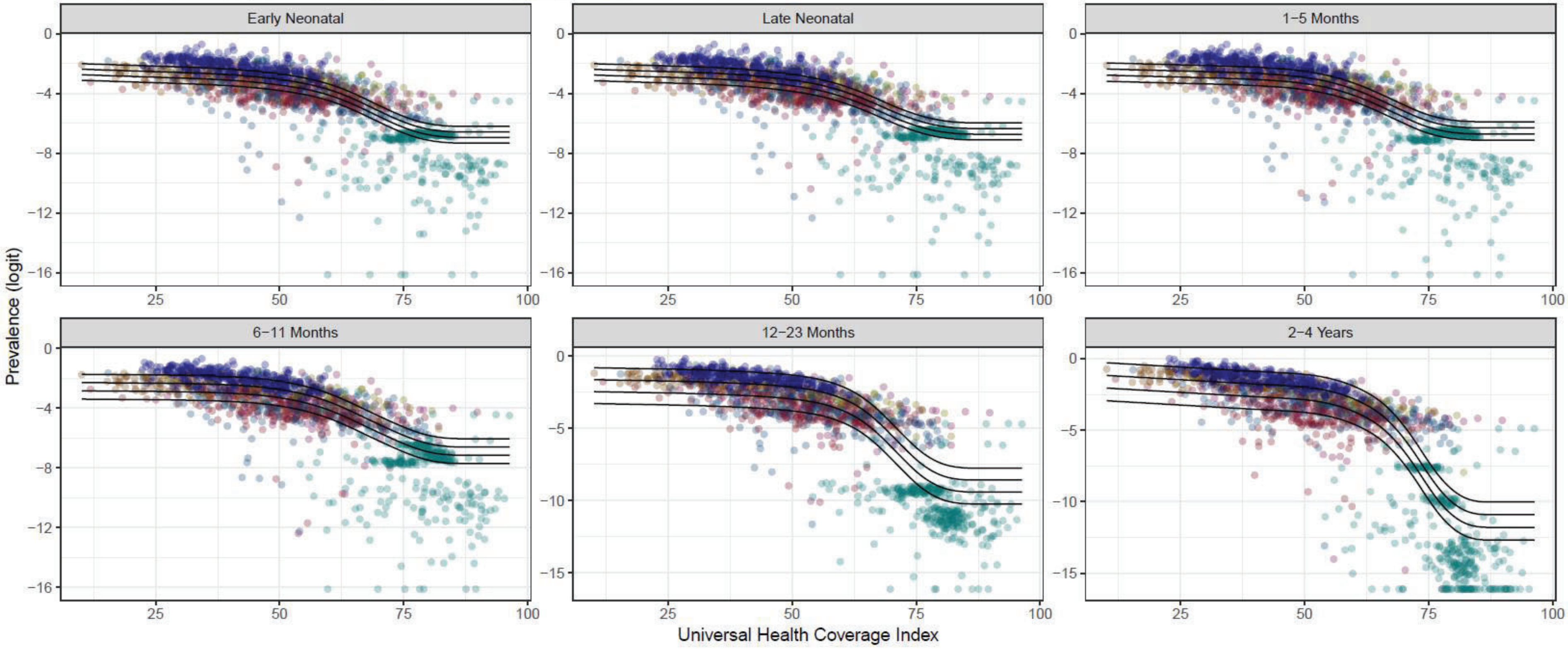

H. Severe Stunting (HAZ < −3 SD) Prevalence among Females, 1990−2020

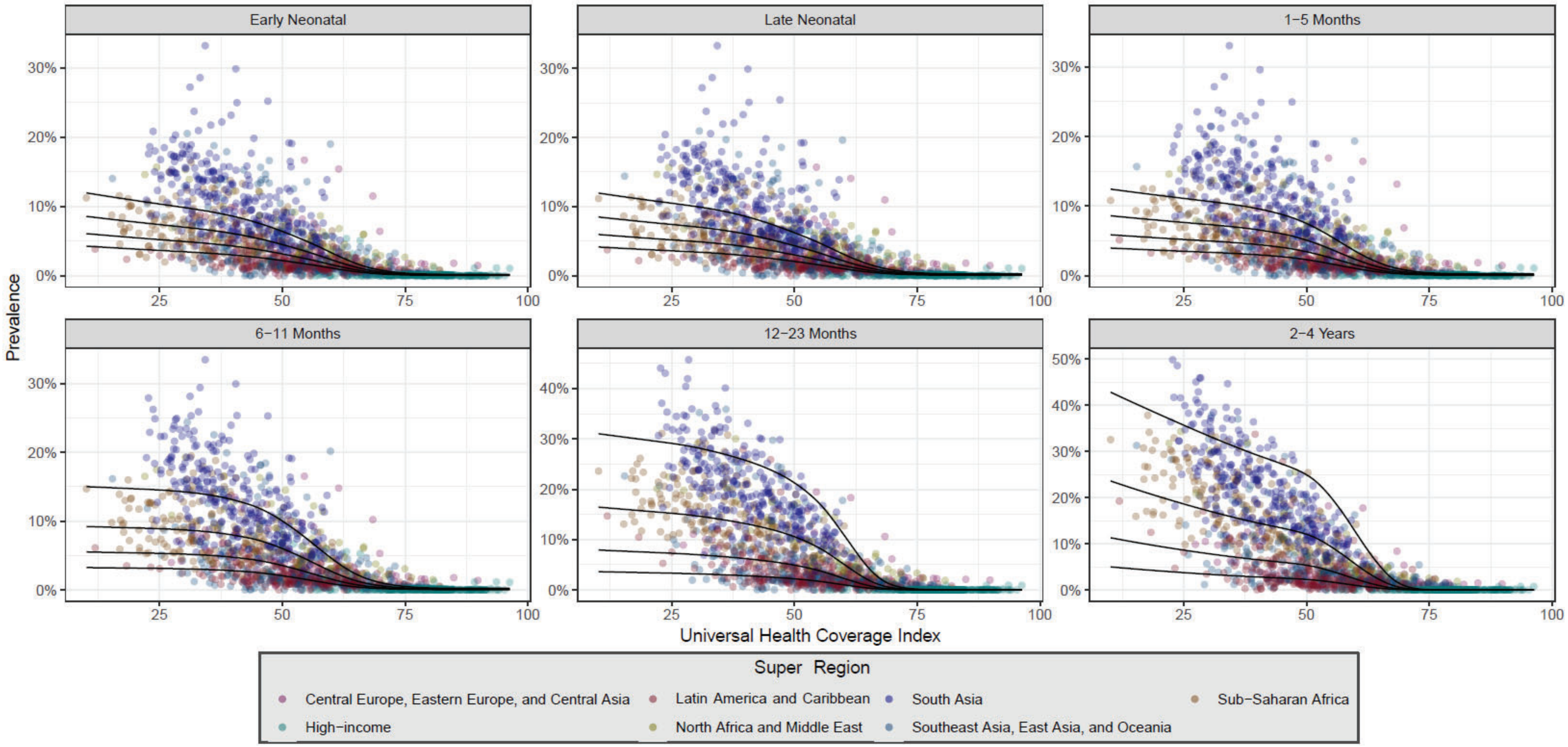

I. Extreme Stunting (HAZ < −4 SD) Prevalence among Males, 1990−2020

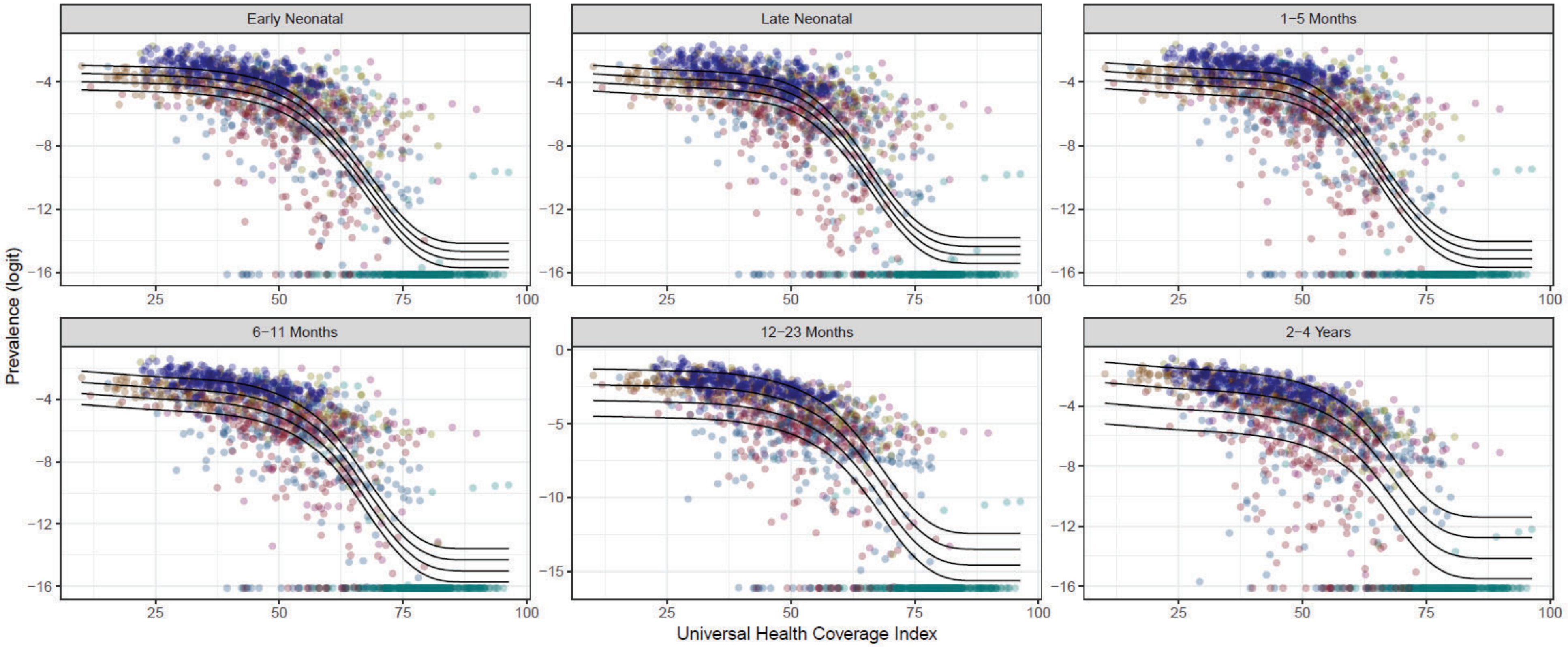

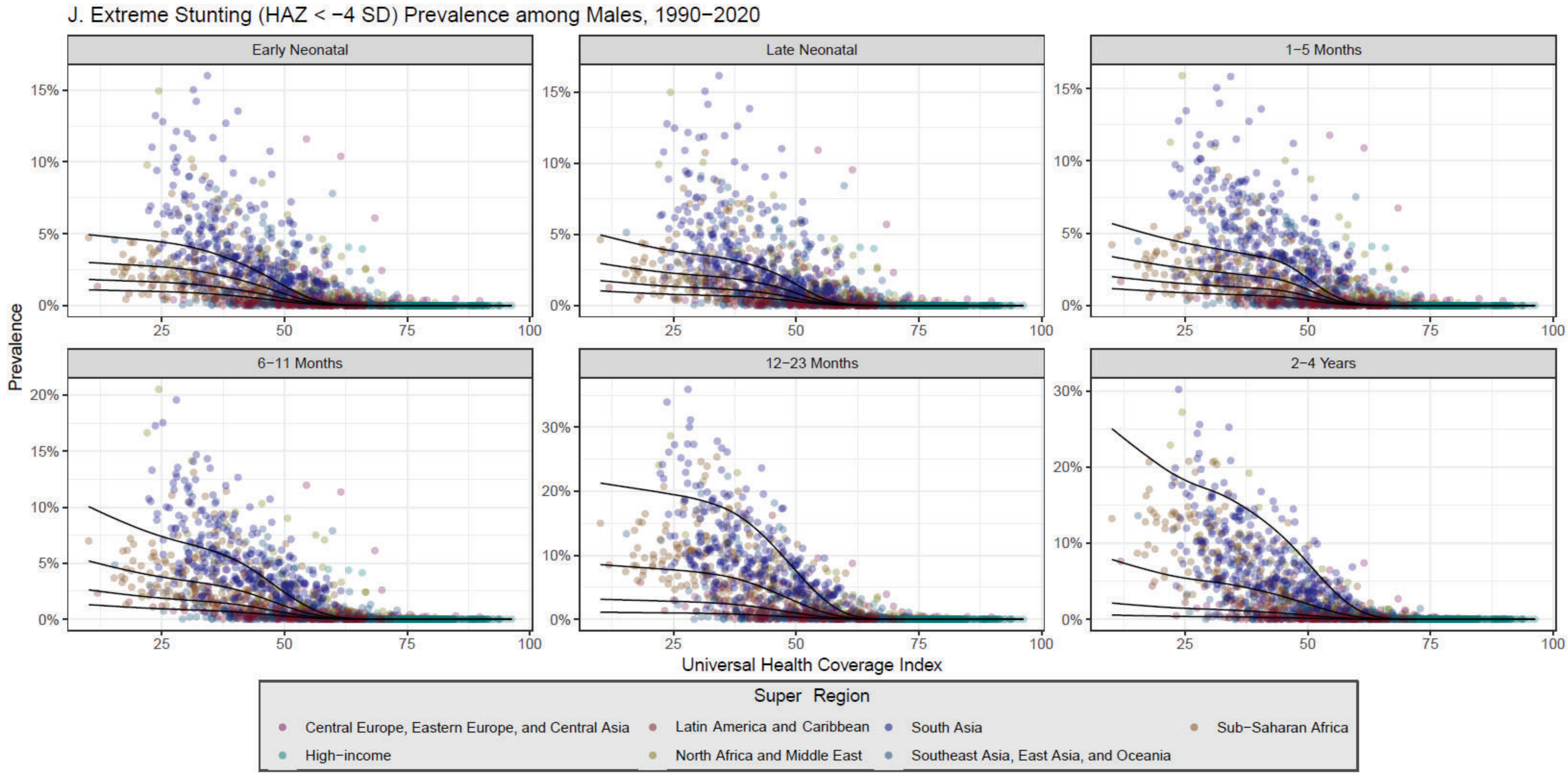
J. Extreme Stunting (HAZ < −4 SD) Prevalence among Males, 1990−2020
Early Neonatal
Late Neonatal
1−5 Months
6−11 Months
12−23 Months
2−4 Years
Prevalence
Universal Health Coverage Index
Super Region
Central Europe, Eastern Europe, and Central Asia
Latin America and Caribbean
South Asia
Sub−Saharan Africa
High−income
North Africa and Middle East
Southeast Asia, East Asia, and Oceania

K. Extreme Stunting (HAZ < −4 SD) Prevalence among Females, 1990−2020

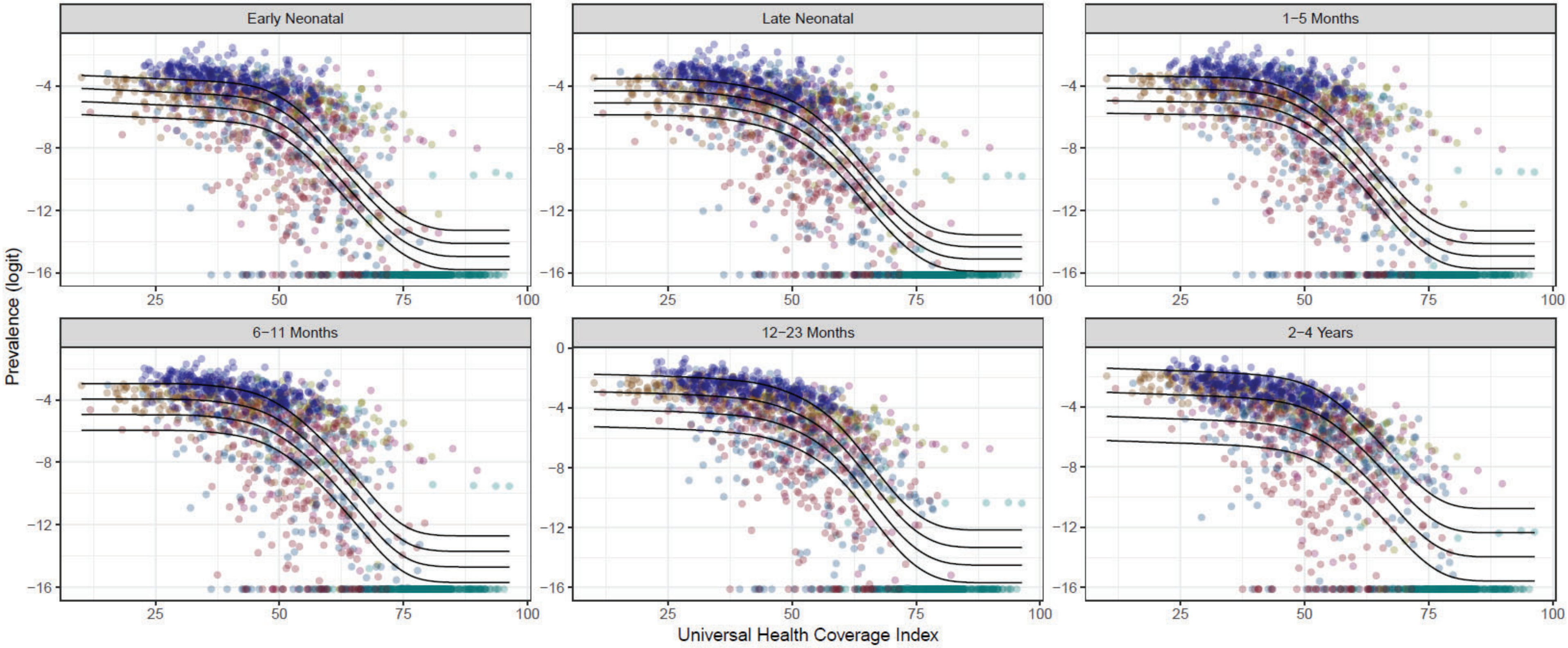

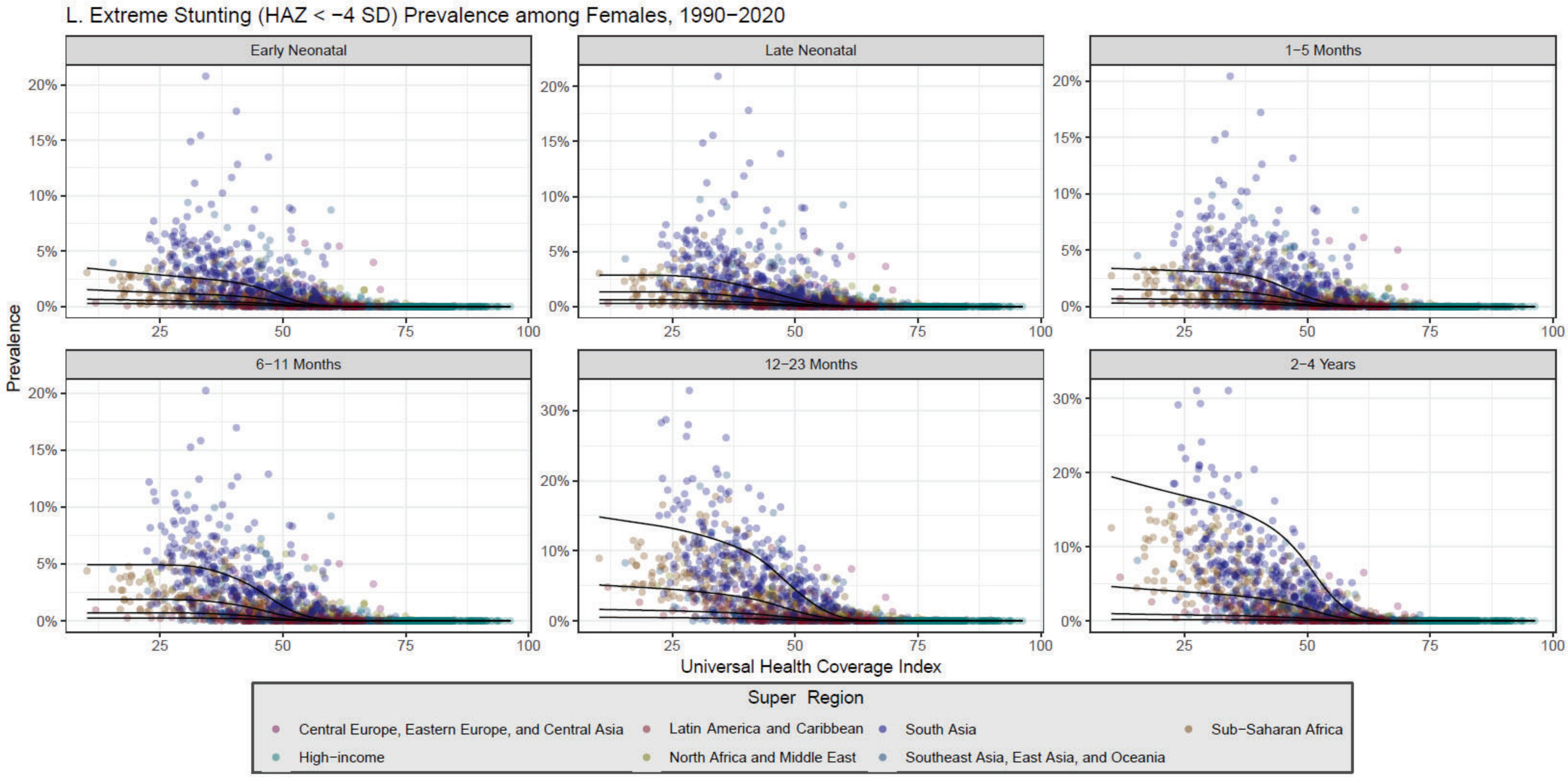

L. Extreme Stunting (HAZ < −4 SD) Prevalence among Females, 1990−2020
Early Neonatal
Late Neonatal
1−5 Months
6−11 Months
12−23 Months
2−4 Years
Prevalence
0%
5%
10%
15%
20%
30%
25
50
75
100
Universal Health Coverage Index
Super Region
Central Europe, Eastern Europe, and Central Asia
Latin America and Caribbean
South Asia
Sub−Saharan Africa
High−income
North Africa and Middle East
Southeast Asia, East Asia, and Oceania

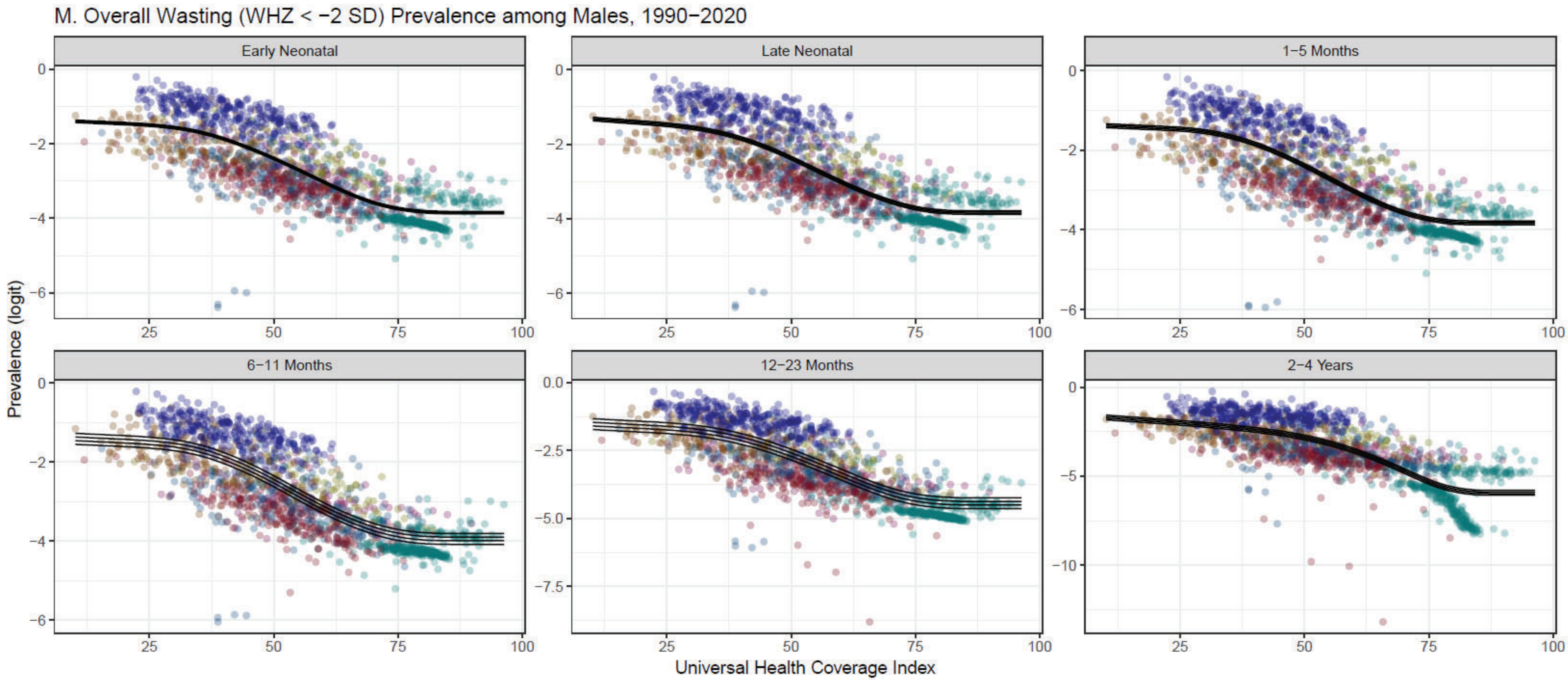
M. Overall Wasting (WHZ < −2 SD) Prevalence among Males, 1990−2020
Early Neonatal
Late Neonatal
1−5 Months
6−11 Months
12−23 Months
2−4 Years
Prevalence (logit)
Universal Health Coverage Index

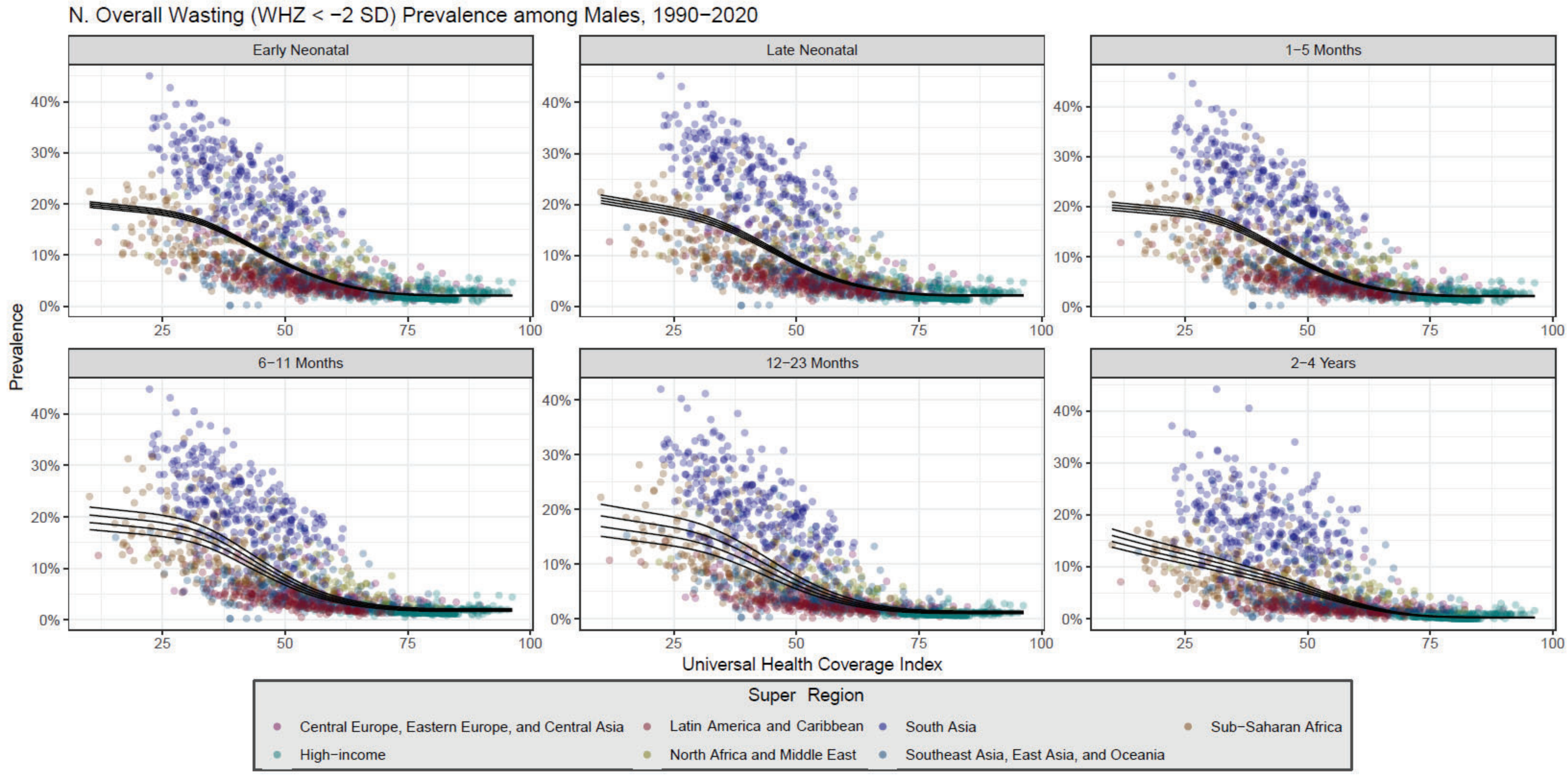
N. Overall Wasting (WHZ < −2 SD) Prevalence among Males, 1990−2020
Early Neonatal
Late Neonatal
1−5 Months
6−11 Months
12−23 Months
2−4 Years
Prevalence
0%
10%
20%
30%
40%
25
50
75
100
Universal Health Coverage Index
Super Region
Central Europe, Eastern Europe, and Central Asia
High−income
Latin America and Caribbean
North Africa and Middle East
South Asia
Southeast Asia, East Asia, and Oceania
Sub−Saharan Africa

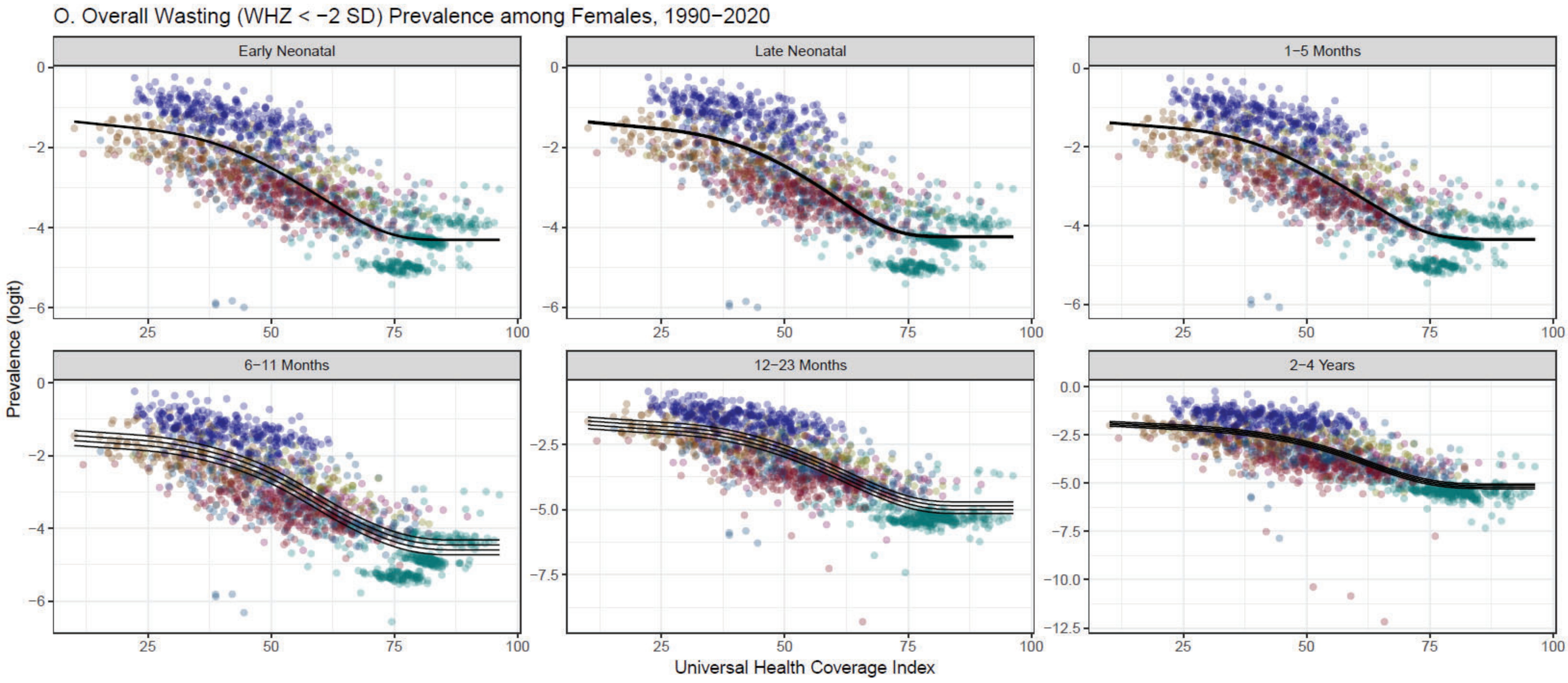

O. Overall Wasting (WHZ < −2 SD) Prevalence among Females, 1990−2020
Early Neonatal
Late Neonatal
1−5 Months
6−11 Months
12−23 Months
2−4 Years
Prevalence (logit)
Universal Health Coverage Index

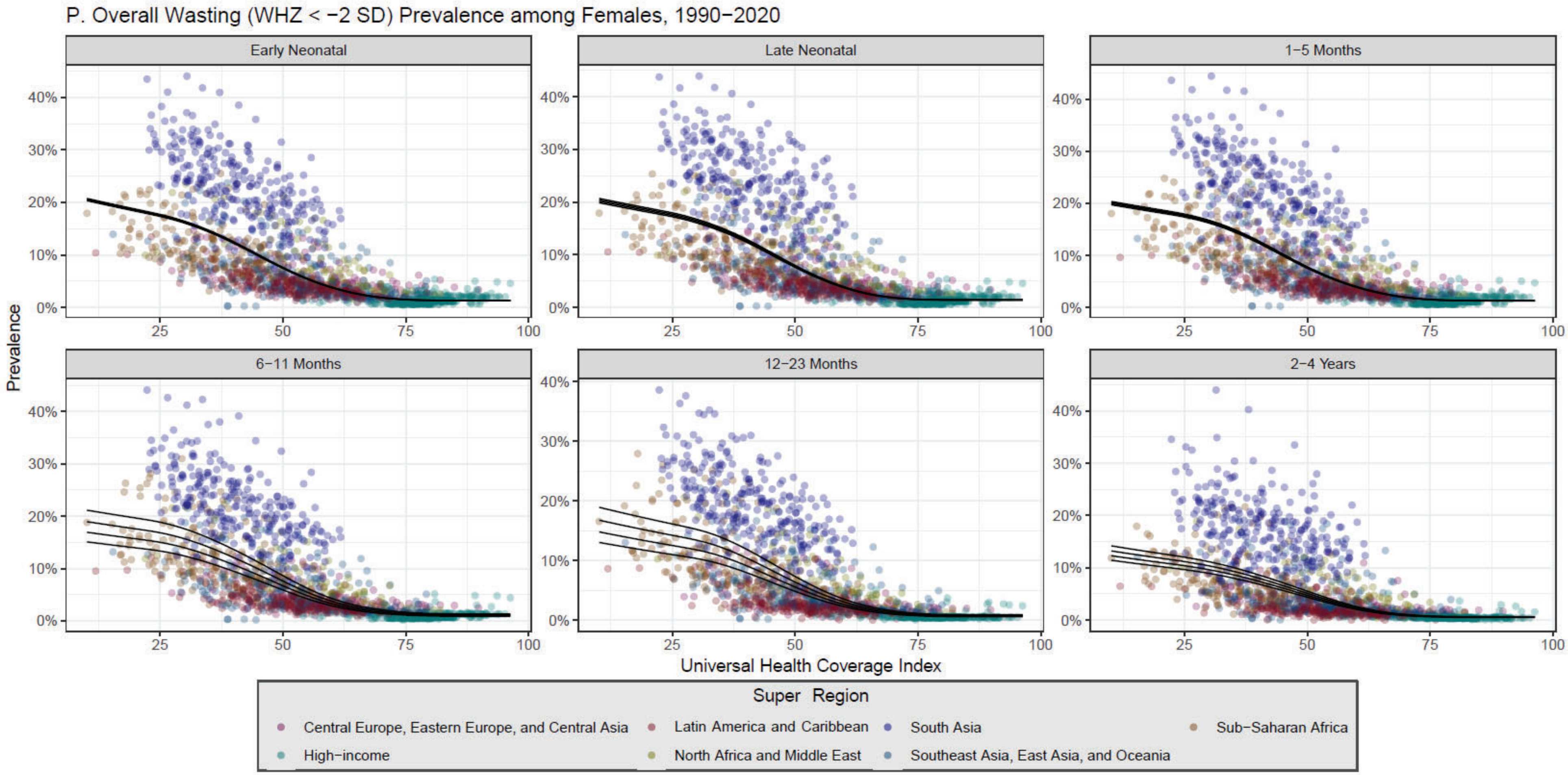

P. Overall Wasting (WHZ < −2 SD) Prevalence among Females, 1990−2020
Early Neonatal
Late Neonatal
1−5 Months
6−11 Months
12−23 Months
2−4 Years
Prevalence
0%
10%
20%
30%
40%
25
50
75
100
Universal Health Coverage Index
Super Region
Central Europe, Eastern Europe, and Central Asia
Latin America and Caribbean
South Asia
Sub−Saharan Africa
High−income
North Africa and Middle East
Southeast Asia, East Asia, and Oceania

Q. Severe Wasting (WHZ < −3 SD) Prevalence among Males, 1990−2020

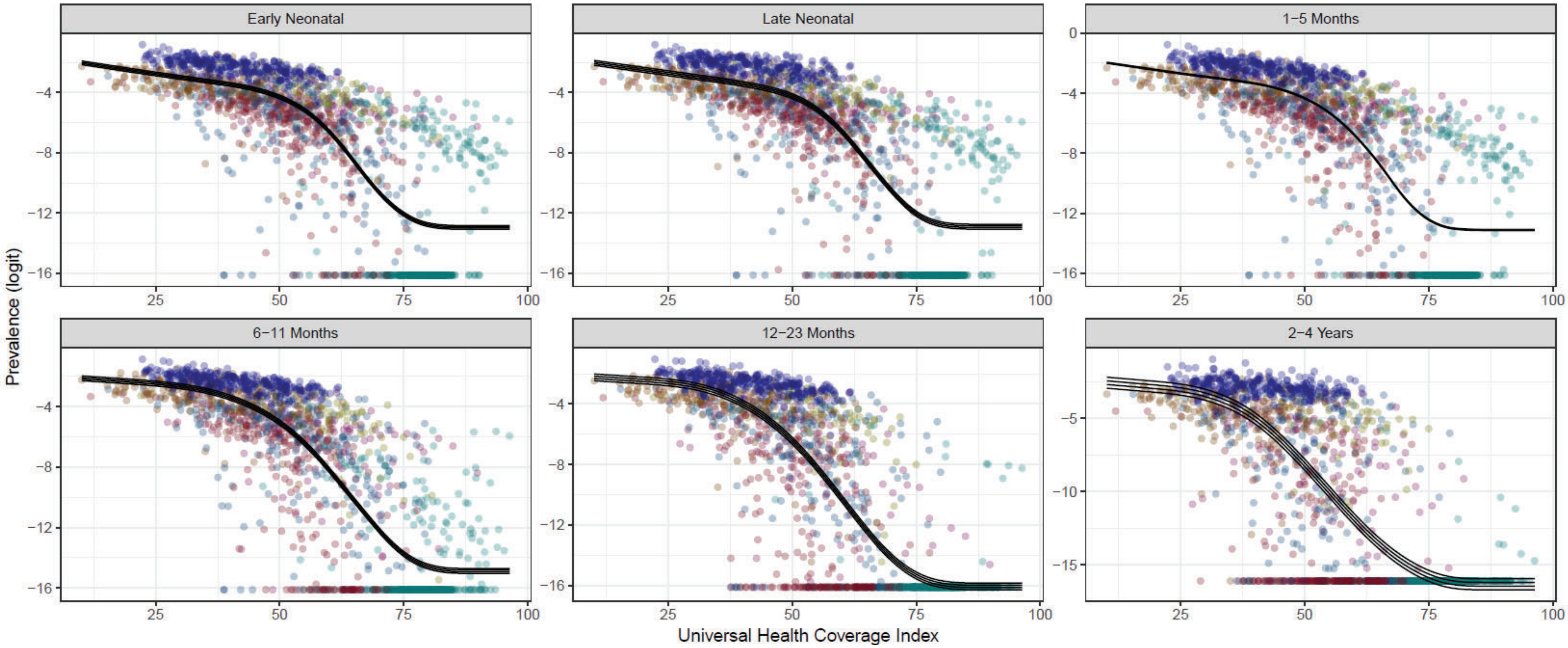

R. Severe Wasting (WHZ < −3 SD) Prevalence among Males, 1990−2020

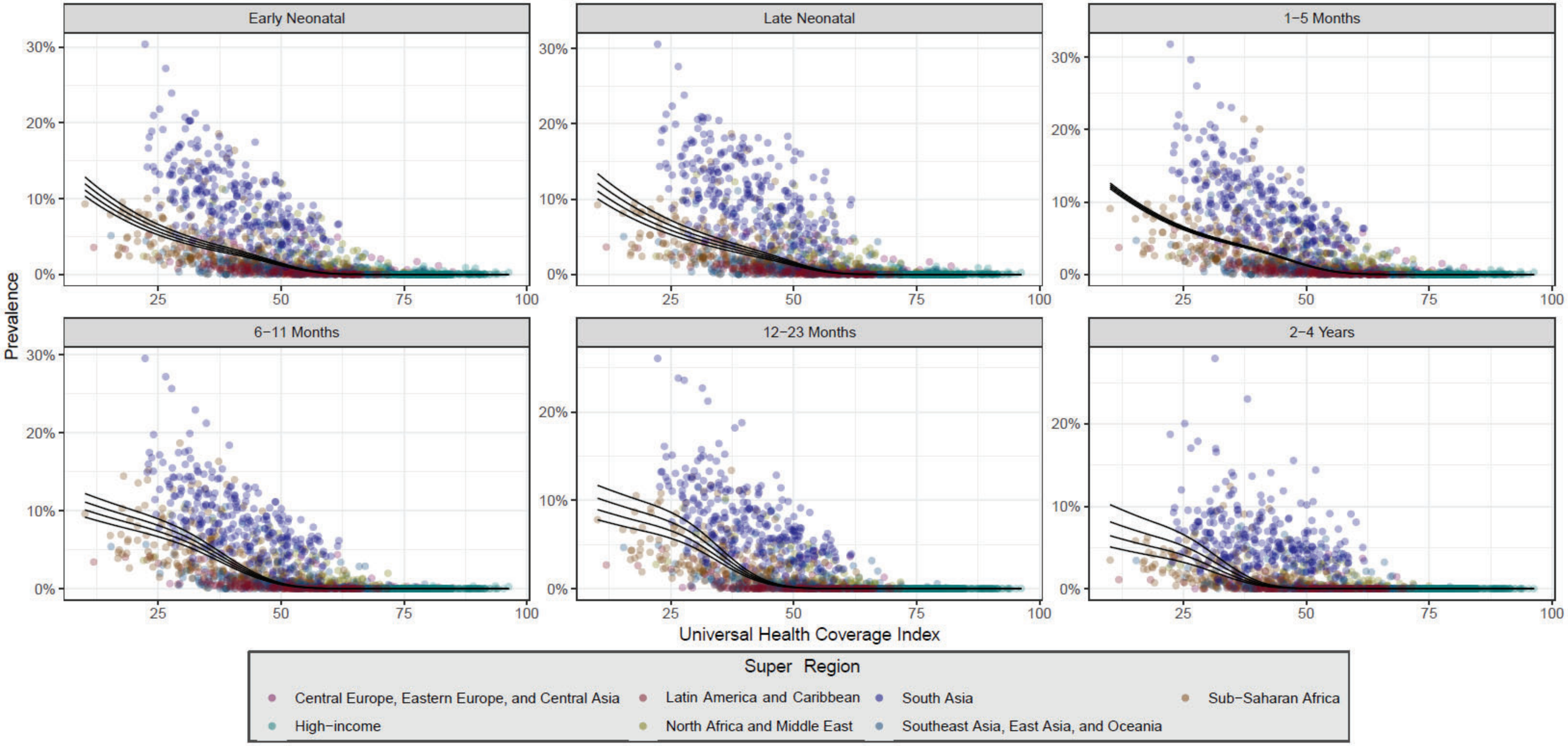

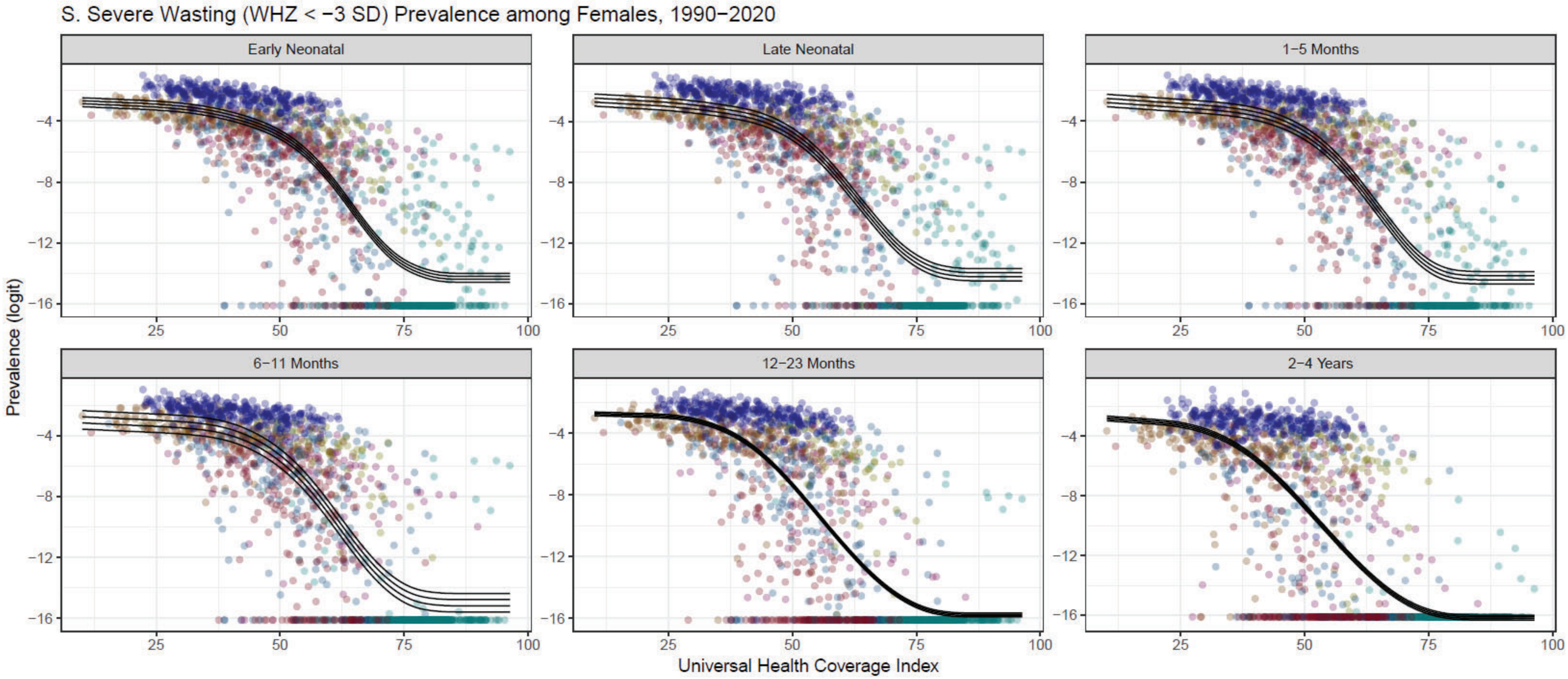

S. Severe Wasting (WHZ < −3 SD) Prevalence among Females, 1990−2020
Early Neonatal
Late Neonatal
1−5 Months
6−11 Months
12−23 Months
2−4 Years
Prevalence (logit)
Universal Health Coverage Index
-4
-8
-12
-16
25
50
75
100

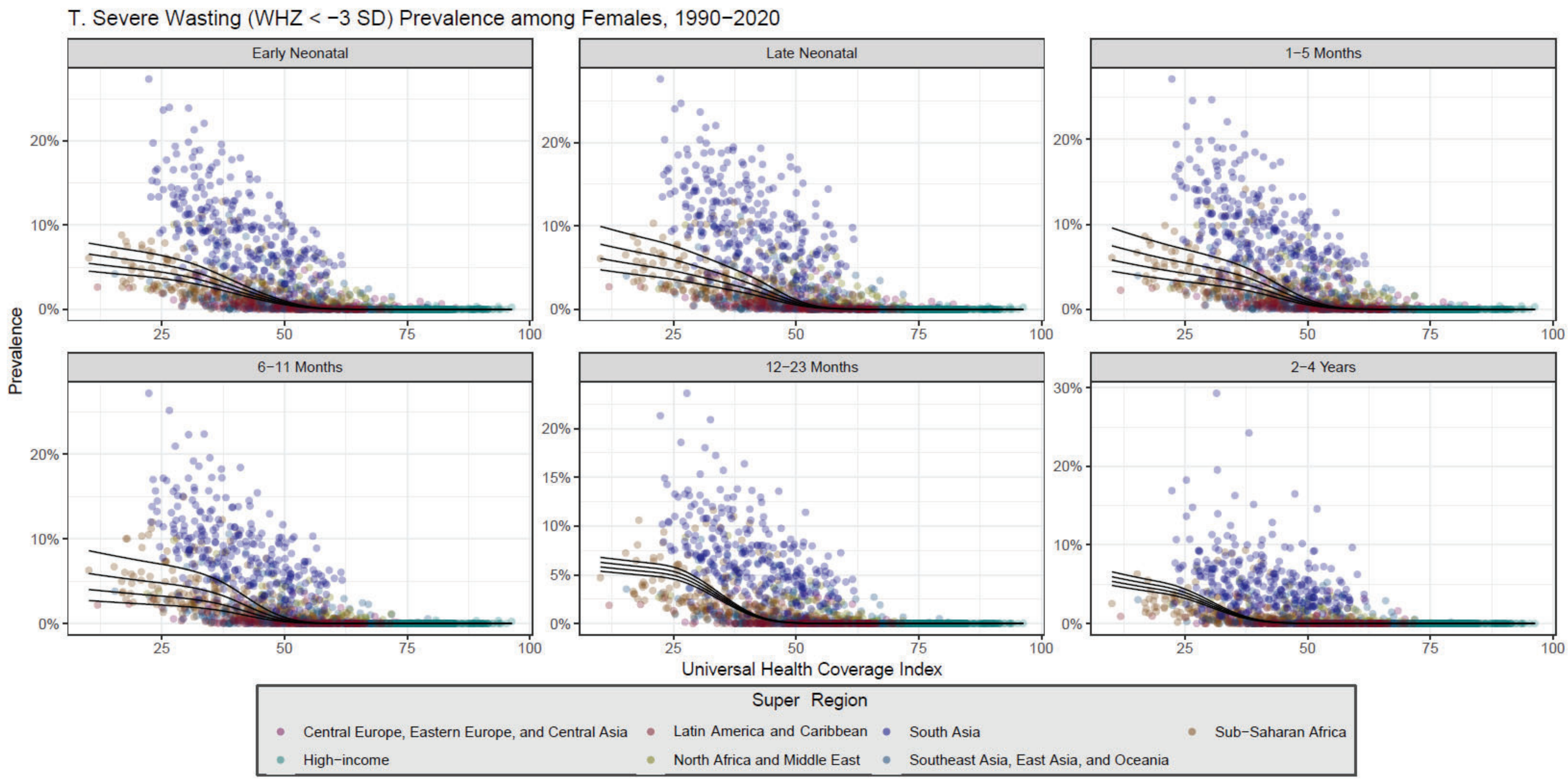

T. Severe Wasting (WHZ < −3 SD) Prevalence among Females, 1990−2020
Early Neonatal
Late Neonatal
1−5 Months
6−11 Months
12−23 Months
2−4 Years
Prevalence
Universal Health Coverage Index
0%
5%
10%
15%
20%
30%
25
50
75
100
Super Region
Central Europe, Eastern Europe, and Central Asia
Latin America and Caribbean
South Asia
Sub−Saharan Africa
High−income
North Africa and Middle East
Southeast Asia, East Asia, and Oceania

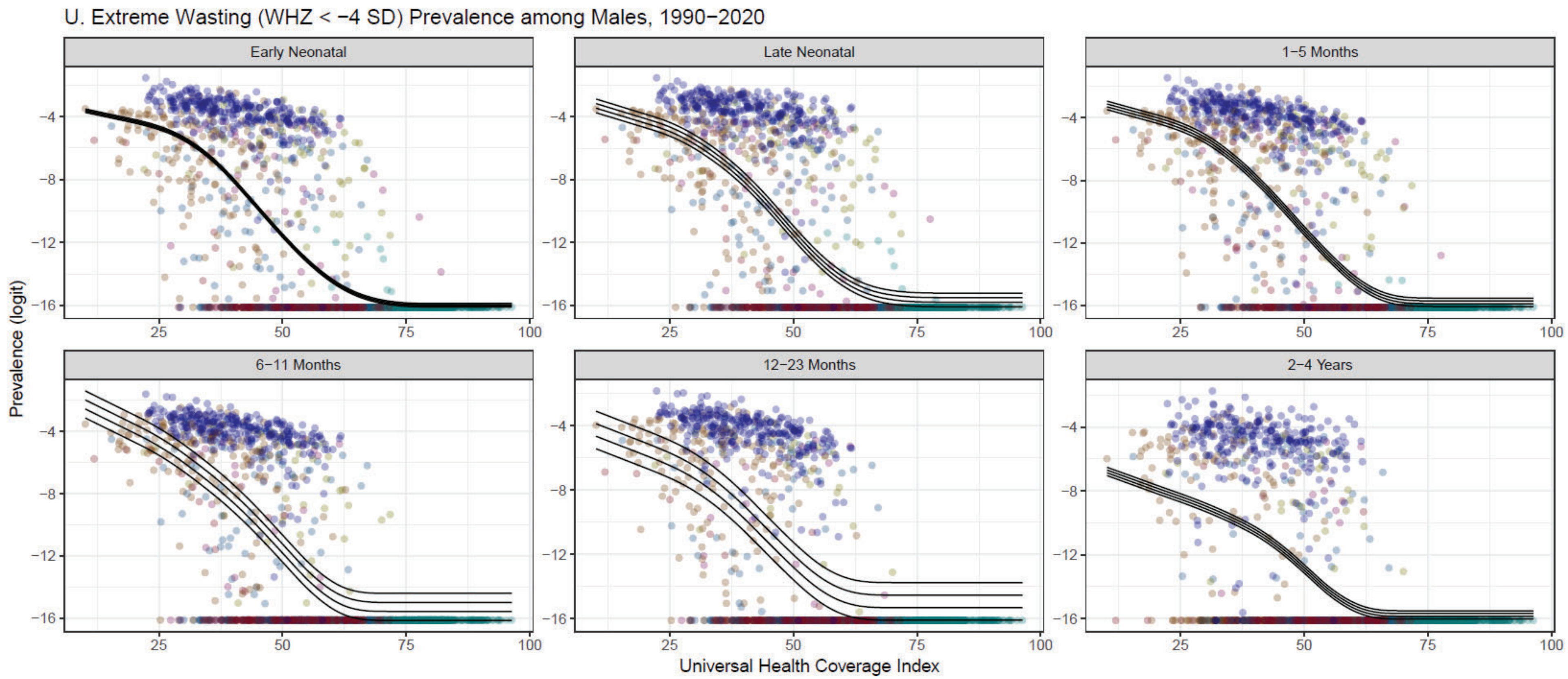

U. Extreme Wasting (WHZ < −4 SD) Prevalence among Males, 1990−2020
Early Neonatal
Late Neonatal
1−5 Months
6−11 Months
12−23 Months
2−4 Years
Prevalence (logit)
Universal Health Coverage Index
-4
-8
-12
-16
25
50
75
100

V. Extreme Wasting (WHZ < −4 SD) Prevalence among Males, 1990−2020

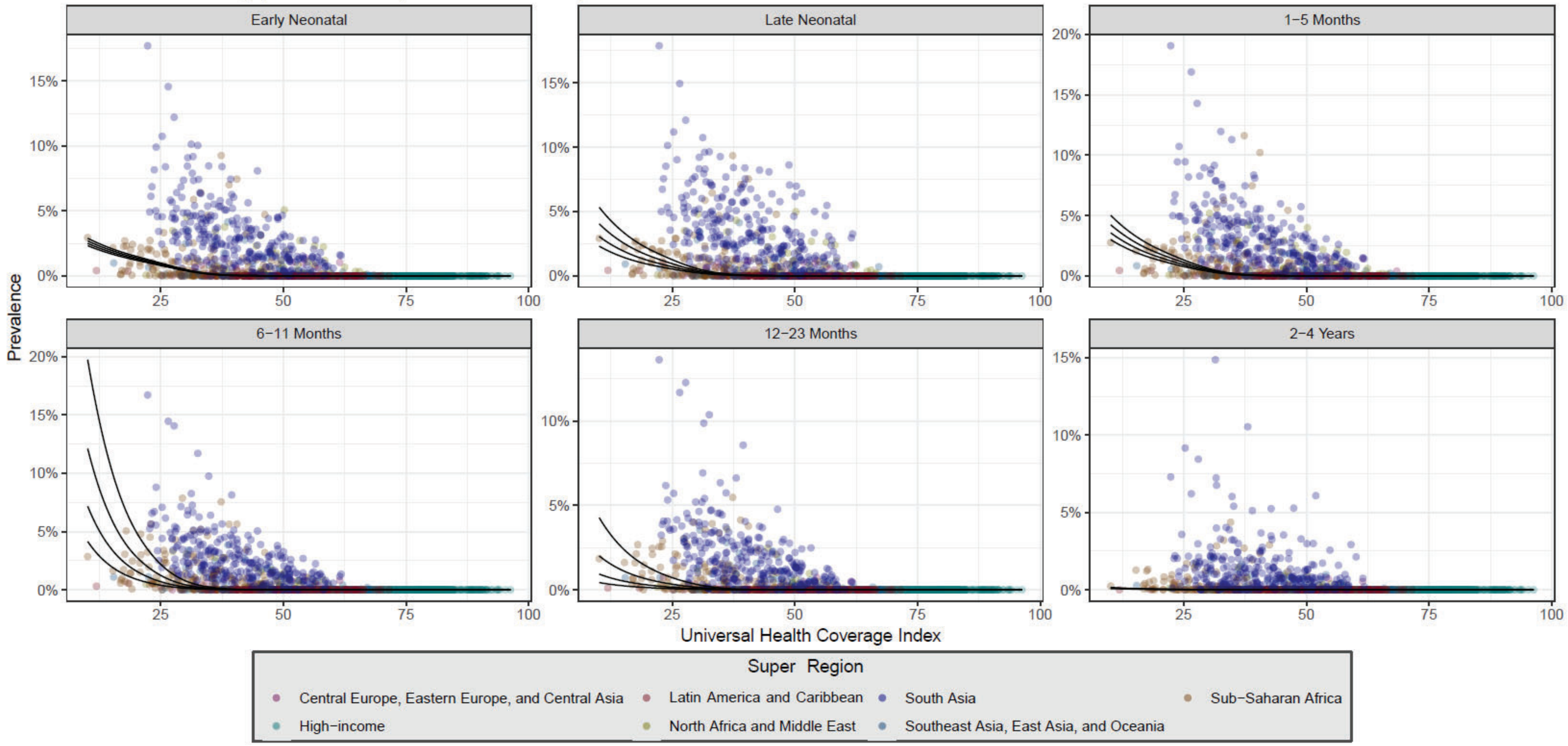

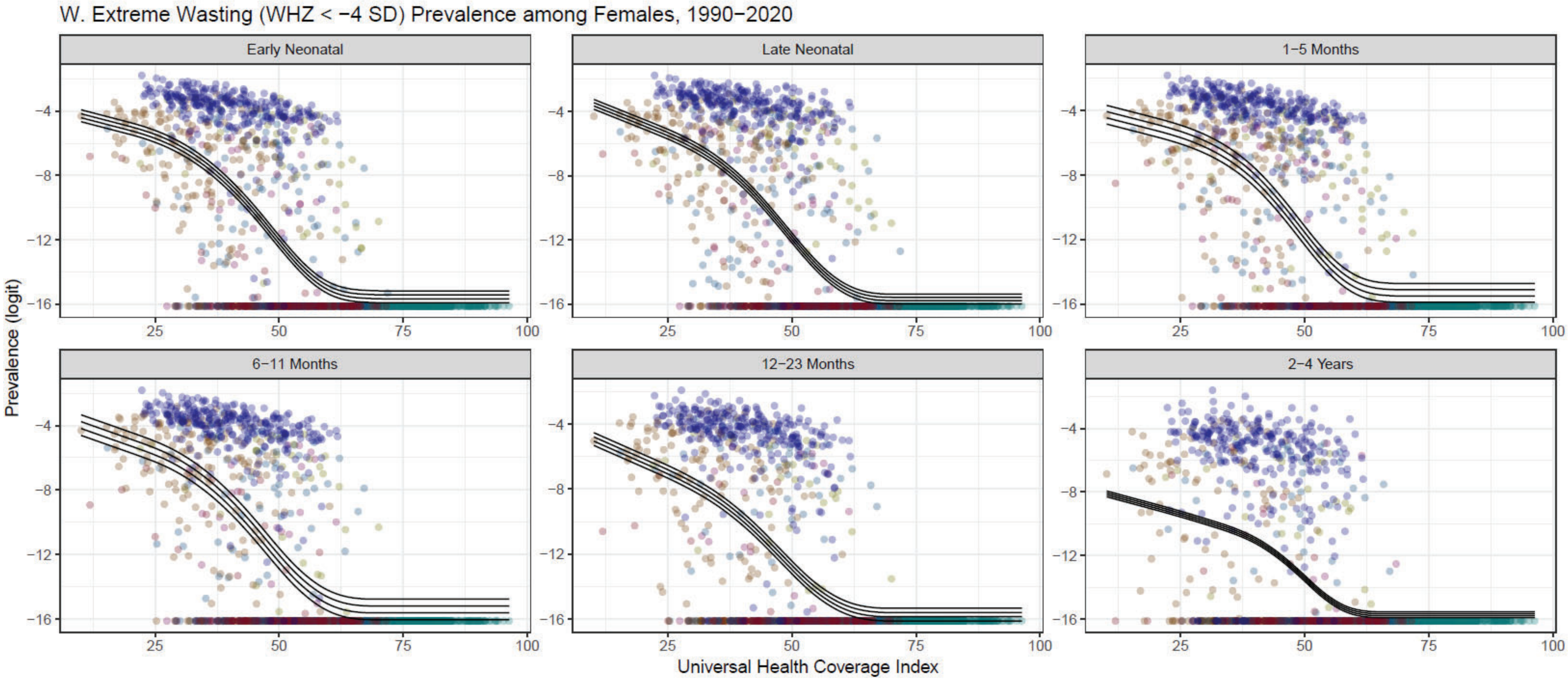

W. Extreme Wasting (WHZ < −4 SD) Prevalence among Females, 1990−2020
Early Neonatal
Late Neonatal
1−5 Months
6−11 Months
12−23 Months
2−4 Years
Prevalence (logit)
Universal Health Coverage Index
-4
-8
-12
-16
25
50
75
100

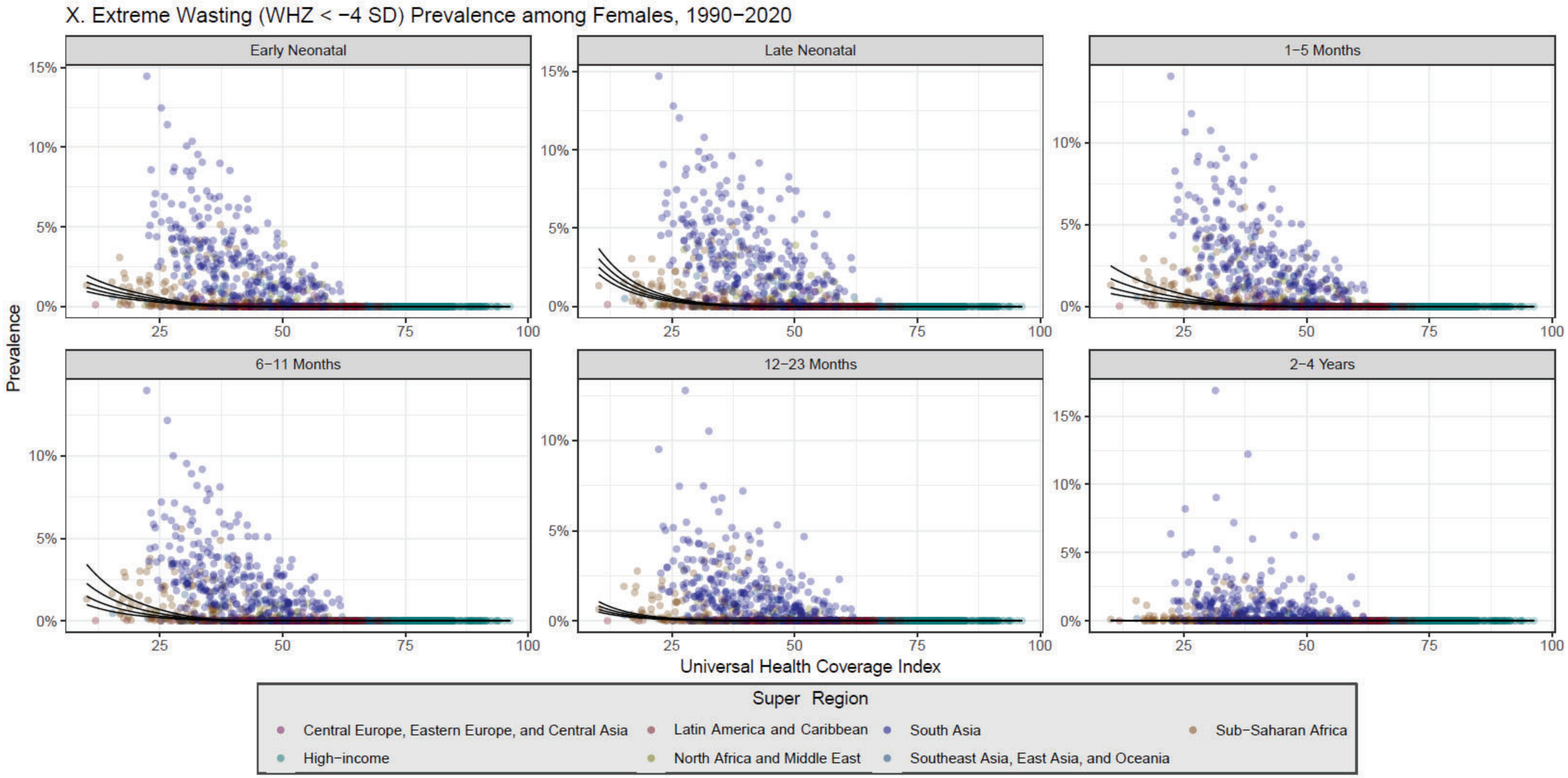
X. Extreme Wasting (WHZ < −4 SD) Prevalence among Females, 1990−2020
Early Neonatal
Late Neonatal
1−5 Months
6−11 Months
12−23 Months
2−4 Years
Prevalence
15%
10%
5%
0%
25
50
75
100
Universal Health Coverage Index
Super Region
Central Europe, Eastern Europe, and Central Asia
Latin America and Caribbean
South Asia
Sub−Saharan Africa
High−income
North Africa and Middle East
Southeast Asia, East Asia, and Oceania

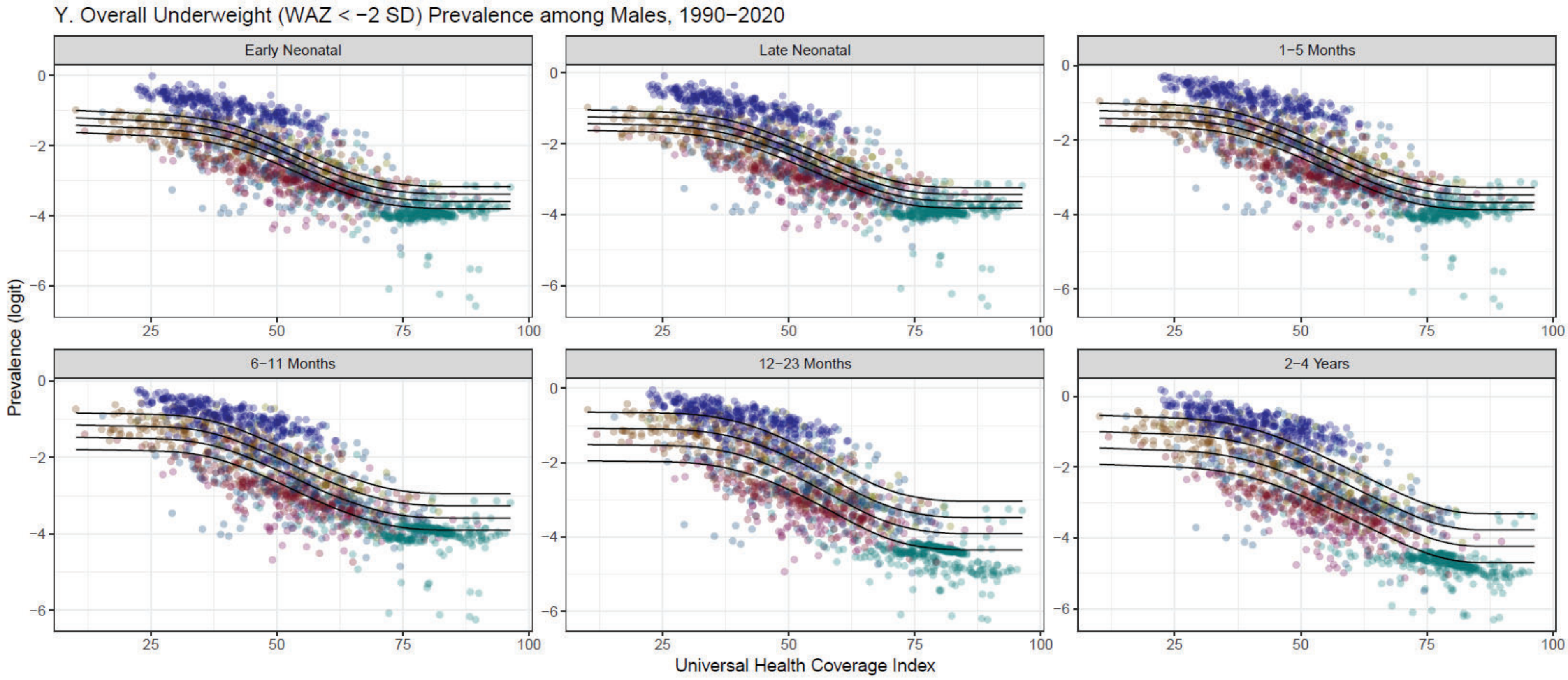

Y. Overall Underweight (WAZ < −2 SD) Prevalence among Males, 1990−2020
Early Neonatal
Late Neonatal
1−5 Months
6−11 Months
12−23 Months
2−4 Years
Prevalence (logit)
Universal Health Coverage Index
0
−2
−4
−6
25
50
75
100

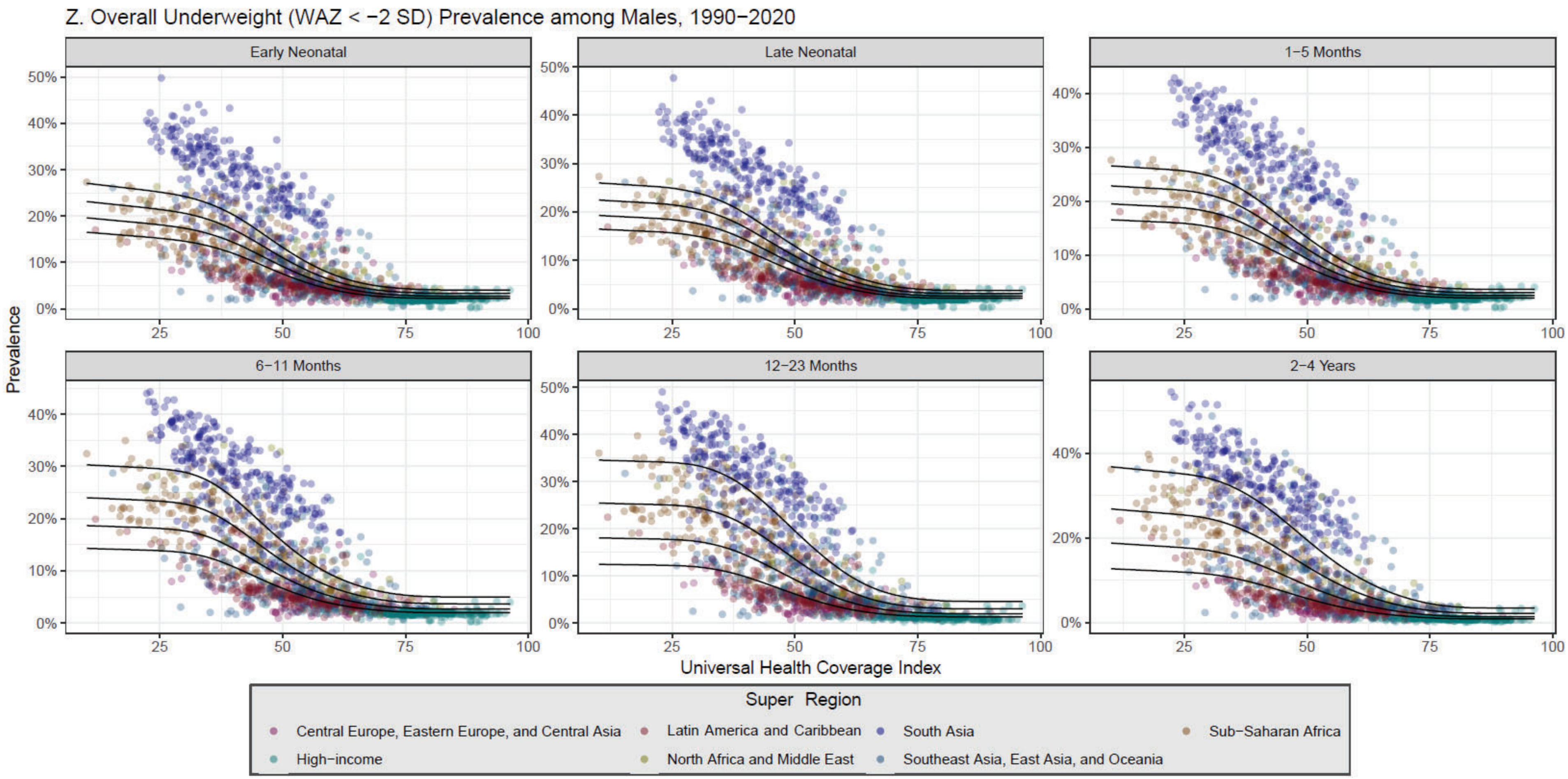

Z. Overall Underweight (WAZ < −2 SD) Prevalence among Males, 1990−2020
Early Neonatal
Late Neonatal
1−5 Months
6−11 Months
12−23 Months
2−4 Years
Prevalence
Universal Health Coverage Index
Super Region
Central Europe, Eastern Europe, and Central Asia
Latin America and Caribbean
South Asia
Sub−Saharan Africa
High−income
North Africa and Middle East
Southeast Asia, East Asia, and Oceania

AA. Overall Underweight (WAZ < −2 SD) Prevalence among Females, 1990−2020

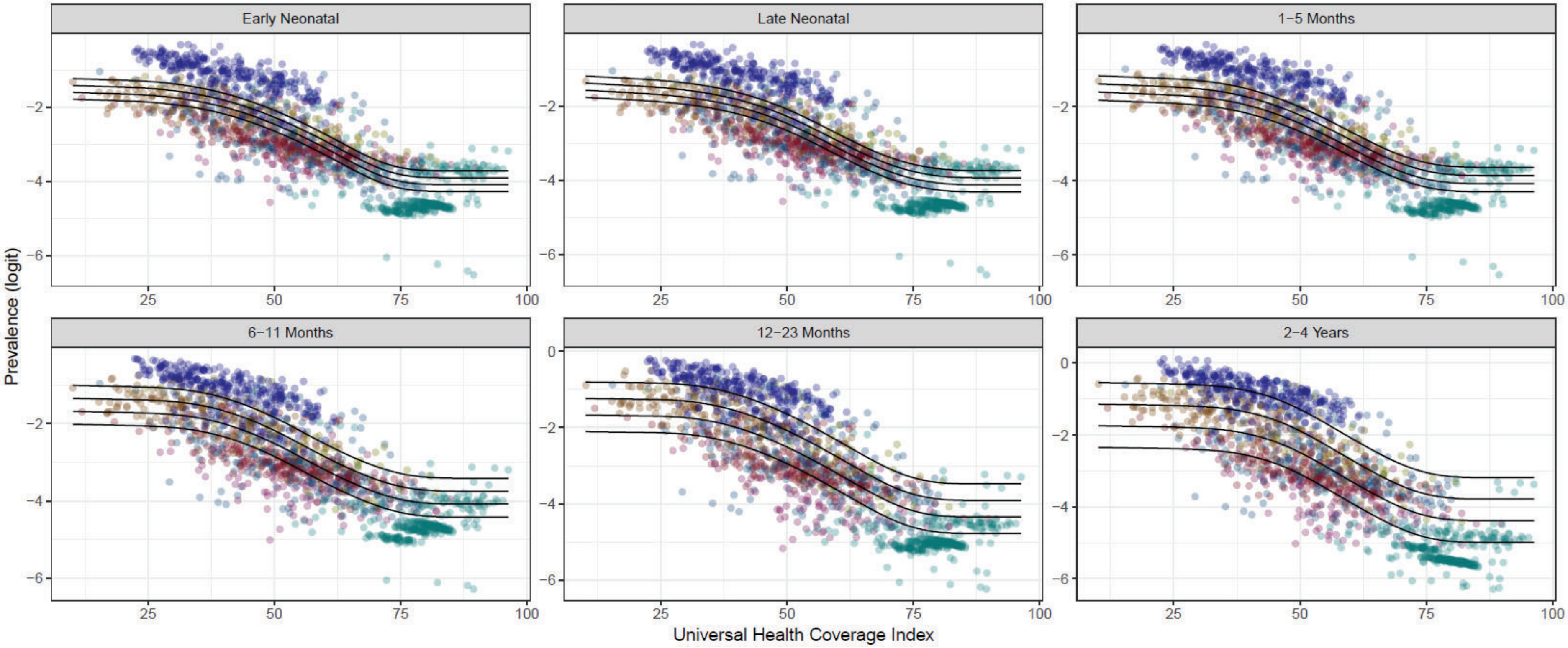

BB. Overall Underweight (WAZ < −2 SD) Prevalence among Females, 1990−2020

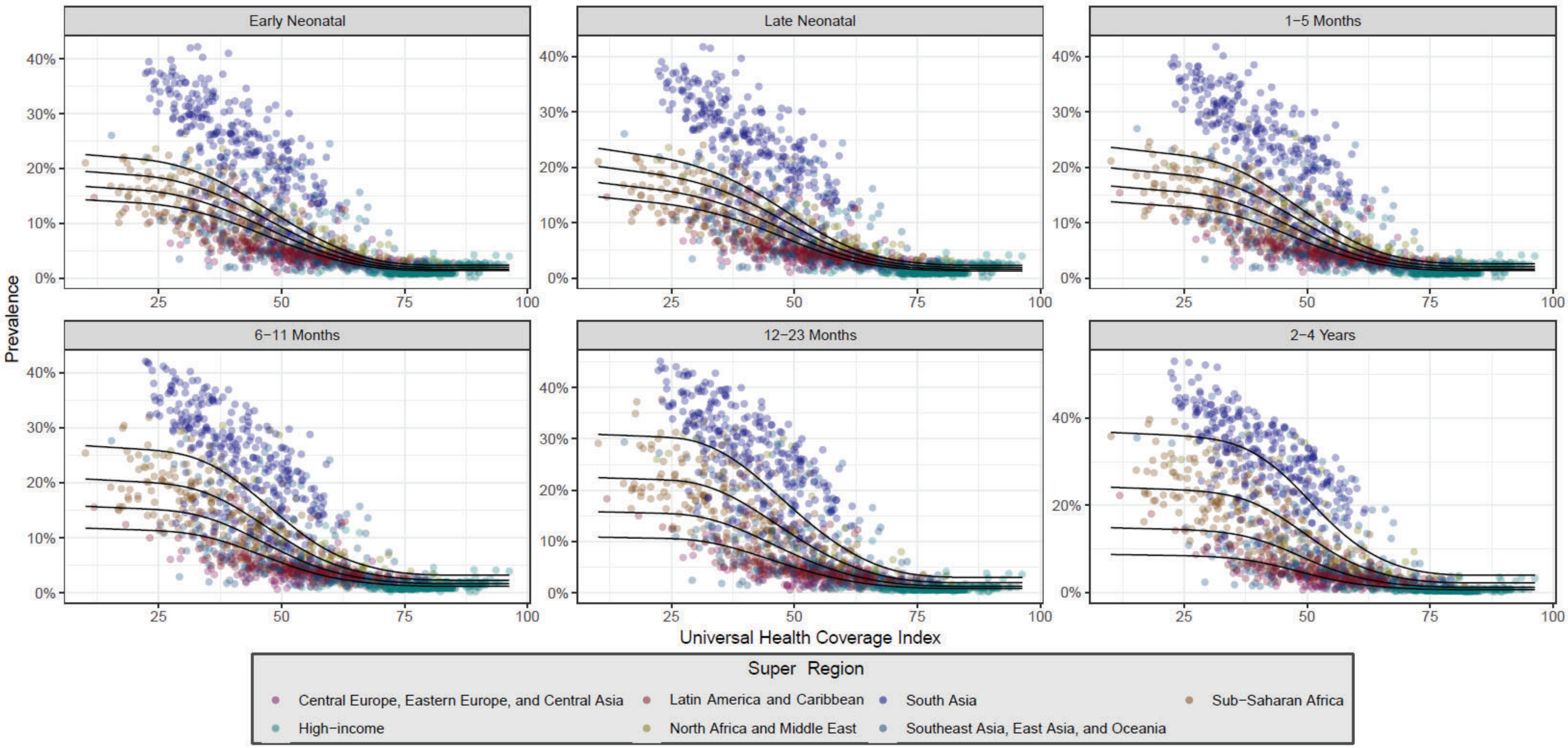

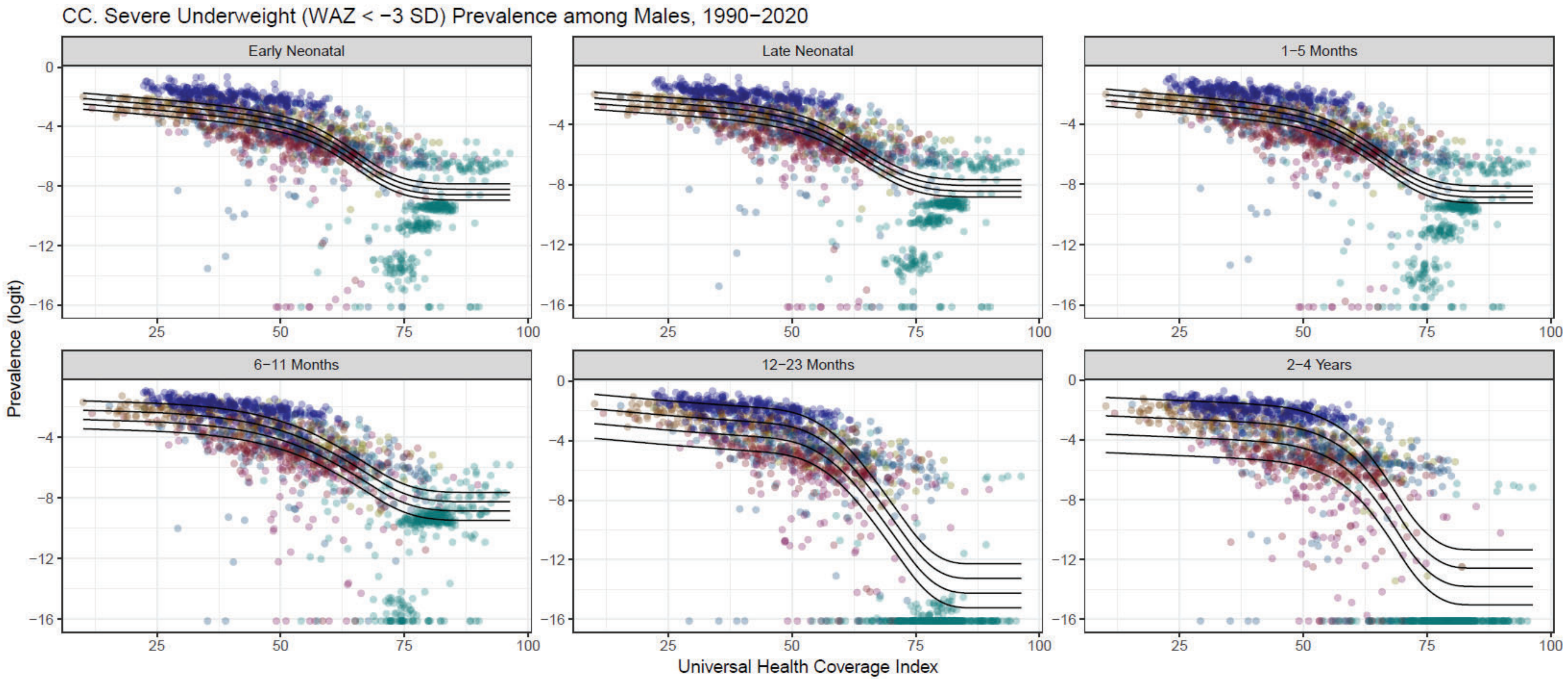
CC. Severe Underweight (WAZ < −3 SD) Prevalence among Males, 1990−2020
Early Neonatal
Late Neonatal
1−5 Months
6−11 Months
12−23 Months
2−4 Years
Prevalence (logit)
Universal Health Coverage Index
0
−4
−8
−12
−16
25
50
75
100

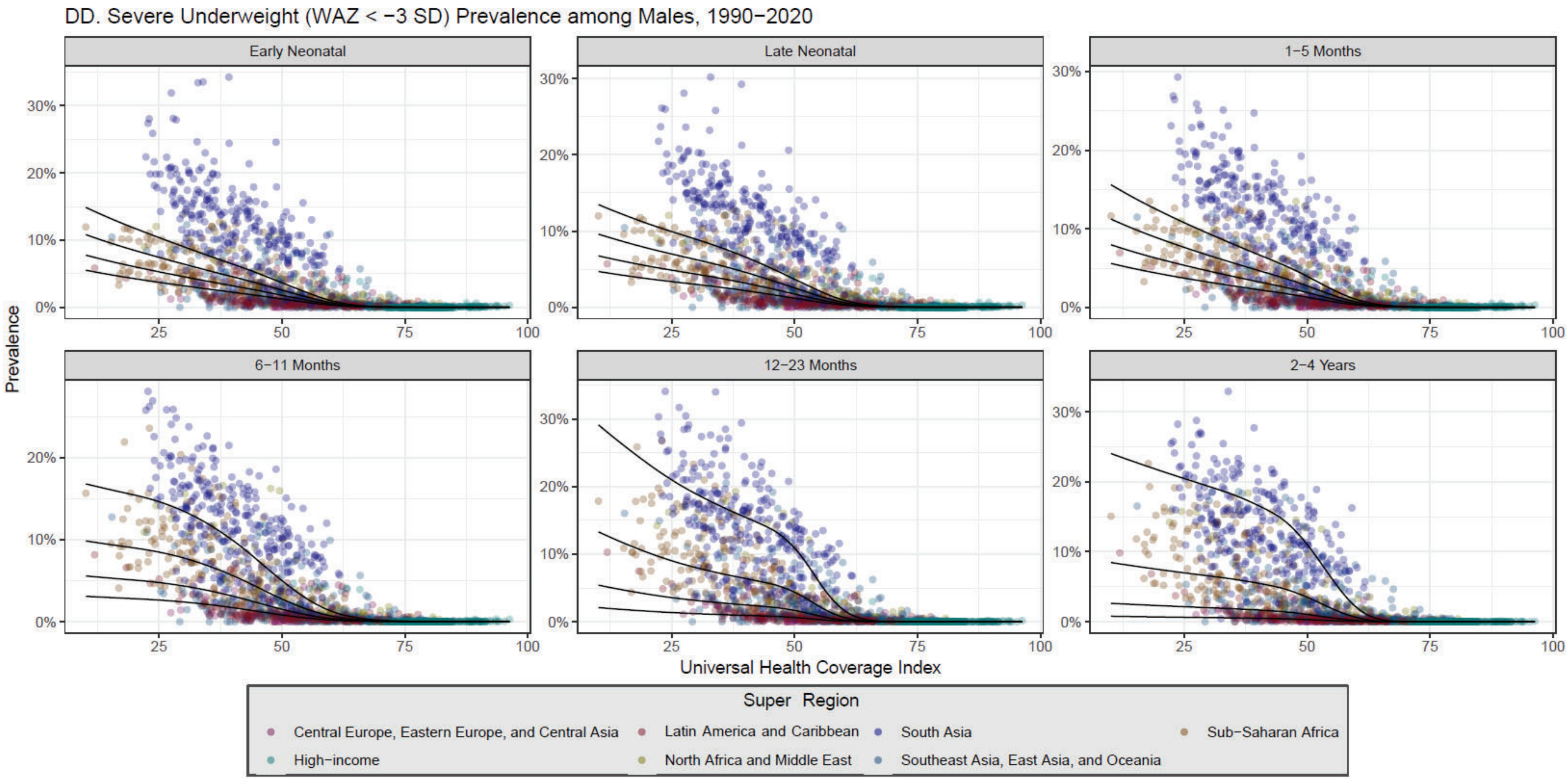
DD. Severe Underweight (WAZ < −3 SD) Prevalence among Males, 1990−2020
Early Neonatal
Late Neonatal
1−5 Months
6−11 Months
12−23 Months
2−4 Years
Prevalence
Universal Health Coverage Index
0%
10%
20%
30%
25
50
75
100
Super Region
Central Europe, Eastern Europe, and Central Asia
Latin America and Caribbean
South Asia
Sub−Saharan Africa
High−income
North Africa and Middle East
Southeast Asia, East Asia, and Oceania

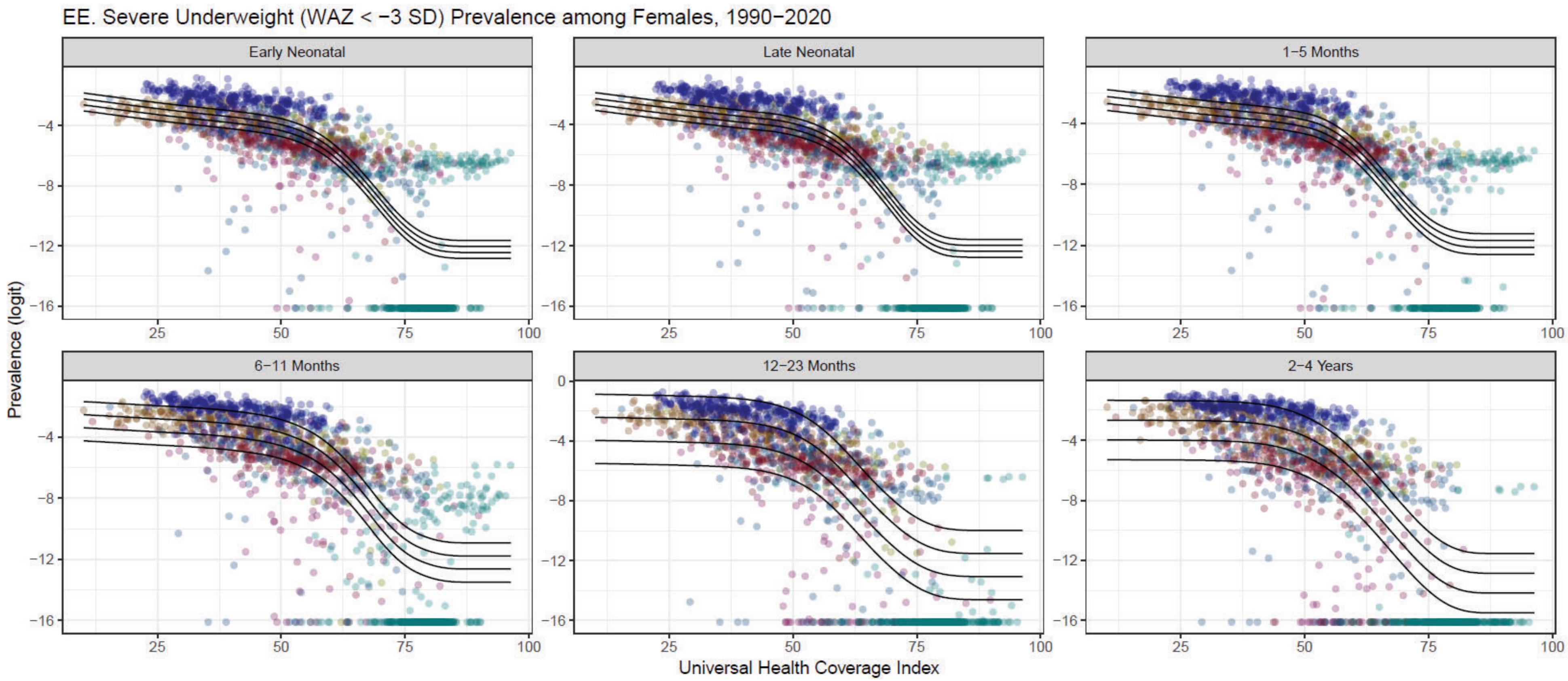
EE. Severe Underweight (WAZ < −3 SD) Prevalence among Females, 1990−2020
Early Neonatal
Late Neonatal
1−5 Months
6−11 Months
12−23 Months
2−4 Years
Prevalence (logit)
Universal Health Coverage Index
0
−4
−8
−12
−16
25
50
75
100

FF. Severe Underweight (WAZ < −3 SD) Prevalence among Females, 1990−2020

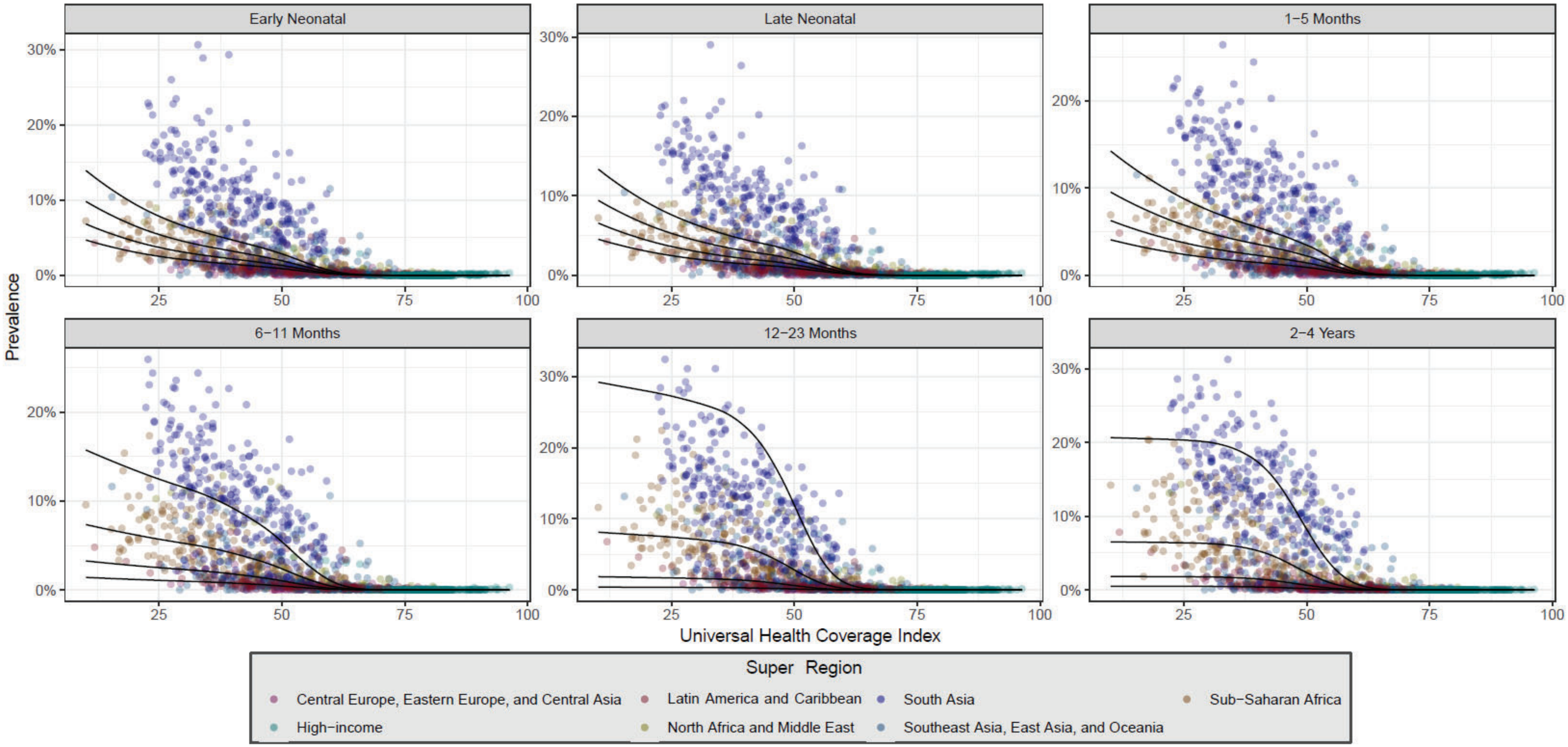

GG. Extreme Underweight (WAZ < −4 SD) Prevalence among Males, 1990−2020

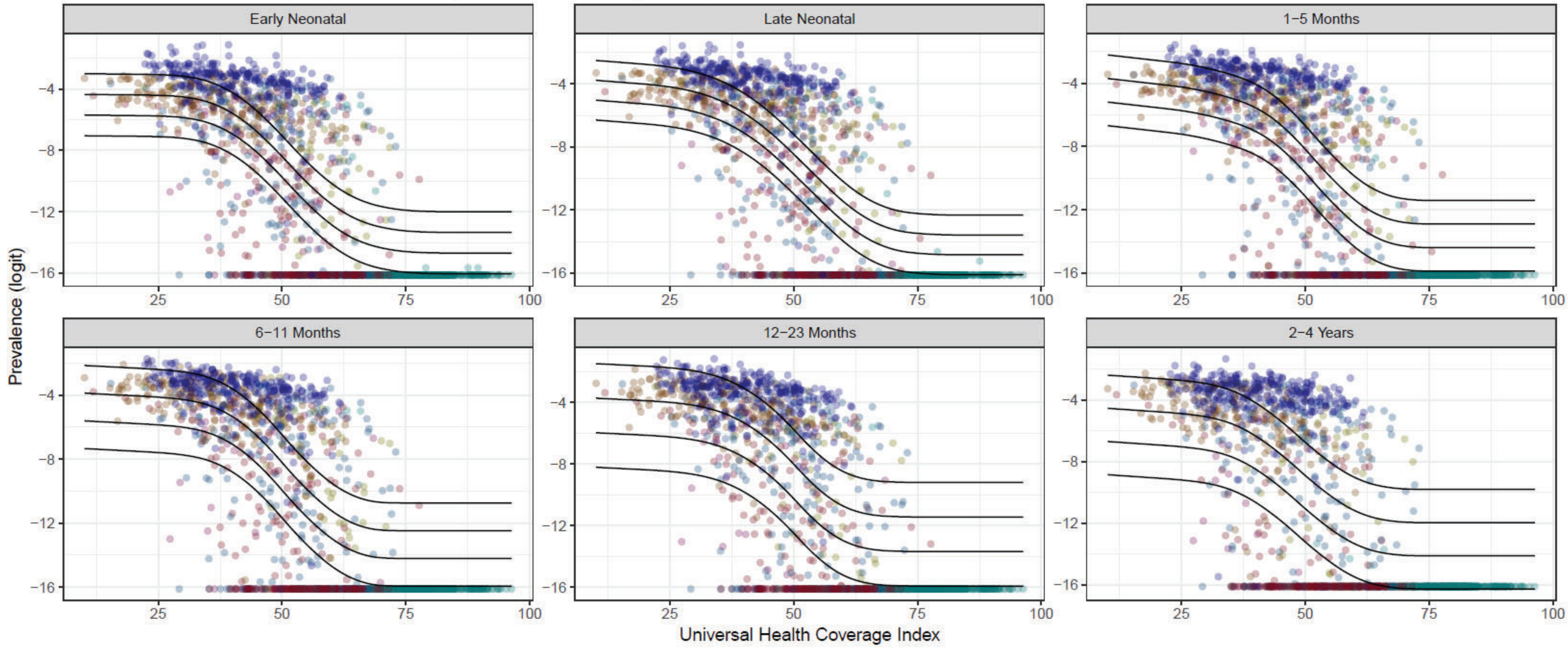

HH. Extreme Underweight (WAZ < −4 SD) Prevalence among Males, 1990−2020

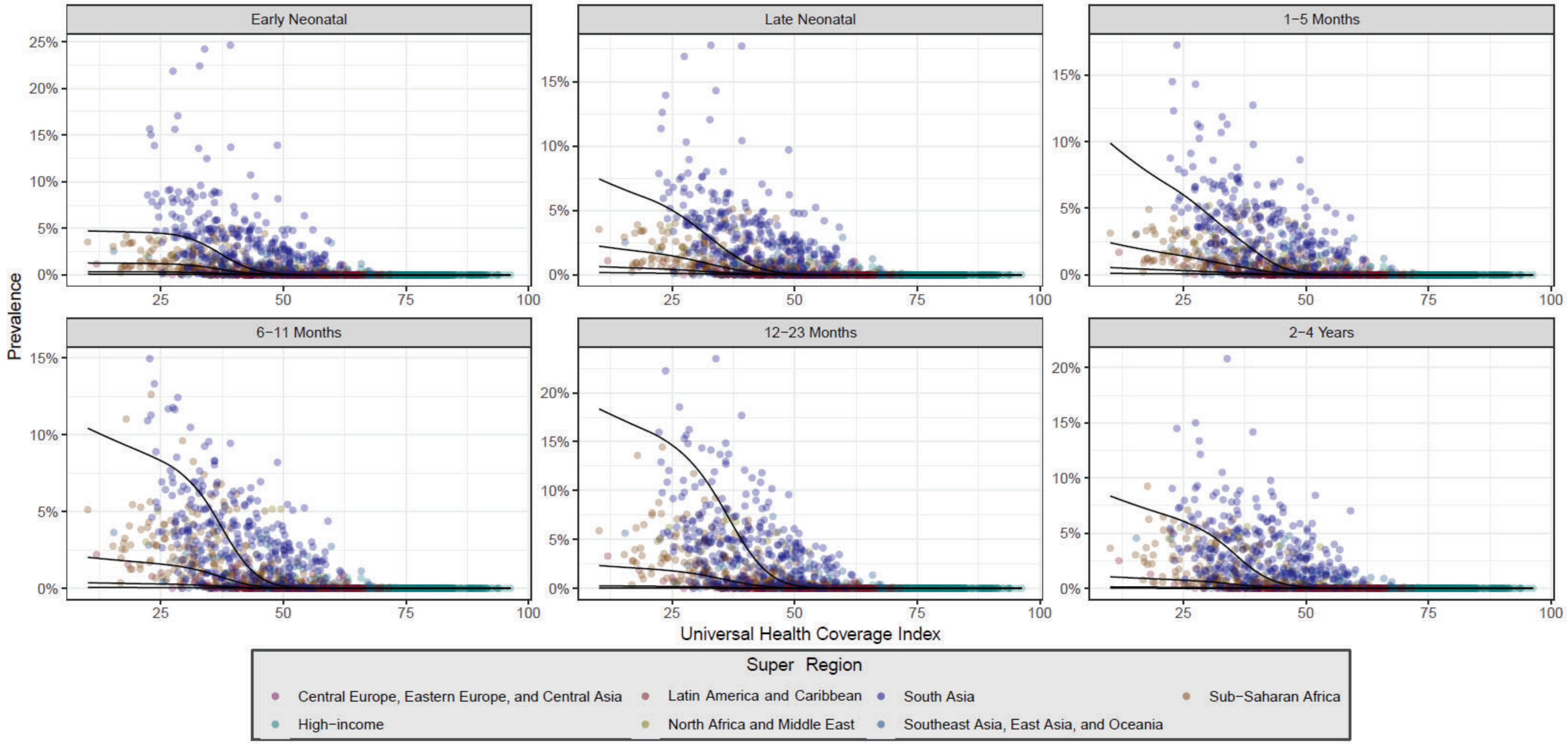

II. Extreme Underweight (WAZ < −4 SD) Prevalence among Females, 1990−2020

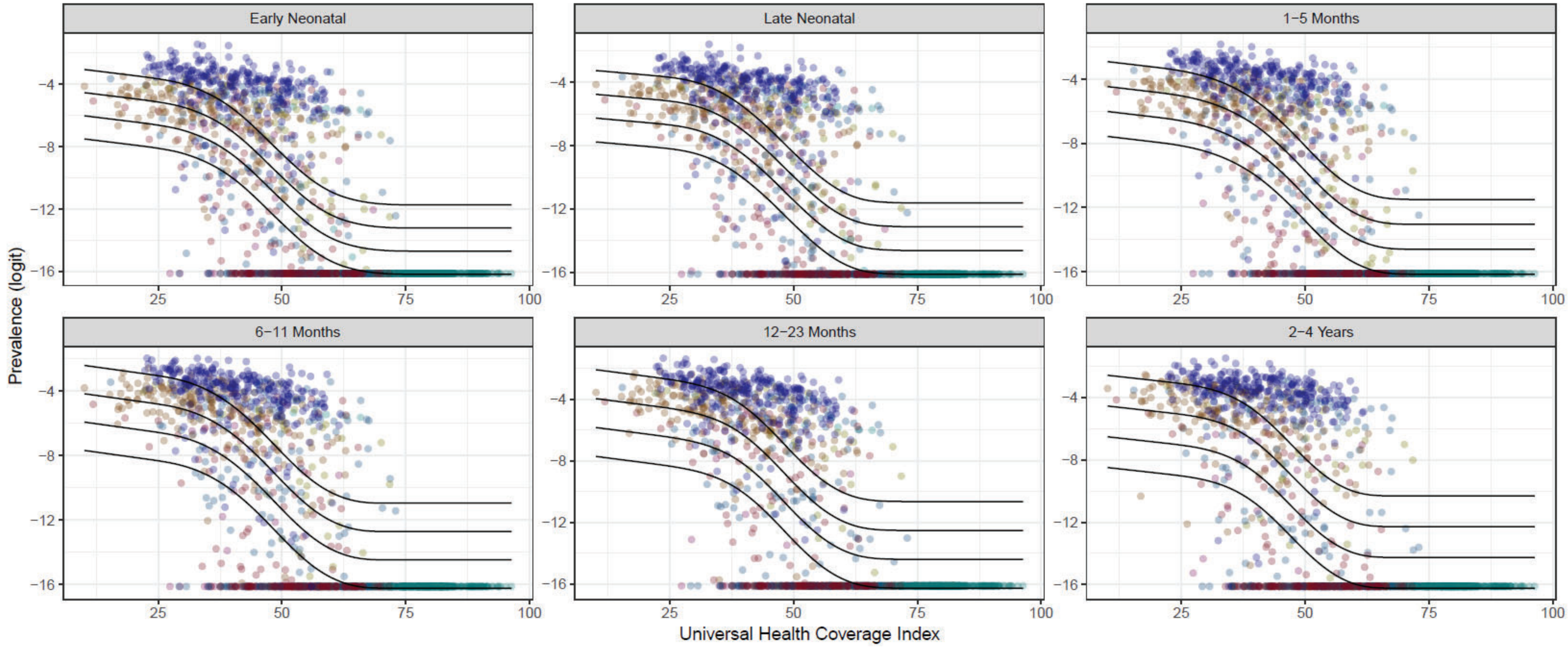

JJ. Extreme Underweight (WAZ < −4 SD) Prevalence among Females, 1990−2020

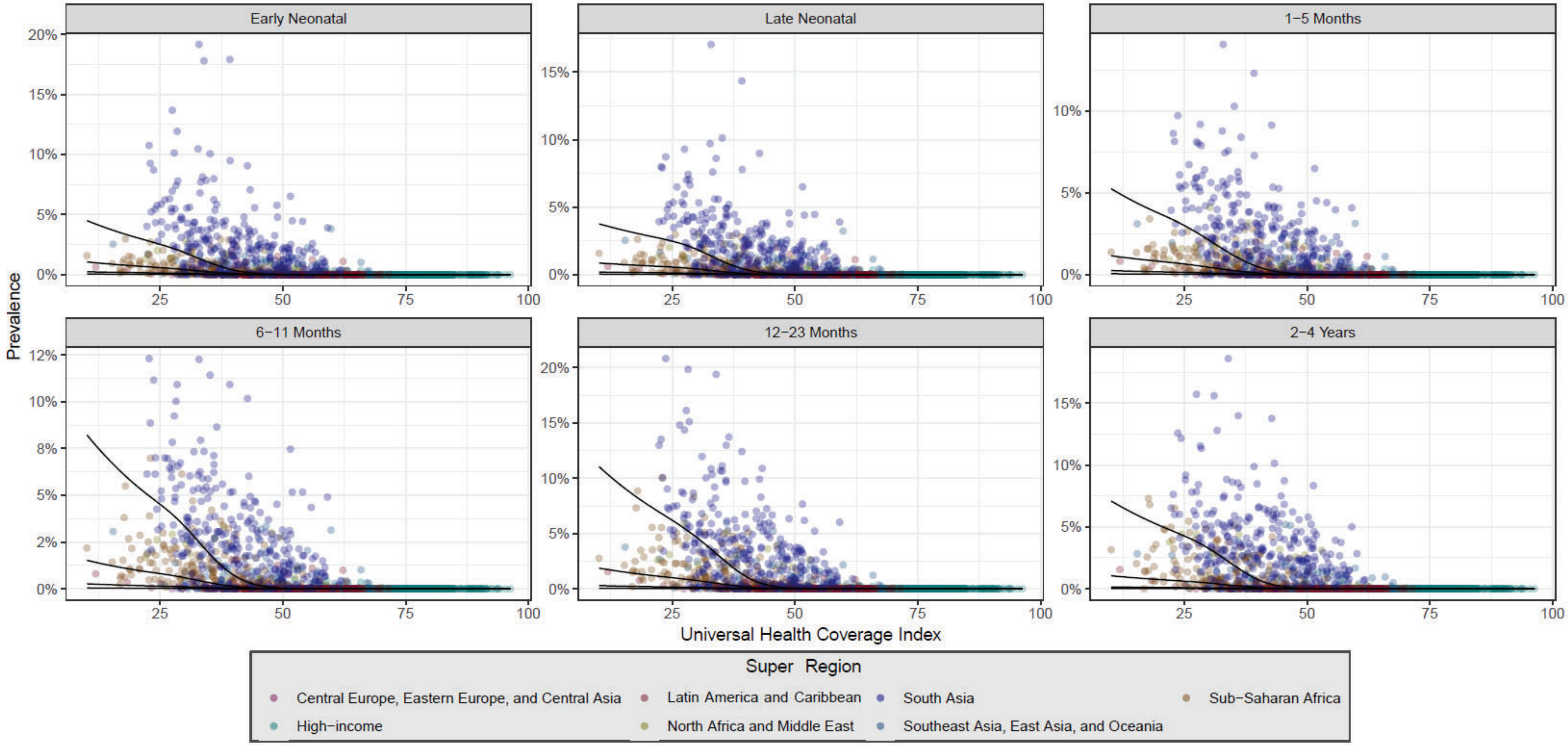

Supplement: Supplementary file 2 — Data S1 to S4 [file sciadv.abm8954_data_files_s1_to_s4.zip › sciadv.abm8954_data_file_s4.pdf]
